# Taxonomic and Environmental Variation of Metabolite Profiles in Marine Dinoflagellates of the Genus *Symbiodinium*

**DOI:** 10.3390/metabo5010074

**Published:** 2015-02-16

**Authors:** Anke Klueter, Jesse B. Crandall, Frederick I. Archer, Mark A. Teece, Mary Alice Coffroth

**Affiliations:** 1 SUNY—University at Buffalo, Graduate Program in Evolution, Ecology and Behavior and Department of Geology, Buffalo, NY 14260, USA; E-Mail: coffroth@buffalo.edu; 2 SUNY—College of Environmental Science and Forestry, Department of Chemistry, Syracuse, NY 13210, USA; E-Mails: jessecrandall@yahoo.com (J.B.C.); mteece@esf.edu (M.A.T.); 3 Southwest Fisheries Science Center, Marine Mammal and Turtle Division, La Jolla, CA 92037, USA; E-Mail: eric.archer@noaa.gov

**Keywords:** metabolism and metabolomics, metabolite profiling, phototrophic algae, marine dinoflagellates, *Symbiodinium*, bioinformatics and systems biology, Random Forests analysis

## Abstract

Microorganisms in terrestrial and marine ecosystems are essential to environmental sustainability. In the marine environment, invertebrates often depend on metabolic cooperation with their endosymbionts. Coral reefs, one of the most important marine ecosystems, are based on the symbiosis between a broad diversity of dinoflagellates of the genus *Symbiodinium* and a wide phyletic diversity of hosts (*i.e*., cnidarian, molluscan, poriferan). This diversity is reflected in the ecology and physiology of the symbionts, yet the underlying biochemical mechanisms are still poorly understood. We examined metabolite profiles of four cultured species of *Symbiodinium* known to form viable symbioses with reef-building corals, *S. microadriaticum* (cp-type A194), *S. minutum* (cp-type B184), *S. psygmophilum* (cp-type B224) and *S. trenchii* (cp-type D206). Metabolite profiles were shown to differ among *Symbiodinium* species and were found to be affected by their physiological response to growth in different temperatures and light regimes. A combined Random Forests and Bayesian analysis revealed that the four *Symbiodinium* species examined primarily differed in their production of sterols and sugars, including a C_29_ stanol and the two sterols C_28_Δ^5^ and C_28_Δ^5,22^, as well as differences in metabolite abundances of a hexose and inositol. Inositol levels were also strongly affected by changes in temperature across all *Symbiodinium* species. Our results offer a detailed view of the metabolite profile characteristic of marine symbiotic dinoflagellates of the genus *Symbiodinium,* and identify patterns of metabolites related to several growth conditions.

## 1. Introduction

Microorganisms serve a critical role in both terrestrial and marine ecosystems and are essential to environmental sustainability. Symbiosis between the microalga *Symbiodinium* spp. (Alveolata: Dinophycea: *Symbiodinium*) and a diverse group of cnidarians enables productivity through the mutual exchange of nutrients in the otherwise oligotrophic tropical seas. The endosymbiotic algae reside within the gastroderm of the anthozoan host and utilize metabolic waste to produce photosynthetically derived carbon metabolites that are then shared with the host [1,2,3]. It is the mutualistic exchange of metabolites between the cnidarian host, the algal symbiont and their associated microbial assemblages (the holobiont), that provides the foundation of the modern coral reef ecosystem and makes significant contributions to global carbon and biogeochemical cycles [4,5]. Yet these ecosystems are being lost at an alarming rate owing to mounting levels of environmental pressures (e.g., [6,7]). To fully understand and predict the impact of environmental change on the future resilience of global coral reef ecosystems we will require a more comprehensive understanding of how these important endosymbiosis are regulated and maintained.

Algal symbionts within the genus *Symbiodinium* are a heterogeneous group of many clades, many of which are presently of uncertain taxonomic status [8,9,10,11,12,13]. Diversity within the genus has been characterized at many taxonomic levels and the genus has been partitioned into multiple clades (A-I) [8,9,10,12]. This taxonomic diversity is reflected in the ecology and physiology of these symbionts. We now recognize that symbionts within a single host are both diverse and dynamic, changing in response to environmental conditions as well as ontogenetically ([14,15,16]). Significant biological and physiological differences exist among distantly, as well as, closely related *Symbiodinium* (growth rates in cultures, [17,18]; photo-adaptation, (e.g., [19,20]); response to increased or decreased temperature, (e.g., [21,22,23,24,25,26,27]); chlorophyll fluorescence, (e.g., [28]), and other physiological traits, (e.g., [29,30,31,32]). Moreover, although different symbiont isolates produce similar metabolites, their proportions shift under different environmental conditions (e.g., [33,34,35,36]). A growing body of research suggests that a corals response to environmental change may not be due to the plasticity of the coral or the algal symbiont within the coral, but due to plasticity in the holobiont itself, so that the same host can manifest different responses depending on the host-symbiont pairing (e.g., [37,38,39,40]). While much progress has been made in understanding the interaction between host and symbiont, we need to gain a better understanding of *Symbiodinium*-cnidarian interactions with an aim at identifying the processes that maintain a viable symbiosis.

Metabolomics is the detection and quantification of the suite of metabolites that are produced and transformed by an organism. This snapshot of the complete set of metabolites, or metabolome, of an organism contains a wide range of biochemicals including secondary natural products that may serve specific roles in physiology, communication, or defense, or are responses to changes in the environment. Other metabolites are involved in energy metabolism, protein and DNA synthesis, and cell wall structure [41]. The large structural diversity and chemical complexity of metabolites can make analysis of the metabolome an analytical challenge. However, significant strides have been made in instrumentation and analysis of the large amounts of metabolomic data [42,43] and metabolomics studies are expanding into new fields [44,45]. Metabolomics has proven valuable in the marine sciences [46]. For example, studies of major bloom forming marine diatoms revealed distinct patterns of metabolite release depending on algal growth phases and the release of some of these compounds resemble signal molecules potentially involved in quorum sensing by bacteria [47,48]. Other metabolomic studies have helped to identify cryptic species in the sea slug *Doris kerguelenensis* [49] or demonstrated that ocean acidification and increased water temperatures affect metabolic function and energy metabolism of oysters [50]. Organismal studies on Atlantic salmon [51], red abalone [52,53], and marine mussels [46] have shown significant metabolic responses to bacterial or anthropogenic influences, and provided substantial insight into mechanisms of response to external environmental factors [54].

In this study we examine the metabolite profiles of the marine dinoflagellate *Symbiodinium* spp. and identify similarities, as well as differences in the composition and abundance of metabolites among different *Symbiodinium* species. Given the direct effect that environmental parameters such as temperature and light have on the photobiology of these marine dinoflagellates (e.g., [25,27,55,56,57,58,59]), we were also interested in understanding how metabolite profiles are affected by changes in these factors. Therefore, we grew four different species of cultured *Symbiodinium* under a variety of temperature and light conditions to directly measure the effects of environmental growth conditions on the metabolome.

## 2. Results and Discussion

To investigate patterns in the algal metabolome related to individual *Symbiodinium* species or environmental conditions such as different temperatures and light intensities, four different species of cultured *Symbiodinium* were grown to exponential phase and subjected to gas-chromatography mass spectrometry (GC-MS). The resulting metabolite profiles of theses cultures were then analyzed using GC-MS analysis. All four species used in this study are known to form viable symbioses with cnidarian hosts. The *Symbiodinium* species used in this study were *S. microadriaticum*, (classified as a *Symbiodinium* cp-type A194 based on clade (A) and fragment length (194bp) of the hypervariable region of domain V in the chloroplast 23S rDNA [cp-type]), *S. minutum* (*Symbiodinium* cp-type B184), *S. psygmophilum* (*Symbiodinium* cp-type B224) and *S. trenchii* (*Symbiodinium* cp-type D206). For the sake of brevity and consistency with other studies, in this study, we refer to these species by their type designation.

GC-MS analyses of the free metabolites of *Symbiodinium* samples detected hydrophilic as well as lipophilic components (Supplementary Information Table S1). A range of sugars, sterols, amino acids, organic acids, phosphoric compounds, glycerol, and fatty acids were identified. In total, 188 individual metabolites were detected, of which 33 were missing in an excessive number of samples, leaving 155 individual metabolites suitable for statistical analyses (Supplementary Information Table S1).

To uncover patterns of metabolite profiles that are characteristic of *Symbiodinium* species or environmental growth conditions, we conducted a Random Forests analysis, which has rapidly become a popular method for mining large datasets due to its ability to quantify patterns of overlap among groups of samples in an unbiased manner and simultaneously identify and rank the variables most responsible for group discrimination [60,61]. We then constructed a multifactorial Bayesian model to estimate the magnitude of differentiation among samples for metabolites identified as being important predictors in the Random Forests analysis. Bayesian models are preferred over standard frequentist methods because of their ability to accurately communicate the uncertainty around estimates rather than reducing the results to presentations of *p*-values, which are often misunderstood and can be misleading [62,63,64]. This combination of analyses provides for a sophisticated question-driven method that, to our knowledge, has not yet been applied in similar studies of the effects of environmental changes on the metabolome of marine dinoflagellates.

### 2.1 Patterns of Metabolite Variability

**Figure 1:**
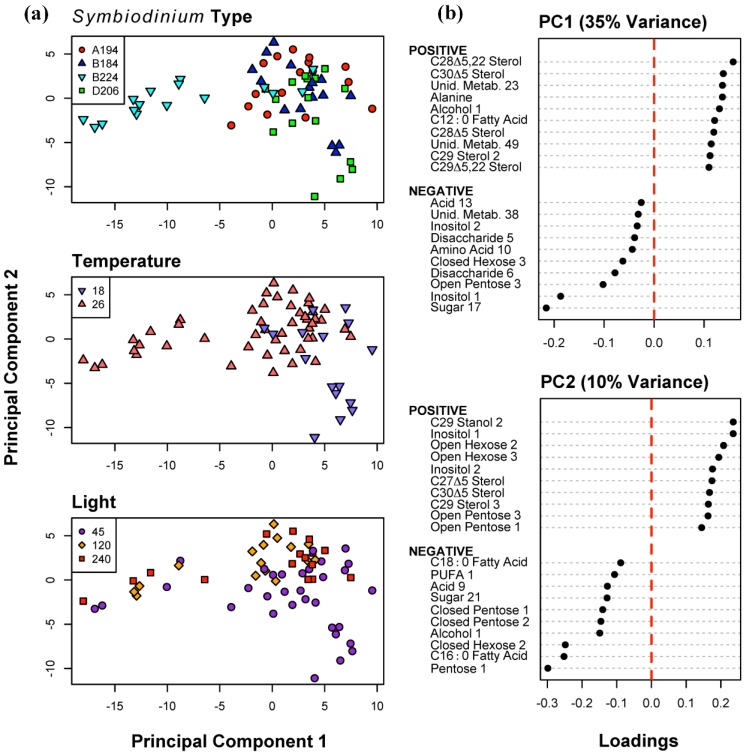
Principal Components Analysis of 155 metabolites for all 64 samples. (**a**) Distribution of sample scores on Principal Components (PC) 1 and 2, identified to *Symbiodinium* type, temperature (18 and 26 °C), and light intensity (45, 120 and 240 μmol photons m^−20^ s^−1^) algae were cultured at; (**b**) The ten most positive and negative metabolite loadings on PC 1 and 2.

To visualize the main sources of variability in the metabolite profiles across all samples, and their distribution relative to (i) *Symbiodinium* type, (ii) differences in temperatures, and (iii) different light intensities, we conducted a Principal Components Analysis (PCA) (Figure 1). Over 90% of the variation in the data could be accounted for by the first 24 components (Supplementary Information Figure S2). The 13th component was the first that explained less than 1/64th of the variance (*i.e*., explained as much variance as one sample). The first three components accounted for over 50% of the variance. The first component (PC1—35% of the variance; Figure 1) was defined as a contrast primarily between two sterols (e.g., C_28_Δ^5.22^ and C_30_Δ^5^), and three sugars (e.g., Sugar 17, Inositol 1, Open Pentose 3). The second component PC2 (10% of the variance) was primarily defined by a contrast between some sterols and sugars (e.g., C_29_ Stanol 2, Inositol 1, Open Hexose 2) and sugars and fatty acids (e.g., Pentose 1, Fatty Acid C_16:0_, Closed Hexose 2). The variability along PC1 is primarily due to the strong differentiation of *Symbiodinium* type B224 at 26 °C from the remaining samples (Figure 1). At 18 °C and the lowest light intensity (45 μmol photons m^−2^ s^−1^), all four samples of *Symbiodinium* type D206, and three samples of *Symbiodinium* type B184 form a cluster in the negative region of PC2. There is also some separation of samples at 45 μmol photons m^−2^ s^−1^ from those at 120 and 240 μmol photons m^−2^ s^−1^ on PC2.

### 2.2. Classification of Symbiotic Dinoflagellates and Environmental Conditions based on Metabolite Profiles

**Figure 2:**
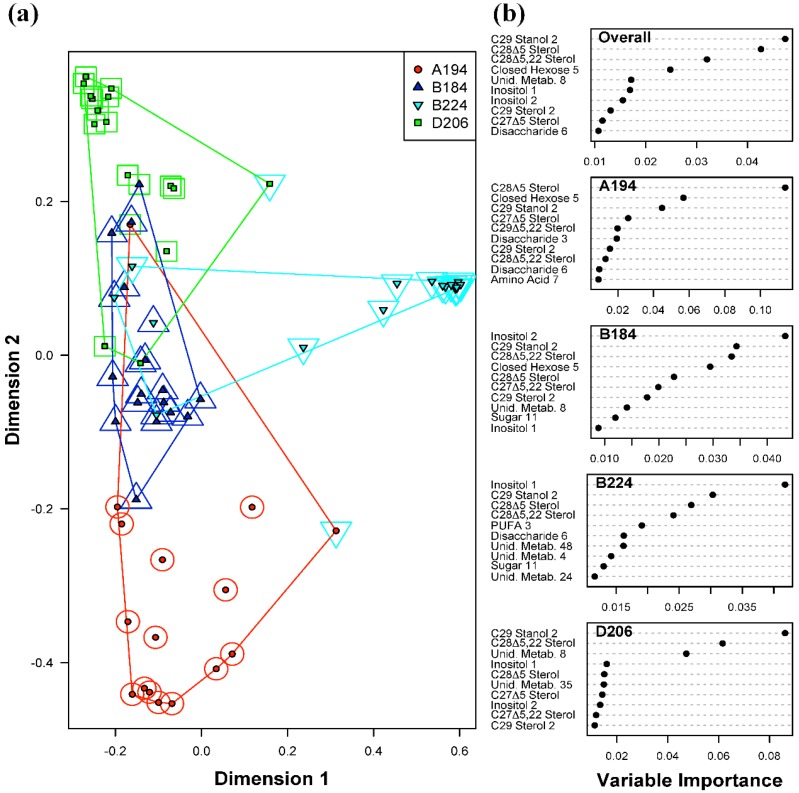
Random Forests classification of *Symbiodinium* type (A194, B184, B224 and D206). (**a**) Plot of proximities of all 64 samples from 5000 Random Forests trees as projected on the first two Principal Coordinate dimensions. Color and shape of the central filled points indicate the observed *Symbiodinium* type of the sample (A194, B184, B224 and D206), while the surrounding color and shape indicate the type predicted by Random Forests. Colored lines are convex hulls linking the outermost points of each type; (**b**) Distribution of importance scores for the 10 most important predictor metabolites (based on mean decrease in prediction accuracy). Plot labeled “Overall” depicts metabolites found to be important for overall prediction accuracy. Other plots show metabolites important for classifying each particular *Symbiodinium* type.

The overall error rate (fraction of all samples misclassified) for the Random Forests classification of *Symbiodinium* types was 12.5% (Table 1a). In comparison to an expected error rate of 75% based on random assignment of samples, this indicates that the model is relatively good at distinguishing types. Each *Symbiodinium* type had between 1 and 3 samples out of 16 misclassified, suggesting strong diagnostic features of their metabolite profiles. As can be seen in Figure 2, *Symbiodinium* type B224 is the most differentiated type out of the four, forming a distinct cluster; however, B184 had only a single misclassification (Table 1a), suggesting that it had the most diagnostic metabolite profiles. The most important predictor metabolites for all four *Symbiodinium* types were C_29_ Stanol 2, and sterols C_28_Δ^5^ and C_28_Δ^5.22^ (“Overall” Figure 2). There is some overlap between these metabolites and those found to be highly loading in the PCA analysis above. This further indicates that the *Symbiodinium* types primarily differed in their sterol levels and as a consequence much of the variability in metabolite profiles was due to these differences. However, the two most important metabolite predictors for *Symbiodinium* types B184 and B224 were the sugars Inositol 1 and 2. In other words, for these two *Symbiodinium* types, the levels of inositol were distinguishing features.

**Figure 3:**
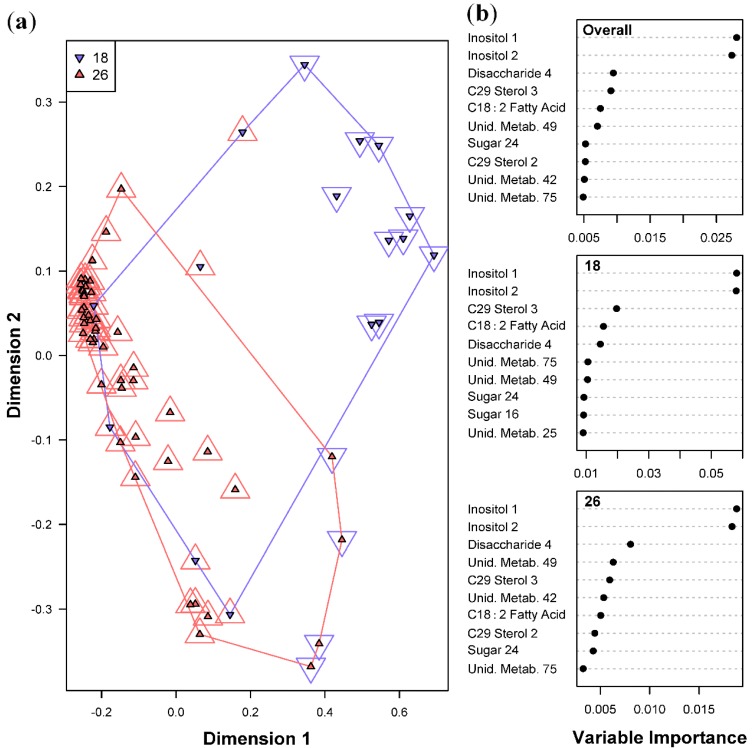
Random Forests classification of temperature (18 and 26 °C). (**a**) Plot of proximities of all 64 samples from 5000 Random Forests trees as projected on the first two Principal Coordinate dimensions. Color and shape of the central filled points indicate the temperature the sample was cultured in (18 and 26 °C), while the surrounding color and shape indicate the temperature predicted by Random Forests. Colored lines are convex hulls linking the outermost points of each temperature; (**b**) Distribution of importance scores for the 10 most important predictor metabolites (based on mean decrease in prediction accuracy). Plot labeled “Overall” depicts metabolites found to be important for overall prediction accuracy. Other plots show metabolites important for classifying each temperature.

Metabolite profiles were found to be indicative of a temperature that the *Symbiodinium* cultures were subjected to. Such metabolites diagnostic of a given temperature condition across all samples is shown in Figure 3. The overall error for the Random Forests model classifying temperature was slightly higher than that for *Symbiodinium* types at 15.6% (Table 1b), with 6 samples at 18 °C, and 4 samples at 26 °C misclassified. Due to experimental limitations, samples placed in 18 °C could only be grown at 45 μmol photons m^−2^ s^−1^. This caused a data imbalance for the light intensities at 120 and 240 μmol photons m^−2^ s^−1^. Given the imbalance in sample size between the two temperature treatments, the expected error rate for samples at 18 °C based on chance alone would be 75%. Therefore, the observed error rate of 37.5% for the samples at 18 °C demonstrates a considerable improvement in classification performance of the model over random chance. The sugars Inositol 1 and 2 were found to be the most important predictor metabolites of temperature and light intensities across all four *Symbiodinium* types, indicating that these two environmental treatments strongly affected the concentrations of these sugars. Lesser, but still significant, important predictor metabolites for temperature include a disaccharide, C_29_ Sterol 3, and fatty acid C_18:2_.

**Figure 4:**
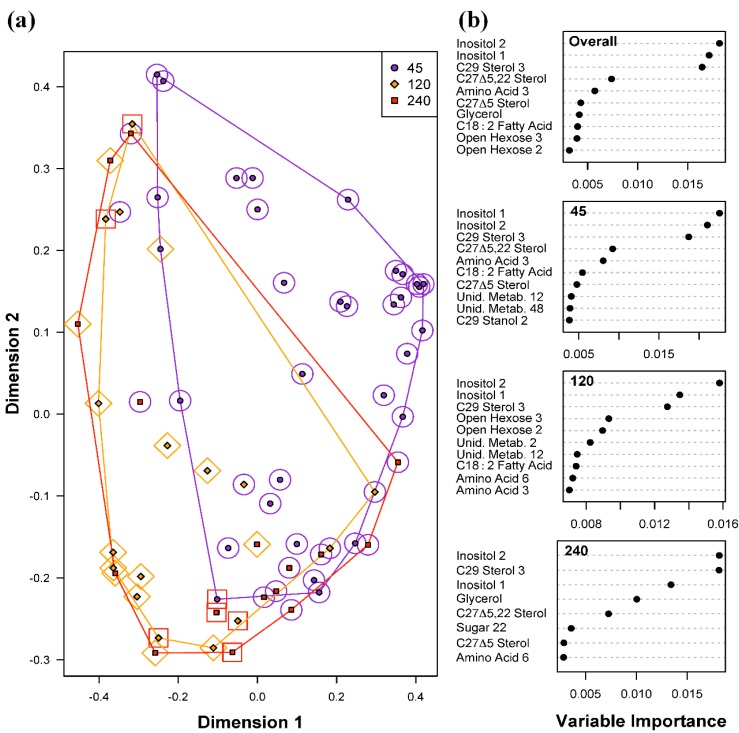
Random Forests classification of light intensities (45, 120 and 240 μmol photons m^−20^ s^−1^). (**a**) Plot of proximities of all 64 samples from 5000 Random Forests trees as projected on the first two Principal Coordinate dimensions. Color and shape of the central filled points indicate the light level the sample was cultured in (45, 120 and 240 μmol photons m^−20^ s^−1^), while the surrounding color and shape indicate the light intensity predicted by Random Forests. Colored lines are convex hulls linking the outermost points of each temperature; (**b**) Distribution of importance scores for the 10 most important predictor metabolites (based on mean decrease in prediction accuracy). Plot labeled “Overall” depicts metabolites found to be important for overall prediction accuracy. Other plots show metabolites important for classifying light intensity.

Random Forests models for classifying the three levels of light intensities had the highest overall error rate of 37.5% (Table 1c), suggesting that variations in light intensities did not exert as strong an effect on the metabolite profiles as was seen for the two temperature levels examined. Given the distribution of samples among the three light intensities, the overall error rate due to random chance would be 62.5%, meaning that even with the high observed error rate of 37.5%, the model results indicate a small effect on the metabolite profiles. The high overall error rate was largely due to misclassification of samples grown at 120 and 240 μmol photons m^−20^ s^−1^ (error rates of 50% and 87.5% respectively). There were only two misclassifications of samples at 45 μmol photons m^−20^ s^−1^, producing a classification error of 6.3%. Similar to the Random Forests results for temperature differences, both inositol metabolites (Inositol 1 and 2) were the most important predictors for light intensities (Figure 4). However, C_29_ Sterol 3 was also identified as an important predictor in the overall model, as well as individually for each light intensity. Glycerol is an important predictor metabolite of light intensity due to its importance in distinguishing samples at 240 μmol photons m^−20^ s^−1^ from those at 45 and 120 μmol photons m^−20^ s^−1^. Whether or not this difference is biologically significant needs to be evaluated carefully due to the large error rate for this class, and the relative importance scores for glycerol.

**Table 1:** Confusion matrices from Random Forests classification of experimental factors (**a**) *Symbiodinium* type; (**b**) temperature (°C), and (**c**) light levels (μmol photons m^−20^ s^−1^). Rows are *a priori* designated levels and columns are predicted levels. Classification Error is the percent of misclassified samples in each category, and the final row, “Overall”, is the total percent of samples misclassified. Random Error is the classification error expected by random assignment based on the distribution of sample sizes among levels.

**(a)**	***Symbiodinium* Type**	**A194**	**B184**	**B224**	**D206**	**Classification Error**	**Random Error**
	A194	14	0	1	1	12.5	75
	B184	0	15	0	1	6.3	75
	B224	0	3	13	0	18.8	75
	D206	0	1	1	14	12.5	75
	Overall					12.5	75
**(b)**	**Temperature**	**18**	**26**	****	****	**Classification Error**	**Random Error**
	18	10	6			37.5	75
	26	4	44			8.3	25
	Overall					15.6	37.5
**(c)**	**Light**	**45**	**120**	**240**	****	**Classification Error**	**Random Error**
	45	30	1	1		6.3	50
	120	4	8	4		50	75
	240	9	5	2		87.5	75
	Overall					37.5	62.5

### 2.3. Differences among Diagnostic Metabolite Profiles for Symbiotic Dinoflagellates and Environmental Conditions

The Random Forests results described above were used to identify and rank metabolites that showed significant differentiation among levels for each of the three main factors (*Symbiodinium* type, temperature, and light intensities). In order to estimate the magnitude of differences between these important metabolites, a multi-factorial Bayesian model was then constructed. Estimates of the relative metabolite abundance value from these Bayesian models are represented by posterior probability distributions, which incorporate the uncertainty resulting from small sample sizes within a specific treatment as well as variability in response across treatments. Based on their ability to distinguish *Symbiodinium* types, temperatures, or light intensities (Figure 2, Figure 3 and Figure 4), we examined differences in the relative abundance of the most diagnostic metabolites for each of these three factors. Estimates of metabolite abundance for all 155 metabolites are presented in Supplementary Information Figures S3–5. Unidentified metabolites (*n* = 47) were omitted from the illustrations and discussions due to inability to resolve their identifications making a presentation of their results of limited utility.

#### 2.3.1. Metabolite Profiles Specific of Different Symbiotic Dinoflagellates

**Figure 5:**
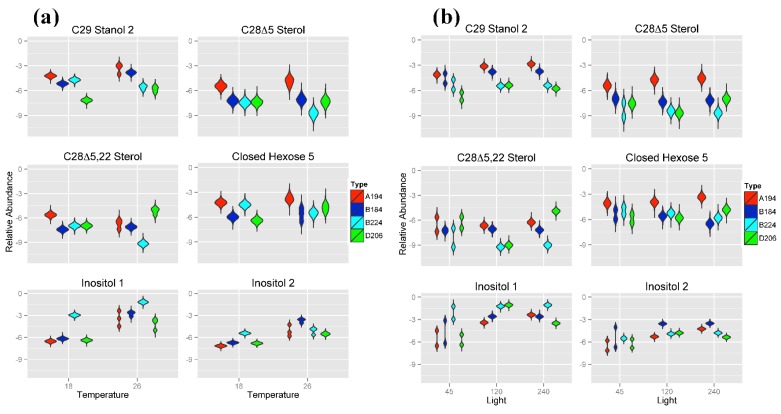
Violin plots showing posterior distributions of estimates of the top six significant predictor metabolites of *Symbiodinium* type. Metabolite abundance values are Area Under the Curve (AUC)-normalized. Predictor metabolites are sorted from top left to lower right according to their relative importance scores derived from Random Forests analysis. Posterior distributions are grouped by (**a**) temperature (°C) and (**b**) light intensity (μmol photons m^−20^ s^−1^). The width of each figure in the violin plot is proportional to the relative frequency of values from the posterior distribution.

As can be seen from the Random Forests results (Figure 2) and the distributions in Figure 5a,b, *Symbiodinium* type A194 was primarily characterized by a higher metabolite abundance of two steroidal alcohols (C_29_ Stanol 2 and sterol C_28_Δ^5^ Sterol) as well as higher metabolite abundances of Closed Hexose 5 at each temperature and light intensity. Conversely, *Symbiodinium* type D206 had a low metabolite abundance of the sterols C_29_ Stanol 2 and a higher metabolite abundance of C_28_Δ^5.22^ Sterol at 26 °C although differentiation from B224 at 18 °C or 120 μmol photons m^−20^ s^−1^ was minimal. The two *Symbiodinium* types of Clade B (B184 and B224) were primarily distinguished by their distributions of the two Inositol compounds wherein B184 had a higher abundance of Inositol 2 at 26 °C and B224 had a higher abundance of Inositol 1 than the other three *Symbiodinium* types at both temperatures. Metabolite abundance values for the sterols C_29_ Stanol 2, C_28_Δ^5^ and C_28_Δ^5.22^ at 26 °C tended to be greater in *Symbiodinium* type B184 than they are in type B224.

#### 2.3.2. Effect of Temperatures on Metabolite Profiles of Symbiotic Dinoflagellates

**Figure 6:**
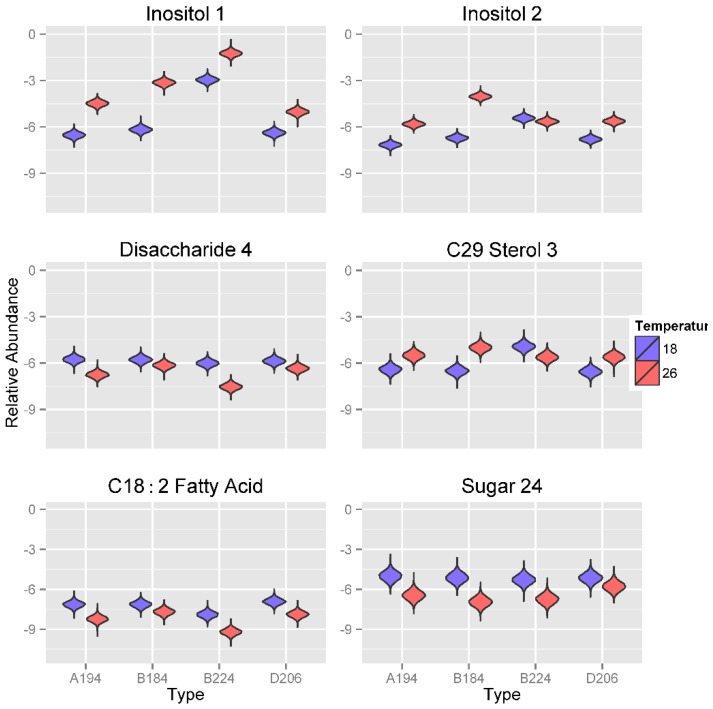
Violin plots showing posterior distributions of estimates of the top six significant predictor metabolites of temperature (°C) for each *Symbiodinium* type. Metabolite abundance values are Area Under the Curve (AUC)-normalized. Predictor metabolites are sorted from top left to lower right according to their relative importance scores derived from Random Forests analysis. The width of each figure in the violin plot is proportional to the relative frequency of values from the posterior distribution.

Metabolite abundance values of the sugars Inositol 1 and 2 respond strongest to changes in temperature (Figure 6). The abundance values of these two metabolites are significantly lower at 18 °C compared to 26 °C, usually by two orders of magnitude. The only exception to this pattern is the metabolite abundance of Inositol 2 for *Symbiodinium* type B224, which shows similar expression at both temperatures. Furthermore, metabolite abundance of Inositol 1 is significantly greater in *Symbiodinium* type B224 compared to all other three *Symbiodinium* types. Interestingly, *Symbiodinium* type B224 is more commonly found in temperate environments whereas *Symbiodinium* types A194, B184 and D206 are commonly found in tropical and sub-tropical environments. Such adaptation is likely to be advantageous to unique symbiont-host combinations and interactions (e.g., [65,66,67]). *Symbiodinium* types cultured at 18 °C show significantly greater abundance values of Disaccharide 4, with the greatest difference being observed for *Symbiodinium* type B224. For C_29_ Sterol 3, *Symbiodinium* types cultured at 18 °C had significantly lower metabolite abundance values in all *Symbiodinium* types except for *Symbiodinium* type B224, for which relative concentrations were significantly larger at 18 °C compared to the three other *Symbiodinium* types. All *Symbiodinium* types cultured at 18 °C were also characterized by greater metabolite abundances of the di-unsaturated fatty acid C_18:2_ and an unknown sugar, (Sugar 24).

#### 2.3.3. Effect of Variation in Light Intensity on Metabolic Profiles of Symbiotic Dinoflagellates

**Figure 7:**
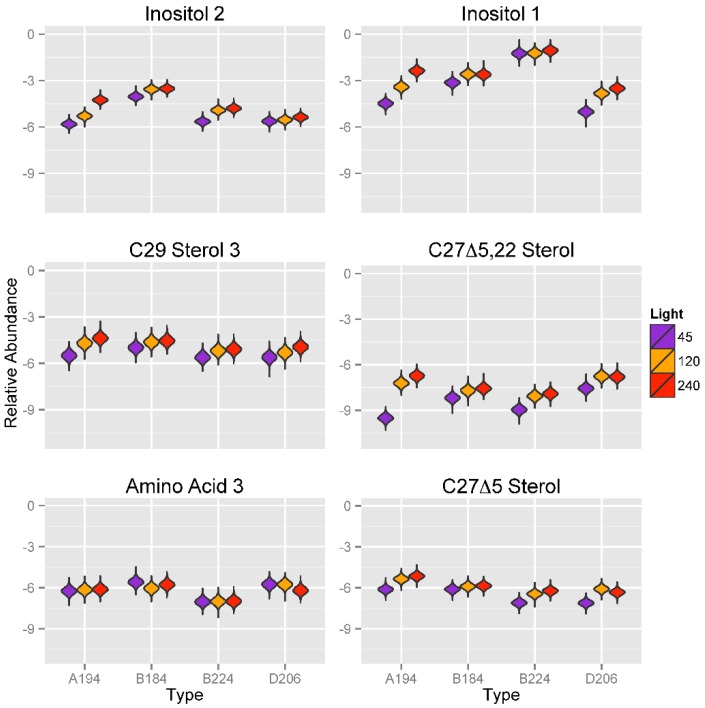
Violin plots showing posterior distributions of estimates of the top six significant predictor metabolites of light intensity (μmol photons m^−2^ s^−1^) for each *Symbiodinium* type. Metabolite abundance values are Area Under the Curve (AUC)-normalized. Predictor metabolites are sorted from top left to lower right according to their relative importance scores derived from Random Forests analysis. The width of each figure in the violin plot is proportional to the relative frequency of values from the posterior distribution.

A strong effect of light intensities on the expression of two inositol compounds was identified (Figure 7). Except as noted below, for most *Symbiodinium* types, the metabolite abundance values of inositol compounds (Inositol 1 and 2) was significantly lower at 45 μmol photons m^−20^ s^−1^ than it was at the higher light intensities of 120 or 240 μmol photons m^−20^ s^−1^, at which abundance values tended to be similar. There were three exceptions to this pattern; *Symbiodinium* type A194 showed an increase for both inositol compounds from 45 to 240 μmol photons m^−20^ s^−1^, which was of approximately two orders of magnitude for Inositol 1. Metabolite abundances of Inositol 2 in *Symbiodinium* type D206, and Inositol 1 in *Symbiodinium* type B224 are similar at 45 and 120 μmol photons m^−20^ s^−1^ and only slightly elevated in 240 μmol photons m^−20^ s^−1^. A similar pattern of lower relative concentrations at lower light intensities was also seen for the three sterols, C_29_ Sterol 3, C_27_Δ^5.220^ Sterol 2, and C_27_Δ^5^ Sterol. For C_27_Δ^5.22^ Sterol, *Symbiodinium* type A194 had a dramatic increase in metabolite abundance of more than two orders of magnitude between 45 and 240 μmol photons m^−20^ s^−1^, greater than that seen for Inositol 1.

### 2.4. Physiological and Environmental Correlates of Metabolite Expression in Symbiotic Dinoflagellates

Using GC-MS analysis we identified metabolite patterns in four phylogenetically distinct *Symbiodinium* spp., *S. microadriaticum*, (*Symbiodinium* cp-type A194), *S. minutum* (*Symbiodinium* cp-type B184), *S. psygmophilum* (*Symbiodinium* cp-type B224) and *S. trenchii* (*Symbiodinium* cp-type D206). All four *Symbiodinium* types showed significant differences in the production of a suite of sterols and sugars, as well as fatty acids and amino acids. Withers *et al.* [1] investigated sterol patterns of cultured symbiotic dinoflagellates that had been isolated from marine invertebrates and found that *Symbiodinium* spp. from different hosts were separated into three distinct groups based on the chemical structures of two major free sterols that are synthesized by each of the symbiotic dinoflagellate. While these studies provide significant insight into metabolite patterns characteristic for an individual *Symbiodinium* type, their physiological and ecological implications are not immediately apparent.

In order to adapt to or respond to environmental changes most biological systems adjust through regulation of their biochemical and cellular processes [68]. Coral-algal symbioses are found across tropical, subtropical and temperate latitudes, where temperature and light regimes vary considerably ([18,69] and references within). They particularly thrive in the nutrient-poor waters of the tropics where they are most ecologically relevant, but are also commonly found in temperate environments. These symbioses produce a range of lipophilic and hydrophilic compounds including glucose, organic acids, fatty acids, triglycerides and wax esters [25,33,34,35,36,70,71,72]. Translocation of energy and organic nutrients between the symbiont and host include transfer of glycerol [4,73,74,75], amino acids [36], fatty acids [71,76] and sterols [76]. In our study we detected many of these compounds in the four *Symbiodinium* species, validating that these compounds are indeed synthesized by the symbiont*.* Individual metabolites or metabolic groups are shown to hold distinctive functions in the coral-algal relationship, where some will have nutritional roles and others are involved in fatty acid or lipid synthesis, protein formation or photosynthesis to give just a few examples ([74] and references within). Given the differences in metabolite abundances seen in our study, it is likely that the unique metabolite patterns of the individual *Symbiodinium* types affect the symbiotic relationship between these symbionts and their coral hosts and may also lead to host-symbiont specificity [18].

Physiological tolerances of reef-building corals to changing environmental conditions result from a complex interaction of the physiological characteristics of both the symbiotic dinoflagellates and their coral hosts [32]. Coral species that are adapted to different light regimes must have photo-physiological strategies that will allow the coral-algal symbiosis to acclimate to differences in the surrounding light environment (e.g., [77,78]). A study by Cooper *et al.* [79] demonstrated that different *Symbiodinium* types have the ability to control the quality and quantity of lipids over a large-scale depth gradient that causes significant light alterations. A strong correlation between light and dark photoperiods and the production of lipids was also found in free-living *Symbiodinium* as well as in *Symbiodinium*-coral symbiosis [70,72]. High-temperature in conjunction with high-irradiance can disrupt the *Symbiodinium-*cnidarian symbiosis and varies either bathymetrically or among illuminated and shaded locations within a single coral colony. Furthermore, the thermal tolerance of *Symbiodinium* types influences the dynamics of *Symbiodinium-*coral communities during disturbance events [80]. Low temperature stress has been shown to affect the maximum photochemical efficiency of photosystem II in isolated *Symbiodinium* [27] as well as *Symbiodinium in hospite* [24,81]. The extent of this effect, however, correlates strongly with the thermal tolerance of the individual *Symbiodinium* type [24,27,81]. *Symbiodinium* type B224 is commonly associated with cnidarian hosts from the temperate habitats of the western Atlantic, although it is found in warmer habitats as well. Growth rates and metabolite profiles of *Symbiodinium* type B224 have been shown to be significantly different from *Symbiodinium* type B184 [18]. Similar inter- and intraspecific differences in metabolite patterns of *Symbiodinium* types were identified in this study highlighting the need to further characterize metabolites in future analyses. As the *Symbiodinium* cultures in our study experienced a range of temperatures and light intensities that are within the thresholds of a healthy *Symbiodinium*-cnidarian association, the variability in metabolite profiles that we observed is unlikely to cause a disruption of the symbiosis. Thus, the shifts in the metabolite profiles we observed here are potentially related to normal homeostatic metabolic pathways distinctive for the specific *Symbiodinium* type.

Although, with these data, it is not possible to directly link our findings to specific metabolic processes, some assumptions may be permitted. A variety of studies have shown that symbiotic dinoflagellates within a single host species as well as free-living dinoflagellates are genetically and physiologically diverse and dynamic and as such, are capable of responding to their distinctive environmental conditions (e.g., [14,25,27,79,80,82]). For example, measurements of the melting curves of photosynthetic membranes from different symbiotic dinoflagellates demonstrated sensitivity to high temperature that was specific to individual *Symbiodinium* types [58]. Associated with the observed membrane sensitivity, the authors also observed variations in fatty acid composition under high temperature and suggested a complex adaptation process in which various modifications in lipid composition may be involved [58]. Tchernov *et al.* [25] showed that reduced membrane fluidity correlates with a greater amount of higher unsaturated fatty acid. The authors found that this partially correlated with a higher temperature tolerance for selected *Symbiodinium* types. Perhaps the increase in abundance of the unsaturated fatty acid, such as the di-unsaturated fatty acid C_18:2_, in cultures grown at 18 °C in our study reflect such a modification in lipid metabolism as a result of the growth temperature. Our study also identified that relative metabolite abundance of two inositol compounds were found to respond strongest to changes in temperature across all four *Symbiodinium* cultures examined. A well-recognized physiological response of marine dinoflagellate to environmental stresses is the temporary cyst formation [83,84,85]. Although the exact molecular underpinnings of the encystment process in dinoflagellates are still unclear, it was found that a relationship exists between the induction of encystment and the formation of inositol phosphates [83,84,85]. While the exact chemical identity of the two inositol compounds found in our study could not be resolved, their change in relative abundance in response to physiological changes could be an indication that those two inositol compounds are inositol phosphates involved in Ca^2+^ influx signalling pathways.

Generally, the approaches to identify metabolite patterns in *Symbiodinium* spp. used here are sensitive and reliable methods for the determination of relative metabolite abundances among individuals as well as metabolite patterns. However, these methods are currently not suitable for the identification of specific biochemical pathways or cellular mechanisms to form hypotheses about activated or de-activated metabolic pathways due to the limited number of metabolites identified from individual and specific biochemical pathways.

Employing metabolomics to investigate symbiotic interactions and to compare and assess entire coral metabolomes [86] imposes exciting opportunities as well as considerable challenges. A recent review by Chasten and Douglas [44] details approaches that should guide the research of metabolomics of symbiotic interactions. The authors recommend including analyses of genomic data, the investigation of metabolic networks, and metabolic modeling considering metabolism genes, which will allow for the identification and quantification of nutritional resources that are being used as well as metabolic adaptations for the release of nutrients to the symbiotic partner [18,86].

By using metabolite profiling in combination with Random Forests classification and Bayesian estimation we demonstrate metabolite profiles of *Symbiodinium* spp*.* to be species-specific as well as temperature- and light-dependent. Given the taxonomic and physiological diversity of *Symbiodinium* spp. we suggest the specificity of the algae’s metabolism have a role in host-symbiont interactions. However, further elucidation of metabolic responses specific to either of these factors will require future studies to characterise and measure metabolites in more detail within both partners.

## 3. Experimental Section

### 3.1. Experimental Design and Sample Preparation

Four different species of *Symbiodinium* were used, *S. microadriaticum*, (*Symbiodinium* cp-type A194, Culture ID 04-503, [87]), *S. minutum* (*Symbiodinium* cp-type B184, Culture ID Mf 1.05b.01.SCI.01, [11]), *S. psygmophilum* (*Symbiodinium* cp-type B224, Culture ID Mf 11.05b.01, [11]) and *S. trenchii* (*Symbiodinium* cp-type D206, Culture ID Mf 2.2b, [13]). These *Symbiodinium* species were originally isolated from the scleractinian coral *Orbicella faveolata*, Florida Keys, USA [88,89]. These original isolates were incidental isolates from *O. faveolata* and are not necessarily the representative symbionts of this host (Coffroth, personal communication). A detailed description of culture conditions has been described by Santos *et al.* [90]. Cultures were maintained in f/2 medium [91], 38 ppt. salinity at 26 °C under a 14:10 h 1ight: dark regime (70–90 μmol photons m^−20^ s^−1^, from 34 W fluorescent lights). Clones were transferred from highly concentrated reference cultures and divided into test tubes containing 10 mL f/2 media. All four *Symbiodinium* species were maintained in four different combinations of light and temperature, biologically replicated four times (*n* = 4/treatment) and grown until they had reached exponential phase. Experimental treatments were (i) 48.6 (±4.4) μmol photons m^−2^ s^−1^ at 26 °C; (ii) 116.6 (±6.01) μmol photons m^−20^ s^−1^ at 26 °C, (iii) 230.6 (±26.57) μmol photons m^−2^ s^−1^ at 26 °C and (iv) 48.6 (±4.4) μmol photons m^−20^ s^−1^ at 18 °C. Light exposure followed a 14:10 h 1ight:dark regime. Light measurements were taken using a handheld light meter unit (LI-250A, Li-COR Inc., Biosciences, Lincoln, NE, USA), with a terrestrial radiation sensor (LI-190, Li-COR Inc., Biosciences, Lincoln, NE, USA). All test tubes were arranged randomly and rotated every 2 days to ensure similar light exposure for all clones of all *Symbiodinium* species.

Samples were harvested within 2 h at midpoint of the light cycle. To ensure an even distribution of algal cells within the f/2 media, samples were mixed thoroughly using a Pasteur pipette. Two aliquots of 1 mL were taken from each sample, to confirm genetic identity as well as cell density. Remaining algal cells were concentrated by centrifugation for 10 min at 4 °C and 750 *g*. The supernatant was discarded and the pellet containing the *Symbiodinium* cells was re-suspended in 1 mL f/2 media, transferred into an Eppendorf tube and centrifuged again for 10 min at 13,000 *g*. The supernatant was removed and the algal pellet flash frozen in liquid nitrogen and stored at −80 °C.

### 3.2. Identification of Symbiotic Dinoflagellates

Total genomic nucleic acids were extracted using a modification of the 2 *x* hexadecyltrimethylammonium bromide (CTAB) extraction protocol [92]. For characterization of the symbiont taxa (here referred to as *Symbiodinium* or symbiont types), a variable region in domain V of the chloroplast large subunit (23S) rDNA molecule was amplified using the polymerase chain reaction as described by Santos *et al.* [93]. Amplified DNA fragments were separated according to molecular weight on a 6.5% non-denaturing polyacrylamide gel (LI-COR 4200 NEN^®^ Global IR2 DNA sequencing system, LI-COR Biosciences, Lincoln, NE, USA). Identification of individual DNA fragments was achieved using a metric ruler with DNA ladders as size standards based on clade and fragment length nomenclature previously established [93].

### 3.3. GC/MS Chromatography and Compound Identification

Metabolites in *Symbiodinium* samples were extracted and analyzed according to established protocols [94,95]. Samples were extracted with chilled methanol, water, and chloroform (2.5:1:1) and repeatedly sonicated. The mixture was centrifuged (12,000 *g*, 2 min), and the supernatant (polar phase) was removed and dried under vacuum. When dry, an internal standard mixture of alkanes at 0.8 mg mL^−1^ in pyridine (decane, dodecane, pentadecane, octadecane, nonadecane, docosane, octacosane, dotriacontane, and hexatriacontane; Sigma-Aldrich, St Louis, MO, USA) was added, and extracts were reacted with methoxyamine hydrochloride (20 μL of 20 mg mL^−1^; Sigma-Aldrich, St Louis, MO, USA) for 90 min at 30 °C to protect aldehydes, ketones, and prevent ring closing of reducing sugars and then trimethyl-sylilated with *N*-methyl-*N*-trimethylsilyl-trifluoroacetamide with 1% trimethylchlorosilane (MSTFA/1% TCMS, 90 μL; Sigma-Aldrich, St Louis, MO, USA) for 30 min at 37 °C to derivatize acidic protons. The reaction mixture was transferred to a 2 mL clear glass autosampler vial with micro-insert and sealed with a 11 mm T/S/T crimp cap. The derivatized samples were analyzed using a Thermo Trace Gas Chromatograph coupled to a Thermo Finnigan MAT 95XP mass spectrometer. A 1 μL sample was taken by autosampler at 1 μL sec^−1^ filling speed and injected at 10 μL sec^−1^ into a clean liner at 250 °C. A new liner was installed after every 20 samples. The GC was operated in split less mode and the split vent was opened after 25 s. The separation was performed using a 30 m long, 0.25 mm internal diameter RTX-5Sil MS column with 0.25 µm 95% dimethyl/5% diphenyl polysiloxane film and an additional 10 m integrated guard column using high purity helium as a carrier gas at a constant flow of 1 mL min^−1^. Oven temperature started at 50 °C for 1 min and was increased at 5 °C min^−1^ to 330 °C, and held constant for 5 min. Samples were ionized using electron impact ionization at 80 V at an ion source temperature of 250 °C. Mass spectra were obtained following a 480 second solvent delay and masses were scanned from m/z 50–500 for 63 min at 2 spectra sec^−1^. Along with samples, a series of known metabolite mixtures, and procedural blanks were analyzed. The metabolite mixtures contained the major compound classes including amino acids, fatty acids, sugars, and sterols and were analyzed to ensure that the method extracted the analyte compounds and these compounds could be identified using GC-MS. 

Mass spectra of metabolite compounds were identified by comparison with known standard mixtures prepared using identical methods and by comparison to the NIST mass spectral library using Xcalibur 2.0 software (Thermo Scientific, Waltham, MA, USA). Retention indices for peaks were calculated for secondary identification. Areas of chromatogram peaks were integrated using the Genesis algorithm in the Xcalibur software. Metabolites were named with the greatest precision possible. Where compounds could not be uniquely identified, they were named according to compound class followed by a sequential number incremented in the order they were found on the chromatogram (e.g., “Amino Acid 1” appears before “Amino Acid 2”).

### 3.4. Data Formatting

Preliminary analyses revealed that samples with low maximum peak abundances (measured as total ion current from the mass spectrometer) also tended to have more metabolites recorded as absent or missing (peak of zero abundance) (Supplementary Information Figure S6). This pattern suggests that these metabolites were at levels below a detection threshold (missing peaks), and thus samples with low overall peak profiles may have informative data missing. Given this, missing peaks were not treated as indicative that the metabolite was absent from the sample. Rather, values of those metabolites that were missing in fewer than 58 (~90%) samples were imputed. Those missing in 58 or more samples were eliminated from the analyses.

Of the 188 metabolites identified, 33 were missing in 58 or more samples and were thus eliminated from all analyses, as imputation would not be able to ensure sufficient confidence. Each sample had from 18 to 102 metabolites missing with a median of 51 (Supplementary Information Figure S7). There were only 23 metabolites that had no missing peaks across all samples. In total, approximately 34% of the metabolite peaks in these 155 metabolites across the 64 samples were missing.

In order to obtain a complete dataset for further analyses, missing values were imputed using the k-Nearest Neighbors algorithm as implemented in the *kNN* function in the *VIM* package [96] in *R* v.3.0.2 (R Core Team 2013). Missing values were imputed based on the five most similar samples. All other parameters were left at their defaults. Peaks were normalized relative to their fraction of the area under the curve (AUC). For each sample, each peak was divided by the sum of all peaks in a sample, and then log-odds-transformed. The transformations were performed so that relative metabolite concentrations would be linearly scaled.

A Principal Components Analysis (PCA) was conducted in order to reduce the number of dimensions being examined and to explore the variance structure of the data. Because there were more variables than samples, the *prcomp* function in *R* v.3.0.2 (R Core Team 2013) was used to compute components.

### 3.5. Random Forests Analysis

Random Forests [60] models were constructed to classify levels within each of the three main experimental factors of: (i) *Symbiodinium* type (A194, B184, B224, D206); (ii) temperature (18 and 26 °C); and (iii) light intensity (45, 120, and 240 μmol photons m^−20^ s^−1^). Each of the three models was built out of 5000 trees. For each tree, samples were randomly chosen with replacement. Those samples not chosen in each tree (the Out-Of-Bag or OOB samples) were then used to estimate the classification error rate. The OOB classification error rates are compared to error rates expected by random assignment, based on the distribution of sample sizes. As an example, if there were two classes, one with 60 samples, and one with 30, then one would correctly classify 2/3^rds^ of the first class (40 samples) and 1/3rd of the second class (10 samples) by chance alone. This would make for an overall error rate of 44% (40/90) based on random assignment rather than the 50% one would expect for equal-sized classes. The number of predictors randomly chosen at each node (*mtry)* was left at the default value for the function (*n* = 13). For this analysis, predictor importance was examined as measured by Mean Decrease in Accuracy [97]. Significance of predictor importance in each model was assessed with 1000 permutations using the *rfPermute* package for *R*. Predictor metabolites are reported as significant, when permutation test *p*-values are ≤0.05.

We also examined whether patterns of missing data were related to any of the experimental factors by constructing a data set where the values for each metabolite were recoded simply as present (1) or absent (0). These presence/absence data were then used to predict the experimental factors by running the same Random Forests models described above. Metabolites found to be significant predictors in any of these models were then removed from AUC-normalized data in the corresponding Random Forests model and that model was re-run with the censored list of predictors.

### 3.6. Bayesian Estimation of Means

To estimate how different relative abundances of metabolites were between experimental effects, we first constructed a multifactorial Bayesian model to estimate the posterior distribution of the mean value for each metabolite for each set of four replicates from every unique type x temperature x light intensity experiment. The model was based on a standard design matrix, **X**, with the model reference arbitrarily set as *Symbiodinium* type A194, temperature 18 °C, and light intensity of 45 μmol photons m^−20^ s^−1^. Coefficients for other main effects, two- and three-way interactions were estimated as offsets from this reference. The likelihood model was specified as

$$y_{i}\;\sim \;Normal\left(\mu_{i},\;\tau \right)$$

$$\mu_{i}=\underset{i}{\sum }X_{i}\cdot \beta$$

where ***X_i_*** is the row in the design matrix for the *i-*th sample, and ***β*** is the vector of parameters (reference mean and offsets) being estimated. The prior distribution for ***β*** was drawn from *Normal* (0, 10^−4^), and the precision parameter, τ, was drawn from *Gamma* (10^−3^, 10^−3^).

The Bayesian models were run using the *rjags* package in *R* v.3.0.2 (R Core Team 2013). For each model, a total of 10 chains were run, each consisting of 5000 iterations for model adaptation, 5000 iterations for burn-in, and 10,000 sampling iterations, with every 10th sample from the posterior saved. Each chain was examined visually to ensure adequate mixing and stability. Posterior samples of the means for each set of interactions (each set of four replicates) were constructed by summing the posterior on *β_1_* (the reference) with the posteriors from those offset *β*’s specifying each interaction. Posteriors for marginal effects were then created by combining all posteriors involving the effect of interest (Supplementary Information Figure S8). Posteriors on differences between effects were calculated by subtracting one posterior sample from the other (delta-posterior) (Supplementary Information Figures S3–5). The biological importance of these differences was evaluated by calculating the fraction of the delta-posterior that was greater than zero.

## 4. Conclusions

Metabolite profiles of each *Symbiodinium* type examined in this study revealed characteristics in the production of a variety of sugars and sterols, as well as fatty acids and amino acids. A Bayesian model identified significant differences in the production of inositol, selected sterols and also glycerol. This was observed across all *Symbiodinium* types as a physiological response to growth in different temperatures and light regimes. Out of all four *Symbiodinium* types that were investigated, *Symbiodinium* type B224 (*S. psygmophilum*) showed the most distinctive metabolite profile, defined by high concentrations of inositol and low concentrations of the sterol C_28_Δ^5.22^. At present, it is beyond the scope of this study to describe detailed cellular mechanisms that would help to better understand metabolic pathways that lead to physiological and ecological differences of different *Symbiodinium* types, however, our study does determine metabolite profiles distinctive for taxonomic and environmental variation and highlight the immense potential that high throughput technologies such as metabolomics hold for studies concerned about dinoflagellate diversity, both free-living and endosymbiotic *Symbiodinium*. As demonstrated in this study, metabolite profiling is a promising technique that in combination with powerful bioinformatics has the potential to unravel some of the more pressing questions related to *Symbiodinium* ecology and evolution and how these algae form one of the most successful symbioses of modern coral reefs.

## Supplementary Files

Supplementary File 1
