# Supplementary material for: Taxonomic and Environmental Variation of Metabolite Profiles in Marine Dinoflagellates of the Genus Symbiodinium
Source: Metabolites. 2015 Feb 16;5(1):74–99. doi: 10.3390/metabo5010074 (PMC4381291; doi:10.3390/metabo5010074)
Supplement: Supplementary File 1 [file metabolites-05-00074-s001.zip › Supplementary Information/Supplementary Information - Captions.docx]

**Supplementary Information for:**

Taxonomic and Environmental Variation of Metabolite Profiles in Marine Dinoflagellates of the genus *Symbiodinium*

Anke Klueter, Jesse B. Crandall, Frederick I. Archer, Mark A. Teece and Mary Alice Coffroth

**Supplementary Information Table S1.** Overview of metabolite data.

**Supplementary Information Figure S2.** PCA-Component-Variance. Cumulative per cent of variation accounted for in Principal Components Analysis of all metabolites using all samples.

**Supplementary Information Figure S3.** Metabolite Concentration Posterior; Type. Histograms of Bayesian posterior for marginal effects and differences between effects (delta posteriors) for Type:Temperature and Type:Light Level. For each metabolite, first figure shows marginal effects posteriors and the following figure shows posteriors of differences between effects.

**Supplementary Information Figure S4.** Metabolite Concentration Posterior; Temperature. Histograms of Bayesian posterior for marginal effects and differences between effects (delta posteriors) for Temperature:Type. For each metabolite, first figure shows marginal effects posteriors and the following figure shows posteriors of differences between effects.

**Supplementary Information Figure S5.** Metabolite Concentration Posterior; Light Intensity. Histograms of Bayesian posterior for marginal effects and differences between effects (delta posteriors) for Light Level:Type. For each metabolite, first figure shows marginal effects posteriors and the following figure shows posteriors of differences between effects.

**Supplementary Information Figure S6.** Missing Data. Distribution of the per cent of metabolites in each sample with peaks missing (recorded as 0) as a function of the maximum peak height in the chromatogram of each sample.

**Supplementary Information Figure S7.** Histogram of the number of peaks missing (recorded as 0) in chromatograms for each sample out of a total of 155 identified peaks.

**Supplementary Information Figure S8.** Metabolite Concentration Posterior. Histograms of Bayesian posterior distributions for relative metabolite concentrations for each combination of Type:Temperature:Light Level. Each page is for one of 155 metabolites.
