# Supplementary material for: Taxonomic and Environmental Variation of Metabolite Profiles in Marine Dinoflagellates of the Genus Symbiodinium
Source: Metabolites. 2015 Feb 16;5(1):74–99. doi: 10.3390/metabo5010074 (PMC4381291; doi:10.3390/metabo5010074)
Supplement: Supplementary File 1 [file metabolites-05-00074-s001.zip › Supplementary Information/Supplementary Information - Captions.pdf]

Supplementary Information for:

Taxonomic and Environmental Variation of Metabolite Profiles in Marine Dinoflagellates of the genus *Symbiodinium*

Anke Klueter, Jesse B. Crandall, Frederick I. Archer, Mark A. Teece and Mary Alice Coffroth

Supplementary Information Table S1. Overview of metabolite data.

Supplementary Information Figure S2. PCA-Component-Variance. Cumulative percent of variation accounted for in Principal Components Analysis of all metabolites using all samples.

Supplementary Information Figure S3. Metabolite Concentration Posterior; Type. Histograms of Bayesian posterior for marginal effects and differences between effects (delta posteriors) for Type:Temperature and Type:Light Level. For each metabolite, first figure shows marginal effects posteriors and the following figure shows posteriors of differences between effects.

Supplementary Information Figure S4. Metabolite Concentration Posterior; Temperature. Histograms of Bayesian posterior for marginal effects and differences between effects (delta posteriors) for Temperature:Type. For each metabolite, first figure shows marginal effects posteriors and the following figure shows posteriors of differences between effects.

Supplementary Information Figure S5. Metabolite Concentration Posterior; Light Intensity. Histograms of Bayesian posterior for marginal effects and differences between effects (delta posteriors) for Light Level:Type. For each metabolite, first figure shows marginal effects posteriors and the following figure shows posteriors of differences between effects.

Supplementary Information Figure S6. Missing Data. Distribution of the percent of metabolites in each sample with peaks missing (recorded as 0) as a function of the maximum peak height in the chromatogram of each sample.

Supplementary Information Figure S7. Histogram of the number of peaks missing (recorded as 0) in chromatograms for each sample out of a total of 155 identified peaks.

Supplementary Information Figure S8. Metabolite Concentration Posterior. Histograms of Bayesian posterior distributions for relative metabolite concentrations for each combination of Type:Temperature:Light Level. Each page is for one of 155 metabolites.
