# Supplementary material for: Taxonomic and Environmental Variation of Metabolite Profiles in Marine Dinoflagellates of the Genus Symbiodinium
Source: Metabolites. 2015 Feb 16;5(1):74–99. doi: 10.3390/metabo5010074 (PMC4381291; doi:10.3390/metabo5010074)
Supplement: Supplementary File 1 [file metabolites-05-00074-s001.zip › Supplementary Information/Supplementary Information Figure S3a - type.pdf]

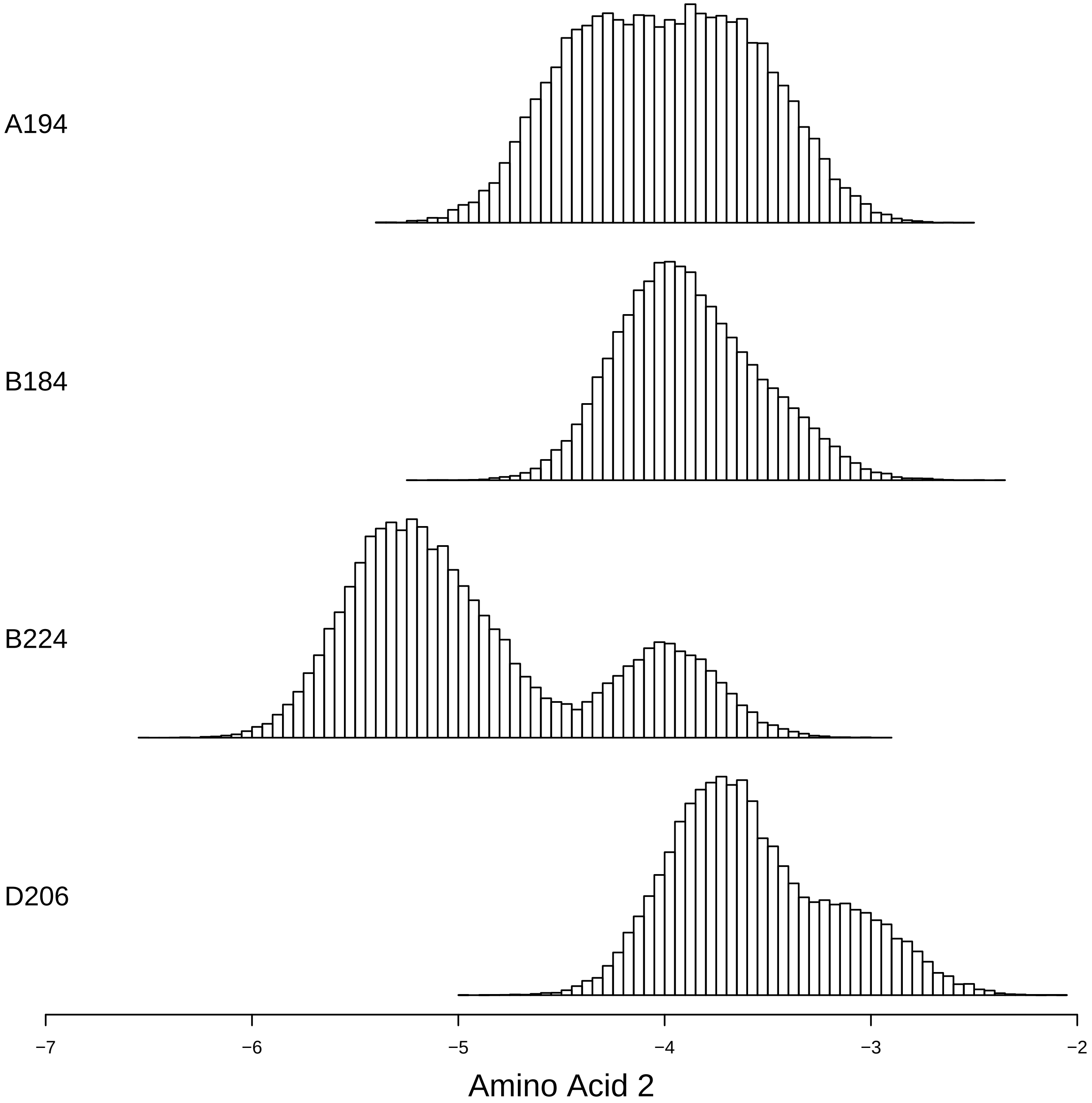

A194
B184
B224
D206
−7
−6
−5
−4
−3
−2
Amino Acid 2

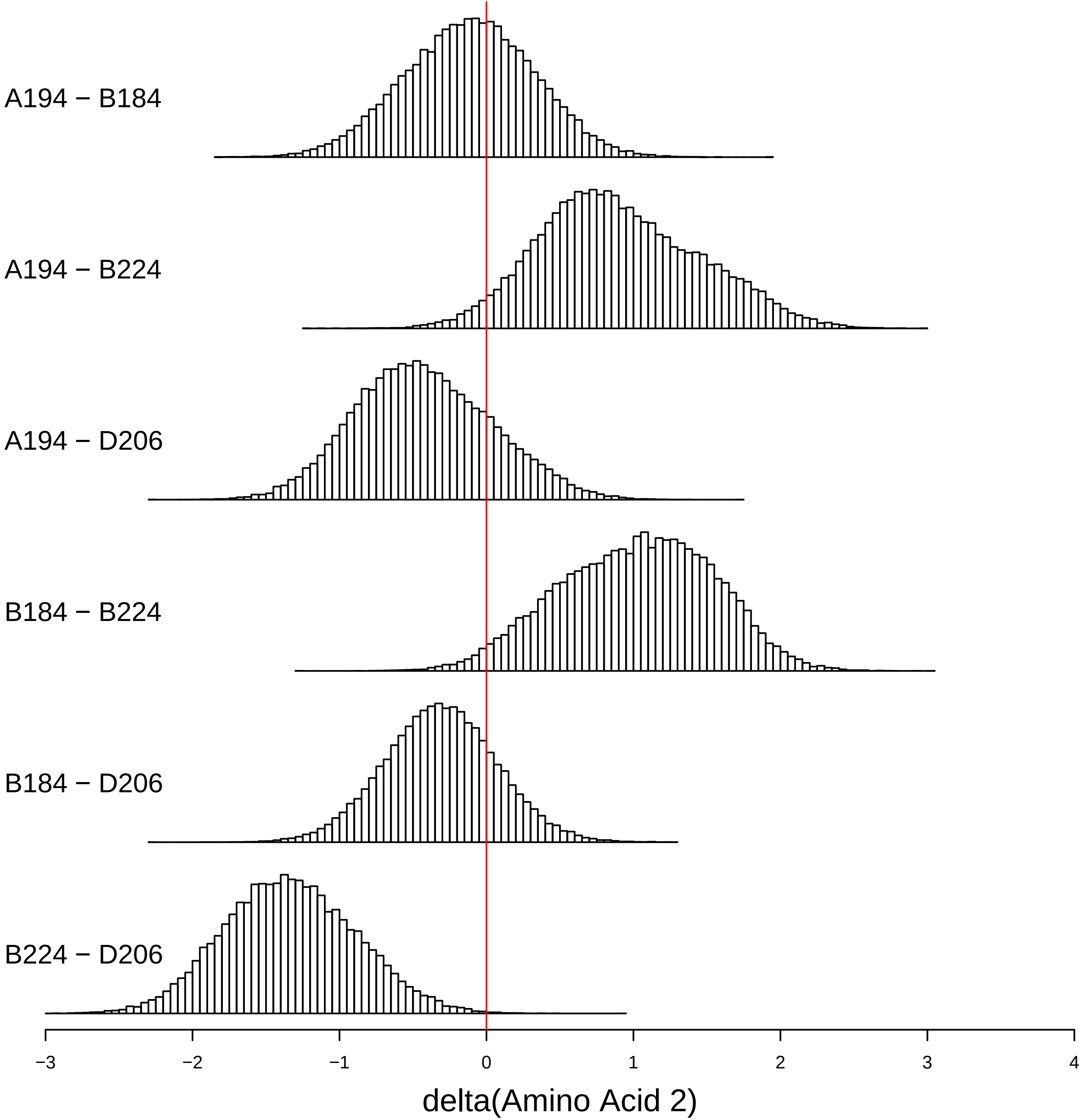

A194 − B184
A194 − B224
A194 − D206
B184 − B224
B184 − D206
B224 − D206
−3
−2
−1
0
1
2
3
4
delta(Amino Acid 2)

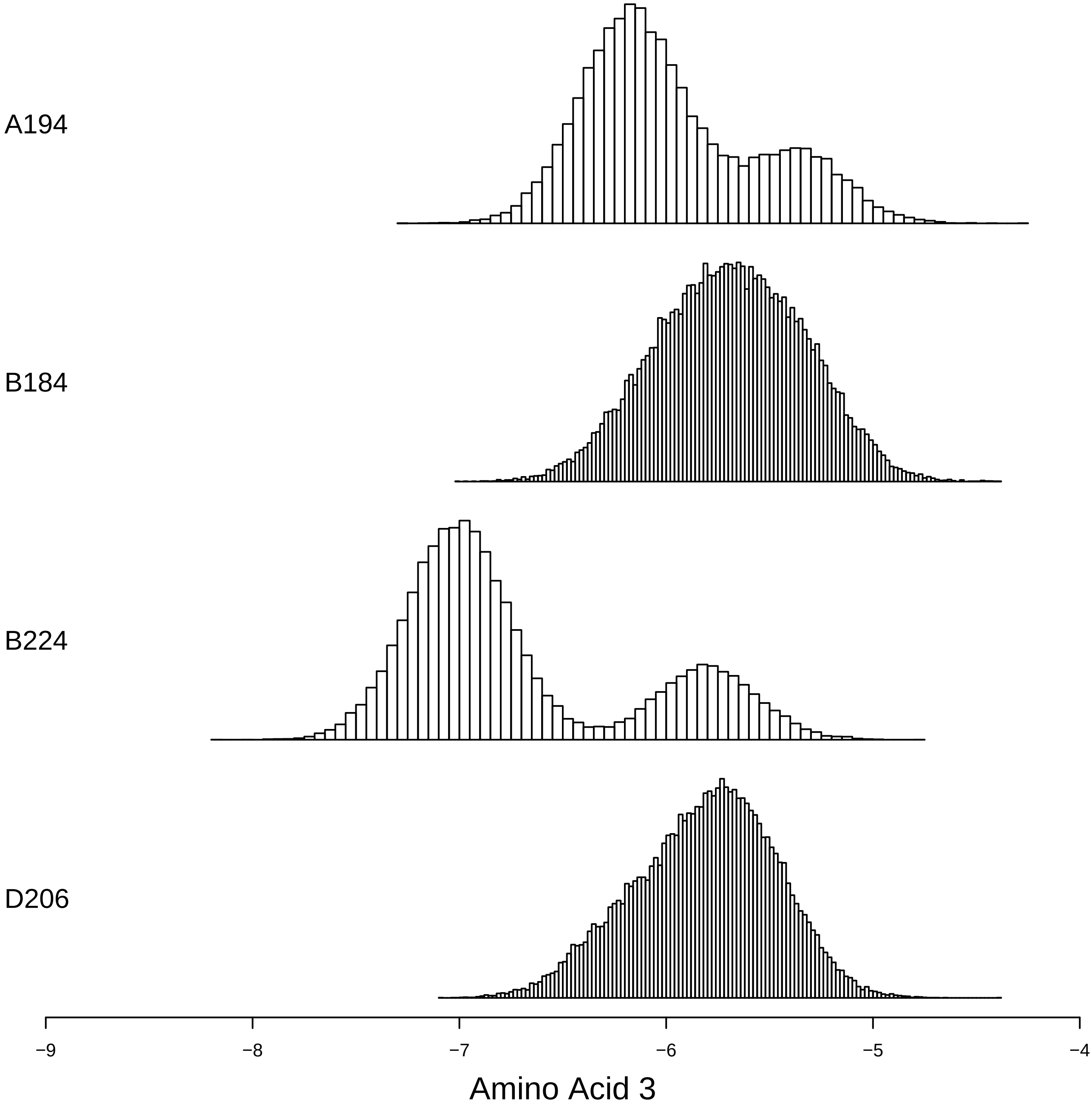

A194
B184
B224
D206
−9
−8
−7
−6
−5
−4
Amino Acid 3

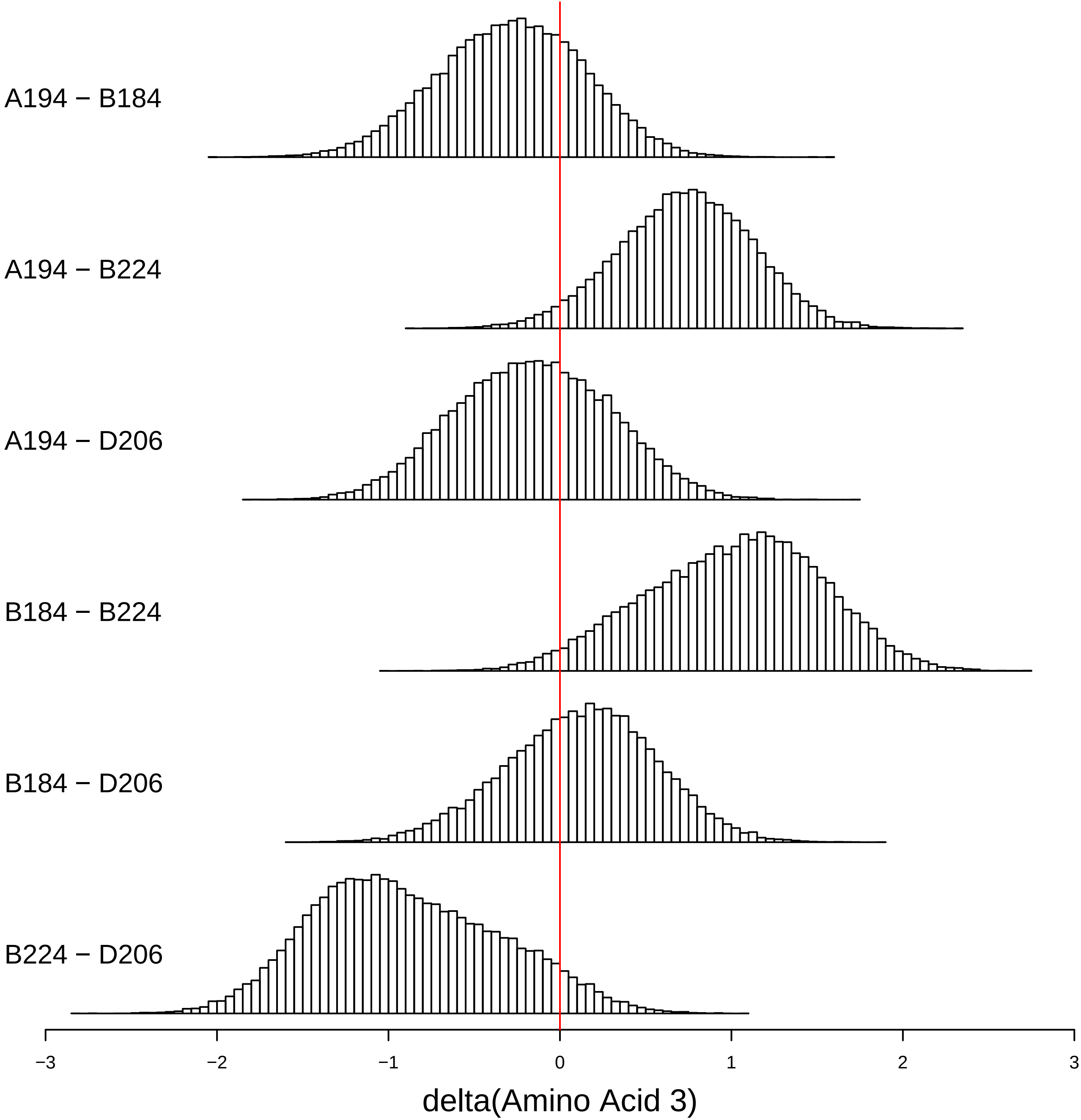

A194 − B184
A194 − B224
A194 − D206
B184 − B224
B184 − D206
B224 − D206
−3
−2
−1
0
1
2
3
delta(Amino Acid 3)

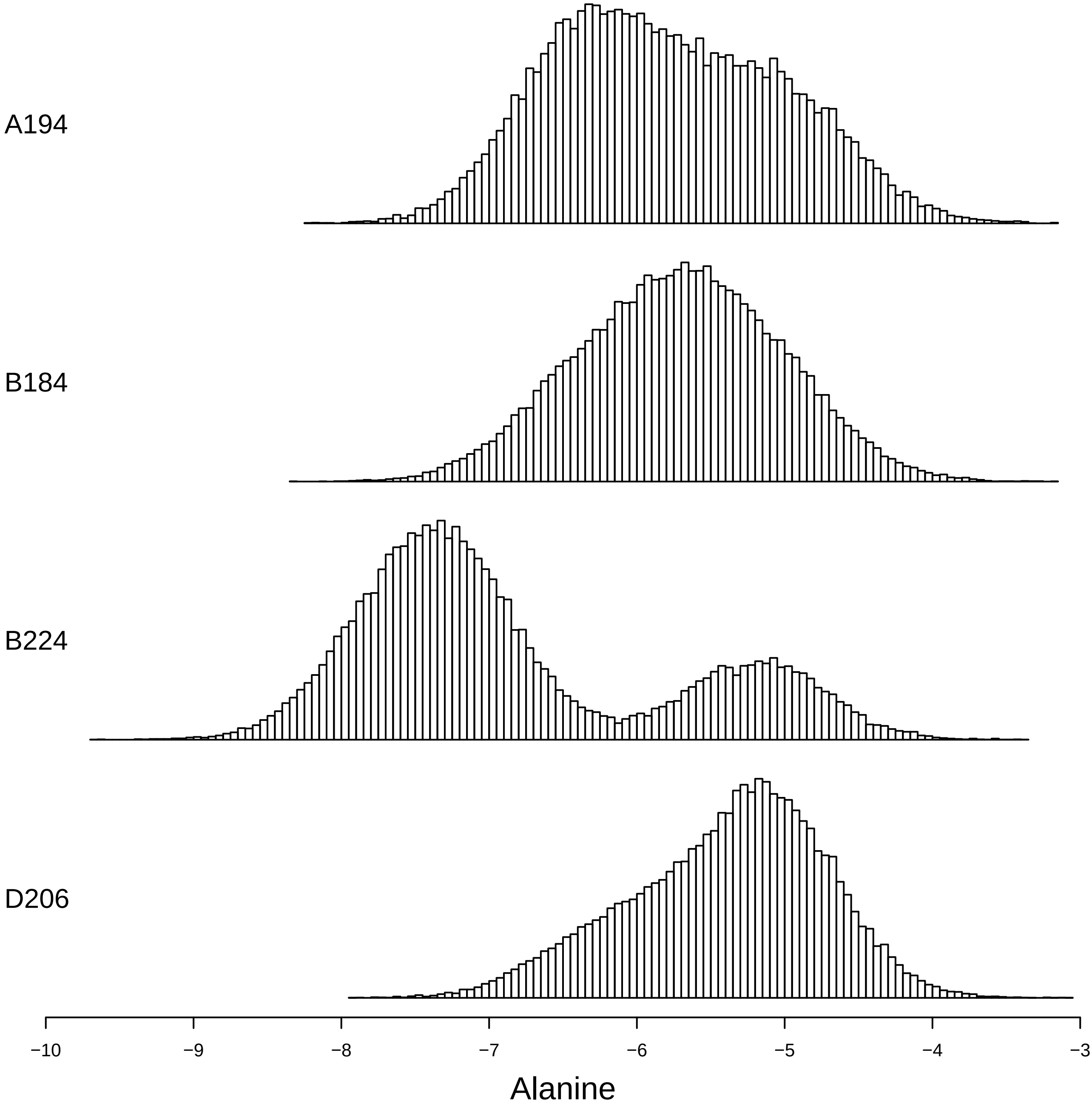
A194
B184
B224
D206
−10
−9
−8
−7
−6
−5
−4
−3
Alanine

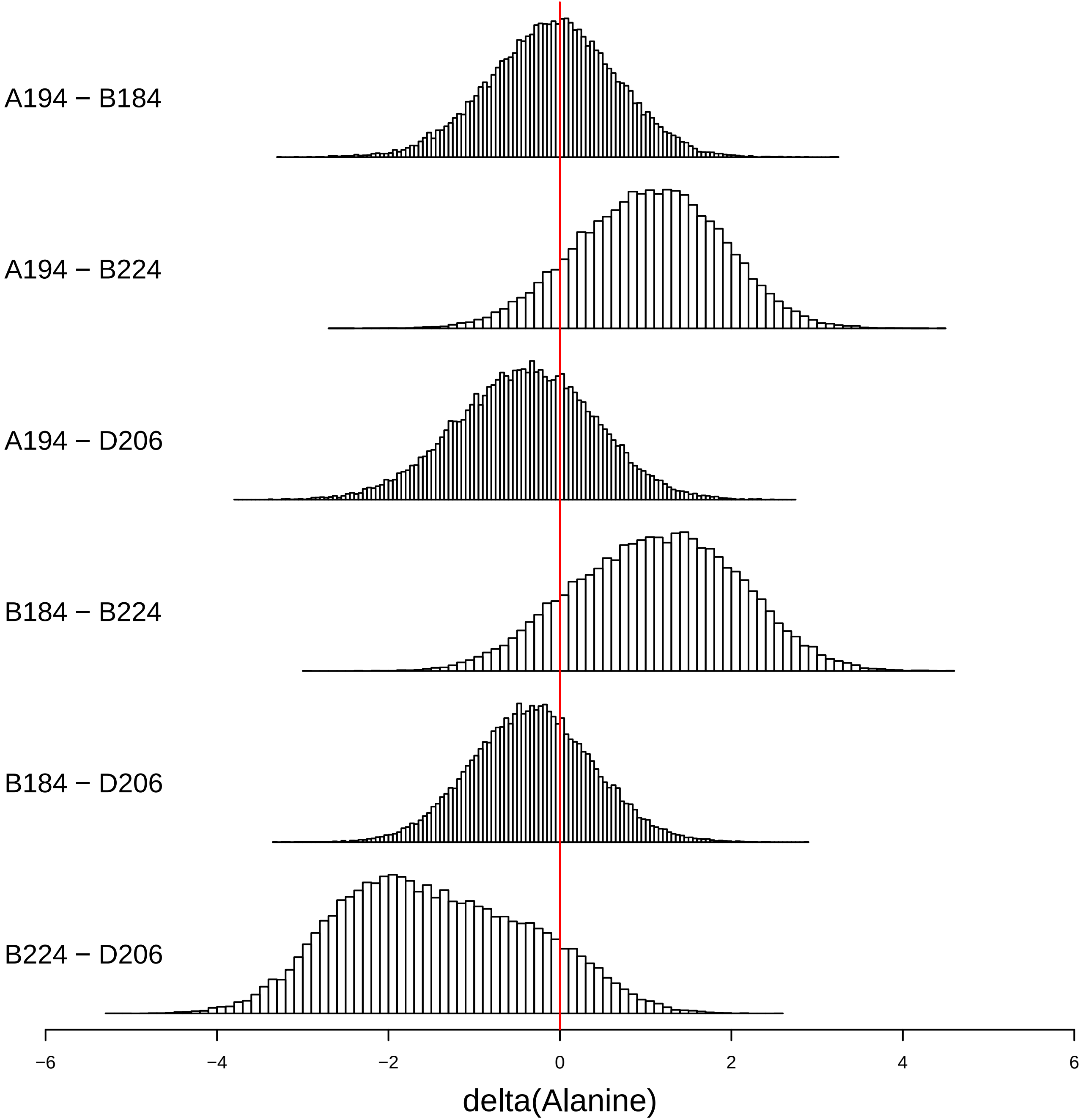
A194 − B184
A194 − B224
A194 − D206
B184 − B224
B184 − D206
B224 − D206
−6
−4
−2
0
2
4
6
delta(Alanine)

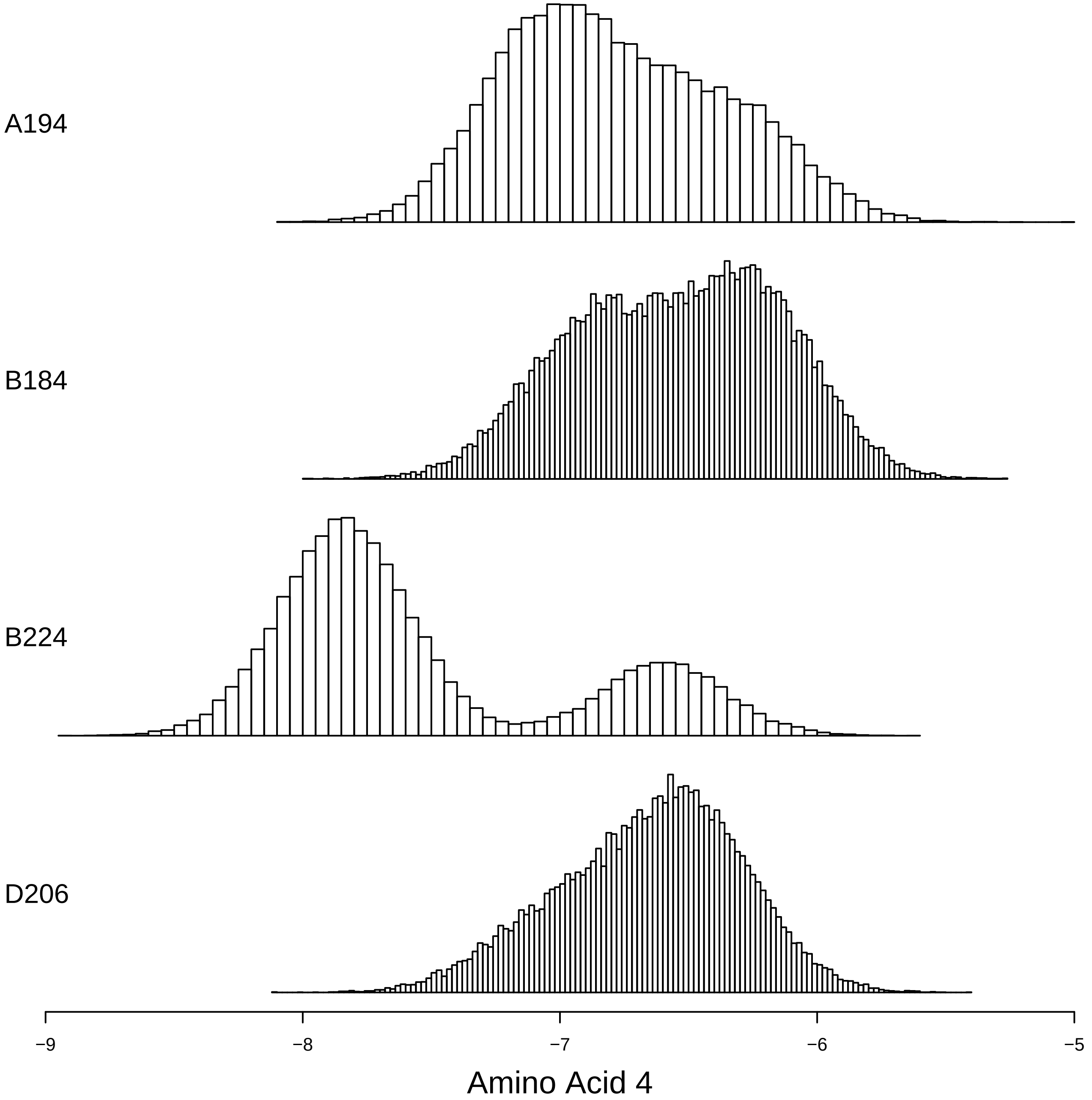
A194
B184
B224
D206
−9
−8
−7
−6
−5
Amino Acid 4

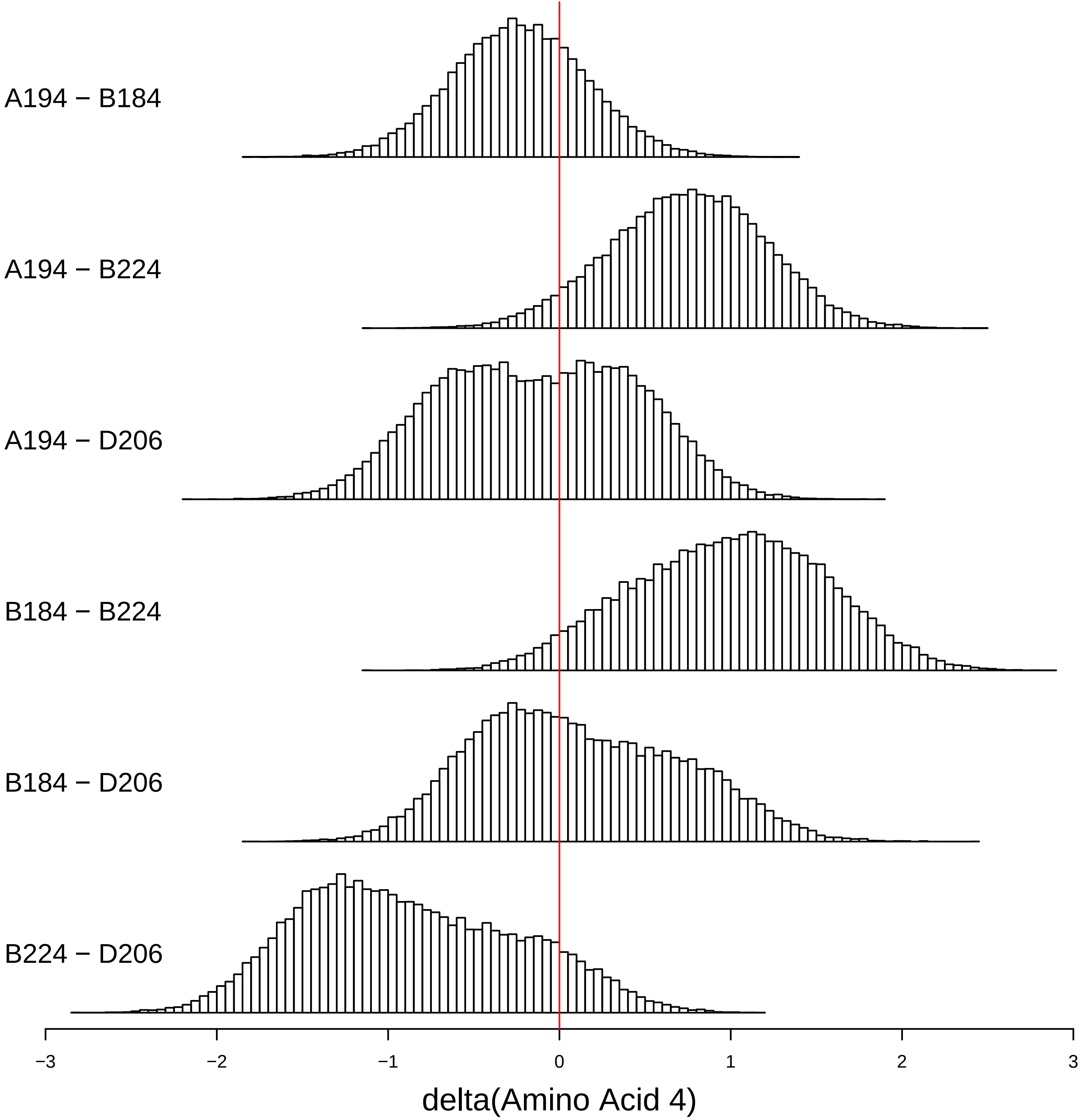

A194 − B184
A194 − B224
A194 − D206
B184 − B224
B184 − D206
B224 − D206
−3
−2
−1
0
1
2
3
delta(Amino Acid 4)

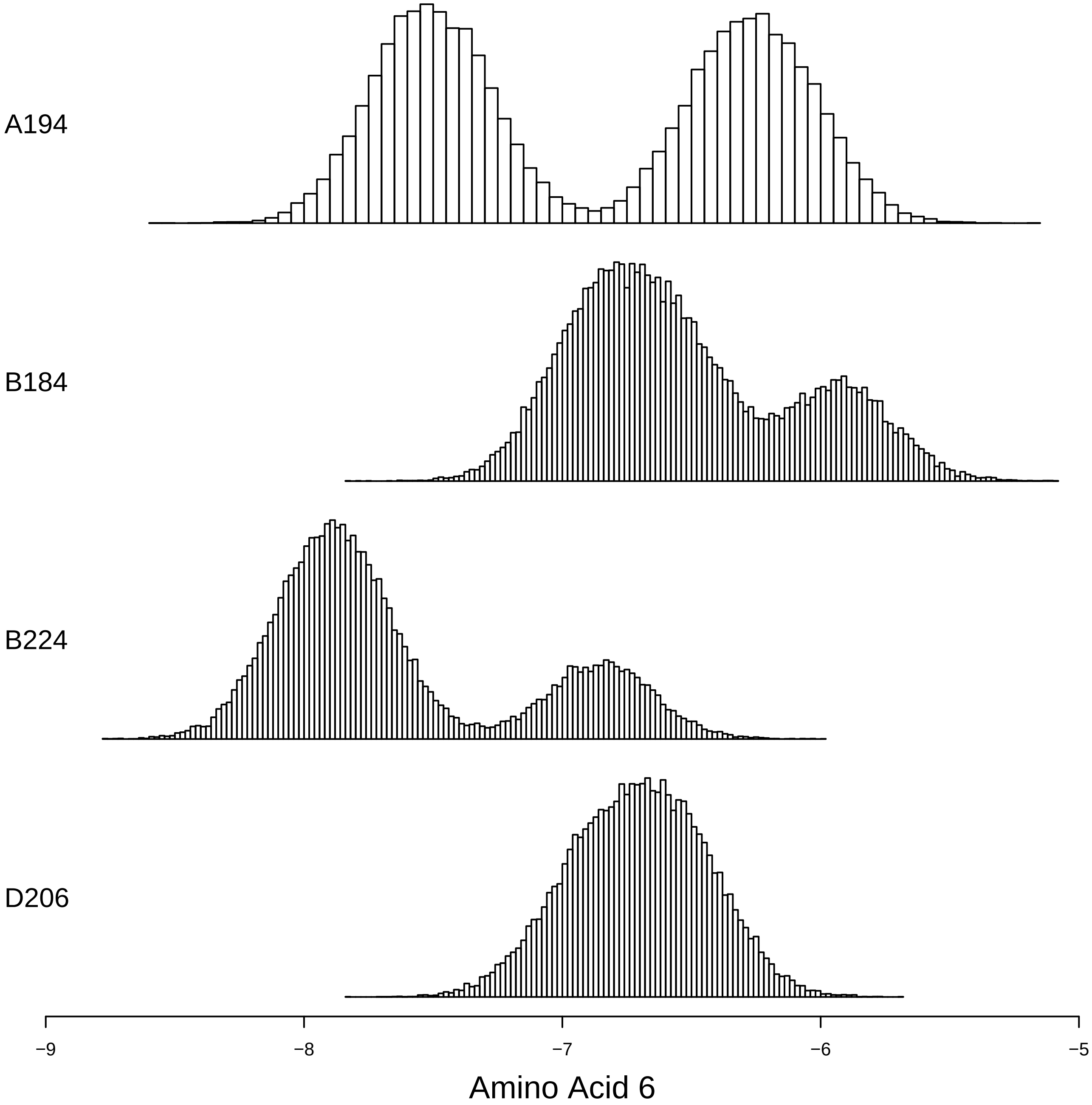
A194
B184
B224
D206
−9
−8
−7
−6
−5
Amino Acid 6

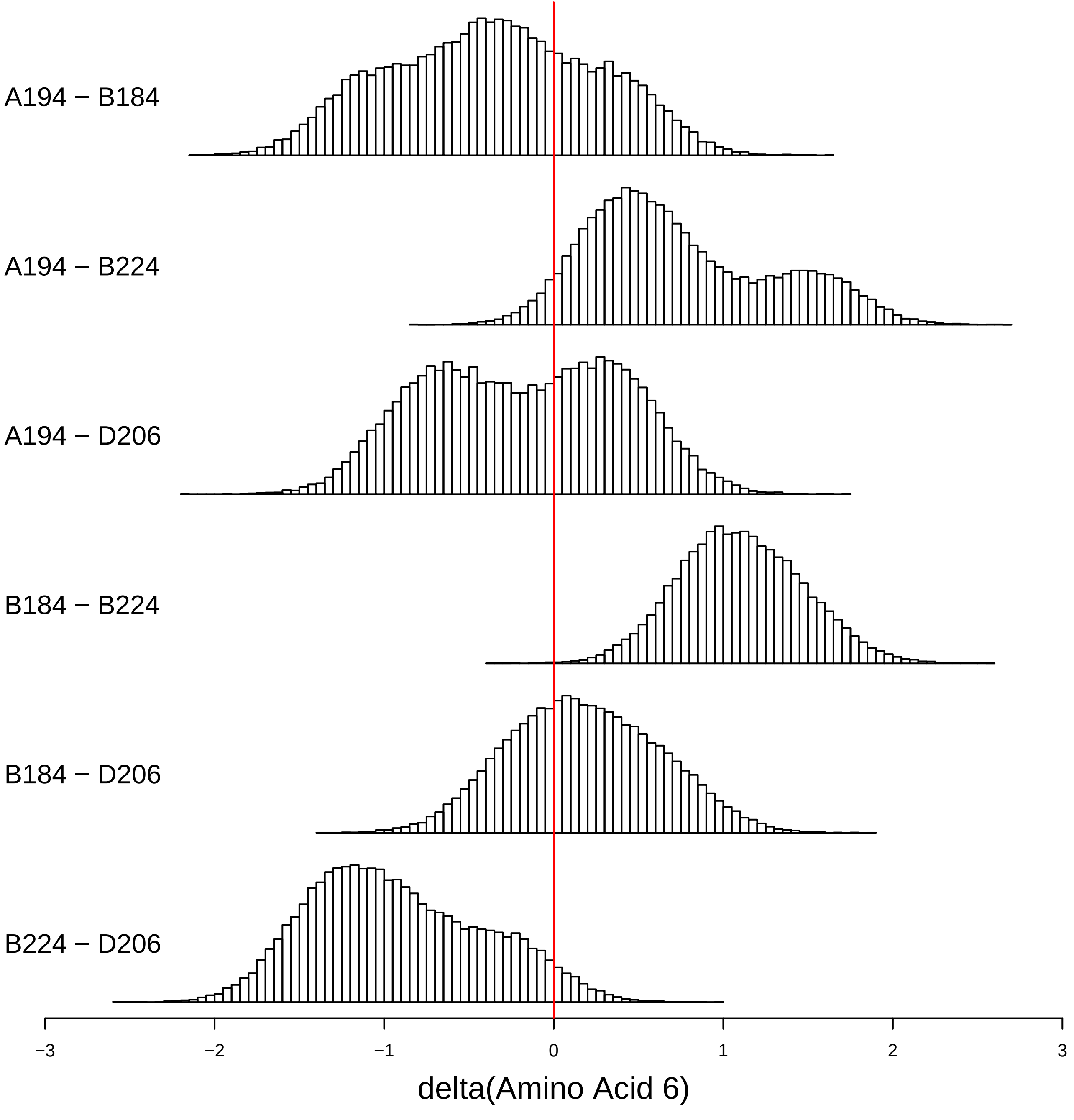
A194 − B184
A194 − B224
A194 − D206
B184 − B224
B184 − D206
B224 − D206
−3
−2
−1
0
1
2
3
delta(Amino Acid 6)

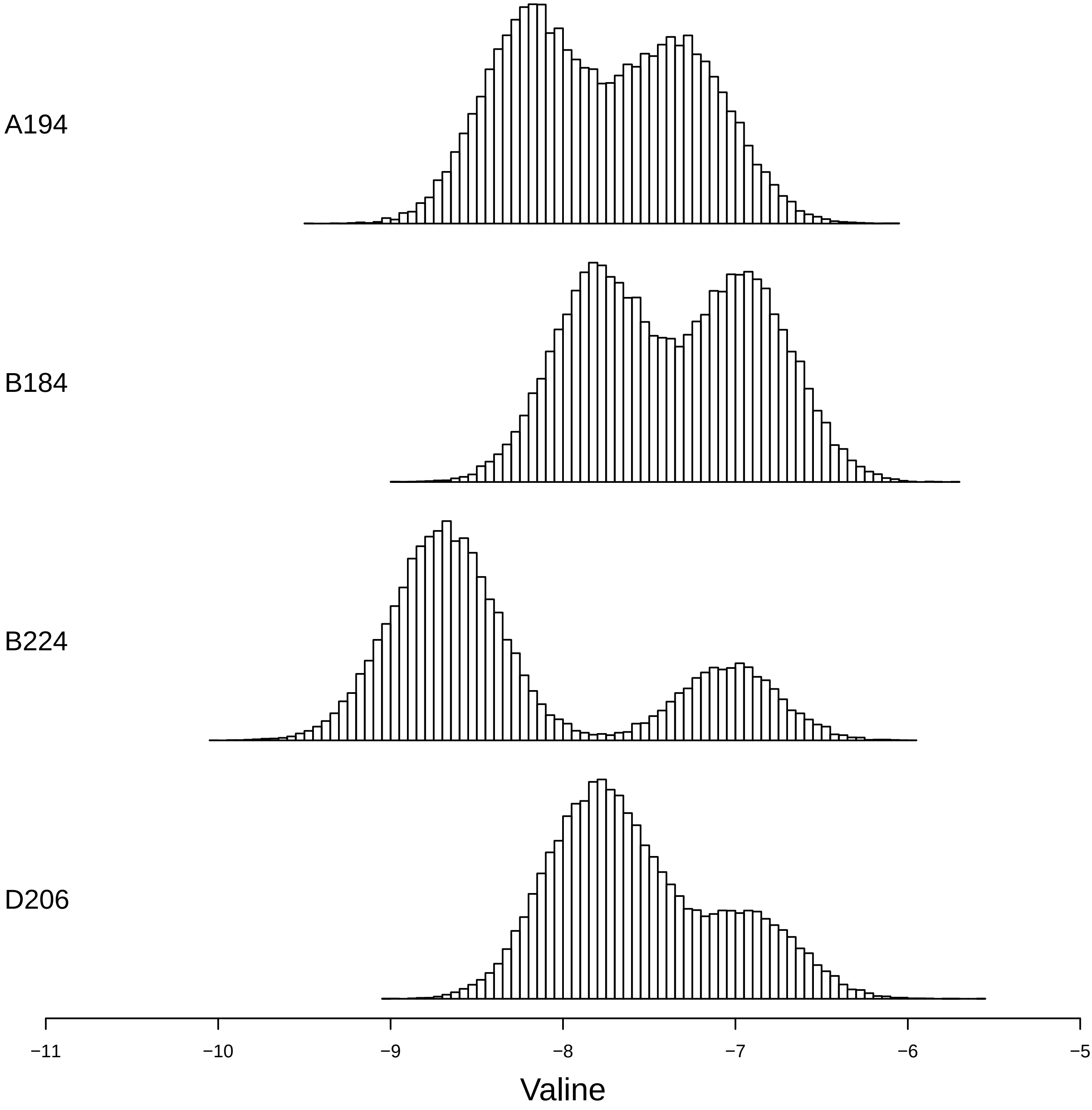
A194
B184
B224
D206
−11
−10
−9
−8
−7
−6
−5
Valine

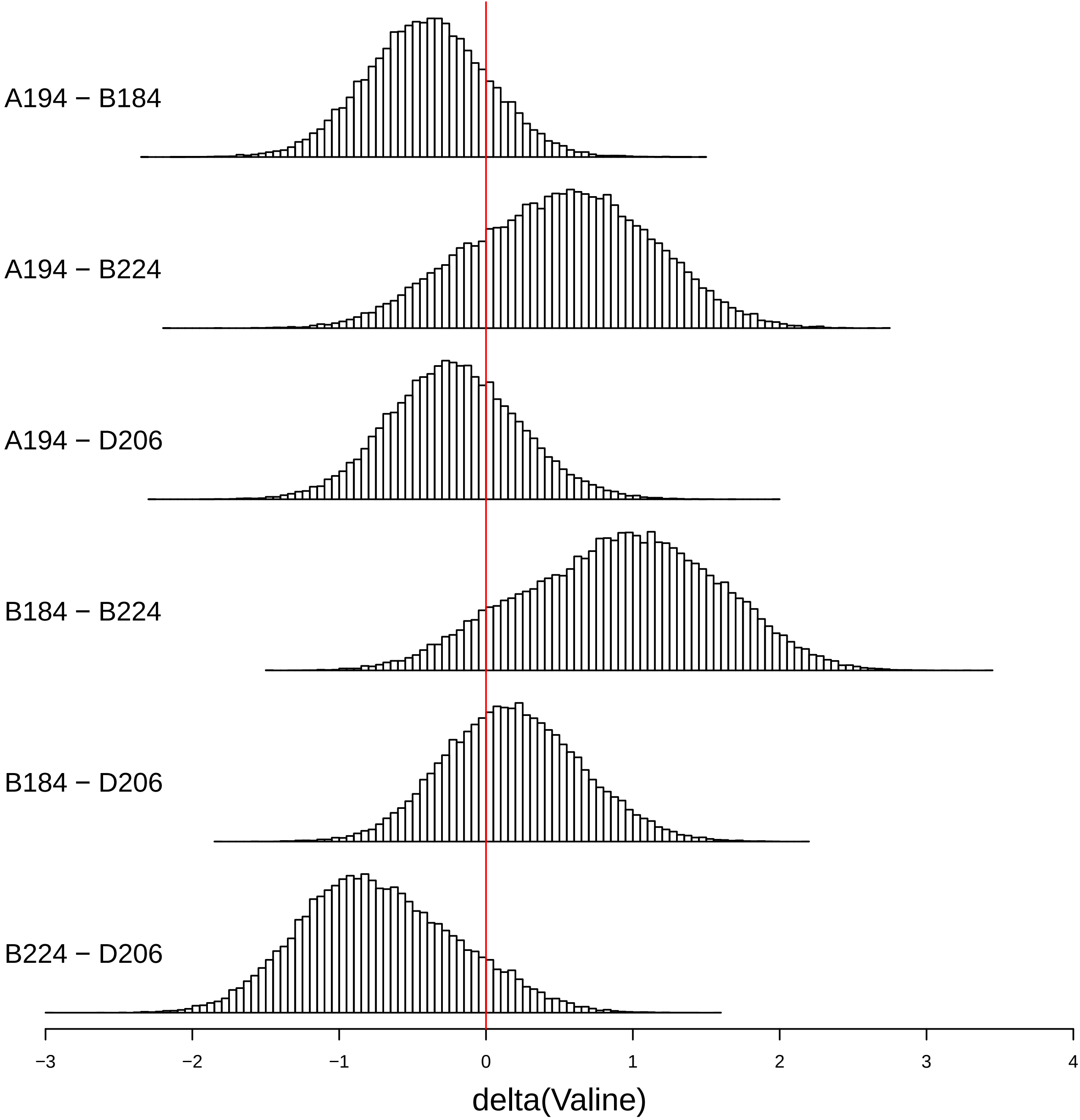
A194 − B184
A194 − B224
A194 − D206
B184 − B224
B184 − D206
B224 − D206
−3
−2
−1
0
1
2
3
4
delta(Valine)

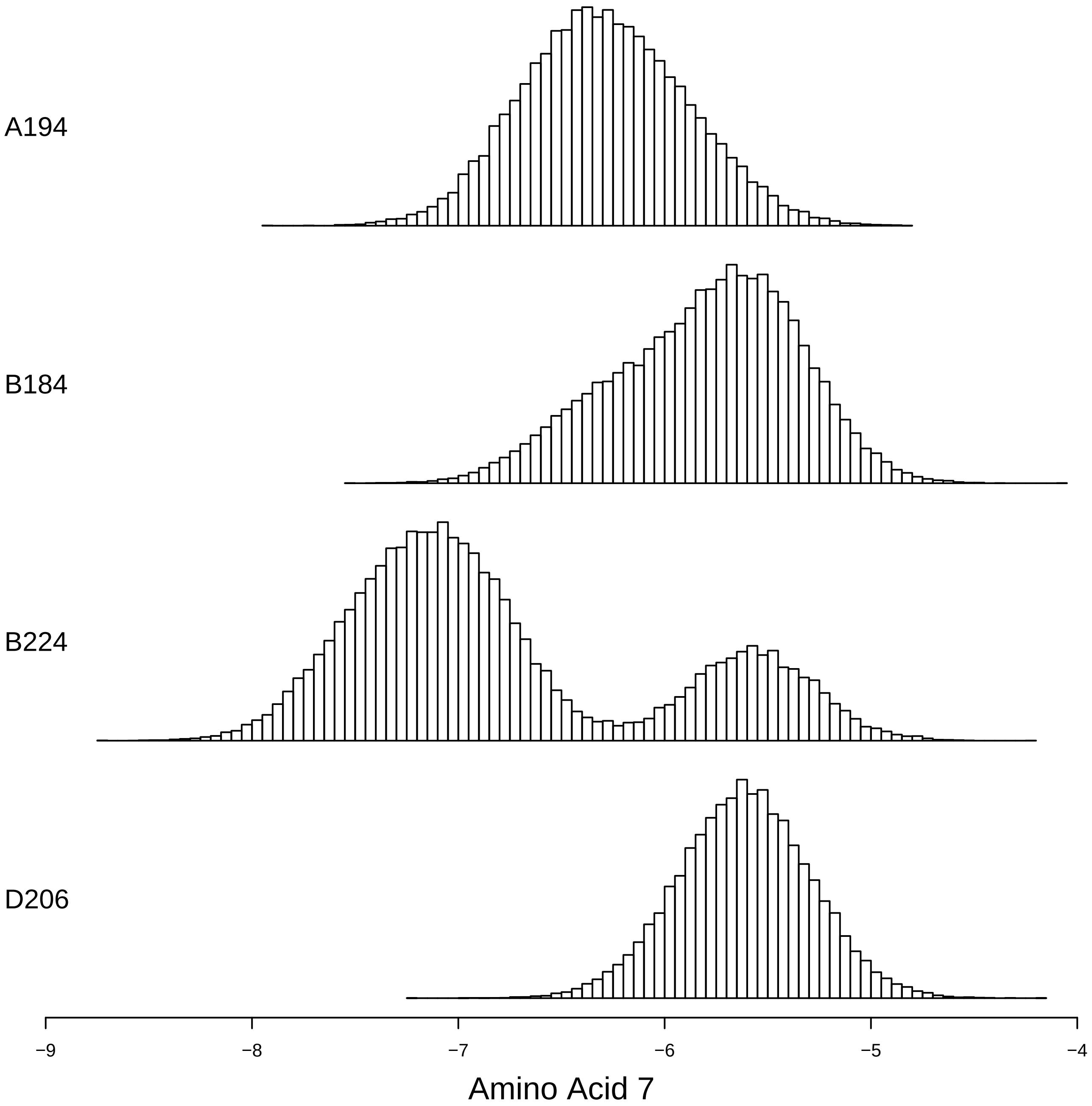
A194
B184
B224
D206
−9
−8
−7
−6
−5
−4
Amino Acid 7

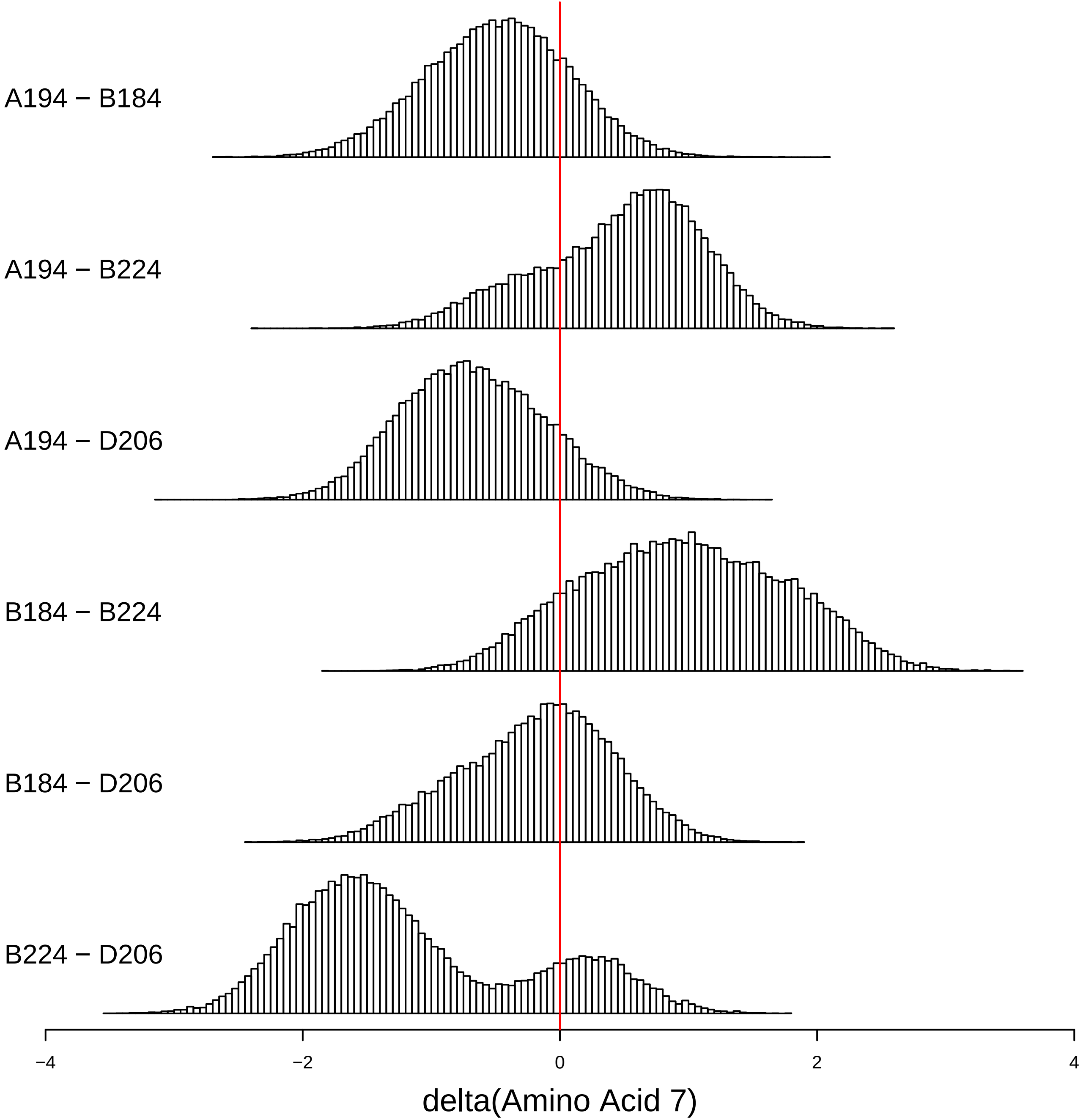
A194 − B184
A194 − B224
A194 − D206
B184 − B224
B184 − D206
B224 − D206
−4
−2
0
2
4
delta(Amino Acid 7)

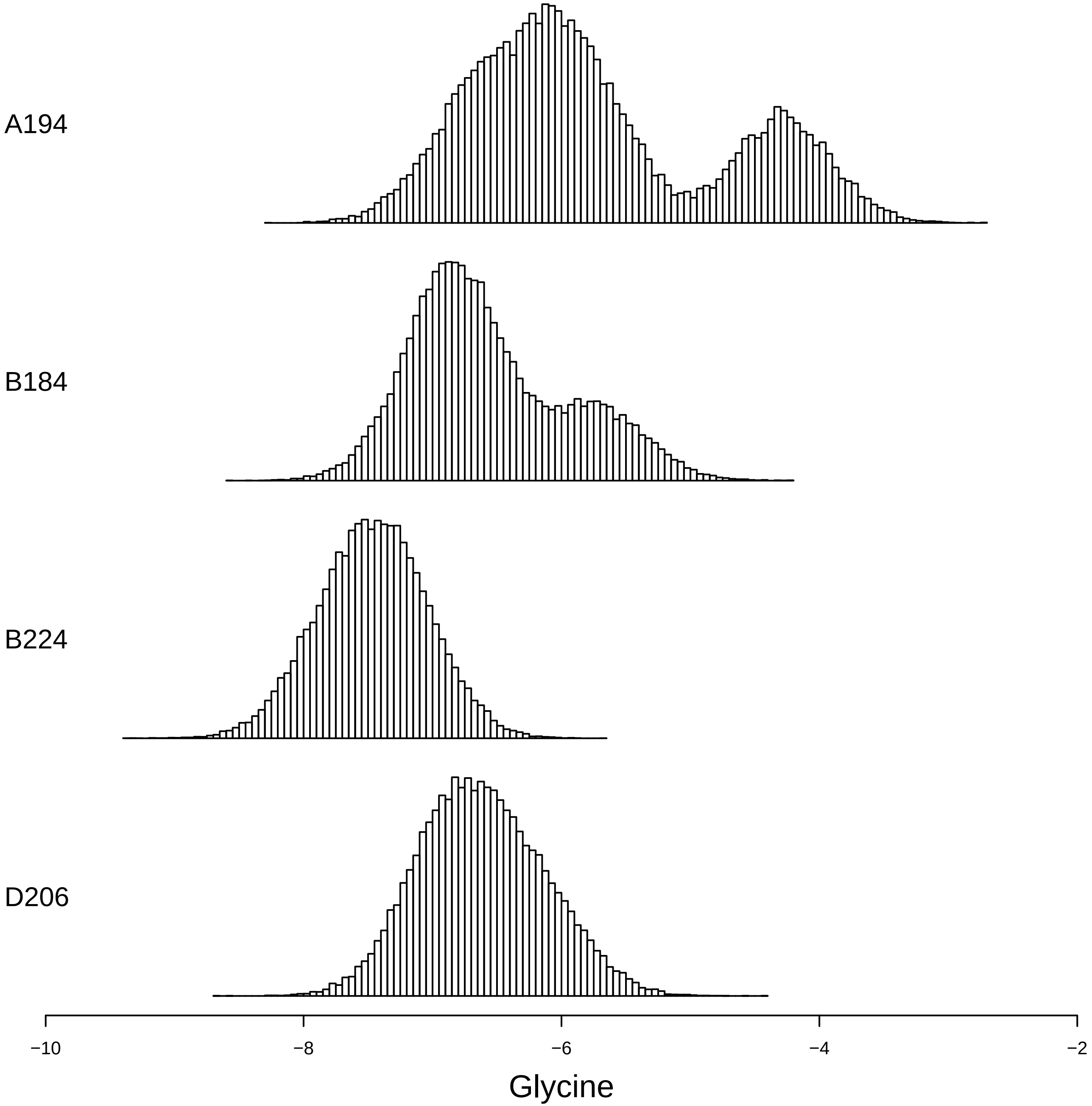
A194
B184
B224
D206
−10
−8
−6
−4
−2
Glycine

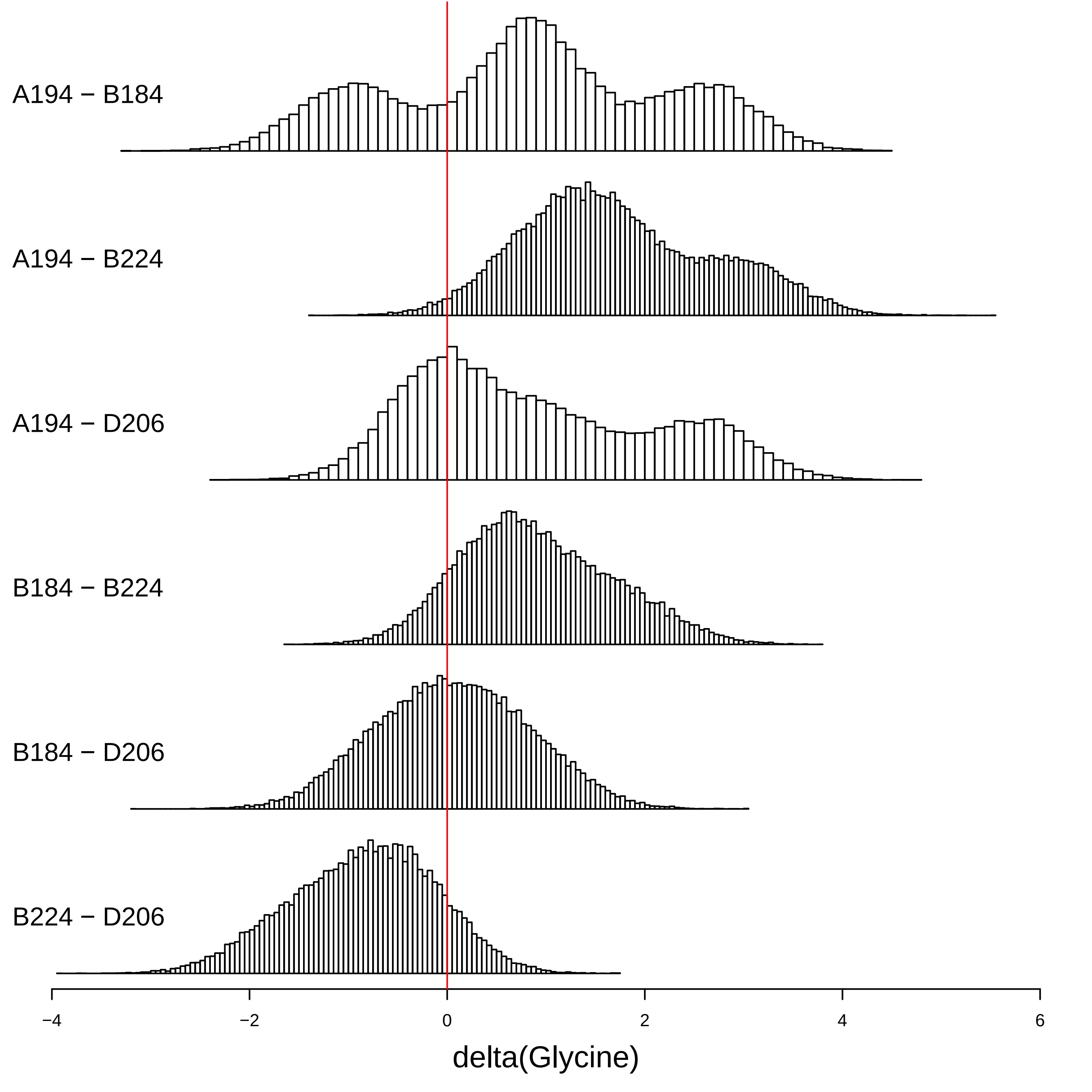

A194 − B184
A194 − B224
A194 − D206
B184 − B224
B184 − D206
B224 − D206
−4
−2
0
2
4
6
delta(Glycine)

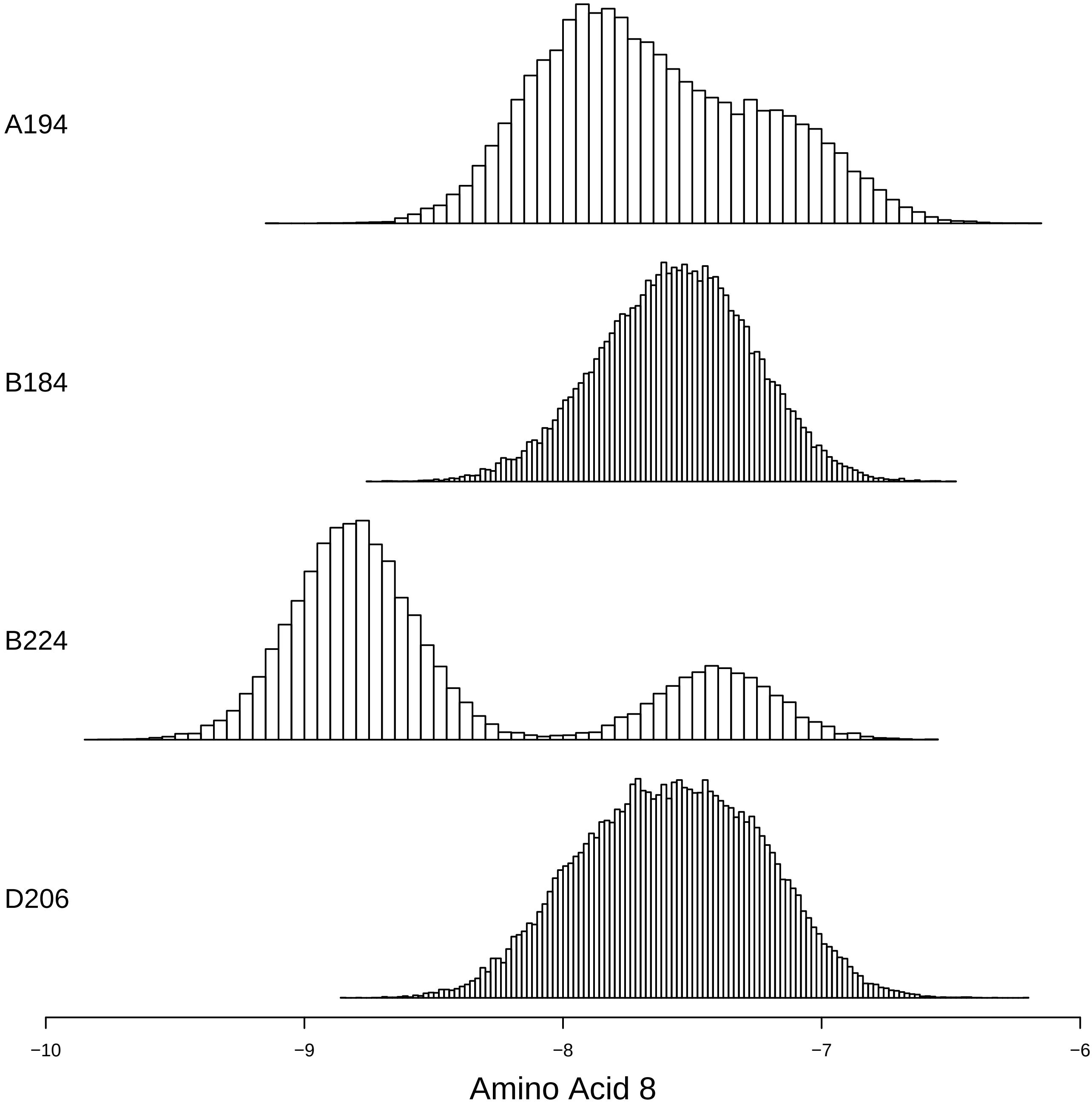
A194
B184
B224
D206
−10
−9
−8
−7
−6
Amino Acid 8

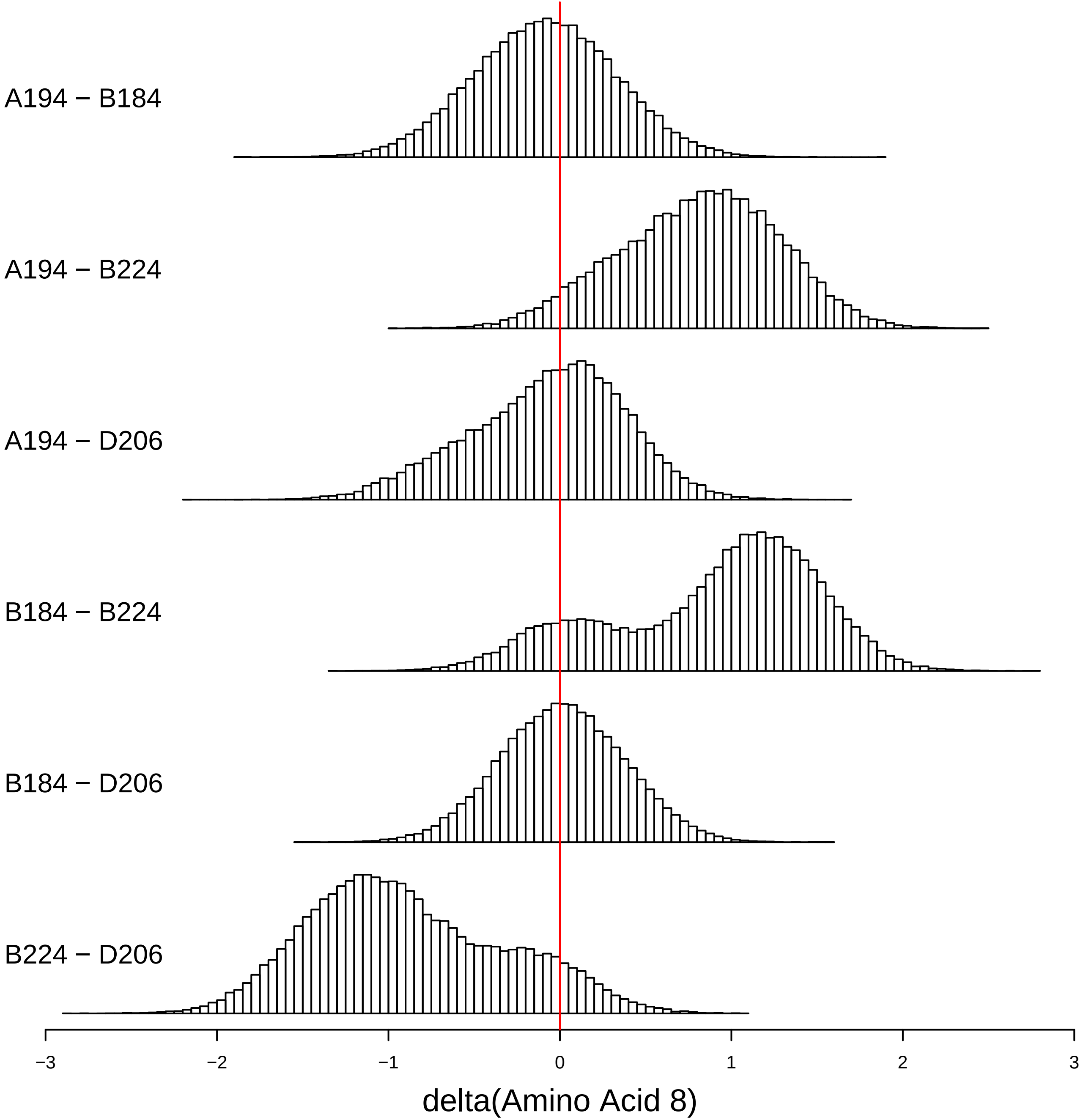

A194 − B184
A194 − B224
A194 − D206
B184 − B224
B184 − D206
B224 − D206
−3
−2
−1
0
1
2
3
delta(Amino Acid 8)

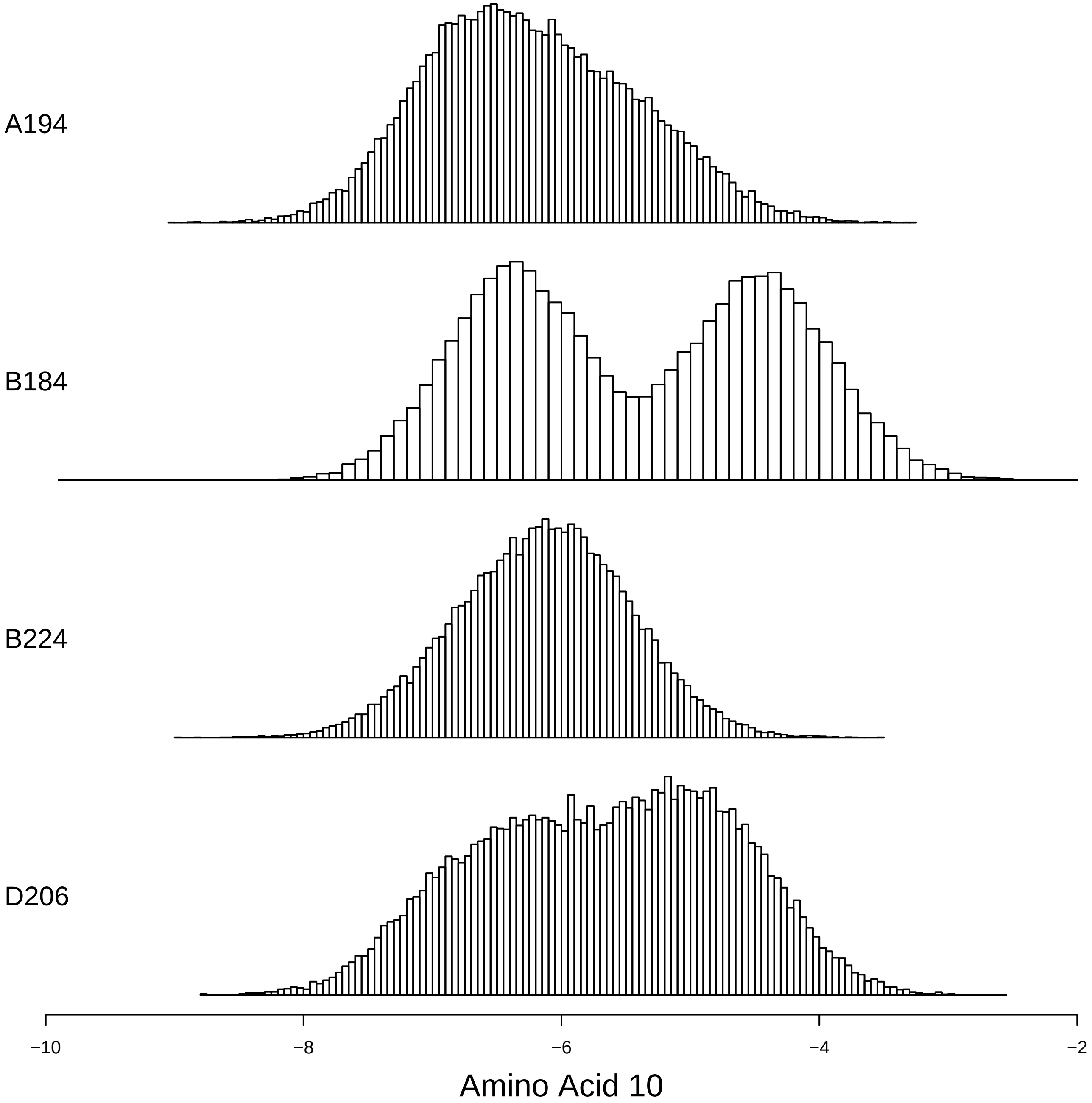

A194
B184
B224
D206
−10
−8
−6
−4
−2
Amino Acid 10

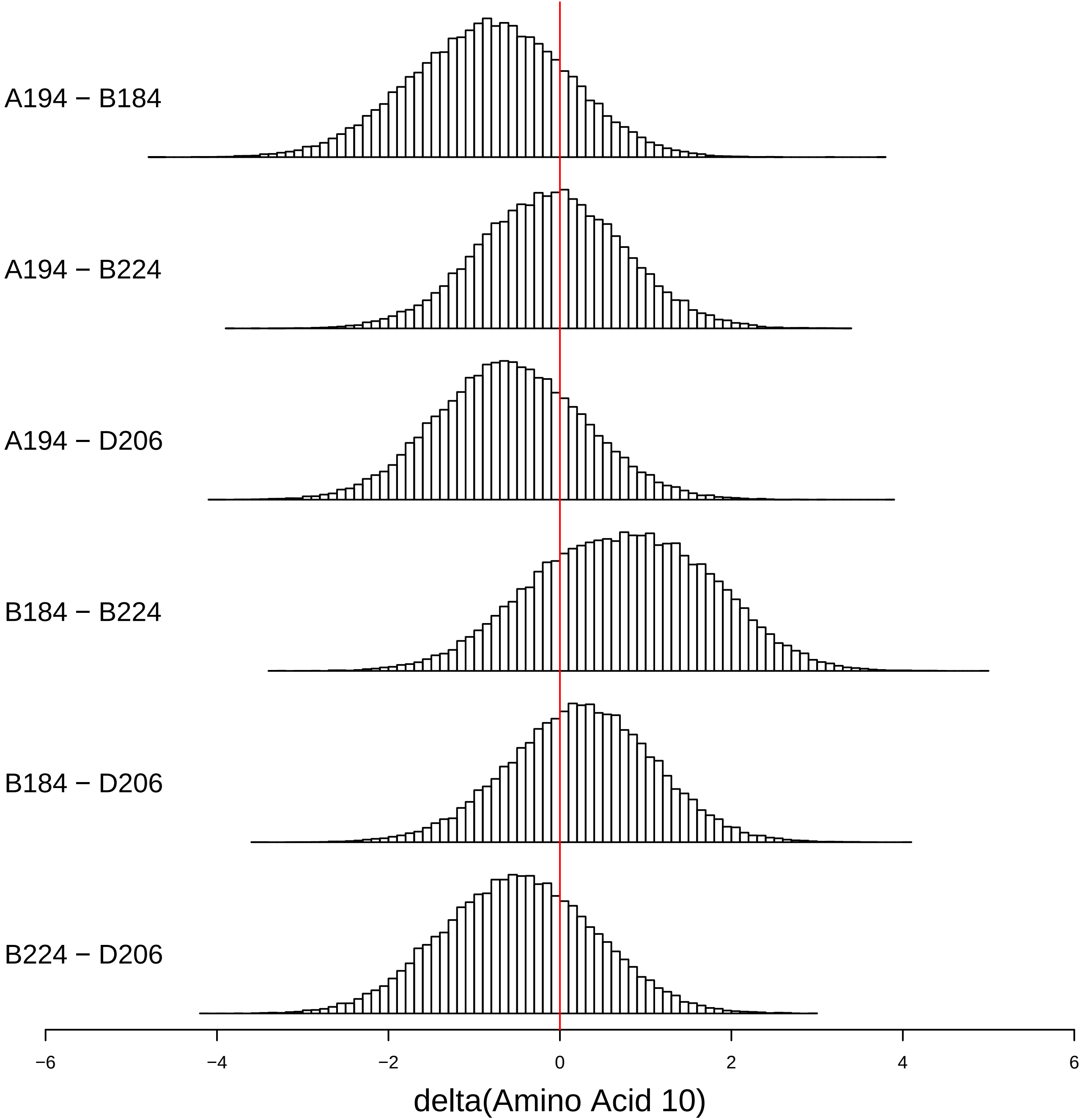

A194 − B184
A194 − B224
A194 − D206
B184 − B224
B184 − D206
B224 − D206
−6
−4
−2
0
2
4
6
delta(Amino Acid 10)

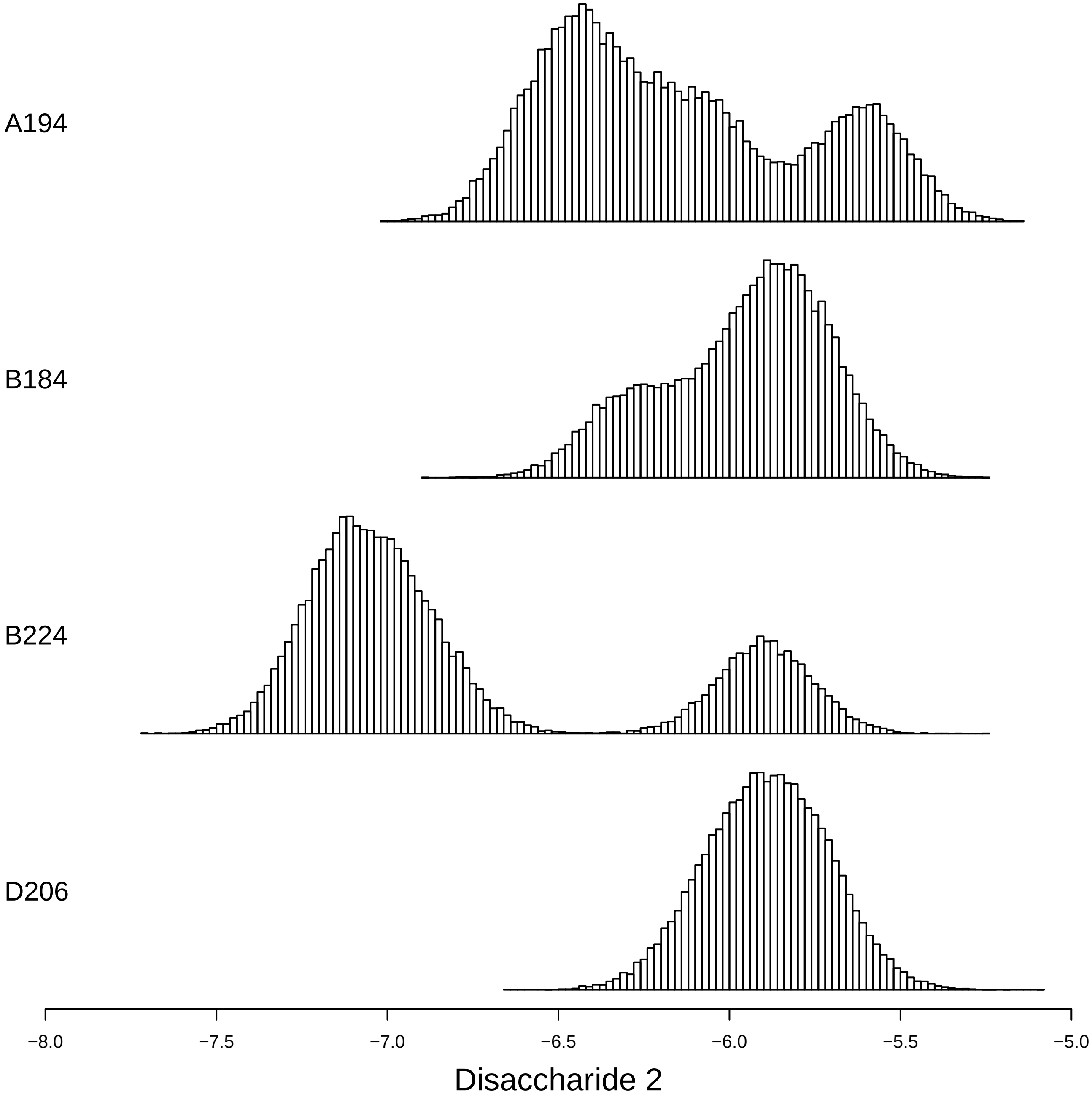

A194
B184
B224
D206
−8.0
−7.5
−7.0
−6.5
−6.0
−5.5
−5.0
Disaccharide 2

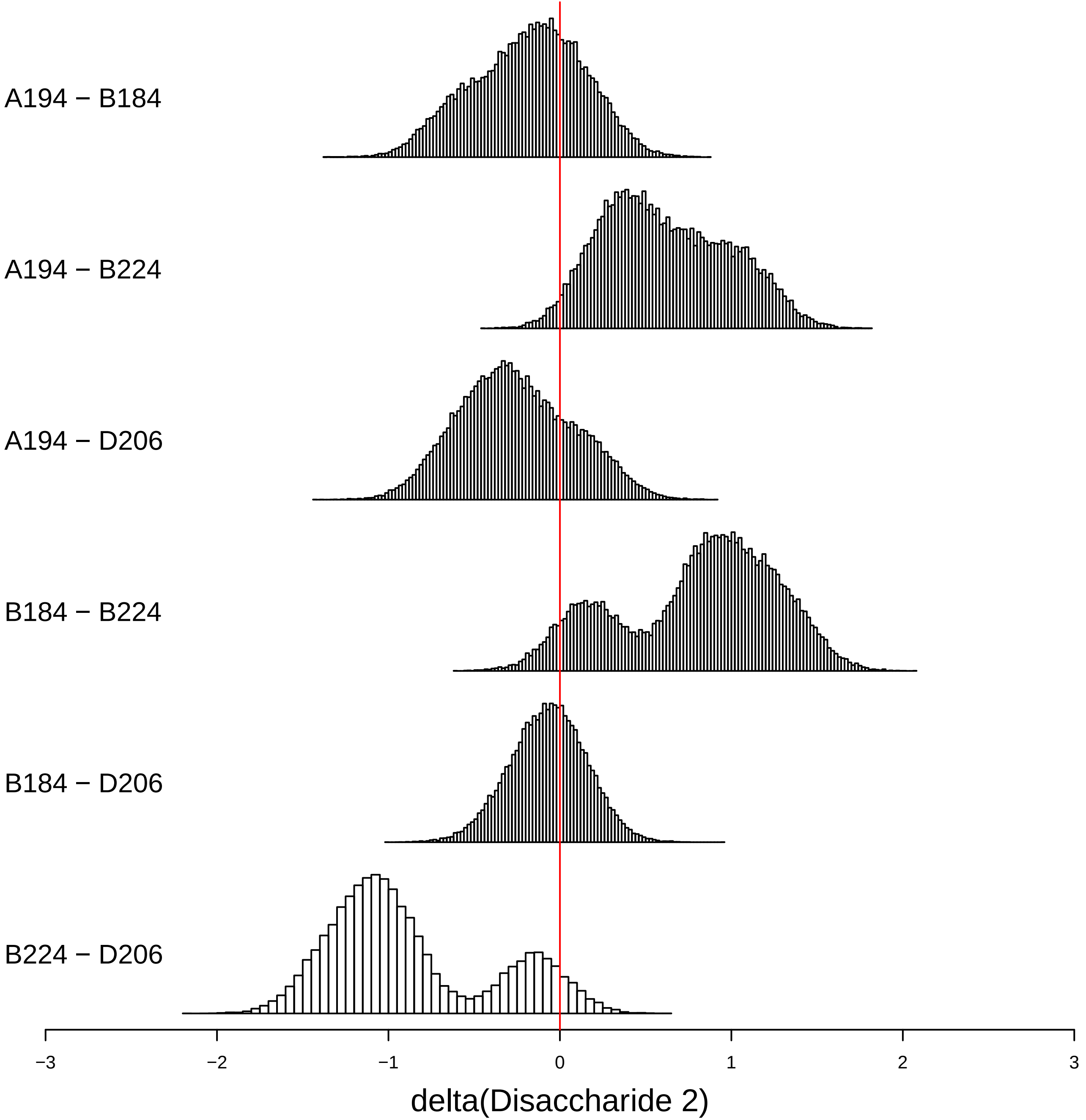
A194 − B184
A194 − B224
A194 − D206
B184 − B224
B184 − D206
B224 − D206
−3
−2
−1
0
1
2
3
delta(Disaccharide 2)

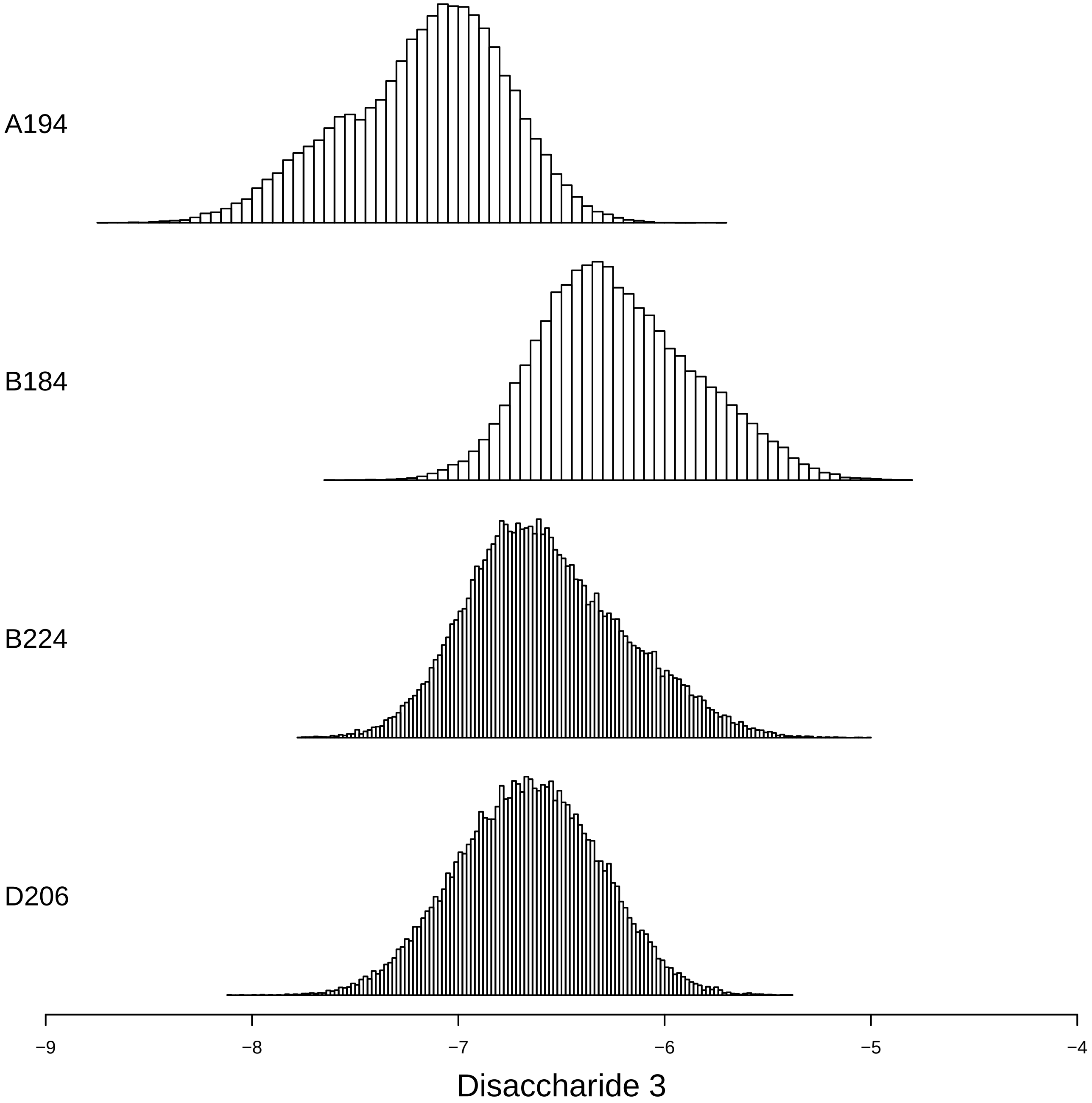

A194
B184
B224
D206
−9
−8
−7
−6
−5
−4
Disaccharide 3

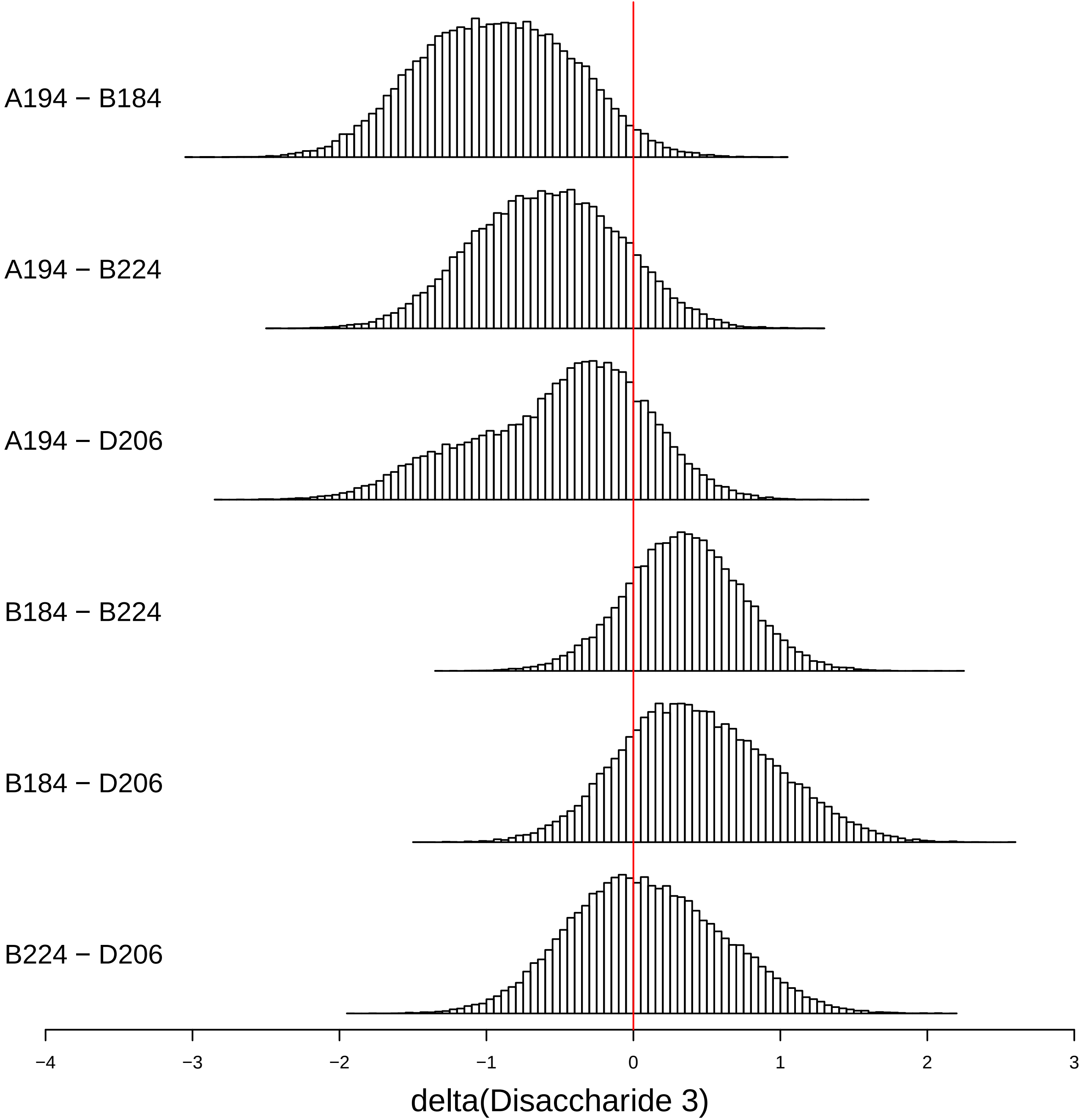
A194 − B184
A194 − B224
A194 − D206
B184 − B224
B184 − D206
B224 − D206
−4
−3
−2
−1
0
1
2
3
delta(Disaccharide 3)

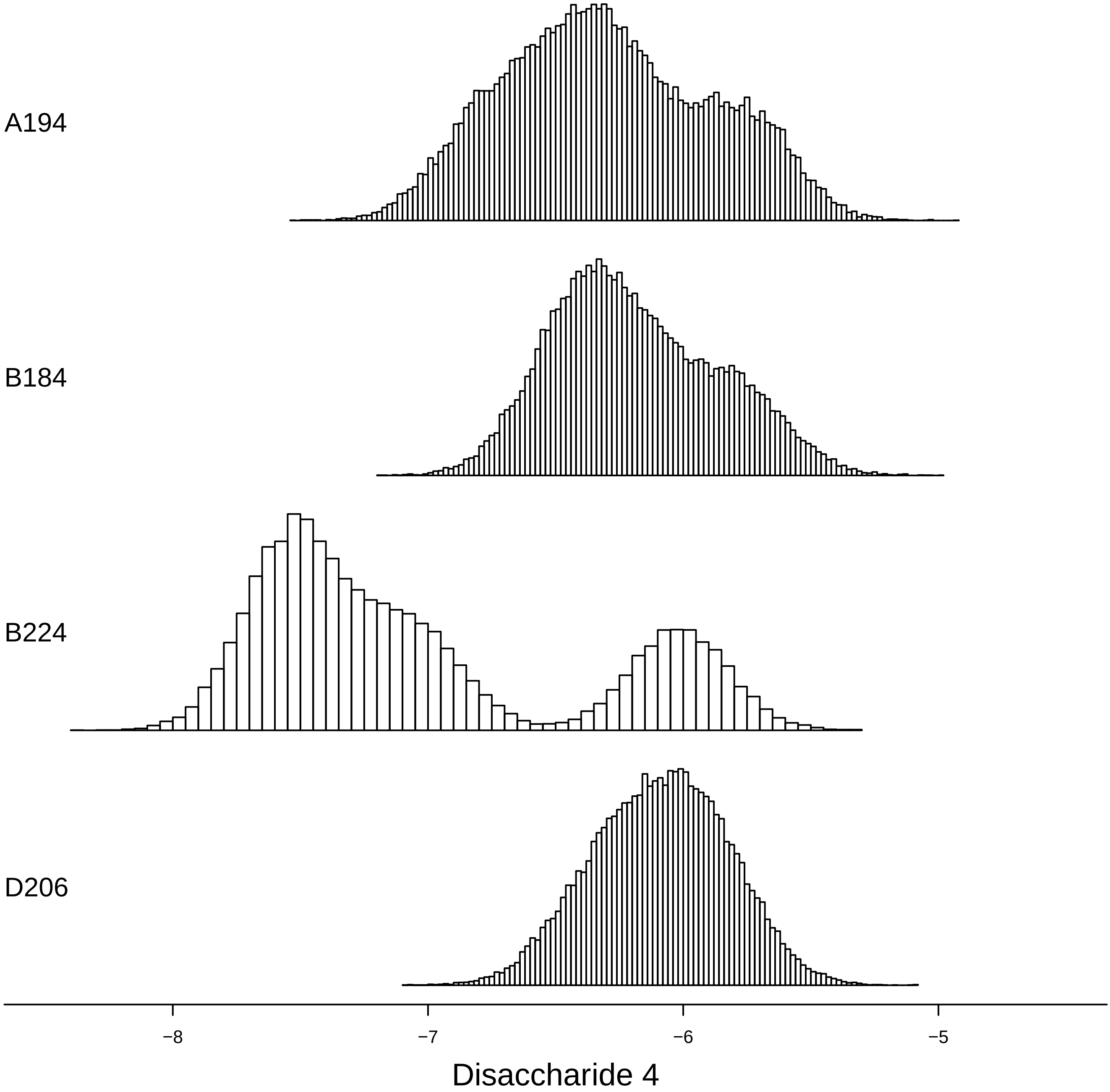
A194
B184
B224
D206
−8
−7
−6
−5
Disaccharide 4

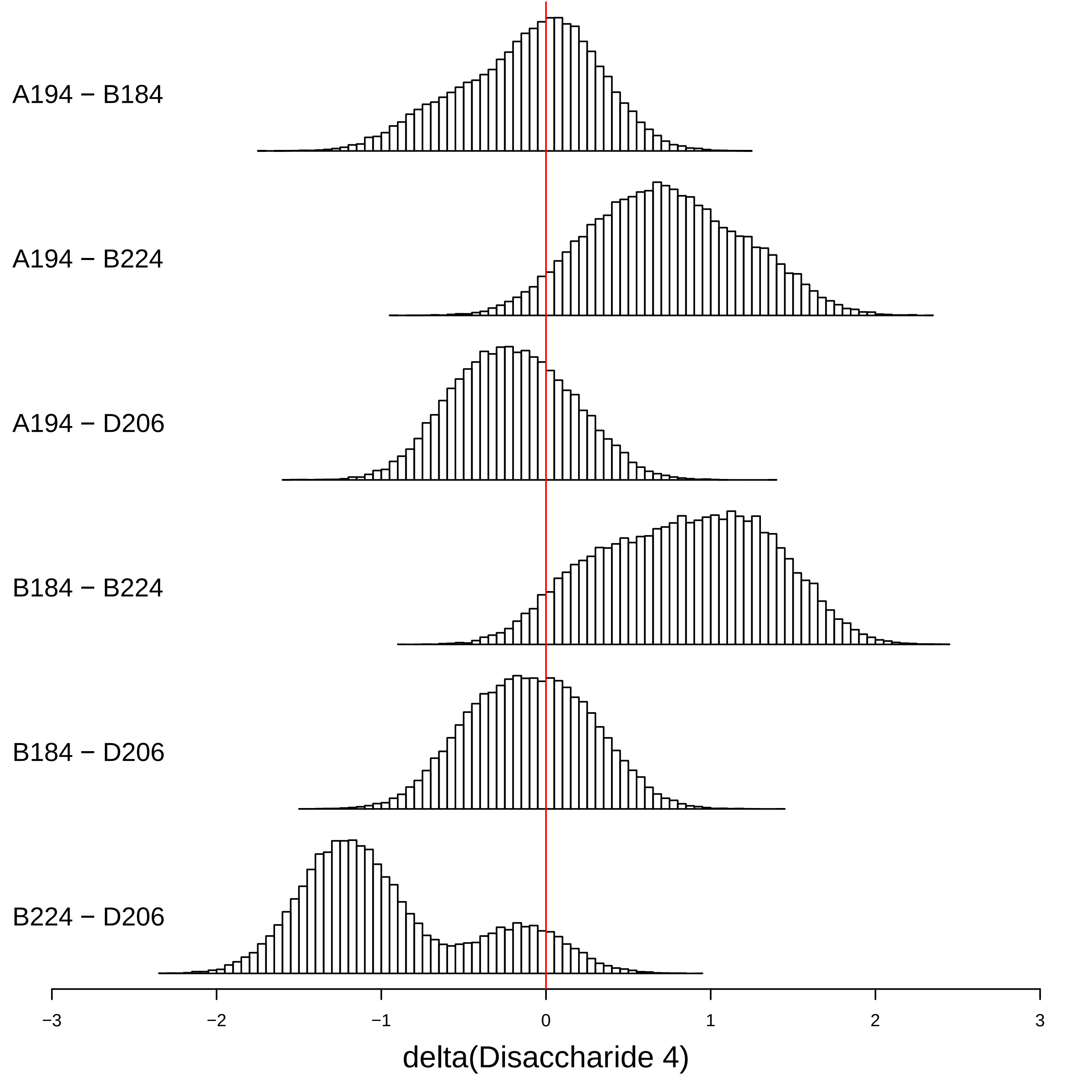

A194 − B184
A194 − B224
A194 − D206
B184 − B224
B184 − D206
B224 − D206
−3
−2
−1
0
1
2
3
delta(Disaccharide 4)

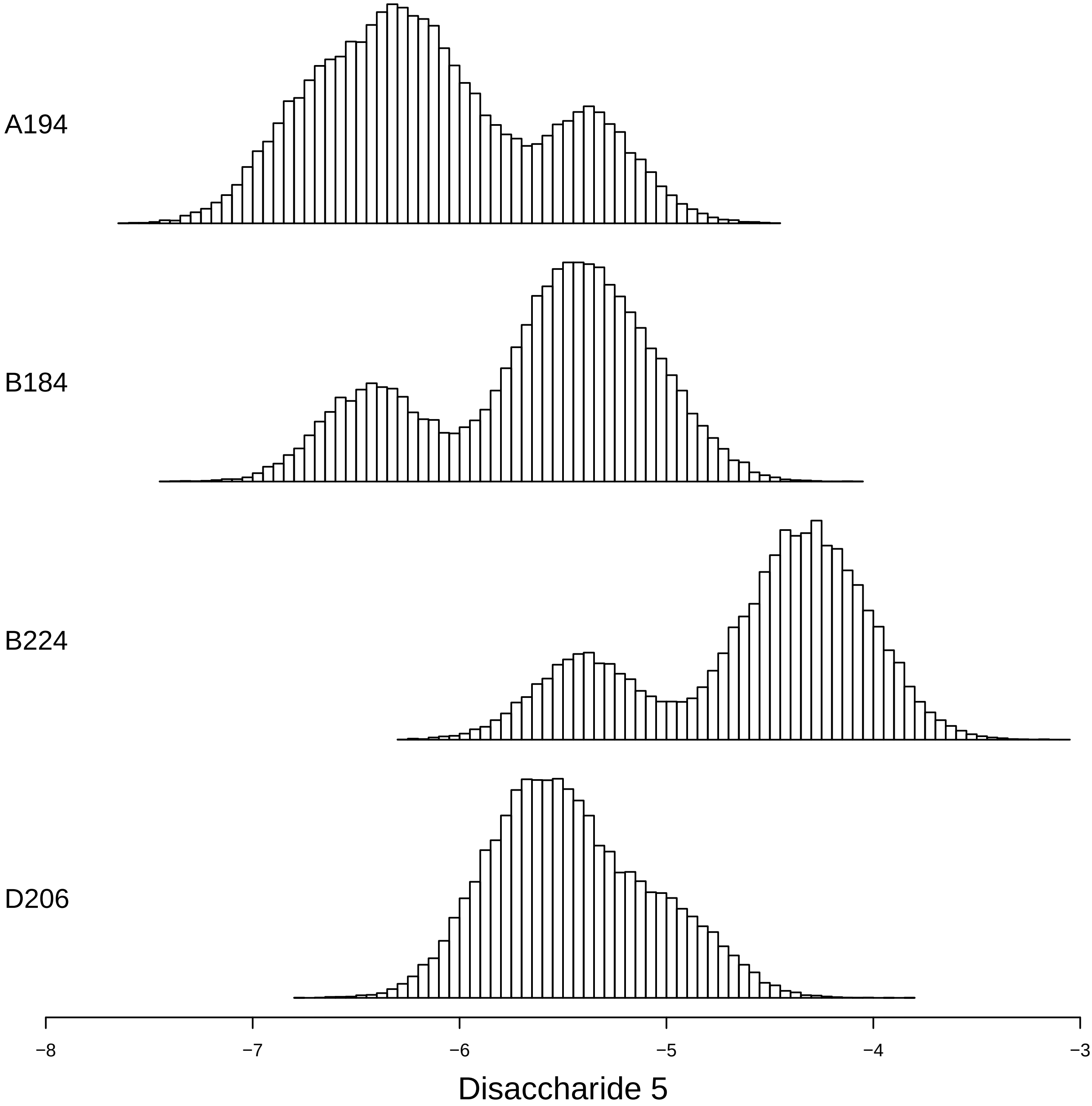
A194
B184
B224
D206
−8
−7
−6
−5
−4
−3
Disaccharide 5

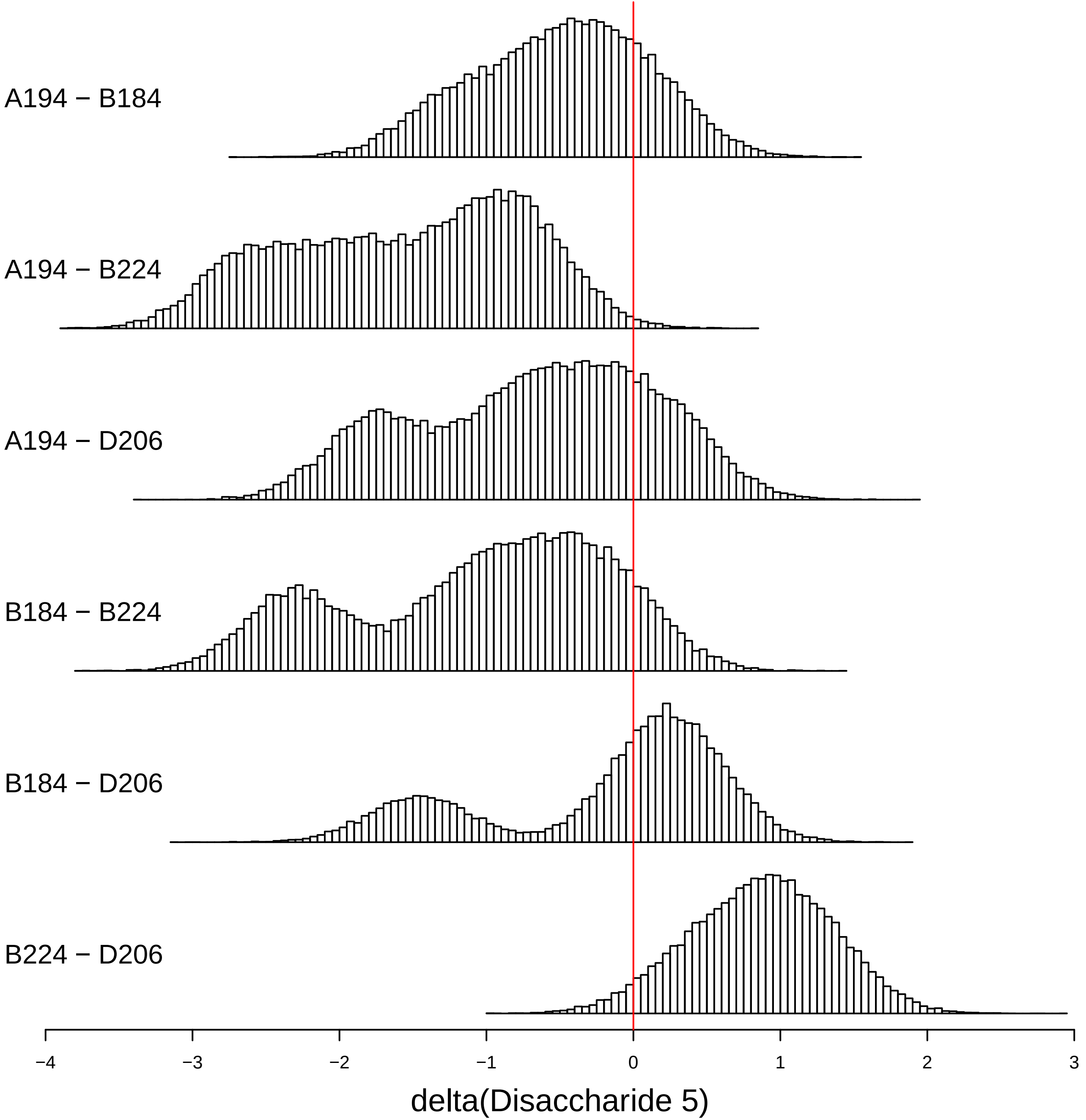
A194 − B184
A194 − B224
A194 − D206
B184 − B224
B184 − D206
B224 − D206
−4
−3
−2
−1
0
1
2
3
delta(Disaccharide 5)

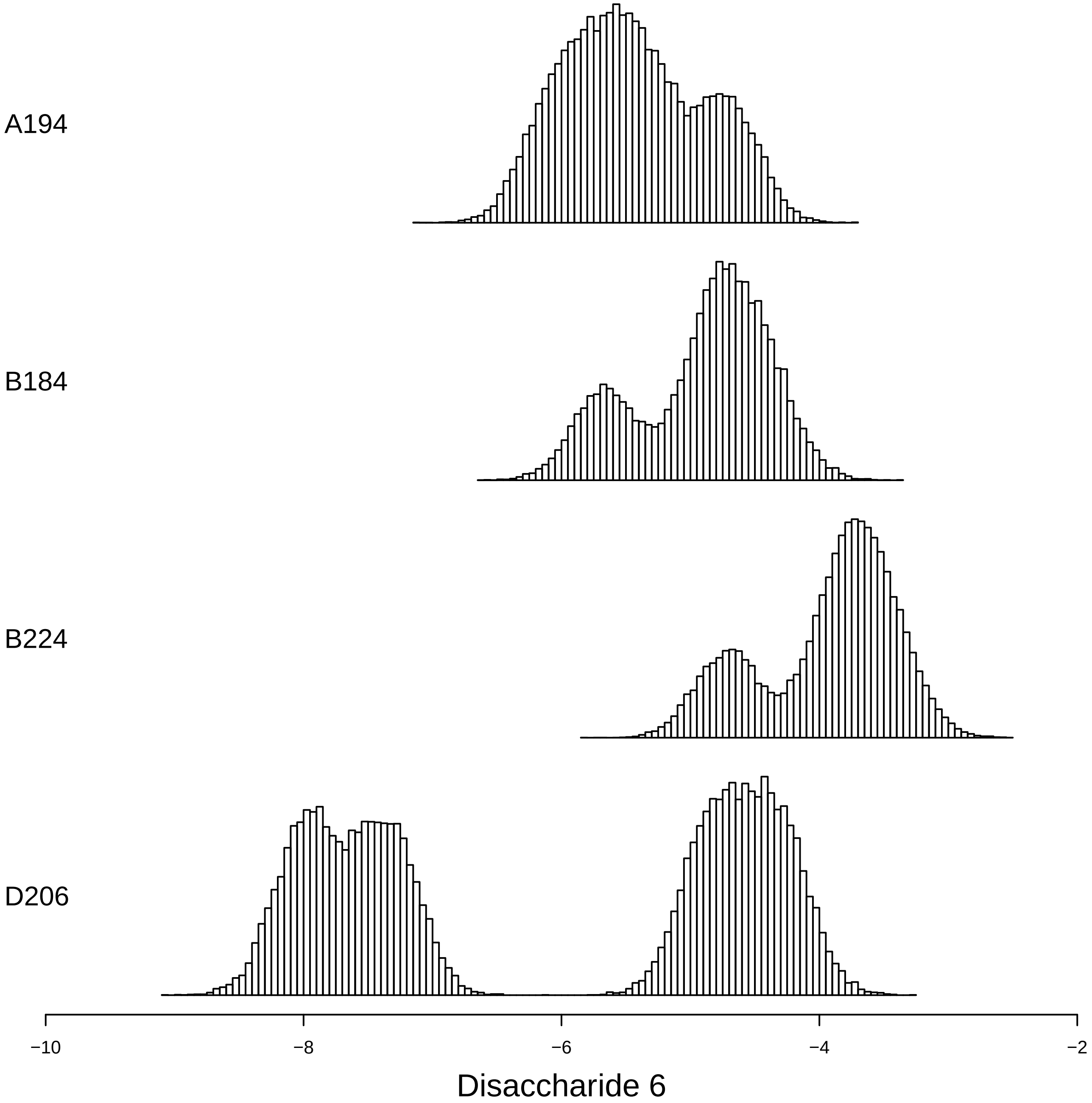
A194
B184
B224
D206
−10
−8
−6
−4
−2
Disaccharide 6

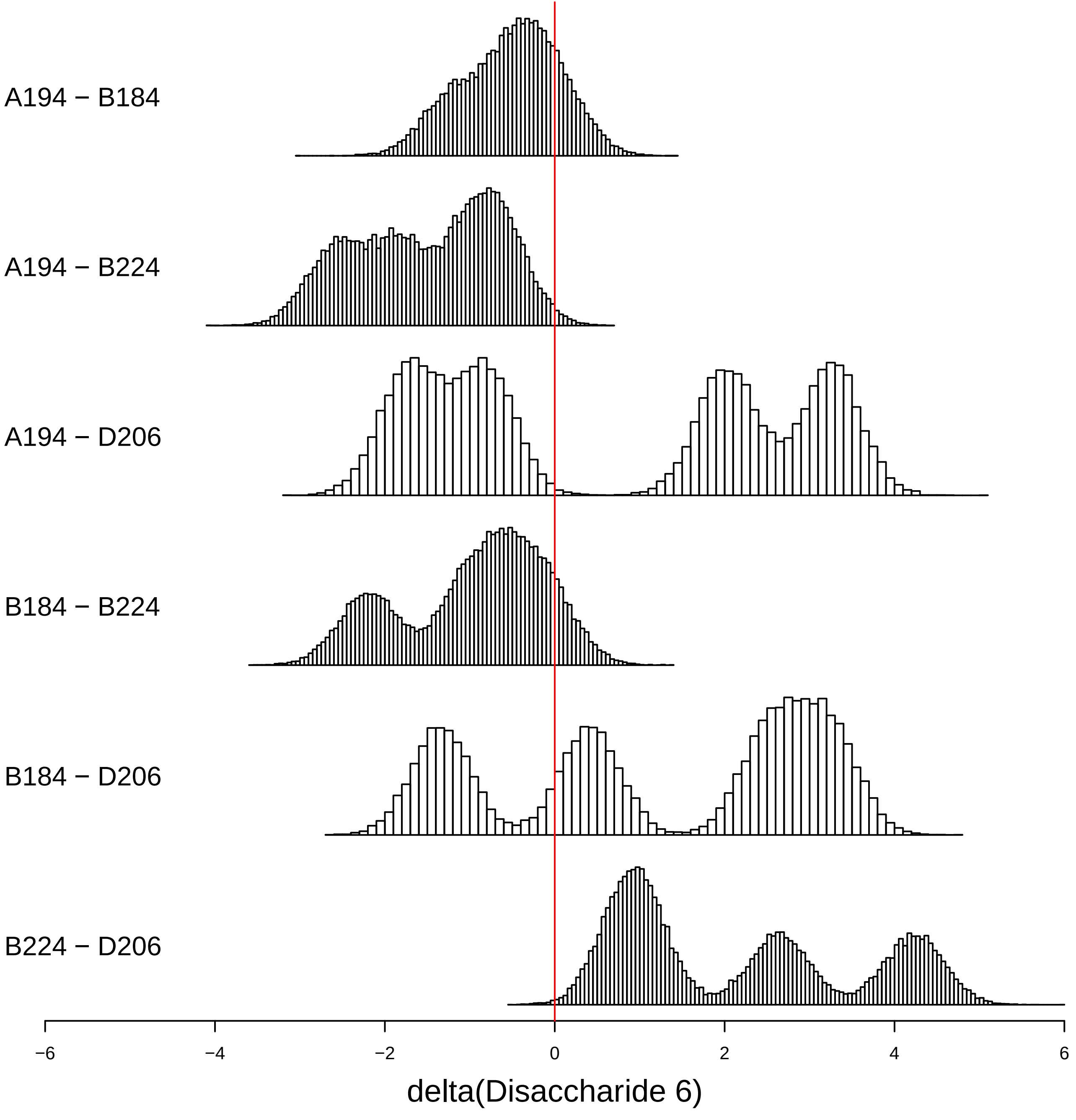

A194 − B184
A194 − B224
A194 − D206
B184 − B224
B184 − D206
B224 − D206
−6
−4
−2
0
2
4
6
delta(Disaccharide 6)

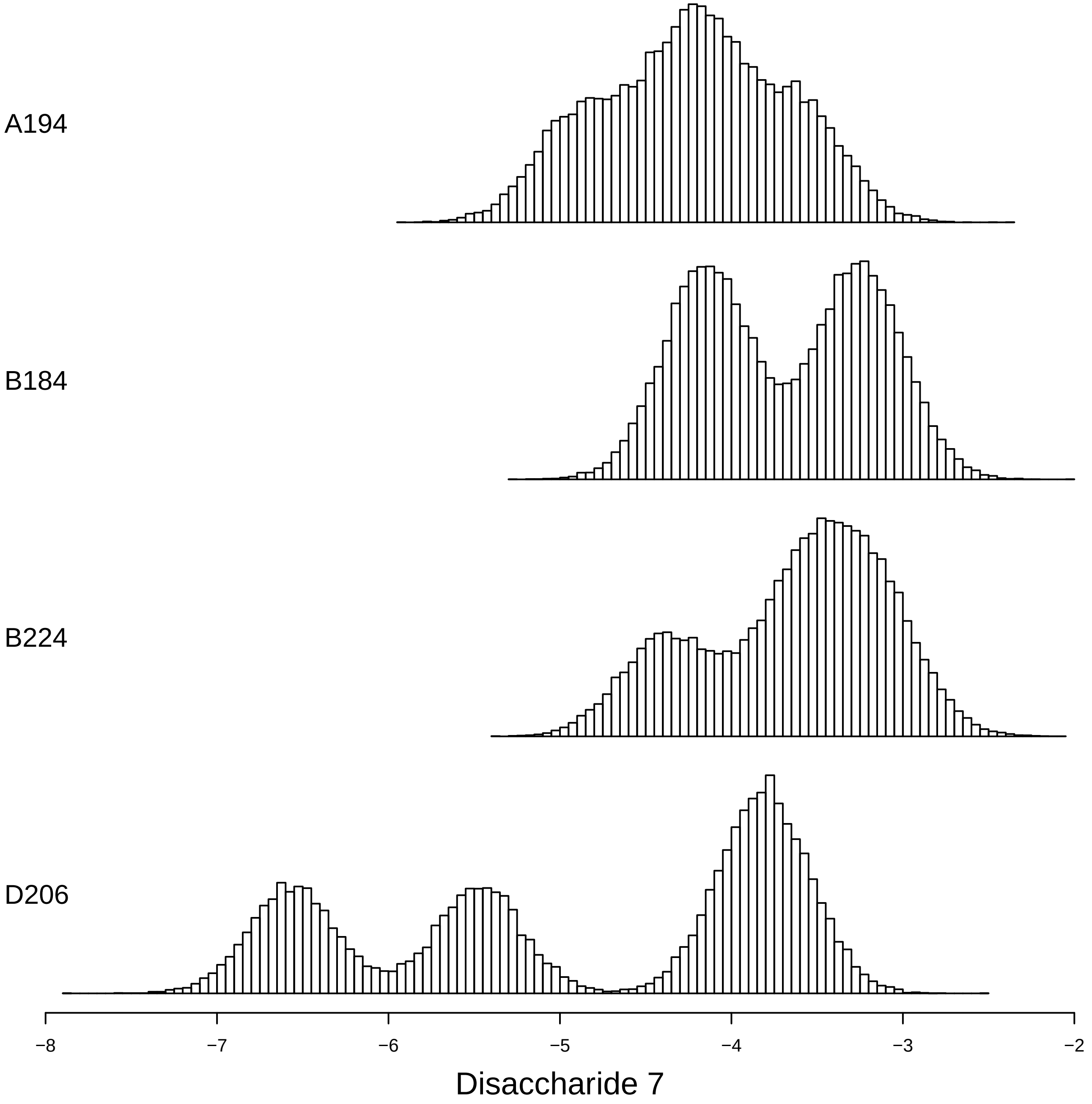

A194
B184
B224
D206
−8
−7
−6
−5
−4
−3
−2
Disaccharide 7

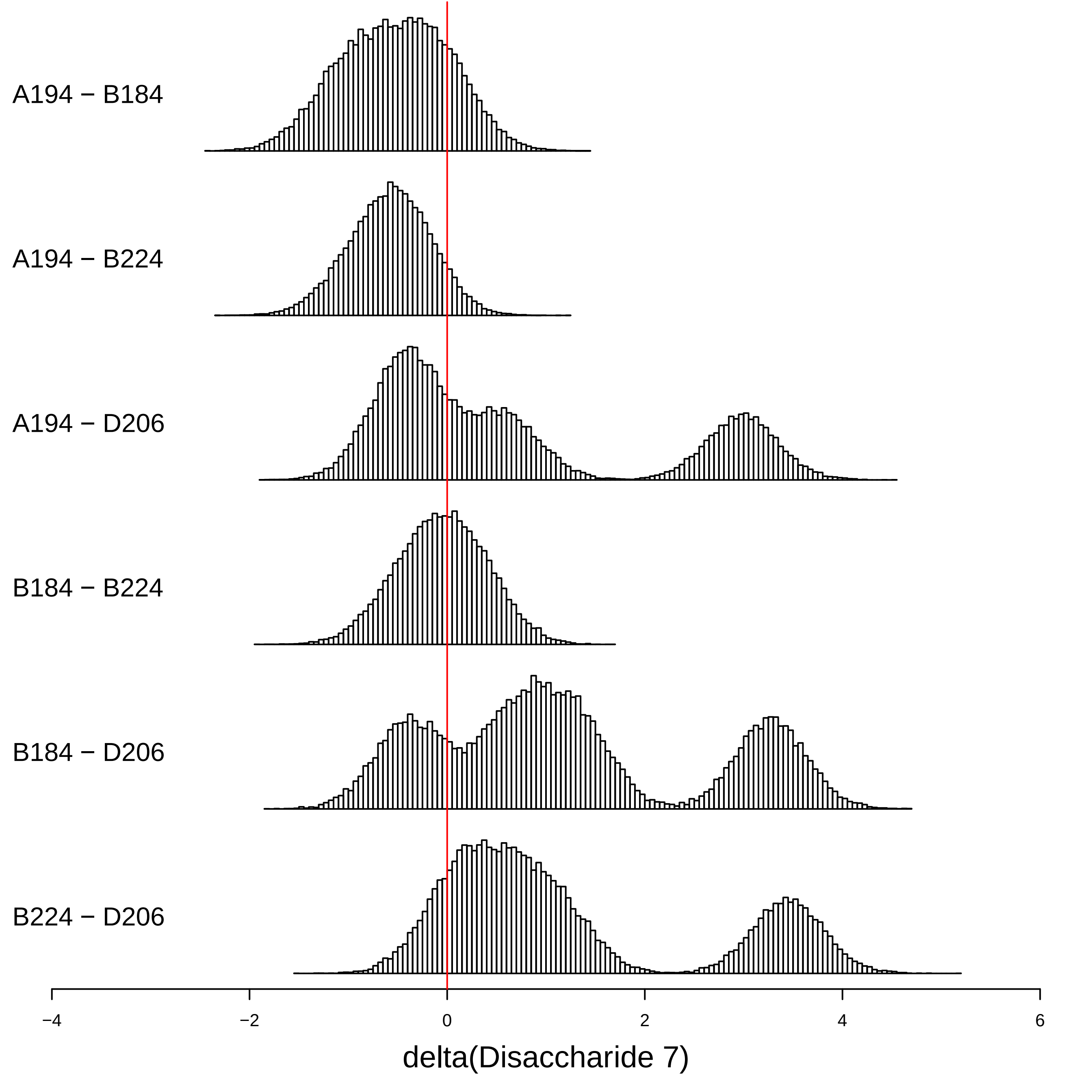
A194 − B184
A194 − B224
A194 − D206
B184 − B224
B184 − D206
B224 − D206
−4
−2
0
2
4
6
delta(Disaccharide 7)

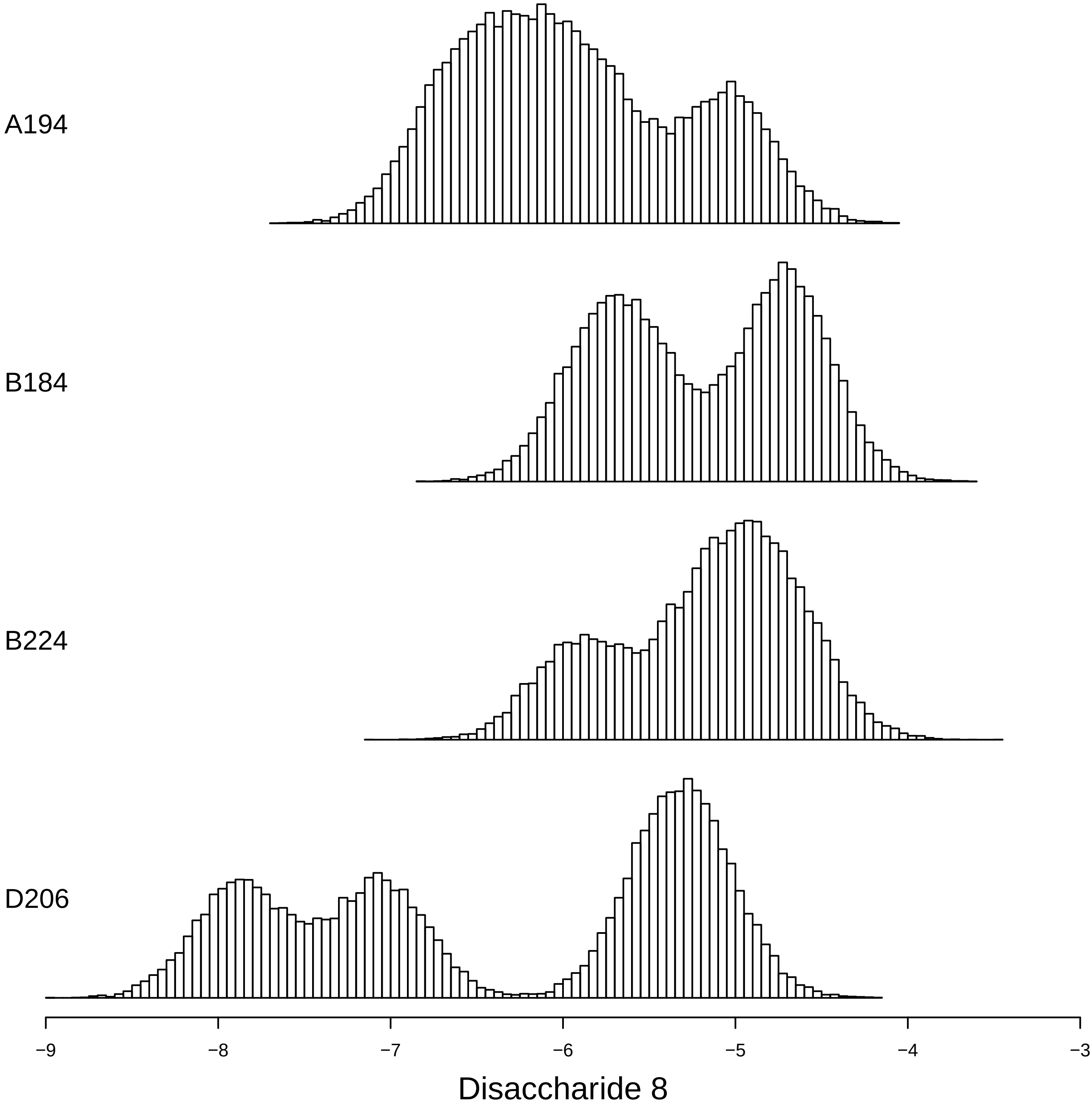

A194
B184
B224
D206
−9
−8
−7
−6
−5
−4
−3
Disaccharide 8

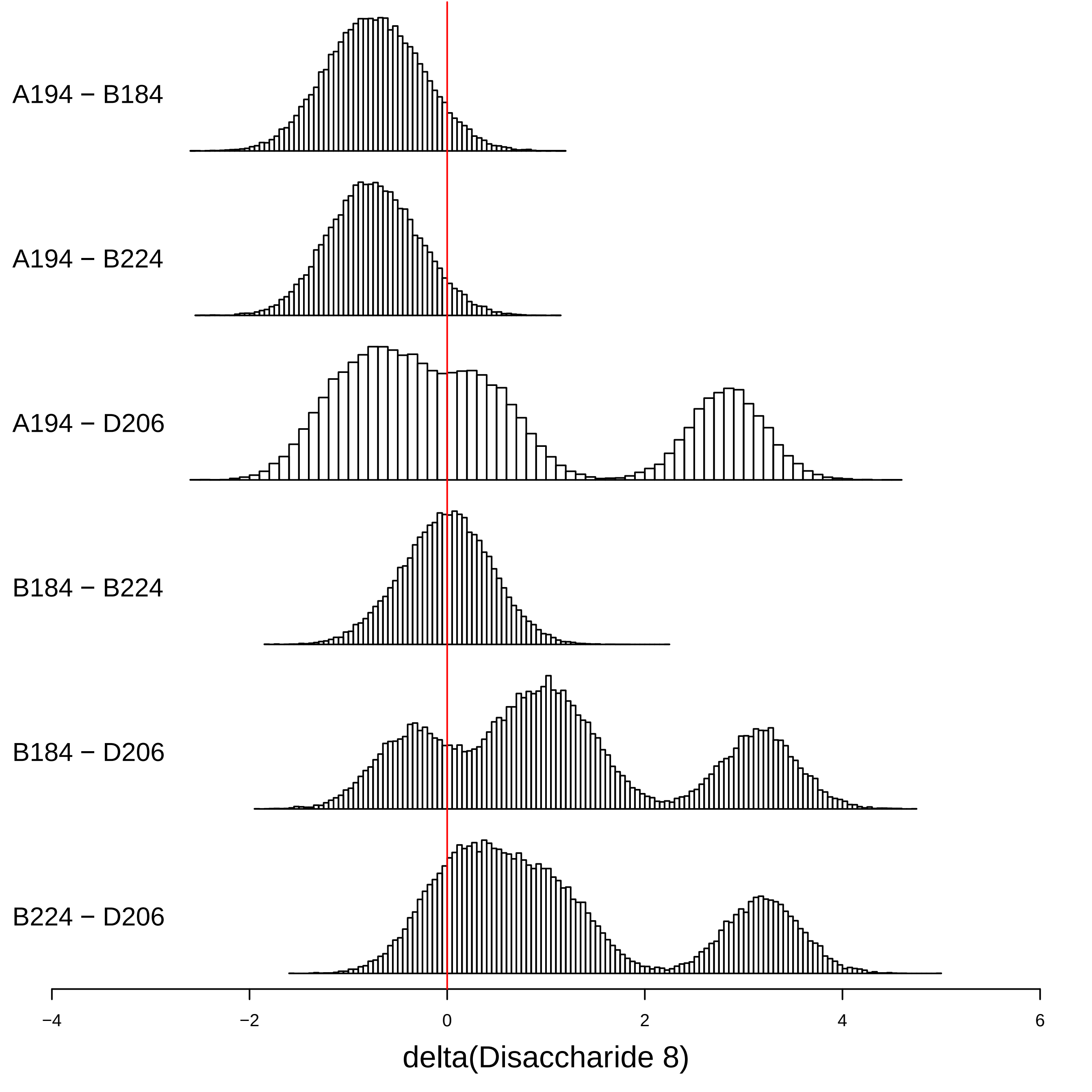
A194 − B184
A194 − B224
A194 − D206
B184 − B224
B184 − D206
B224 − D206
−4
−2
0
2
4
6
delta(Disaccharide 8)

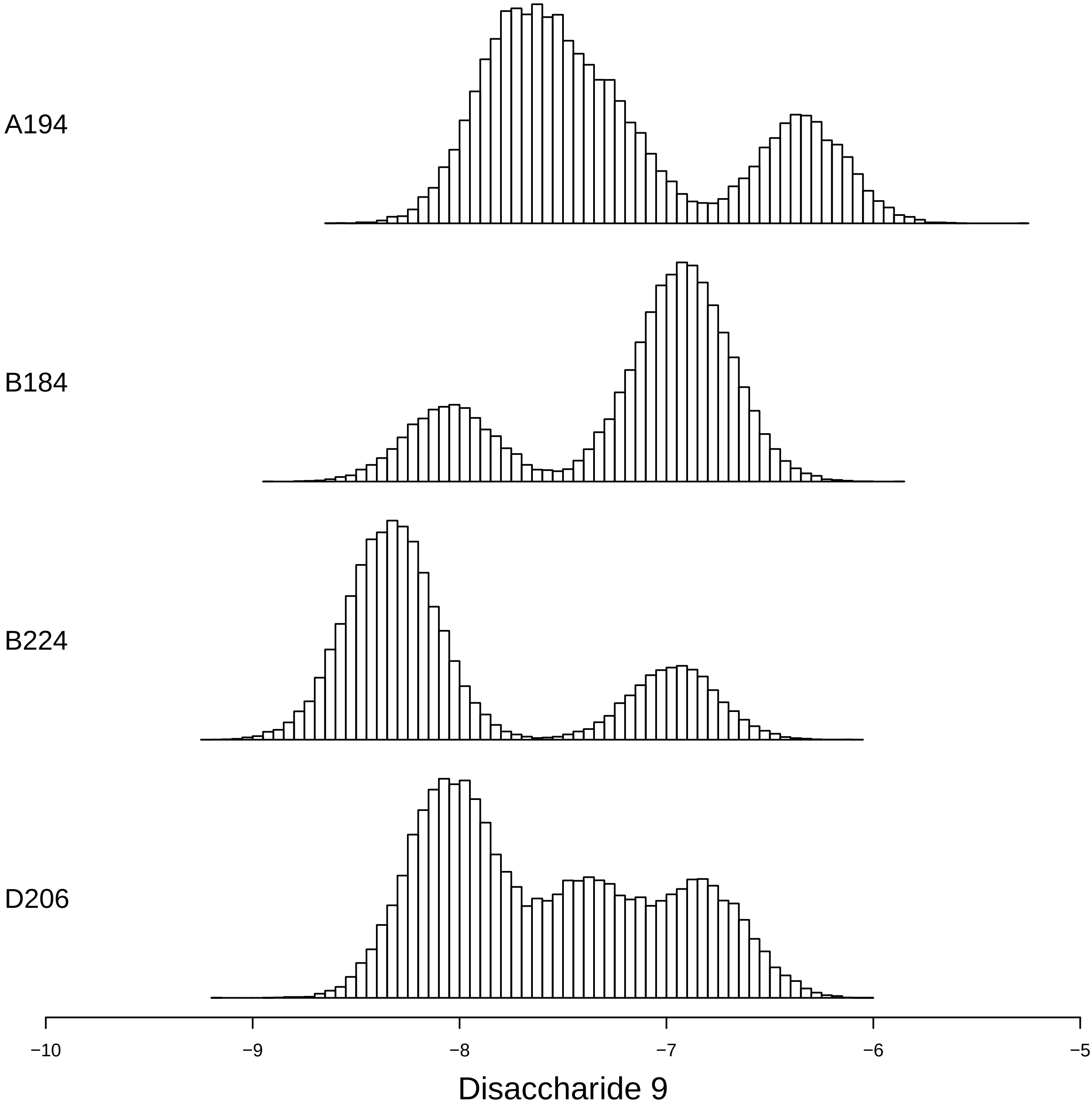
A194
B184
B224
D206
−10
−9
−8
−7
−6
−5
Disaccharide 9

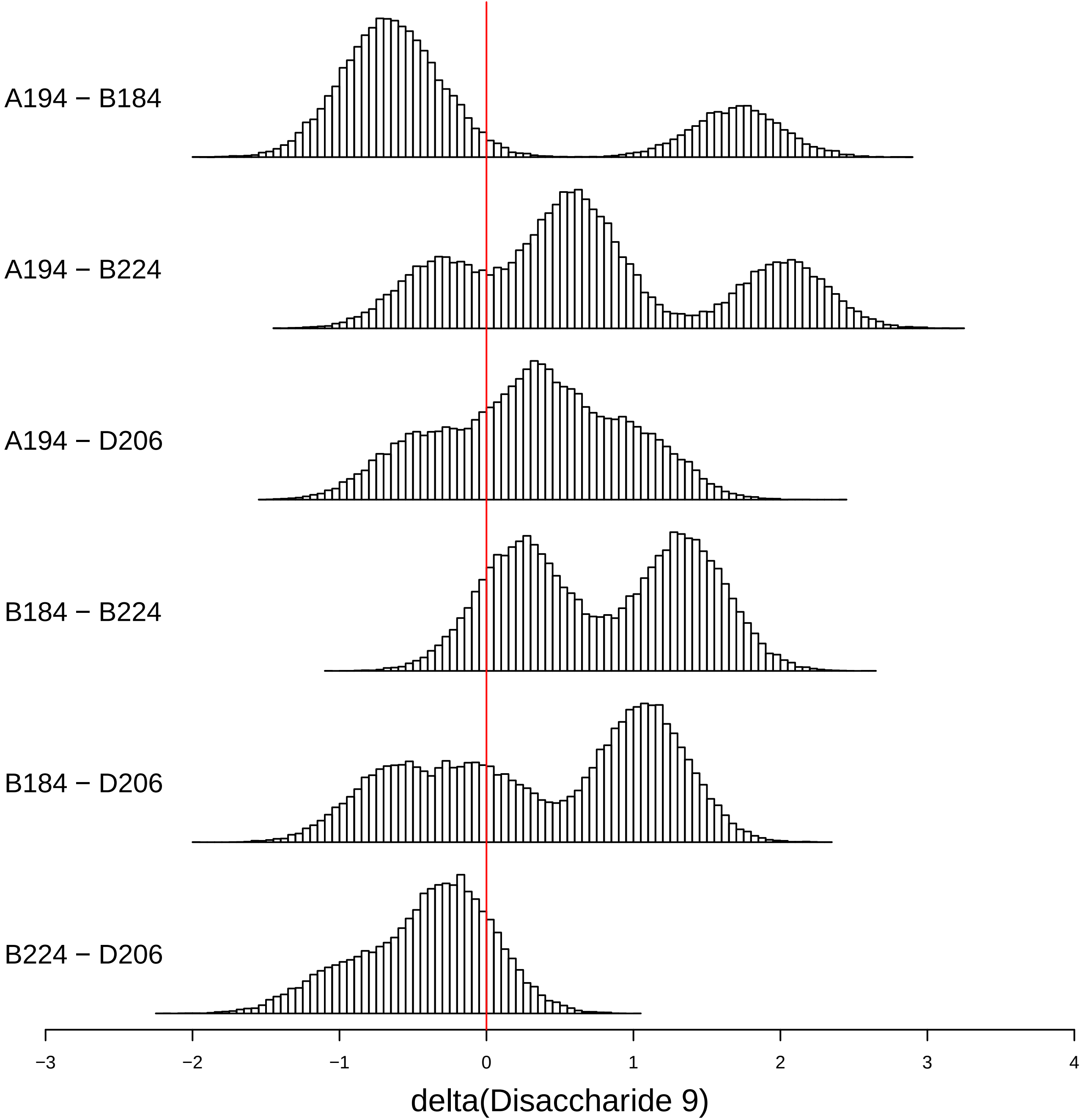
A194 − B184
A194 − B224
A194 − D206
B184 − B224
B184 − D206
B224 − D206
−3
−2
−1
0
1
2
3
4
delta(Disaccharide 9)

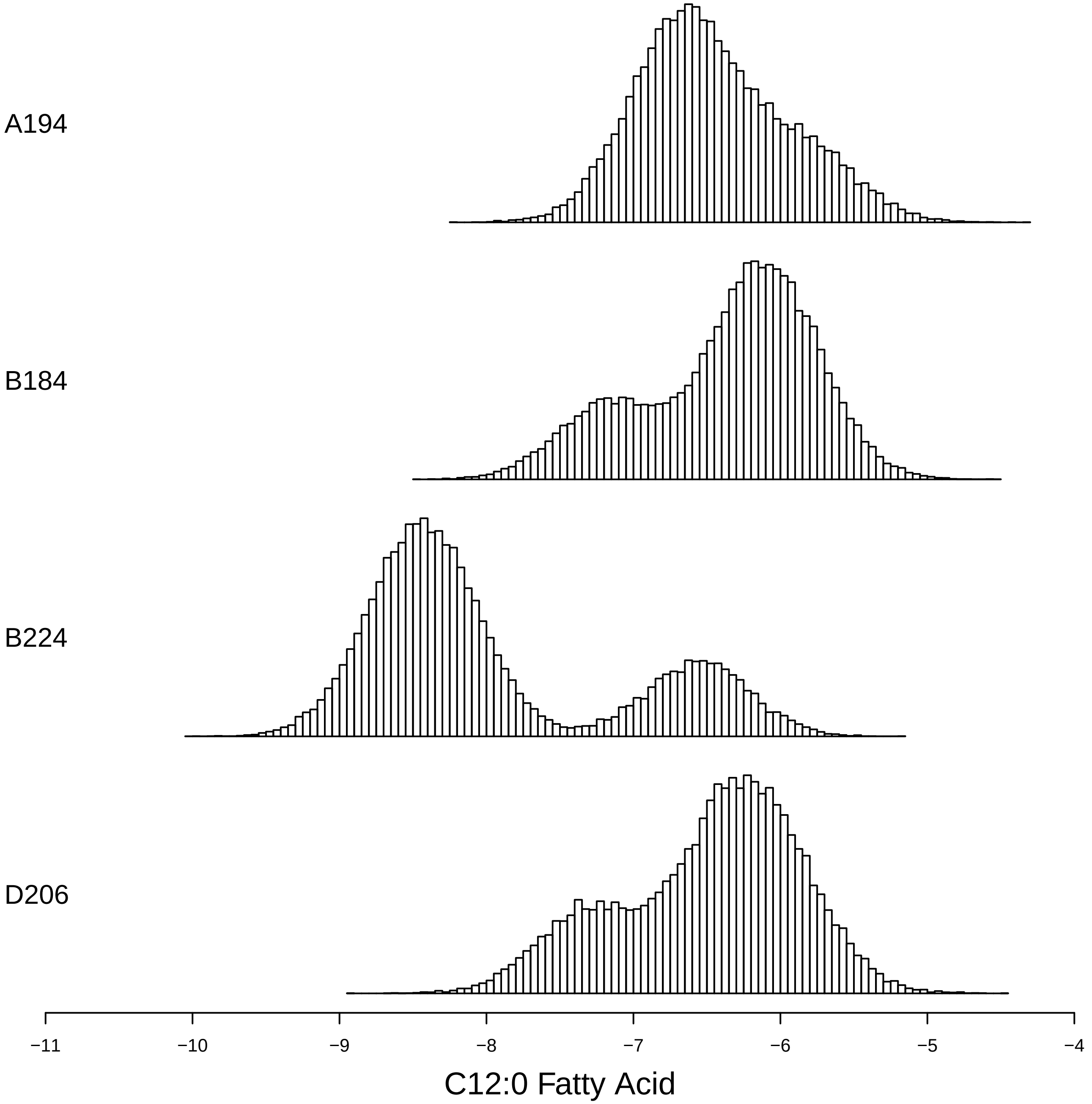
A194
B184
B224
D206
−11
−10
−9
−8
−7
−6
−5
−4
C12:0 Fatty Acid

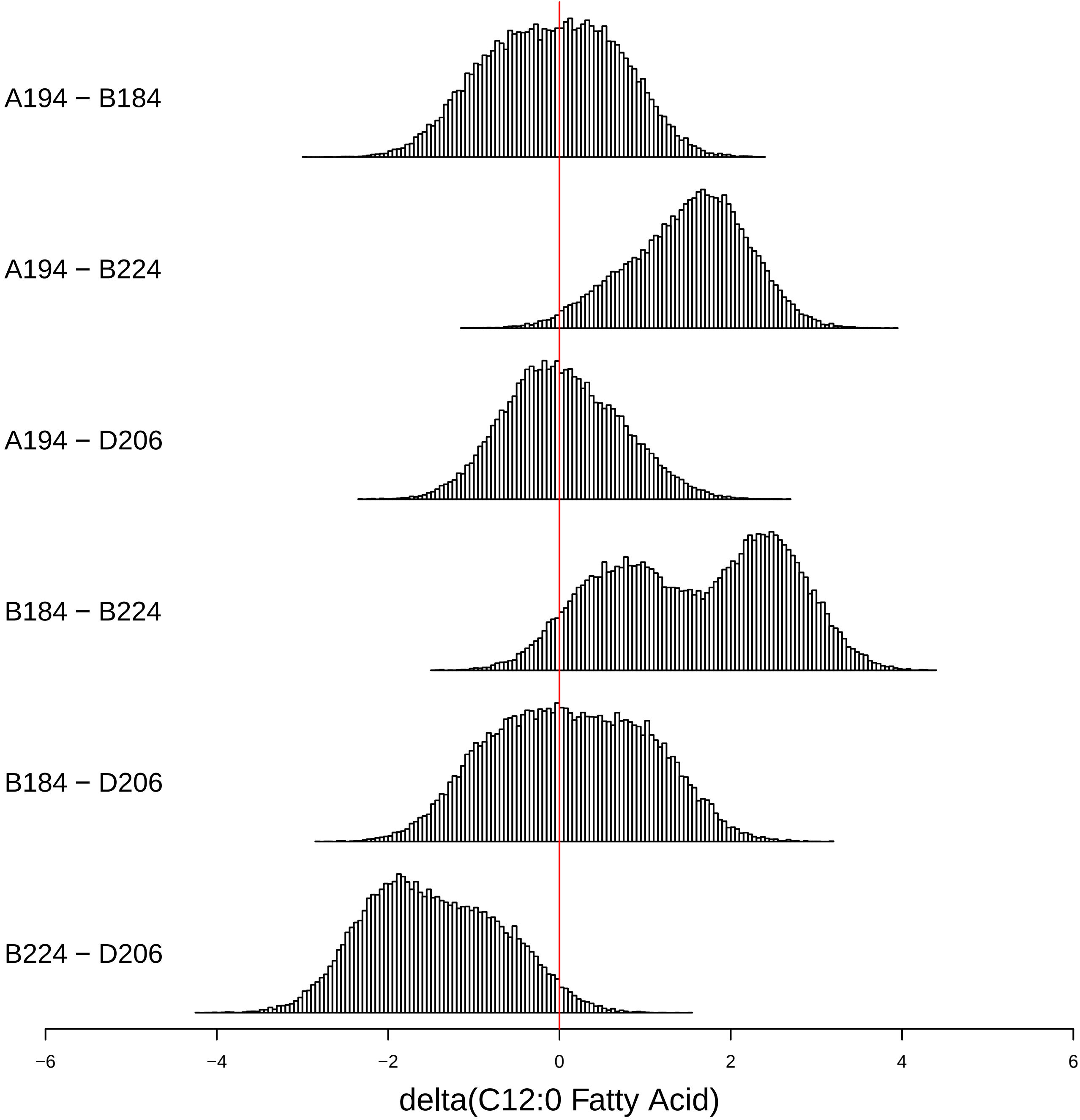

A194 − B184
A194 − B224
A194 − D206
B184 − B224
B184 − D206
B224 − D206
−6
−4
−2
0
2
4
6
delta(C12:0 Fatty Acid)

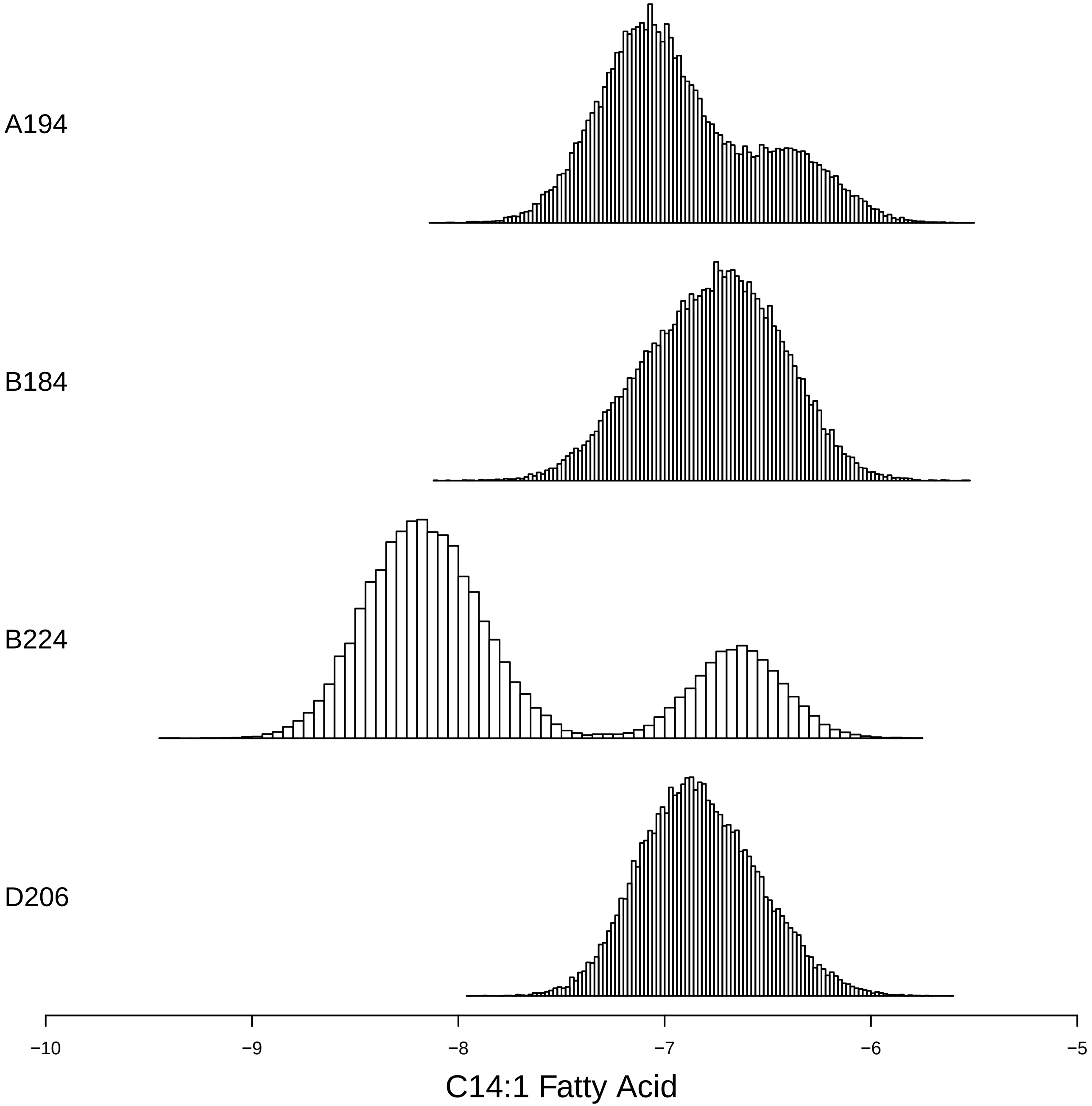
A194
B184
B224
D206
−10
−9
−8
−7
−6
−5
C14:1 Fatty Acid

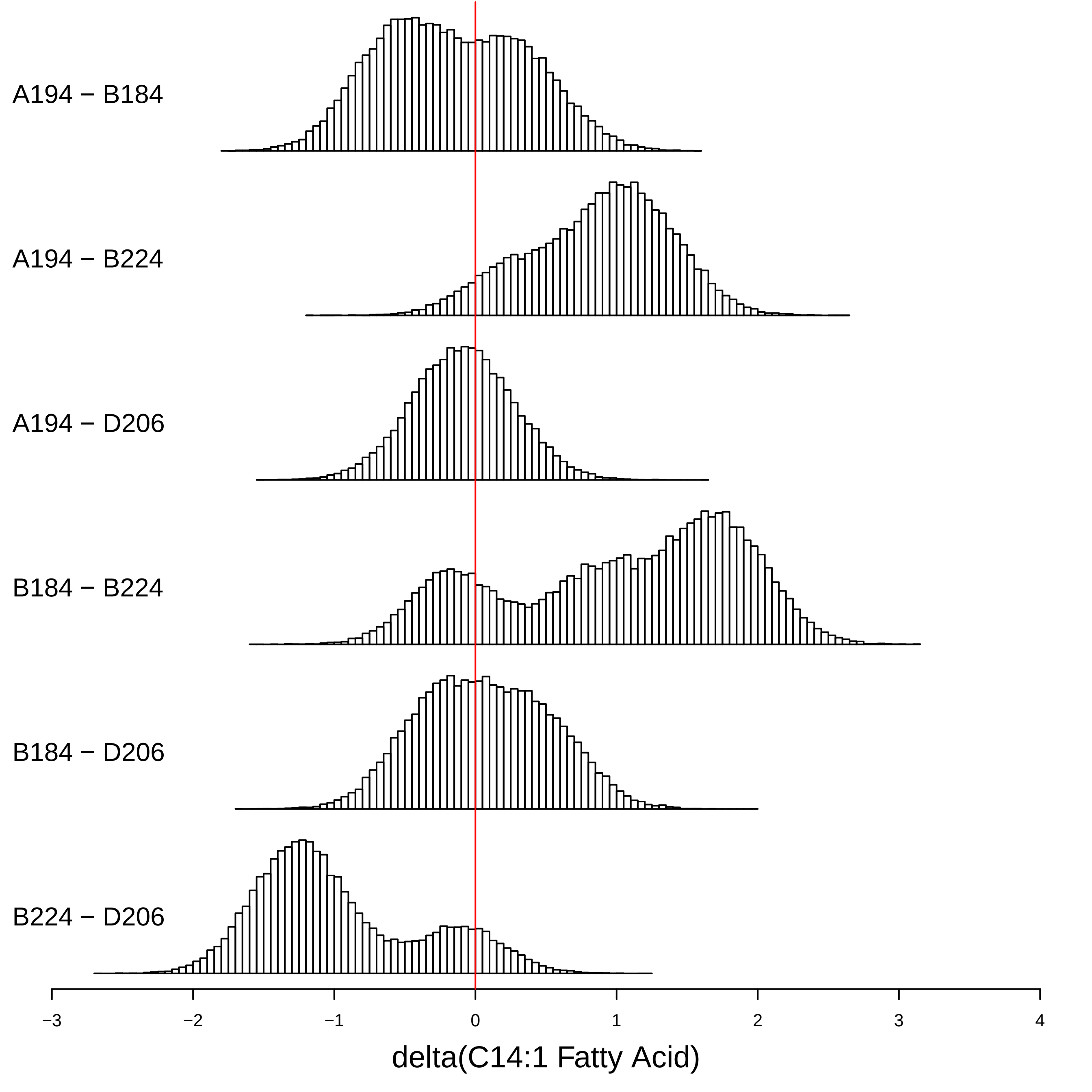

A194 − B184
A194 − B224
A194 − D206
B184 − B224
B184 − D206
B224 − D206
−3
−2
−1
0
1
2
3
4
delta(C14:1 Fatty Acid)

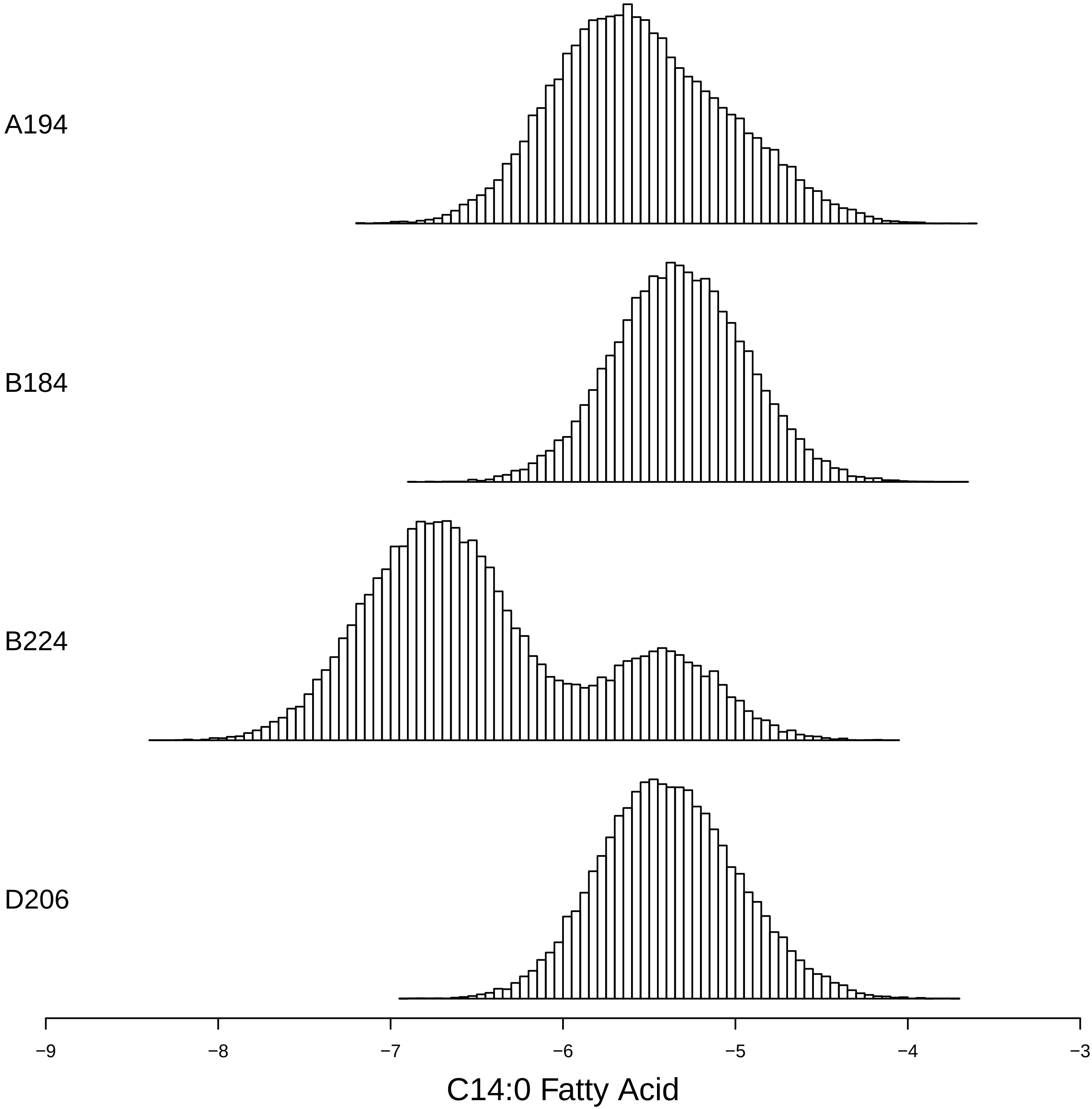
A194
B184
B224
D206
−9
−8
−7
−6
−5
−4
−3
C14:0 Fatty Acid

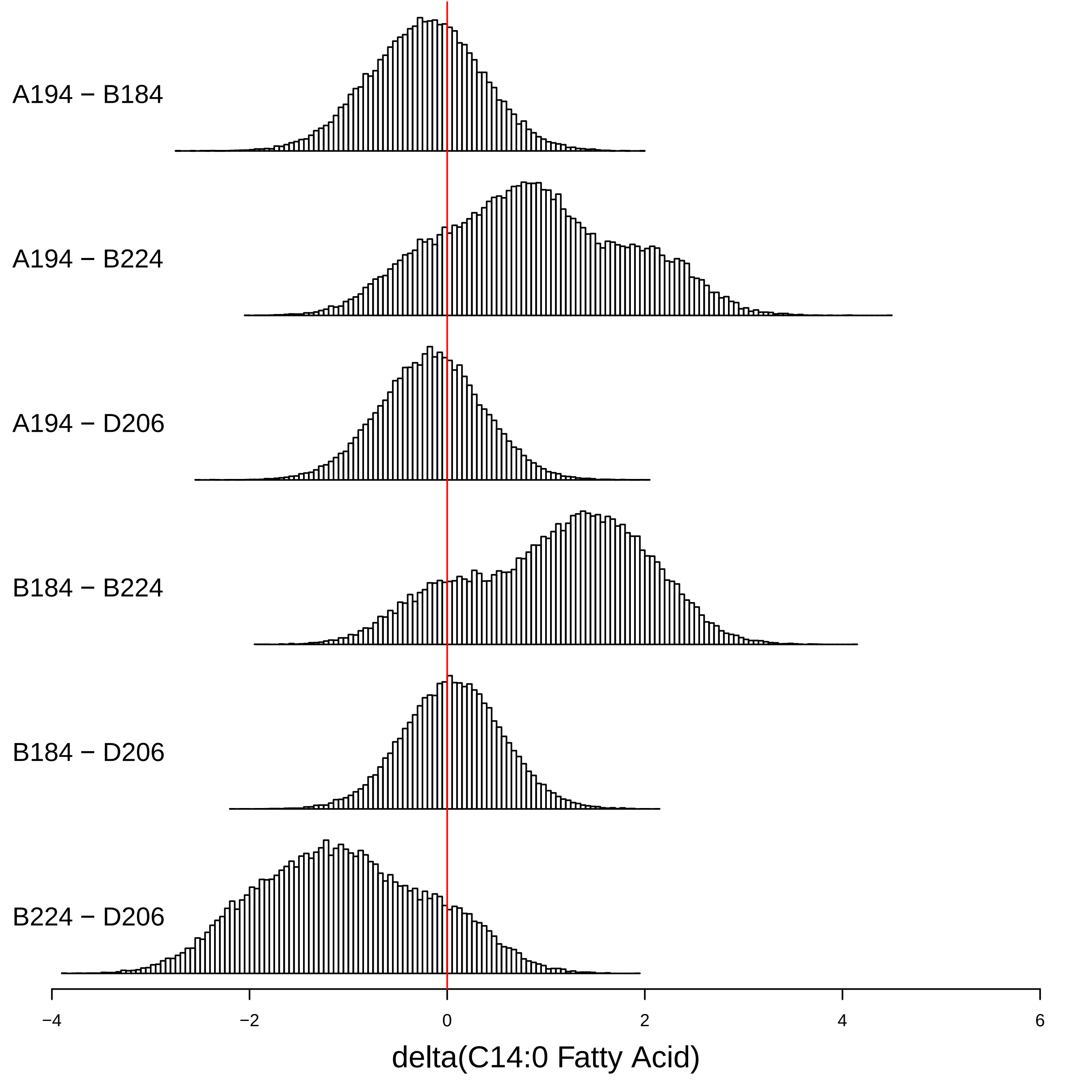
A194 − B184
A194 − B224
A194 − D206
B184 − B224
B184 − D206
B224 − D206
−4
−2
0
2
4
6
delta(C14:0 Fatty Acid)

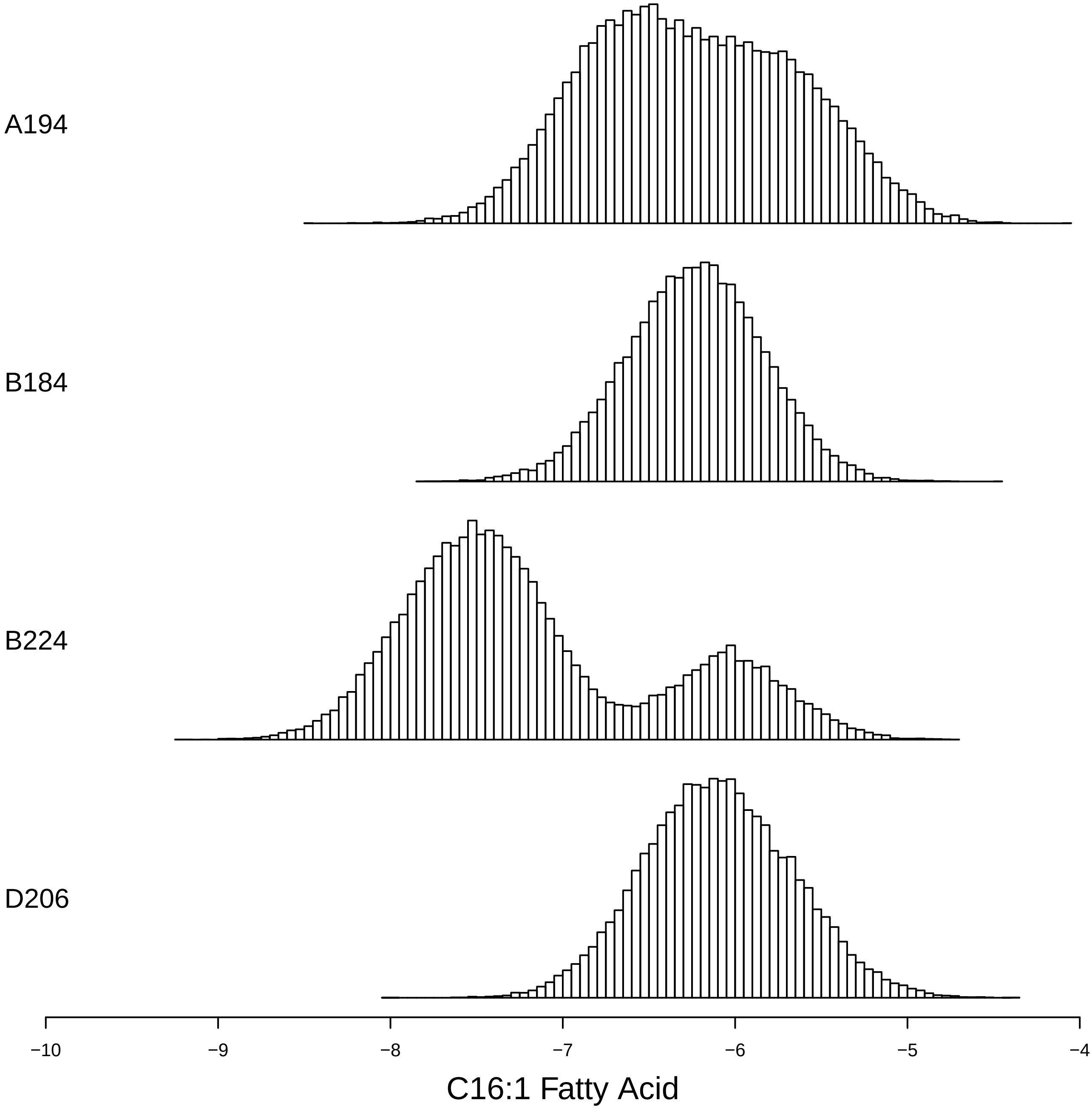
A194
B184
B224
D206
−10
−9
−8
−7
−6
−5
−4
C16:1 Fatty Acid

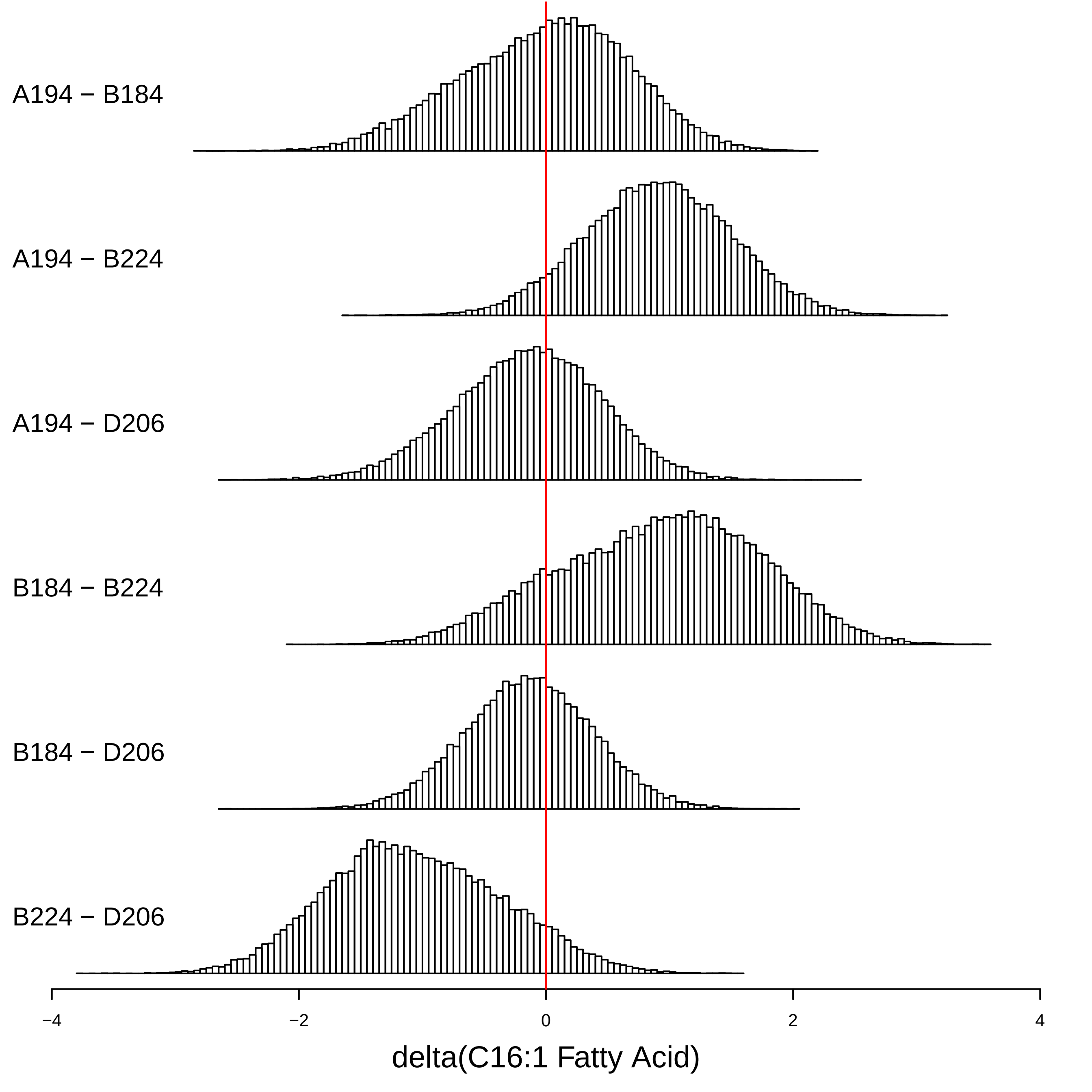

A194 − B184
A194 − B224
A194 − D206
B184 − B224
B184 − D206
B224 − D206
−4
−2
0
2
4
delta(C16:1 Fatty Acid)

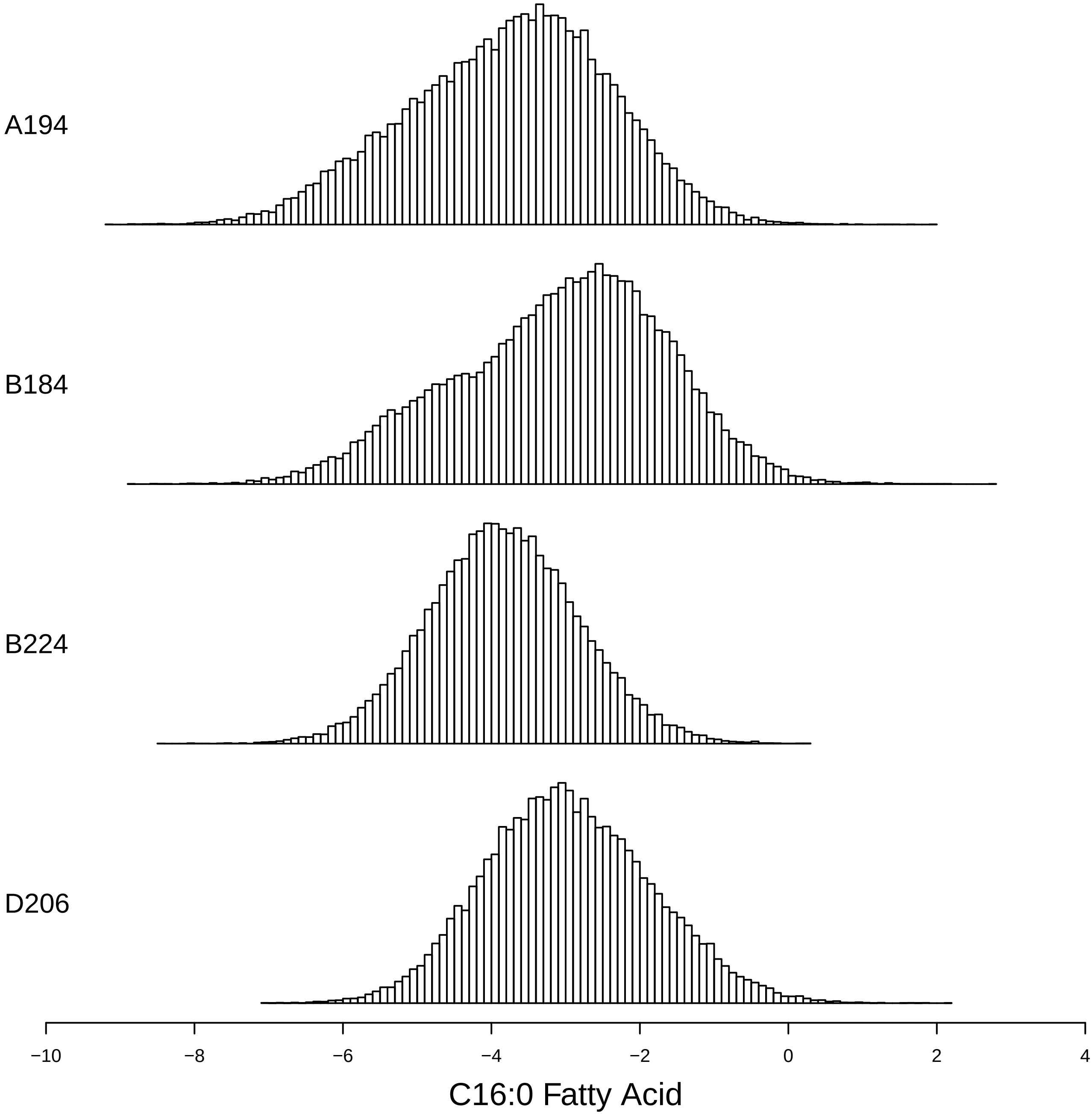
A194
B184
B224
D206
−10
−8
−6
−4
−2
0
2
4
C16:0 Fatty Acid

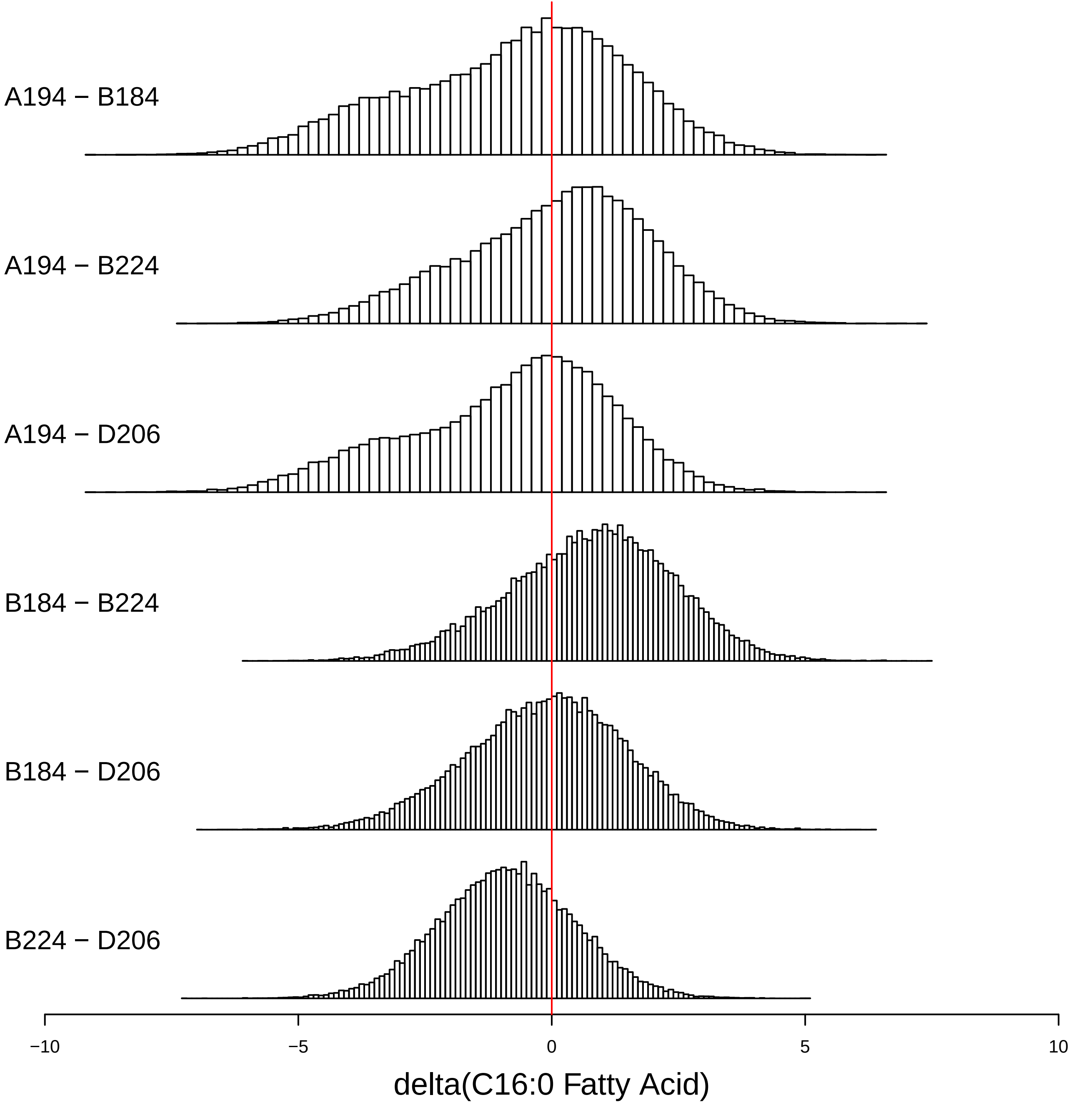
A194 − B184
A194 − B224
A194 − D206
B184 − B224
B184 − D206
B224 − D206
−10
−5
0
5
10
delta(C16:0 Fatty Acid)

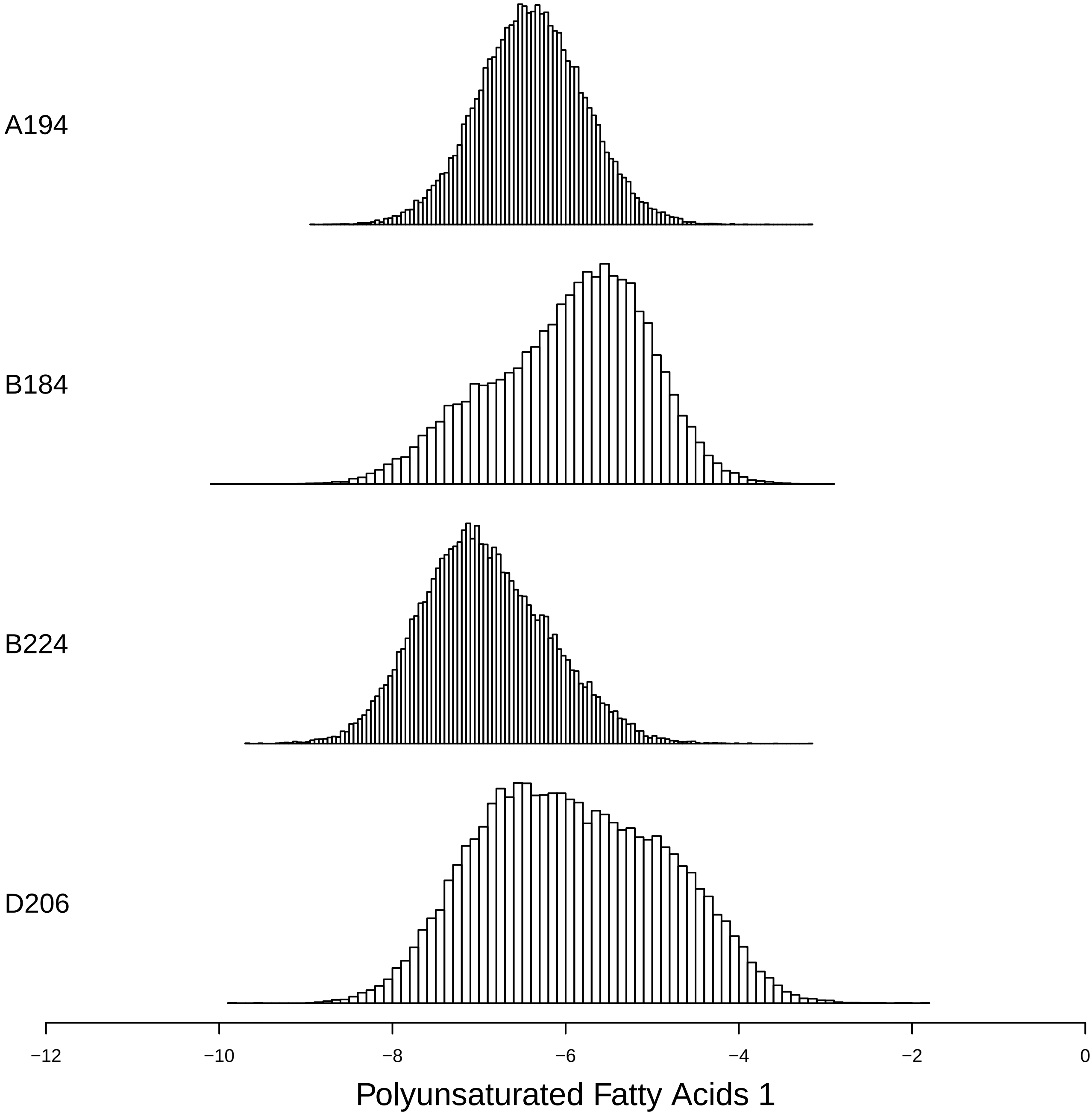
A194
B184
B224
D206
−12
−10
−8
−6
−4
−2
0
Polyunsaturated Fatty Acids 1

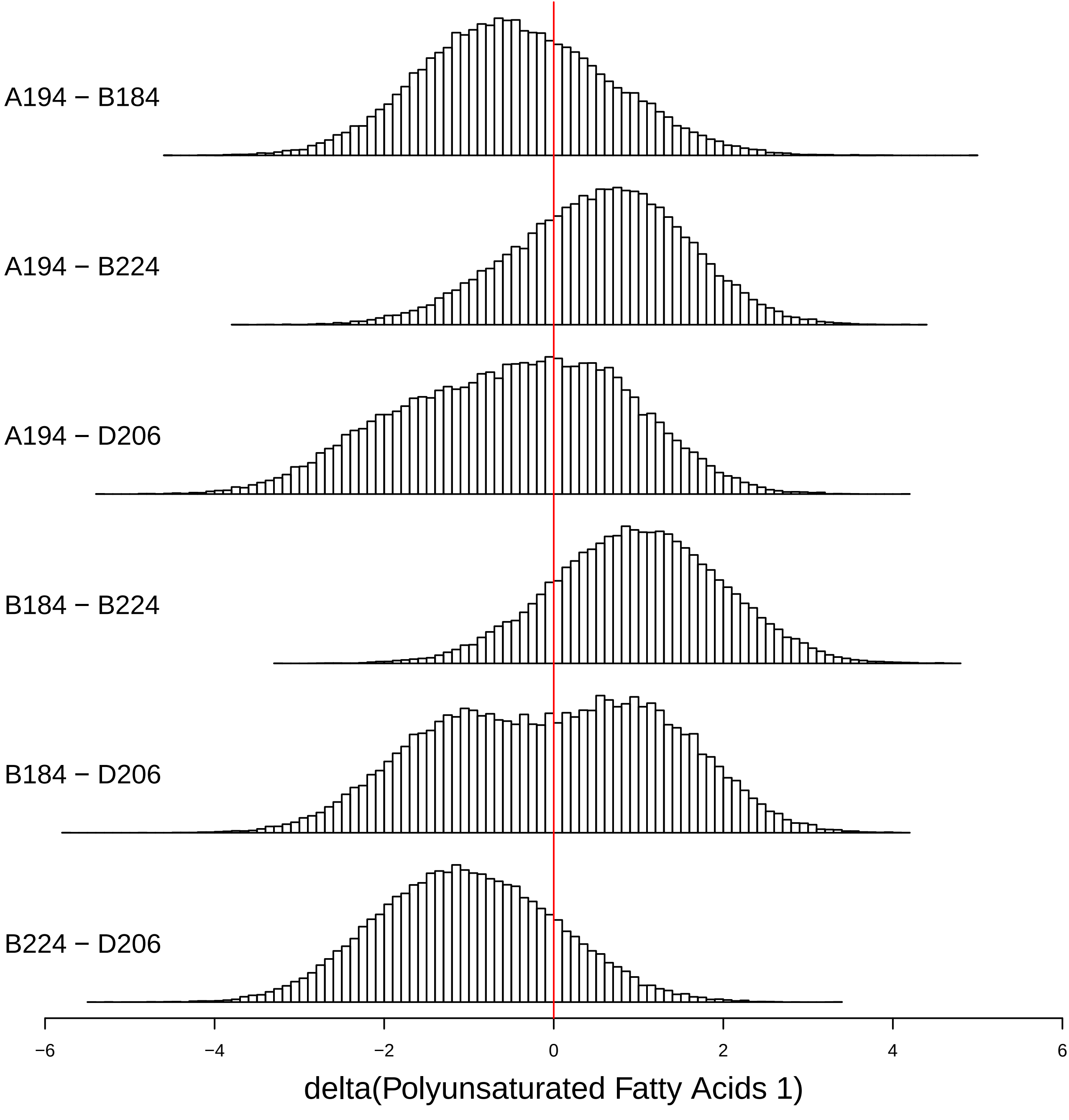

A194 − B184
A194 − B224
A194 − D206
B184 − B224
B184 − D206
B224 − D206
−6
−4
−2
0
2
4
6
delta(Polyunsaturated Fatty Acids 1)

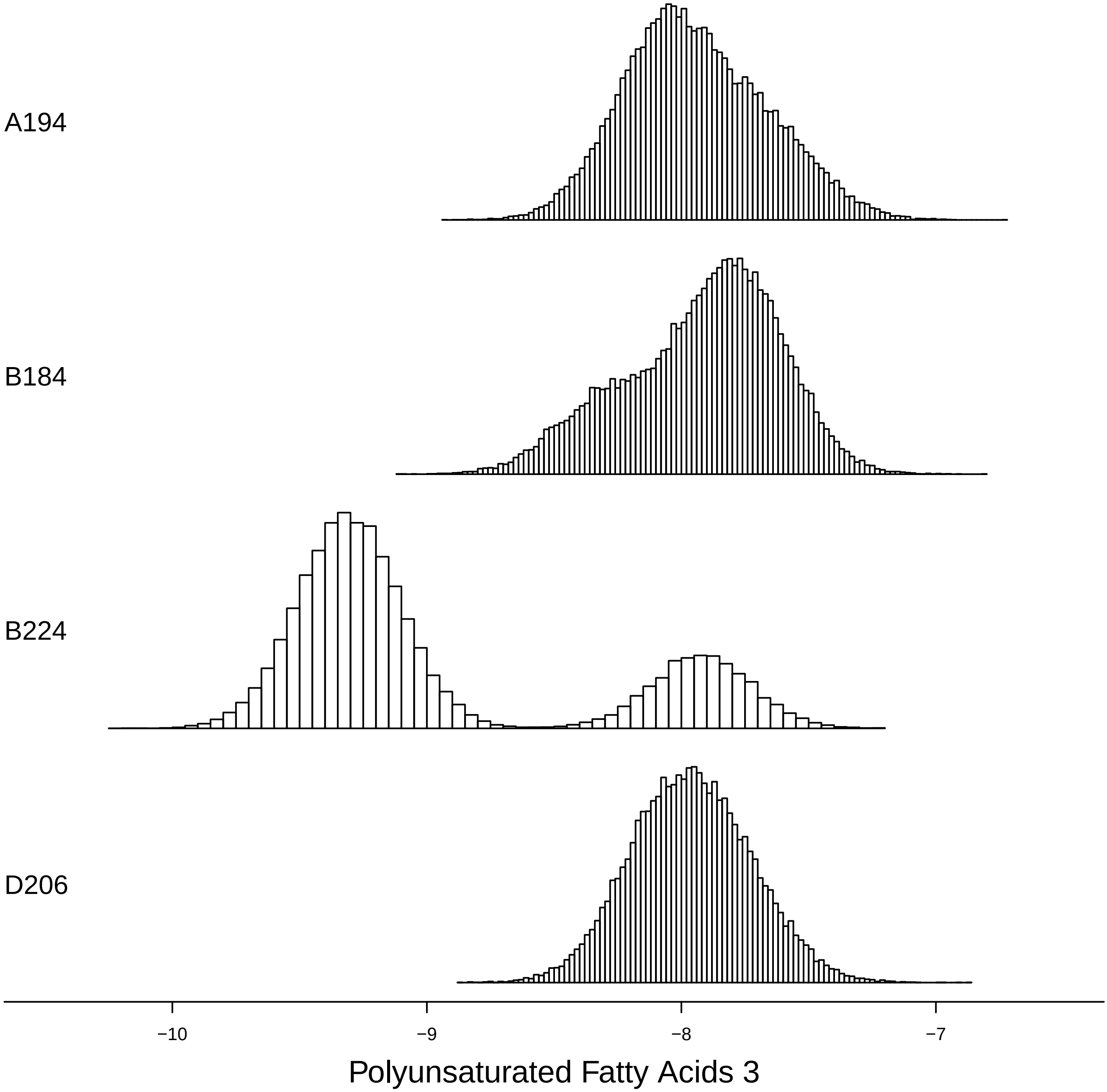
A194
B184
B224
D206
−10
−9
−8
−7
Polyunsaturated Fatty Acids 3

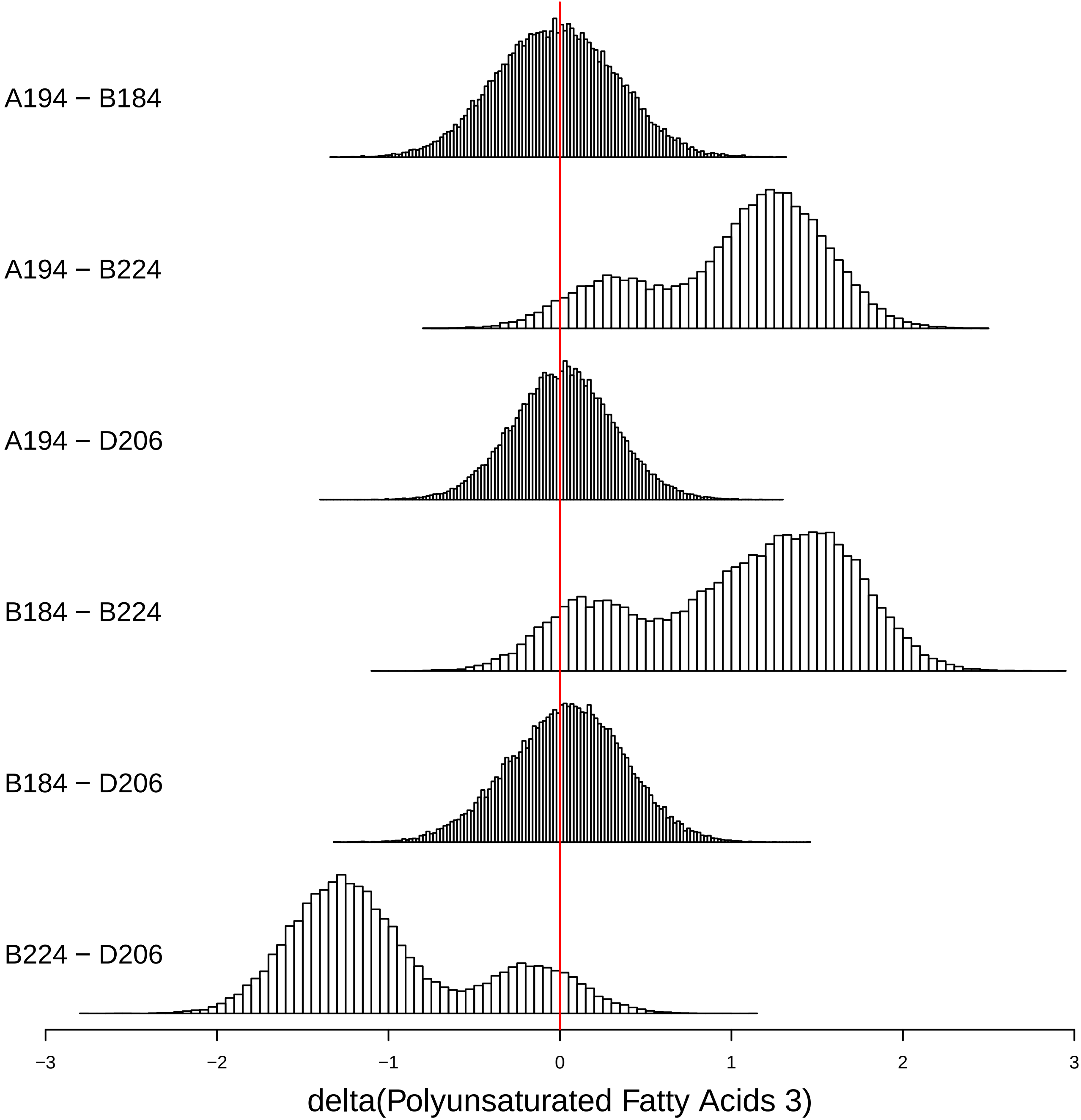

A194 − B184
A194 − B224
A194 − D206
B184 − B224
B184 − D206
B224 − D206
−3
−2
−1
0
1
2
3
delta(Polyunsaturated Fatty Acids 3)

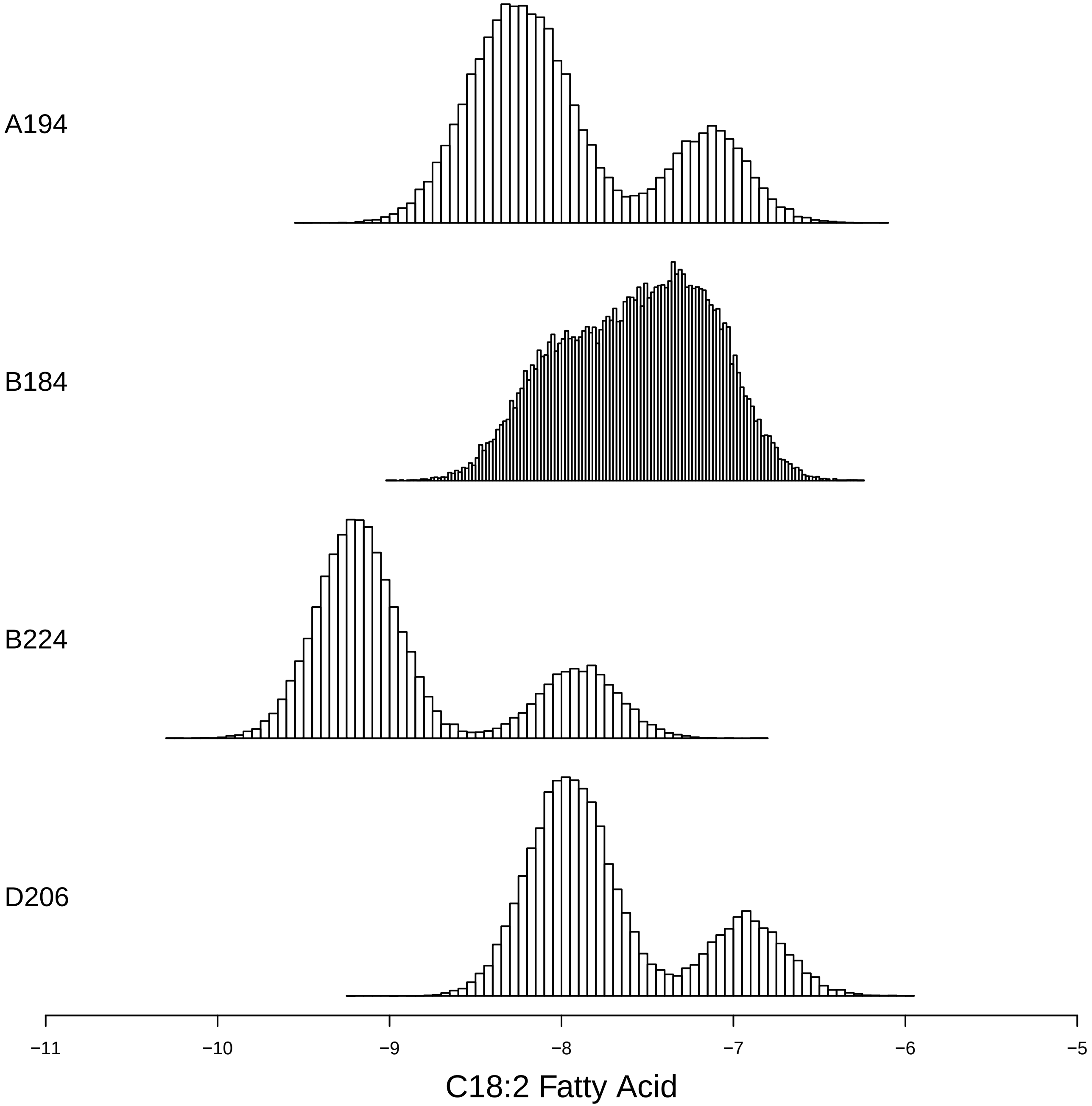
A194
B184
B224
D206
−11
−10
−9
−8
−7
−6
−5
C18:2 Fatty Acid

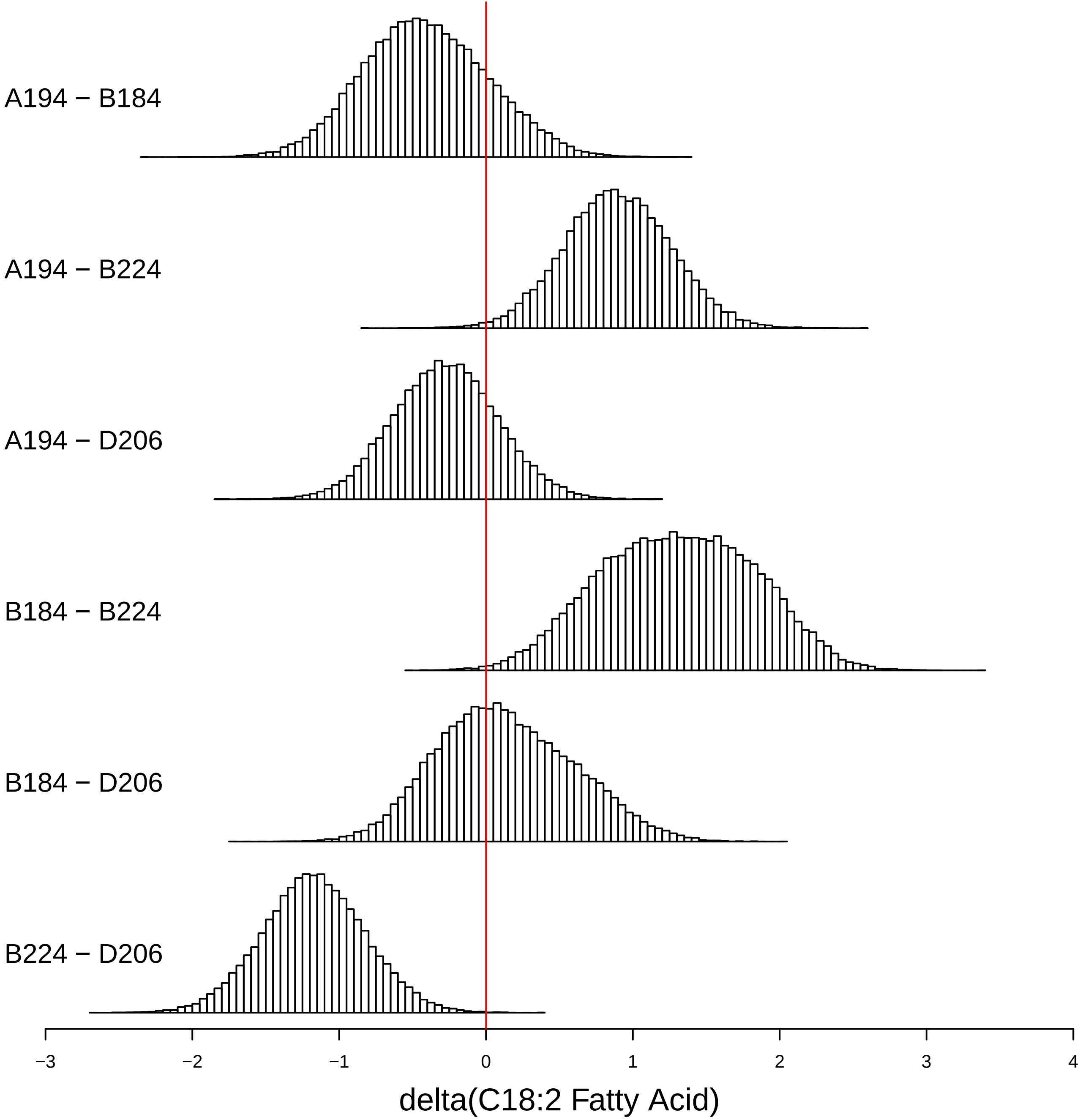

A194 − B184
A194 − B224
A194 − D206
B184 − B224
B184 − D206
B224 − D206
−3
−2
−1
0
1
2
3
4
delta(C18:2 Fatty Acid)

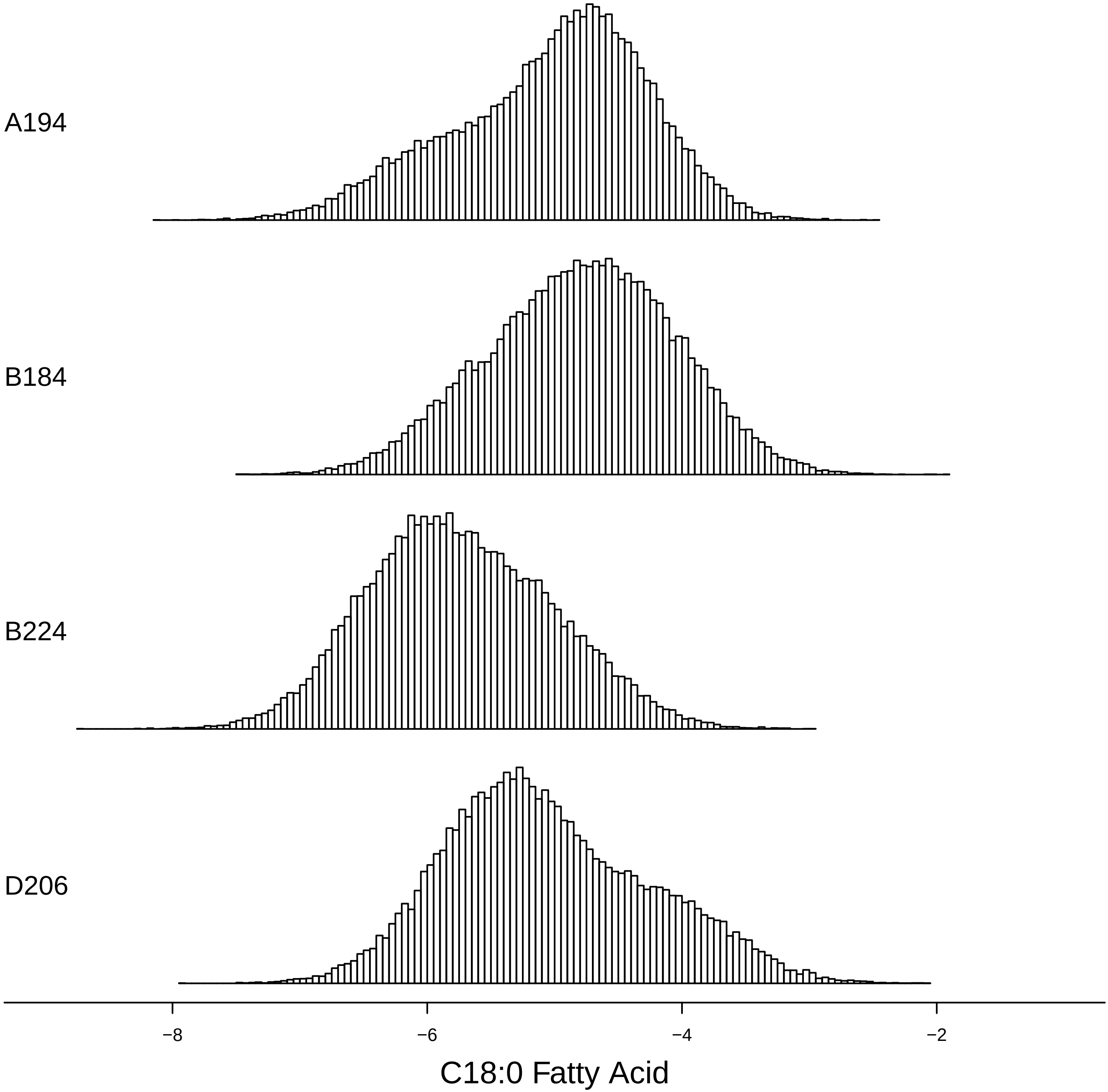
A194
B184
B224
D206
−8
−6
−4
−2
C18:0 Fatty Acid

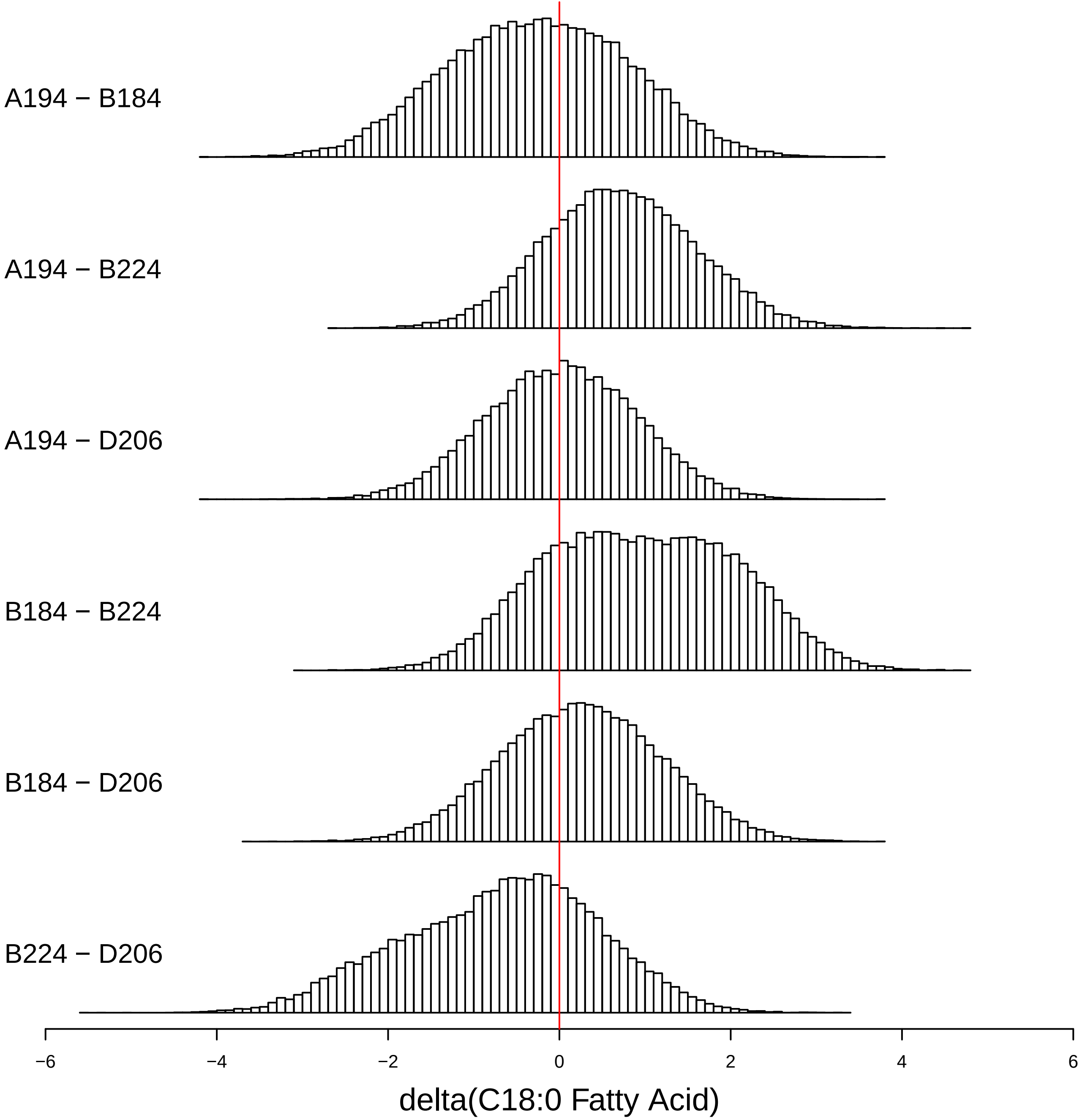
A194 − B184
A194 − B224
A194 − D206
B184 − B224
B184 − D206
B224 − D206
−6
−4
−2
0
2
4
6
delta(C18:0 Fatty Acid)

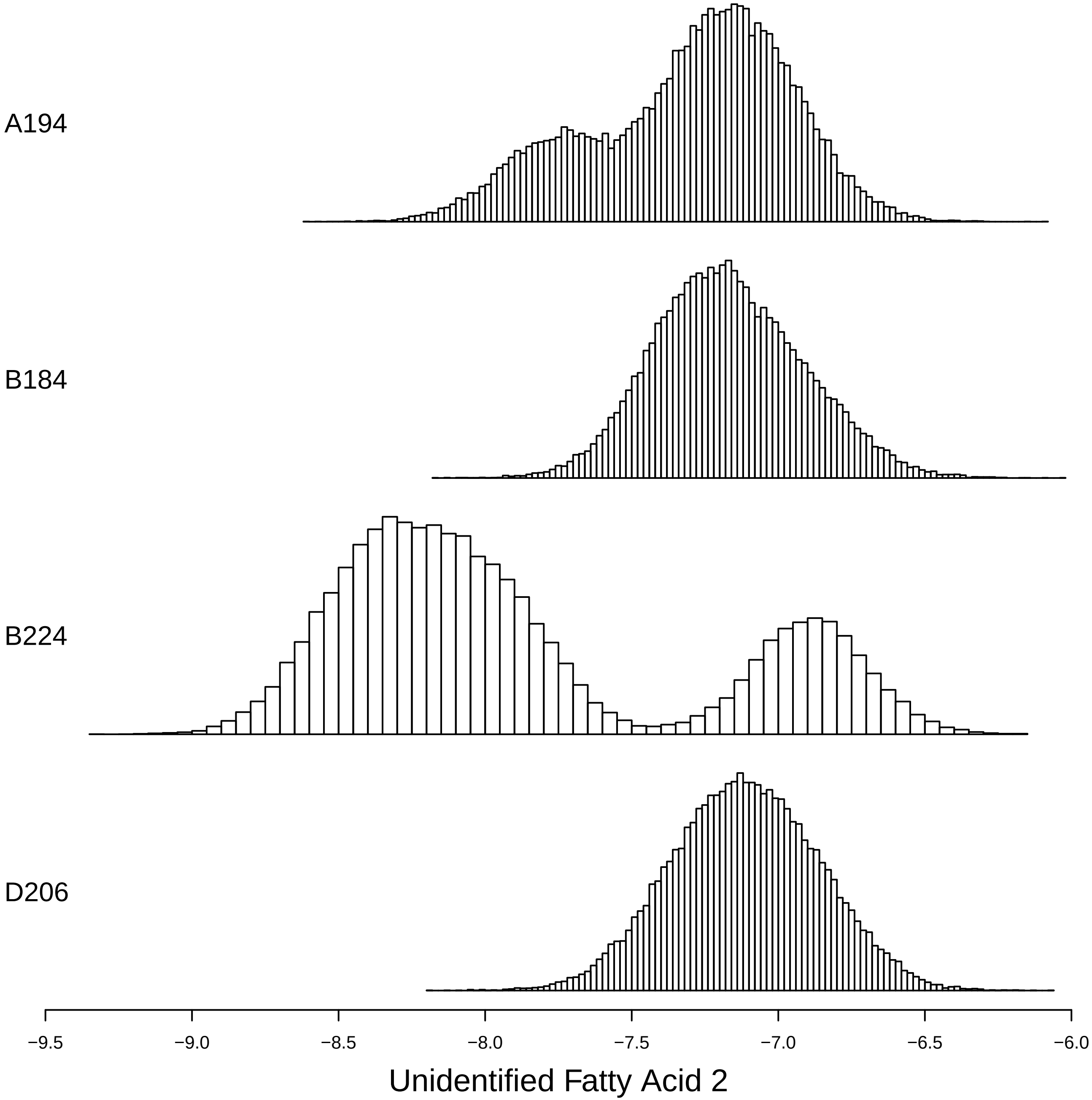
A194
B184
B224
D206
−9.5
−9.0
−8.5
−8.0
−7.5
−7.0
−6.5
−6.0
Unidentified Fatty Acid 2

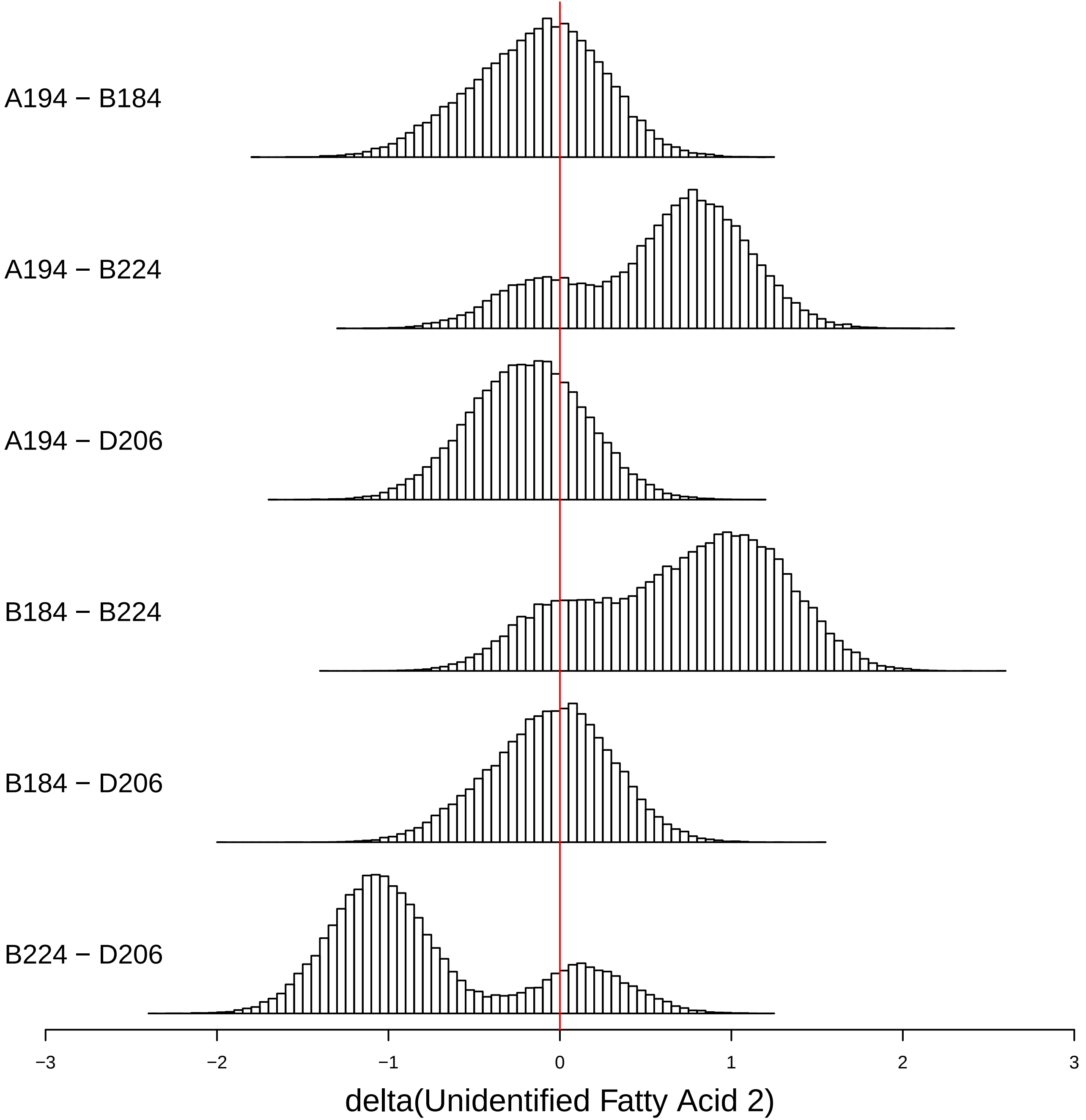
A194 − B184
A194 − B224
A194 − D206
B184 − B224
B184 − D206
B224 − D206
−3
−2
−1
0
1
2
3
delta(Unidentified Fatty Acid 2)

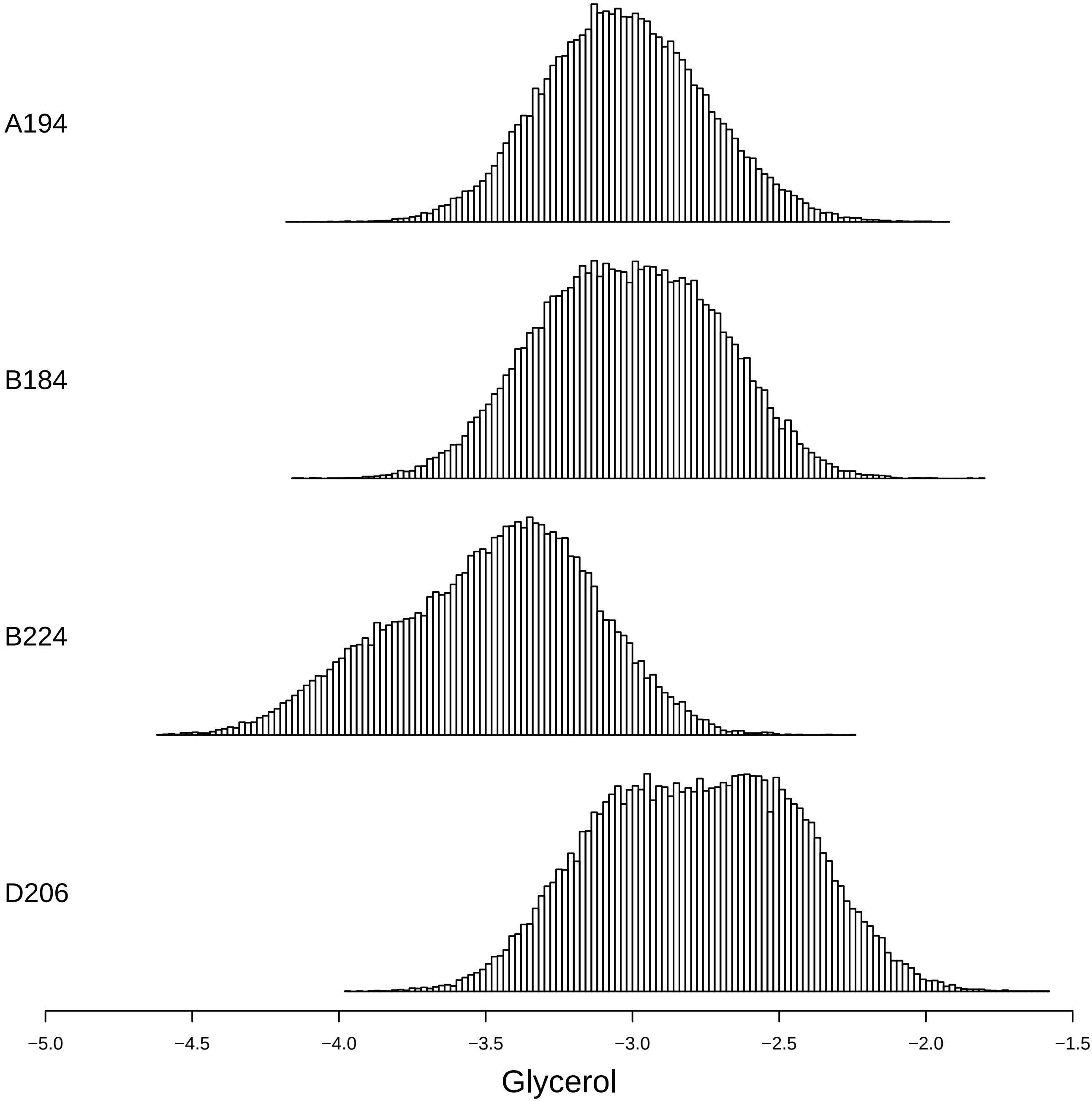

A194
B184
B224
D206
−5.0
−4.5
−4.0
−3.5
−3.0
−2.5
−2.0
−1.5
Glycerol

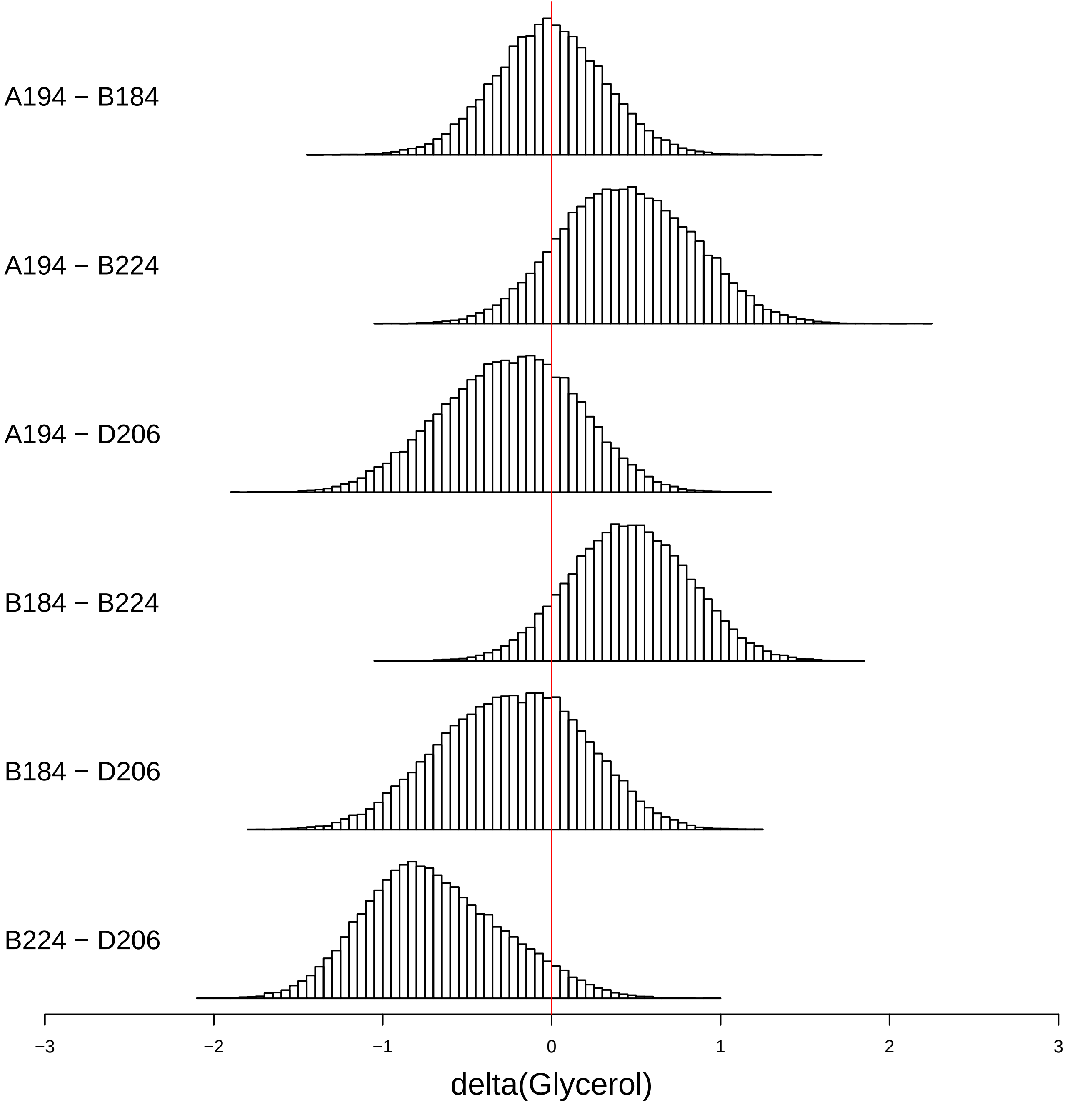
A194 − B184
A194 − B224
A194 − D206
B184 − B224
B184 − D206
B224 − D206
−3
−2
−1
0
1
2
3
delta(Glycerol)

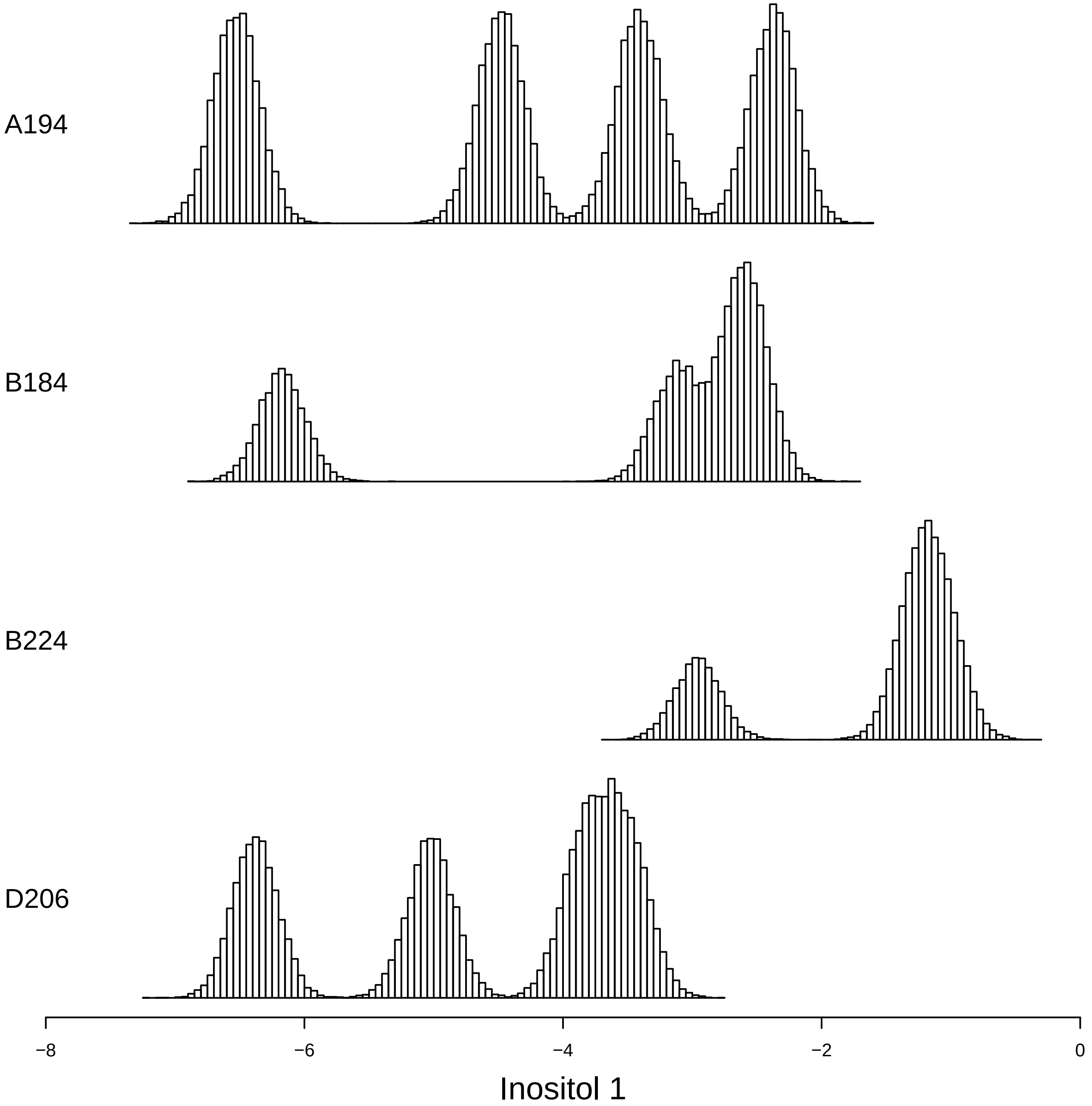

A194
B184
B224
D206
−8
−6
−4
−2
0
Inositol 1

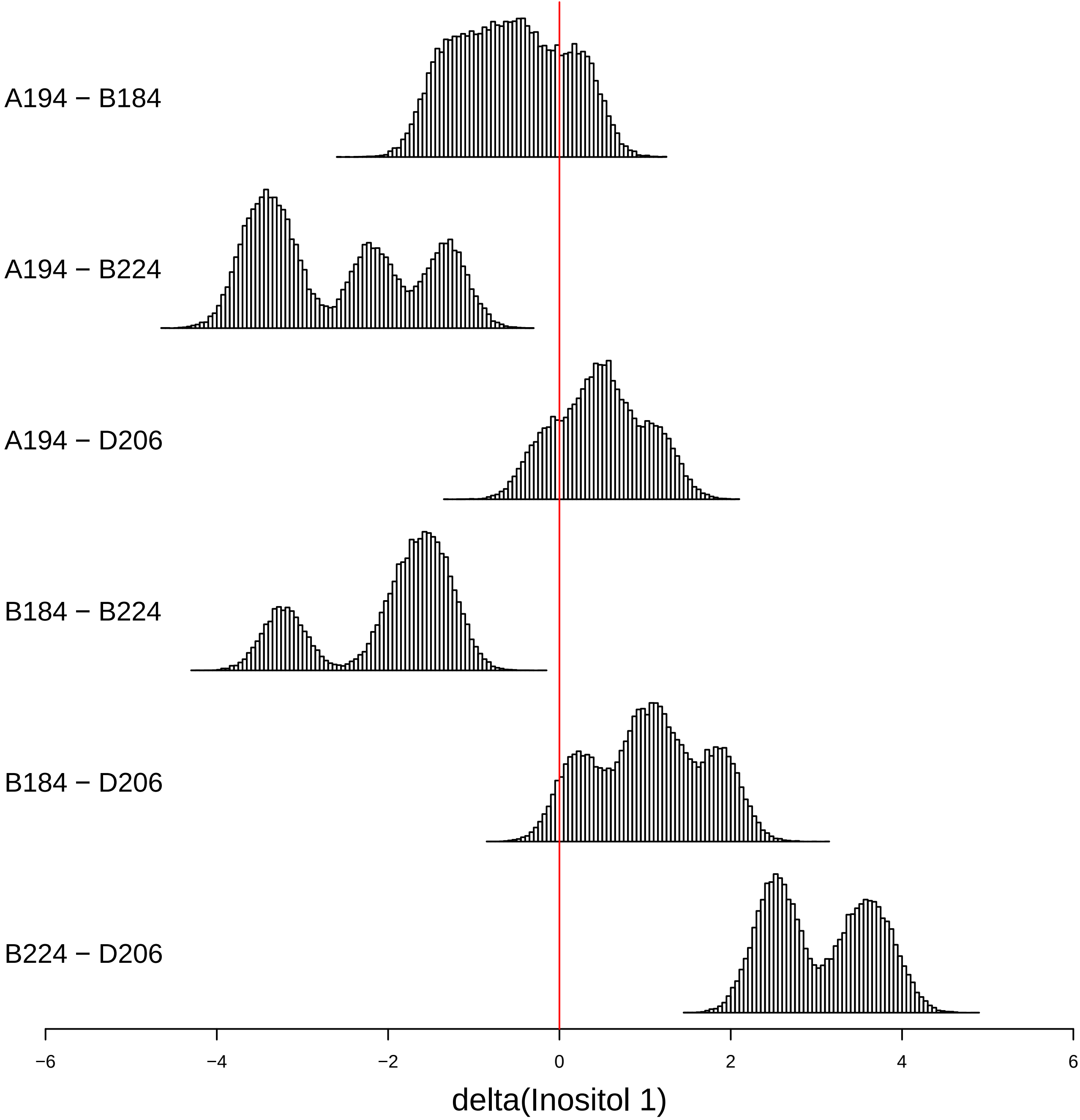
A194 − B184
A194 − B224
A194 − D206
B184 − B224
B184 − D206
B224 − D206
−6
−4
−2
0
2
4
6
delta(Inositol 1)

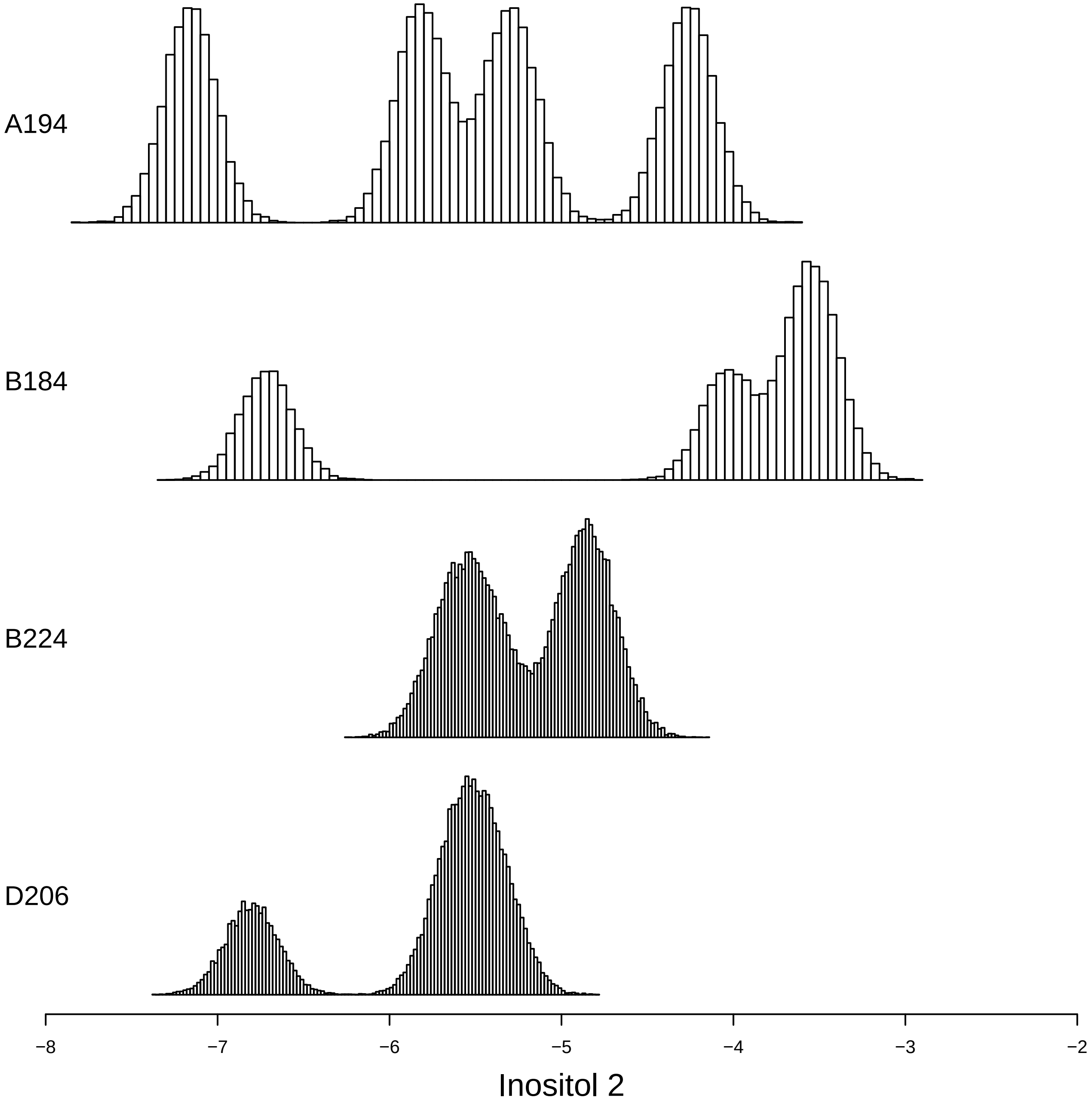
A194
B184
B224
D206
−8
−7
−6
−5
−4
−3
−2
Inositol 2

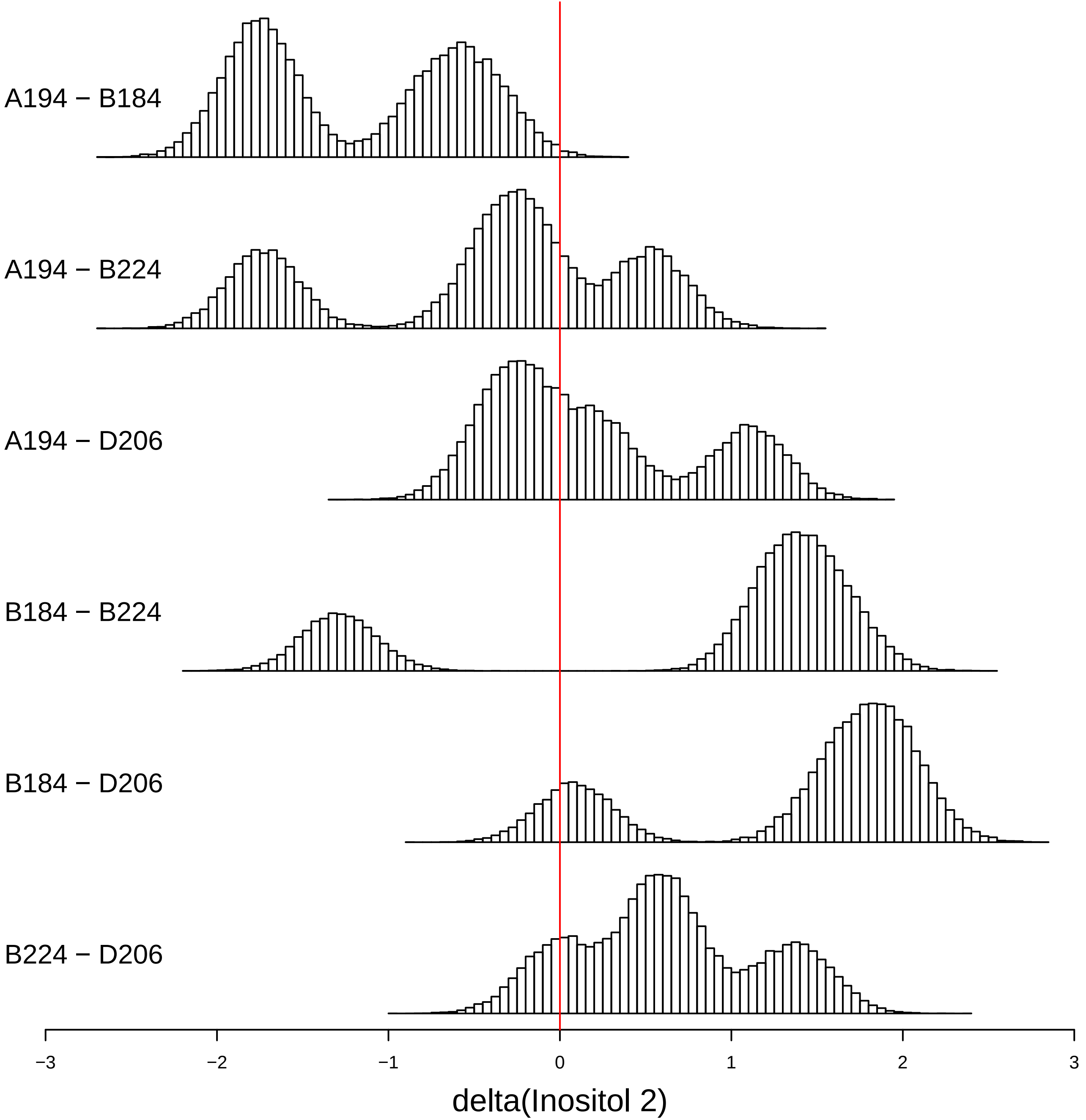

A194 − B184
A194 − B224
A194 − D206
B184 − B224
B184 − D206
B224 − D206
−3
−2
−1
0
1
2
3
delta(Inositol 2)

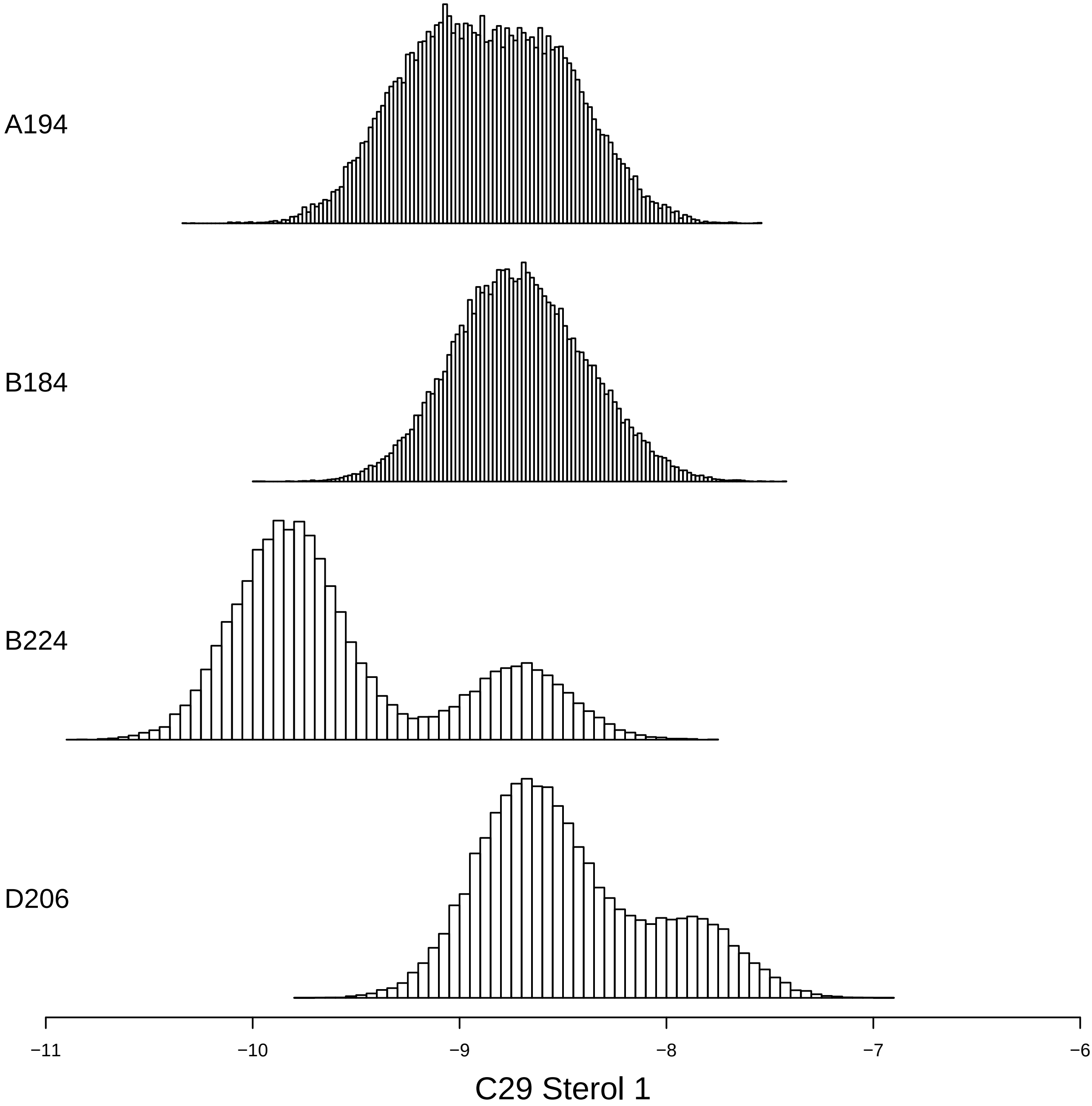
A194
B184
B224
D206
−11
−10
−9
−8
−7
−6
C29 Sterol 1

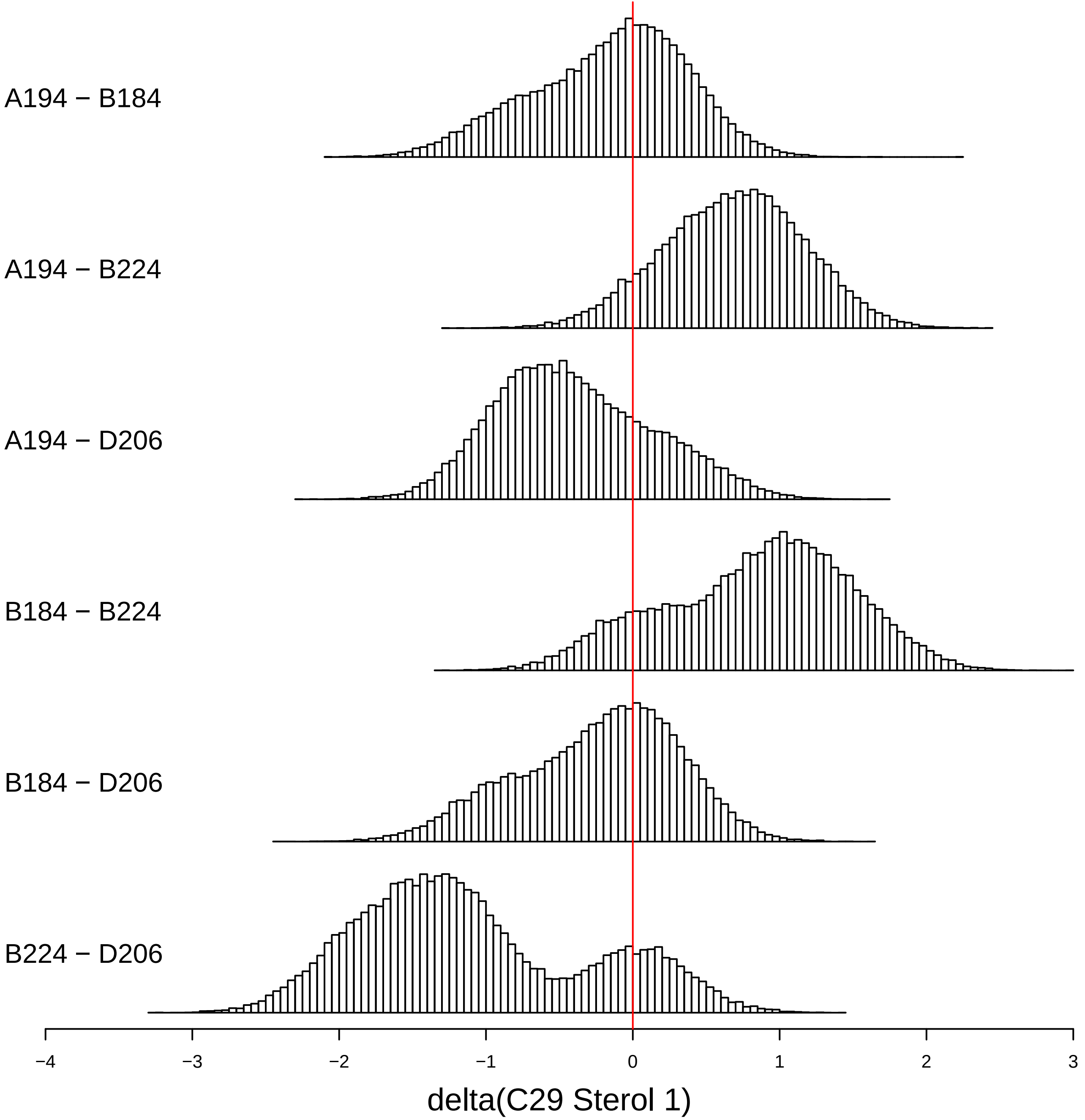
A194 − B184
A194 − B224
A194 − D206
B184 − B224
B184 − D206
B224 − D206
−4
−3
−2
−1
0
1
2
3
delta(C29 Sterol 1)

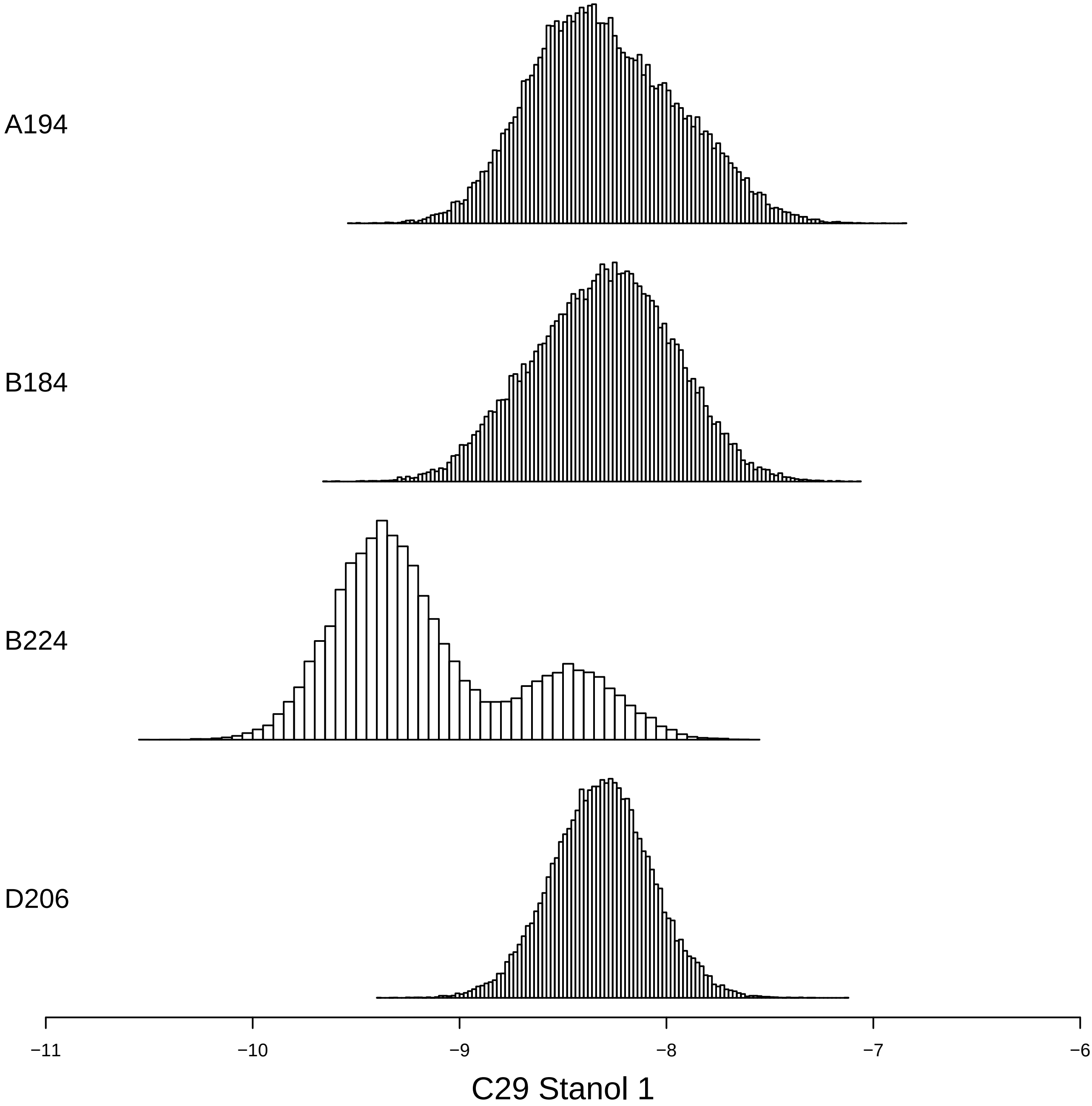
A194
B184
B224
D206
−11
−10
−9
−8
−7
−6
C29 Stanol 1

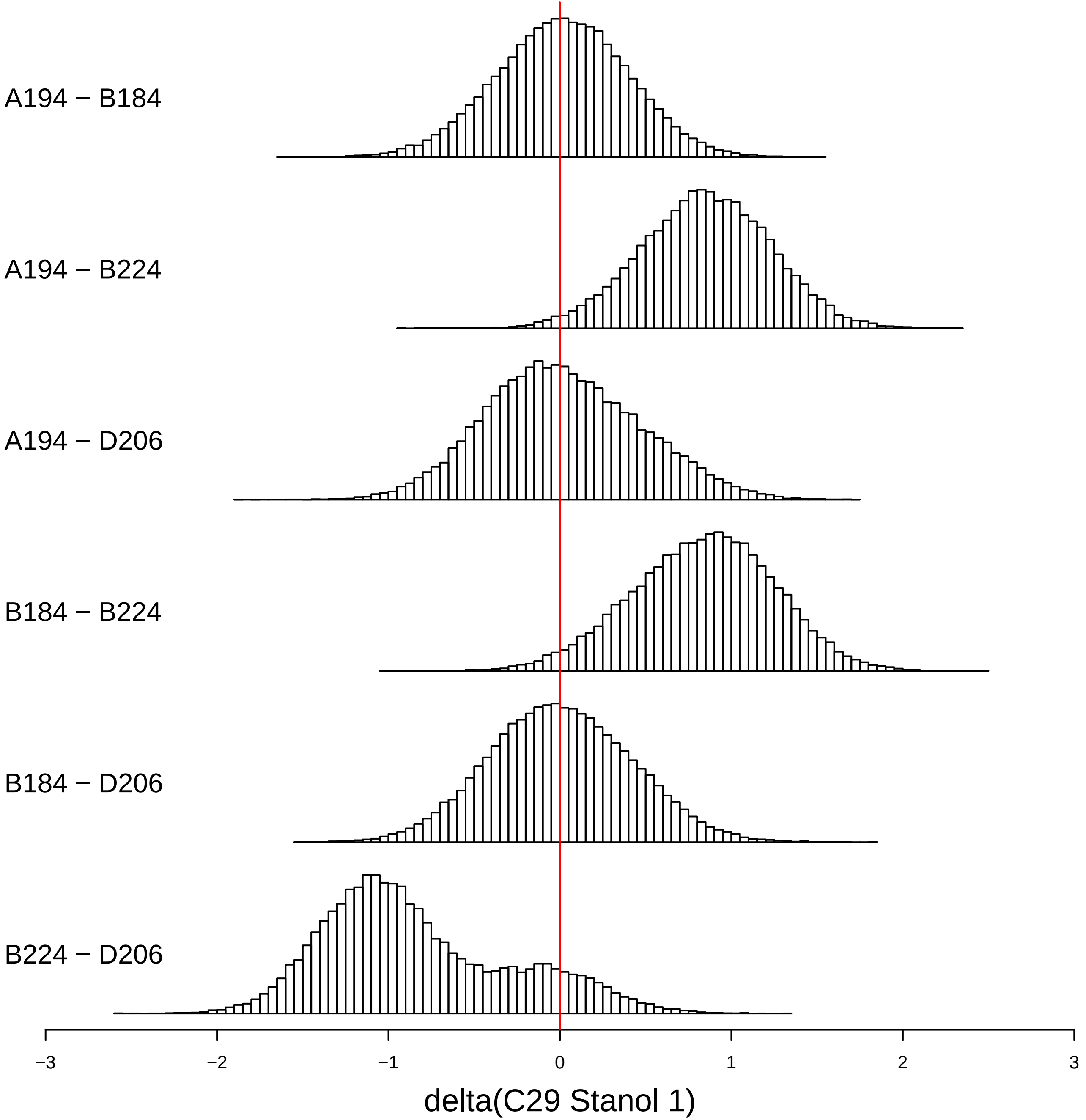
A194 − B184
A194 − B224
A194 − D206
B184 − B224
B184 − D206
B224 − D206
−3
−2
−1
0
1
2
3
delta(C29 Stanol 1)

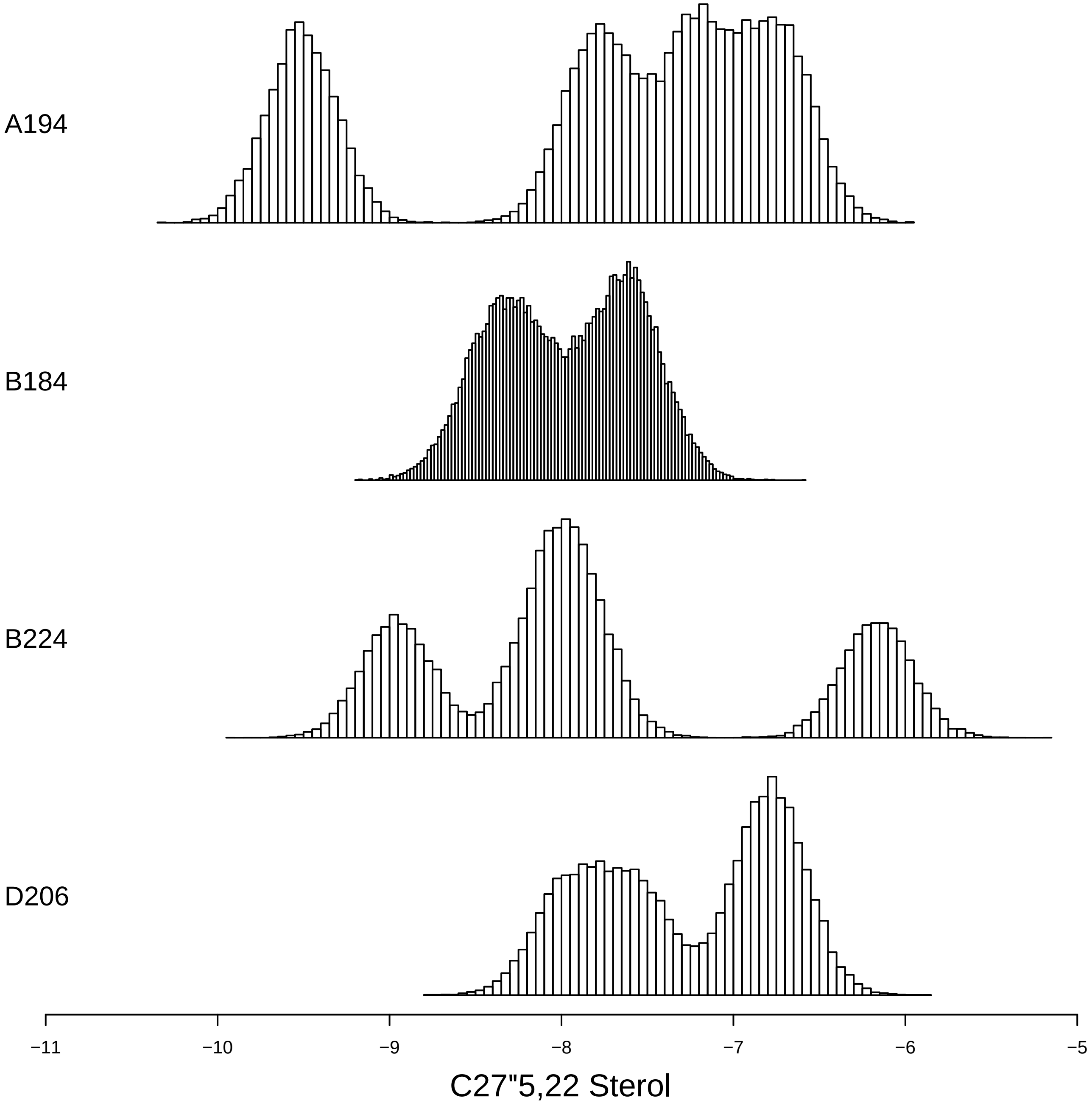
A194
B184
B224
D206
−11
−10
−9
−8
−7
−6
−5
C27"5,22 Sterol

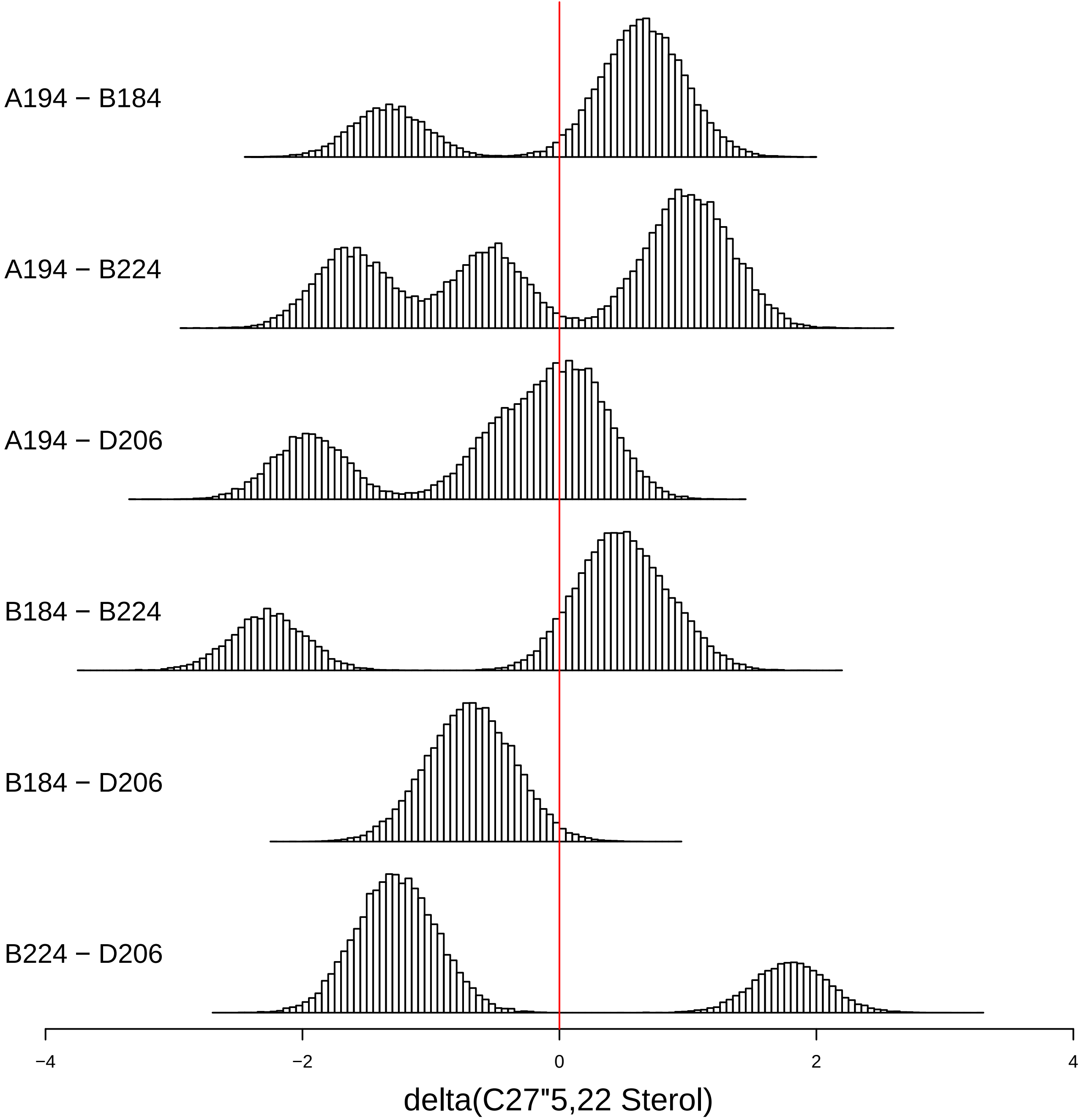
A194 − B184
A194 − B224
A194 − D206
B184 − B224
B184 − D206
B224 − D206
−4
−2
0
2
4
delta(C27"5,22 Sterol)

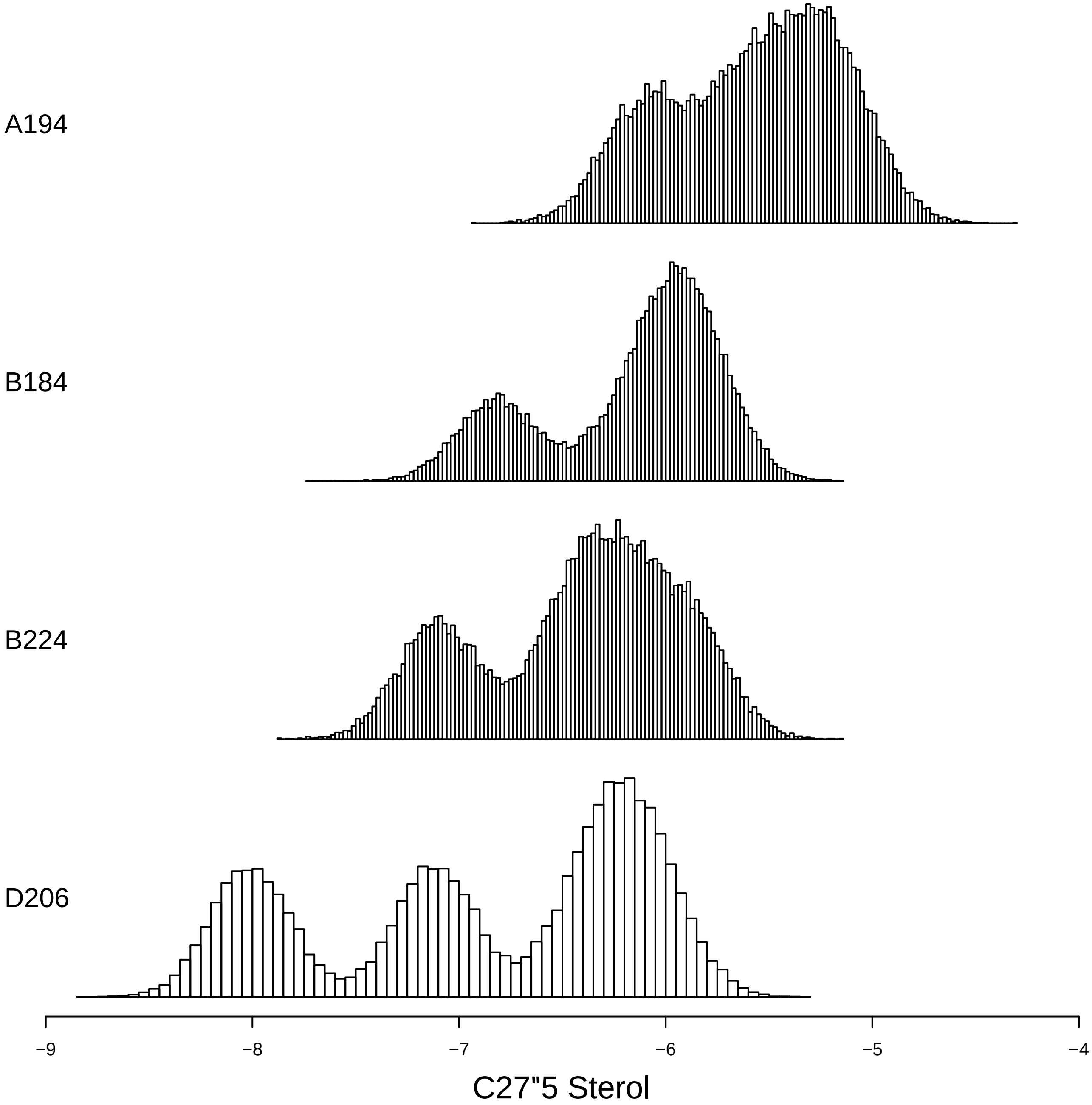
A194
B184
B224
D206
−9
−8
−7
−6
−5
−4
C27"5 Sterol

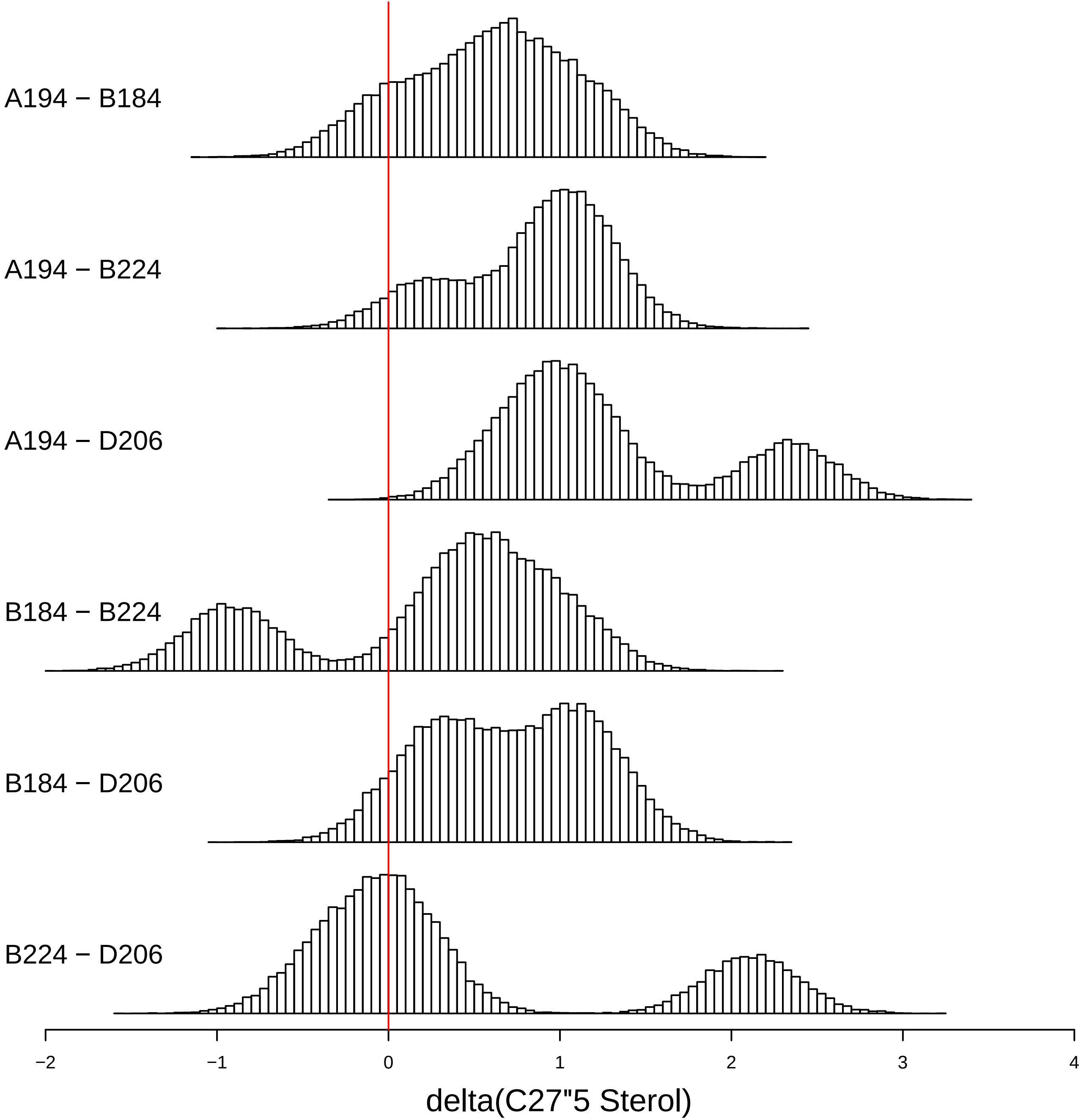
A194 − B184
A194 − B224
A194 − D206
B184 − B224
B184 − D206
B224 − D206
−2
−1
0
1
2
3
4
delta(C27"5 Sterol)

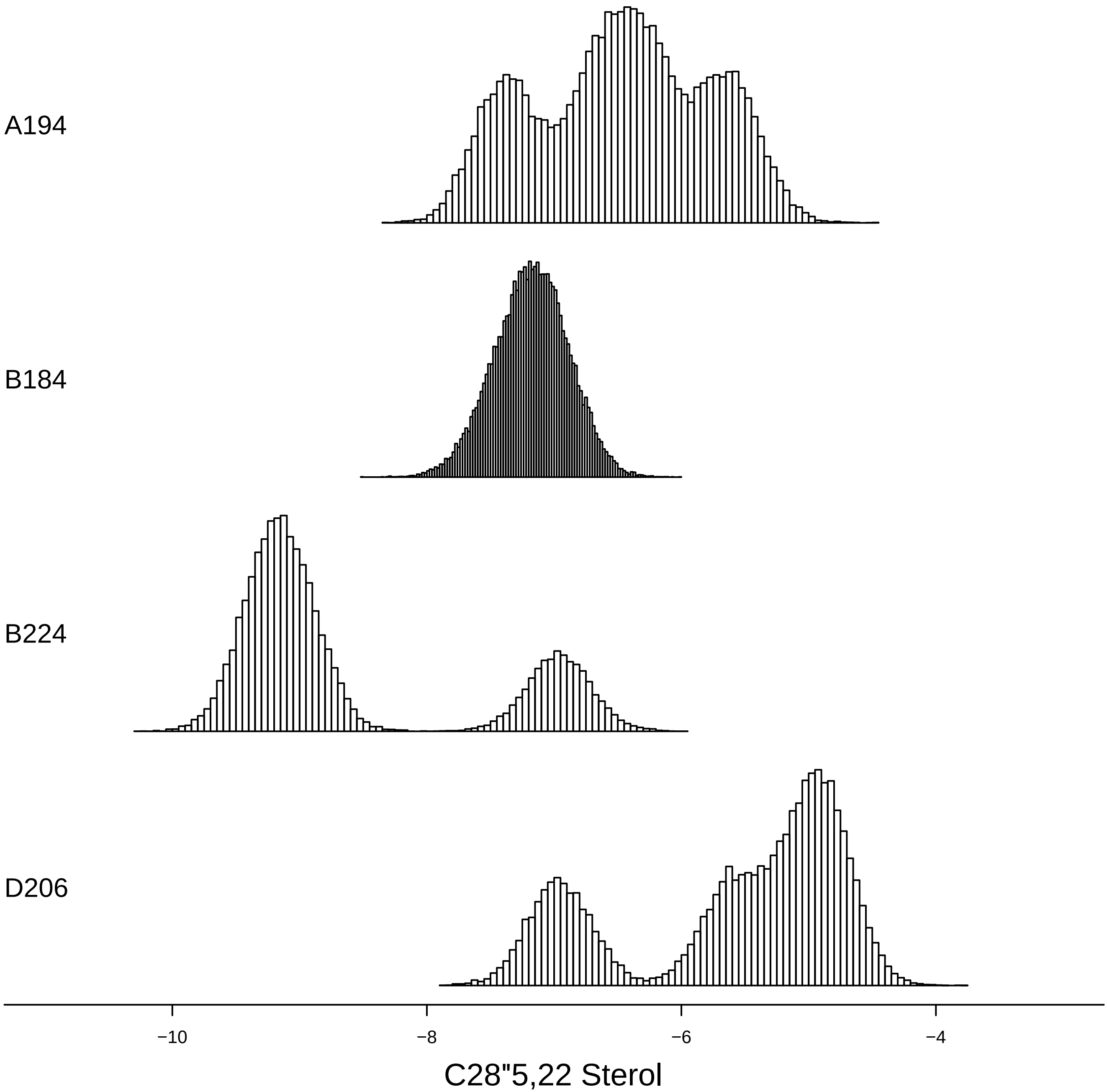
A194
B184
B224
D206
−10
−8
−6
−4
C28"5,22 Sterol

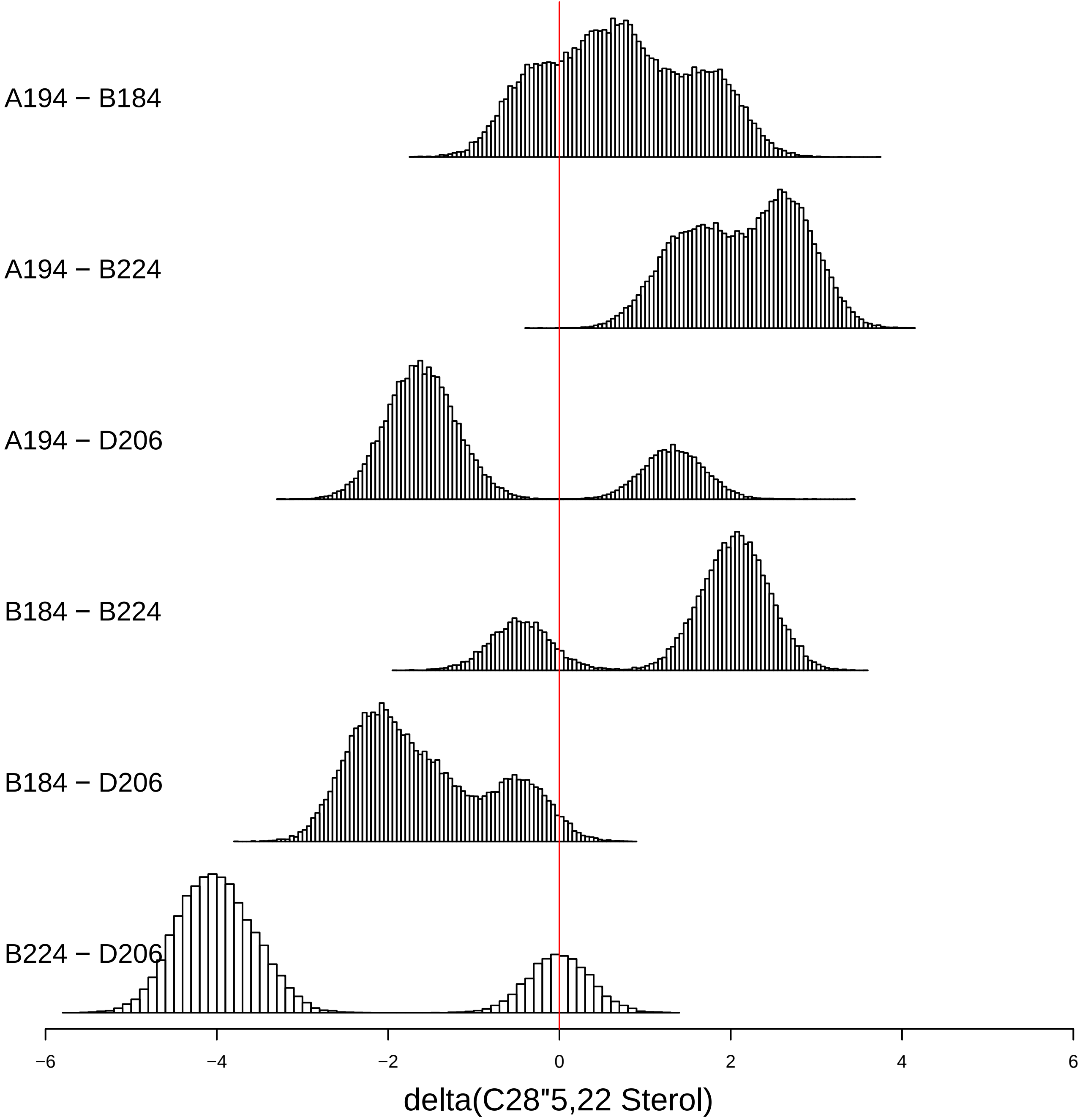

A194 − B184
A194 − B224
A194 − D206
B184 − B224
B184 − D206
B224 − D206
−6
−4
−2
0
2
4
6
delta(C28"5,22 Sterol)

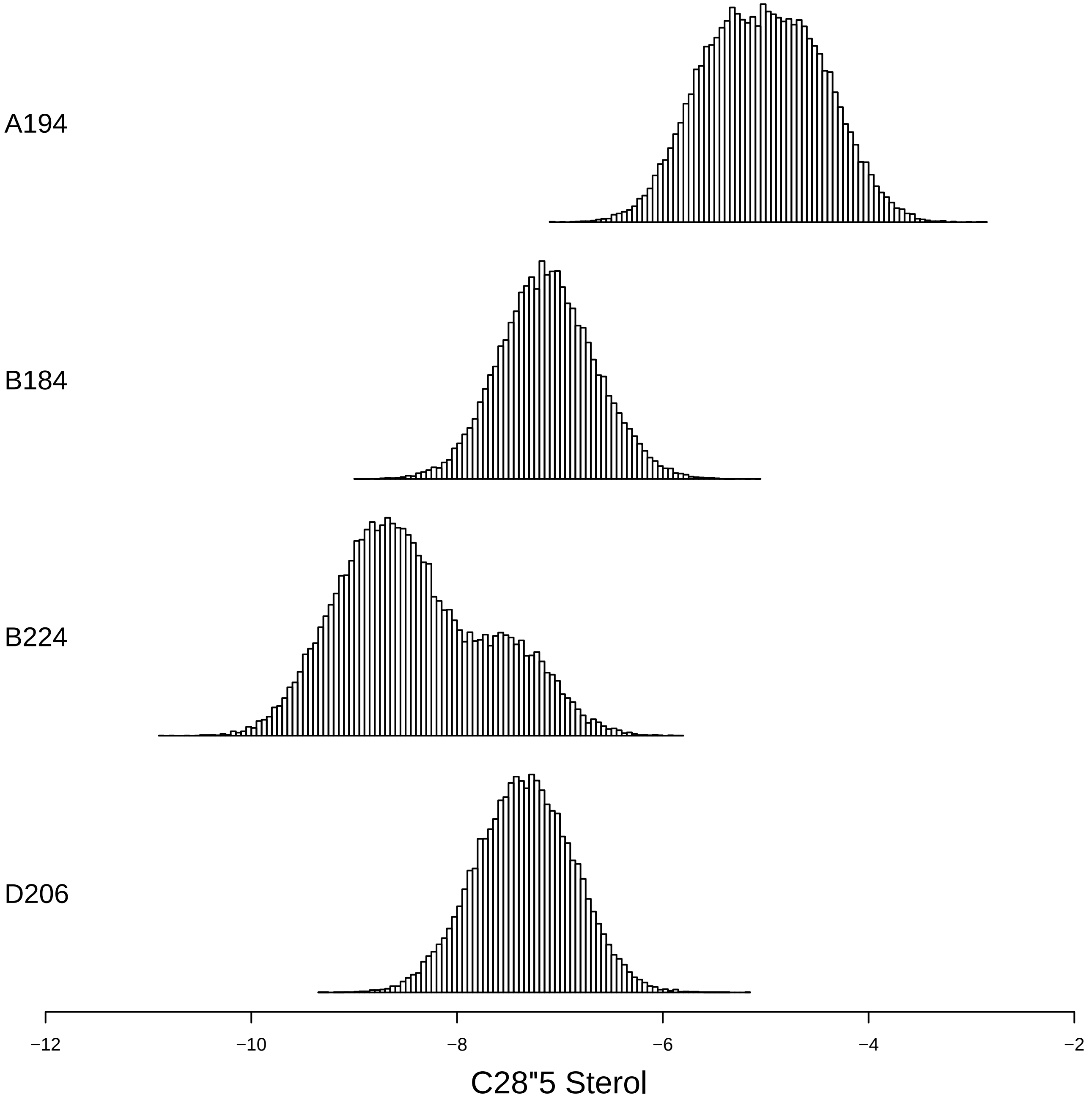
A194
B184
B224
D206
−12
−10
−8
−6
−4
−2
C28"5 Sterol

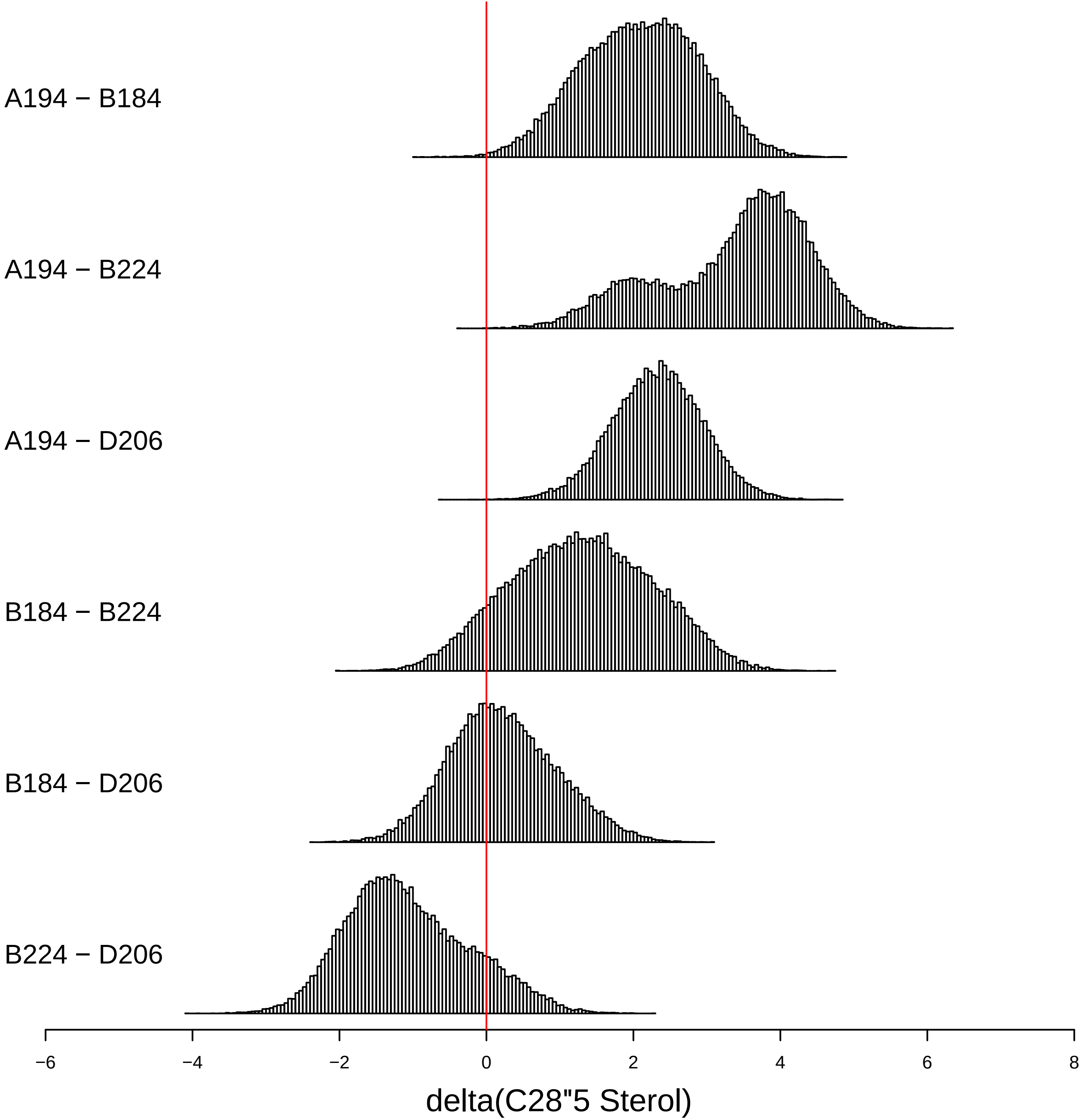
A194 − B184
A194 − B224
A194 − D206
B184 − B224
B184 − D206
B224 − D206
−6
−4
−2
0
2
4
6
8
delta(C28"5 Sterol)

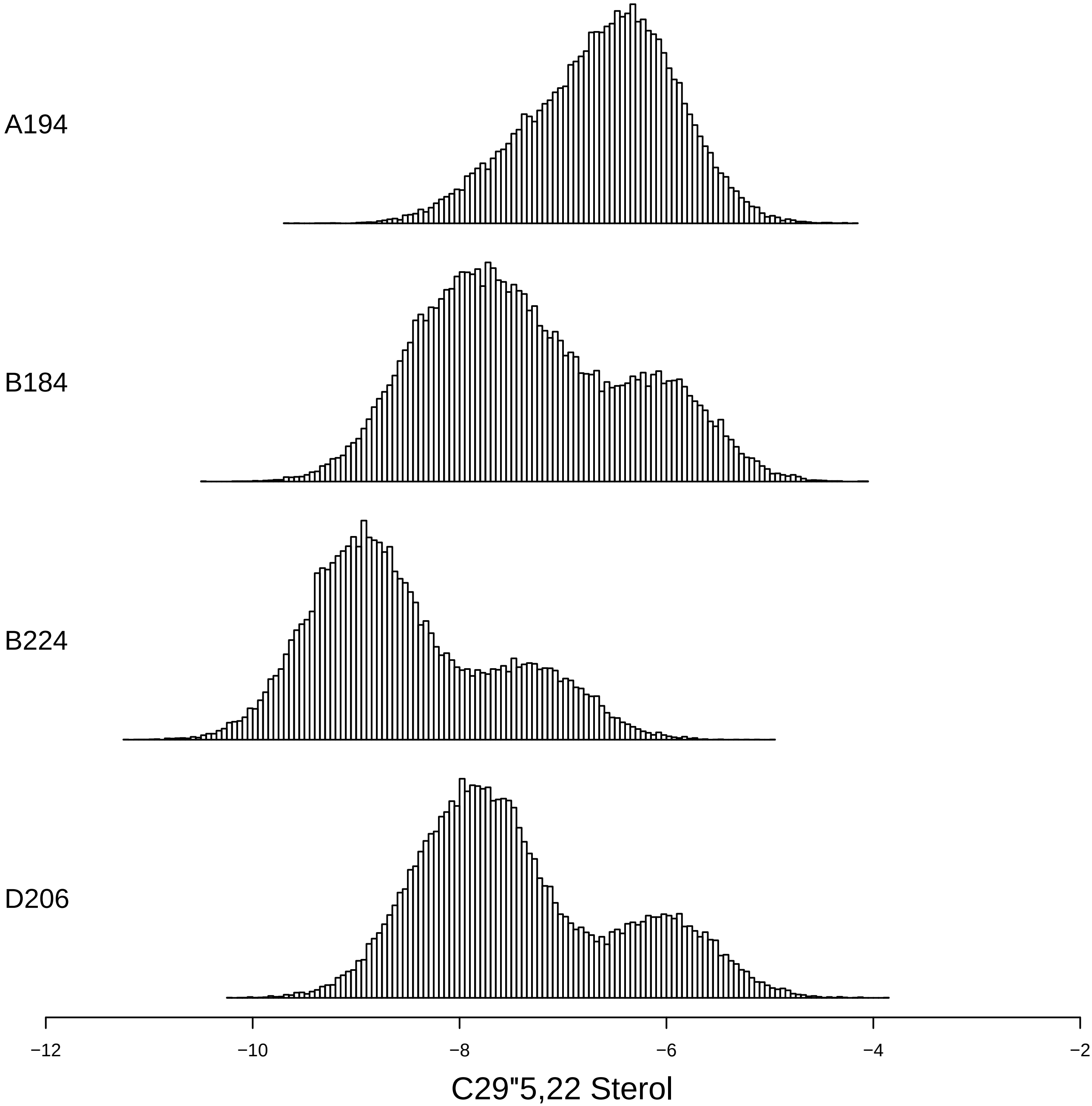

A194
B184
B224
D206
−12
−10
−8
−6
−4
−2
C29"5,22 Sterol

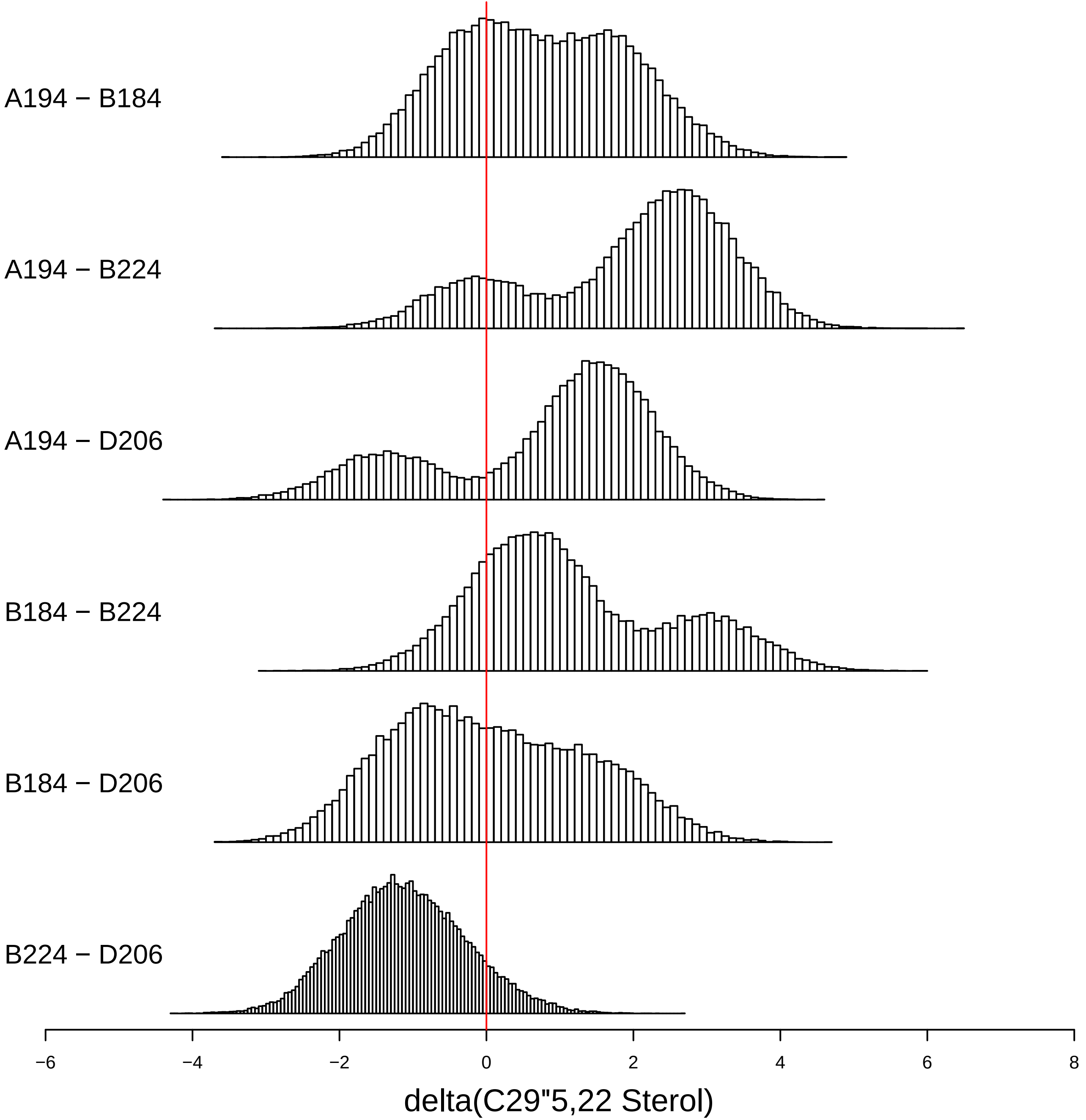
A194 − B184
A194 − B224
A194 − D206
B184 − B224
B184 − D206
B224 − D206
−6
−4
−2
0
2
4
6
8
delta(C29"5,22 Sterol)

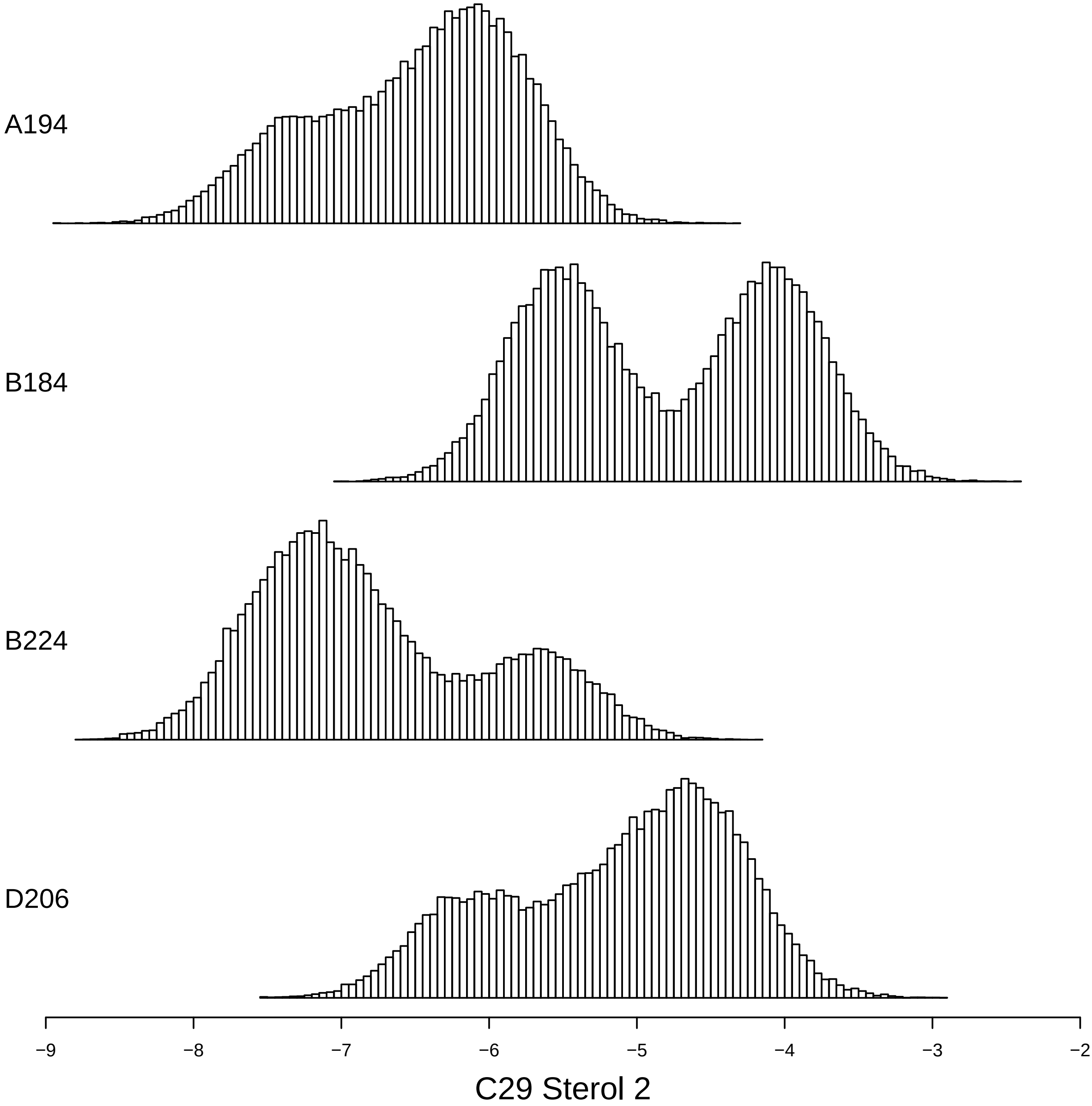
A194
B184
B224
D206
−9
−8
−7
−6
−5
−4
−3
−2
C29 Sterol 2

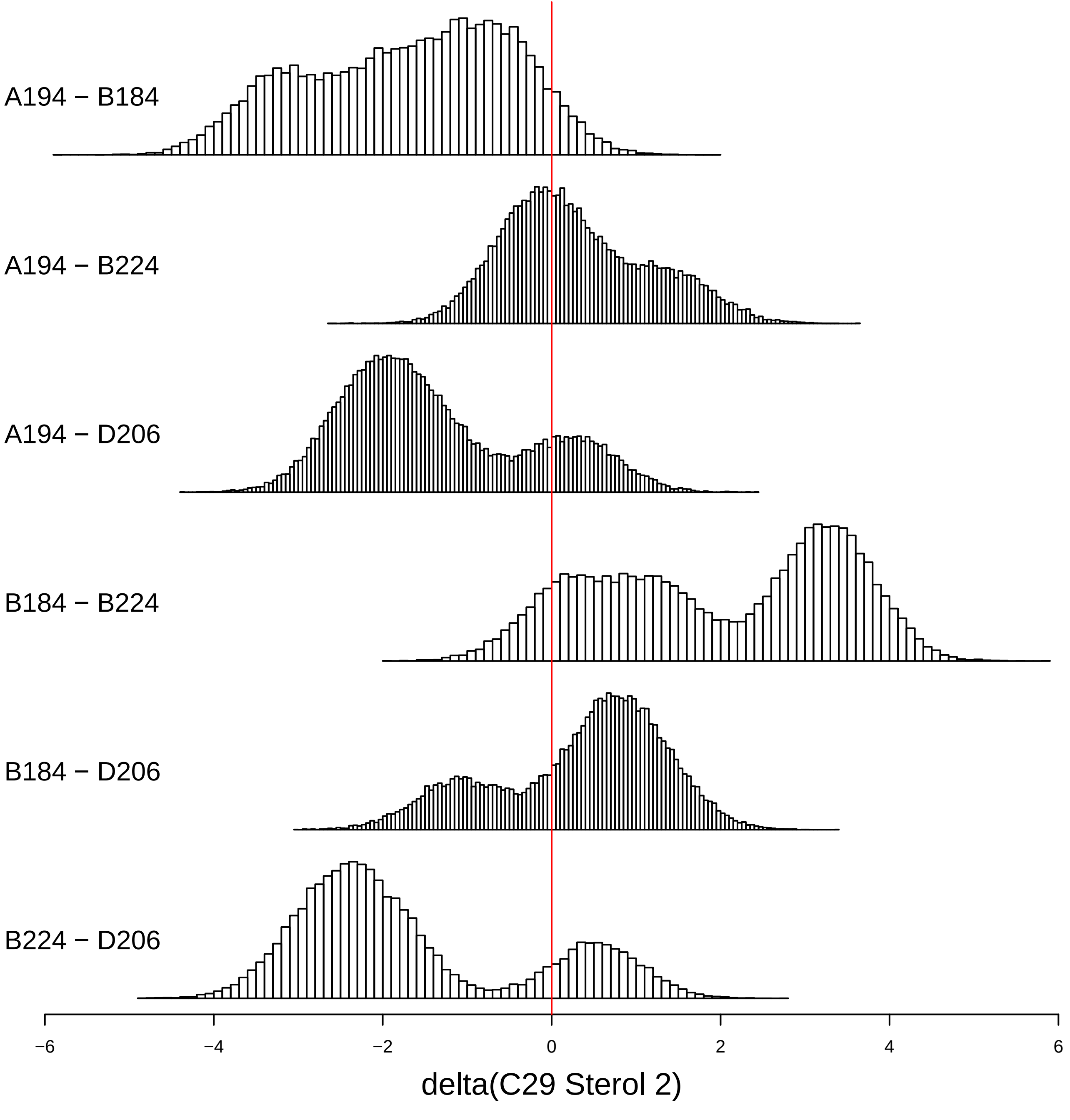
A194 − B184
A194 − B224
A194 − D206
B184 − B224
B184 − D206
B224 − D206
−6
−4
−2
0
2
4
6
delta(C29 Sterol 2)

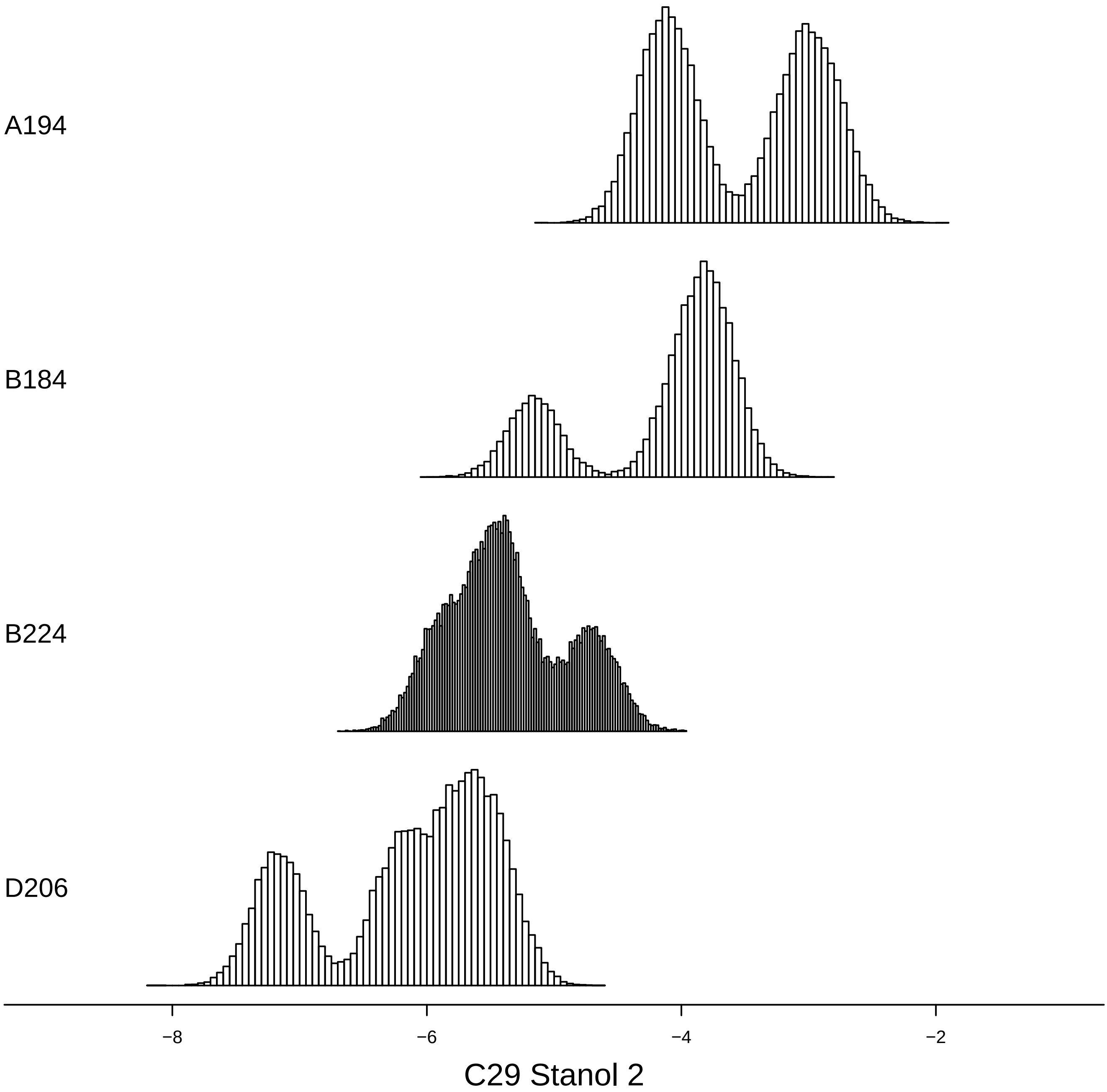
A194
B184
B224
D206
−8
−6
−4
−2
C29 Stanol 2

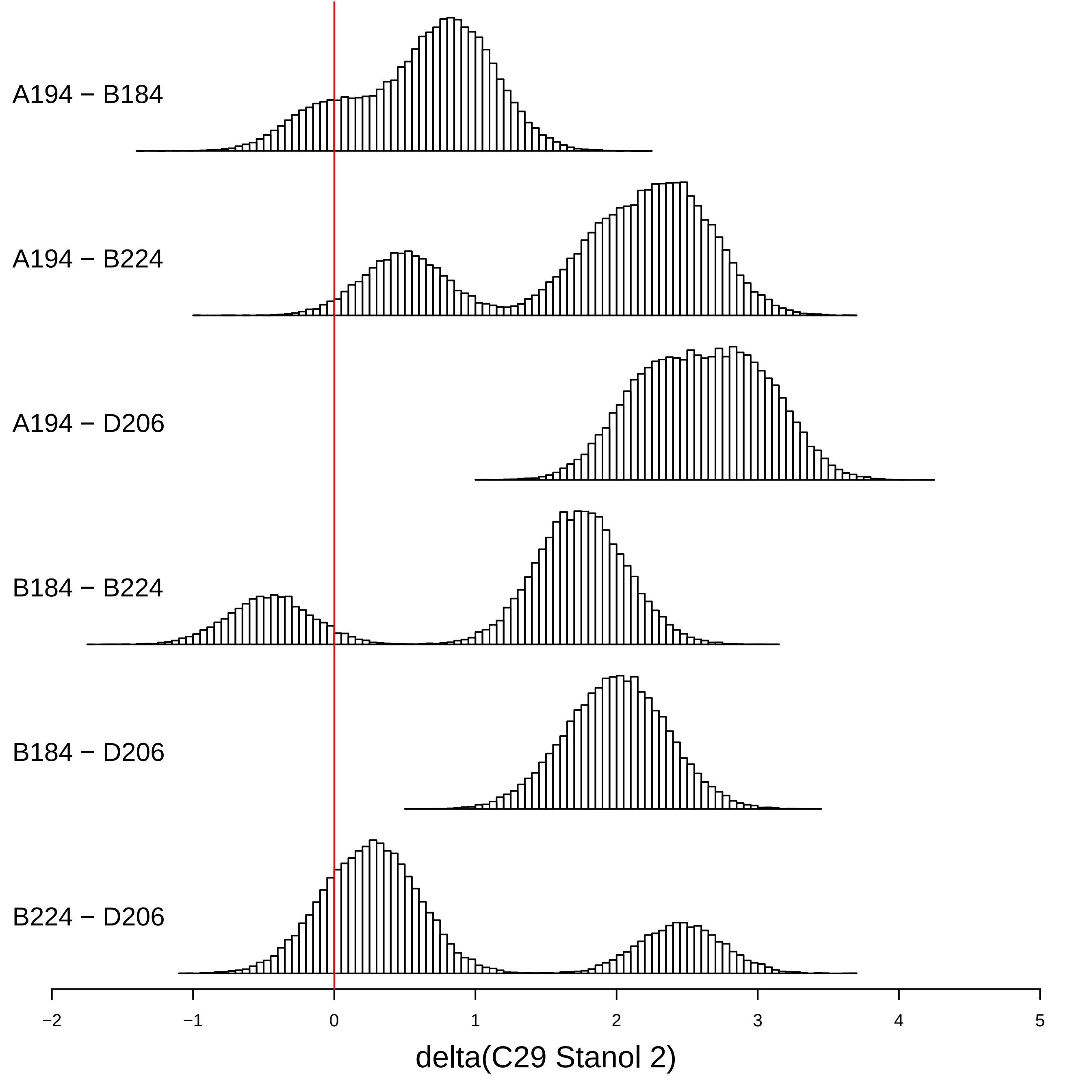

A194 − B184
A194 − B224
A194 − D206
B184 − B224
B184 − D206
B224 − D206
−2
−1
0
1
2
3
4
5
delta(C29 Stanol 2)

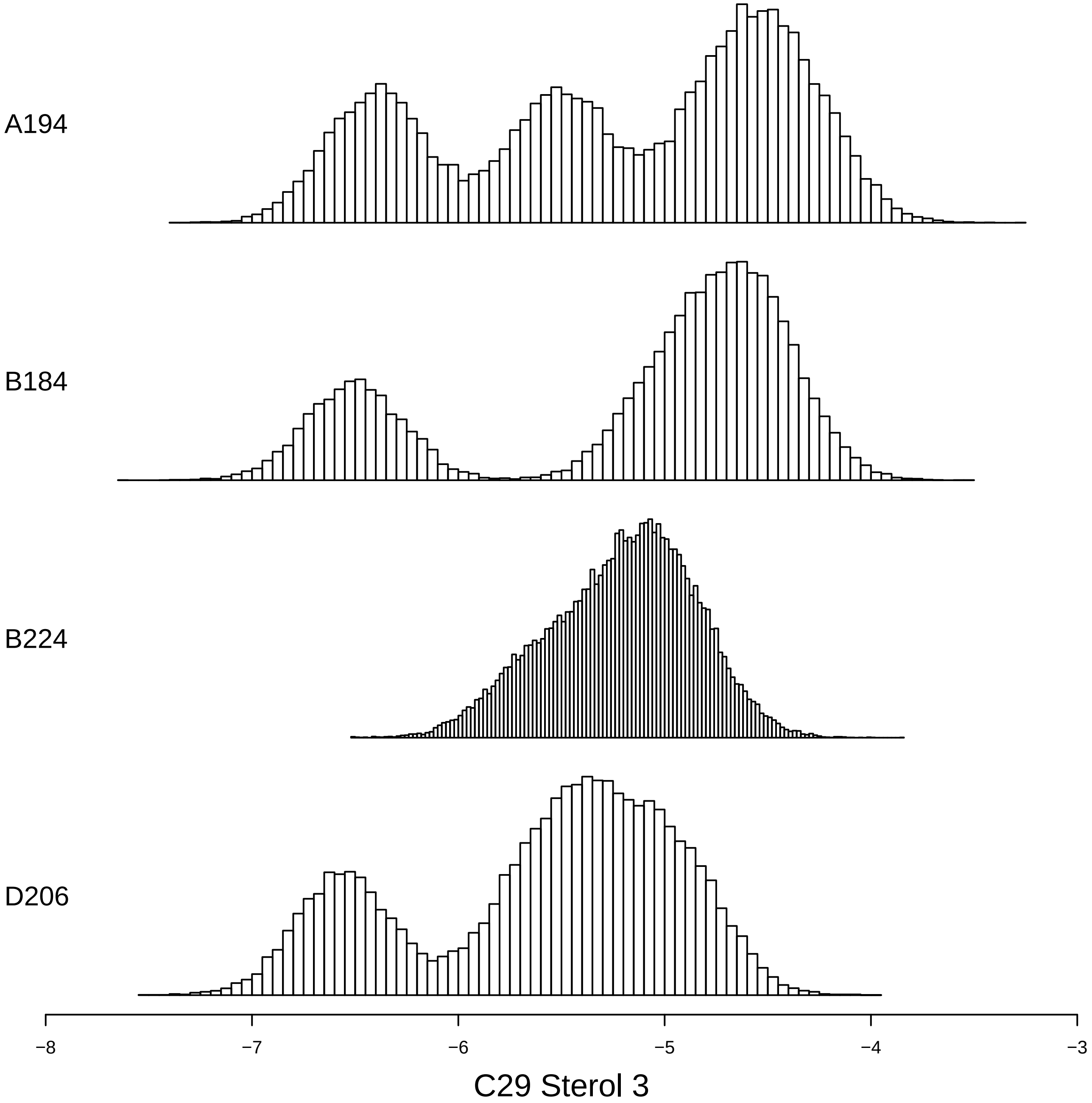
A194
B184
B224
D206
−8
−7
−6
−5
−4
−3
C29 Sterol 3

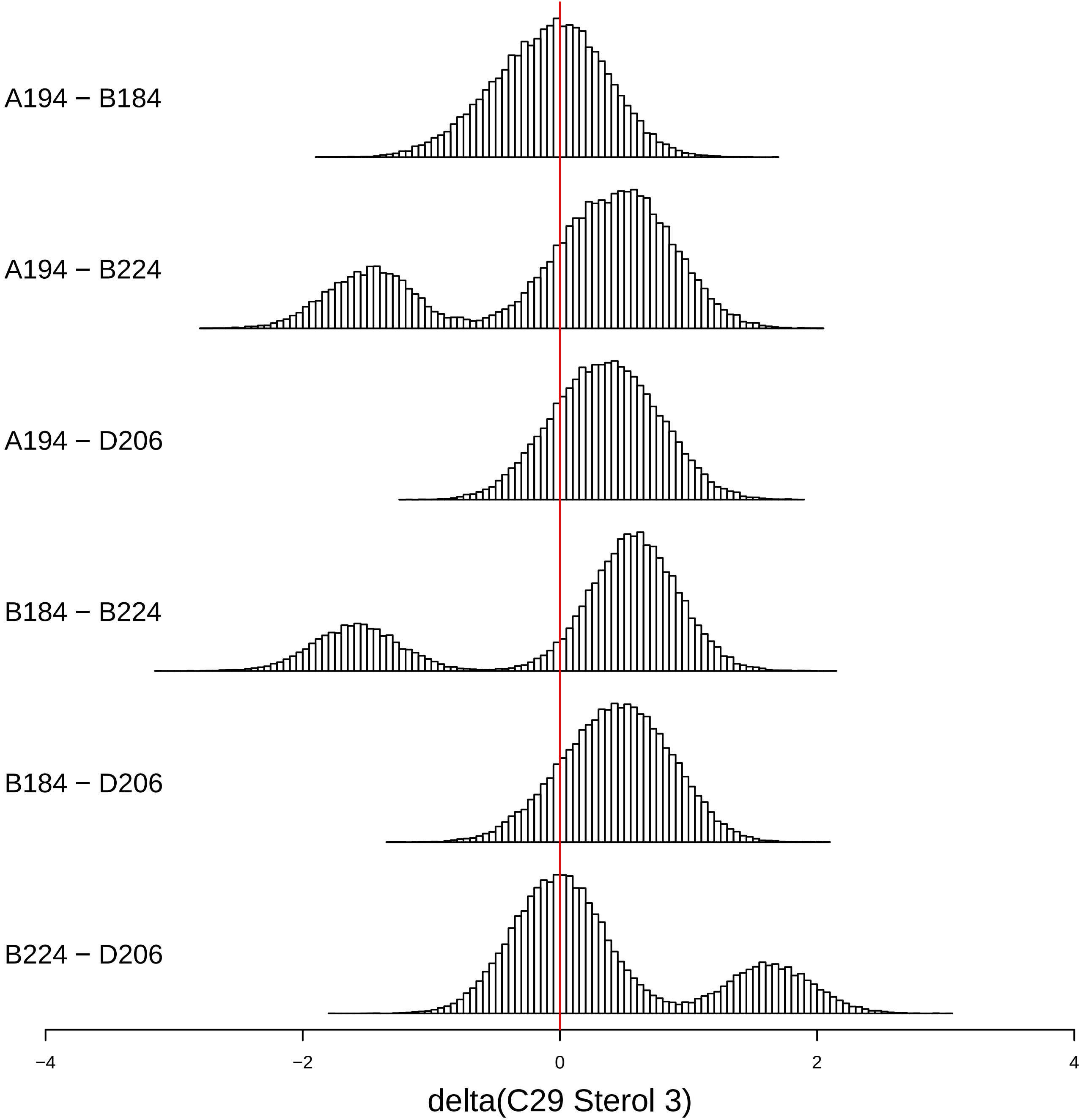

A194 − B184
A194 − B224
A194 − D206
B184 − B224
B184 − D206
B224 − D206
−4
−2
0
2
4
delta(C29 Sterol 3)

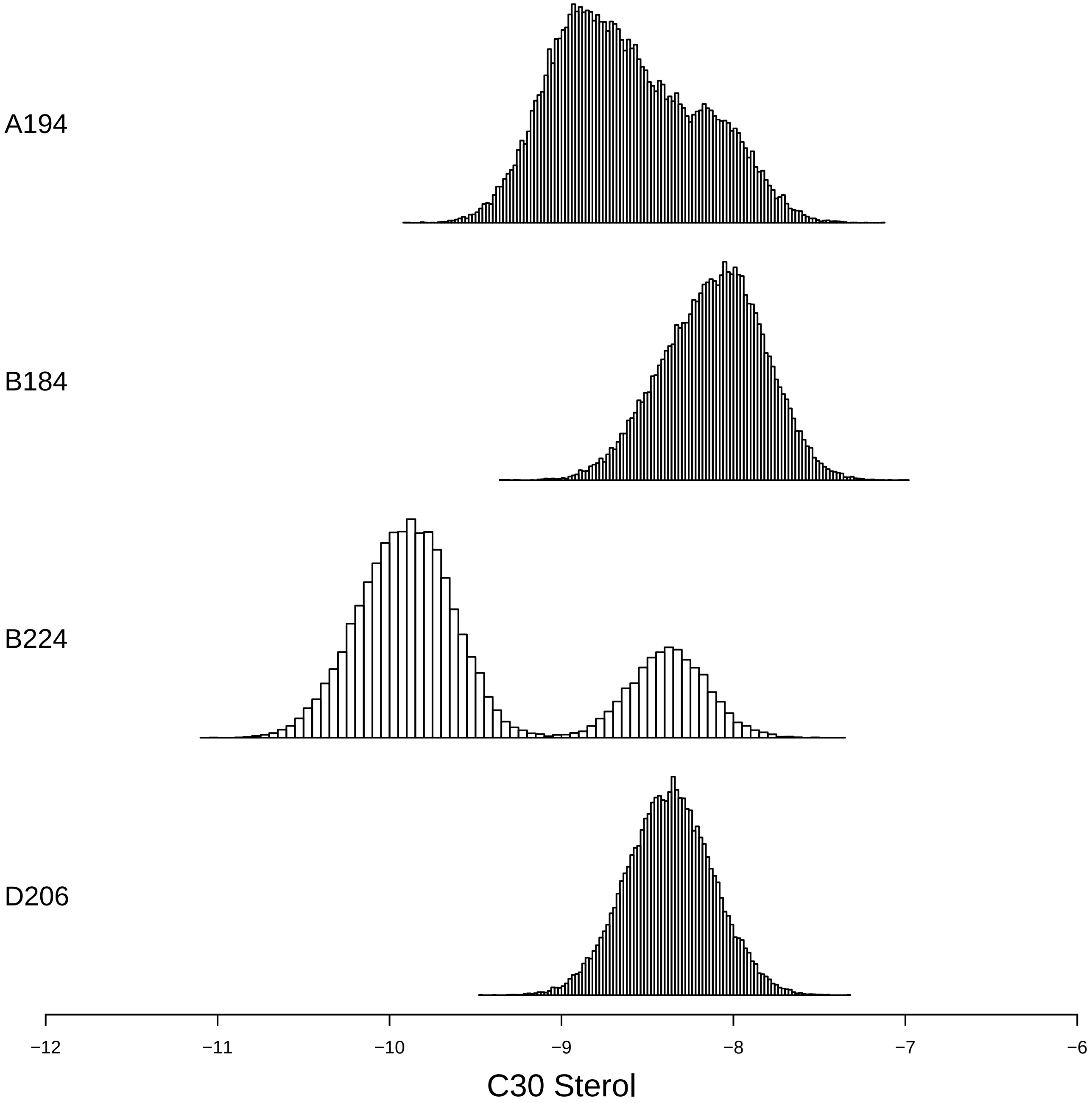
A194
B184
B224
D206
−12
−11
−10
−9
−8
−7
−6
C30 Sterol

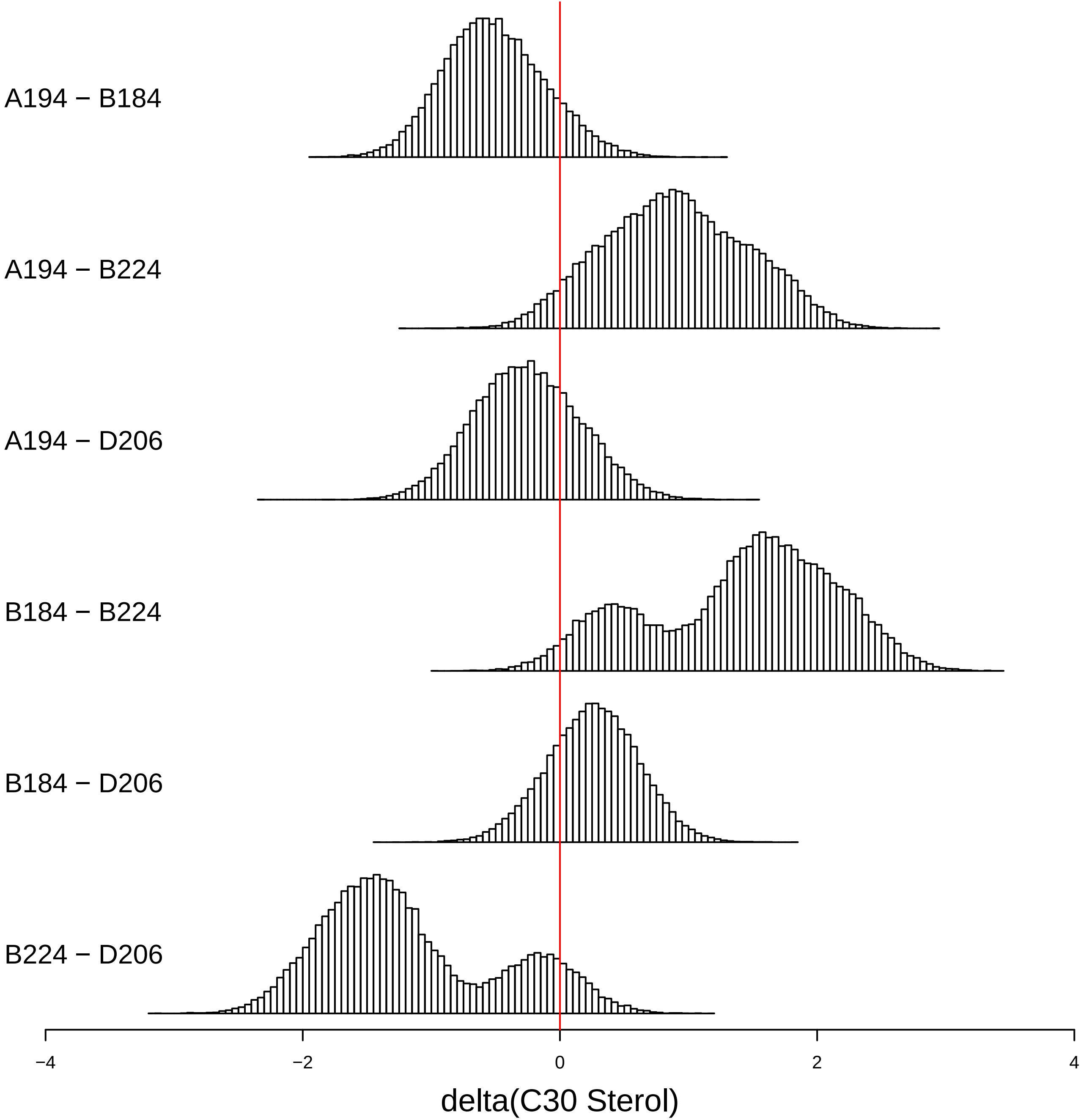
A194 − B184
A194 − B224
A194 − D206
B184 − B224
B184 − D206
B224 − D206
−4
−2
0
2
4
delta(C30 Sterol)

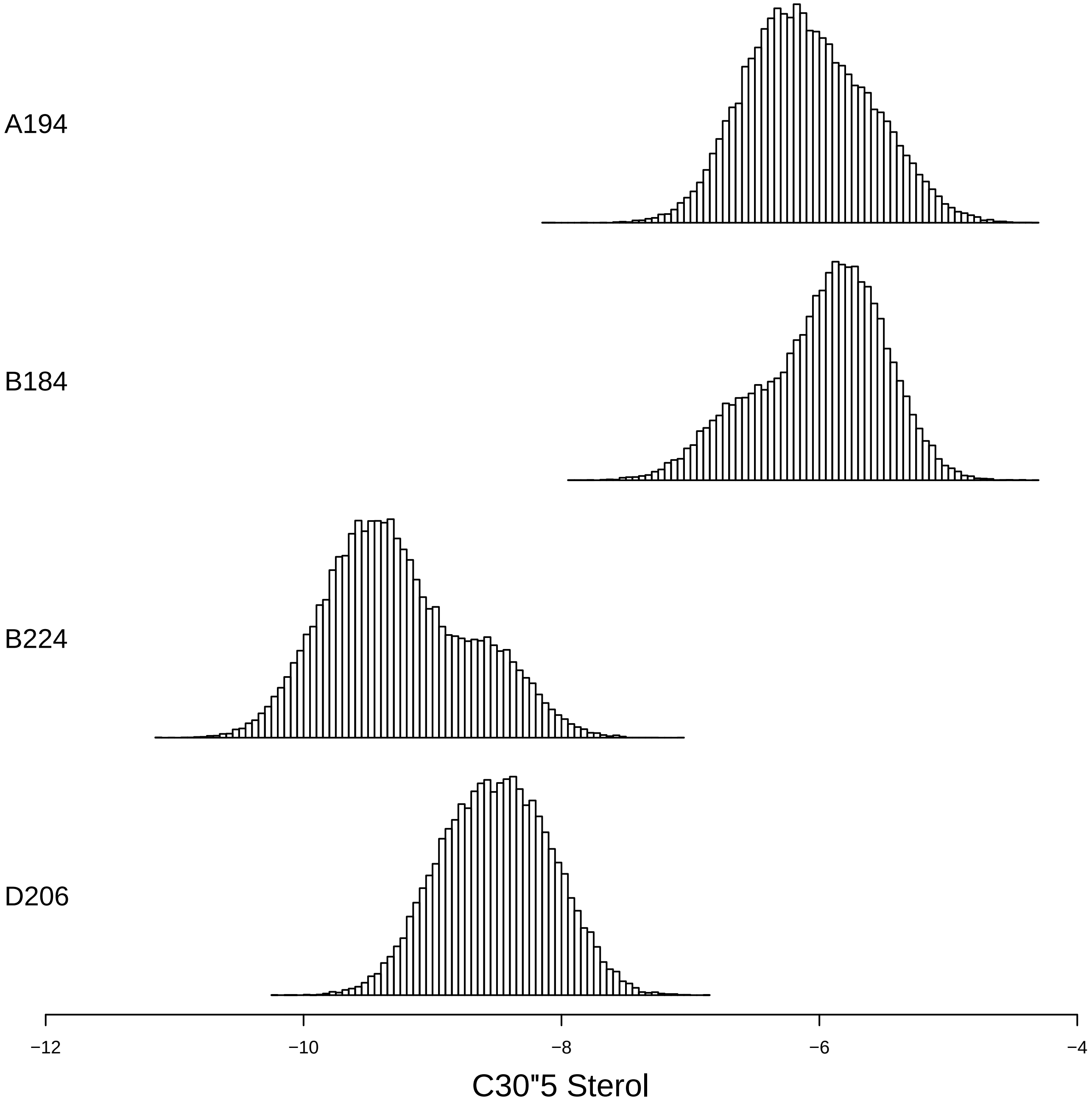

A194
B184
B224
D206
−12
−10
−8
−6
−4
C30"5 Sterol

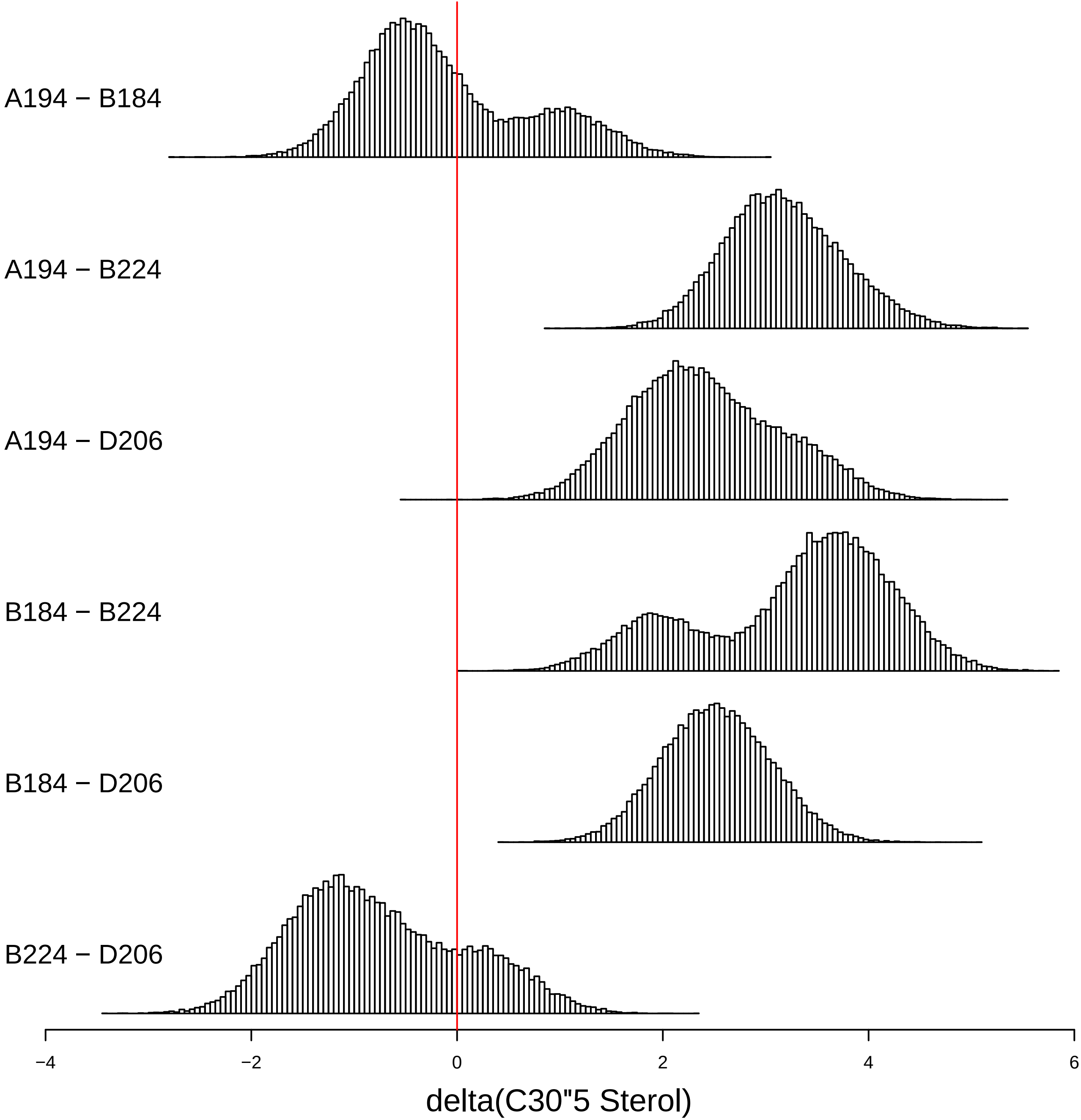

A194 − B184
A194 − B224
A194 − D206
B184 − B224
B184 − D206
B224 − D206
−4
−2
0
2
4
6
delta(C30"5 Sterol)

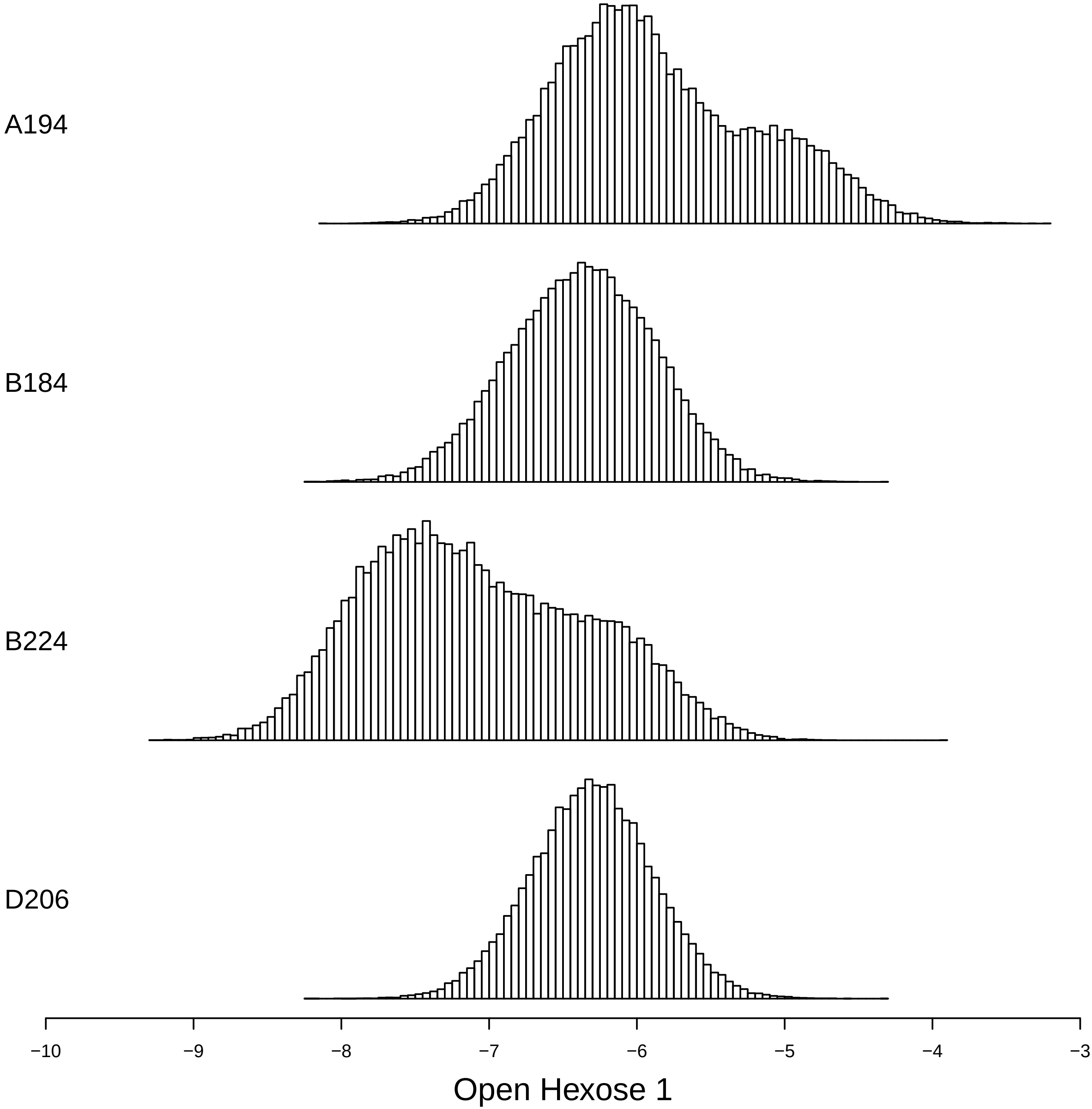
A194
B184
B224
D206
−10
−9
−8
−7
−6
−5
−4
−3
Open Hexose 1

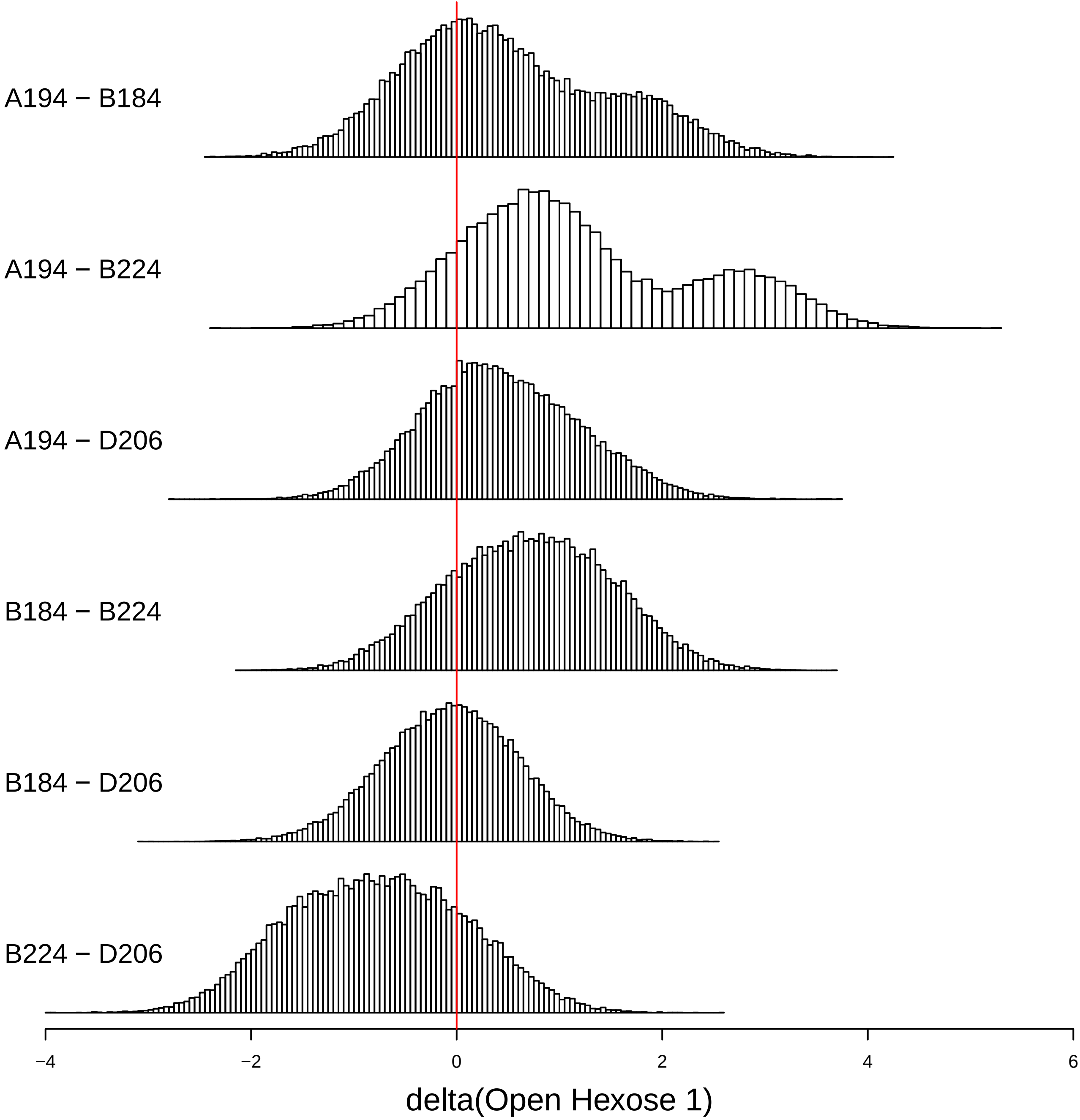
A194 − B184
A194 − B224
A194 − D206
B184 − B224
B184 − D206
B224 − D206
−4
−2
0
2
4
6
delta(Open Hexose 1)

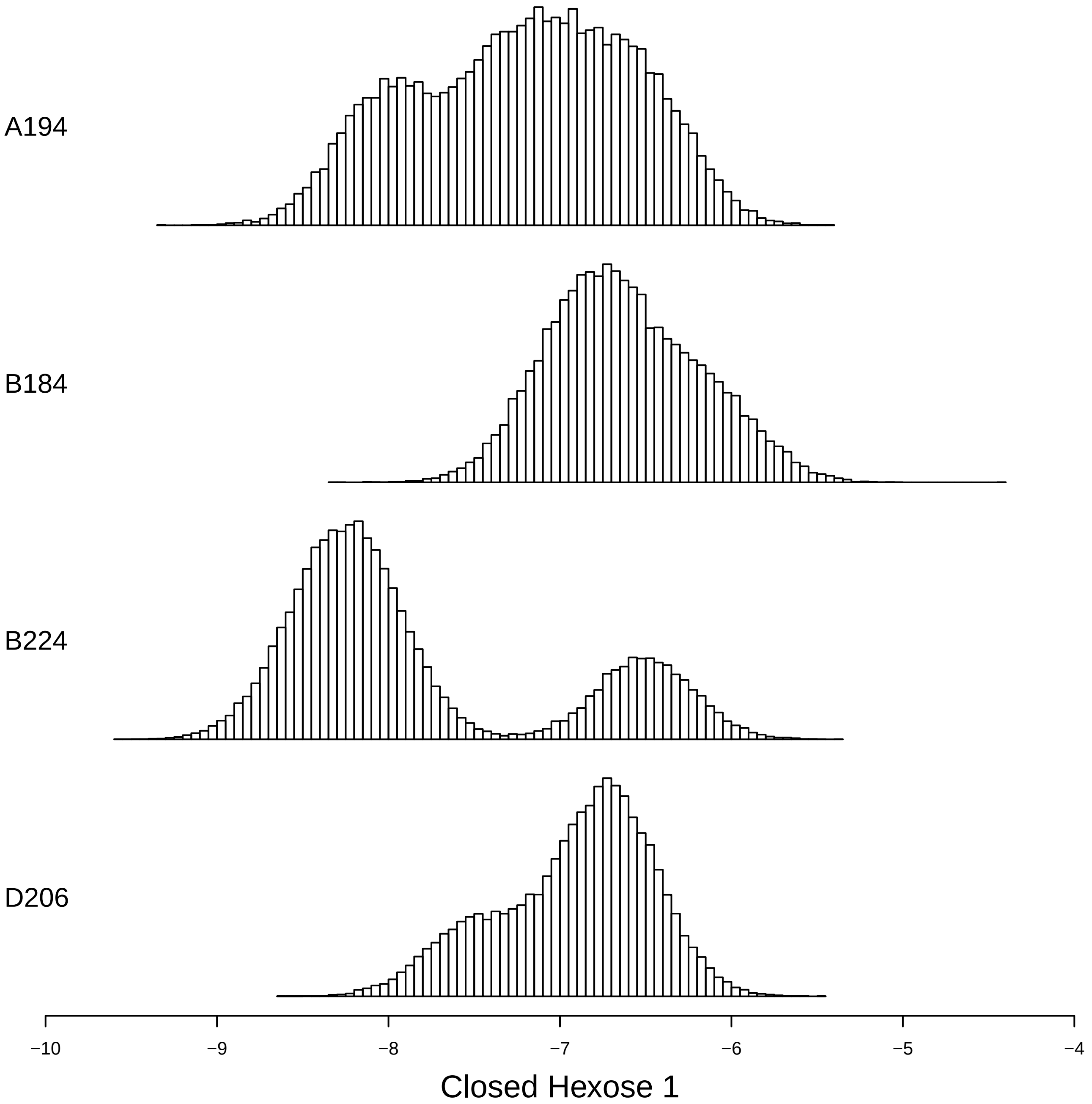
A194
B184
B224
D206
−10
−9
−8
−7
−6
−5
−4
Closed Hexose 1

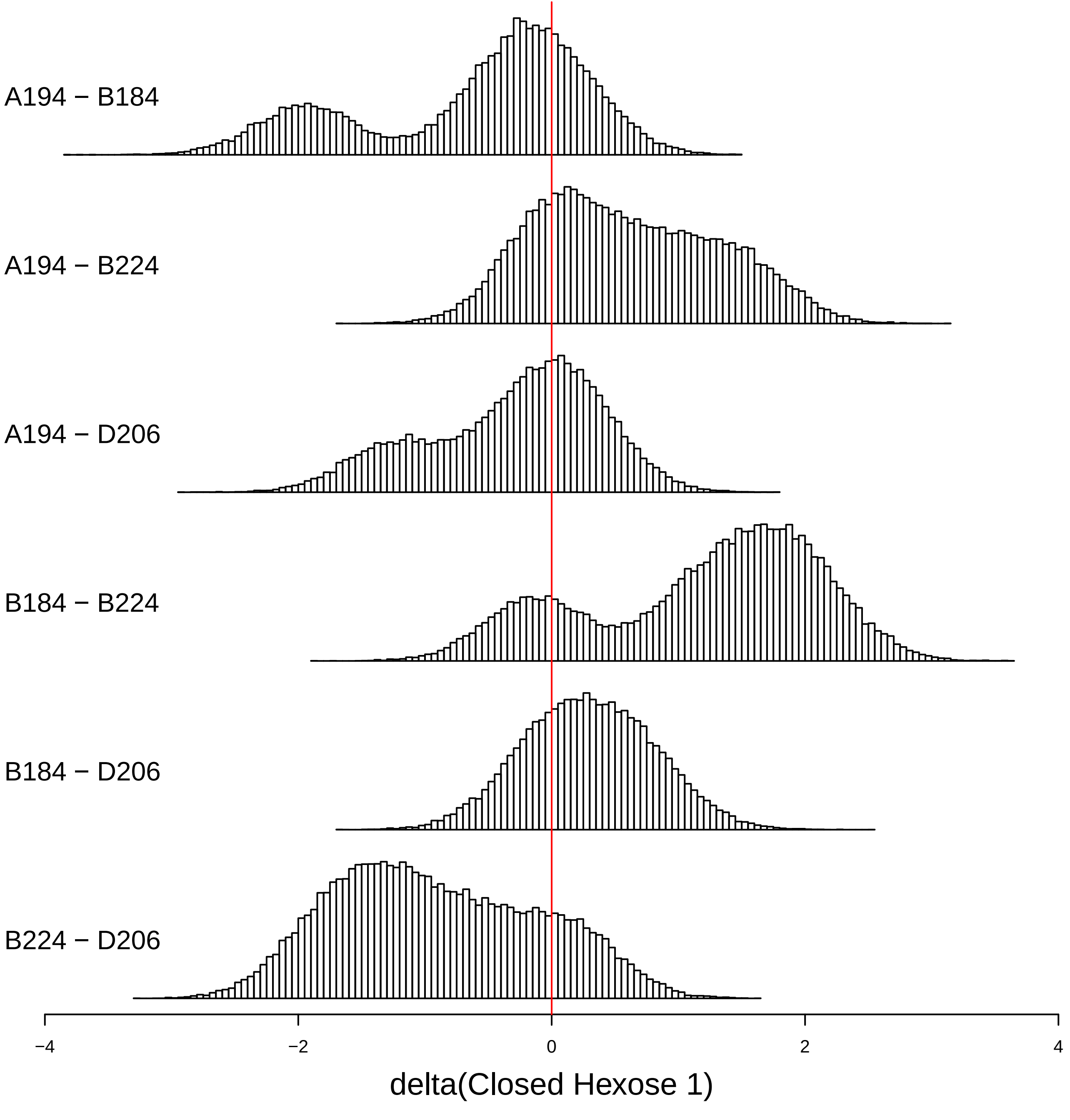
A194 − B184
A194 − B224
A194 − D206
B184 − B224
B184 − D206
B224 − D206
−4
−2
0
2
4
delta(Closed Hexose 1)

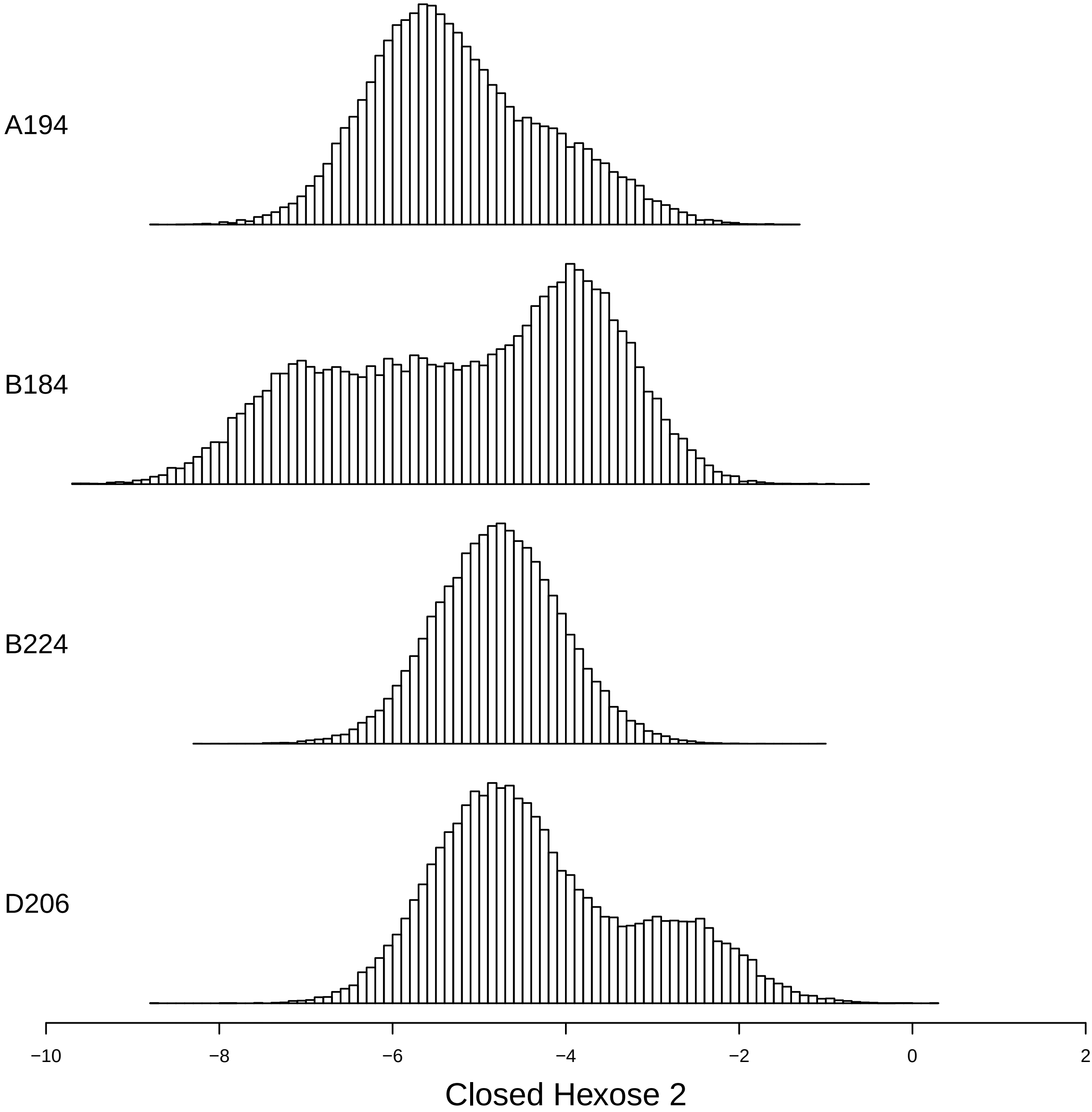
A194
B184
B224
D206
−10
−8
−6
−4
−2
0
2
Closed Hexose 2

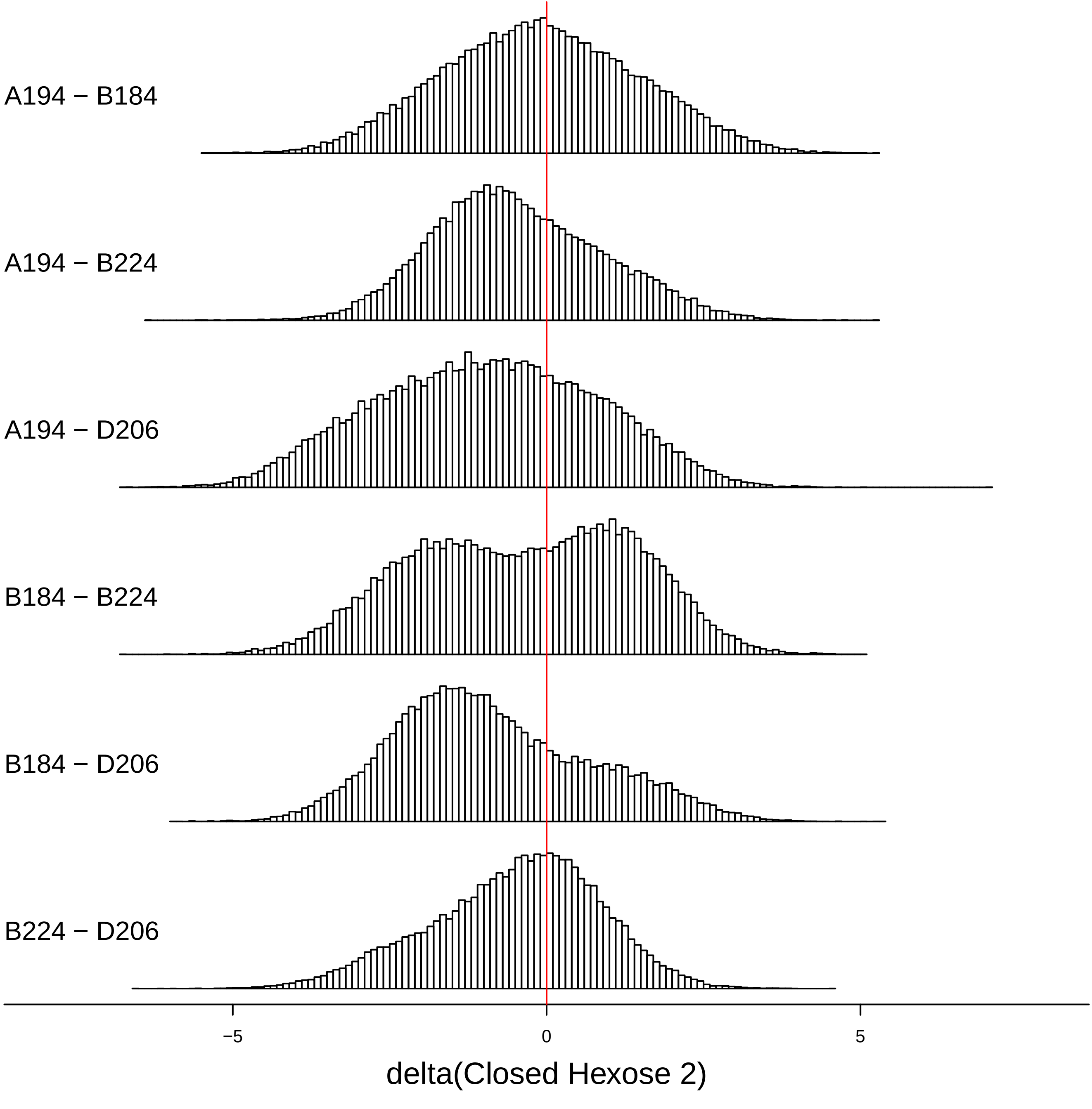
A194 − B184
A194 − B224
A194 − D206
B184 − B224
B184 − D206
B224 − D206
−5
0
5
delta(Closed Hexose 2)

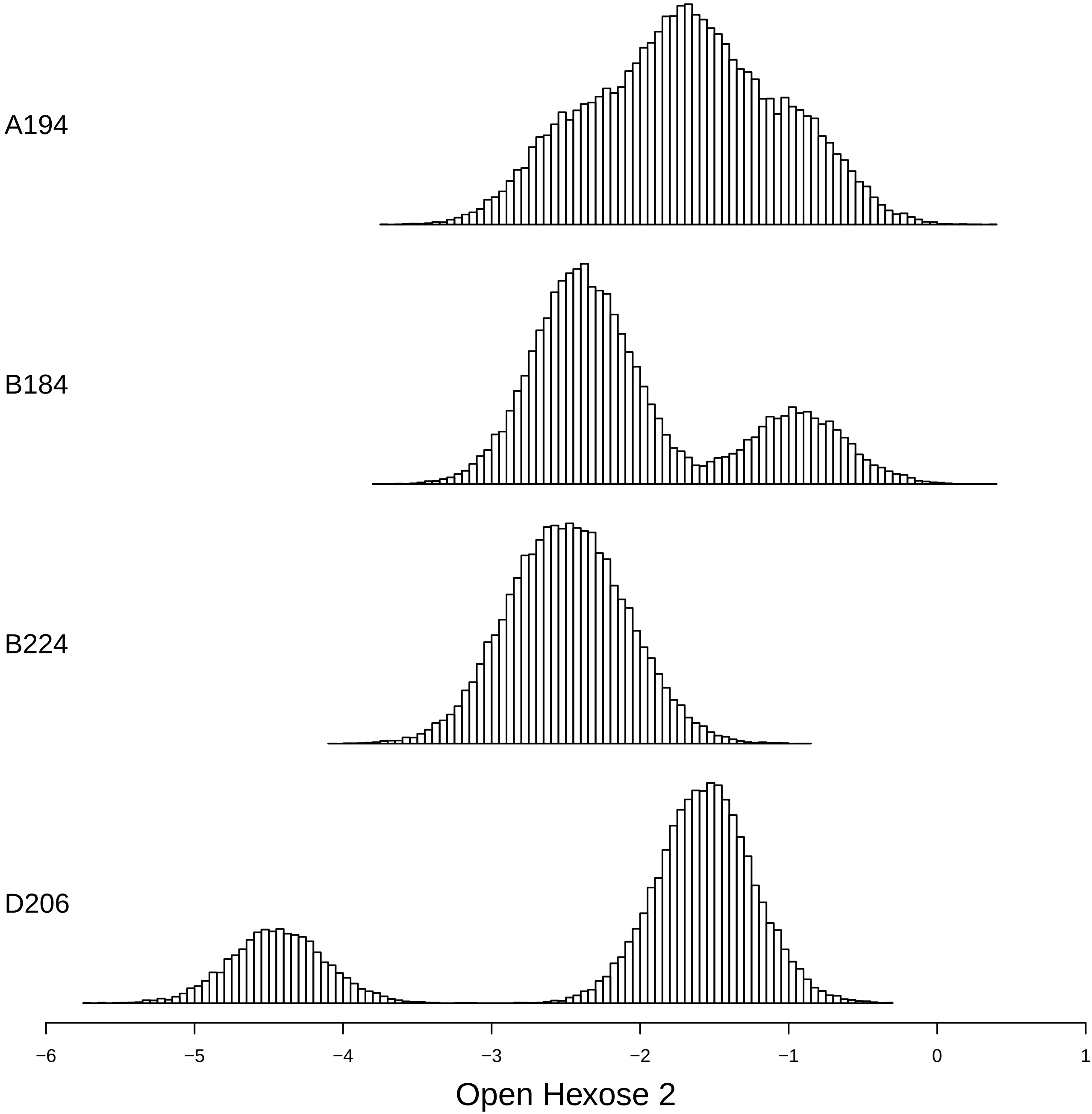
A194
B184
B224
D206
−6
−5
−4
−3
−2
−1
0
1
Open Hexose 2

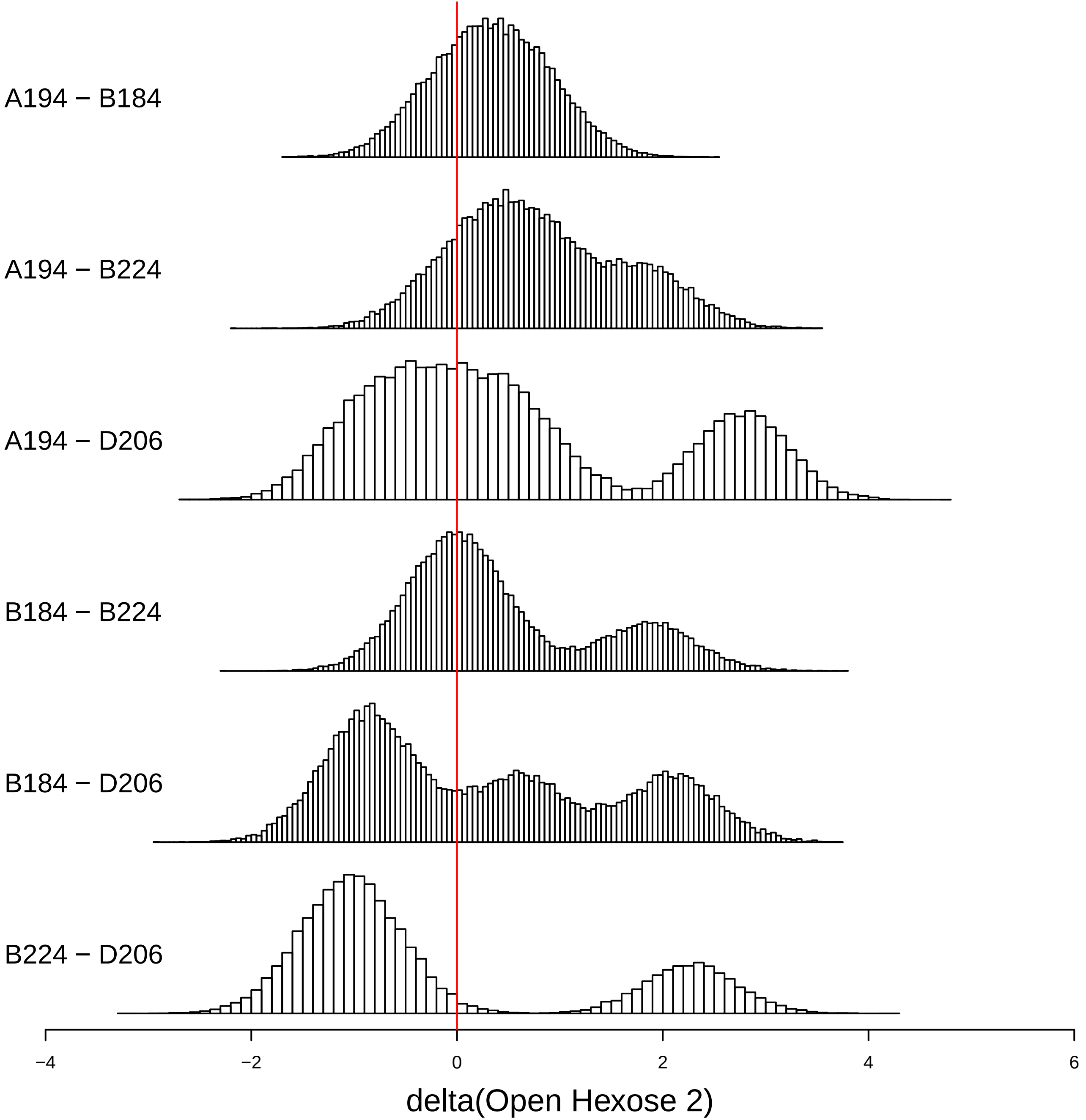
A194 − B184
A194 − B224
A194 − D206
B184 − B224
B184 − D206
B224 − D206
−4
−2
0
2
4
6
delta(Open Hexose 2)

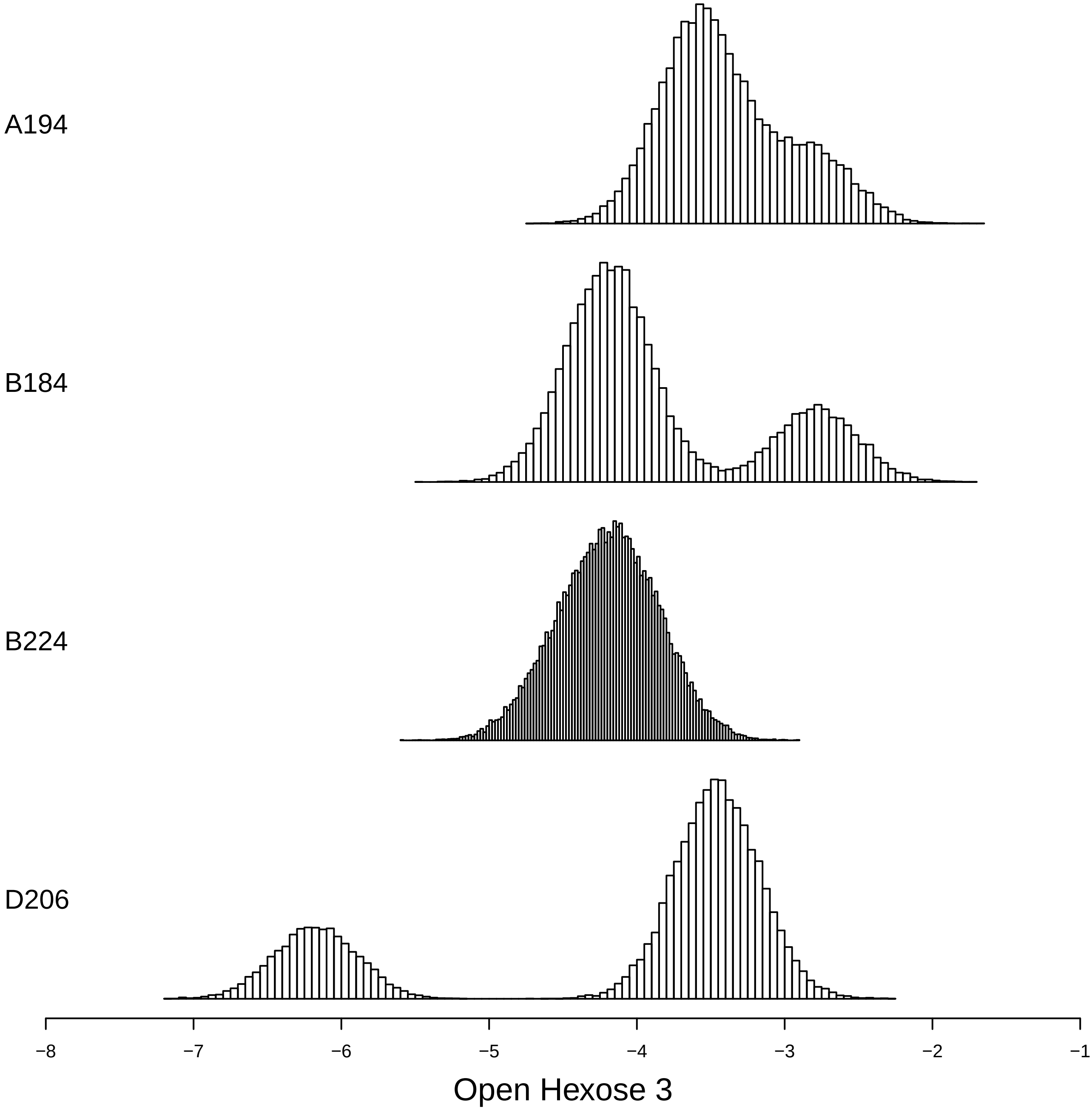

A194
B184
B224
D206
−8
−7
−6
−5
−4
−3
−2
−1
Open Hexose 3

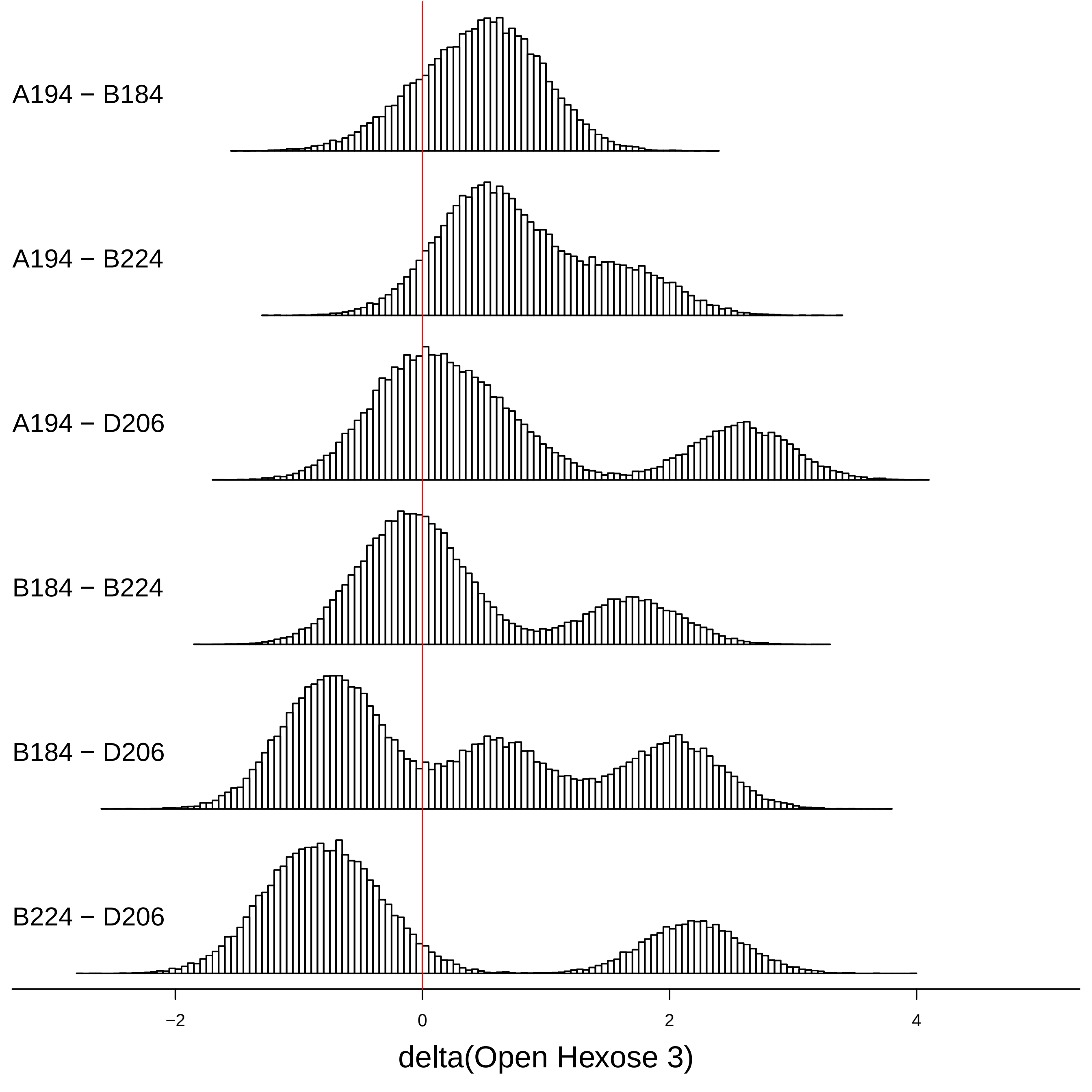
A194 − B184
A194 − B224
A194 − D206
B184 − B224
B184 − D206
B224 − D206
−2
0
2
4
delta(Open Hexose 3)

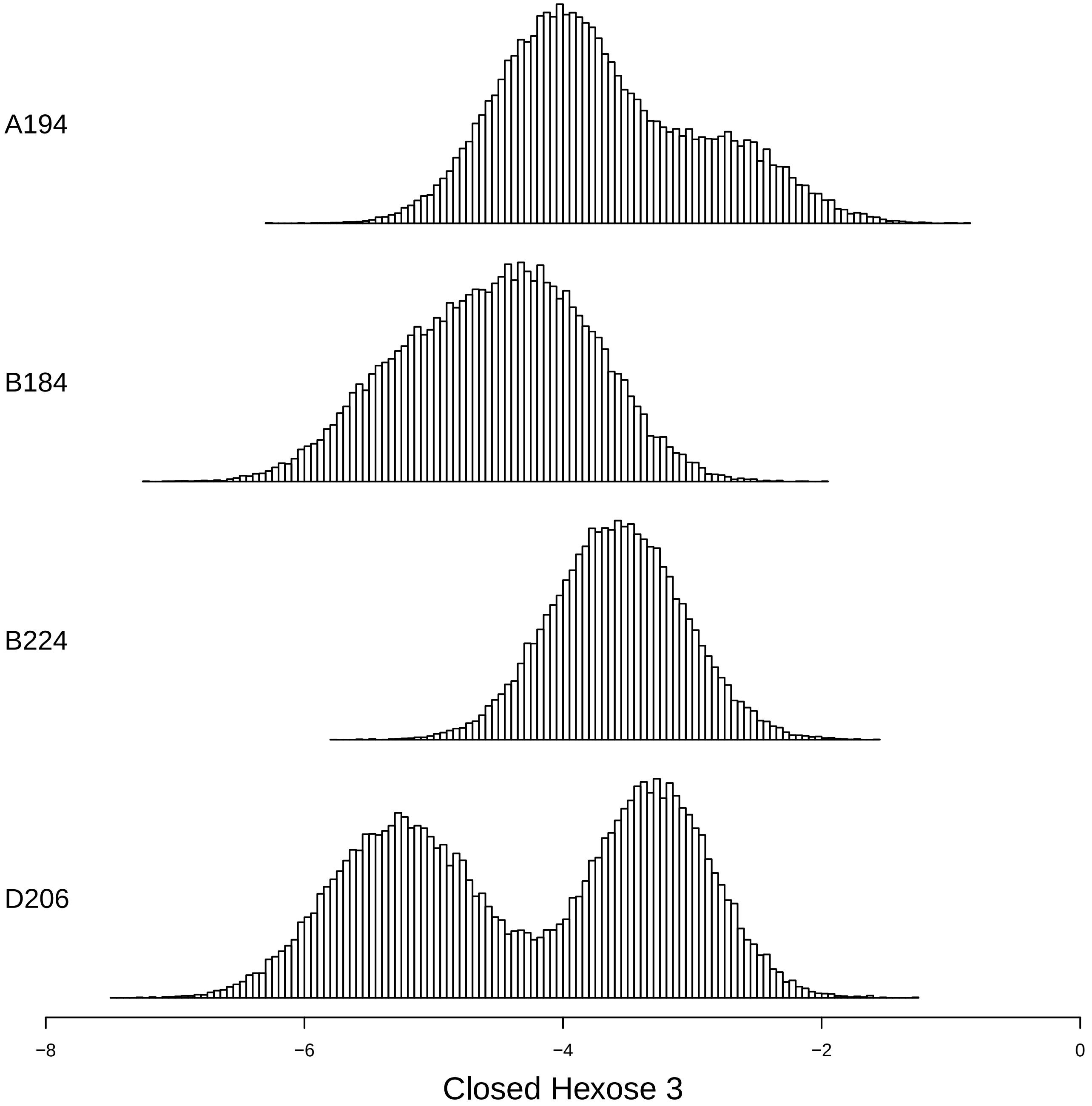
A194
B184
B224
D206
−8
−6
−4
−2
0
Closed Hexose 3

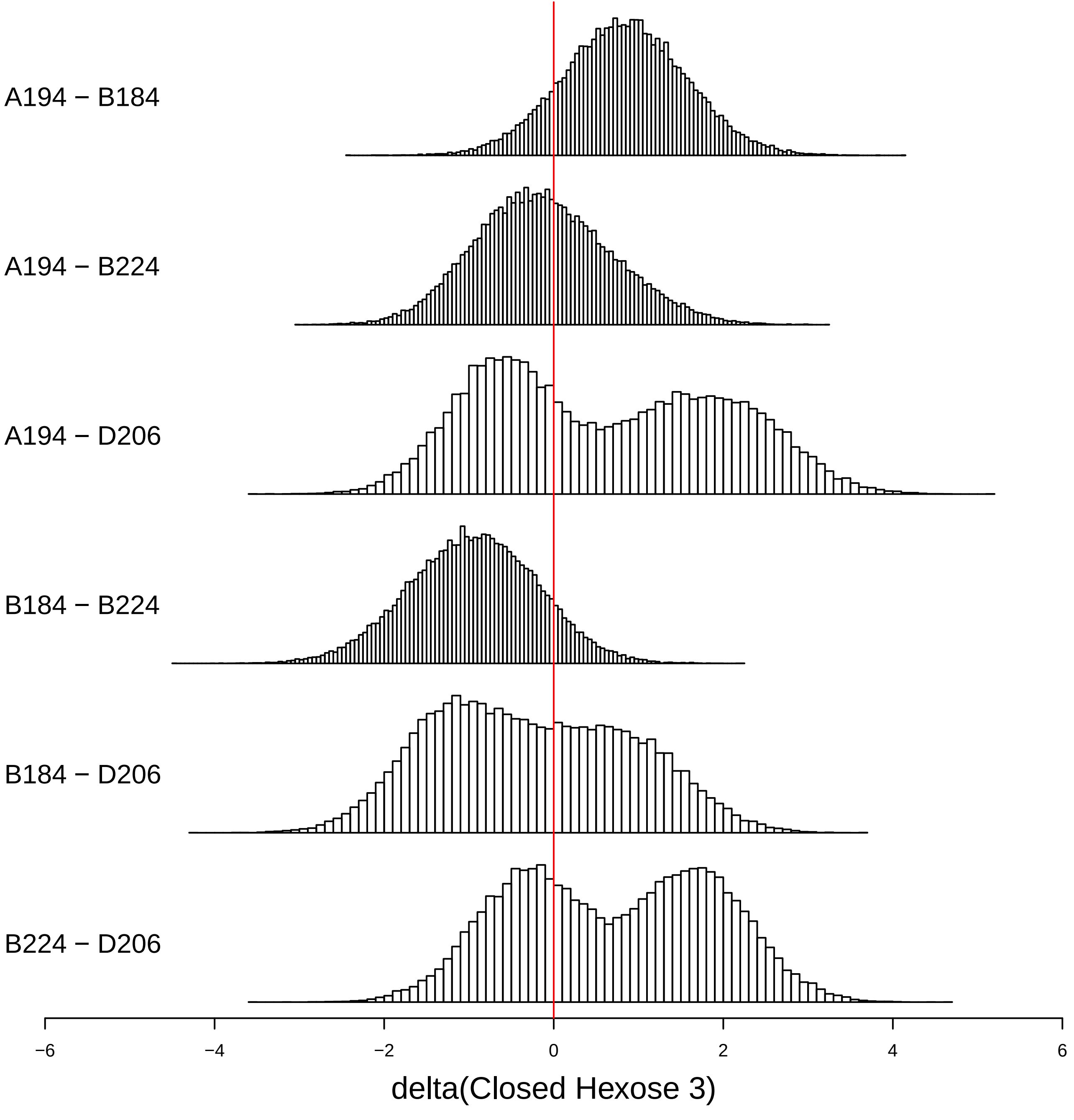
A194 − B184
A194 − B224
A194 − D206
B184 − B224
B184 − D206
B224 − D206
−6
−4
−2
0
2
4
6
delta(Closed Hexose 3)

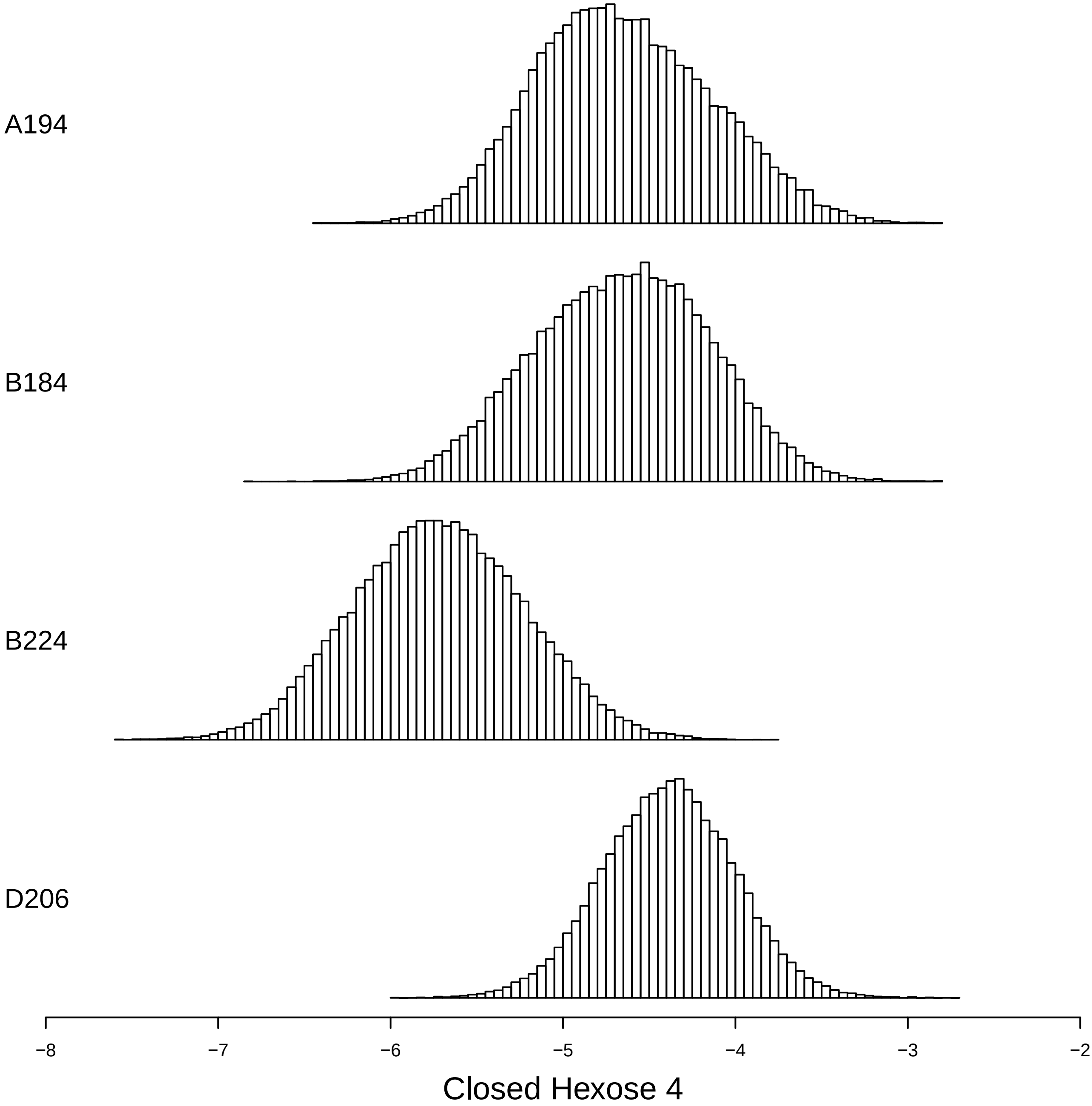
A194
B184
B224
D206
−8
−7
−6
−5
−4
−3
−2
Closed Hexose 4

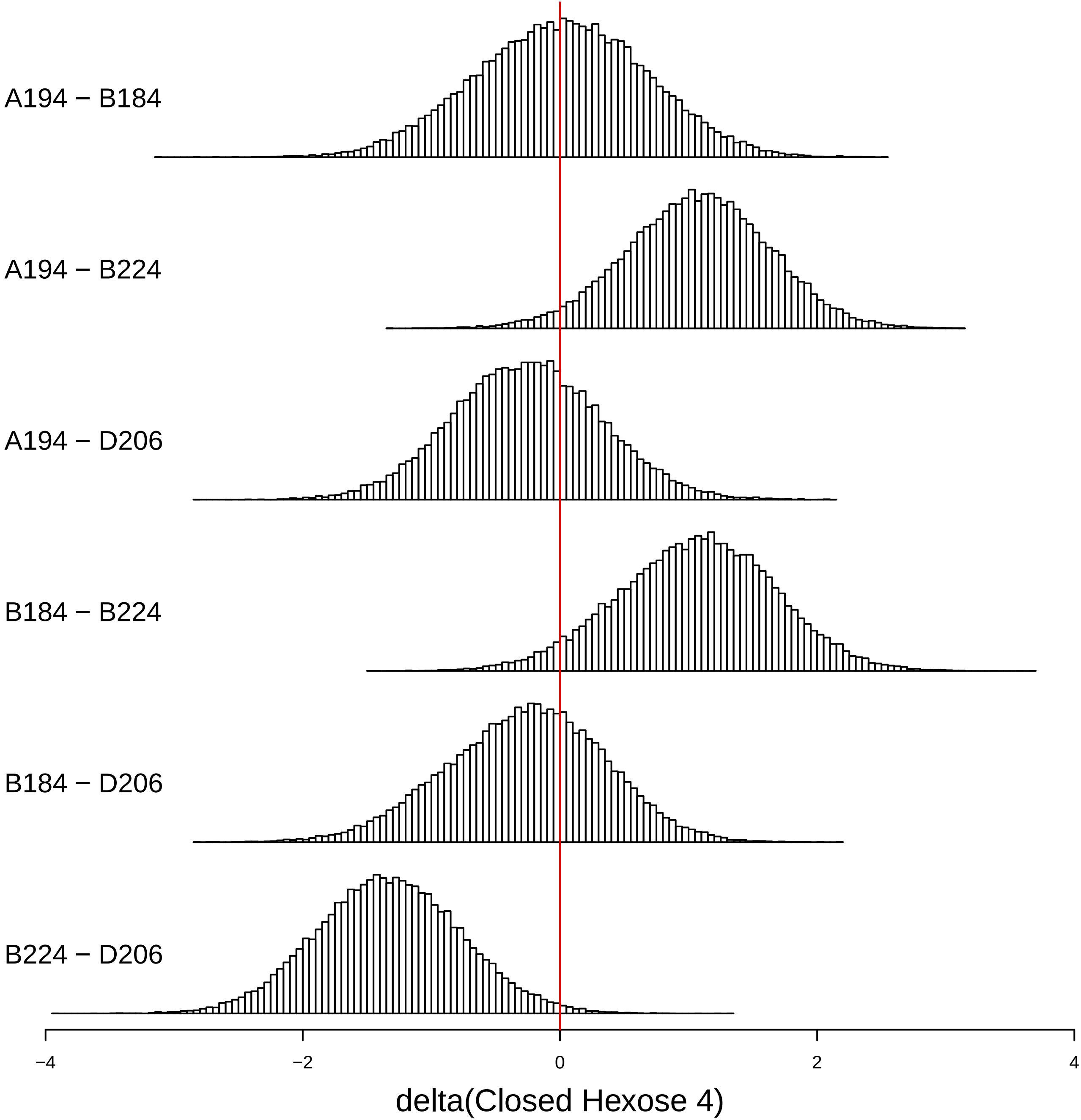

A194 − B184
A194 − B224
A194 − D206
B184 − B224
B184 − D206
B224 − D206
−4
−2
0
2
4
delta(Closed Hexose 4)

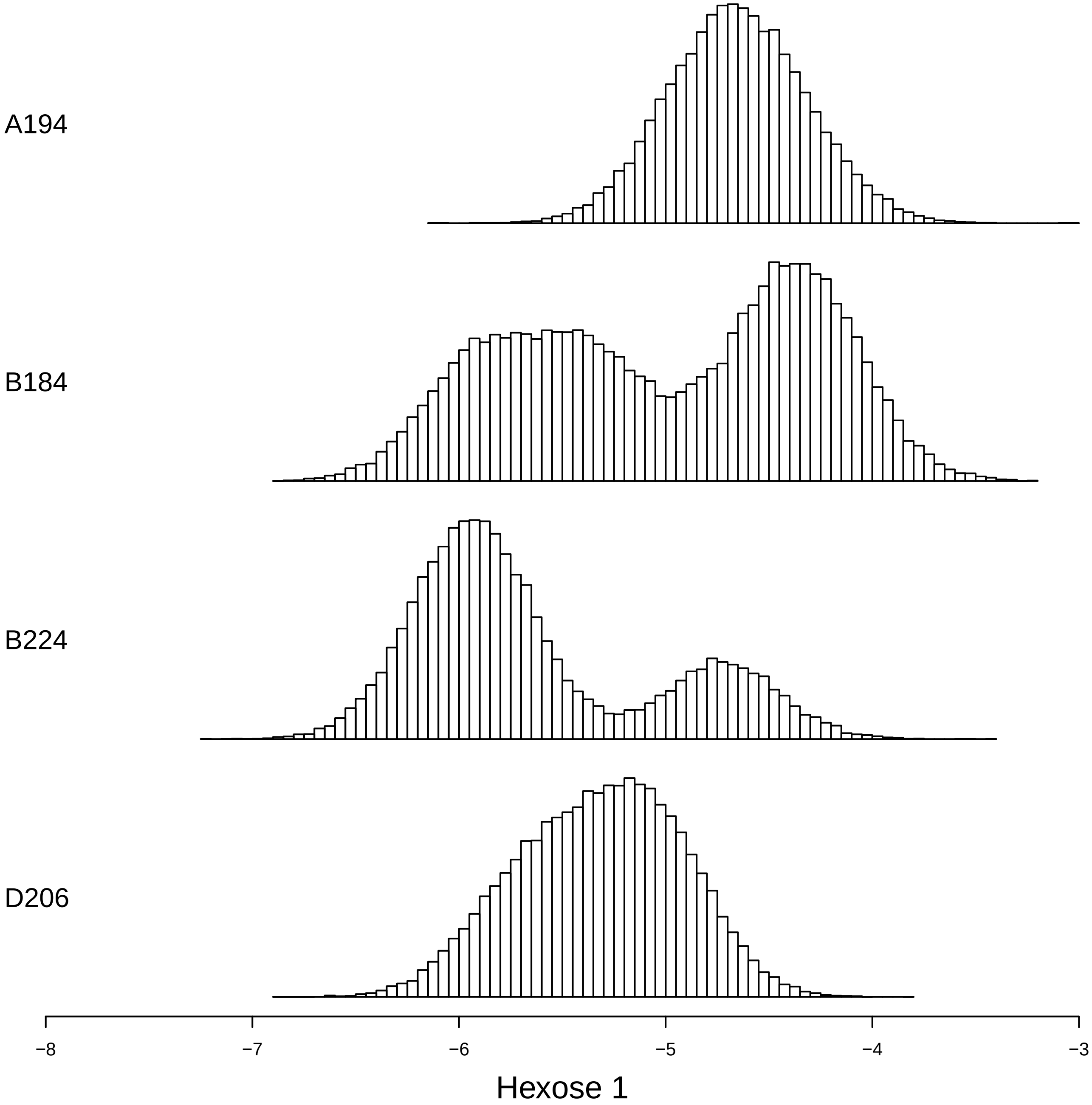
A194
B184
B224
D206
−8
−7
−6
−5
−4
−3
Hexose 1

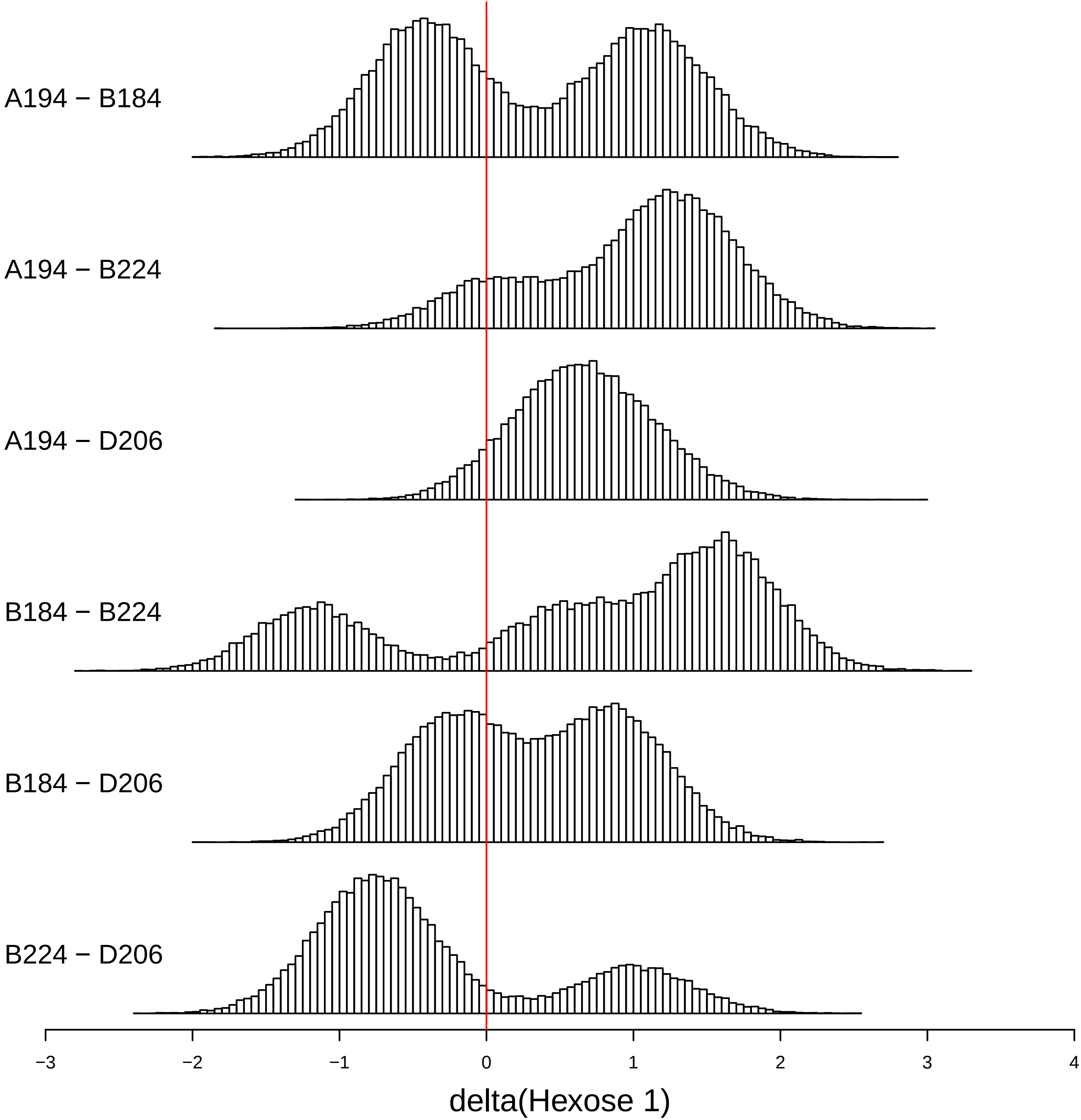

A194 − B184
A194 − B224
A194 − D206
B184 − B224
B184 − D206
B224 − D206
−3
−2
−1
0
1
2
3
4
delta(Hexose 1)

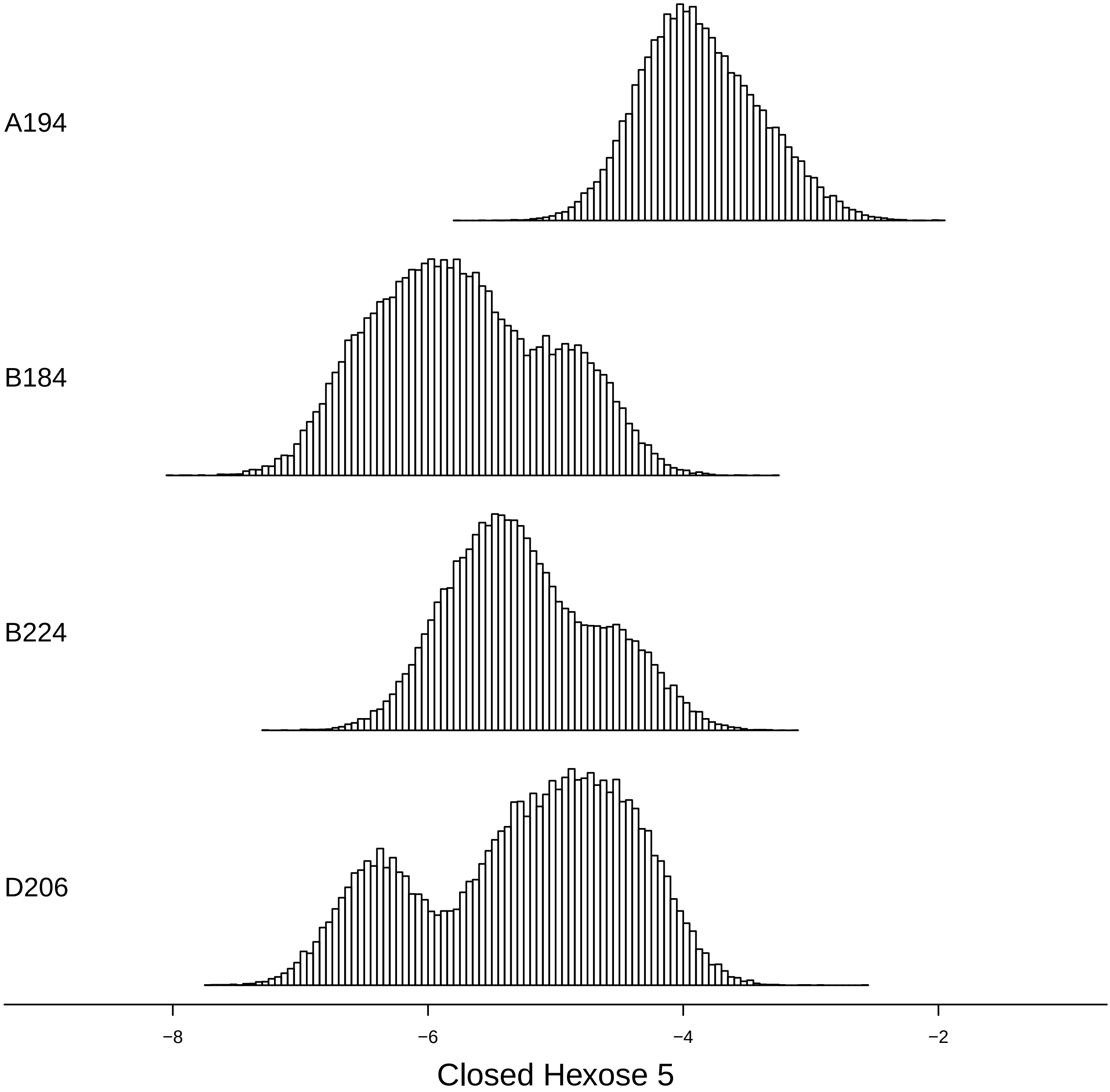
A194
B184
B224
D206
−8
−6
−4
−2
Closed Hexose 5

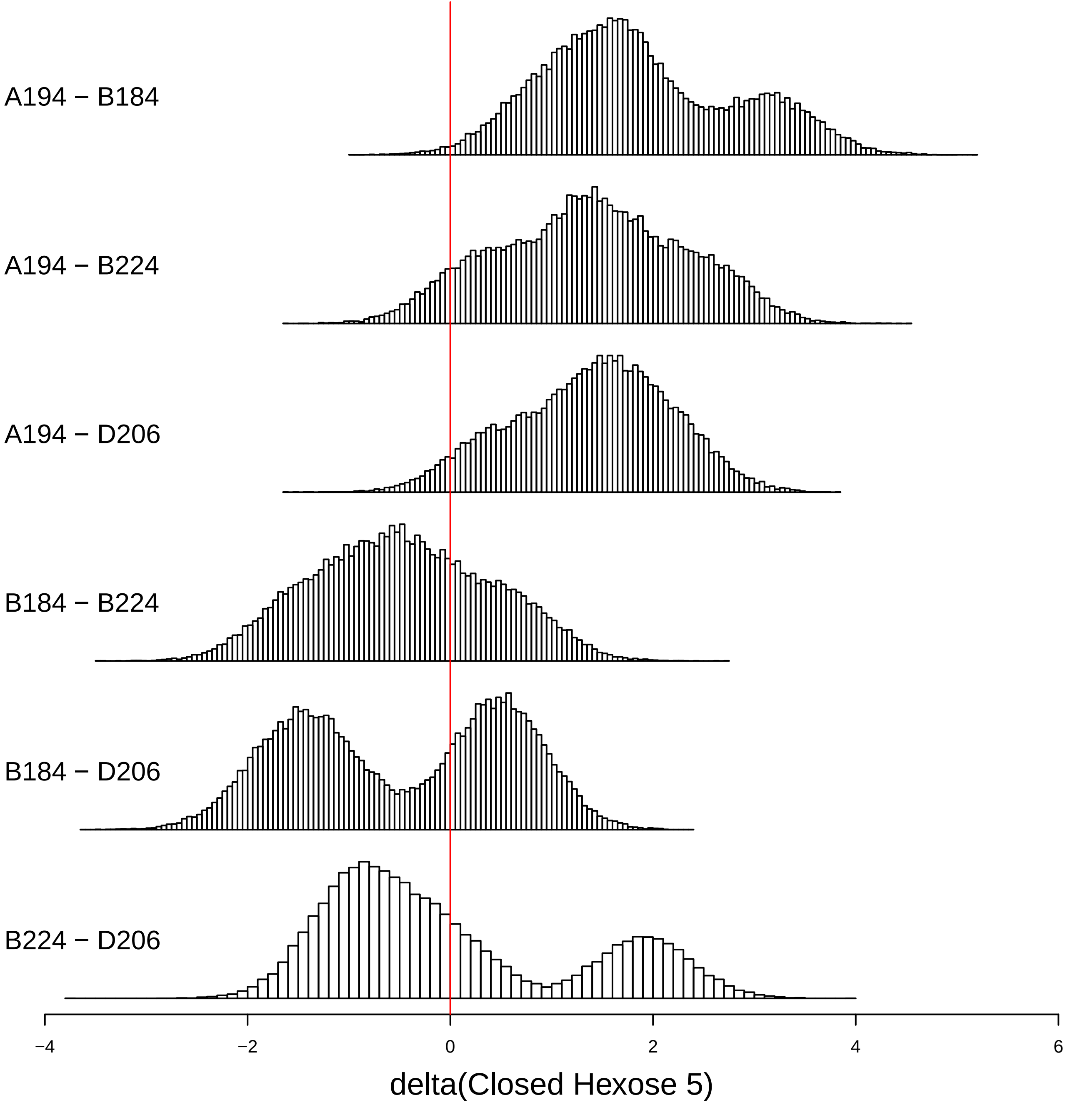
A194 − B184
A194 − B224
A194 − D206
B184 − B224
B184 − D206
B224 − D206
−4
−2
0
2
4
6
delta(Closed Hexose 5)

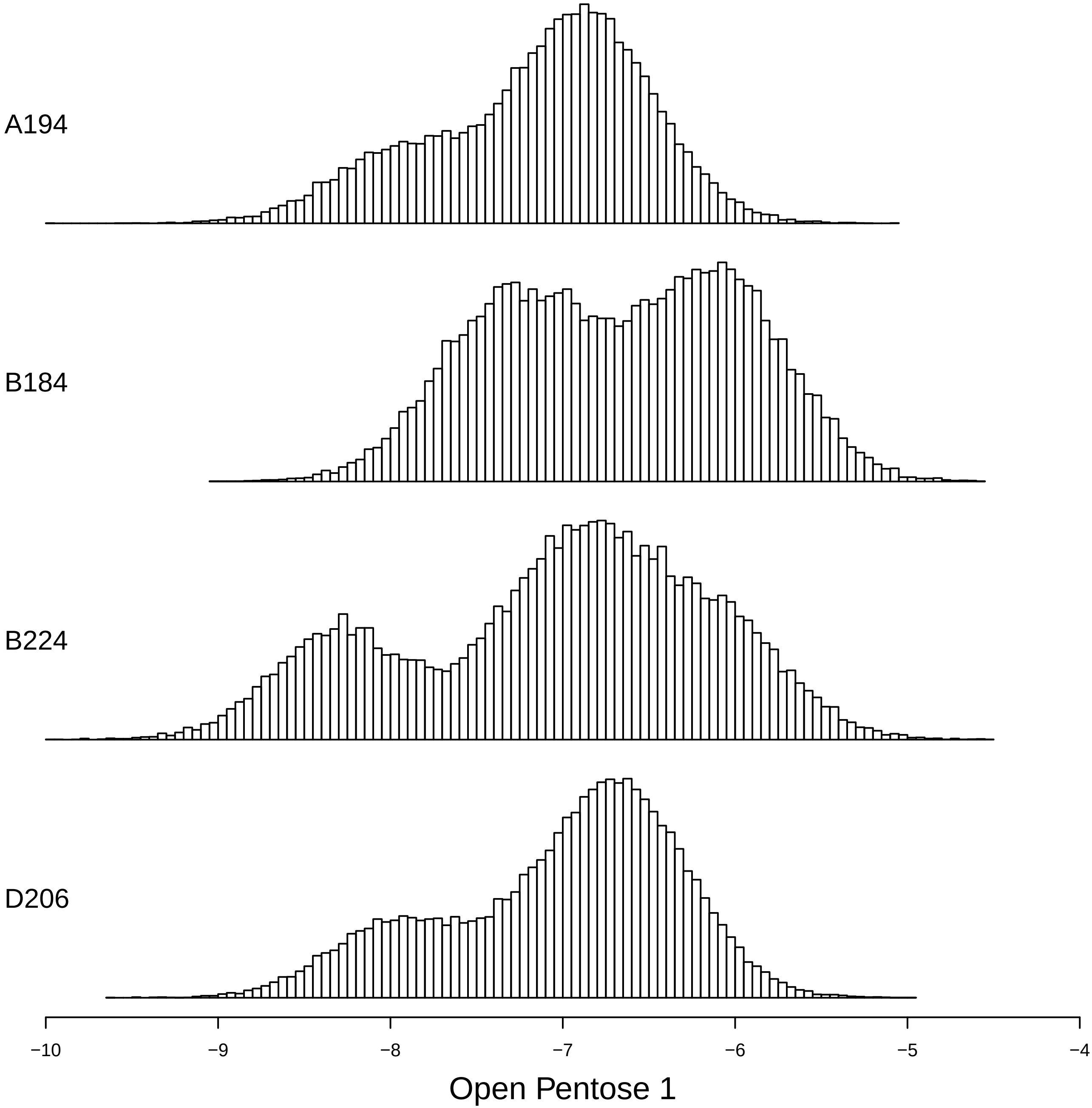

A194
B184
B224
D206
−10
−9
−8
−7
−6
−5
−4
Open Pentose 1

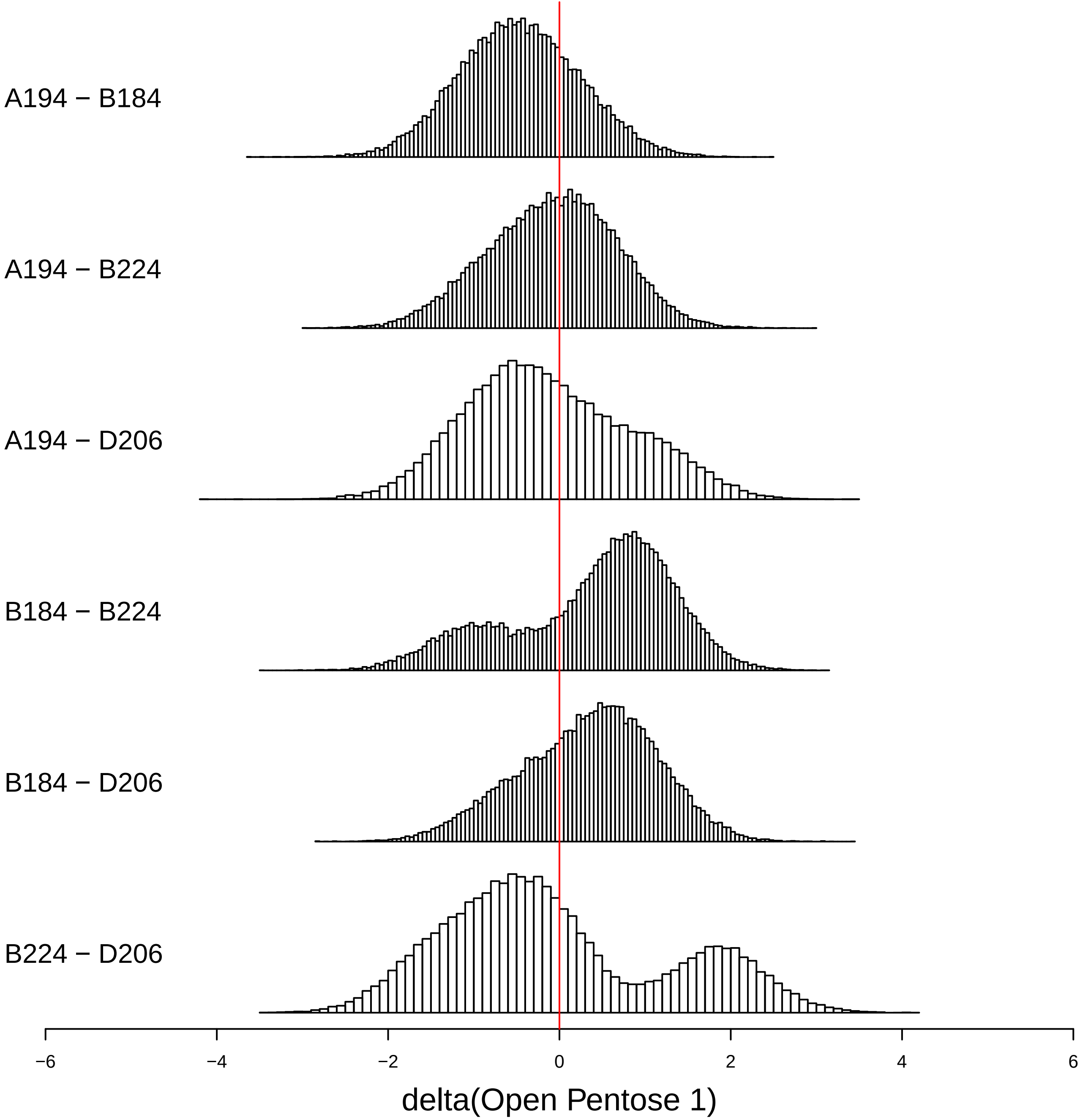
A194 − B184
A194 − B224
A194 − D206
B184 − B224
B184 − D206
B224 − D206
−6
−4
−2
0
2
4
6
delta(Open Pentose 1)

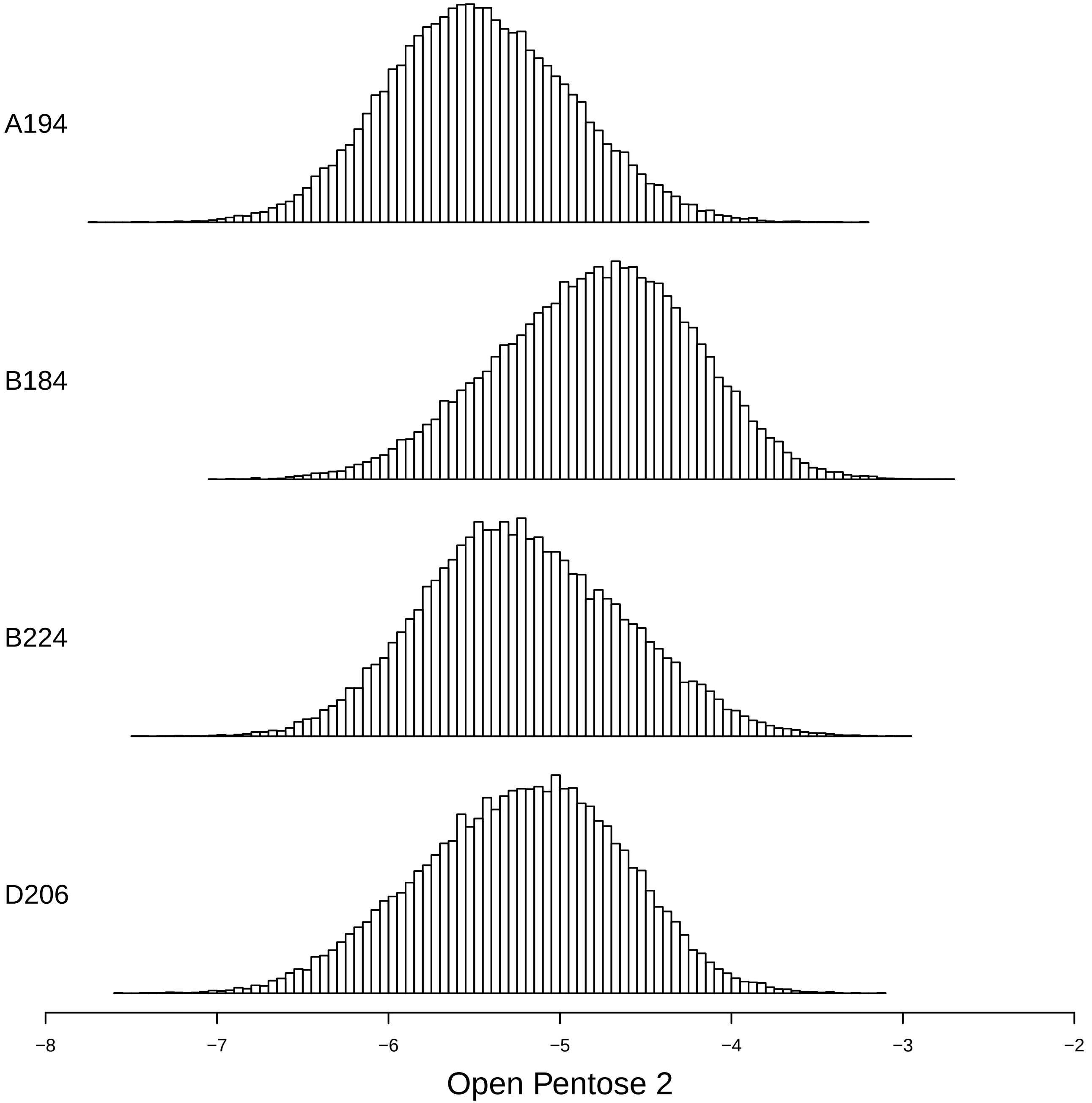
A194
B184
B224
D206
−8
−7
−6
−5
−4
−3
−2
Open Pentose 2

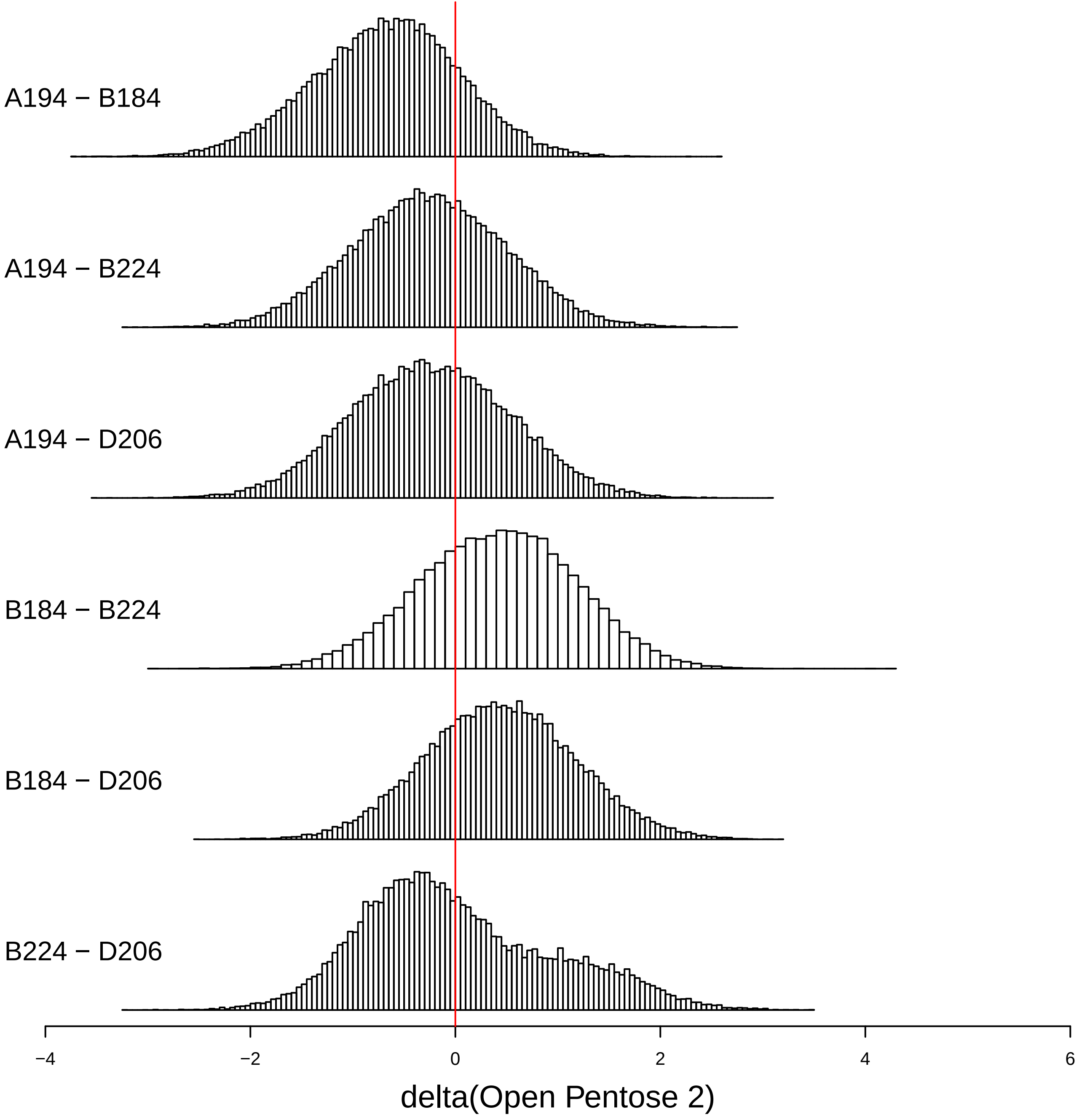
A194 − B184
A194 − B224
A194 − D206
B184 − B224
B184 − D206
B224 − D206
−4
−2
0
2
4
6
delta(Open Pentose 2)

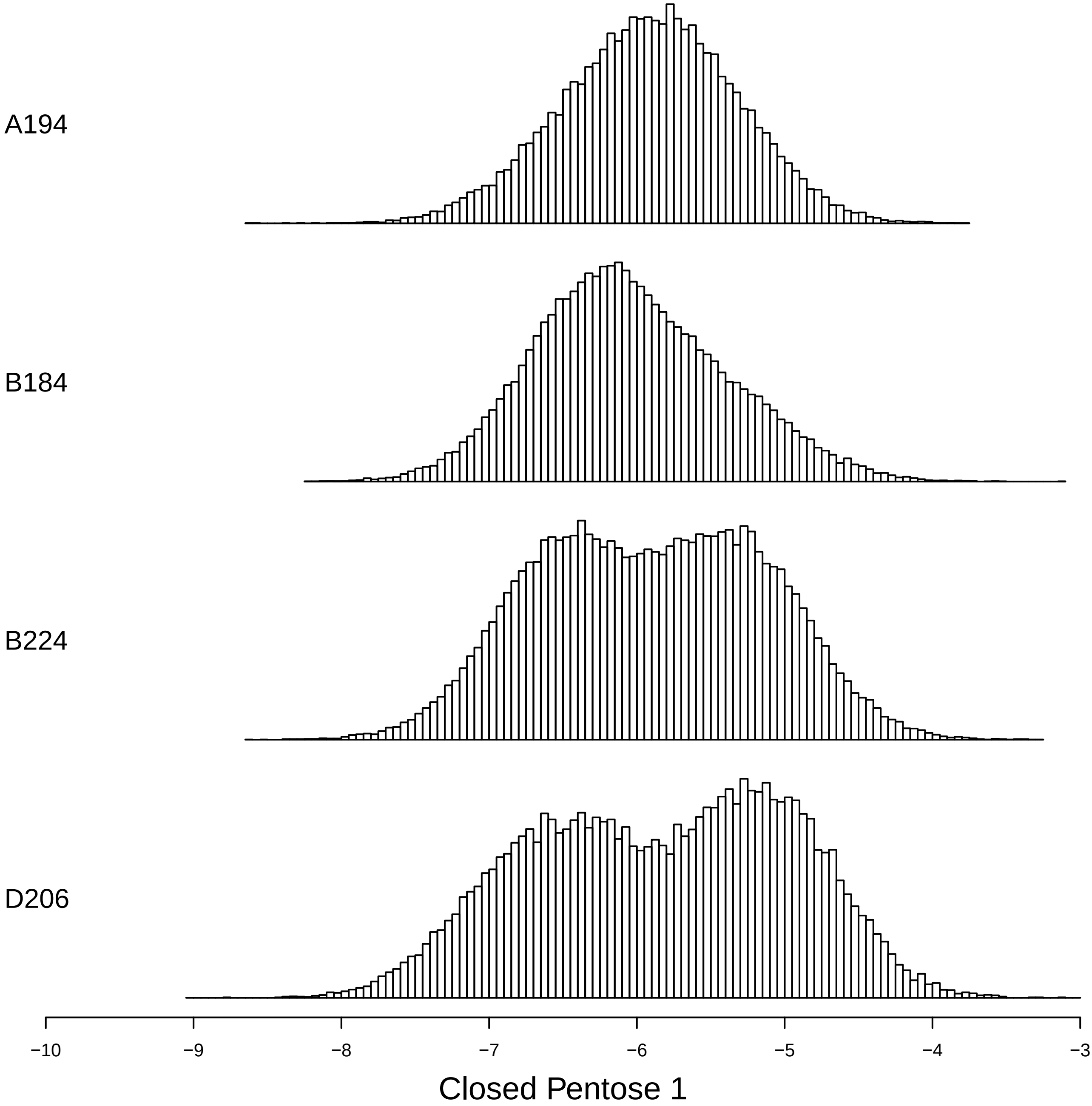
A194
B184
B224
D206
−10
−9
−8
−7
−6
−5
−4
−3
Closed Pentose 1

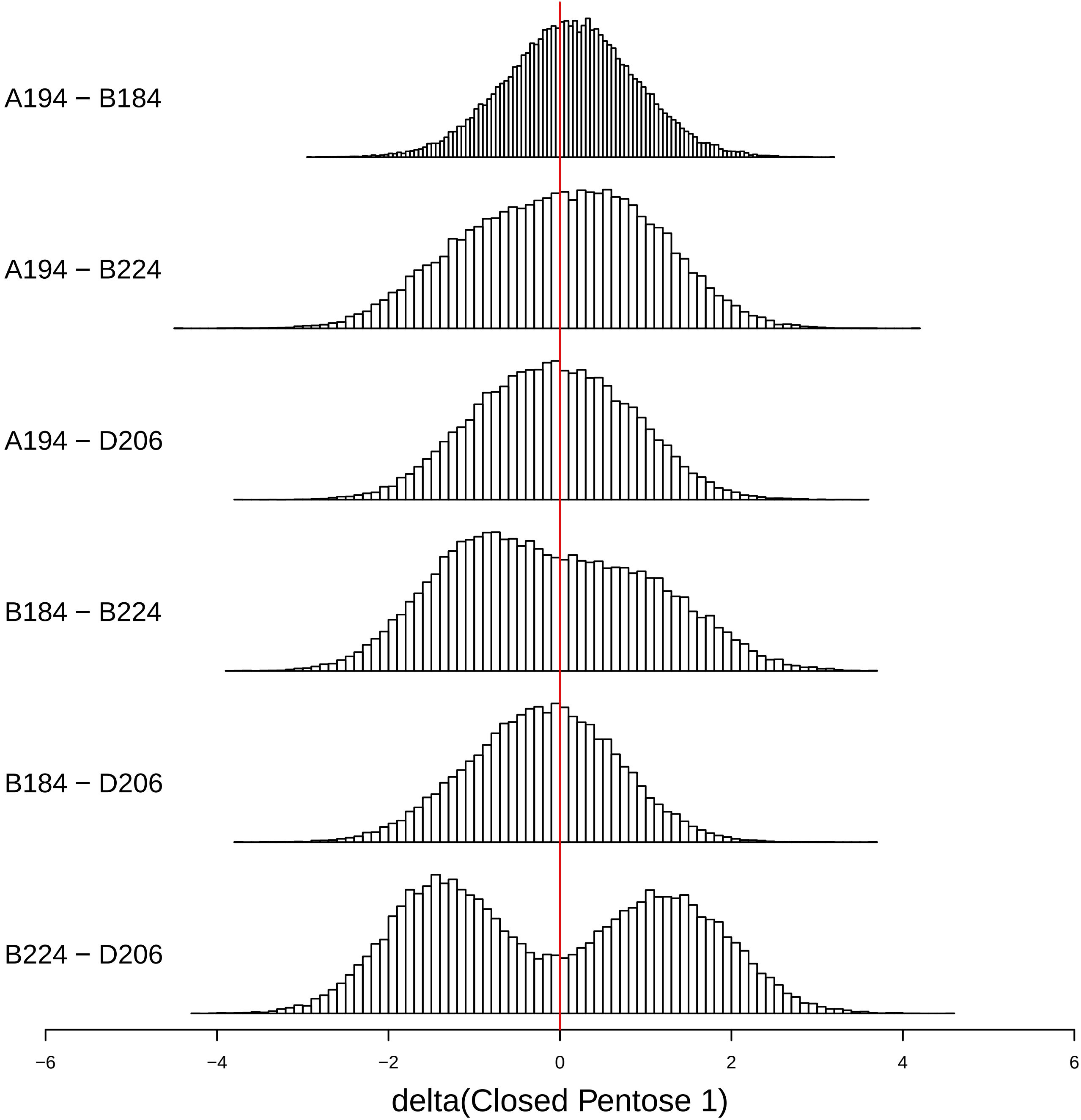

A194 − B184
A194 − B224
A194 − D206
B184 − B224
B184 − D206
B224 − D206
−6
−4
−2
0
2
4
6
delta(Closed Pentose 1)

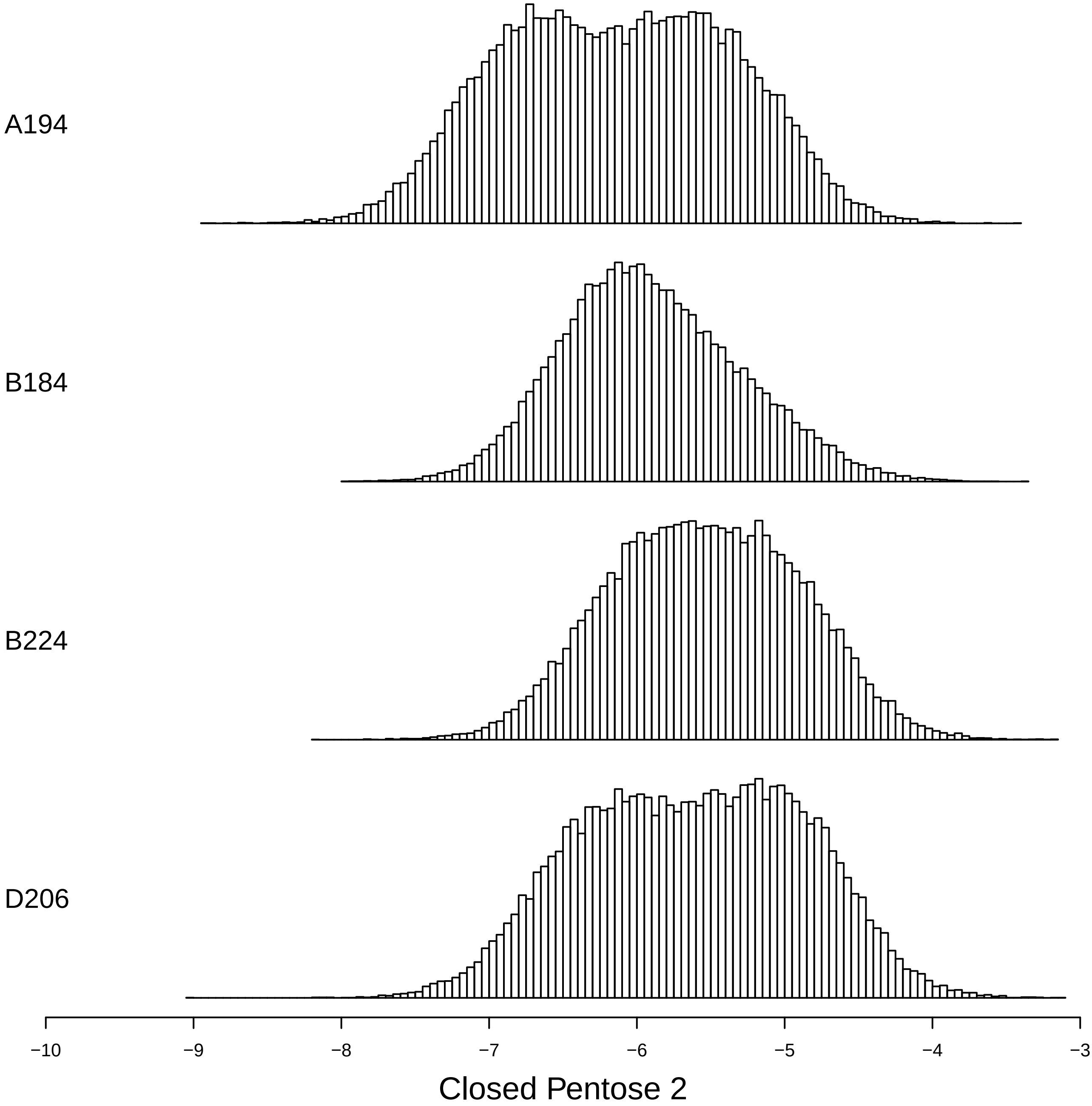
A194
B184
B224
D206
−10
−9
−8
−7
−6
−5
−4
−3
Closed Pentose 2

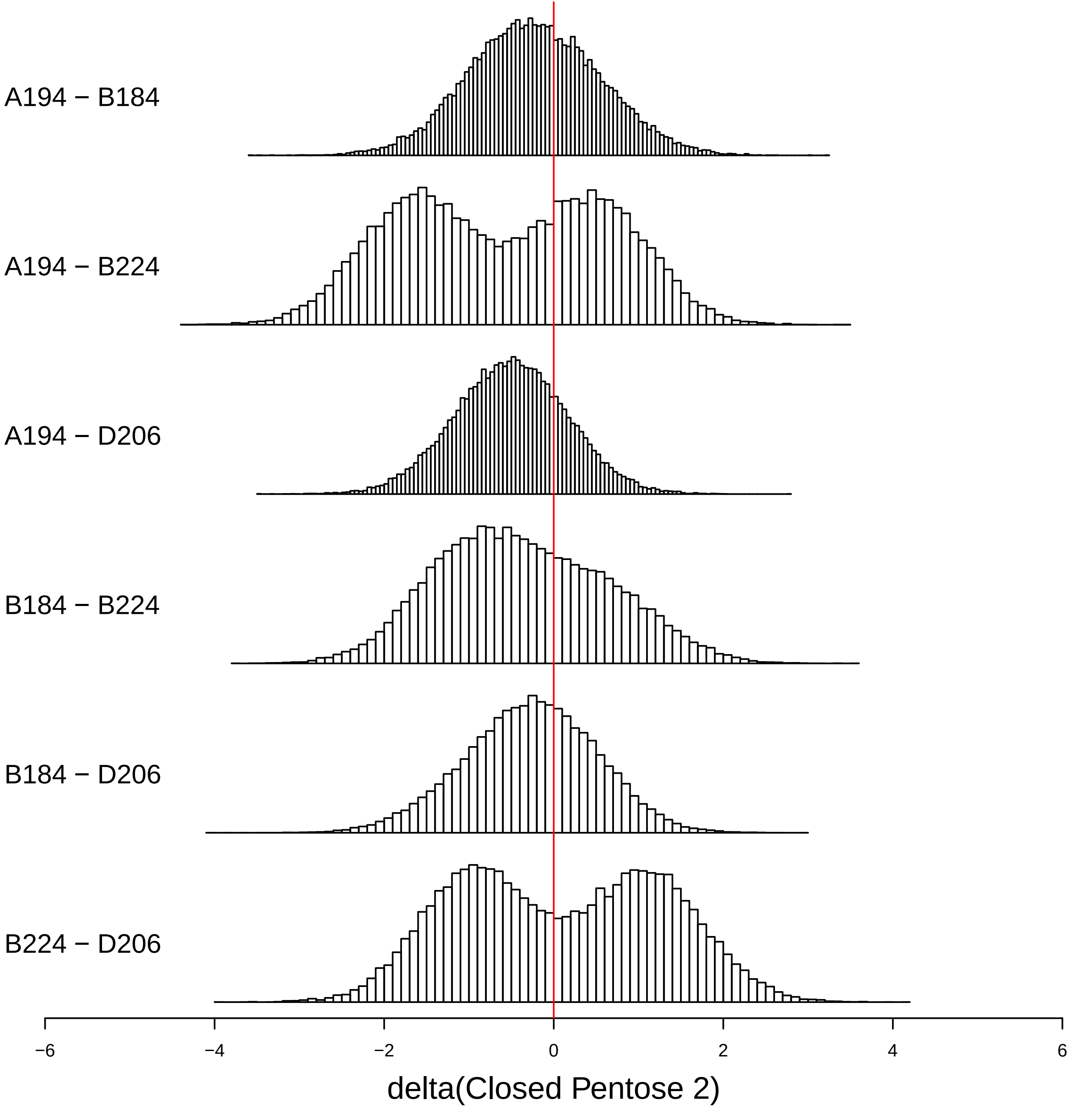
A194 − B184
A194 − B224
A194 − D206
B184 − B224
B184 − D206
B224 − D206
−6
−4
−2
0
2
4
6
delta(Closed Pentose 2)

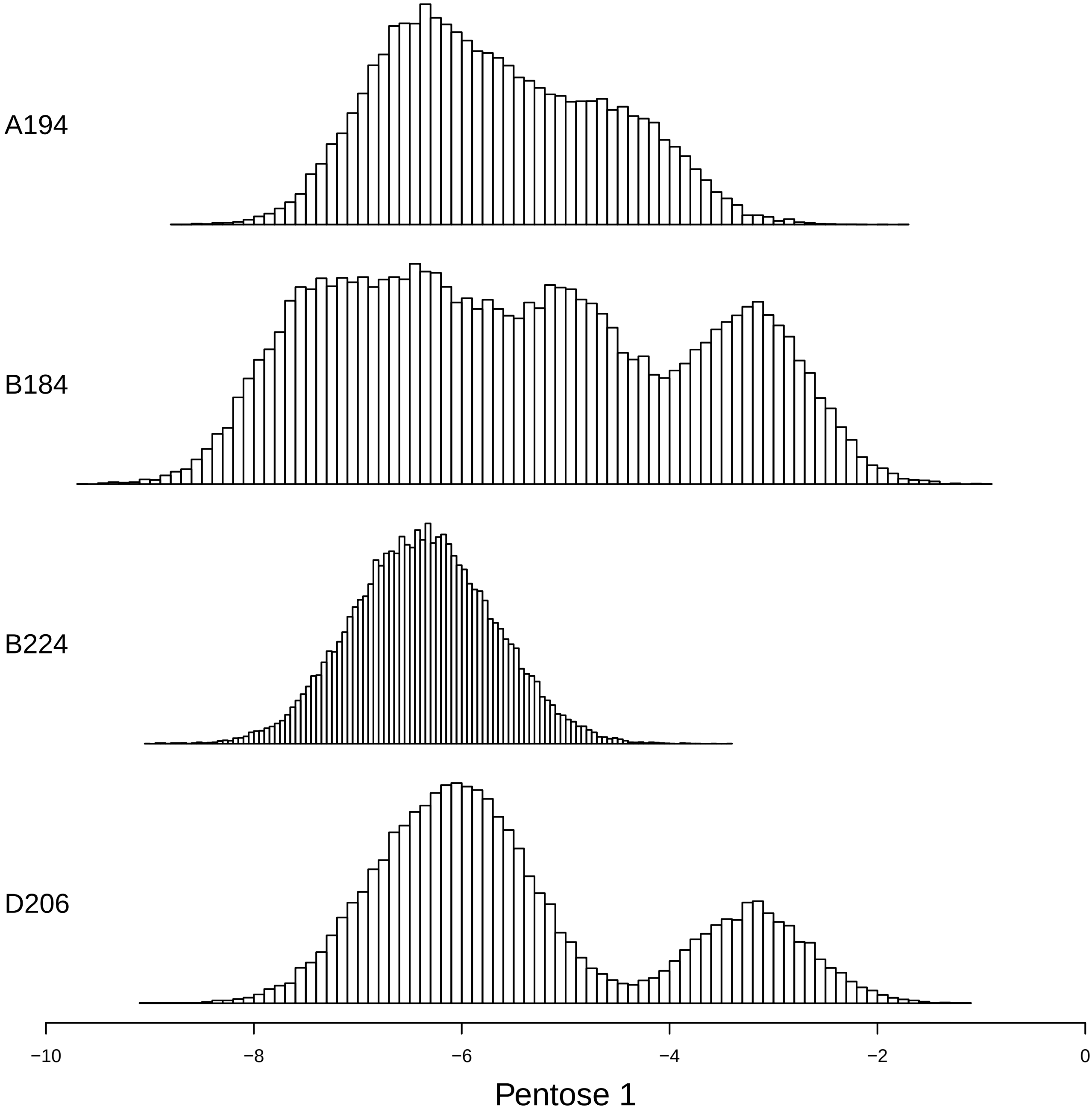
A194
B184
B224
D206
−10
−8
−6
−4
−2
0
Pentose 1

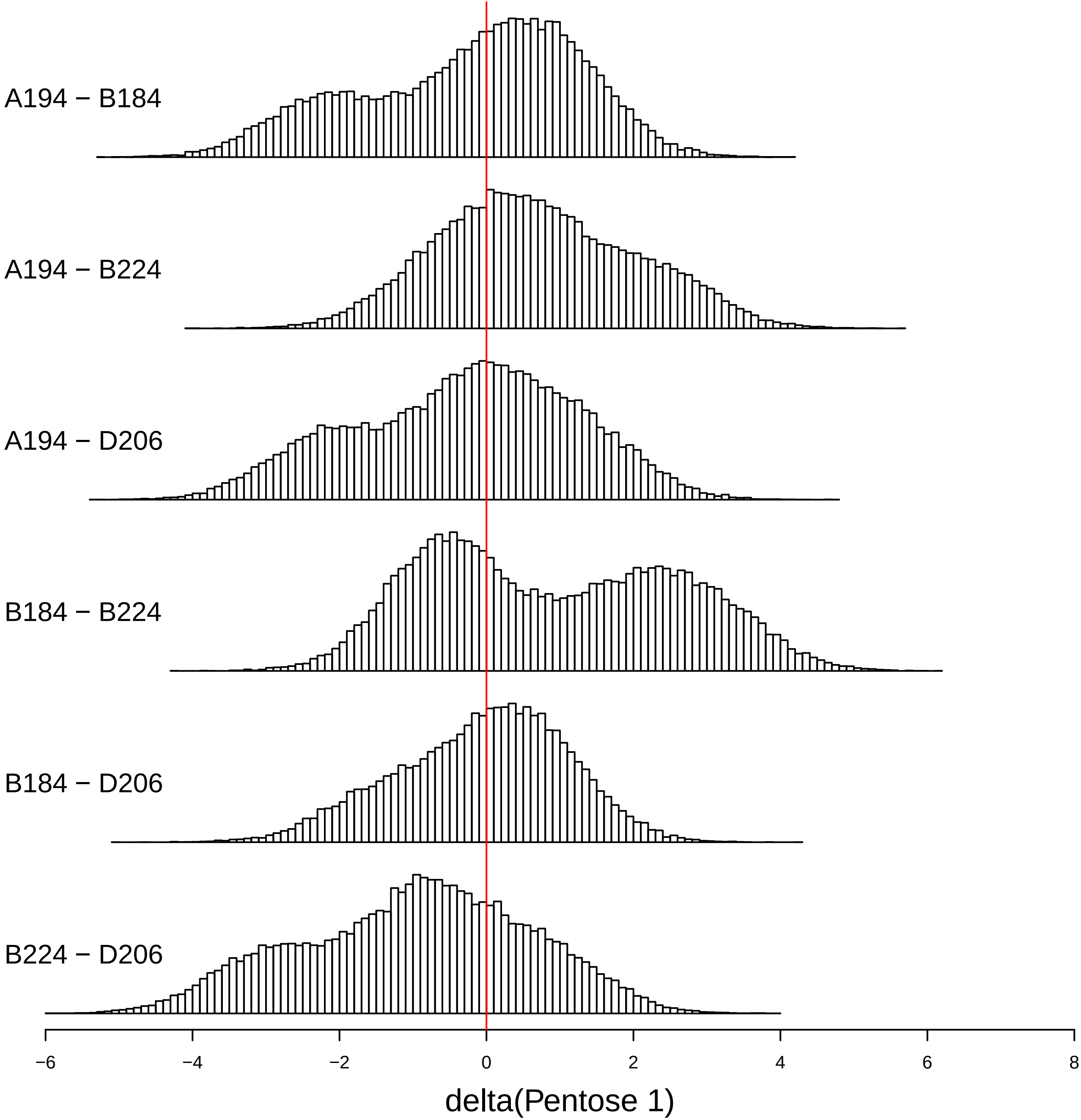
A194 − B184
A194 − B224
A194 − D206
B184 − B224
B184 − D206
B224 − D206
−6
−4
−2
0
2
4
6
8
delta(Pentose 1)

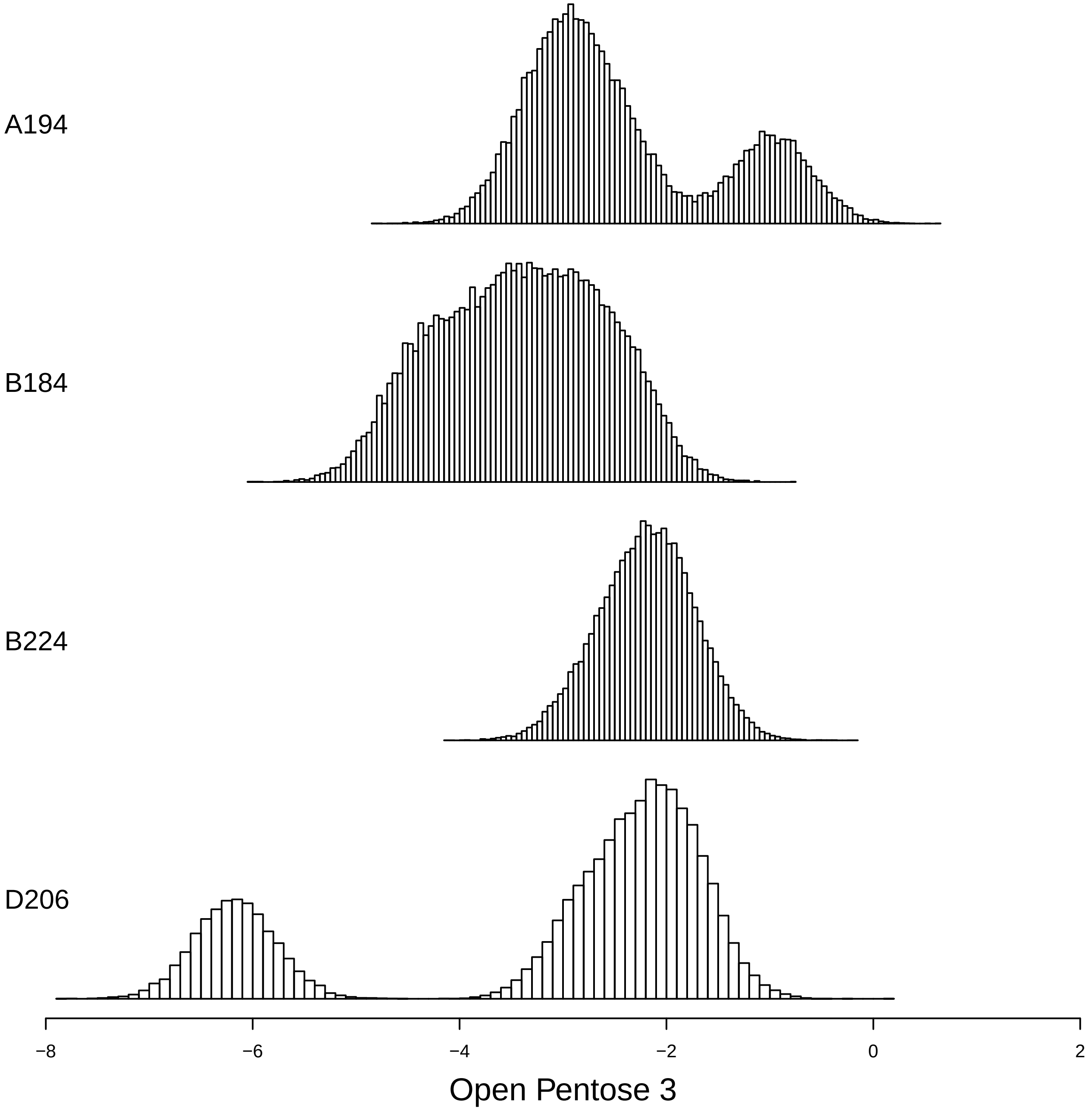
A194
B184
B224
D206
−8
−6
−4
−2
0
2
Open Pentose 3

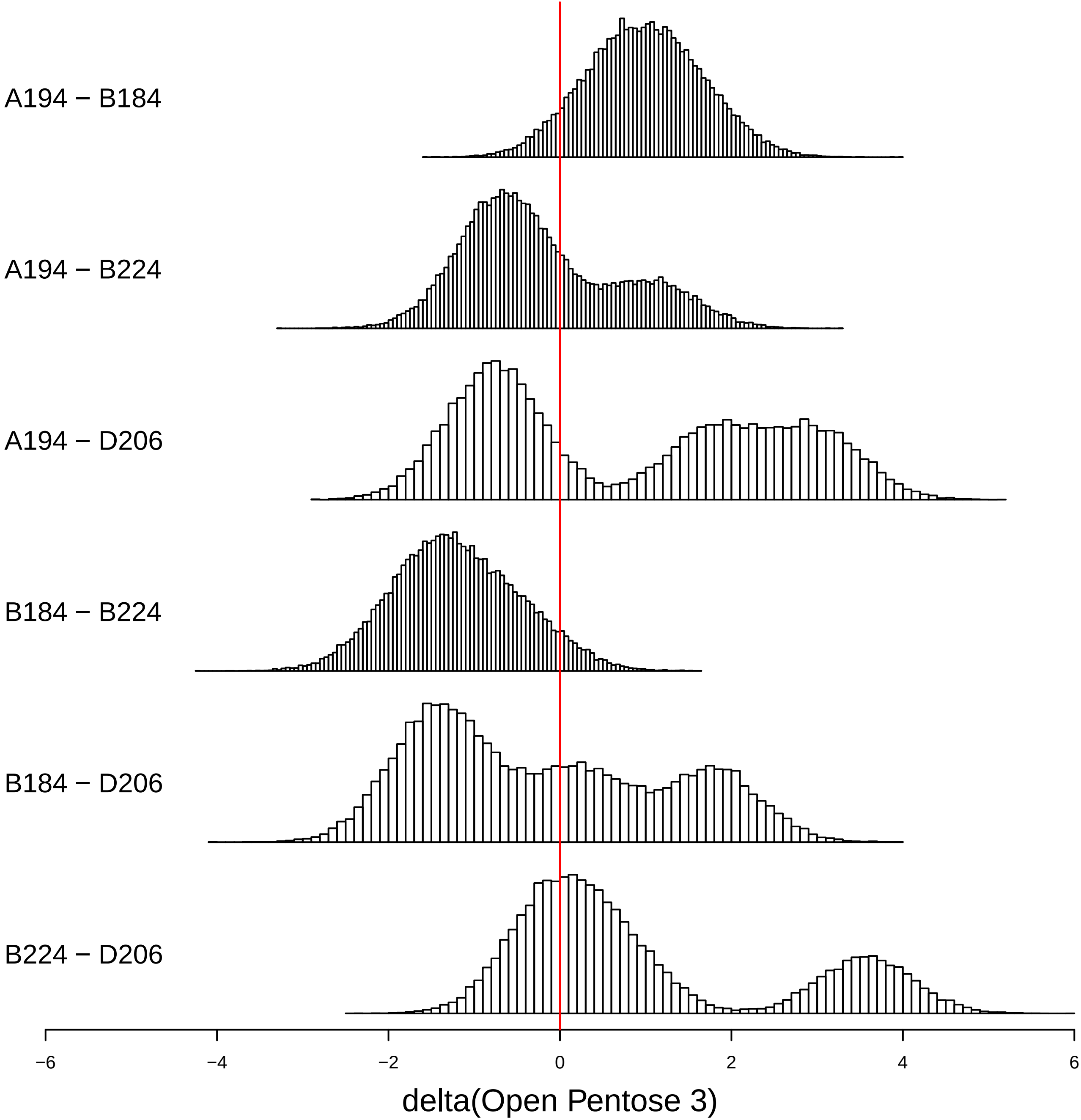
A194 − B184
A194 − B224
A194 − D206
B184 − B224
B184 − D206
B224 − D206
−6
−4
−2
0
2
4
6
delta(Open Pentose 3)

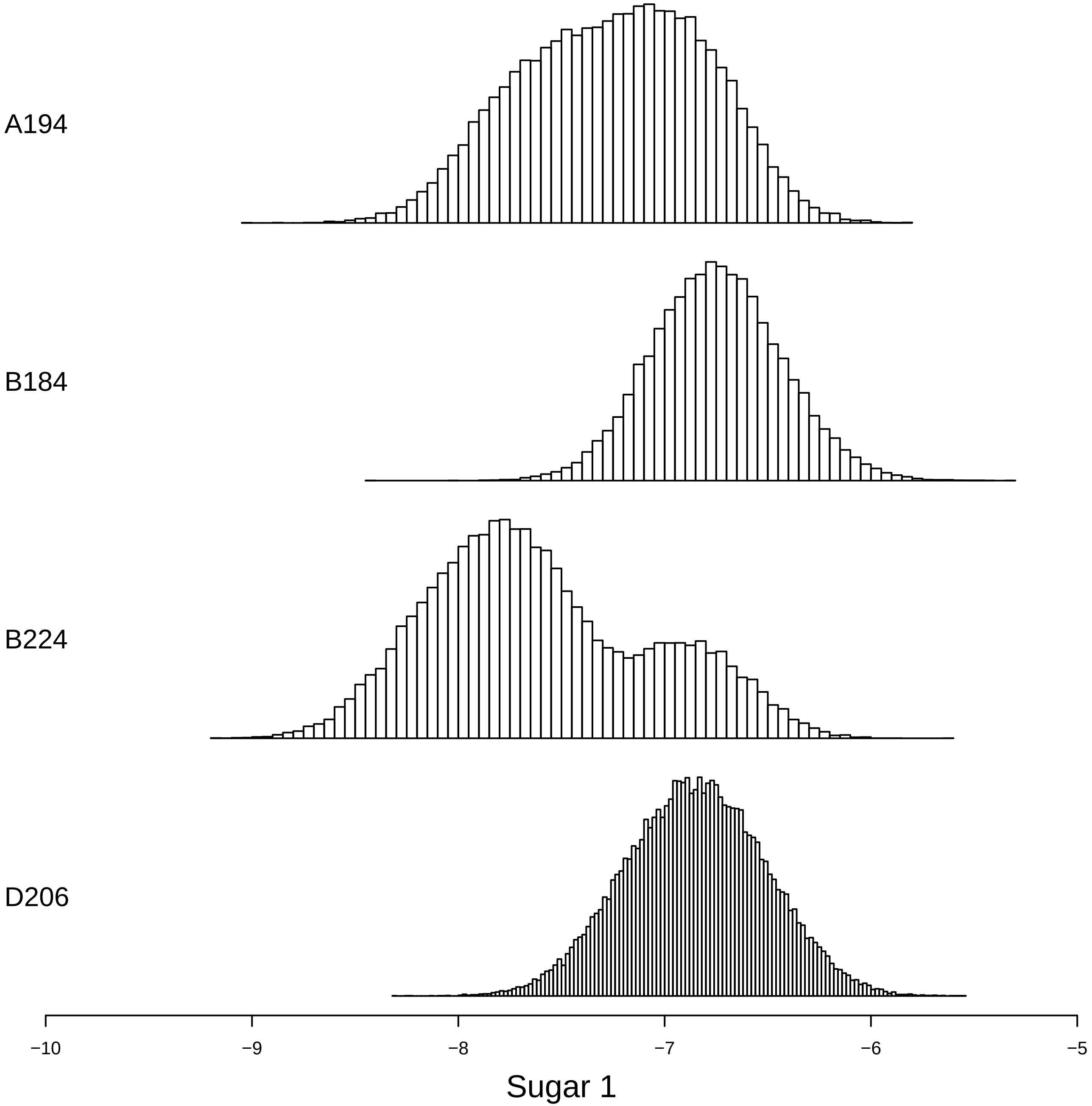
A194
B184
B224
D206
−10
−9
−8
−7
−6
−5
Sugar 1

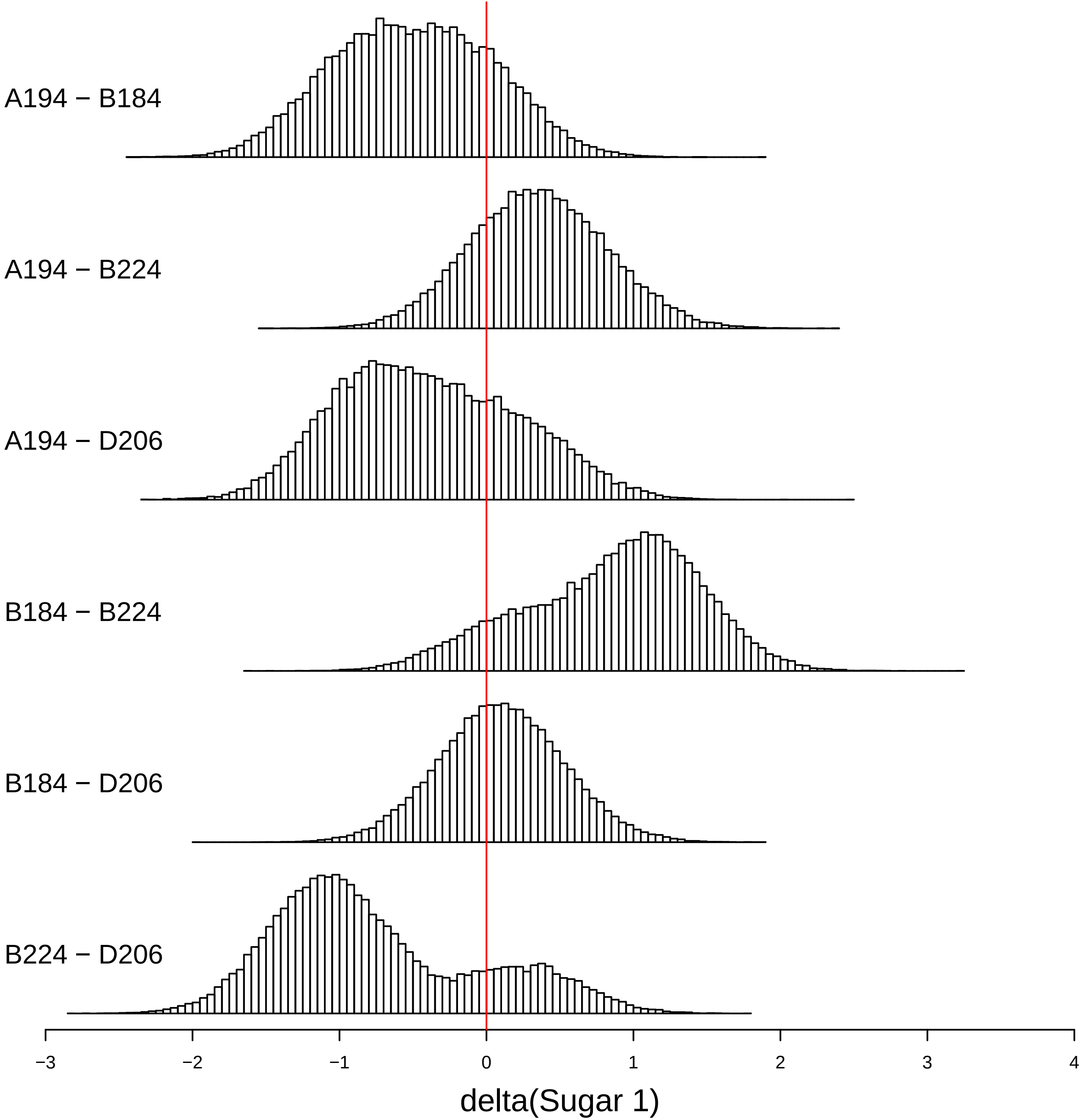
A194 − B184
A194 − B224
A194 − D206
B184 − B224
B184 − D206
B224 − D206
−3
−2
−1
0
1
2
3
4
delta(Sugar 1)

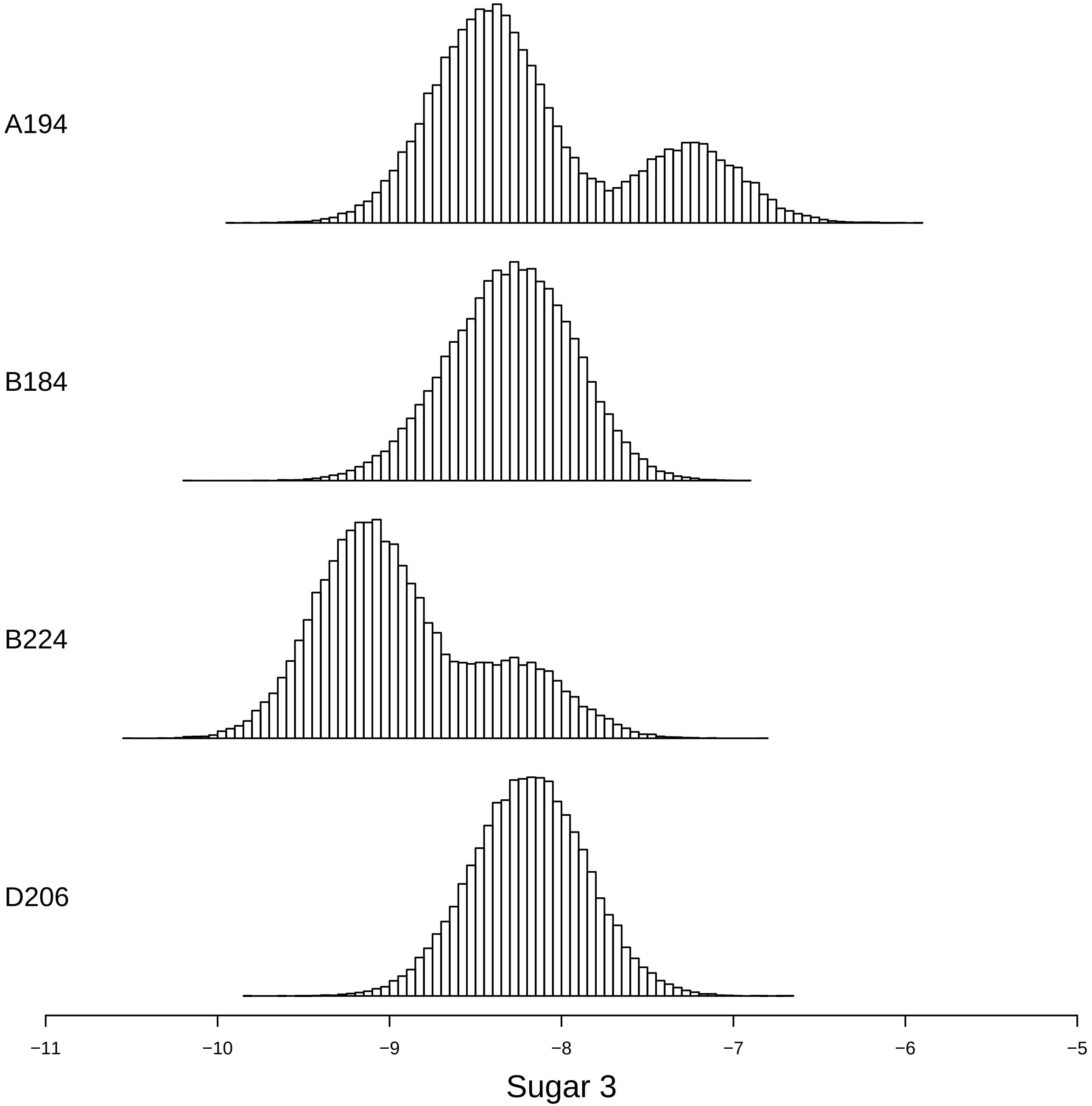
A194
B184
B224
D206
−11
−10
−9
−8
−7
−6
−5
Sugar 3

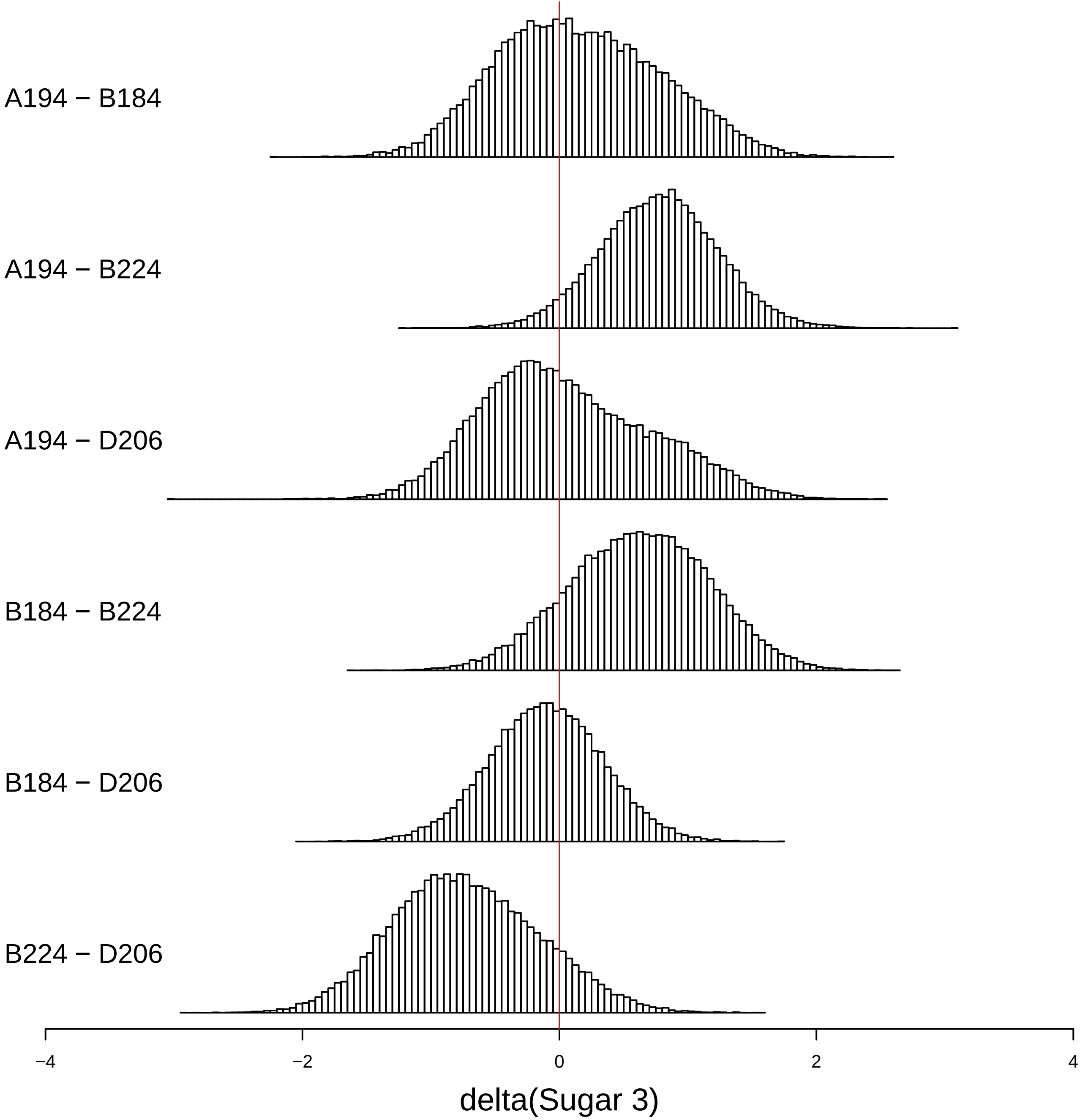
A194 − B184
A194 − B224
A194 − D206
B184 − B224
B184 − D206
B224 − D206
−4
−2
0
2
4
delta(Sugar 3)

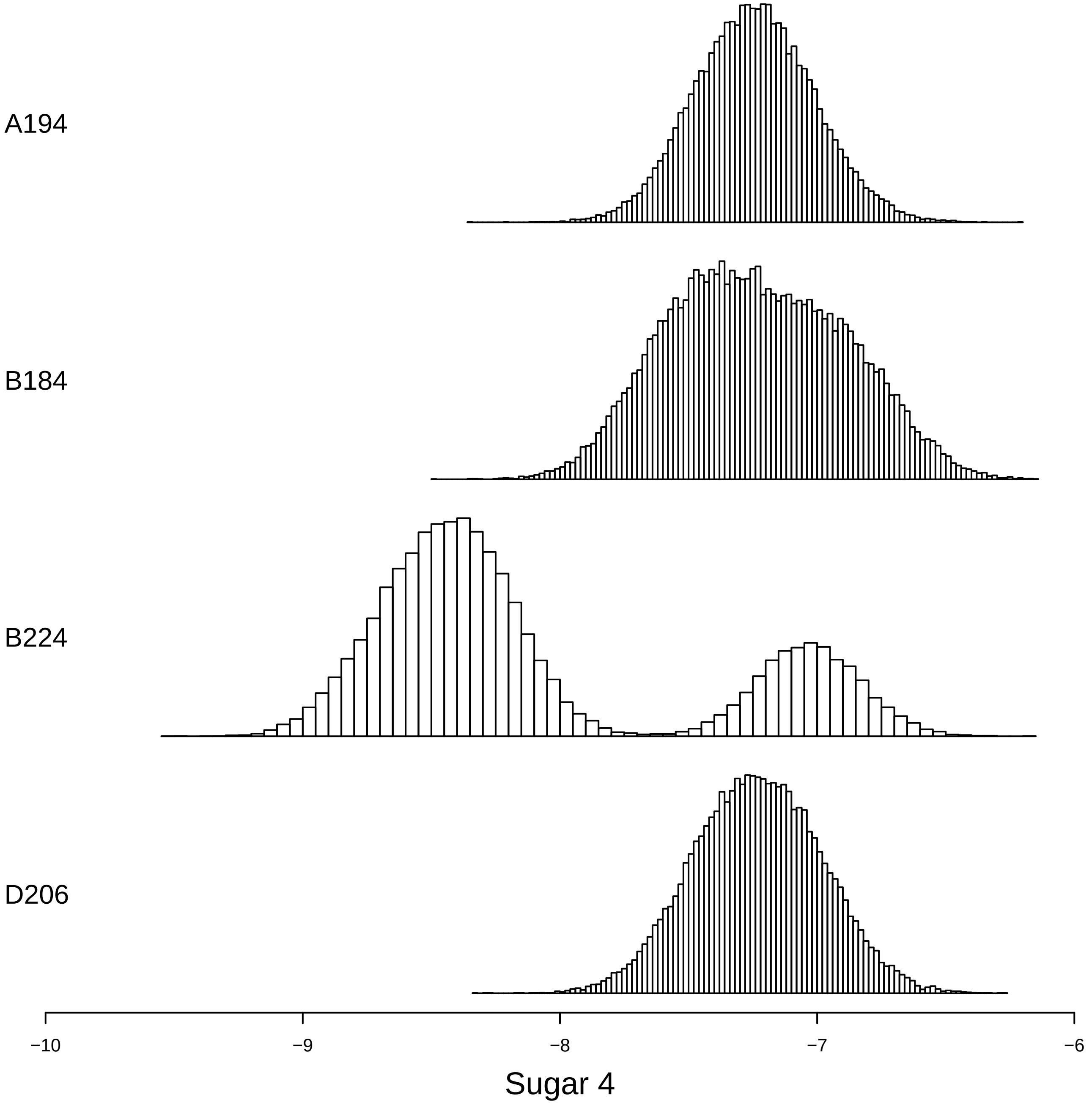
A194
B184
B224
D206
−10
−9
−8
−7
−6
Sugar 4

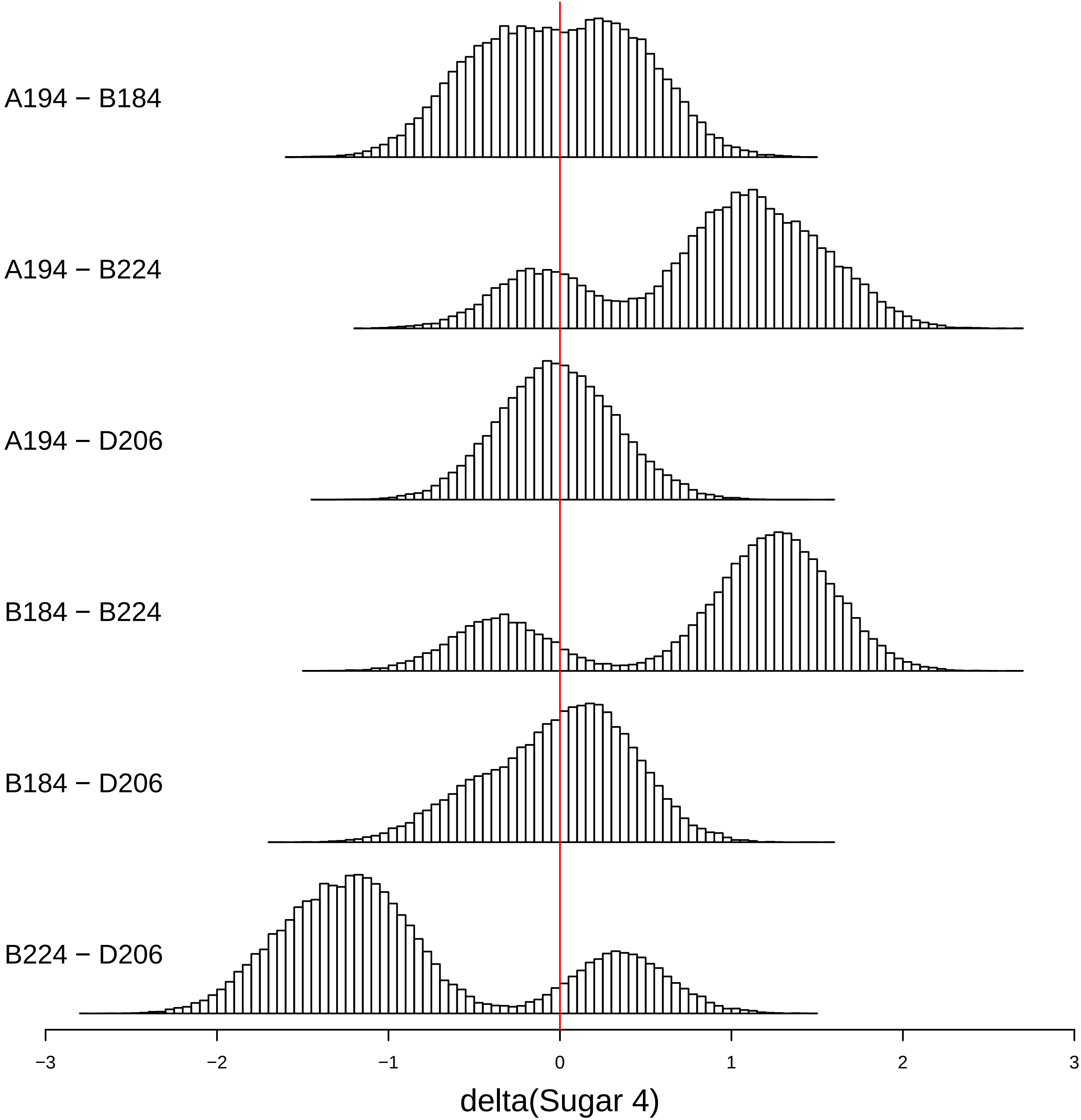

A194 − B184
A194 − B224
A194 − D206
B184 − B224
B184 − D206
B224 − D206
−3
−2
−1
0
1
2
3
delta(Sugar 4)

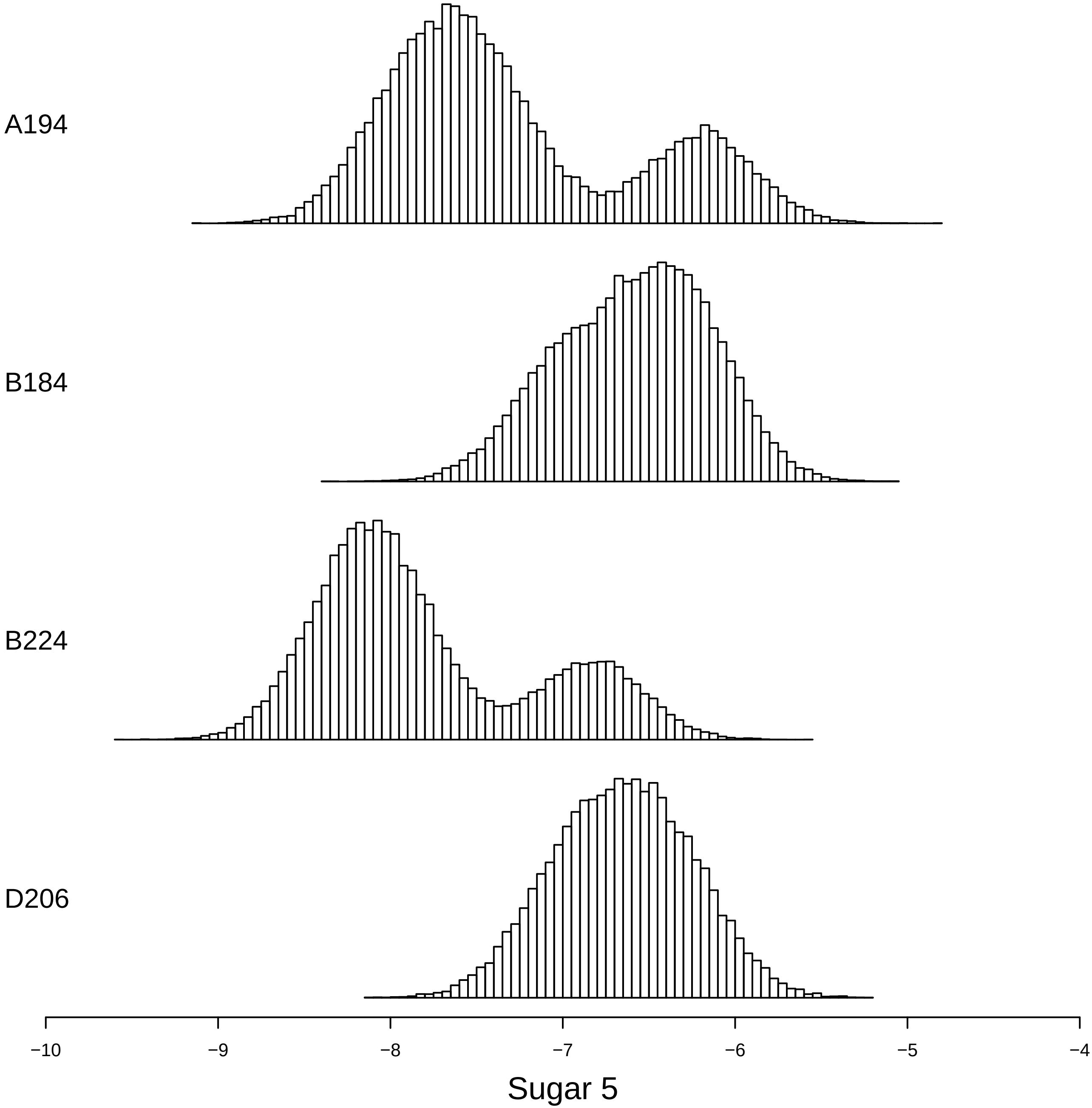

A194
B184
B224
D206
−10
−9
−8
−7
−6
−5
−4
Sugar 5

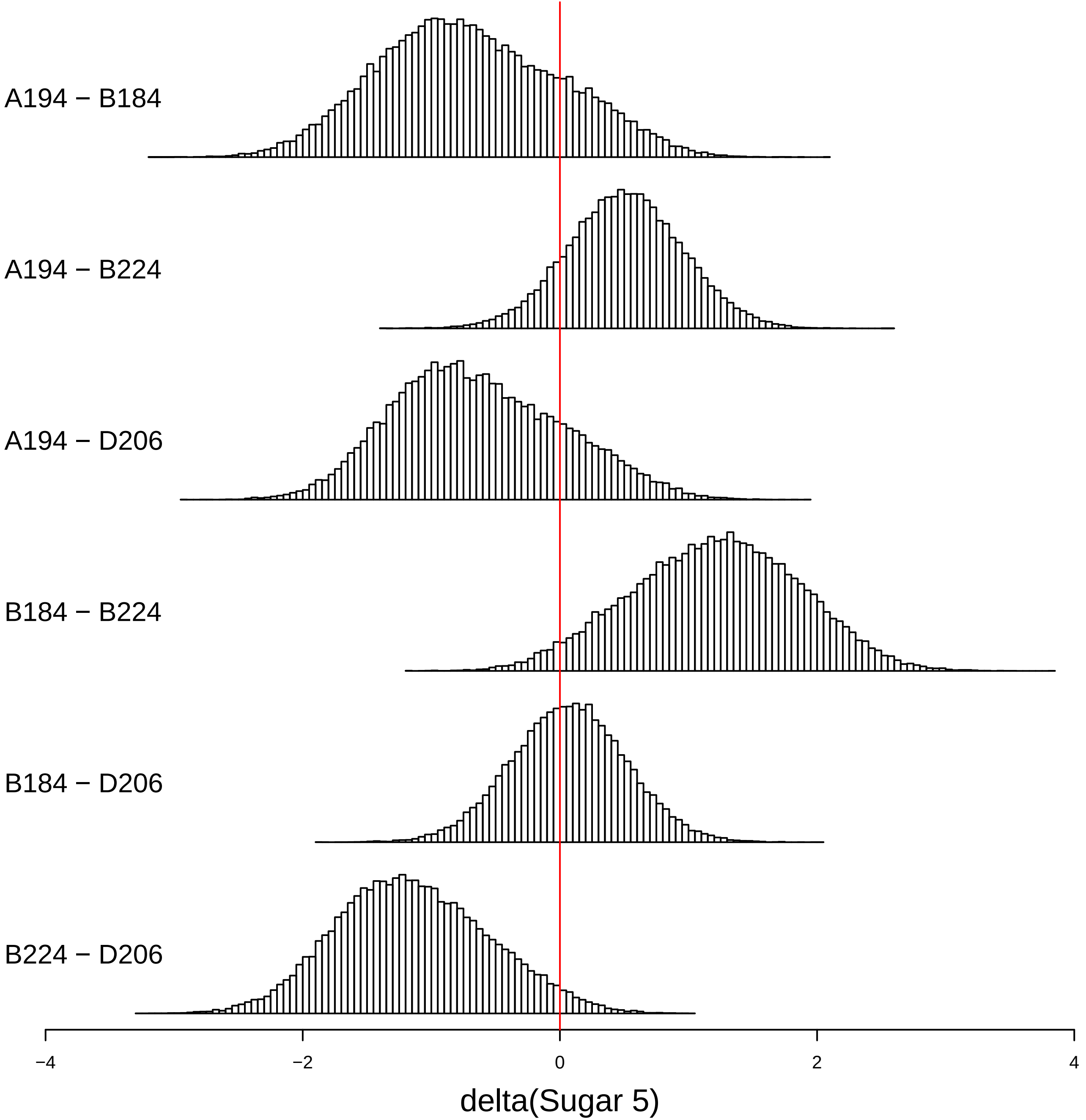
A194 − B184
A194 − B224
A194 − D206
B184 − B224
B184 − D206
B224 − D206
−4
−2
0
2
4
delta(Sugar 5)

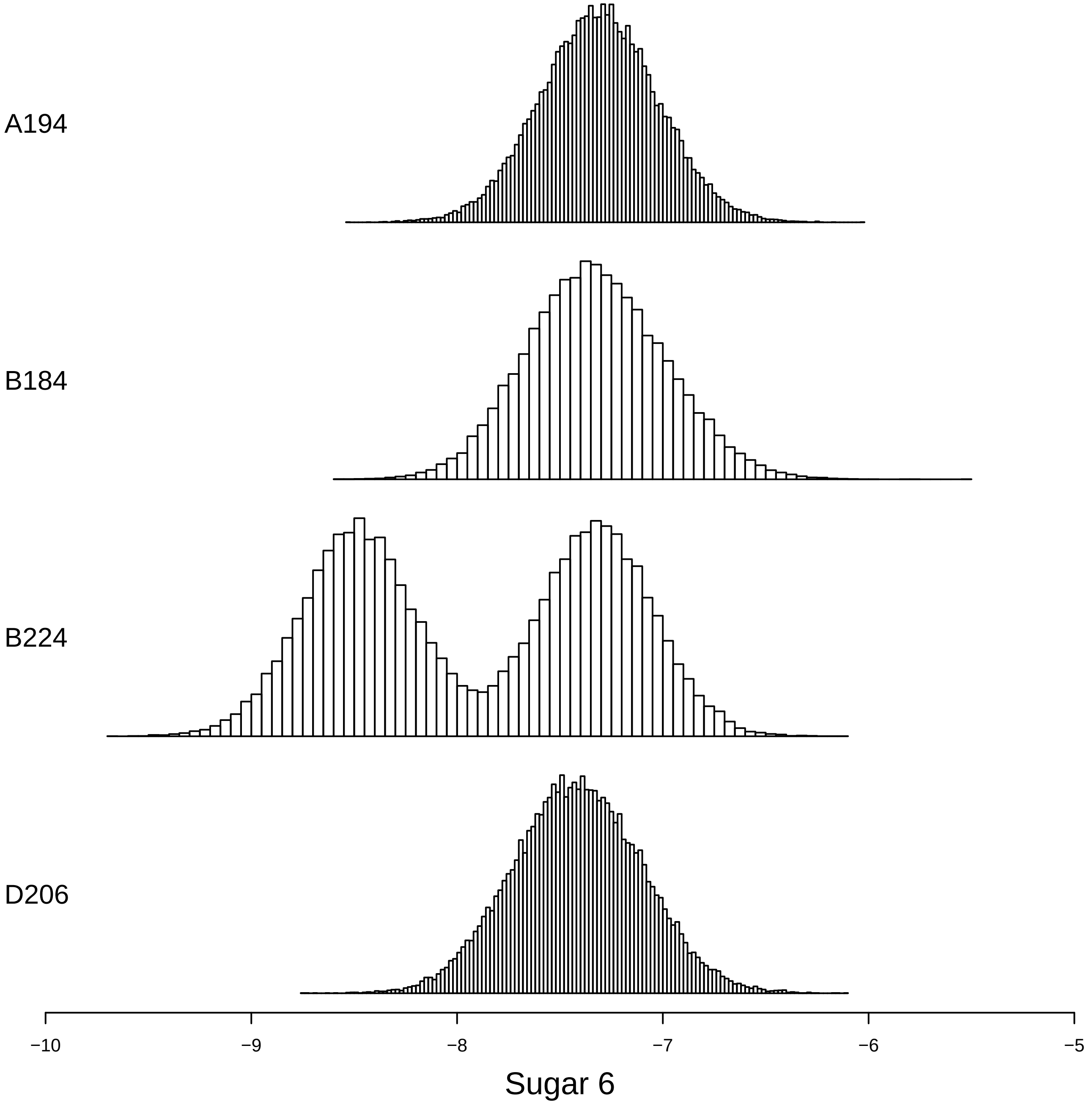

A194
B184
B224
D206
−10
−9
−8
−7
−6
−5
Sugar 6

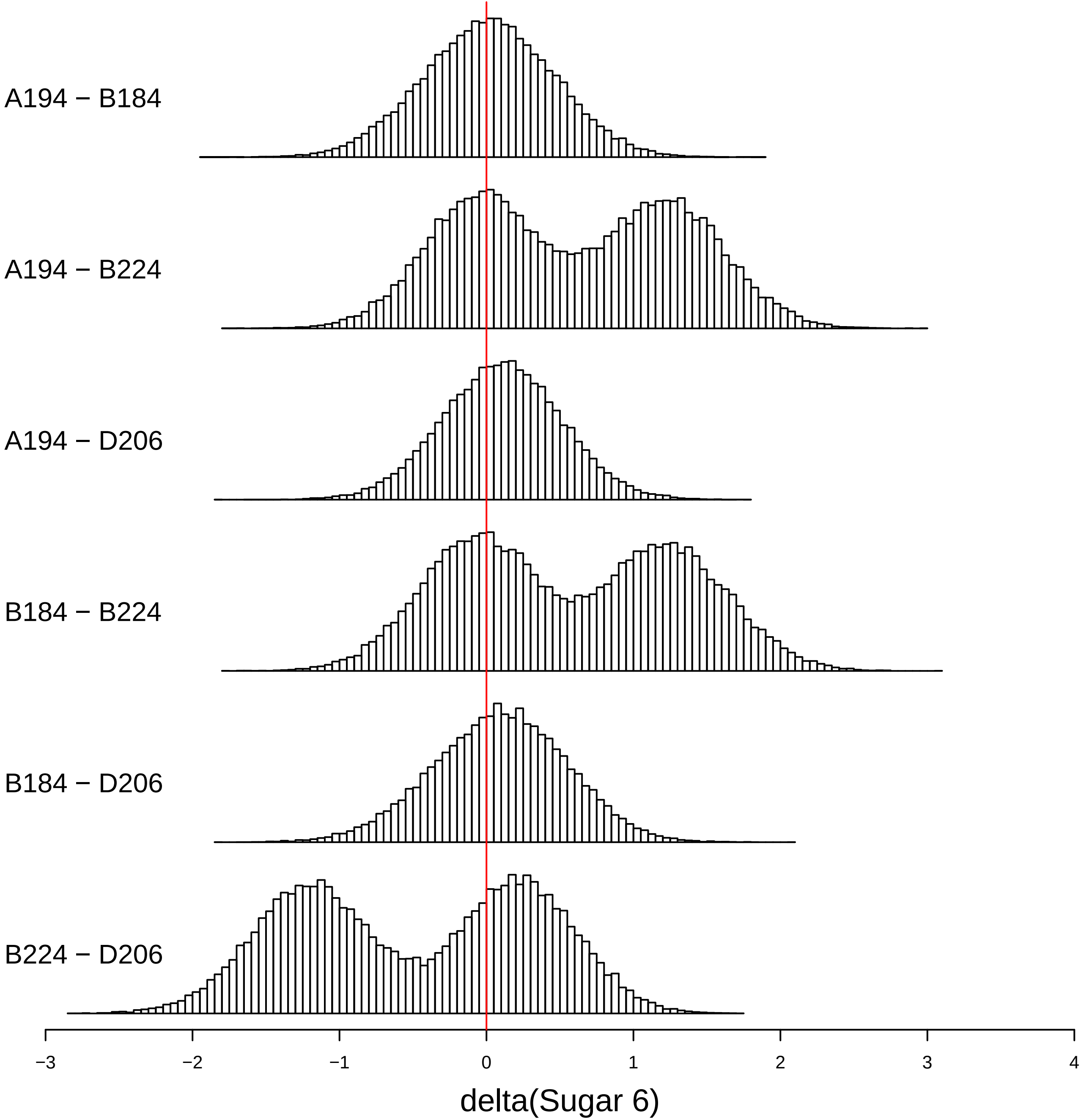
A194 − B184
A194 − B224
A194 − D206
B184 − B224
B184 − D206
B224 − D206
−3
−2
−1
0
1
2
3
4
delta(Sugar 6)

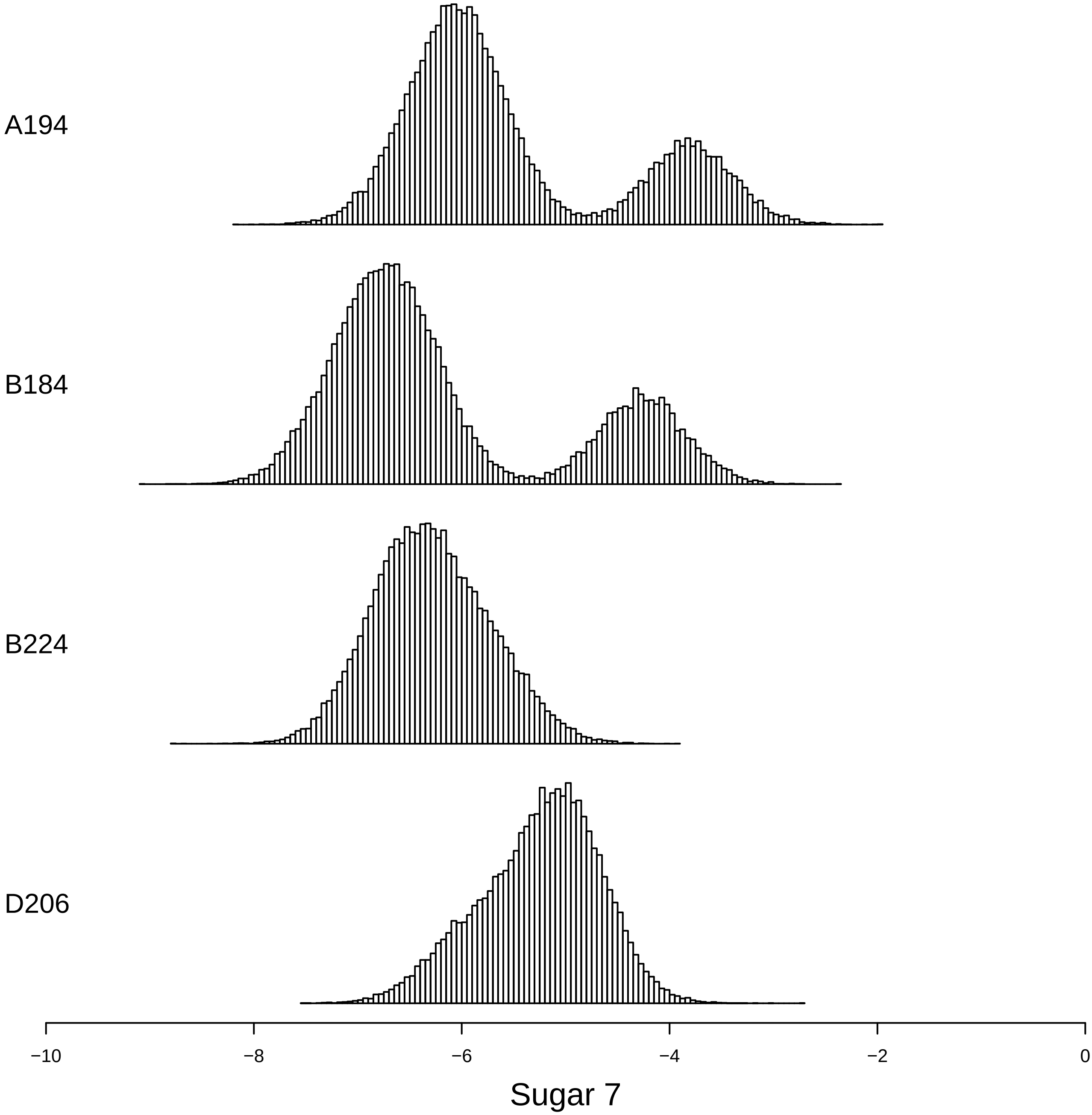
A194
B184
B224
D206
−10
−8
−6
−4
−2
0
Sugar 7

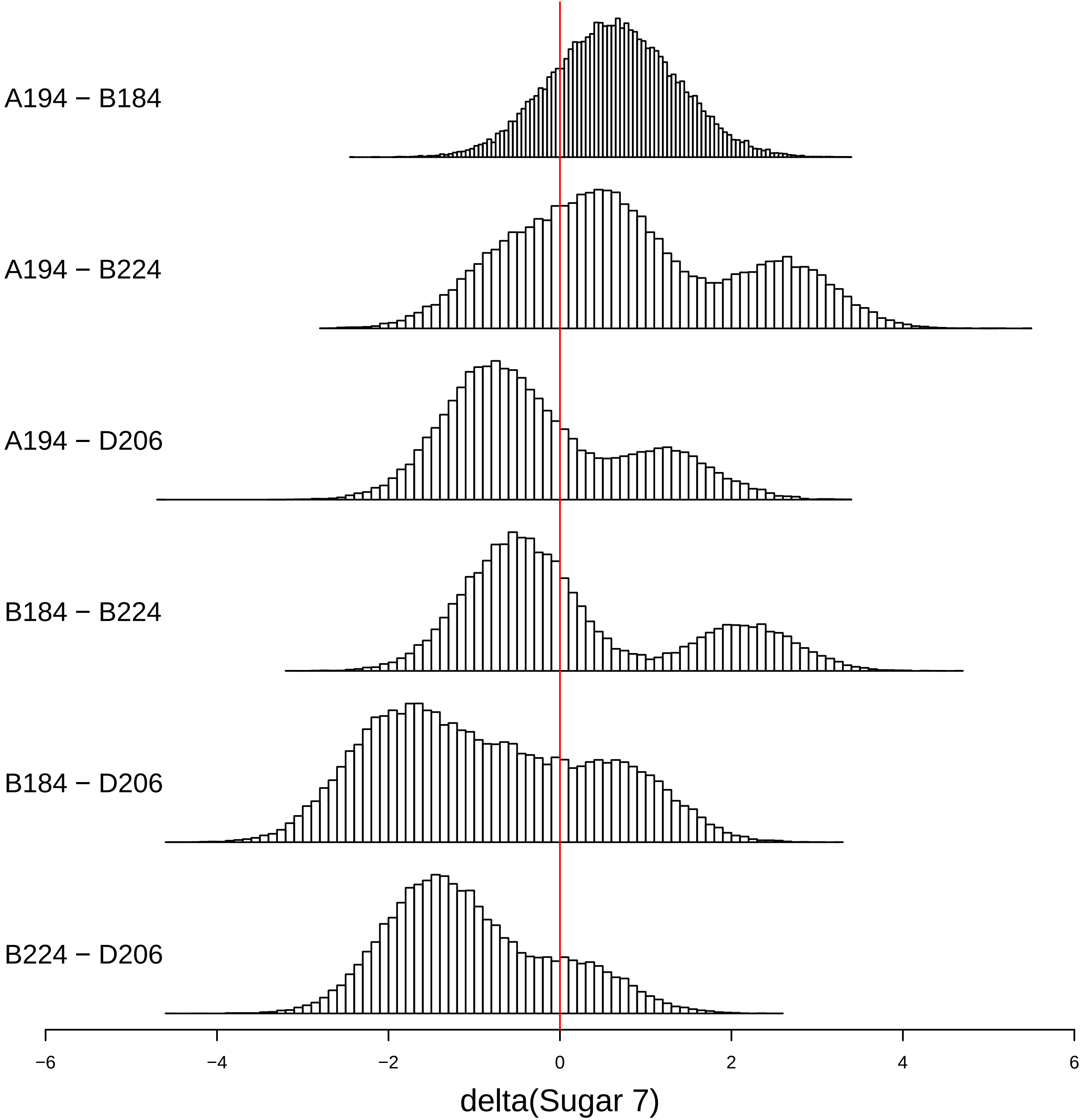

A194 − B184
A194 − B224
A194 − D206
B184 − B224
B184 − D206
B224 − D206
−6
−4
−2
0
2
4
6
delta(Sugar 7)

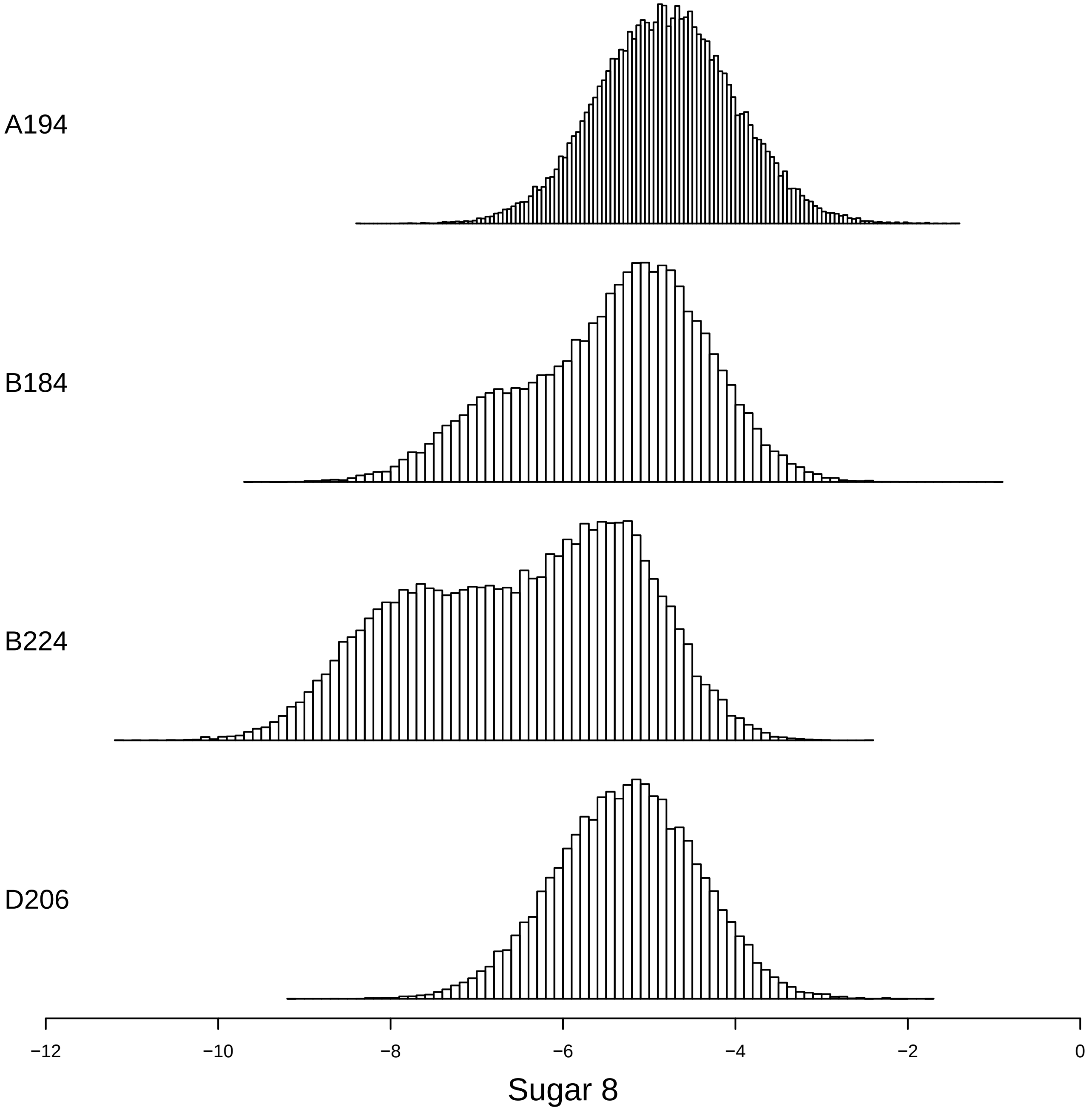
A194
B184
B224
D206
−12
−10
−8
−6
−4
−2
0
Sugar 8

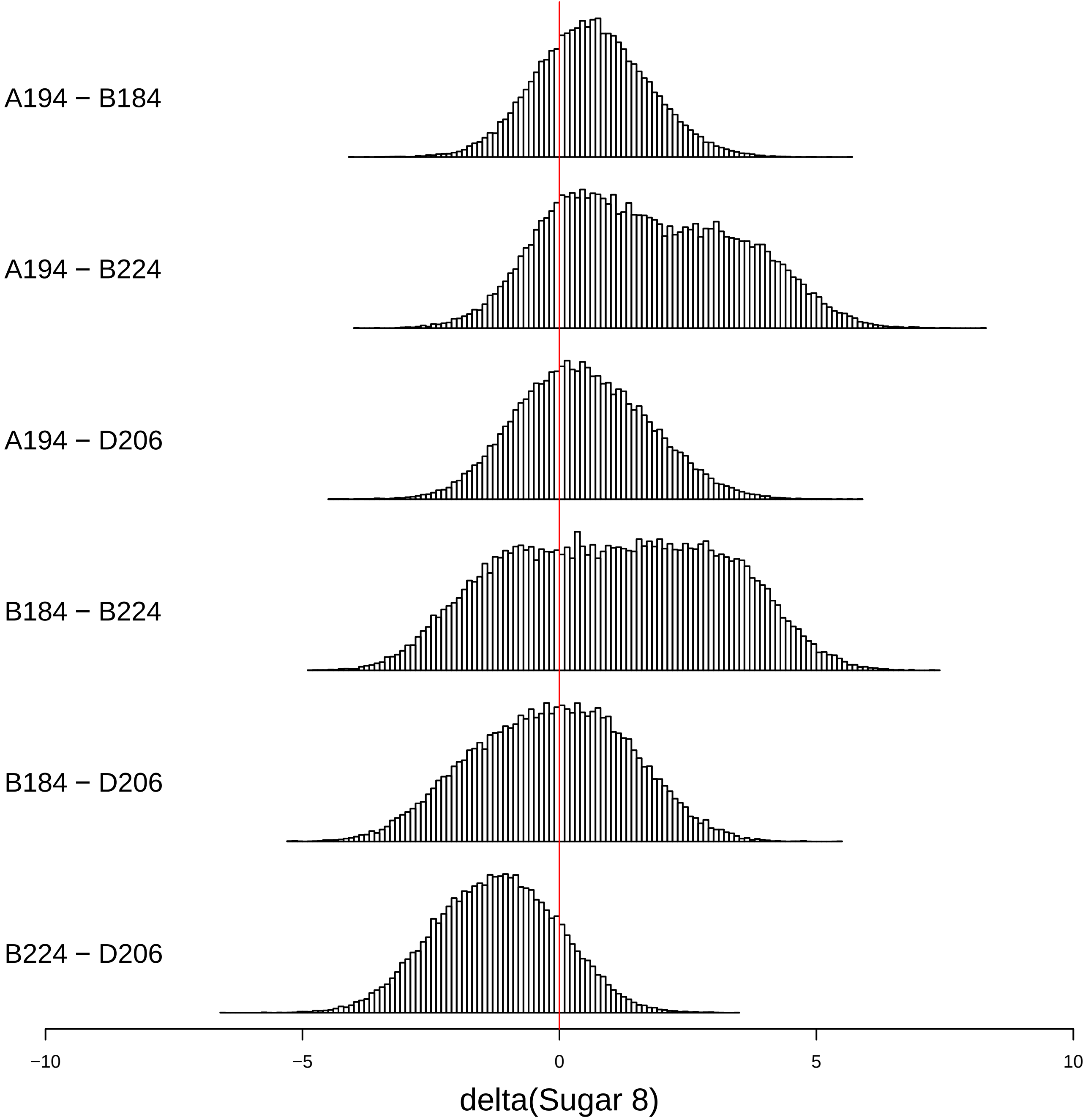
A194 − B184
A194 − B224
A194 − D206
B184 − B224
B184 − D206
B224 − D206
−10
−5
0
5
10
delta(Sugar 8)

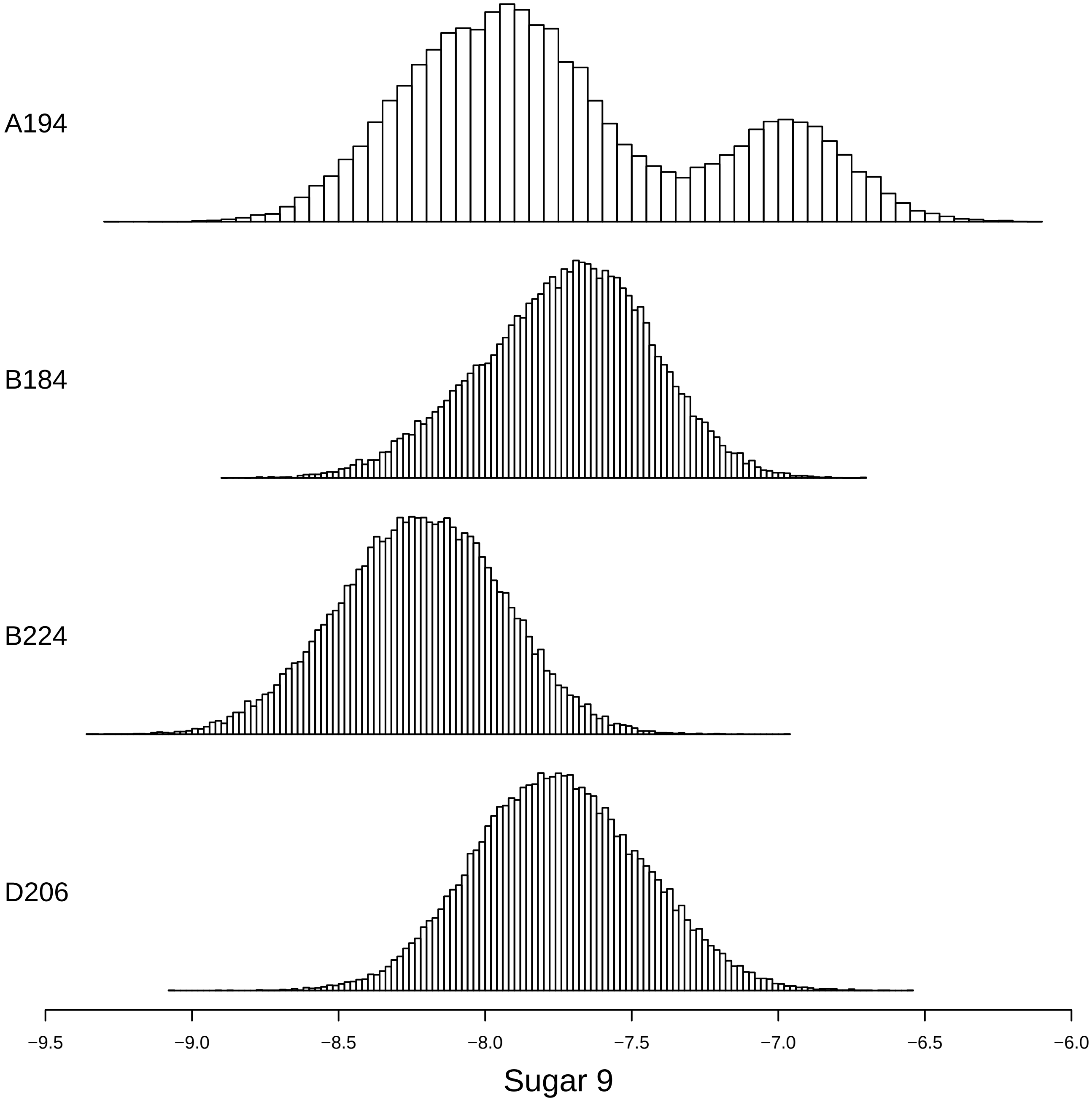
A194
B184
B224
D206
−9.5
−9.0
−8.5
−8.0
−7.5
−7.0
−6.5
−6.0
Sugar 9

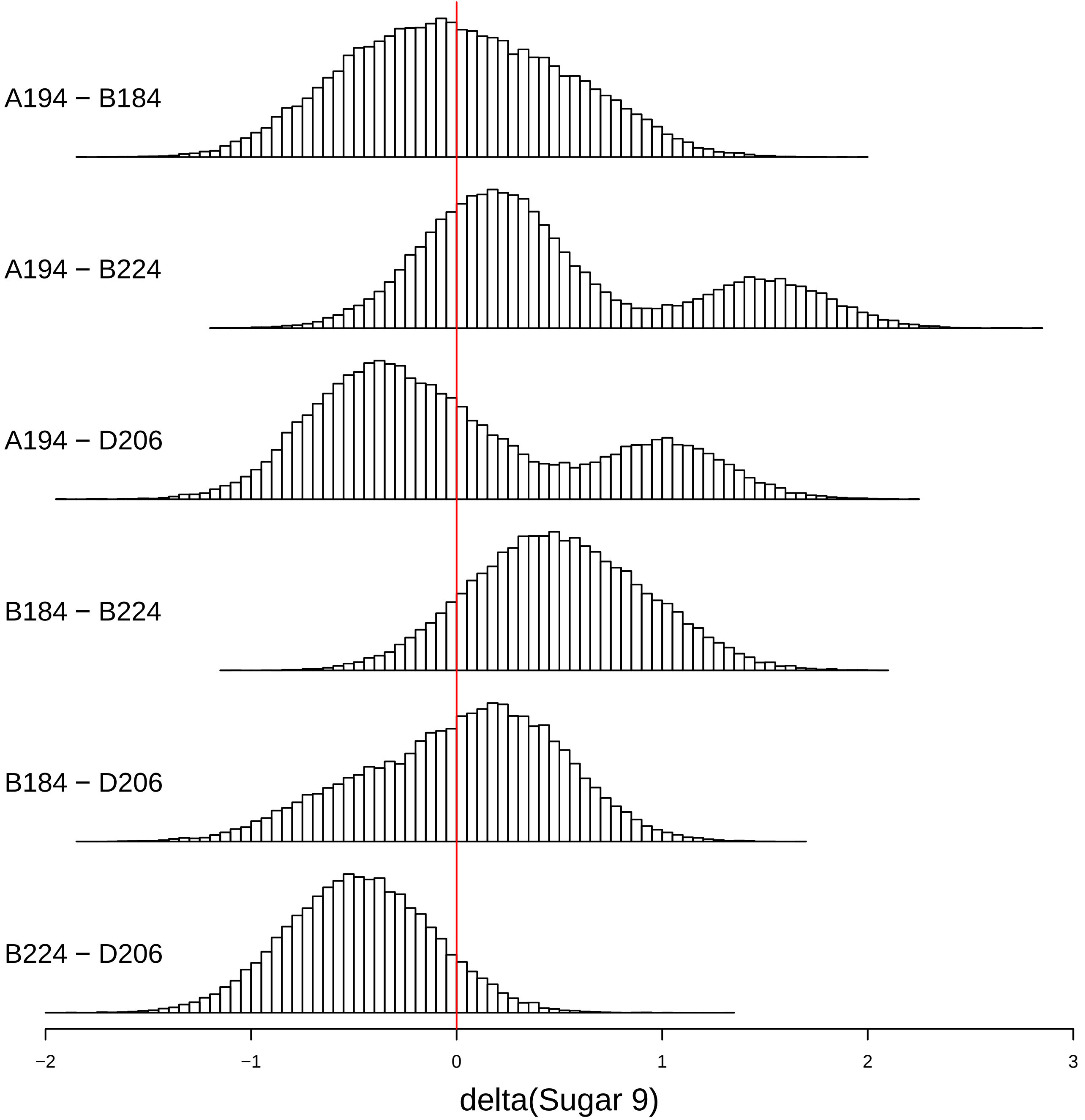
A194 − B184
A194 − B224
A194 − D206
B184 − B224
B184 − D206
B224 − D206
−2
−1
0
1
2
3
delta(Sugar 9)

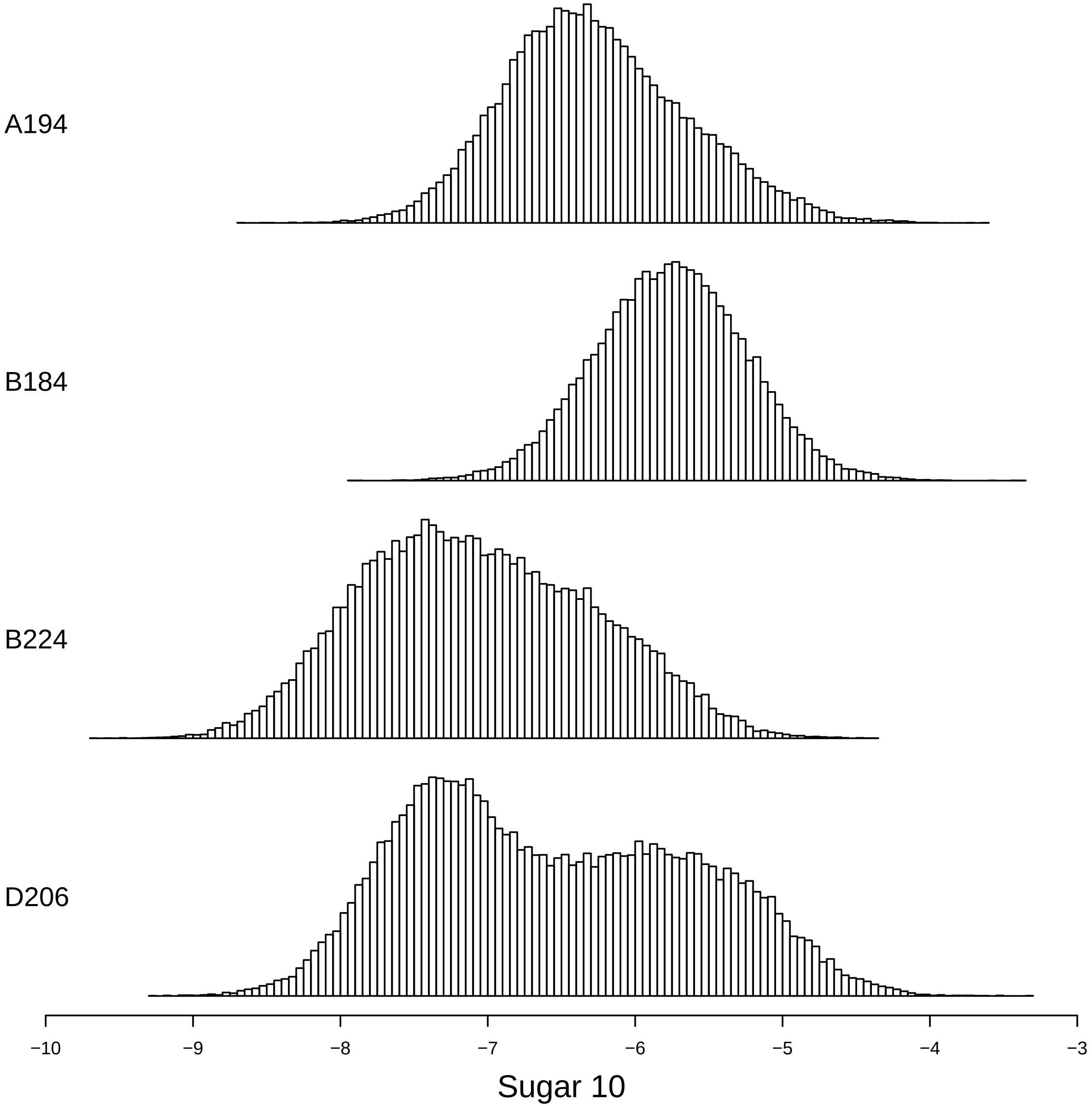
A194
B184
B224
D206
−10
−9
−8
−7
−6
−5
−4
−3
Sugar 10

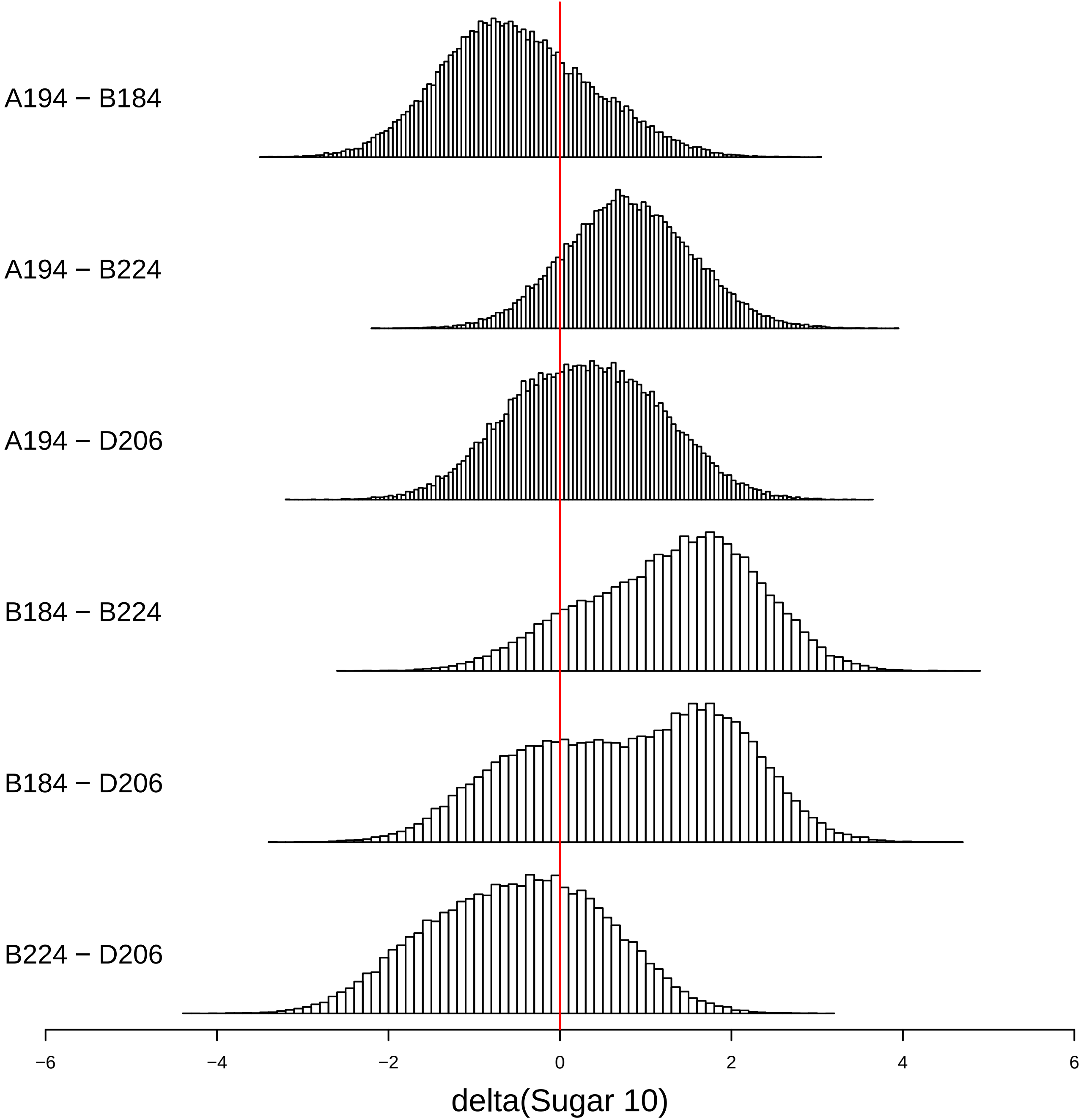
A194 − B184
A194 − B224
A194 − D206
B184 − B224
B184 − D206
B224 − D206
−6
−4
−2
0
2
4
6
delta(Sugar 10)

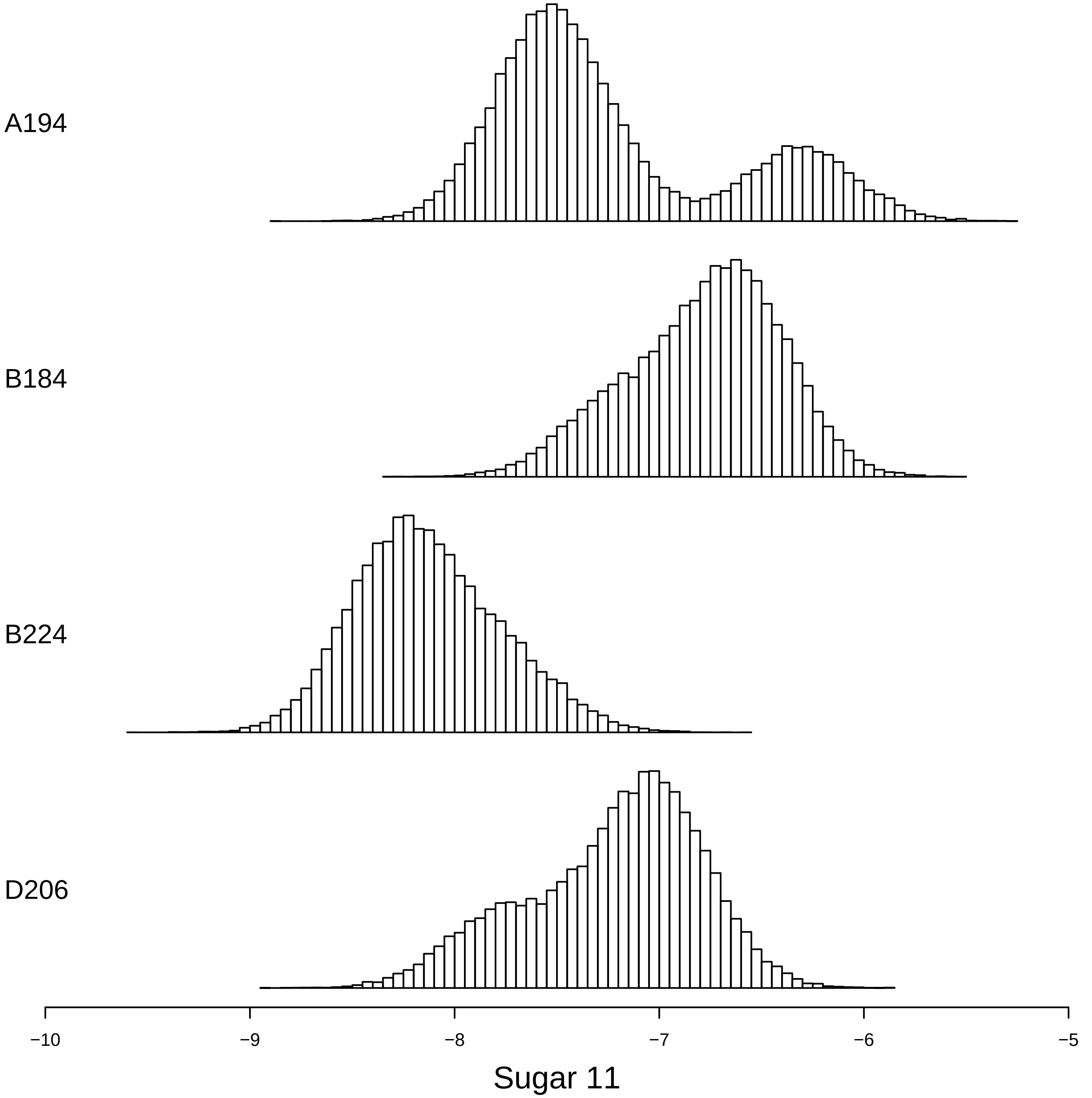
A194
B184
B224
D206
−10
−9
−8
−7
−6
−5
Sugar 11

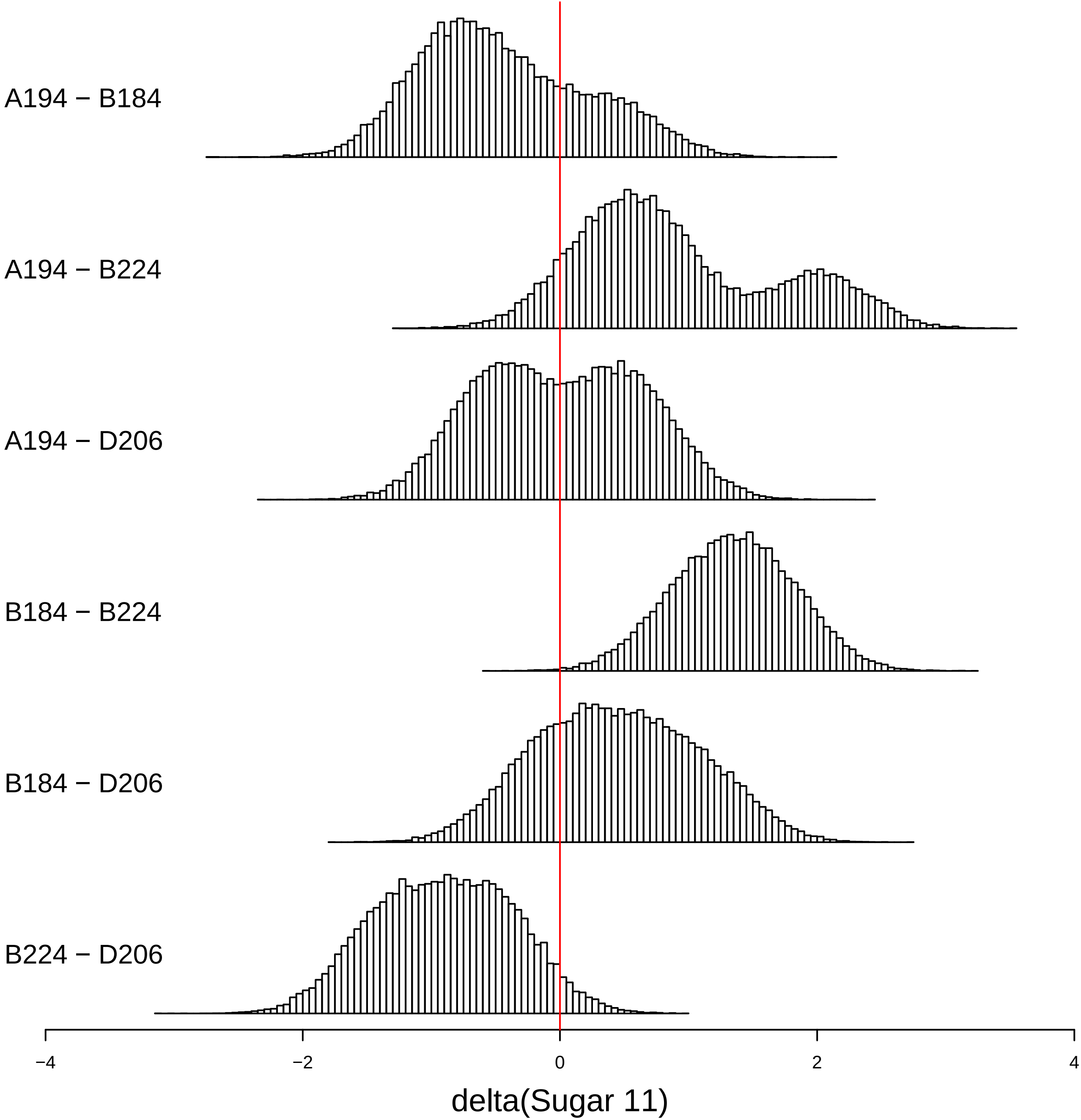
A194 − B184
A194 − B224
A194 − D206
B184 − B224
B184 − D206
B224 − D206
−4
−2
0
2
4
delta(Sugar 11)

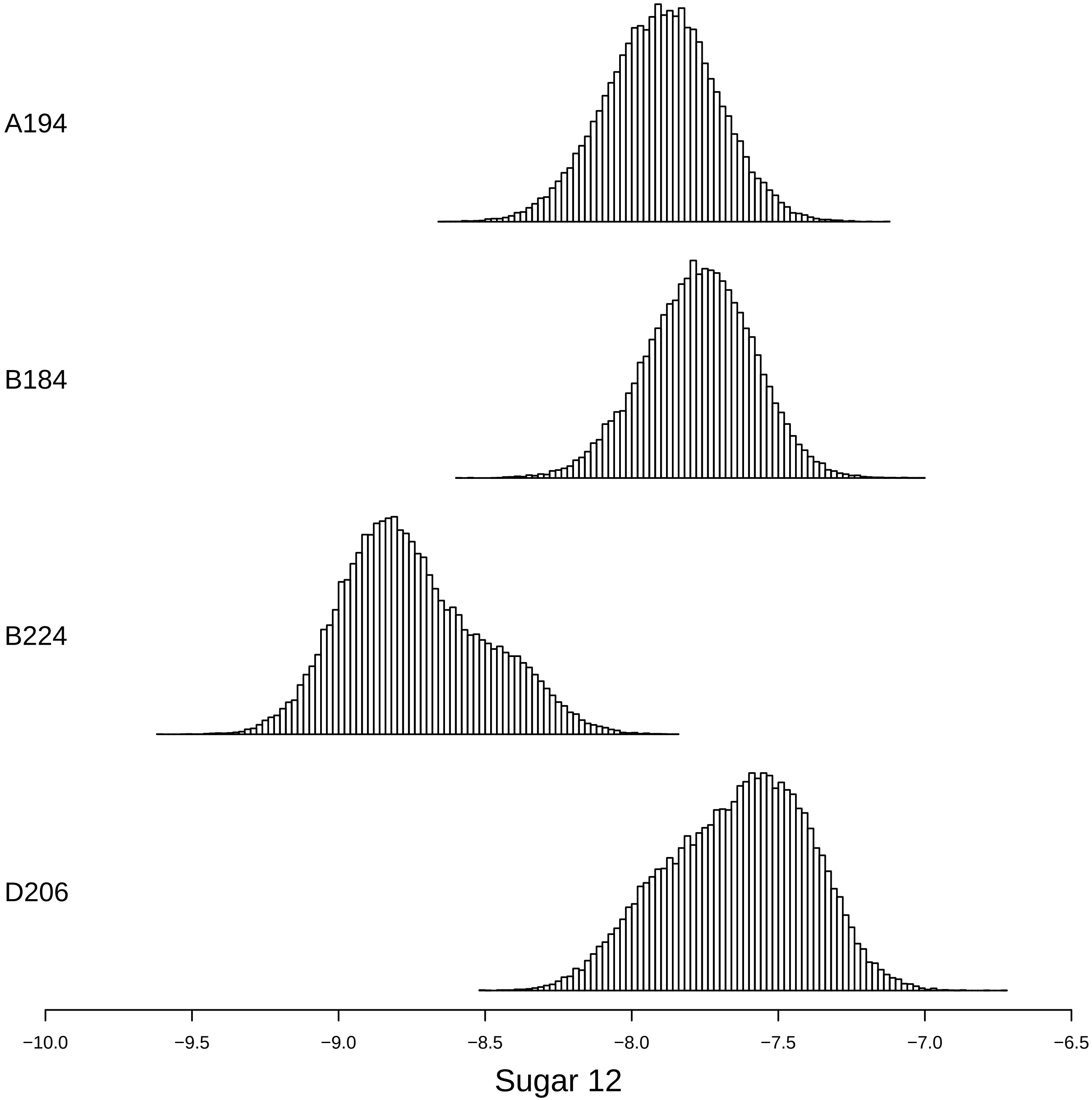
A194
B184
B224
D206
−10.0
−9.5
−9.0
−8.5
−8.0
−7.5
−7.0
−6.5
Sugar 12

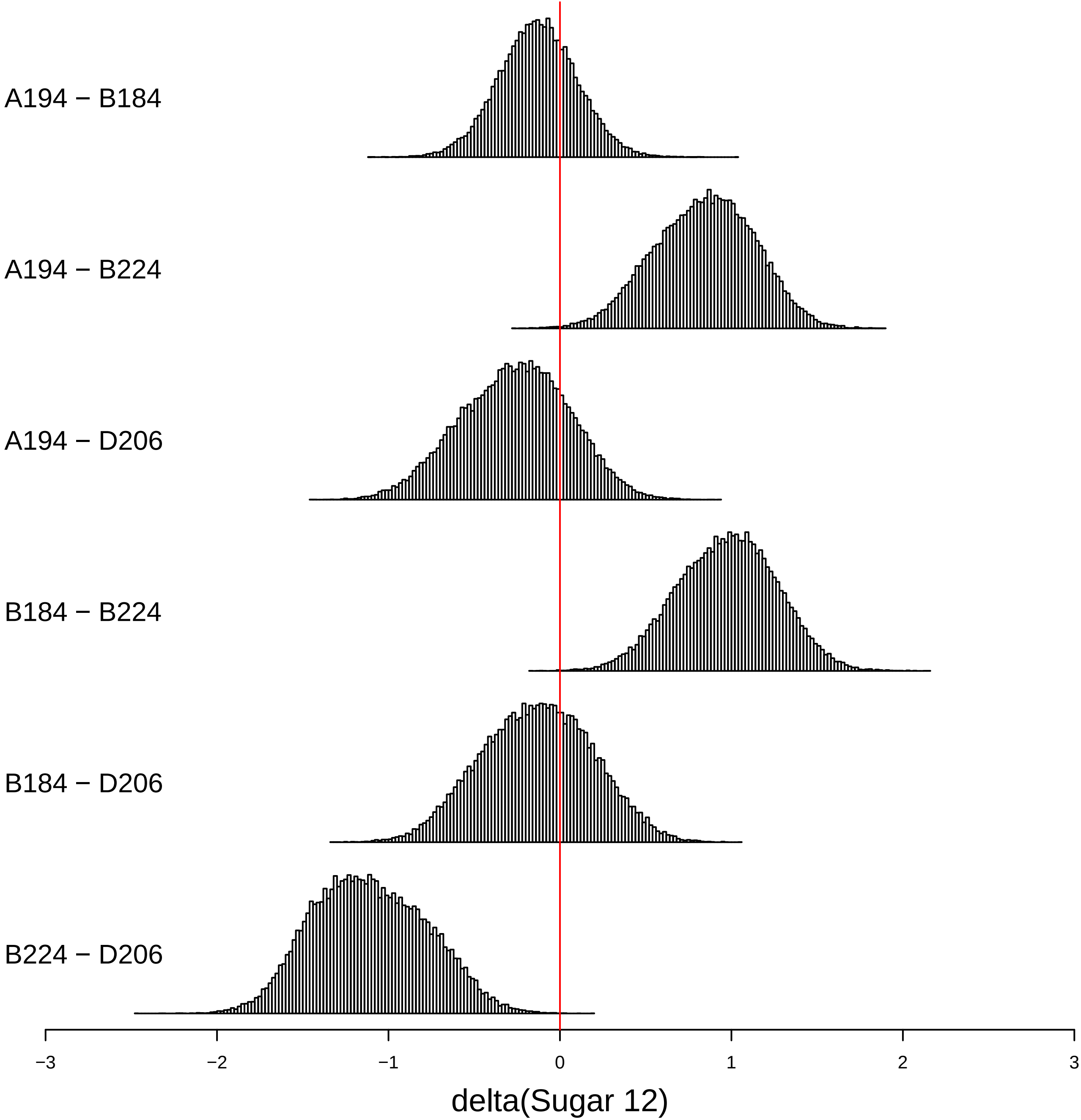
A194 − B184
A194 − B224
A194 − D206
B184 − B224
B184 − D206
B224 − D206
−3
−2
−1
0
1
2
3
delta(Sugar 12)

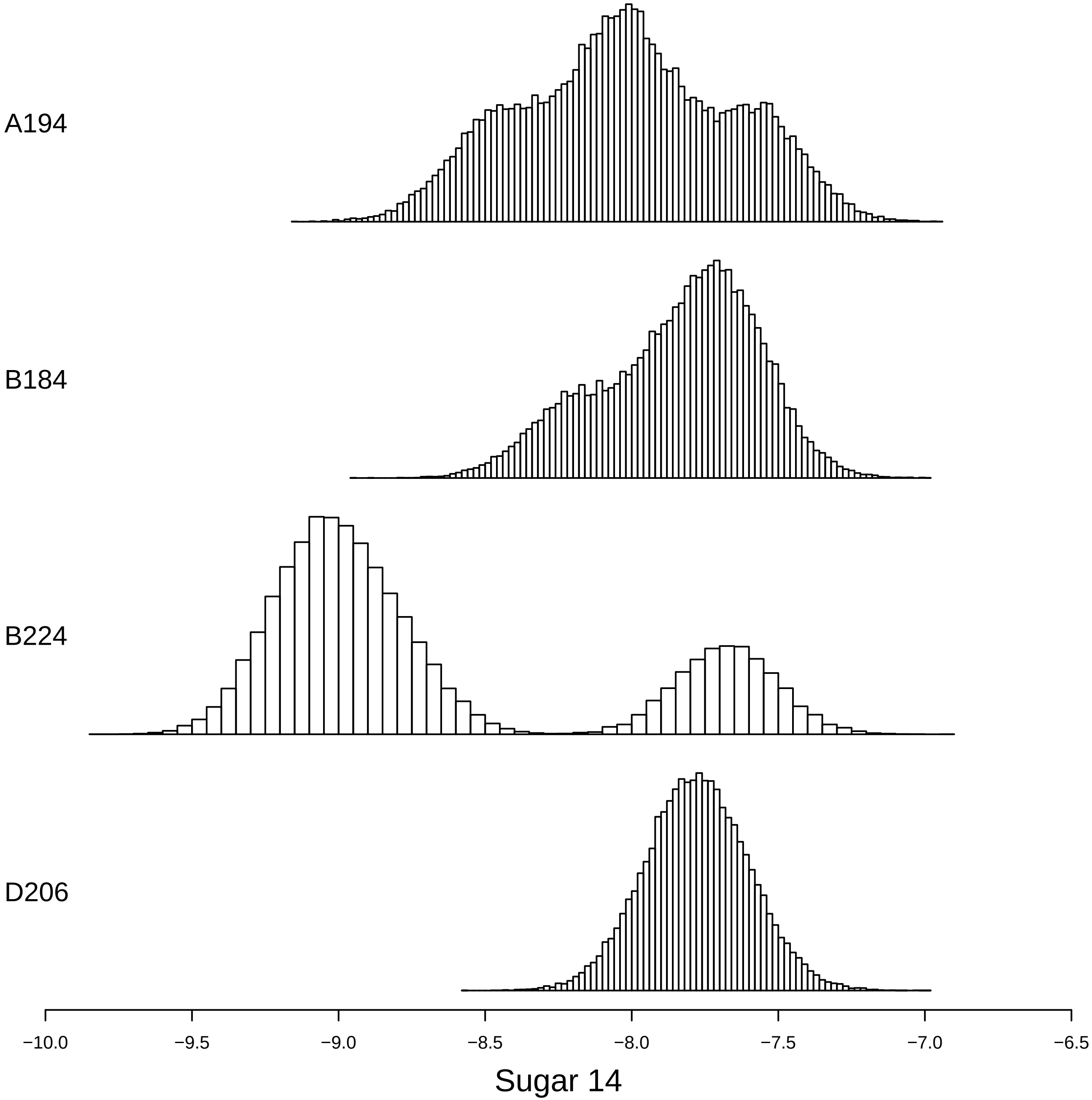
A194
B184
B224
D206
−10.0
−9.5
−9.0
−8.5
−8.0
−7.5
−7.0
−6.5
Sugar 14

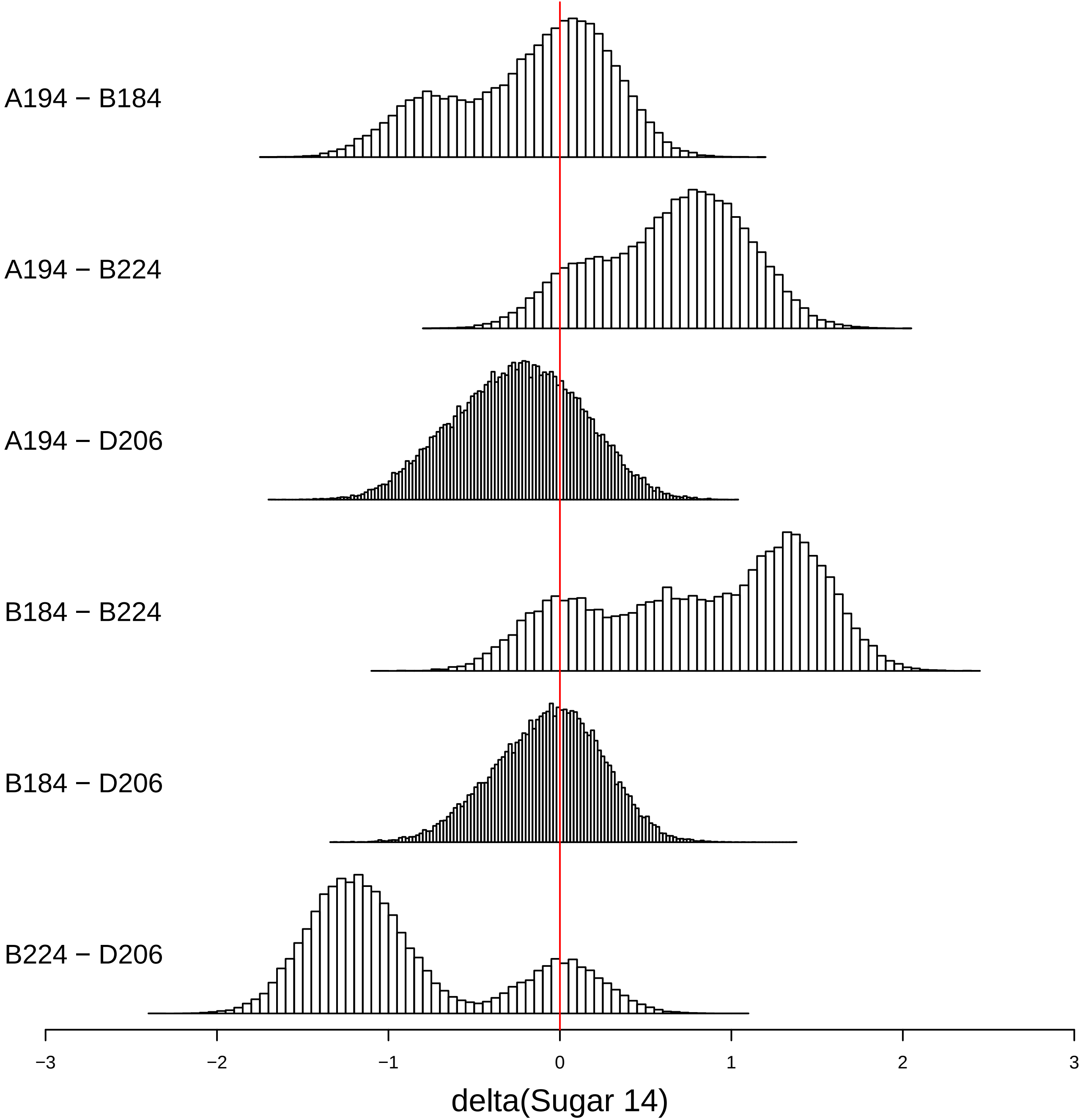
A194 − B184
A194 − B224
A194 − D206
B184 − B224
B184 − D206
B224 − D206
−3
−2
−1
0
1
2
3
delta(Sugar 14)

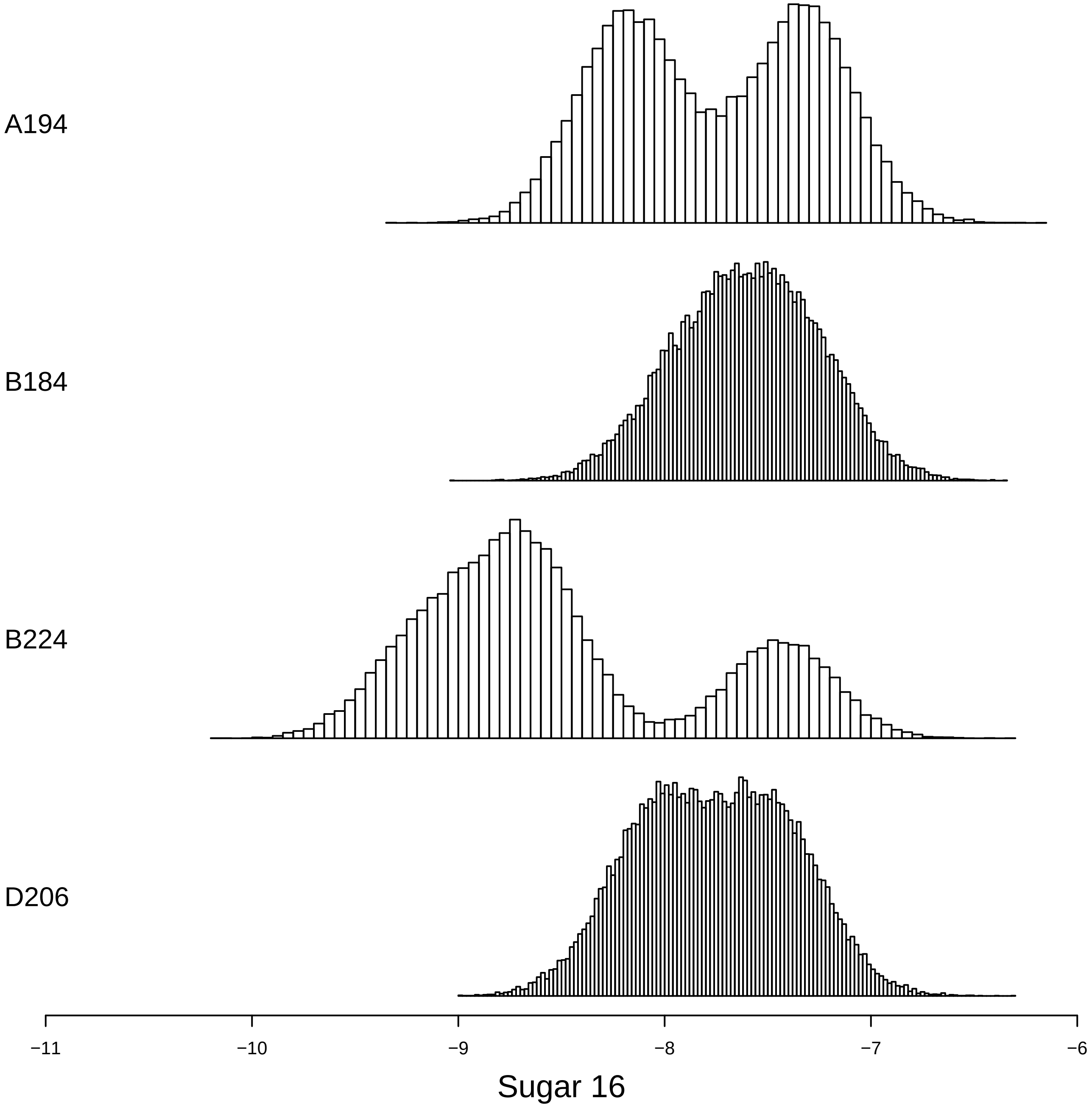

A194
B184
B224
D206
−11
−10
−9
−8
−7
−6
Sugar 16

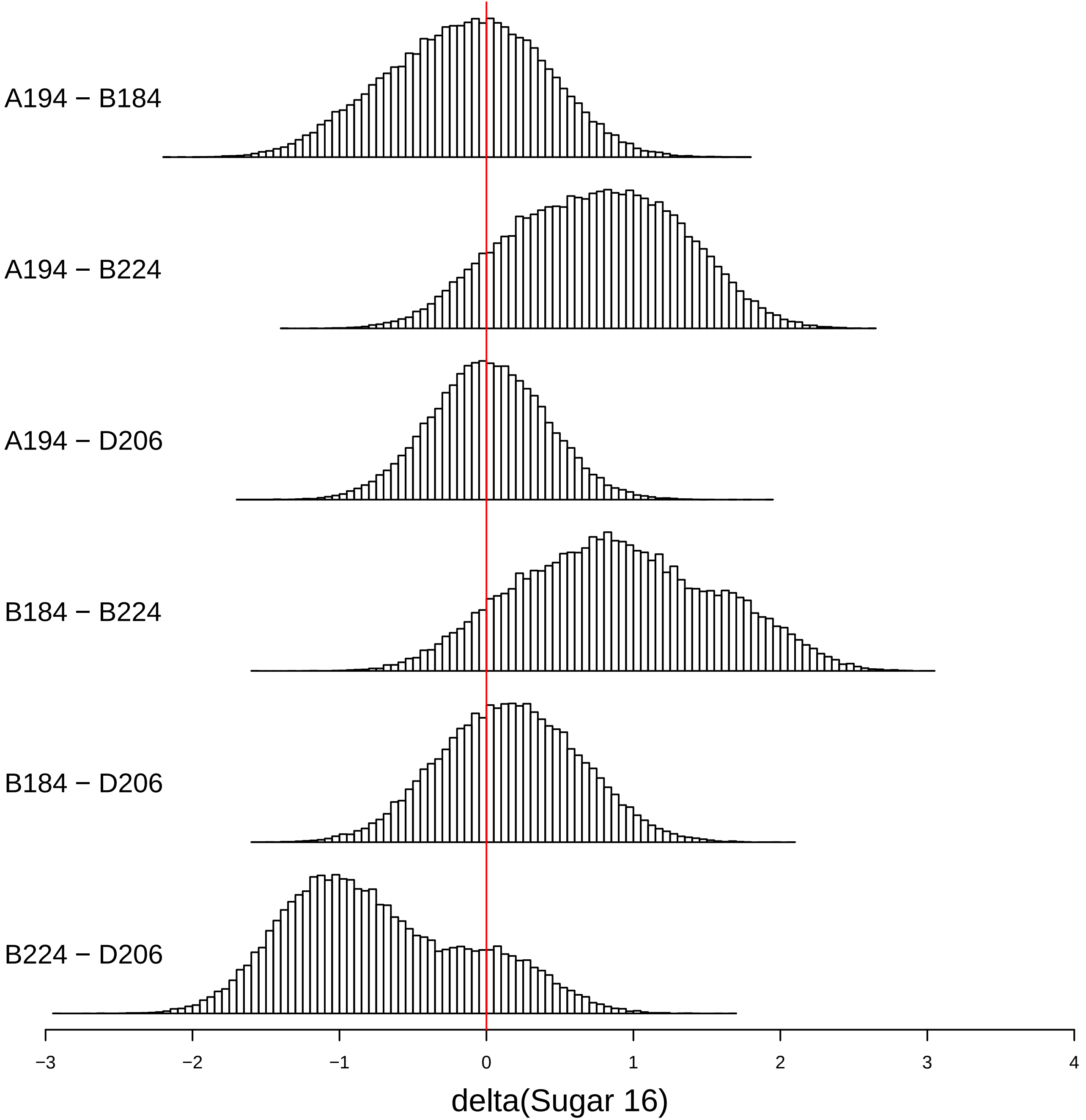
A194 − B184
A194 − B224
A194 − D206
B184 − B224
B184 − D206
B224 − D206
−3
−2
−1
0
1
2
3
4
delta(Sugar 16)

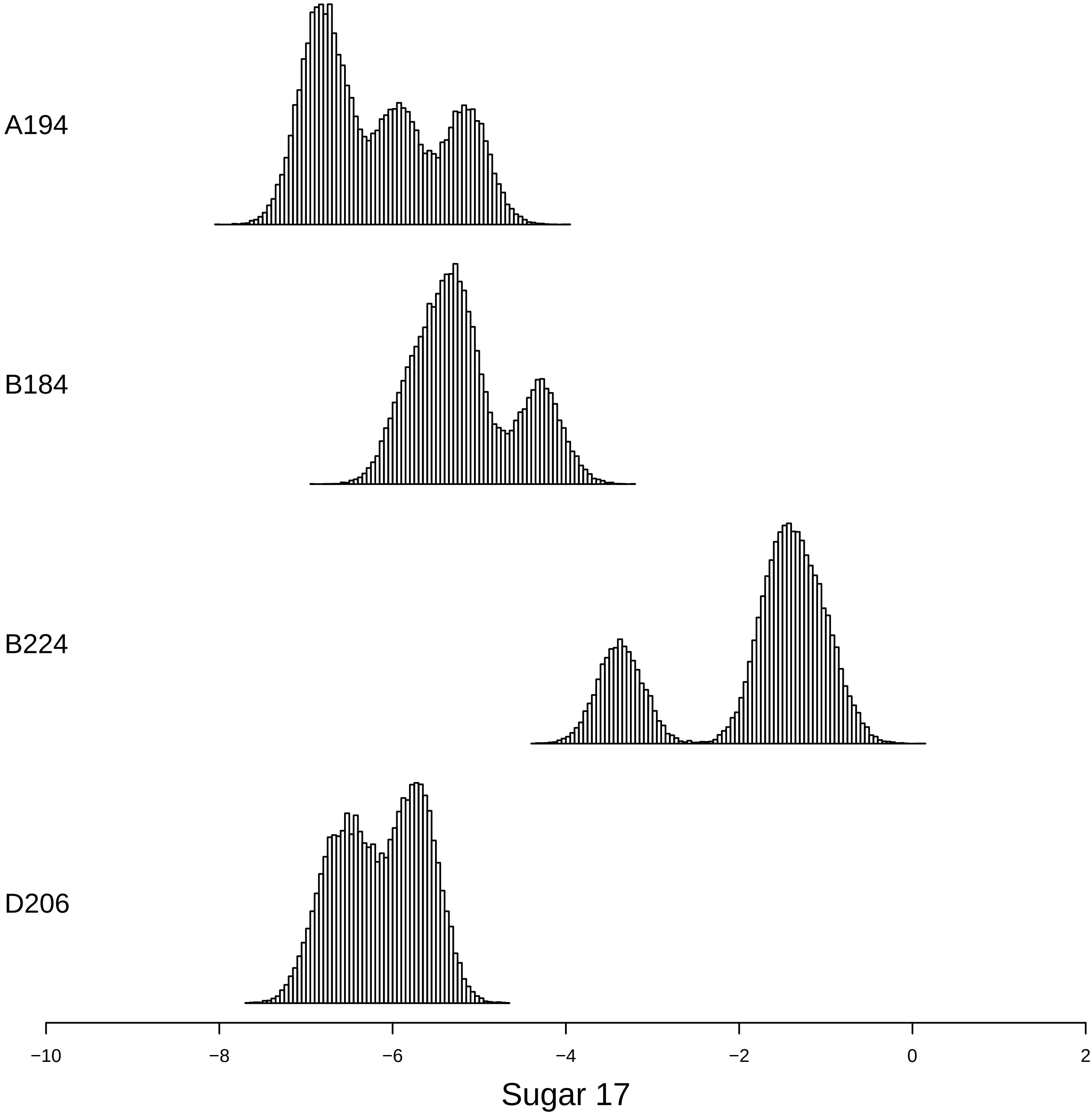

A194
B184
B224
D206
−10
−8
−6
−4
−2
0
2
Sugar 17

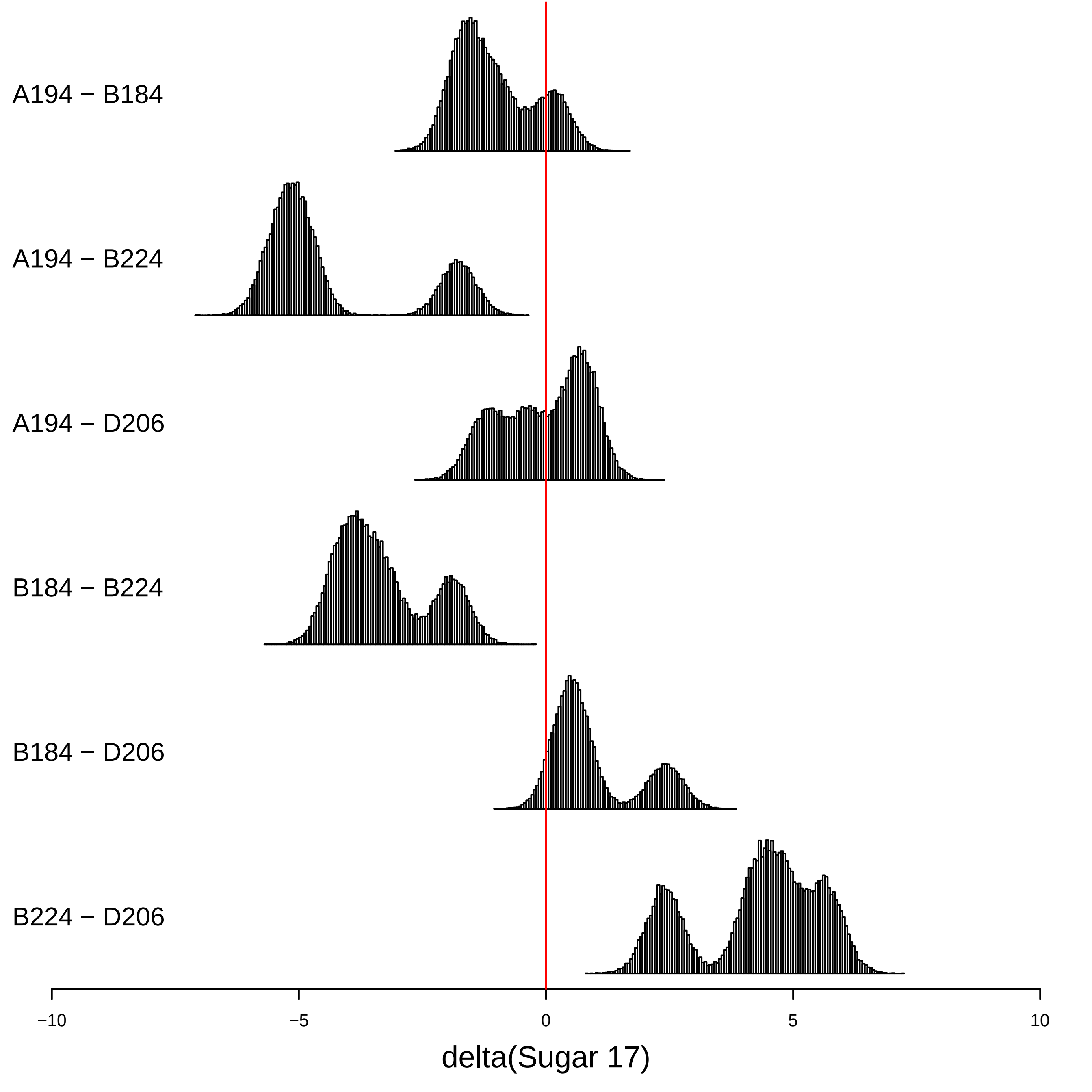

A194 − B184
A194 − B224
A194 − D206
B184 − B224
B184 − D206
B224 − D206
−10
−5
0
5
10
delta(Sugar 17)

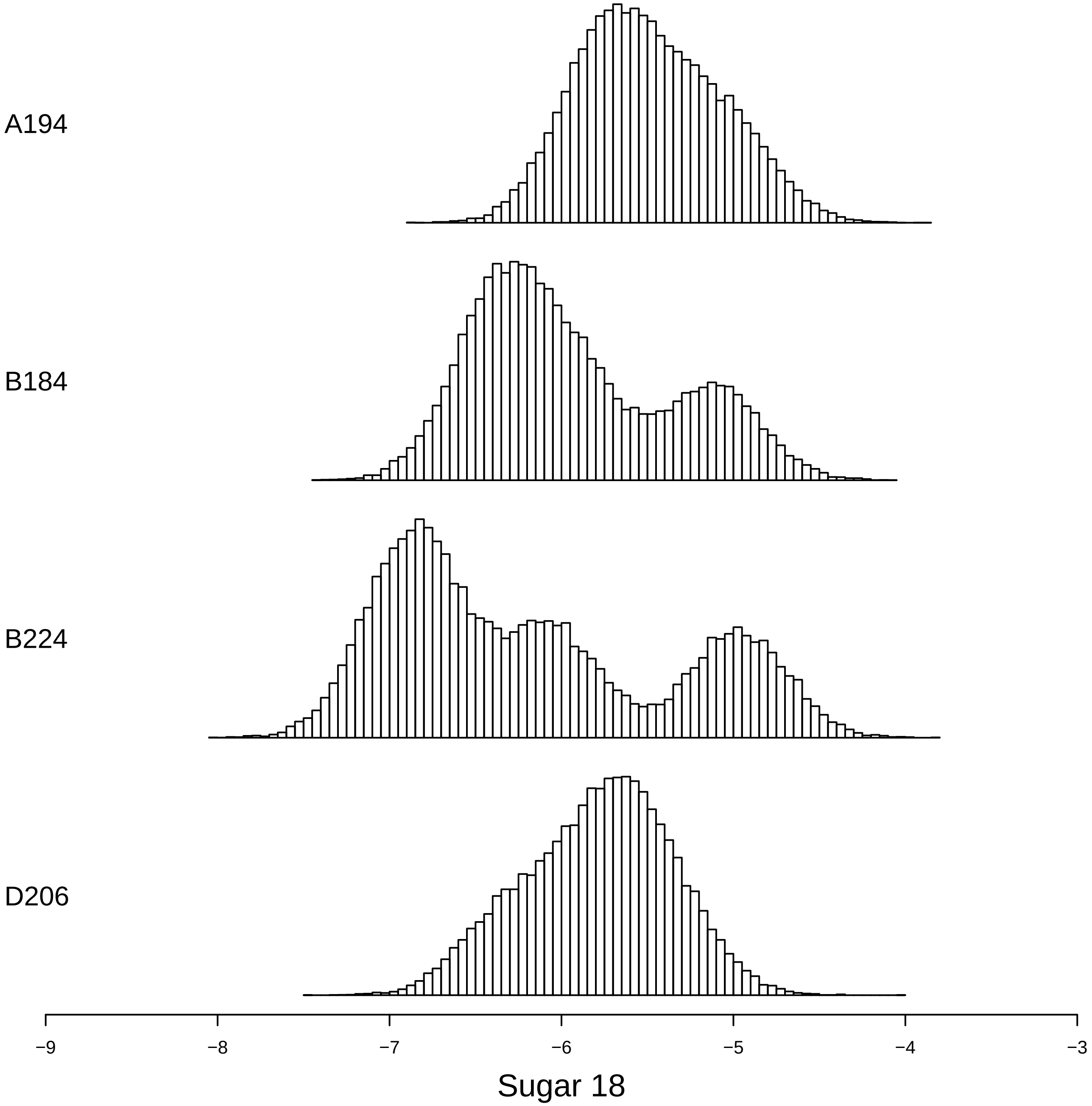
A194
B184
B224
D206
−9
−8
−7
−6
−5
−4
−3
Sugar 18

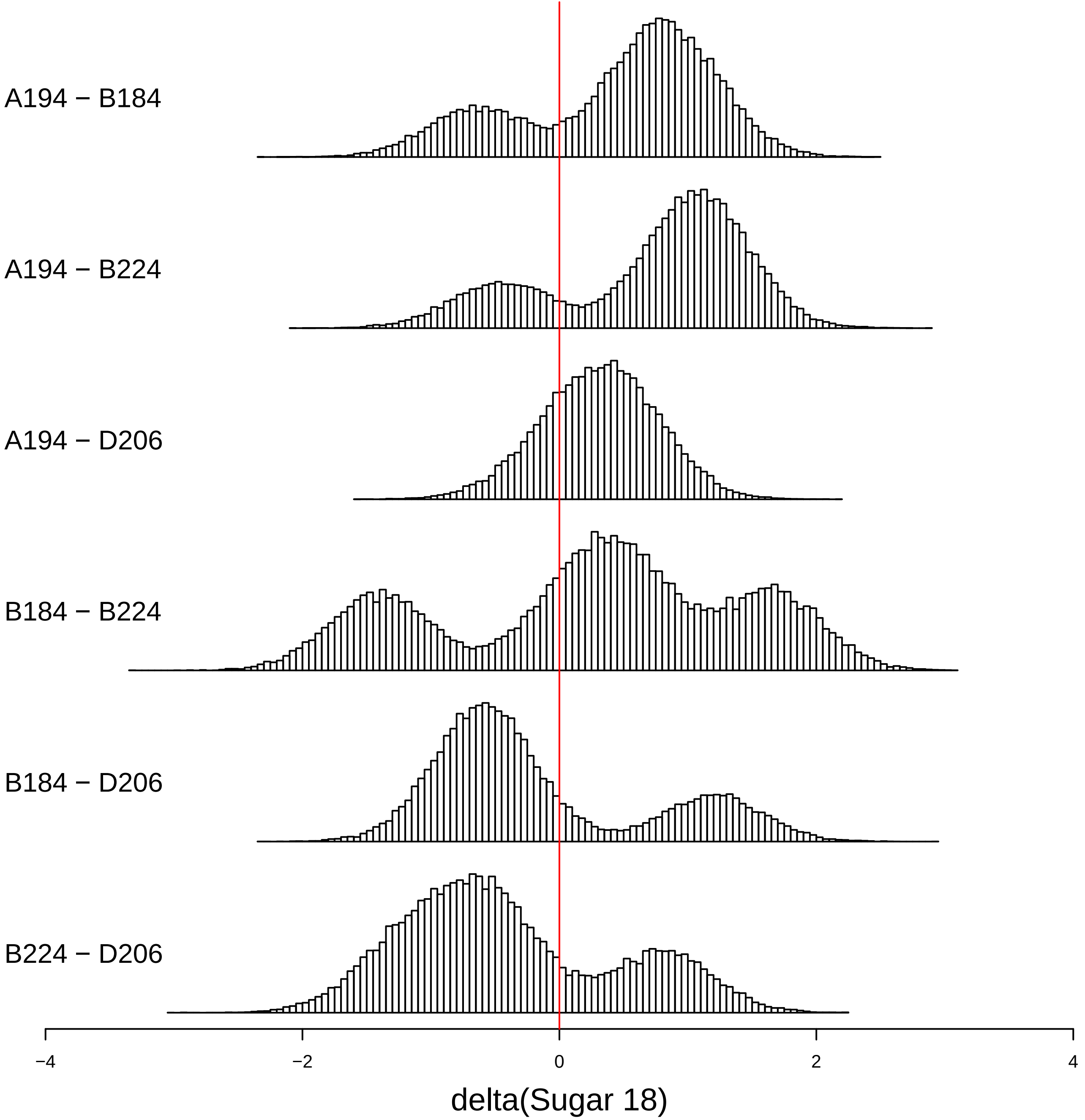
A194 − B184
A194 − B224
A194 − D206
B184 − B224
B184 − D206
B224 − D206
−4
−2
0
2
4
delta(Sugar 18)

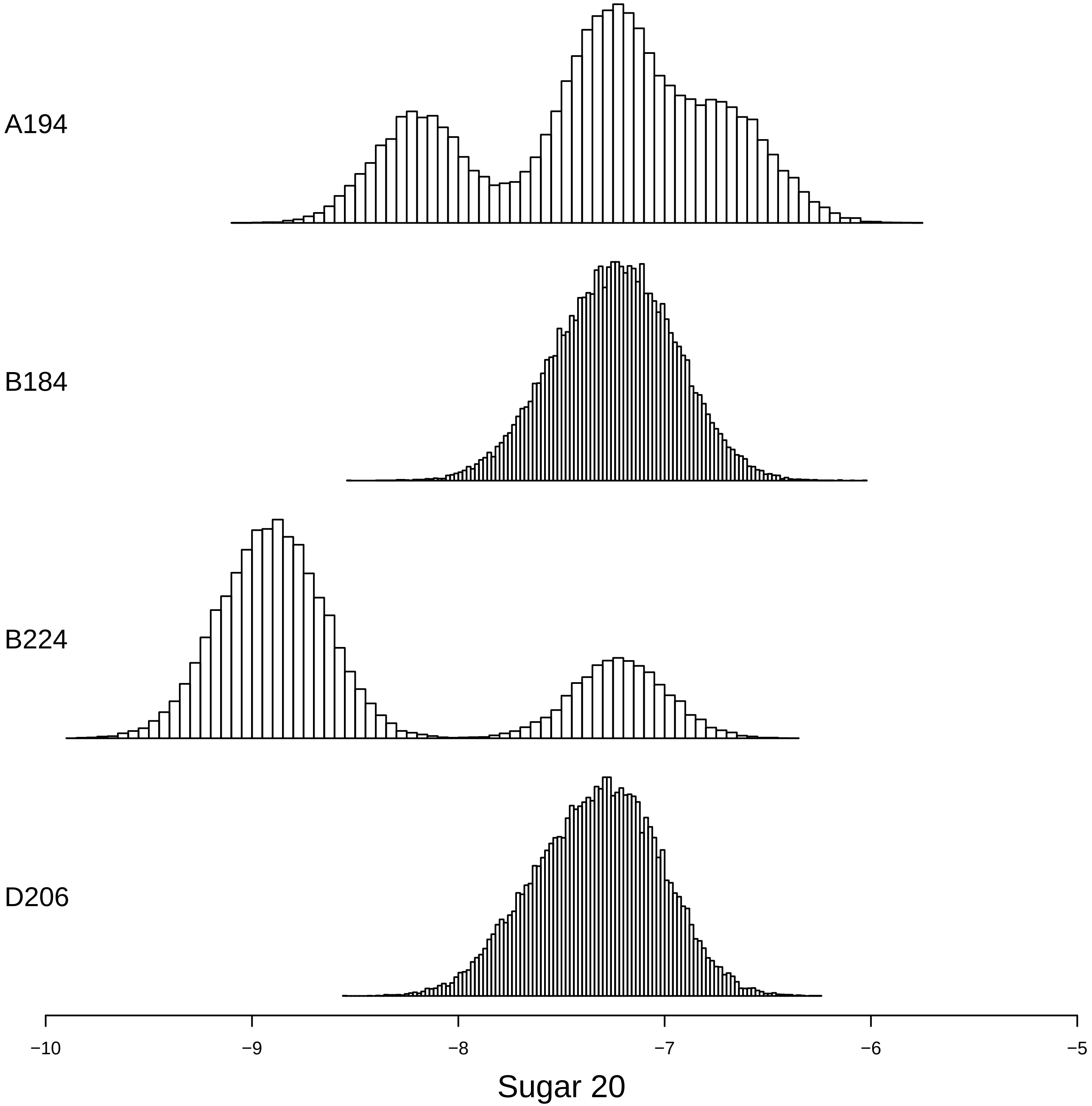
A194
B184
B224
D206
−10
−9
−8
−7
−6
−5
Sugar 20

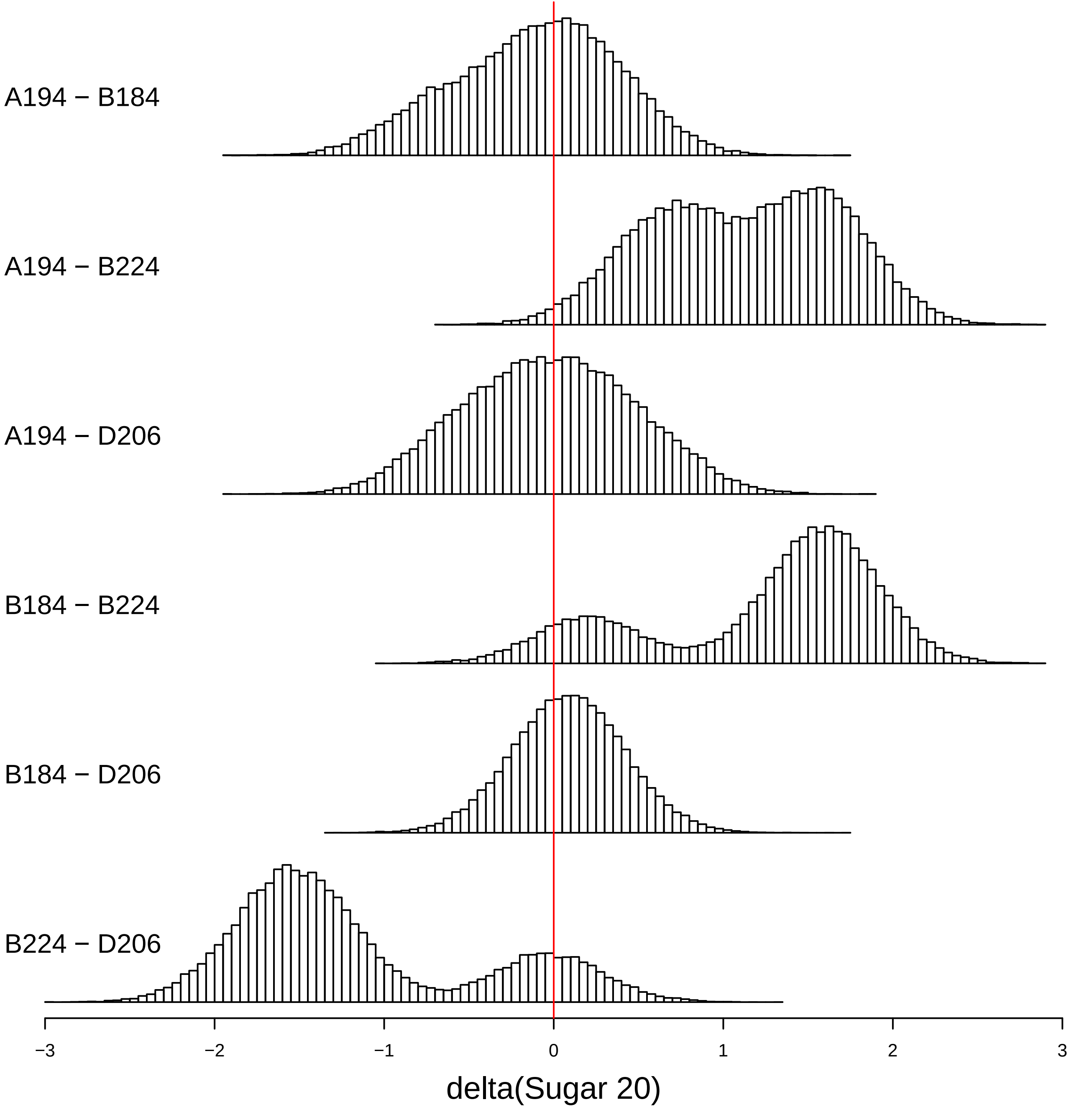
A194 − B184
A194 − B224
A194 − D206
B184 − B224
B184 − D206
B224 − D206
−3
−2
−1
0
1
2
3
delta(Sugar 20)

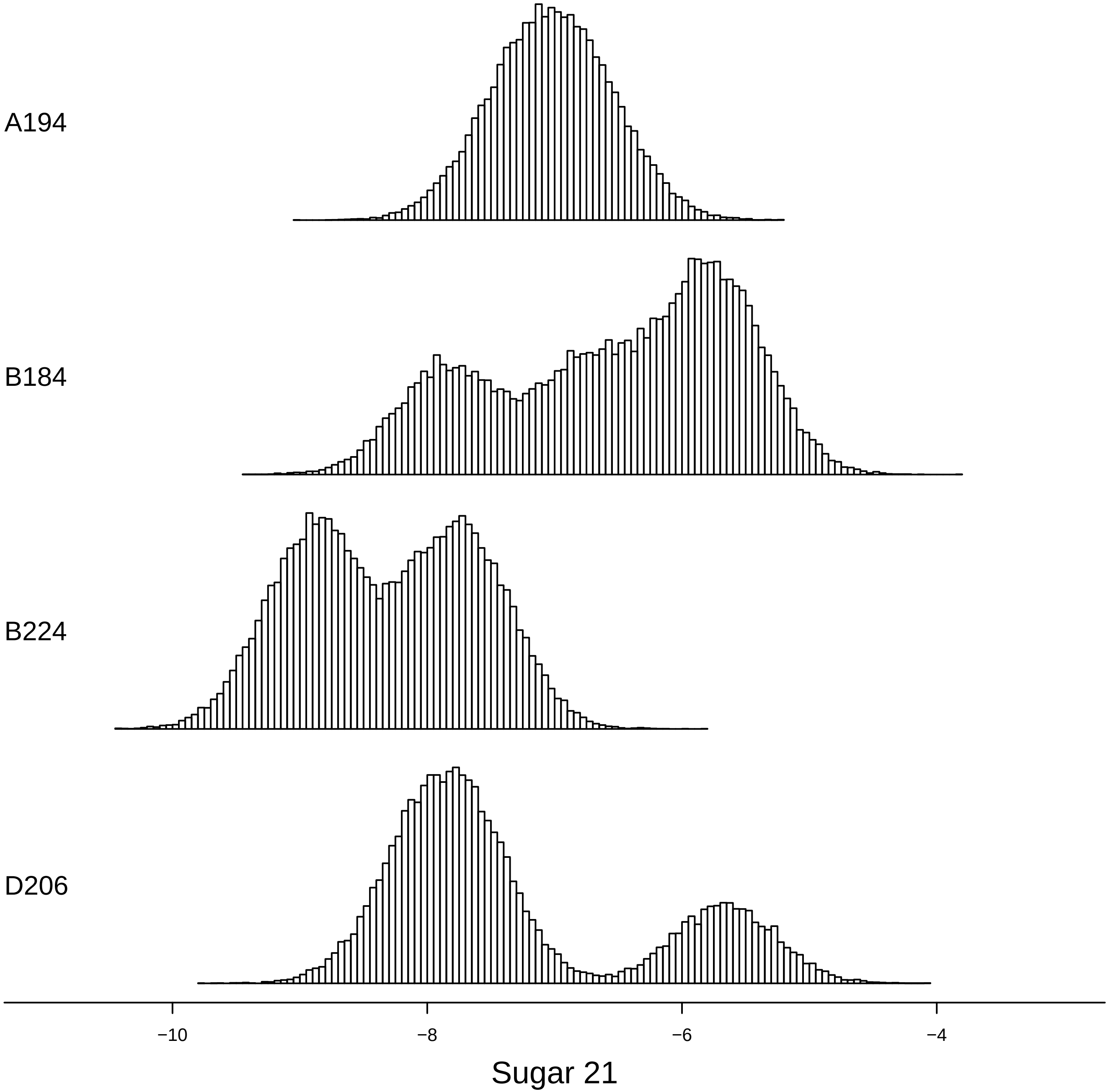
A194
B184
B224
D206
−10
−8
−6
−4
Sugar 21

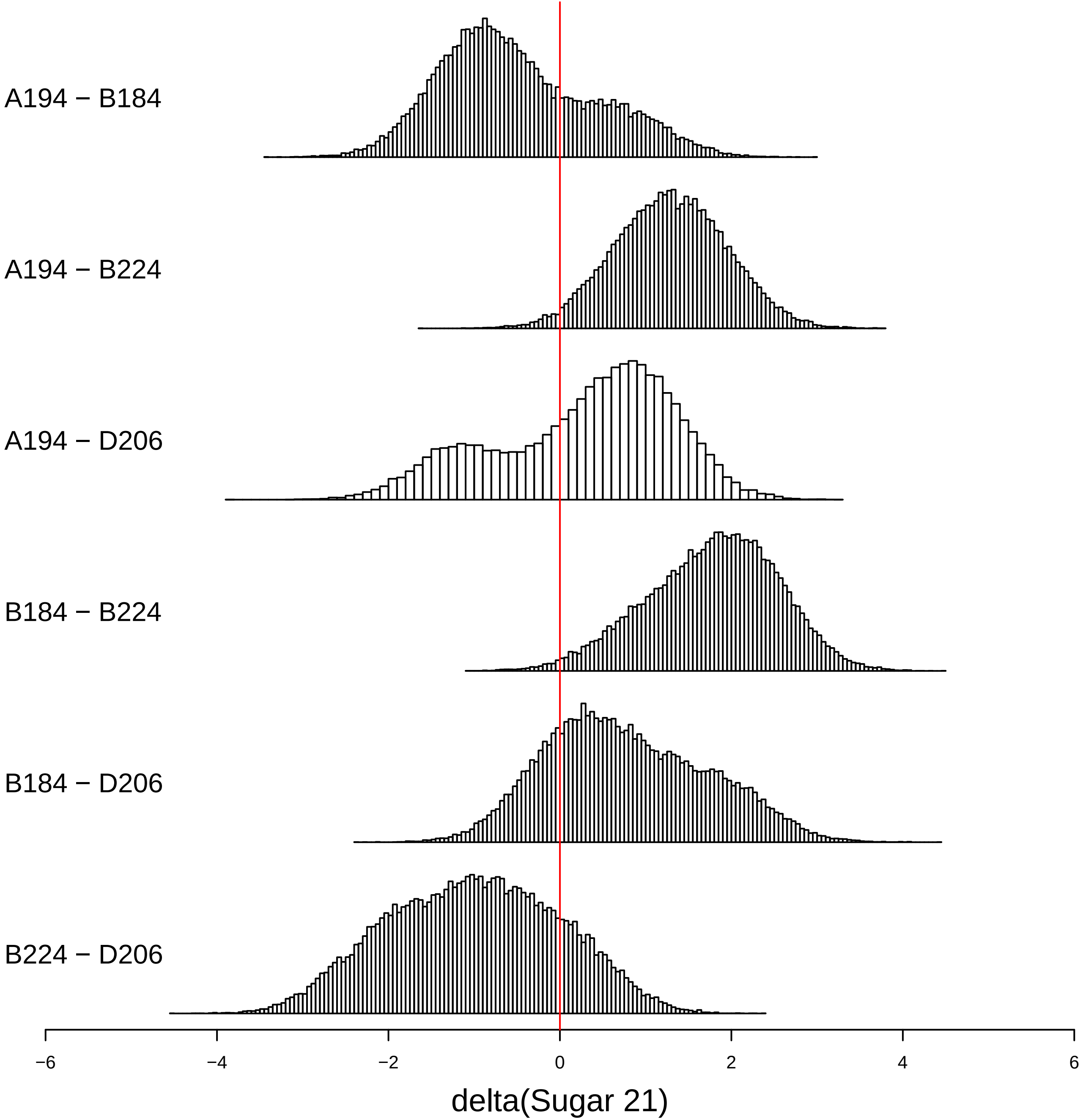

A194 − B184
A194 − B224
A194 − D206
B184 − B224
B184 − D206
B224 − D206
−6
−4
−2
0
2
4
6
delta(Sugar 21)

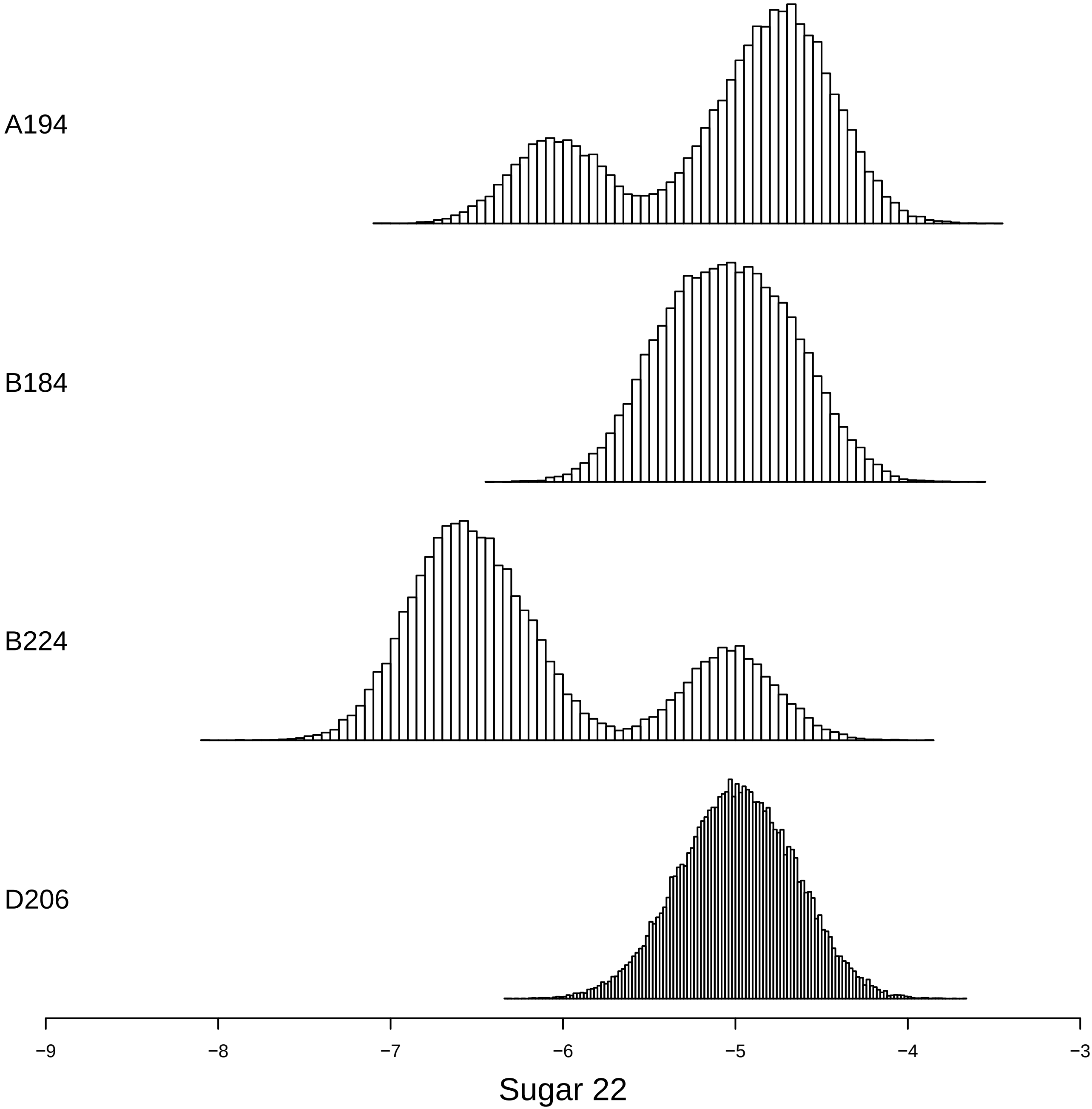
A194
B184
B224
D206
−9
−8
−7
−6
−5
−4
−3
Sugar 22

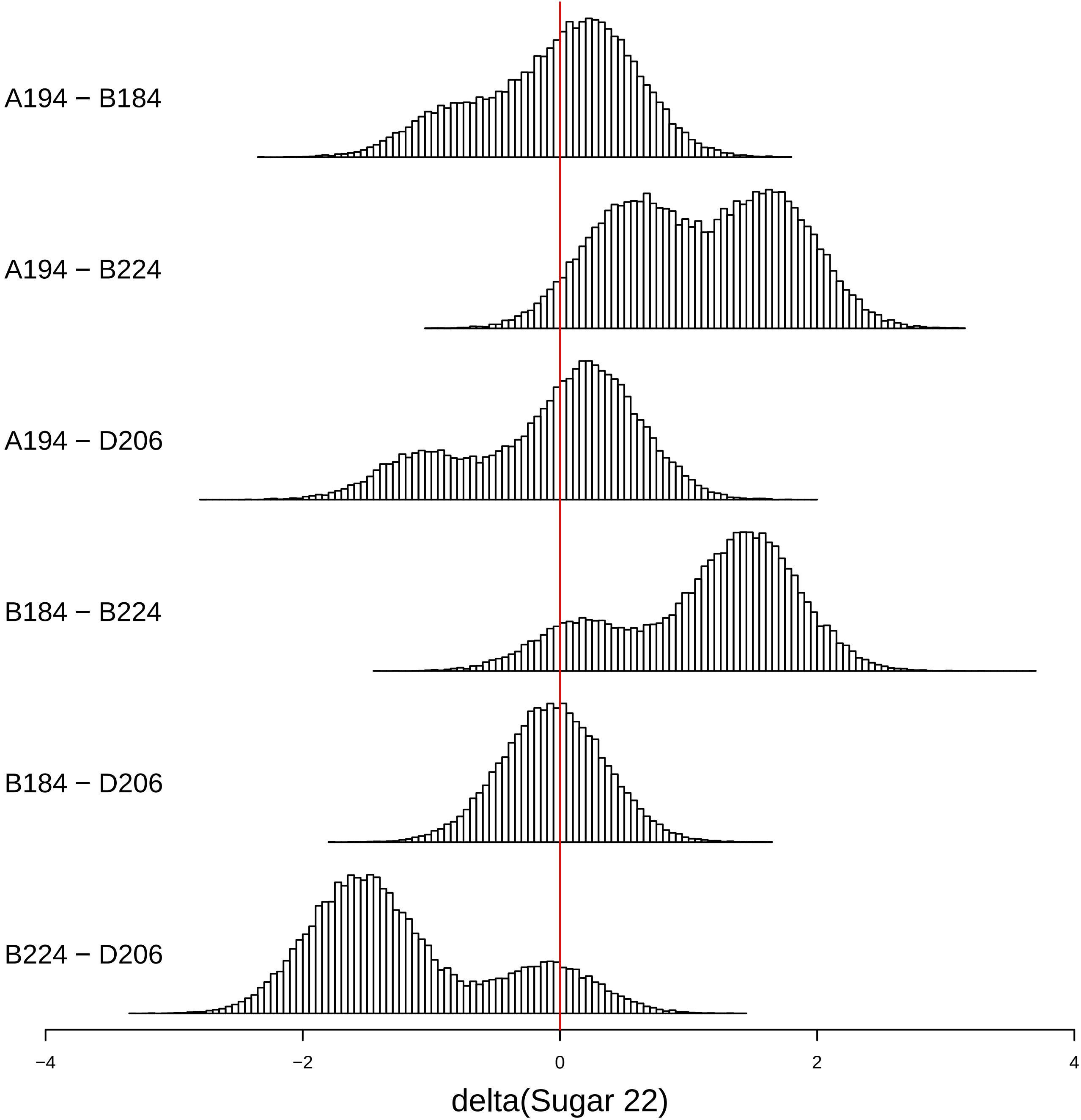
A194 − B184
A194 − B224
A194 − D206
B184 − B224
B184 − D206
B224 − D206
−4
−2
0
2
4
delta(Sugar 22)

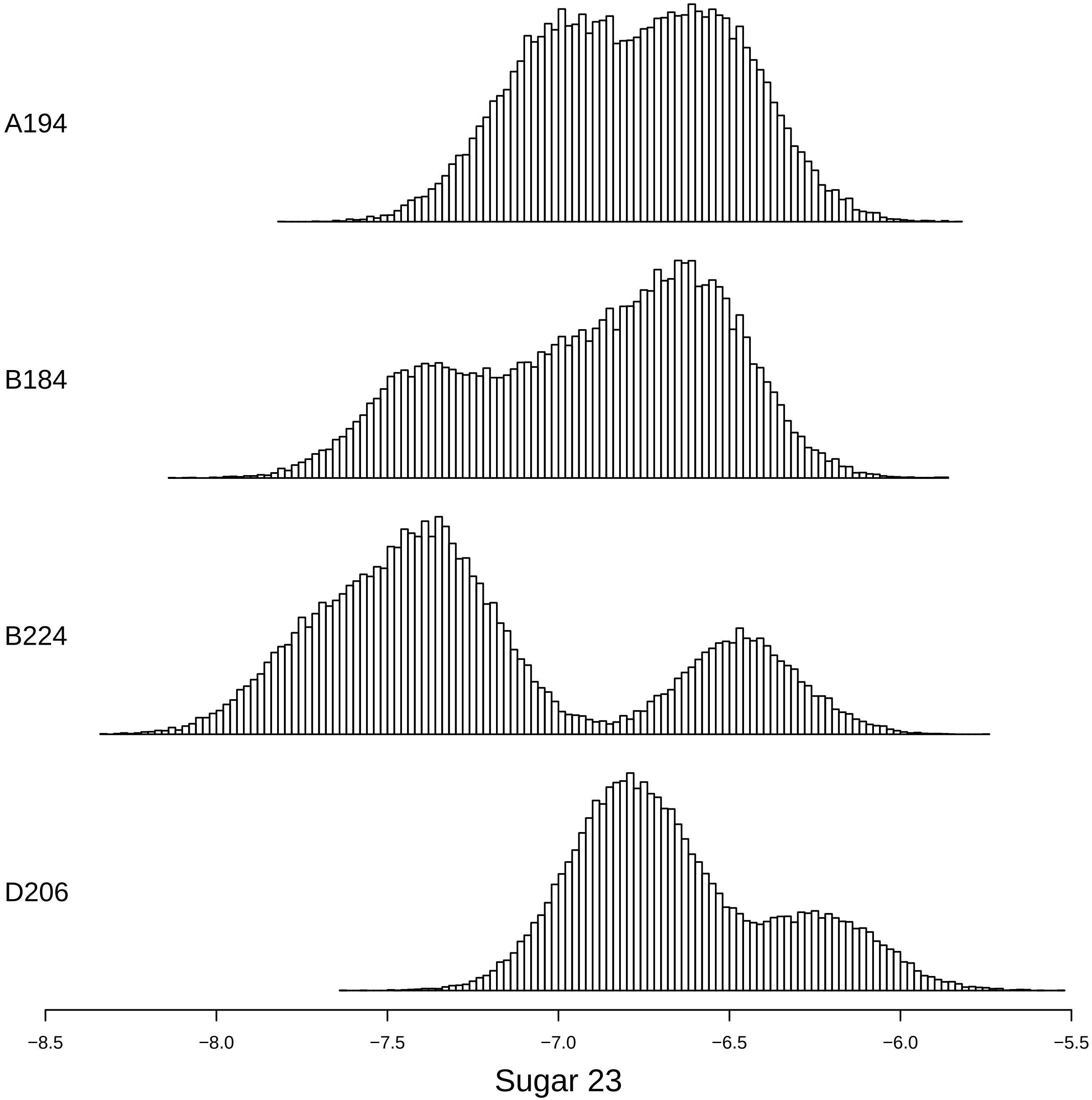

A194
B184
B224
D206
−8.5
−8.0
−7.5
−7.0
−6.5
−6.0
−5.5
Sugar 23

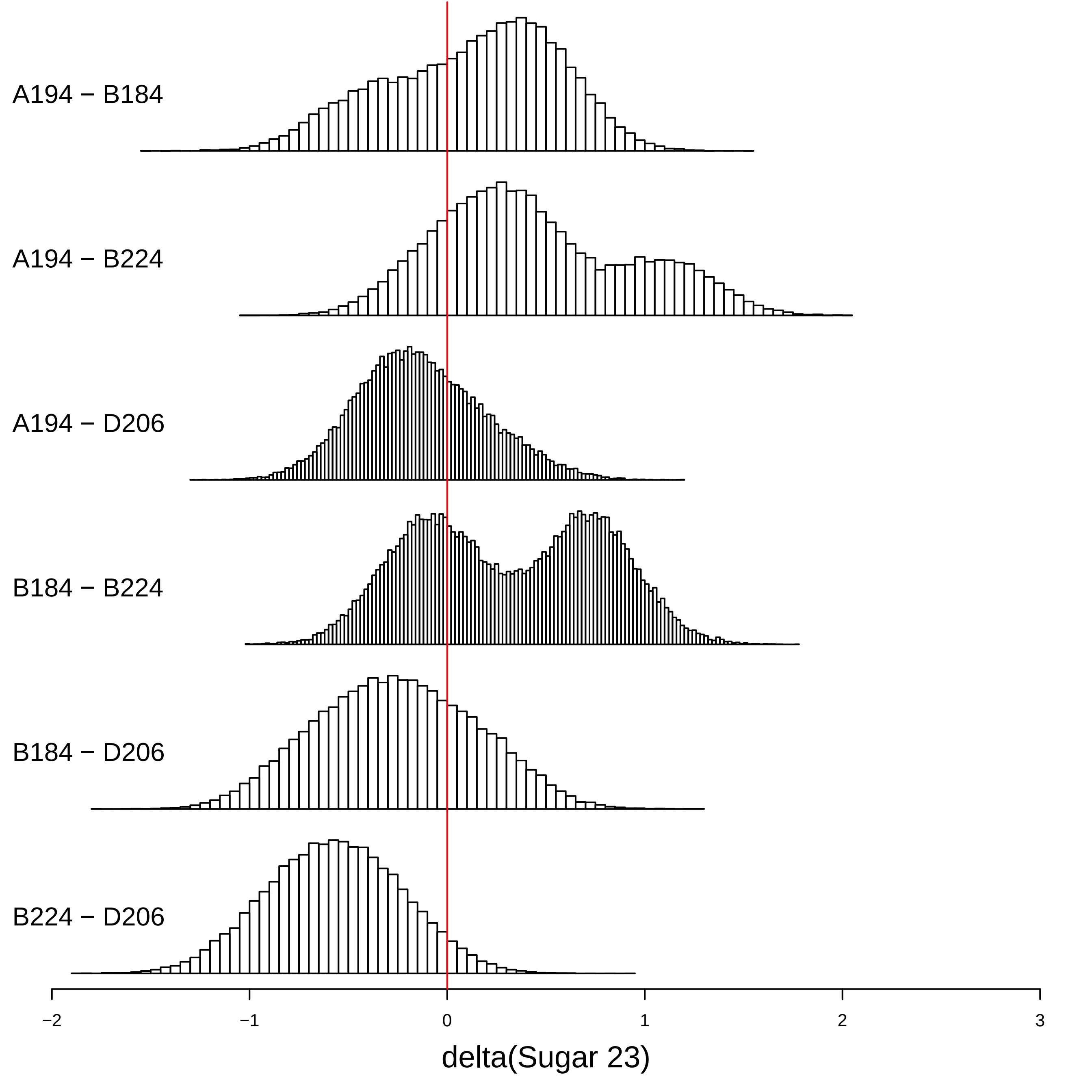

A194 − B184
A194 − B224
A194 − D206
B184 − B224
B184 − D206
B224 − D206
−2
−1
0
1
2
3
delta(Sugar 23)

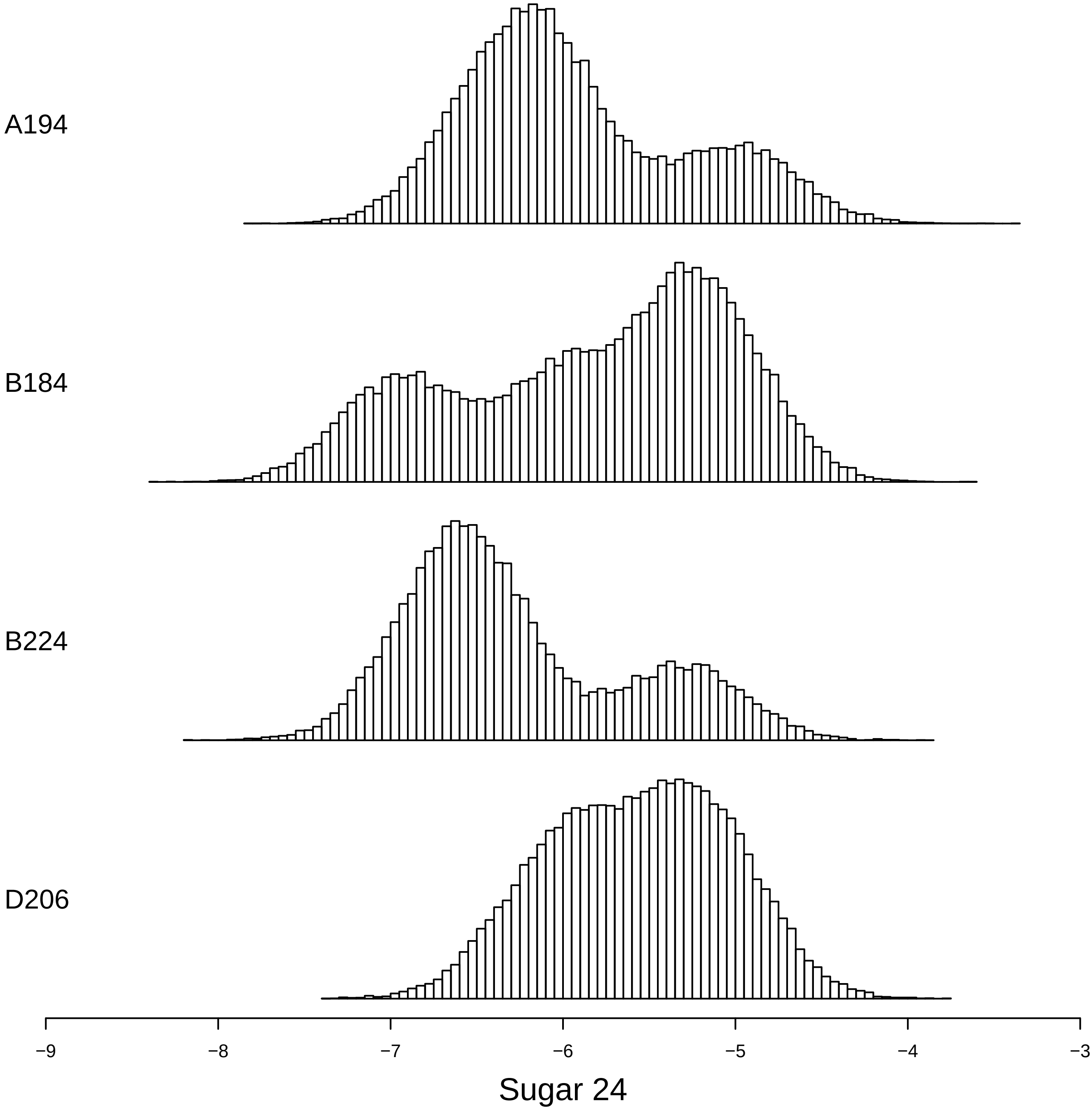

A194
B184
B224
D206
−9
−8
−7
−6
−5
−4
−3
Sugar 24

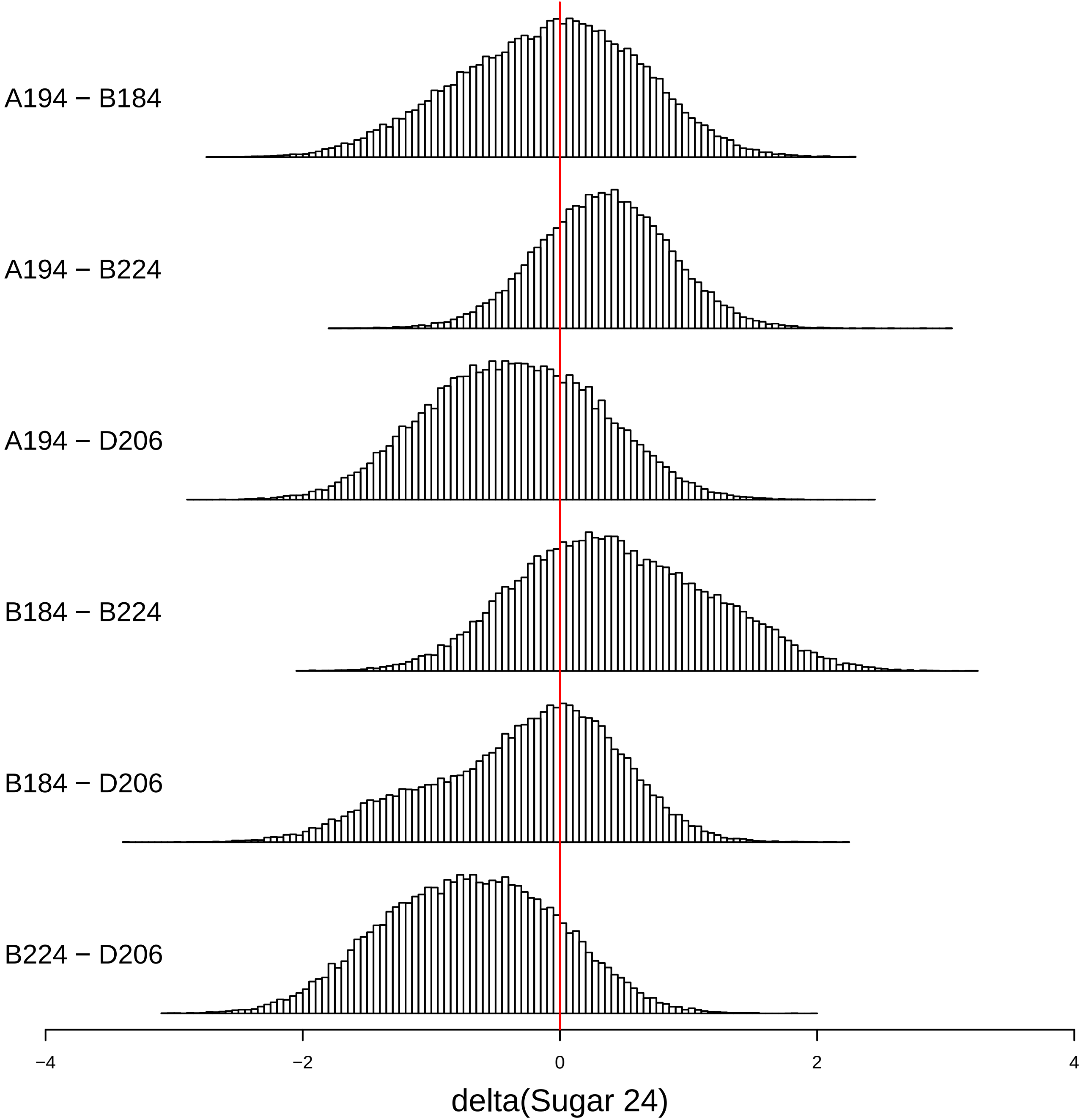
A194 − B184
A194 − B224
A194 − D206
B184 − B224
B184 − D206
B224 − D206
−4
−2
0
2
4
delta(Sugar 24)

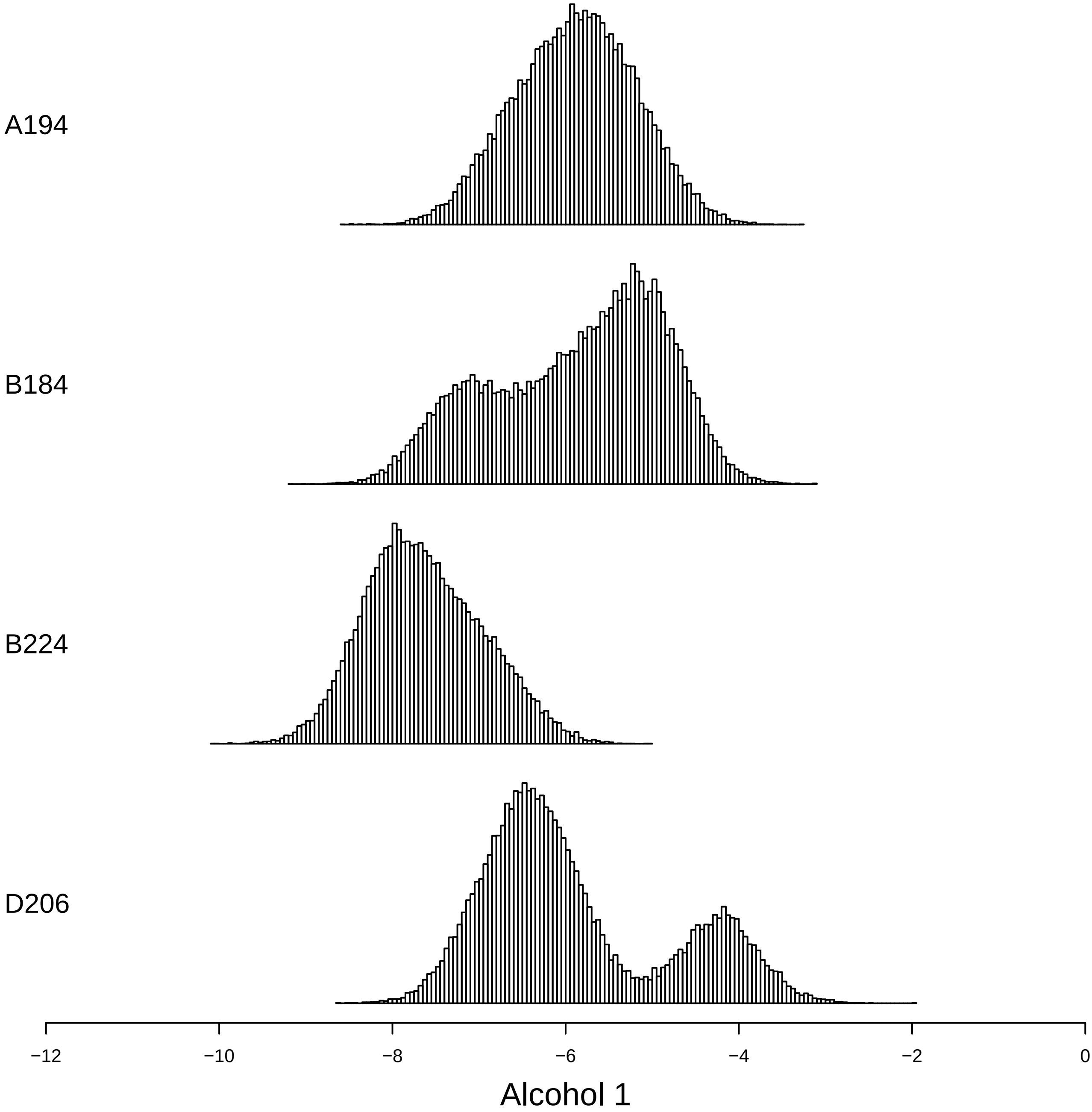

A194
B184
B224
D206
−12
−10
−8
−6
−4
−2
0
Alcohol 1

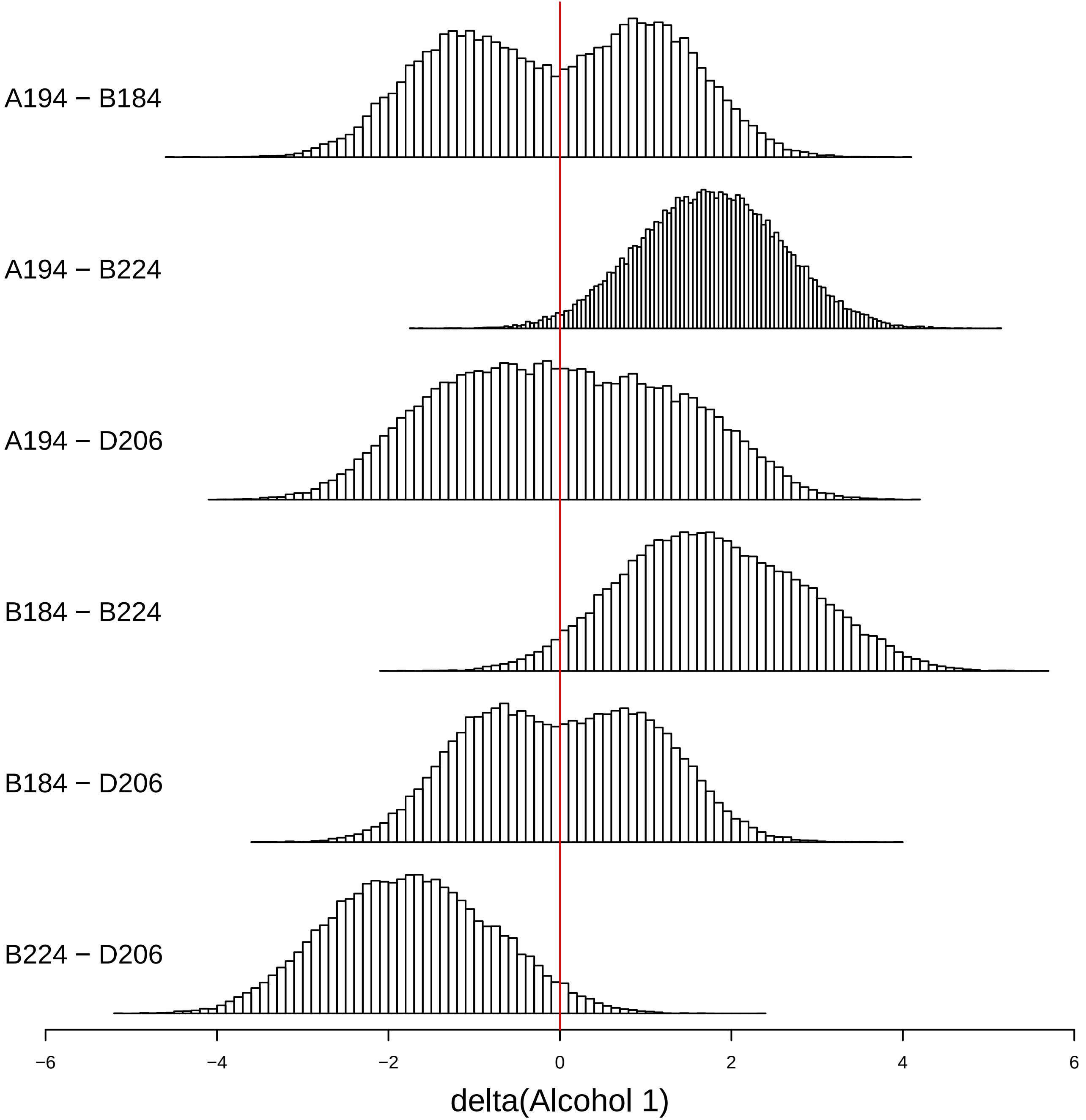

A194 − B184
A194 − B224
A194 − D206
B184 − B224
B184 − D206
B224 − D206
−6
−4
−2
0
2
4
6
delta(Alcohol 1)

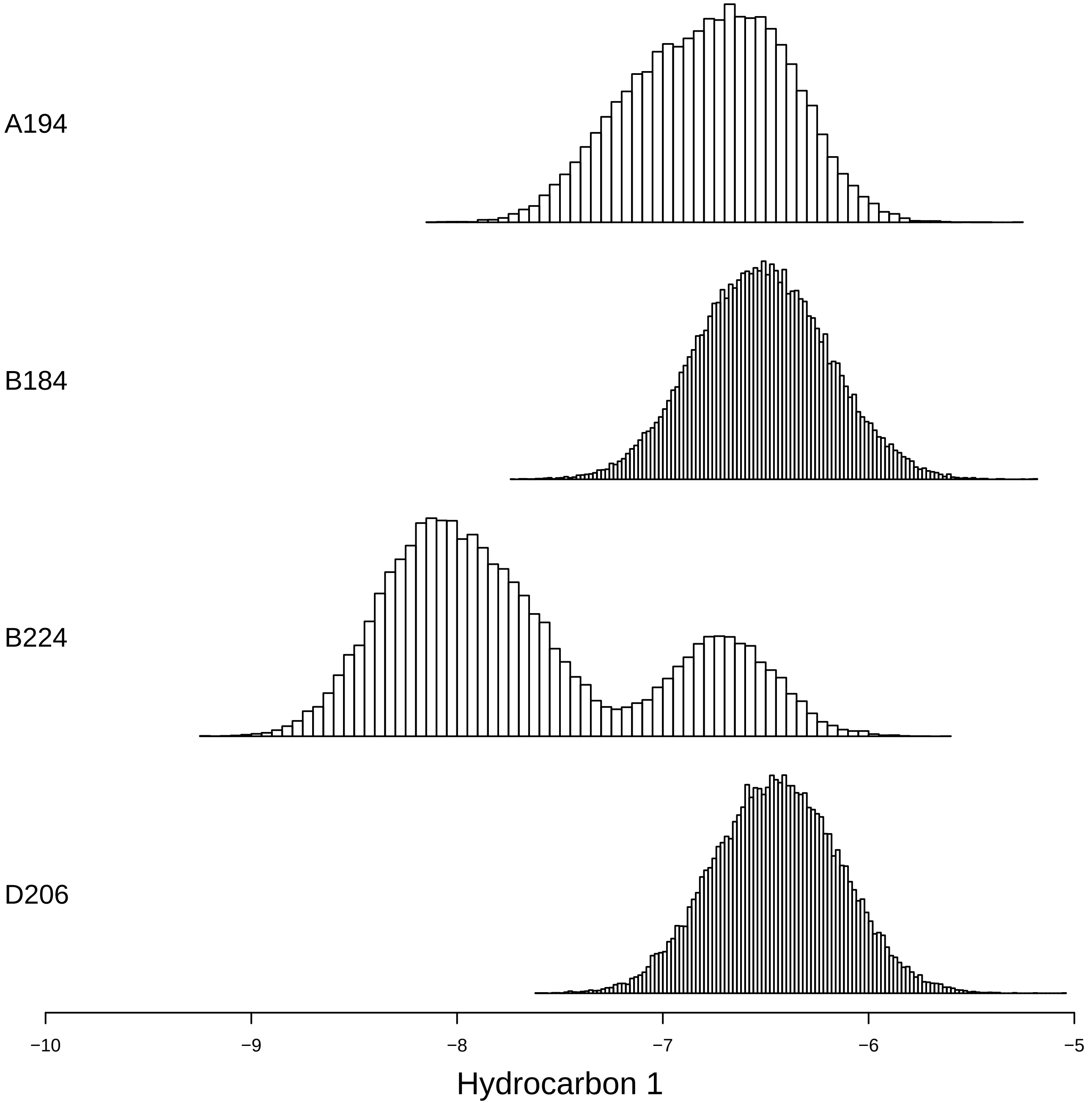

A194
B184
B224
D206
−10
−9
−8
−7
−6
−5
Hydrocarbon 1

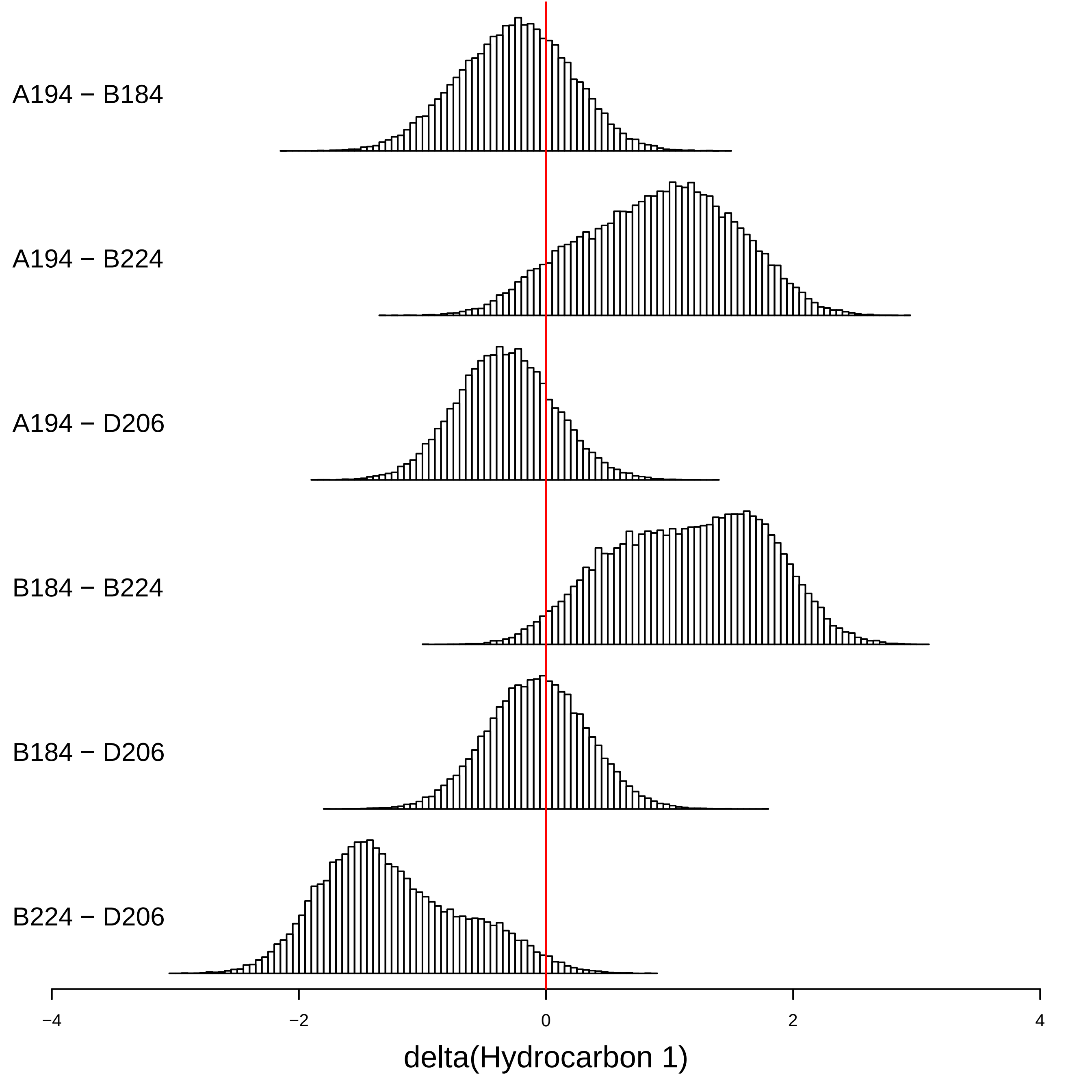

A194 − B184
A194 − B224
A194 − D206
B184 − B224
B184 − D206
B224 − D206
−4
−2
0
2
4
delta(Hydrocarbon 1)

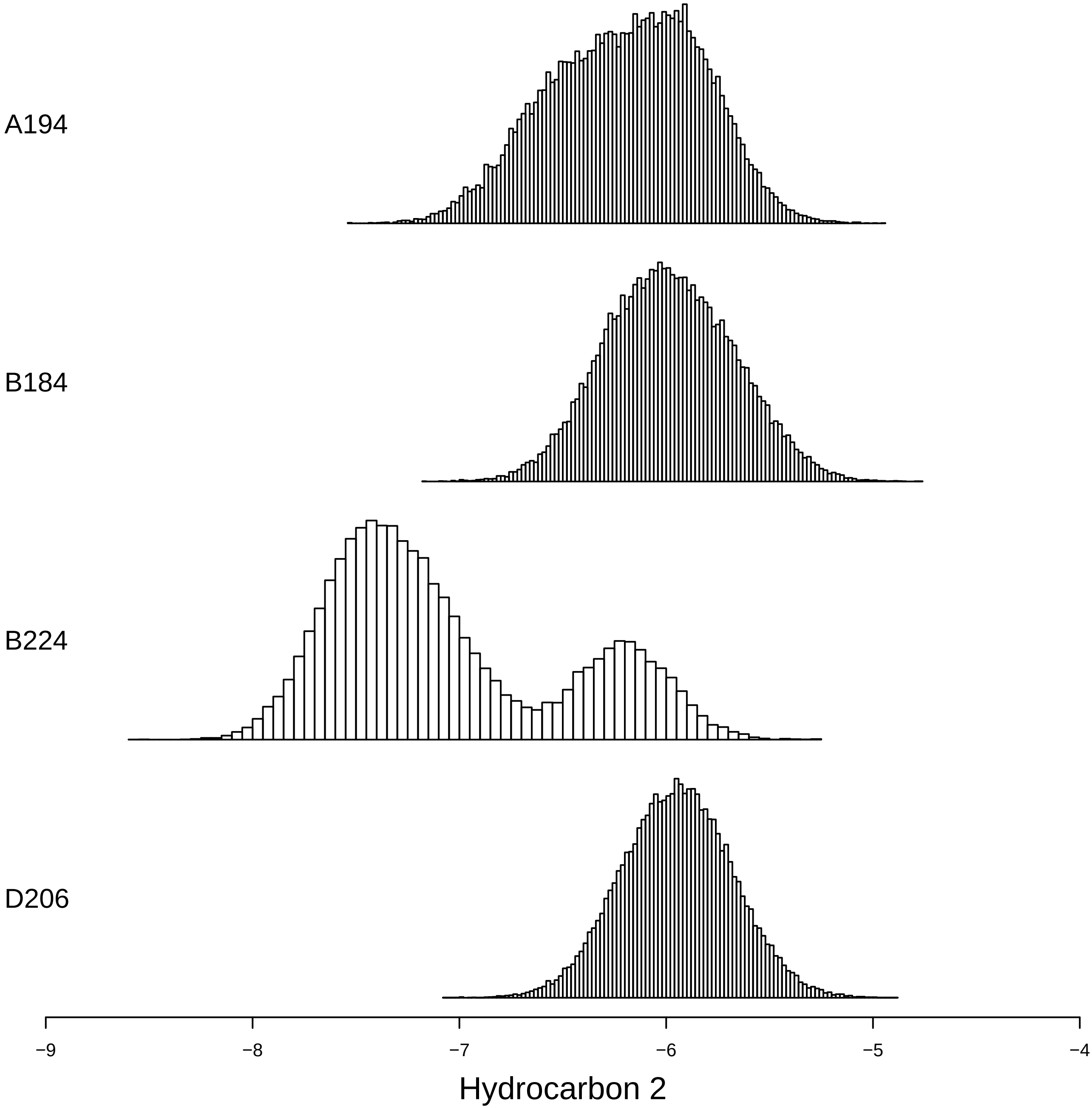

A194
B184
B224
D206
−9
−8
−7
−6
−5
−4
Hydrocarbon 2

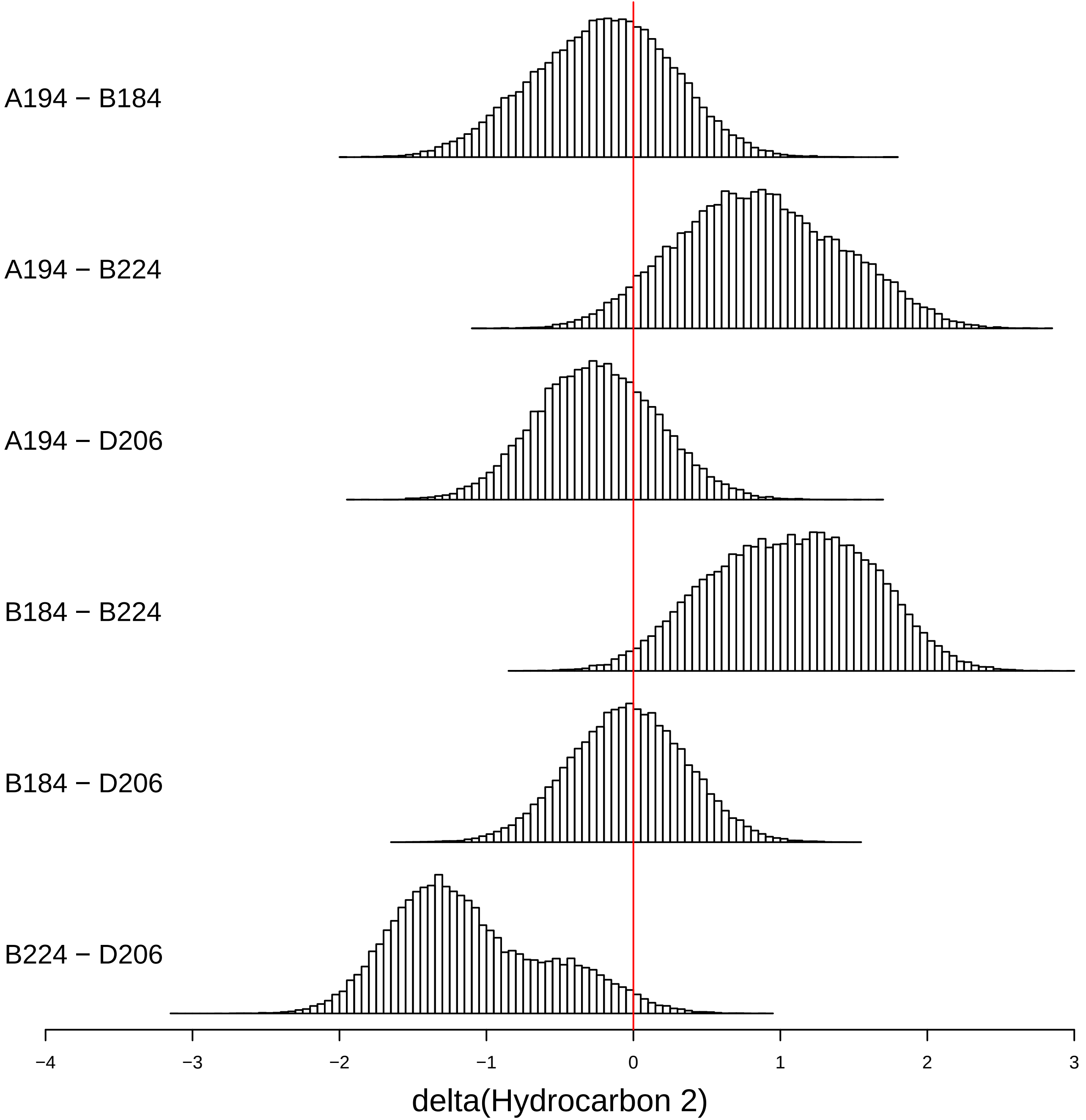

A194 − B184
A194 − B224
A194 − D206
B184 − B224
B184 − D206
B224 − D206
−4
−3
−2
−1
0
1
2
3
delta(Hydrocarbon 2)

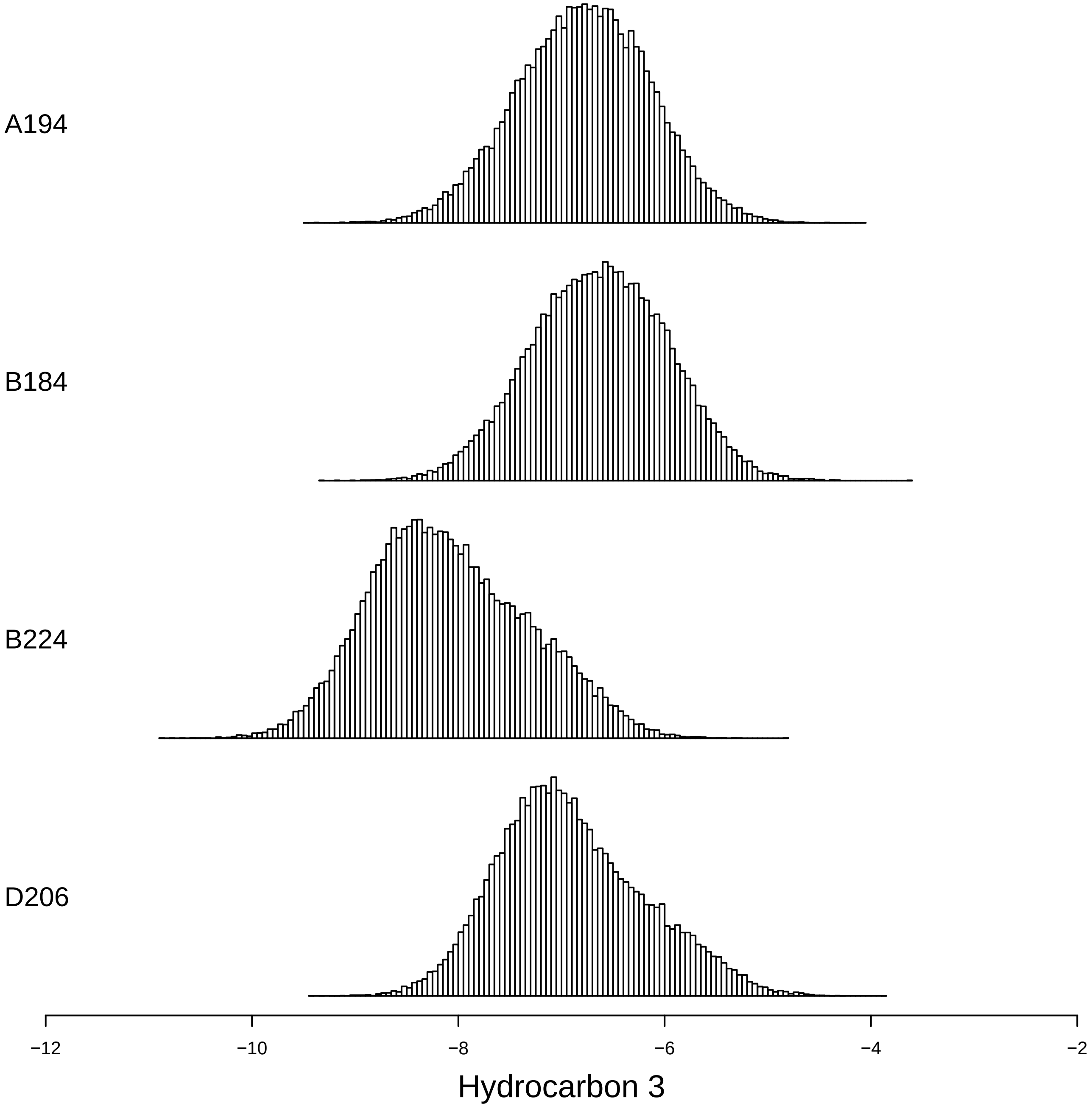
A194
B184
B224
D206
−12
−10
−8
−6
−4
−2
Hydrocarbon 3

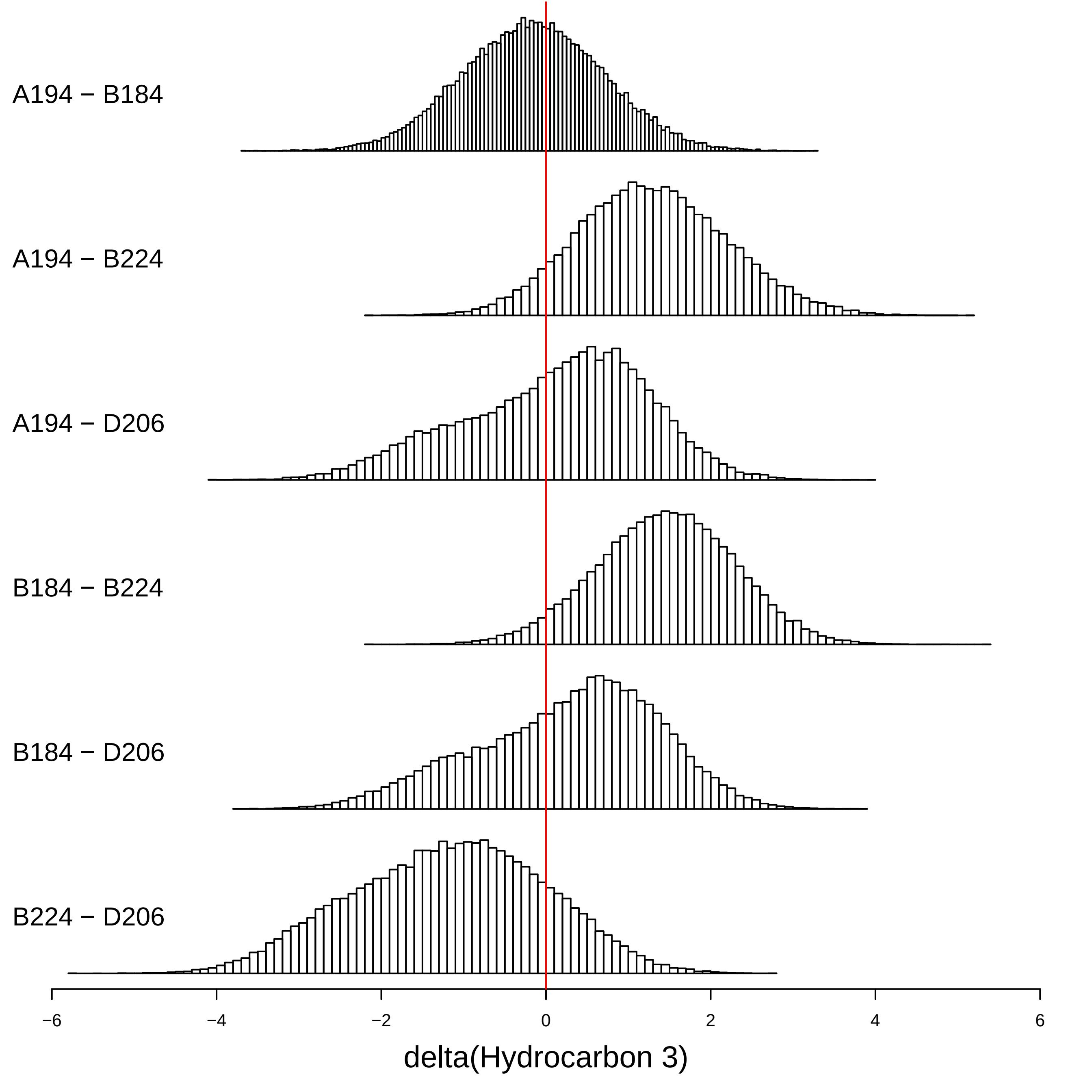

A194 − B184
A194 − B224
A194 − D206
B184 − B224
B184 − D206
B224 − D206
−6
−4
−2
0
2
4
6
delta(Hydrocarbon 3)

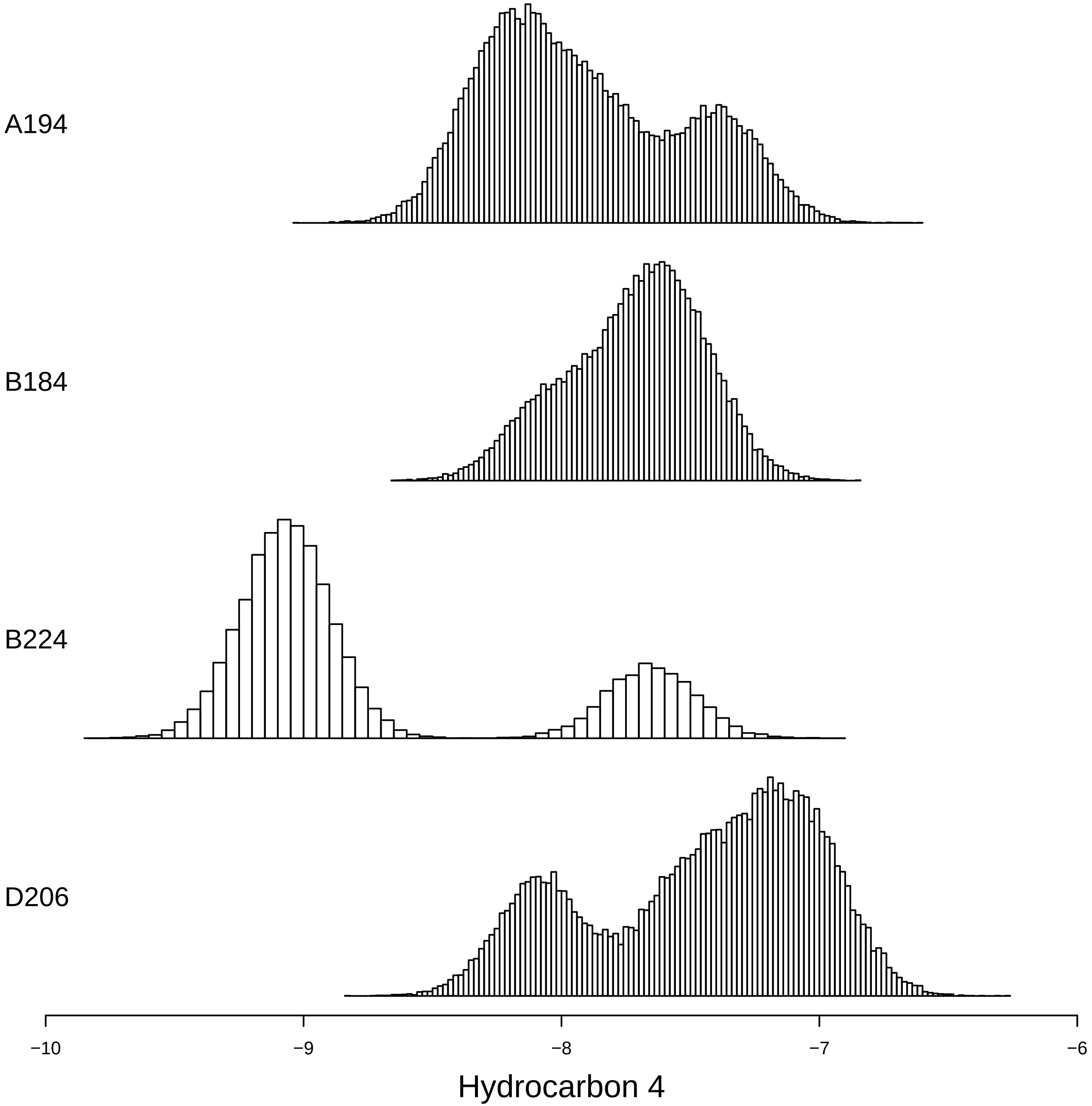
A194
B184
B224
D206
−10
−9
−8
−7
−6
Hydrocarbon 4

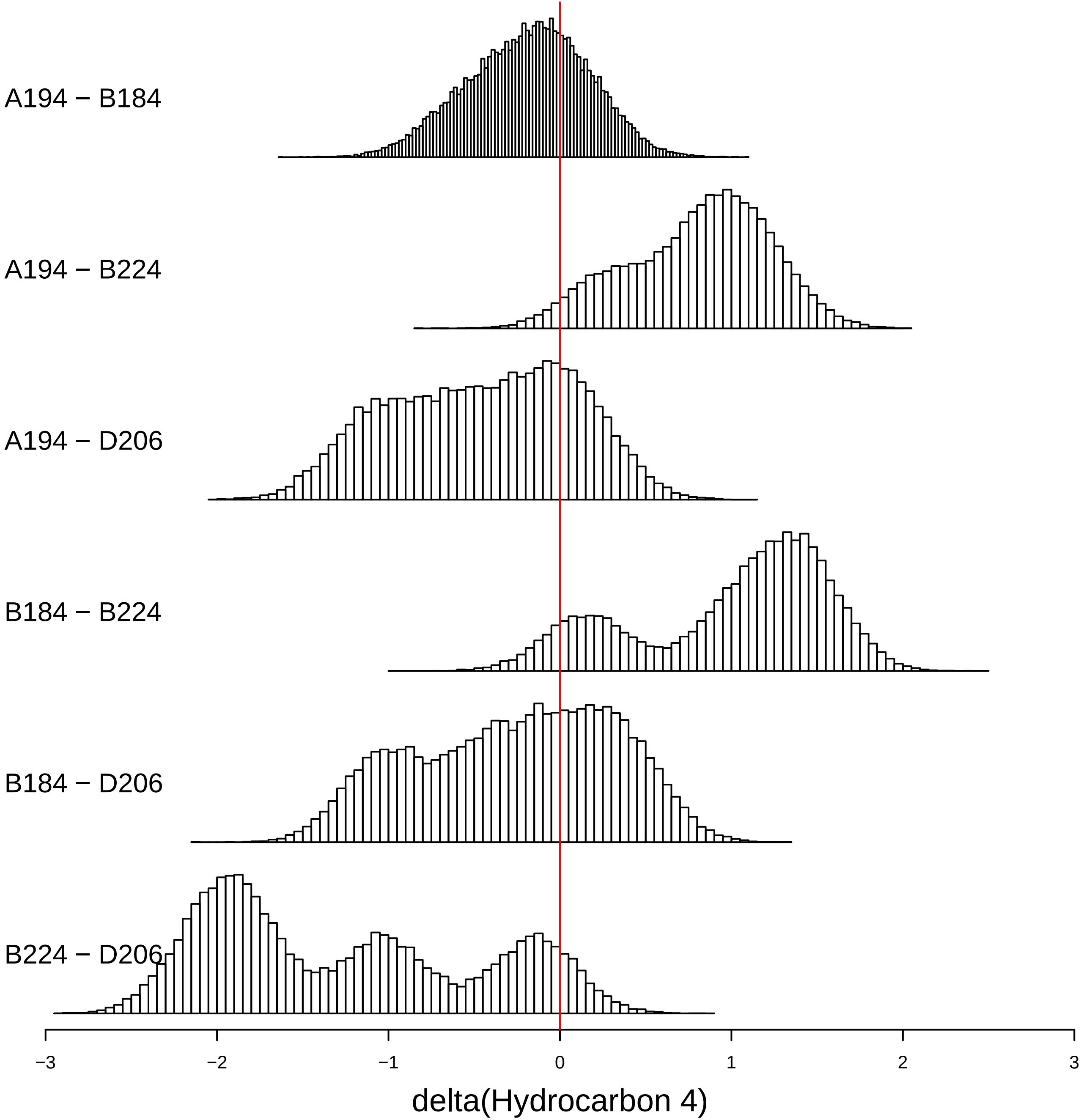

A194 − B184
A194 − B224
A194 − D206
B184 − B224
B184 − D206
B224 − D206
−3
−2
−1
0
1
2
3
delta(Hydrocarbon 4)

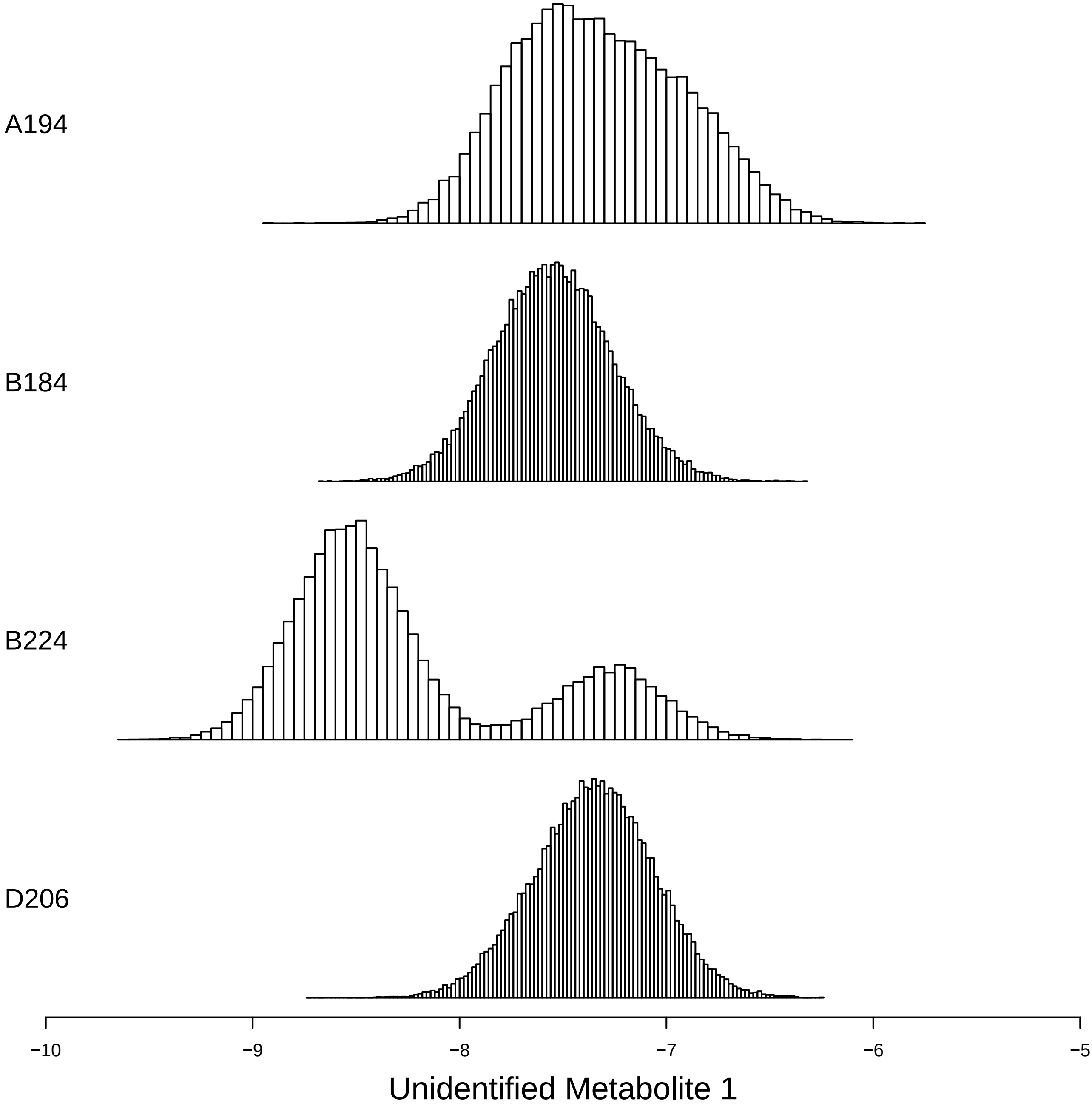

A194
B184
B224
D206
−10
−9
−8
−7
−6
−5
Unidentified Metabolite 1

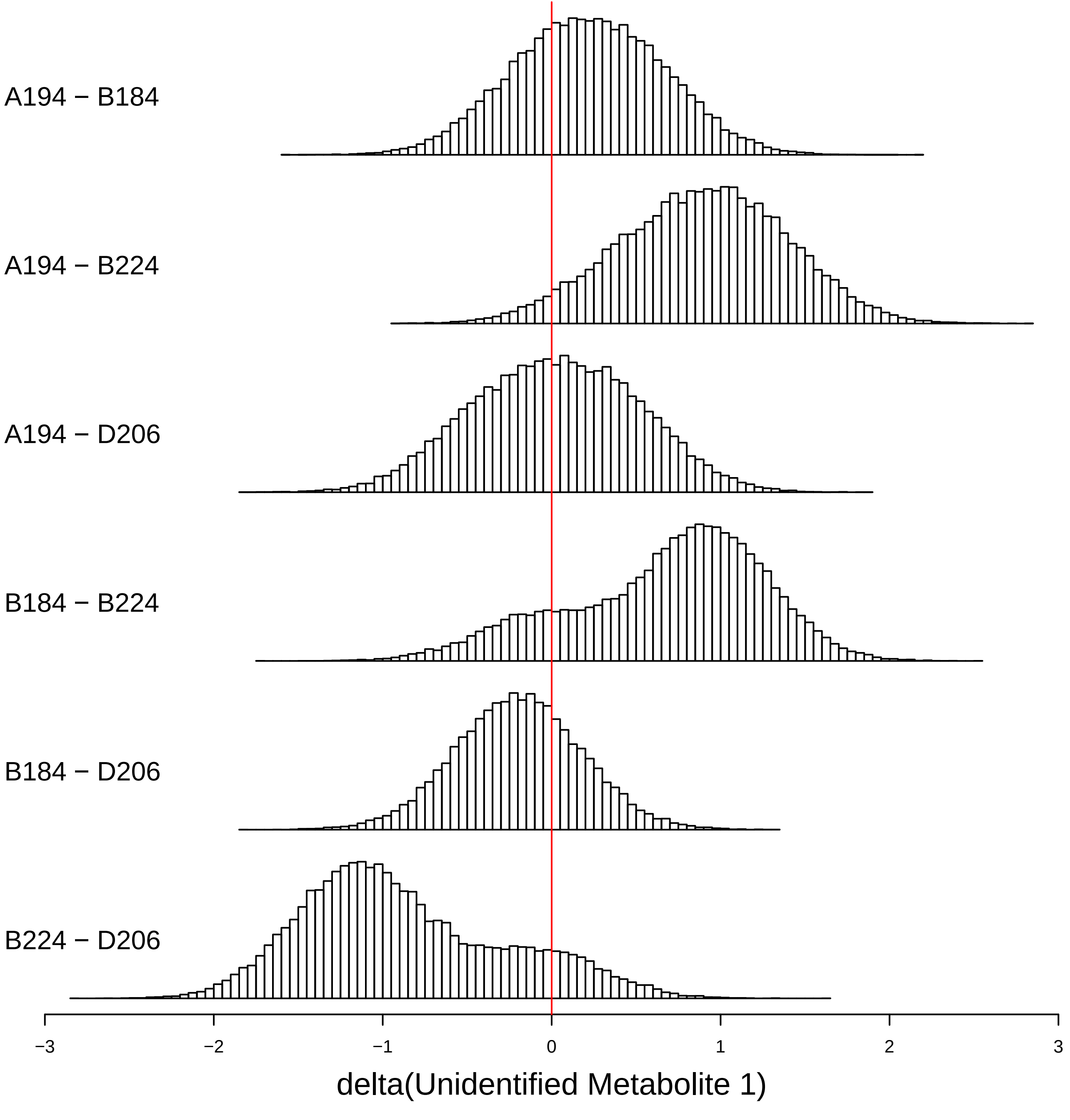
A194 − B184
A194 − B224
A194 − D206
B184 − B224
B184 − D206
B224 − D206
−3
−2
−1
0
1
2
3
delta(Unidentified Metabolite 1)

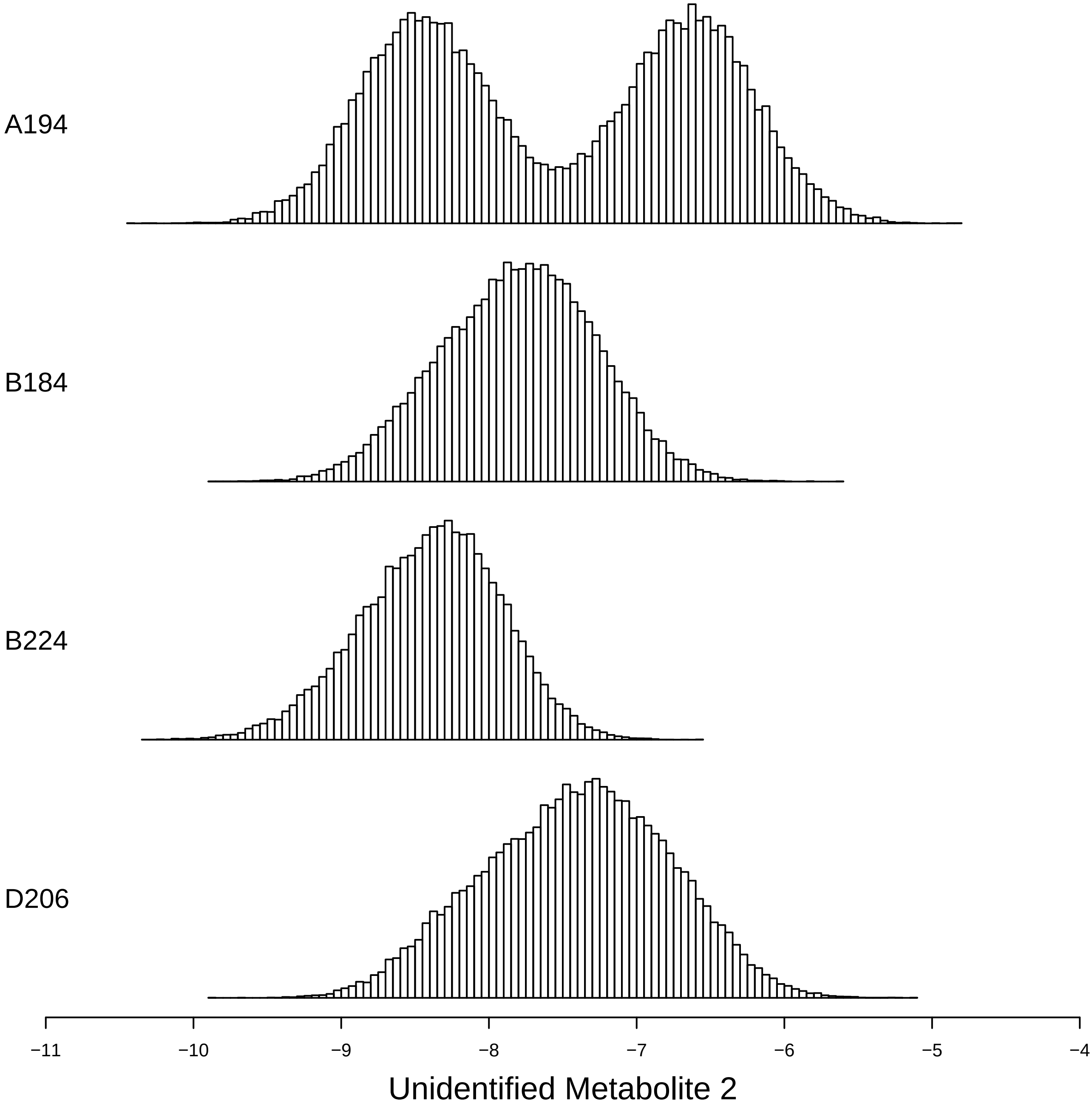
A194
B184
B224
D206
−11
−10
−9
−8
−7
−6
−5
−4
Unidentified Metabolite 2

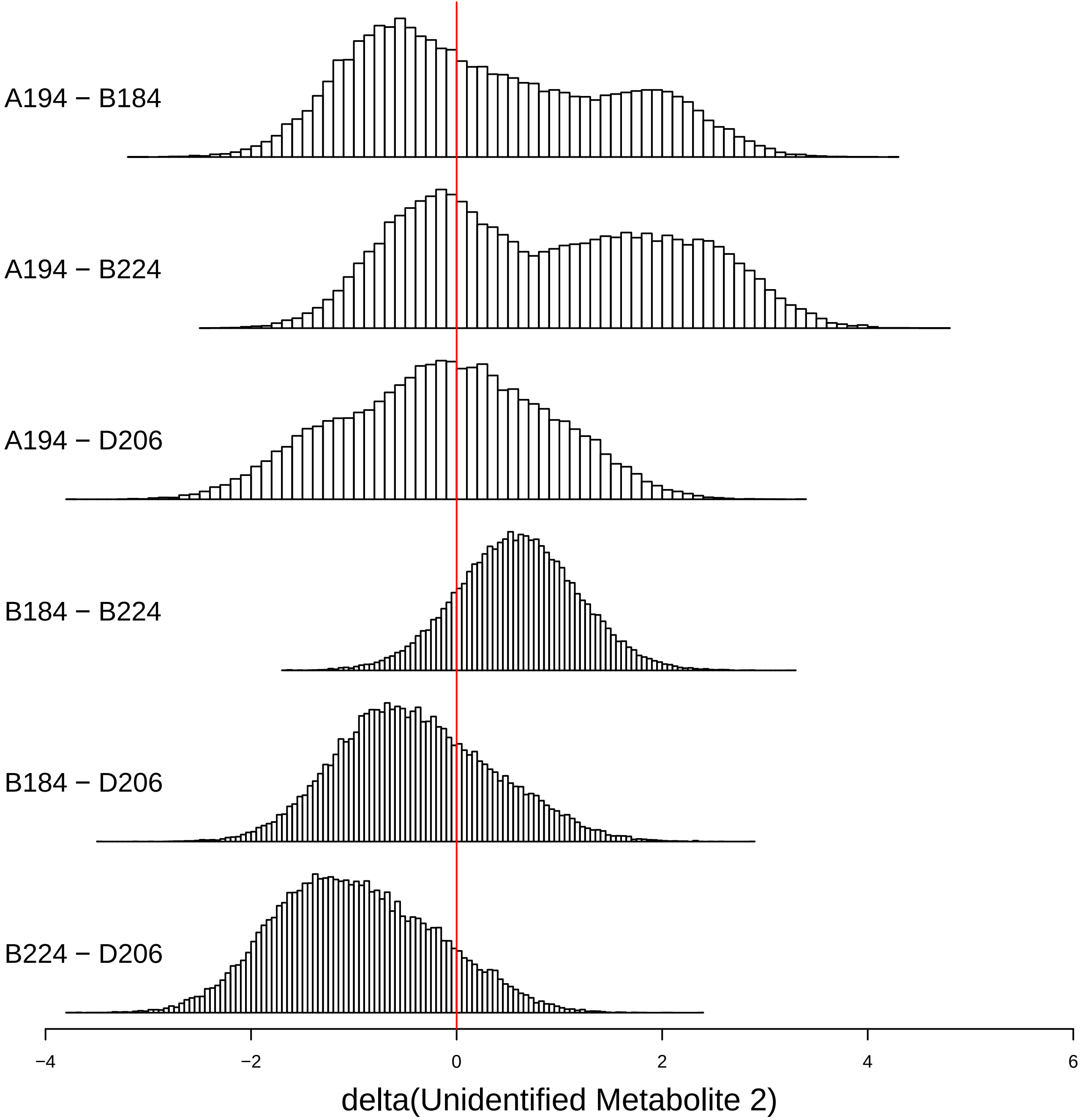
A194 − B184
A194 − B224
A194 − D206
B184 − B224
B184 − D206
B224 − D206
−4
−2
0
2
4
6
delta(Unidentified Metabolite 2)

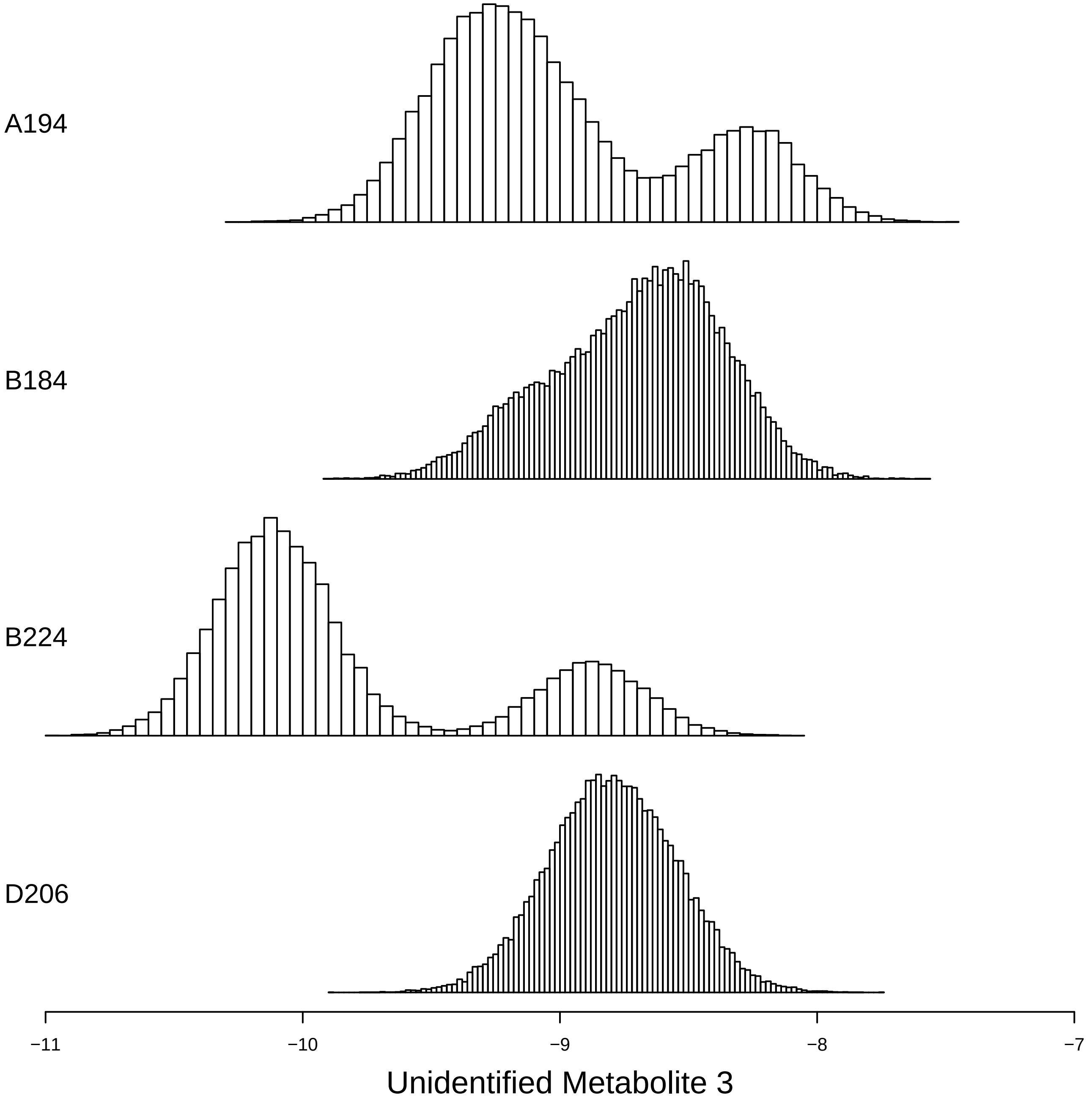

A194
B184
B224
D206
−11
−10
−9
−8
−7
Unidentified Metabolite 3

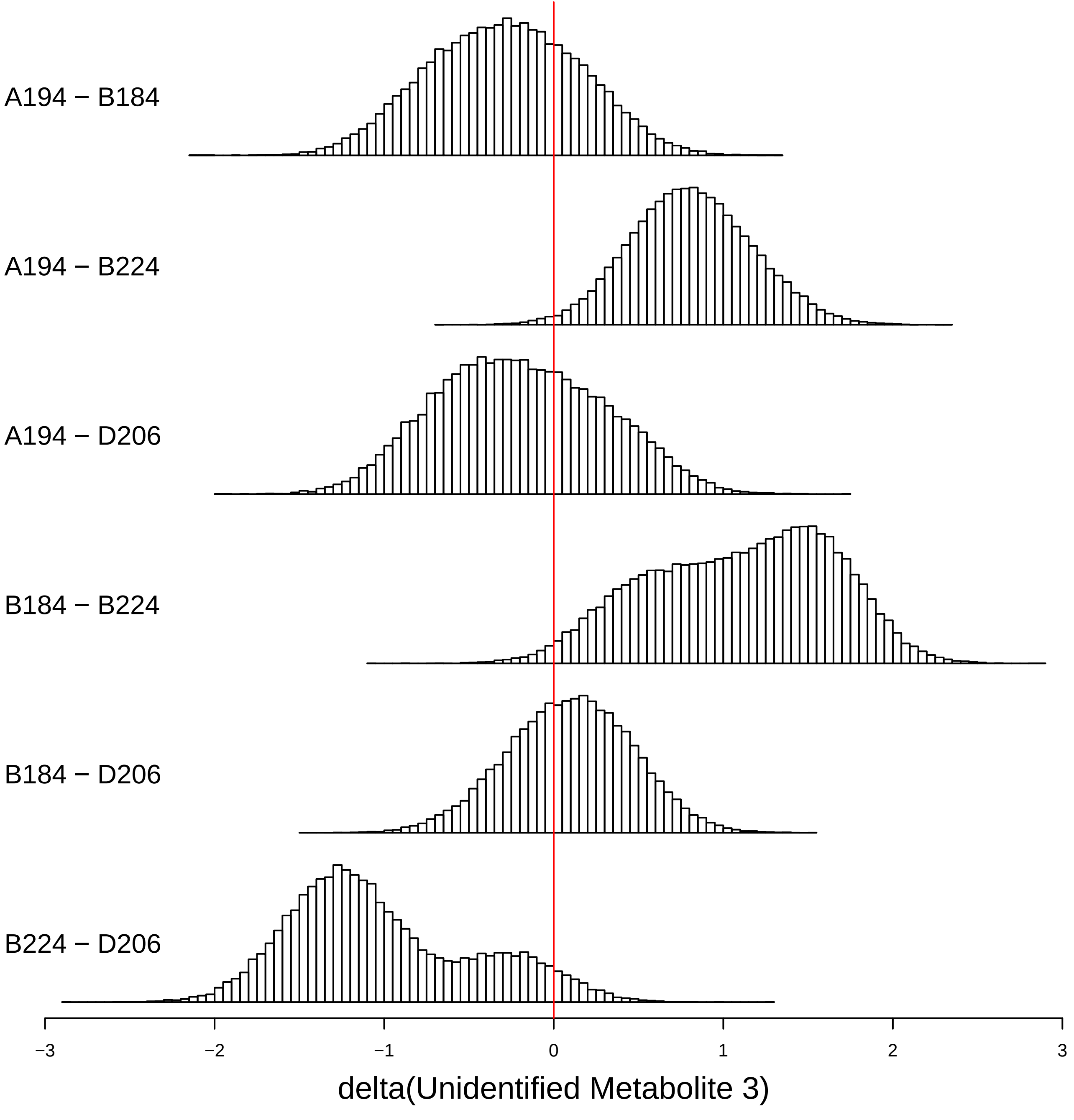
A194 − B184
A194 − B224
A194 − D206
B184 − B224
B184 − D206
B224 − D206
−3
−2
−1
0
1
2
3
delta(Unidentified Metabolite 3)

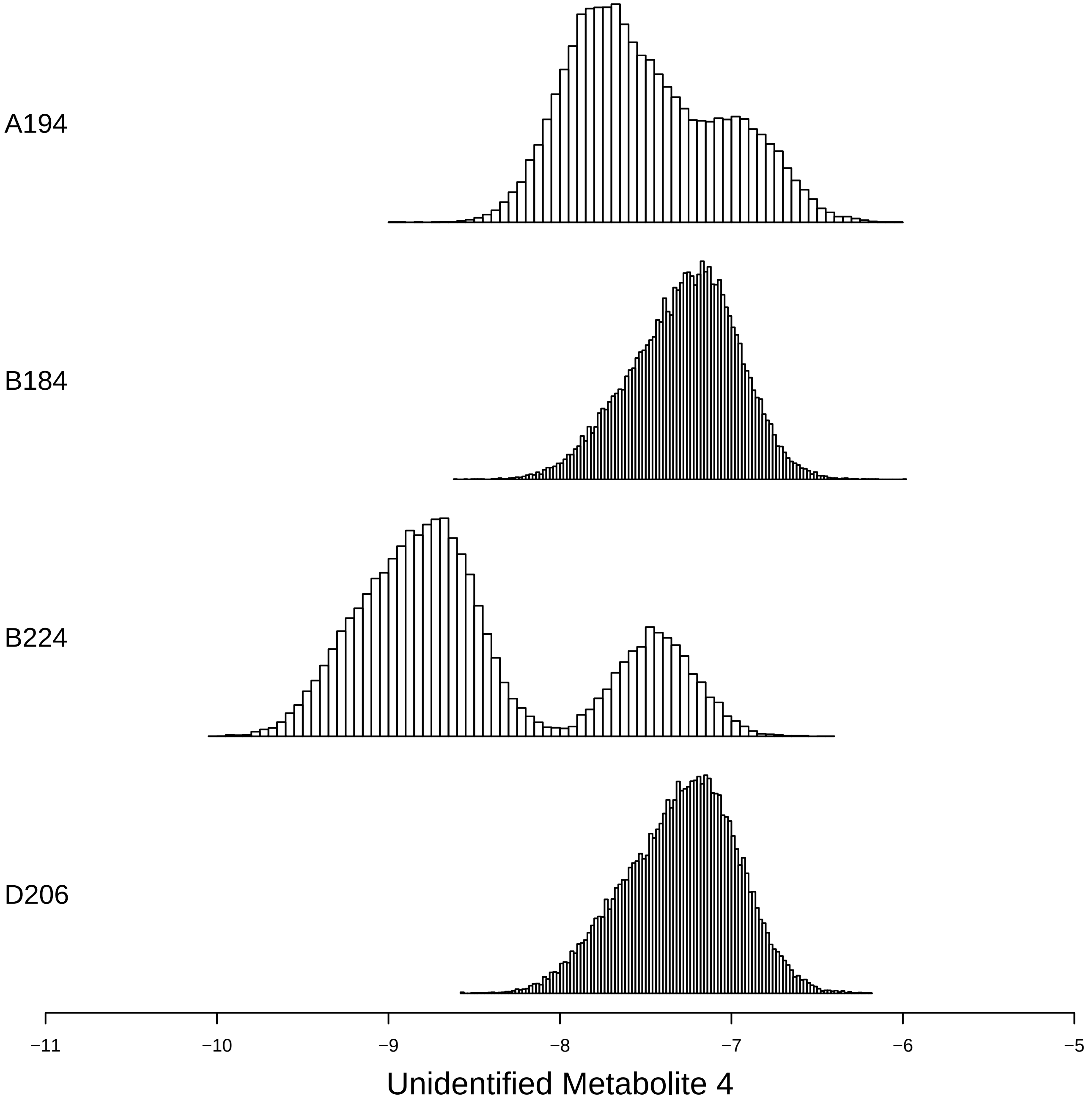
A194
B184
B224
D206
−11
−10
−9
−8
−7
−6
−5
Unidentified Metabolite 4

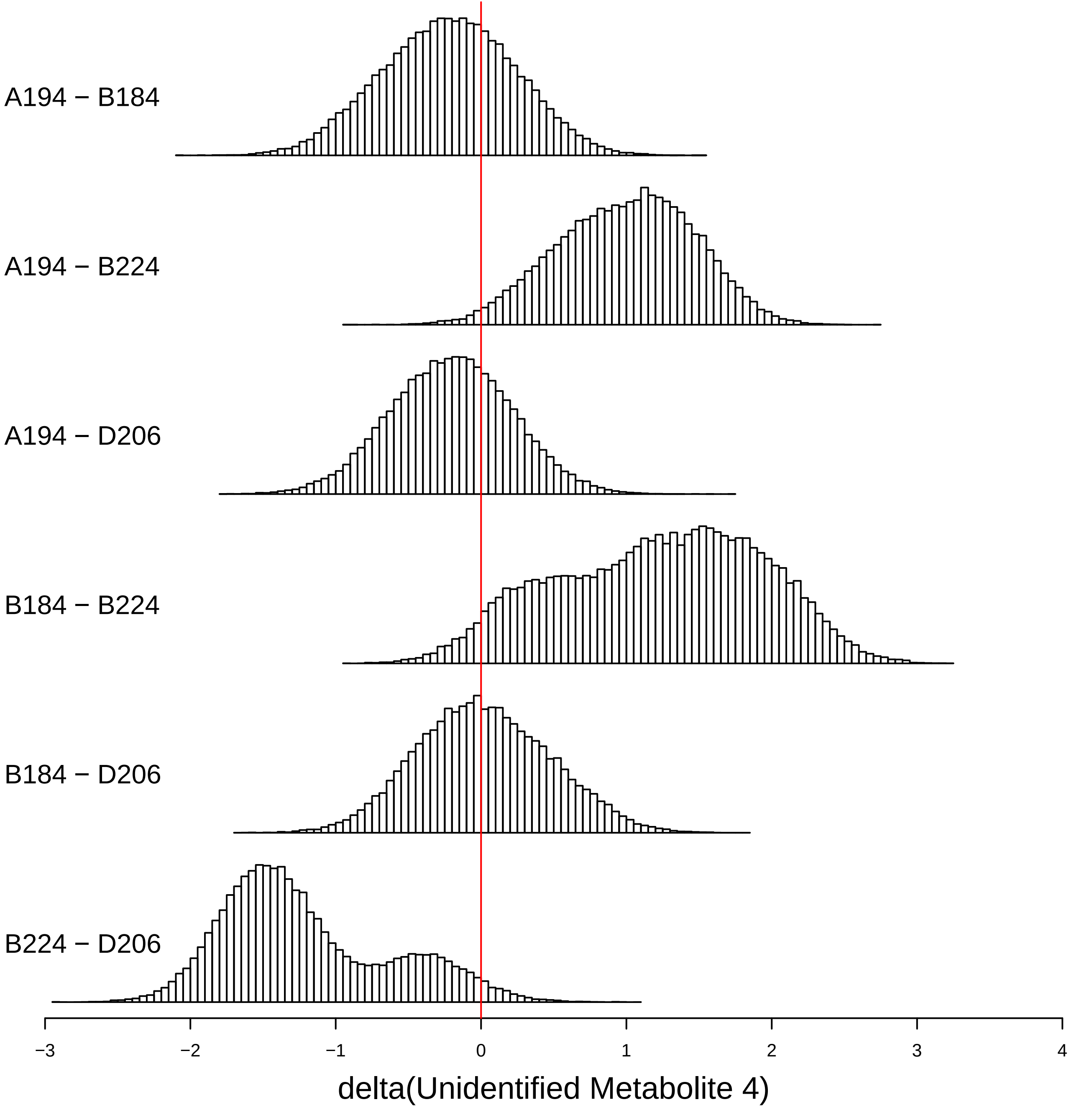
A194 − B184
A194 − B224
A194 − D206
B184 − B224
B184 − D206
B224 − D206
−3
−2
−1
0
1
2
3
4
delta(Unidentified Metabolite 4)

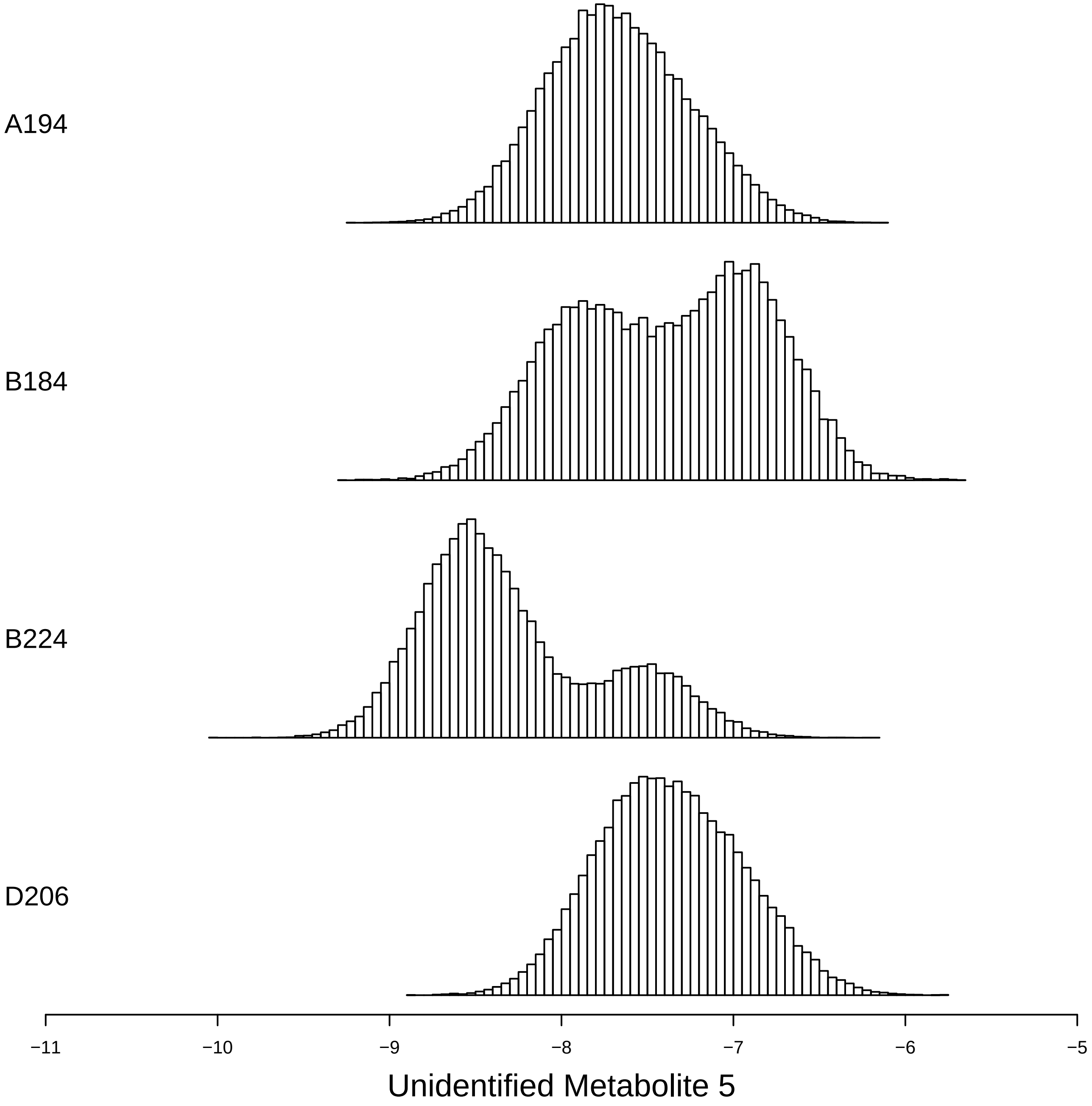
A194
B184
B224
D206
−11
−10
−9
−8
−7
−6
−5
Unidentified Metabolite 5

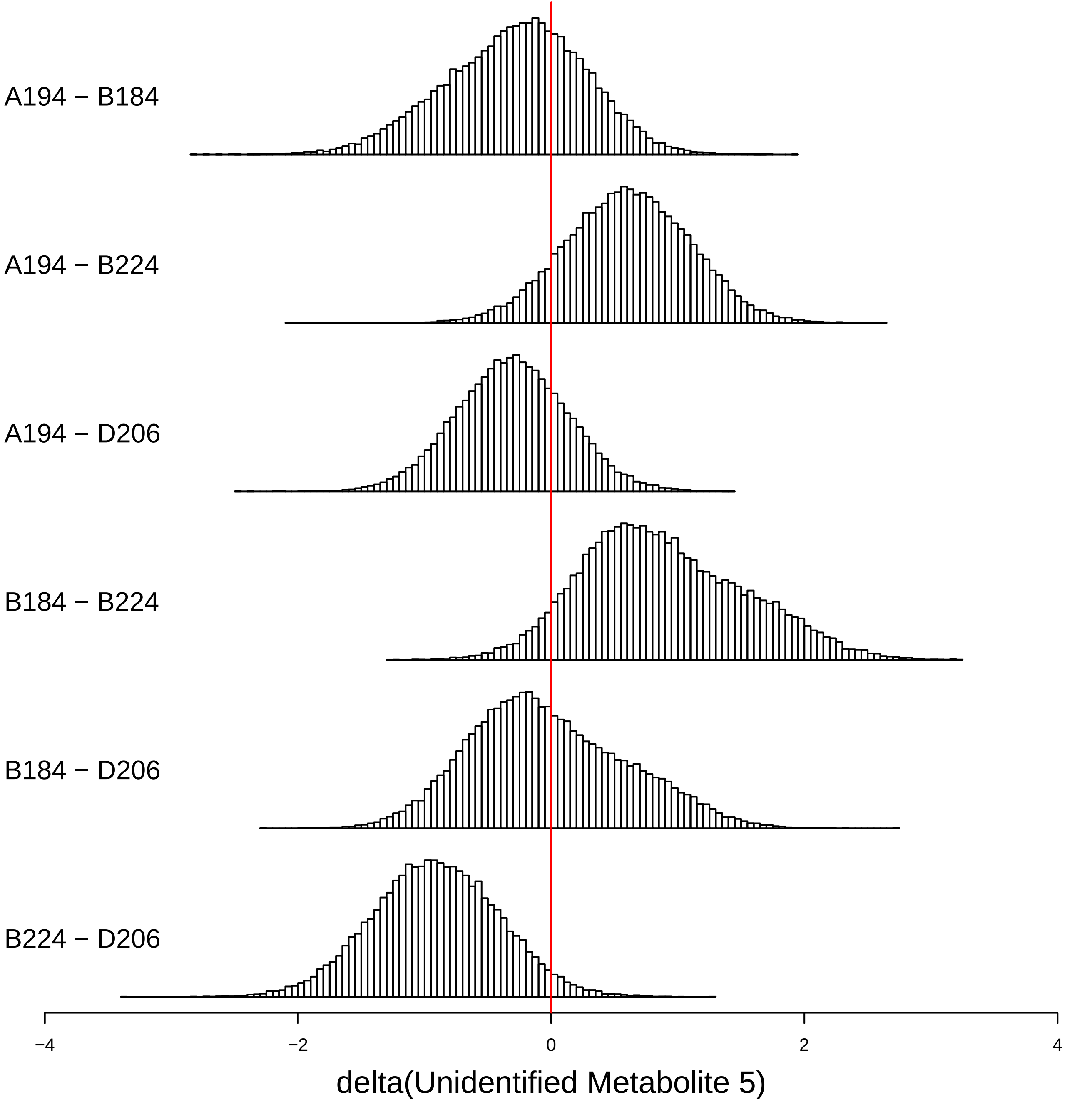
A194 − B184
A194 − B224
A194 − D206
B184 − B224
B184 − D206
B224 − D206
−4
−2
0
2
4
delta(Unidentified Metabolite 5)

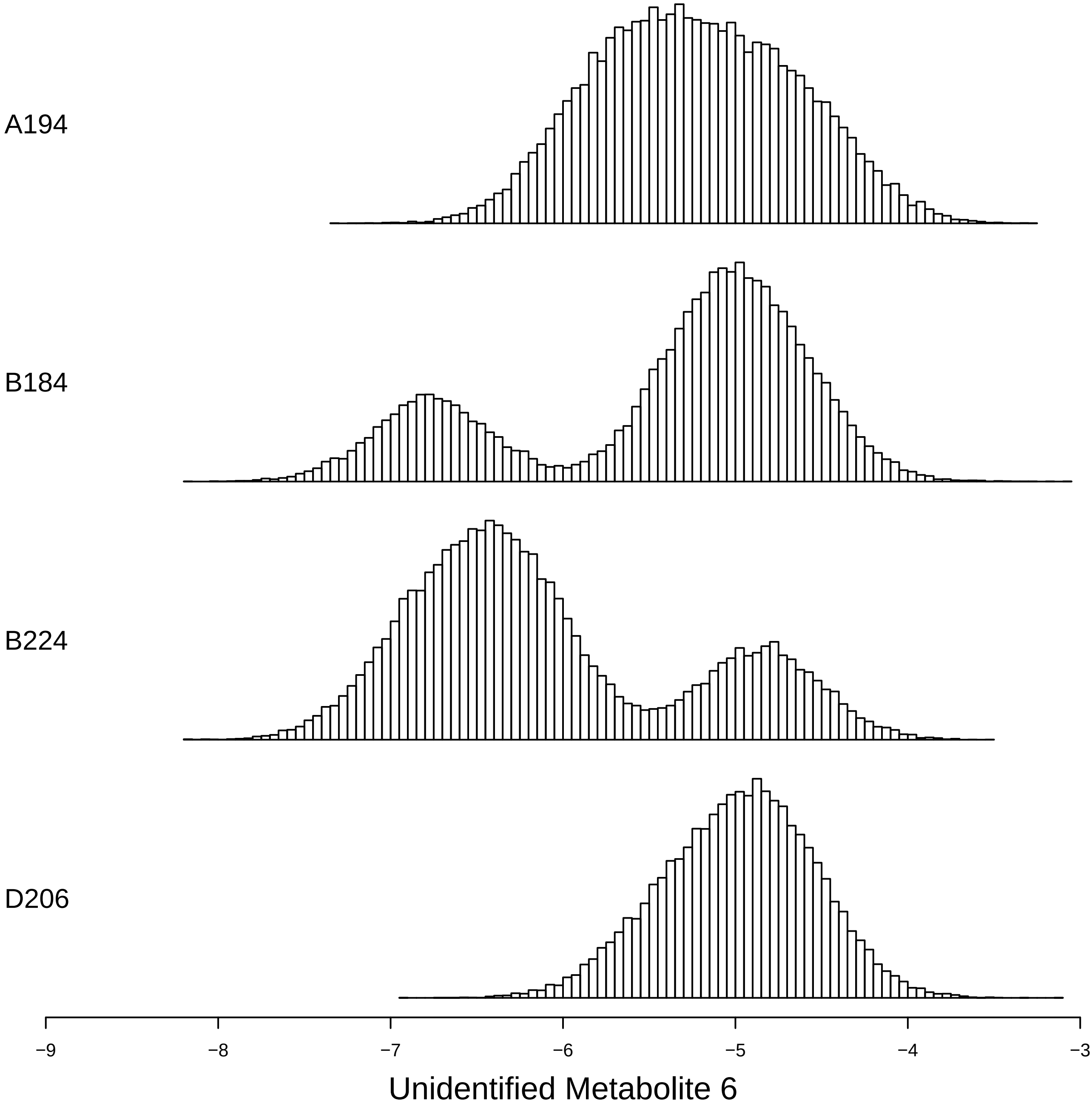
A194
B184
B224
D206
−9
−8
−7
−6
−5
−4
−3
Unidentified Metabolite 6

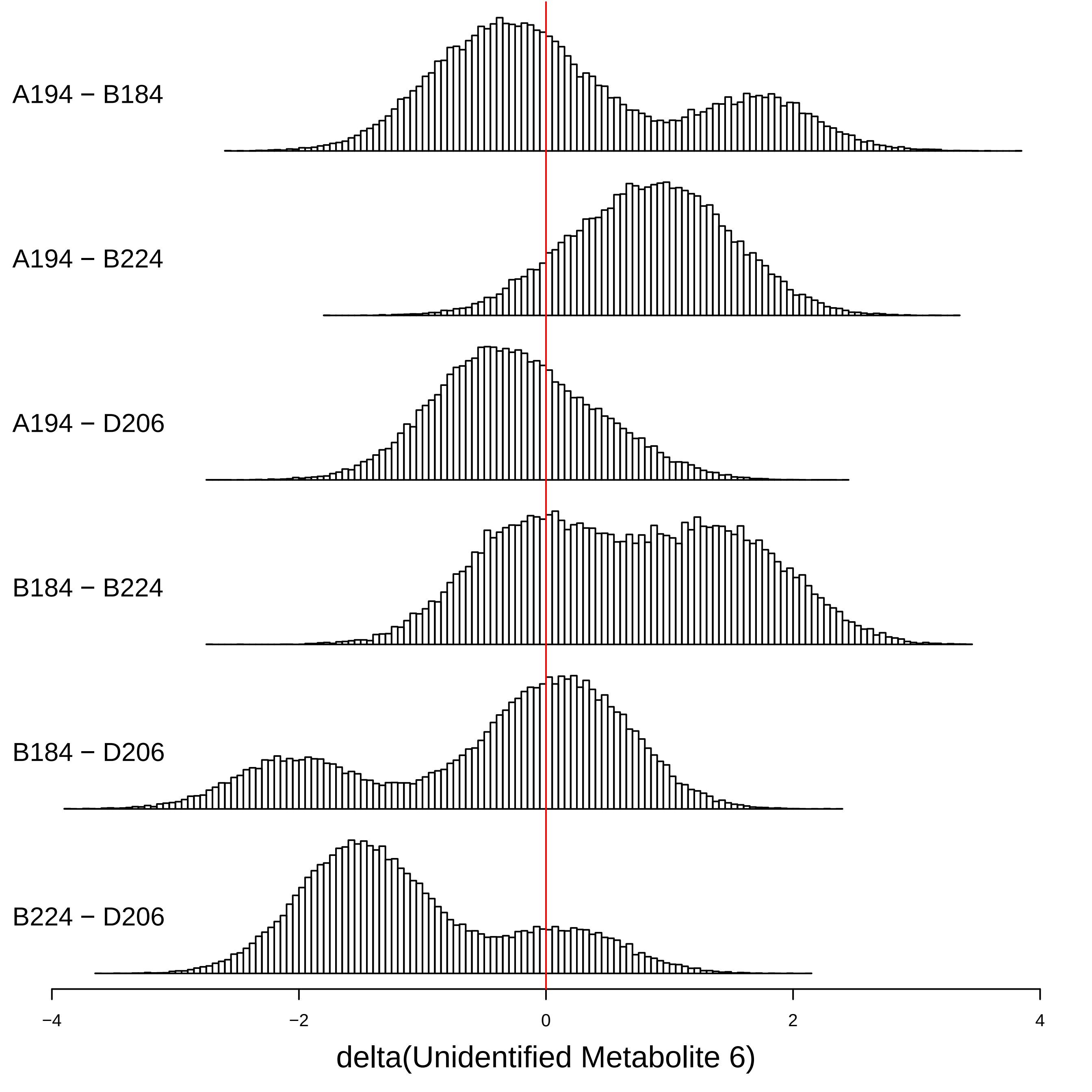
A194 − B184
A194 − B224
A194 − D206
B184 − B224
B184 − D206
B224 − D206
−4
−2
0
2
4
delta(Unidentified Metabolite 6)

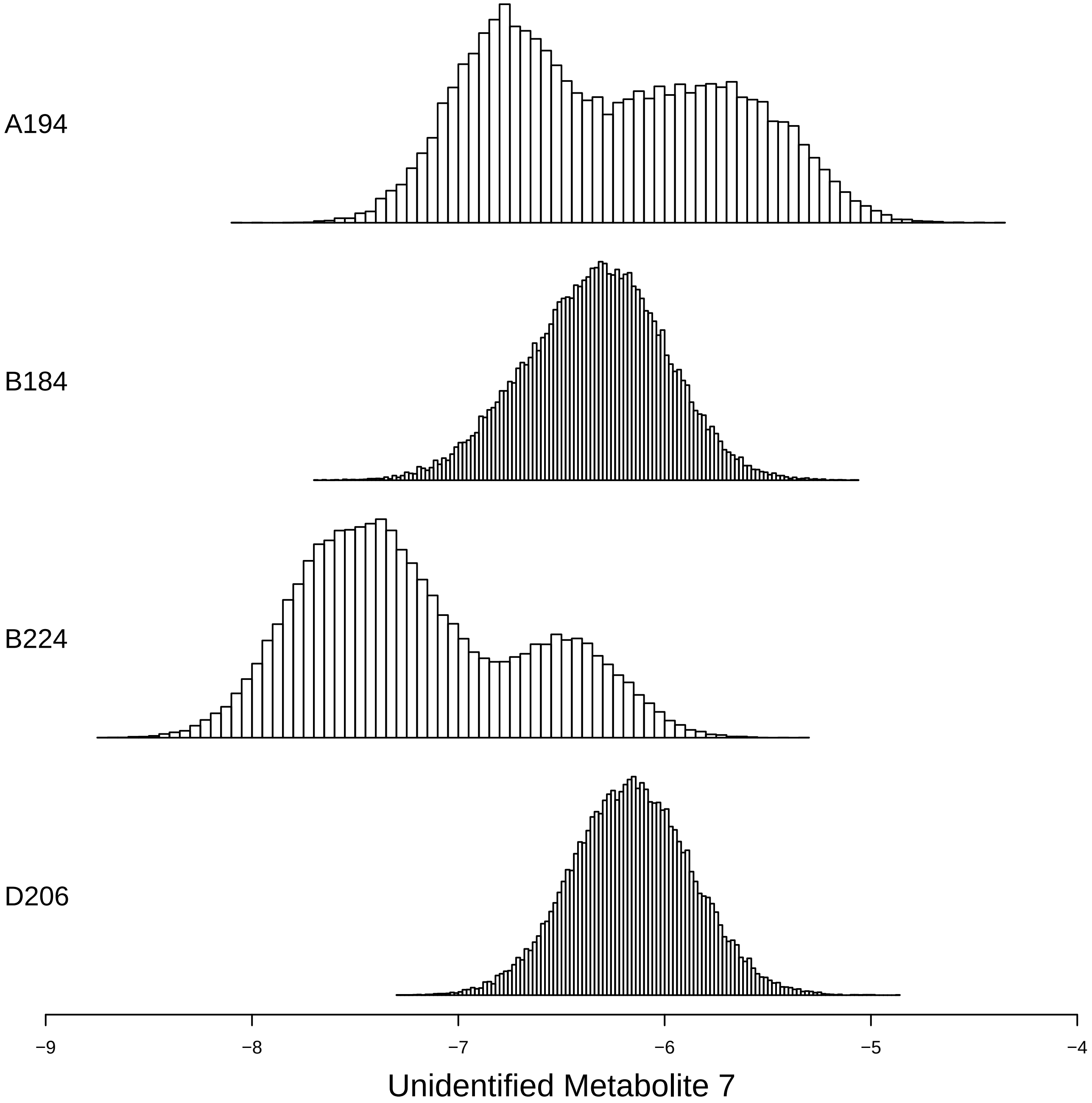

A194
B184
B224
D206
−9
−8
−7
−6
−5
−4
Unidentified Metabolite 7

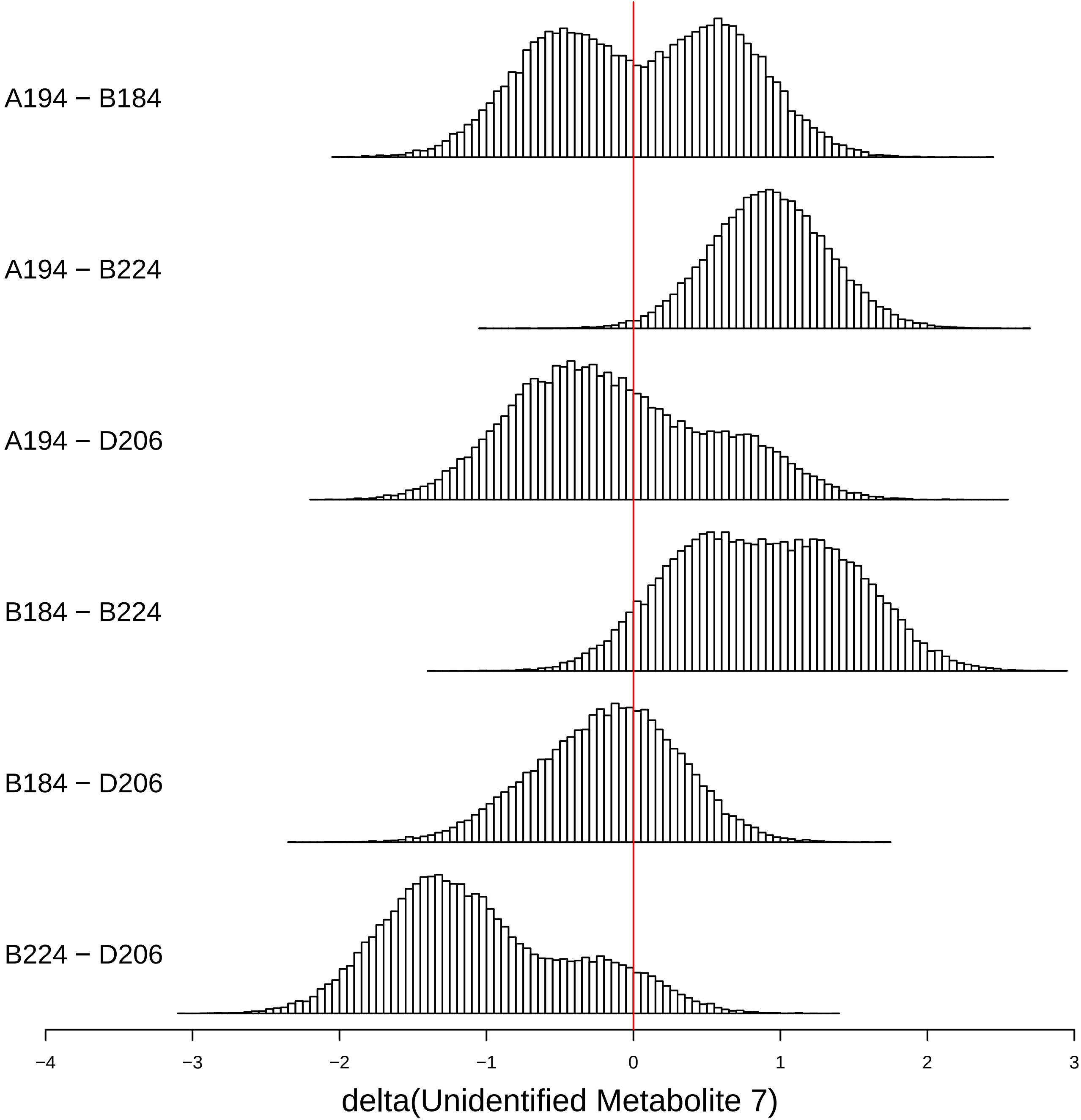
A194 − B184
A194 − B224
A194 − D206
B184 − B224
B184 − D206
B224 − D206
−4
−3
−2
−1
0
1
2
3
delta(Unidentified Metabolite 7)

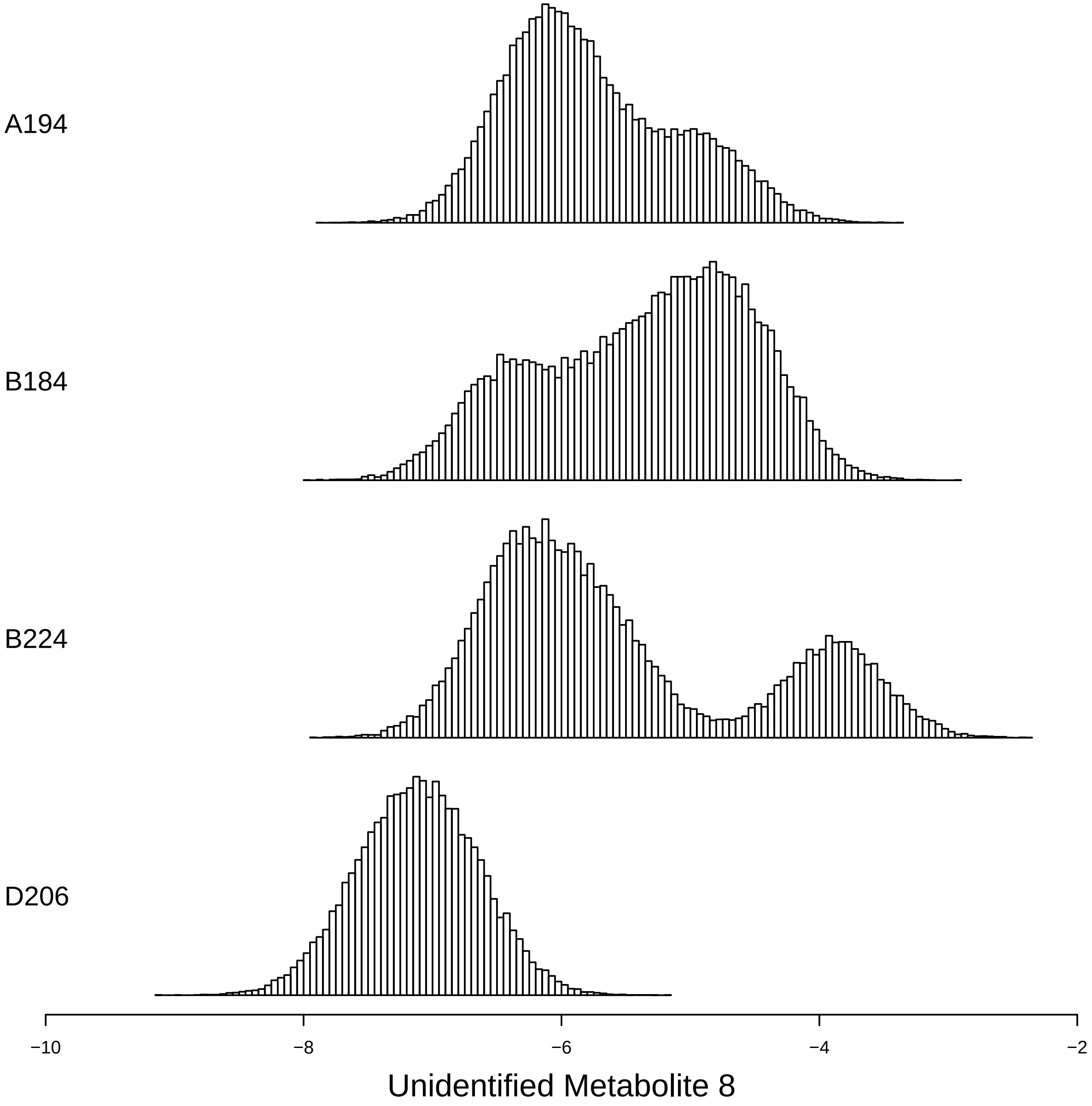
A194
B184
B224
D206
−10
−8
−6
−4
−2
Unidentified Metabolite 8

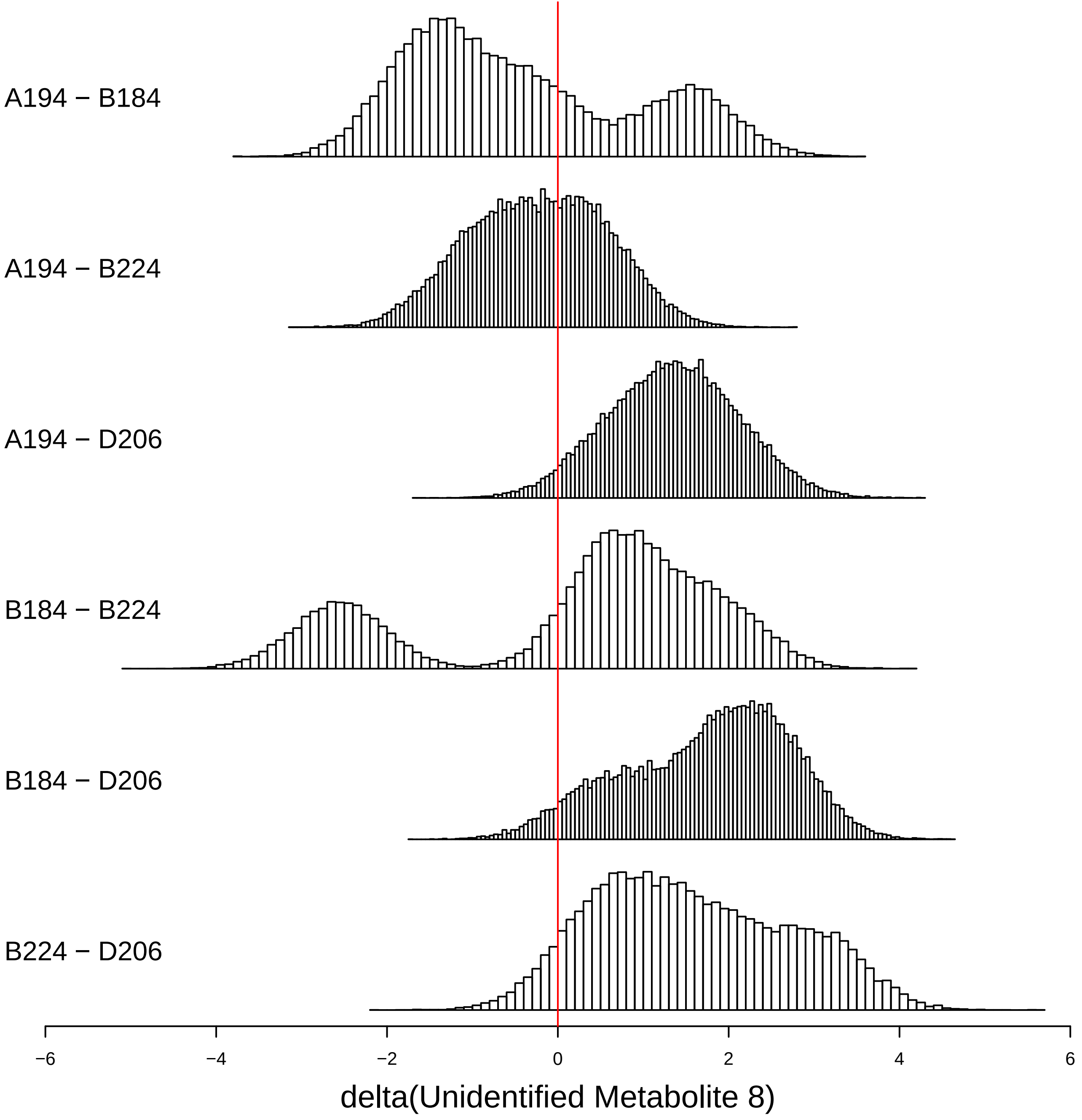
A194 − B184
A194 − B224
A194 − D206
B184 − B224
B184 − D206
B224 − D206
−6
−4
−2
0
2
4
6
delta(Unidentified Metabolite 8)

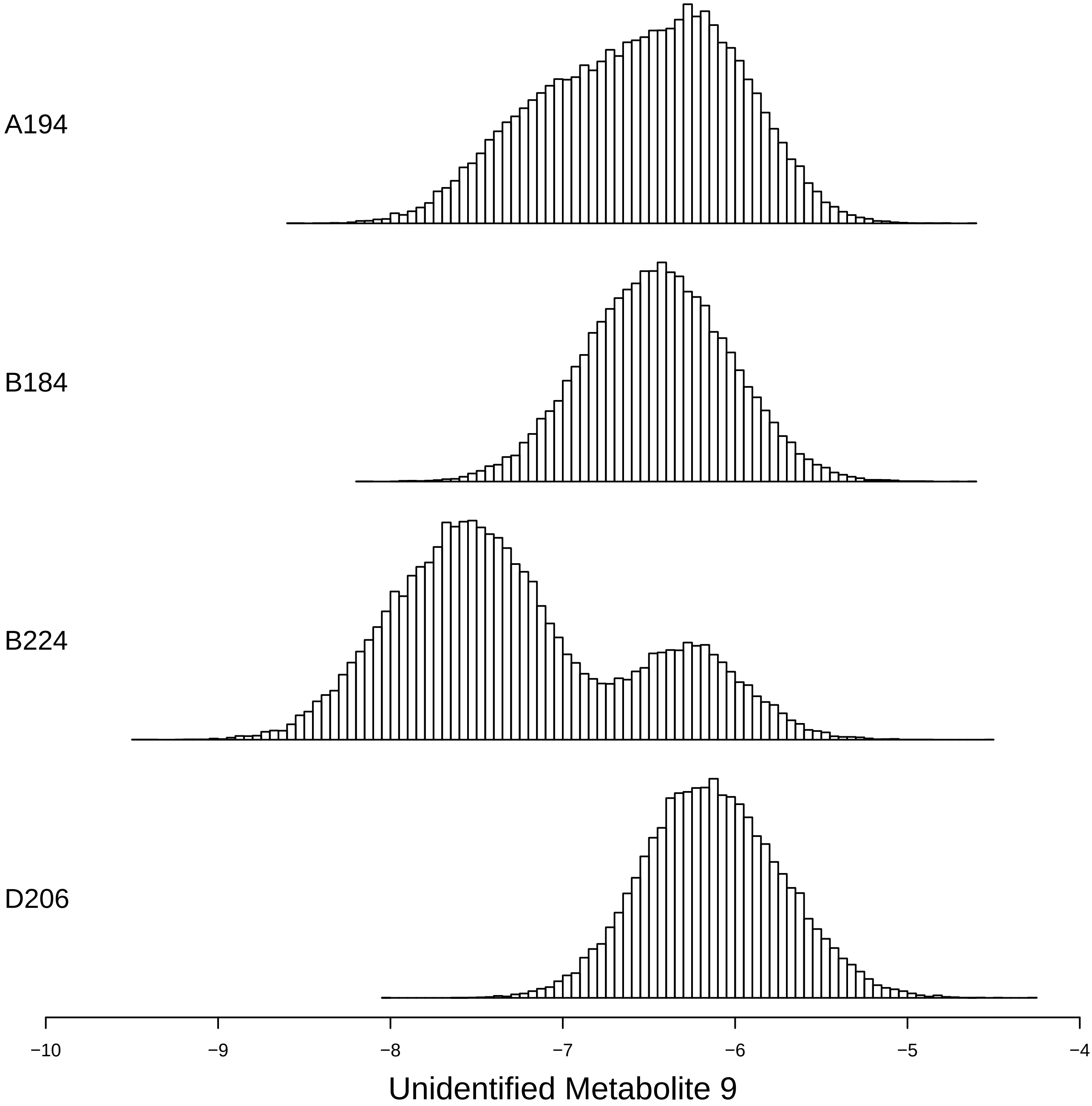
A194
B184
B224
D206
−10
−9
−8
−7
−6
−5
−4
Unidentified Metabolite 9

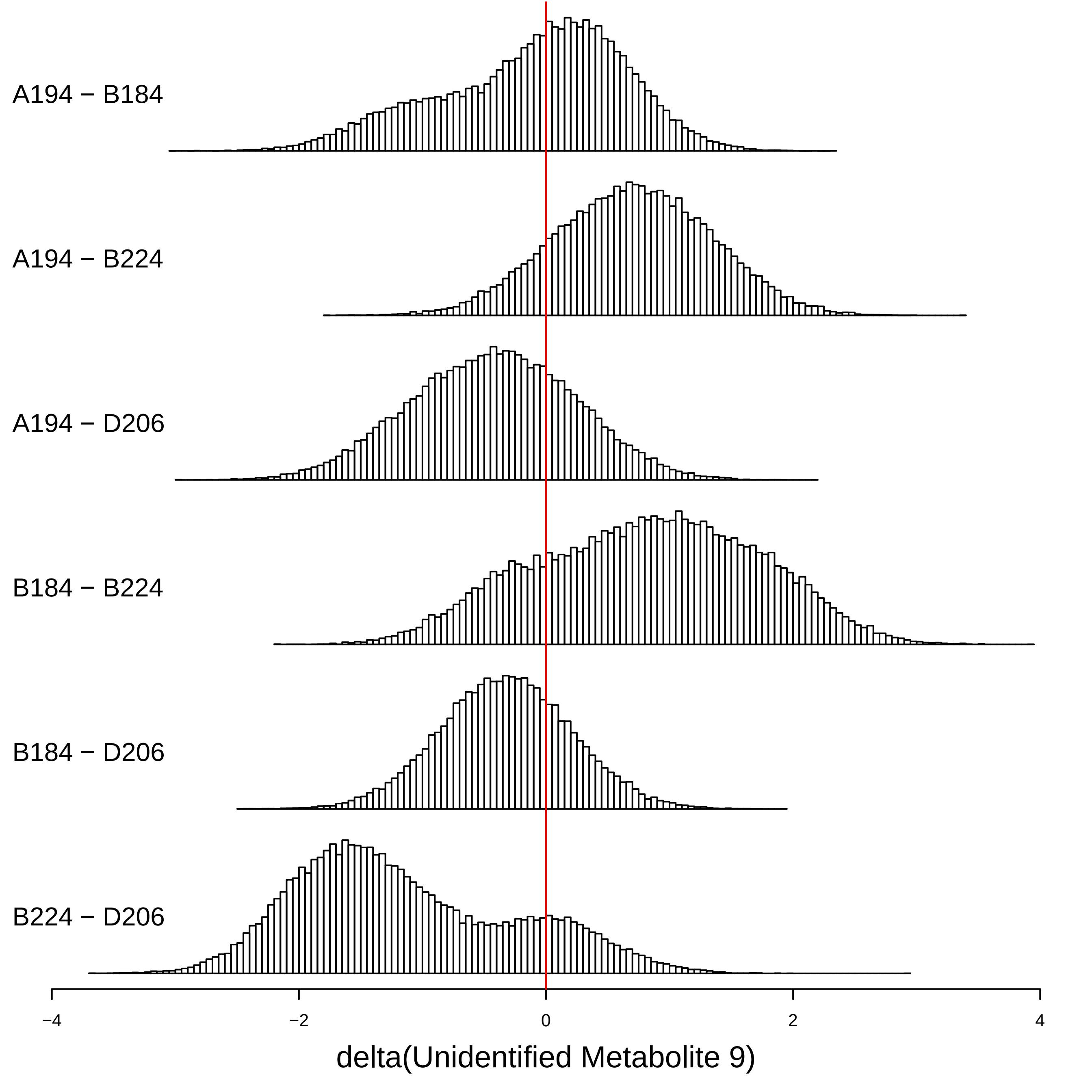

A194 − B184
A194 − B224
A194 − D206
B184 − B224
B184 − D206
B224 − D206
−4
−2
0
2
4
delta(Unidentified Metabolite 9)

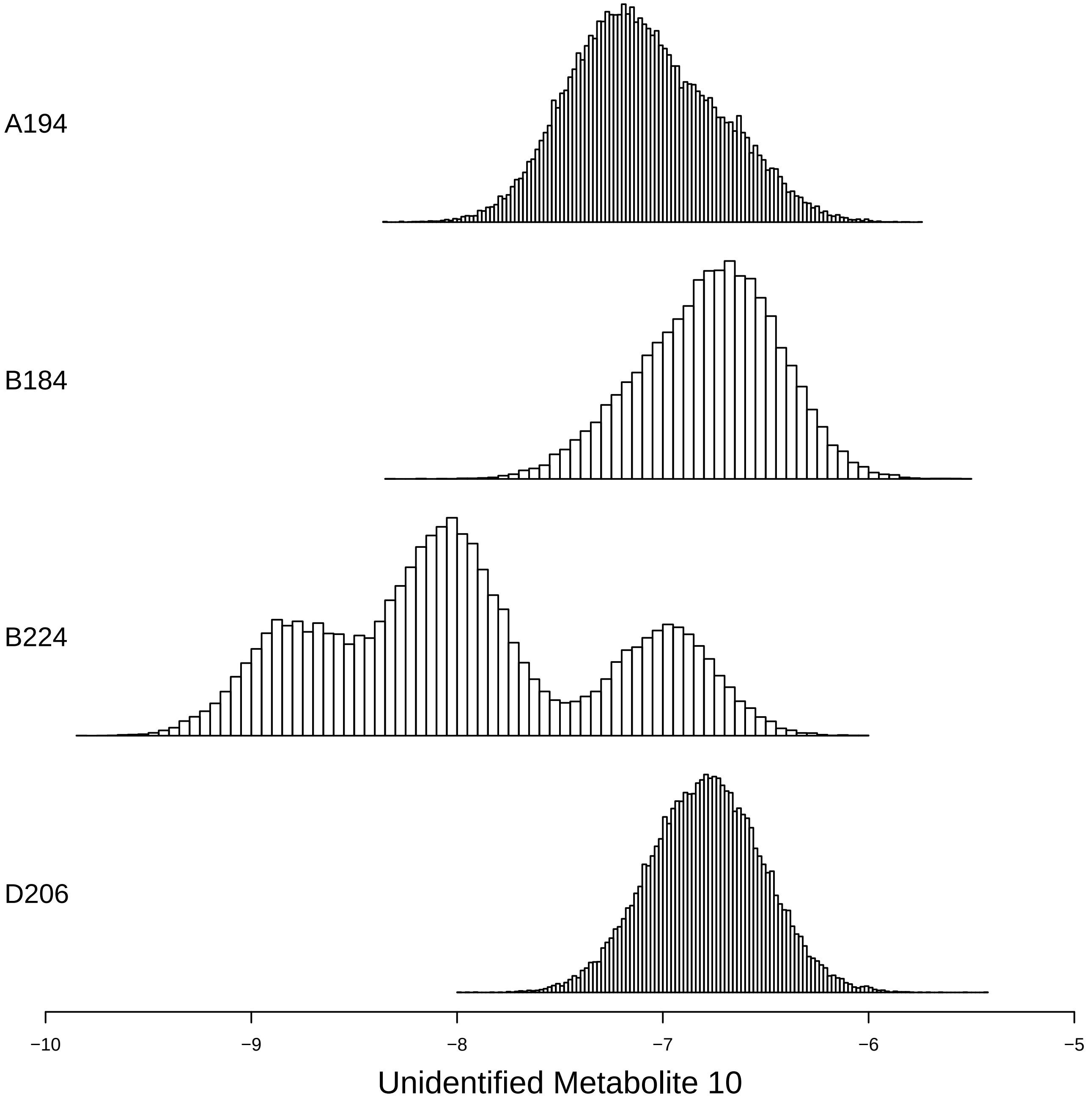
A194
B184
B224
D206
−10
−9
−8
−7
−6
−5
Unidentified Metabolite 10

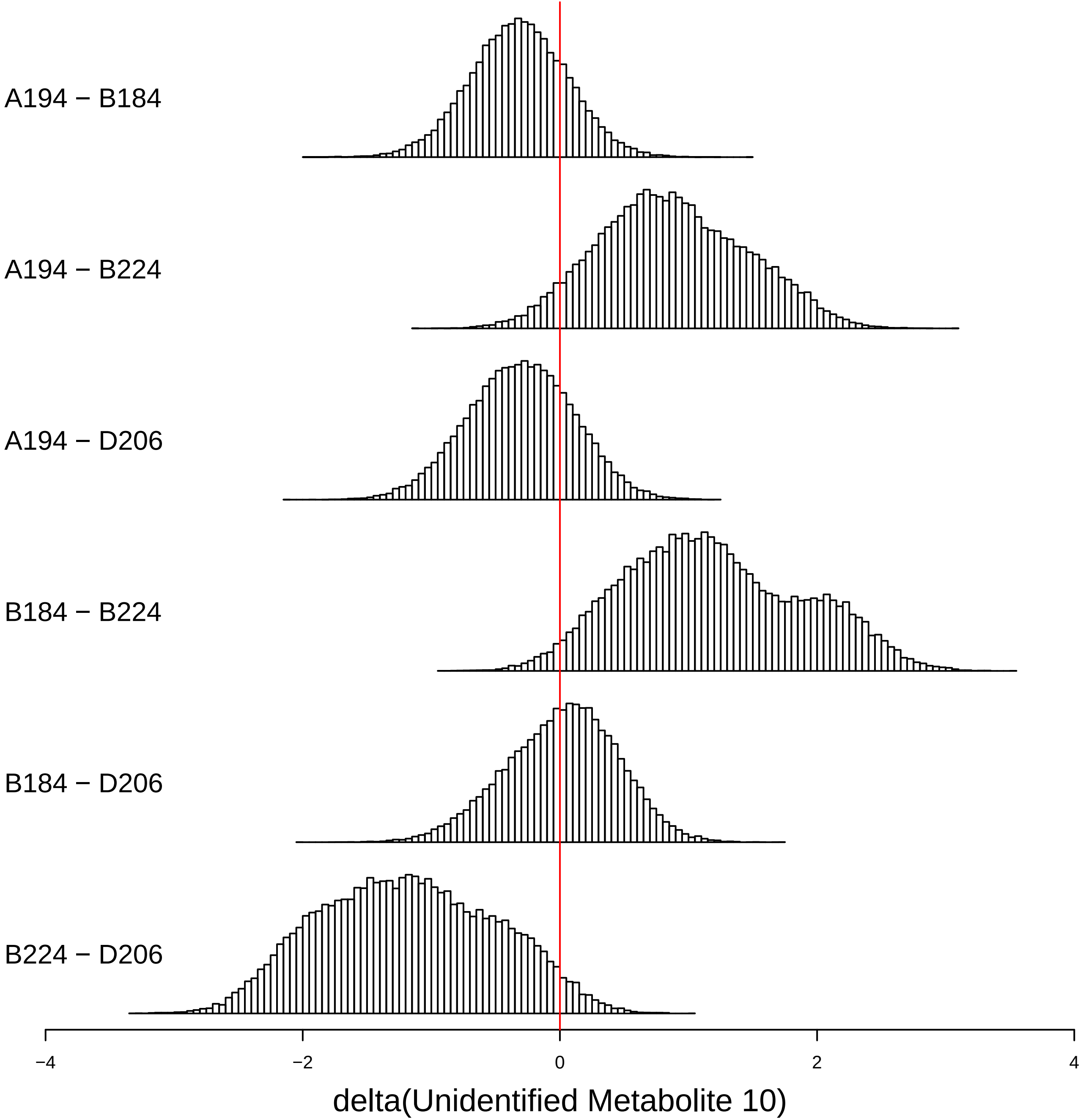
A194 − B184
A194 − B224
A194 − D206
B184 − B224
B184 − D206
B224 − D206
−4
−2
0
2
4
delta(Unidentified Metabolite 10)

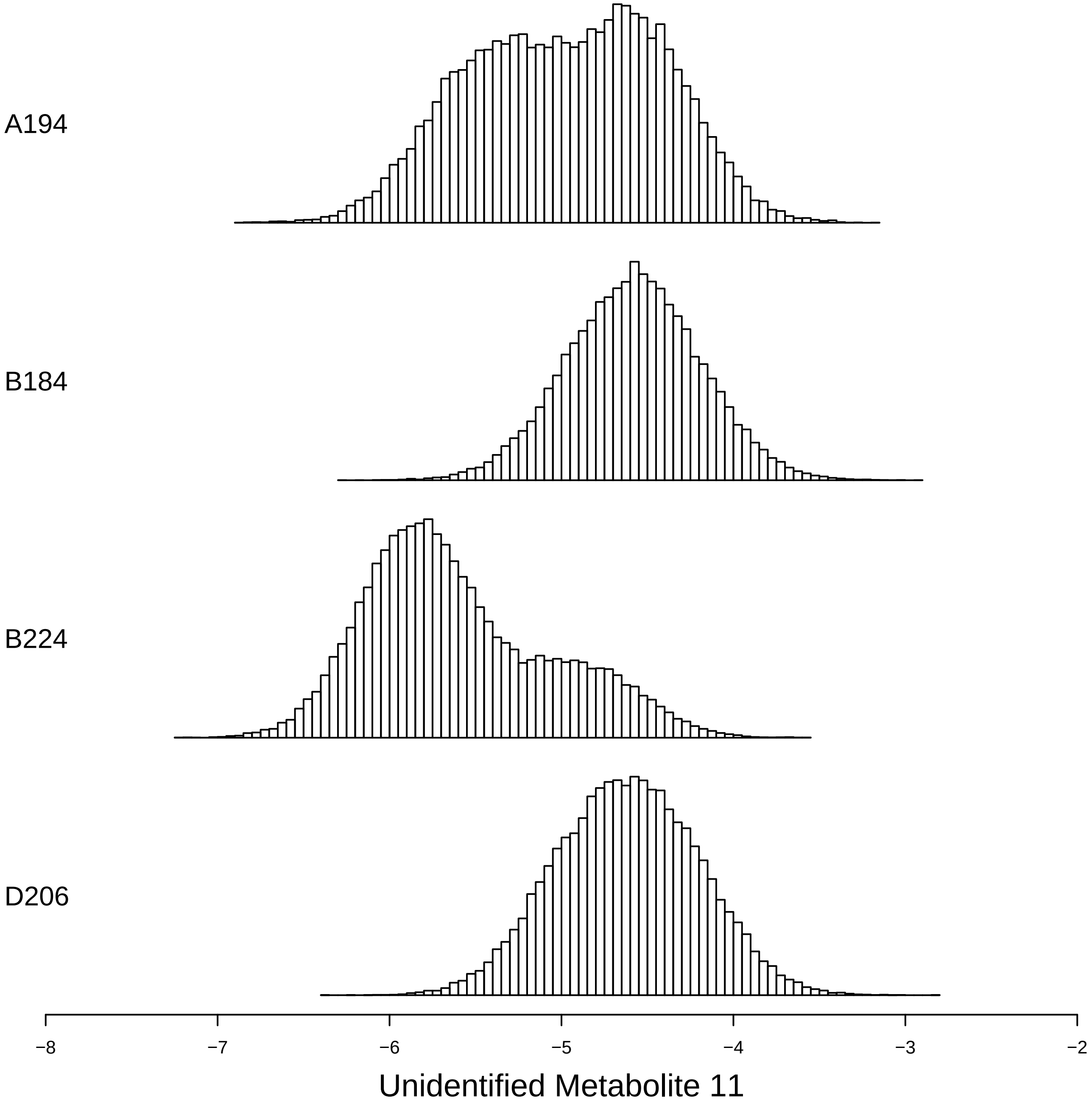
A194
B184
B224
D206
−8
−7
−6
−5
−4
−3
−2
Unidentified Metabolite 11

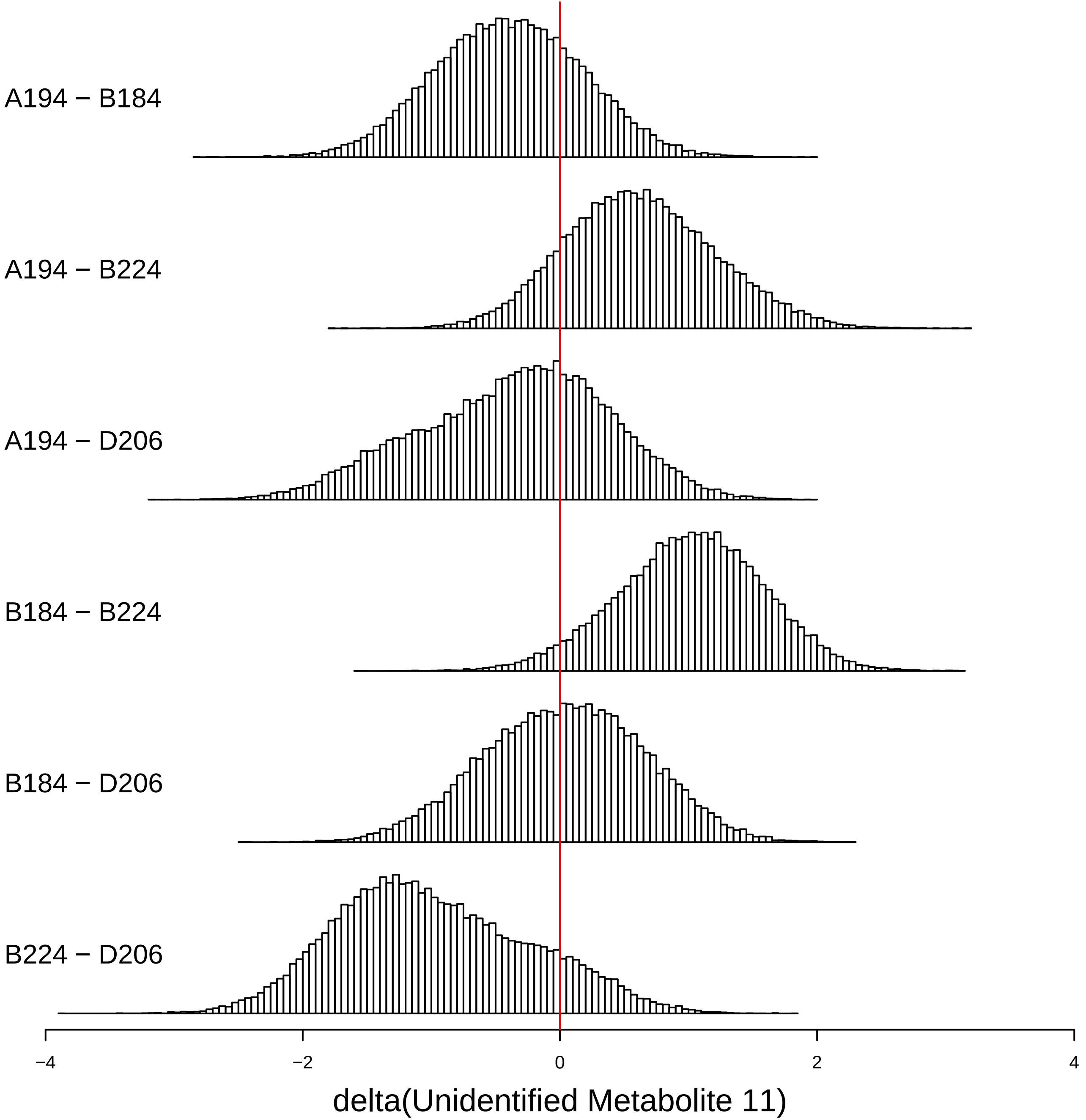

A194 − B184
A194 − B224
A194 − D206
B184 − B224
B184 − D206
B224 − D206
−4
−2
0
2
4
delta(Unidentified Metabolite 11)

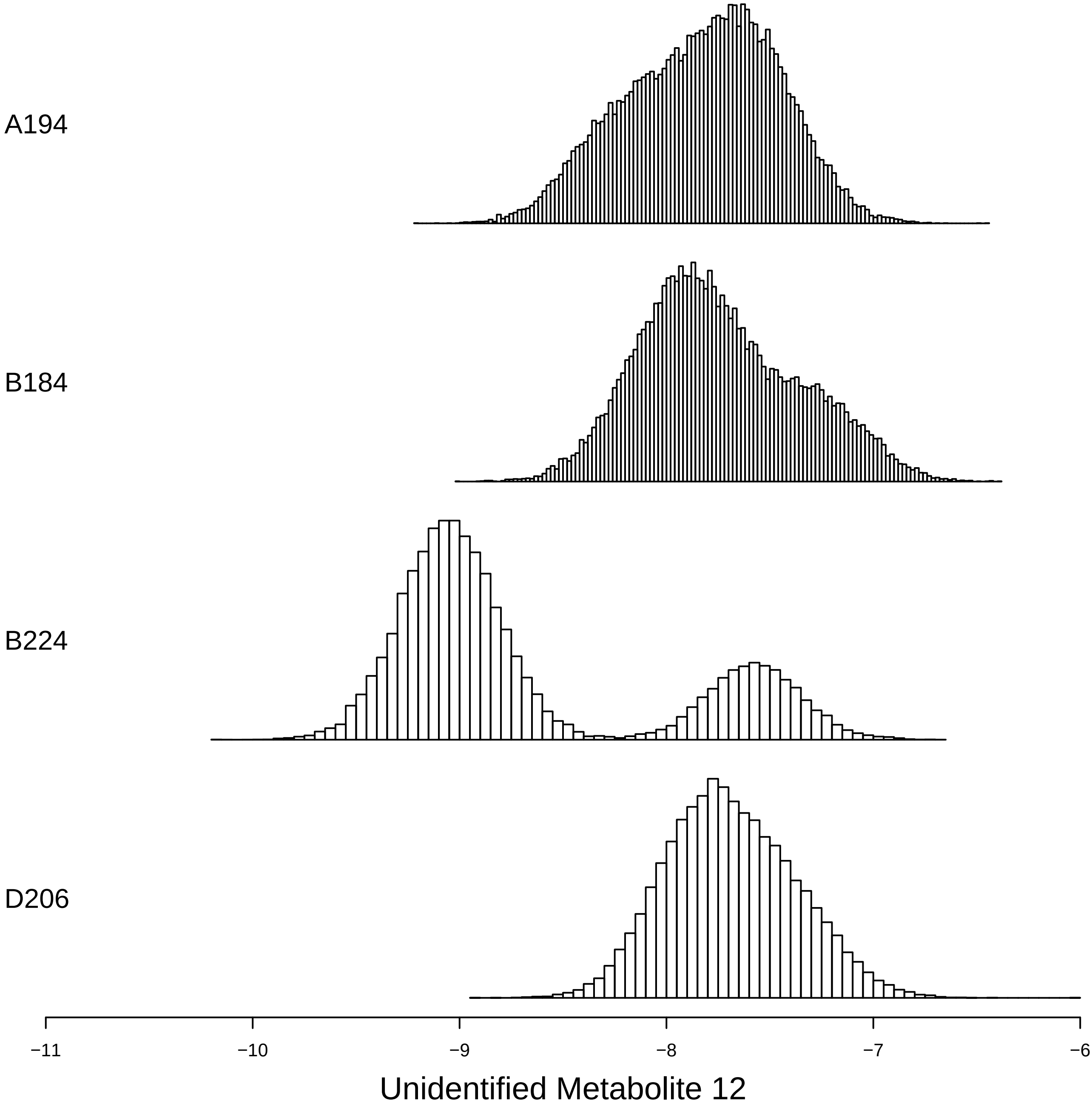
A194
B184
B224
D206
−11
−10
−9
−8
−7
−6
Unidentified Metabolite 12

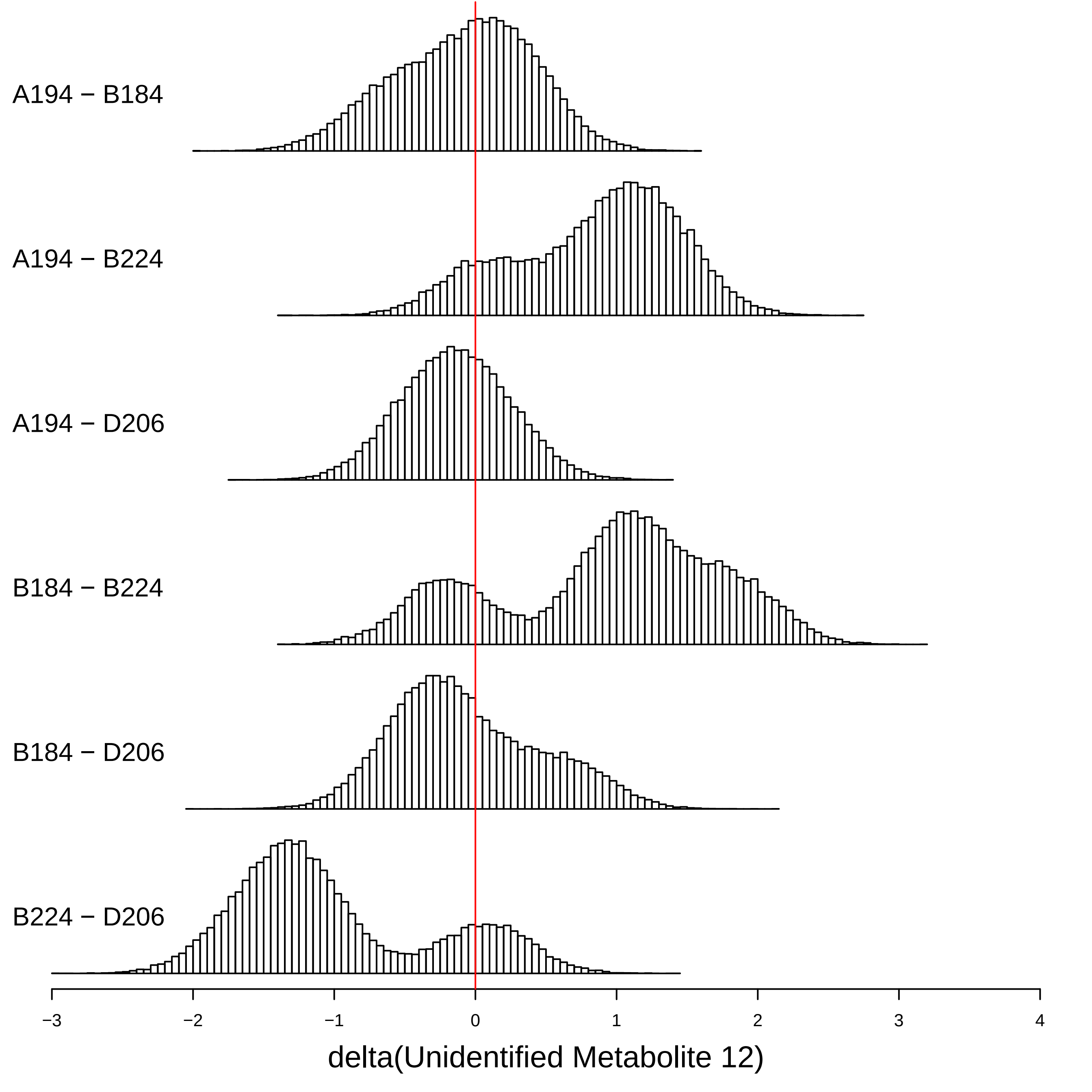

A194 − B184
A194 − B224
A194 − D206
B184 − B224
B184 − D206
B224 − D206
−3
−2
−1
0
1
2
3
4
delta(Unidentified Metabolite 12)

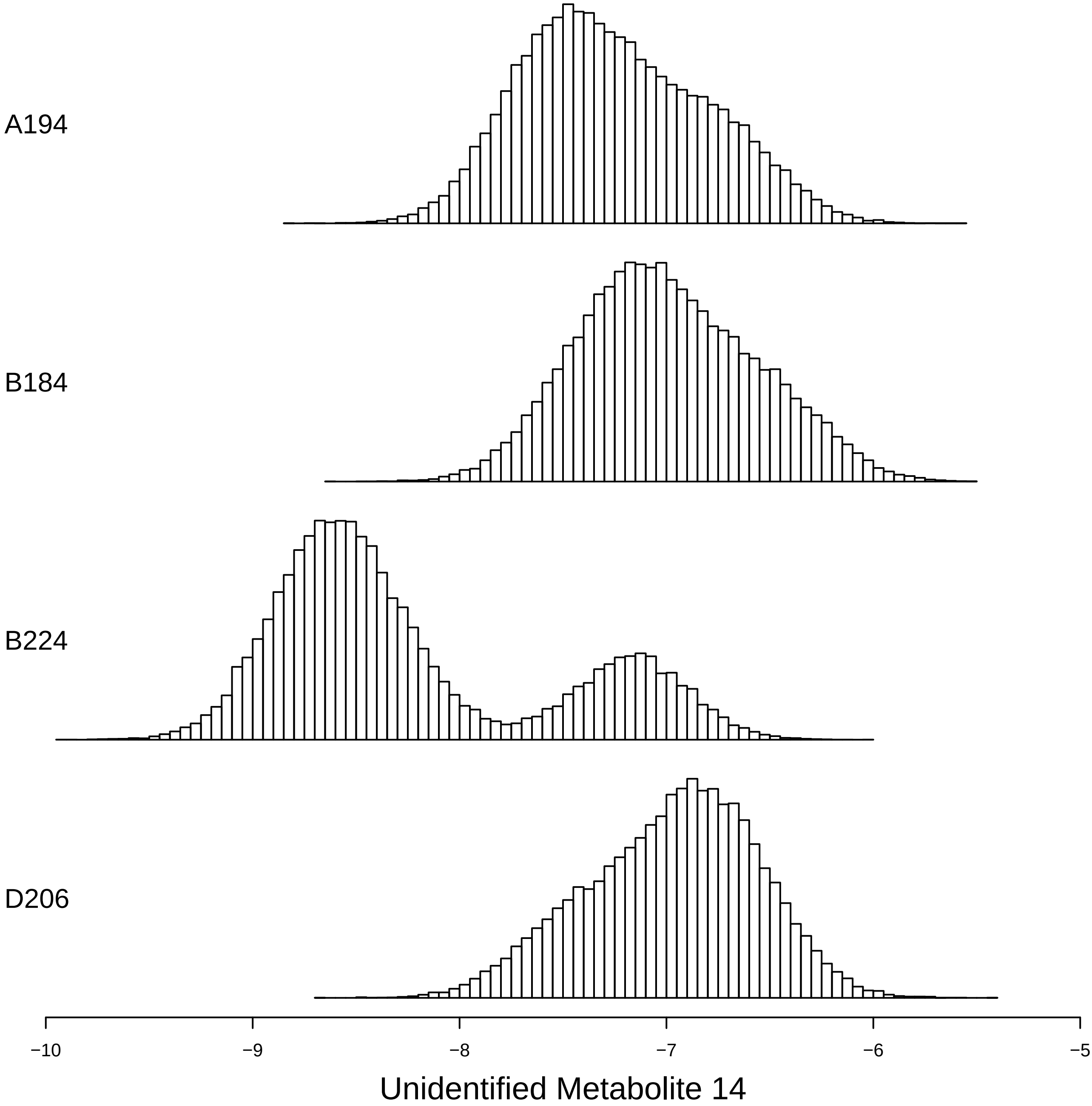
A194
B184
B224
D206
−10
−9
−8
−7
−6
−5
Unidentified Metabolite 14

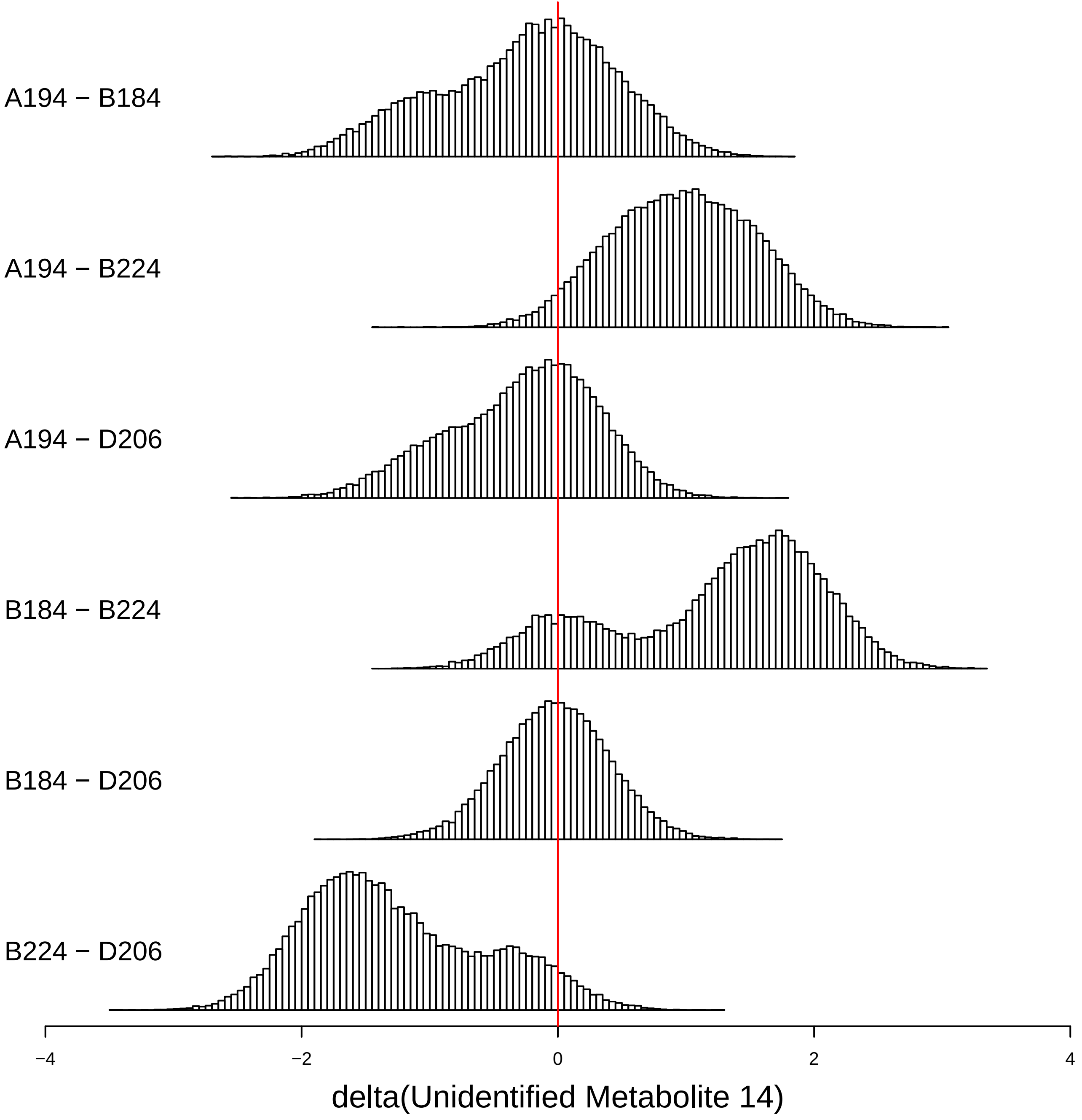

A194 − B184
A194 − B224
A194 − D206
B184 − B224
B184 − D206
B224 − D206
−4
−2
0
2
4
delta(Unidentified Metabolite 14)

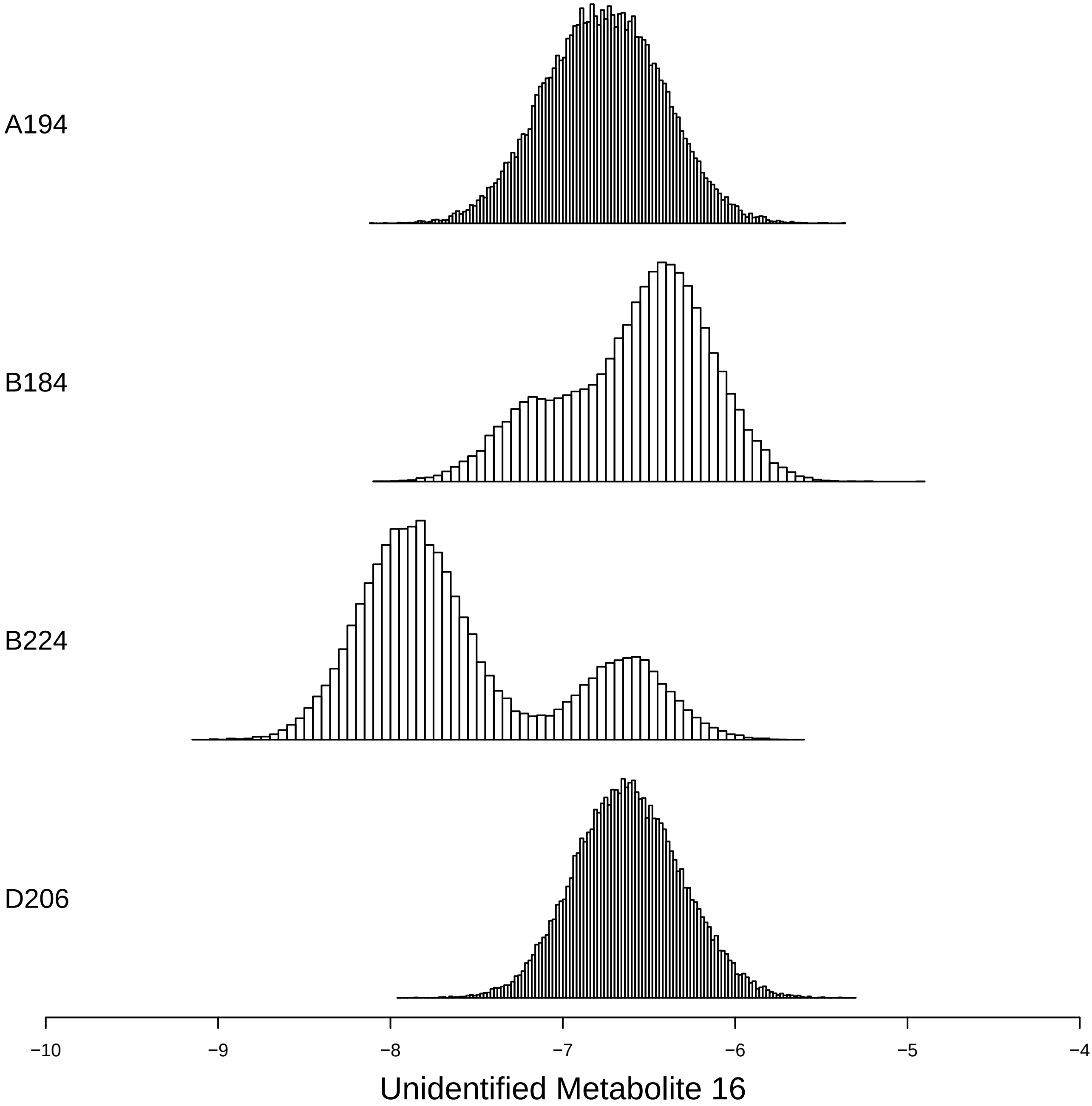
A194
B184
B224
D206
−10
−9
−8
−7
−6
−5
−4
Unidentified Metabolite 16

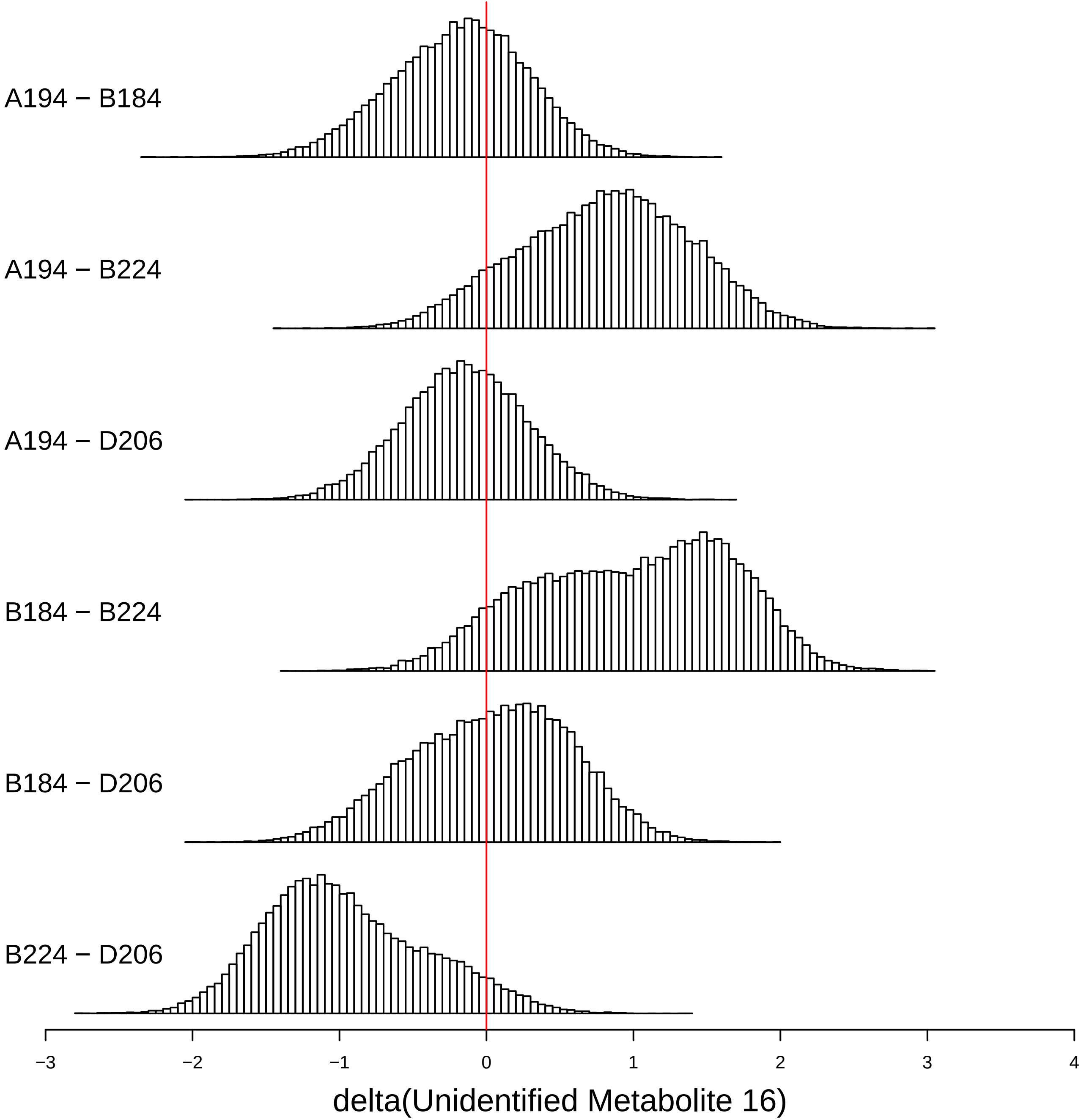

A194 − B184
A194 − B224
A194 − D206
B184 − B224
B184 − D206
B224 − D206
−3
−2
−1
0
1
2
3
4
delta(Unidentified Metabolite 16)

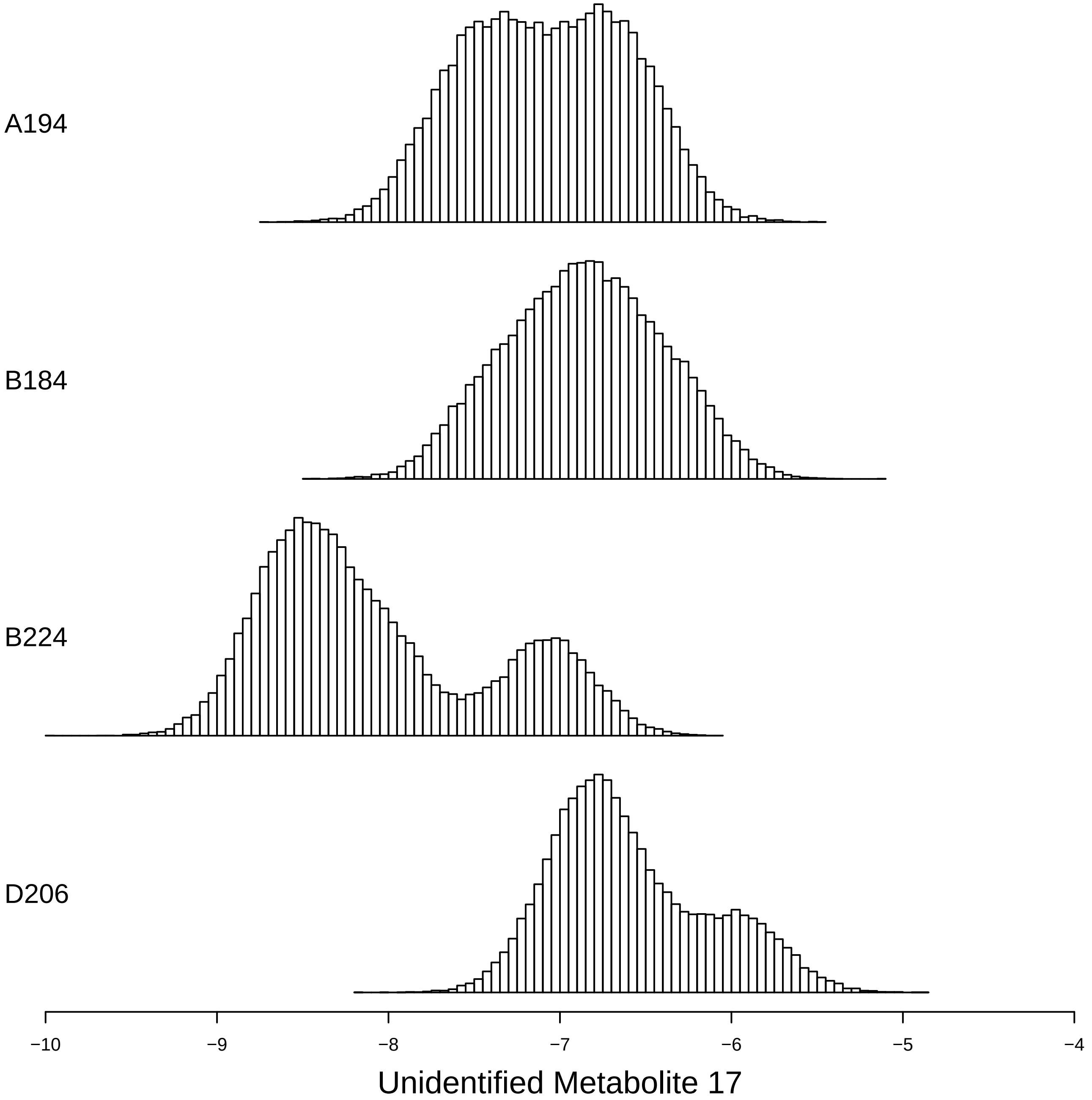
A194
B184
B224
D206
−10
−9
−8
−7
−6
−5
−4
Unidentified Metabolite 17

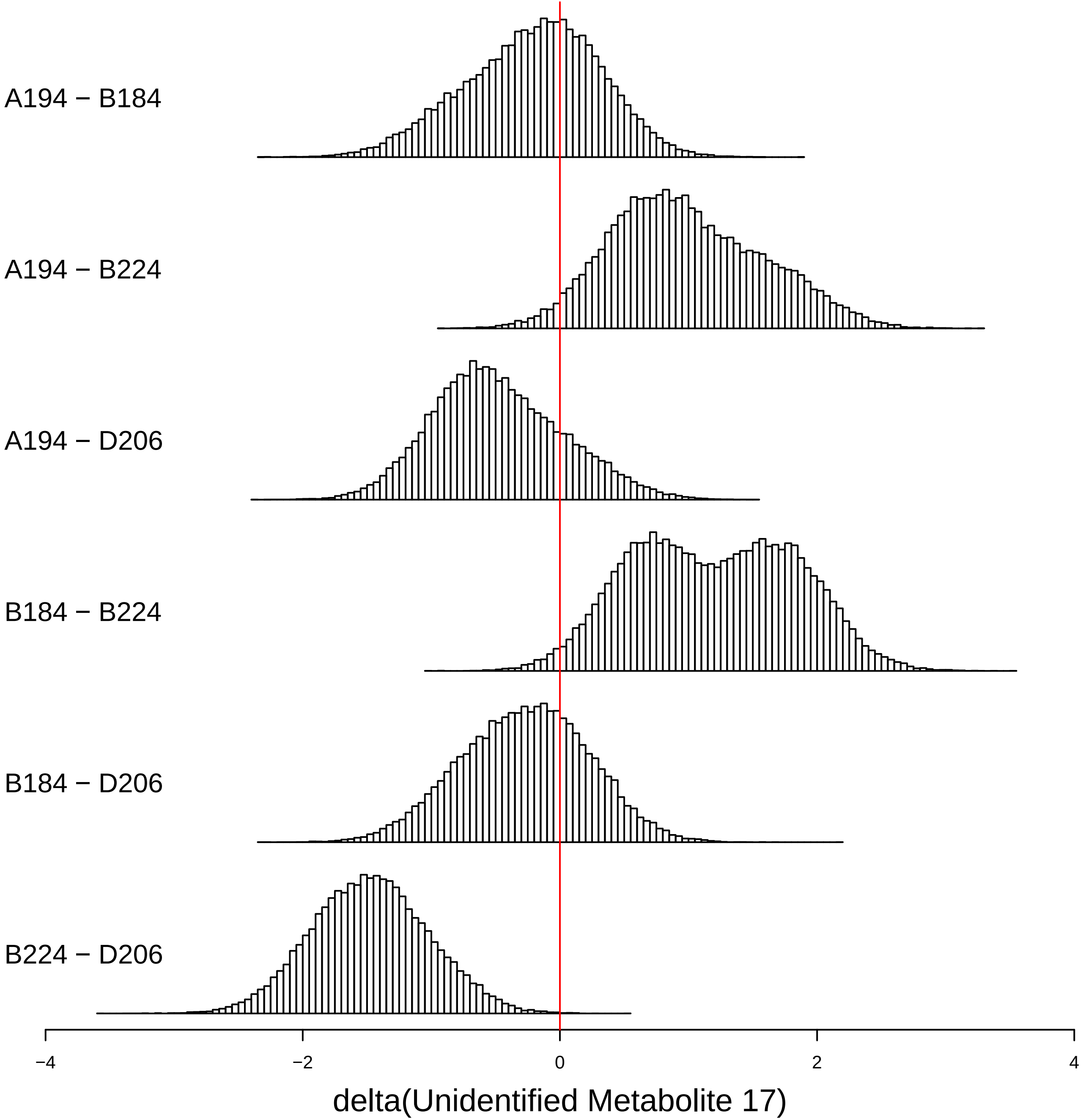

A194 − B184
A194 − B224
A194 − D206
B184 − B224
B184 − D206
B224 − D206
−4
−2
0
2
4
delta(Unidentified Metabolite 17)

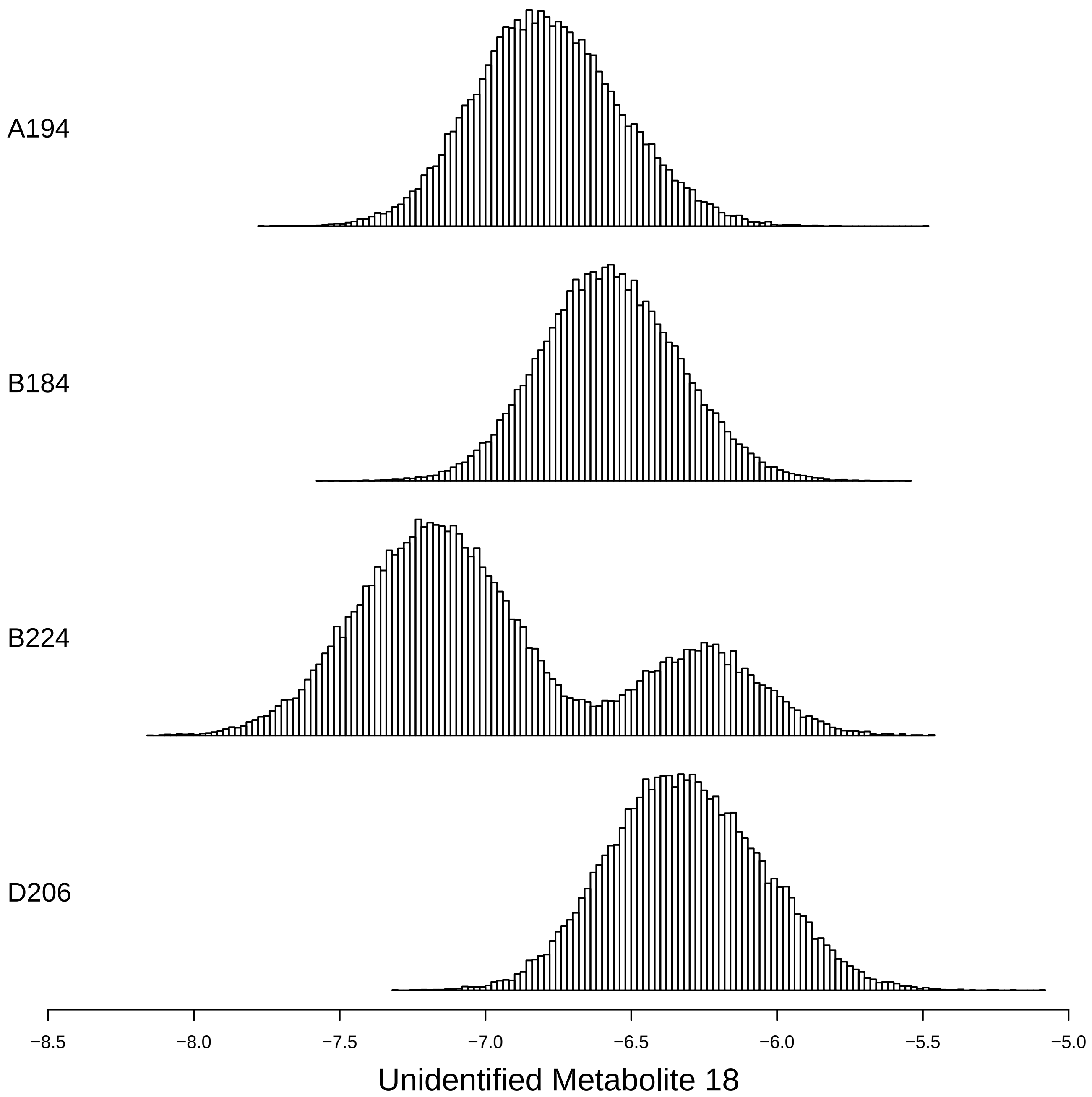

A194
B184
B224
D206
−8.5
−8.0
−7.5
−7.0
−6.5
−6.0
−5.5
−5.0
Unidentified Metabolite 18

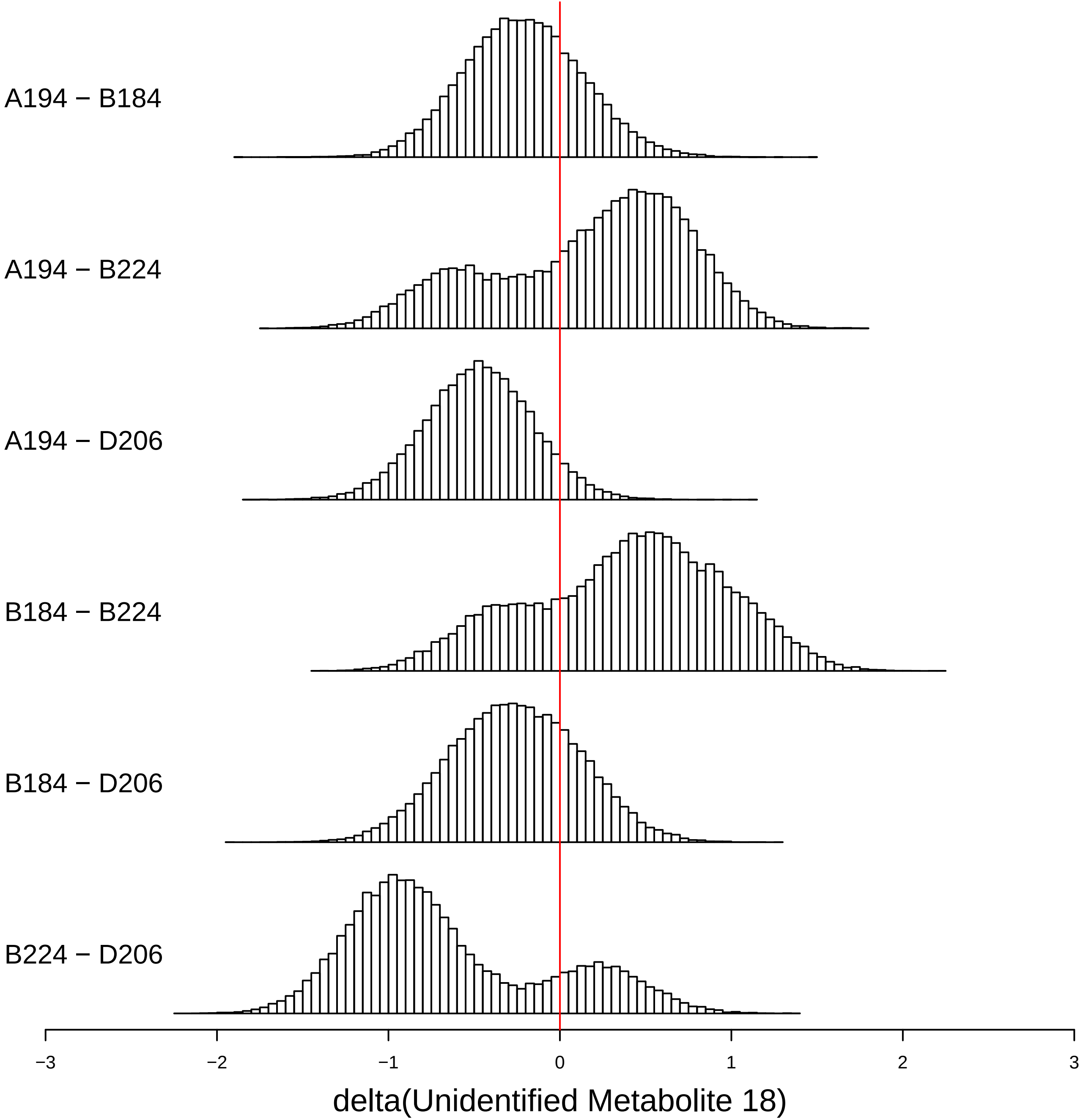

A194 − B184
A194 − B224
A194 − D206
B184 − B224
B184 − D206
B224 − D206
−3
−2
−1
0
1
2
3
delta(Unidentified Metabolite 18)

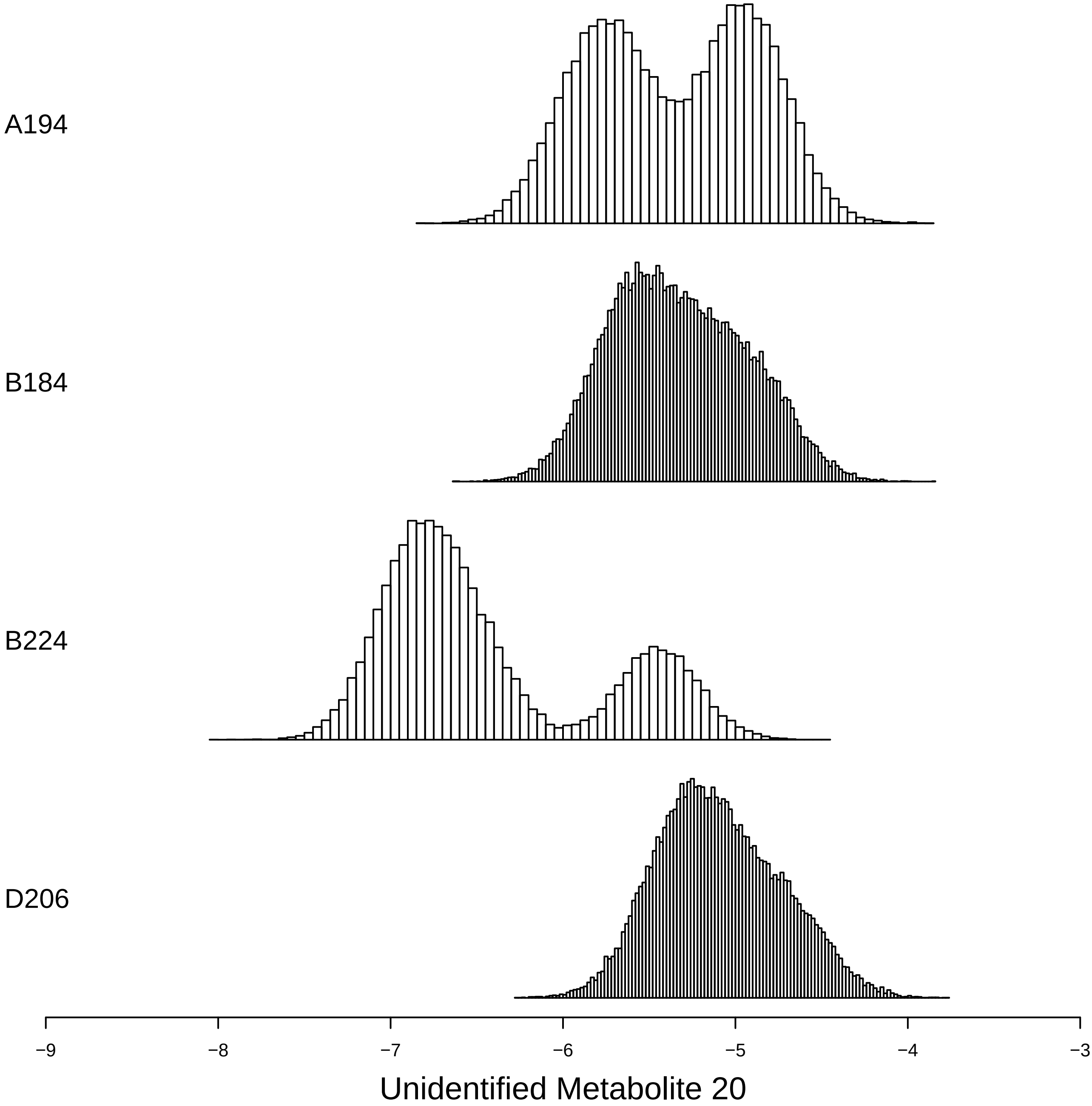
A194
B184
B224
D206
−9
−8
−7
−6
−5
−4
−3
Unidentified Metabolite 20

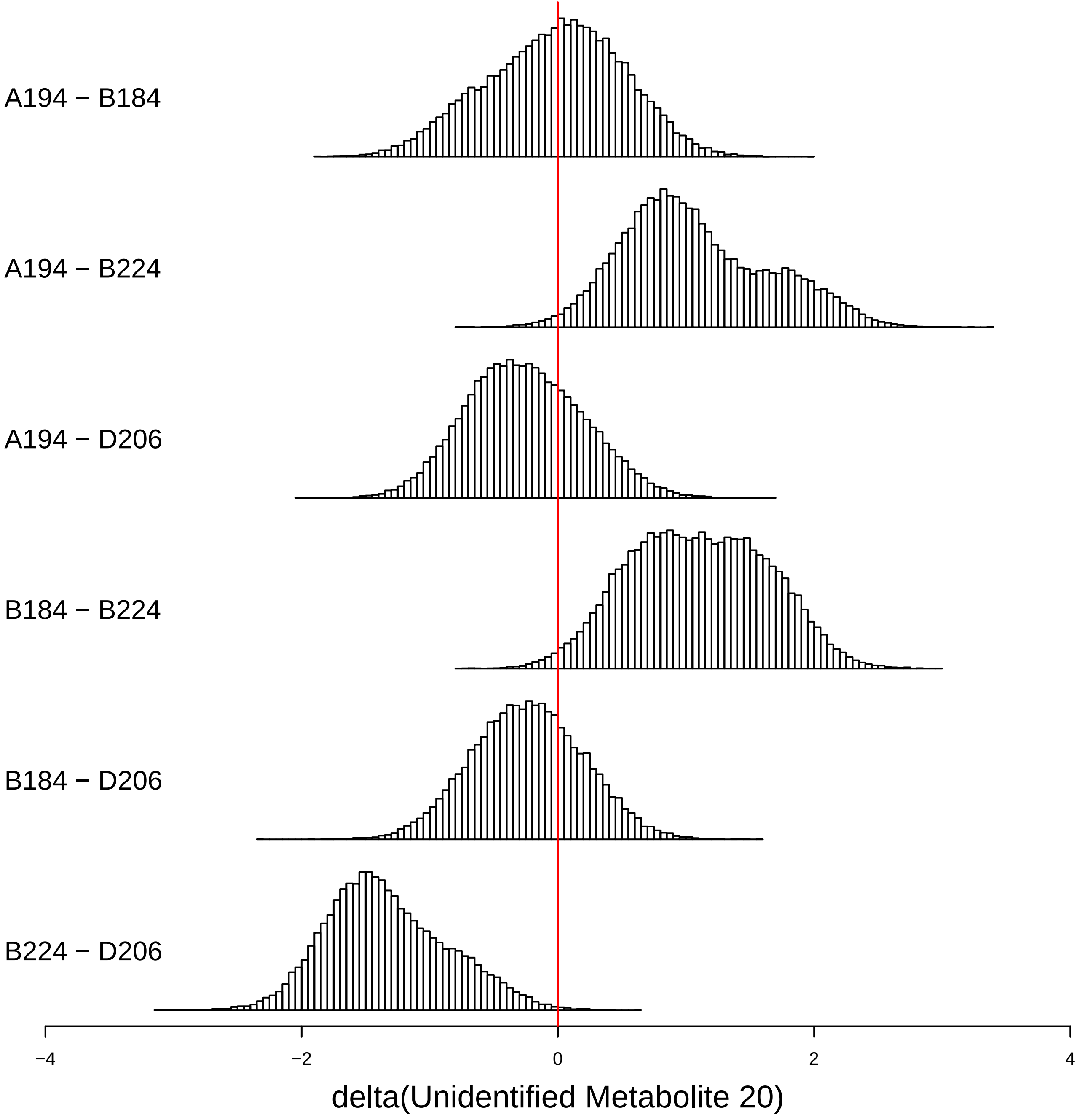

A194 − B184
A194 − B224
A194 − D206
B184 − B224
B184 − D206
B224 − D206
−4
−2
0
2
4
delta(Unidentified Metabolite 20)

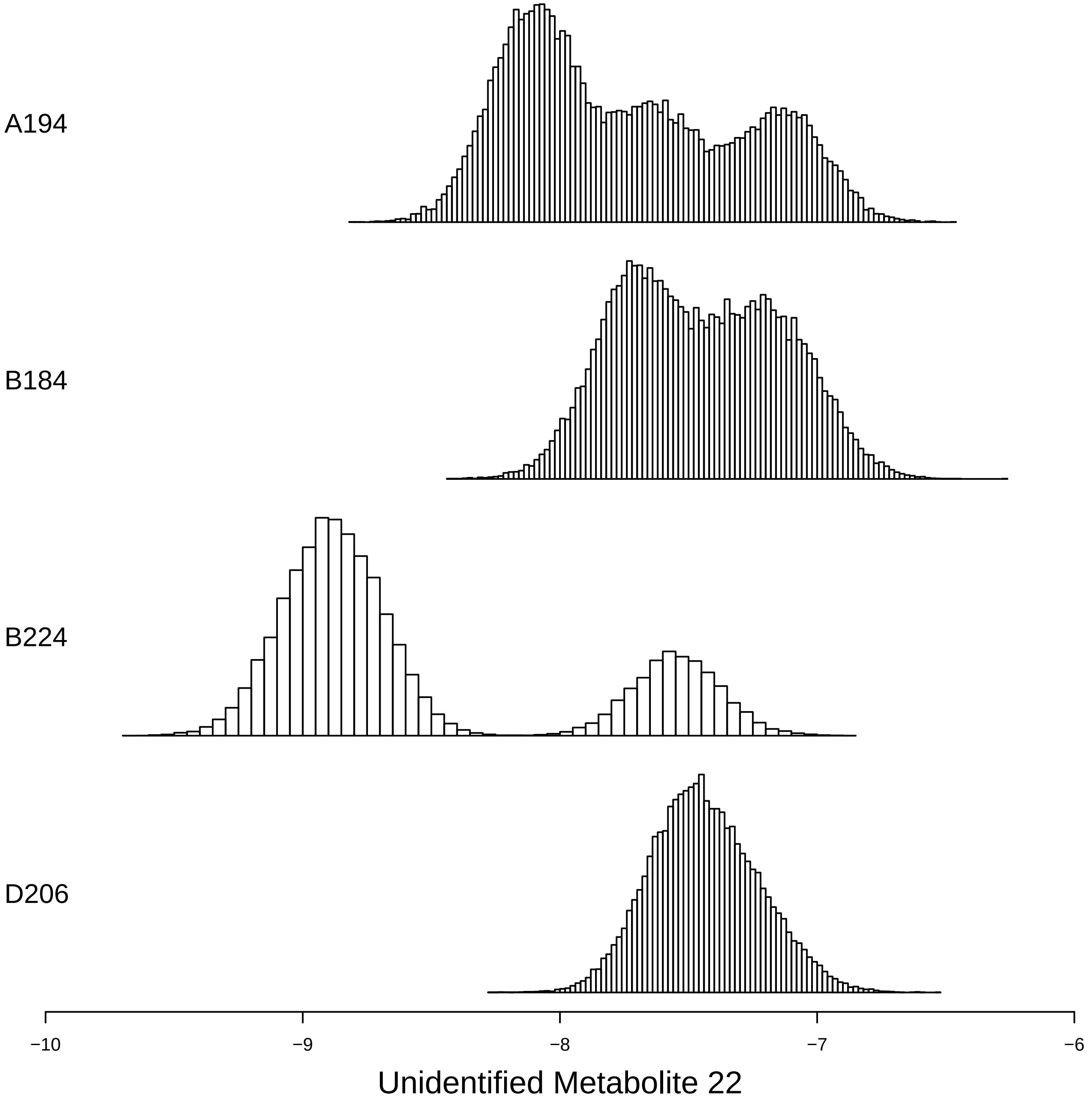
A194
B184
B224
D206
−10
−9
−8
−7
−6
Unidentified Metabolite 22

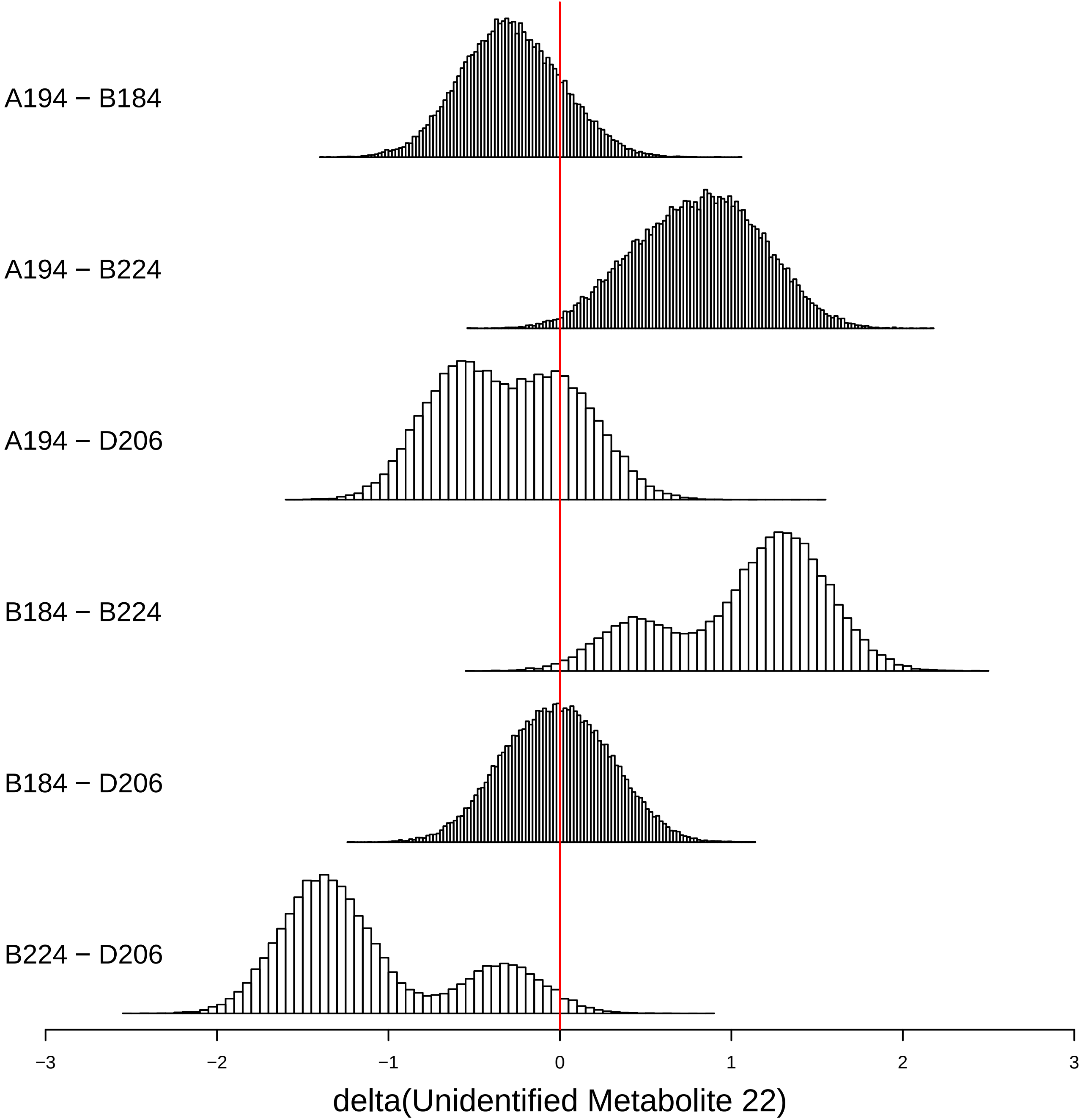

A194 − B184
A194 − B224
A194 − D206
B184 − B224
B184 − D206
B224 − D206
−3
−2
−1
0
1
2
3
delta(Unidentified Metabolite 22)

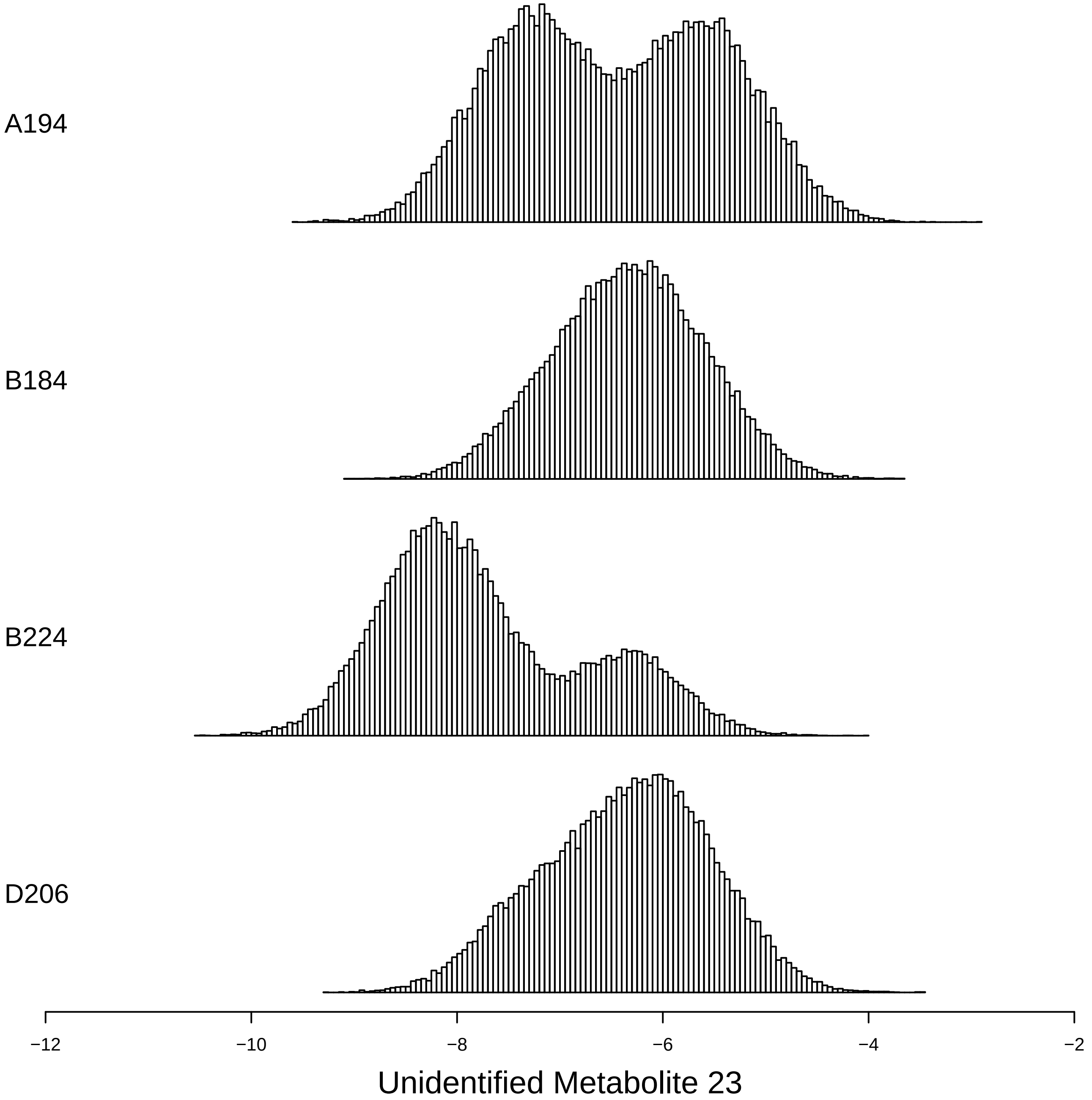
A194
B184
B224
D206
−12
−10
−8
−6
−4
−2
Unidentified Metabolite 23

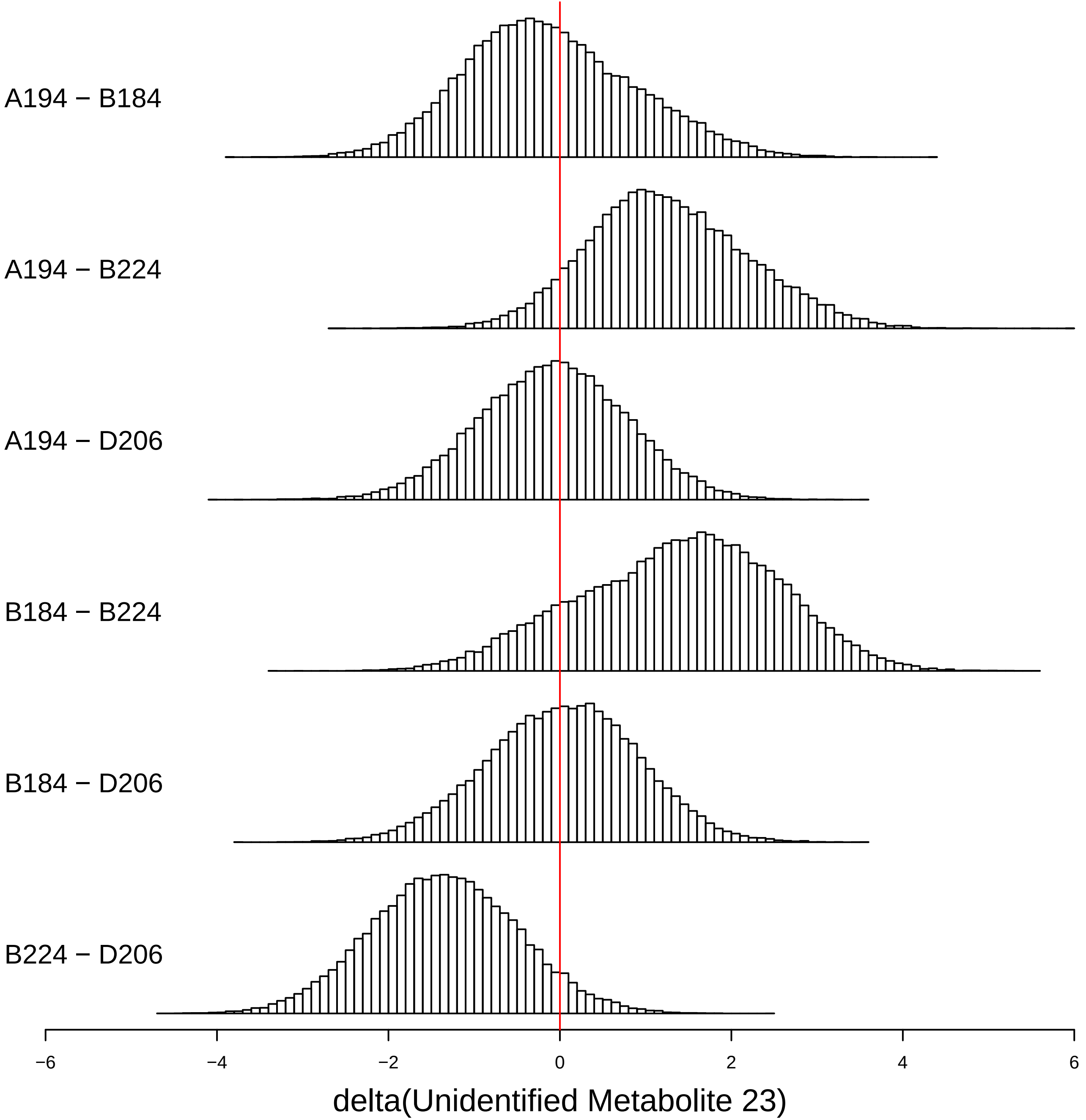

A194 − B184
A194 − B224
A194 − D206
B184 − B224
B184 − D206
B224 − D206
−6
−4
−2
0
2
4
6
delta(Unidentified Metabolite 23)

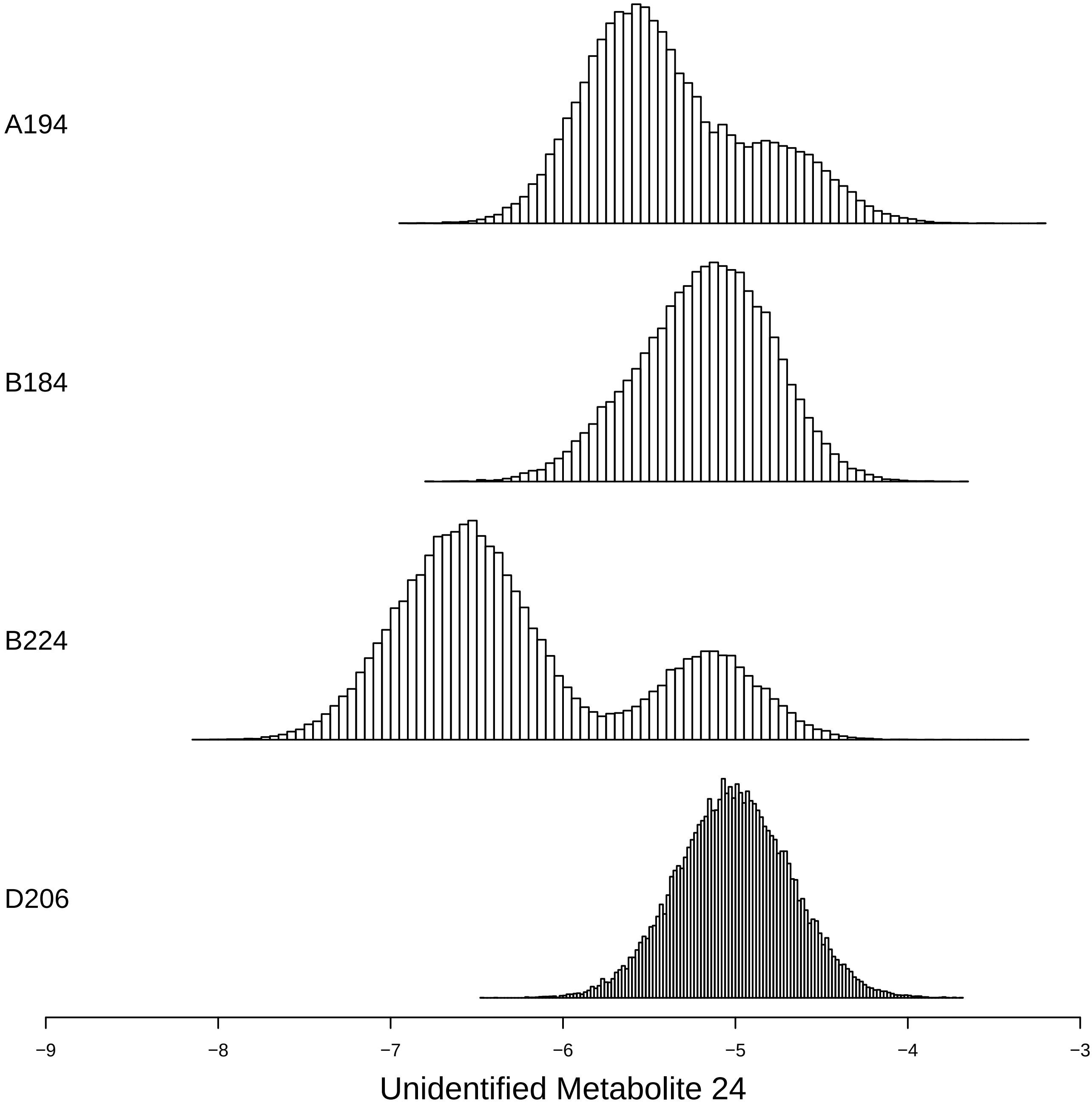
A194
B184
B224
D206
−9
−8
−7
−6
−5
−4
−3
Unidentified Metabolite 24

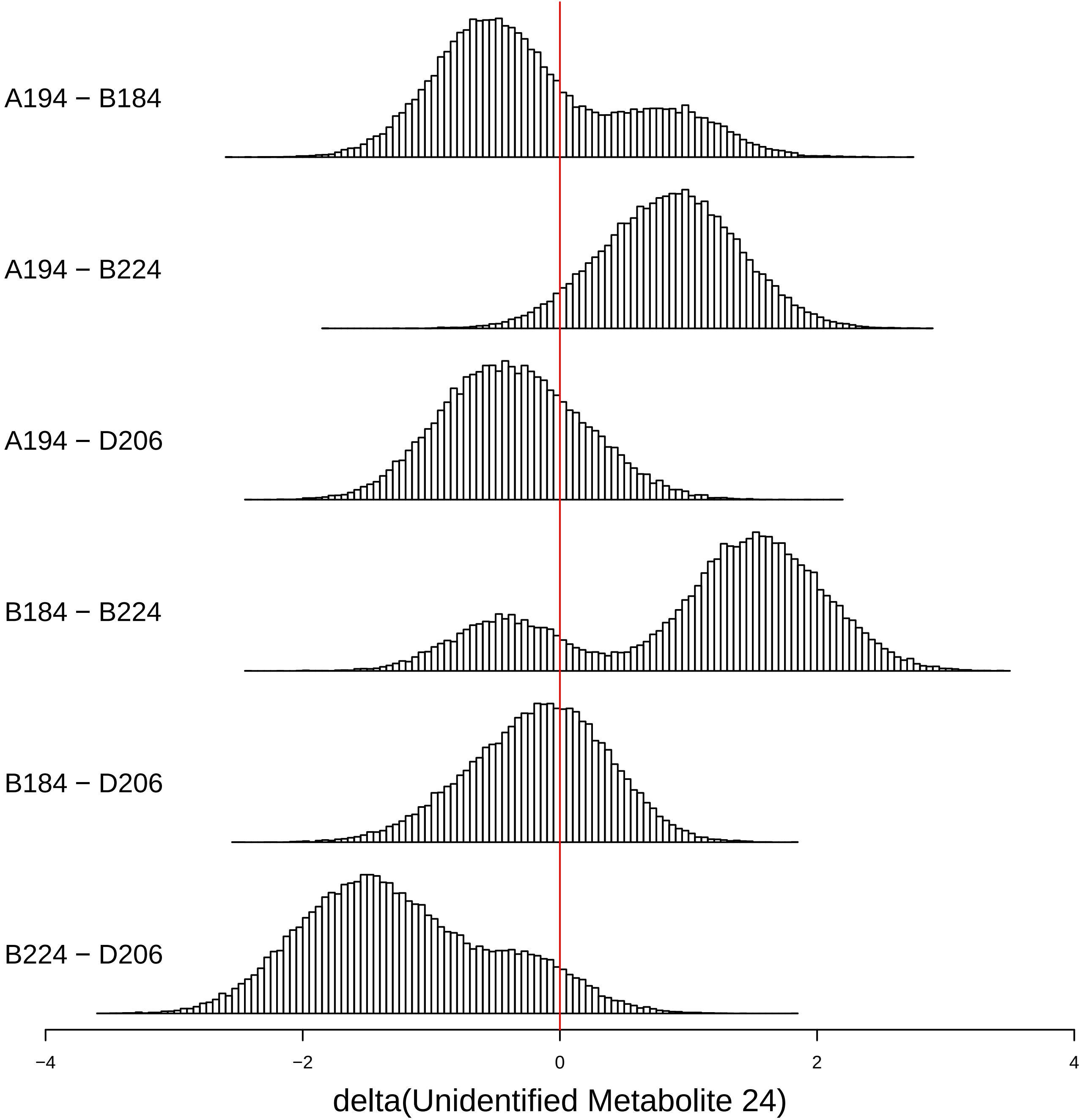
A194 − B184
A194 − B224
A194 − D206
B184 − B224
B184 − D206
B224 − D206
−4
−2
0
2
4
delta(Unidentified Metabolite 24)

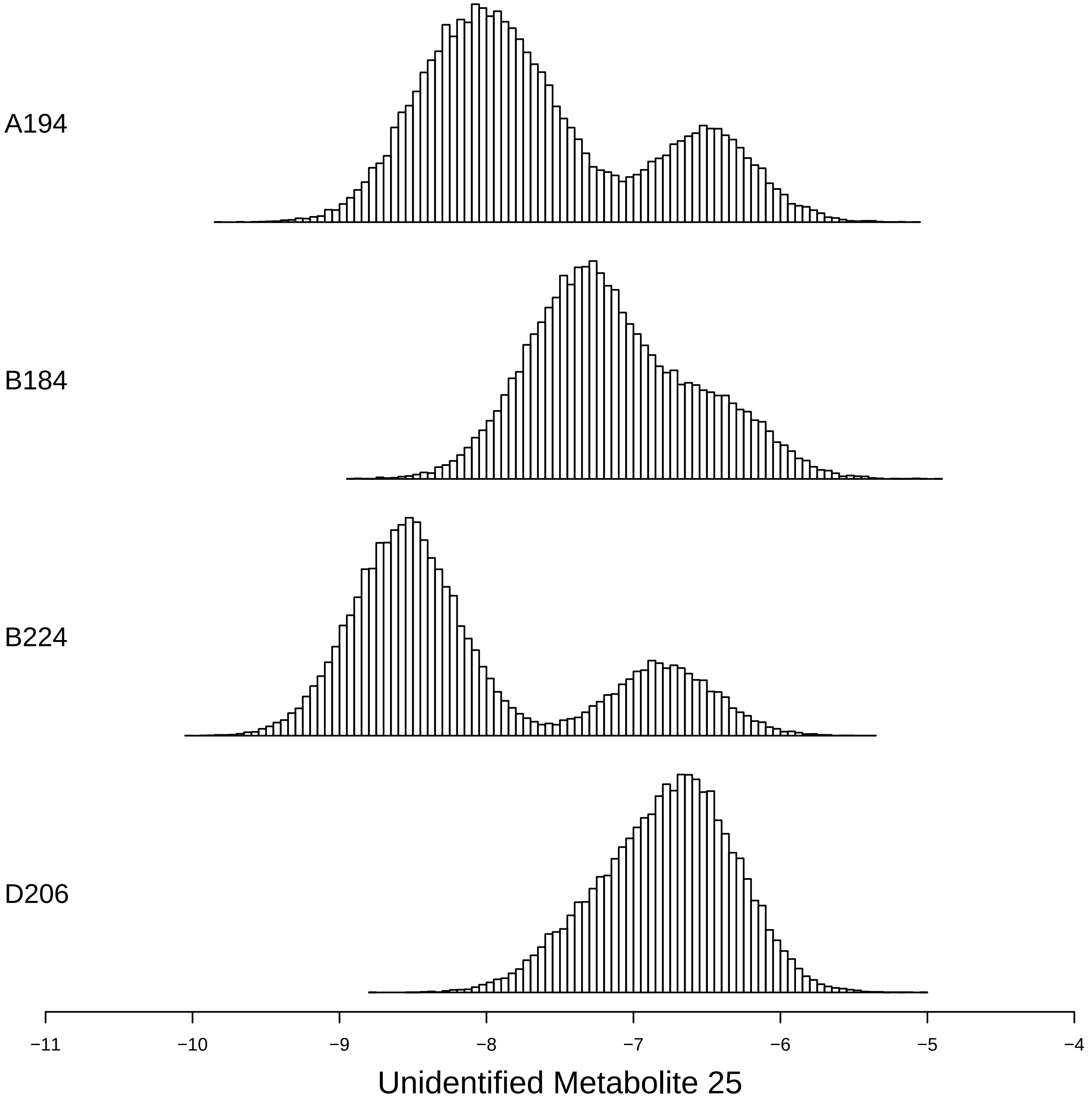
A194
B184
B224
D206
−11
−10
−9
−8
−7
−6
−5
−4
Unidentified Metabolite 25

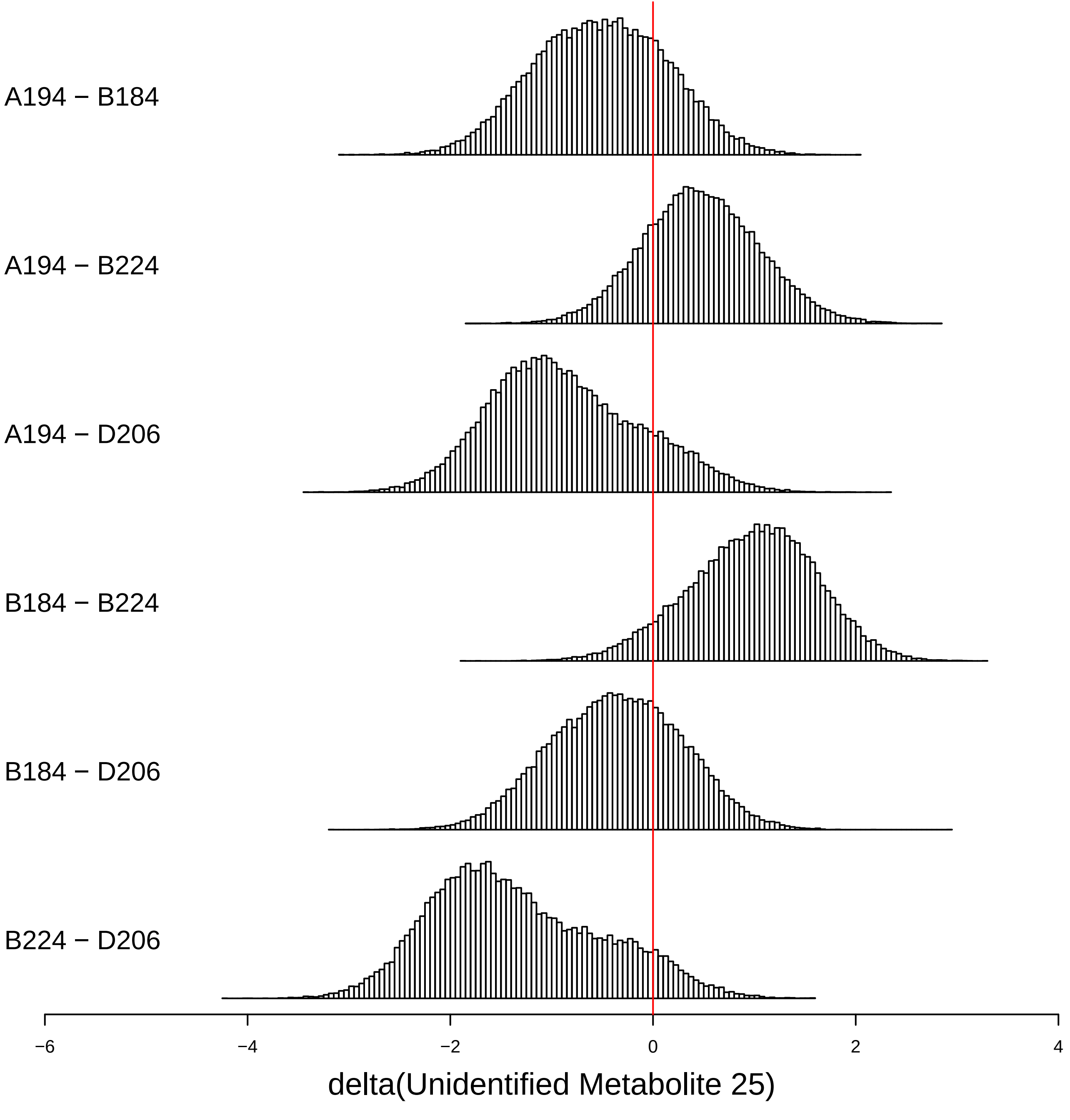

A194 − B184
A194 − B224
A194 − D206
B184 − B224
B184 − D206
B224 − D206
−6
−4
−2
0
2
4
delta(Unidentified Metabolite 25)

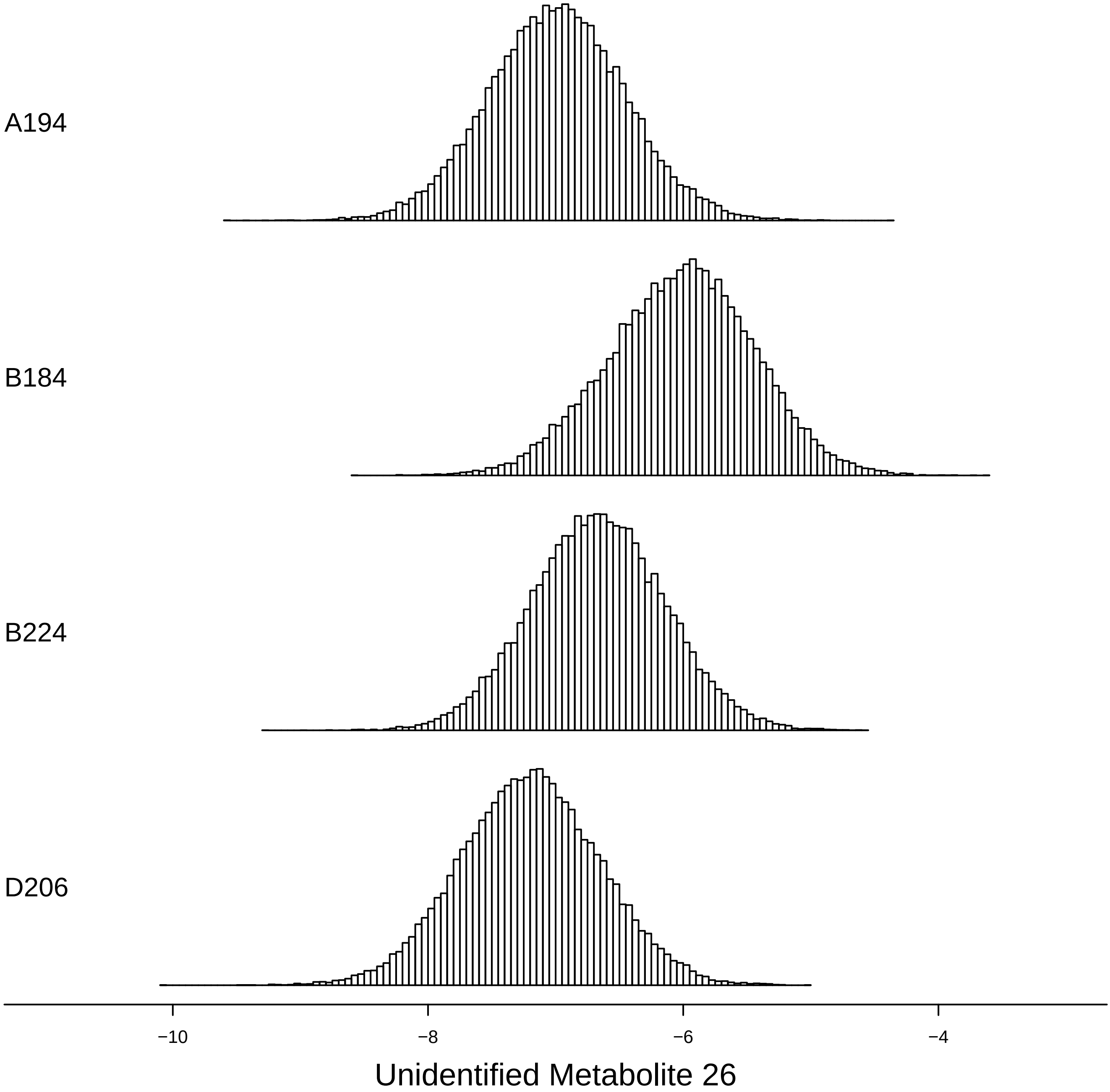
A194
B184
B224
D206
−10
−8
−6
−4
Unidentified Metabolite 26

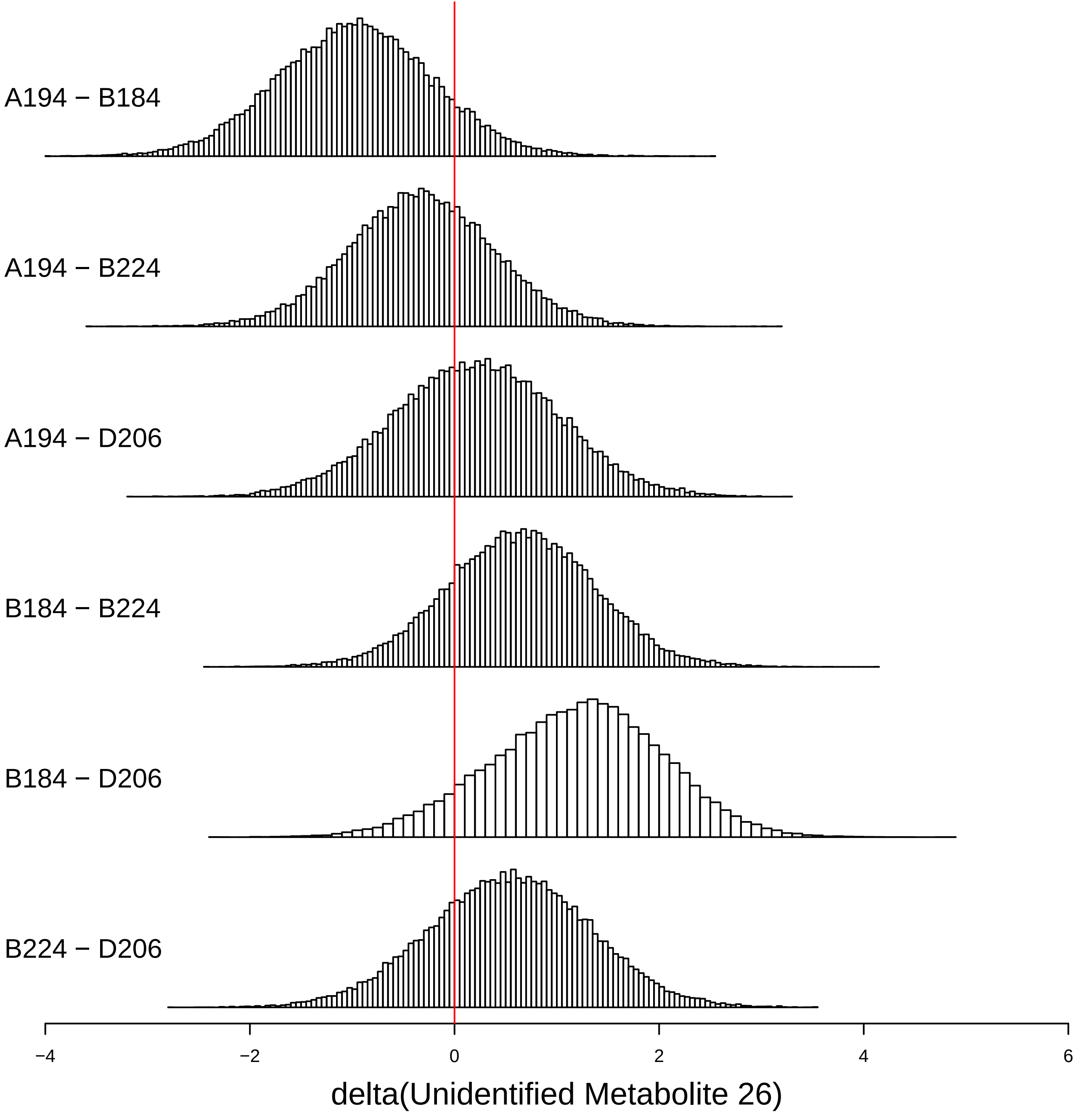

A194 − B184
A194 − B224
A194 − D206
B184 − B224
B184 − D206
B224 − D206
−4
−2
0
2
4
6
delta(Unidentified Metabolite 26)

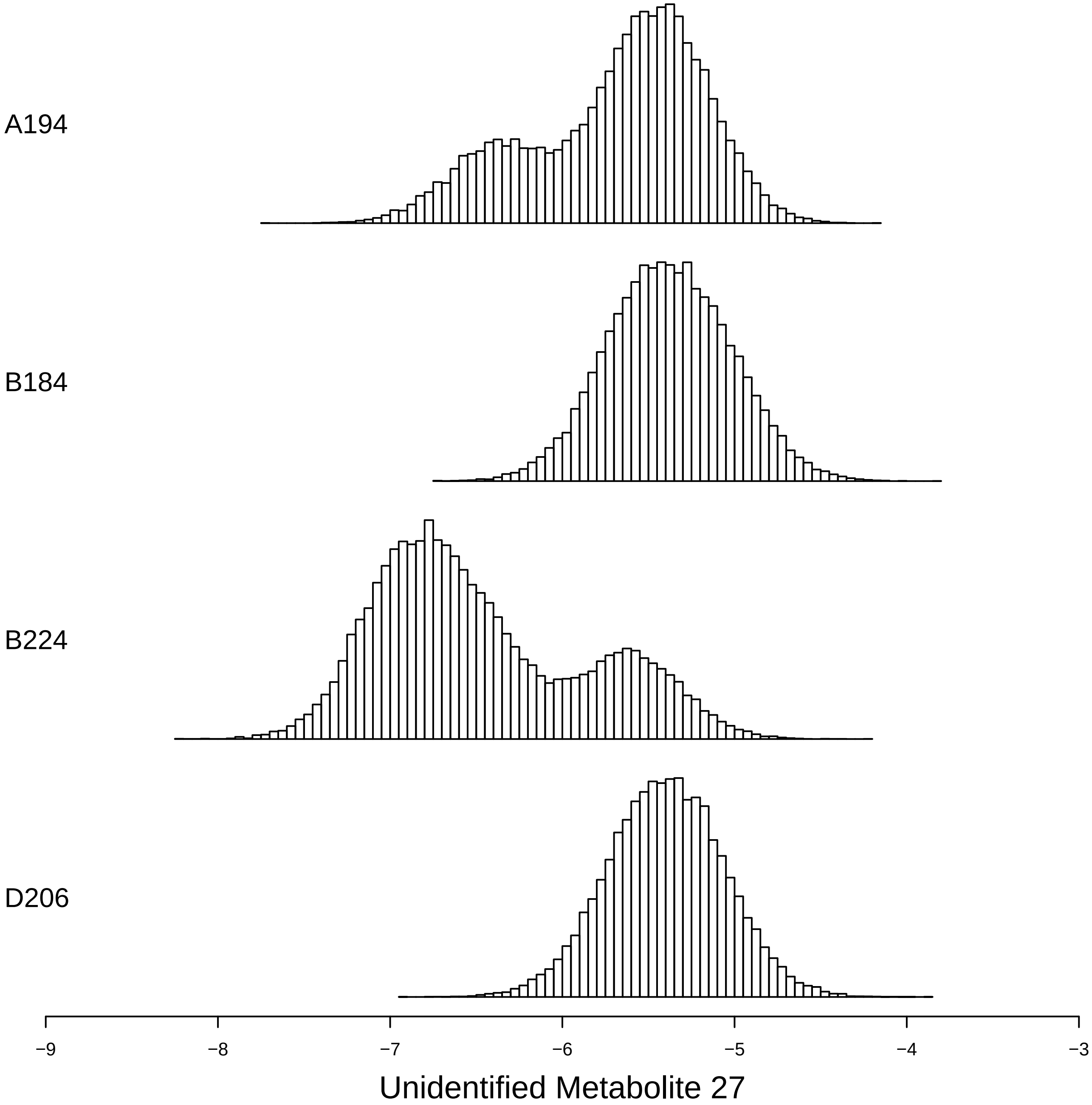
A194
B184
B224
D206
−9
−8
−7
−6
−5
−4
−3
Unidentified Metabolite 27

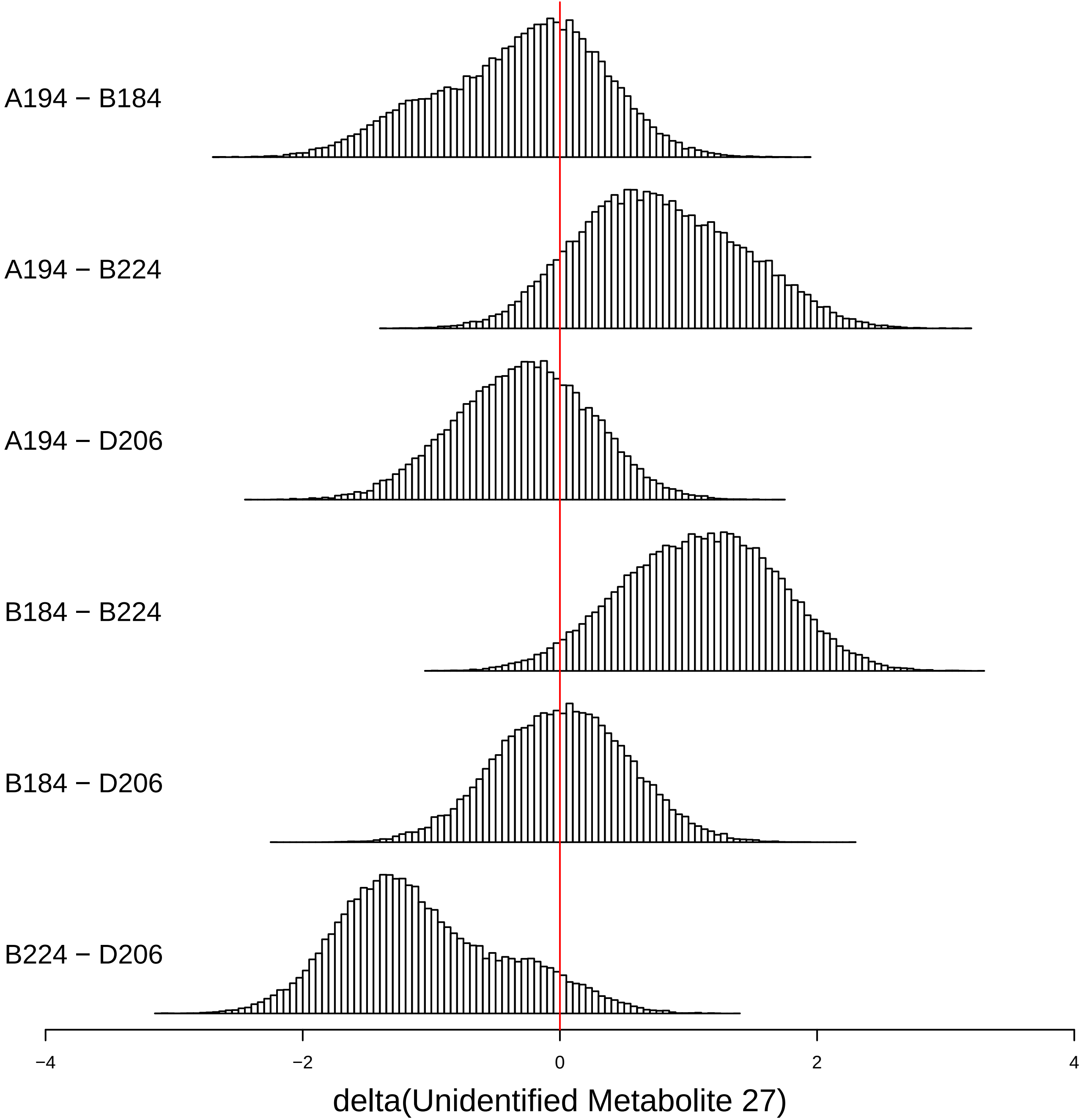
A194 − B184
A194 − B224
A194 − D206
B184 − B224
B184 − D206
B224 − D206
−4
−2
0
2
4
delta(Unidentified Metabolite 27)

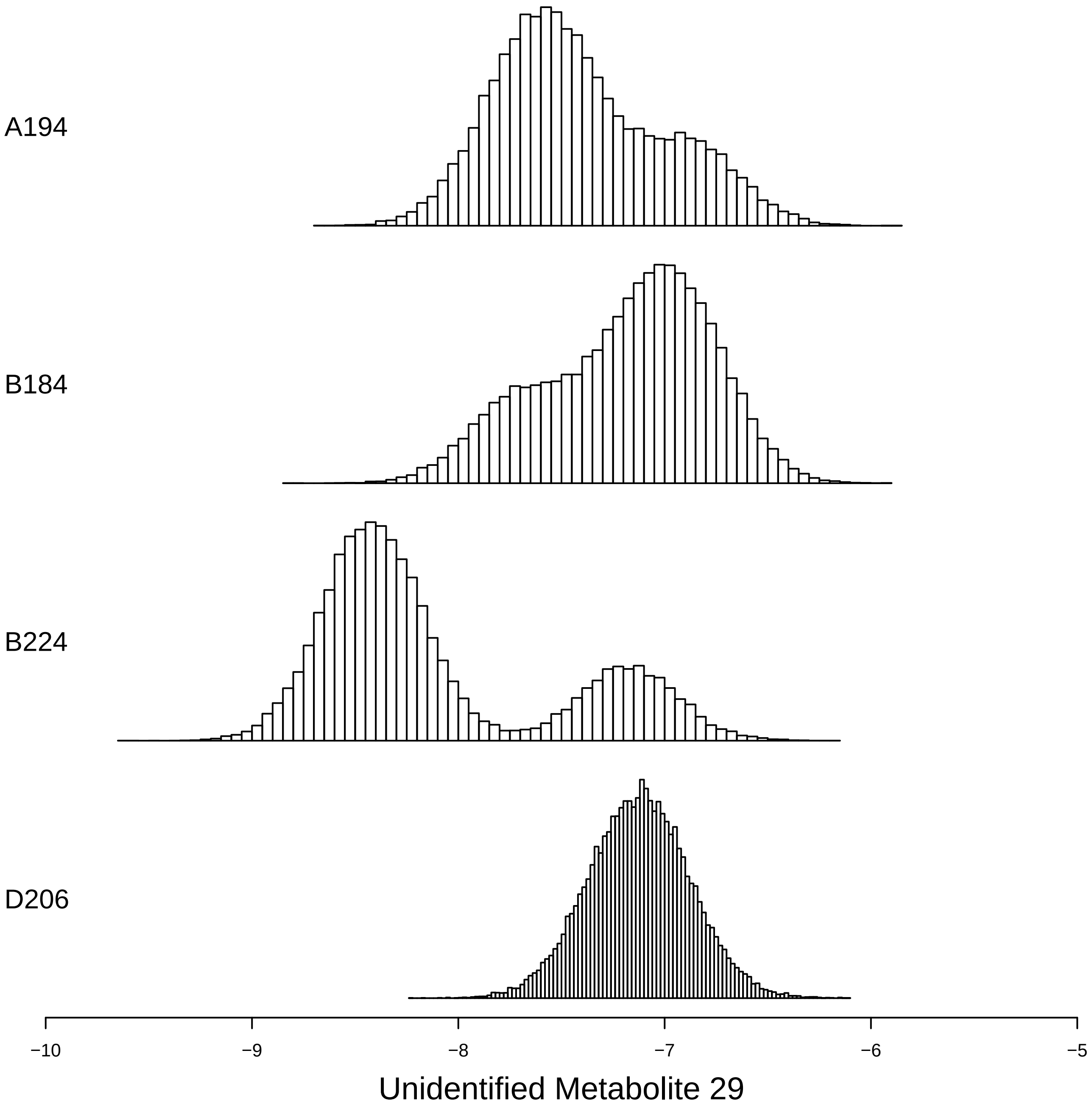
A194
B184
B224
D206
−10
−9
−8
−7
−6
−5
Unidentified Metabolite 29

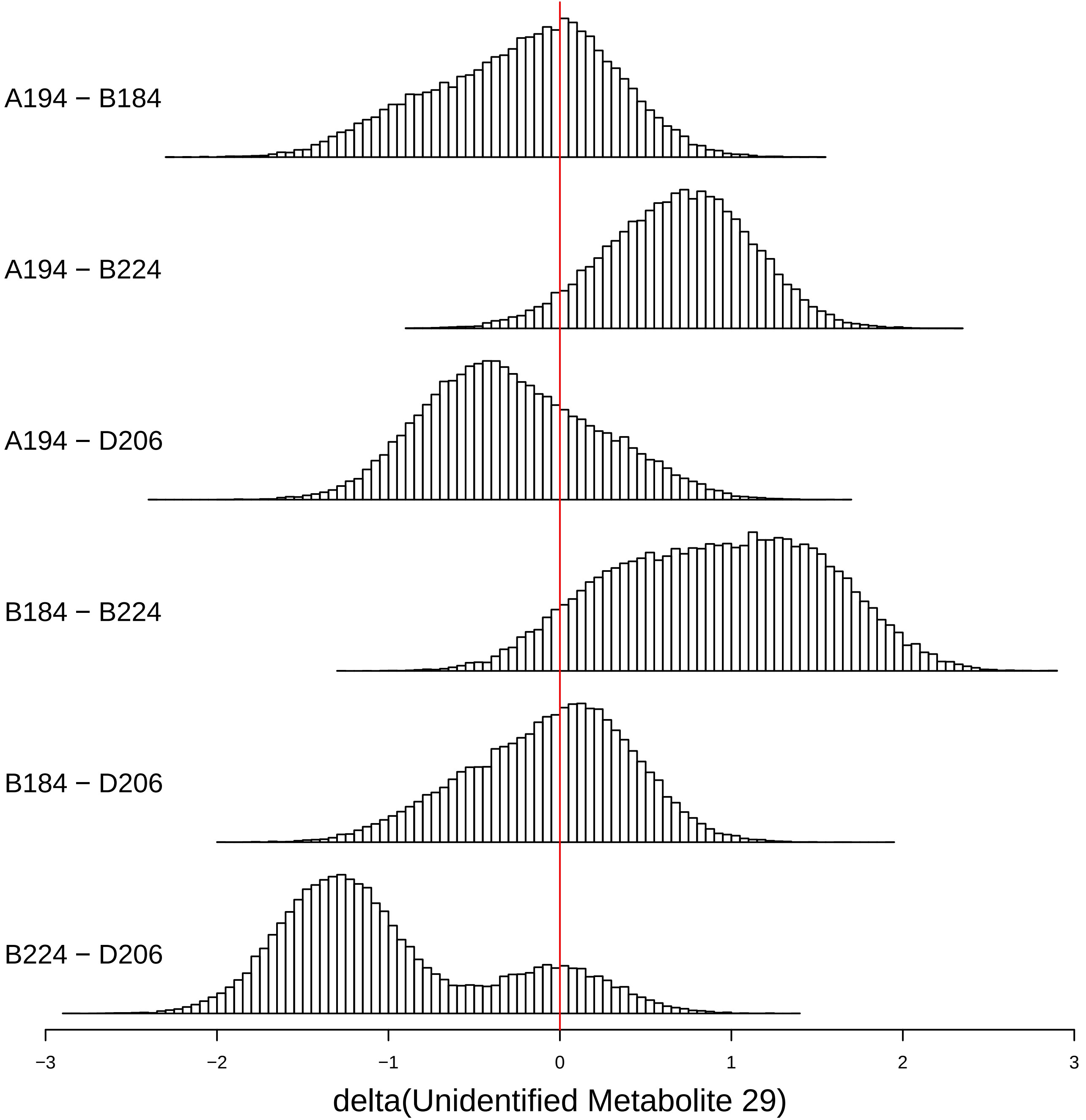

A194 − B184
A194 − B224
A194 − D206
B184 − B224
B184 − D206
B224 − D206
−3
−2
−1
0
1
2
3
delta(Unidentified Metabolite 29)

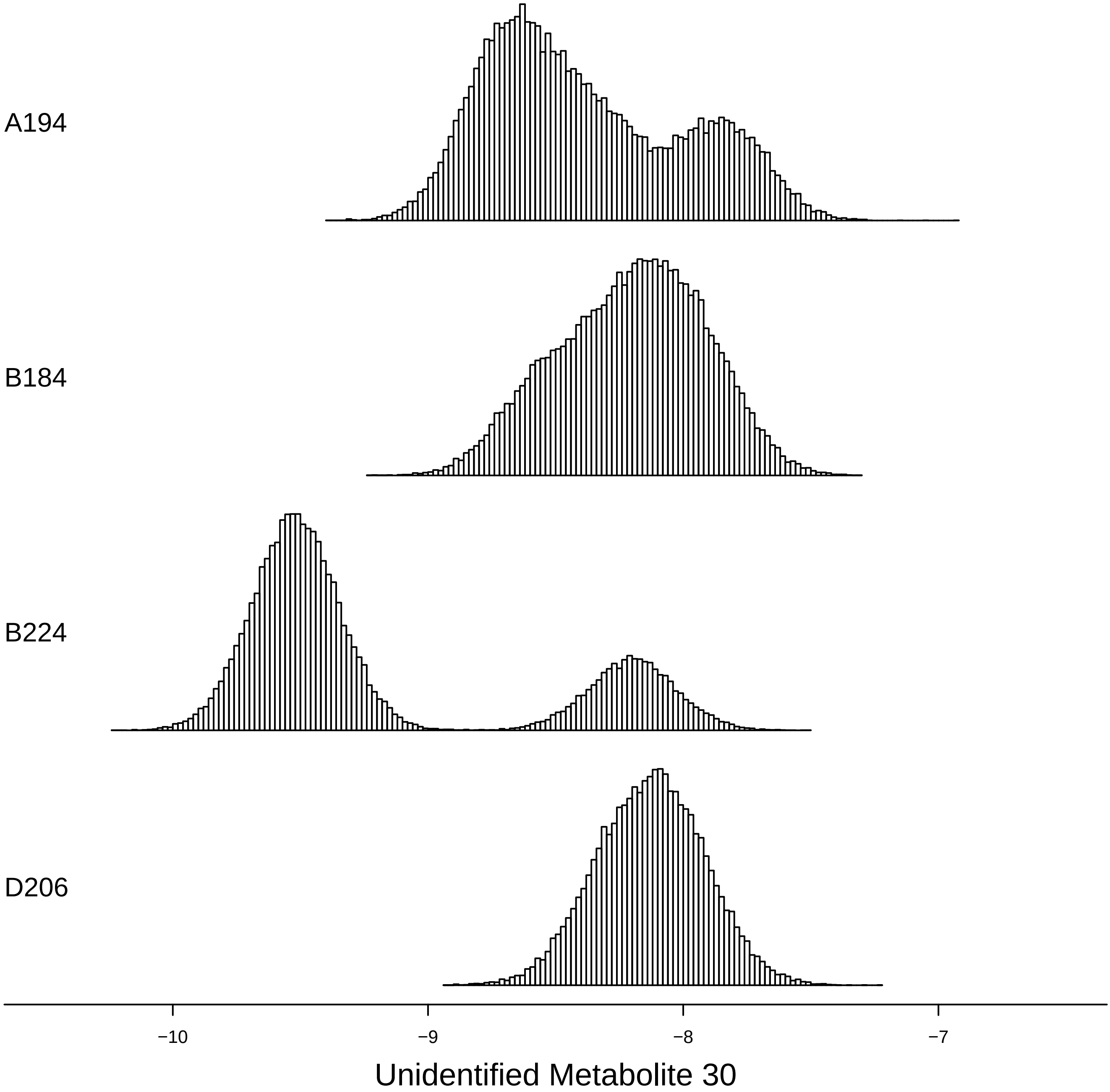
A194
B184
B224
D206
−10
−9
−8
−7
Unidentified Metabolite 30

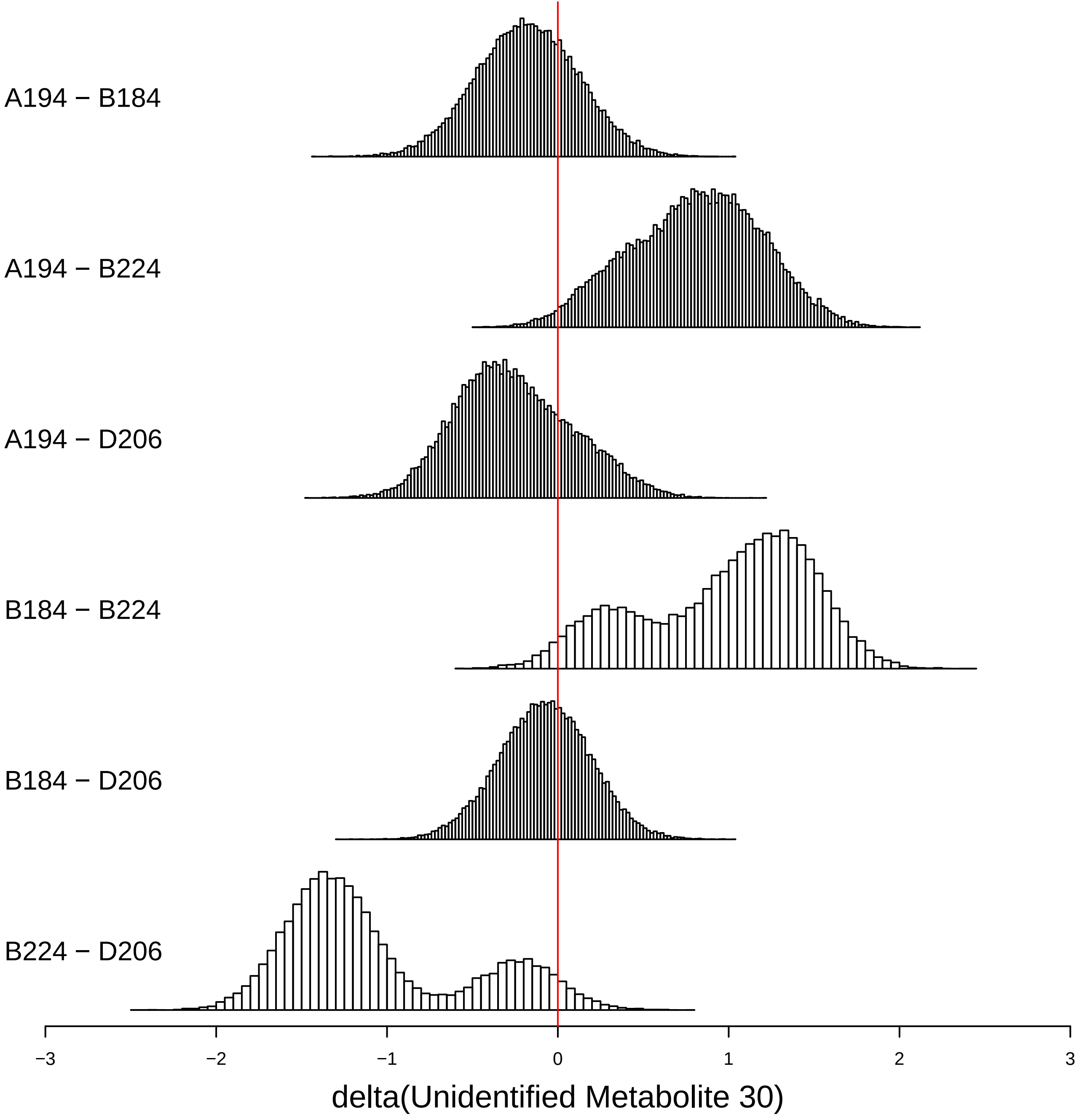
A194 − B184
A194 − B224
A194 − D206
B184 − B224
B184 − D206
B224 − D206
−3
−2
−1
0
1
2
3
delta(Unidentified Metabolite 30)

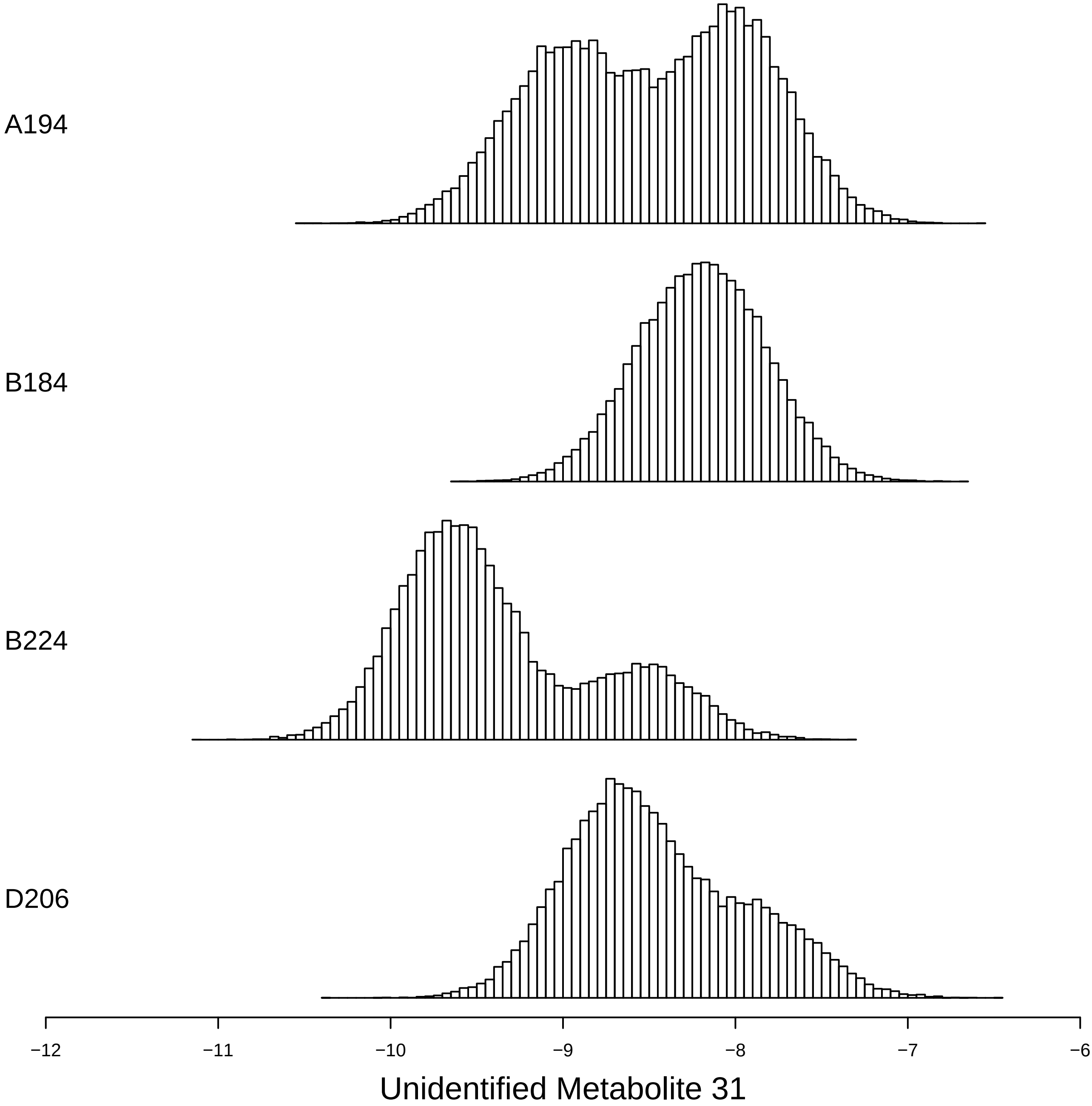
A194
B184
B224
D206
−12
−11
−10
−9
−8
−7
−6
Unidentified Metabolite 31

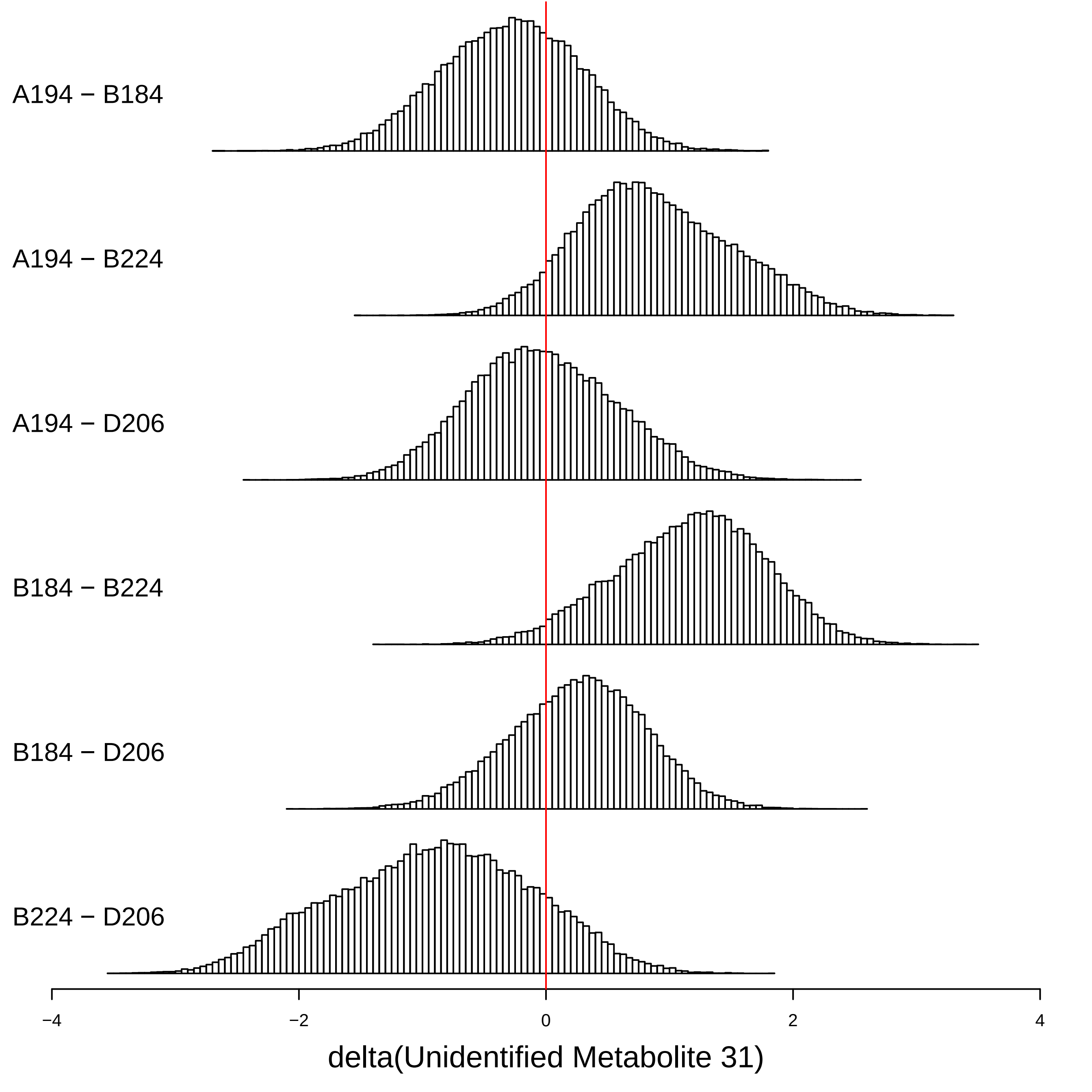
A194 − B184
A194 − B224
A194 − D206
B184 − B224
B184 − D206
B224 − D206
−4
−2
0
2
4
delta(Unidentified Metabolite 31)

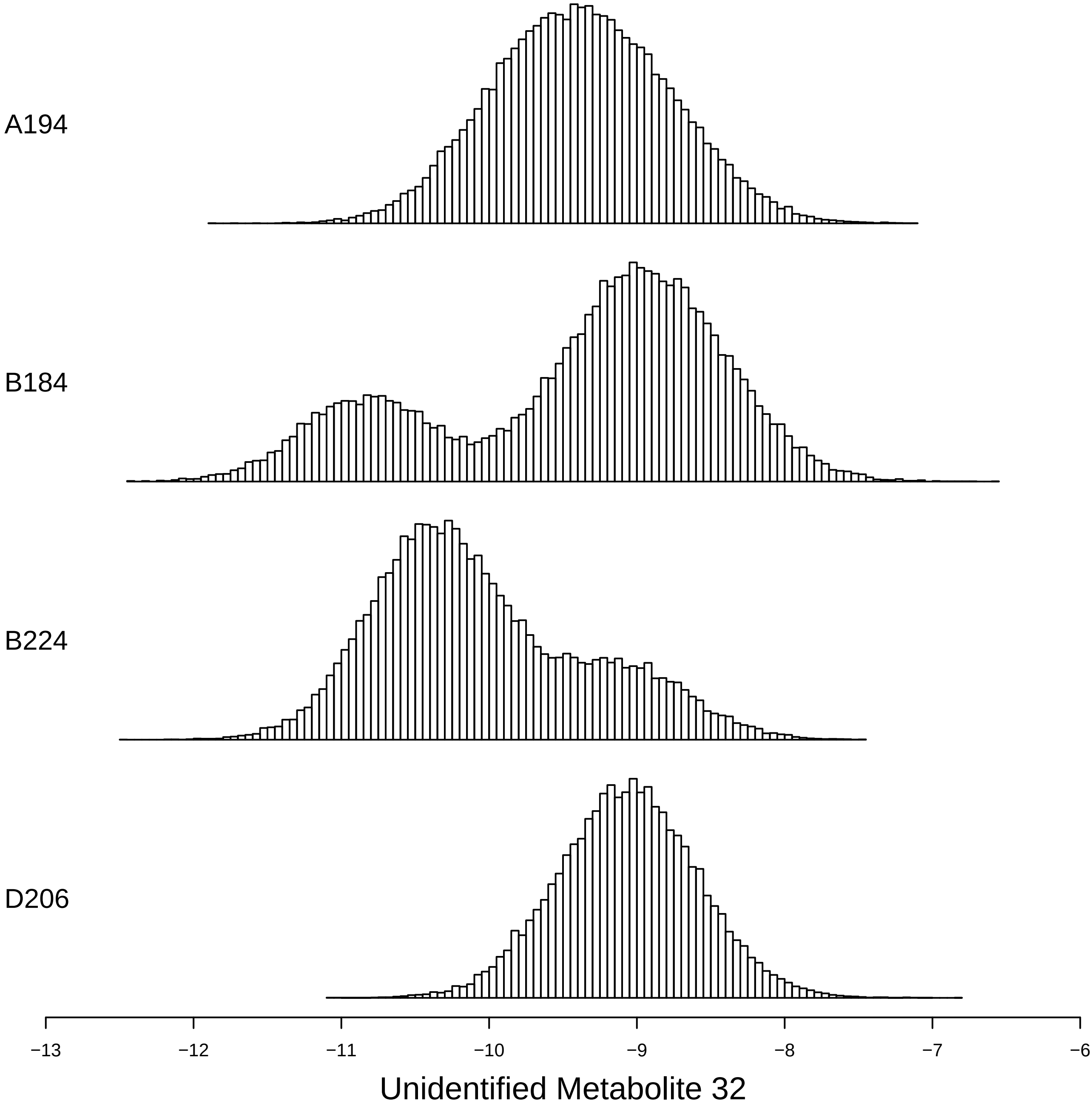

A194
B184
B224
D206
−13
−12
−11
−10
−9
−8
−7
−6
Unidentified Metabolite 32

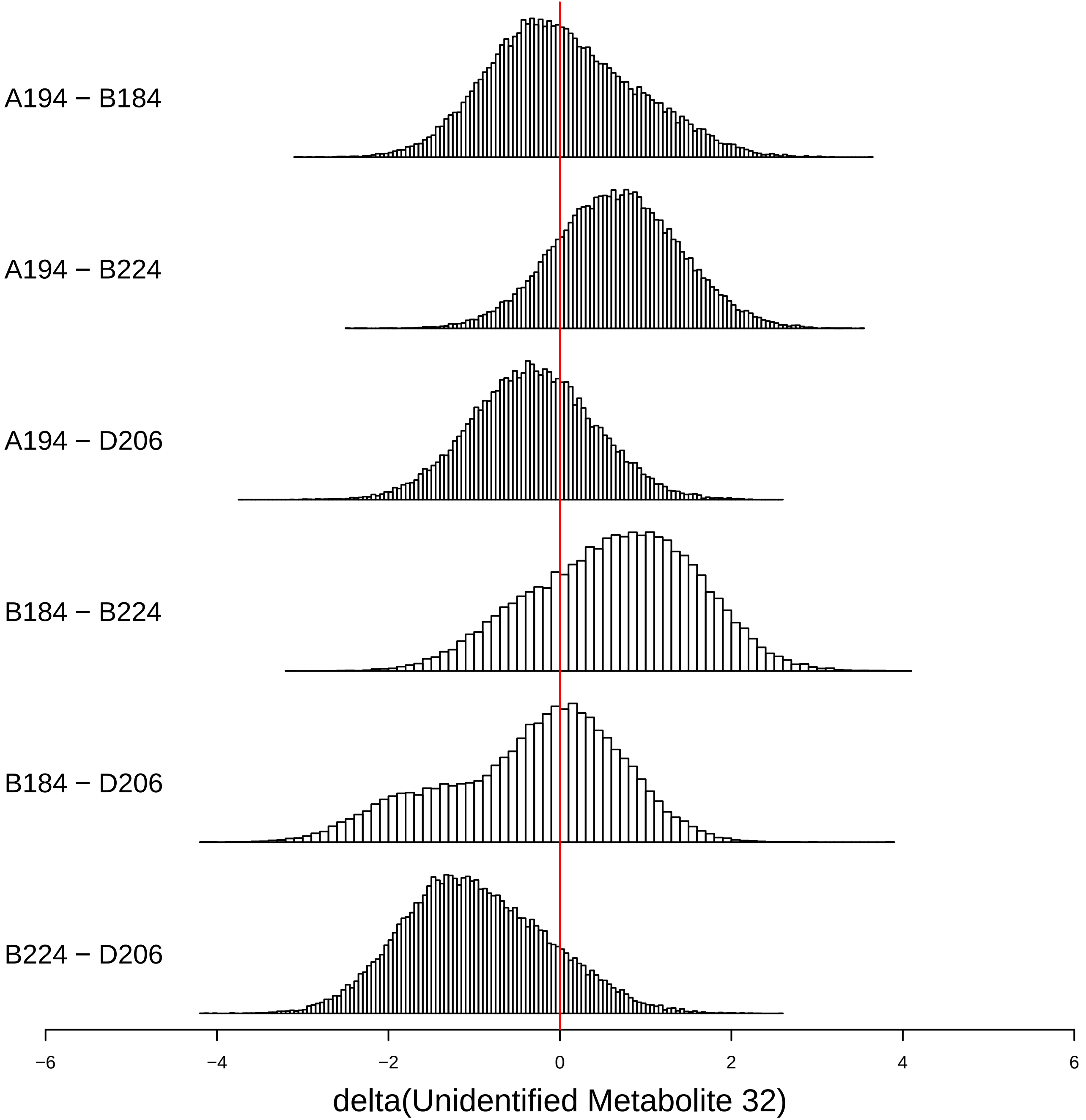

A194 − B184
A194 − B224
A194 − D206
B184 − B224
B184 − D206
B224 − D206
−6
−4
−2
0
2
4
6
delta(Unidentified Metabolite 32)

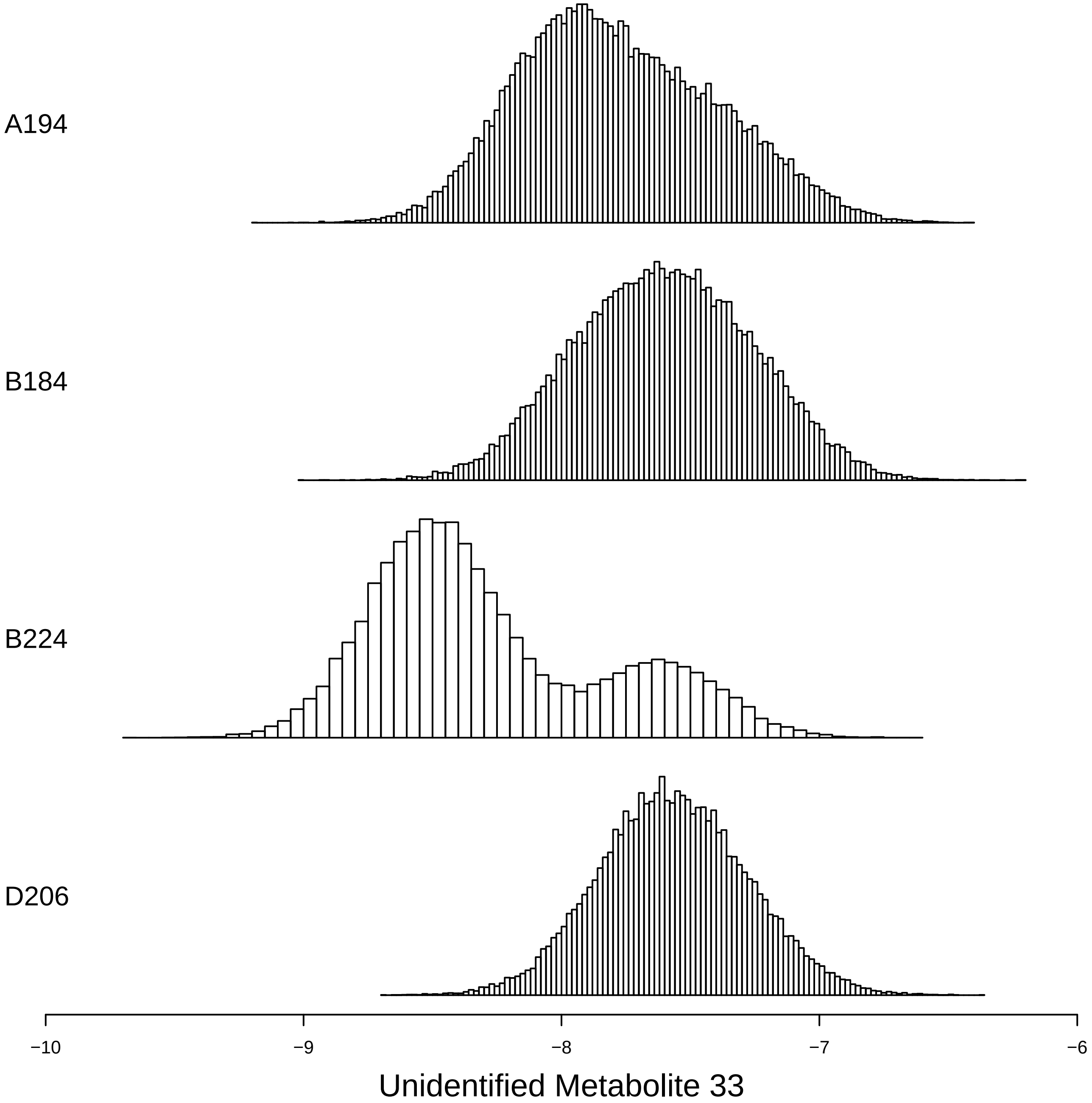

A194
B184
B224
D206
−10
−9
−8
−7
−6
Unidentified Metabolite 33

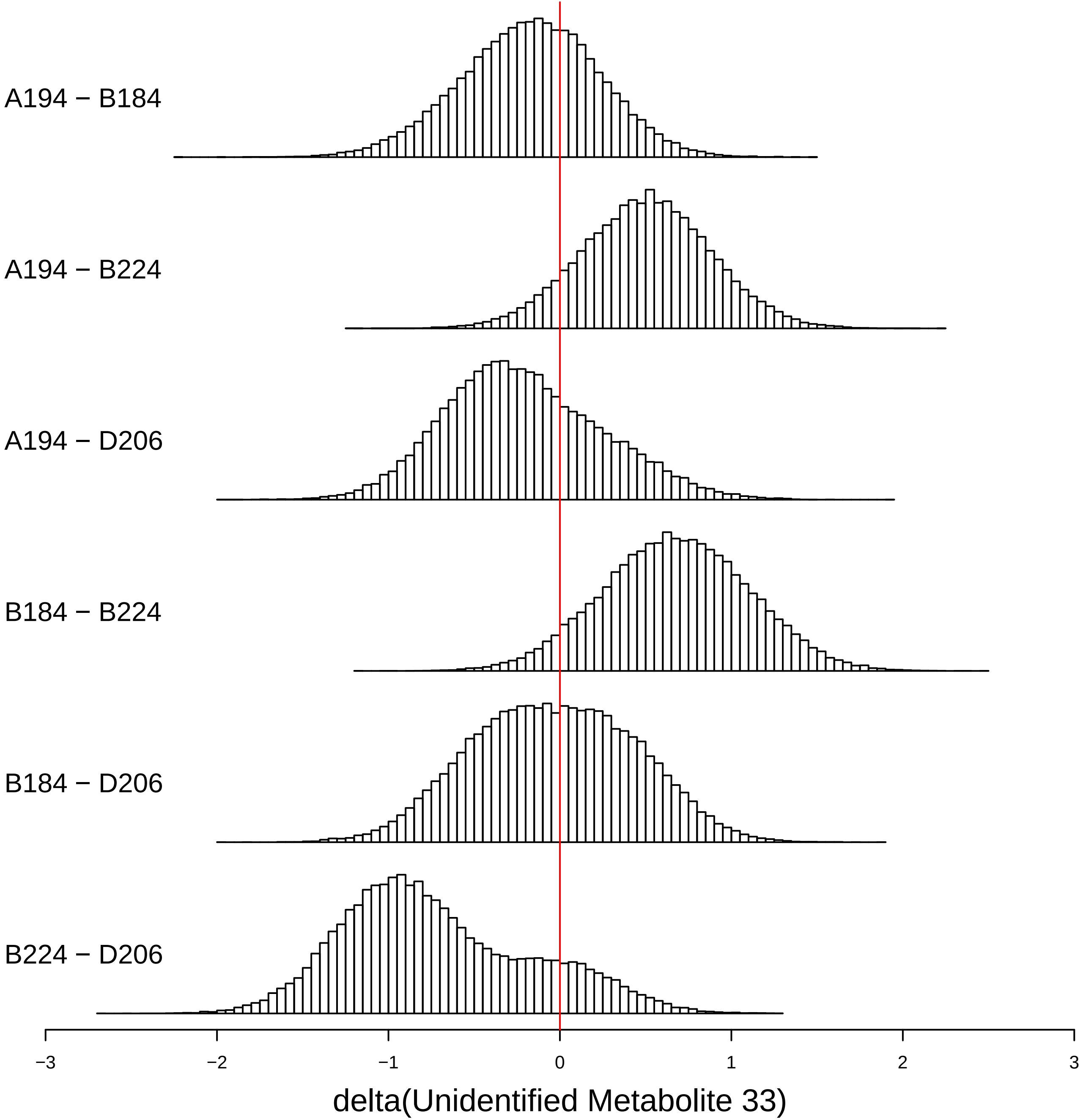
A194 − B184
A194 − B224
A194 − D206
B184 − B224
B184 − D206
B224 − D206
−3
−2
−1
0
1
2
3
delta(Unidentified Metabolite 33)

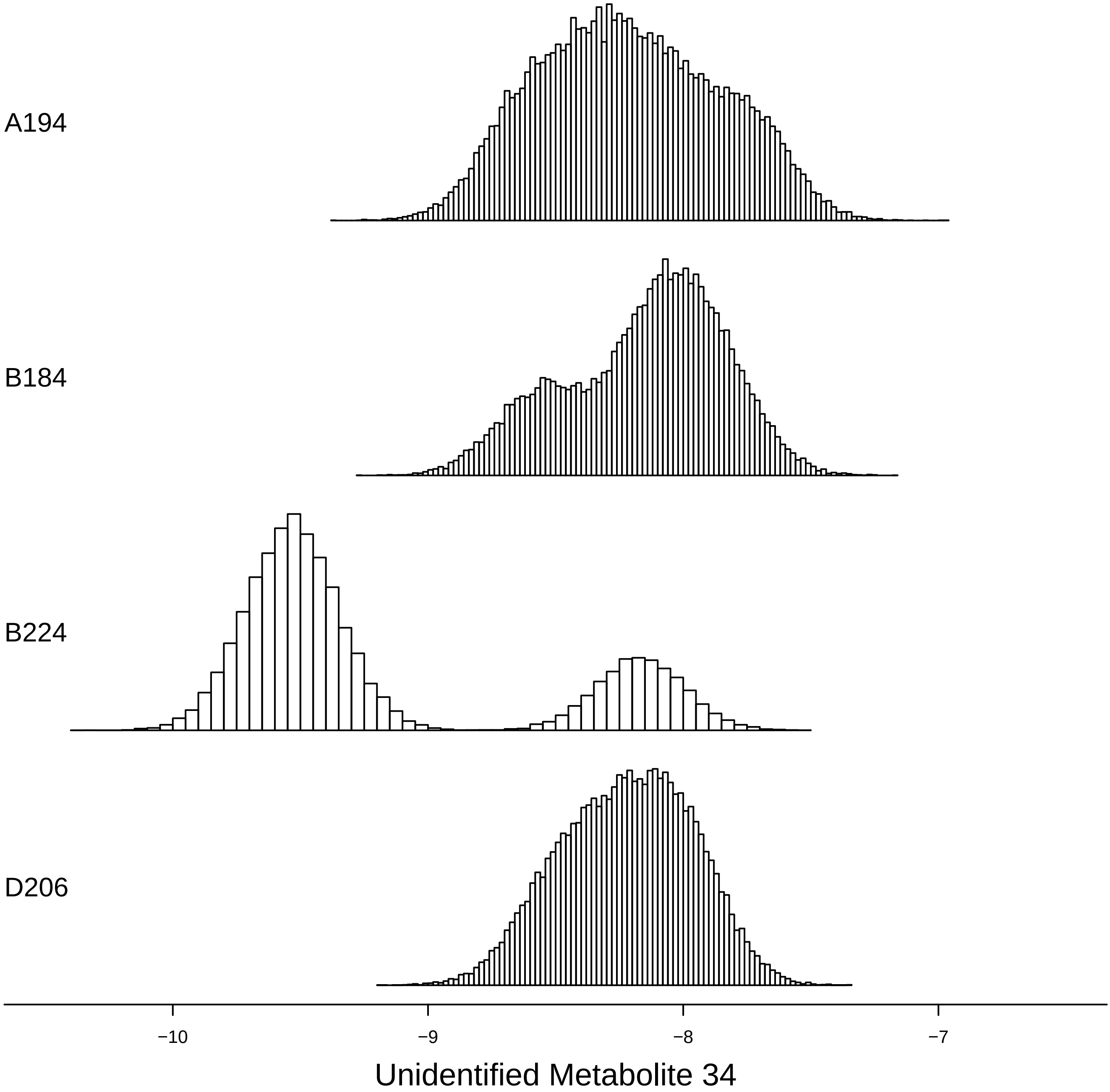
A194
B184
B224
D206
−10
−9
−8
−7
Unidentified Metabolite 34

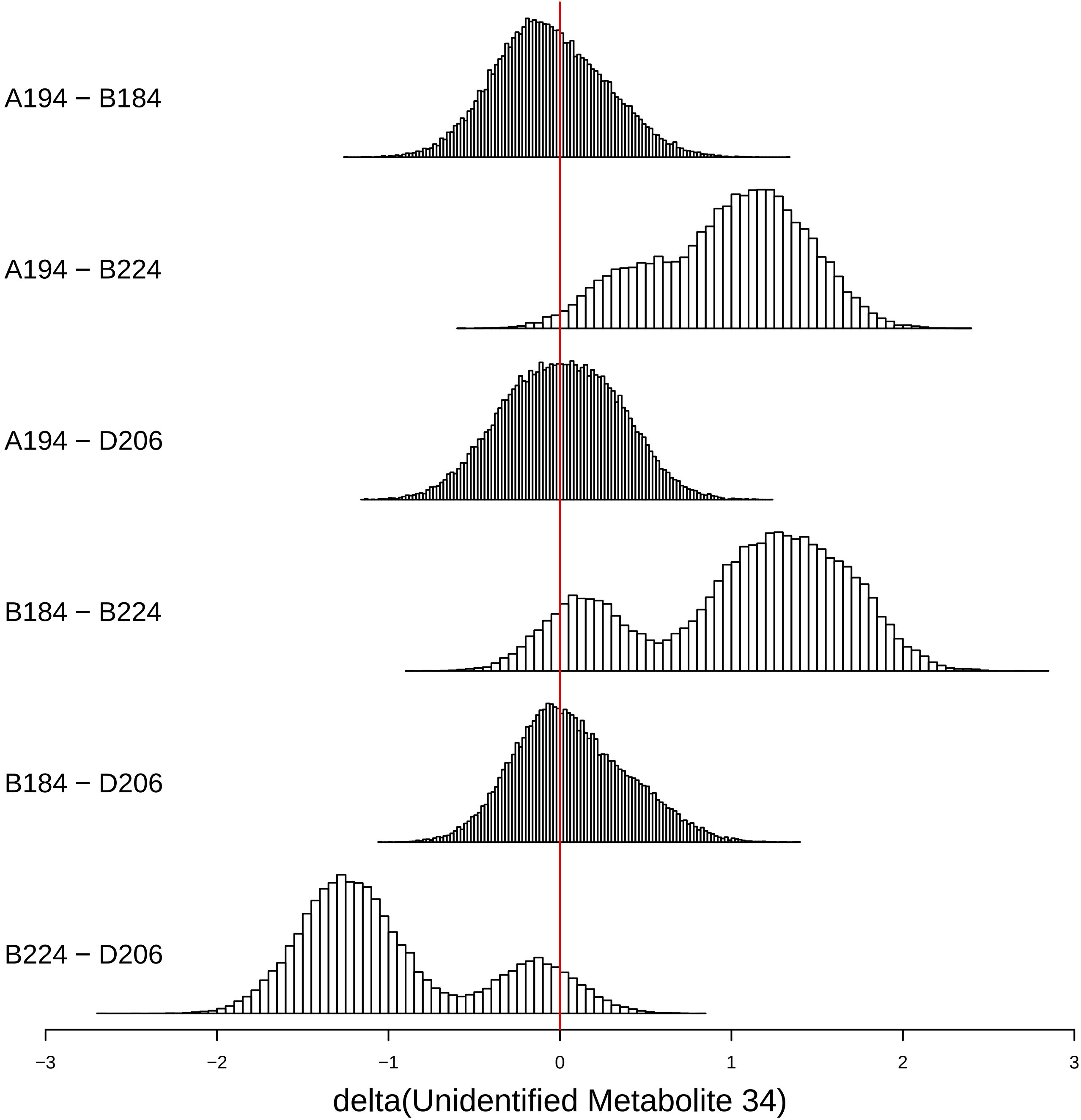
A194 − B184
A194 − B224
A194 − D206
B184 − B224
B184 − D206
B224 − D206
−3
−2
−1
0
1
2
3
delta(Unidentified Metabolite 34)

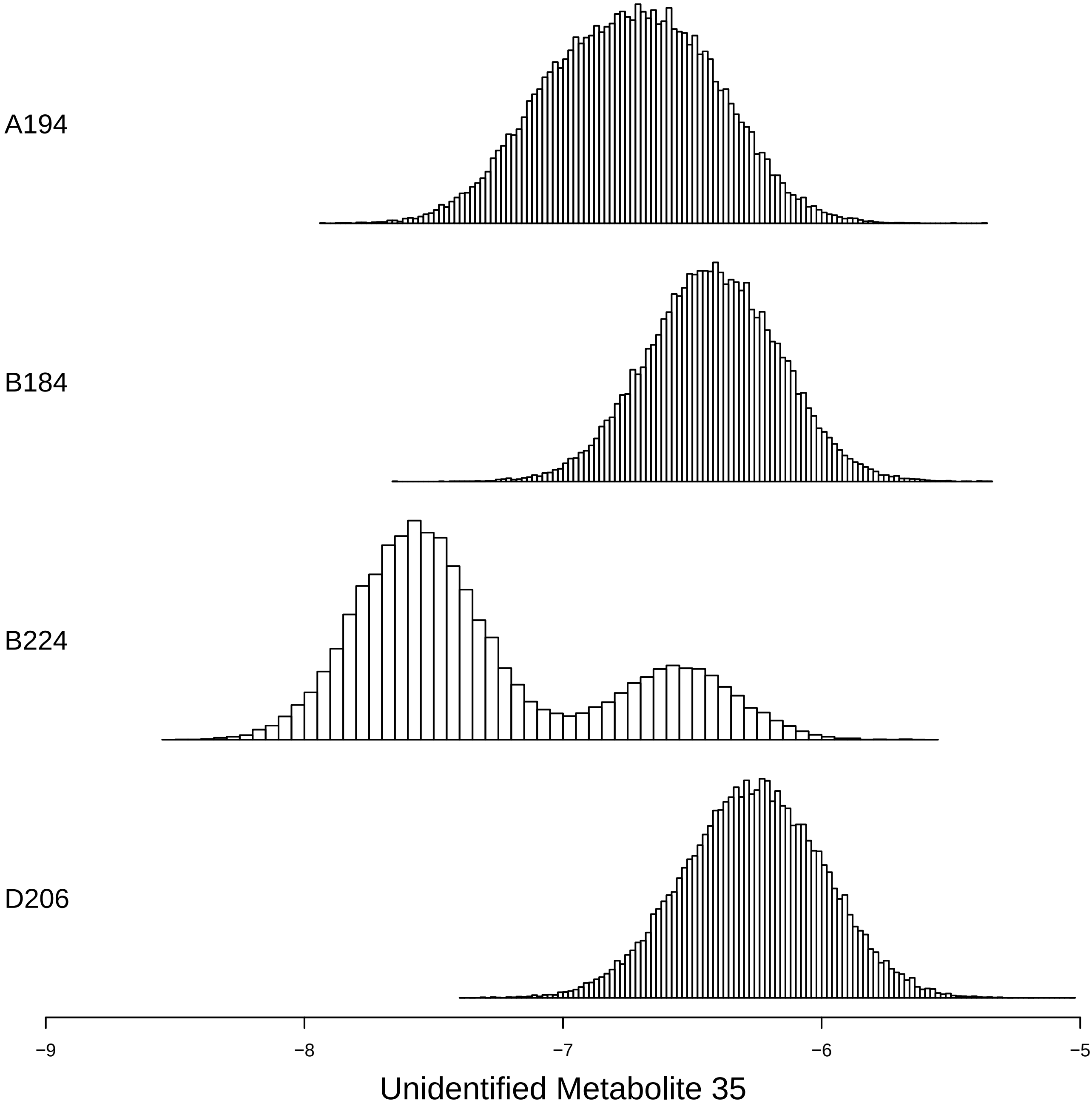

A194
B184
B224
D206
−9
−8
−7
−6
−5
Unidentified Metabolite 35

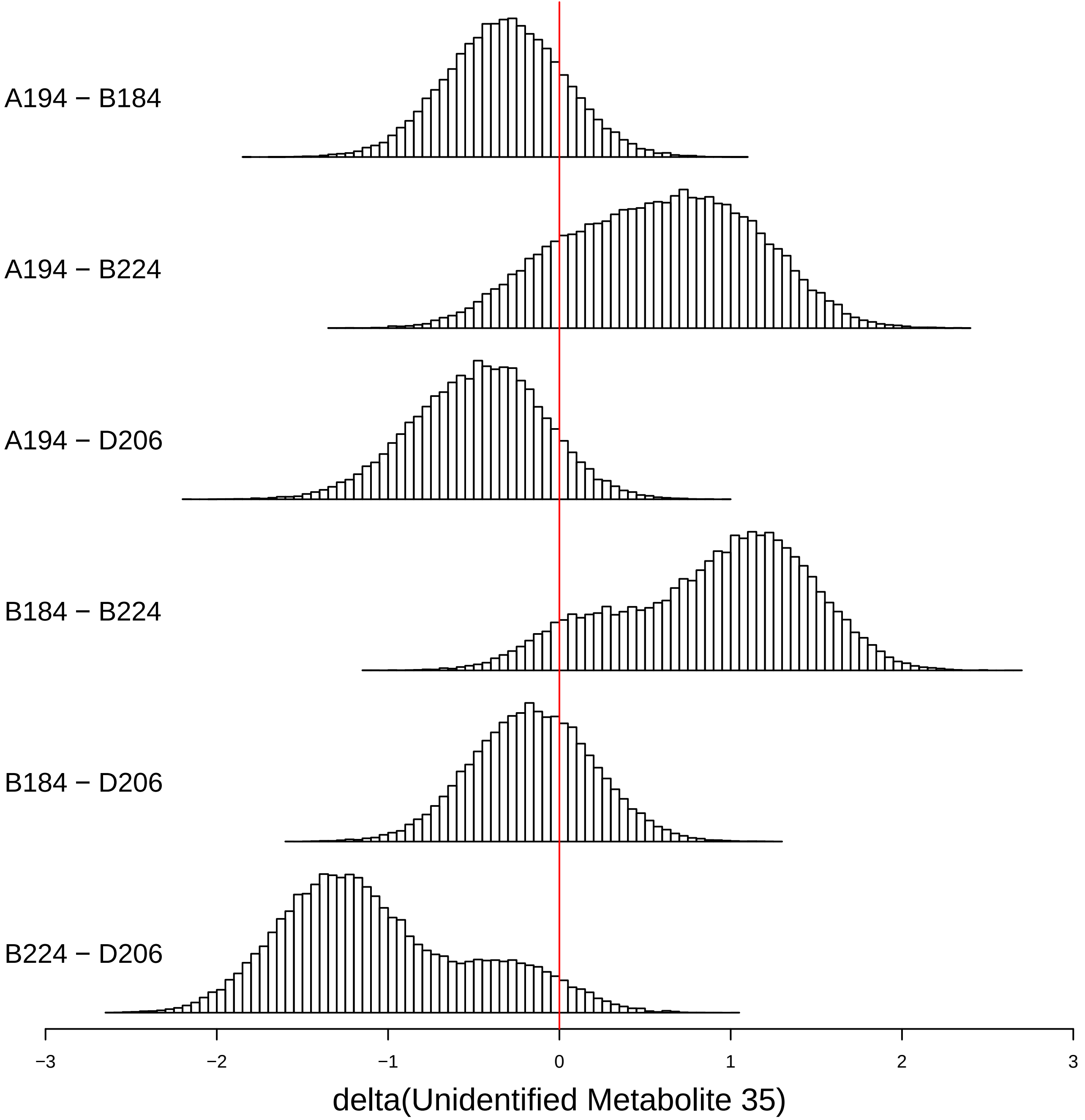

A194 − B184
A194 − B224
A194 − D206
B184 − B224
B184 − D206
B224 − D206
−3
−2
−1
0
1
2
3
delta(Unidentified Metabolite 35)

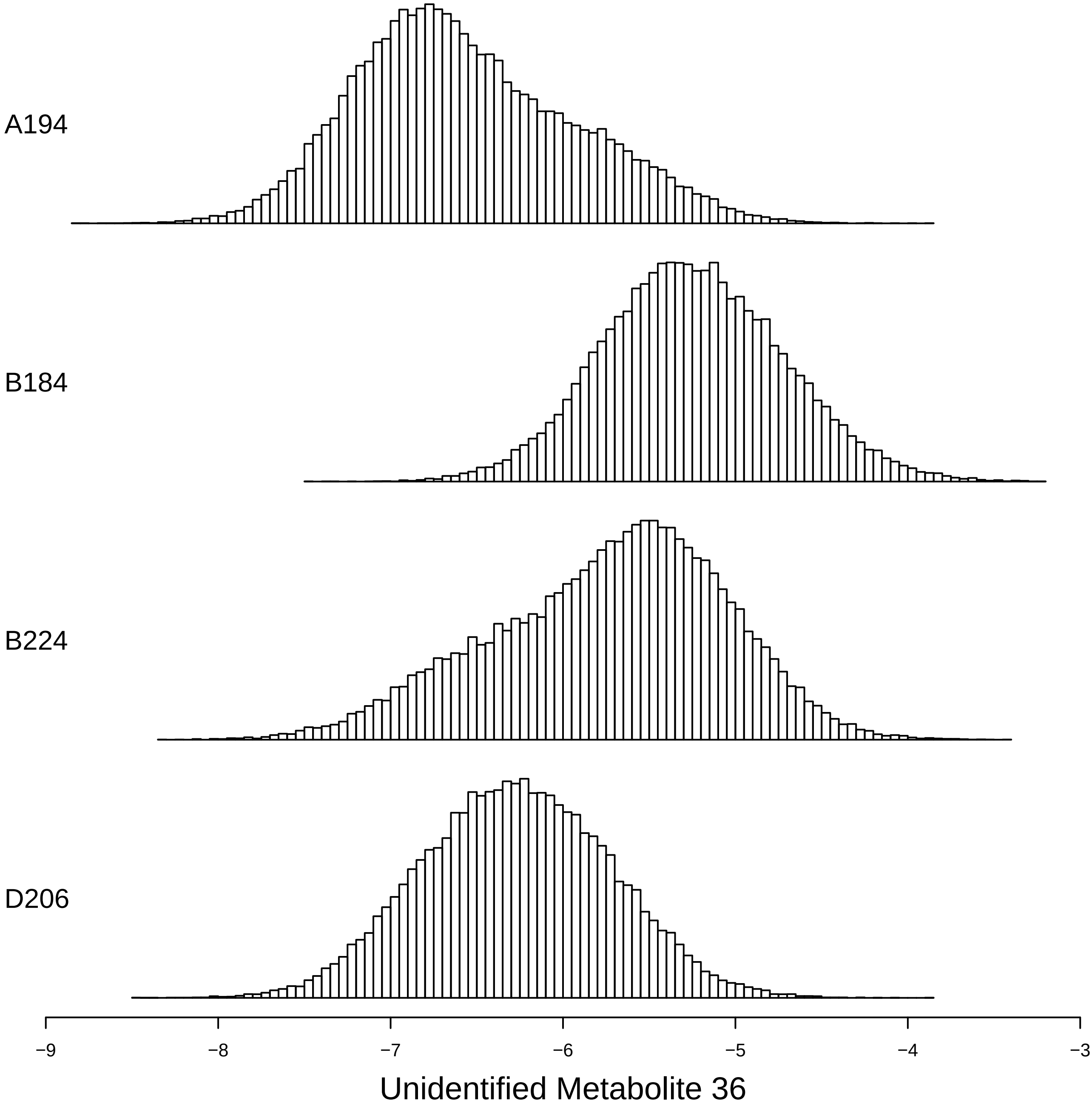
A194
B184
B224
D206
−9
−8
−7
−6
−5
−4
−3
Unidentified Metabolite 36

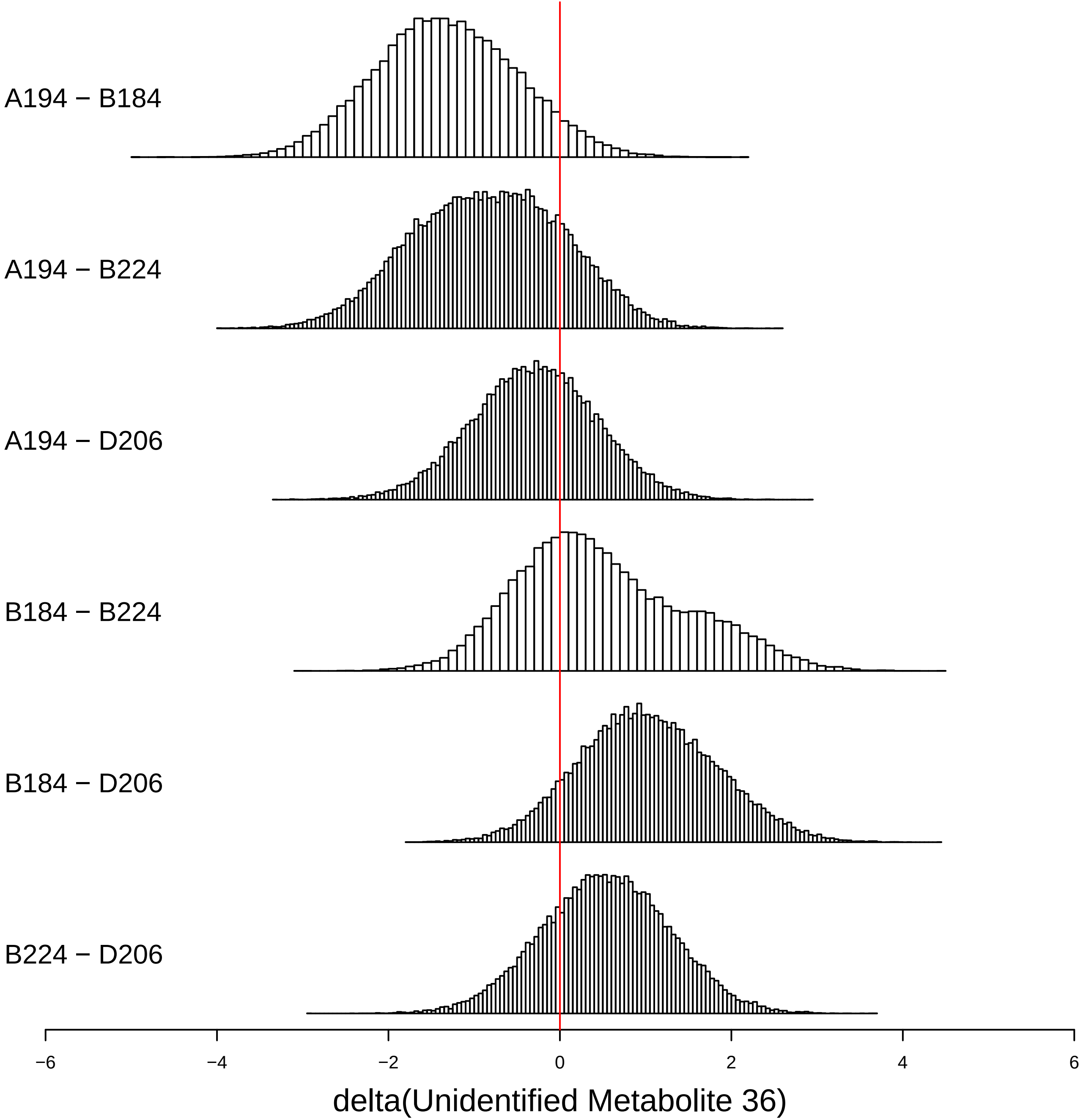
A194 − B184
A194 − B224
A194 − D206
B184 − B224
B184 − D206
B224 − D206
−6
−4
−2
0
2
4
6
delta(Unidentified Metabolite 36)

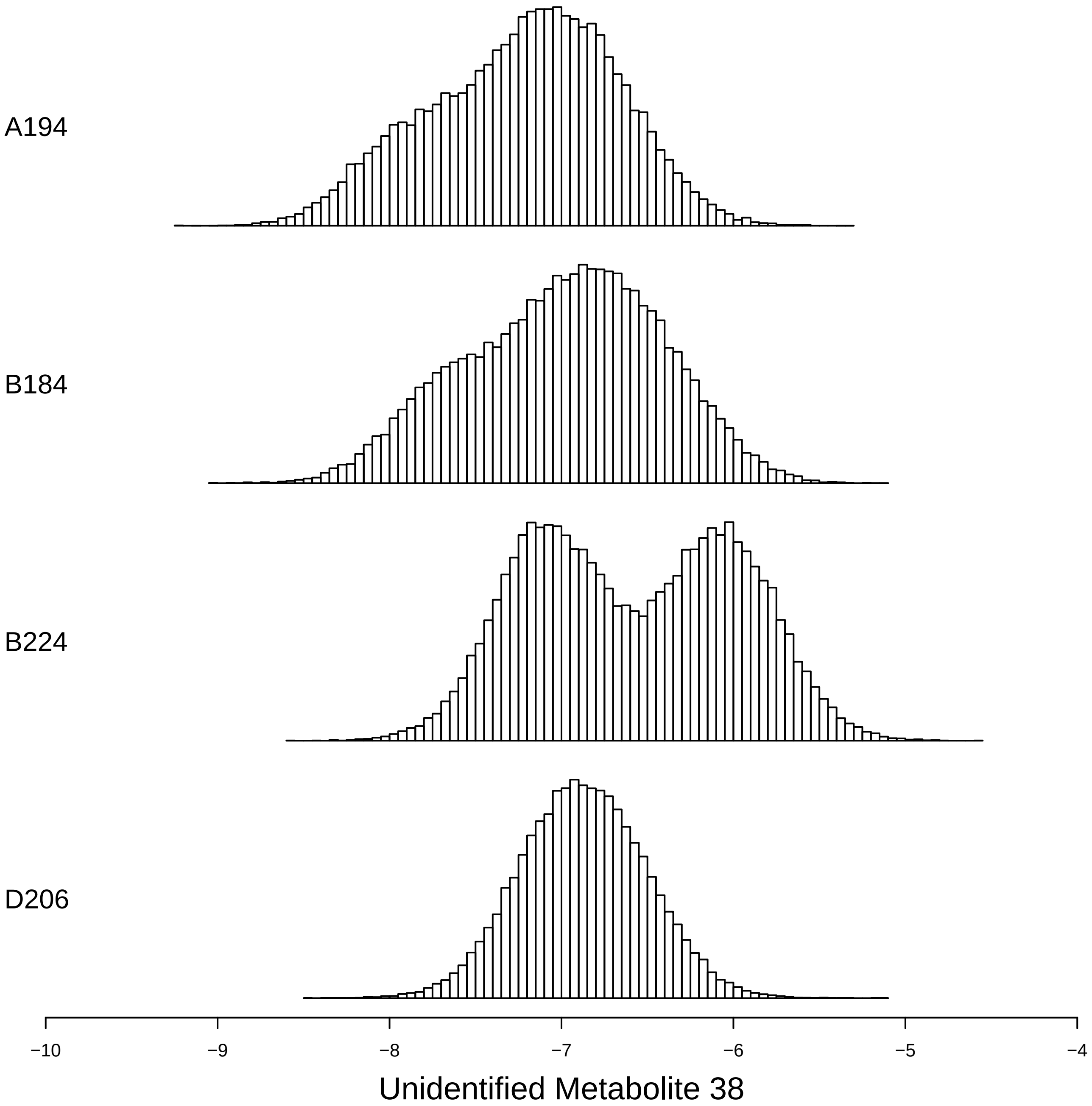

A194
B184
B224
D206
−10
−9
−8
−7
−6
−5
−4
Unidentified Metabolite 38

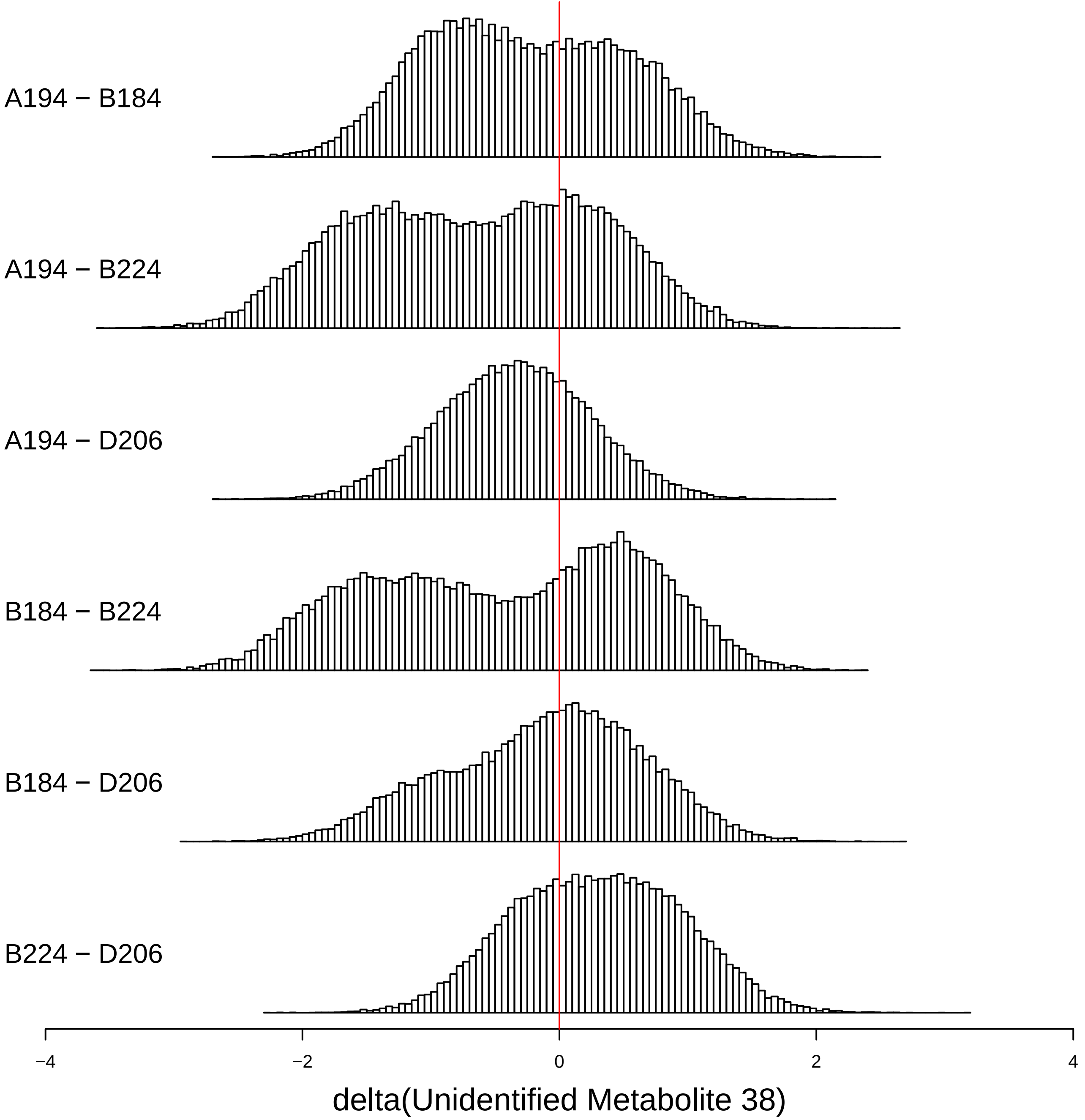
A194 − B184
A194 − B224
A194 − D206
B184 − B224
B184 − D206
B224 − D206
−4
−2
0
2
4
delta(Unidentified Metabolite 38)

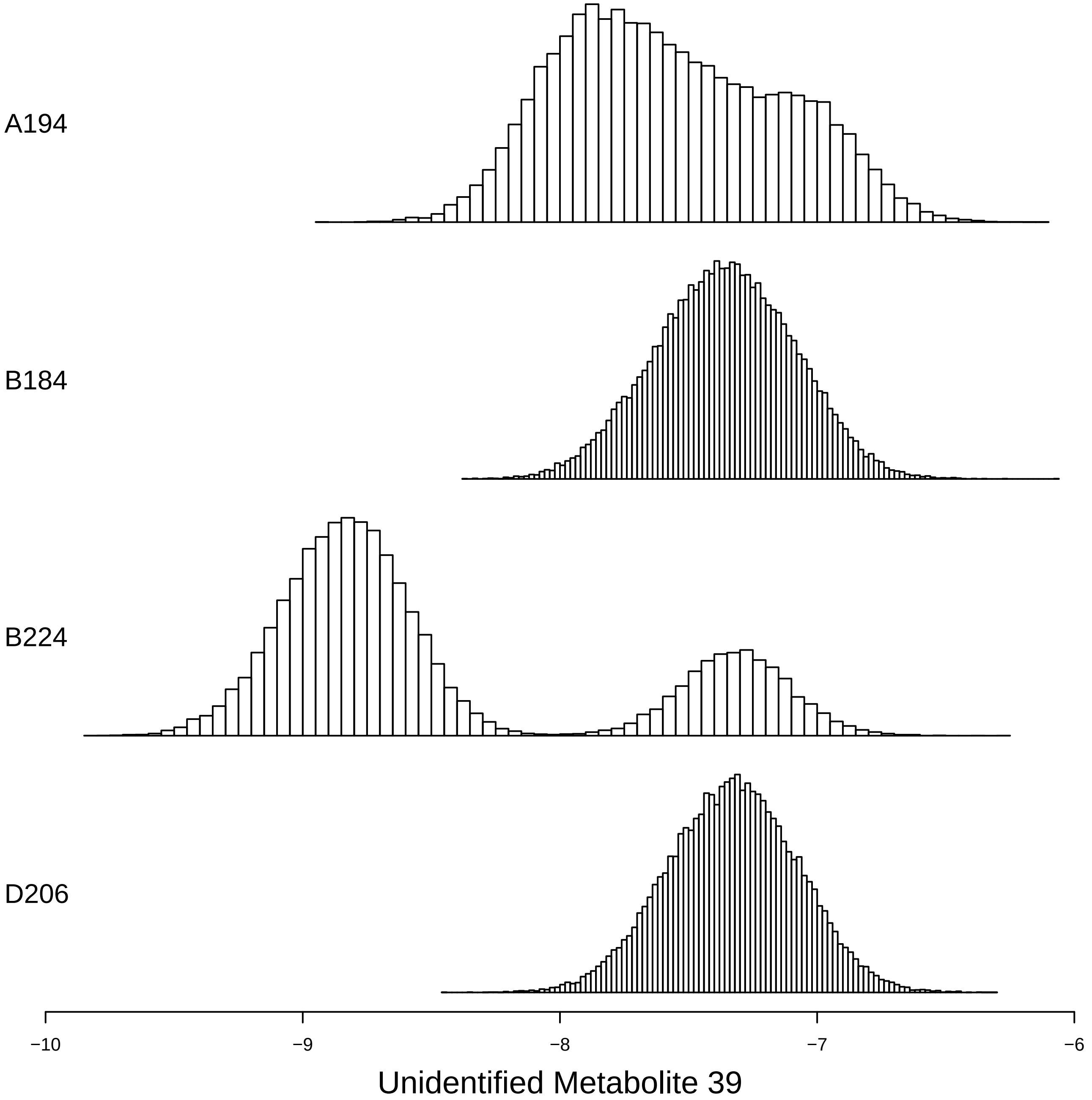
A194
B184
B224
D206
−10
−9
−8
−7
−6
Unidentified Metabolite 39

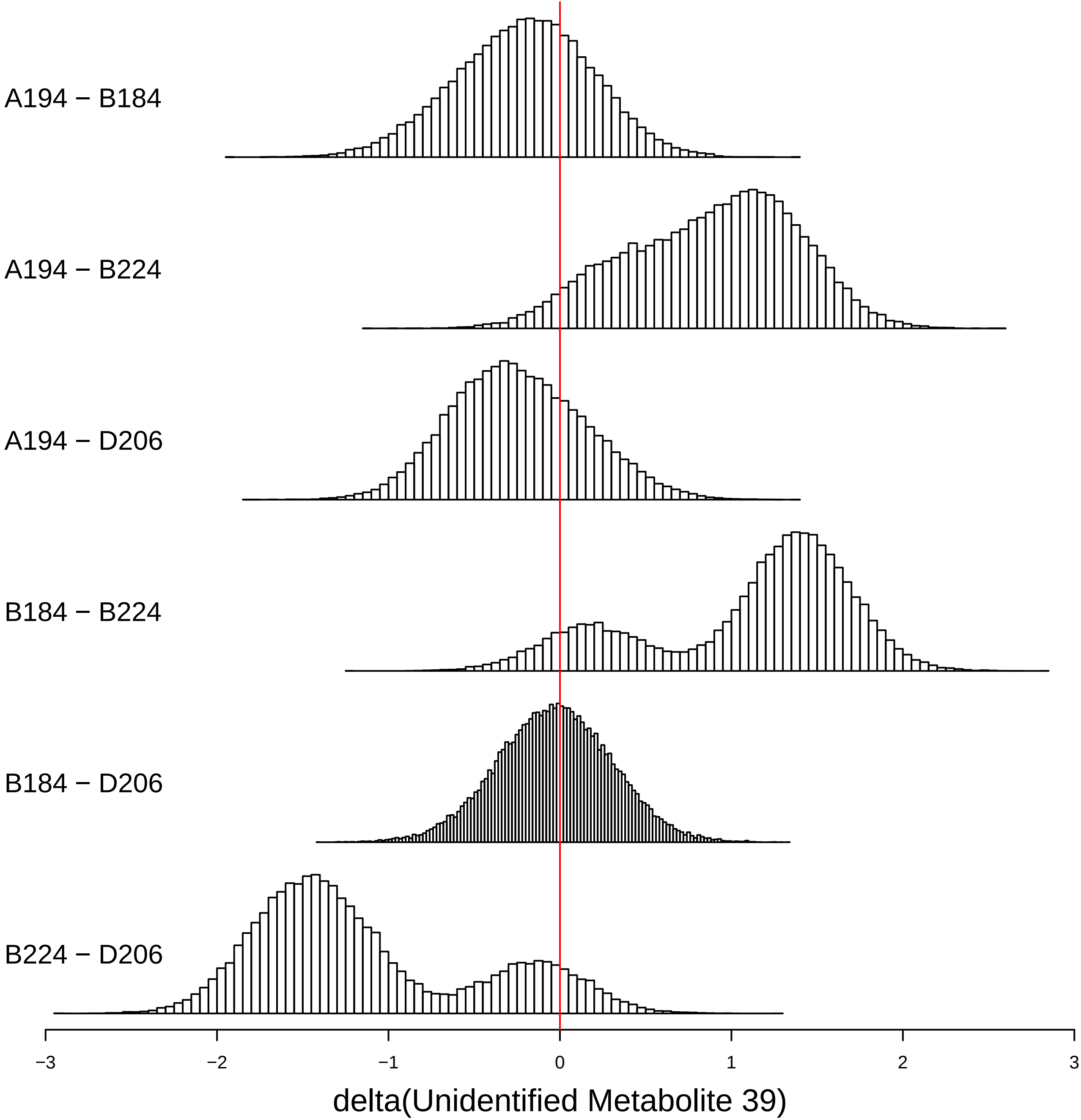
A194 − B184
A194 − B224
A194 − D206
B184 − B224
B184 − D206
B224 − D206
−3
−2
−1
0
1
2
3
delta(Unidentified Metabolite 39)

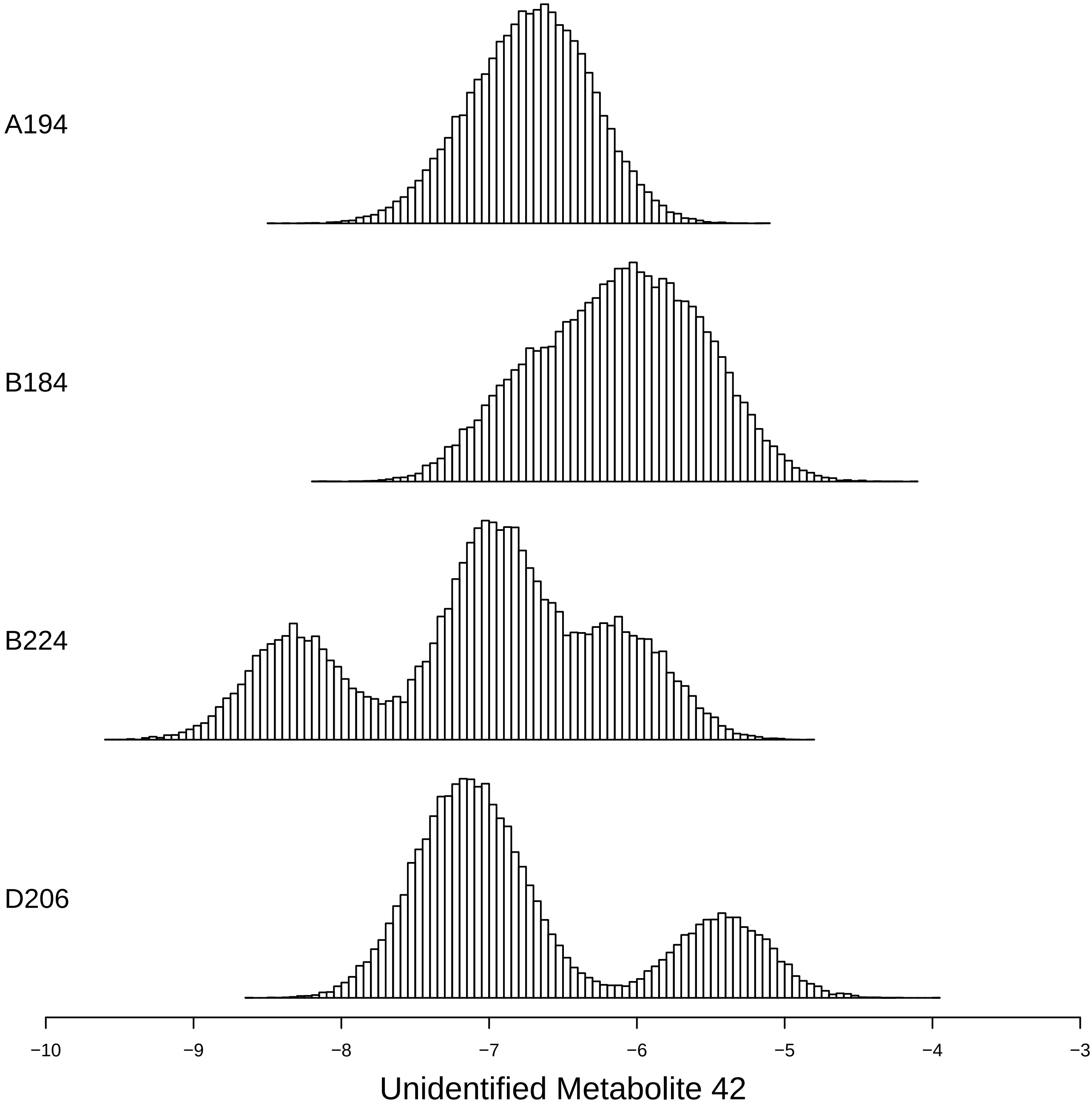
A194
B184
B224
D206
−10
−9
−8
−7
−6
−5
−4
−3
Unidentified Metabolite 42

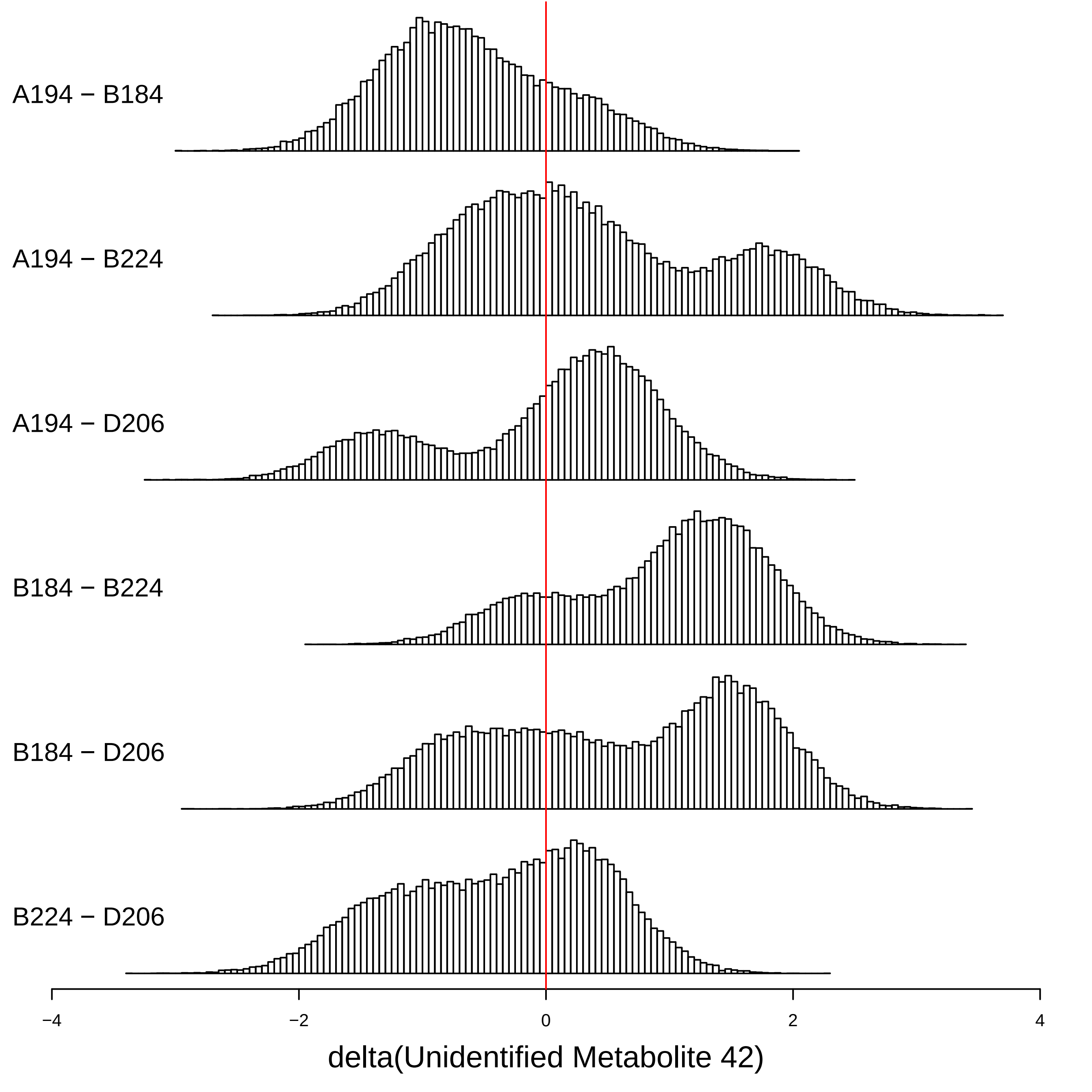
A194 − B184
A194 − B224
A194 − D206
B184 − B224
B184 − D206
B224 − D206
−4
−2
0
2
4
delta(Unidentified Metabolite 42)

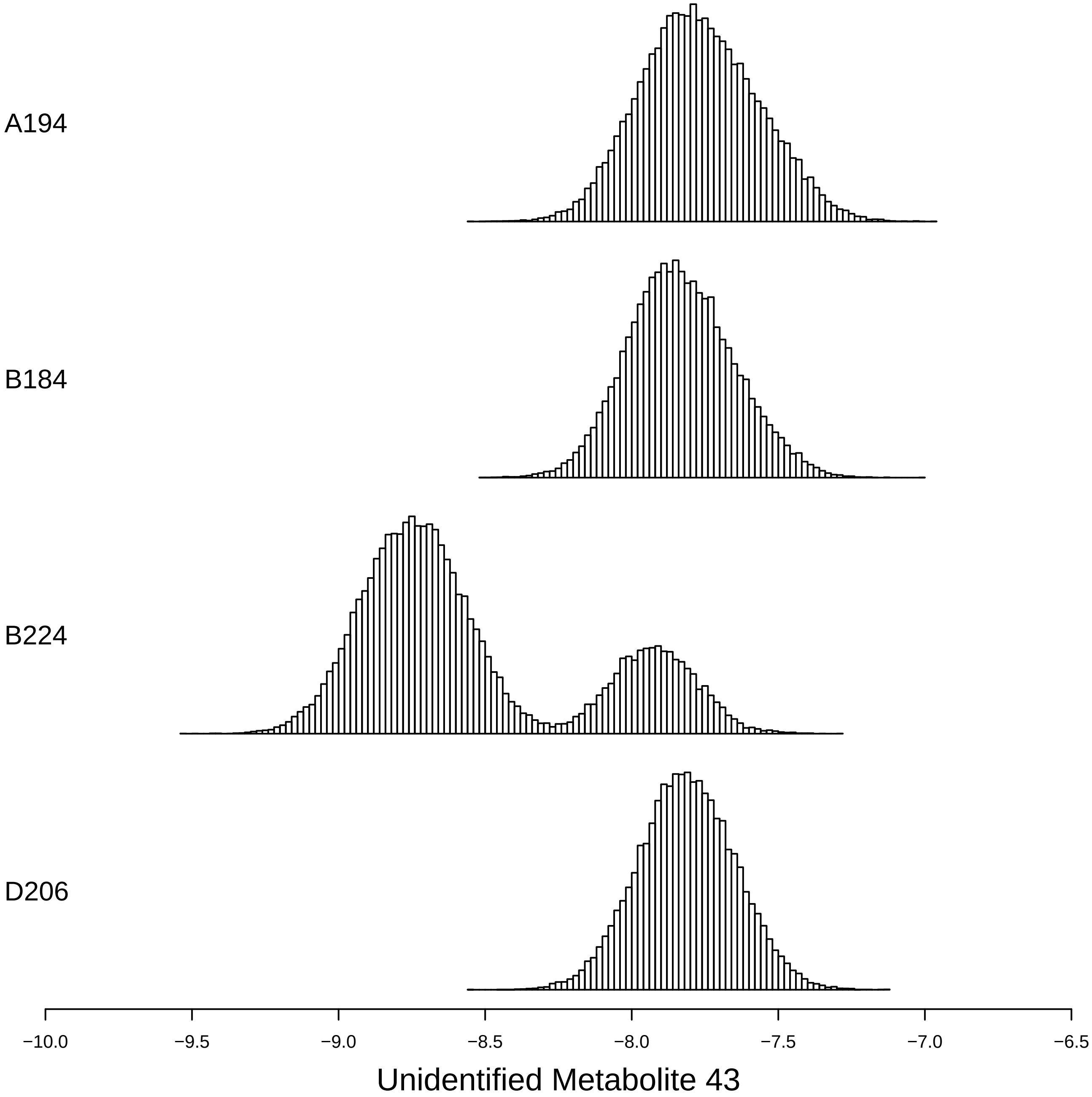
A194
B184
B224
D206
−10.0
−9.5
−9.0
−8.5
−8.0
−7.5
−7.0
−6.5
Unidentified Metabolite 43

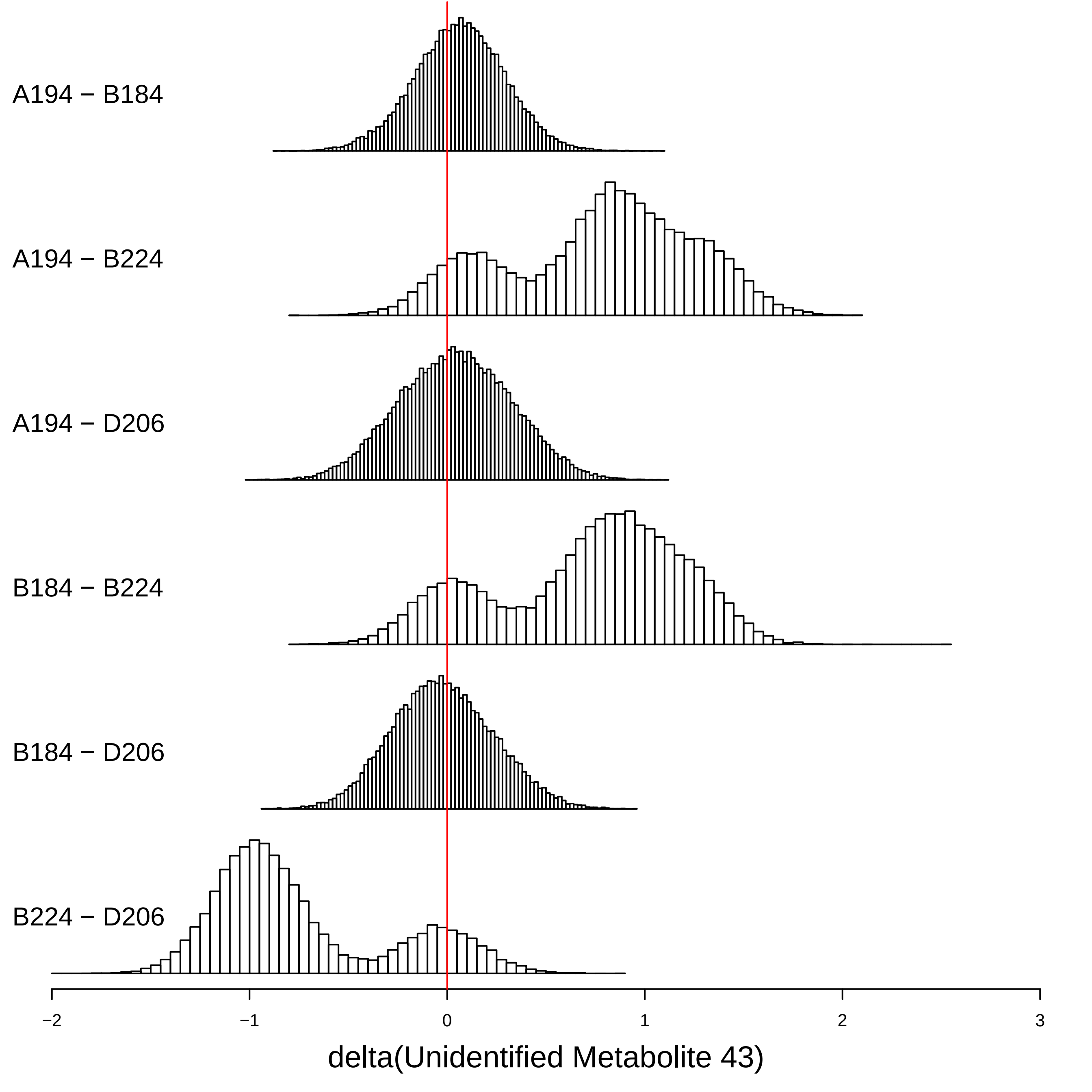
A194 − B184
A194 − B224
A194 − D206
B184 − B224
B184 − D206
B224 − D206
−2
−1
0
1
2
3
delta(Unidentified Metabolite 43)

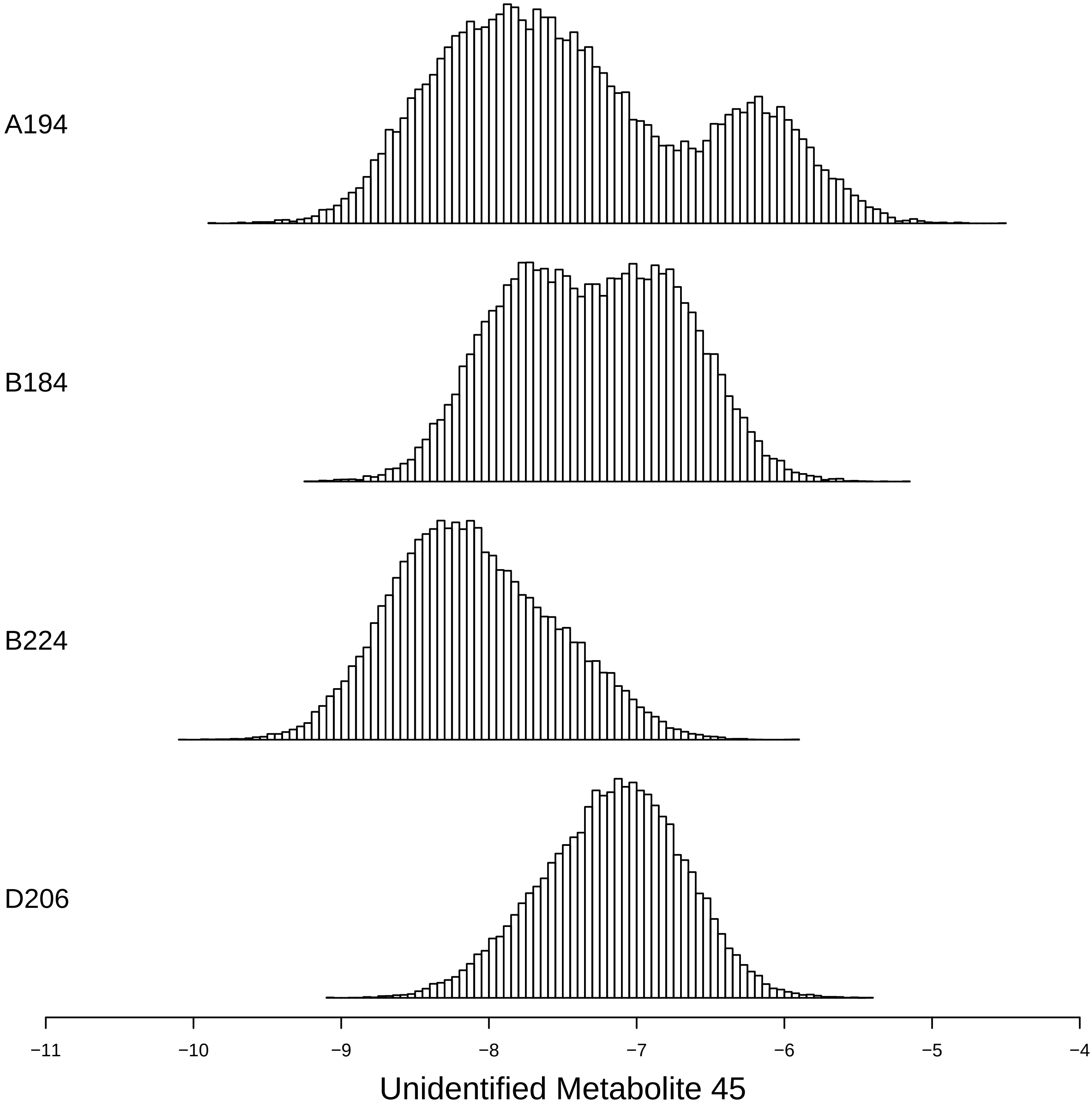
A194
B184
B224
D206
−11
−10
−9
−8
−7
−6
−5
−4
Unidentified Metabolite 45

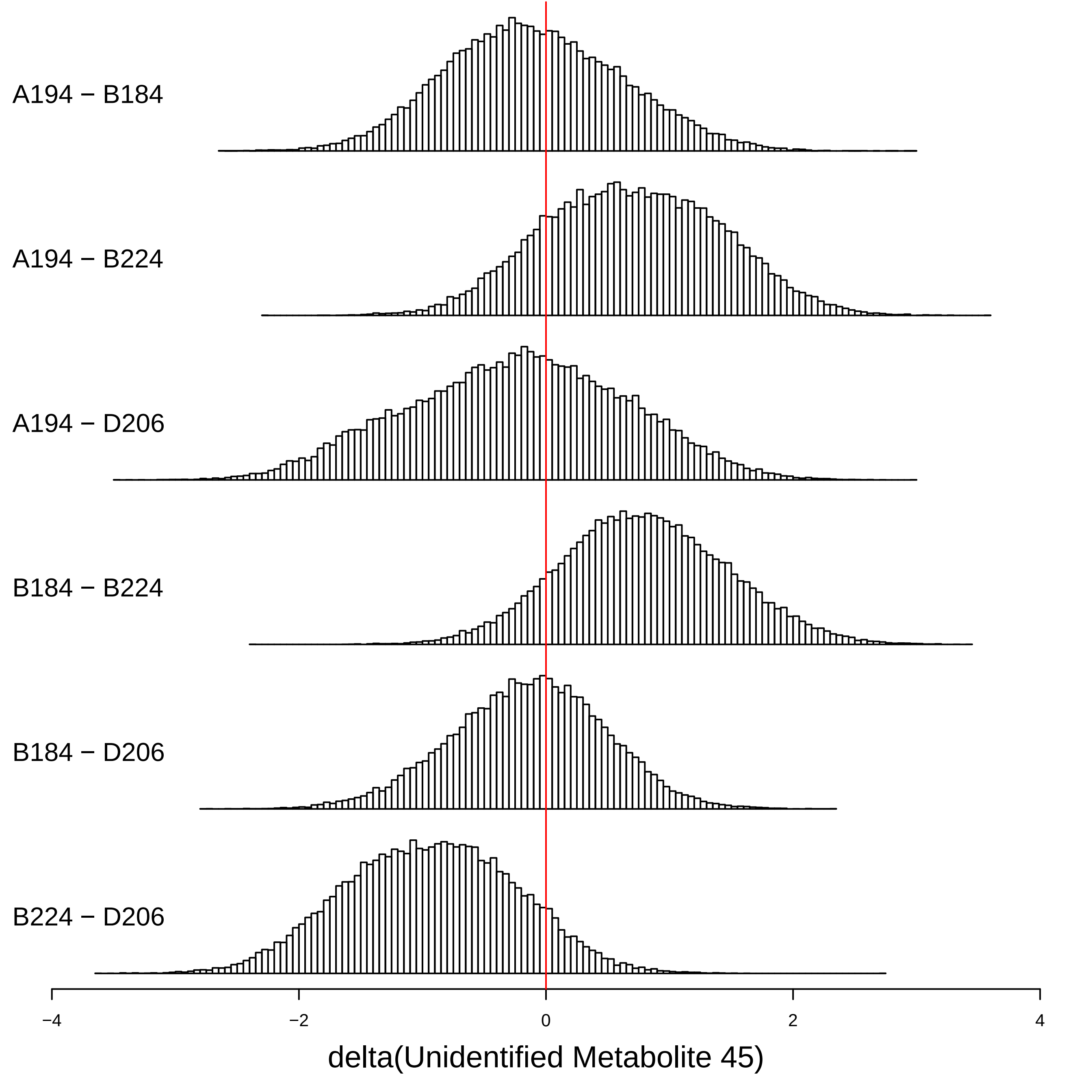
A194 − B184
A194 − B224
A194 − D206
B184 − B224
B184 − D206
B224 − D206
−4
−2
0
2
4
delta(Unidentified Metabolite 45)

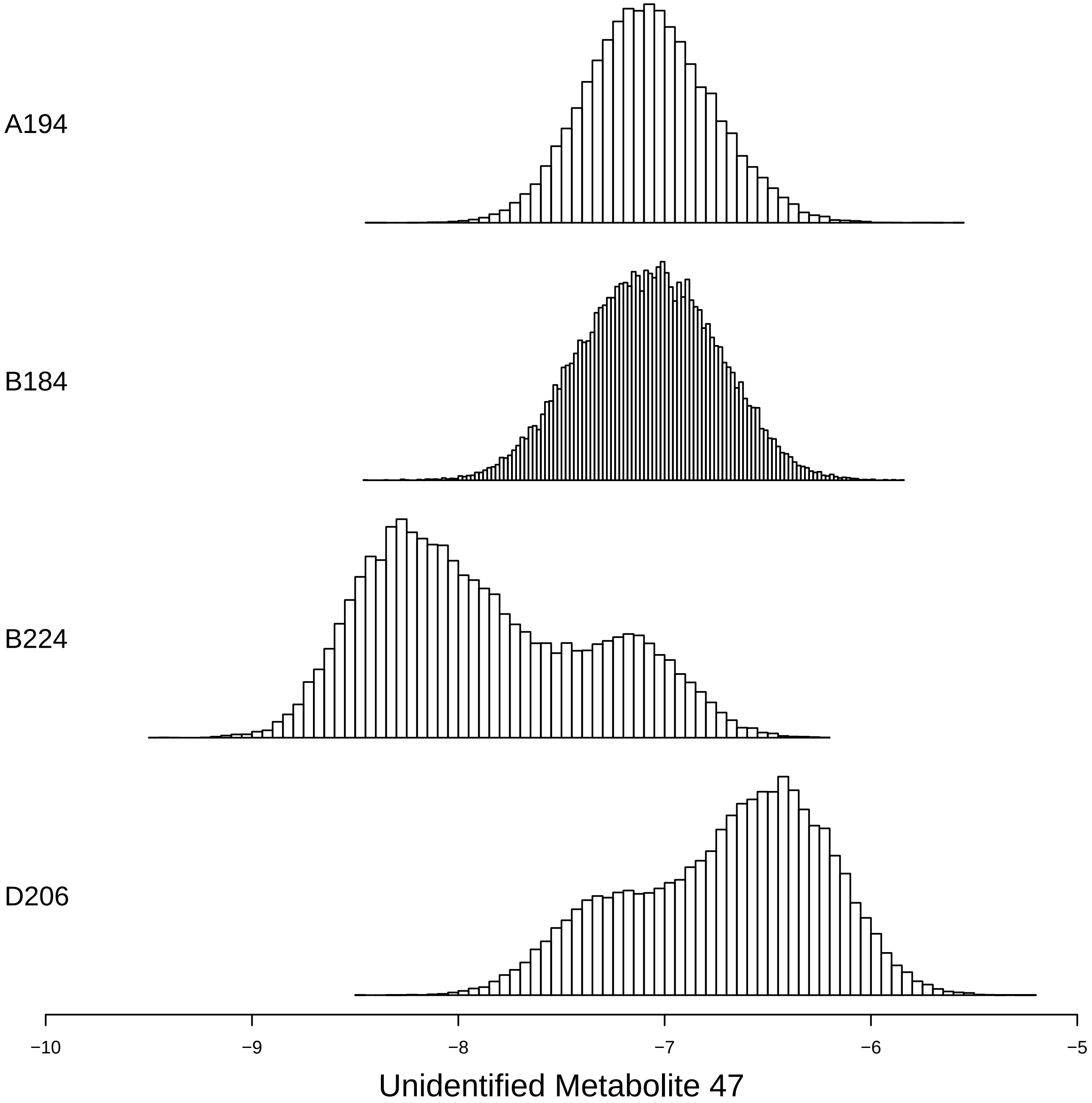
A194
B184
B224
D206
−10
−9
−8
−7
−6
−5
Unidentified Metabolite 47

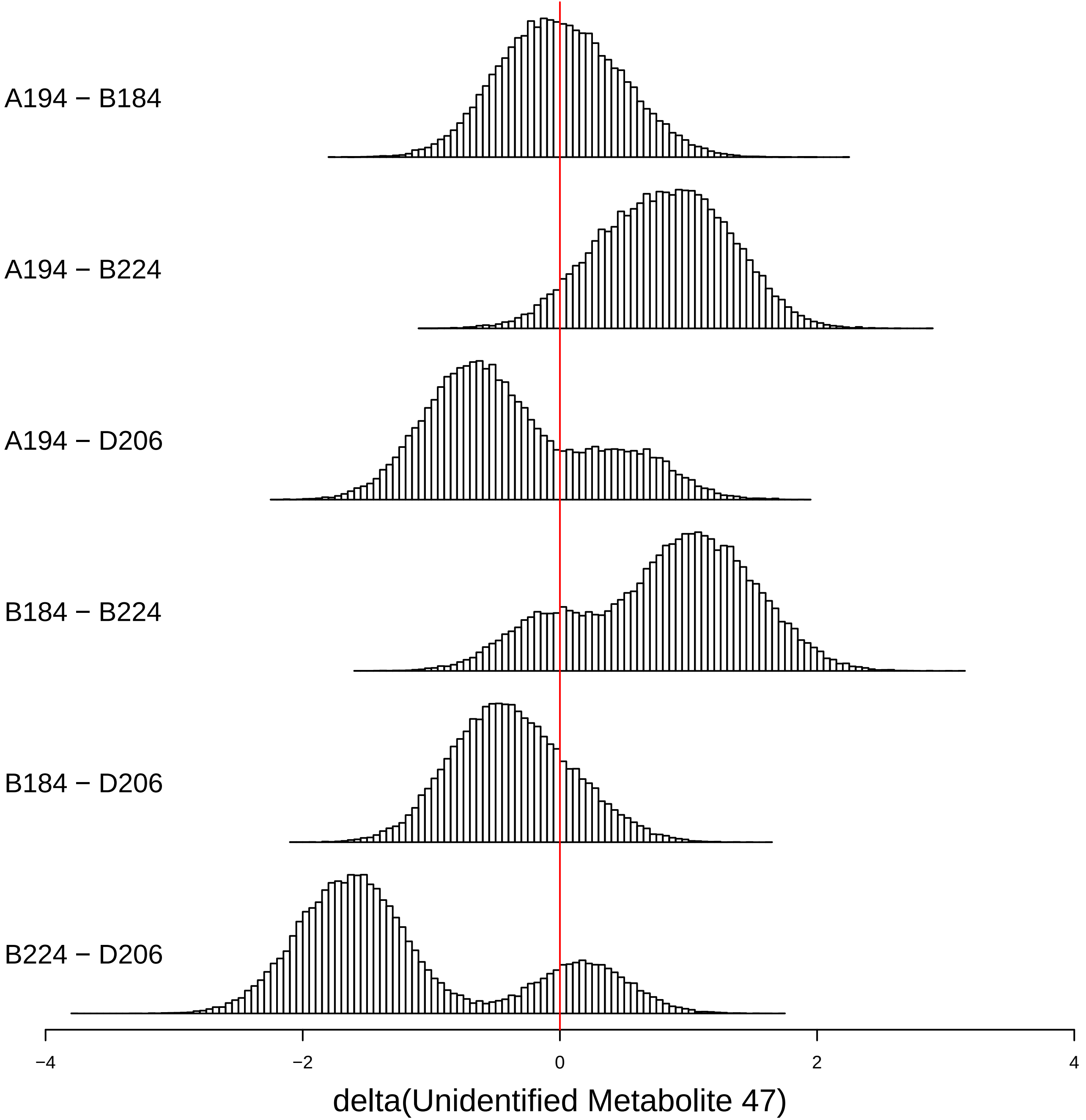
A194 − B184
A194 − B224
A194 − D206
B184 − B224
B184 − D206
B224 − D206
−4
−2
0
2
4
delta(Unidentified Metabolite 47)

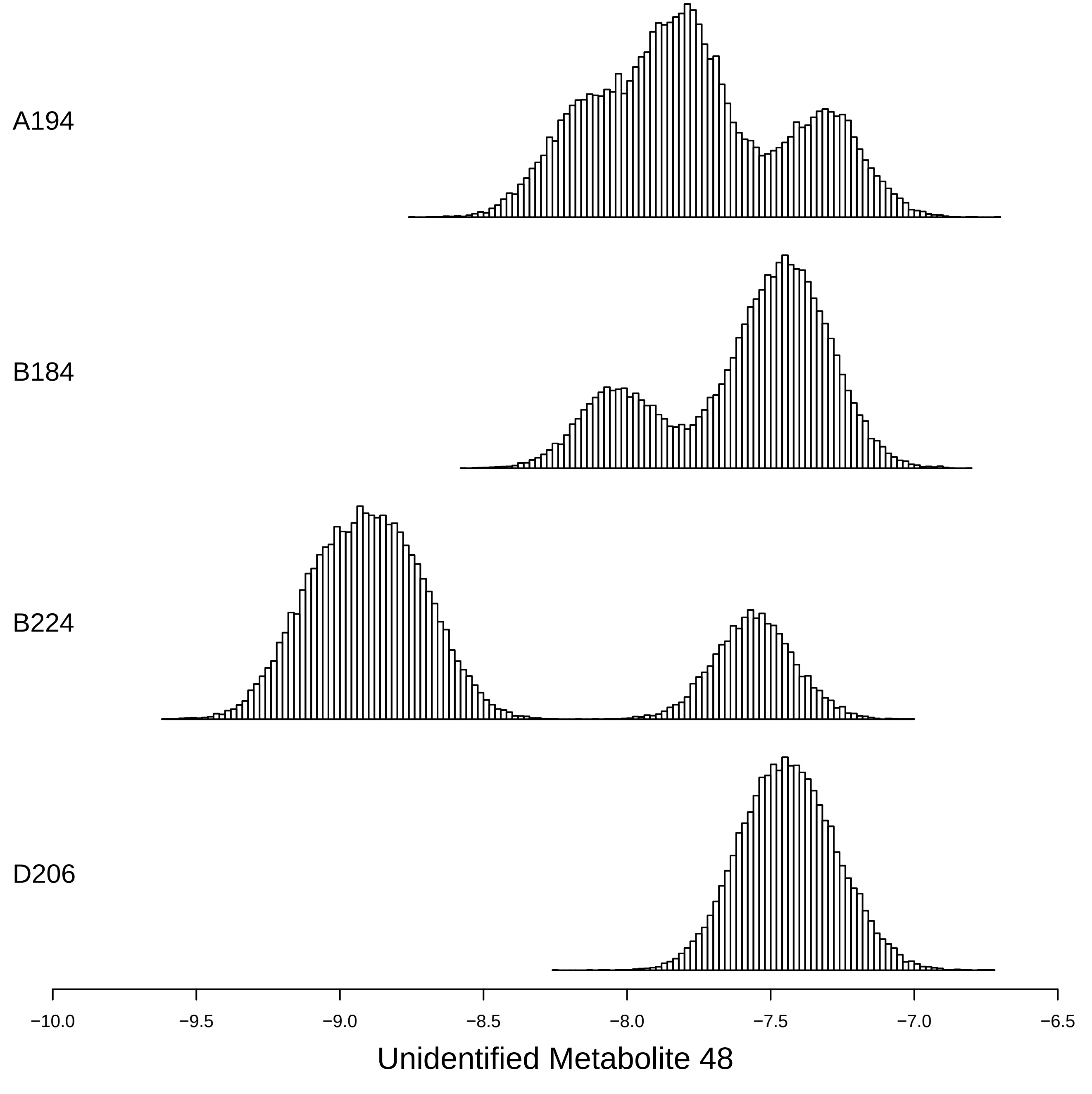
A194
B184
B224
D206
−10.0
−9.5
−9.0
−8.5
−8.0
−7.5
−7.0
−6.5
Unidentified Metabolite 48

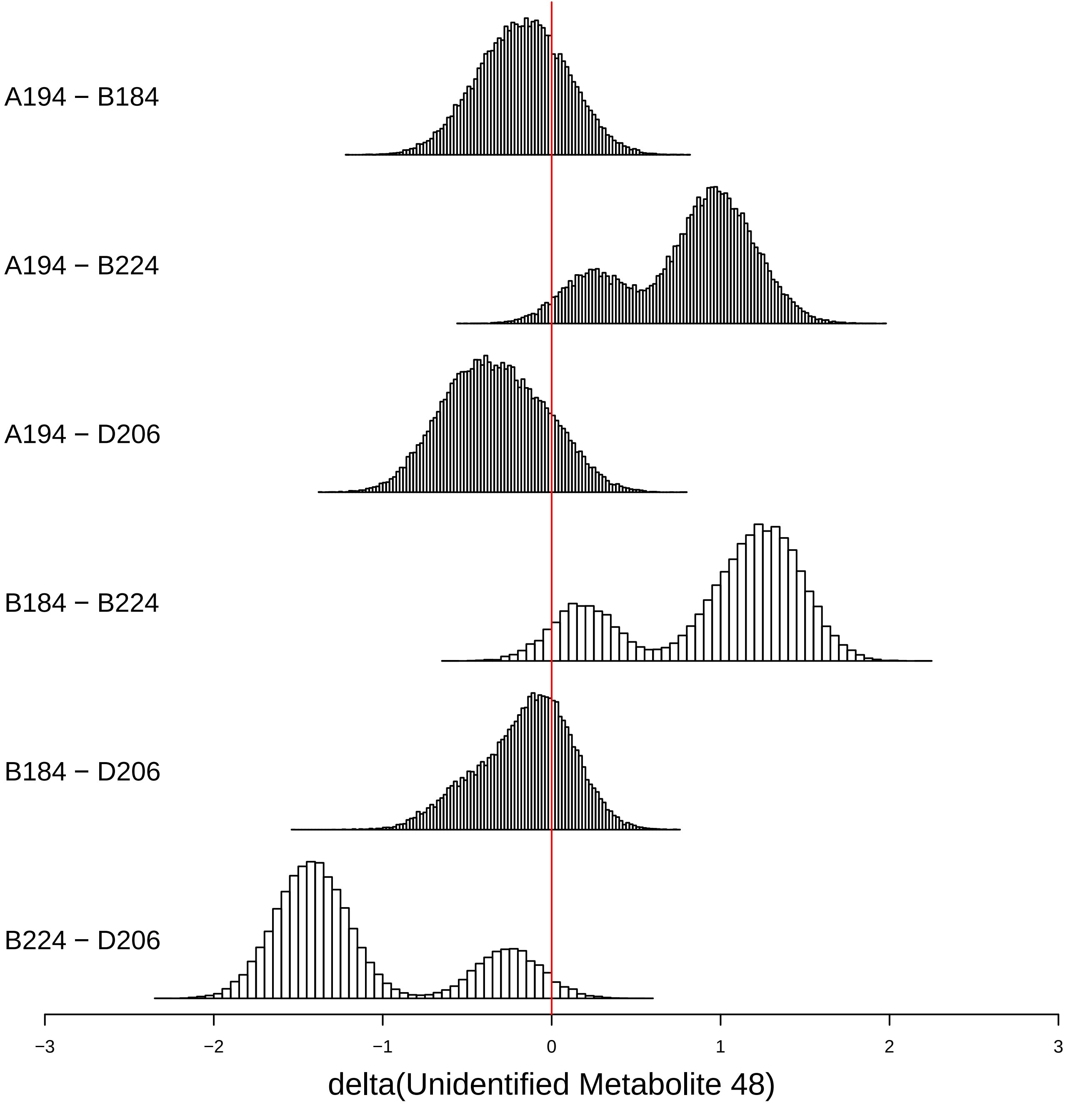
A194 − B184
A194 − B224
A194 − D206
B184 − B224
B184 − D206
B224 − D206
−3
−2
−1
0
1
2
3
delta(Unidentified Metabolite 48)

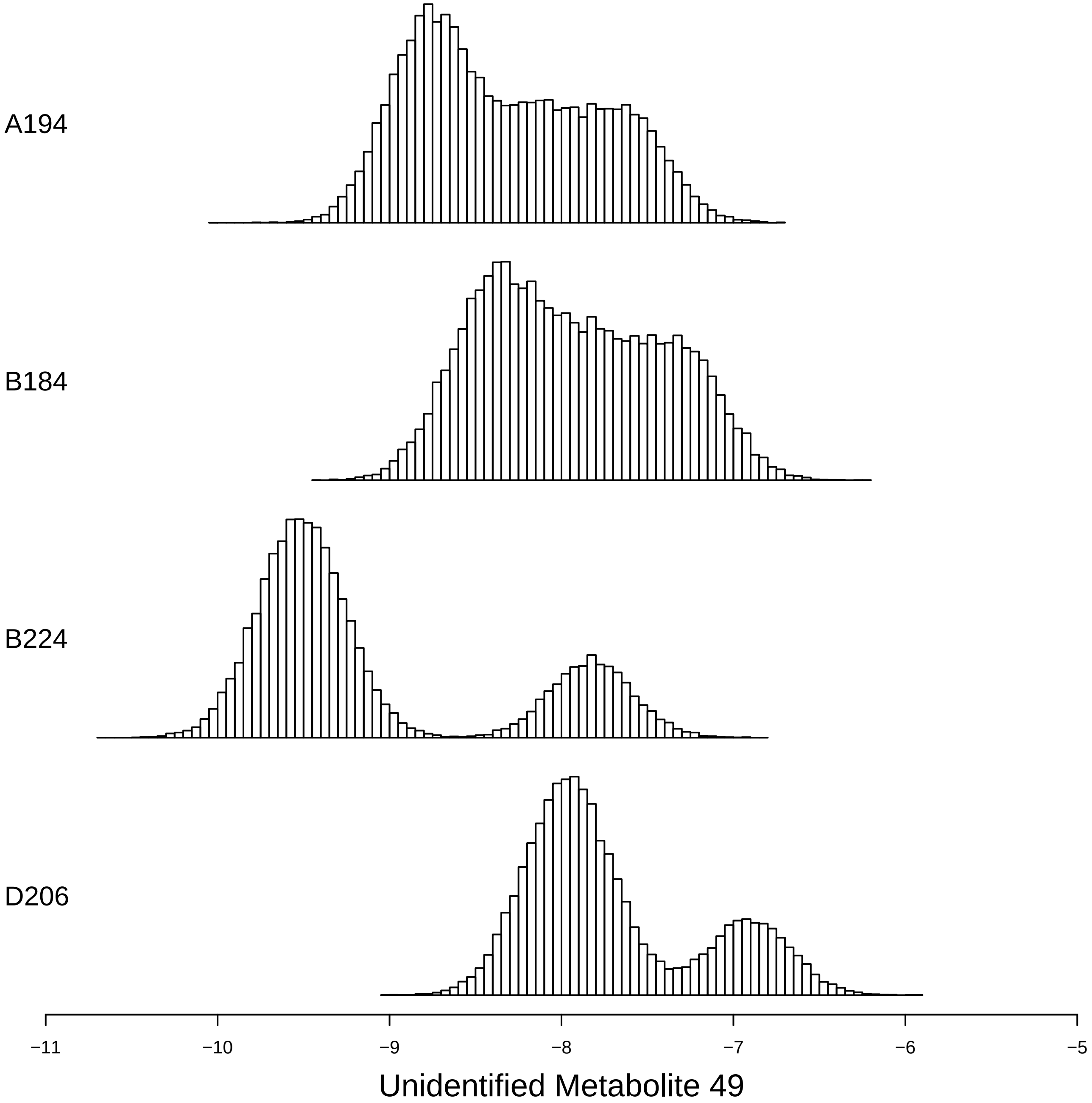

A194
B184
B224
D206
−11
−10
−9
−8
−7
−6
−5
Unidentified Metabolite 49

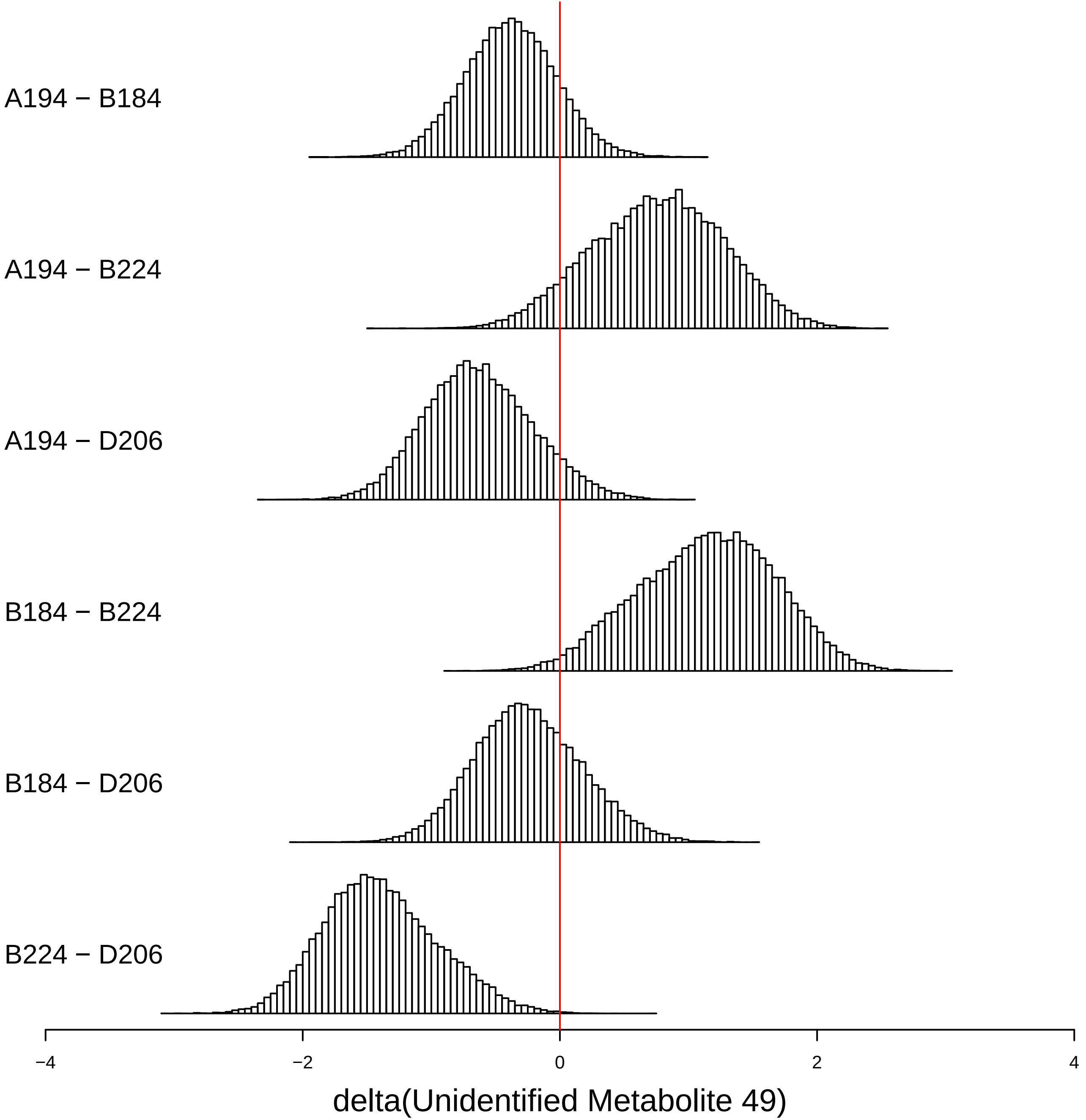

A194 − B184
A194 − B224
A194 − D206
B184 − B224
B184 − D206
B224 − D206
−4
−2
0
2
4
delta(Unidentified Metabolite 49)

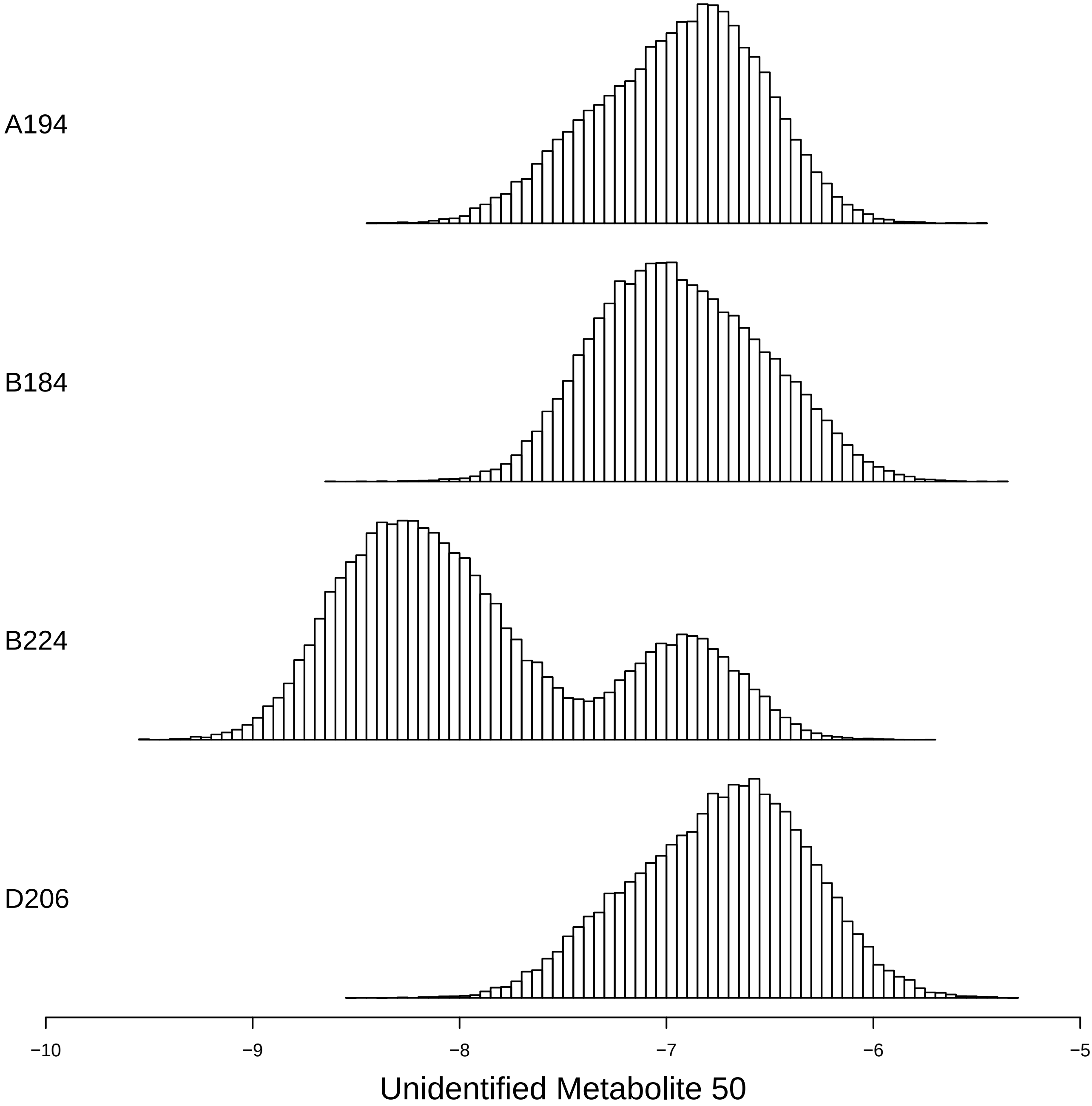
A194
B184
B224
D206
−10
−9
−8
−7
−6
−5
Unidentified Metabolite 50

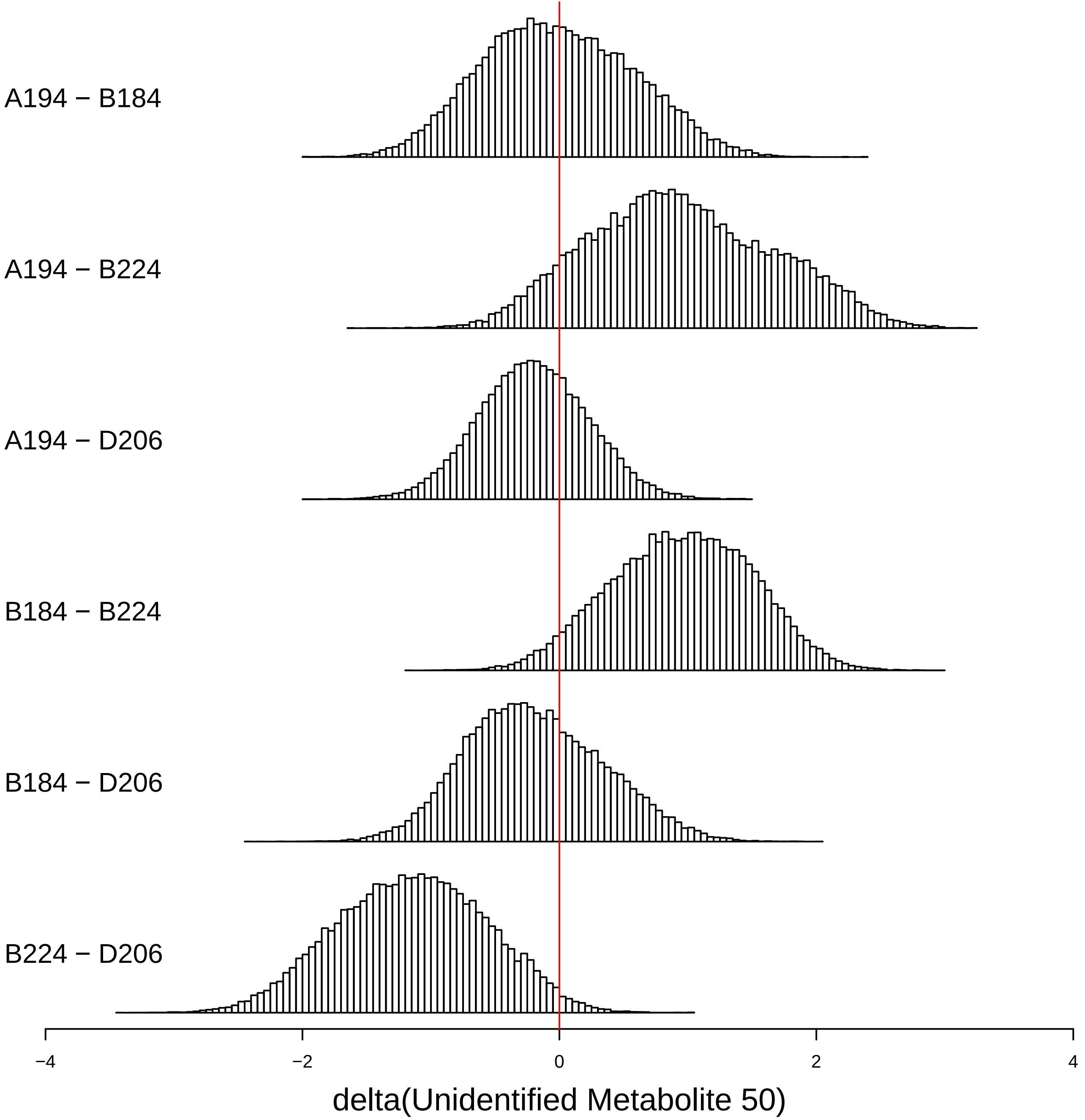
A194 − B184
A194 − B224
A194 − D206
B184 − B224
B184 − D206
B224 − D206
−4
−2
0
2
4
delta(Unidentified Metabolite 50)

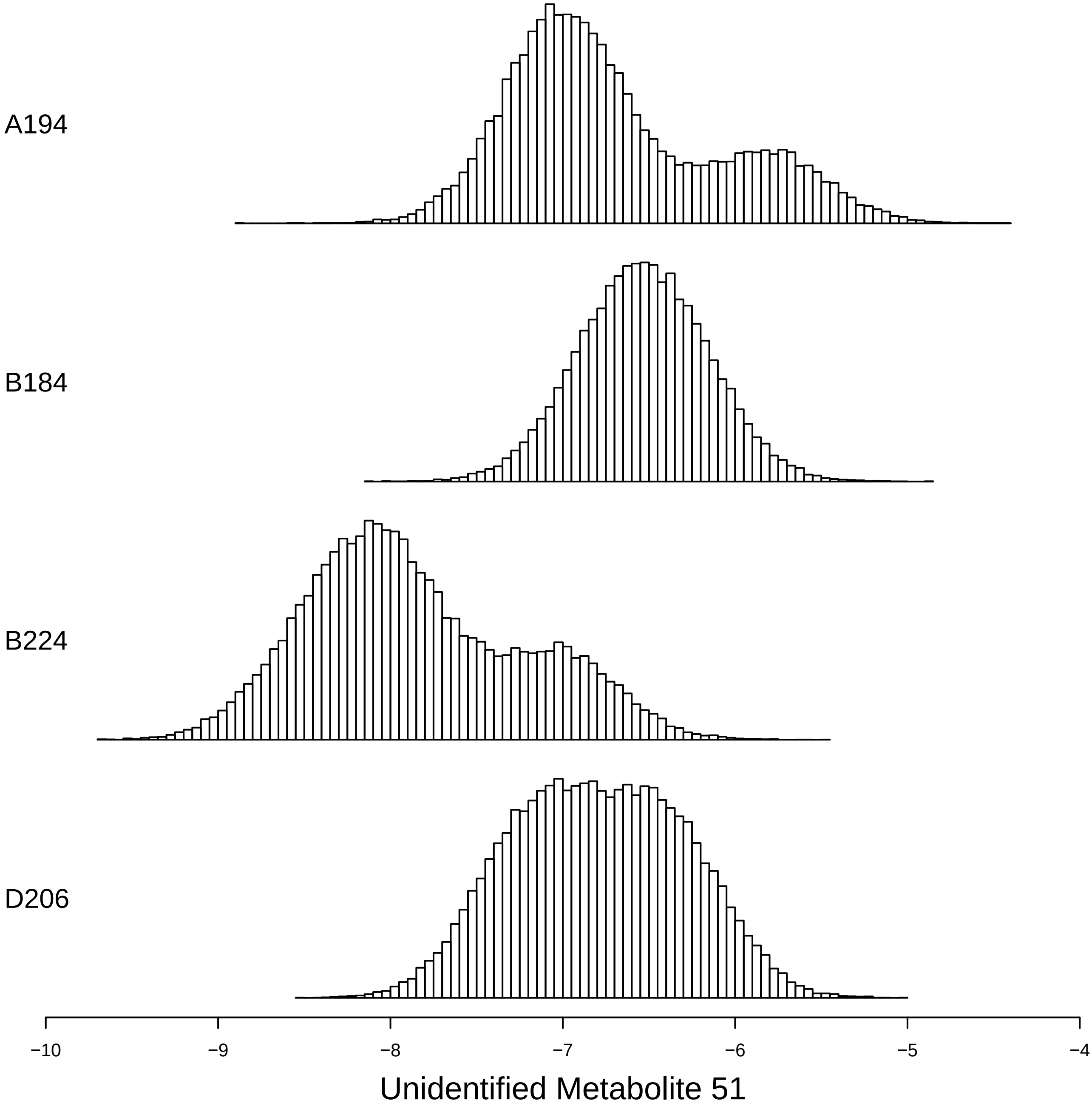
A194
B184
B224
D206
−10
−9
−8
−7
−6
−5
−4
Unidentified Metabolite 51

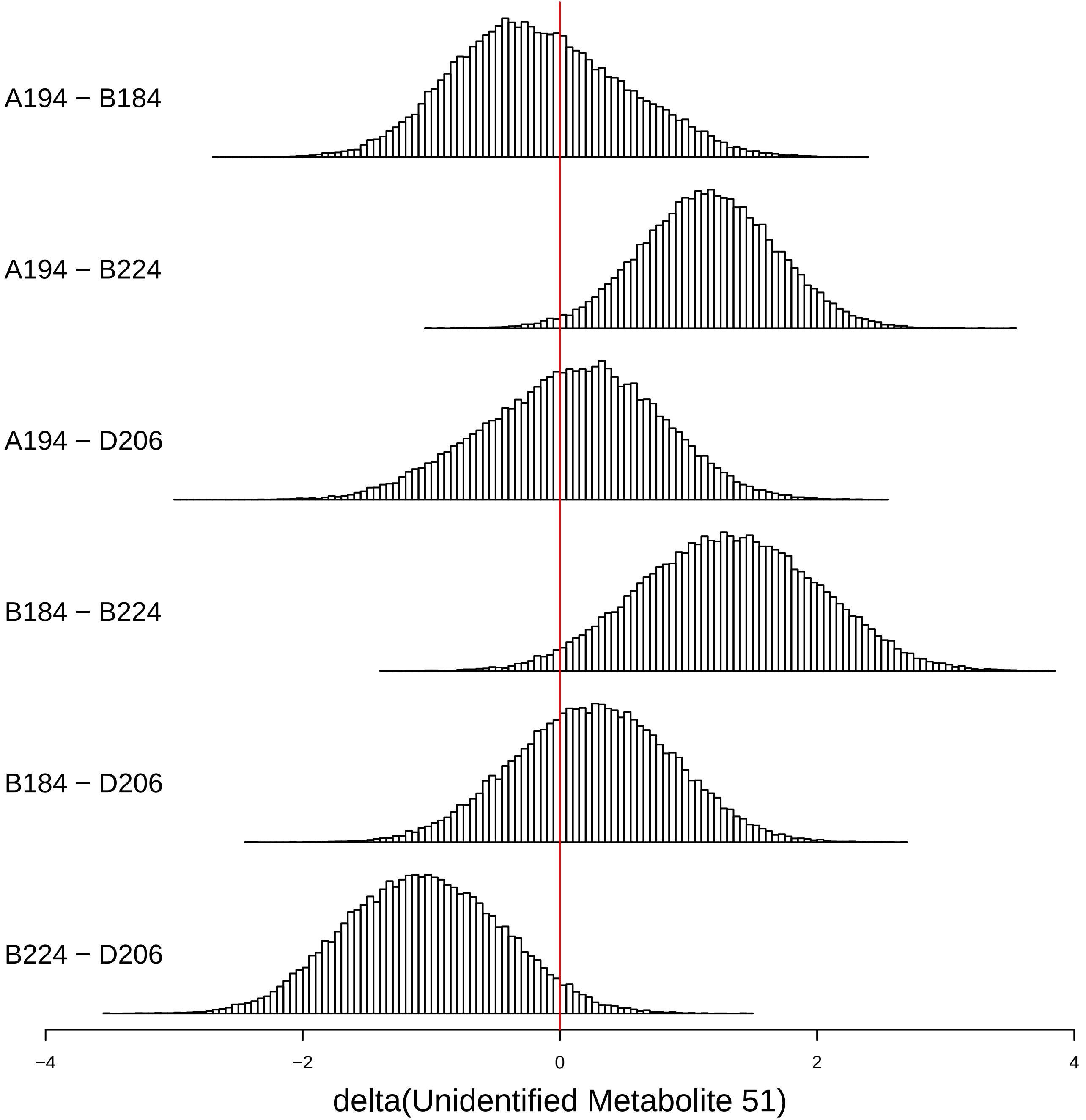
A194 − B184
A194 − B224
A194 − D206
B184 − B224
B184 − D206
B224 − D206
−4
−2
0
2
4
delta(Unidentified Metabolite 51)

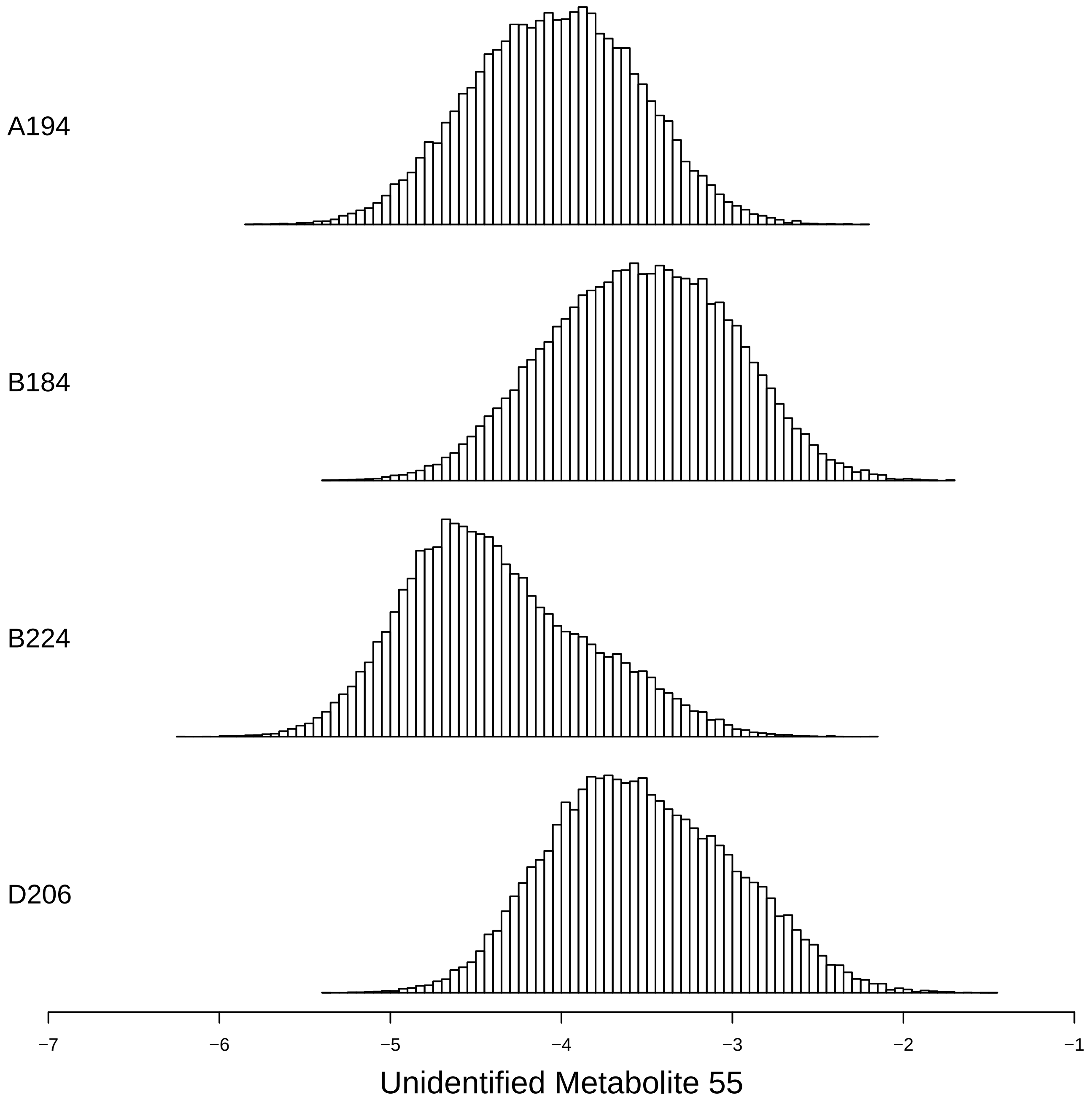

A194
B184
B224
D206
−7
−6
−5
−4
−3
−2
−1
Unidentified Metabolite 55

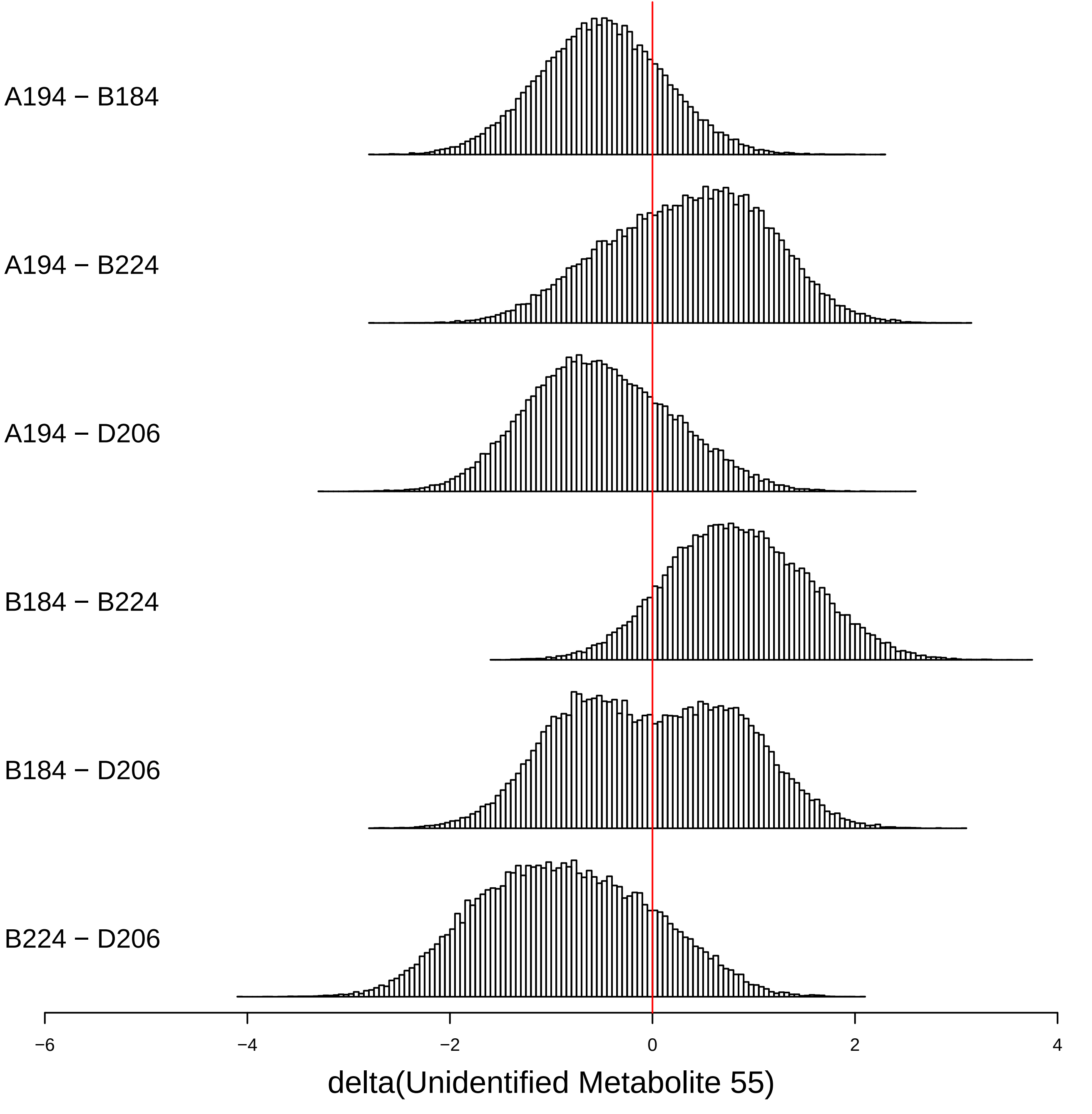
A194 − B184
A194 − B224
A194 − D206
B184 − B224
B184 − D206
B224 − D206
−6
−4
−2
0
2
4
delta(Unidentified Metabolite 55)

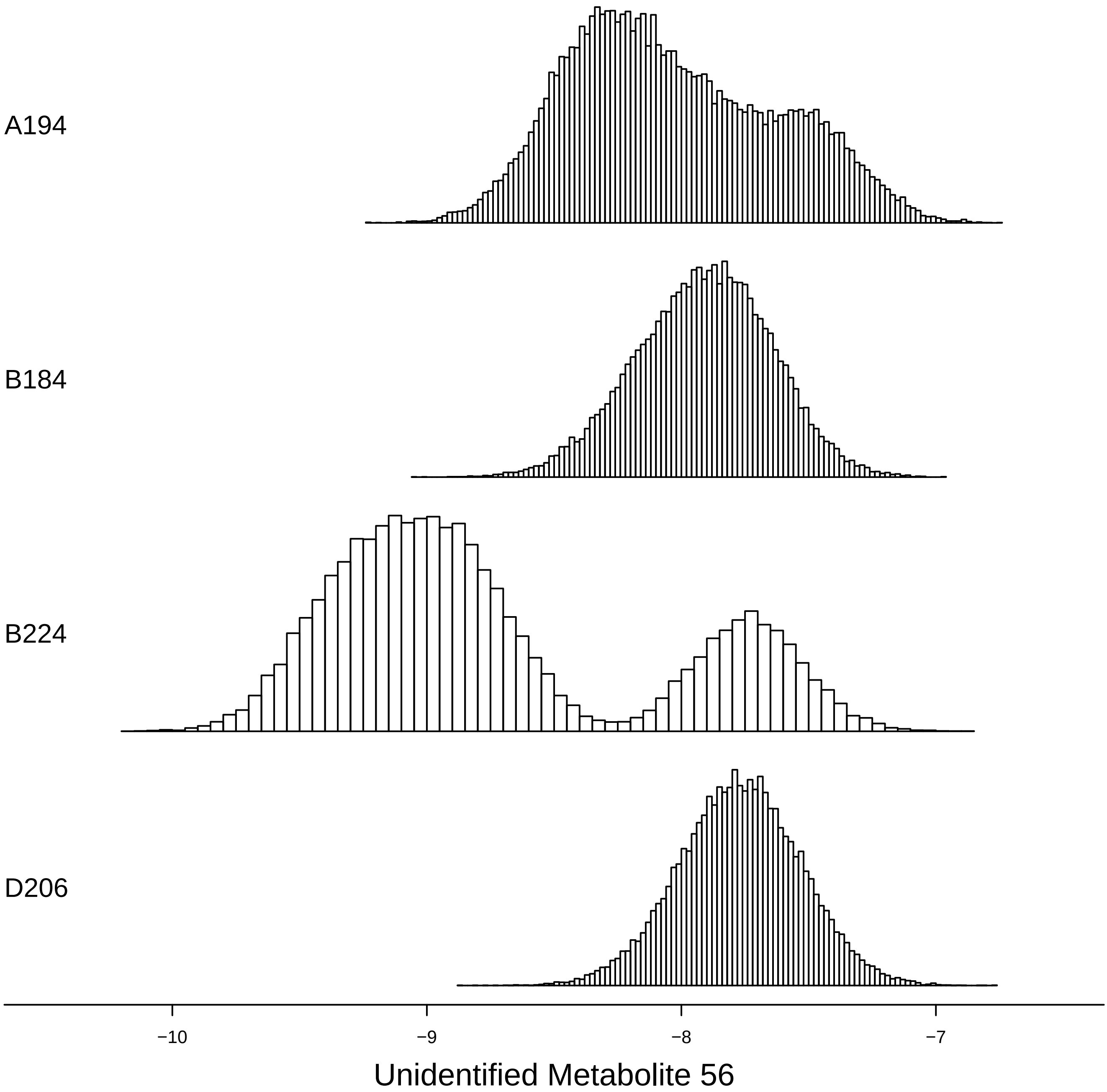
A194
B184
B224
D206
−10
−9
−8
−7
Unidentified Metabolite 56

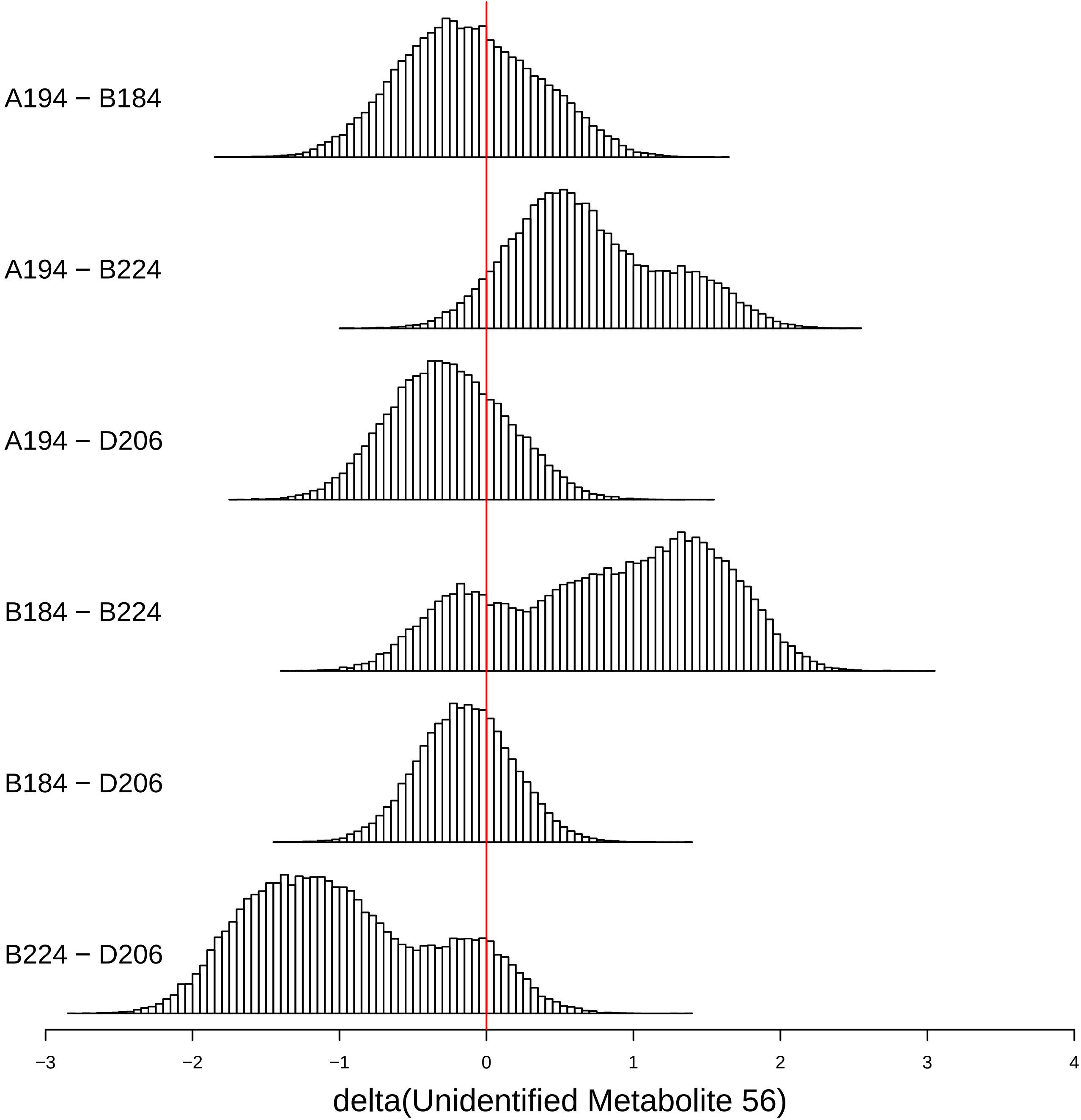
A194 − B184
A194 − B224
A194 − D206
B184 − B224
B184 − D206
B224 − D206
−3
−2
−1
0
1
2
3
4
delta(Unidentified Metabolite 56)

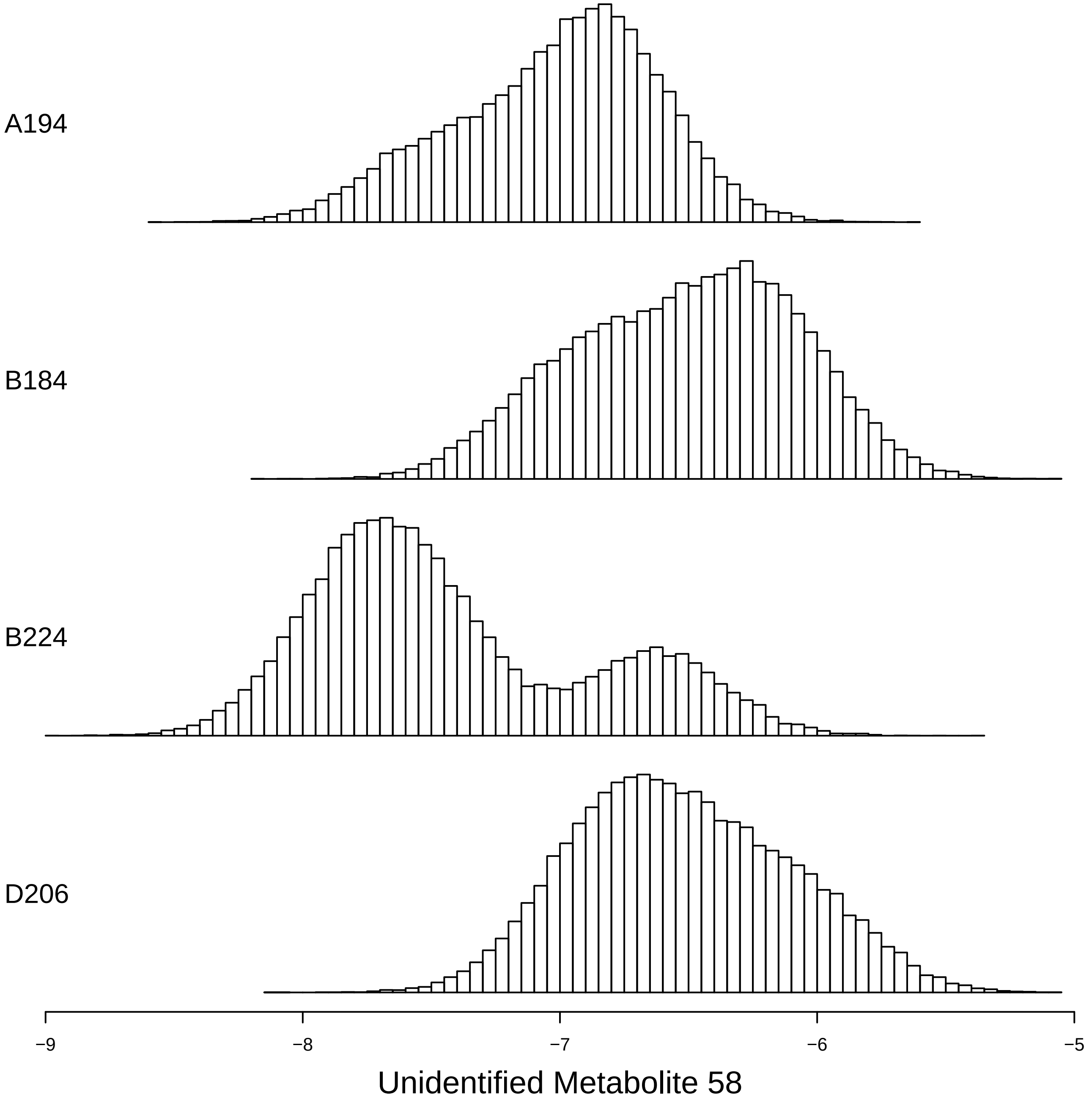

A194
B184
B224
D206
−9
−8
−7
−6
−5
Unidentified Metabolite 58

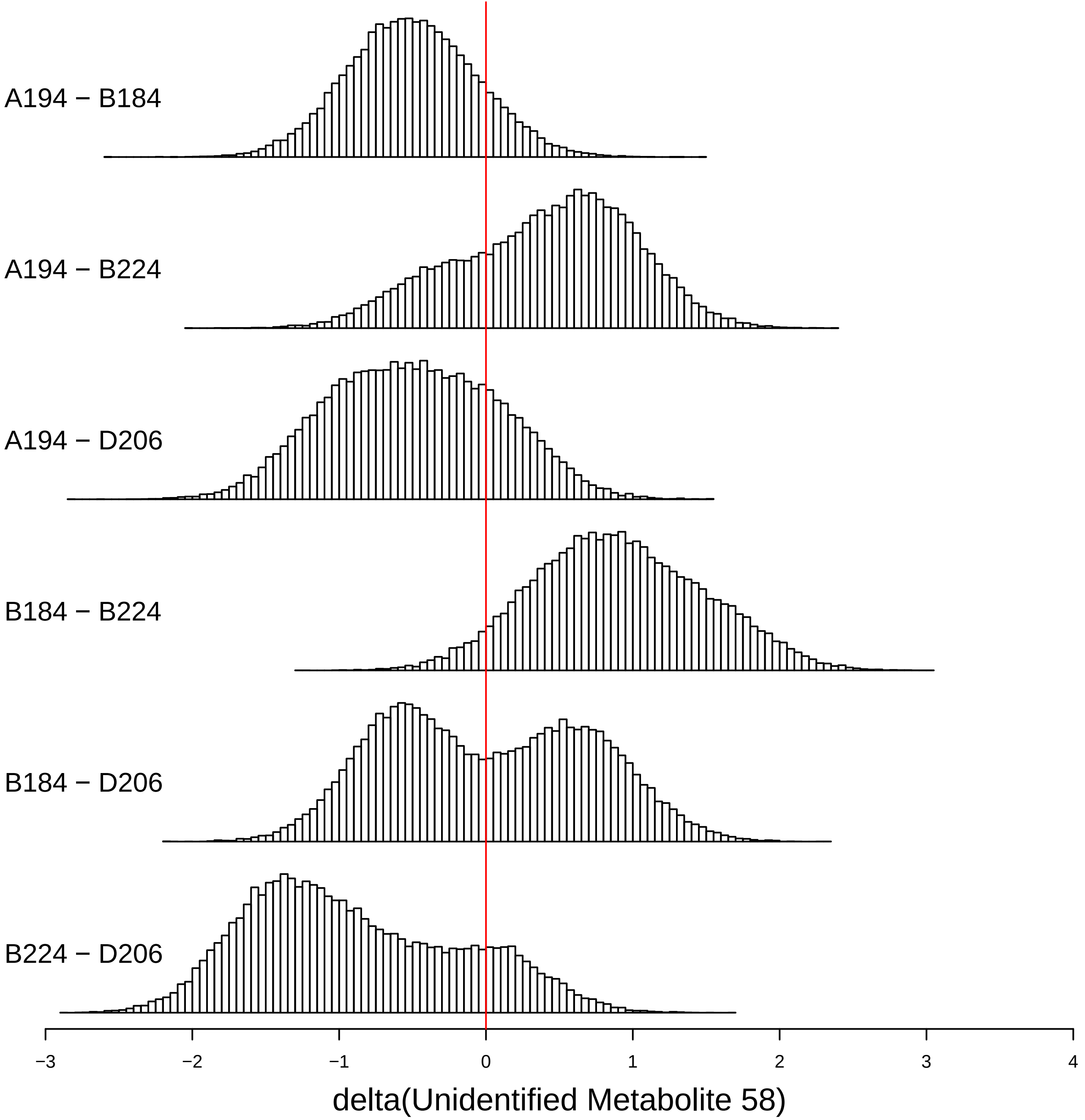

A194 − B184
A194 − B224
A194 − D206
B184 − B224
B184 − D206
B224 − D206
−3
−2
−1
0
1
2
3
4
delta(Unidentified Metabolite 58)

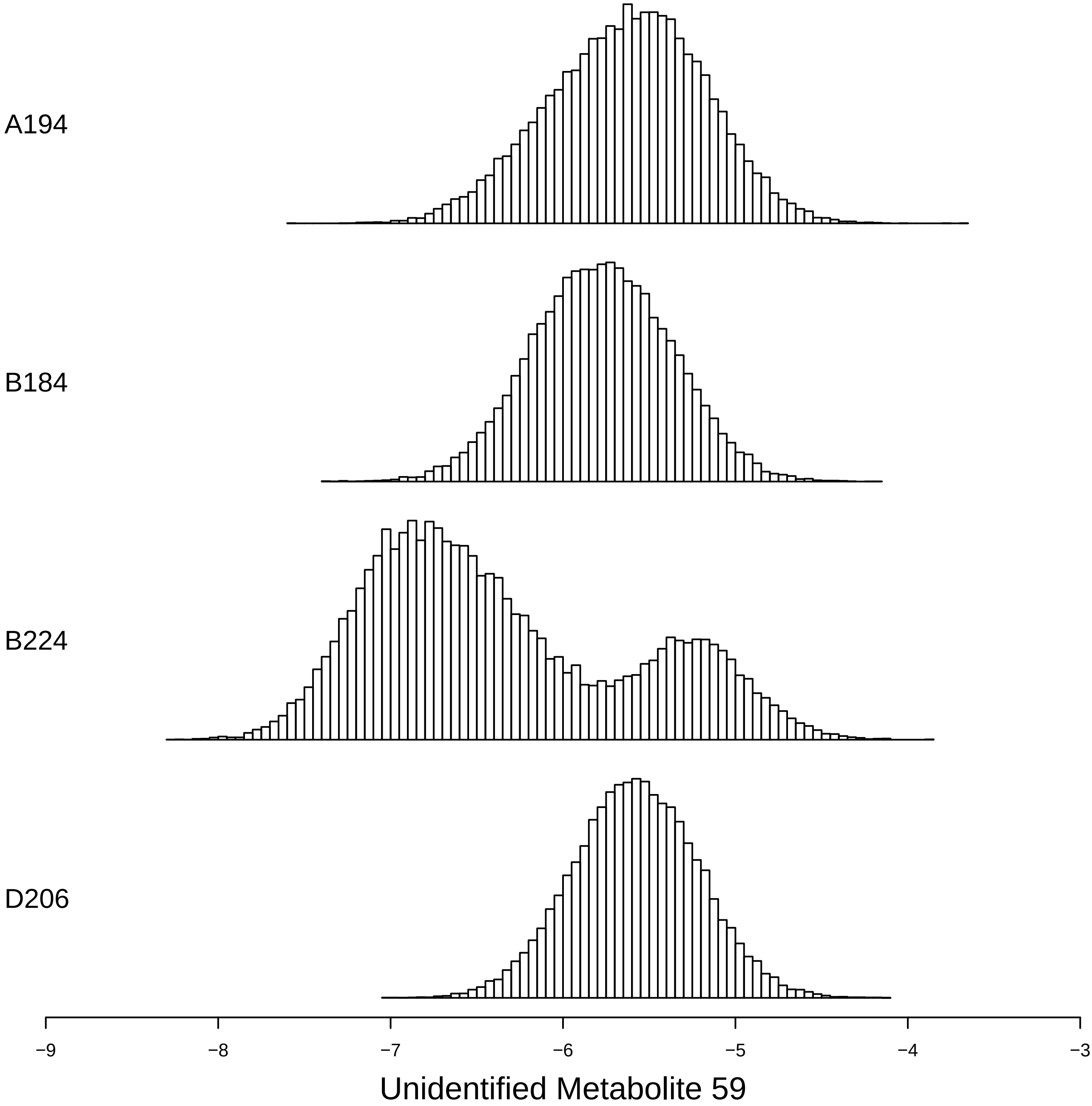

A194
B184
B224
D206
−9
−8
−7
−6
−5
−4
−3
Unidentified Metabolite 59

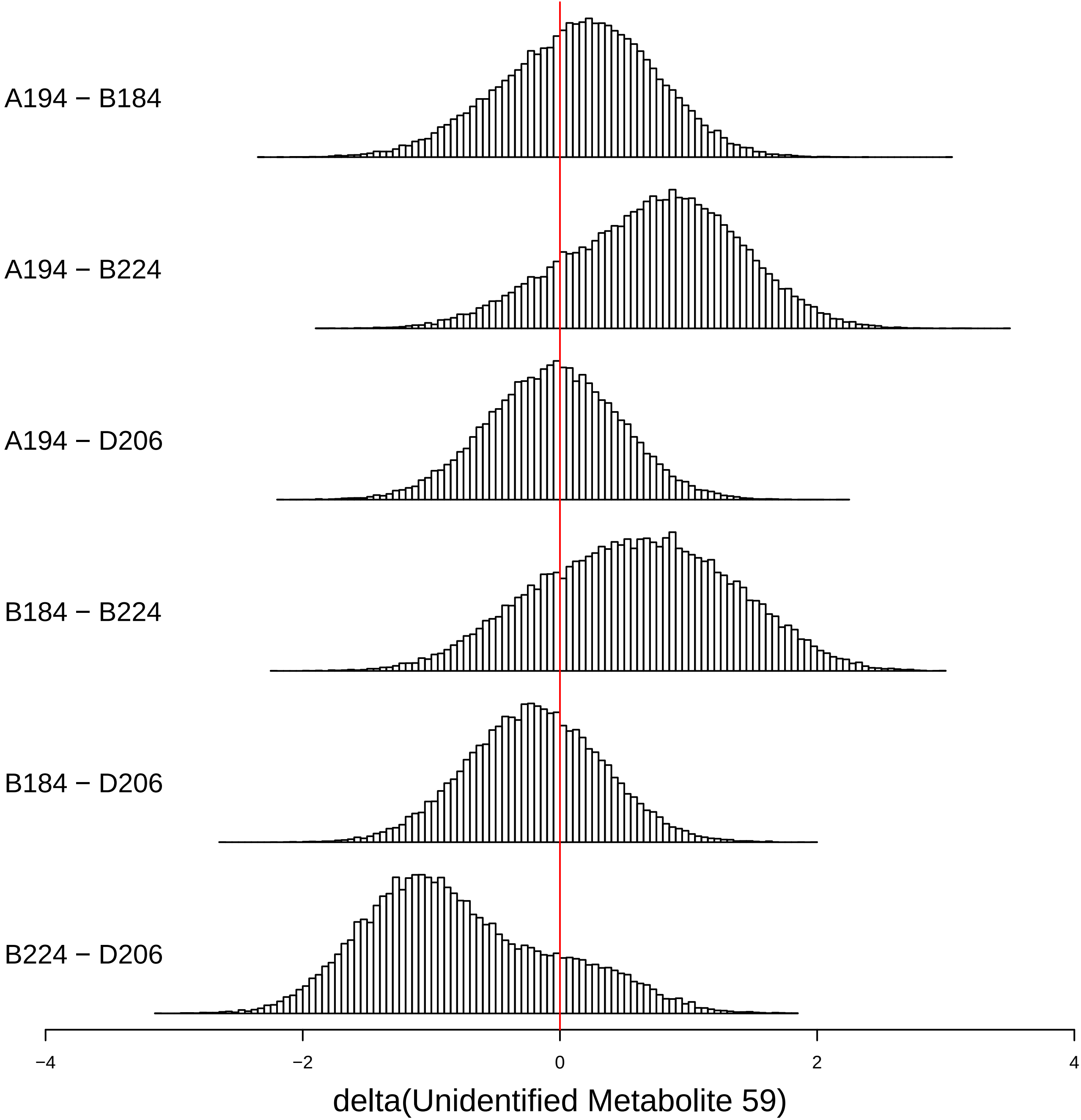

A194 − B184
A194 − B224
A194 − D206
B184 − B224
B184 − D206
B224 − D206
−4
−2
0
2
4
delta(Unidentified Metabolite 59)

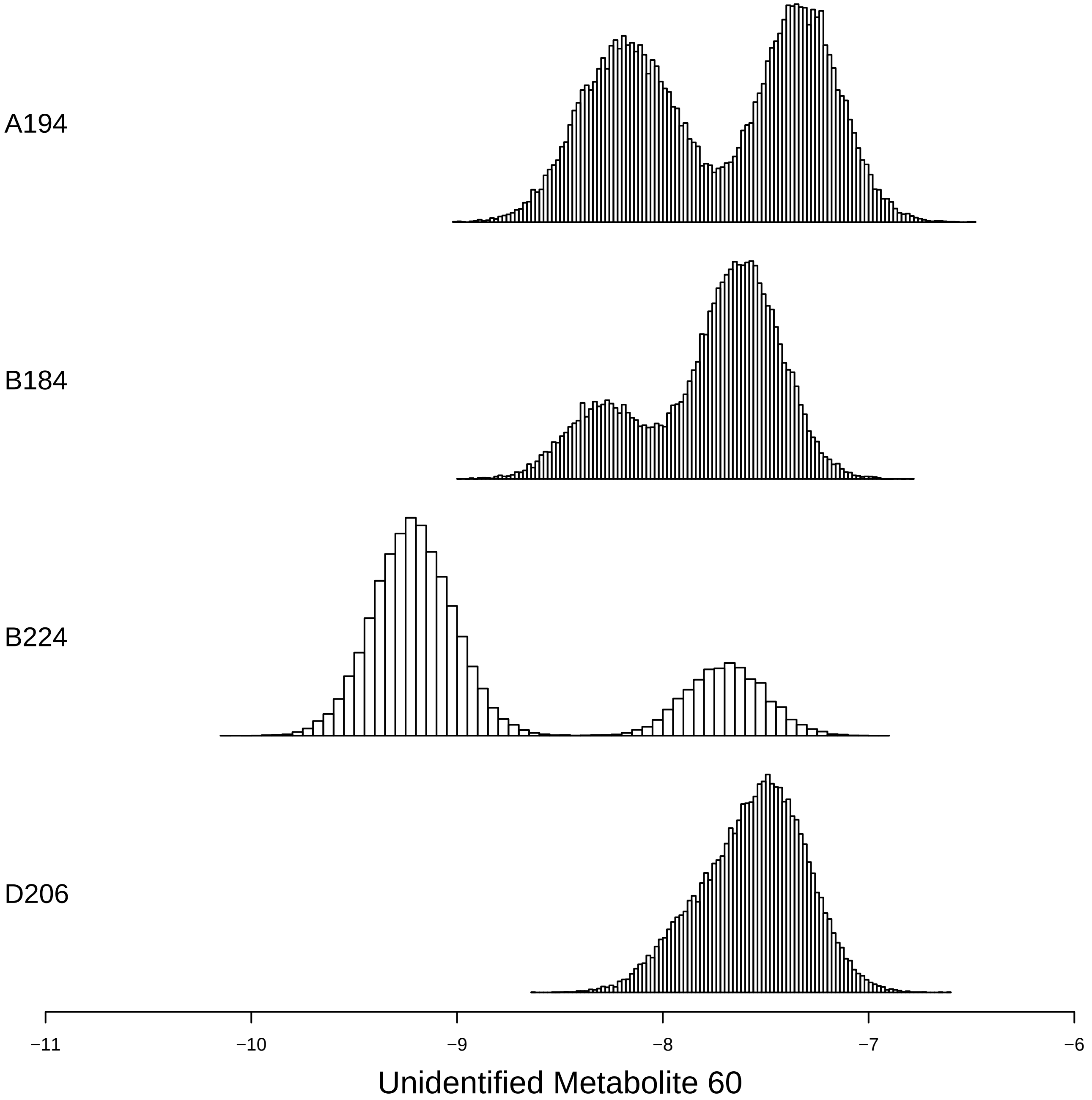
A194
B184
B224
D206
−11
−10
−9
−8
−7
−6
Unidentified Metabolite 60

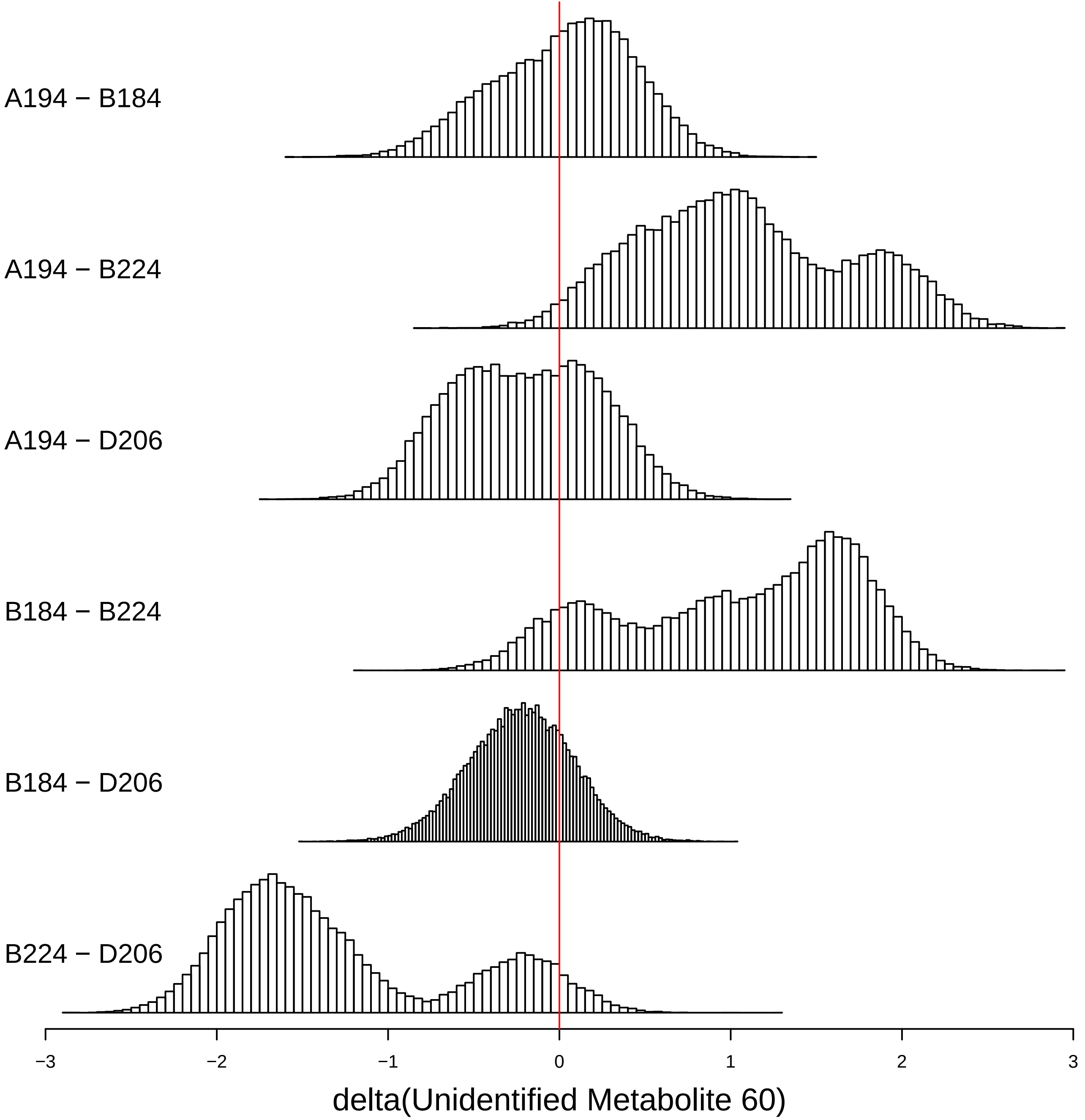

A194 − B184
A194 − B224
A194 − D206
B184 − B224
B184 − D206
B224 − D206
−3
−2
−1
0
1
2
3
delta(Unidentified Metabolite 60)

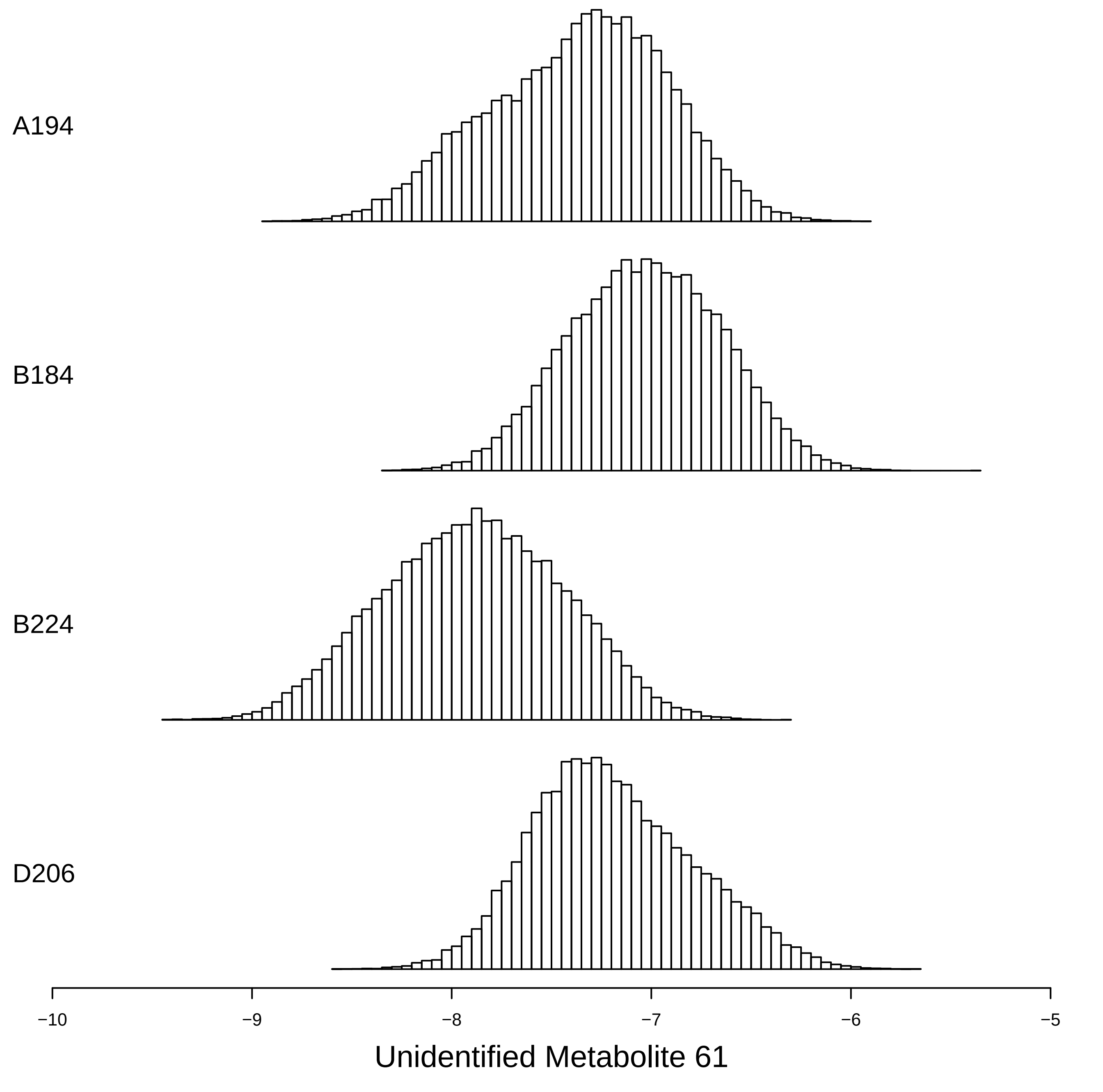
A194
B184
B224
D206
−10
−9
−8
−7
−6
−5
Unidentified Metabolite 61

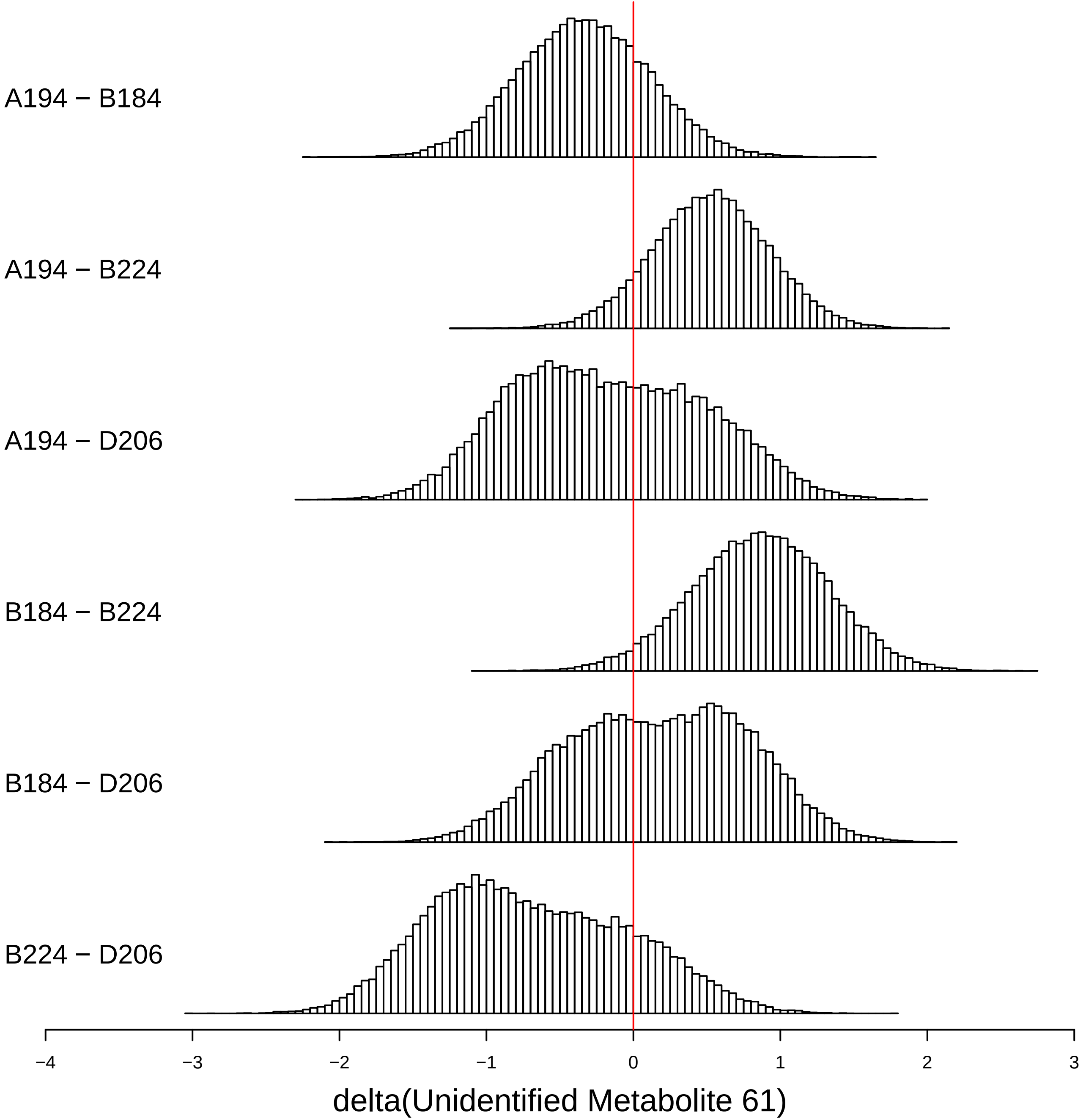
A194 − B184
A194 − B224
A194 − D206
B184 − B224
B184 − D206
B224 − D206
−4
−3
−2
−1
0
1
2
3
delta(Unidentified Metabolite 61)

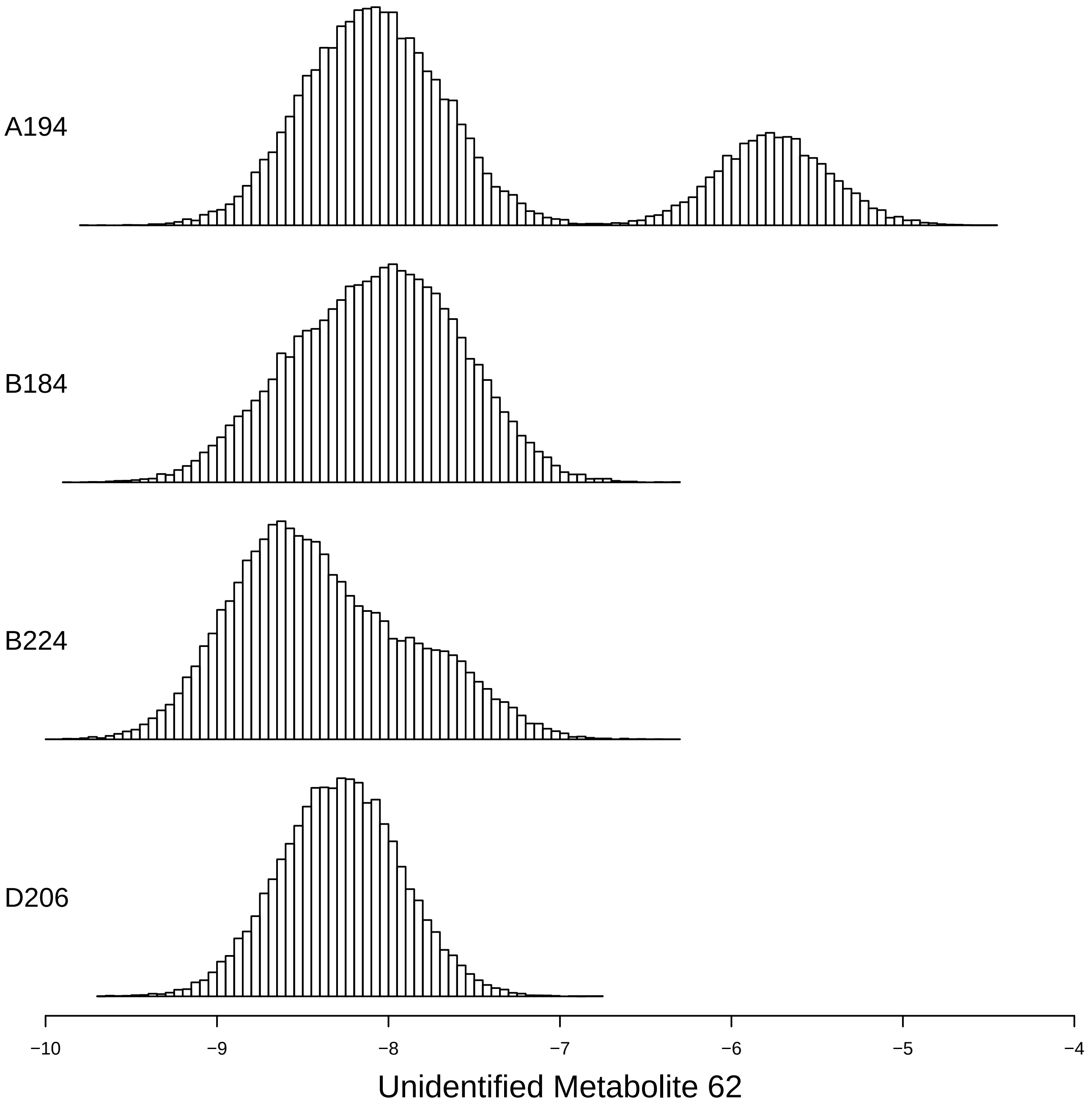
A194
B184
B224
D206
−10
−9
−8
−7
−6
−5
−4
Unidentified Metabolite 62

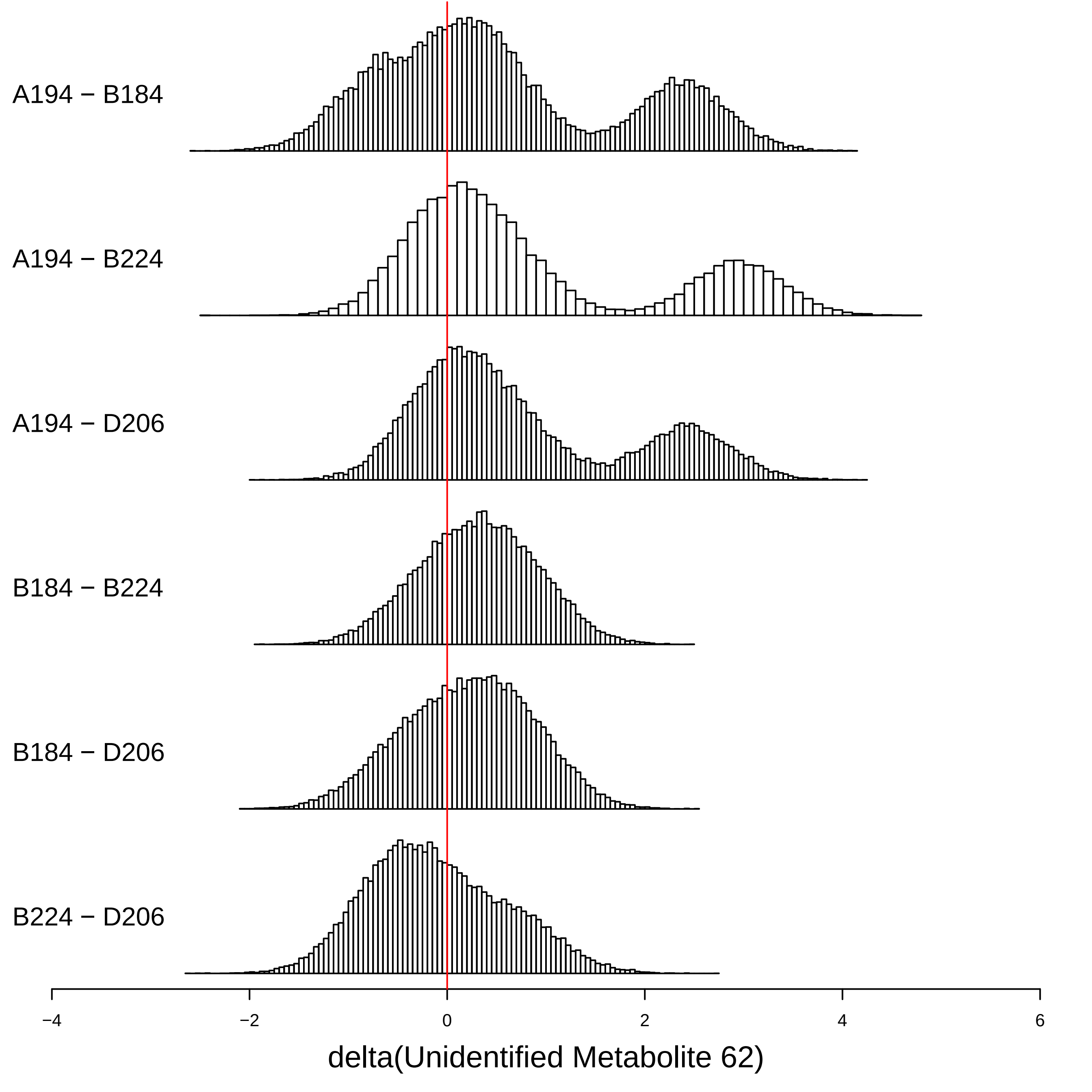

A194 − B184
A194 − B224
A194 − D206
B184 − B224
B184 − D206
B224 − D206
−4
−2
0
2
4
6
delta(Unidentified Metabolite 62)

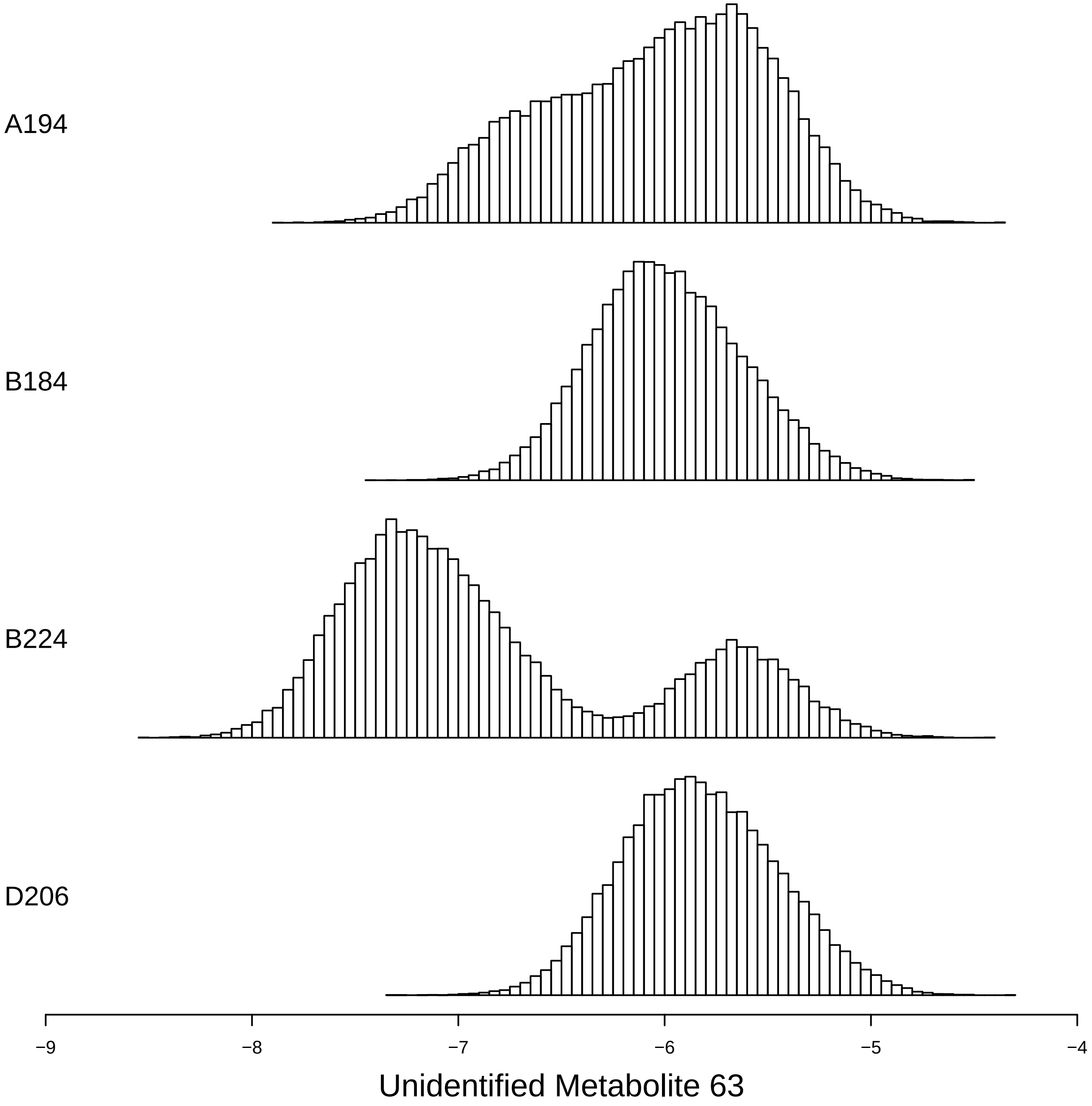

A194
B184
B224
D206
−9
−8
−7
−6
−5
−4
Unidentified Metabolite 63

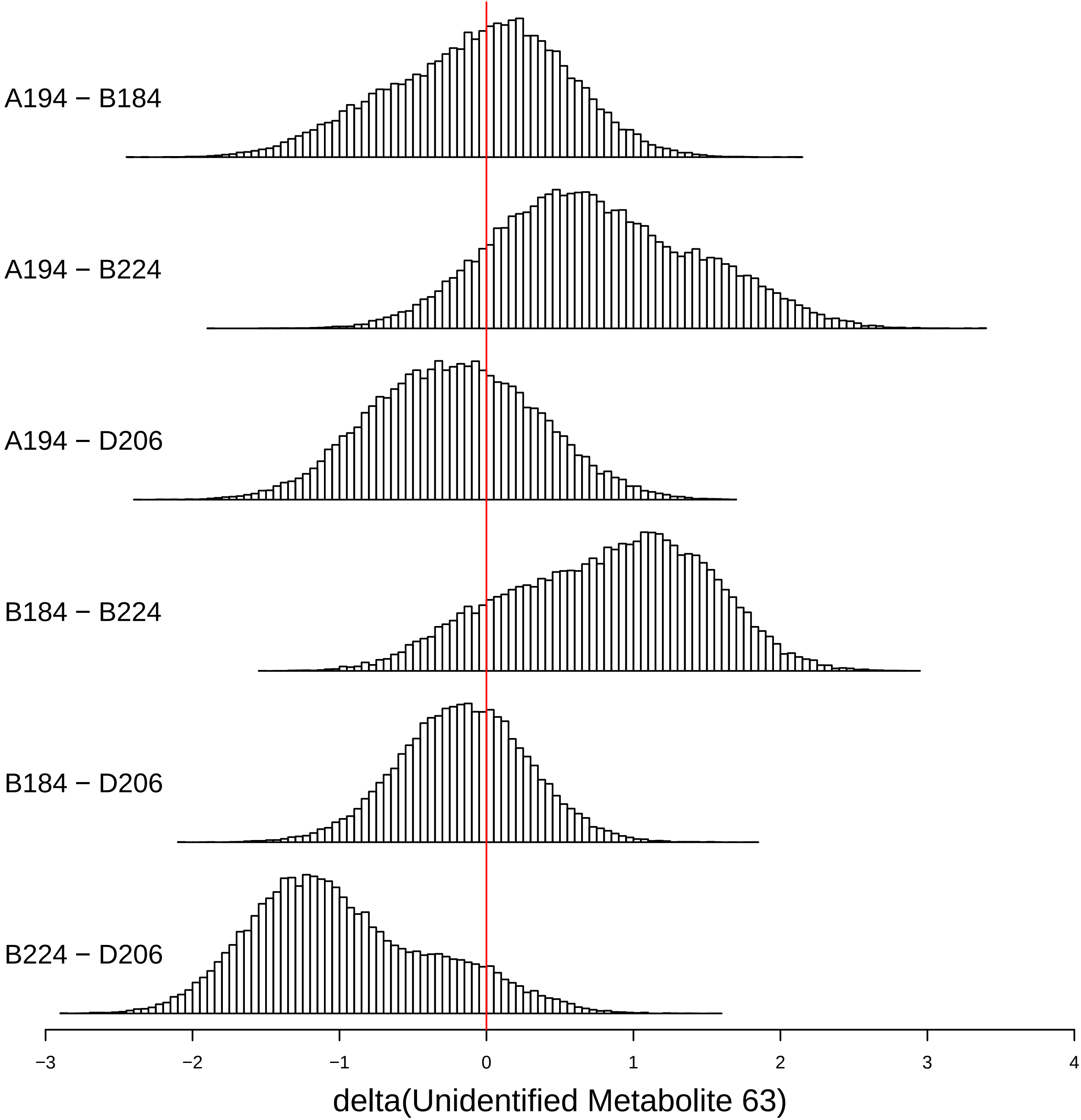

A194 − B184
A194 − B224
A194 − D206
B184 − B224
B184 − D206
B224 − D206
−3
−2
−1
0
1
2
3
4
delta(Unidentified Metabolite 63)

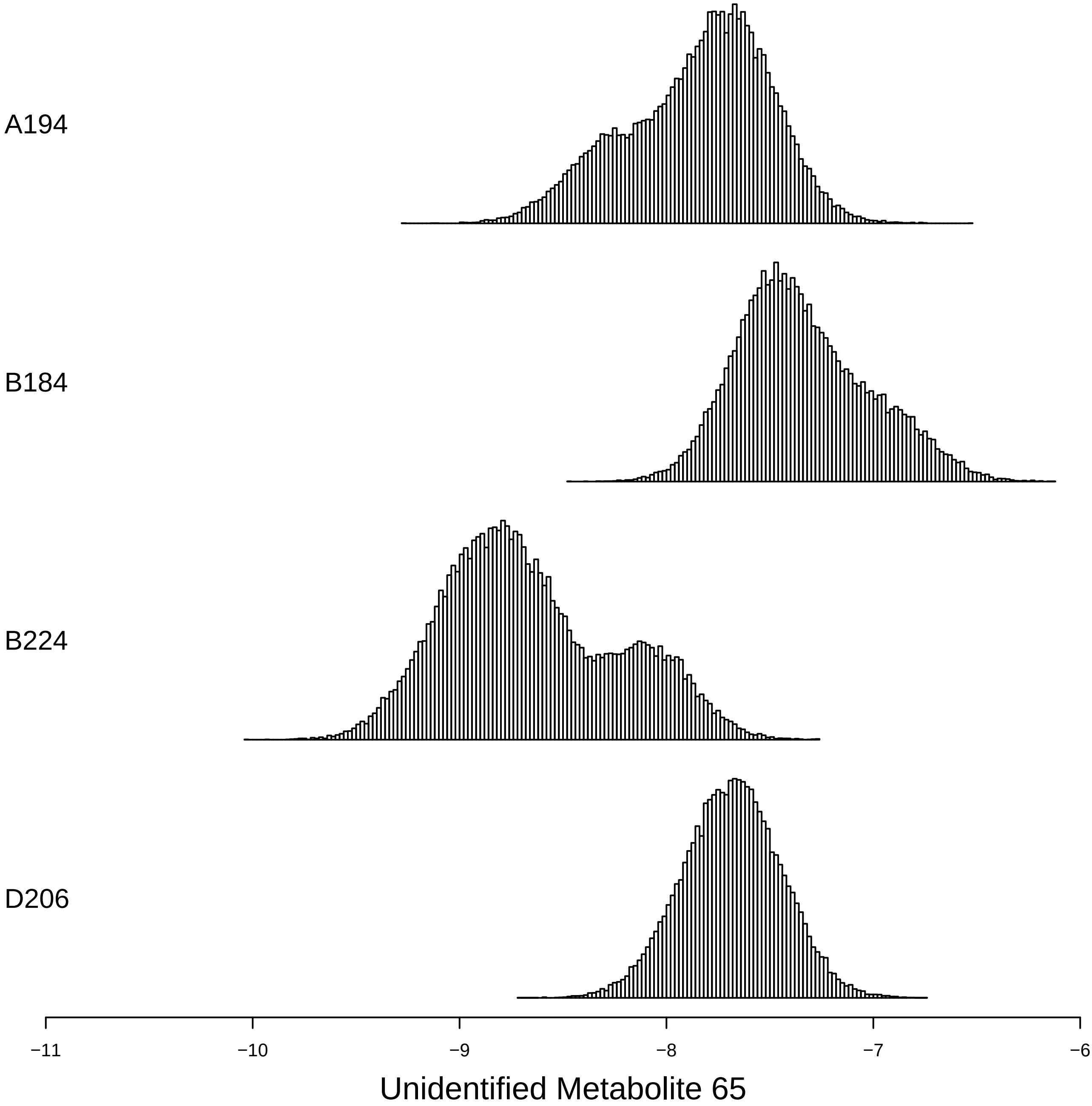
A194
B184
B224
D206
−11
−10
−9
−8
−7
−6
Unidentified Metabolite 65

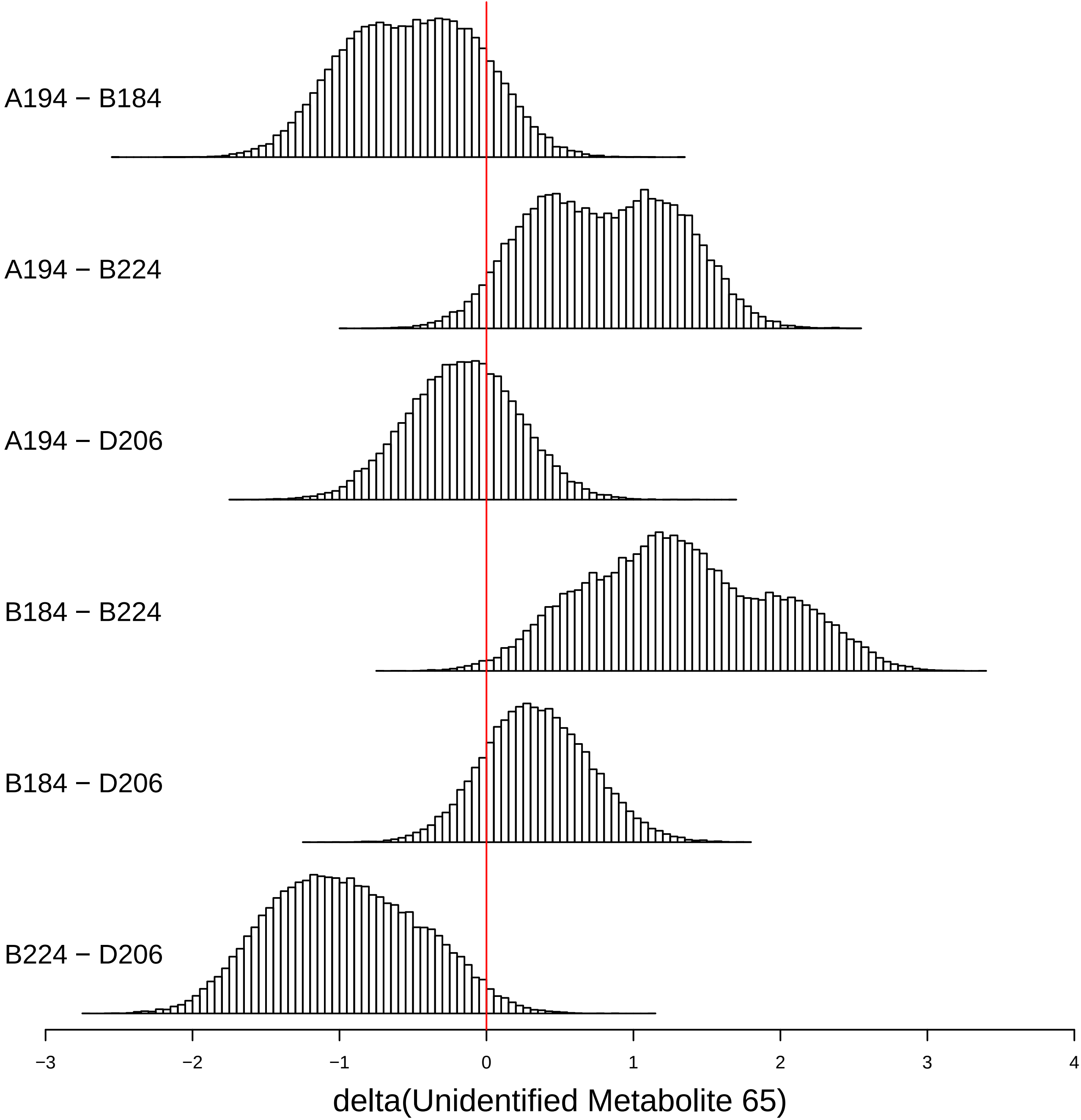
A194 − B184
A194 − B224
A194 − D206
B184 − B224
B184 − D206
B224 − D206
−3
−2
−1
0
1
2
3
4
delta(Unidentified Metabolite 65)

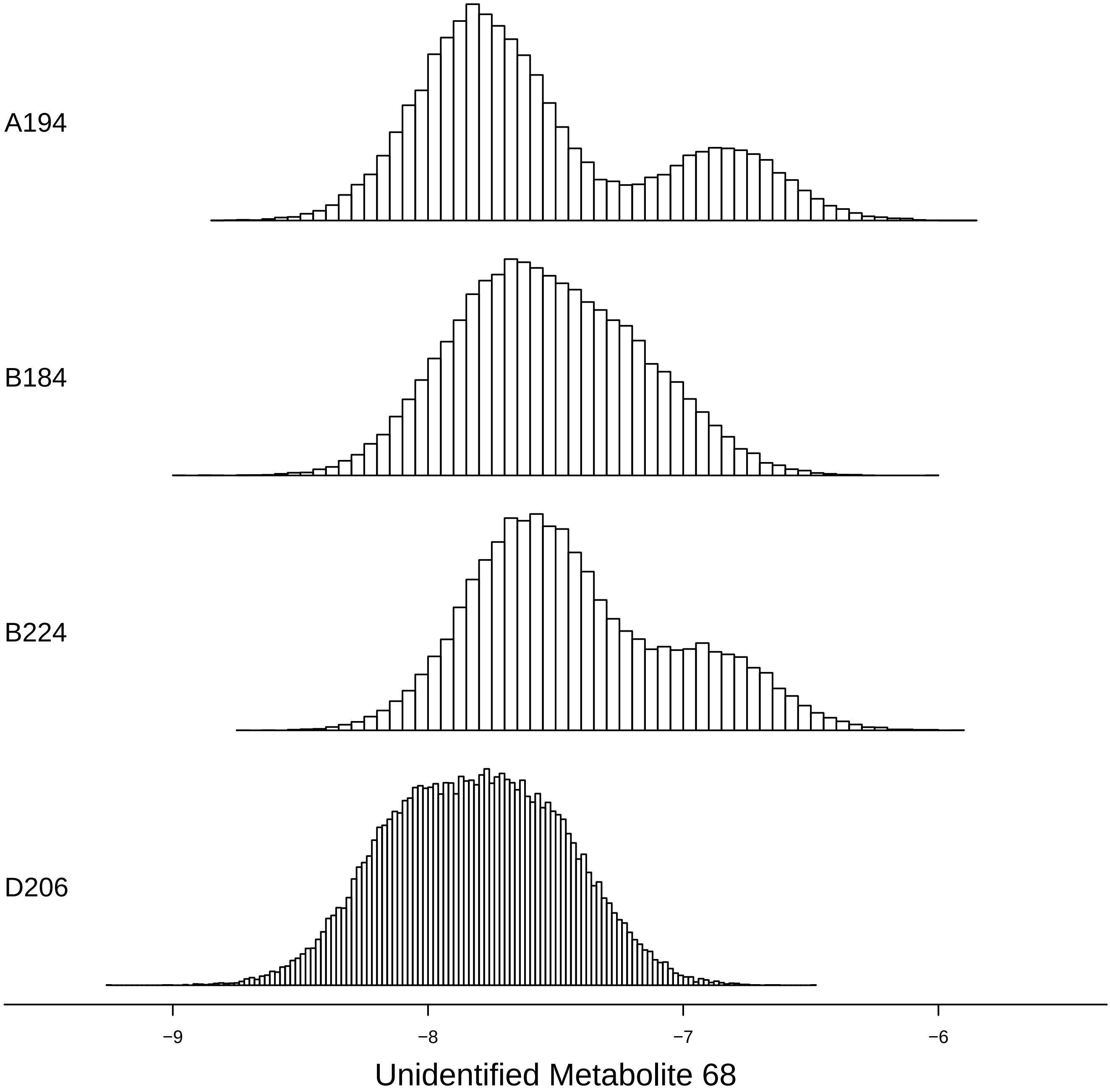
A194
B184
B224
D206
−9
−8
−7
−6
Unidentified Metabolite 68

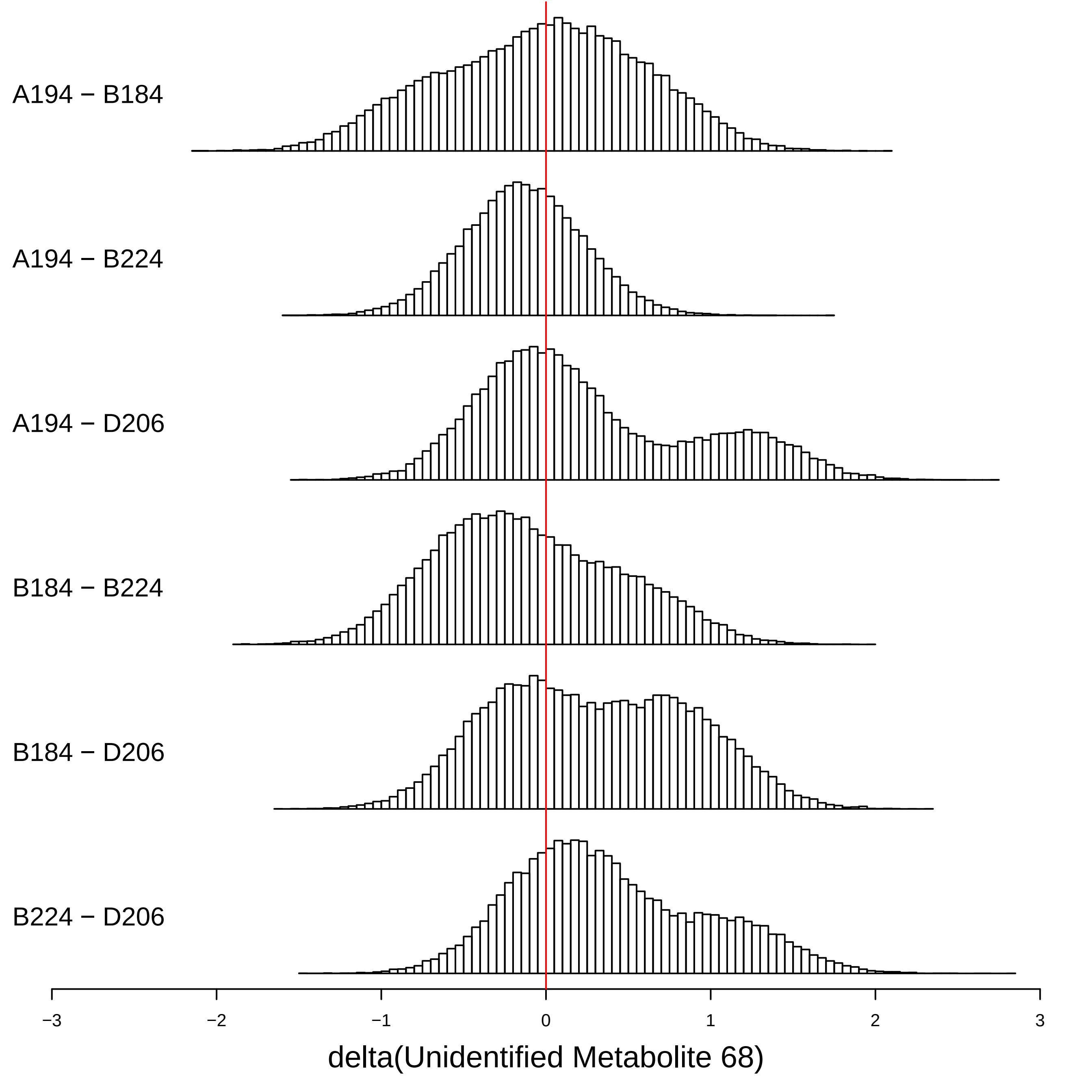

A194 − B184
A194 − B224
A194 − D206
B184 − B224
B184 − D206
B224 − D206
−3
−2
−1
0
1
2
3
delta(Unidentified Metabolite 68)

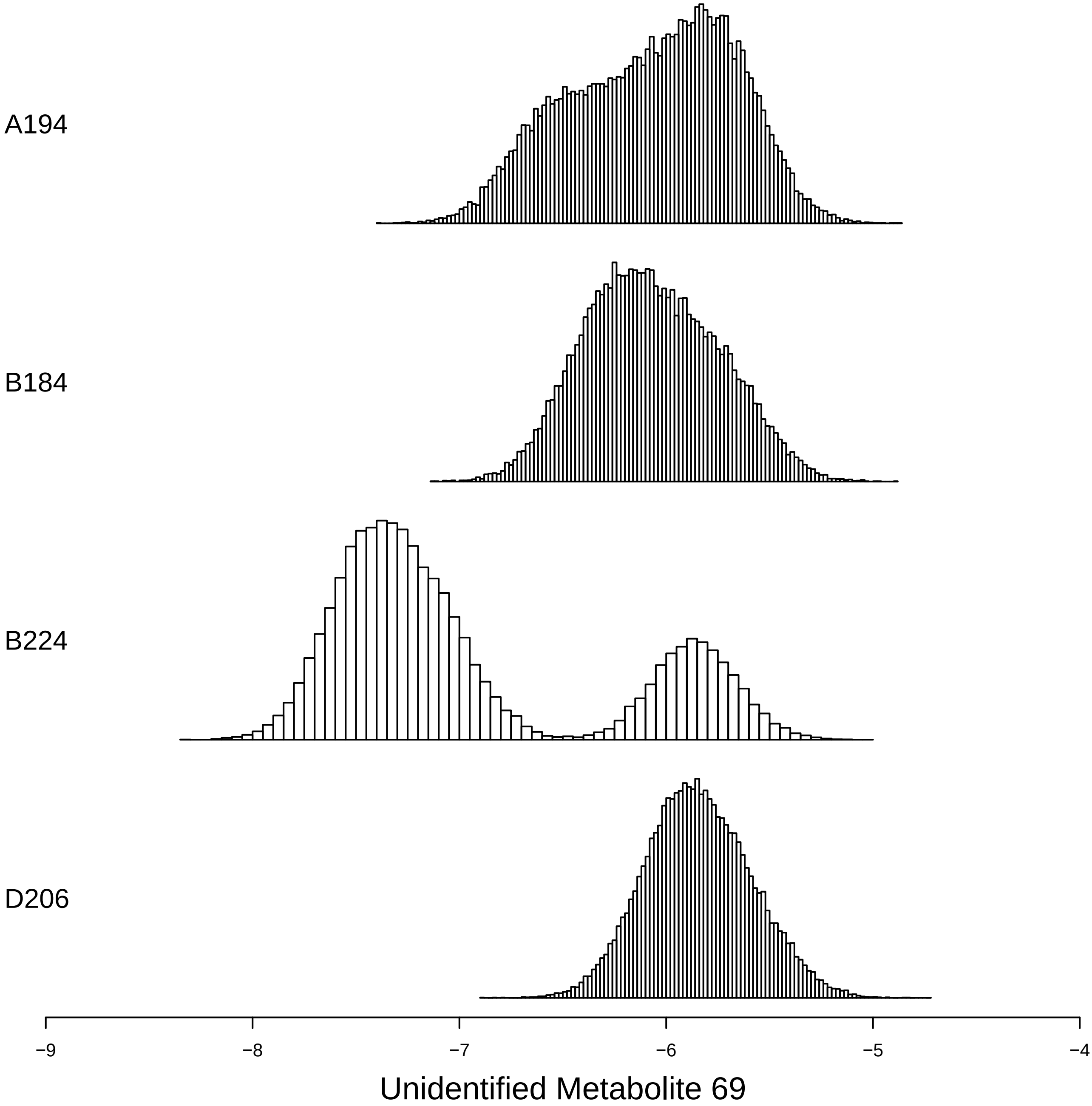
A194
B184
B224
D206
−9
−8
−7
−6
−5
−4
Unidentified Metabolite 69

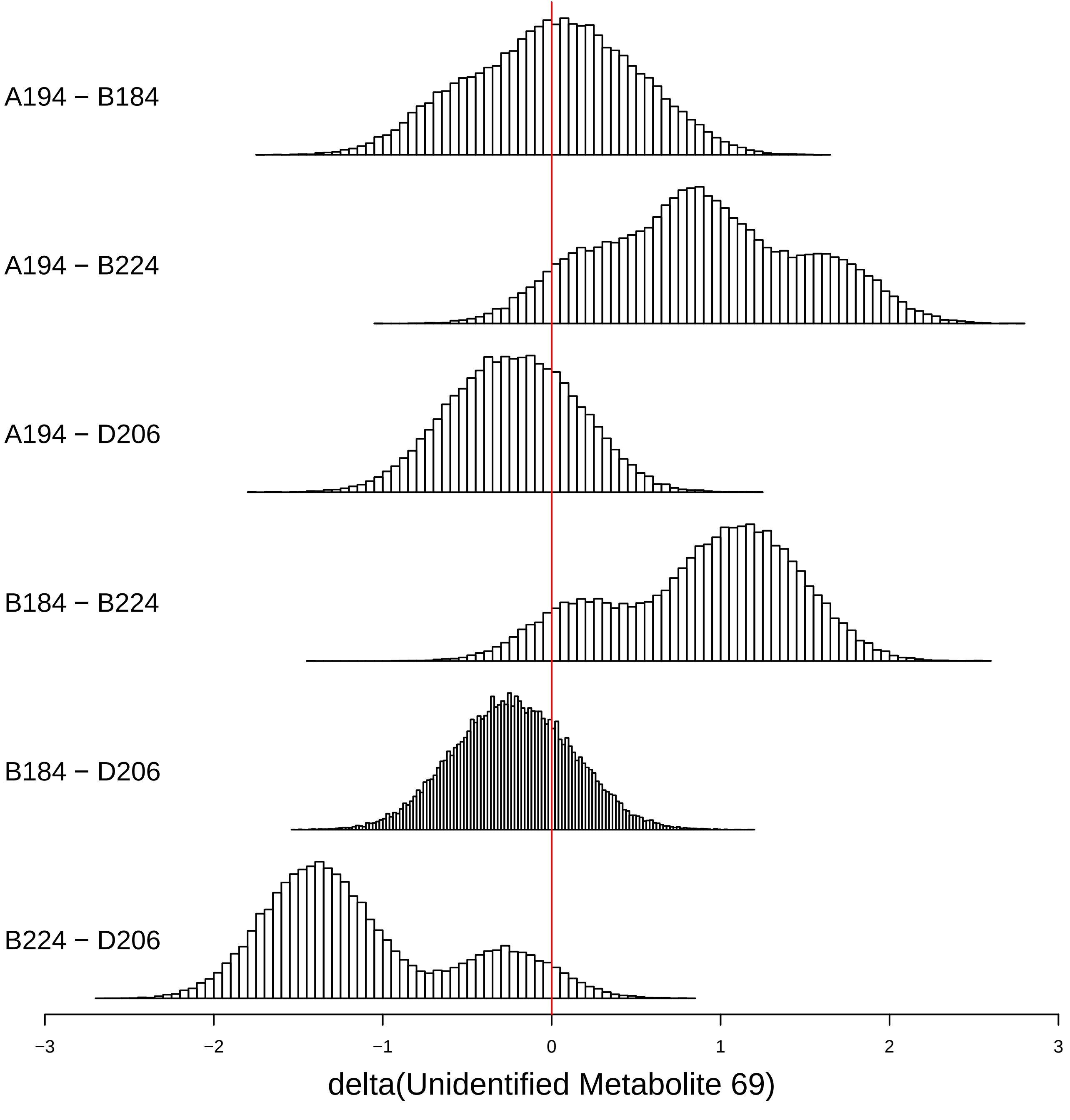
A194 − B184
A194 − B224
A194 − D206
B184 − B224
B184 − D206
B224 − D206
−3
−2
−1
0
1
2
3
delta(Unidentified Metabolite 69)

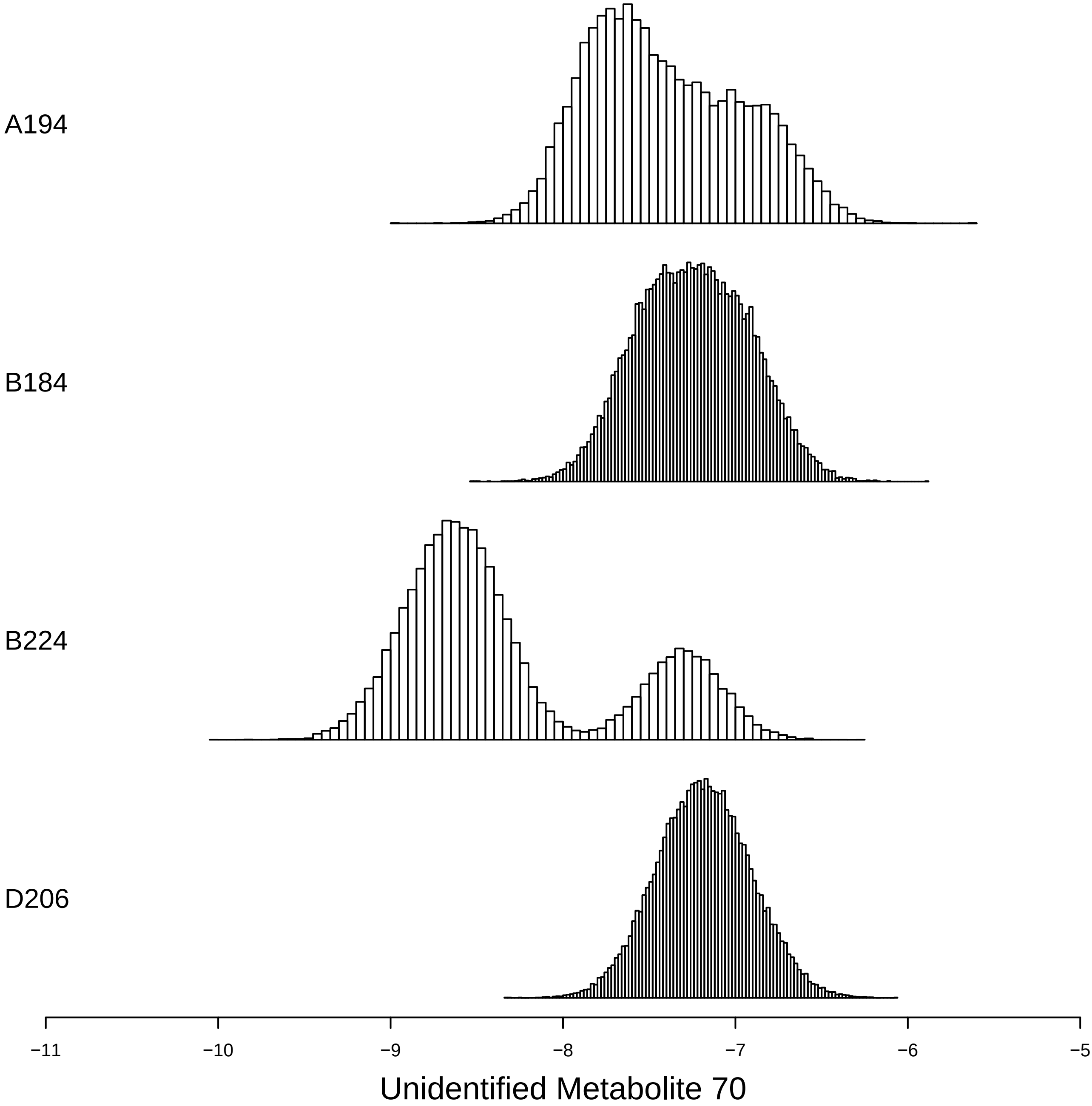
A194
B184
B224
D206
−11
−10
−9
−8
−7
−6
−5
Unidentified Metabolite 70

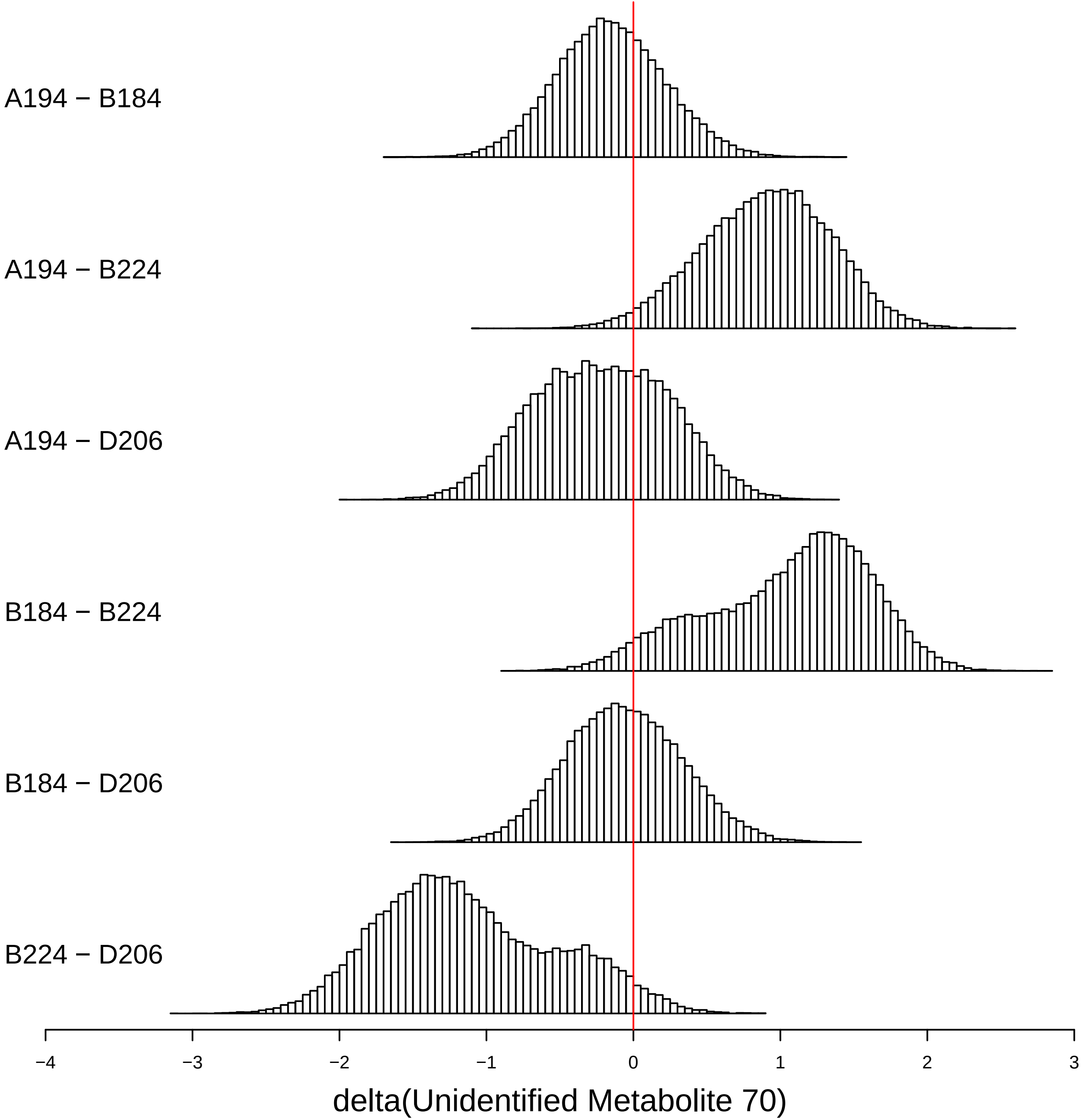
A194 − B184
A194 − B224
A194 − D206
B184 − B224
B184 − D206
B224 − D206
−4
−3
−2
−1
0
1
2
3
delta(Unidentified Metabolite 70)

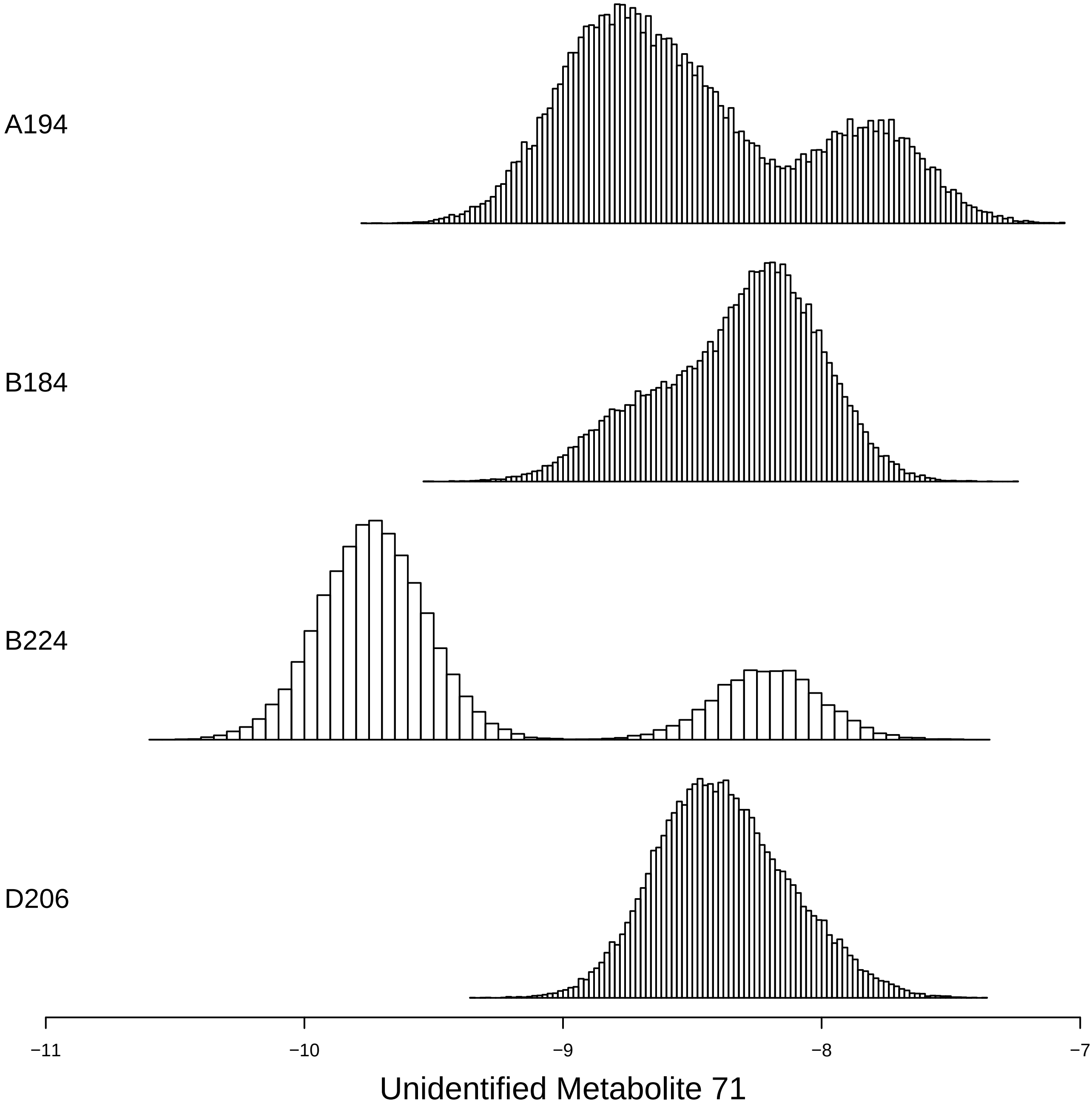

A194
B184
B224
D206
−11
−10
−9
−8
−7
Unidentified Metabolite 71

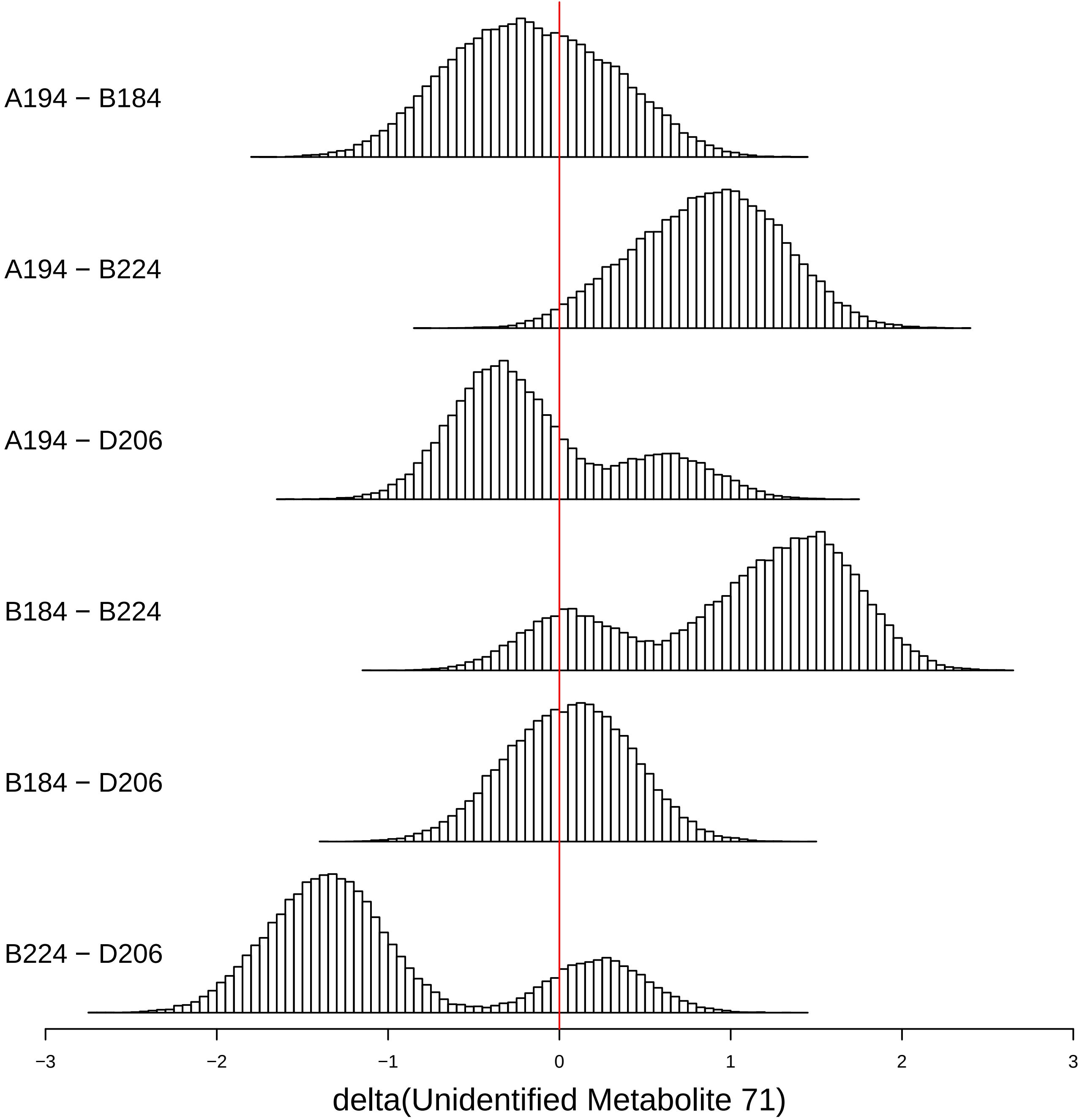

A194 − B184
A194 − B224
A194 − D206
B184 − B224
B184 − D206
B224 − D206
−3
−2
−1
0
1
2
3
delta(Unidentified Metabolite 71)

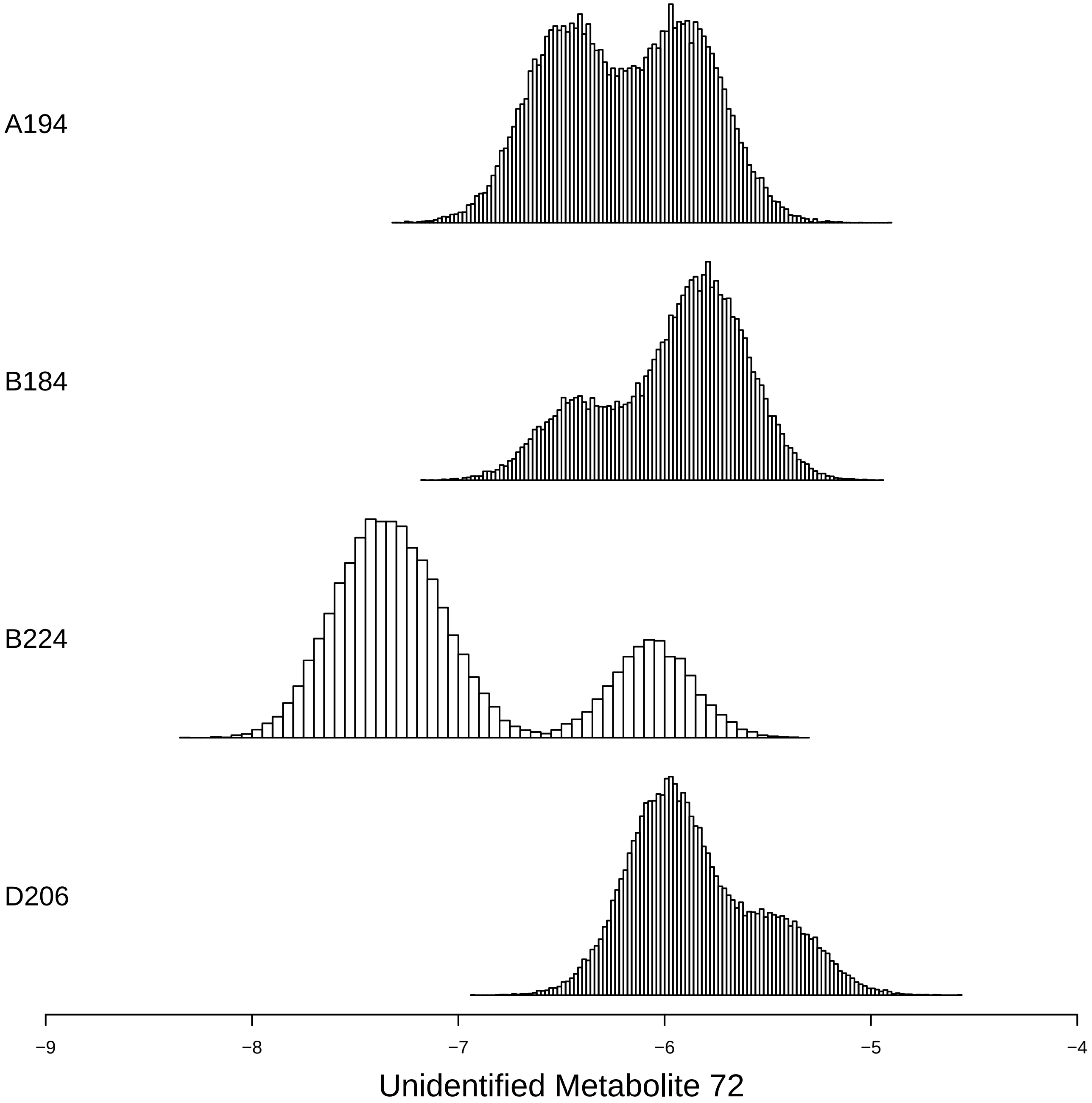
A194
B184
B224
D206
−9
−8
−7
−6
−5
−4
Unidentified Metabolite 72

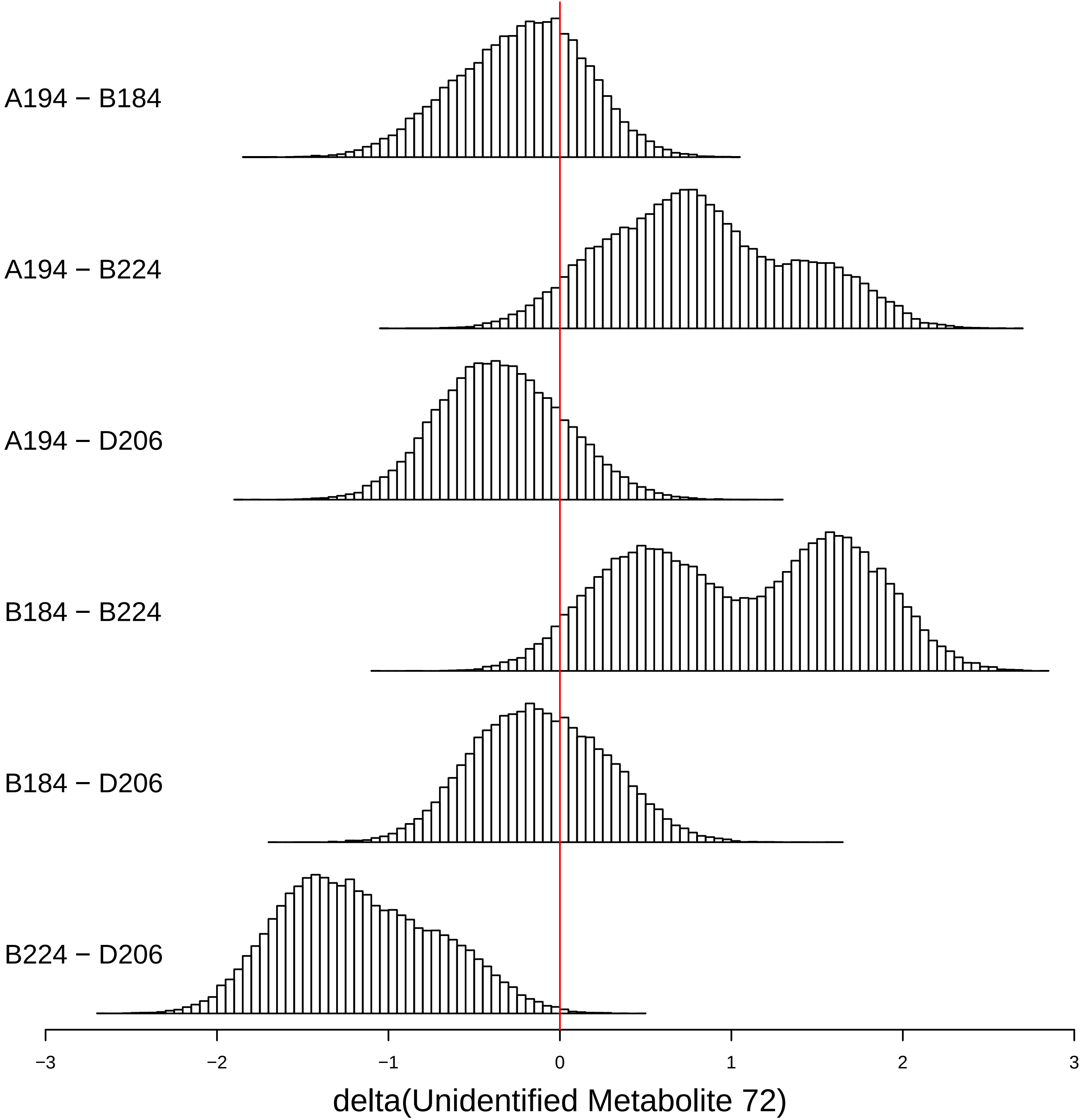
A194 − B184
A194 − B224
A194 − D206
B184 − B224
B184 − D206
B224 − D206
−3
−2
−1
0
1
2
3
delta(Unidentified Metabolite 72)

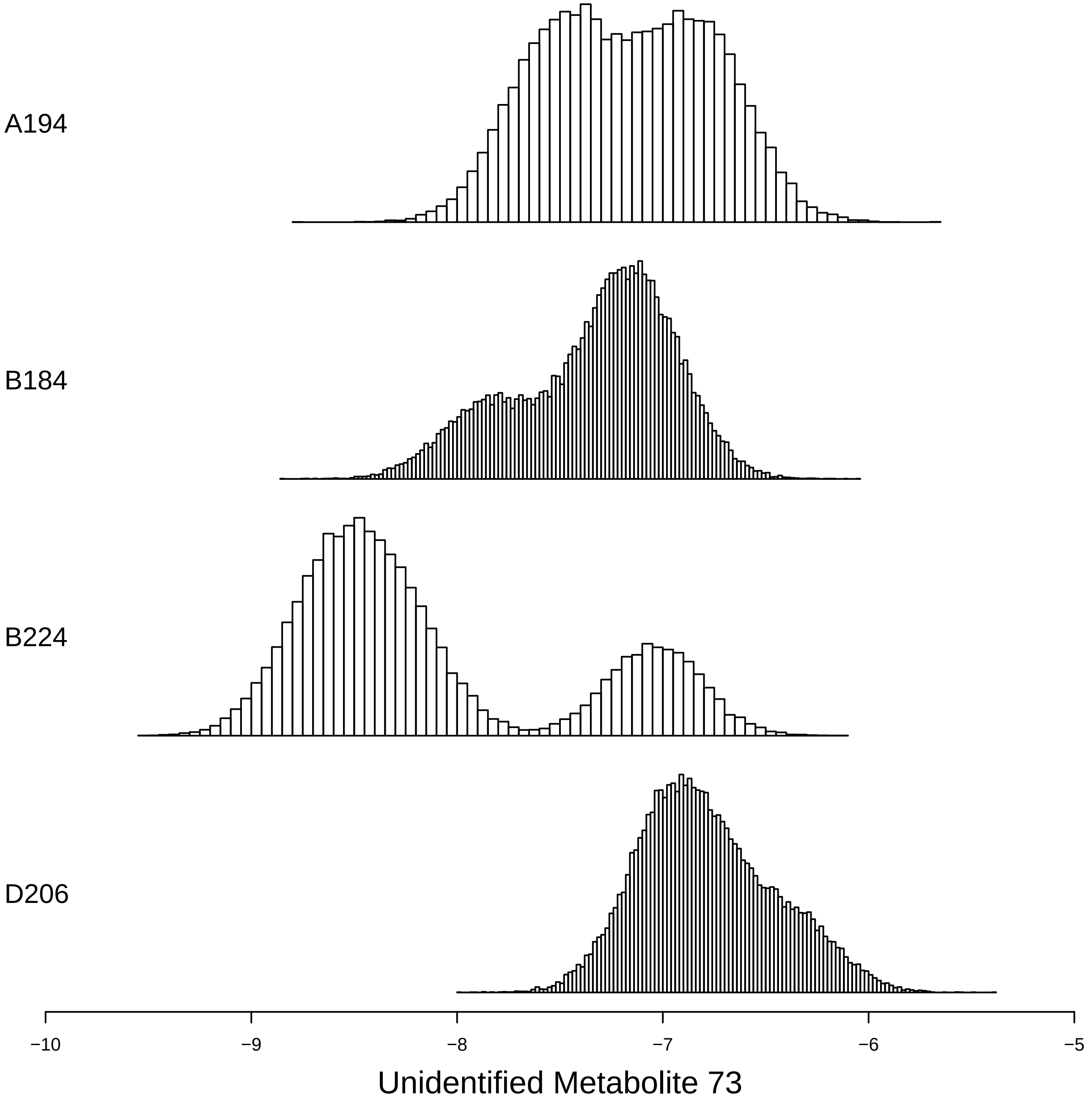
A194
B184
B224
D206
−10
−9
−8
−7
−6
−5
Unidentified Metabolite 73

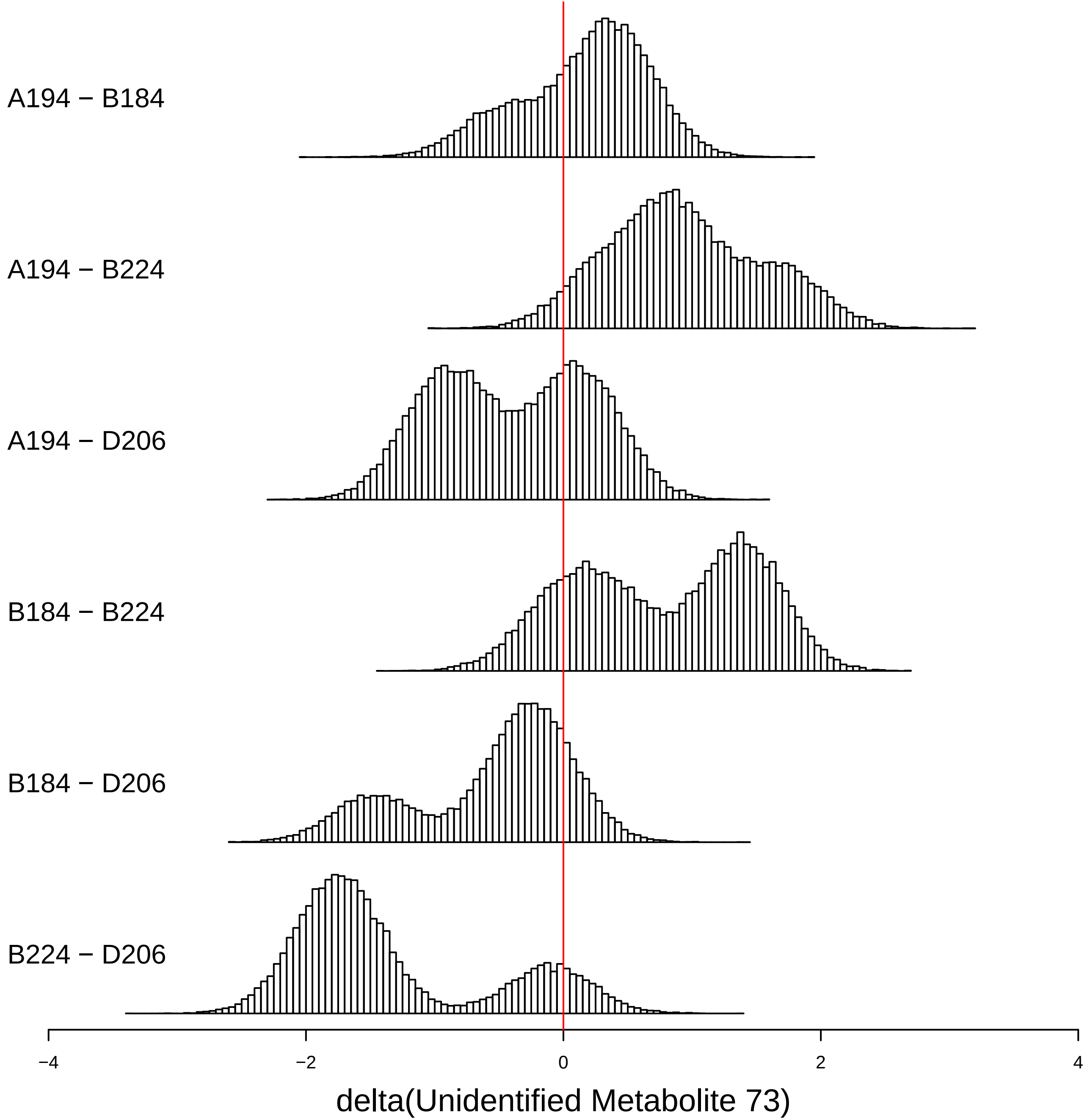
A194 − B184
A194 − B224
A194 − D206
B184 − B224
B184 − D206
B224 − D206
−4
−2
0
2
4
delta(Unidentified Metabolite 73)

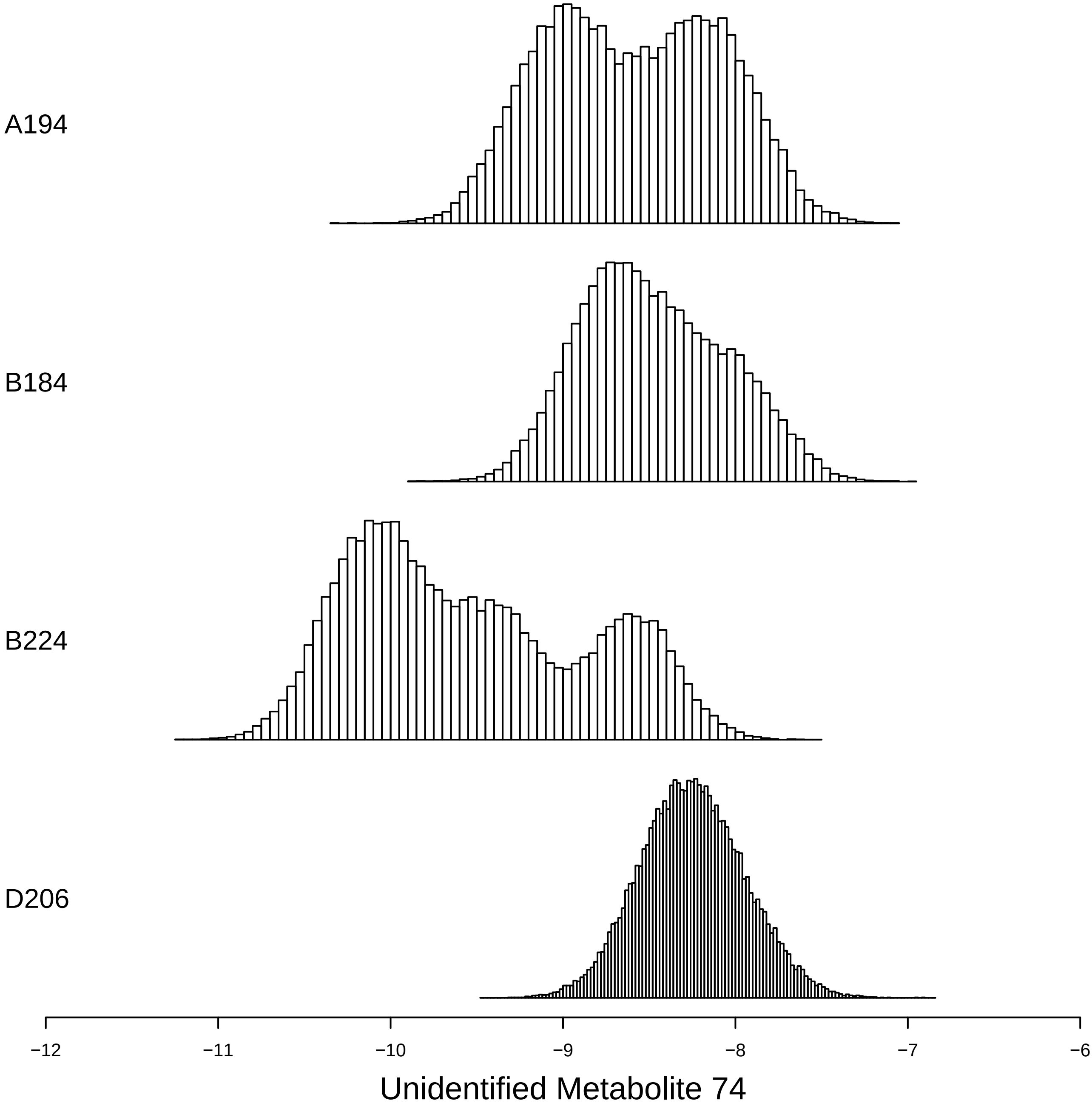
A194
B184
B224
D206
−12
−11
−10
−9
−8
−7
−6
Unidentified Metabolite 74

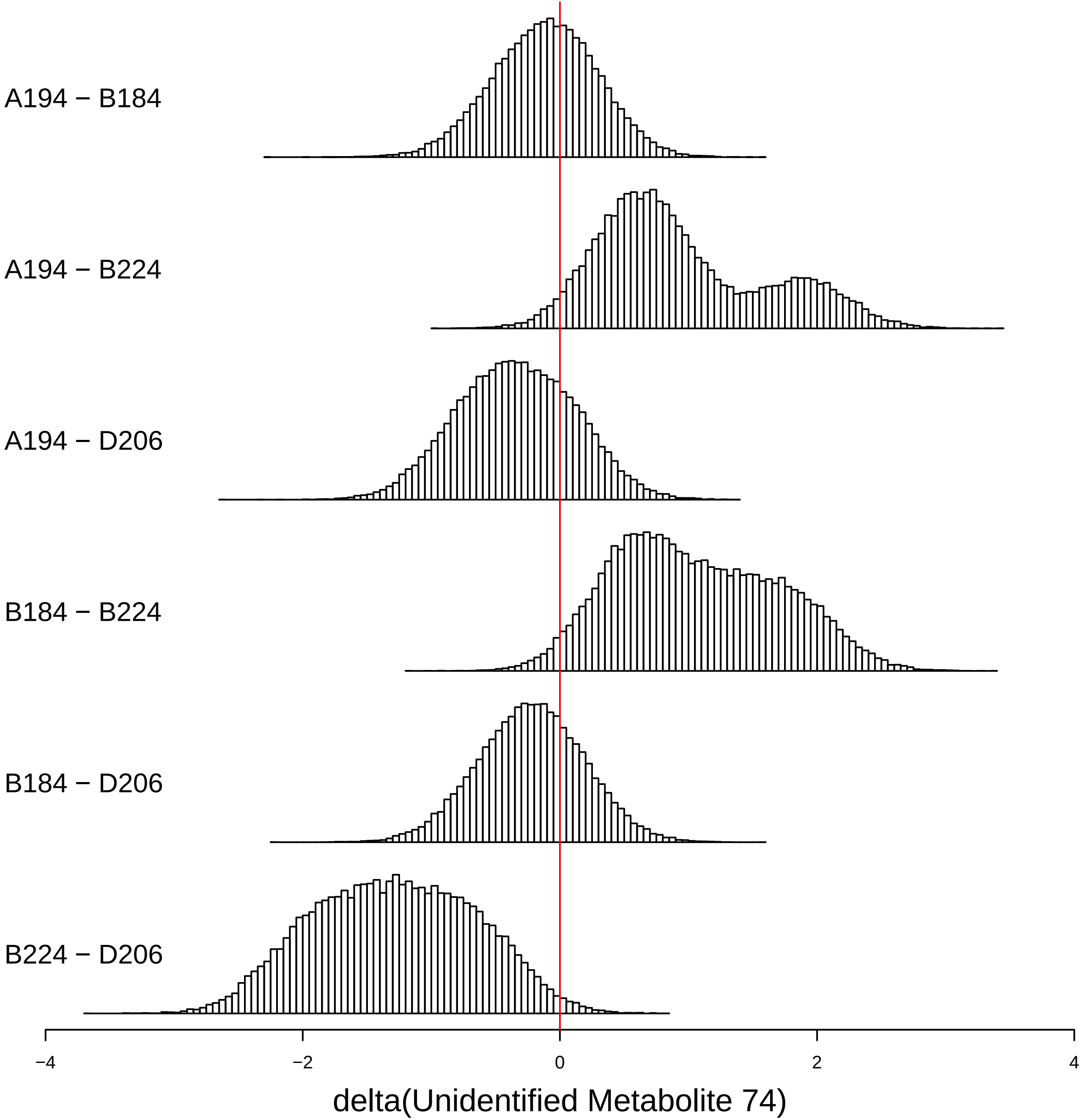

A194 − B184
A194 − B224
A194 − D206
B184 − B224
B184 − D206
B224 − D206
−4
−2
0
2
4
delta(Unidentified Metabolite 74)

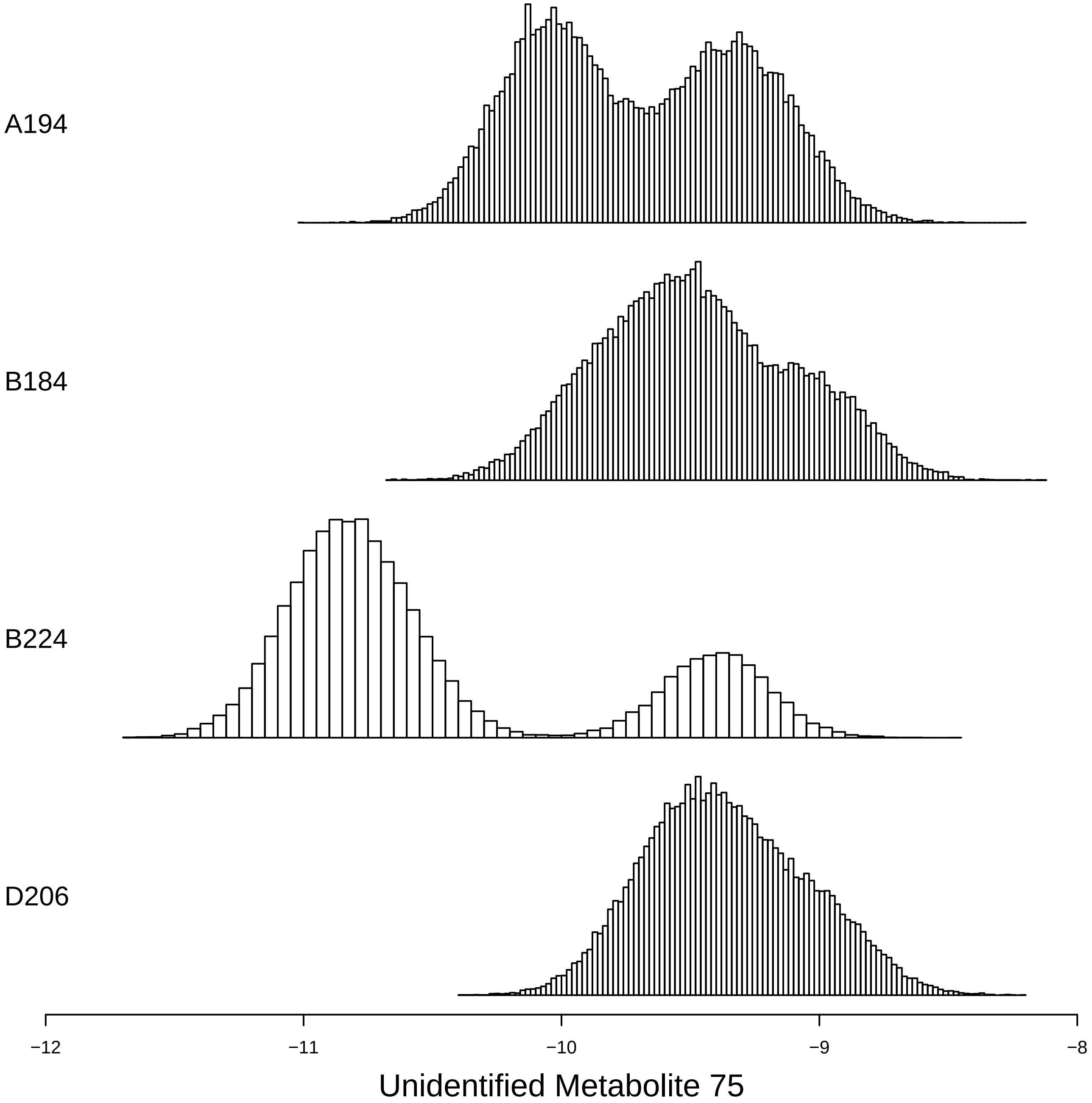
A194
B184
B224
D206
−12
−11
−10
−9
−8
Unidentified Metabolite 75

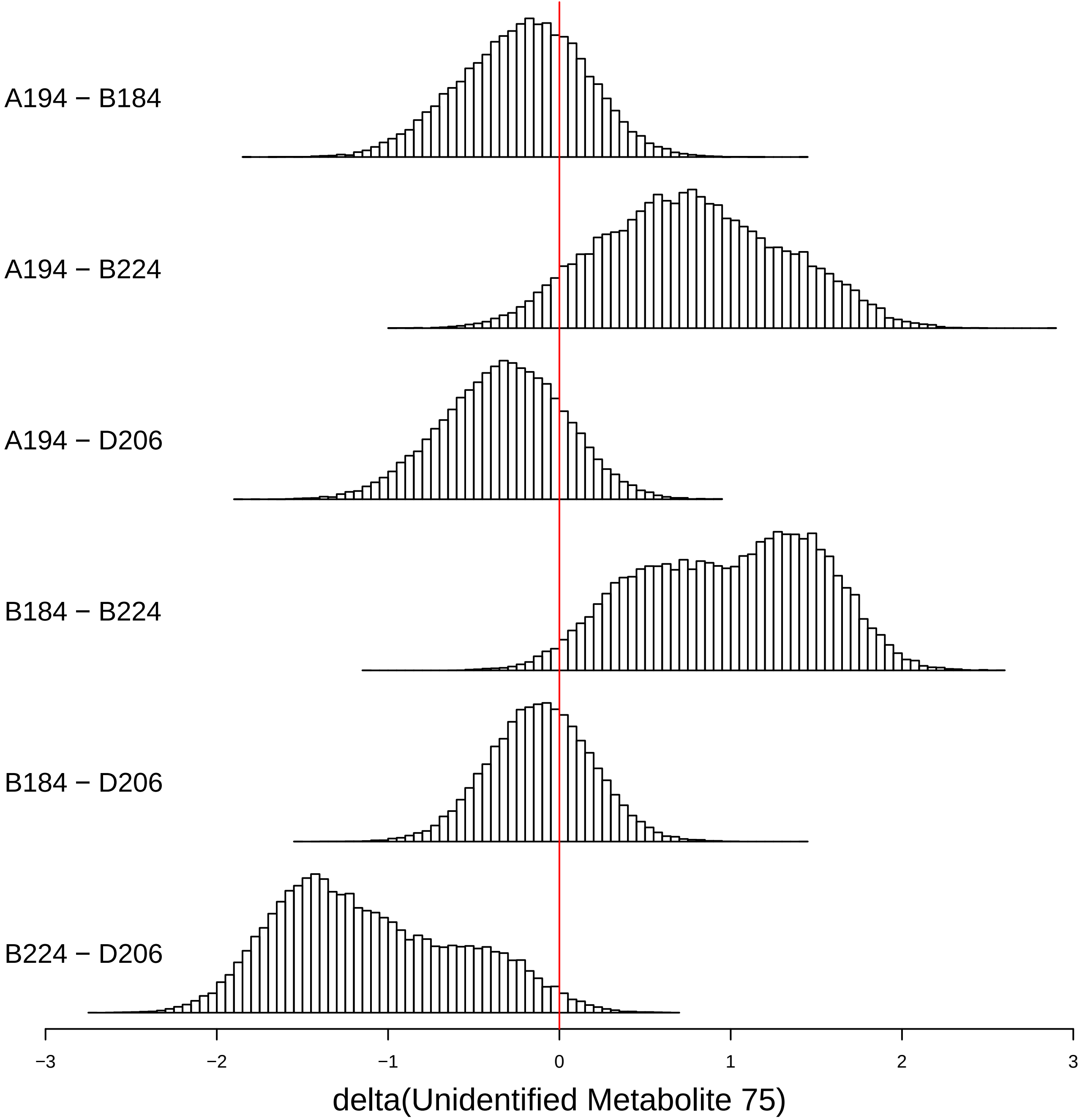

A194 − B184
A194 − B224
A194 − D206
B184 − B224
B184 − D206
B224 − D206
−3
−2
−1
0
1
2
3
delta(Unidentified Metabolite 75)

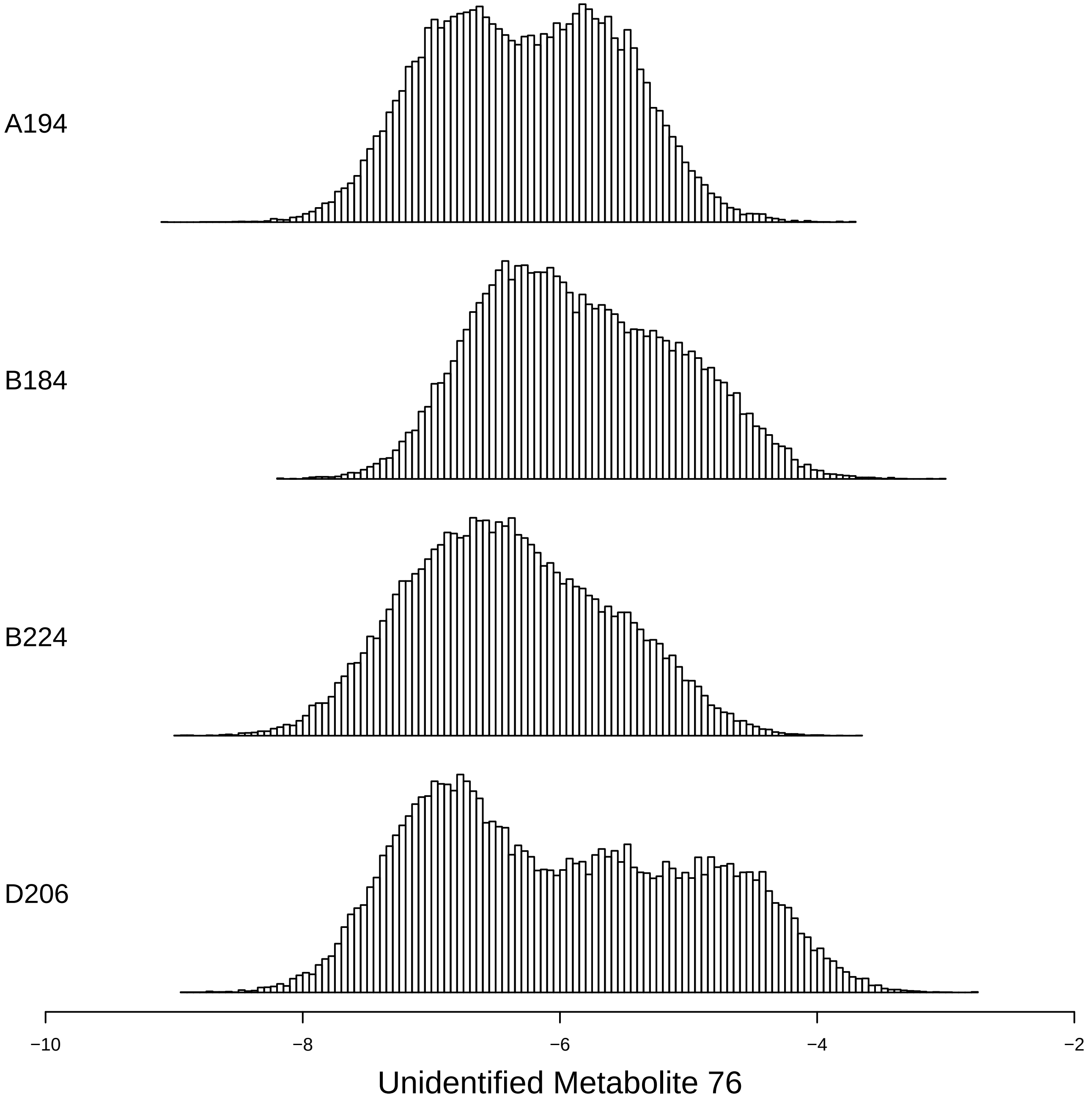

A194
B184
B224
D206
−10
−8
−6
−4
−2
Unidentified Metabolite 76

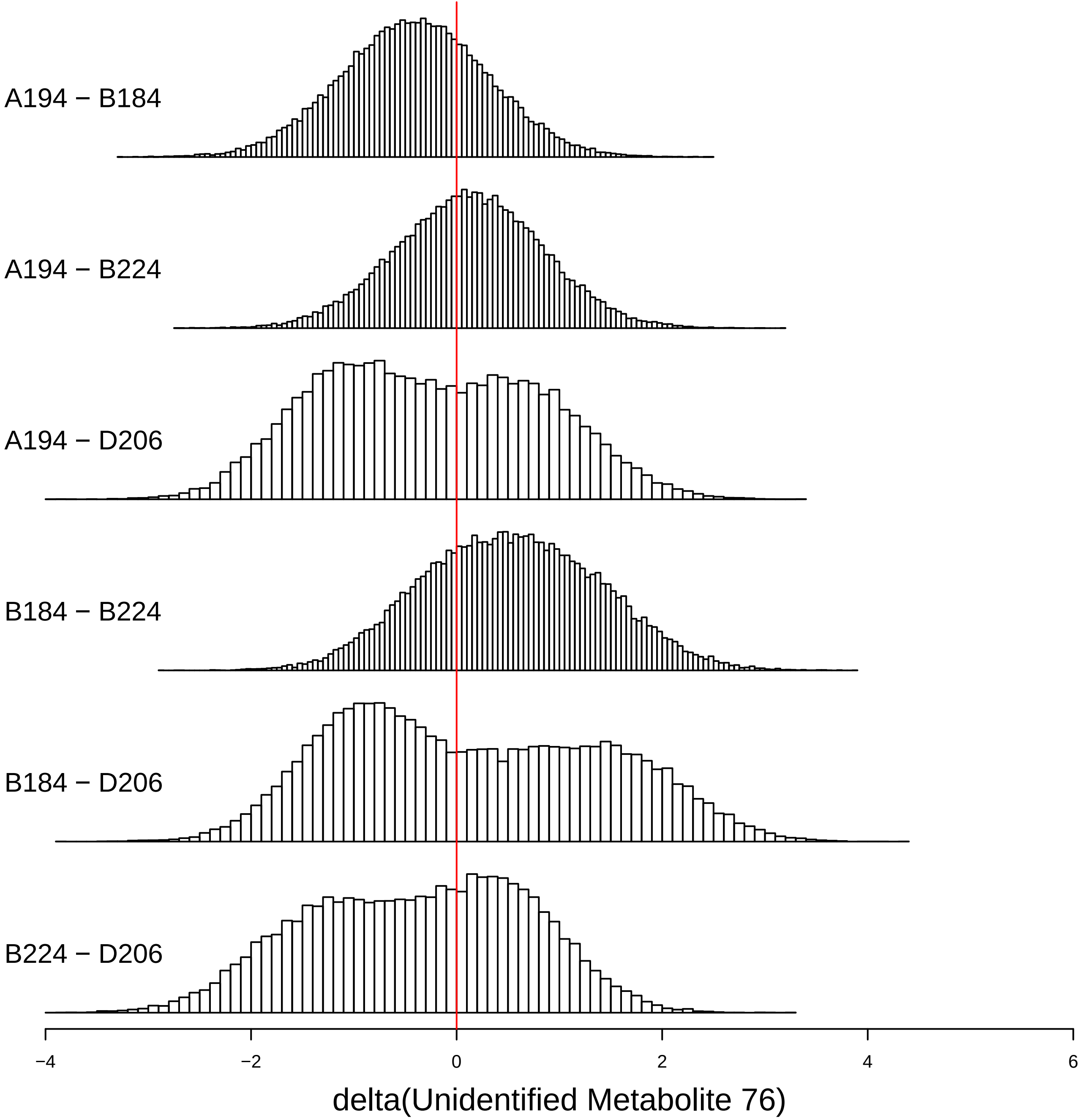

A194 − B184
A194 − B224
A194 − D206
B184 − B224
B184 − D206
B224 − D206
−4
−2
0
2
4
6
delta(Unidentified Metabolite 76)

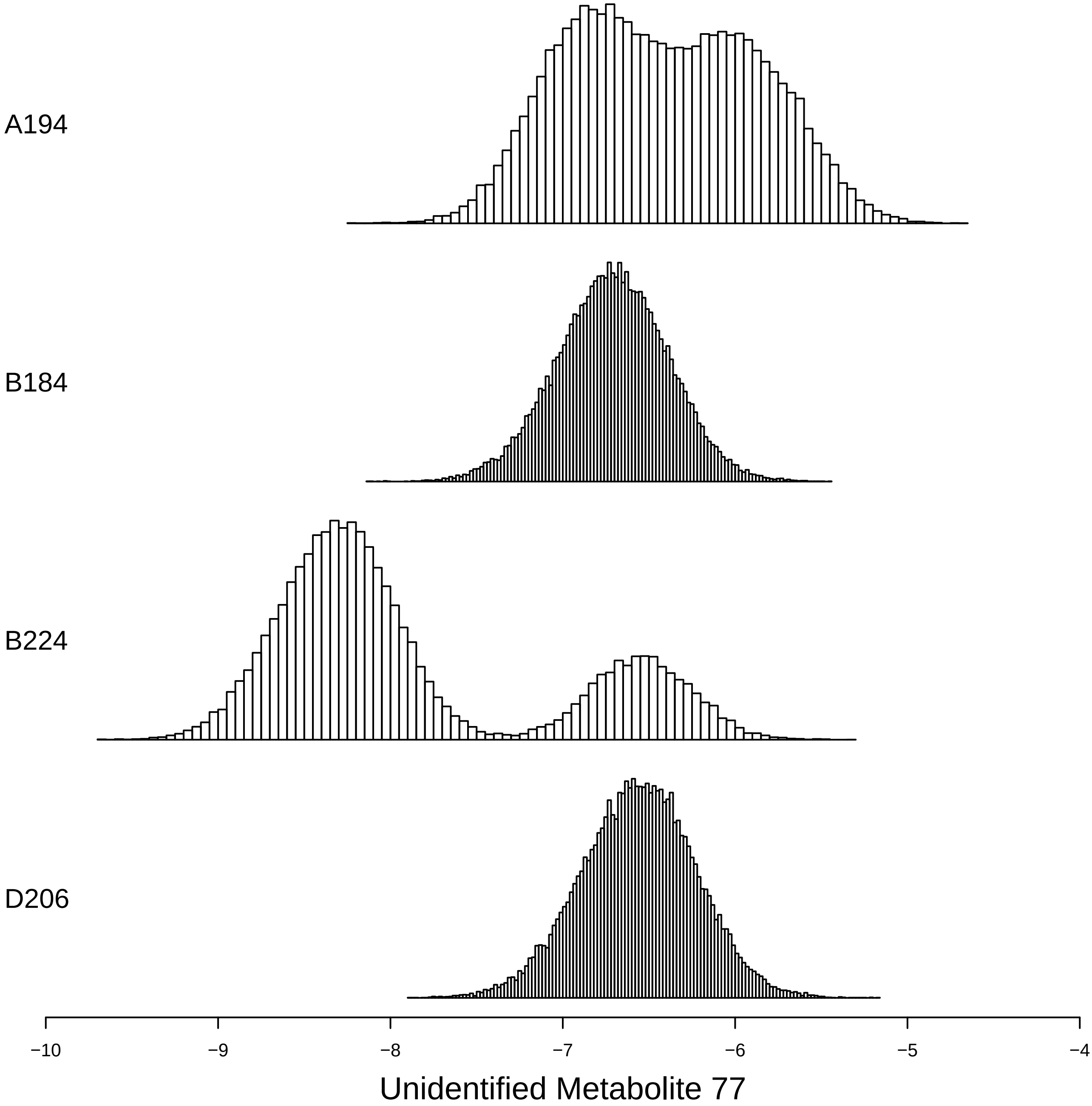
A194
B184
B224
D206
−10
−9
−8
−7
−6
−5
−4
Unidentified Metabolite 77

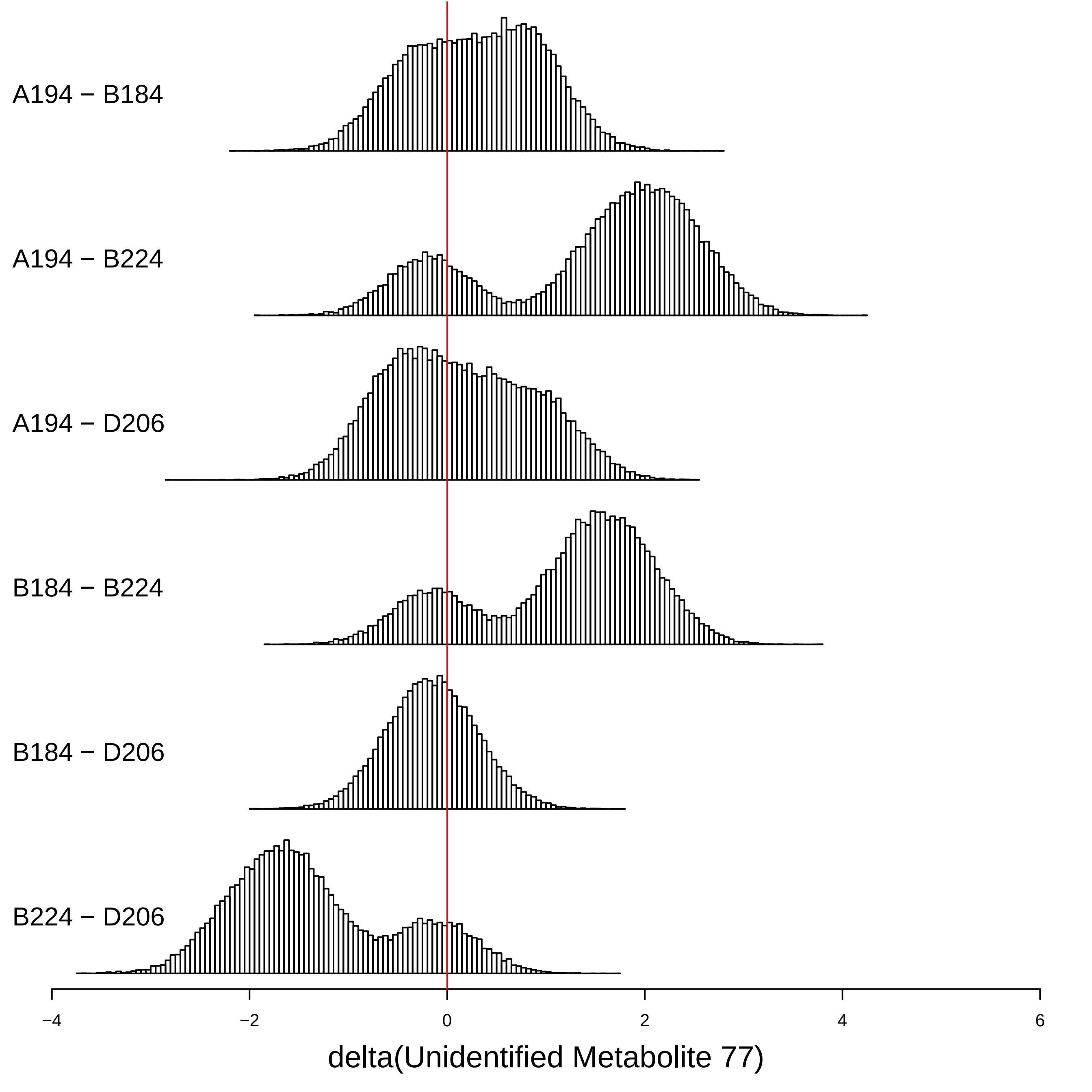
A194 − B184
A194 − B224
A194 − D206
B184 − B224
B184 − D206
B224 − D206
−4
−2
0
2
4
6
delta(Unidentified Metabolite 77)

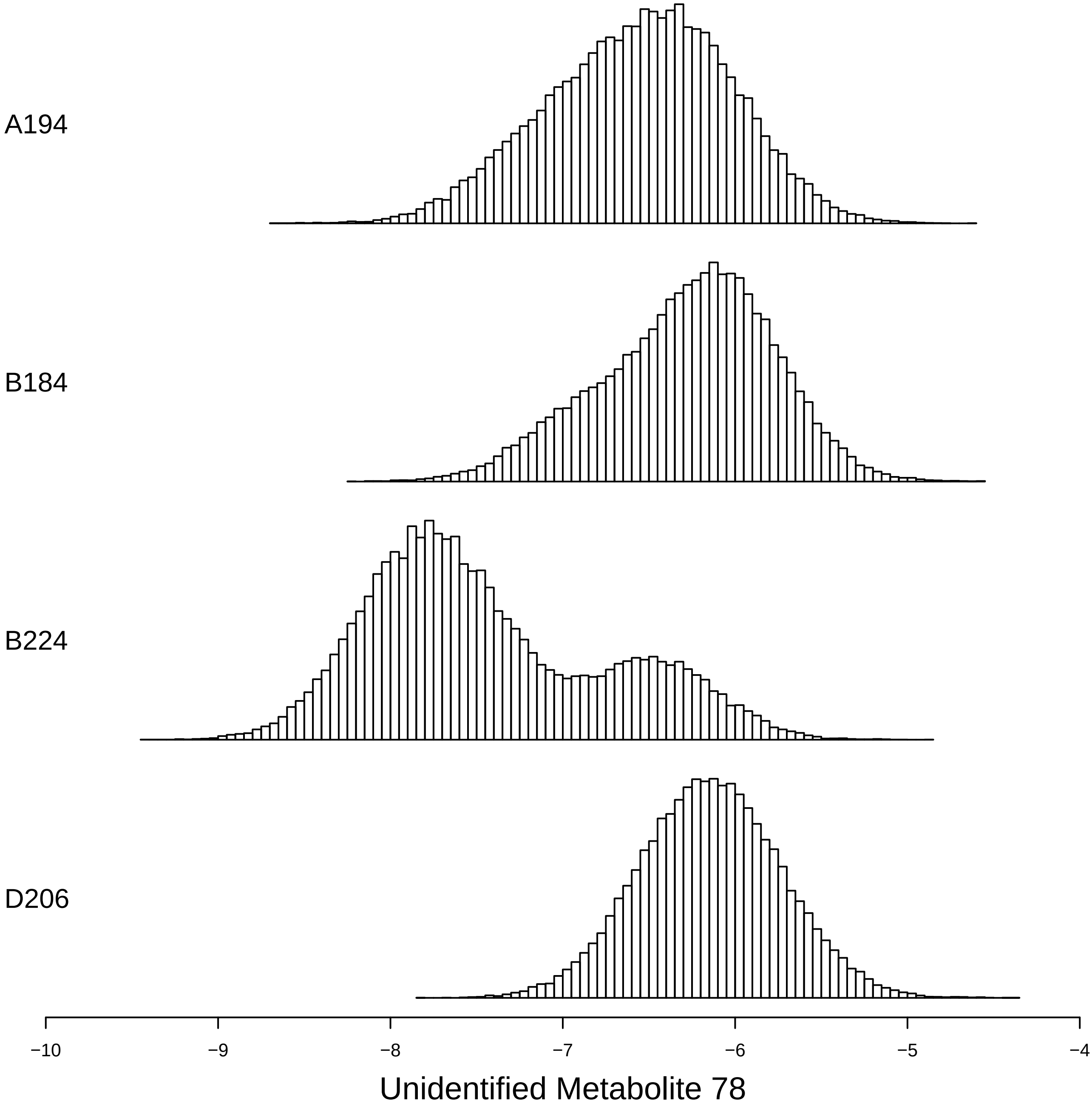
A194
B184
B224
D206
−10
−9
−8
−7
−6
−5
−4
Unidentified Metabolite 78

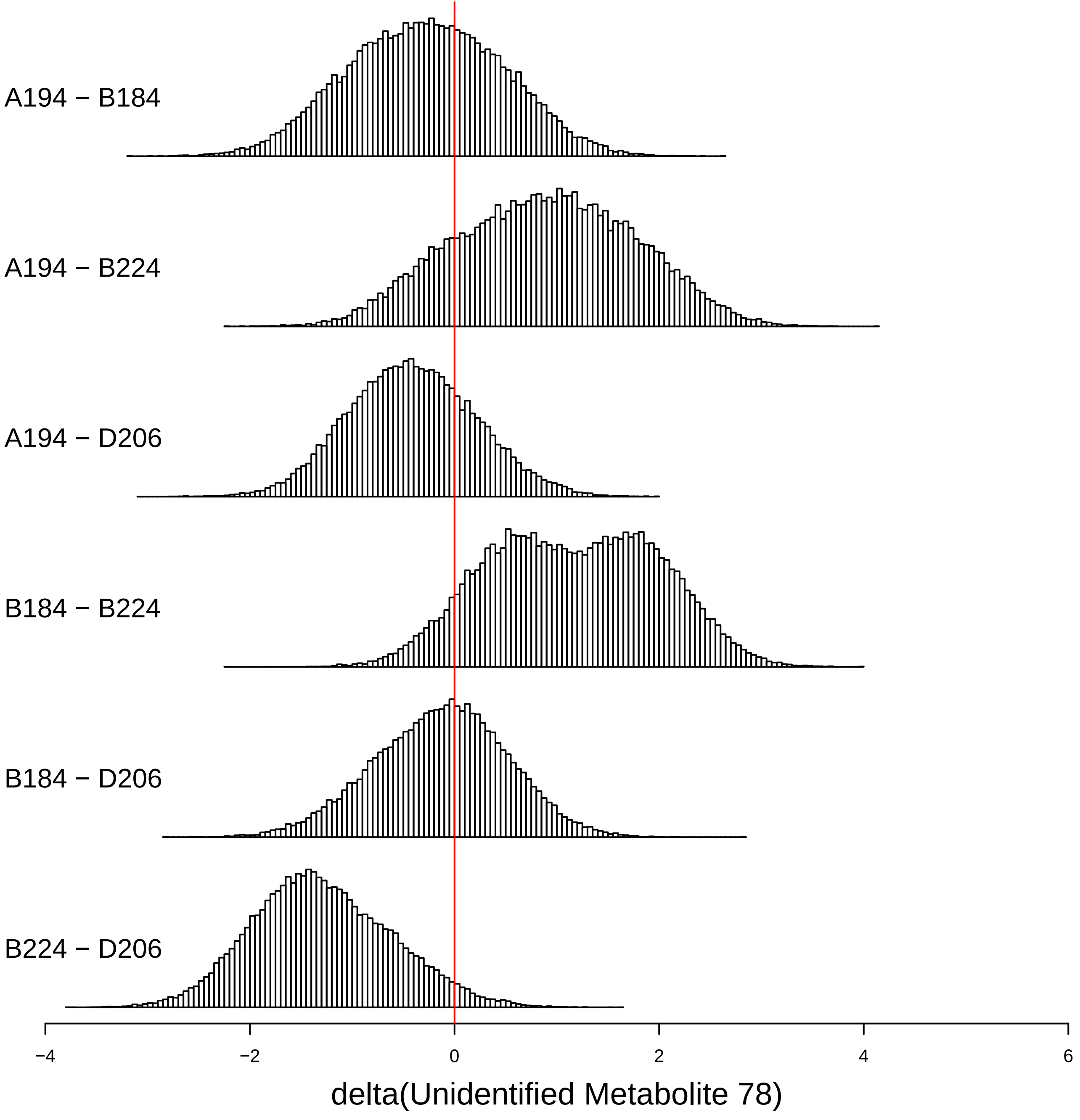
A194 − B184
A194 − B224
A194 − D206
B184 − B224
B184 − D206
B224 − D206
−4
−2
0
2
4
6
delta(Unidentified Metabolite 78)

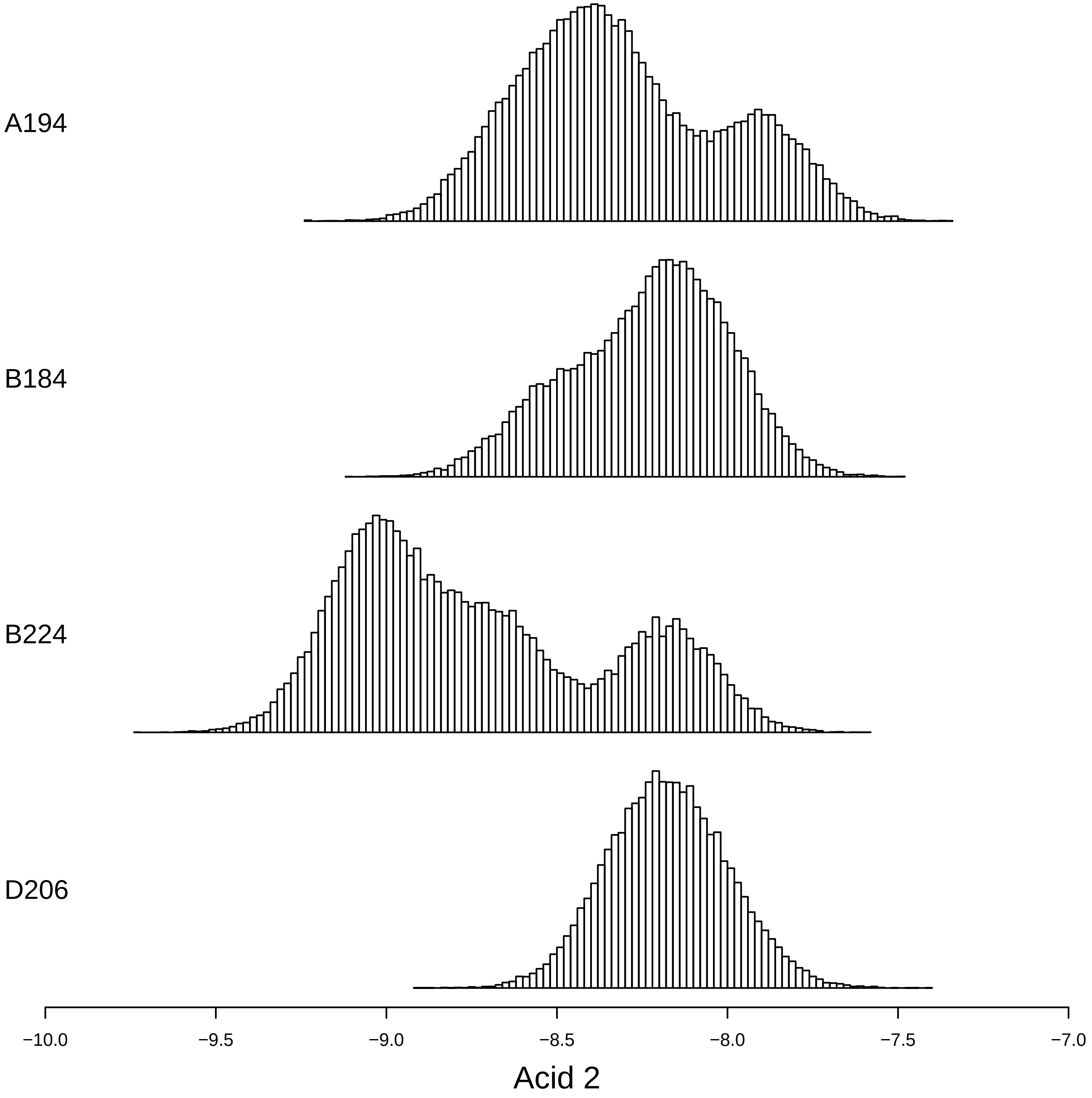
A194
B184
B224
D206
−10.0
−9.5
−9.0
−8.5
−8.0
−7.5
−7.0
Acid 2

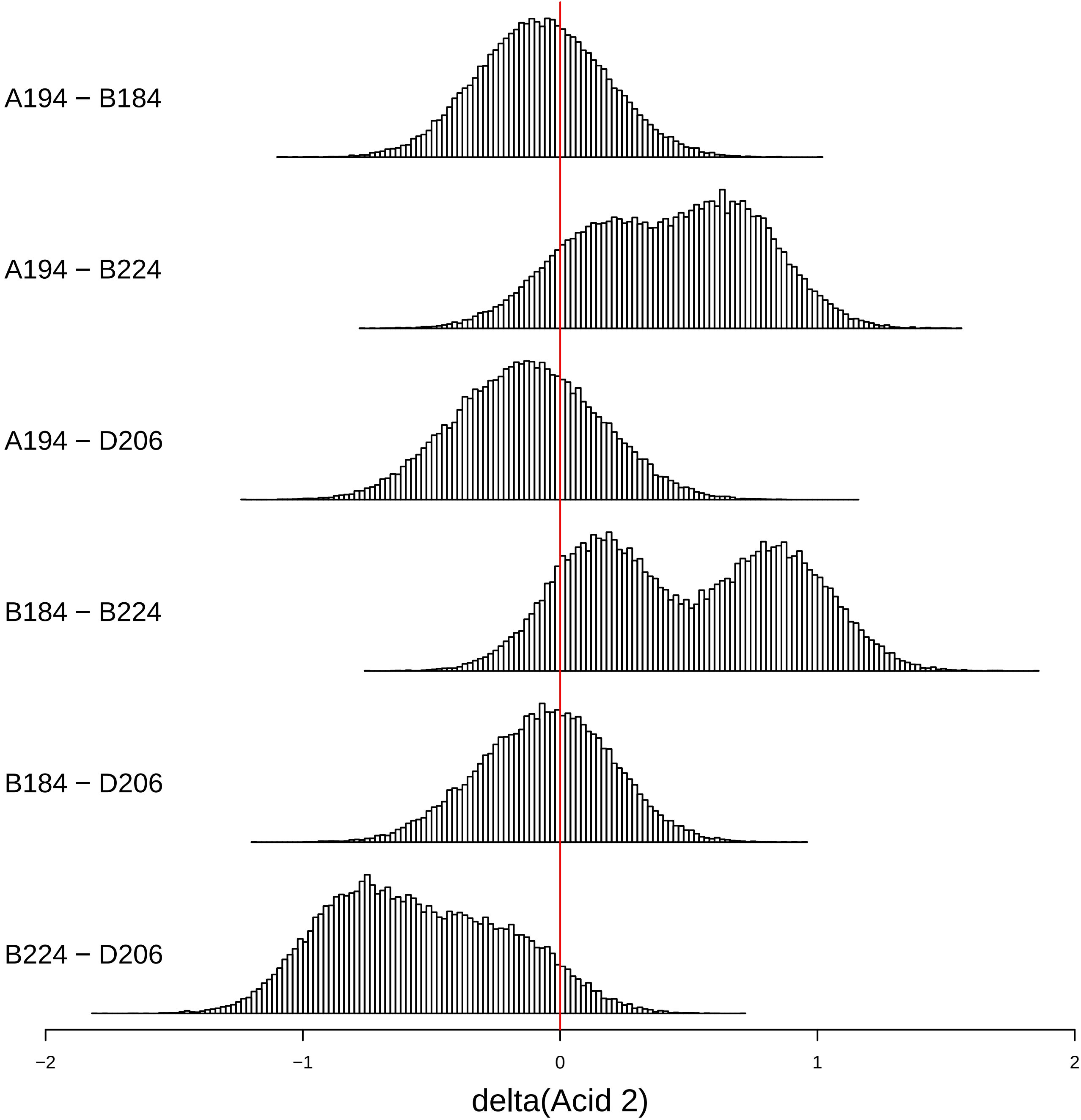
A194 − B184
A194 − B224
A194 − D206
B184 − B224
B184 − D206
B224 − D206
−2
−1
0
1
2
delta(Acid 2)

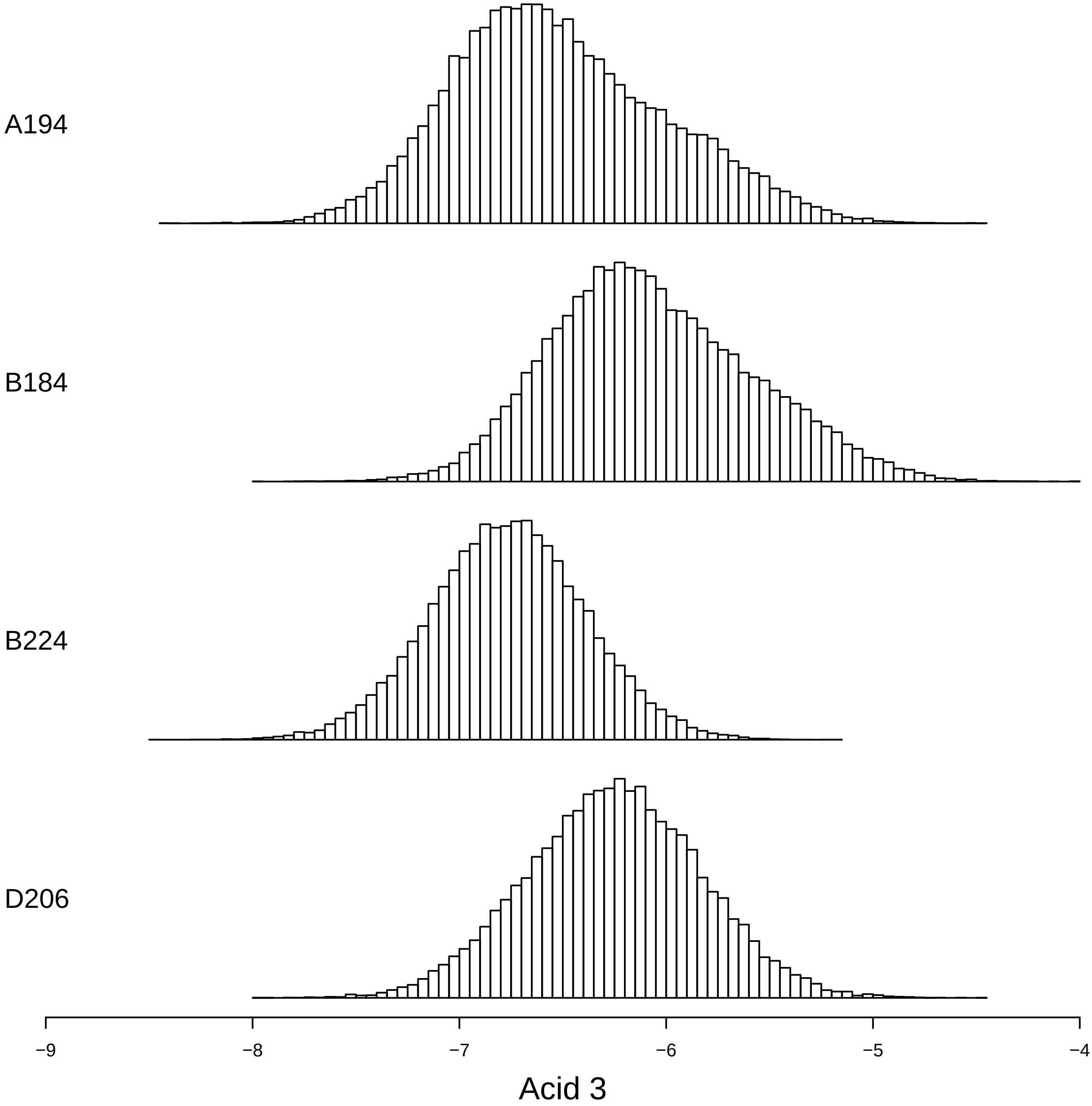
A194
B184
B224
D206
−9
−8
−7
−6
−5
−4
Acid 3

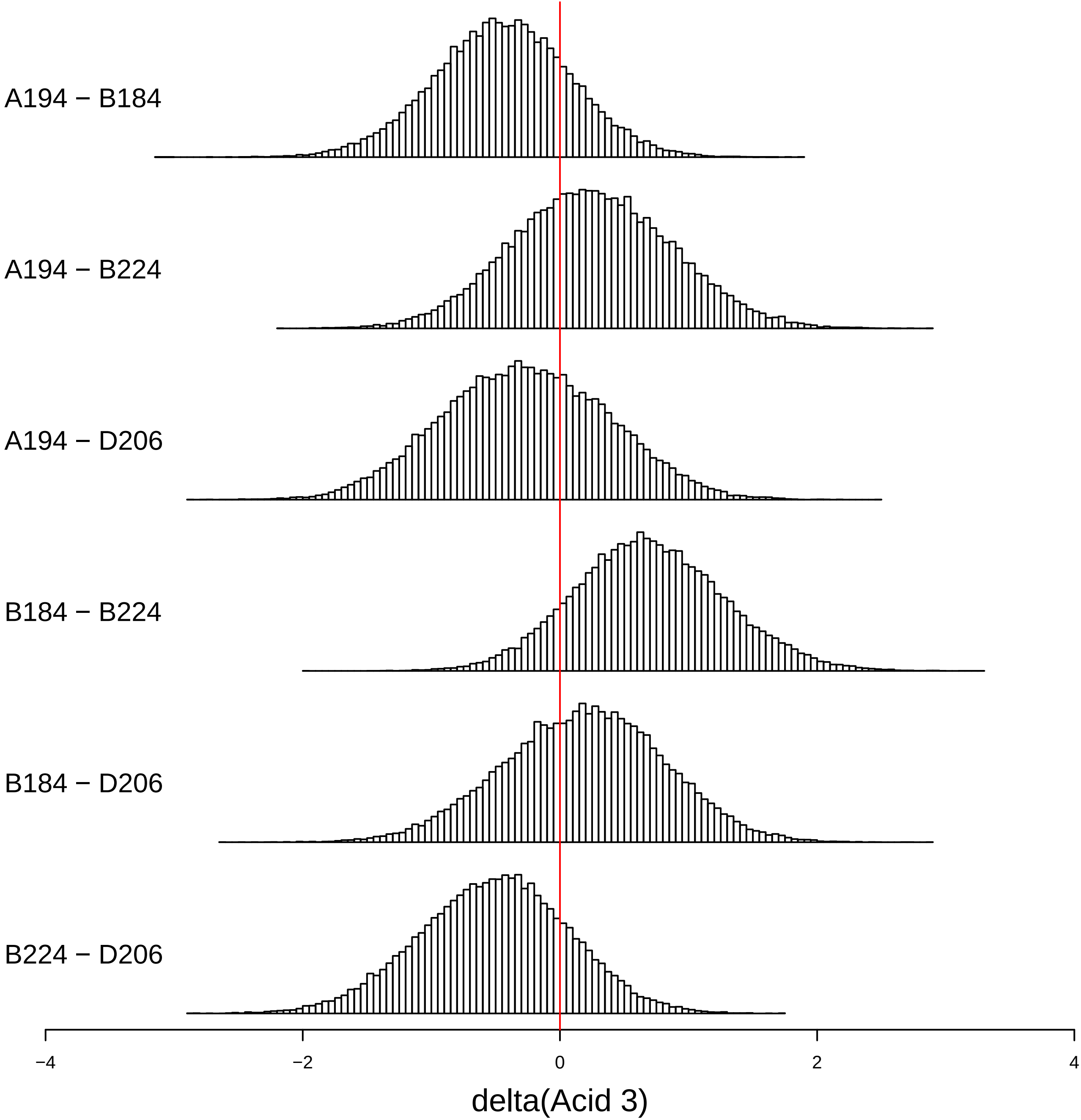

A194 − B184
A194 − B224
A194 − D206
B184 − B224
B184 − D206
B224 − D206
−4
−2
0
2
4
delta(Acid 3)

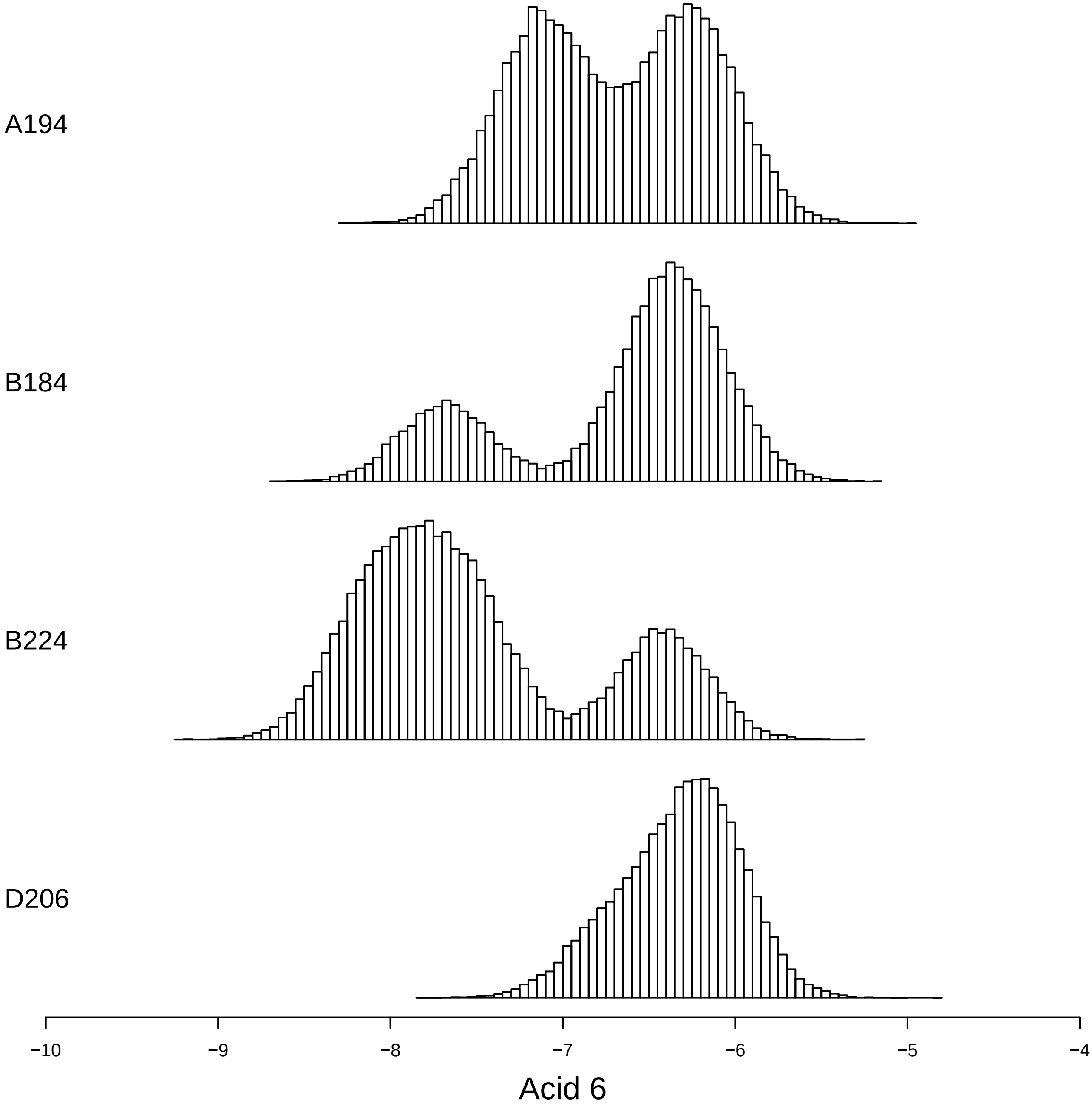
A194
B184
B224
D206
−10
−9
−8
−7
−6
−5
−4
Acid 6

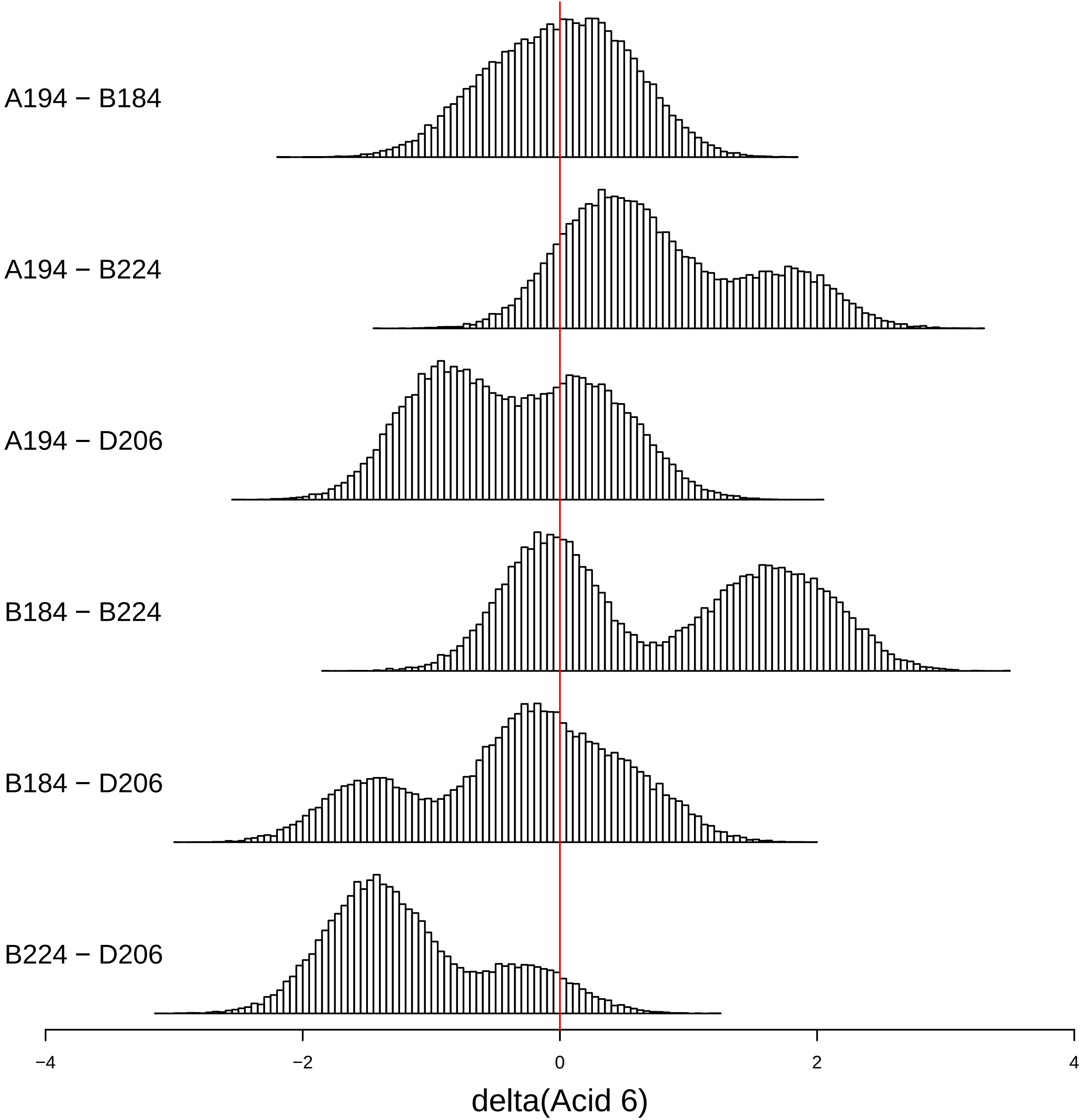
A194 − B184
A194 − B224
A194 − D206
B184 − B224
B184 − D206
B224 − D206
−4
−2
0
2
4
delta(Acid 6)

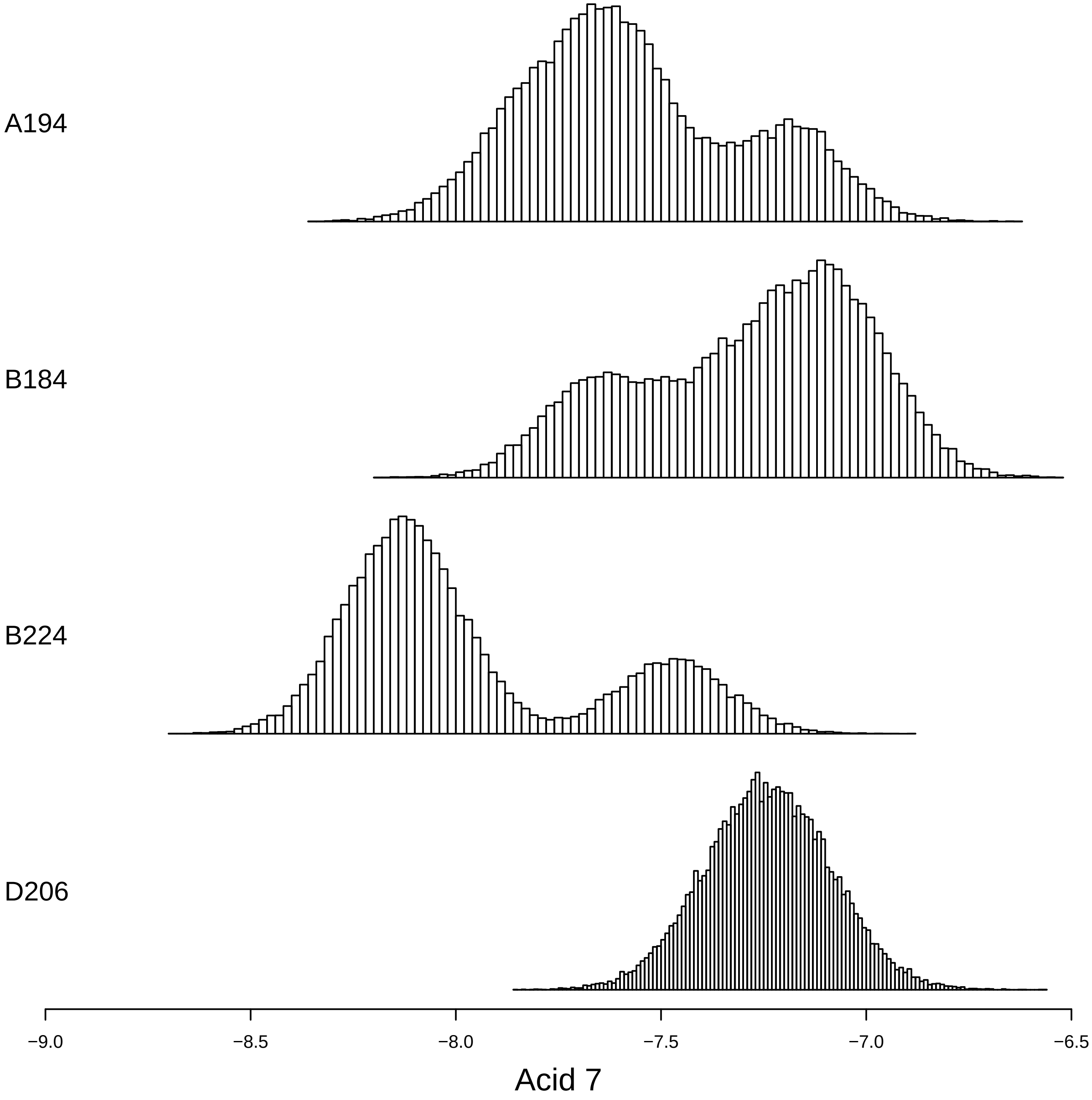
A194
B184
B224
D206
−9.0
−8.5
−8.0
−7.5
−7.0
−6.5
Acid 7

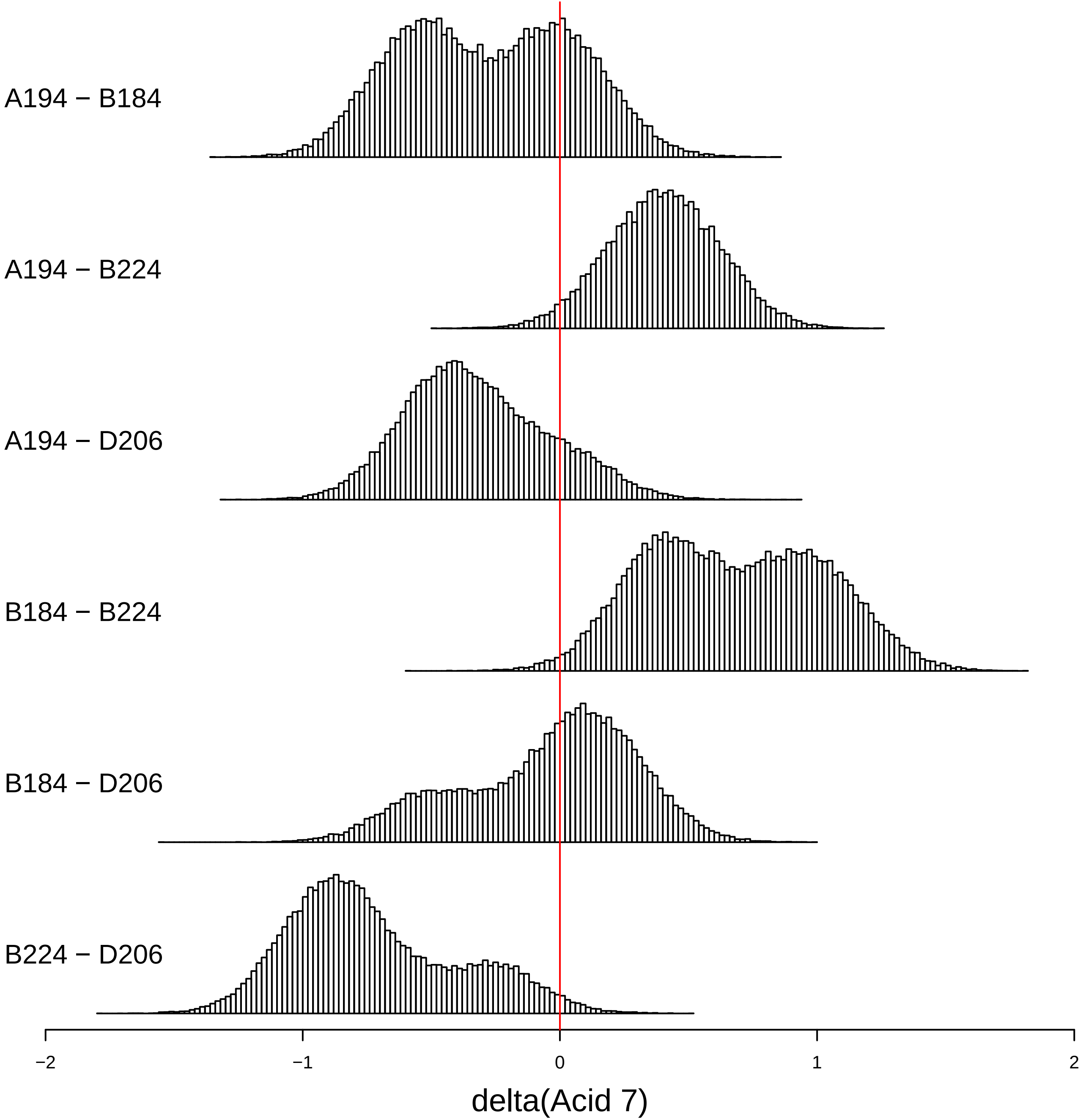
A194 − B184
A194 − B224
A194 − D206
B184 − B224
B184 − D206
B224 − D206
−2
−1
0
1
2
delta(Acid 7)

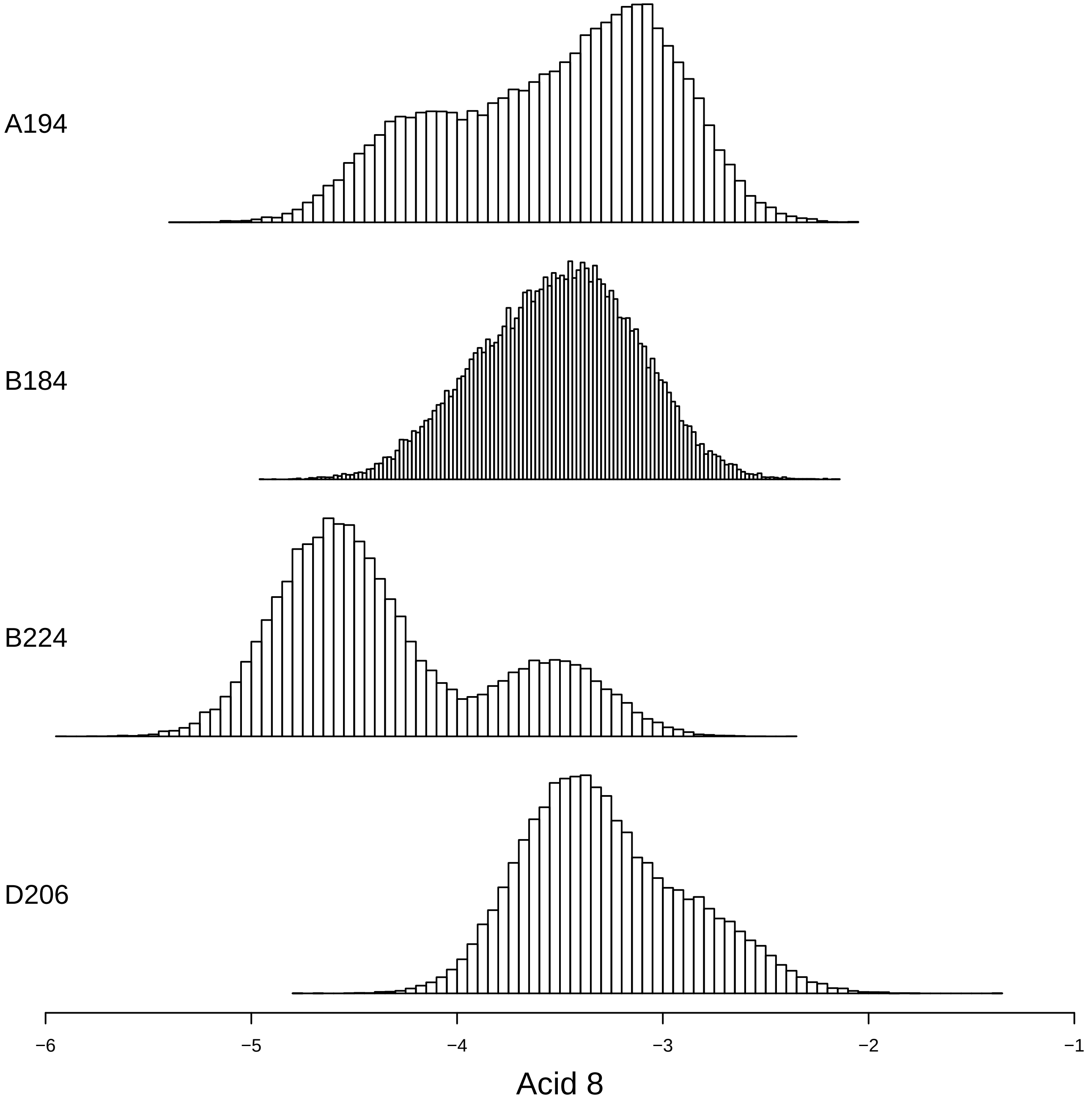
A194
B184
B224
D206
−6
−5
−4
−3
−2
−1
Acid 8

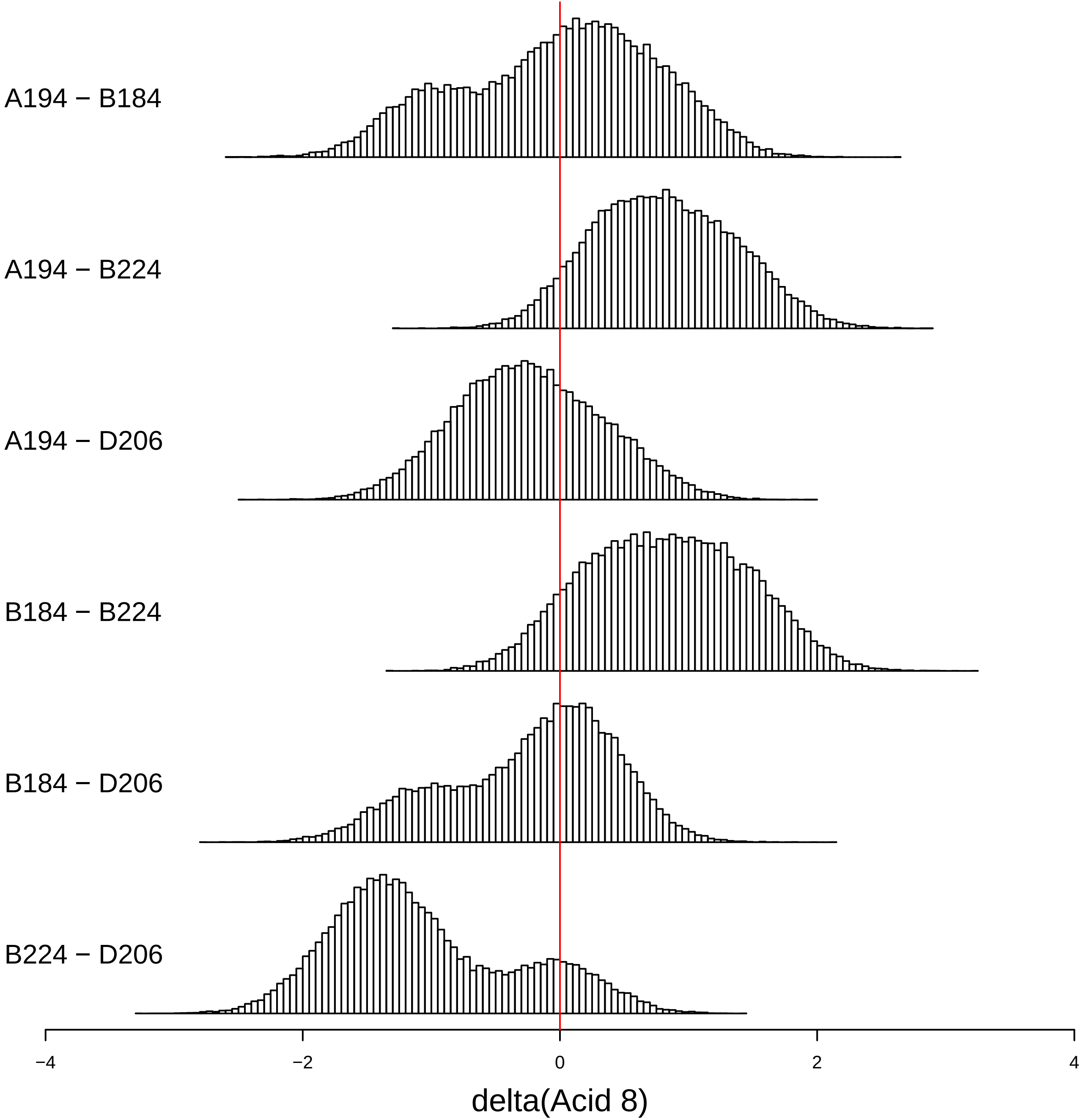
A194 − B184
A194 − B224
A194 − D206
B184 − B224
B184 − D206
B224 − D206
−4
−2
0
2
4
delta(Acid 8)

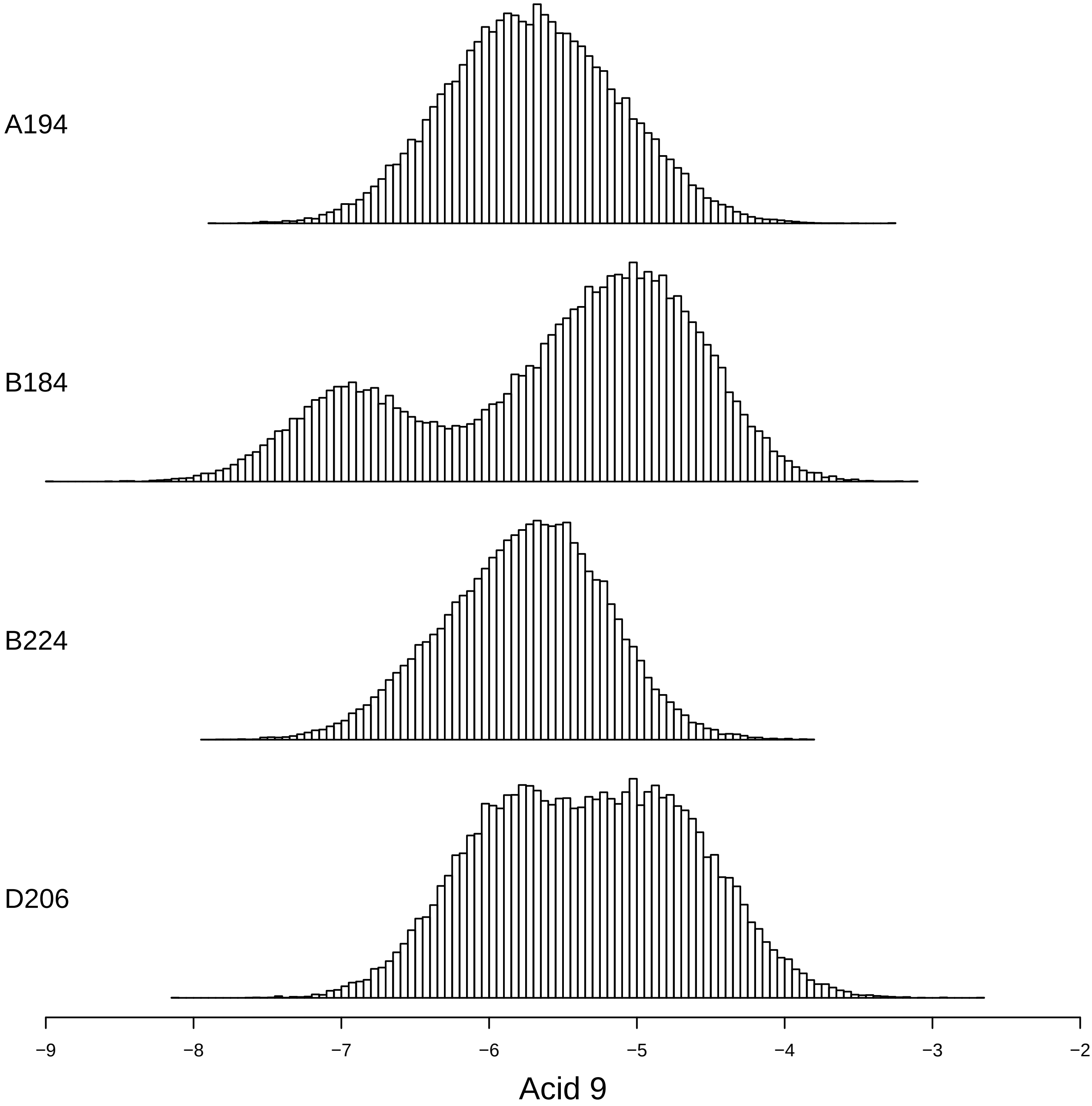
A194
B184
B224
D206
−9
−8
−7
−6
−5
−4
−3
−2
Acid 9

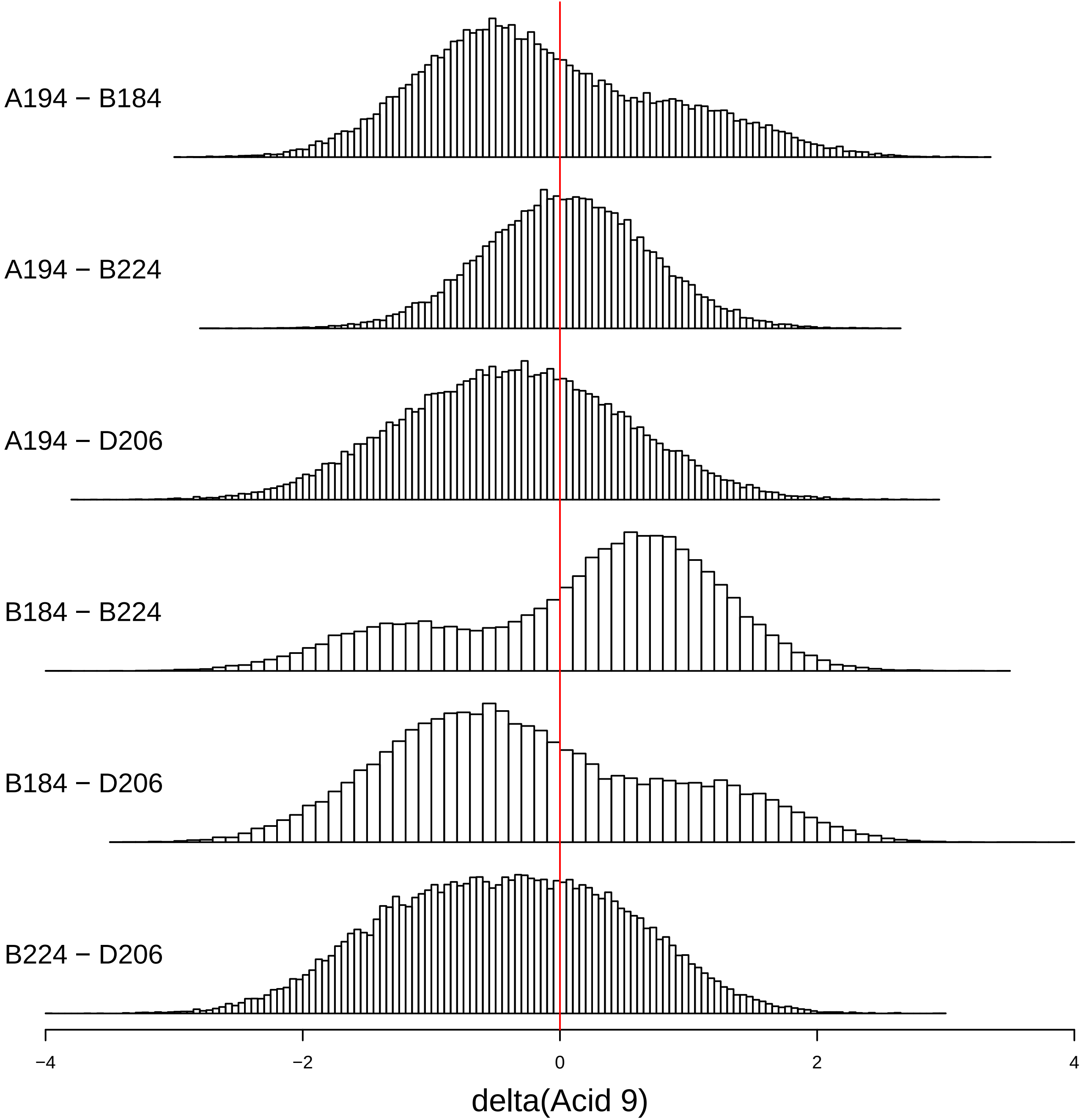

A194 − B184
A194 − B224
A194 − D206
B184 − B224
B184 − D206
B224 − D206
−4
−2
0
2
4
delta(Acid 9)

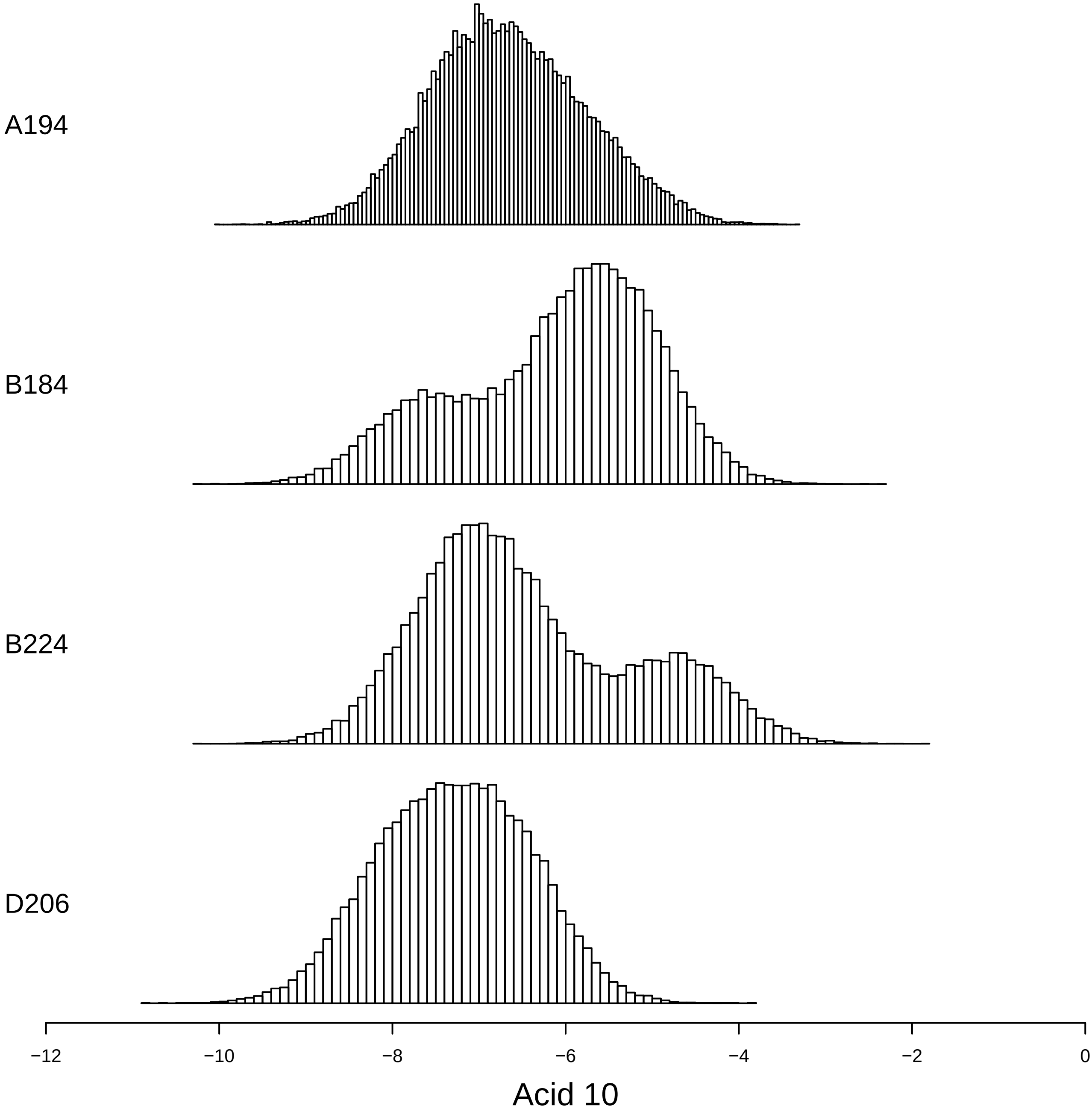

A194
B184
B224
D206
−12
−10
−8
−6
−4
−2
0
Acid 10

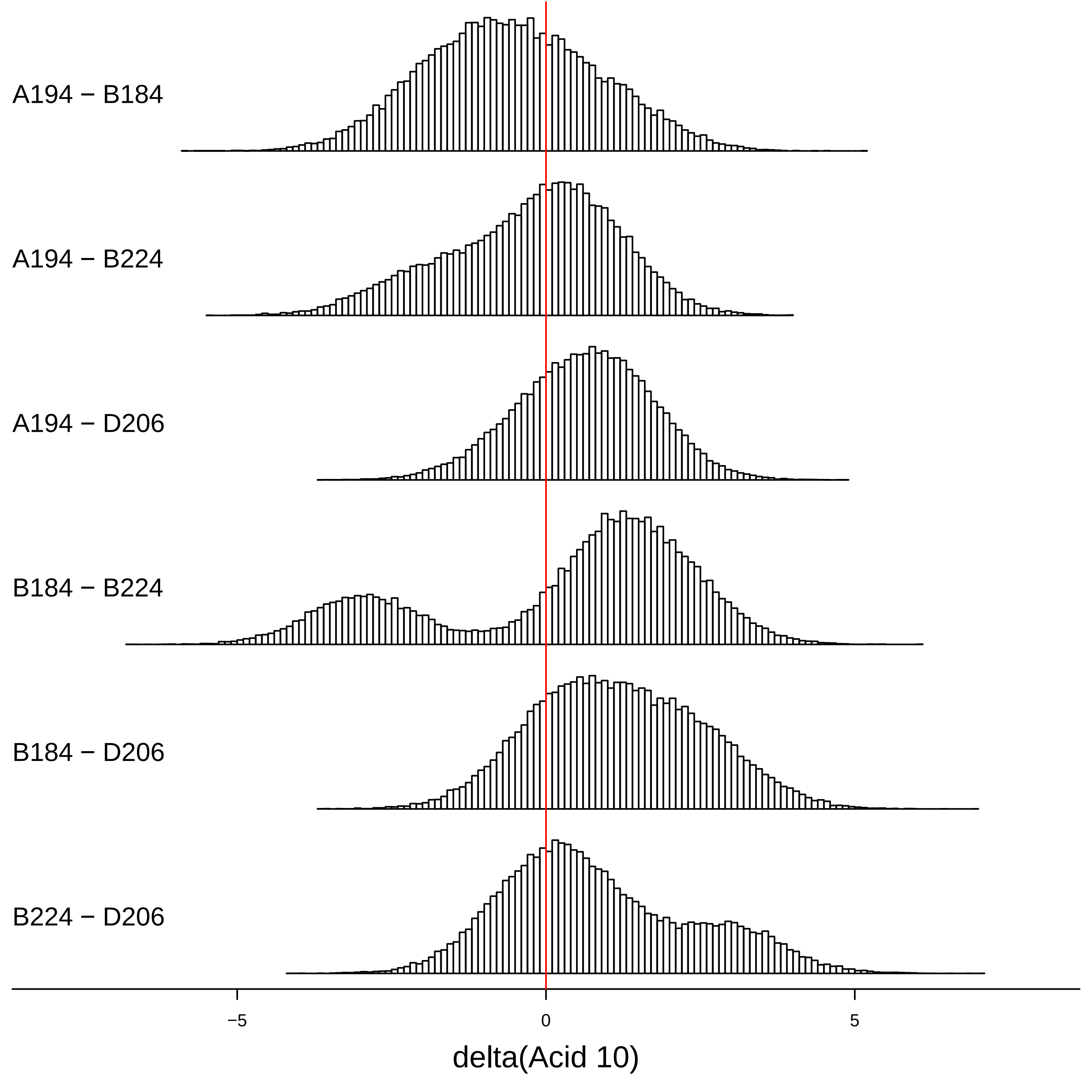
A194 − B184
A194 − B224
A194 − D206
B184 − B224
B184 − D206
B224 − D206
−5
0
5
delta(Acid 10)

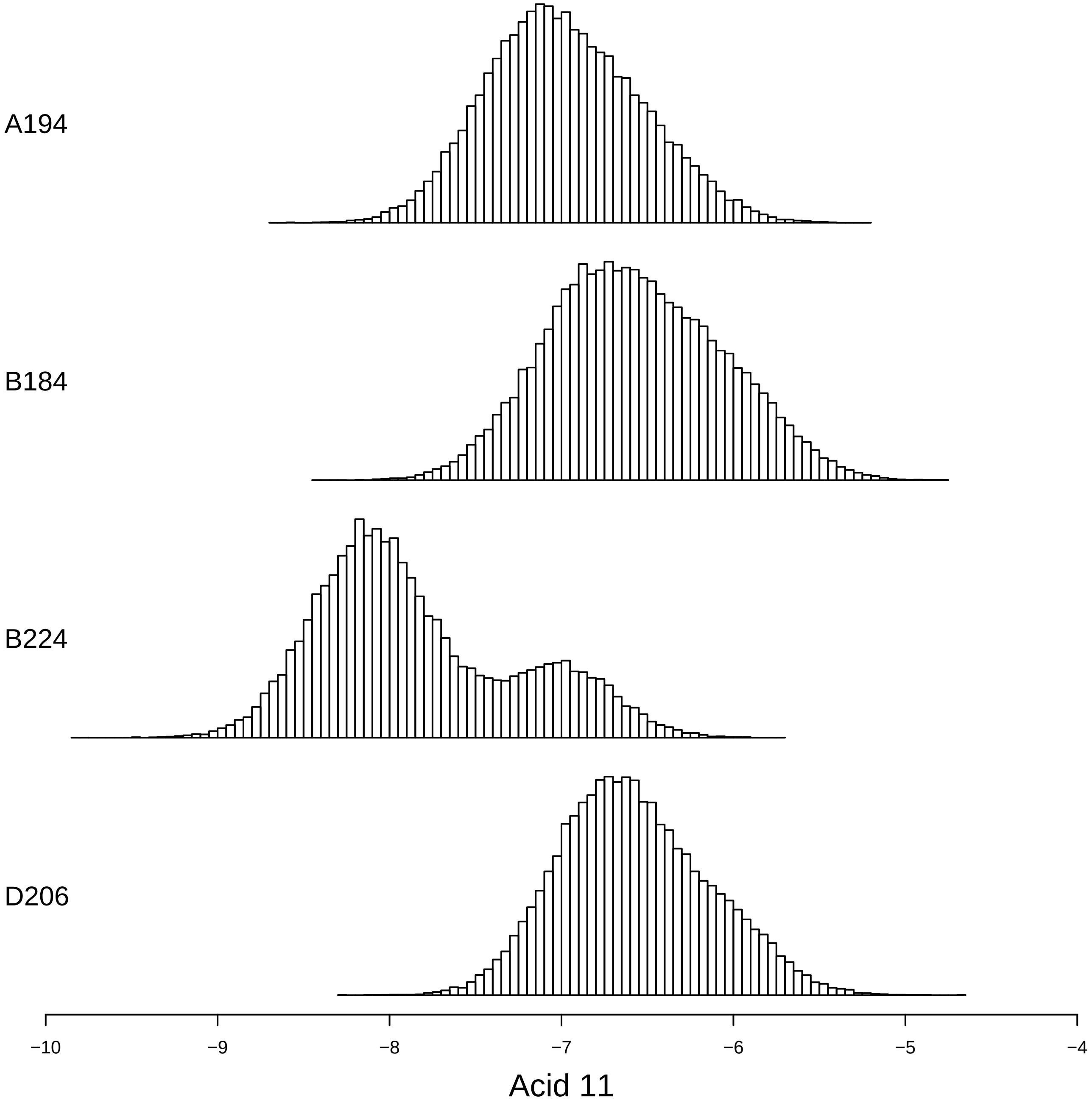

A194
B184
B224
D206
−10
−9
−8
−7
−6
−5
−4
Acid 11

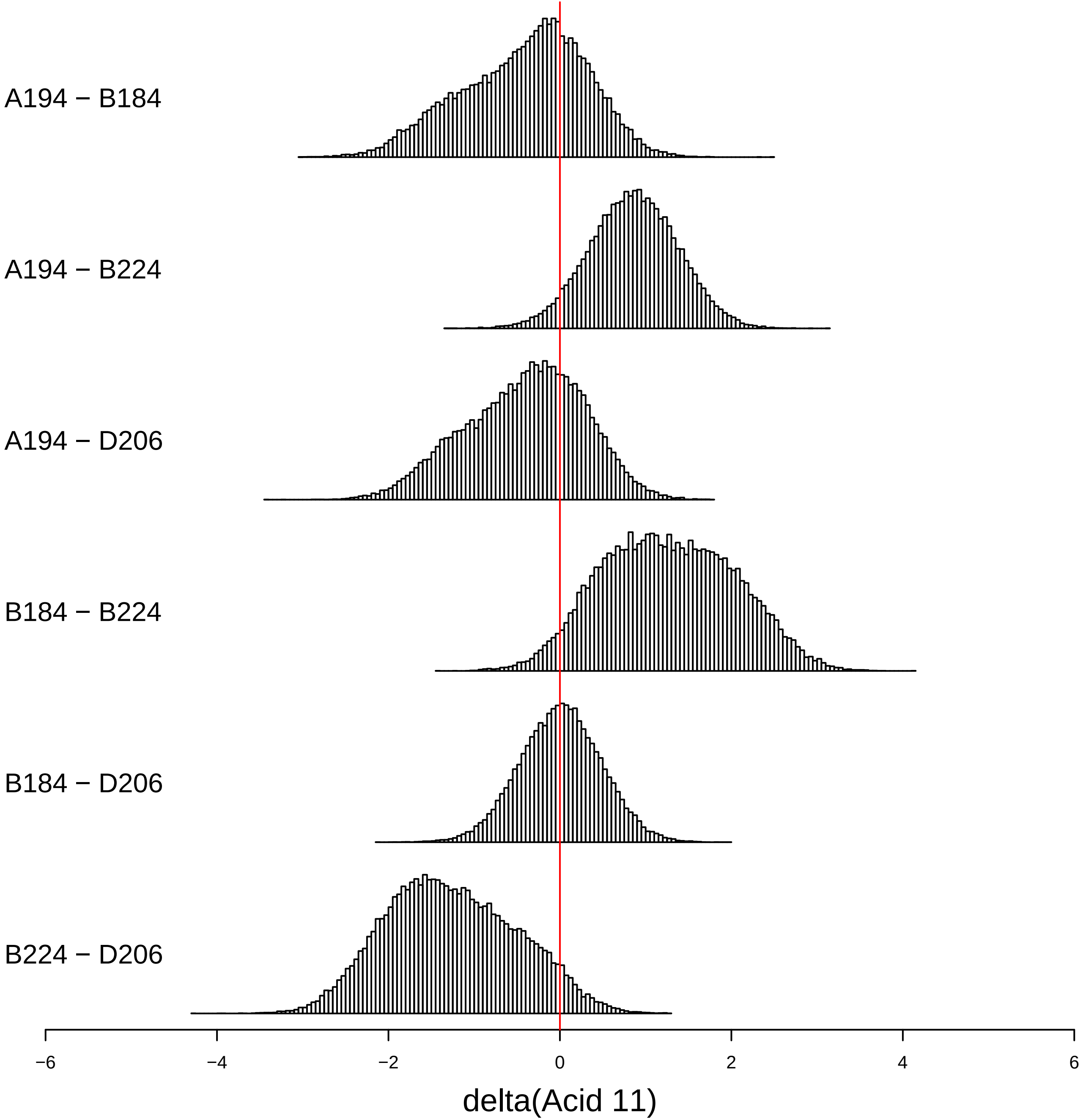
A194 − B184
A194 − B224
A194 − D206
B184 − B224
B184 − D206
B224 − D206
−6
−4
−2
0
2
4
6
delta(Acid 11)

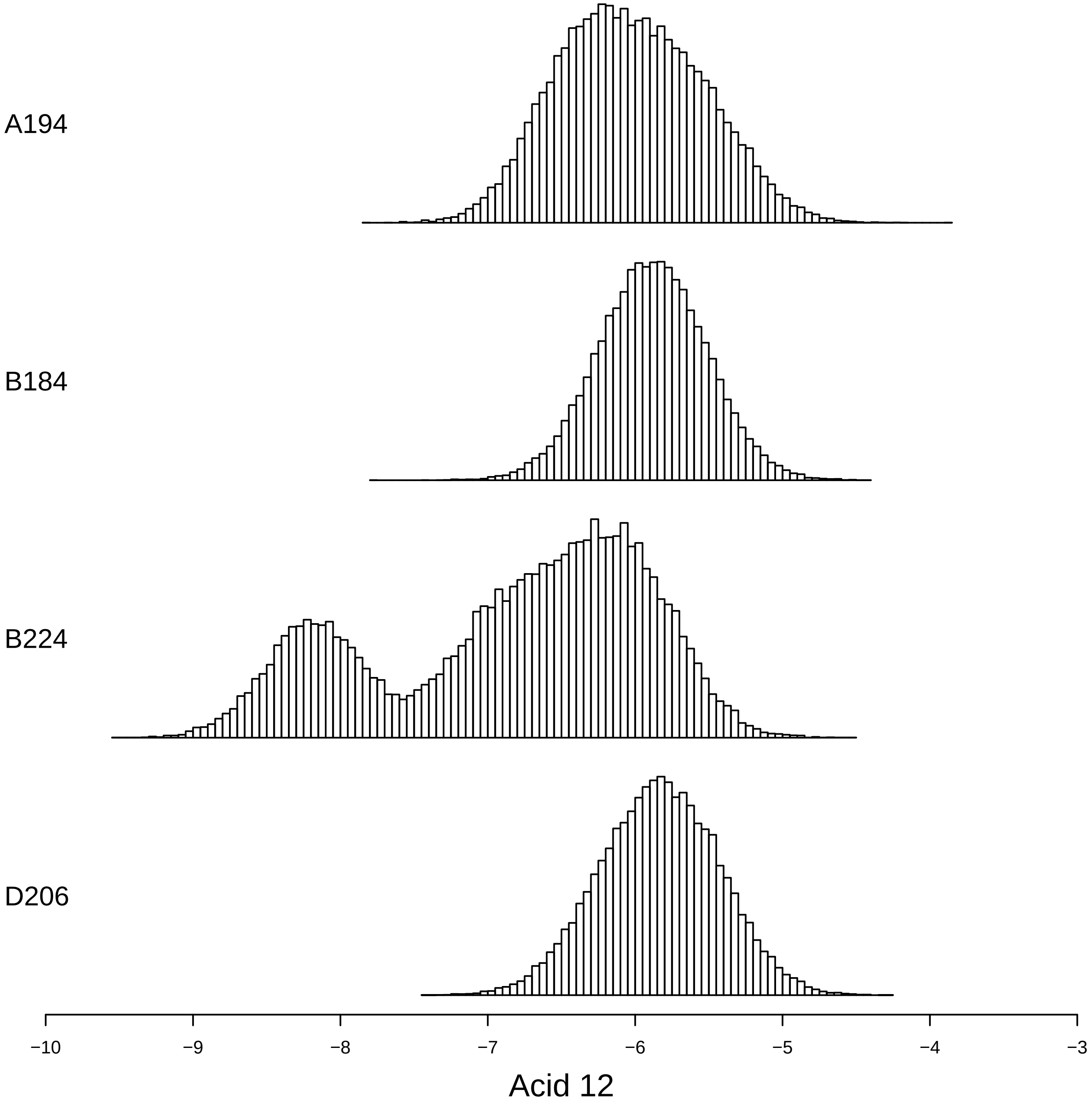
A194
B184
B224
D206
−10
−9
−8
−7
−6
−5
−4
−3
Acid 12

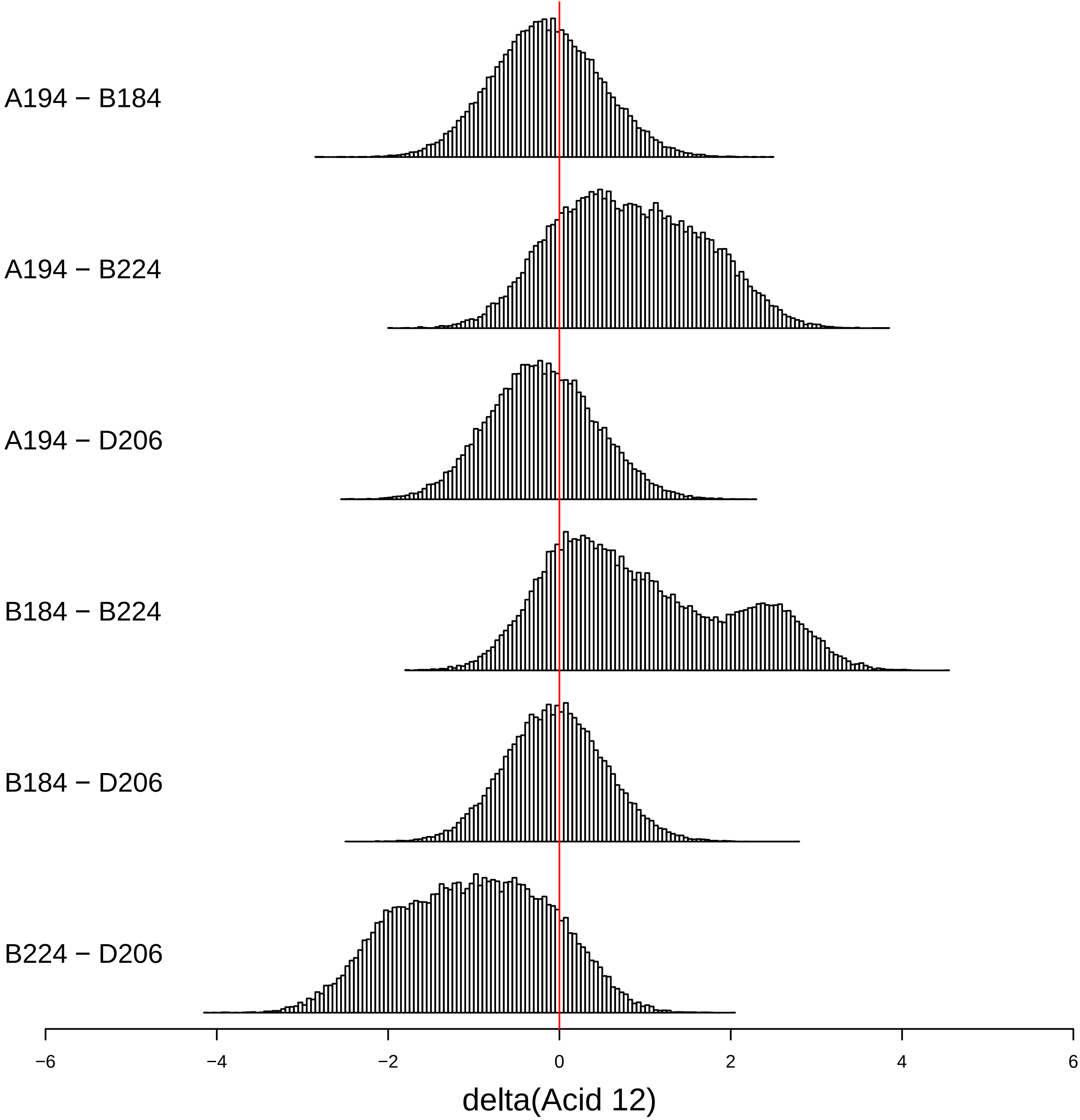
A194 − B184
A194 − B224
A194 − D206
B184 − B224
B184 − D206
B224 − D206
−6
−4
−2
0
2
4
6
delta(Acid 12)

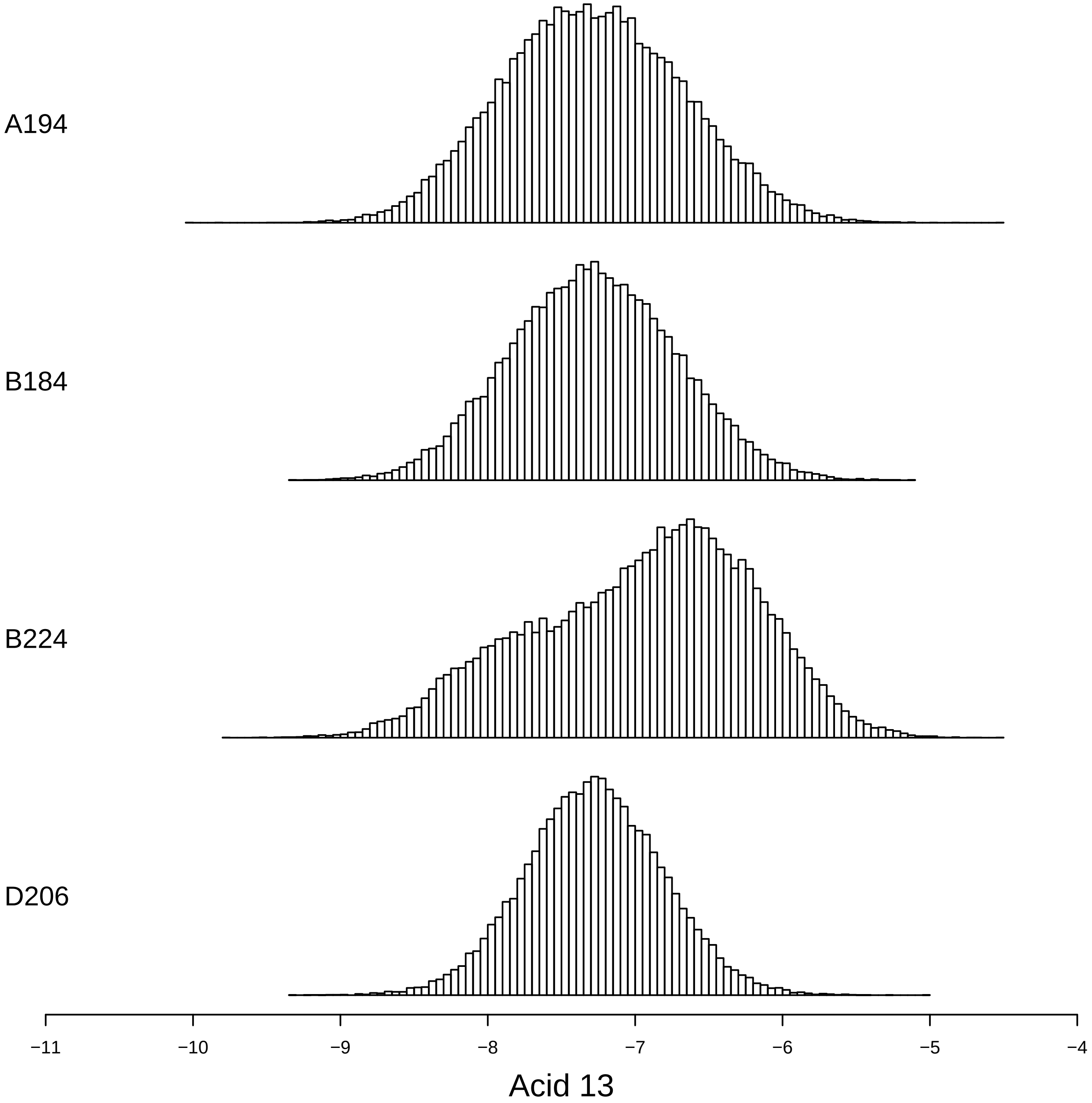
A194
B184
B224
D206
−11
−10
−9
−8
−7
−6
−5
−4
Acid 13

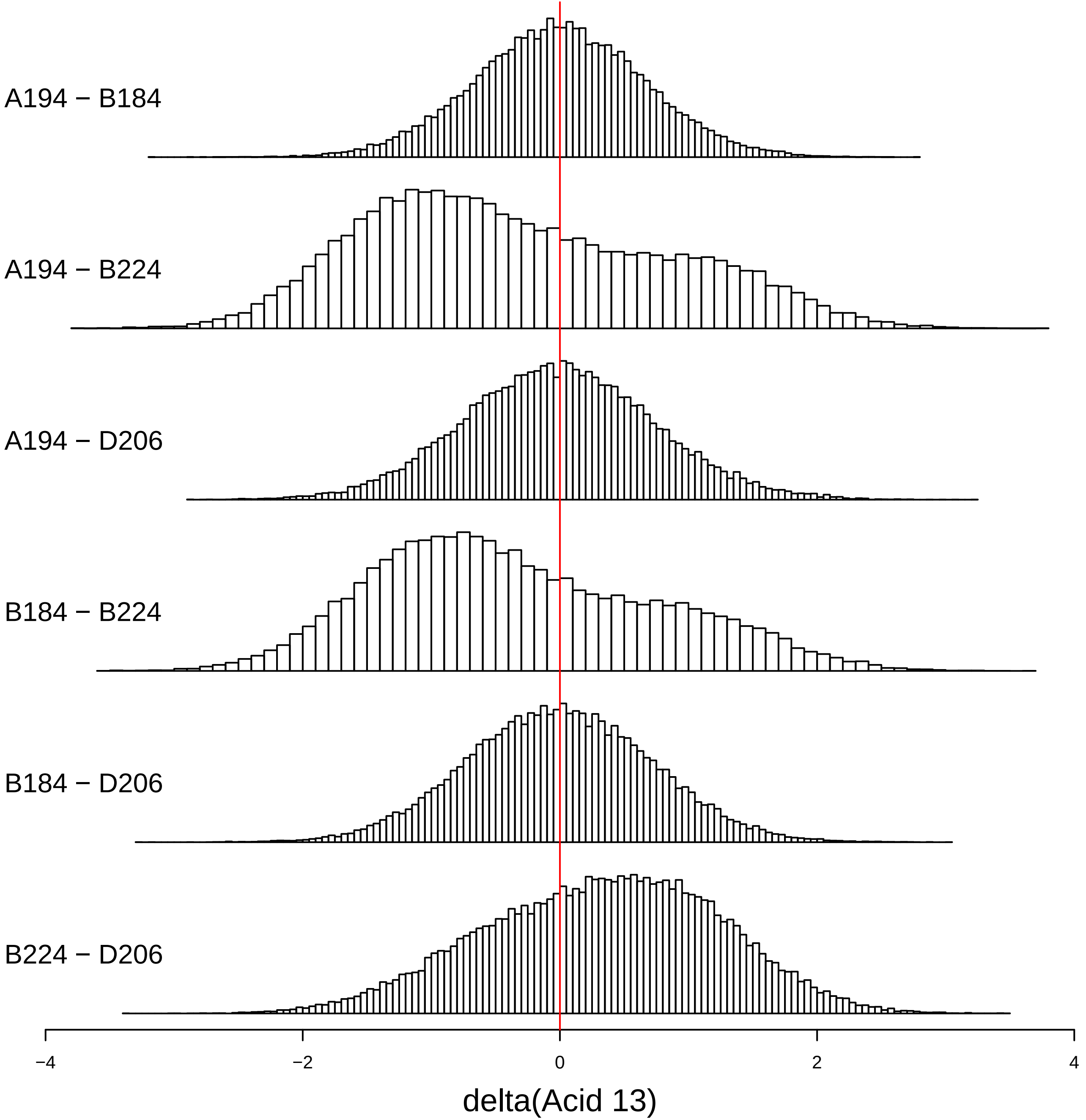
A194 − B184
A194 − B224
A194 − D206
B184 − B224
B184 − D206
B224 − D206
−4
−2
0
2
4
delta(Acid 13)

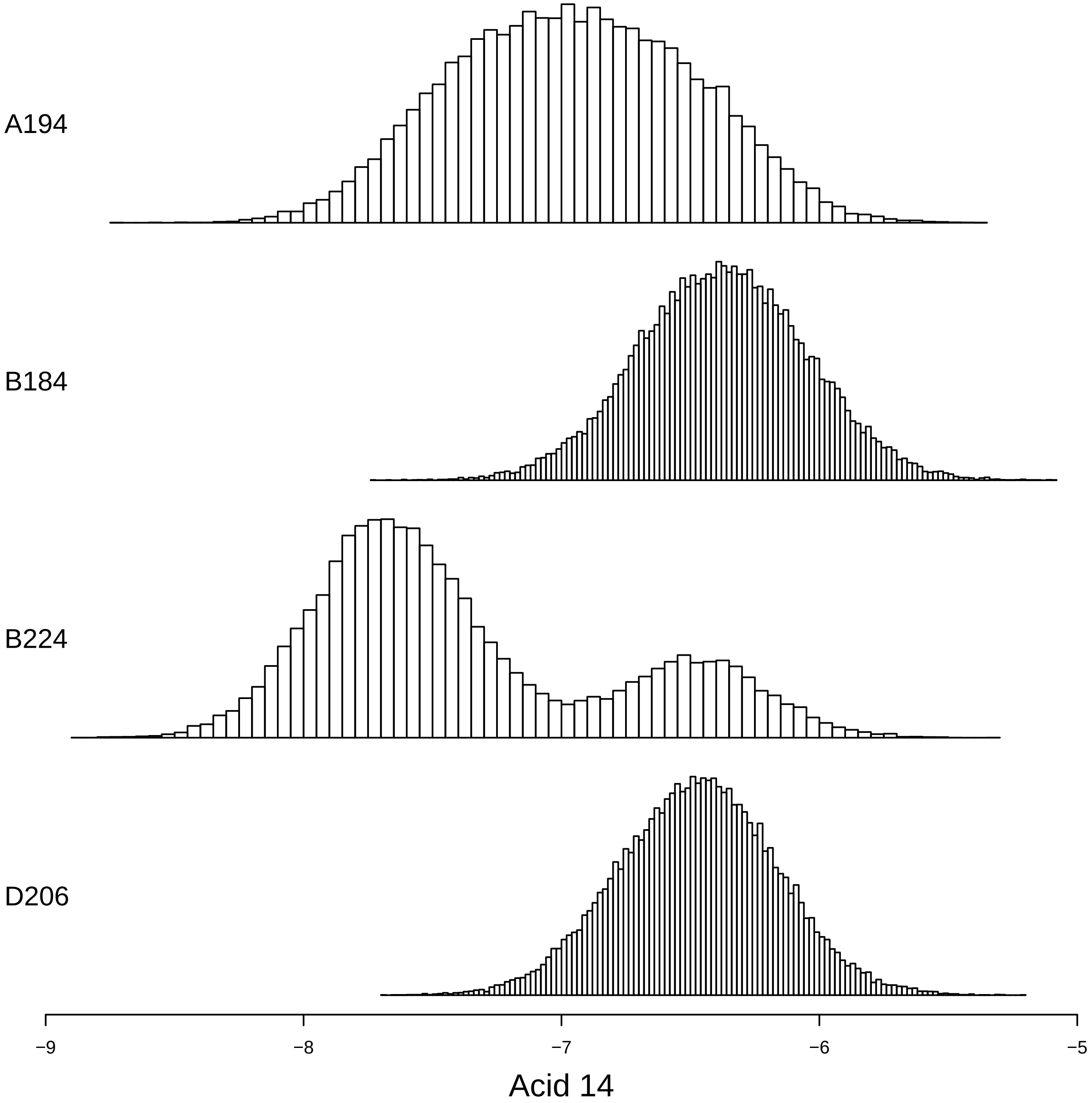
A194
B184
B224
D206
−9
−8
−7
−6
−5
Acid 14

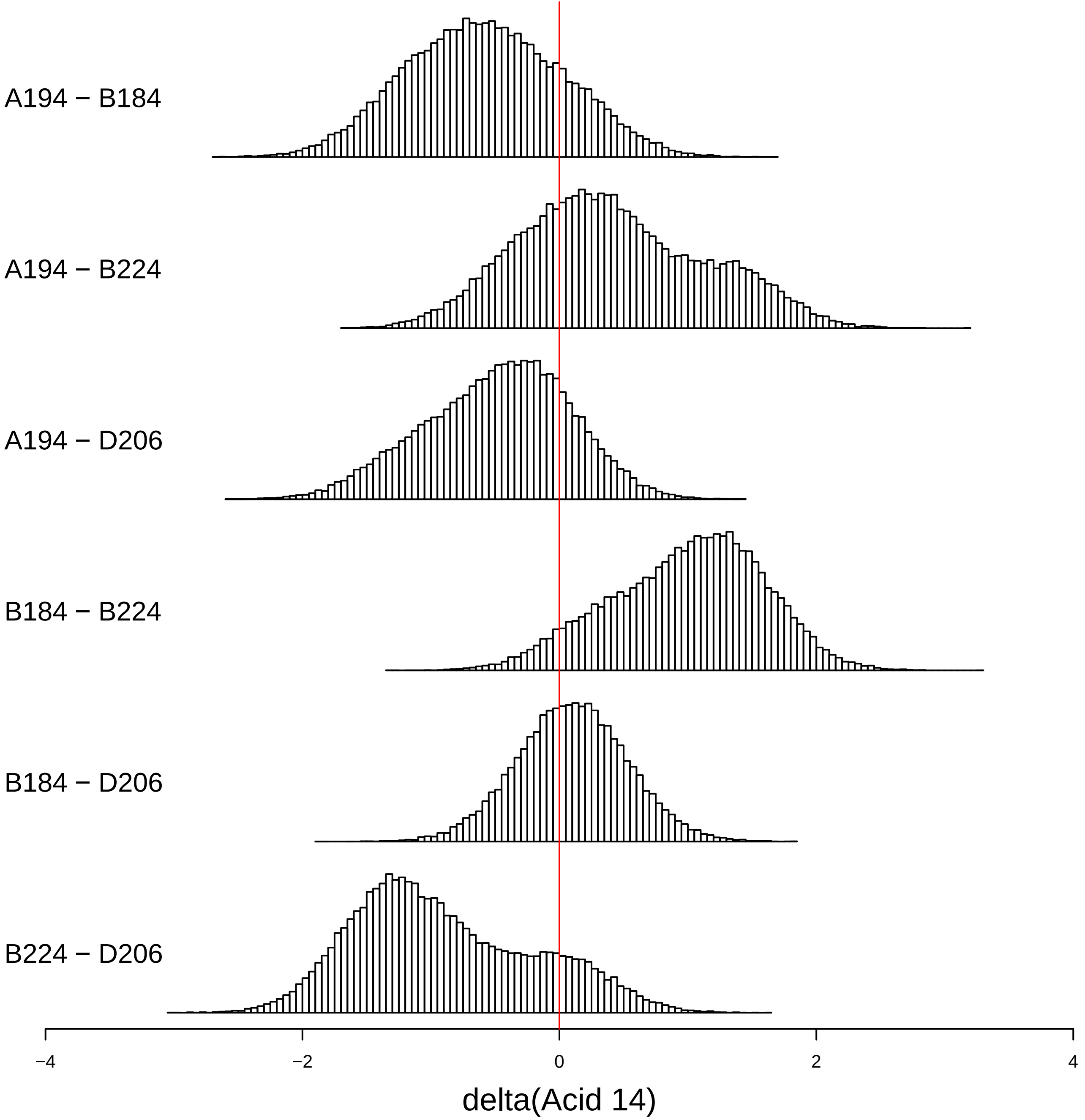
A194 − B184
A194 − B224
A194 − D206
B184 − B224
B184 − D206
B224 − D206
−4
−2
0
2
4
delta(Acid 14)
