# Supplementary material for: Taxonomic and Environmental Variation of Metabolite Profiles in Marine Dinoflagellates of the Genus Symbiodinium
Source: Metabolites. 2015 Feb 16;5(1):74–99. doi: 10.3390/metabo5010074 (PMC4381291; doi:10.3390/metabo5010074)
Supplement: Supplementary File 1 [file metabolites-05-00074-s001.zip › Supplementary Information/Supplementary Information Figure S4a - temp.A194.pdf]

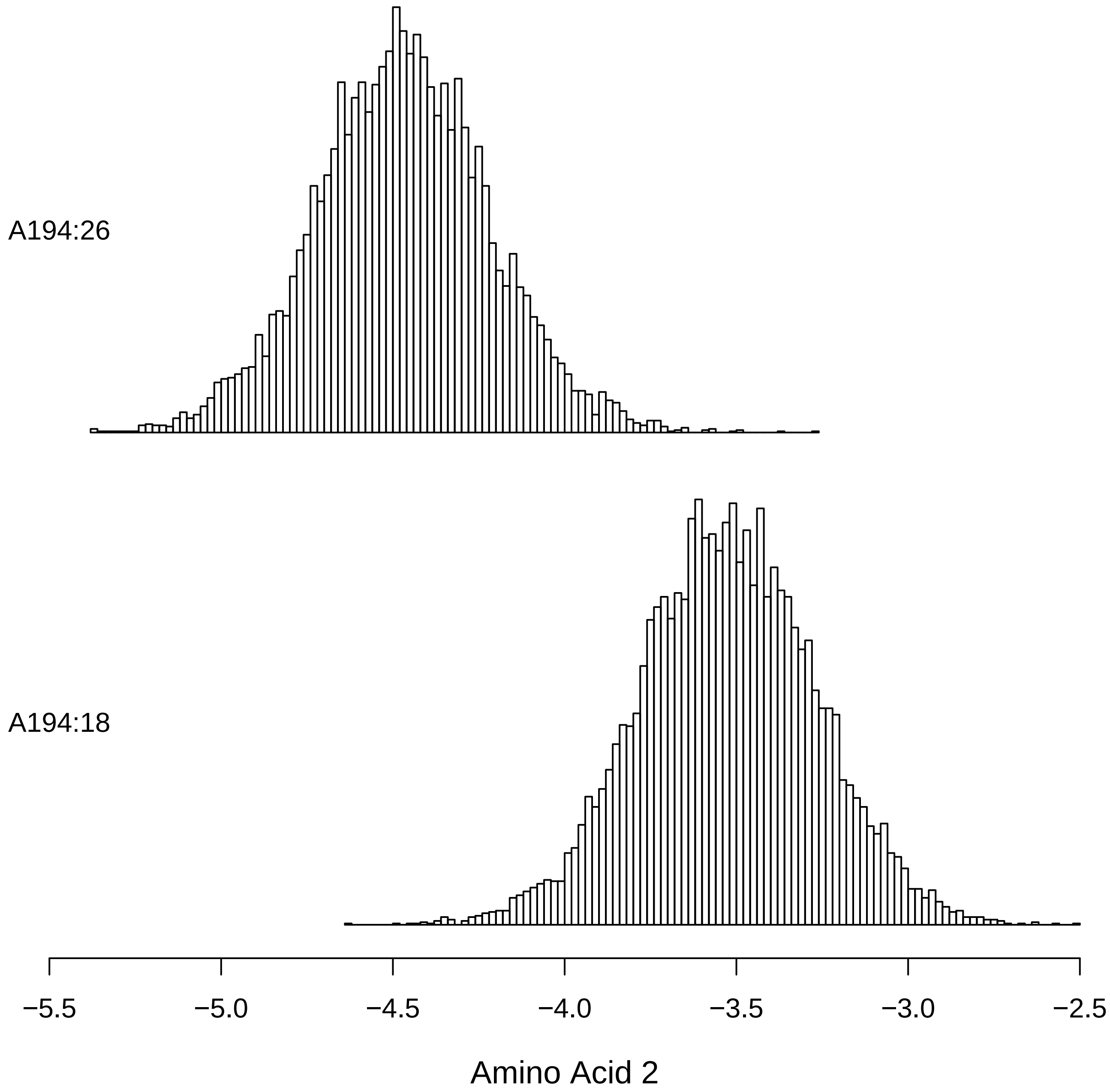

A194:26
A194:18
−5.5
−5.0
−4.5
−4.0
−3.5
−3.0
−2.5
Amino Acid 2

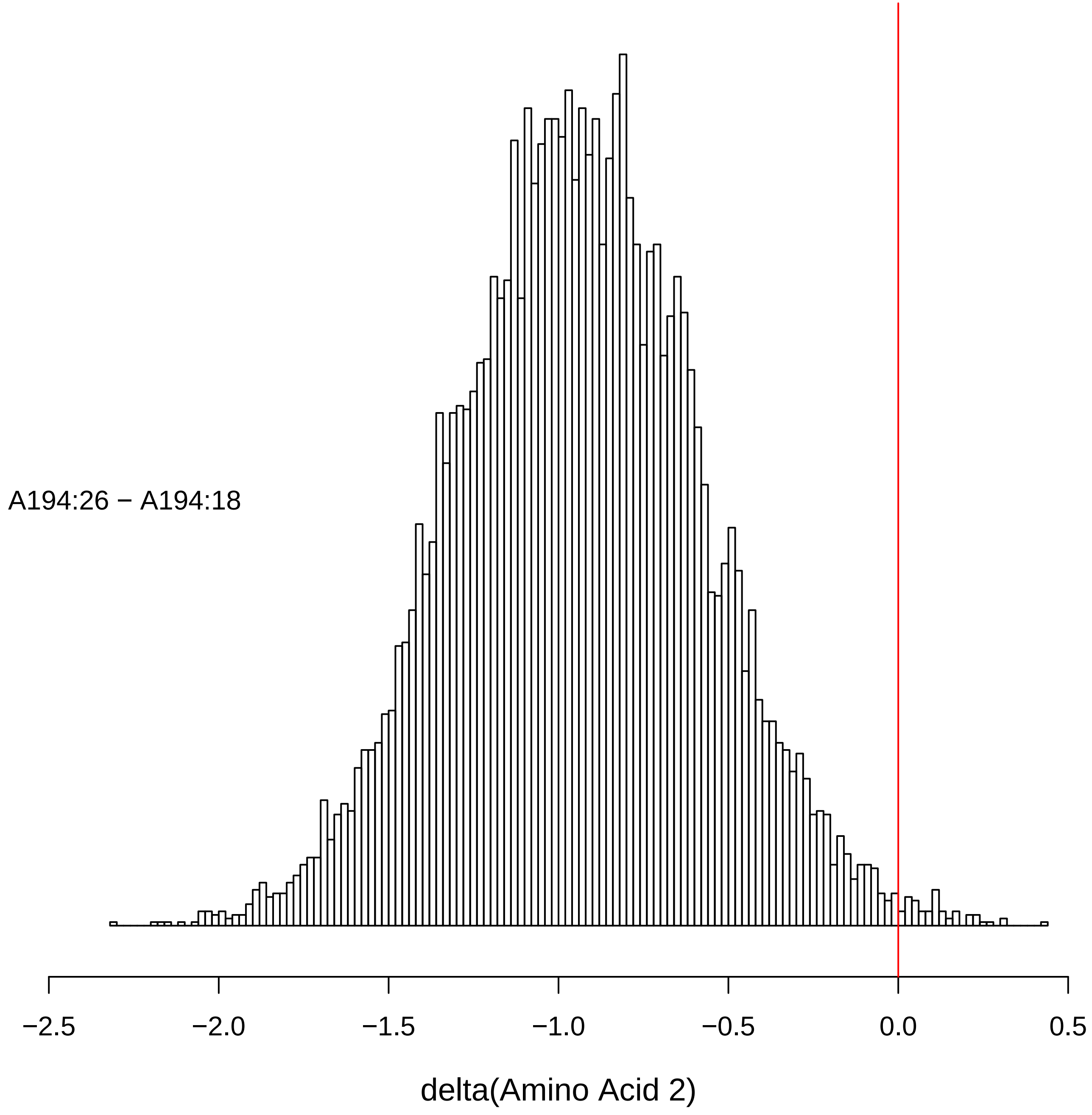
A194:26 − A194:18
−2.5
−2.0
−1.5
−1.0
−0.5
0.0
0.5
delta(Amino Acid 2)

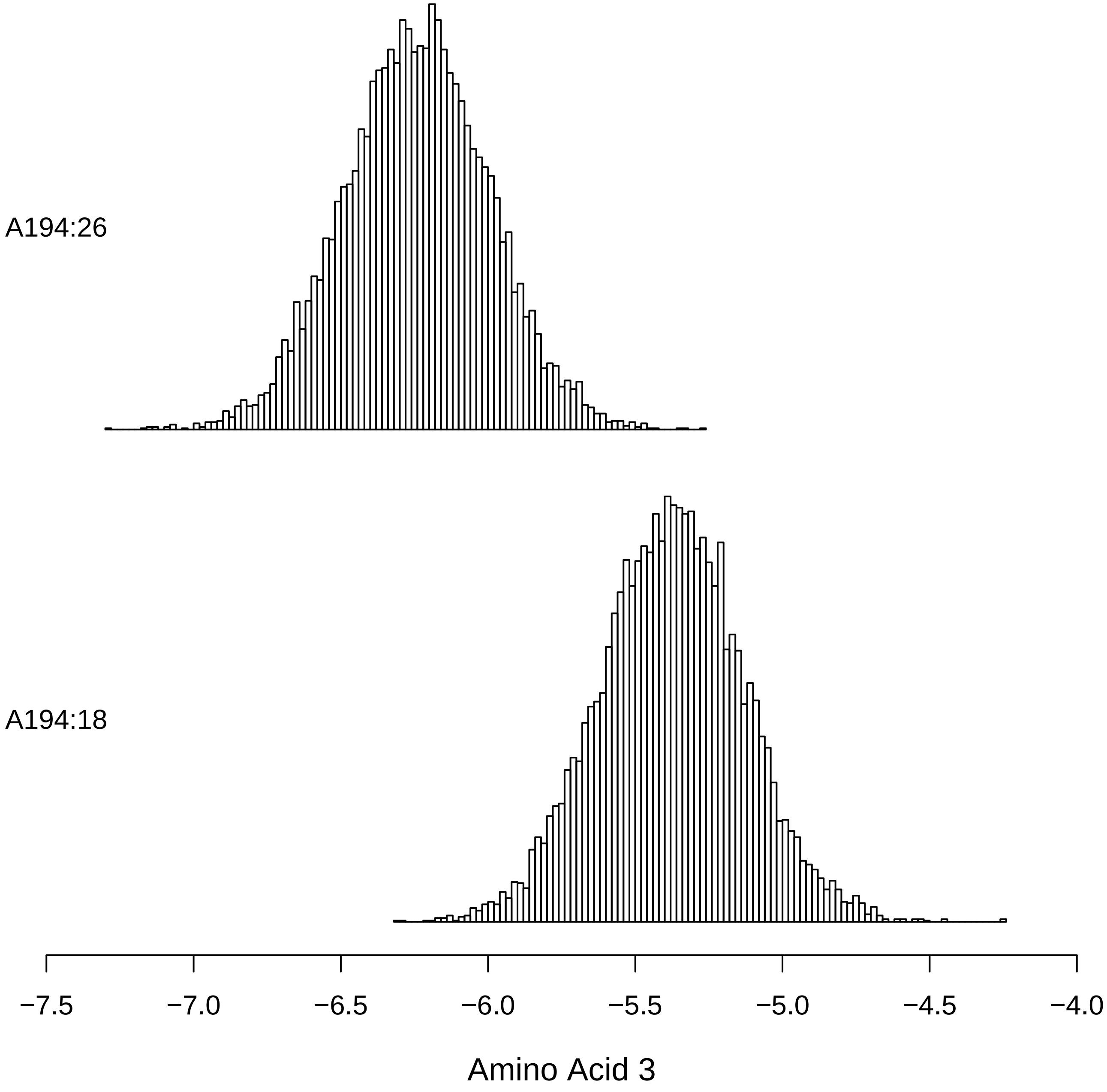
A194:26
A194:18
−7.5
−7.0
−6.5
−6.0
−5.5
−5.0
−4.5
−4.0
Amino Acid 3

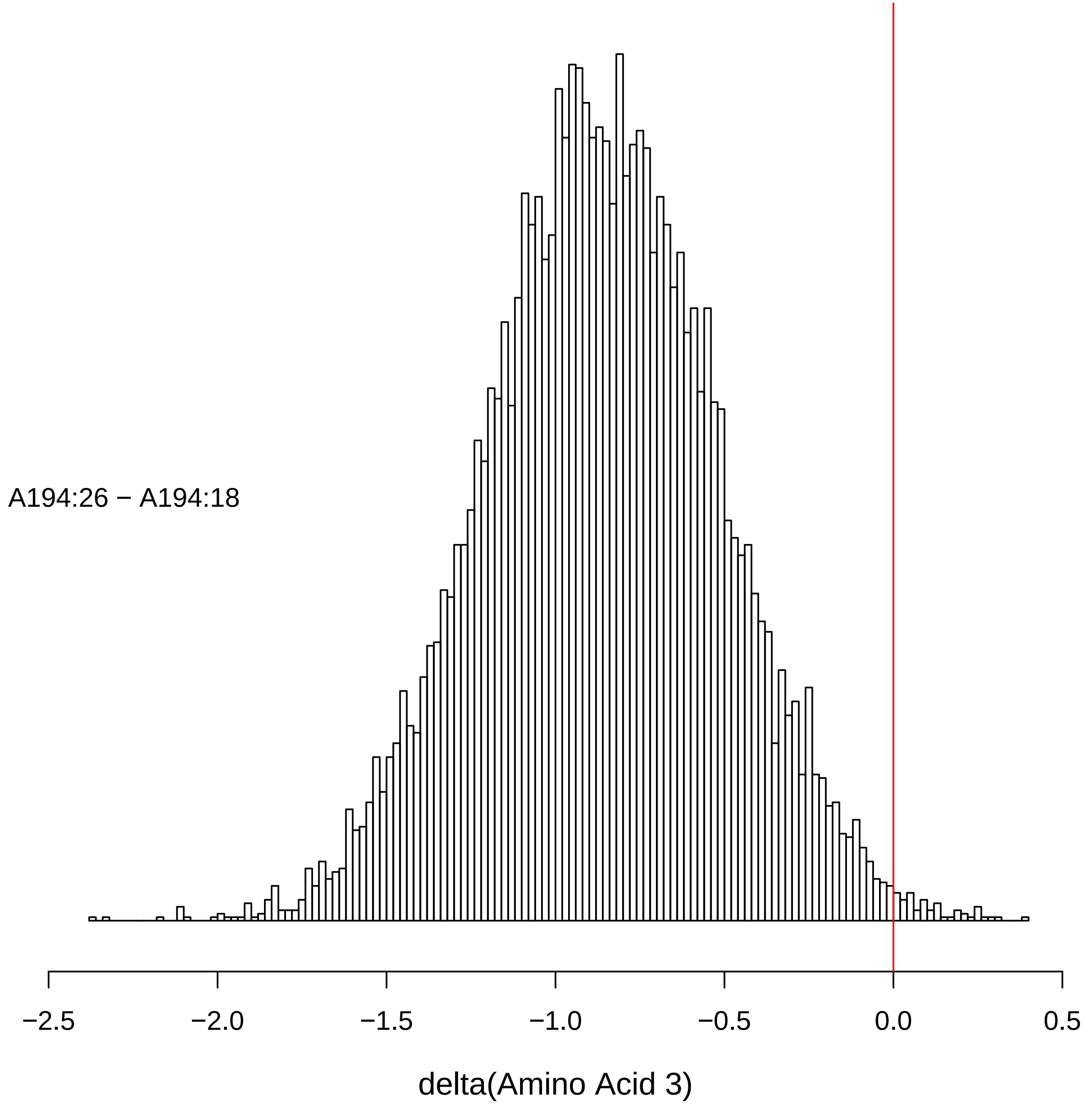
A194:26 − A194:18
−2.5
−2.0
−1.5
−1.0
−0.5
0.0
0.5
delta(Amino Acid 3)

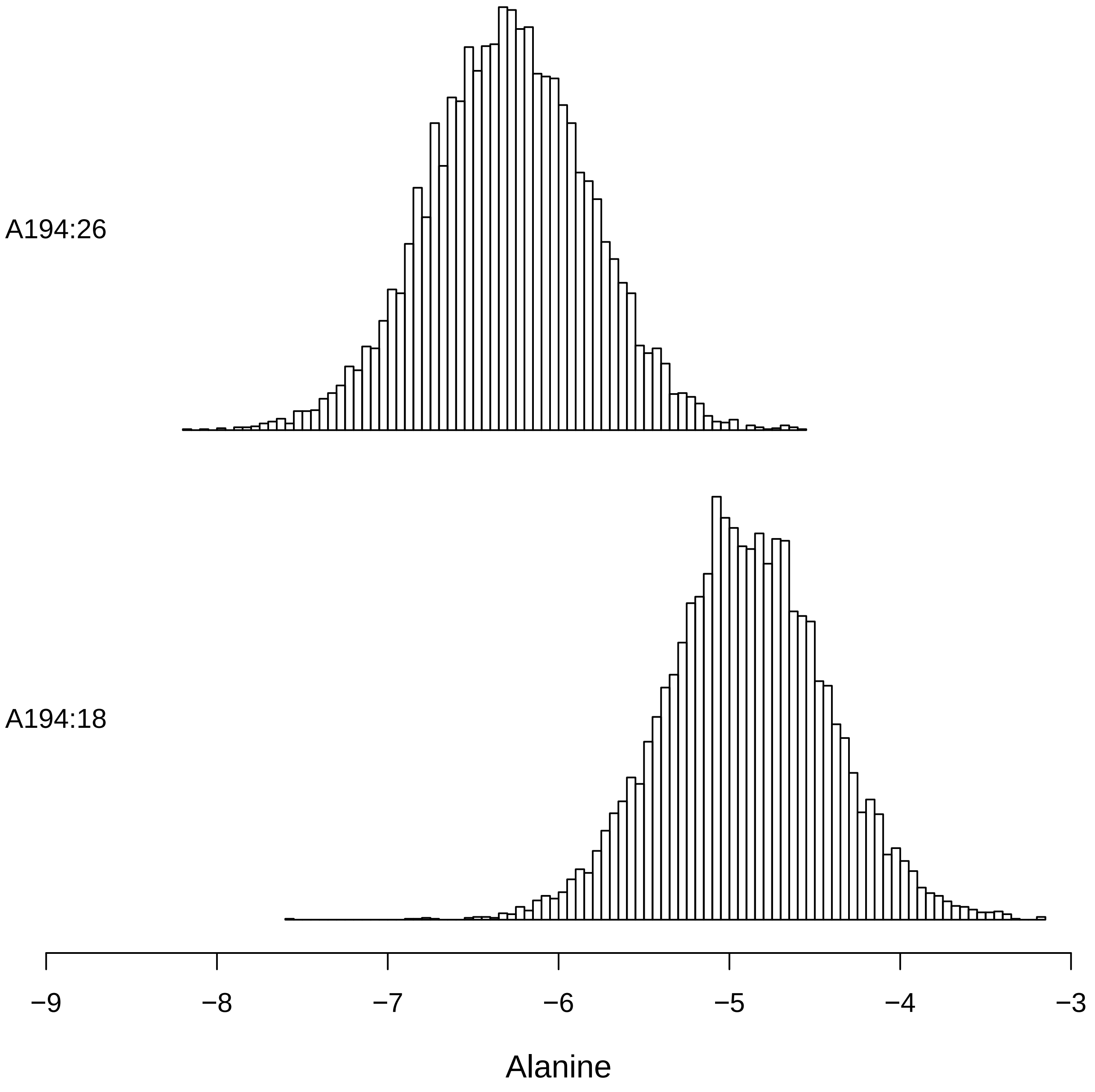
A194:26
A194:18
−9
−8
−7
−6
−5
−4
−3
Alanine

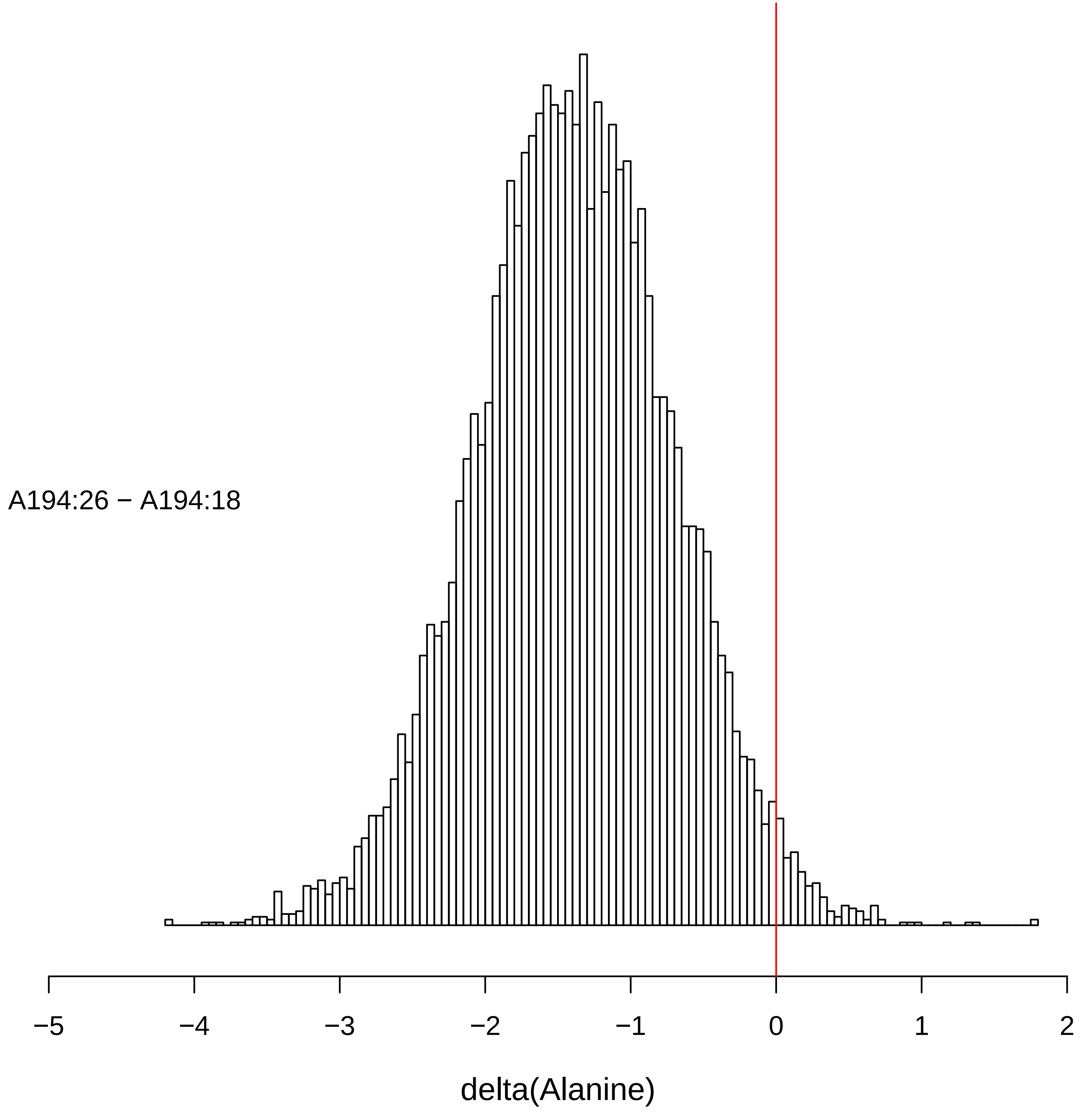
A194:26 − A194:18
−5
−4
−3
−2
−1
0
1
2
delta(Alanine)

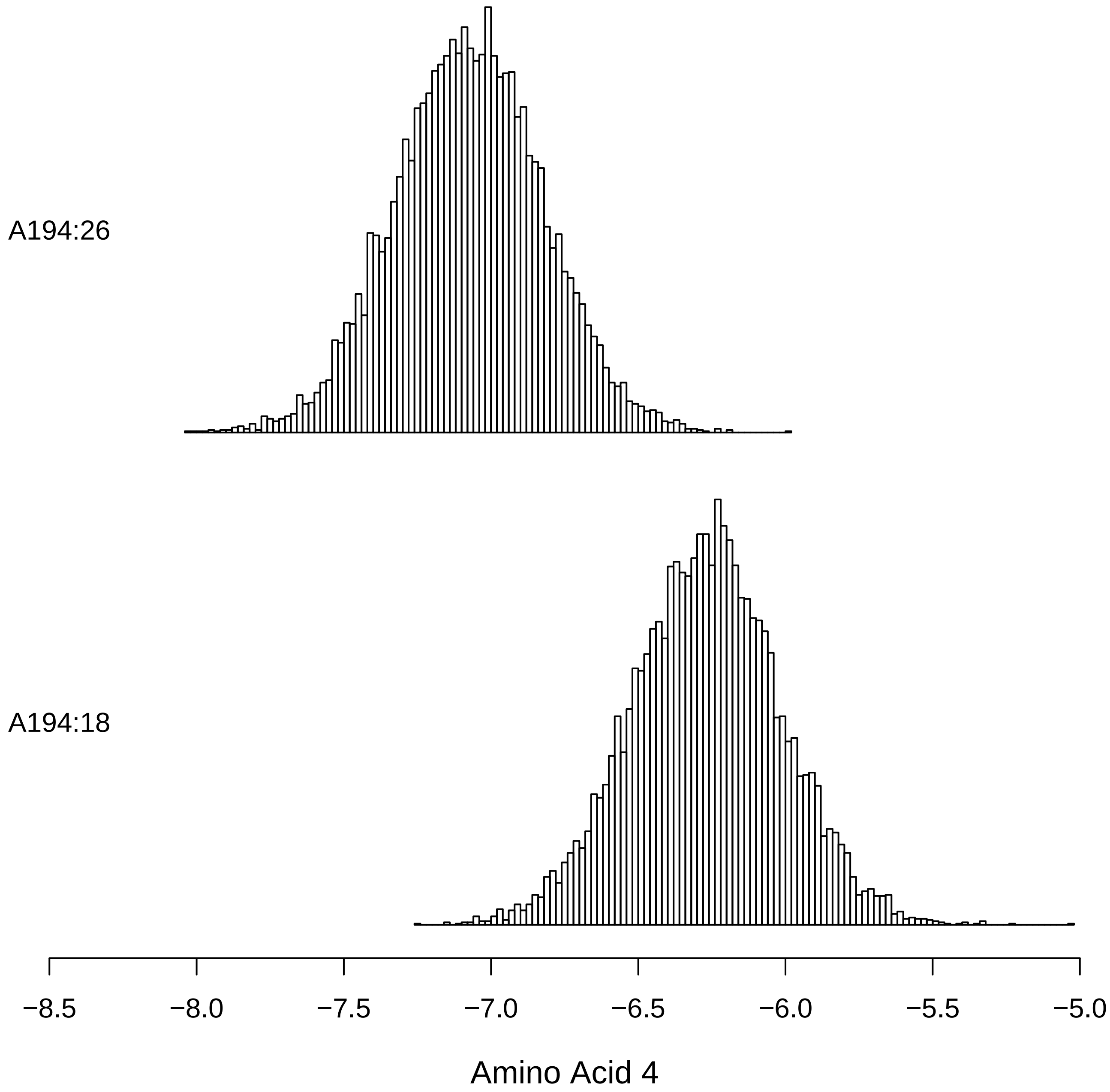
A194:26
A194:18
−8.5
−8.0
−7.5
−7.0
−6.5
−6.0
−5.5
−5.0
Amino Acid 4

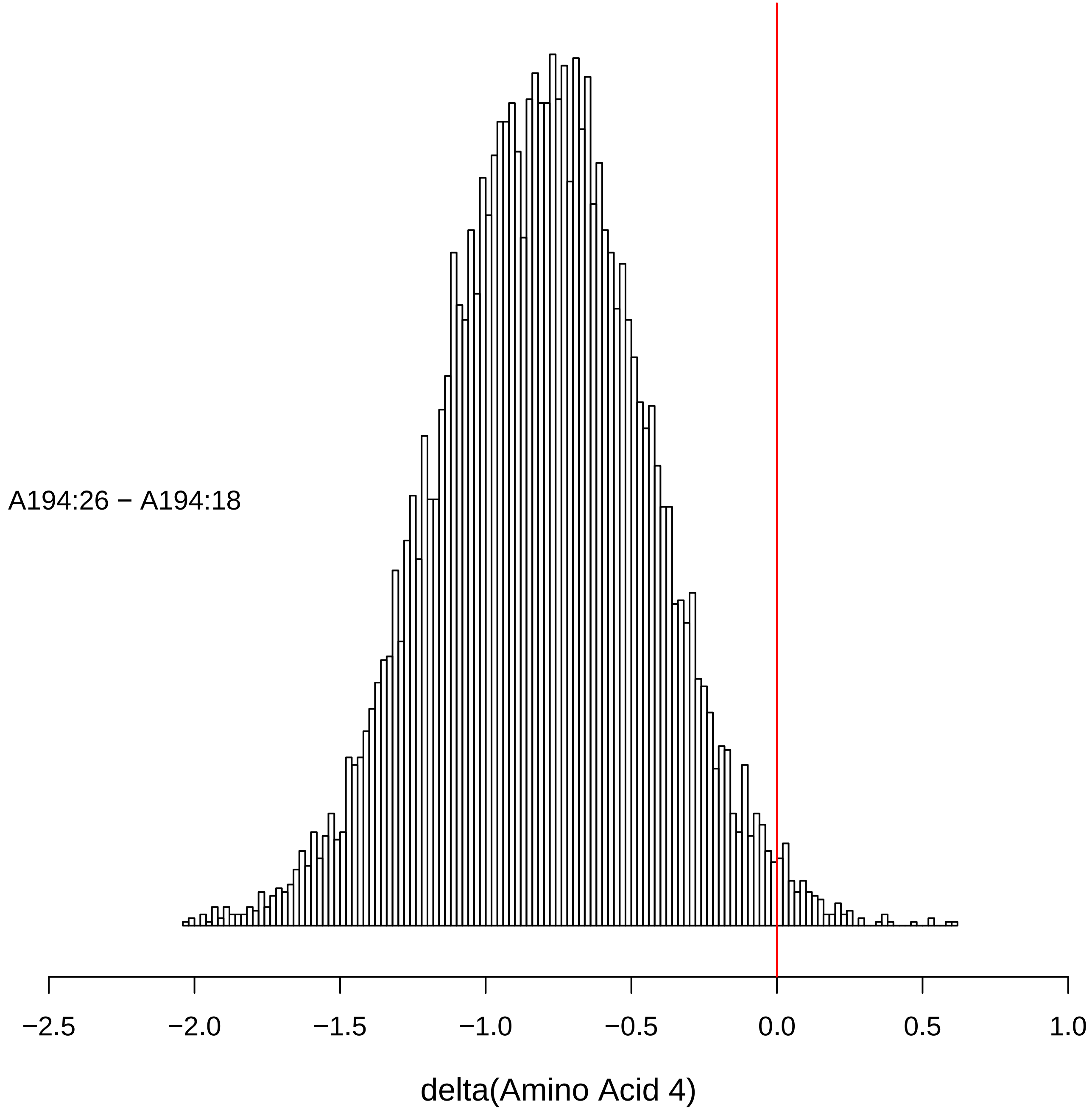

A194:26 − A194:18
−2.5
−2.0
−1.5
−1.0
−0.5
0.0
0.5
1.0
delta(Amino Acid 4)

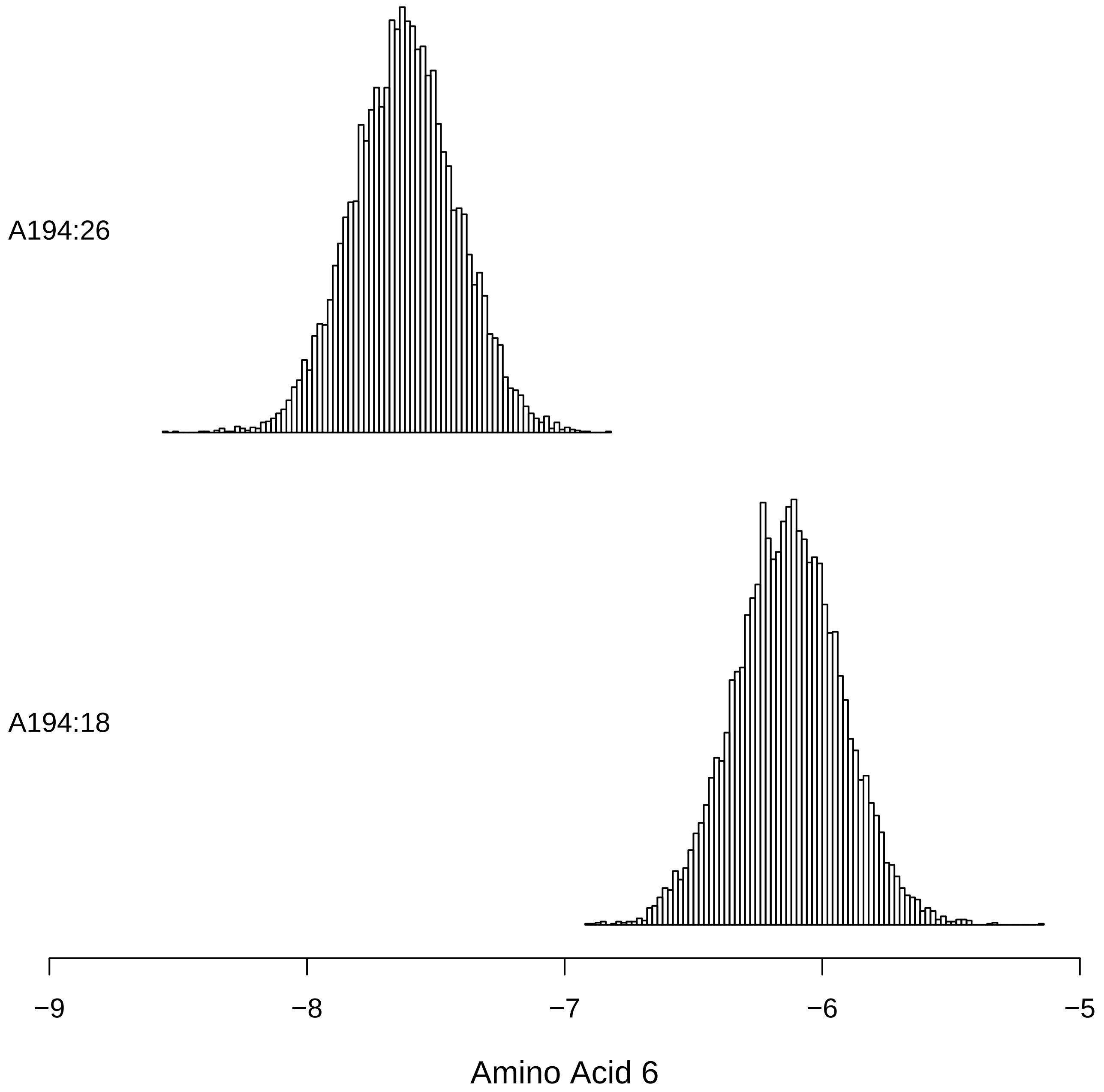

A194:26
A194:18
−9
−8
−7
−6
−5
Amino Acid 6

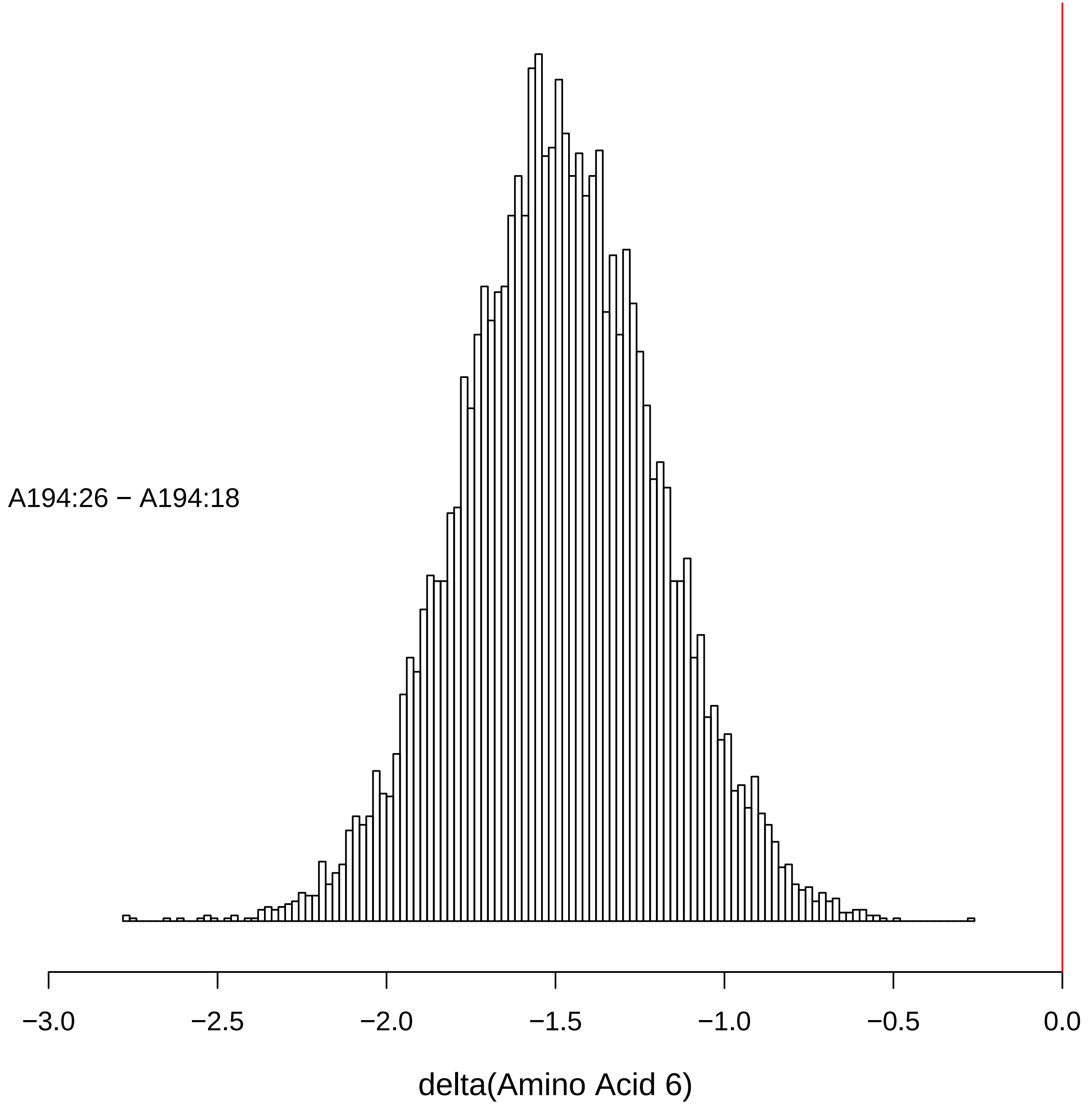
A194:26 − A194:18
−3.0
−2.5
−2.0
−1.5
−1.0
−0.5
0.0
delta(Amino Acid 6)

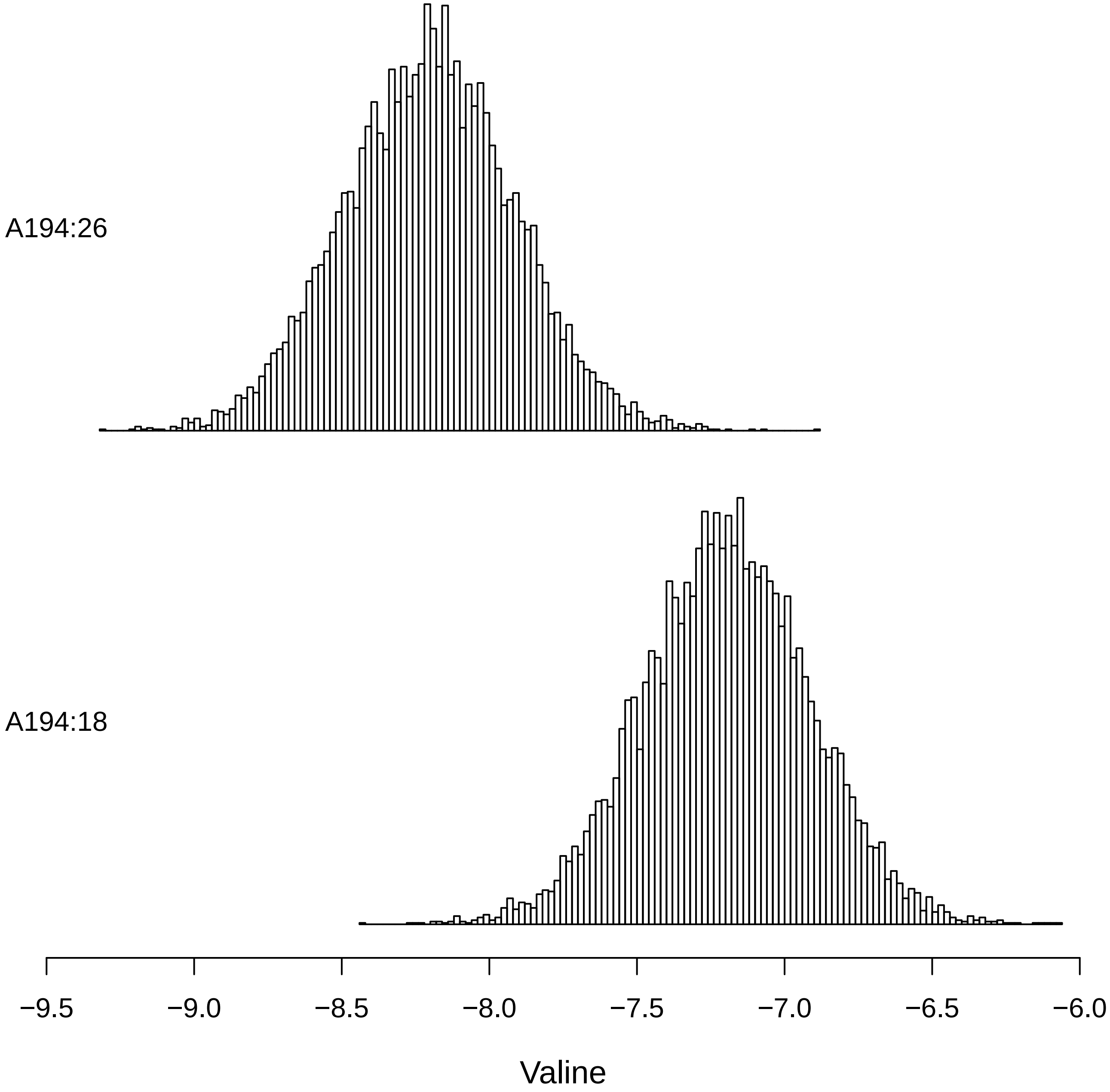

A194:26
A194:18
−9.5
−9.0
−8.5
−8.0
−7.5
−7.0
−6.5
−6.0
Valine

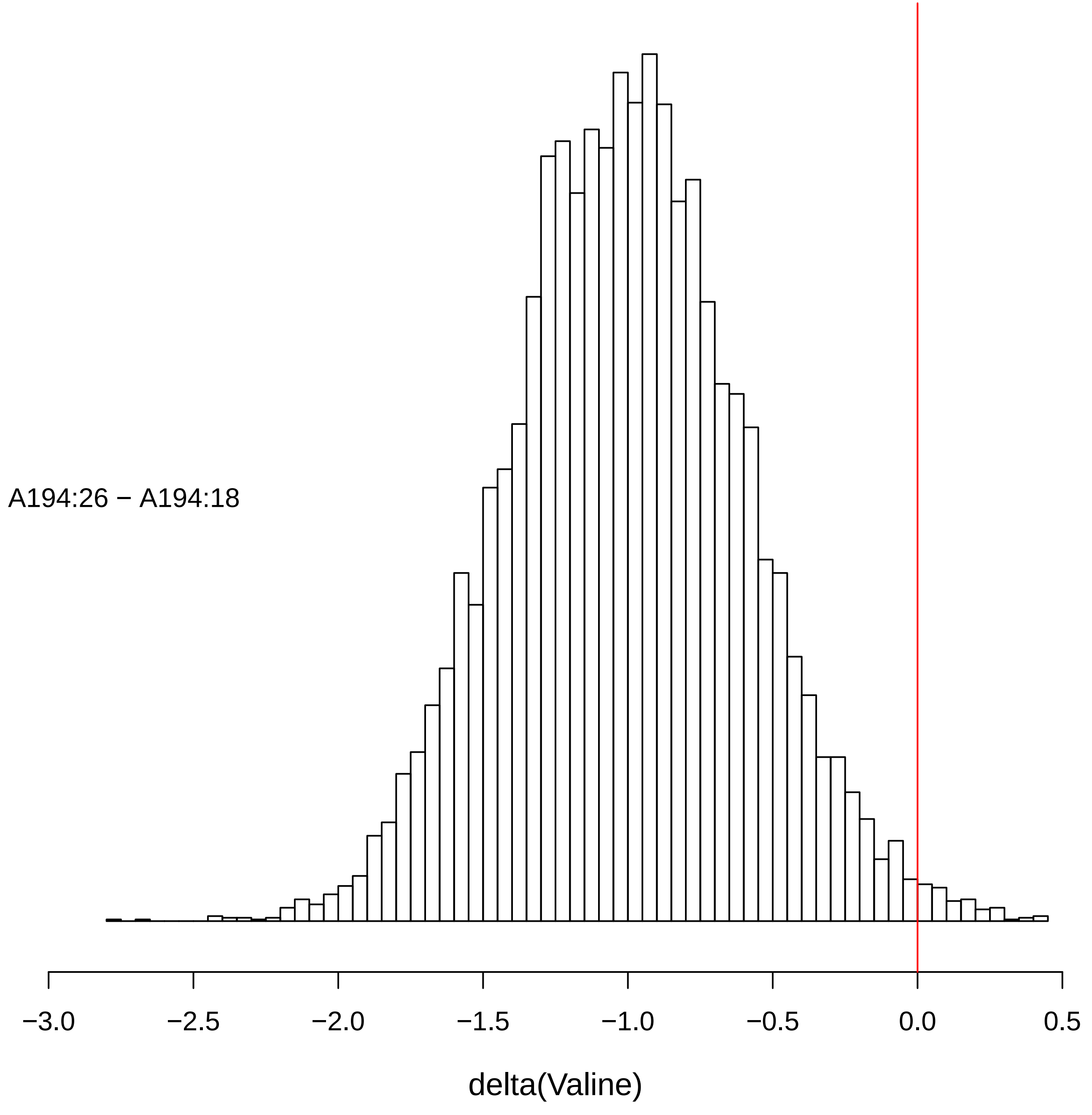
A194:26 − A194:18
−3.0
−2.5
−2.0
−1.5
−1.0
−0.5
0.0
0.5
delta(Valine)

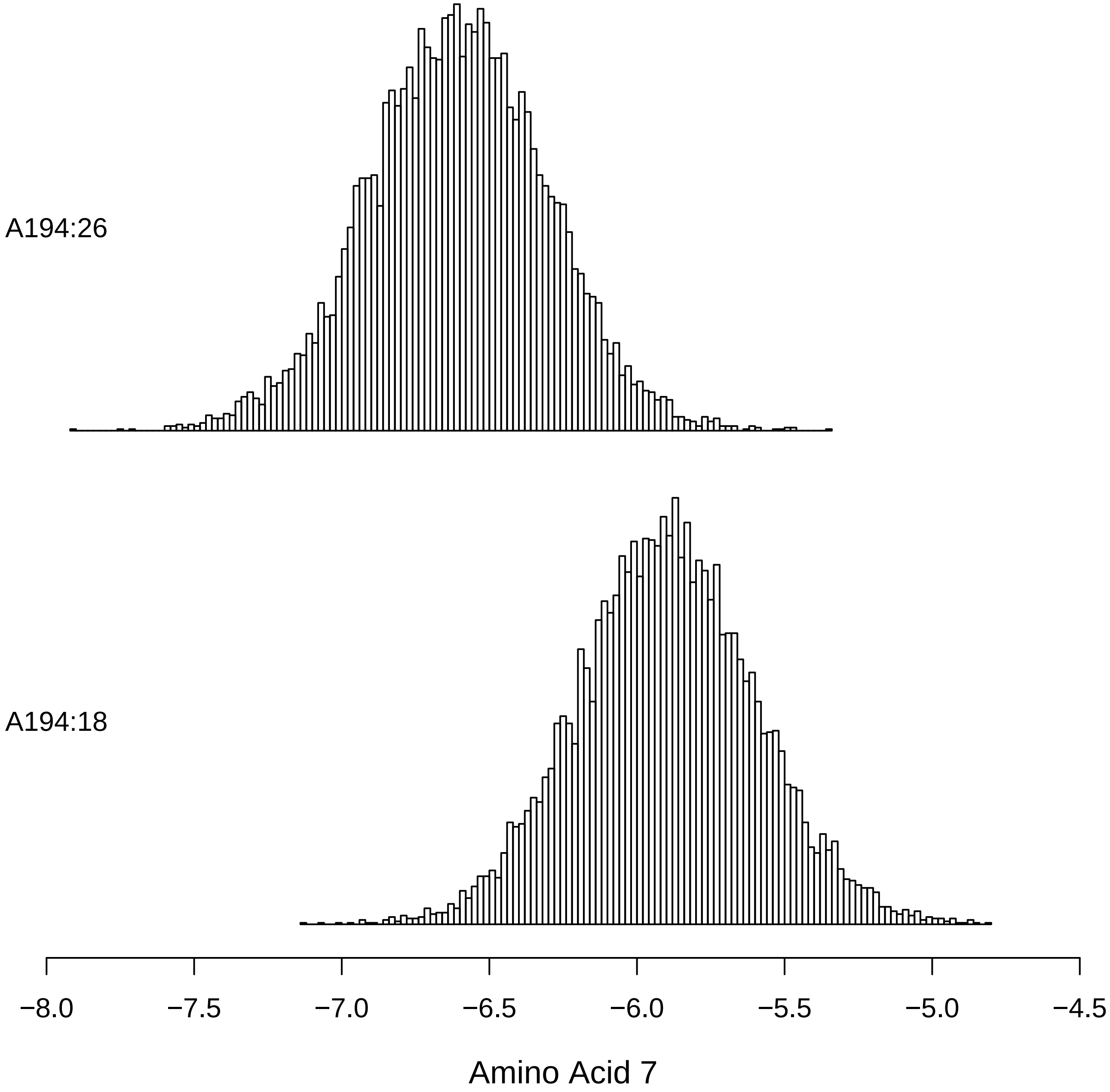
A194:26
A194:18
−8.0
−7.5
−7.0
−6.5
−6.0
−5.5
−5.0
−4.5
Amino Acid 7

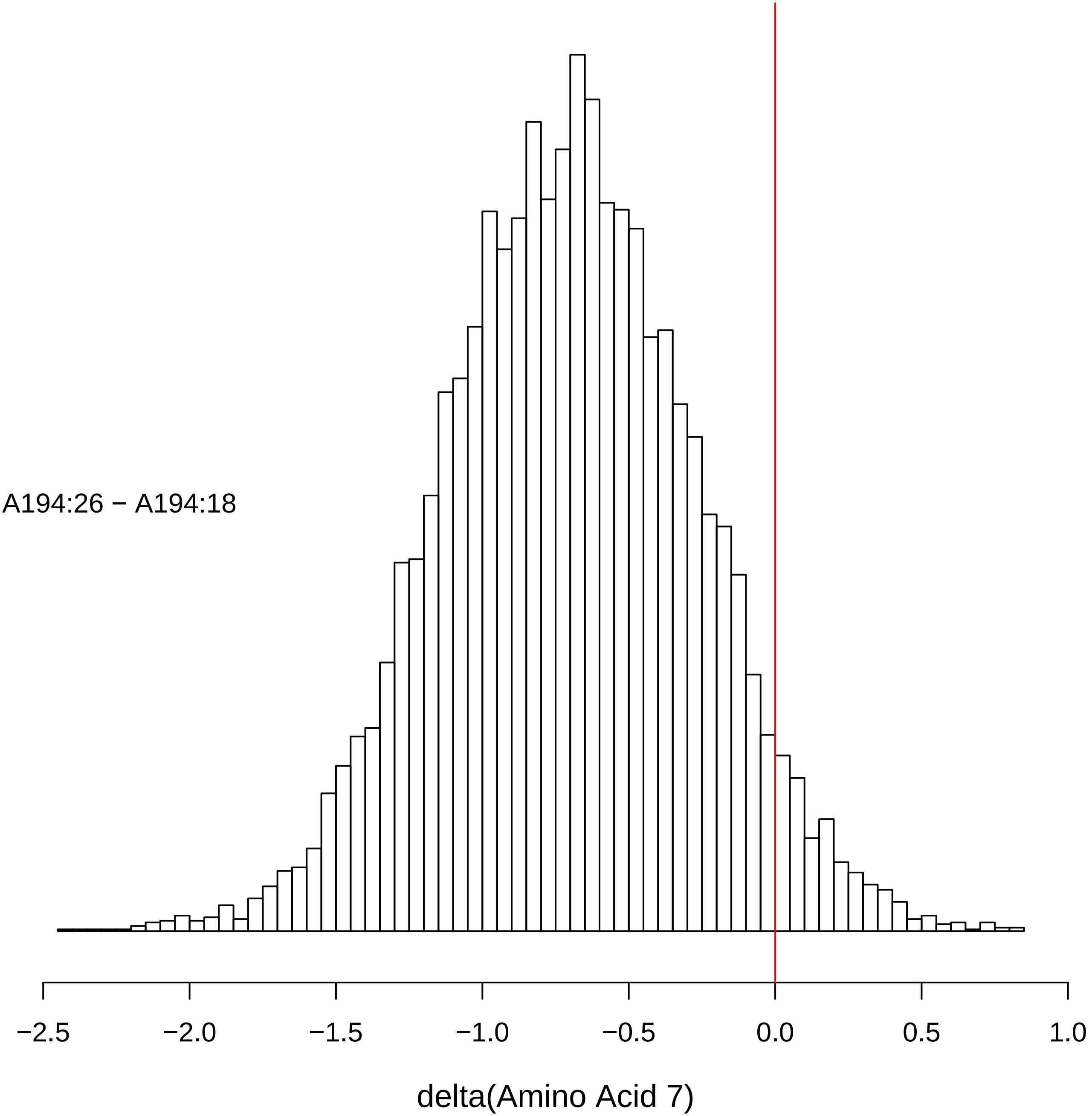

A194:26 − A194:18
−2.5
−2.0
−1.5
−1.0
−0.5
0.0
0.5
1.0
delta(Amino Acid 7)

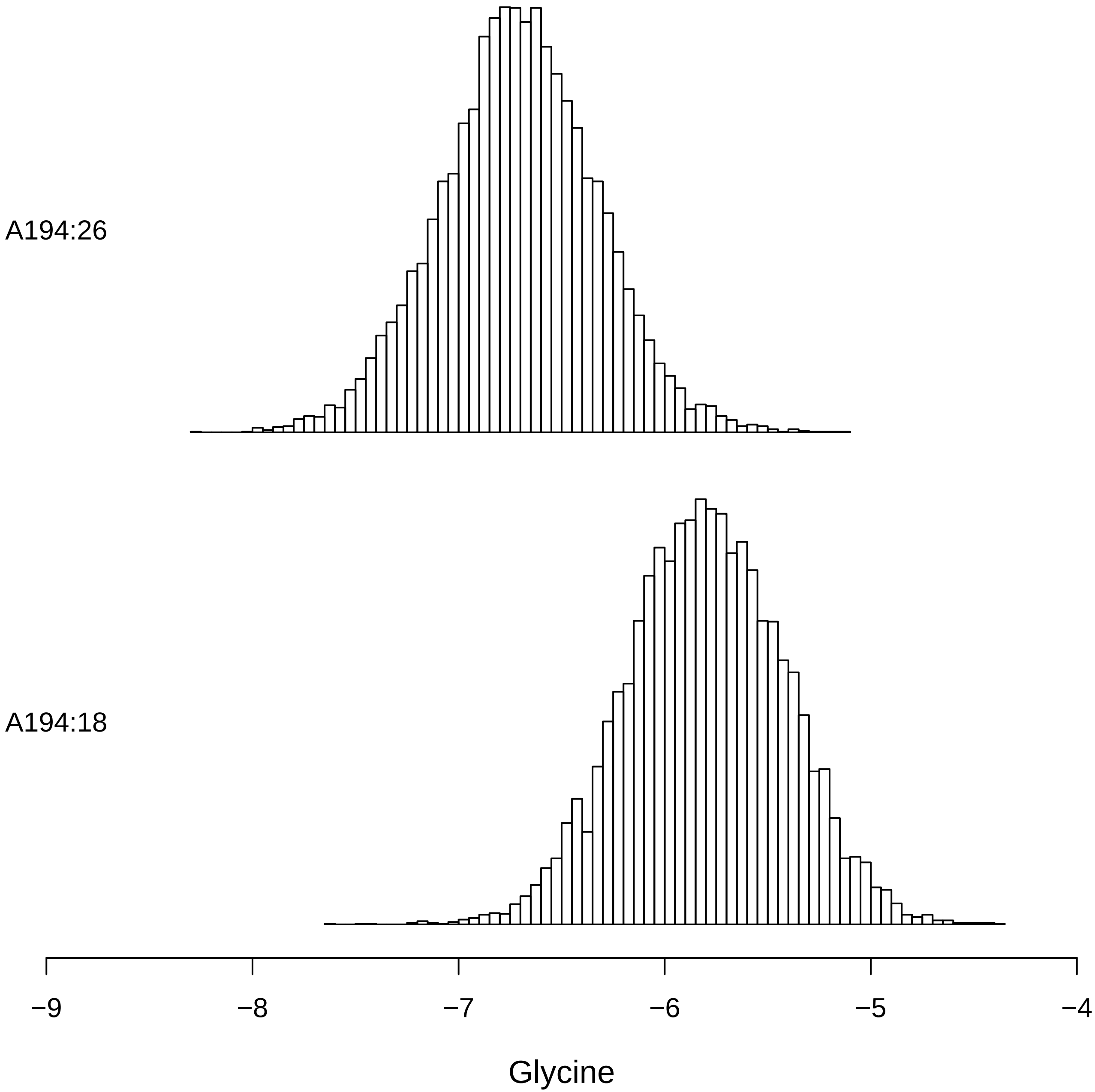
A194:26
A194:18
−9
−8
−7
−6
−5
−4
Glycine

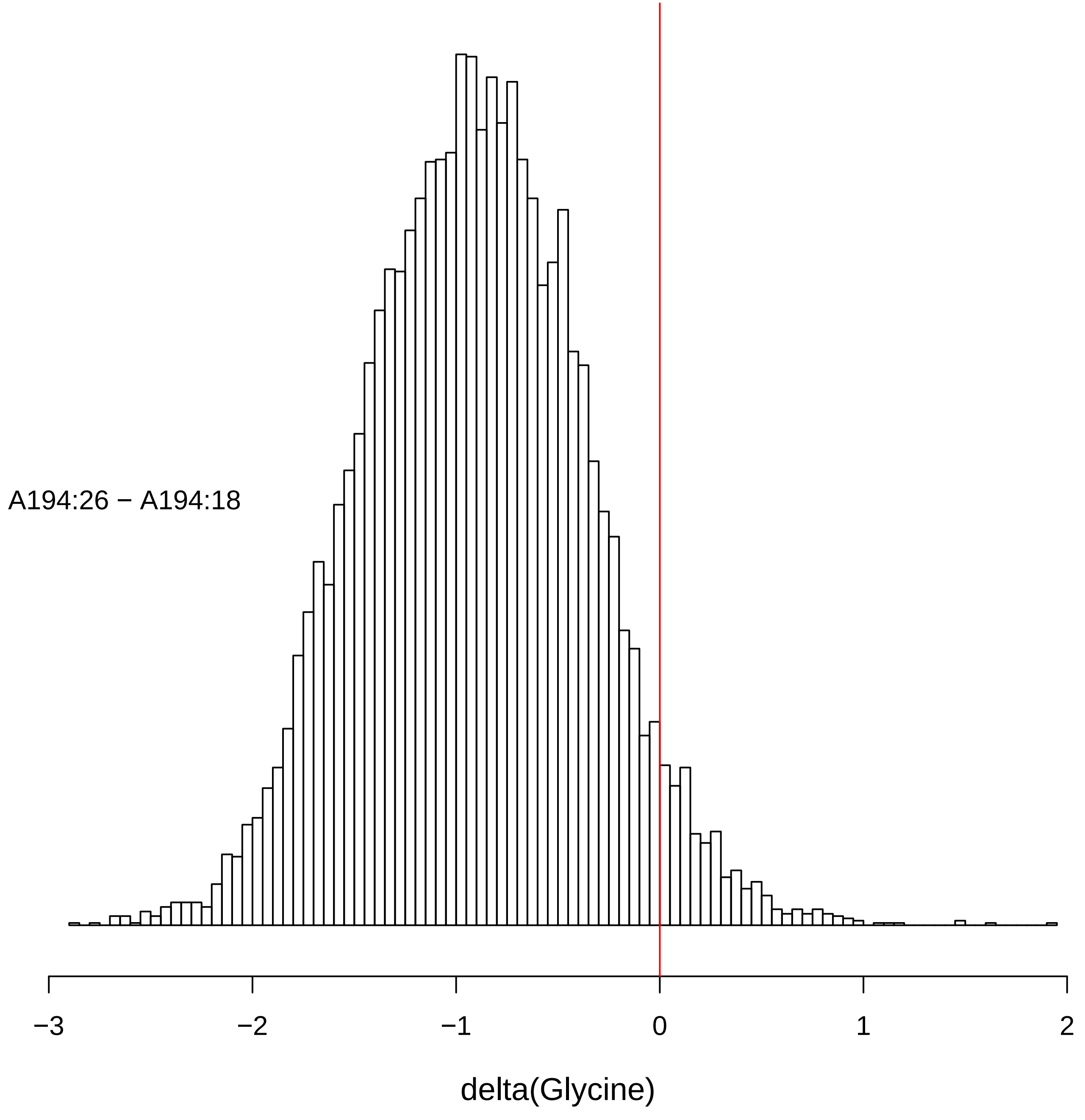
A194:26 − A194:18
−3
−2
−1
0
1
2
delta(Glycine)

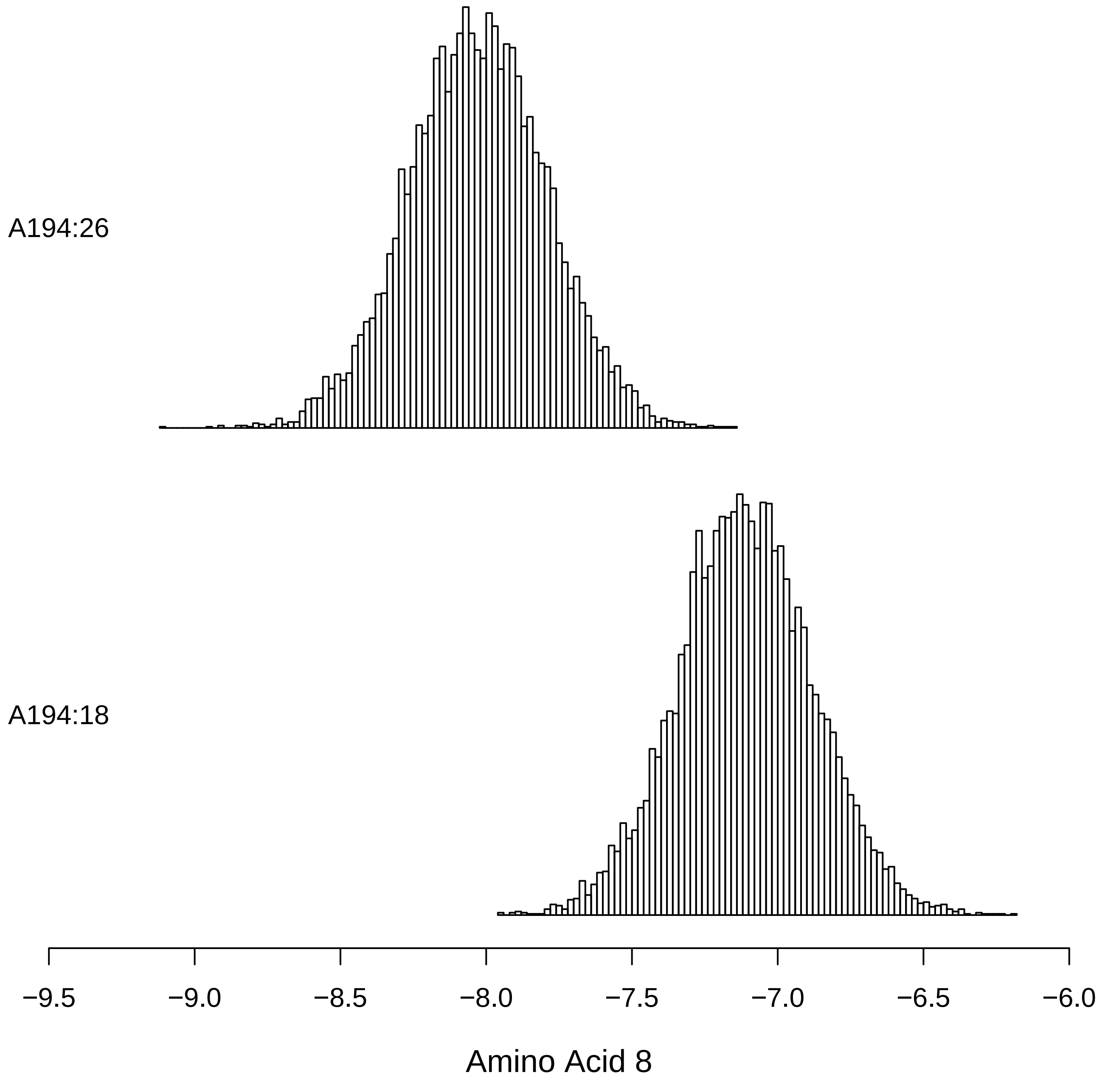
A194:26
A194:18
−9.5
−9.0
−8.5
−8.0
−7.5
−7.0
−6.5
−6.0
Amino Acid 8

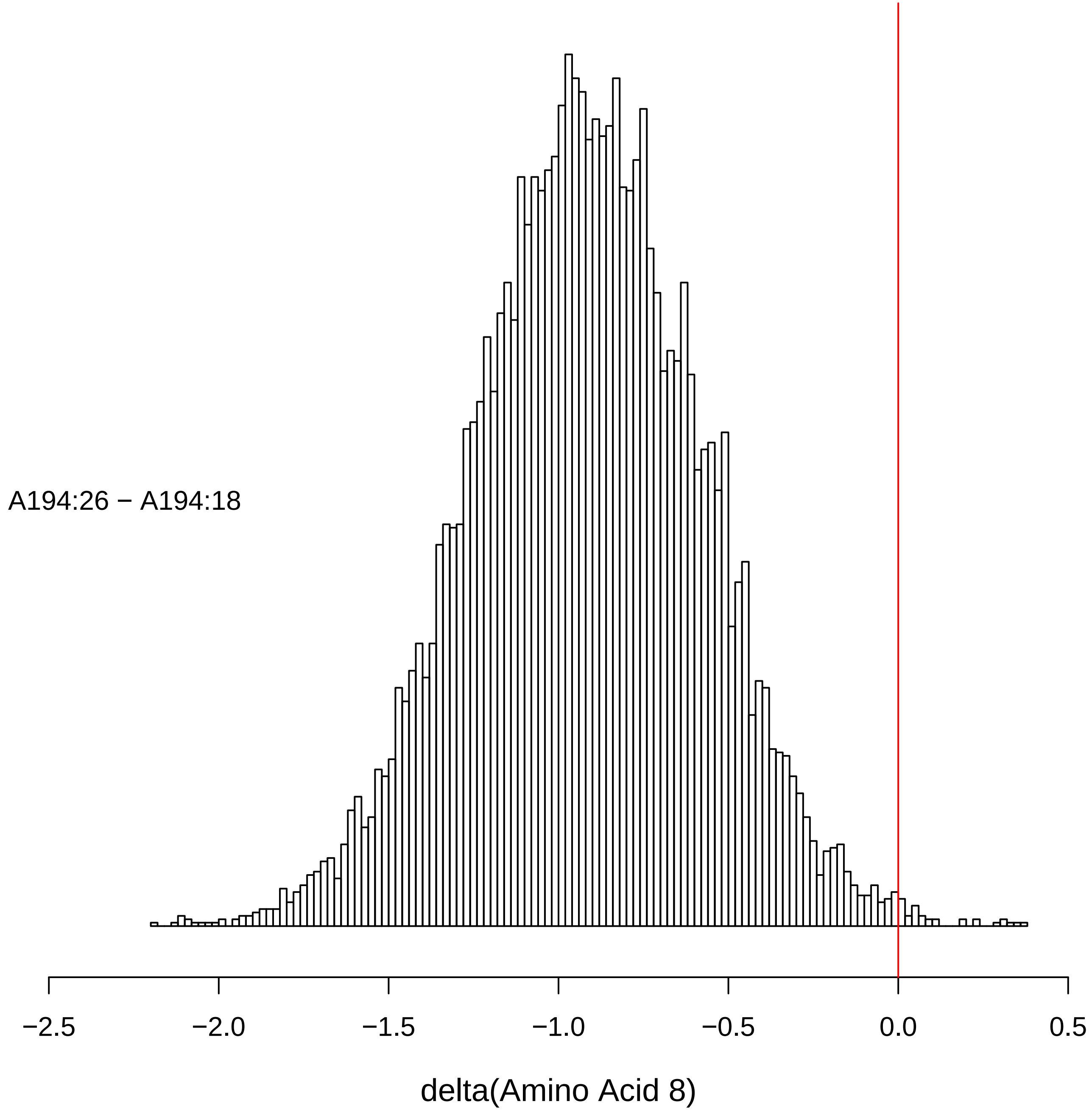
A194:26 − A194:18
−2.5
−2.0
−1.5
−1.0
−0.5
0.0
0.5
delta(Amino Acid 8)

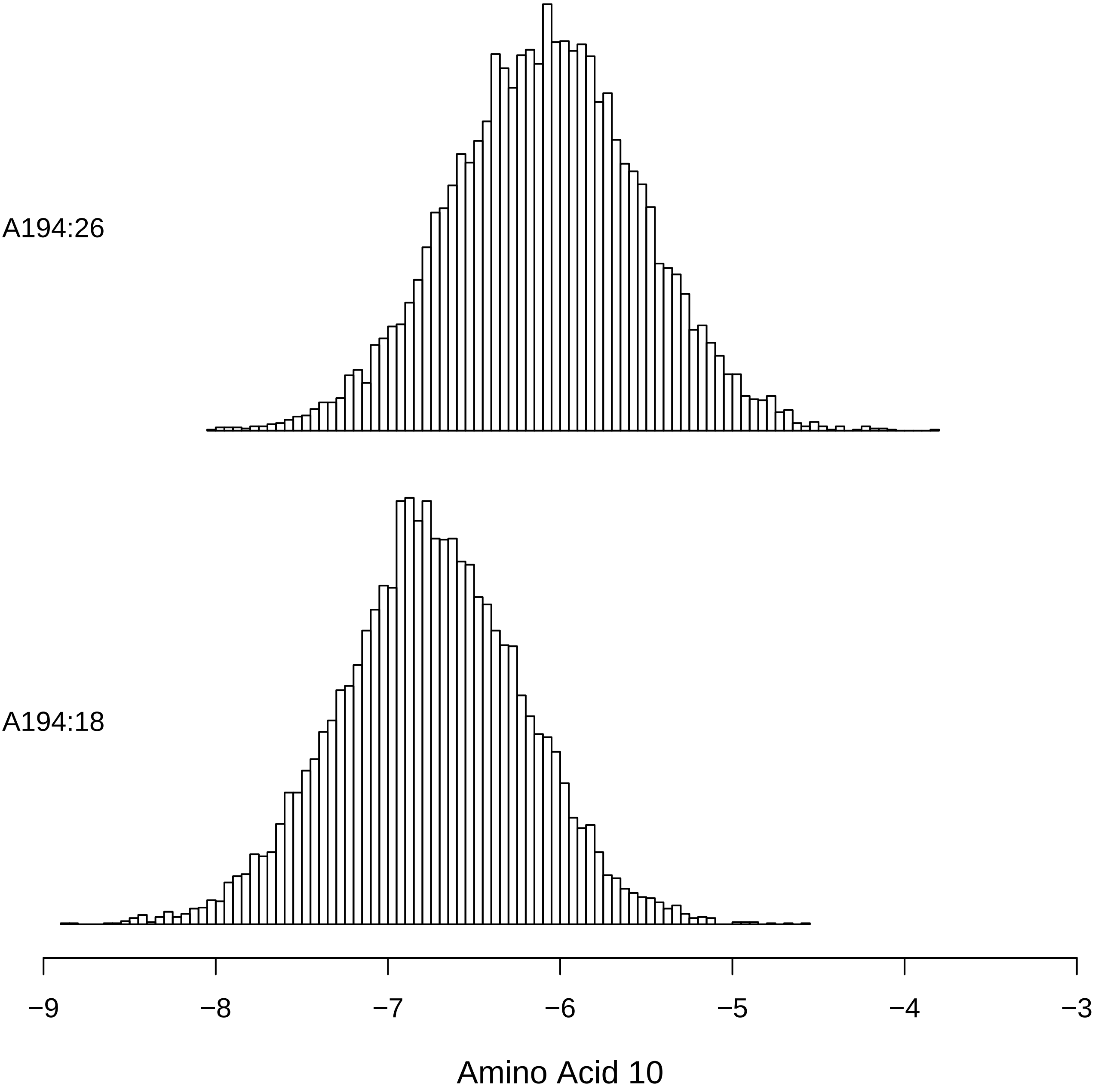
A194:26
A194:18
−9
−8
−7
−6
−5
−4
−3
Amino Acid 10

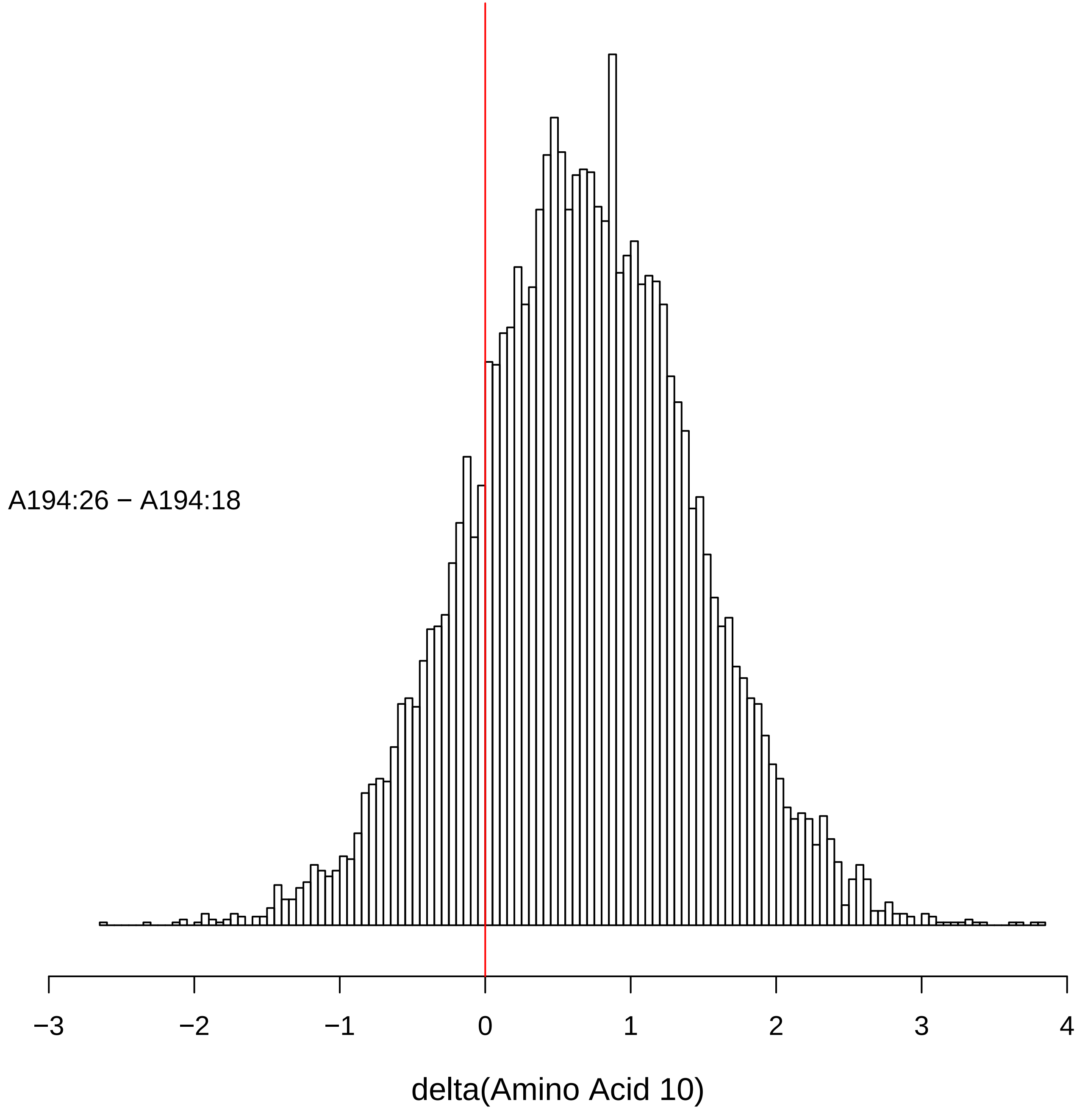
A194:26 − A194:18
−3
−2
−1
0
1
2
3
4
delta(Amino Acid 10)

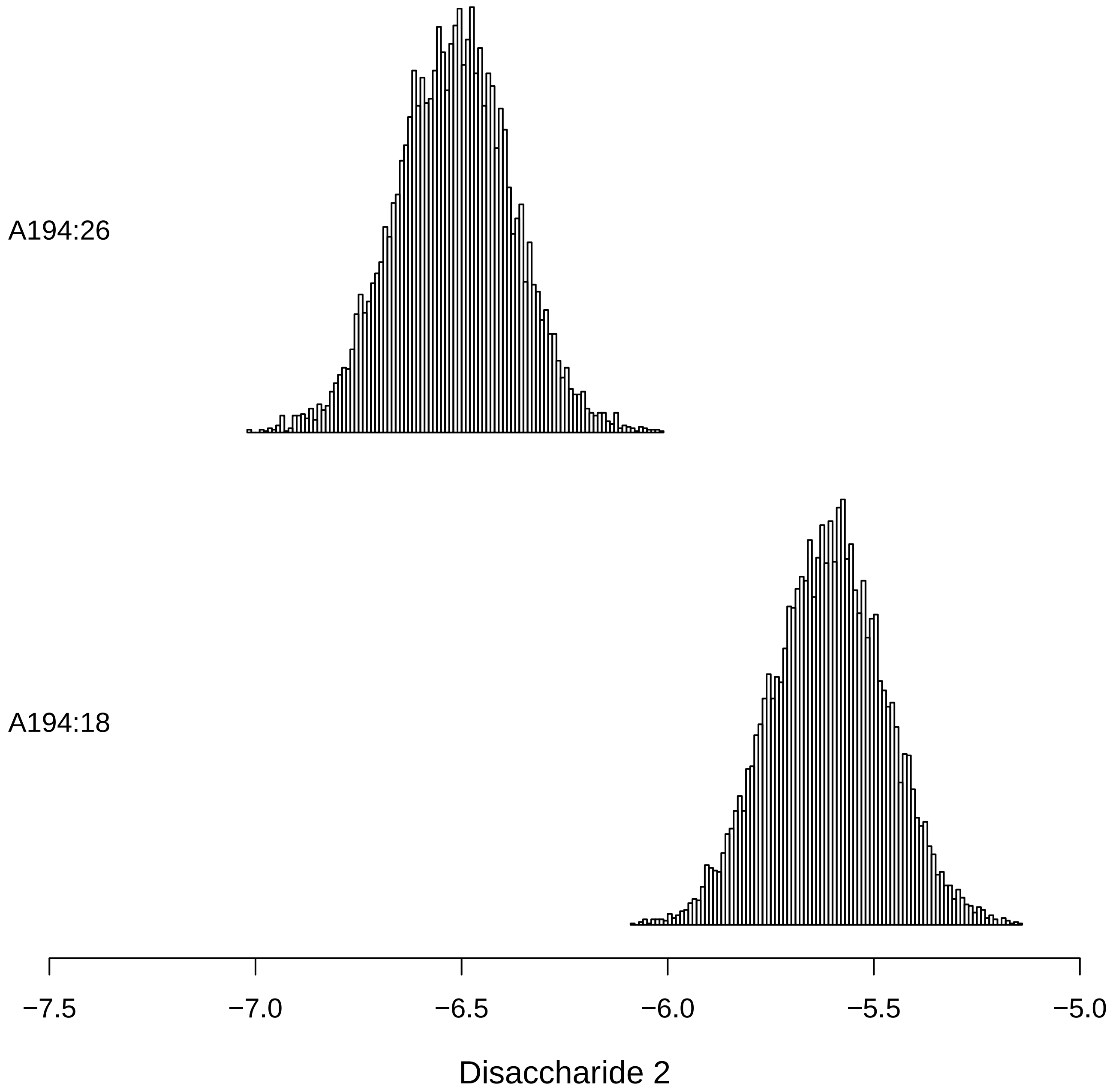
A194:26
A194:18
−7.5
−7.0
−6.5
−6.0
−5.5
−5.0
Disaccharide 2

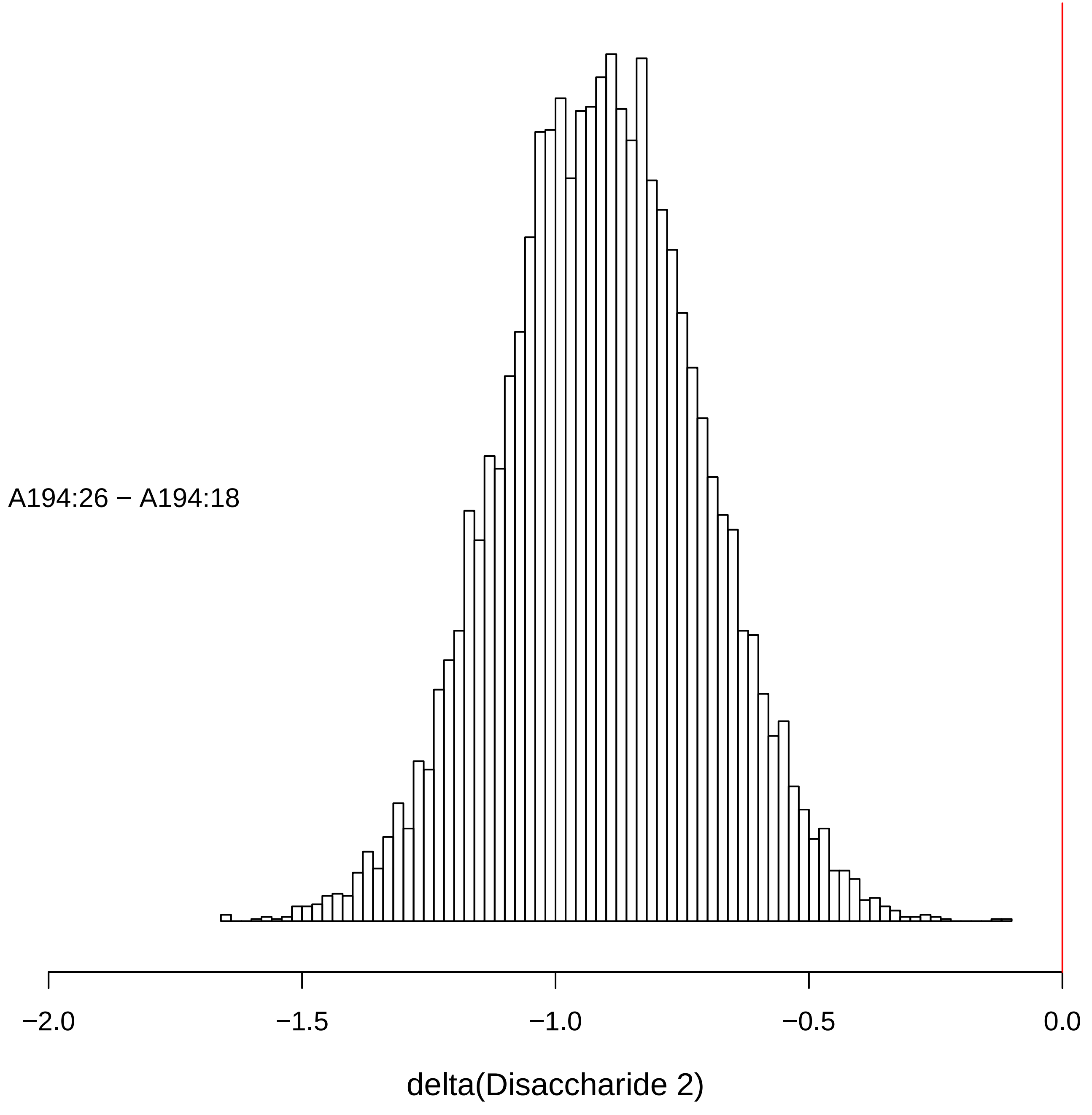
A194:26 − A194:18
−2.0
−1.5
−1.0
−0.5
0.0
delta(Disaccharide 2)

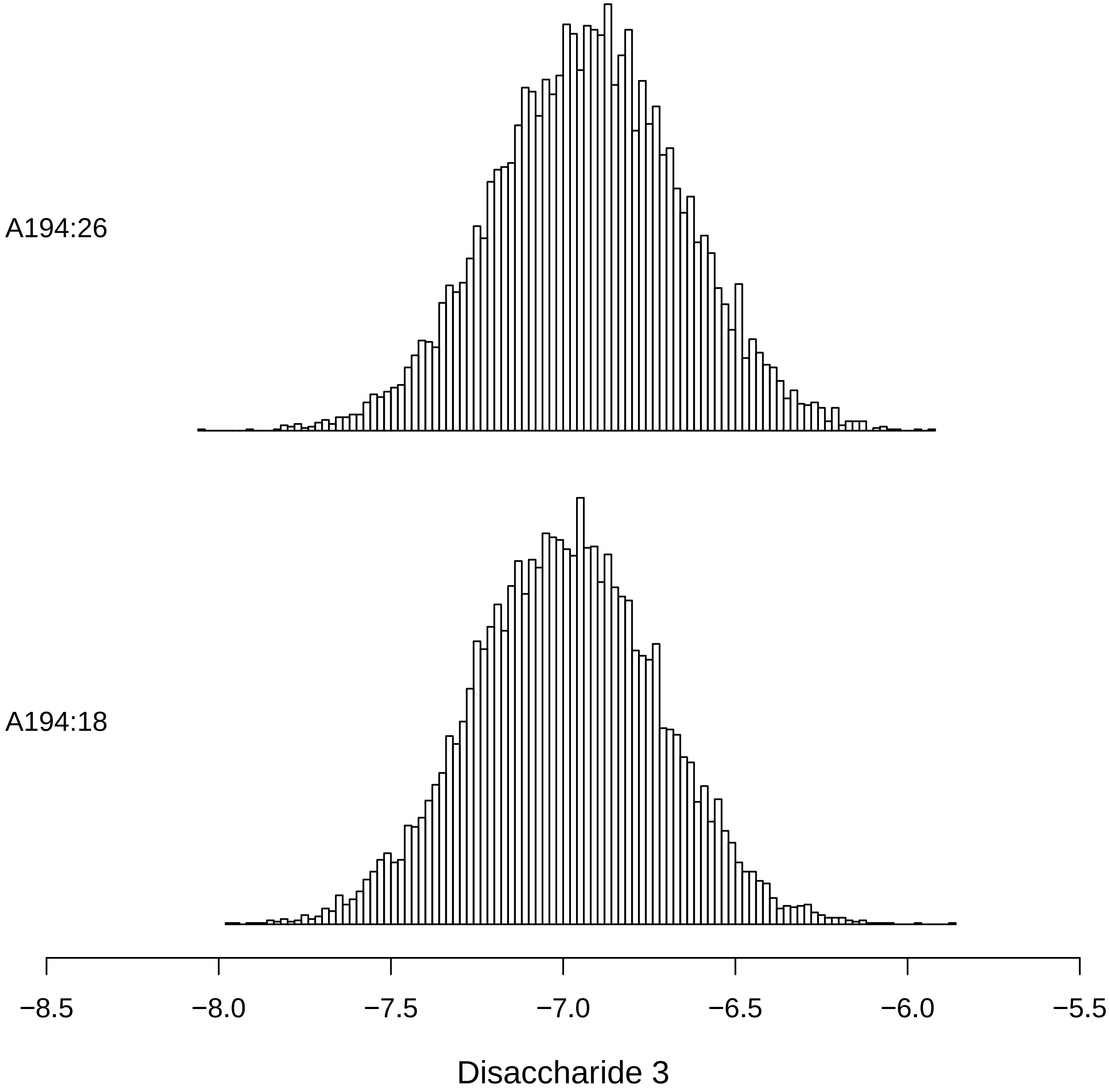
A194:26
A194:18
−8.5
−8.0
−7.5
−7.0
−6.5
−6.0
−5.5
Disaccharide 3

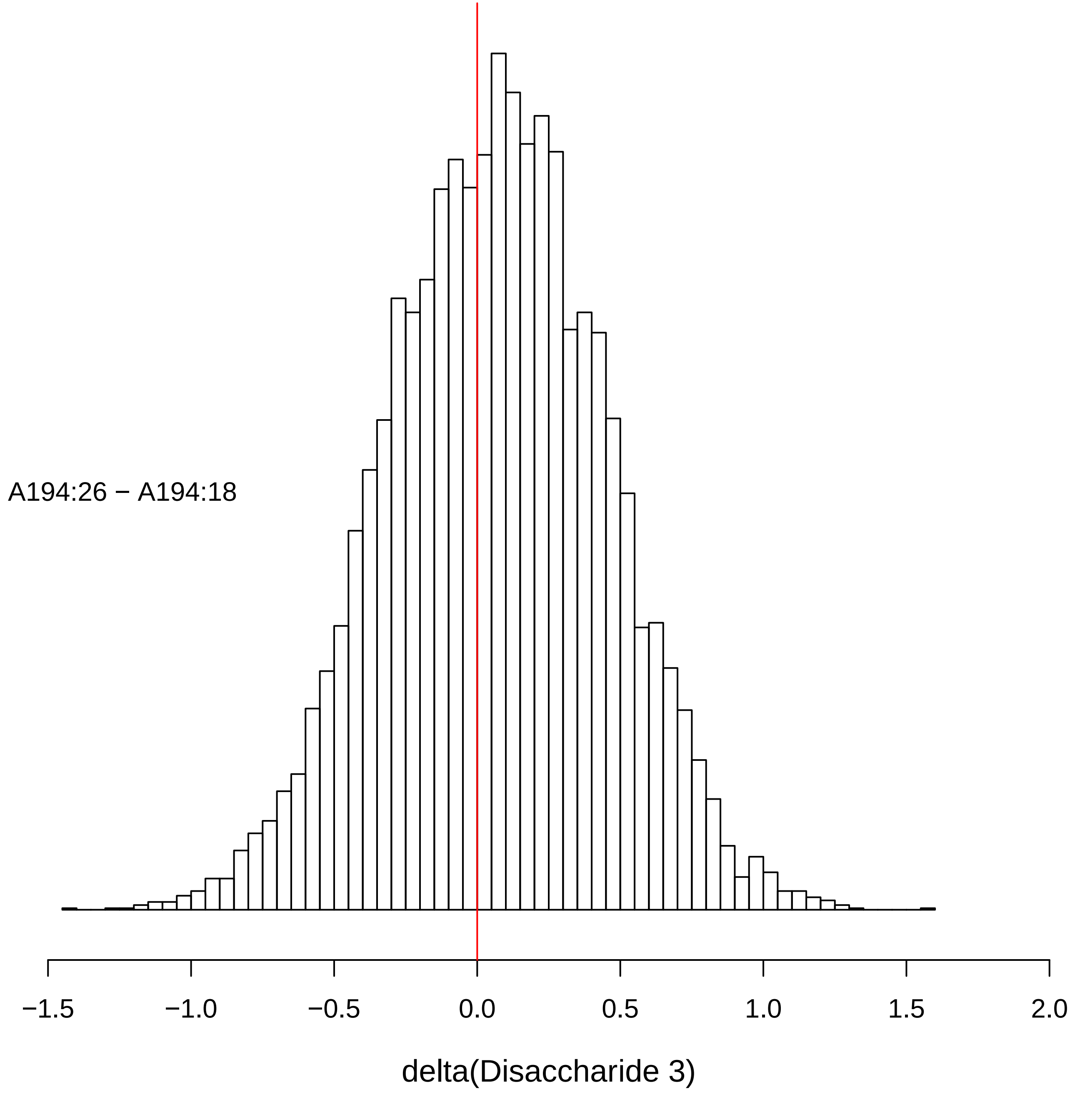

A194:26 − A194:18
−1.5
−1.0
−0.5
0.0
0.5
1.0
1.5
2.0
delta(Disaccharide 3)

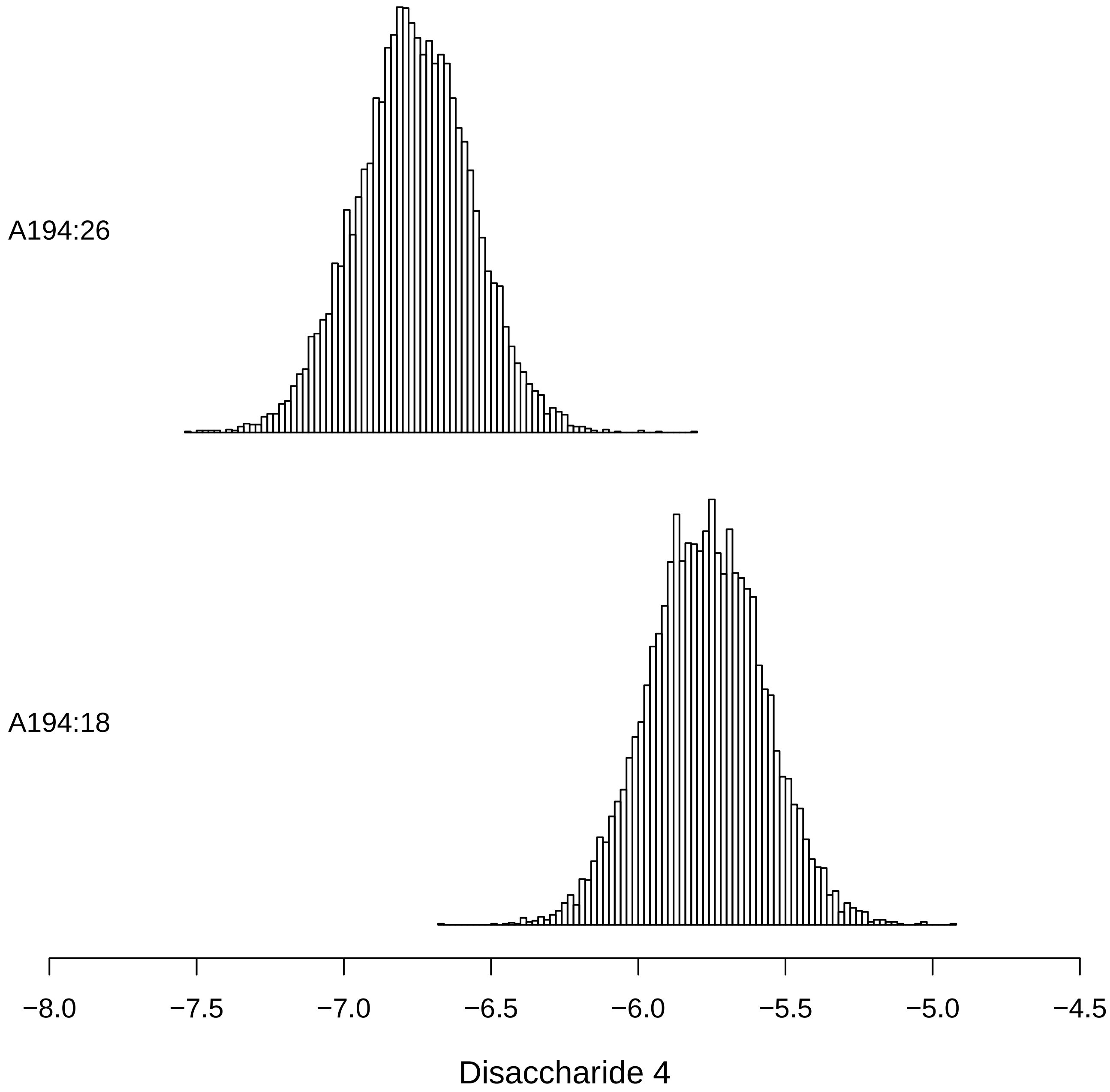
A194:26
A194:18
−8.0
−7.5
−7.0
−6.5
−6.0
−5.5
−5.0
−4.5
Disaccharide 4

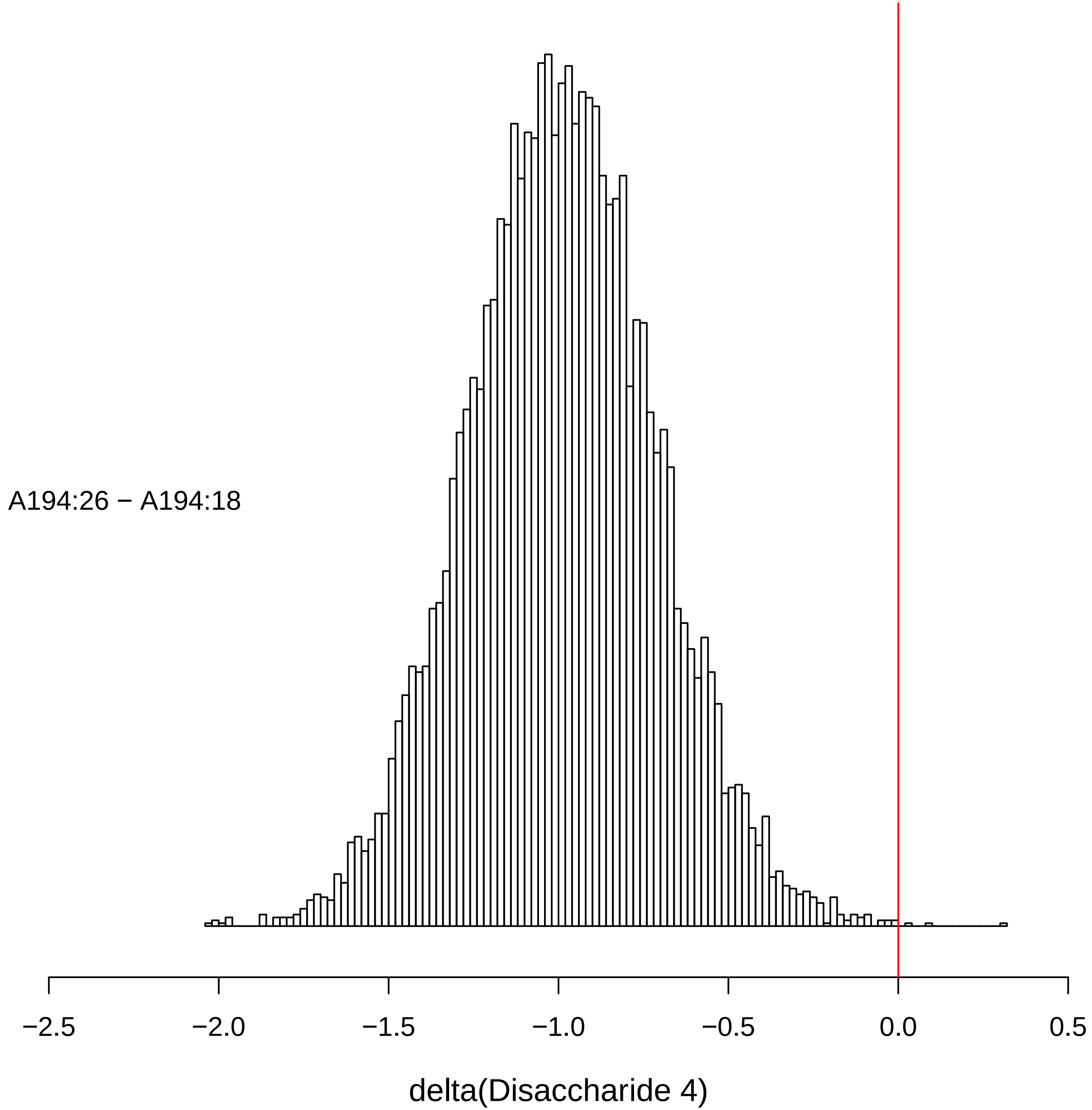
A194:26 − A194:18
−2.5
−2.0
−1.5
−1.0
−0.5
0.0
0.5
delta(Disaccharide 4)

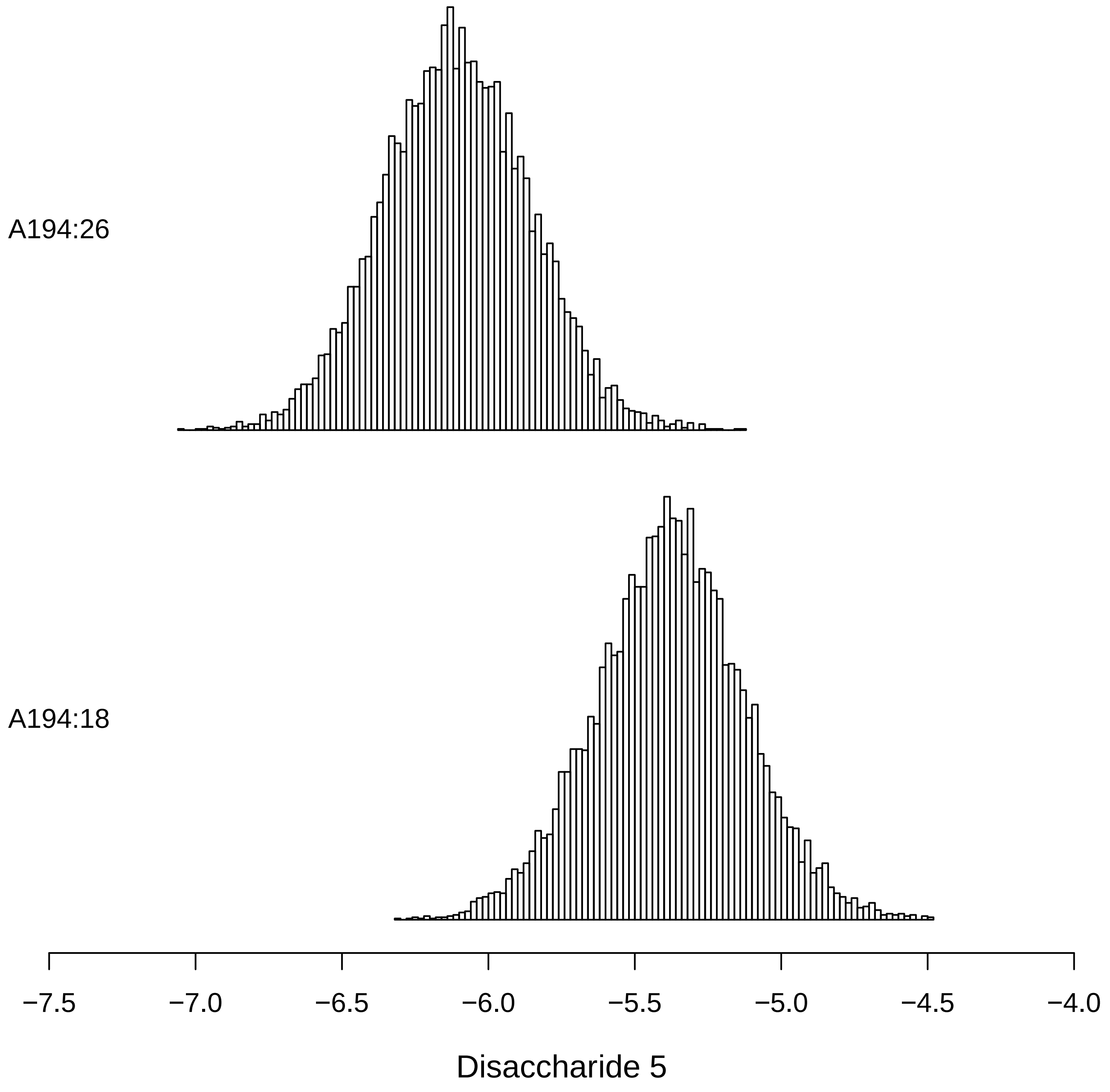
A194:26
A194:18
−7.5
−7.0
−6.5
−6.0
−5.5
−5.0
−4.5
−4.0
Disaccharide 5

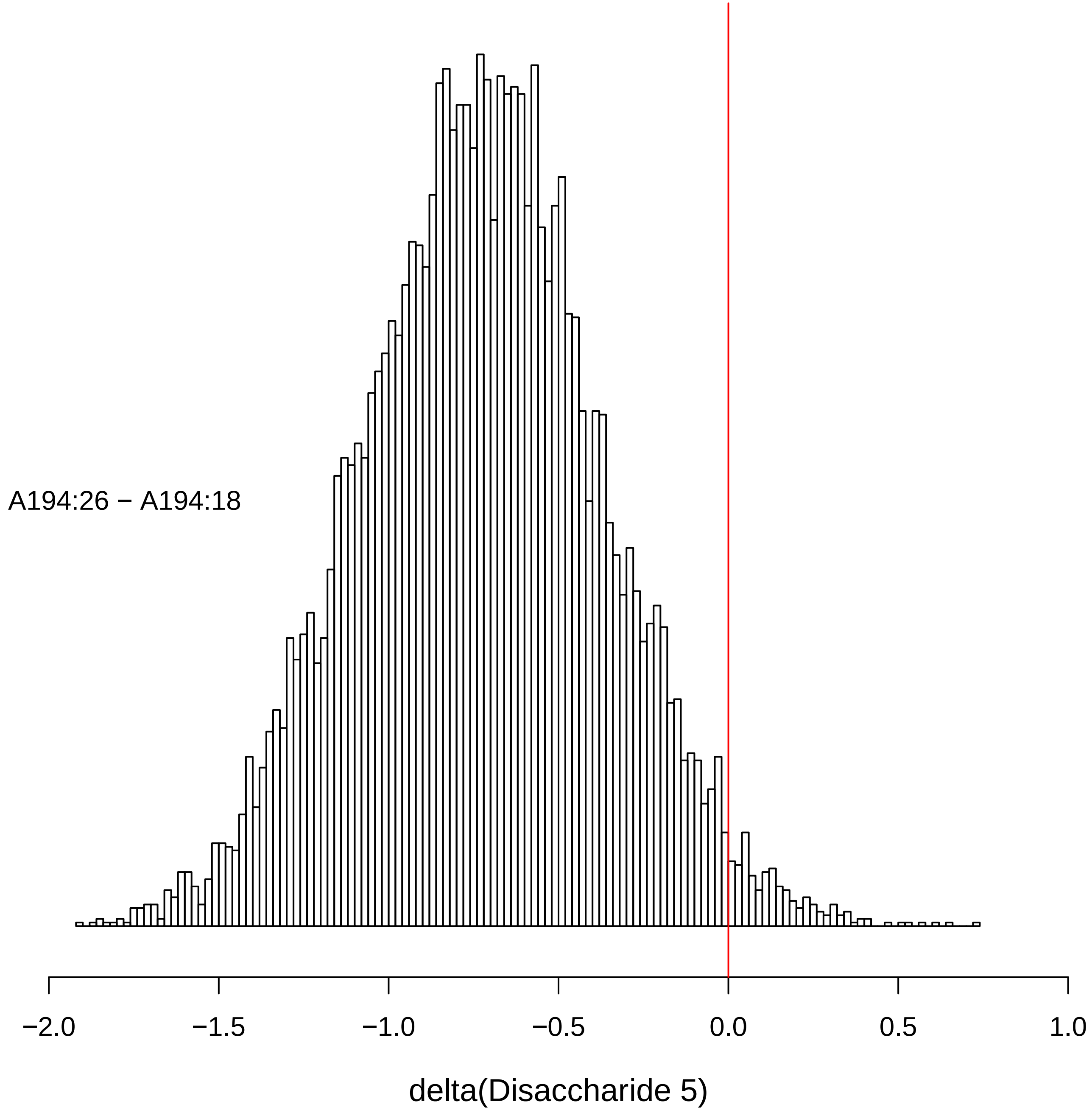
A194:26 − A194:18
−2.0
−1.5
−1.0
−0.5
0.0
0.5
1.0
delta(Disaccharide 5)

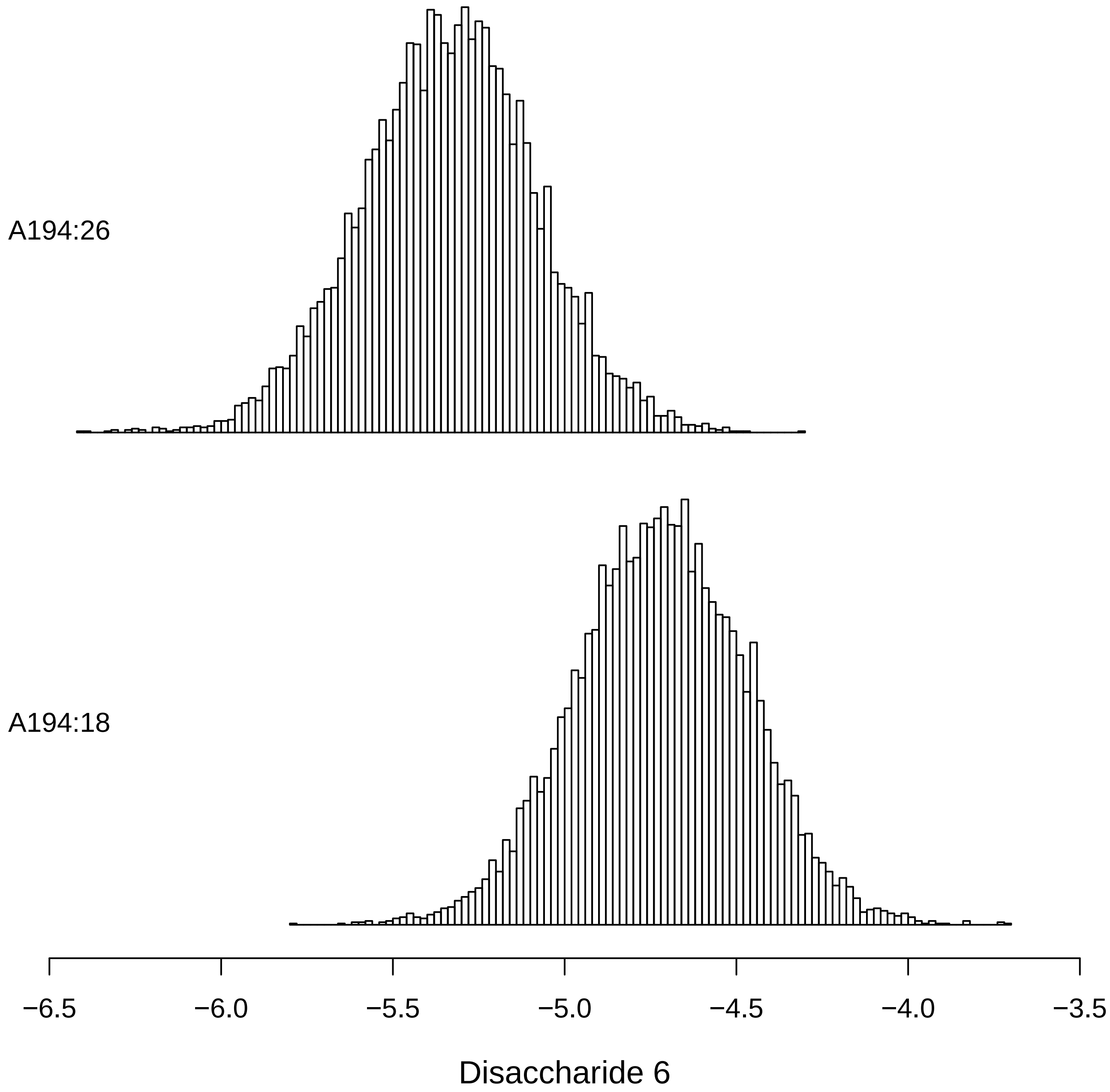
A194:26
A194:18
−6.5
−6.0
−5.5
−5.0
−4.5
−4.0
−3.5
Disaccharide 6

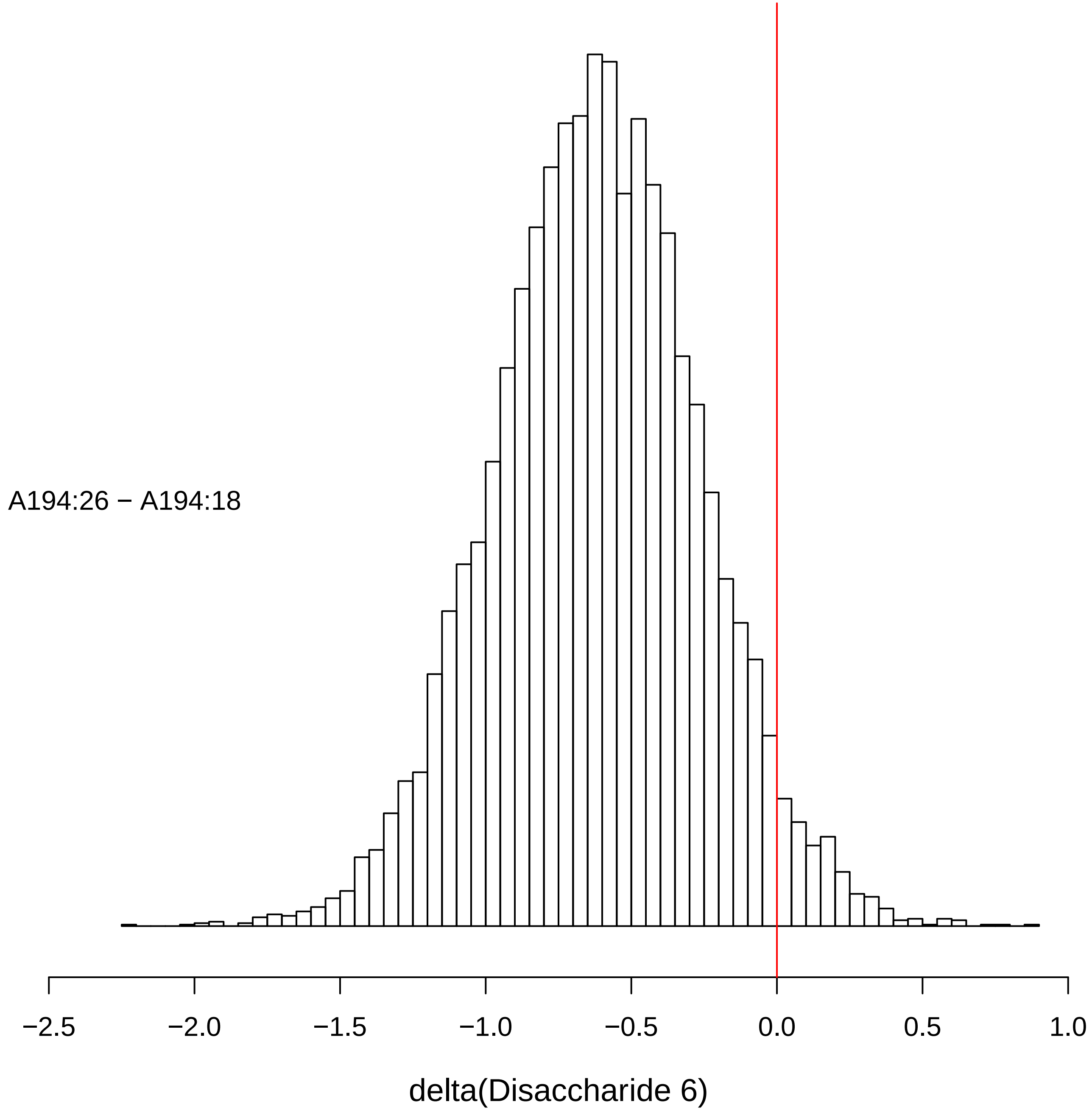
A194:26 − A194:18
−2.5
−2.0
−1.5
−1.0
−0.5
0.0
0.5
1.0
delta(Disaccharide 6)

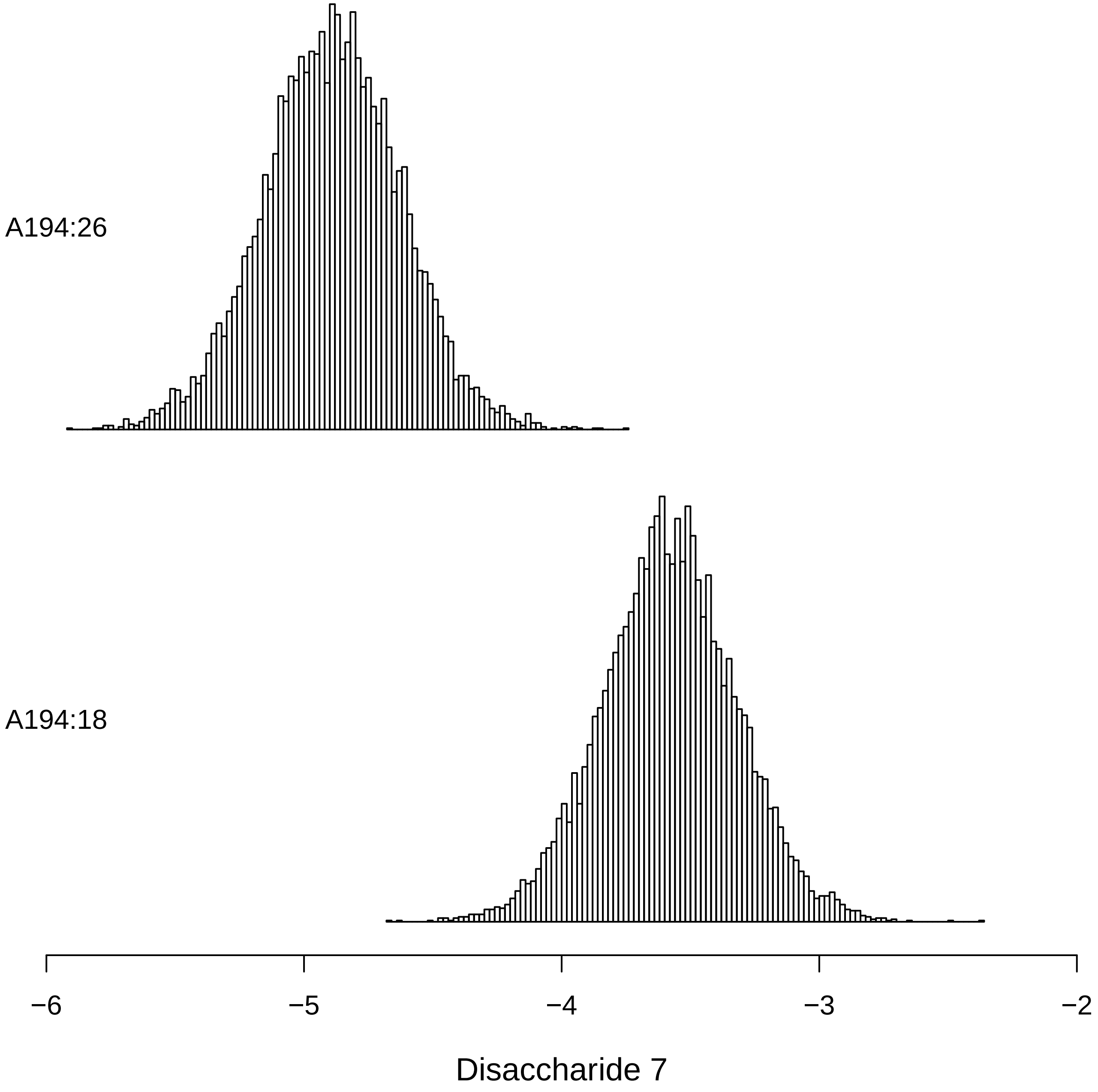
A194:26
A194:18
−6
−5
−4
−3
−2
Disaccharide 7

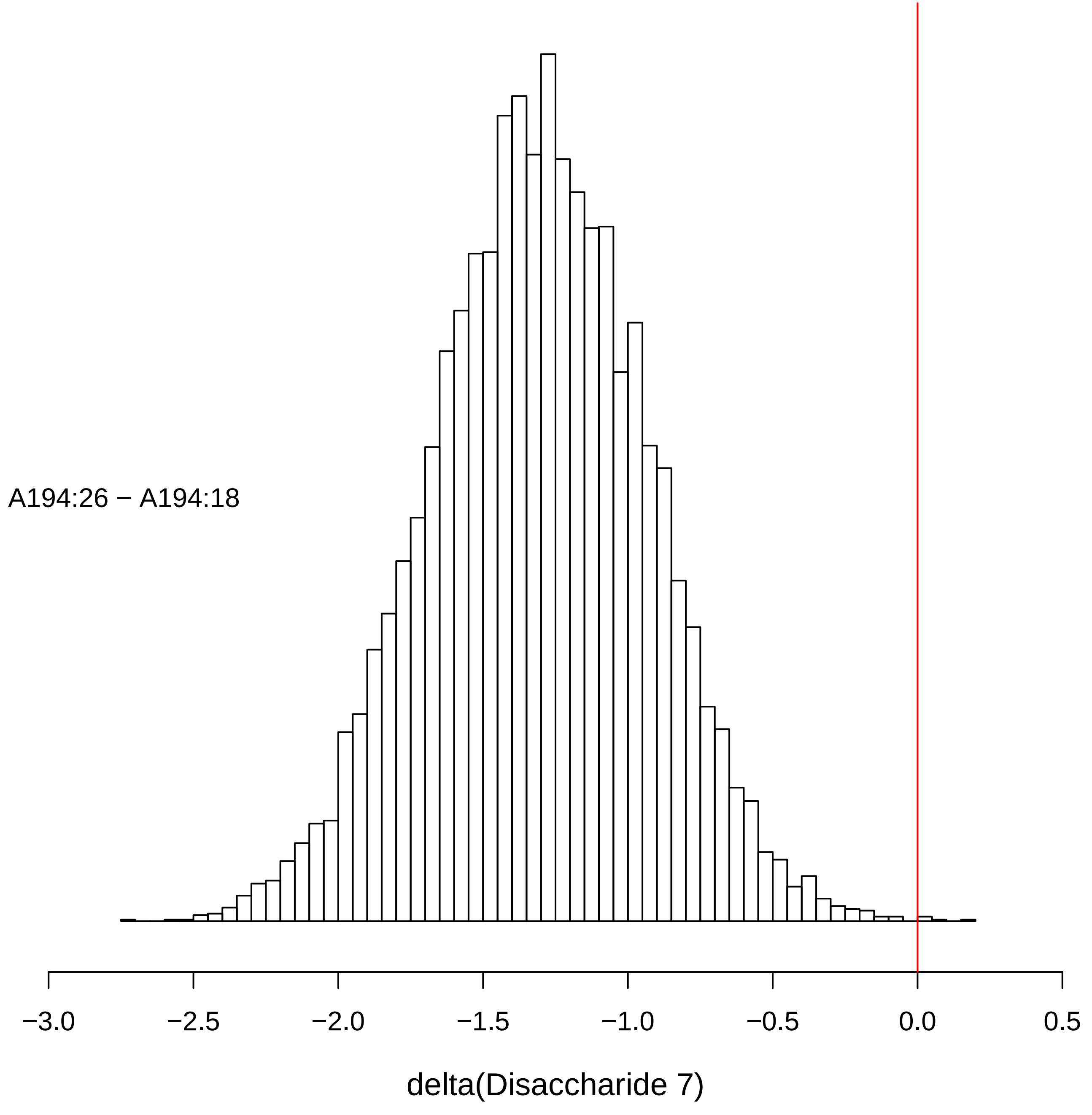

A194:26 − A194:18
−3.0
−2.5
−2.0
−1.5
−1.0
−0.5
0.0
0.5
delta(Disaccharide 7)

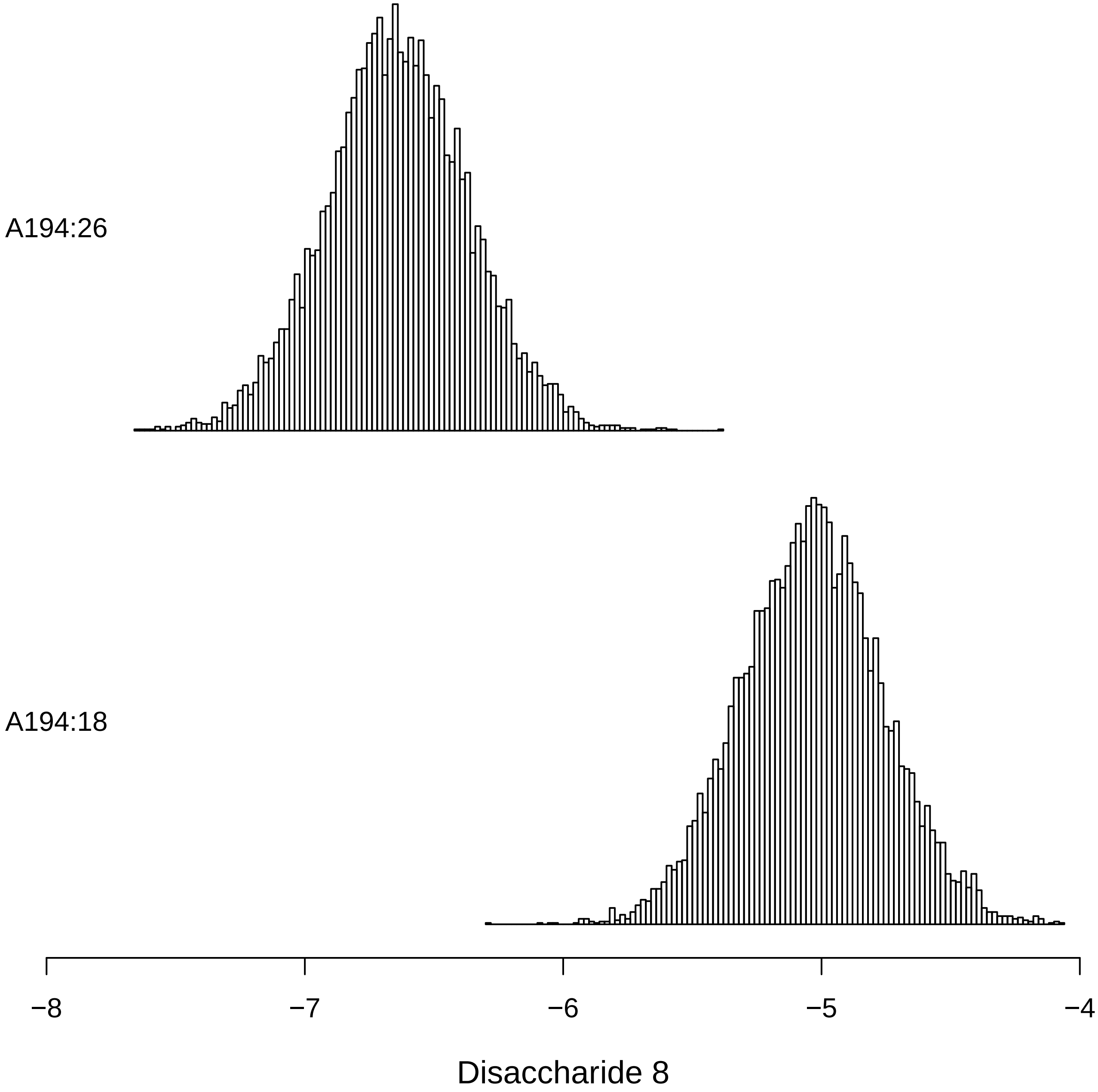
A194:26
A194:18
−8
−7
−6
−5
−4
Disaccharide 8

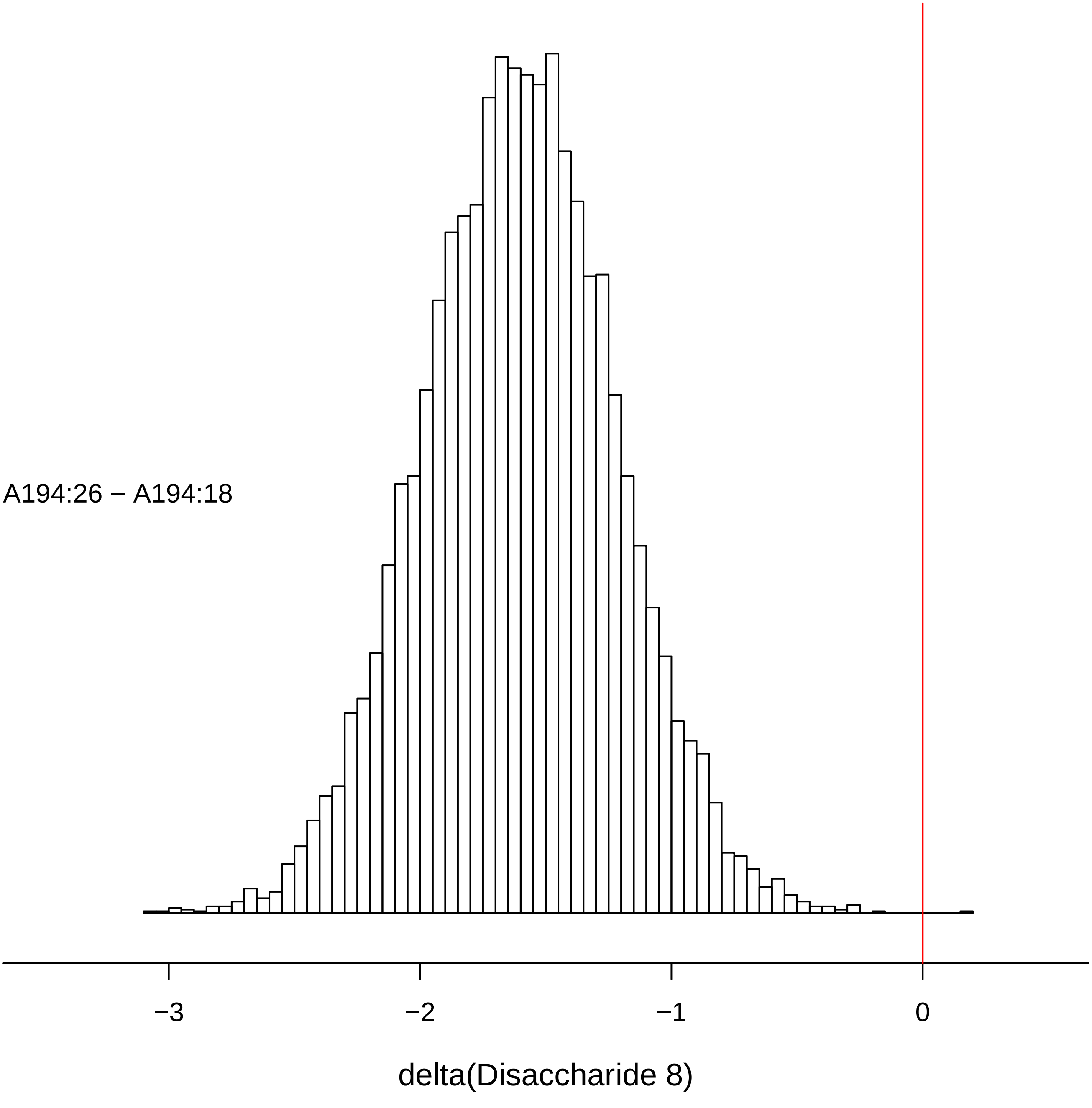
A194:26 − A194:18
−3
−2
−1
0
delta(Disaccharide 8)

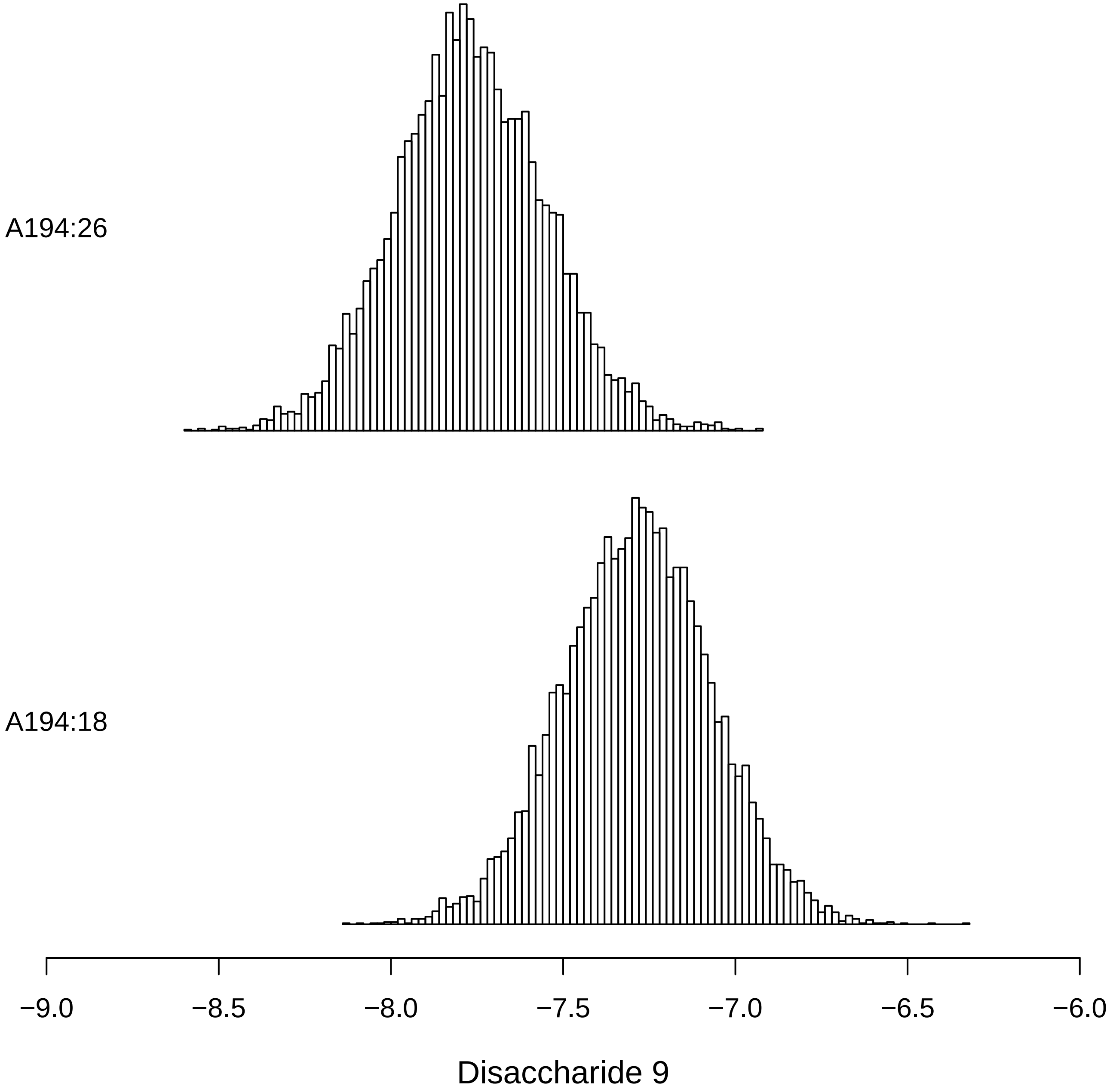
A194:26
A194:18
−9.0
−8.5
−8.0
−7.5
−7.0
−6.5
−6.0
Disaccharide 9

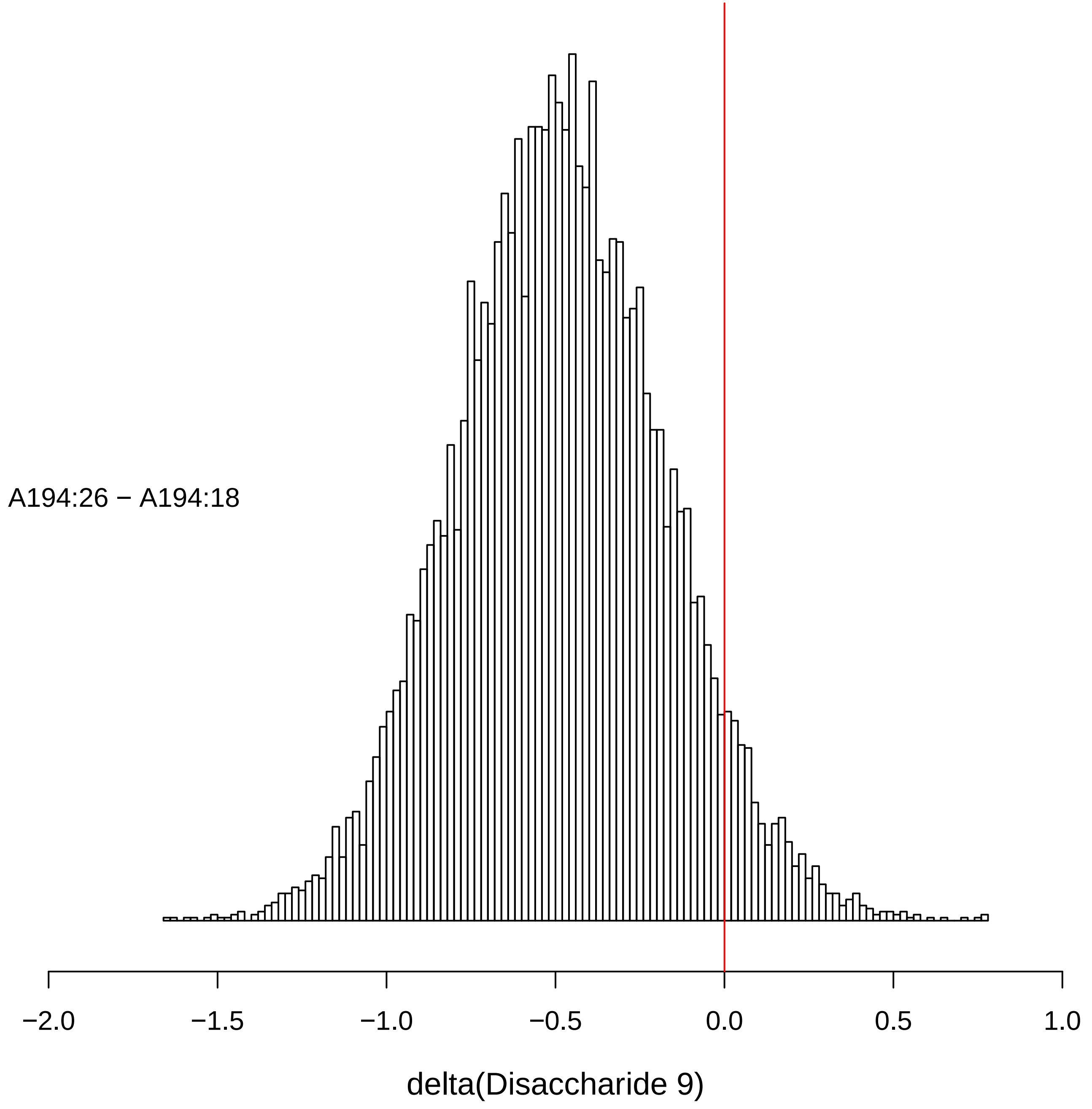
A194:26 − A194:18
−2.0
−1.5
−1.0
−0.5
0.0
0.5
1.0
delta(Disaccharide 9)

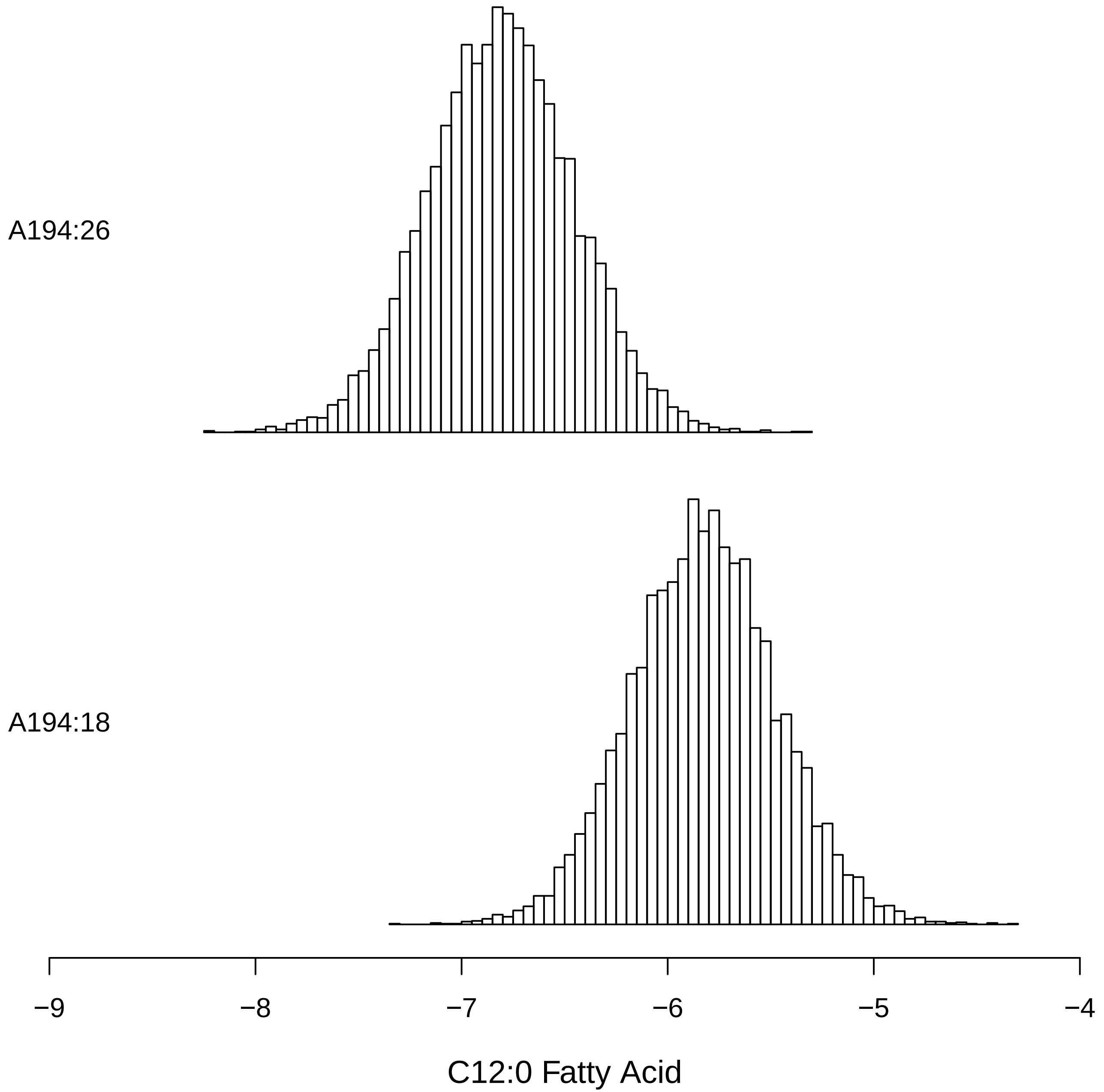
A194:26
A194:18
−9
−8
−7
−6
−5
−4
C12:0 Fatty Acid

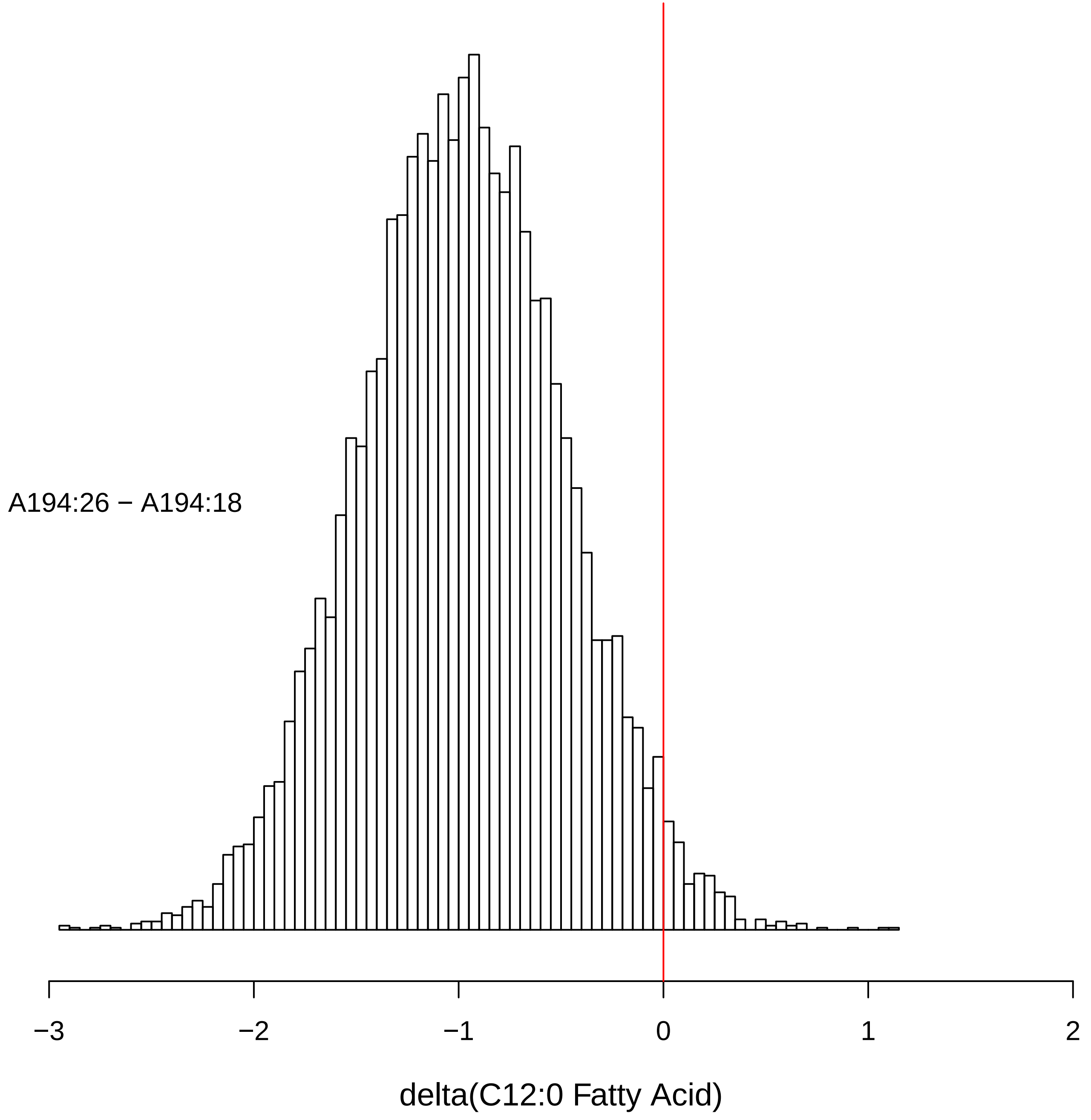
A194:26 − A194:18
−3
−2
−1
0
1
2
delta(C12:0 Fatty Acid)

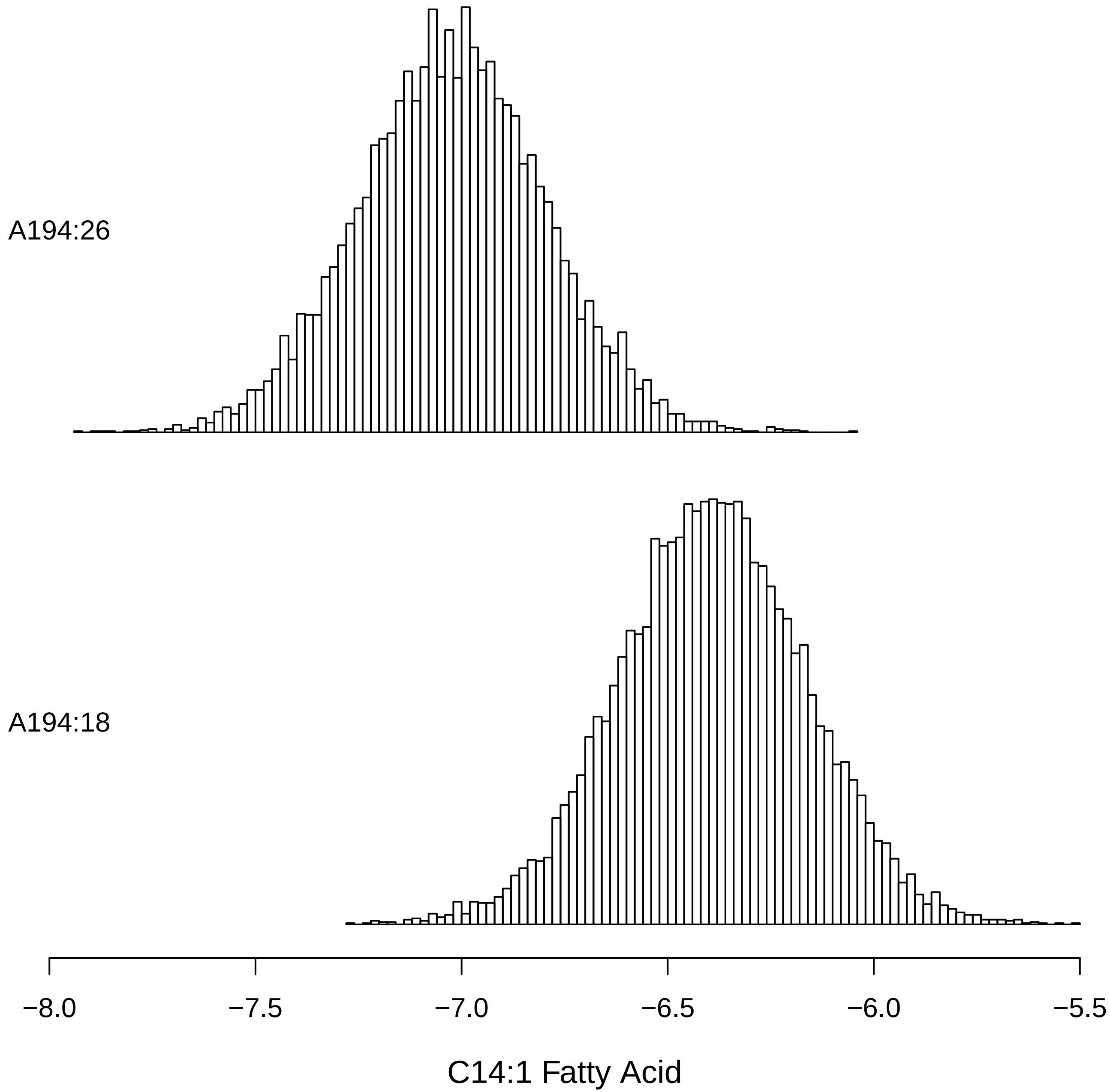
A194:26
A194:18
−8.0
−7.5
−7.0
−6.5
−6.0
−5.5
C14:1 Fatty Acid

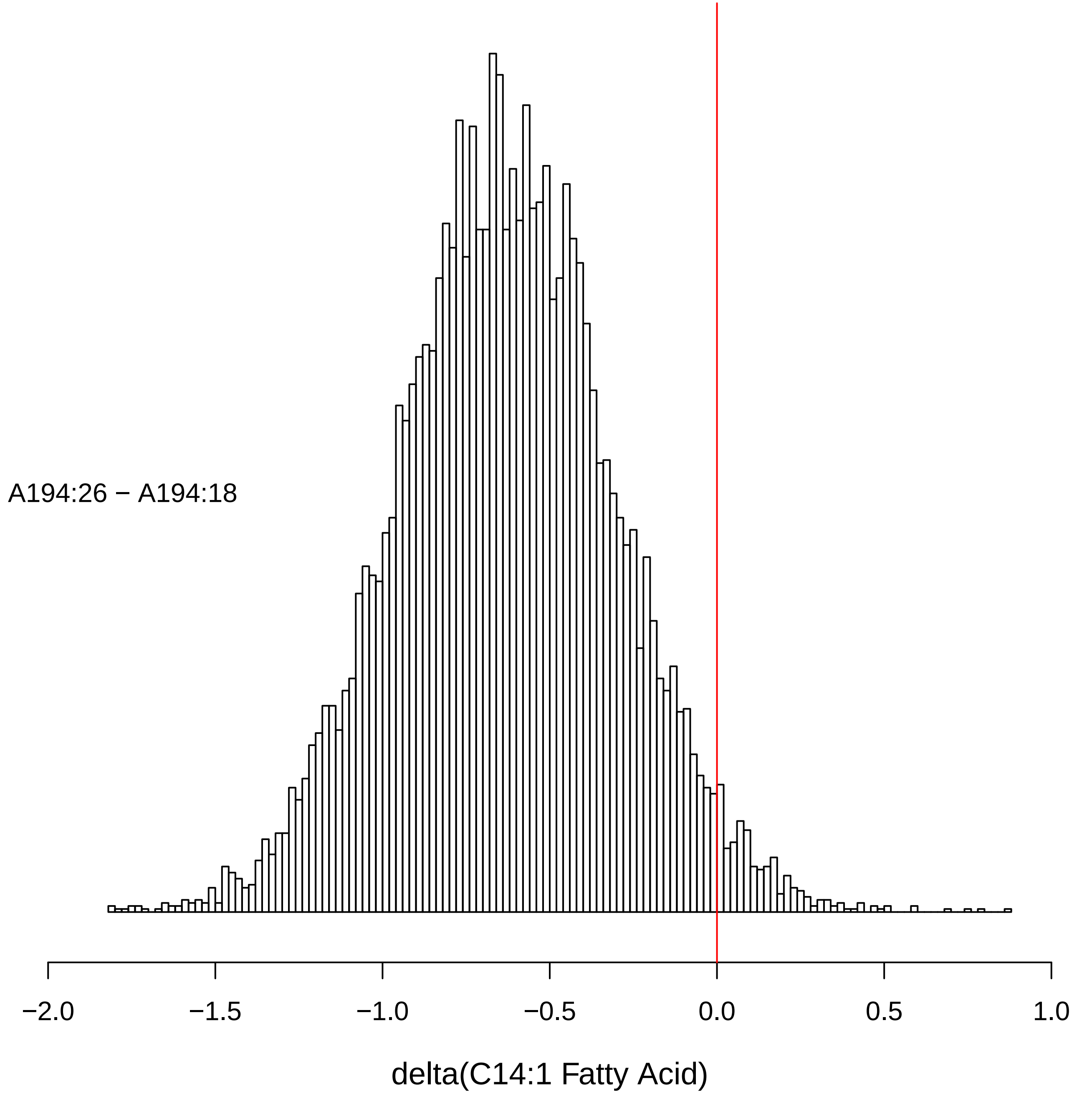
A194:26 − A194:18
−2.0
−1.5
−1.0
−0.5
0.0
0.5
1.0
delta(C14:1 Fatty Acid)

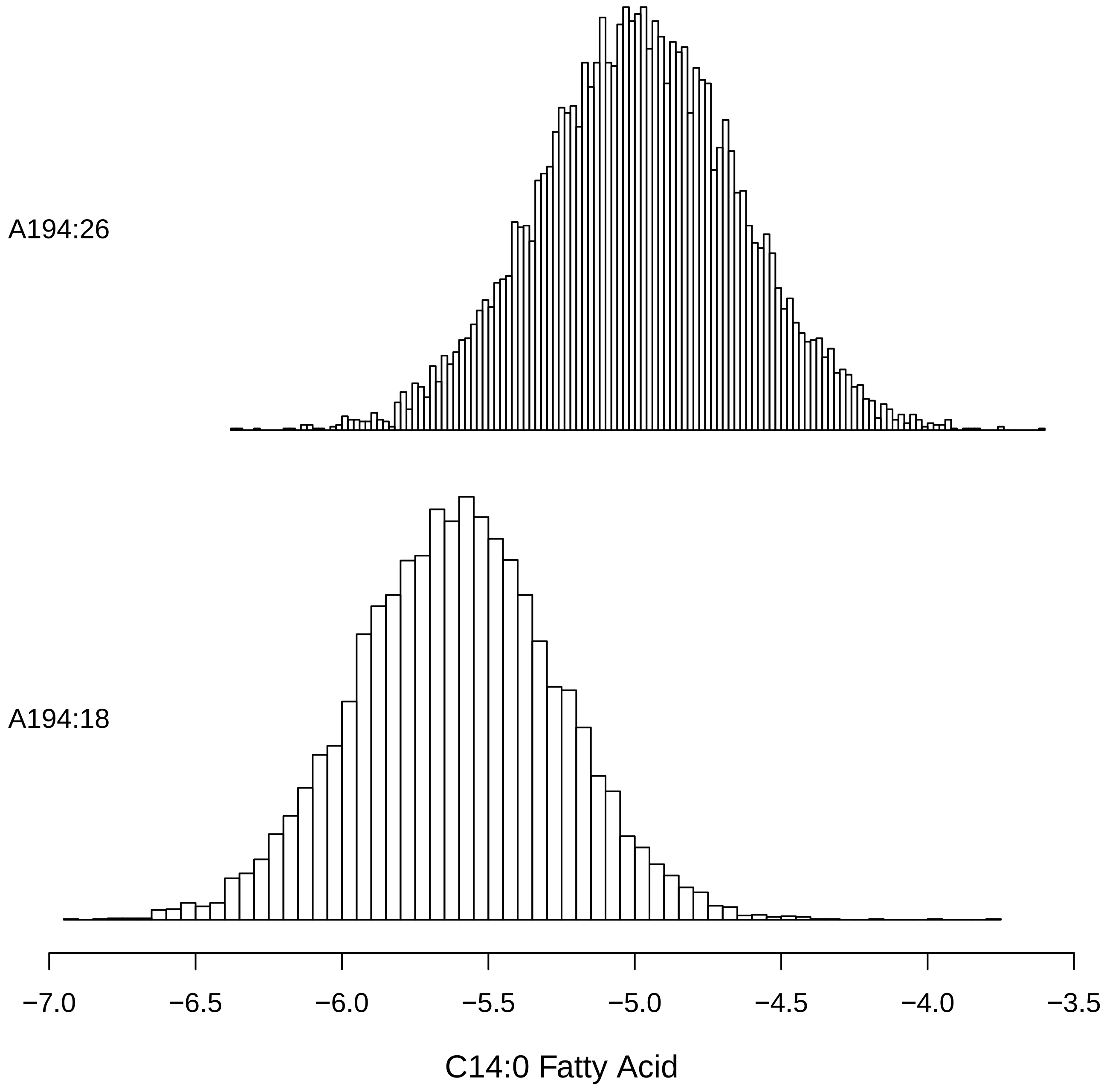
A194:26
A194:18
−7.0
−6.5
−6.0
−5.5
−5.0
−4.5
−4.0
−3.5
C14:0 Fatty Acid

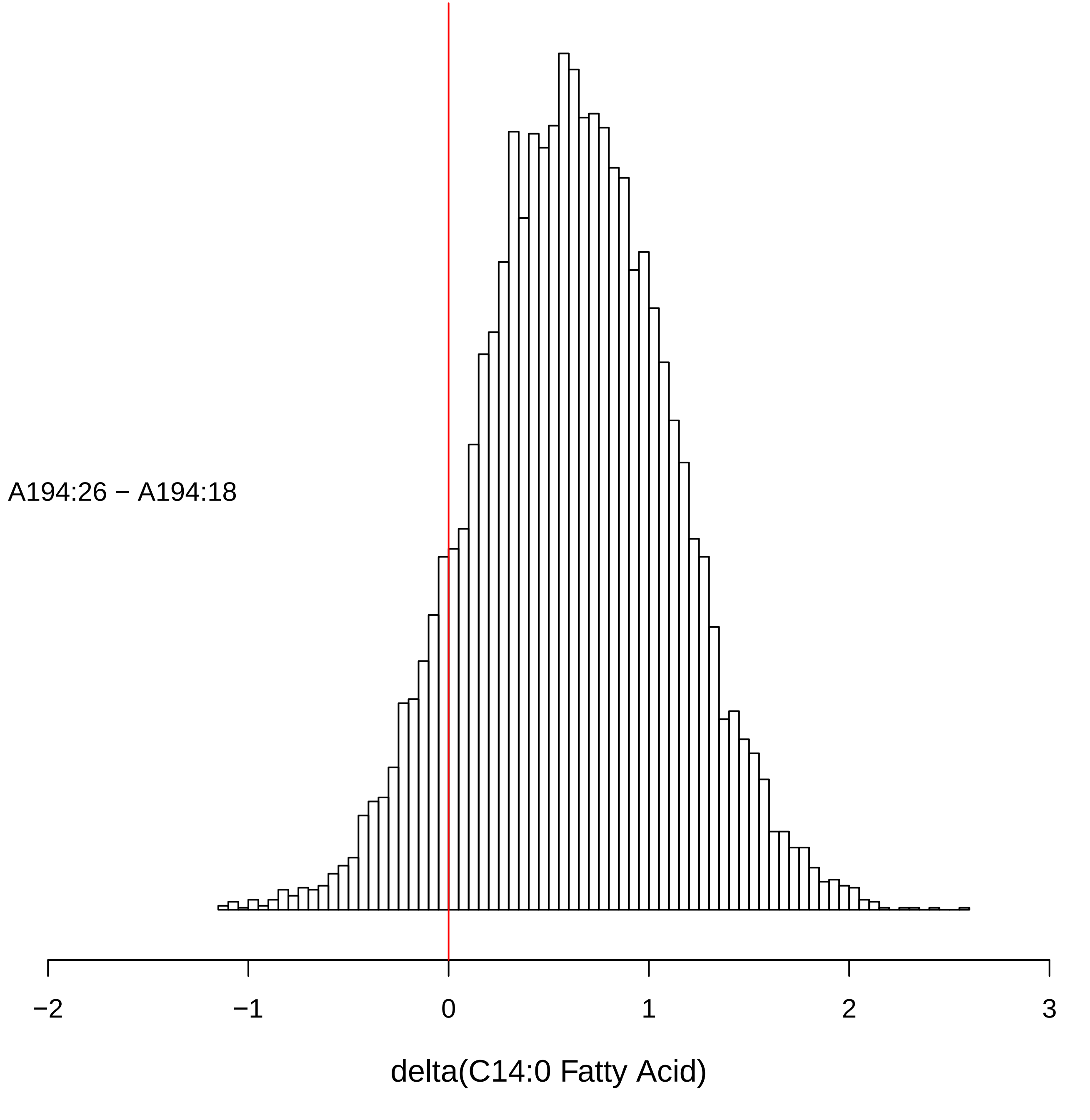
A194:26 − A194:18
−2
−1
0
1
2
3
delta(C14:0 Fatty Acid)

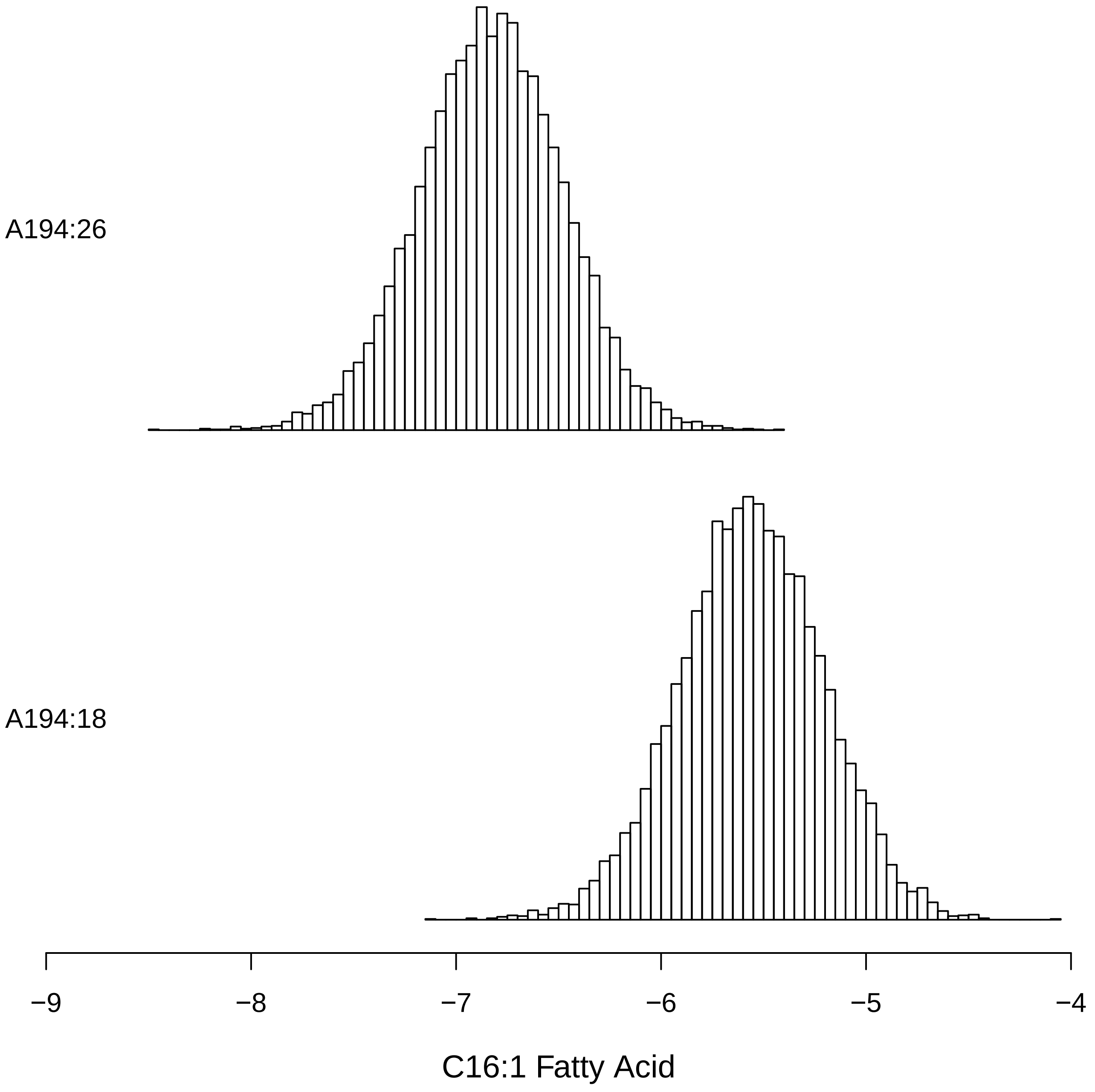
A194:26
A194:18
−9
−8
−7
−6
−5
−4
C16:1 Fatty Acid

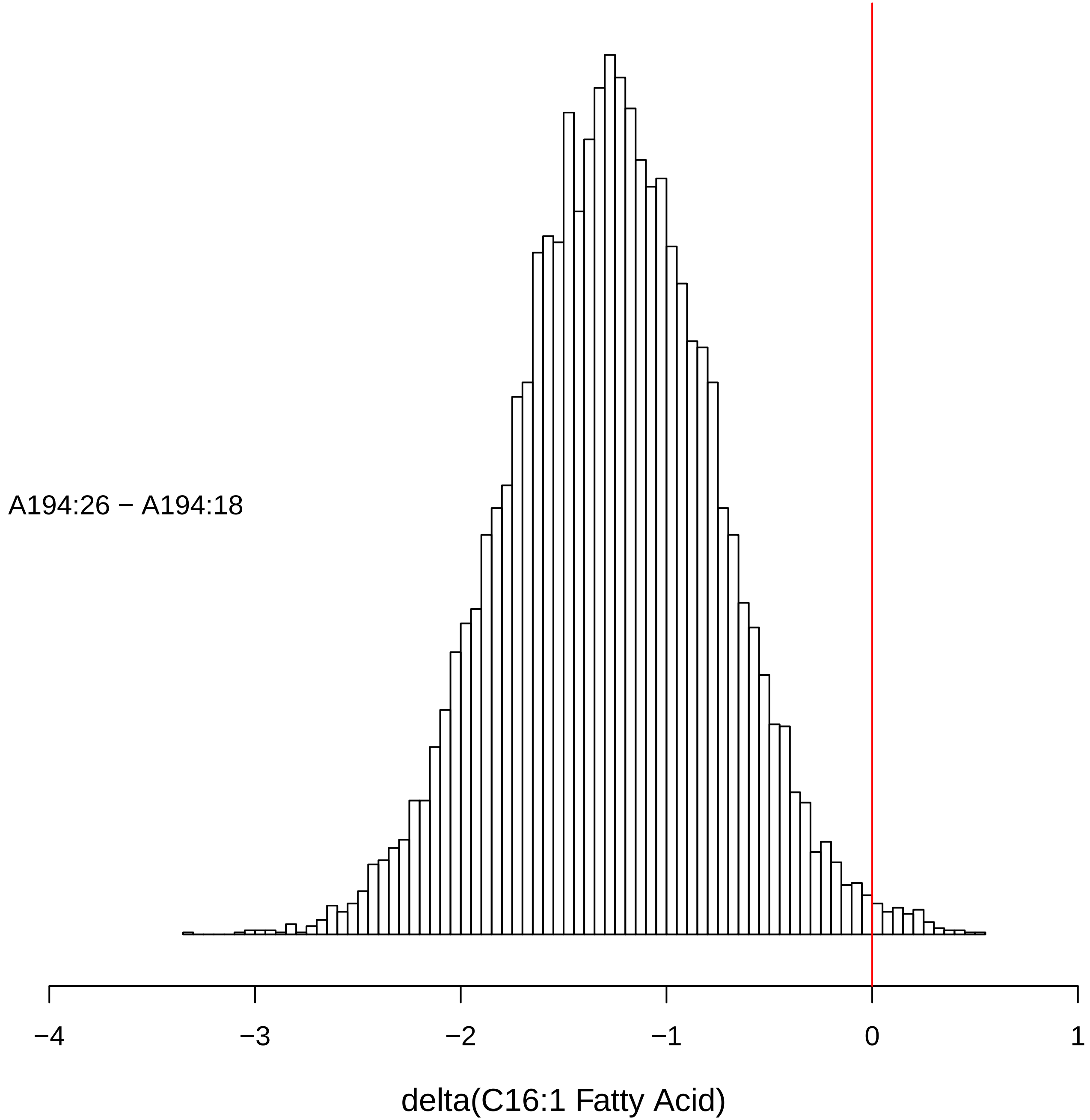
A194:26 − A194:18
−4
−3
−2
−1
0
1
delta(C16:1 Fatty Acid)

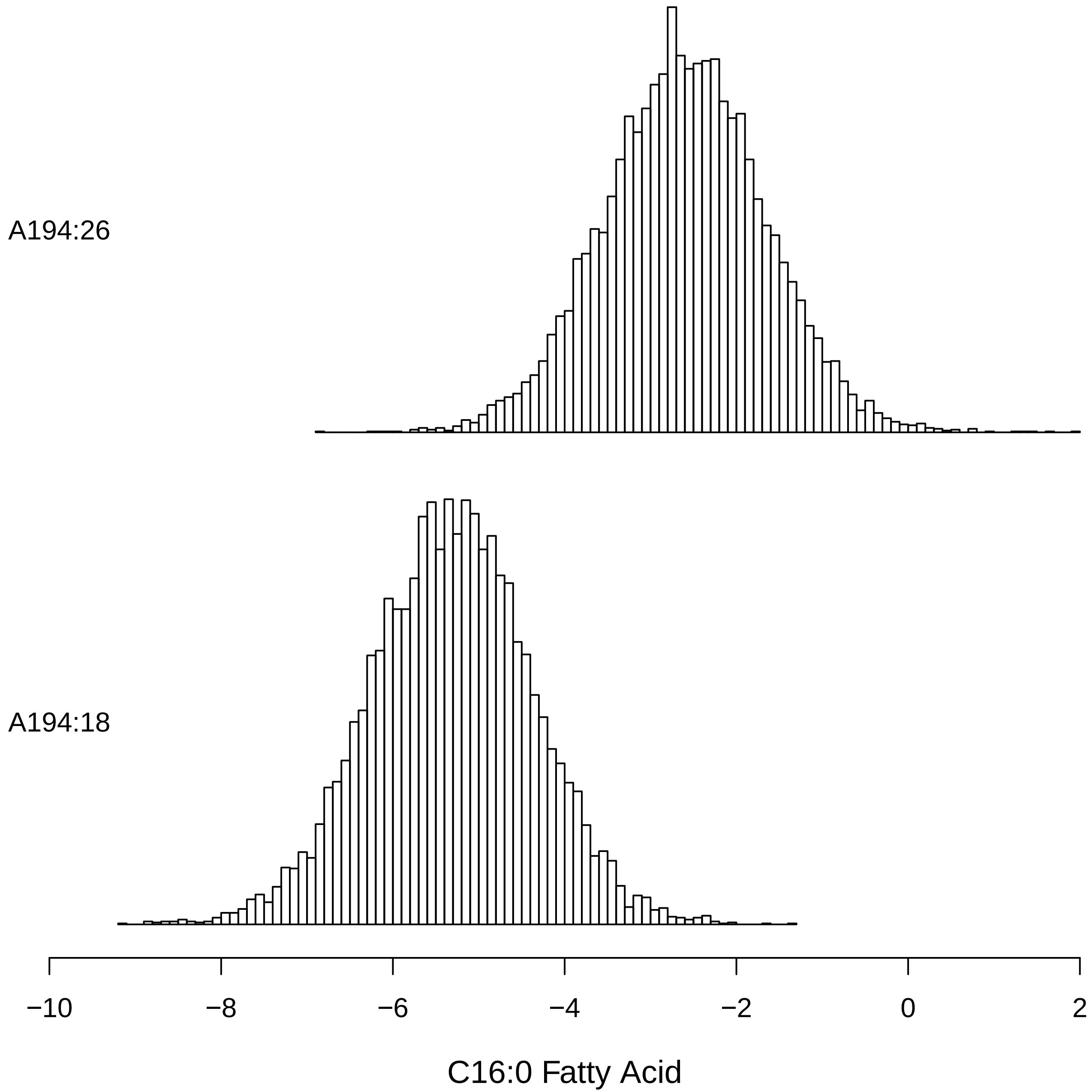
A194:26
A194:18
−10
−8
−6
−4
−2
0
2
C16:0 Fatty Acid

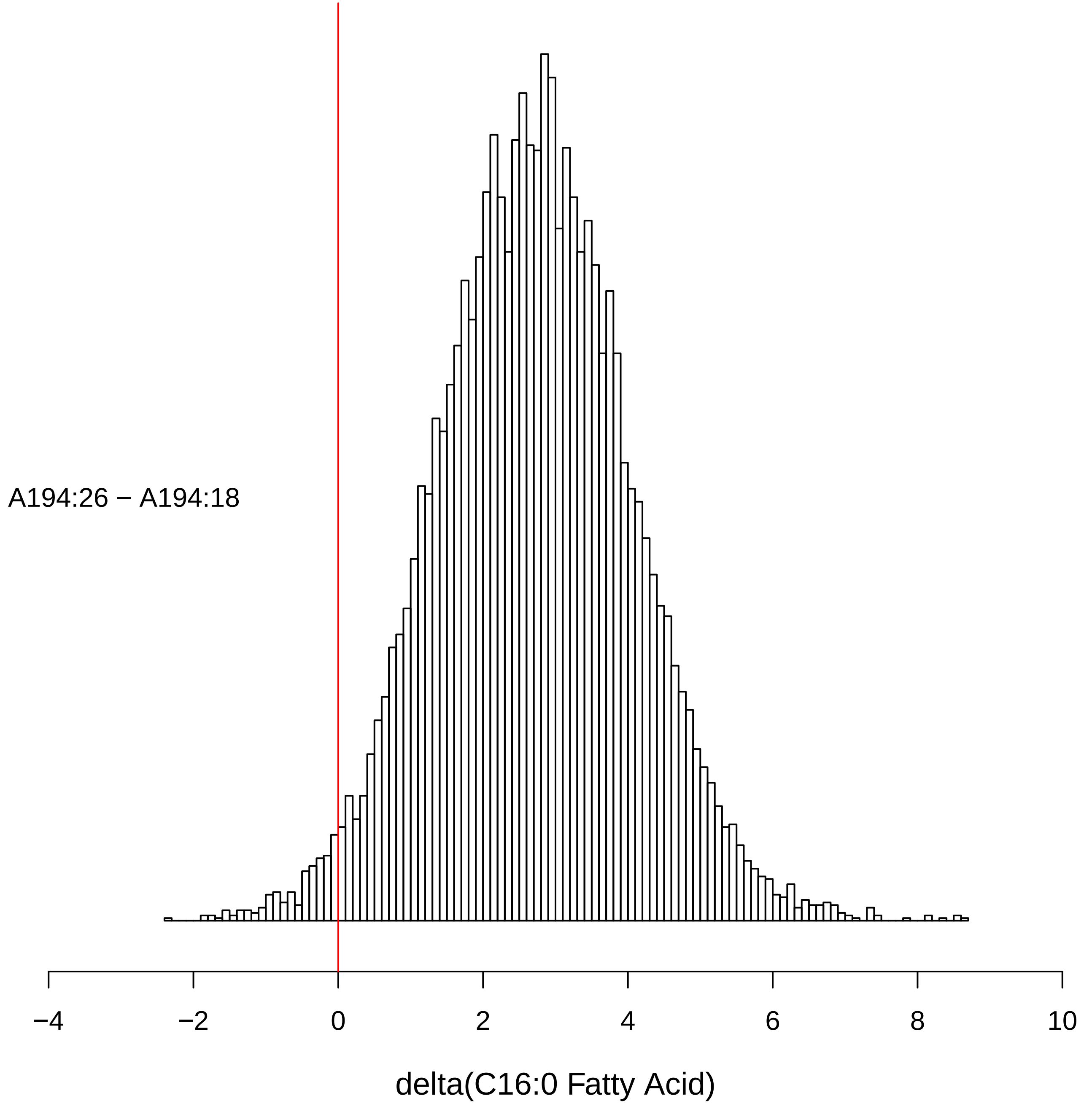
A194:26 − A194:18
−4
−2
0
2
4
6
8
10
delta(C16:0 Fatty Acid)

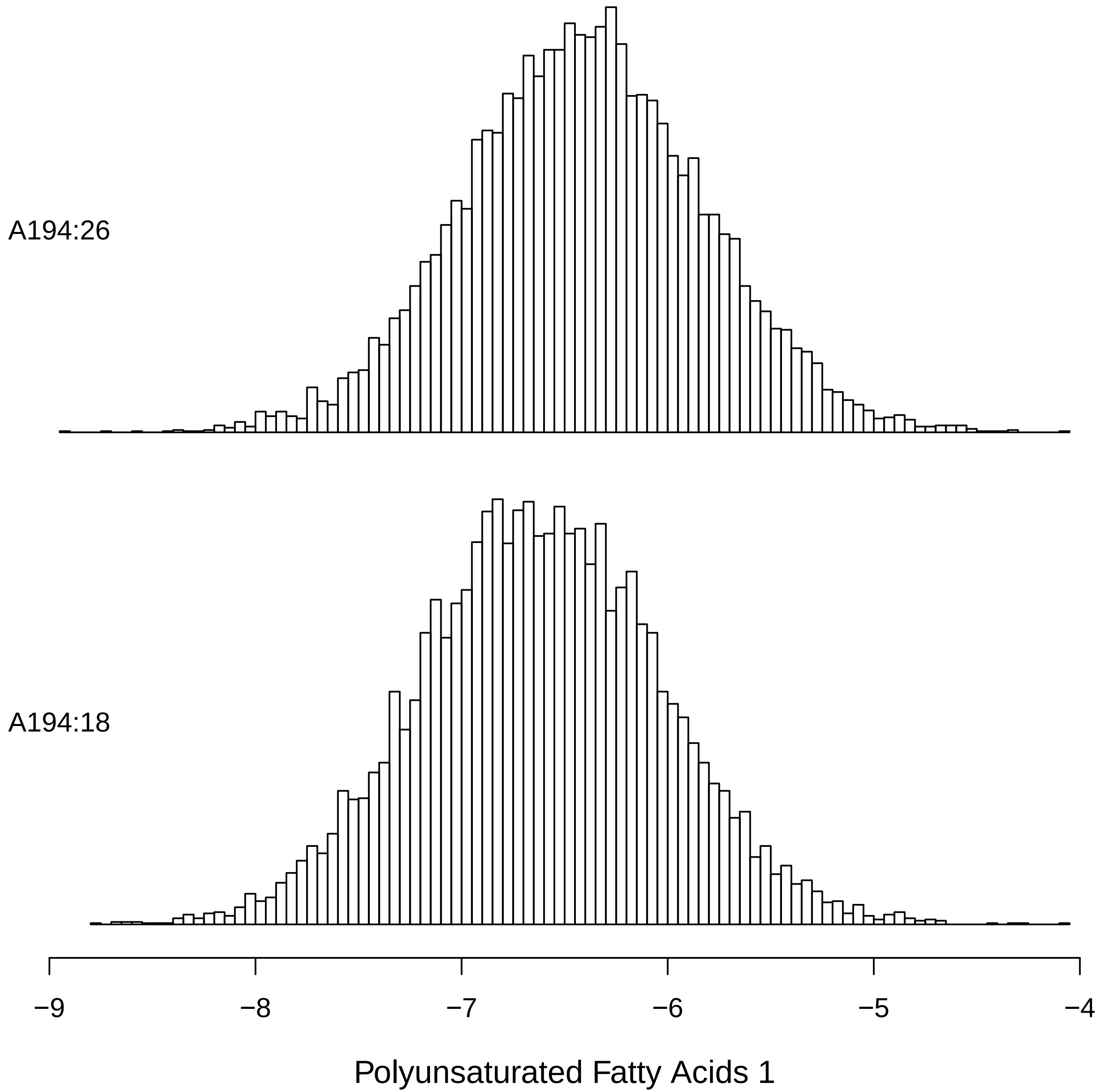
A194:26
A194:18
−9
−8
−7
−6
−5
−4
Polyunsaturated Fatty Acids 1

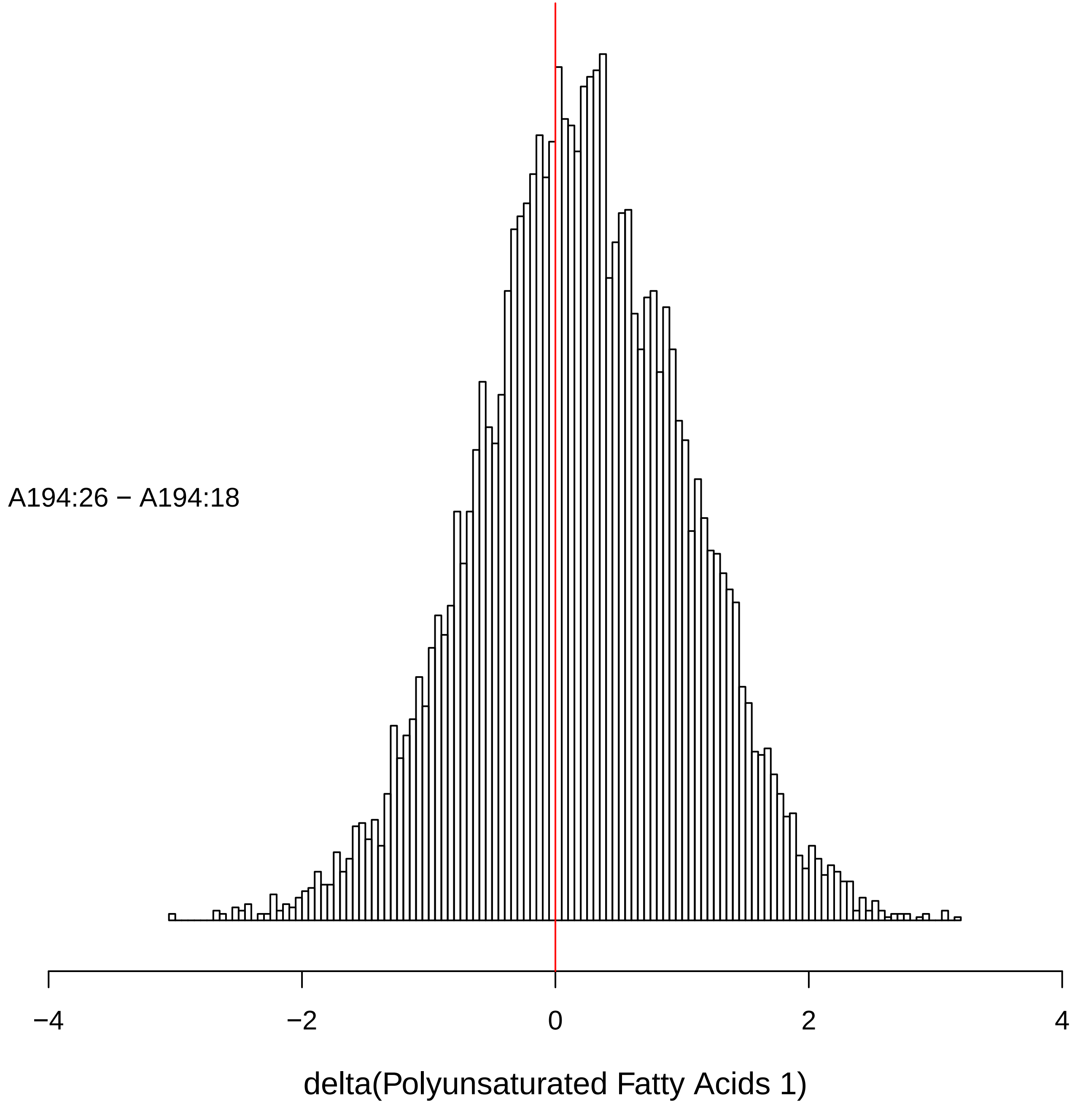
A194:26 − A194:18
−4
−2
0
2
4
delta(Polyunsaturated Fatty Acids 1)

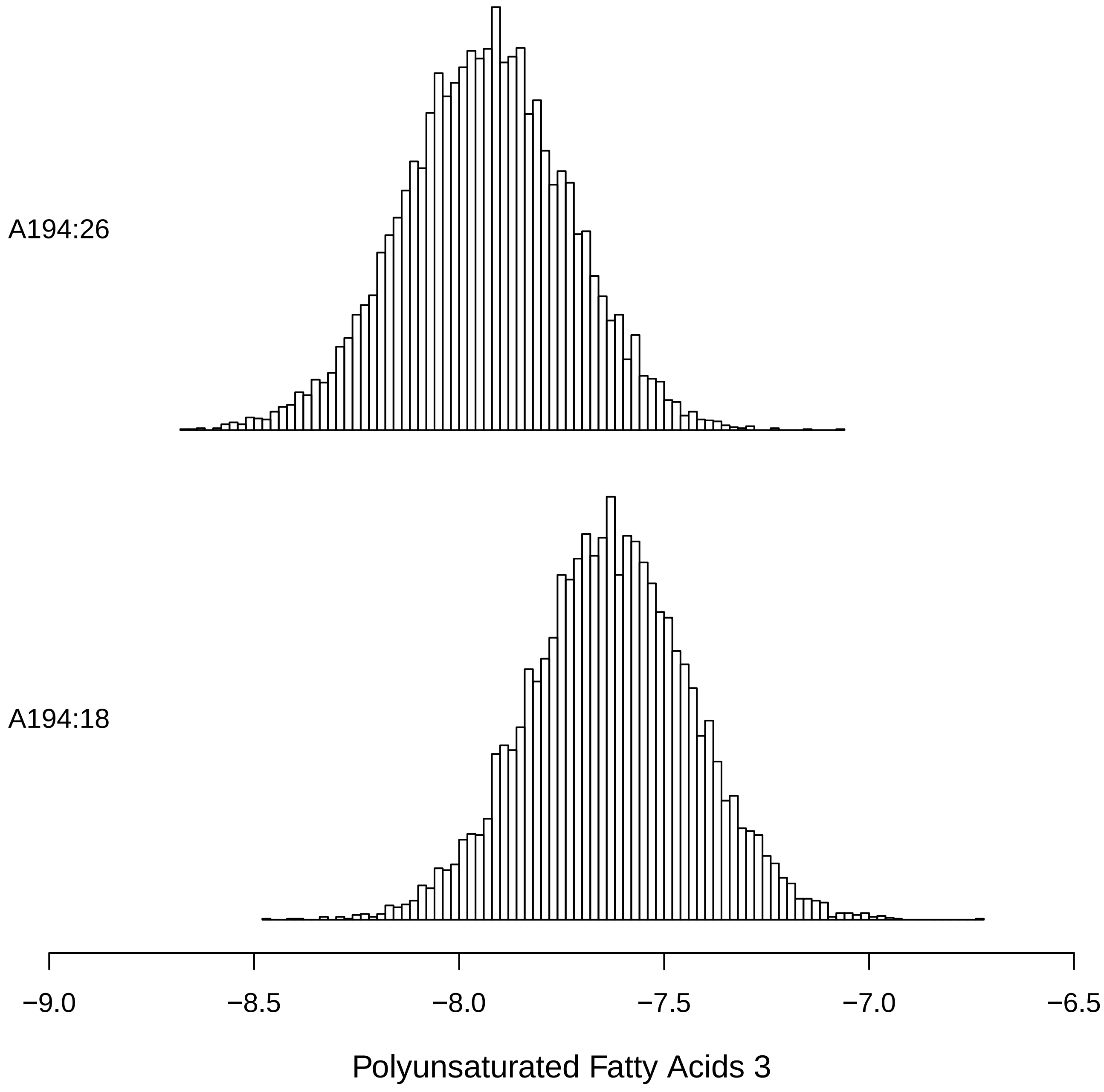
A194:26
A194:18
−9.0
−8.5
−8.0
−7.5
−7.0
−6.5
Polyunsaturated Fatty Acids 3

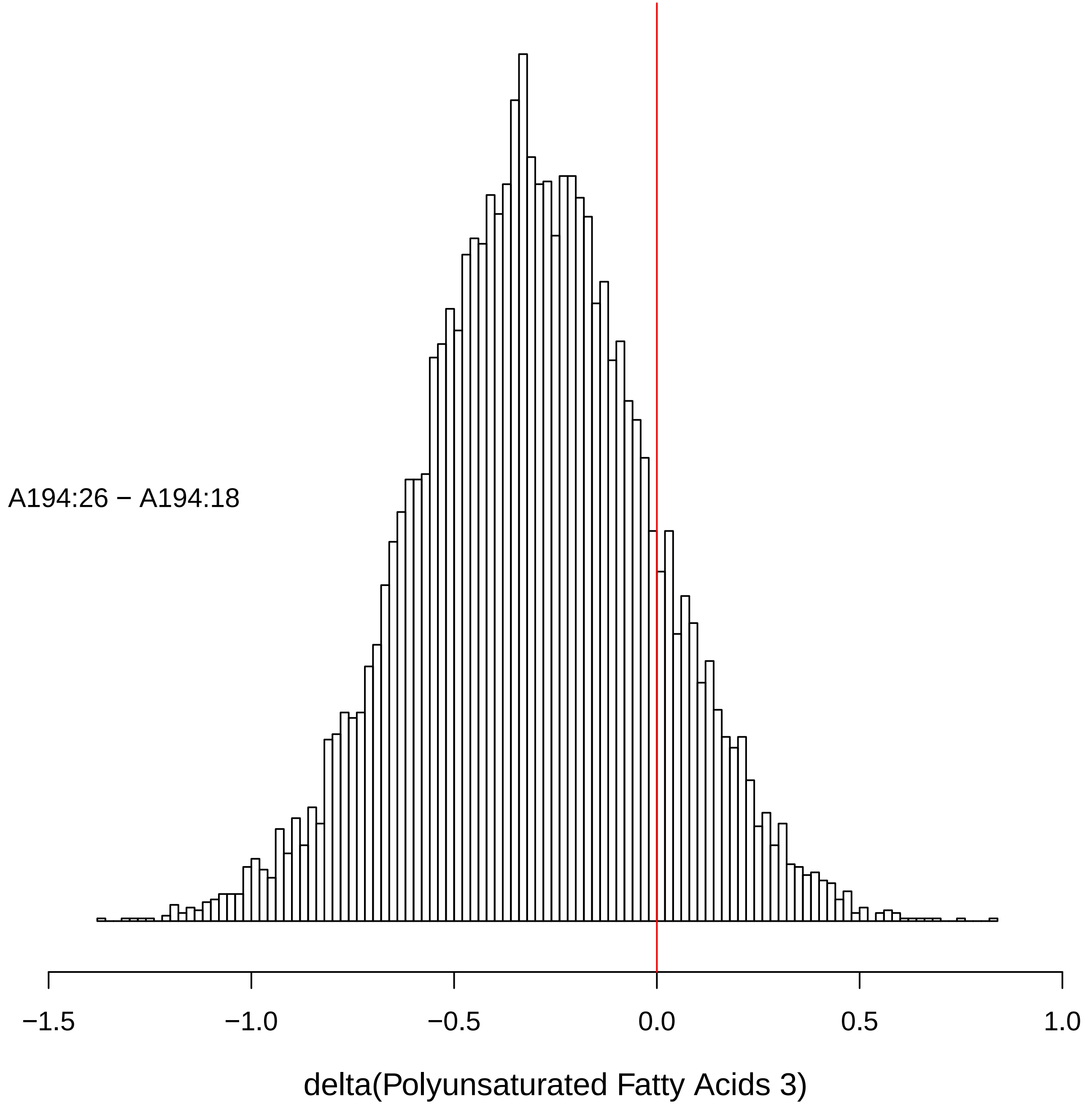

A194:26 − A194:18
−1.5
−1.0
−0.5
0.0
0.5
1.0
delta(Polyunsaturated Fatty Acids 3)

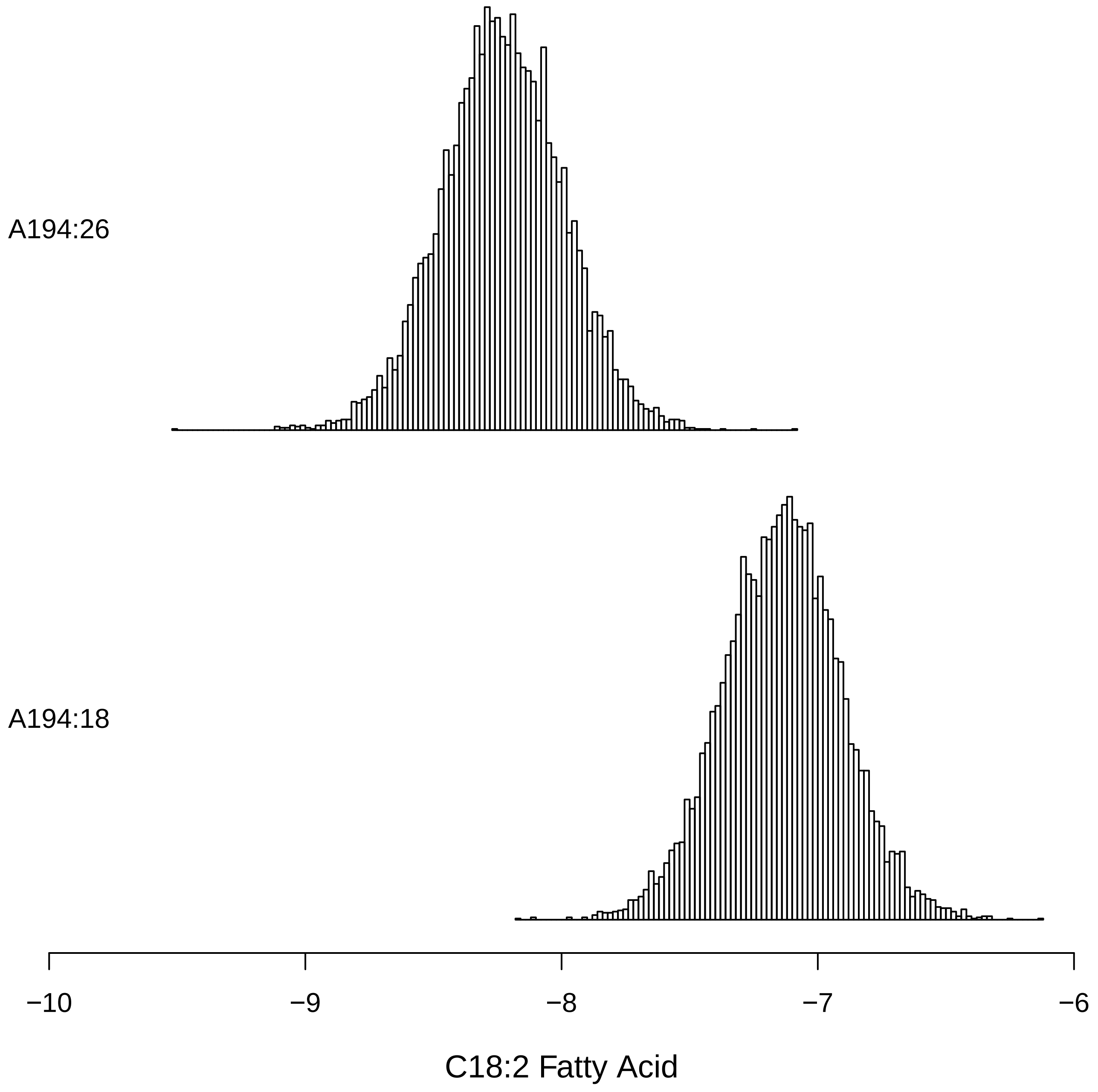
A194:26
A194:18
−10
−9
−8
−7
−6
C18:2 Fatty Acid

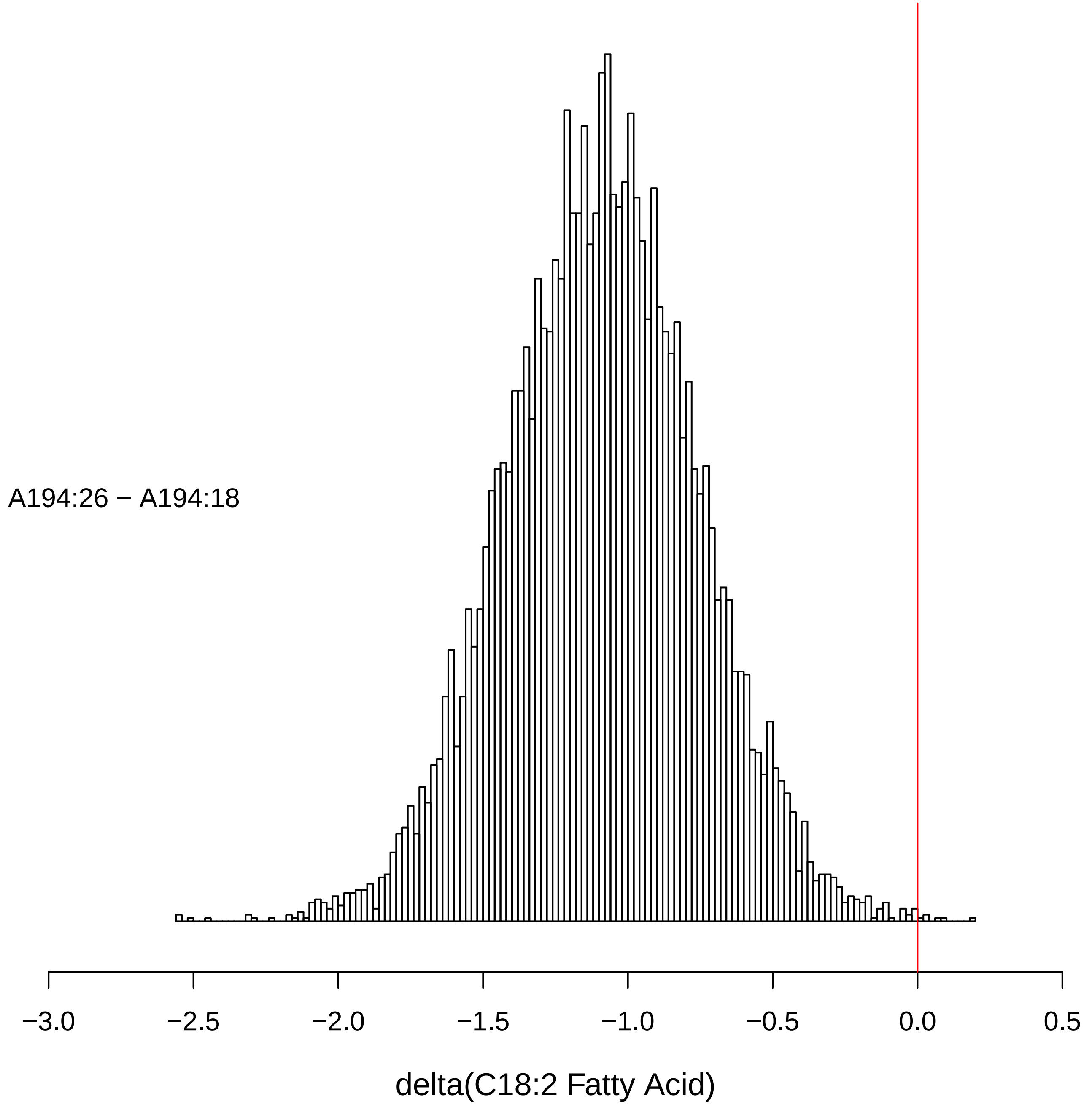
A194:26 − A194:18
−3.0
−2.5
−2.0
−1.5
−1.0
−0.5
0.0
0.5
delta(C18:2 Fatty Acid)

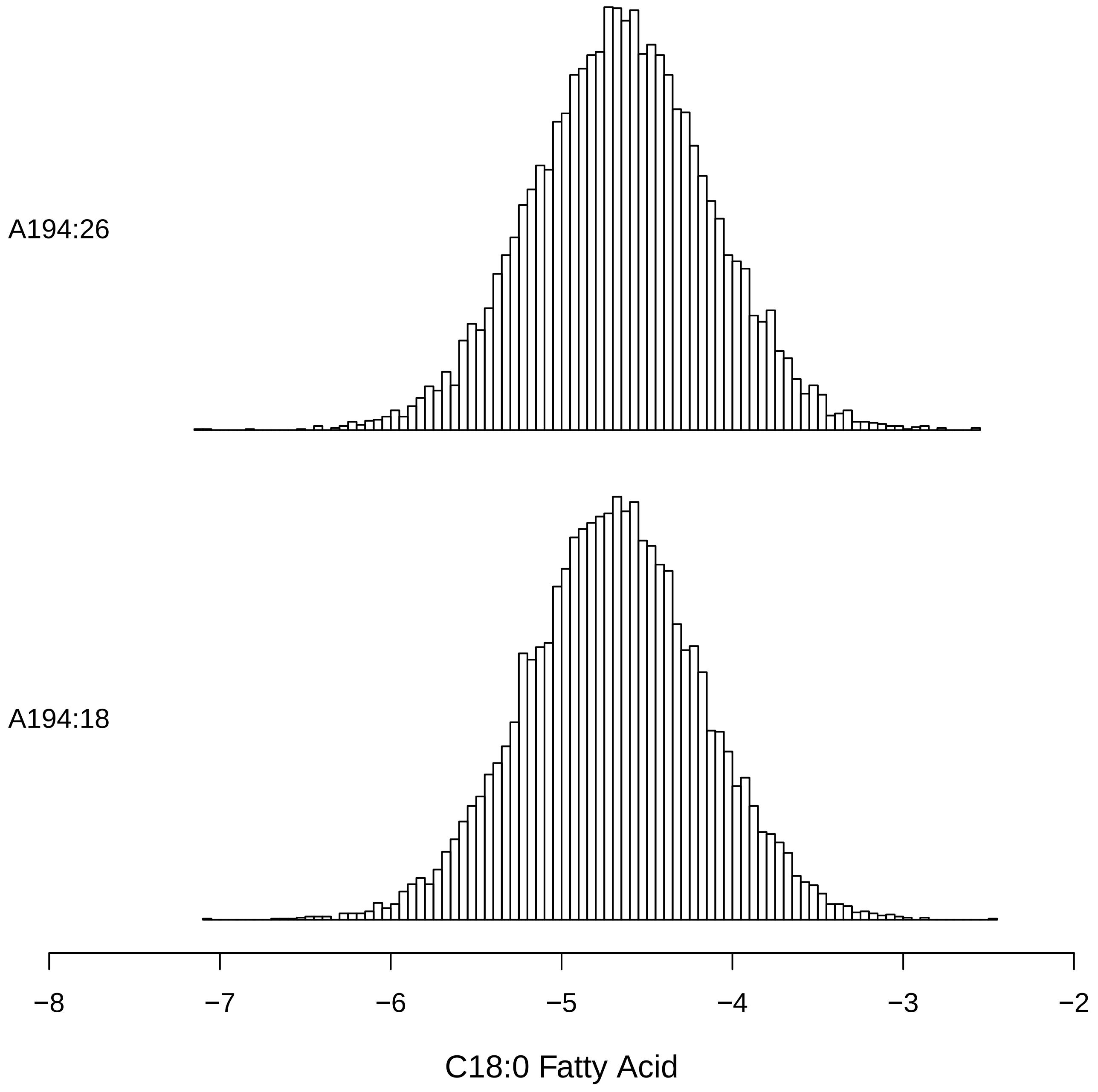
A194:26
A194:18
−8
−7
−6
−5
−4
−3
−2
C18:0 Fatty Acid

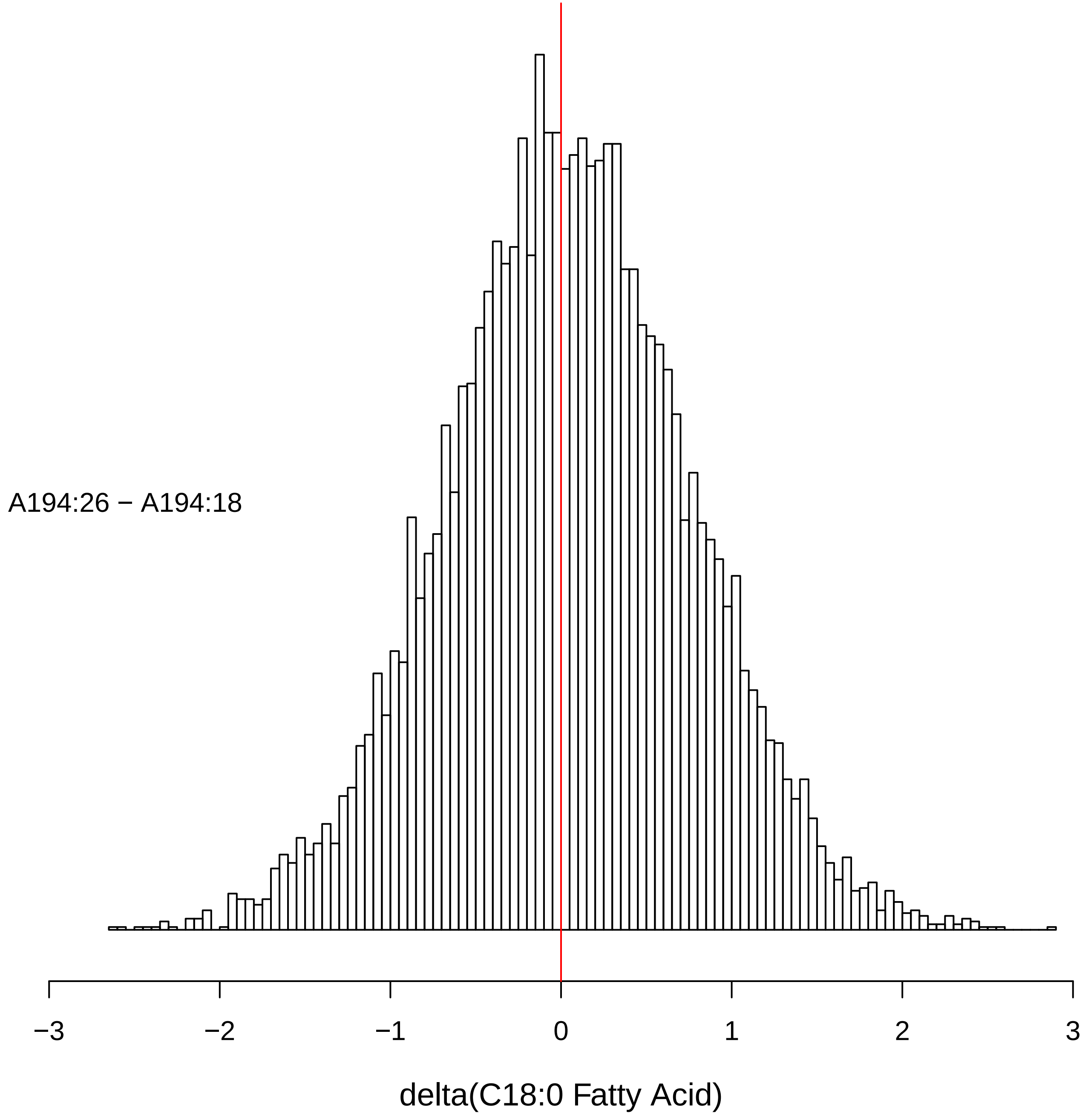
A194:26 − A194:18
−3
−2
−1
0
1
2
3
delta(C18:0 Fatty Acid)

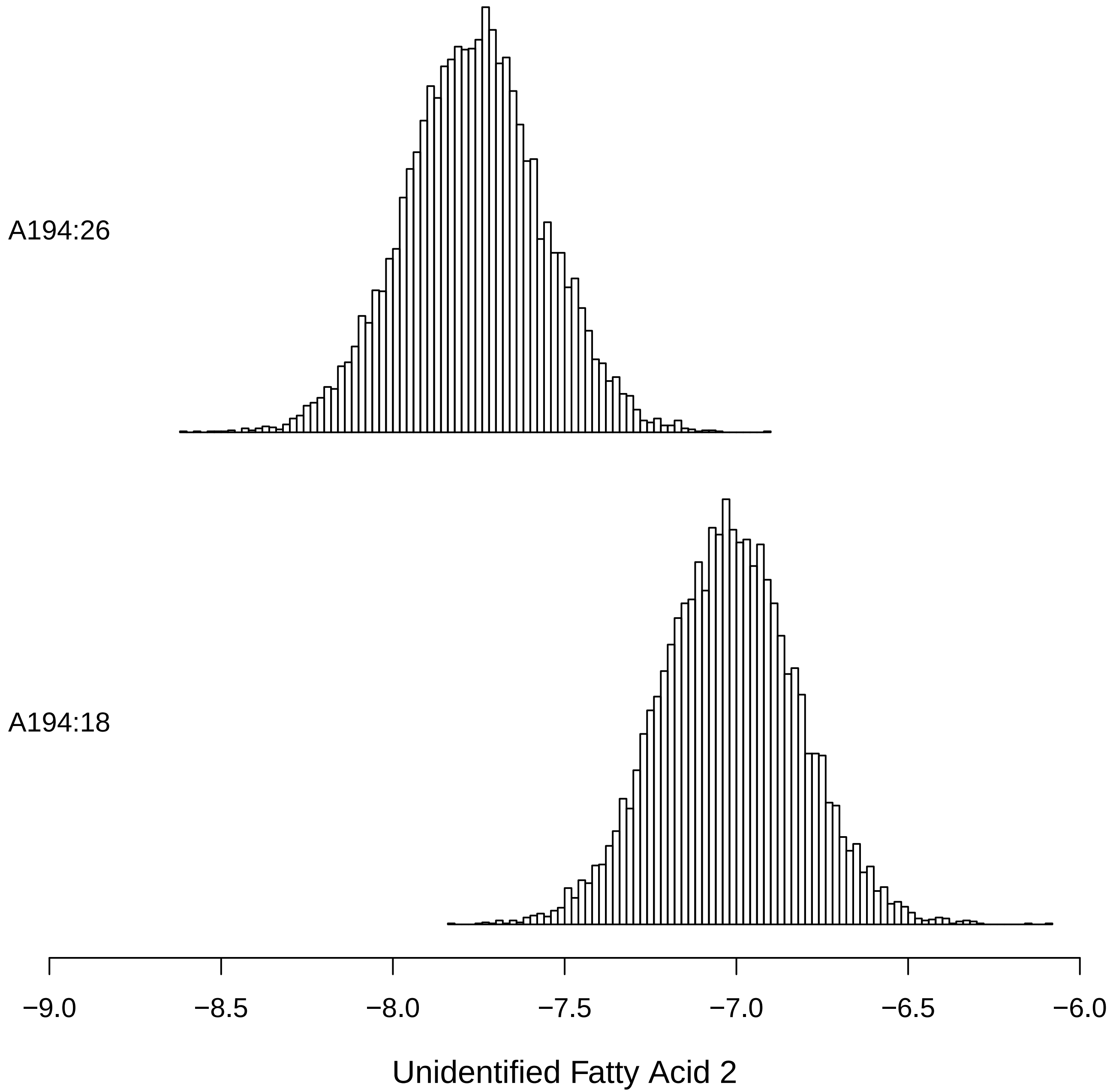
A194:26
A194:18
−9.0
−8.5
−8.0
−7.5
−7.0
−6.5
−6.0
Unidentified Fatty Acid 2

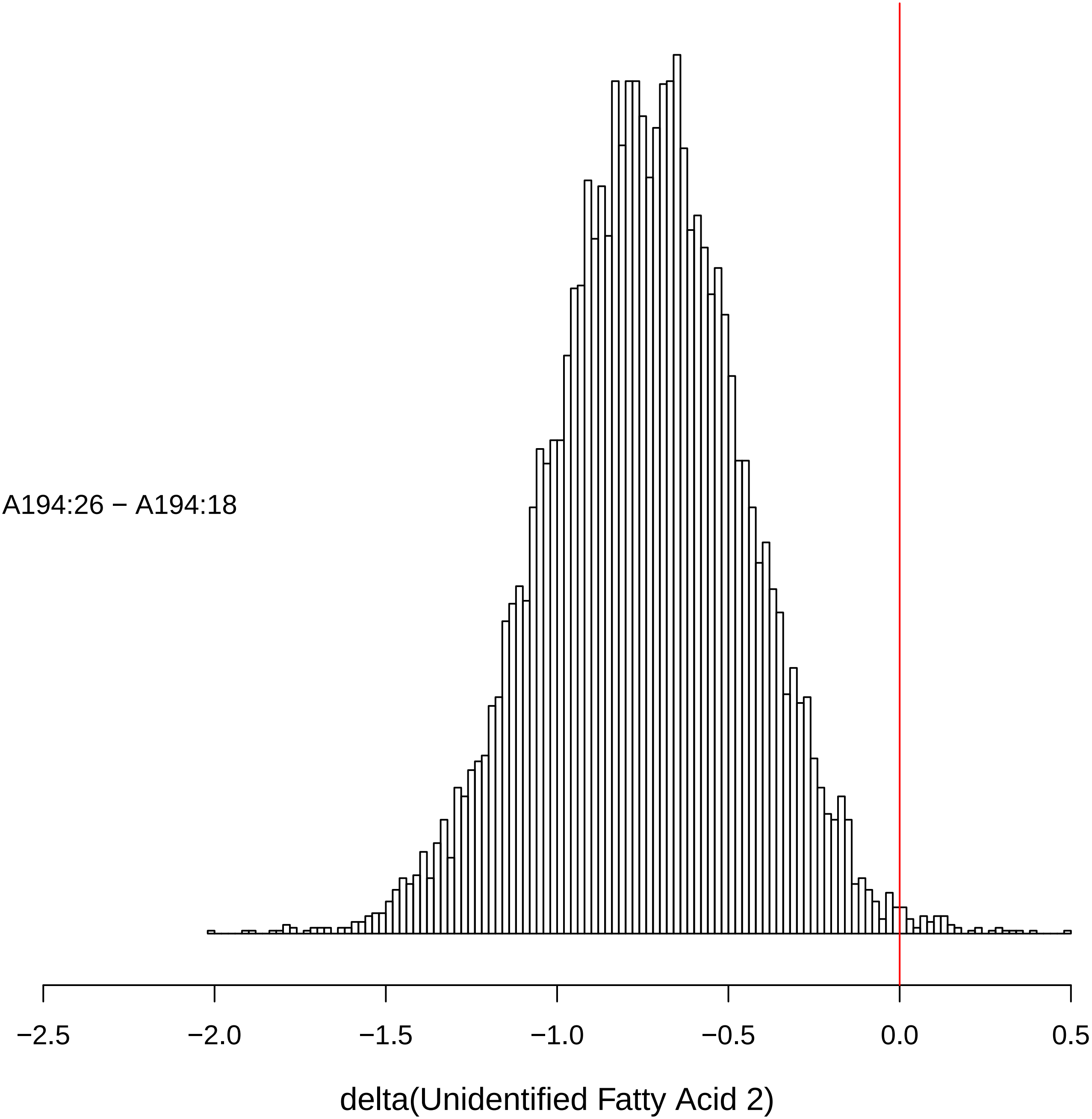
A194:26 − A194:18
−2.5
−2.0
−1.5
−1.0
−0.5
0.0
0.5
delta(Unidentified Fatty Acid 2)

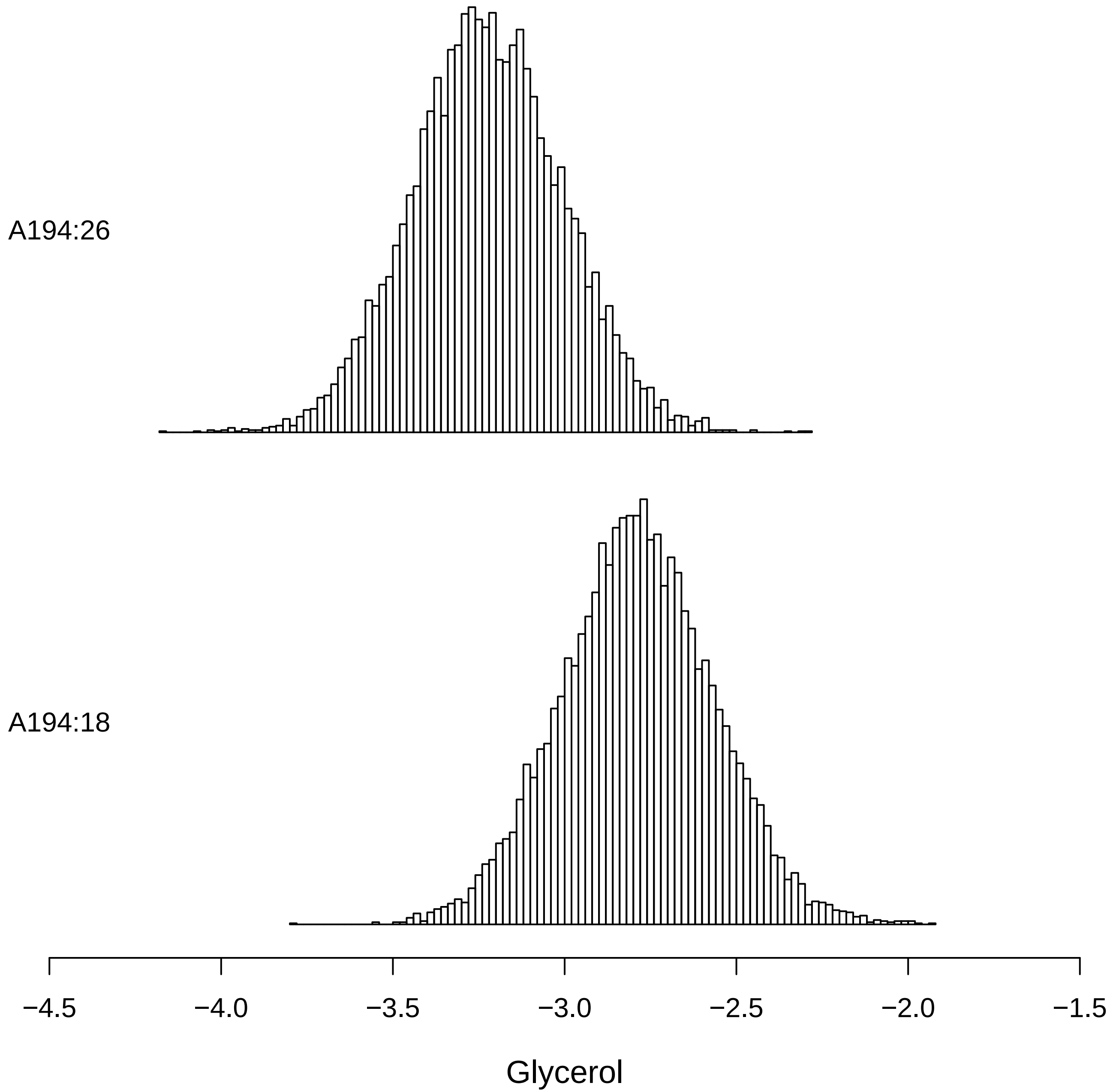
A194:26
A194:18
−4.5
−4.0
−3.5
−3.0
−2.5
−2.0
−1.5
Glycerol

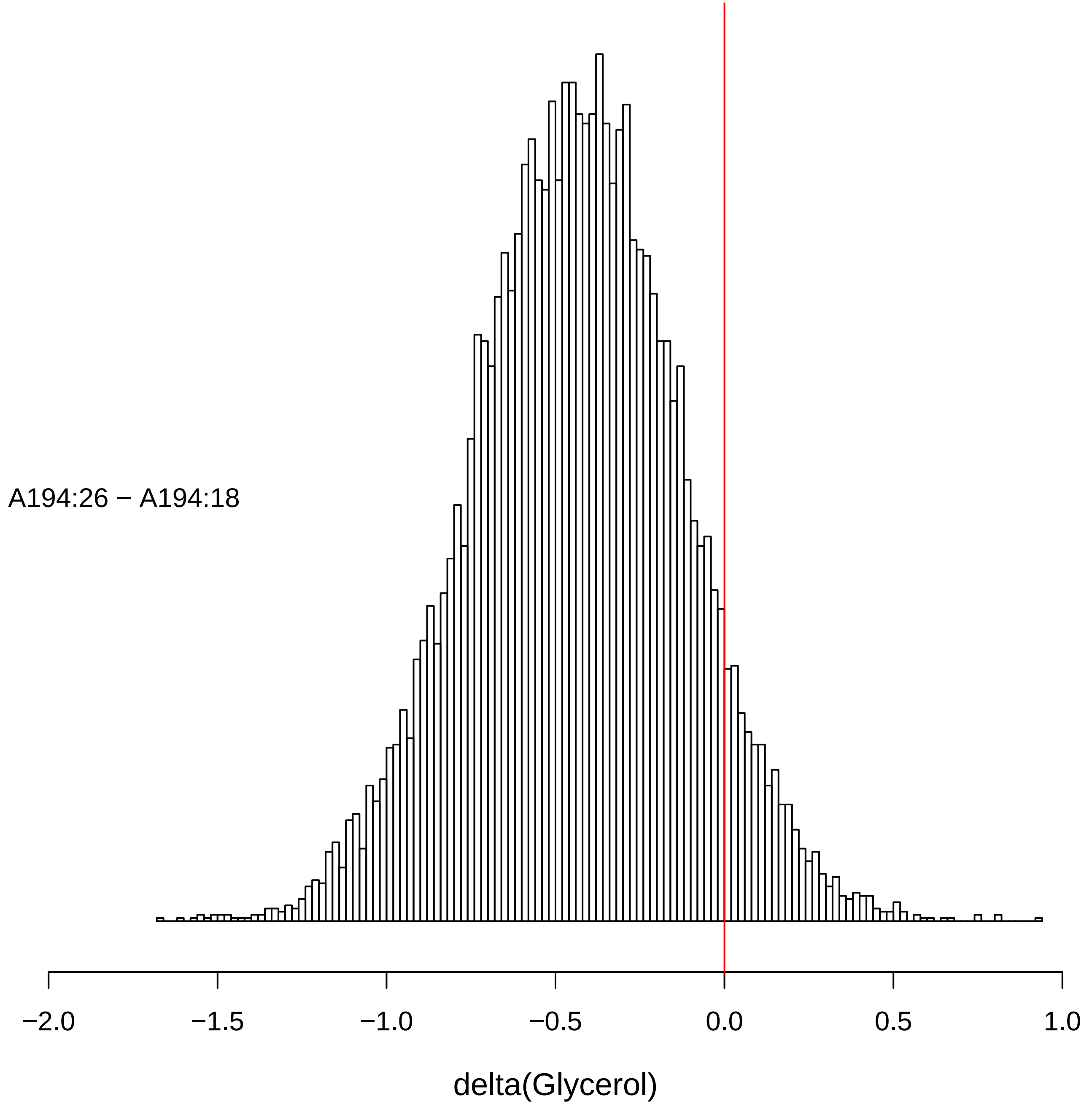
A194:26 − A194:18
−2.0
−1.5
−1.0
−0.5
0.0
0.5
1.0
delta(Glycerol)

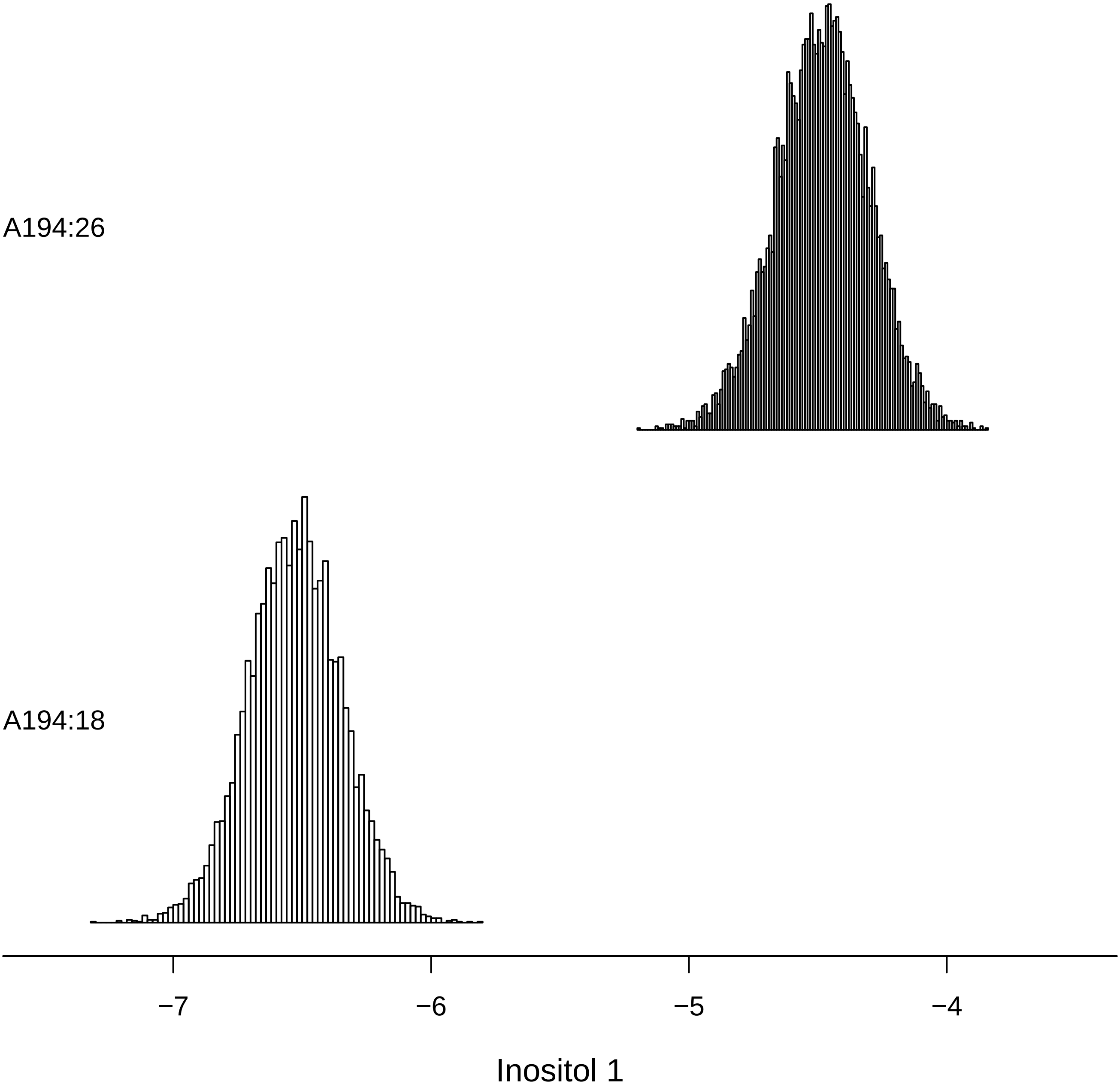
A194:26
A194:18
−7
−6
−5
−4
Inositol 1

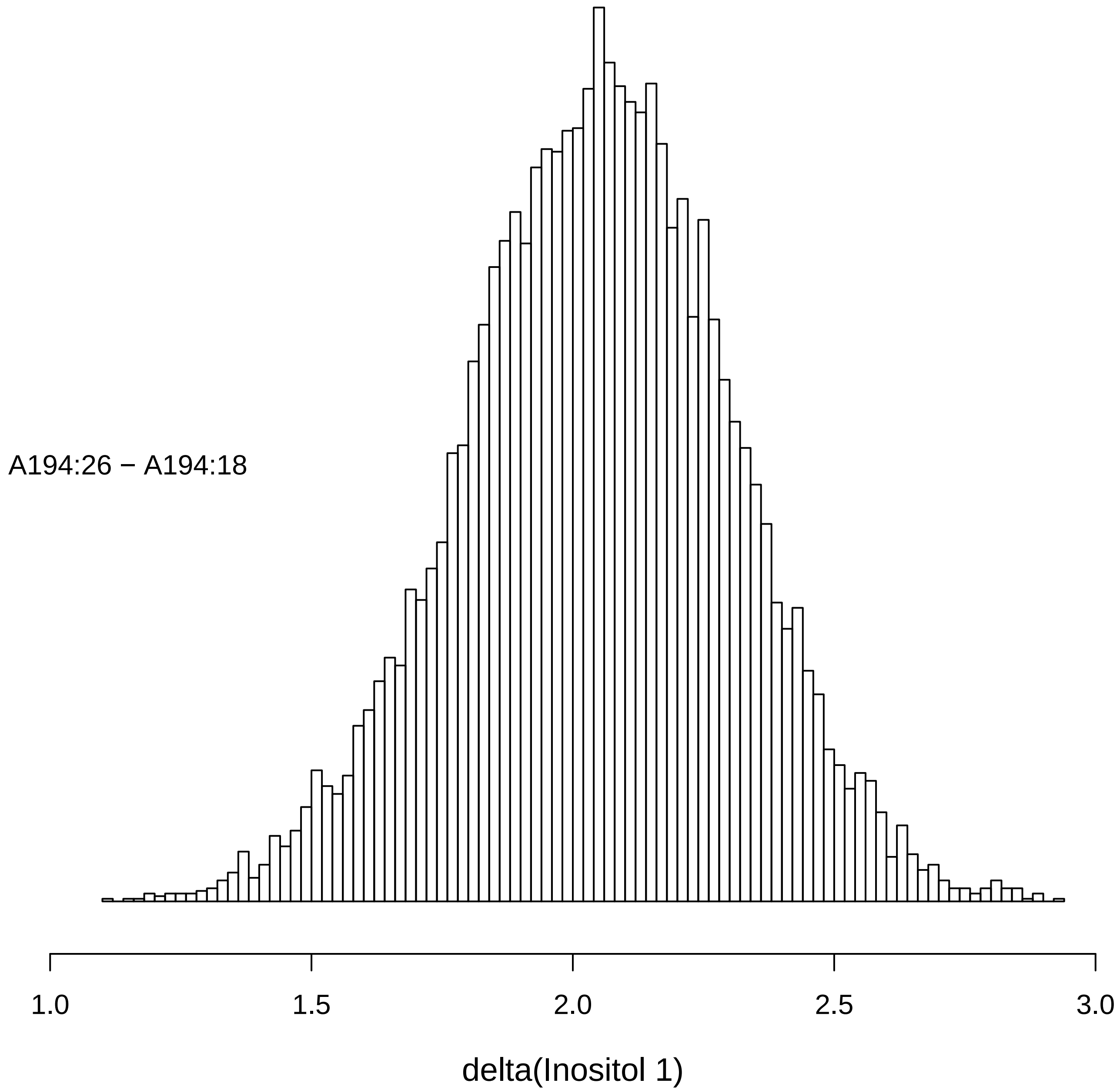

A194:26 − A194:18
1.0
1.5
2.0
2.5
3.0
delta(Inositol 1)

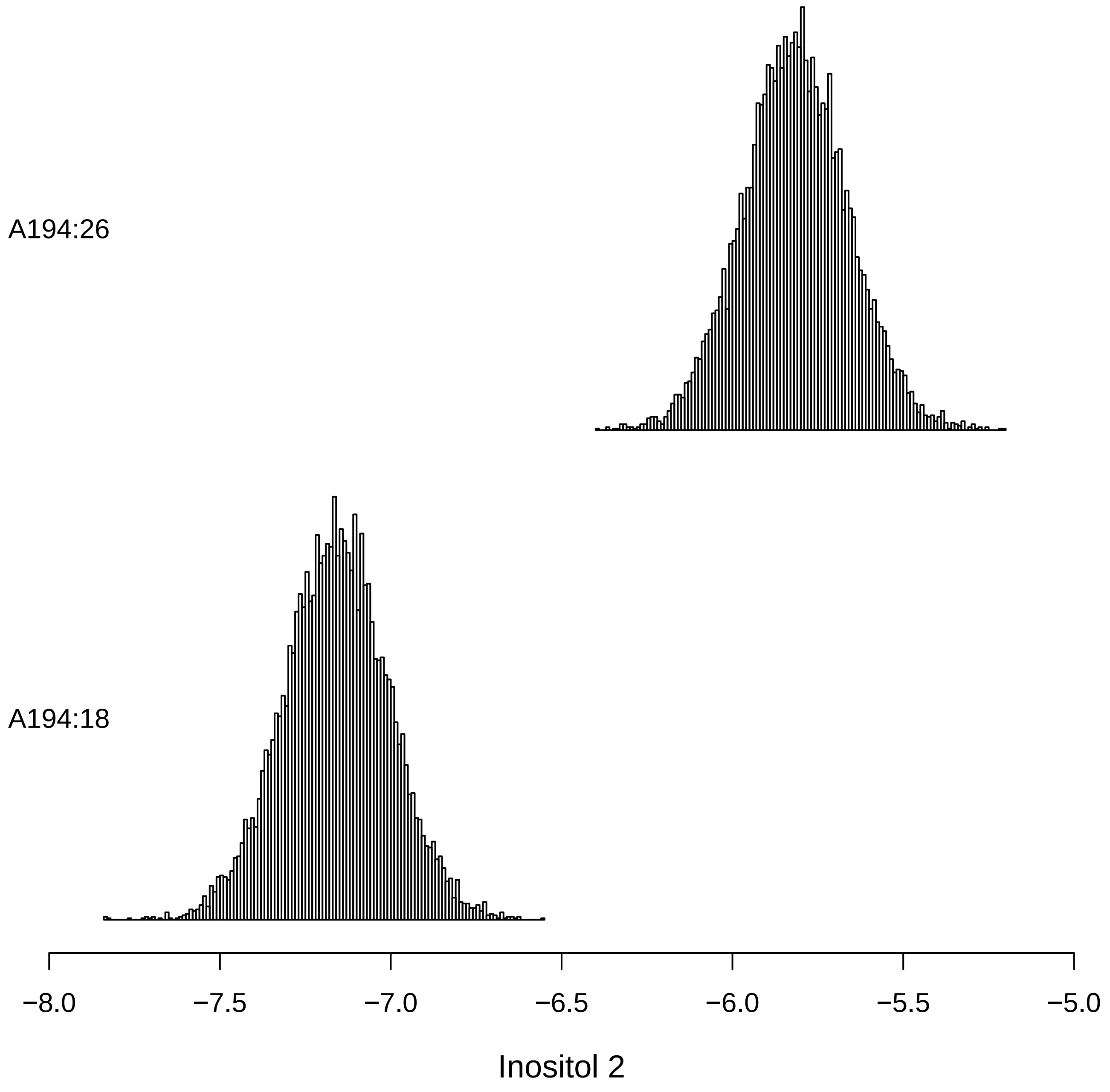
A194:26
A194:18
−8.0
−7.5
−7.0
−6.5
−6.0
−5.5
−5.0
Inositol 2

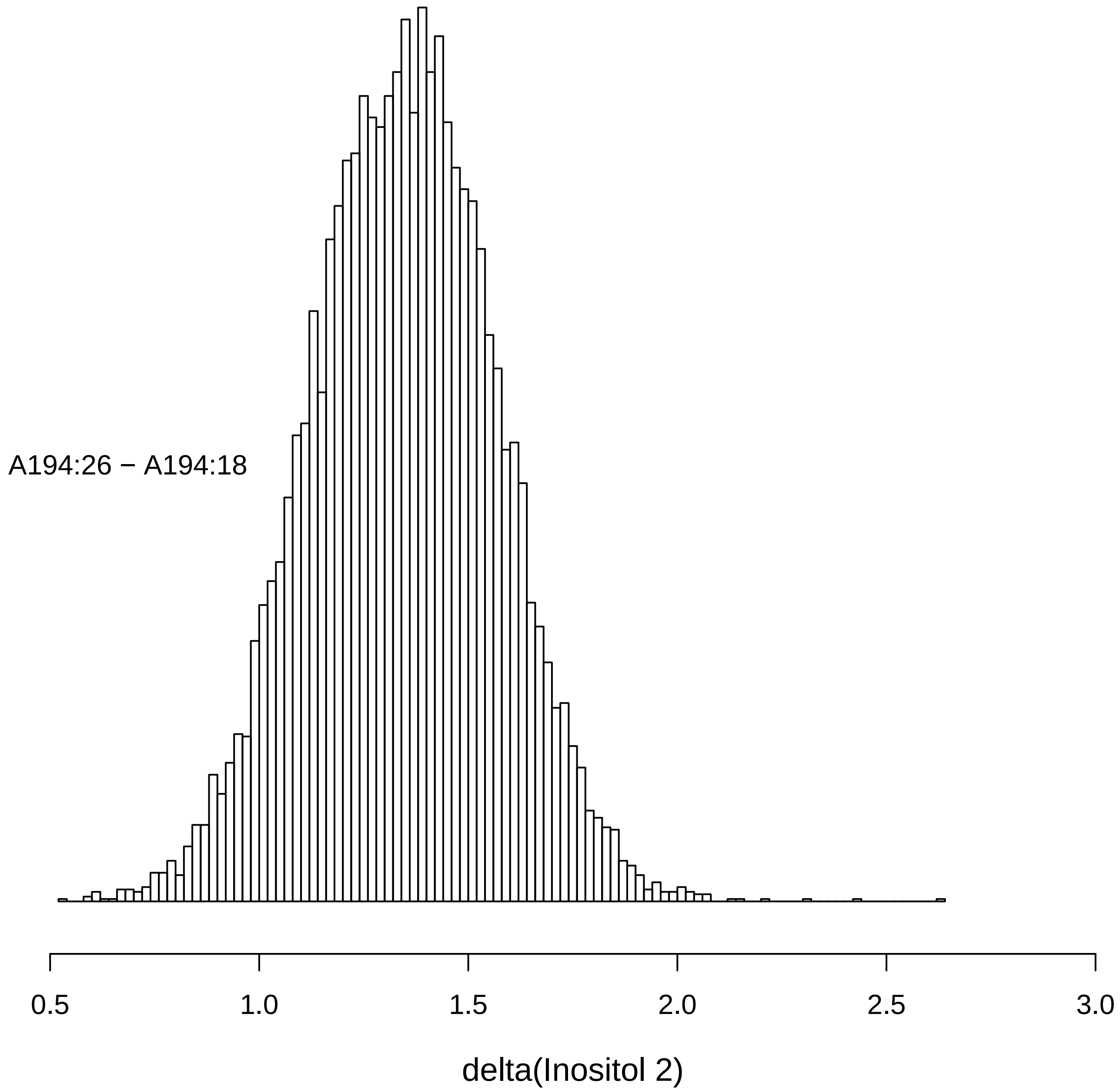

A194:26 − A194:18
0.5
1.0
1.5
2.0
2.5
3.0
delta(Inositol 2)

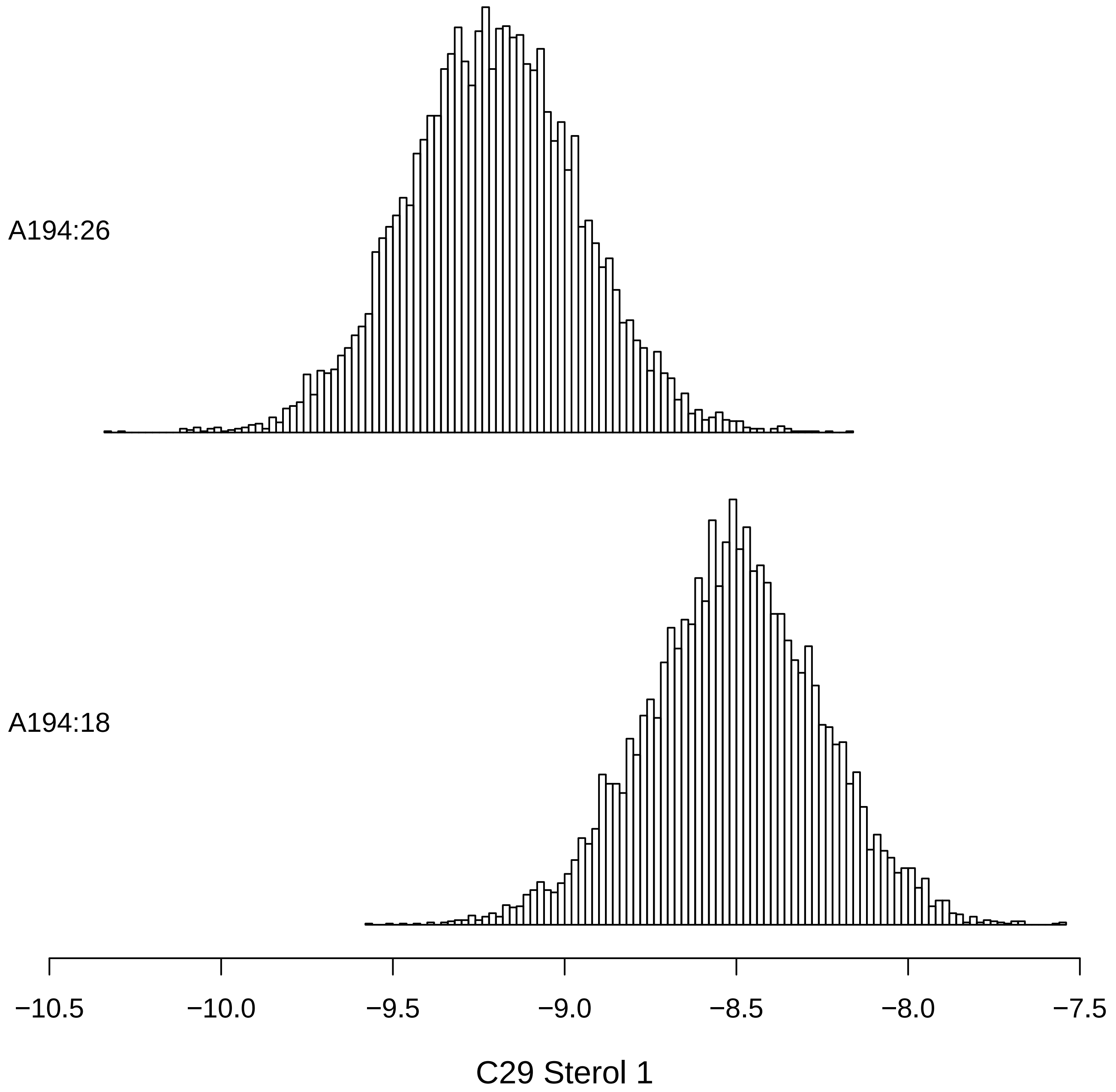
A194:26
A194:18
−10.5
−10.0
−9.5
−9.0
−8.5
−8.0
−7.5
C29 Sterol 1

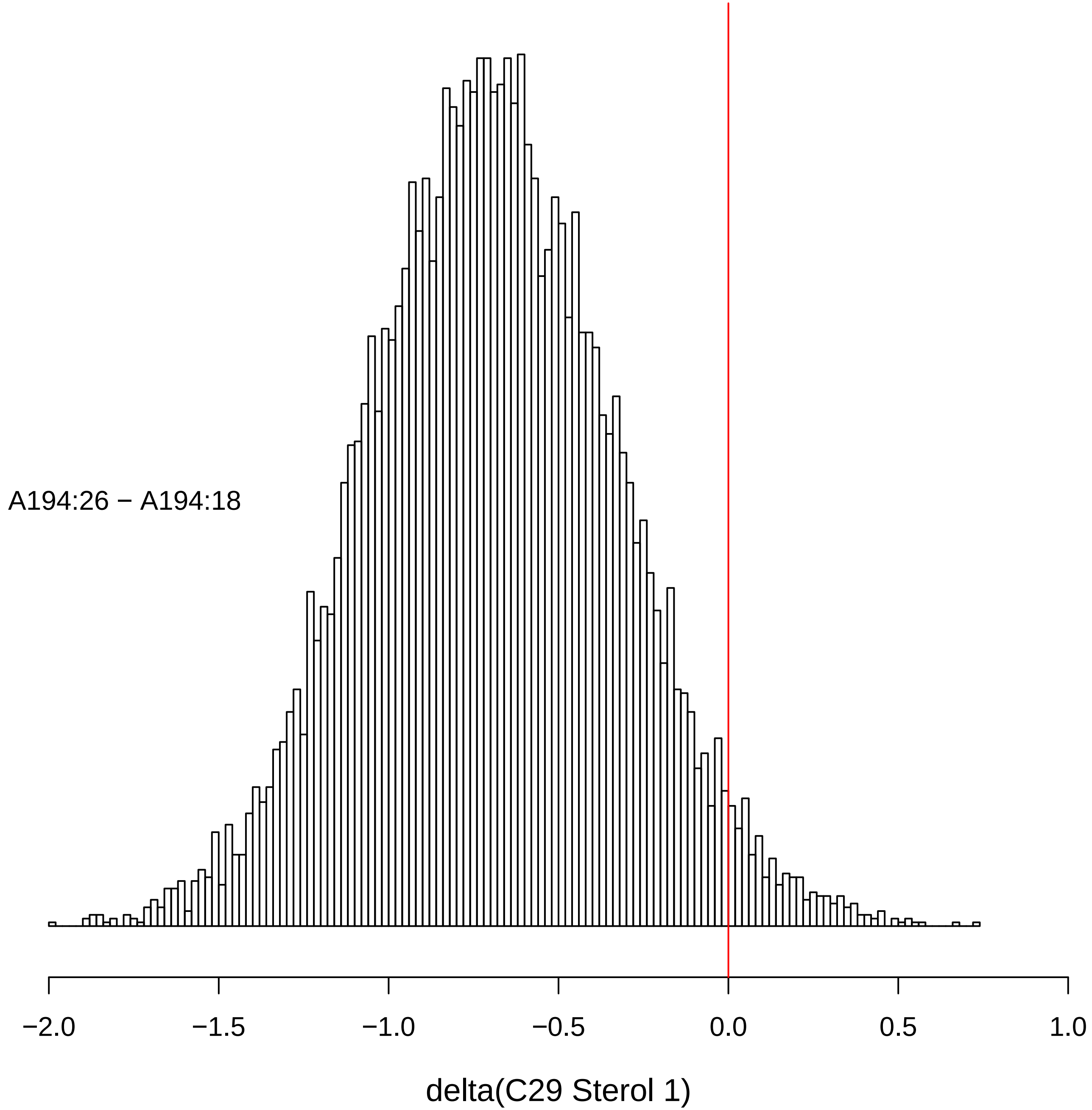

A194:26 − A194:18
−2.0
−1.5
−1.0
−0.5
0.0
0.5
1.0
delta(C29 Sterol 1)

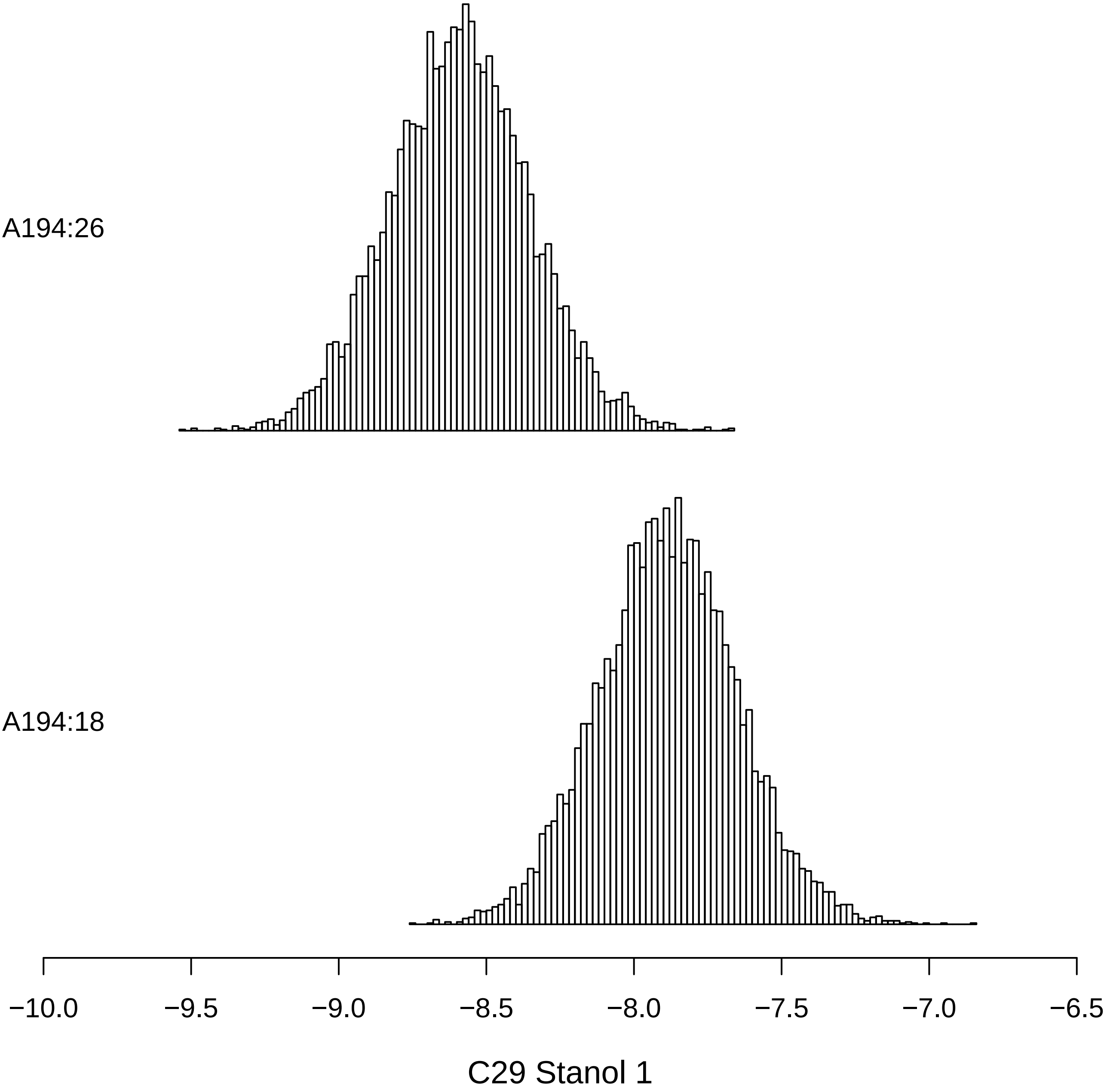
A194:26
A194:18
−10.0
−9.5
−9.0
−8.5
−8.0
−7.5
−7.0
−6.5
C29 Stanol 1

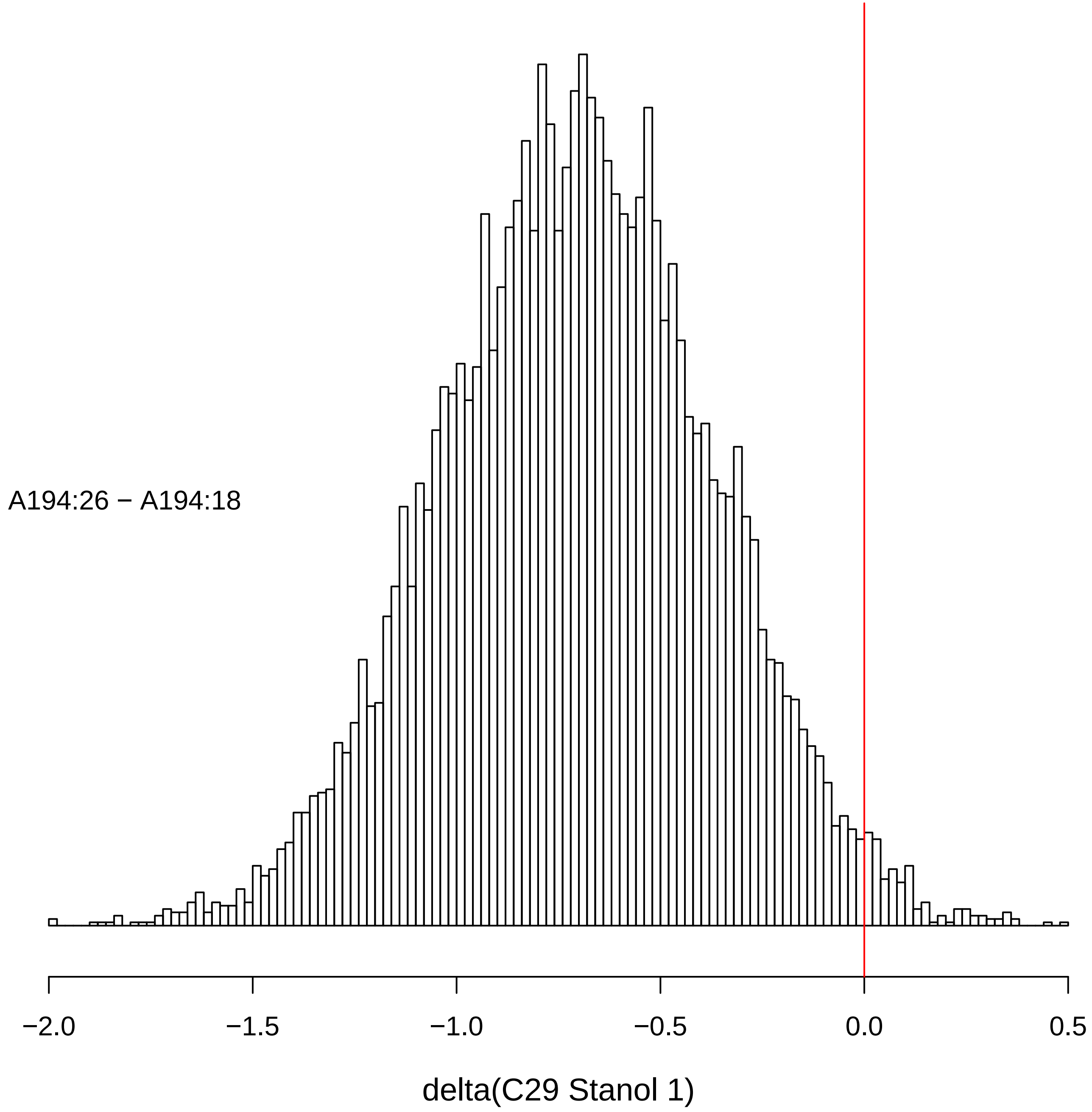
A194:26 − A194:18
−2.0
−1.5
−1.0
−0.5
0.0
0.5
delta(C29 Stanol 1)

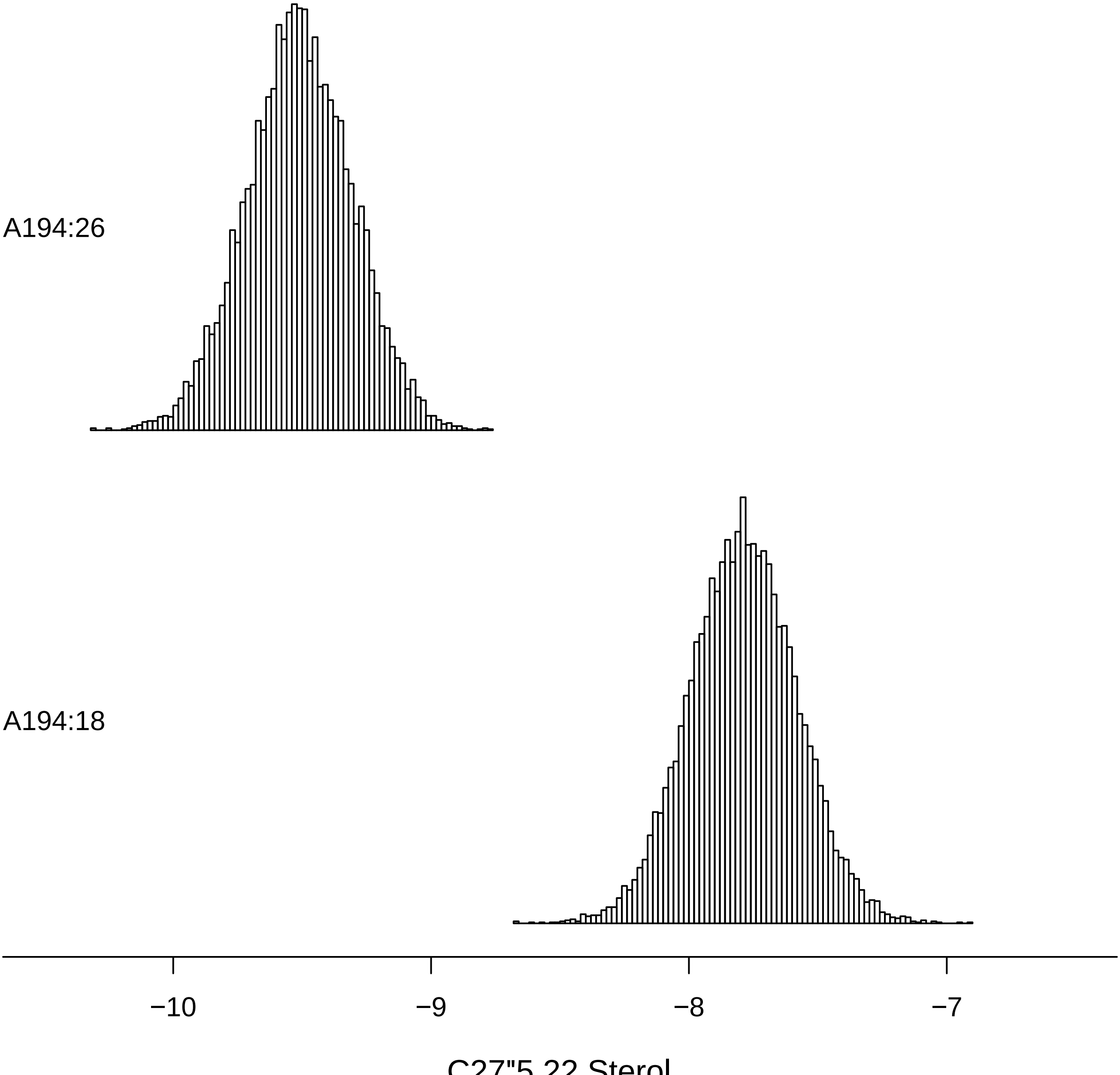
A194:26
A194:18
−10
−9
−8
−7
C27"5,22 Sterol

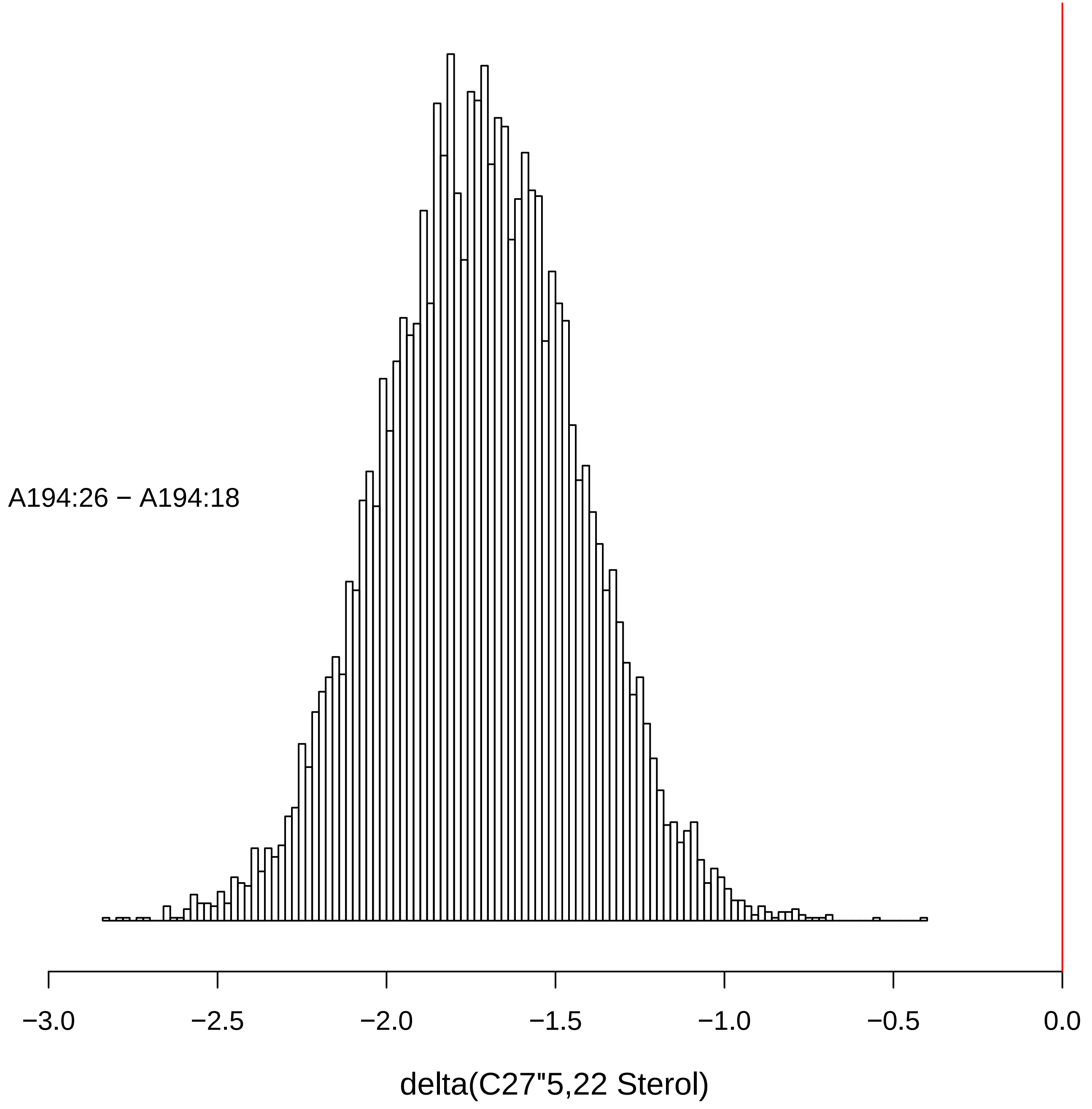
A194:26 − A194:18
−3.0
−2.5
−2.0
−1.5
−1.0
−0.5
0.0
delta(C27"5,22 Sterol)

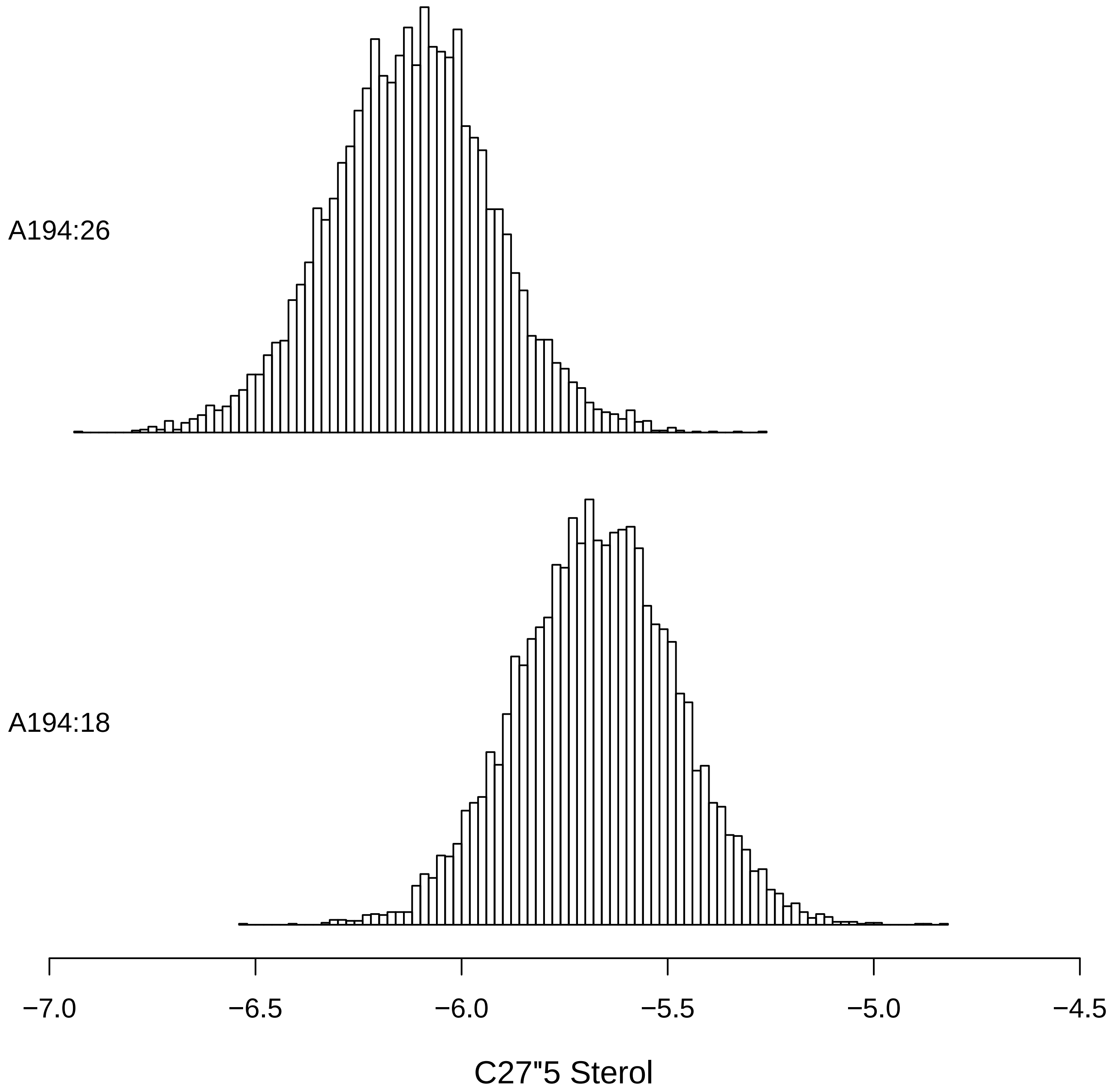
A194:26
A194:18
−7.0
−6.5
−6.0
−5.5
−5.0
−4.5
C27"5 Sterol

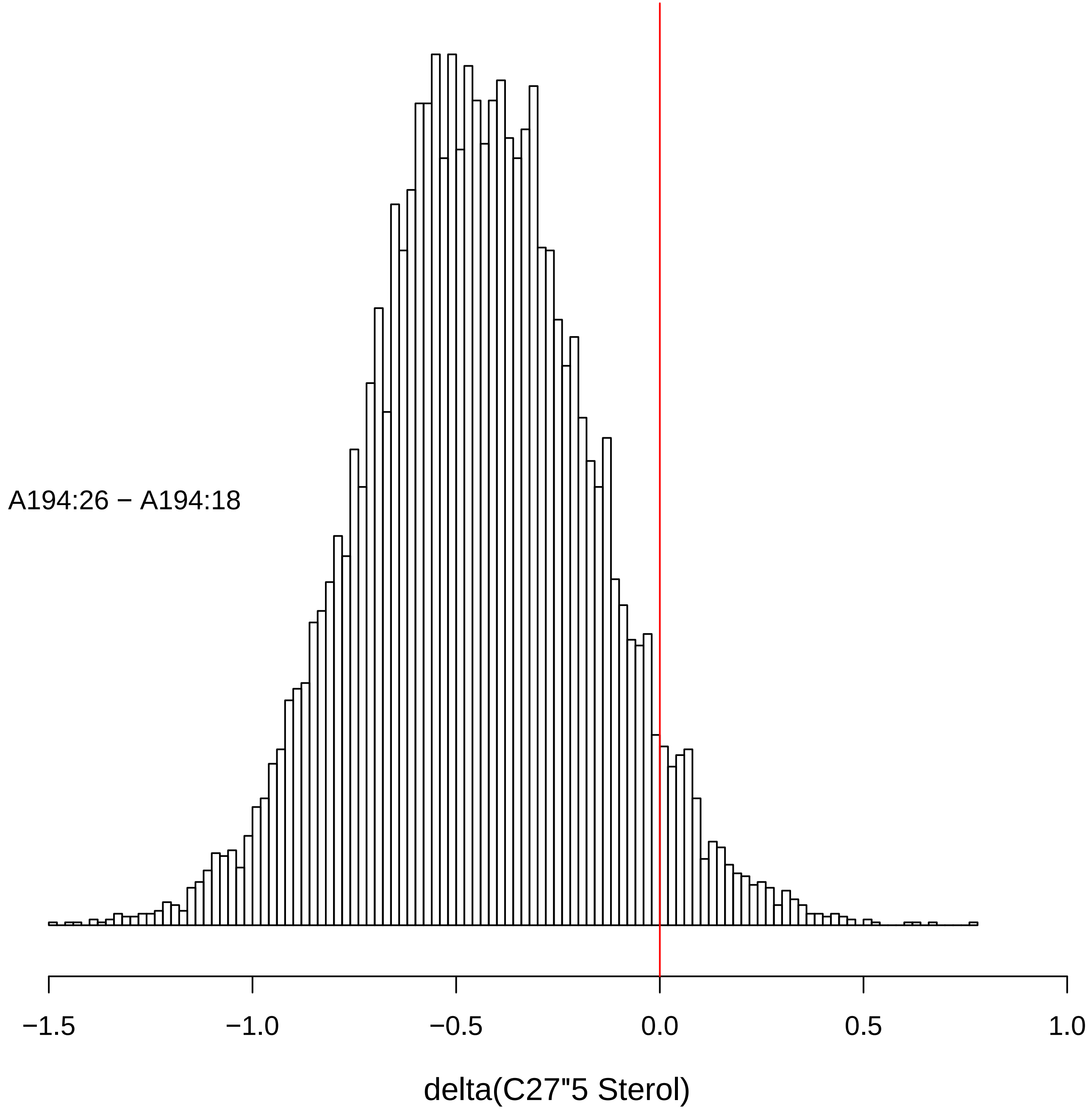
A194:26 − A194:18
−1.5
−1.0
−0.5
0.0
0.5
1.0
delta(C27"5 Sterol)

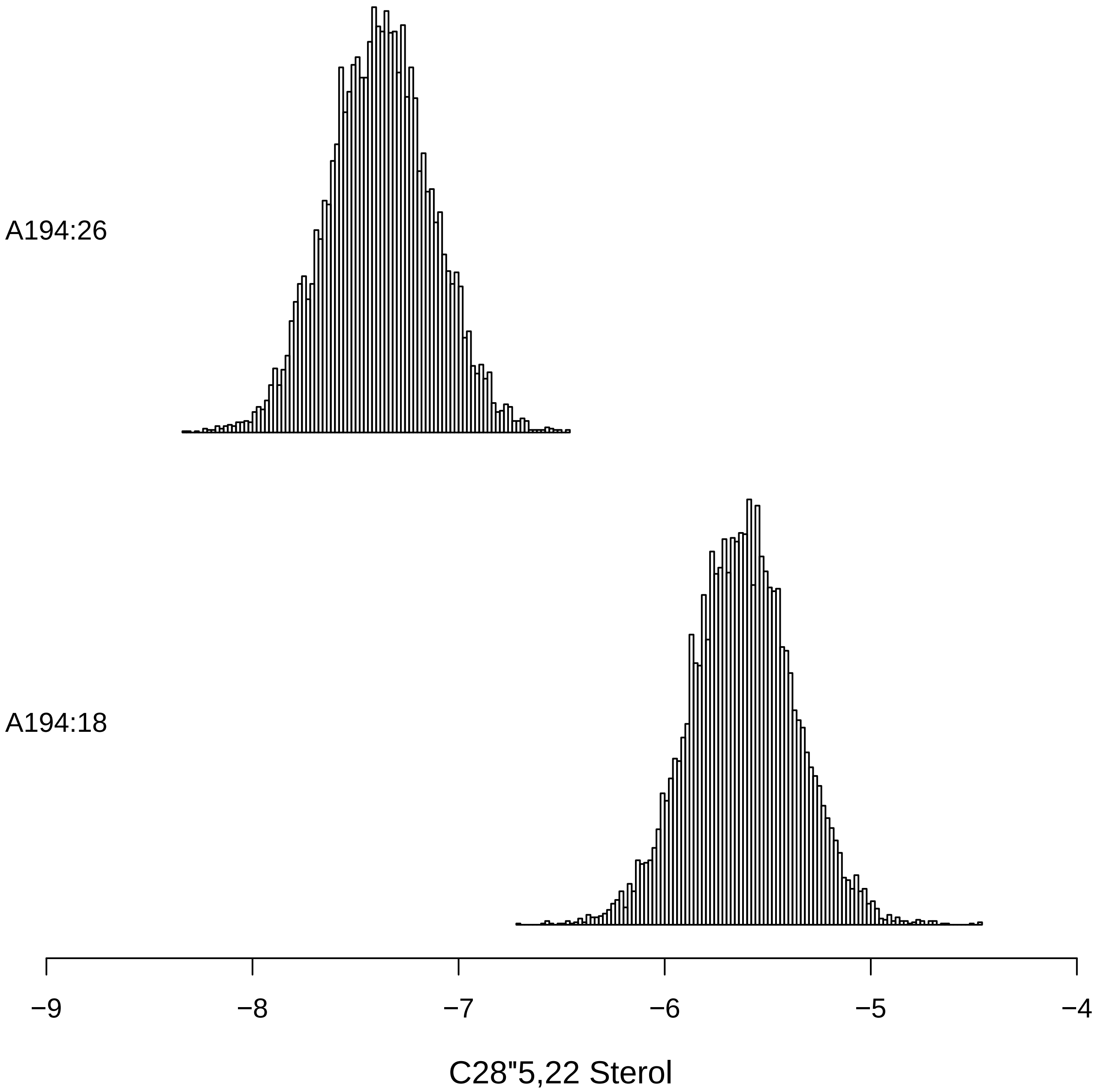

A194:26
A194:18
−9
−8
−7
−6
−5
−4
C28"5,22 Sterol

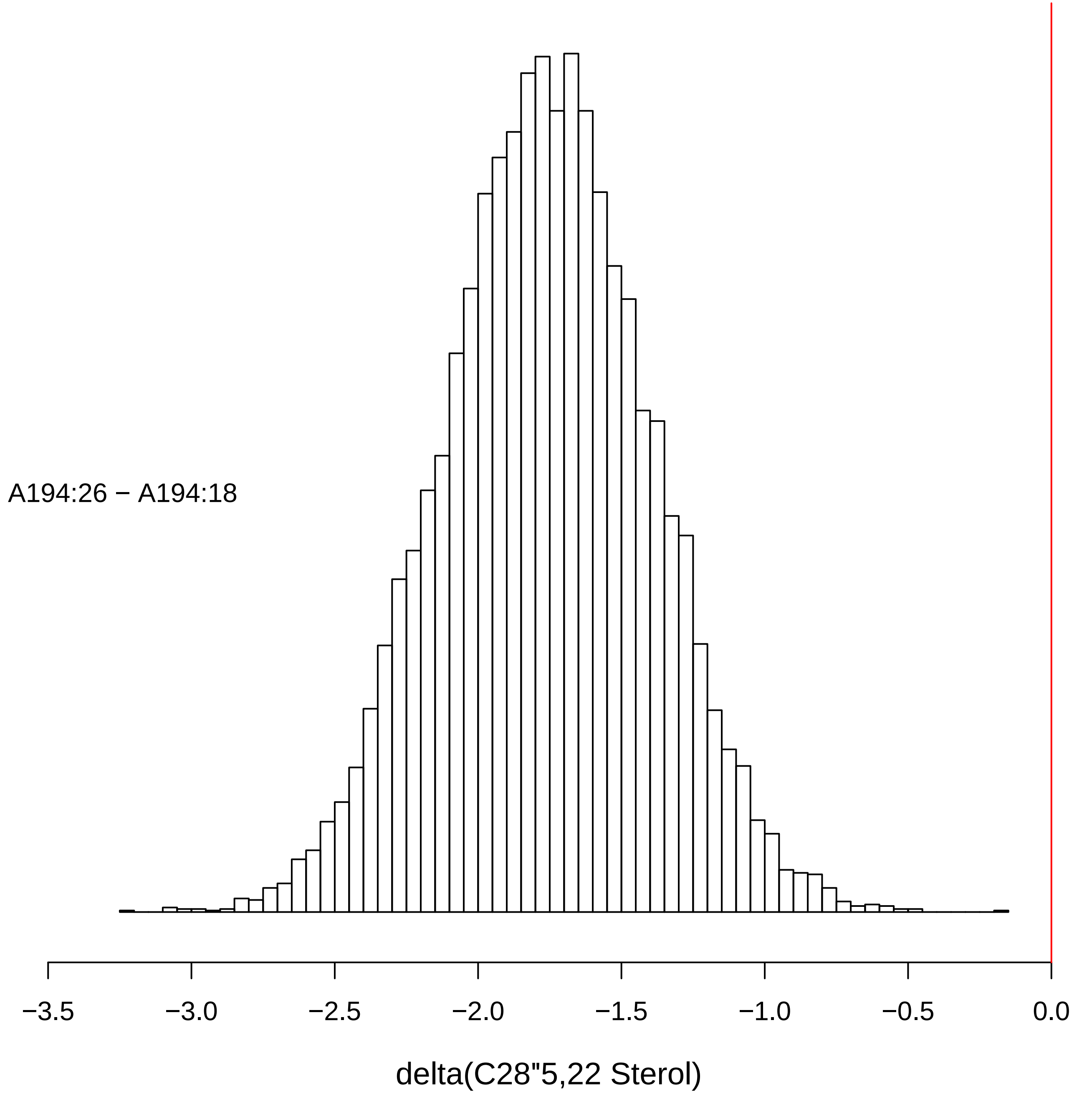
A194:26 − A194:18
−3.5
−3.0
−2.5
−2.0
−1.5
−1.0
−0.5
0.0
delta(C28"5,22 Sterol)

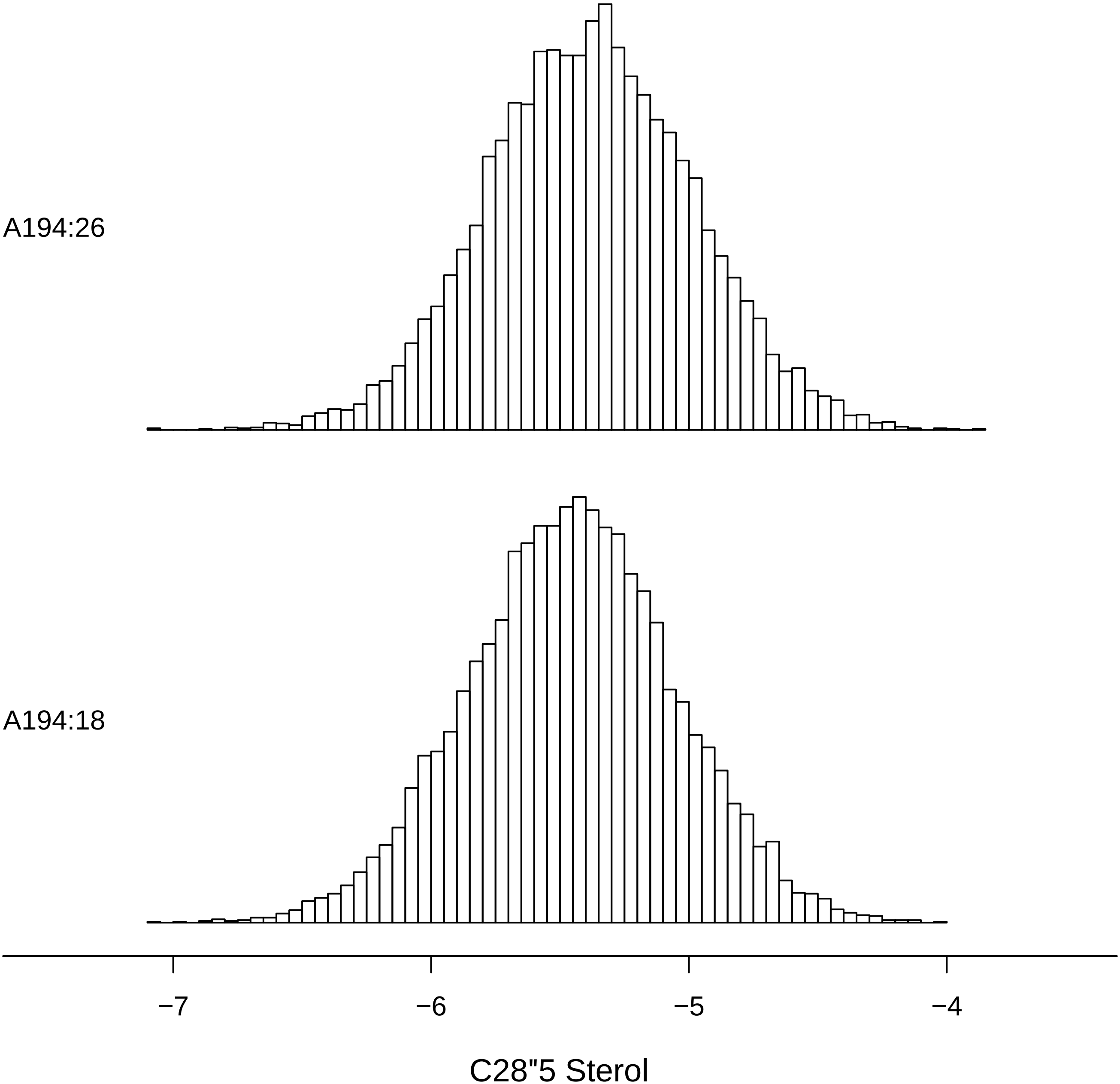
A194:26
A194:18
−7
−6
−5
−4
C28"5 Sterol

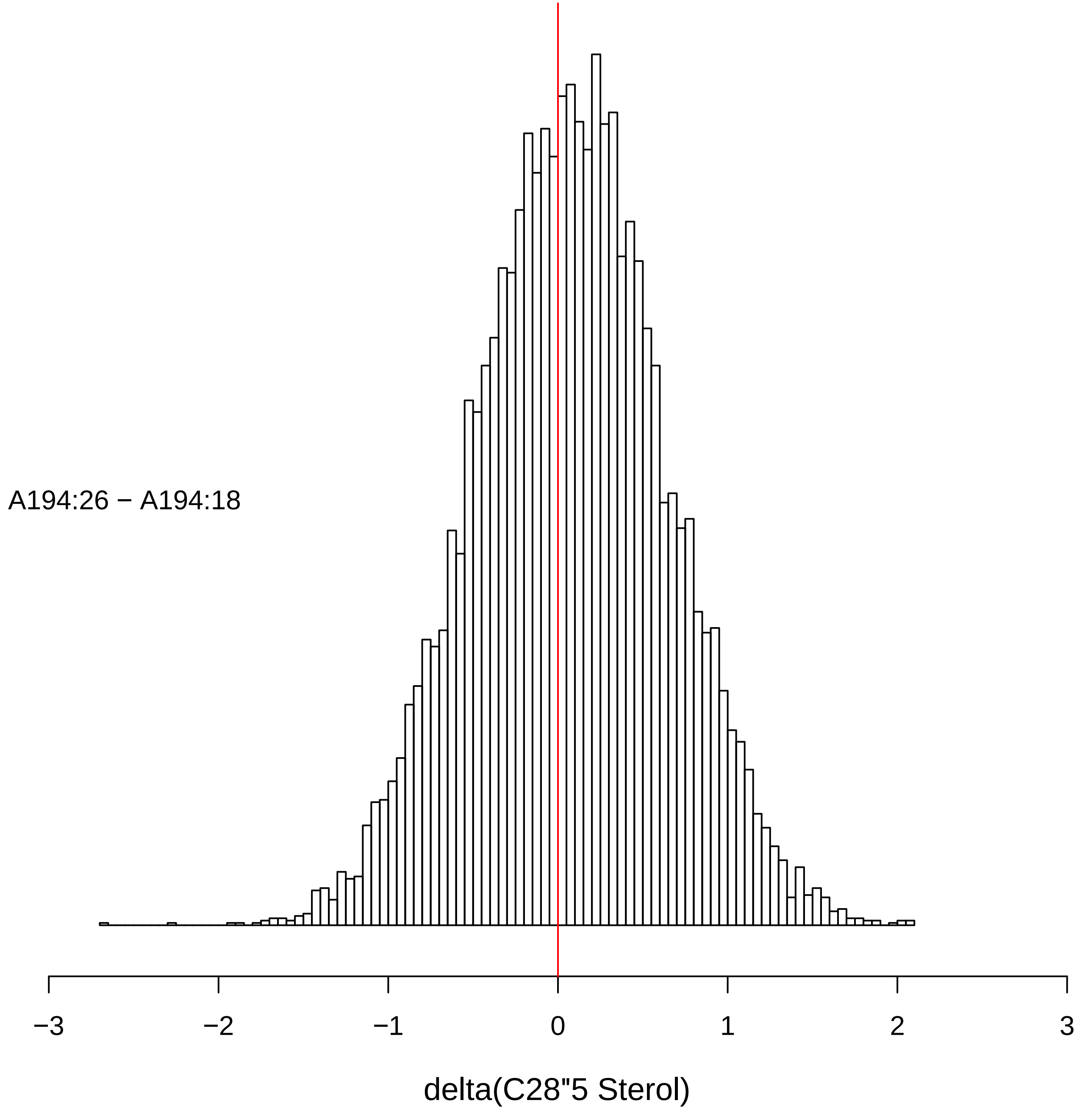
A194:26 − A194:18
−3
−2
−1
0
1
2
3
delta(C28"5 Sterol)

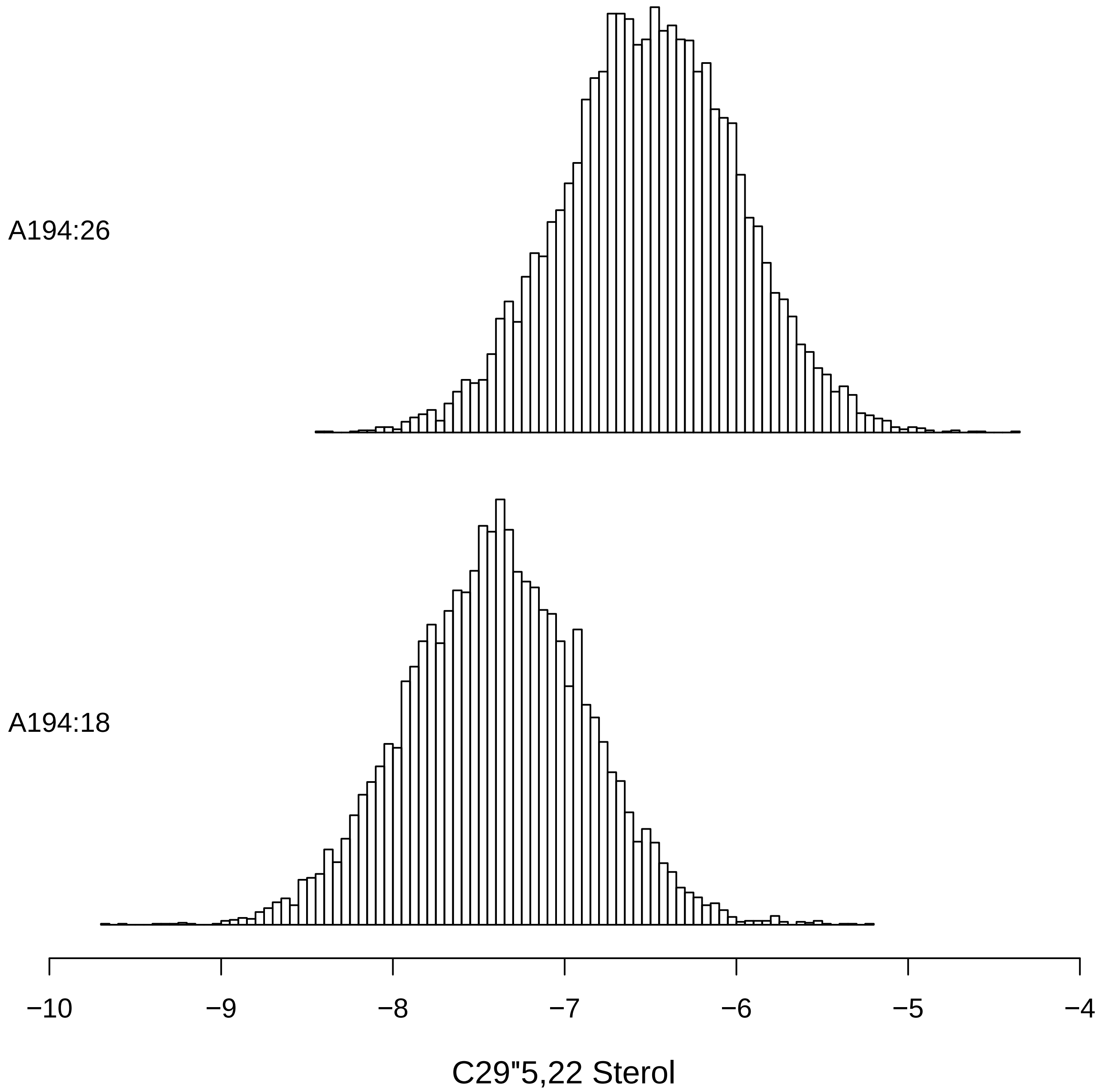

A194:26
A194:18
−10
−9
−8
−7
−6
−5
−4
C29"5,22 Sterol

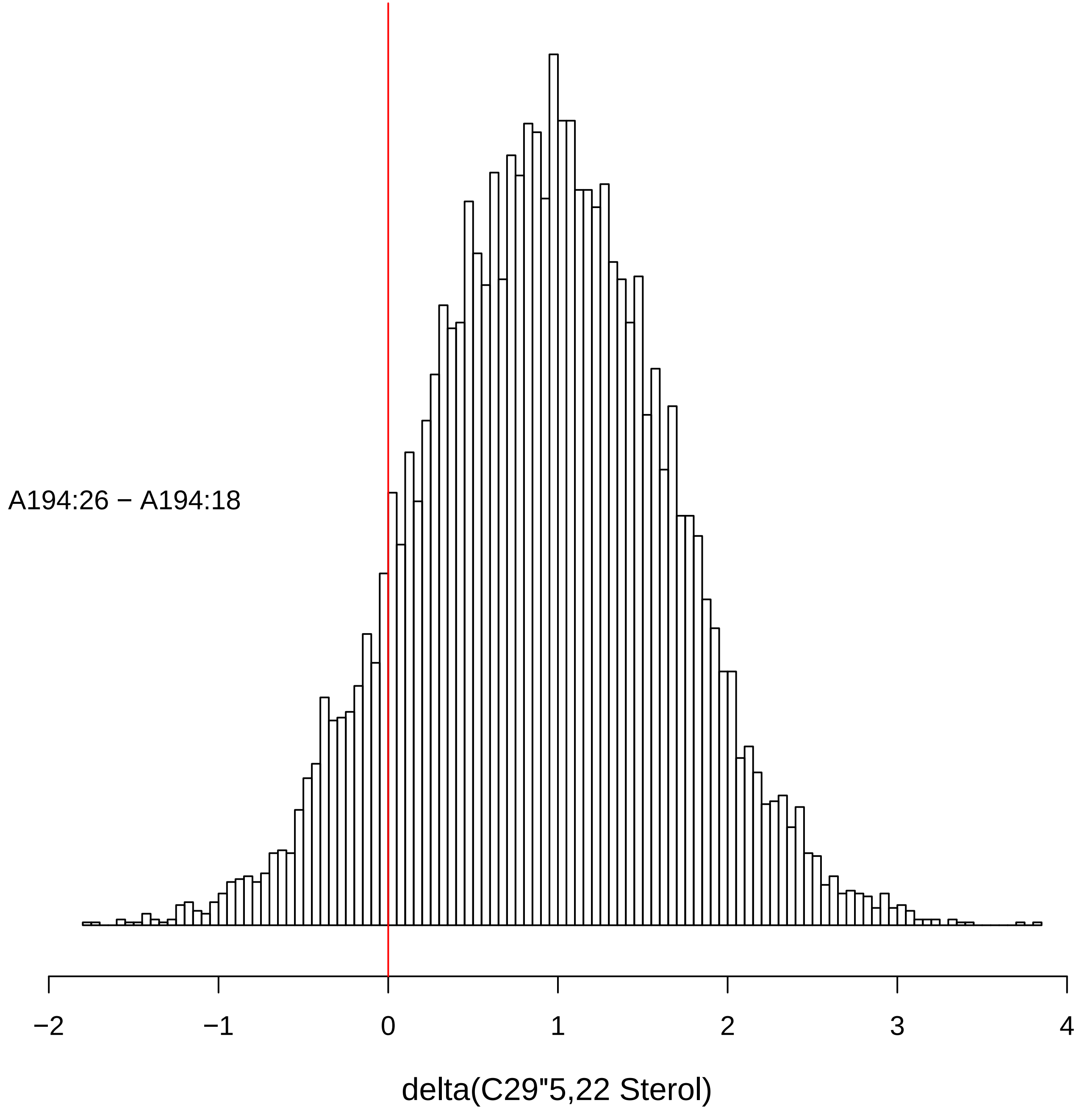
A194:26 − A194:18
−2
−1
0
1
2
3
4
delta(C29"5,22 Sterol)

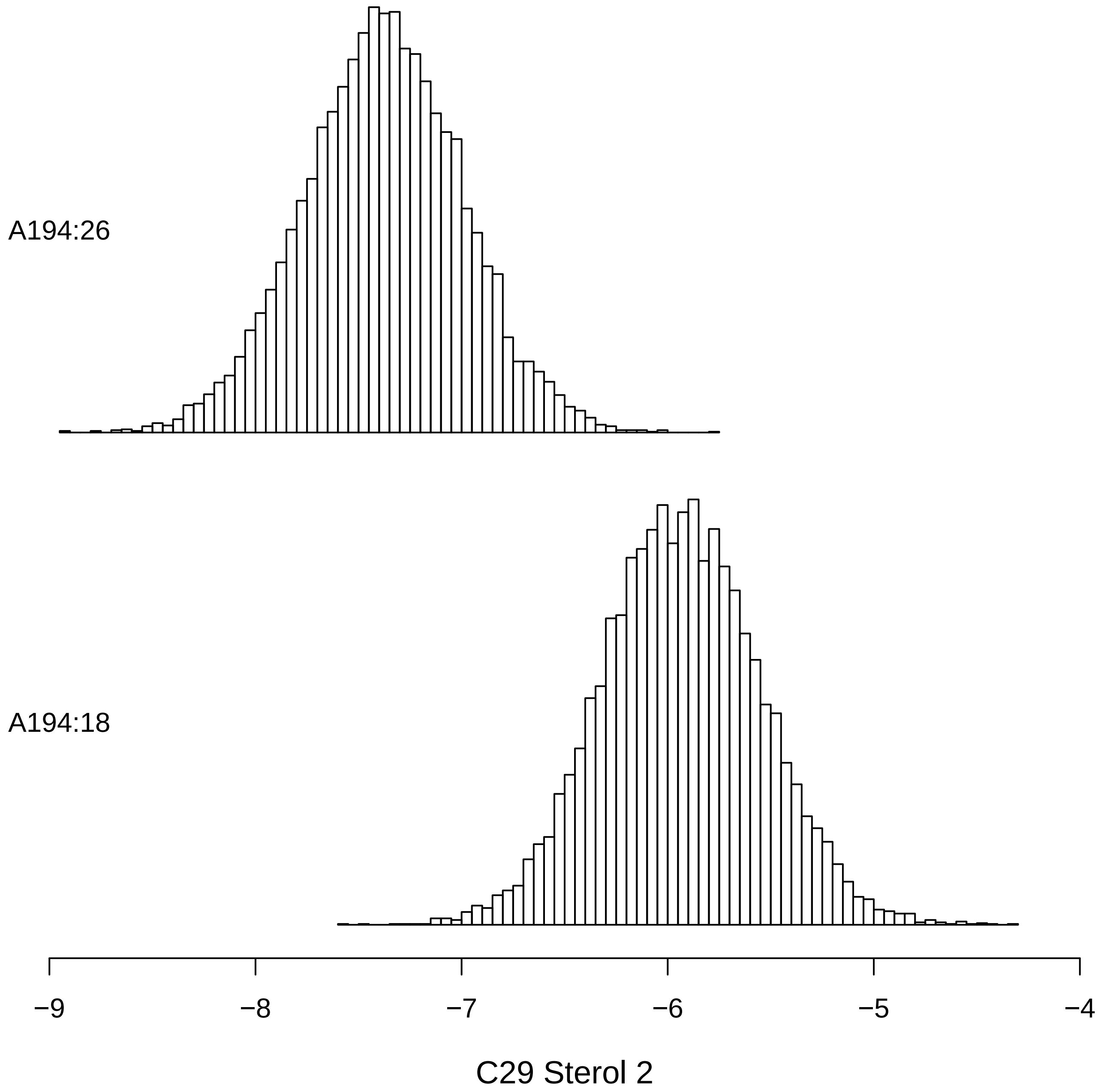

A194:26
A194:18
−9
−8
−7
−6
−5
−4
C29 Sterol 2

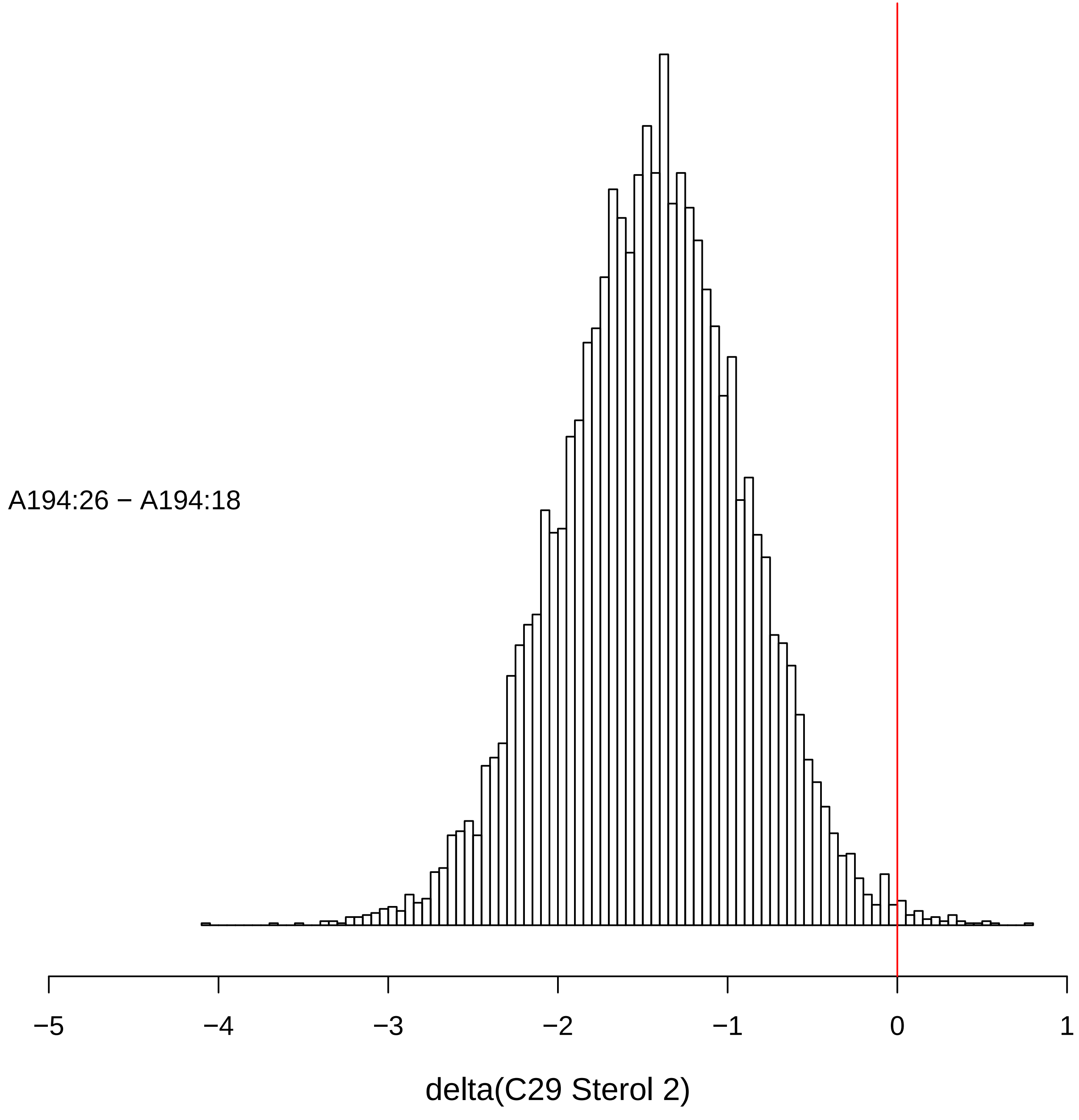
A194:26 − A194:18
−5
−4
−3
−2
−1
0
1
delta(C29 Sterol 2)

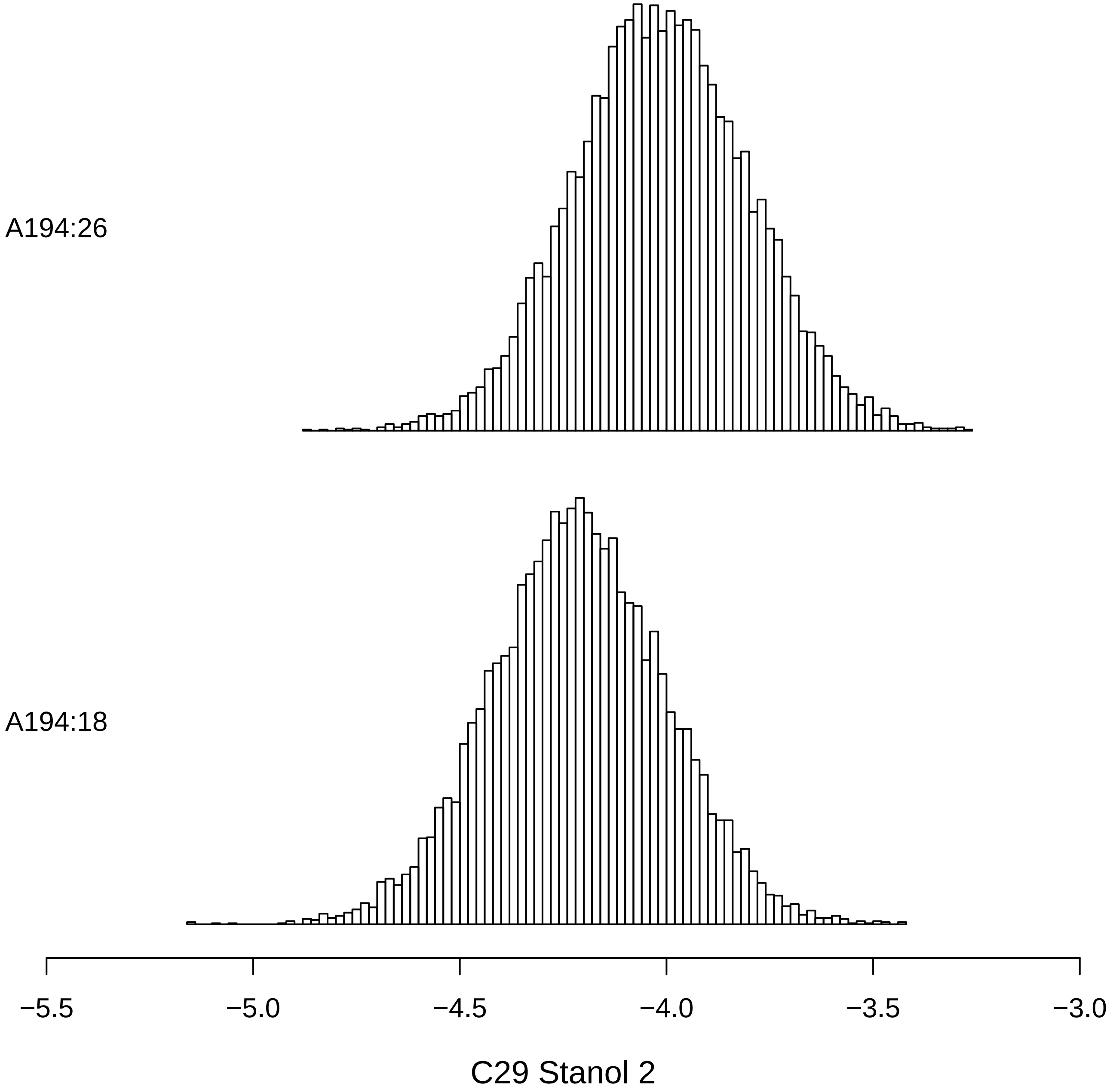
A194:26
A194:18
−5.5
−5.0
−4.5
−4.0
−3.5
−3.0
C29 Stanol 2

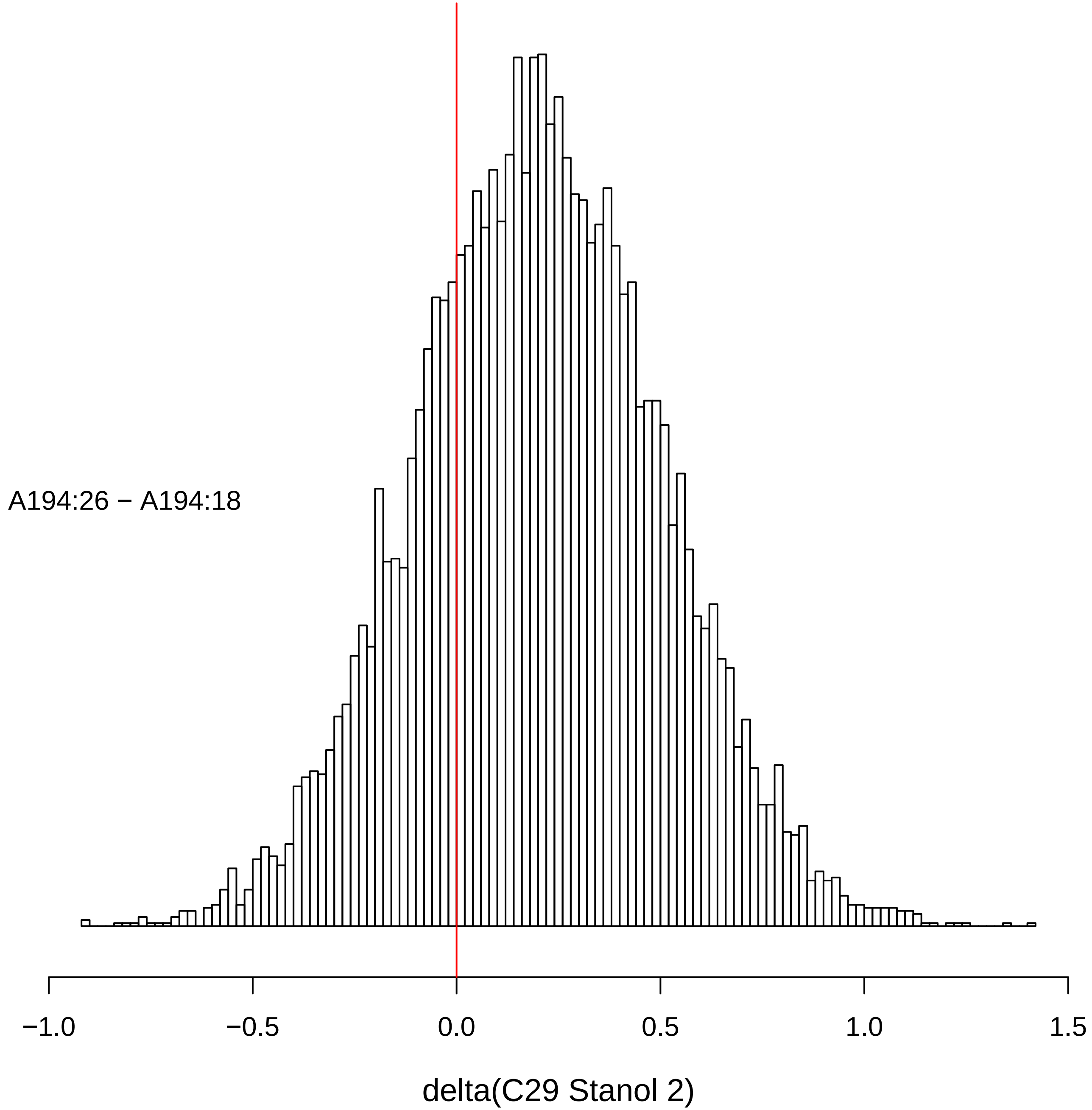

A194:26 − A194:18
−1.0
−0.5
0.0
0.5
1.0
1.5
delta(C29 Stanol 2)

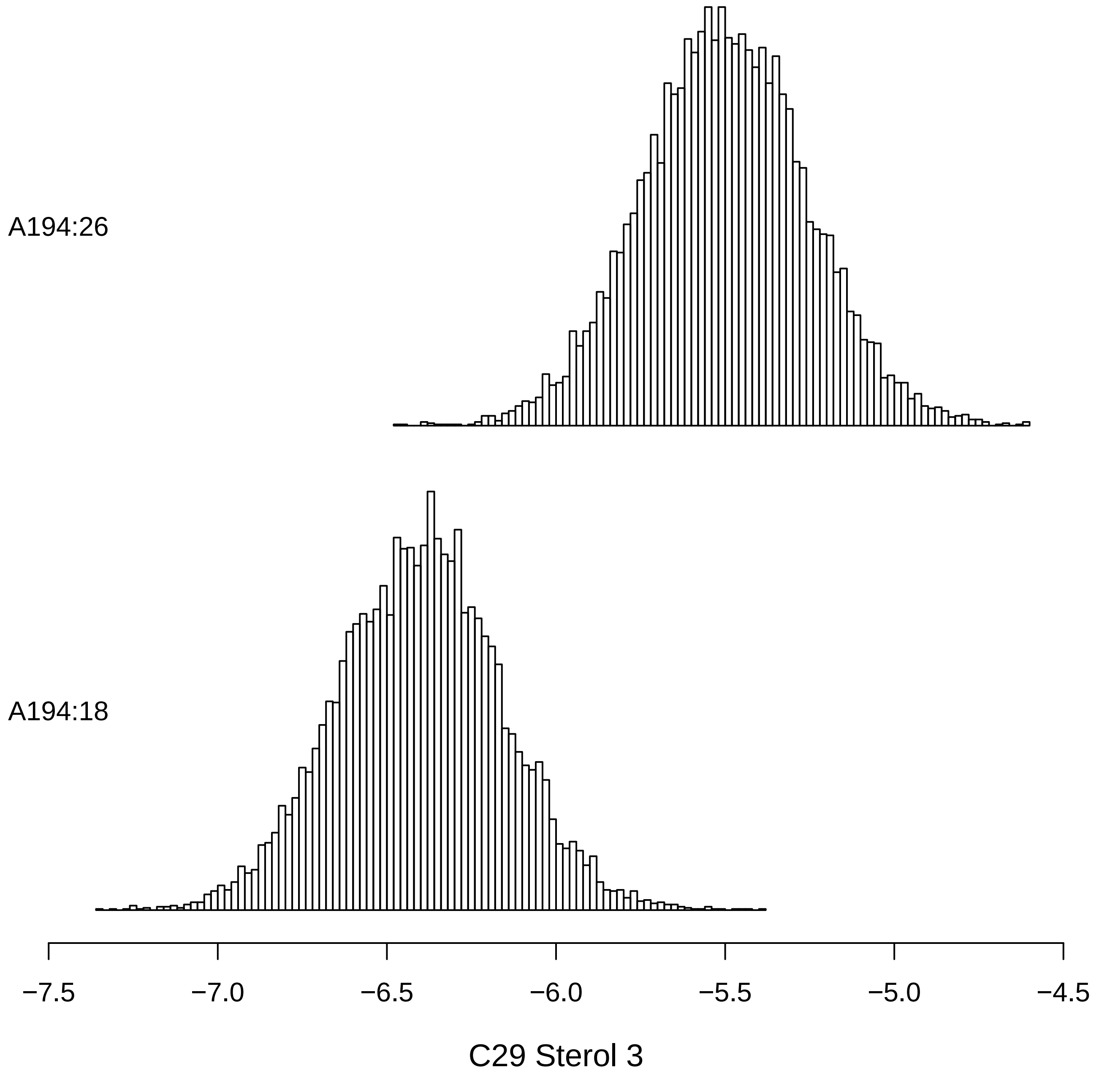
A194:26
A194:18
−7.5
−7.0
−6.5
−6.0
−5.5
−5.0
−4.5
C29 Sterol 3

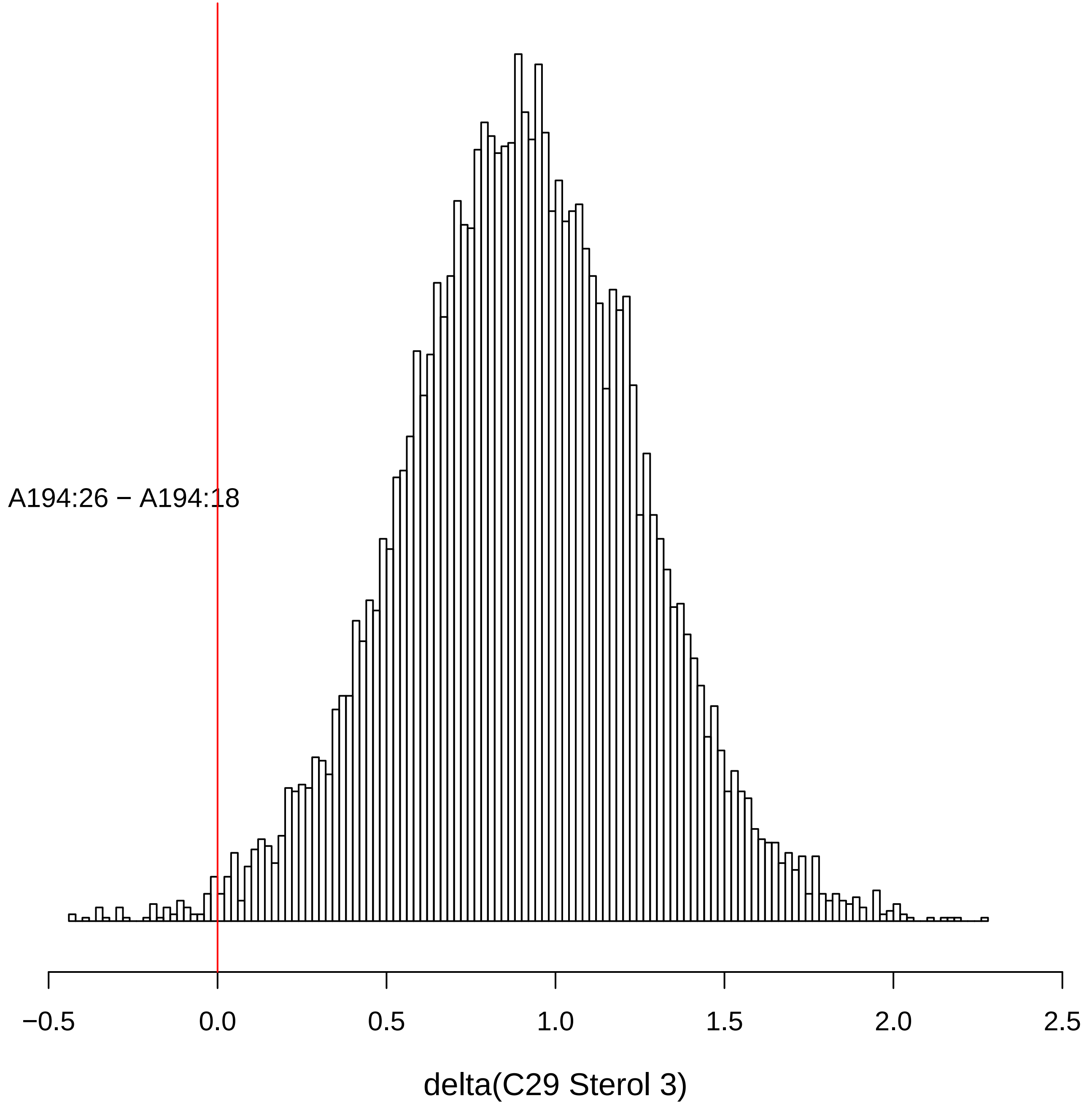
A194:26 − A194:18
−0.5
0.0
0.5
1.0
1.5
2.0
2.5
delta(C29 Sterol 3)

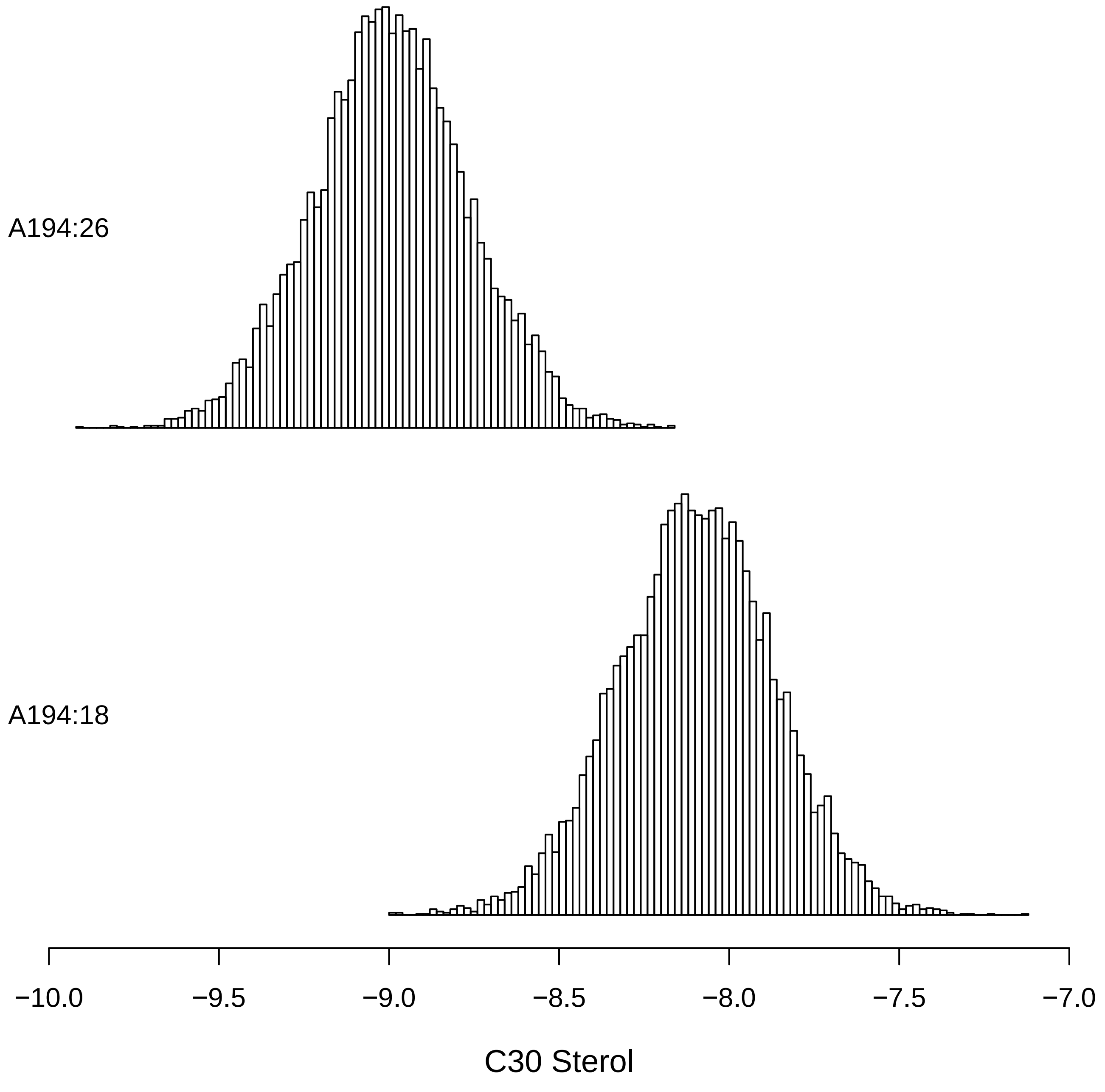
A194:26
A194:18
−10.0
−9.5
−9.0
−8.5
−8.0
−7.5
−7.0
C30 Sterol

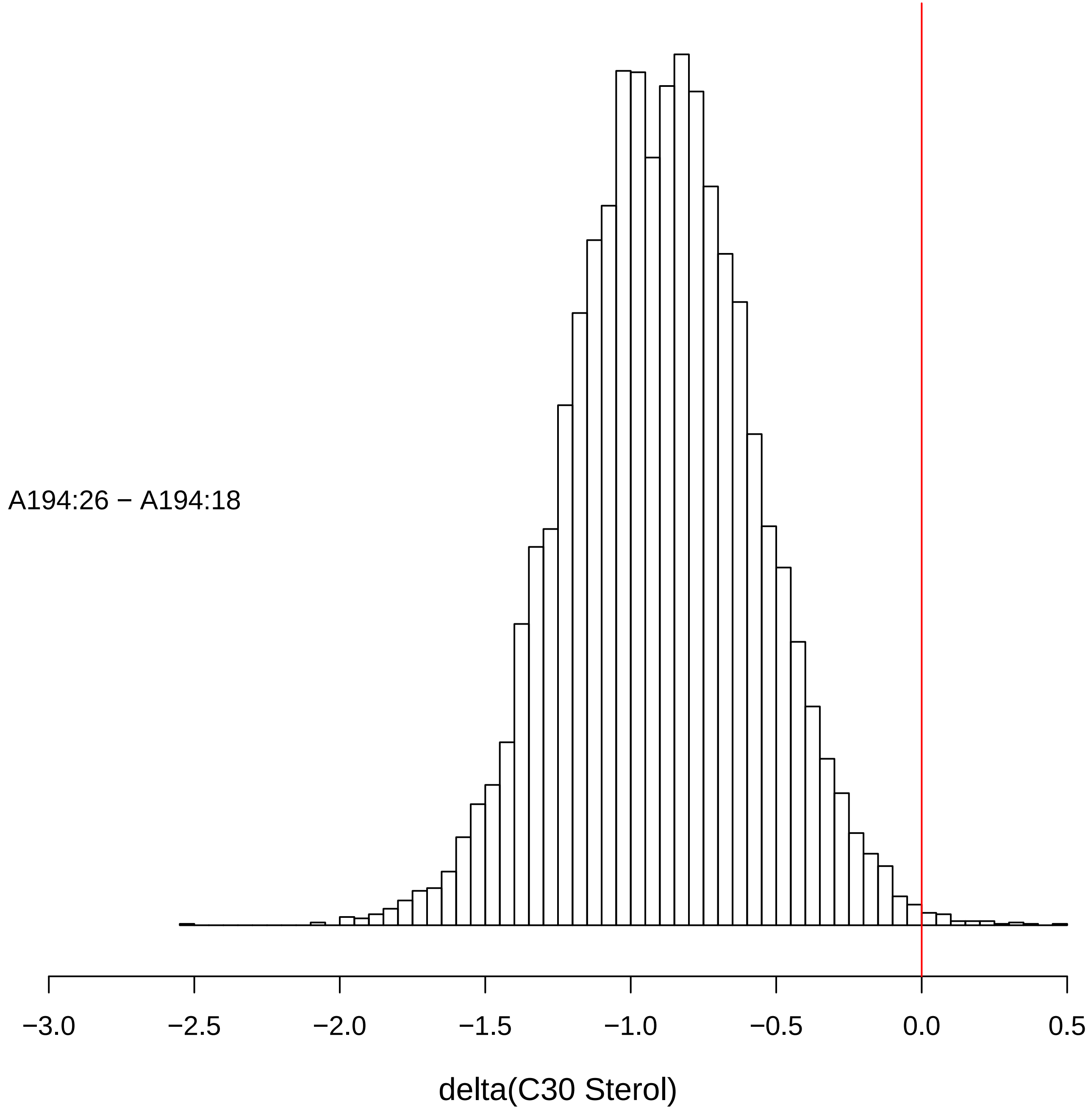

A194:26 − A194:18
−3.0
−2.5
−2.0
−1.5
−1.0
−0.5
0.0
0.5
delta(C30 Sterol)

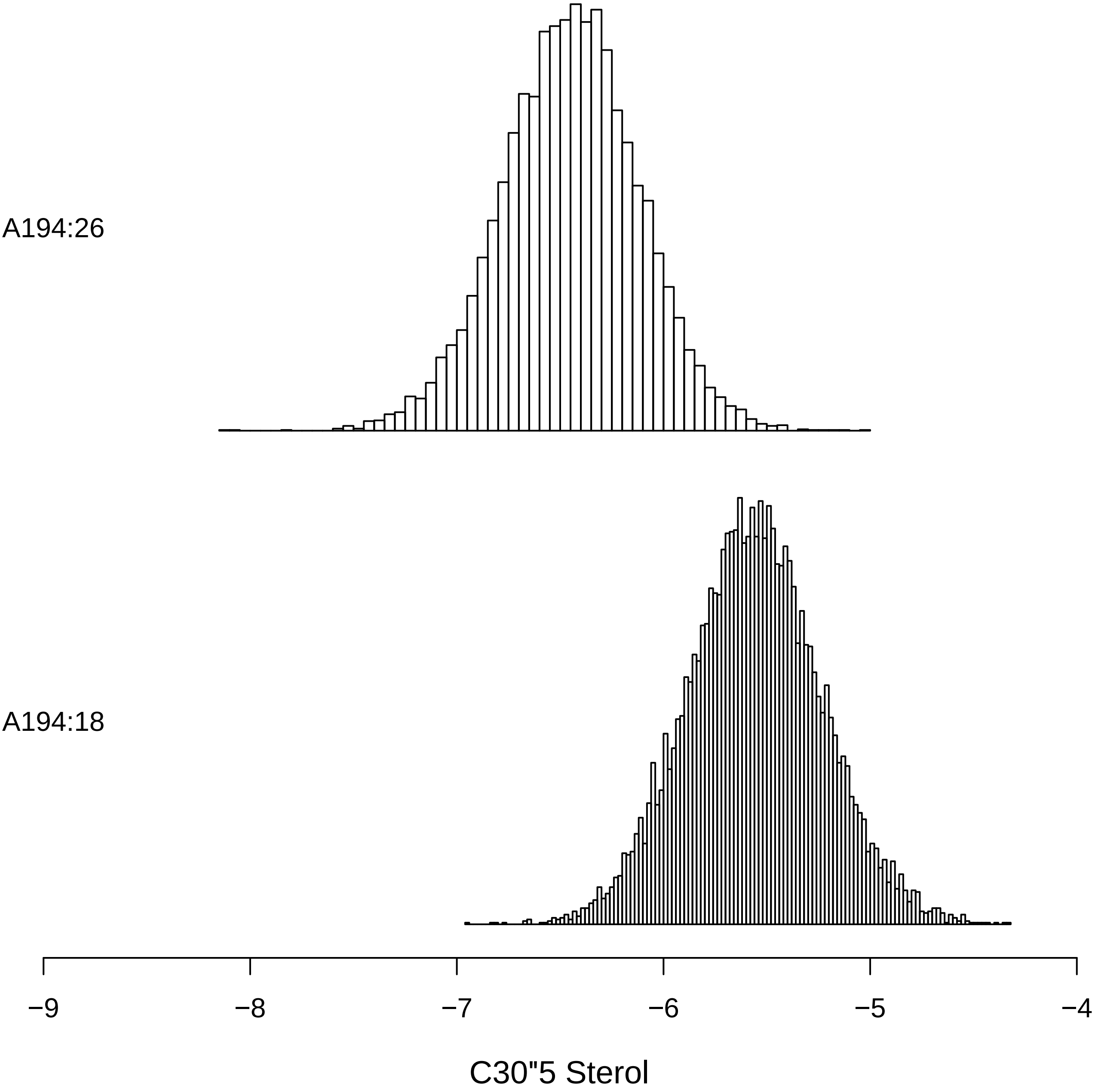
A194:26
A194:18
−9
−8
−7
−6
−5
−4
C30"5 Sterol

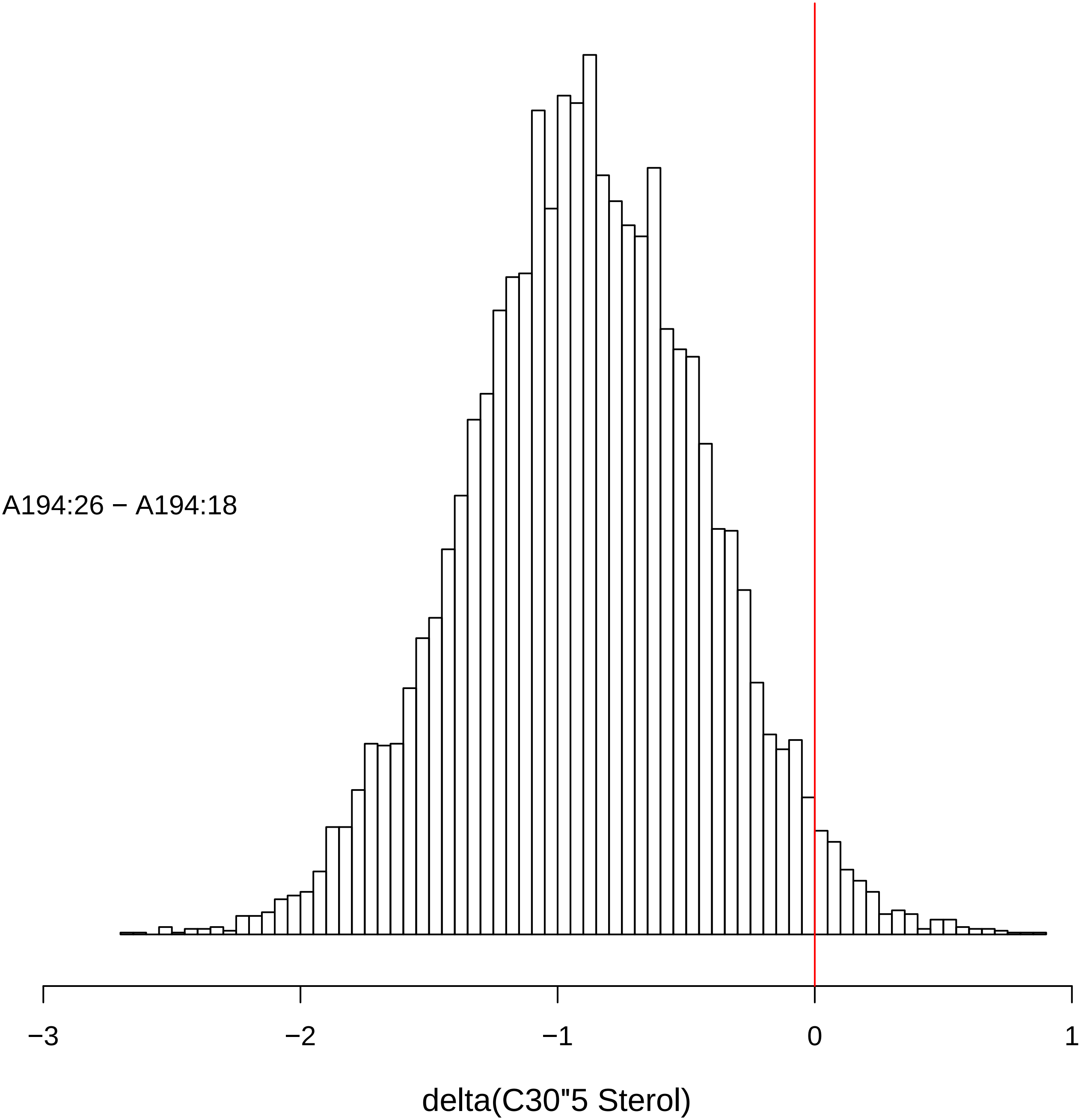
A194:26 − A194:18
−3
−2
−1
0
1
delta(C30"5 Sterol)

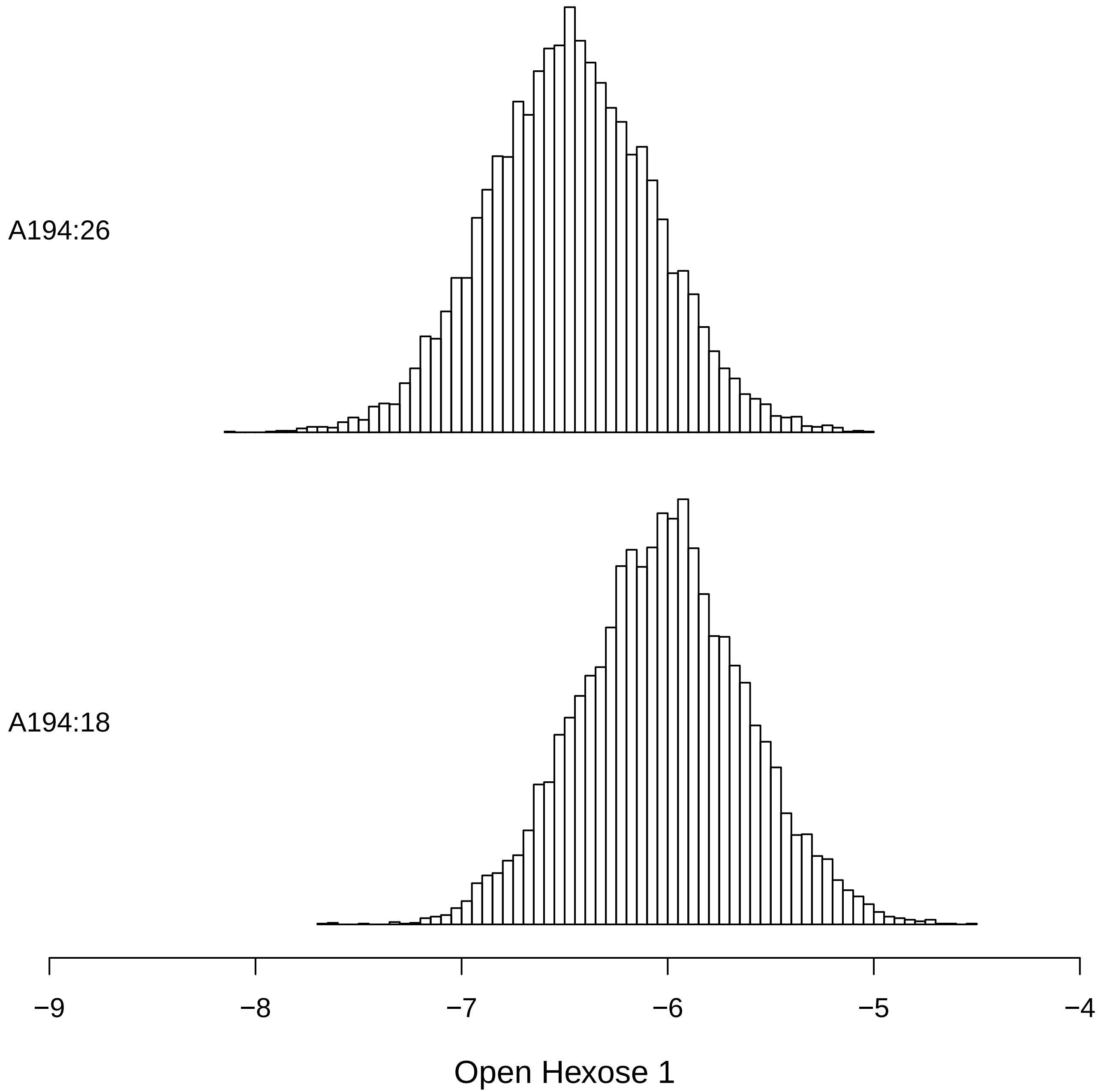

A194:26
A194:18
−9
−8
−7
−6
−5
−4
Open Hexose 1

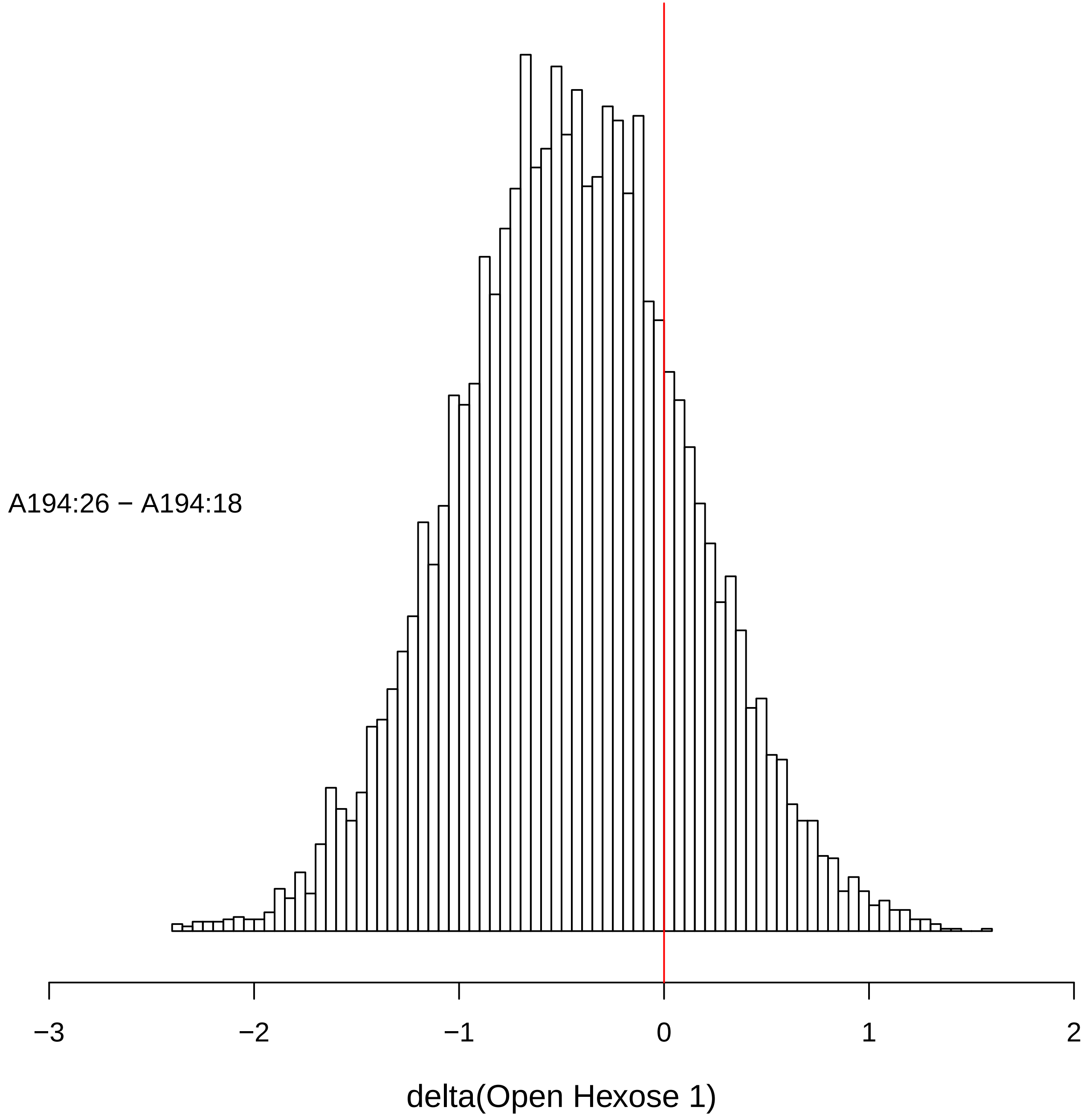
A194:26 − A194:18
−3
−2
−1
0
1
2
delta(Open Hexose 1)

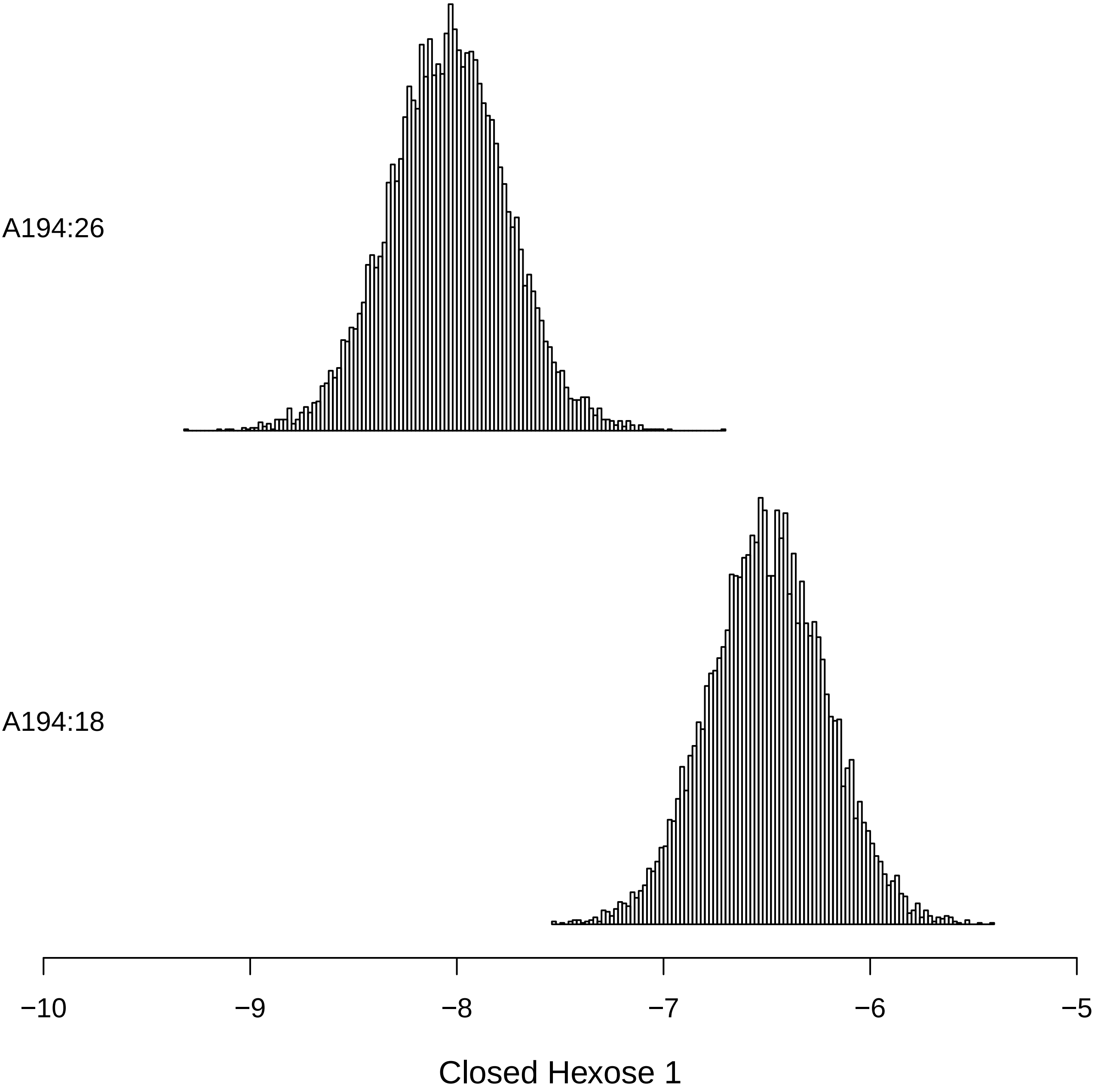
A194:26
A194:18
−10
−9
−8
−7
−6
−5
Closed Hexose 1

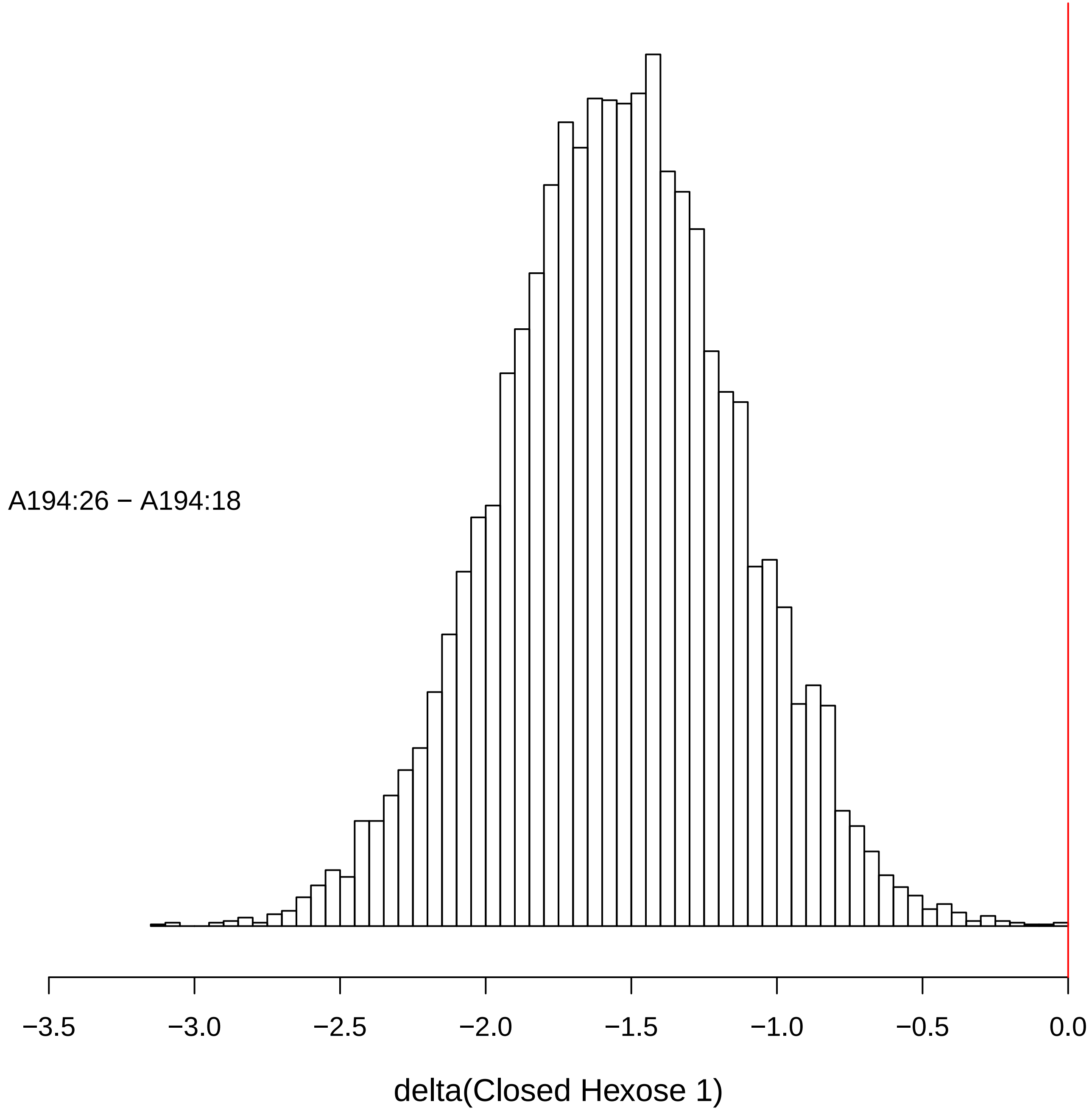
A194:26 − A194:18
−3.5
−3.0
−2.5
−2.0
−1.5
−1.0
−0.5
0.0
delta(Closed Hexose 1)

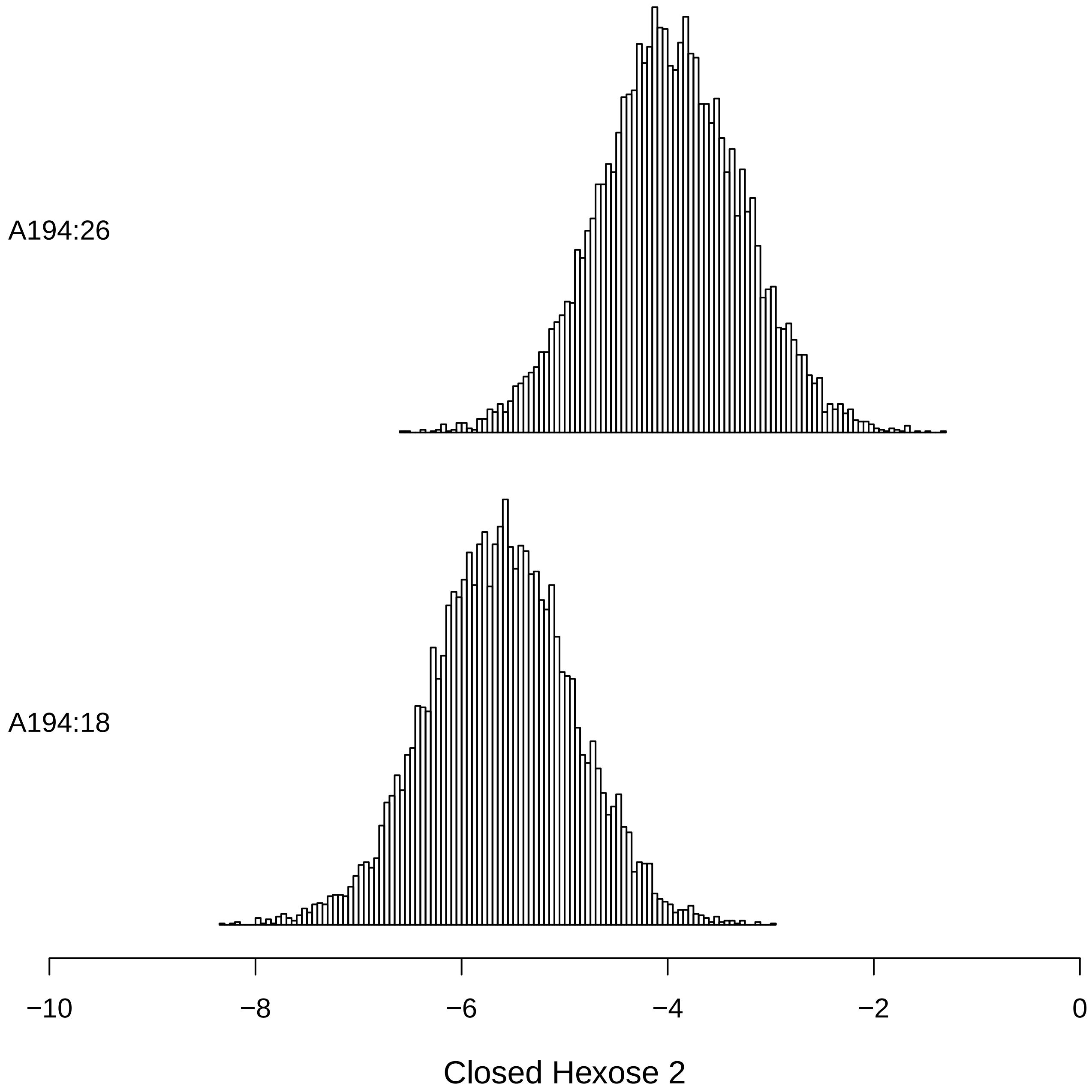
A194:26
A194:18
−10
−8
−6
−4
−2
0
Closed Hexose 2

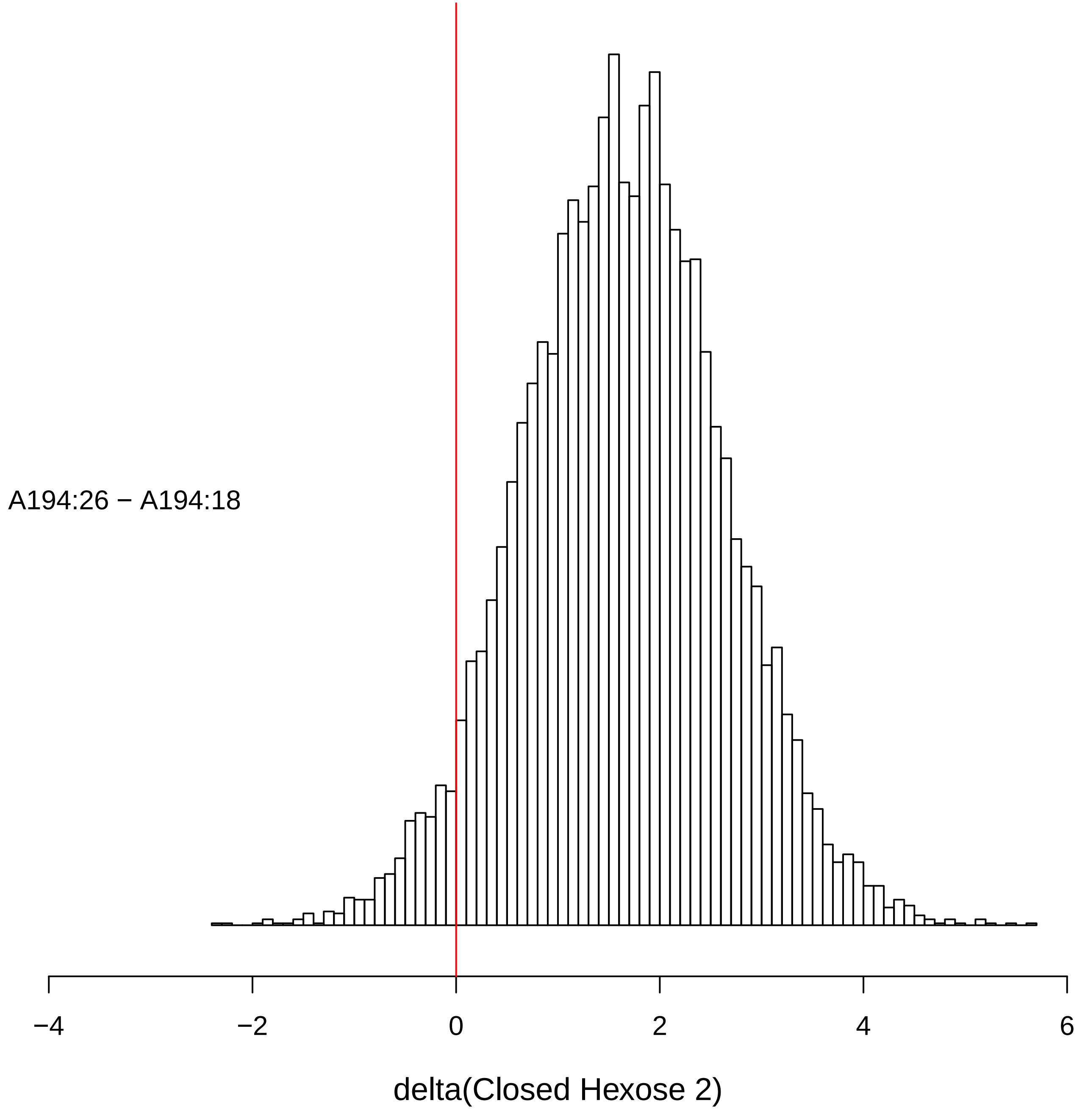
A194:26 − A194:18
−4
−2
0
2
4
6
delta(Closed Hexose 2)

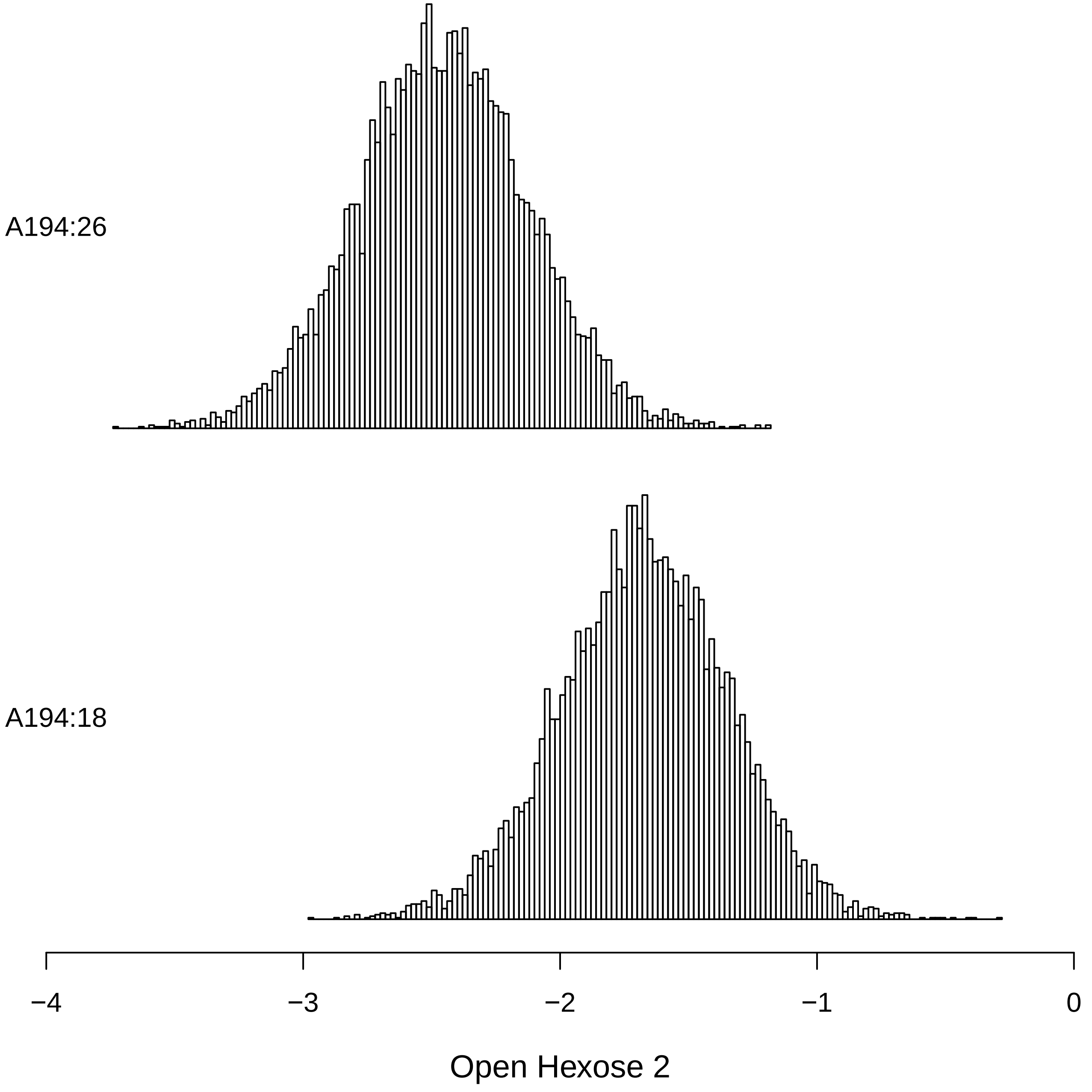
A194:26
A194:18
−4
−3
−2
−1
0
Open Hexose 2

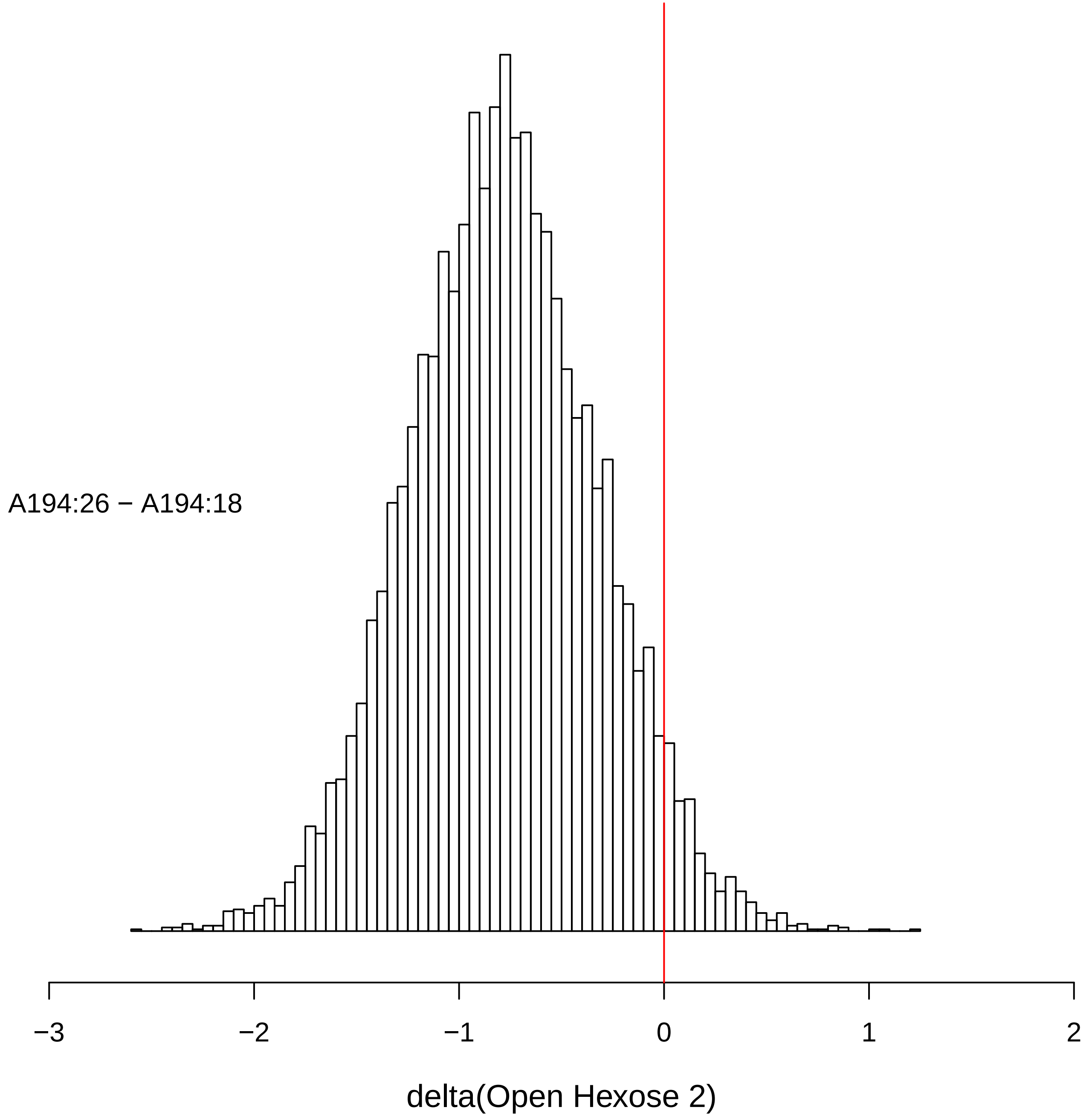
A194:26 − A194:18
−3
−2
−1
0
1
2
delta(Open Hexose 2)

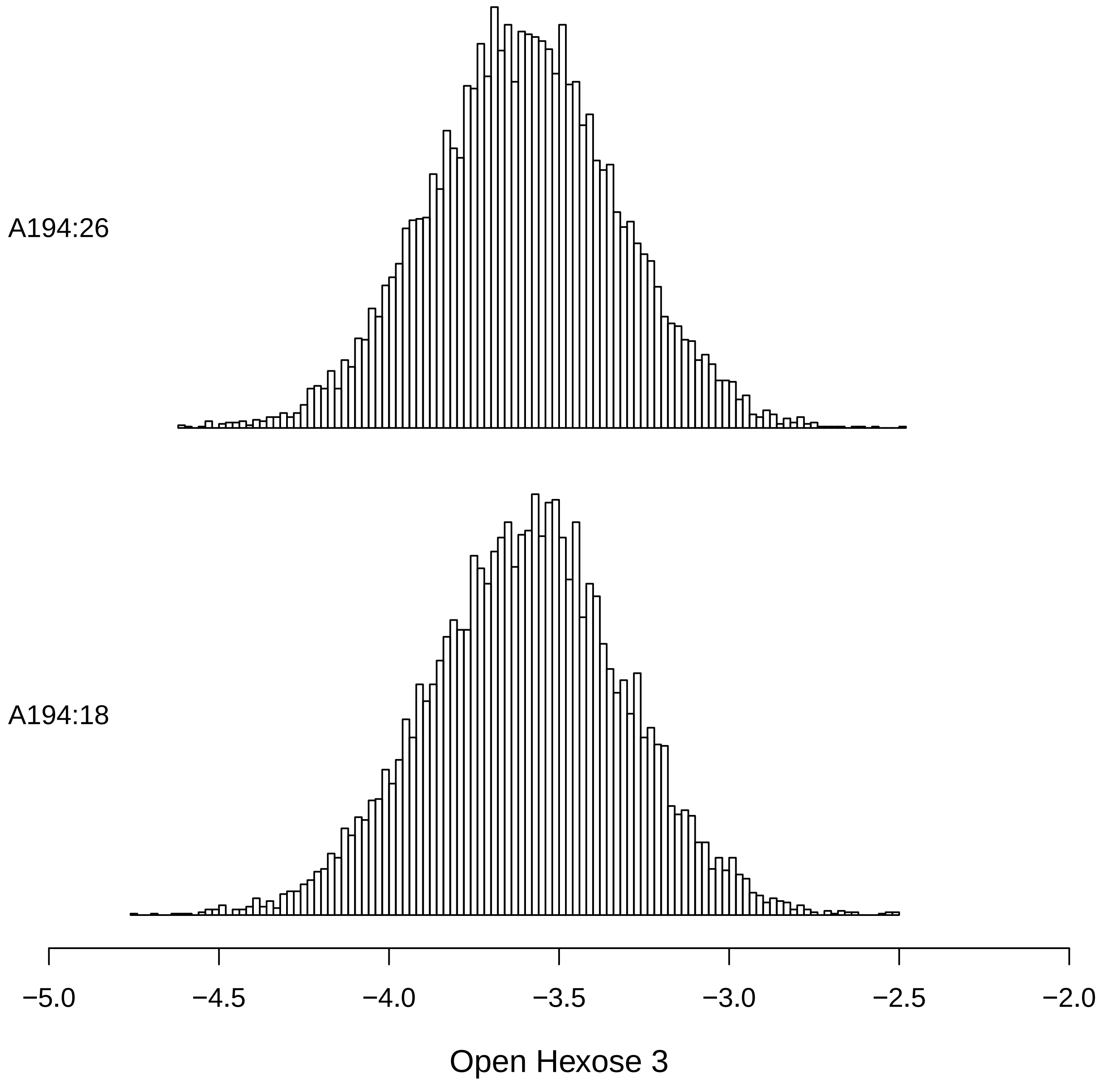

A194:26
A194:18
−5.0
−4.5
−4.0
−3.5
−3.0
−2.5
−2.0
Open Hexose 3

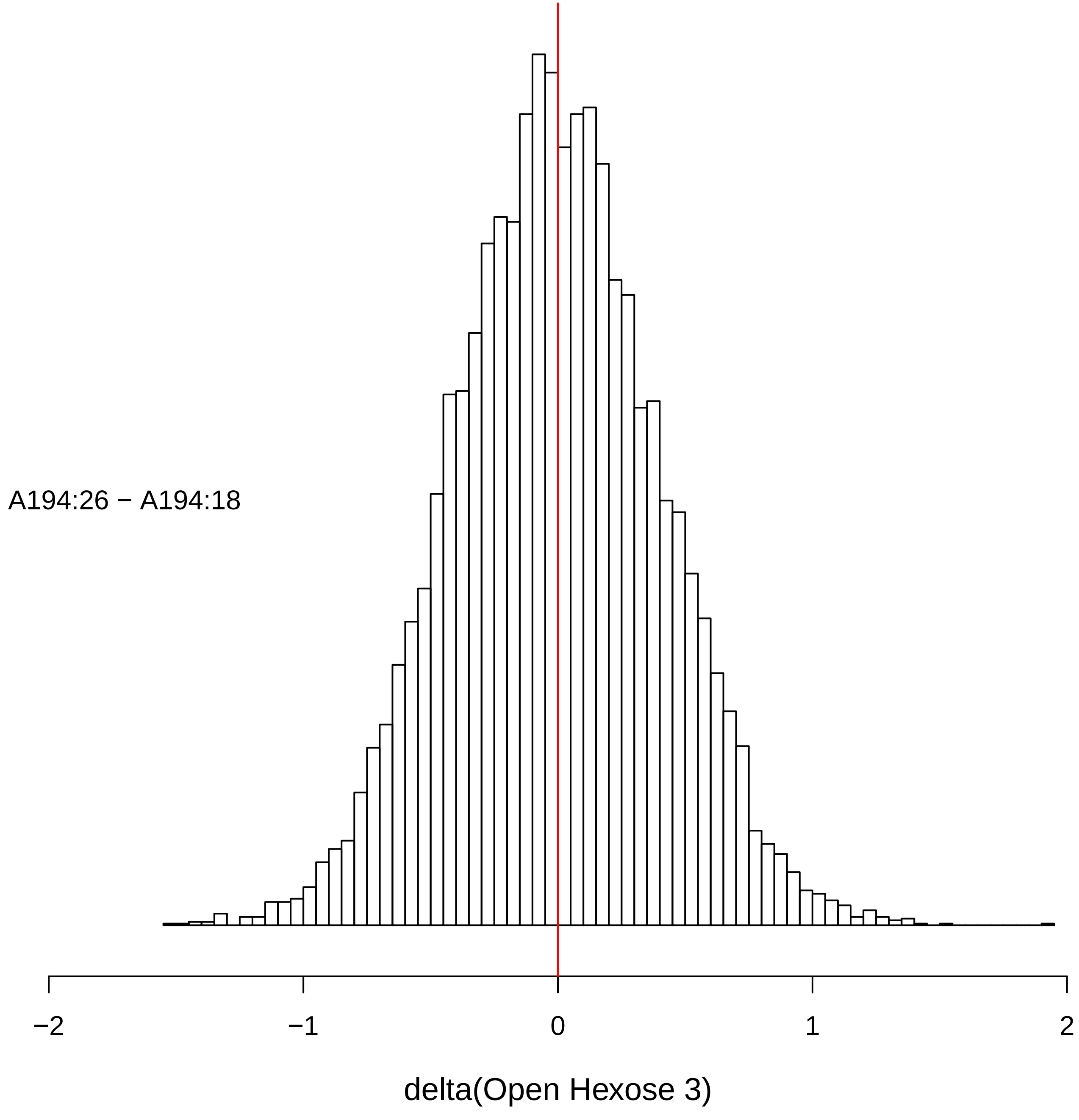

A194:26 − A194:18
−2
−1
0
1
2
delta(Open Hexose 3)

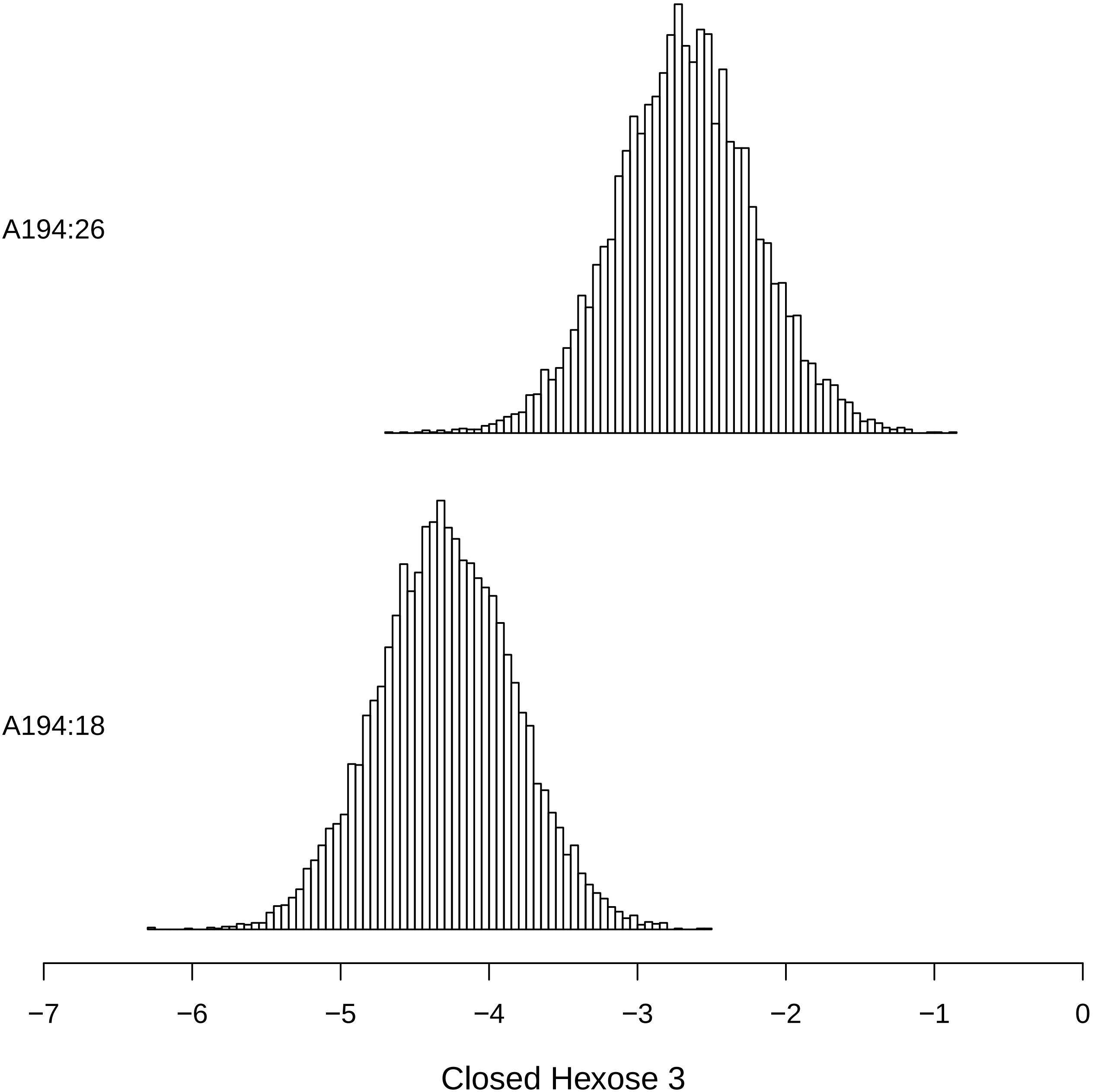

A194:26
A194:18
−7
−6
−5
−4
−3
−2
−1
0
Closed Hexose 3

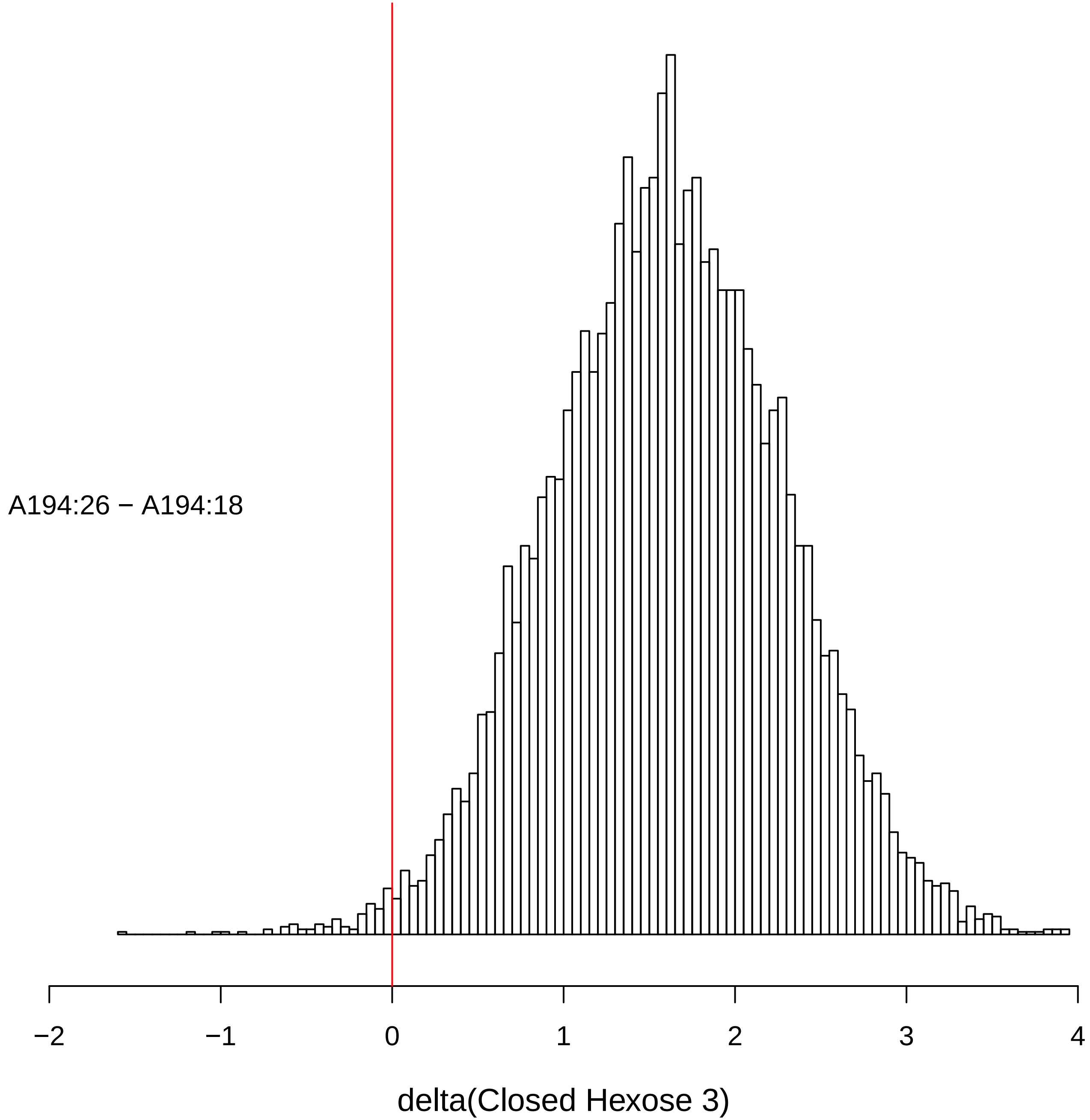
A194:26 − A194:18
−2
−1
0
1
2
3
4
delta(Closed Hexose 3)

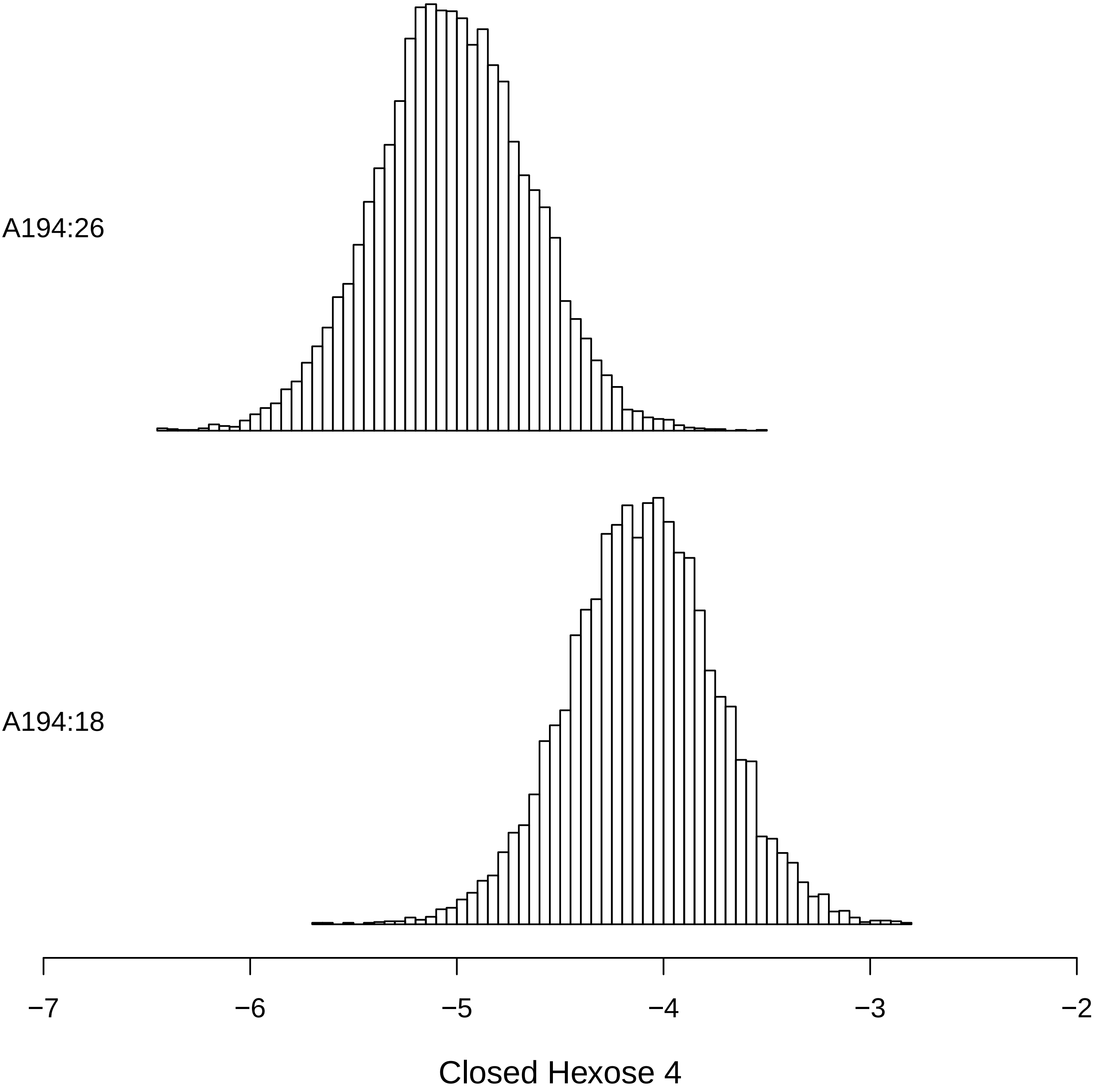

A194:26
A194:18
−7
−6
−5
−4
−3
−2
Closed Hexose 4

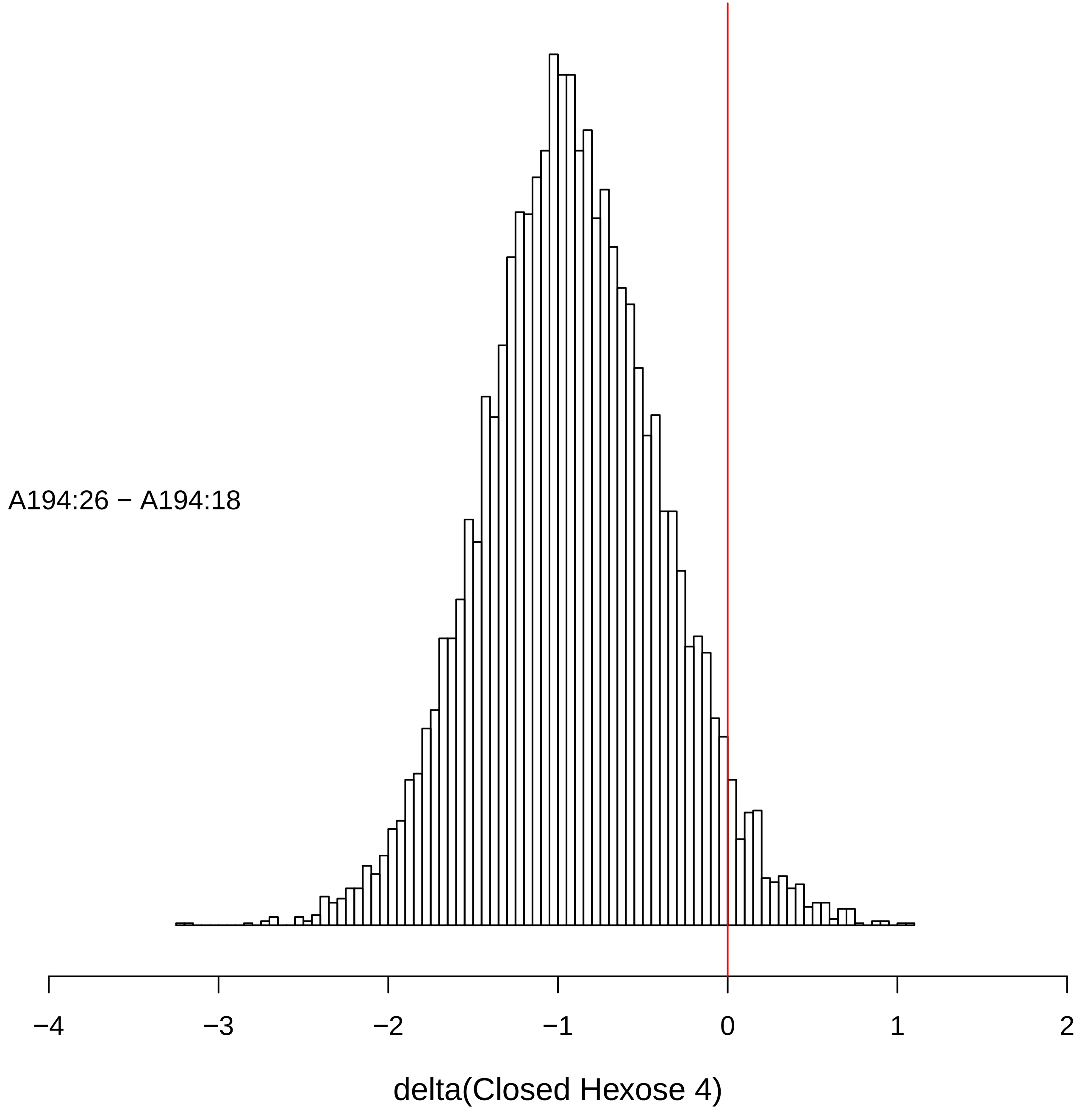
A194:26 − A194:18
−4
−3
−2
−1
0
1
2
delta(Closed Hexose 4)

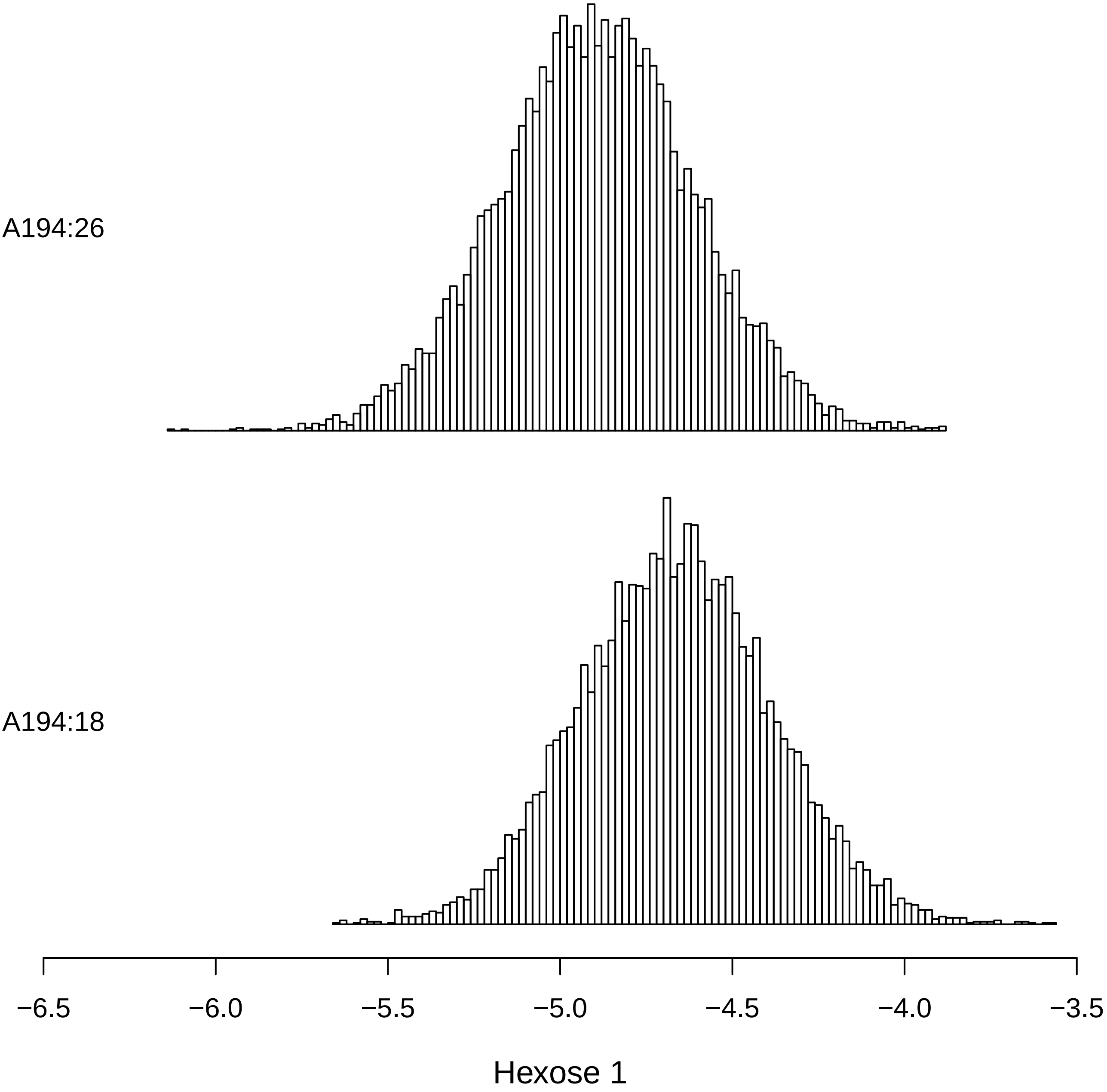

A194:26
A194:18
−6.5
−6.0
−5.5
−5.0
−4.5
−4.0
−3.5
Hexose 1

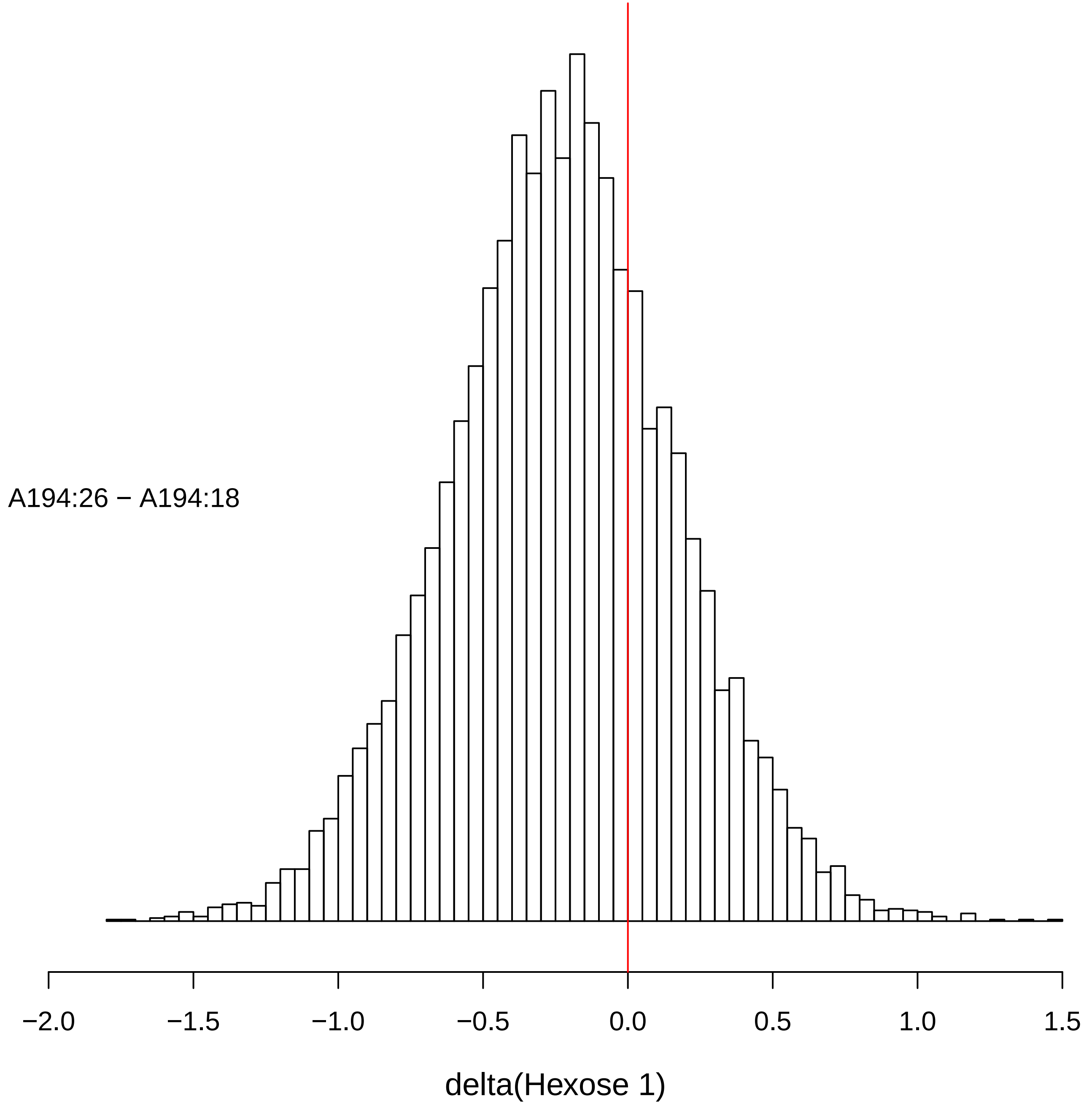
A194:26 − A194:18
−2.0
−1.5
−1.0
−0.5
0.0
0.5
1.0
1.5
delta(Hexose 1)

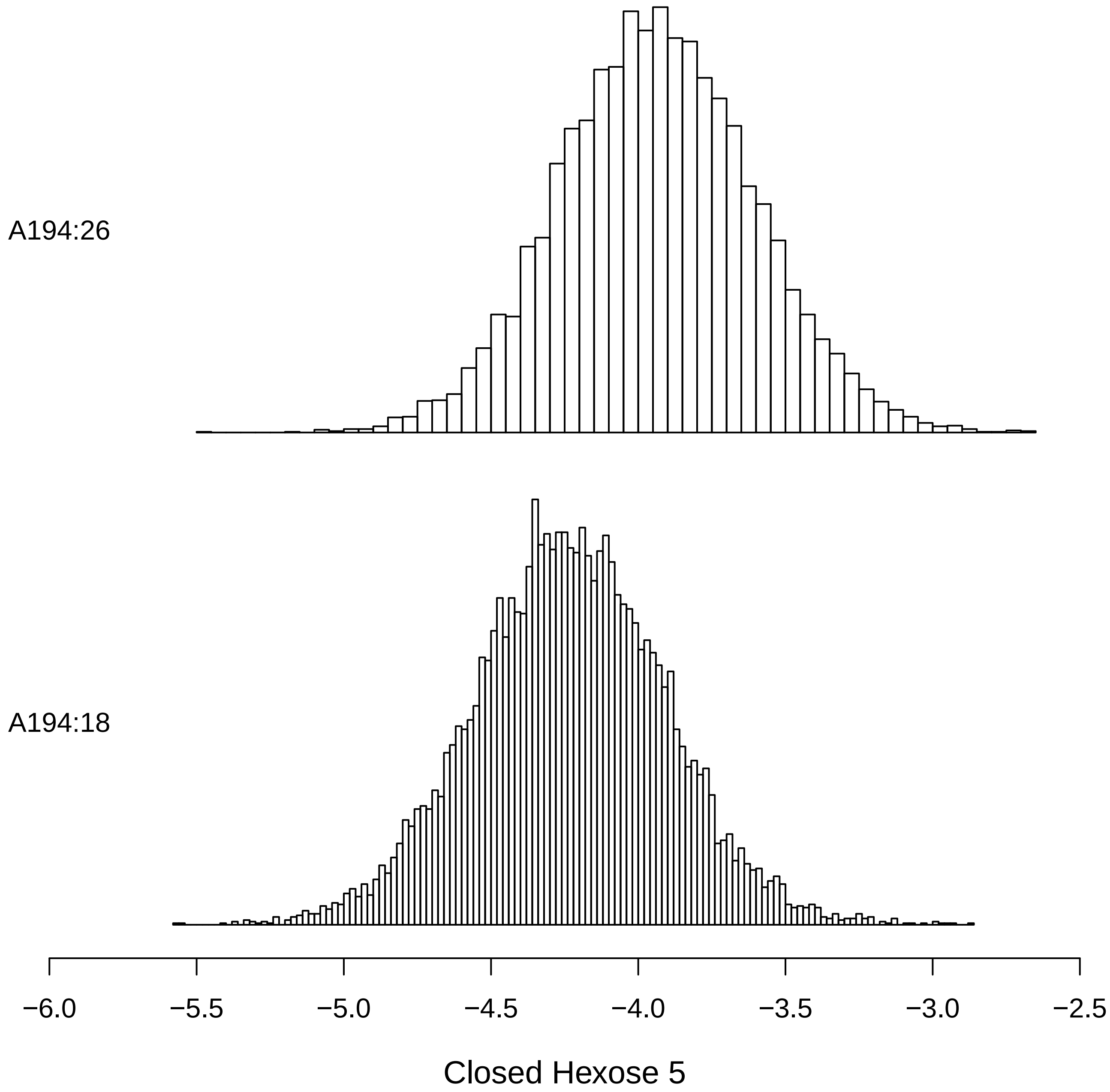
A194:26
A194:18
−6.0
−5.5
−5.0
−4.5
−4.0
−3.5
−3.0
−2.5
Closed Hexose 5

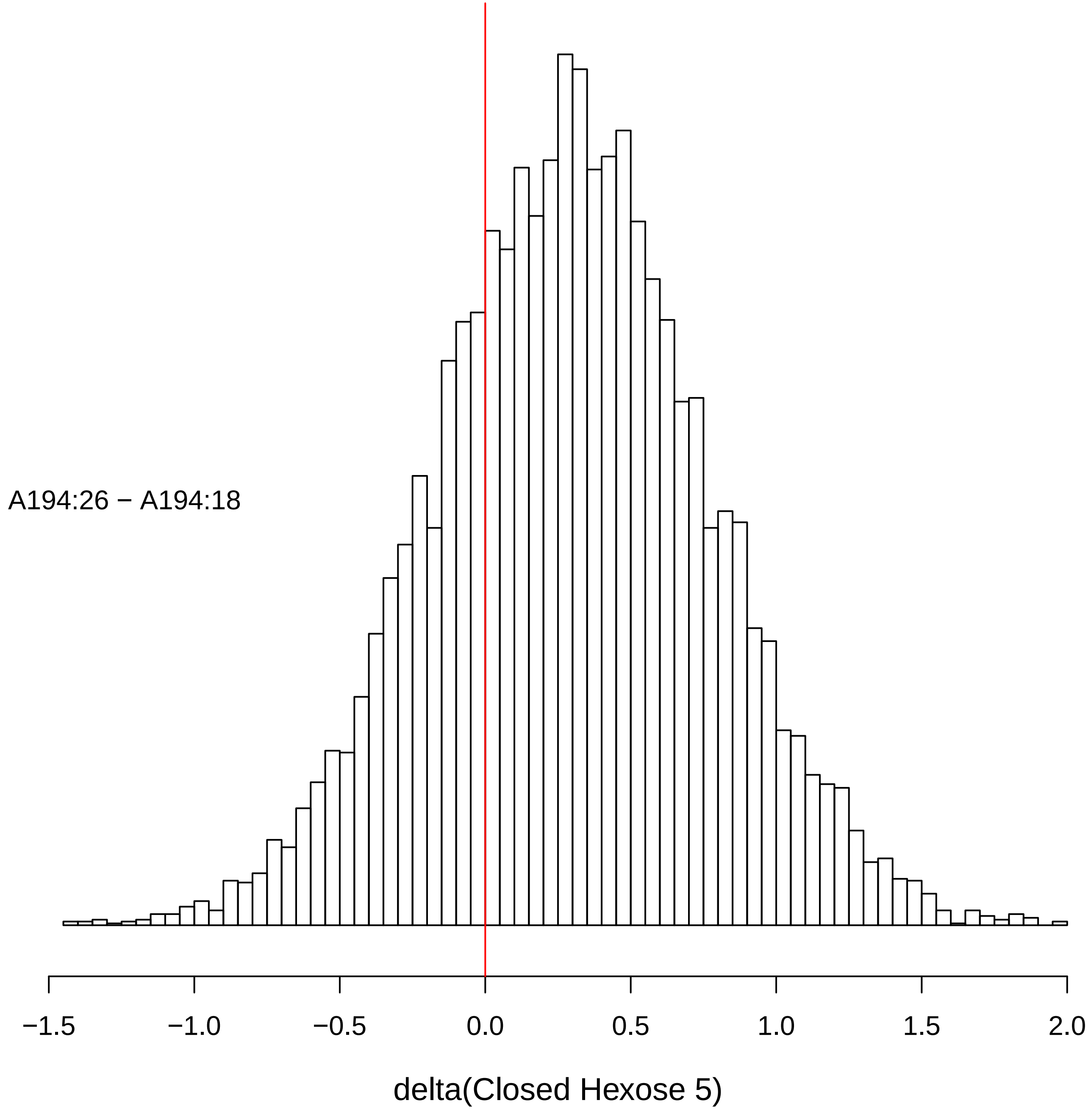
A194:26 − A194:18
−1.5
−1.0
−0.5
0.0
0.5
1.0
1.5
2.0
delta(Closed Hexose 5)

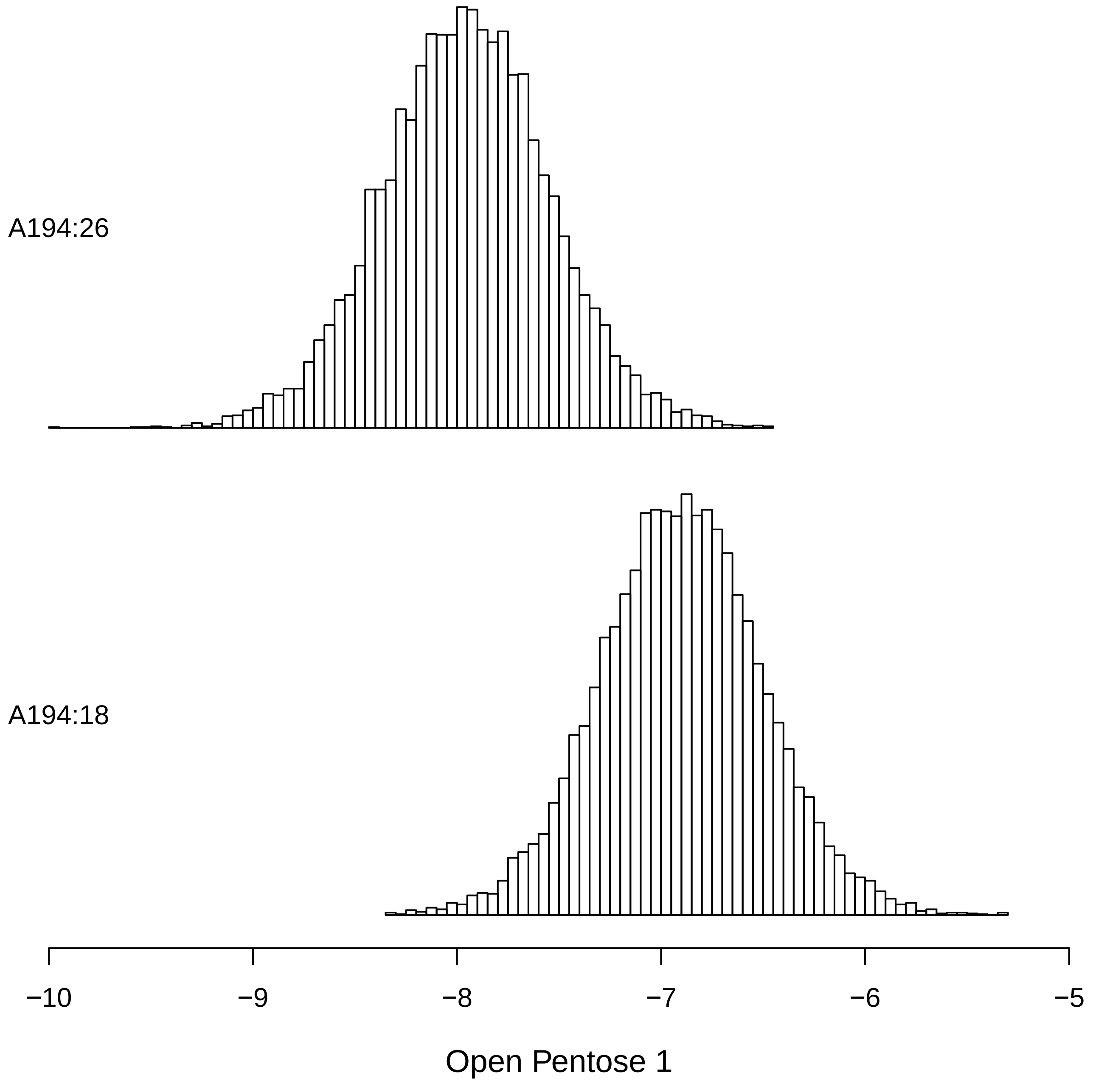
A194:26
A194:18
−10
−9
−8
−7
−6
−5
Open Pentose 1

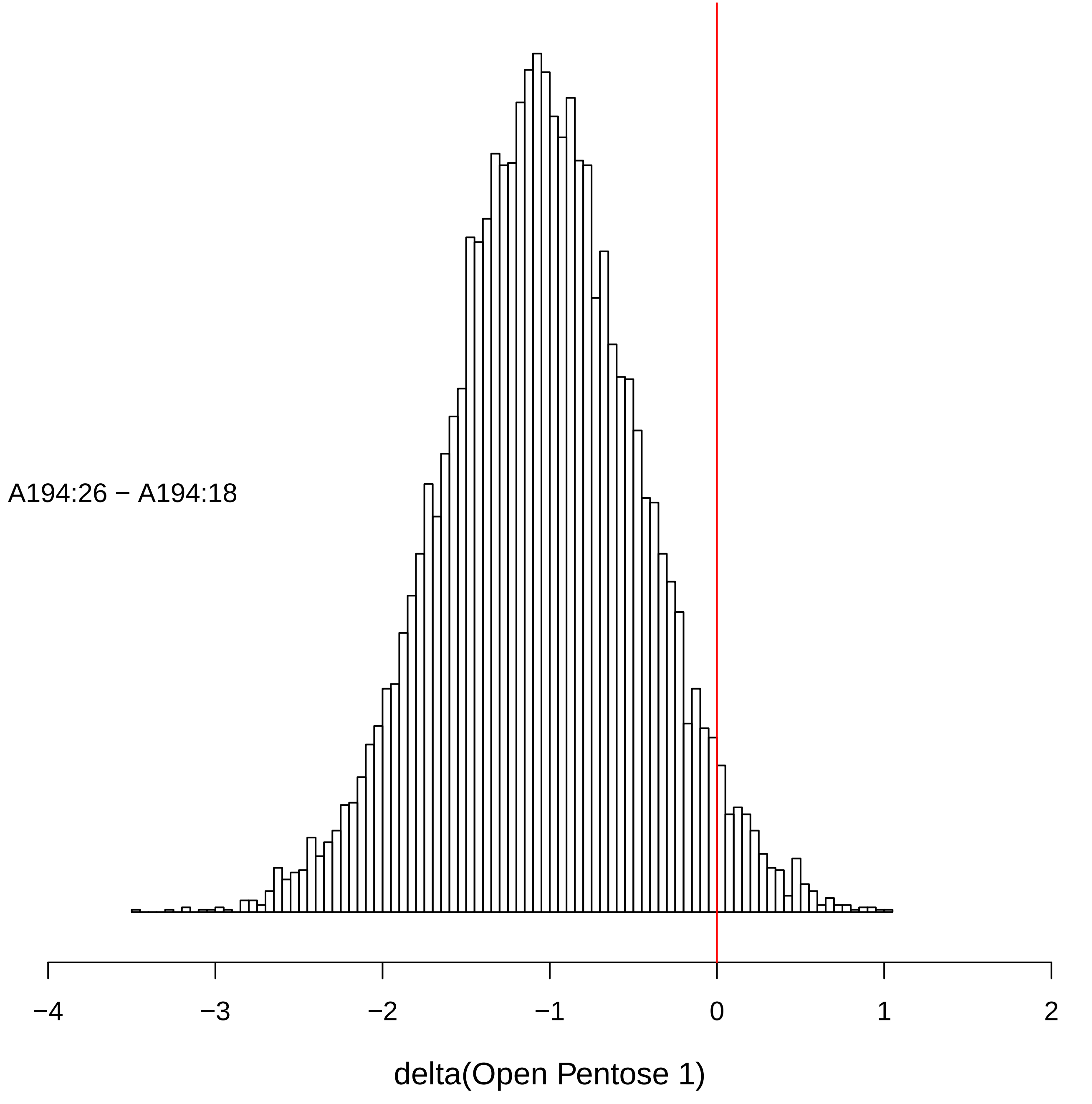
A194:26 − A194:18
−4
−3
−2
−1
0
1
2
delta(Open Pentose 1)

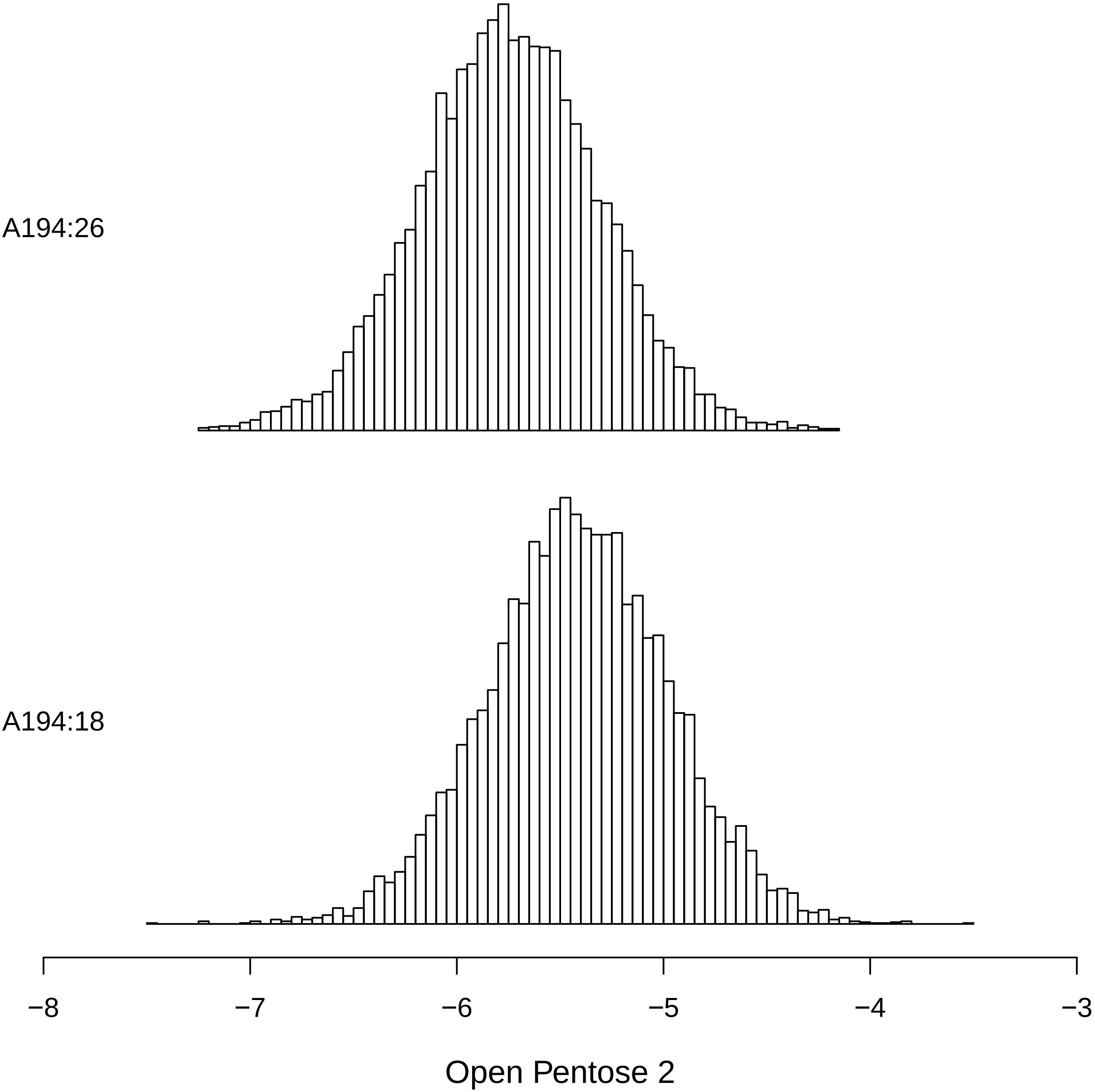

A194:26
A194:18
−8
−7
−6
−5
−4
−3
Open Pentose 2

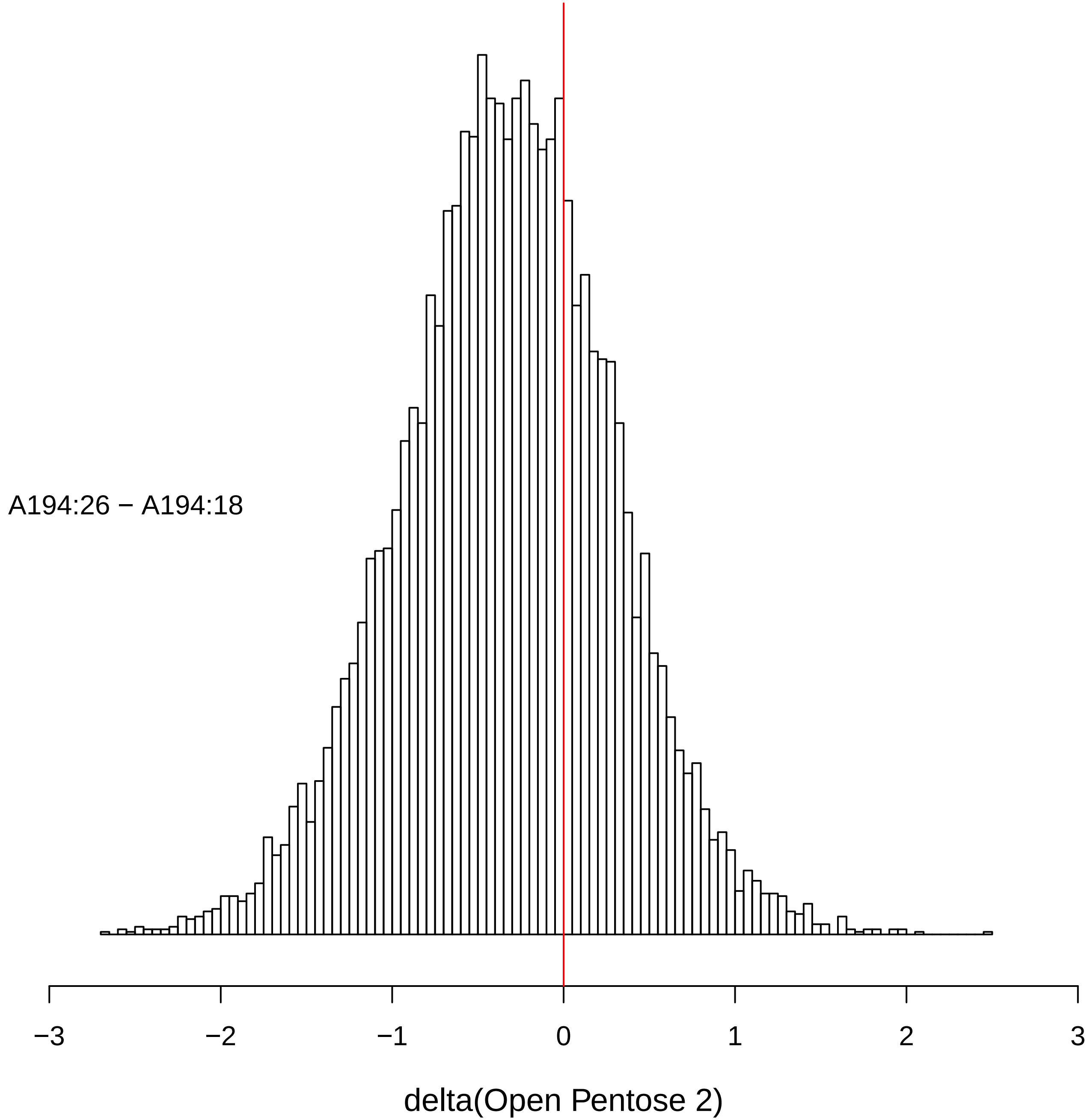

A194:26 − A194:18
−3
−2
−1
0
1
2
3
delta(Open Pentose 2)

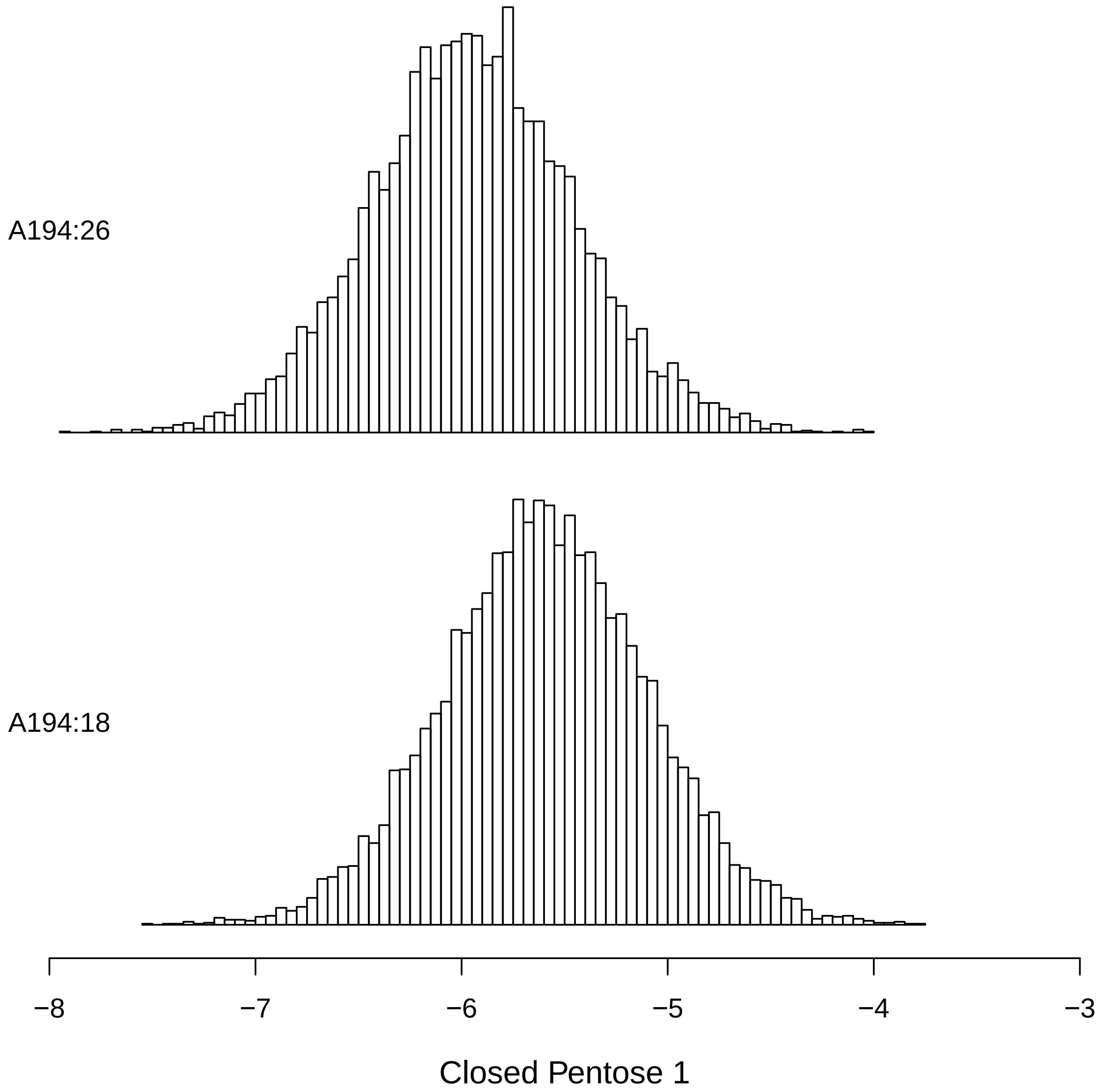
A194:26
A194:18
−8
−7
−6
−5
−4
−3
Closed Pentose 1

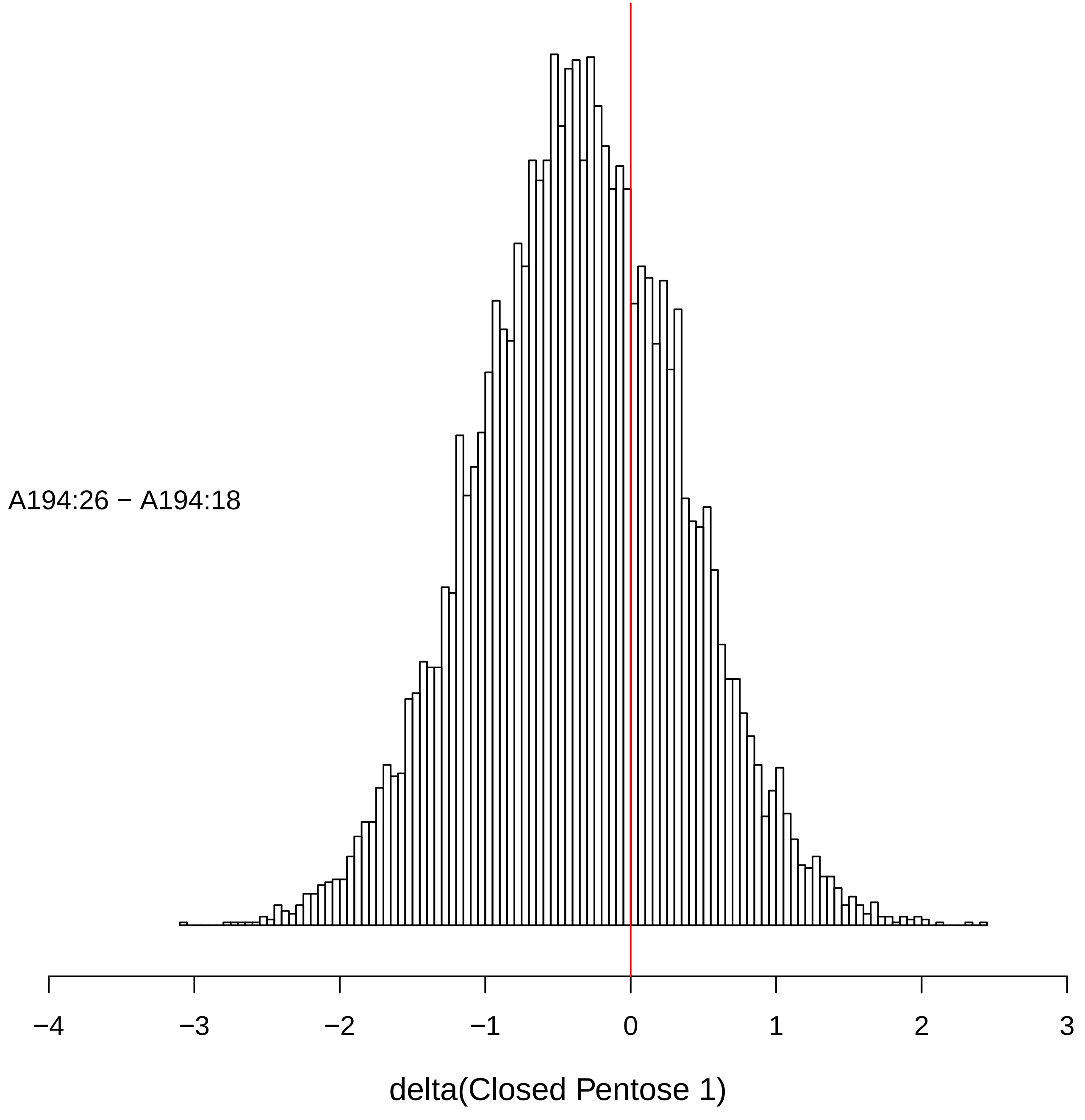

A194:26 − A194:18
−4
−3
−2
−1
0
1
2
3
delta(Closed Pentose 1)

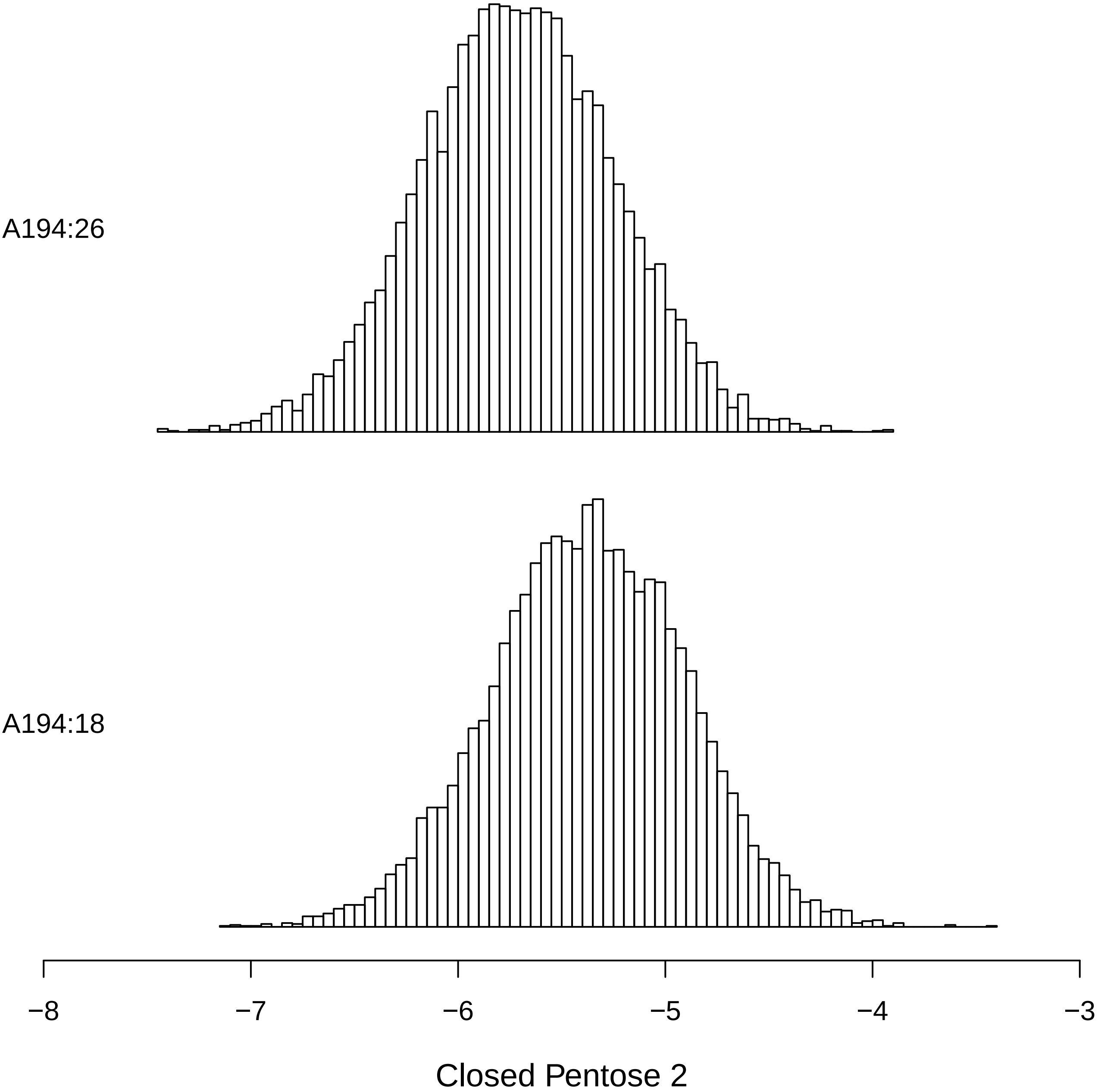
A194:26
A194:18
−8
−7
−6
−5
−4
−3
Closed Pentose 2

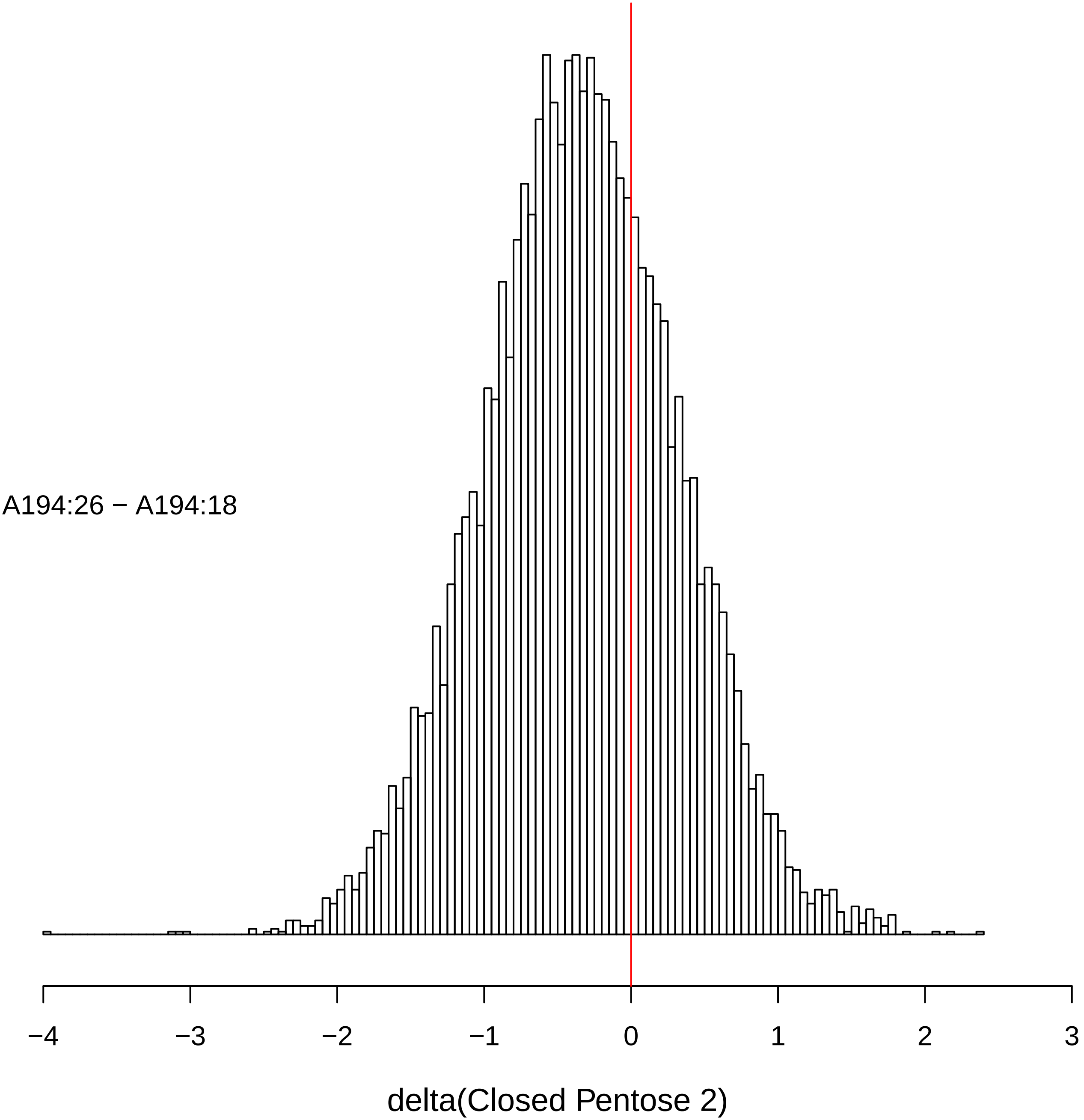
A194:26 − A194:18
−4
−3
−2
−1
0
1
2
3
delta(Closed Pentose 2)

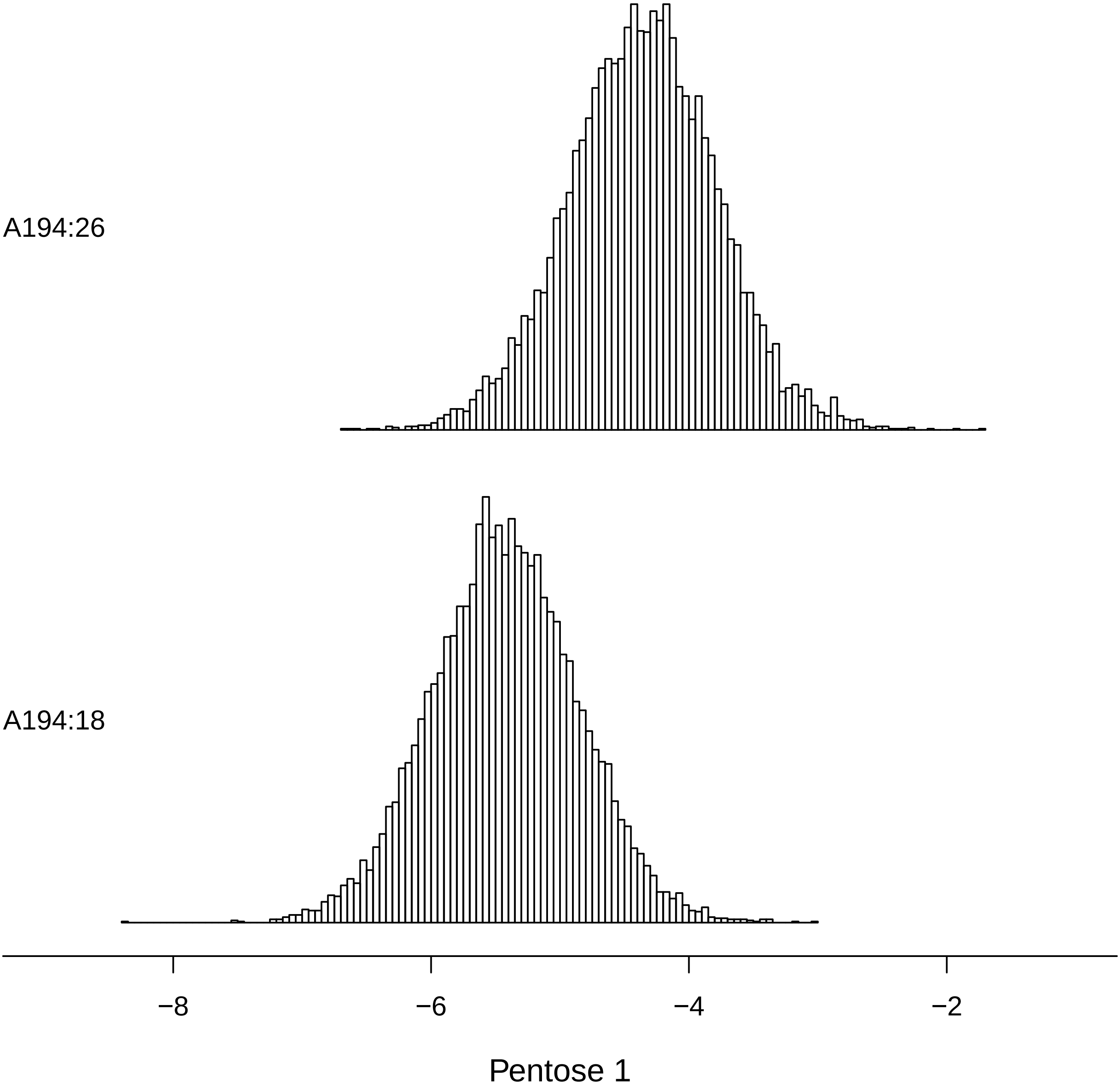
A194:26
A194:18
−8
−6
−4
−2
Pentose 1

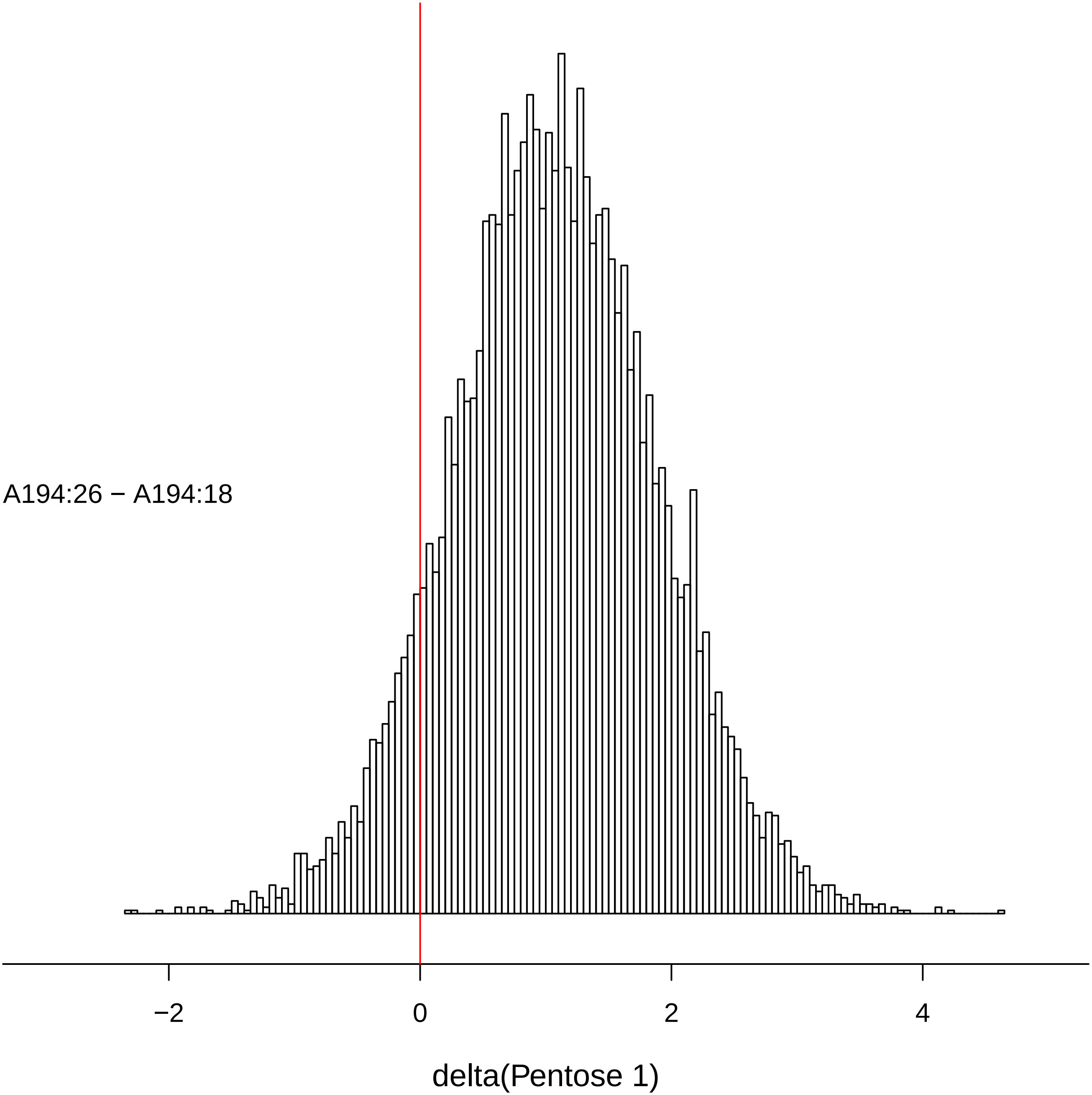
A194:26 − A194:18
−2
0
2
4
delta(Pentose 1)

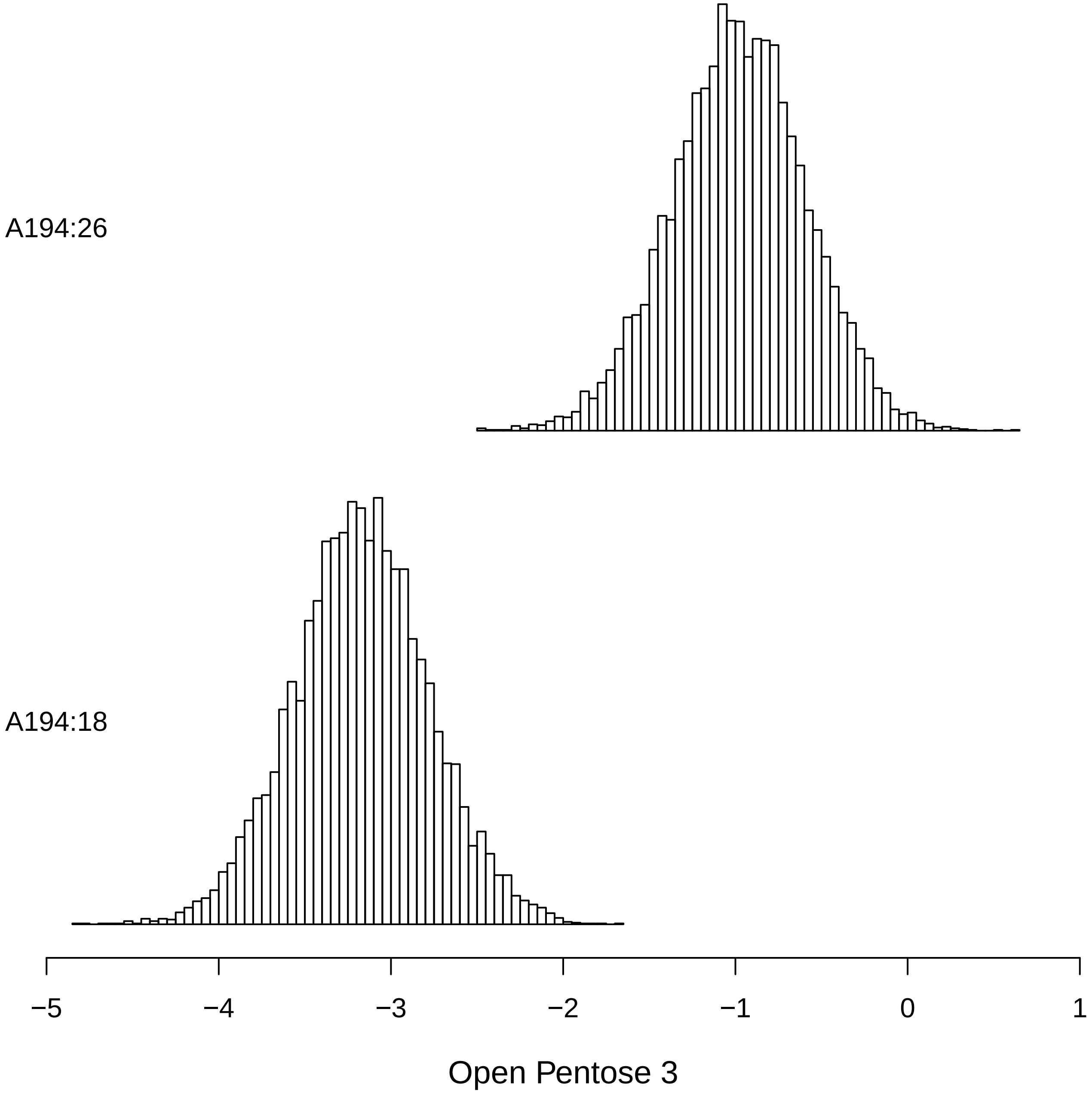

A194:26
A194:18
−5
−4
−3
−2
−1
0
1
Open Pentose 3

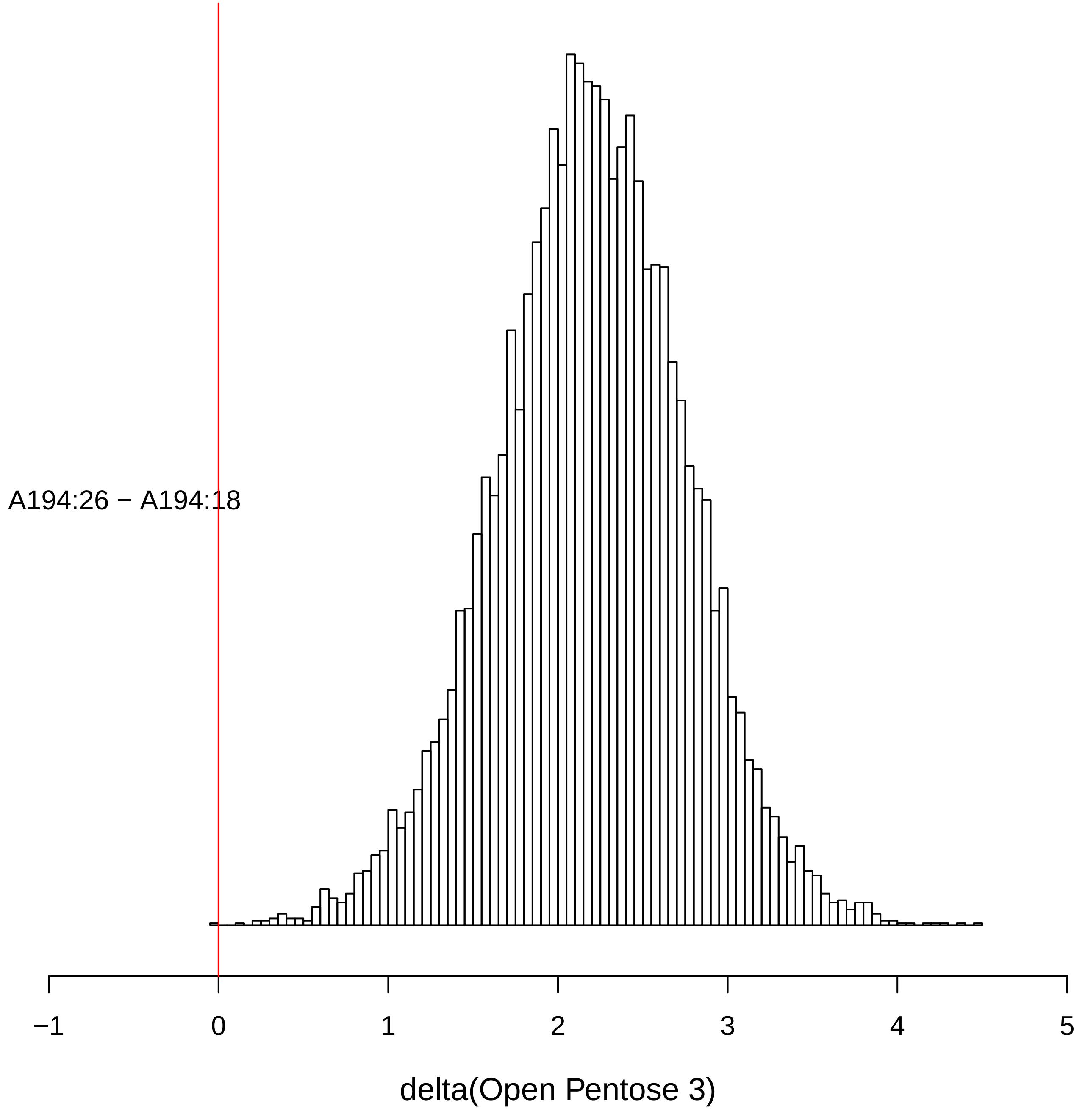
A194:26 − A194:18
−1
0
1
2
3
4
5
delta(Open Pentose 3)

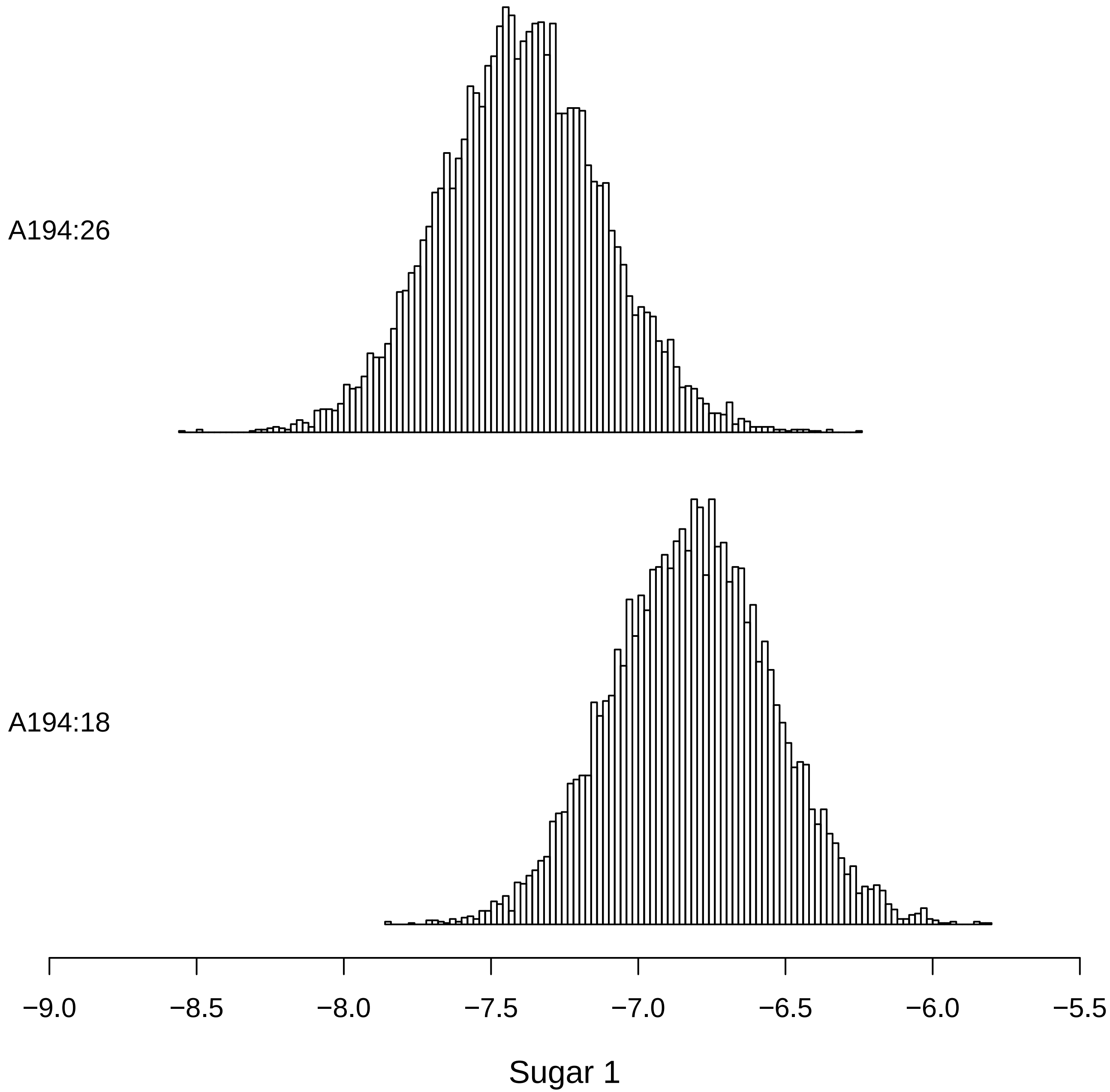
A194:26
A194:18
−9.0
−8.5
−8.0
−7.5
−7.0
−6.5
−6.0
−5.5
Sugar 1

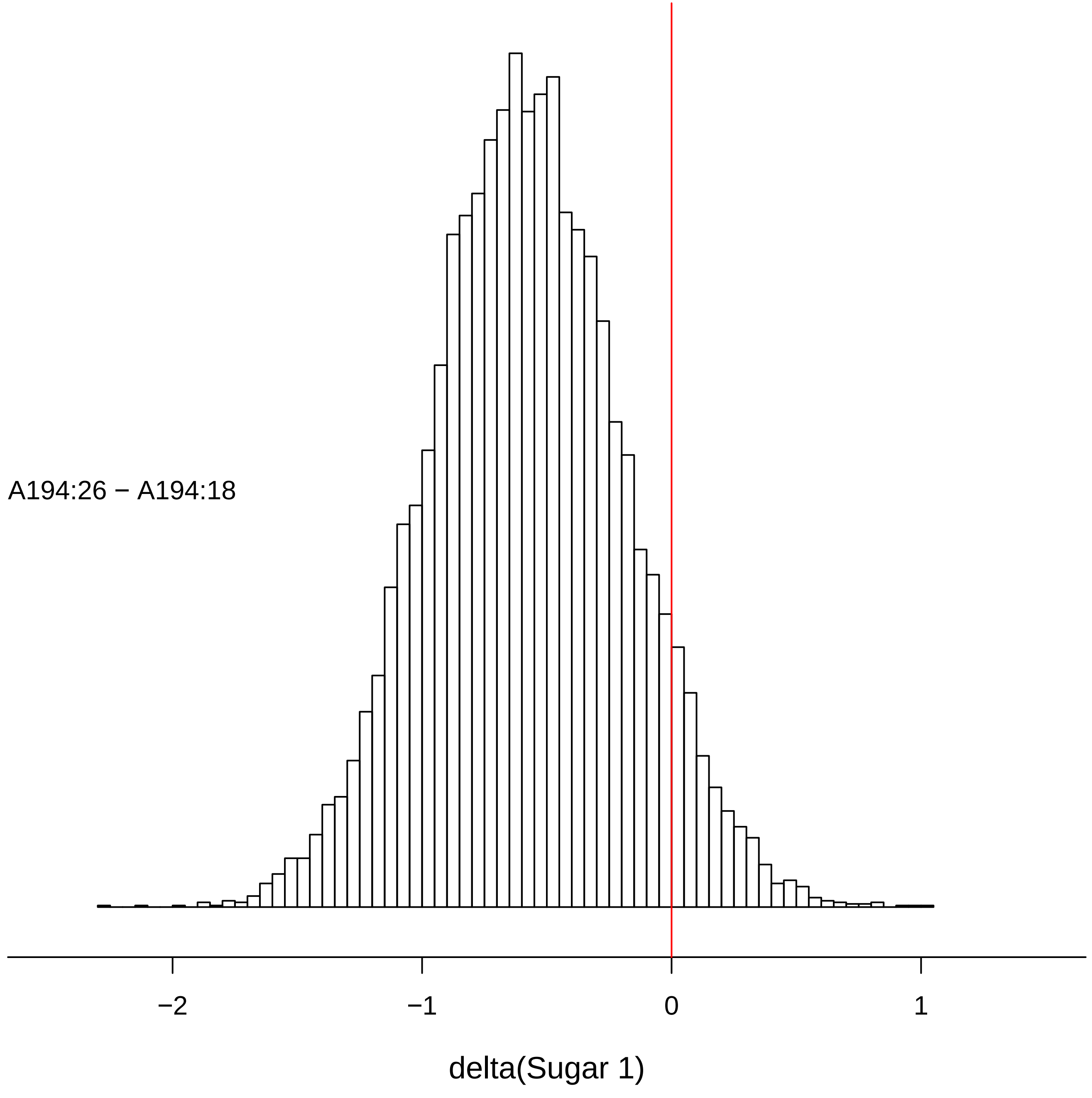
A194:26 − A194:18
−2
−1
0
1
delta(Sugar 1)

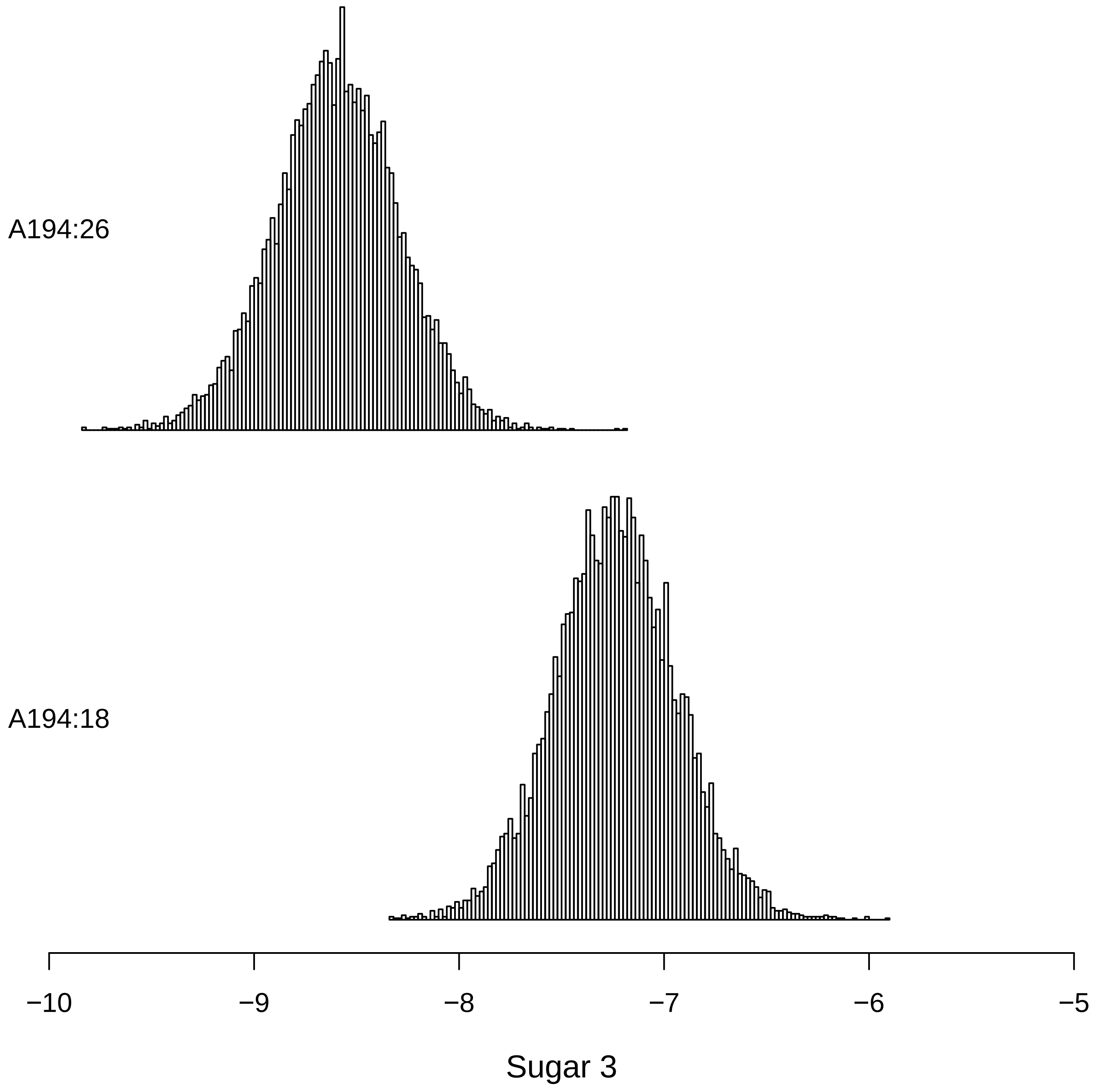

A194:26
A194:18
−10
−9
−8
−7
−6
−5
Sugar 3

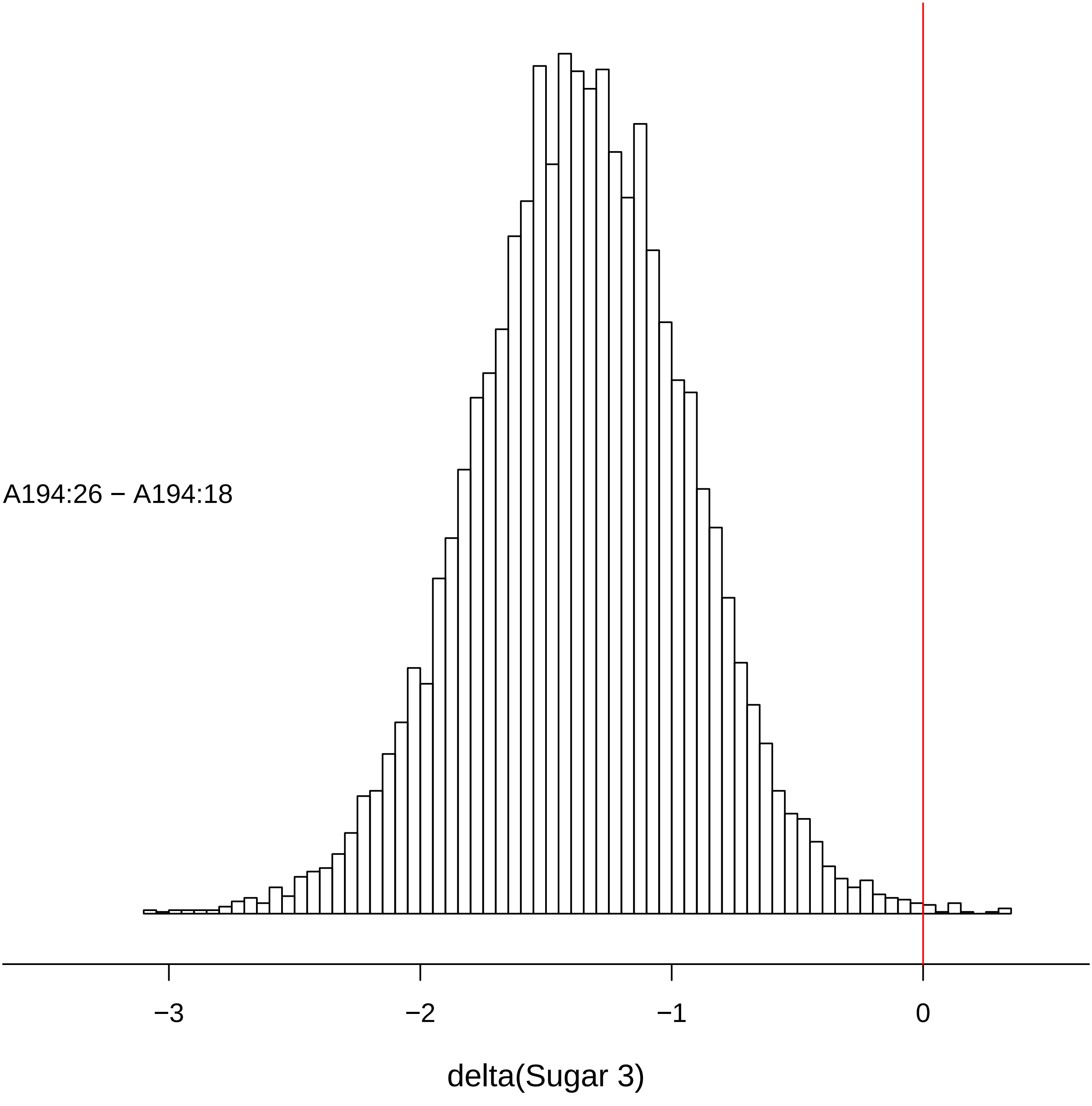

A194:26 − A194:18
−3
−2
−1
0
delta(Sugar 3)

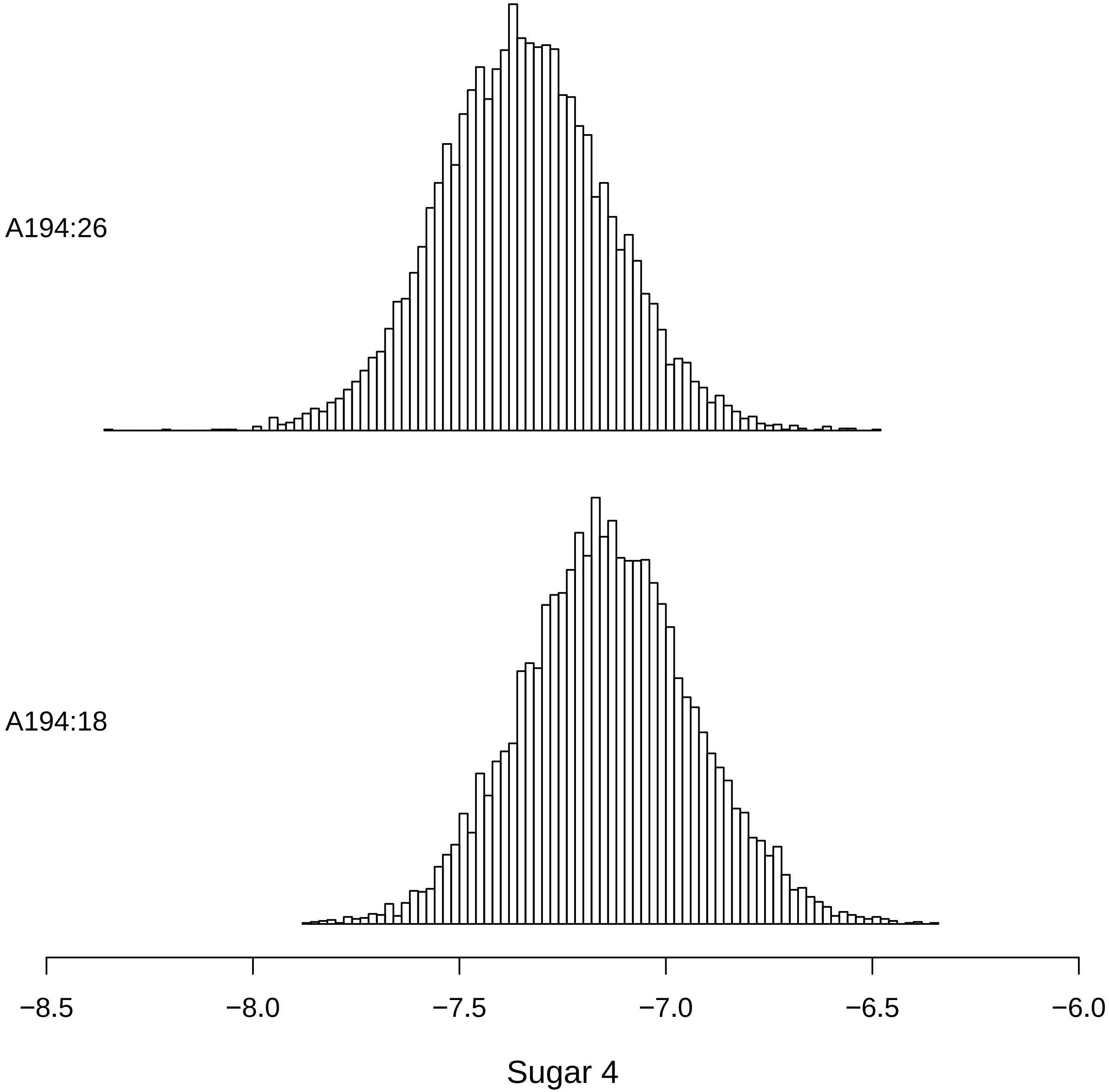

A194:26
A194:18
−8.5
−8.0
−7.5
−7.0
−6.5
−6.0
Sugar 4

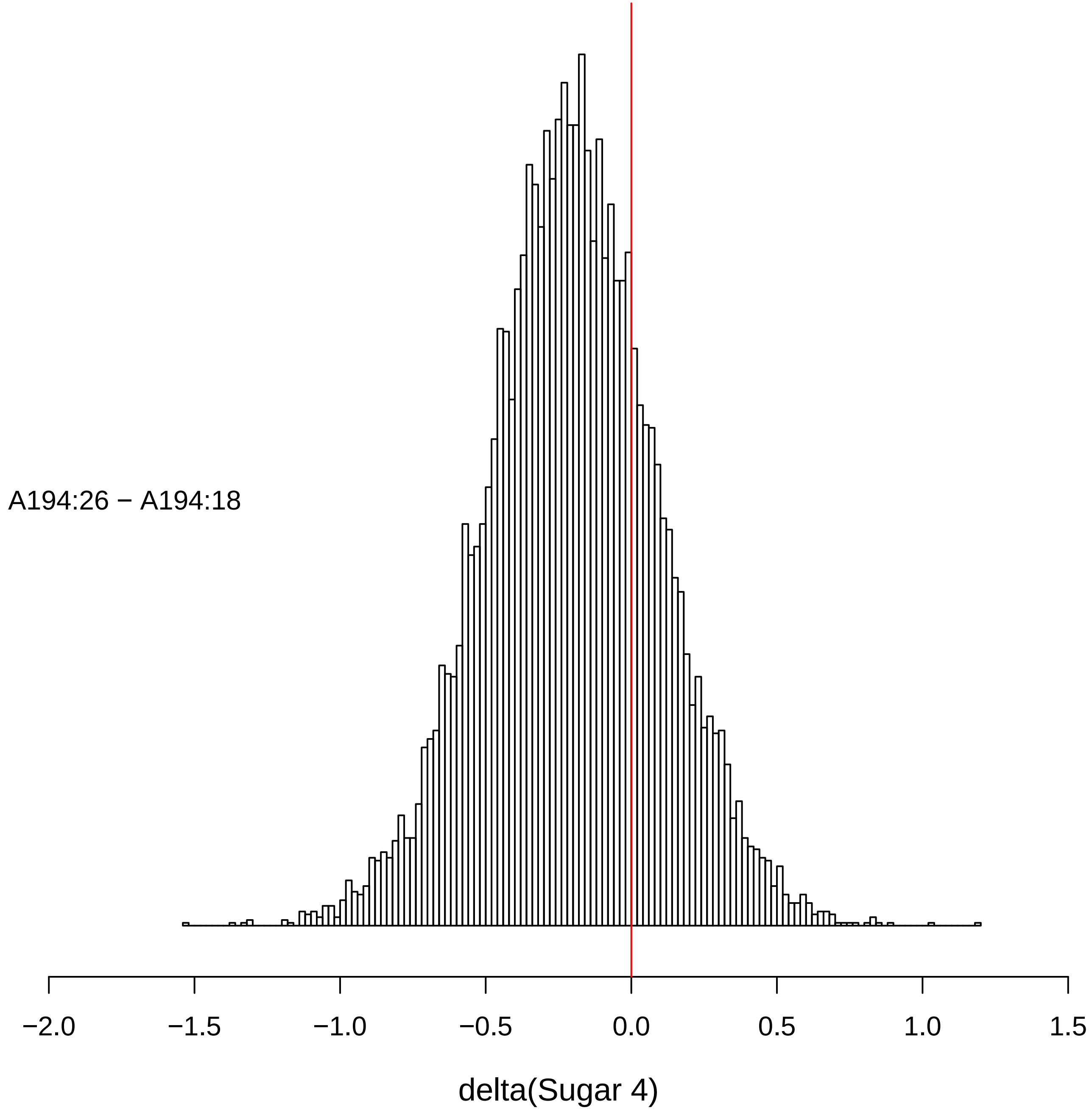
A194:26 − A194:18
−2.0
−1.5
−1.0
−0.5
0.0
0.5
1.0
1.5
delta(Sugar 4)

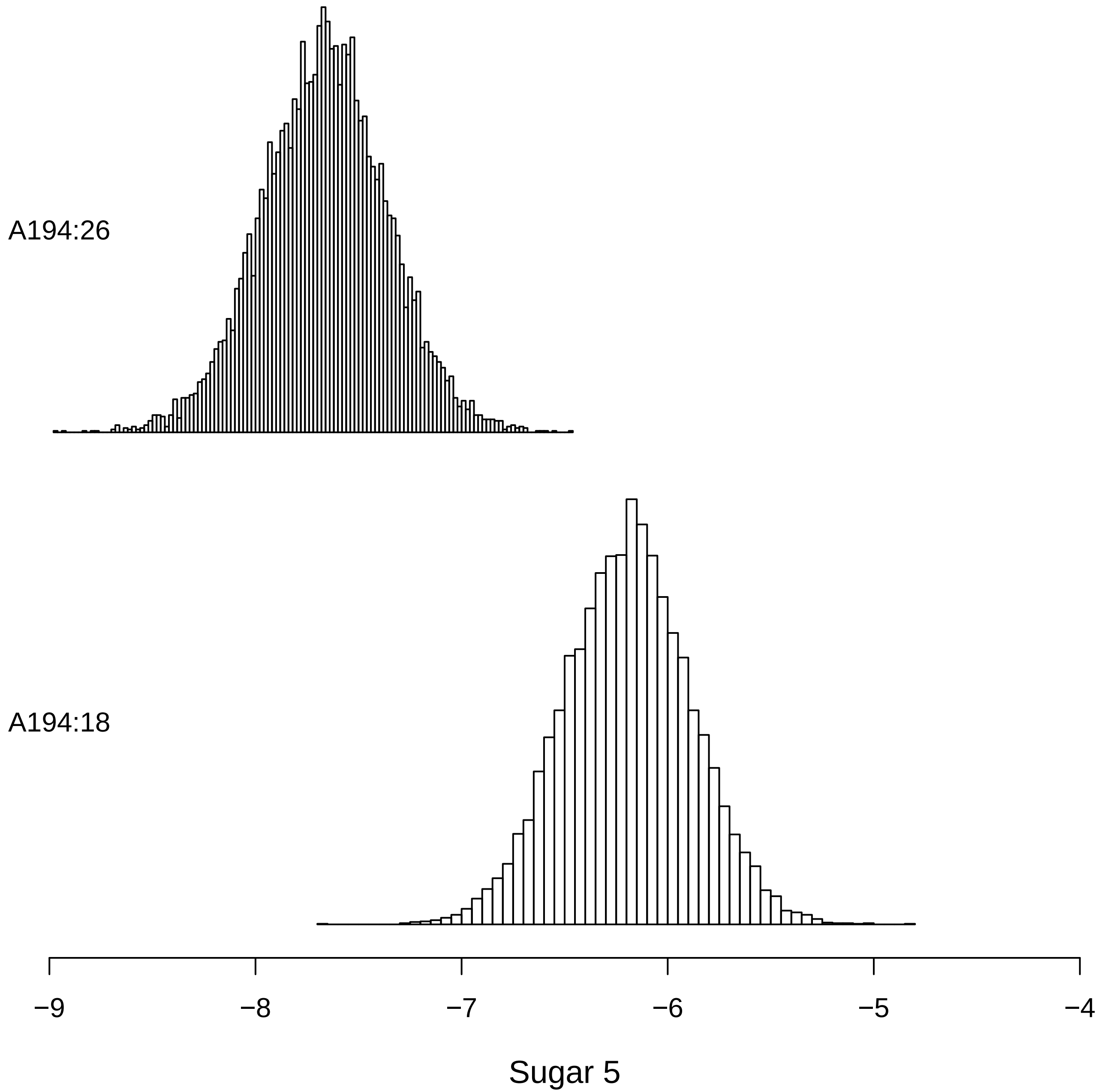
A194:26
A194:18
−9
−8
−7
−6
−5
−4
Sugar 5

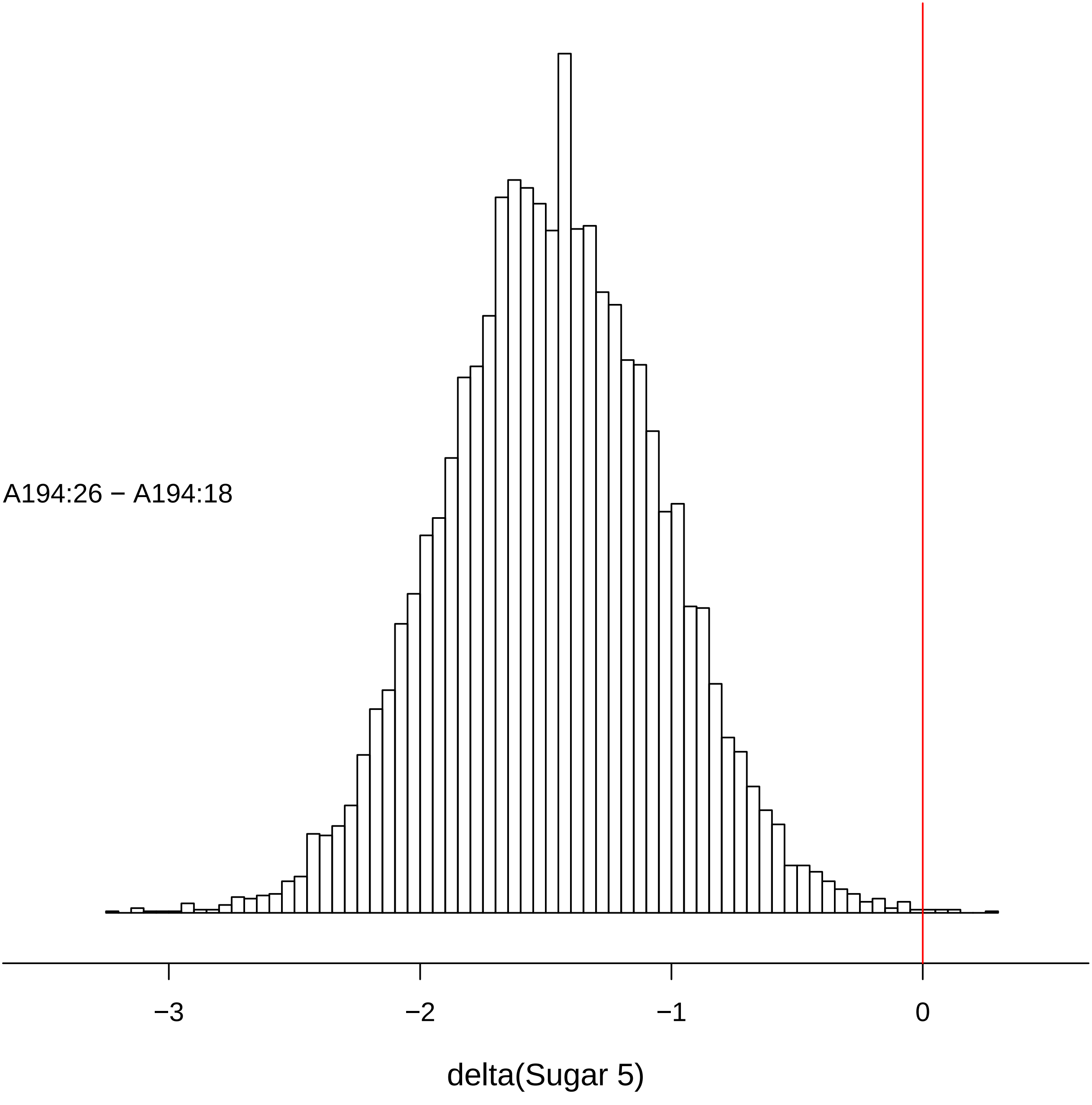
A194:26 − A194:18
−3
−2
−1
0
delta(Sugar 5)

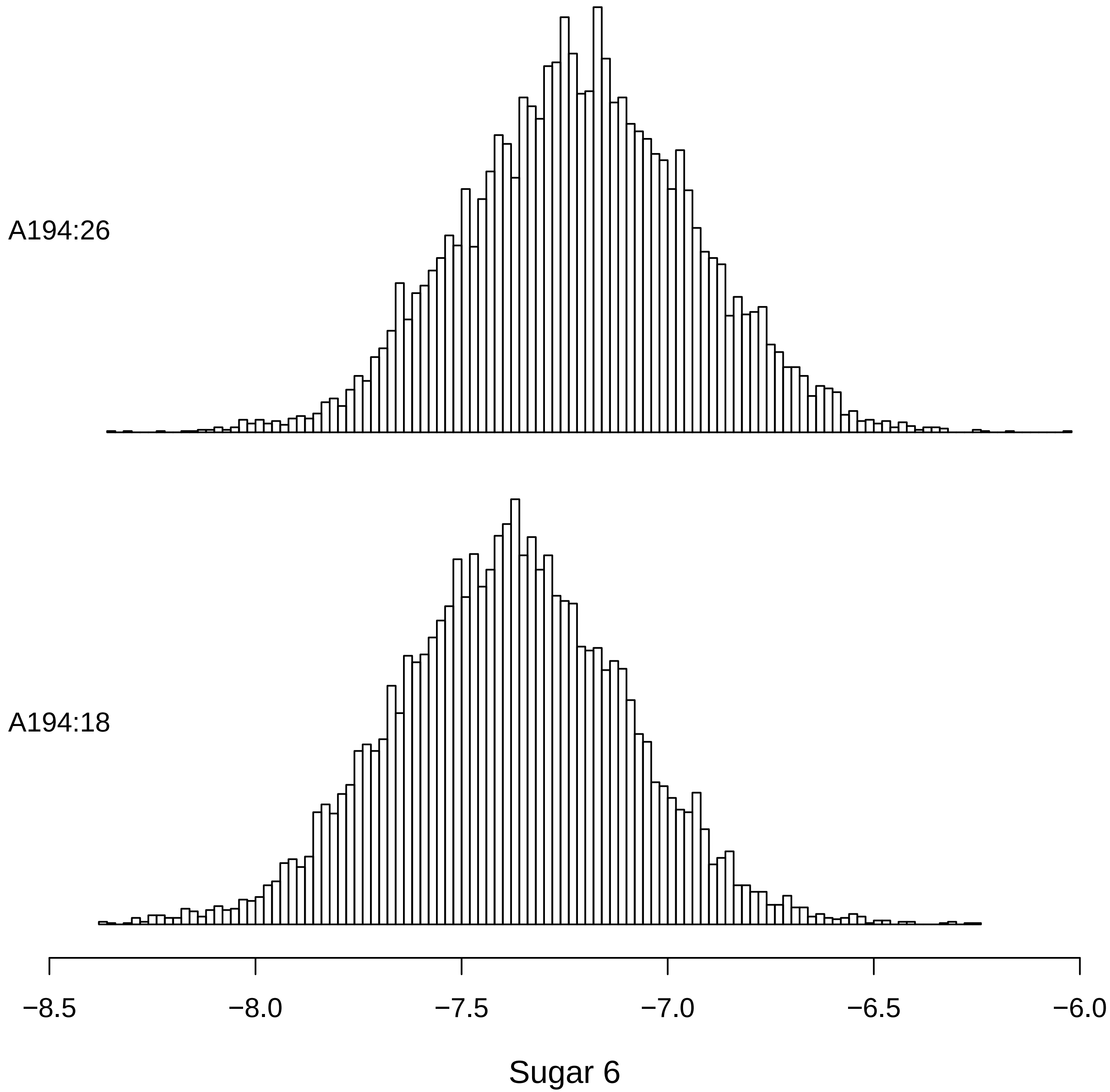
A194:26
A194:18
−8.5
−8.0
−7.5
−7.0
−6.5
−6.0
Sugar 6

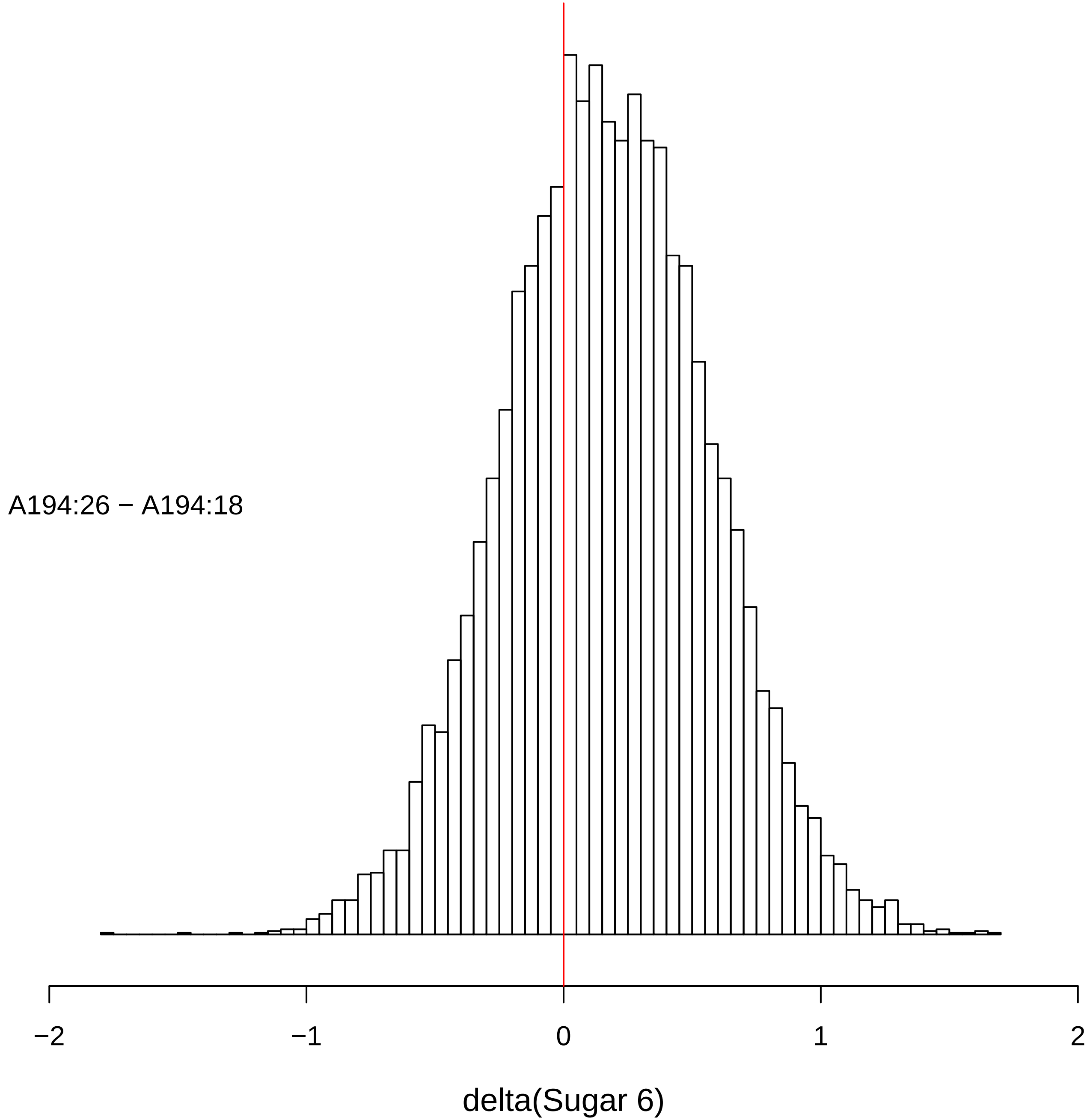
A194:26 − A194:18
−2
−1
0
1
2
delta(Sugar 6)

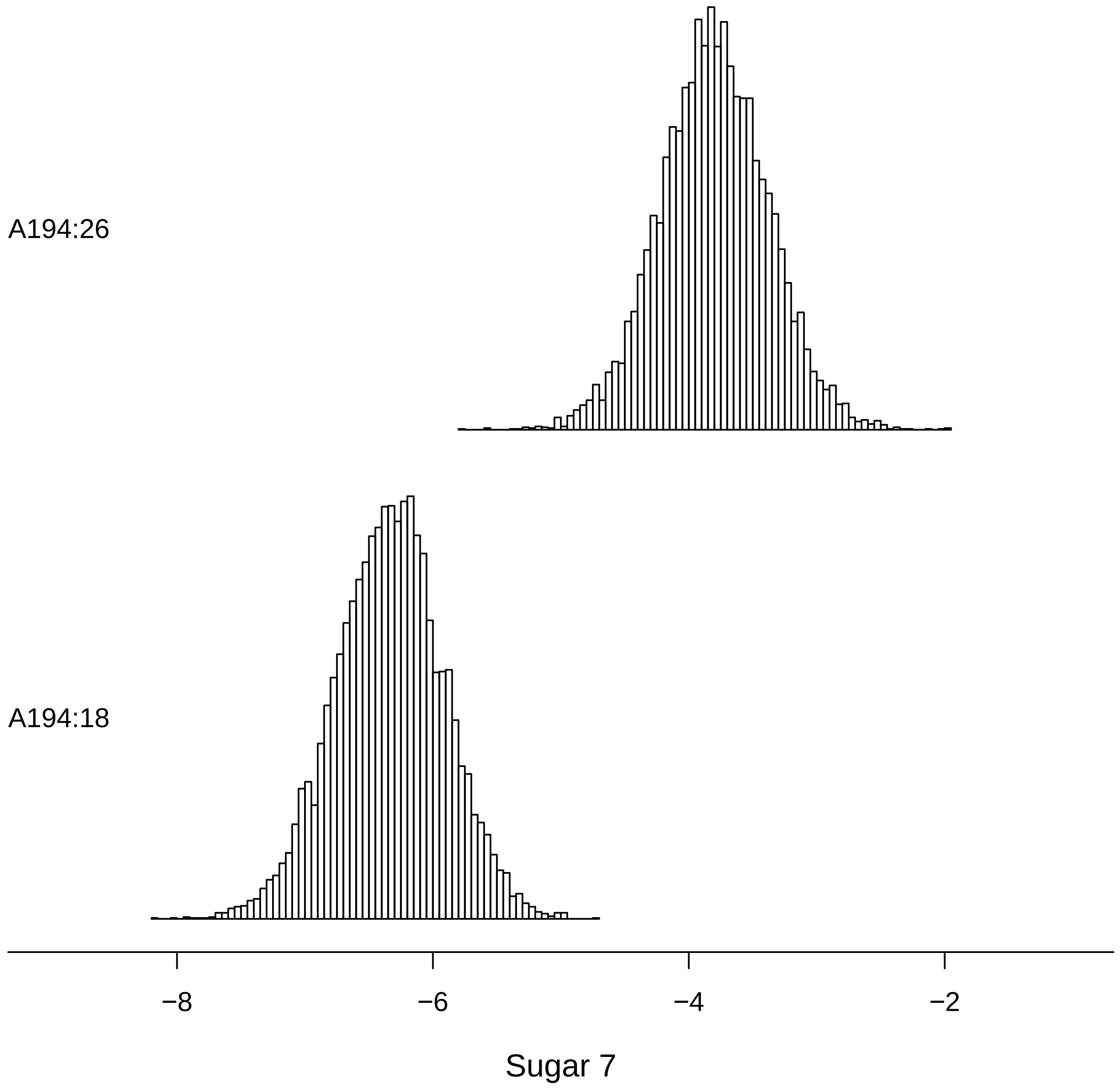

A194:26
A194:18
−8
−6
−4
−2
Sugar 7

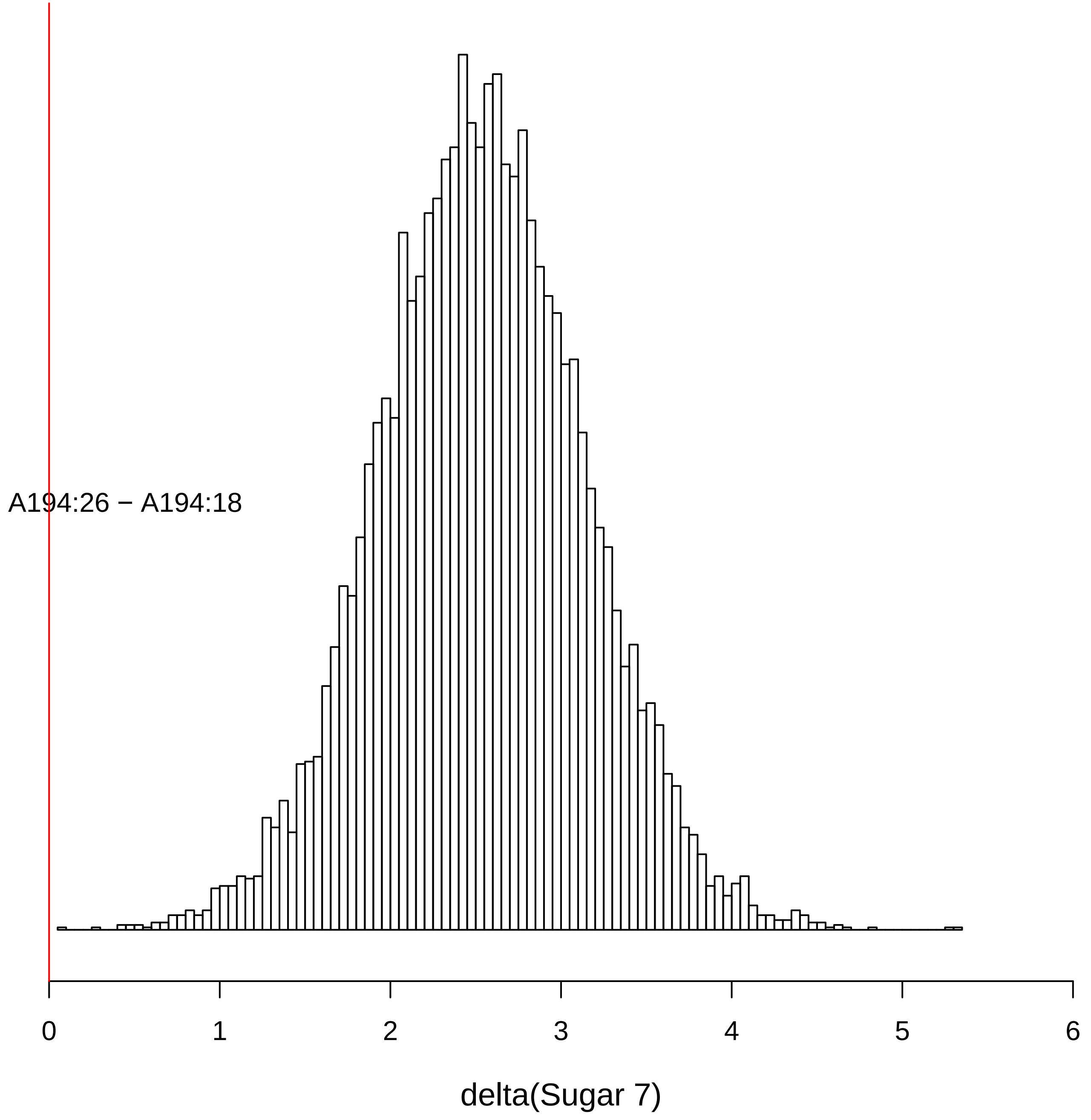

A194:26 − A194:18
0
1
2
3
4
5
6
delta(Sugar 7)

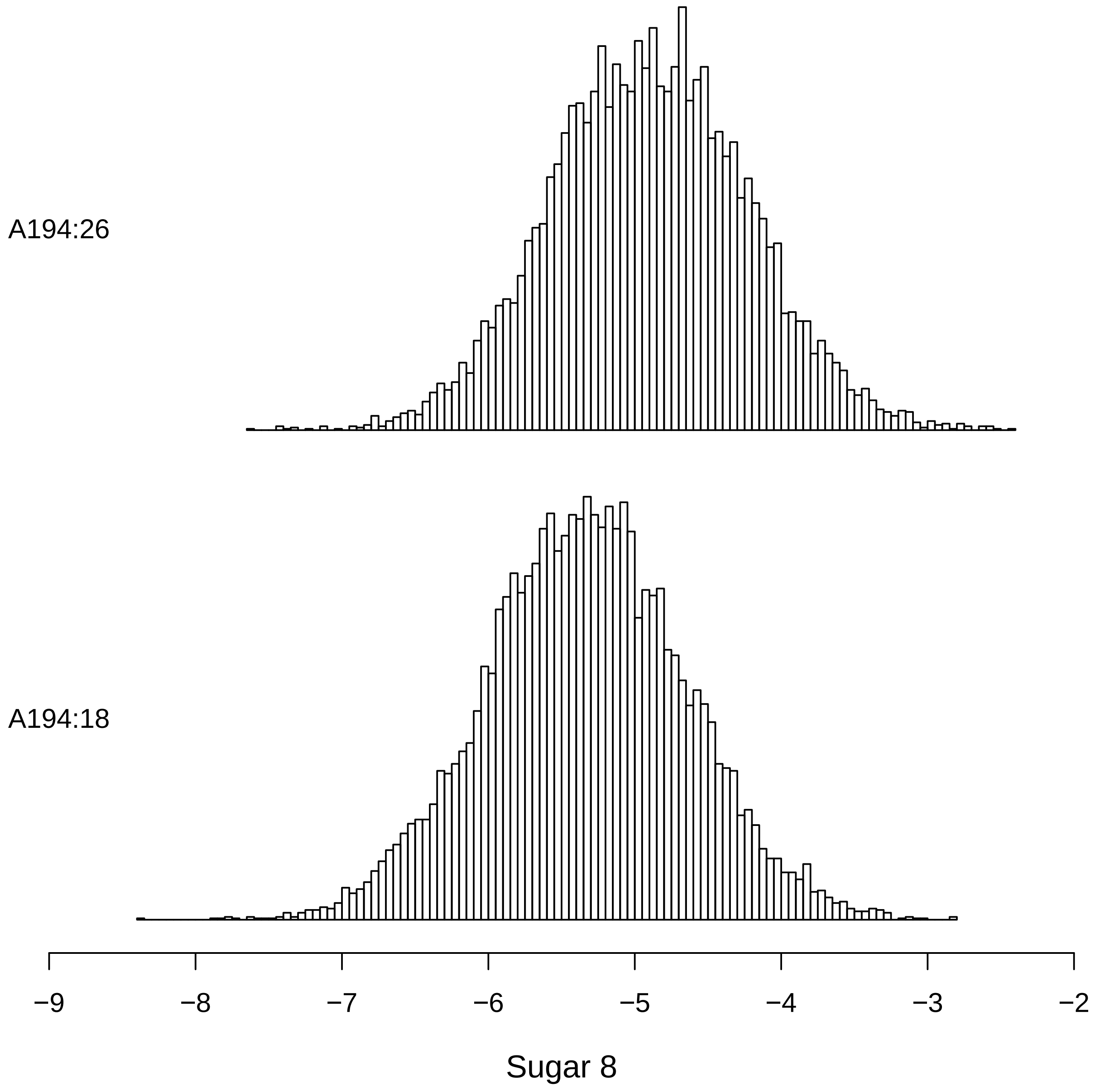
A194:26
A194:18
−9
−8
−7
−6
−5
−4
−3
−2
Sugar 8

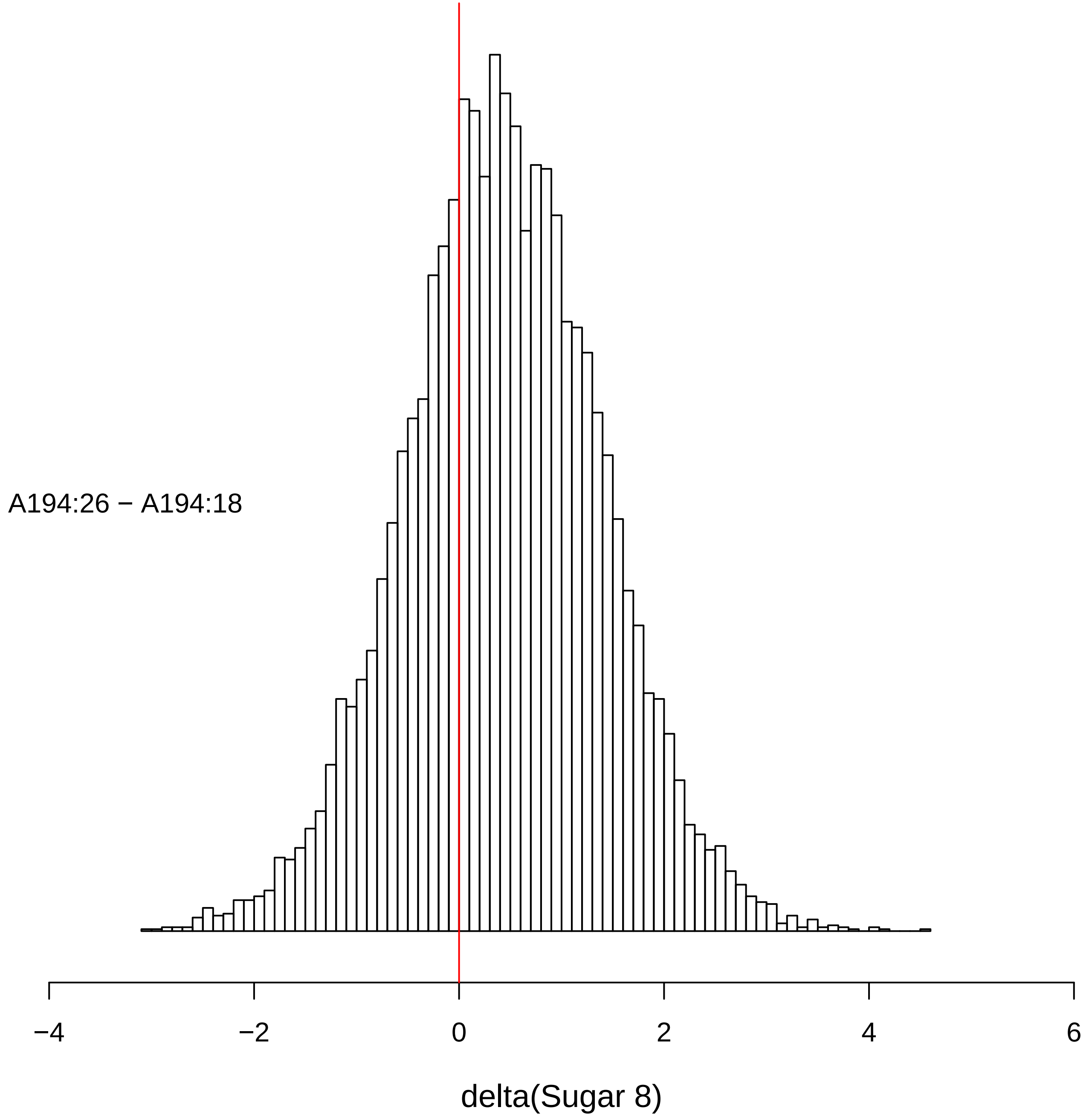
A194:26 − A194:18
−4
−2
0
2
4
6
delta(Sugar 8)

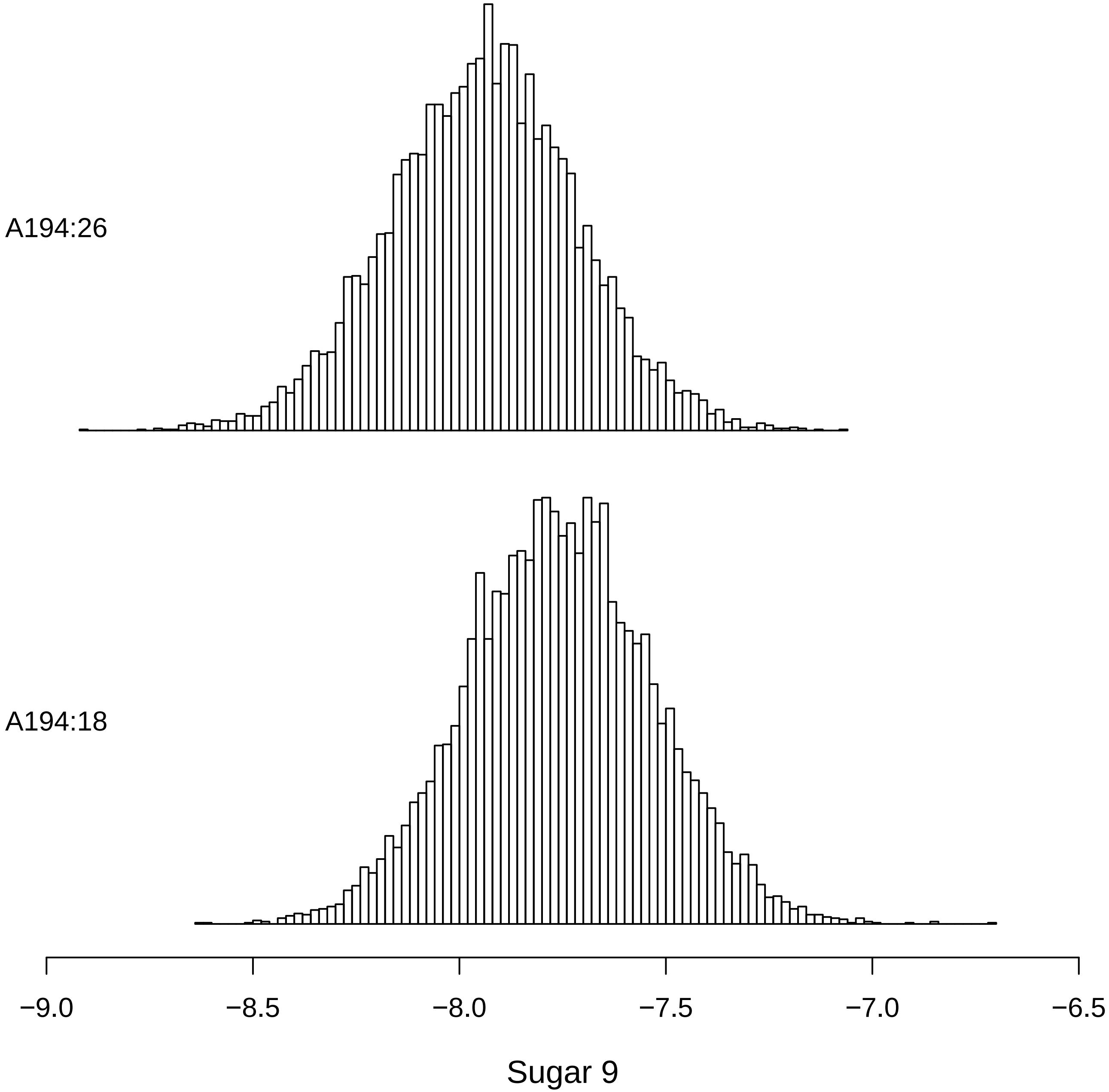
A194:26
A194:18
−9.0
−8.5
−8.0
−7.5
−7.0
−6.5
Sugar 9

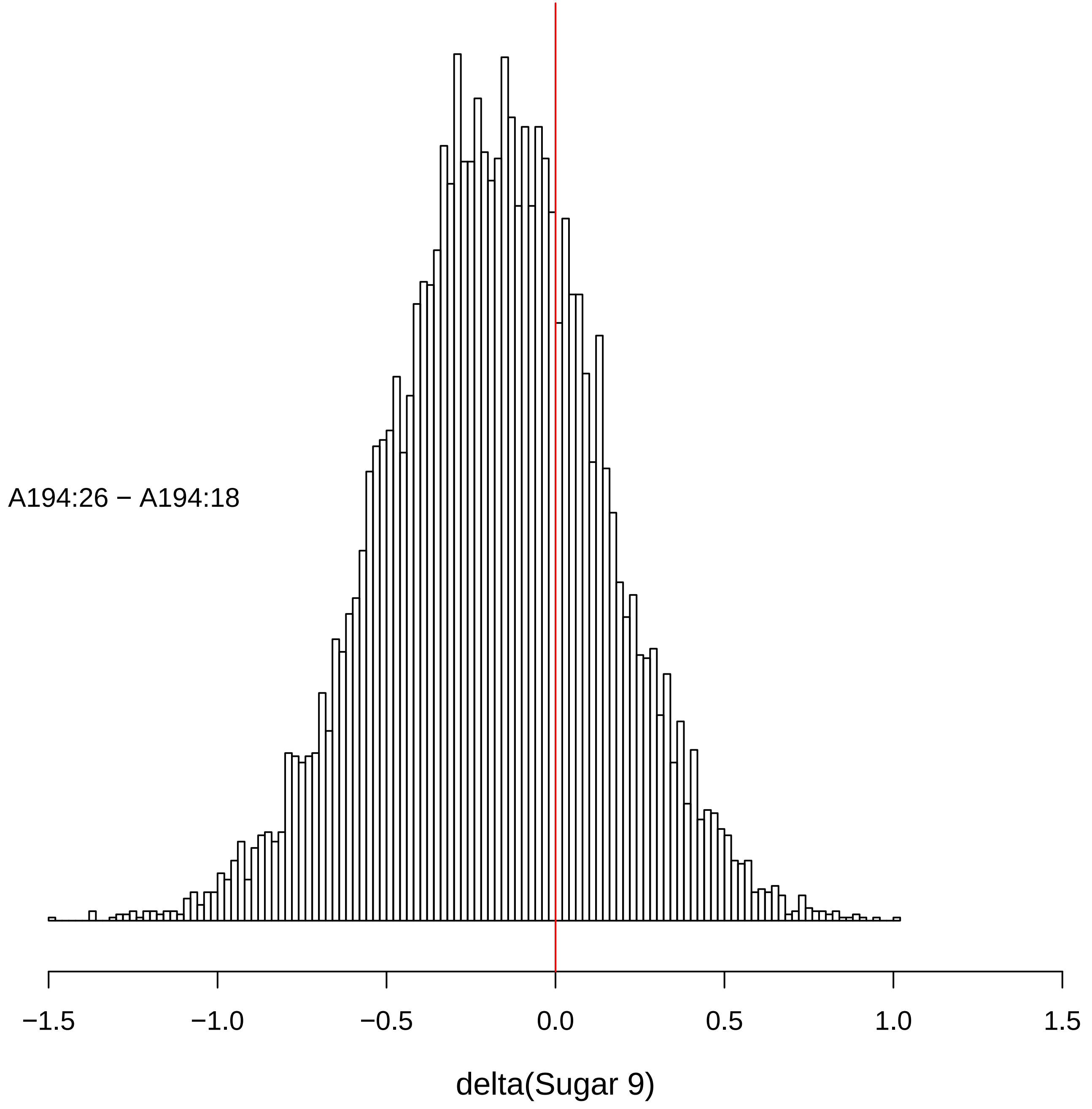
A194:26 − A194:18
−1.5
−1.0
−0.5
0.0
0.5
1.0
1.5
delta(Sugar 9)

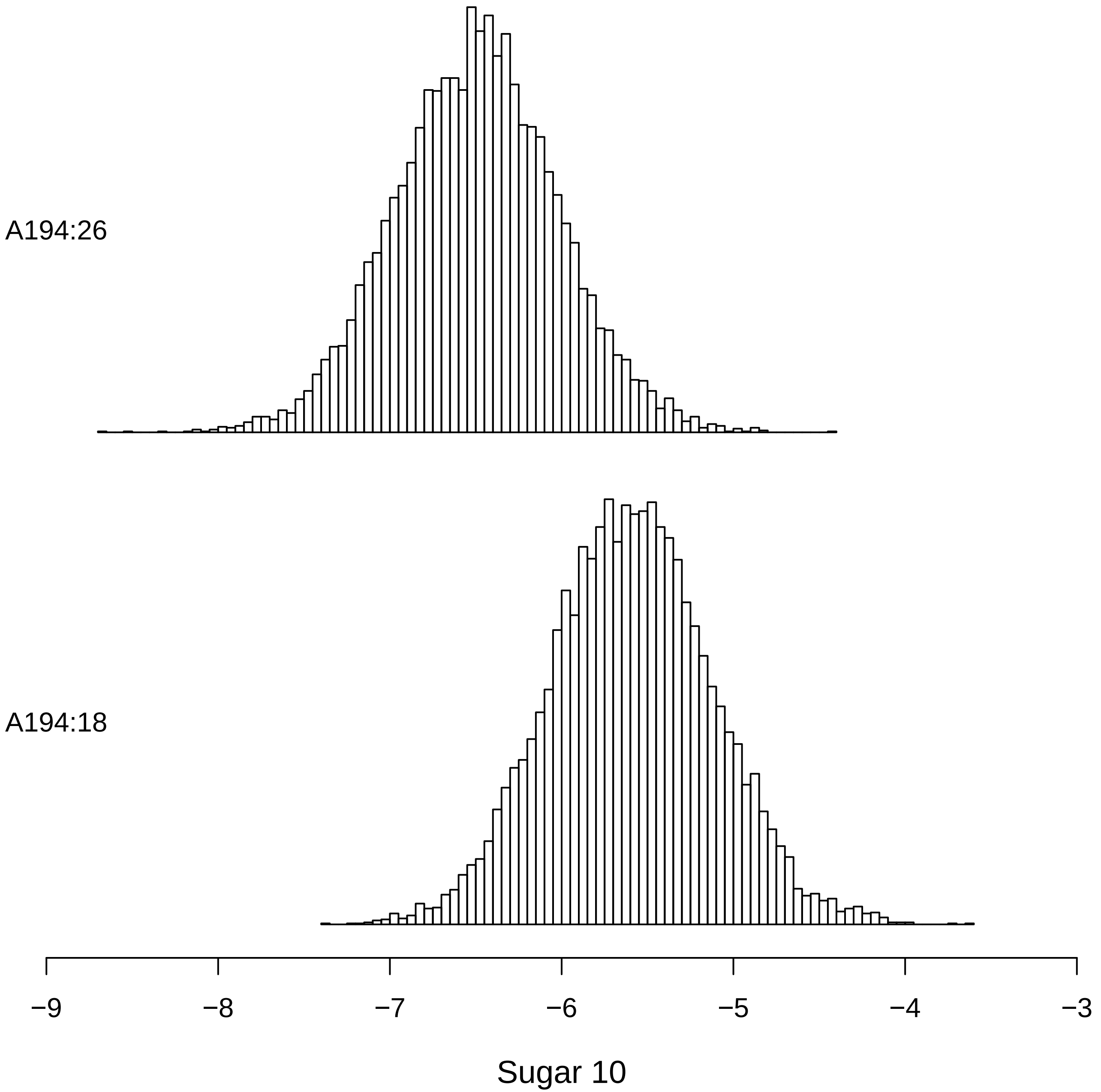
A194:26
A194:18
−9
−8
−7
−6
−5
−4
−3
Sugar 10

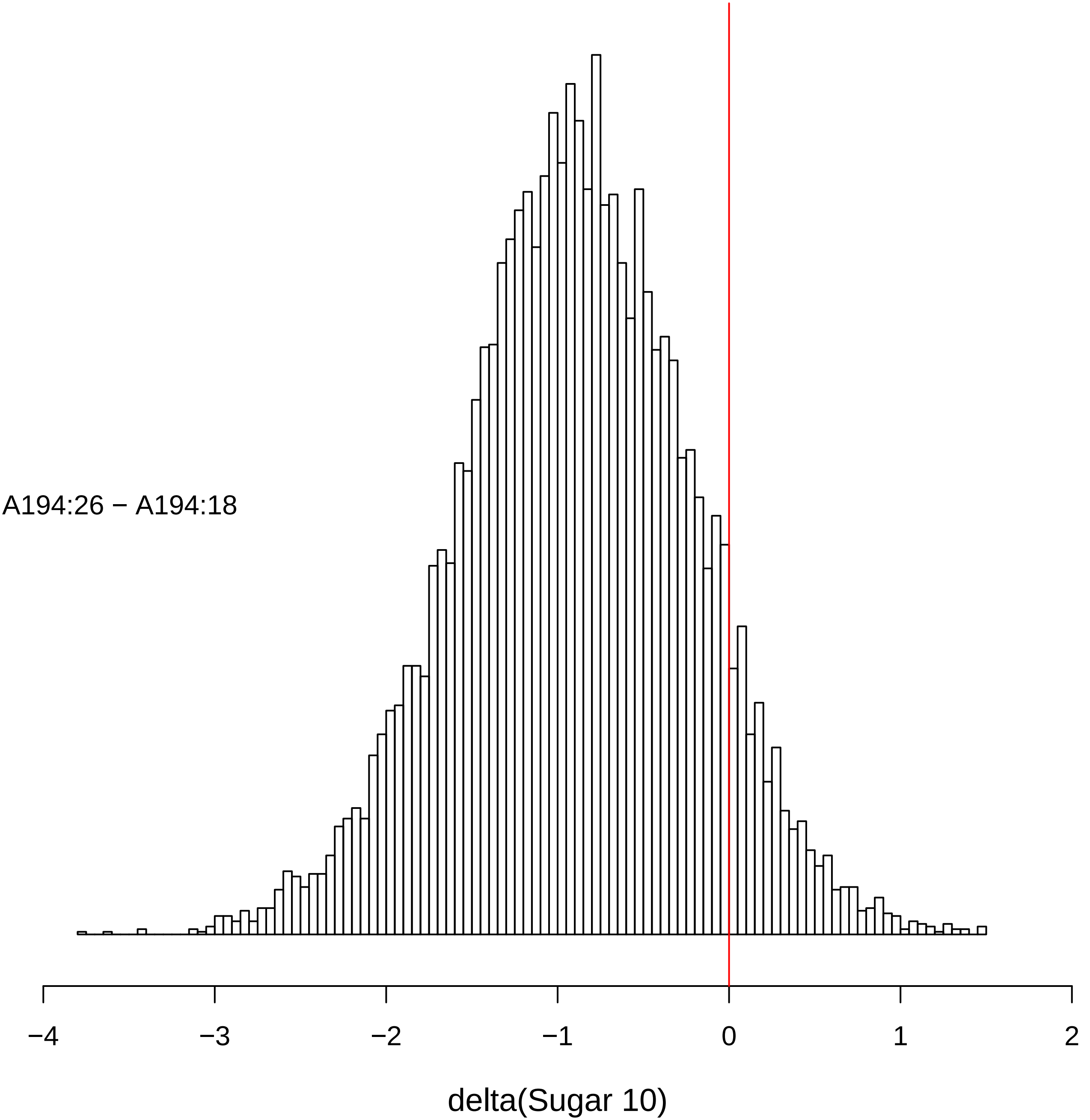
A194:26 − A194:18
−4
−3
−2
−1
0
1
2
delta(Sugar 10)

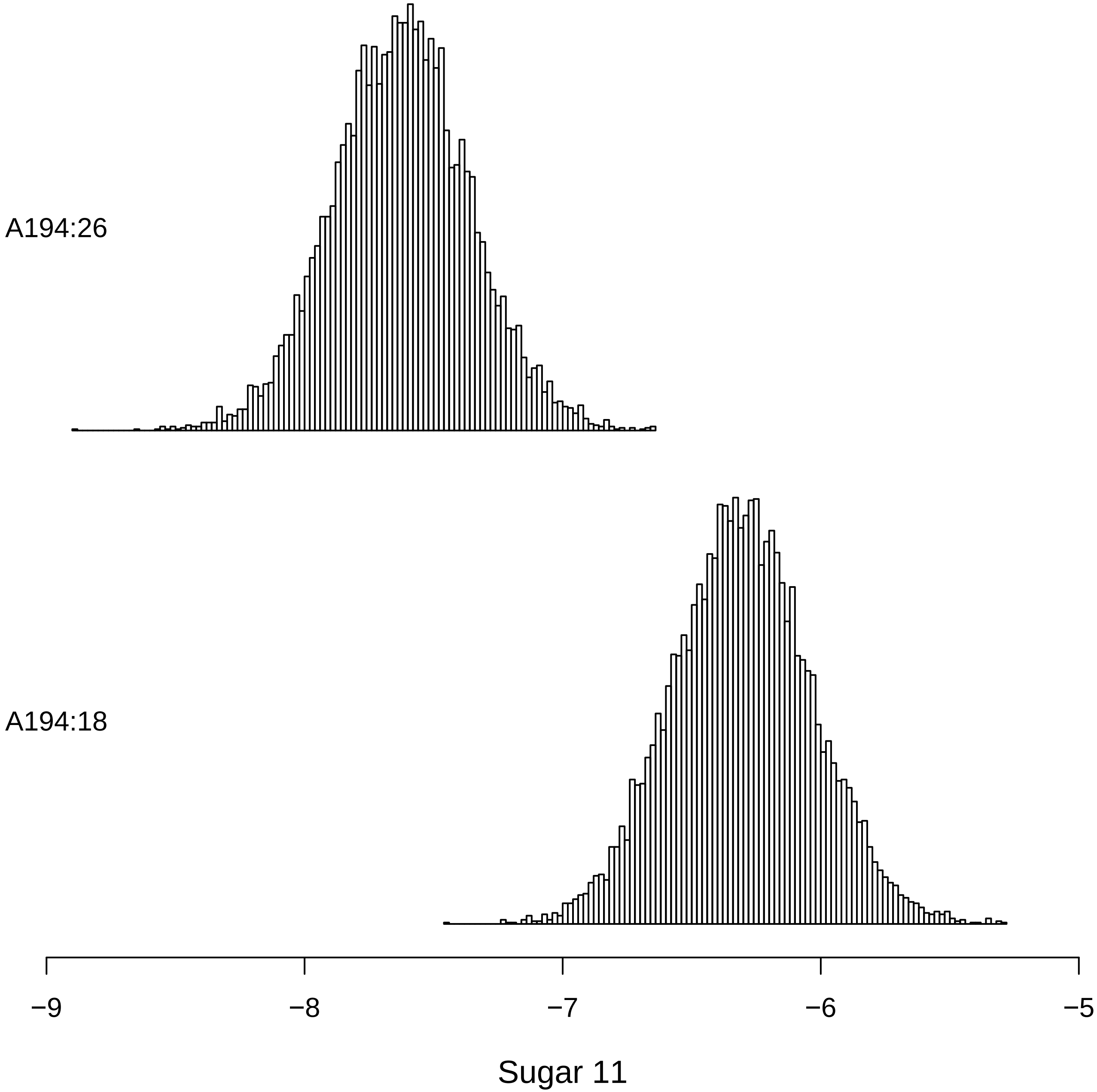
A194:26
A194:18
−9
−8
−7
−6
−5
Sugar 11

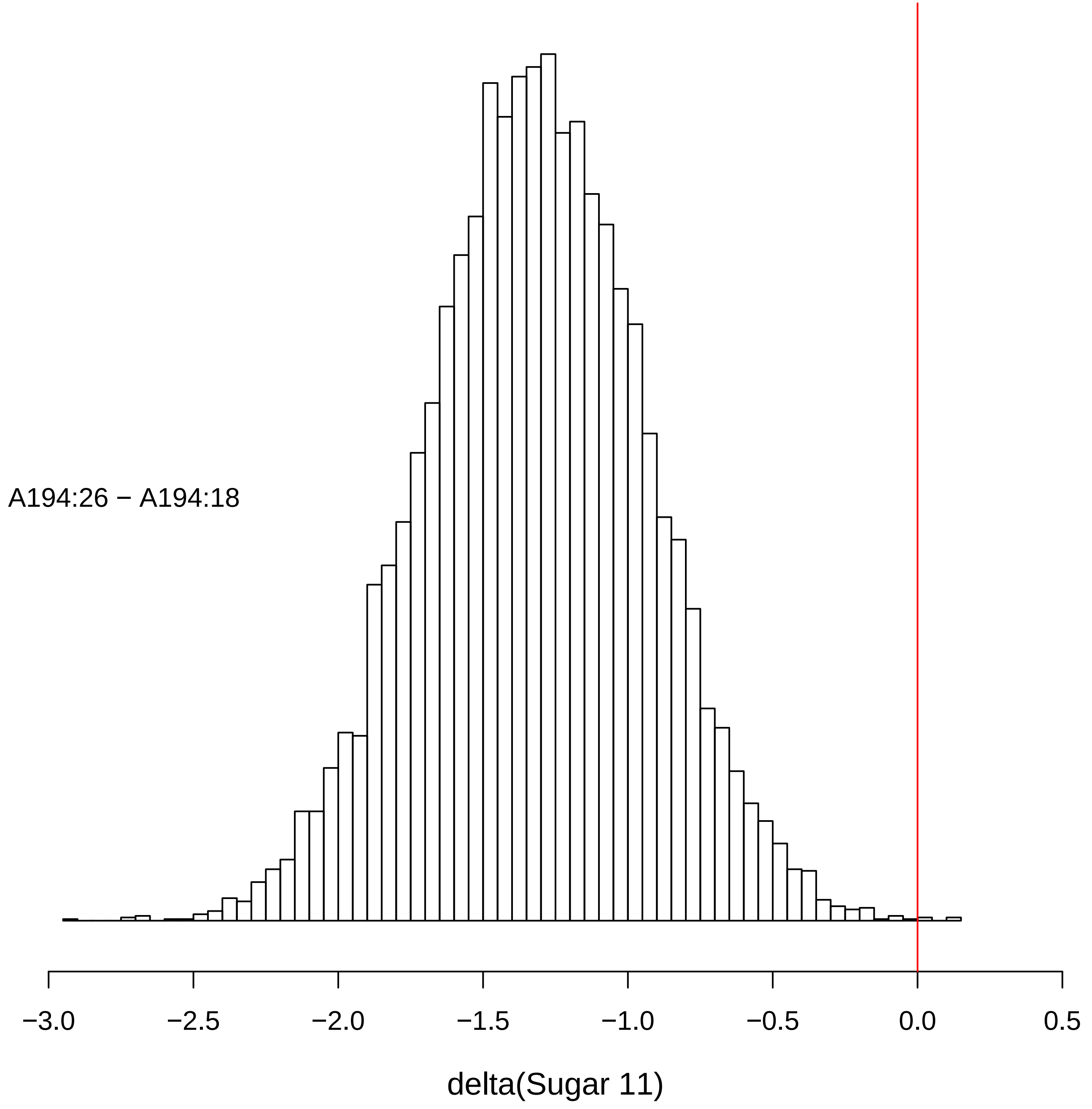
A194:26 − A194:18
−3.0
−2.5
−2.0
−1.5
−1.0
−0.5
0.0
0.5
delta(Sugar 11)

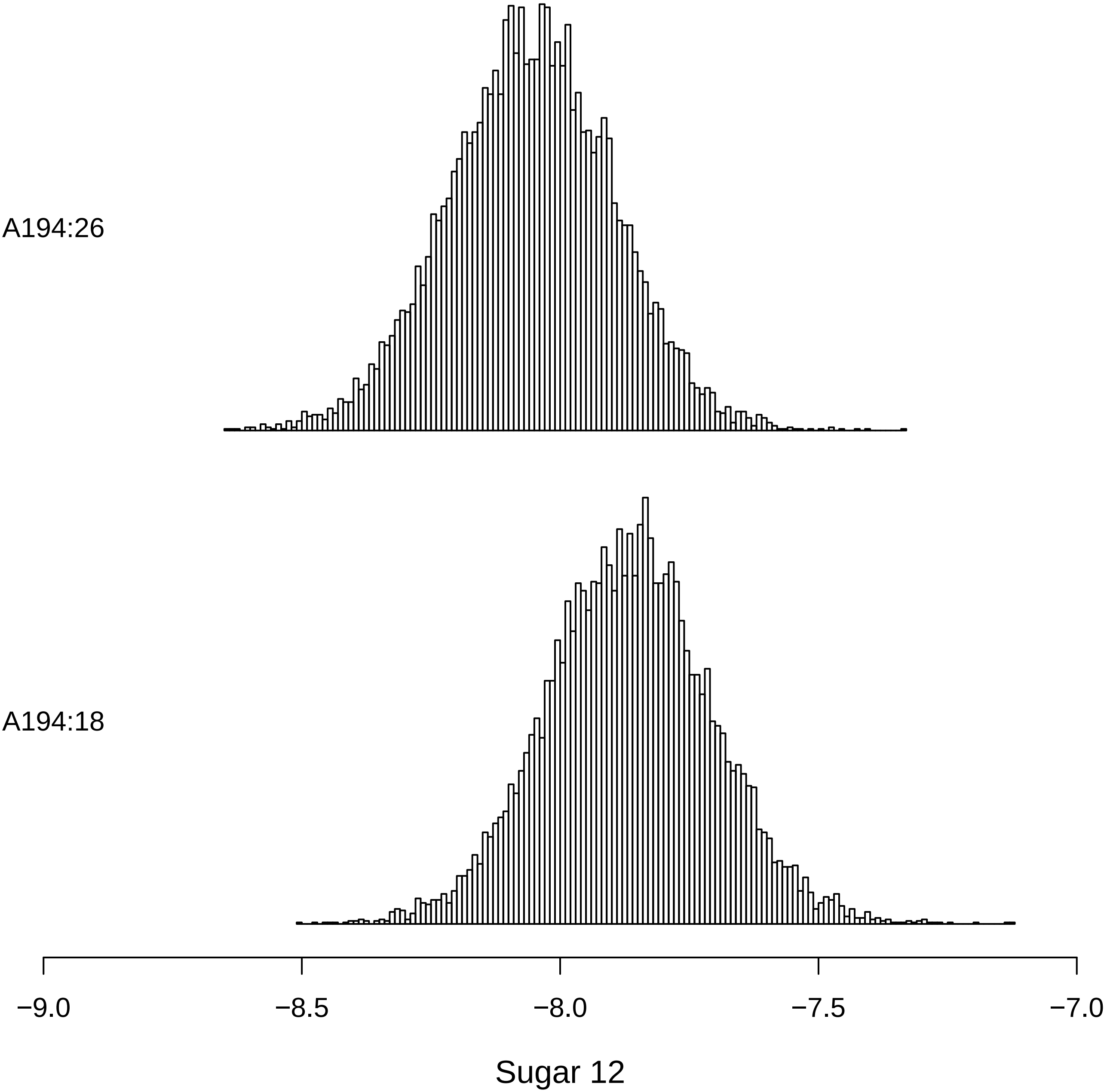

A194:26
A194:18
−9.0
−8.5
−8.0
−7.5
−7.0
Sugar 12

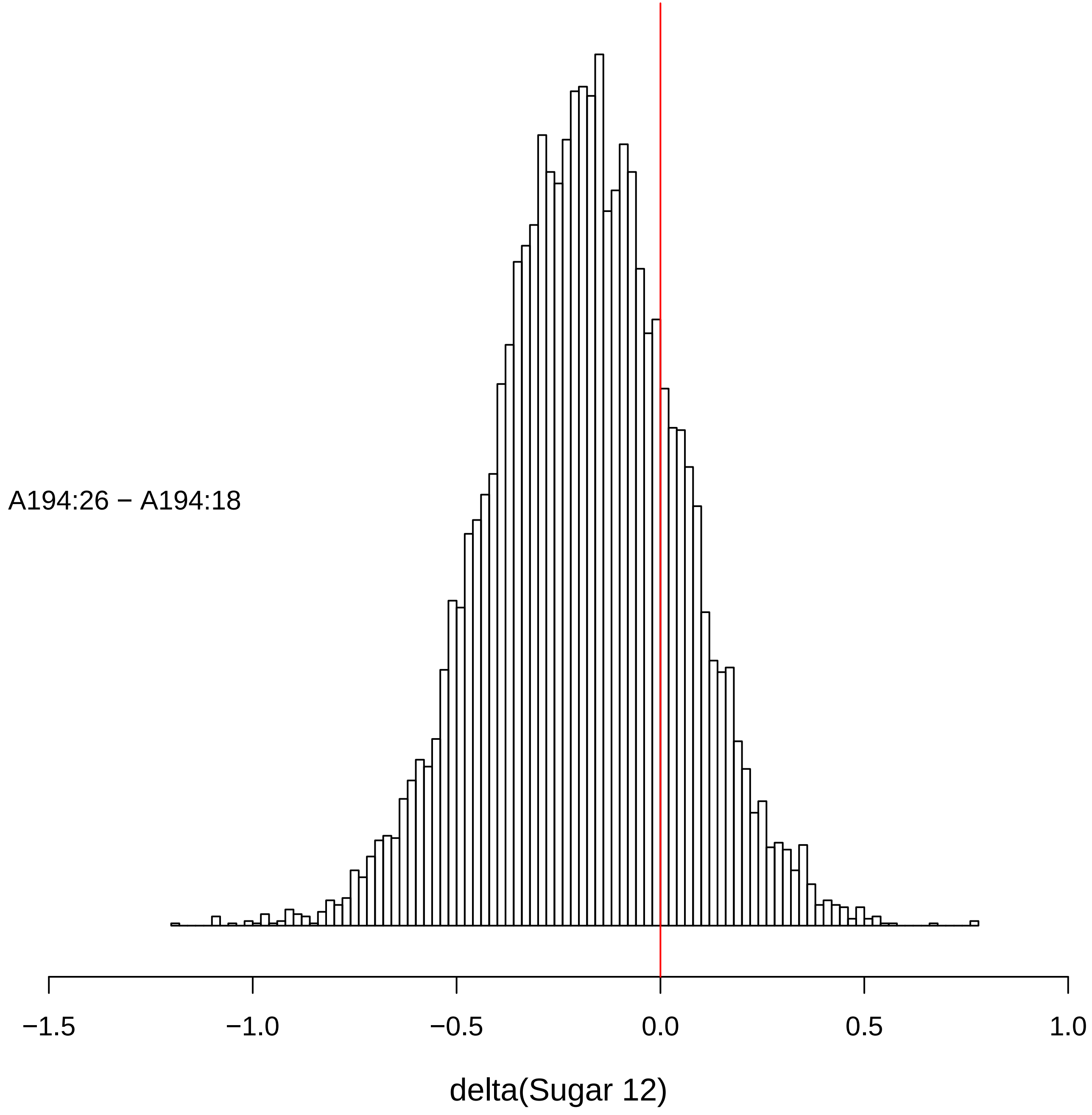
A194:26 − A194:18
−1.5
−1.0
−0.5
0.0
0.5
1.0
delta(Sugar 12)

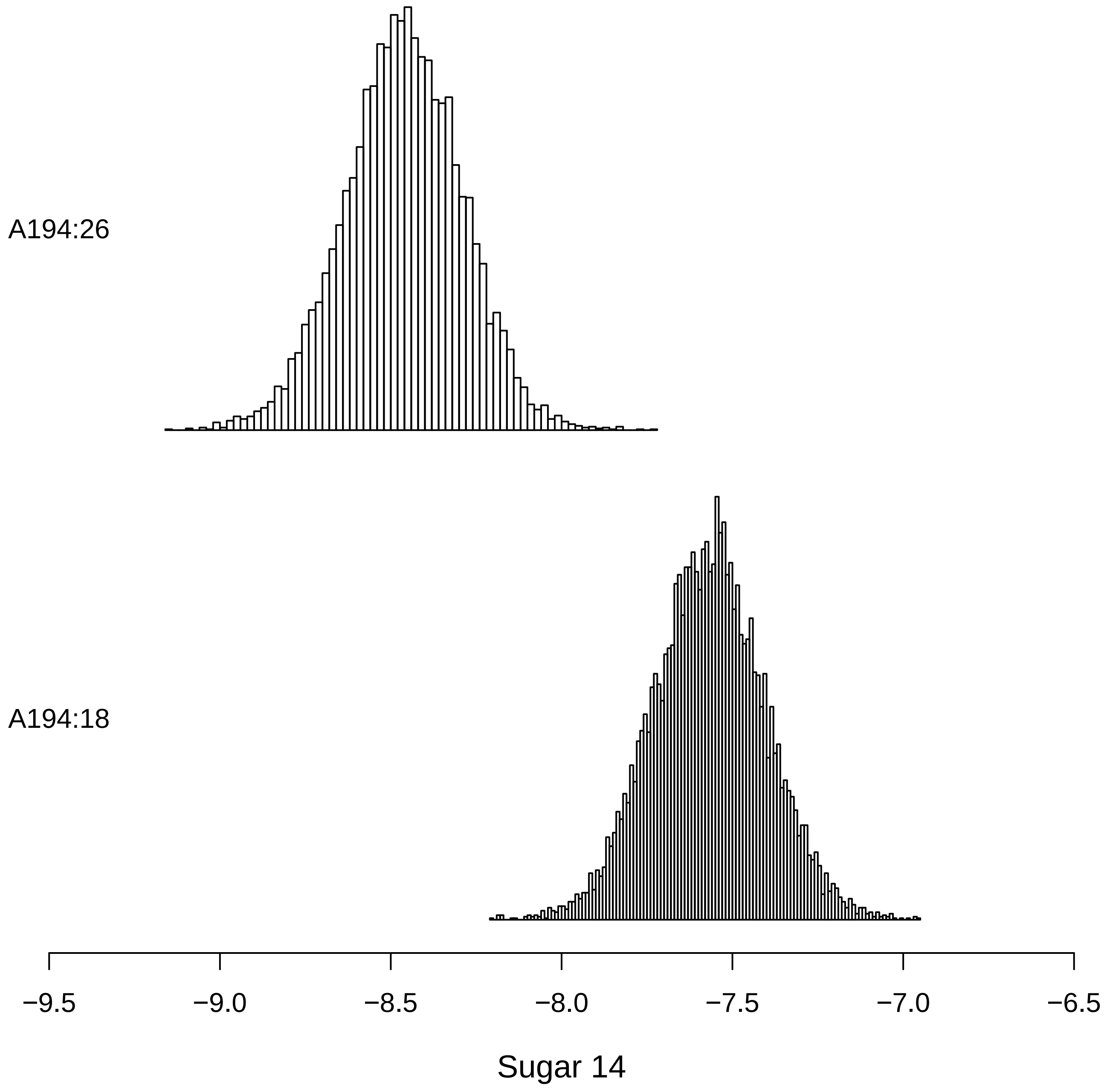
A194:26
A194:18
−9.5
−9.0
−8.5
−8.0
−7.5
−7.0
−6.5
Sugar 14

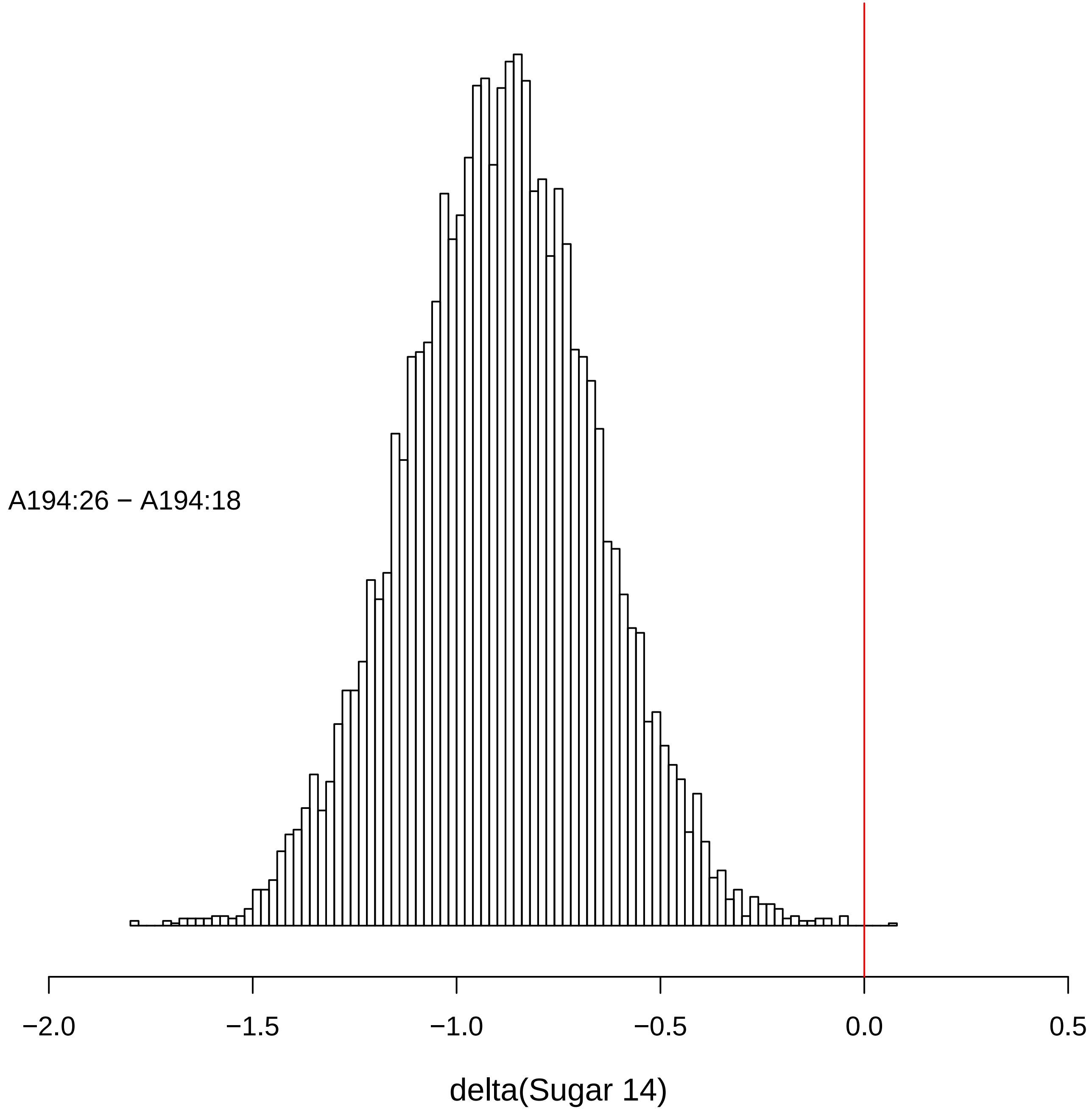

A194:26 − A194:18
−2.0
−1.5
−1.0
−0.5
0.0
0.5
delta(Sugar 14)

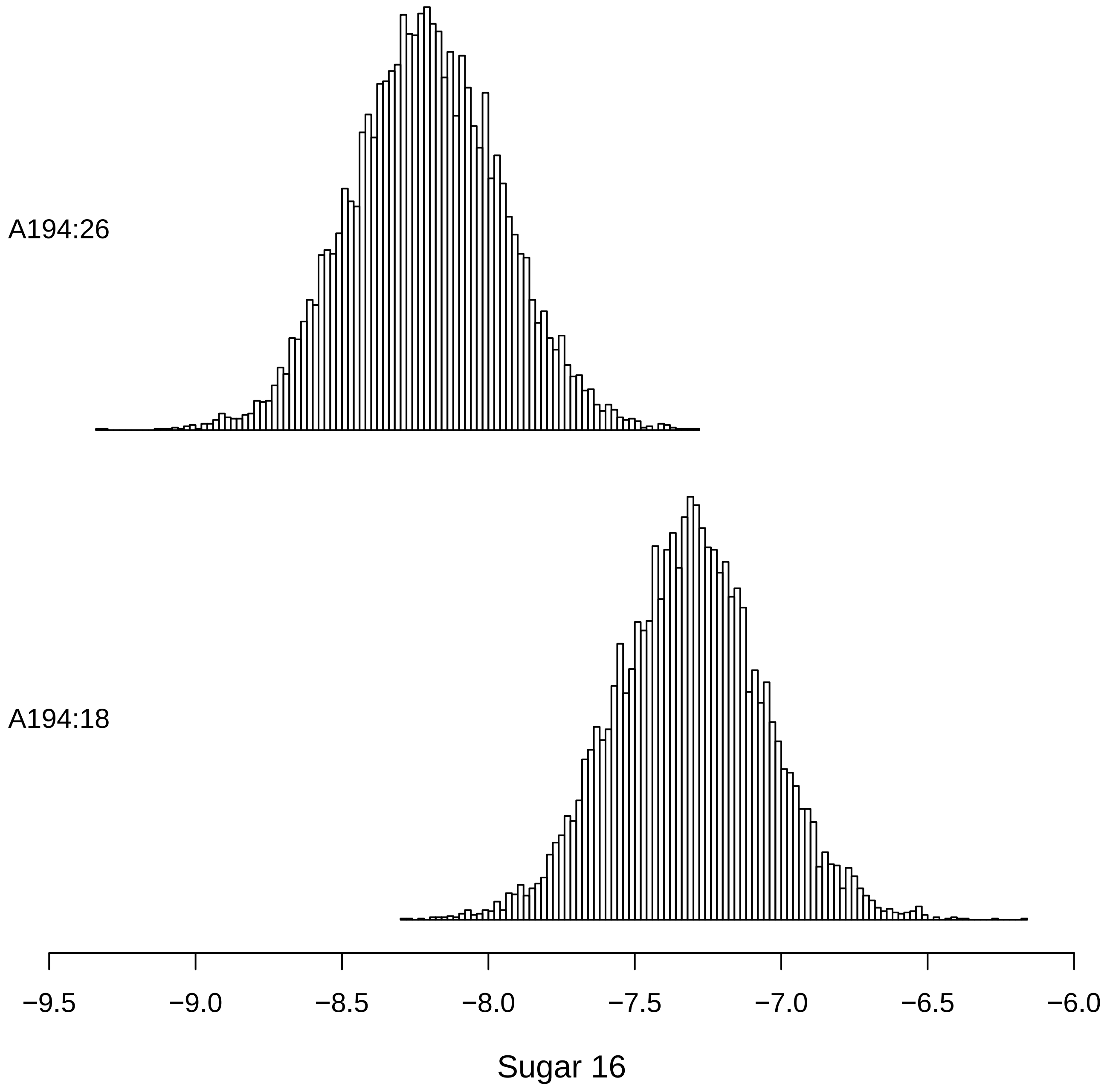
A194:26
A194:18
−9.5
−9.0
−8.5
−8.0
−7.5
−7.0
−6.5
−6.0
Sugar 16

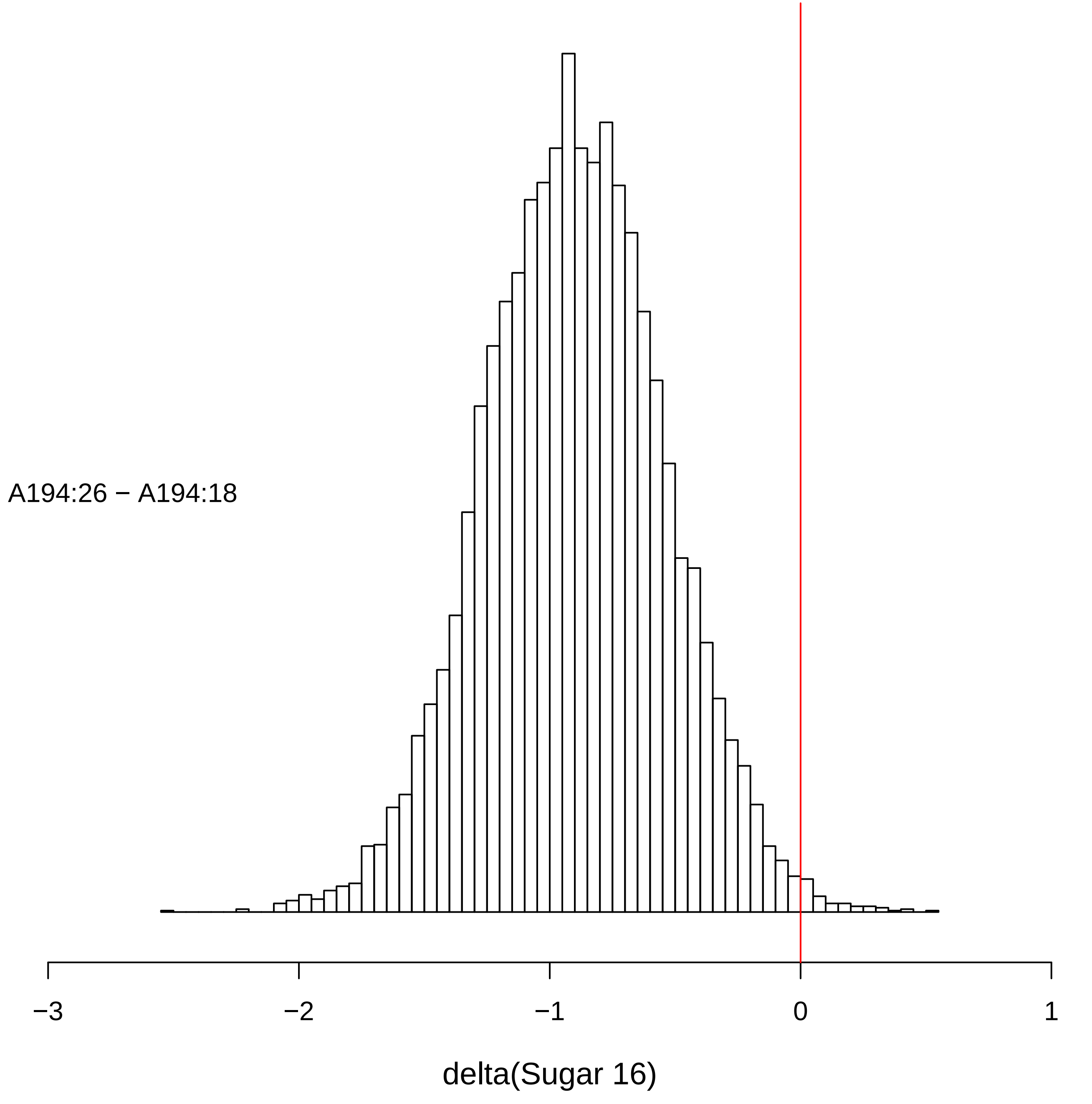
A194:26 − A194:18
−3
−2
−1
0
1
delta(Sugar 16)

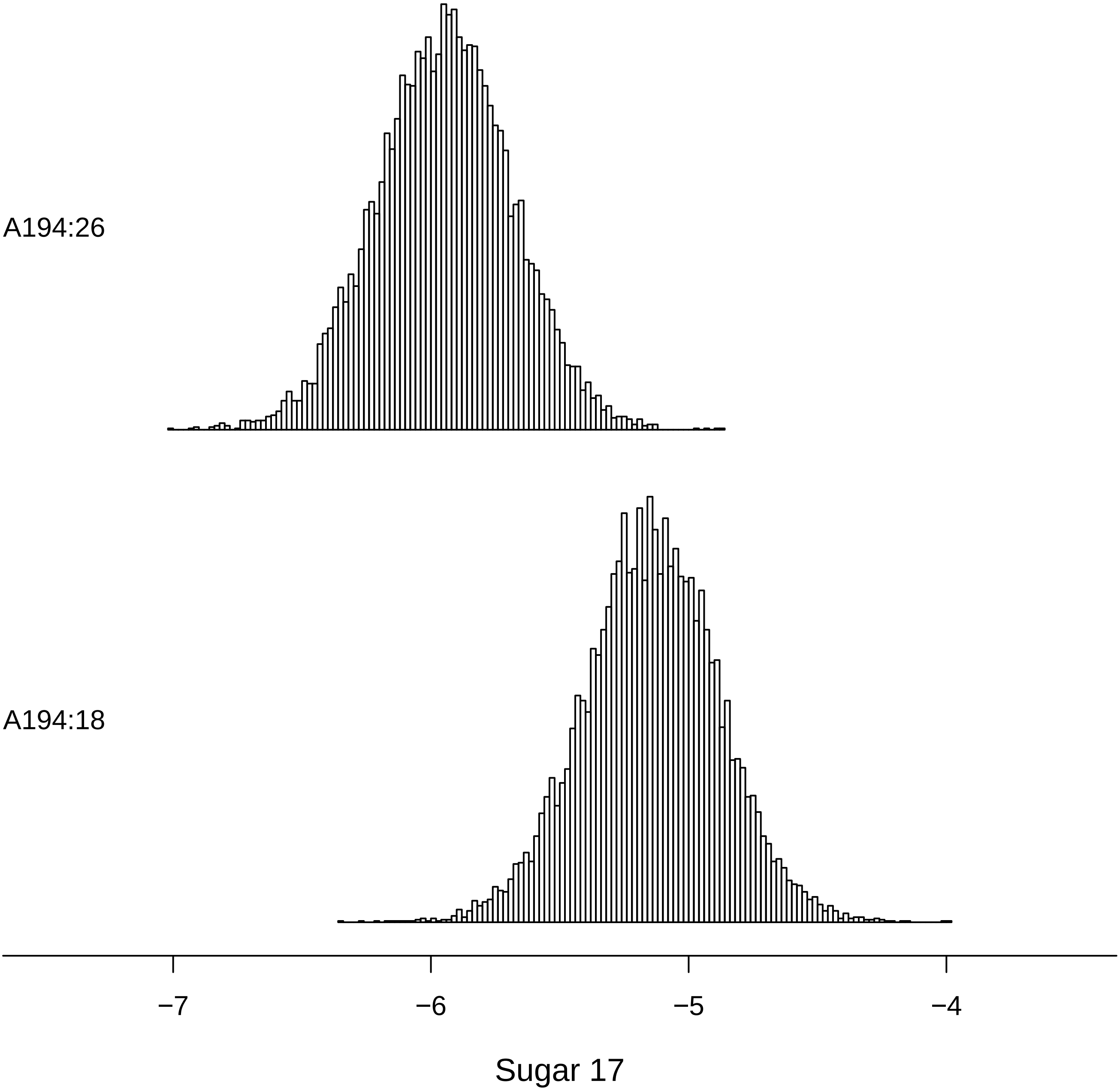
A194:26
A194:18
−7
−6
−5
−4
Sugar 17

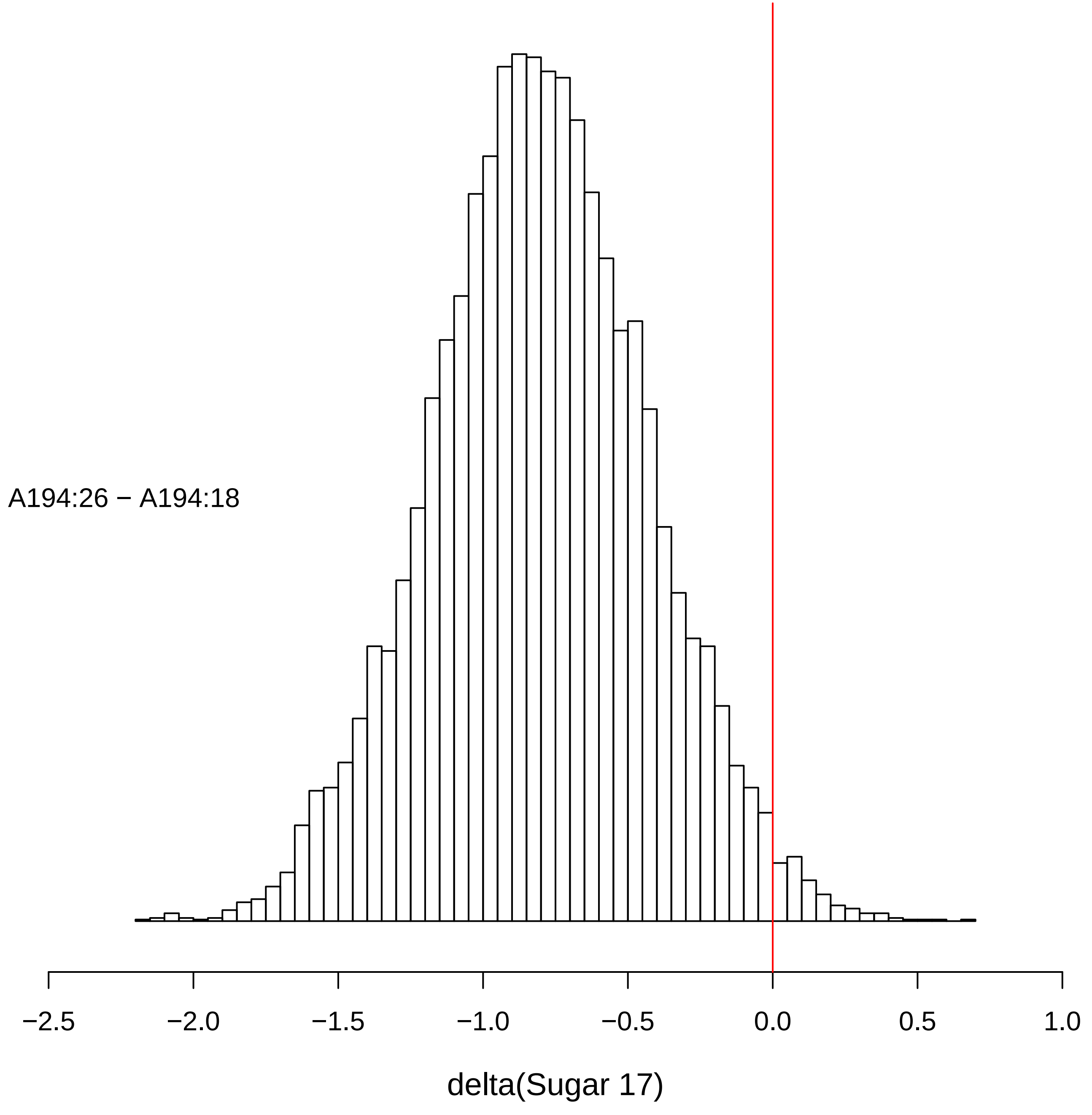
A194:26 − A194:18
−2.5
−2.0
−1.5
−1.0
−0.5
0.0
0.5
1.0
delta(Sugar 17)

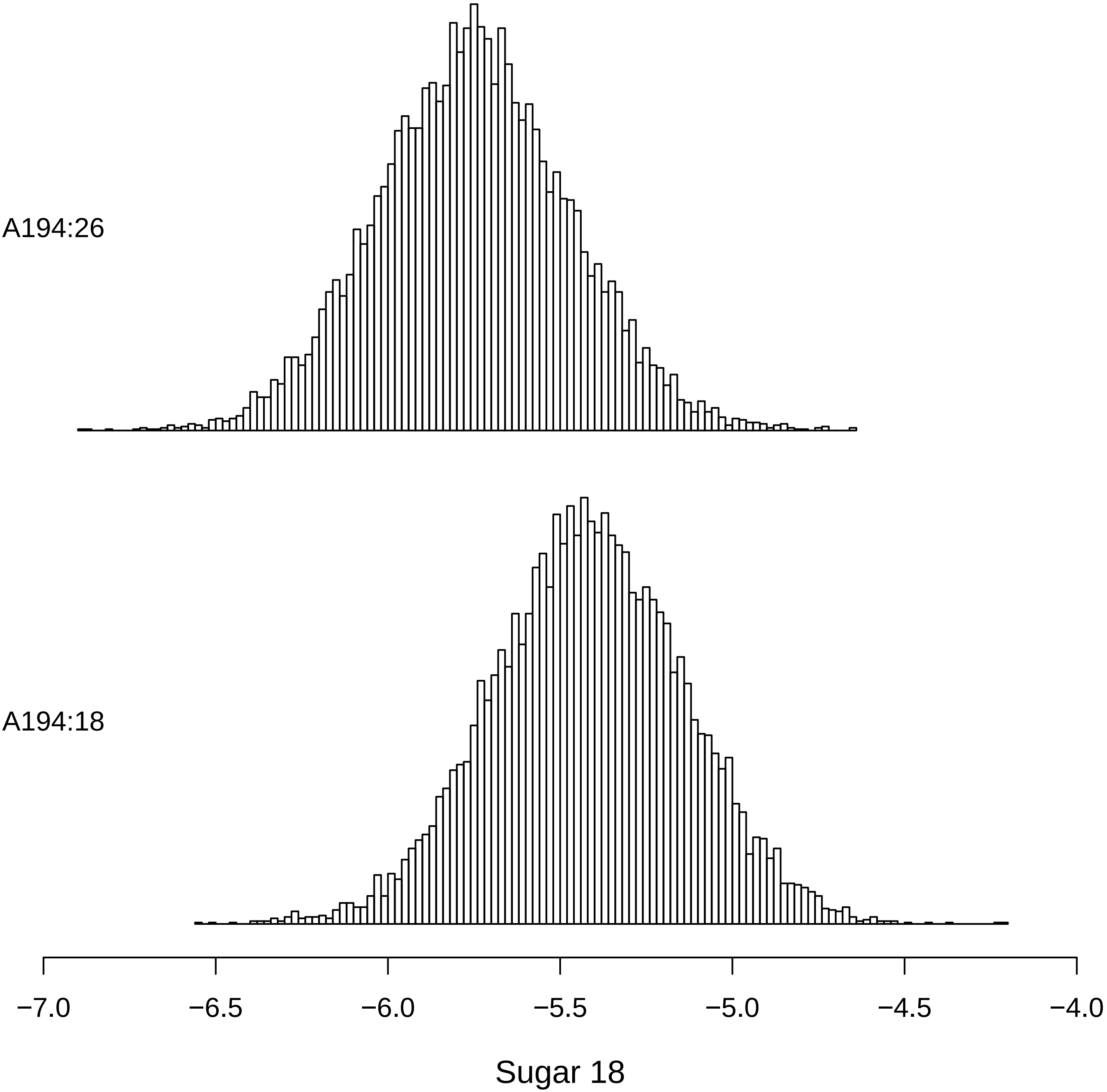
A194:26
A194:18
−7.0
−6.5
−6.0
−5.5
−5.0
−4.5
−4.0
Sugar 18

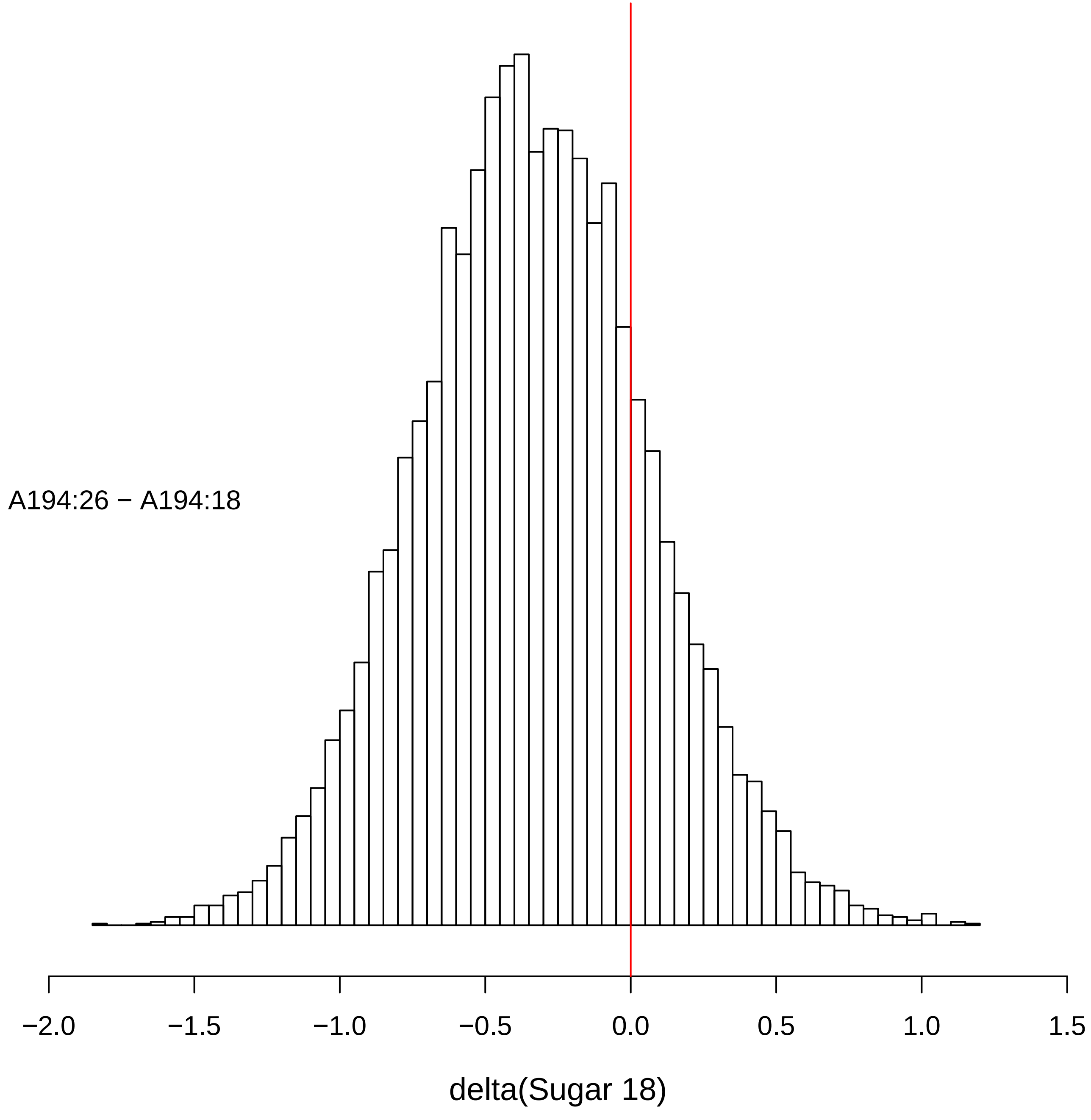
A194:26 − A194:18
−2.0
−1.5
−1.0
−0.5
0.0
0.5
1.0
1.5
delta(Sugar 18)

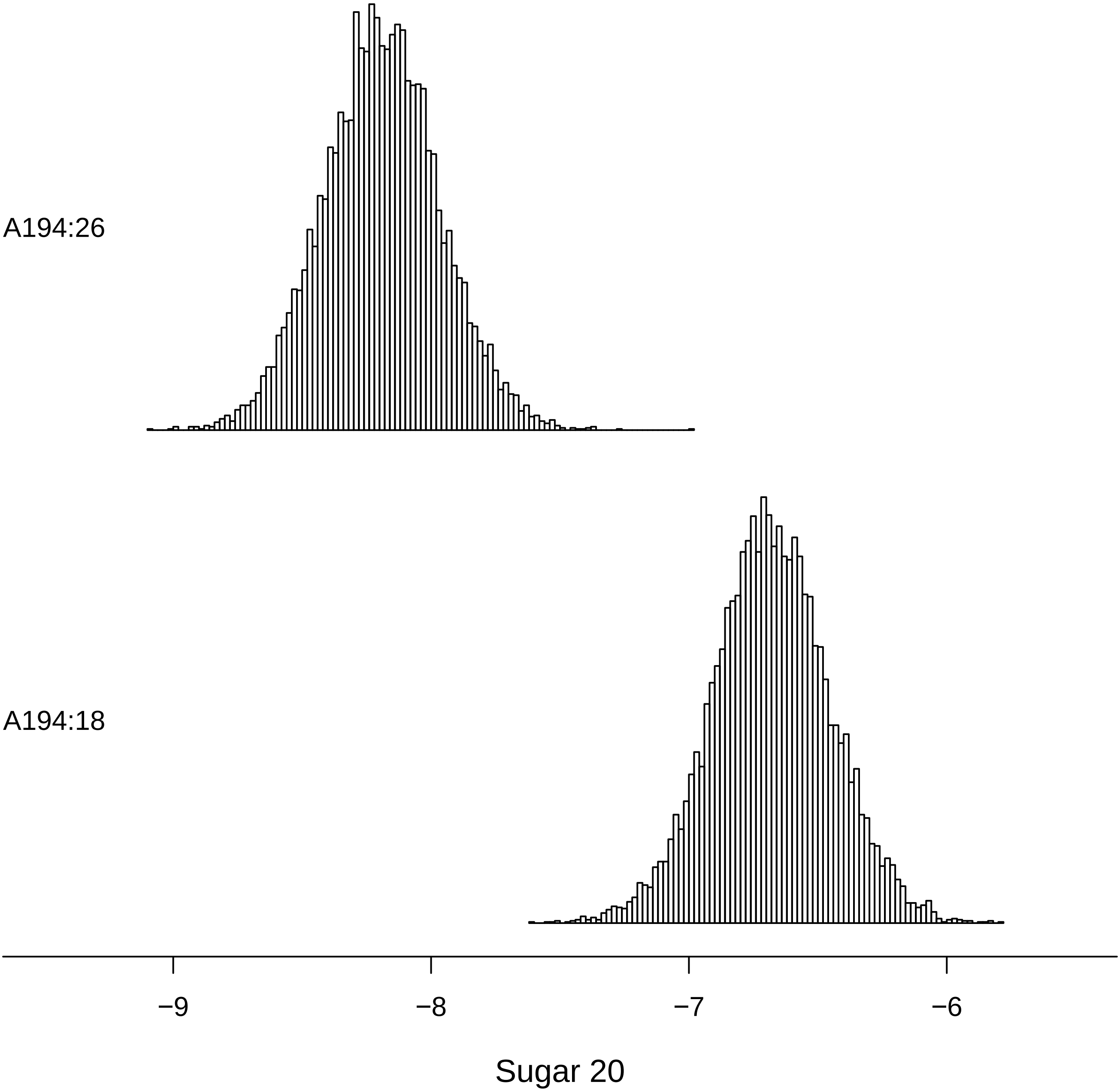
A194:26
A194:18
−9
−8
−7
−6
Sugar 20

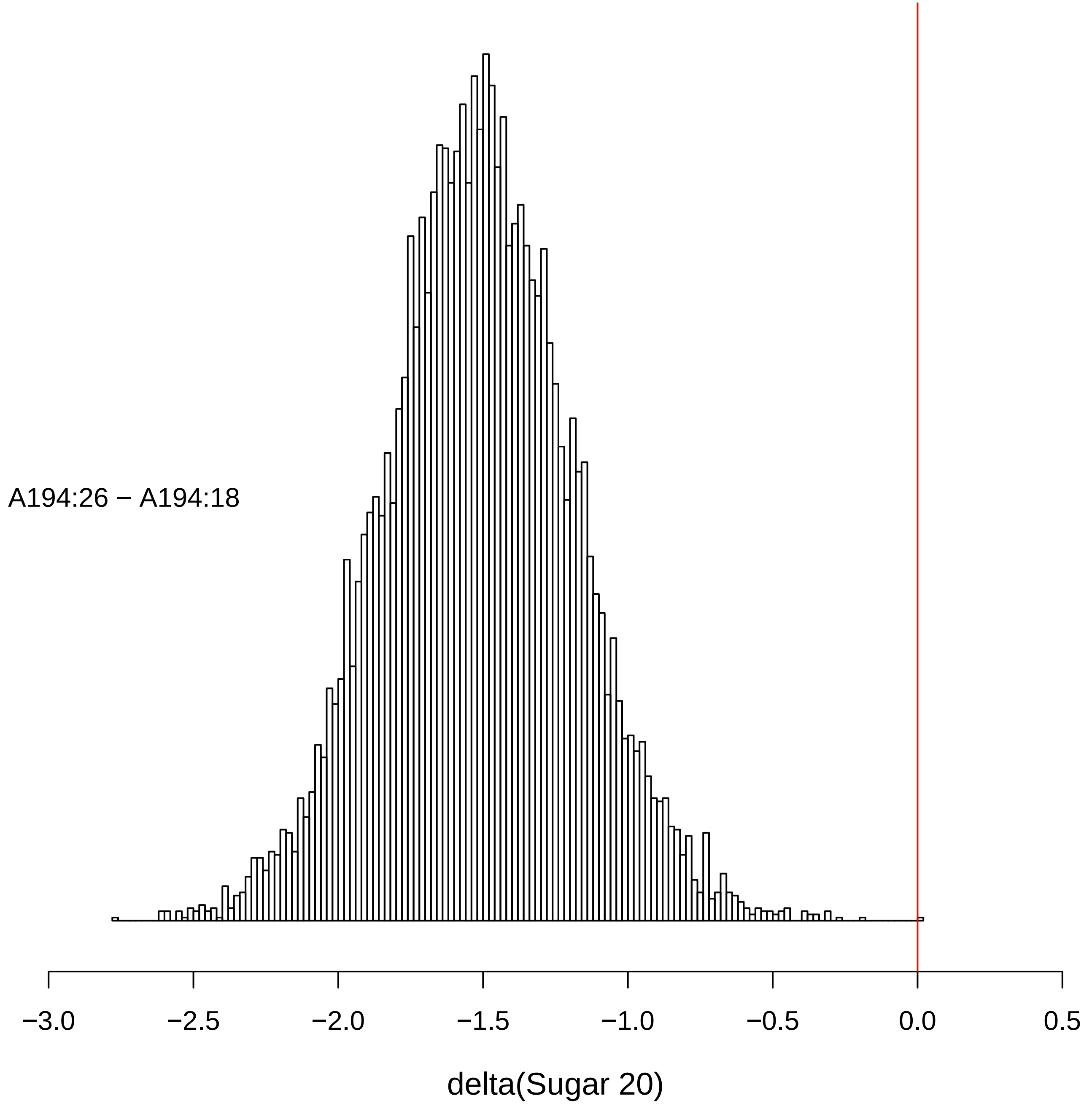
A194:26 − A194:18
−3.0
−2.5
−2.0
−1.5
−1.0
−0.5
0.0
0.5
delta(Sugar 20)

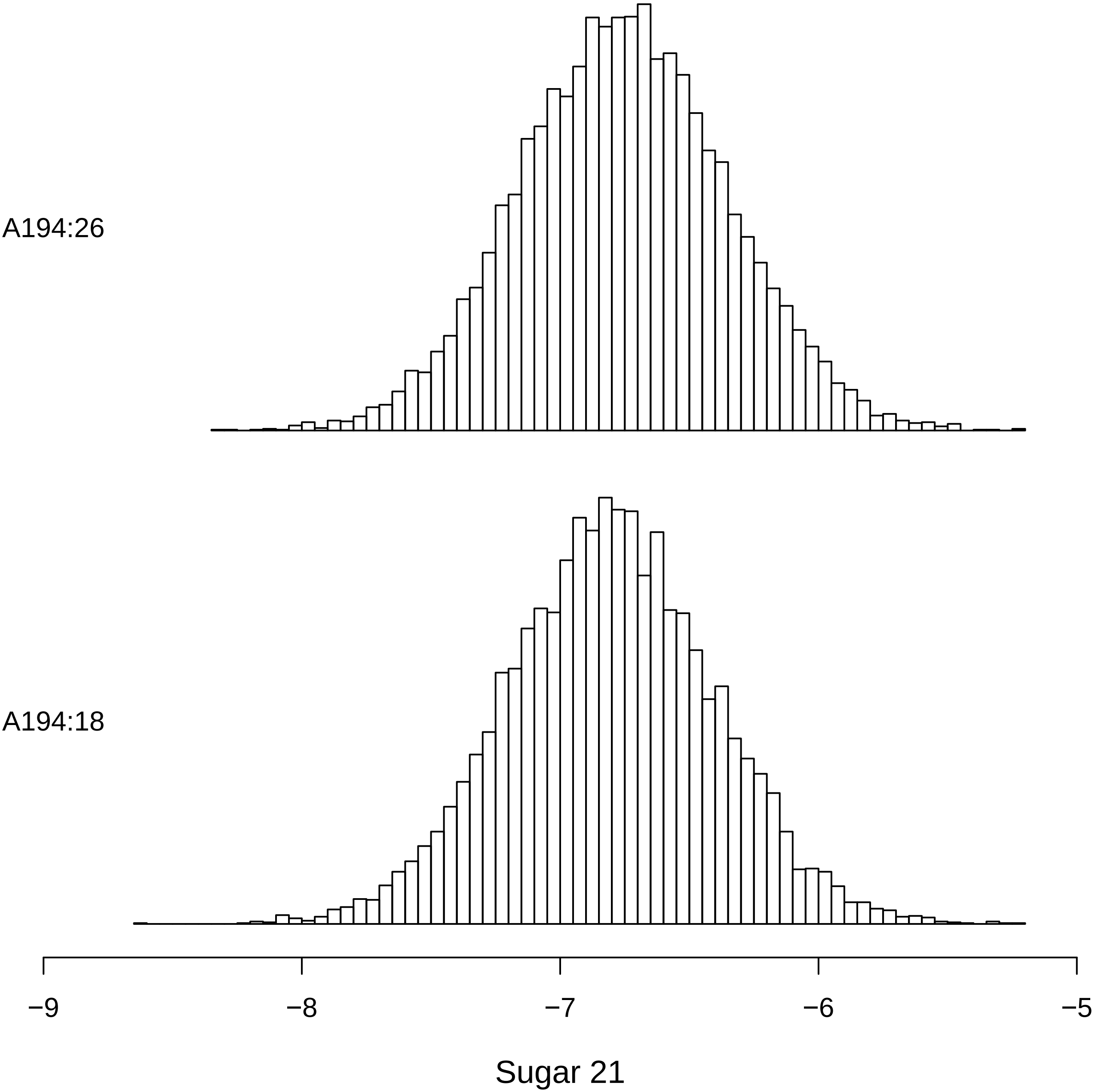
A194:26
A194:18
−9
−8
−7
−6
−5
Sugar 21

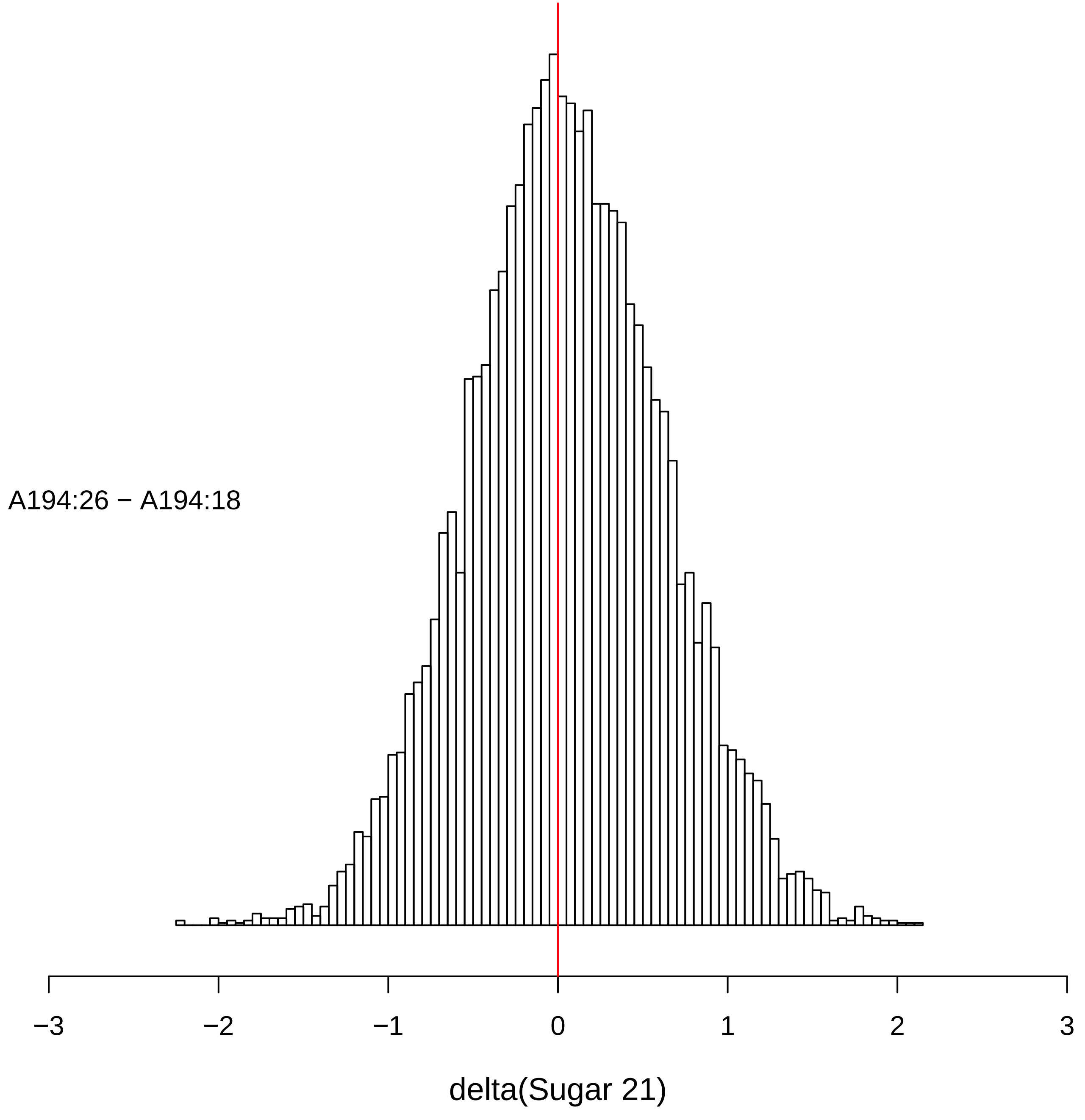
A194:26 − A194:18
−3
−2
−1
0
1
2
3
delta(Sugar 21)

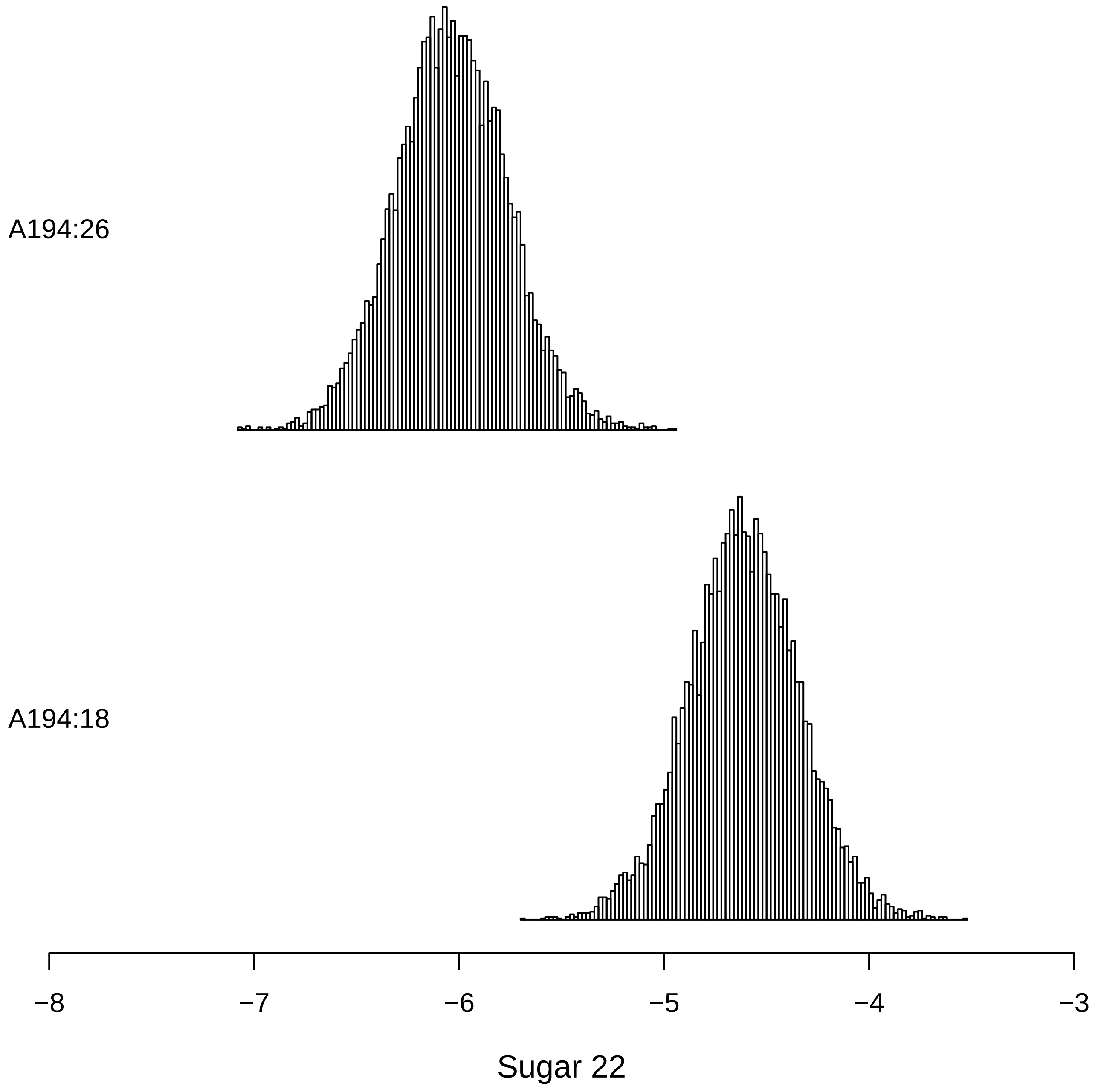
A194:26
A194:18
−8
−7
−6
−5
−4
−3
Sugar 22

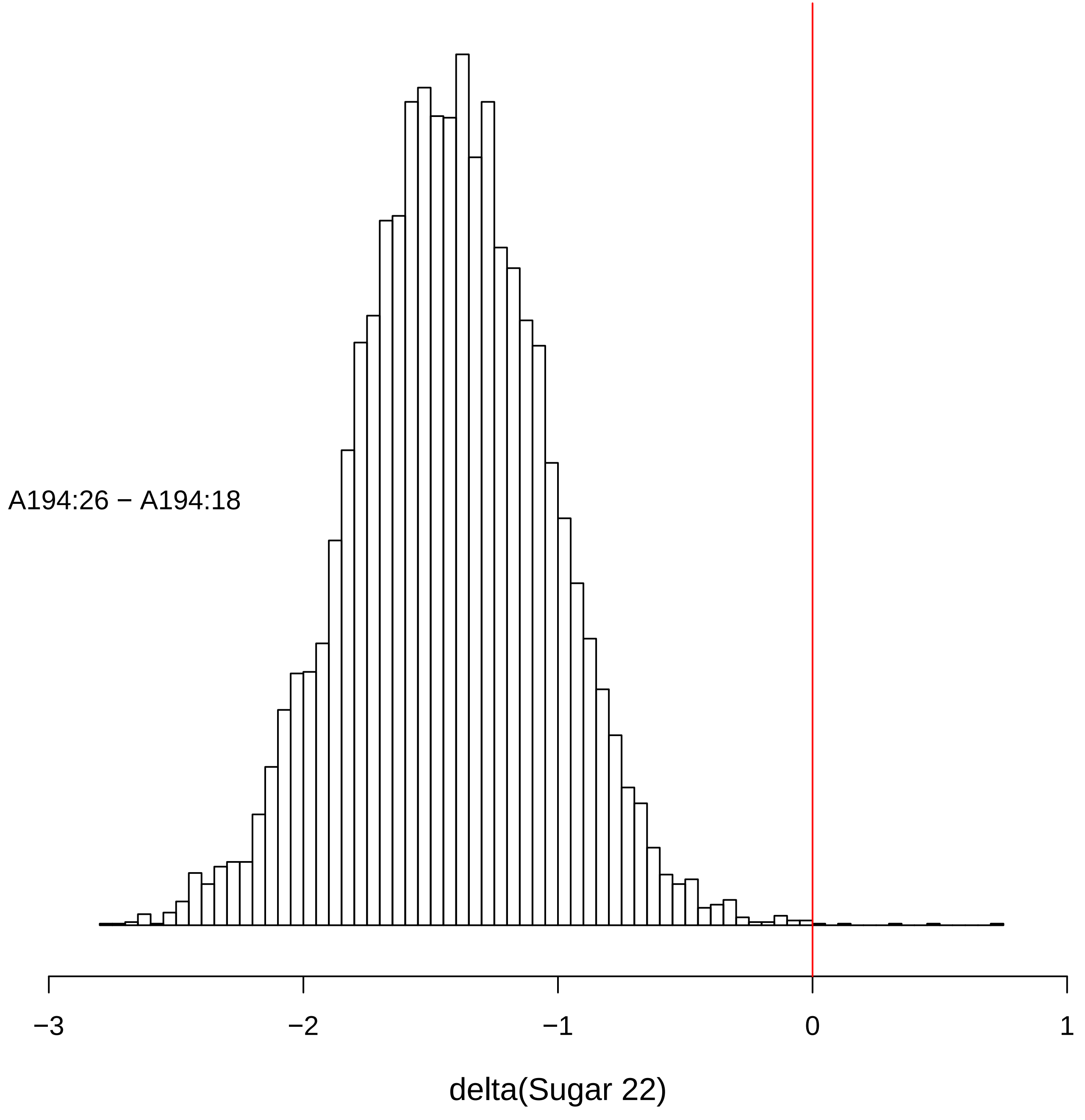
A194:26 − A194:18
−3
−2
−1
0
1
delta(Sugar 22)

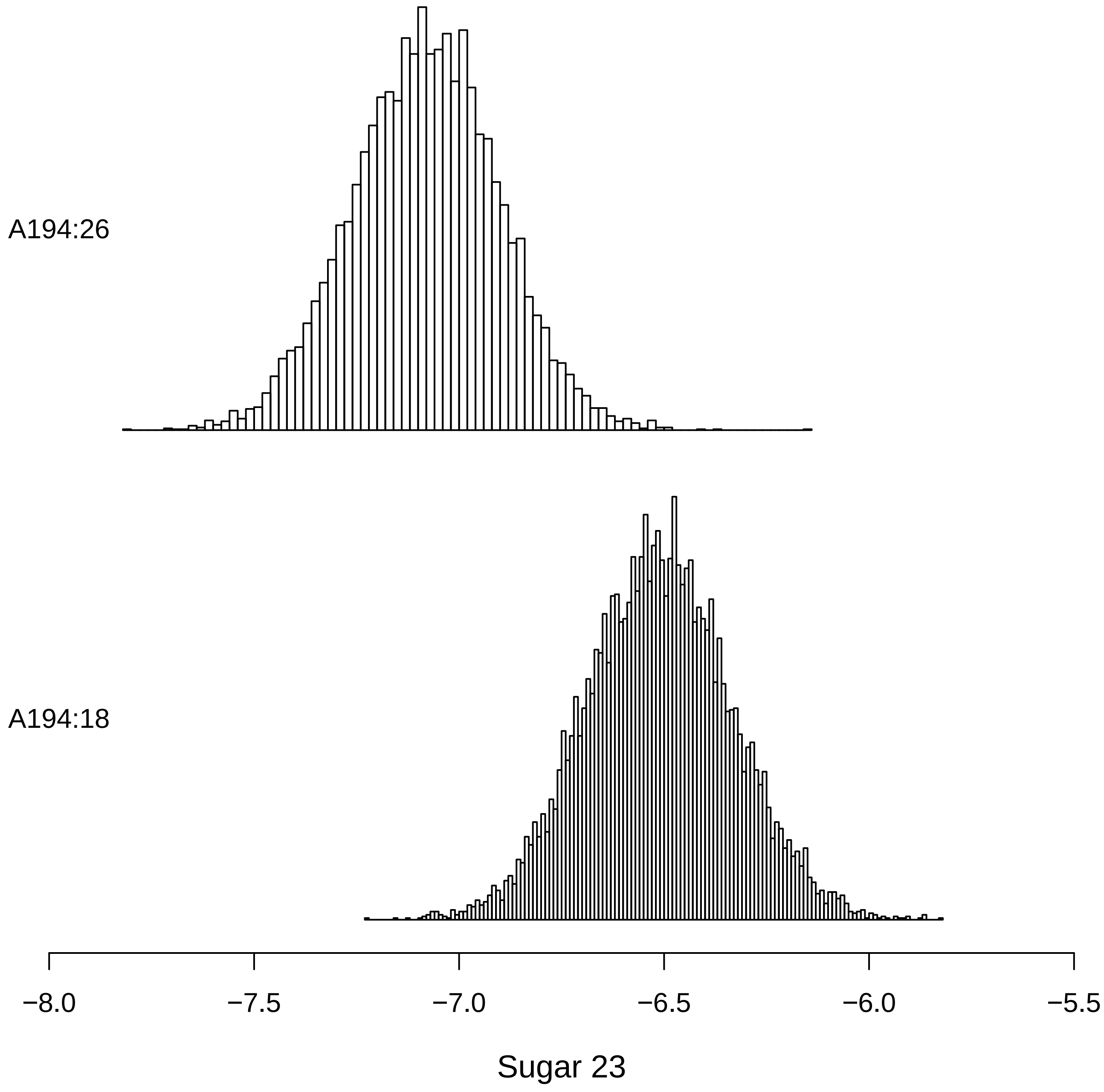
A194:26
A194:18
−8.0
−7.5
−7.0
−6.5
−6.0
−5.5
Sugar 23

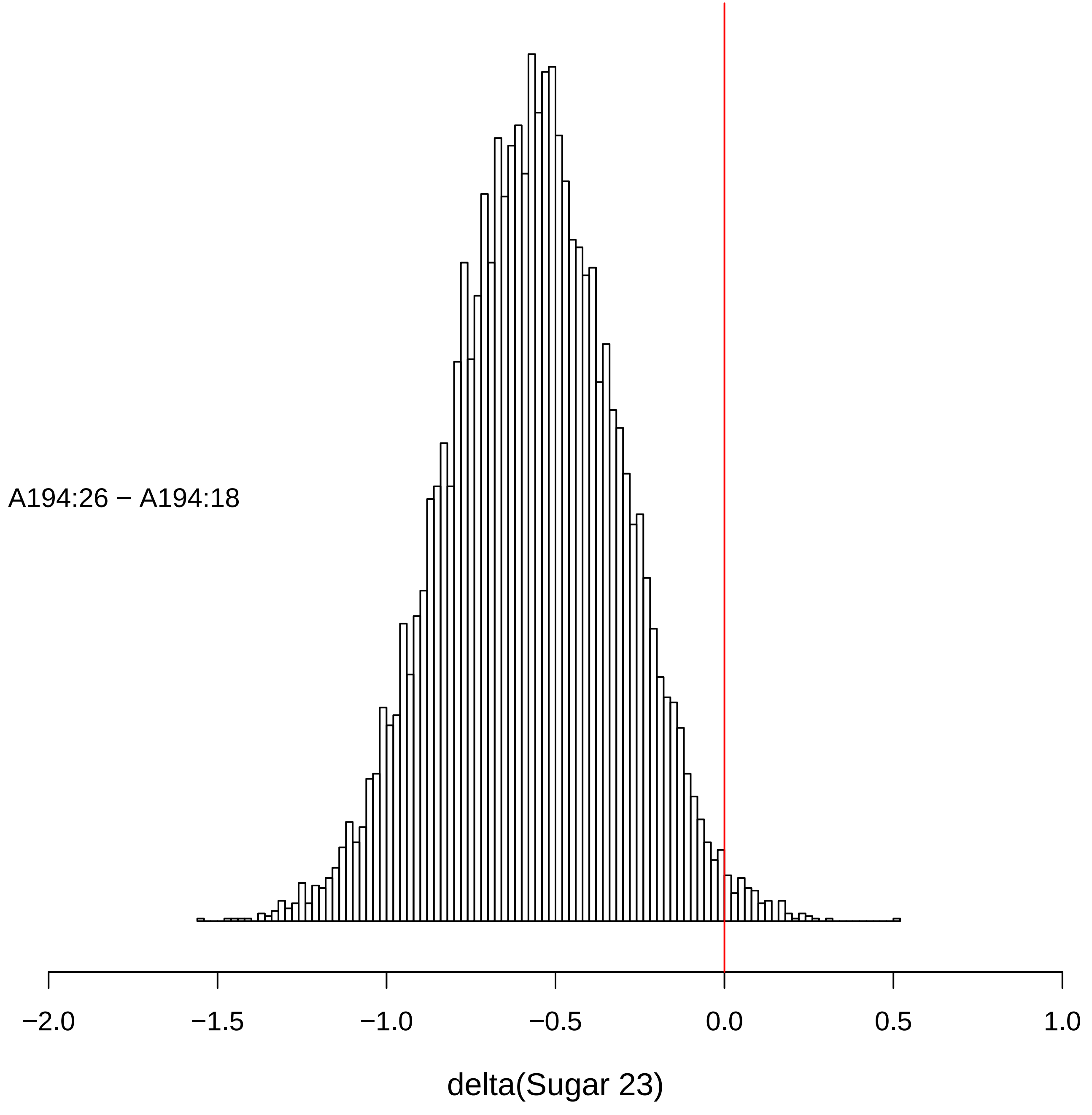
A194:26 − A194:18
−2.0
−1.5
−1.0
−0.5
0.0
0.5
1.0
delta(Sugar 23)

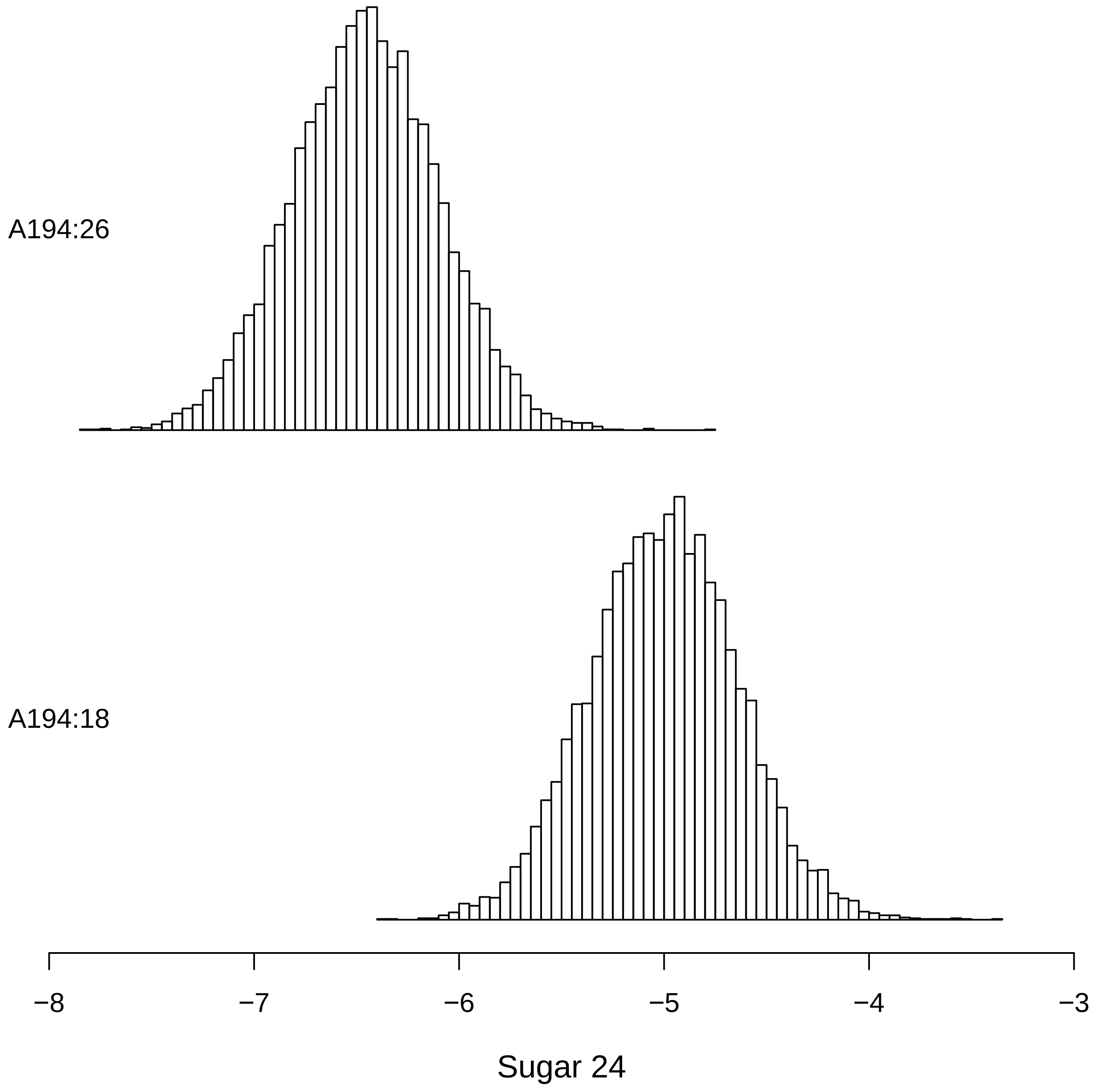
A194:26
A194:18
−8
−7
−6
−5
−4
−3
Sugar 24

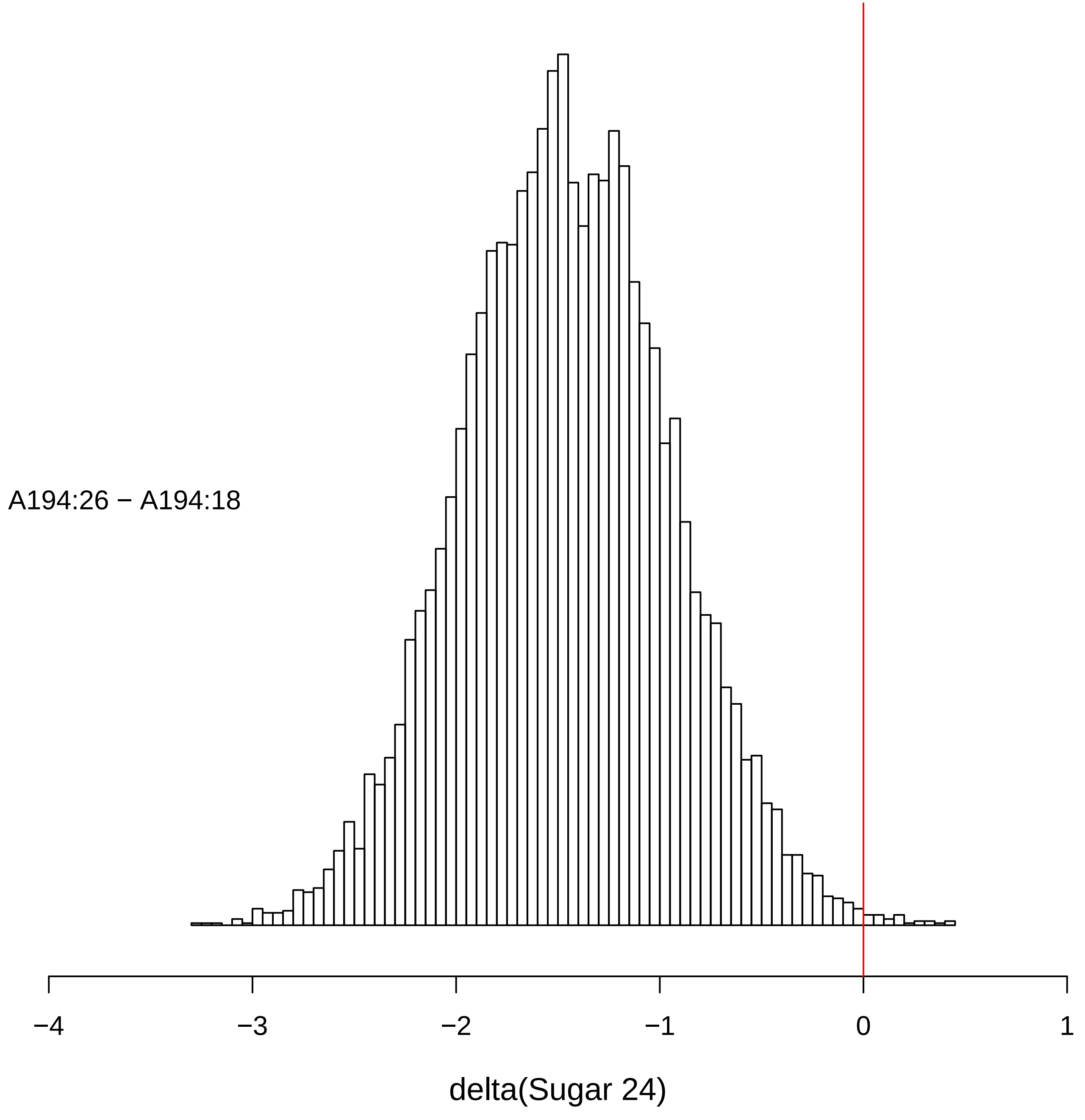
A194:26 − A194:18
−4
−3
−2
−1
0
1
delta(Sugar 24)

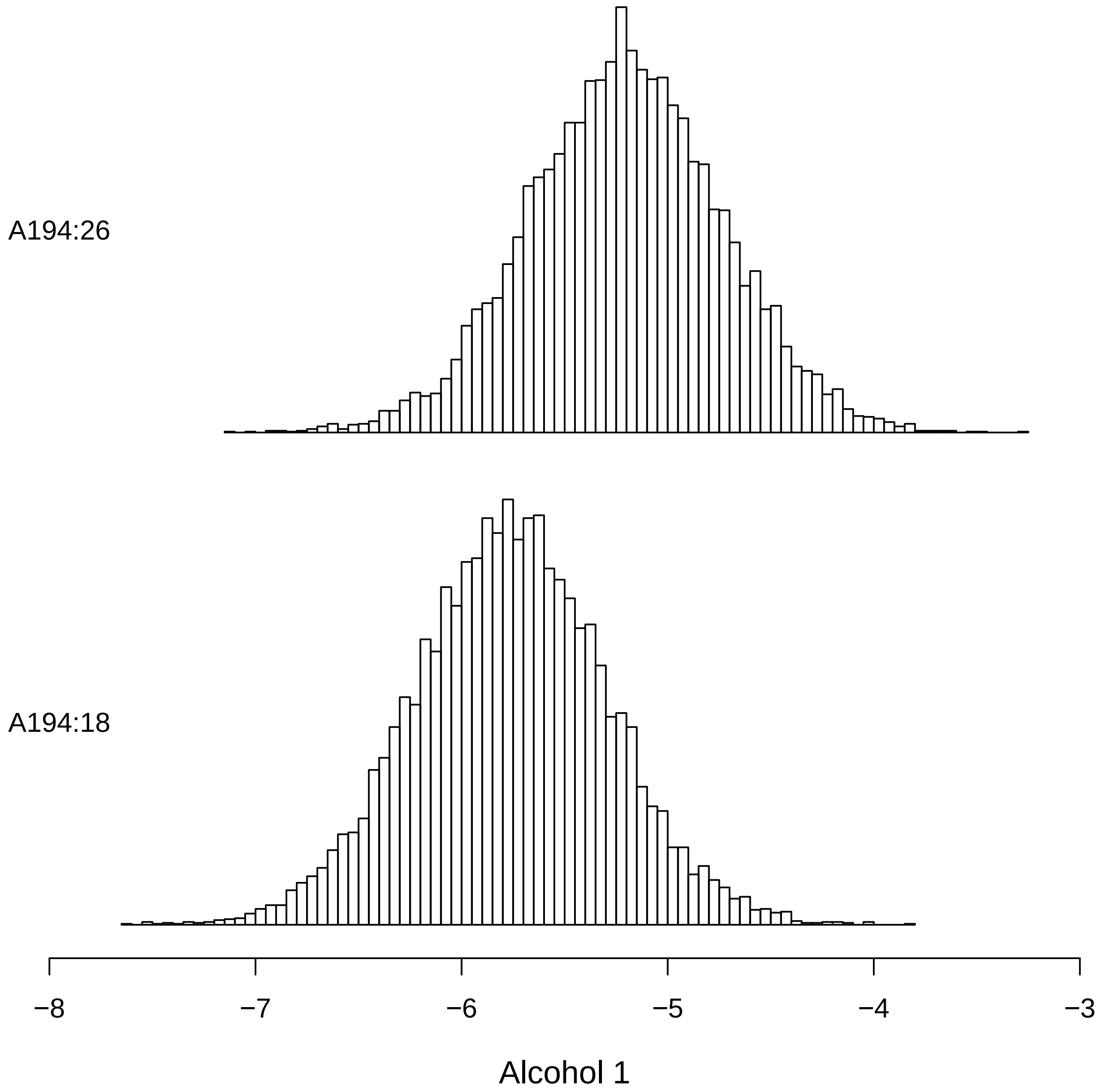
A194:26
A194:18
−8
−7
−6
−5
−4
−3
Alcohol 1

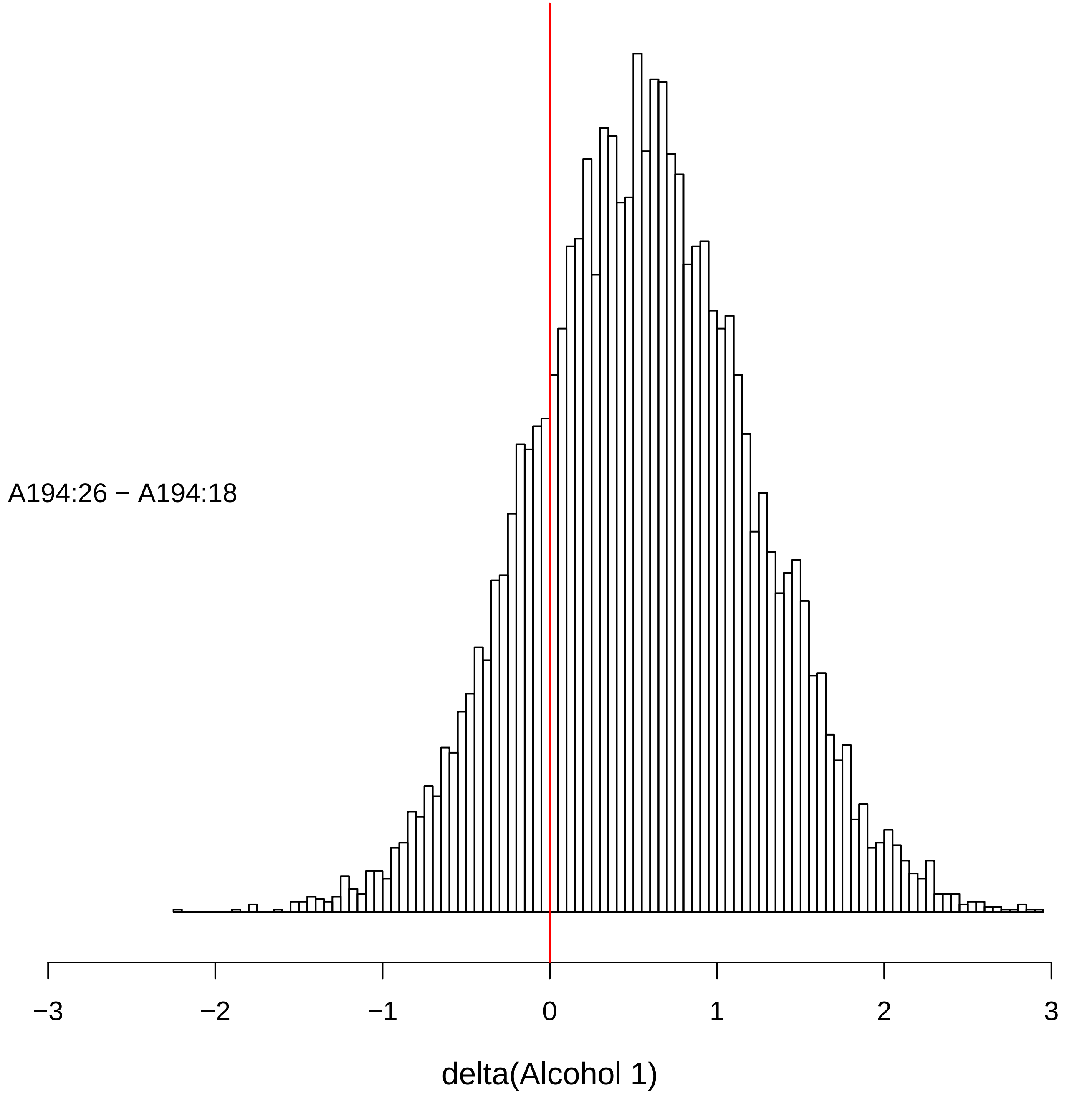
A194:26 − A194:18
−3
−2
−1
0
1
2
3
delta(Alcohol 1)

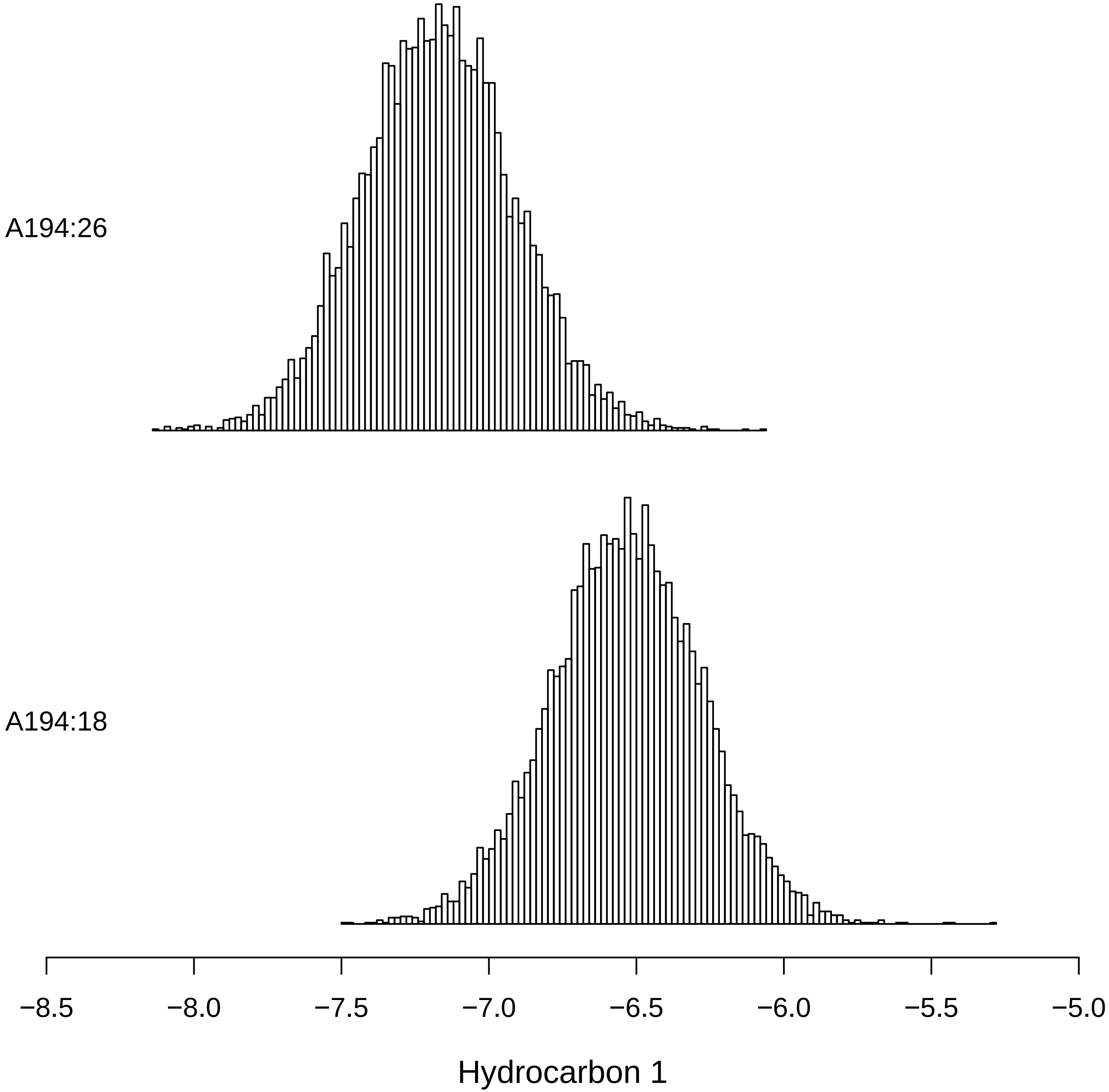
A194:26
A194:18
−8.5
−8.0
−7.5
−7.0
−6.5
−6.0
−5.5
−5.0
Hydrocarbon 1

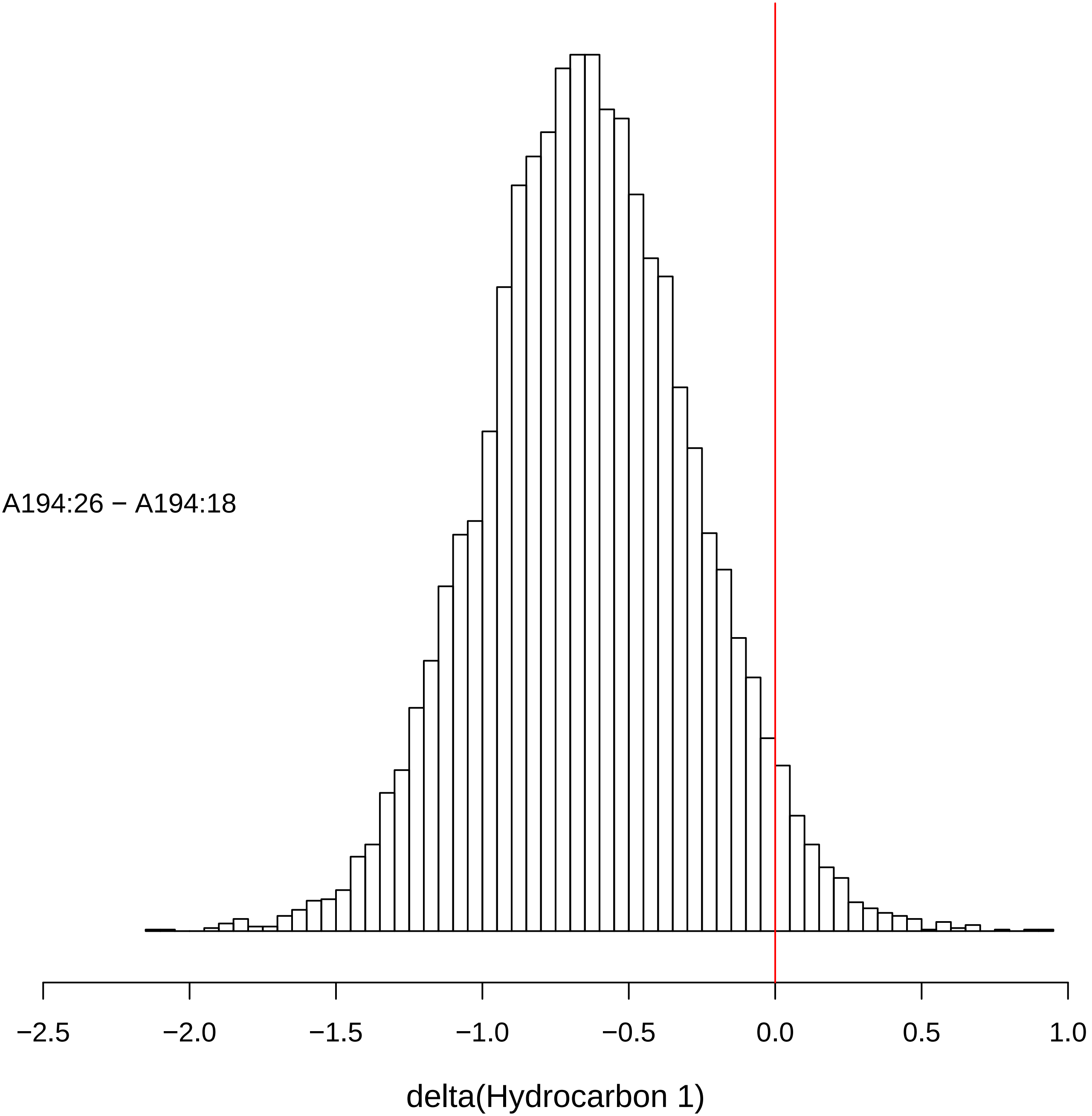
A194:26 − A194:18
−2.5
−2.0
−1.5
−1.0
−0.5
0.0
0.5
1.0
delta(Hydrocarbon 1)

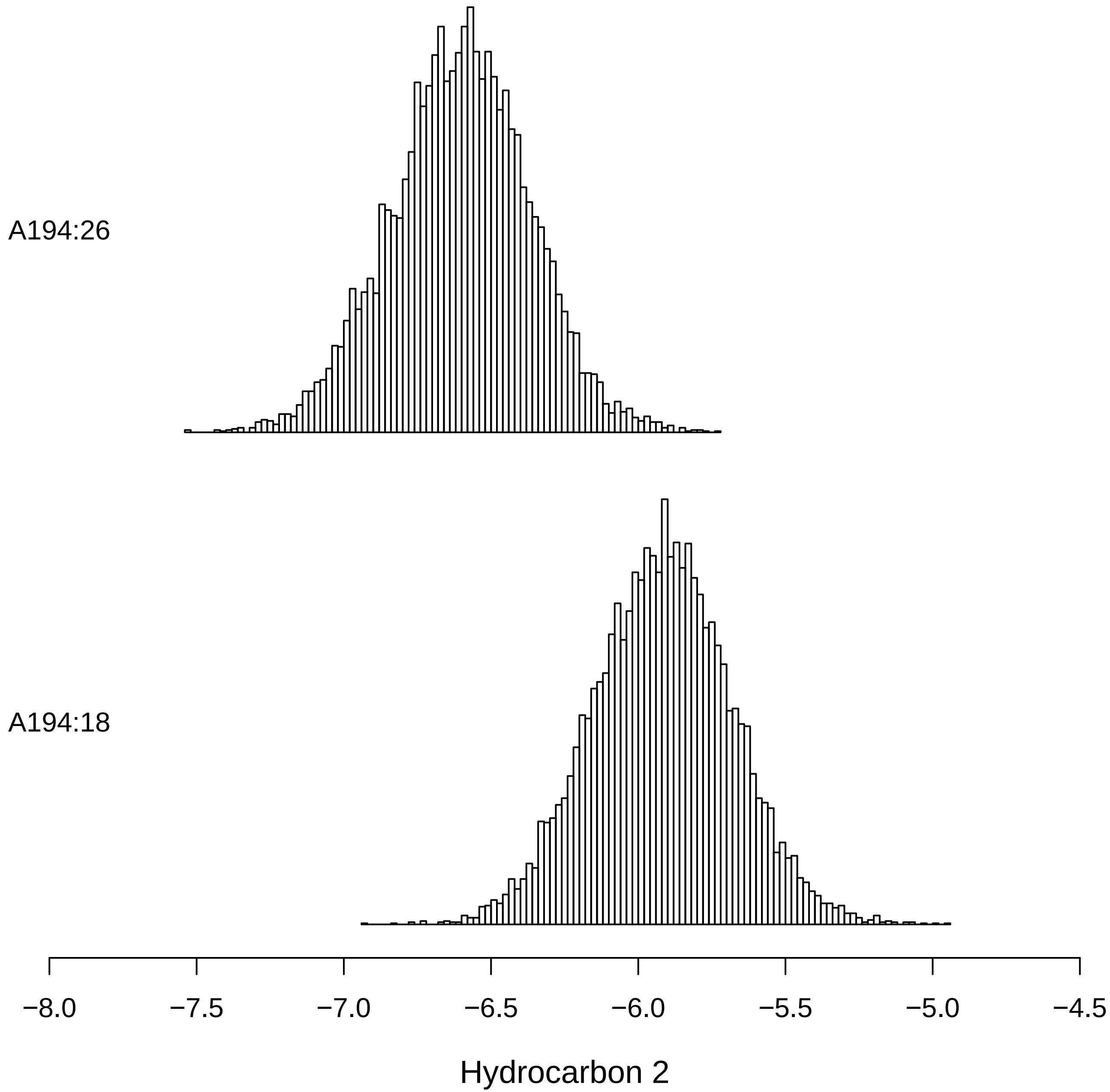
A194:26
A194:18
−8.0
−7.5
−7.0
−6.5
−6.0
−5.5
−5.0
−4.5
Hydrocarbon 2

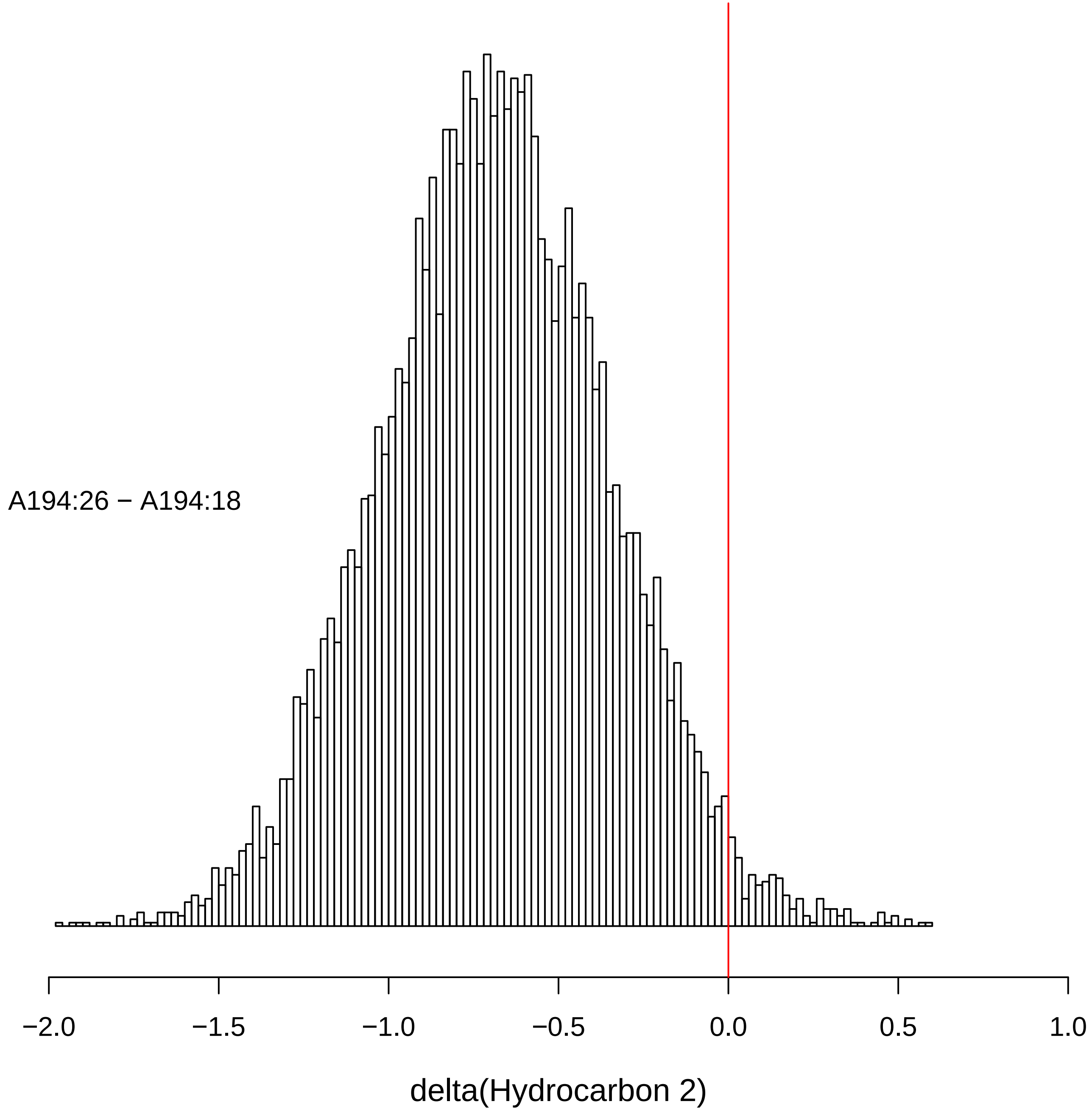

A194:26 − A194:18
−2.0
−1.5
−1.0
−0.5
0.0
0.5
1.0
delta(Hydrocarbon 2)

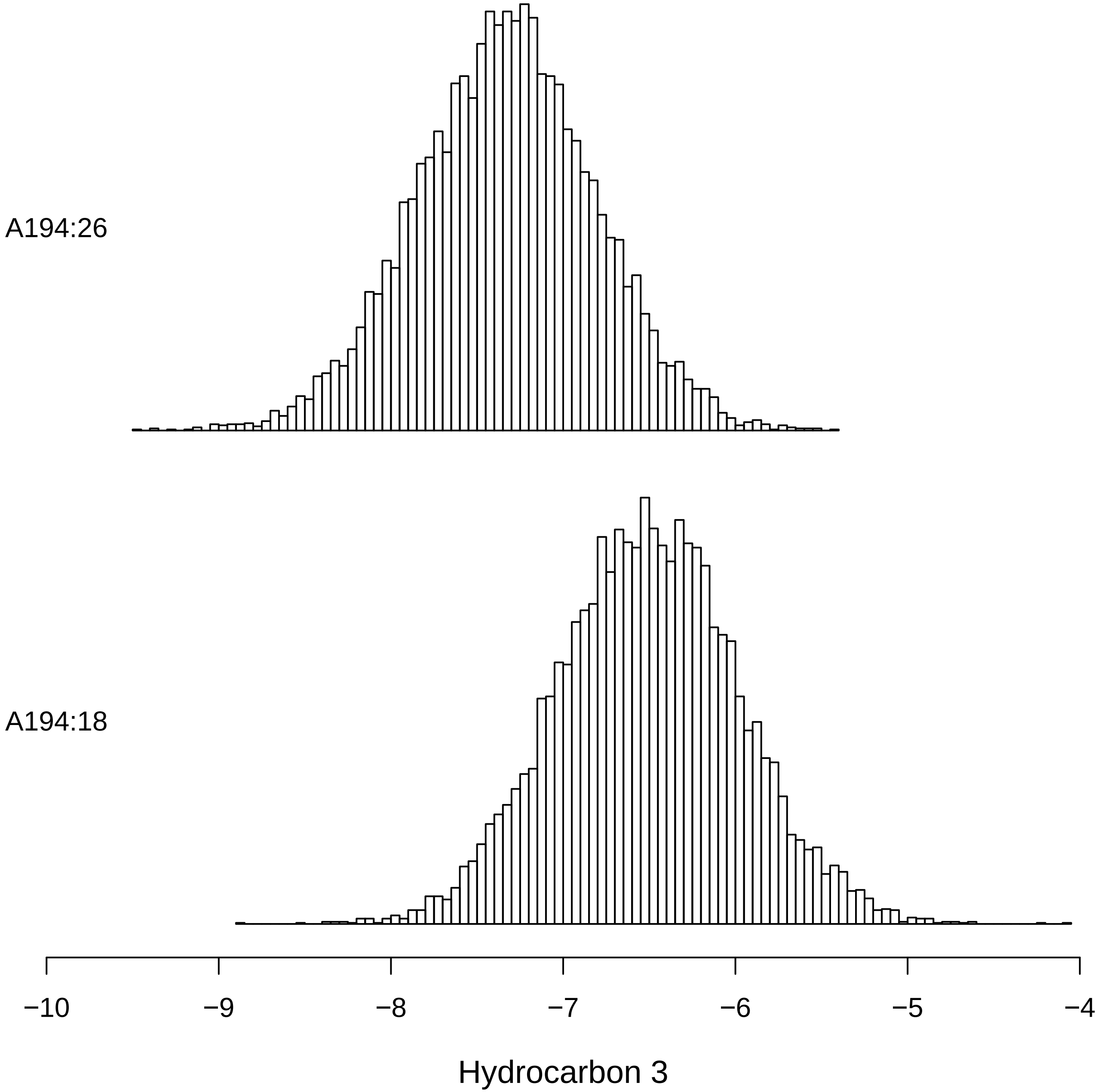
A194:26
A194:18
−10
−9
−8
−7
−6
−5
−4
Hydrocarbon 3

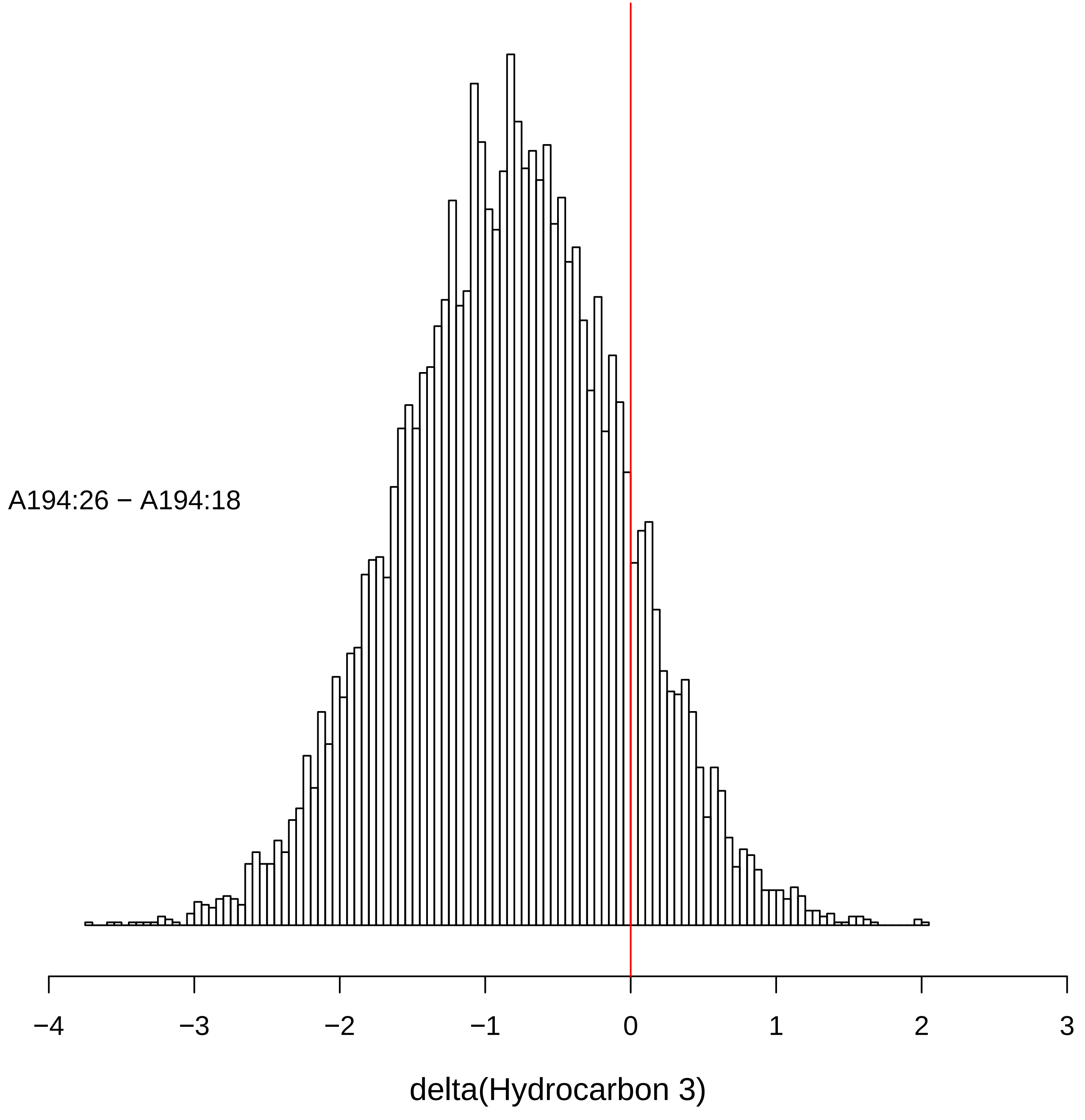
A194:26 − A194:18
−4
−3
−2
−1
0
1
2
3
delta(Hydrocarbon 3)

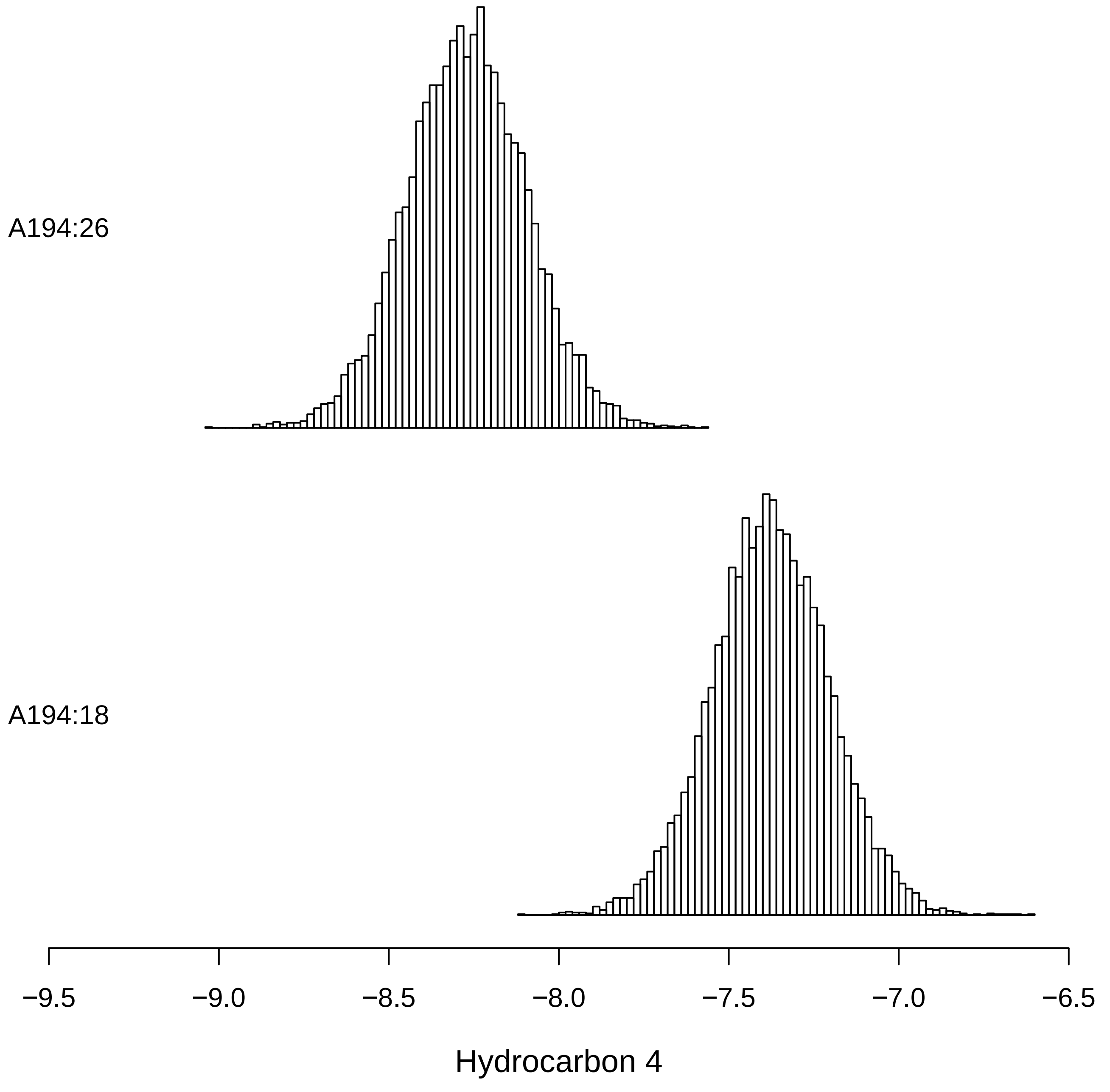
A194:26
A194:18
−9.5
−9.0
−8.5
−8.0
−7.5
−7.0
−6.5
Hydrocarbon 4

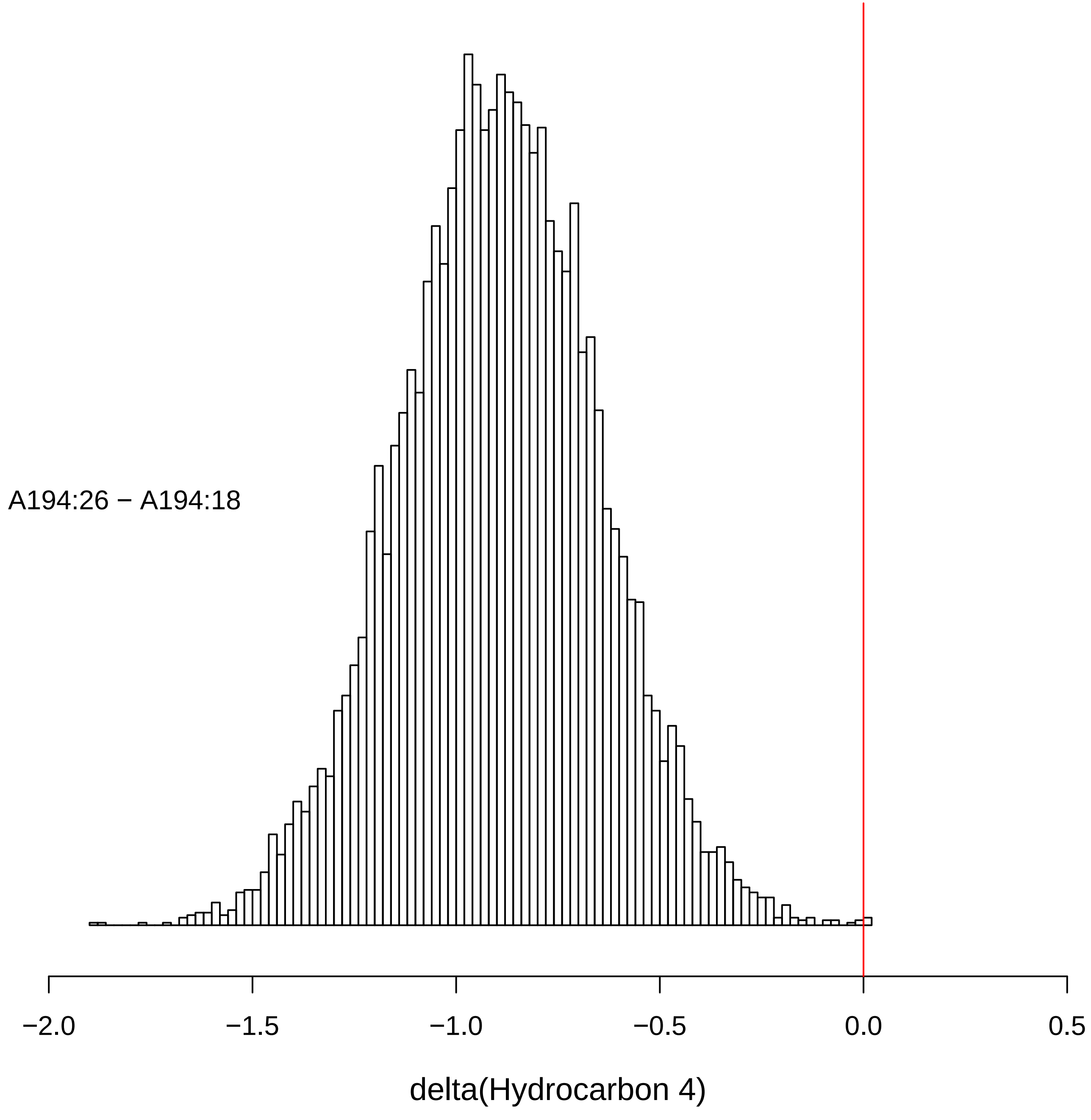

A194:26 − A194:18
−2.0
−1.5
−1.0
−0.5
0.0
0.5
delta(Hydrocarbon 4)

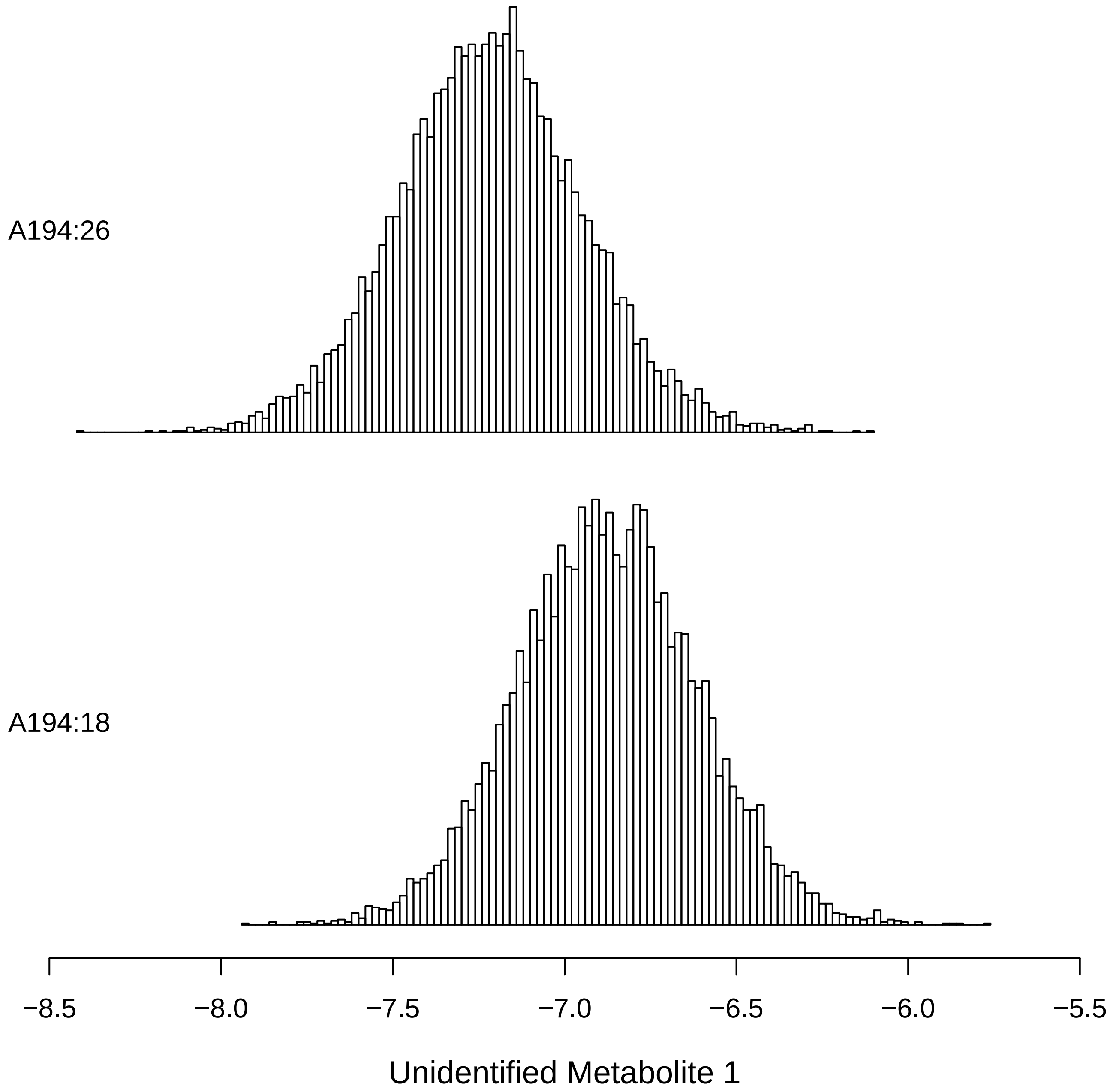
A194:26
A194:18
−8.5
−8.0
−7.5
−7.0
−6.5
−6.0
−5.5
Unidentified Metabolite 1

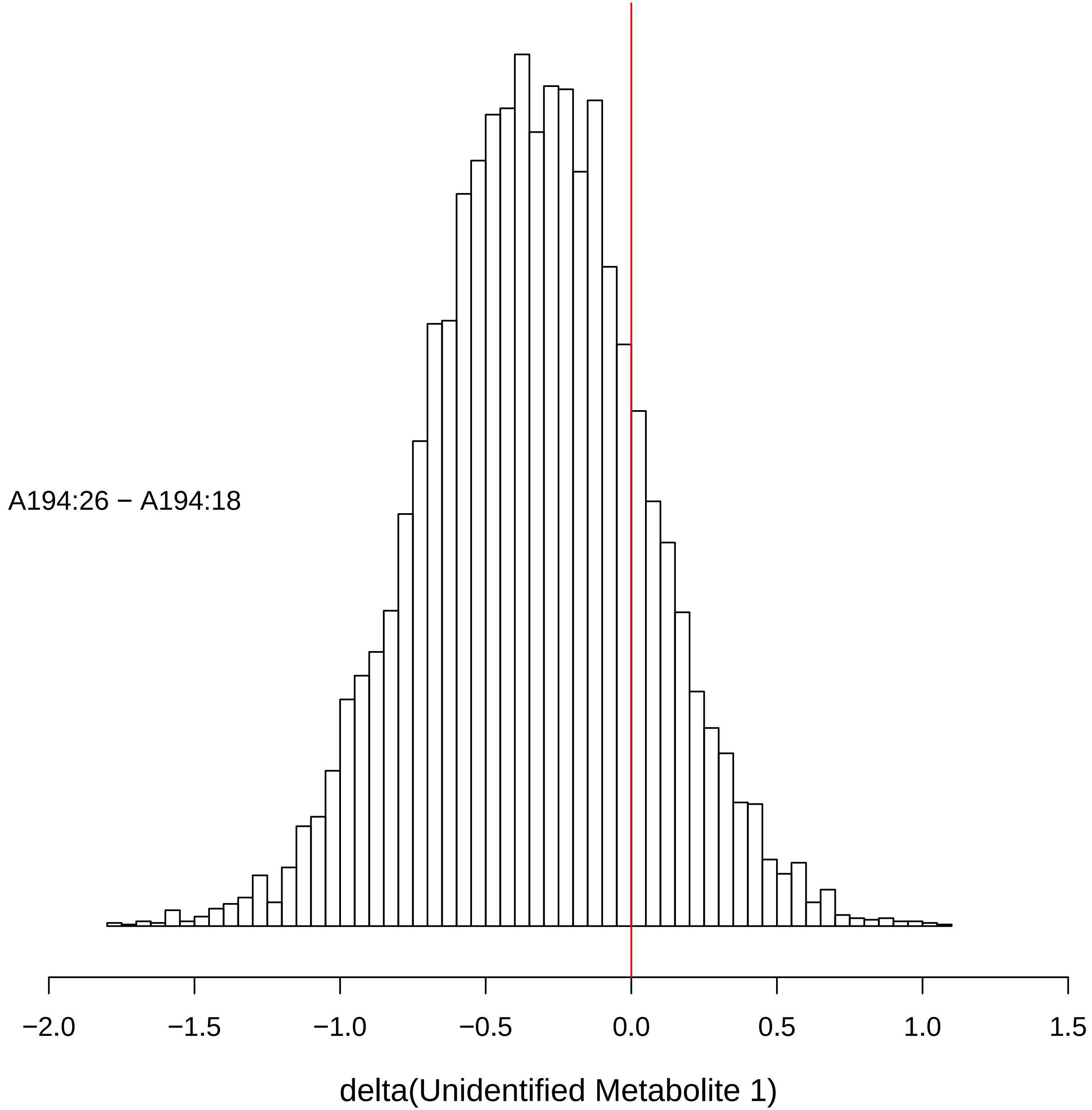
A194:26 − A194:18
−2.0
−1.5
−1.0
−0.5
0.0
0.5
1.0
1.5
delta(Unidentified Metabolite 1)

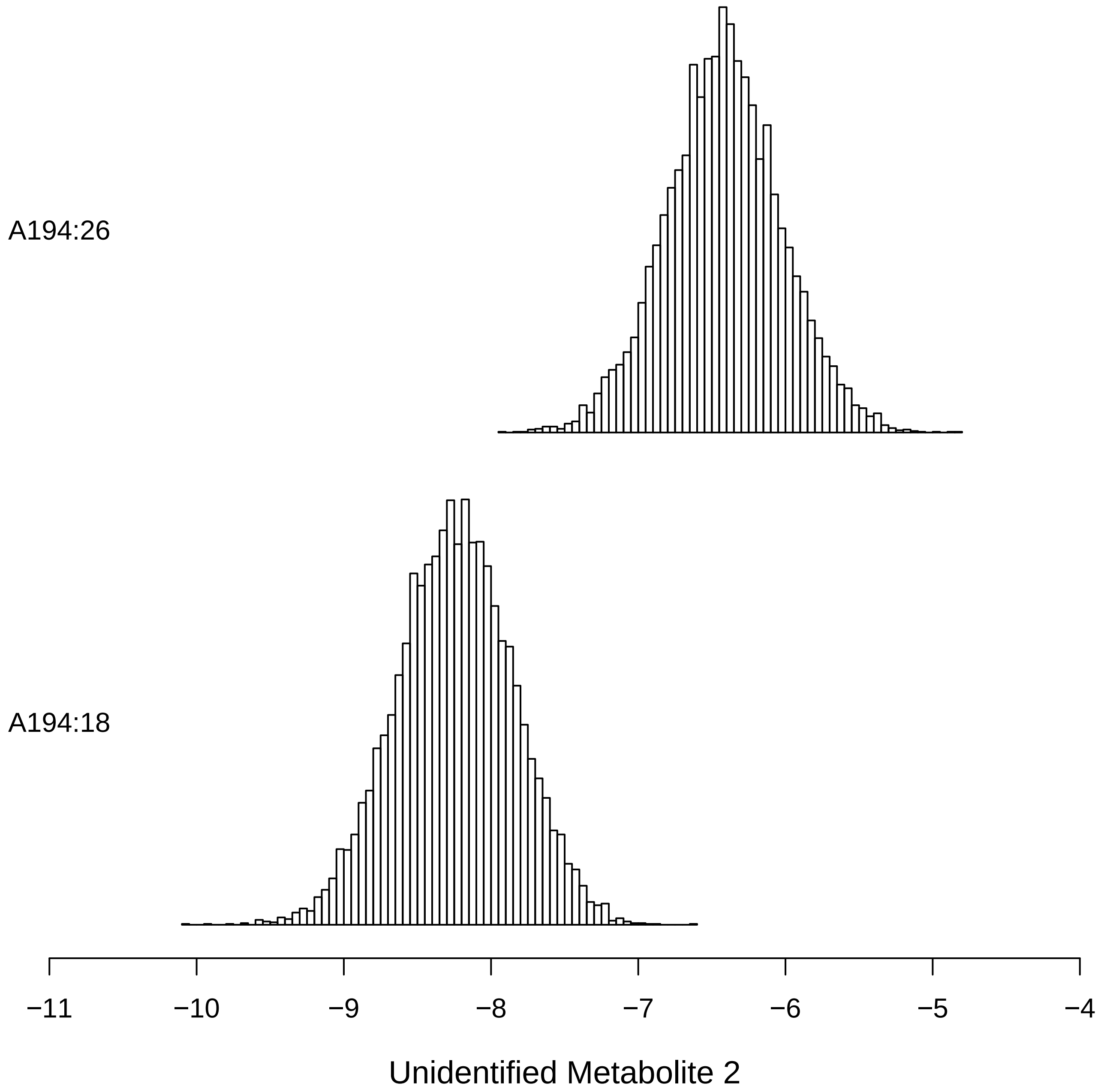

A194:26
A194:18
−11
−10
−9
−8
−7
−6
−5
−4
Unidentified Metabolite 2

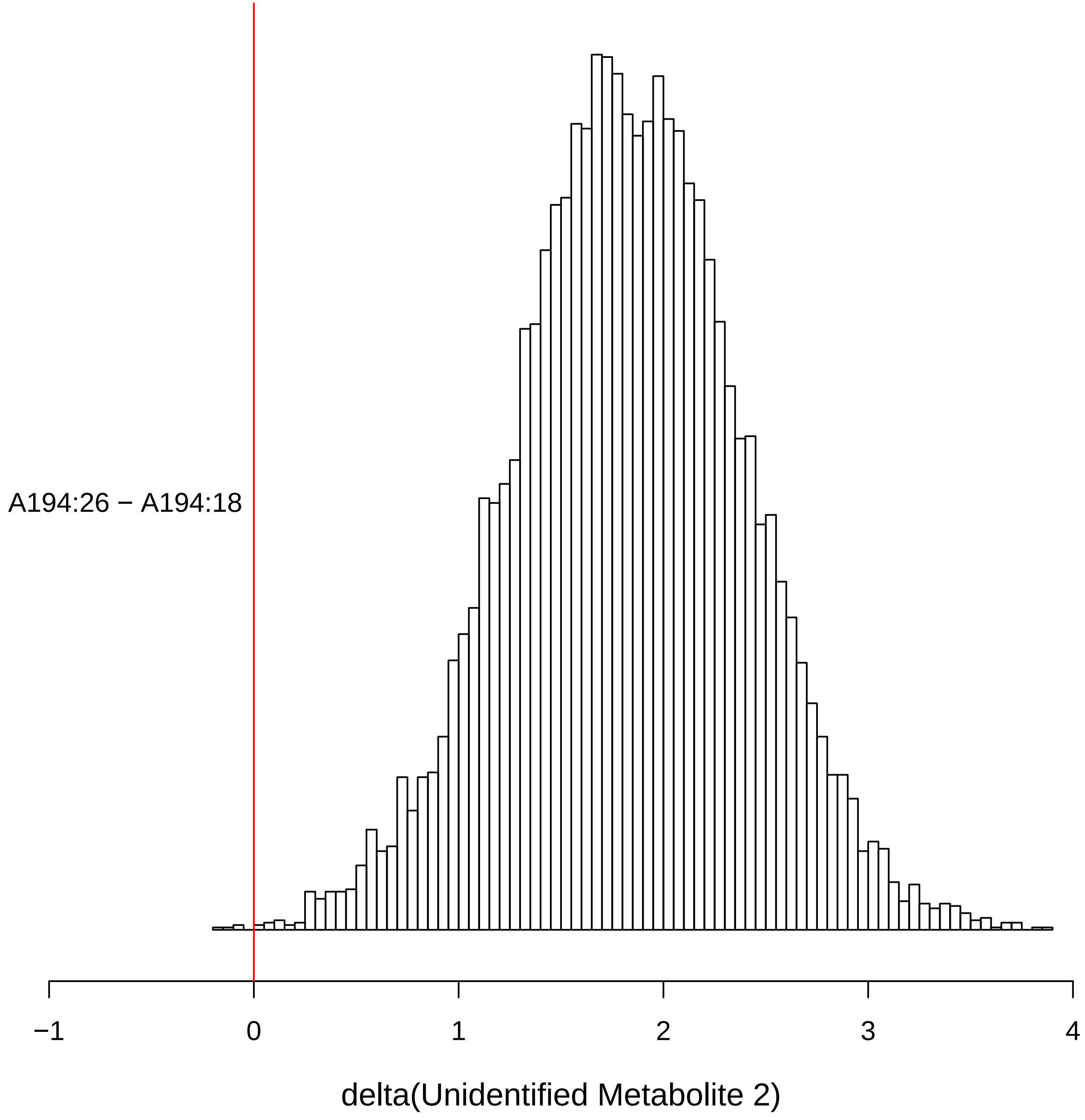
A194:26 − A194:18
−1
0
1
2
3
4
delta(Unidentified Metabolite 2)

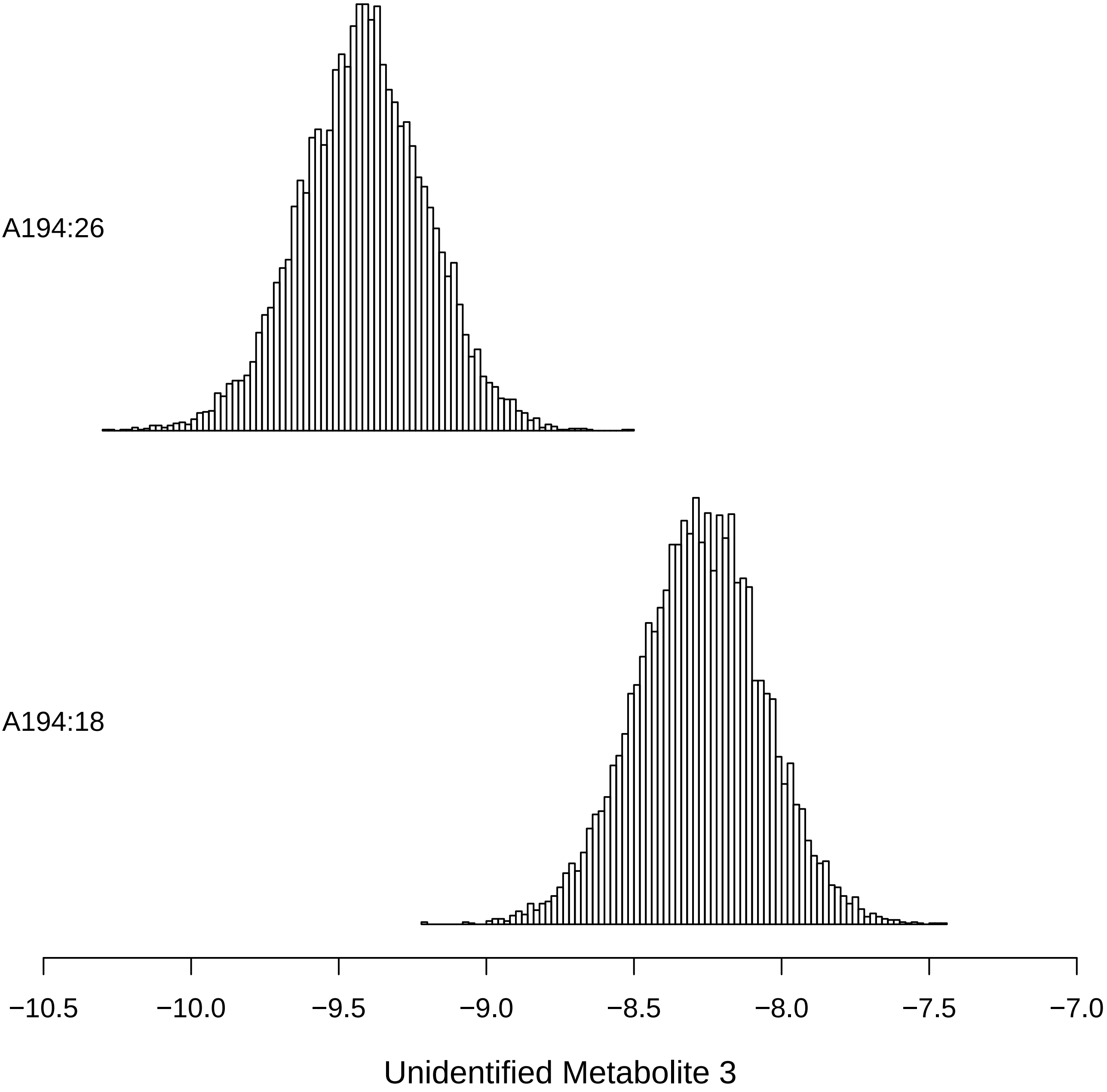

A194:26
A194:18
−10.5
−10.0
−9.5
−9.0
−8.5
−8.0
−7.5
−7.0
Unidentified Metabolite 3

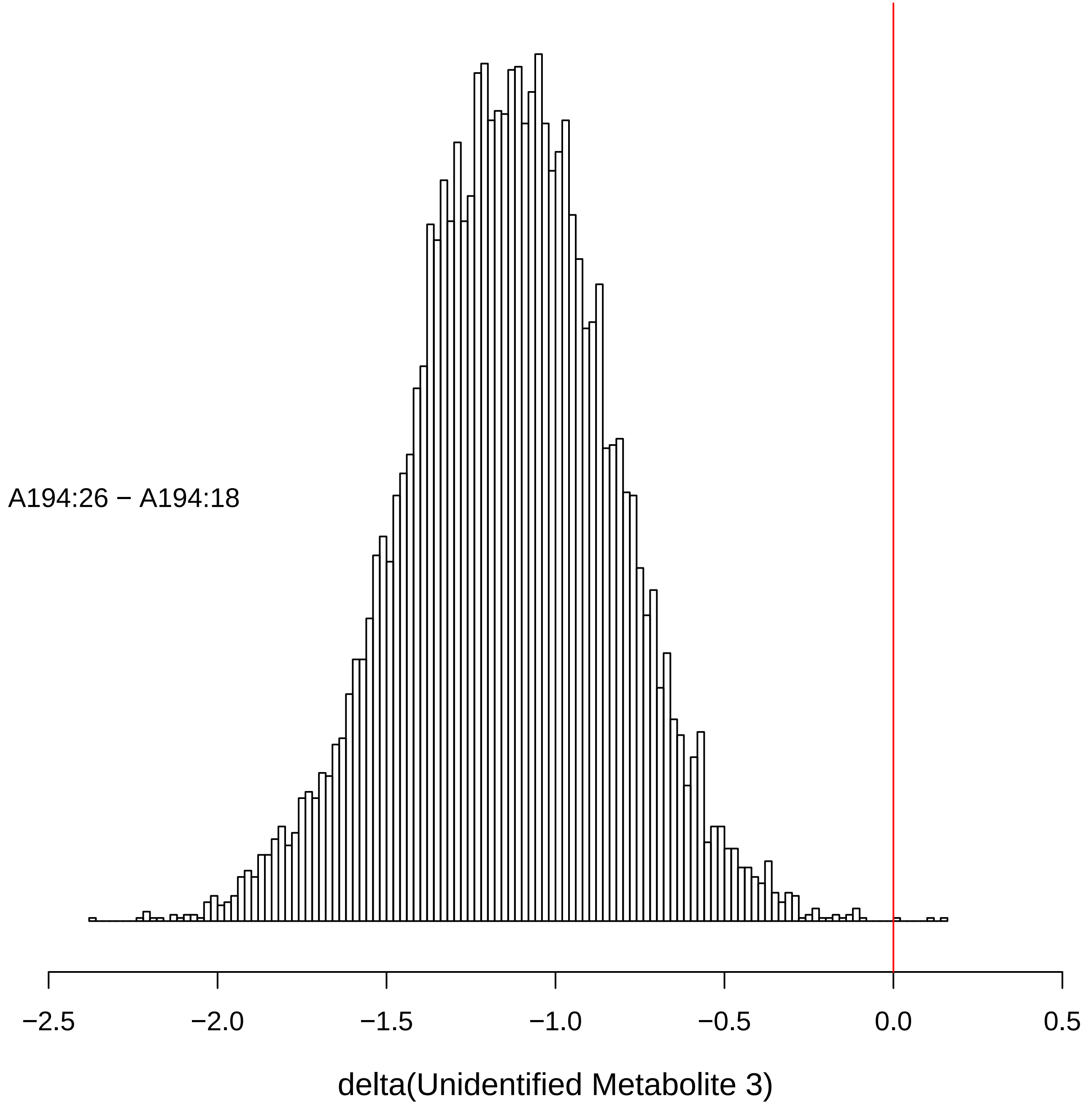
A194:26 − A194:18
−2.5
−2.0
−1.5
−1.0
−0.5
0.0
0.5
delta(Unidentified Metabolite 3)

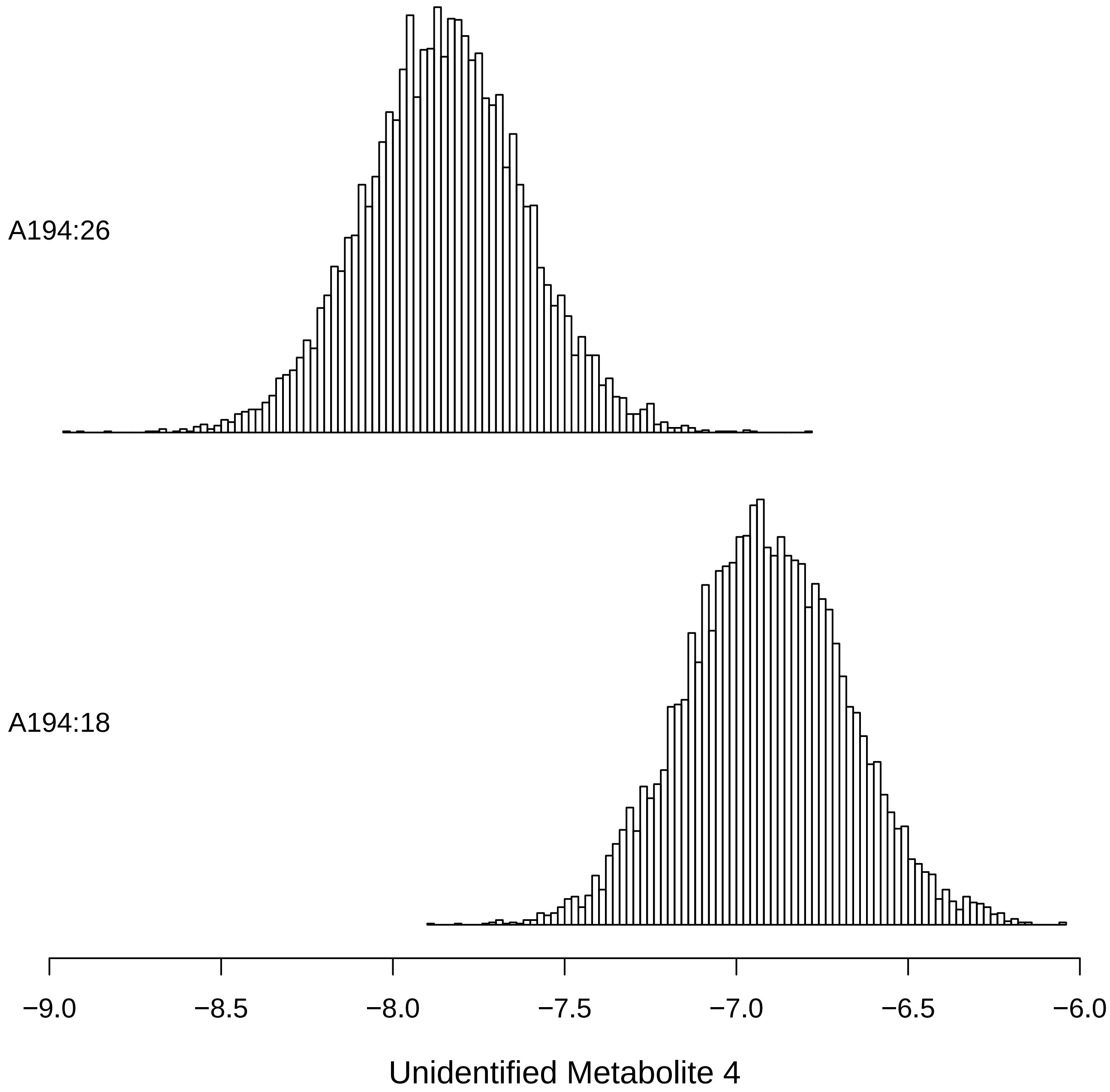
A194:26
A194:18
−9.0
−8.5
−8.0
−7.5
−7.0
−6.5
−6.0
Unidentified Metabolite 4

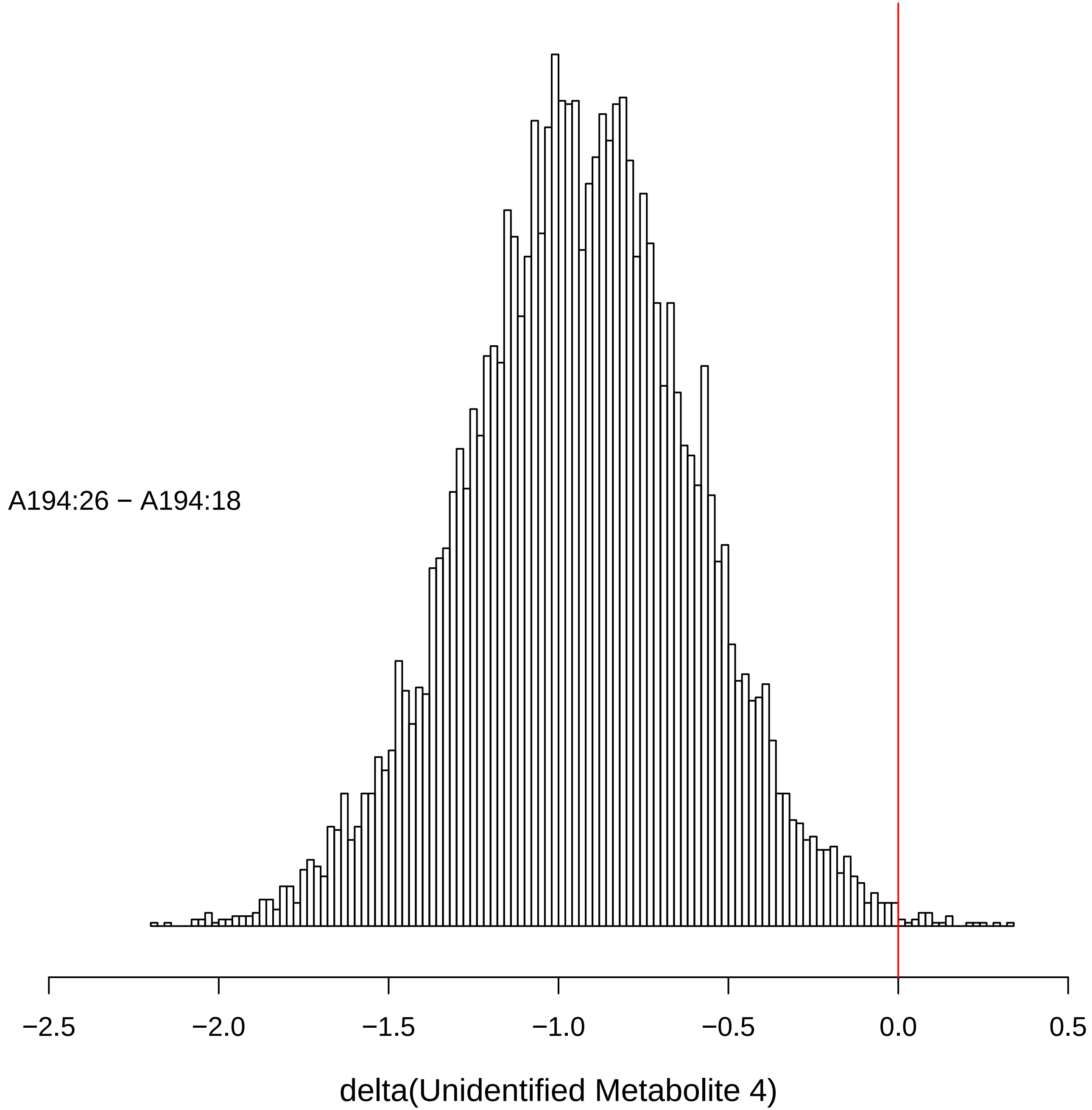
A194:26 − A194:18
−2.5
−2.0
−1.5
−1.0
−0.5
0.0
0.5
delta(Unidentified Metabolite 4)

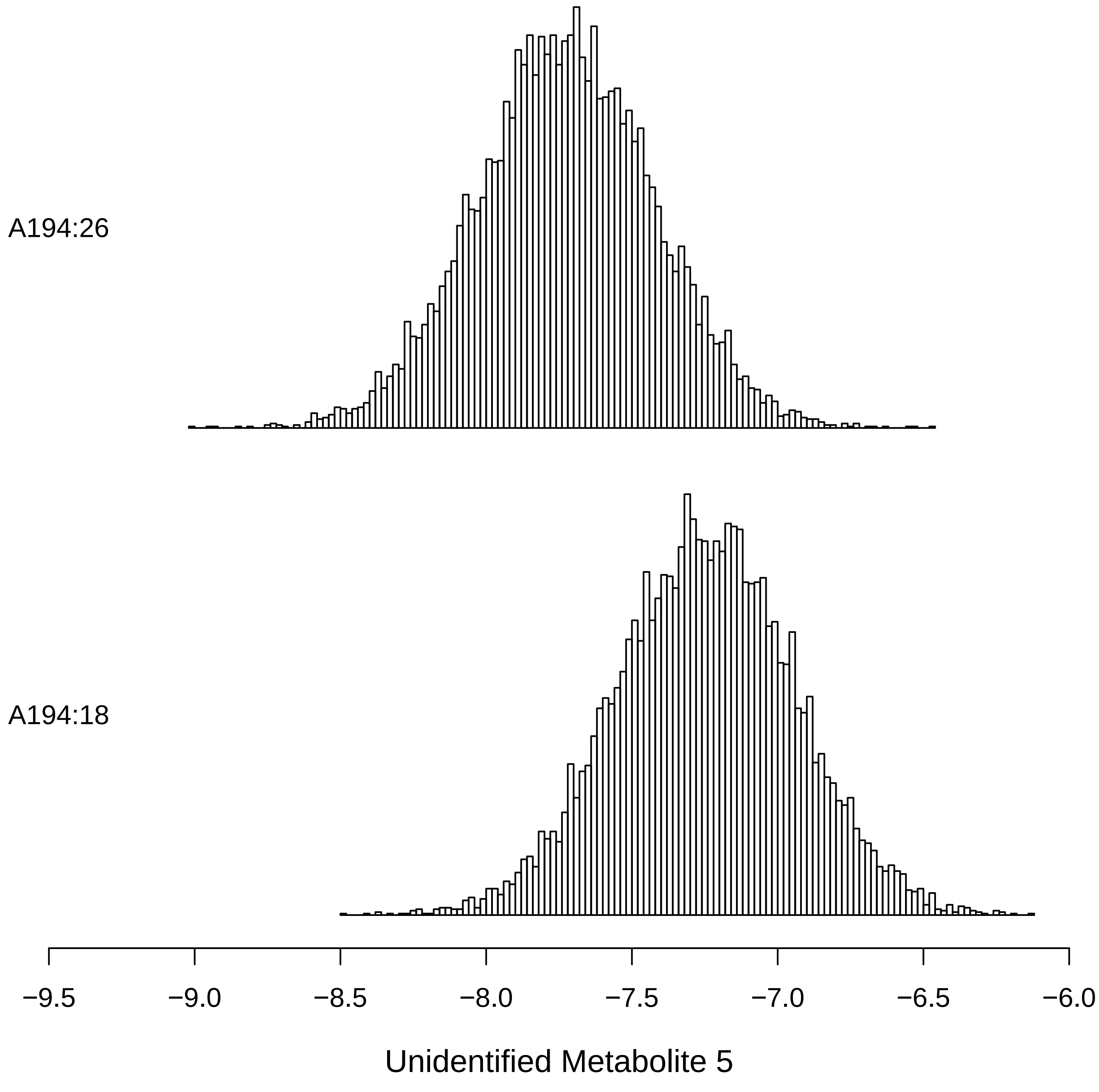
A194:26
A194:18
−9.5
−9.0
−8.5
−8.0
−7.5
−7.0
−6.5
−6.0
Unidentified Metabolite 5

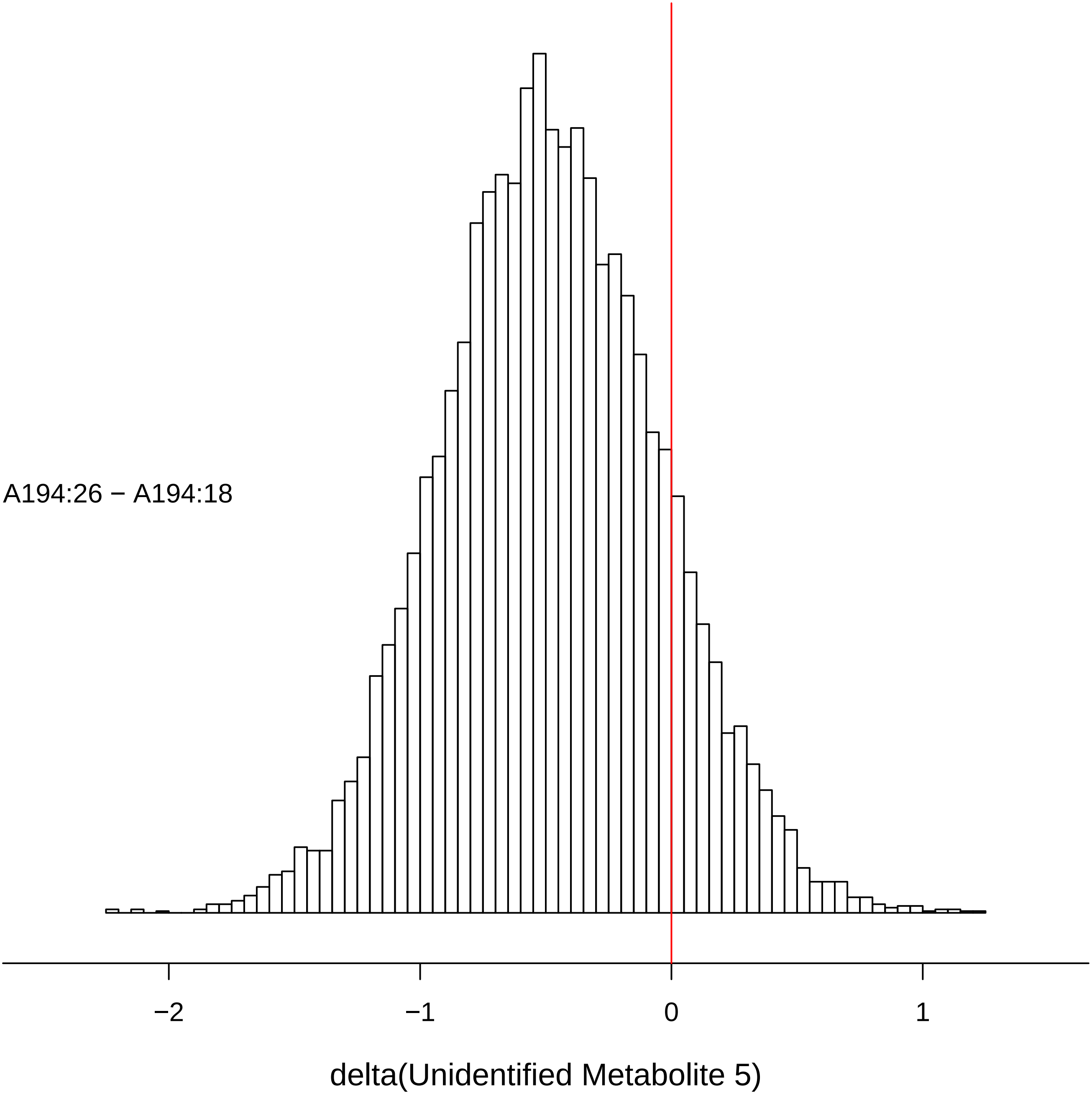
A194:26 − A194:18
−2
−1
0
1
delta(Unidentified Metabolite 5)

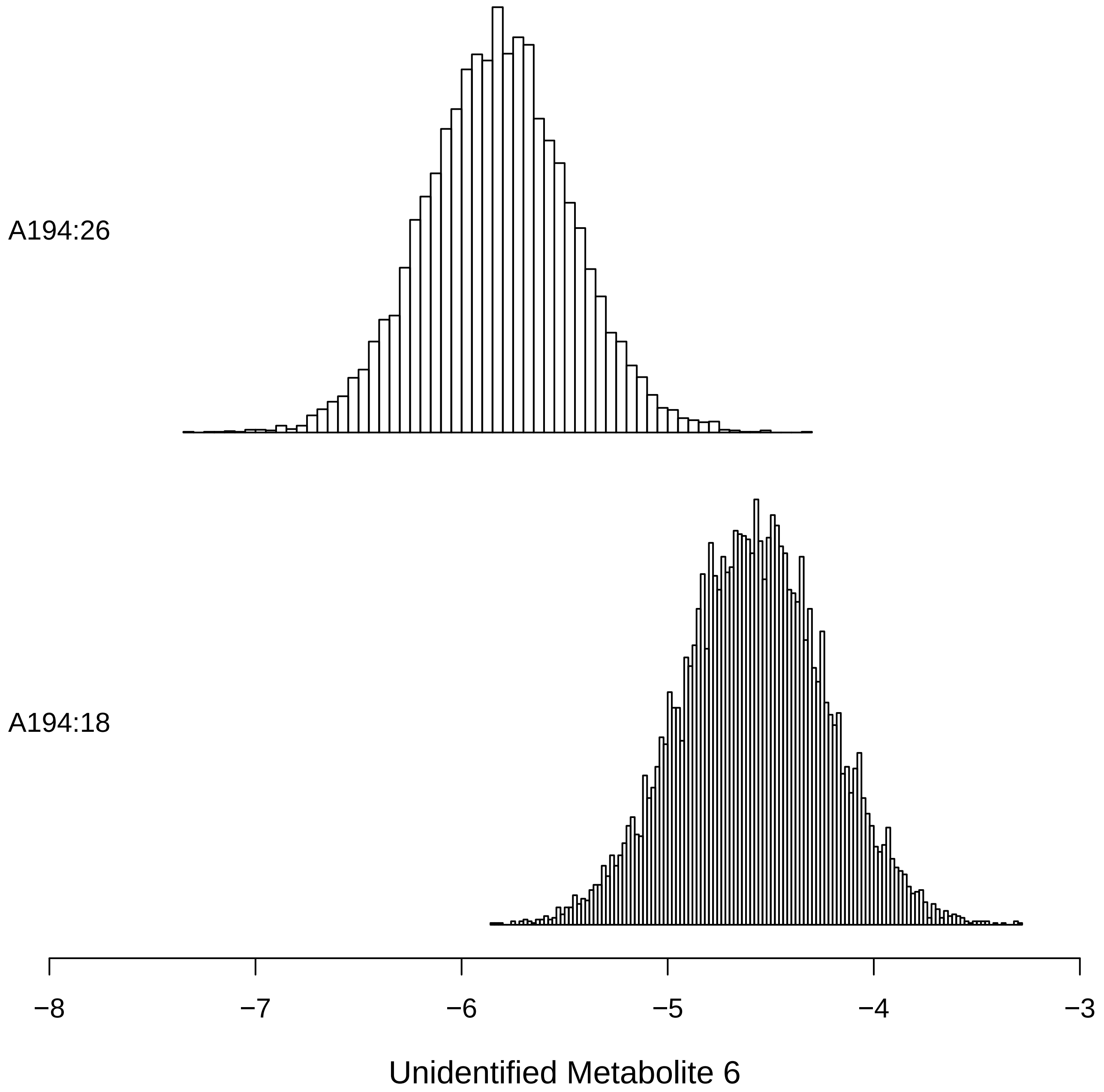
A194:26
A194:18
−8
−7
−6
−5
−4
−3
Unidentified Metabolite 6

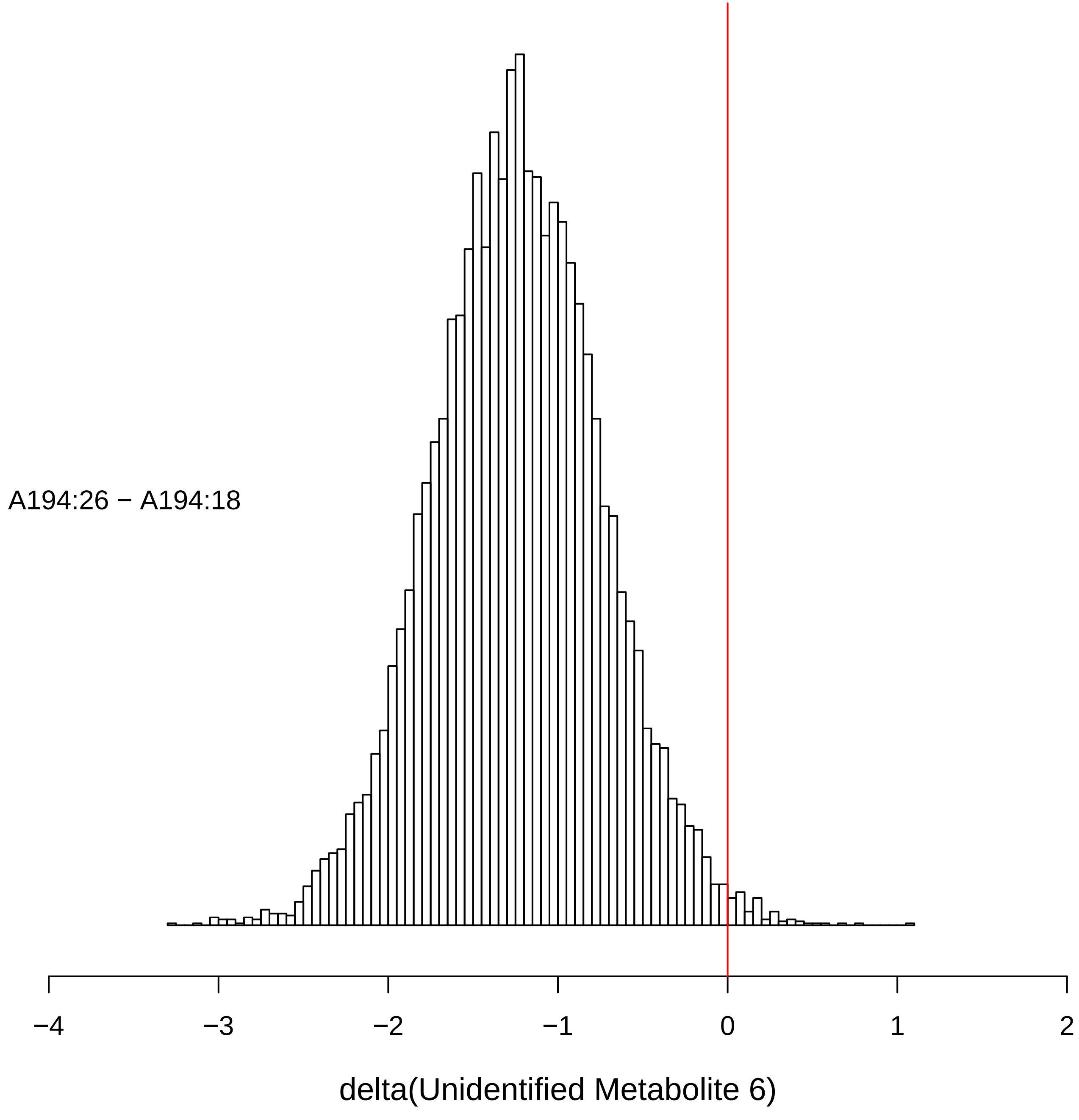
A194:26 − A194:18
−4
−3
−2
−1
0
1
2
delta(Unidentified Metabolite 6)

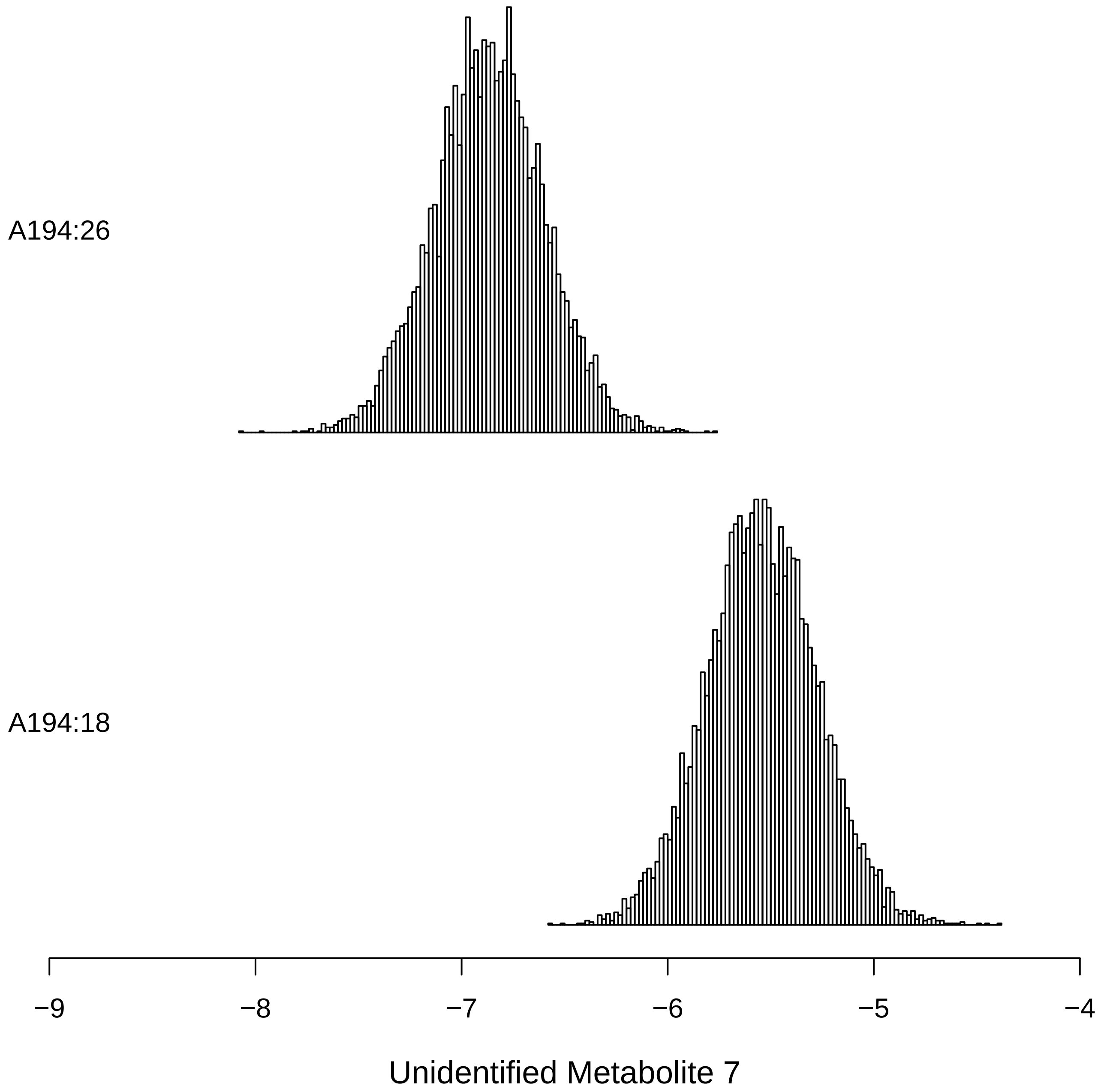
A194:26
A194:18
−9
−8
−7
−6
−5
−4
Unidentified Metabolite 7

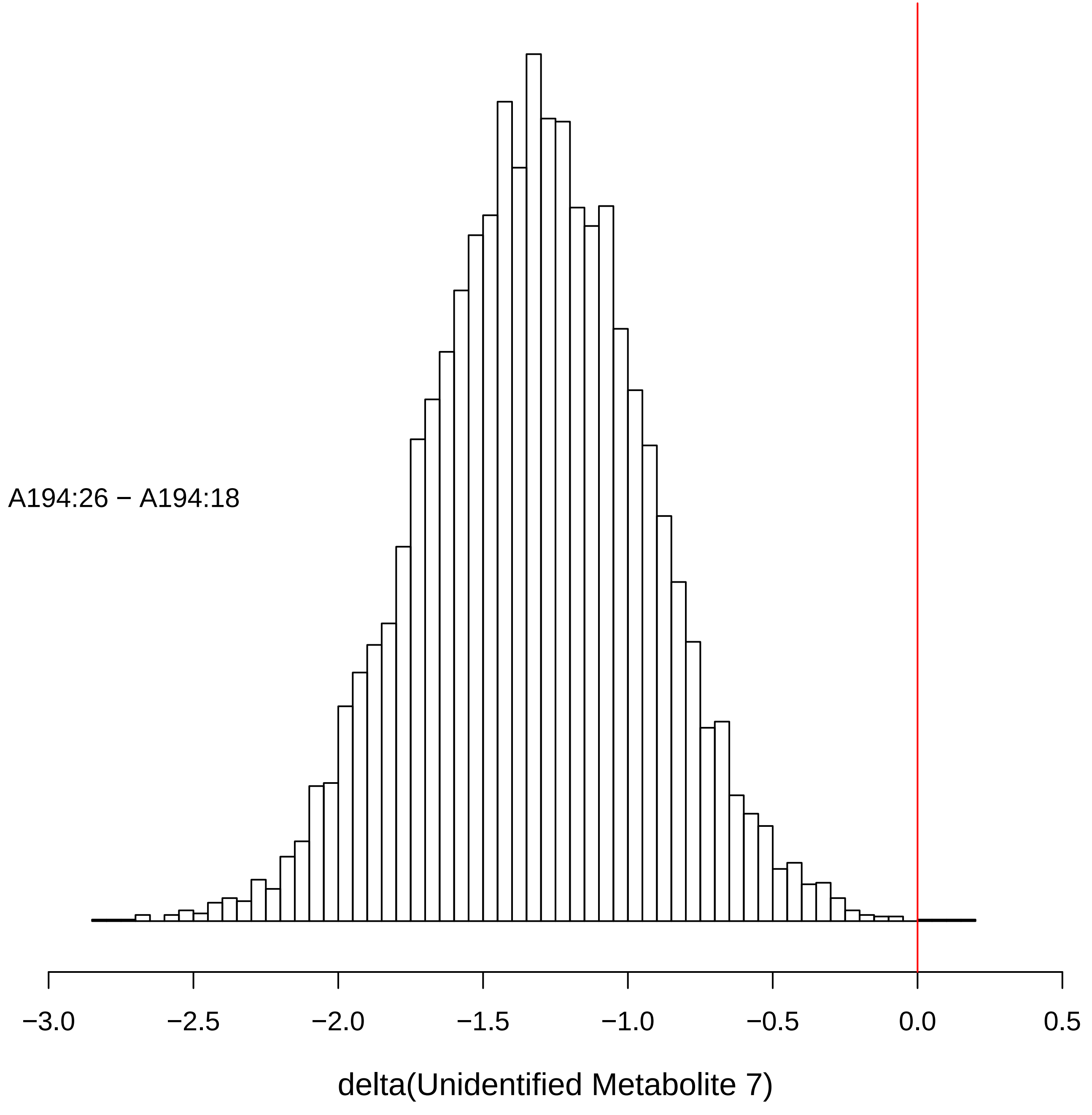
A194:26 − A194:18
−3.0
−2.5
−2.0
−1.5
−1.0
−0.5
0.0
0.5
delta(Unidentified Metabolite 7)

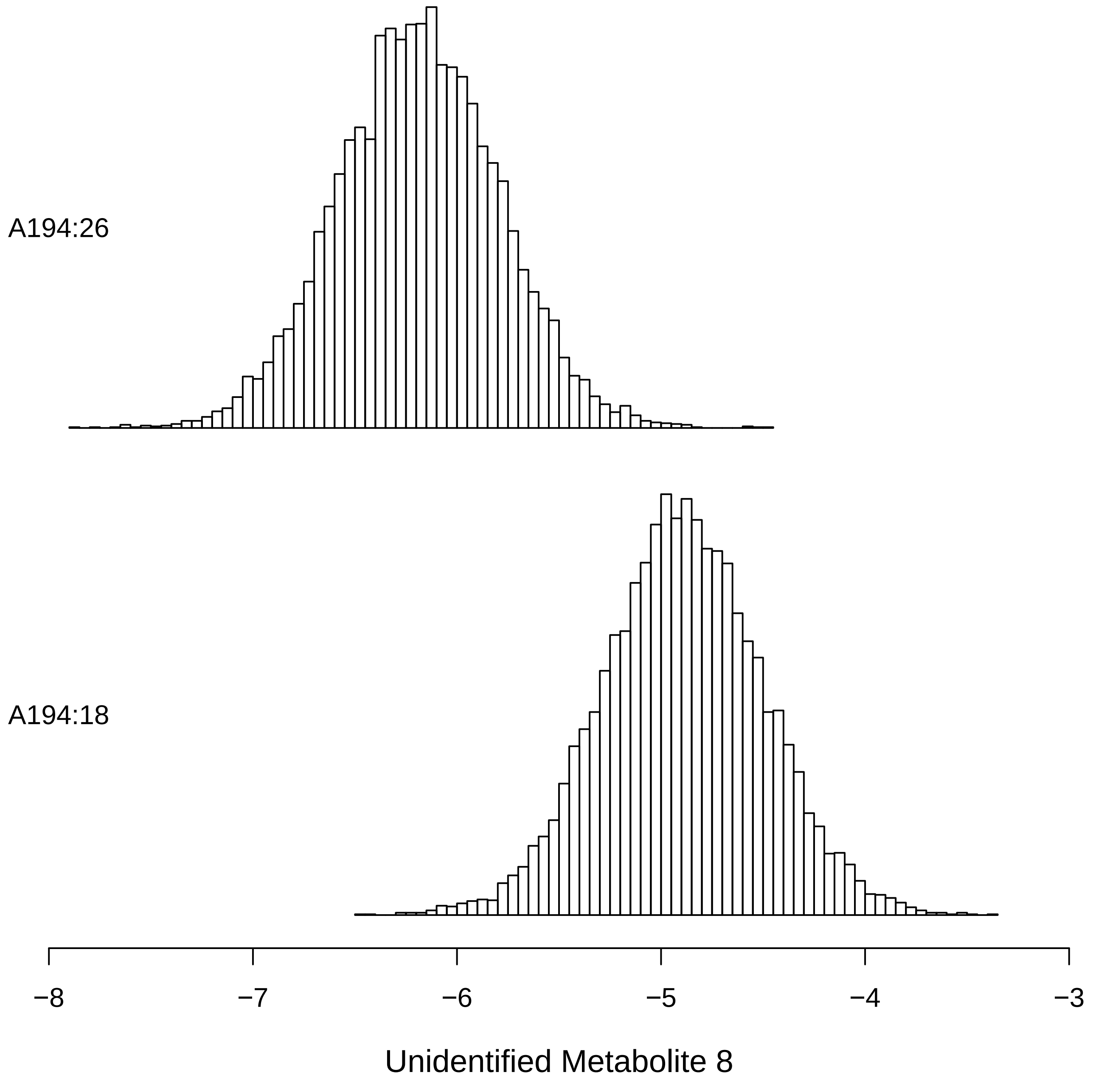
A194:26
A194:18
−8
−7
−6
−5
−4
−3
Unidentified Metabolite 8

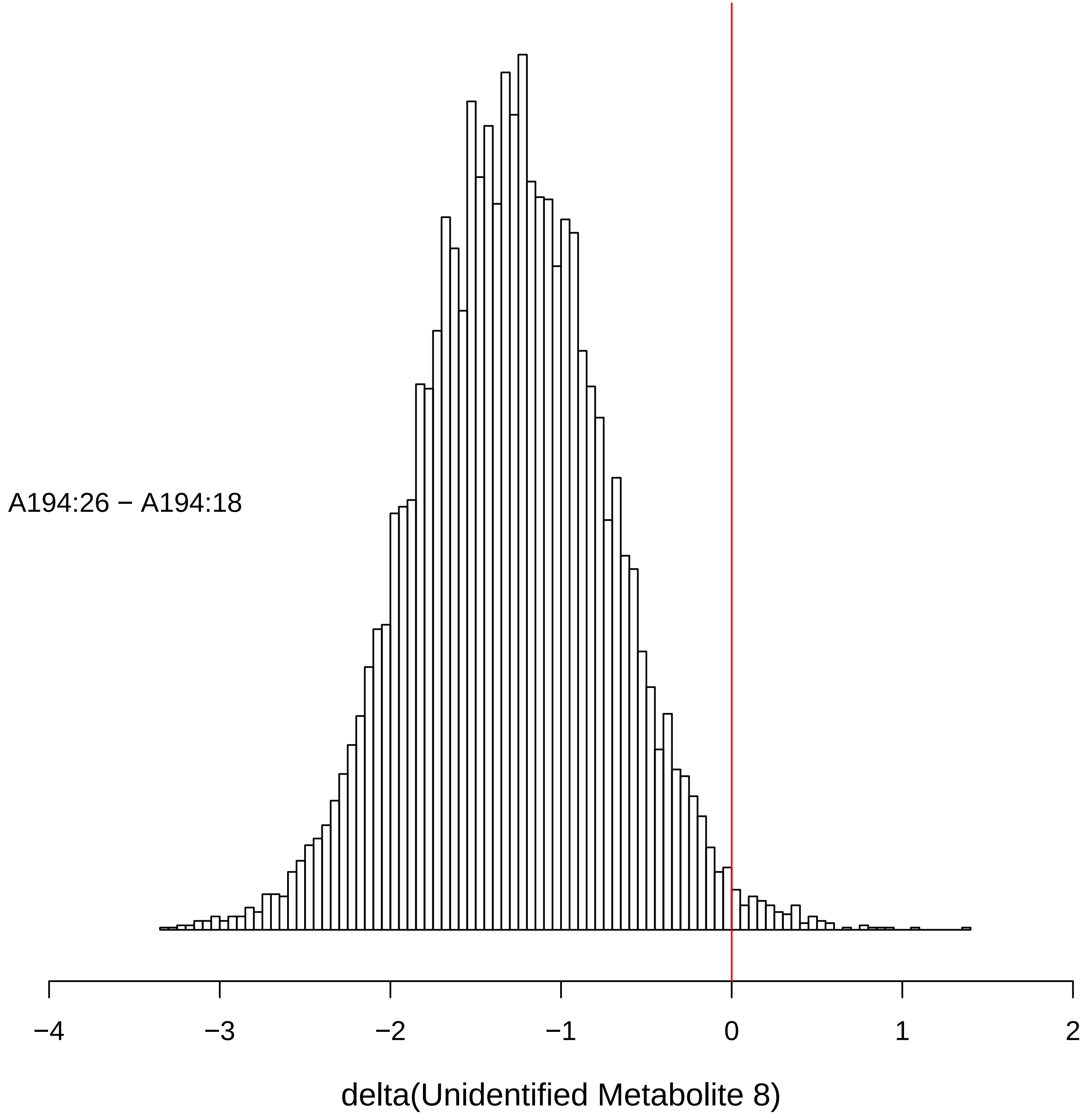

A194:26 − A194:18
−4
−3
−2
−1
0
1
2
delta(Unidentified Metabolite 8)

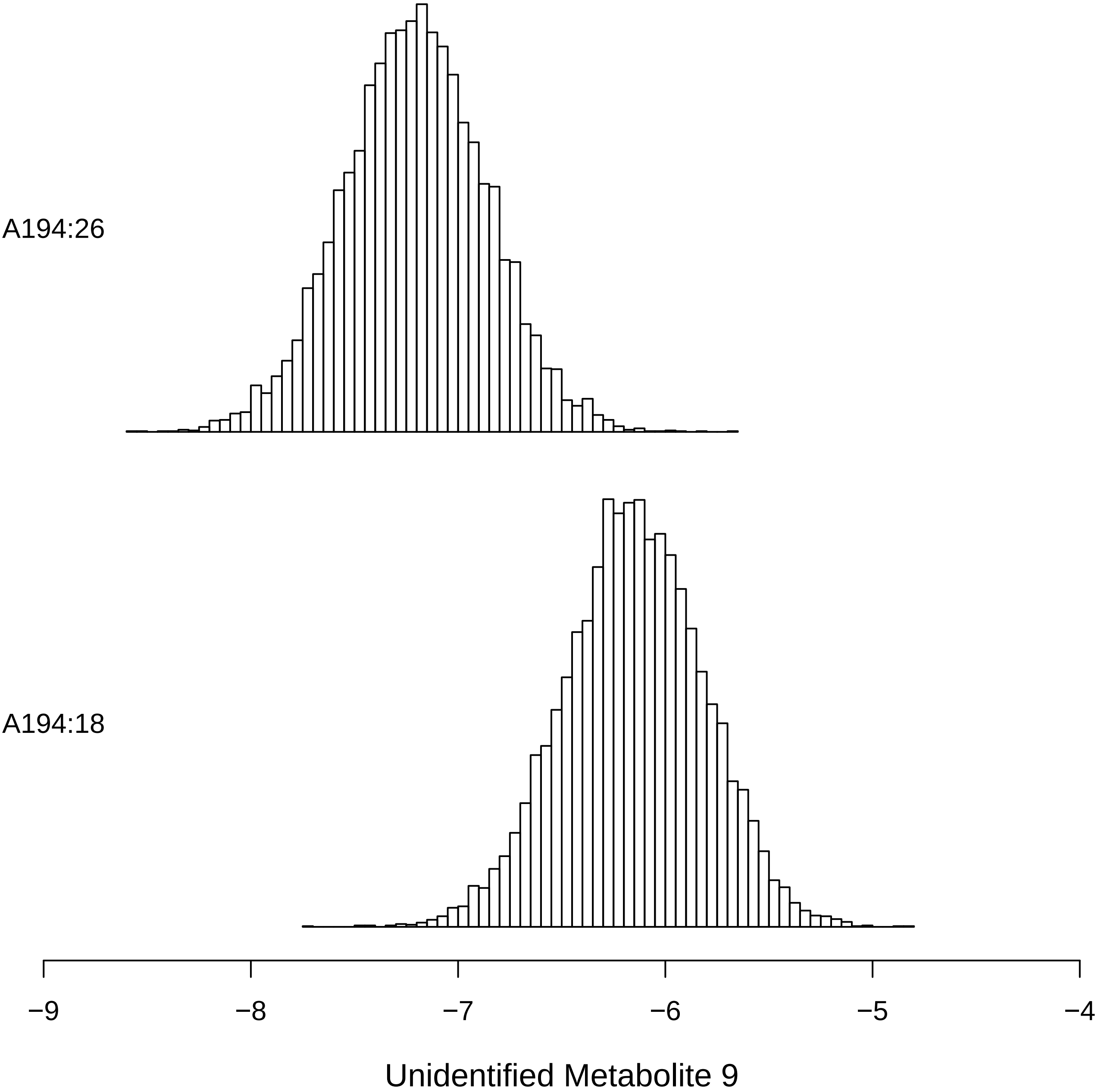

A194:26
A194:18
−9
−8
−7
−6
−5
−4
Unidentified Metabolite 9

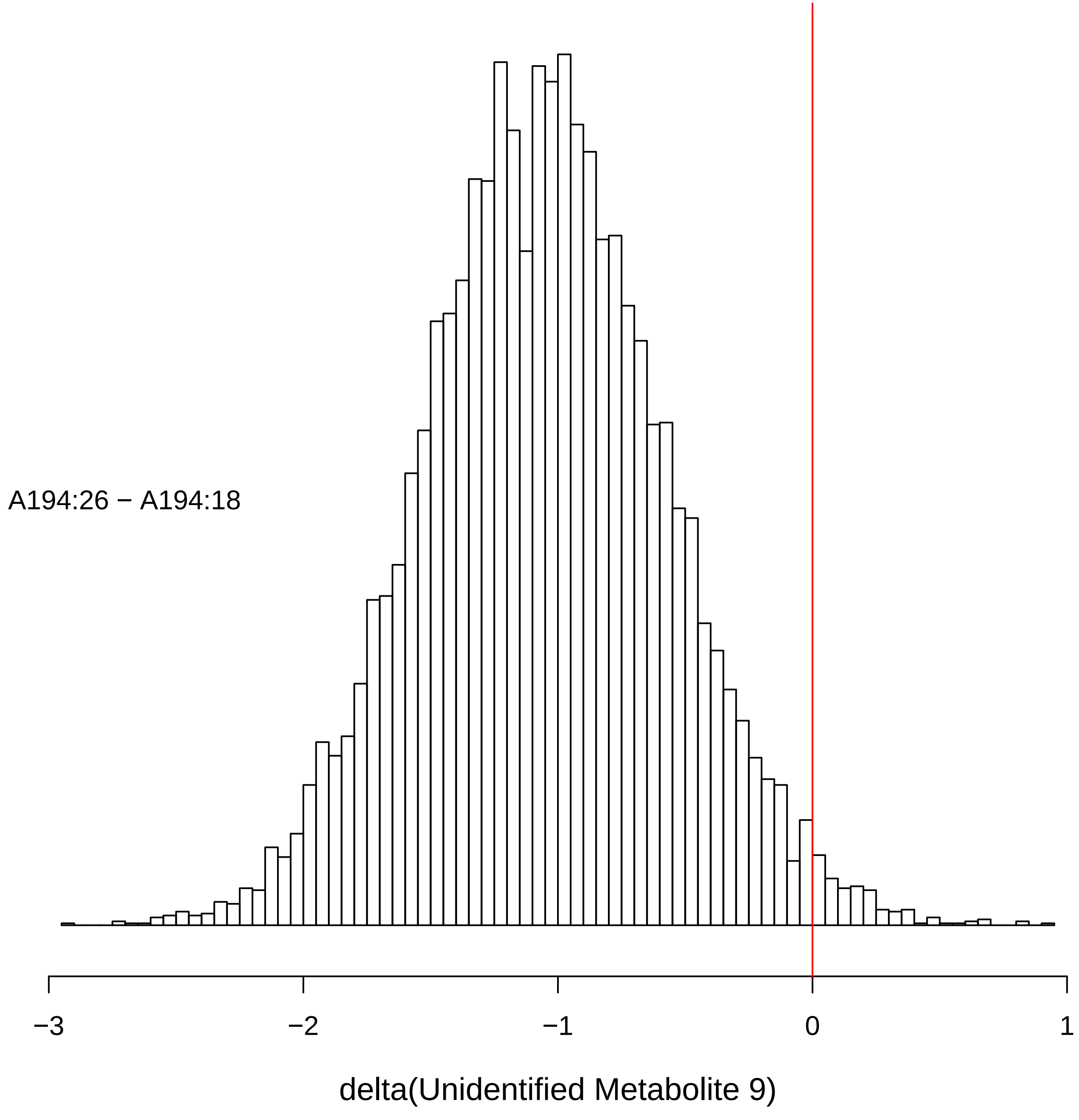
A194:26 − A194:18
−3
−2
−1
0
1
delta(Unidentified Metabolite 9)

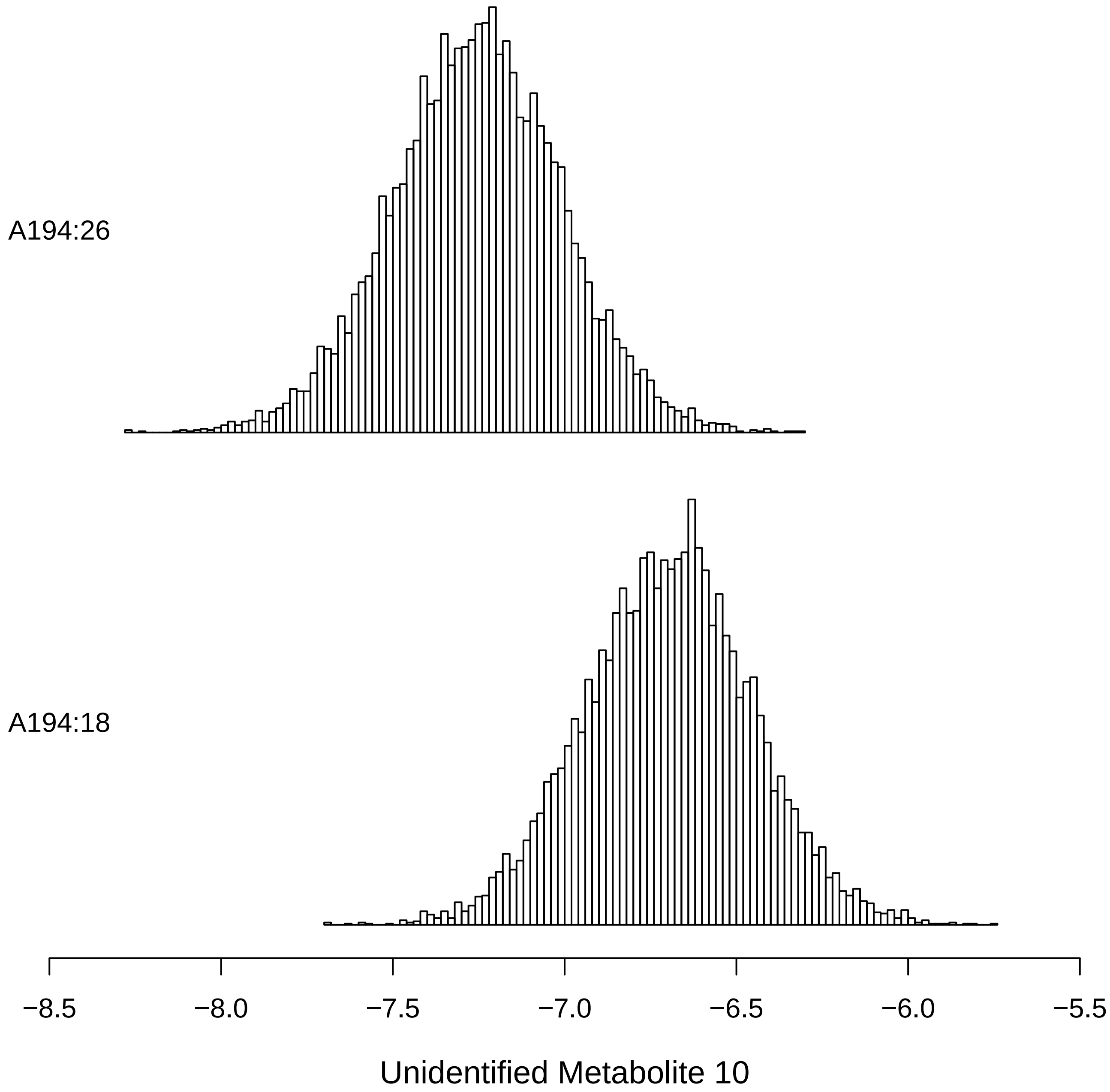
A194:26
A194:18
−8.5
−8.0
−7.5
−7.0
−6.5
−6.0
−5.5
Unidentified Metabolite 10

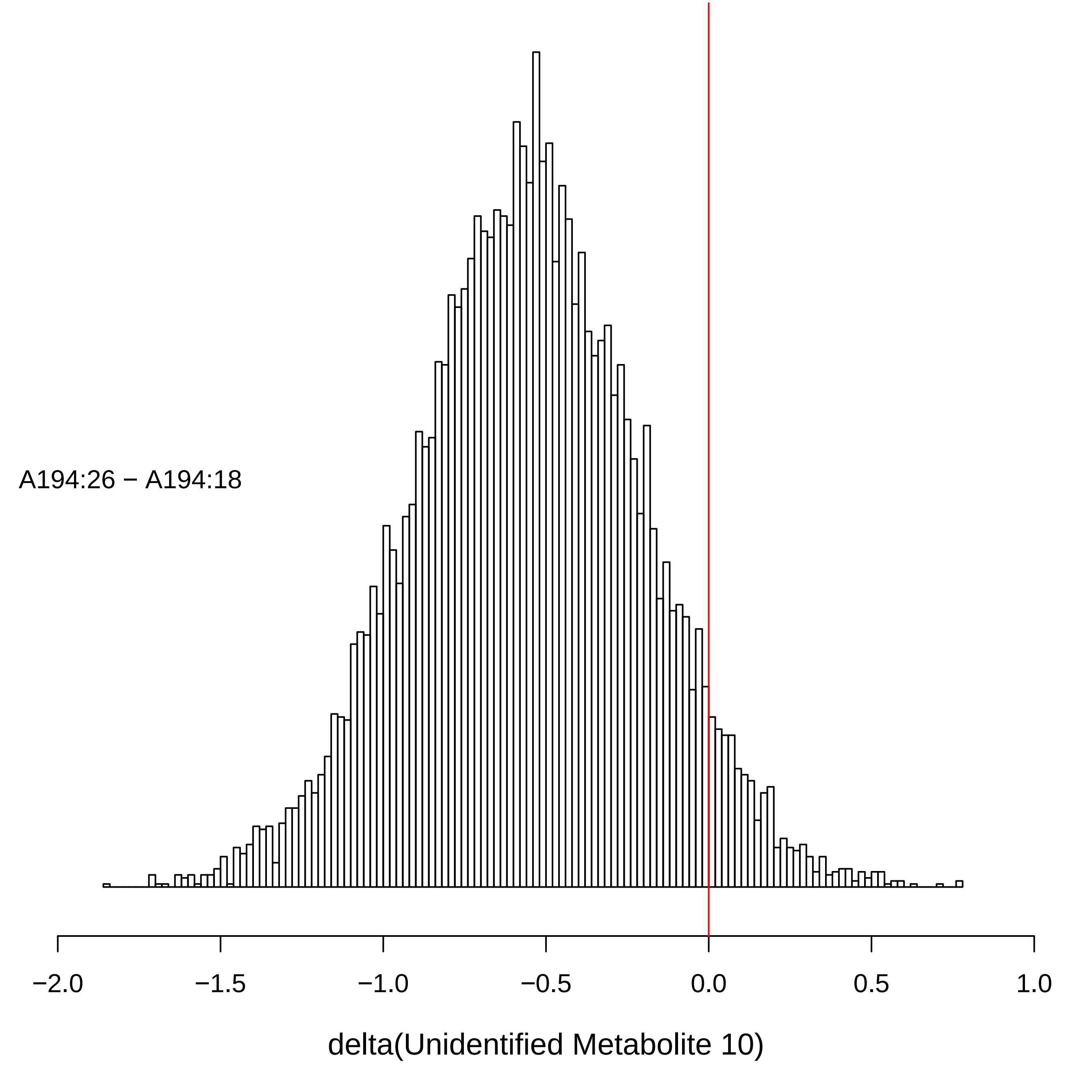

A194:26 − A194:18
−2.0
−1.5
−1.0
−0.5
0.0
0.5
1.0
delta(Unidentified Metabolite 10)

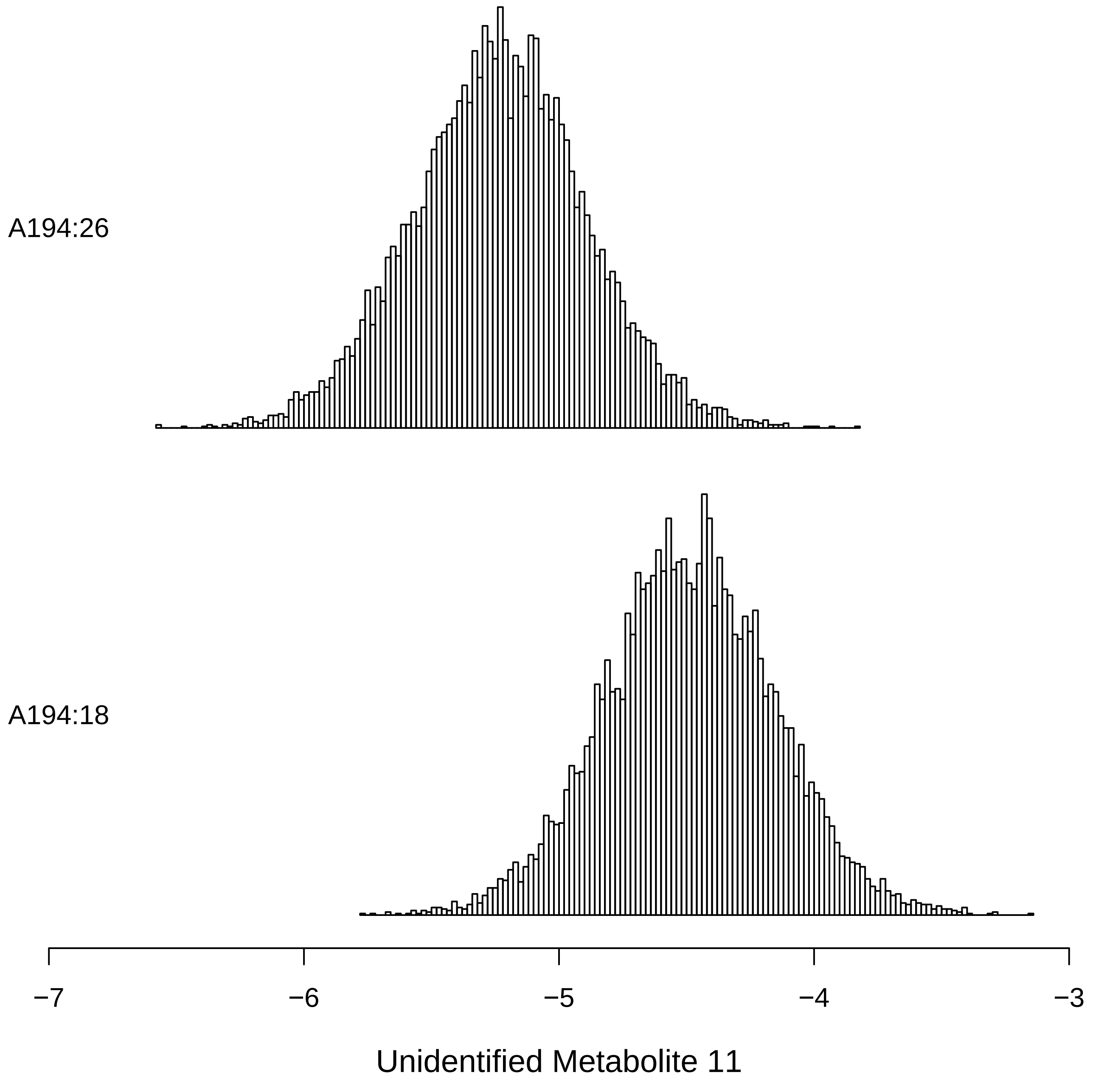

A194:26
A194:18
−7
−6
−5
−4
−3
Unidentified Metabolite 11

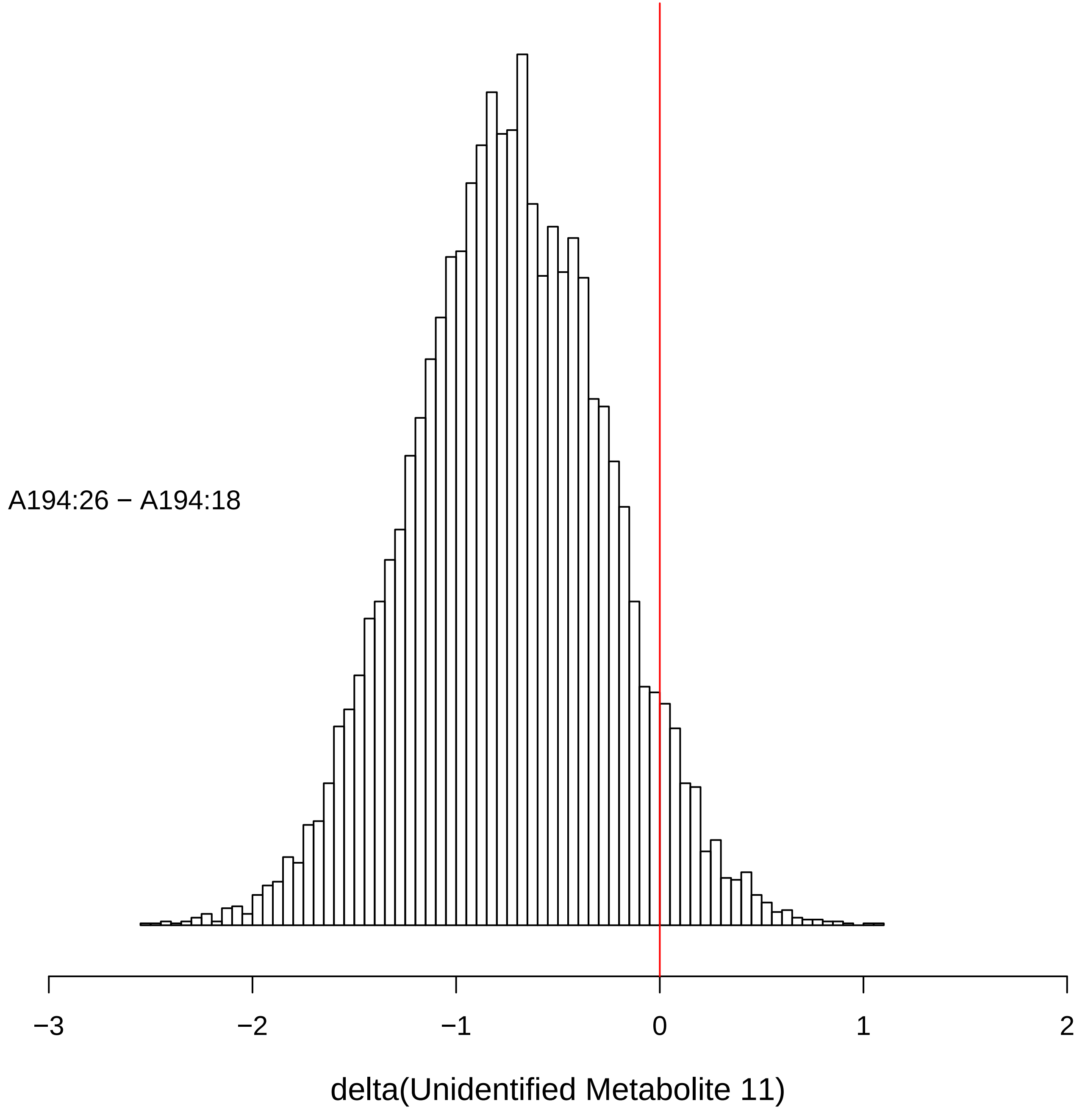
A194:26 − A194:18
−3
−2
−1
0
1
2
delta(Unidentified Metabolite 11)

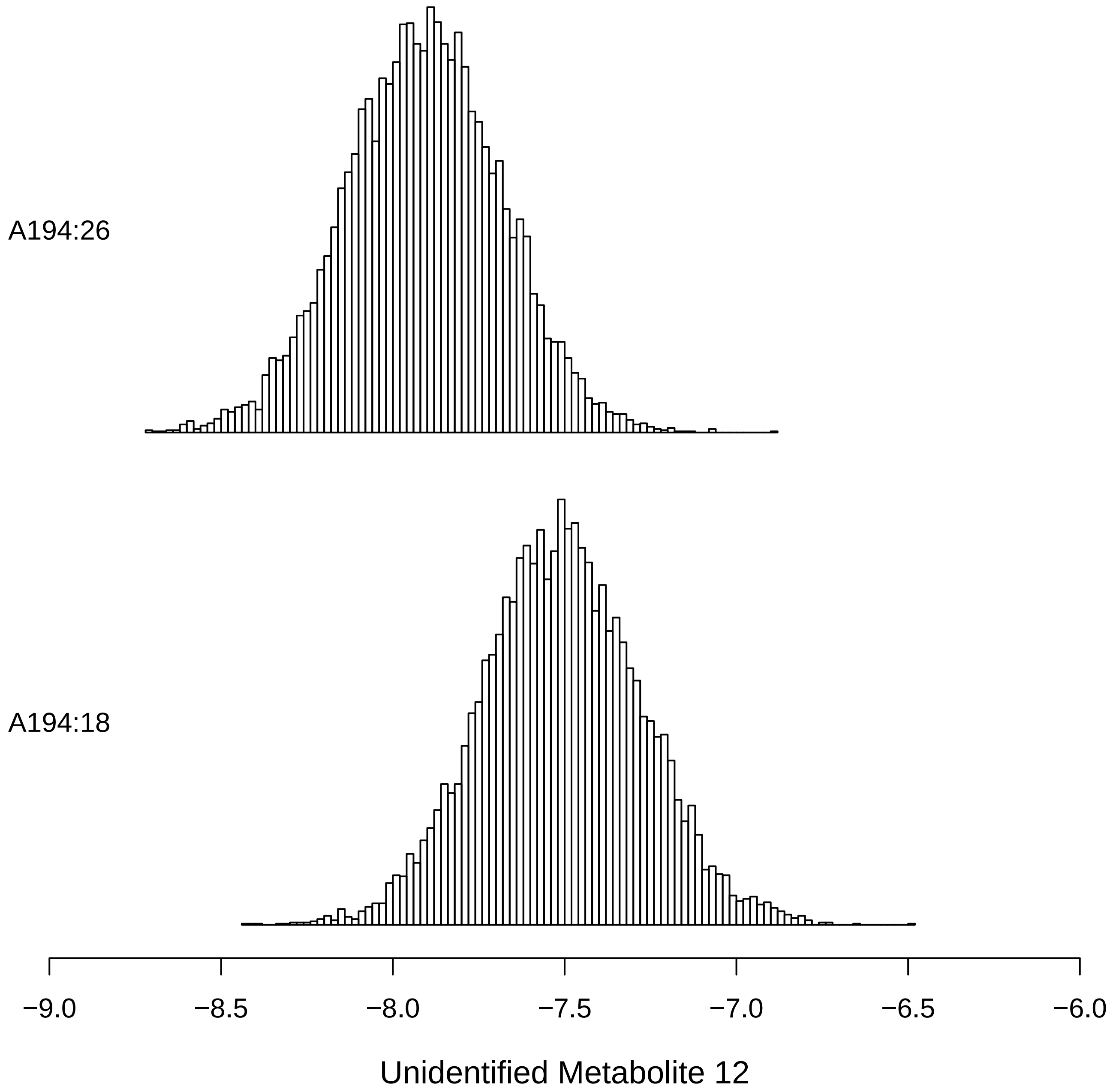
A194:26
A194:18
−9.0
−8.5
−8.0
−7.5
−7.0
−6.5
−6.0
Unidentified Metabolite 12

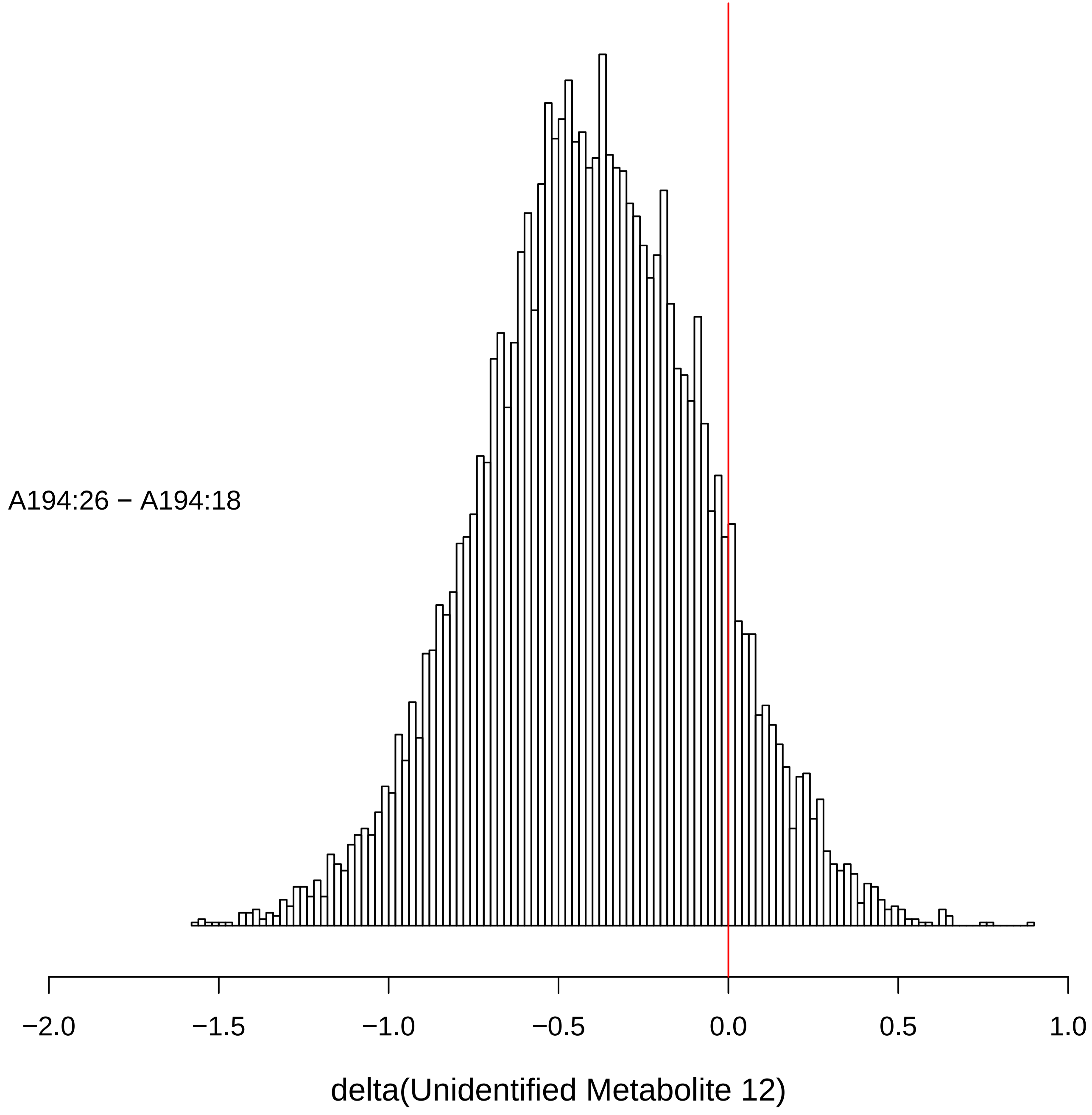
A194:26 − A194:18
−2.0
−1.5
−1.0
−0.5
0.0
0.5
1.0
delta(Unidentified Metabolite 12)

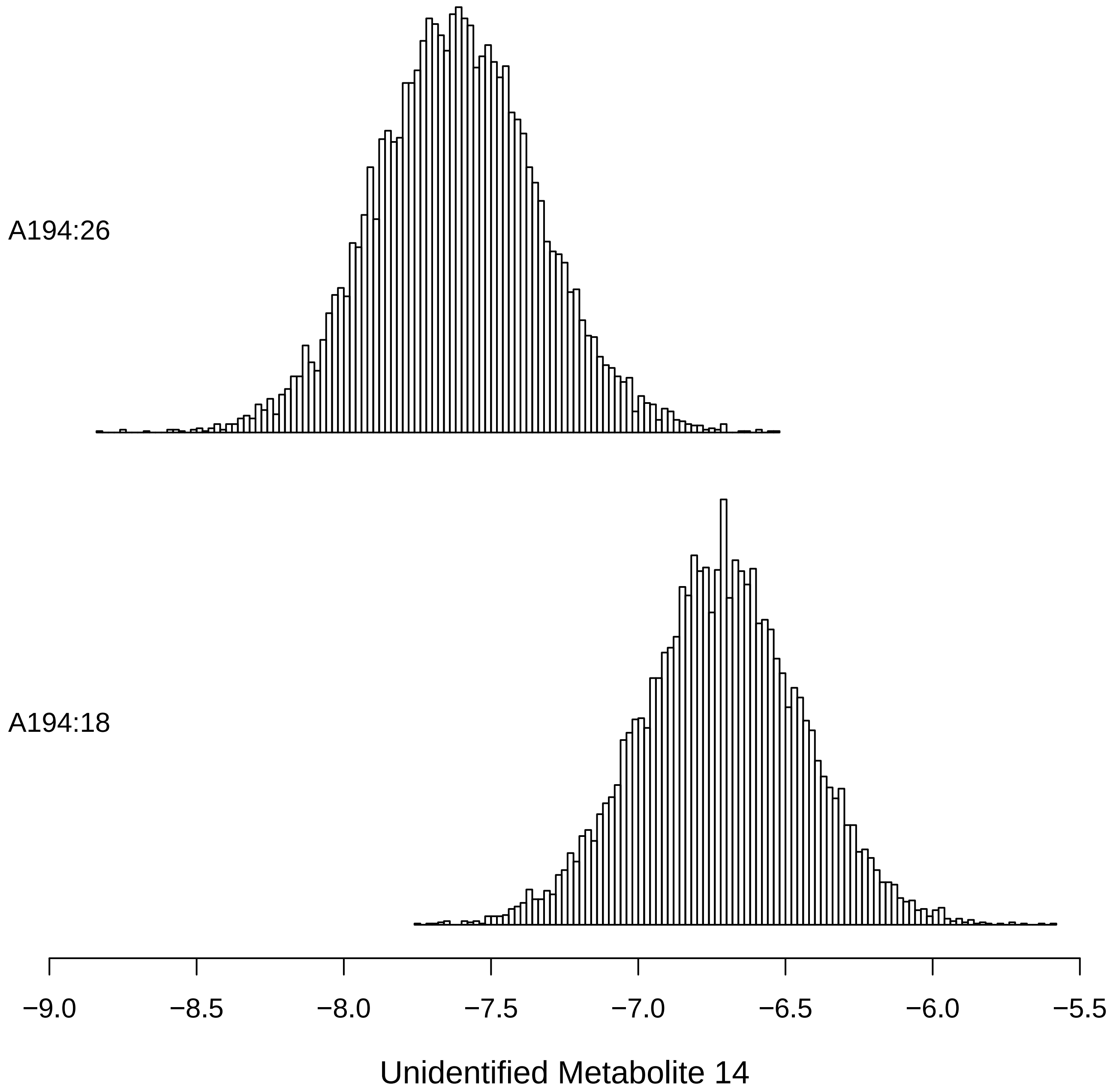
A194:26
A194:18
−9.0
−8.5
−8.0
−7.5
−7.0
−6.5
−6.0
−5.5
Unidentified Metabolite 14

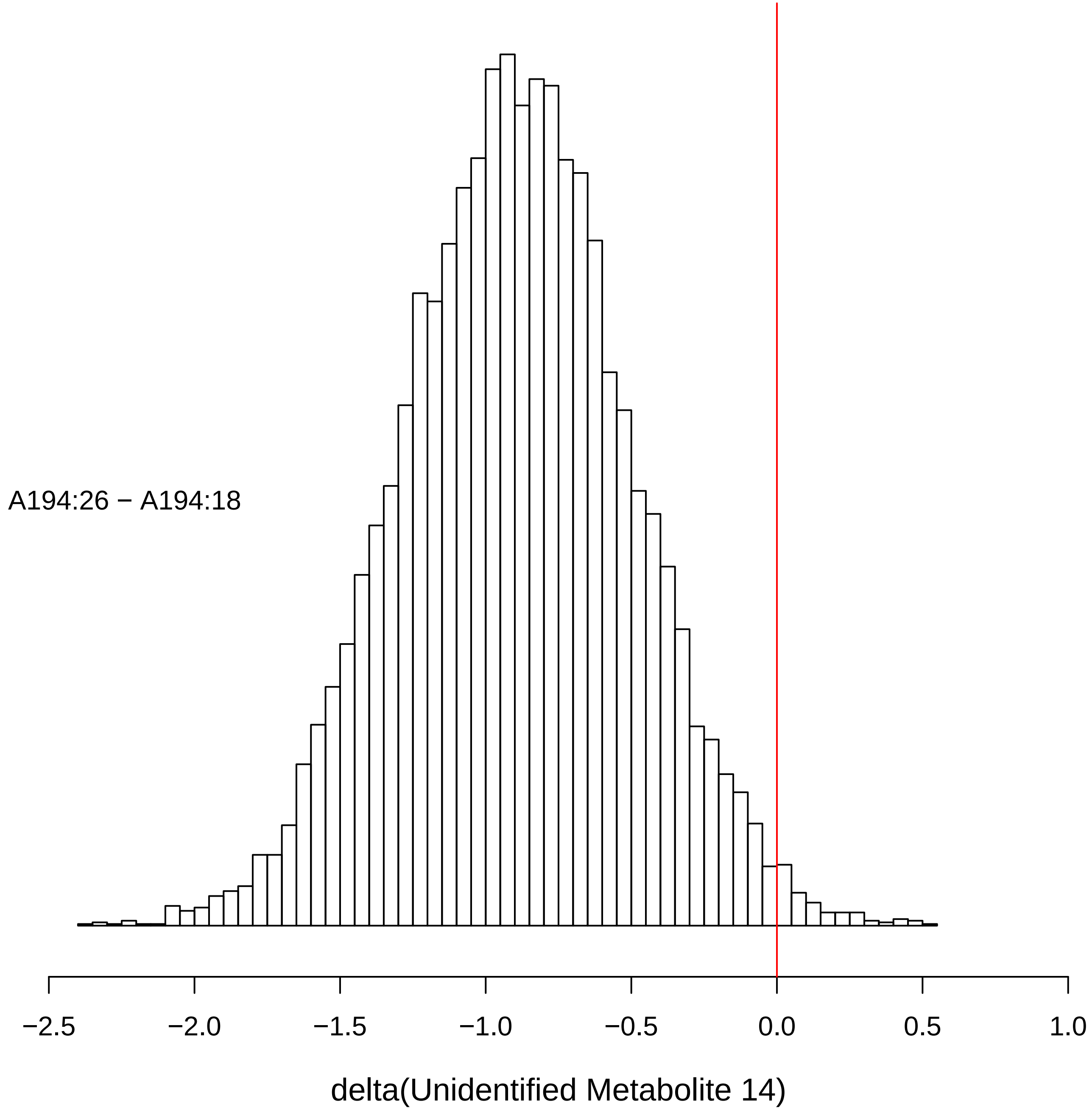

A194:26 − A194:18
−2.5
−2.0
−1.5
−1.0
−0.5
0.0
0.5
1.0
delta(Unidentified Metabolite 14)

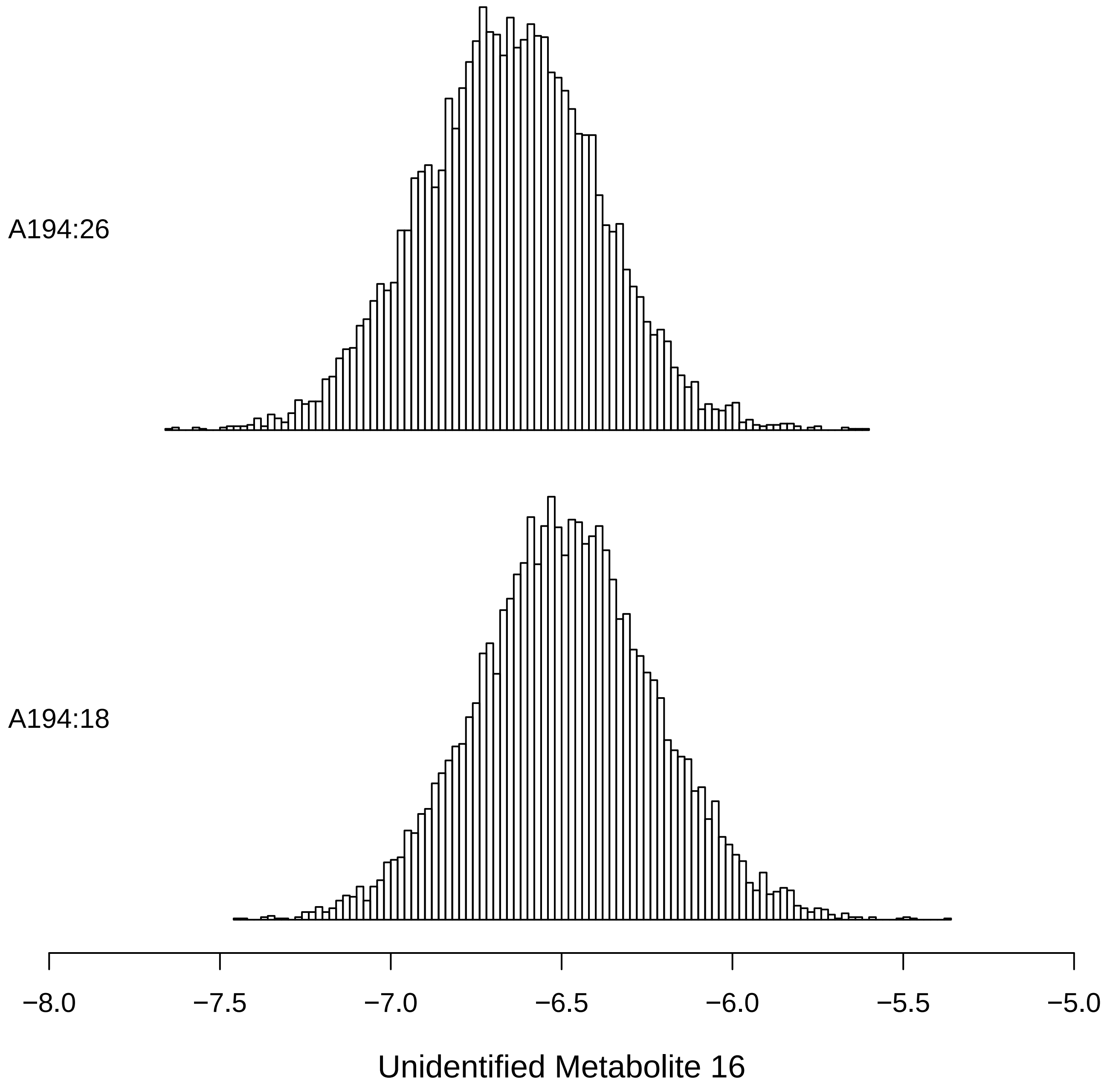
A194:26
A194:18
−8.0
−7.5
−7.0
−6.5
−6.0
−5.5
−5.0
Unidentified Metabolite 16

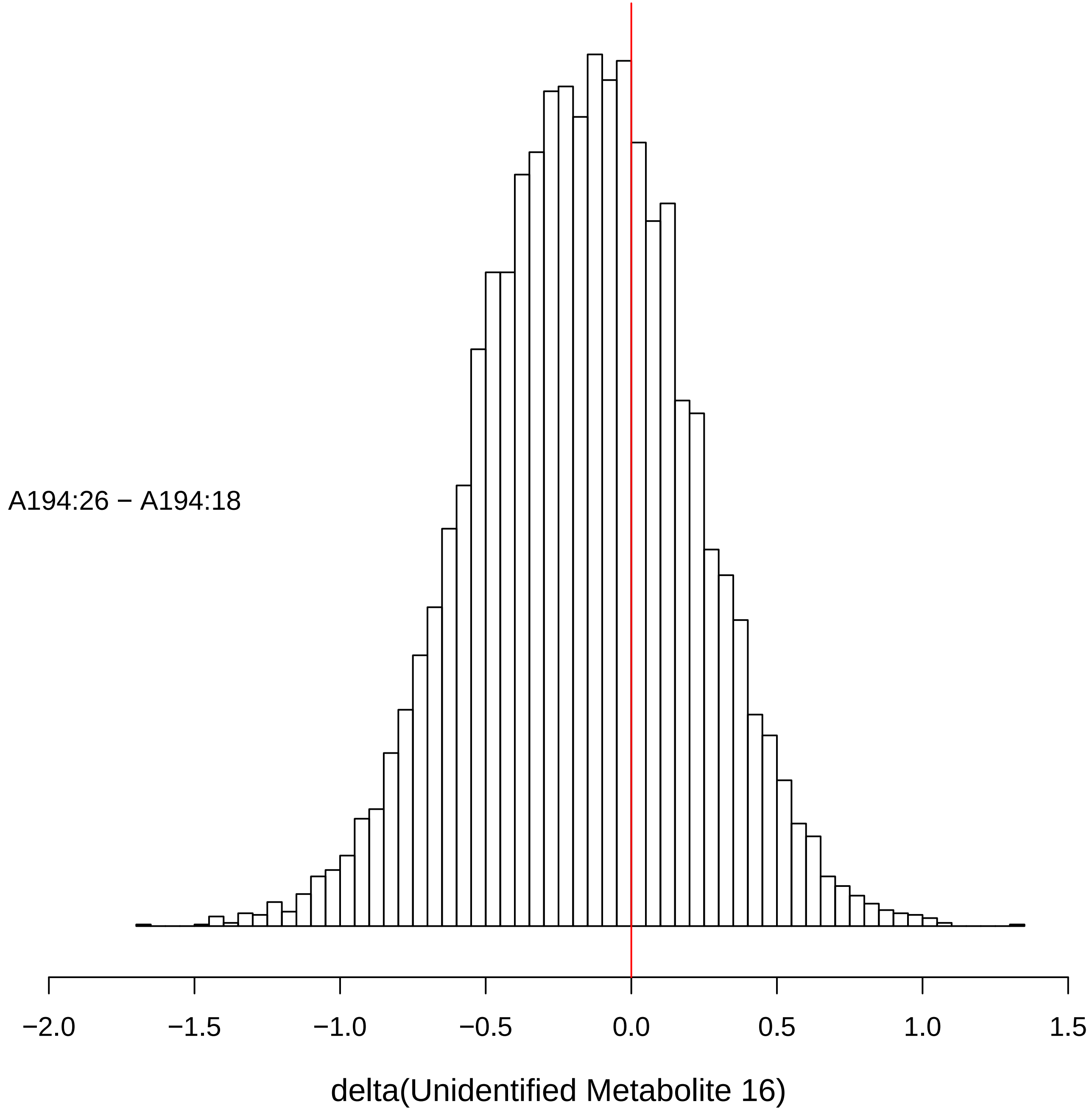
A194:26 − A194:18
−2.0
−1.5
−1.0
−0.5
0.0
0.5
1.0
1.5
delta(Unidentified Metabolite 16)

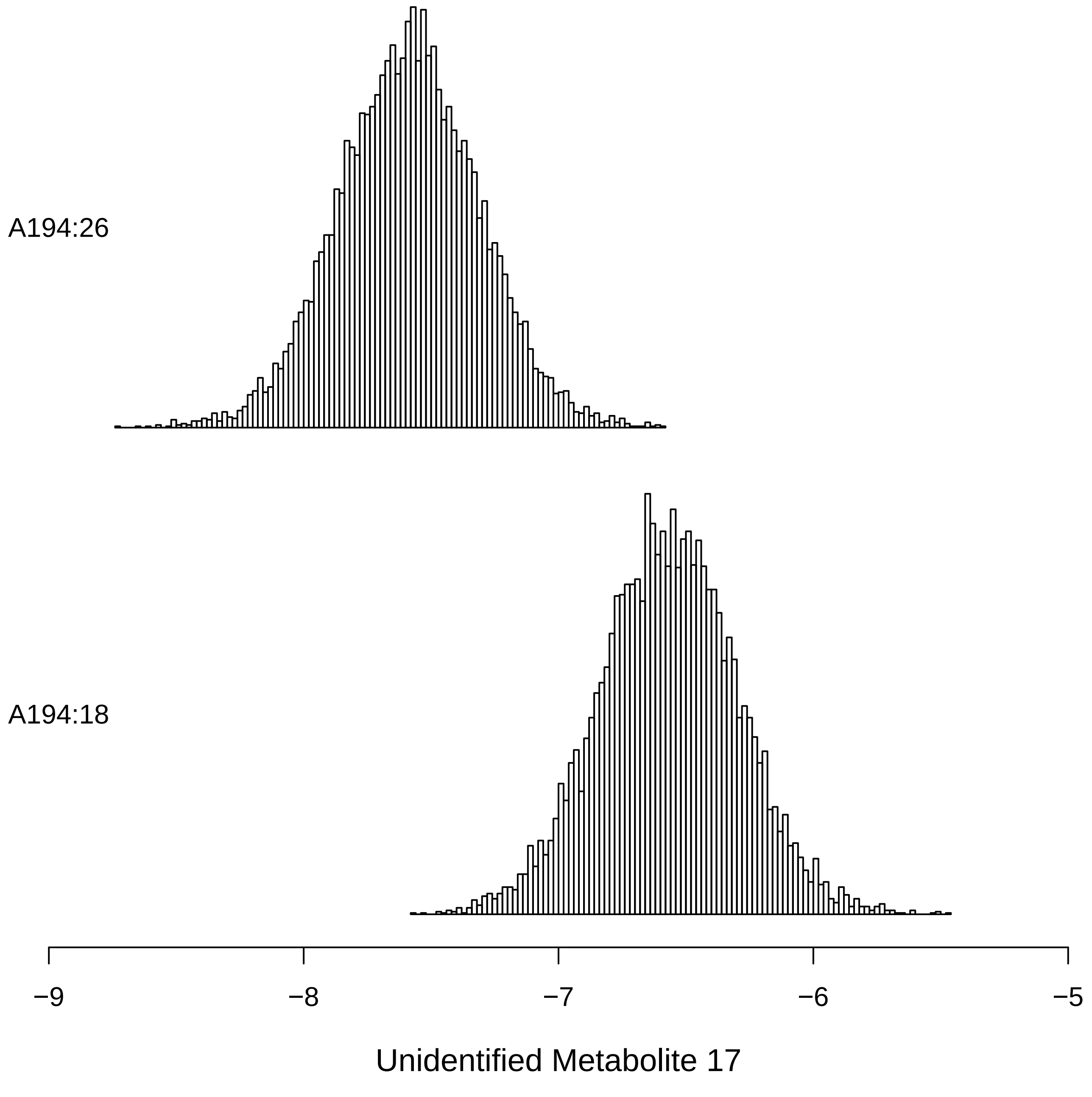
A194:26
A194:18
−9
−8
−7
−6
−5
Unidentified Metabolite 17

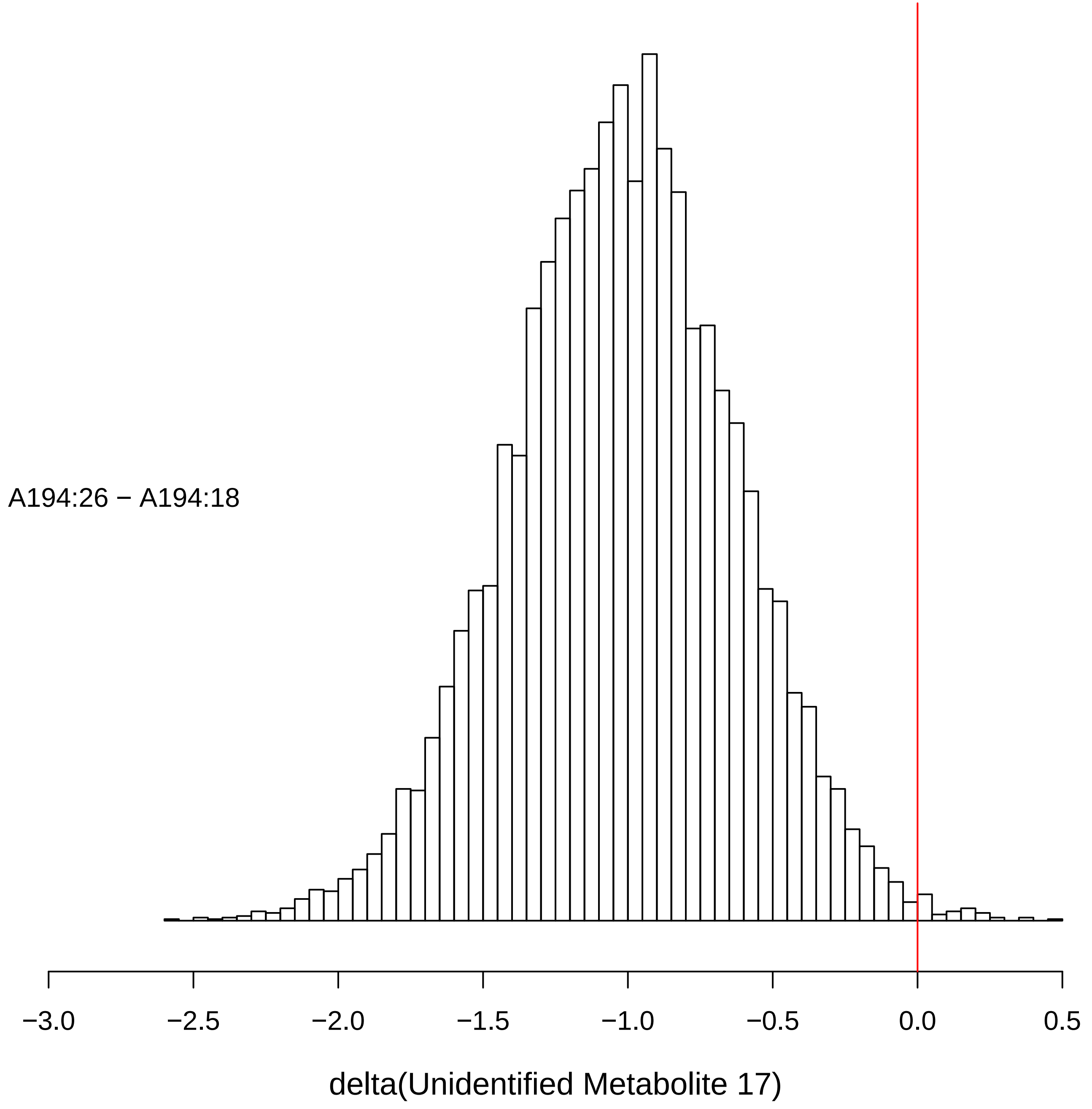
A194:26 − A194:18
−3.0
−2.5
−2.0
−1.5
−1.0
−0.5
0.0
0.5
delta(Unidentified Metabolite 17)

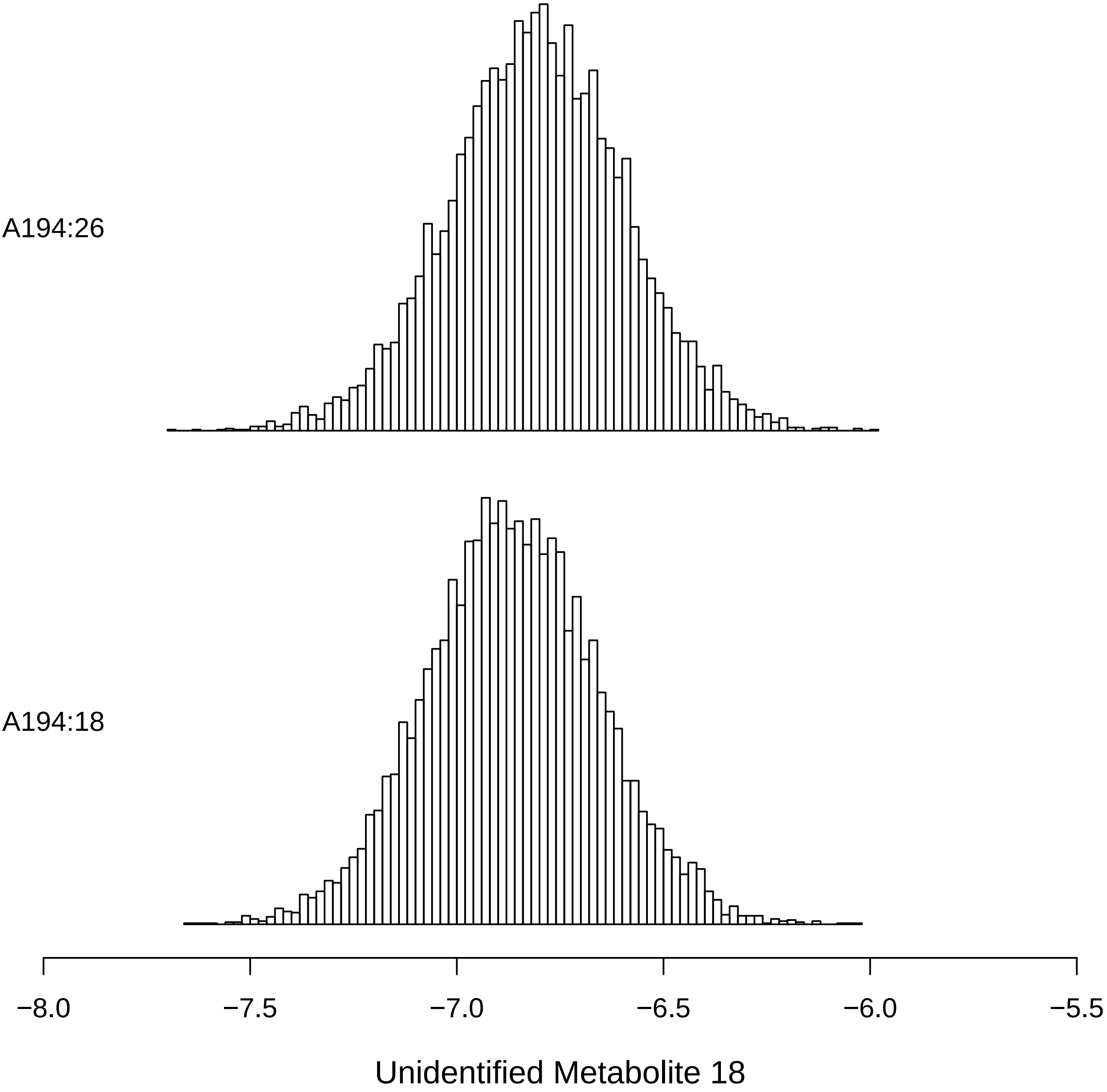
A194:26
A194:18
−8.0
−7.5
−7.0
−6.5
−6.0
−5.5
Unidentified Metabolite 18

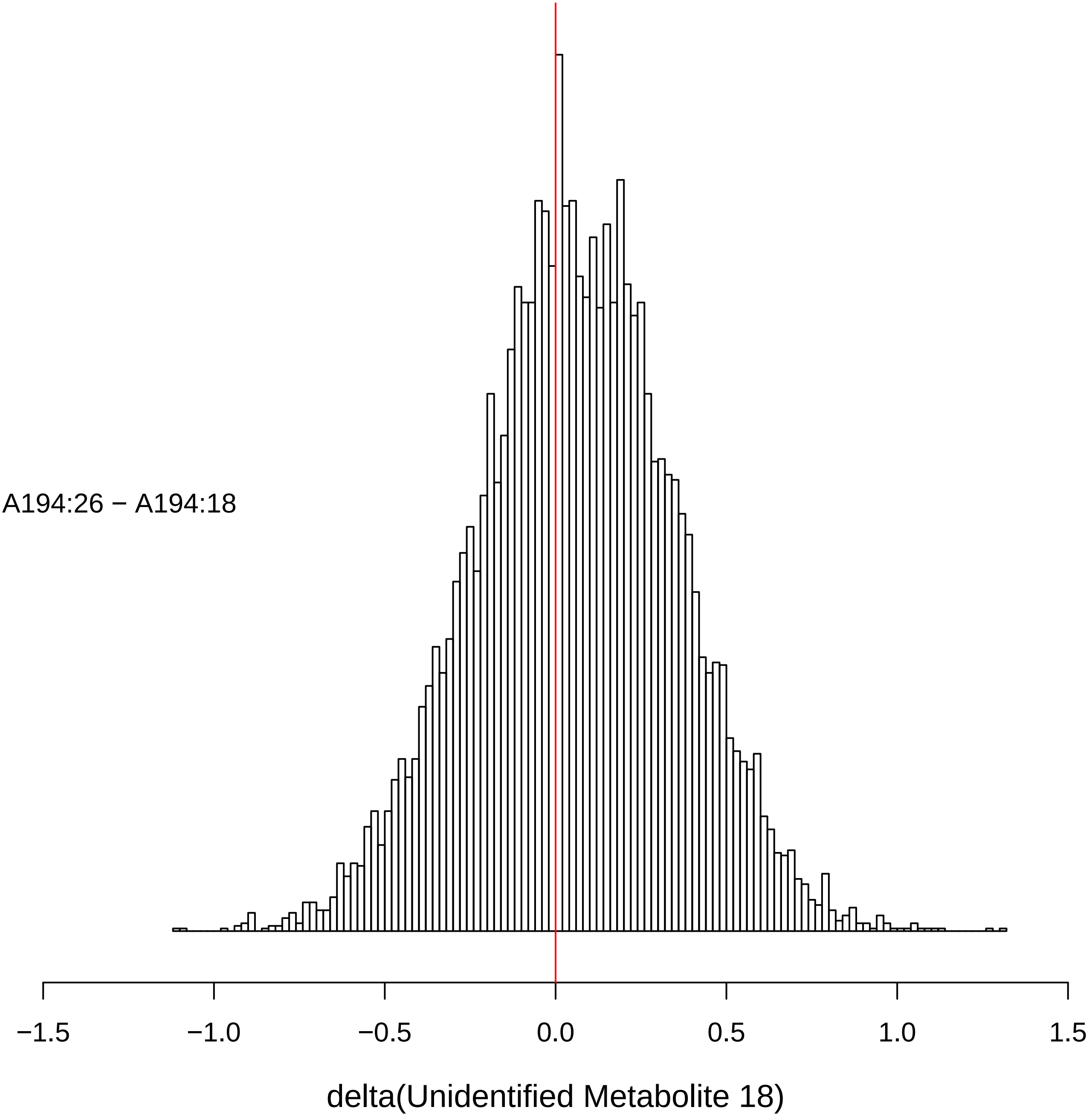
A194:26 − A194:18
−1.5
−1.0
−0.5
0.0
0.5
1.0
1.5
delta(Unidentified Metabolite 18)

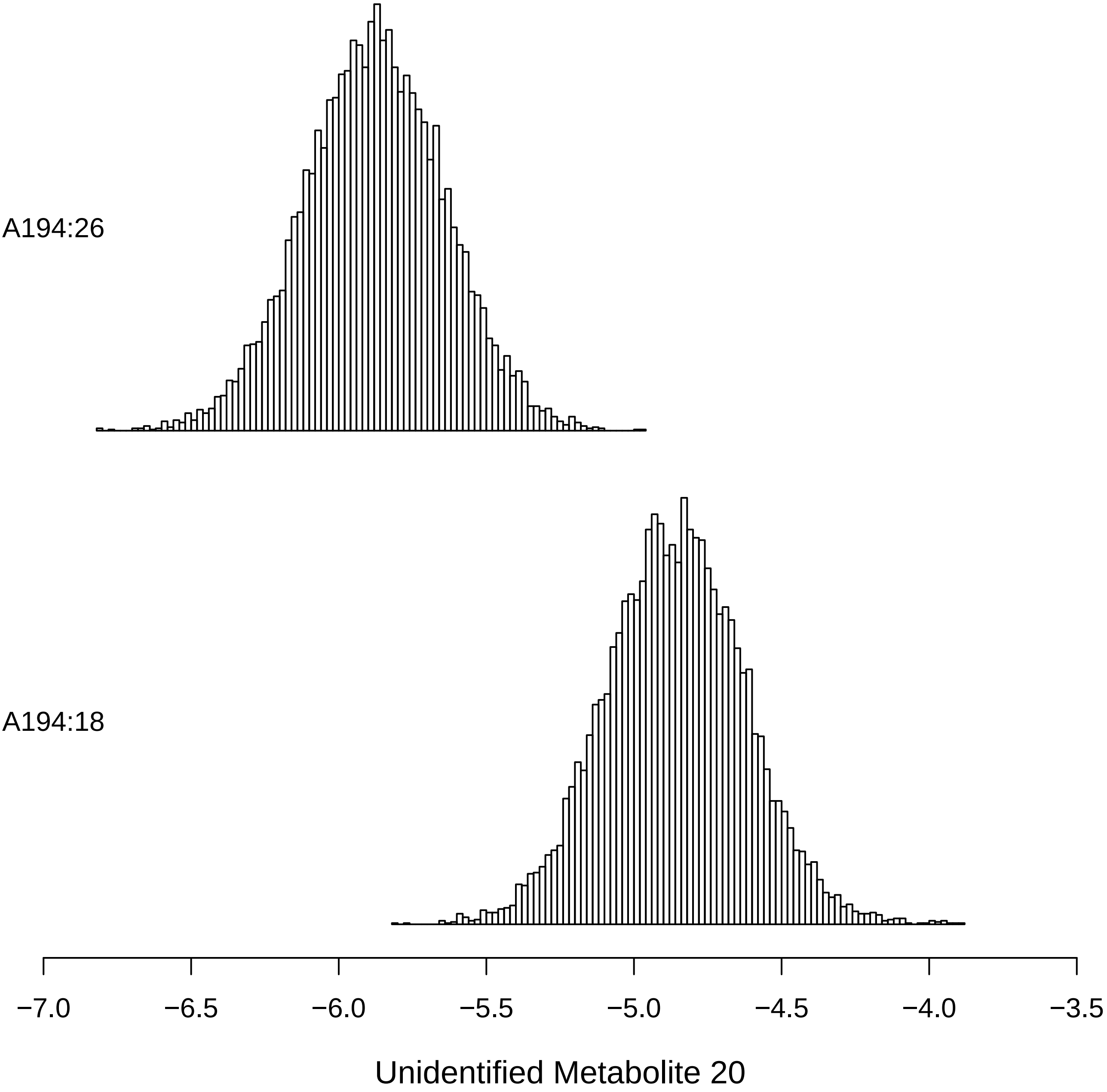

A194:26
A194:18
−7.0
−6.5
−6.0
−5.5
−5.0
−4.5
−4.0
−3.5
Unidentified Metabolite 20

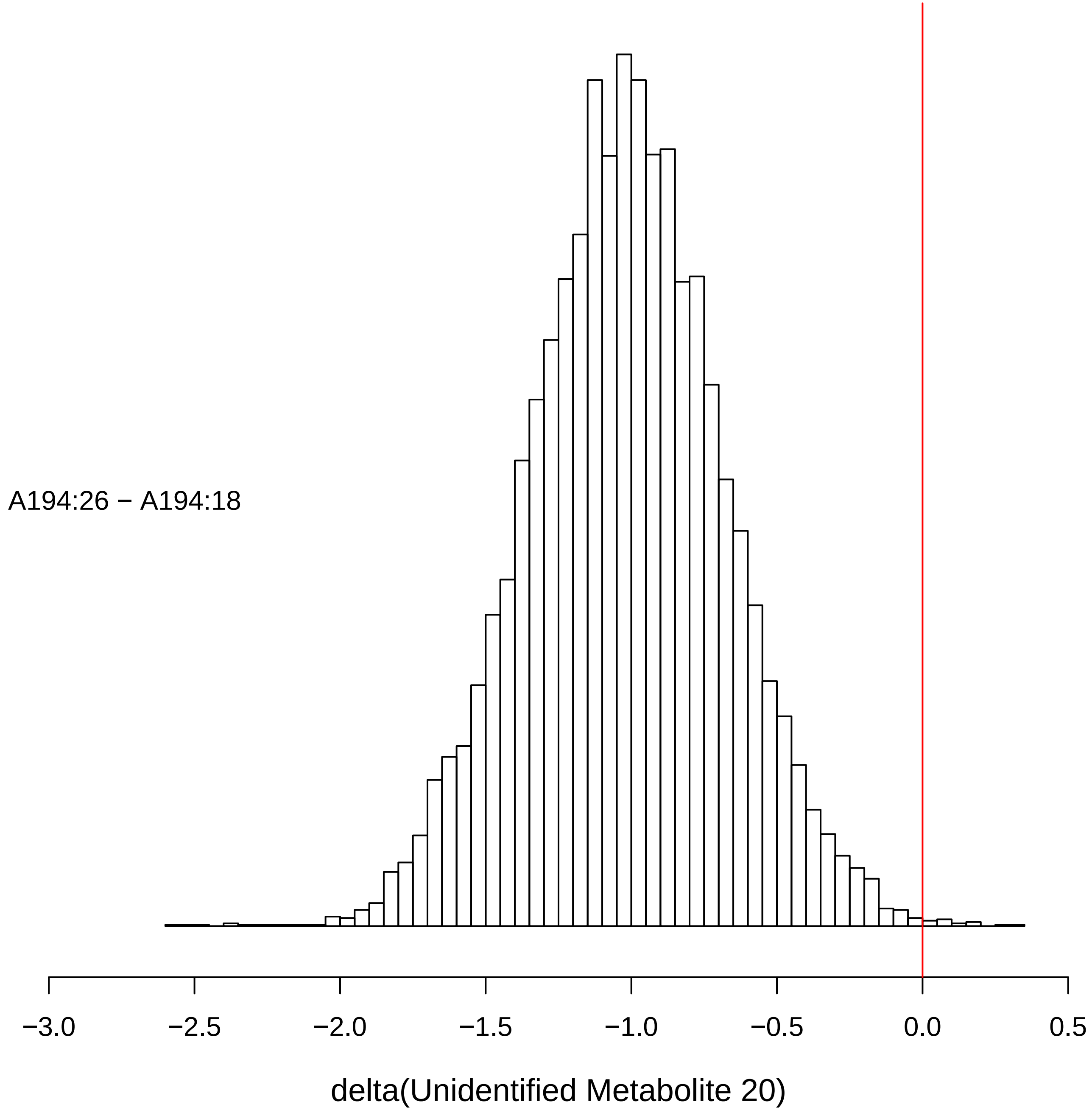
A194:26 − A194:18
−3.0
−2.5
−2.0
−1.5
−1.0
−0.5
0.0
0.5
delta(Unidentified Metabolite 20)

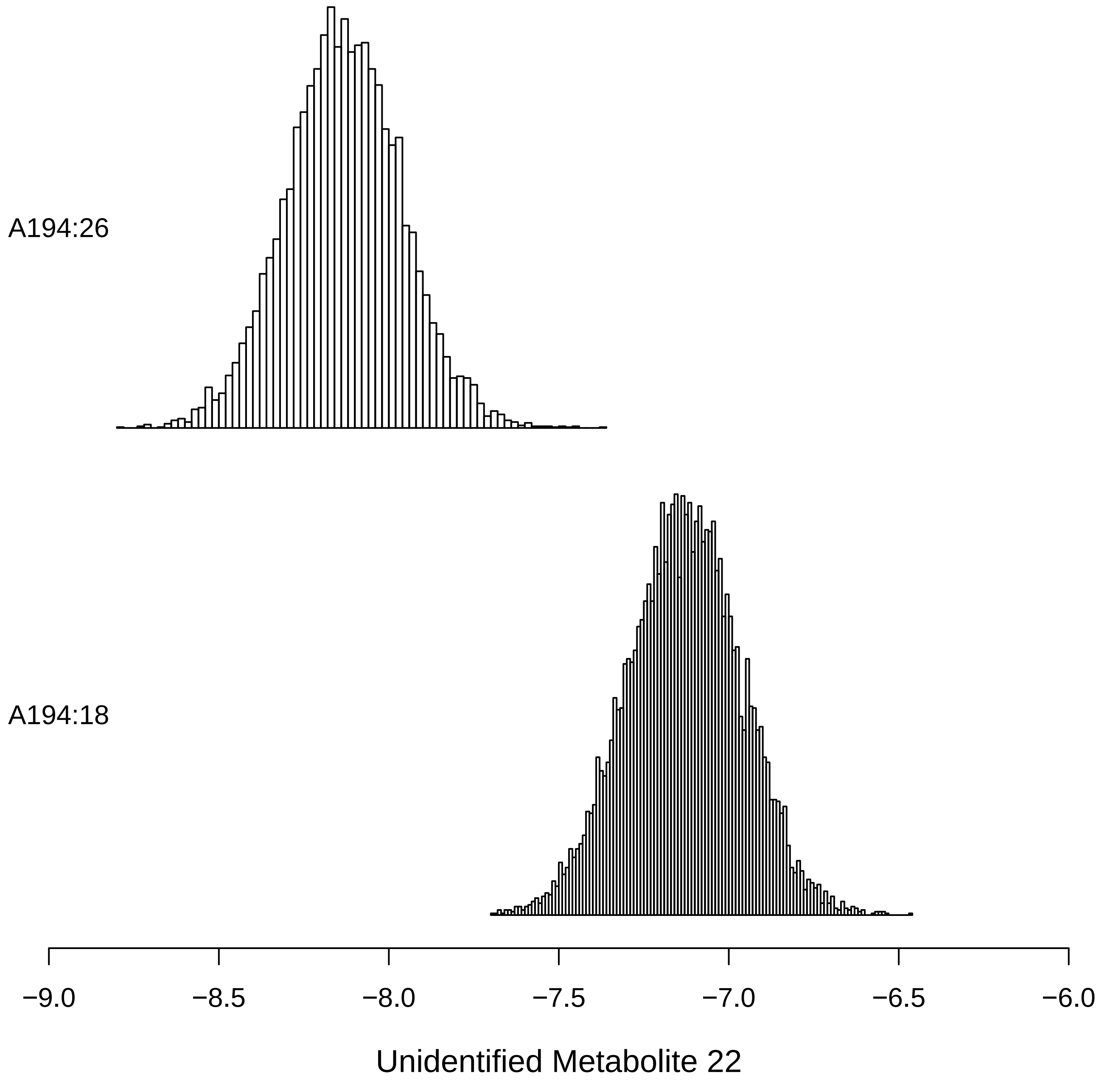
A194:26
A194:18
−9.0
−8.5
−8.0
−7.5
−7.0
−6.5
−6.0
Unidentified Metabolite 22

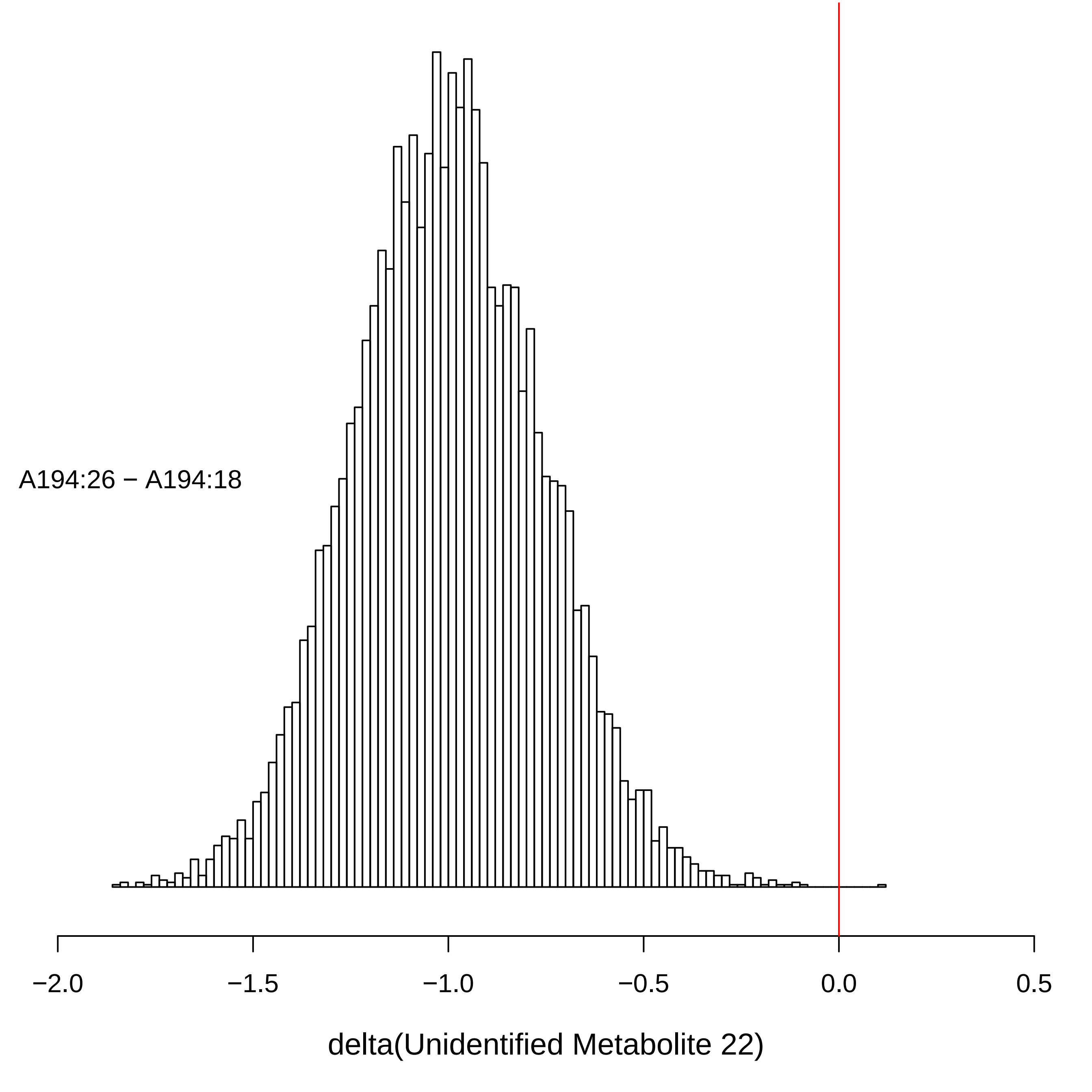
A194:26 − A194:18
−2.0
−1.5
−1.0
−0.5
0.0
0.5
delta(Unidentified Metabolite 22)

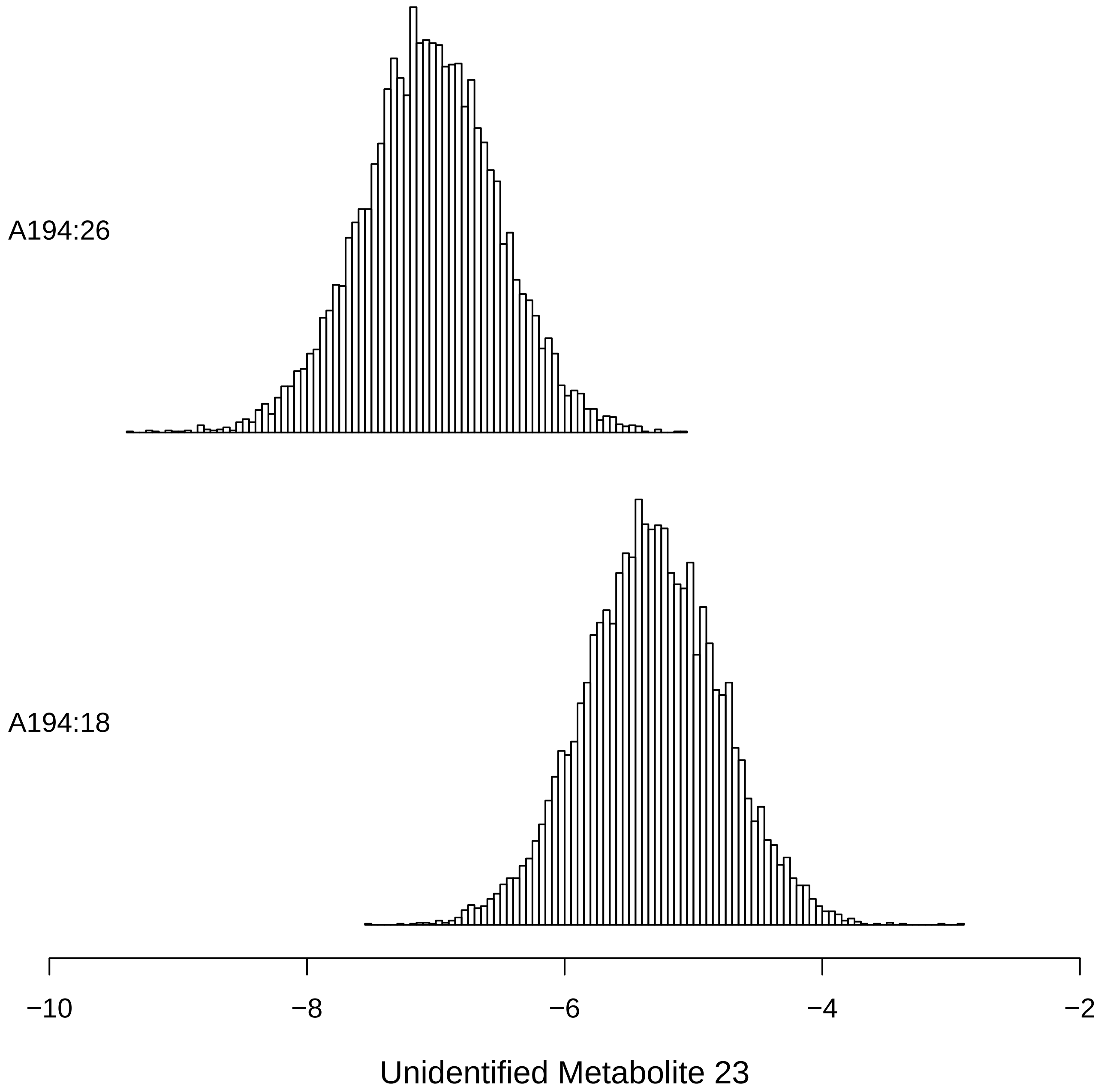
A194:26
A194:18
−10
−8
−6
−4
−2
Unidentified Metabolite 23

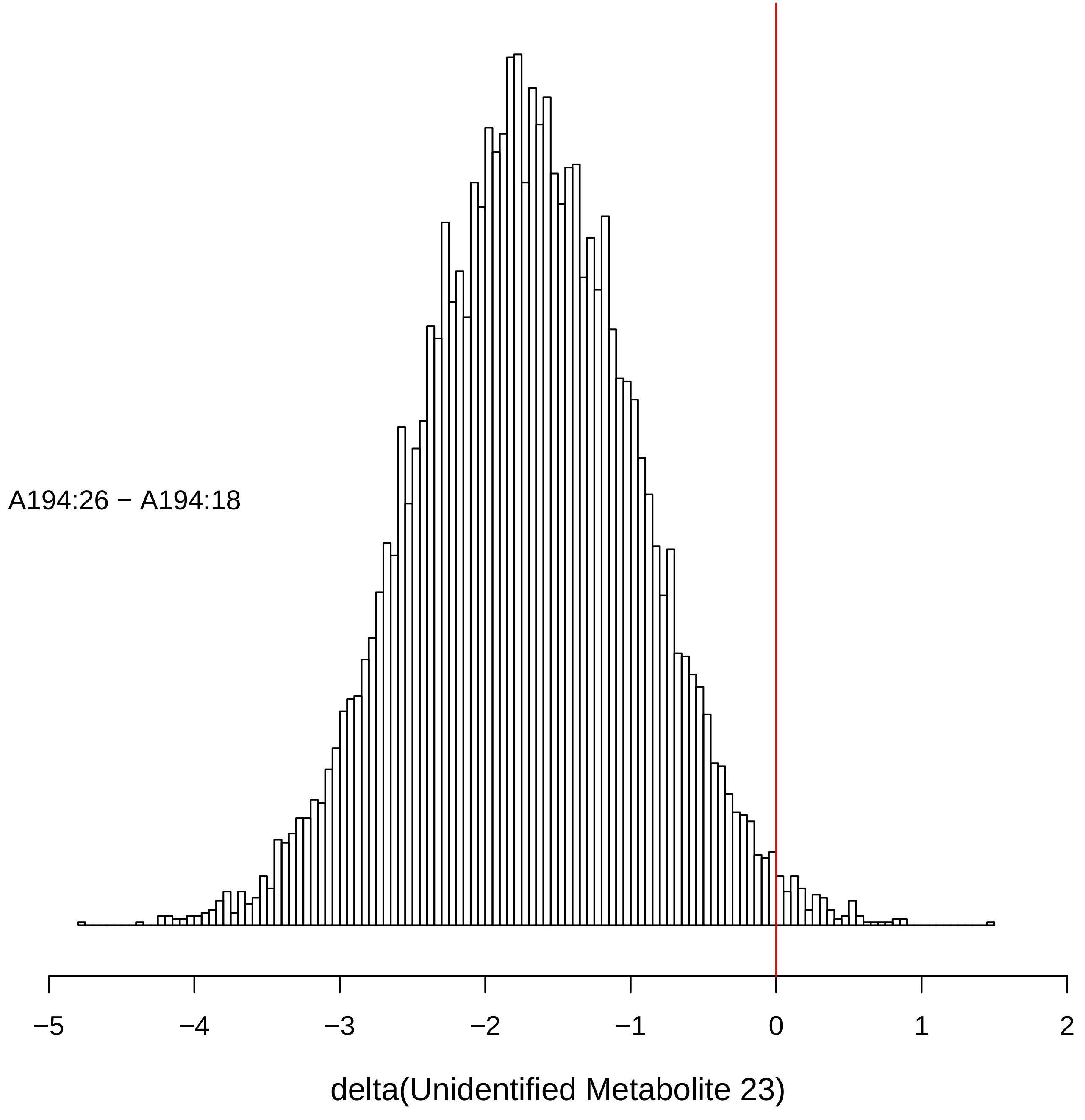
A194:26 − A194:18
−5
−4
−3
−2
−1
0
1
2
delta(Unidentified Metabolite 23)

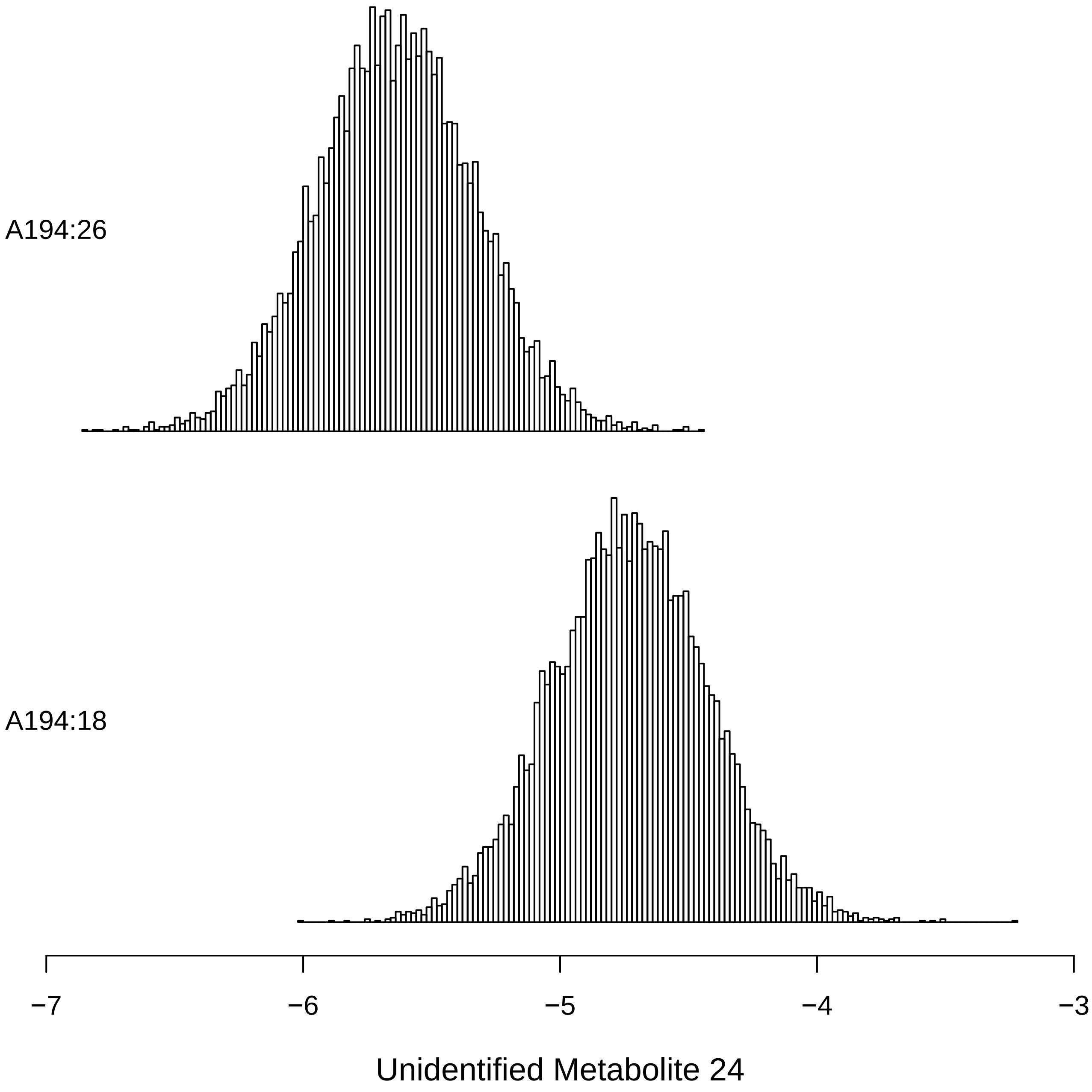

A194:26
A194:18
−7
−6
−5
−4
−3
Unidentified Metabolite 24

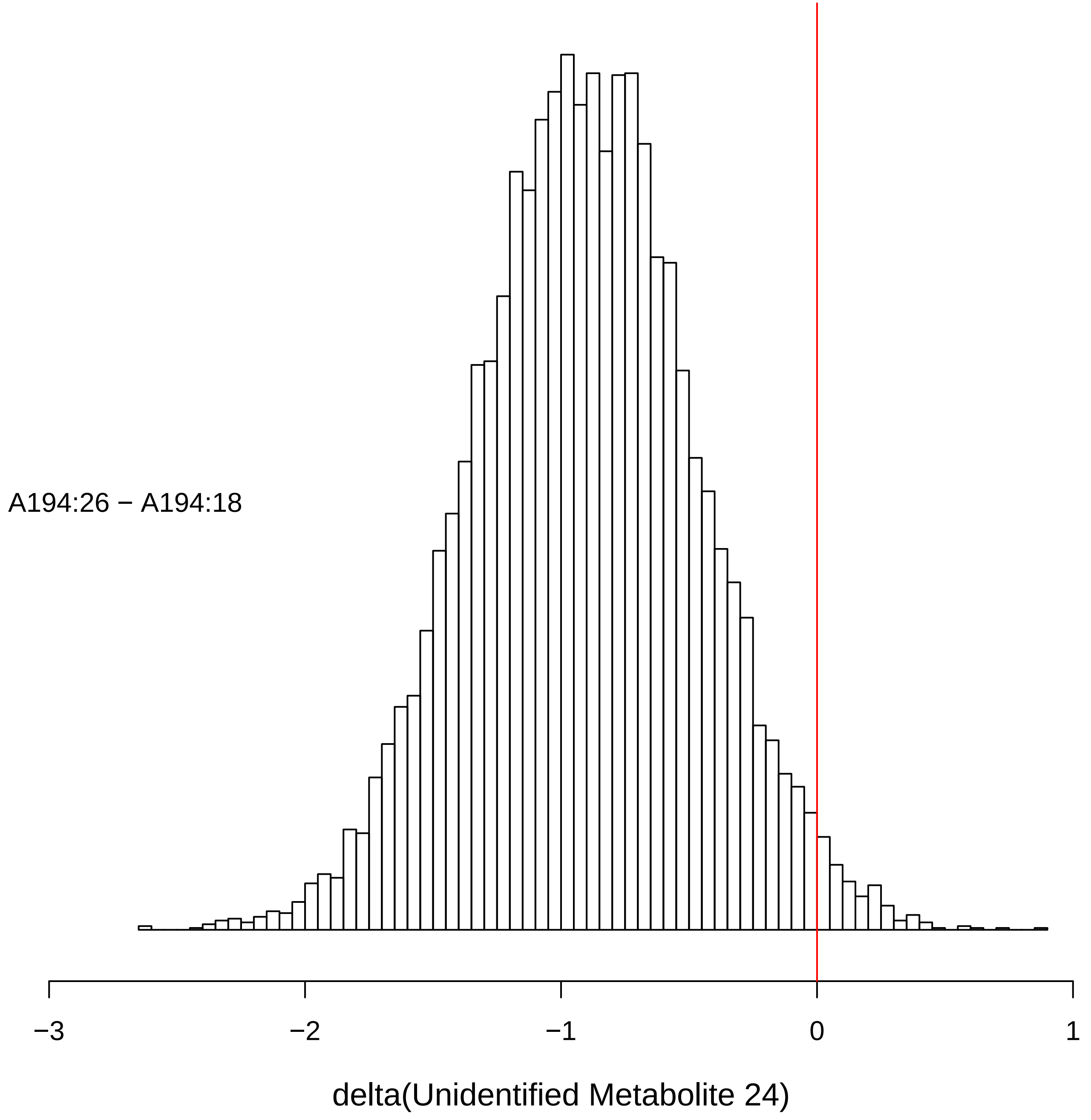
A194:26 − A194:18
−3
−2
−1
0
1
delta(Unidentified Metabolite 24)

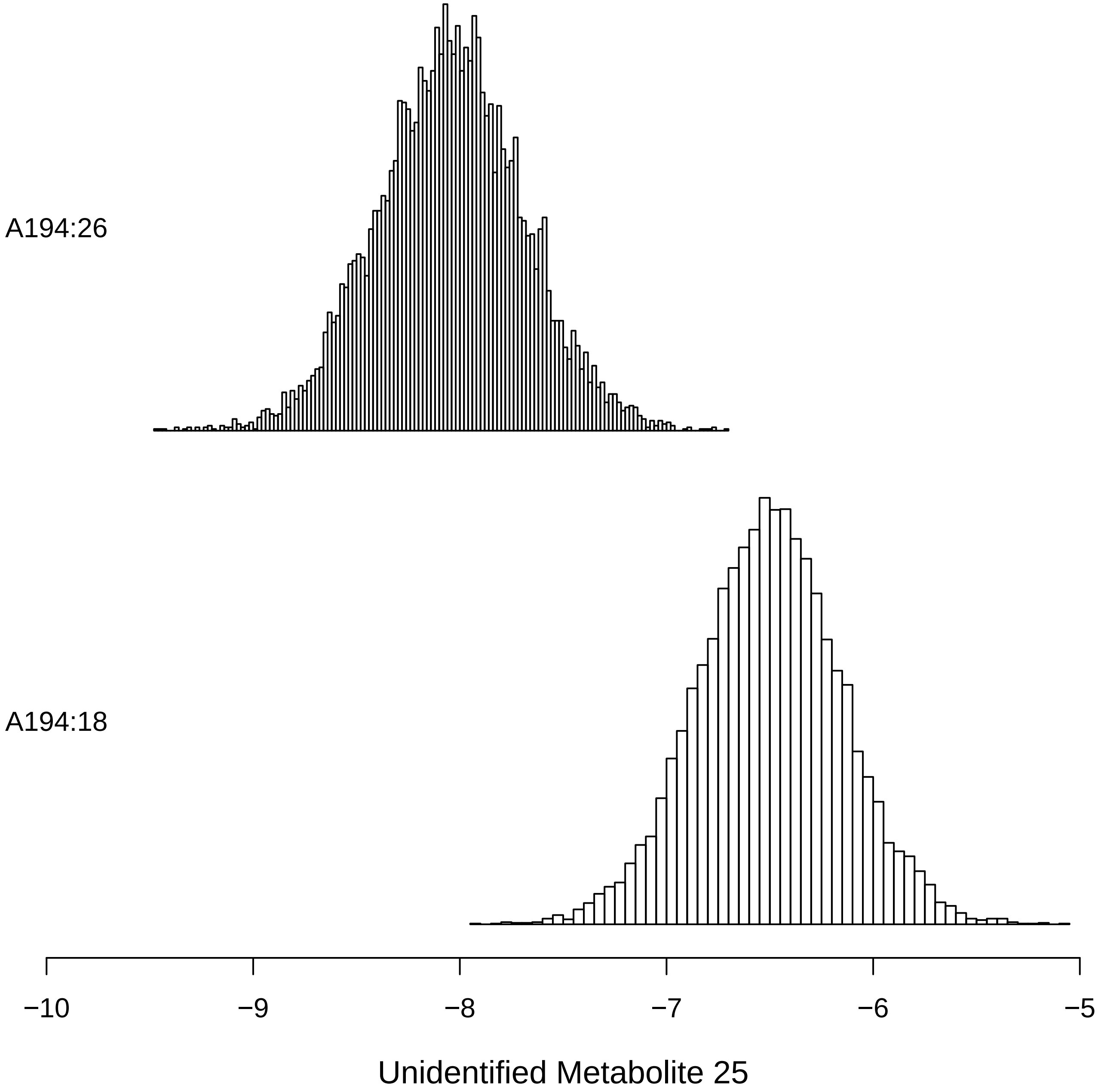
A194:26
A194:18
−10
−9
−8
−7
−6
−5
Unidentified Metabolite 25

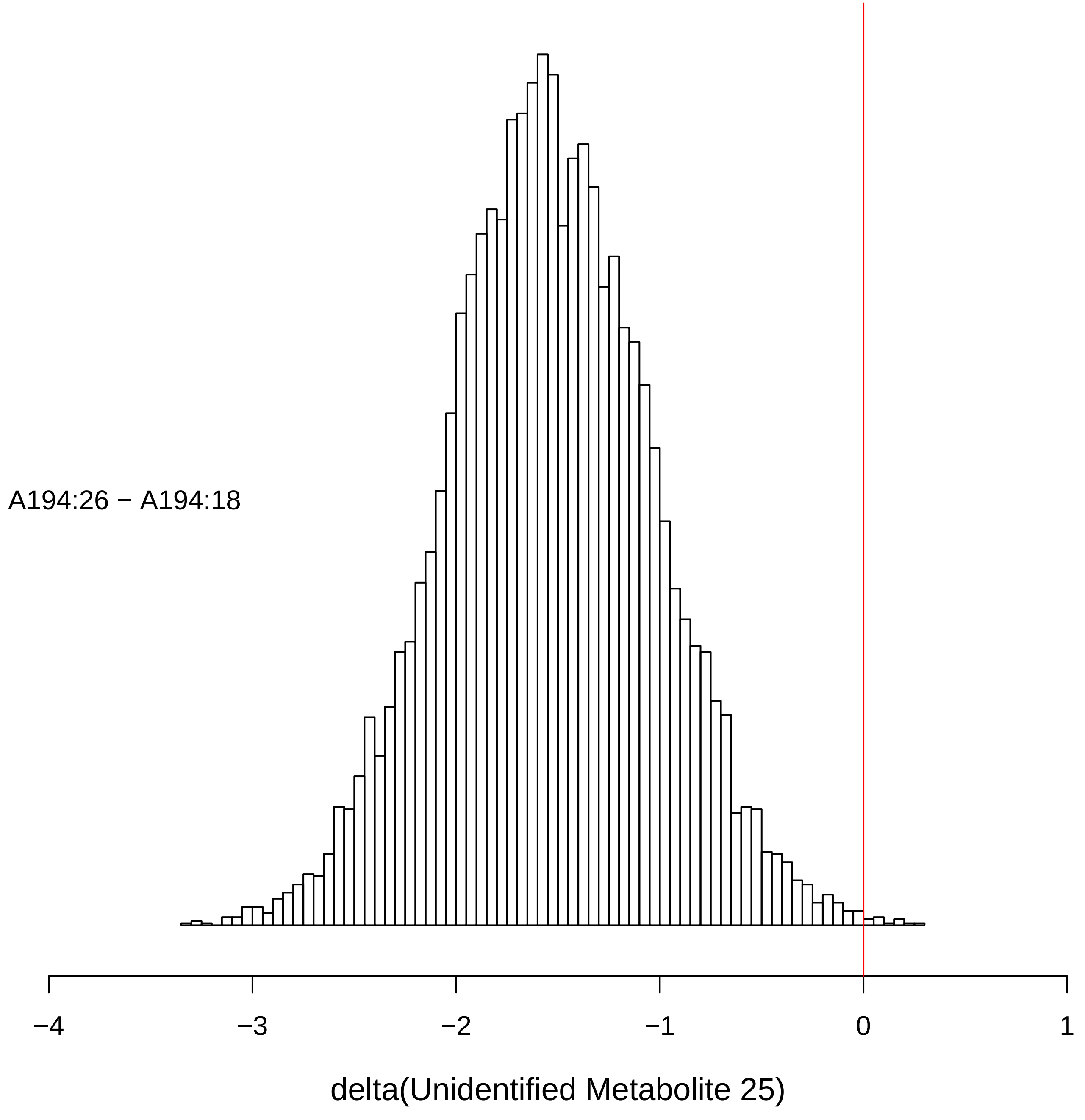
A194:26 − A194:18
−4
−3
−2
−1
0
1
delta(Unidentified Metabolite 25)

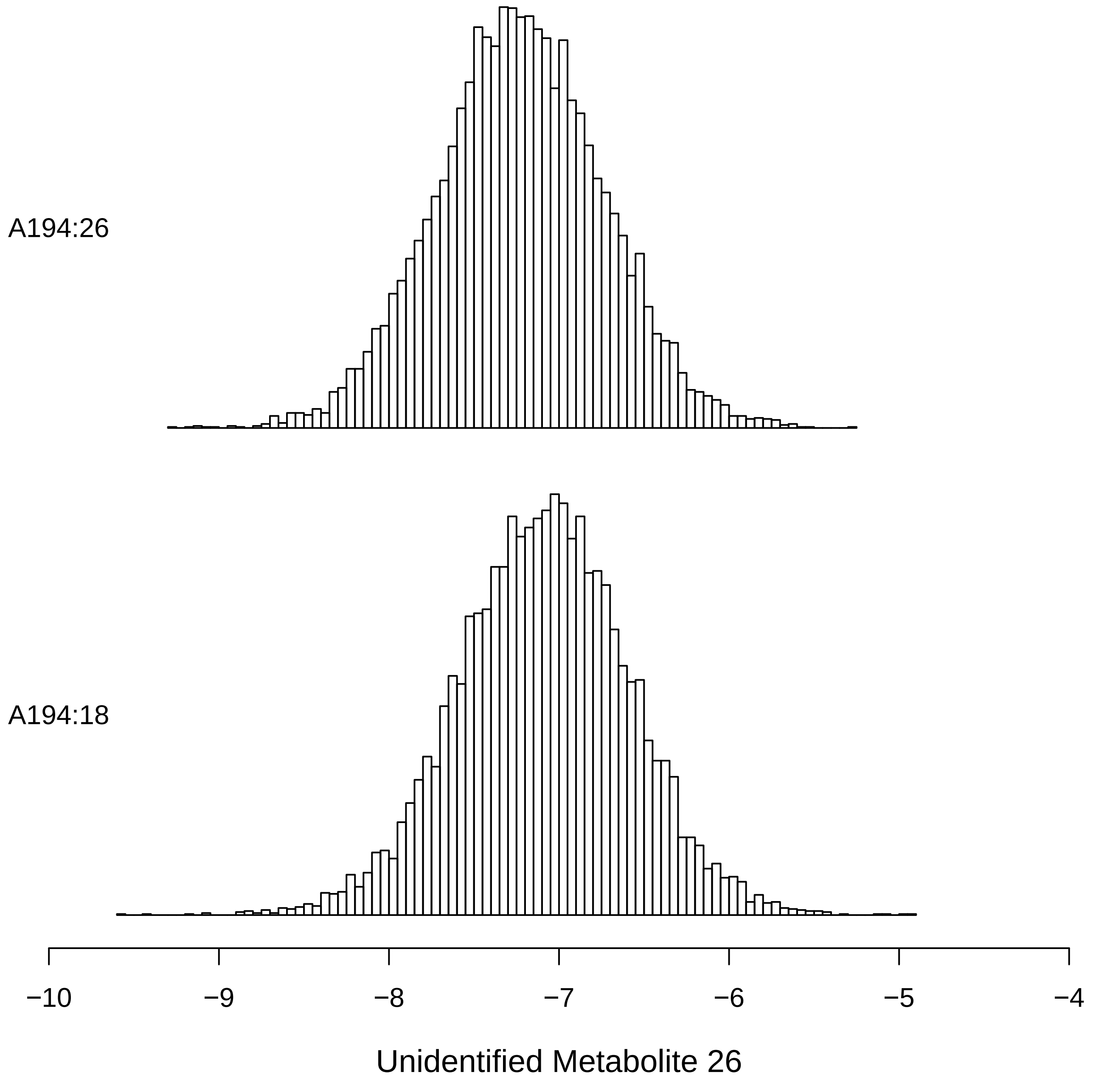
A194:26
A194:18
−10
−9
−8
−7
−6
−5
−4
Unidentified Metabolite 26

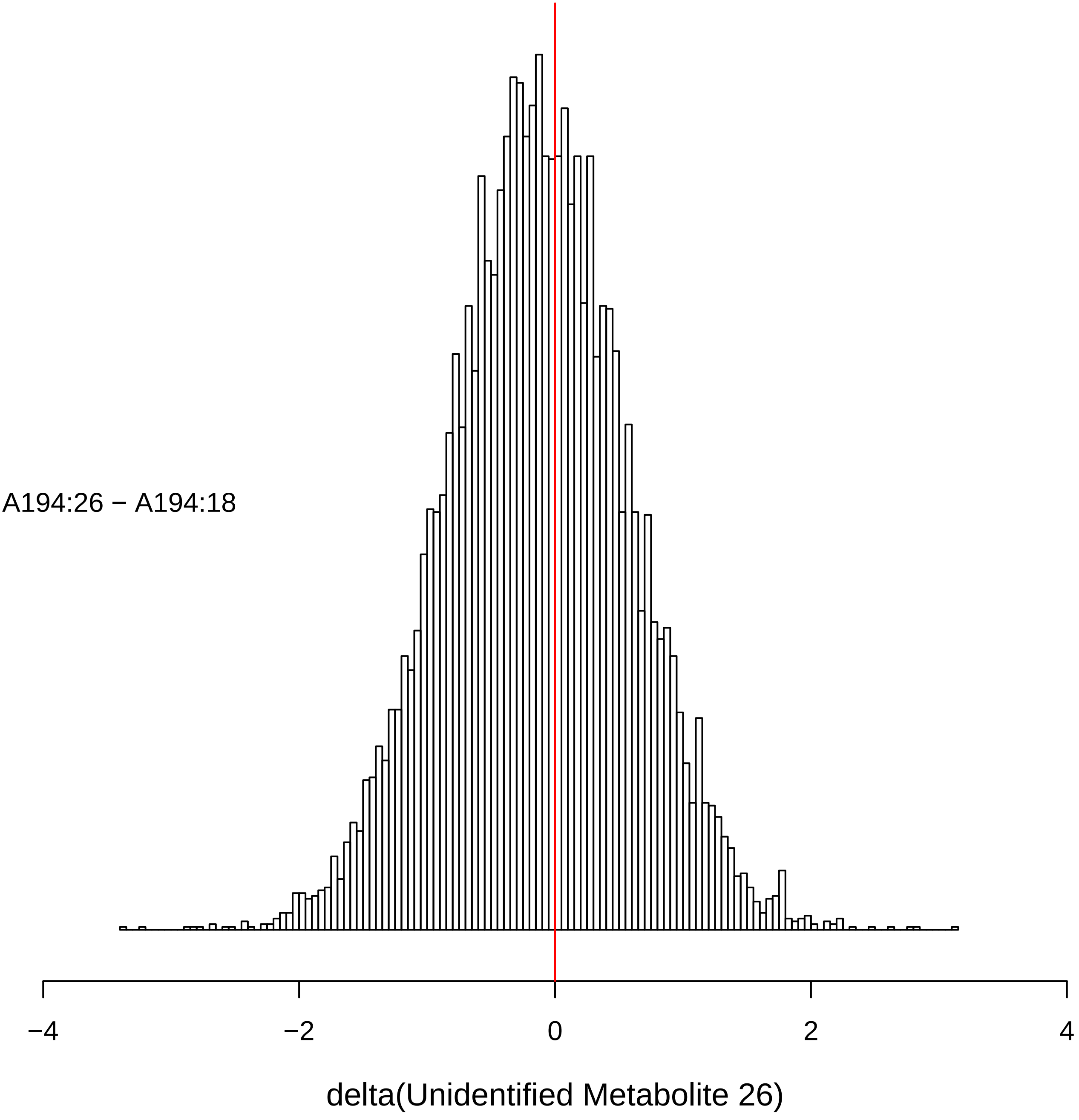
A194:26 − A194:18
−4
−2
0
2
4
delta(Unidentified Metabolite 26)

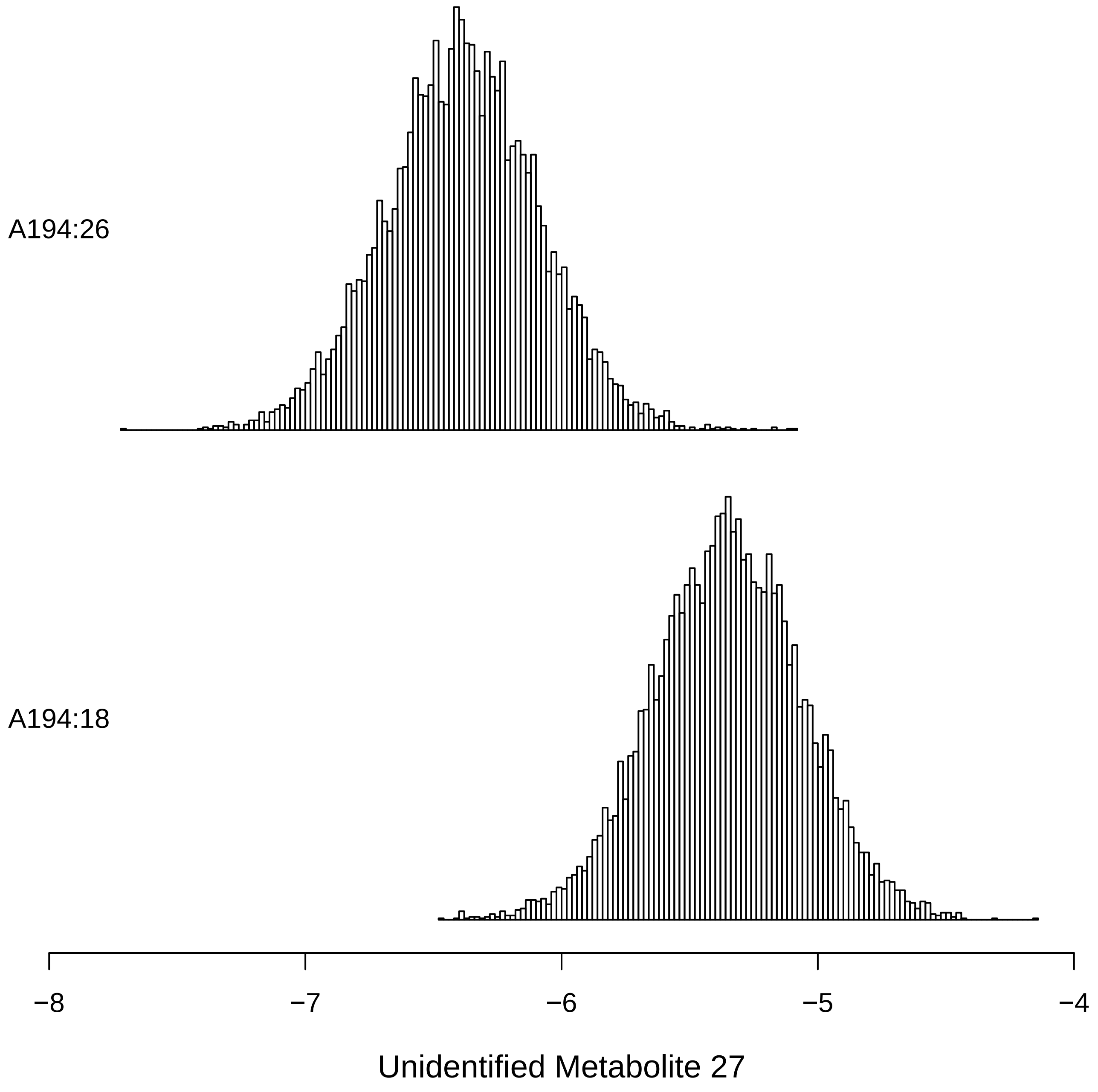
A194:26
A194:18
−8
−7
−6
−5
−4
Unidentified Metabolite 27

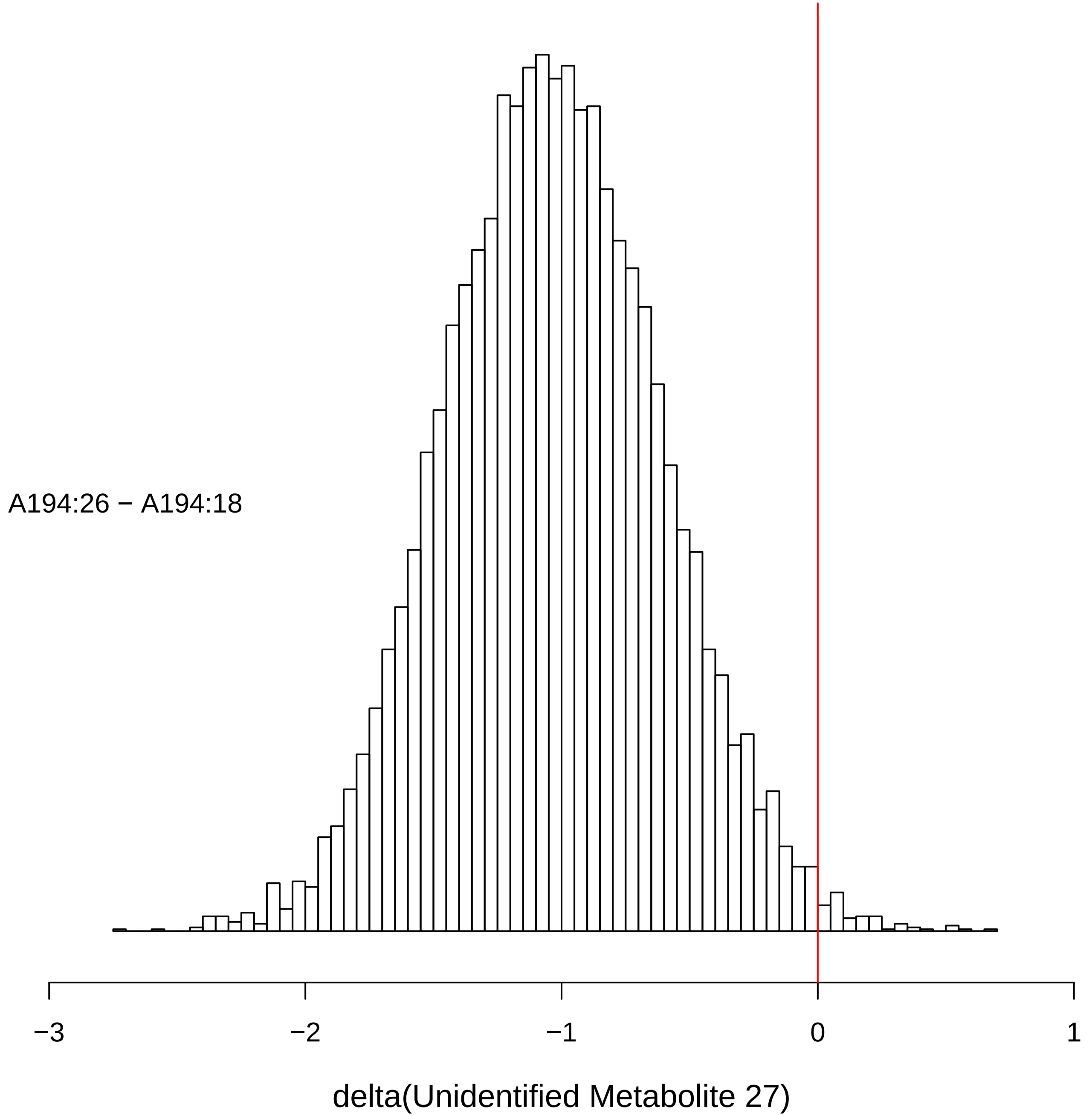
A194:26 − A194:18
−3
−2
−1
0
1
delta(Unidentified Metabolite 27)

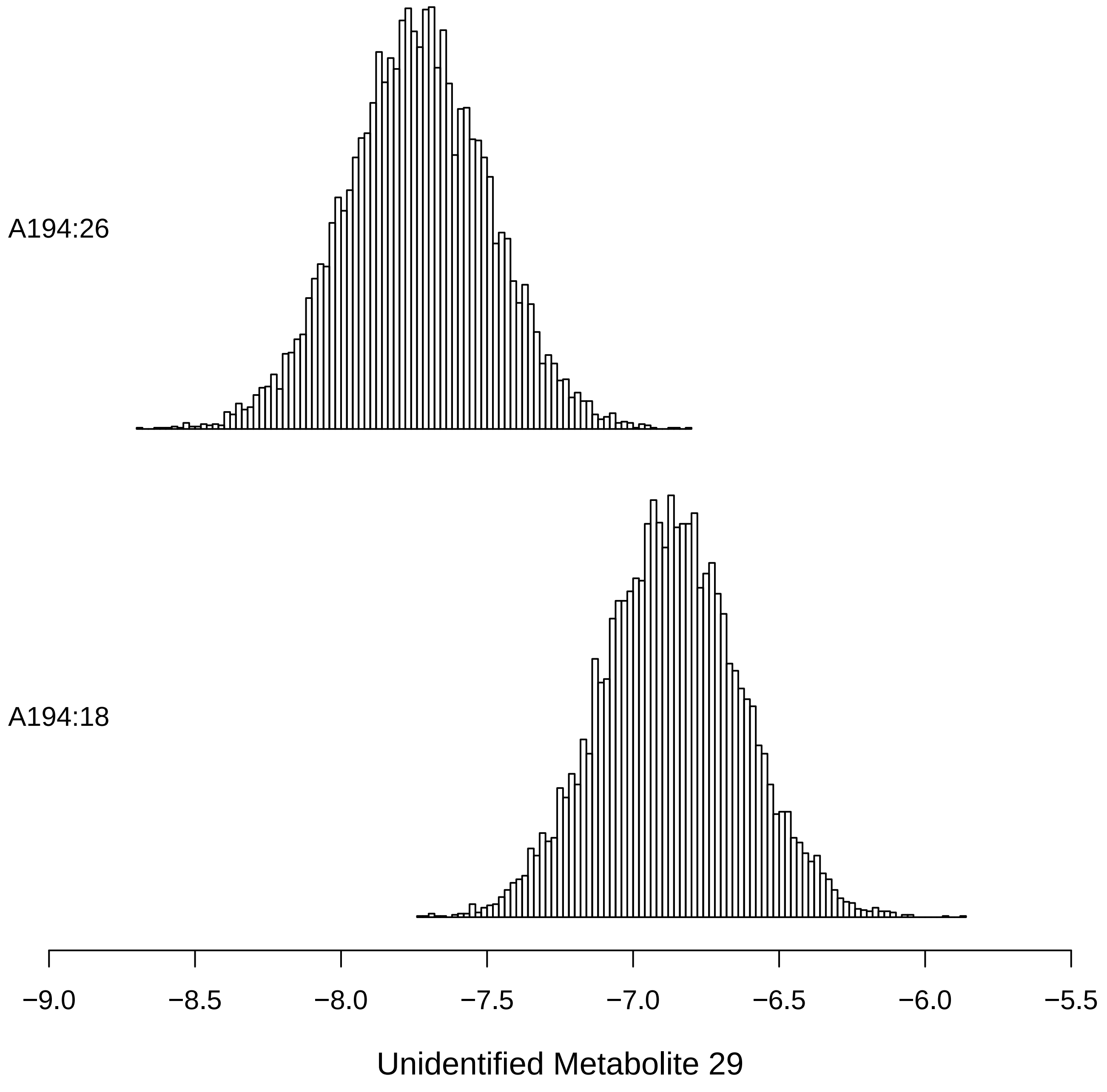
A194:26
A194:18
−9.0
−8.5
−8.0
−7.5
−7.0
−6.5
−6.0
−5.5
Unidentified Metabolite 29

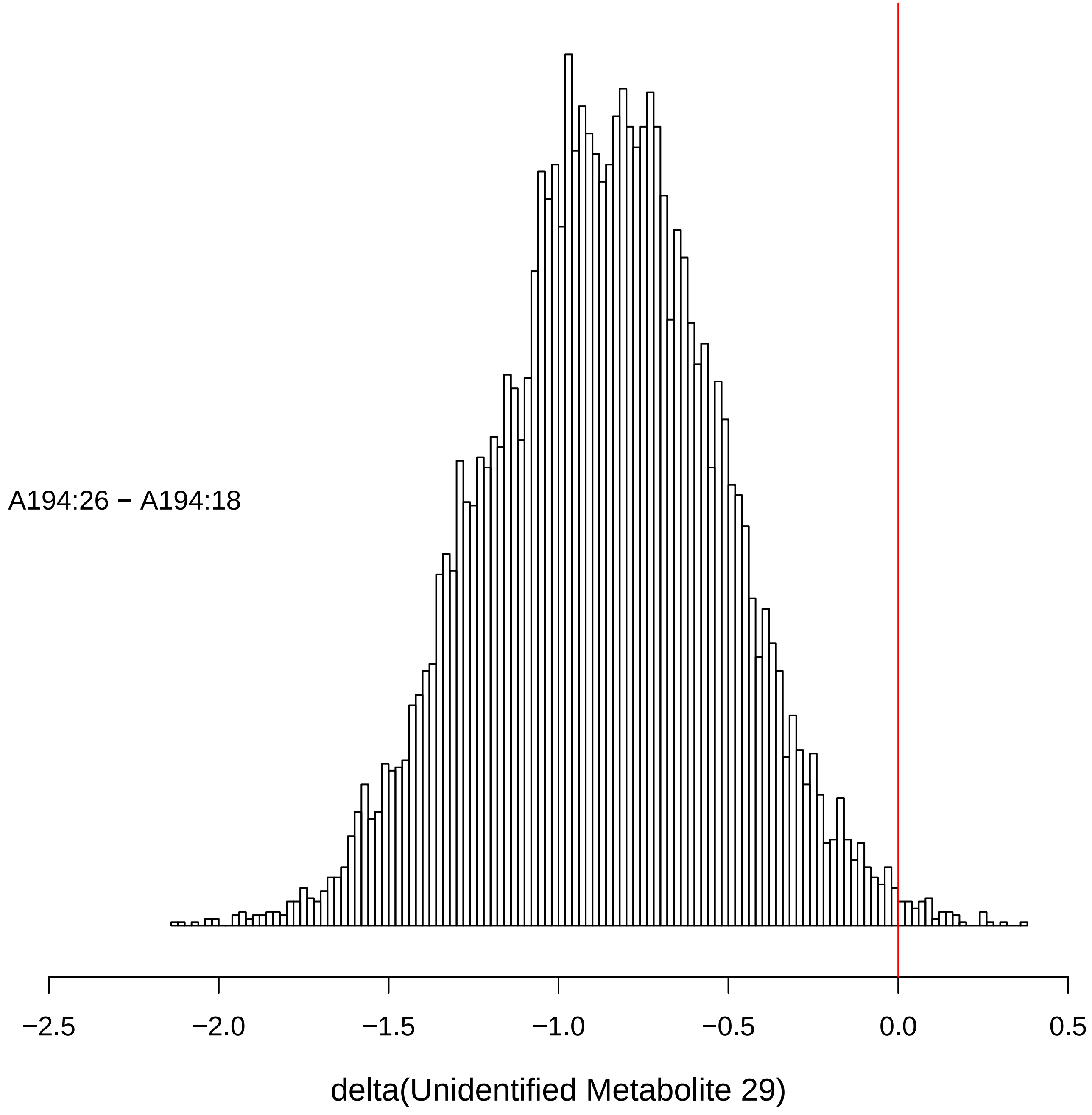
A194:26 − A194:18
−2.5
−2.0
−1.5
−1.0
−0.5
0.0
0.5
delta(Unidentified Metabolite 29)

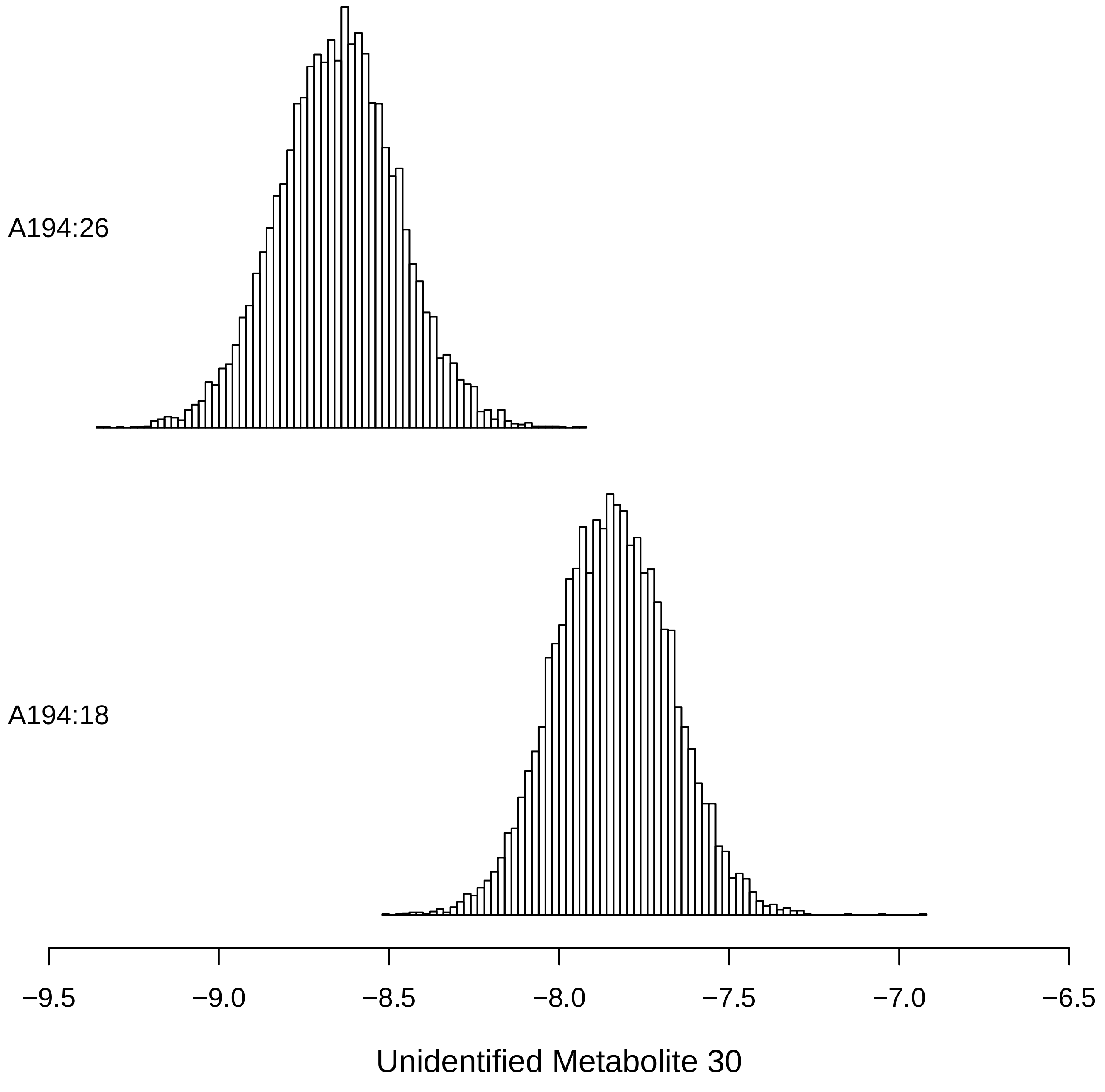

A194:26
A194:18
−9.5
−9.0
−8.5
−8.0
−7.5
−7.0
−6.5
Unidentified Metabolite 30

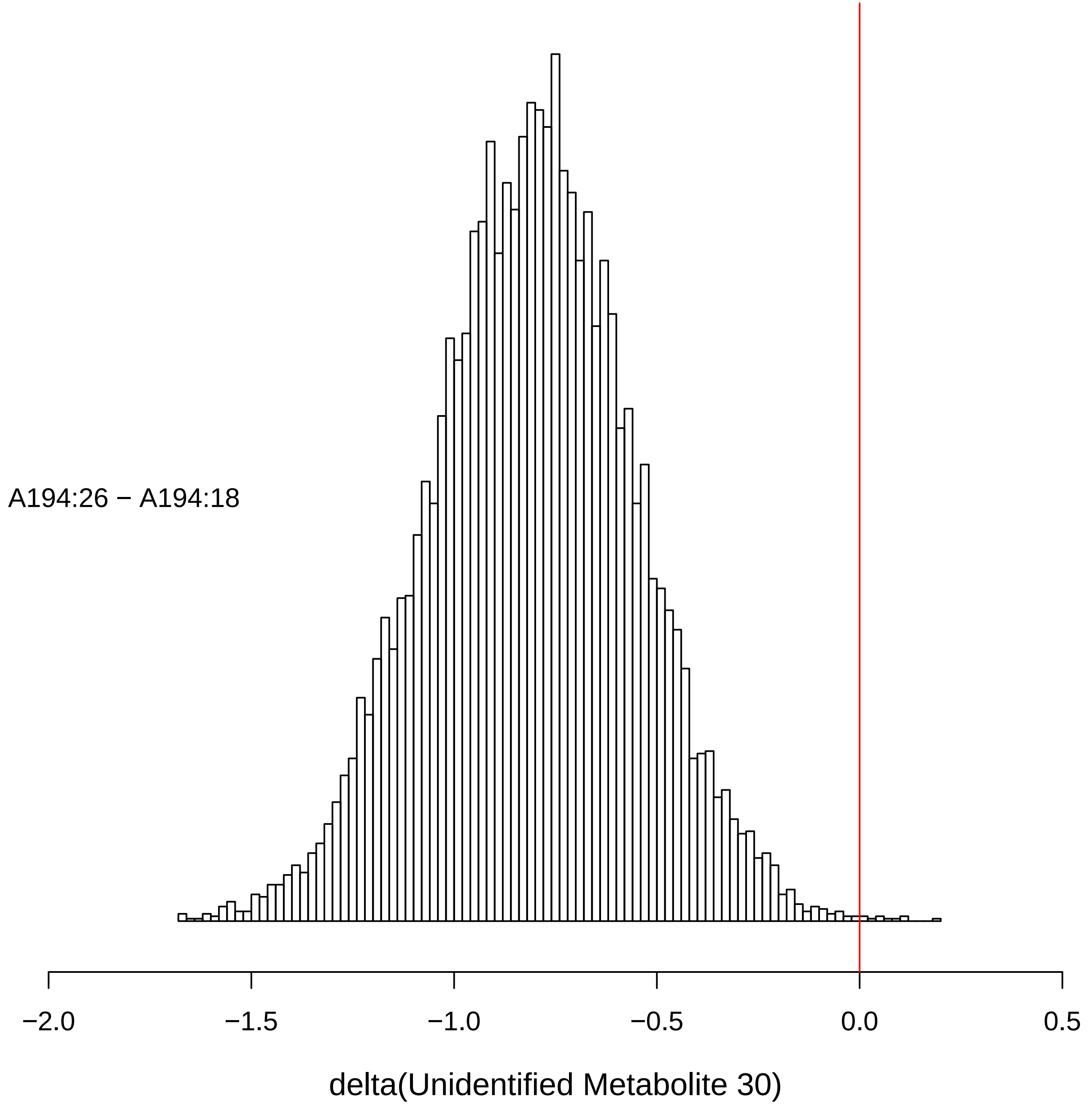
A194:26 − A194:18
−2.0
−1.5
−1.0
−0.5
0.0
0.5
delta(Unidentified Metabolite 30)

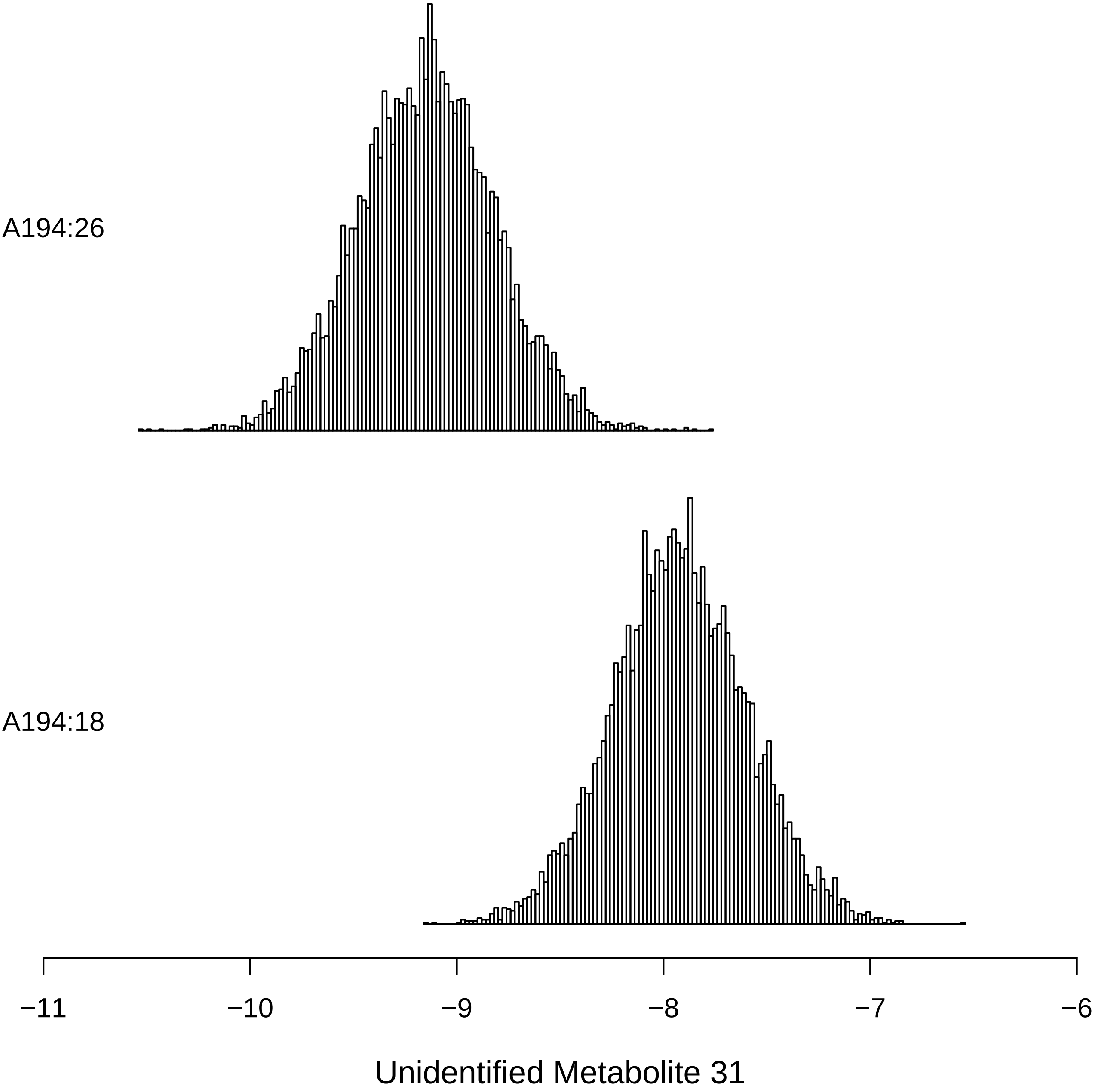

A194:26
A194:18
−11
−10
−9
−8
−7
−6
Unidentified Metabolite 31

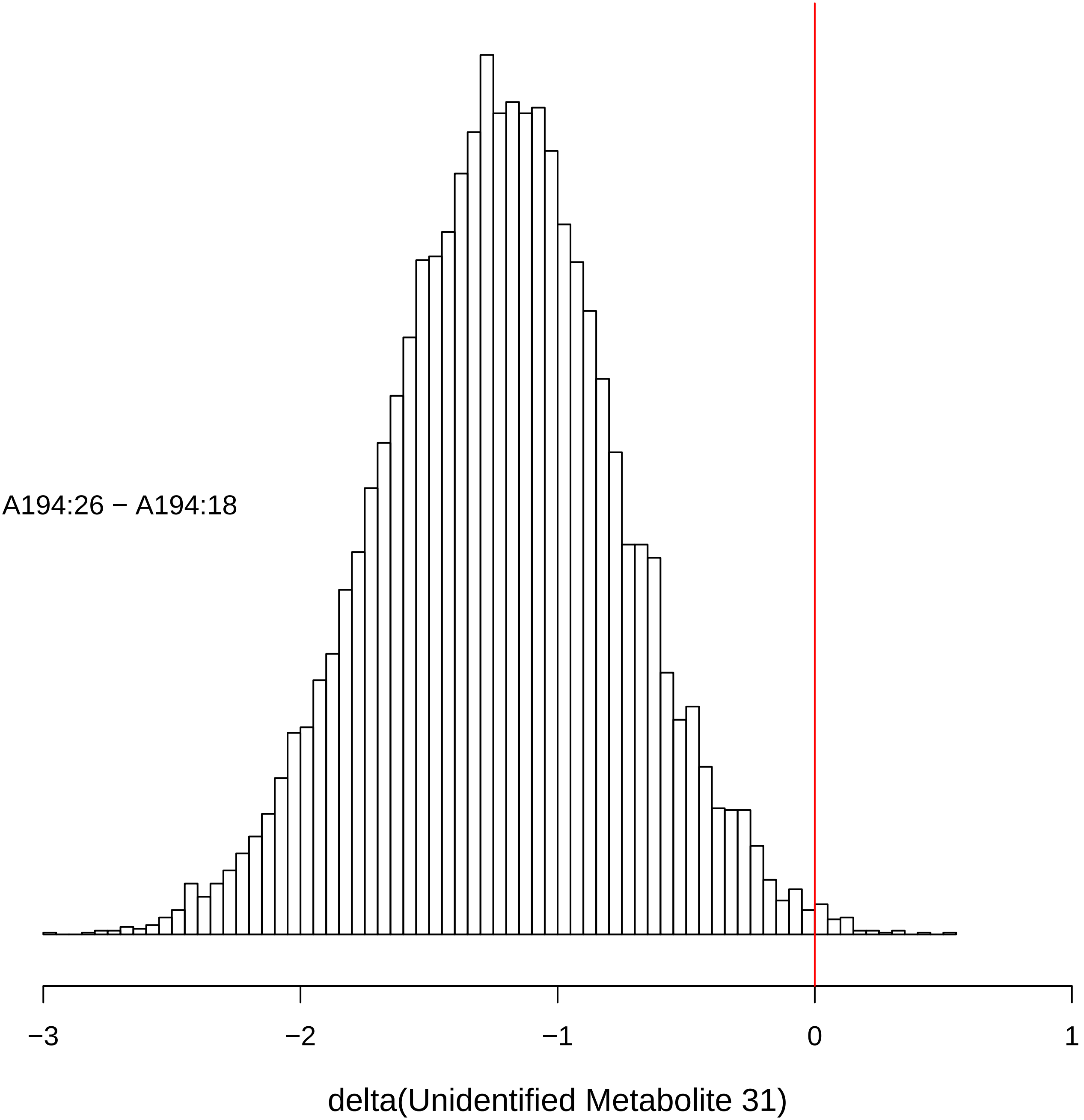
A194:26 − A194:18
−3
−2
−1
0
1
delta(Unidentified Metabolite 31)

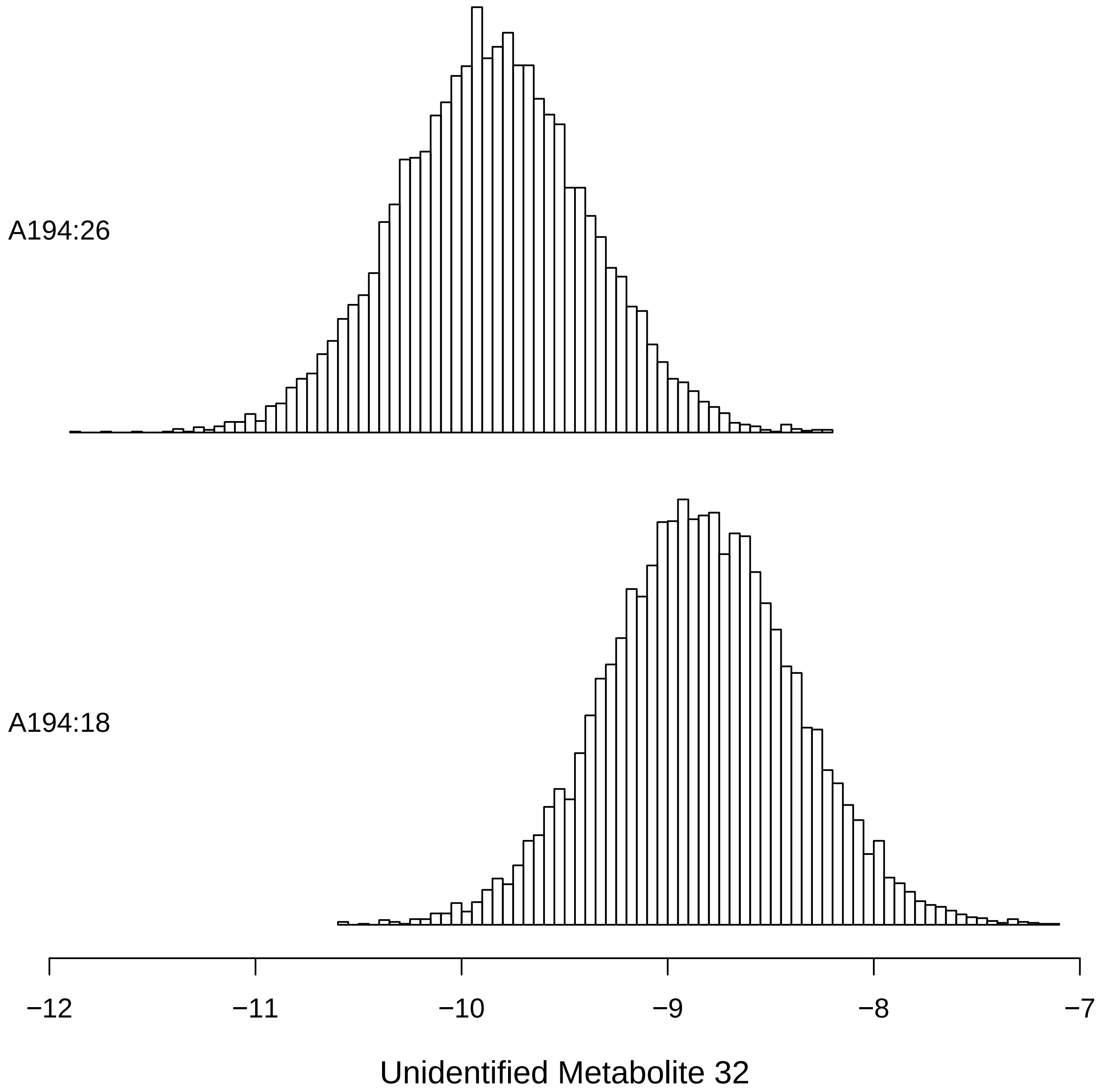
A194:26
A194:18
−12
−11
−10
−9
−8
−7
Unidentified Metabolite 32

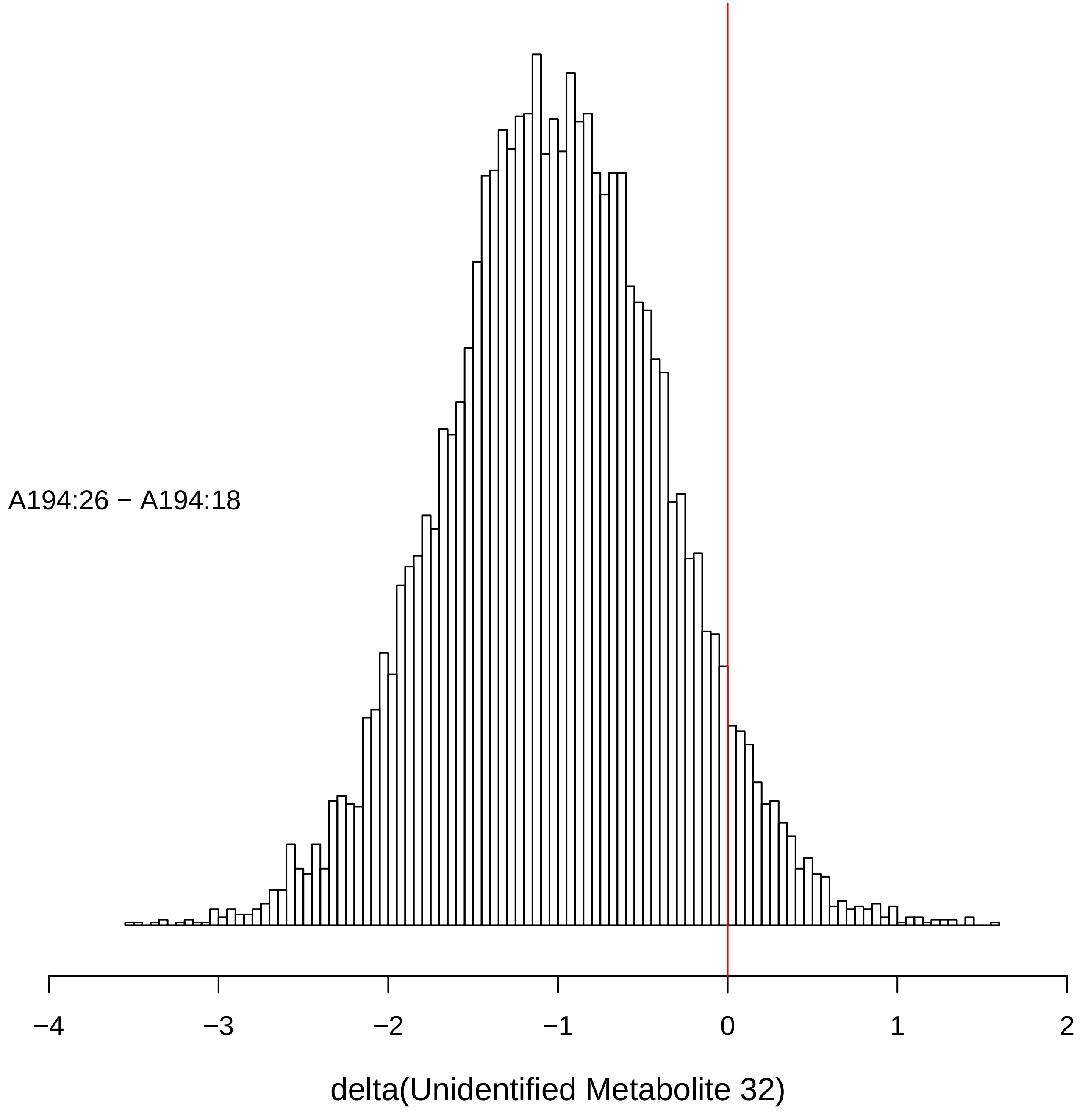
A194:26 − A194:18
−4
−3
−2
−1
0
1
2
delta(Unidentified Metabolite 32)

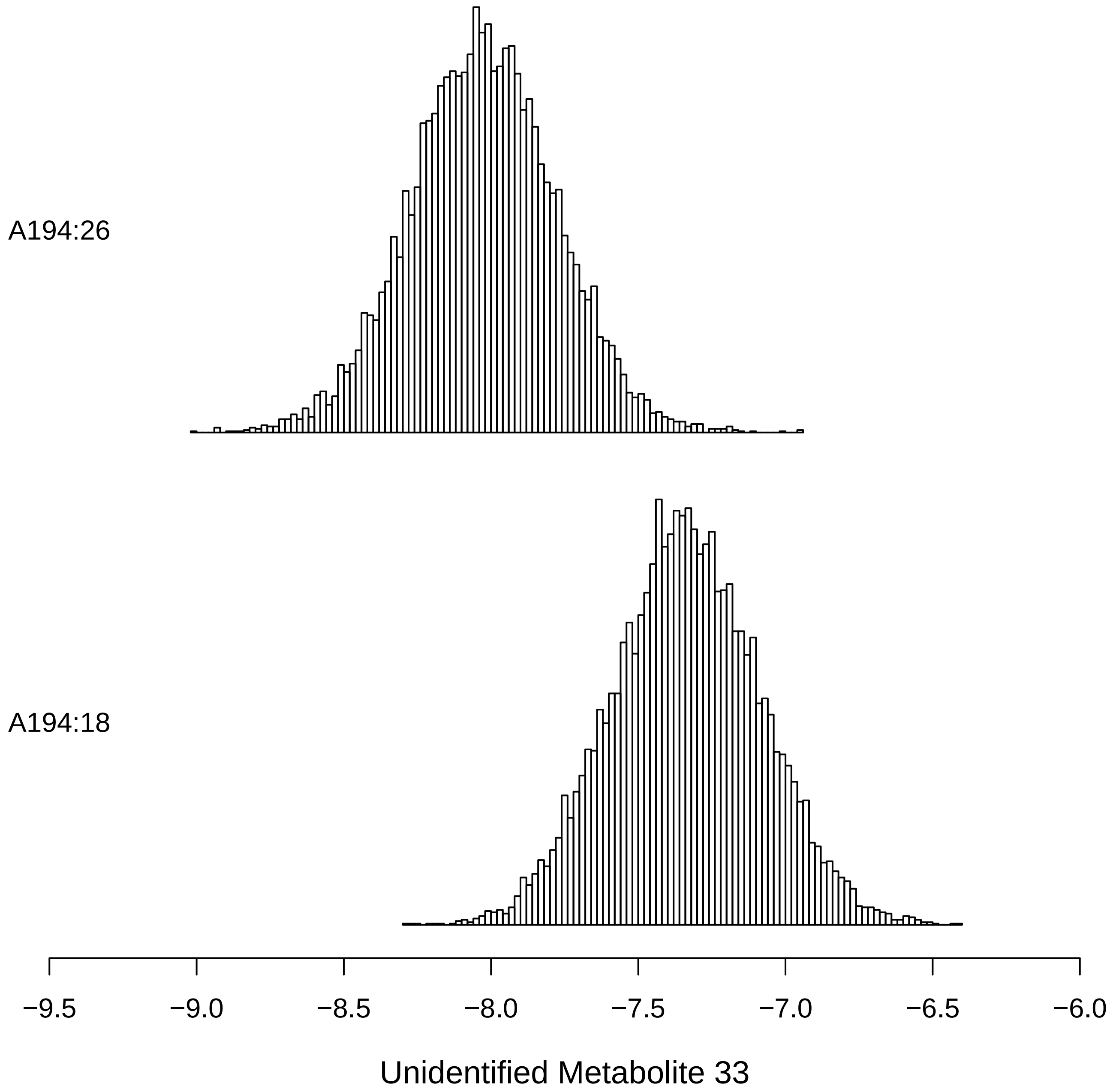
A194:26
A194:18
−9.5
−9.0
−8.5
−8.0
−7.5
−7.0
−6.5
−6.0
Unidentified Metabolite 33

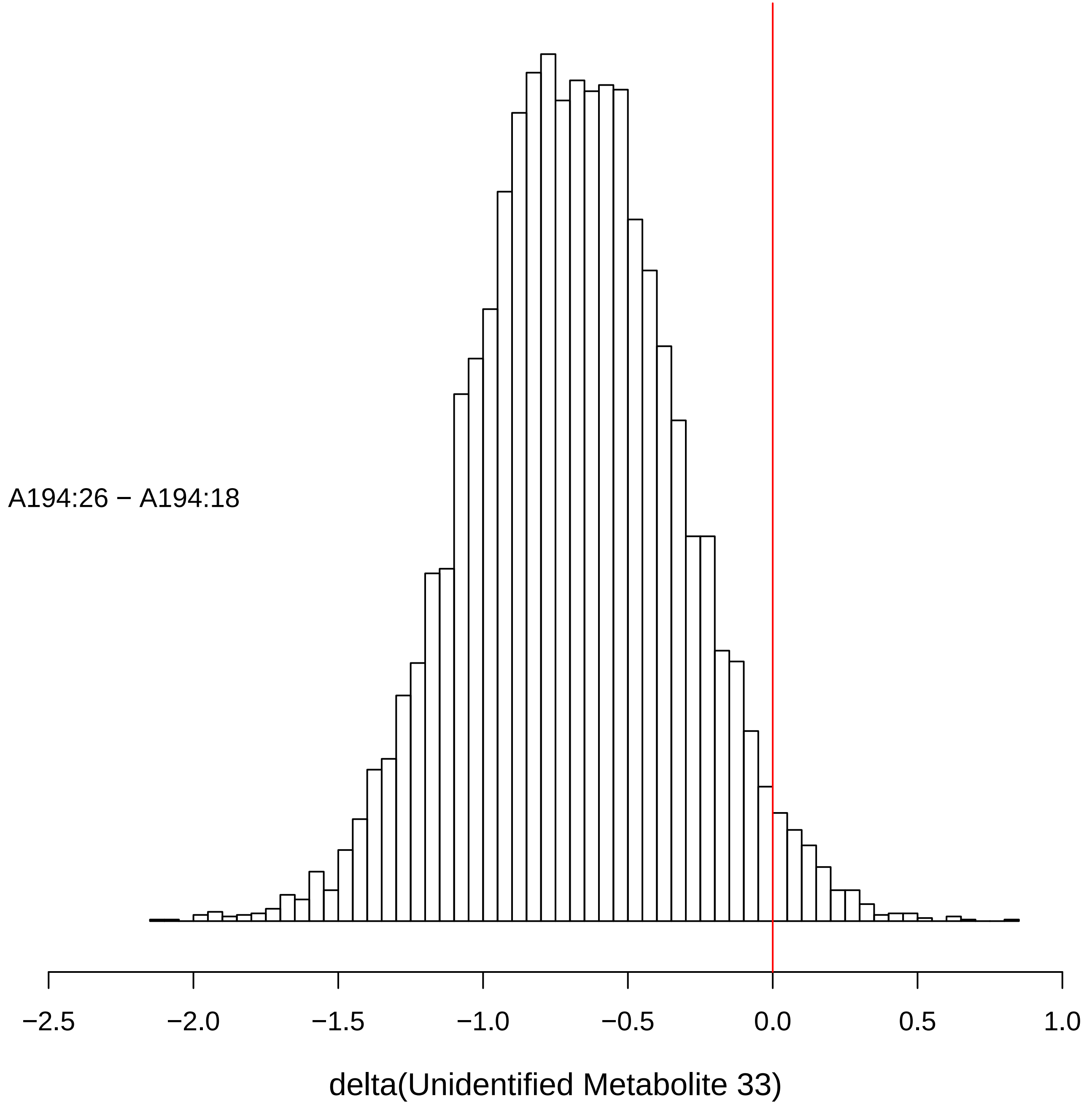
A194:26 − A194:18
−2.5
−2.0
−1.5
−1.0
−0.5
0.0
0.5
1.0
delta(Unidentified Metabolite 33)

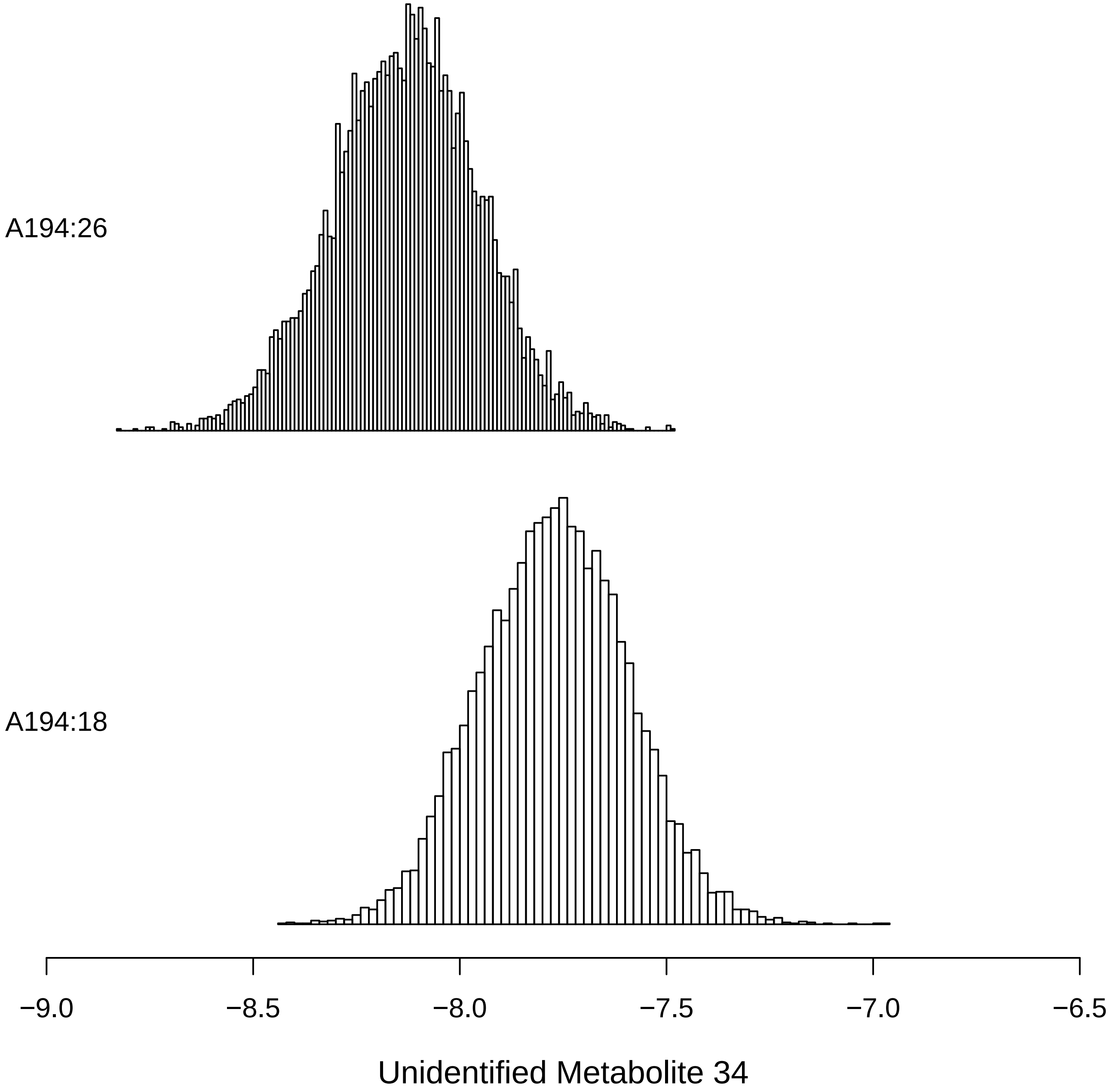
A194:26
A194:18
−9.0
−8.5
−8.0
−7.5
−7.0
−6.5
Unidentified Metabolite 34

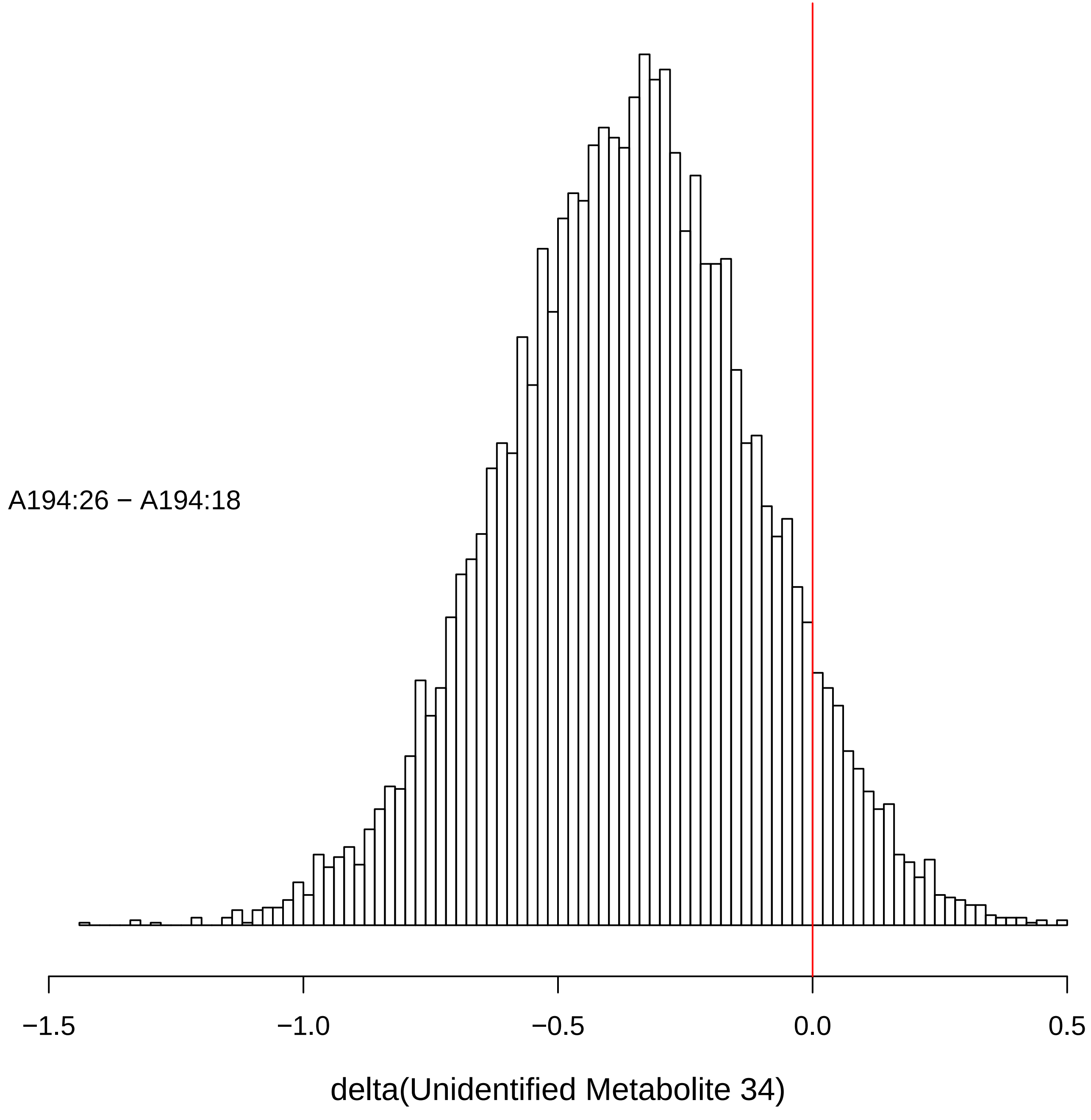
A194:26 − A194:18
−1.5
−1.0
−0.5
0.0
0.5
delta(Unidentified Metabolite 34)

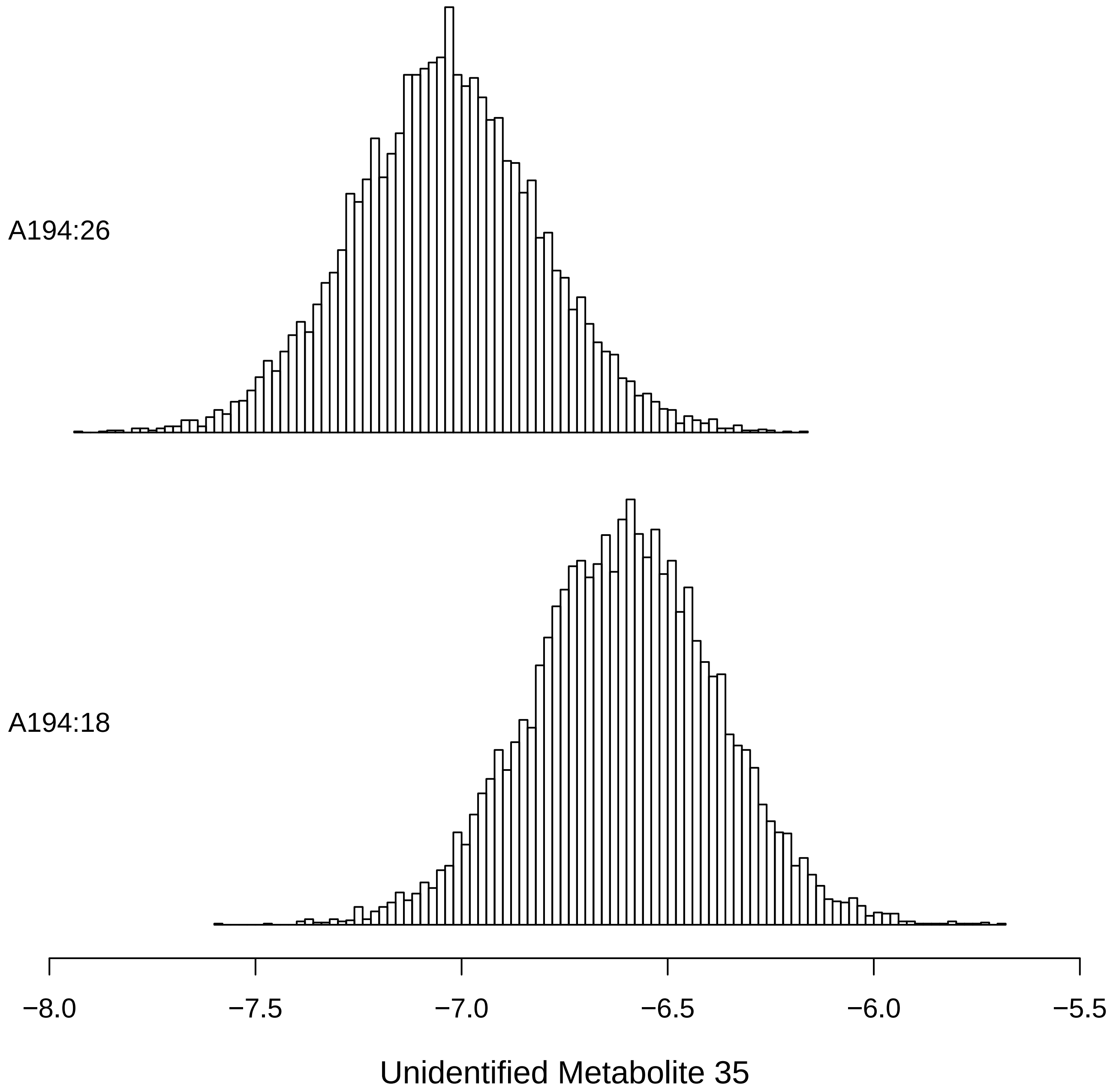
A194:26
A194:18
−8.0
−7.5
−7.0
−6.5
−6.0
−5.5
Unidentified Metabolite 35

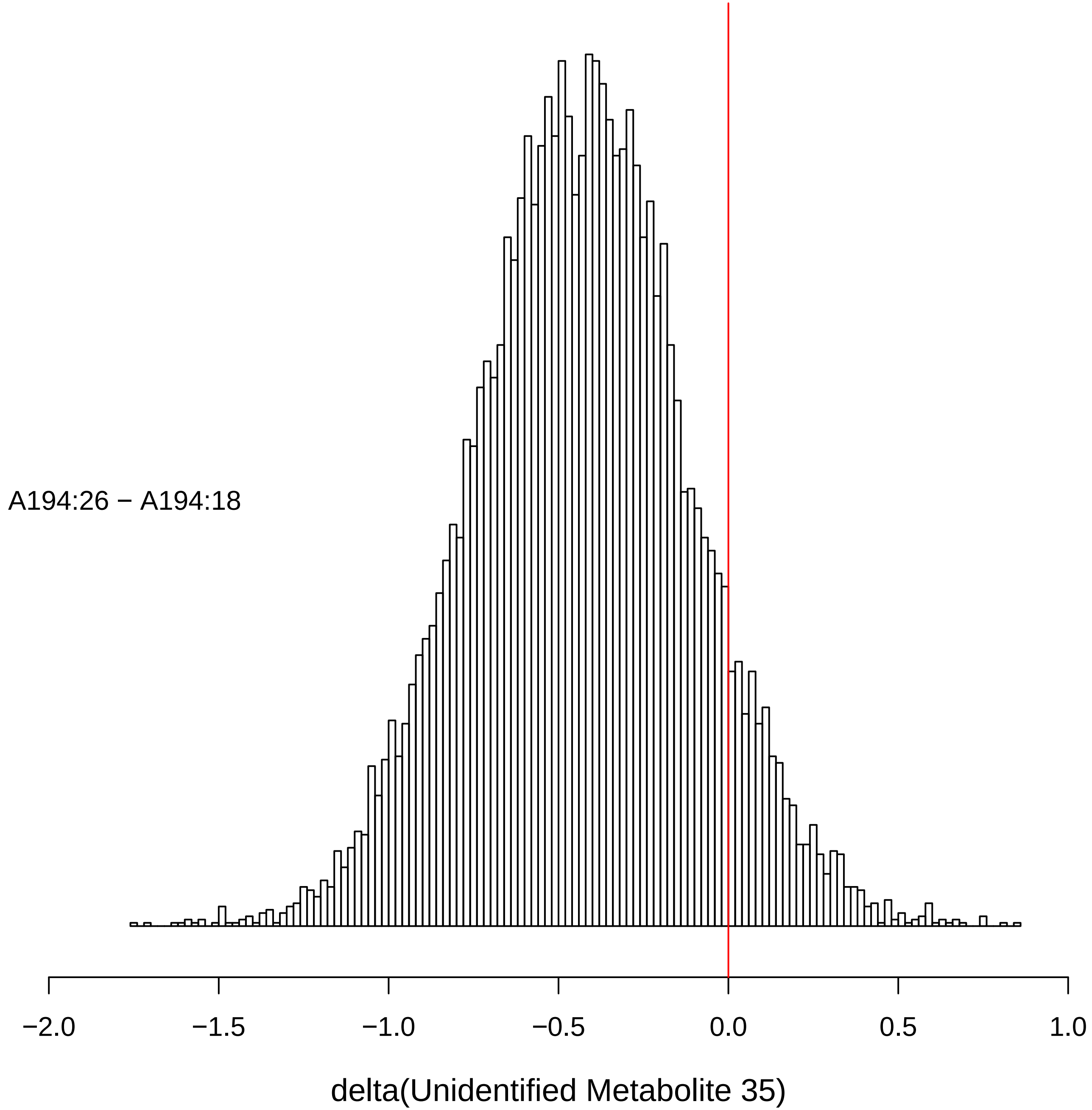

A194:26 − A194:18
−2.0
−1.5
−1.0
−0.5
0.0
0.5
1.0
delta(Unidentified Metabolite 35)

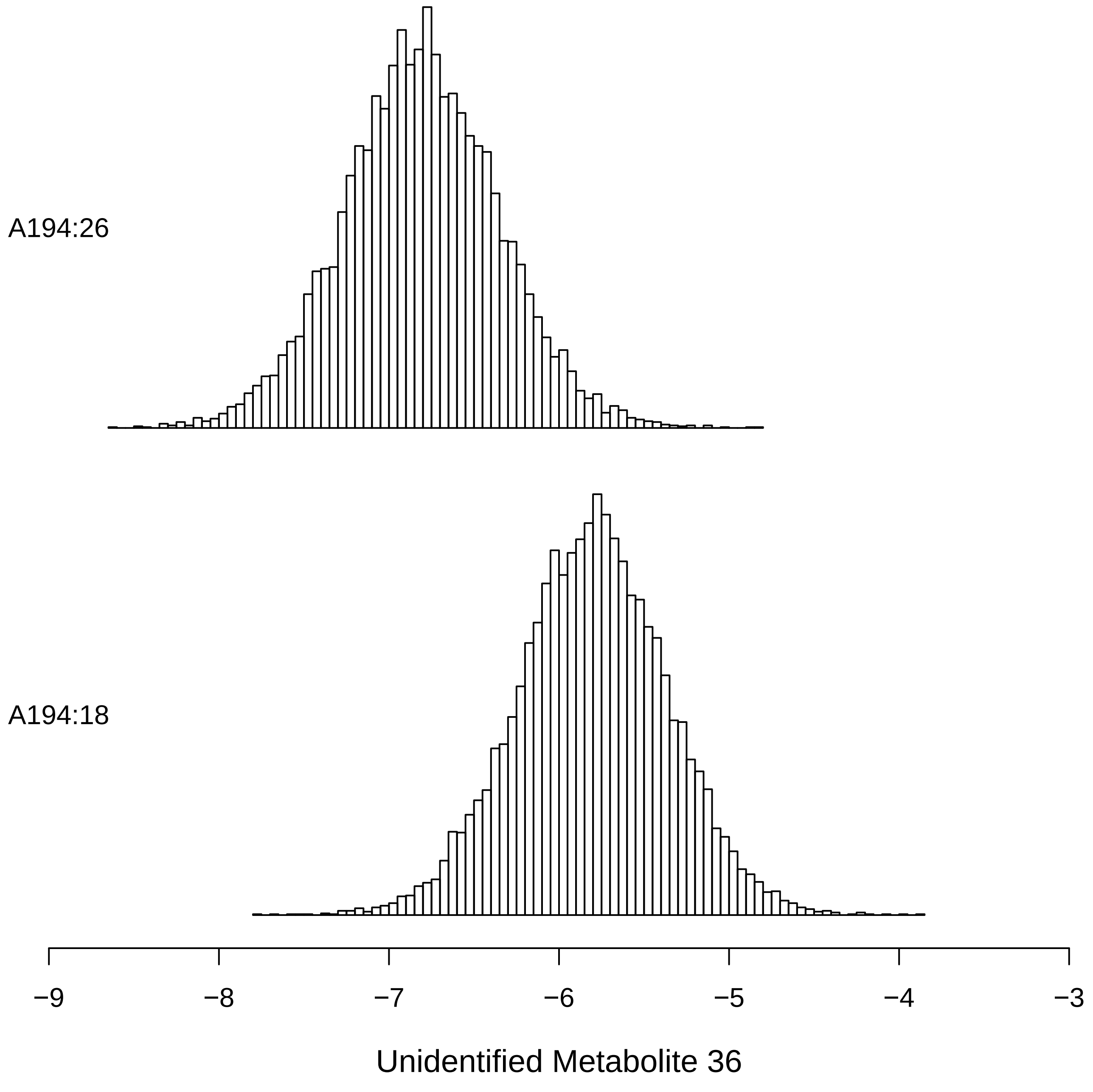
A194:26
A194:18
−9
−8
−7
−6
−5
−4
−3
Unidentified Metabolite 36

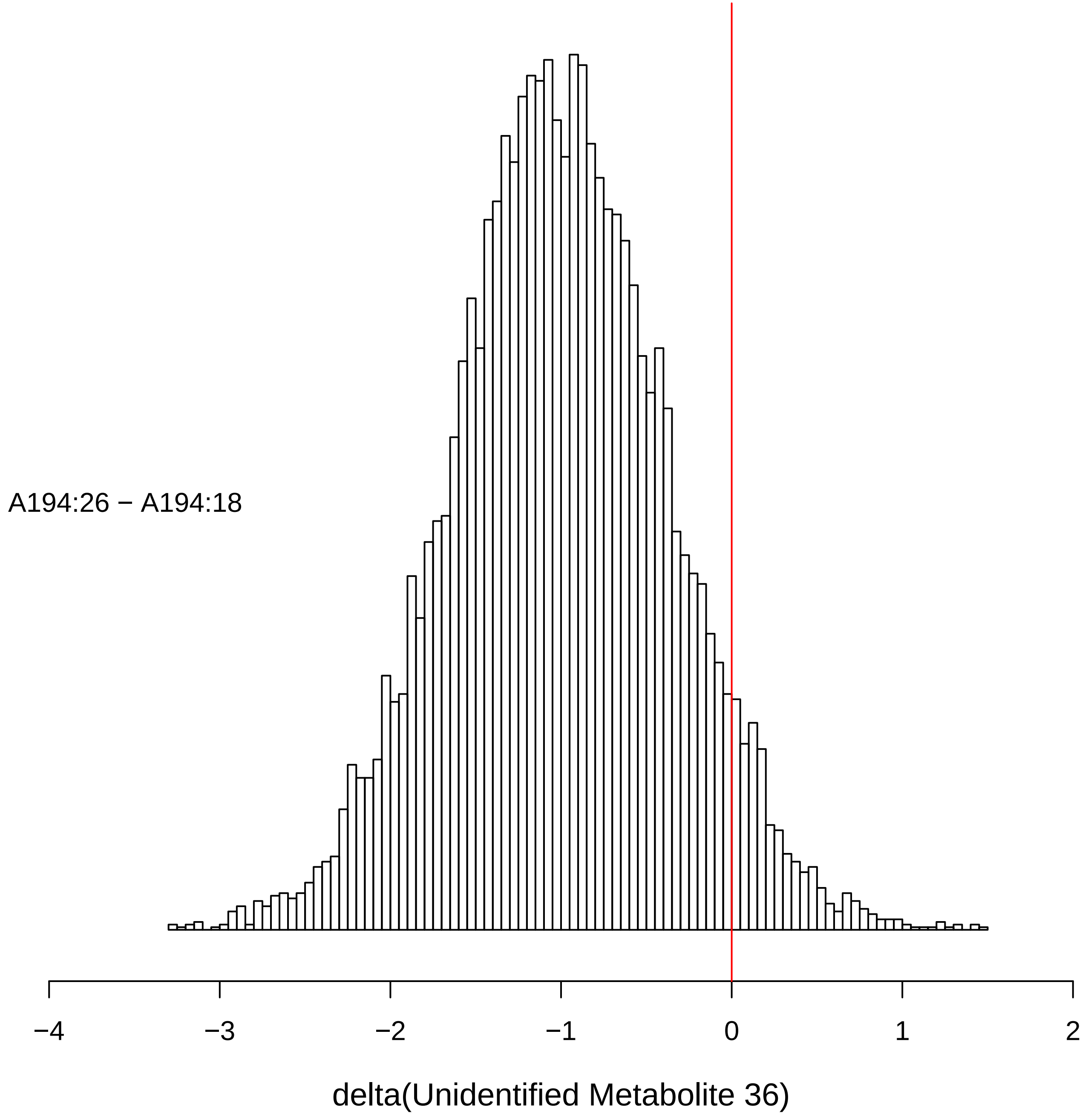
A194:26 − A194:18
−4
−3
−2
−1
0
1
2
delta(Unidentified Metabolite 36)

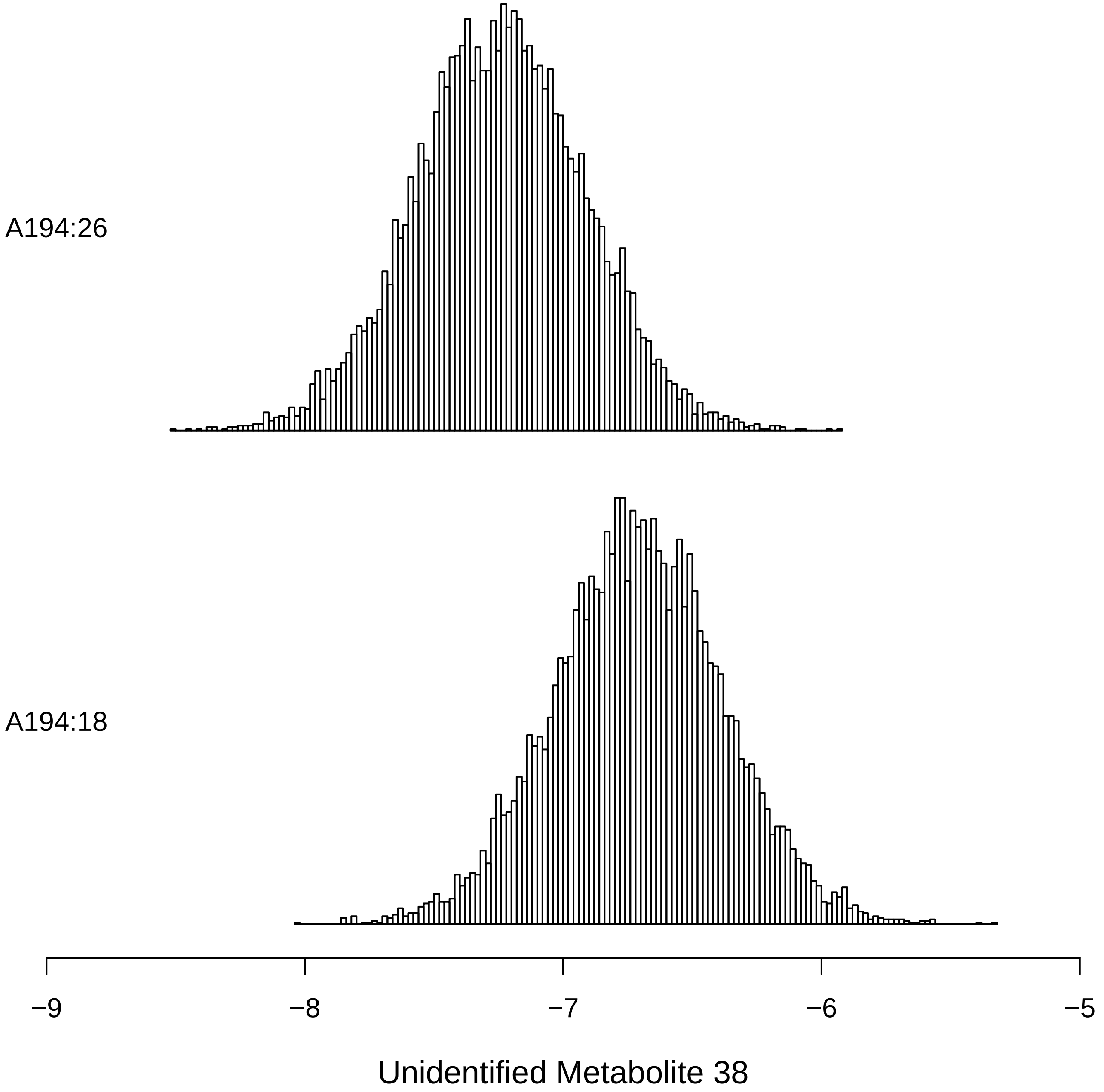
A194:26
A194:18
−9
−8
−7
−6
−5
Unidentified Metabolite 38

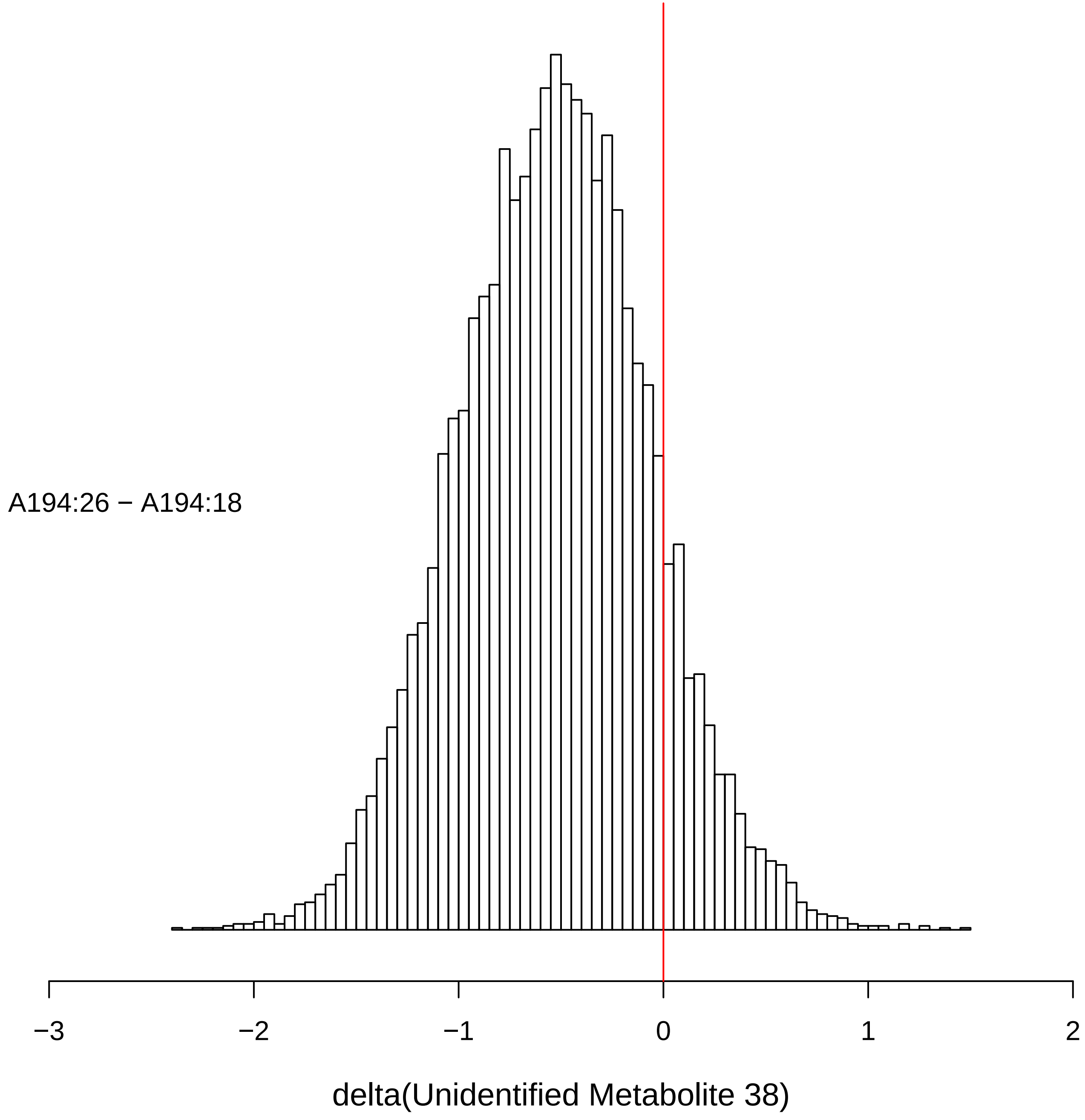
A194:26 − A194:18
−3
−2
−1
0
1
2
delta(Unidentified Metabolite 38)

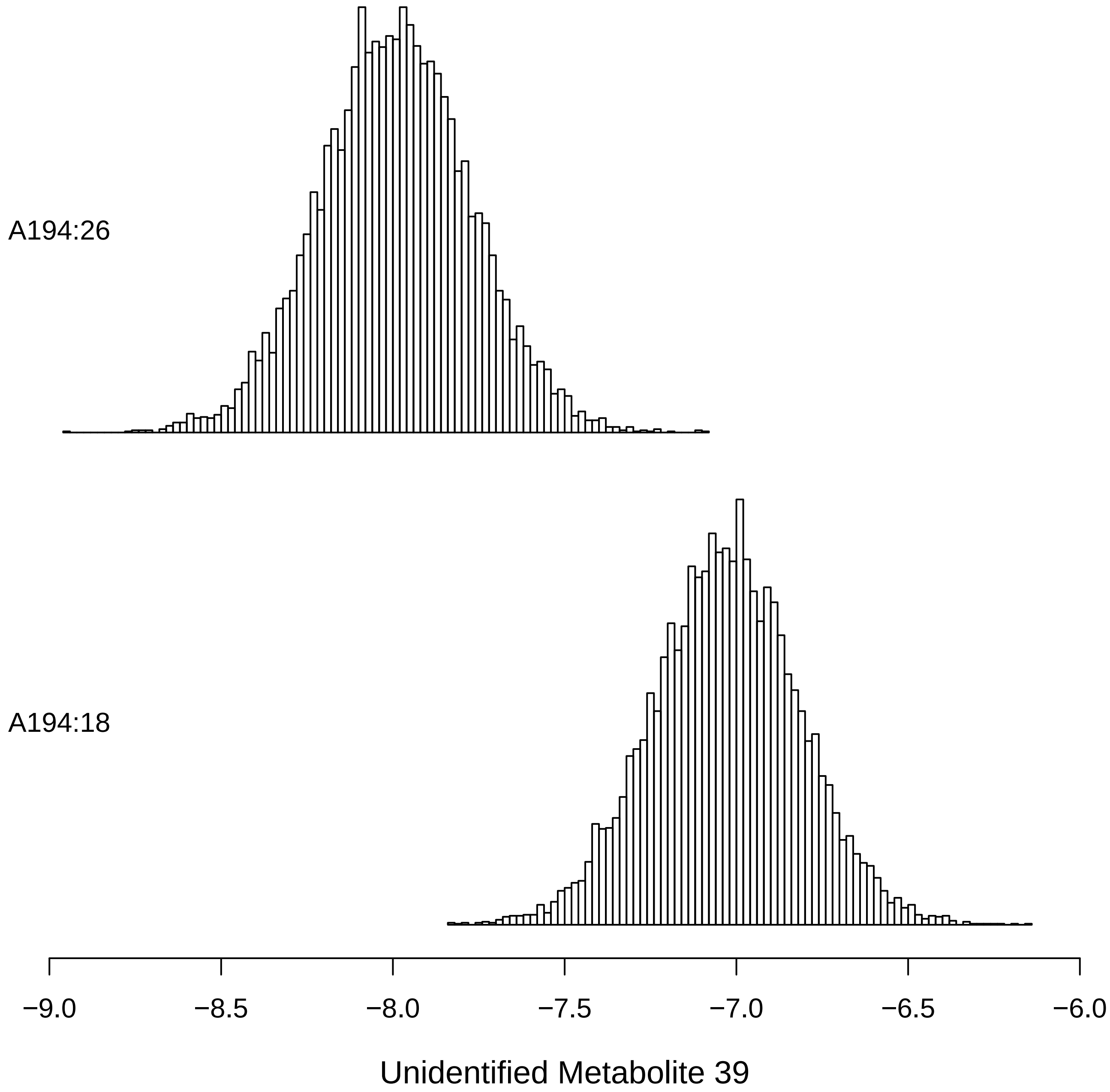
A194:26
A194:18
−9.0
−8.5
−8.0
−7.5
−7.0
−6.5
−6.0
Unidentified Metabolite 39

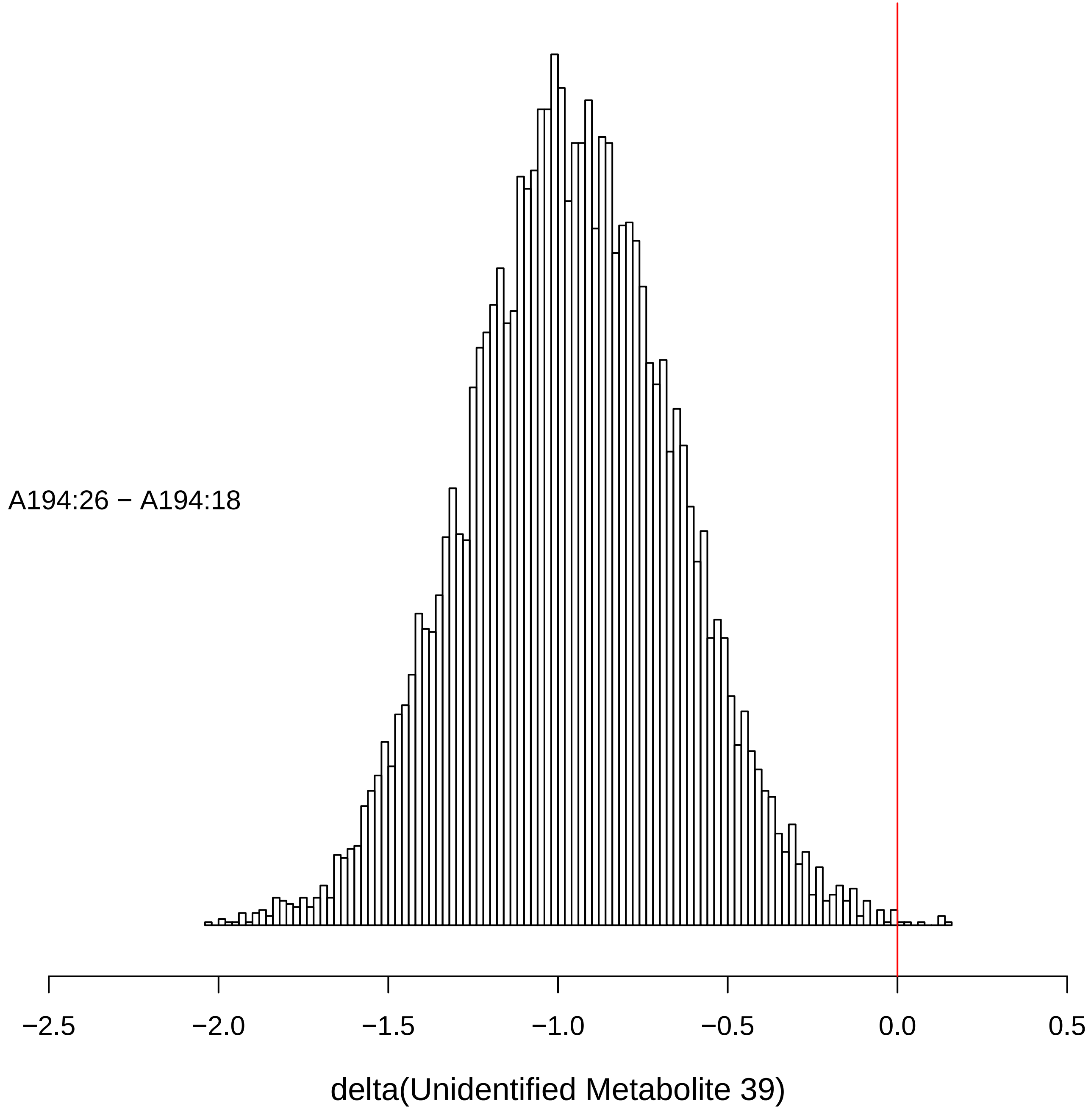
A194:26 − A194:18
−2.5
−2.0
−1.5
−1.0
−0.5
0.0
0.5
delta(Unidentified Metabolite 39)

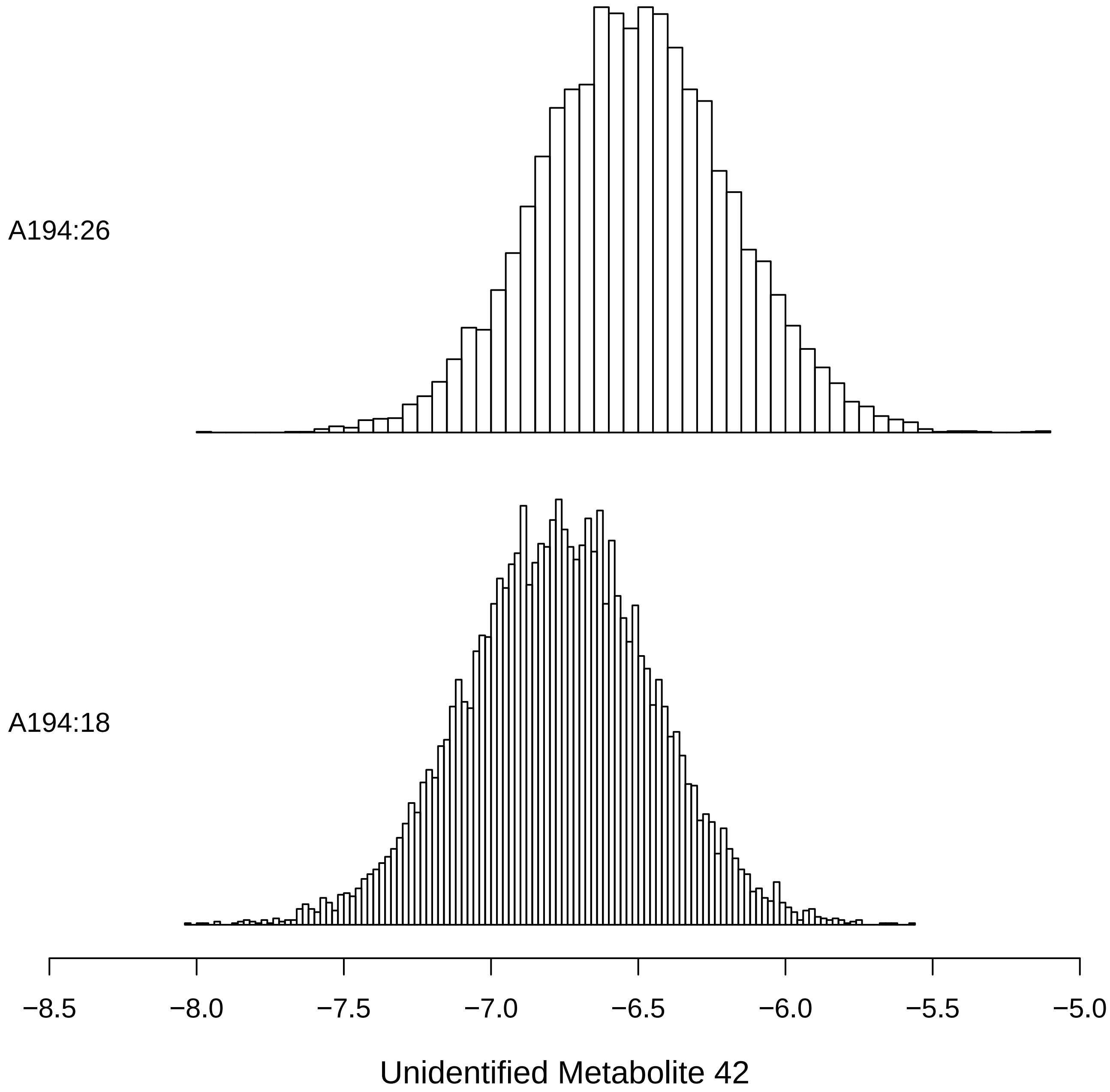

A194:26
A194:18
−8.5
−8.0
−7.5
−7.0
−6.5
−6.0
−5.5
−5.0
Unidentified Metabolite 42

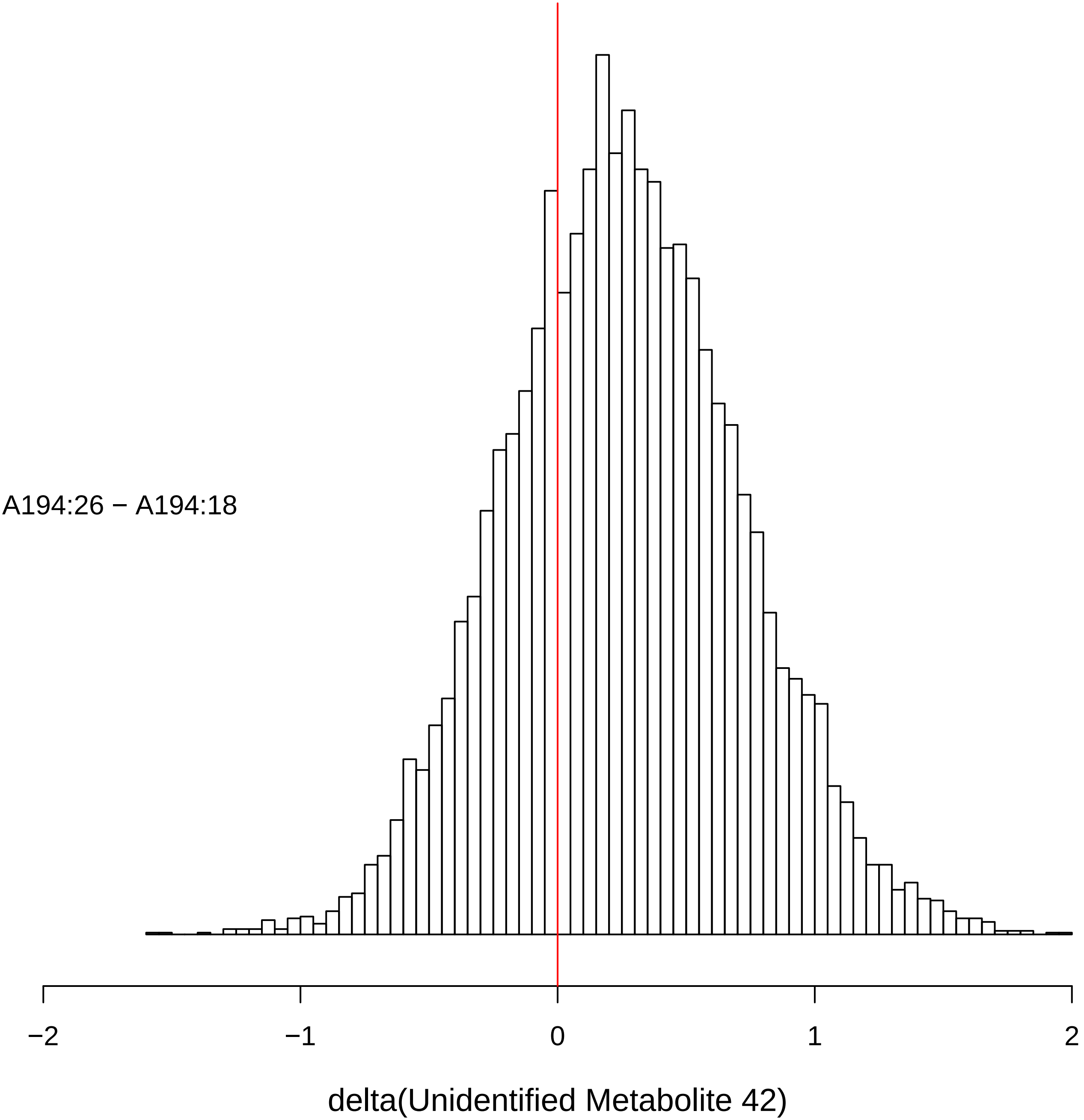

A194:26 − A194:18
−2
−1
0
1
2
delta(Unidentified Metabolite 42)

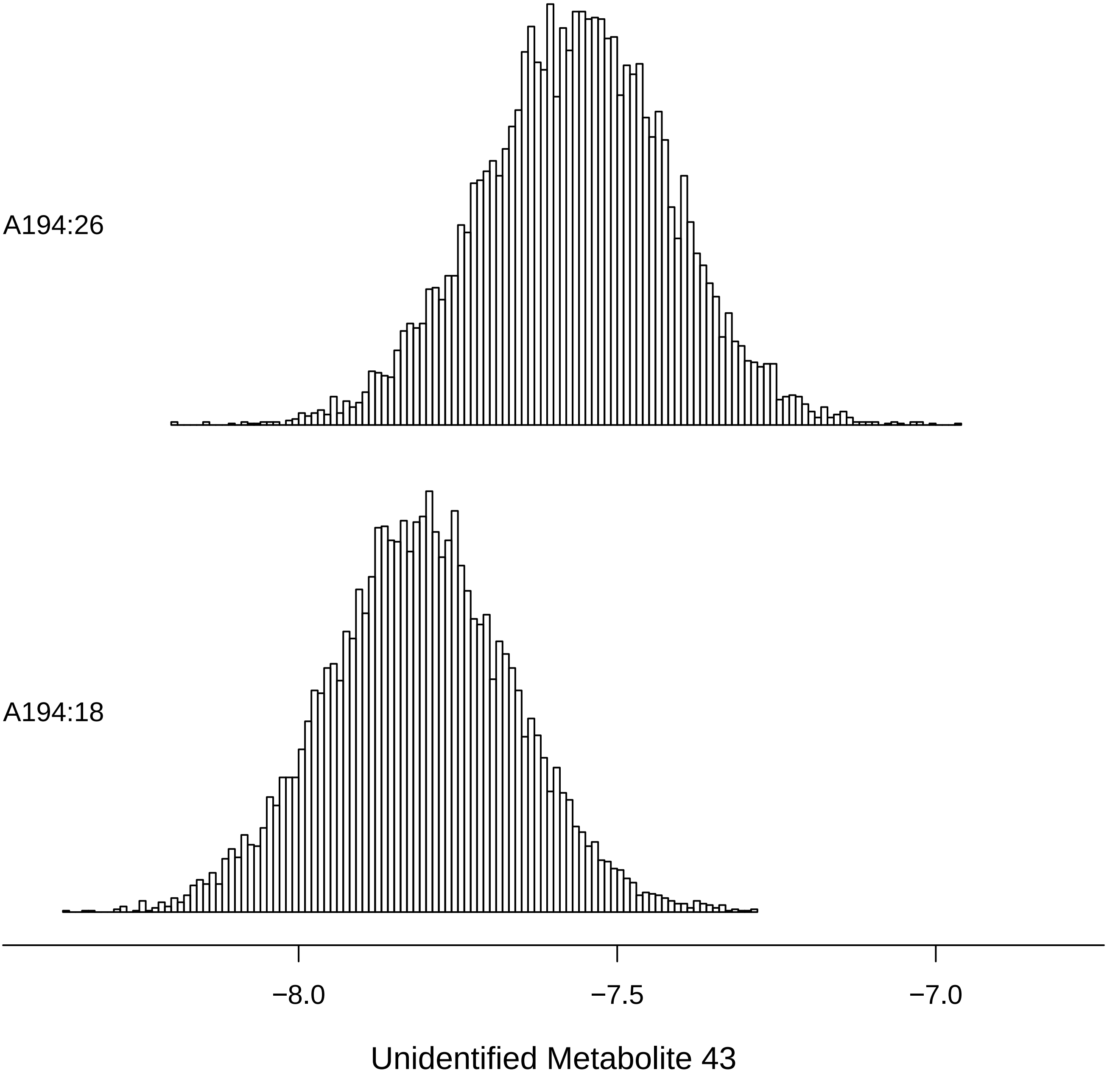
A194:26
A194:18
−8.0
−7.5
−7.0
Unidentified Metabolite 43

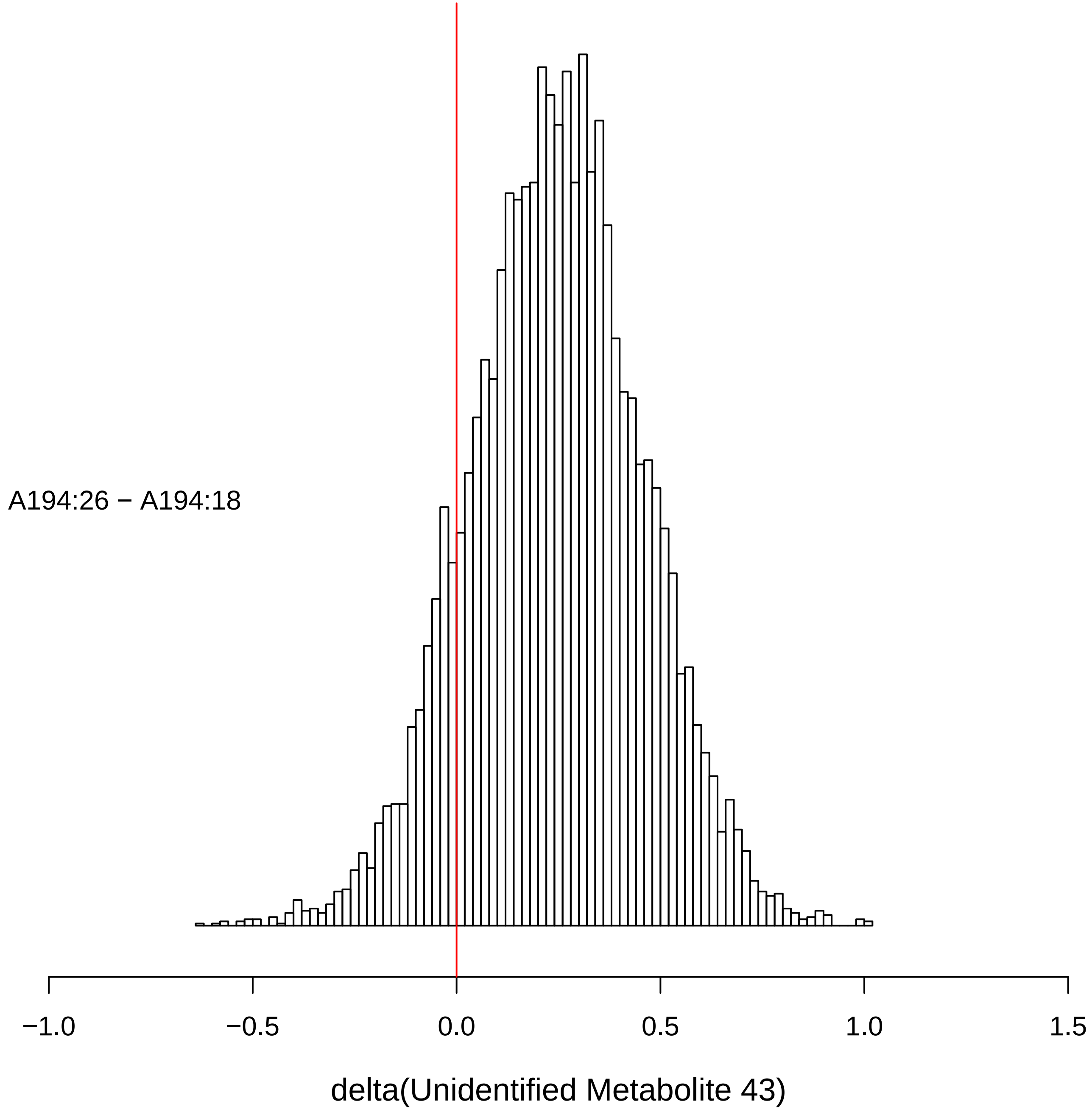
A194:26 − A194:18
−1.0
−0.5
0.0
0.5
1.0
1.5
delta(Unidentified Metabolite 43)

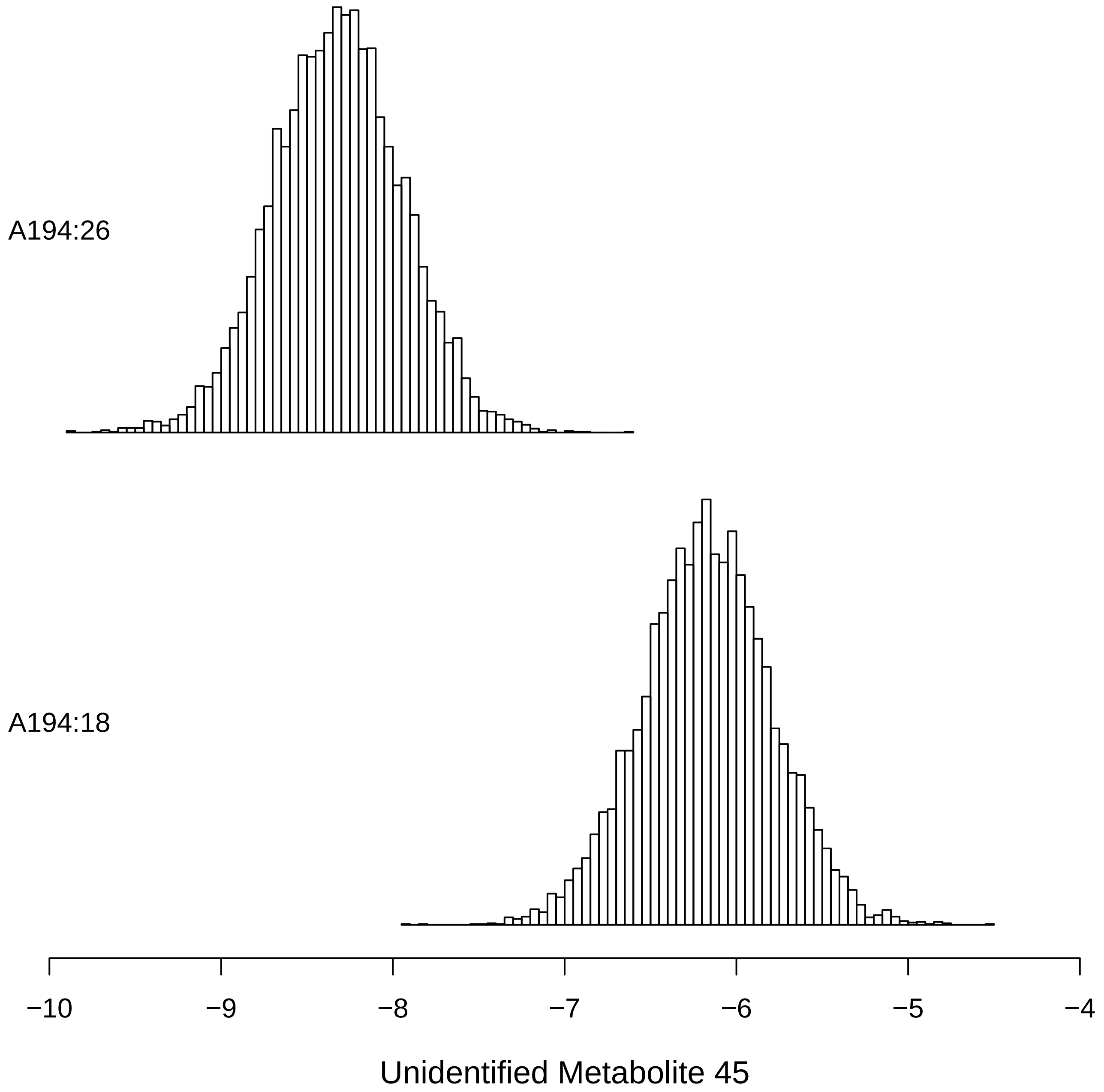
A194:26
A194:18
−10
−9
−8
−7
−6
−5
−4
Unidentified Metabolite 45

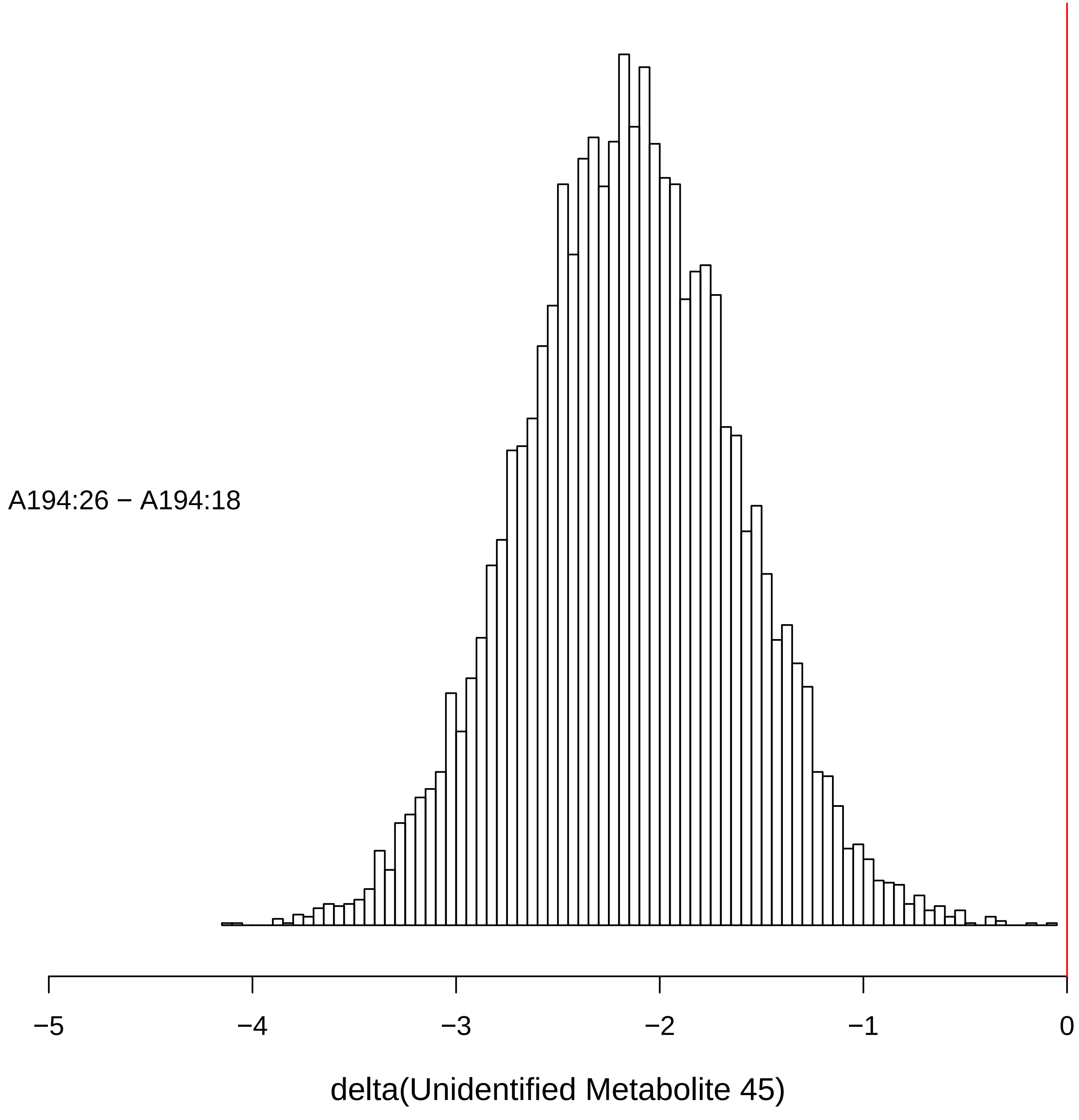
A194:26 − A194:18
−5
−4
−3
−2
−1
0
delta(Unidentified Metabolite 45)

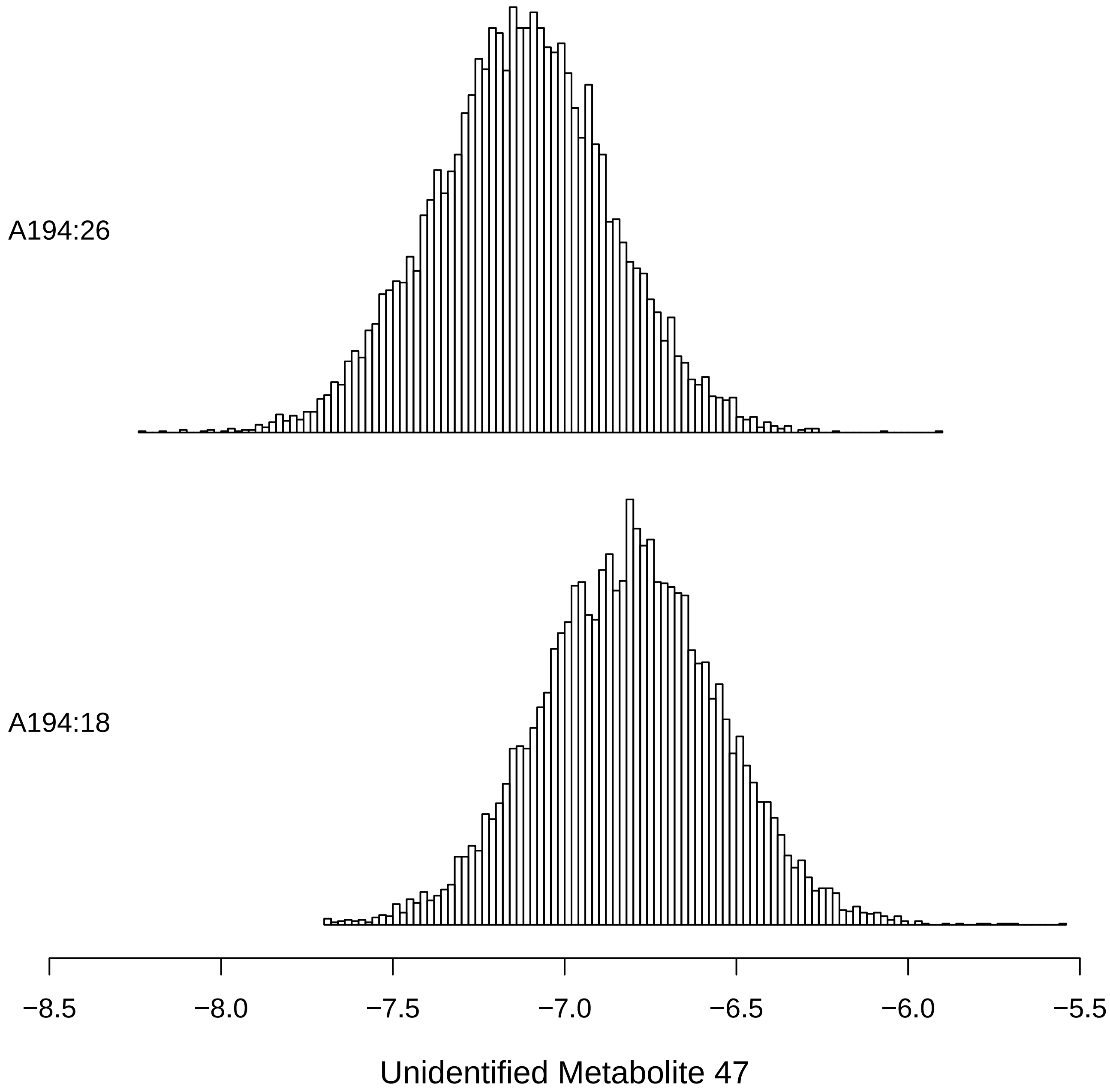
A194:26
A194:18
−8.5
−8.0
−7.5
−7.0
−6.5
−6.0
−5.5
Unidentified Metabolite 47

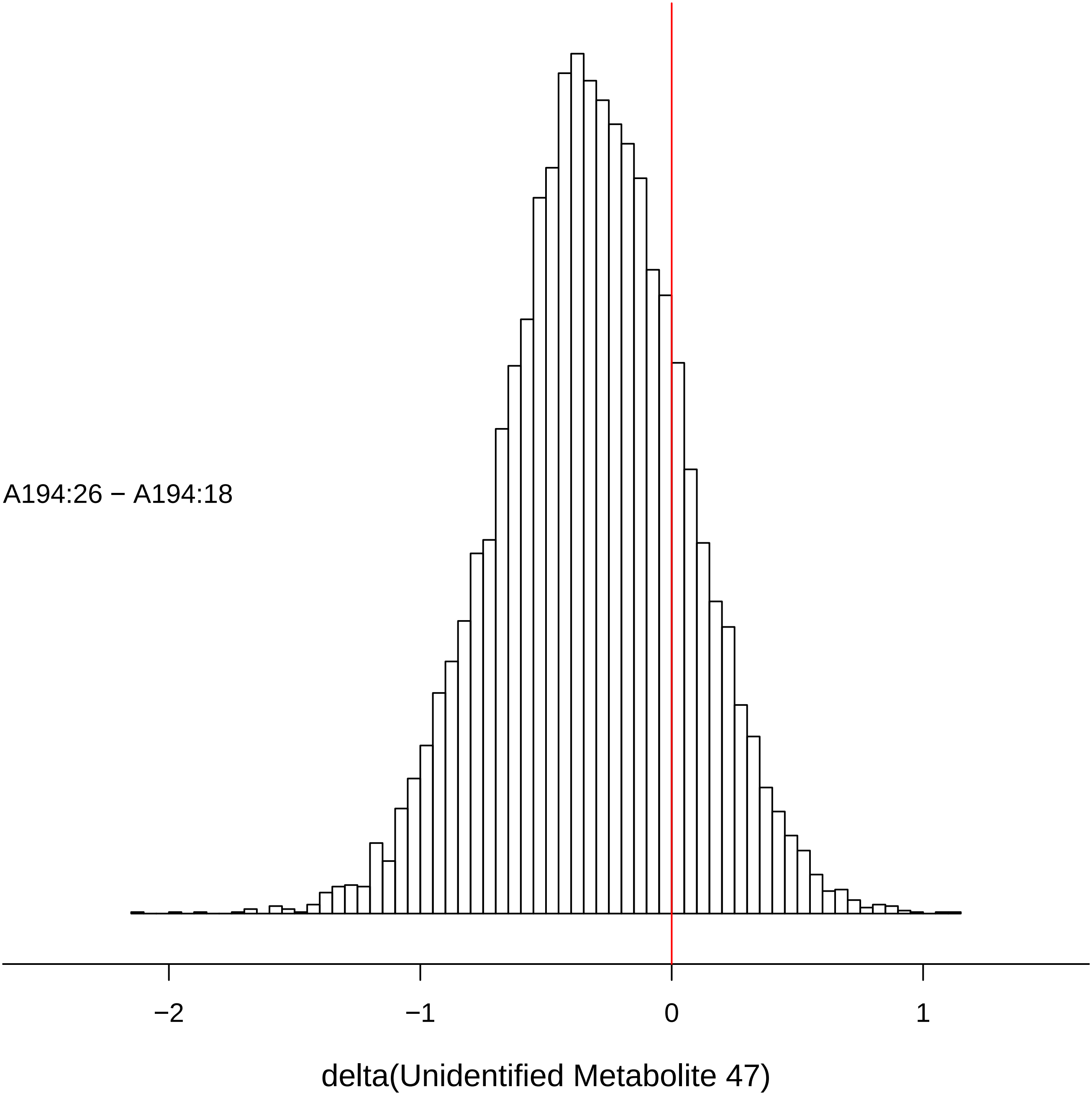

A194:26 − A194:18
−2
−1
0
1
delta(Unidentified Metabolite 47)

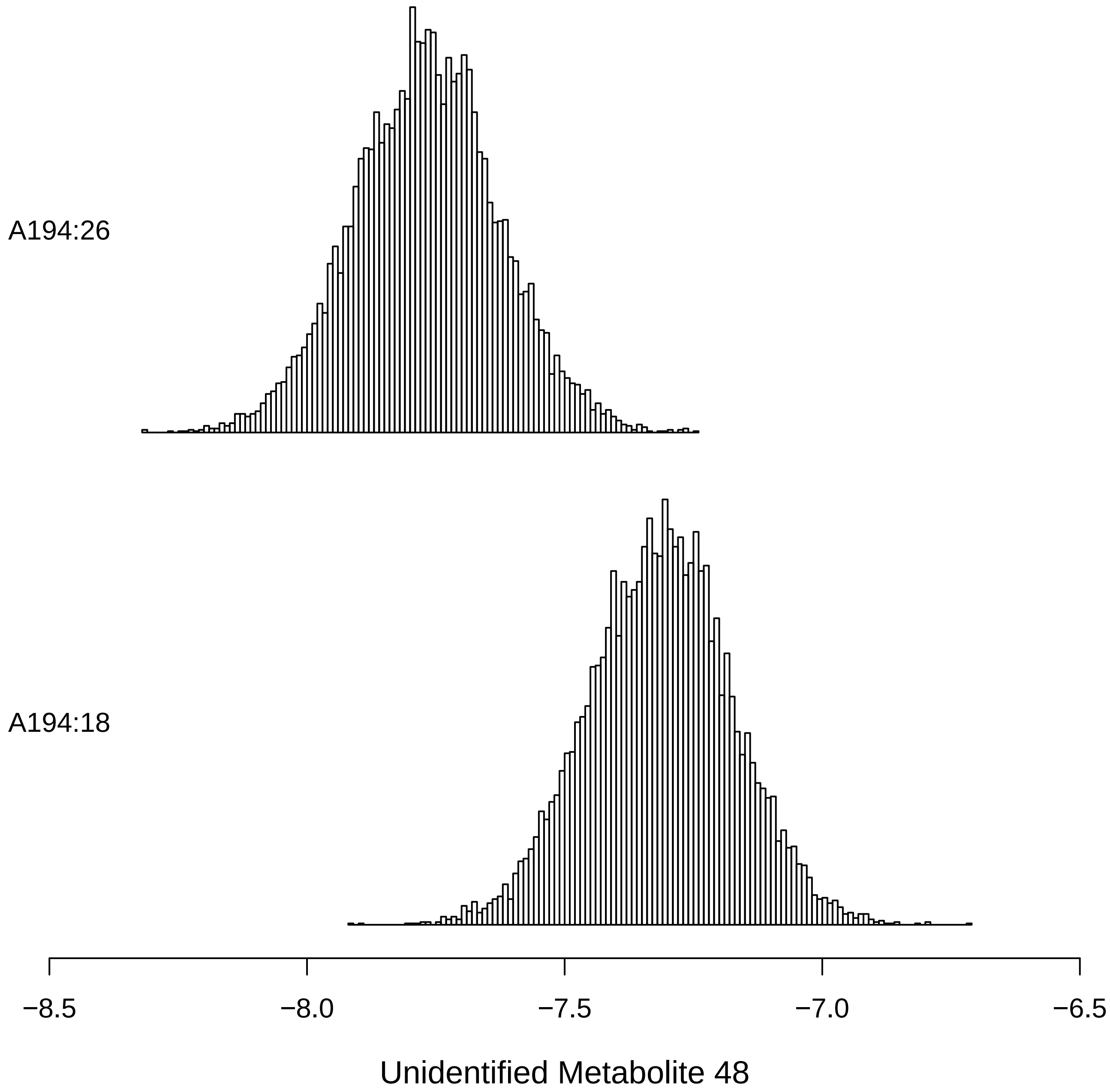
A194:26
A194:18
−8.5
−8.0
−7.5
−7.0
−6.5
Unidentified Metabolite 48

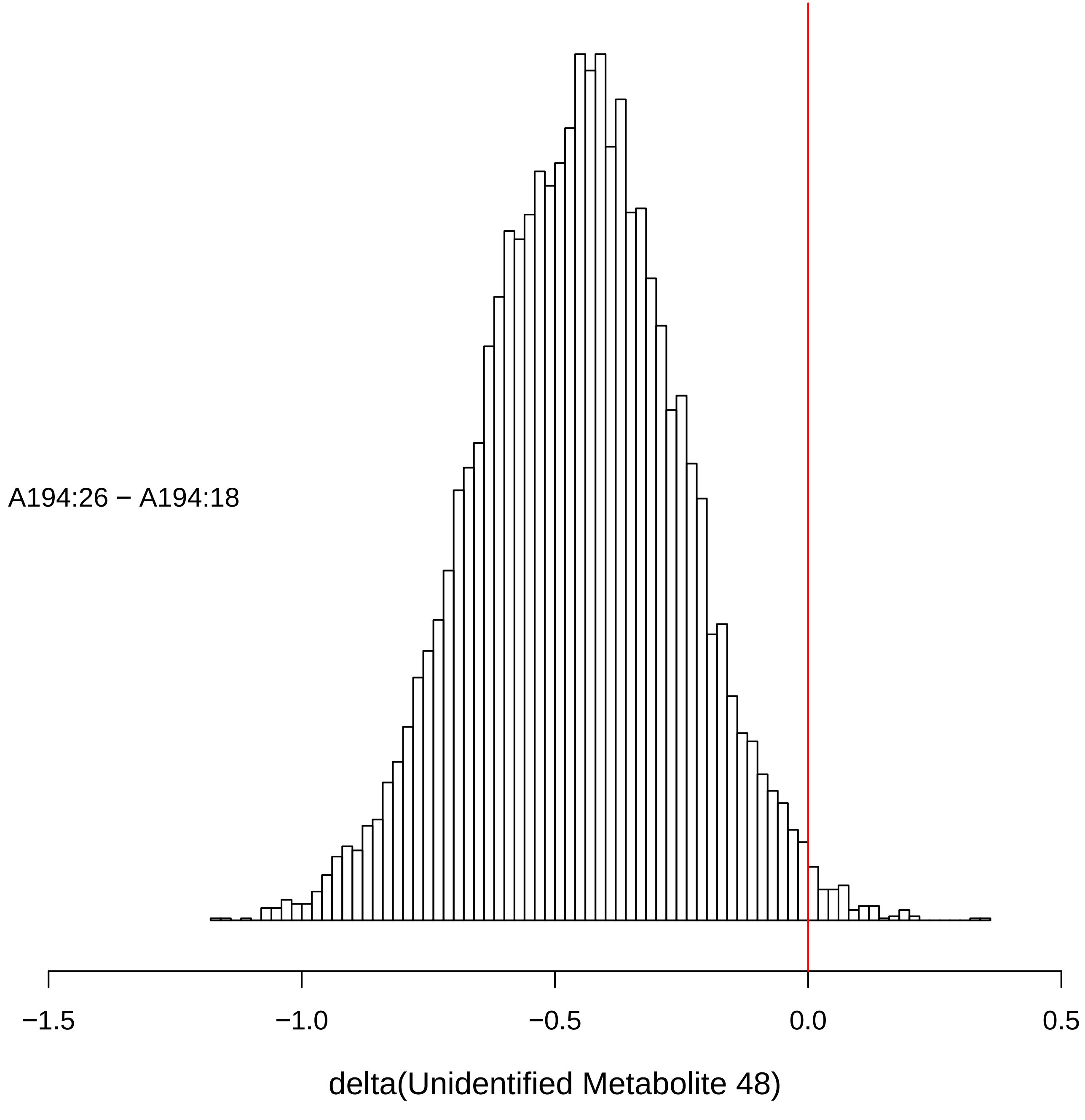
A194:26 − A194:18
−1.5
−1.0
−0.5
0.0
0.5
delta(Unidentified Metabolite 48)

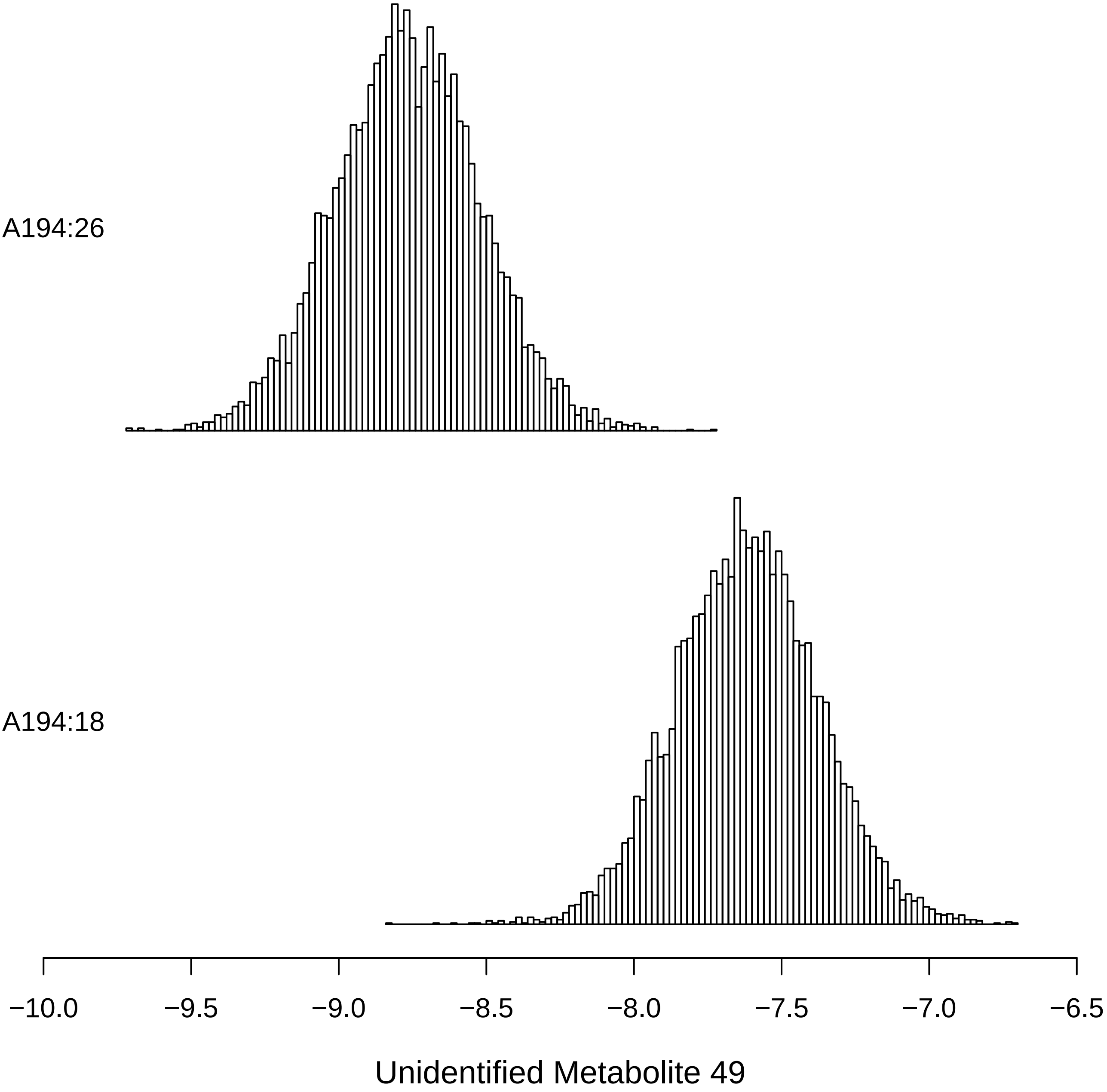

A194:26
A194:18
−10.0
−9.5
−9.0
−8.5
−8.0
−7.5
−7.0
−6.5
Unidentified Metabolite 49

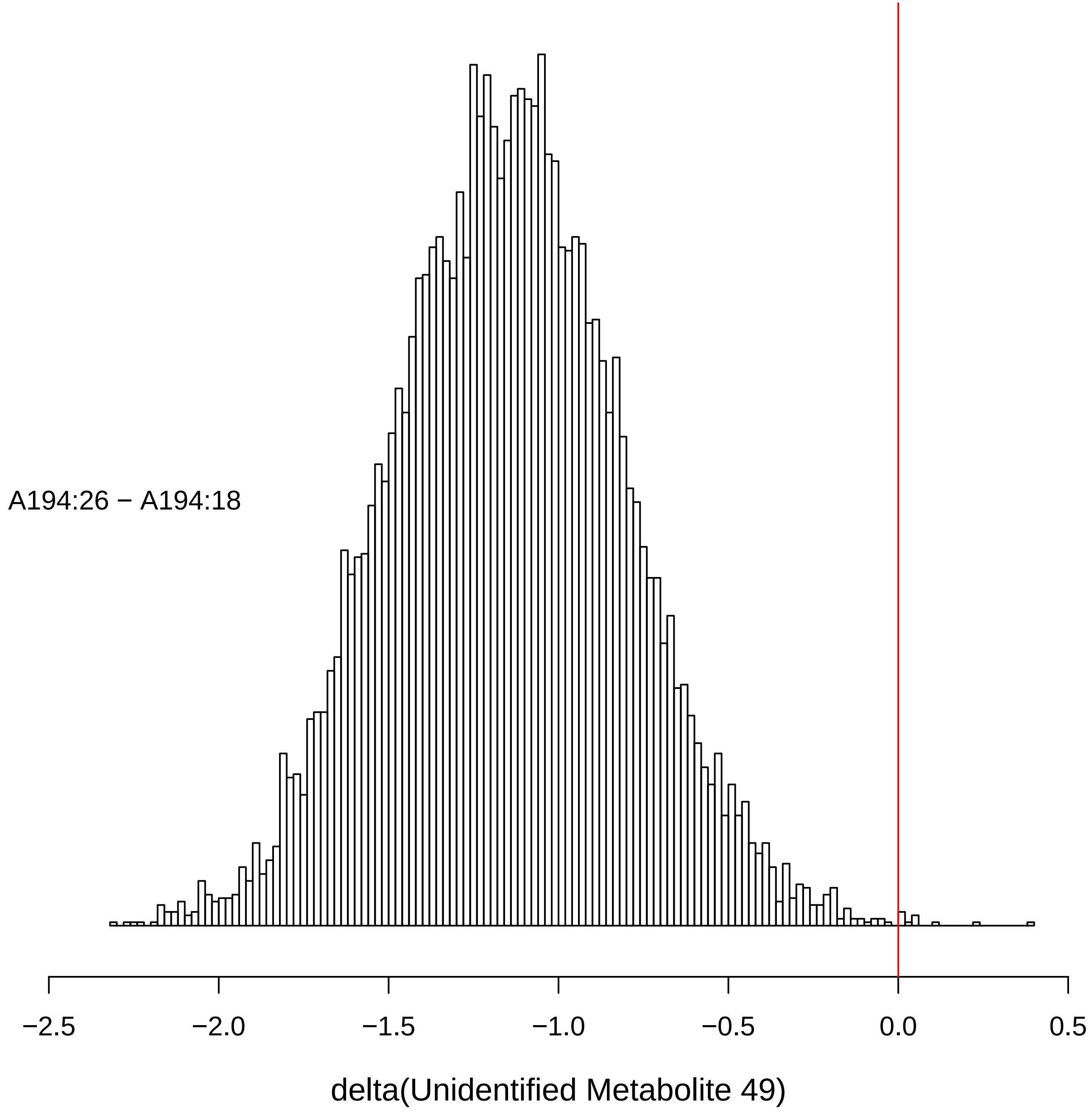
A194:26 − A194:18
−2.5
−2.0
−1.5
−1.0
−0.5
0.0
0.5
delta(Unidentified Metabolite 49)

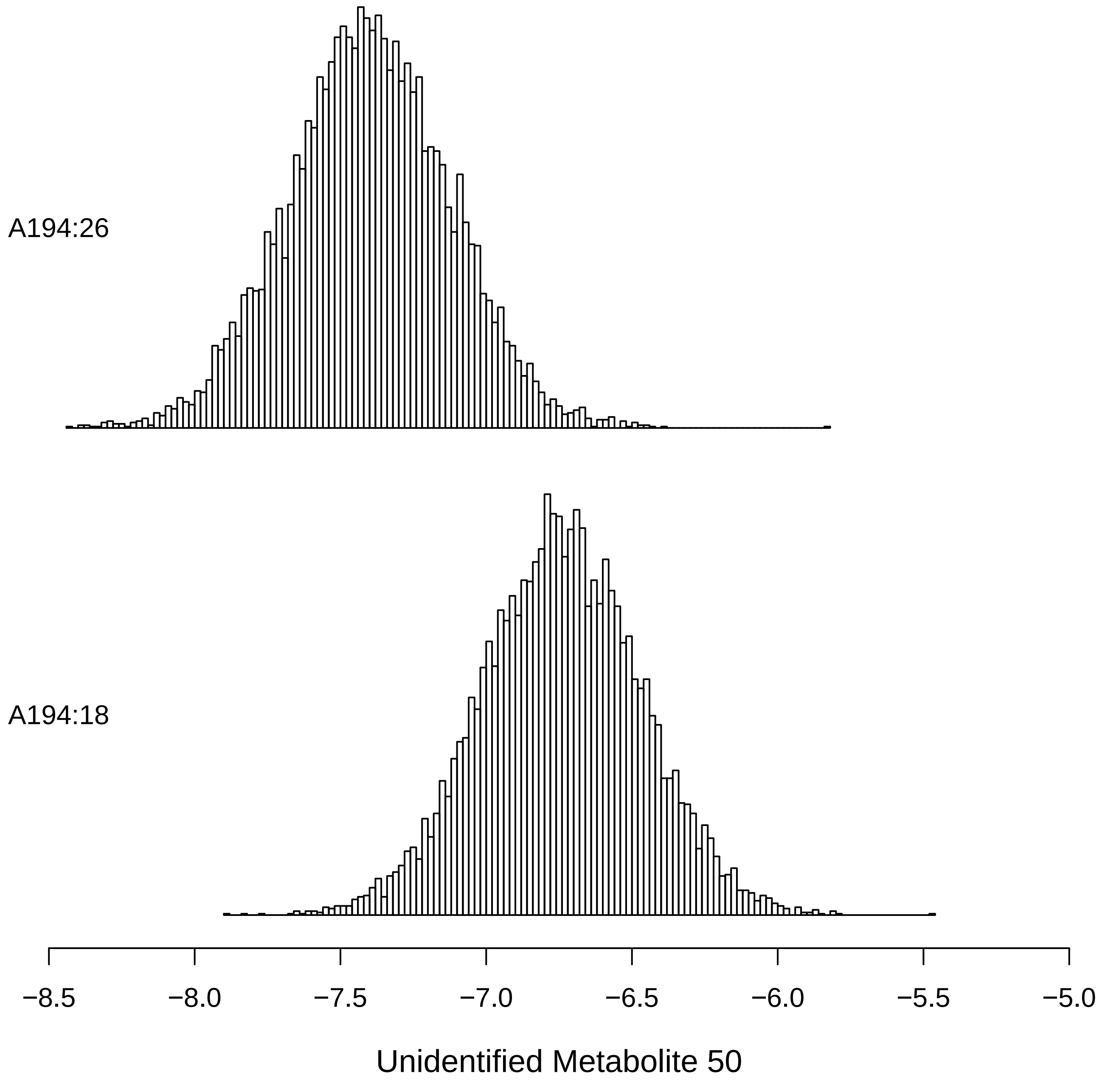
A194:26
A194:18
−8.5
−8.0
−7.5
−7.0
−6.5
−6.0
−5.5
−5.0
Unidentified Metabolite 50

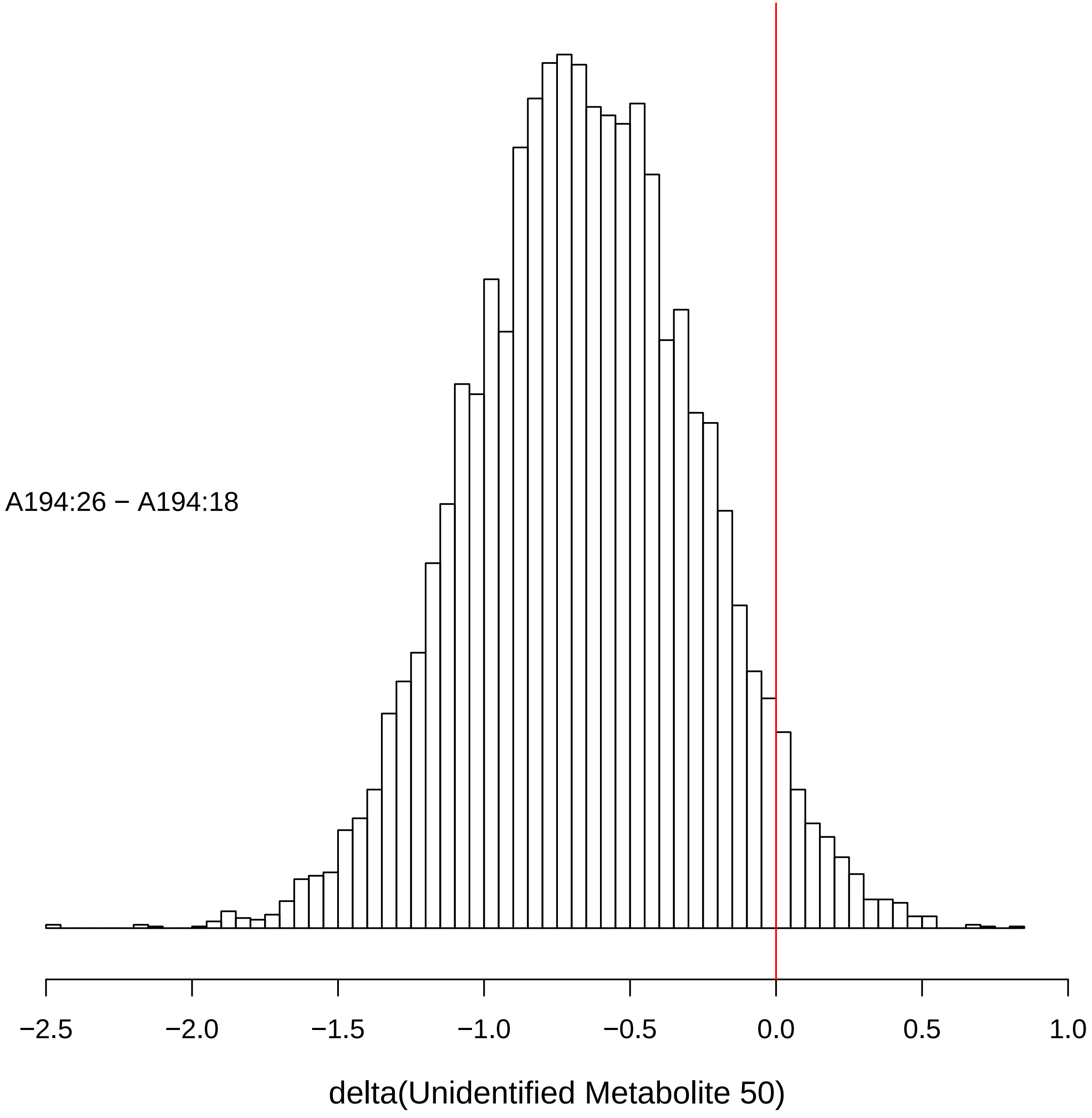
A194:26 − A194:18
−2.5
−2.0
−1.5
−1.0
−0.5
0.0
0.5
1.0
delta(Unidentified Metabolite 50)

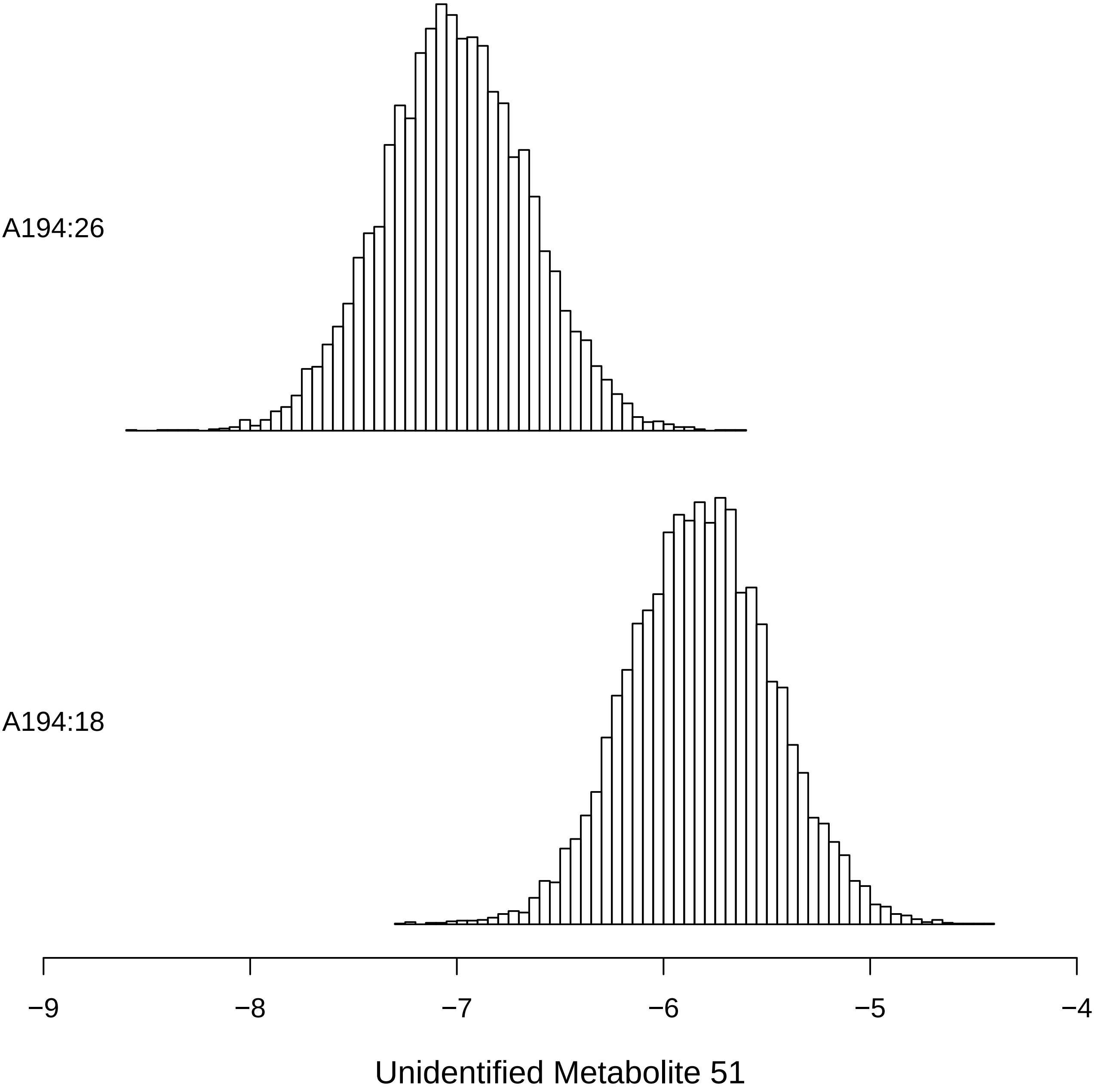
A194:26
A194:18
−9
−8
−7
−6
−5
−4
Unidentified Metabolite 51

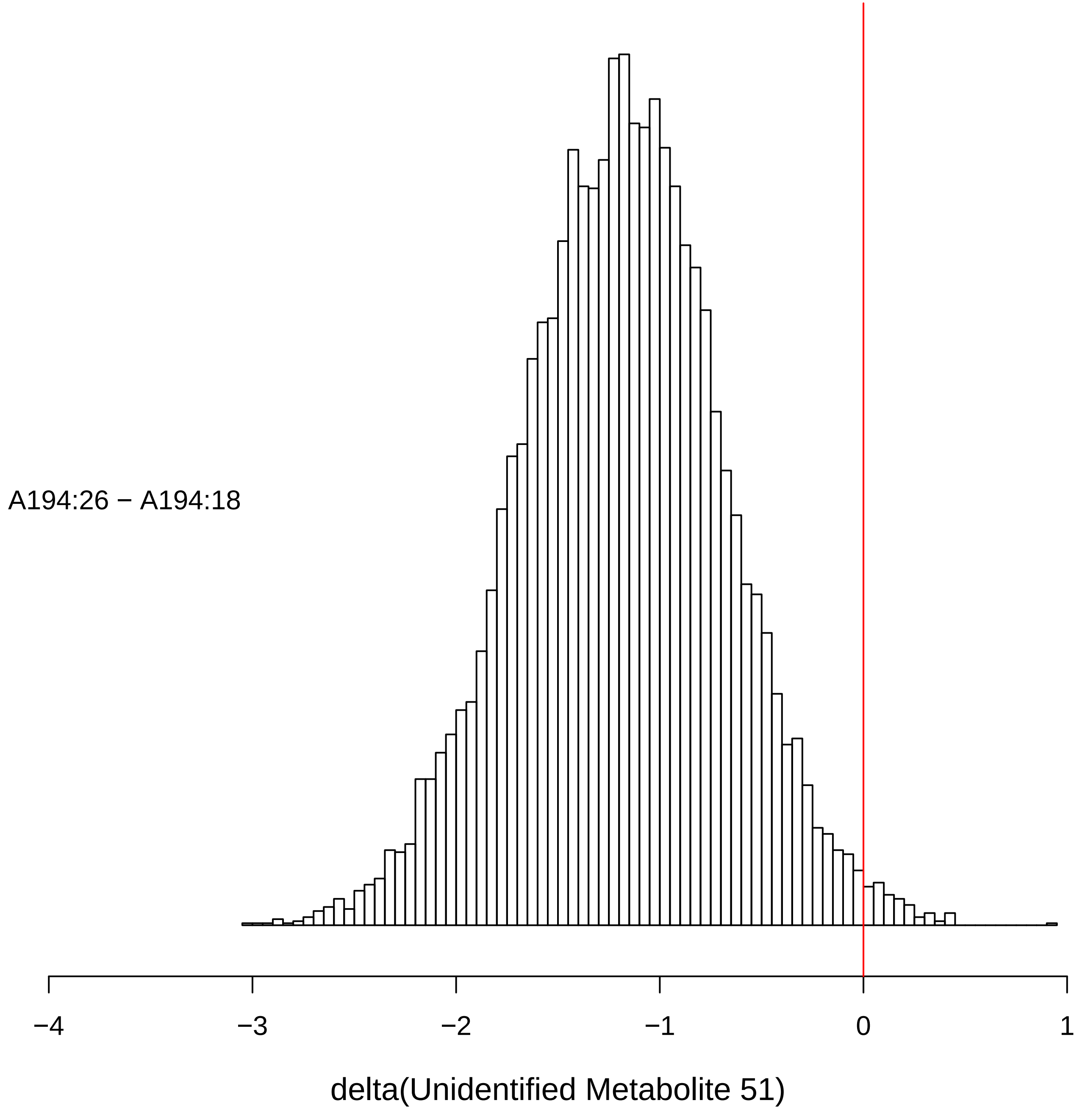

A194:26 − A194:18
−4
−3
−2
−1
0
1
delta(Unidentified Metabolite 51)

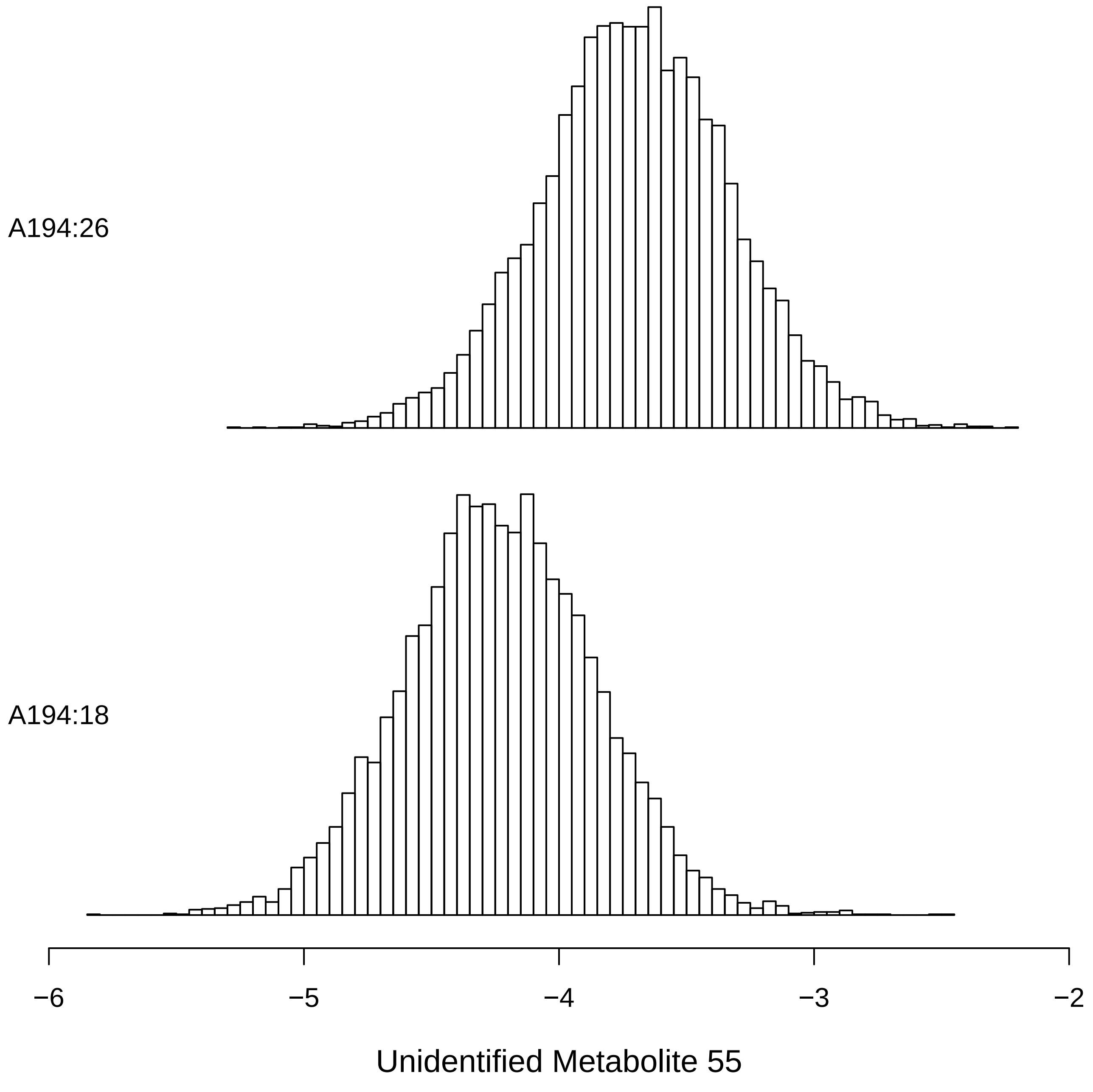
A194:26
A194:18
−6
−5
−4
−3
−2
Unidentified Metabolite 55

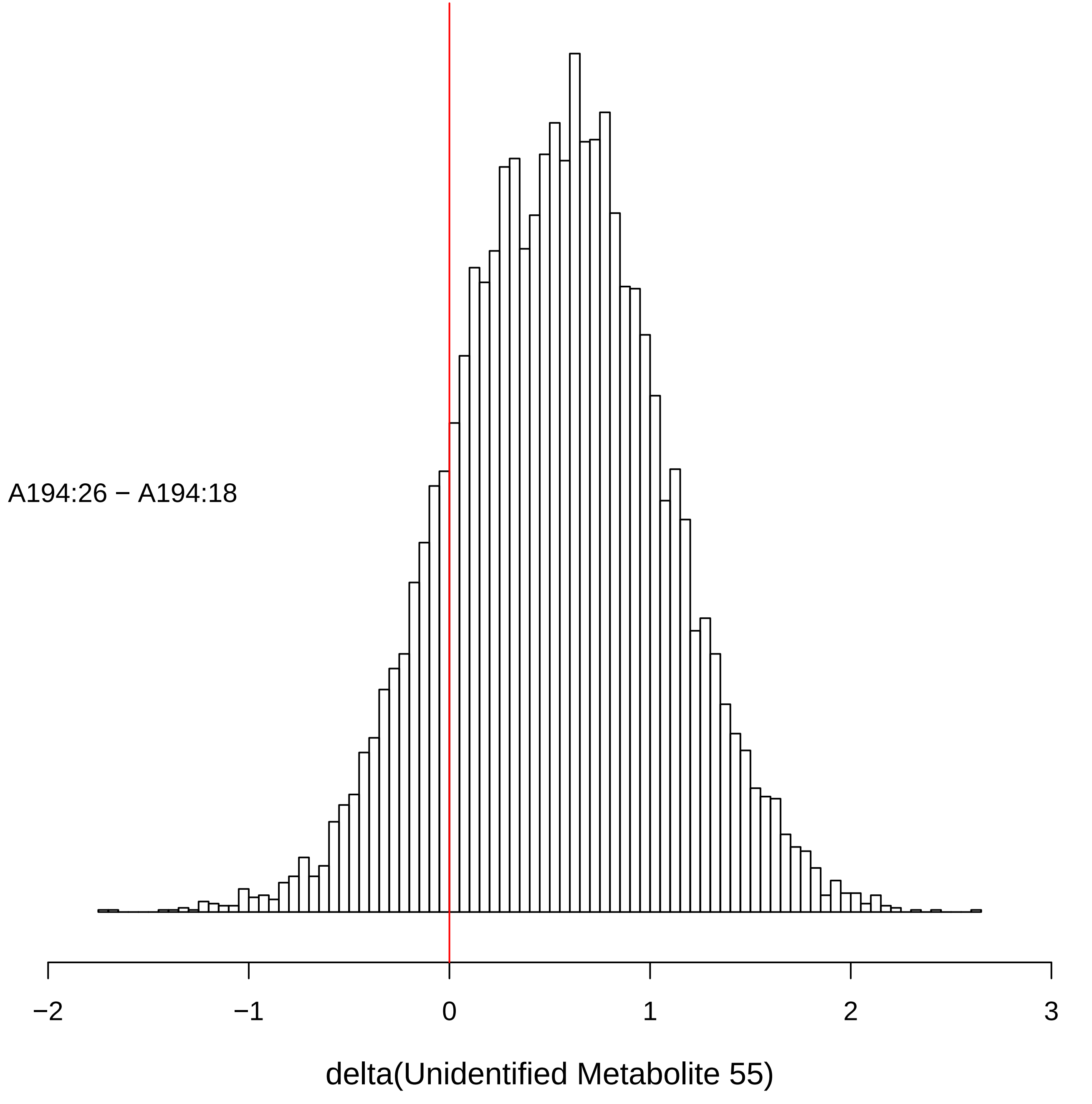

A194:26 − A194:18
−2
−1
0
1
2
3
delta(Unidentified Metabolite 55)

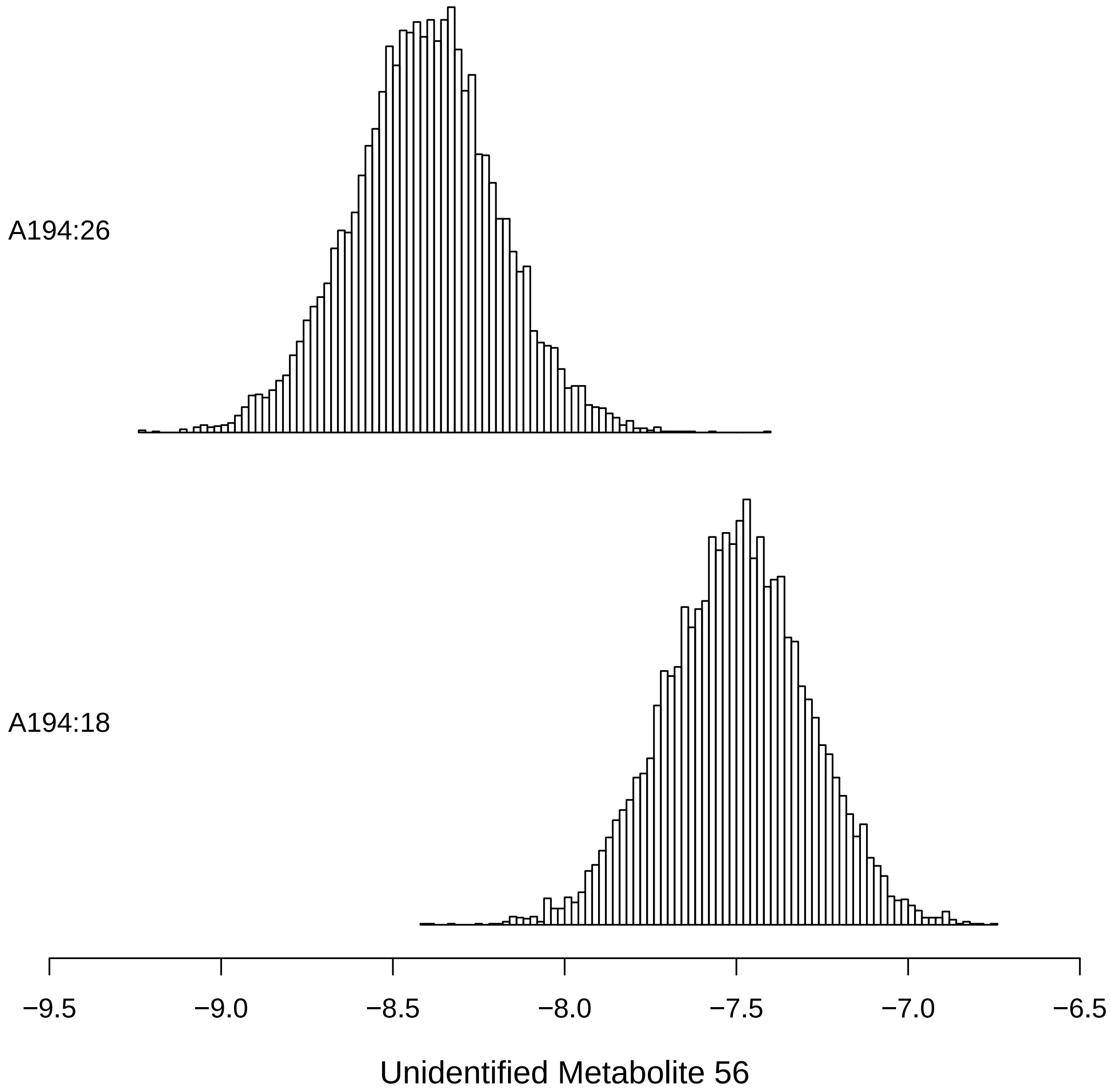
A194:26
A194:18
−9.5
−9.0
−8.5
−8.0
−7.5
−7.0
−6.5
Unidentified Metabolite 56

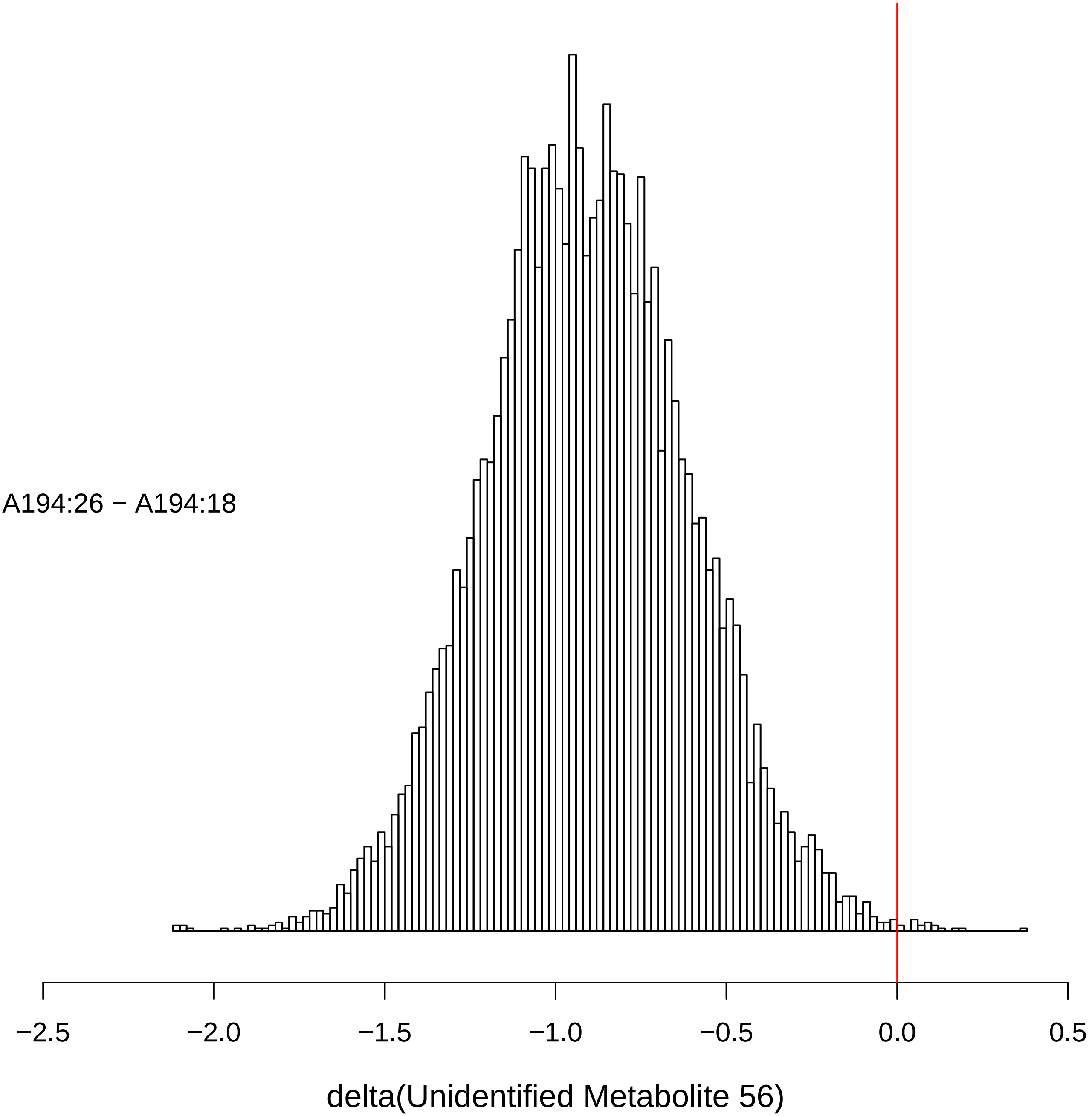

A194:26 − A194:18
−2.5
−2.0
−1.5
−1.0
−0.5
0.0
0.5
delta(Unidentified Metabolite 56)

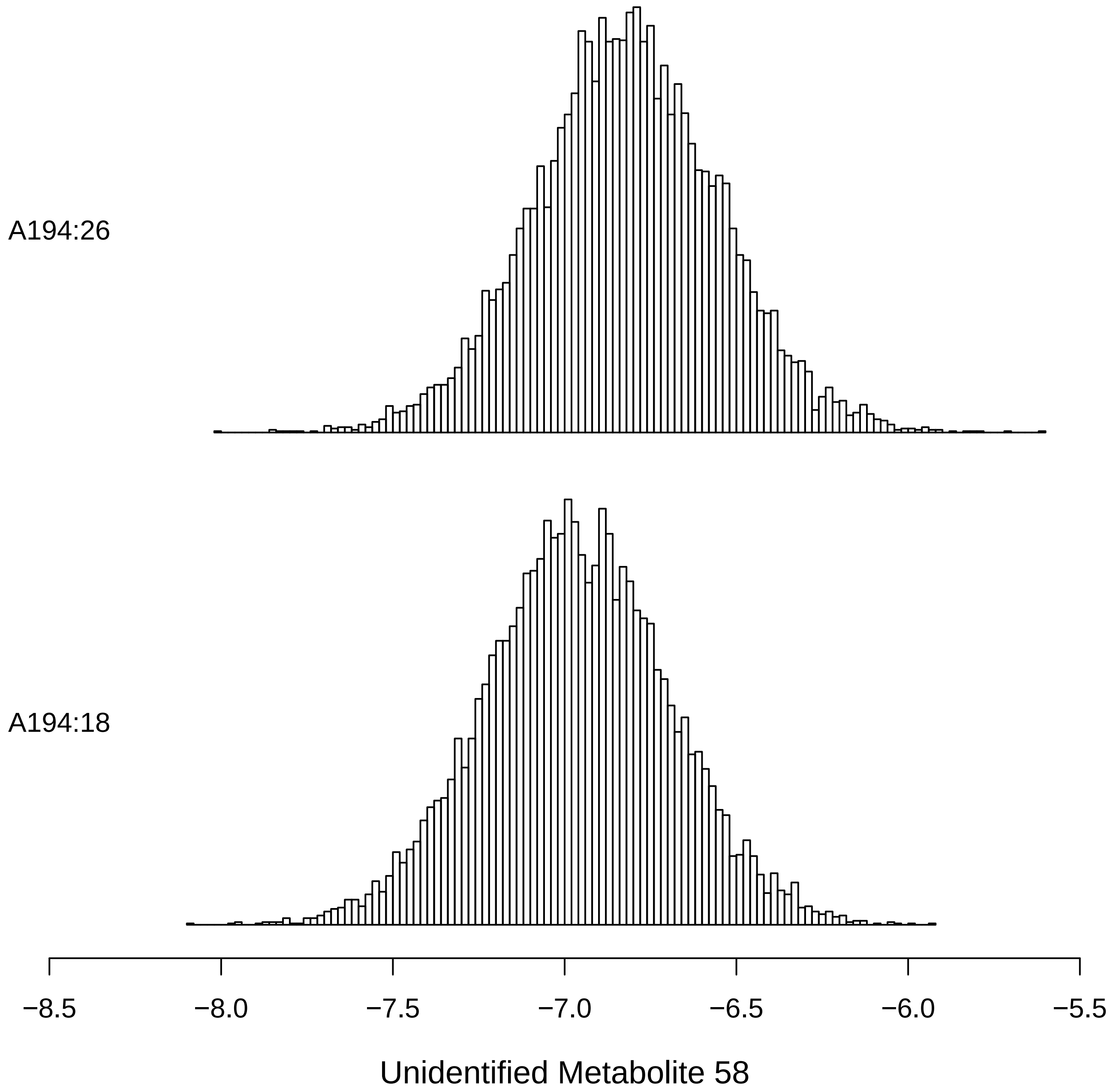

A194:26
A194:18
−8.5
−8.0
−7.5
−7.0
−6.5
−6.0
−5.5
Unidentified Metabolite 58

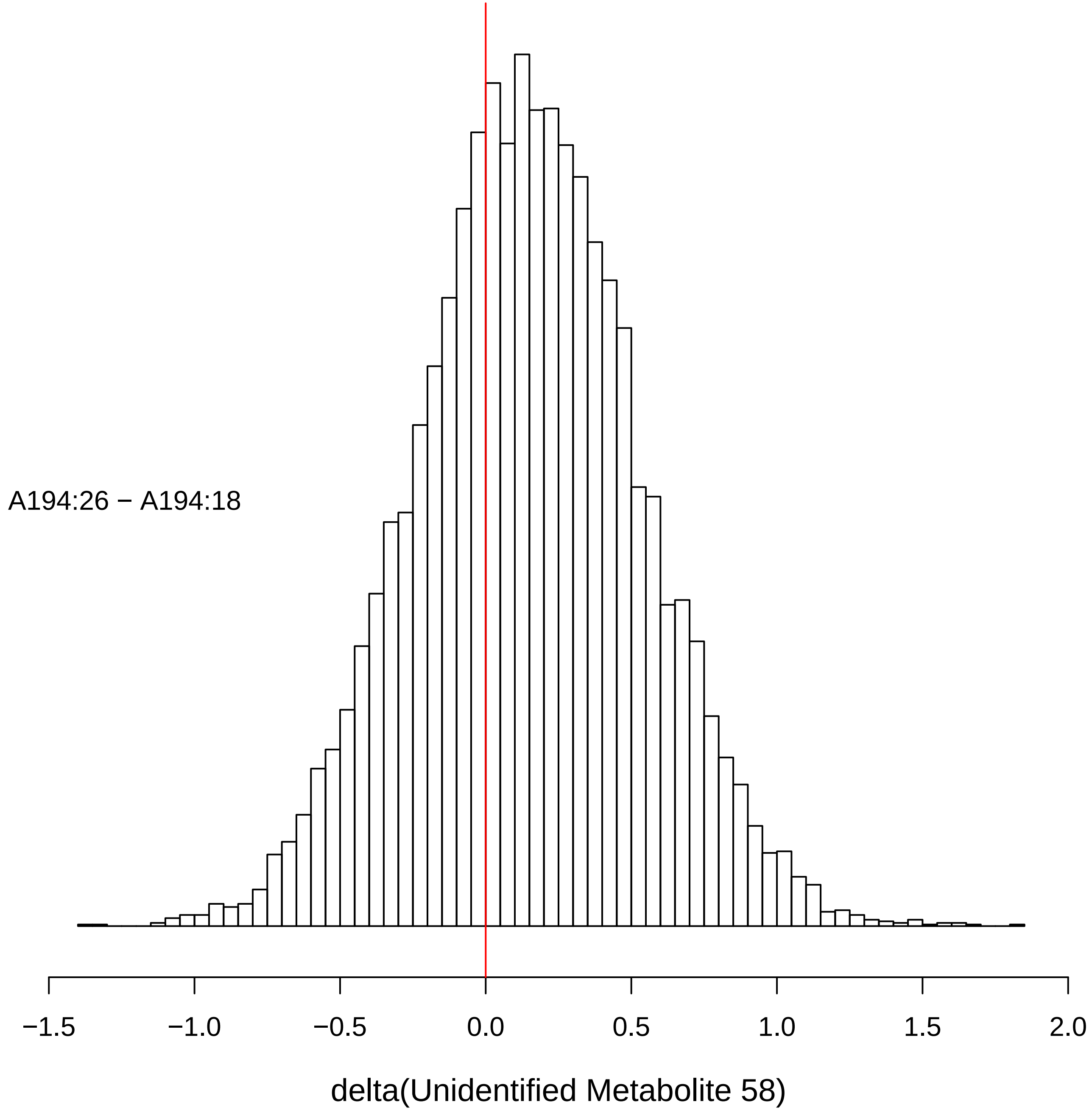
A194:26 − A194:18
−1.5
−1.0
−0.5
0.0
0.5
1.0
1.5
2.0
delta(Unidentified Metabolite 58)

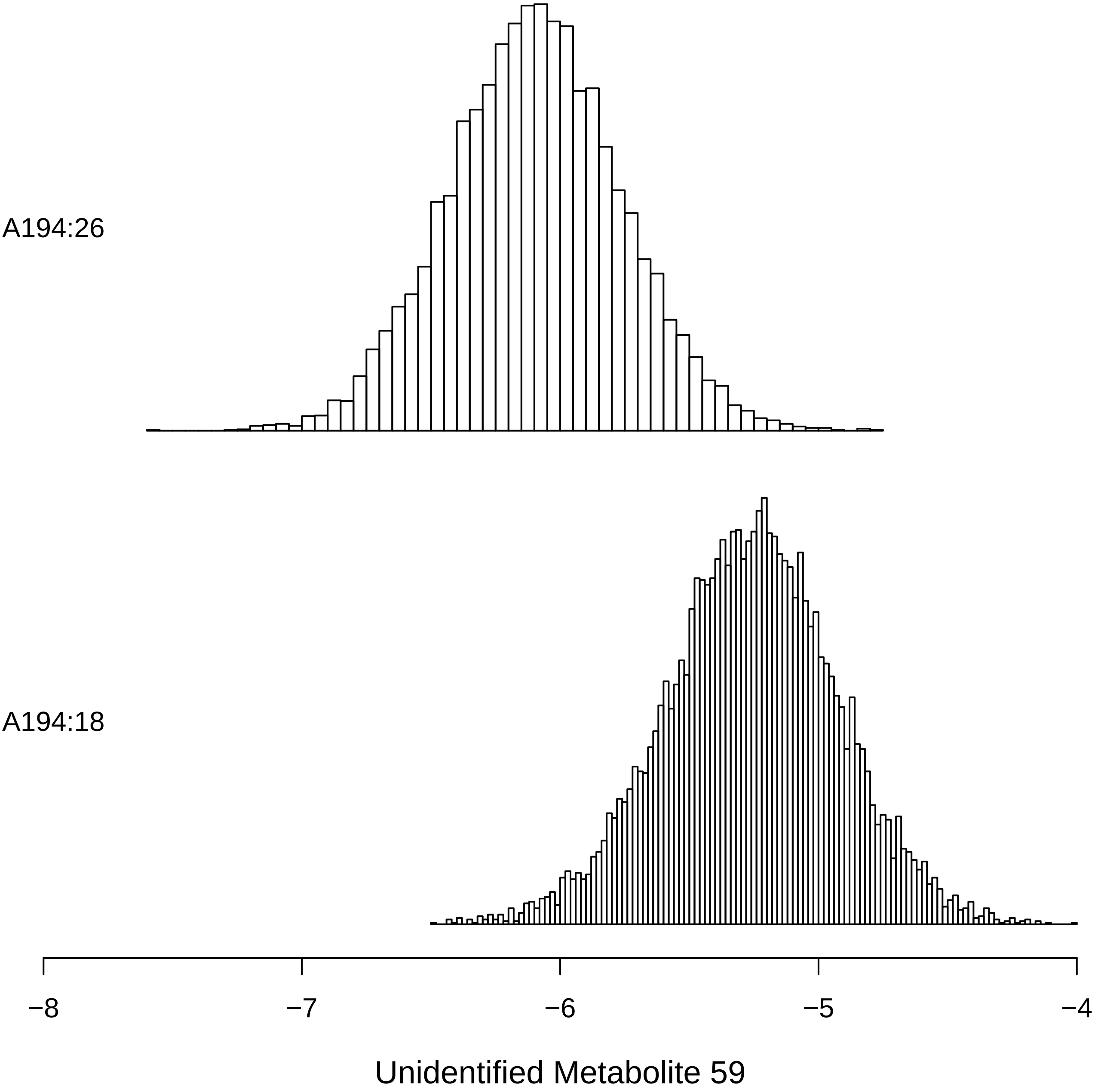

A194:26
A194:18
−8
−7
−6
−5
−4
Unidentified Metabolite 59

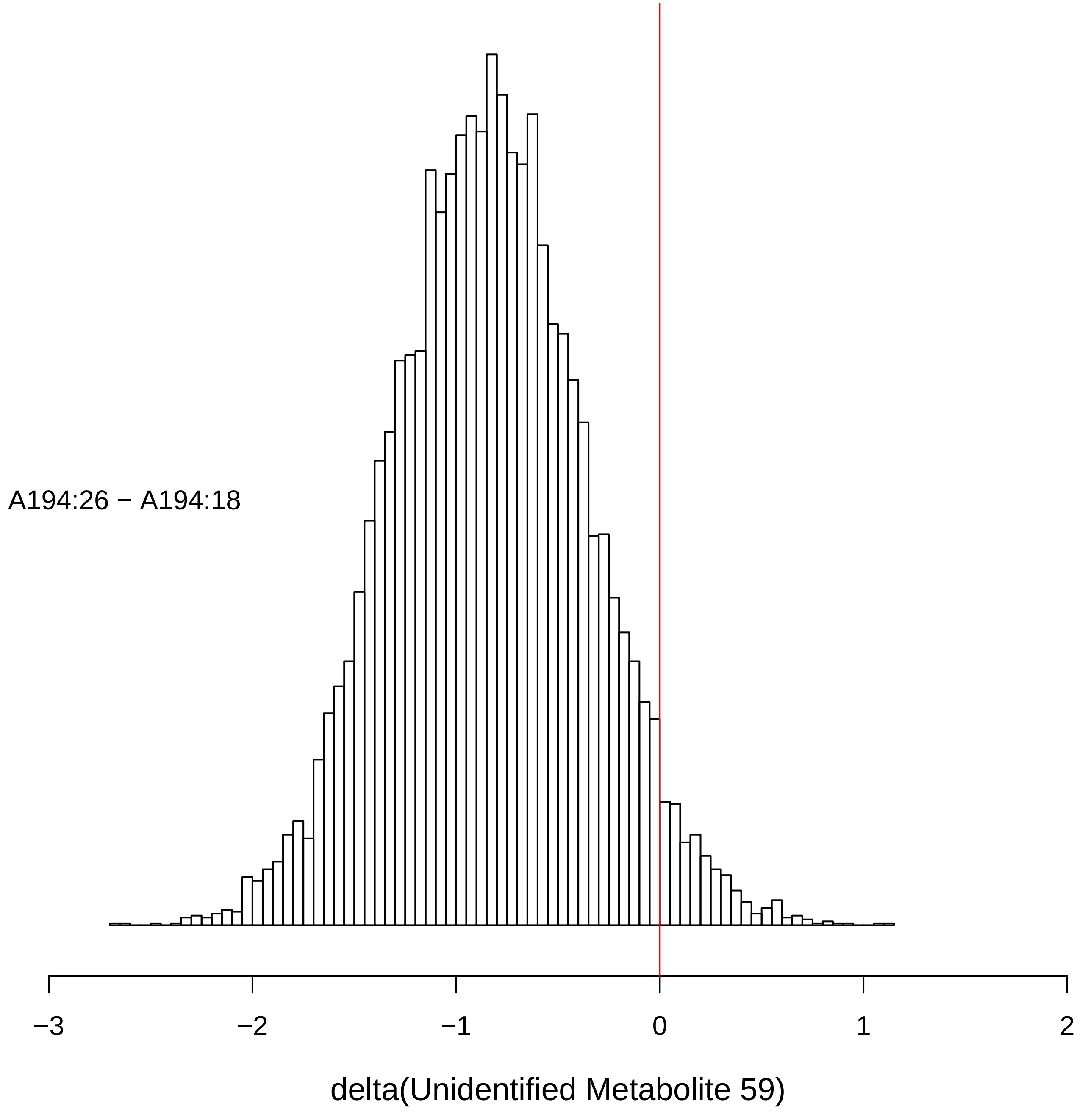
A194:26 − A194:18
−3
−2
−1
0
1
2
delta(Unidentified Metabolite 59)

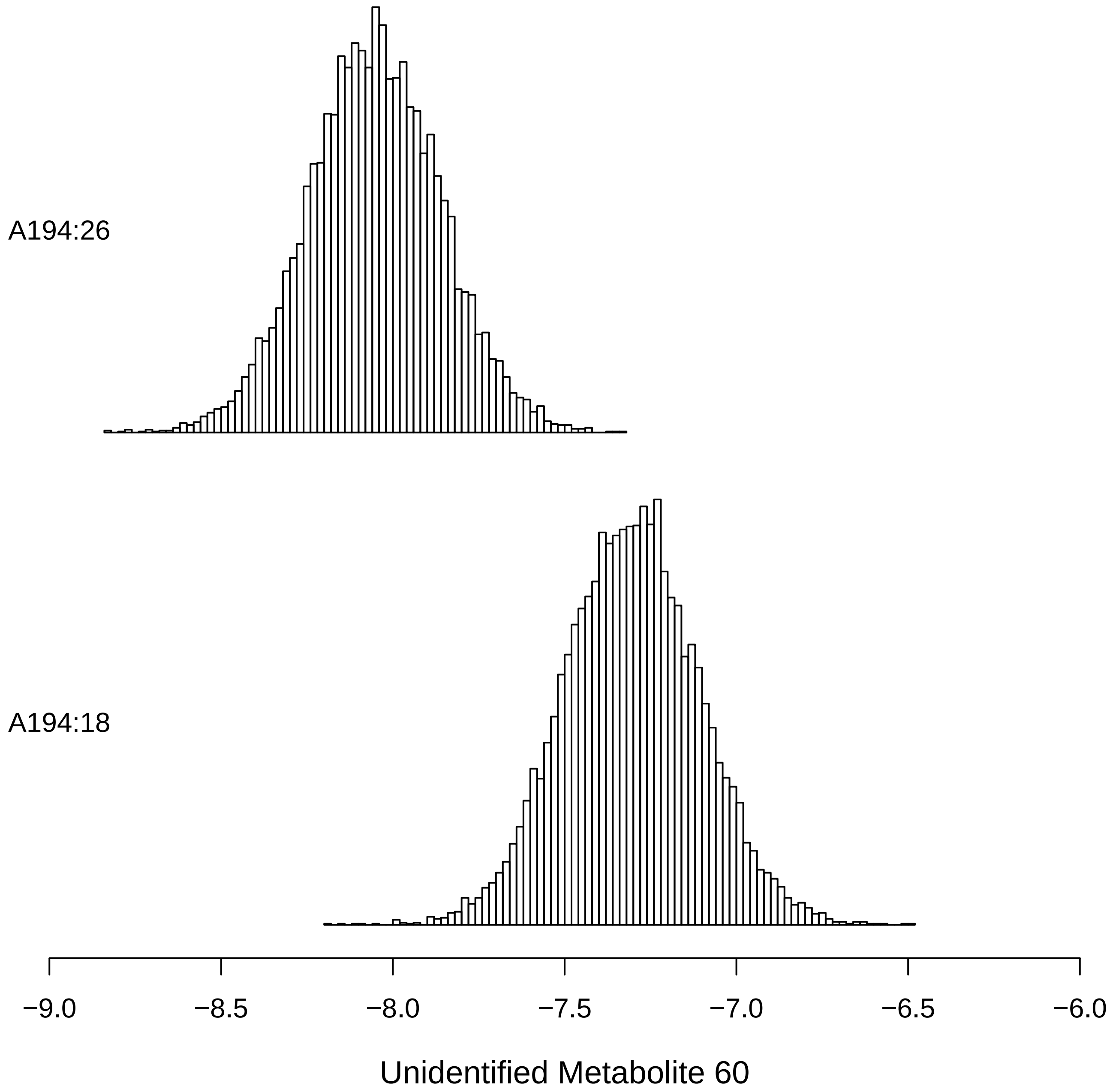

A194:26
A194:18
−9.0
−8.5
−8.0
−7.5
−7.0
−6.5
−6.0
Unidentified Metabolite 60

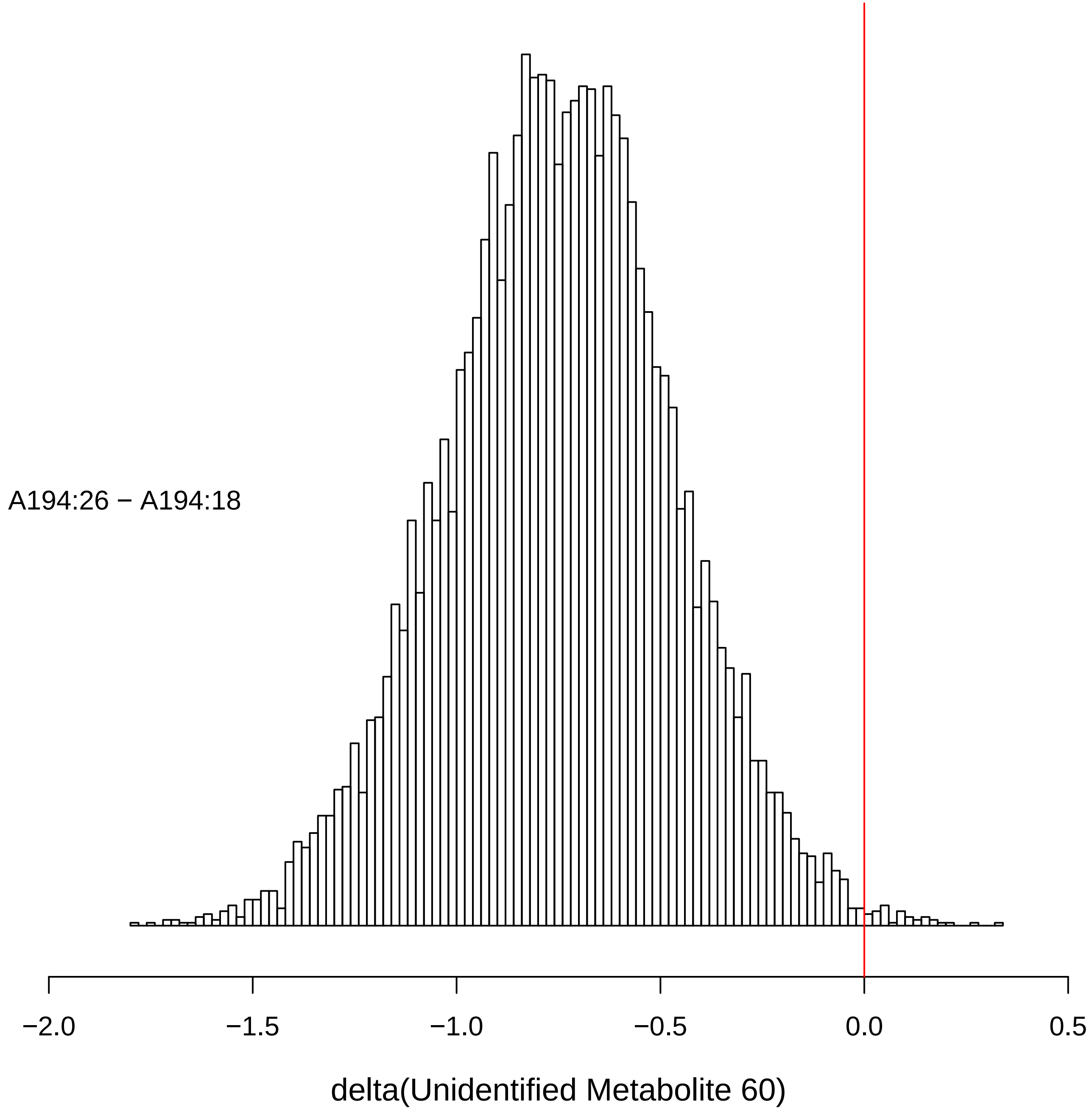

A194:26 − A194:18
−2.0
−1.5
−1.0
−0.5
0.0
0.5
delta(Unidentified Metabolite 60)

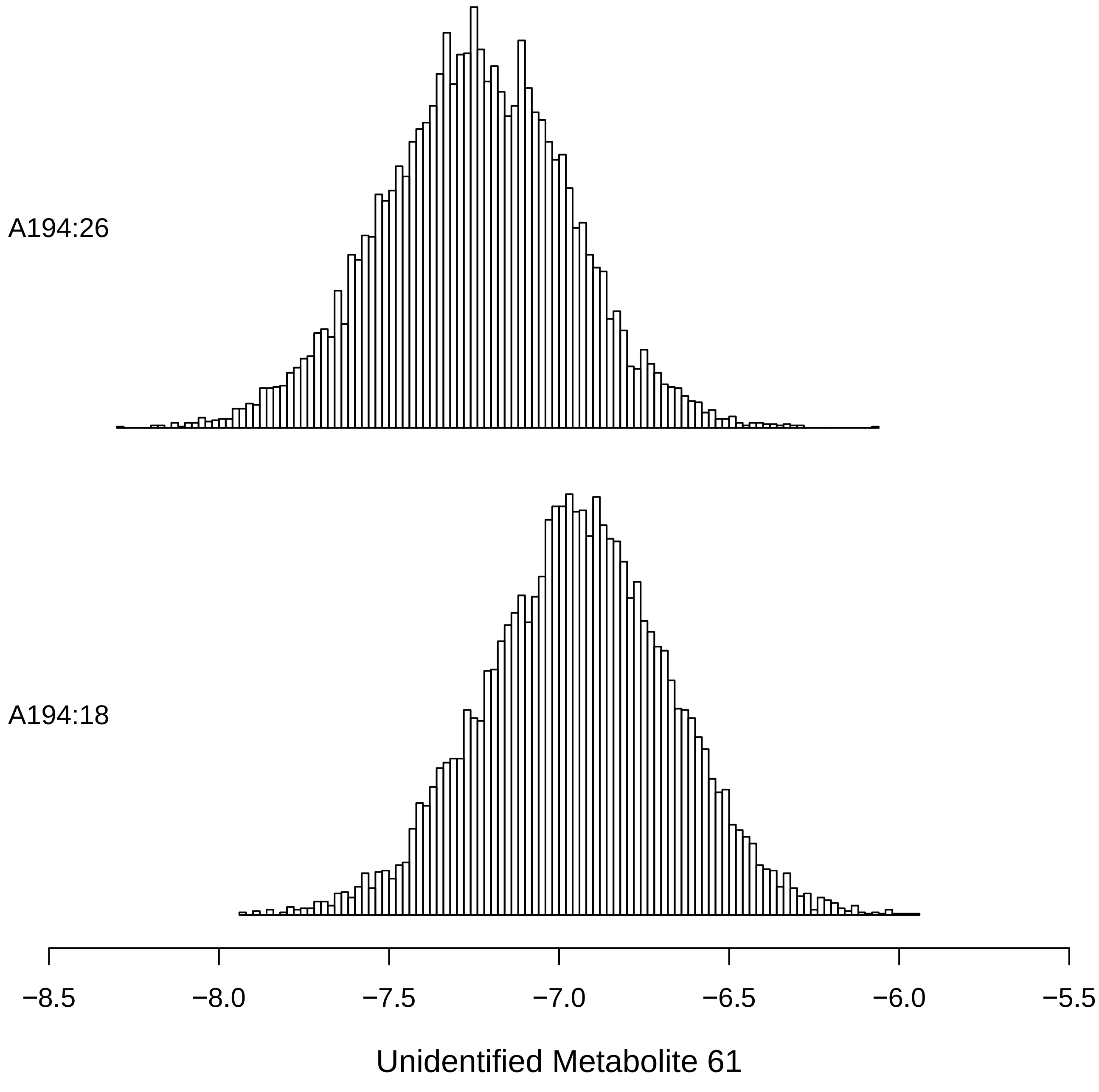

A194:26
A194:18
−8.5
−8.0
−7.5
−7.0
−6.5
−6.0
−5.5
Unidentified Metabolite 61

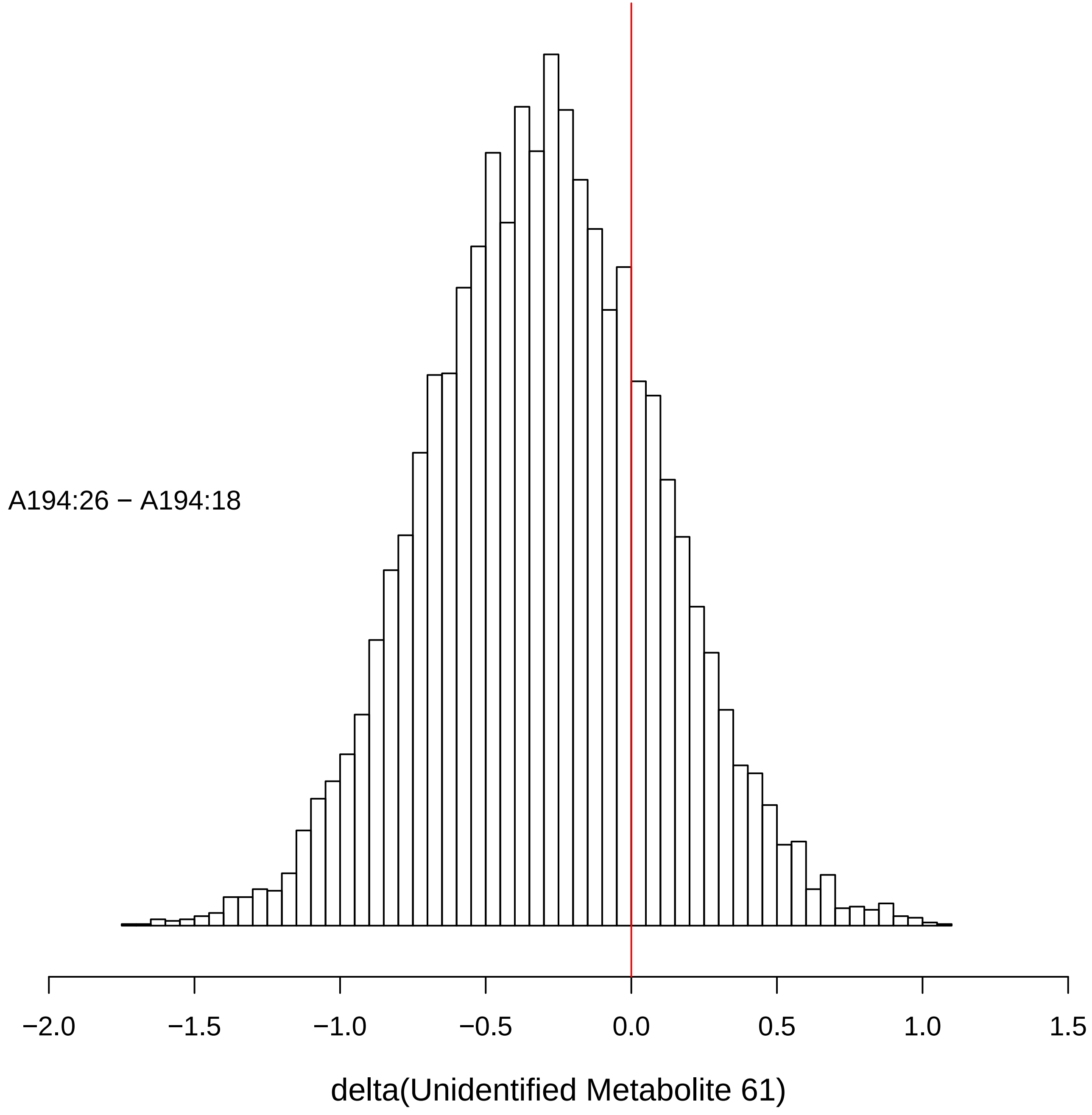
A194:26 − A194:18
−2.0
−1.5
−1.0
−0.5
0.0
0.5
1.0
1.5
delta(Unidentified Metabolite 61)

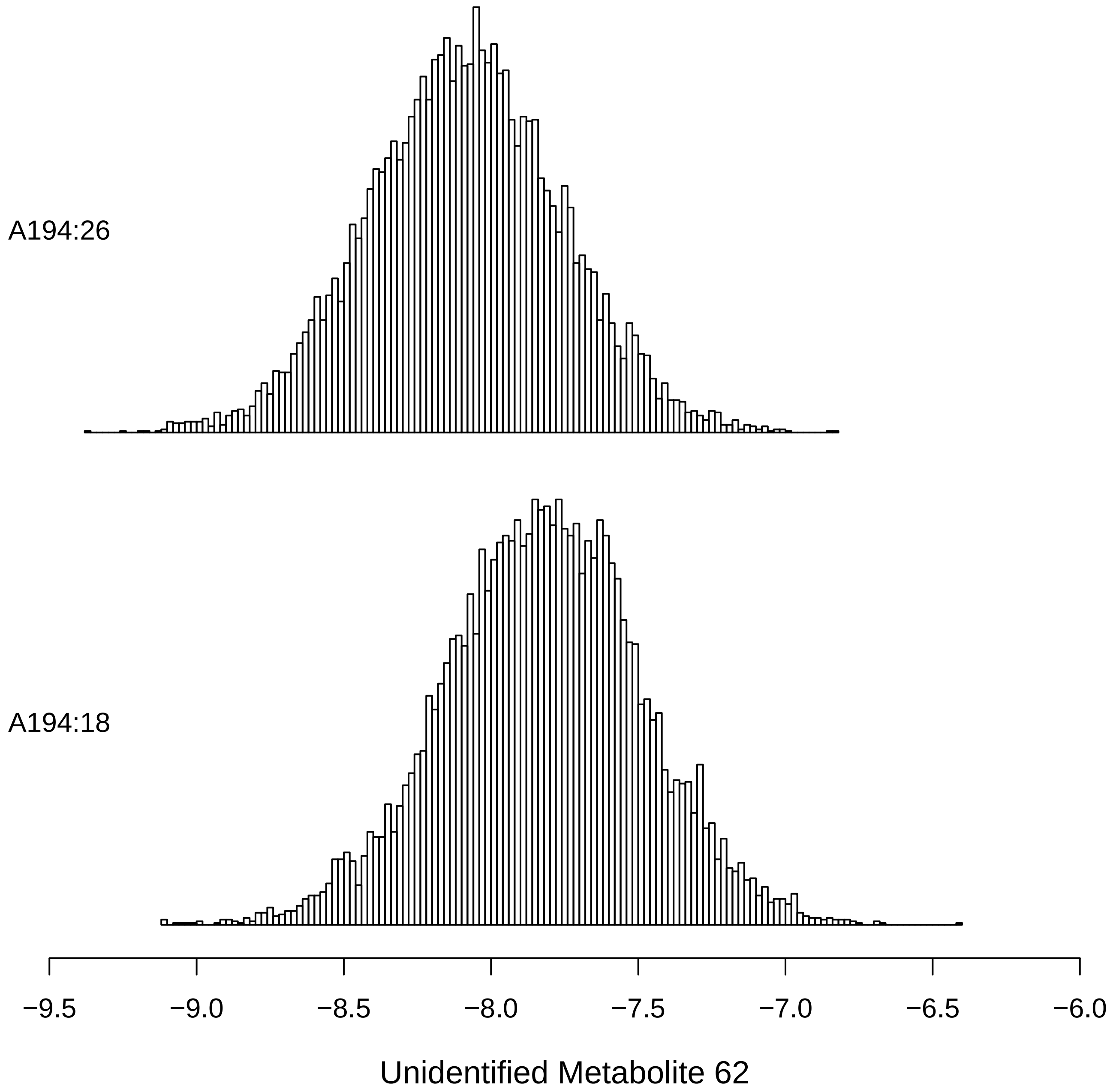
A194:26
A194:18
−9.5
−9.0
−8.5
−8.0
−7.5
−7.0
−6.5
−6.0
Unidentified Metabolite 62

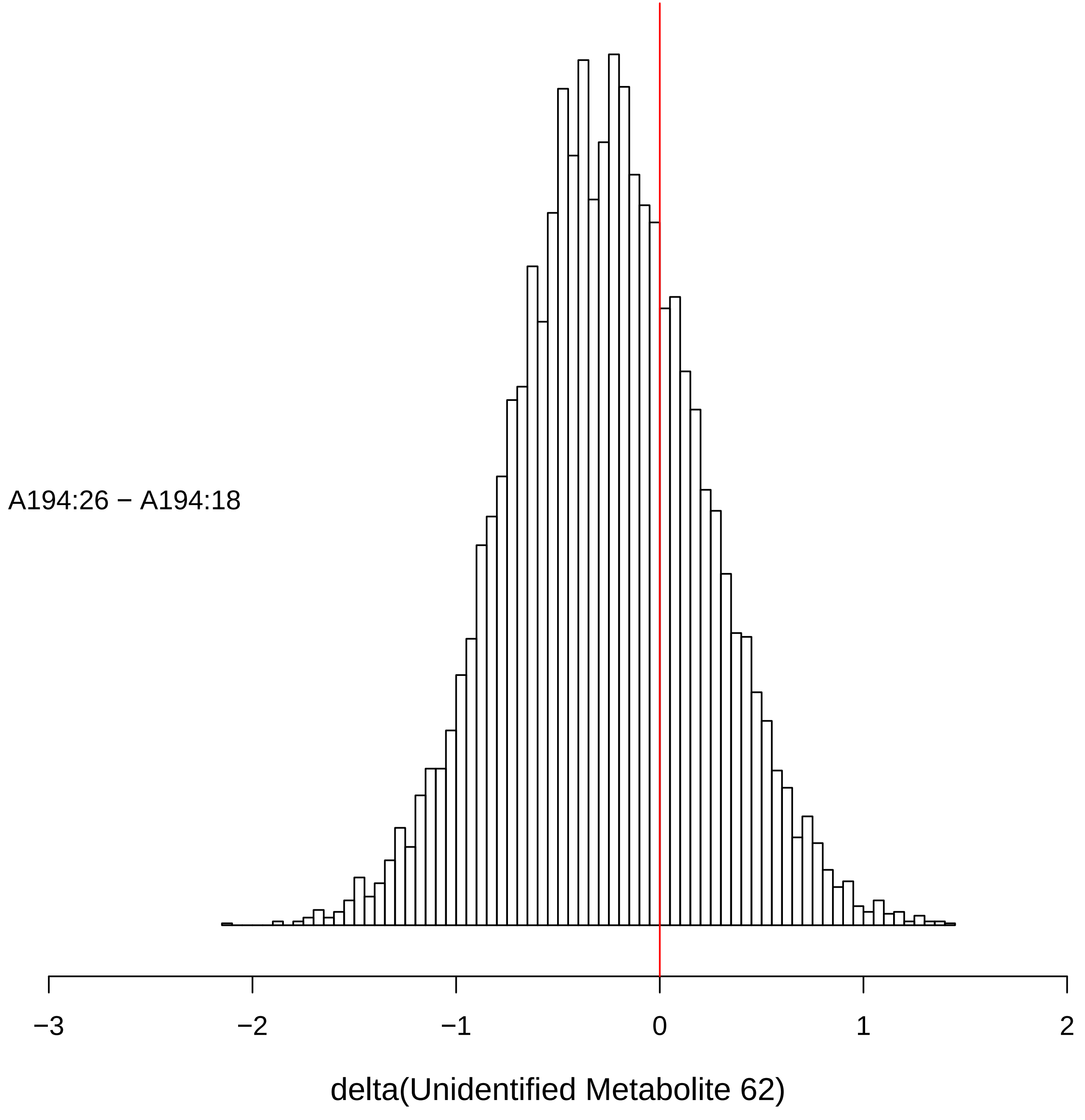
A194:26 − A194:18
−3
−2
−1
0
1
2
delta(Unidentified Metabolite 62)

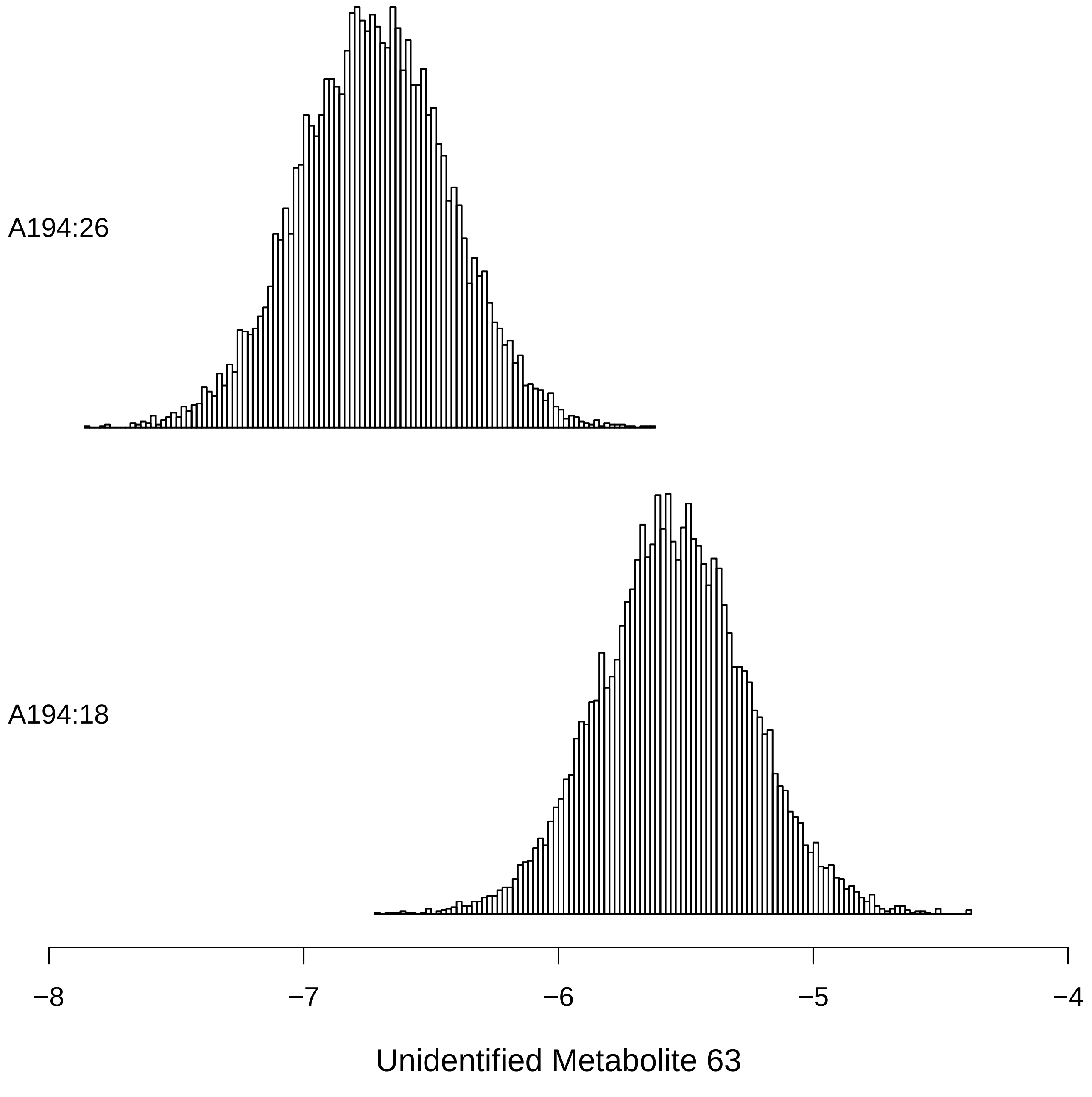

A194:26
A194:18
−8
−7
−6
−5
−4
Unidentified Metabolite 63

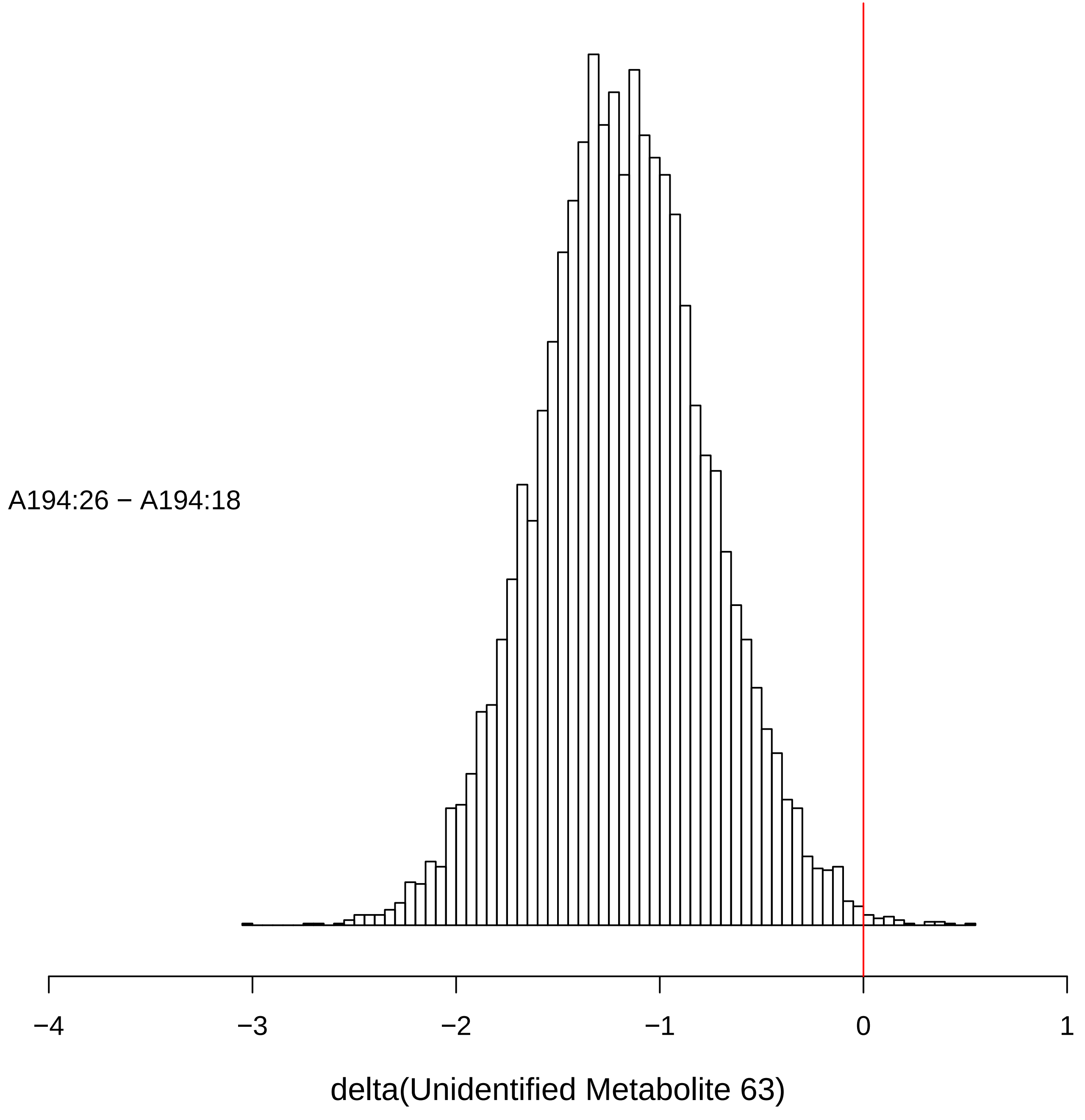
A194:26 − A194:18
−4
−3
−2
−1
0
1
delta(Unidentified Metabolite 63)

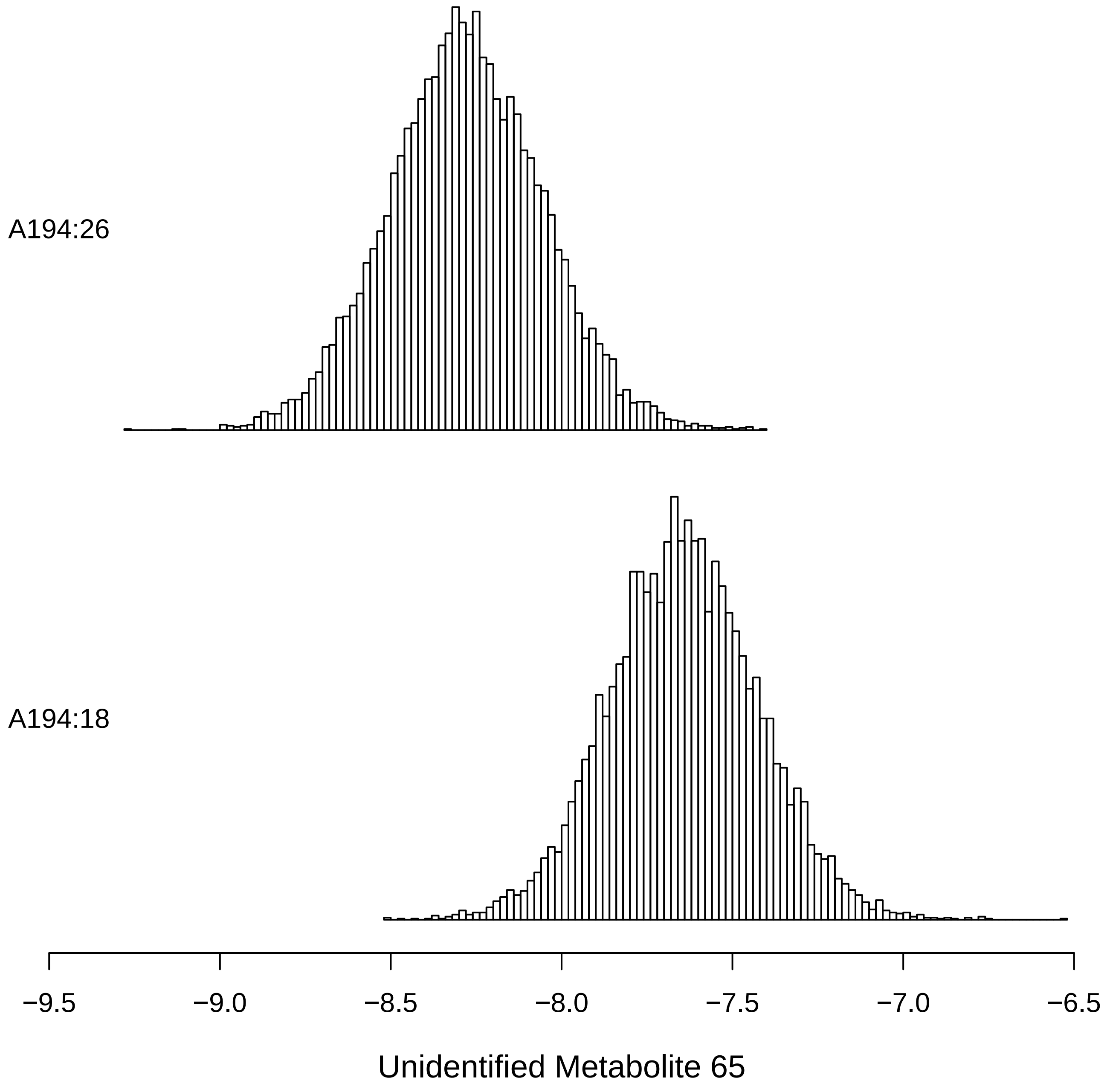

A194:26
A194:18
−9.5
−9.0
−8.5
−8.0
−7.5
−7.0
−6.5
Unidentified Metabolite 65

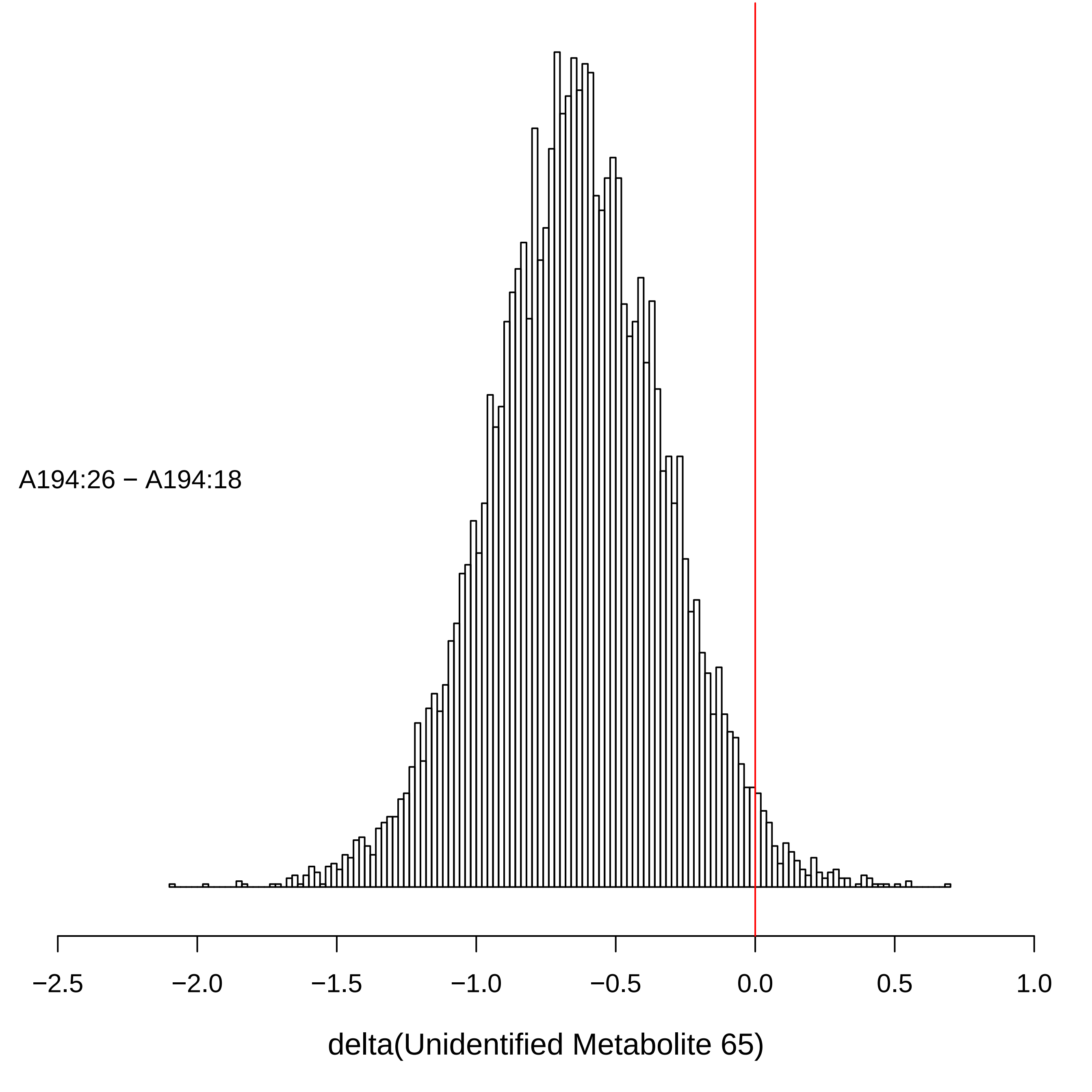
A194:26 − A194:18
−2.5
−2.0
−1.5
−1.0
−0.5
0.0
0.5
1.0
delta(Unidentified Metabolite 65)

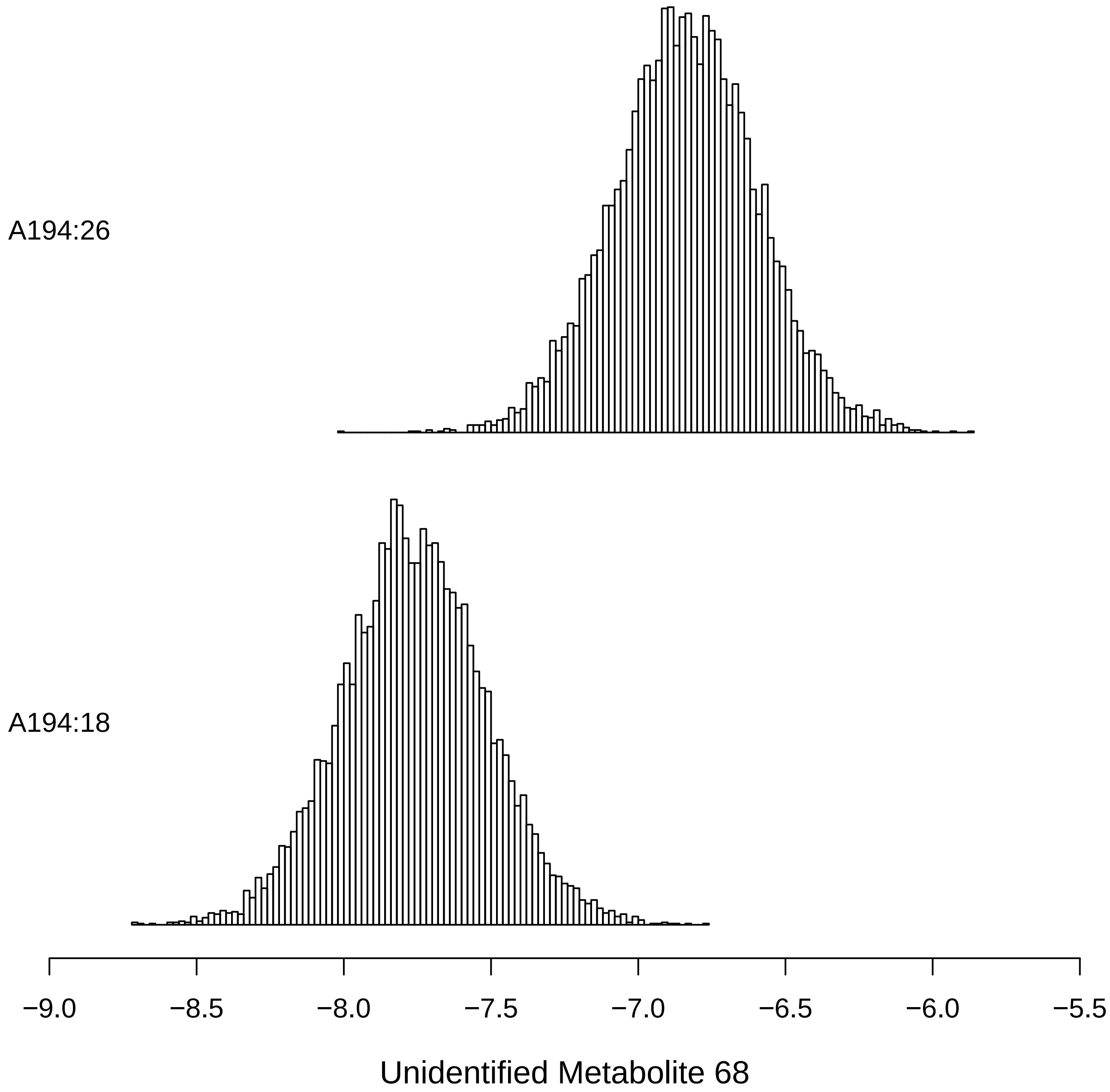
A194:26
A194:18
−9.0
−8.5
−8.0
−7.5
−7.0
−6.5
−6.0
−5.5
Unidentified Metabolite 68

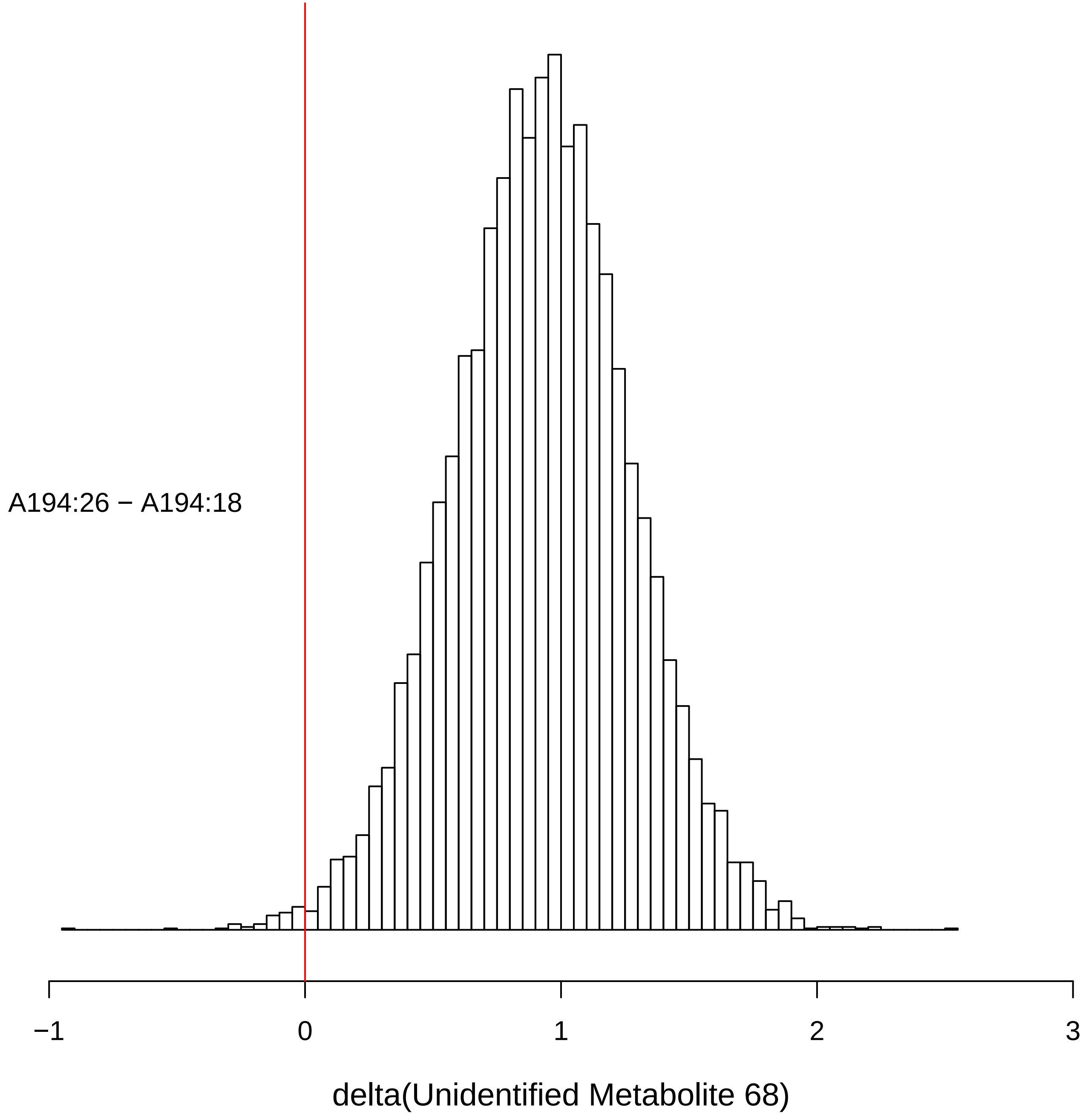

A194:26 − A194:18
−1
0
1
2
3
delta(Unidentified Metabolite 68)

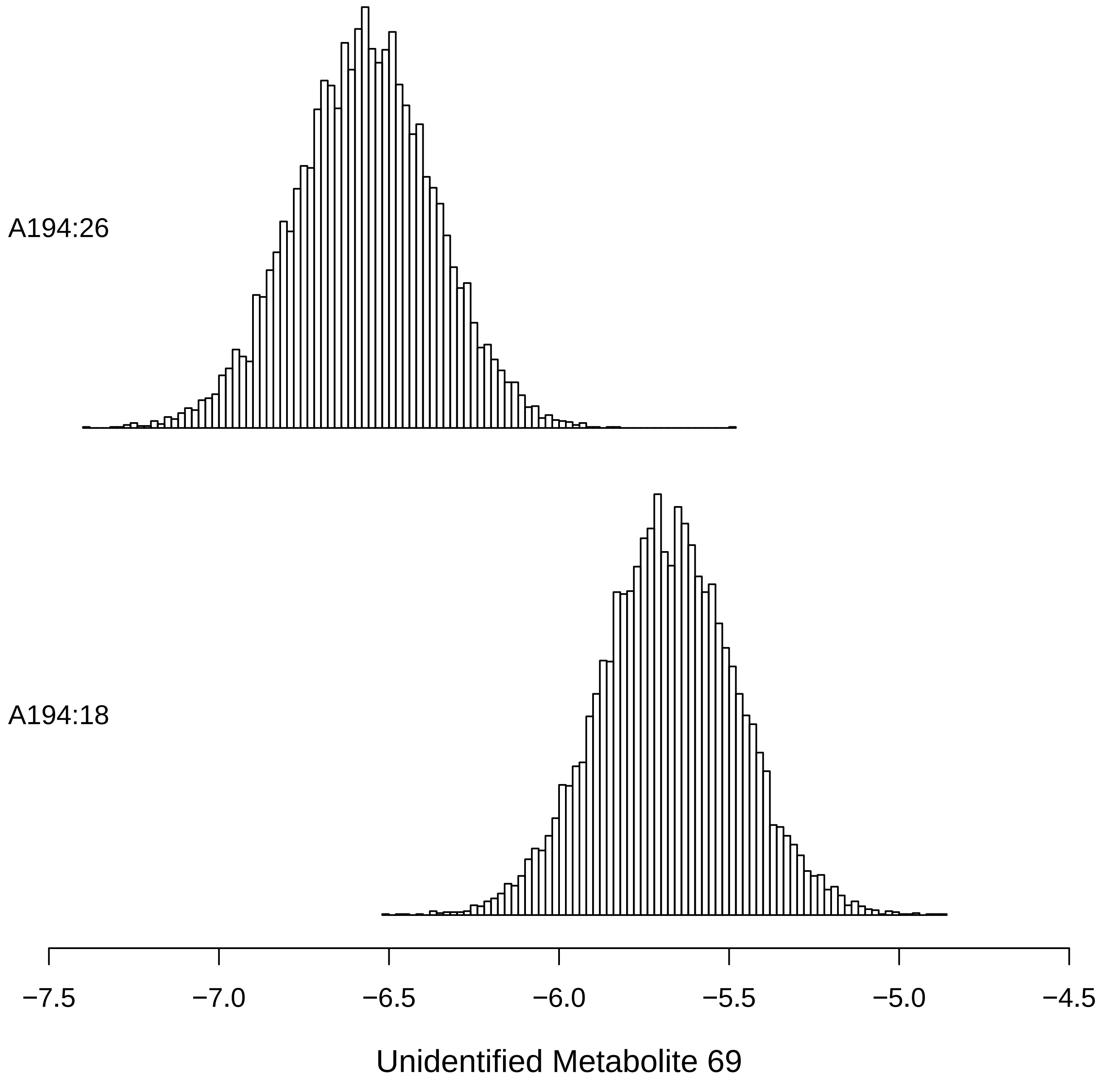
A194:26
A194:18
−7.5
−7.0
−6.5
−6.0
−5.5
−5.0
−4.5
Unidentified Metabolite 69

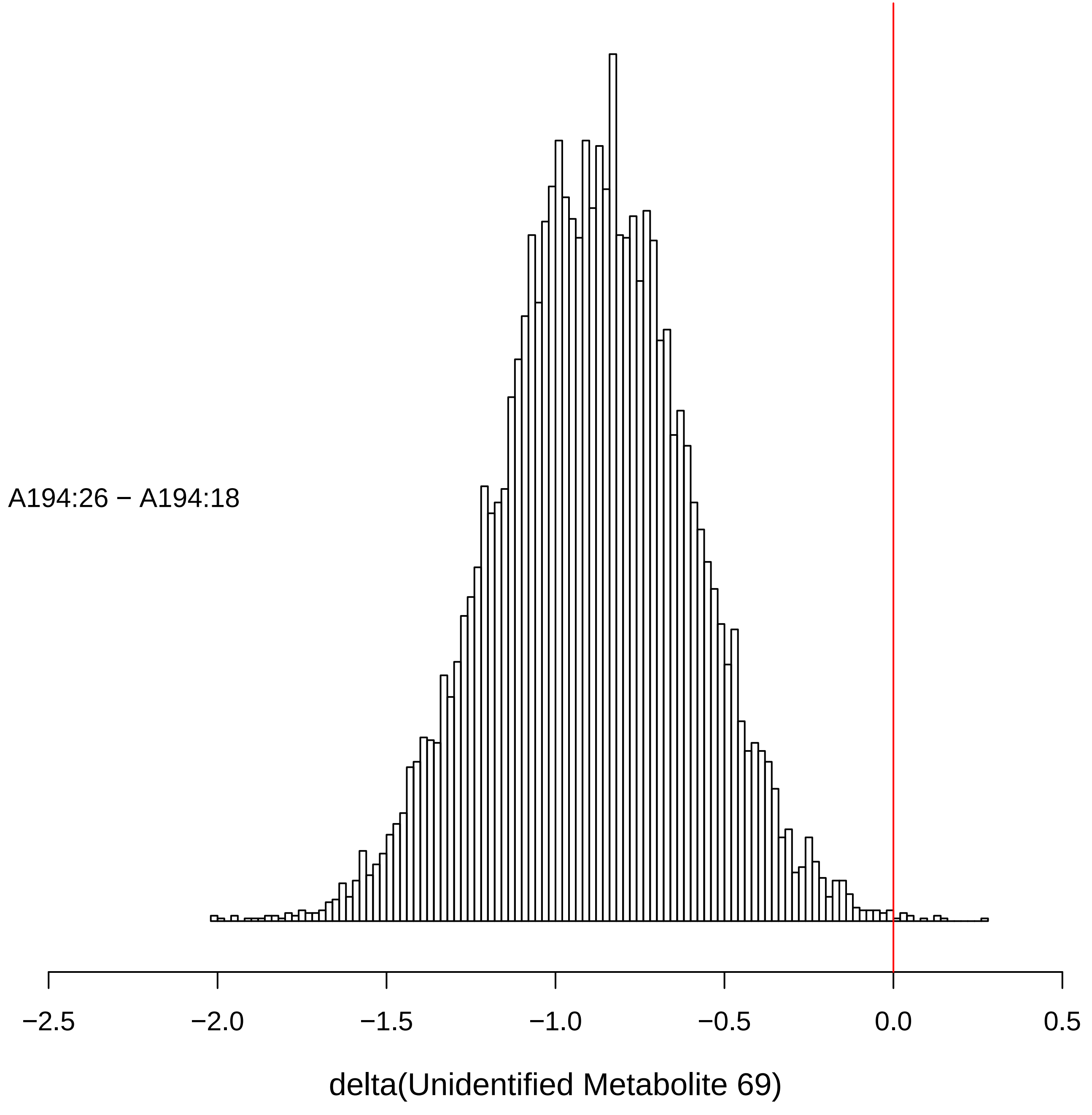
A194:26 − A194:18
−2.5
−2.0
−1.5
−1.0
−0.5
0.0
0.5
delta(Unidentified Metabolite 69)

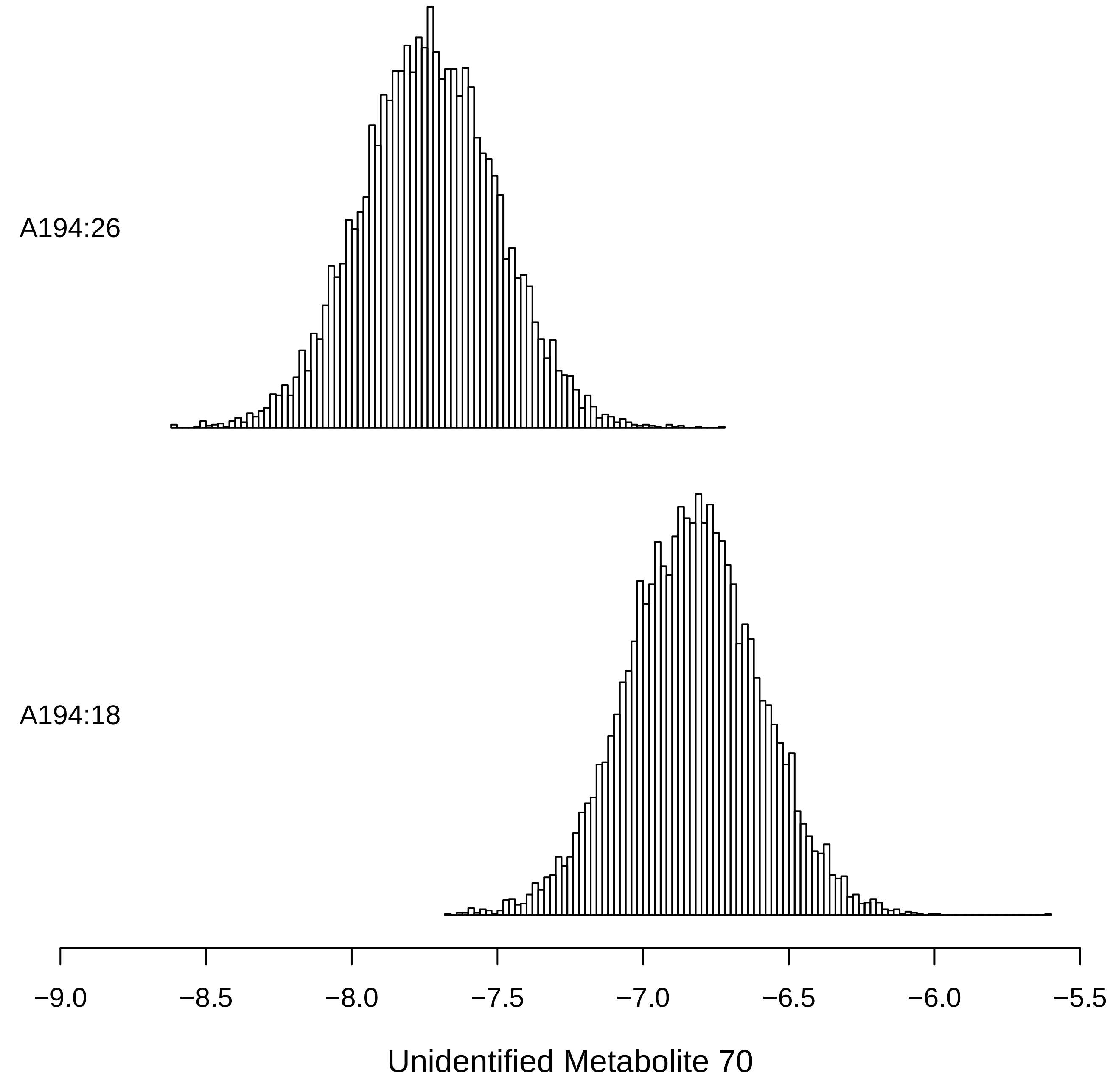
A194:26
A194:18
−9.0
−8.5
−8.0
−7.5
−7.0
−6.5
−6.0
−5.5
Unidentified Metabolite 70

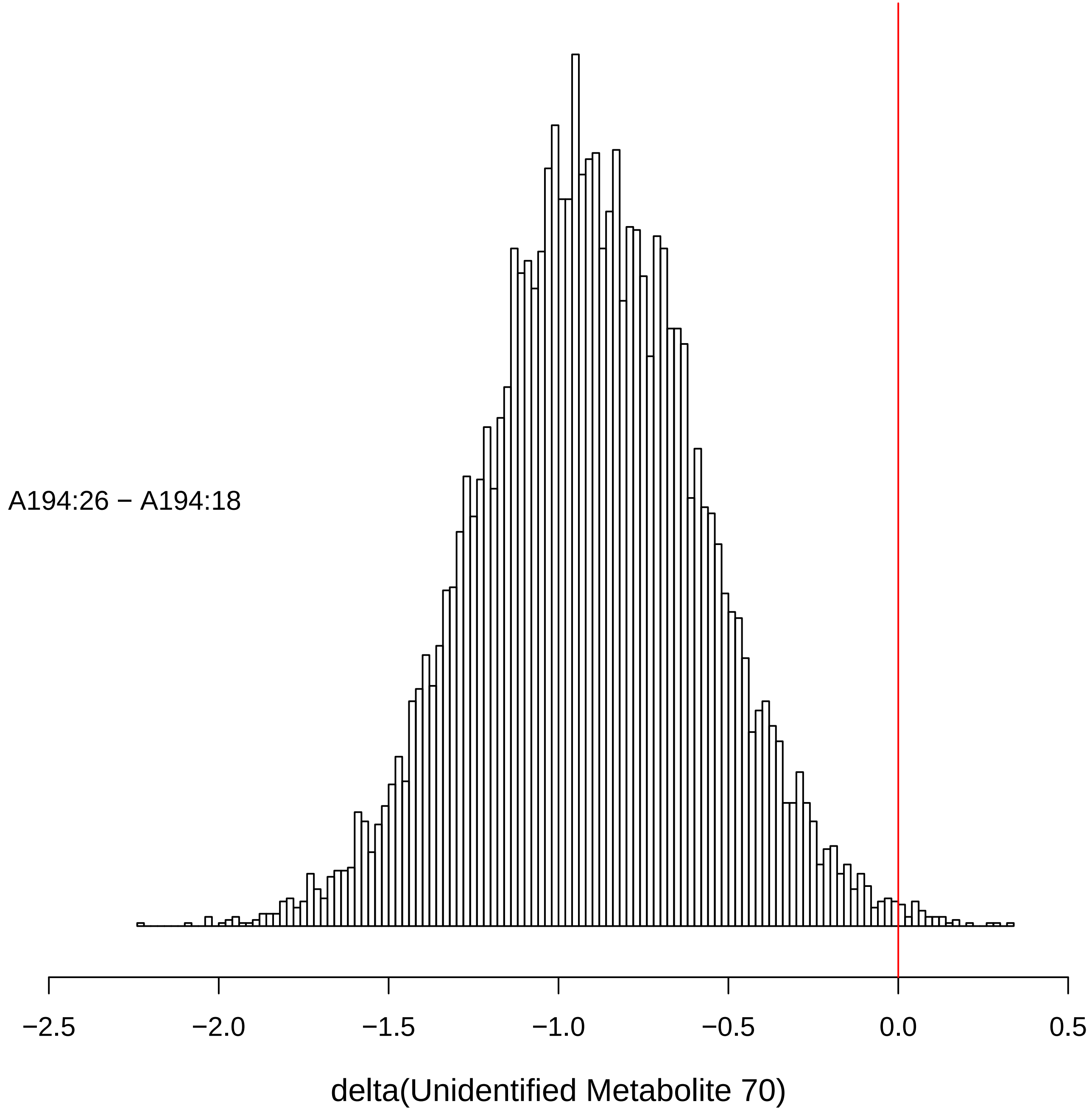
A194:26 − A194:18
−2.5
−2.0
−1.5
−1.0
−0.5
0.0
0.5
delta(Unidentified Metabolite 70)

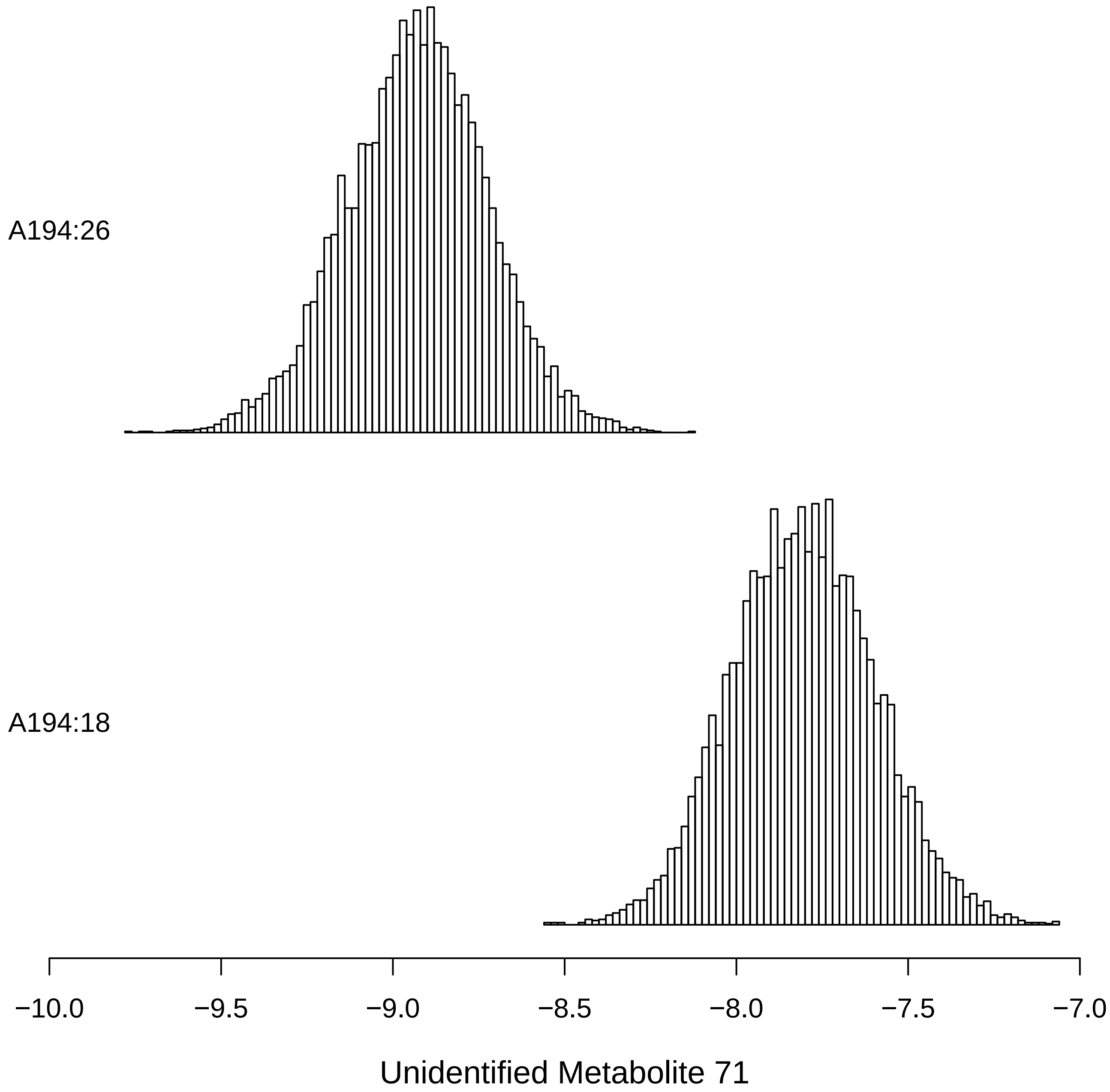
A194:26
A194:18
−10.0
−9.5
−9.0
−8.5
−8.0
−7.5
−7.0
Unidentified Metabolite 71

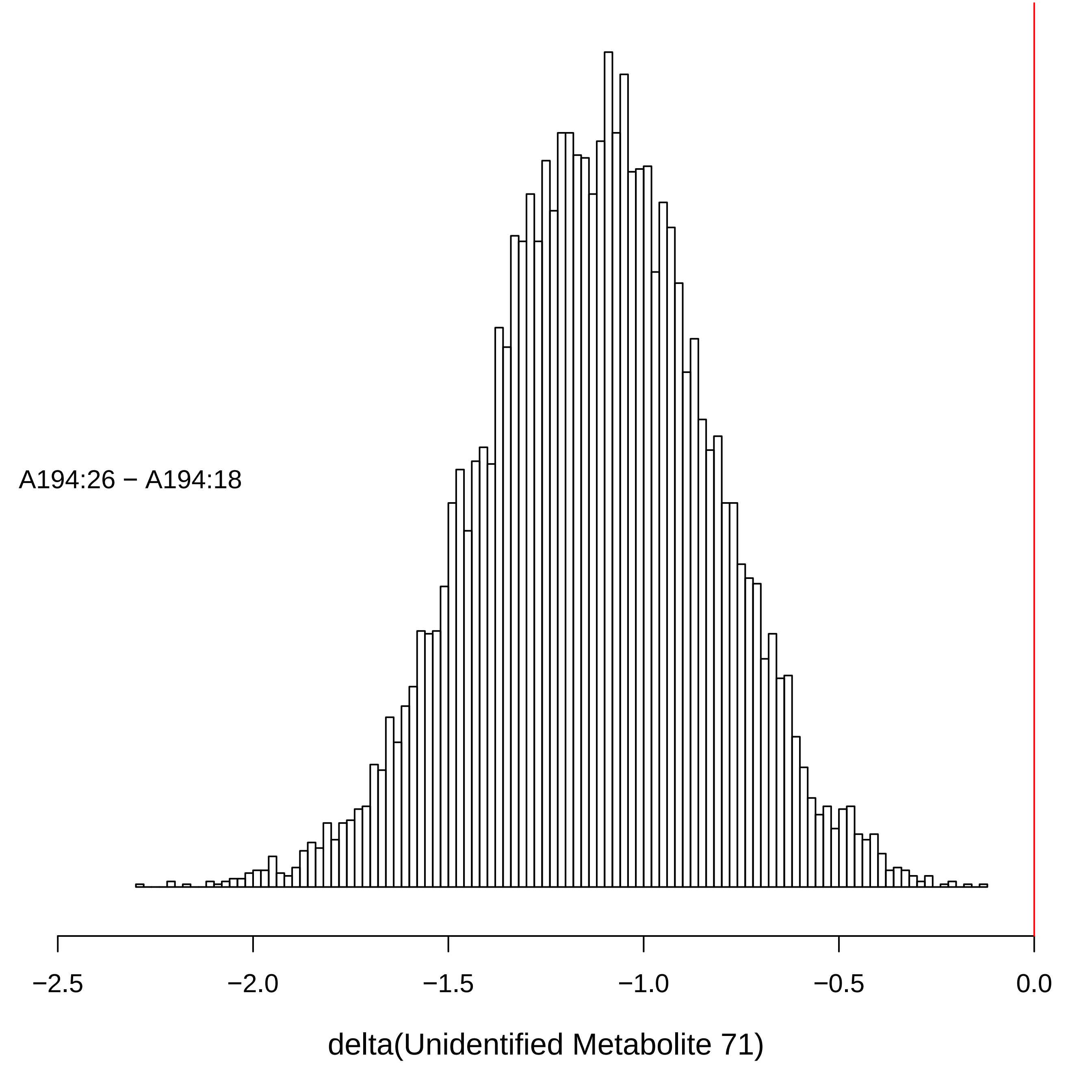
A194:26 − A194:18
−2.5
−2.0
−1.5
−1.0
−0.5
0.0
delta(Unidentified Metabolite 71)

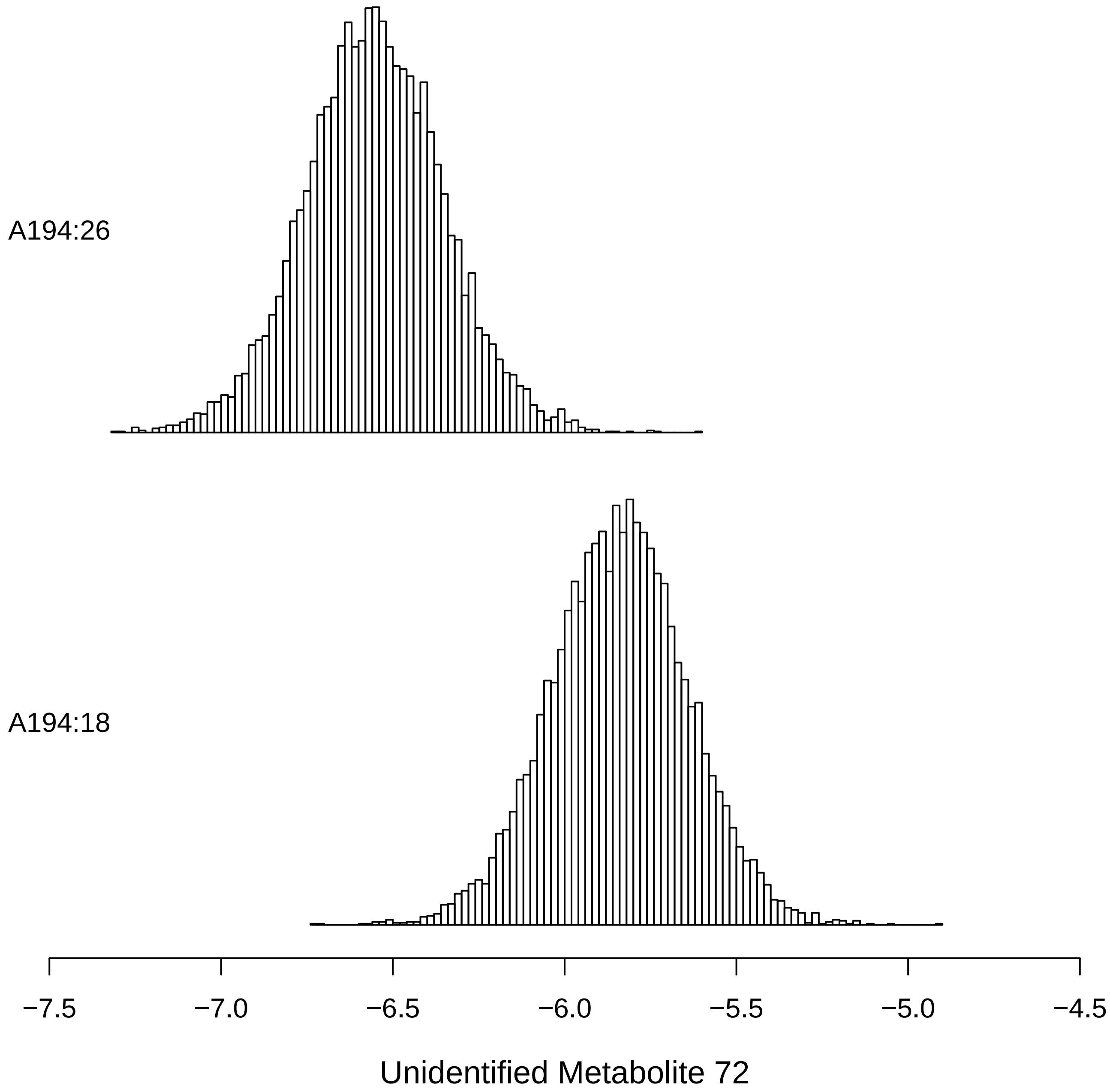

A194:26
A194:18
−7.5
−7.0
−6.5
−6.0
−5.5
−5.0
−4.5
Unidentified Metabolite 72

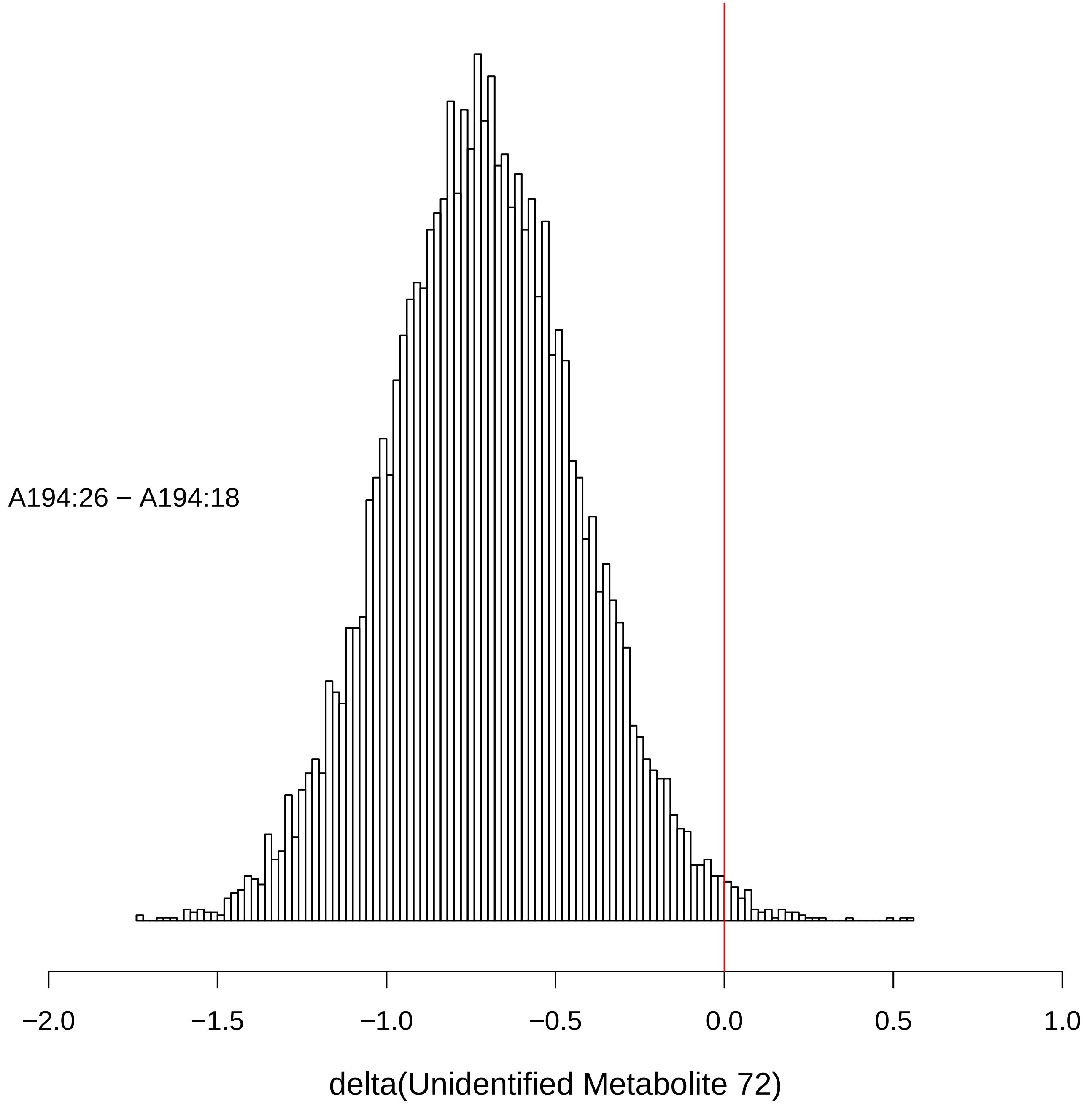
A194:26 − A194:18
−2.0
−1.5
−1.0
−0.5
0.0
0.5
1.0
delta(Unidentified Metabolite 72)

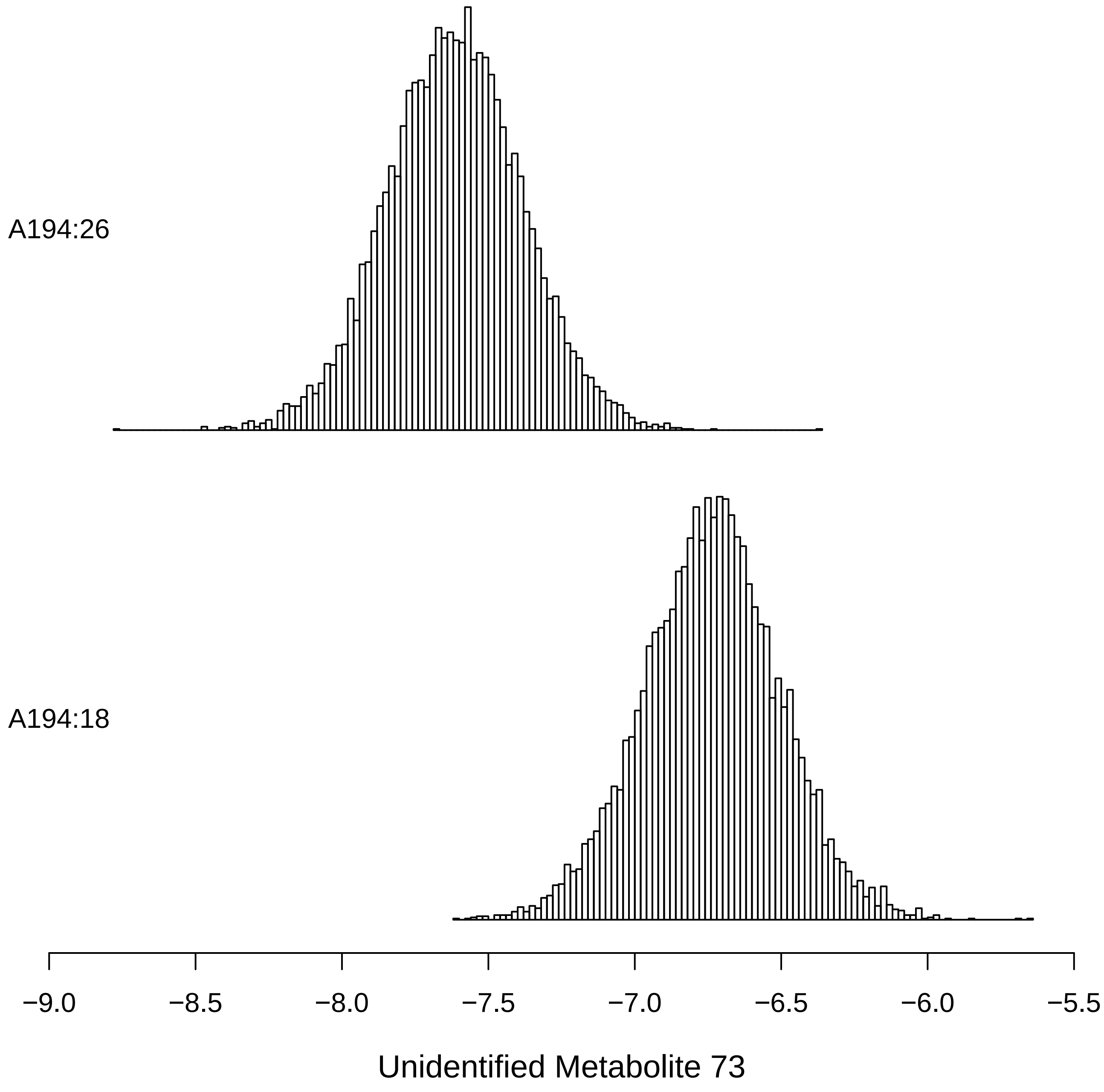
A194:26
A194:18
−9.0
−8.5
−8.0
−7.5
−7.0
−6.5
−6.0
−5.5
Unidentified Metabolite 73

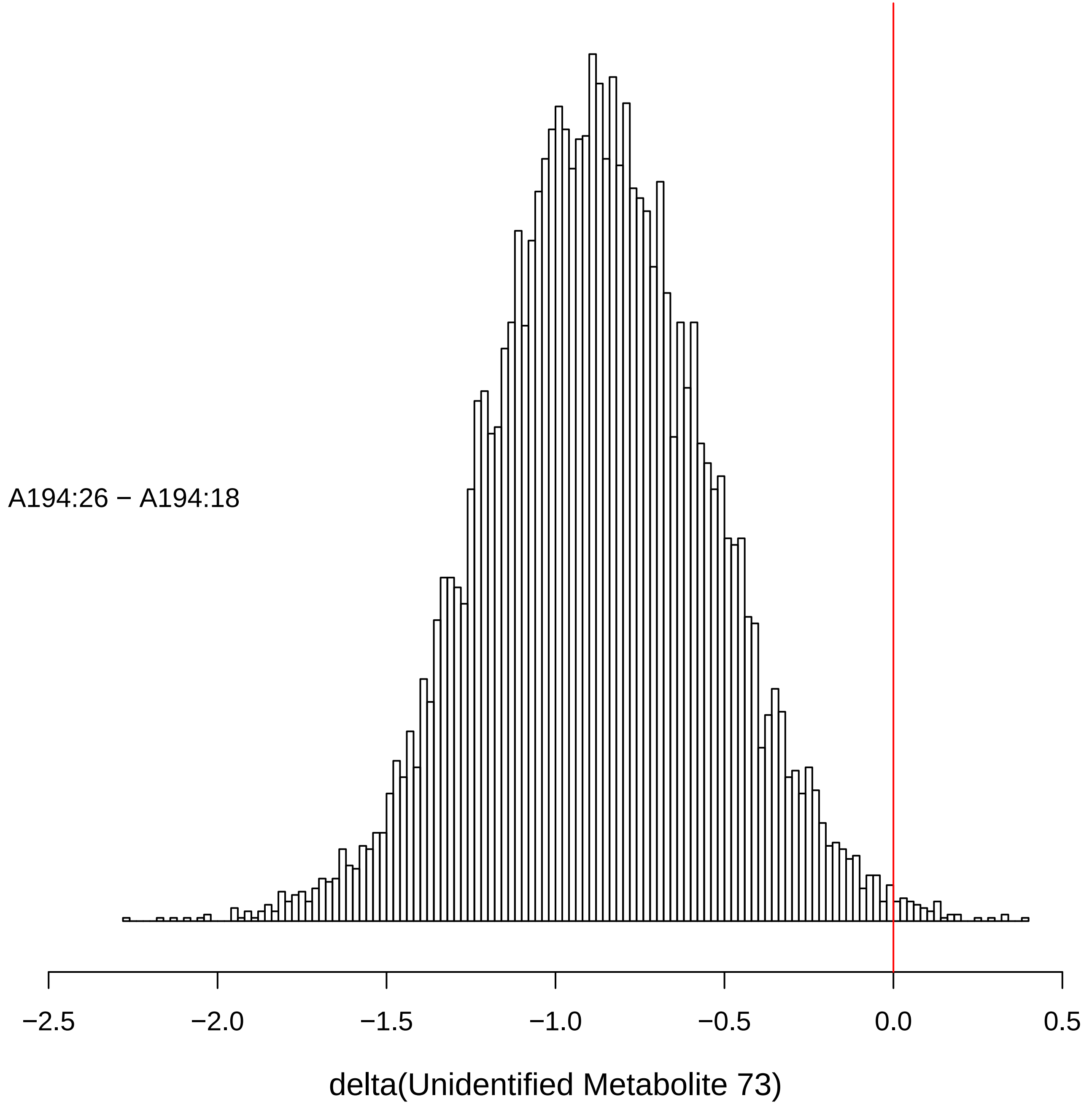
A194:26 − A194:18
−2.5
−2.0
−1.5
−1.0
−0.5
0.0
0.5
delta(Unidentified Metabolite 73)

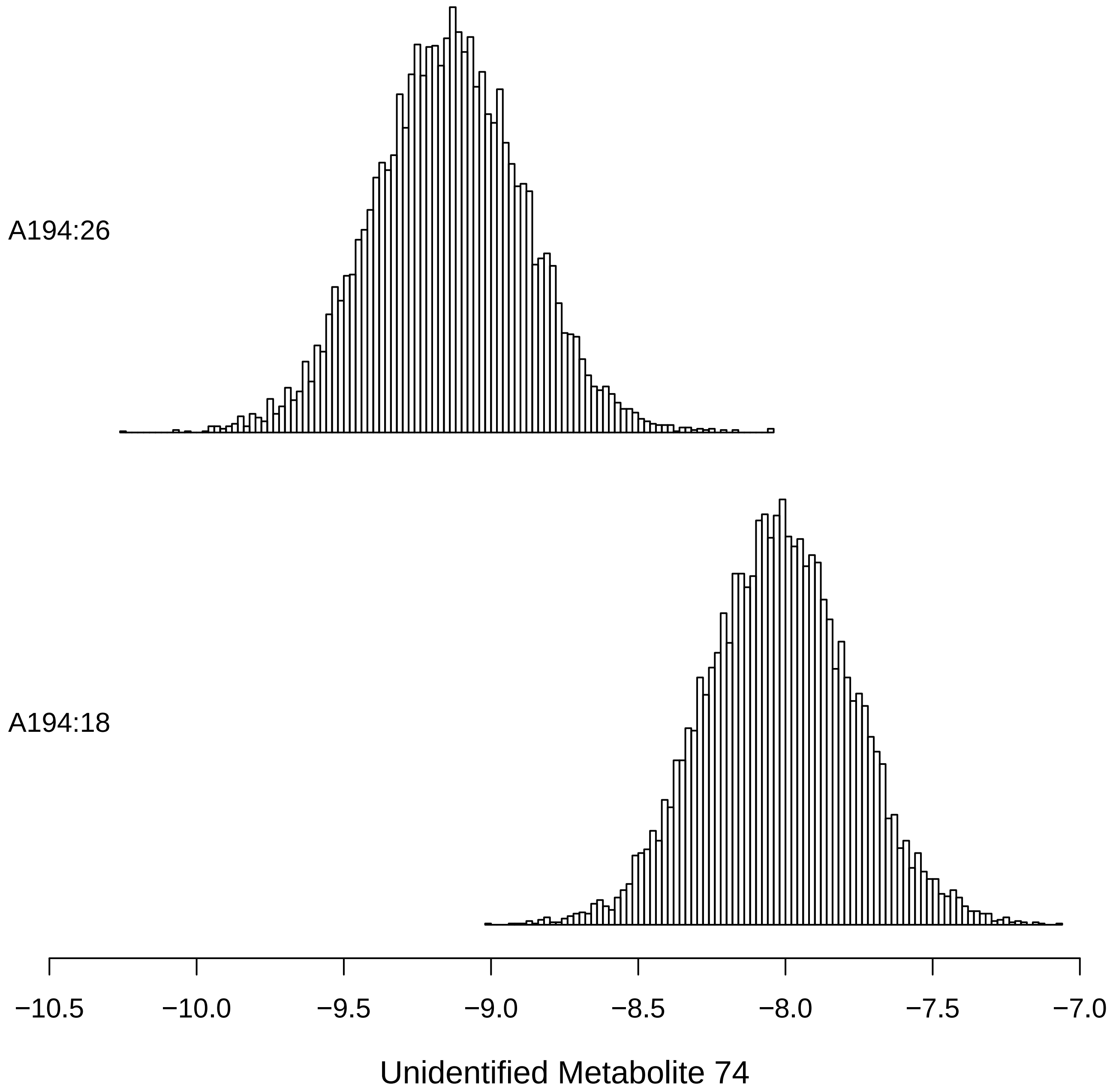

A194:26
A194:18
−10.5
−10.0
−9.5
−9.0
−8.5
−8.0
−7.5
−7.0
Unidentified Metabolite 74

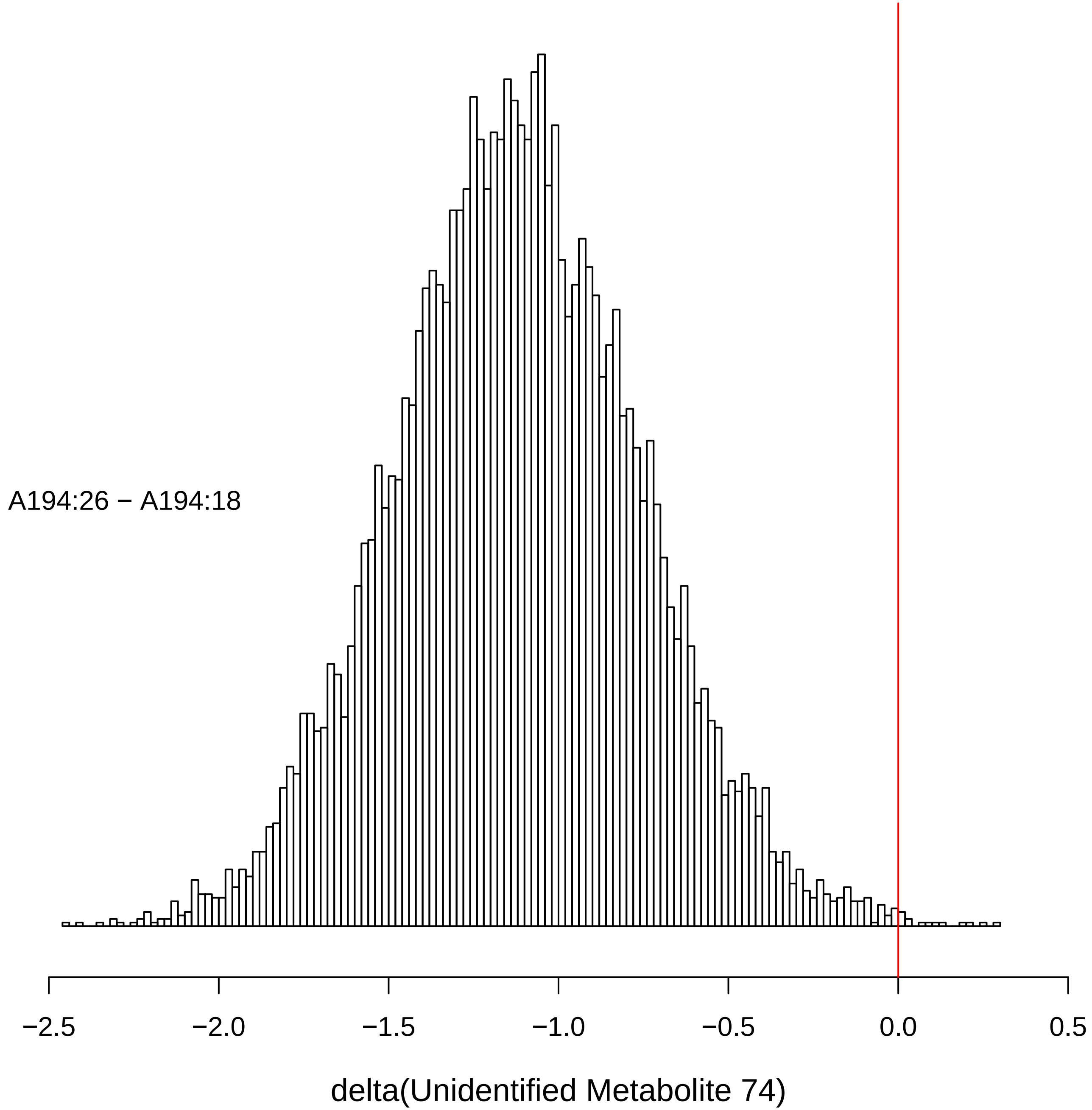
A194:26 − A194:18
−2.5
−2.0
−1.5
−1.0
−0.5
0.0
0.5
delta(Unidentified Metabolite 74)

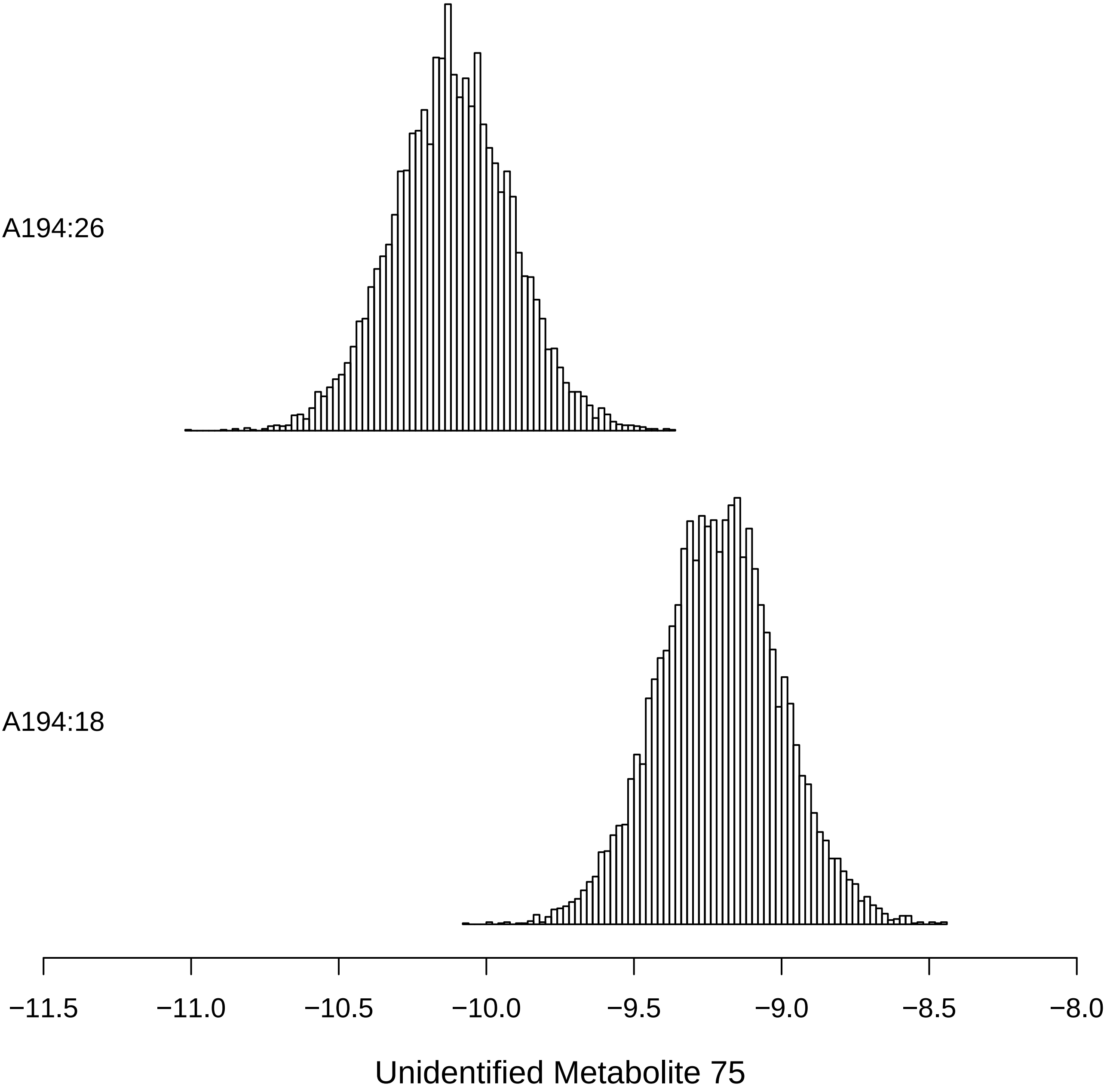
A194:26
A194:18
−11.5
−11.0
−10.5
−10.0
−9.5
−9.0
−8.5
−8.0
Unidentified Metabolite 75

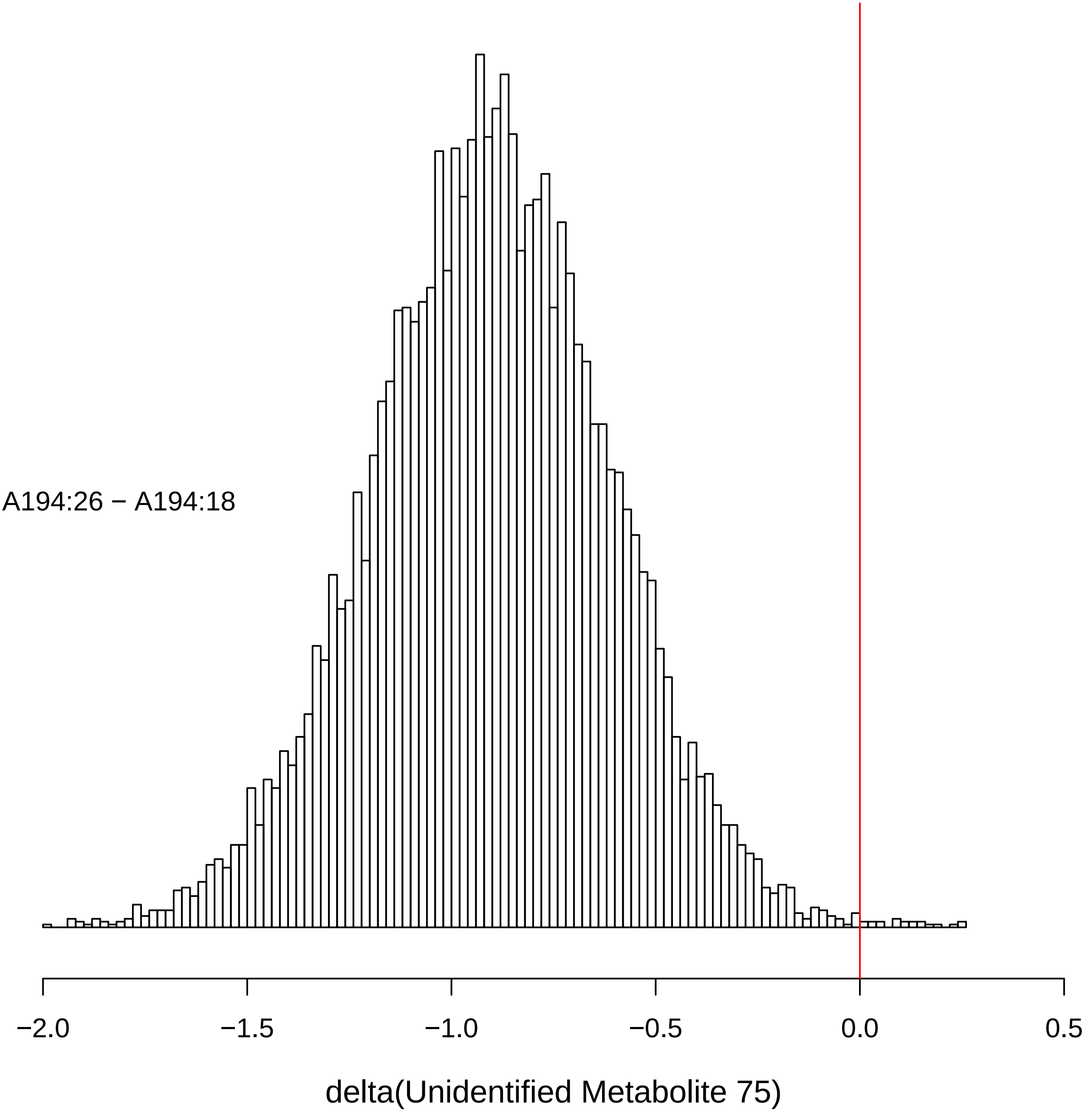
A194:26 − A194:18
−2.0
−1.5
−1.0
−0.5
0.0
0.5
delta(Unidentified Metabolite 75)

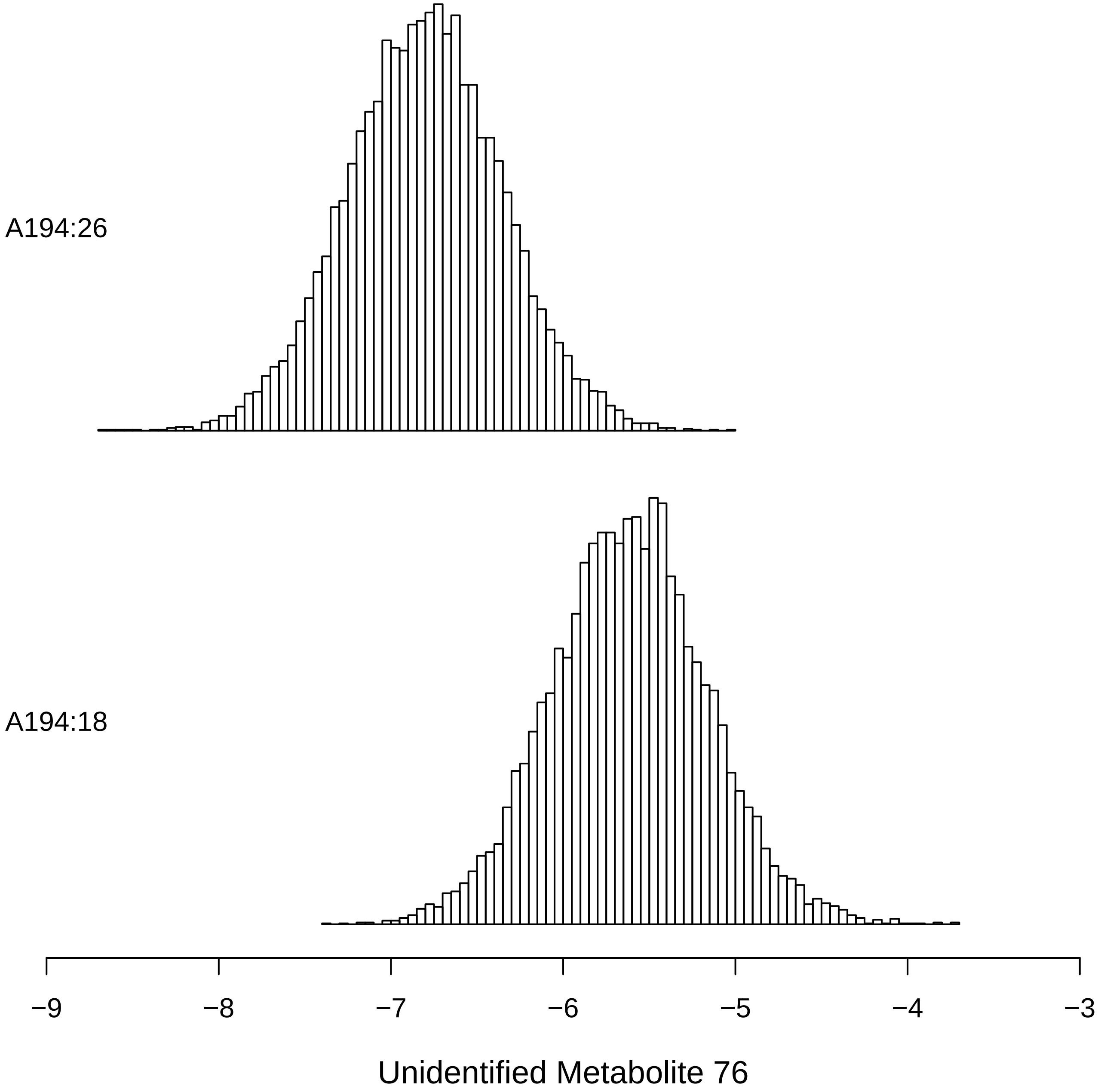

A194:26
A194:18
−9
−8
−7
−6
−5
−4
−3
Unidentified Metabolite 76

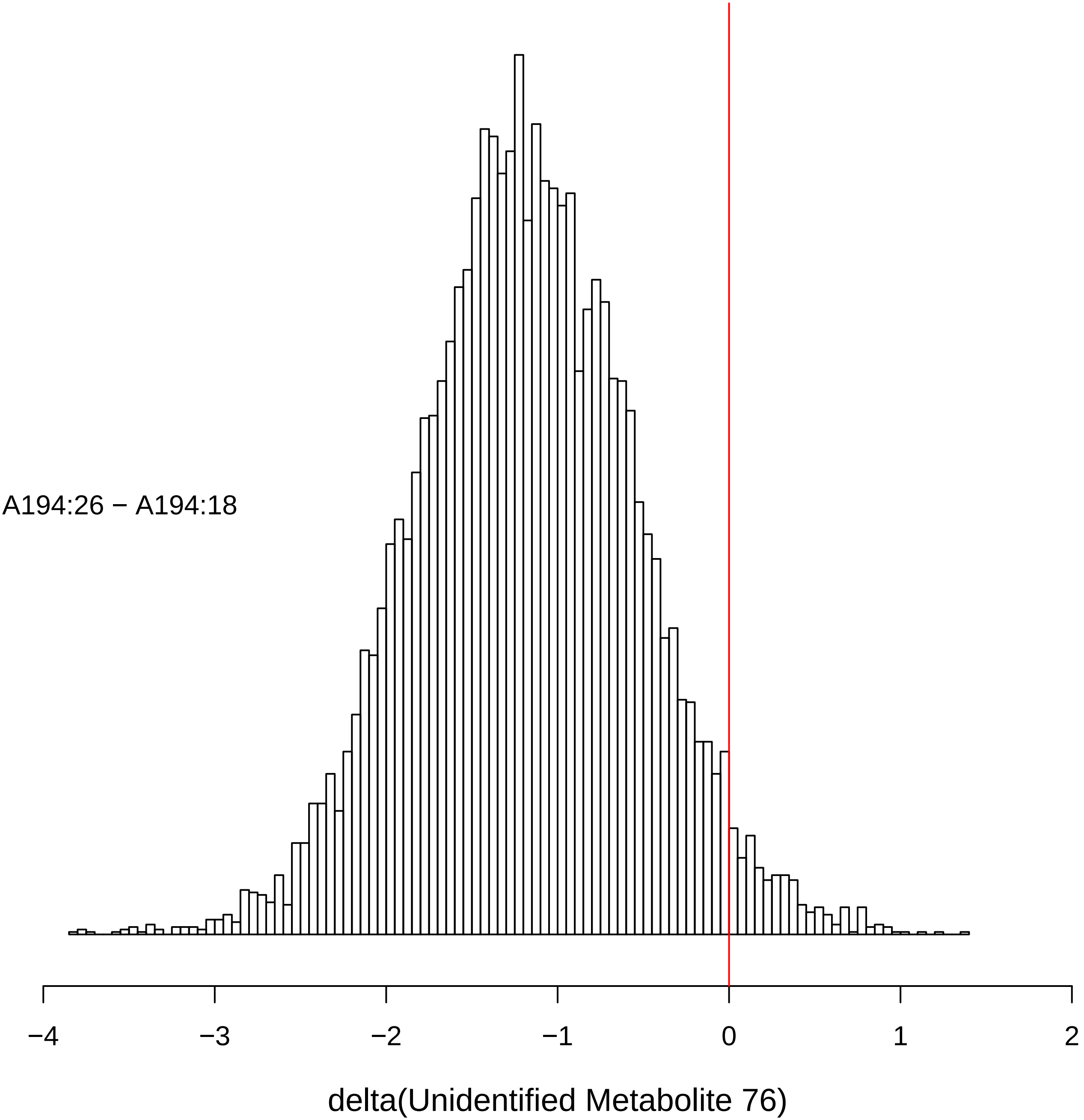

A194:26 − A194:18
−4
−3
−2
−1
0
1
2
delta(Unidentified Metabolite 76)

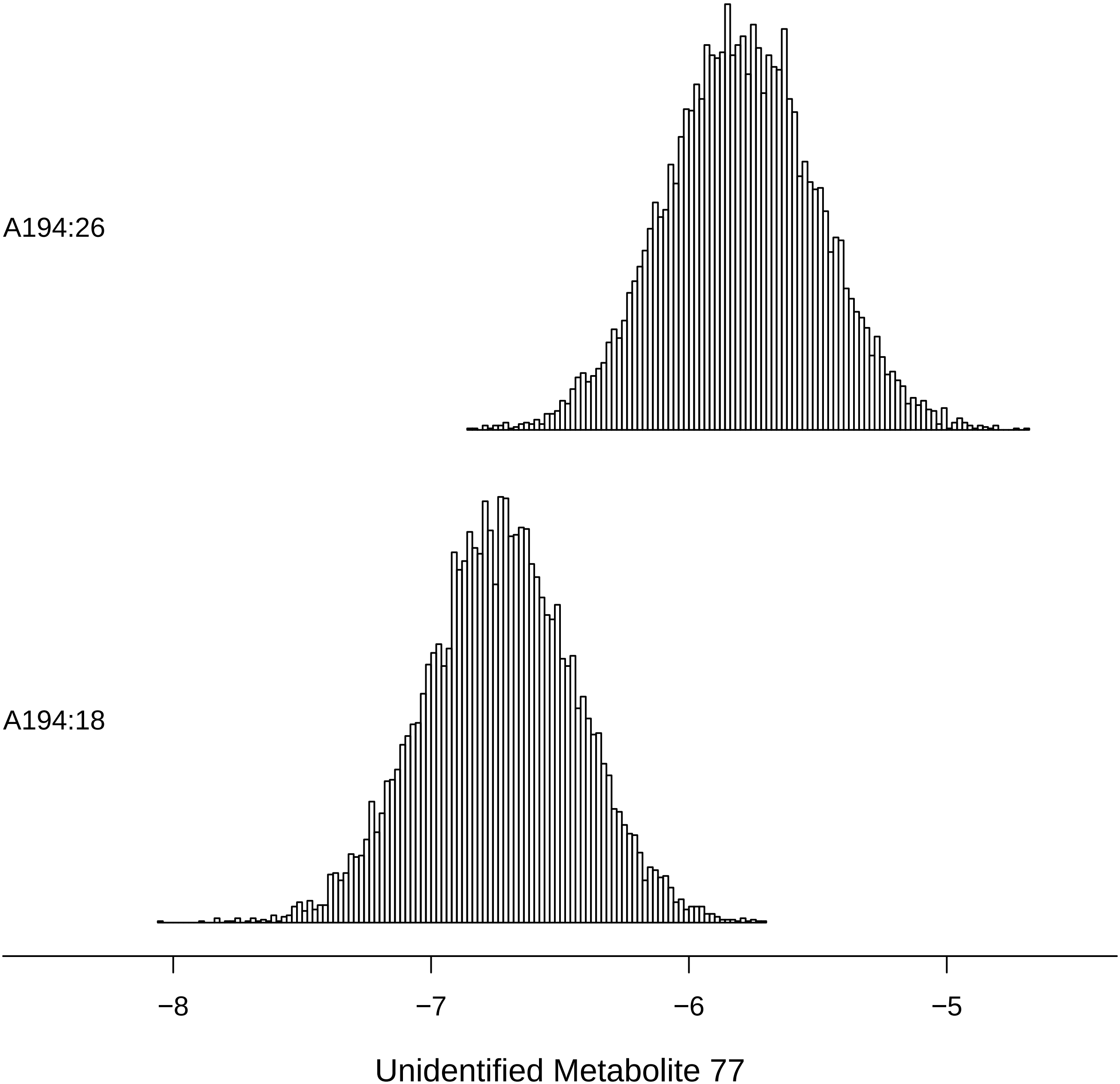
A194:26
A194:18
−8
−7
−6
−5
Unidentified Metabolite 77

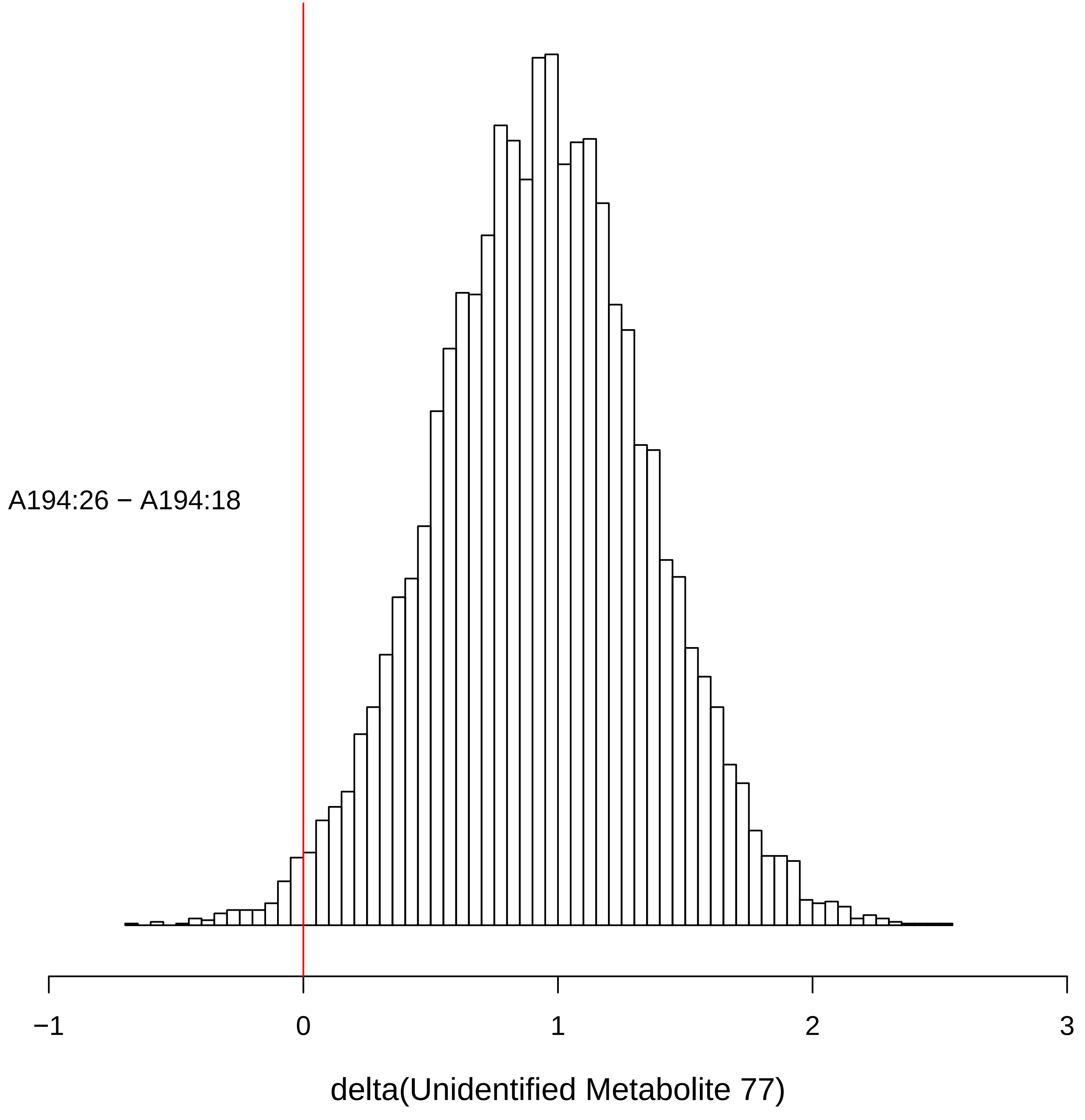
A194:26 − A194:18
−1
0
1
2
3
delta(Unidentified Metabolite 77)

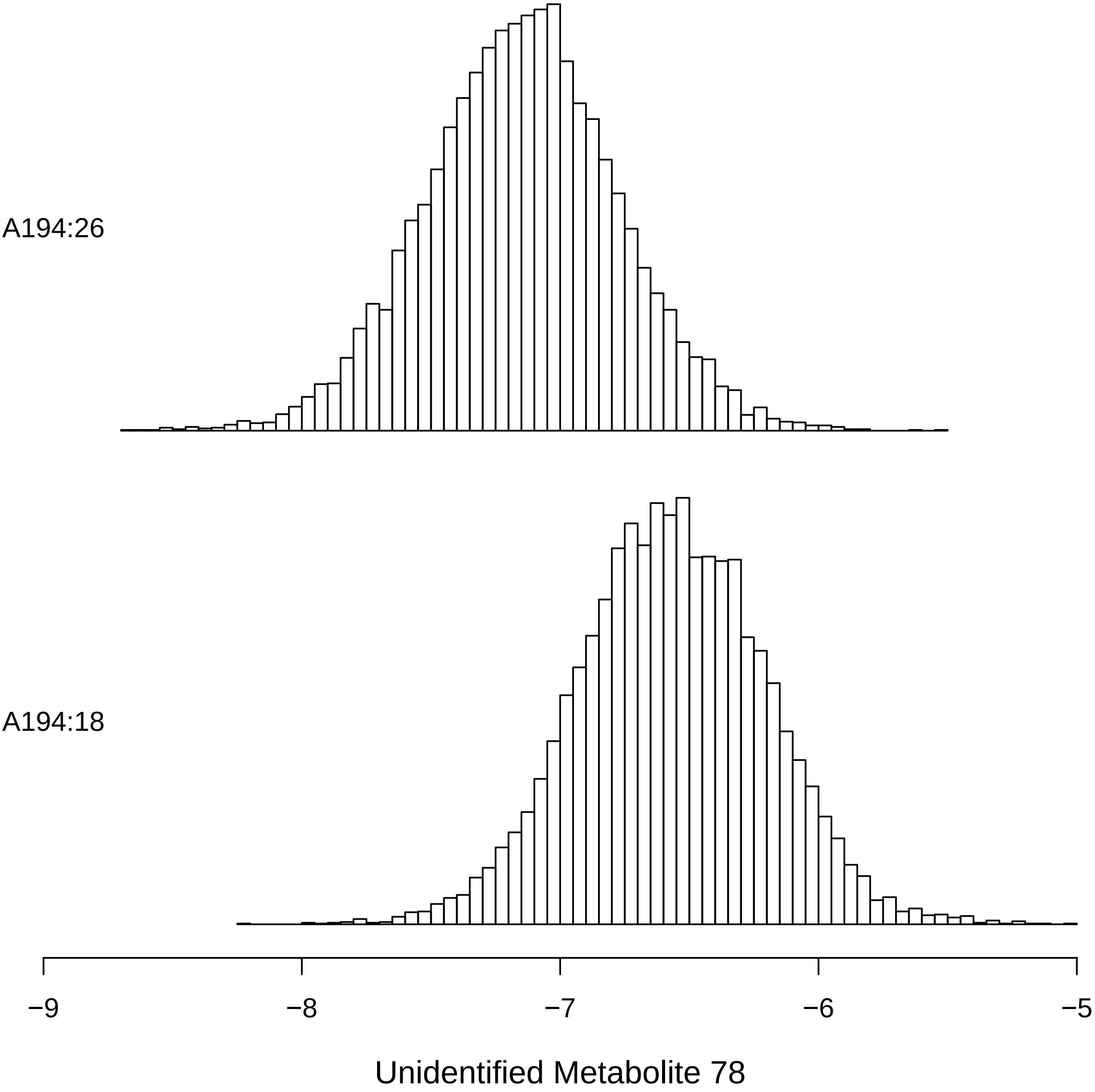
A194:26
A194:18
−9
−8
−7
−6
−5
Unidentified Metabolite 78

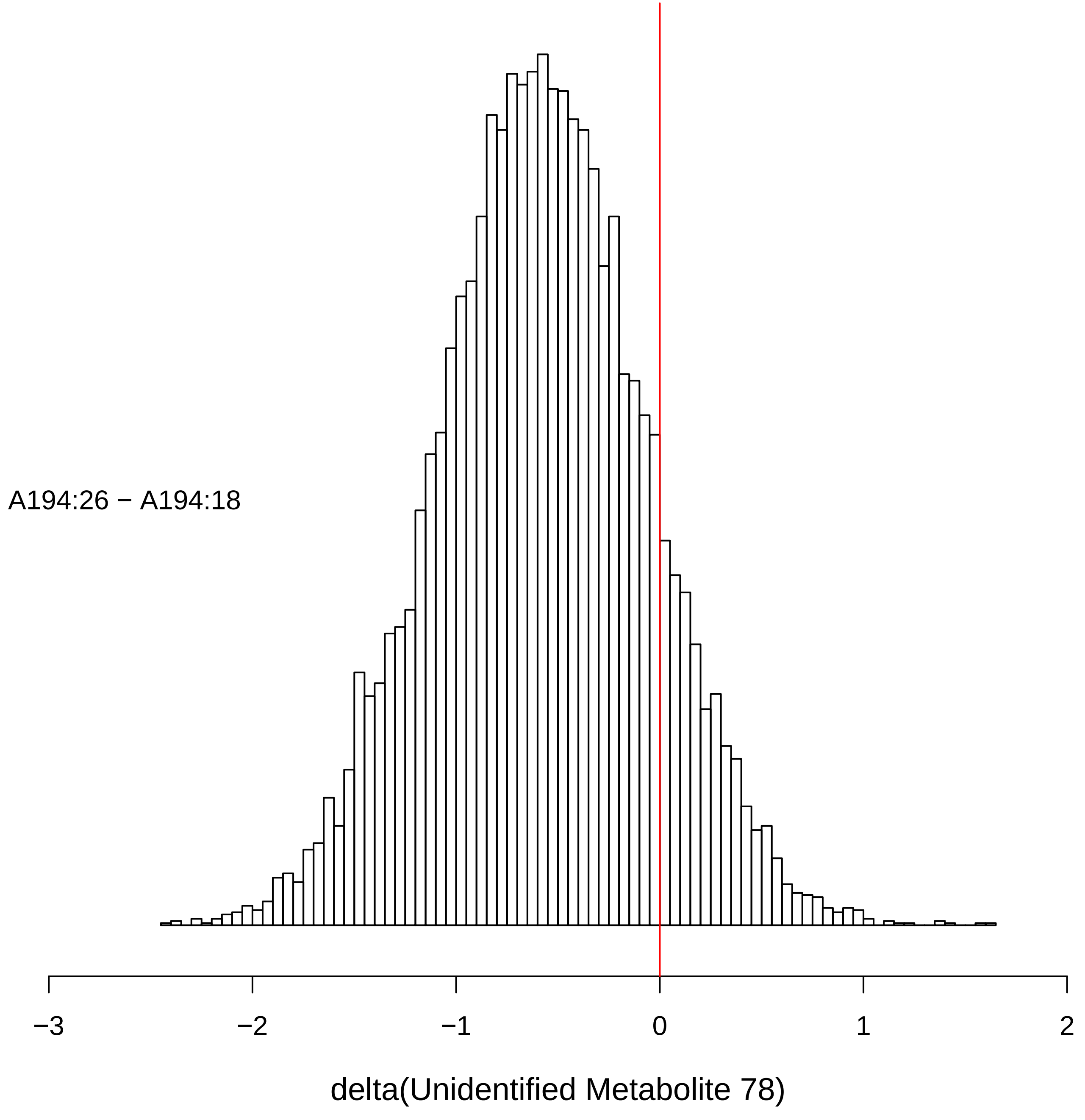
A194:26 − A194:18
−3
−2
−1
0
1
2
delta(Unidentified Metabolite 78)

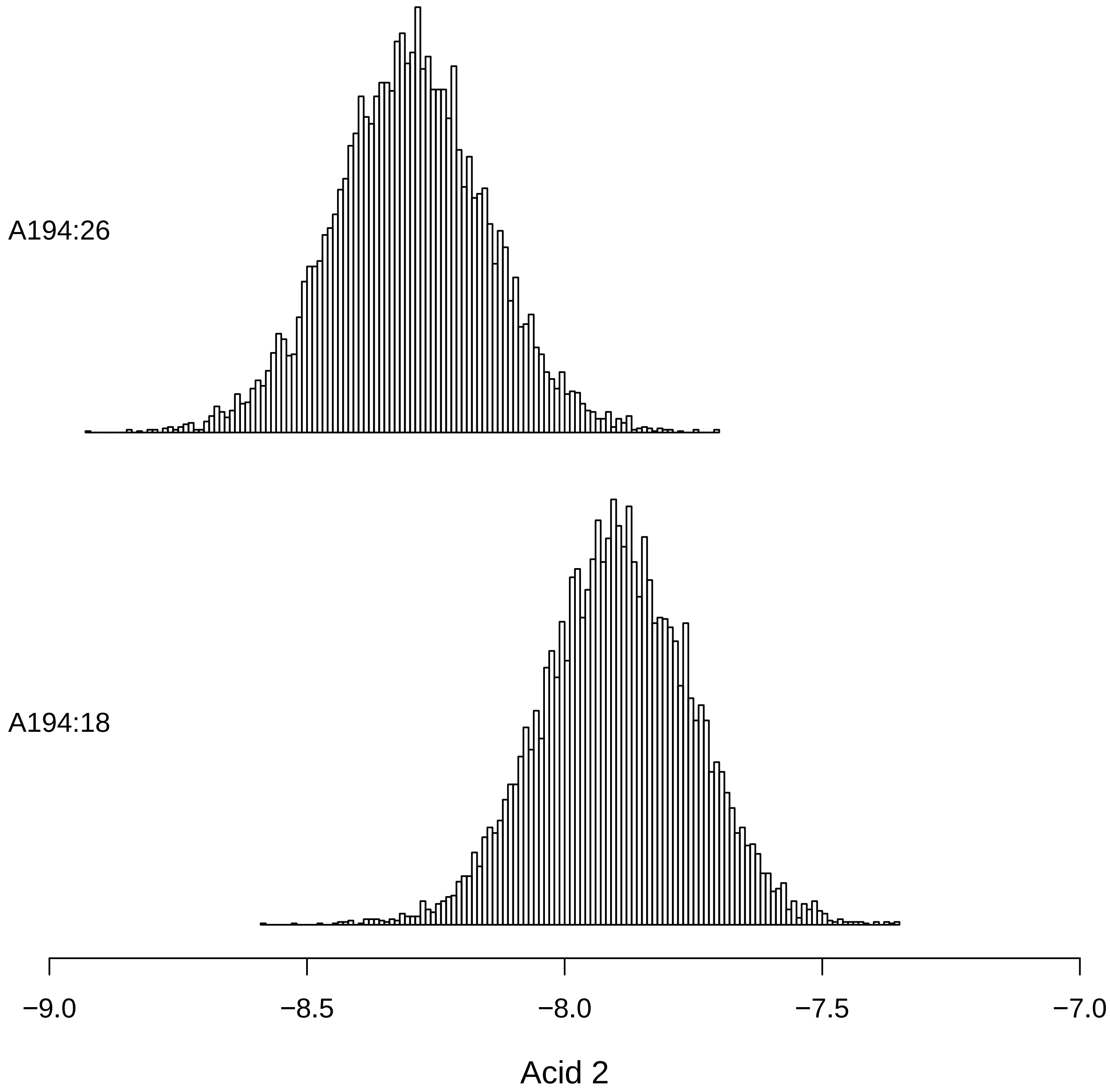
A194:26
A194:18
−9.0
−8.5
−8.0
−7.5
−7.0
Acid 2

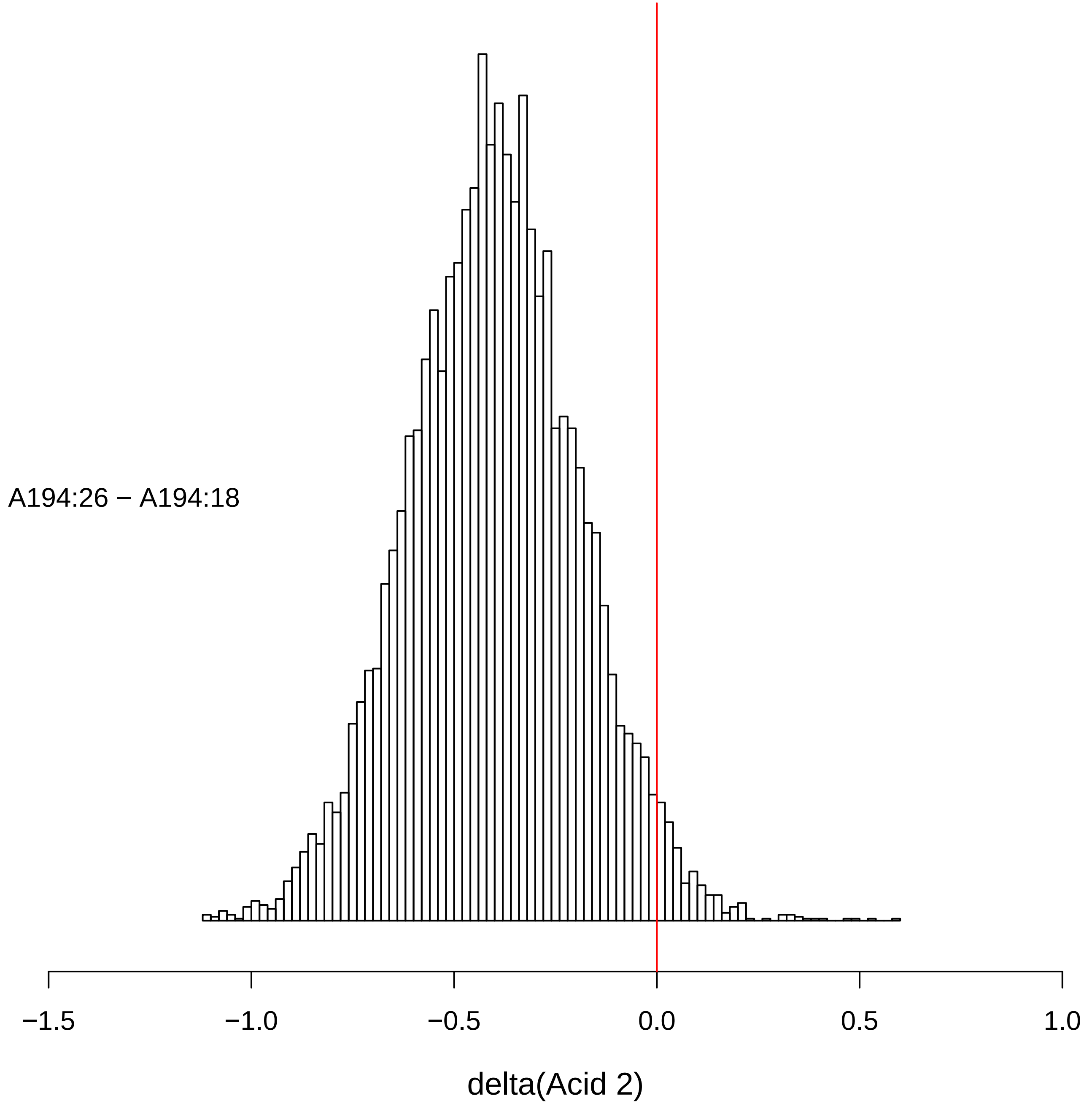
A194:26 − A194:18
−1.5
−1.0
−0.5
0.0
0.5
1.0
delta(Acid 2)

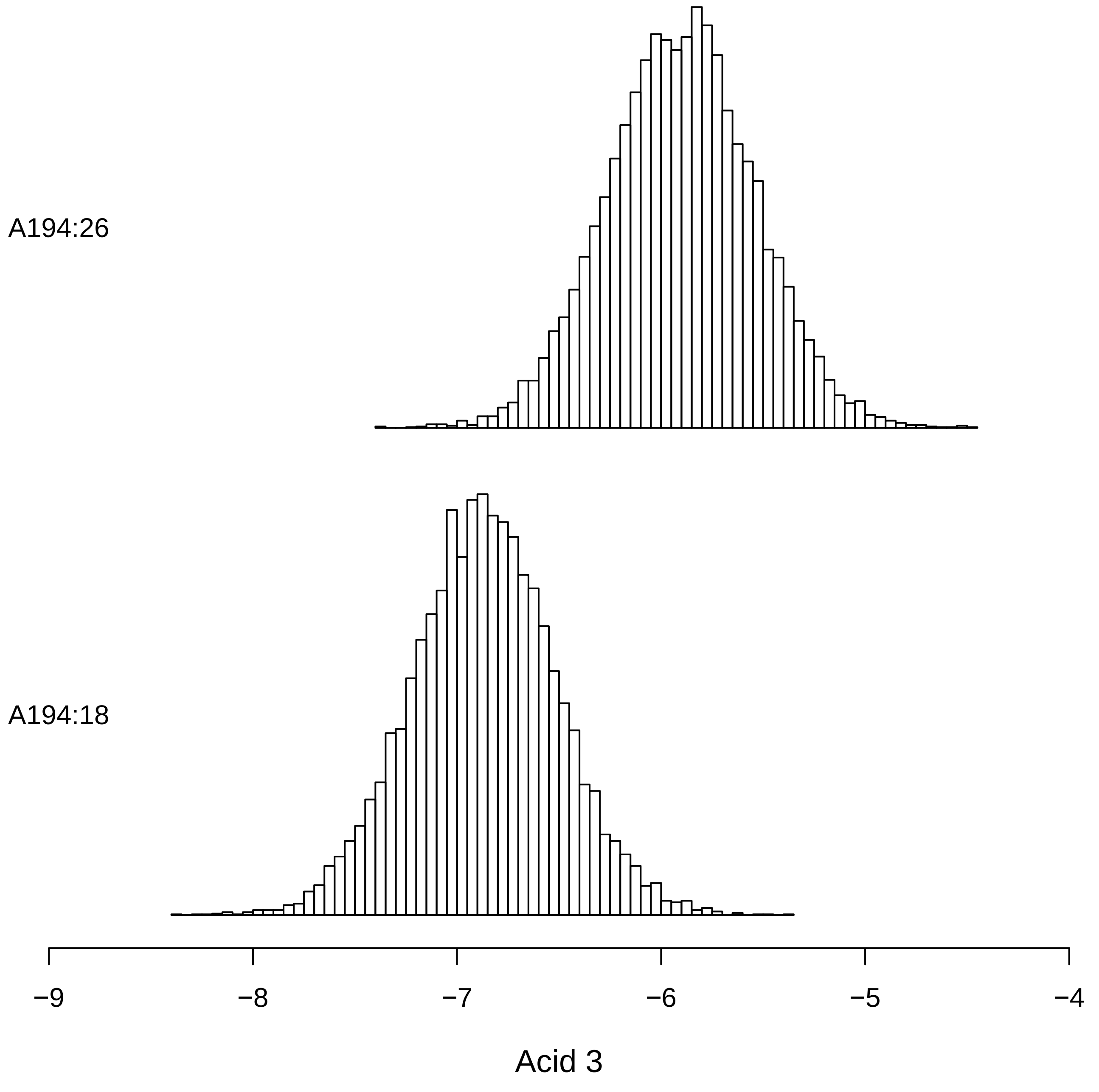
A194:26
A194:18
−9
−8
−7
−6
−5
−4
Acid 3

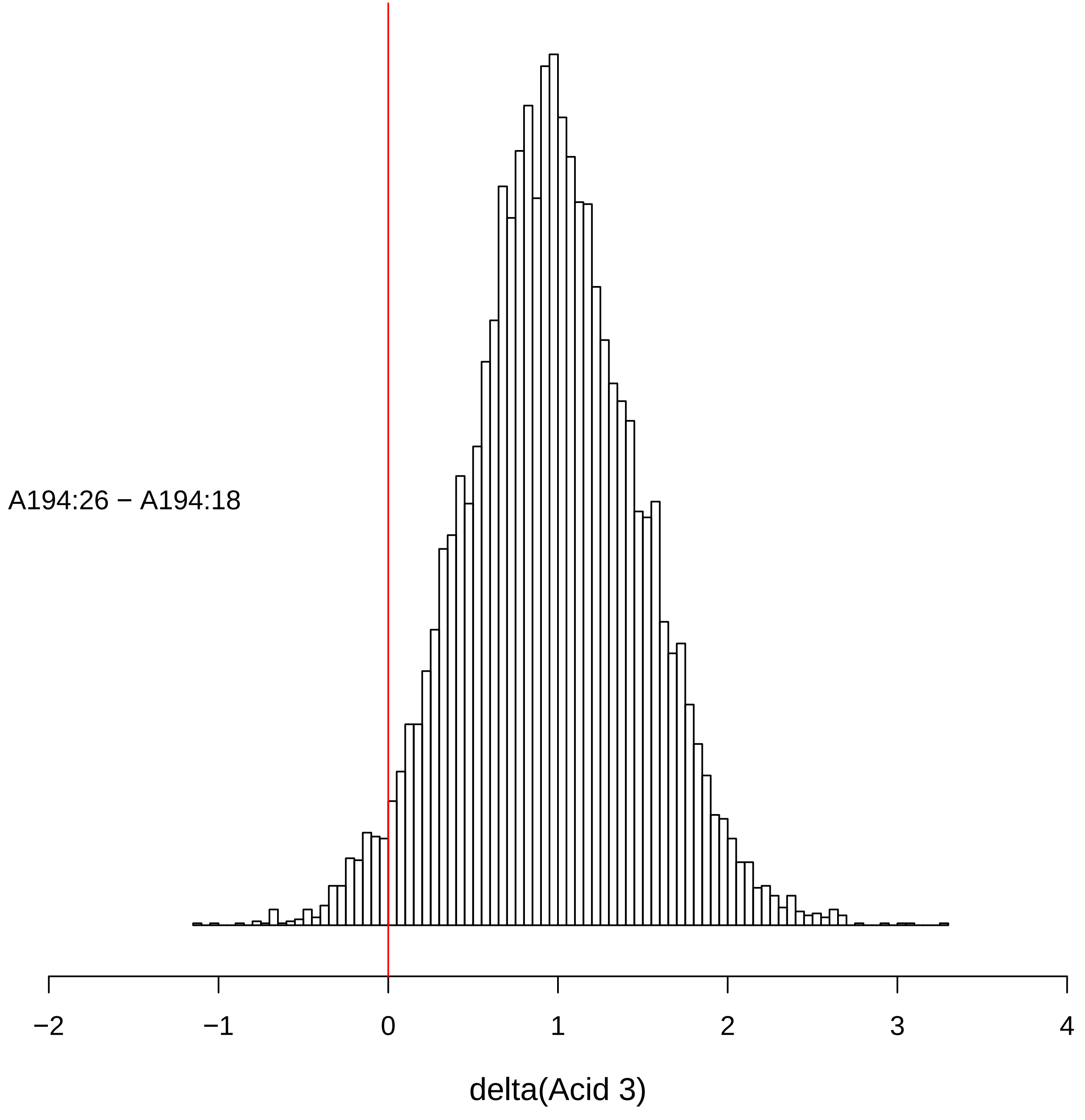
A194:26 − A194:18
−2
−1
0
1
2
3
4
delta(Acid 3)

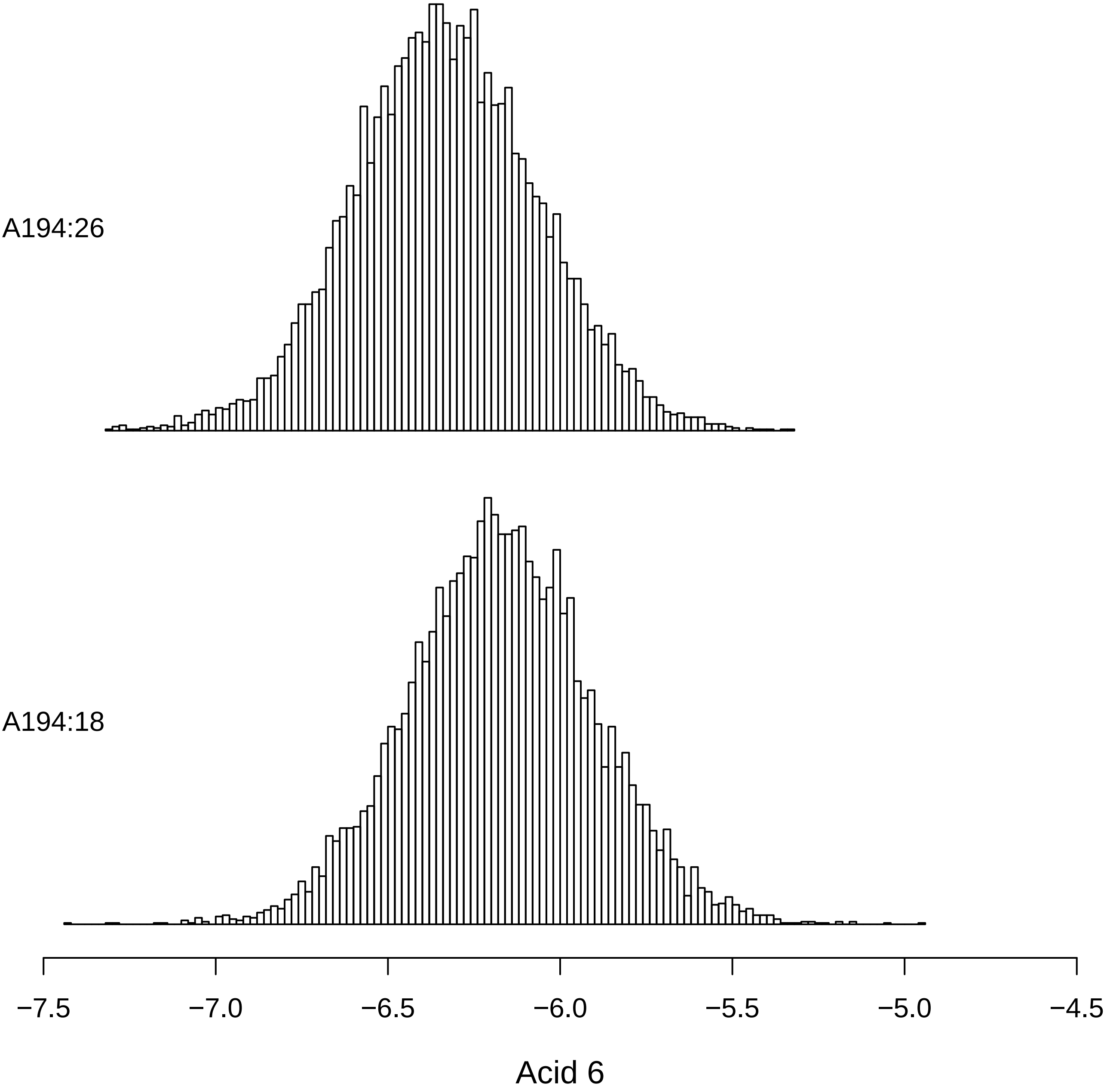
A194:26
A194:18
−7.5
−7.0
−6.5
−6.0
−5.5
−5.0
−4.5
Acid 6

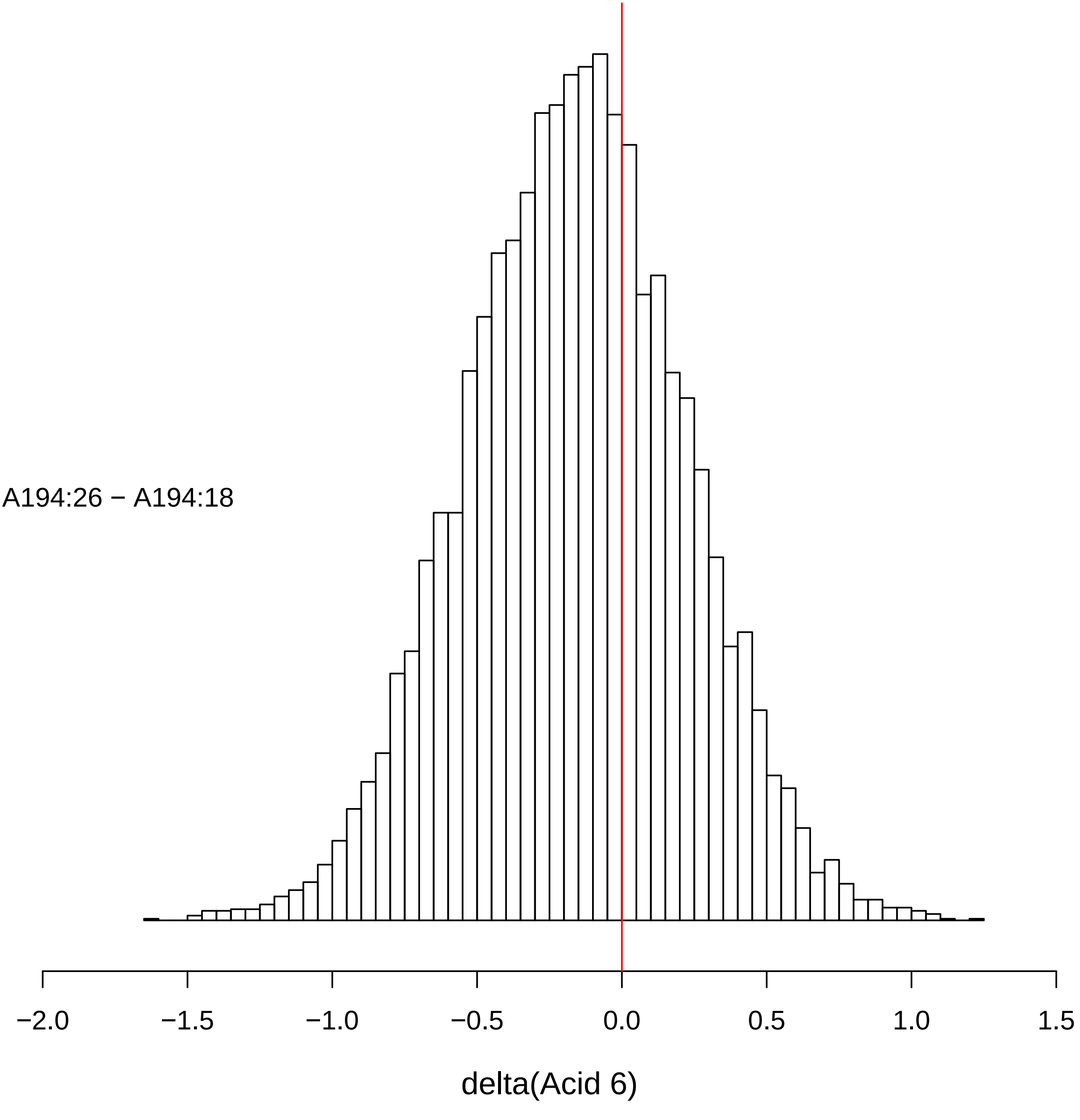
A194:26 − A194:18
−2.0
−1.5
−1.0
−0.5
0.0
0.5
1.0
1.5
delta(Acid 6)

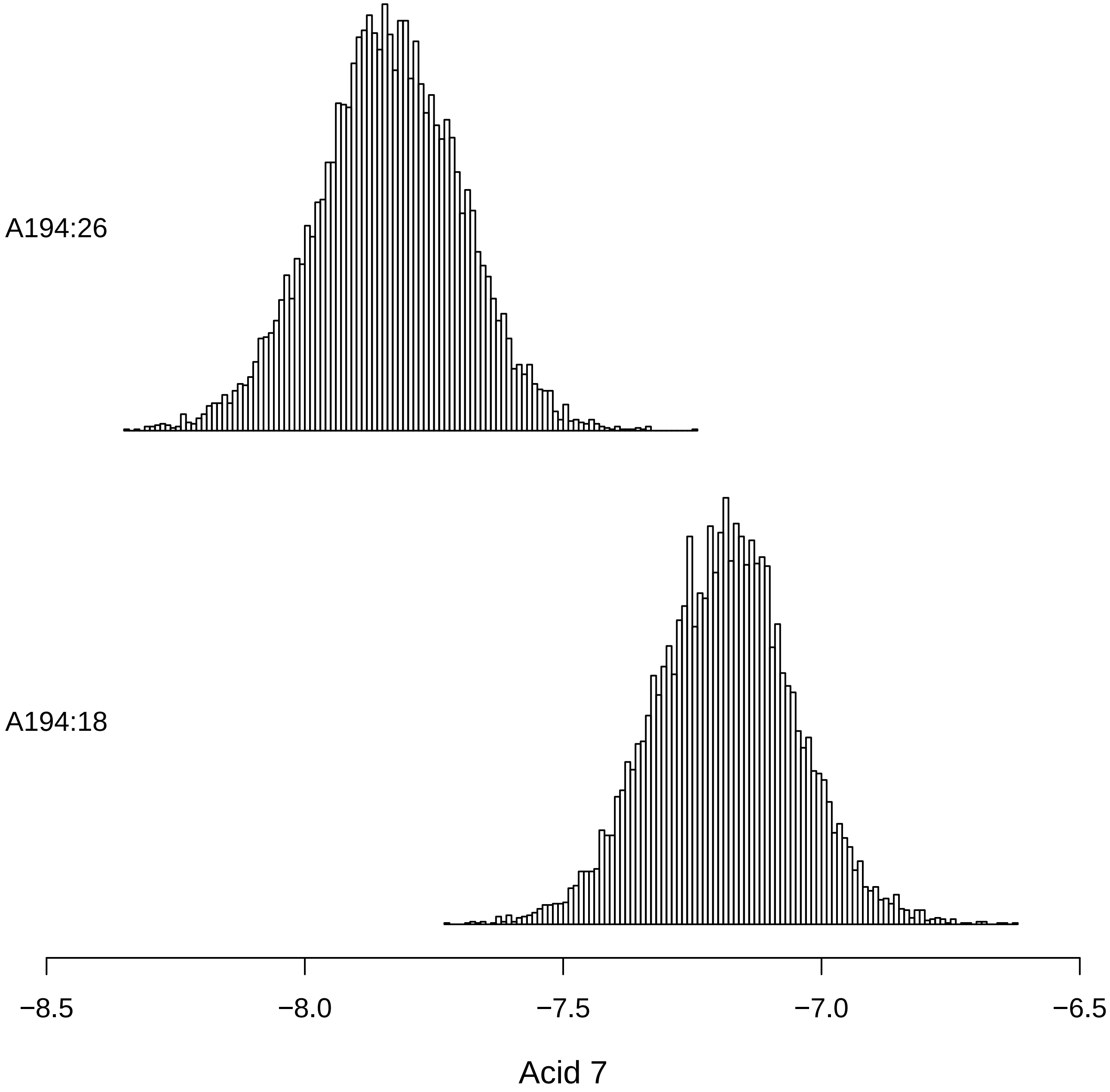
A194:26
A194:18
−8.5
−8.0
−7.5
−7.0
−6.5
Acid 7

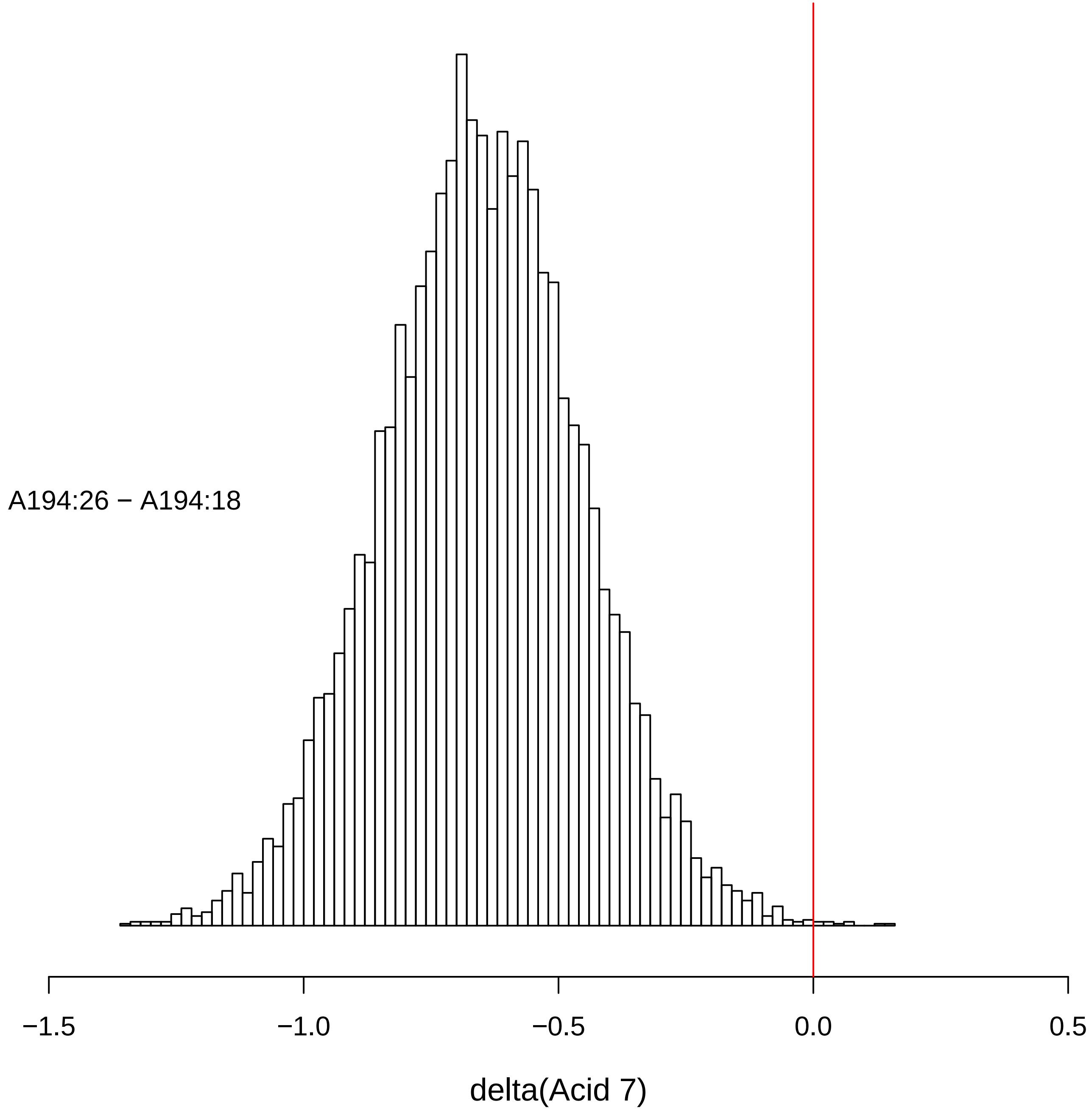

A194:26 − A194:18
−1.5
−1.0
−0.5
0.0
0.5
delta(Acid 7)

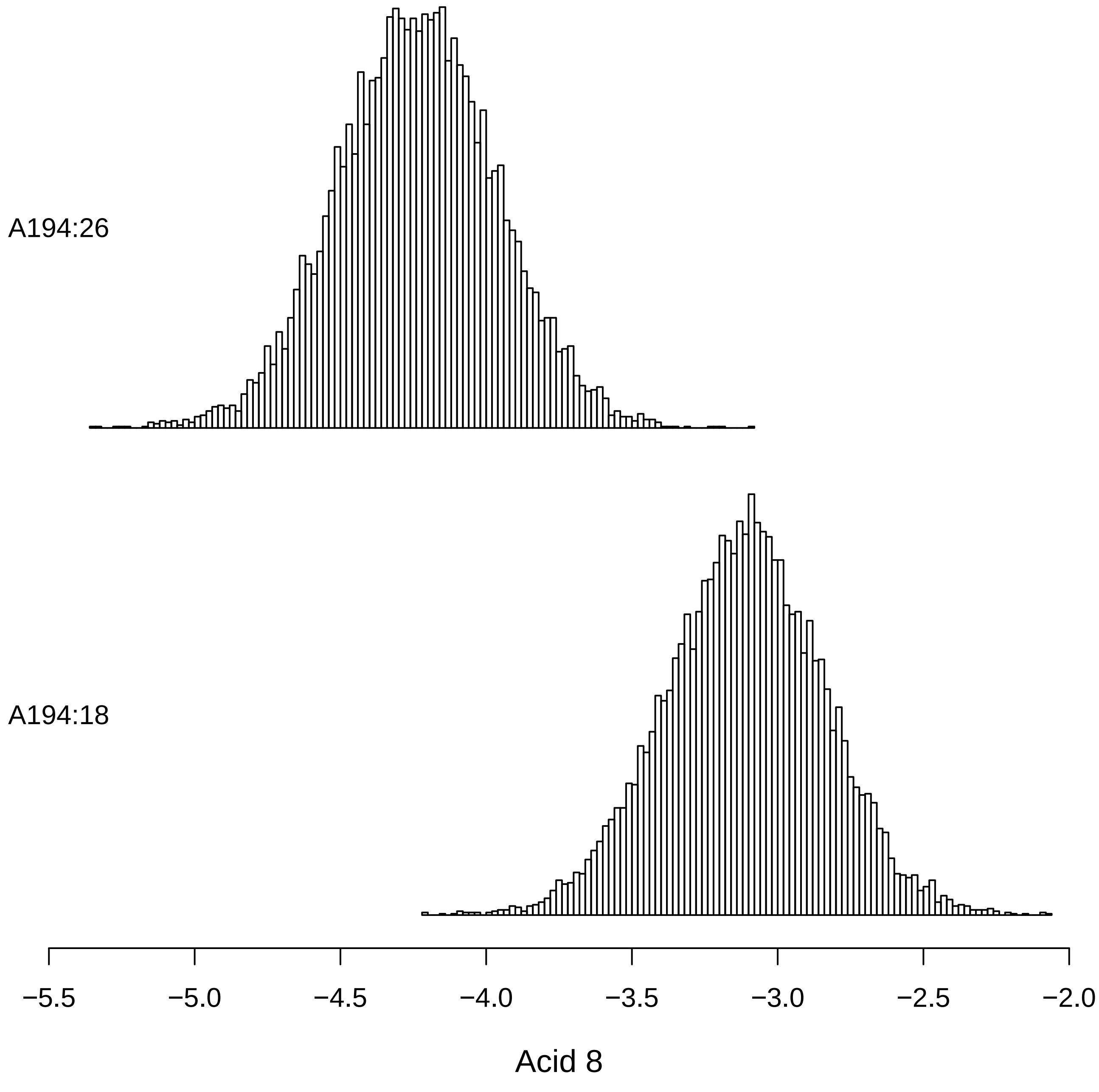
A194:26
A194:18
−5.5
−5.0
−4.5
−4.0
−3.5
−3.0
−2.5
−2.0
Acid 8

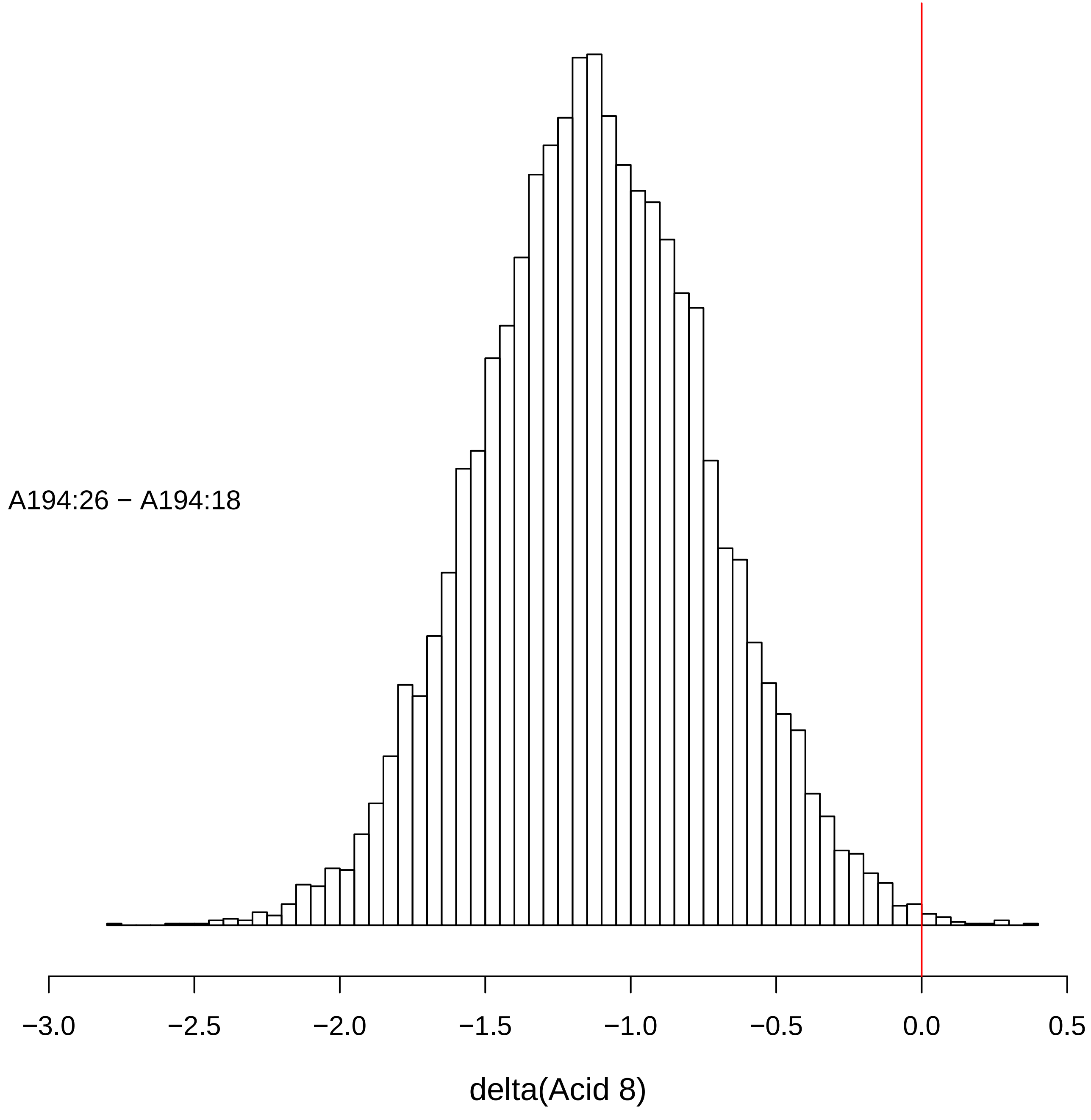
A194:26 − A194:18
−3.0
−2.5
−2.0
−1.5
−1.0
−0.5
0.0
0.5
delta(Acid 8)

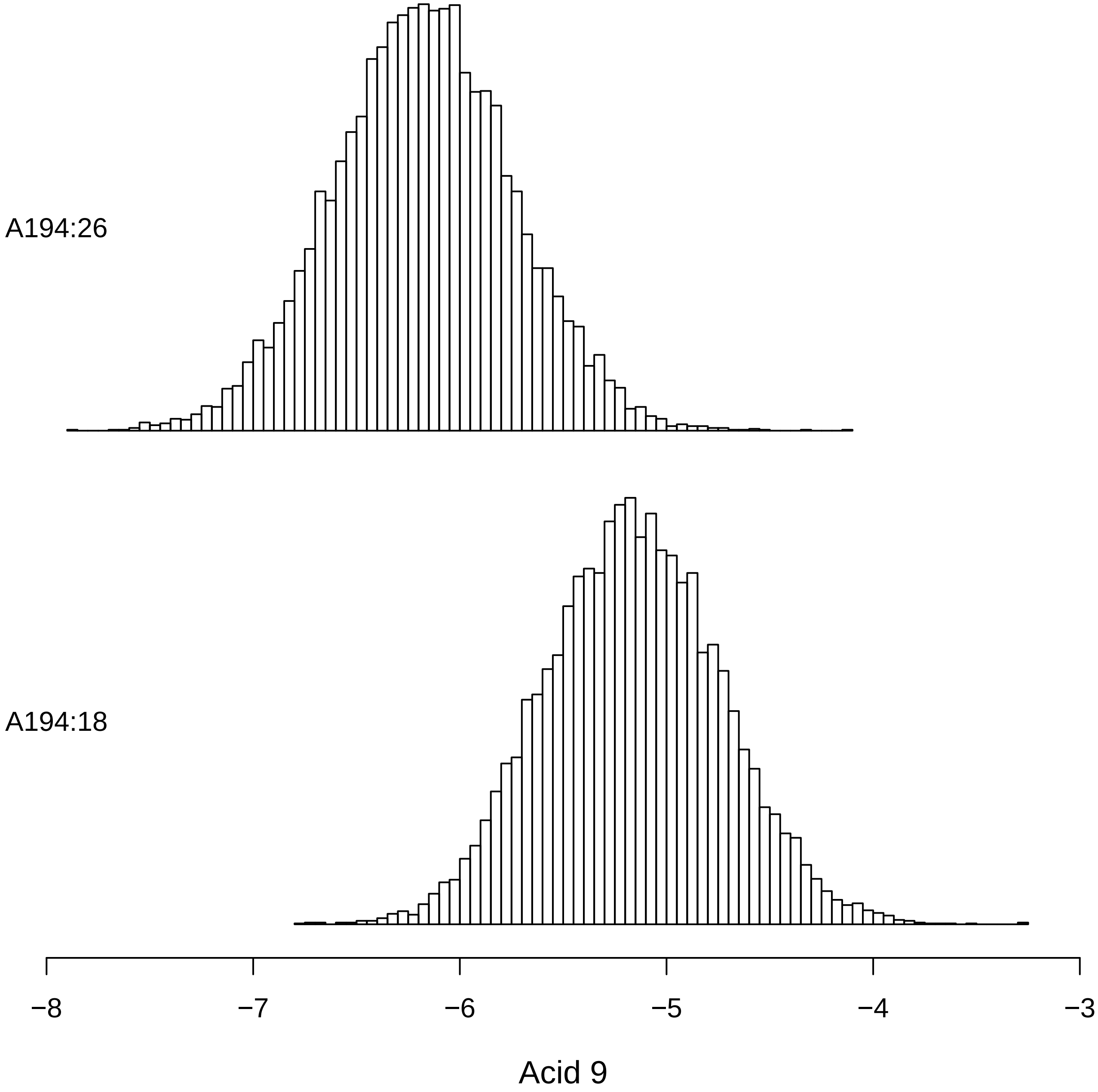
A194:26
A194:18
−8
−7
−6
−5
−4
−3
Acid 9

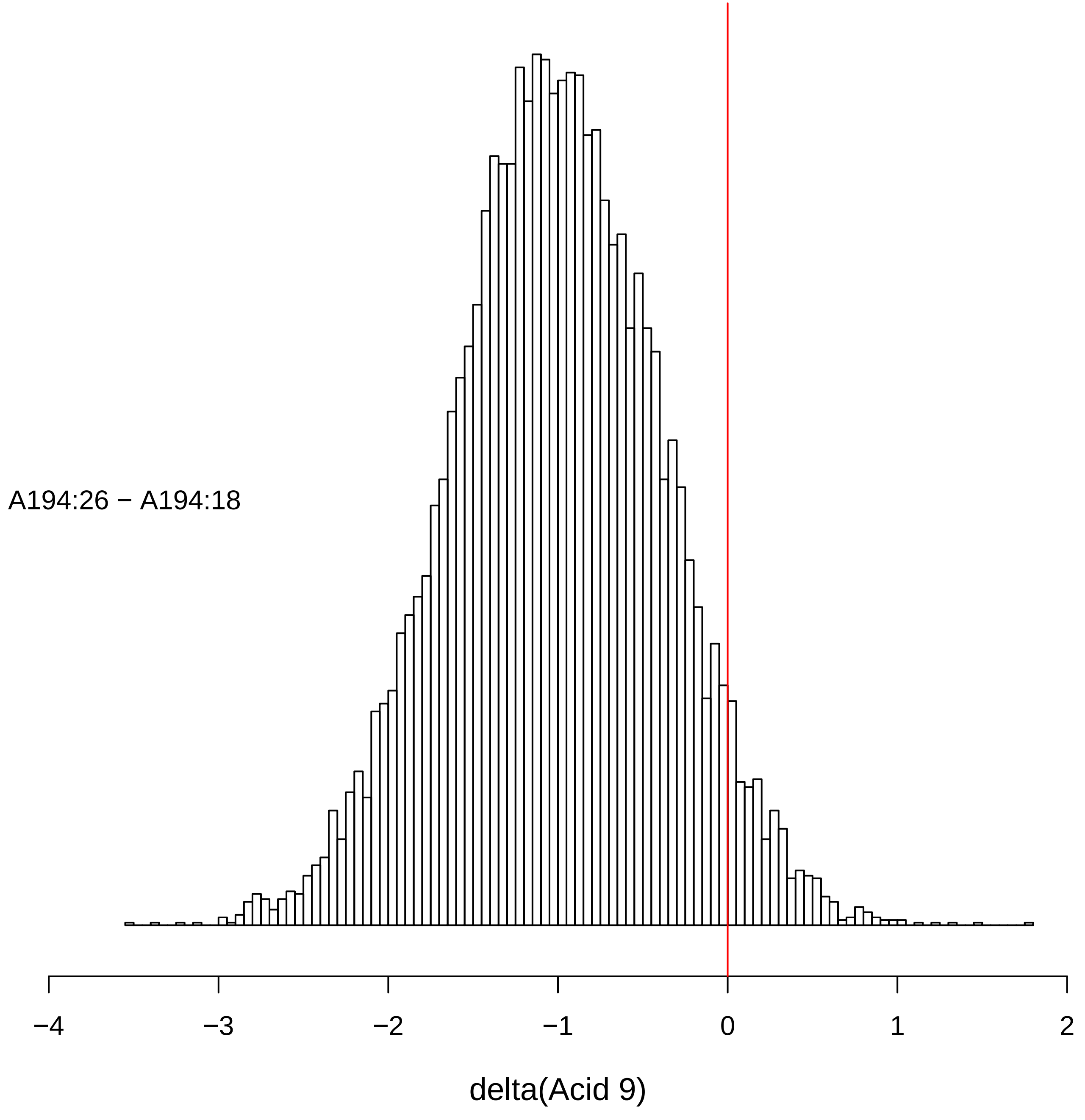
A194:26 − A194:18
−4
−3
−2
−1
0
1
2
delta(Acid 9)

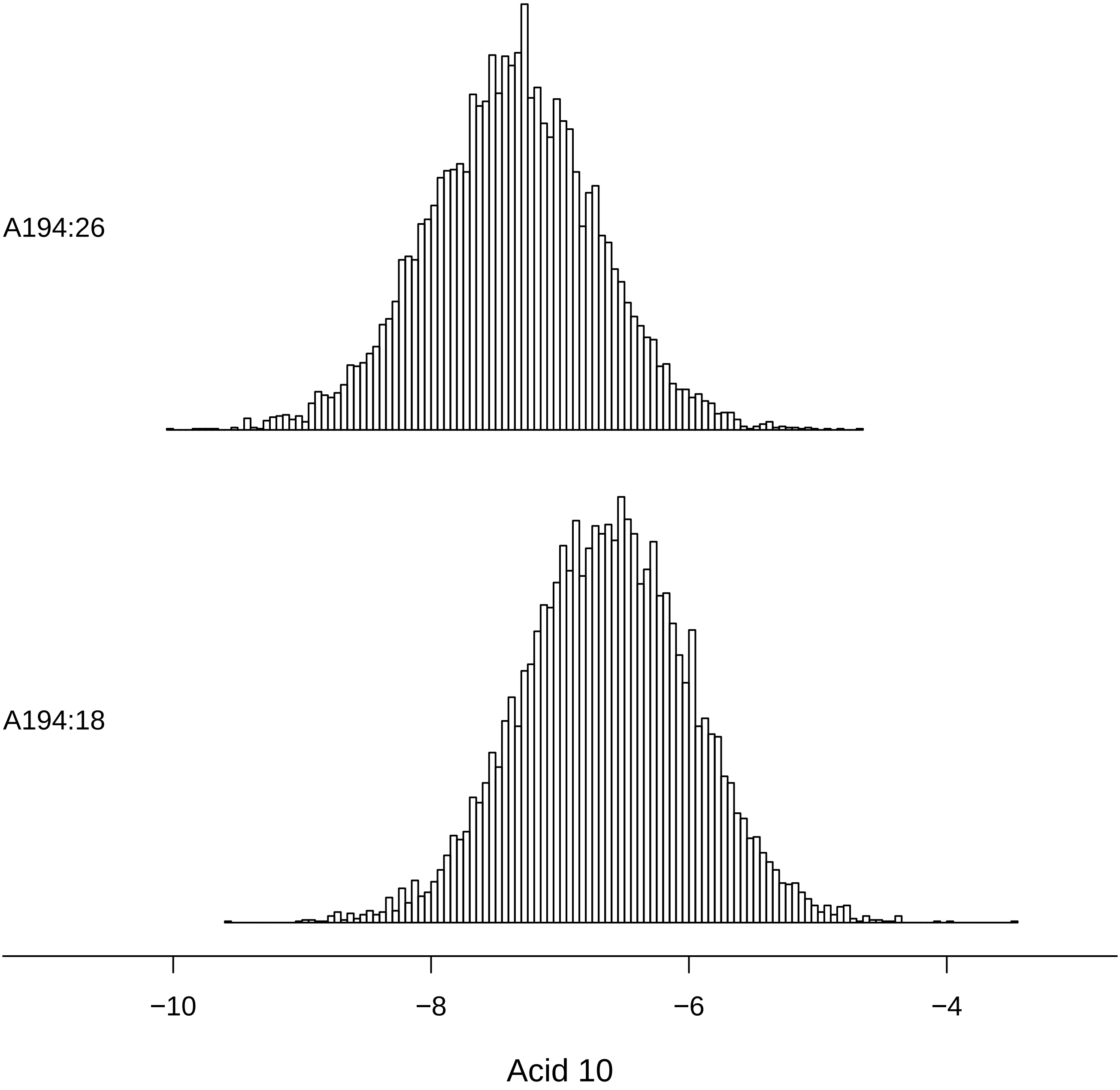
A194:26
A194:18
−10
−8
−6
−4
Acid 10

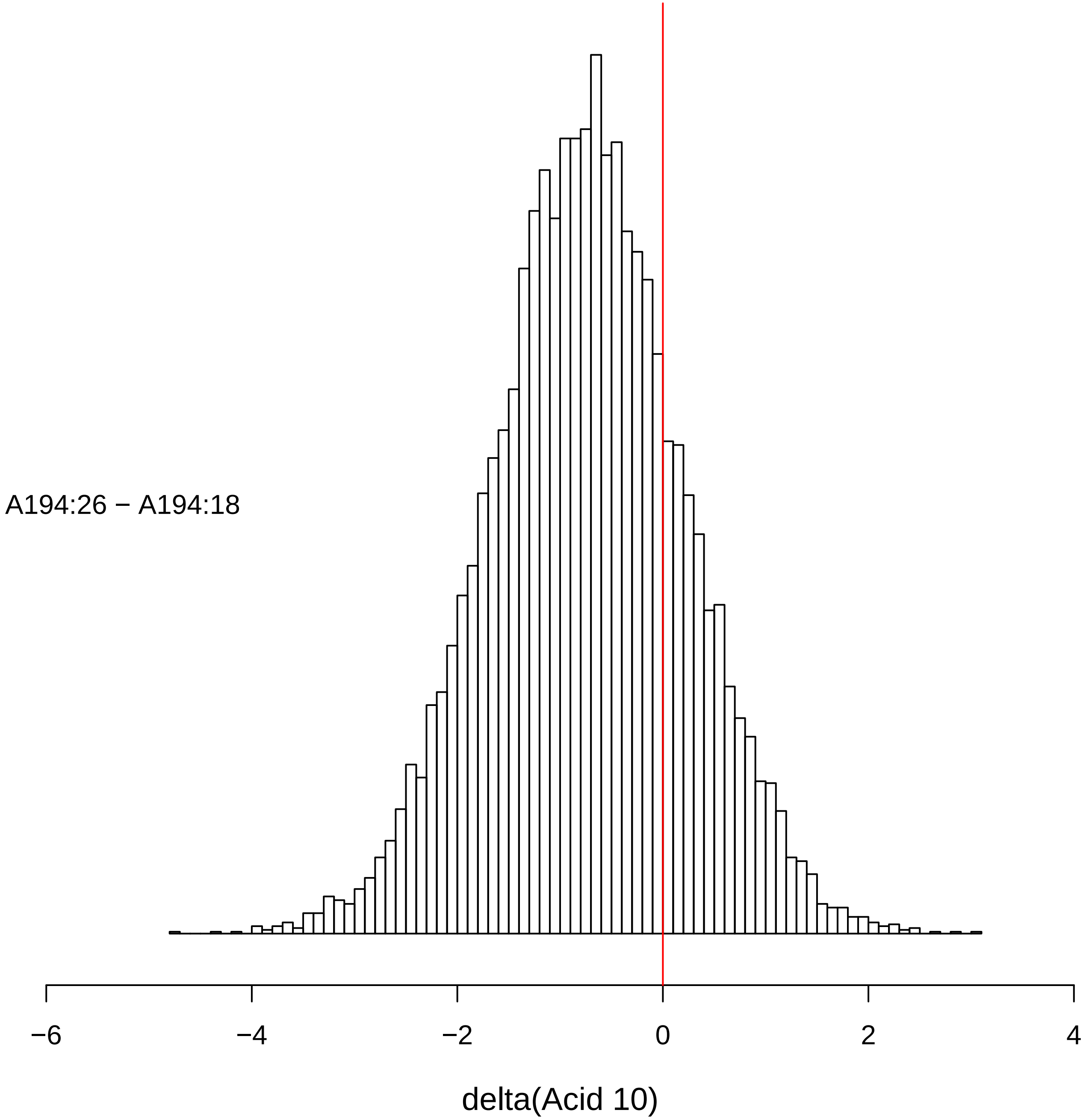
A194:26 − A194:18
−6
−4
−2
0
2
4
delta(Acid 10)

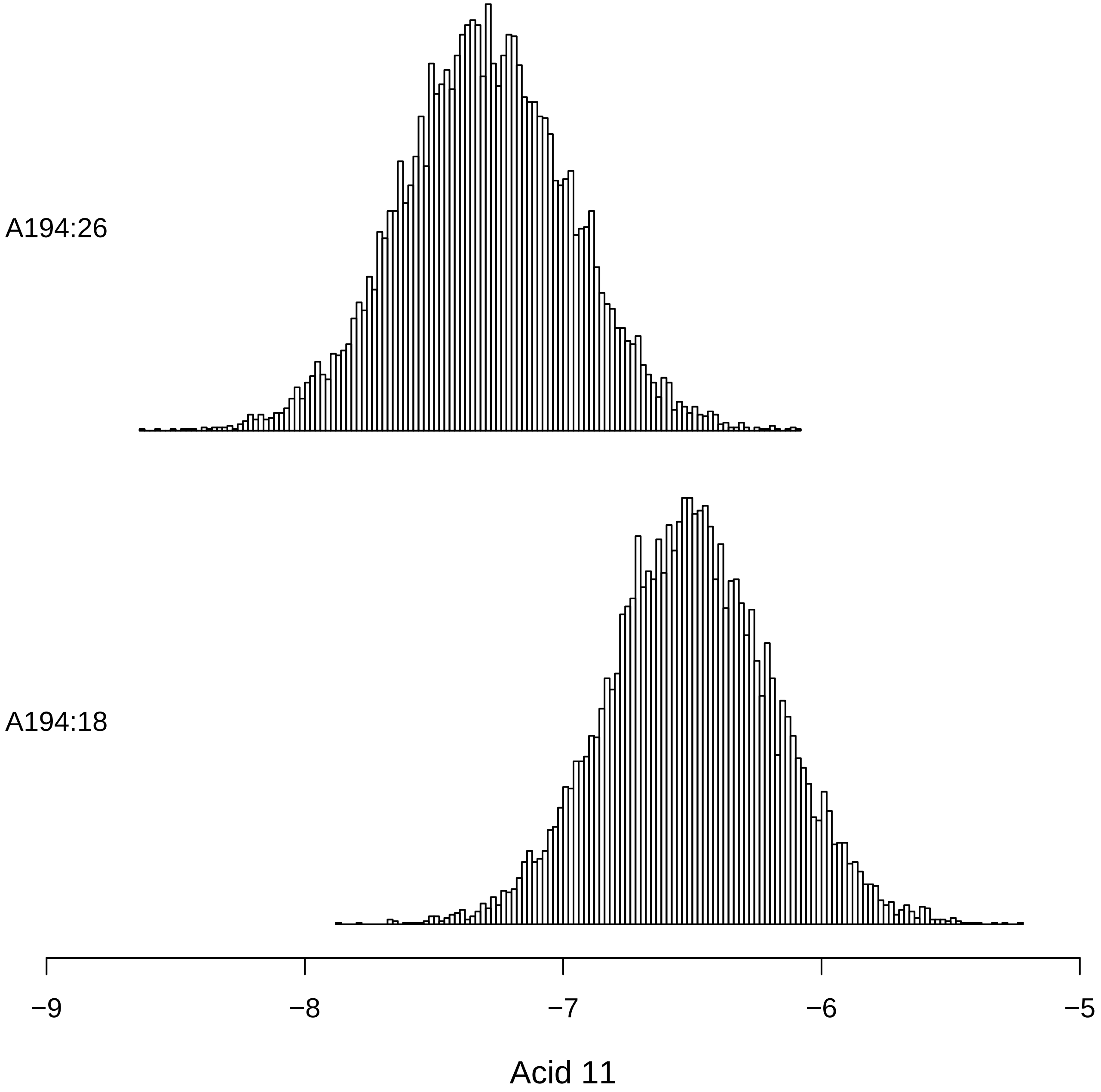
A194:26
A194:18
−9
−8
−7
−6
−5
Acid 11

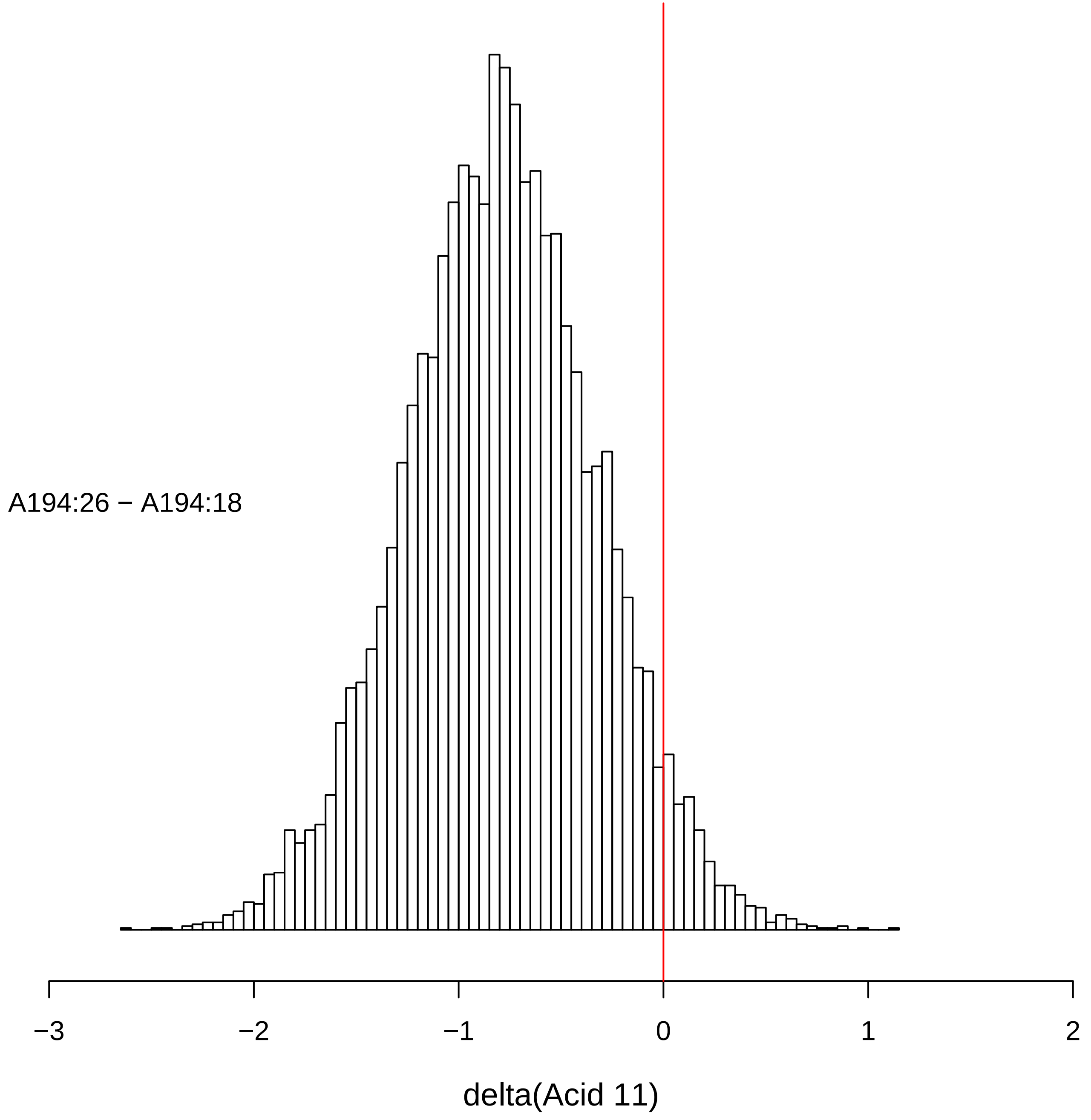
A194:26 − A194:18
−3
−2
−1
0
1
2
delta(Acid 11)

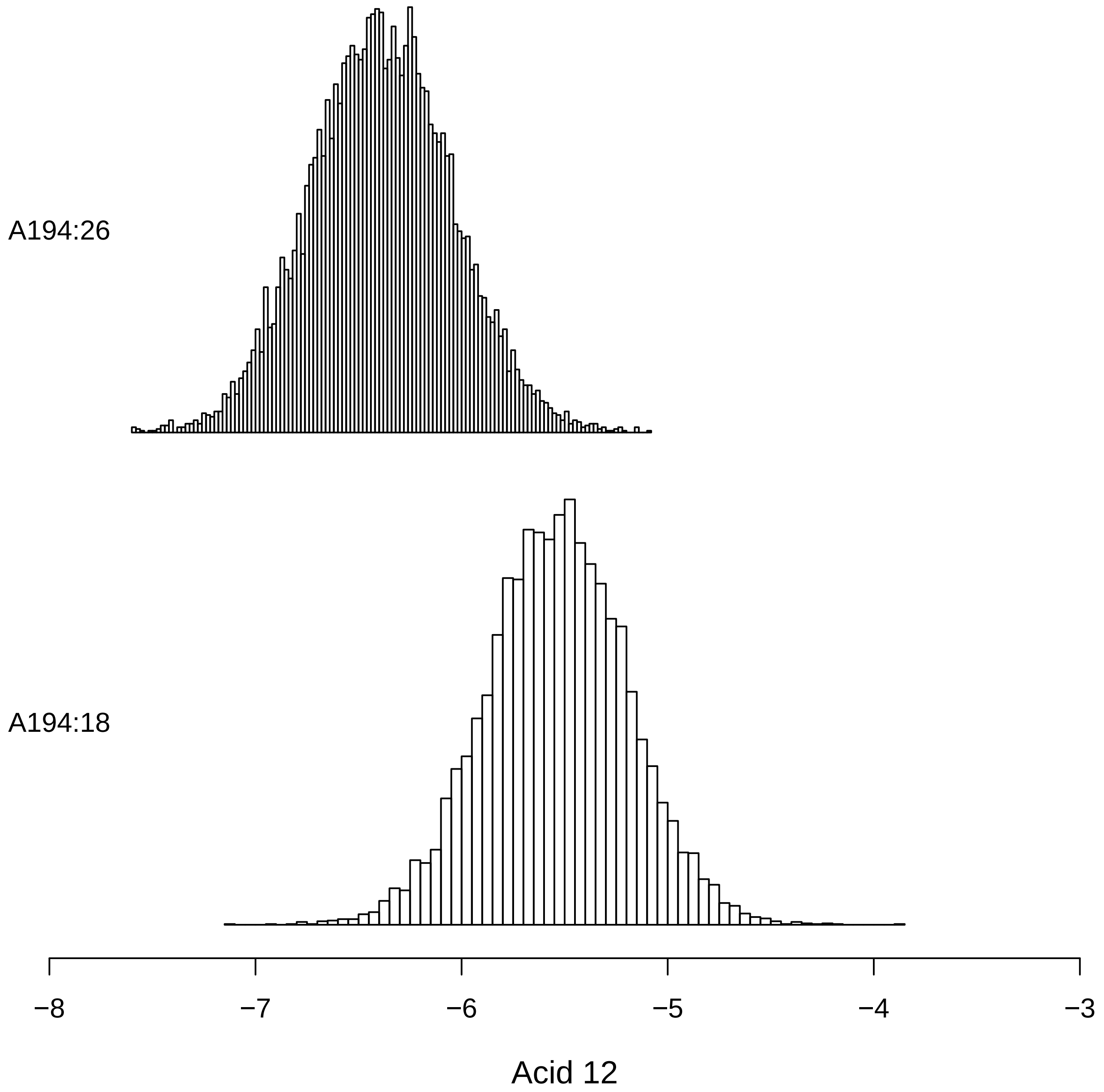
A194:26
A194:18
−8
−7
−6
−5
−4
−3
Acid 12

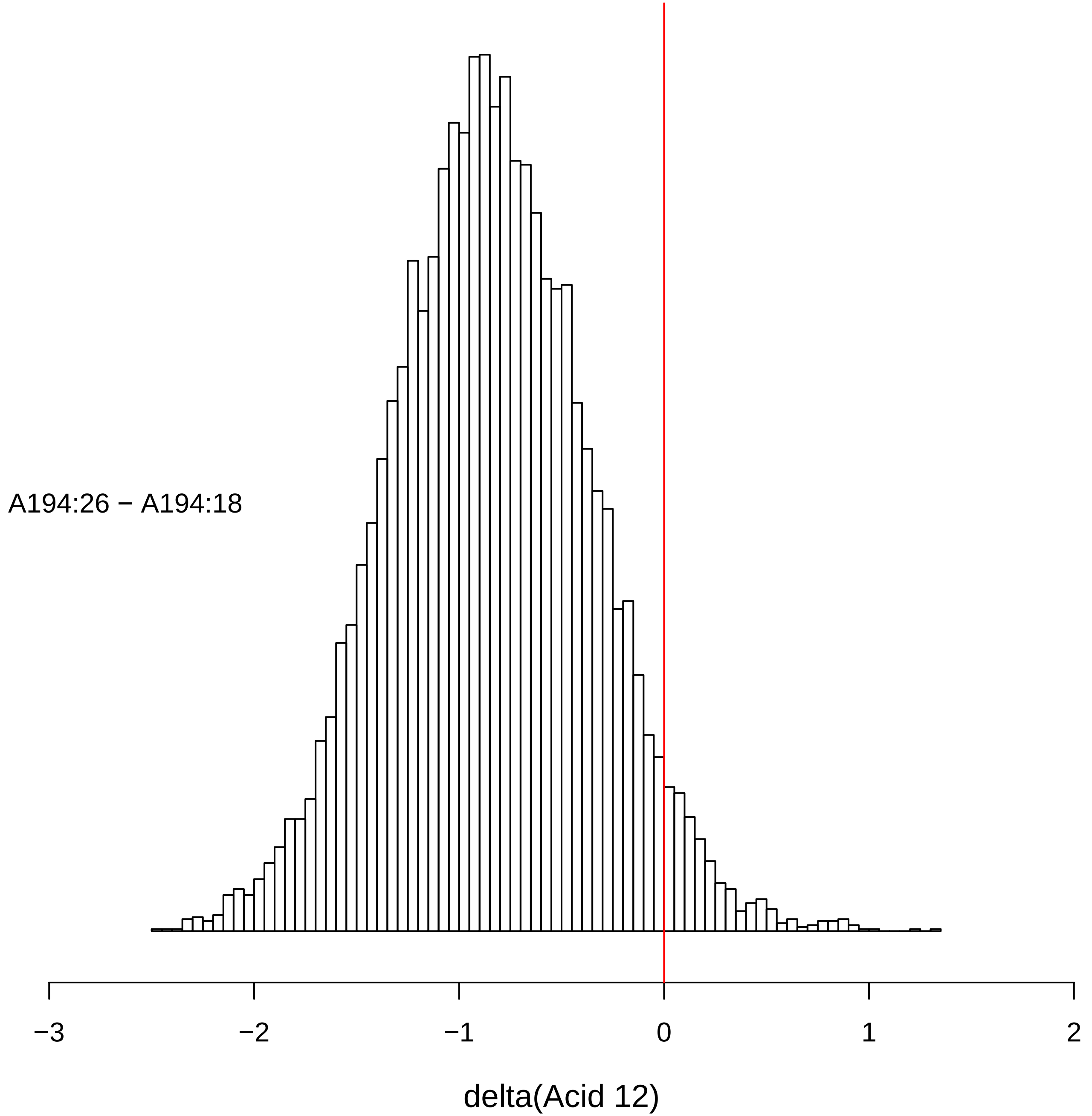
A194:26 − A194:18
−3
−2
−1
0
1
2
delta(Acid 12)

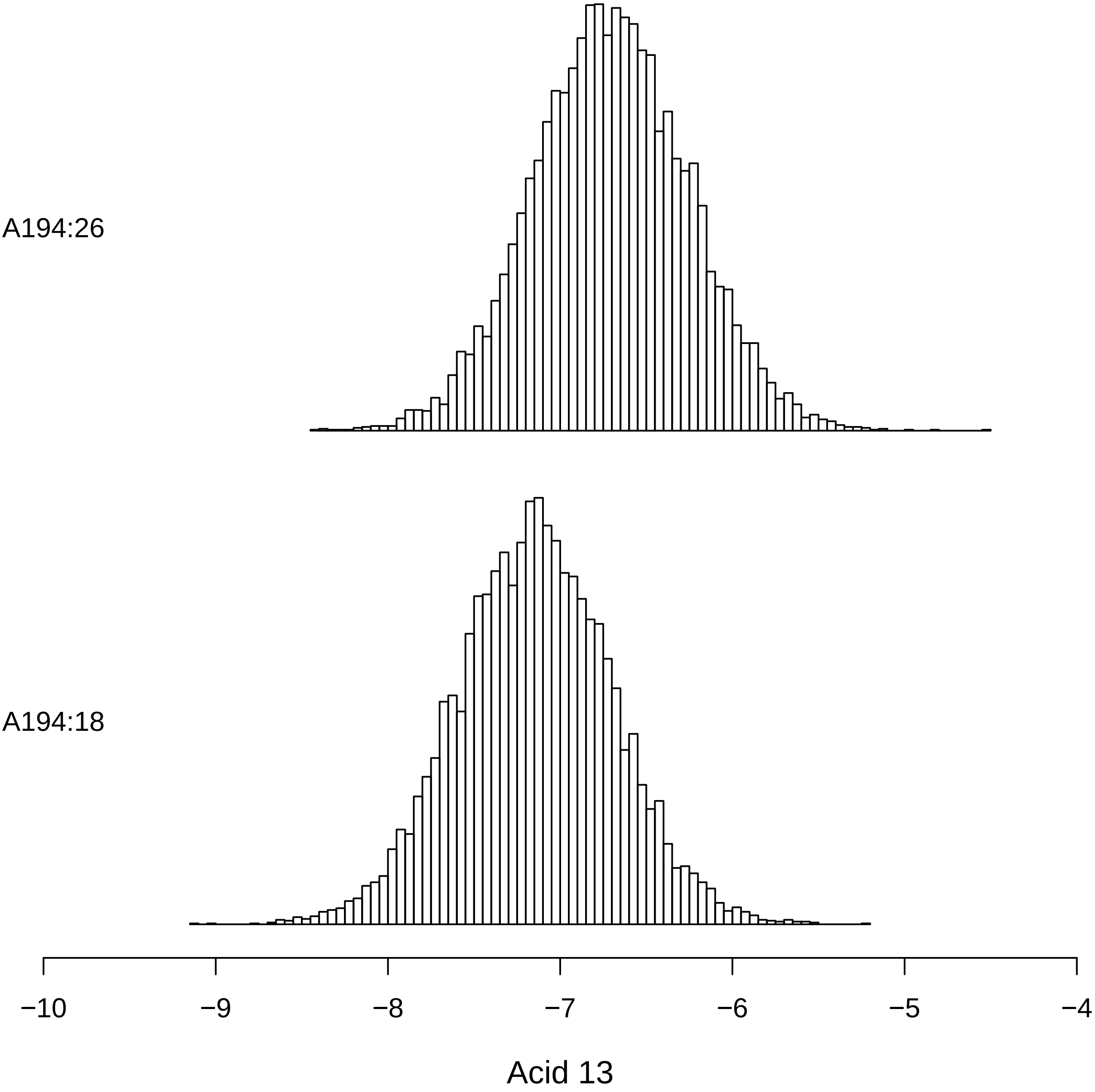
A194:26
A194:18
−10
−9
−8
−7
−6
−5
−4
Acid 13

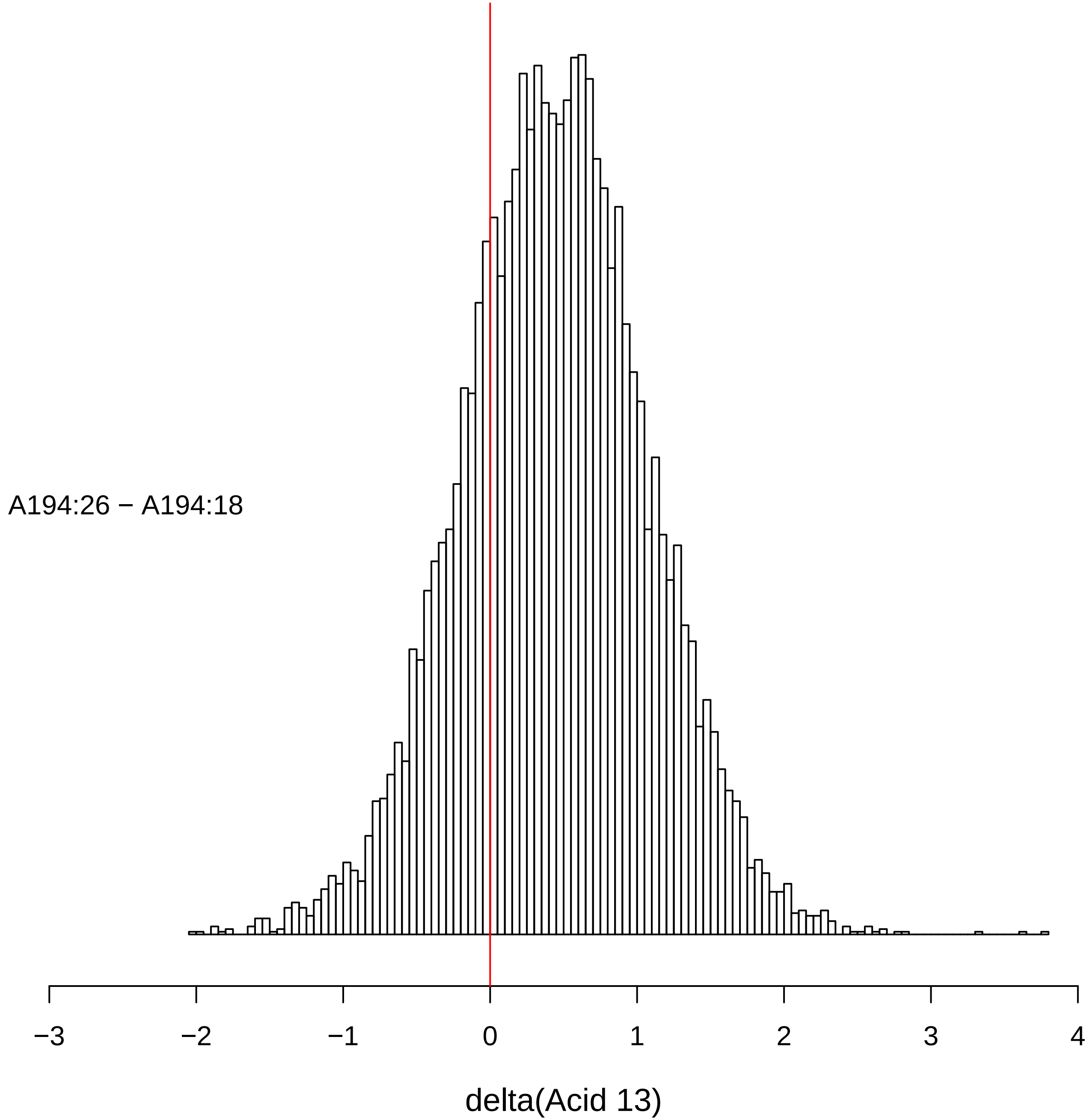
A194:26 − A194:18
−3
−2
−1
0
1
2
3
4
delta(Acid 13)

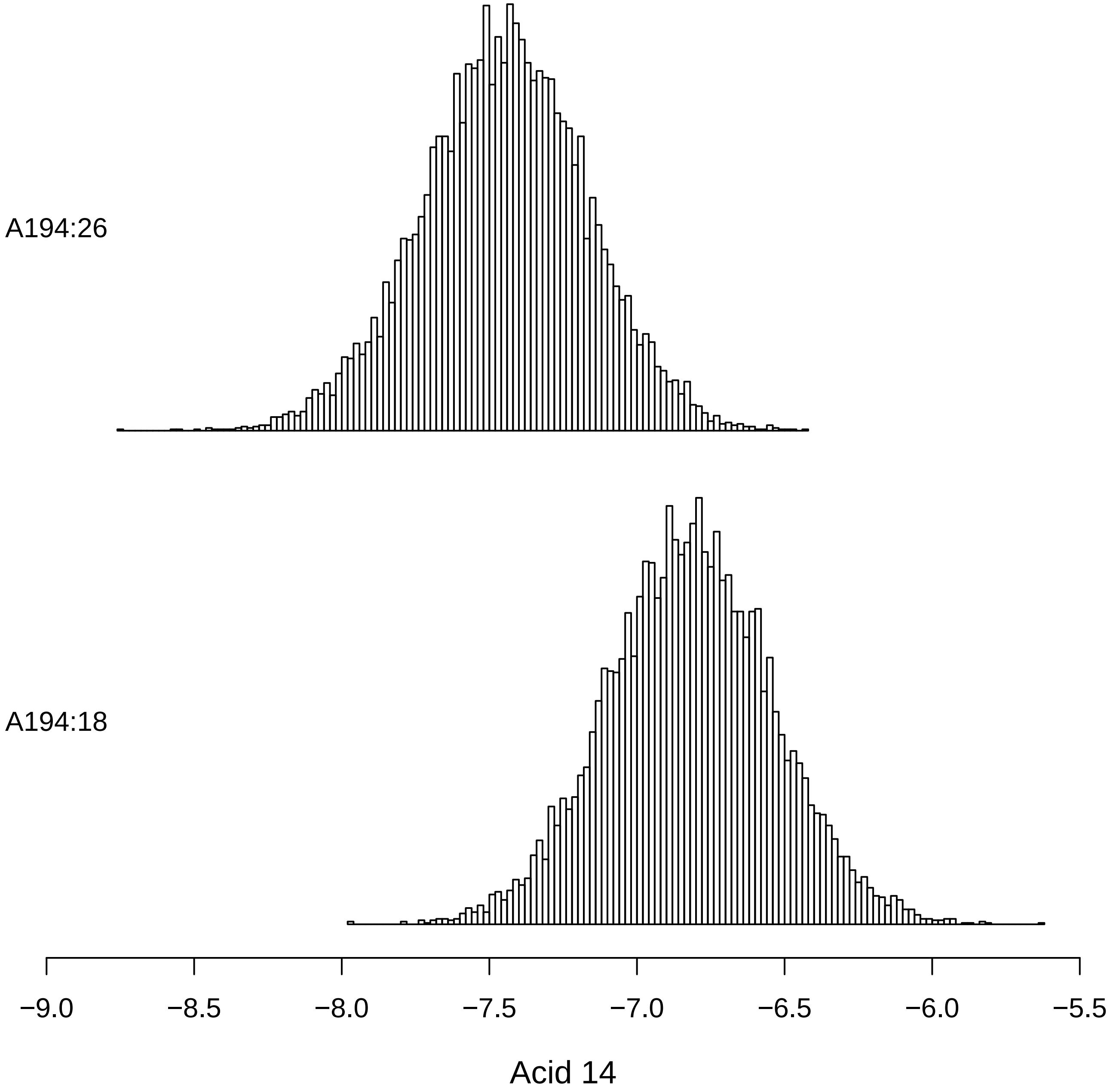
A194:26
A194:18
−9.0
−8.5
−8.0
−7.5
−7.0
−6.5
−6.0
−5.5
Acid 14

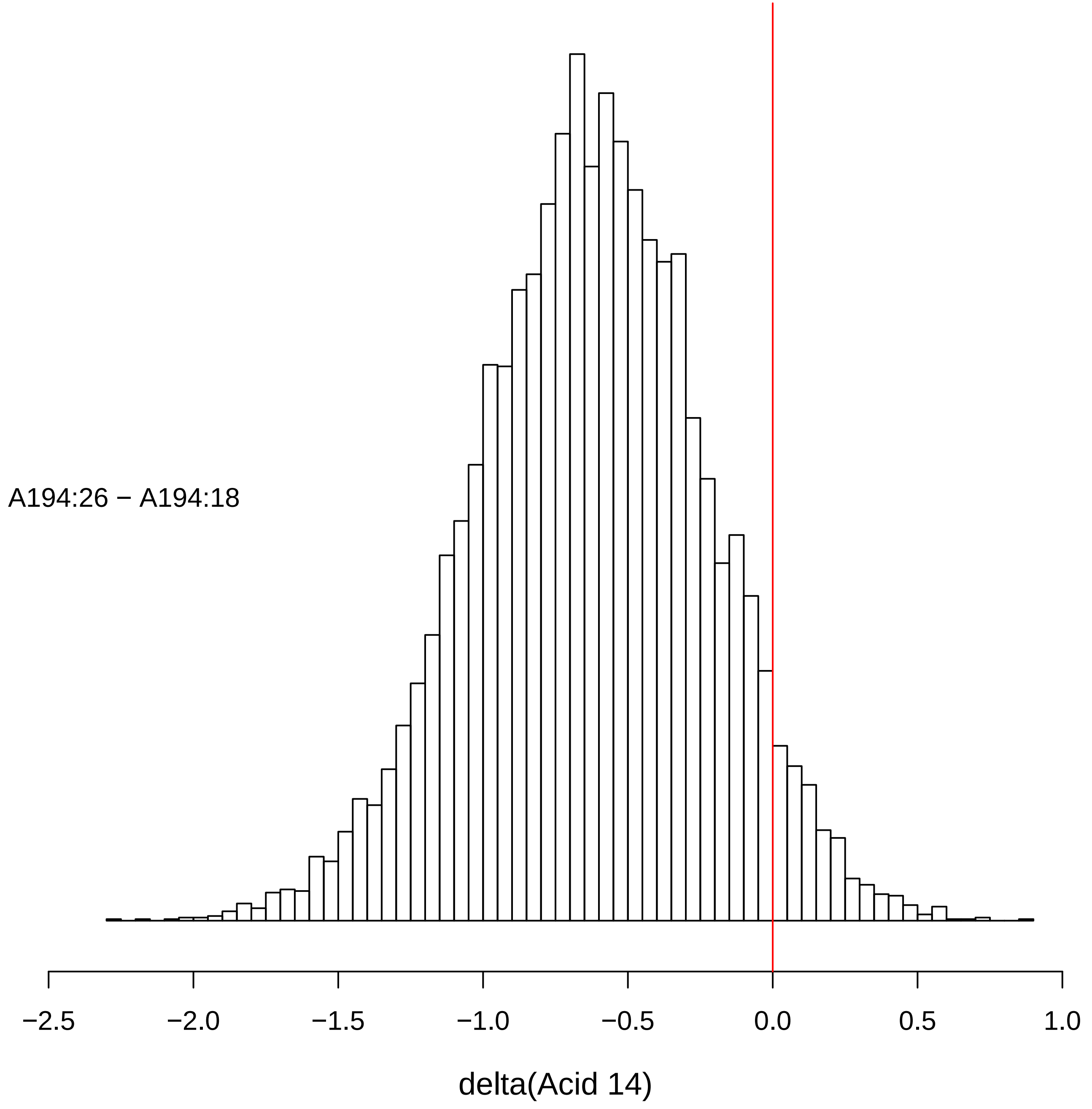
A194:26 − A194:18
−2.5
−2.0
−1.5
−1.0
−0.5
0.0
0.5
1.0
delta(Acid 14)
