# Supplementary material for: Taxonomic and Environmental Variation of Metabolite Profiles in Marine Dinoflagellates of the Genus Symbiodinium
Source: Metabolites. 2015 Feb 16;5(1):74–99. doi: 10.3390/metabo5010074 (PMC4381291; doi:10.3390/metabo5010074)
Supplement: Supplementary File 1 [file metabolites-05-00074-s001.zip › Supplementary Information/Supplementary Information Figure S4b - temp.B184.pdf]

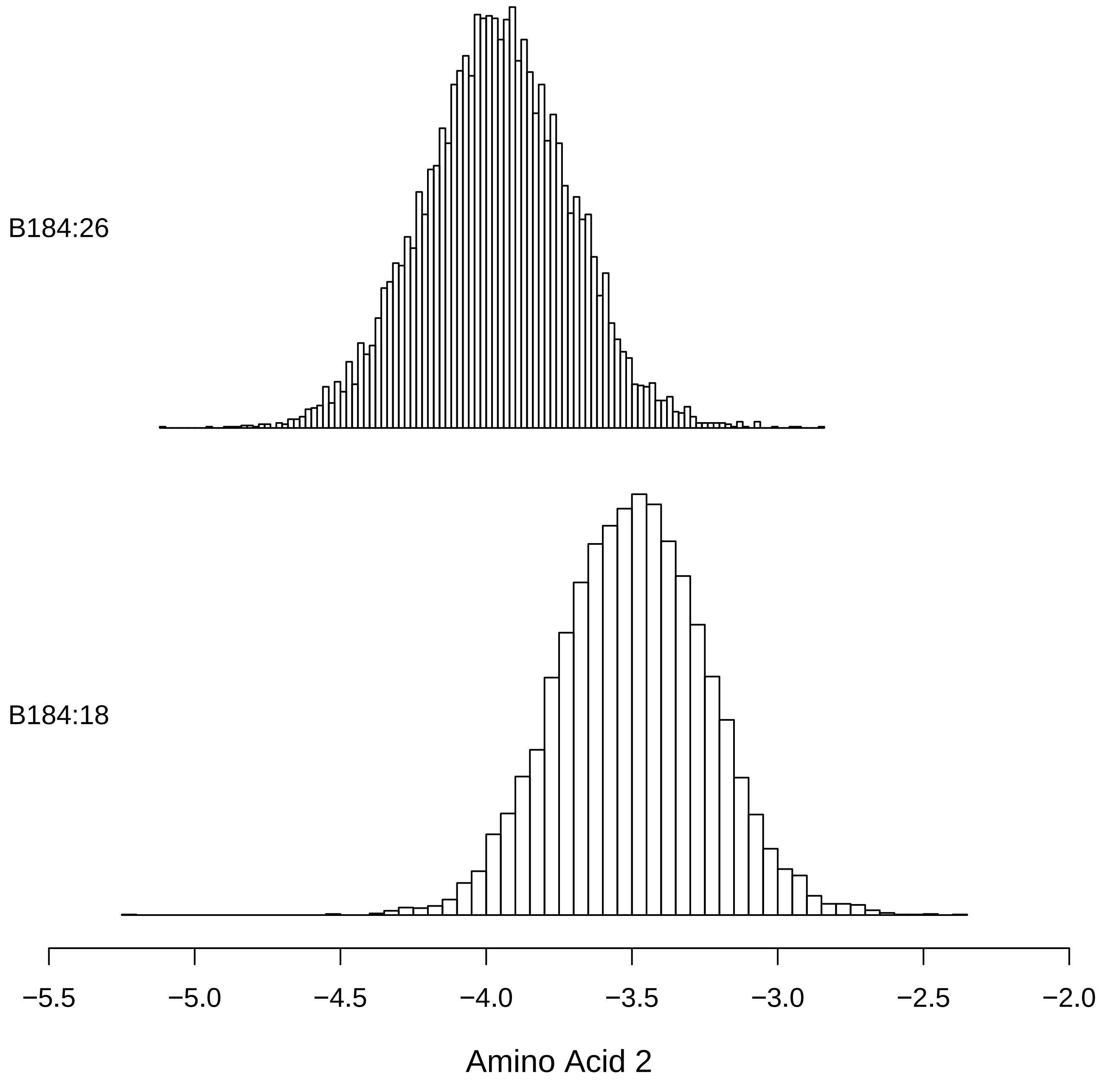
B184:26
B184:18
−5.5
−5.0
−4.5
−4.0
−3.5
−3.0
−2.5
−2.0
Amino Acid 2

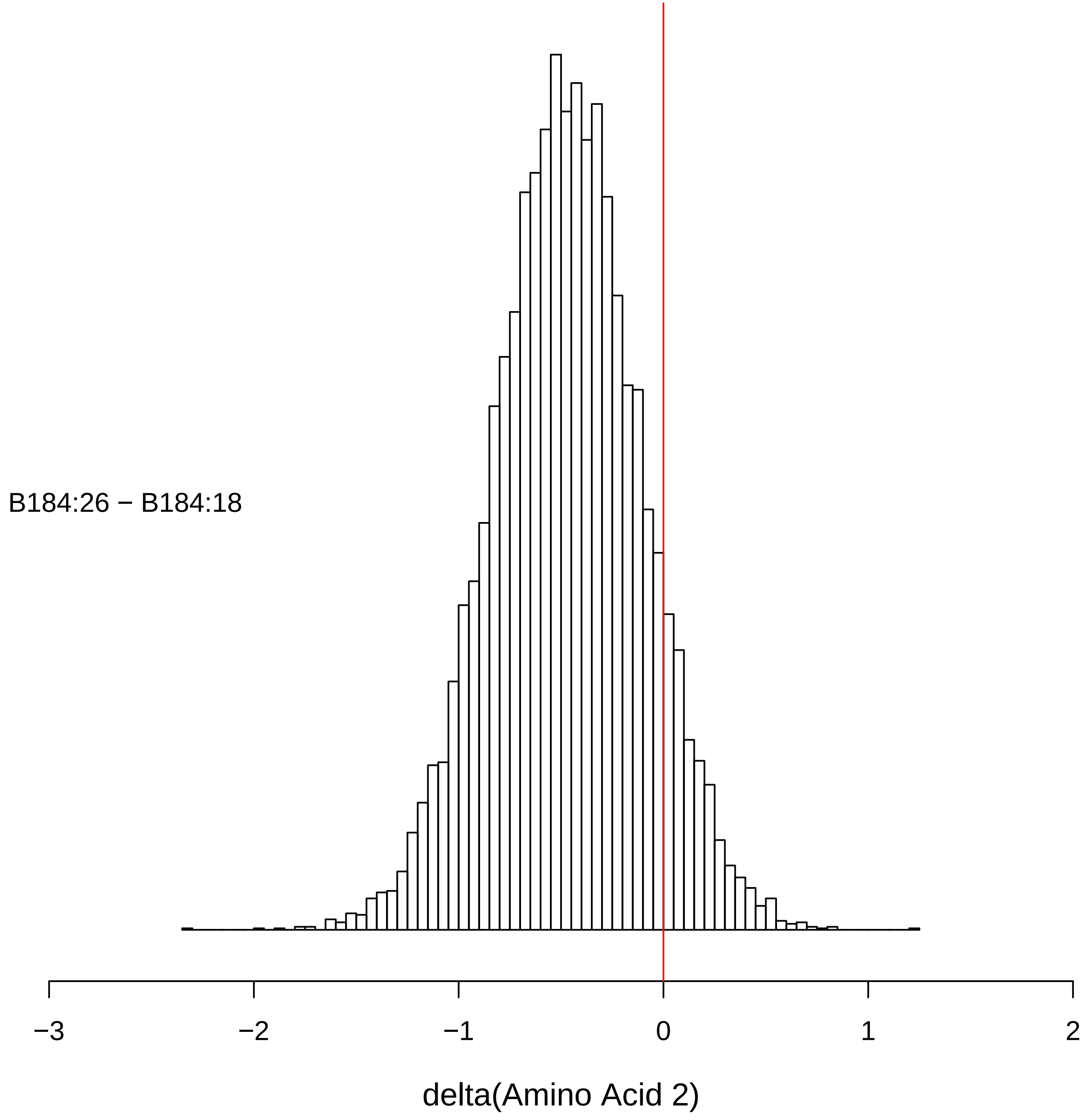

B184:26 − B184:18
−3
−2
−1
0
1
2
delta(Amino Acid 2)

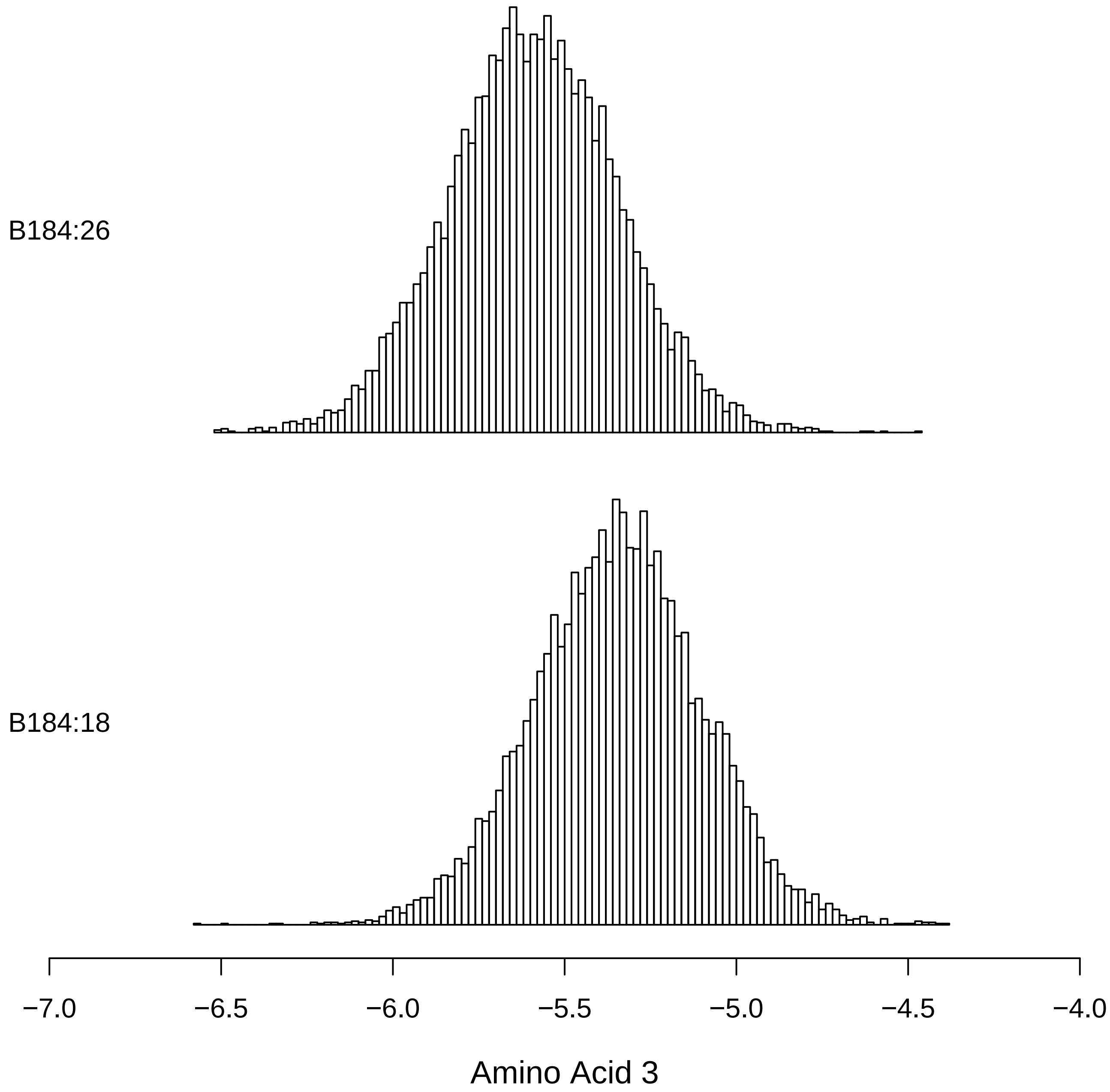

B184:26
B184:18
−7.0
−6.5
−6.0
−5.5
−5.0
−4.5
−4.0
Amino Acid 3

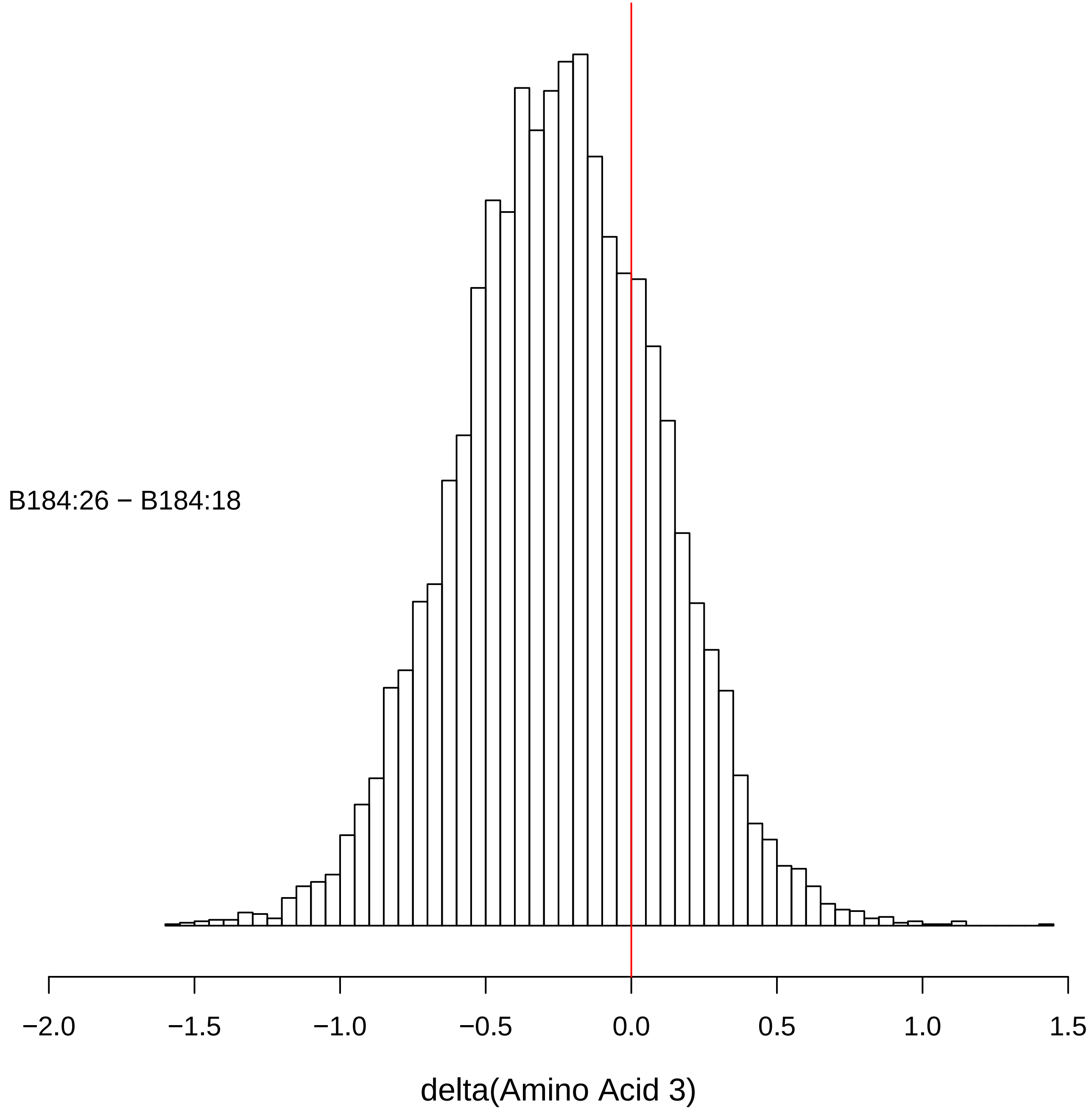
B184:26 − B184:18
−2.0
−1.5
−1.0
−0.5
0.0
0.5
1.0
1.5
delta(Amino Acid 3)

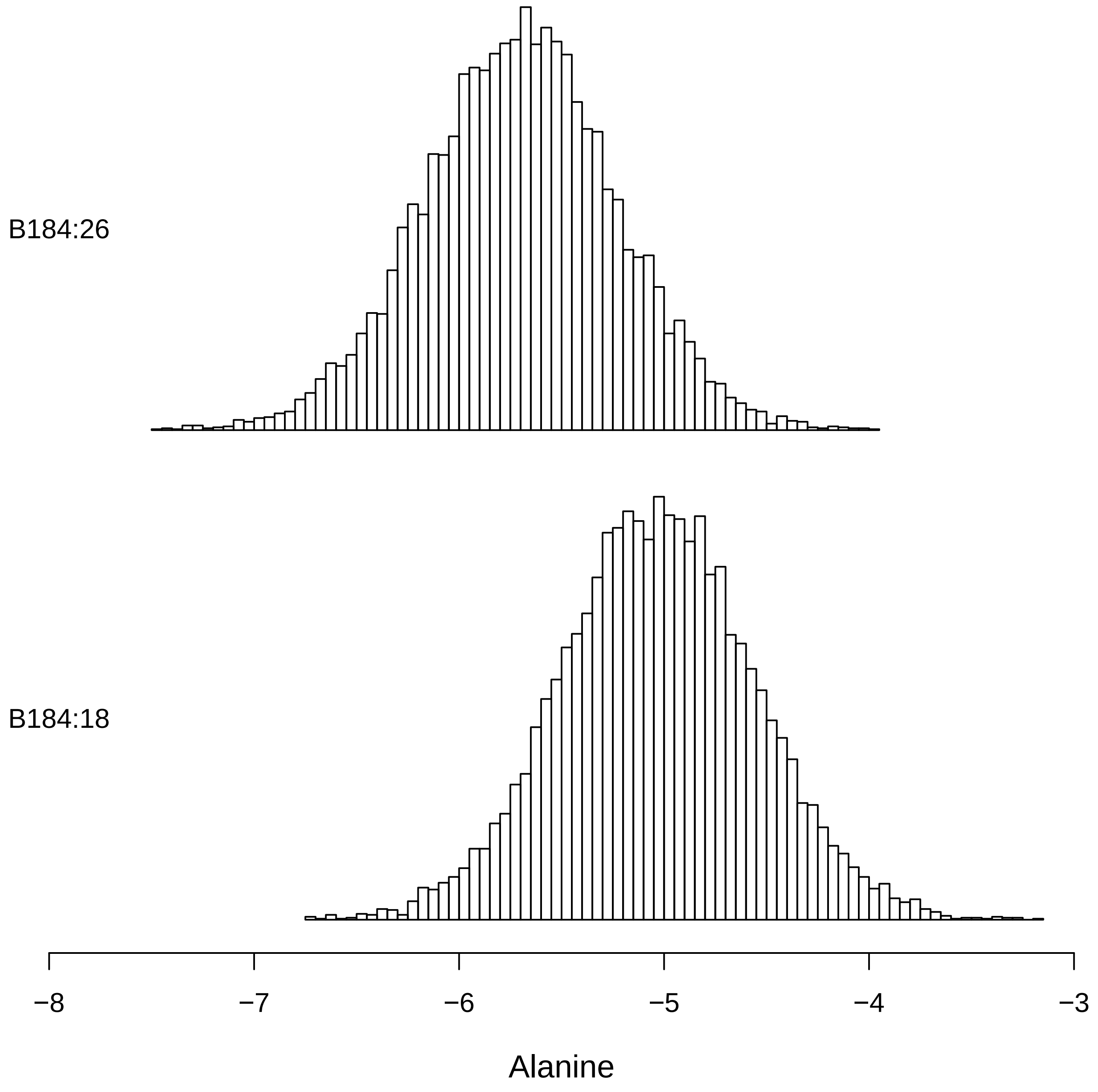
B184:26
B184:18
−8
−7
−6
−5
−4
−3
Alanine

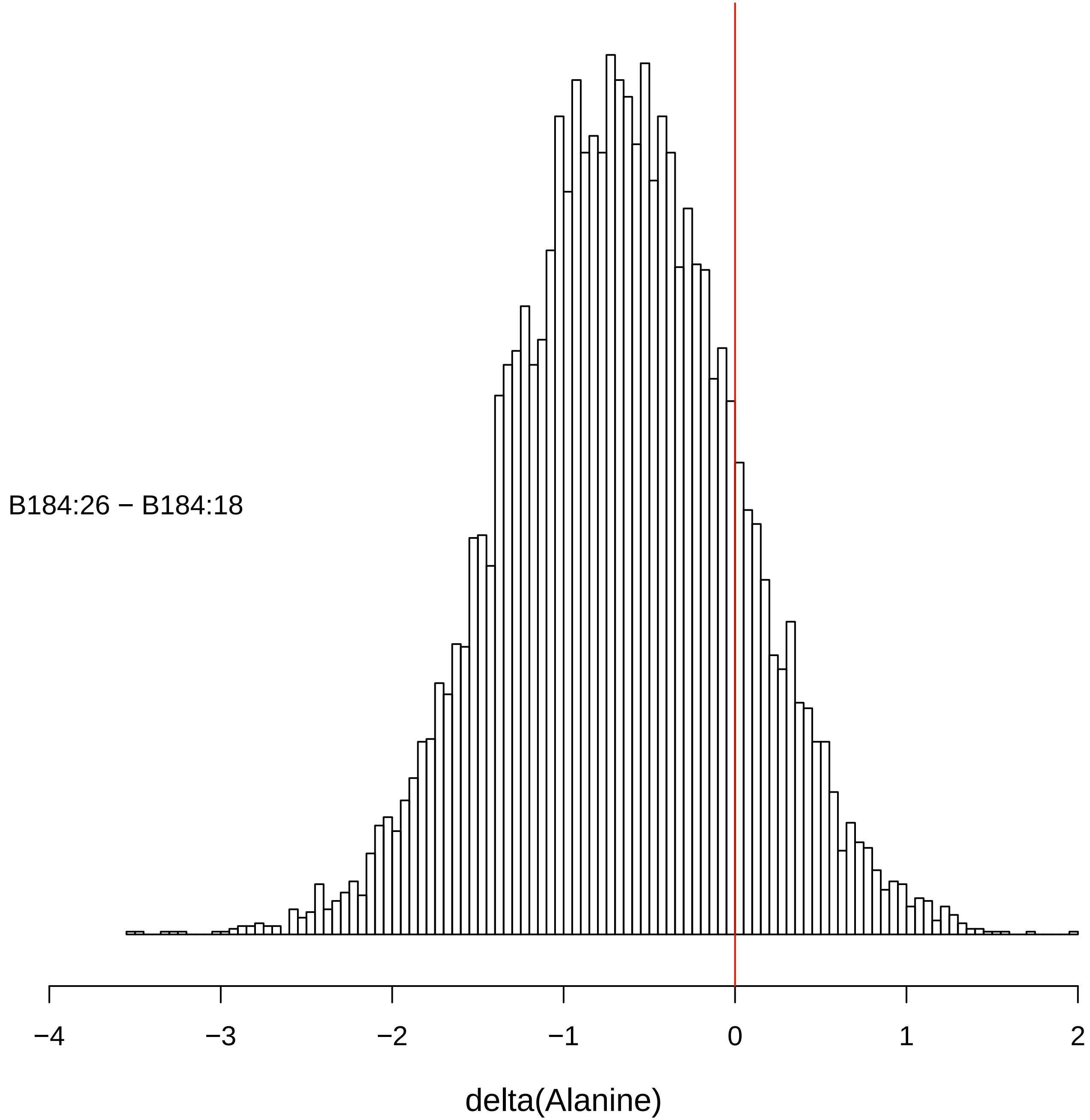
B184:26 − B184:18
−4
−3
−2
−1
0
1
2
delta(Alanine)

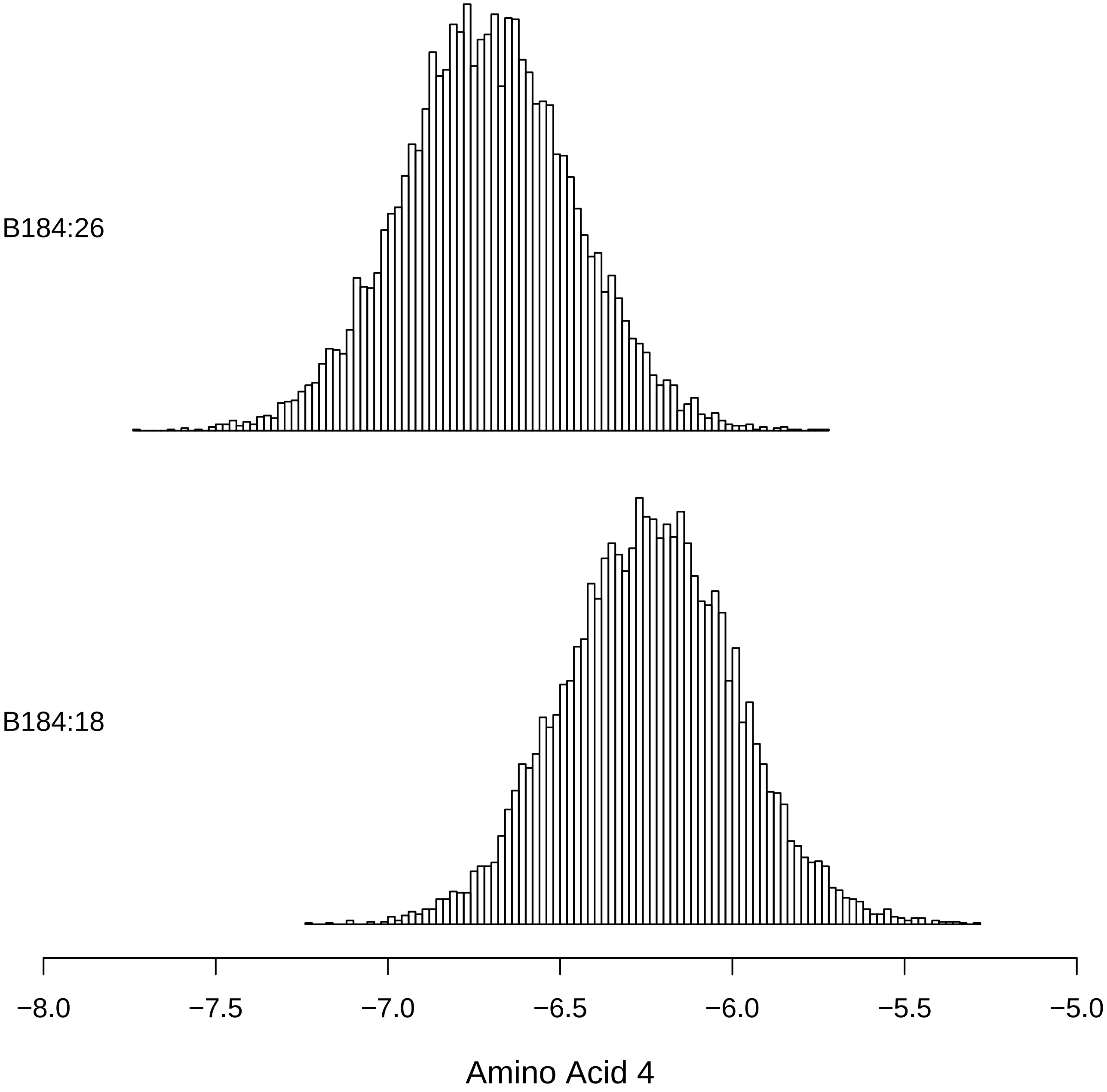
B184:26
B184:18
−8.0
−7.5
−7.0
−6.5
−6.0
−5.5
−5.0
Amino Acid 4

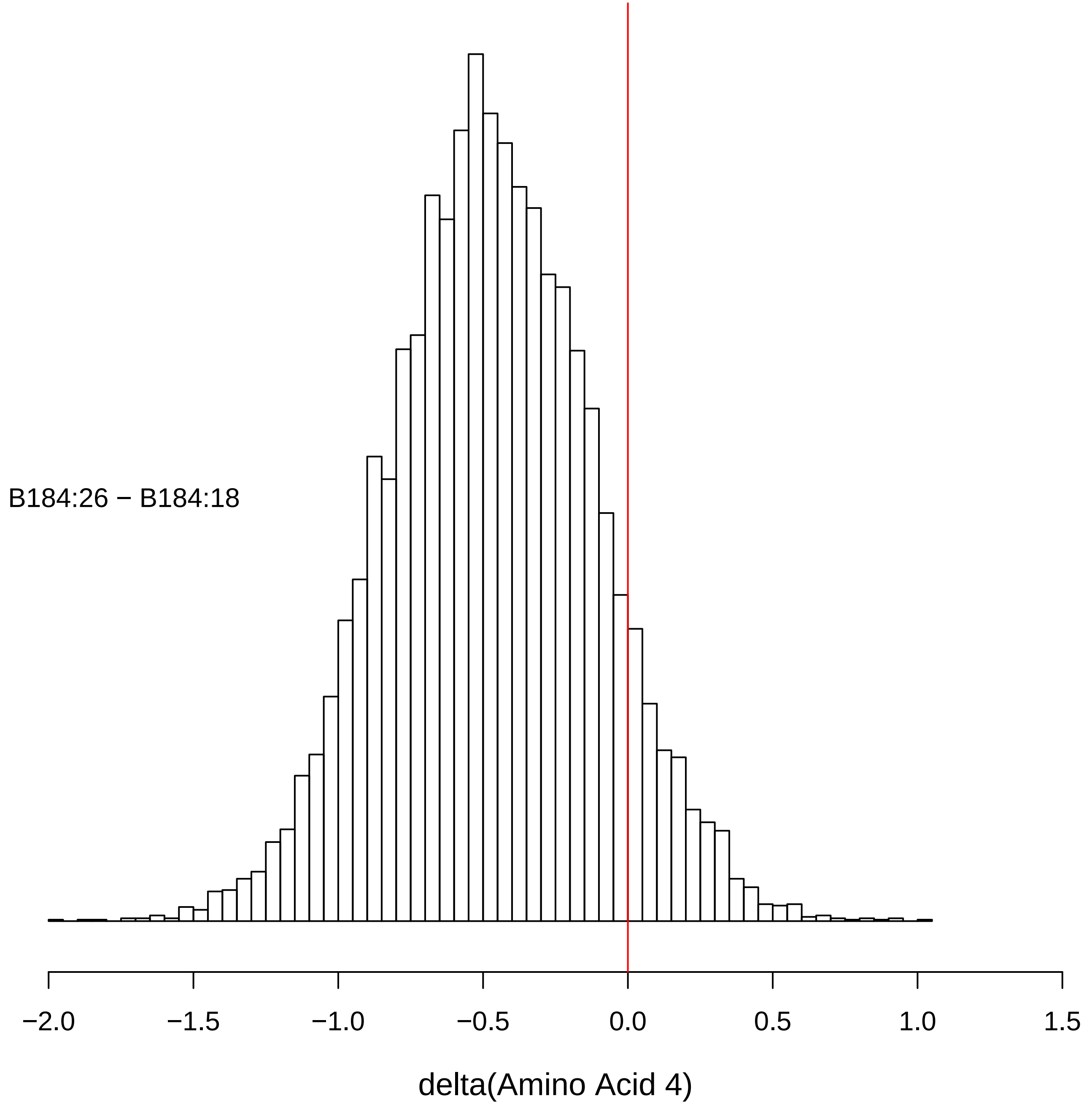

B184:26 − B184:18
−2.0
−1.5
−1.0
−0.5
0.0
0.5
1.0
1.5
delta(Amino Acid 4)

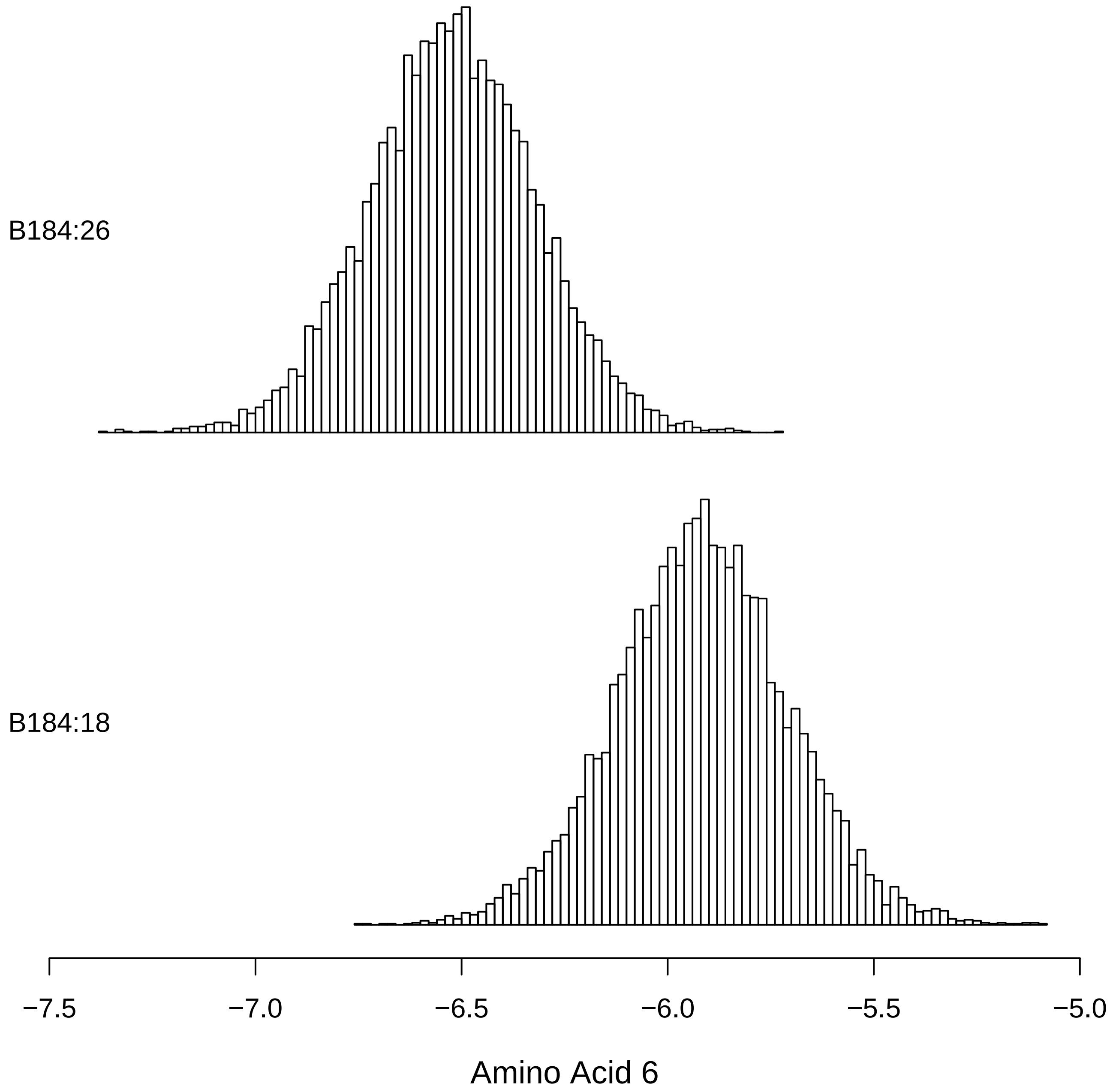
B184:26
B184:18
−7.5
−7.0
−6.5
−6.0
−5.5
−5.0
Amino Acid 6

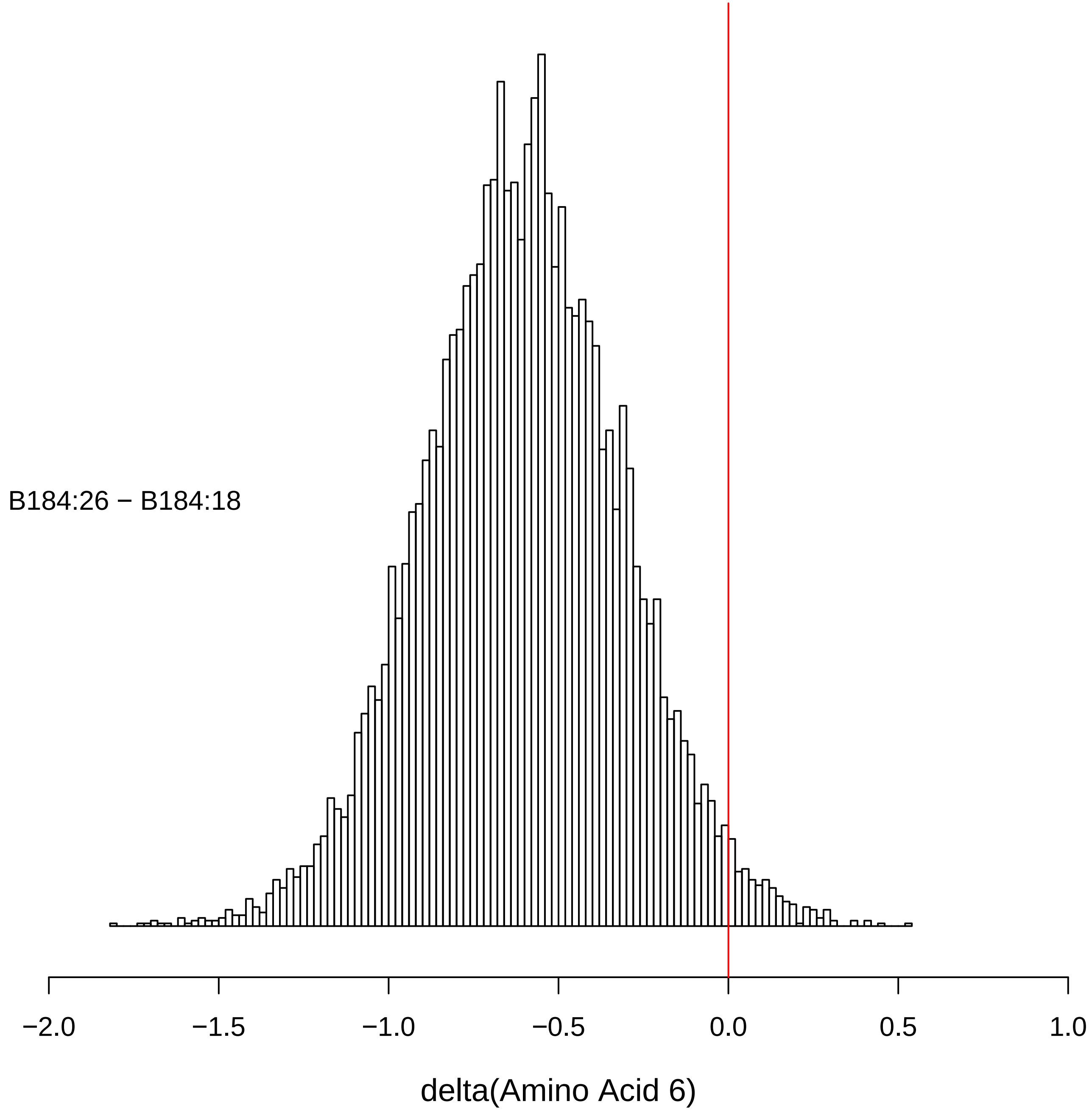
B184:26 − B184:18
−2.0
−1.5
−1.0
−0.5
0.0
0.5
1.0
delta(Amino Acid 6)

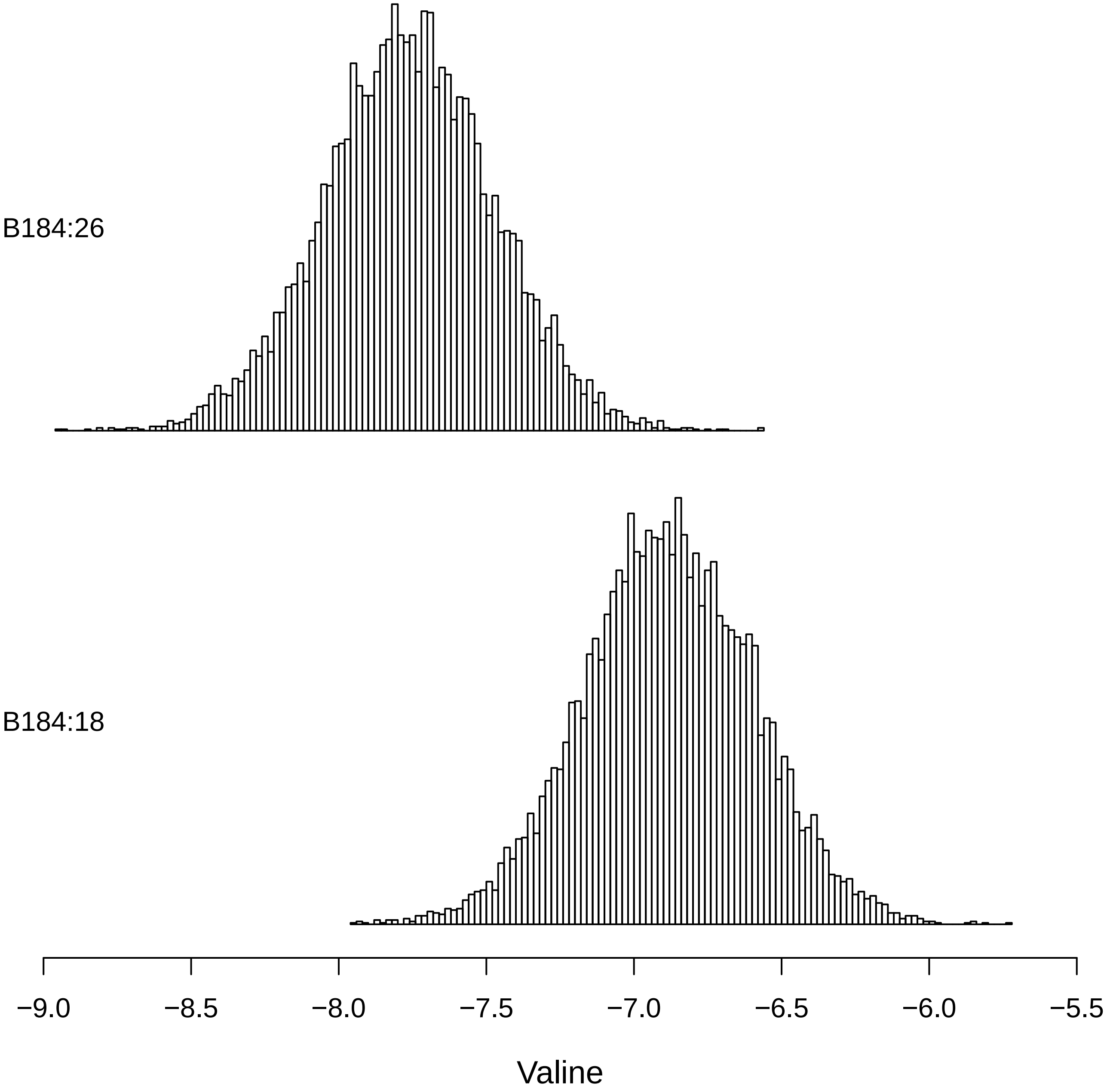

B184:26
B184:18
−9.0
−8.5
−8.0
−7.5
−7.0
−6.5
−6.0
−5.5
Valine

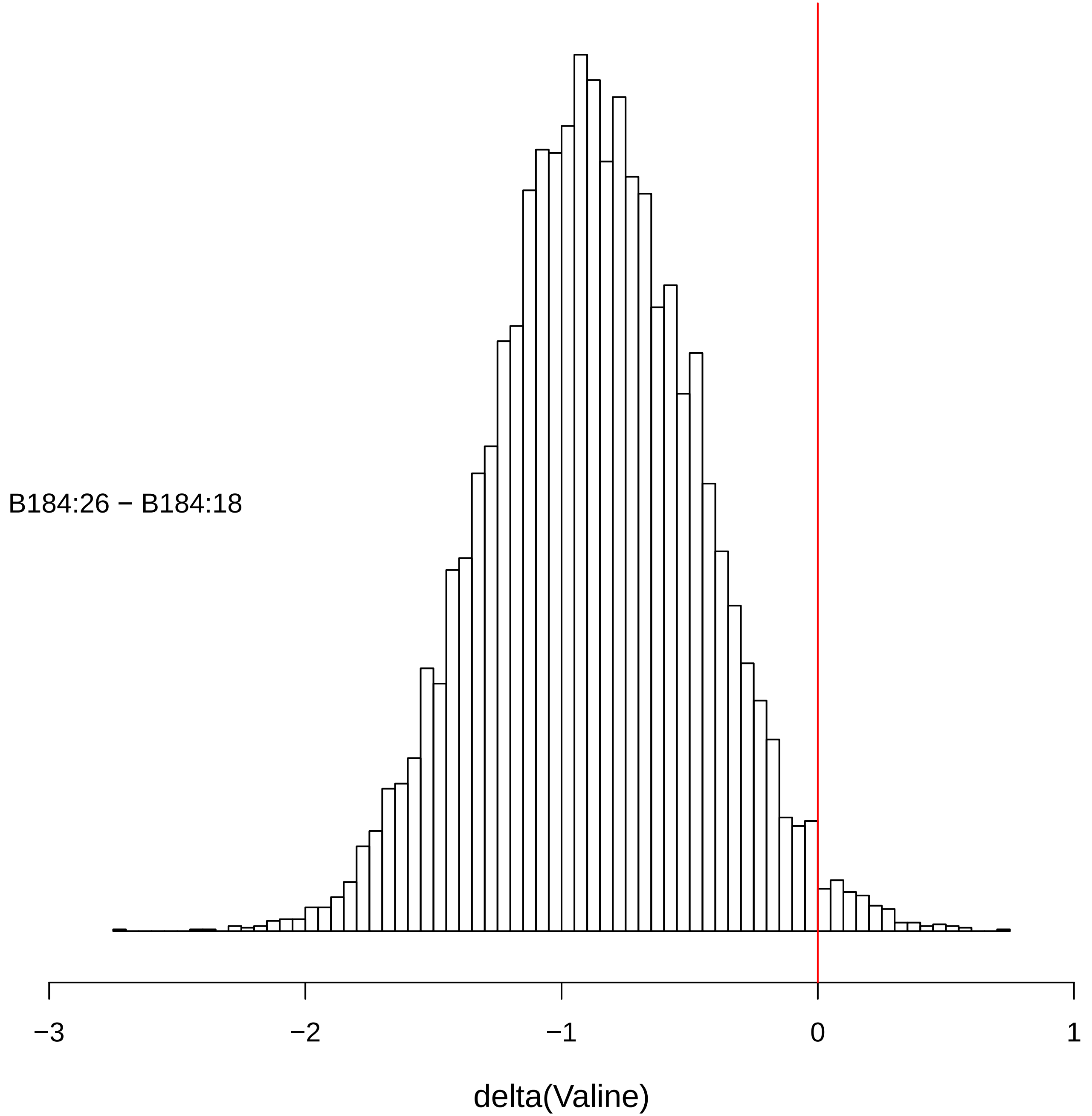

B184:26 − B184:18
−3
−2
−1
0
1
delta(Valine)

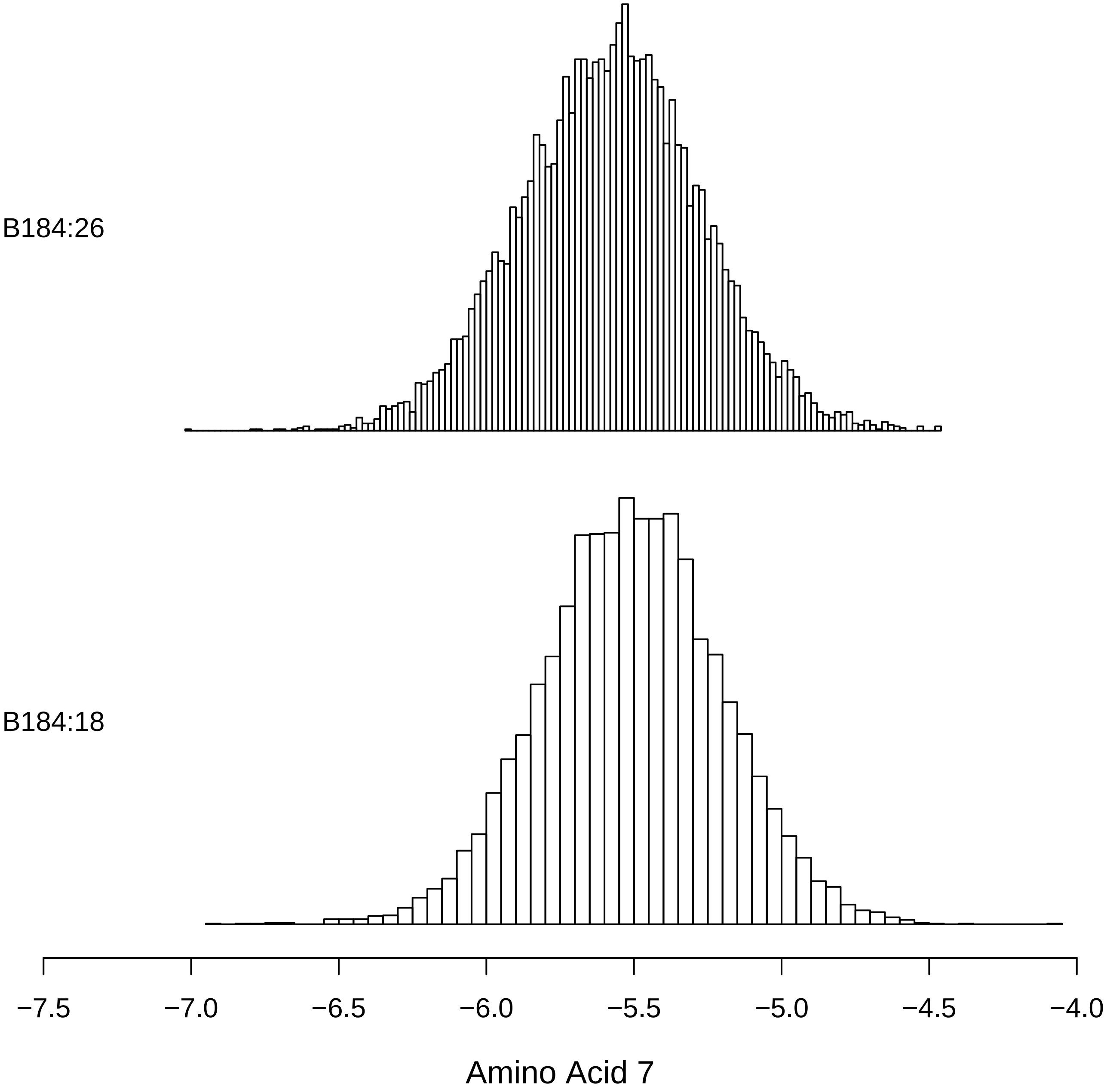
B184:26
B184:18
−7.5
−7.0
−6.5
−6.0
−5.5
−5.0
−4.5
−4.0
Amino Acid 7

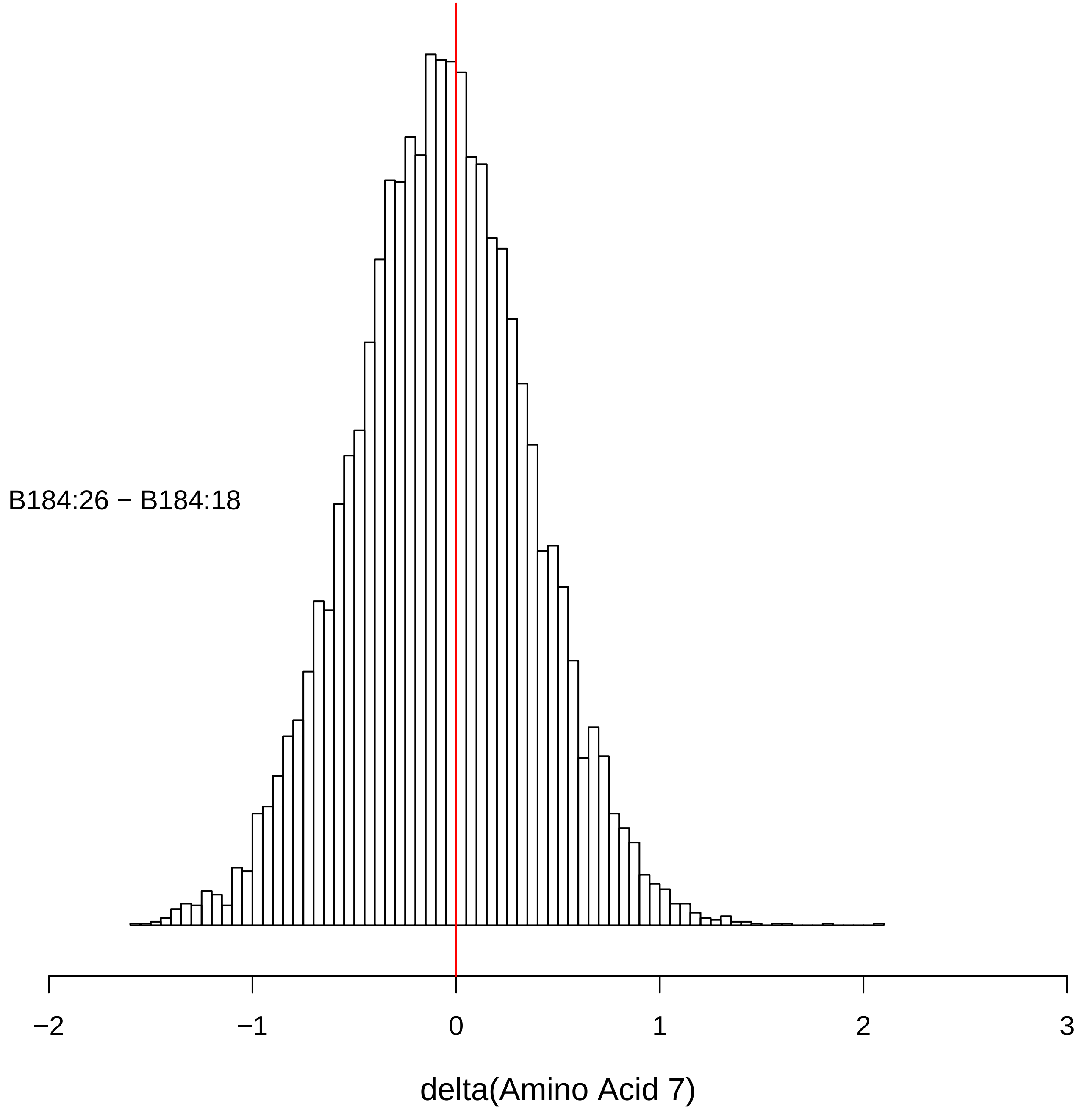
B184:26 − B184:18
−2
−1
0
1
2
3
delta(Amino Acid 7)

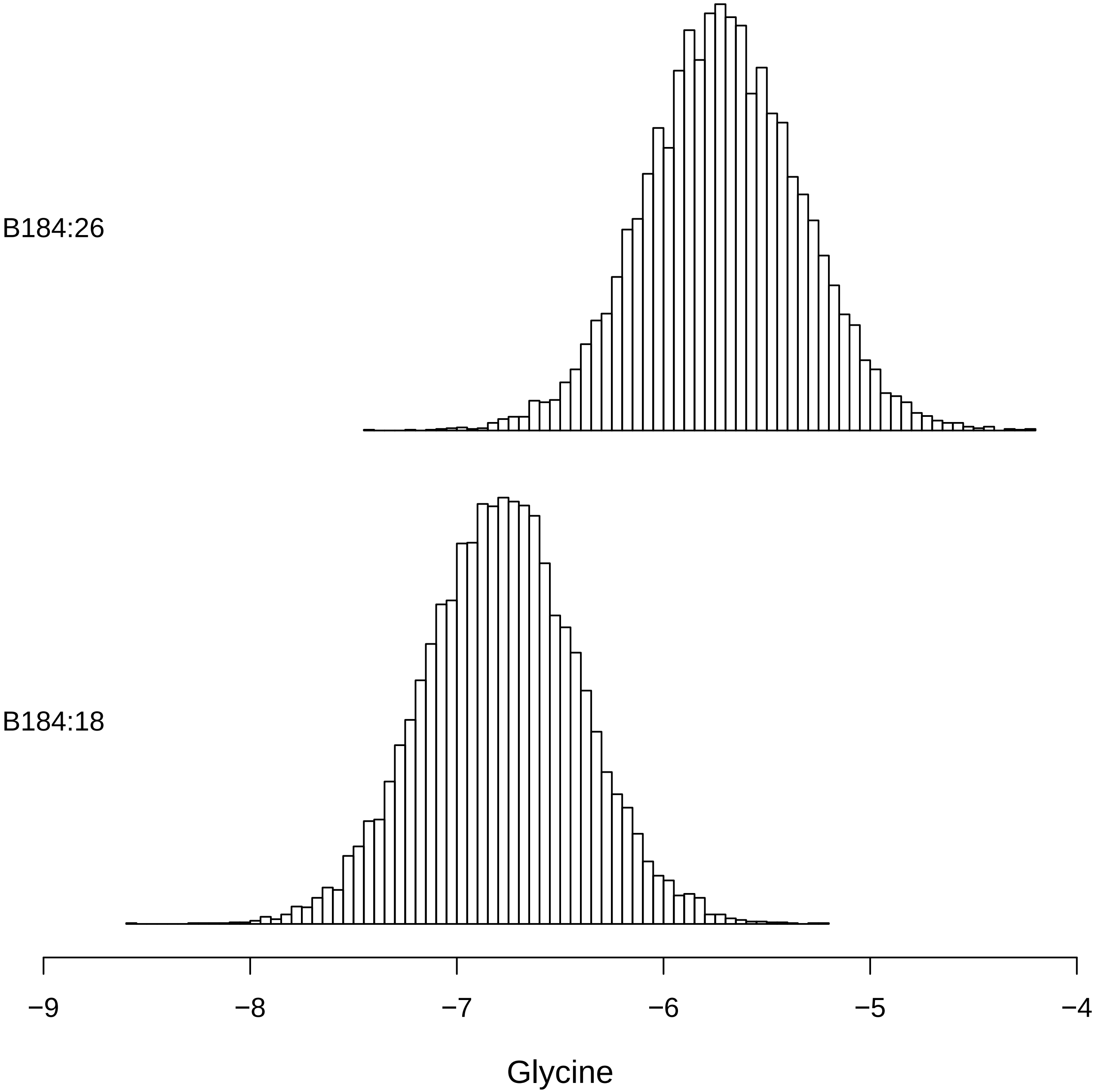
B184:26
B184:18
−9
−8
−7
−6
−5
−4
Glycine

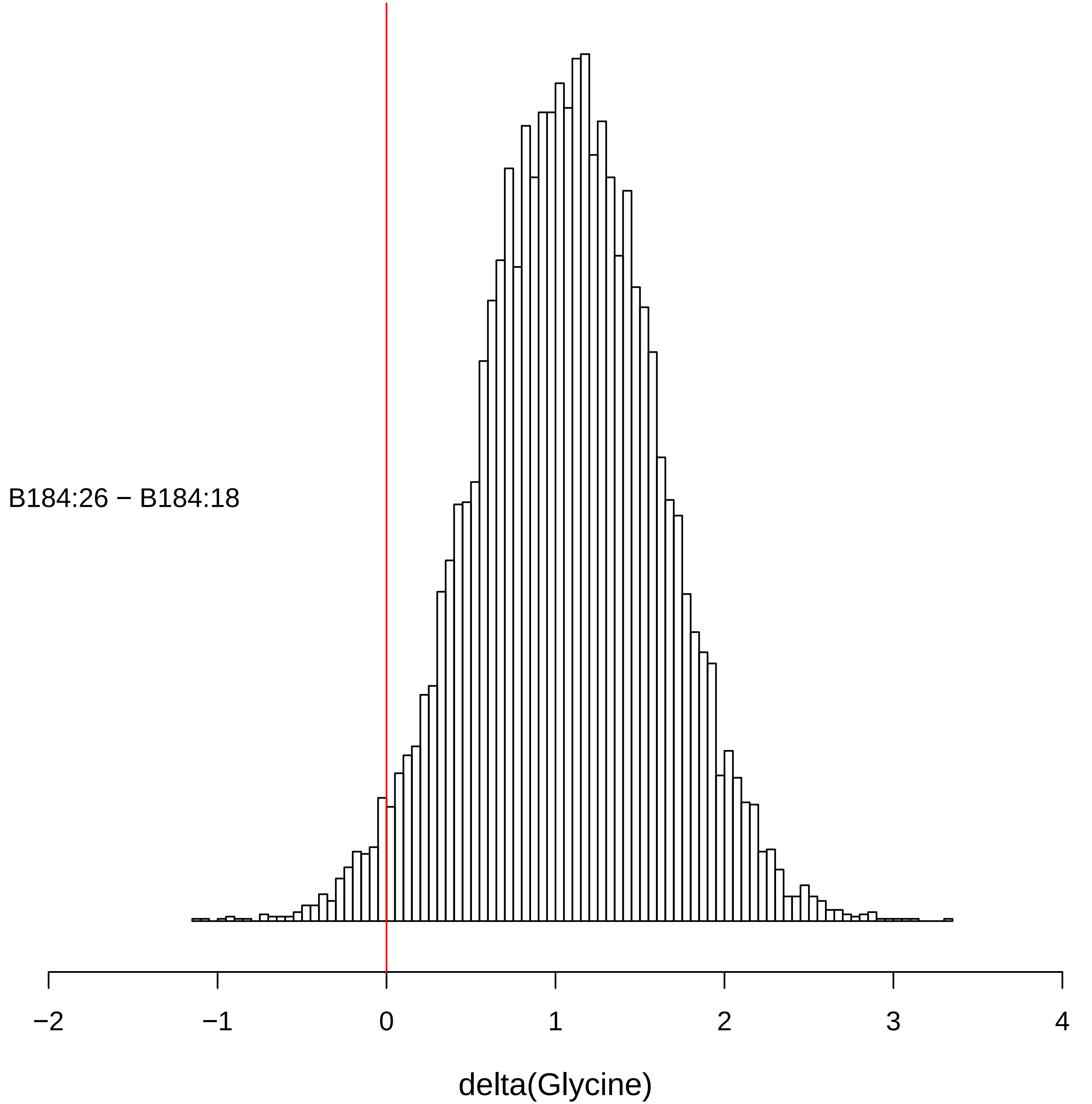
B184:26 − B184:18
−2
−1
0
1
2
3
4
delta(Glycine)

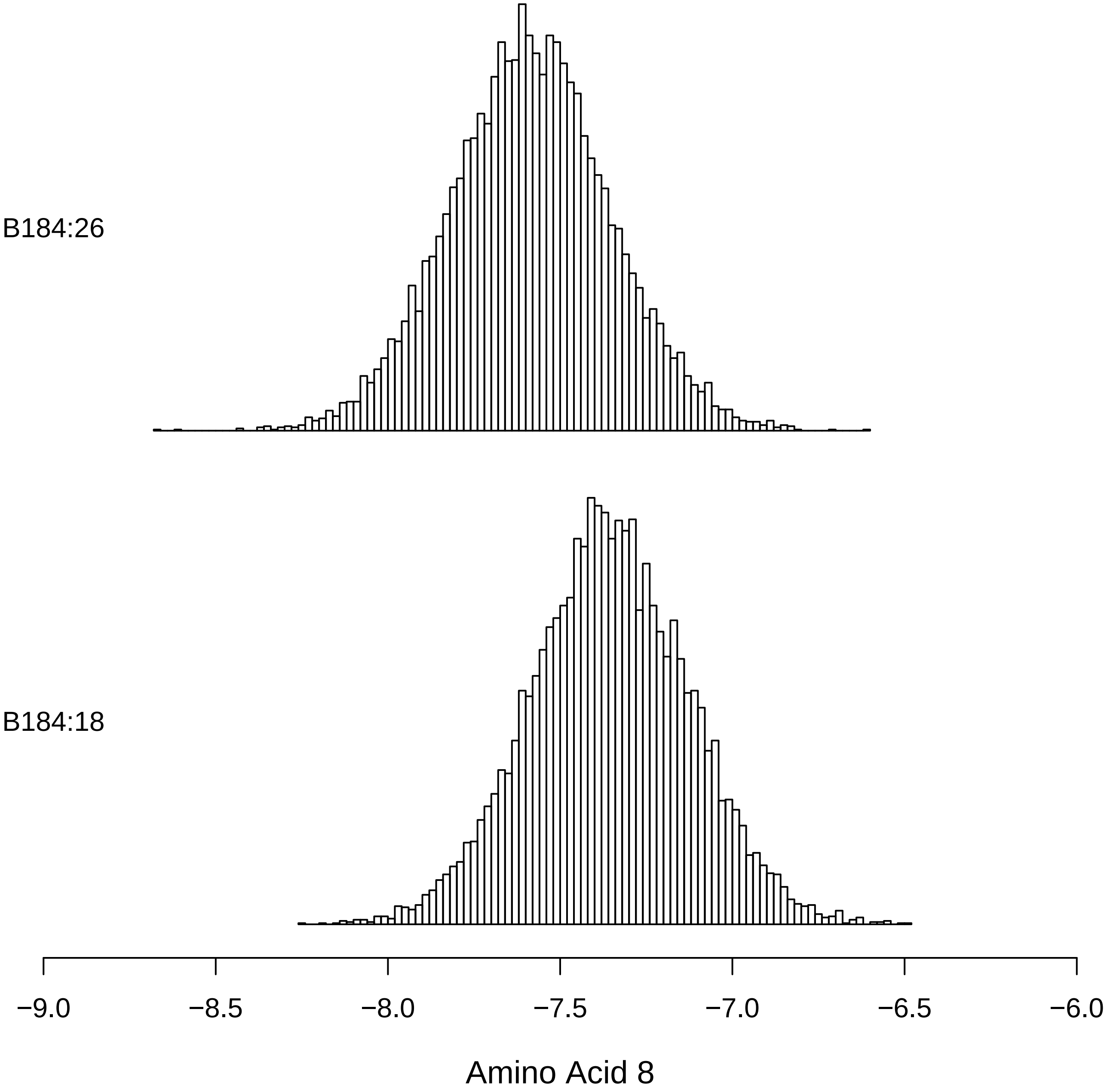
B184:26
B184:18
−9.0
−8.5
−8.0
−7.5
−7.0
−6.5
−6.0
Amino Acid 8

B184:26 − B184:18

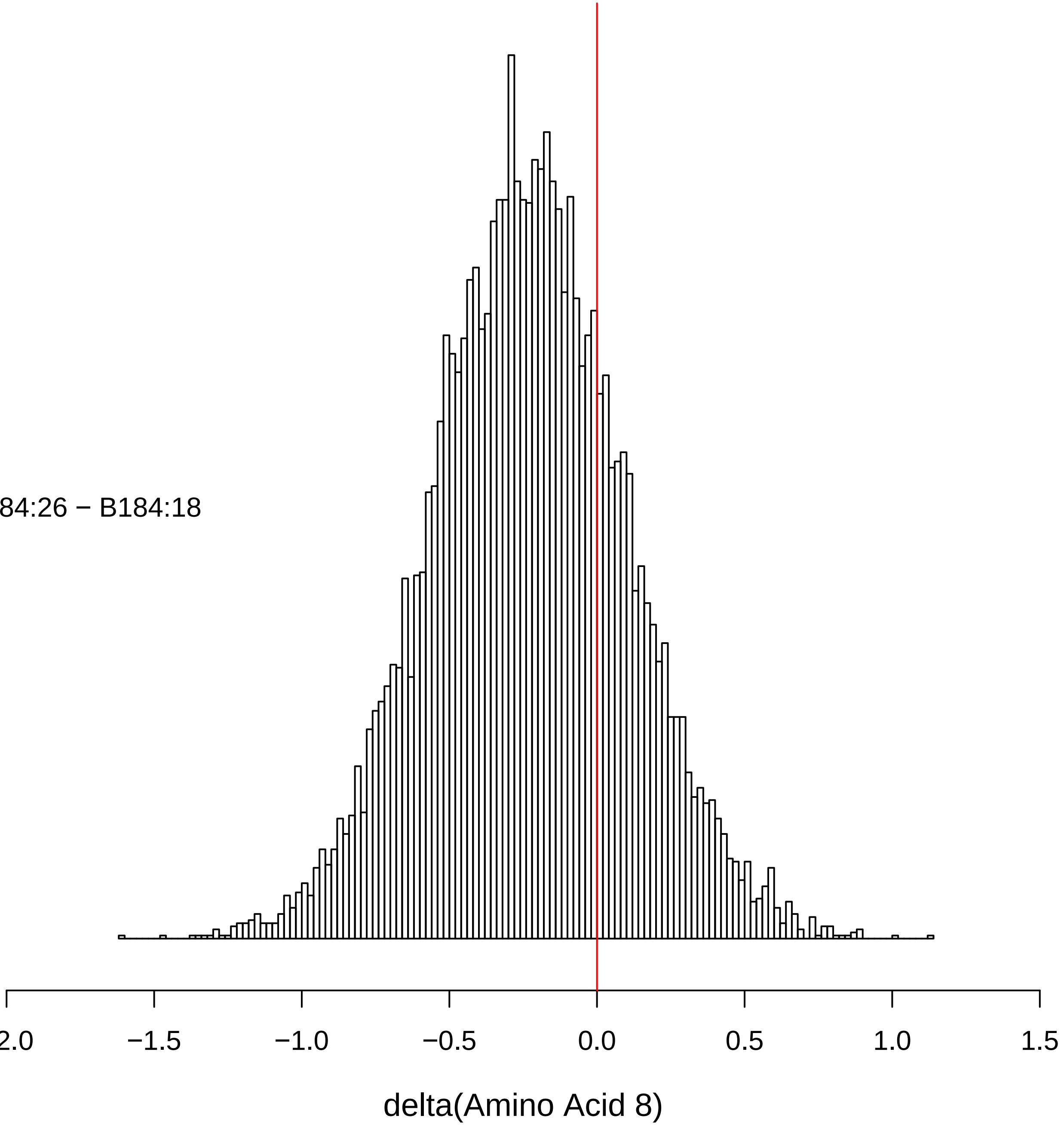

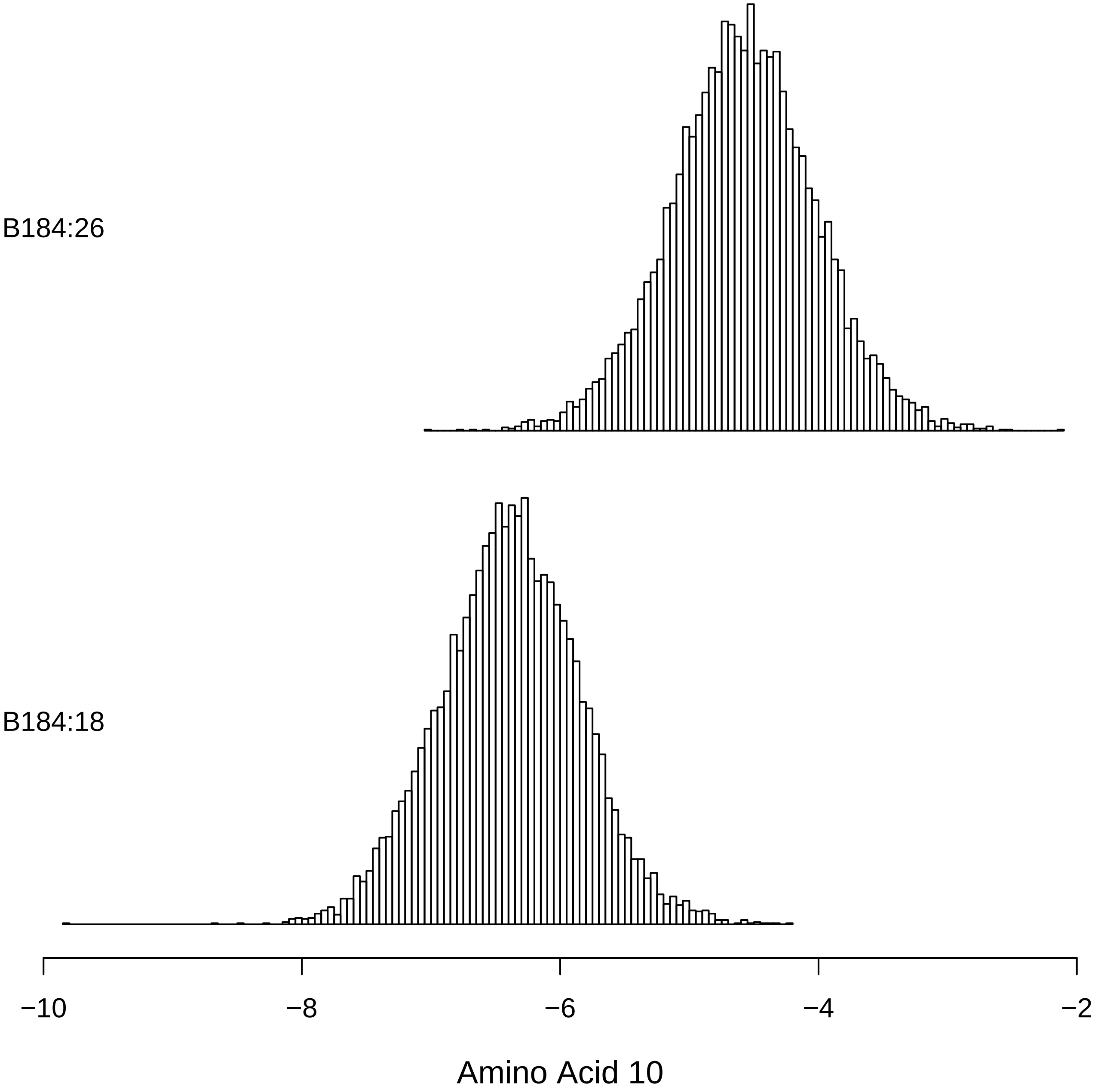

B184:26
B184:18
−10
−8
−6
−4
−2
Amino Acid 10

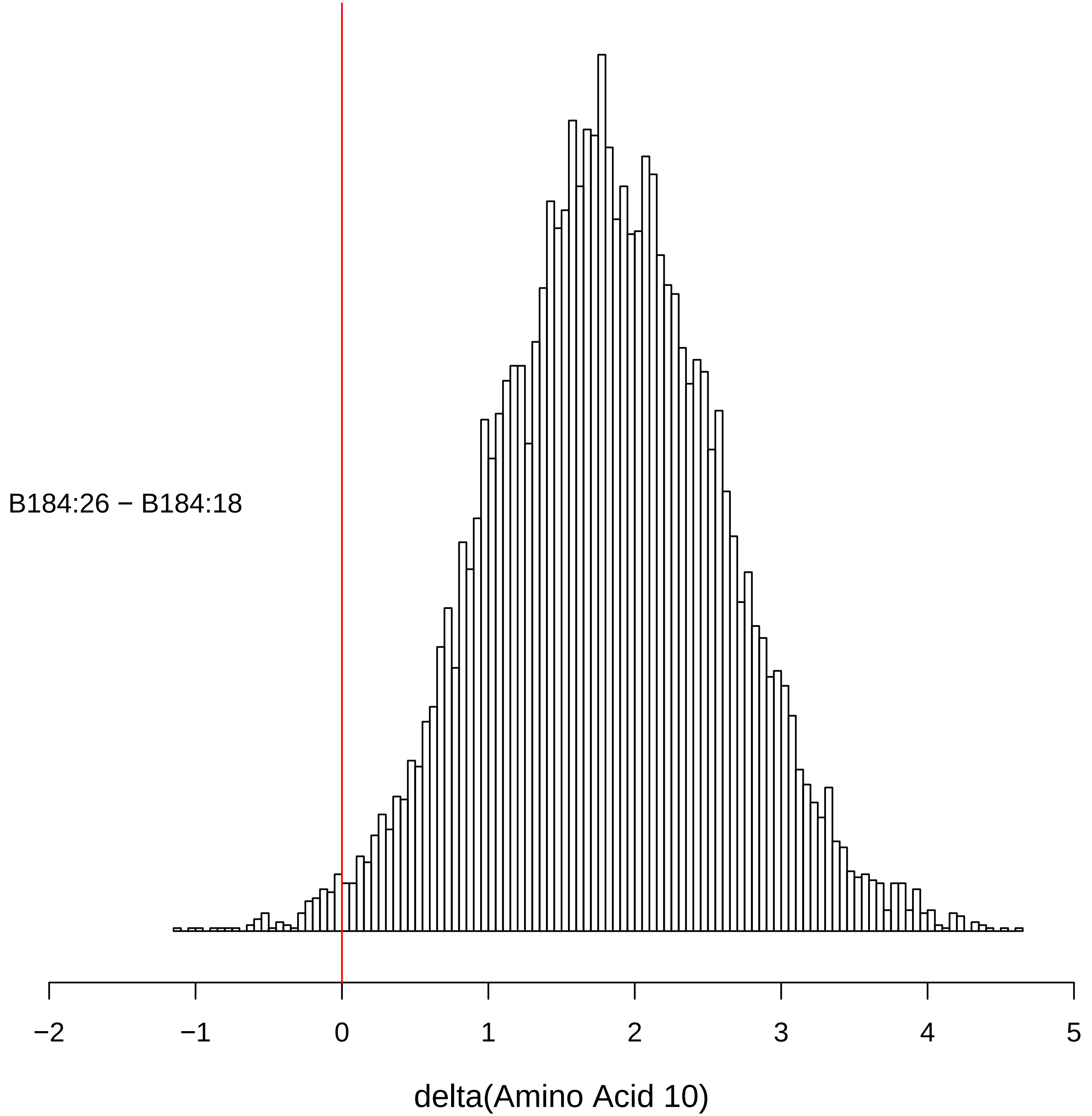
B184:26 − B184:18
−2
−1
0
1
2
3
4
5
delta(Amino Acid 10)

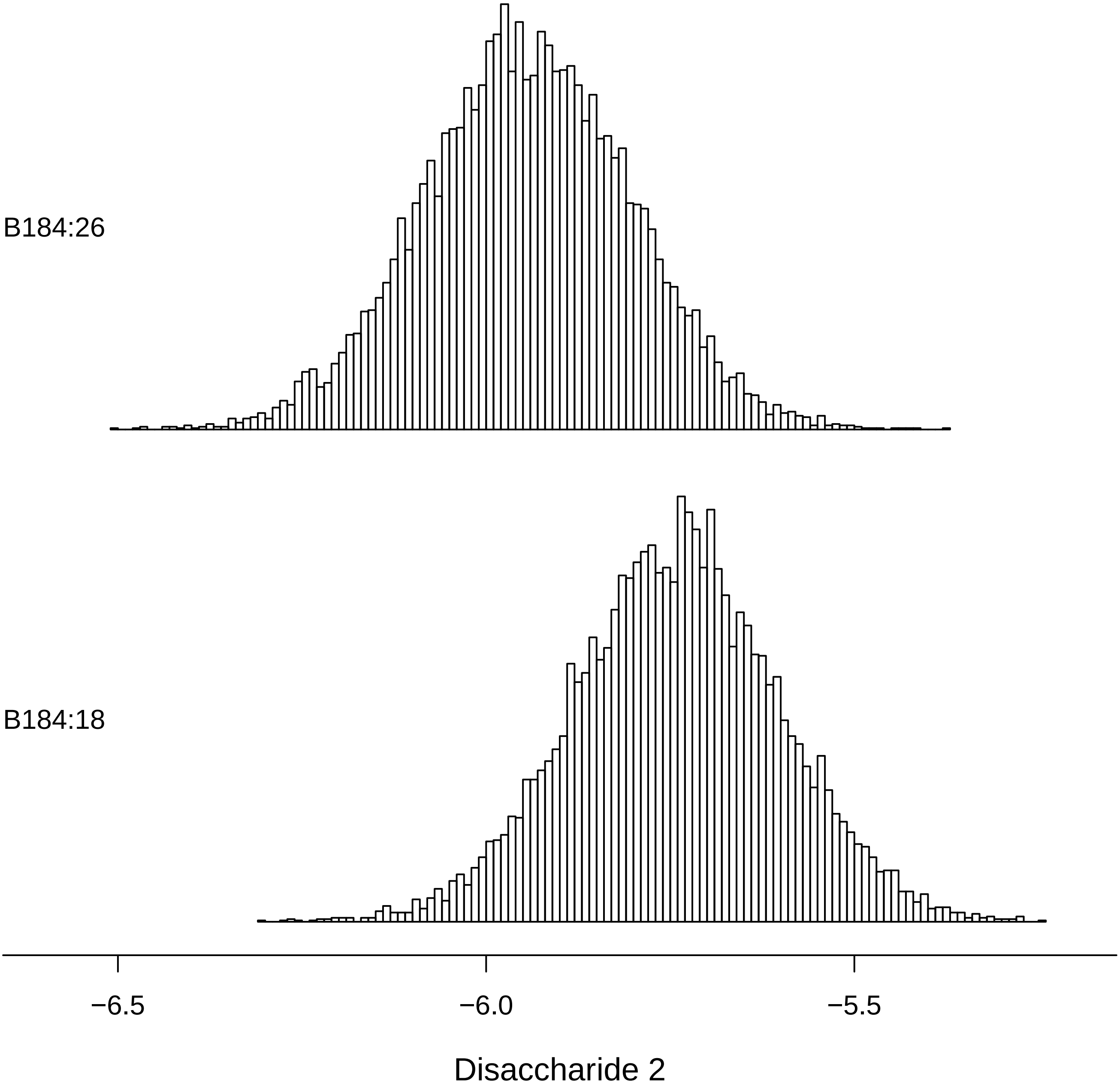
B184:26
B184:18
−6.5
−6.0
−5.5
Disaccharide 2

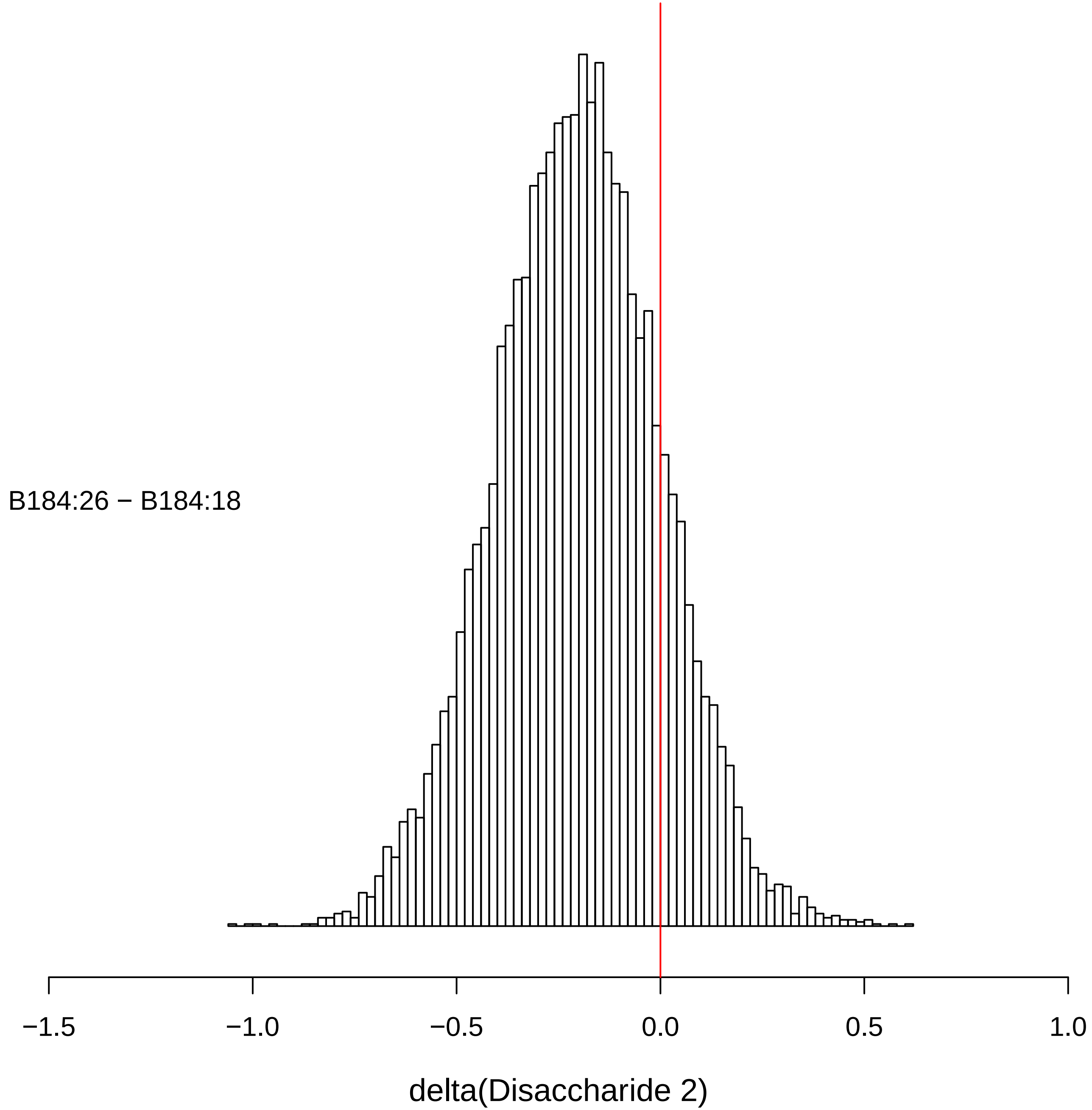
B184:26 − B184:18
−1.5
−1.0
−0.5
0.0
0.5
1.0
delta(Disaccharide 2)

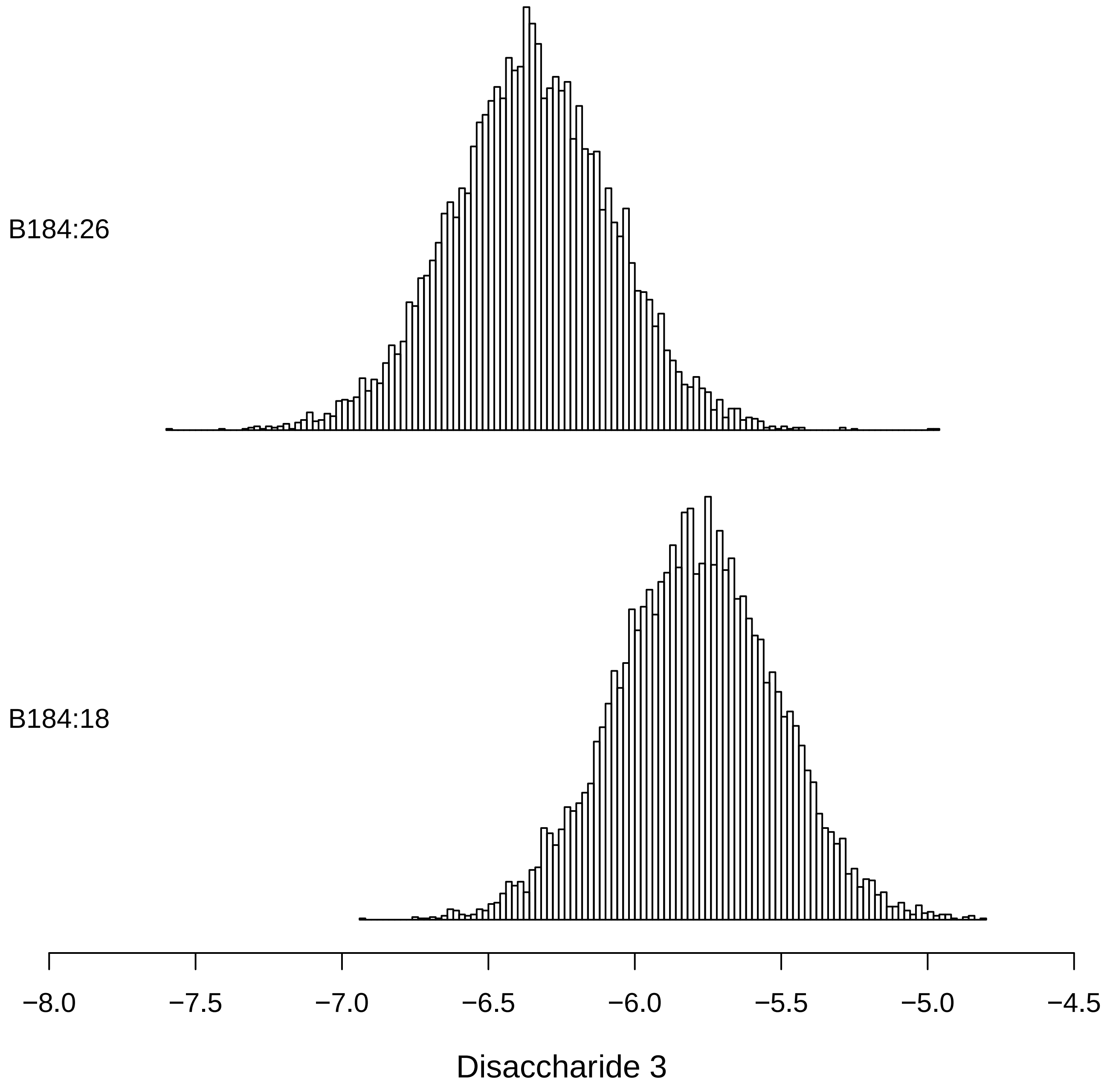
B184:26
B184:18
−8.0
−7.5
−7.0
−6.5
−6.0
−5.5
−5.0
−4.5
Disaccharide 3

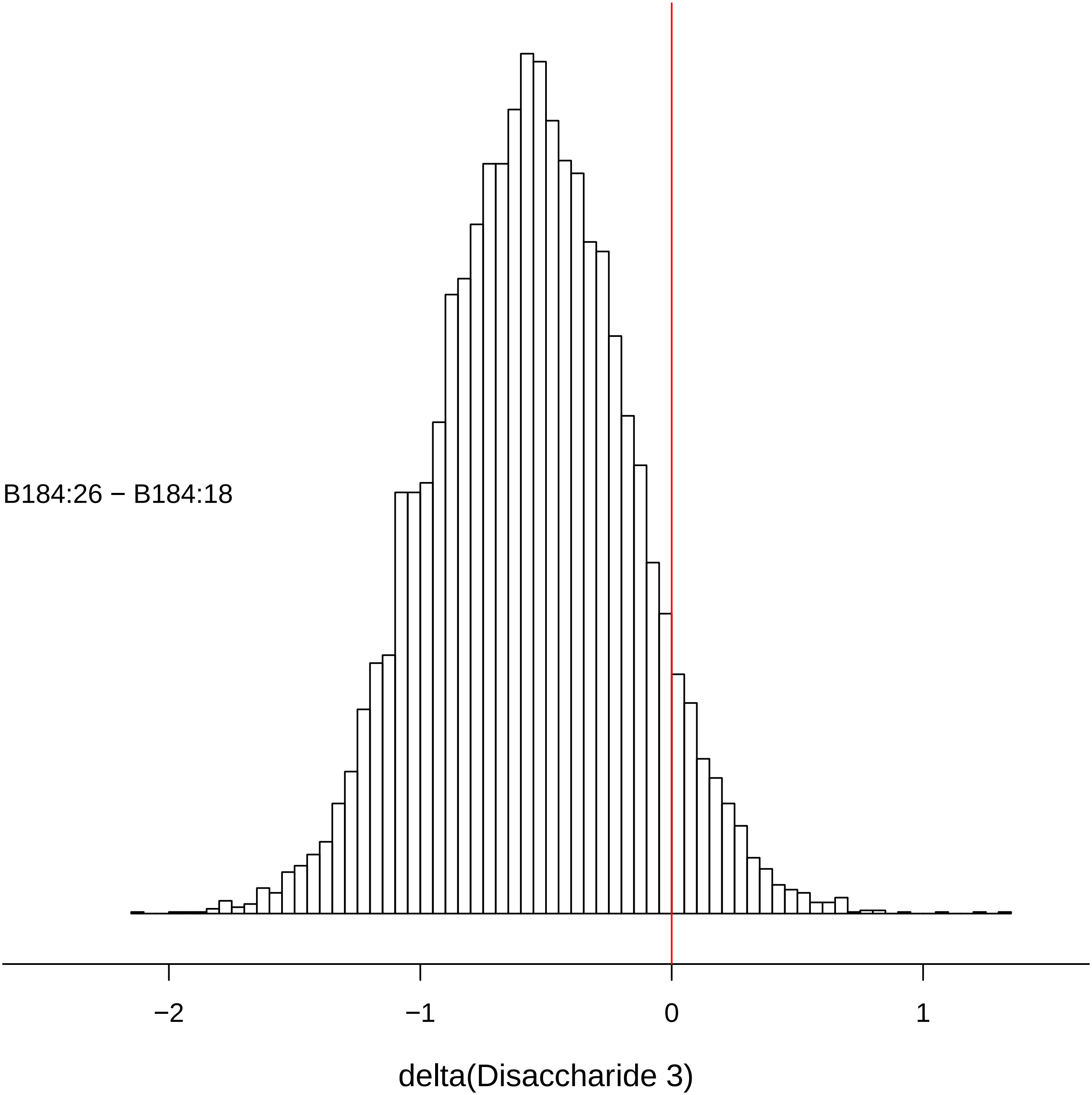
B184:26 − B184:18
−2
−1
0
1
delta(Disaccharide 3)

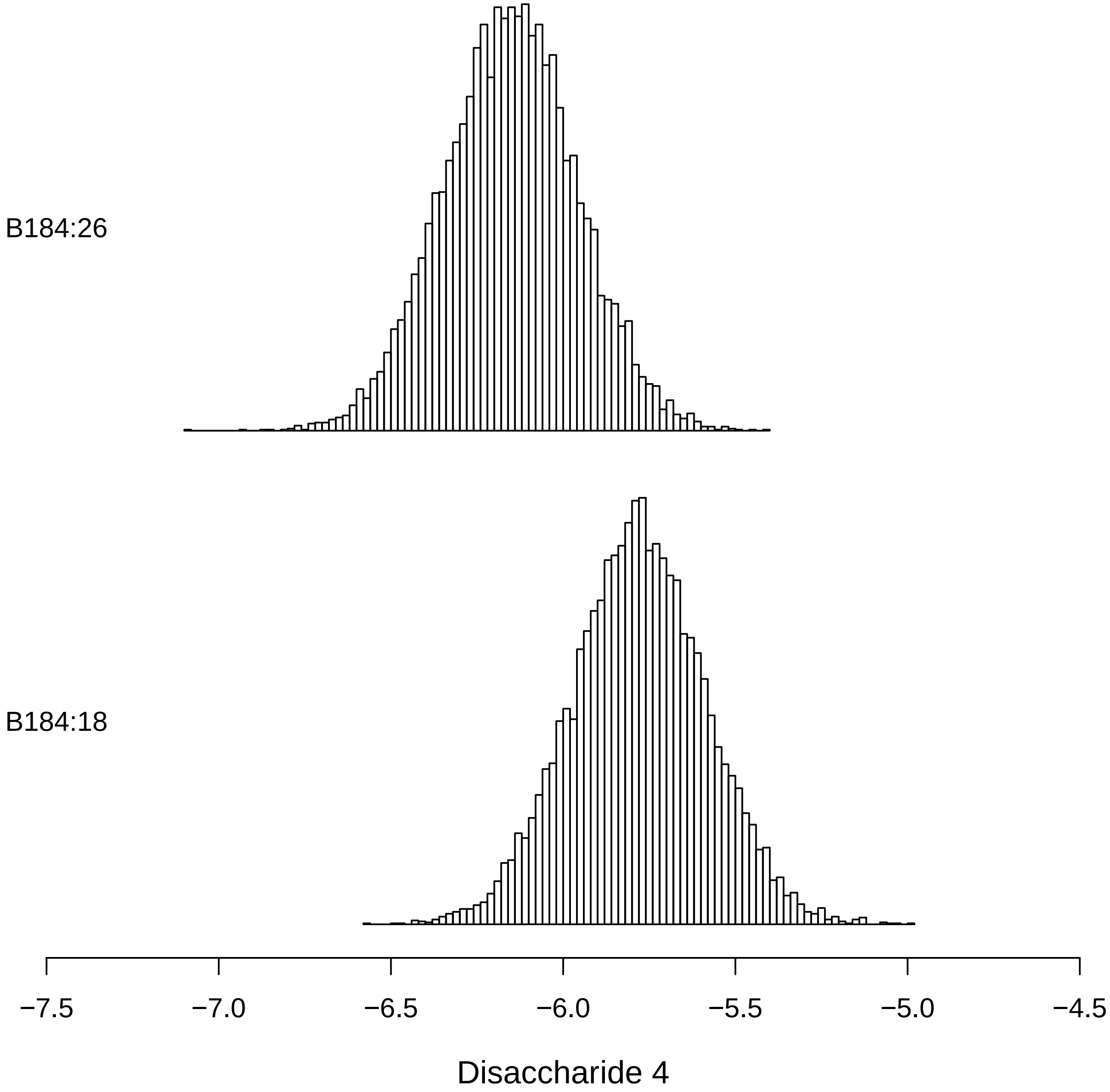
B184:26
B184:18
−7.5
−7.0
−6.5
−6.0
−5.5
−5.0
−4.5
Disaccharide 4

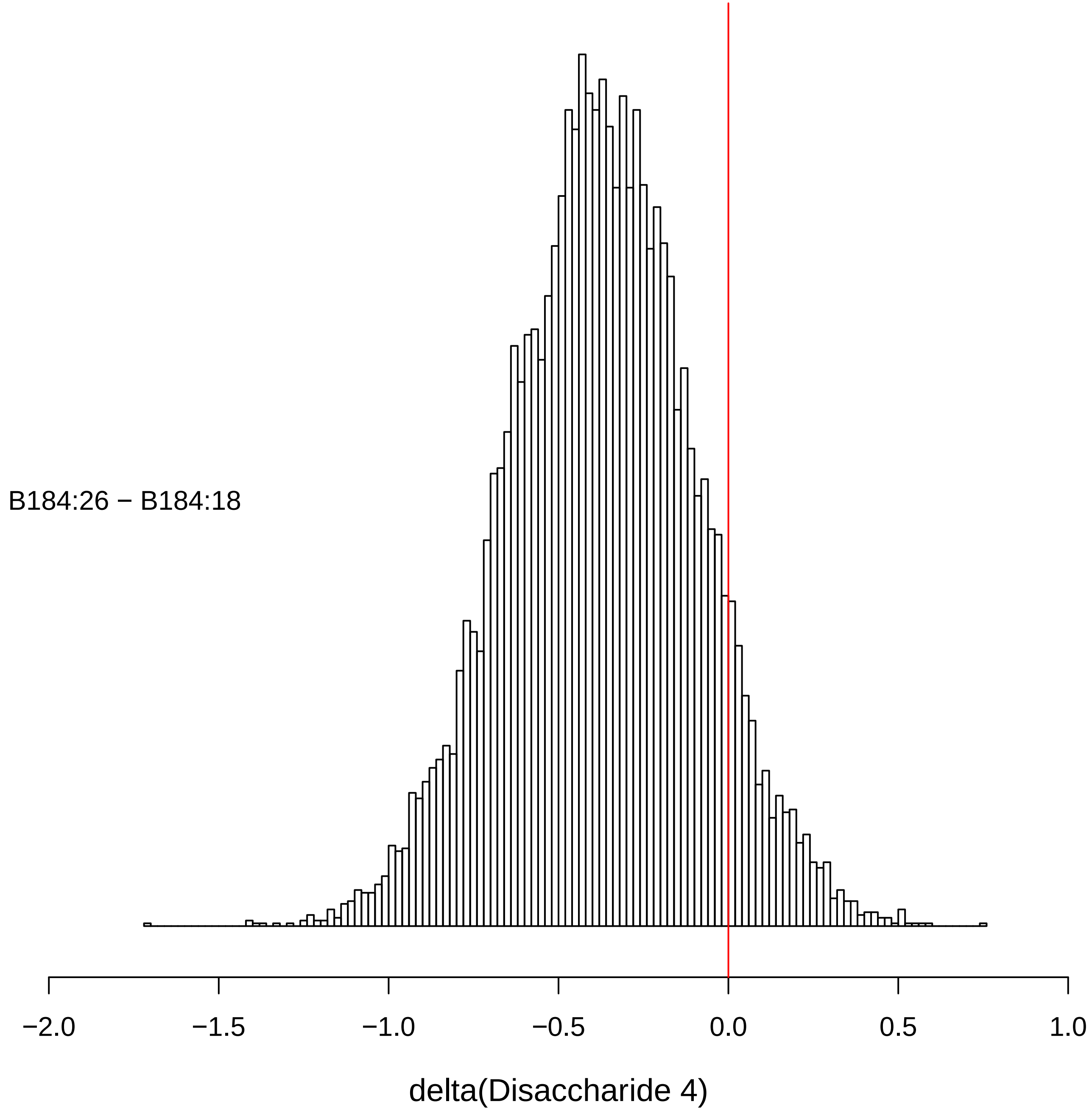
B184:26 − B184:18
−2.0
−1.5
−1.0
−0.5
0.0
0.5
1.0
delta(Disaccharide 4)

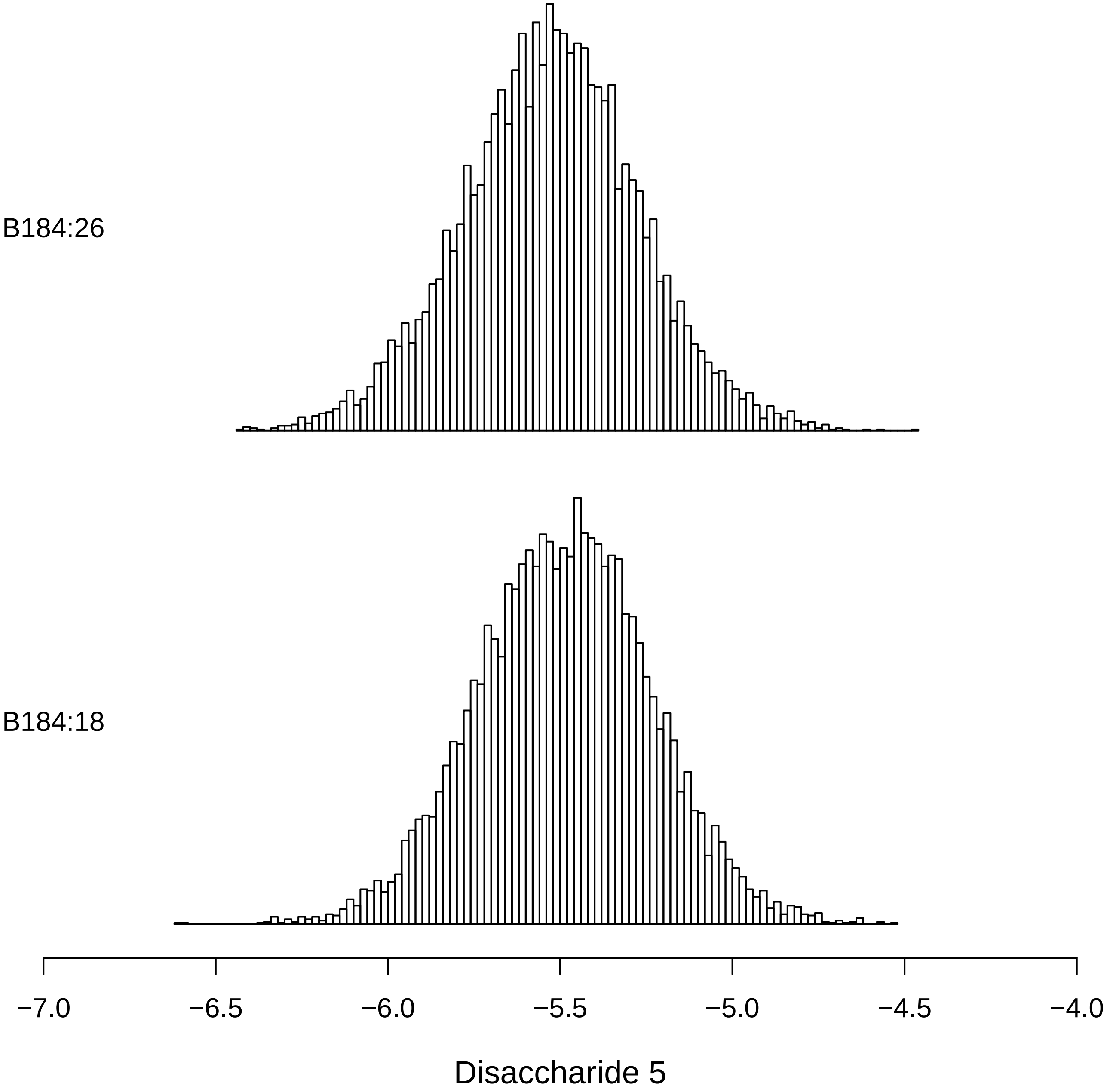
B184:26
B184:18
−7.0
−6.5
−6.0
−5.5
−5.0
−4.5
−4.0
Disaccharide 5

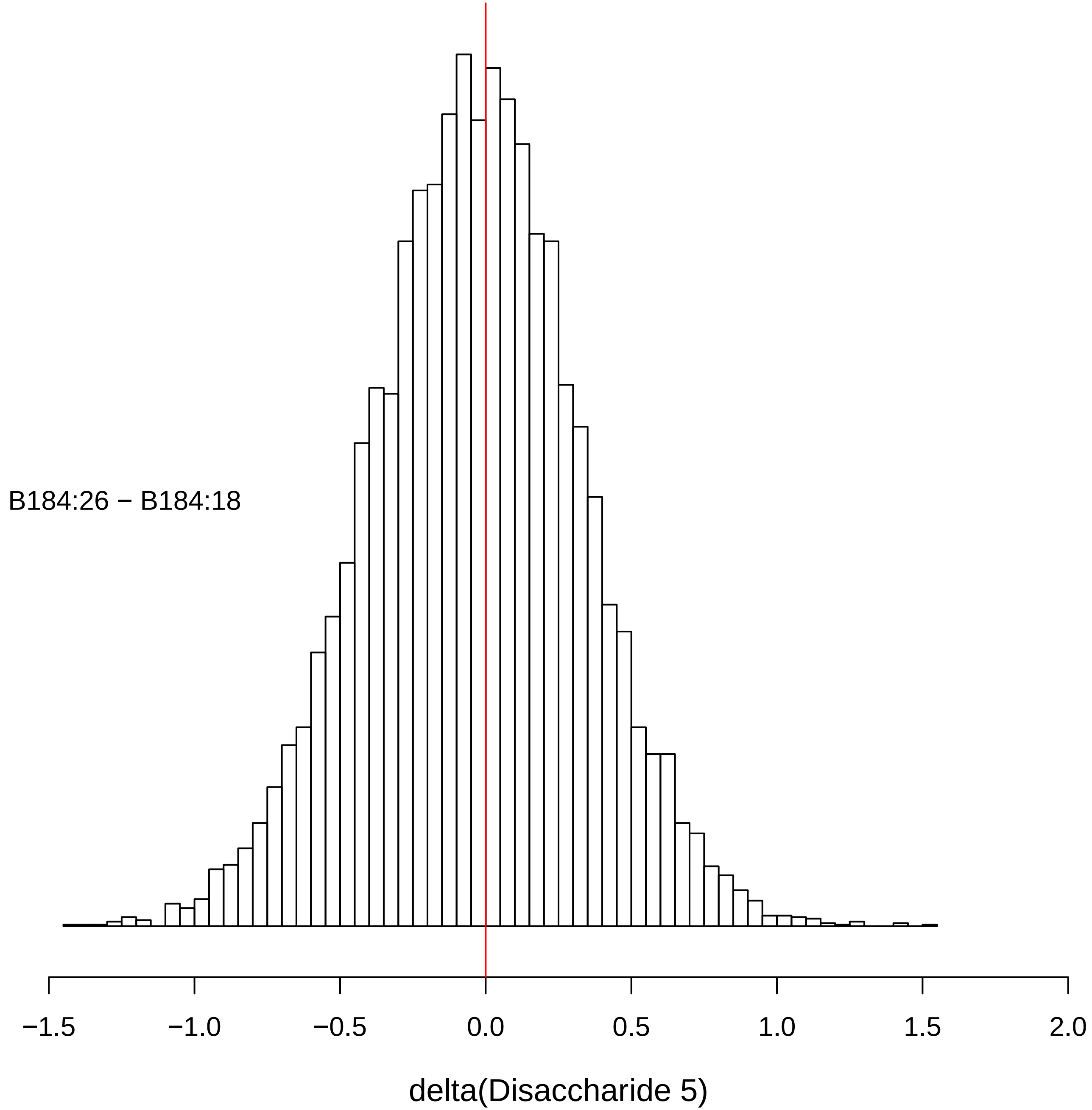

B184:26 − B184:18
−1.5
−1.0
−0.5
0.0
0.5
1.0
1.5
2.0
delta(Disaccharide 5)

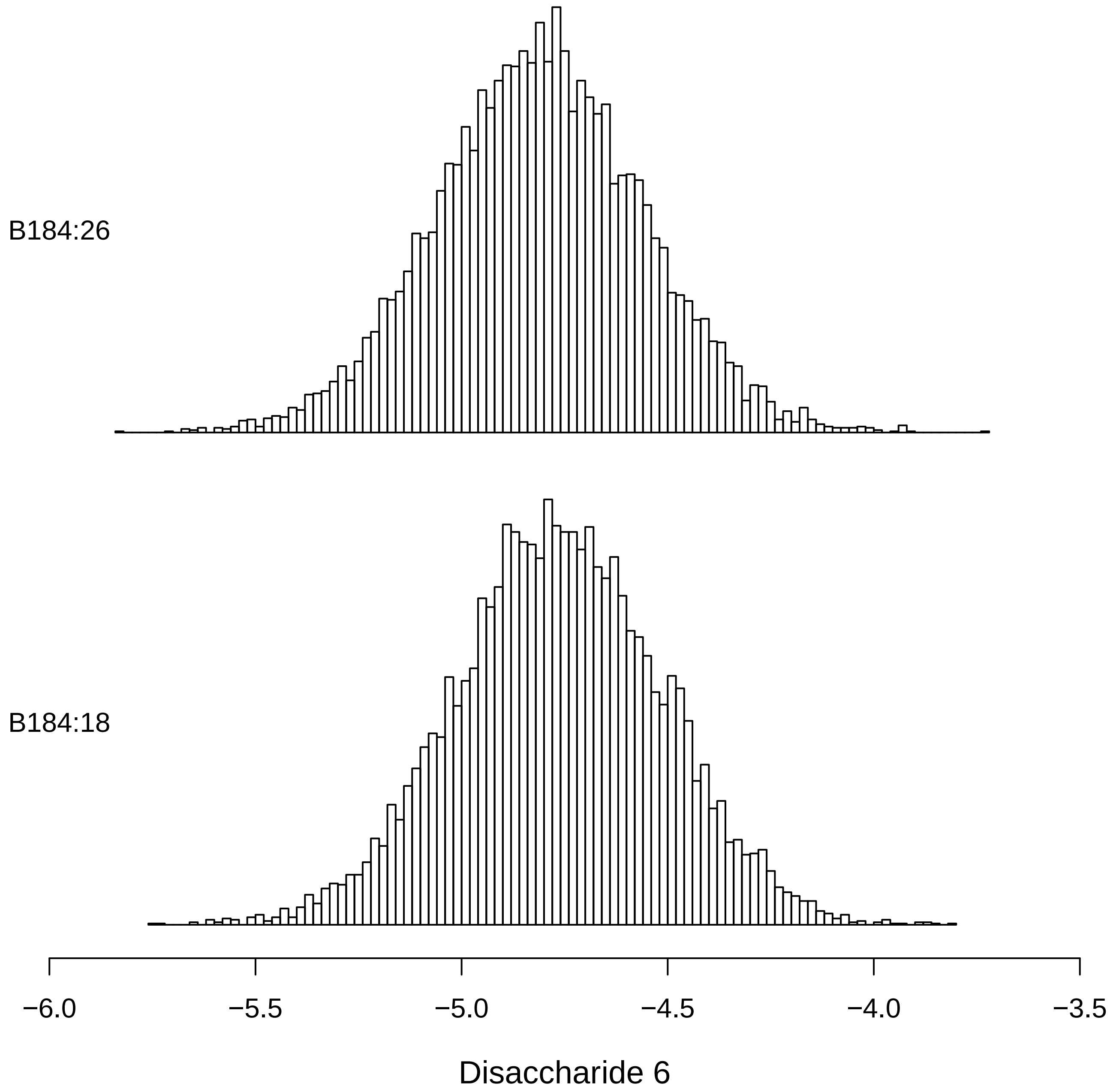

B184:26
B184:18
−6.0
−5.5
−5.0
−4.5
−4.0
−3.5
Disaccharide 6

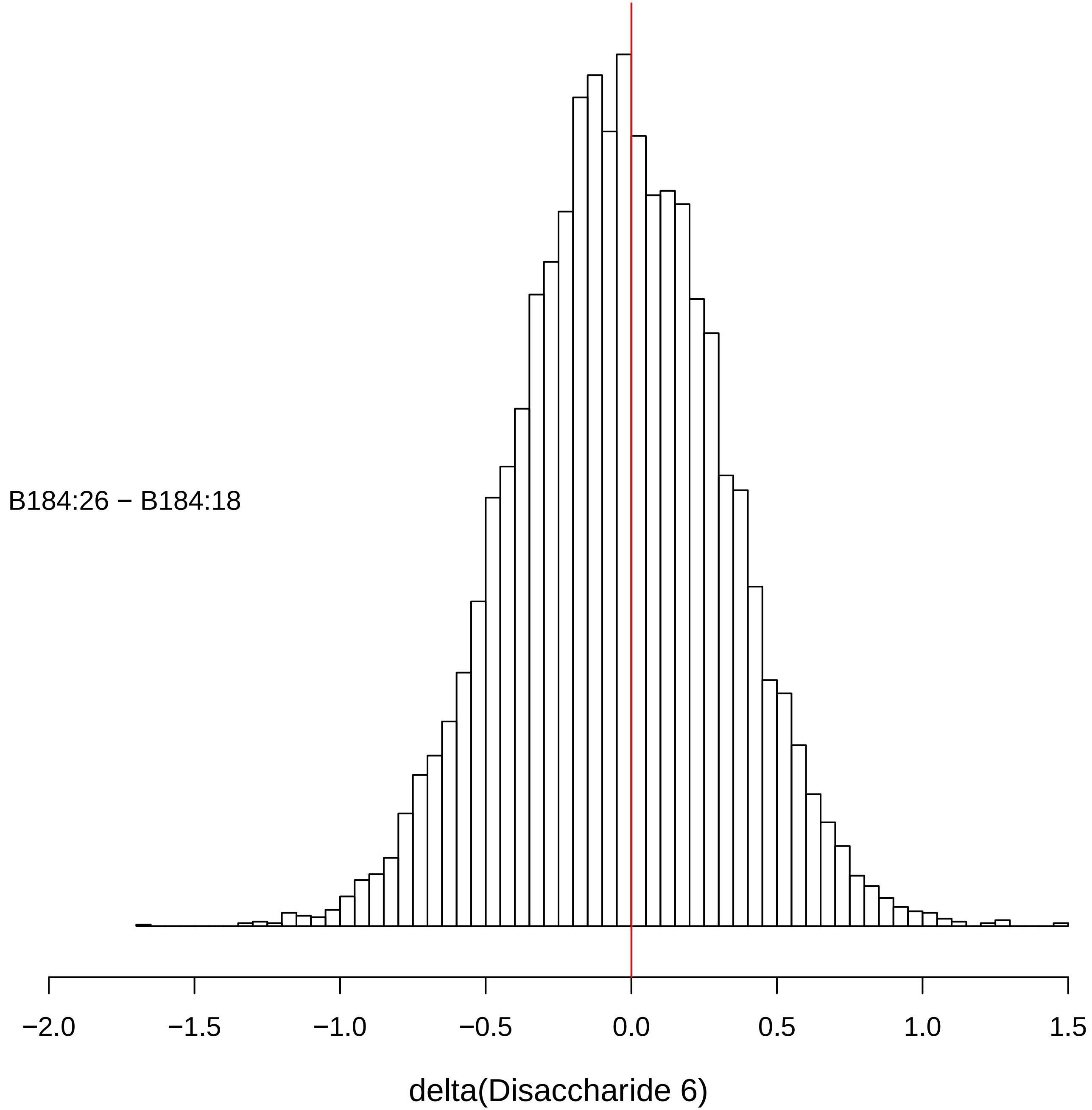
B184:26 − B184:18
−2.0
−1.5
−1.0
−0.5
0.0
0.5
1.0
1.5
delta(Disaccharide 6)

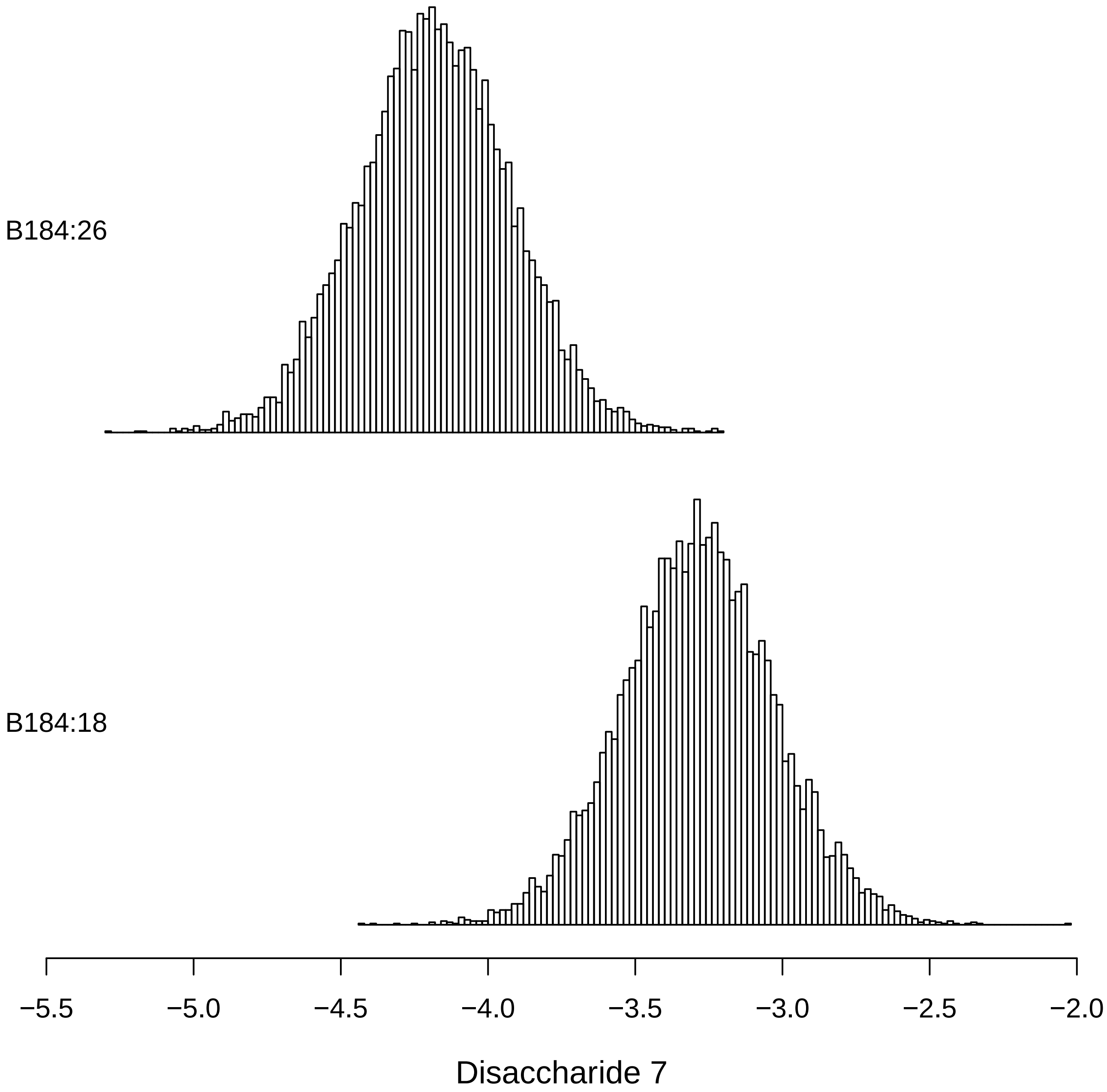
B184:26
B184:18
−5.5
−5.0
−4.5
−4.0
−3.5
−3.0
−2.5
−2.0
Disaccharide 7

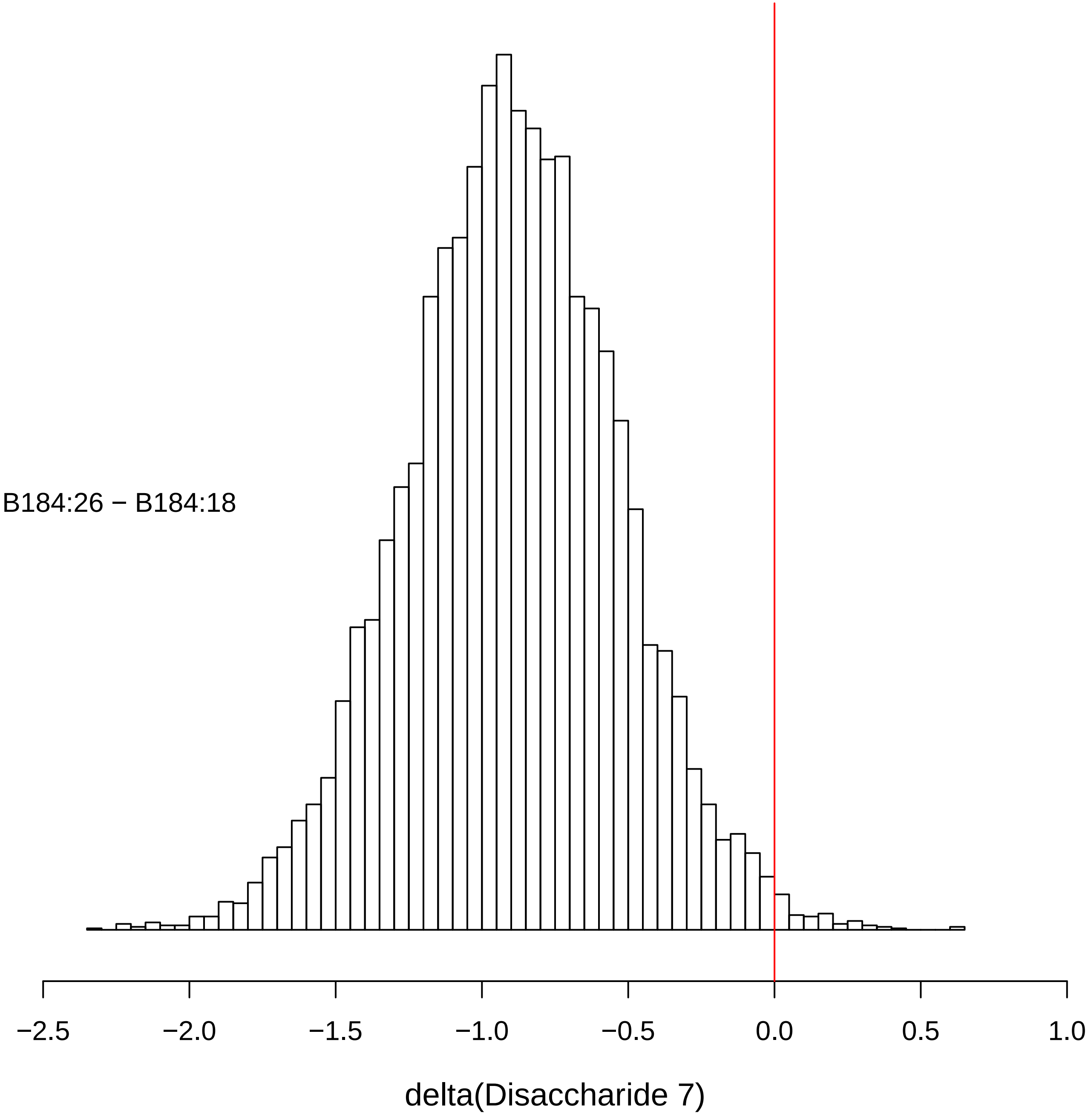

B184:26 − B184:18
−2.5
−2.0
−1.5
−1.0
−0.5
0.0
0.5
1.0
delta(Disaccharide 7)

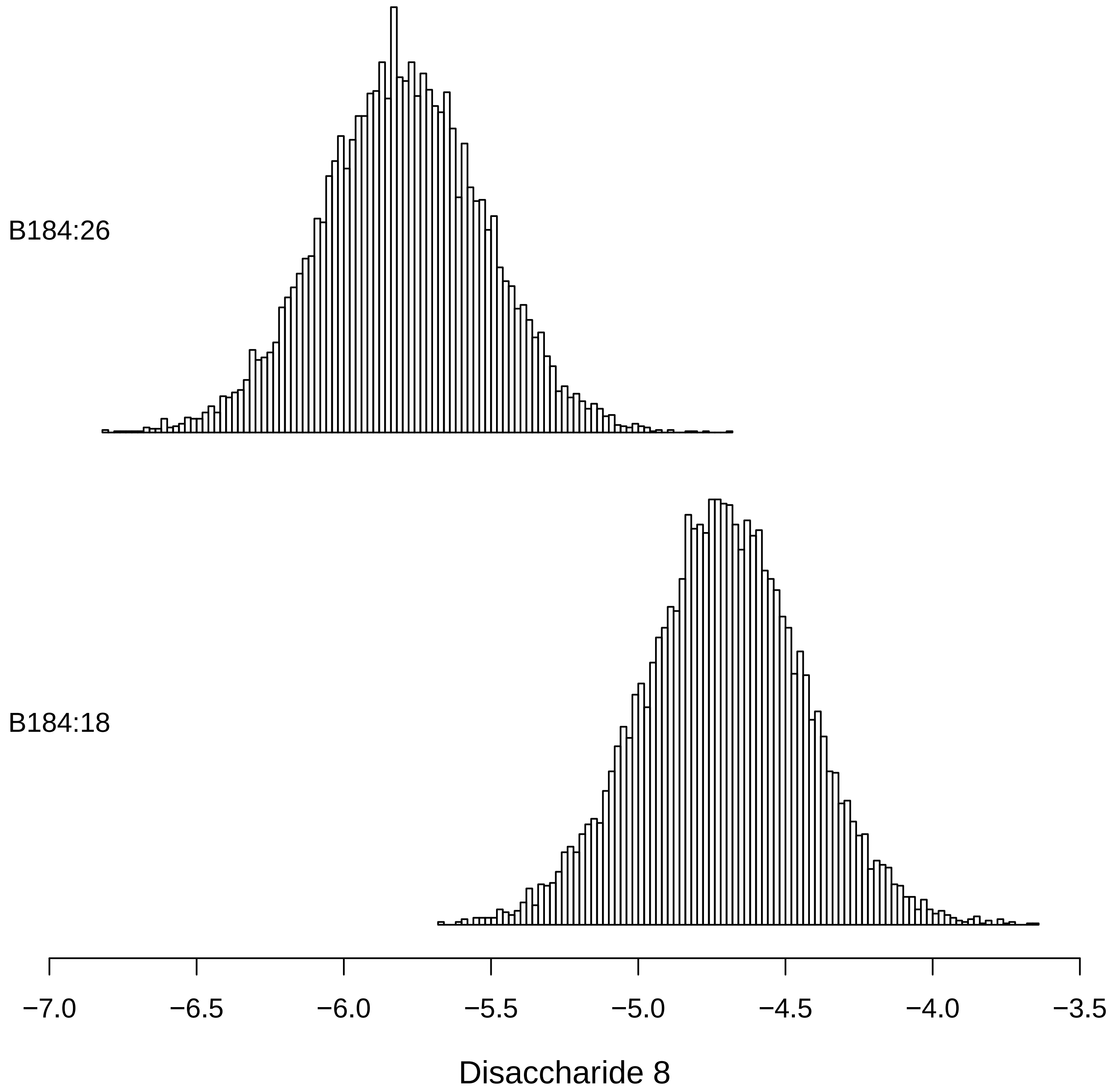
B184:26
B184:18
−7.0
−6.5
−6.0
−5.5
−5.0
−4.5
−4.0
−3.5
Disaccharide 8

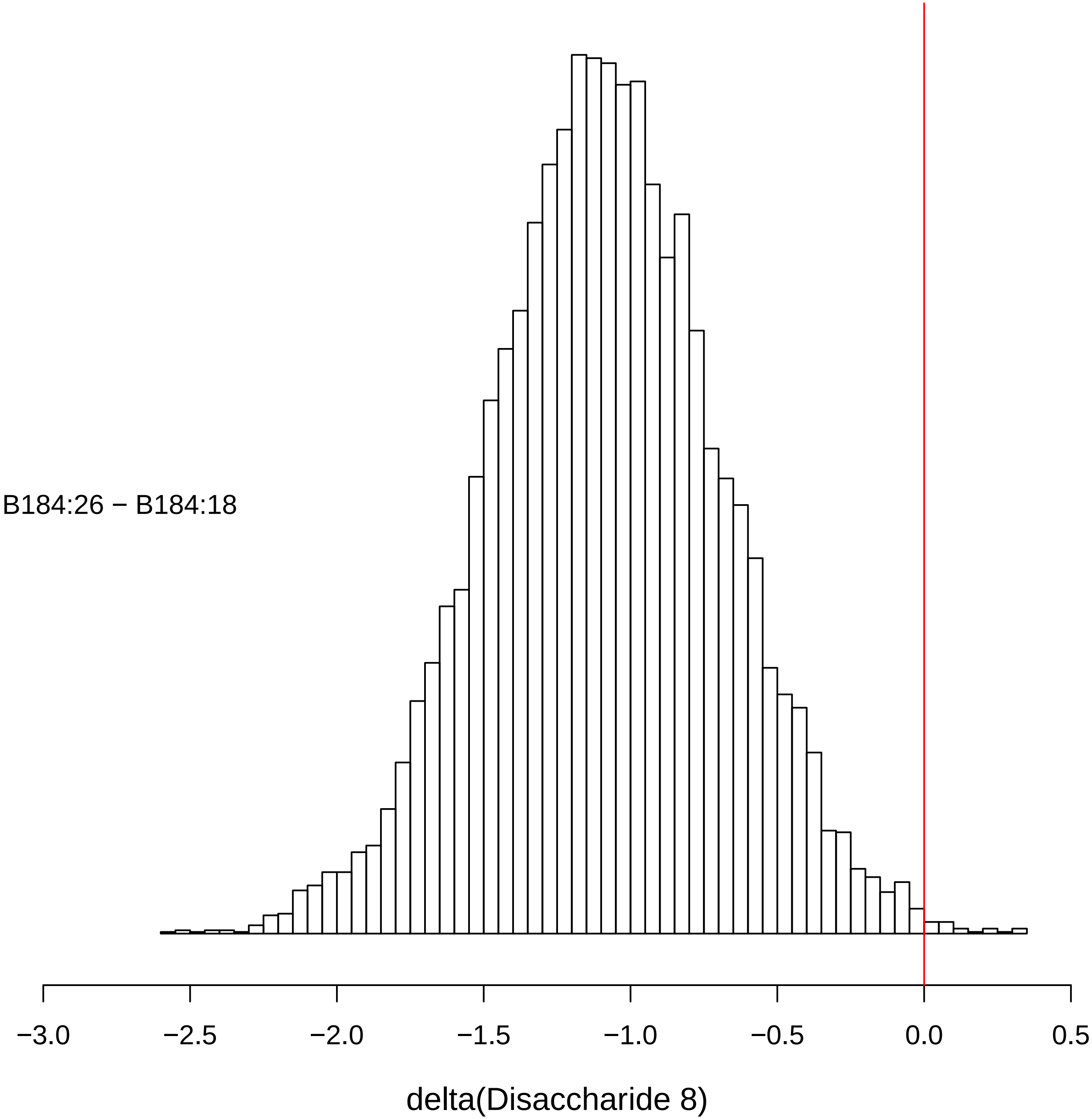
B184:26 − B184:18
−3.0
−2.5
−2.0
−1.5
−1.0
−0.5
0.0
0.5
delta(Disaccharide 8)

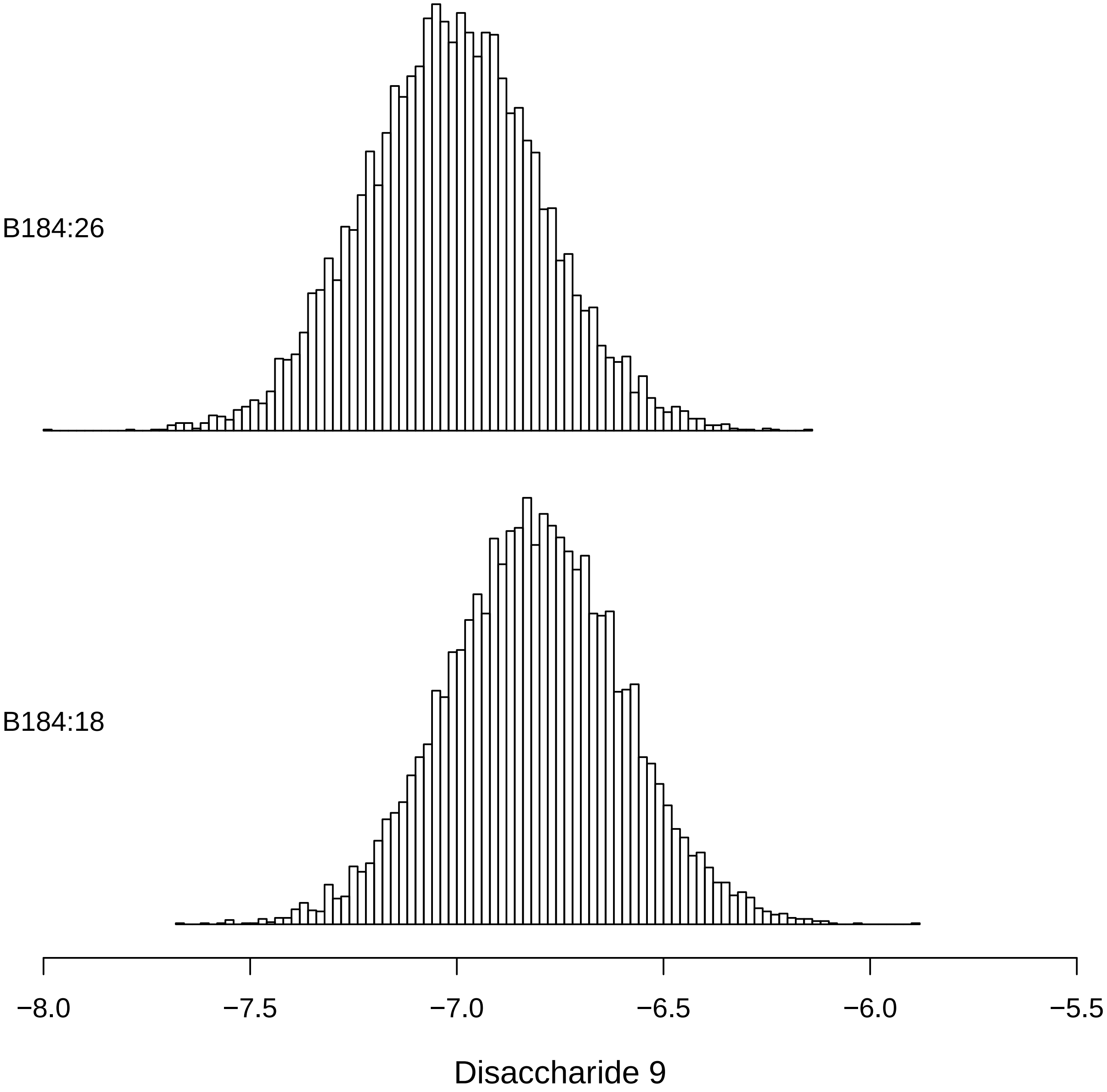
B184:26
B184:18
−8.0
−7.5
−7.0
−6.5
−6.0
−5.5
Disaccharide 9

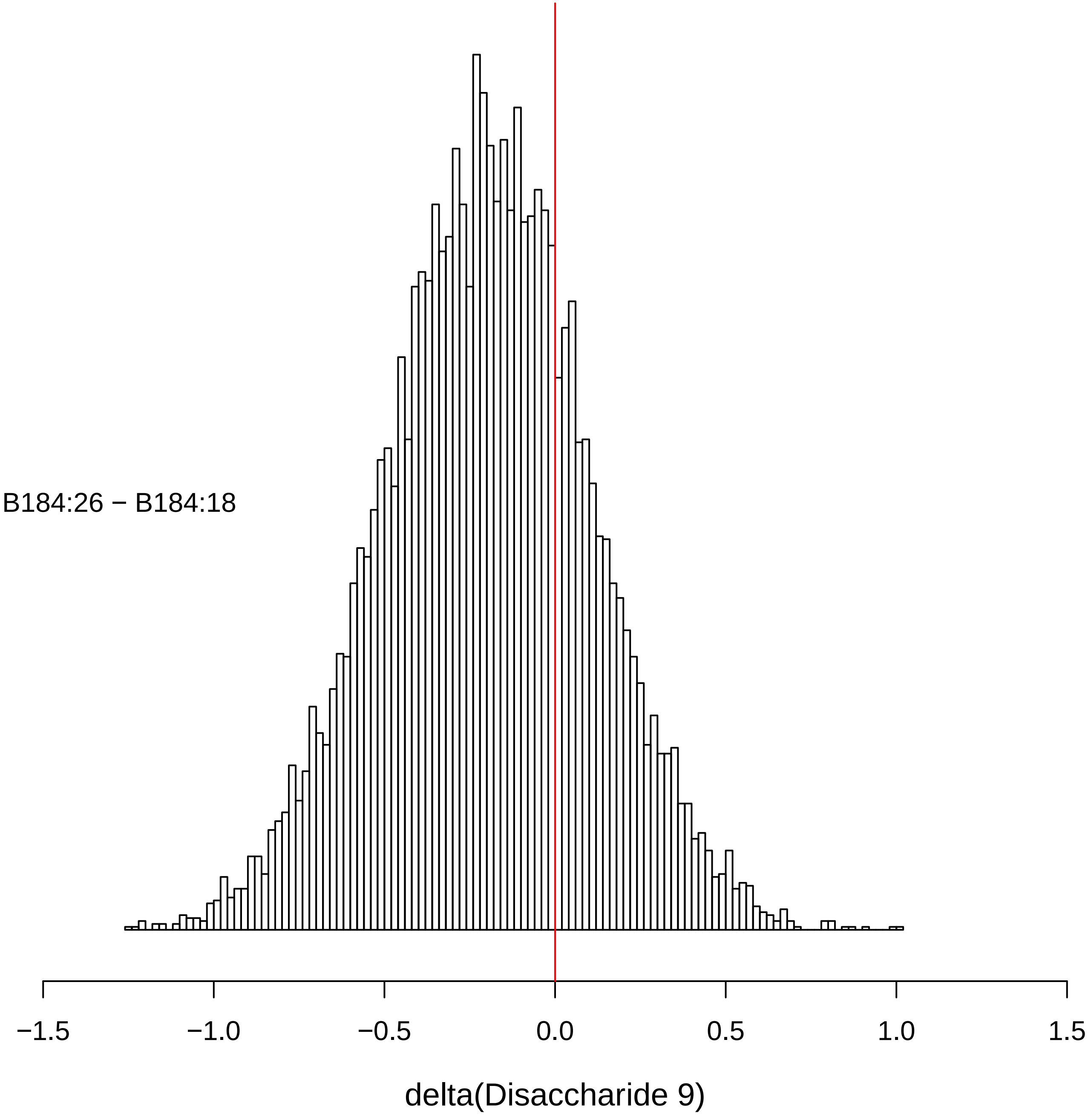
B184:26 − B184:18
−1.5
−1.0
−0.5
0.0
0.5
1.0
1.5
delta(Disaccharide 9)

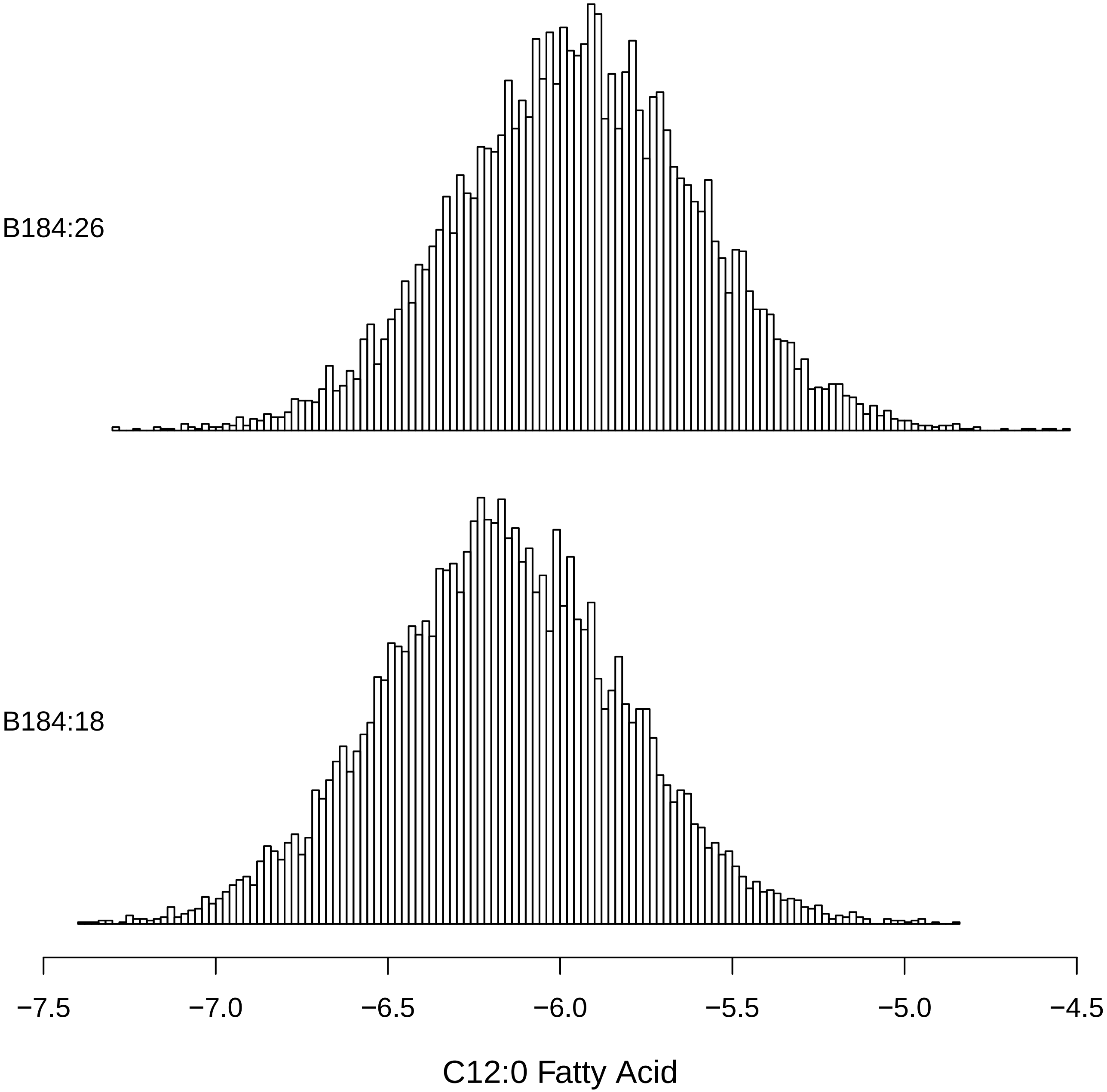
B184:26
B184:18
−7.5
−7.0
−6.5
−6.0
−5.5
−5.0
−4.5
C12:0 Fatty Acid

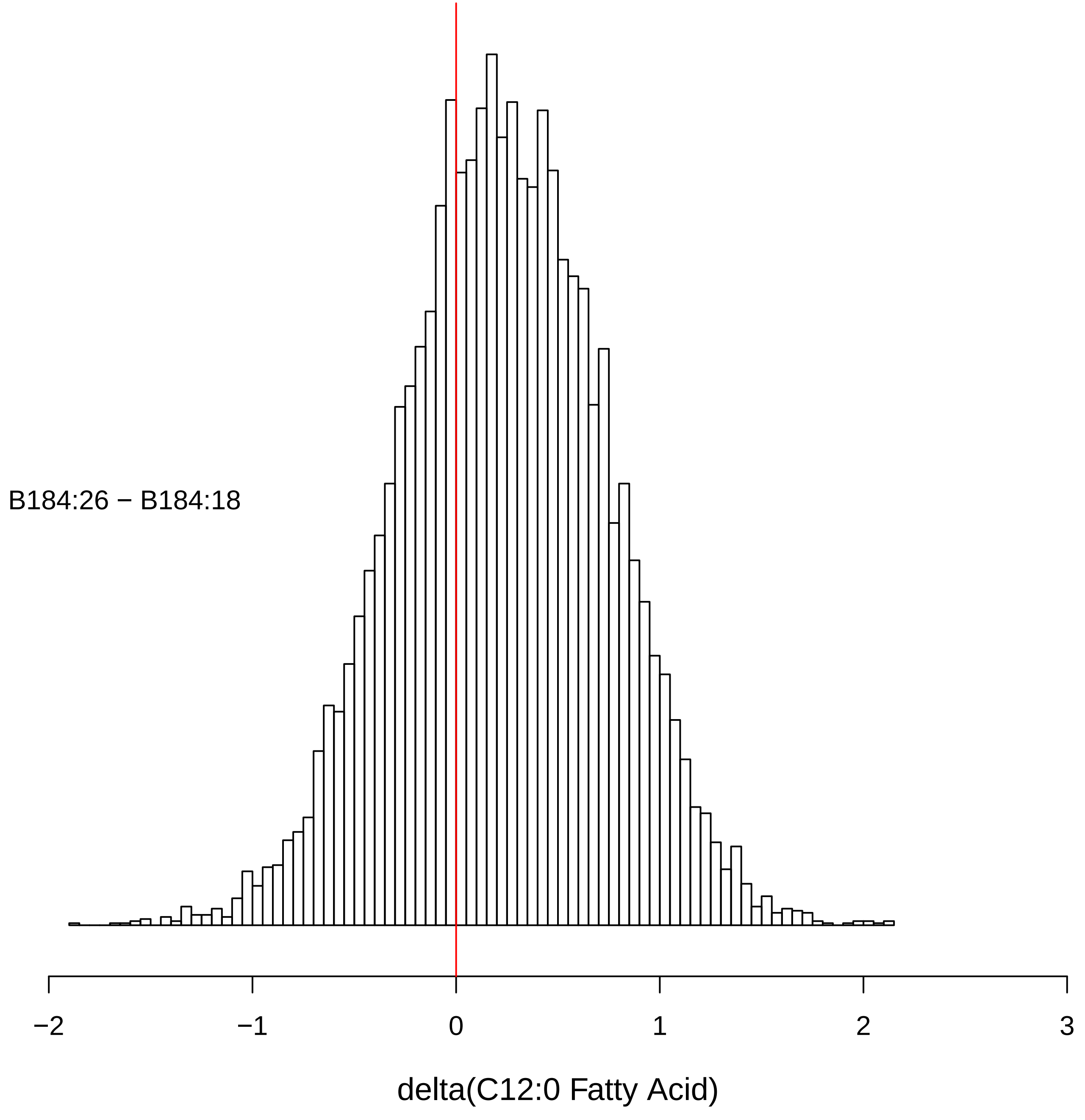
B184:26 − B184:18
−2
−1
0
1
2
3
delta(C12:0 Fatty Acid)

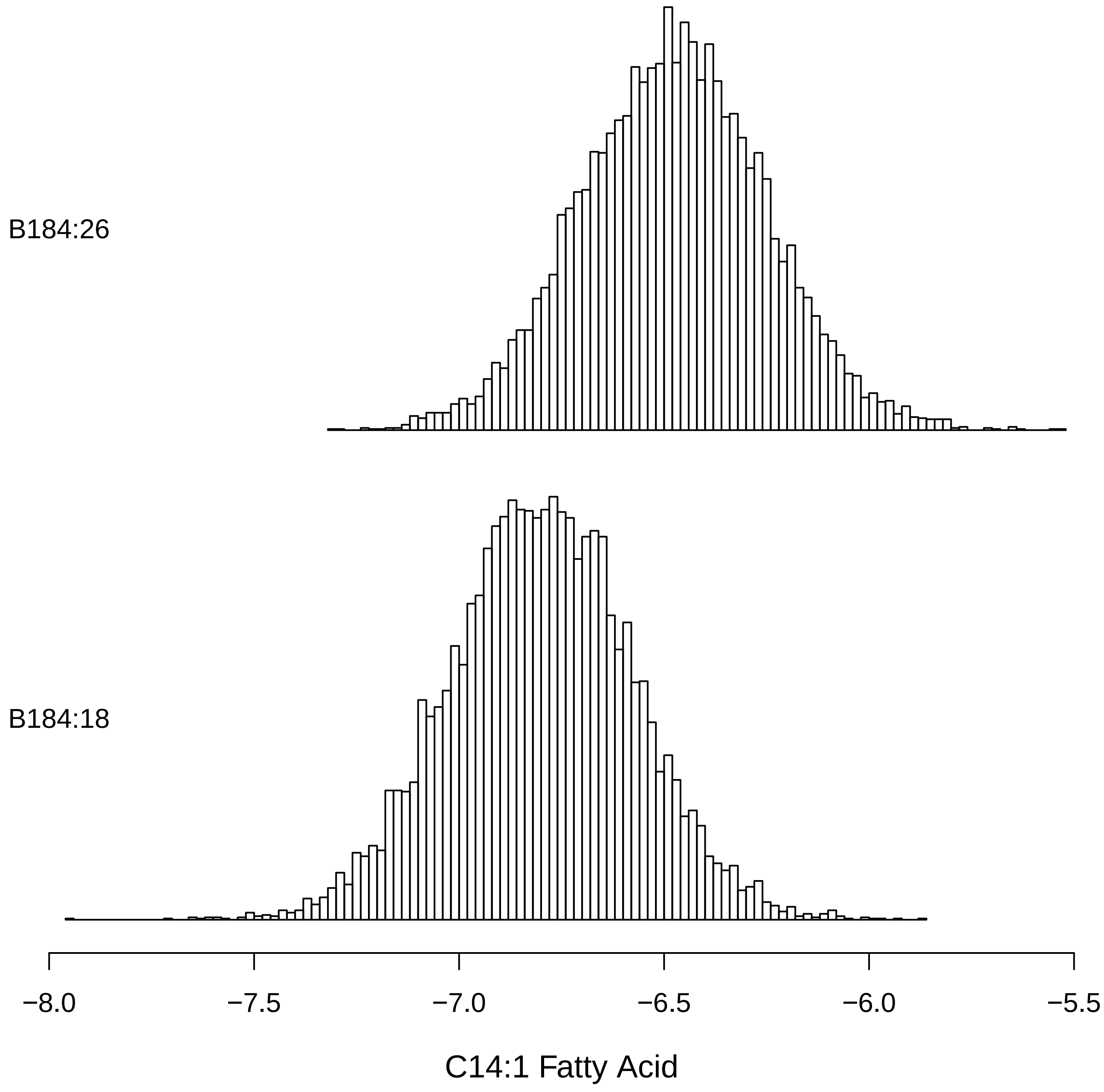

B184:26
B184:18
−8.0
−7.5
−7.0
−6.5
−6.0
−5.5
C14:1 Fatty Acid

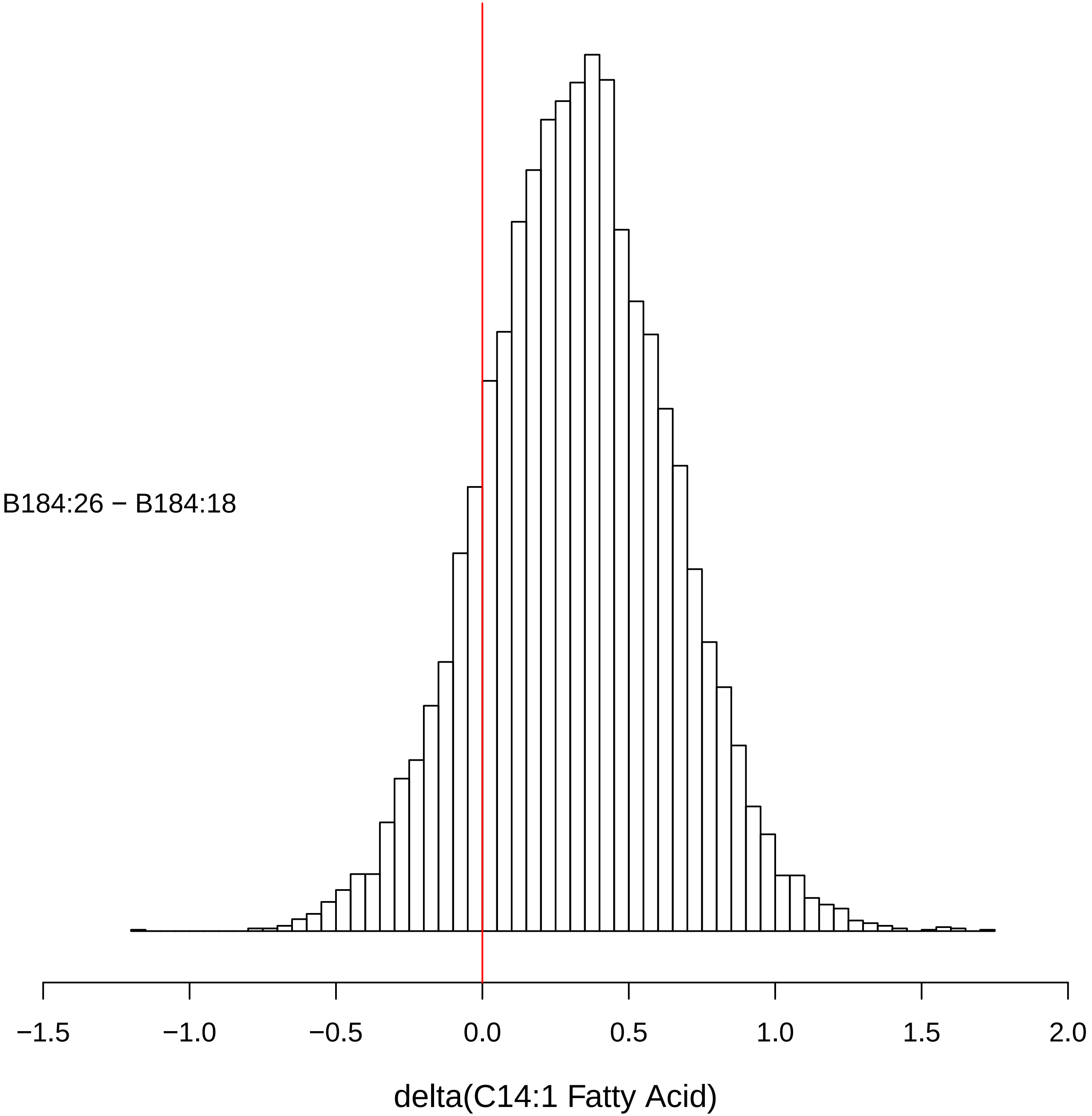
B184:26 − B184:18
−1.5
−1.0
−0.5
0.0
0.5
1.0
1.5
2.0
delta(C14:1 Fatty Acid)

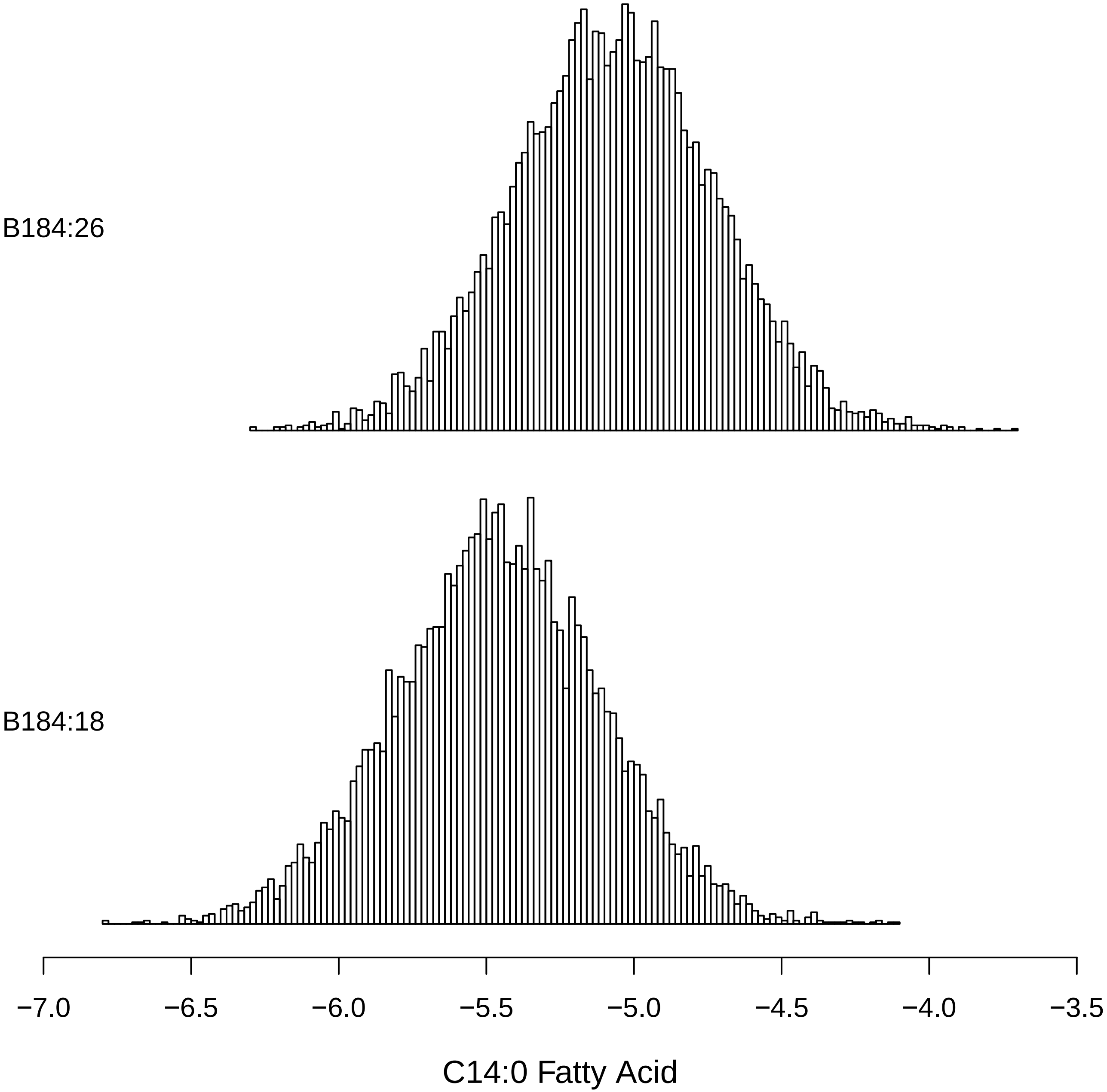
B184:26
B184:18
−7.0
−6.5
−6.0
−5.5
−5.0
−4.5
−4.0
−3.5
C14:0 Fatty Acid

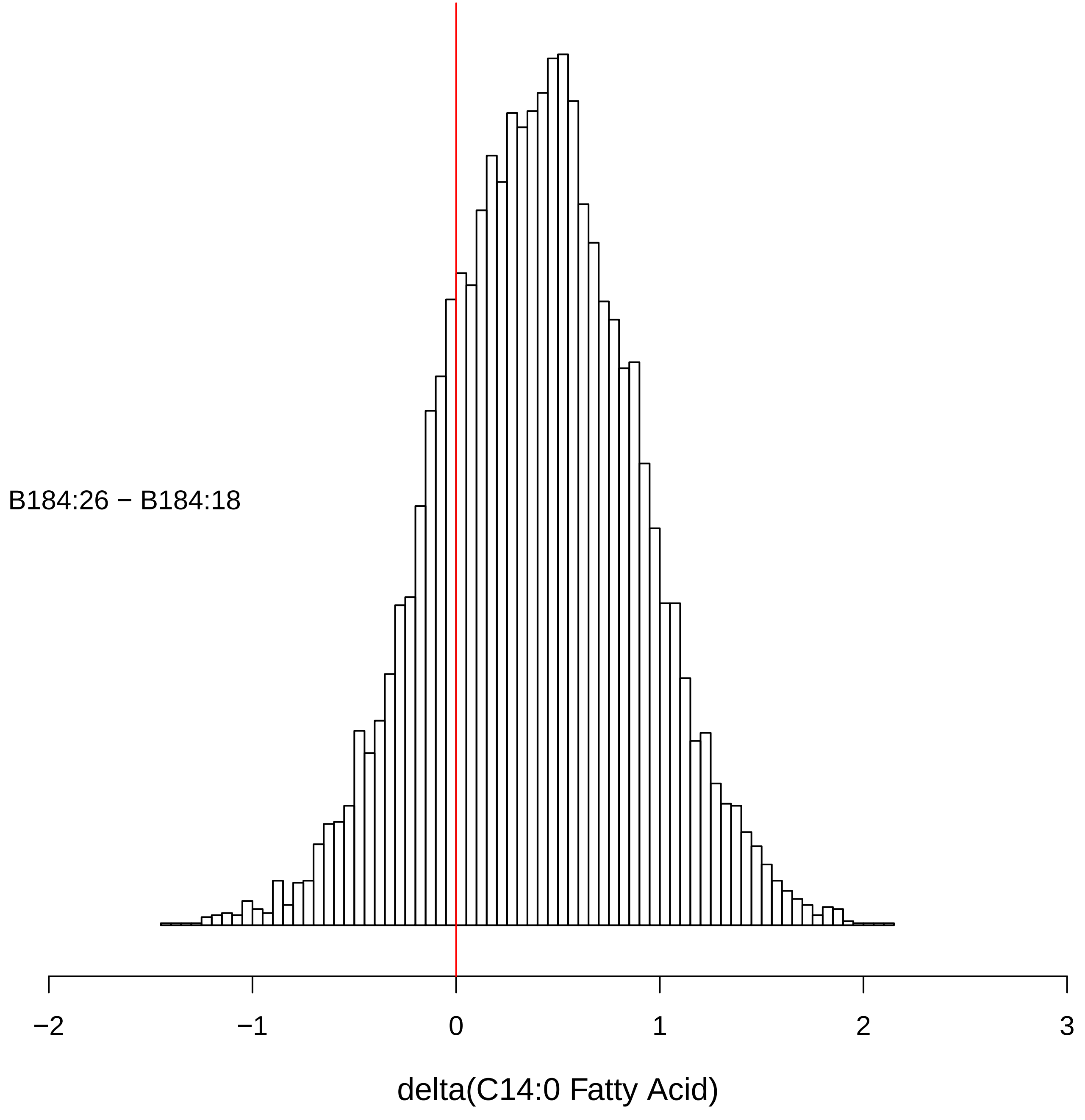

B184:26 − B184:18
−2
−1
0
1
2
3
delta(C14:0 Fatty Acid)

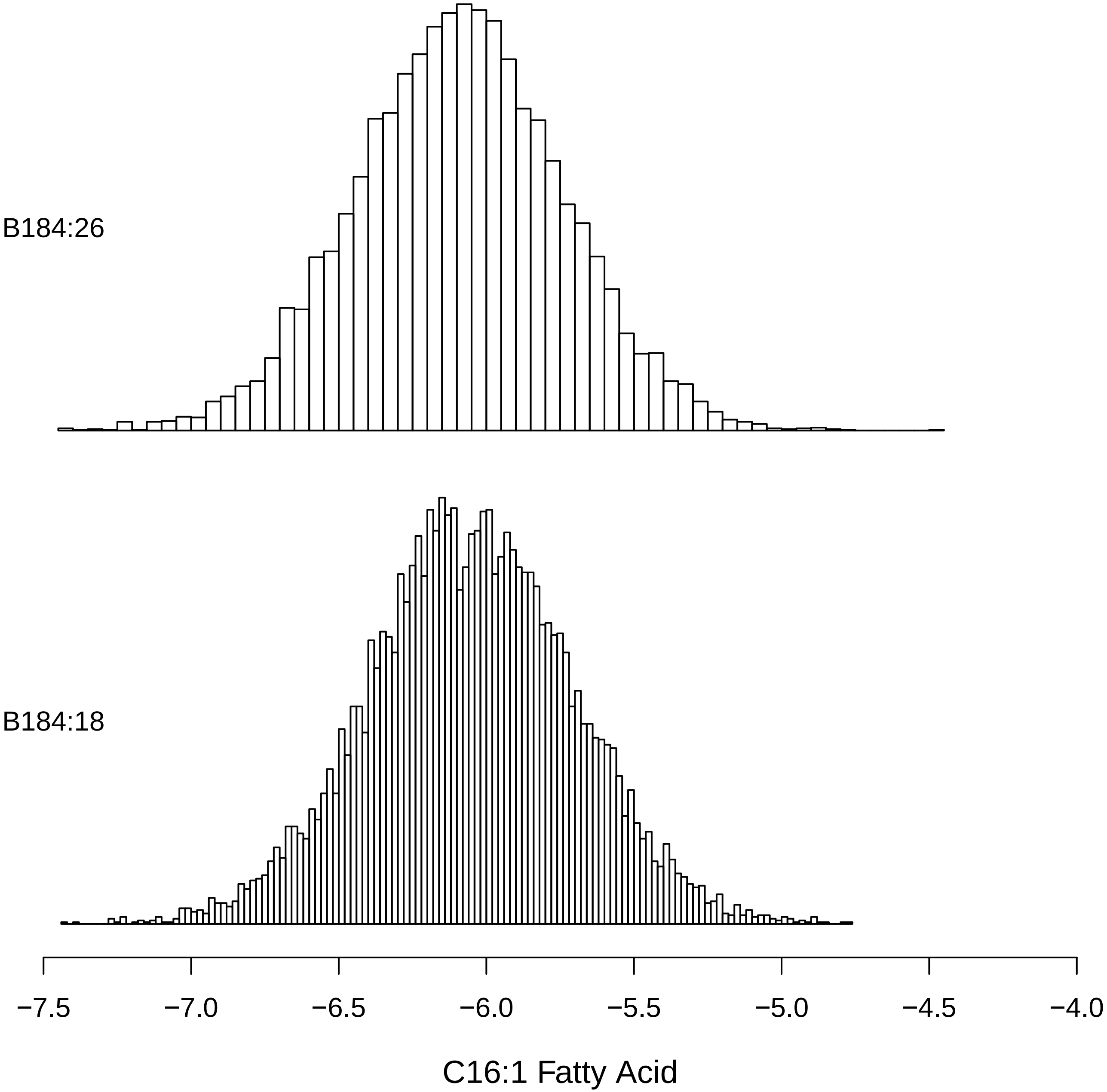
B184:26
B184:18
−7.5
−7.0
−6.5
−6.0
−5.5
−5.0
−4.5
−4.0
C16:1 Fatty Acid

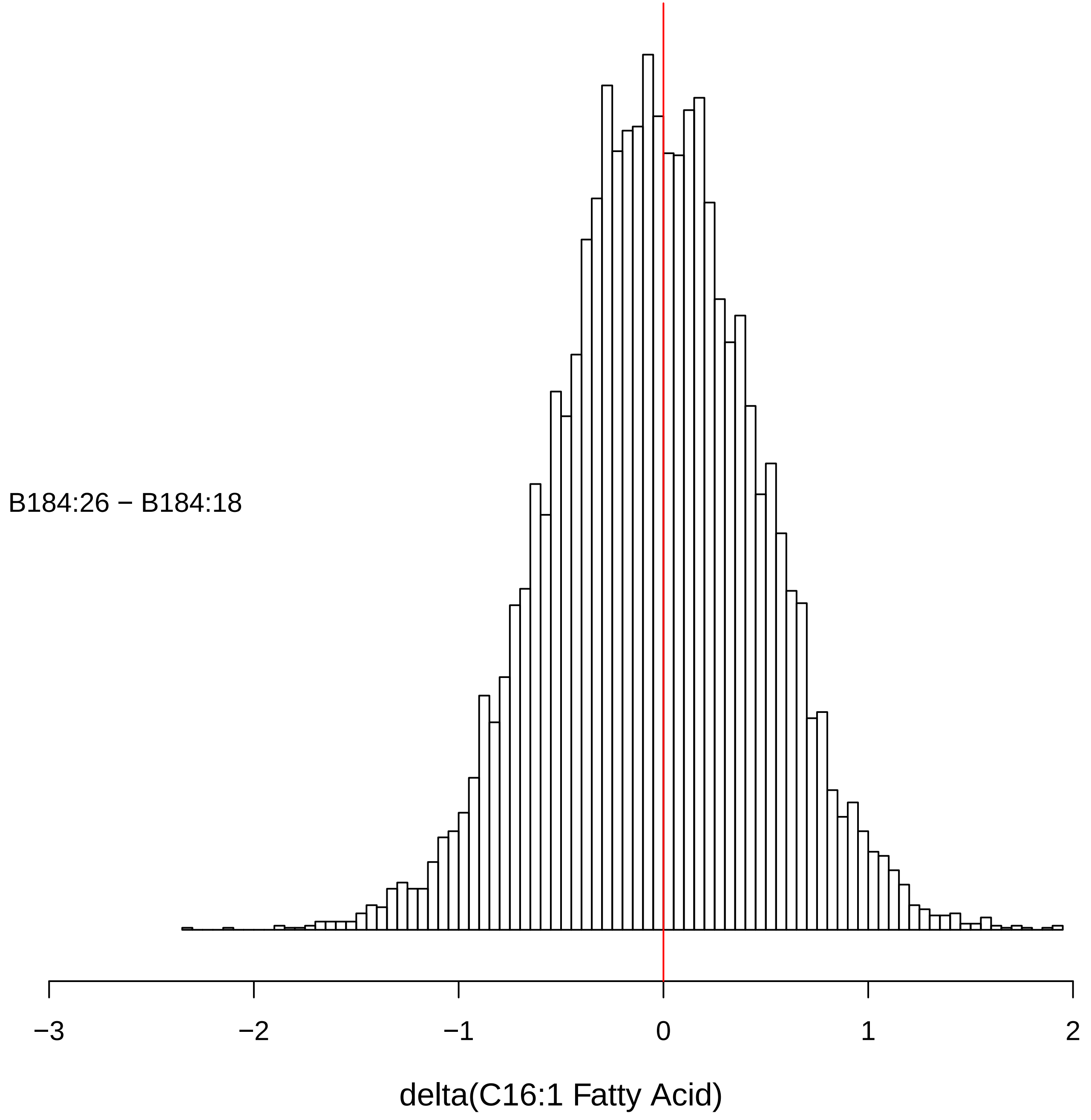

B184:26 − B184:18
−3
−2
−1
0
1
2
delta(C16:1 Fatty Acid)

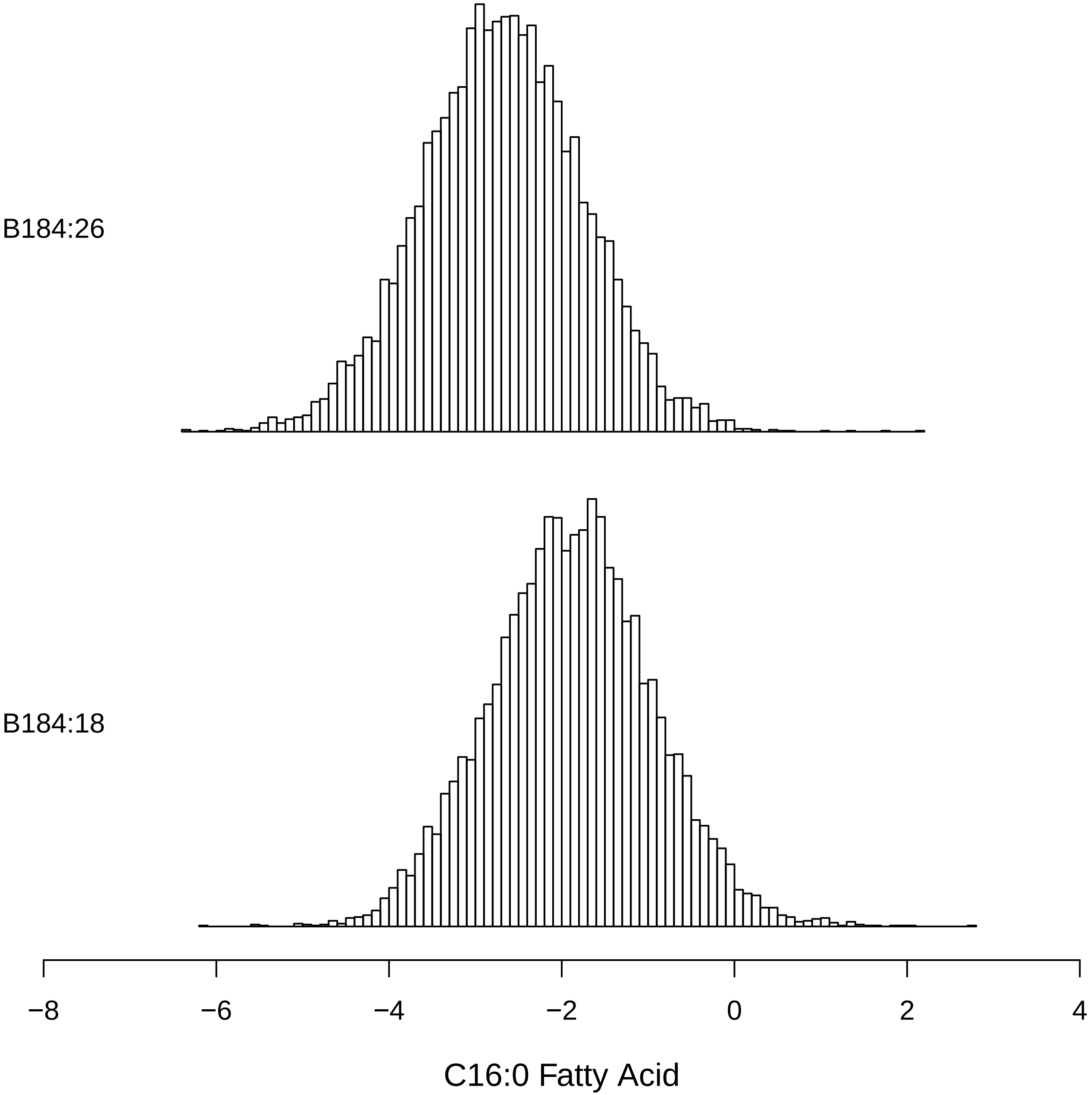
B184:26
B184:18
−8
−6
−4
−2
0
2
4
C16:0 Fatty Acid

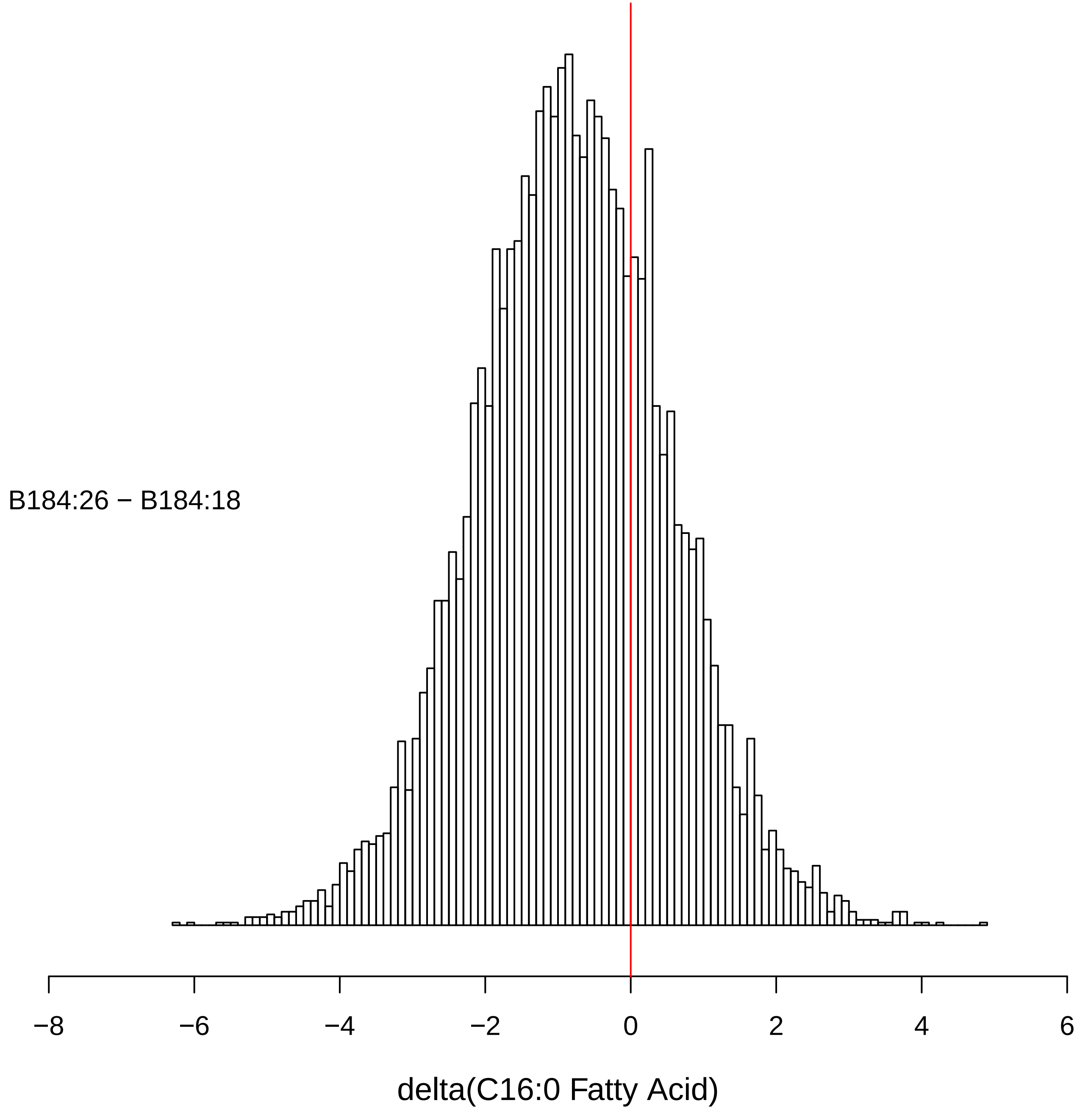
B184:26 − B184:18
−8
−6
−4
−2
0
2
4
6
delta(C16:0 Fatty Acid)

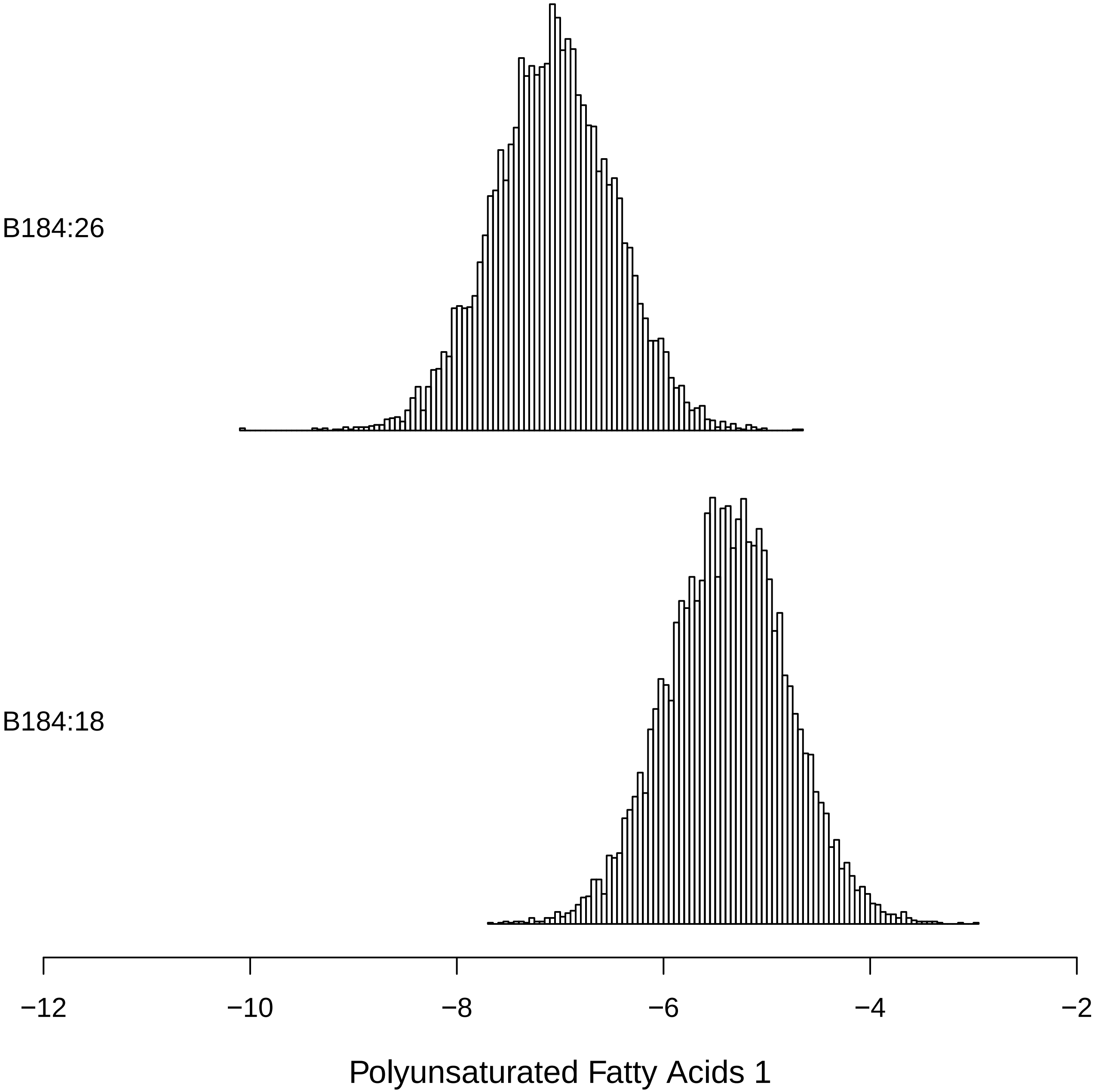
B184:26
B184:18
−12
−10
−8
−6
−4
−2
Polyunsaturated Fatty Acids 1

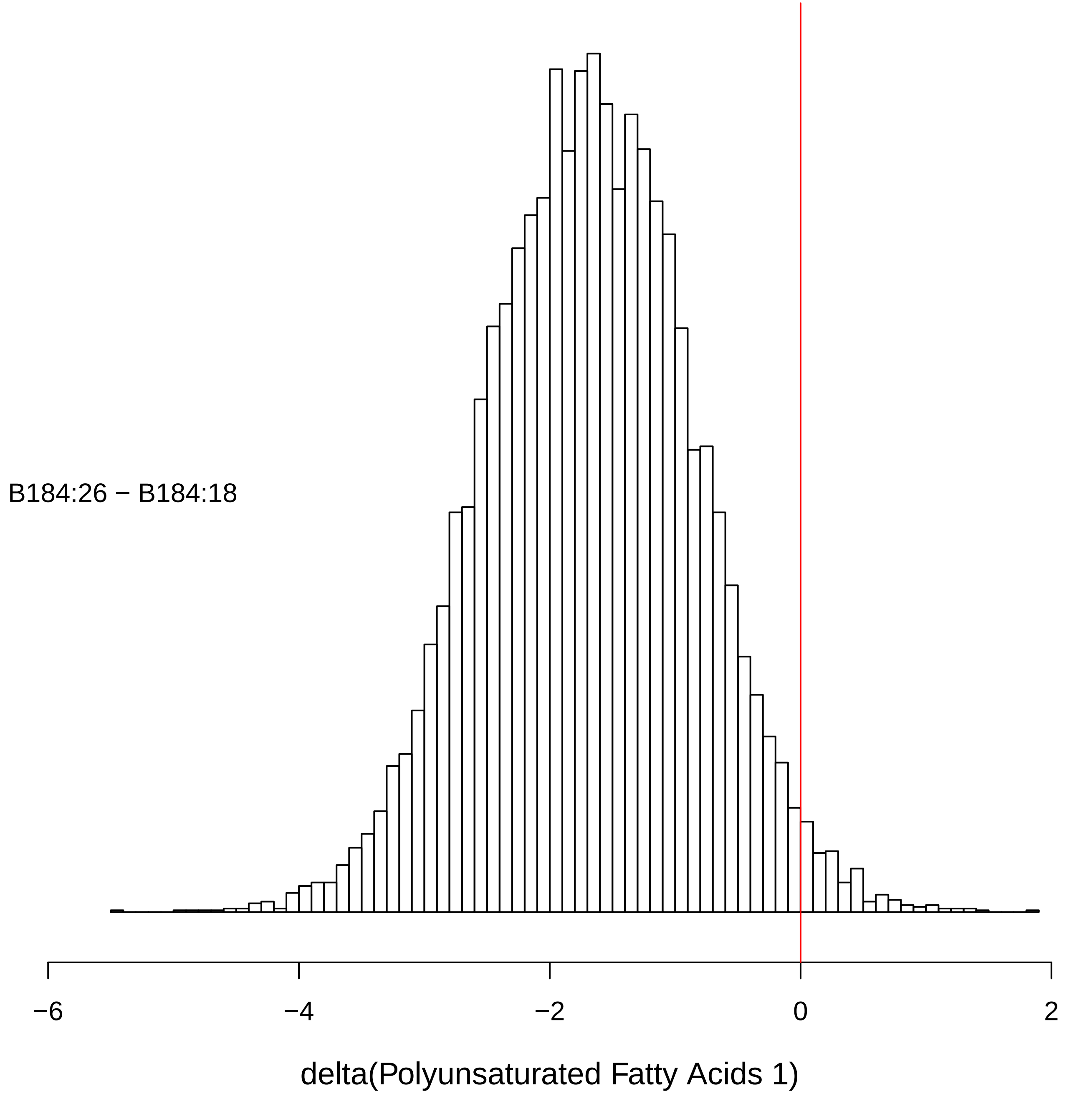
B184:26 − B184:18
−6
−4
−2
0
2
delta(Polyunsaturated Fatty Acids 1)

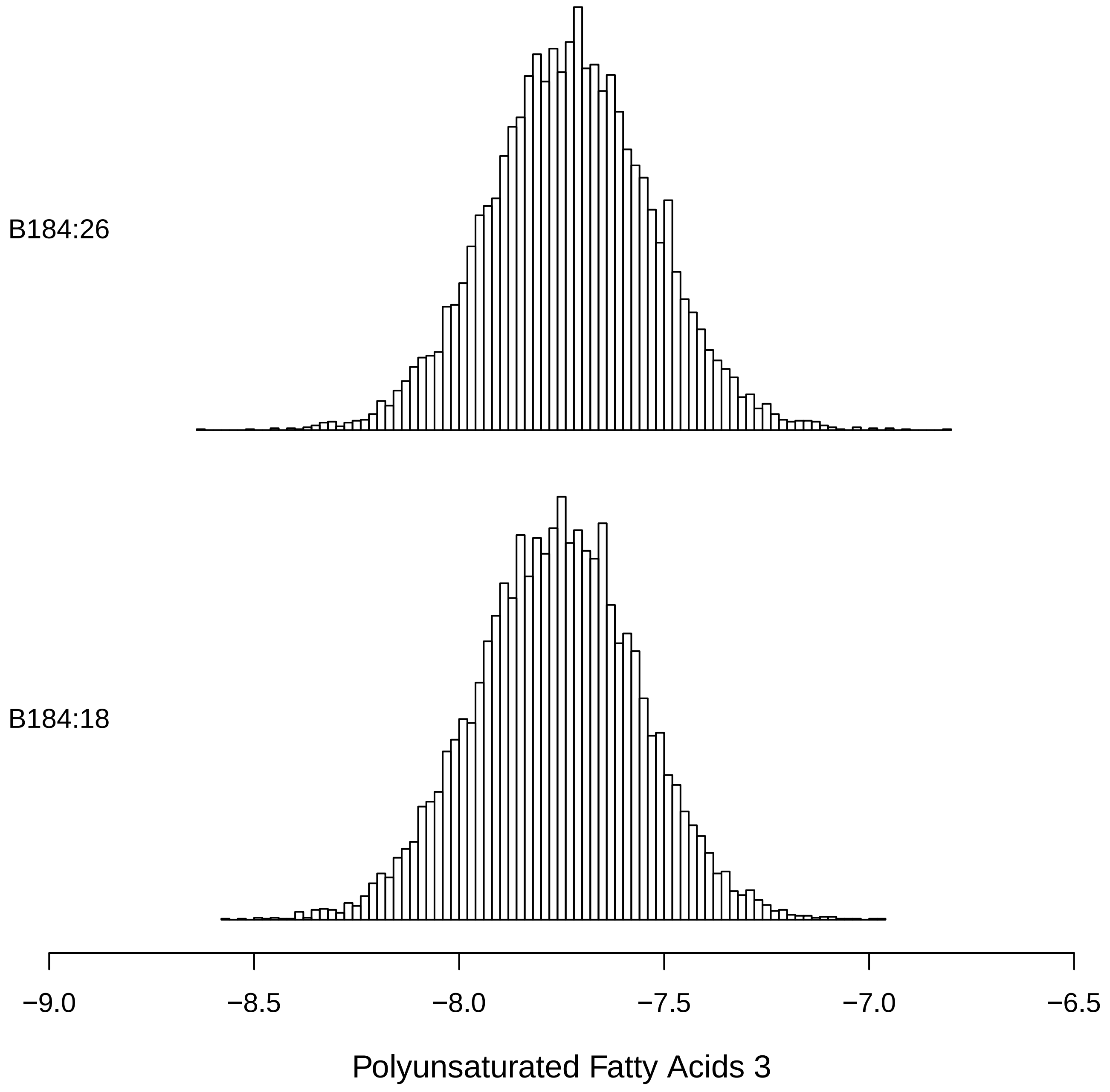
B184:26
B184:18
−9.0
−8.5
−8.0
−7.5
−7.0
−6.5
Polyunsaturated Fatty Acids 3

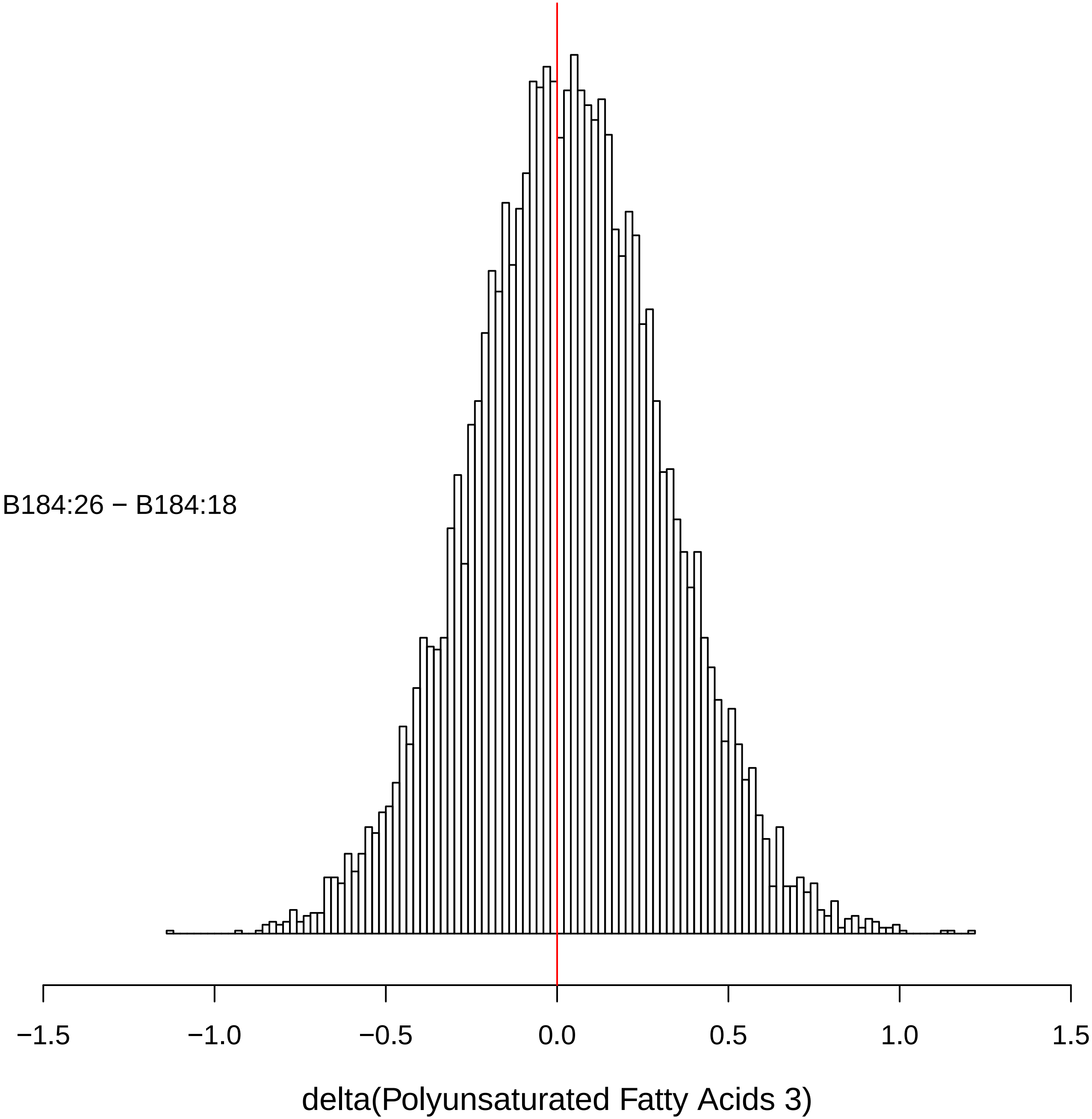

B184:26 − B184:18
−1.5
−1.0
−0.5
0.0
0.5
1.0
1.5
delta(Polyunsaturated Fatty Acids 3)

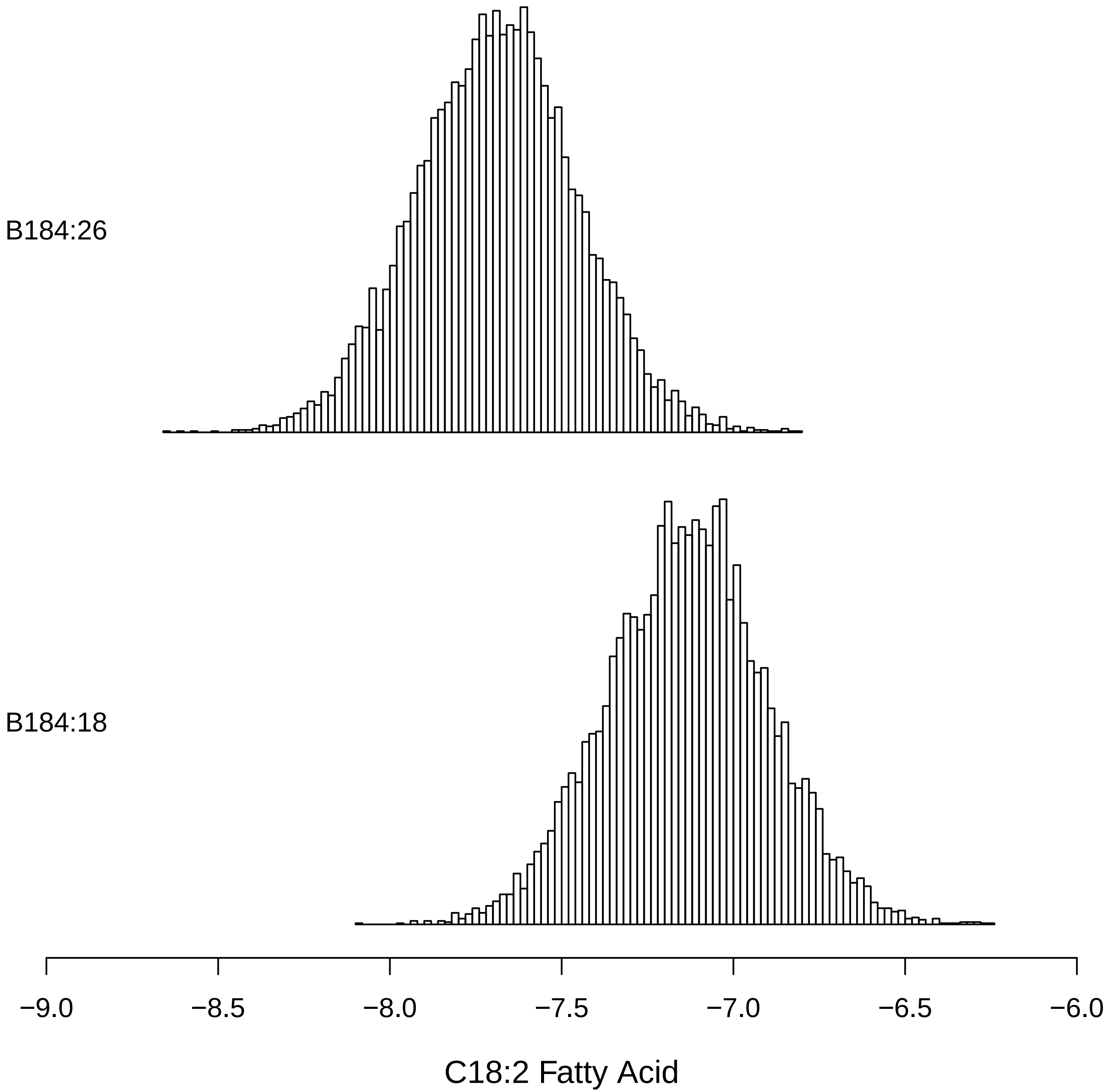
B184:26
B184:18
−9.0
−8.5
−8.0
−7.5
−7.0
−6.5
−6.0
C18:2 Fatty Acid

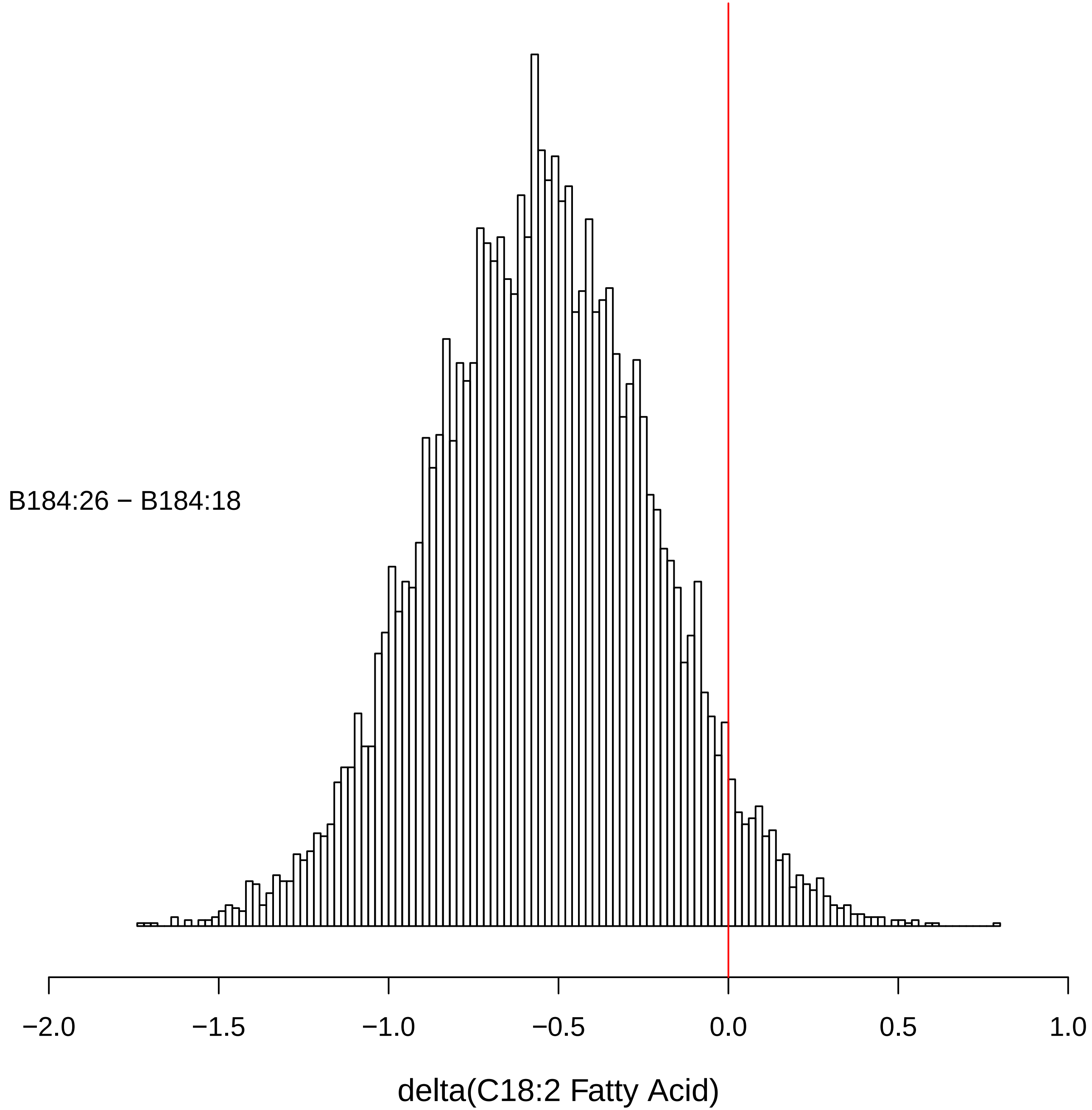
B184:26 − B184:18
−2.0
−1.5
−1.0
−0.5
0.0
0.5
1.0
delta(C18:2 Fatty Acid)

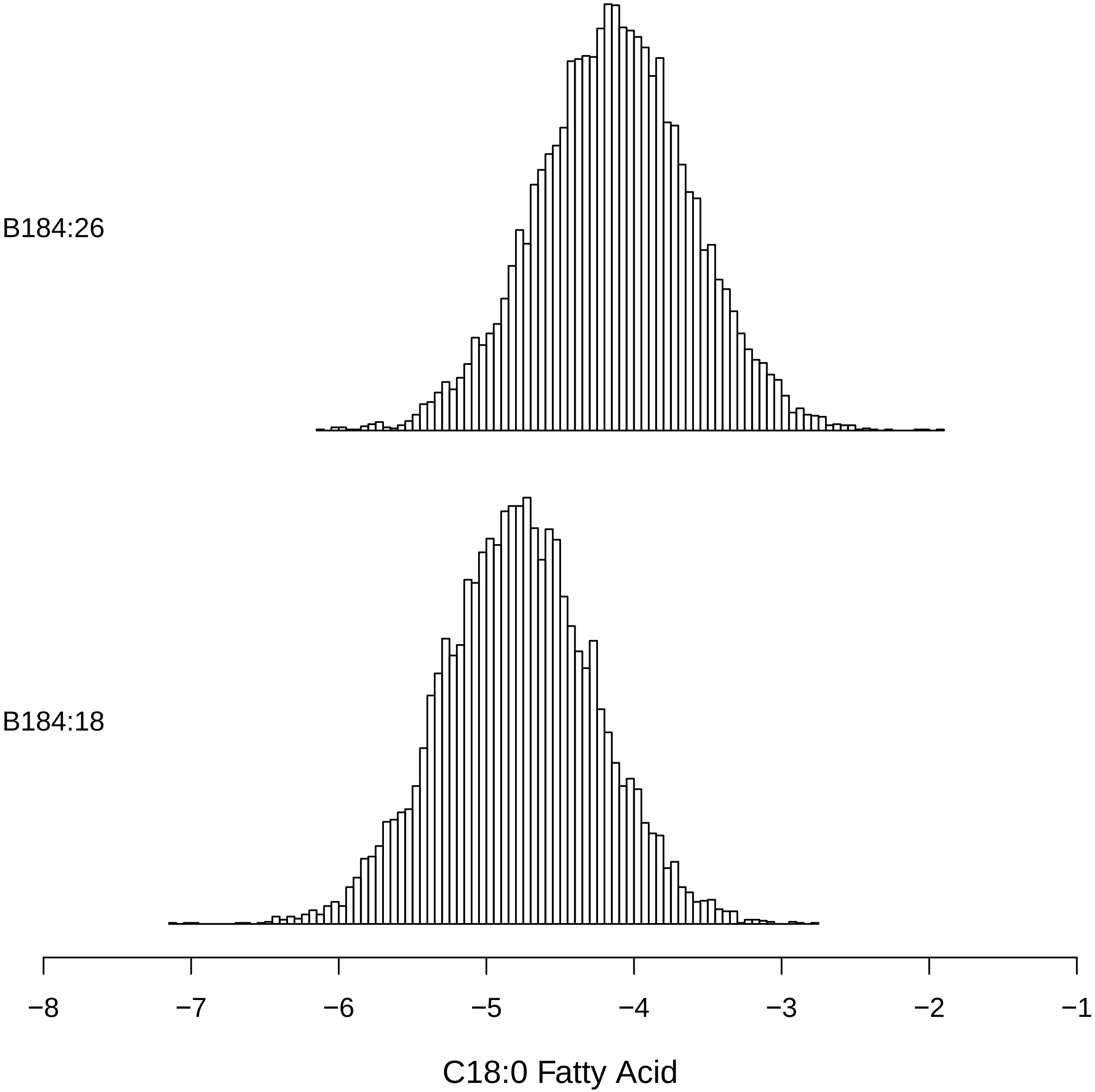

B184:26
B184:18
−8
−7
−6
−5
−4
−3
−2
−1
C18:0 Fatty Acid

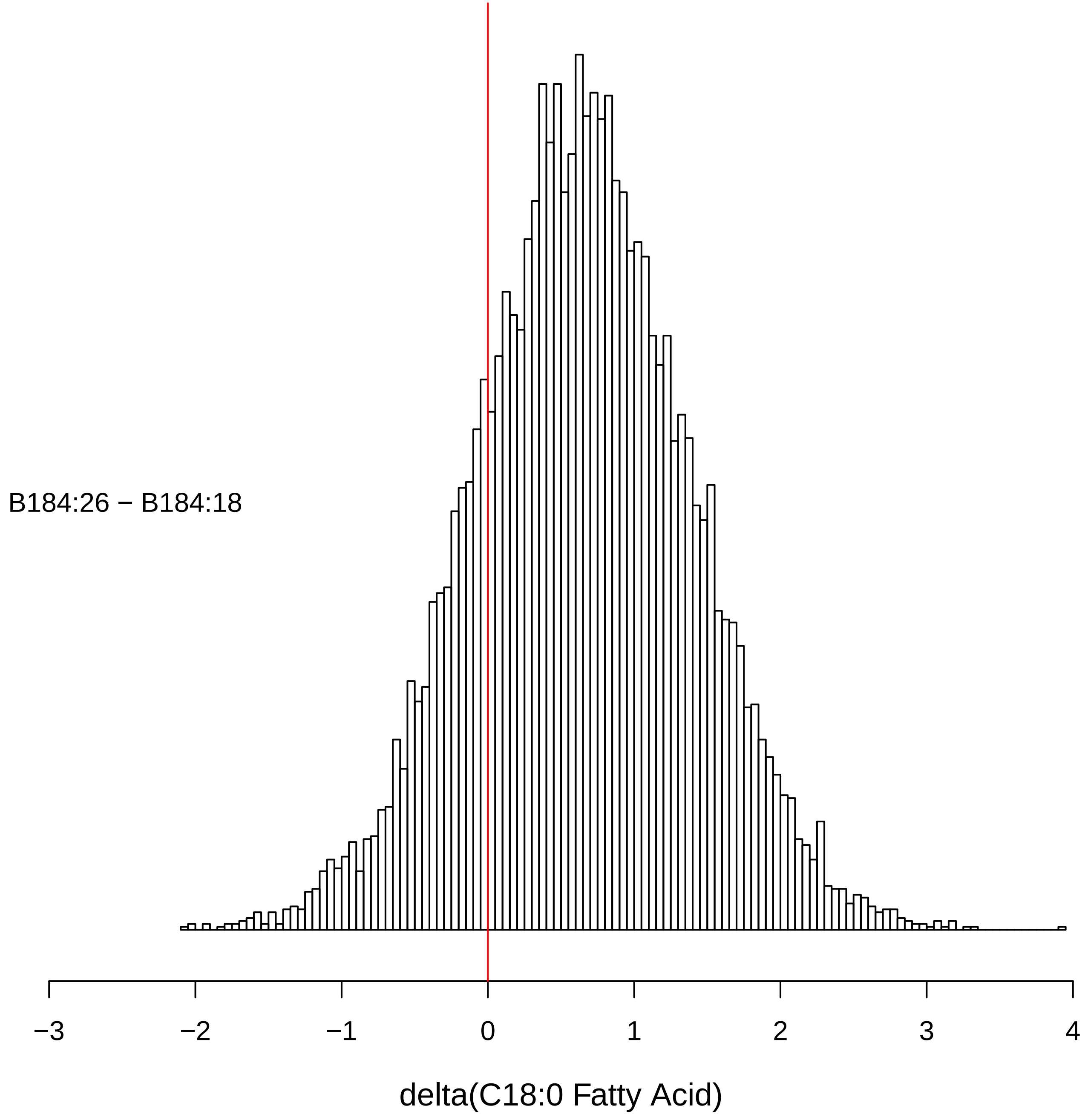
B184:26 − B184:18
−3
−2
−1
0
1
2
3
4
delta(C18:0 Fatty Acid)

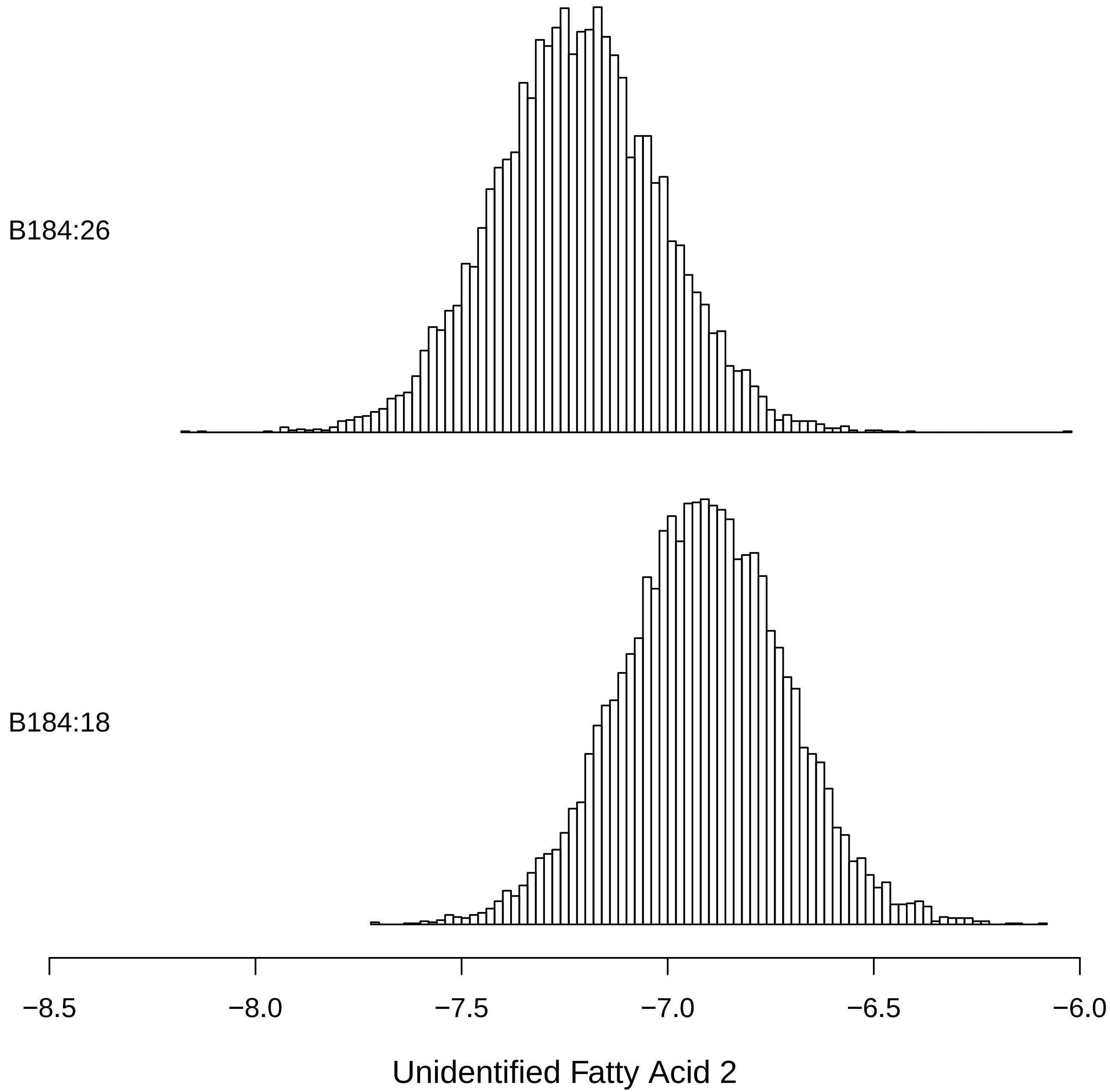
B184:26
B184:18
−8.5
−8.0
−7.5
−7.0
−6.5
−6.0
Unidentified Fatty Acid 2

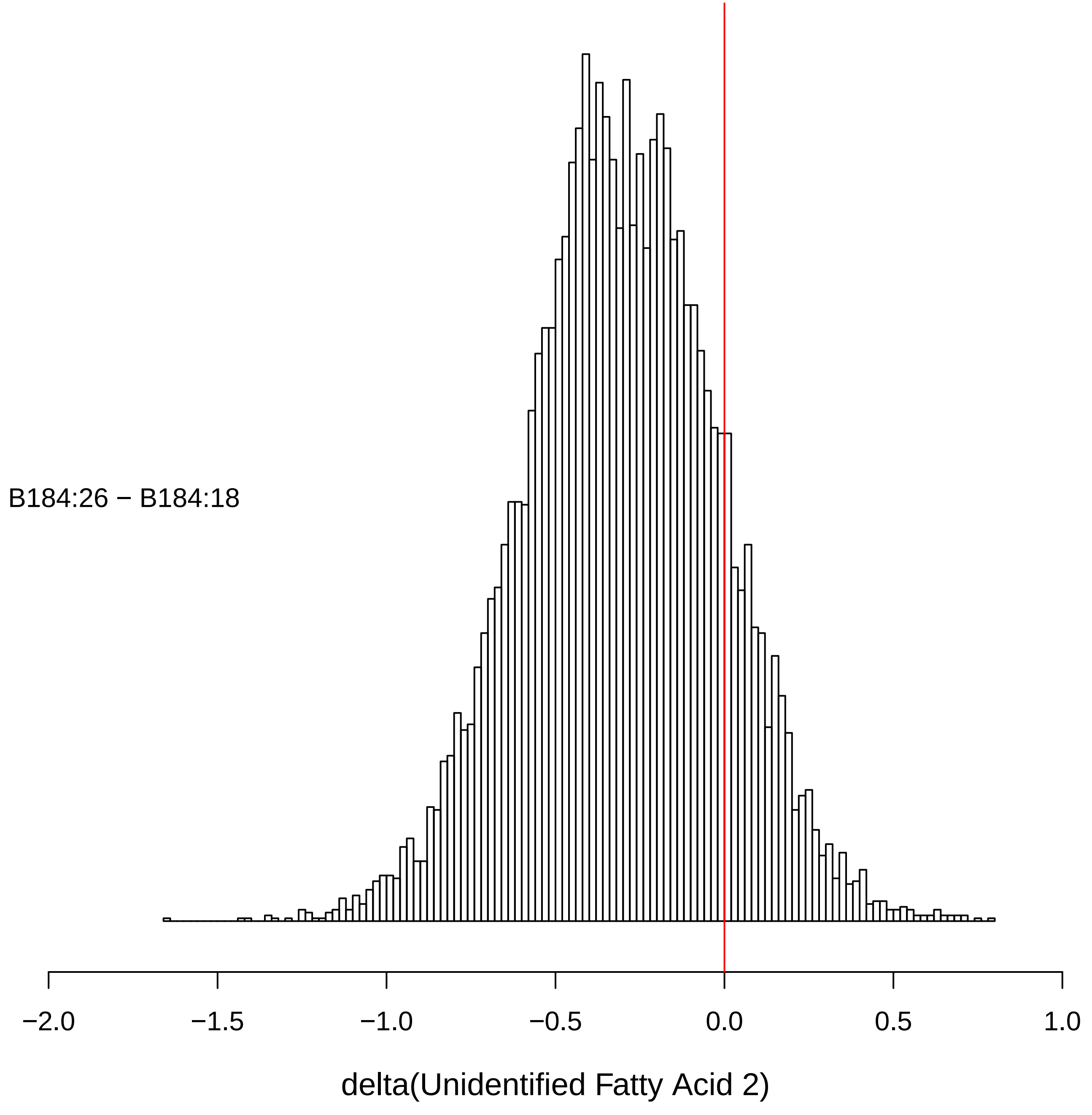
B184:26 − B184:18
−2.0
−1.5
−1.0
−0.5
0.0
0.5
1.0
delta(Unidentified Fatty Acid 2)

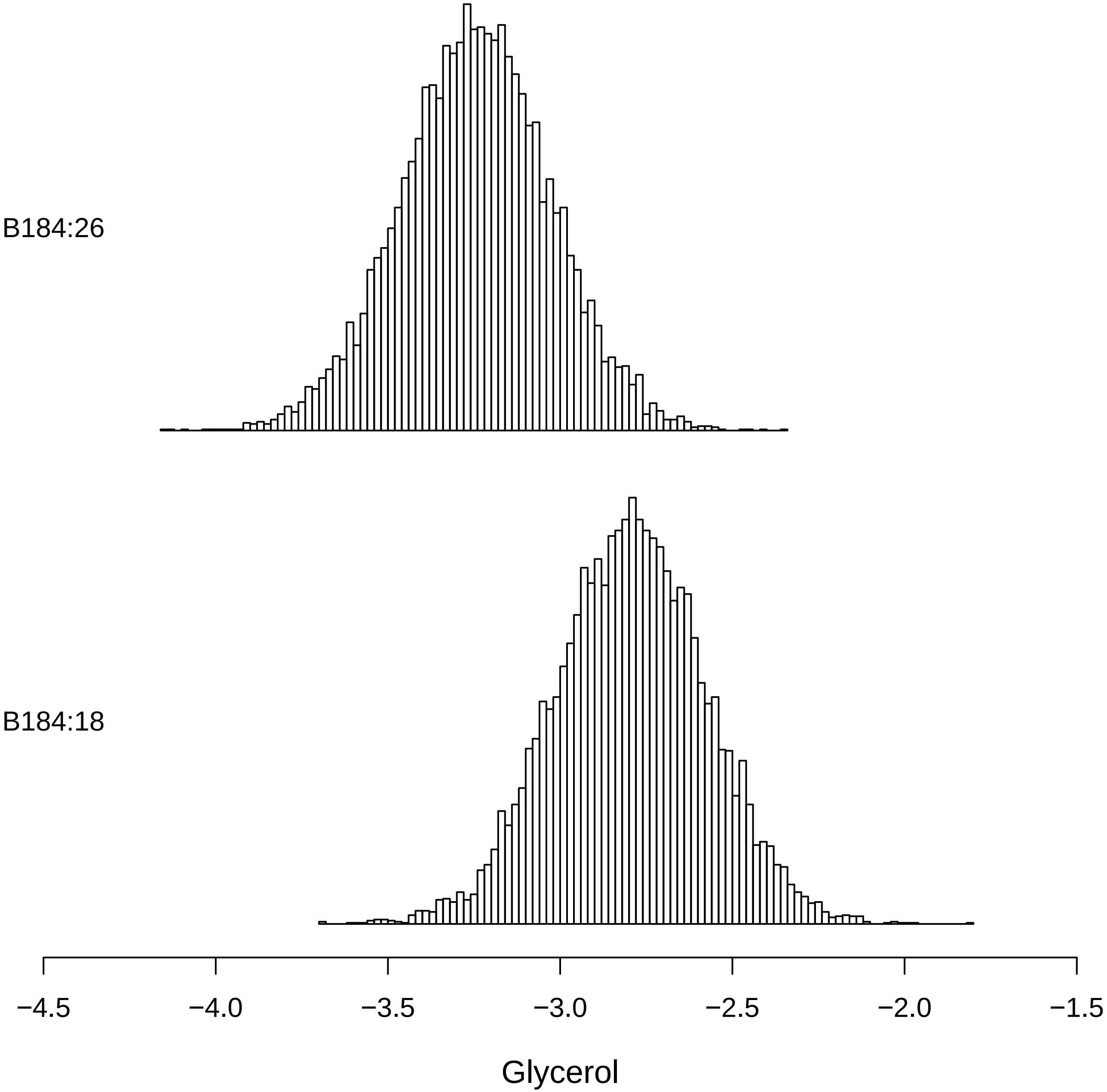

B184:26
B184:18
−4.5
−4.0
−3.5
−3.0
−2.5
−2.0
−1.5
Glycerol

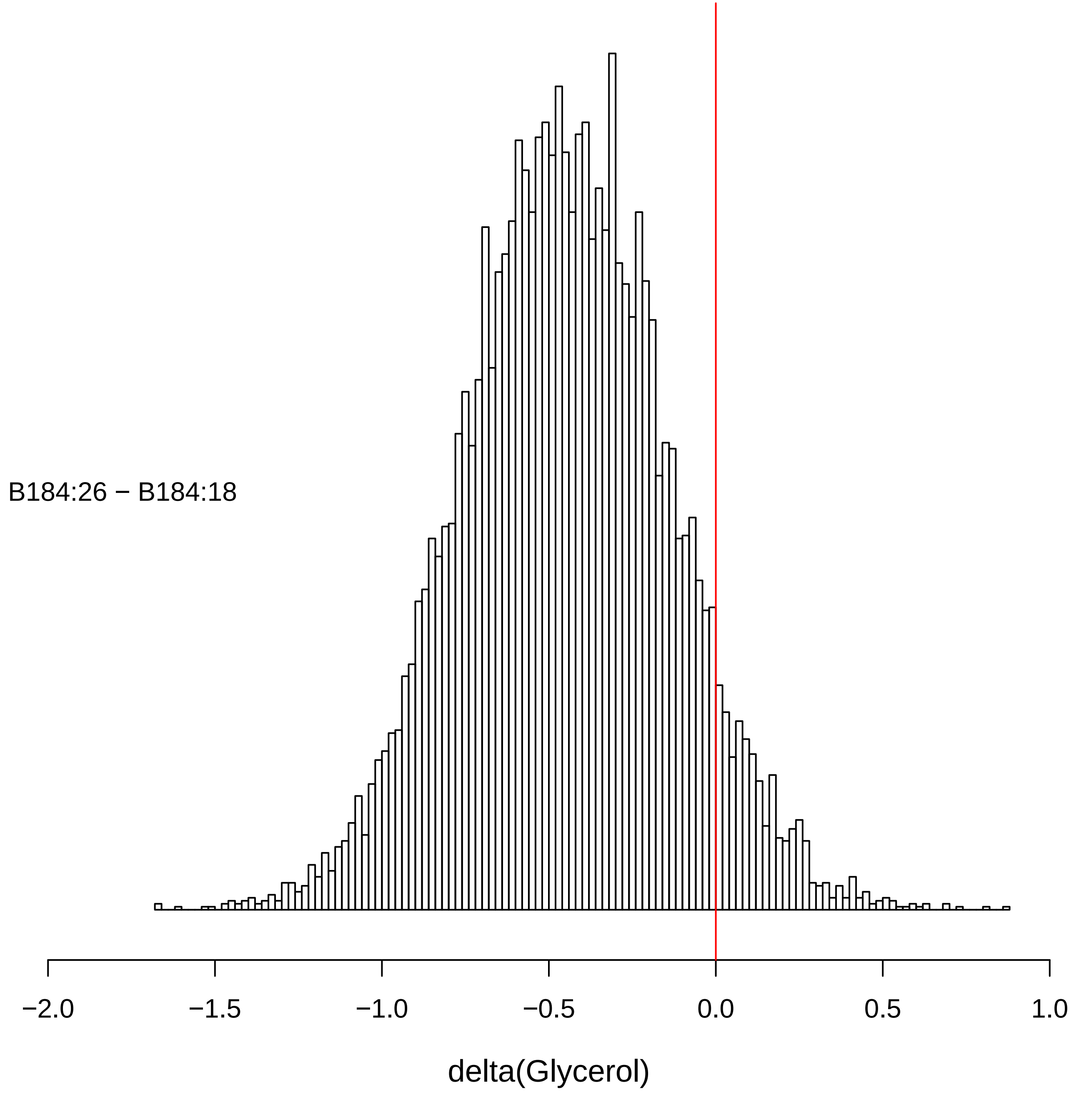
B184:26 − B184:18
−2.0
−1.5
−1.0
−0.5
0.0
0.5
1.0
delta(Glycerol)

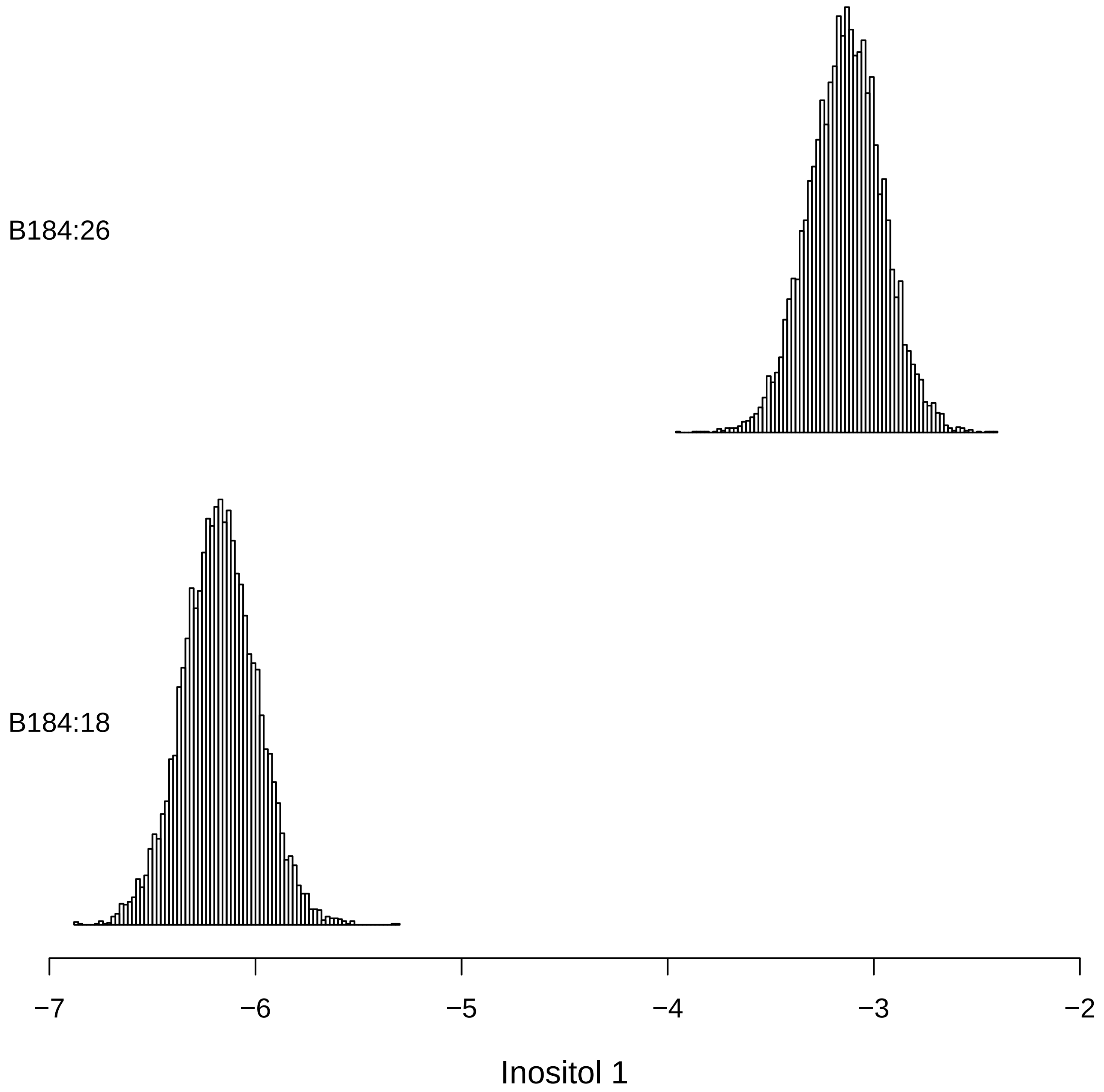
B184:26
B184:18
−7
−6
−5
−4
−3
−2
Inositol 1

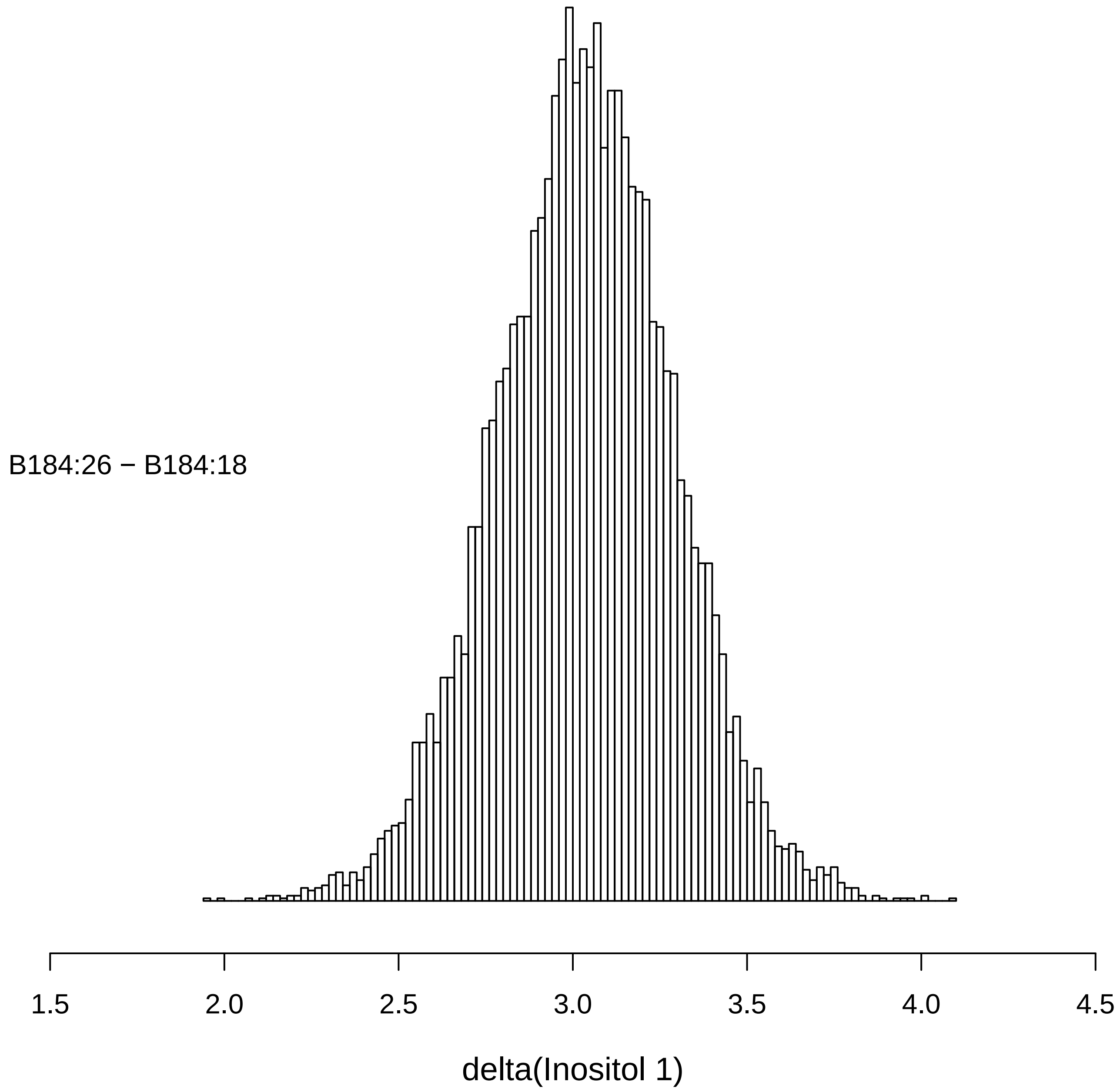
B184:26 − B184:18
1.5
2.0
2.5
3.0
3.5
4.0
4.5
delta(Inositol 1)

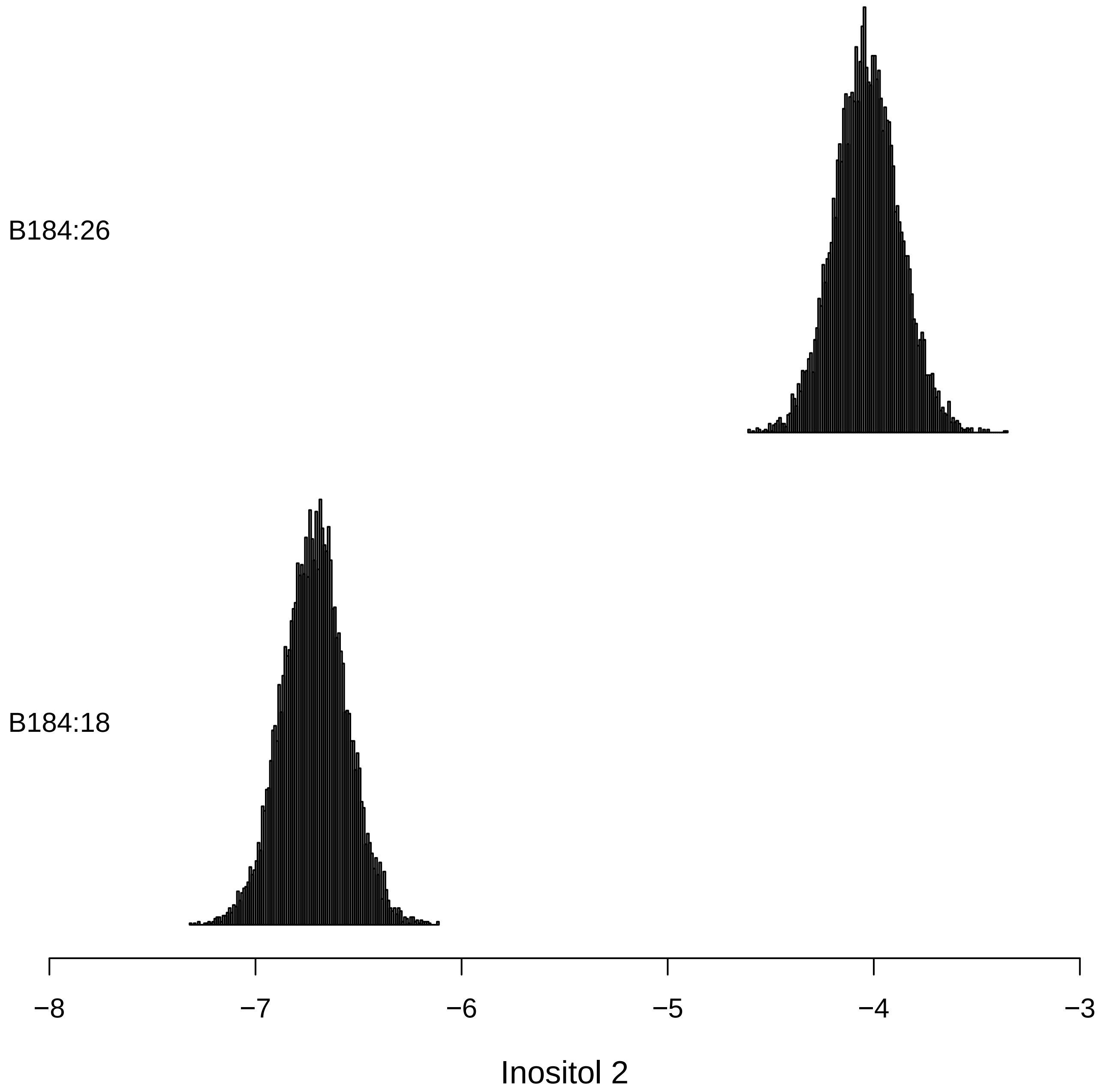
B184:26
B184:18
−8
−7
−6
−5
−4
−3
Inositol 2

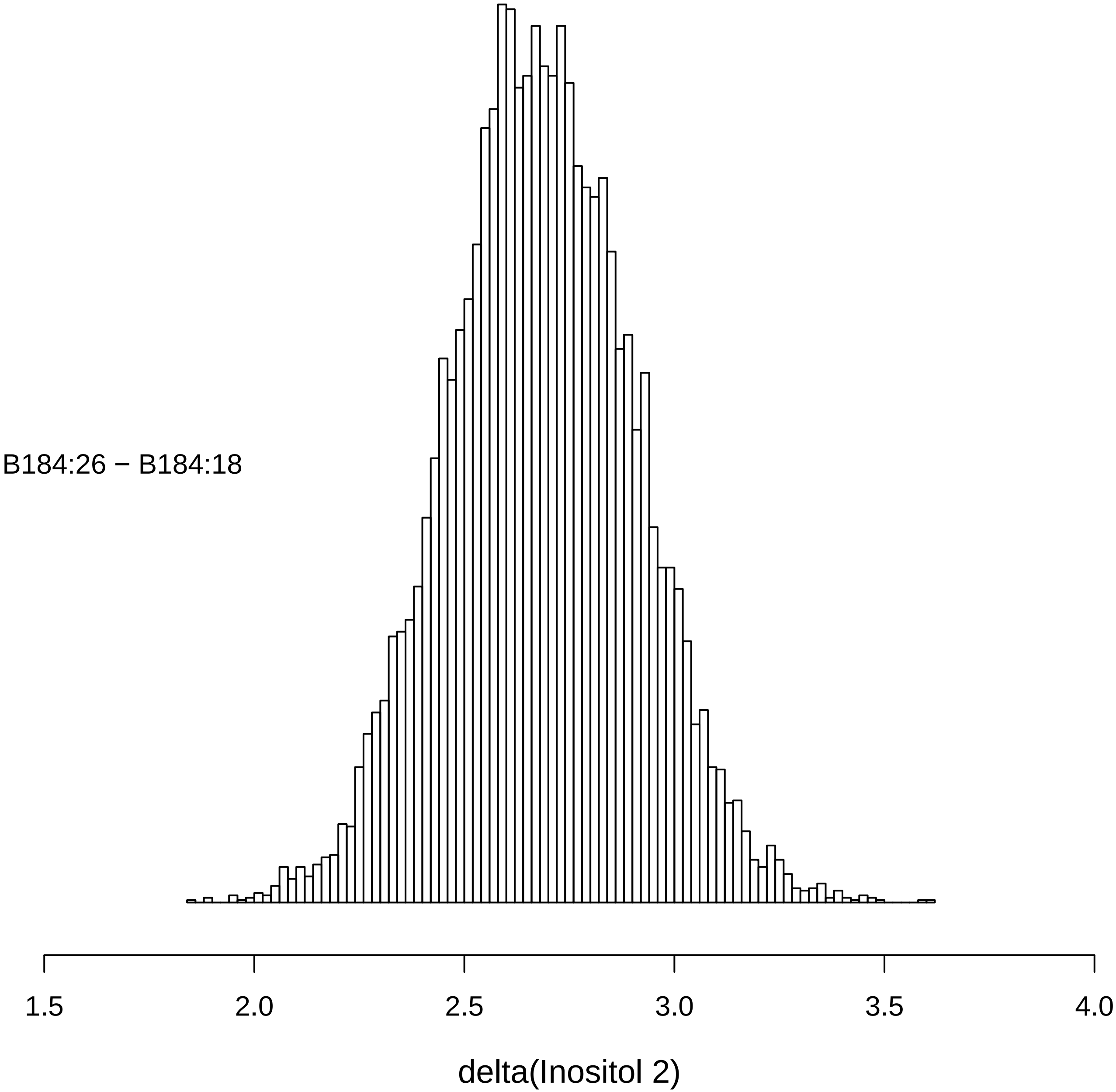

B184:26 − B184:18
1.5
2.0
2.5
3.0
3.5
4.0
delta(Inositol 2)

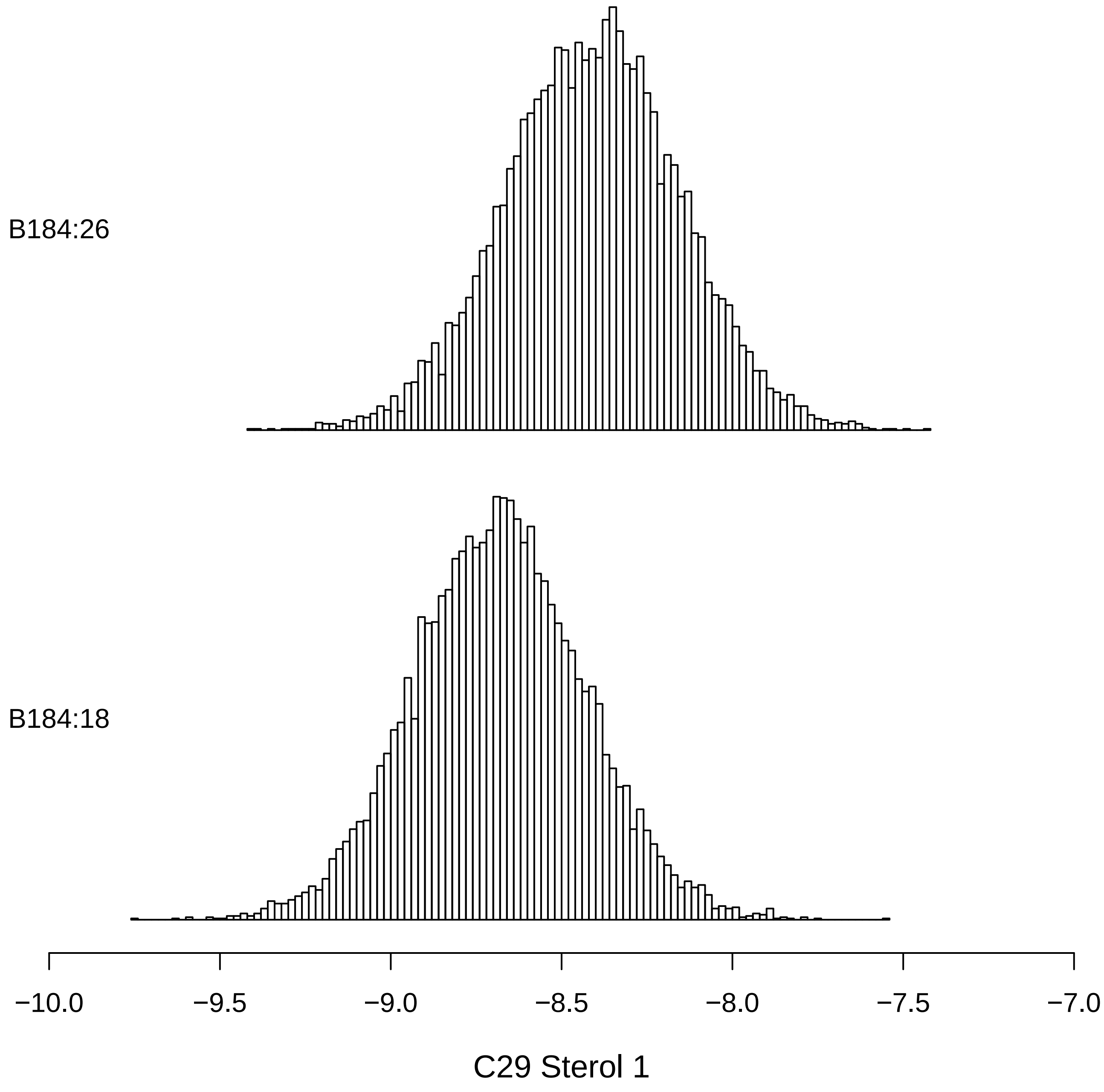
B184:26
B184:18
−10.0
−9.5
−9.0
−8.5
−8.0
−7.5
−7.0
C29 Sterol 1

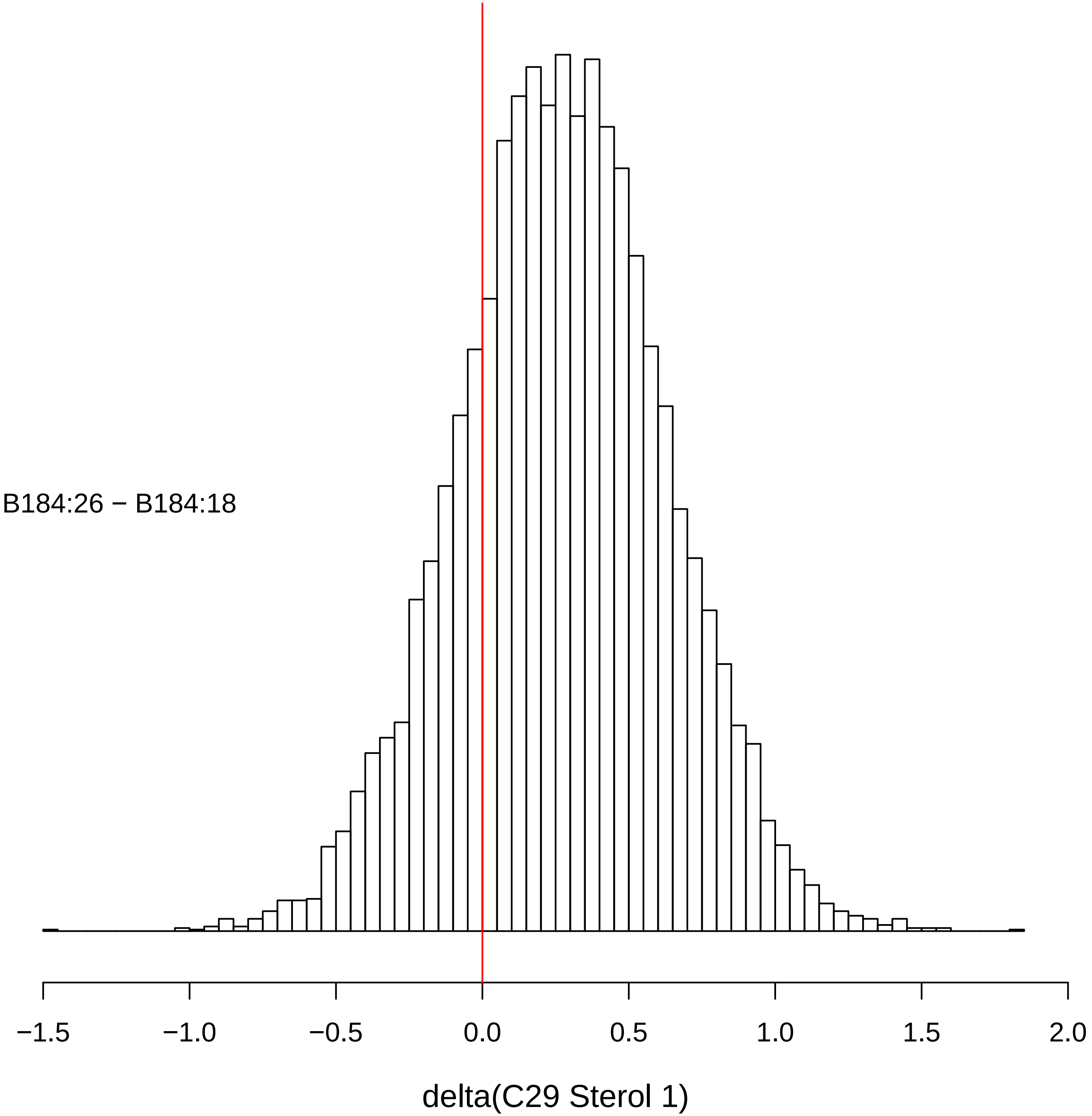
B184:26 − B184:18
−1.5
−1.0
−0.5
0.0
0.5
1.0
1.5
2.0
delta(C29 Sterol 1)

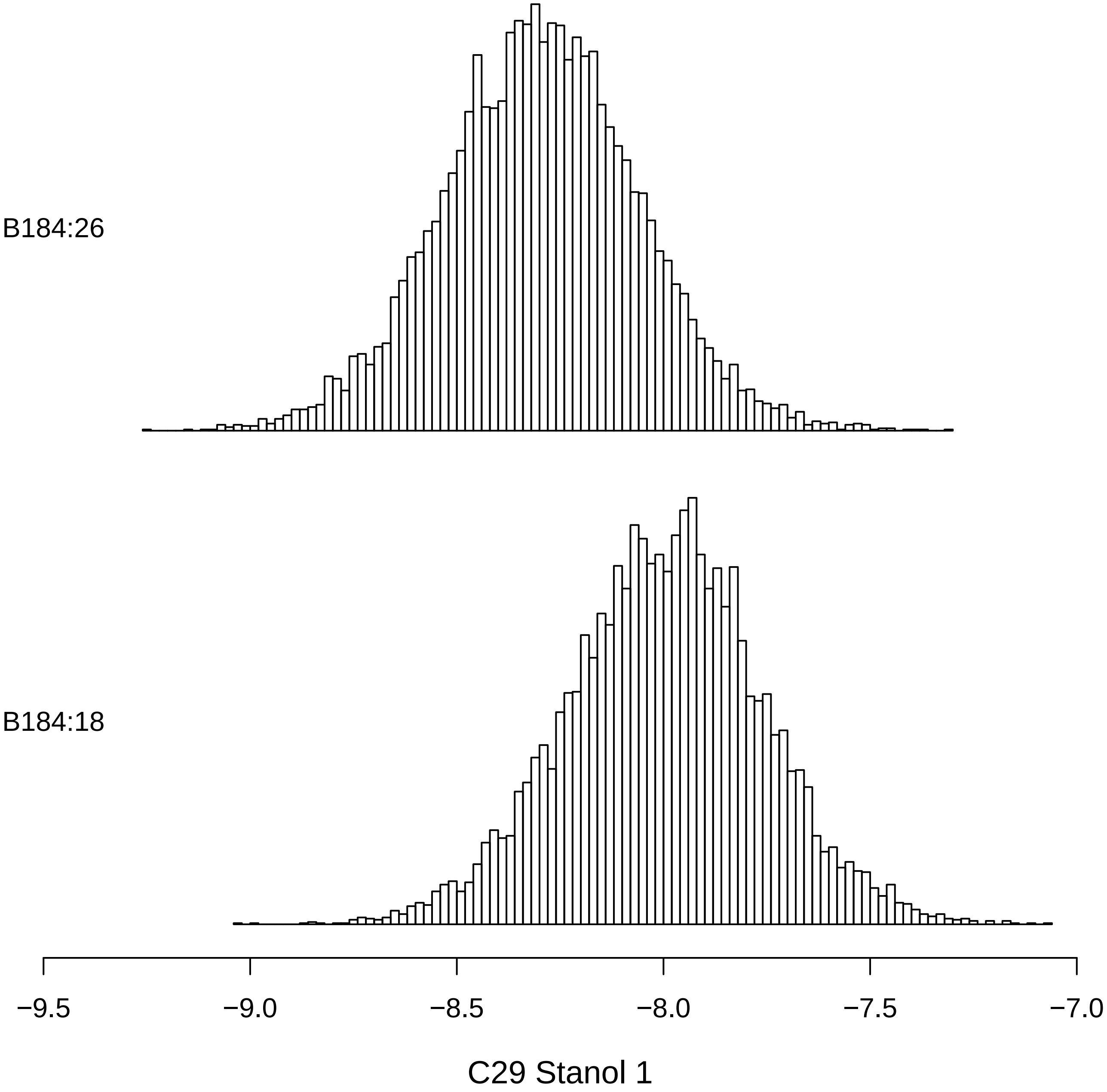

B184:26
B184:18
−9.5
−9.0
−8.5
−8.0
−7.5
−7.0
C29 Stanol 1

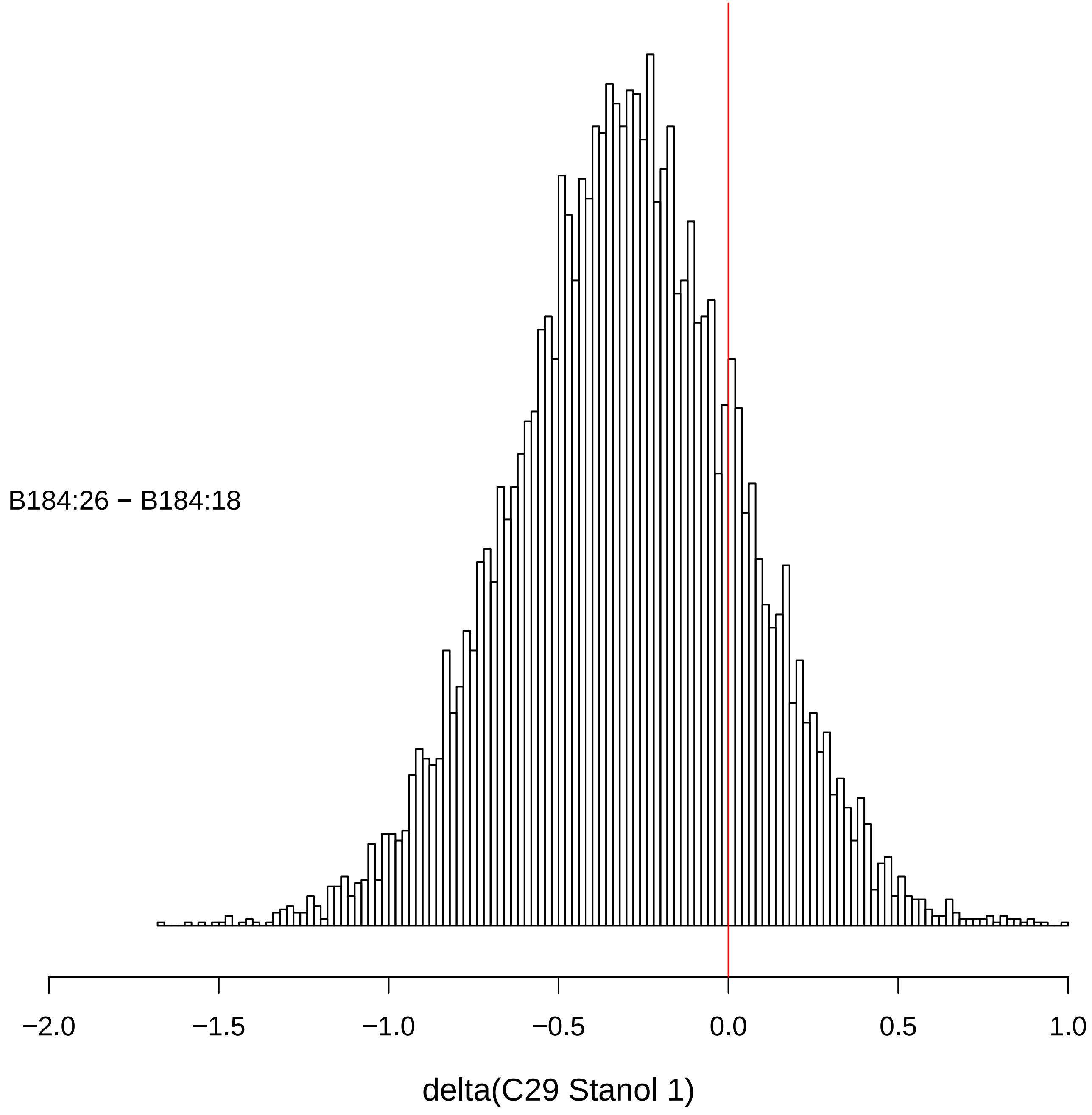
B184:26 − B184:18
−2.0
−1.5
−1.0
−0.5
0.0
0.5
1.0
delta(C29 Stanol 1)

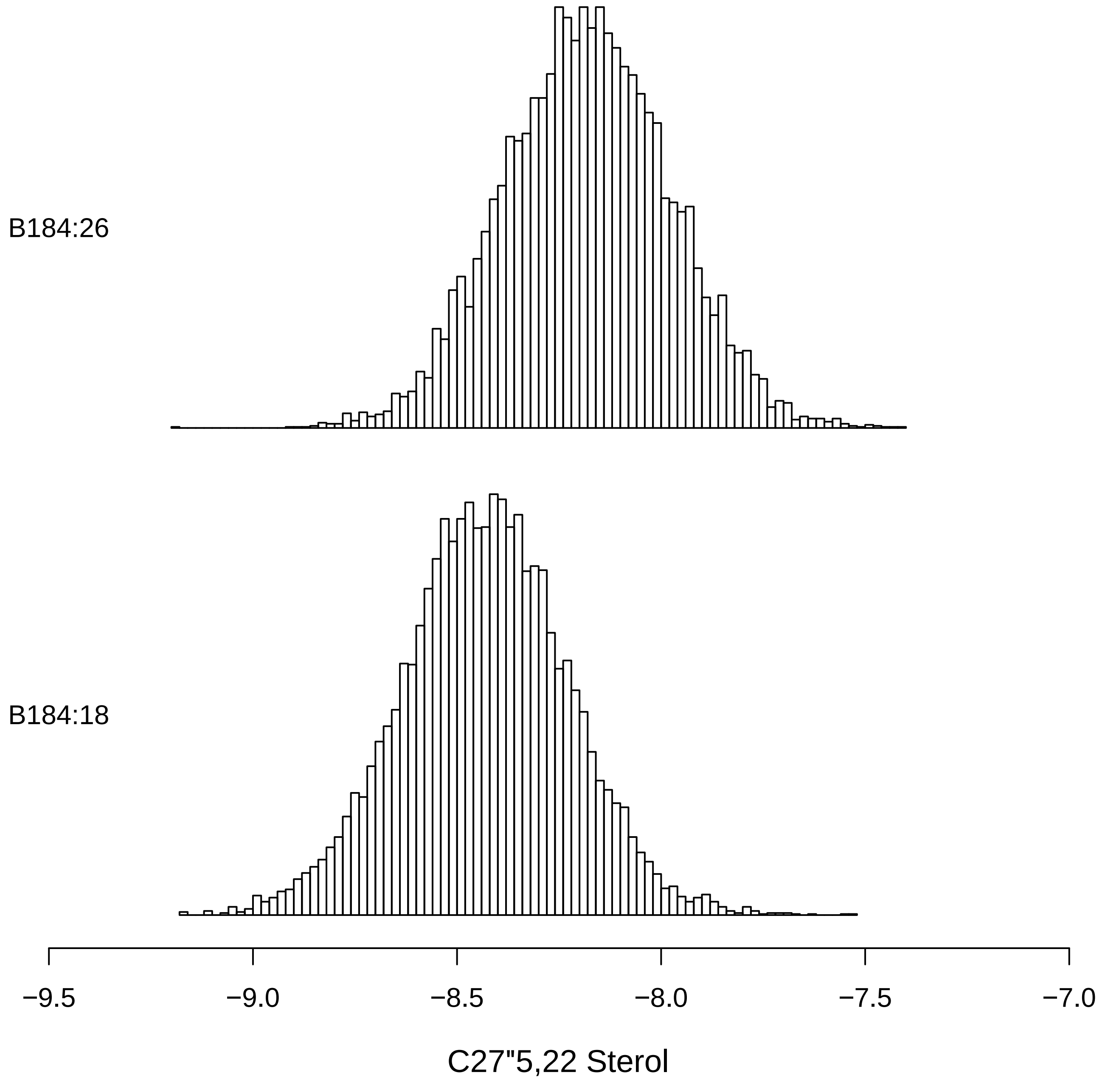
B184:26
B184:18
−9.5
−9.0
−8.5
−8.0
−7.5
−7.0
C27"5,22 Sterol

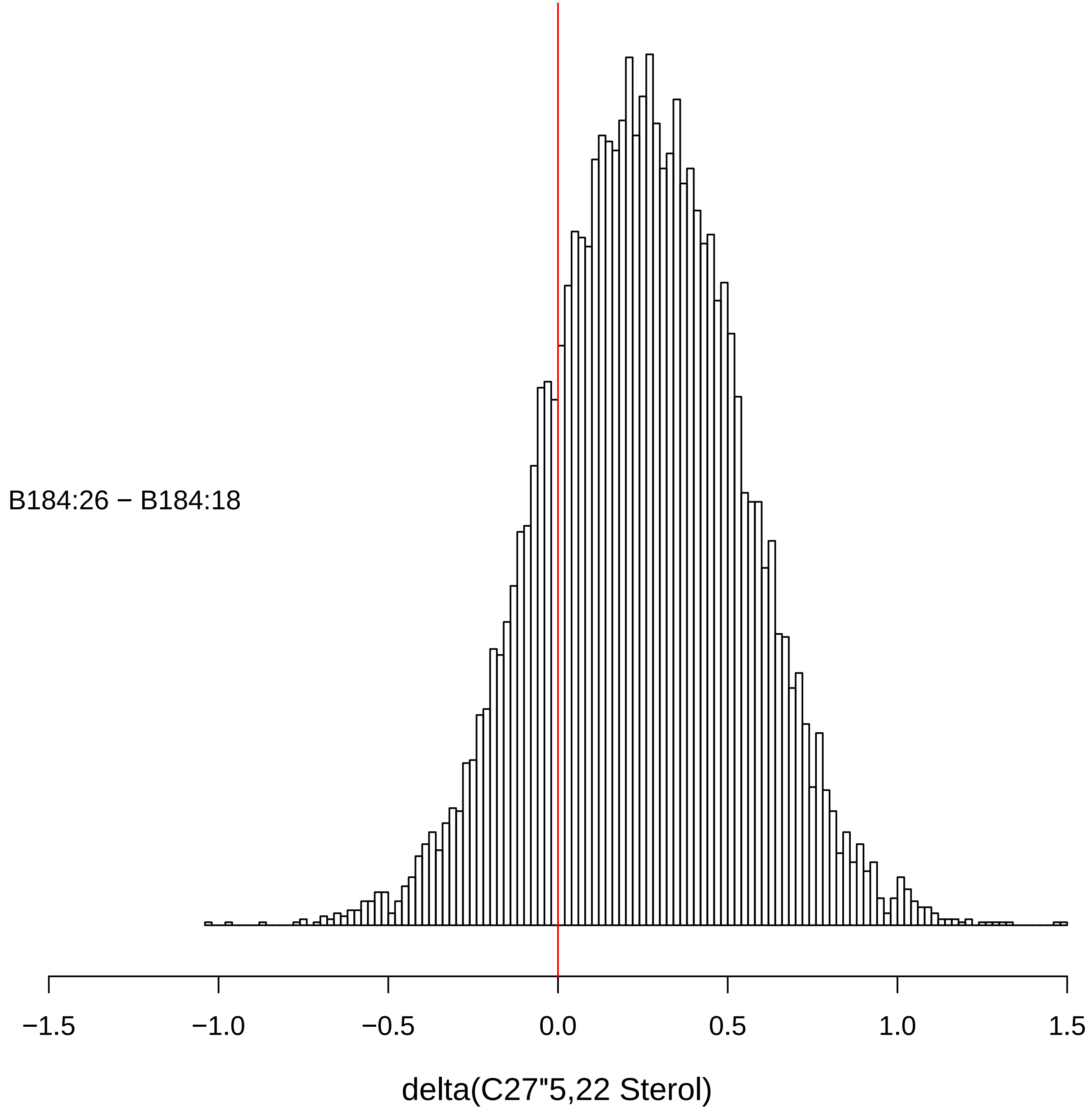
B184:26 − B184:18
−1.5
−1.0
−0.5
0.0
0.5
1.0
1.5
delta(C27"5,22 Sterol)

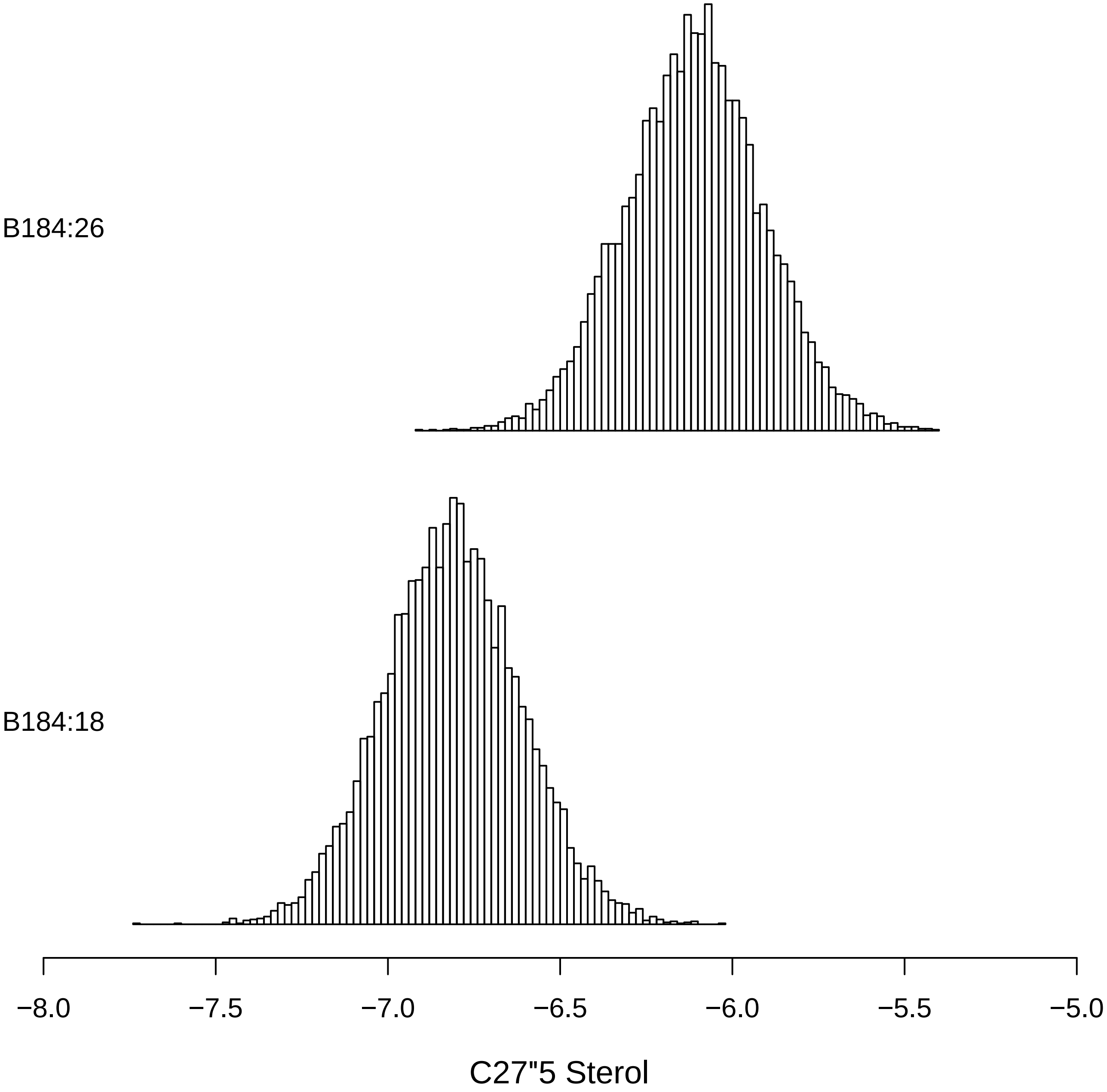
B184:26
B184:18
−8.0
−7.5
−7.0
−6.5
−6.0
−5.5
−5.0
C27"5 Sterol

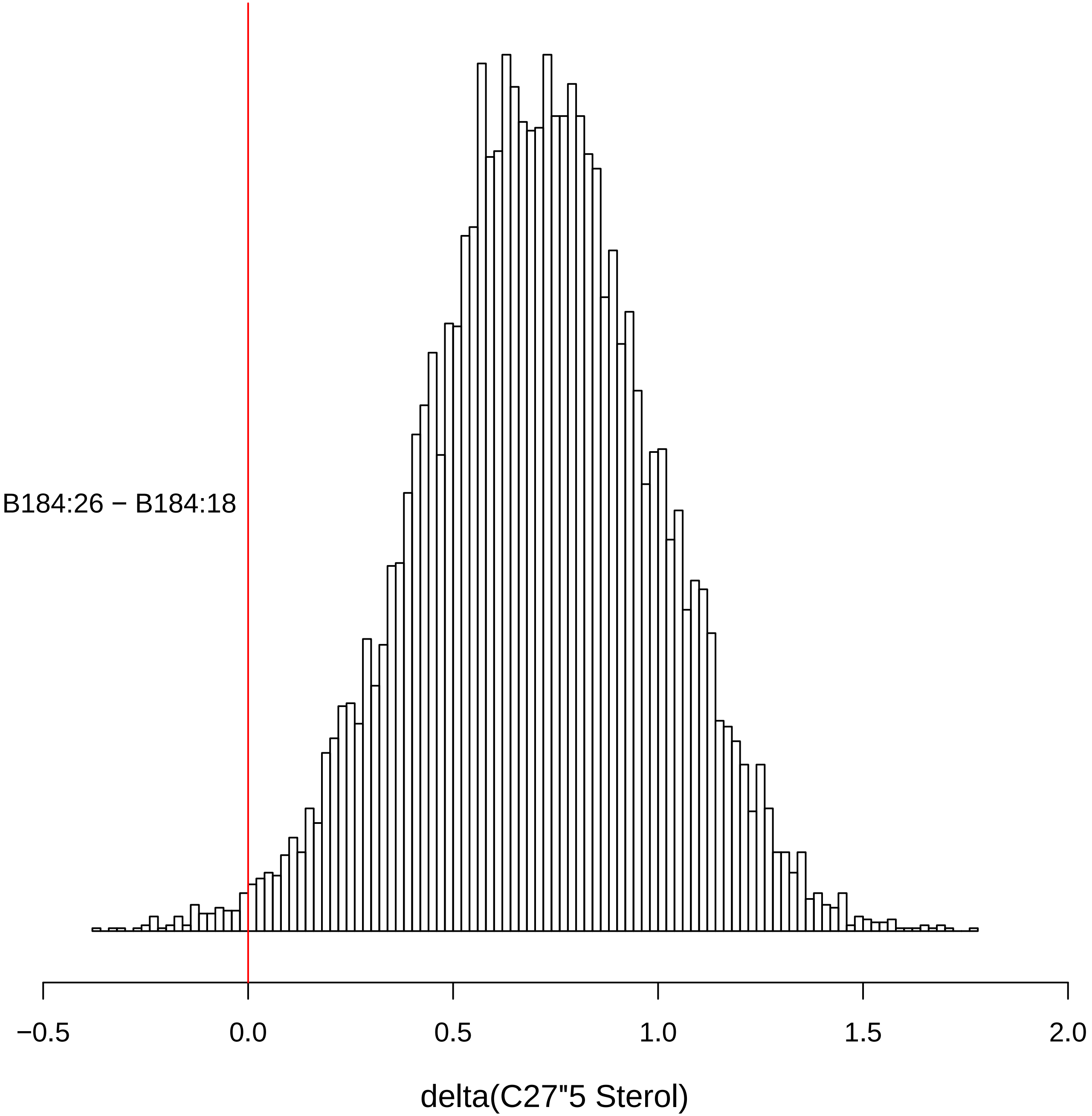

B184:26 − B184:18
−0.5
0.0
0.5
1.0
1.5
2.0
delta(C27"5 Sterol)

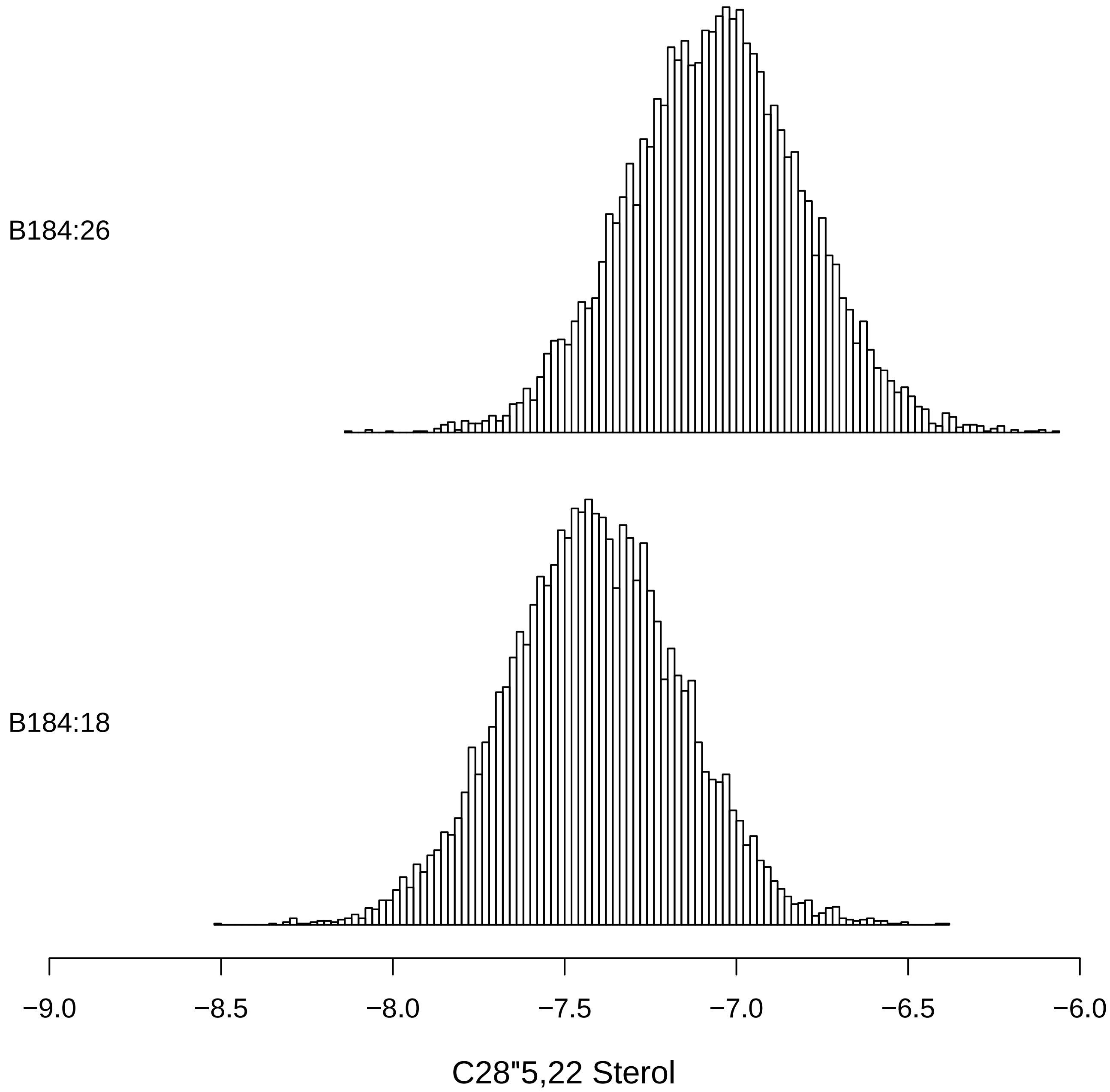
B184:26
B184:18
−9.0
−8.5
−8.0
−7.5
−7.0
−6.5
−6.0
C28"5,22 Sterol

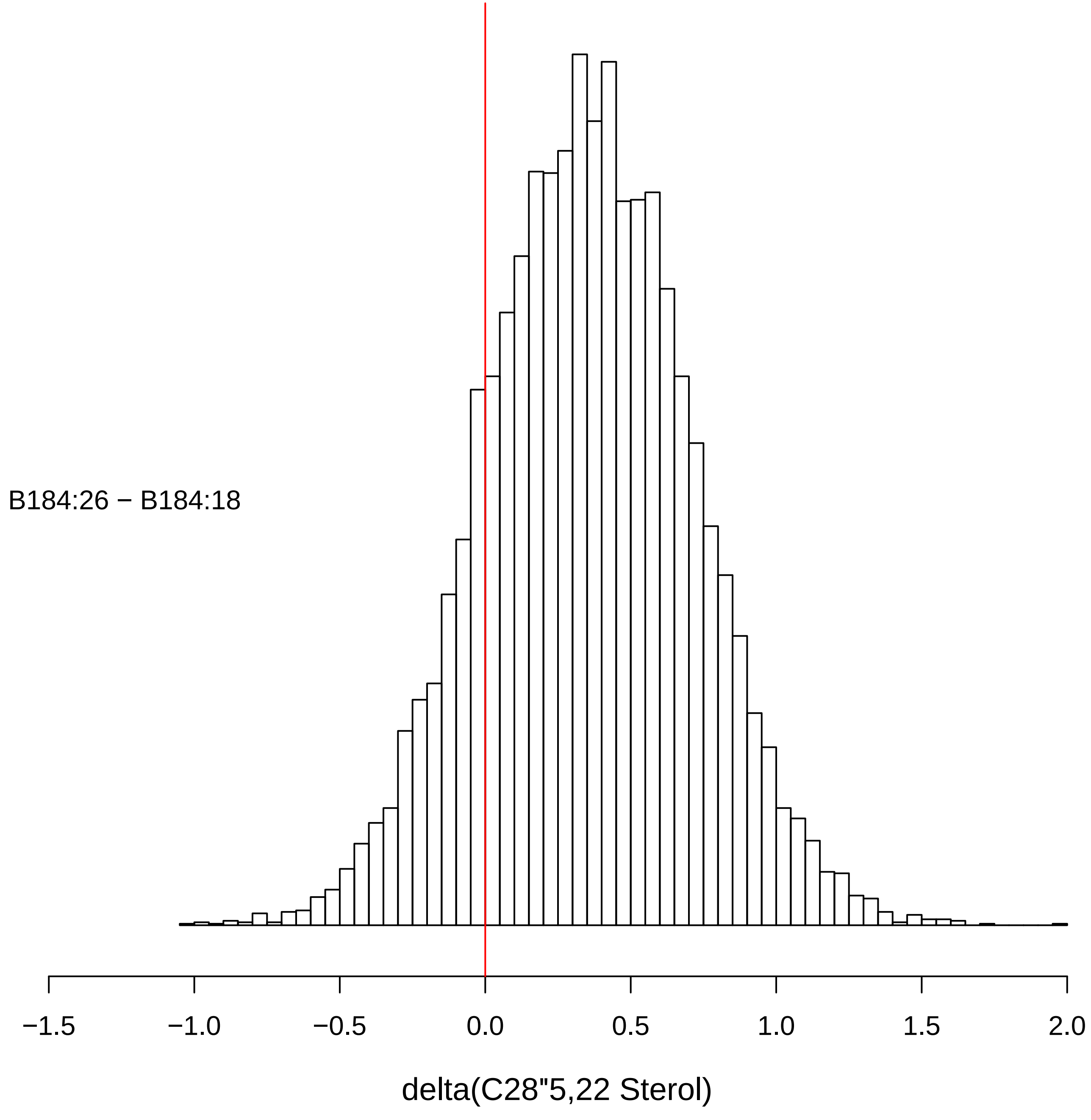
B184:26 − B184:18
−1.5
−1.0
−0.5
0.0
0.5
1.0
1.5
2.0
delta(C28"5,22 Sterol)

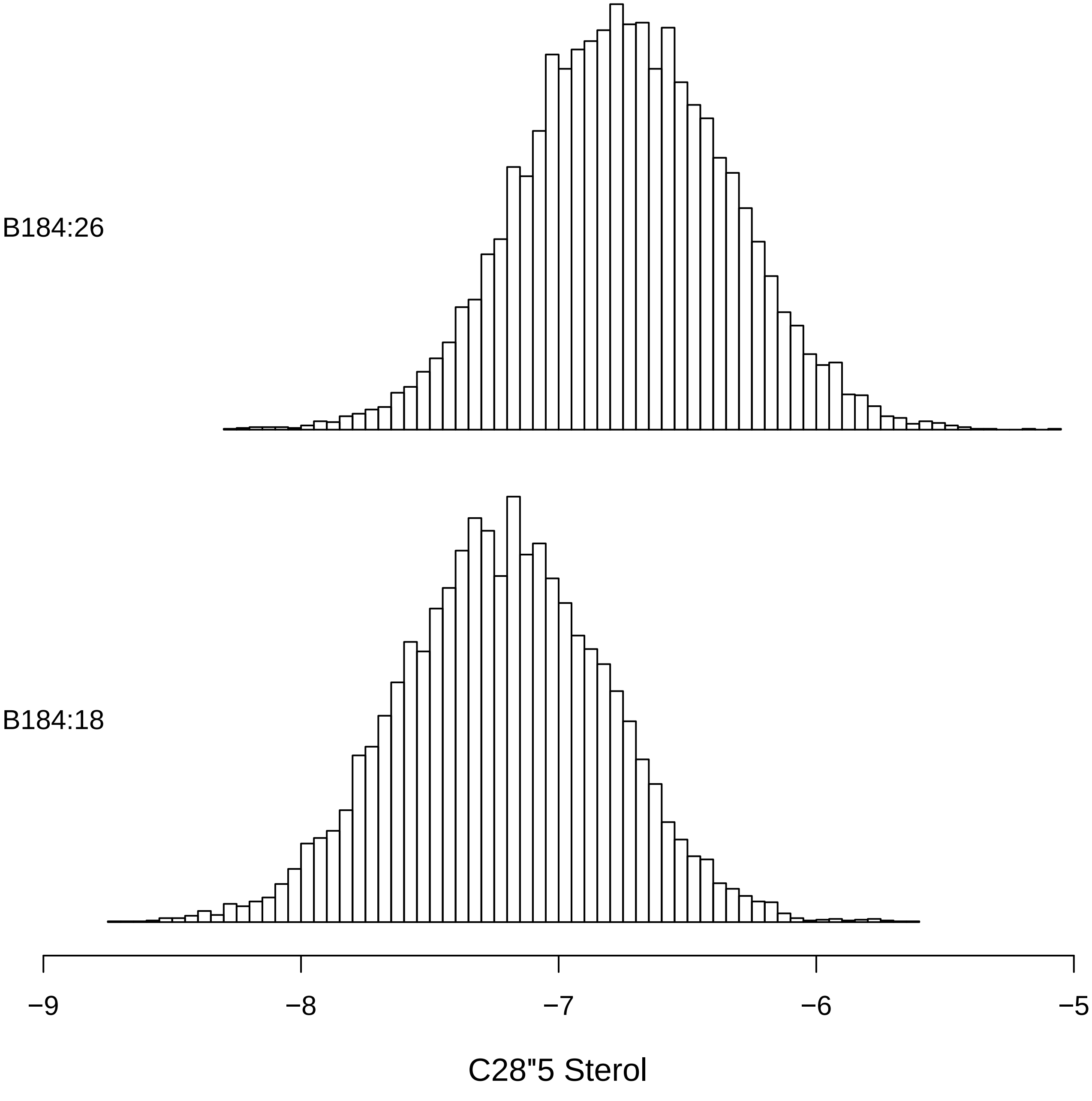

B184:26
B184:18
−9
−8
−7
−6
−5
C28"5 Sterol

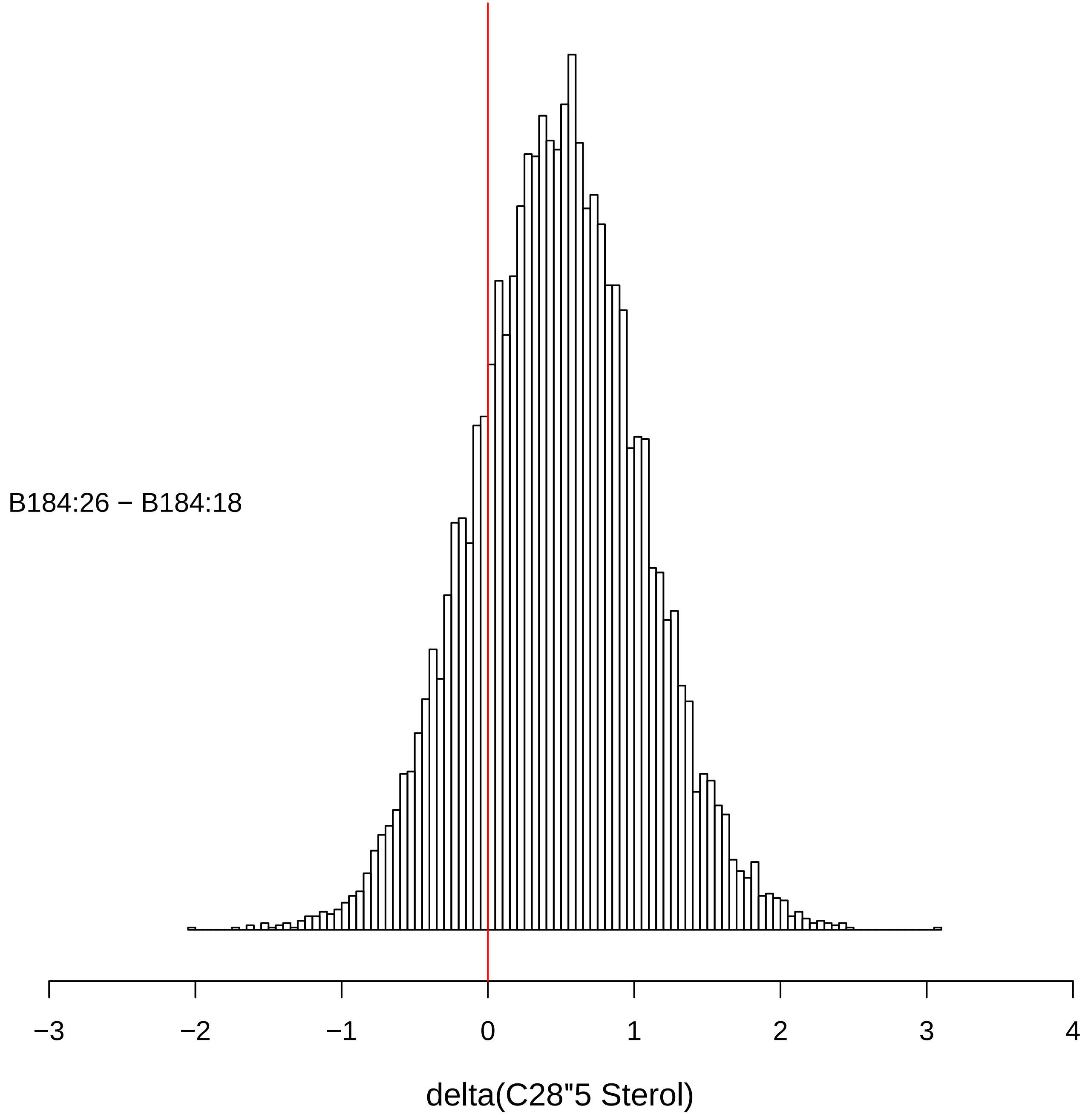

B184:26 − B184:18
−3
−2
−1
0
1
2
3
4
delta(C28"5 Sterol)

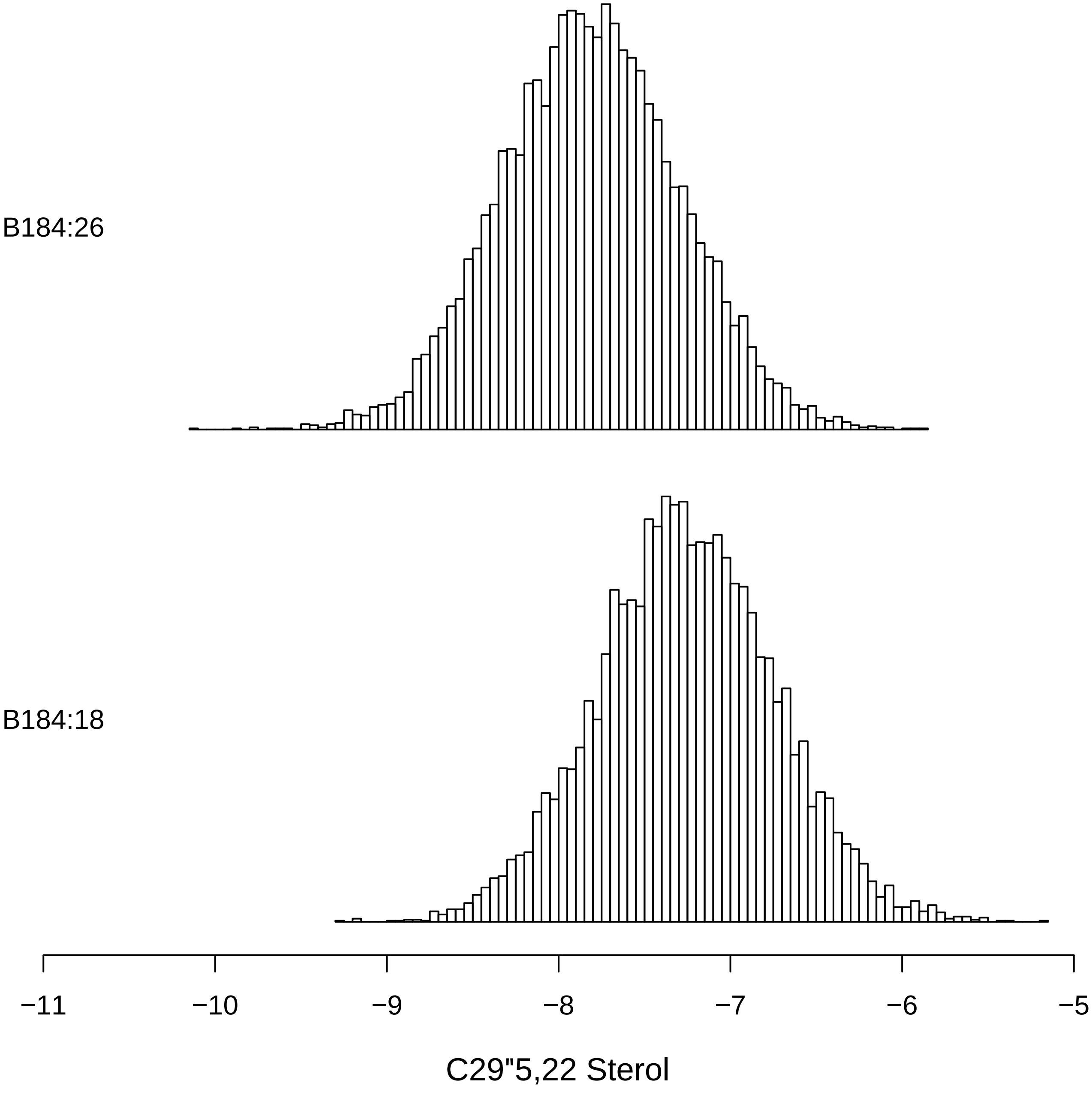
B184:26
B184:18
−11
−10
−9
−8
−7
−6
−5
C29"5,22 Sterol

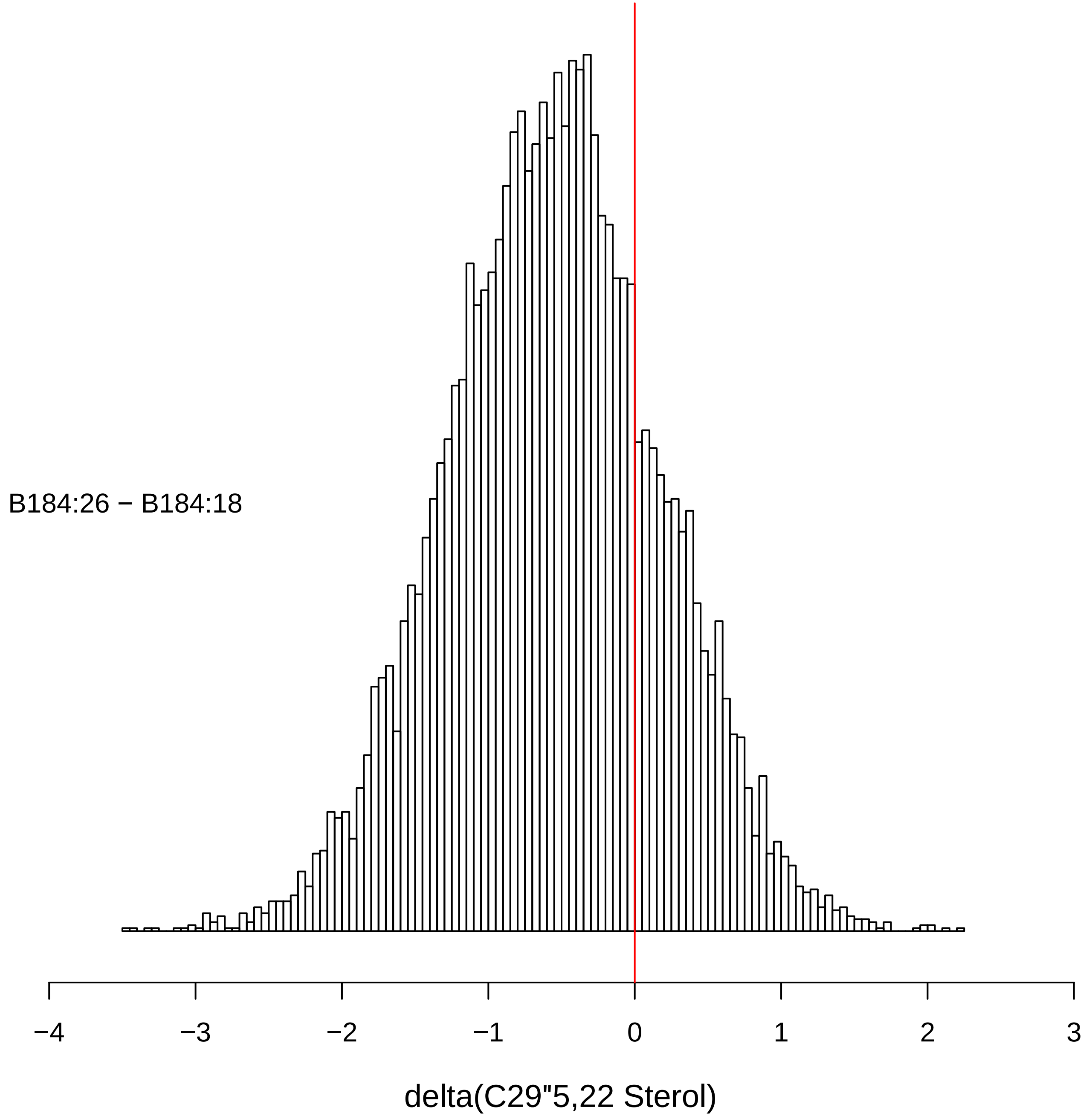
B184:26 − B184:18
−4
−3
−2
−1
0
1
2
3
delta(C29"5,22 Sterol)

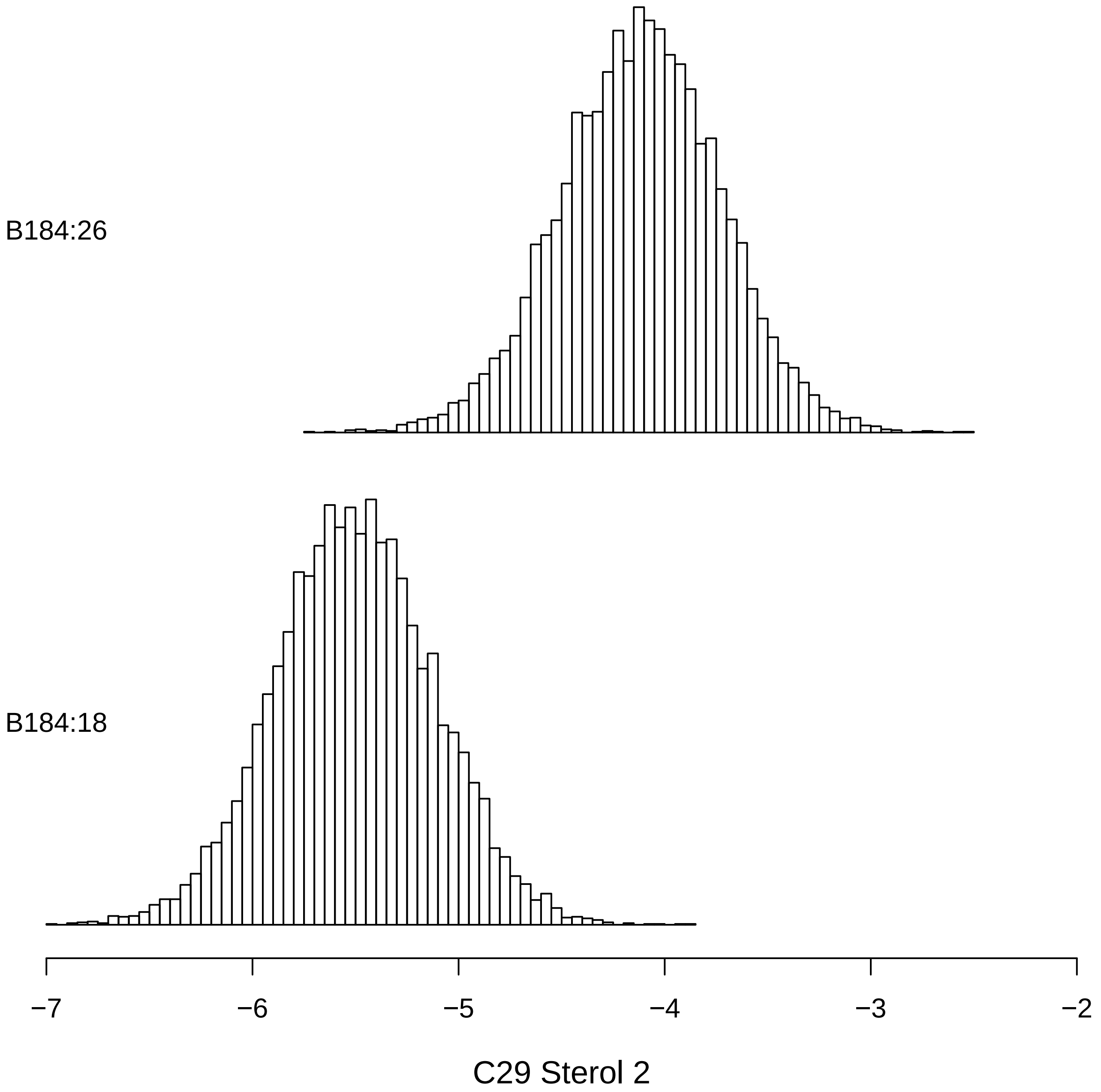

B184:26
B184:18
−7
−6
−5
−4
−3
−2
C29 Sterol 2

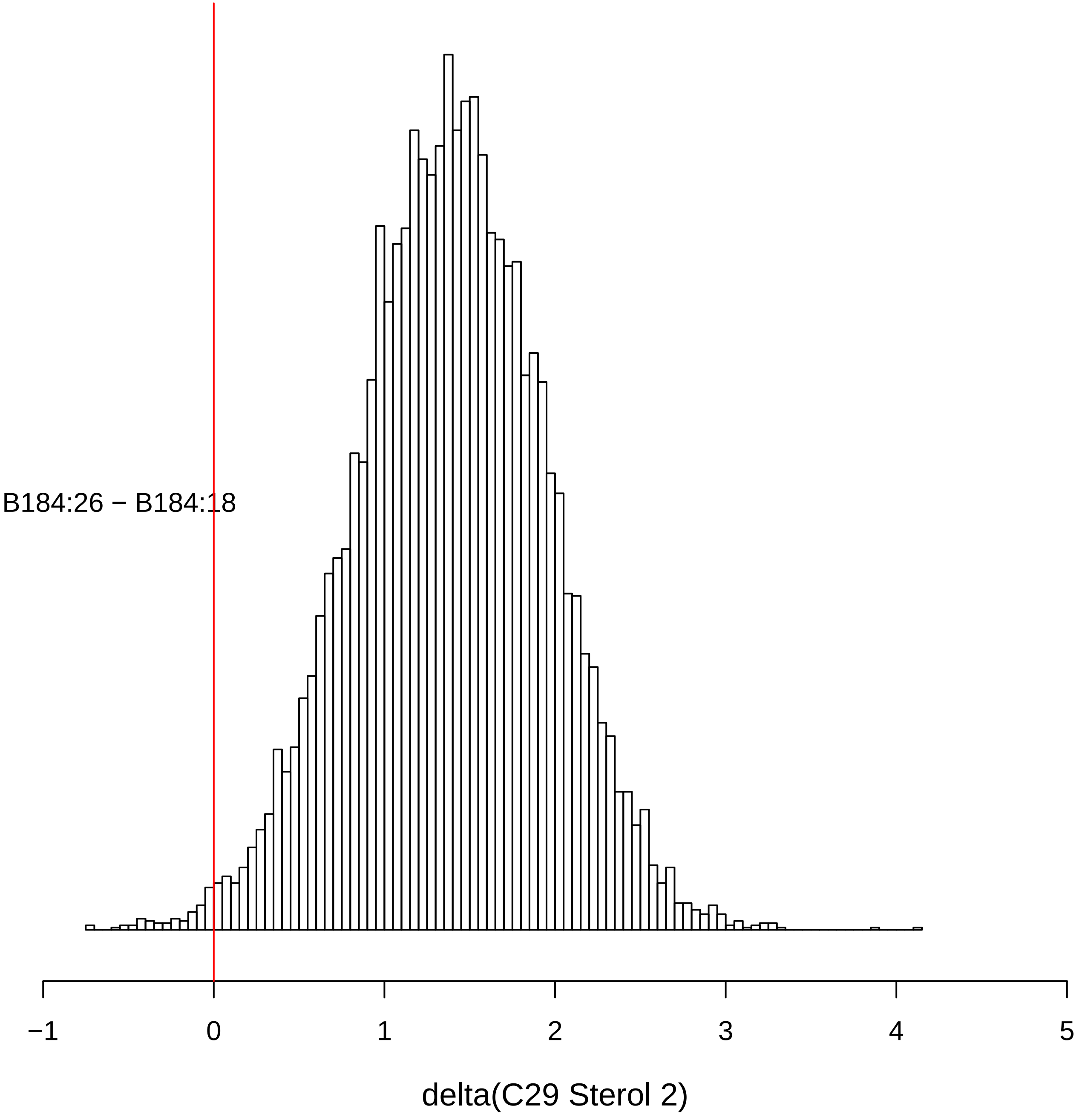
B184:26 − B184:18
−1
0
1
2
3
4
5
delta(C29 Sterol 2)

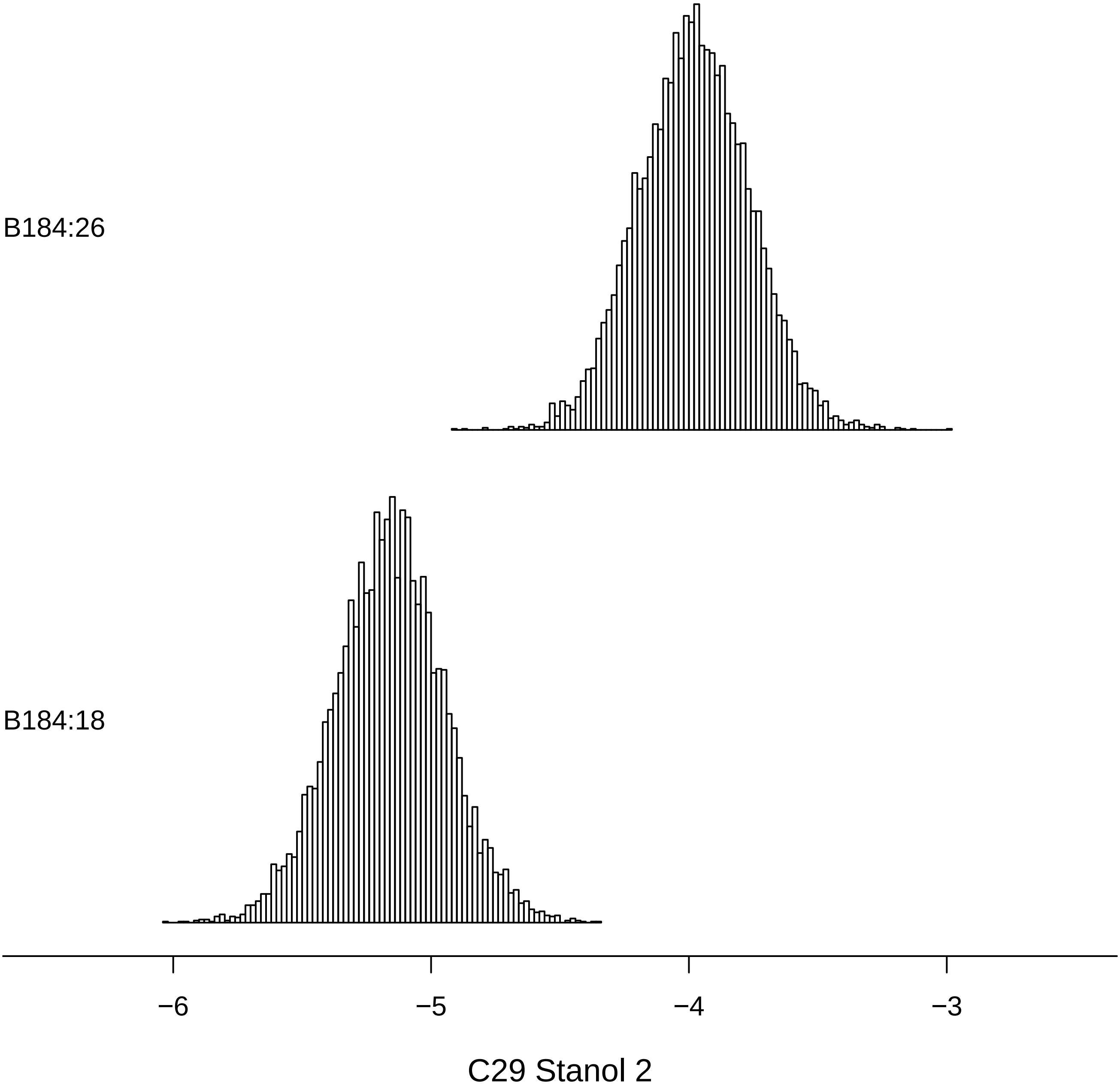

B184:26
B184:18
−6
−5
−4
−3
C29 Stanol 2

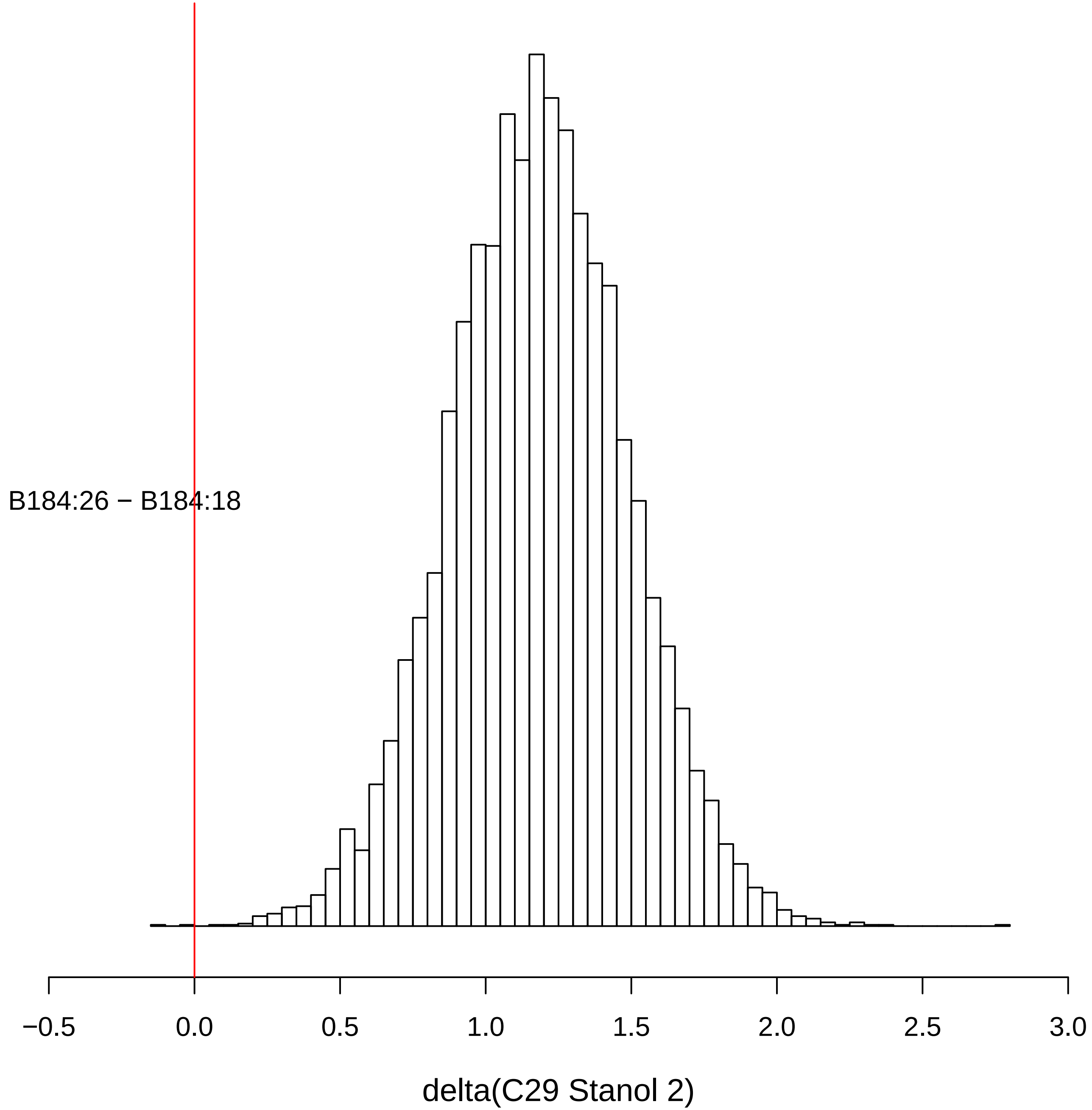
B184:26 − B184:18
−0.5
0.0
0.5
1.0
1.5
2.0
2.5
3.0
delta(C29 Stanol 2)

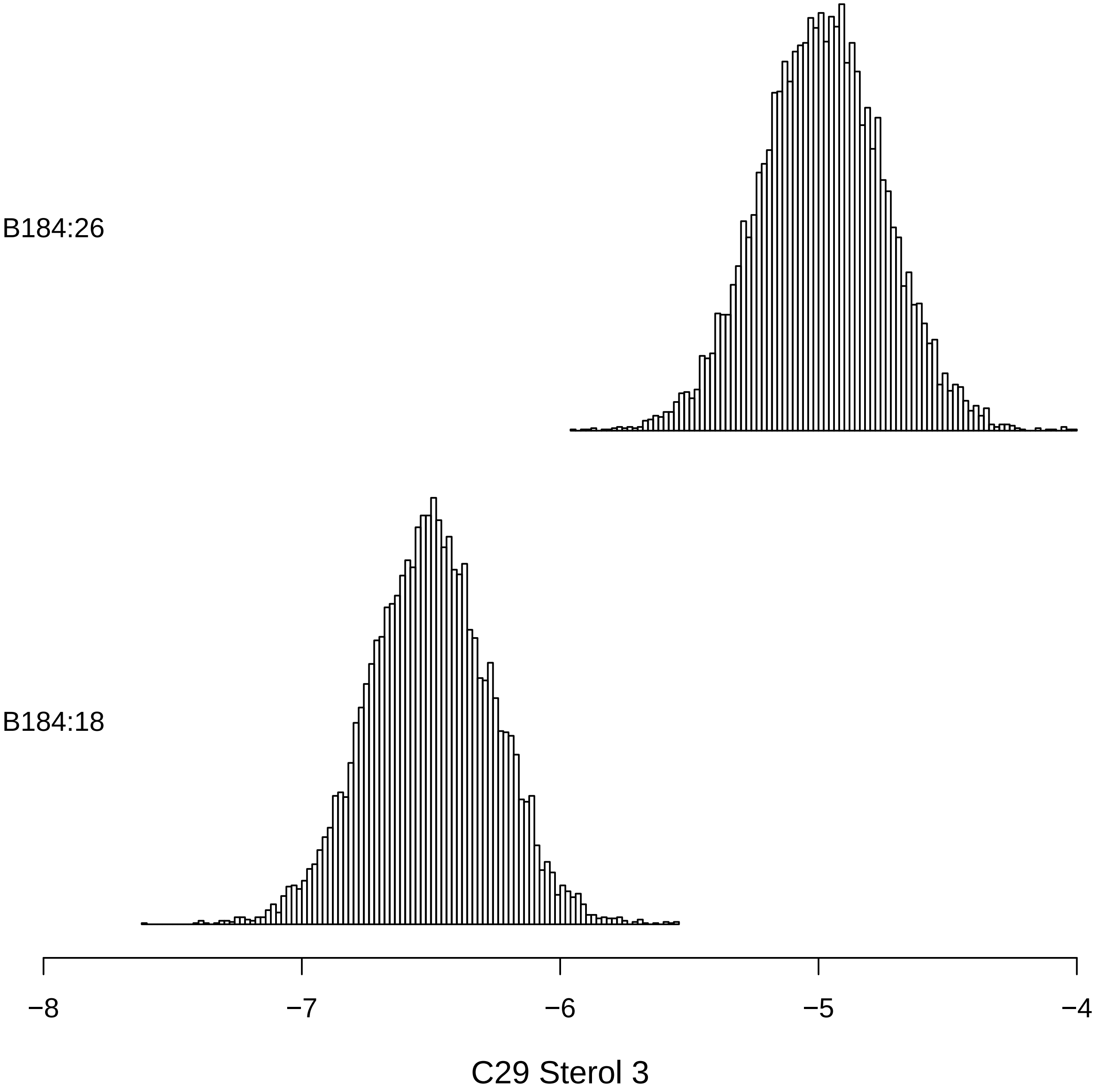

B184:26
B184:18
−8
−7
−6
−5
−4
C29 Sterol 3

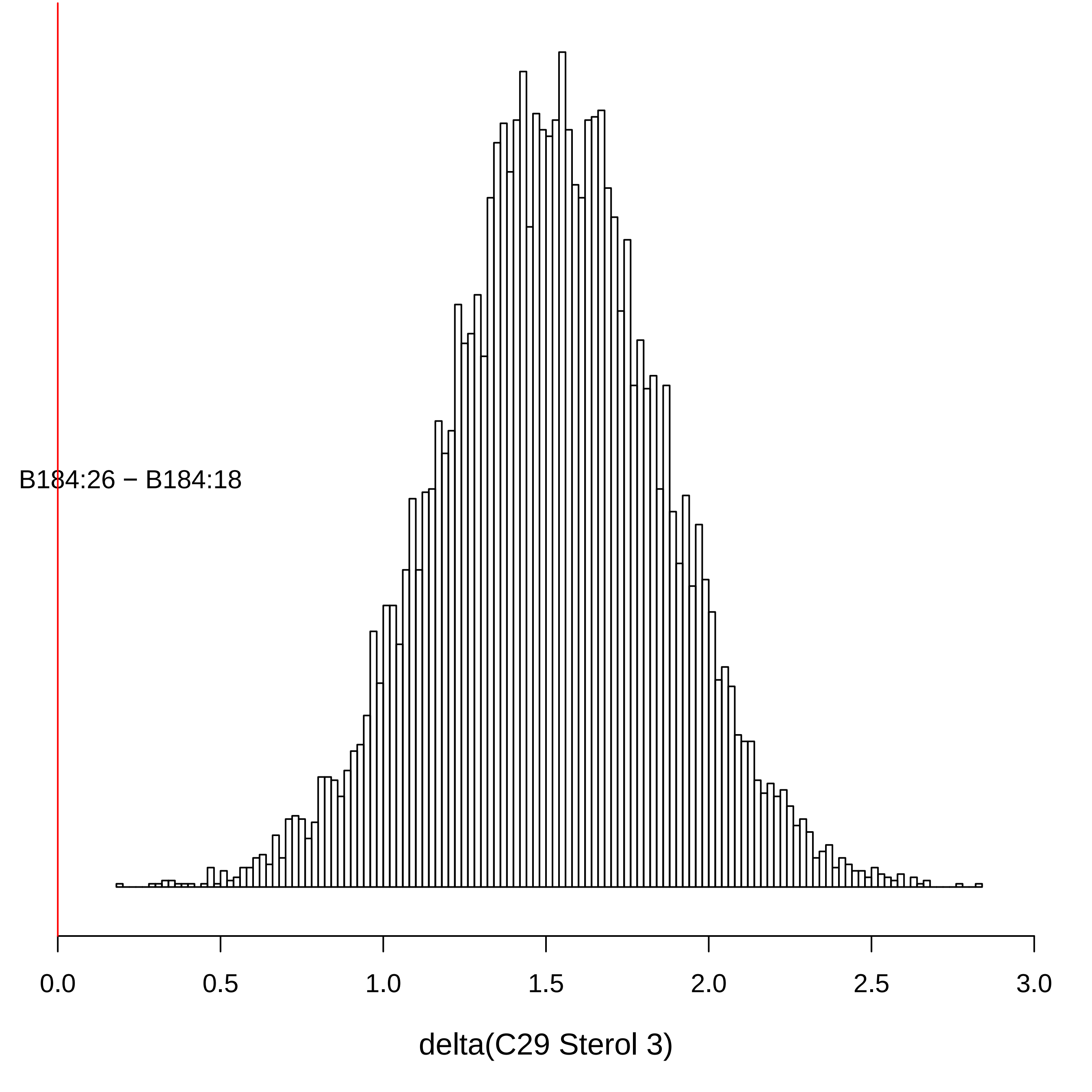
B184:26 − B184:18
0.0
0.5
1.0
1.5
2.0
2.5
3.0
delta(C29 Sterol 3)

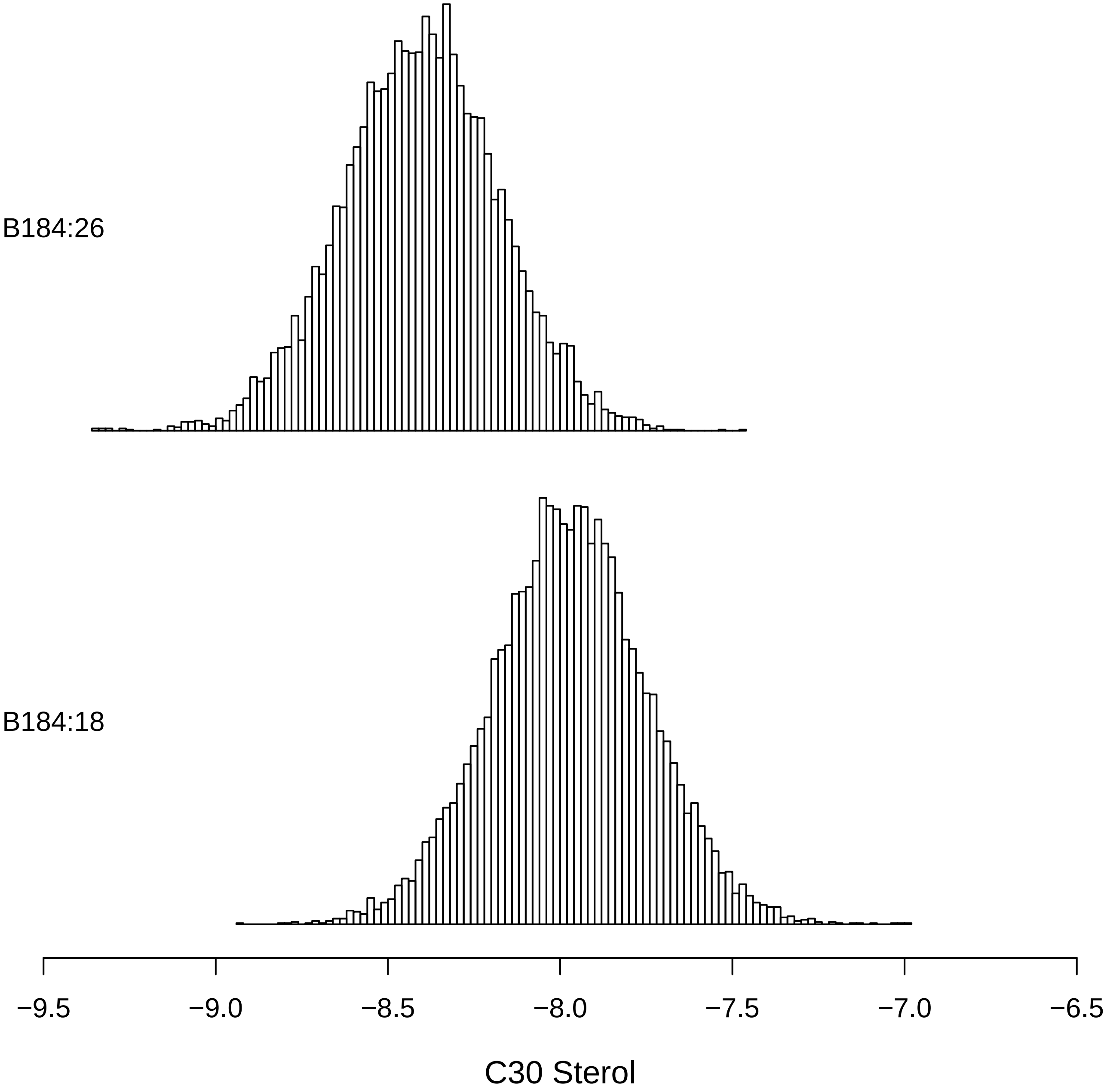
B184:26
B184:18
−9.5
−9.0
−8.5
−8.0
−7.5
−7.0
−6.5
C30 Sterol

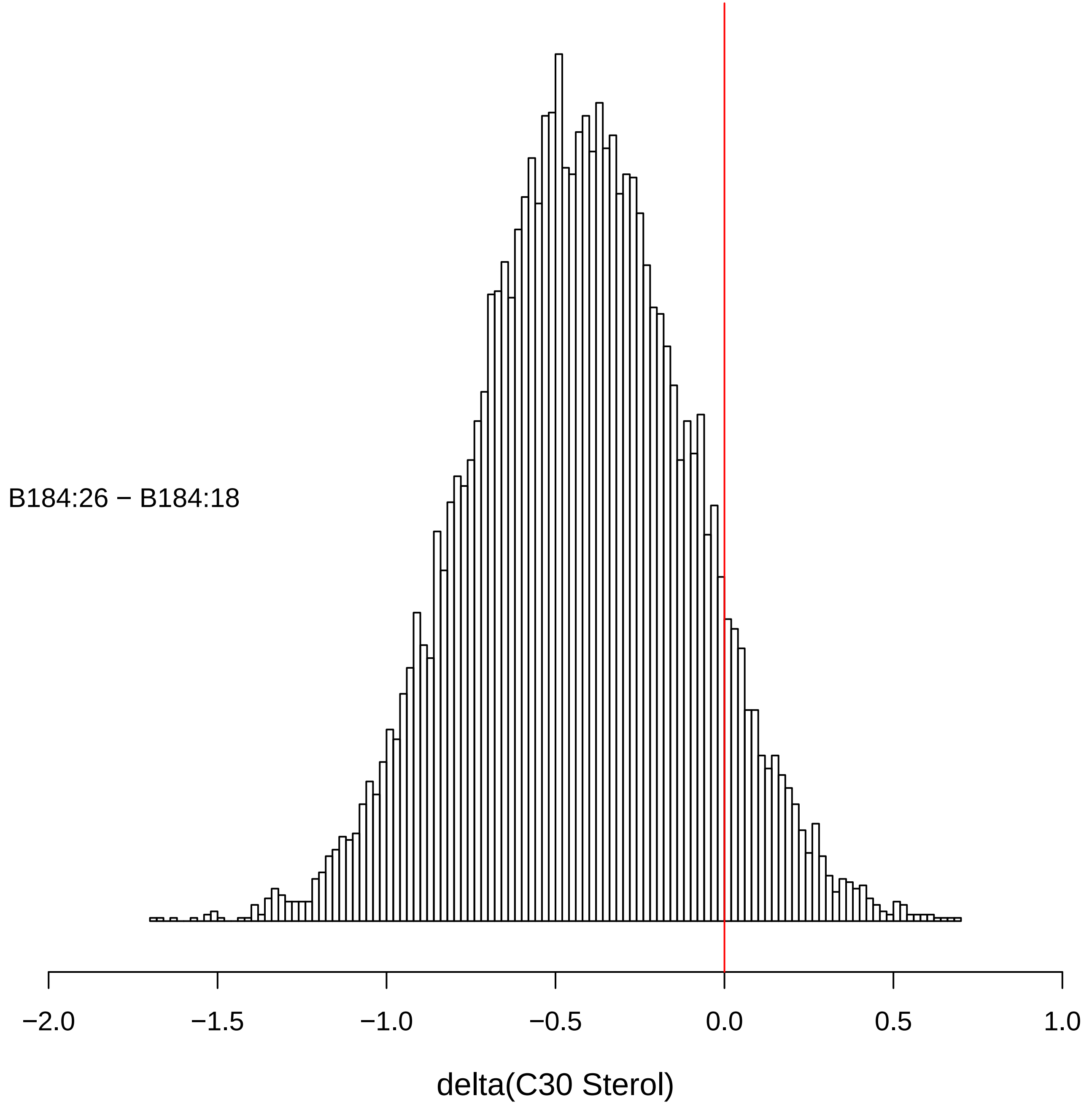

B184:26 − B184:18
−2.0
−1.5
−1.0
−0.5
0.0
0.5
1.0
delta(C30 Sterol)

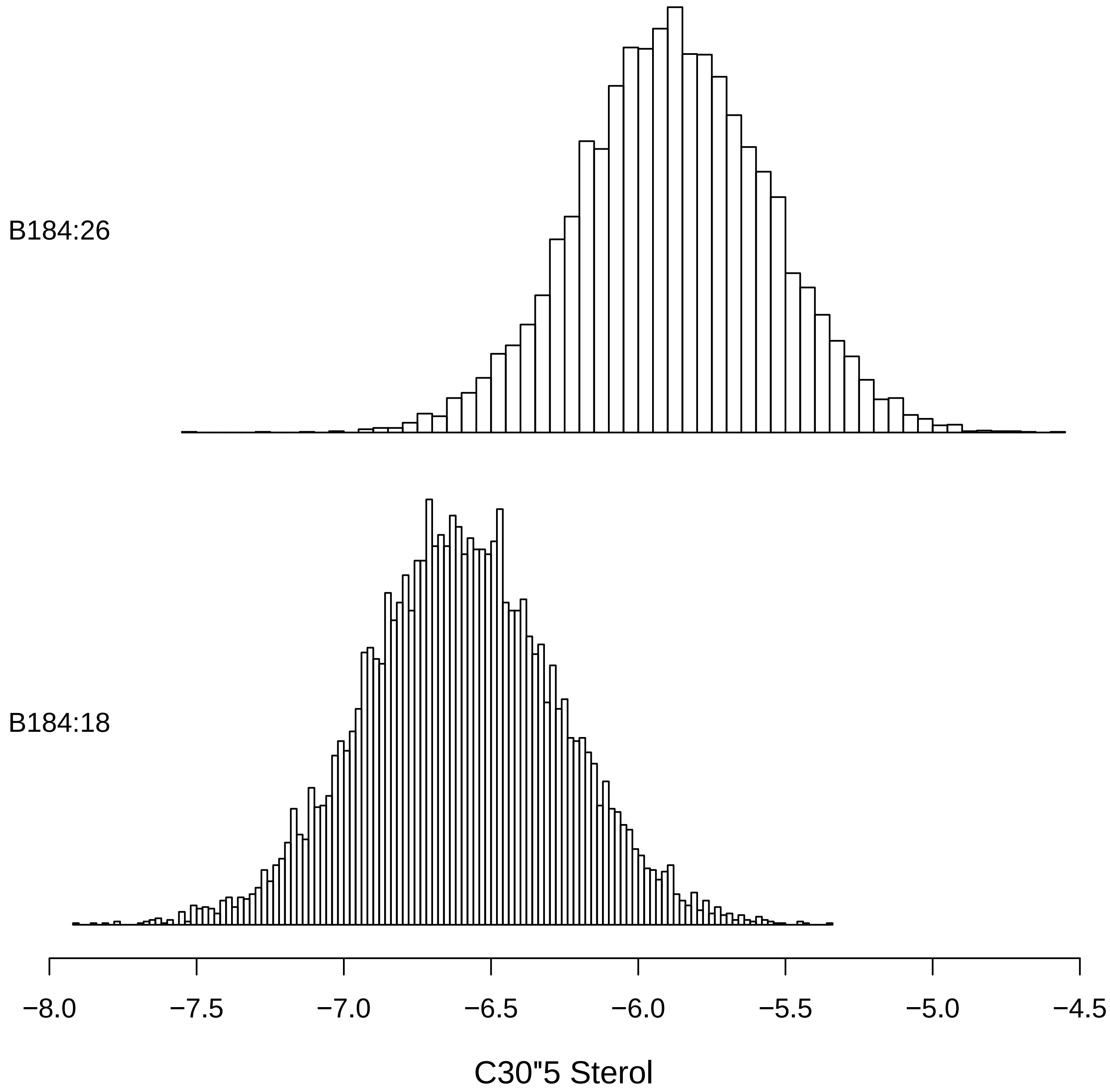
B184:26
B184:18
−8.0
−7.5
−7.0
−6.5
−6.0
−5.5
−5.0
−4.5
C30"5 Sterol

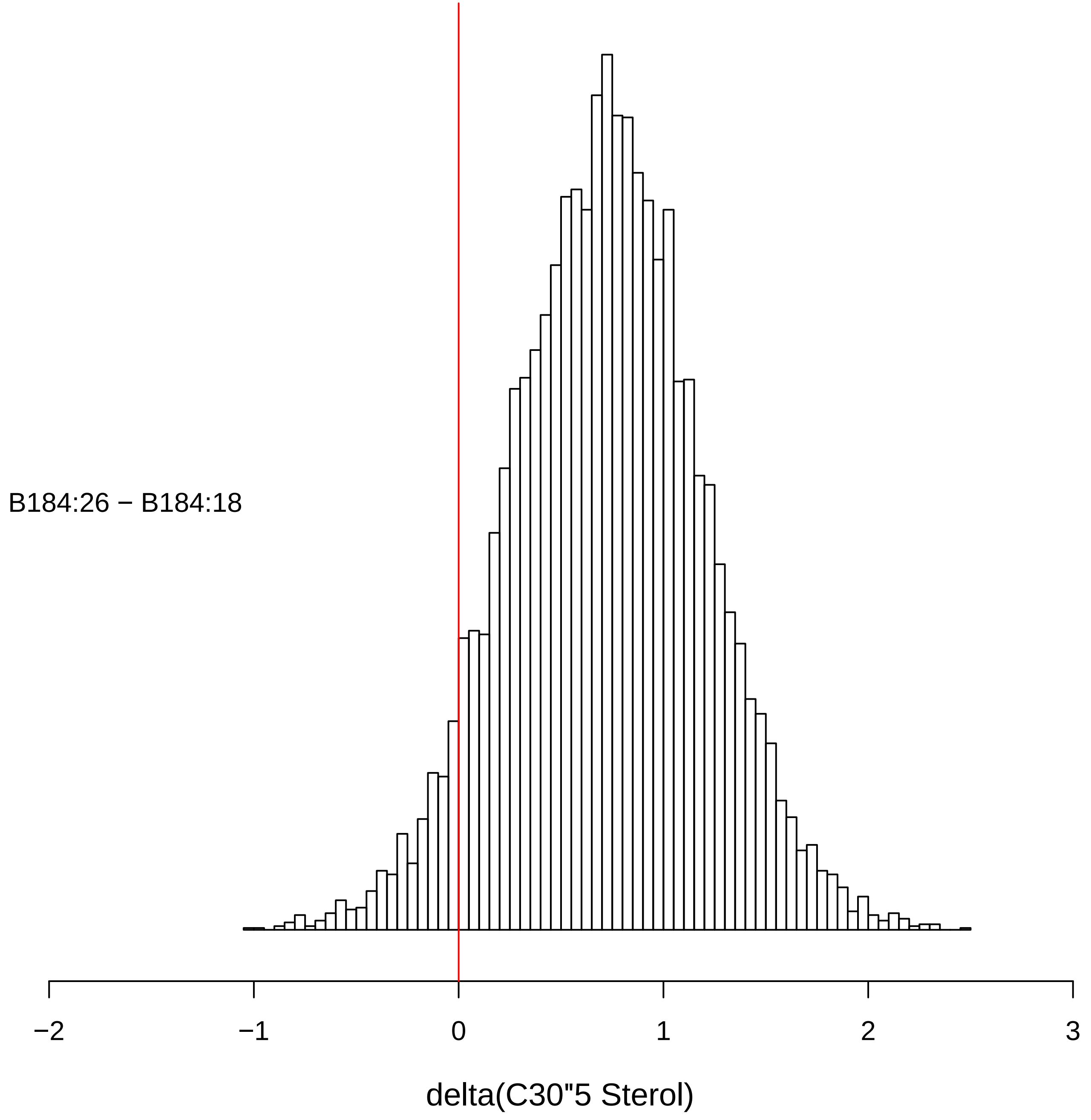
B184:26 − B184:18
−2
−1
0
1
2
3
delta(C30"5 Sterol)

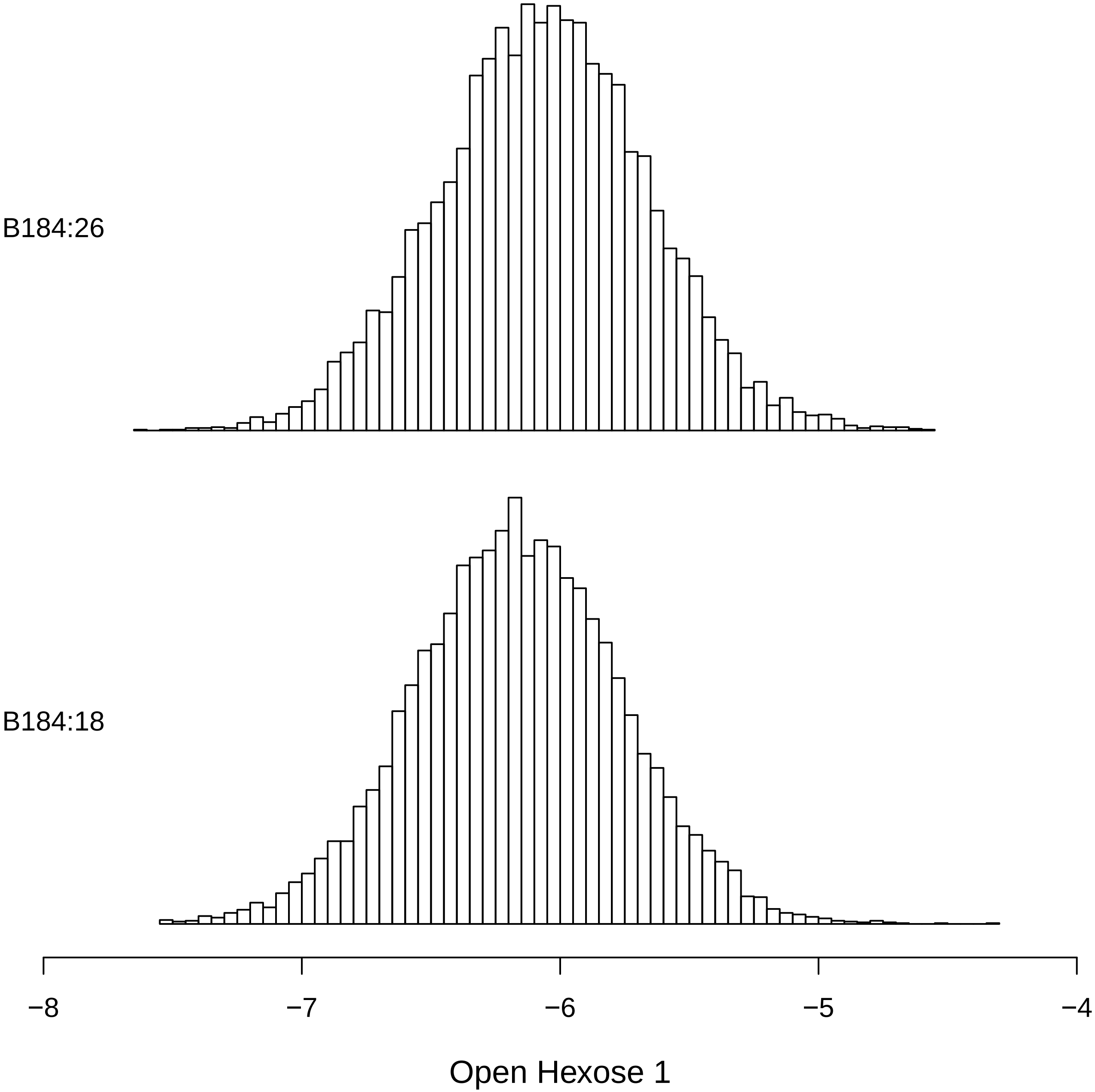
B184:26
B184:18
−8
−7
−6
−5
−4
Open Hexose 1

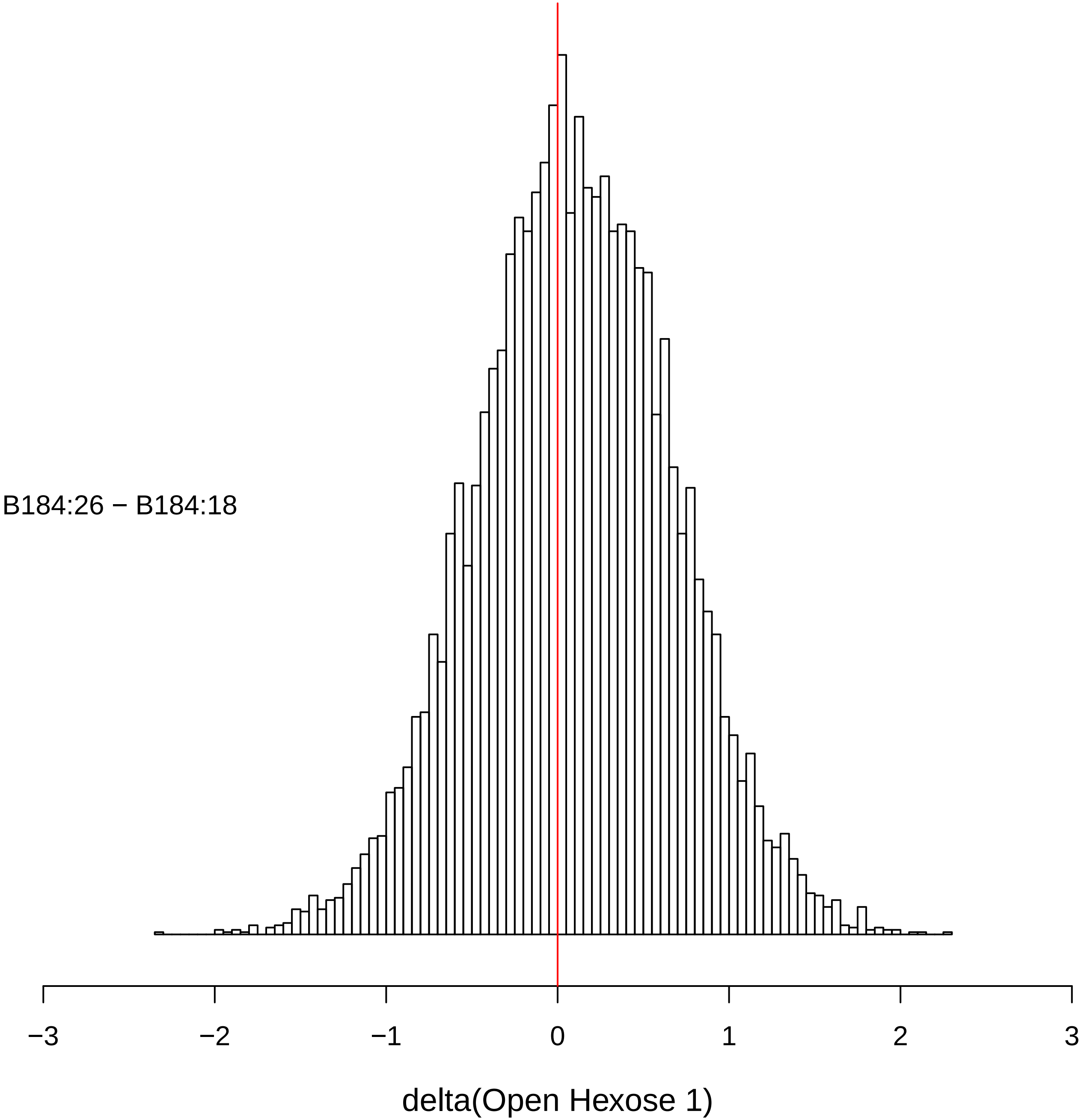

B184:26 − B184:18
−3
−2
−1
0
1
2
3
delta(Open Hexose 1)

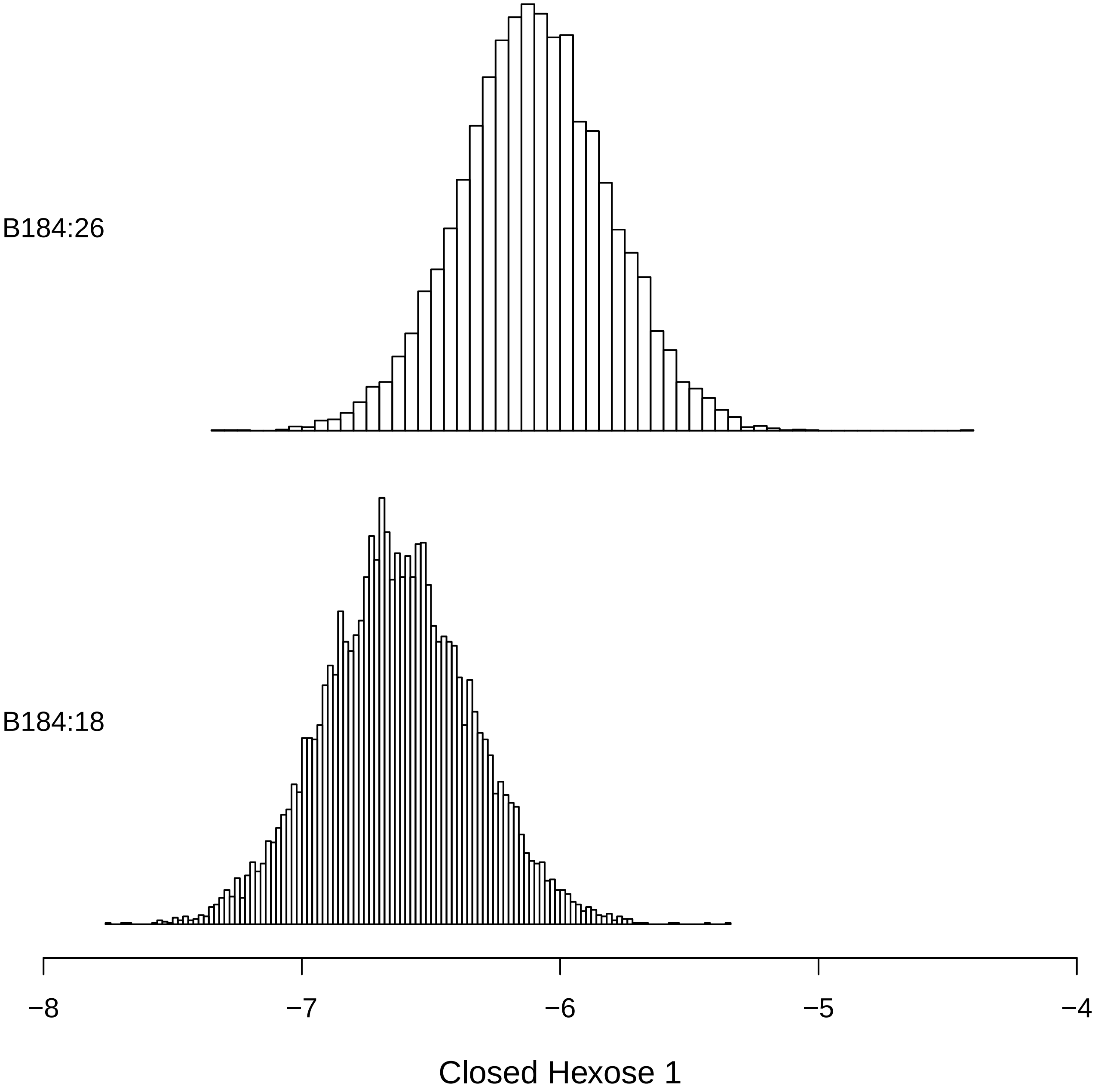
B184:26
B184:18
−8
−7
−6
−5
−4
Closed Hexose 1

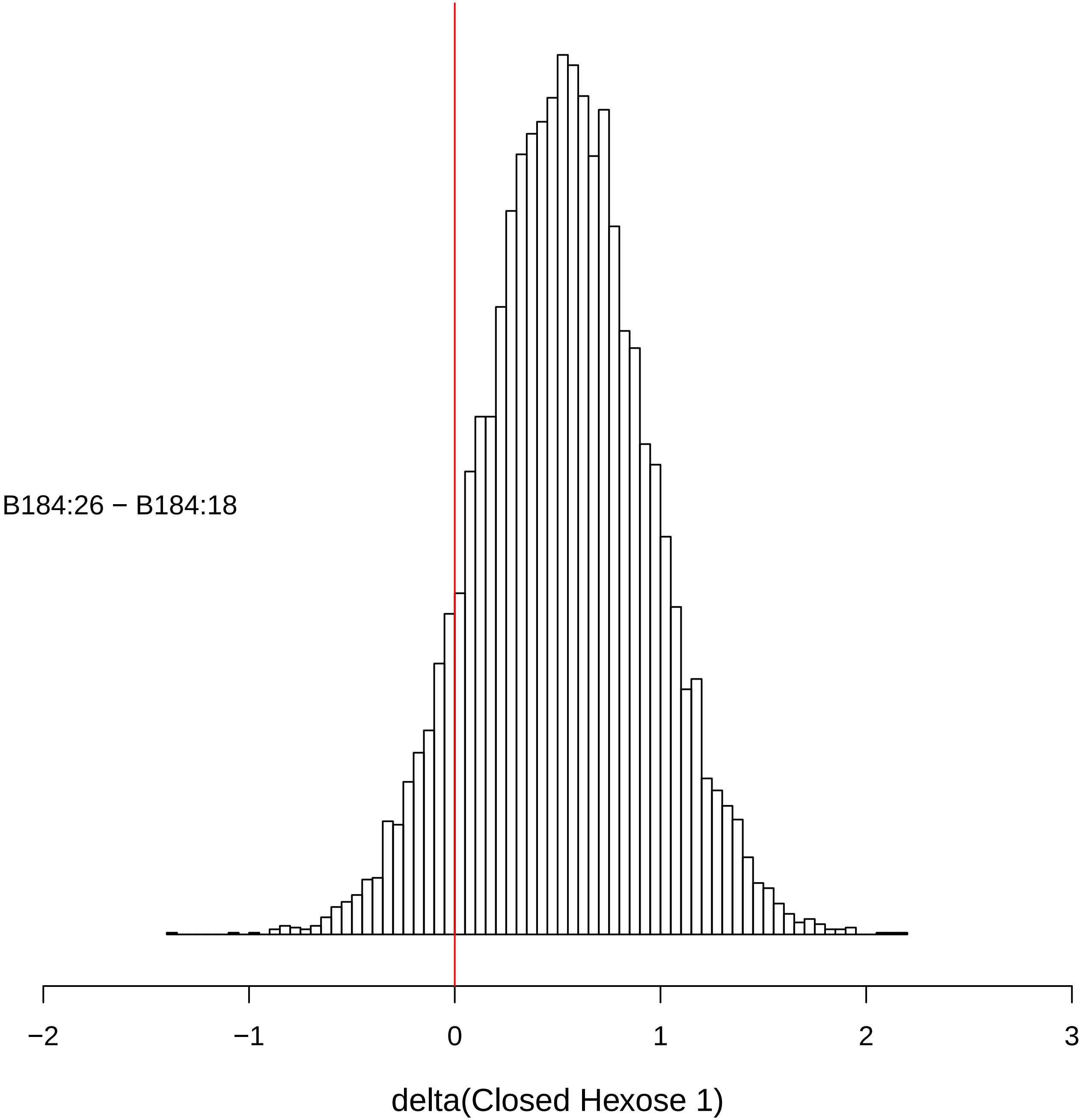

B184:26 − B184:18
−2
−1
0
1
2
3
delta(Closed Hexose 1)

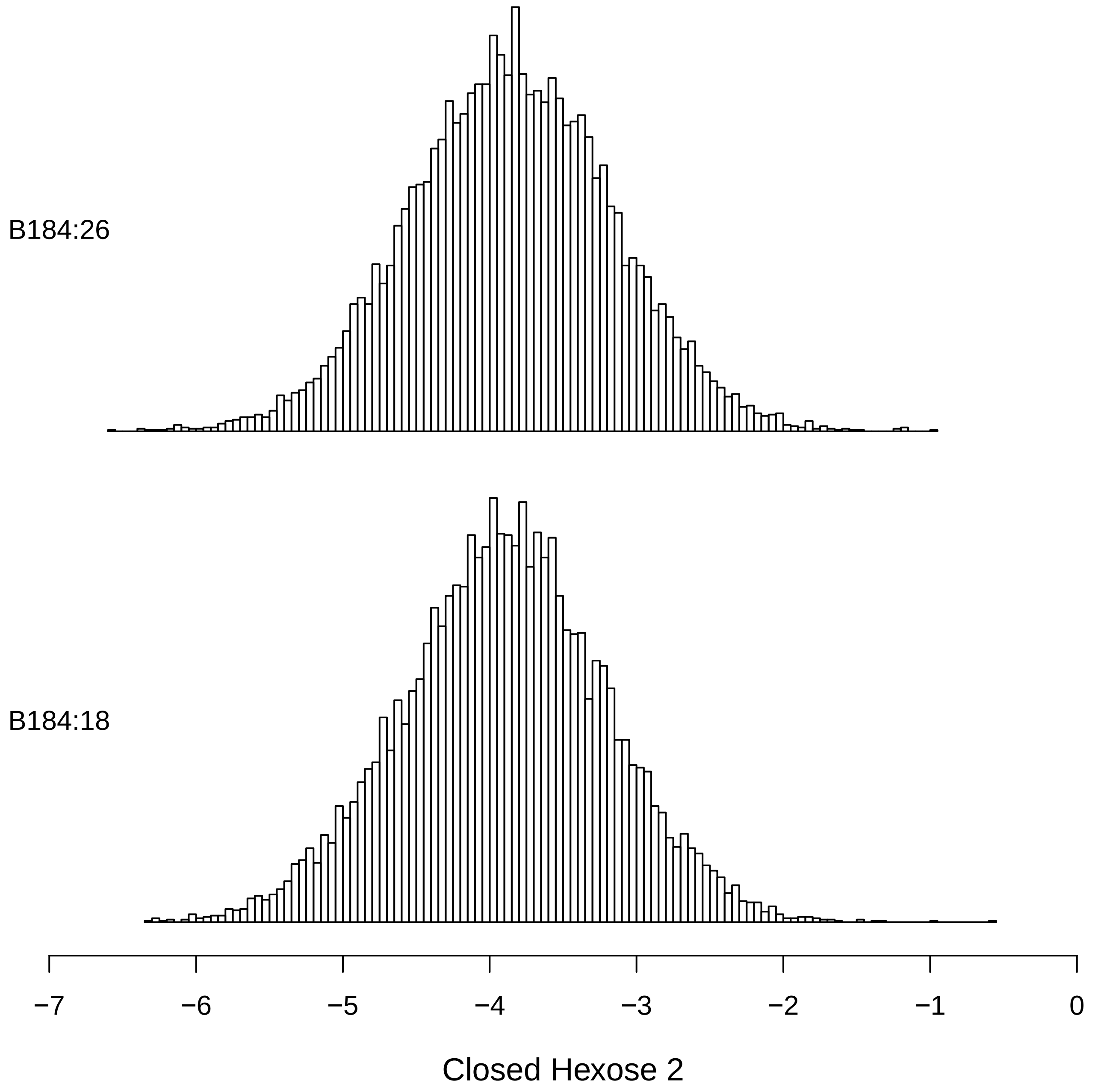
B184:26
B184:18
−7
−6
−5
−4
−3
−2
−1
0
Closed Hexose 2

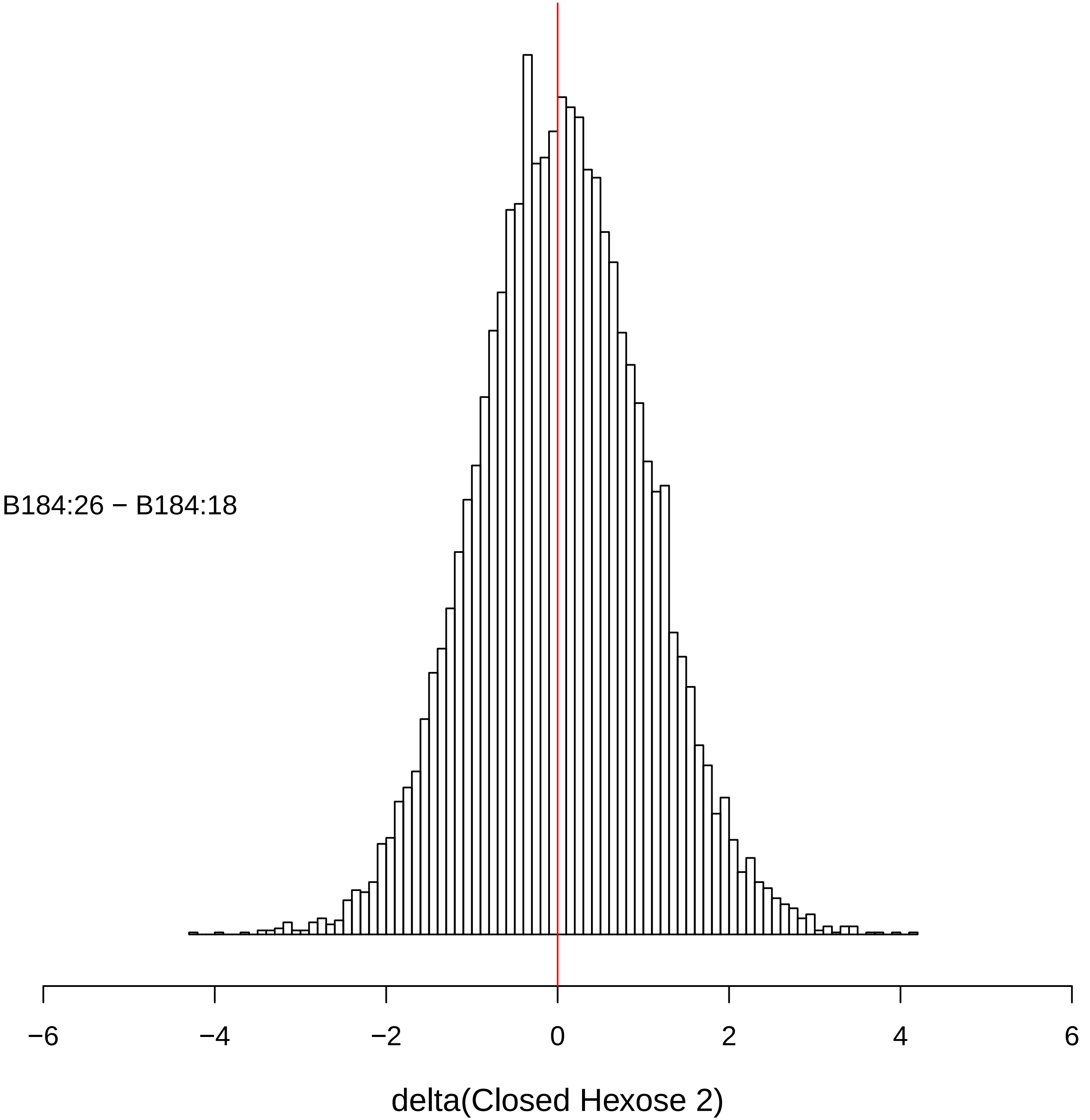
B184:26 − B184:18
−6
−4
−2
0
2
4
6
delta(Closed Hexose 2)

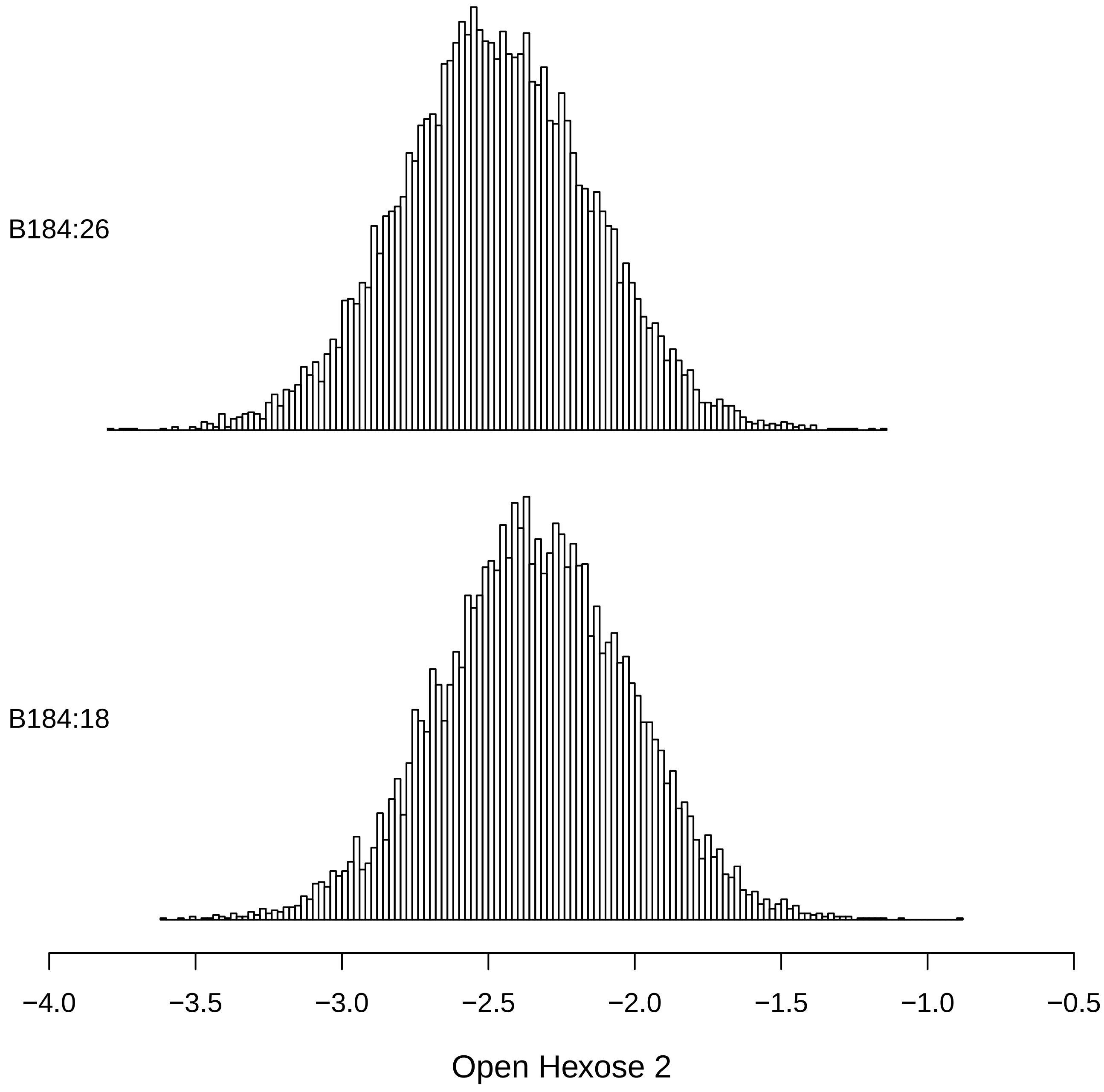
B184:26
B184:18
−4.0
−3.5
−3.0
−2.5
−2.0
−1.5
−1.0
−0.5
Open Hexose 2

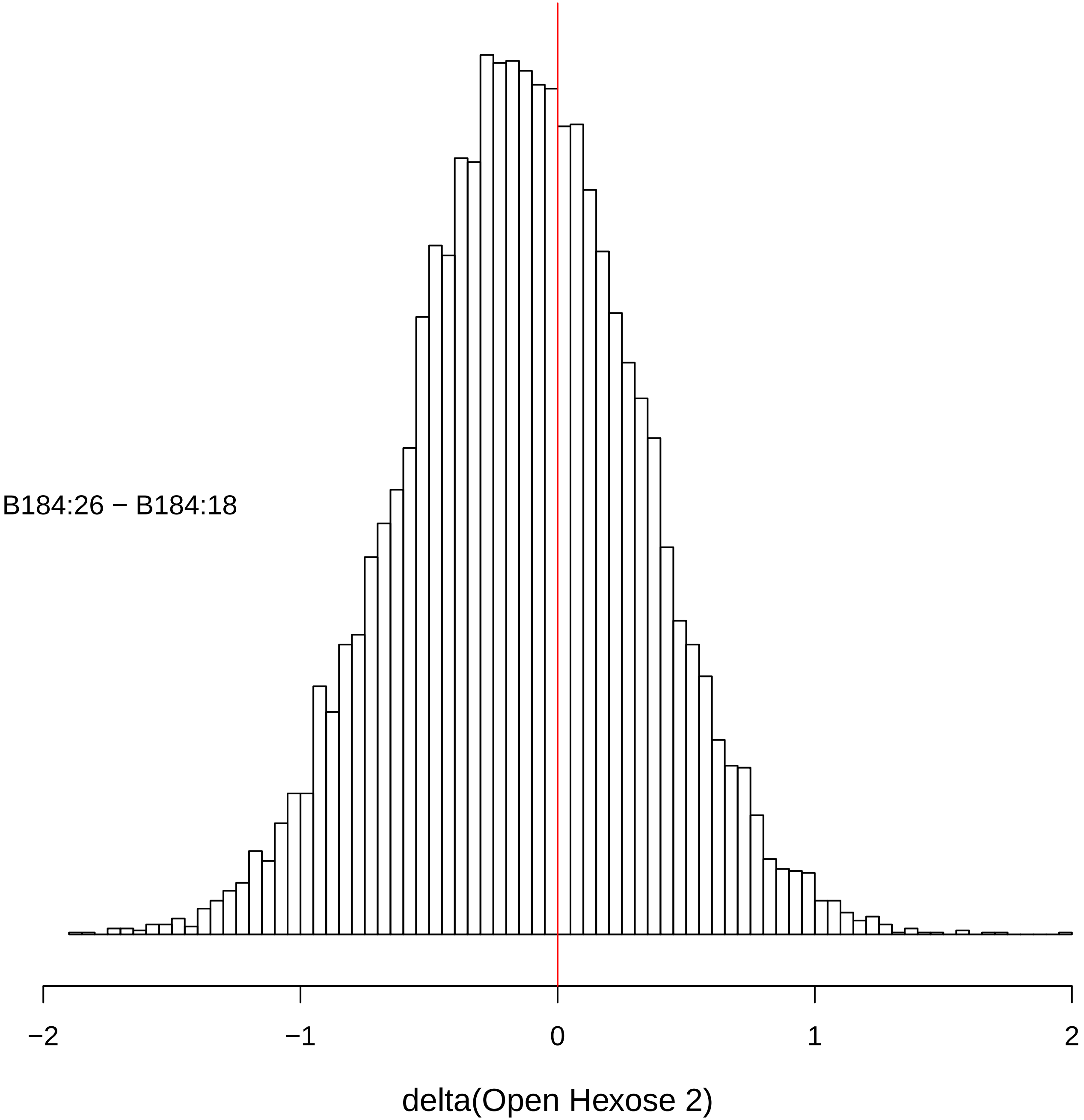
B184:26 − B184:18
−2
−1
0
1
2
delta(Open Hexose 2)

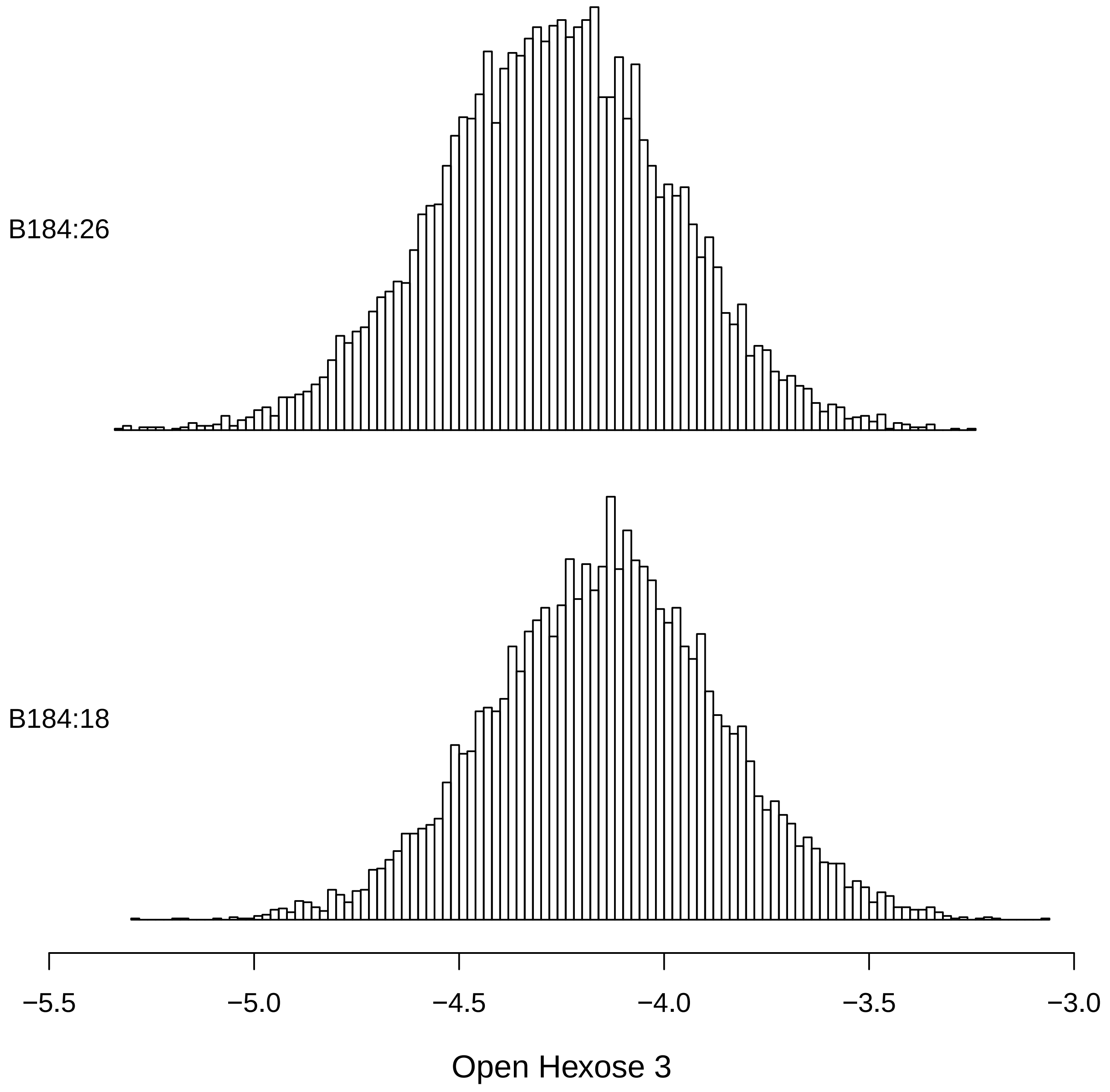
B184:26
B184:18
−5.5
−5.0
−4.5
−4.0
−3.5
−3.0
Open Hexose 3

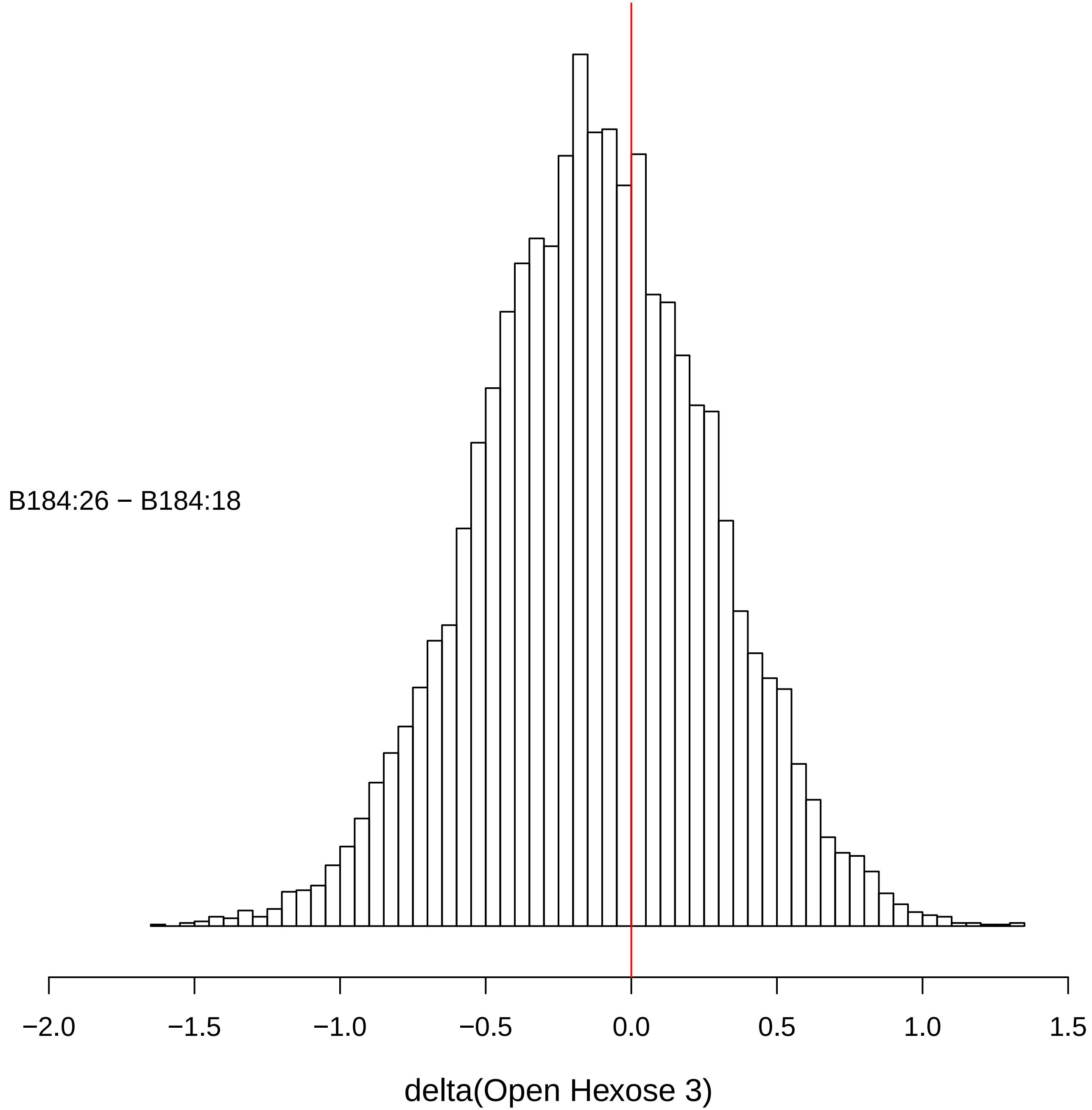
B184:26 − B184:18
−2.0
−1.5
−1.0
−0.5
0.0
0.5
1.0
1.5
delta(Open Hexose 3)

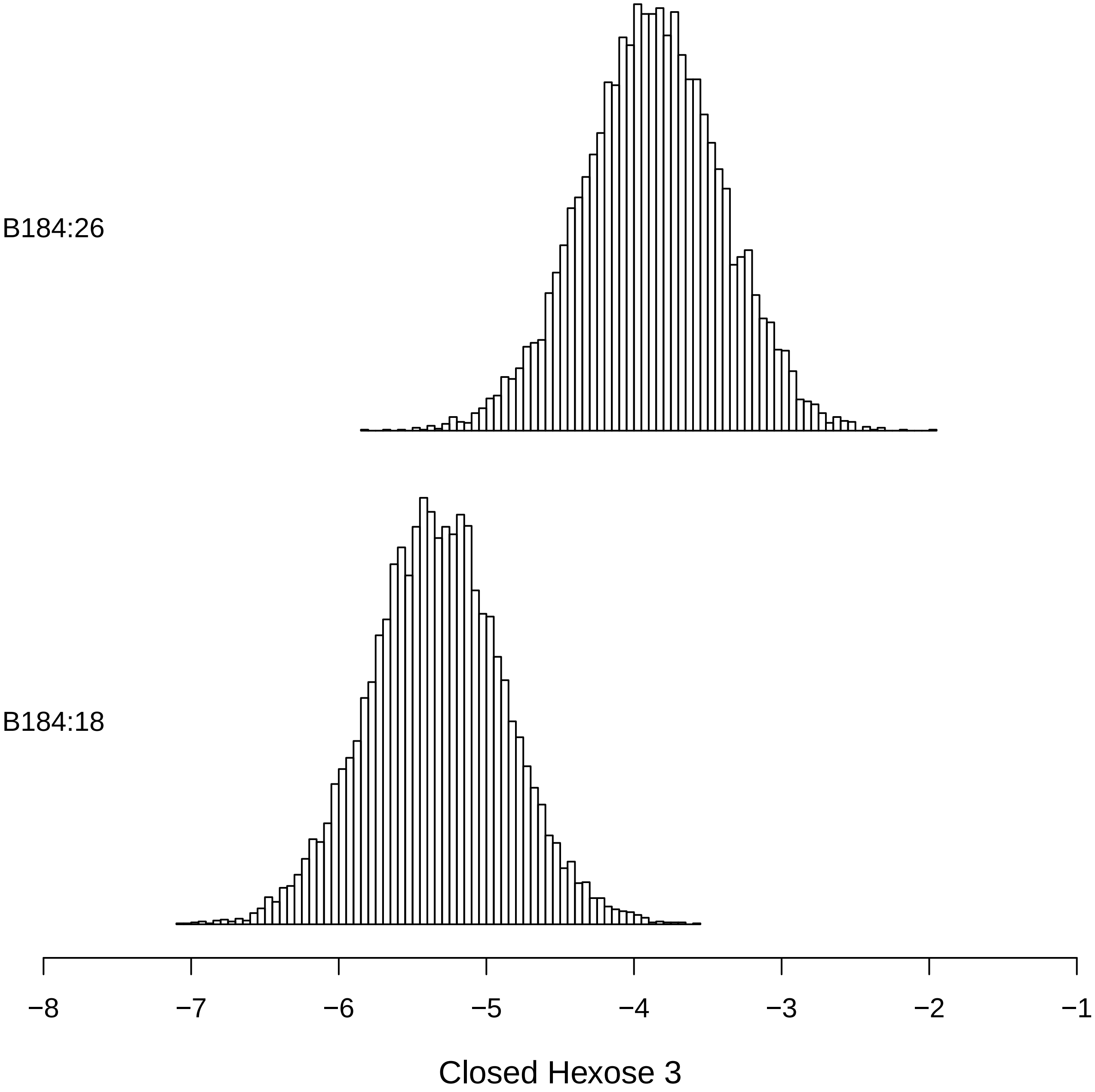
B184:26
B184:18
−8
−7
−6
−5
−4
−3
−2
−1
Closed Hexose 3

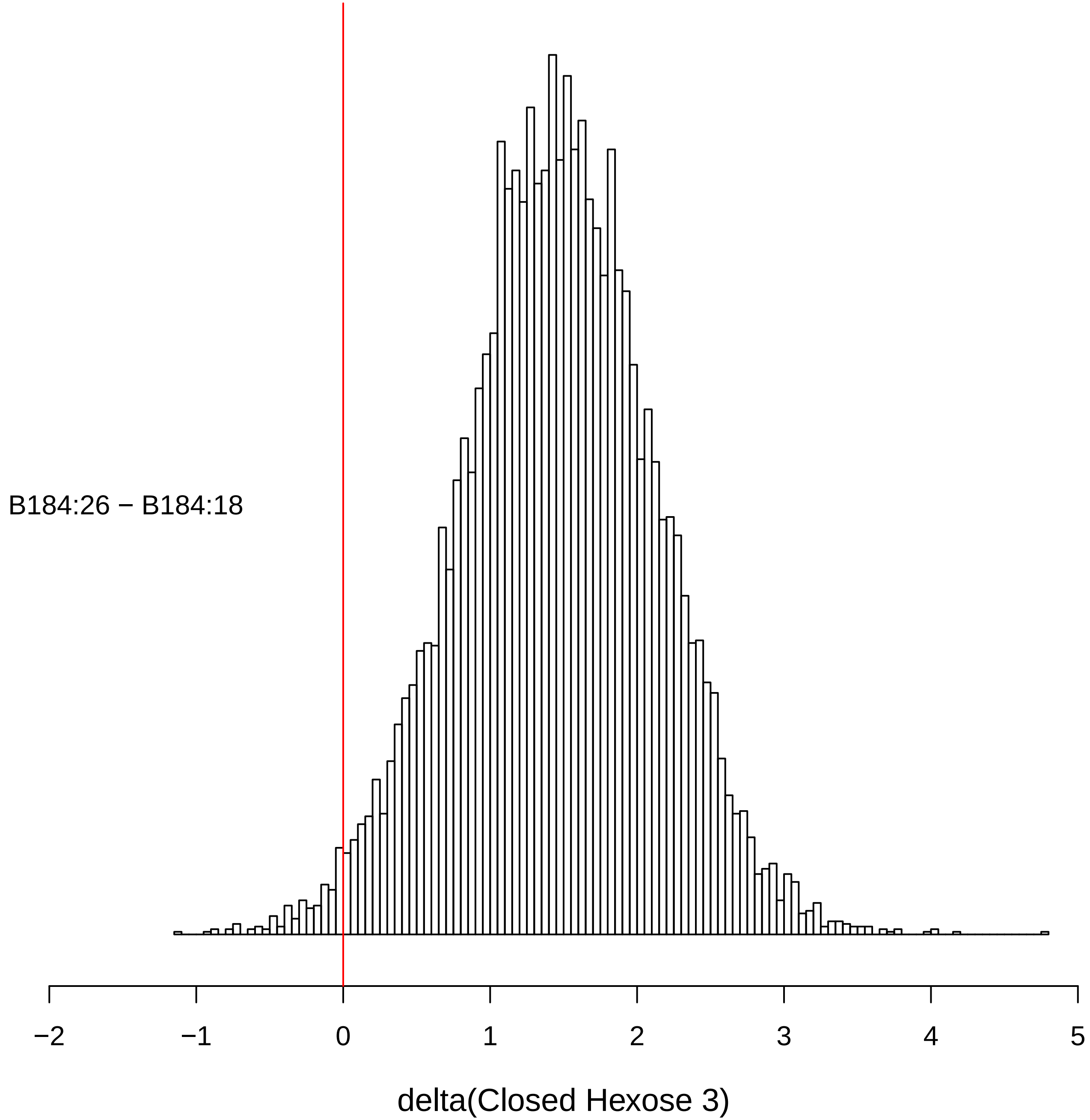

B184:26 − B184:18
−2
−1
0
1
2
3
4
5
delta(Closed Hexose 3)

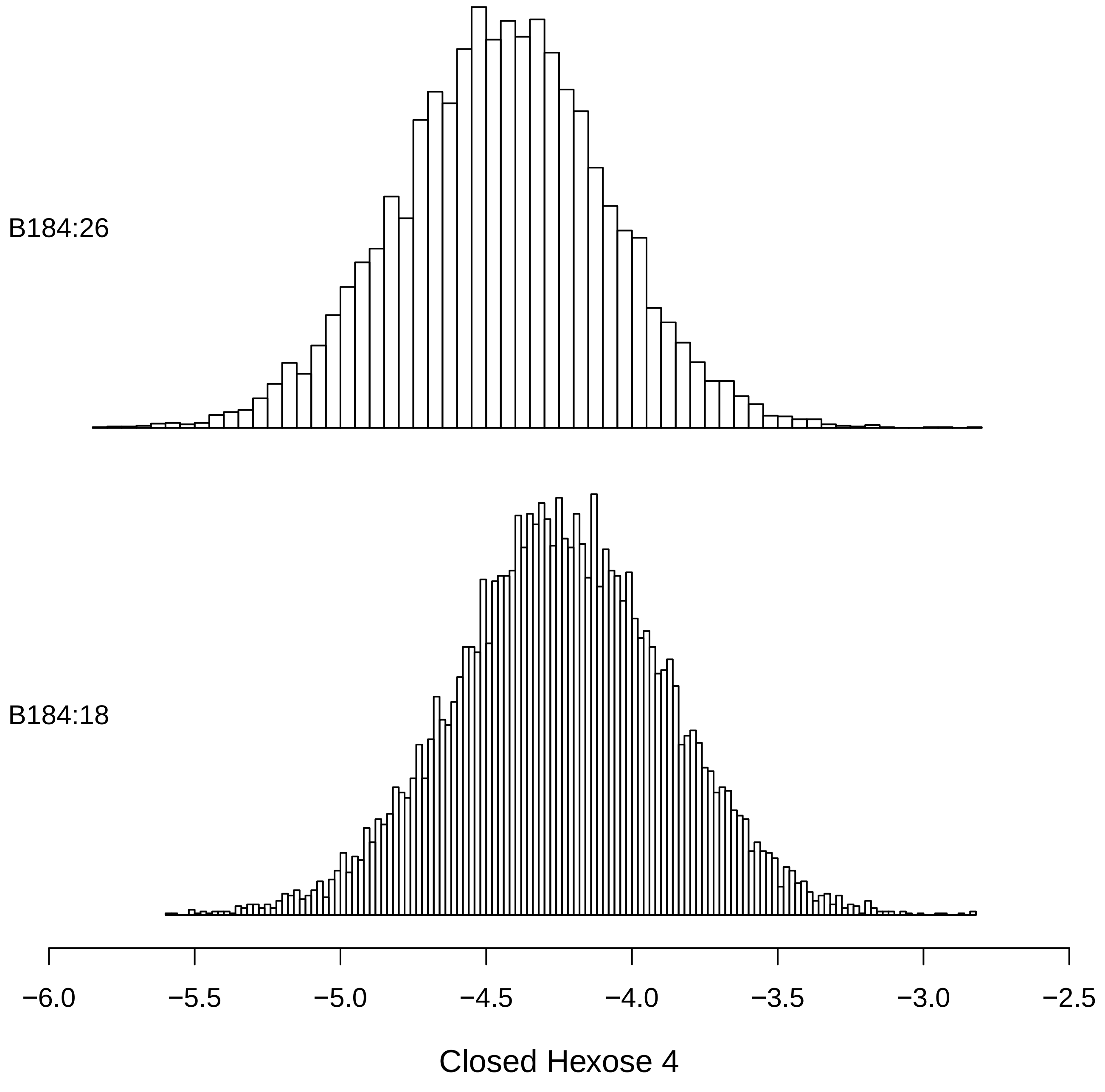

B184:26
B184:18
−6.0
−5.5
−5.0
−4.5
−4.0
−3.5
−3.0
−2.5
Closed Hexose 4

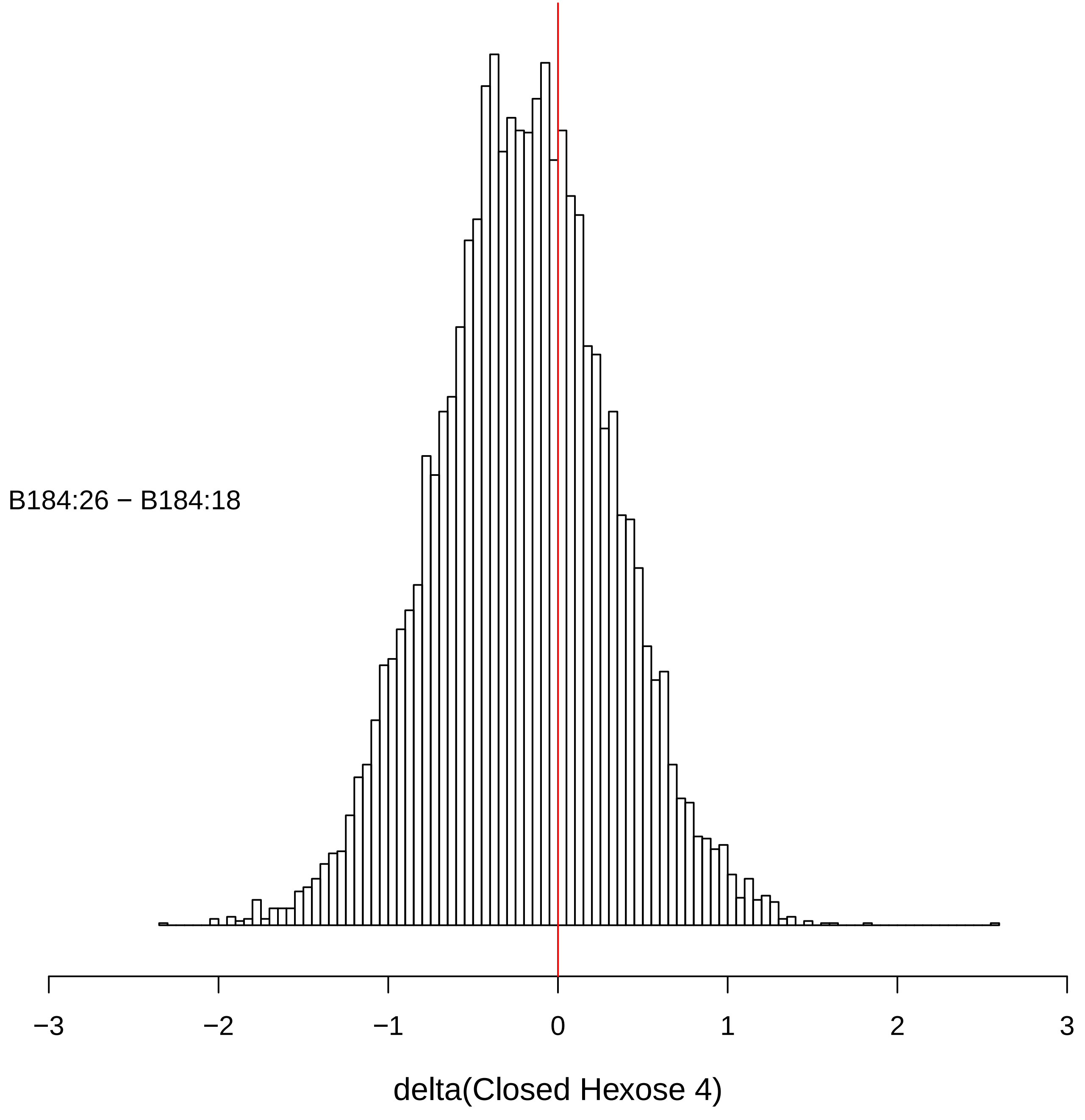
B184:26 − B184:18
−3
−2
−1
0
1
2
3
delta(Closed Hexose 4)

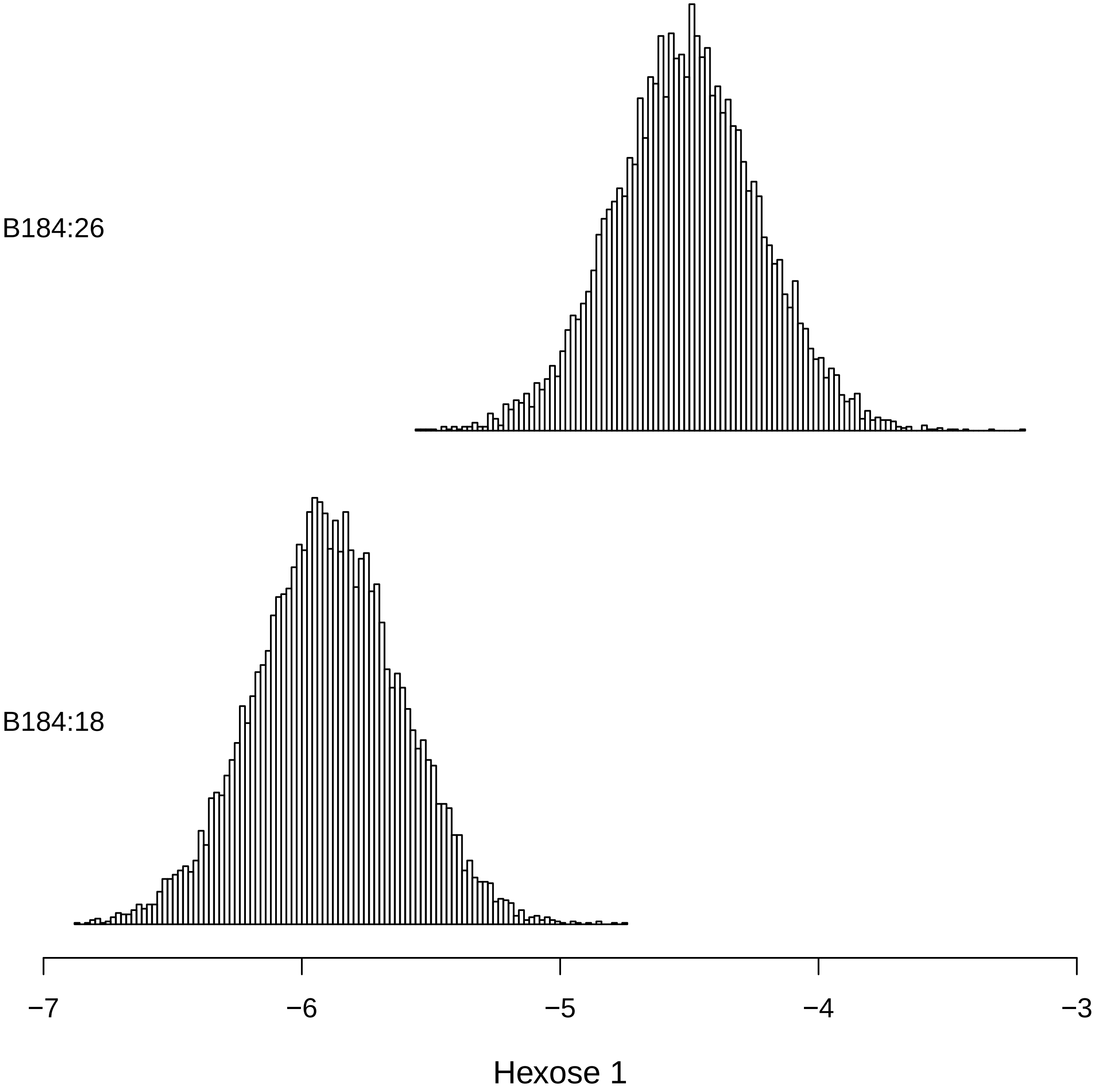

B184:26
B184:18
−7
−6
−5
−4
−3
Hexose 1

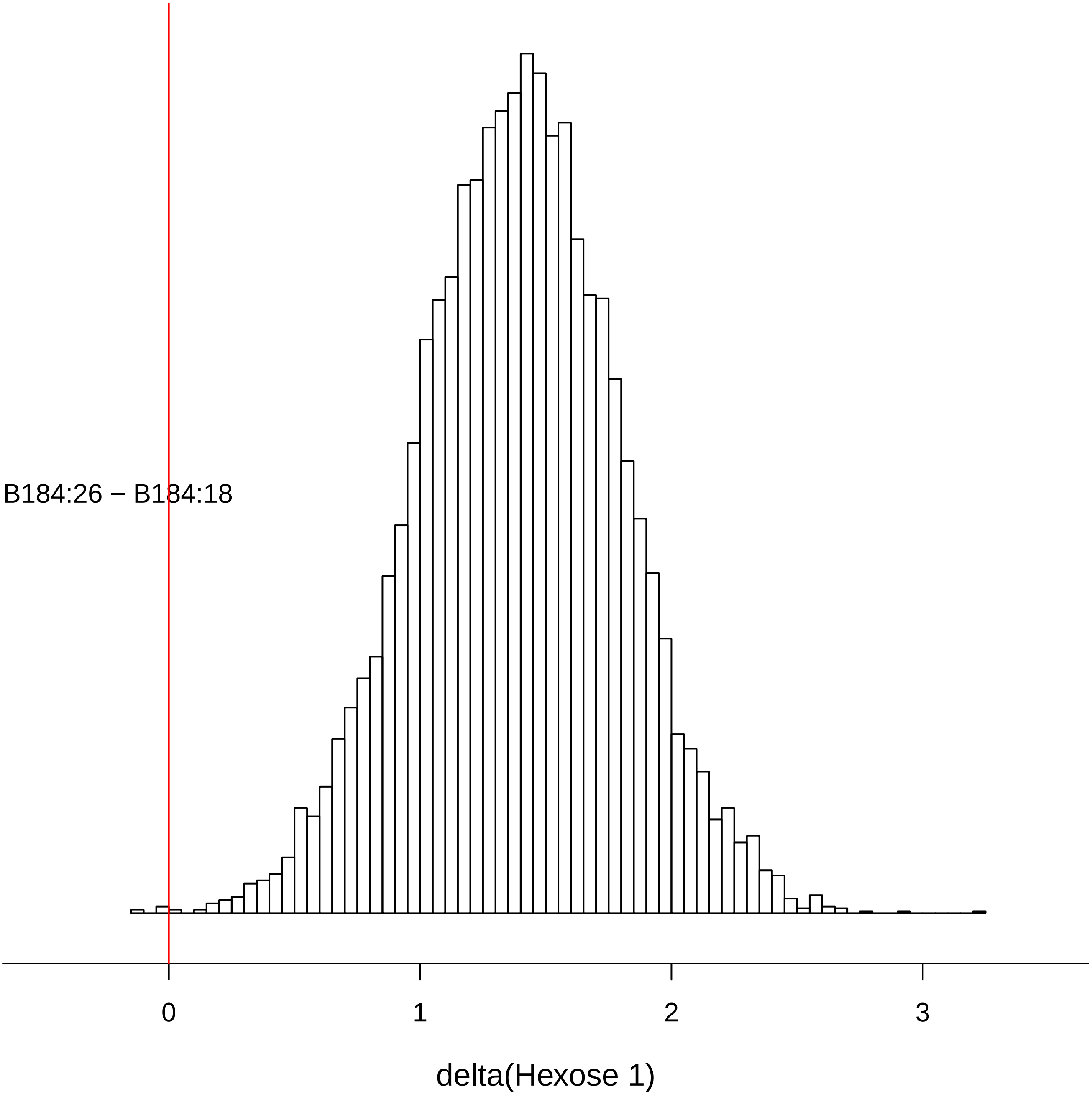
B184:26 − B184:18
0
1
2
3
delta(Hexose 1)

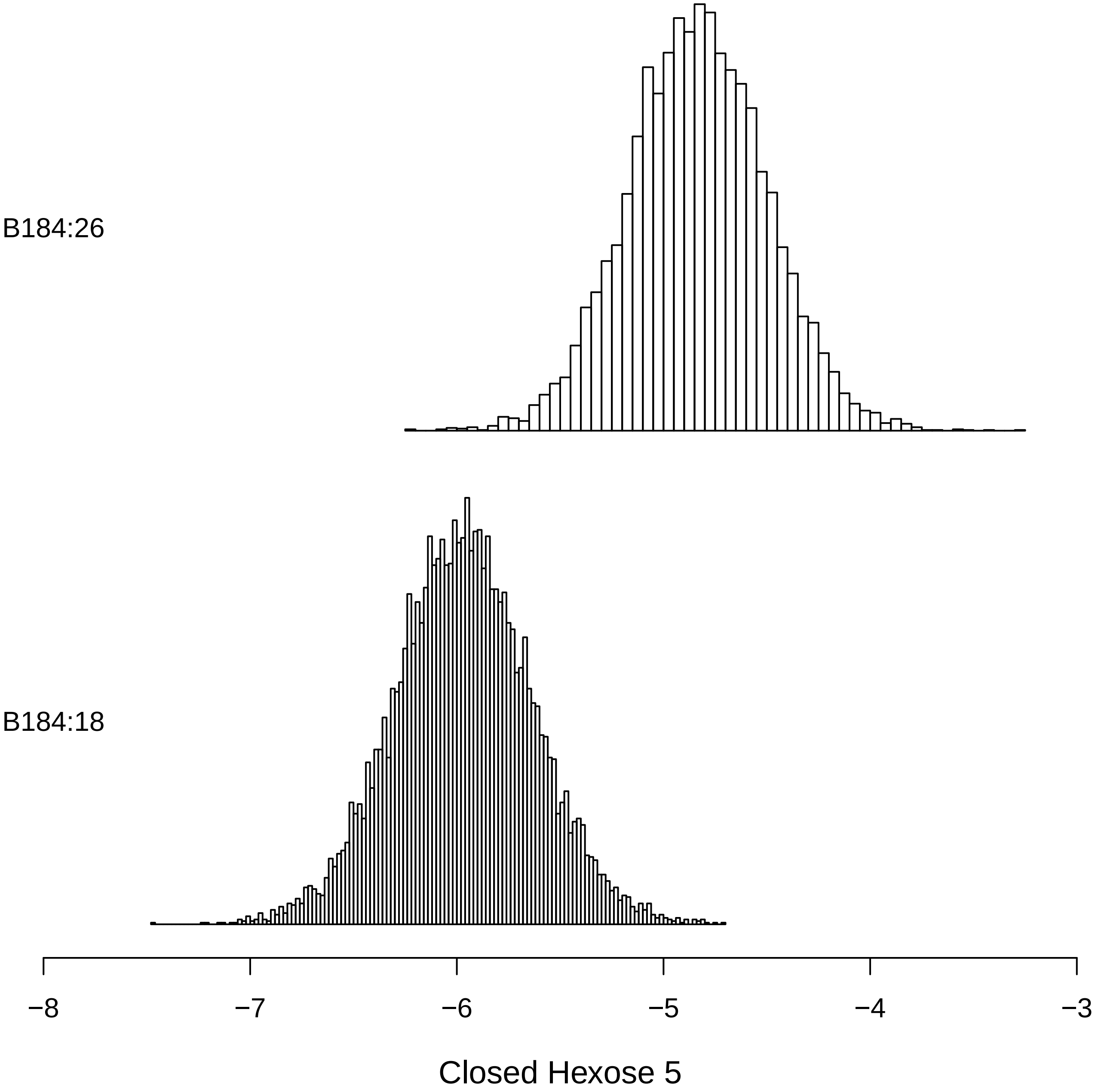

B184:26
B184:18
−8
−7
−6
−5
−4
−3
Closed Hexose 5

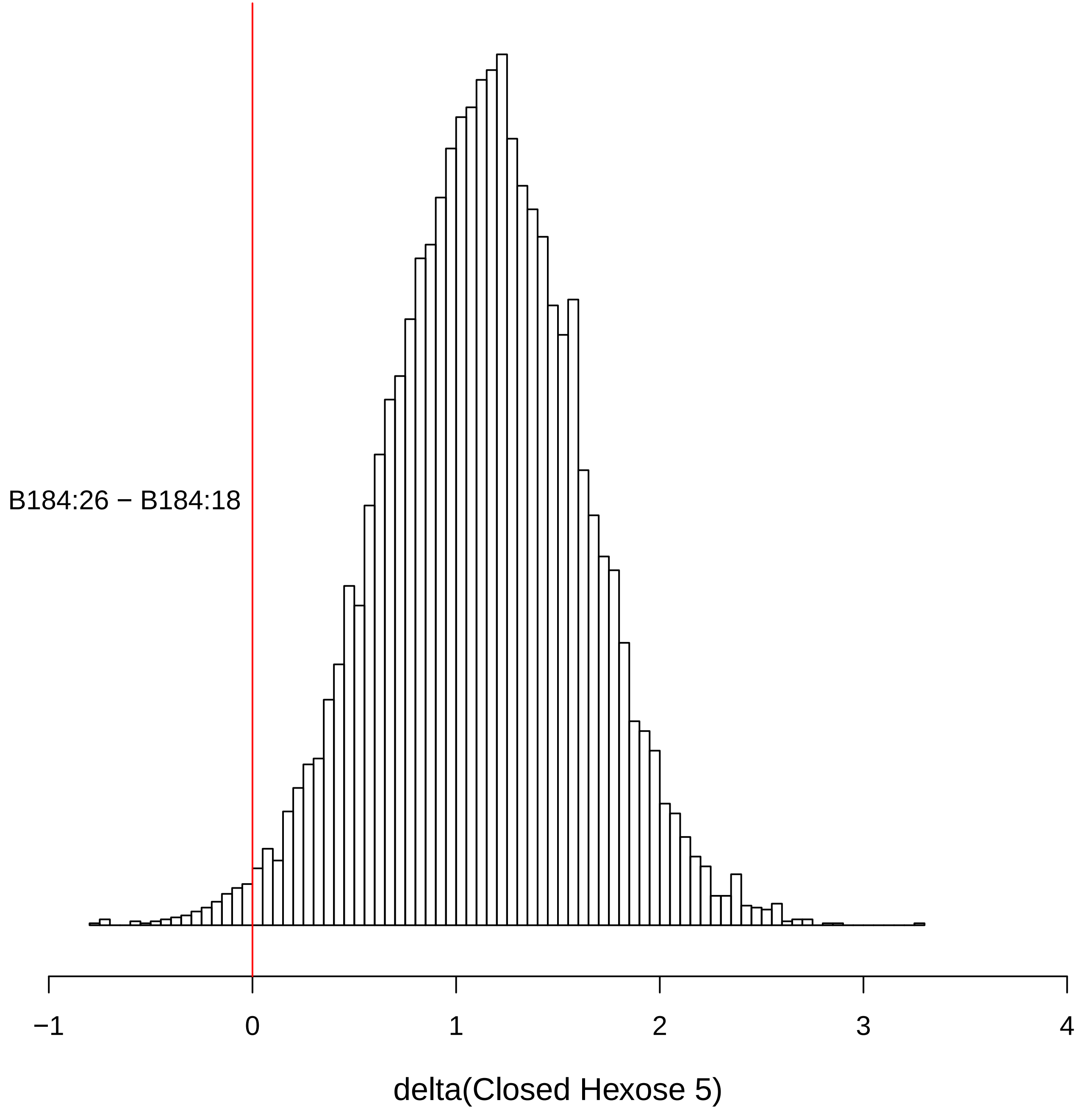
B184:26 − B184:18
−1
0
1
2
3
4
delta(Closed Hexose 5)

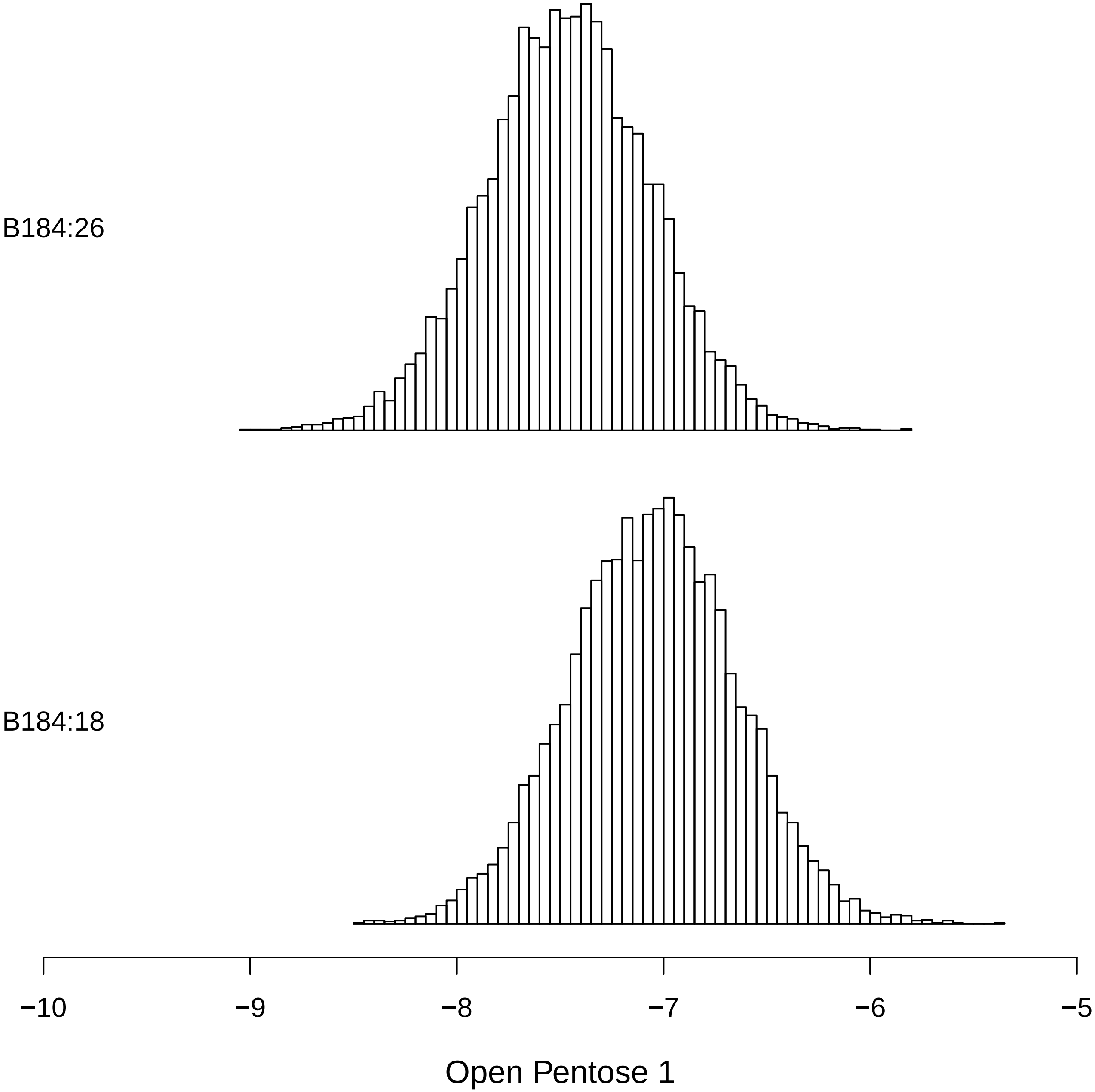
B184:26
B184:18
−10
−9
−8
−7
−6
−5
Open Pentose 1

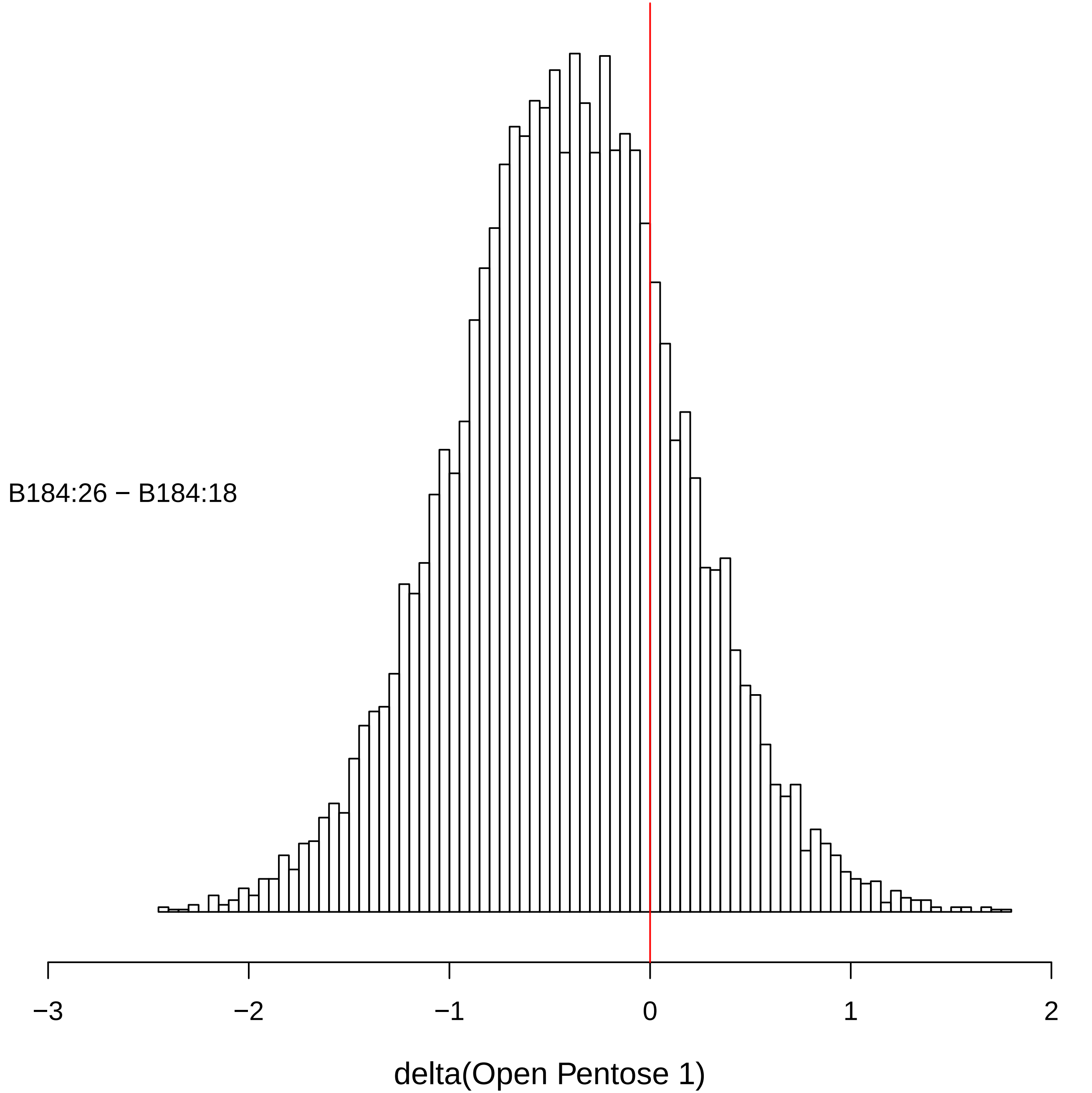
B184:26 − B184:18
−3
−2
−1
0
1
2
delta(Open Pentose 1)

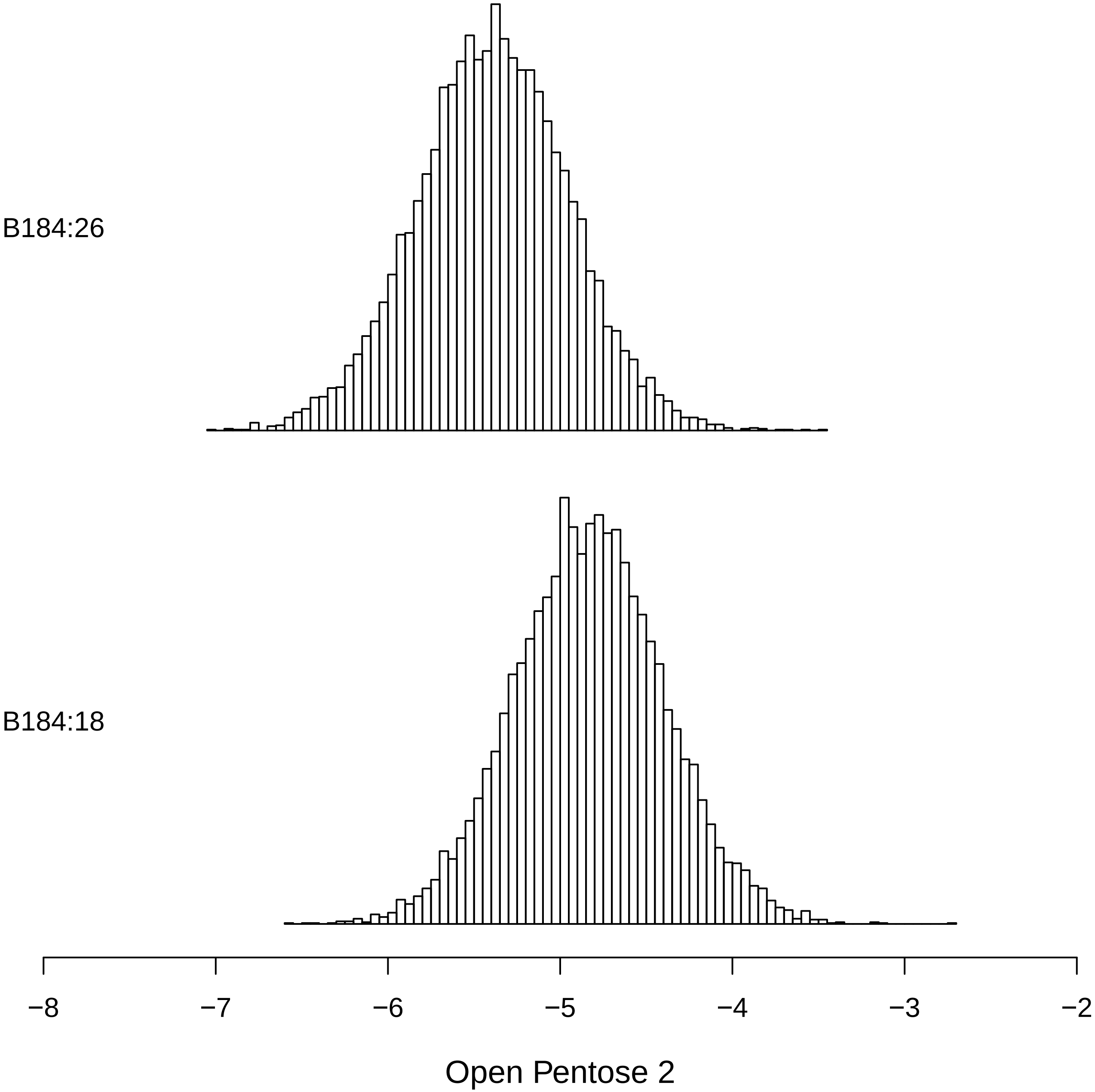

B184:26
B184:18
−8
−7
−6
−5
−4
−3
−2
Open Pentose 2

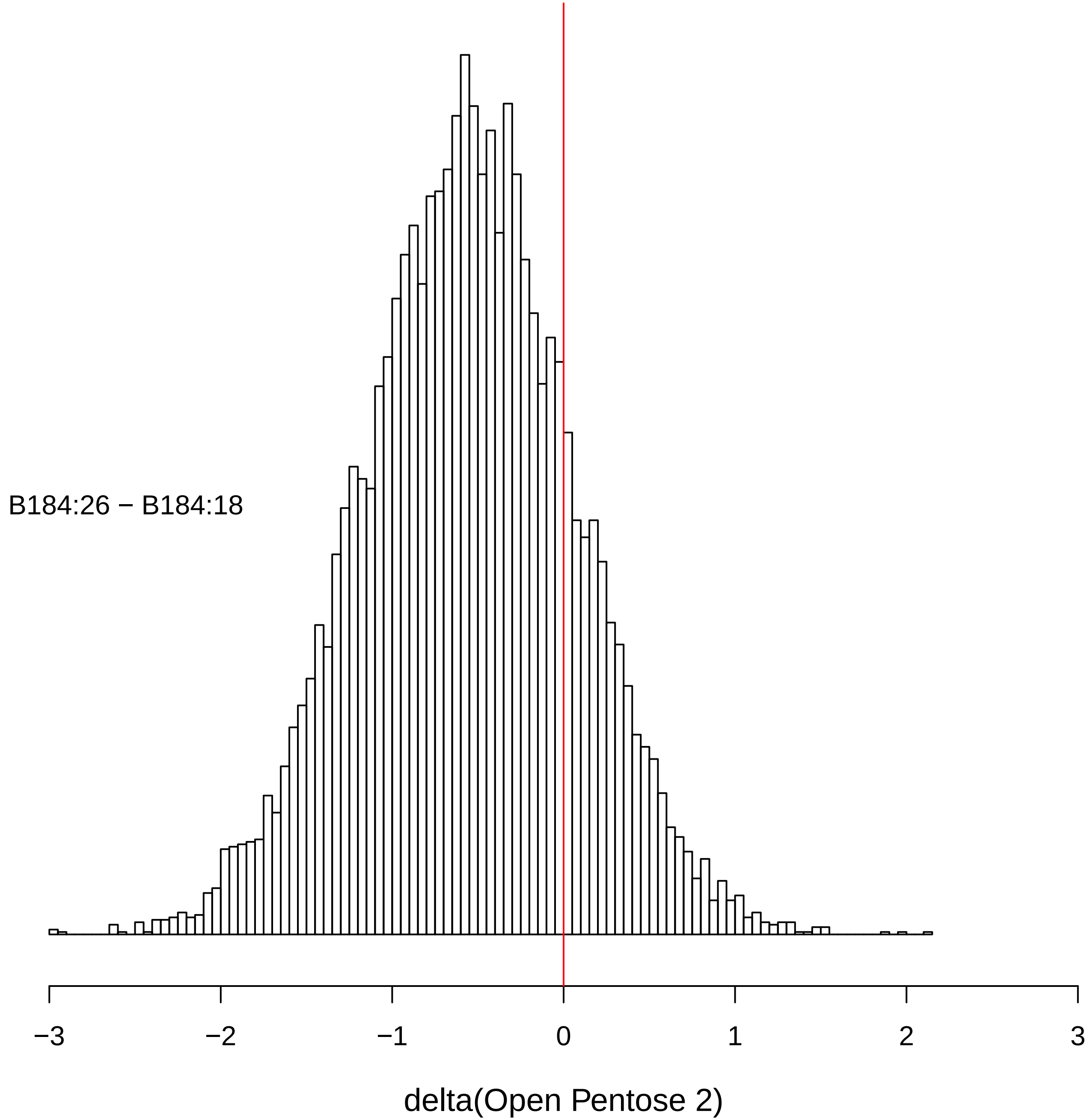
B184:26 − B184:18
−3
−2
−1
0
1
2
3
delta(Open Pentose 2)

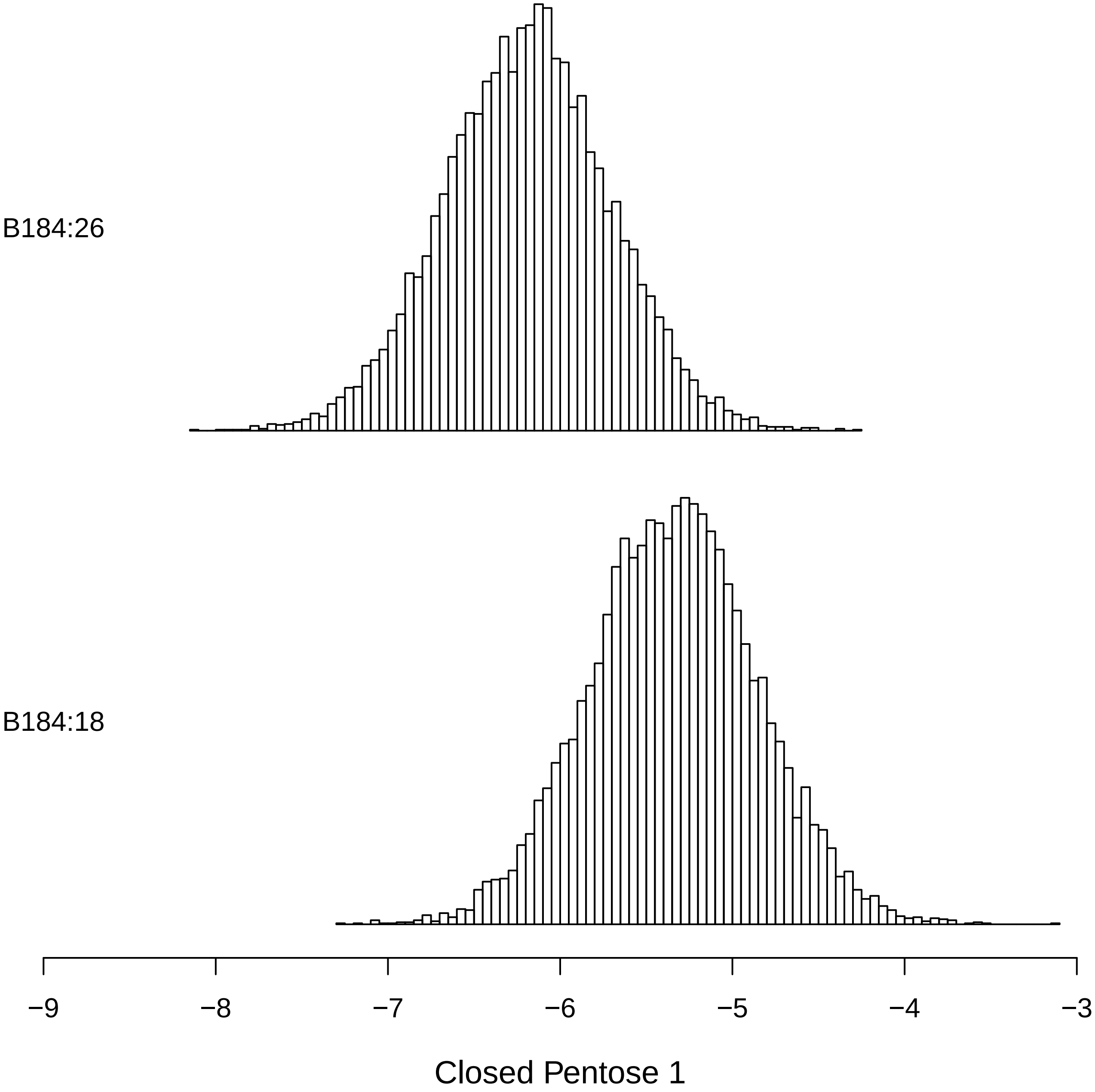
B184:26
B184:18
−9
−8
−7
−6
−5
−4
−3
Closed Pentose 1

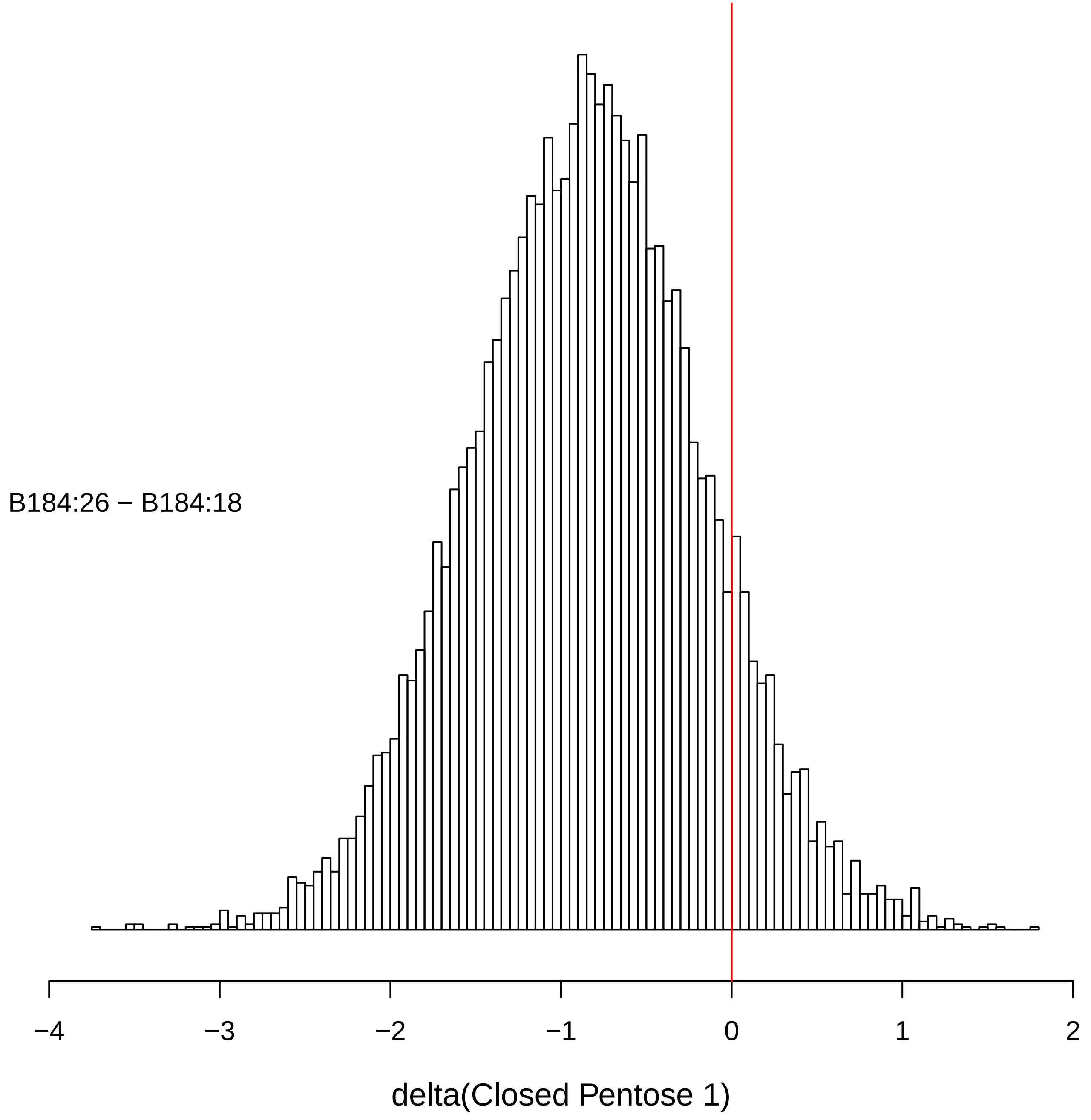

B184:26 − B184:18
−4
−3
−2
−1
0
1
2
delta(Closed Pentose 1)

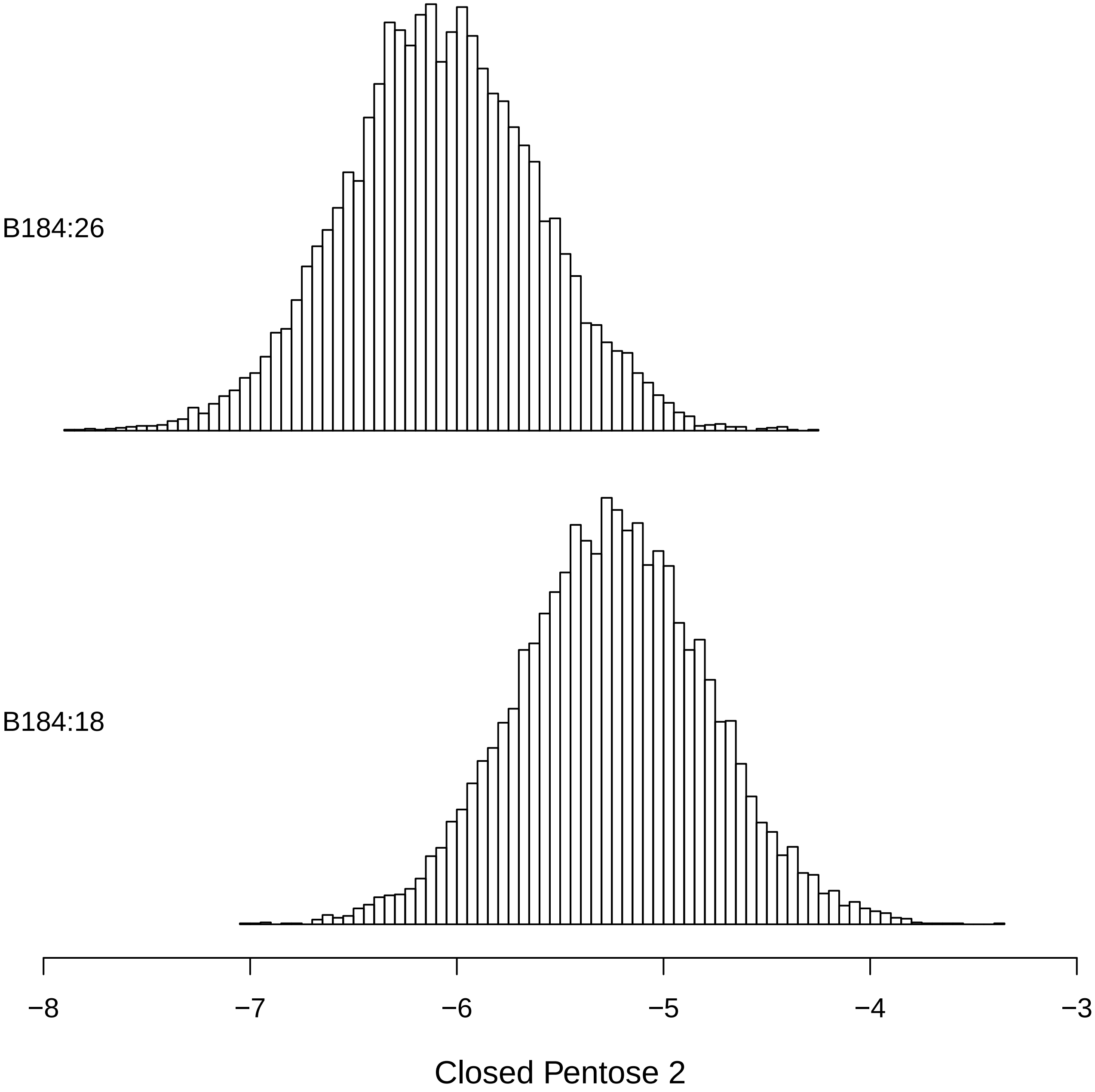
B184:26
B184:18
−8
−7
−6
−5
−4
−3
Closed Pentose 2

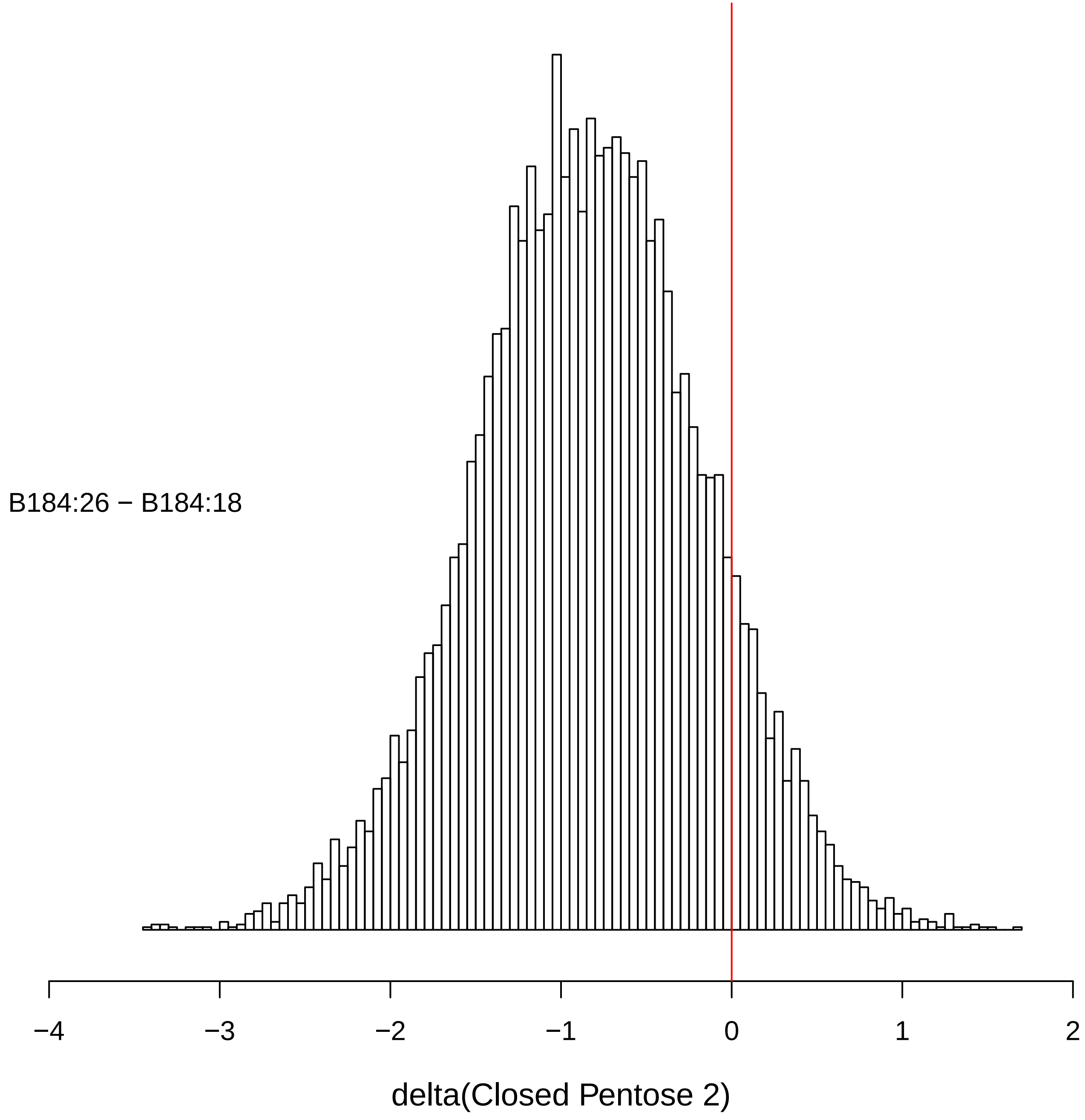

B184:26 − B184:18
−4
−3
−2
−1
0
1
2
delta(Closed Pentose 2)

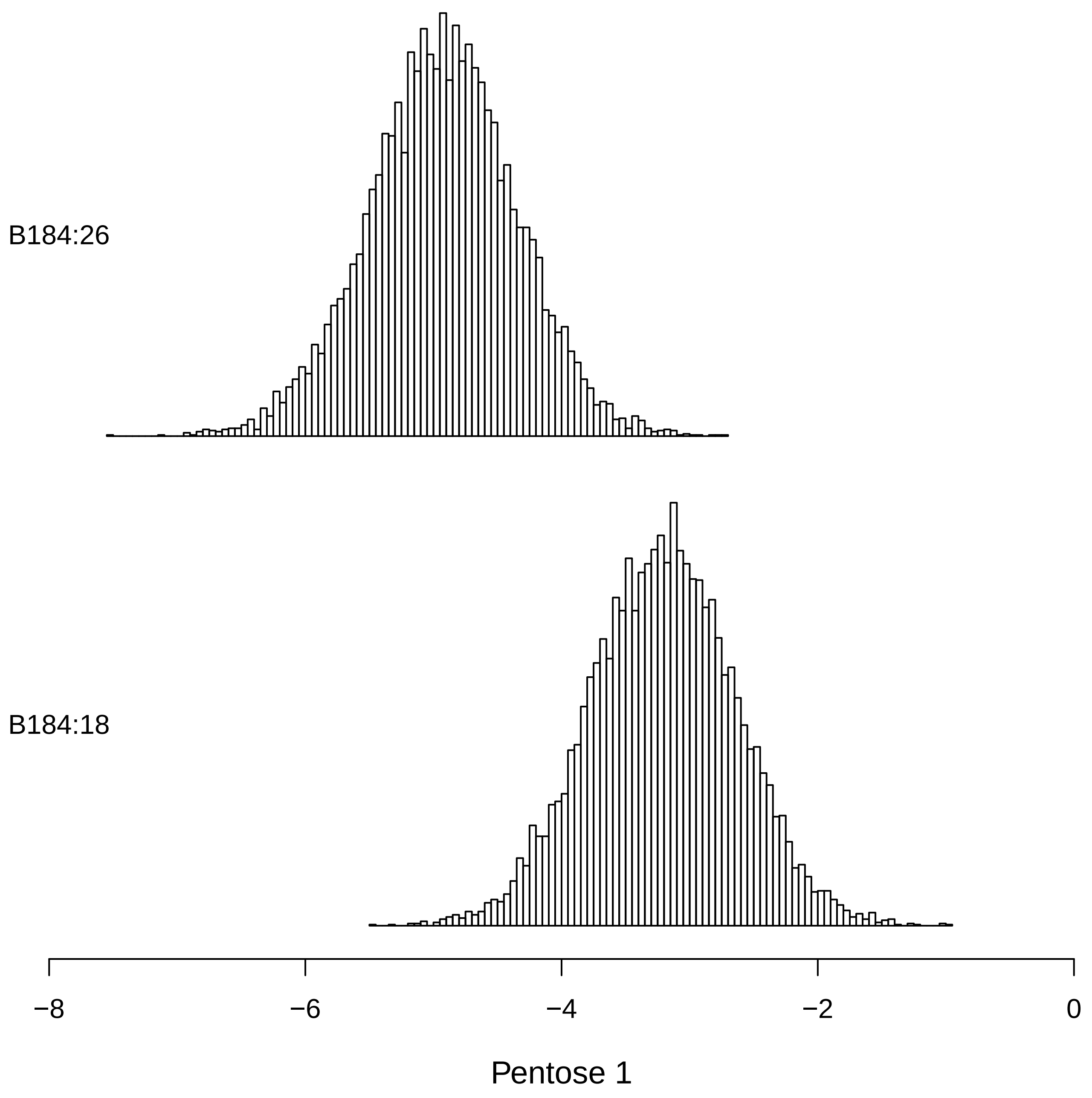
B184:26
B184:18
−8
−6
−4
−2
0
Pentose 1

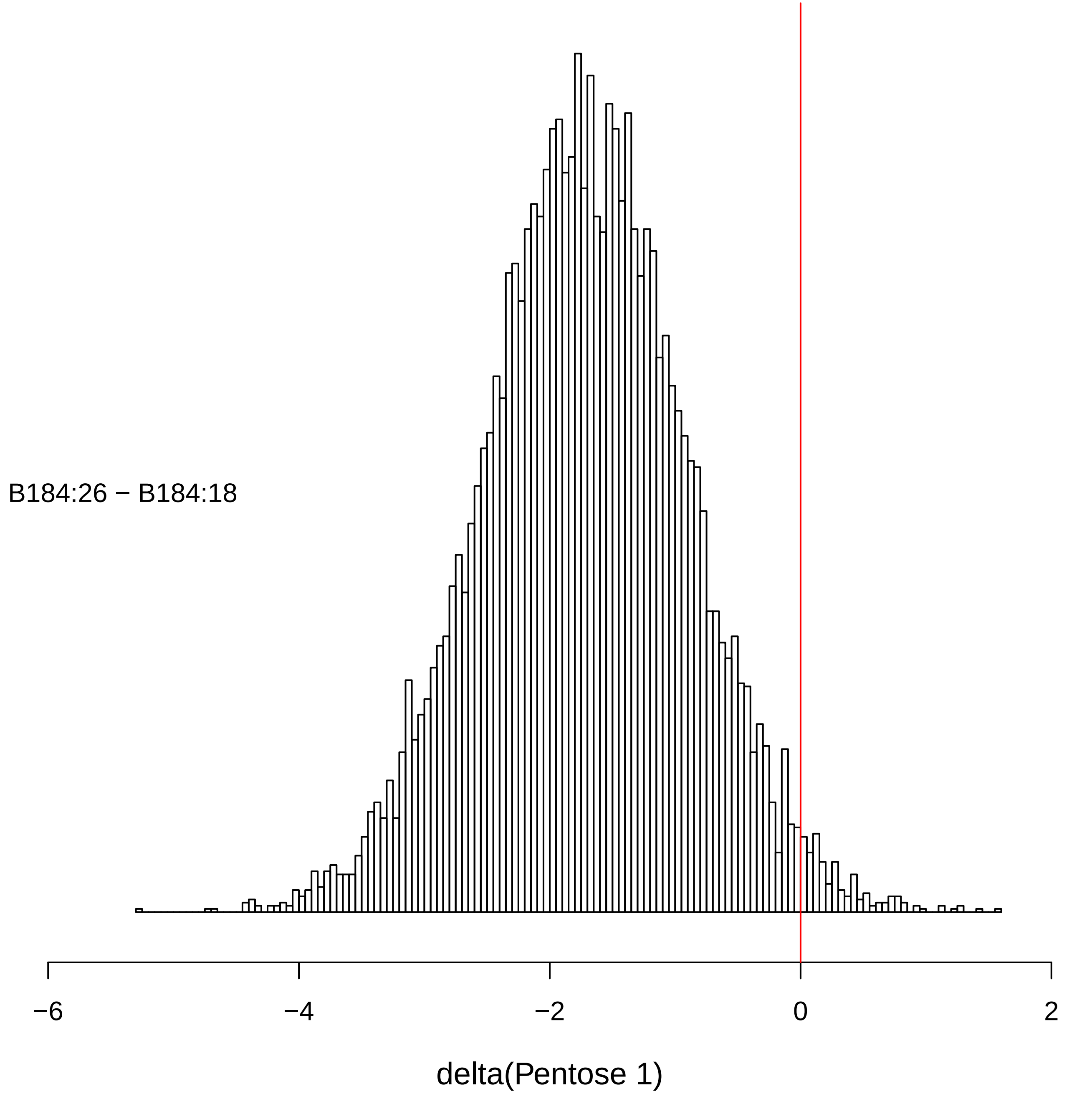
B184:26 − B184:18
−6
−4
−2
0
2
delta(Pentose 1)

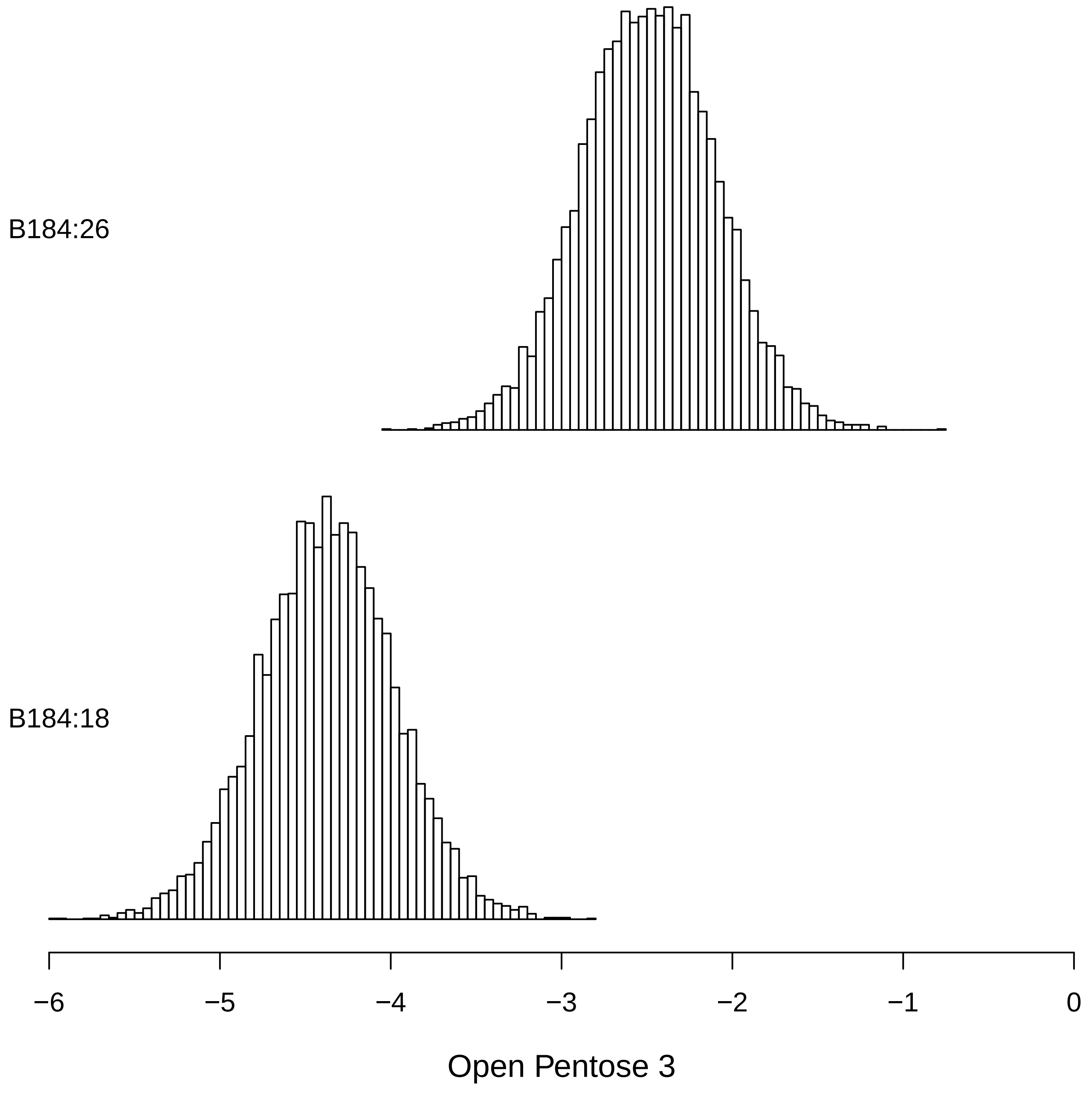

B184:26
B184:18
−6
−5
−4
−3
−2
−1
0
Open Pentose 3

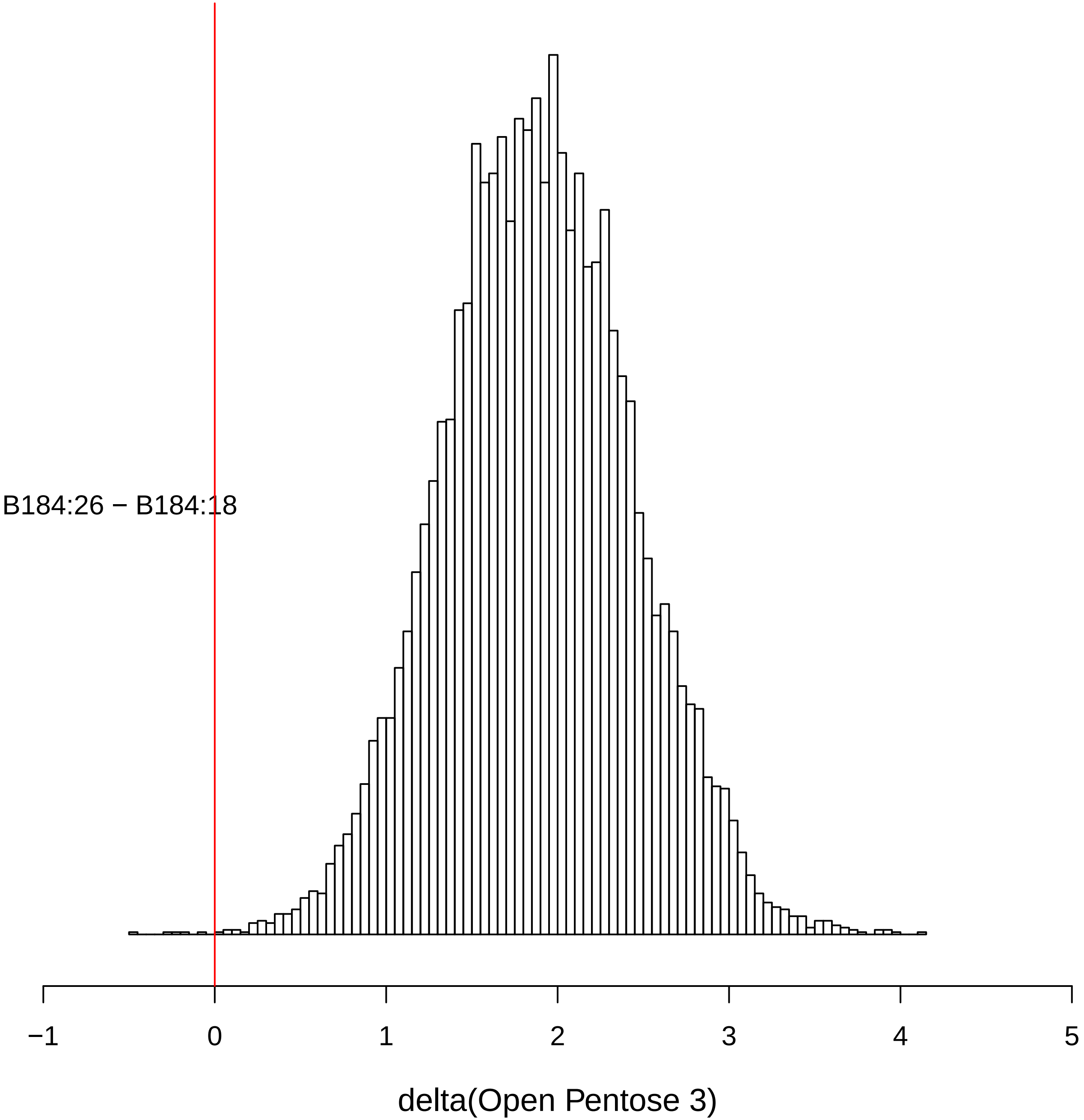

B184:26 − B184:18
−1
0
1
2
3
4
5
delta(Open Pentose 3)

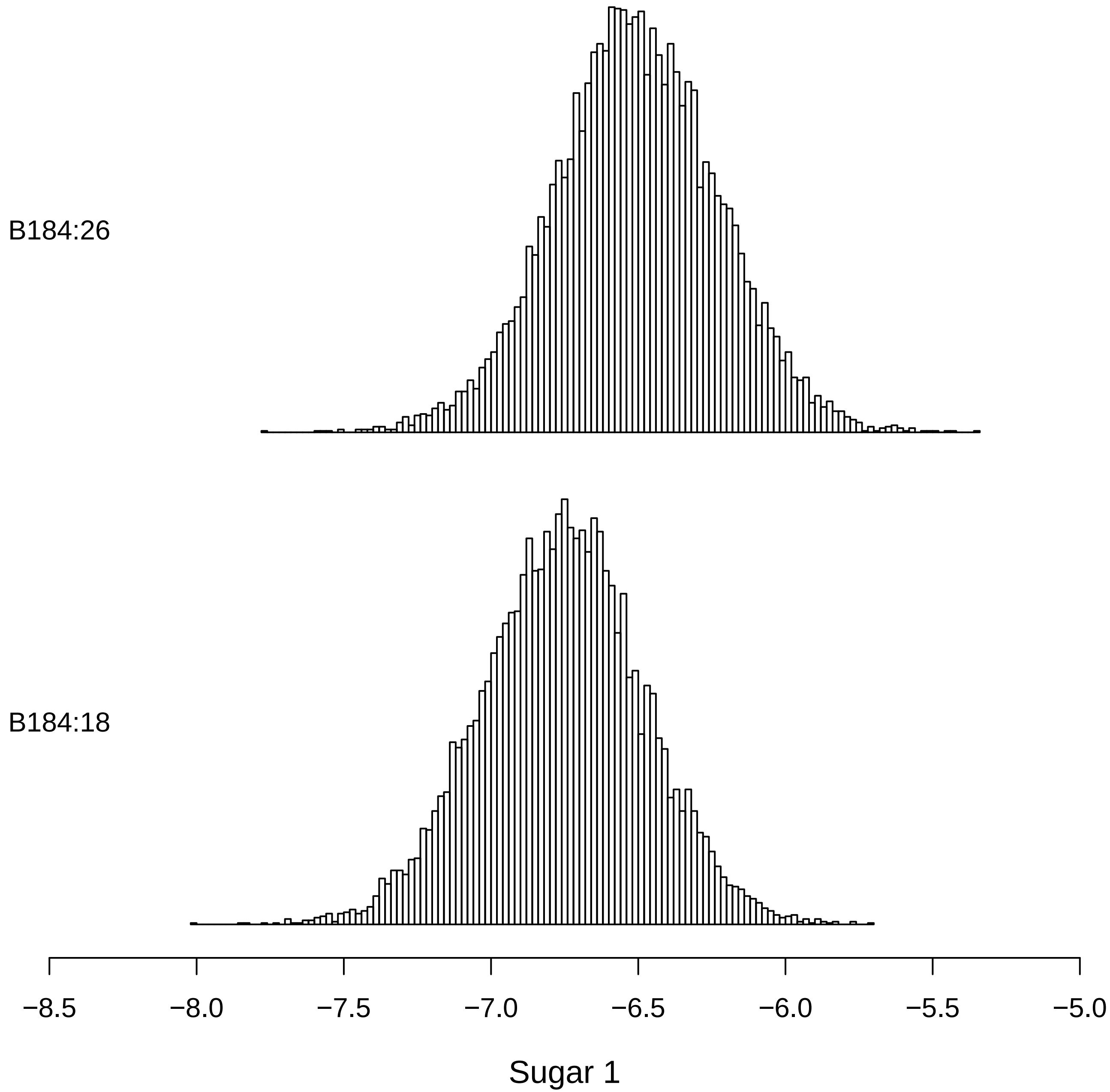

B184:26
B184:18
−8.5
−8.0
−7.5
−7.0
−6.5
−6.0
−5.5
−5.0
Sugar 1

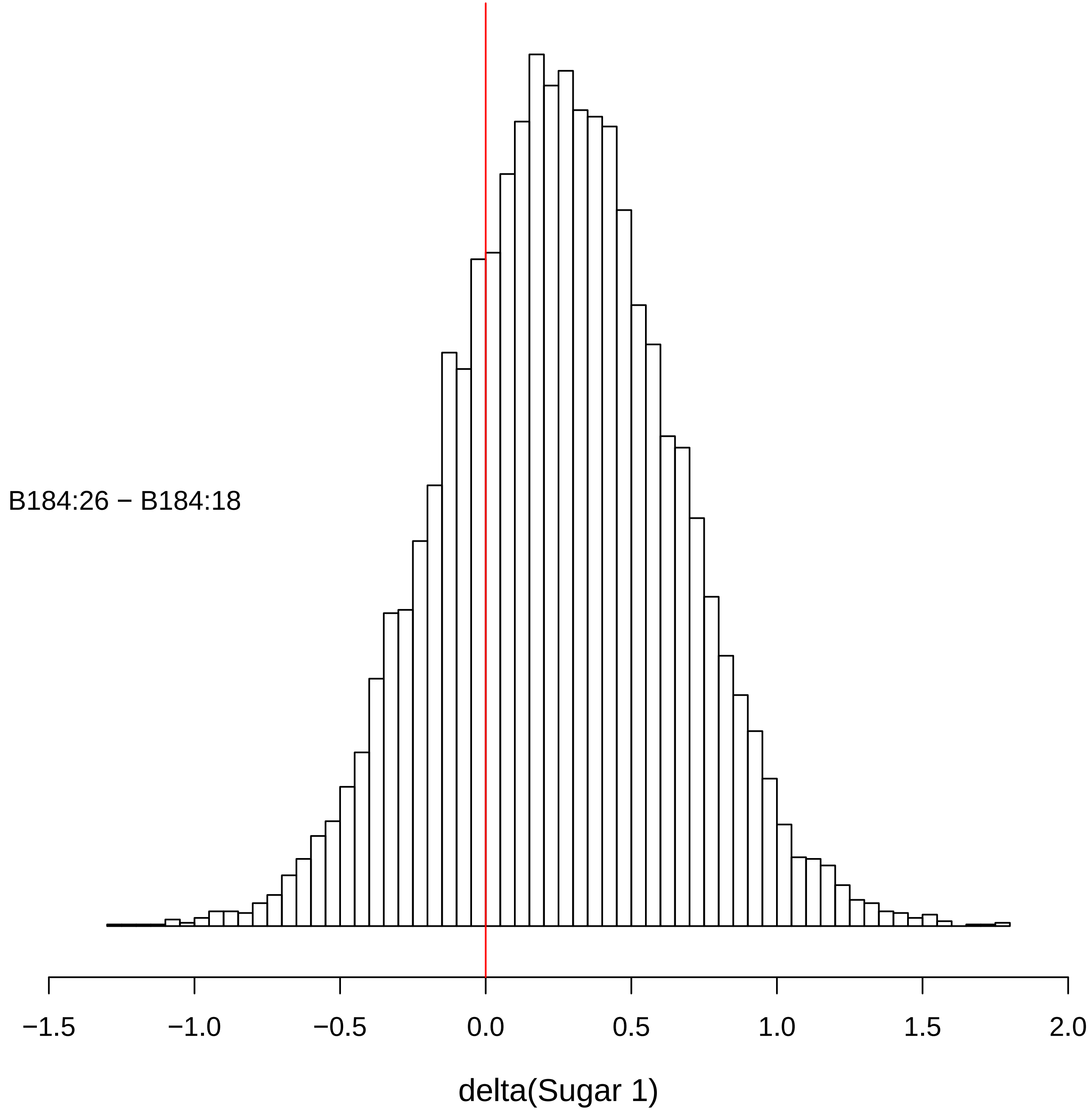

B184:26 − B184:18
−1.5
−1.0
−0.5
0.0
0.5
1.0
1.5
2.0
delta(Sugar 1)

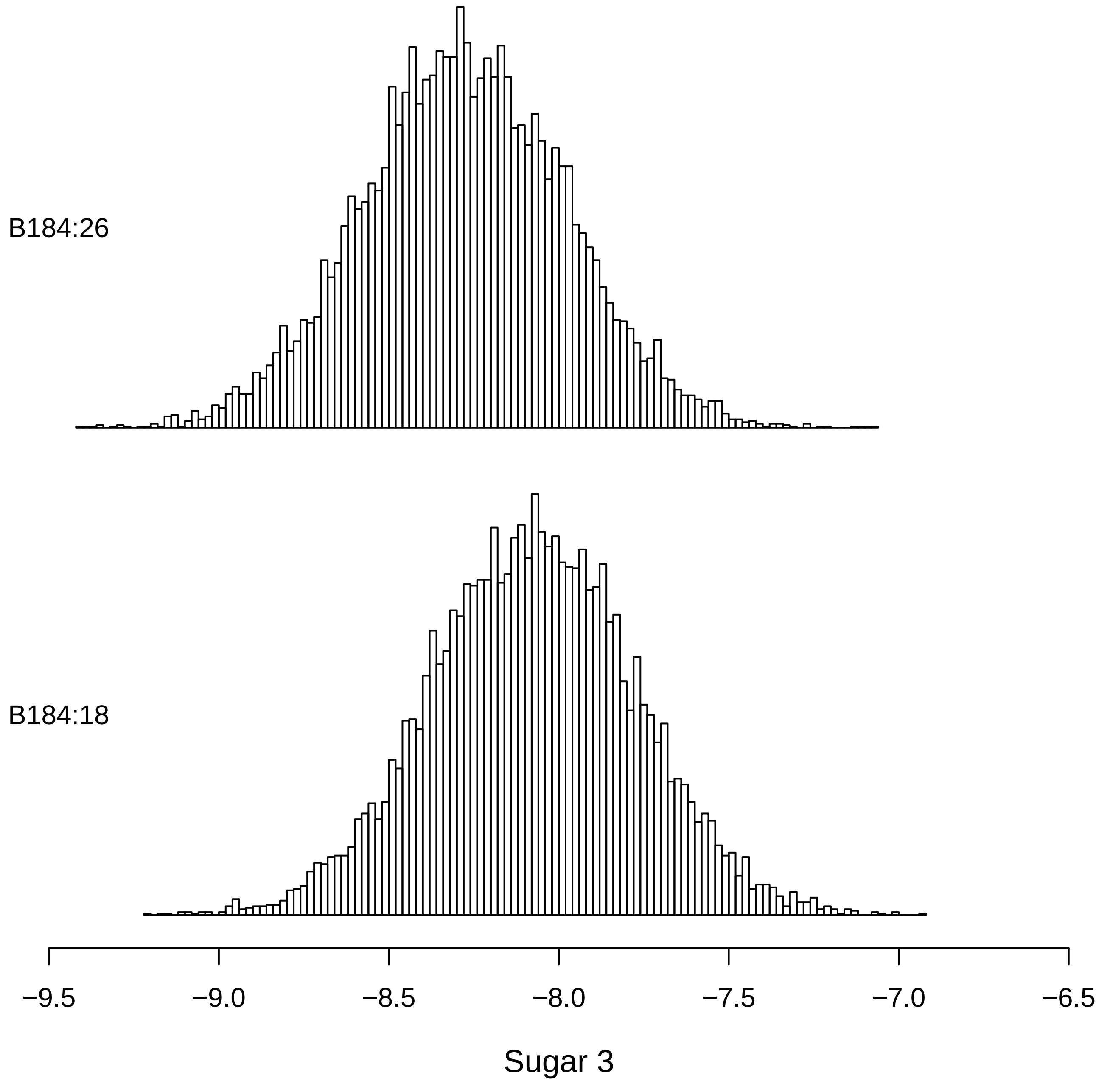
B184:26
B184:18
−9.5
−9.0
−8.5
−8.0
−7.5
−7.0
−6.5
Sugar 3

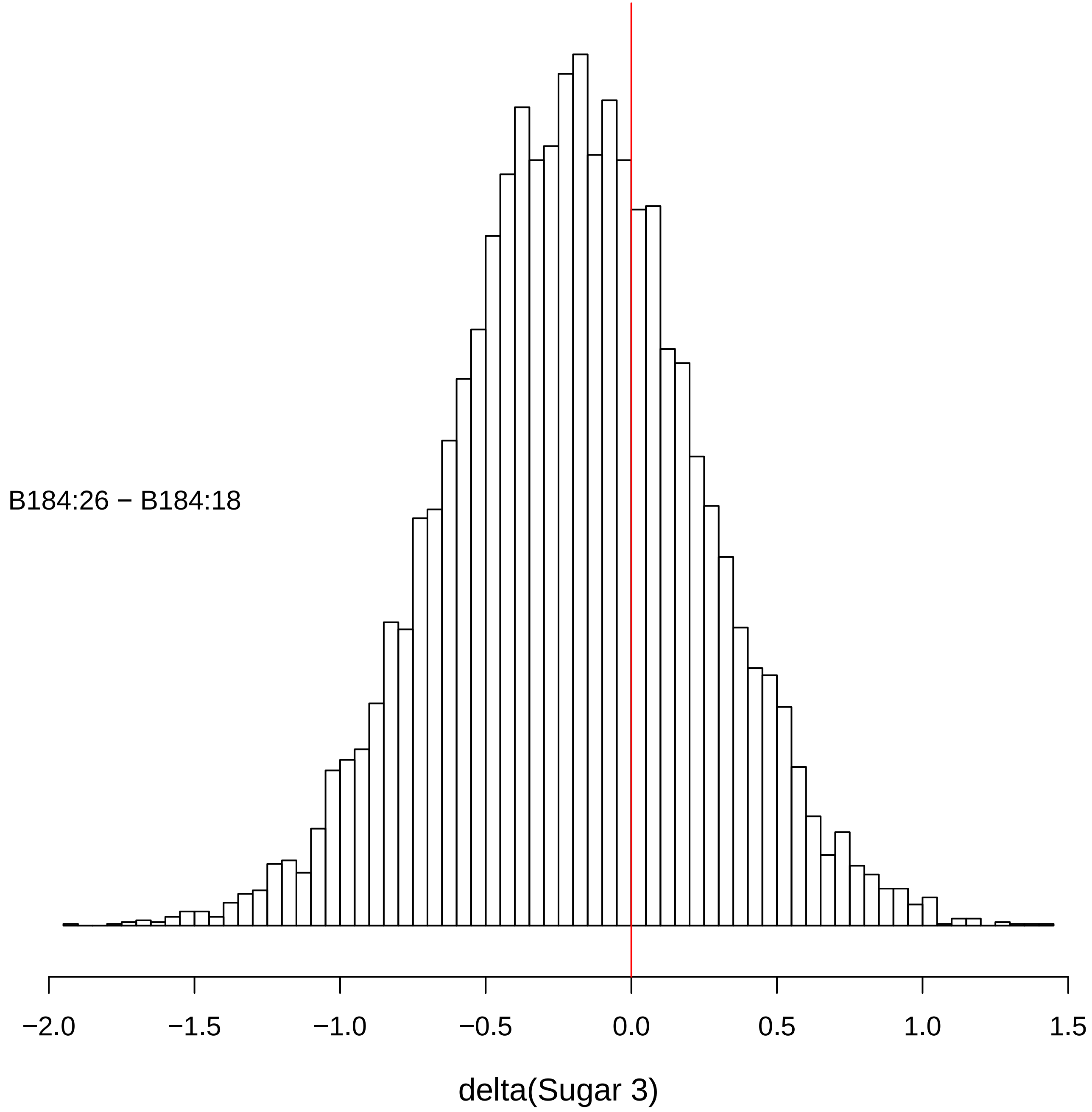
B184:26 − B184:18
−2.0
−1.5
−1.0
−0.5
0.0
0.5
1.0
1.5
delta(Sugar 3)

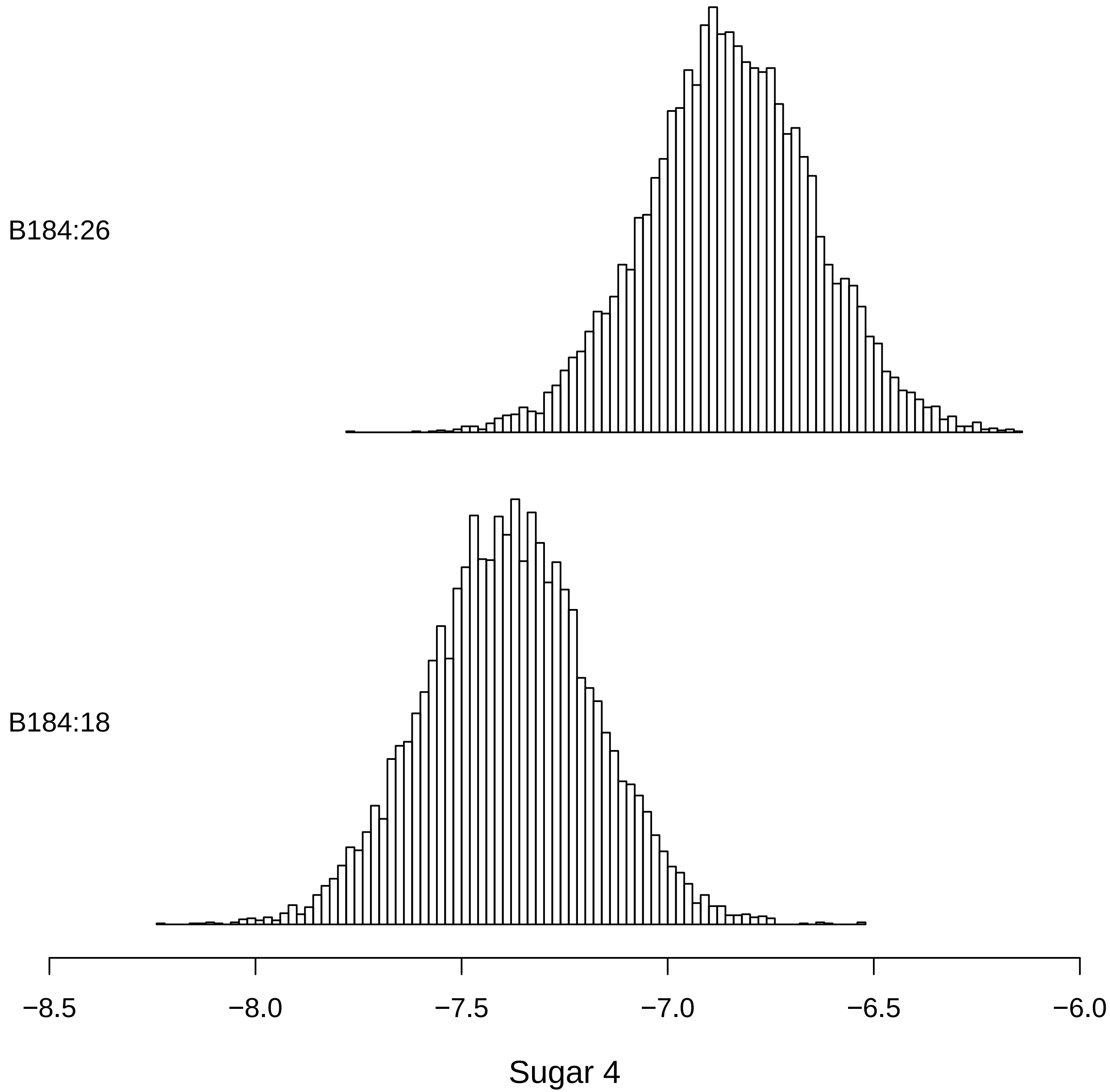
B184:26
B184:18
−8.5
−8.0
−7.5
−7.0
−6.5
−6.0
Sugar 4

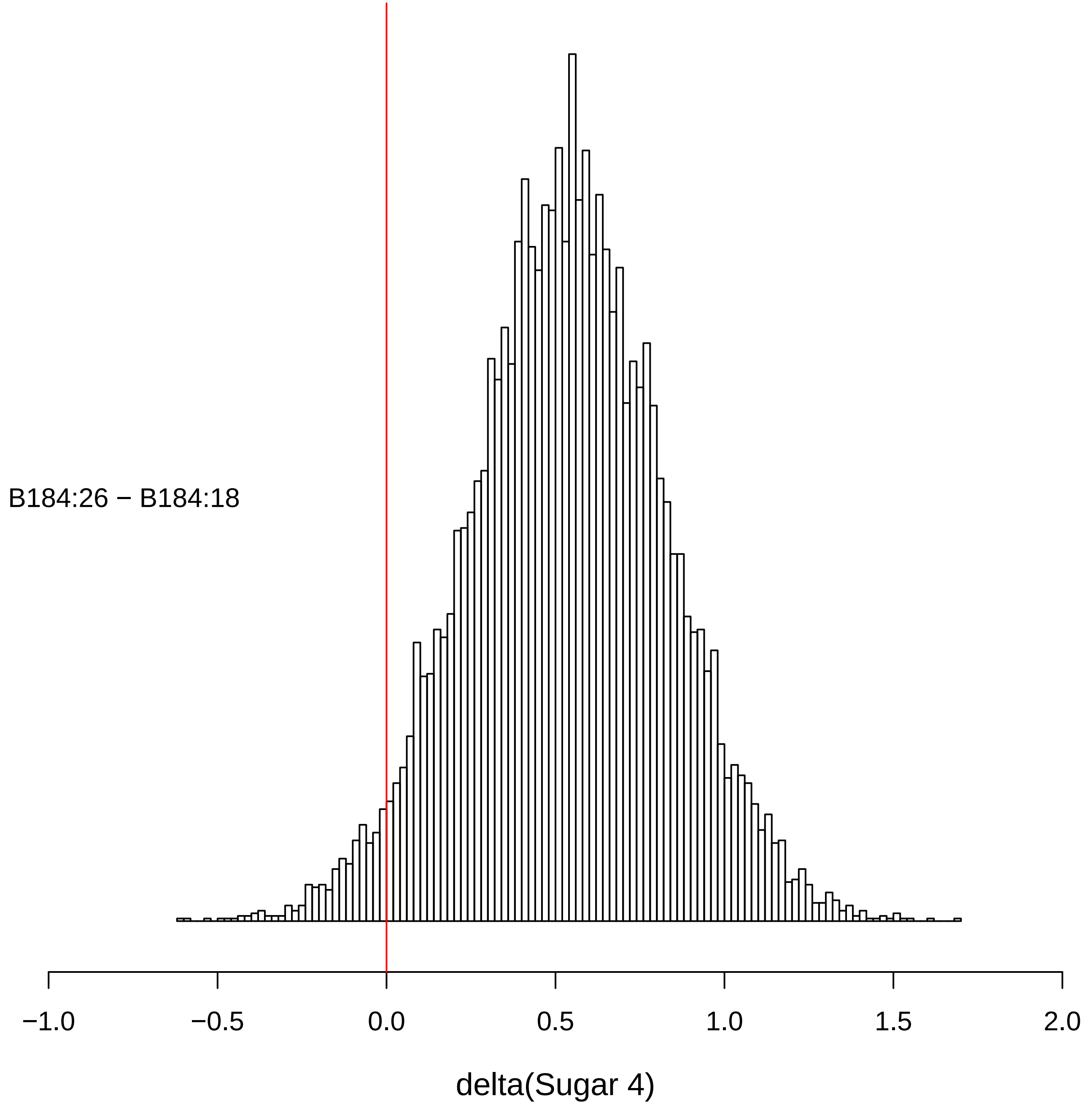
B184:26 − B184:18
−1.0
−0.5
0.0
0.5
1.0
1.5
2.0
delta(Sugar 4)

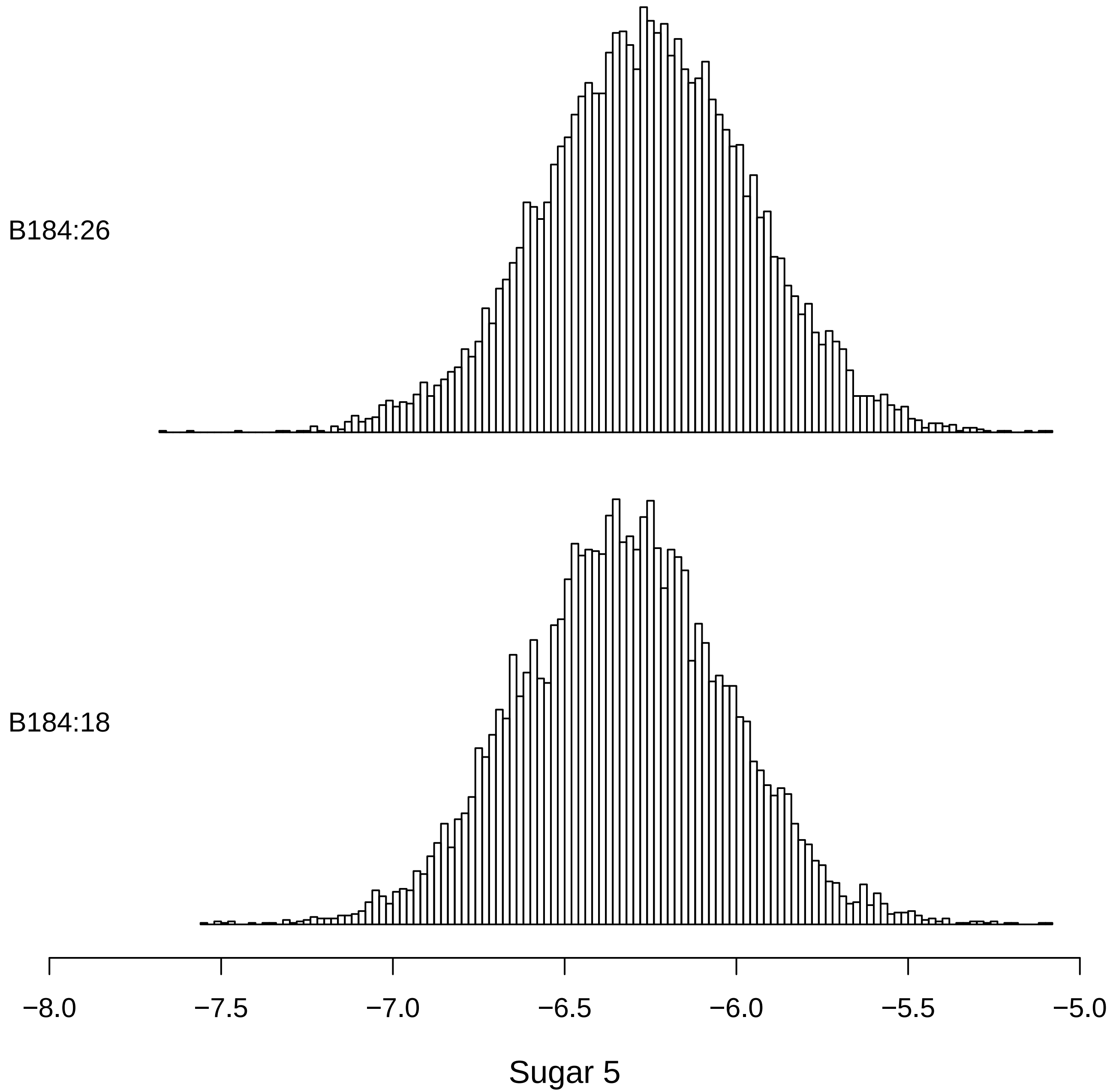
B184:26
B184:18
−8.0
−7.5
−7.0
−6.5
−6.0
−5.5
−5.0
Sugar 5

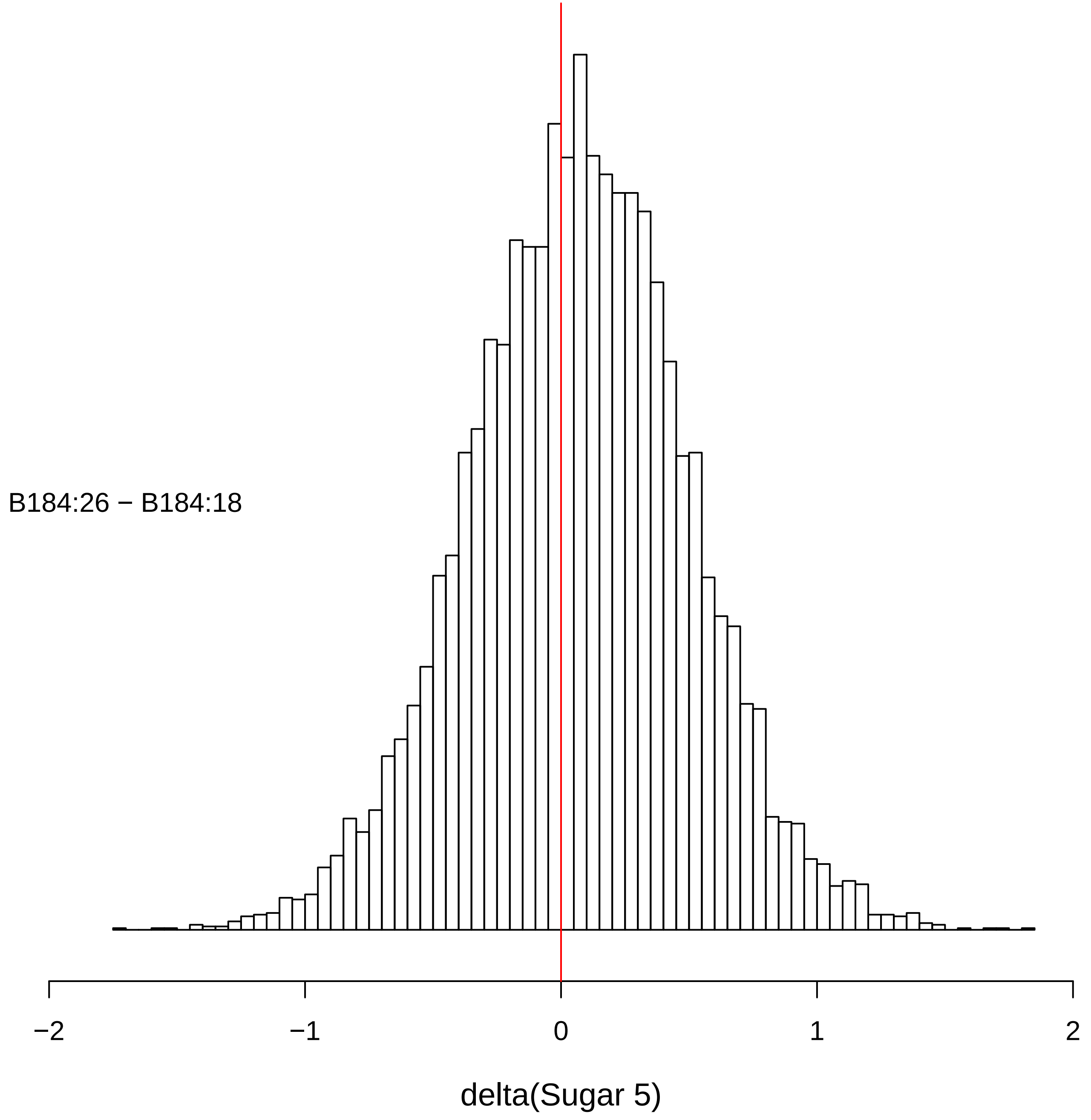
B184:26 − B184:18
−2
−1
0
1
2
delta(Sugar 5)

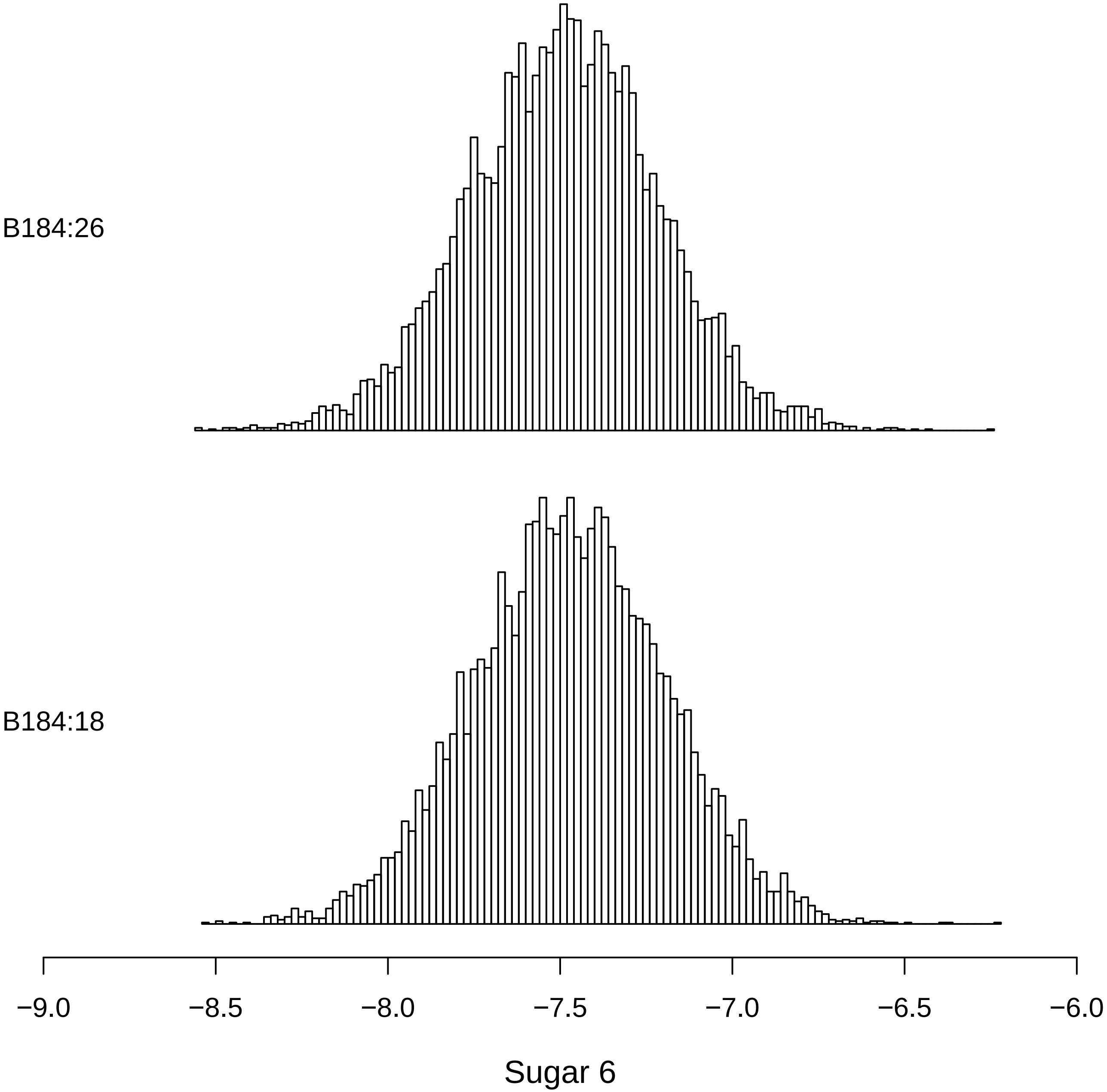

B184:26
B184:18
−9.0
−8.5
−8.0
−7.5
−7.0
−6.5
−6.0
Sugar 6

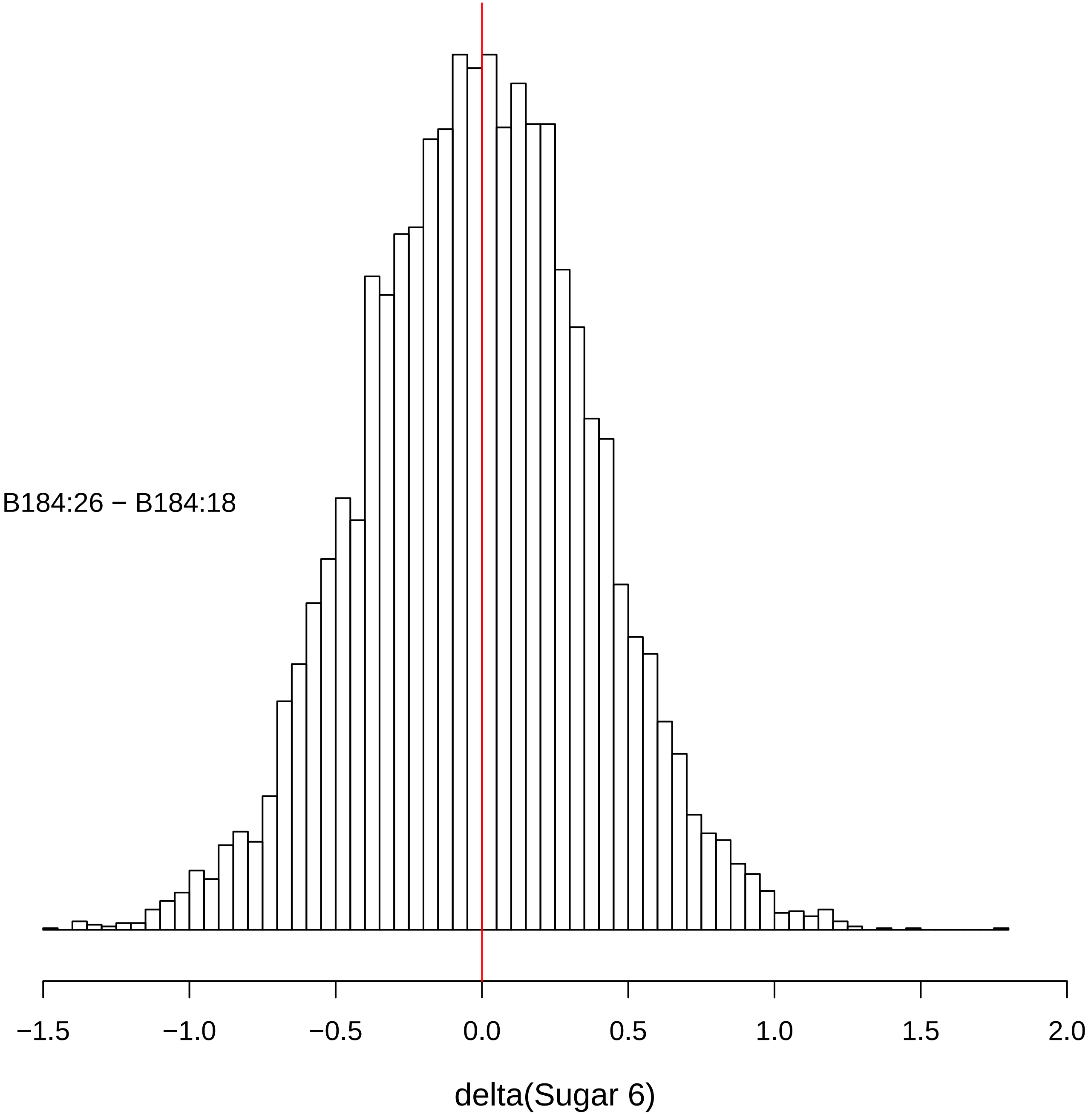
B184:26 − B184:18
−1.5
−1.0
−0.5
0.0
0.5
1.0
1.5
2.0
delta(Sugar 6)

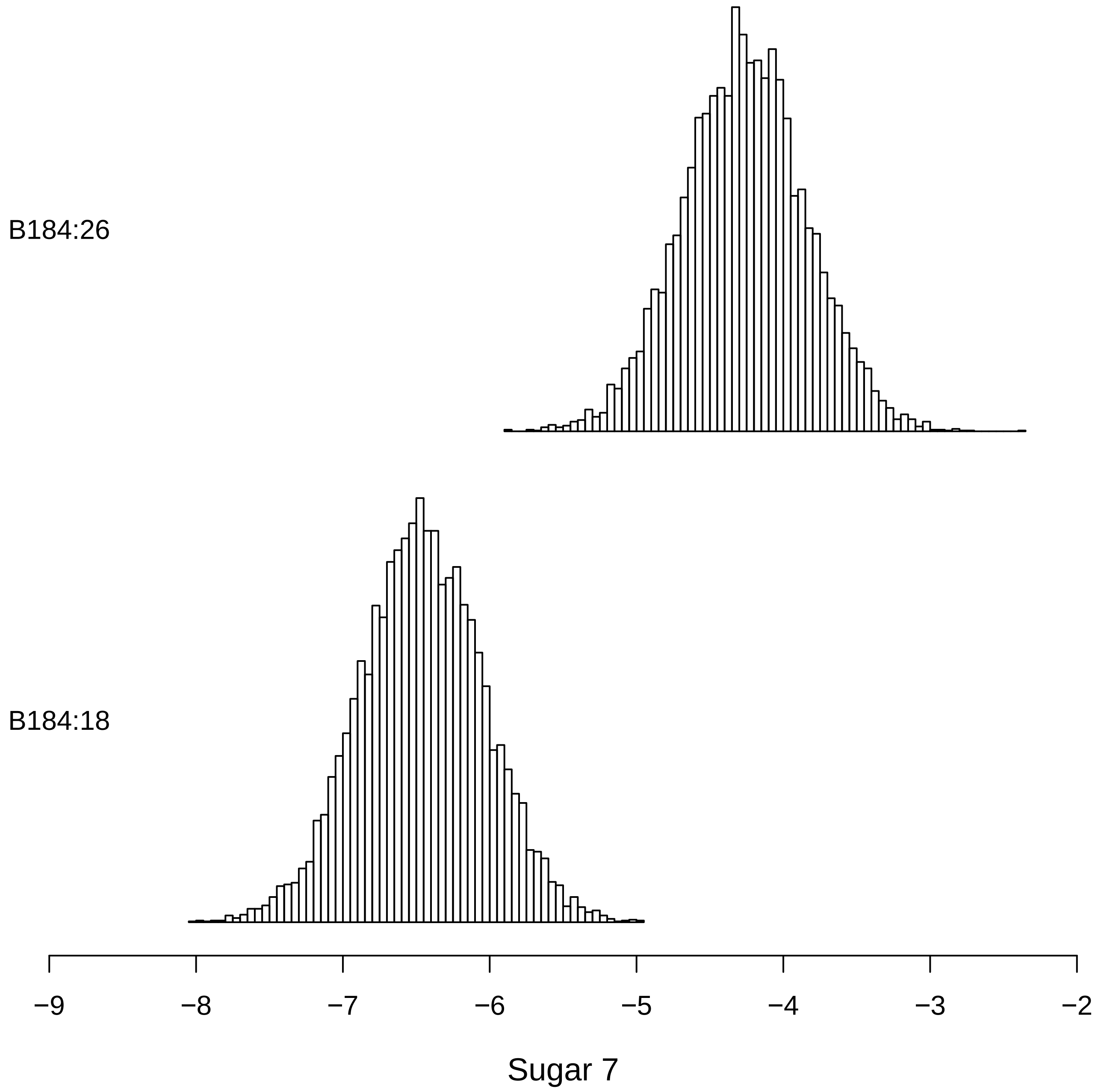
B184:26
B184:18
−9
−8
−7
−6
−5
−4
−3
−2
Sugar 7

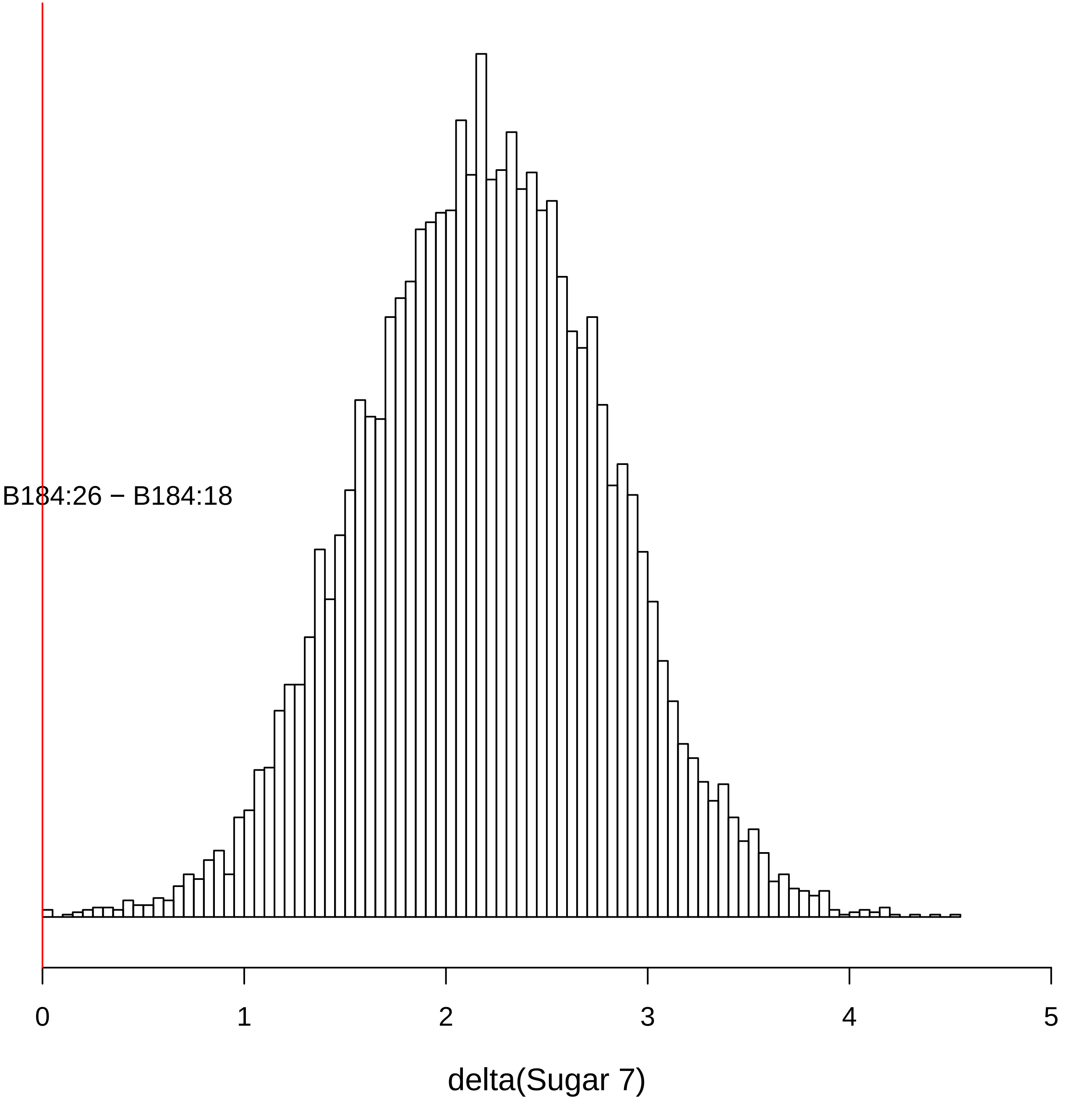
B184:26 − B184:18
0
1
2
3
4
5
delta(Sugar 7)

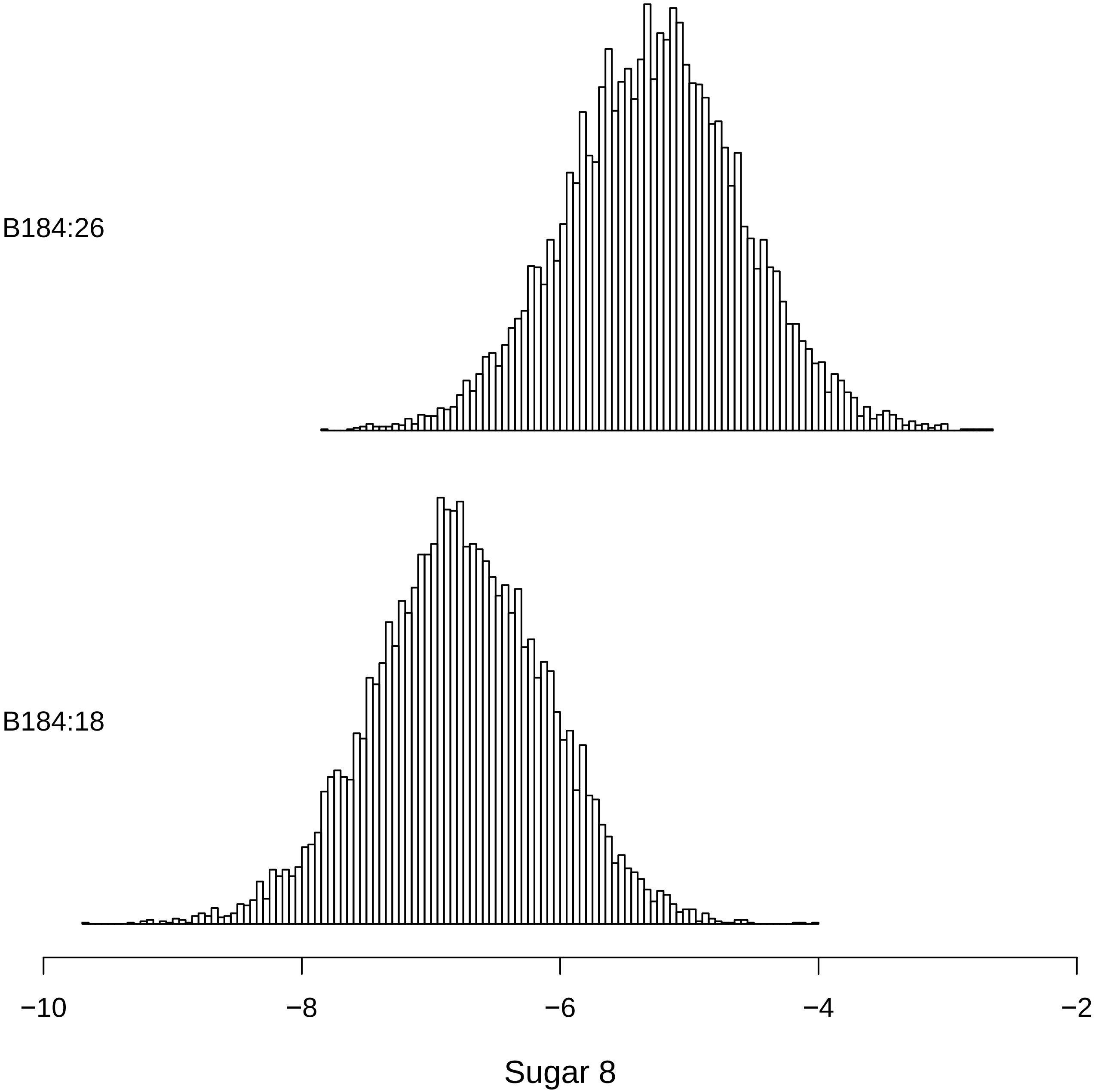
B184:26
B184:18
−10
−8
−6
−4
−2
Sugar 8

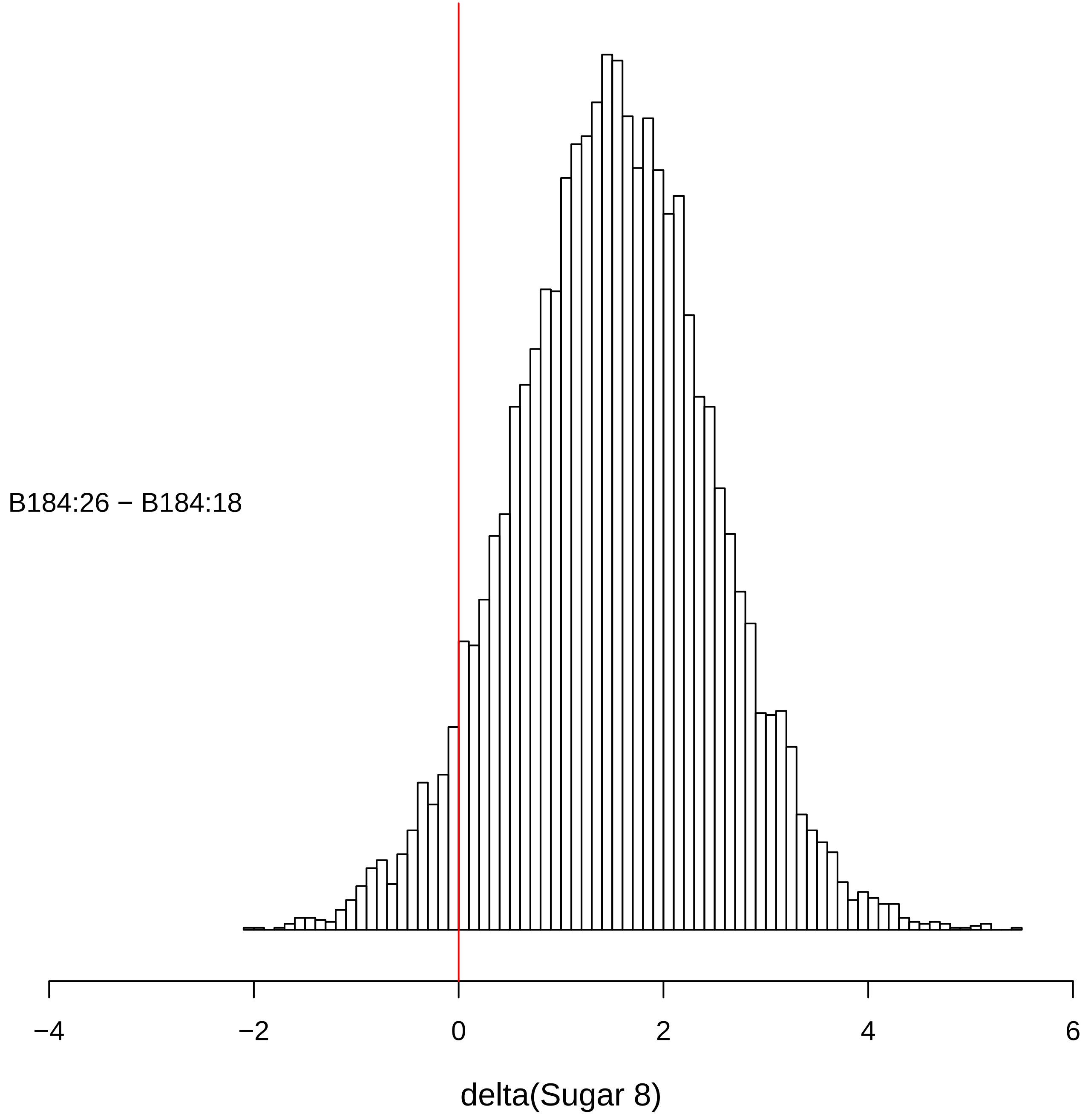
B184:26 − B184:18
−4
−2
0
2
4
6
delta(Sugar 8)

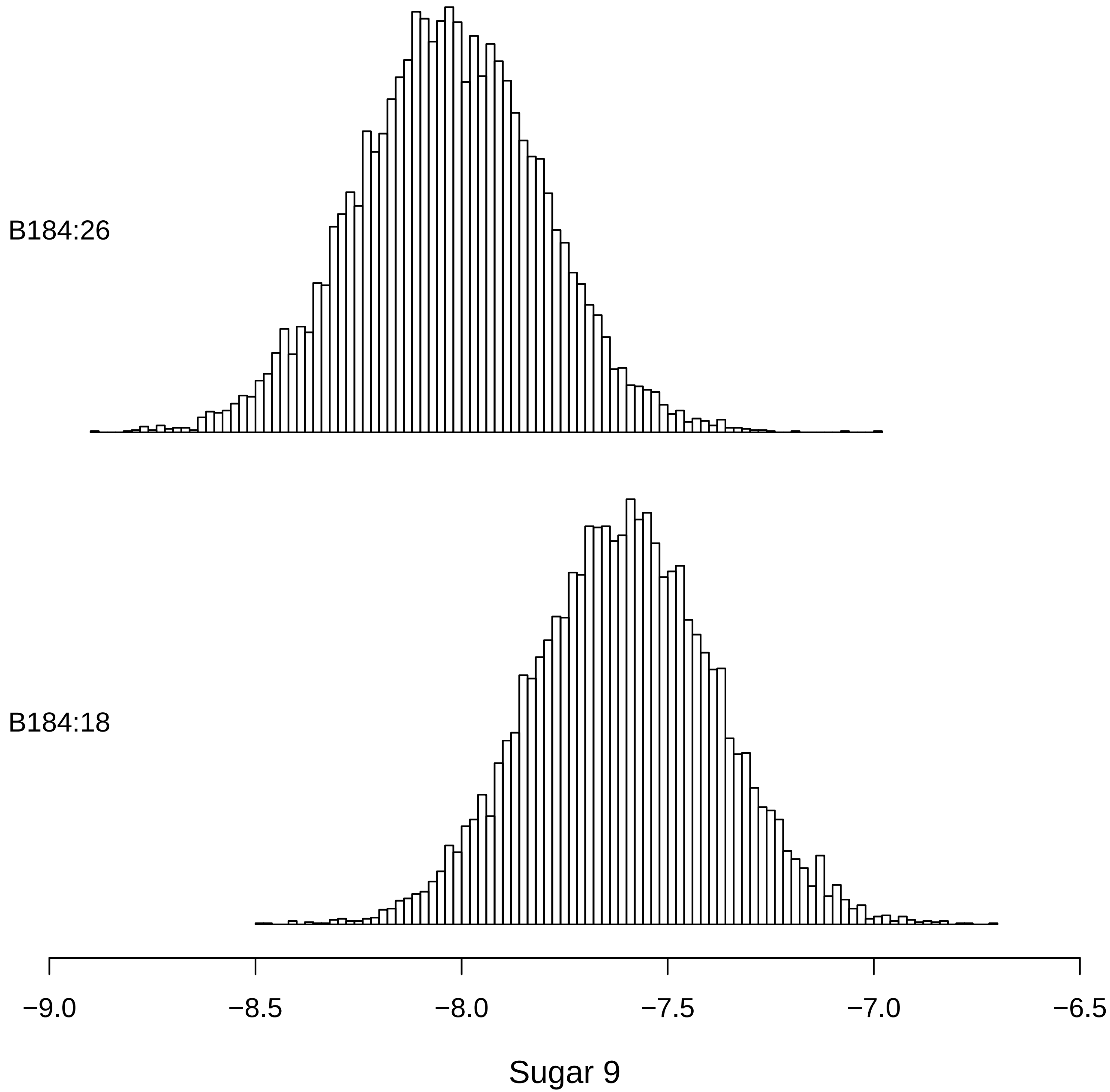
B184:26
B184:18
−9.0
−8.5
−8.0
−7.5
−7.0
−6.5
Sugar 9

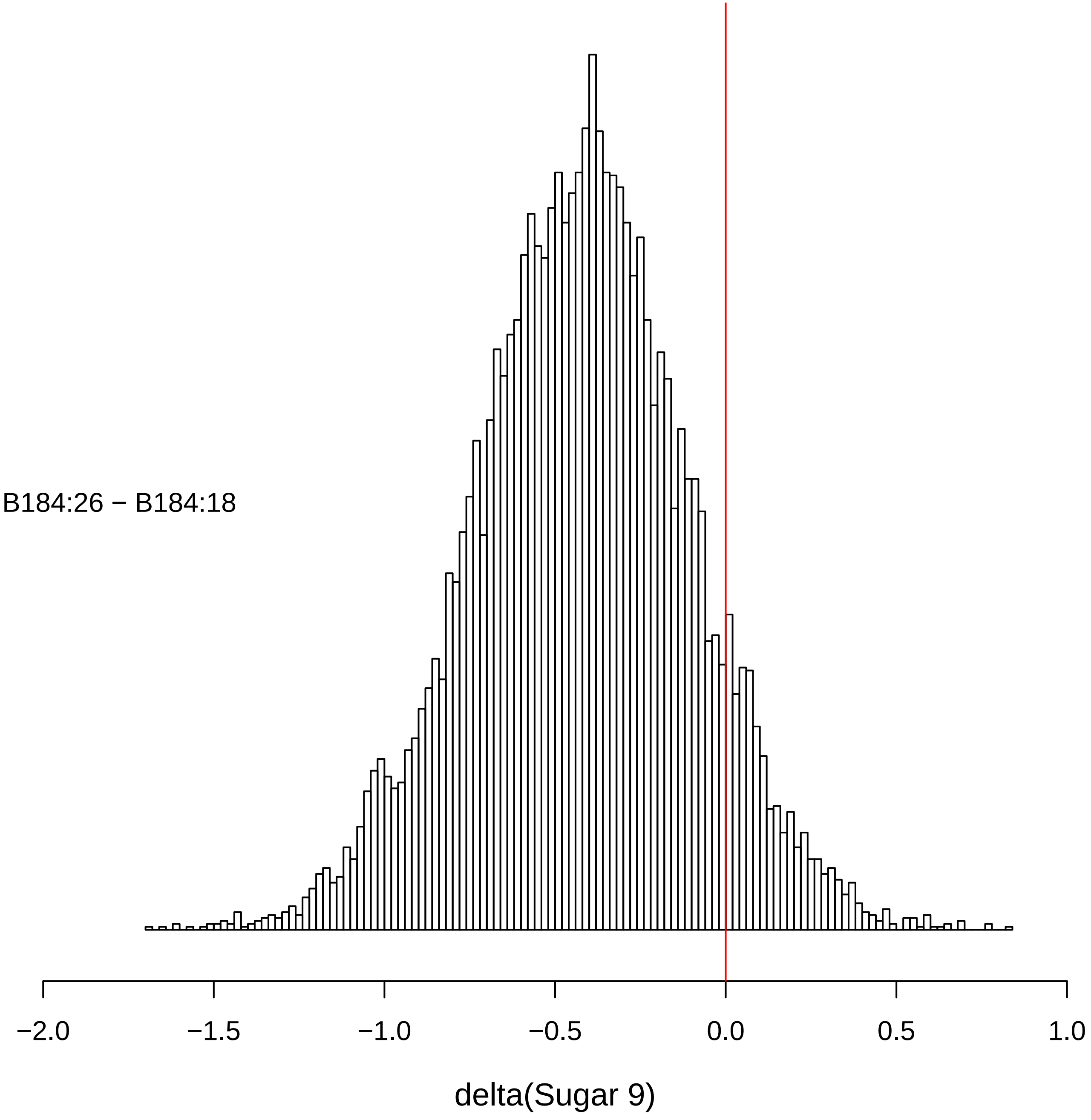
B184:26 − B184:18
−2.0
−1.5
−1.0
−0.5
0.0
0.5
1.0
delta(Sugar 9)

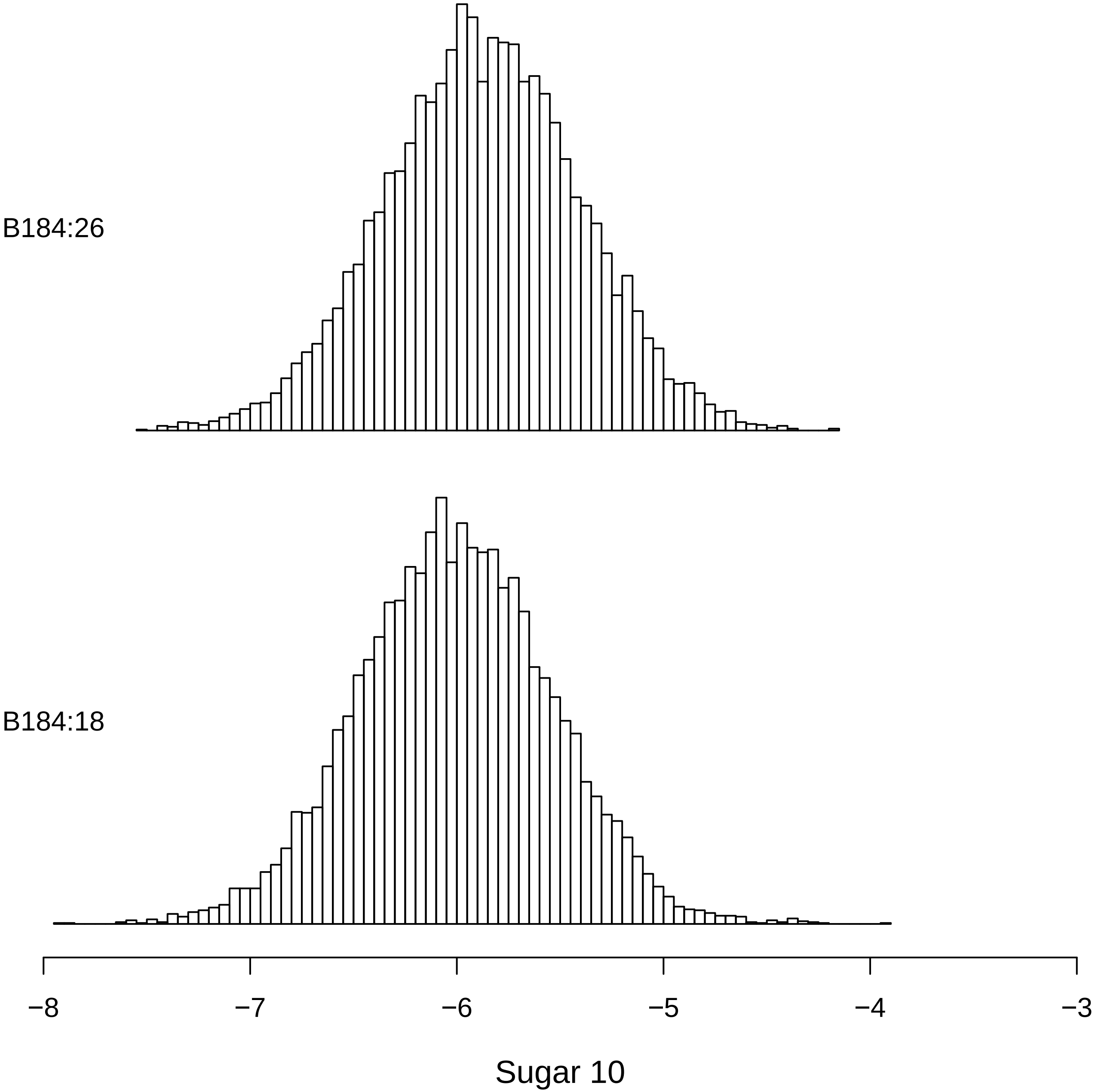
B184:26
B184:18
−8
−7
−6
−5
−4
−3
Sugar 10

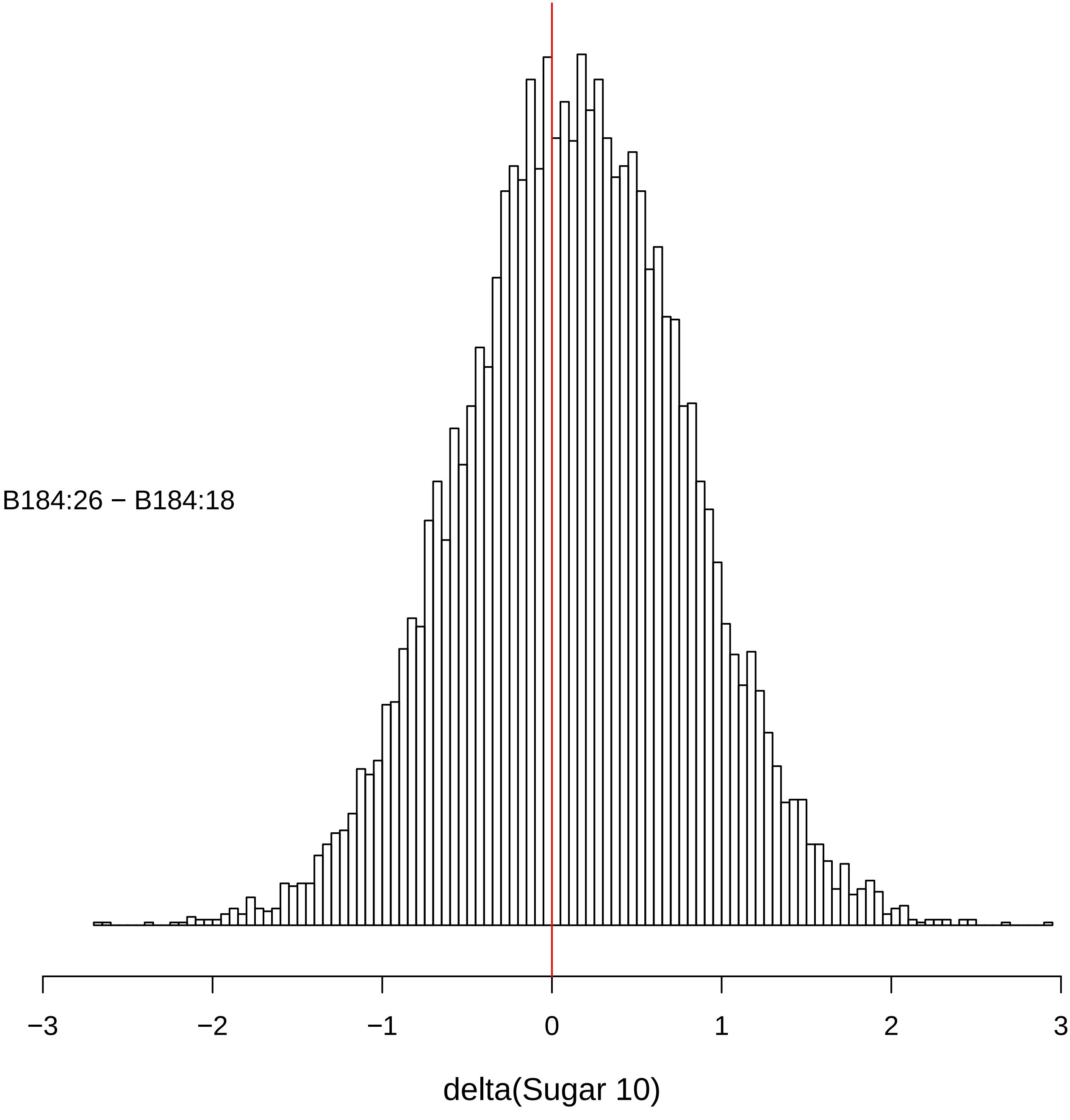
B184:26 − B184:18
−3
−2
−1
0
1
2
3
delta(Sugar 10)

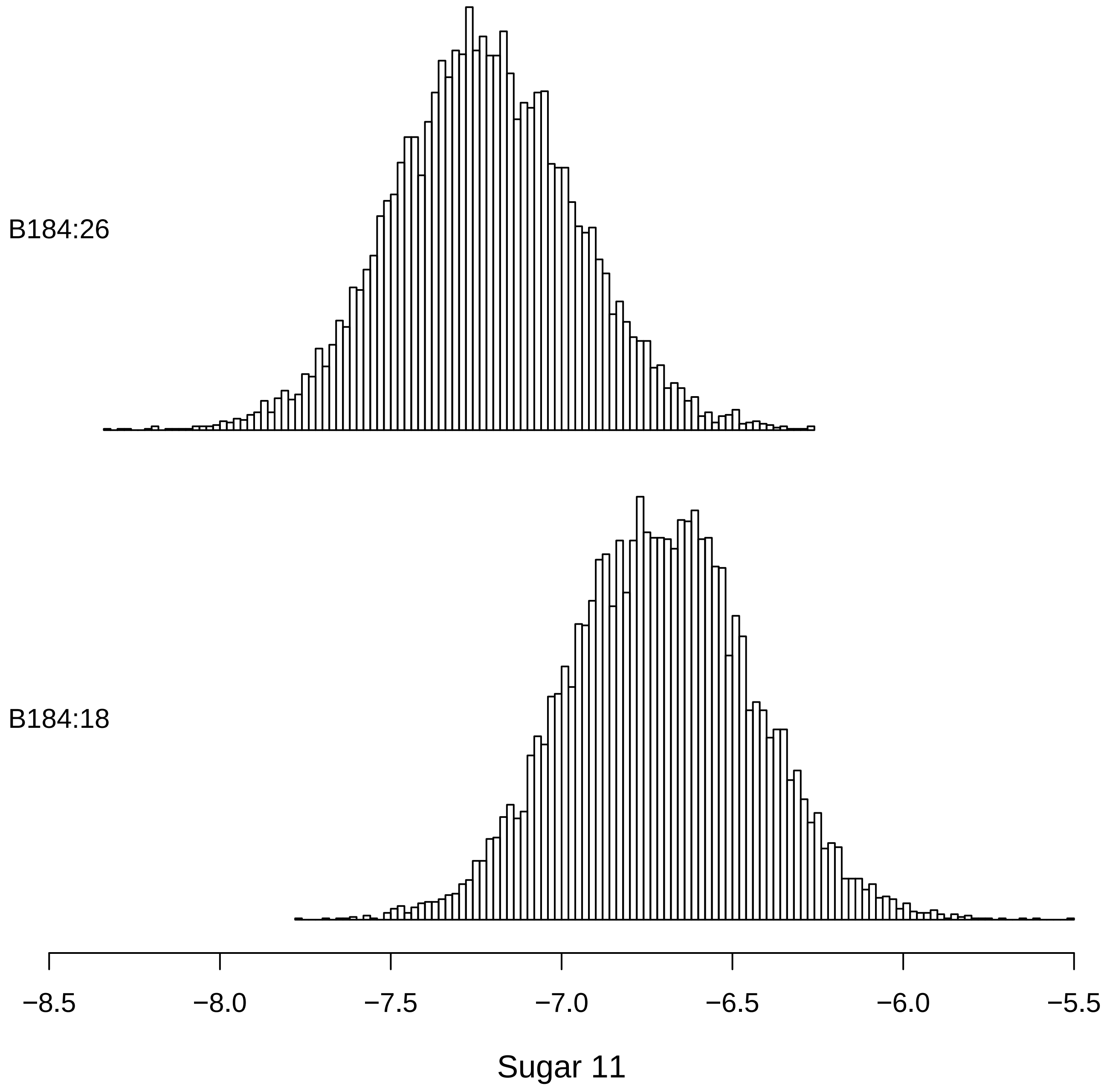
B184:26
B184:18
−8.5
−8.0
−7.5
−7.0
−6.5
−6.0
−5.5
Sugar 11

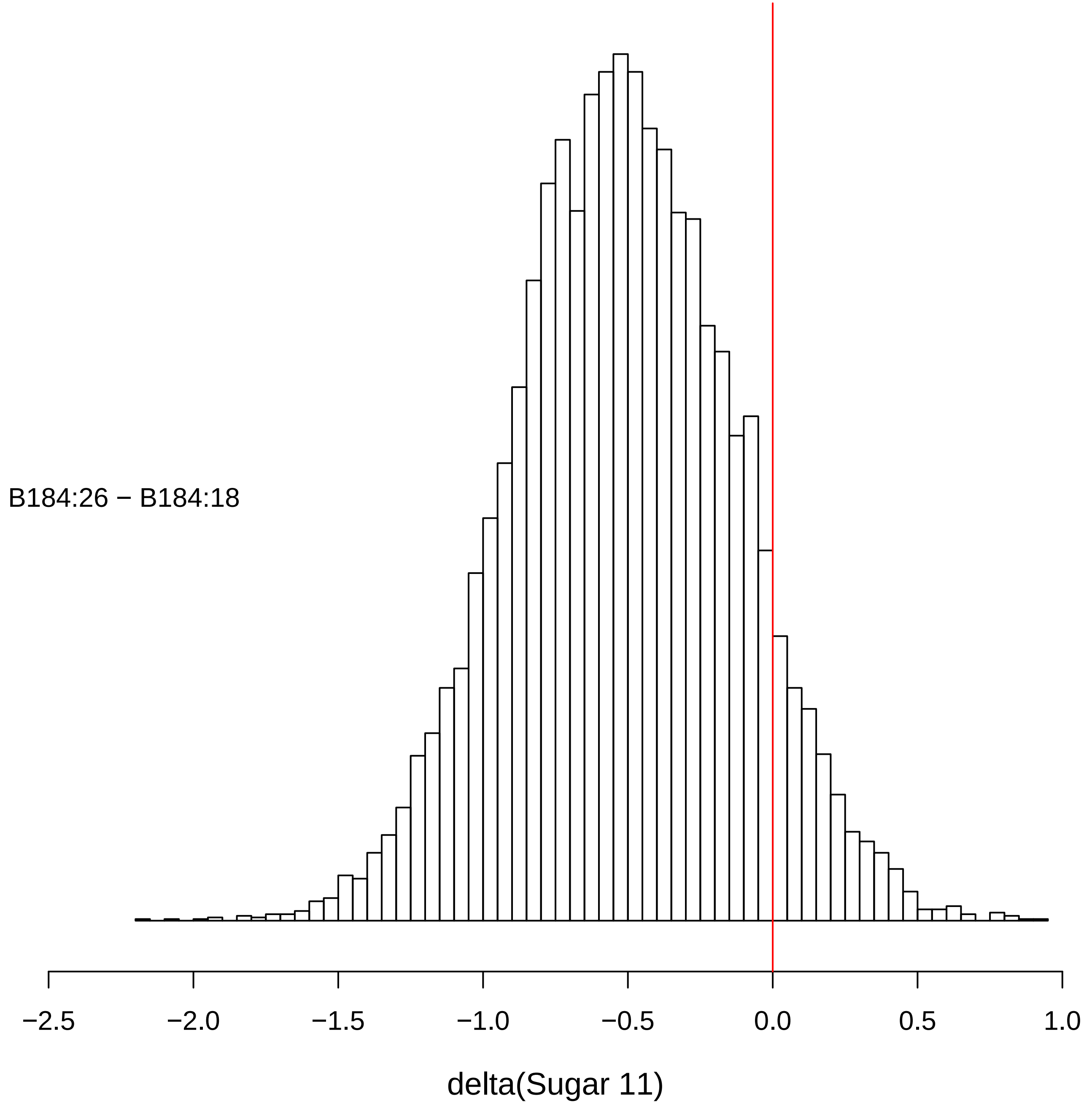

B184:26 − B184:18
−2.5
−2.0
−1.5
−1.0
−0.5
0.0
0.5
1.0
delta(Sugar 11)

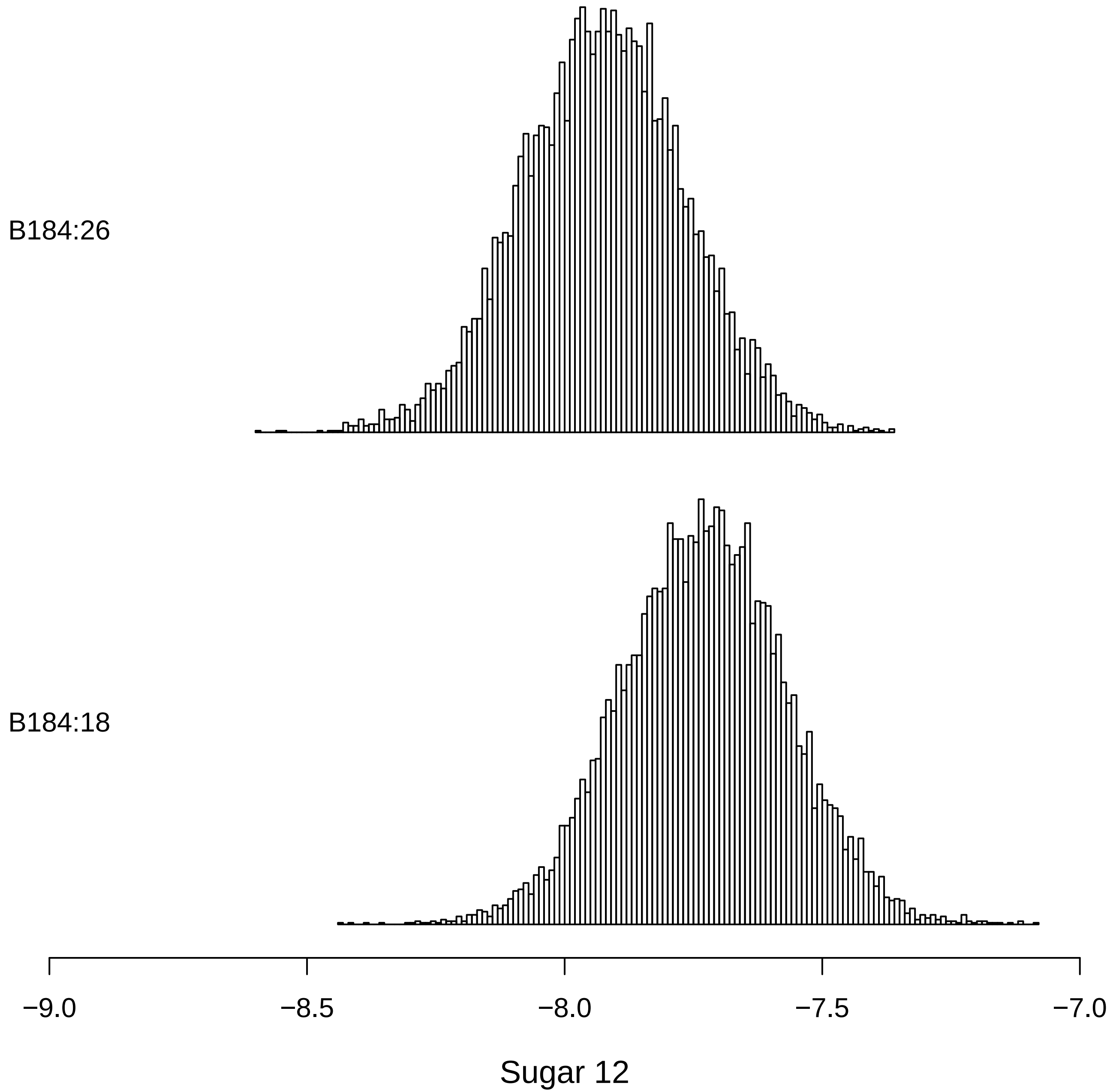
B184:26
B184:18
−9.0
−8.5
−8.0
−7.5
−7.0
Sugar 12

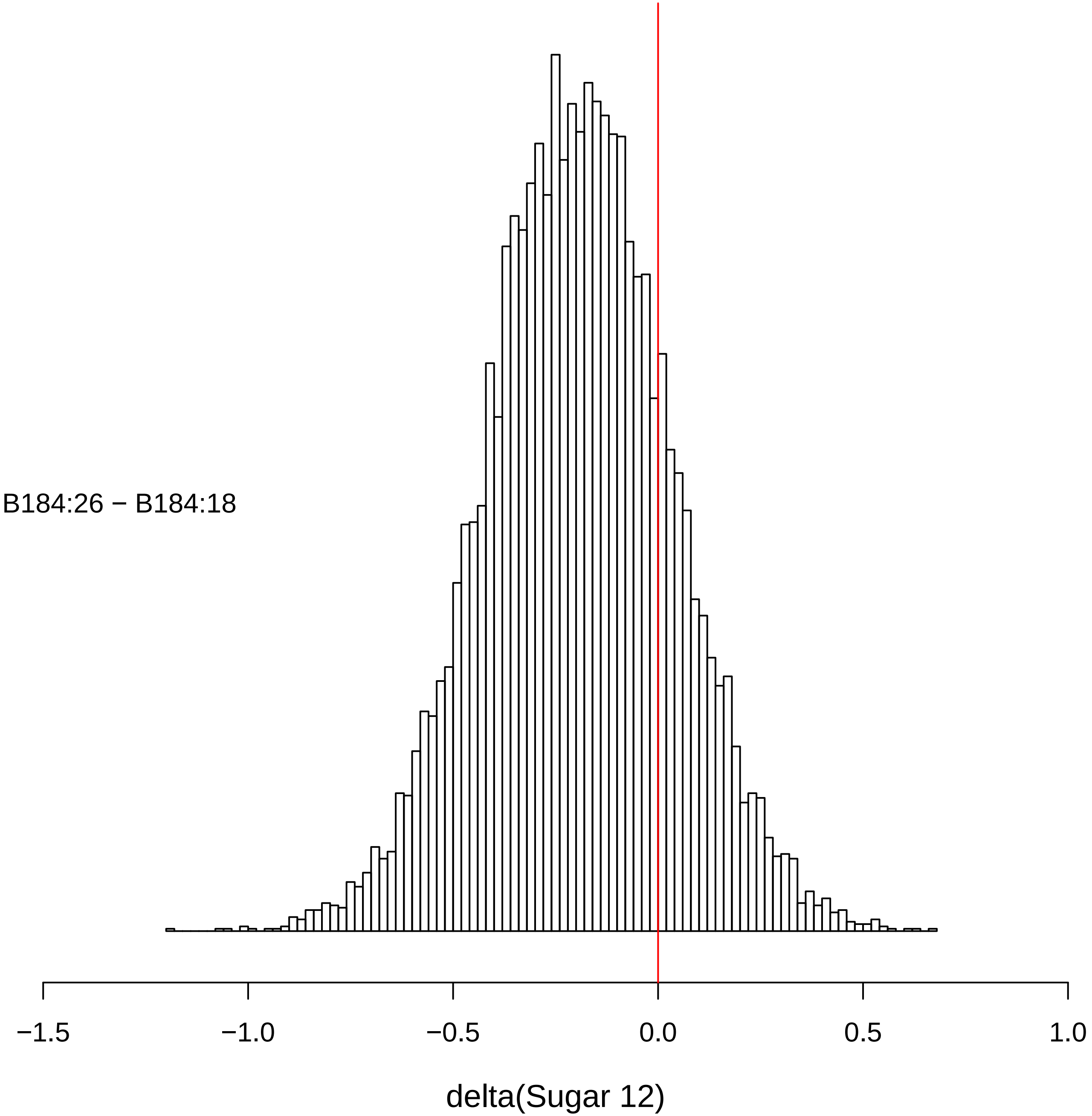
B184:26 − B184:18
−1.5
−1.0
−0.5
0.0
0.5
1.0
delta(Sugar 12)

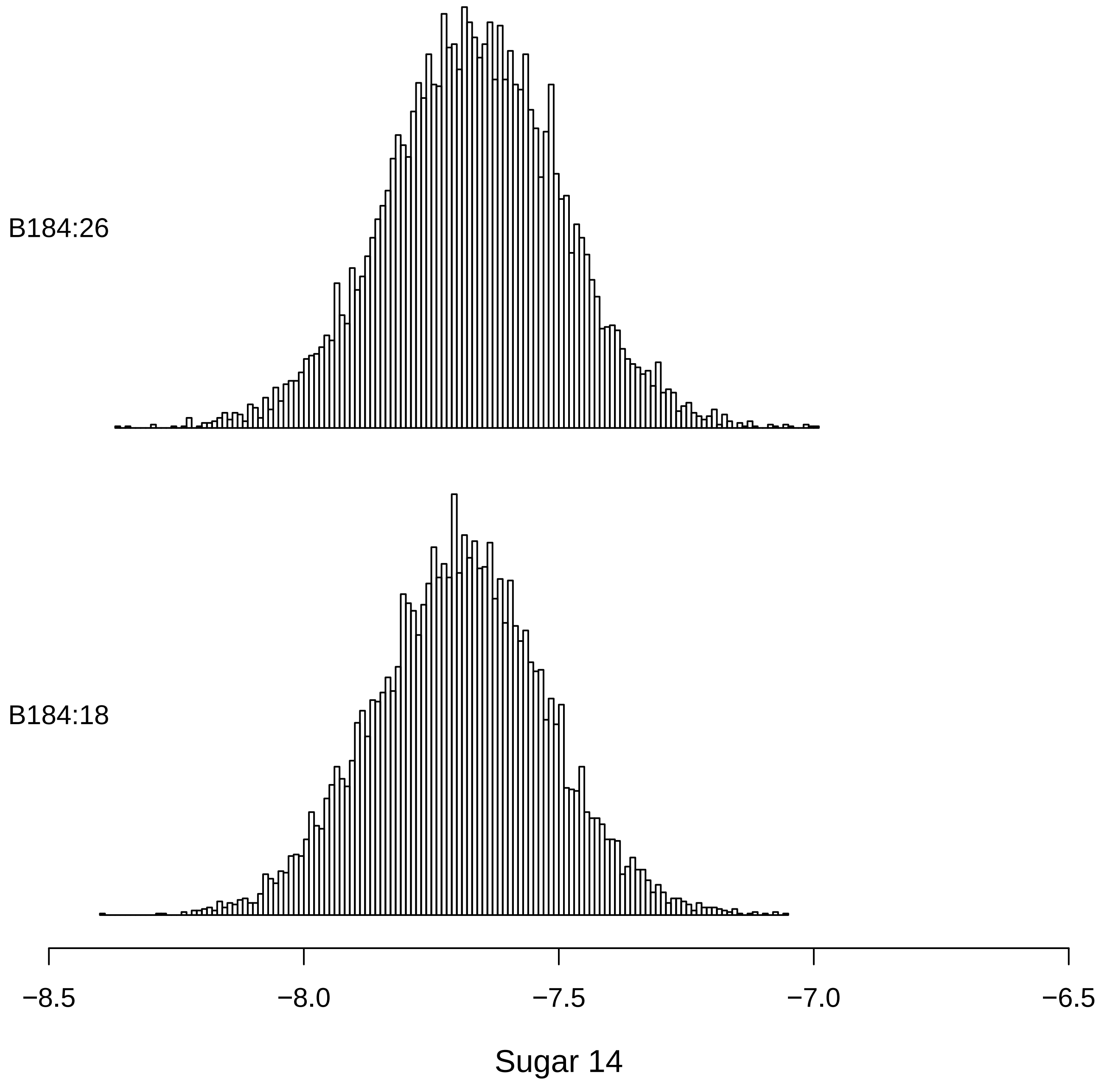

B184:26
B184:18
−8.5
−8.0
−7.5
−7.0
−6.5
Sugar 14

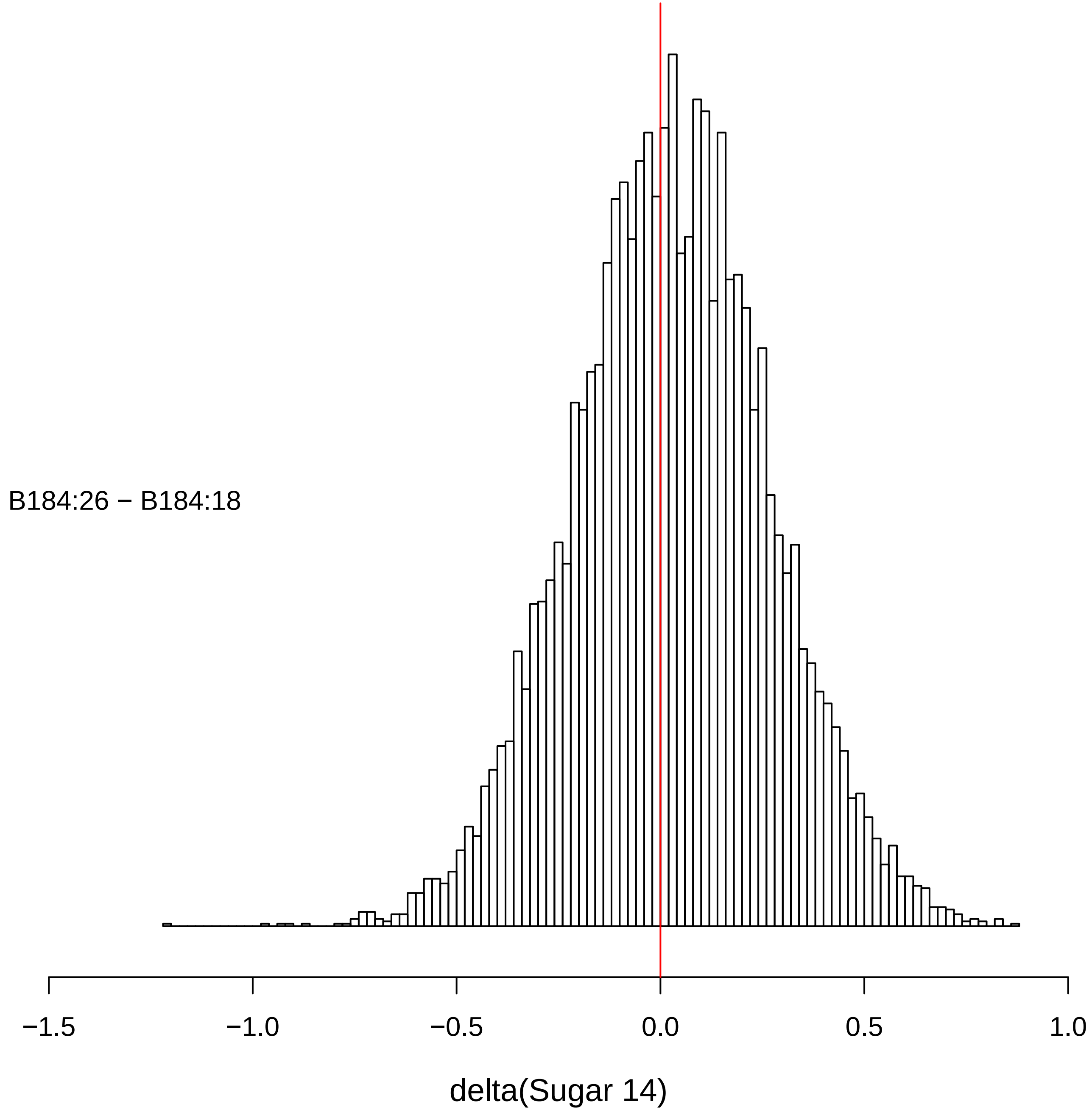
B184:26 − B184:18
−1.5
−1.0
−0.5
0.0
0.5
1.0
delta(Sugar 14)

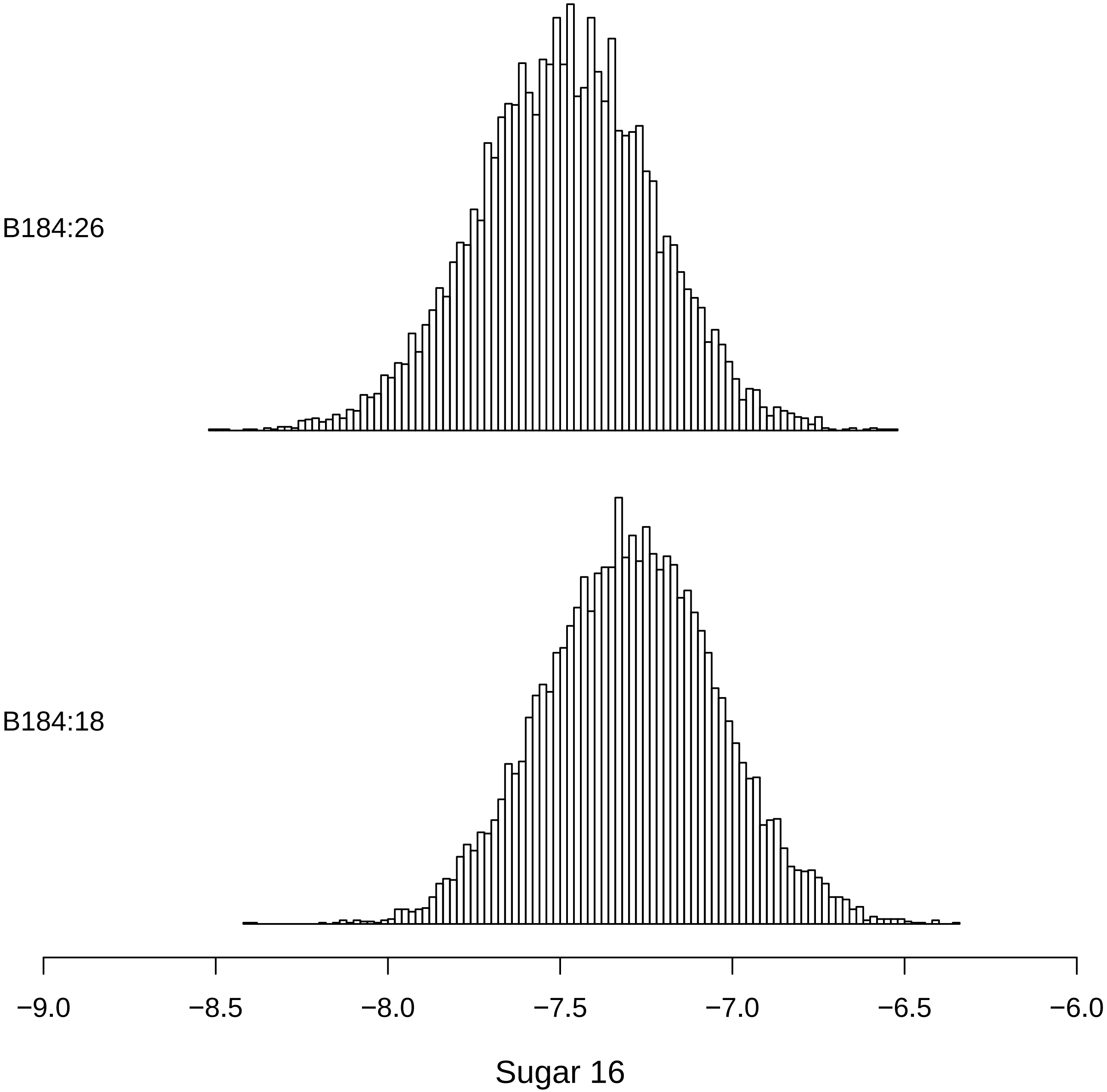

B184:26
B184:18
−9.0
−8.5
−8.0
−7.5
−7.0
−6.5
−6.0
Sugar 16

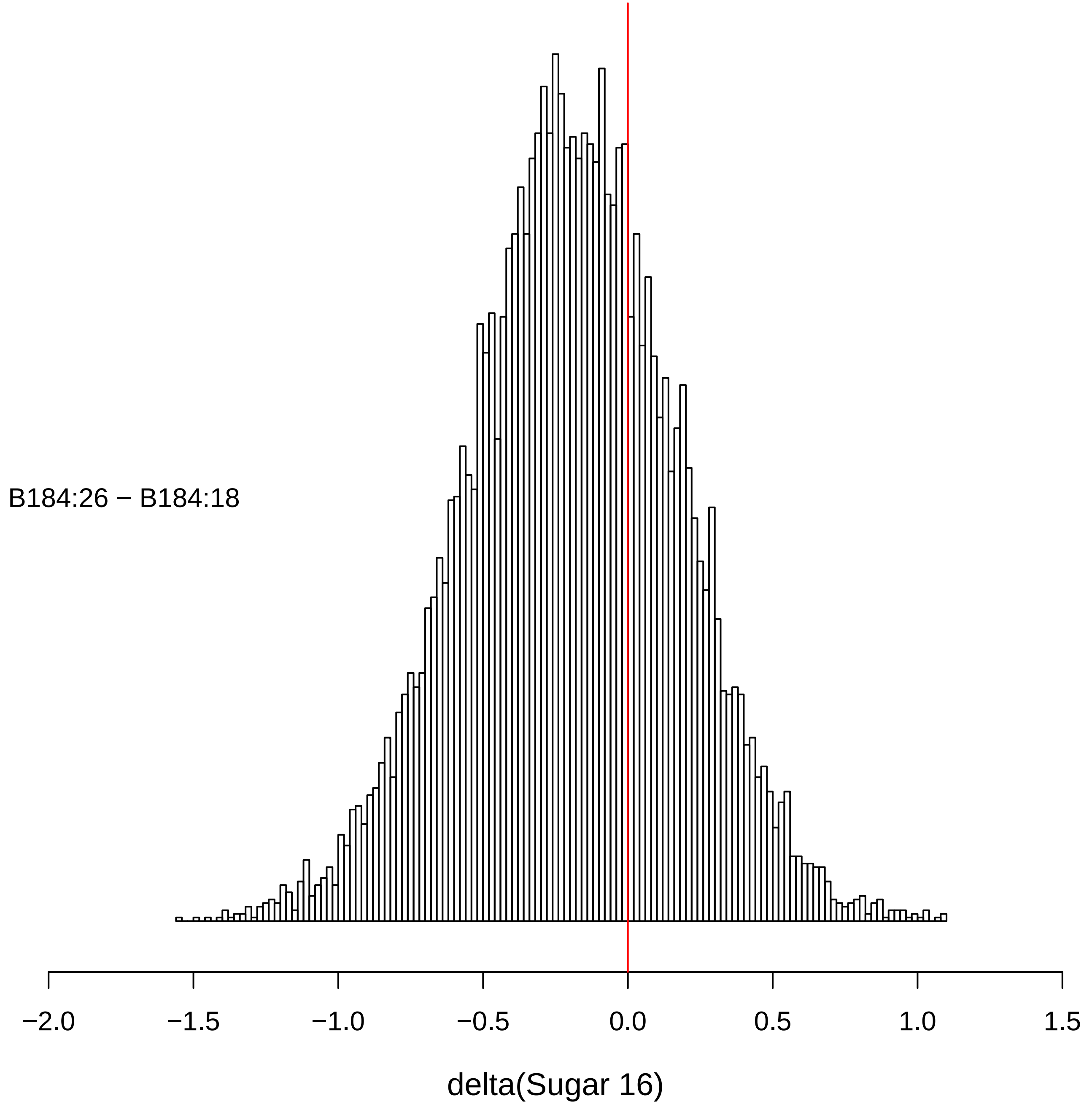
B184:26 − B184:18
−2.0
−1.5
−1.0
−0.5
0.0
0.5
1.0
1.5
delta(Sugar 16)

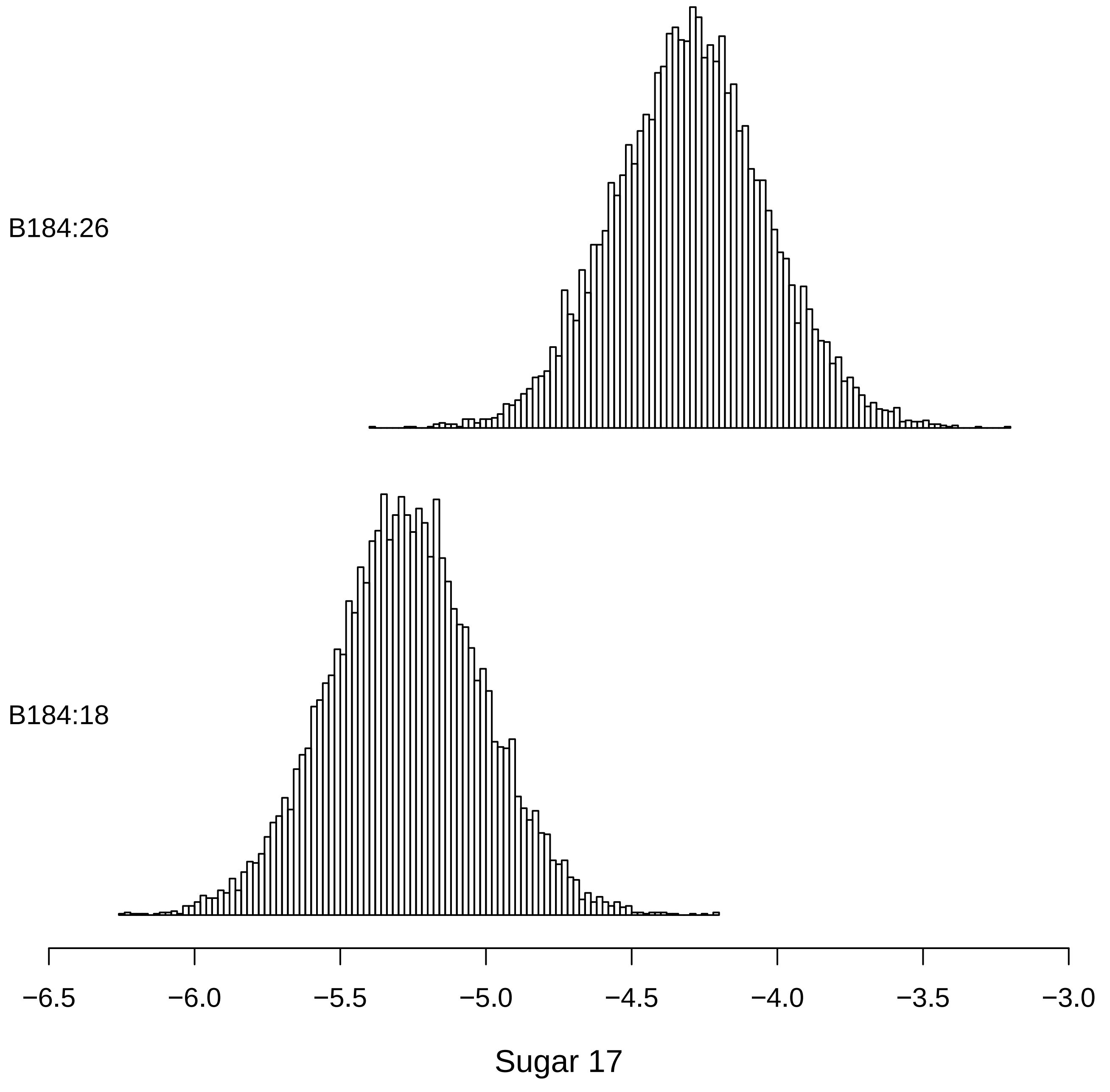
B184:26
B184:18
−6.5
−6.0
−5.5
−5.0
−4.5
−4.0
−3.5
−3.0
Sugar 17

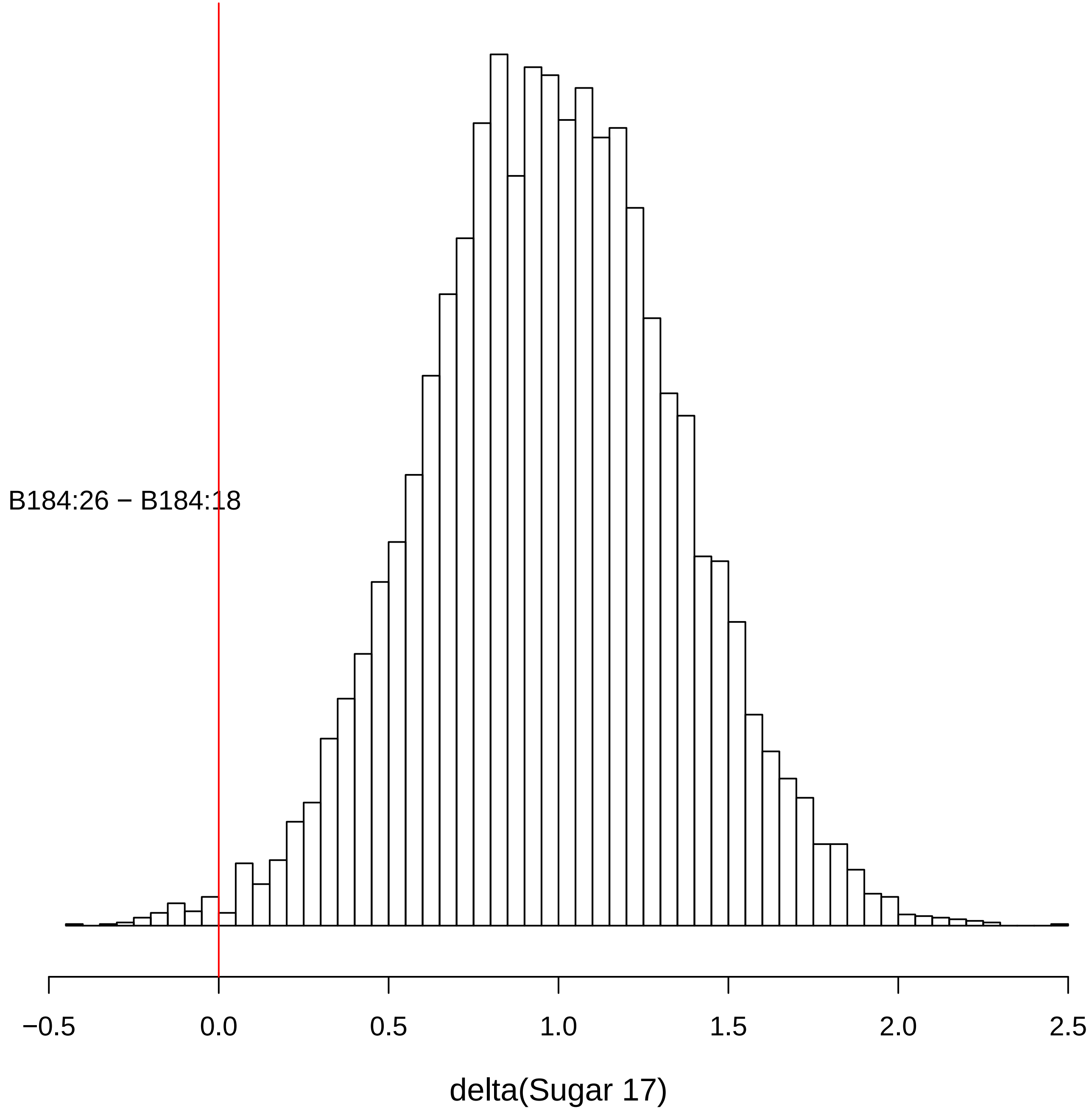
B184:26 − B184:18
−0.5
0.0
0.5
1.0
1.5
2.0
2.5
delta(Sugar 17)

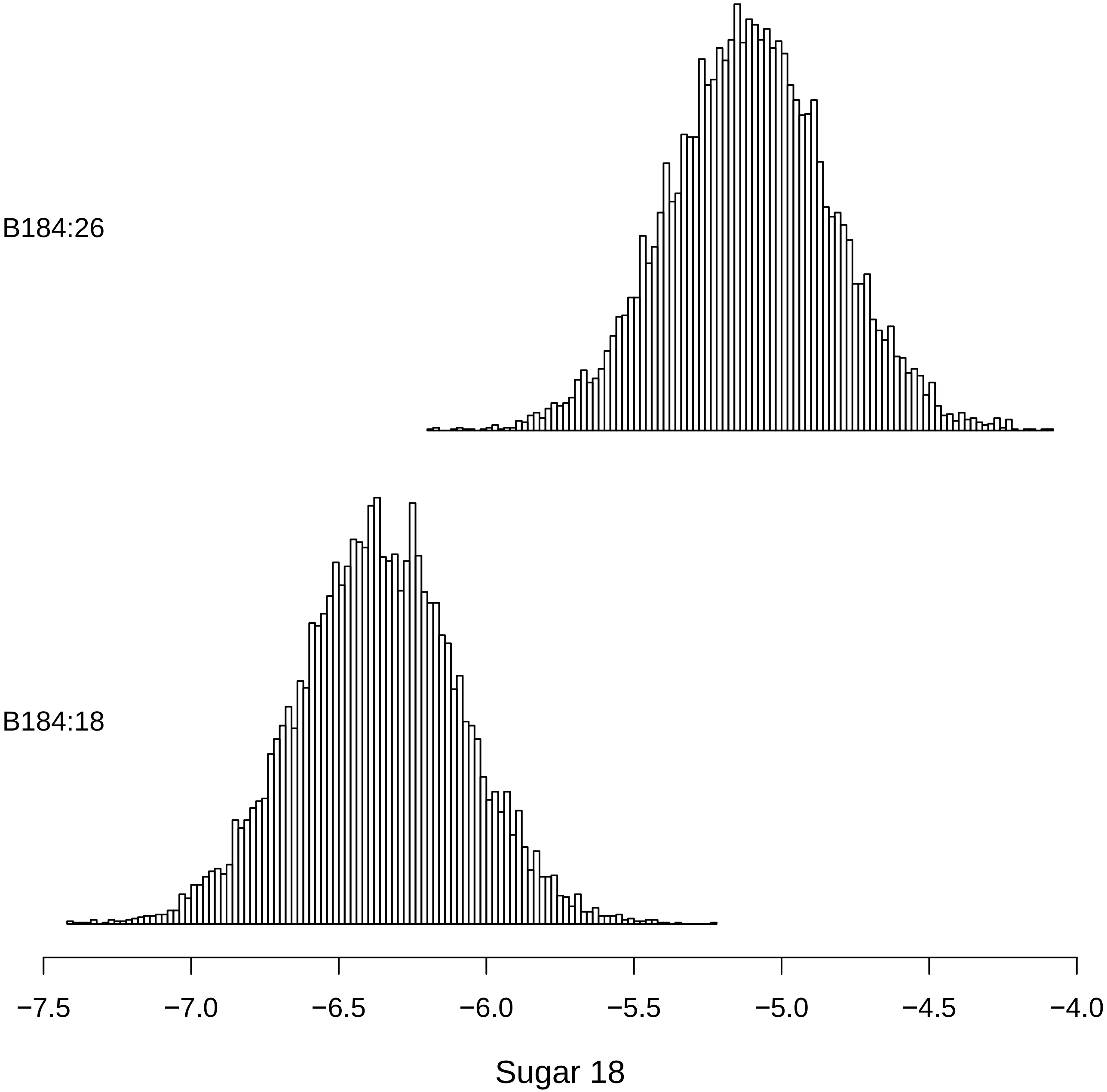

B184:26
B184:18
−7.5
−7.0
−6.5
−6.0
−5.5
−5.0
−4.5
−4.0
Sugar 18

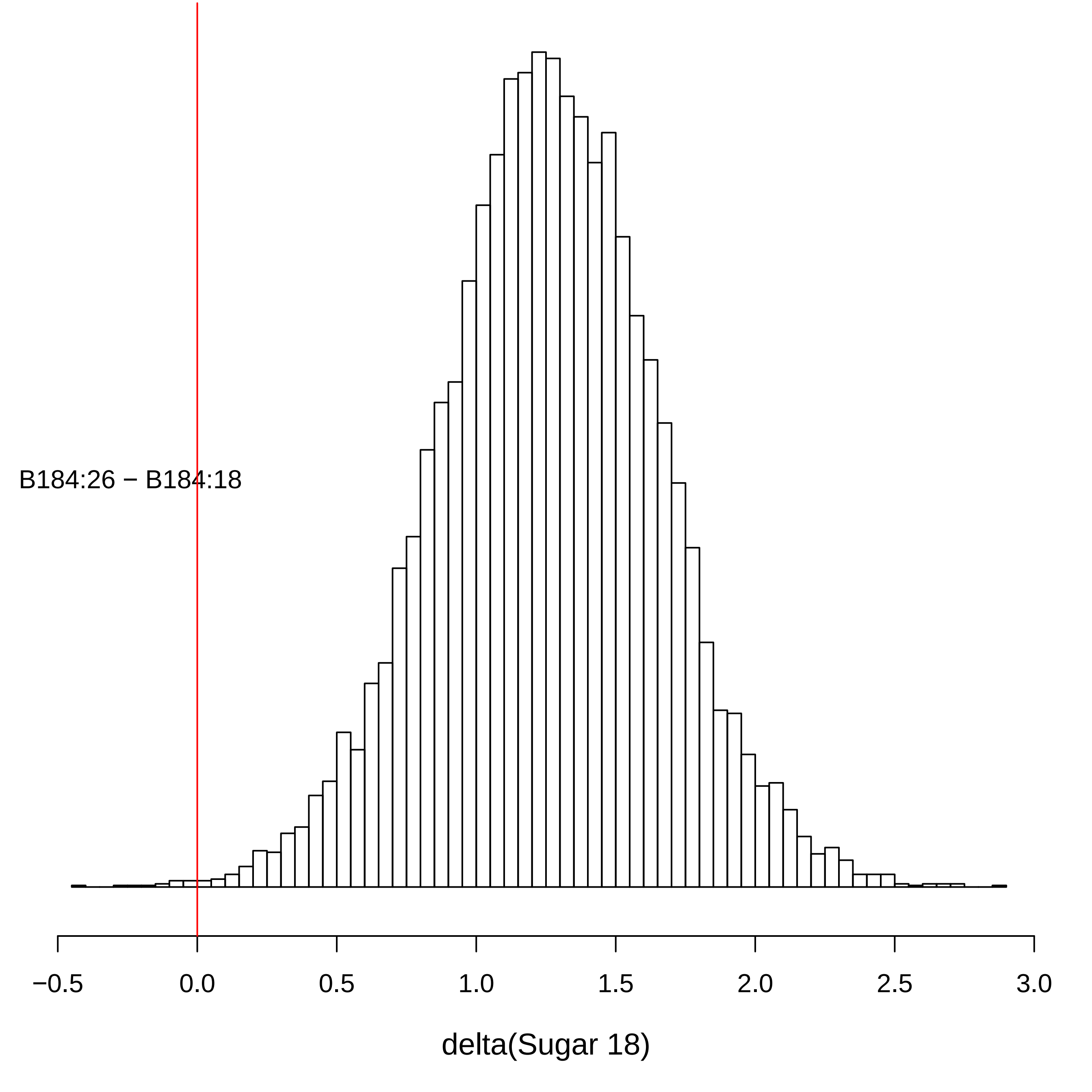

B184:26 − B184:18
−0.5
0.0
0.5
1.0
1.5
2.0
2.5
3.0
delta(Sugar 18)

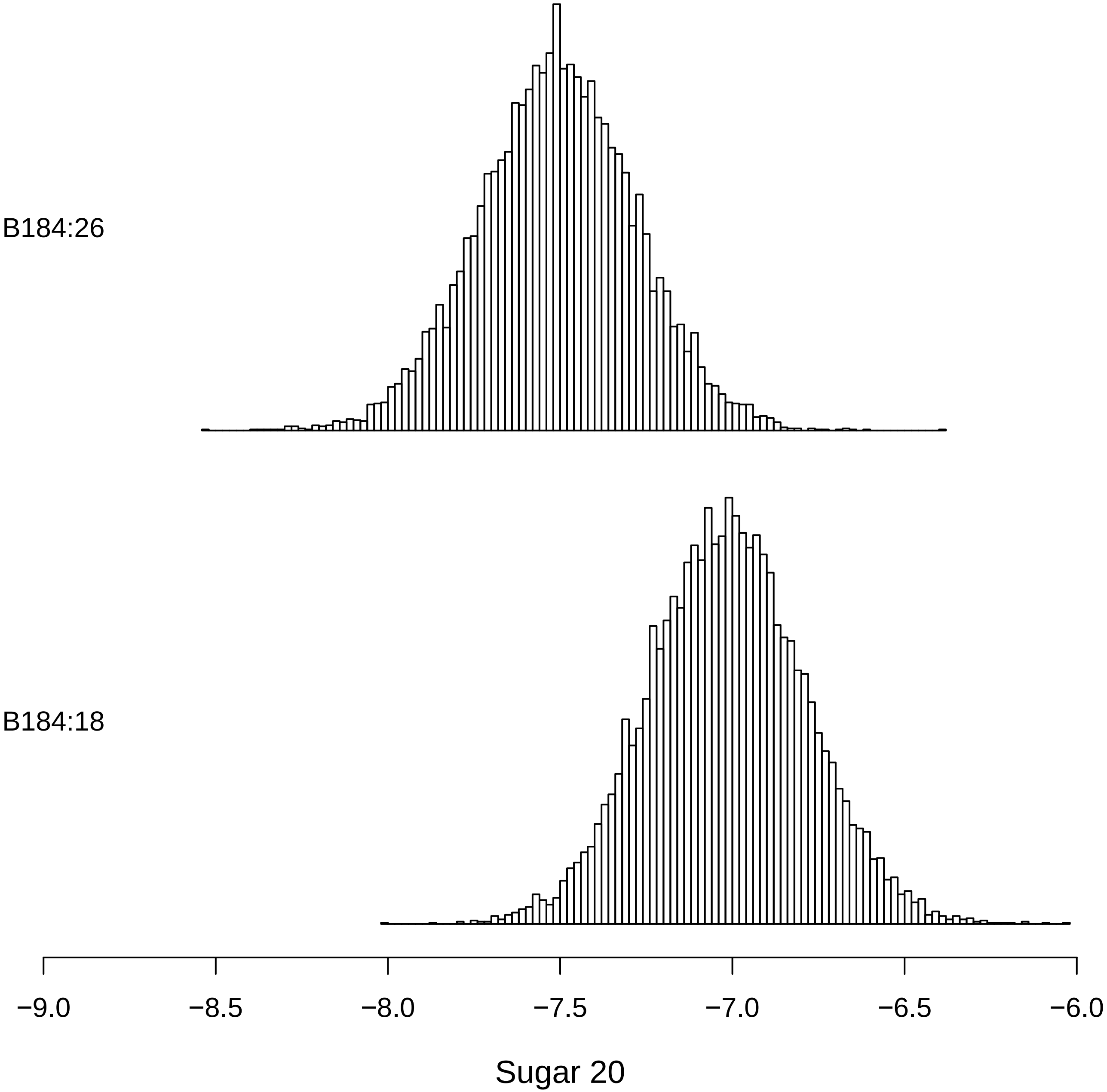
B184:26
B184:18
−9.0
−8.5
−8.0
−7.5
−7.0
−6.5
−6.0
Sugar 20

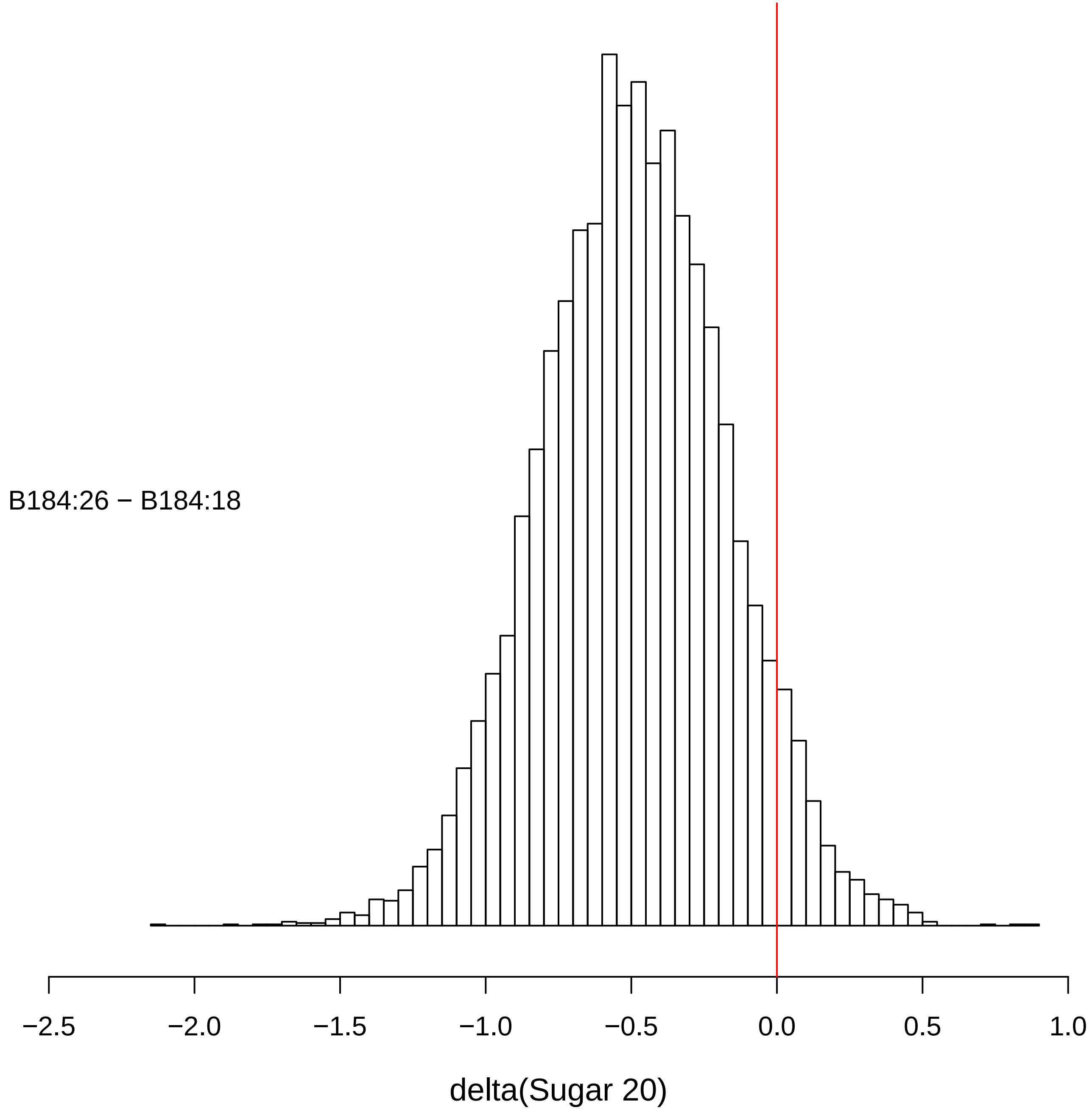

B184:26 − B184:18
−2.5
−2.0
−1.5
−1.0
−0.5
0.0
0.5
1.0
delta(Sugar 20)

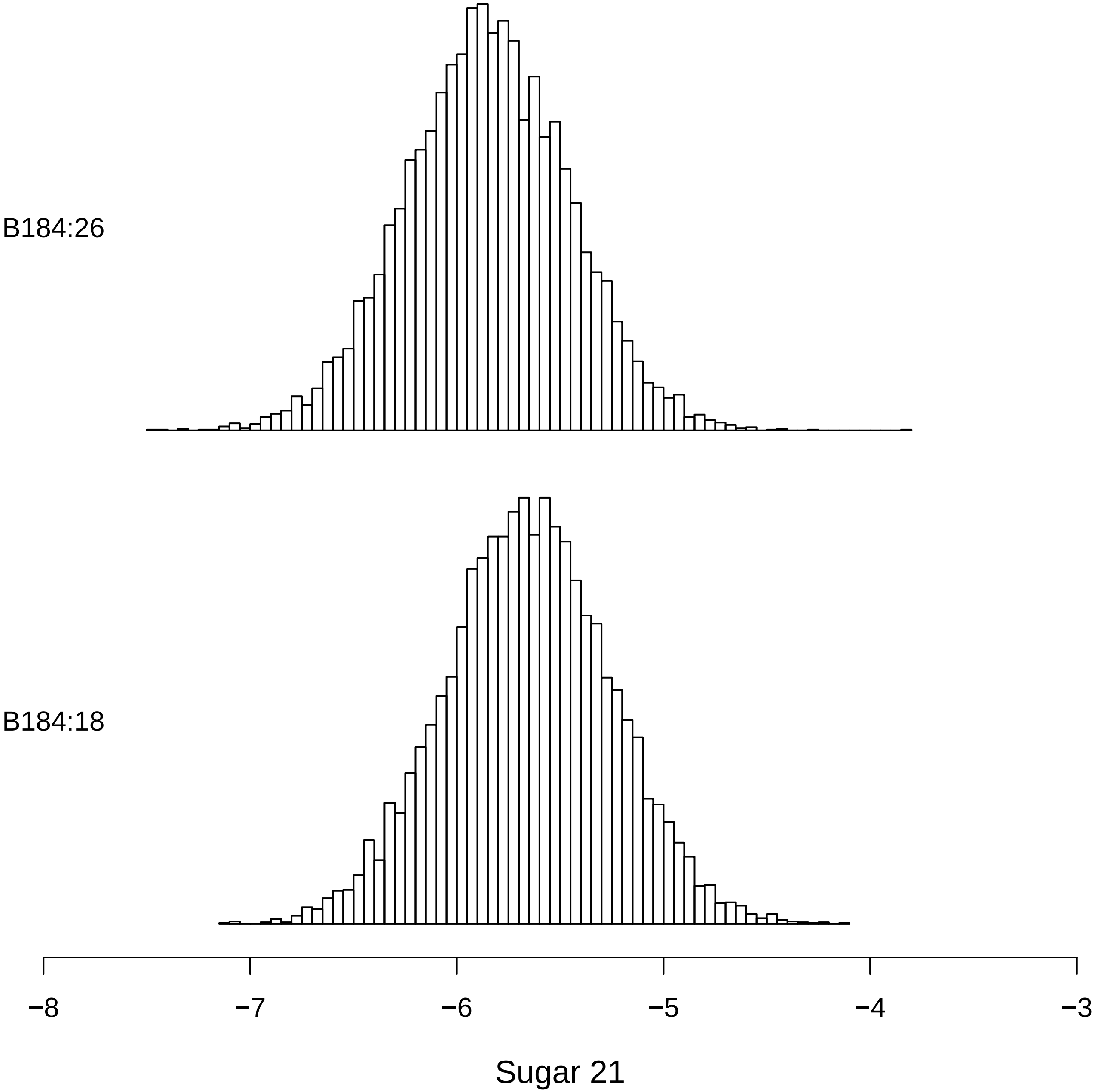
B184:26
B184:18
−8
−7
−6
−5
−4
−3
Sugar 21

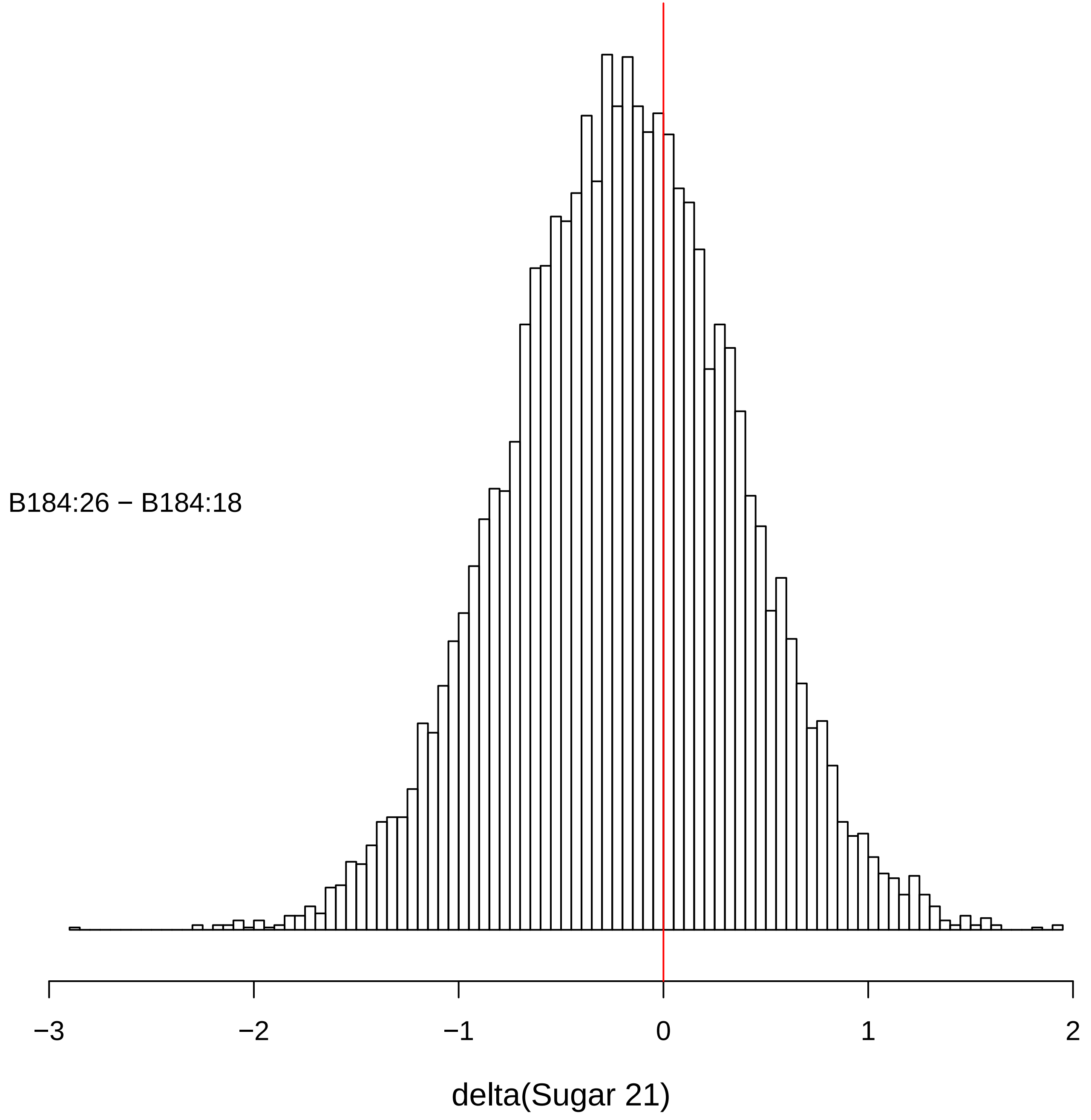
B184:26 − B184:18
−3
−2
−1
0
1
2
delta(Sugar 21)

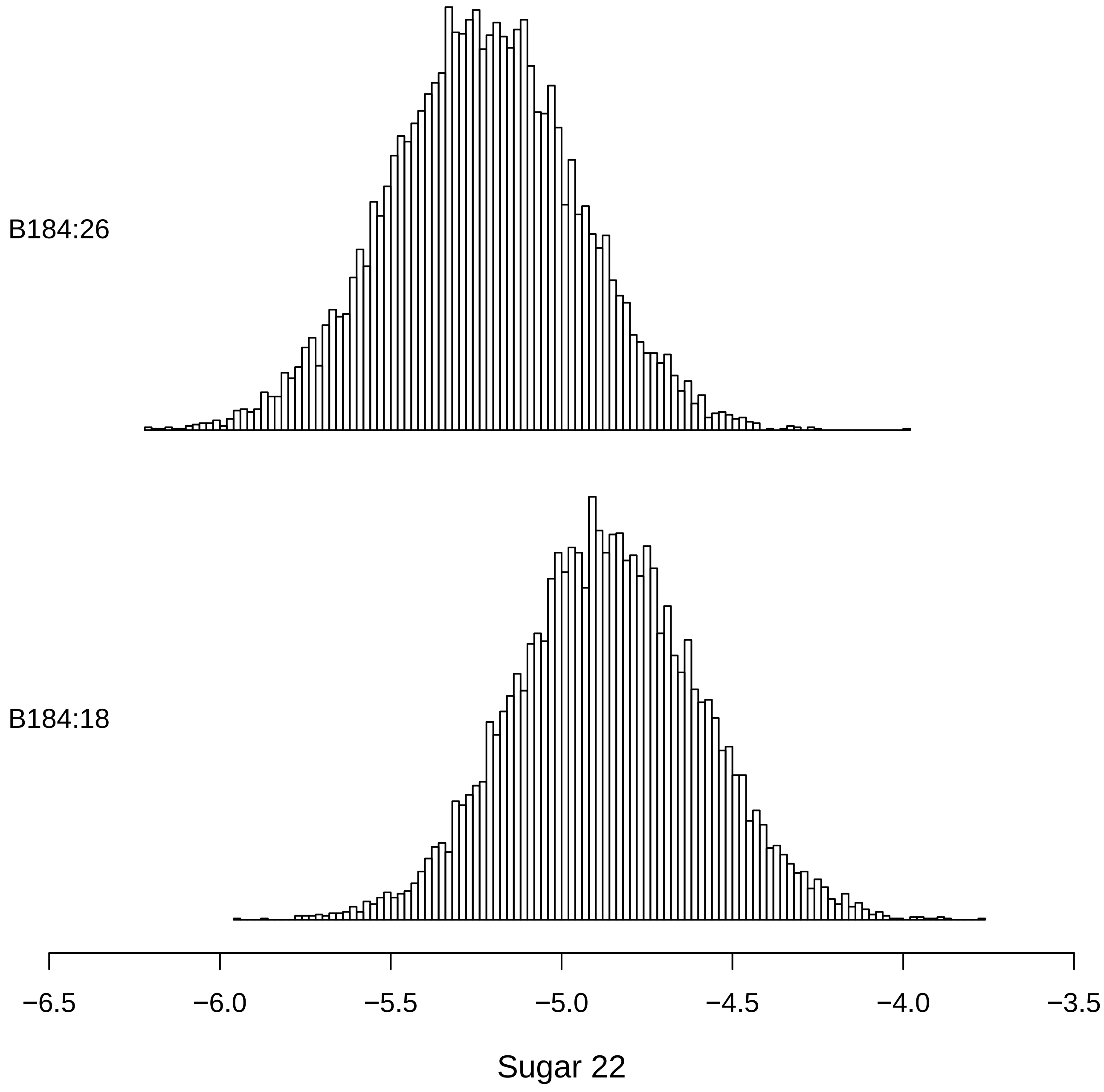
B184:26
B184:18
−6.5
−6.0
−5.5
−5.0
−4.5
−4.0
−3.5
Sugar 22

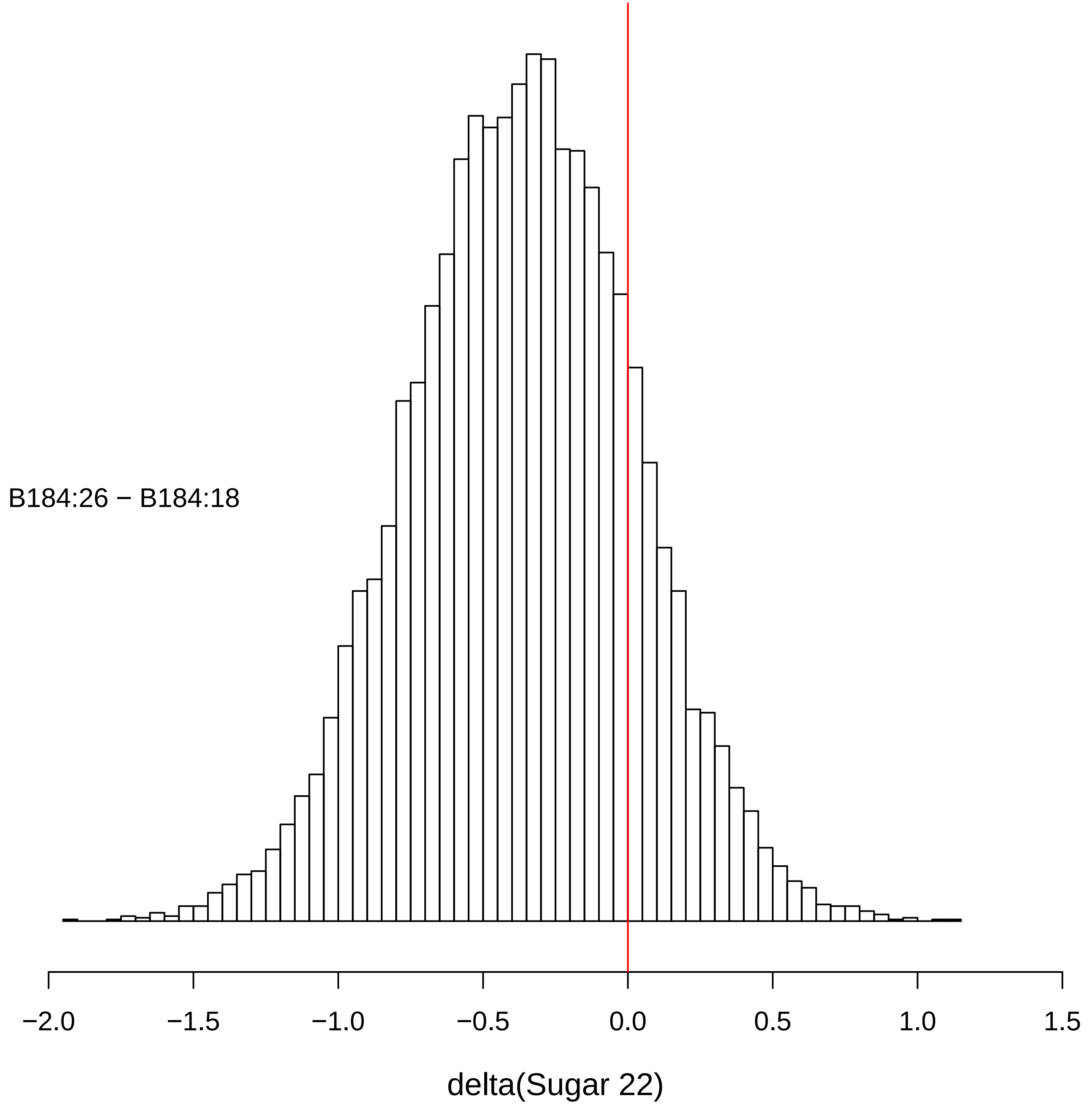
B184:26 − B184:18
−2.0
−1.5
−1.0
−0.5
0.0
0.5
1.0
1.5
delta(Sugar 22)

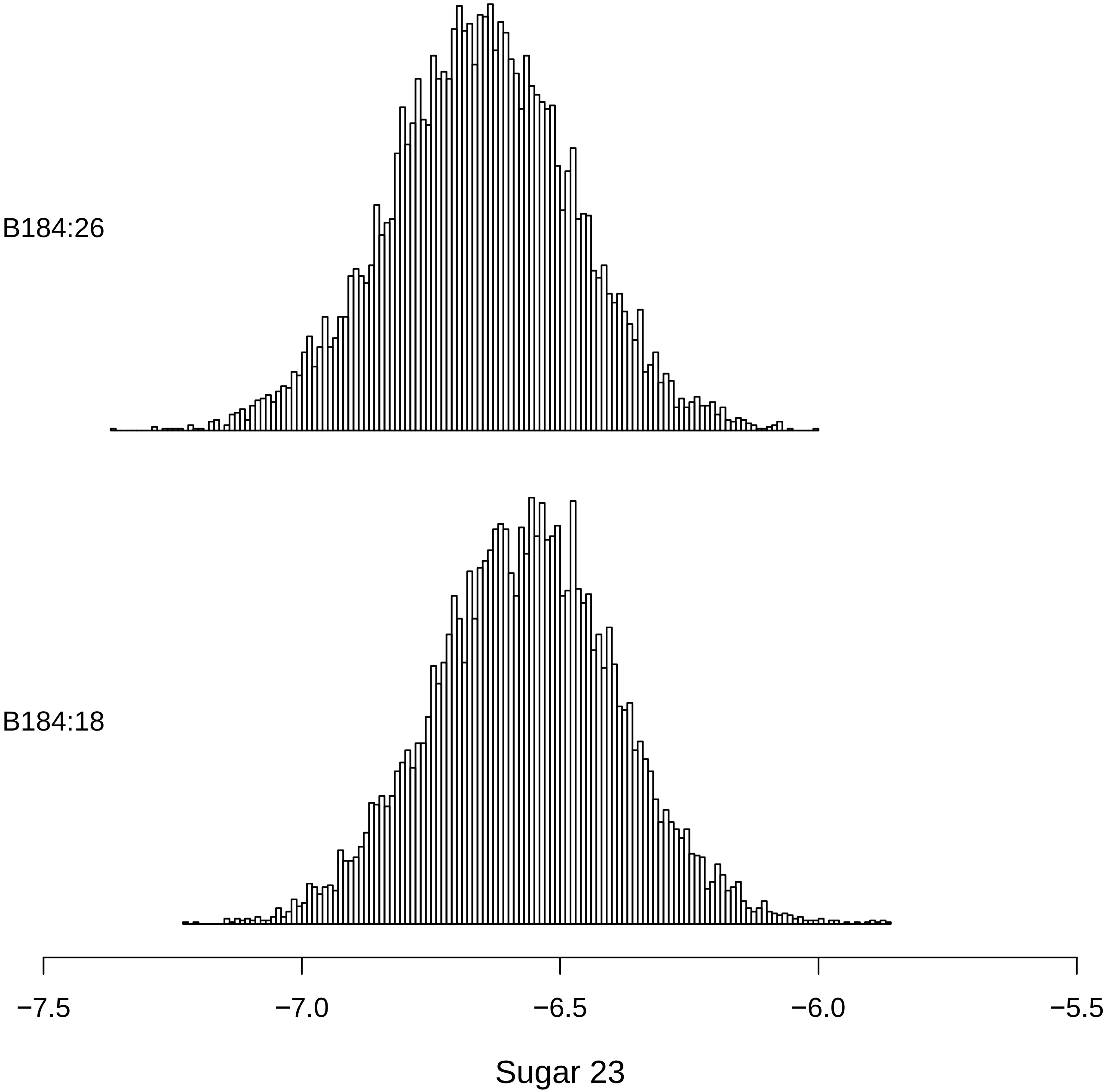
B184:26
B184:18
−7.5
−7.0
−6.5
−6.0
−5.5
Sugar 23

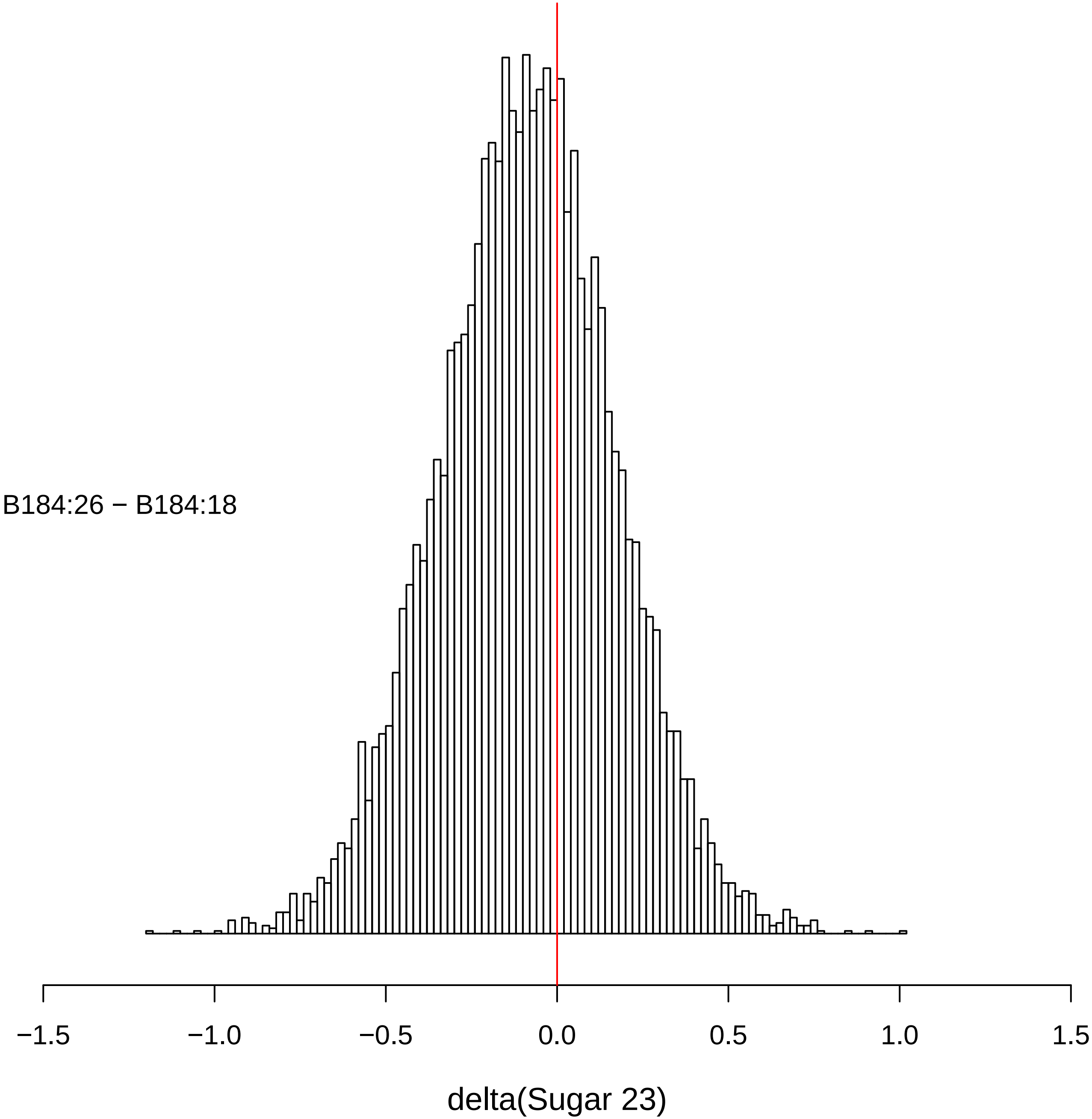
B184:26 − B184:18
−1.5
−1.0
−0.5
0.0
0.5
1.0
1.5
delta(Sugar 23)

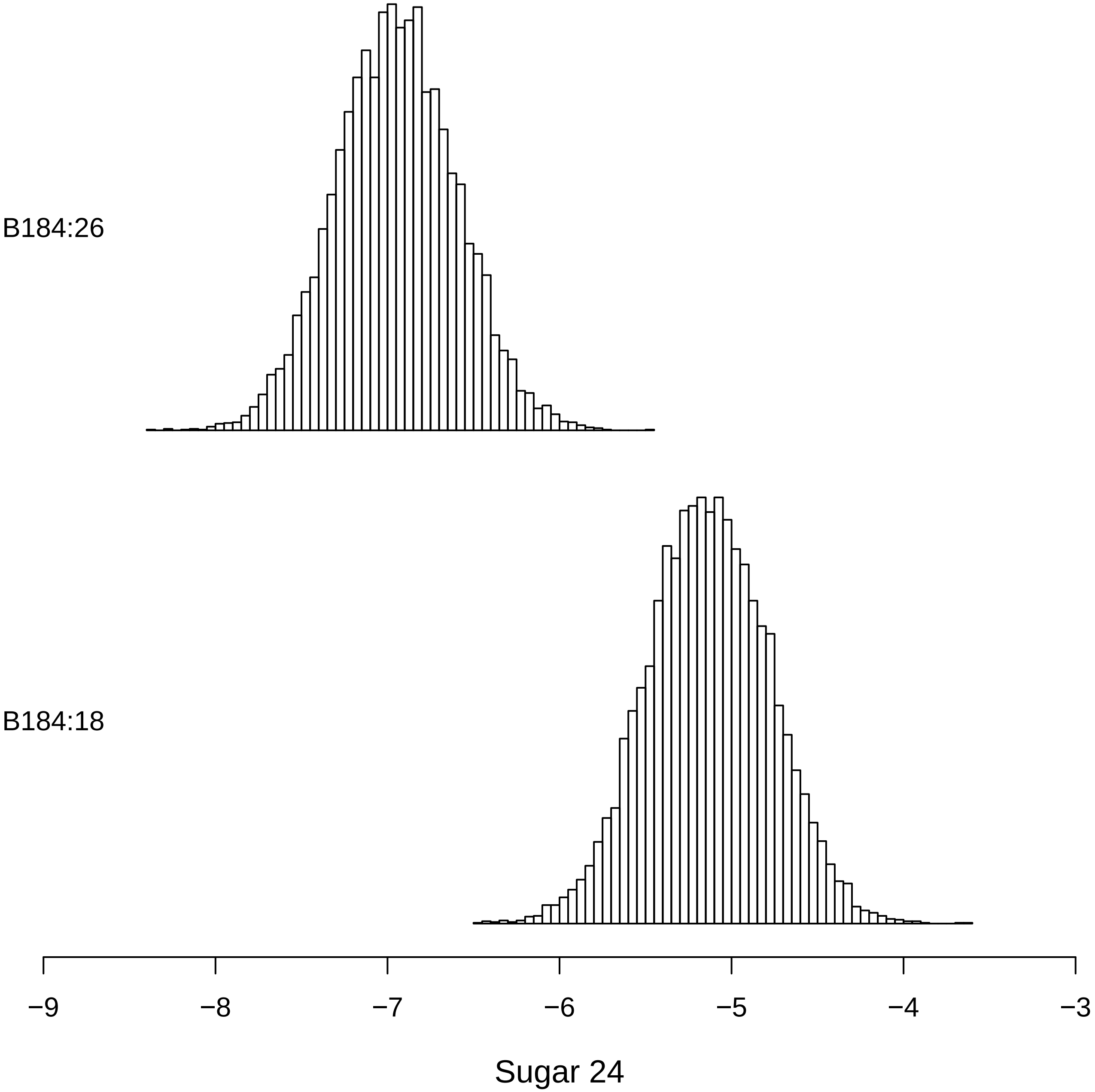

B184:26
B184:18
−9
−8
−7
−6
−5
−4
−3
Sugar 24

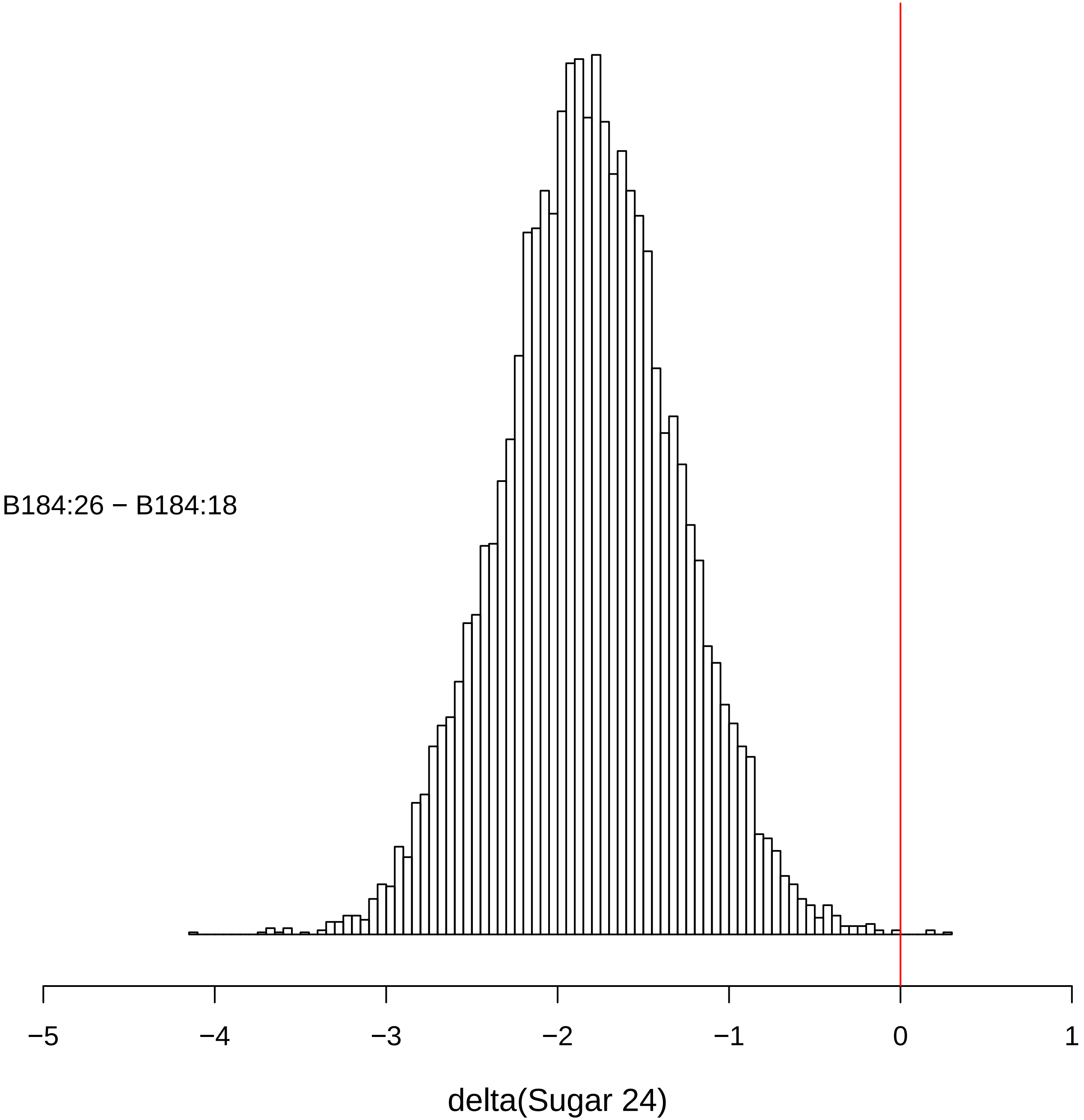
B184:26 − B184:18
−5
−4
−3
−2
−1
0
1
delta(Sugar 24)

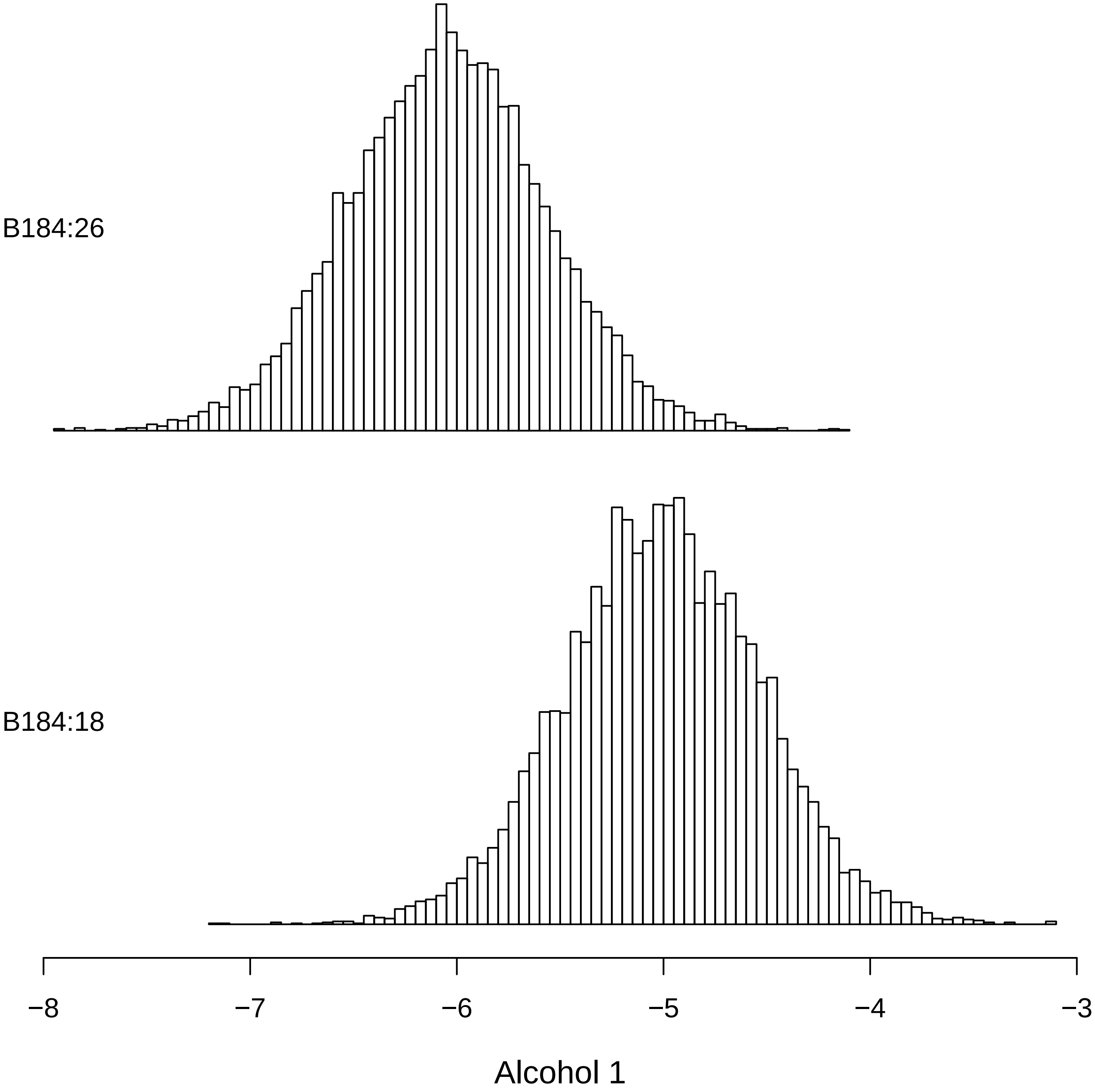
B184:26
B184:18
−8
−7
−6
−5
−4
−3
Alcohol 1

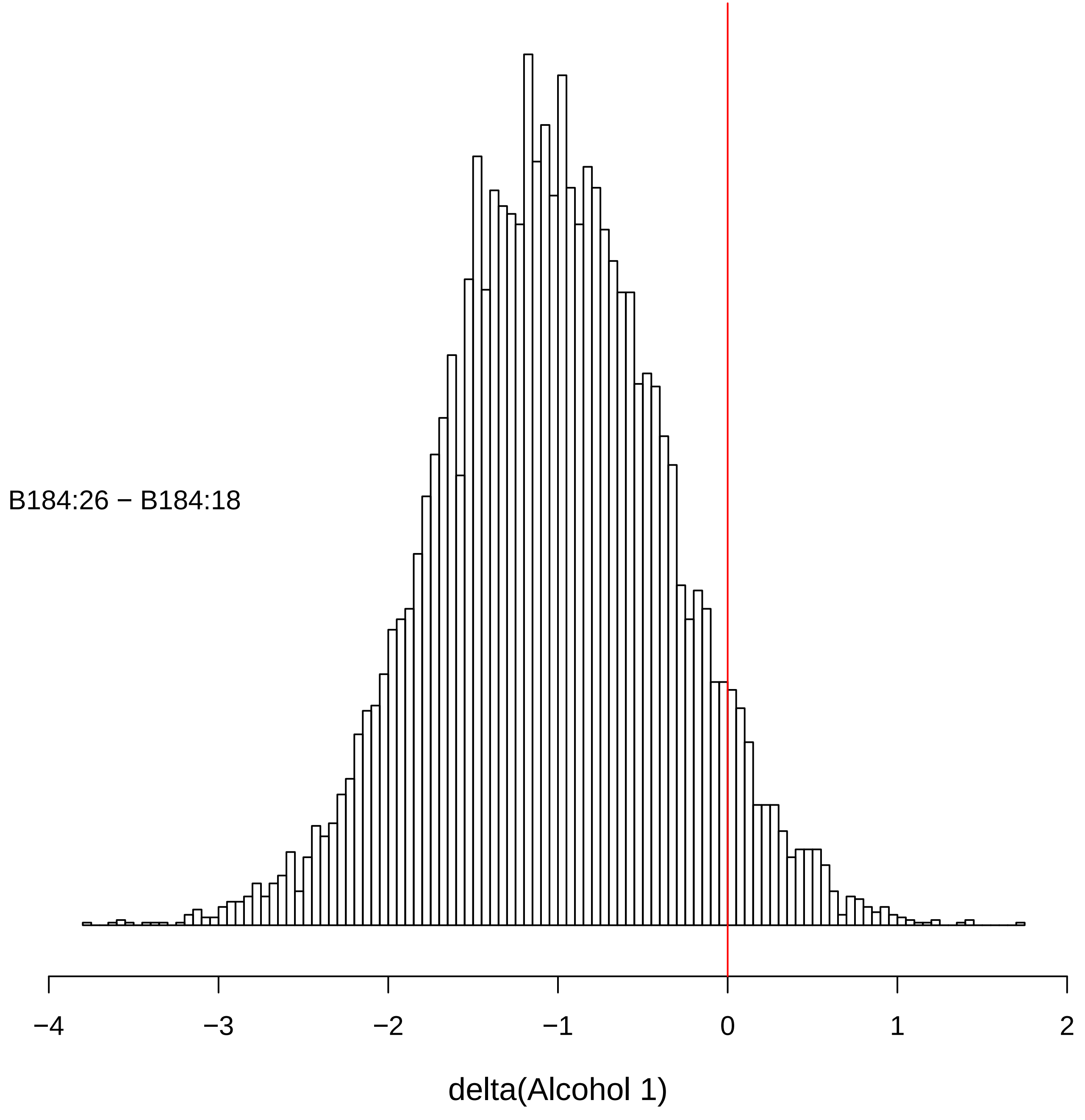

B184:26 − B184:18
−4
−3
−2
−1
0
1
2
delta(Alcohol 1)

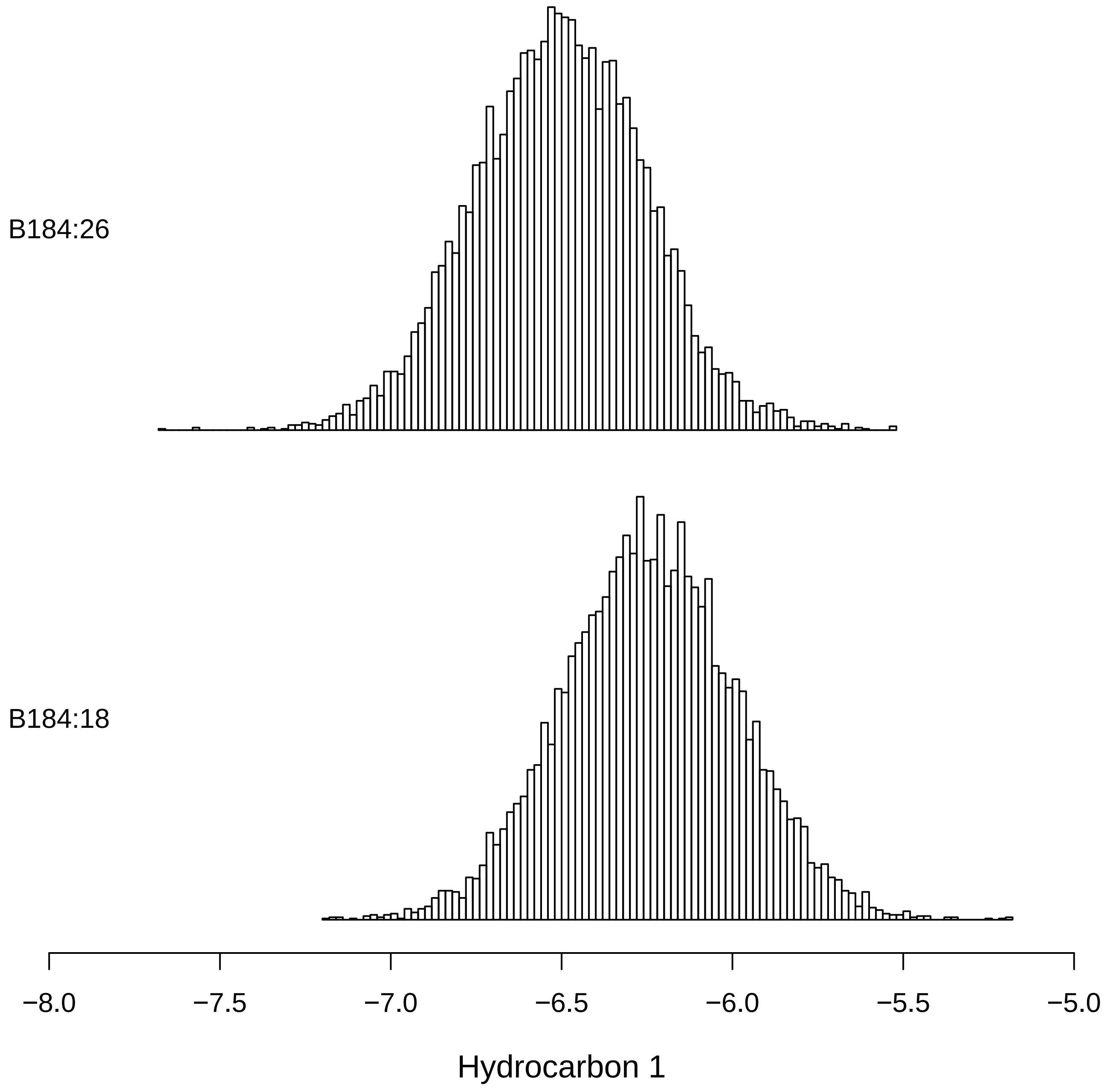
B184:26
B184:18
−8.0
−7.5
−7.0
−6.5
−6.0
−5.5
−5.0
Hydrocarbon 1

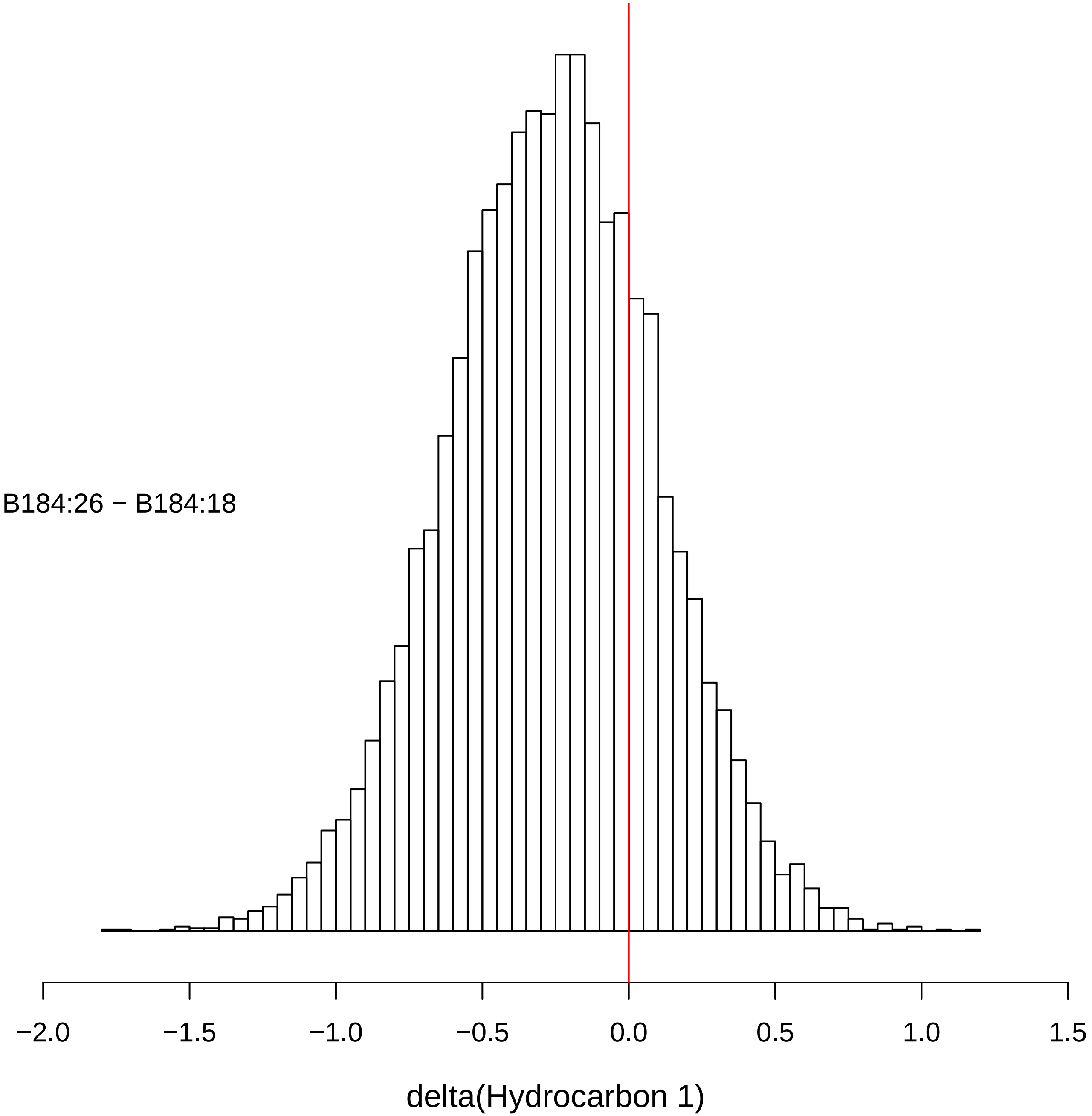

B184:26 − B184:18
−2.0
−1.5
−1.0
−0.5
0.0
0.5
1.0
1.5
delta(Hydrocarbon 1)

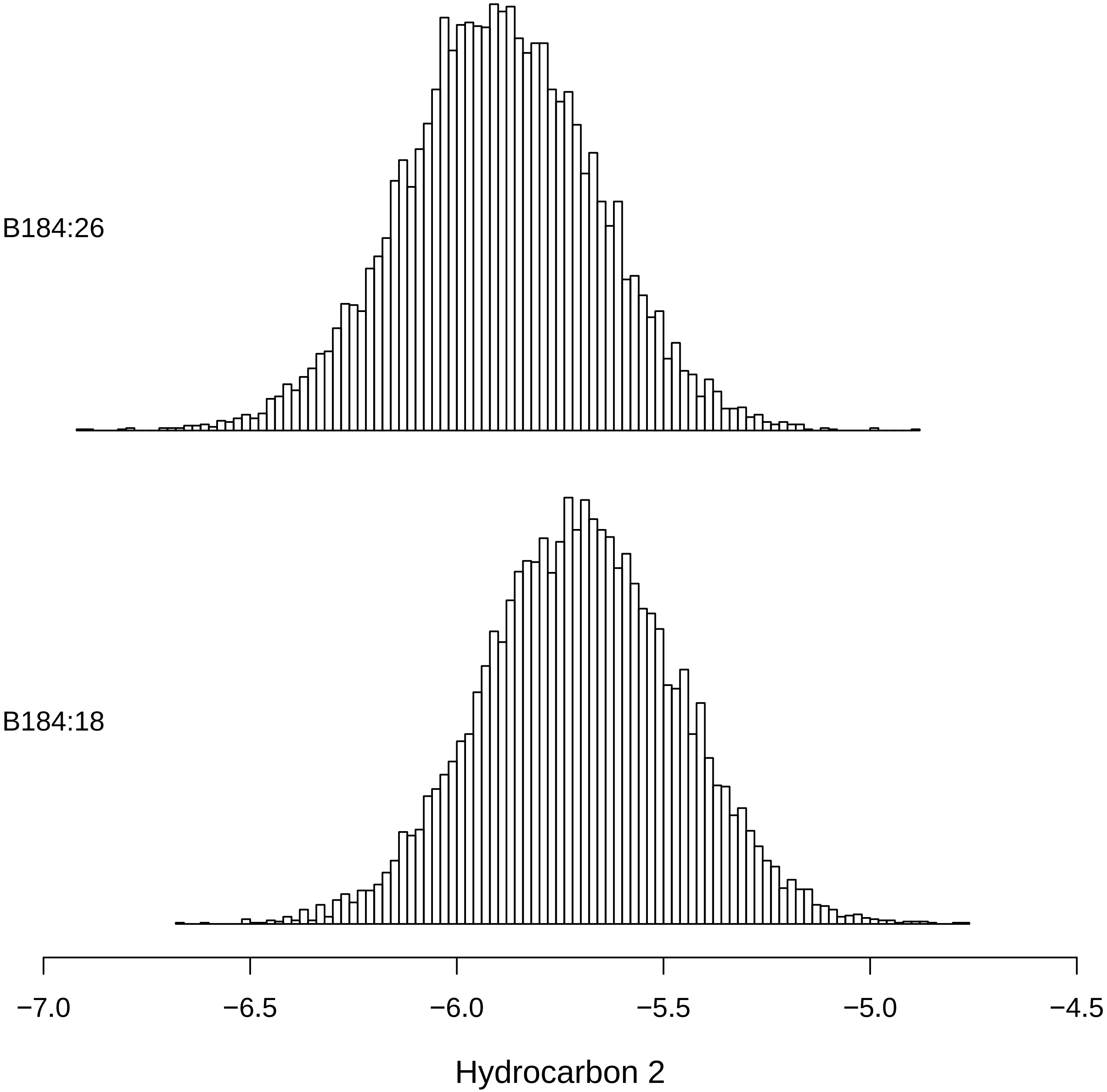

B184:26
B184:18
−7.0
−6.5
−6.0
−5.5
−5.0
−4.5
Hydrocarbon 2

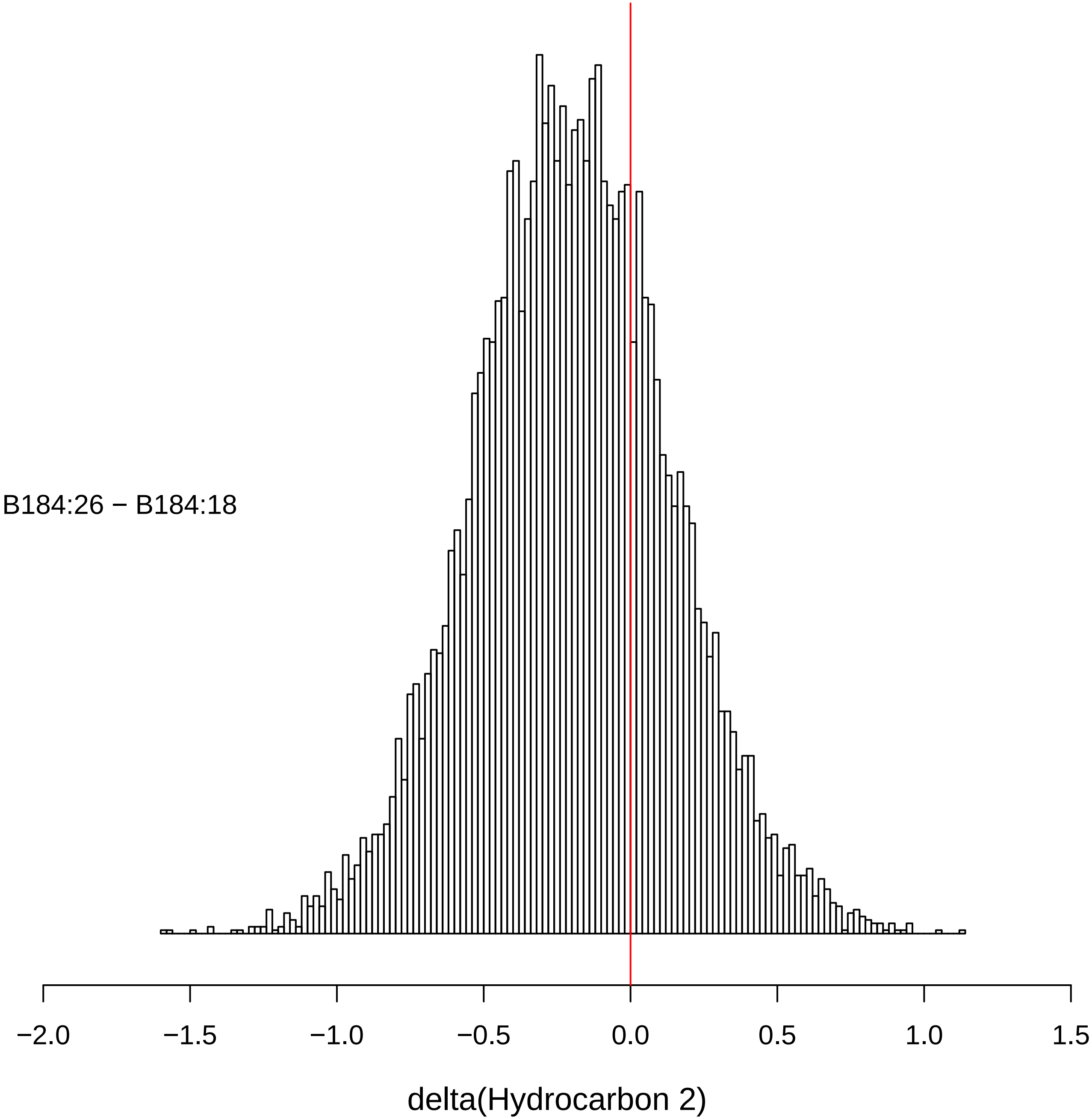

B184:26 − B184:18
−2.0
−1.5
−1.0
−0.5
0.0
0.5
1.0
1.5
delta(Hydrocarbon 2)

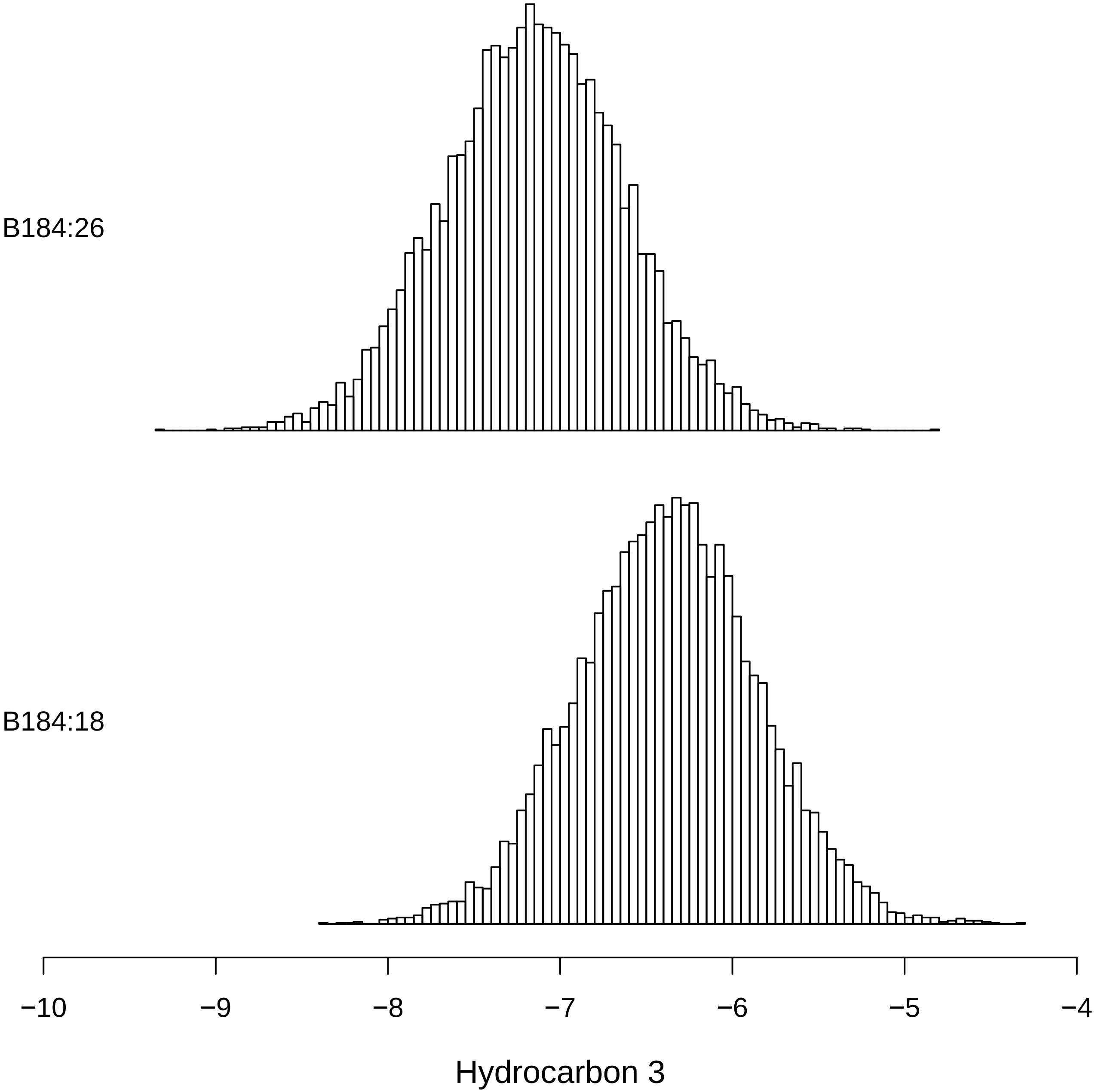
B184:26
B184:18
−10
−9
−8
−7
−6
−5
−4
Hydrocarbon 3

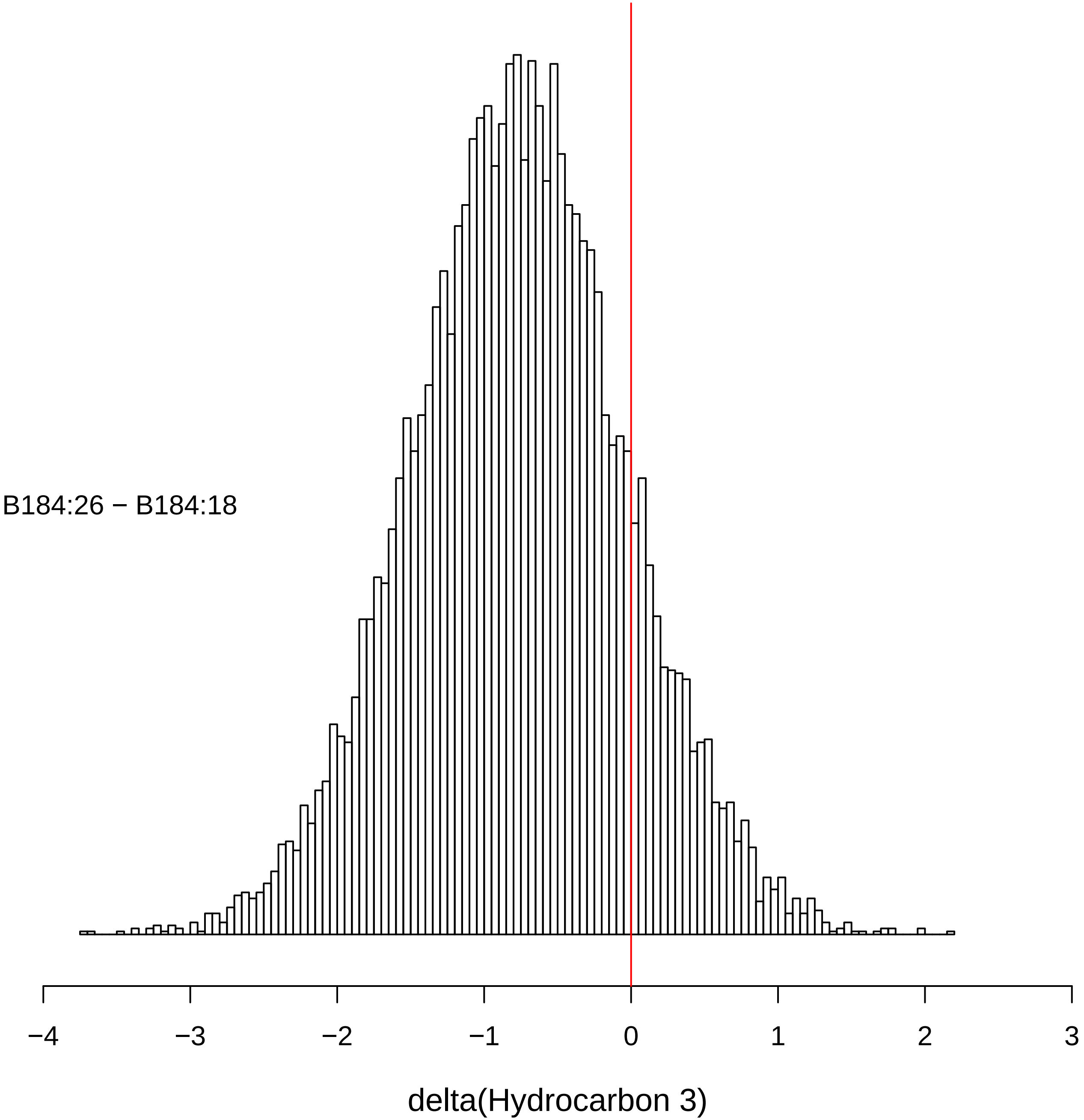
B184:26 − B184:18
−4
−3
−2
−1
0
1
2
3
delta(Hydrocarbon 3)

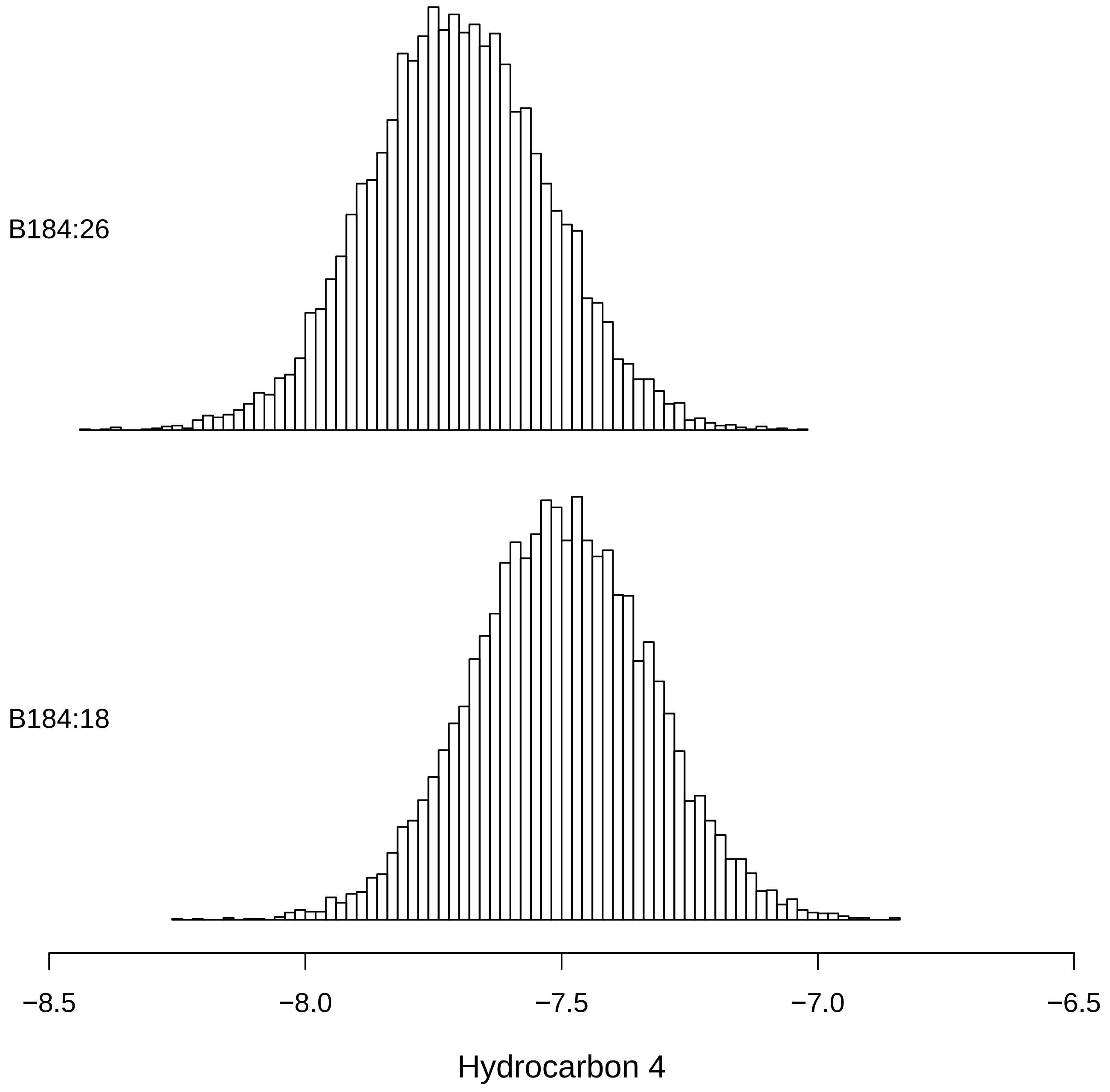
B184:26
B184:18
−8.5
−8.0
−7.5
−7.0
−6.5
Hydrocarbon 4

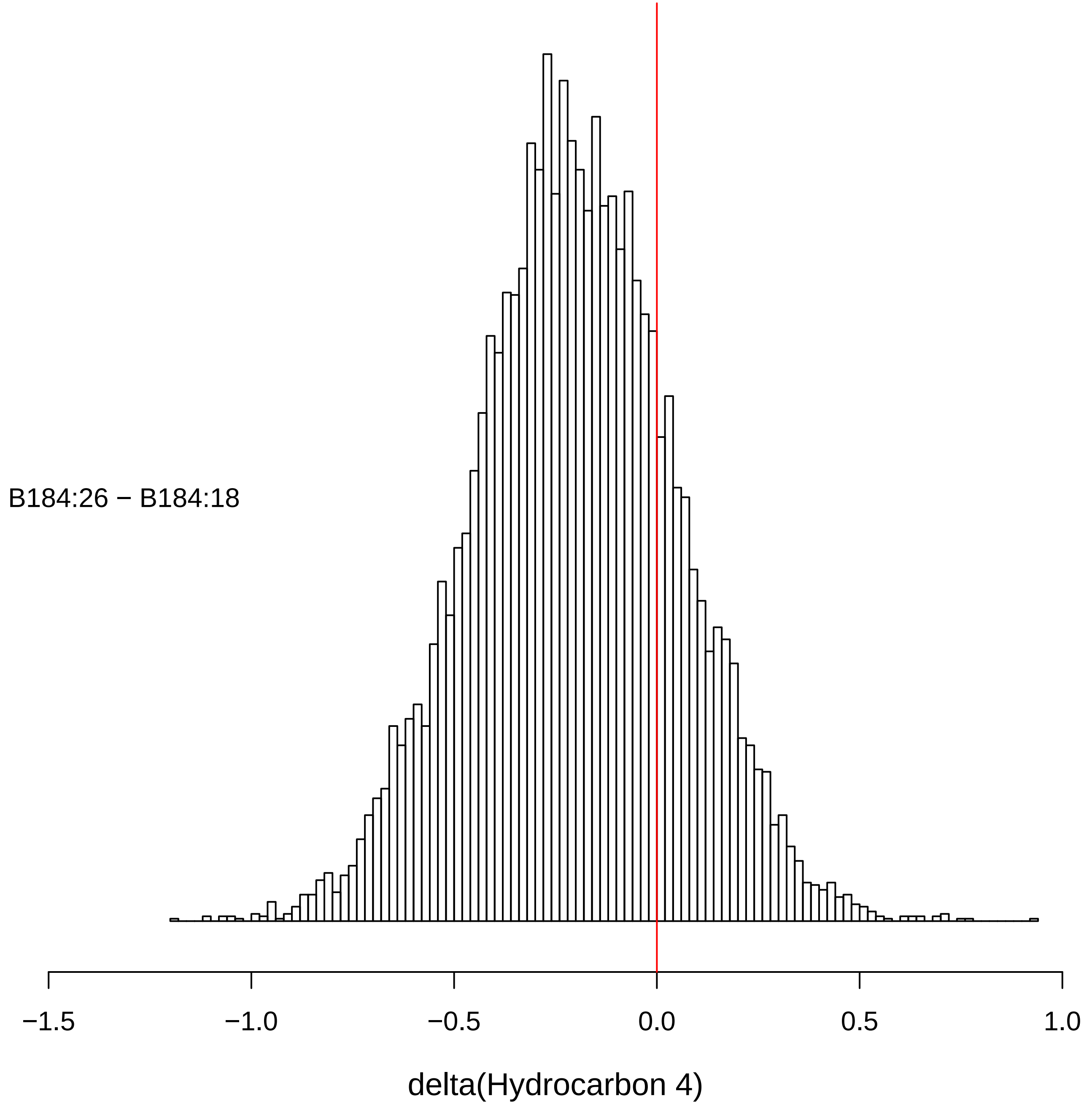

B184:26 − B184:18
−1.5
−1.0
−0.5
0.0
0.5
1.0
delta(Hydrocarbon 4)

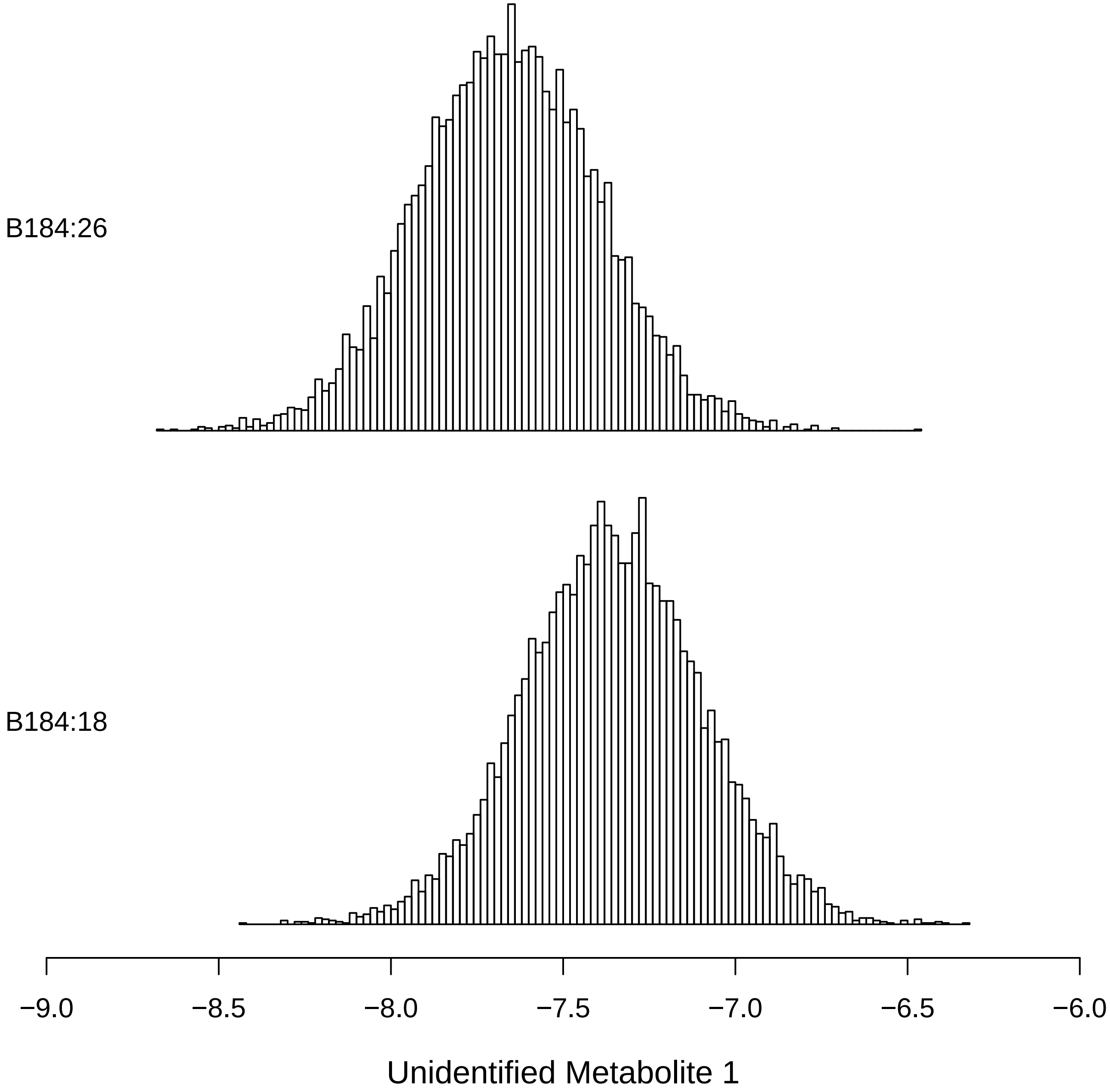
B184:26
B184:18
−9.0
−8.5
−8.0
−7.5
−7.0
−6.5
−6.0
Unidentified Metabolite 1

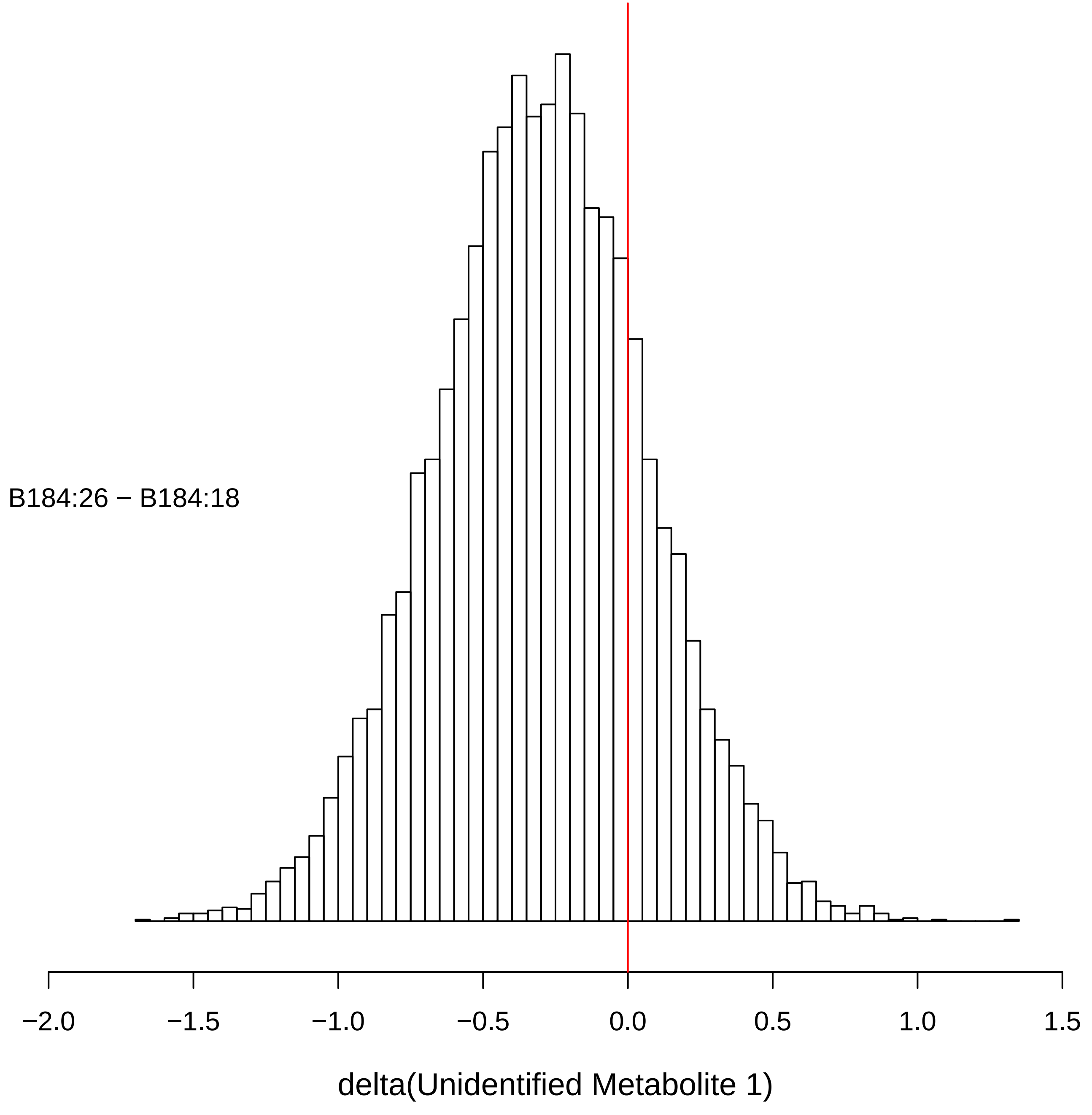
B184:26 − B184:18
−2.0
−1.5
−1.0
−0.5
0.0
0.5
1.0
1.5
delta(Unidentified Metabolite 1)

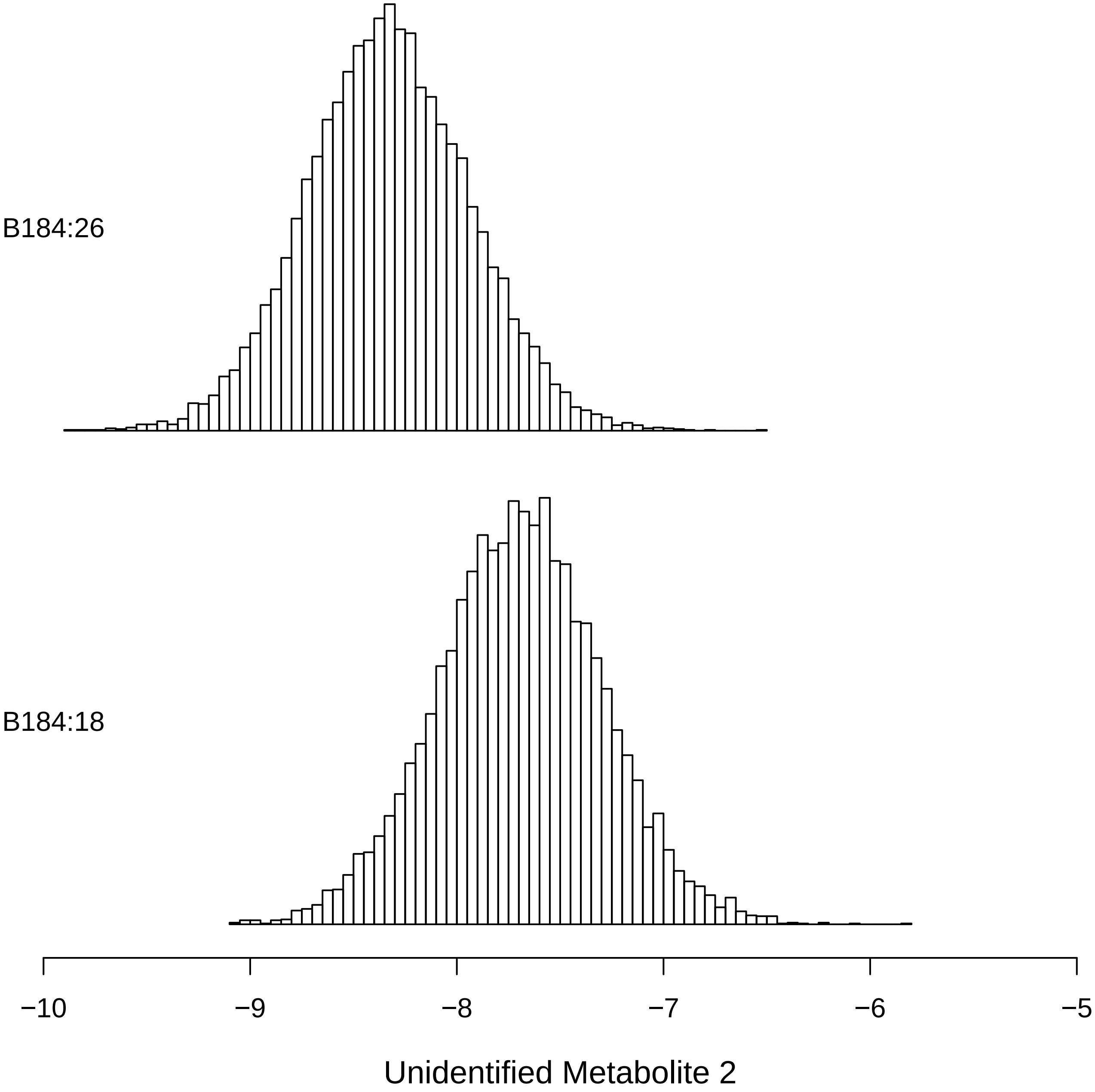
B184:26
B184:18
−10
−9
−8
−7
−6
−5
Unidentified Metabolite 2

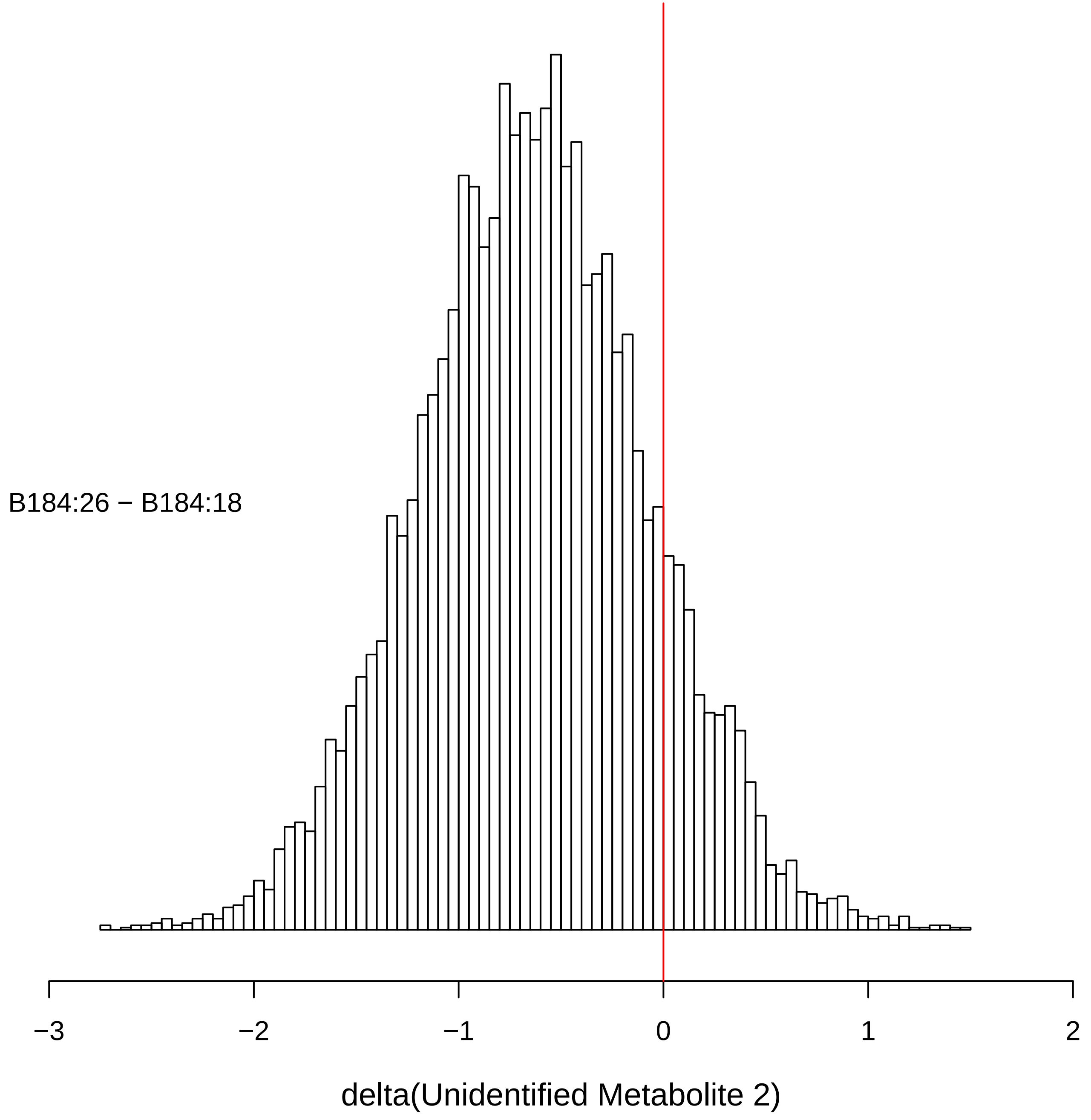
B184:26 − B184:18
−3
−2
−1
0
1
2
delta(Unidentified Metabolite 2)

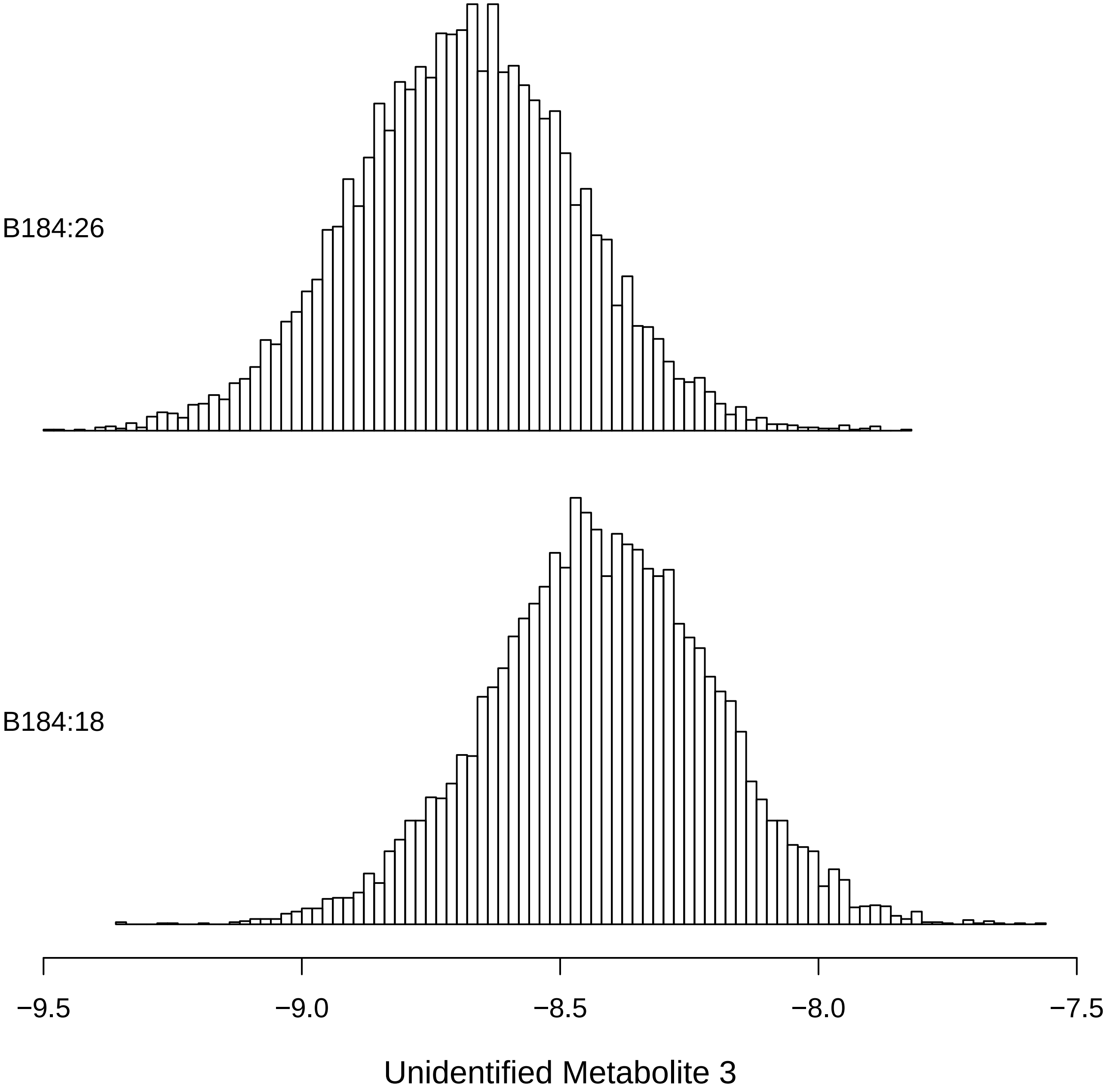

B184:26
B184:18
−9.5
−9.0
−8.5
−8.0
−7.5
Unidentified Metabolite 3

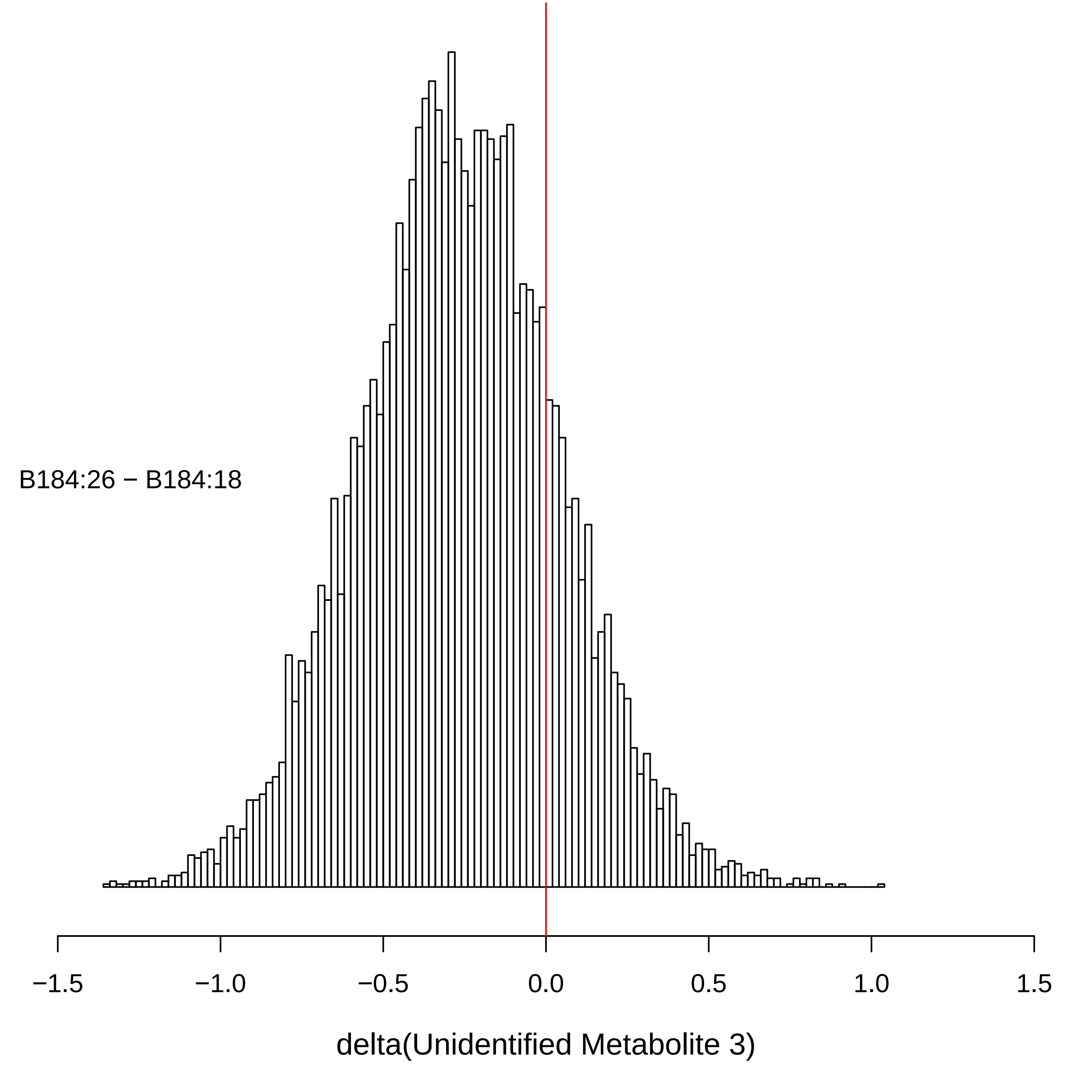

B184:26 − B184:18
−1.5
−1.0
−0.5
0.0
0.5
1.0
1.5
delta(Unidentified Metabolite 3)

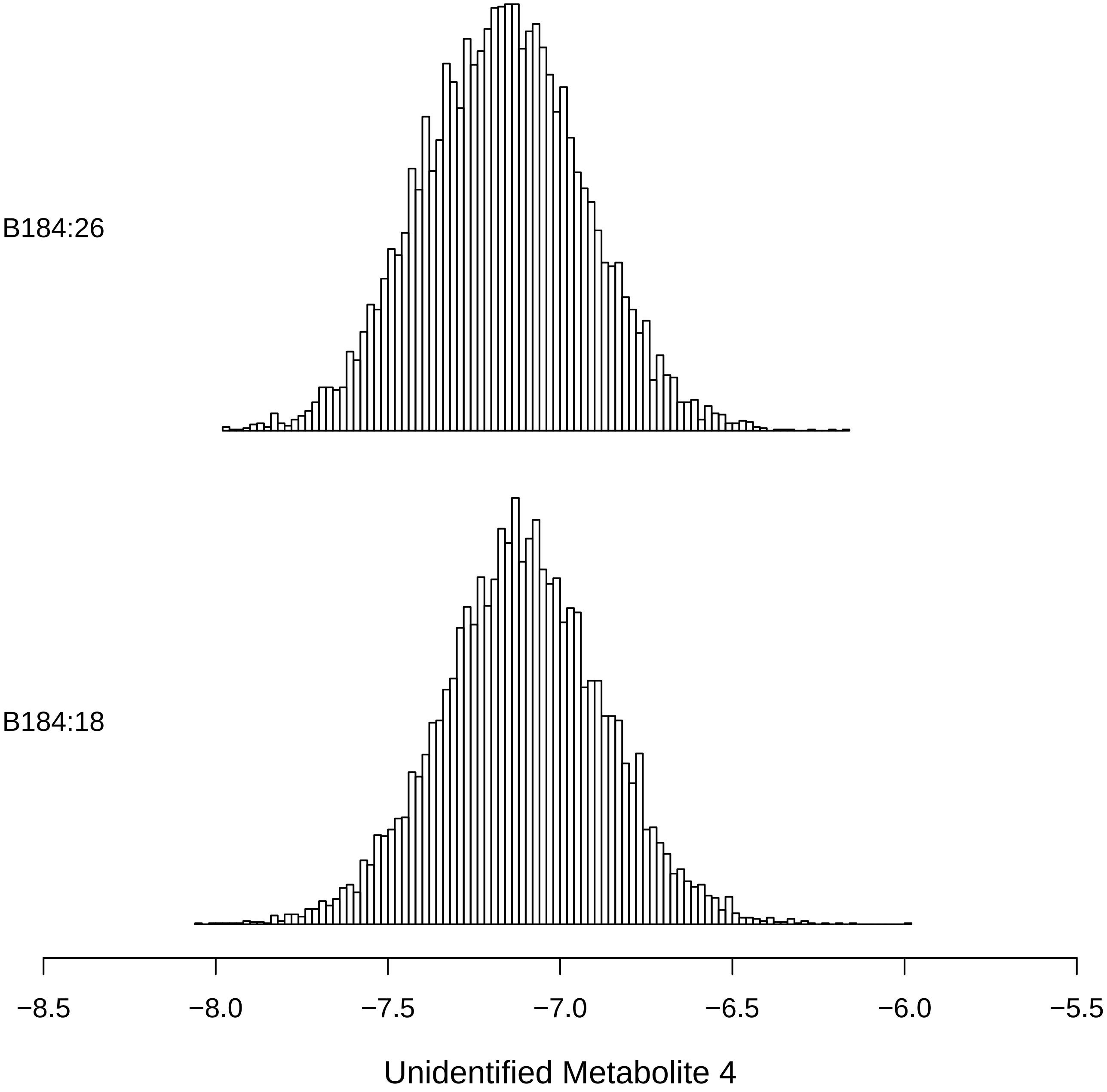
B184:26
B184:18
−8.5
−8.0
−7.5
−7.0
−6.5
−6.0
−5.5
Unidentified Metabolite 4

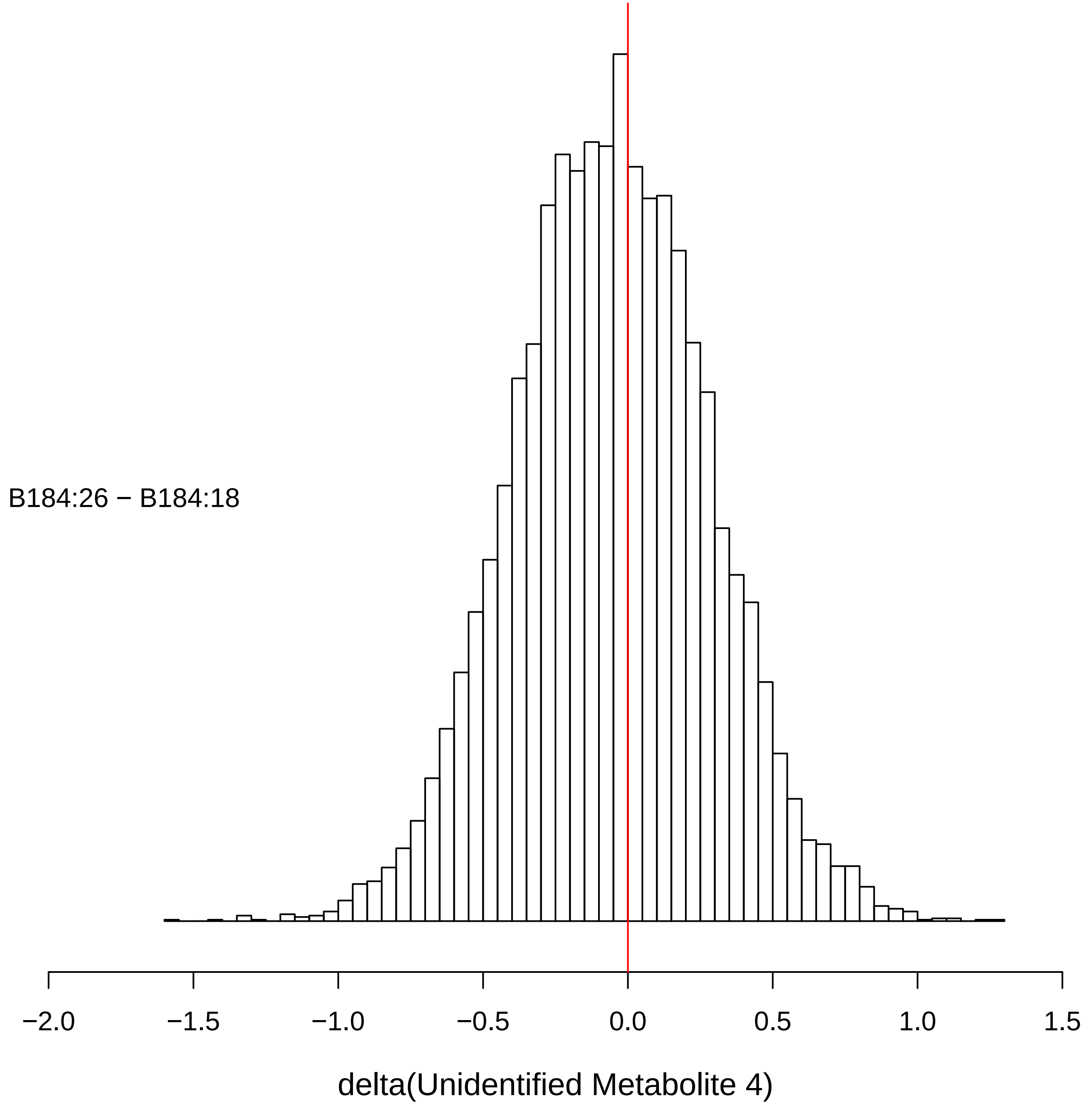
B184:26 − B184:18
−2.0
−1.5
−1.0
−0.5
0.0
0.5
1.0
1.5
delta(Unidentified Metabolite 4)

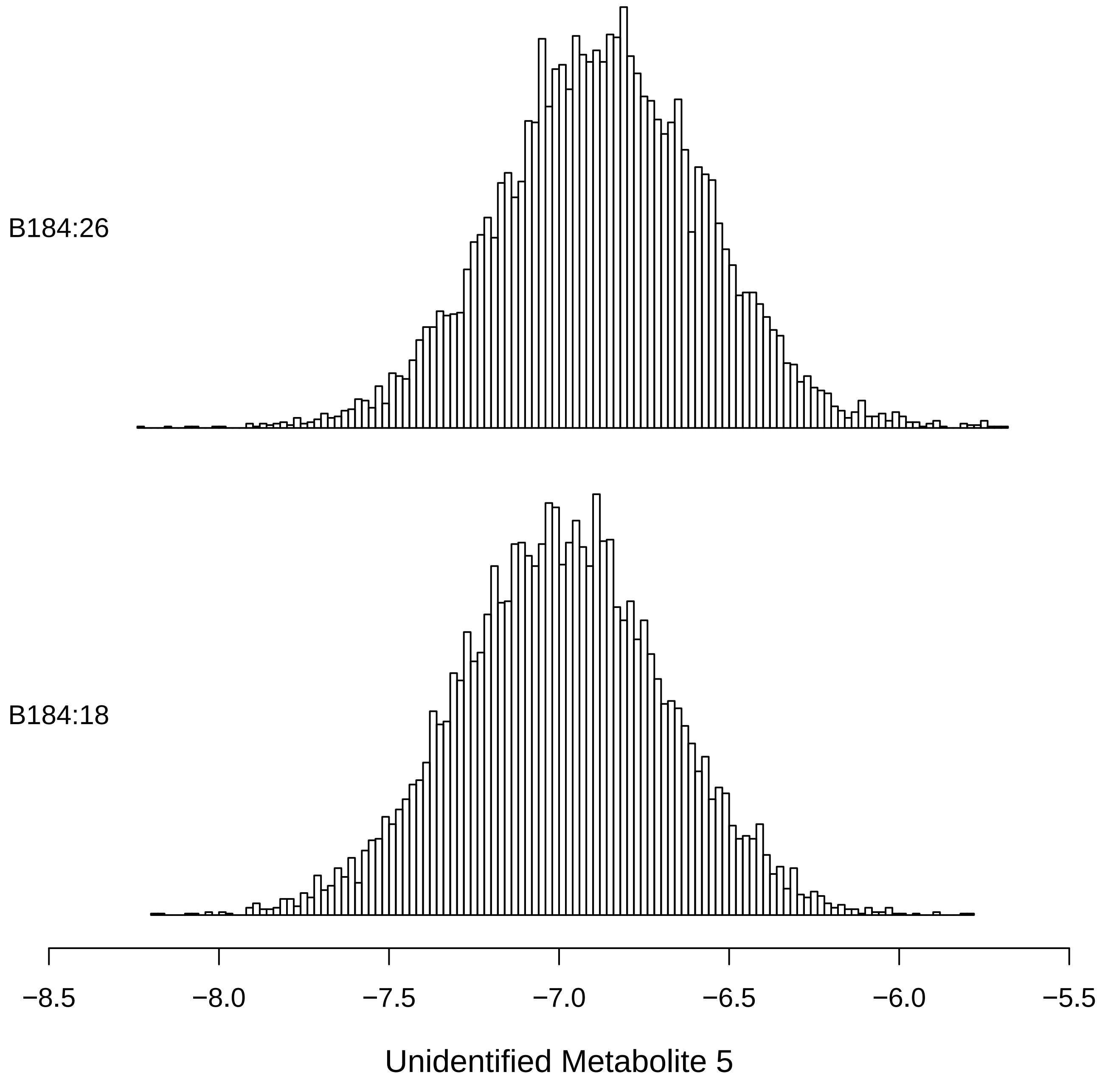
B184:26
B184:18
−8.5
−8.0
−7.5
−7.0
−6.5
−6.0
−5.5
Unidentified Metabolite 5

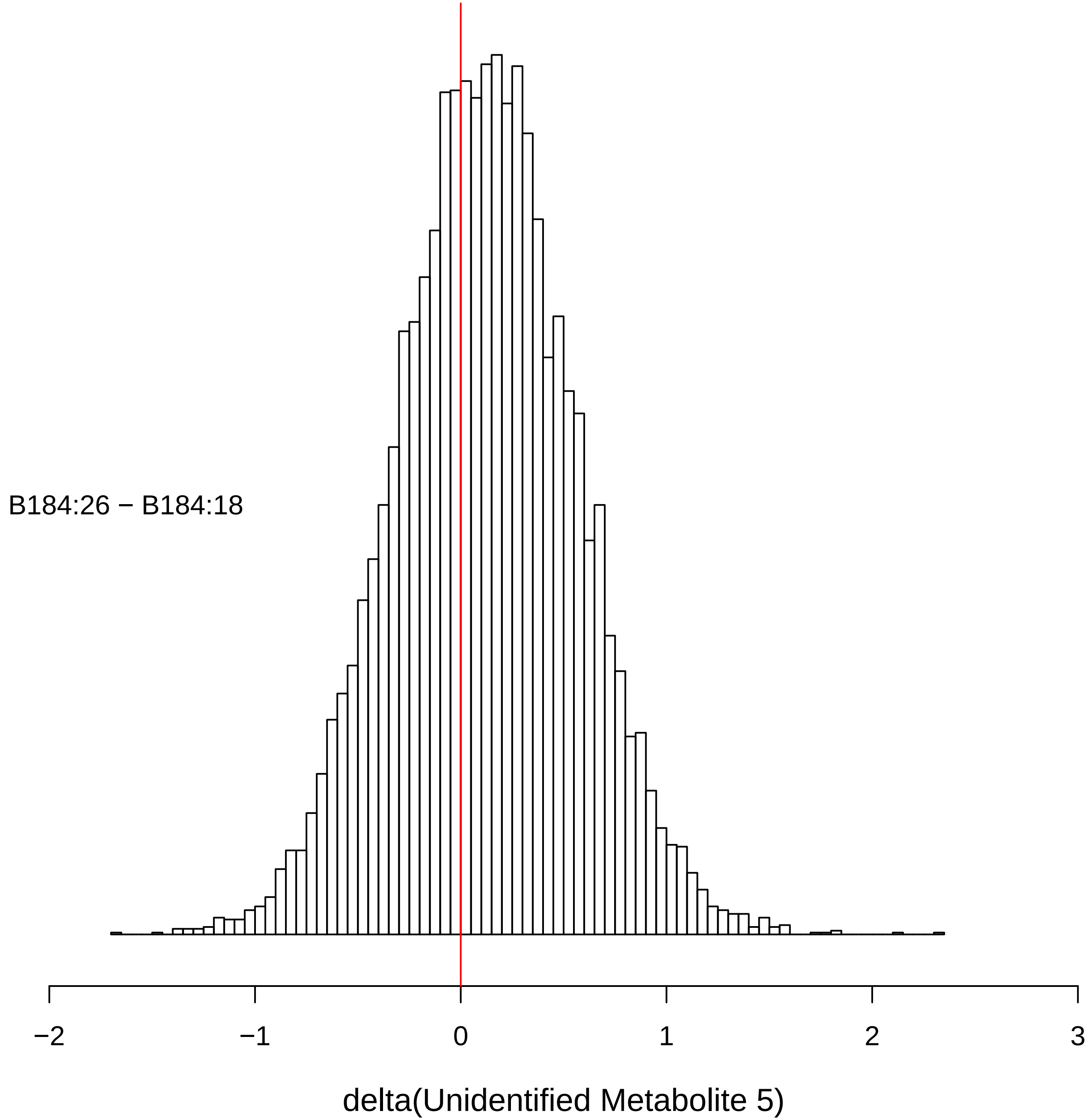
B184:26 − B184:18
−2
−1
0
1
2
3
delta(Unidentified Metabolite 5)

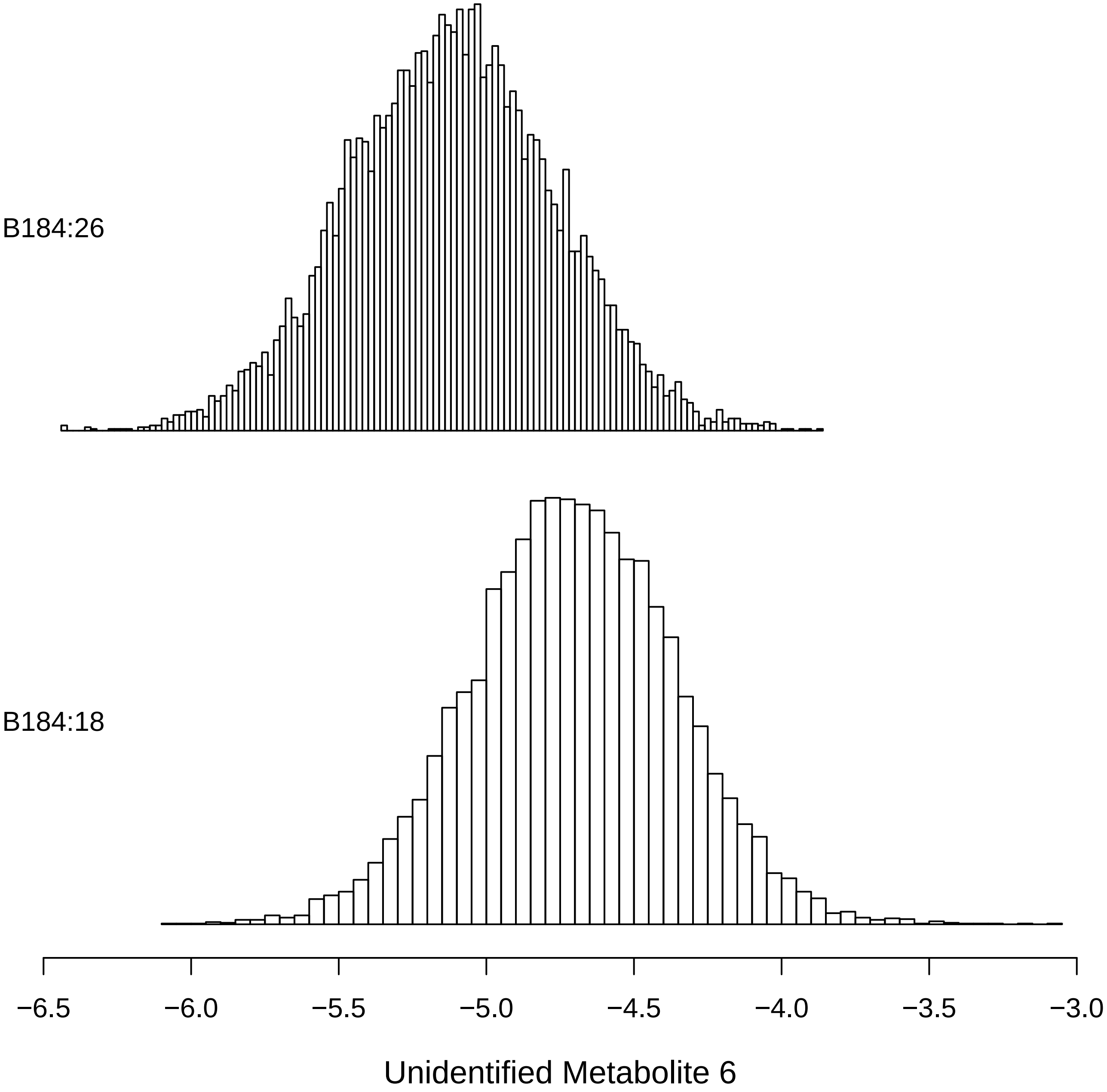
B184:26
B184:18
−6.5
−6.0
−5.5
−5.0
−4.5
−4.0
−3.5
−3.0
Unidentified Metabolite 6

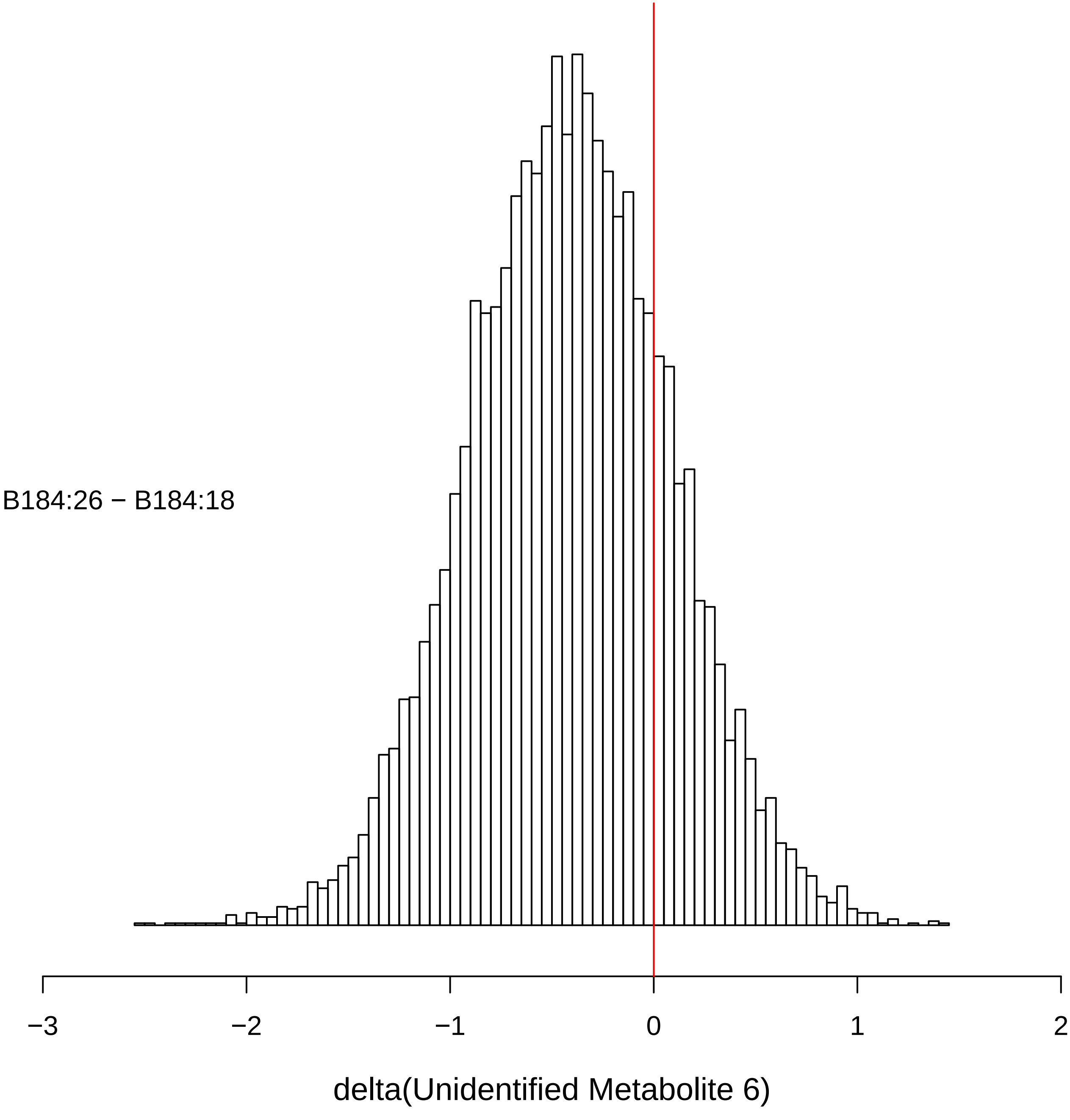
B184:26 − B184:18
−3
−2
−1
0
1
2
delta(Unidentified Metabolite 6)

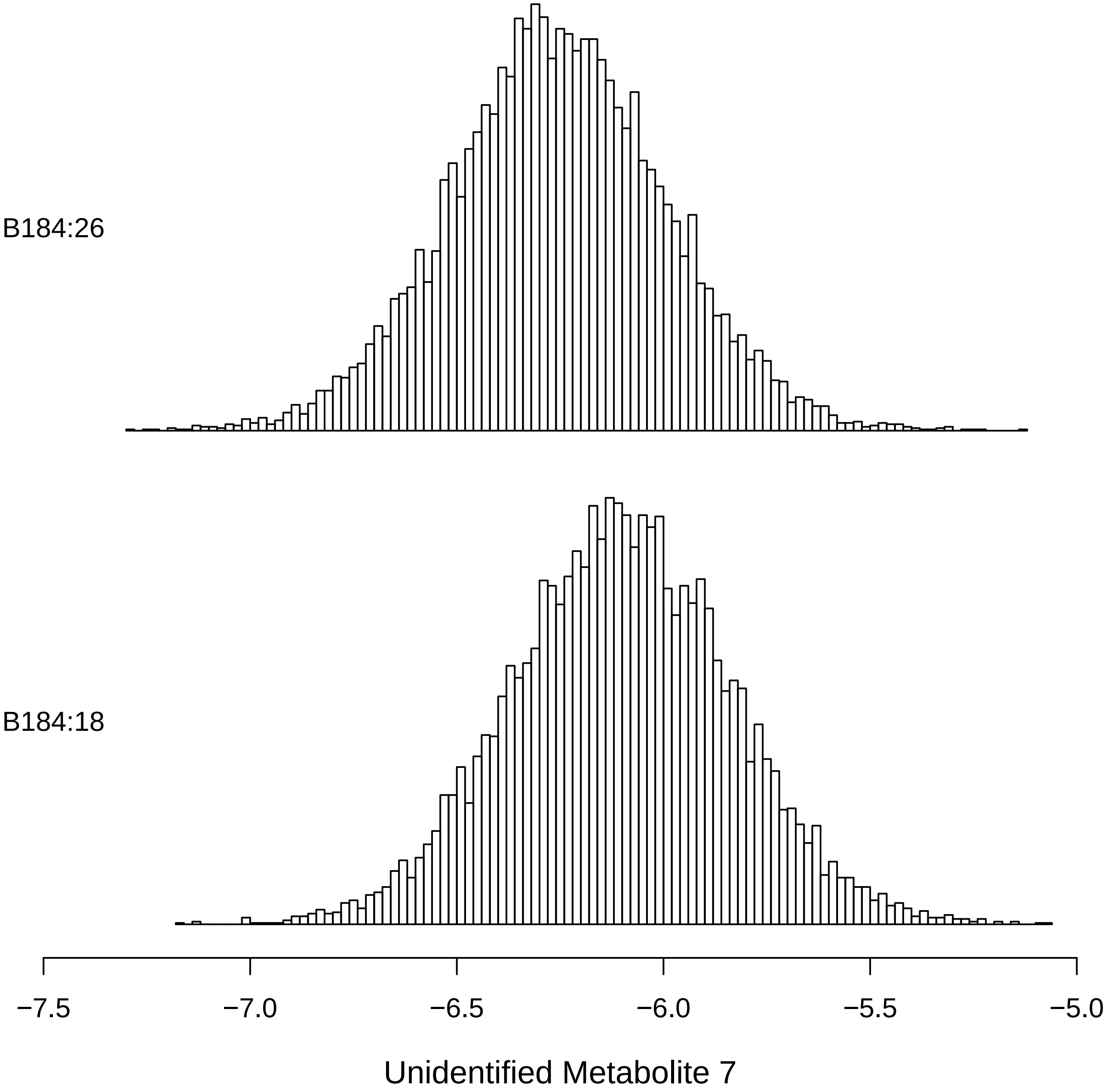
B184:26
B184:18
−7.5
−7.0
−6.5
−6.0
−5.5
−5.0
Unidentified Metabolite 7

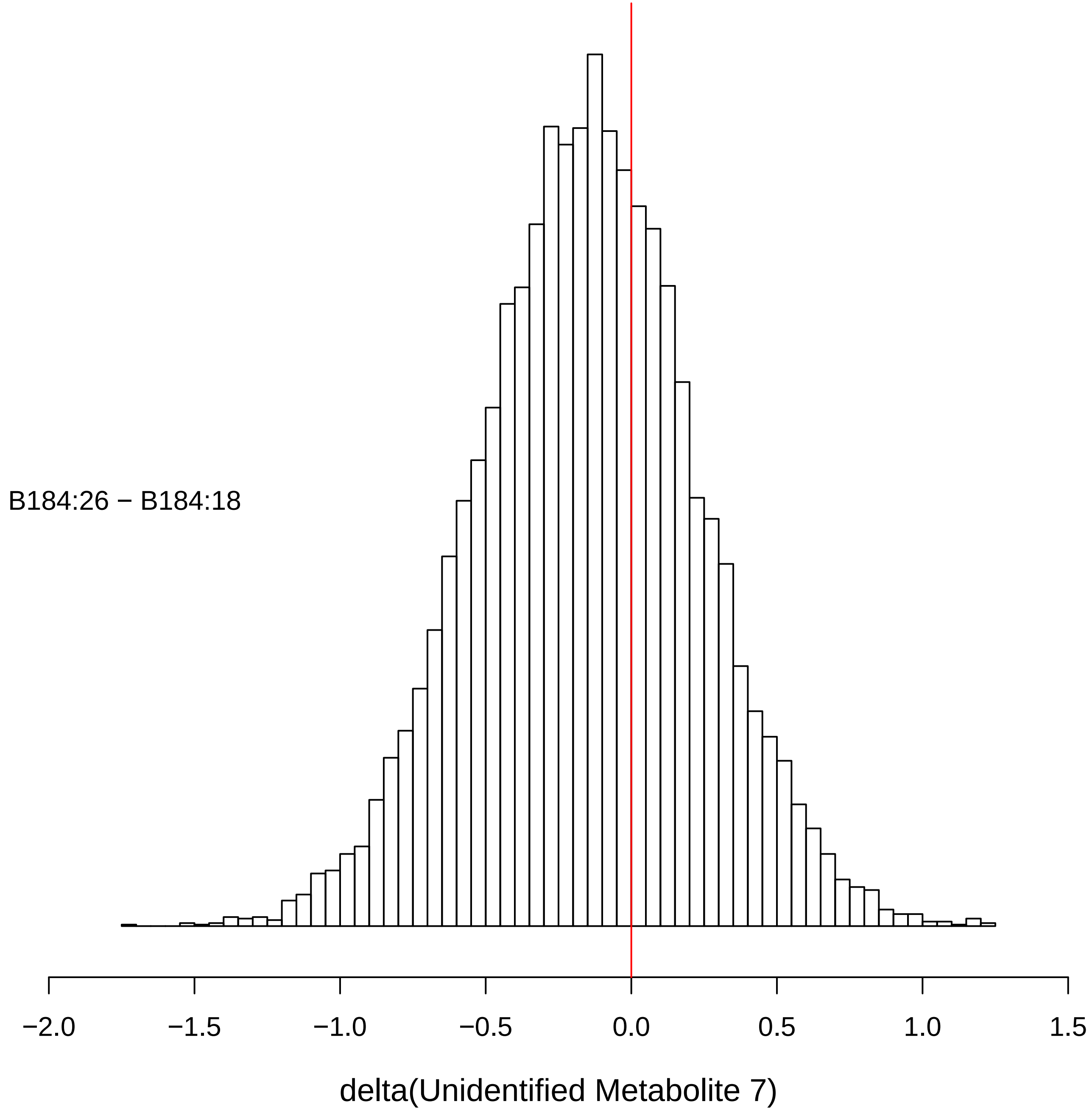
B184:26 − B184:18
−2.0
−1.5
−1.0
−0.5
0.0
0.5
1.0
1.5
delta(Unidentified Metabolite 7)

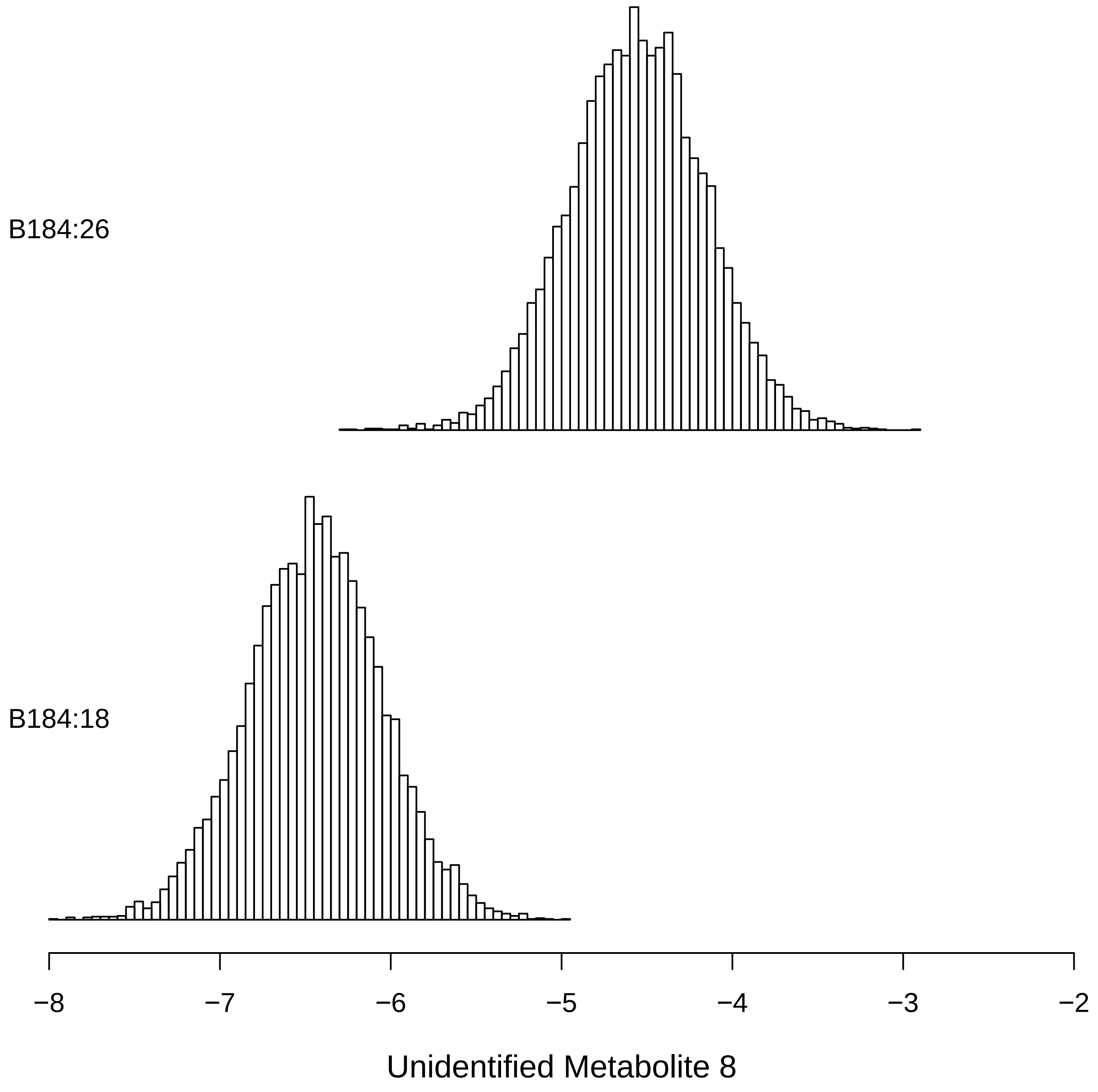
B184:26
B184:18
−8
−7
−6
−5
−4
−3
−2
Unidentified Metabolite 8

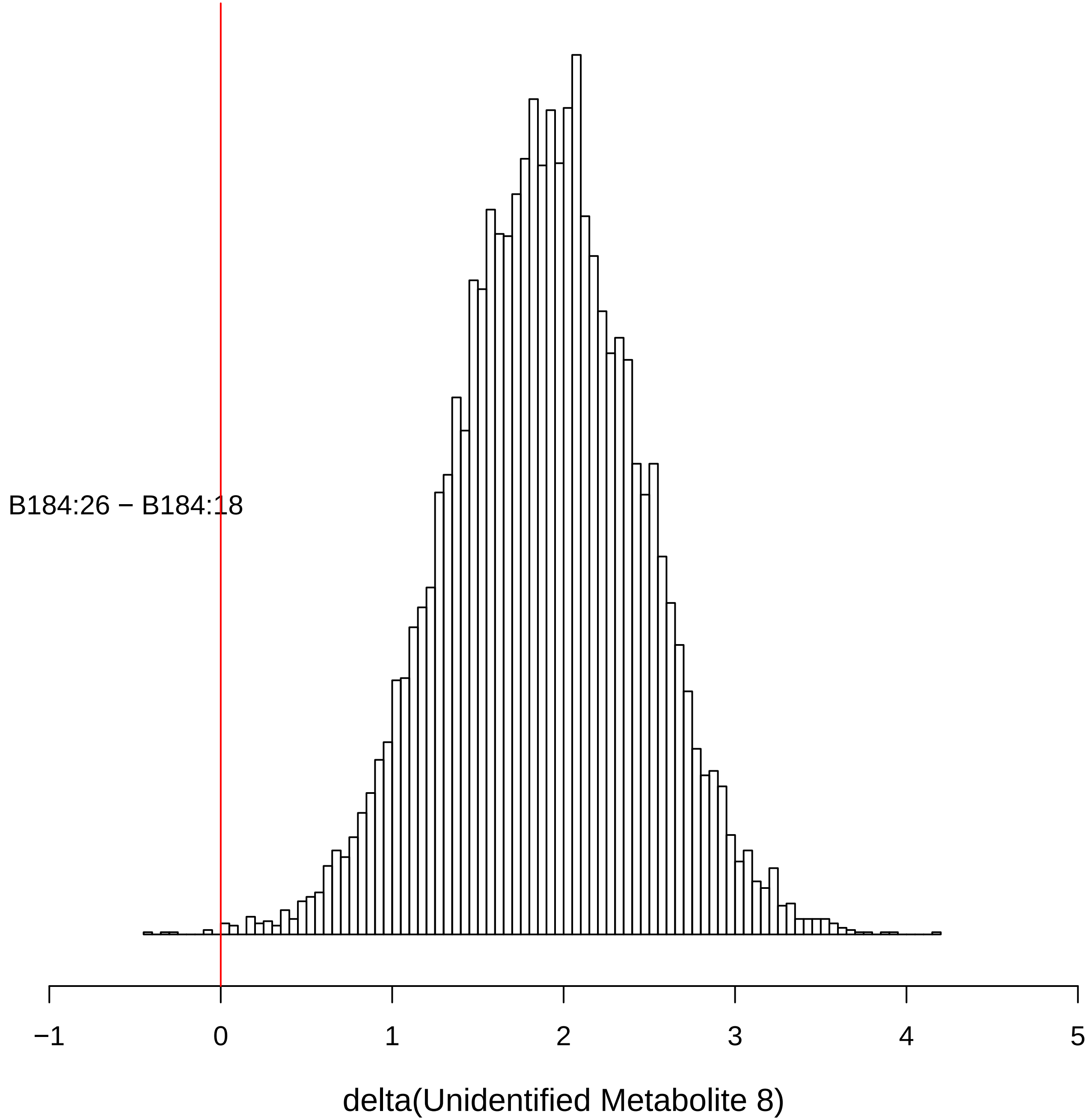
B184:26 − B184:18
−1
0
1
2
3
4
5
delta(Unidentified Metabolite 8)

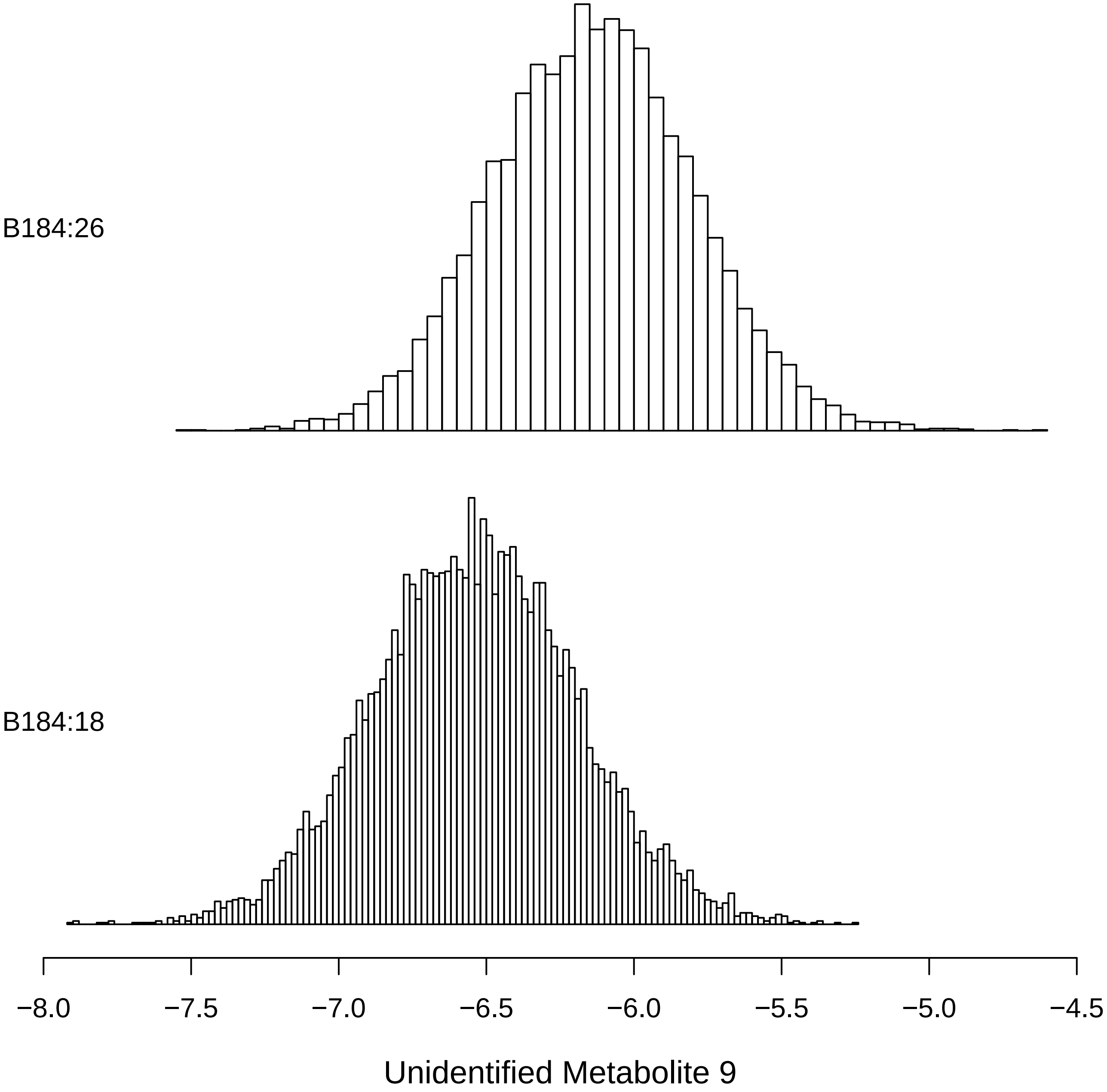
B184:26
B184:18
−8.0
−7.5
−7.0
−6.5
−6.0
−5.5
−5.0
−4.5
Unidentified Metabolite 9

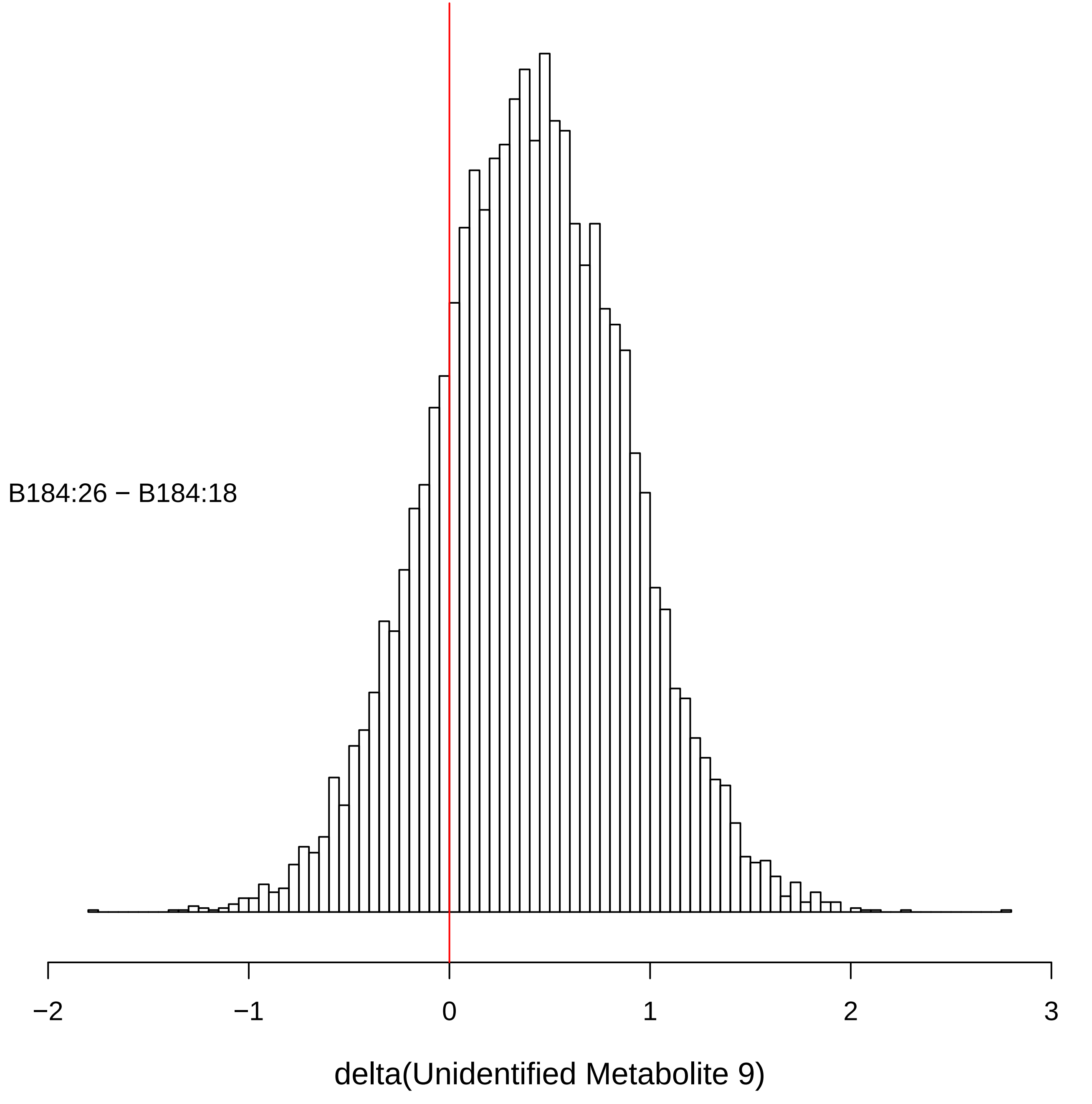
B184:26 − B184:18
−2
−1
0
1
2
3
delta(Unidentified Metabolite 9)

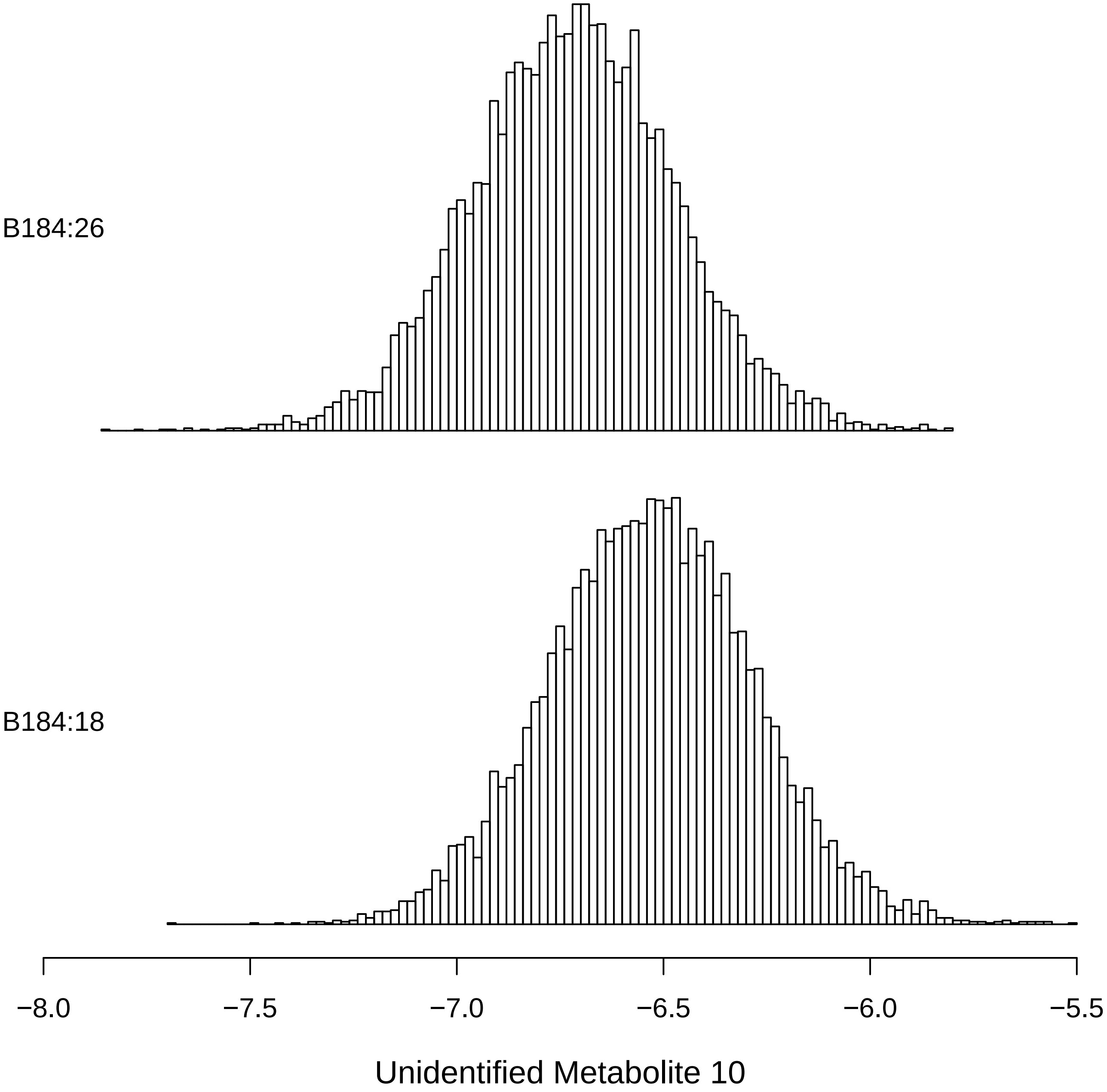

B184:26
B184:18
−8.0
−7.5
−7.0
−6.5
−6.0
−5.5
Unidentified Metabolite 10

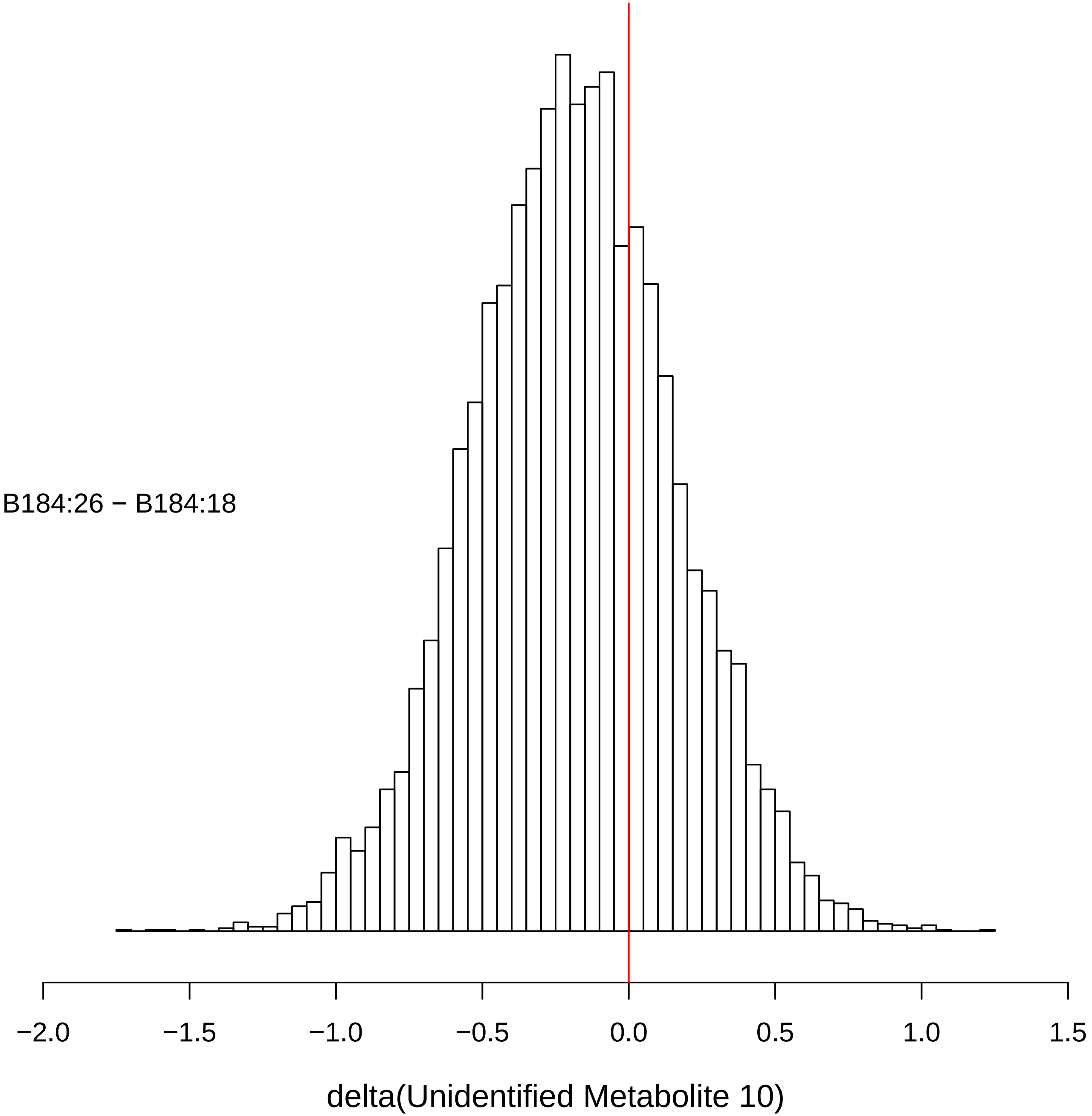

B184:26 − B184:18
−2.0
−1.5
−1.0
−0.5
0.0
0.5
1.0
1.5
delta(Unidentified Metabolite 10)

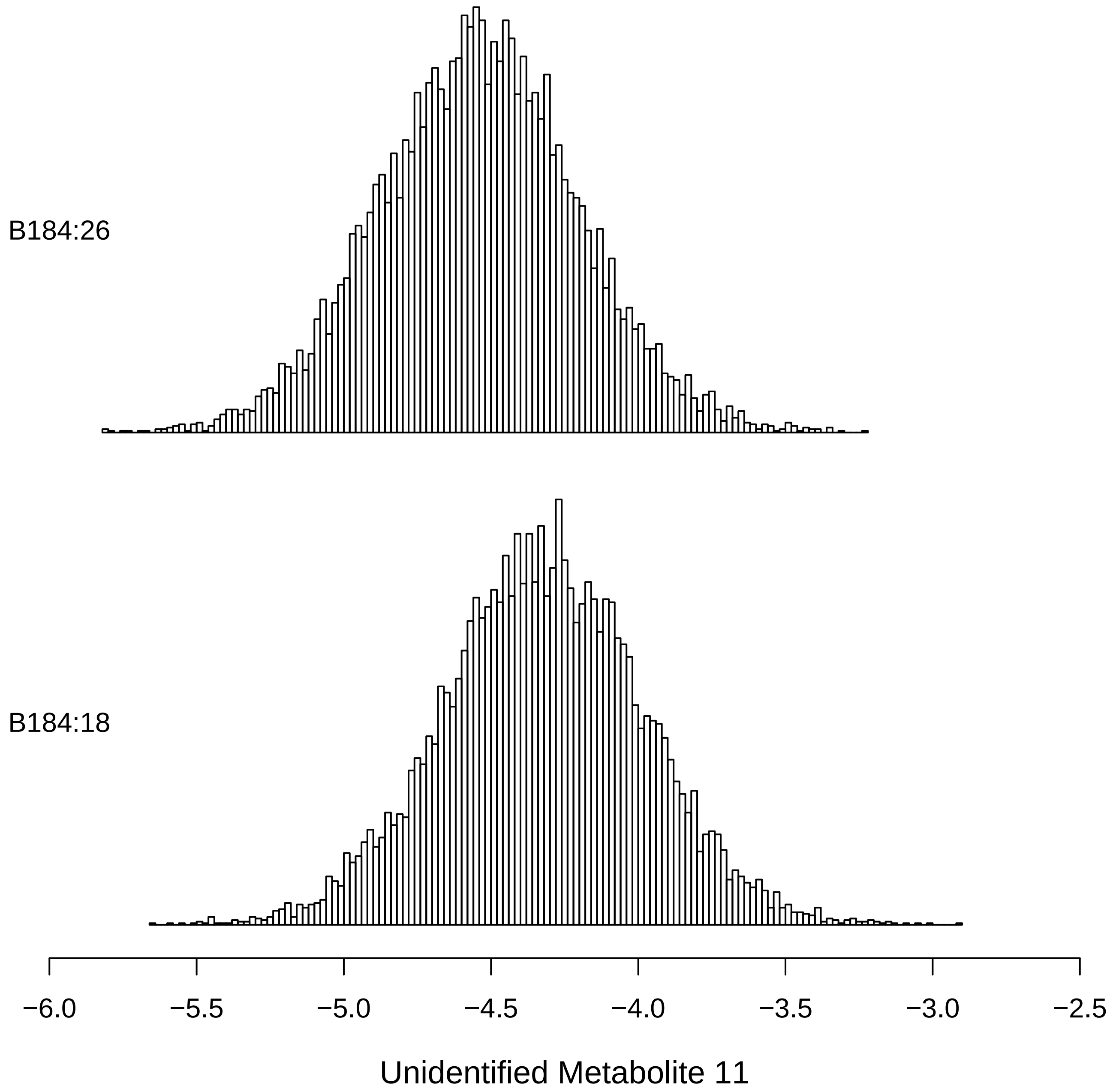
B184:26
B184:18
−6.0
−5.5
−5.0
−4.5
−4.0
−3.5
−3.0
−2.5
Unidentified Metabolite 11

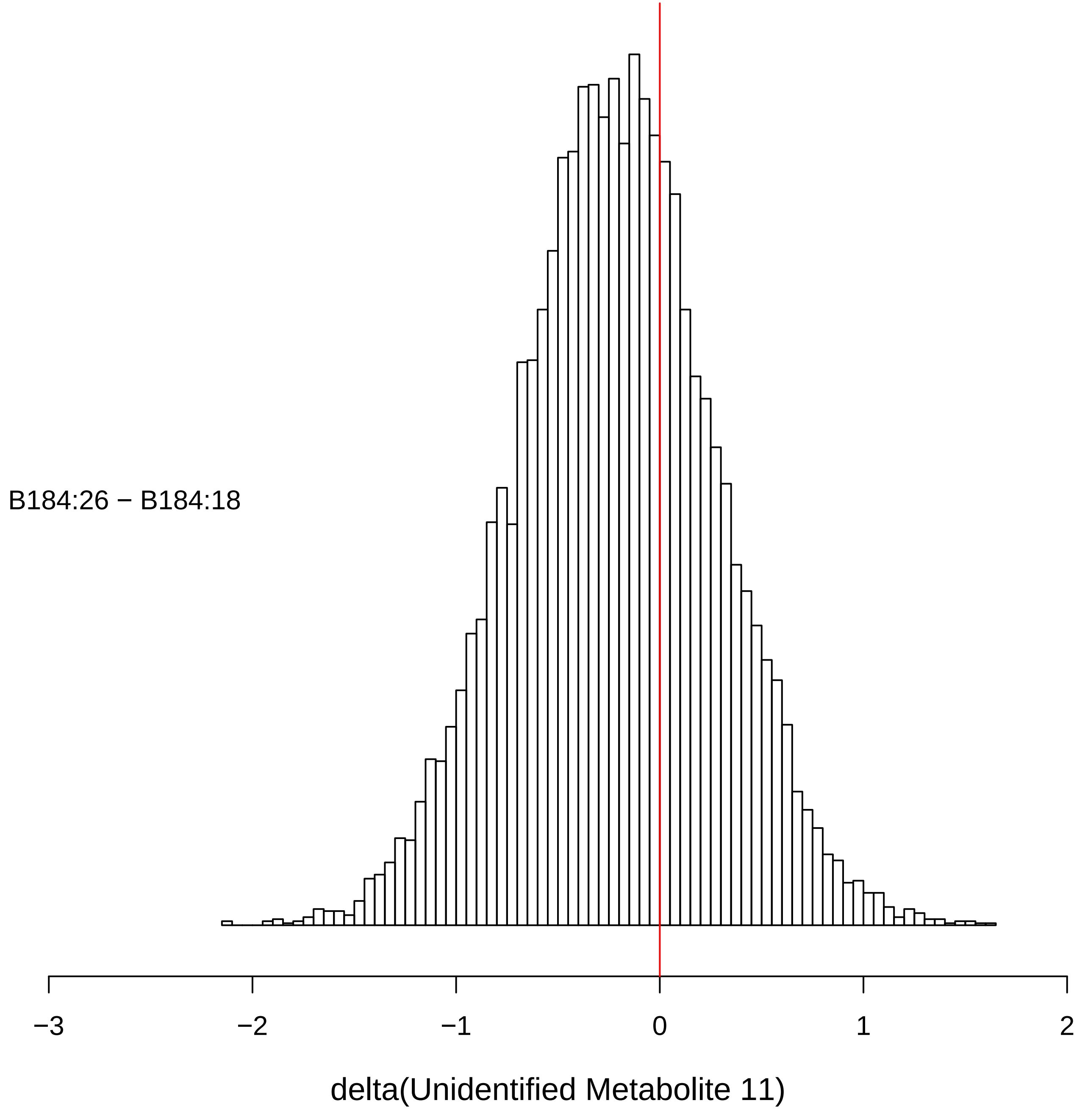
B184:26 − B184:18
−3
−2
−1
0
1
2
delta(Unidentified Metabolite 11)

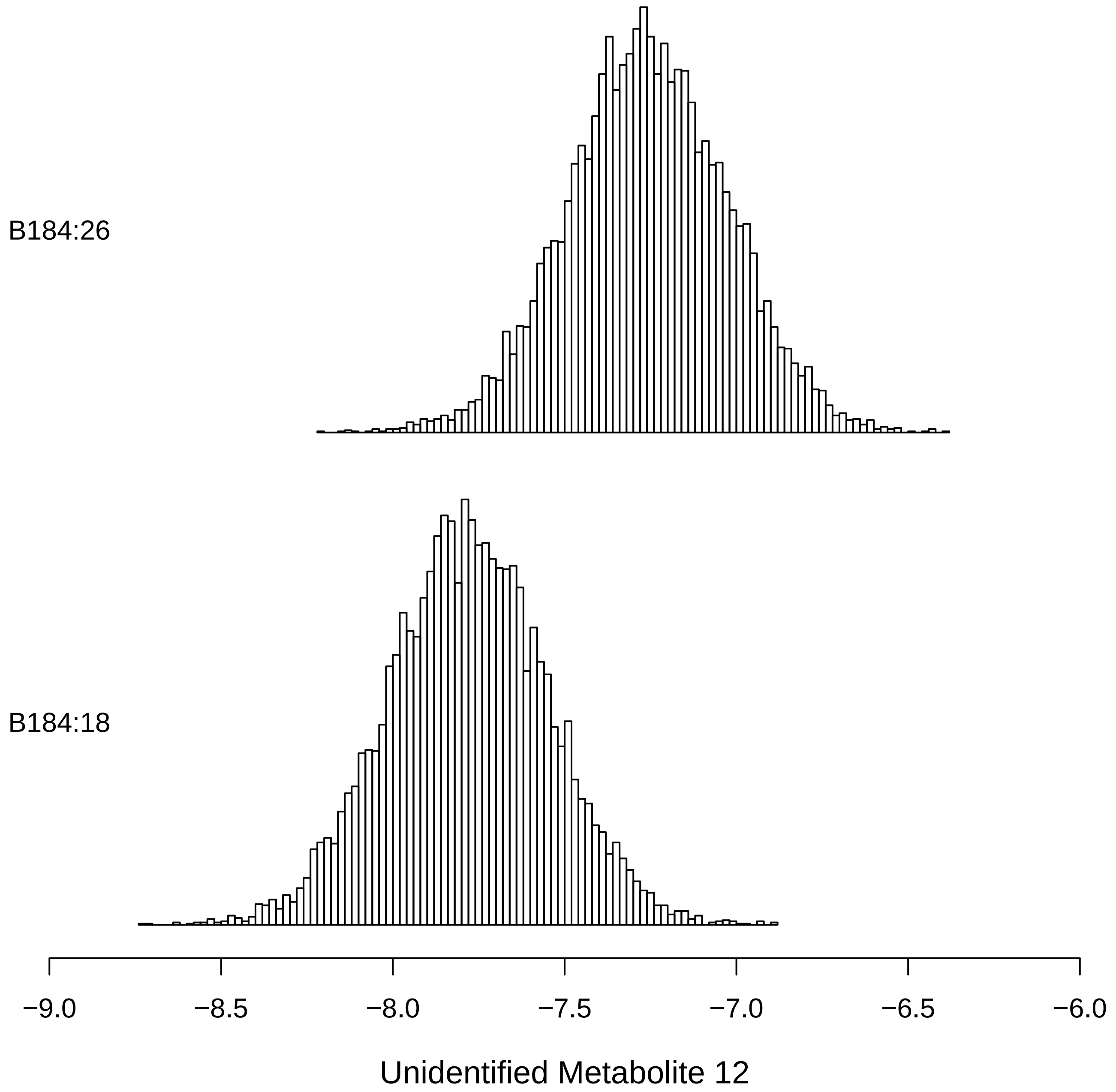
B184:26
B184:18
−9.0
−8.5
−8.0
−7.5
−7.0
−6.5
−6.0
Unidentified Metabolite 12

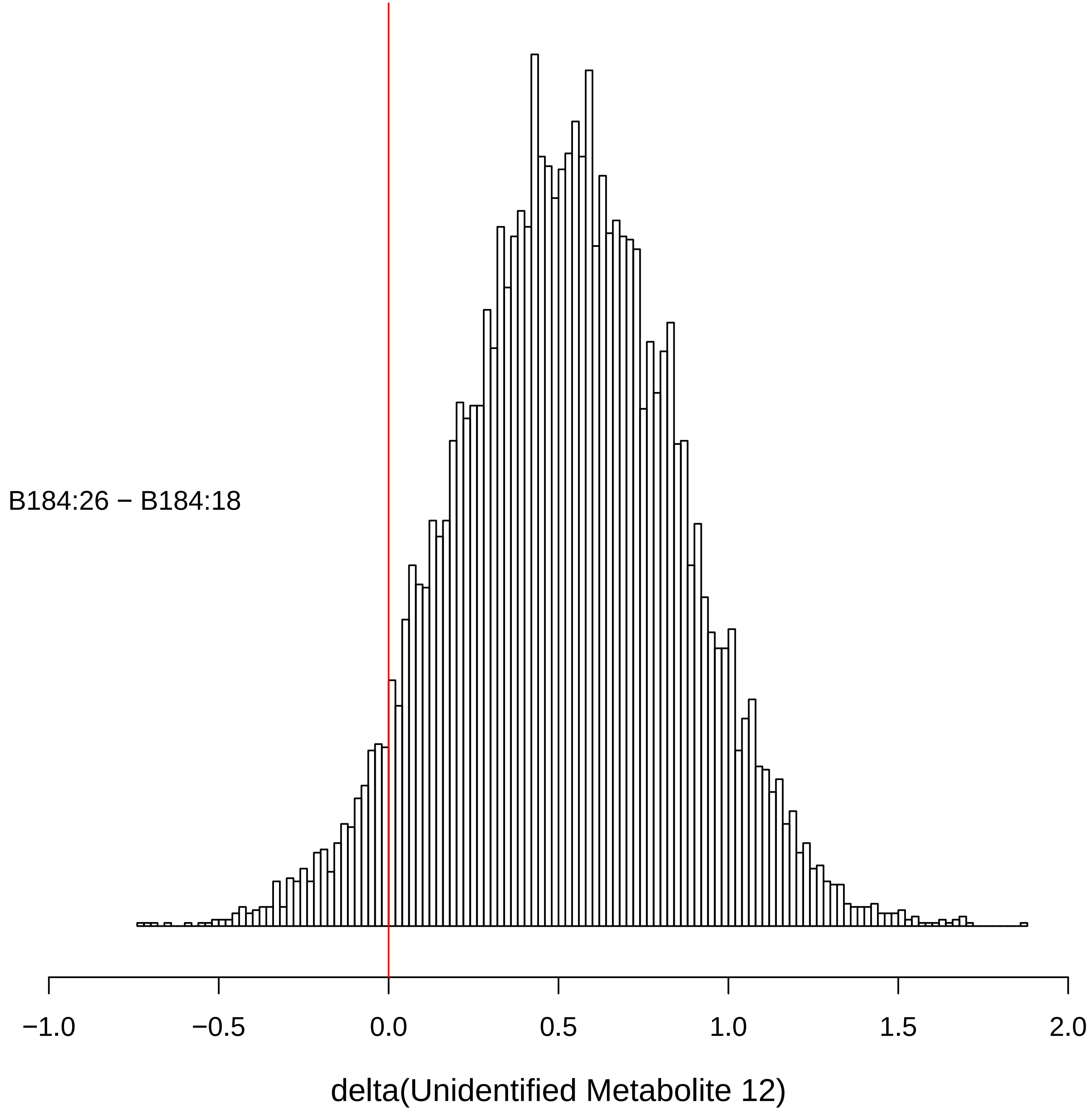
B184:26 − B184:18
−1.0
−0.5
0.0
0.5
1.0
1.5
2.0
delta(Unidentified Metabolite 12)

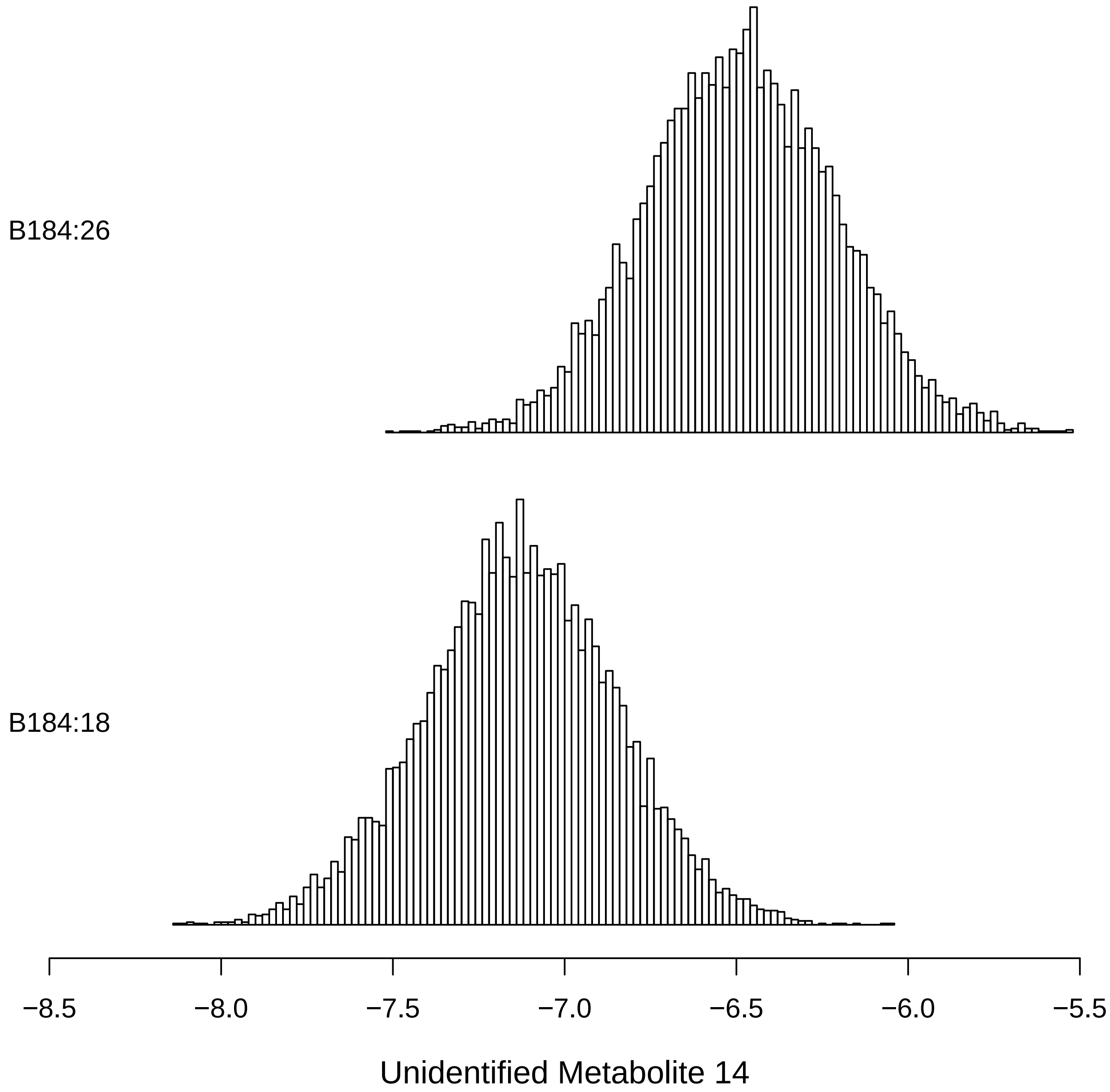

B184:26
B184:18
−8.5
−8.0
−7.5
−7.0
−6.5
−6.0
−5.5
Unidentified Metabolite 14

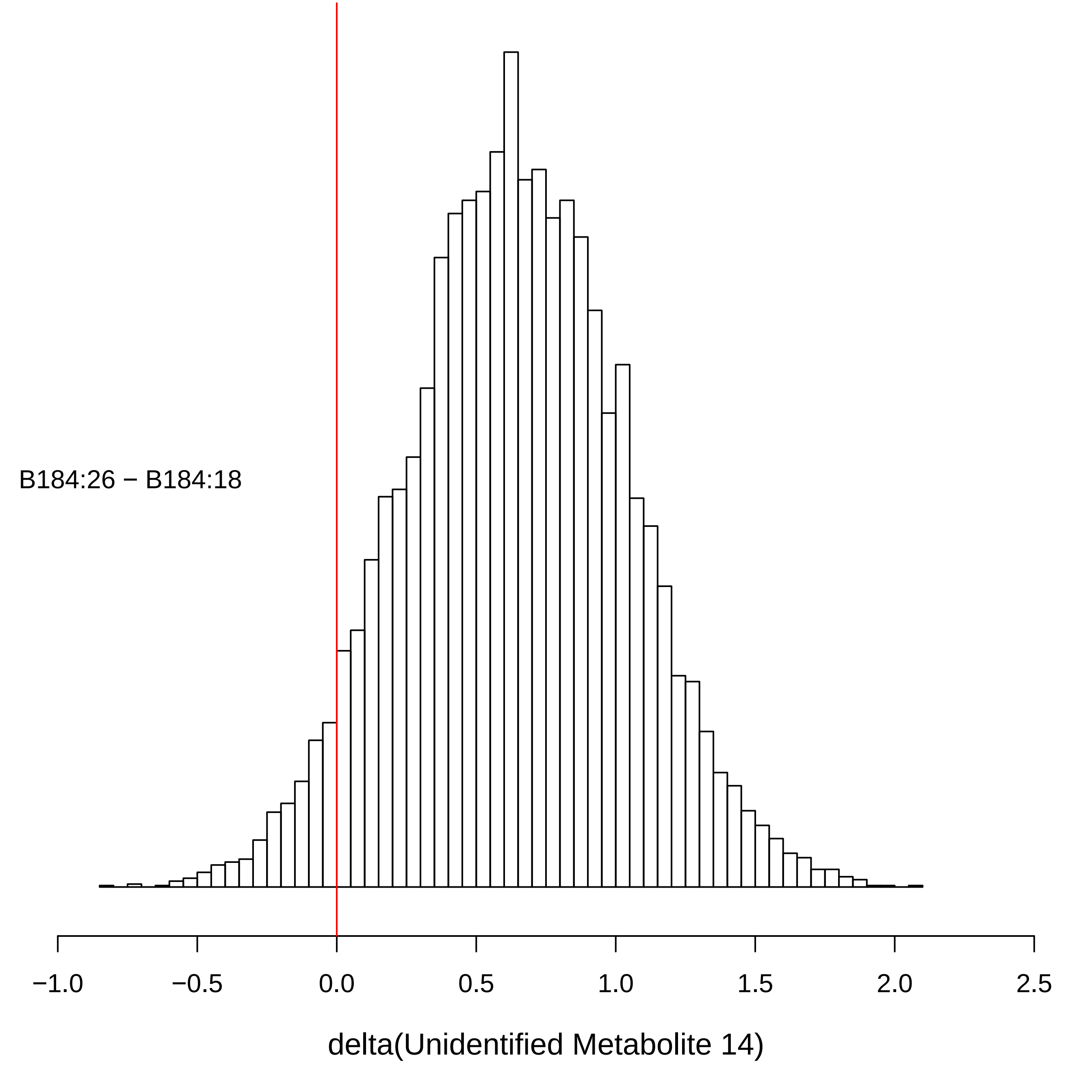

B184:26 − B184:18
−1.0
−0.5
0.0
0.5
1.0
1.5
2.0
2.5
delta(Unidentified Metabolite 14)

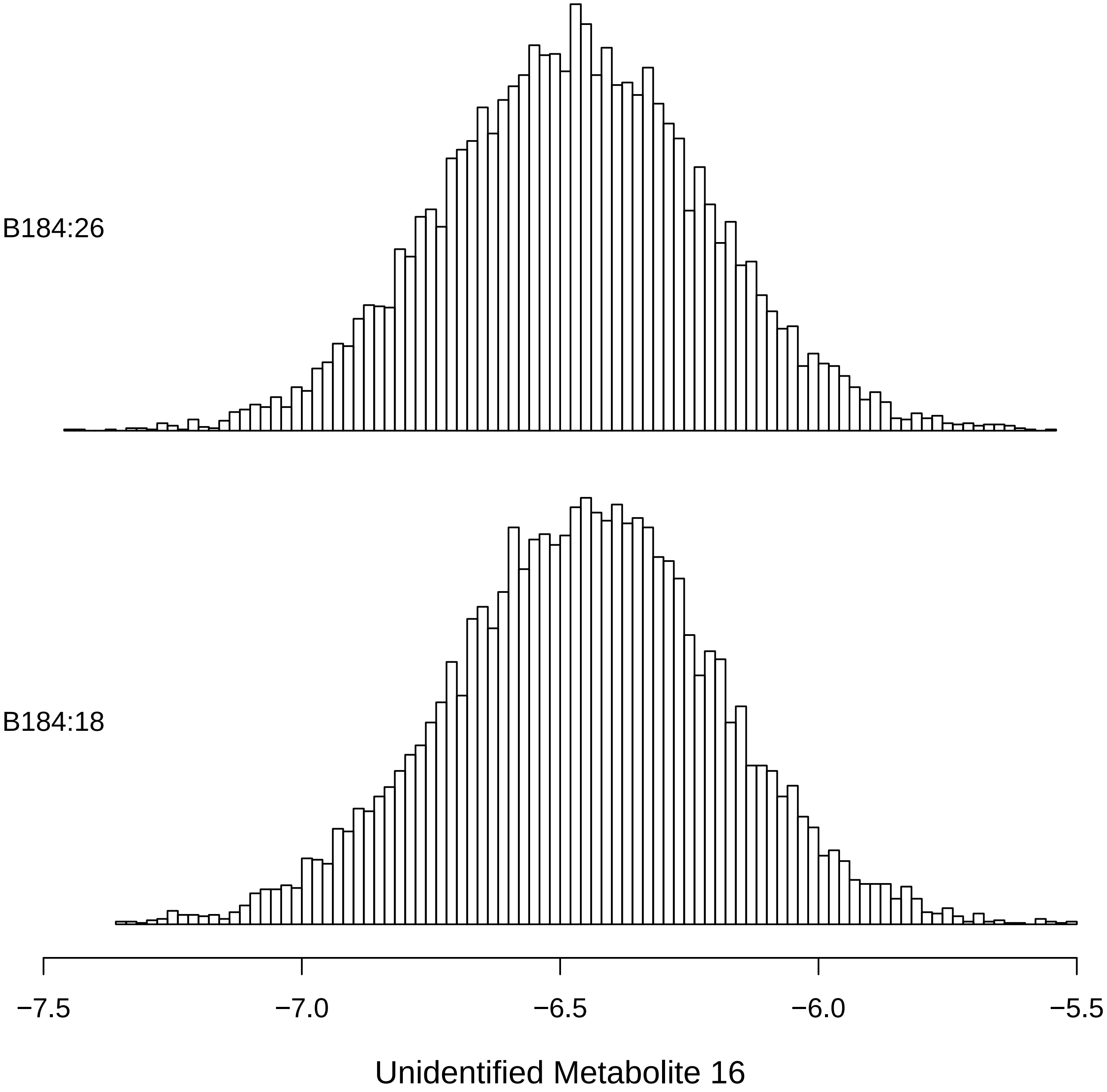
B184:26
B184:18
−7.5
−7.0
−6.5
−6.0
−5.5
Unidentified Metabolite 16

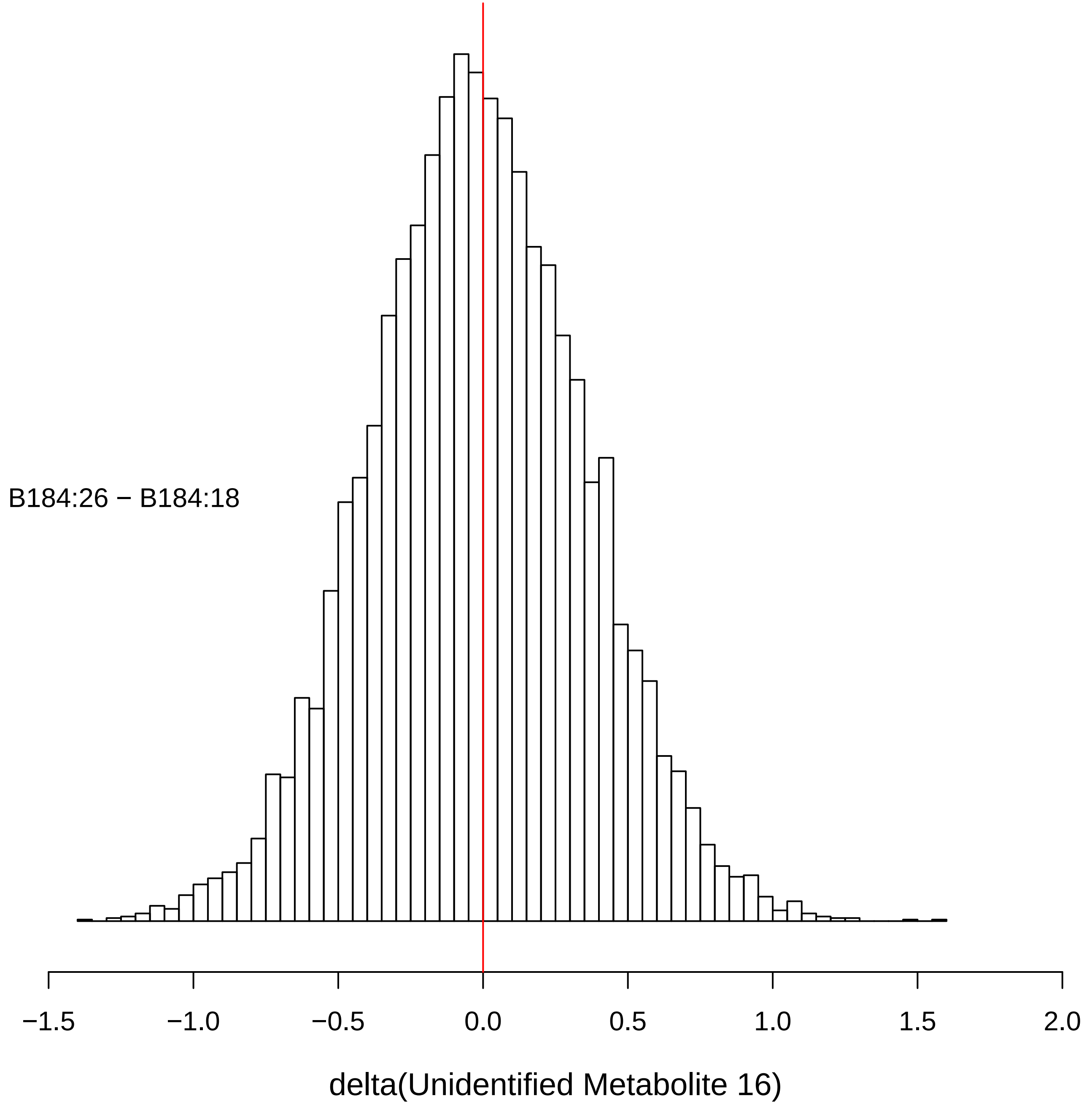
B184:26 − B184:18
−1.5
−1.0
−0.5
0.0
0.5
1.0
1.5
2.0
delta(Unidentified Metabolite 16)

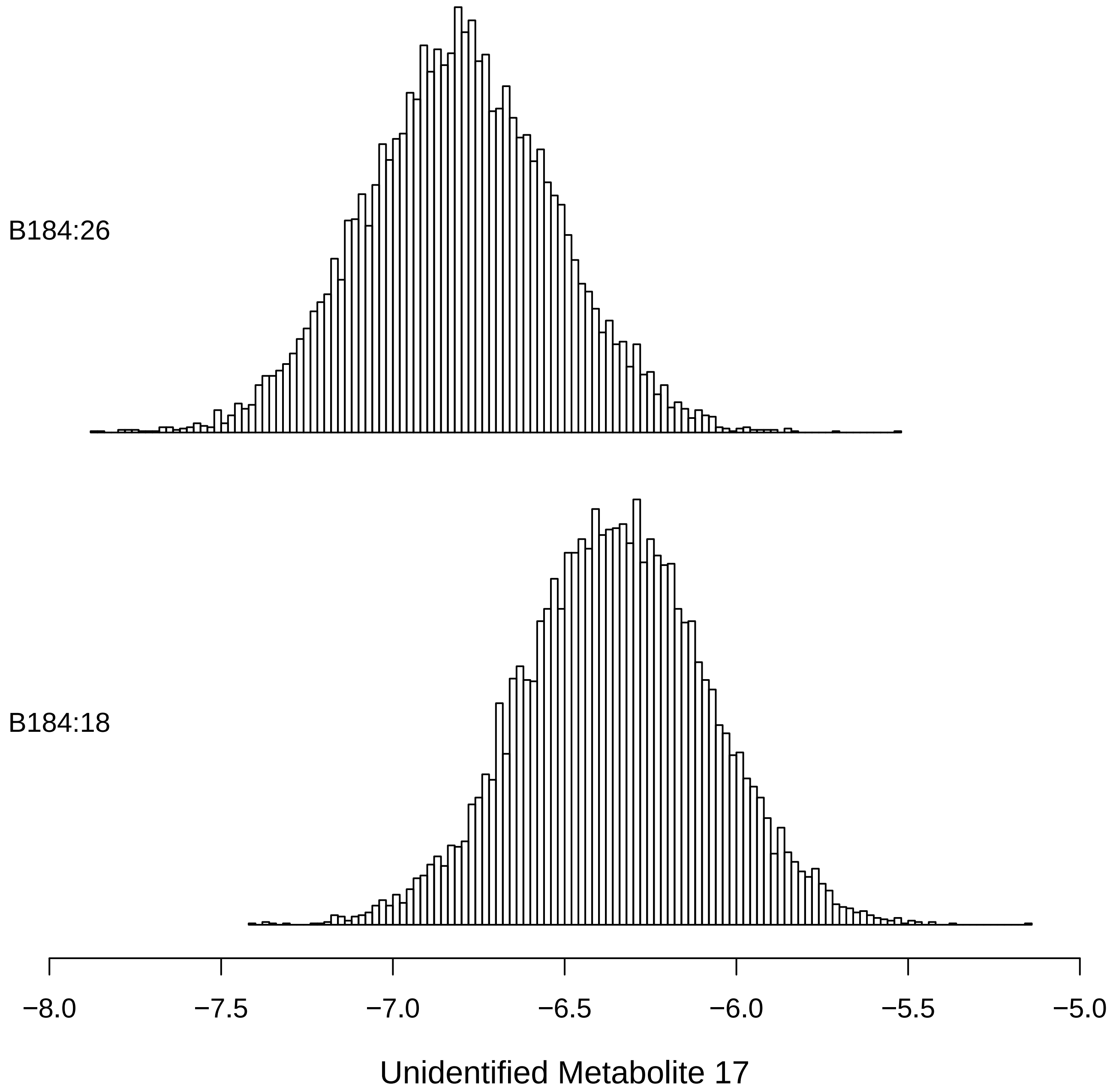
B184:26
B184:18
−8.0
−7.5
−7.0
−6.5
−6.0
−5.5
−5.0
Unidentified Metabolite 17

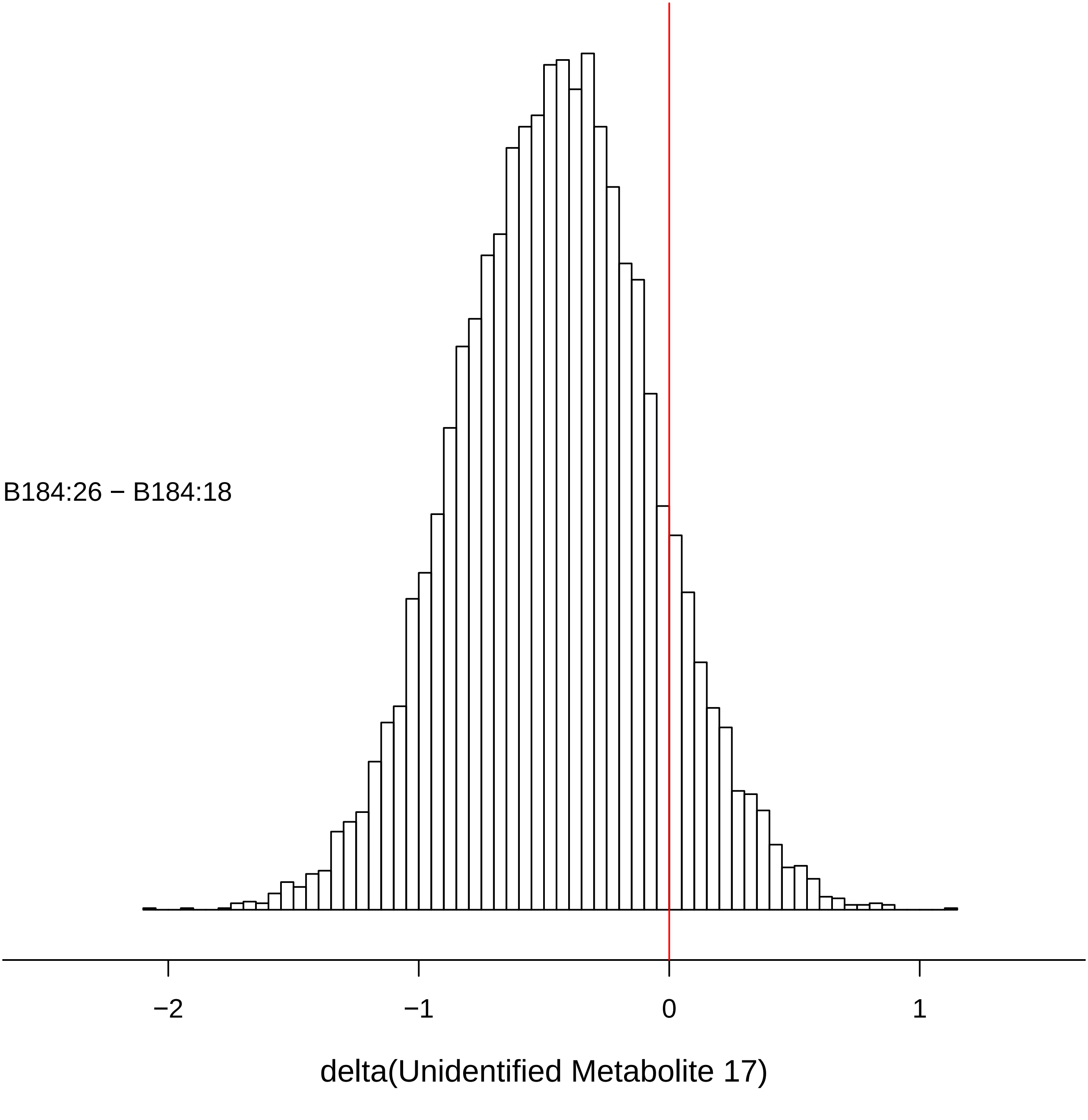
B184:26 − B184:18
−2
−1
0
1
delta(Unidentified Metabolite 17)

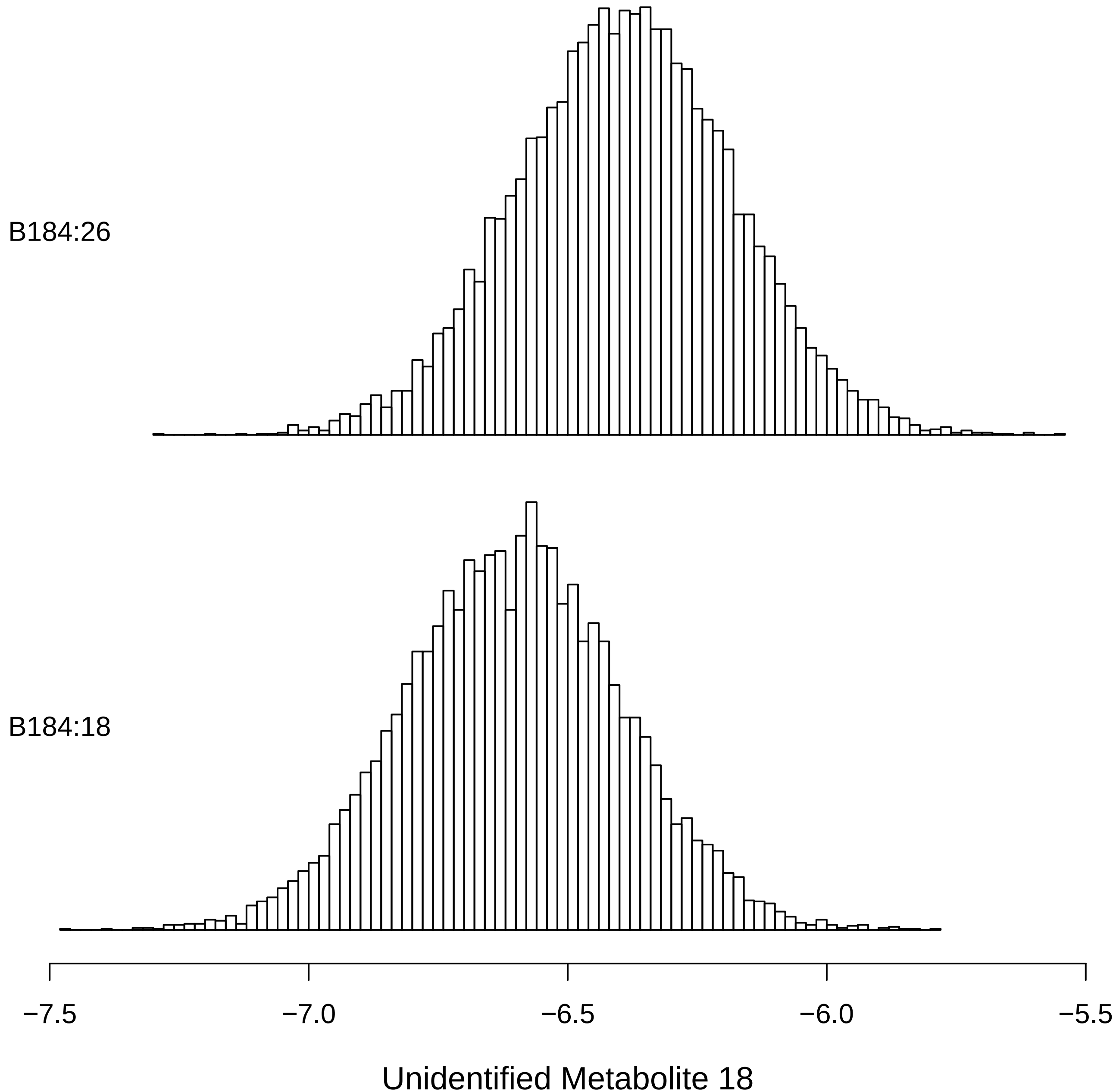

B184:26
B184:18
−7.5
−7.0
−6.5
−6.0
−5.5
Unidentified Metabolite 18

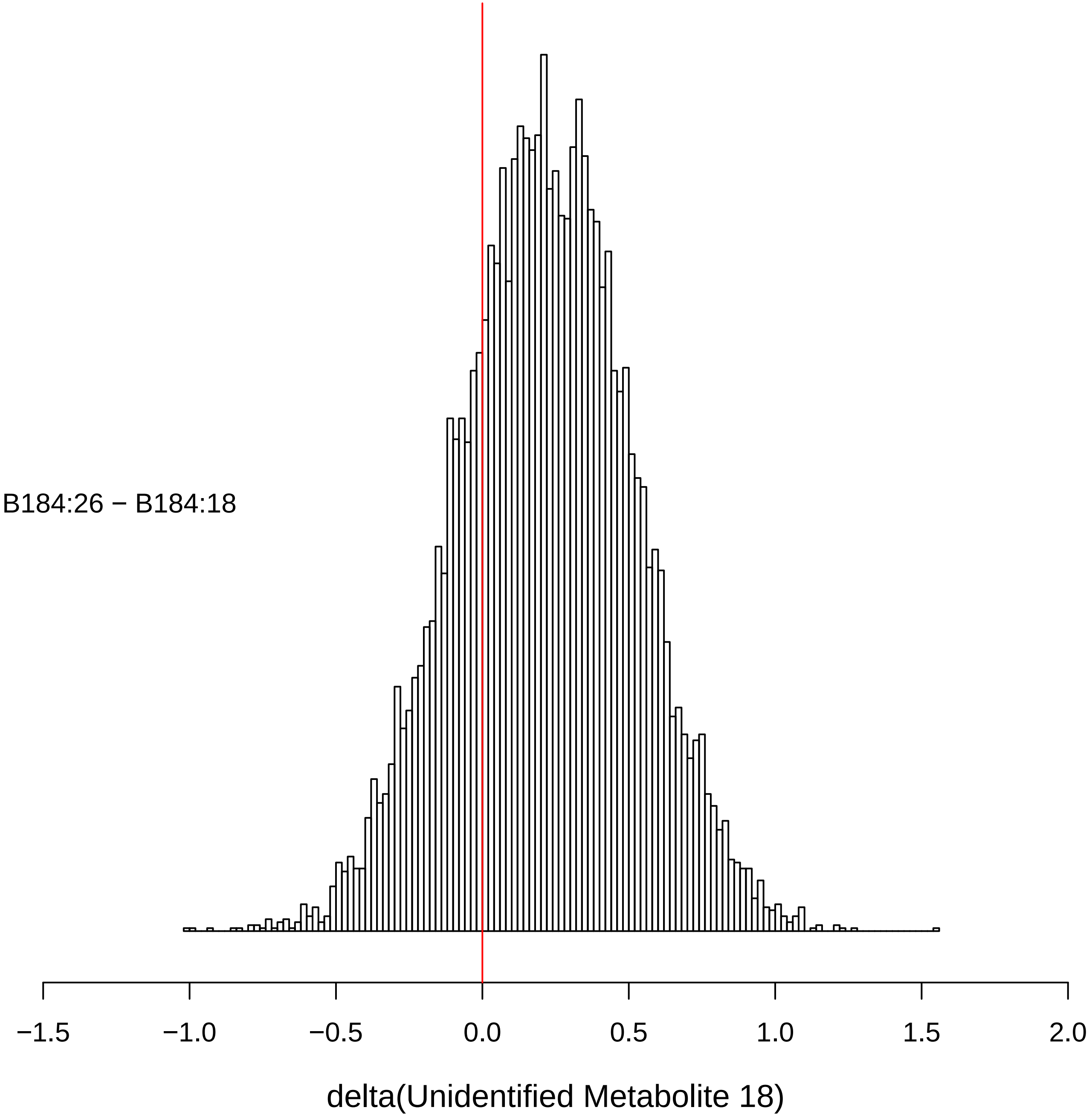
B184:26 − B184:18
−1.5
−1.0
−0.5
0.0
0.5
1.0
1.5
2.0
delta(Unidentified Metabolite 18)

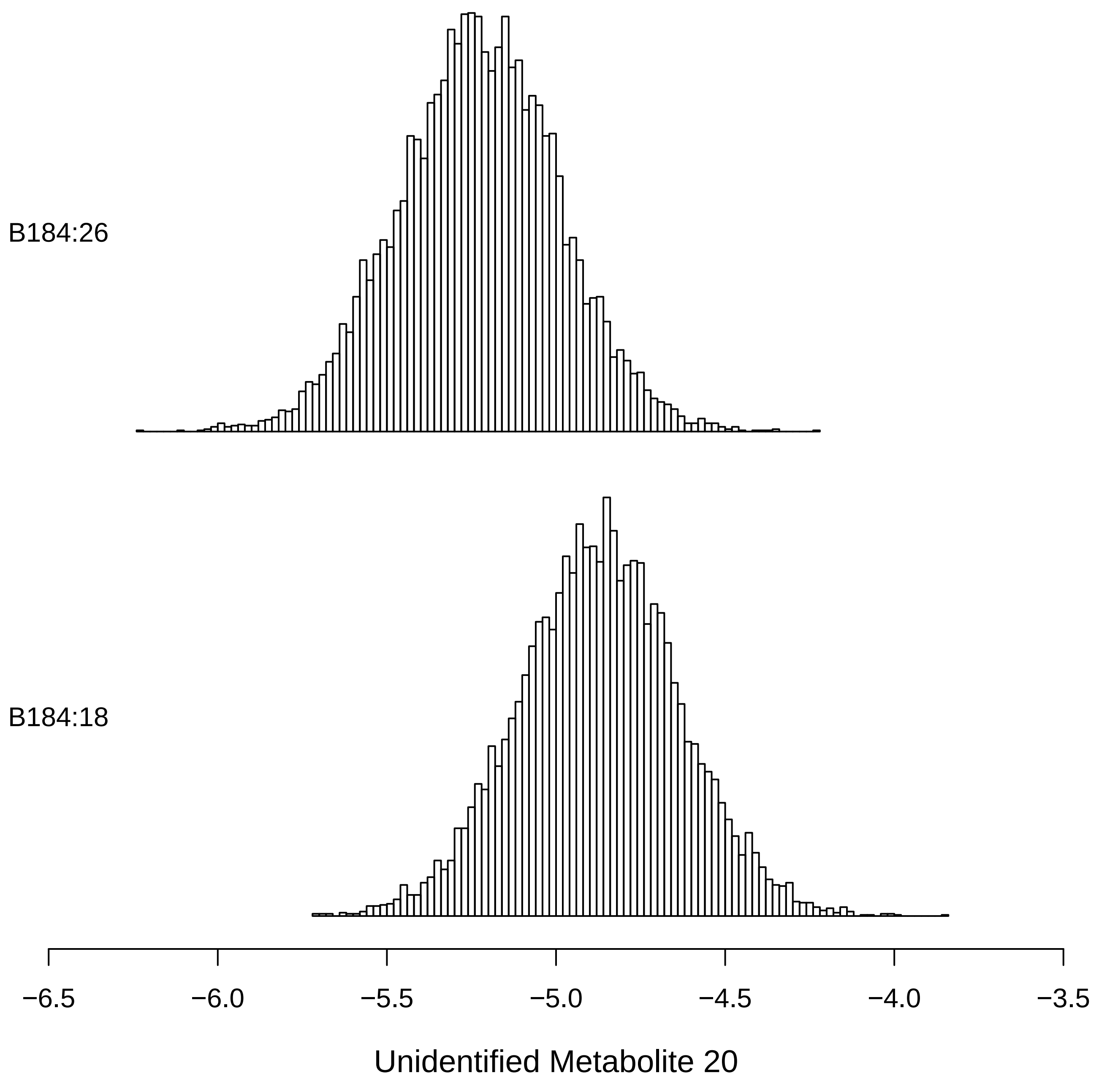
B184:26
B184:18
−6.5
−6.0
−5.5
−5.0
−4.5
−4.0
−3.5
Unidentified Metabolite 20

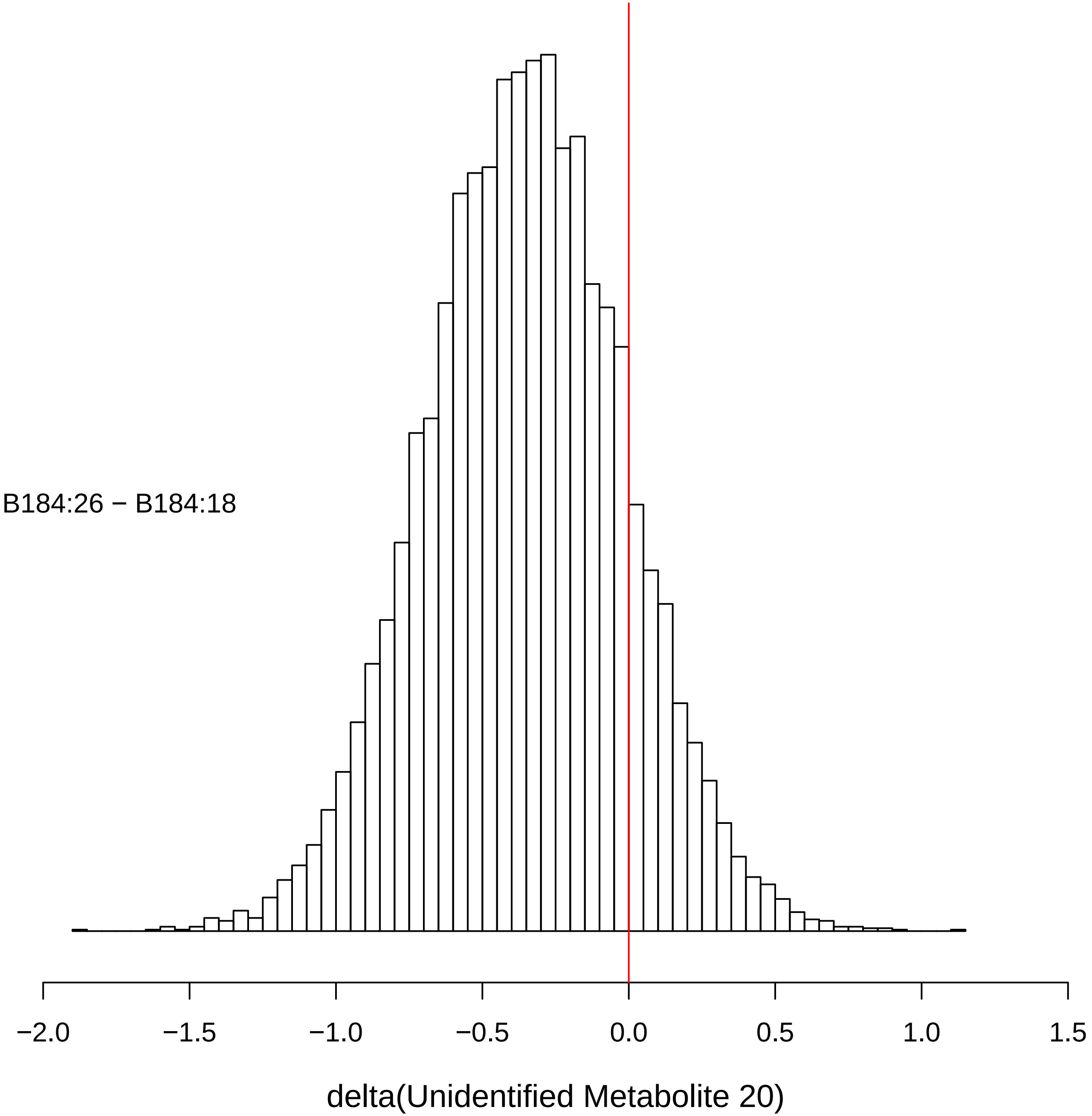
B184:26 − B184:18
−2.0
−1.5
−1.0
−0.5
0.0
0.5
1.0
1.5
delta(Unidentified Metabolite 20)

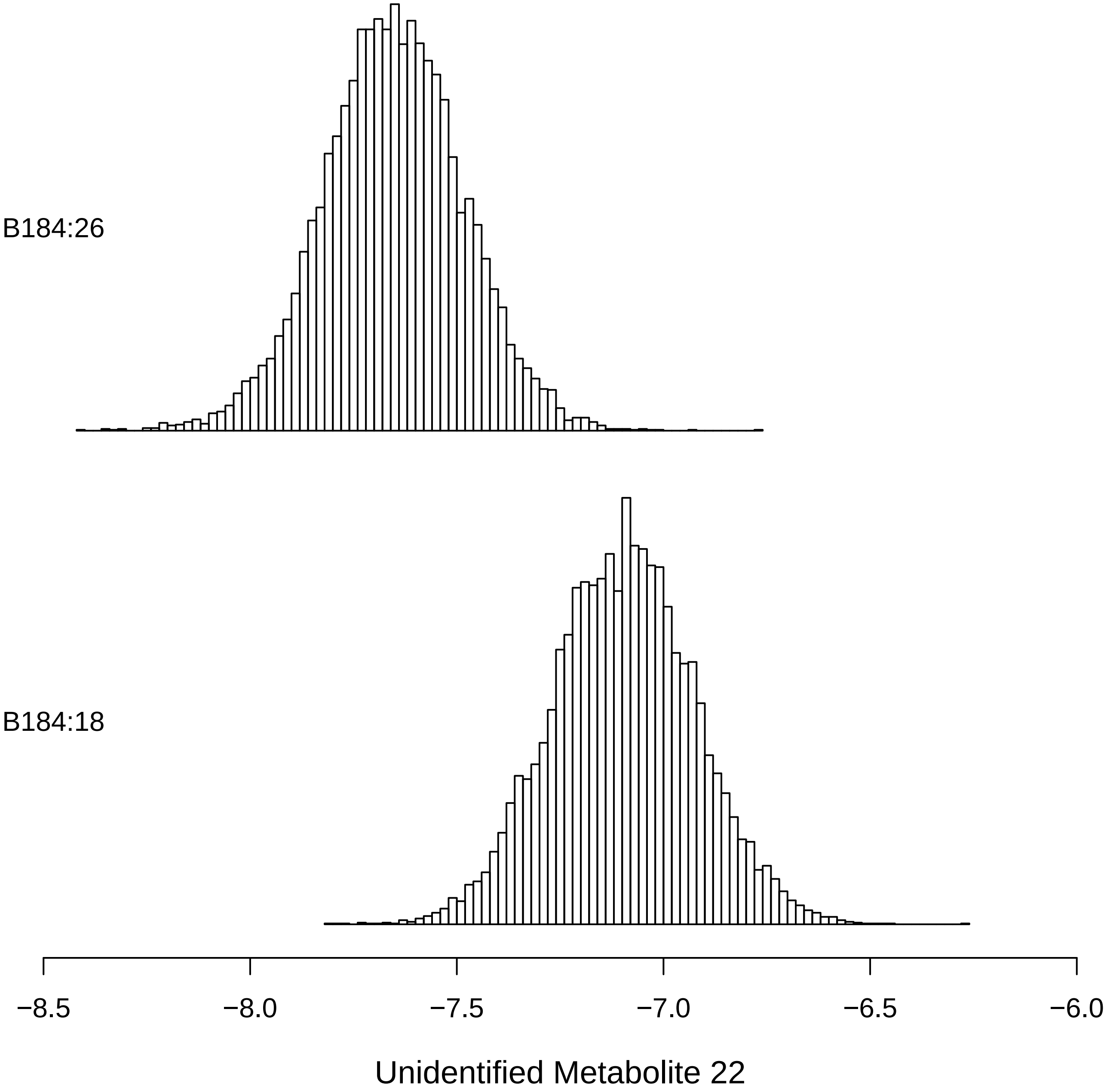
B184:26
B184:18
−8.5
−8.0
−7.5
−7.0
−6.5
−6.0
Unidentified Metabolite 22

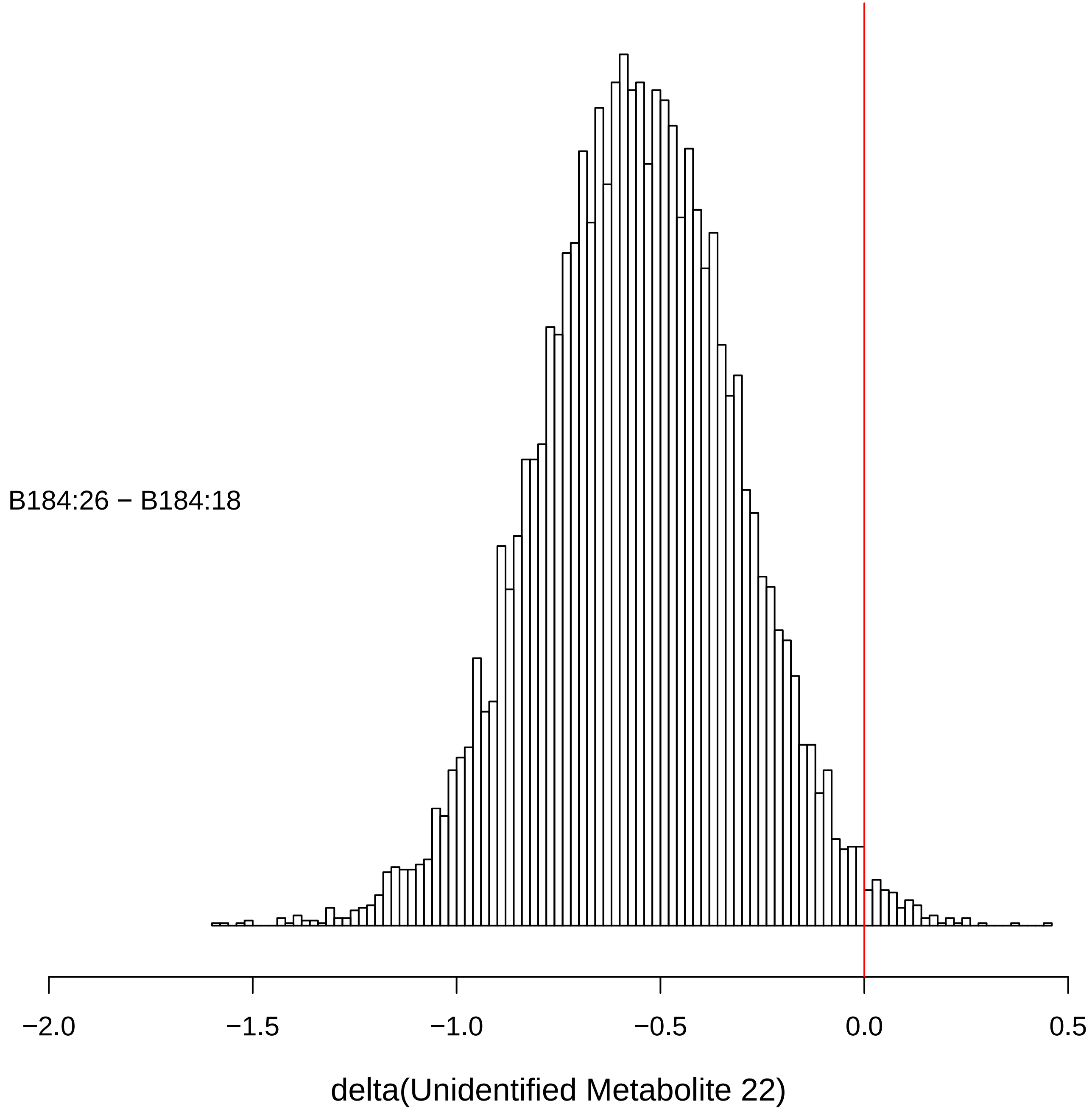

B184:26 − B184:18
−2.0
−1.5
−1.0
−0.5
0.0
0.5
delta(Unidentified Metabolite 22)

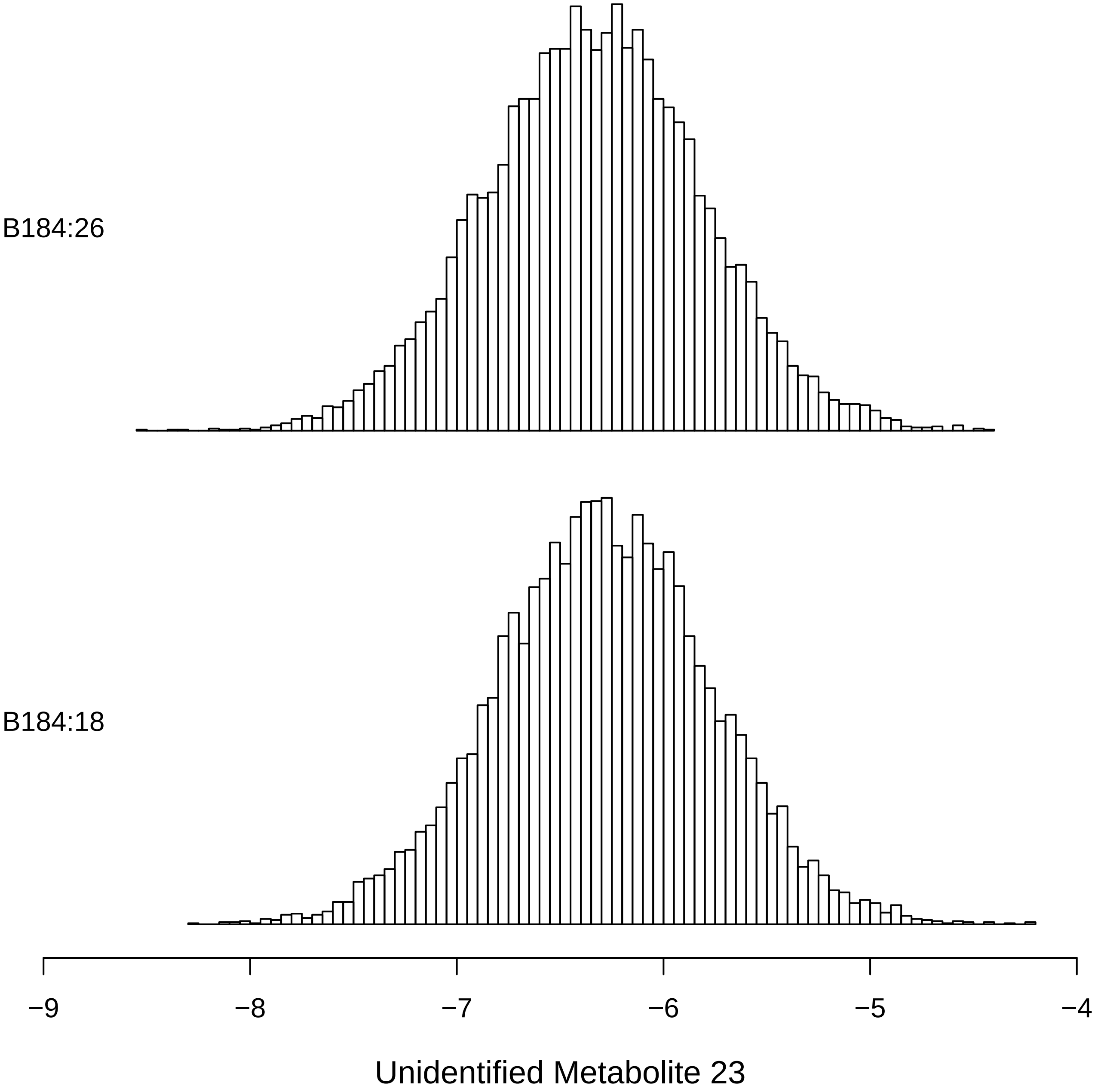

B184:26
B184:18
−9
−8
−7
−6
−5
−4
Unidentified Metabolite 23

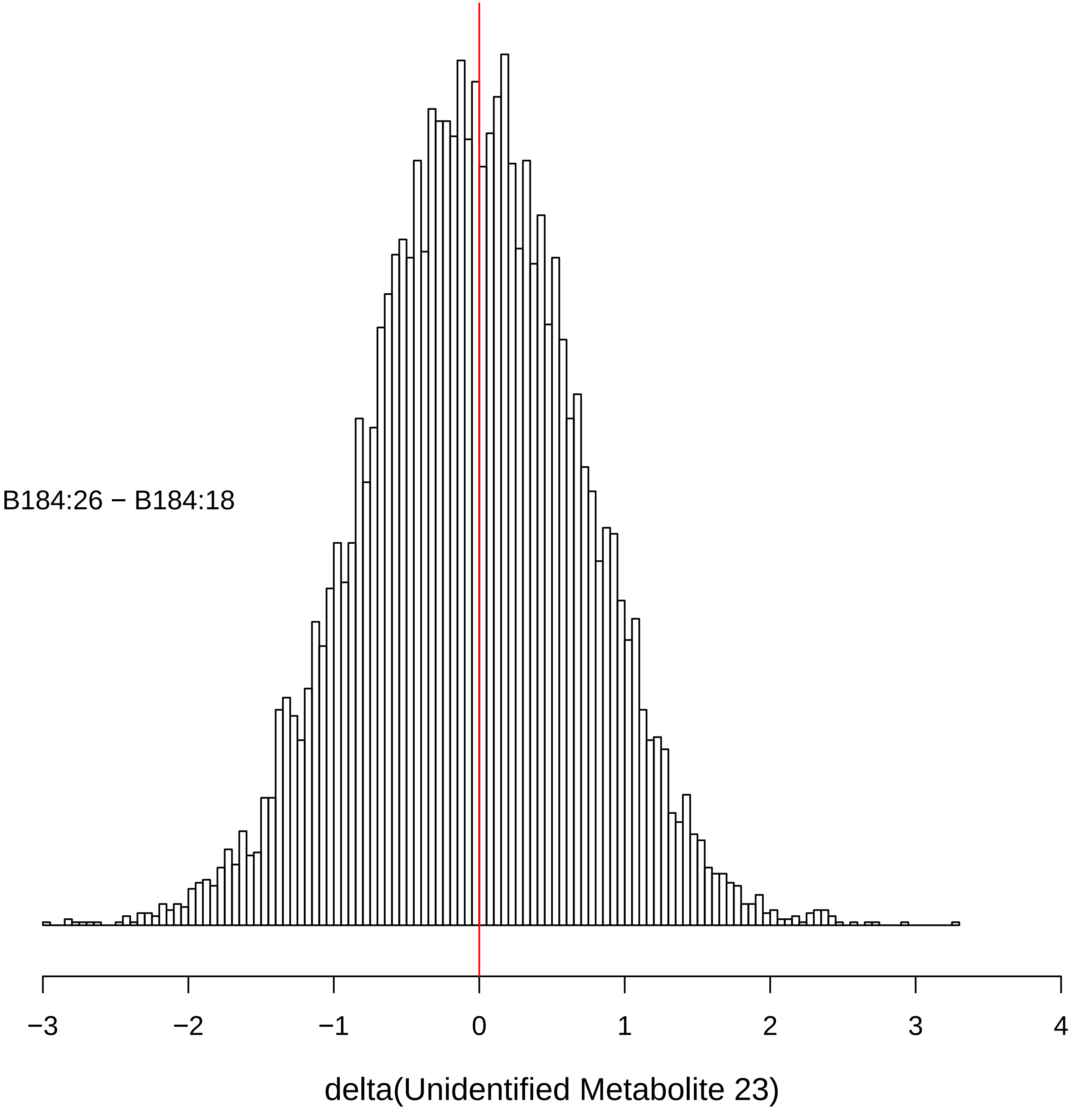

B184:26 − B184:18
−3
−2
−1
0
1
2
3
4
delta(Unidentified Metabolite 23)

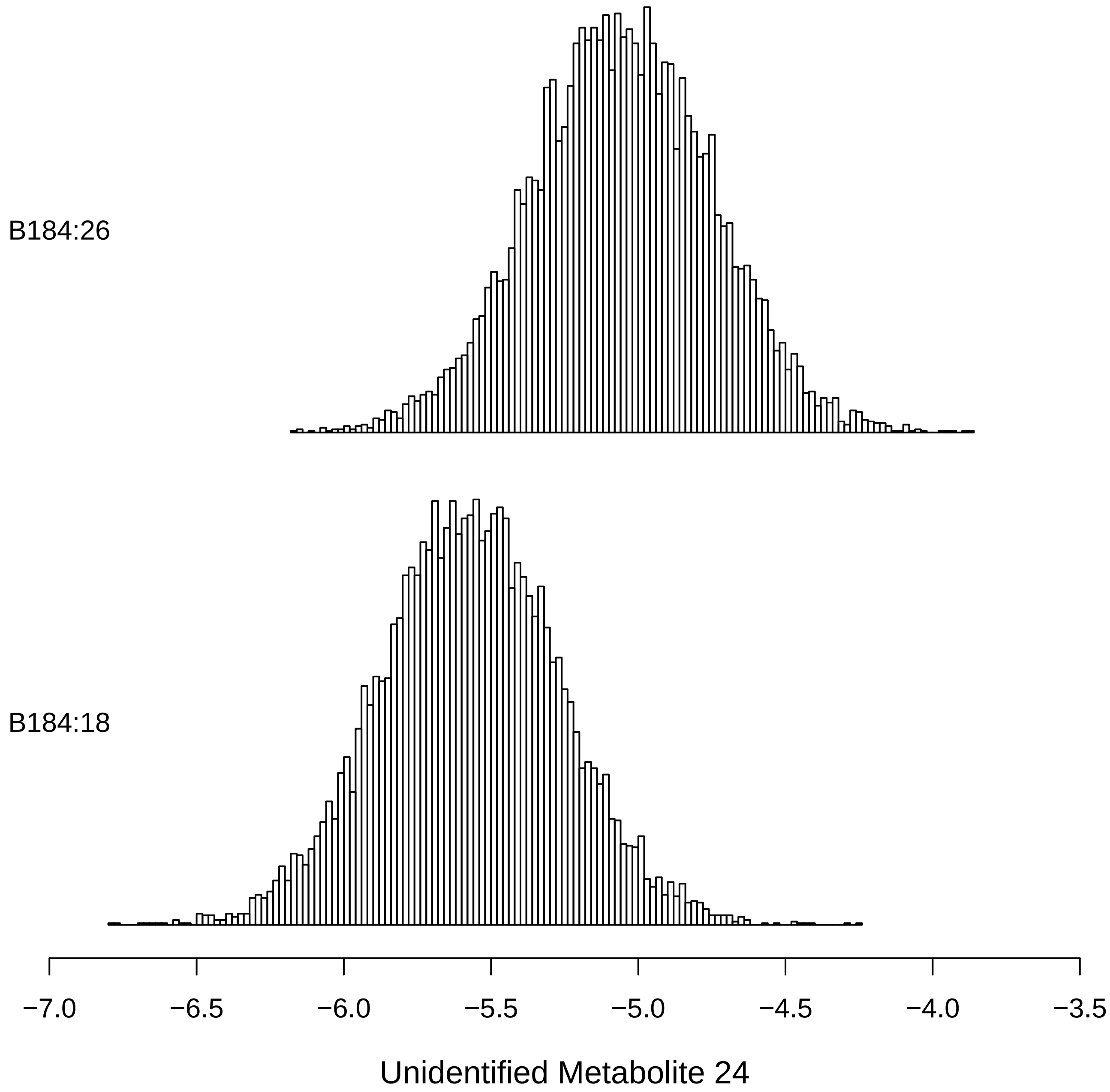
B184:26
B184:18
−7.0
−6.5
−6.0
−5.5
−5.0
−4.5
−4.0
−3.5
Unidentified Metabolite 24

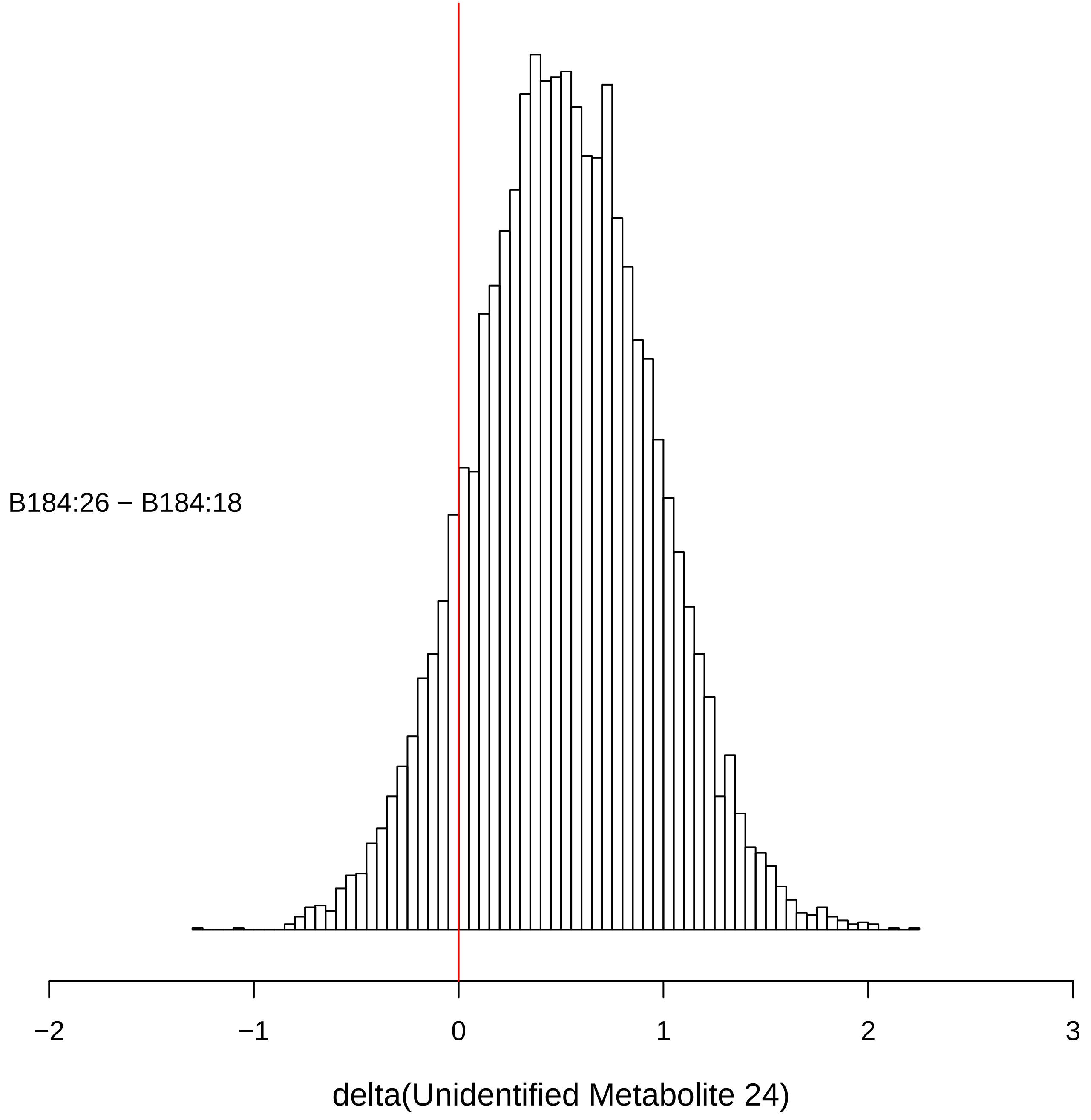
B184:26 − B184:18
−2
−1
0
1
2
3
delta(Unidentified Metabolite 24)

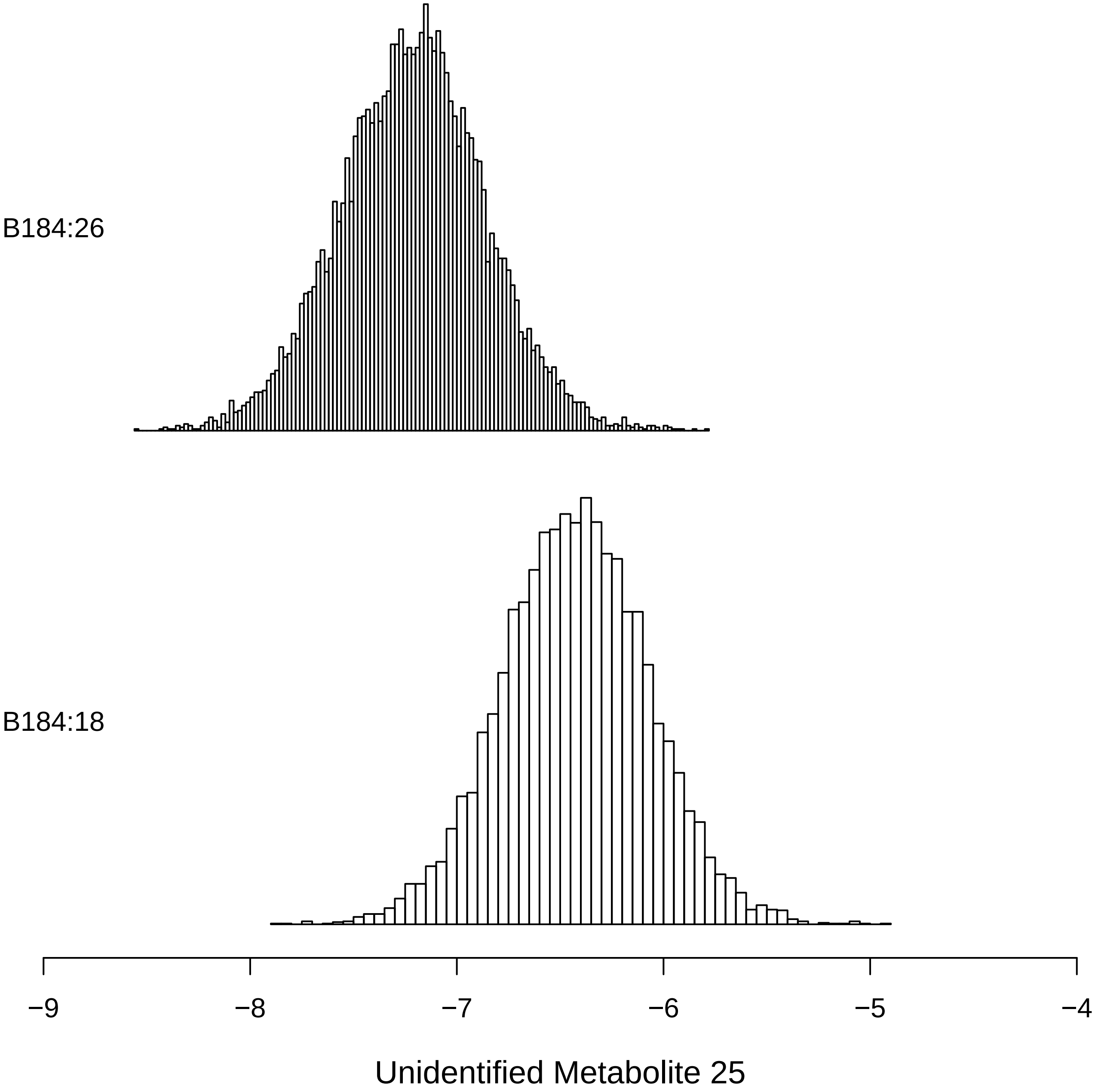
B184:26
B184:18
−9
−8
−7
−6
−5
−4
Unidentified Metabolite 25

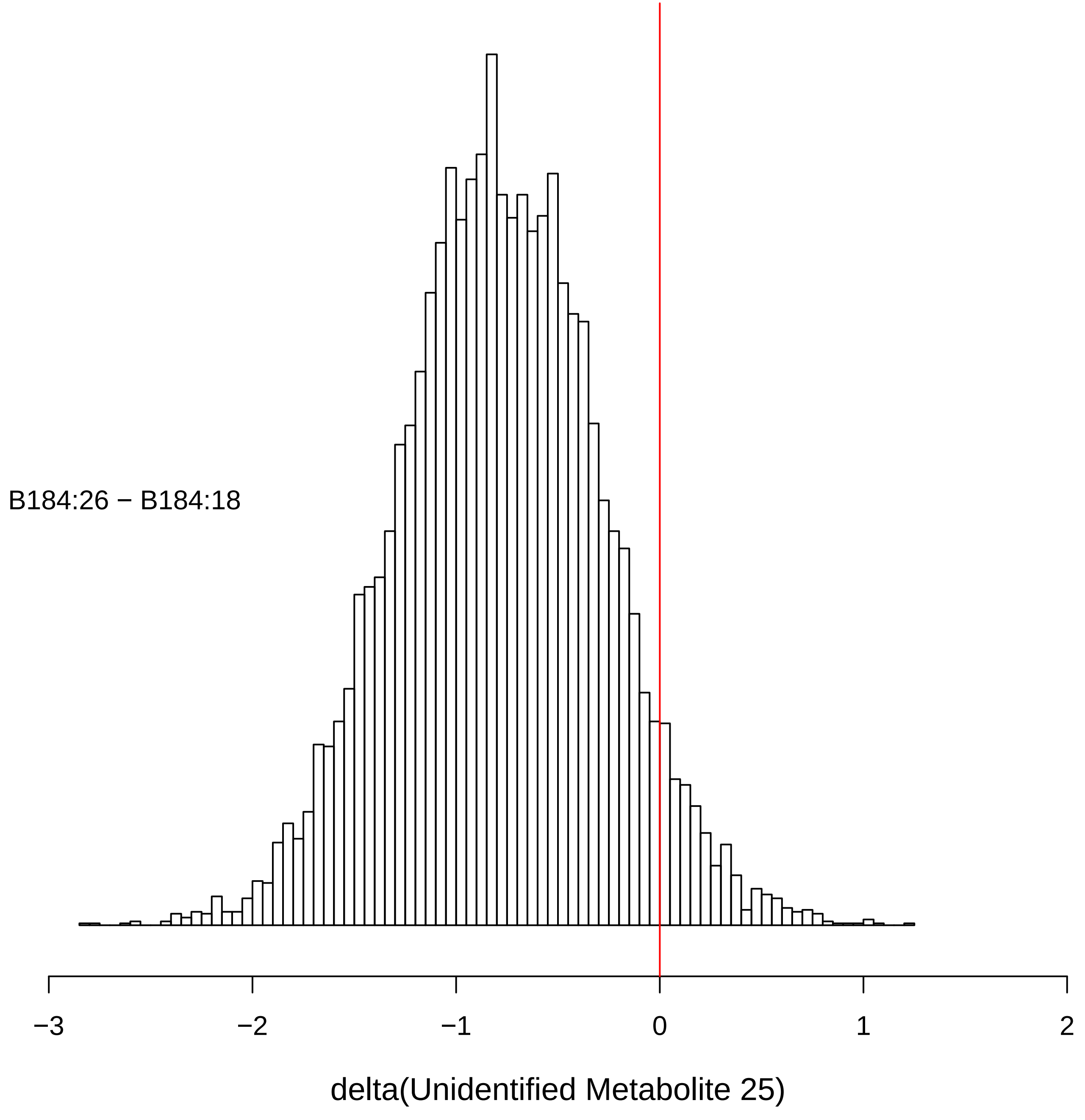

B184:26 − B184:18
−3
−2
−1
0
1
2
delta(Unidentified Metabolite 25)

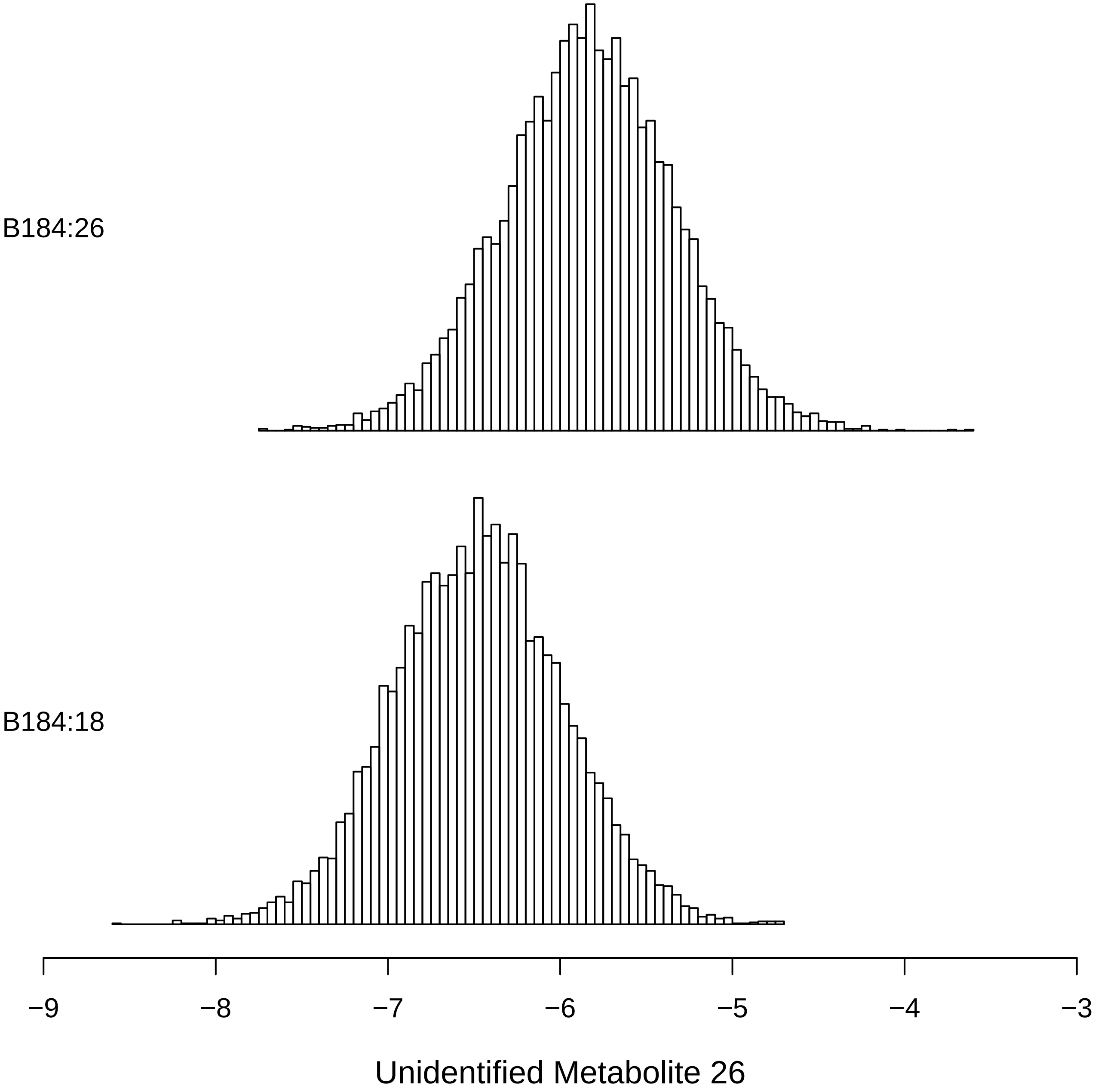
B184:26
B184:18
−9
−8
−7
−6
−5
−4
−3
Unidentified Metabolite 26

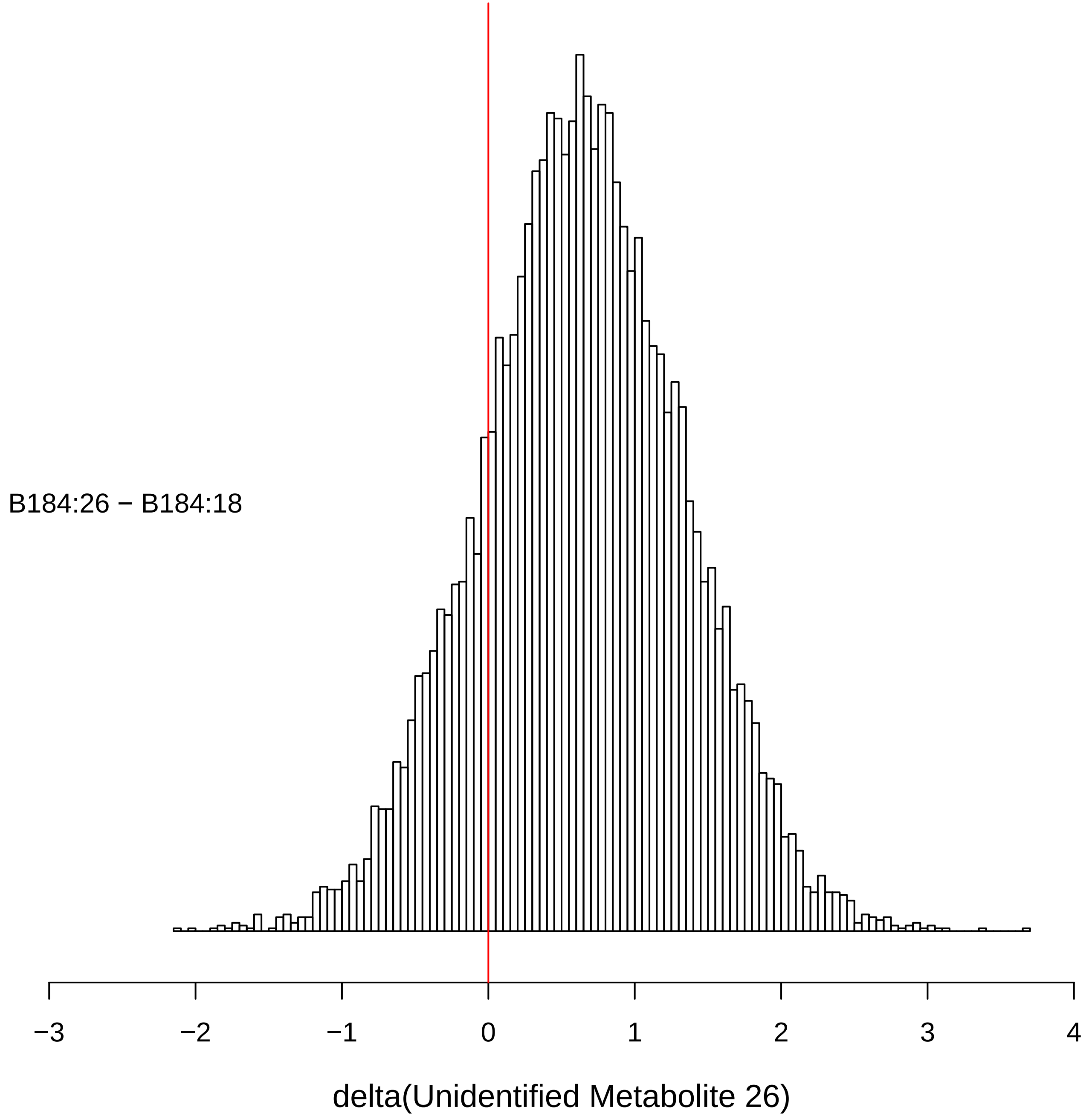

B184:26 − B184:18
−3
−2
−1
0
1
2
3
4
delta(Unidentified Metabolite 26)

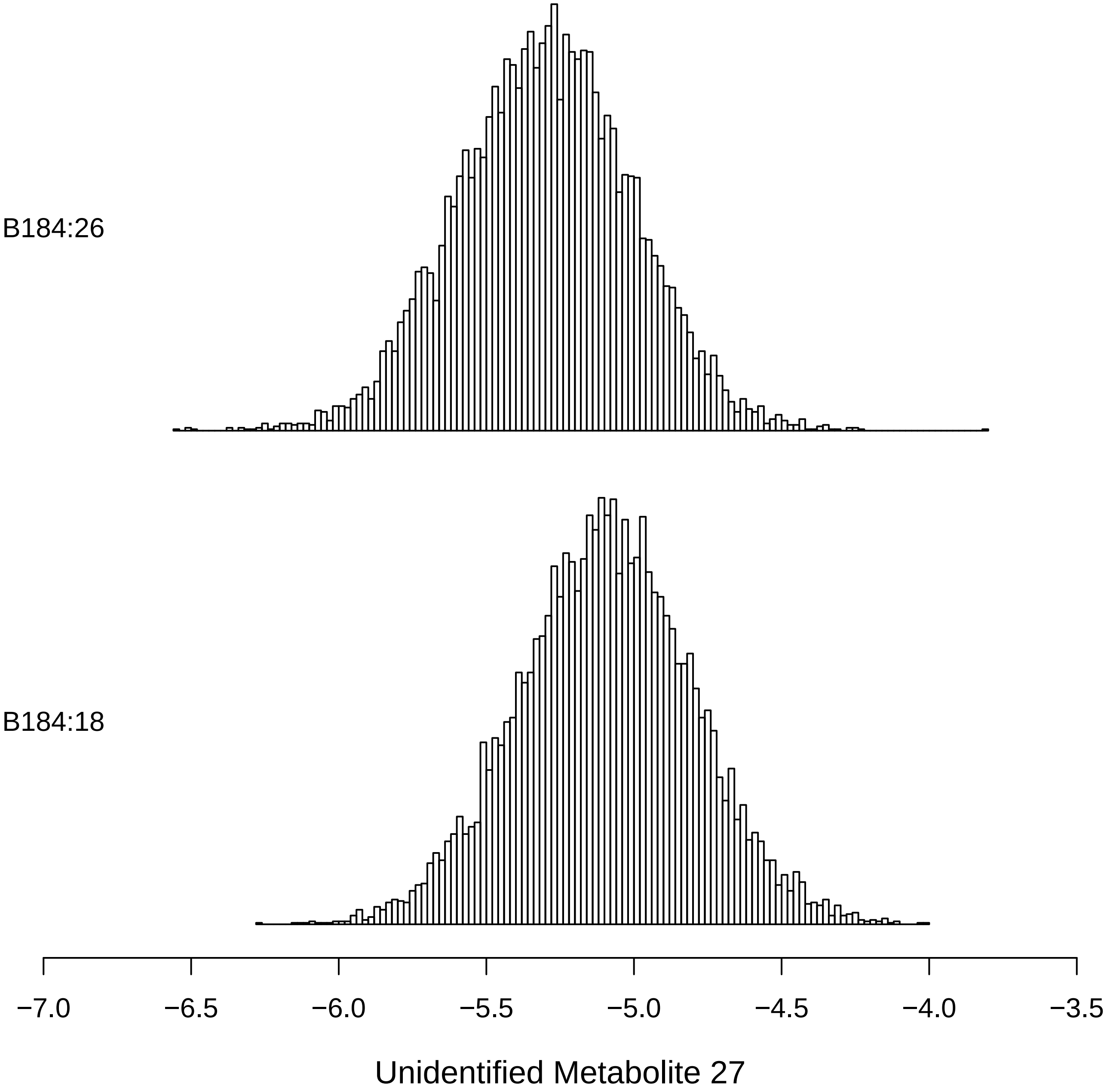

B184:26
B184:18
−7.0
−6.5
−6.0
−5.5
−5.0
−4.5
−4.0
−3.5
Unidentified Metabolite 27

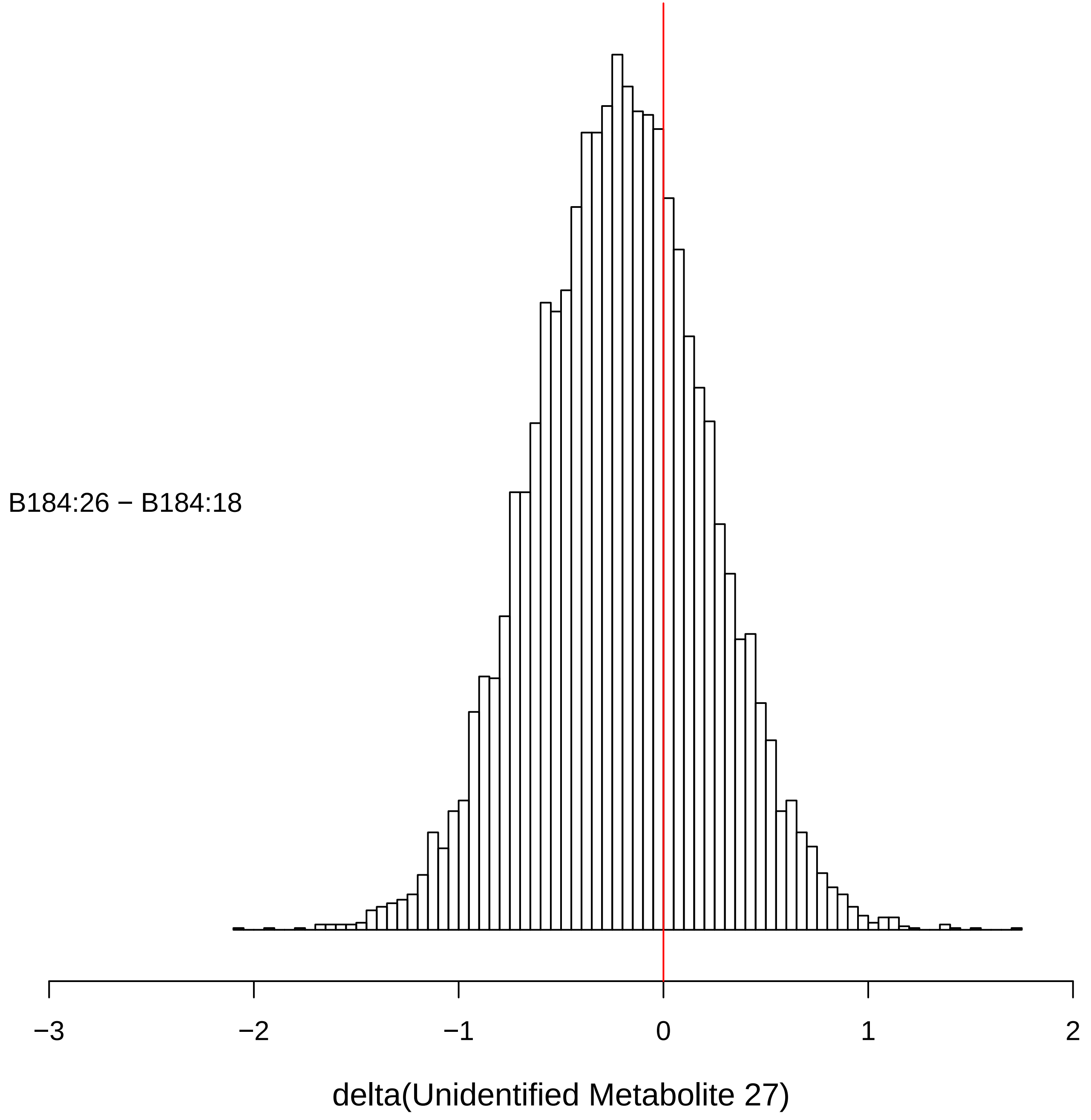
B184:26 − B184:18
−3
−2
−1
0
1
2
delta(Unidentified Metabolite 27)

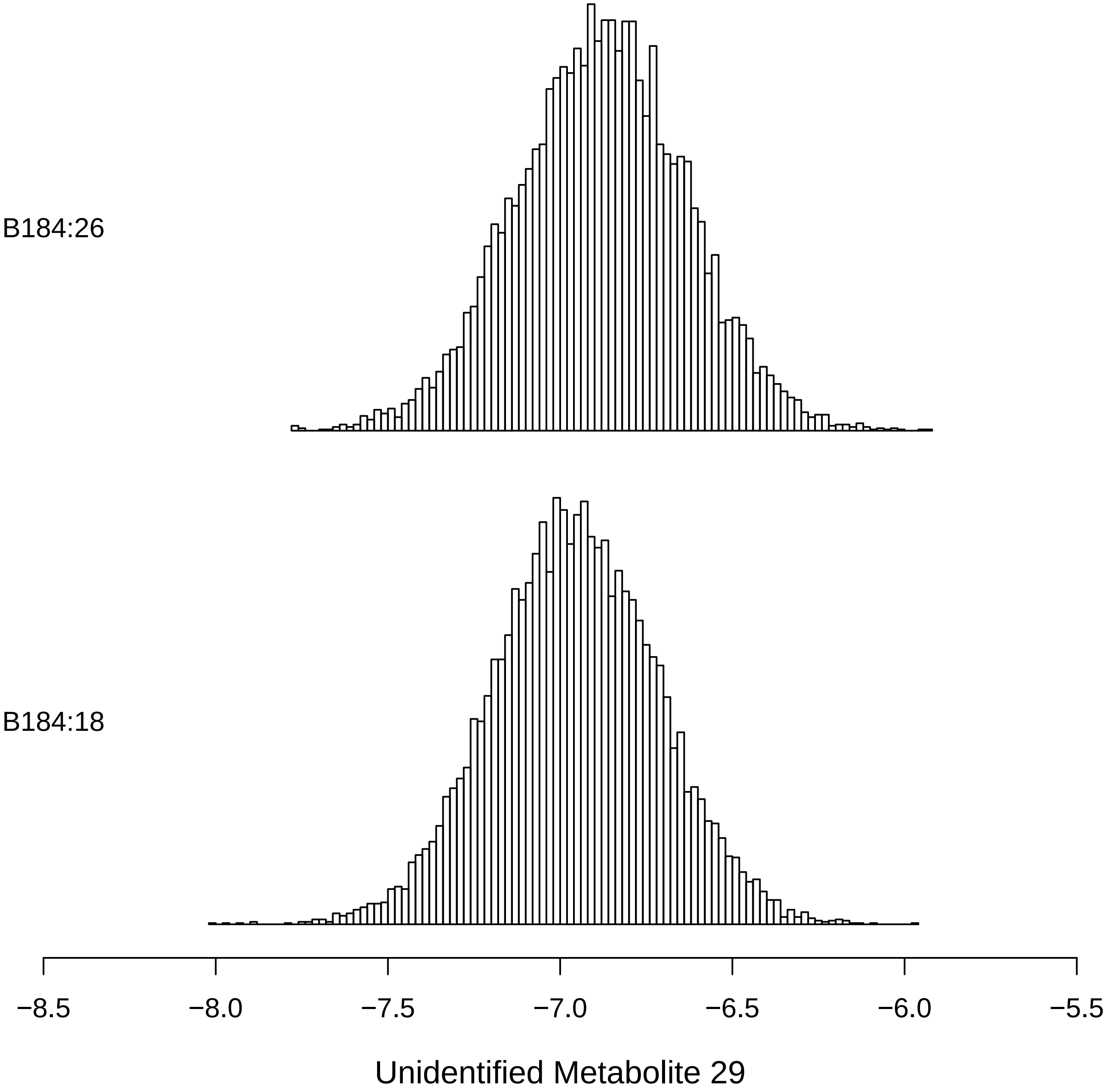
B184:26
B184:18
−8.5
−8.0
−7.5
−7.0
−6.5
−6.0
−5.5
Unidentified Metabolite 29

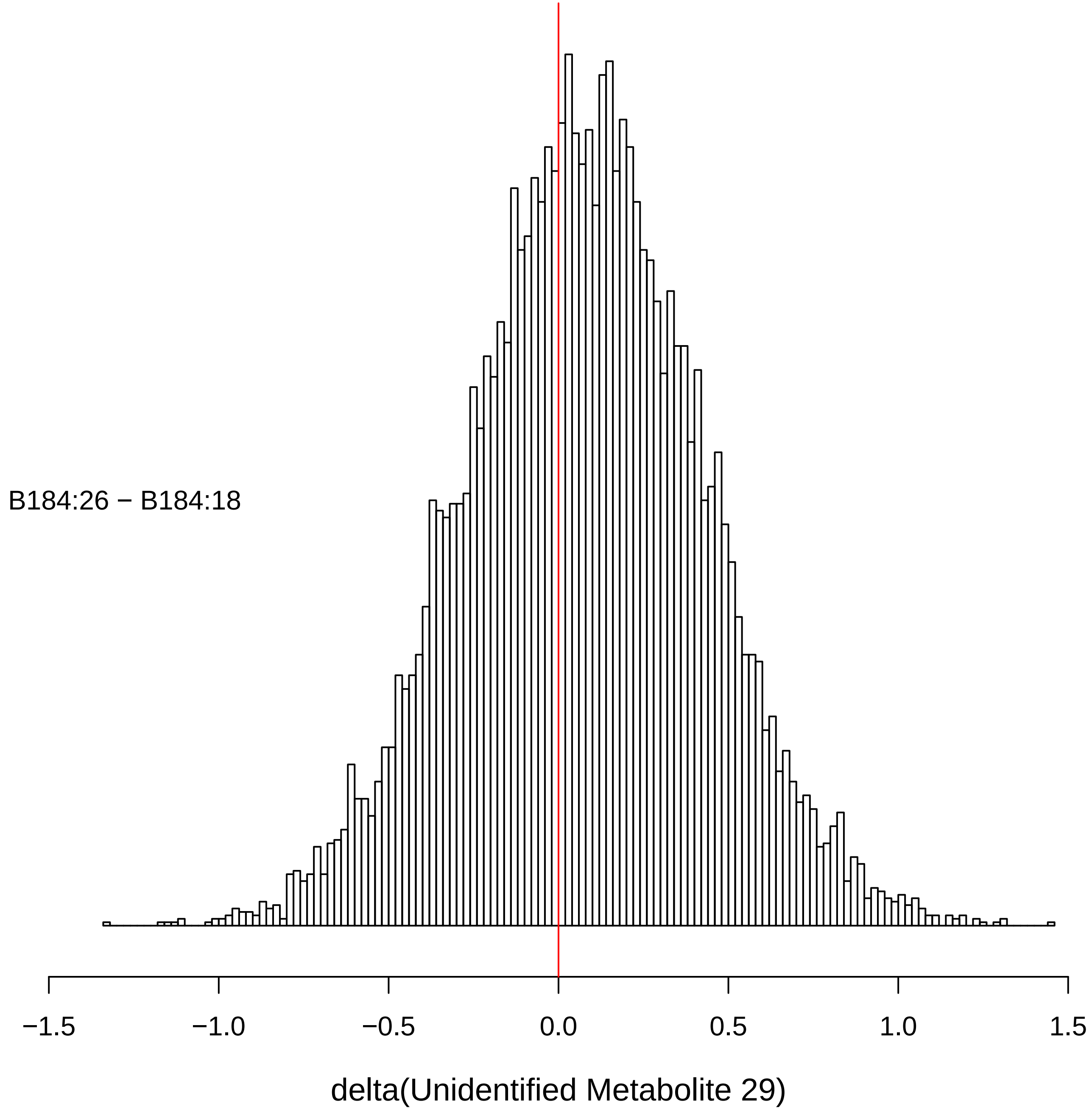
B184:26 − B184:18
−1.5
−1.0
−0.5
0.0
0.5
1.0
1.5
delta(Unidentified Metabolite 29)

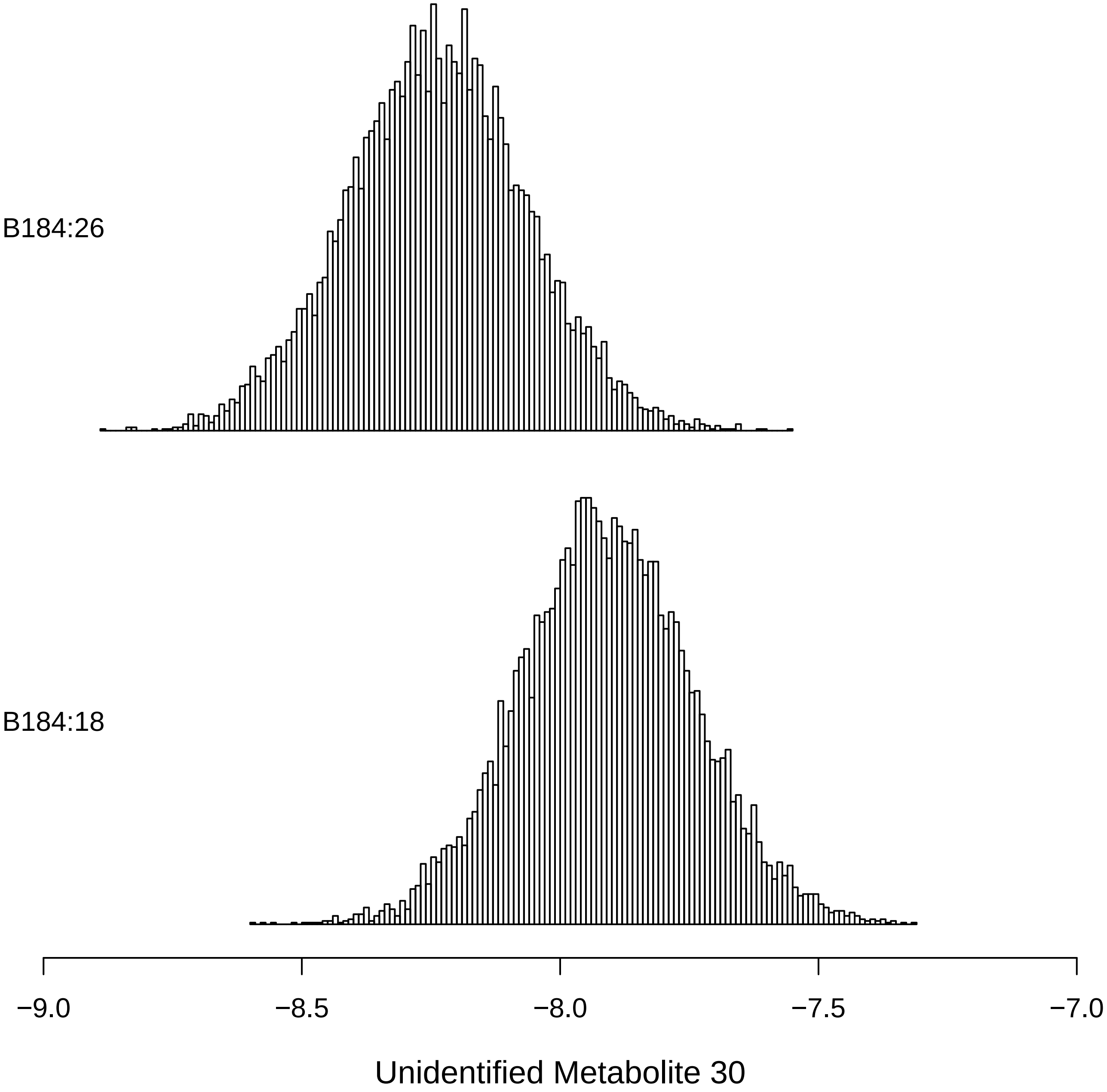

B184:26
B184:18
−9.0
−8.5
−8.0
−7.5
−7.0
Unidentified Metabolite 30

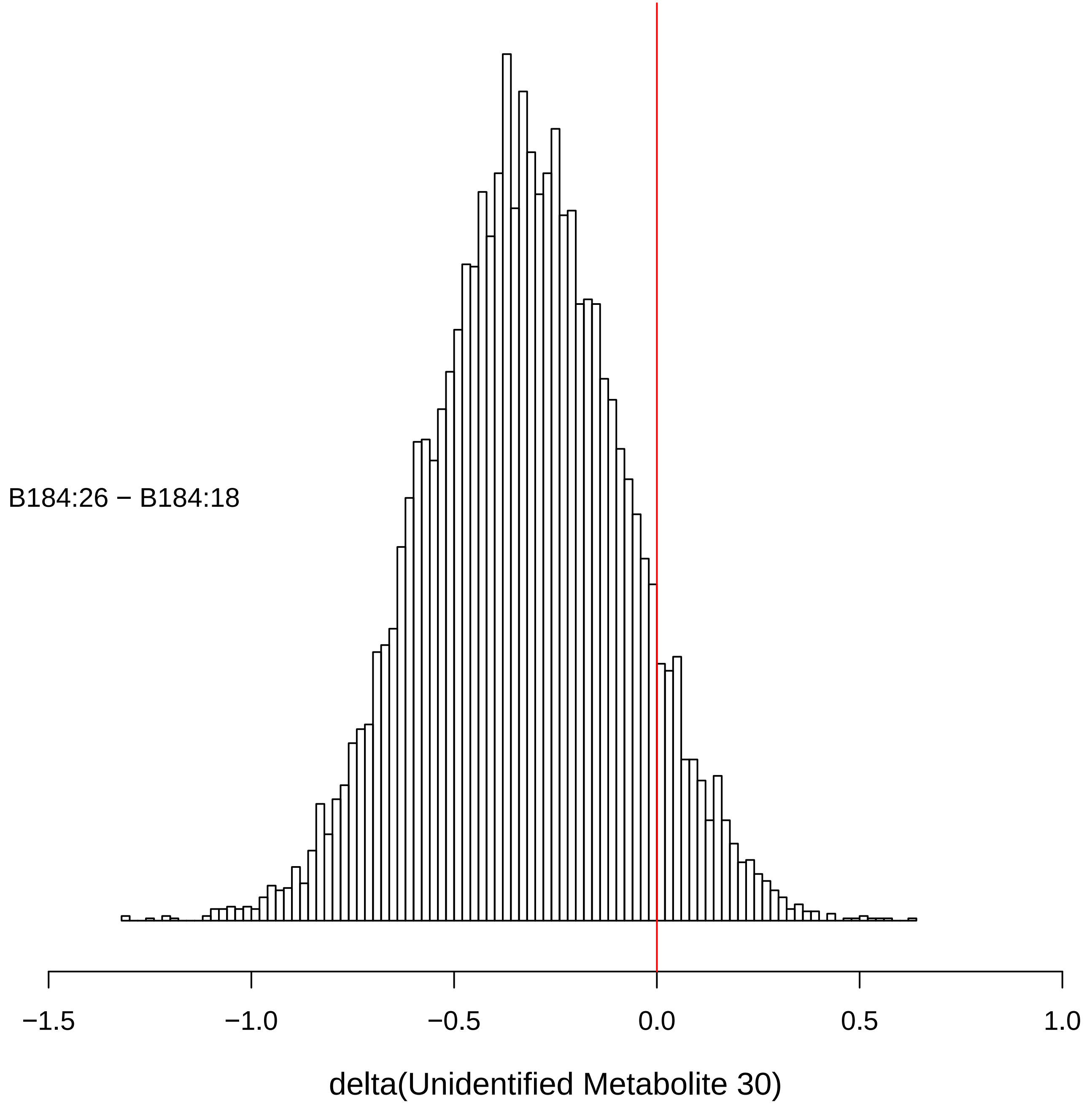
B184:26 − B184:18
−1.5
−1.0
−0.5
0.0
0.5
1.0
delta(Unidentified Metabolite 30)

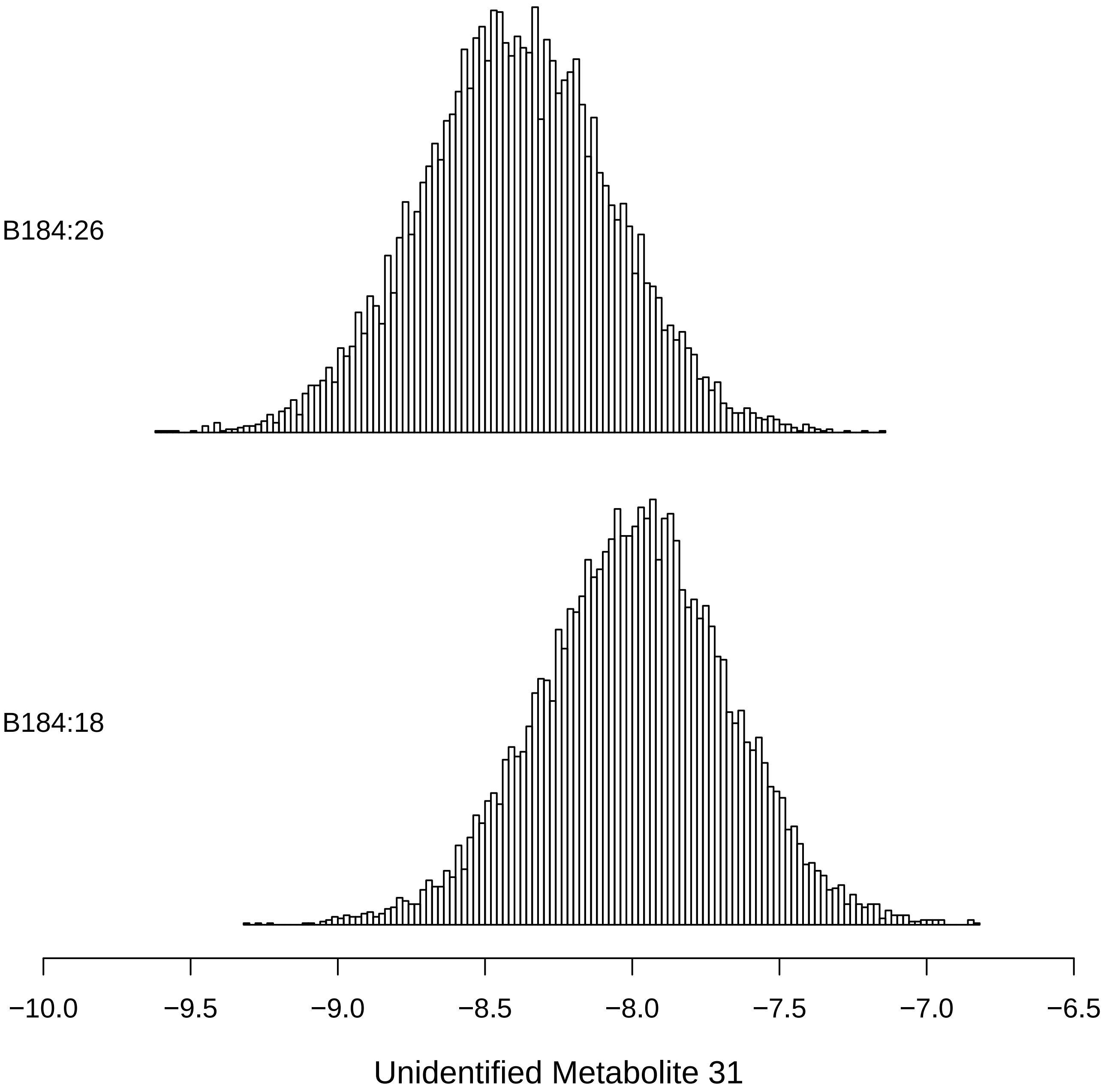
B184:26
B184:18
−10.0
−9.5
−9.0
−8.5
−8.0
−7.5
−7.0
−6.5
Unidentified Metabolite 31

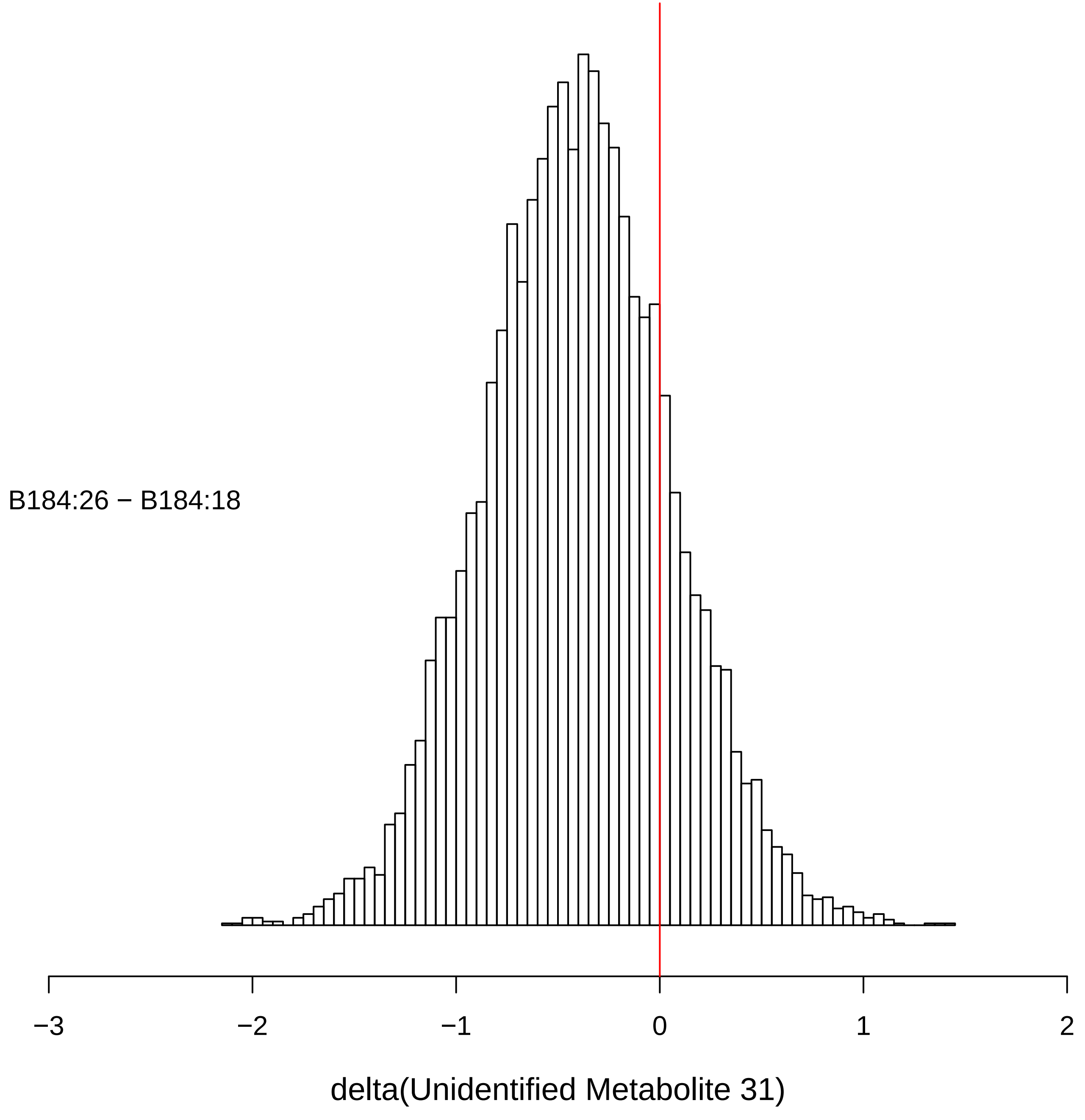

B184:26 − B184:18
−3
−2
−1
0
1
2
delta(Unidentified Metabolite 31)

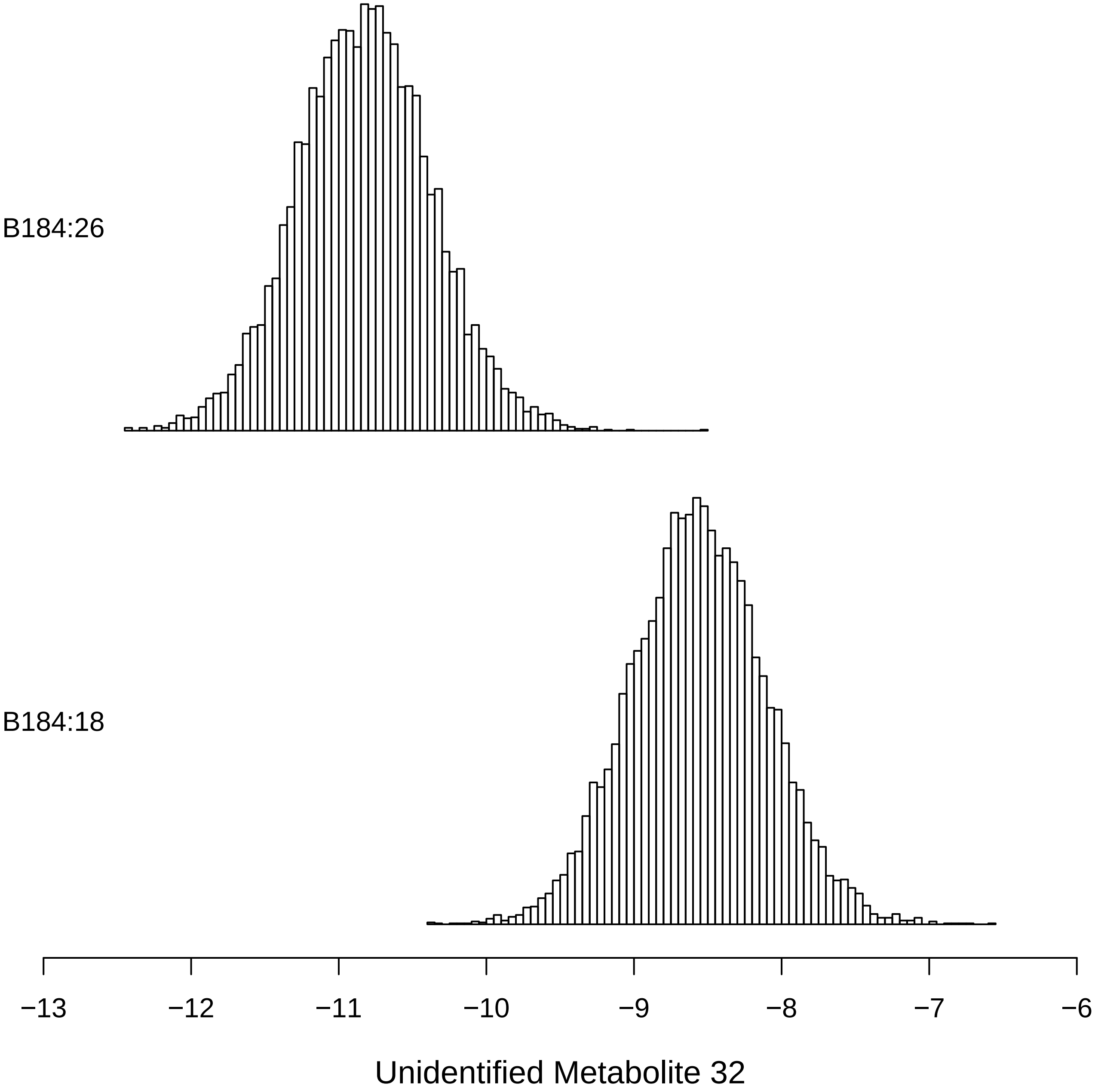

B184:26
B184:18
−13
−12
−11
−10
−9
−8
−7
−6
Unidentified Metabolite 32

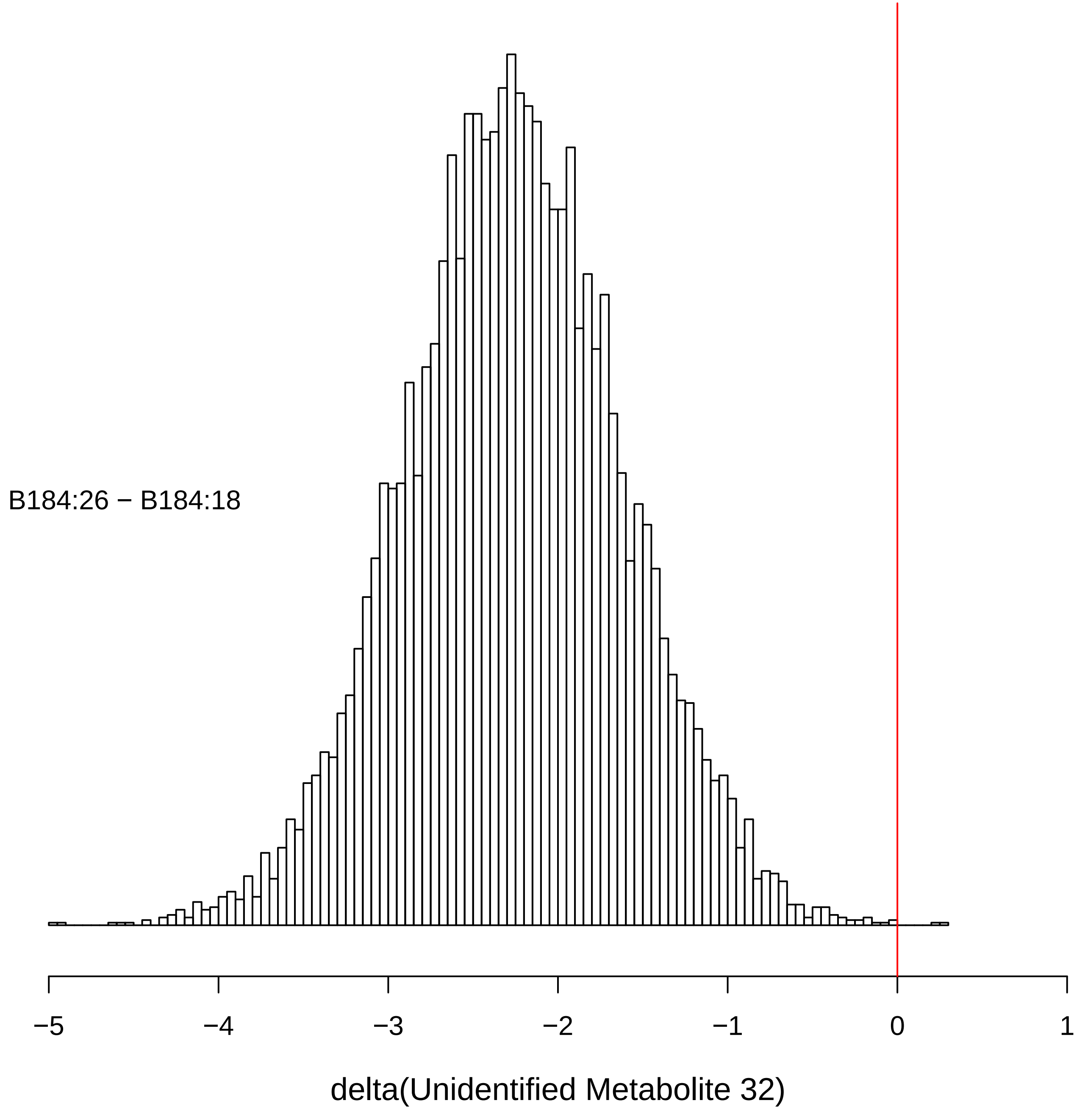
B184:26 − B184:18
−5
−4
−3
−2
−1
0
1
delta(Unidentified Metabolite 32)

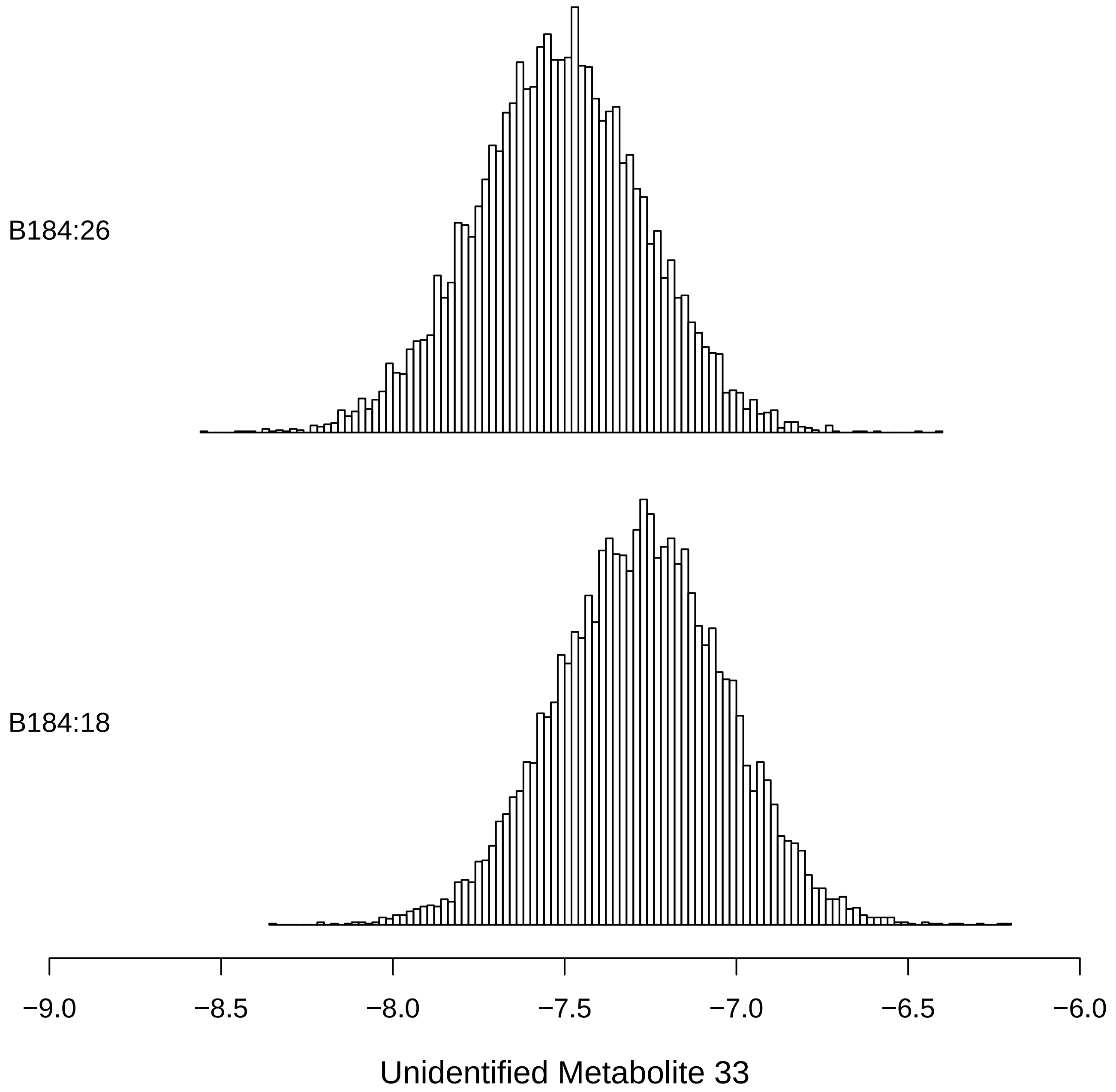
B184:26
B184:18
−9.0
−8.5
−8.0
−7.5
−7.0
−6.5
−6.0
Unidentified Metabolite 33

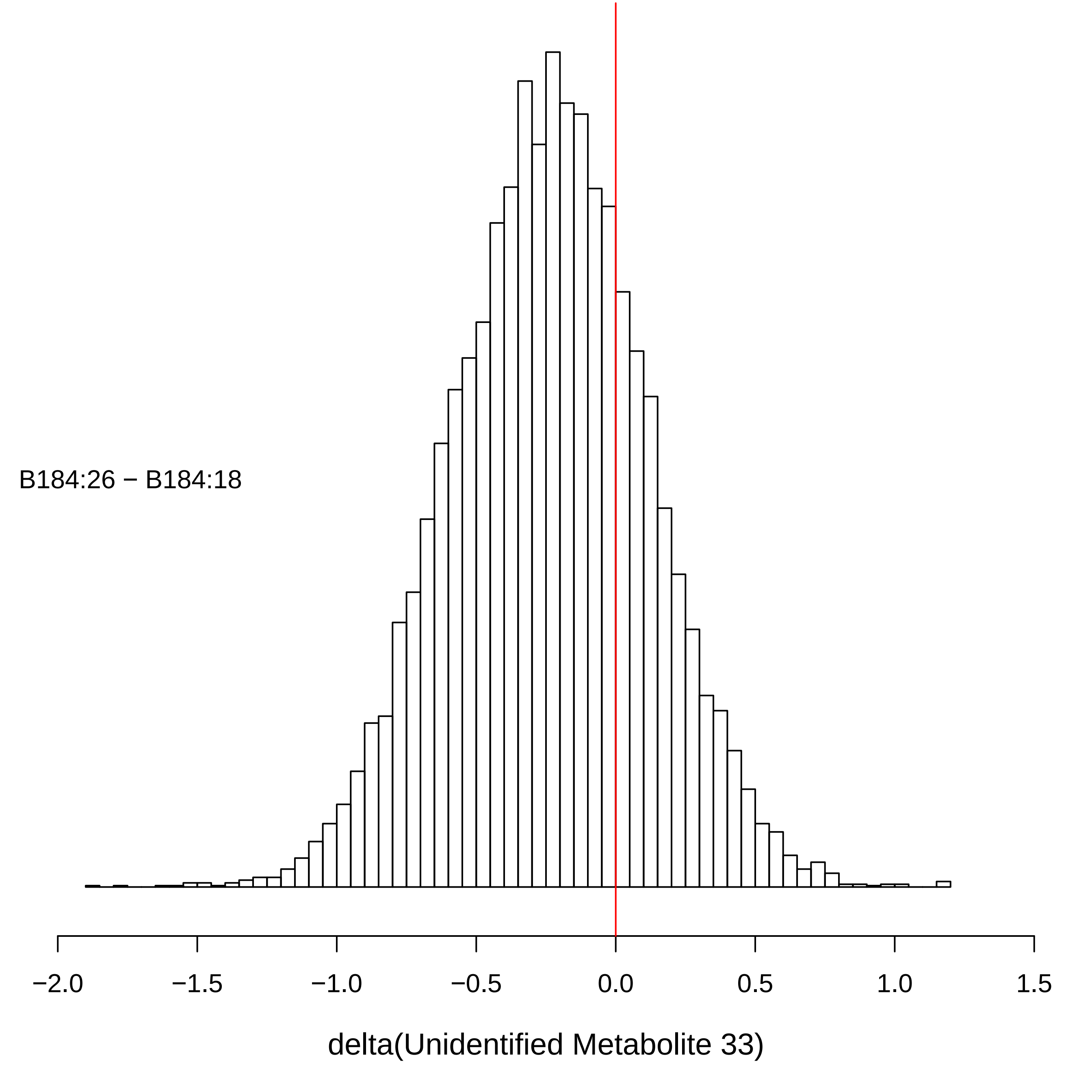

B184:26 − B184:18
−2.0
−1.5
−1.0
−0.5
0.0
0.5
1.0
1.5
delta(Unidentified Metabolite 33)

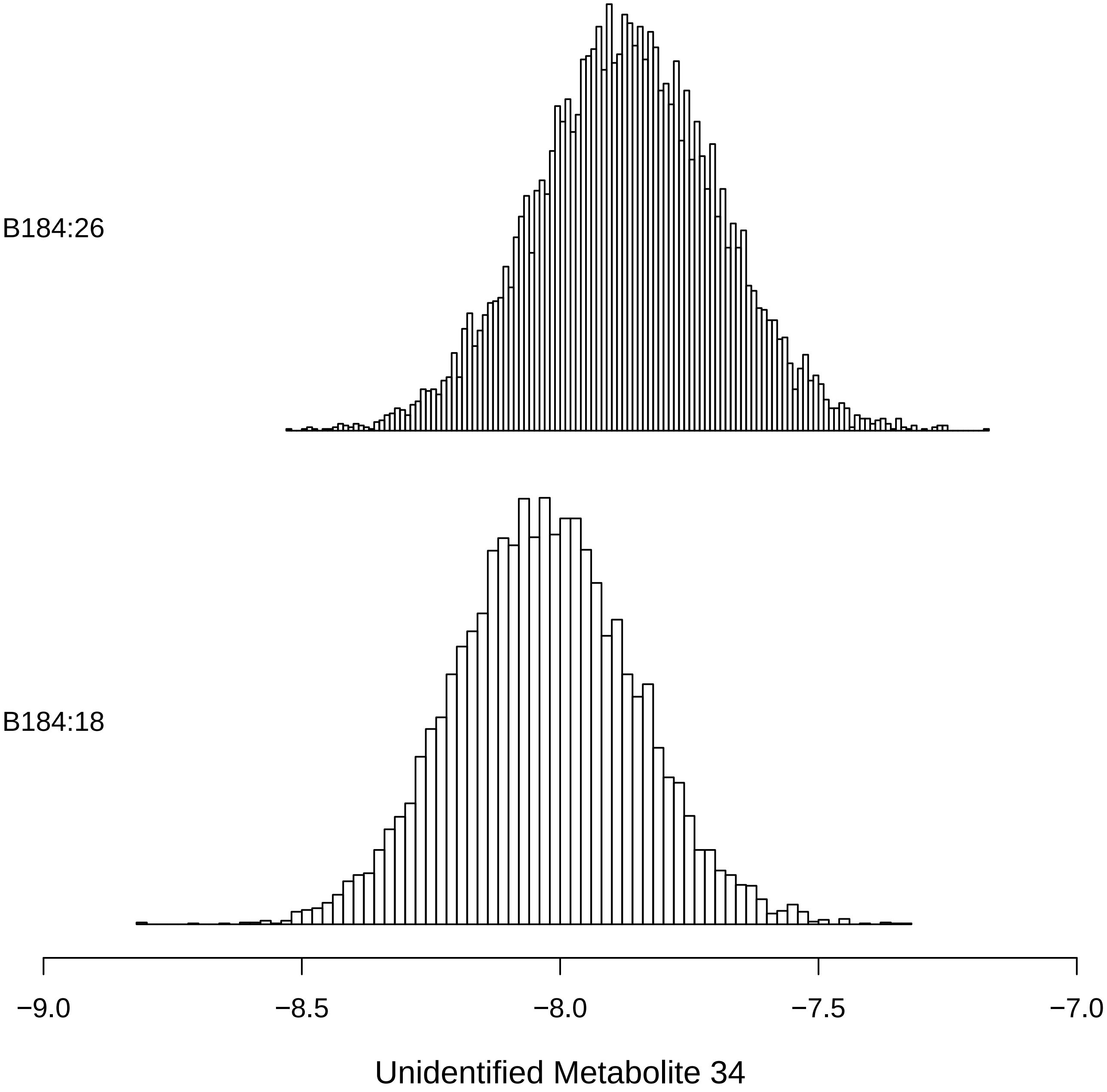

B184:26
B184:18
−9.0
−8.5
−8.0
−7.5
−7.0
Unidentified Metabolite 34

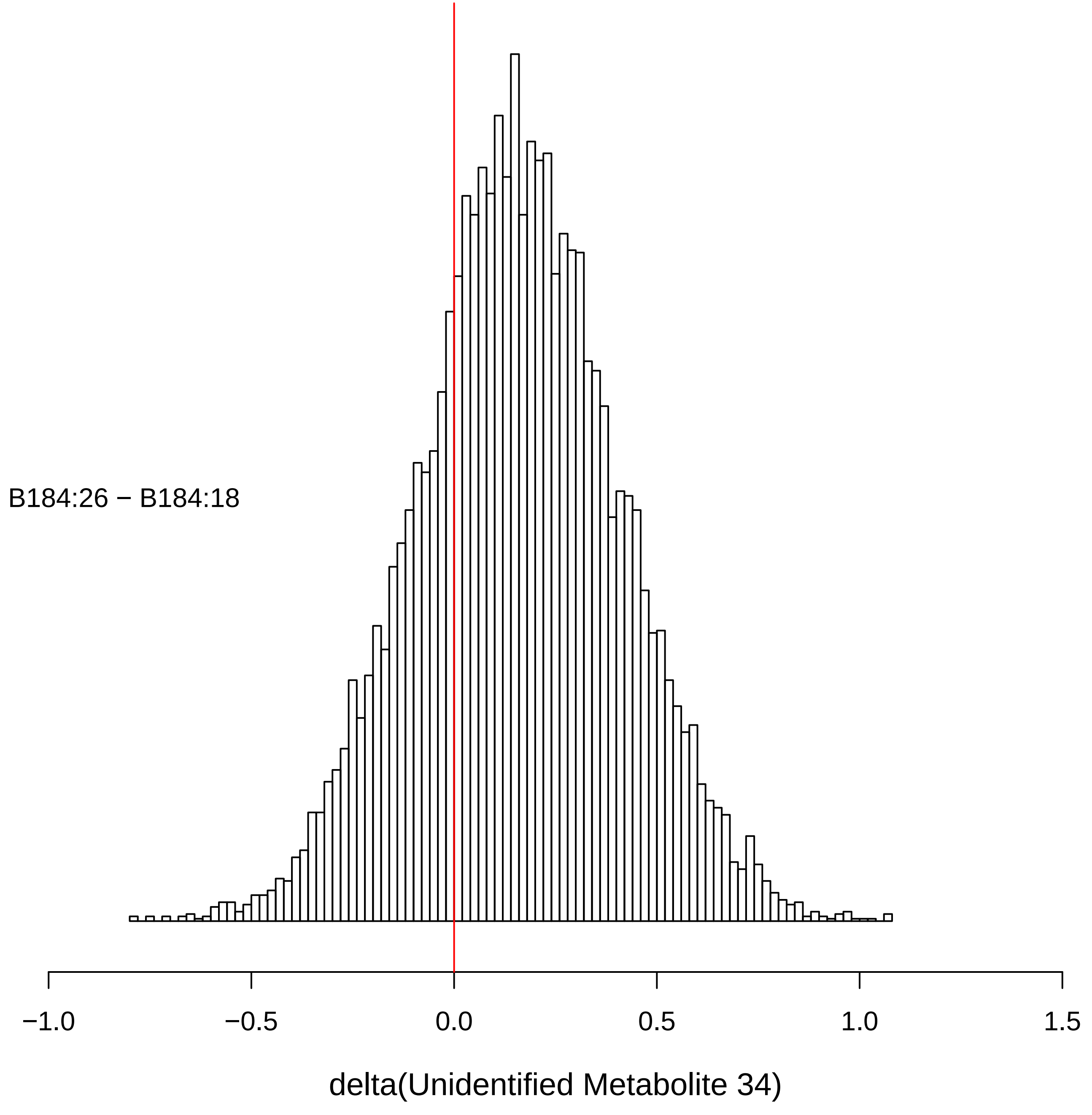

B184:26 − B184:18
−1.0
−0.5
0.0
0.5
1.0
1.5
delta(Unidentified Metabolite 34)

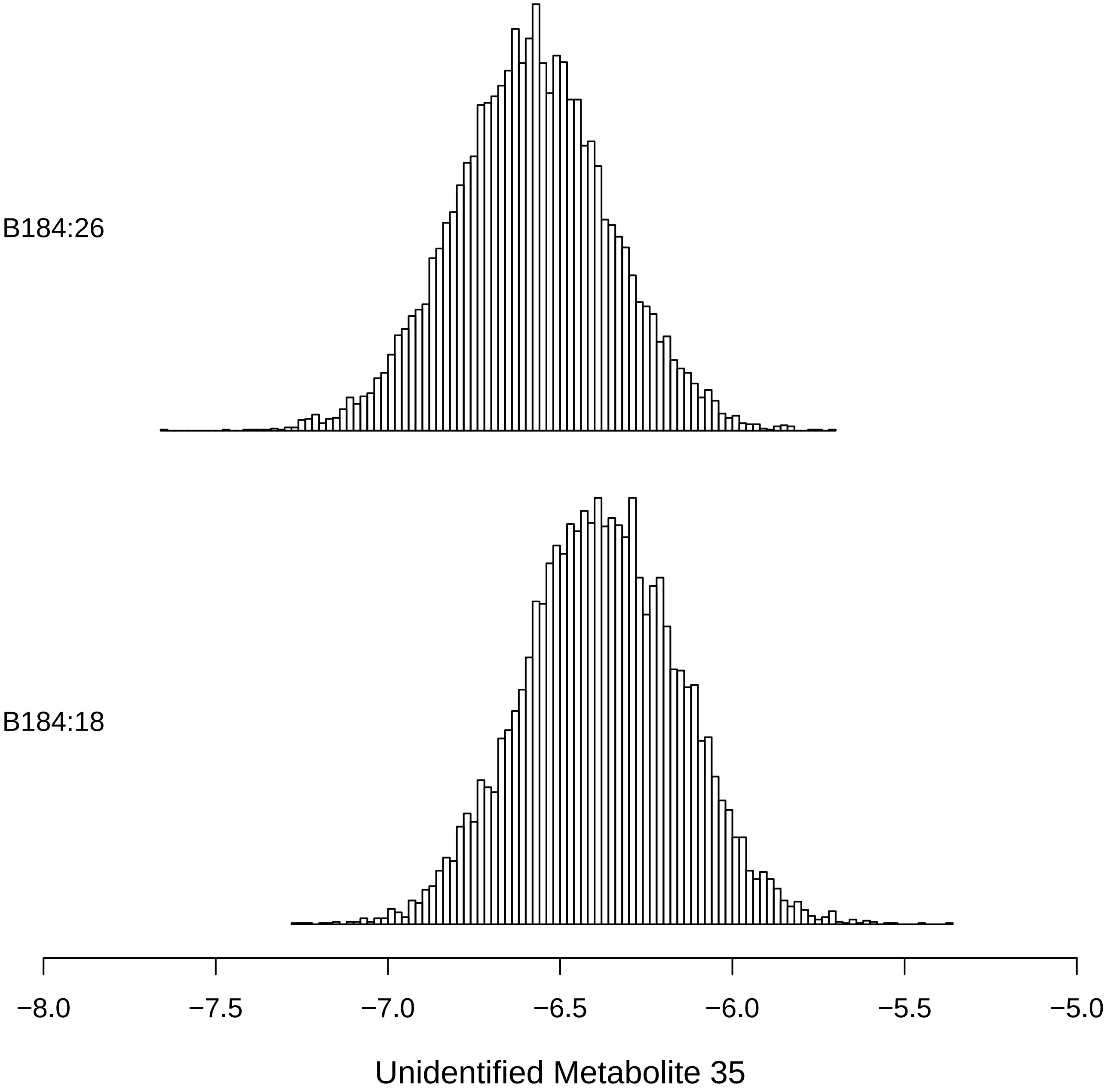

B184:26
B184:18
−8.0
−7.5
−7.0
−6.5
−6.0
−5.5
−5.0
Unidentified Metabolite 35

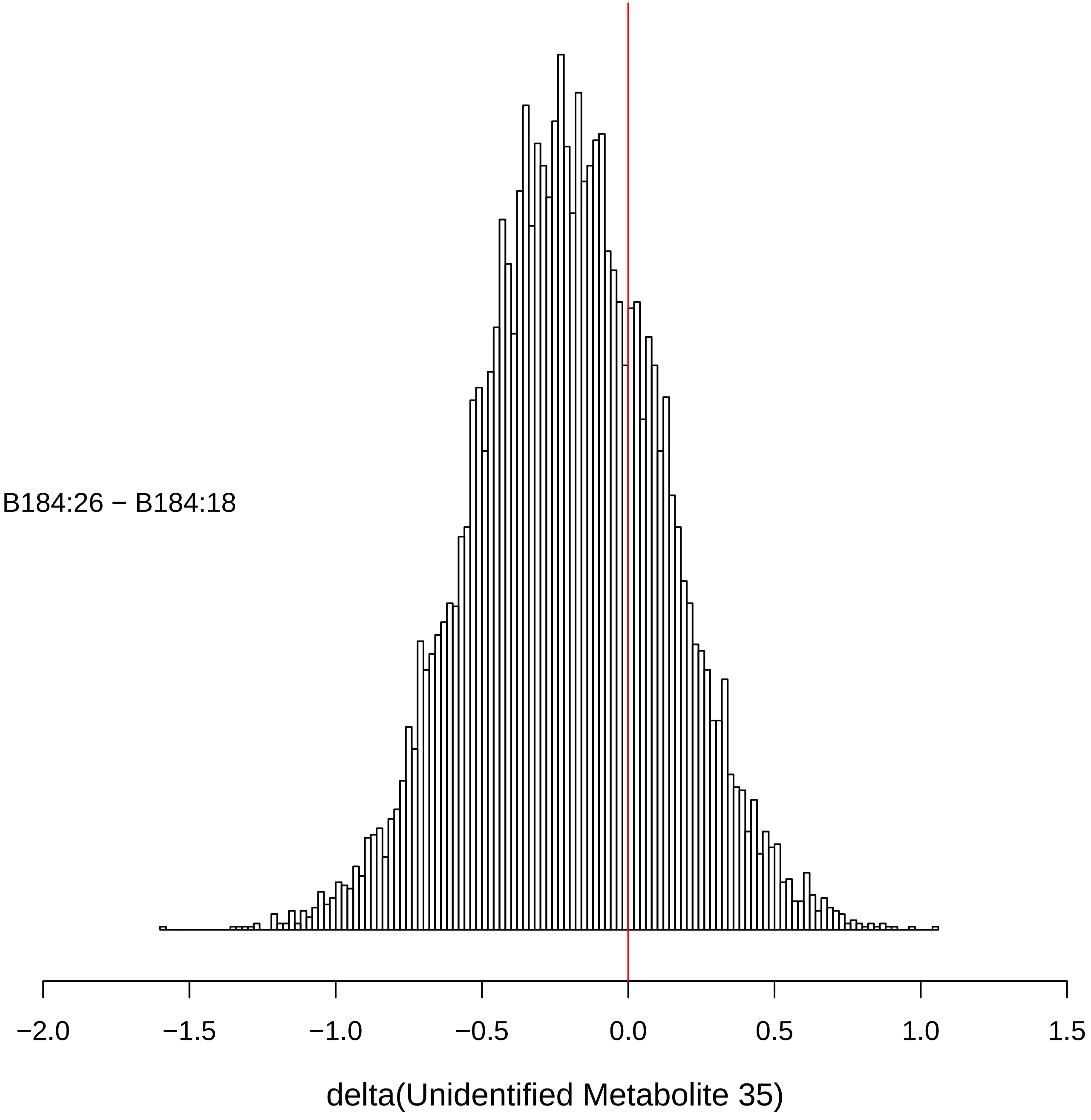

B184:26 − B184:18
−2.0
−1.5
−1.0
−0.5
0.0
0.5
1.0
1.5
delta(Unidentified Metabolite 35)

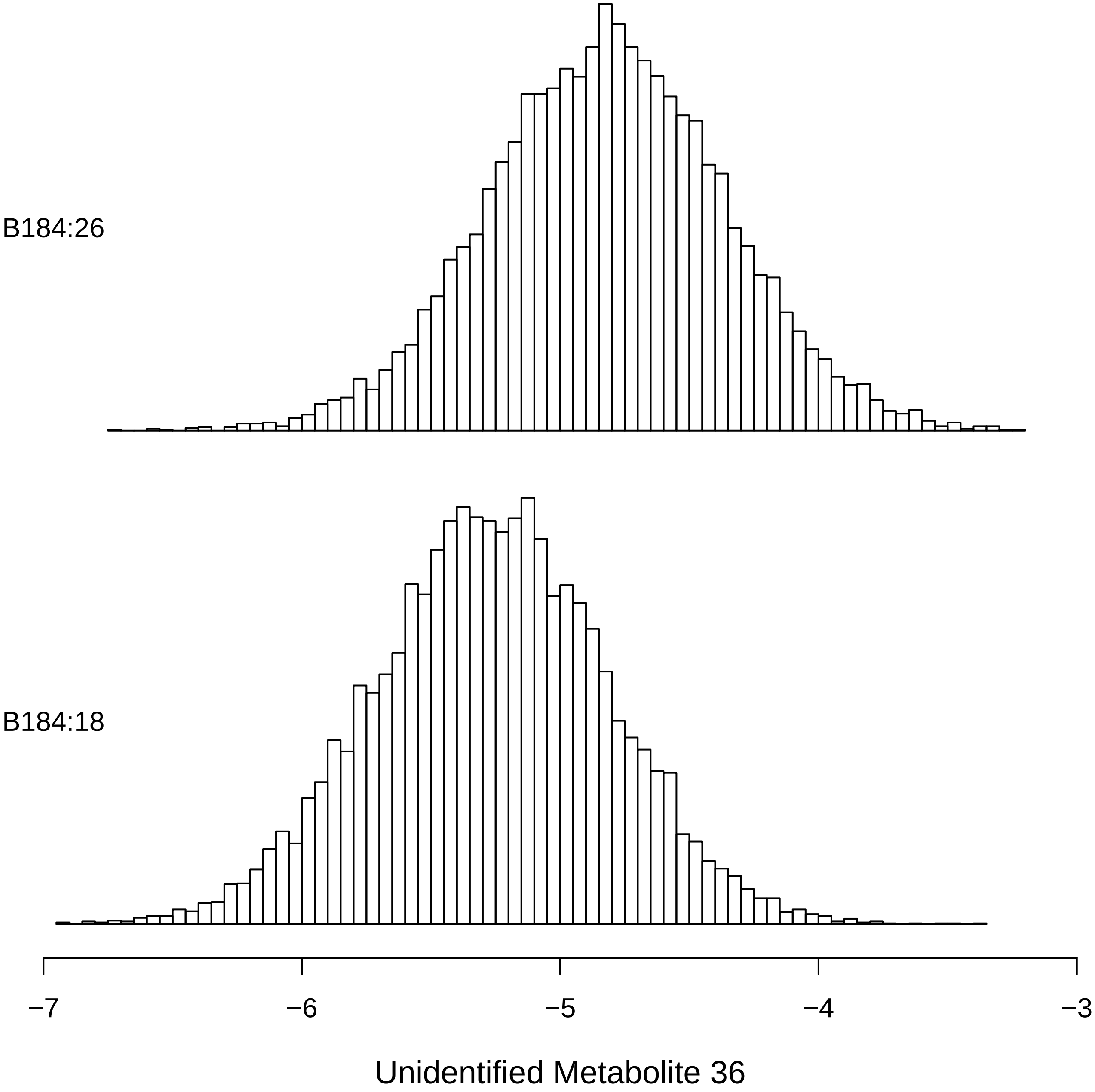
B184:26
B184:18
−7
−6
−5
−4
−3
Unidentified Metabolite 36

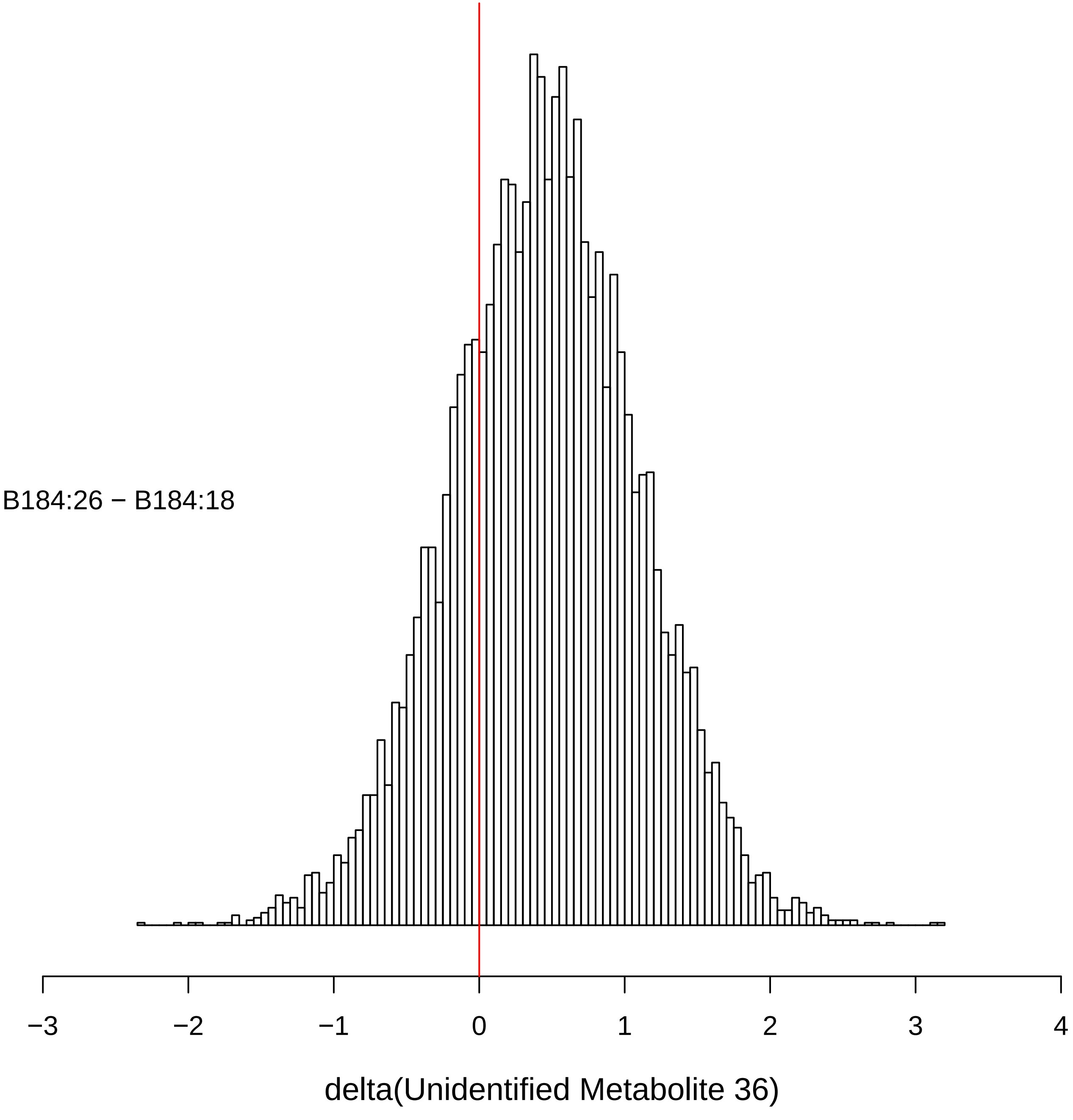
B184:26 − B184:18
−3
−2
−1
0
1
2
3
4
delta(Unidentified Metabolite 36)

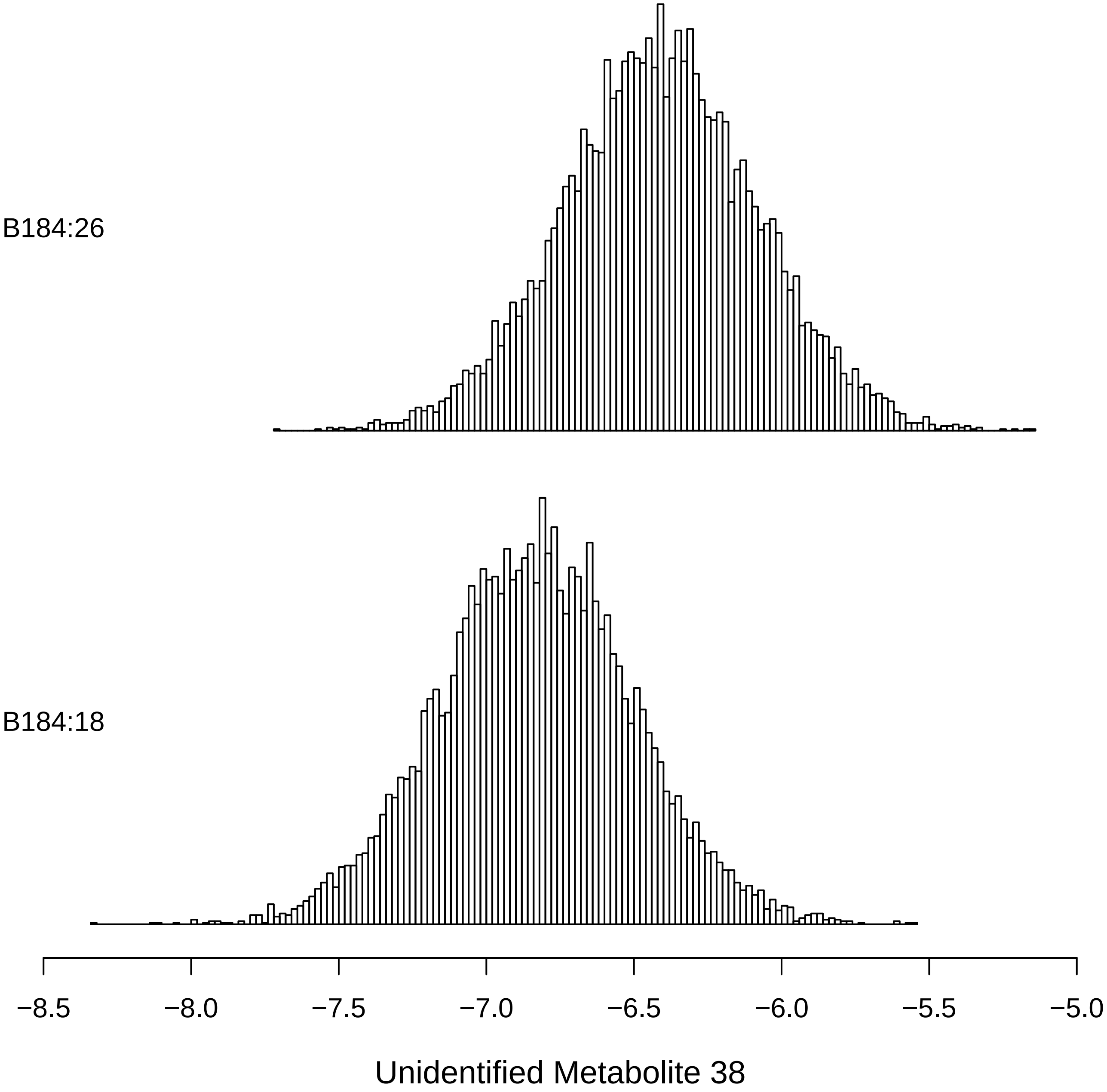
B184:26
B184:18
−8.5
−8.0
−7.5
−7.0
−6.5
−6.0
−5.5
−5.0
Unidentified Metabolite 38

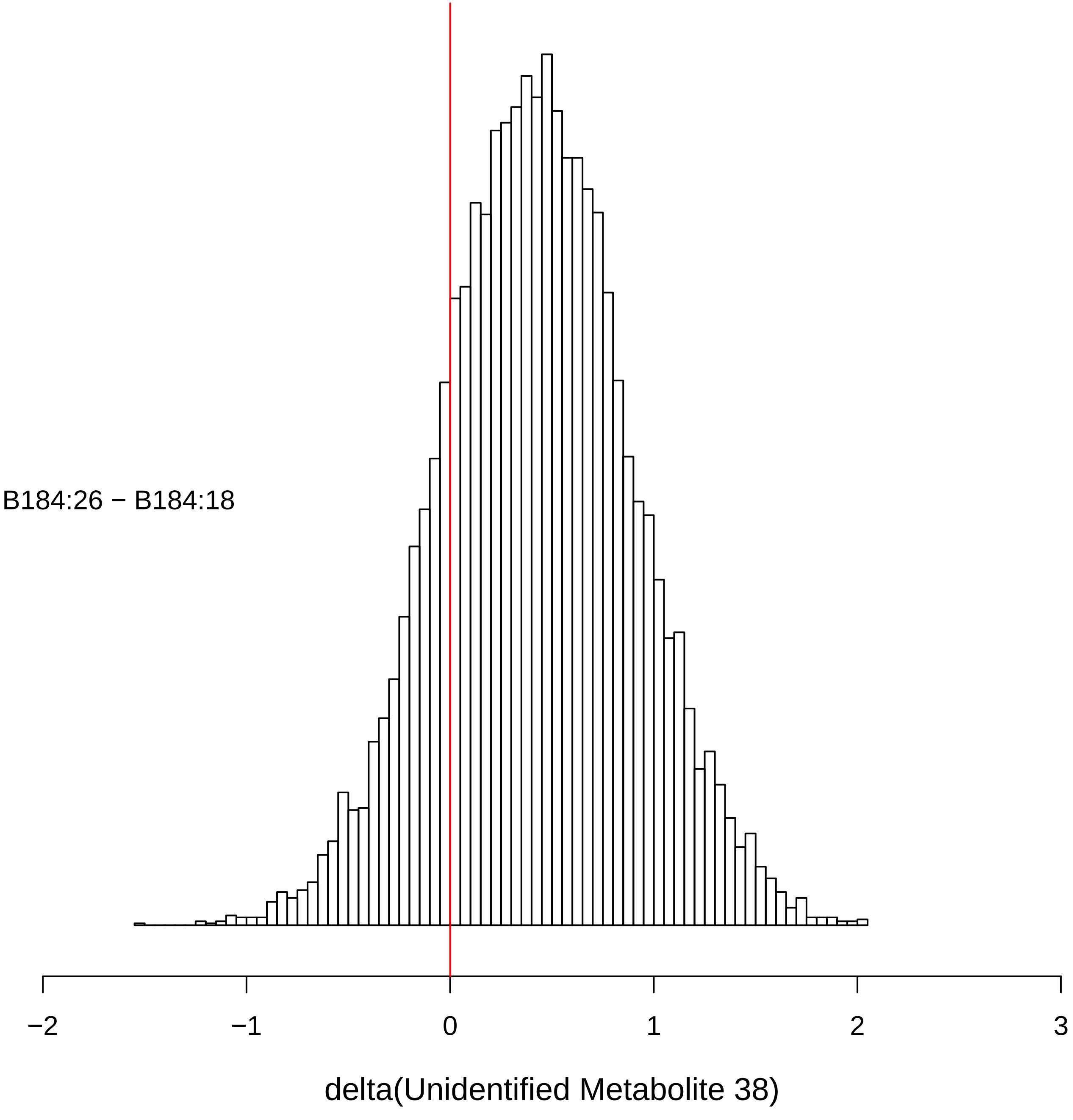

B184:26 − B184:18
−2
−1
0
1
2
3
delta(Unidentified Metabolite 38)

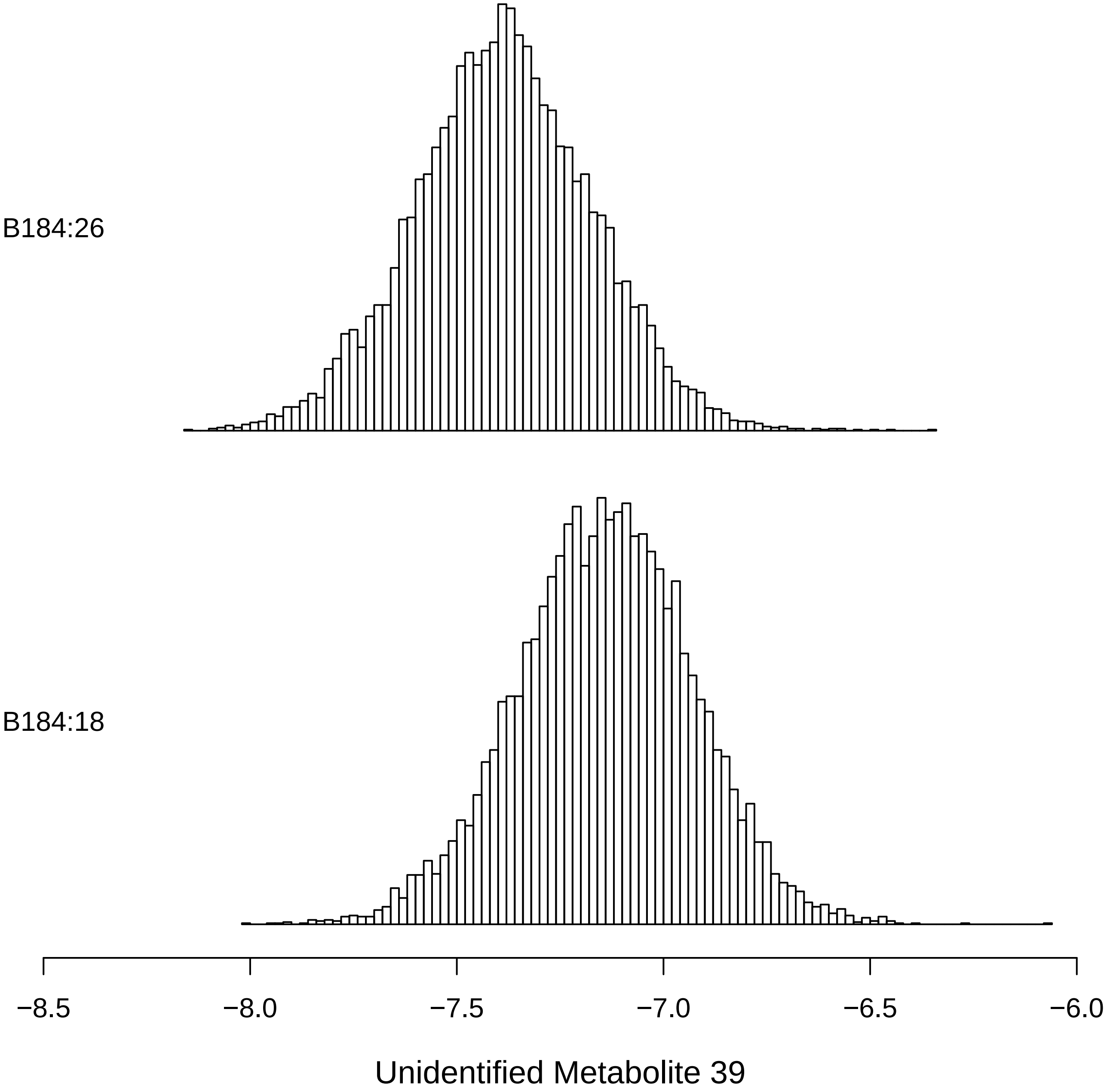

B184:26
B184:18
−8.5
−8.0
−7.5
−7.0
−6.5
−6.0
Unidentified Metabolite 39

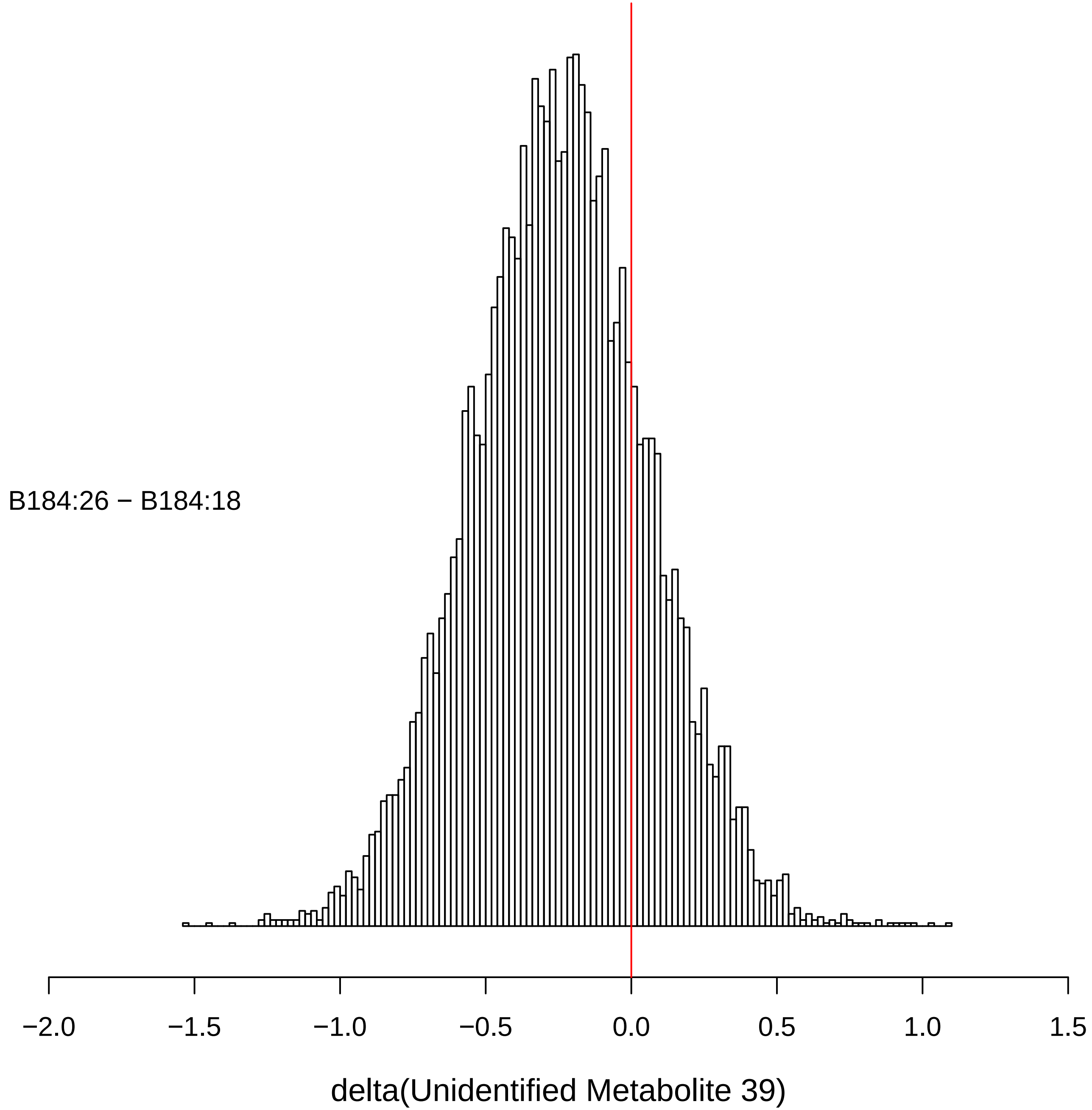
B184:26 − B184:18
−2.0
−1.5
−1.0
−0.5
0.0
0.5
1.0
1.5
delta(Unidentified Metabolite 39)

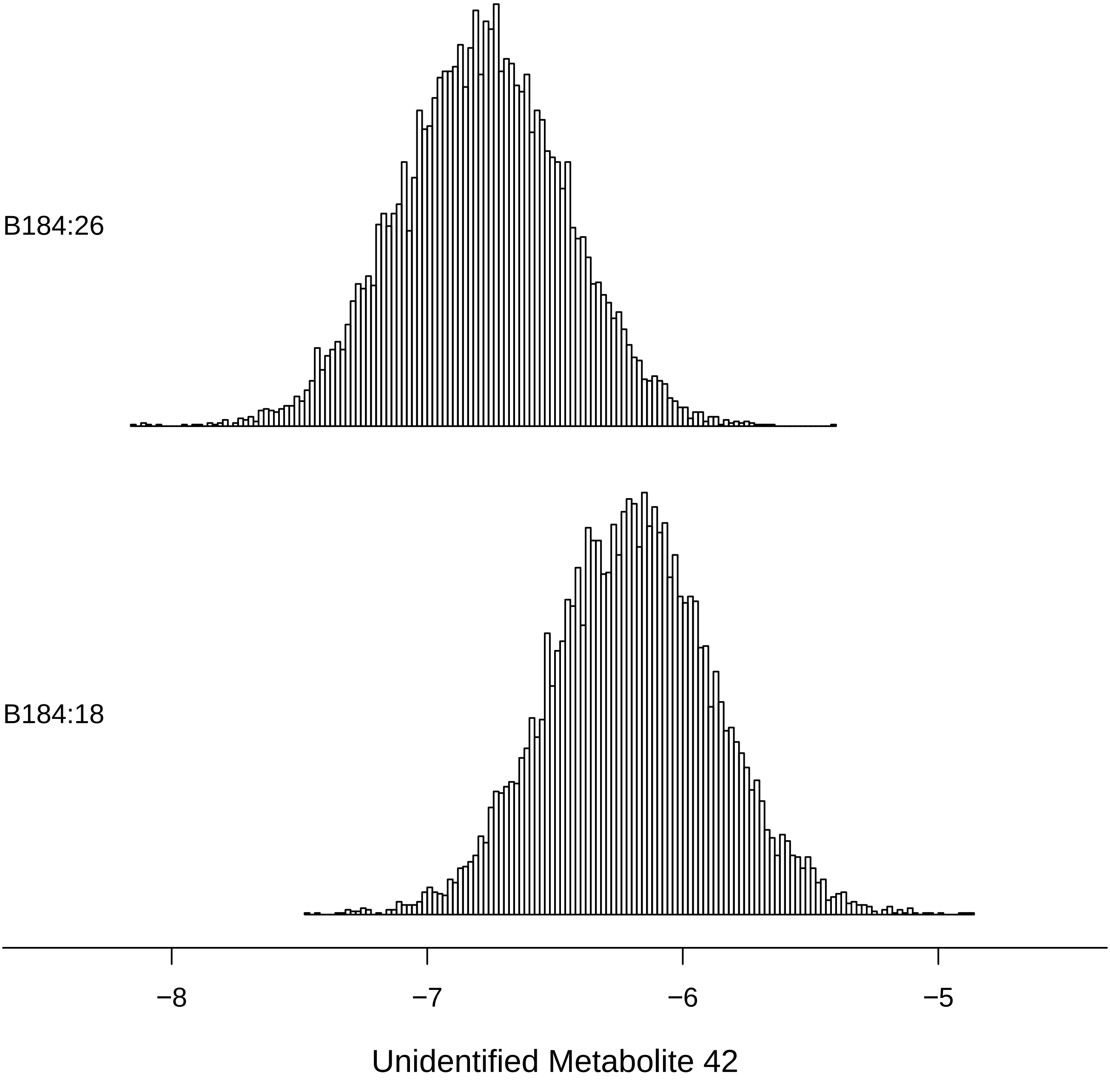

B184:26
B184:18
−8
−7
−6
−5
Unidentified Metabolite 42

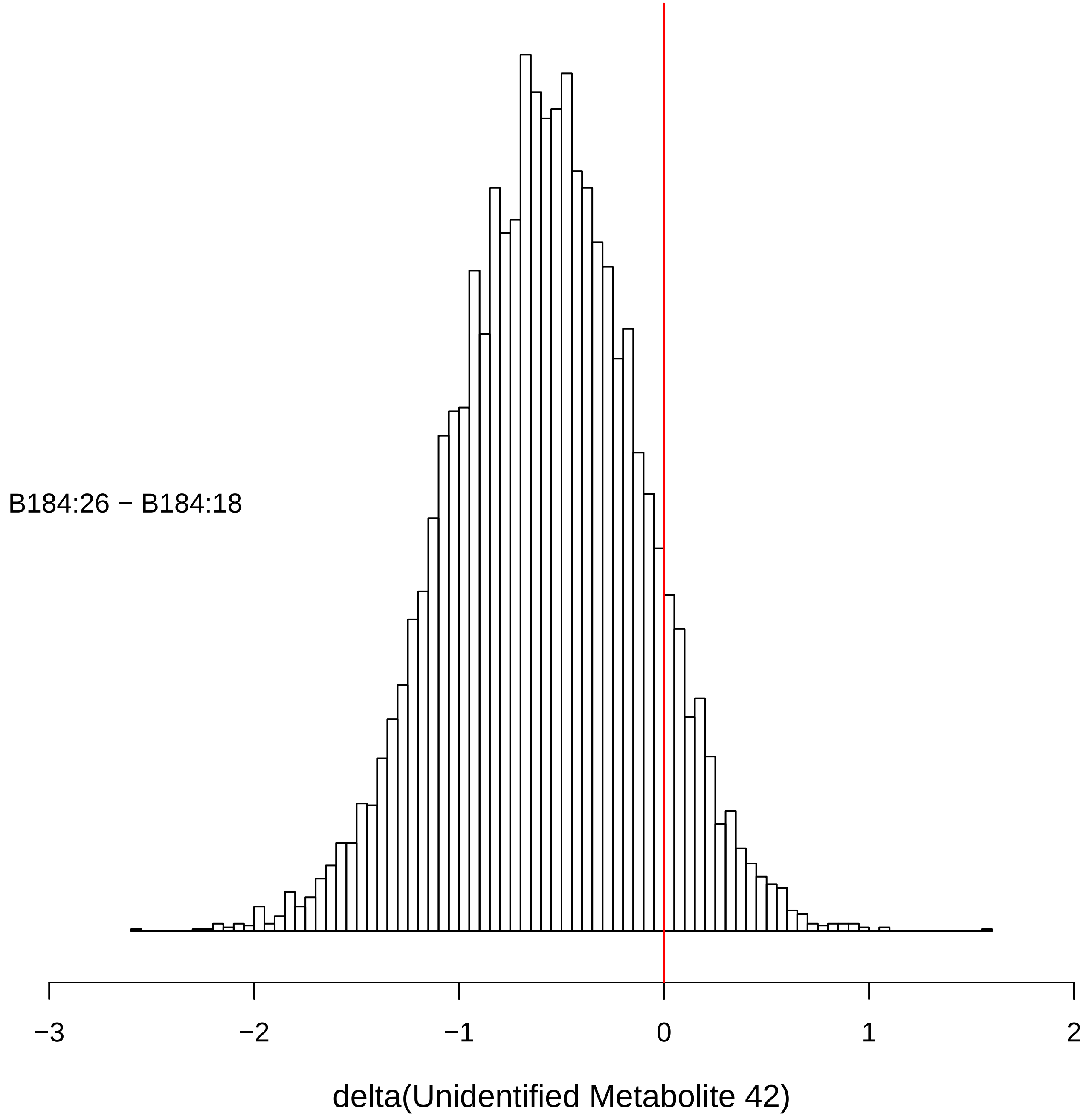

B184:26 − B184:18
−3
−2
−1
0
1
2
delta(Unidentified Metabolite 42)

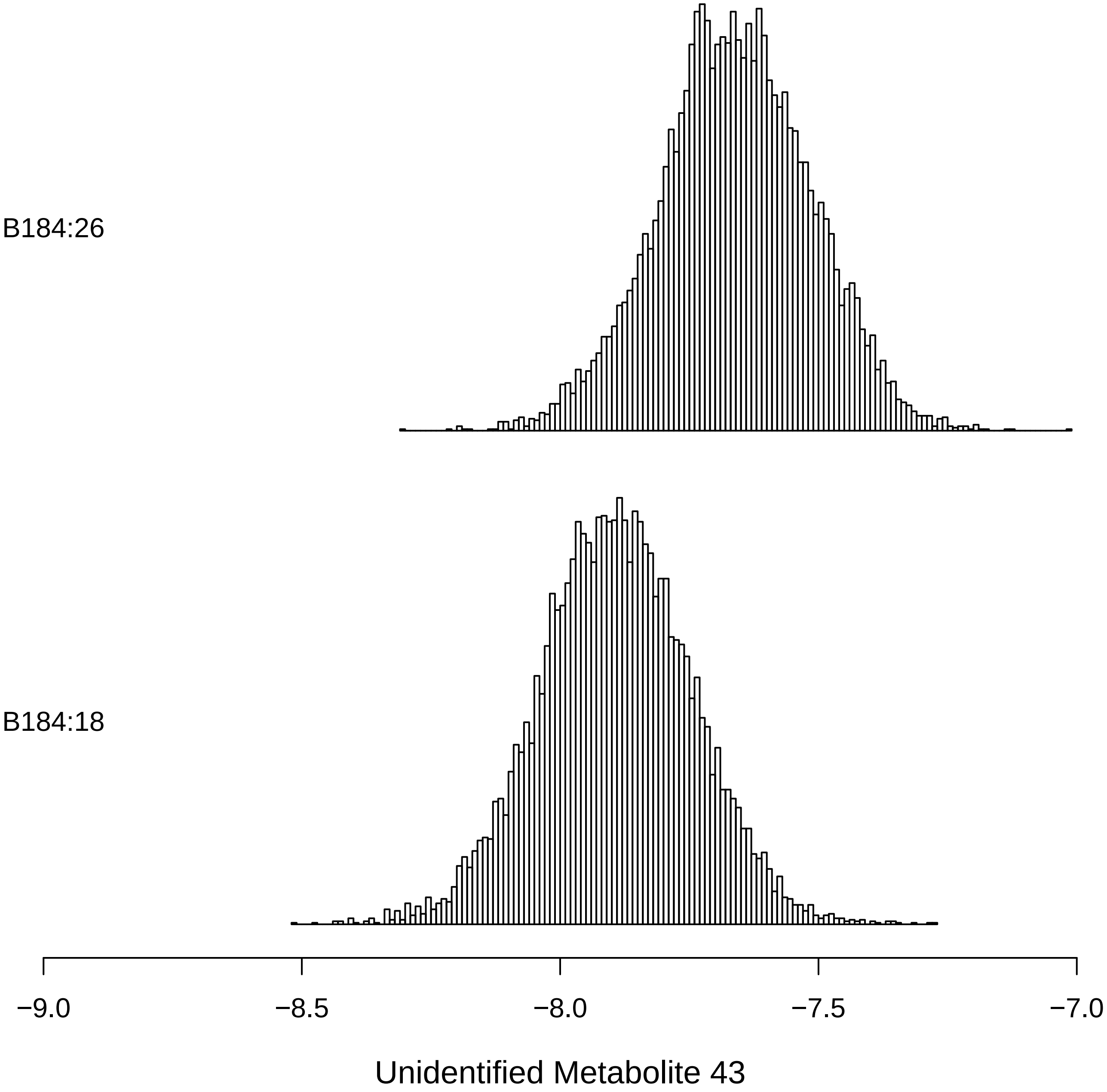
B184:26
B184:18
−9.0
−8.5
−8.0
−7.5
−7.0
Unidentified Metabolite 43

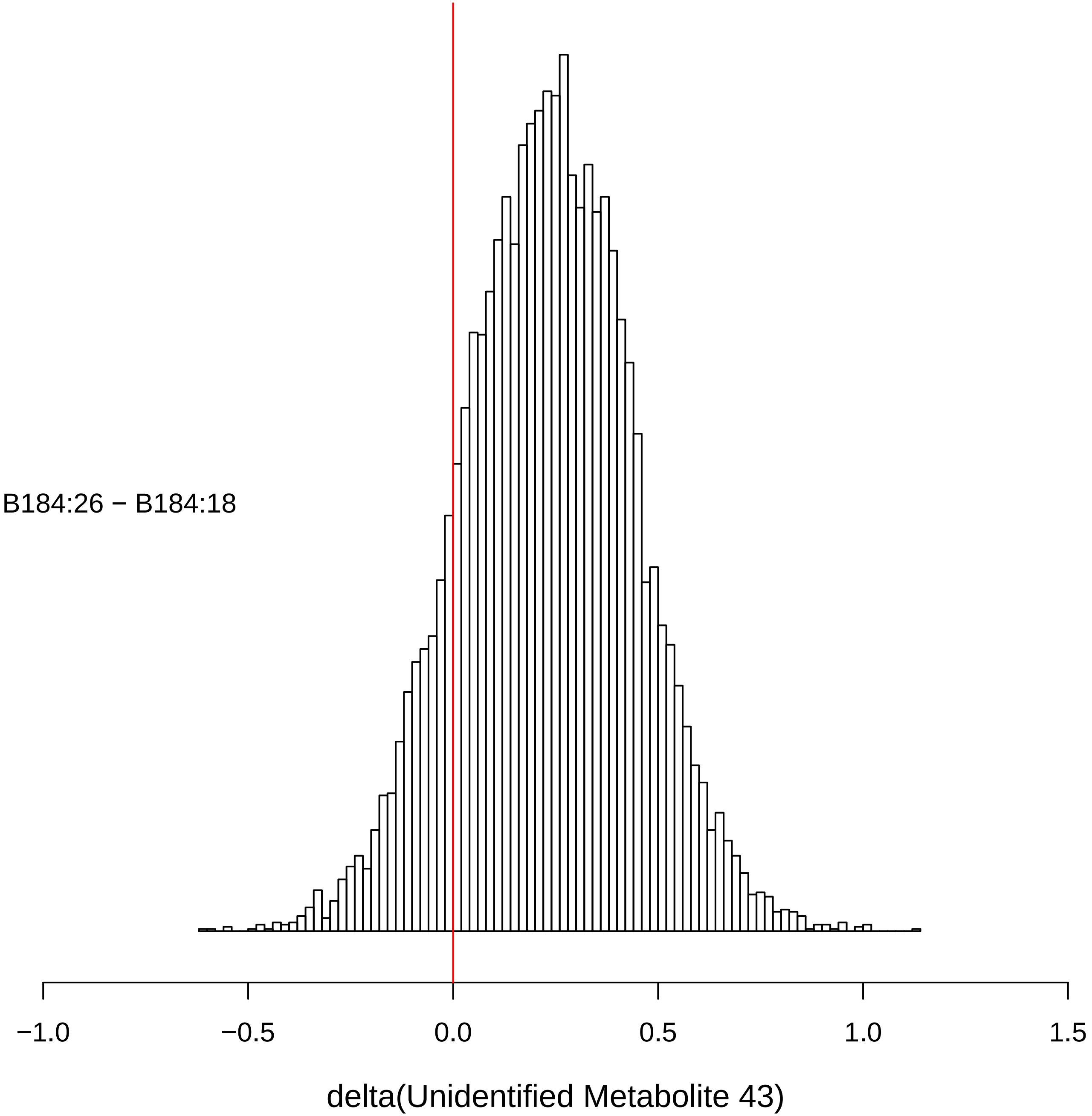
B184:26 − B184:18
−1.0
−0.5
0.0
0.5
1.0
1.5
delta(Unidentified Metabolite 43)

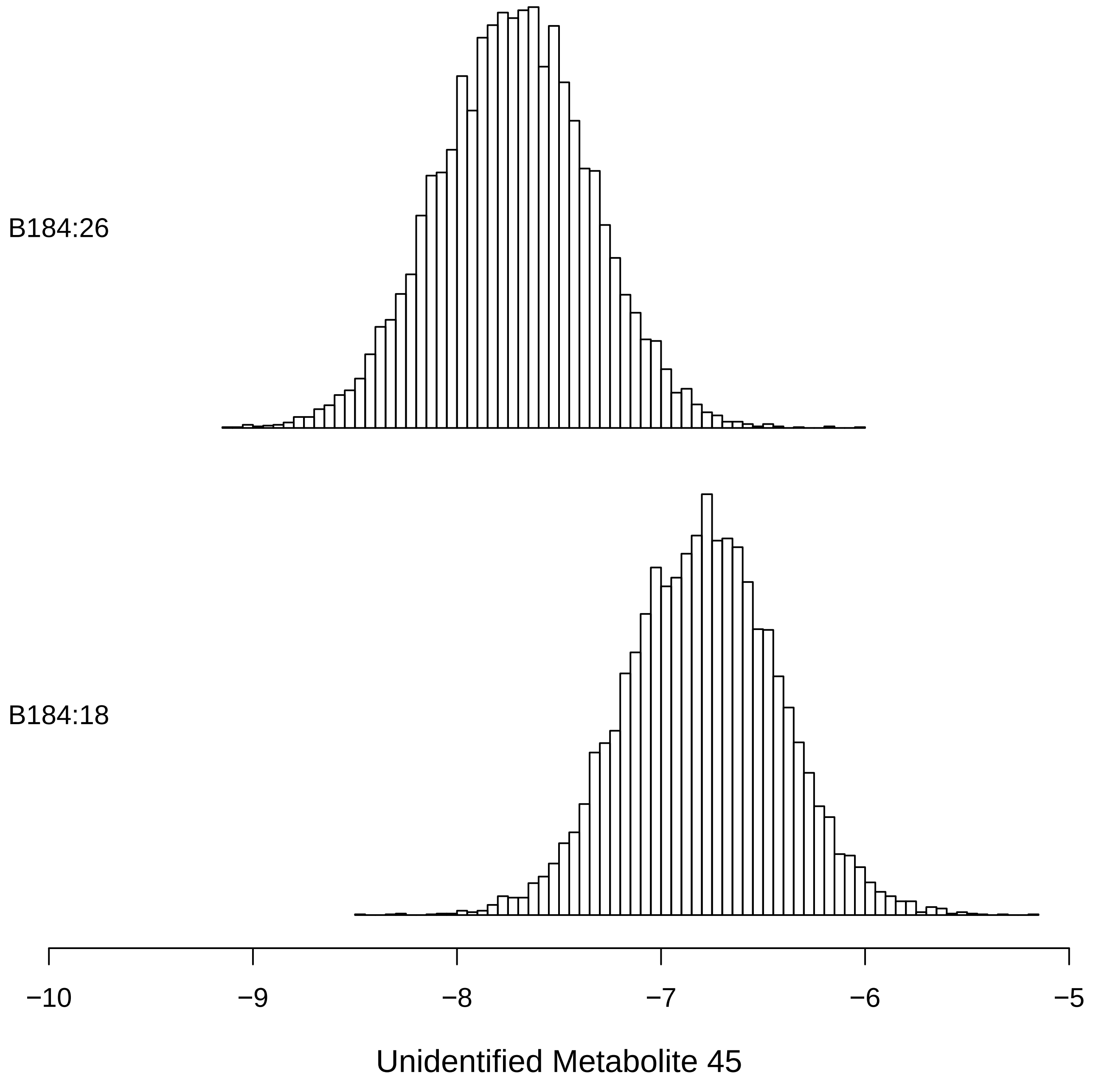
B184:26
B184:18
−10
−9
−8
−7
−6
−5
Unidentified Metabolite 45

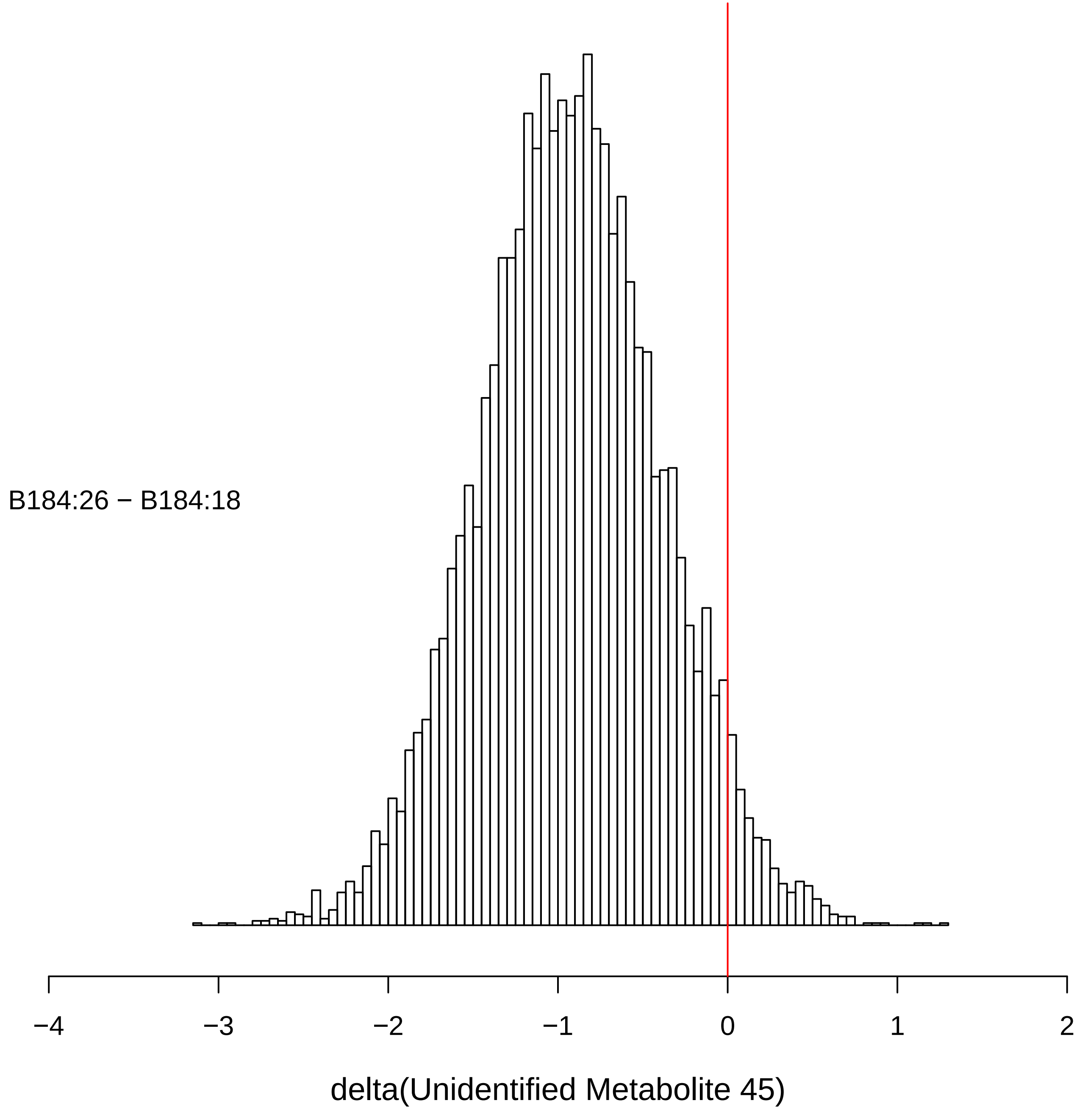
B184:26 − B184:18
−4
−3
−2
−1
0
1
2
delta(Unidentified Metabolite 45)

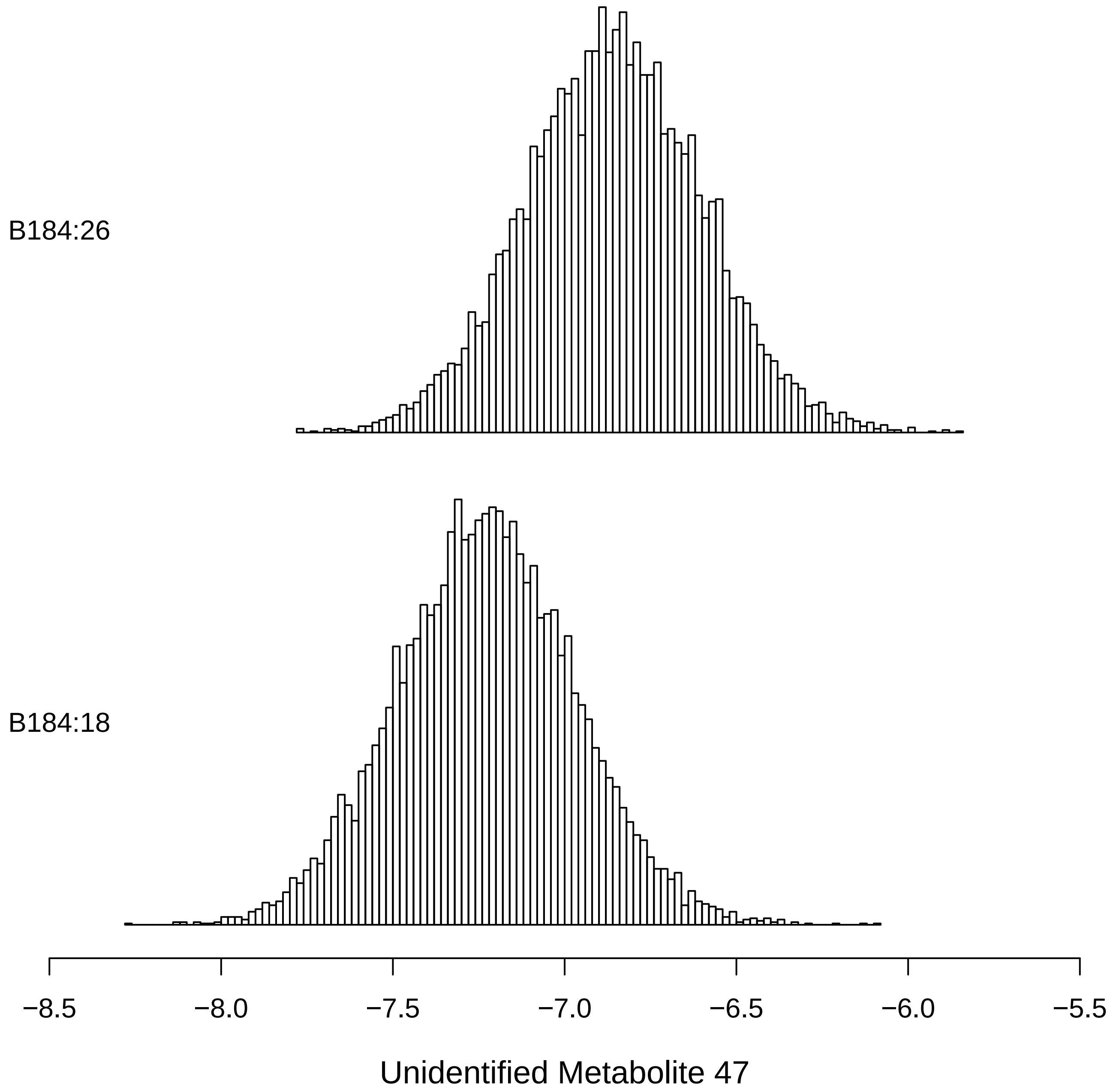

B184:26
B184:18
−8.5
−8.0
−7.5
−7.0
−6.5
−6.0
−5.5
Unidentified Metabolite 47

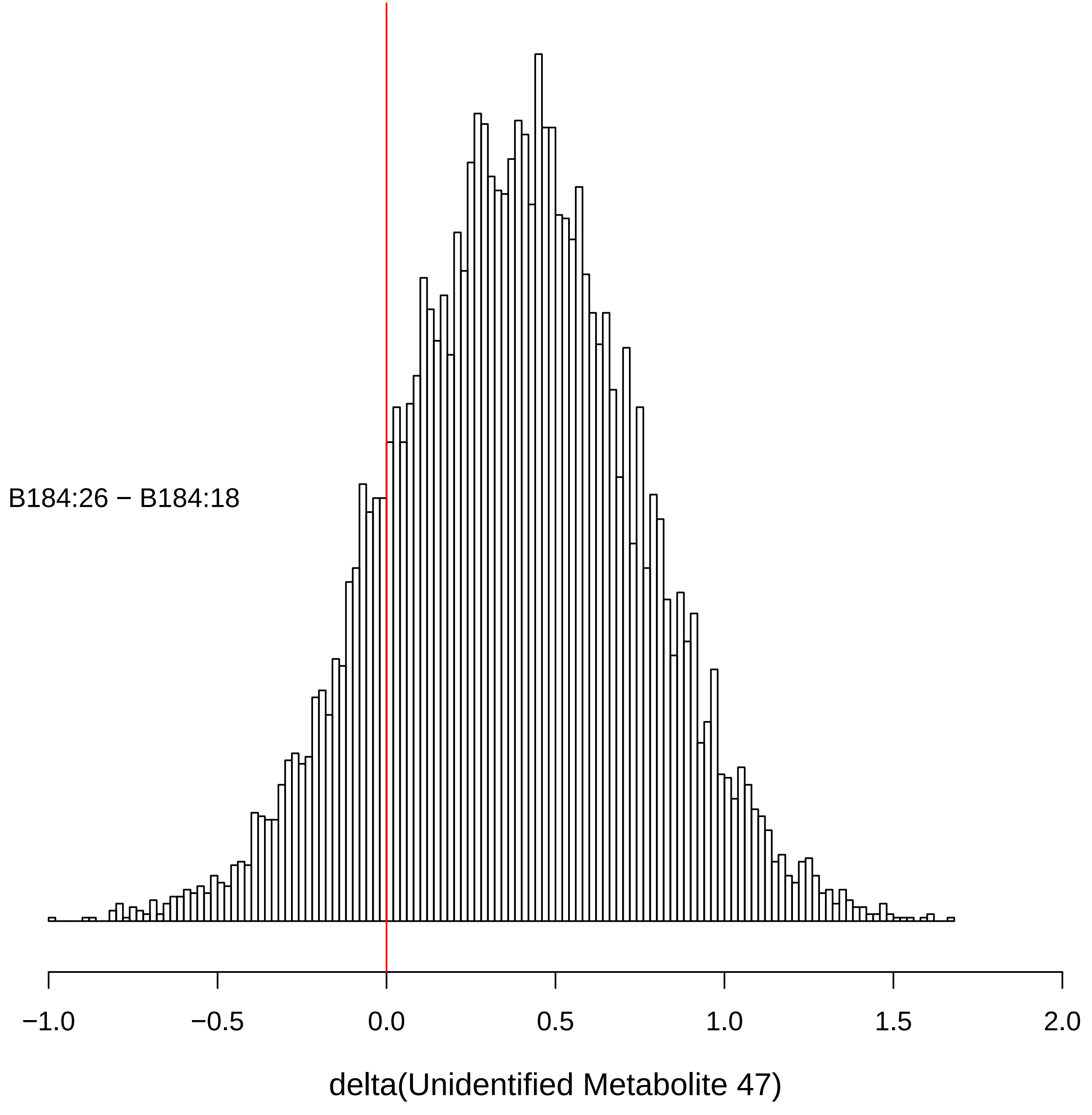
B184:26 − B184:18
−1.0
−0.5
0.0
0.5
1.0
1.5
2.0
delta(Unidentified Metabolite 47)

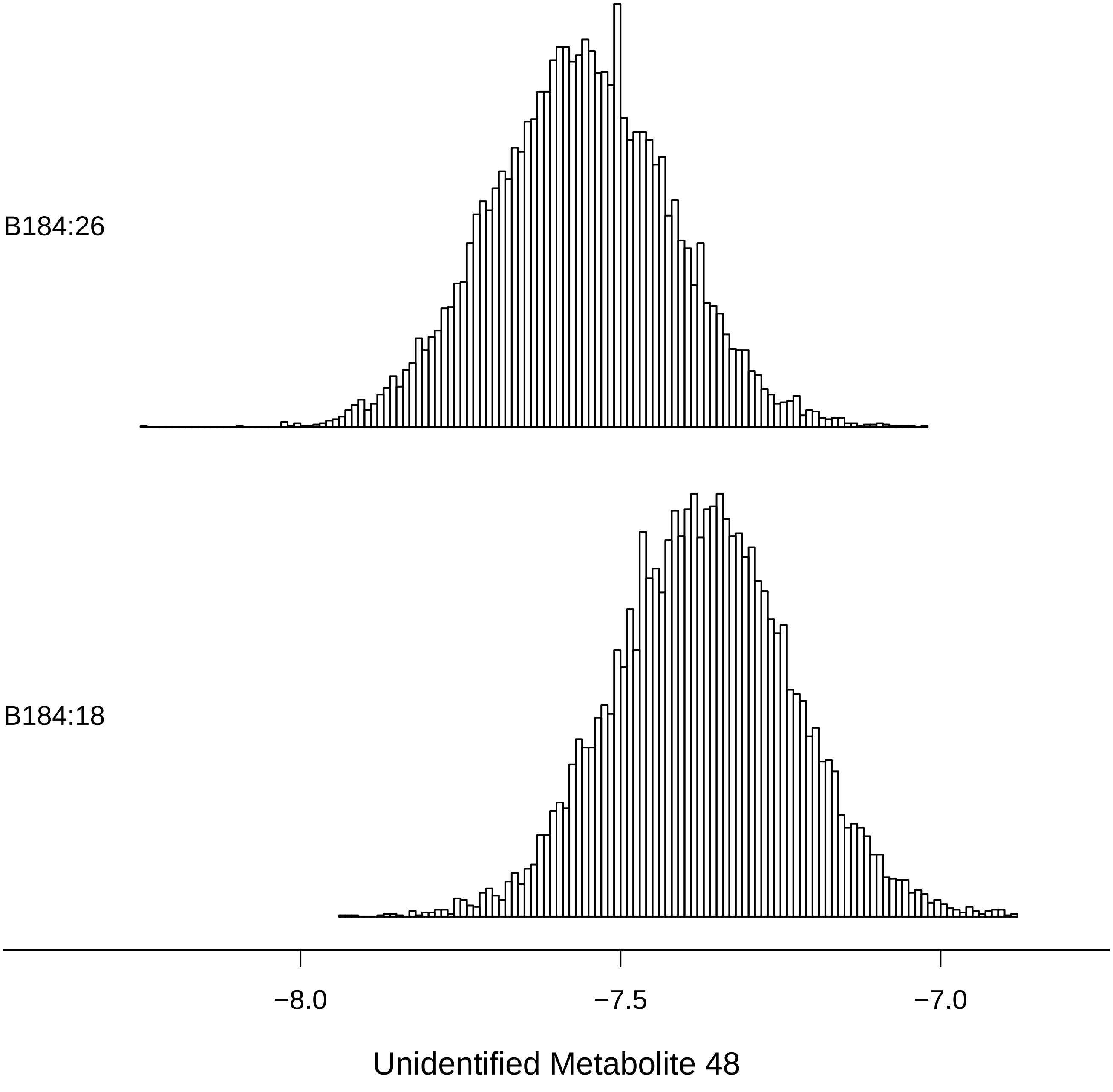

B184:26
B184:18
−8.0
−7.5
−7.0
Unidentified Metabolite 48

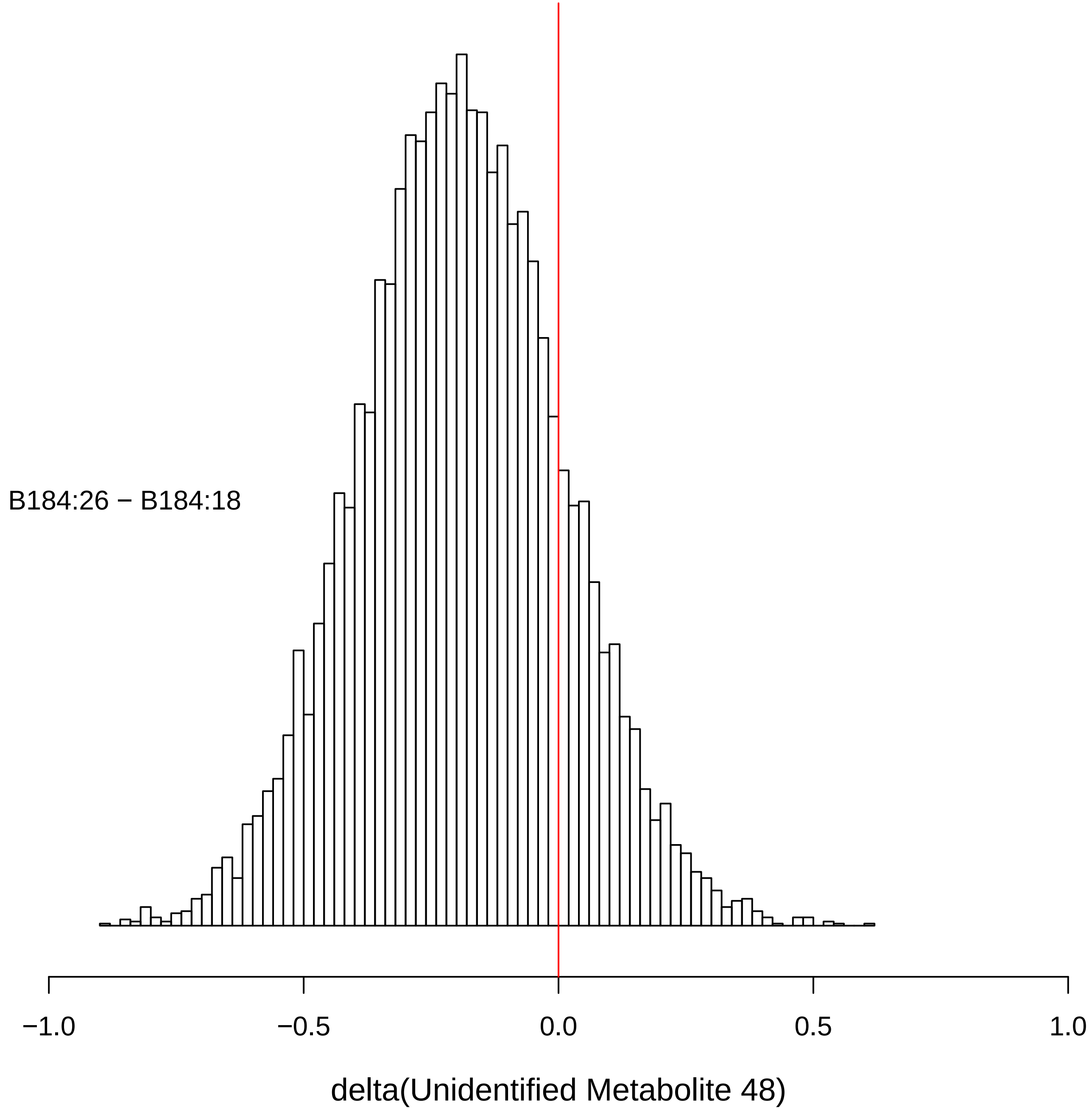
B184:26 − B184:18
−1.0
−0.5
0.0
0.5
1.0
delta(Unidentified Metabolite 48)

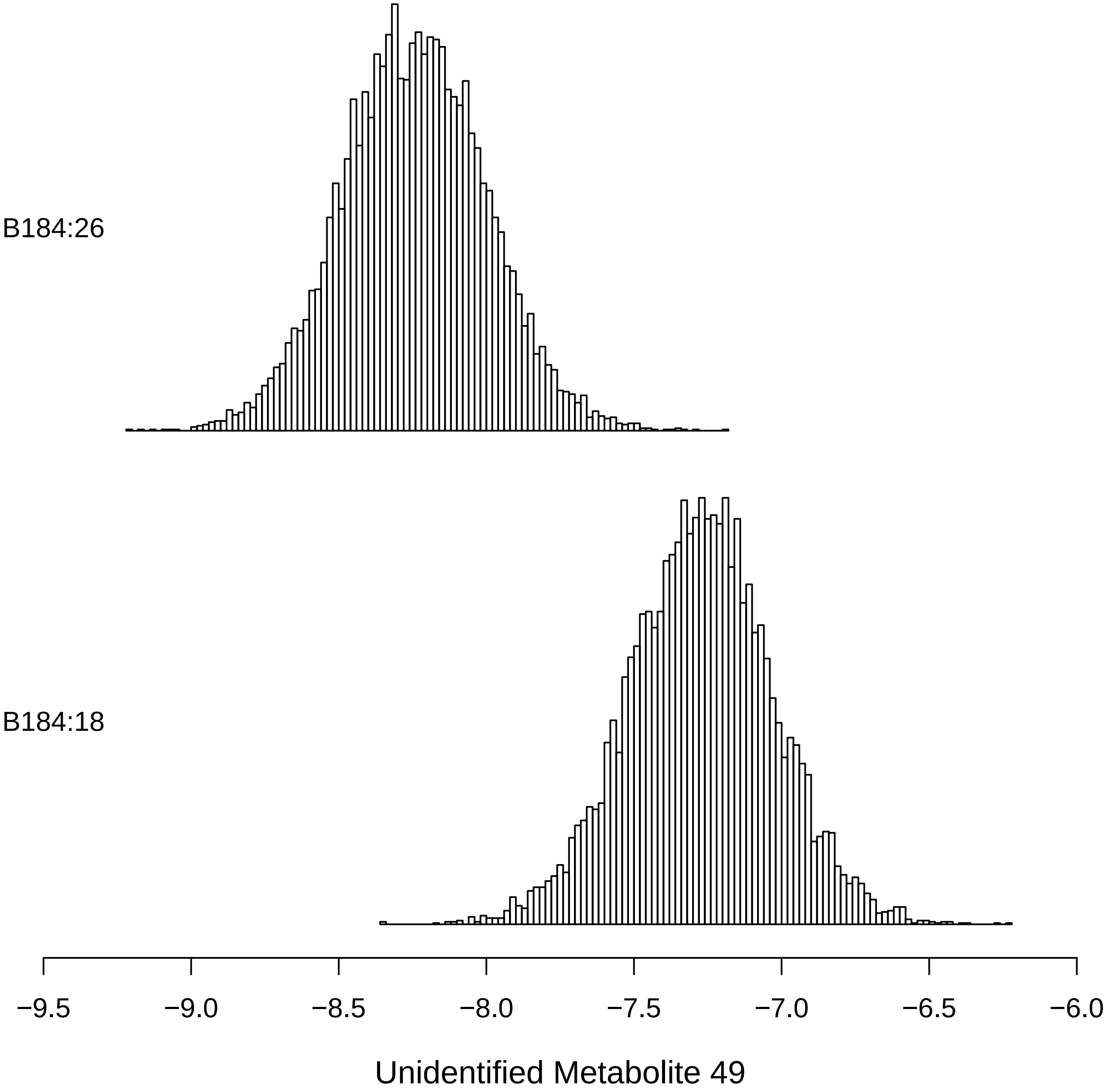
B184:26
B184:18
−9.5
−9.0
−8.5
−8.0
−7.5
−7.0
−6.5
−6.0
Unidentified Metabolite 49

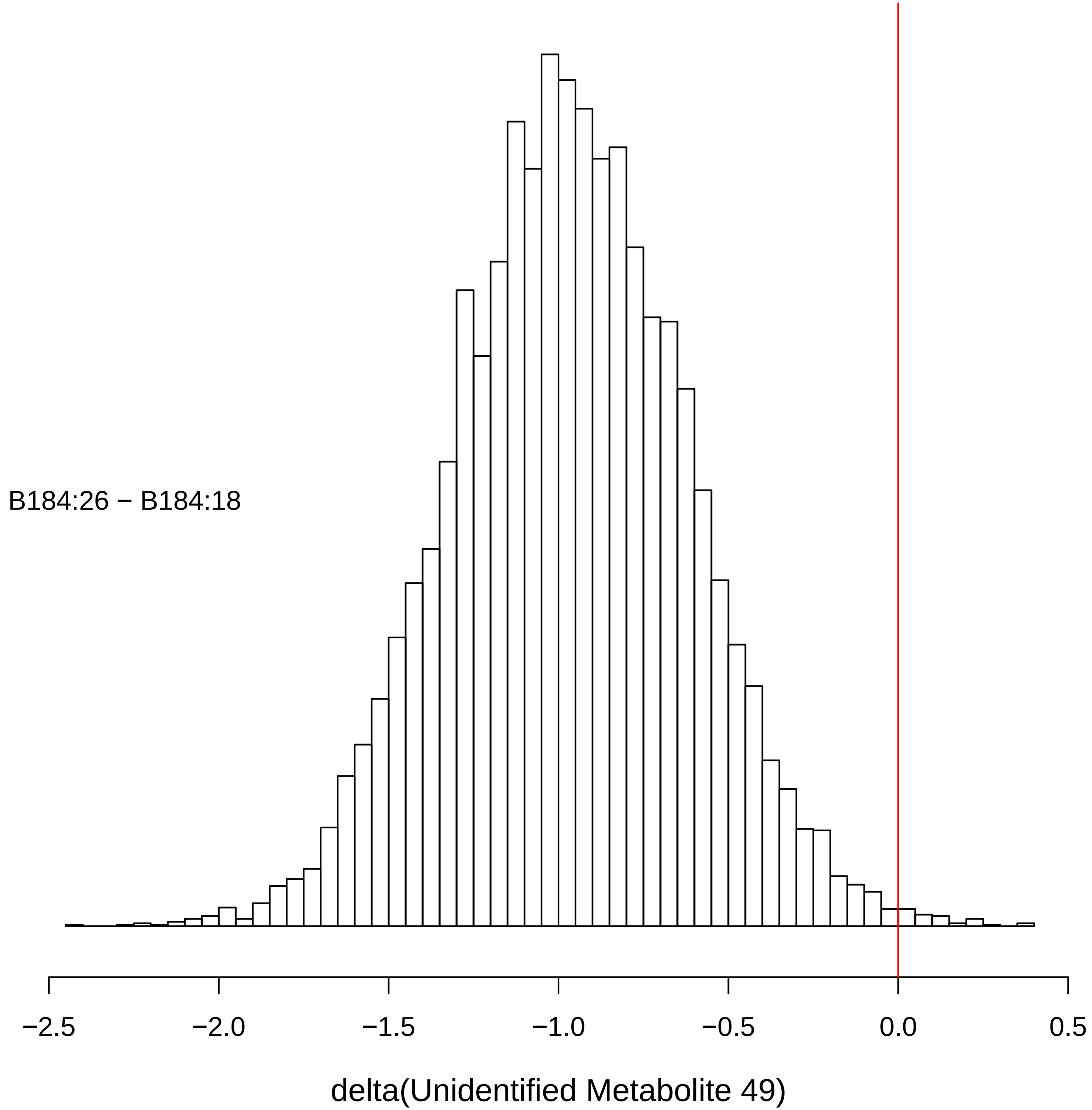
B184:26 − B184:18
−2.5
−2.0
−1.5
−1.0
−0.5
0.0
0.5
delta(Unidentified Metabolite 49)

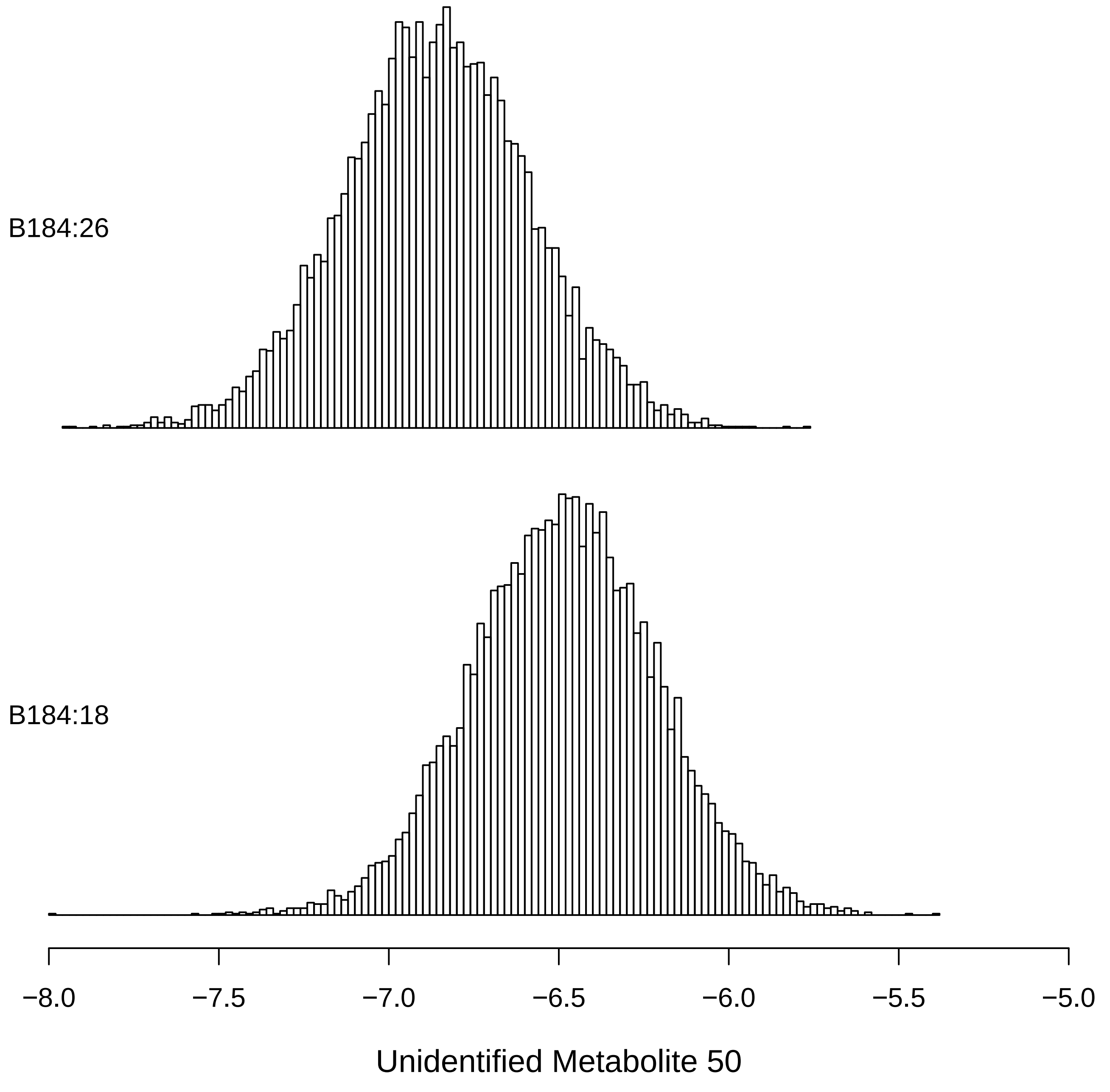
B184:26
B184:18
−8.0
−7.5
−7.0
−6.5
−6.0
−5.5
−5.0
Unidentified Metabolite 50

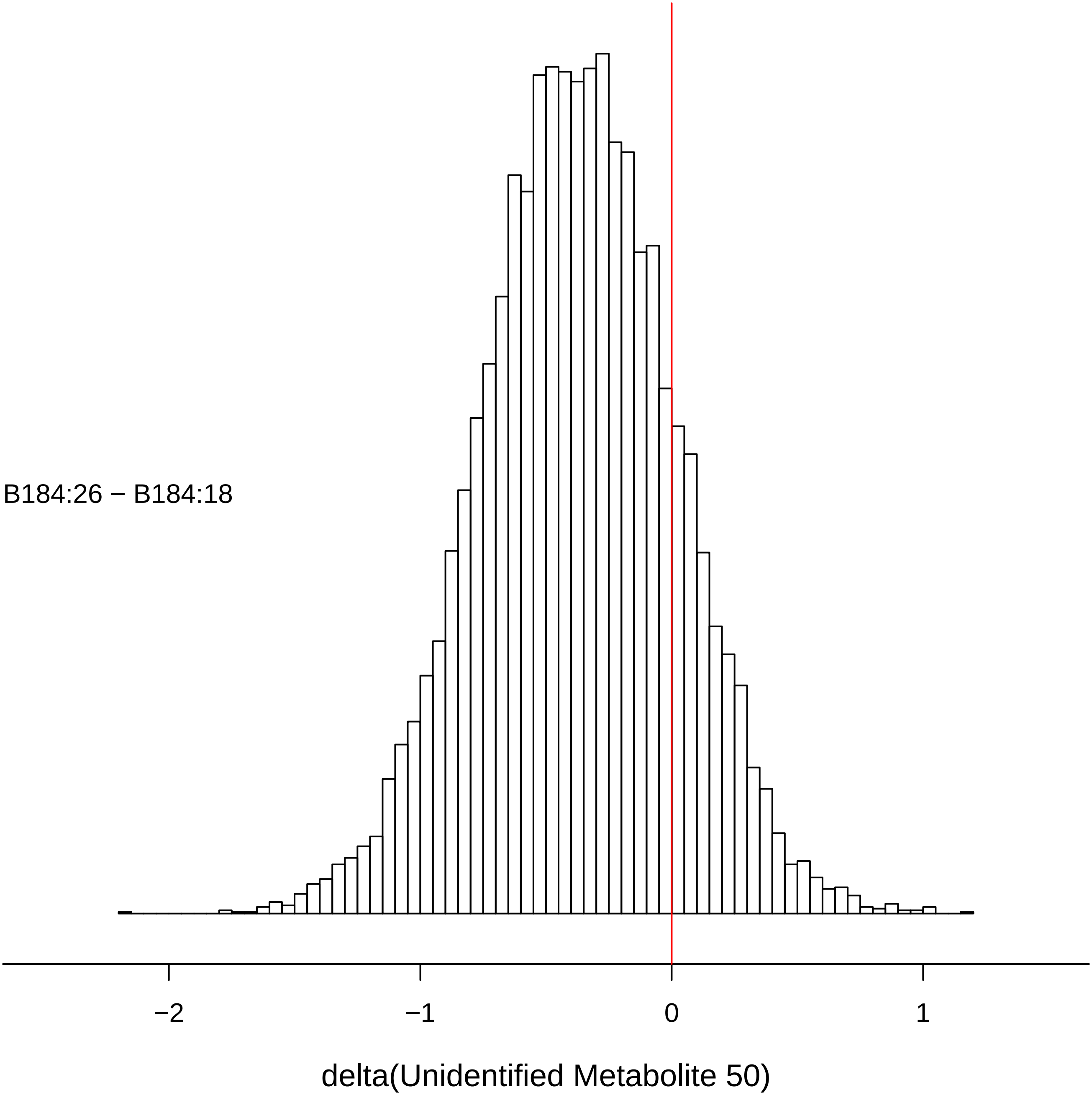

B184:26 − B184:18
−2
−1
0
1
delta(Unidentified Metabolite 50)

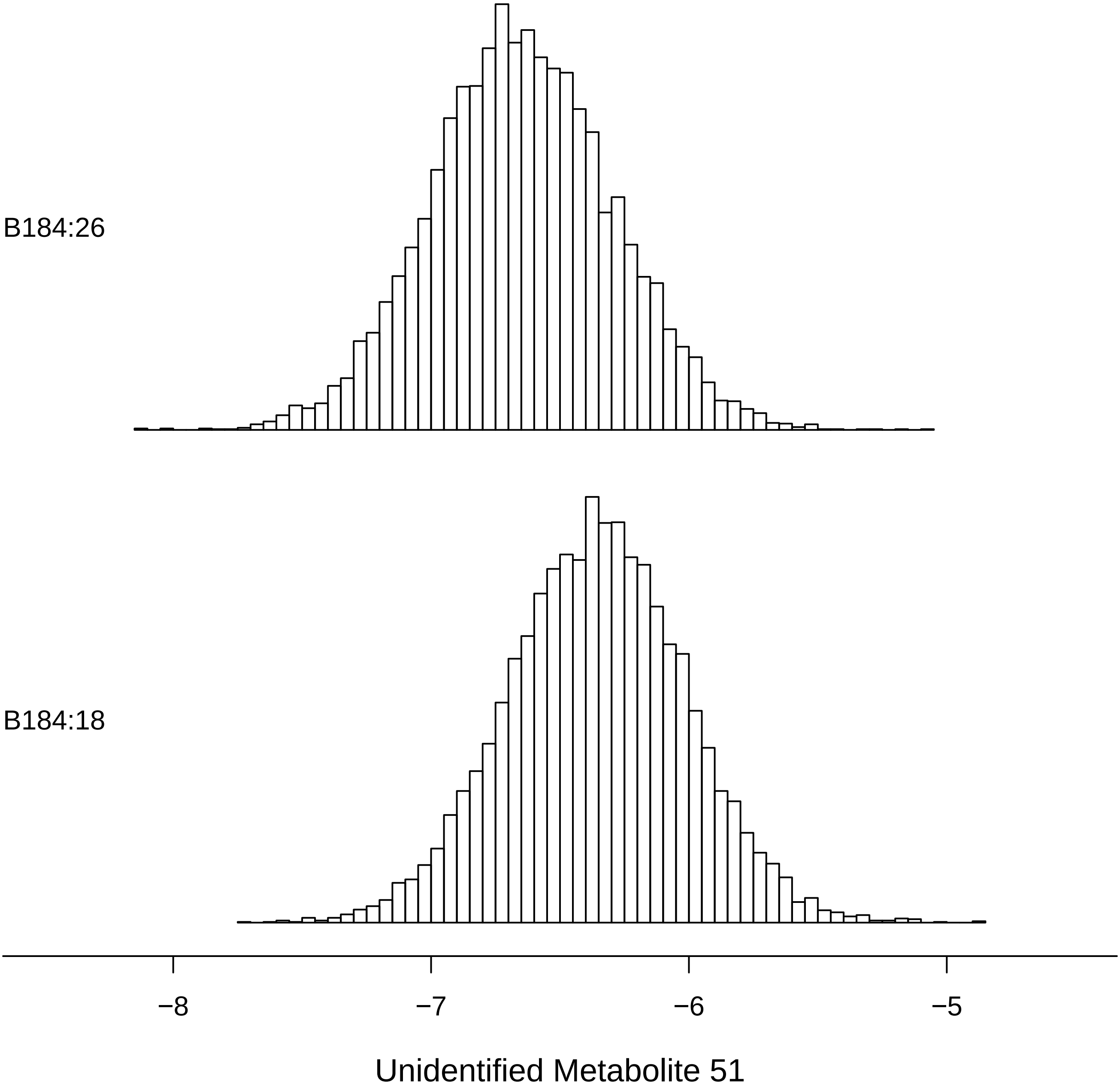

B184:26
B184:18
−8
−7
−6
−5
Unidentified Metabolite 51

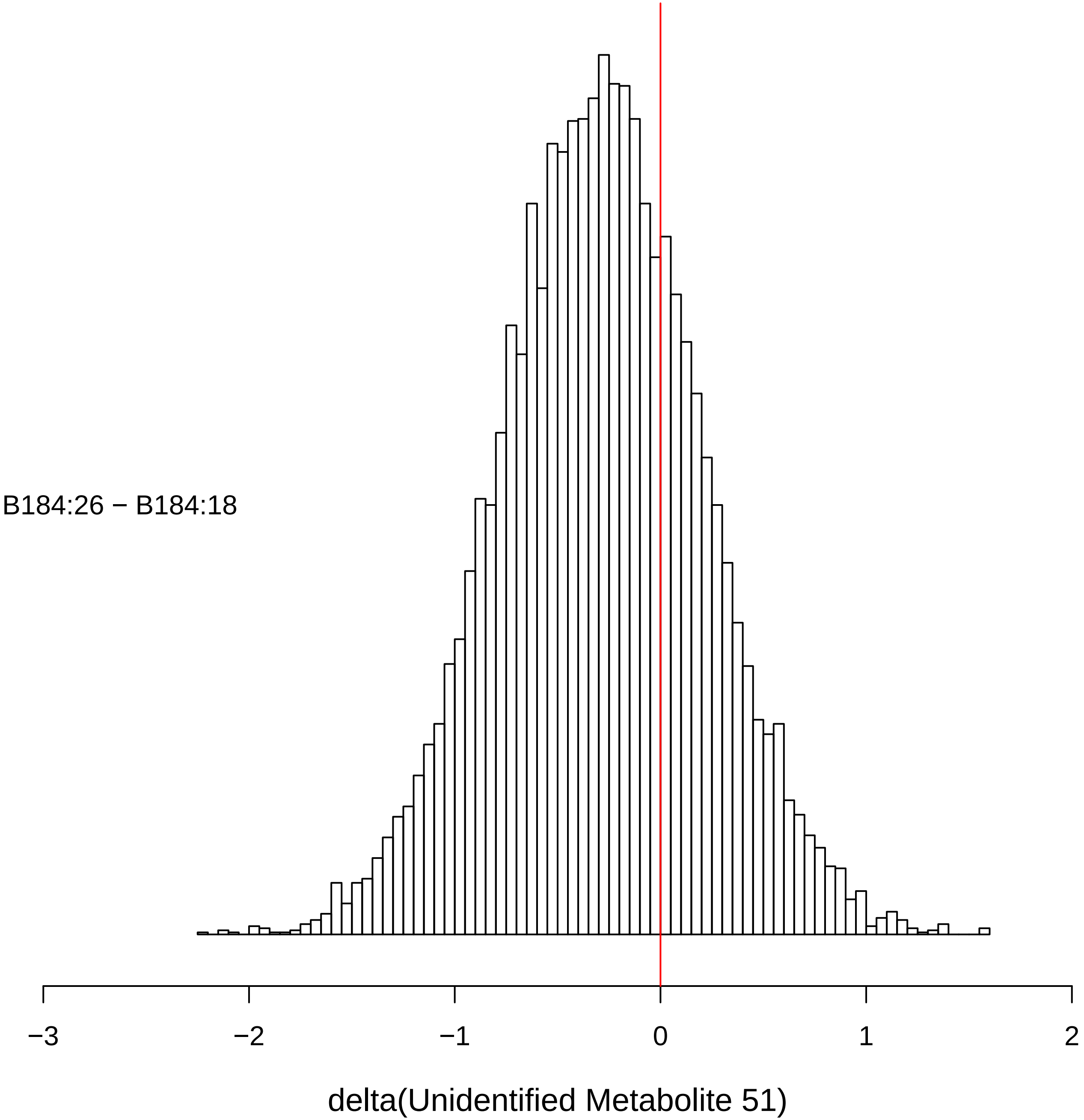
B184:26 − B184:18
−3
−2
−1
0
1
2
delta(Unidentified Metabolite 51)

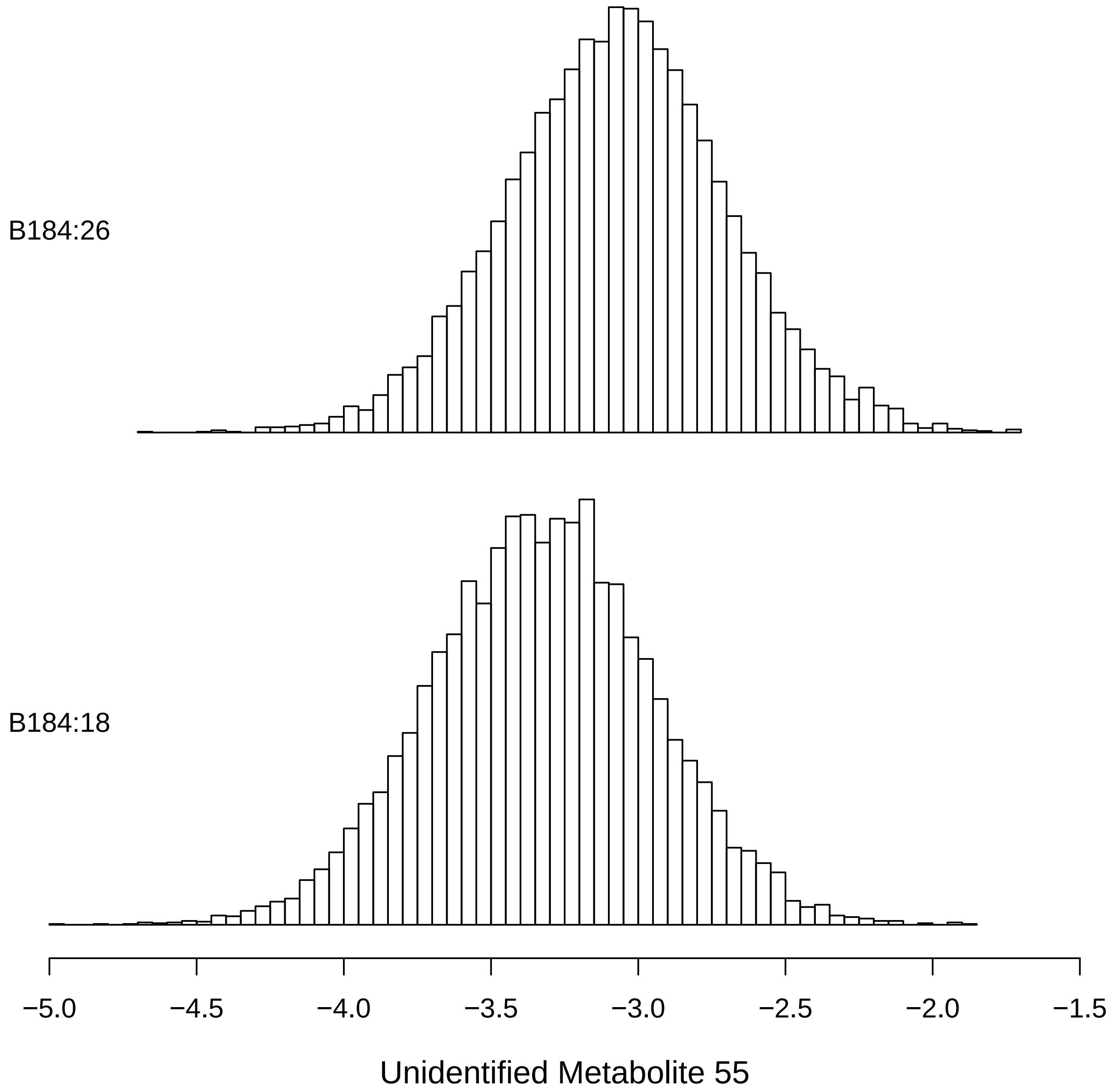
B184:26
B184:18
−5.0
−4.5
−4.0
−3.5
−3.0
−2.5
−2.0
−1.5
Unidentified Metabolite 55

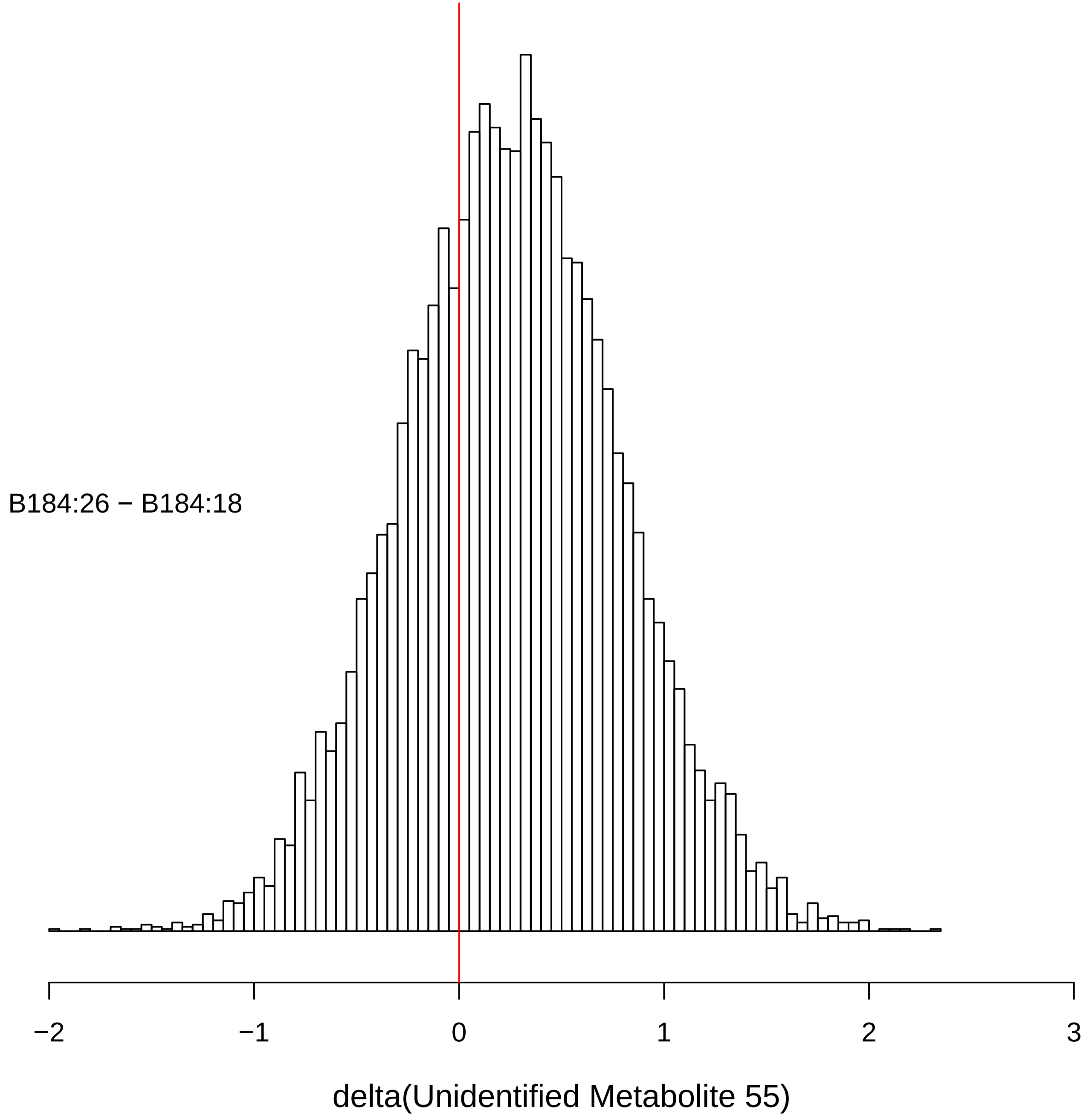

B184:26 − B184:18
−2
−1
0
1
2
3
delta(Unidentified Metabolite 55)

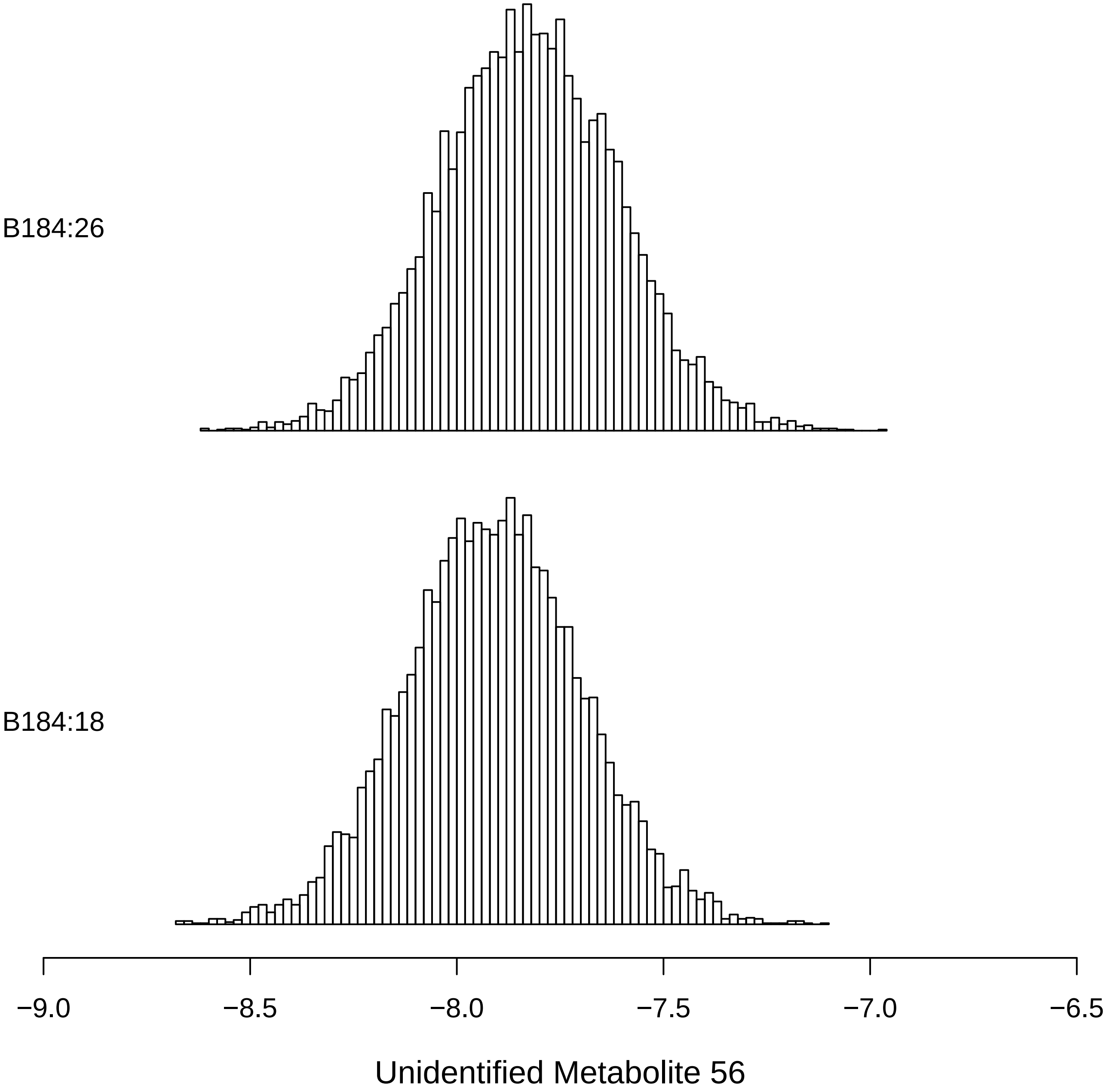
B184:26
B184:18
−9.0
−8.5
−8.0
−7.5
−7.0
−6.5
Unidentified Metabolite 56

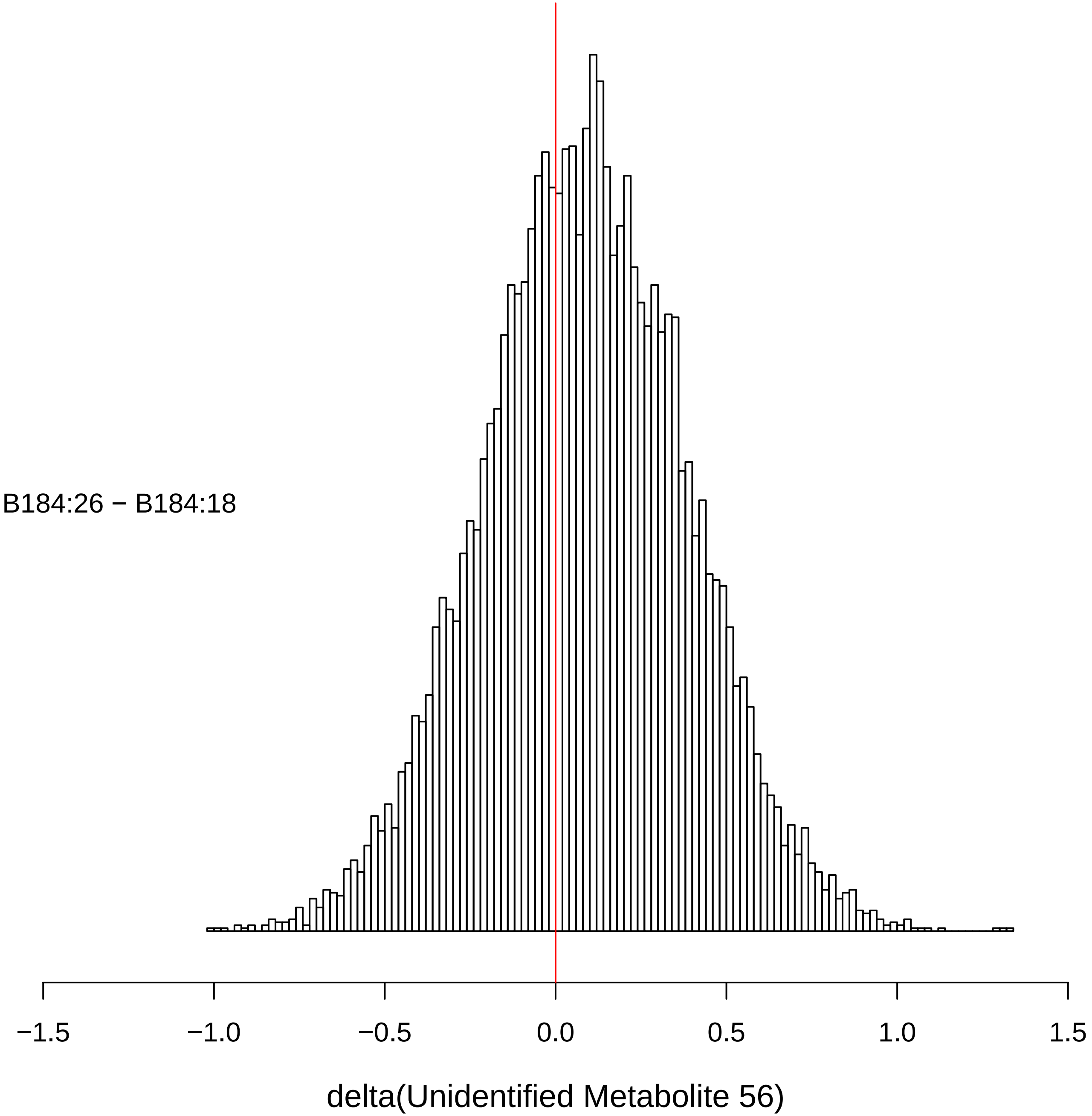
B184:26 − B184:18
−1.5
−1.0
−0.5
0.0
0.5
1.0
1.5
delta(Unidentified Metabolite 56)

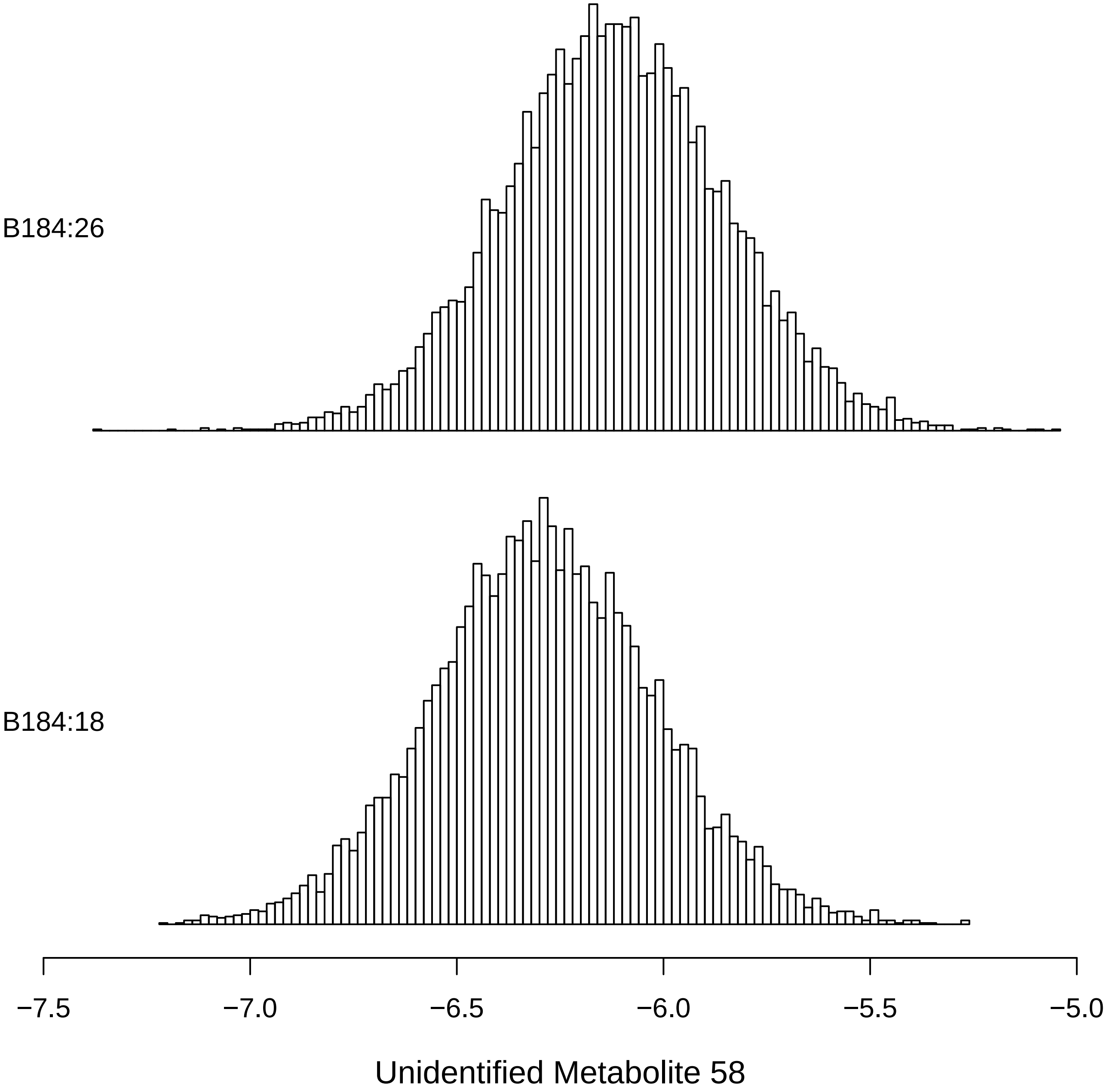
B184:26
B184:18
−7.5
−7.0
−6.5
−6.0
−5.5
−5.0
Unidentified Metabolite 58

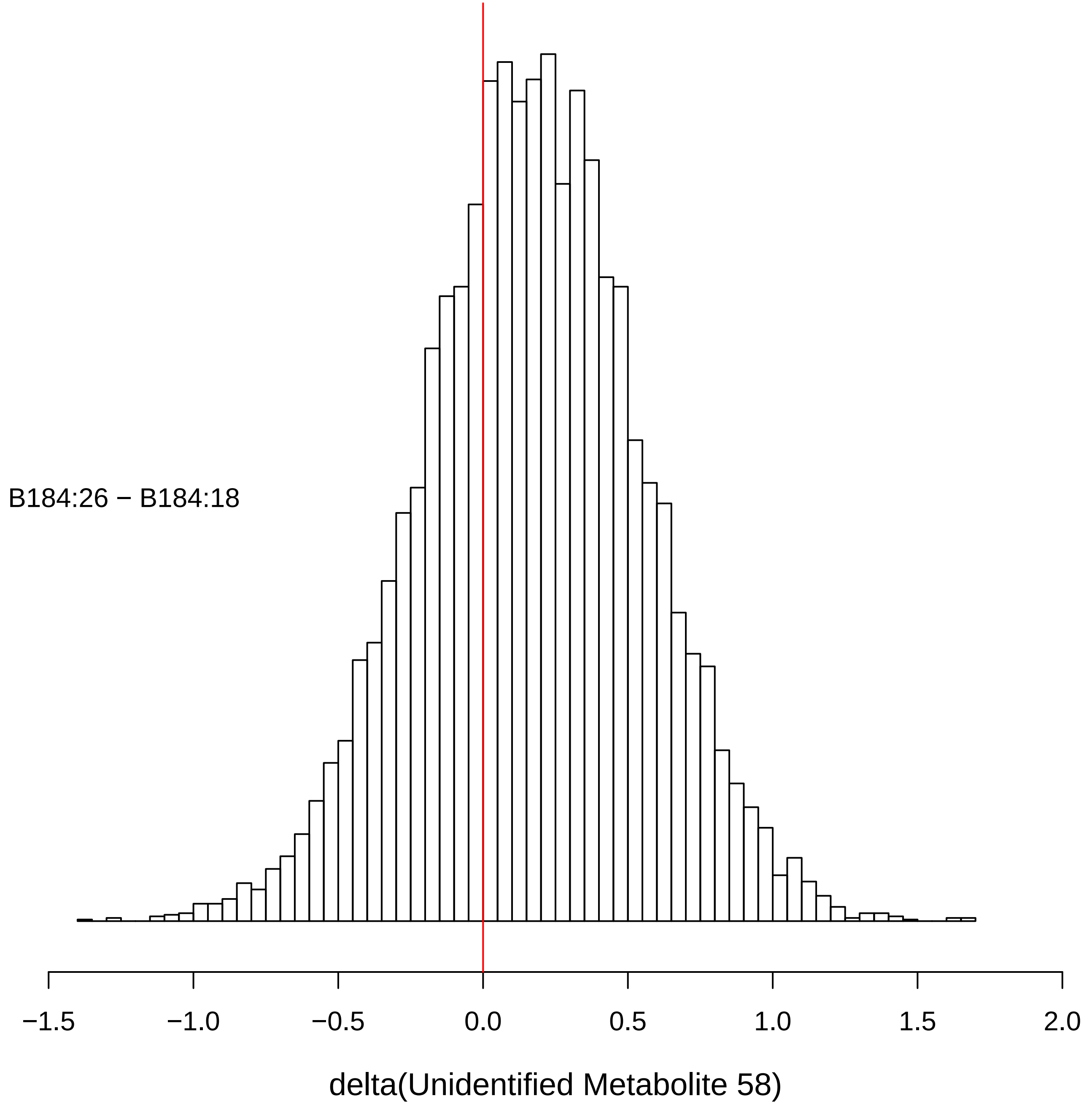
B184:26 − B184:18
−1.5
−1.0
−0.5
0.0
0.5
1.0
1.5
2.0
delta(Unidentified Metabolite 58)

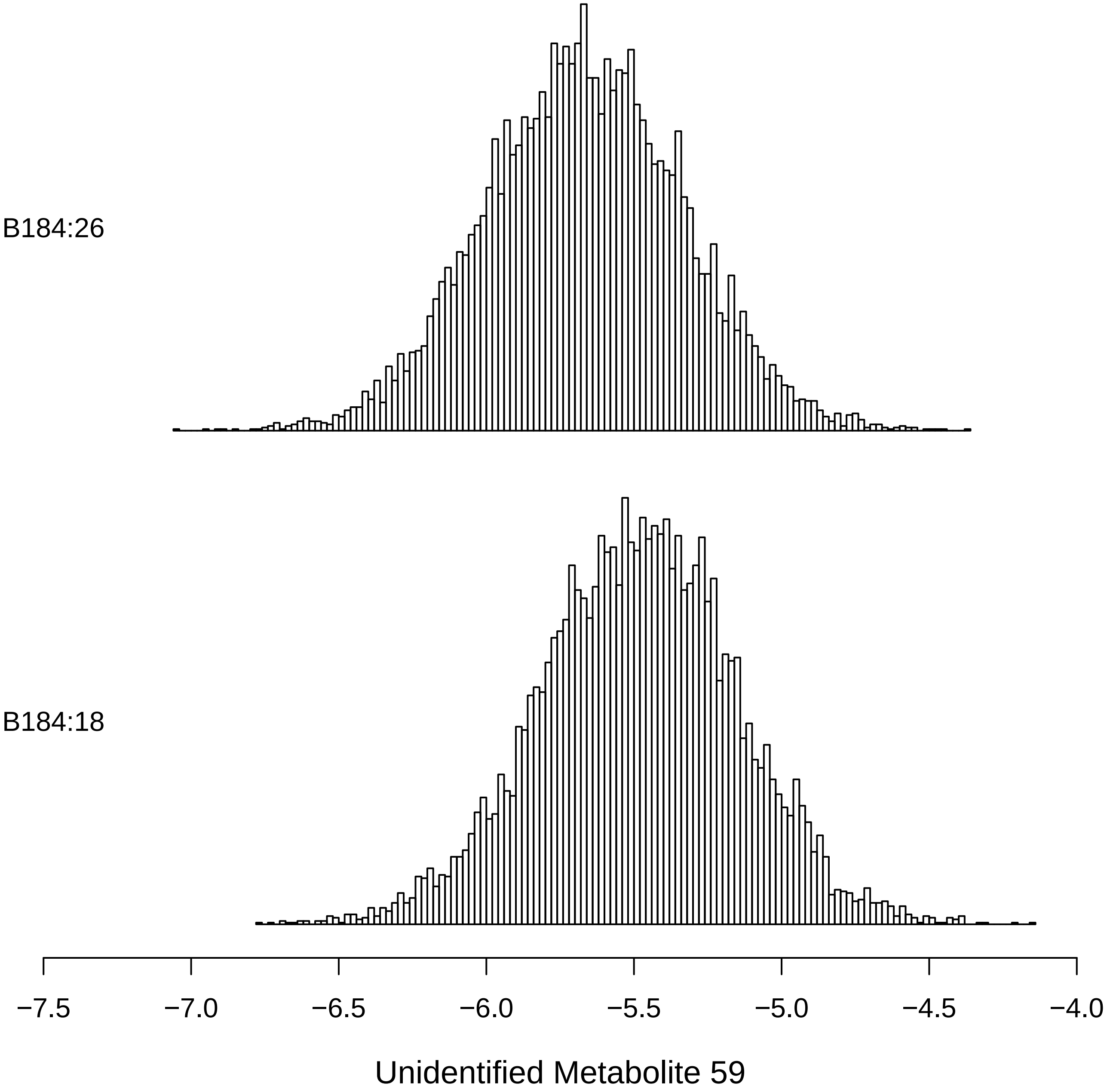

B184:26
B184:18
−7.5
−7.0
−6.5
−6.0
−5.5
−5.0
−4.5
−4.0
Unidentified Metabolite 59

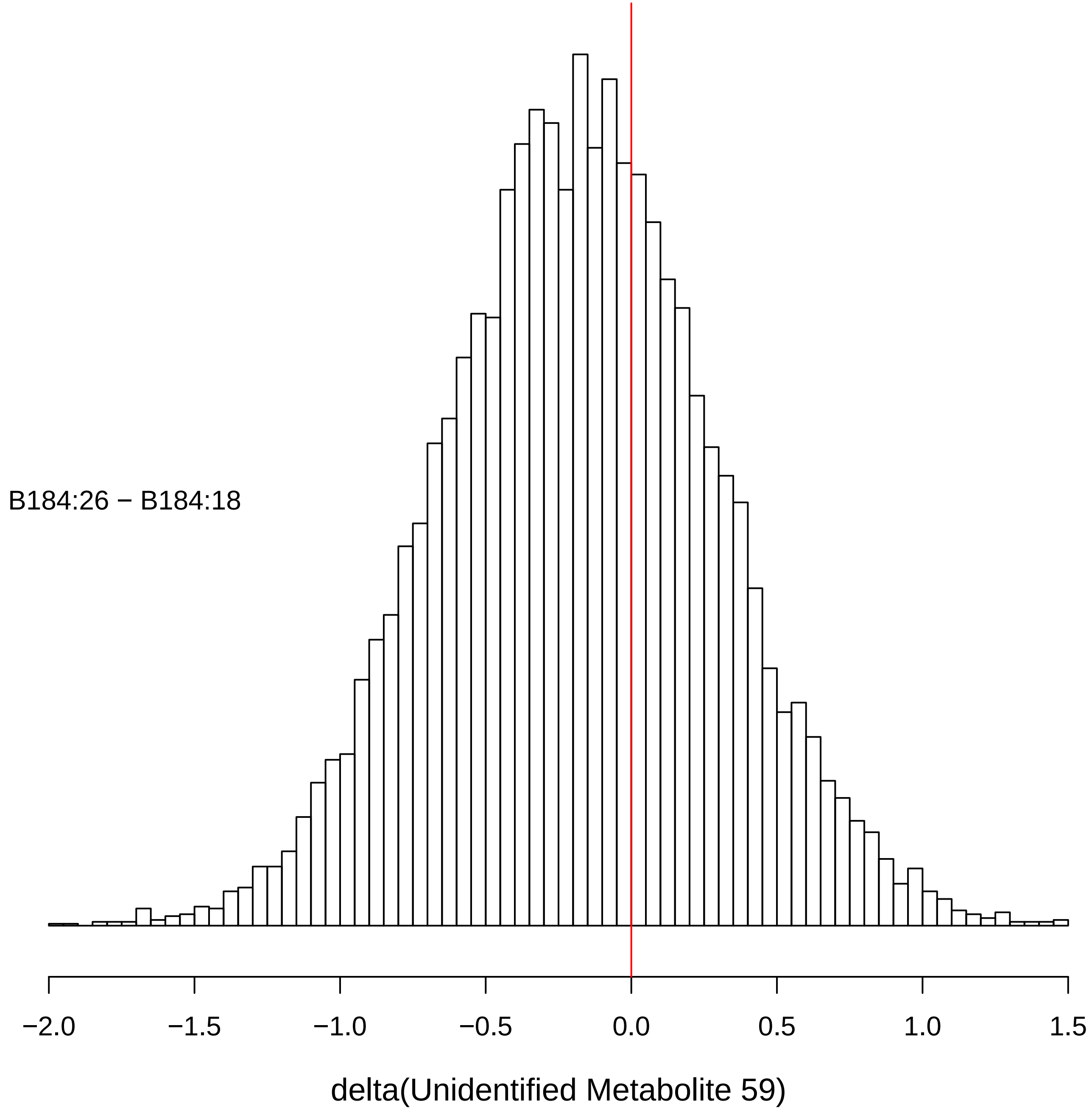
B184:26 − B184:18
−2.0
−1.5
−1.0
−0.5
0.0
0.5
1.0
1.5
delta(Unidentified Metabolite 59)

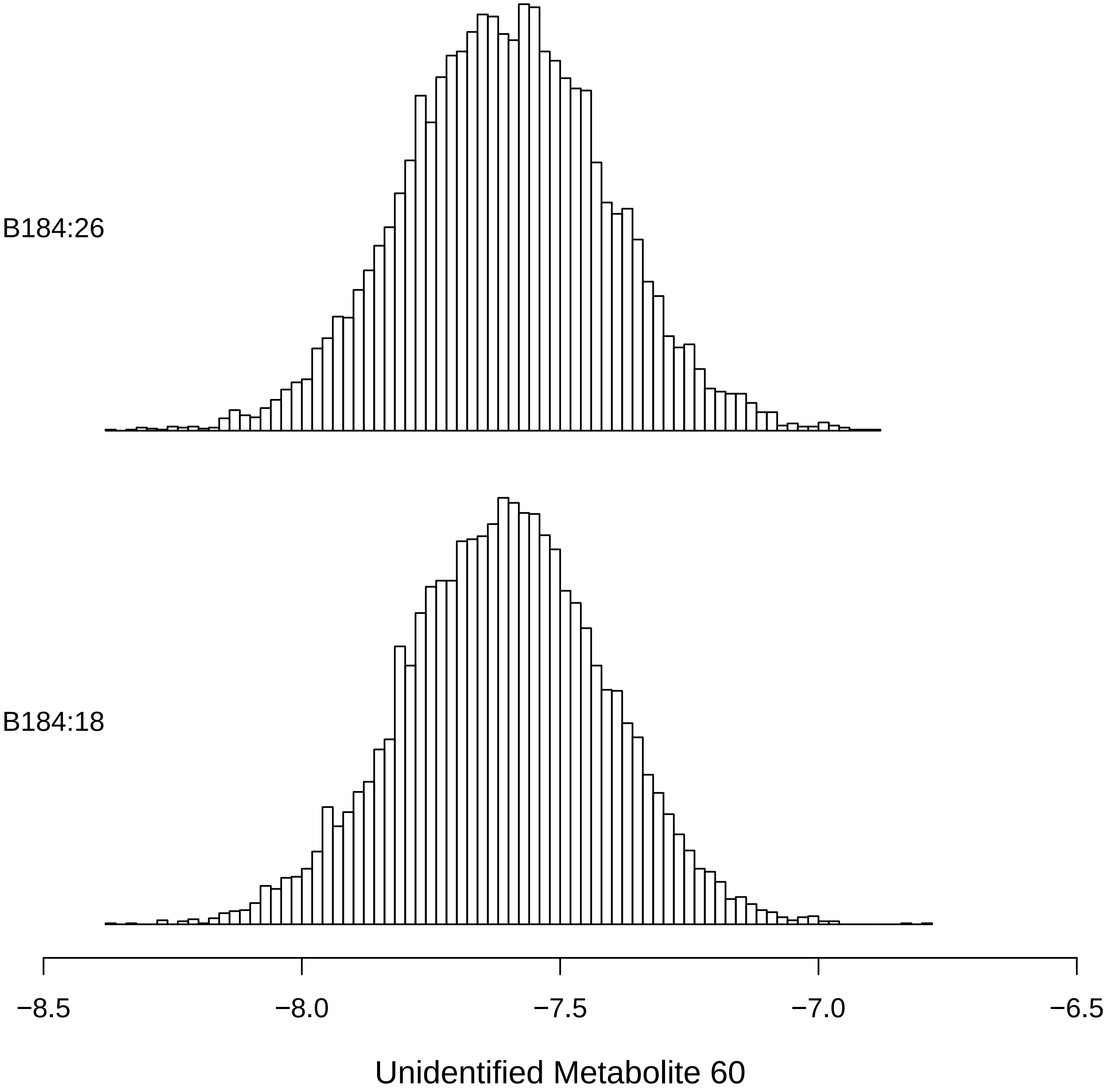
B184:26
B184:18
−8.5
−8.0
−7.5
−7.0
−6.5
Unidentified Metabolite 60

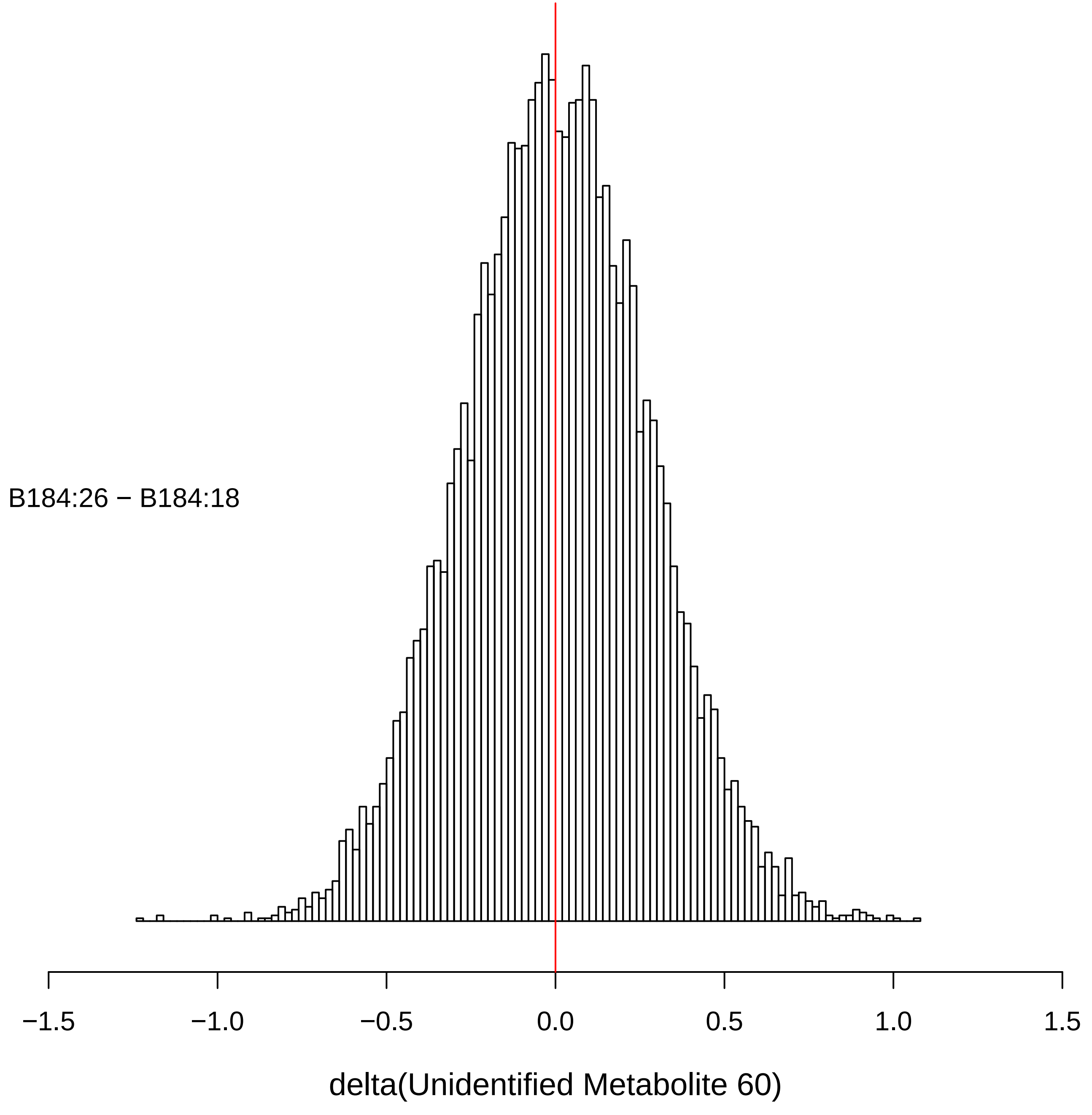
B184:26 − B184:18
−1.5
−1.0
−0.5
0.0
0.5
1.0
1.5
delta(Unidentified Metabolite 60)

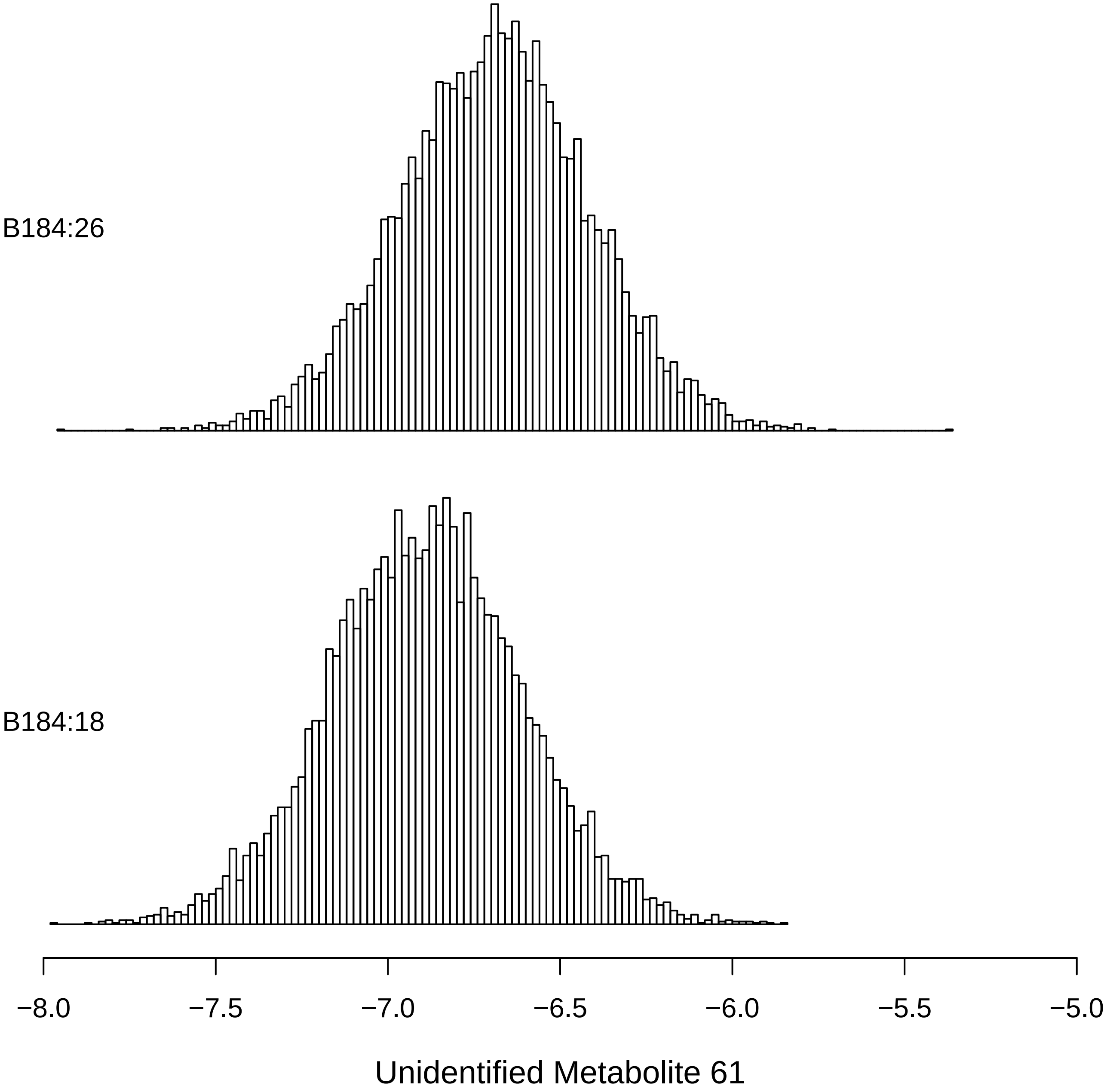

B184:26
B184:18
−8.0
−7.5
−7.0
−6.5
−6.0
−5.5
−5.0
Unidentified Metabolite 61

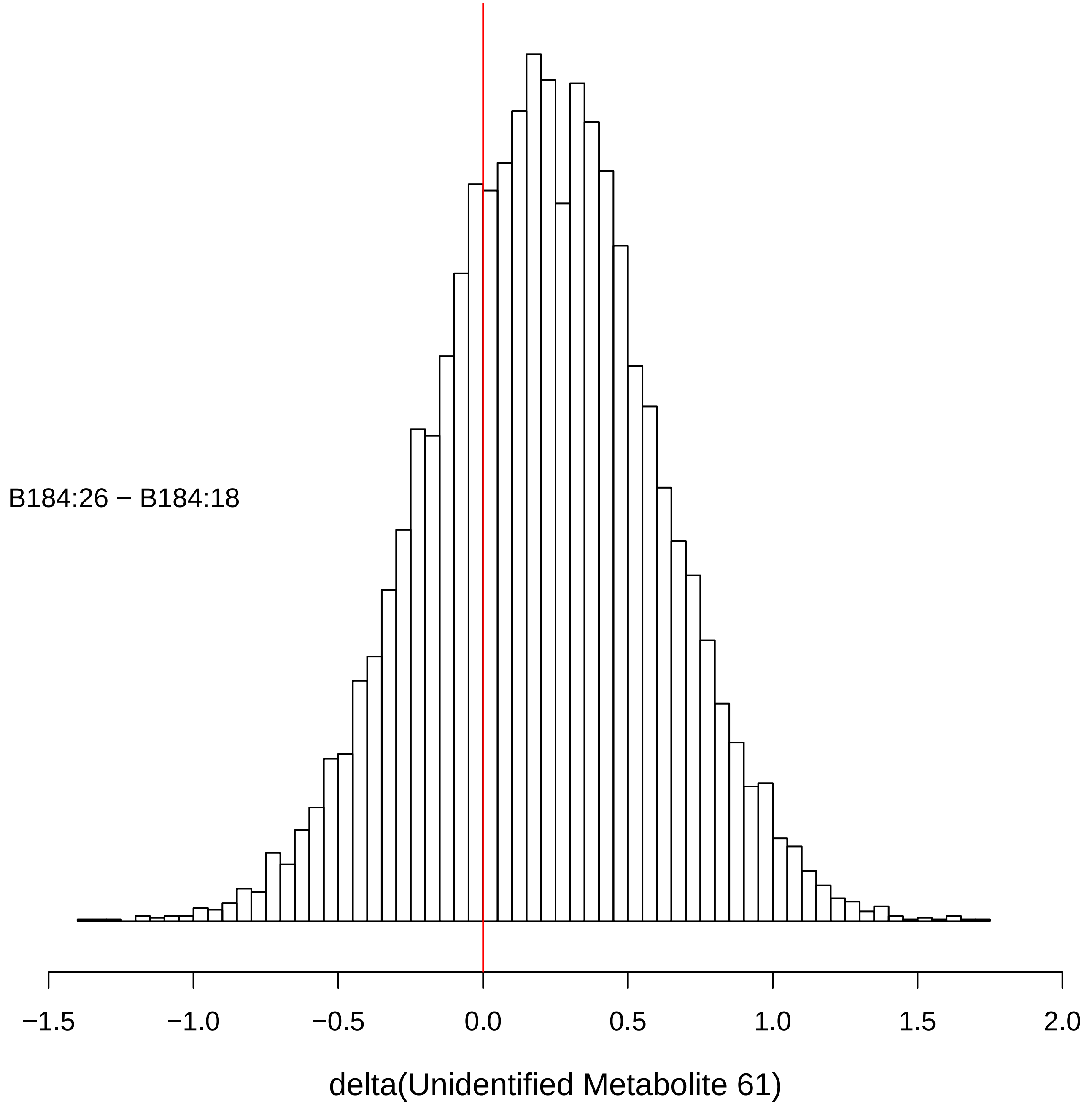

B184:26 − B184:18
−1.5
−1.0
−0.5
0.0
0.5
1.0
1.5
2.0
delta(Unidentified Metabolite 61)

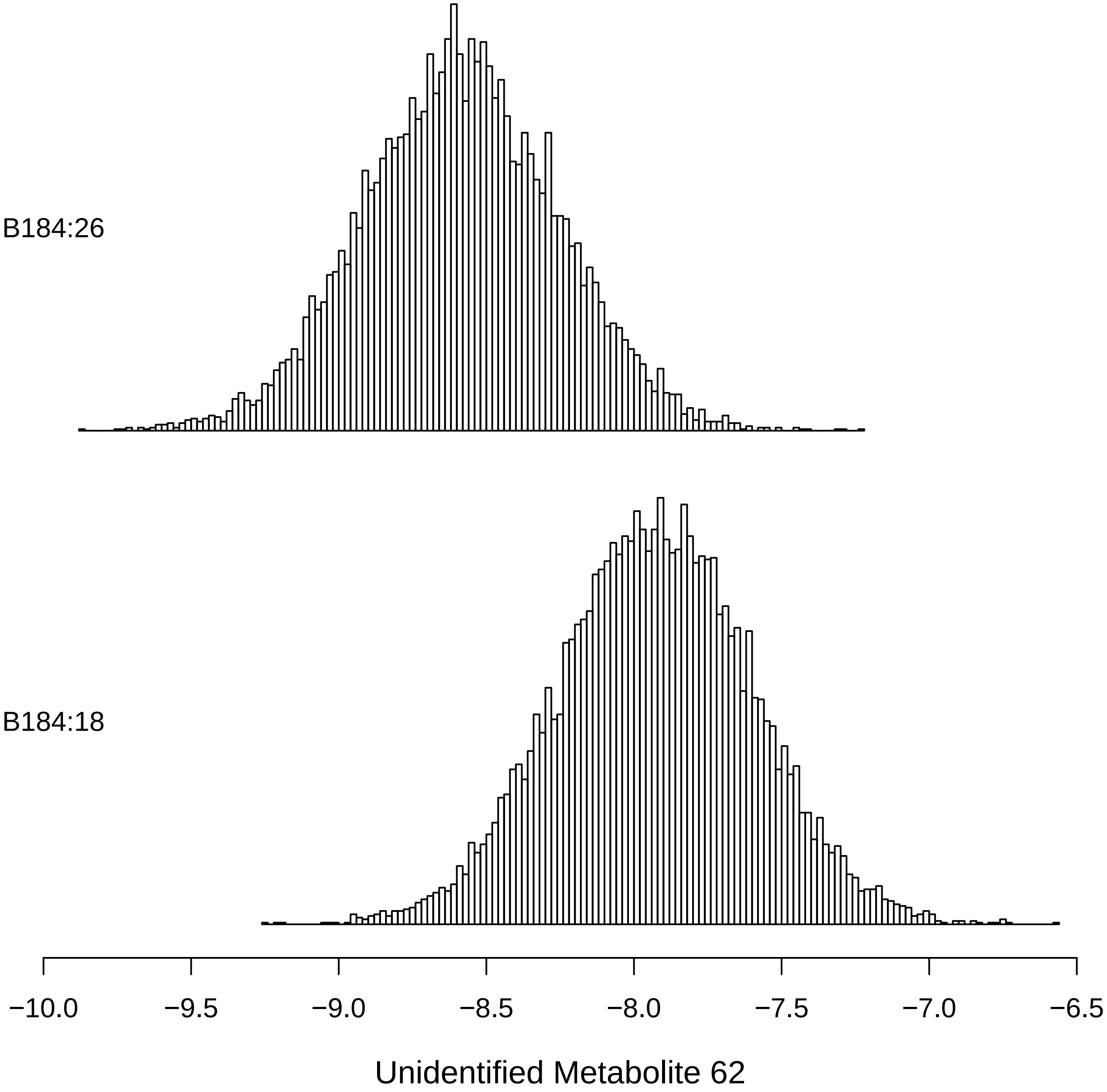
B184:26
B184:18
−10.0
−9.5
−9.0
−8.5
−8.0
−7.5
−7.0
−6.5
Unidentified Metabolite 62

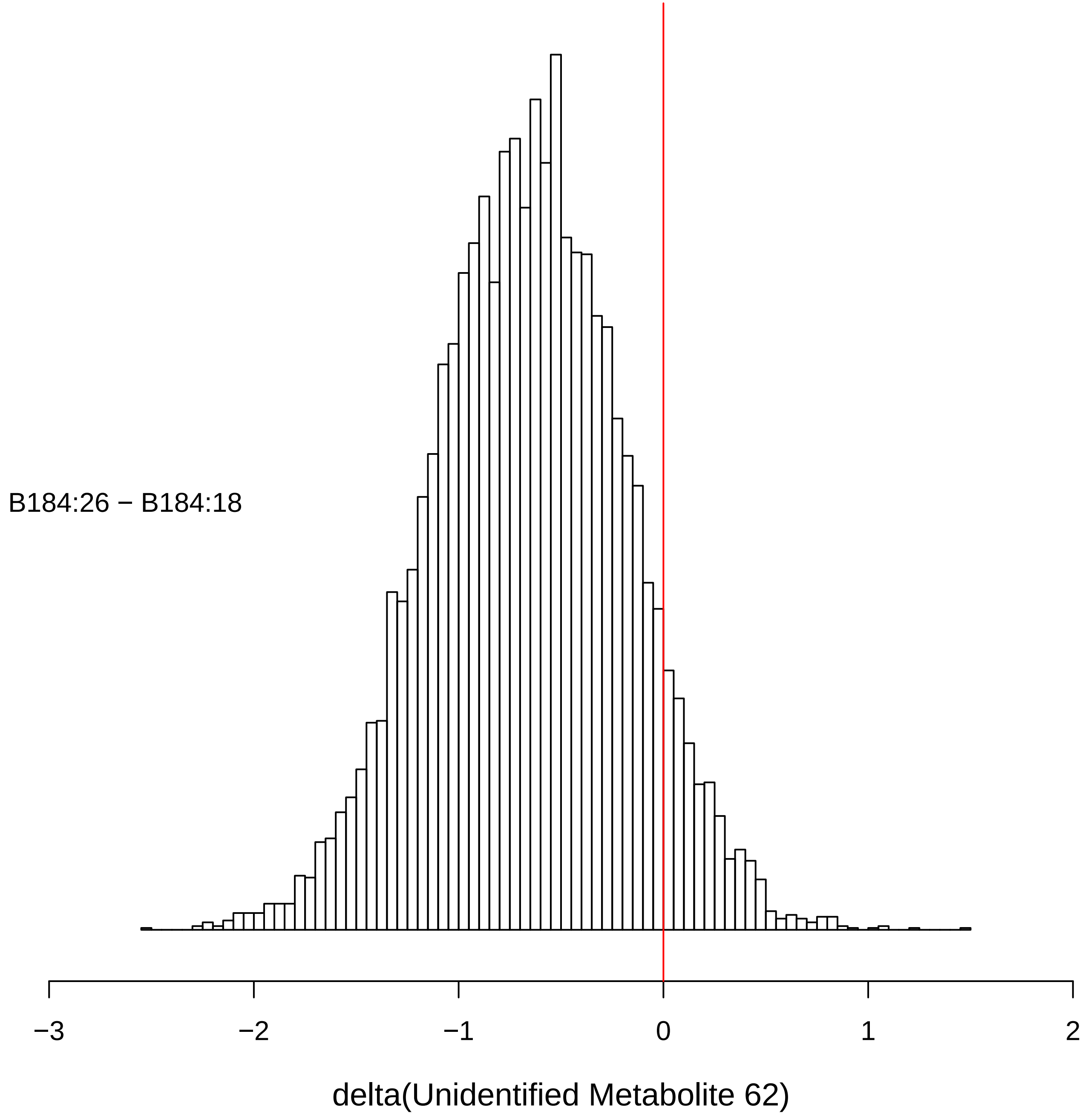

B184:26 − B184:18
−3
−2
−1
0
1
2
delta(Unidentified Metabolite 62)

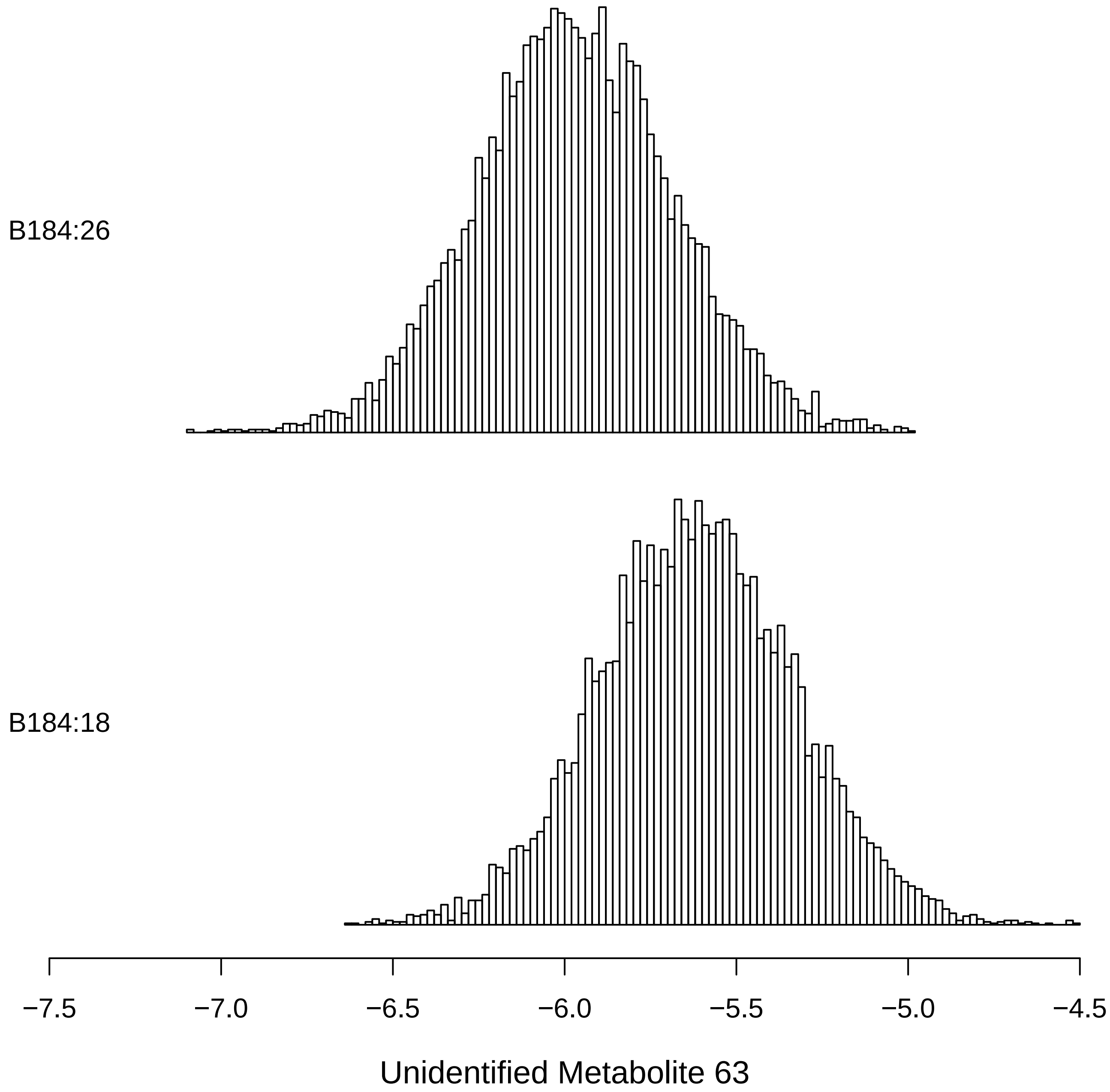
B184:26
B184:18
−7.5
−7.0
−6.5
−6.0
−5.5
−5.0
−4.5
Unidentified Metabolite 63

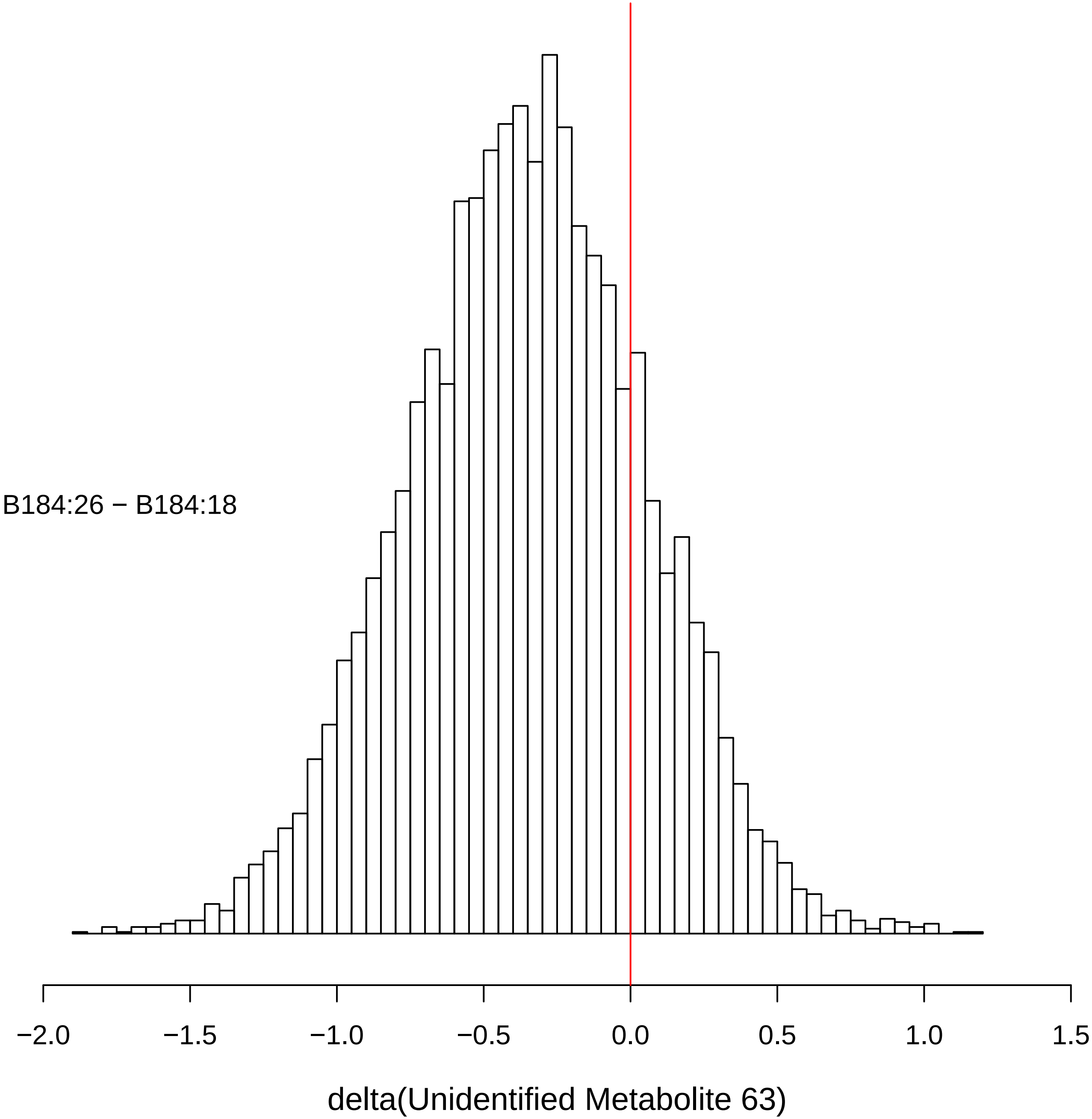
B184:26 − B184:18
−2.0
−1.5
−1.0
−0.5
0.0
0.5
1.0
1.5
delta(Unidentified Metabolite 63)

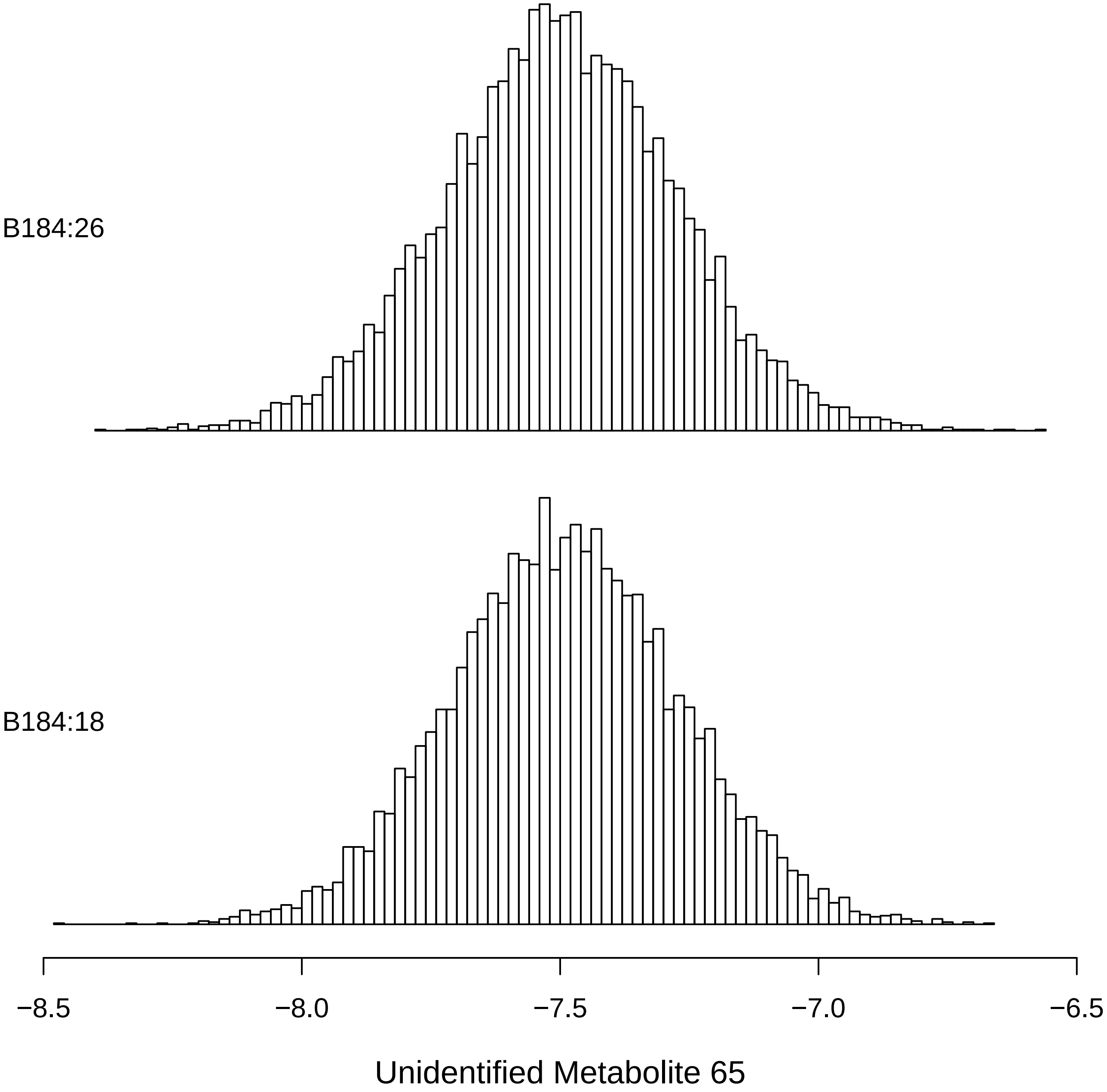
B184:26
B184:18
−8.5
−8.0
−7.5
−7.0
−6.5
Unidentified Metabolite 65

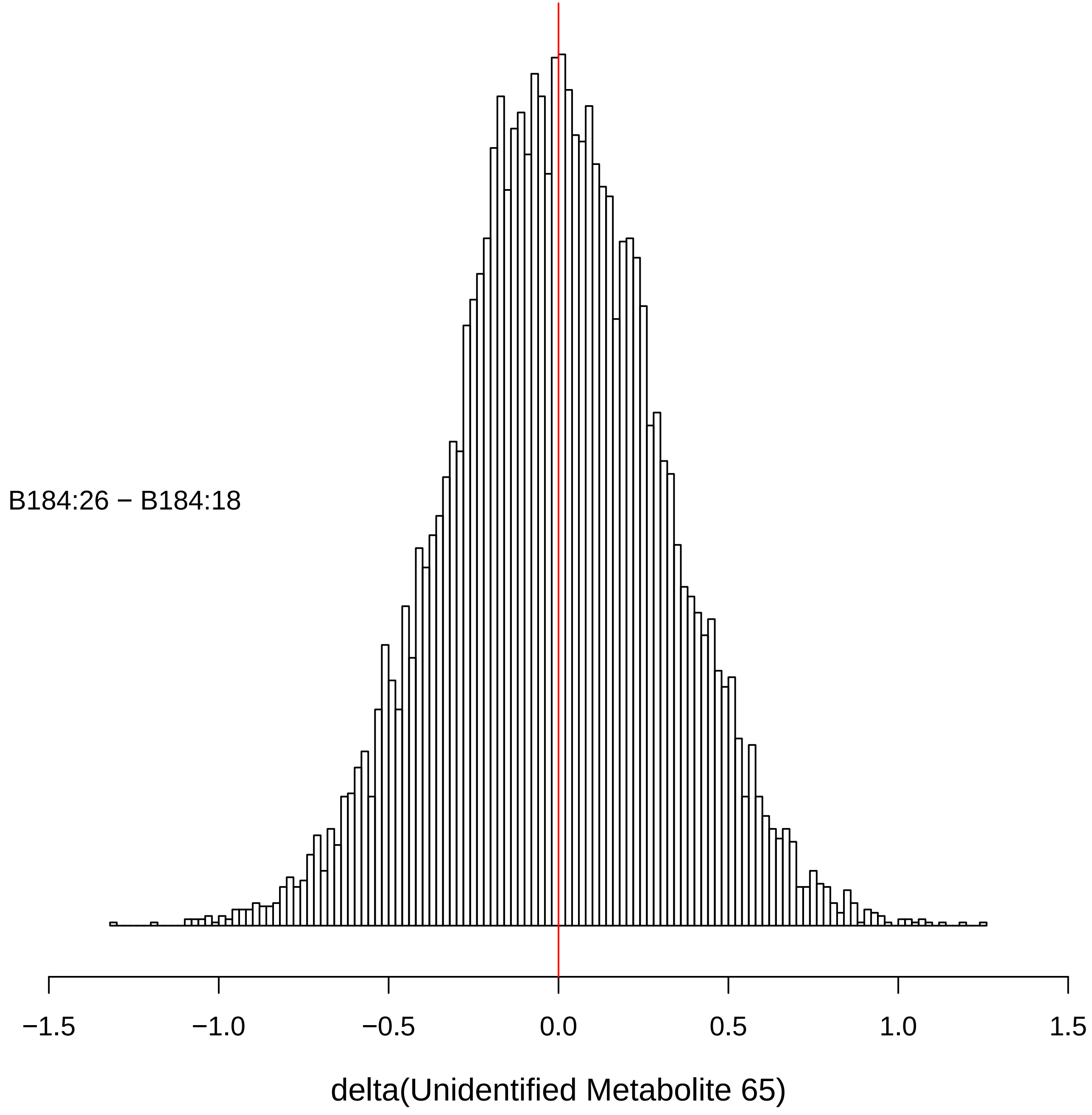
B184:26 − B184:18
−1.5
−1.0
−0.5
0.0
0.5
1.0
1.5
delta(Unidentified Metabolite 65)

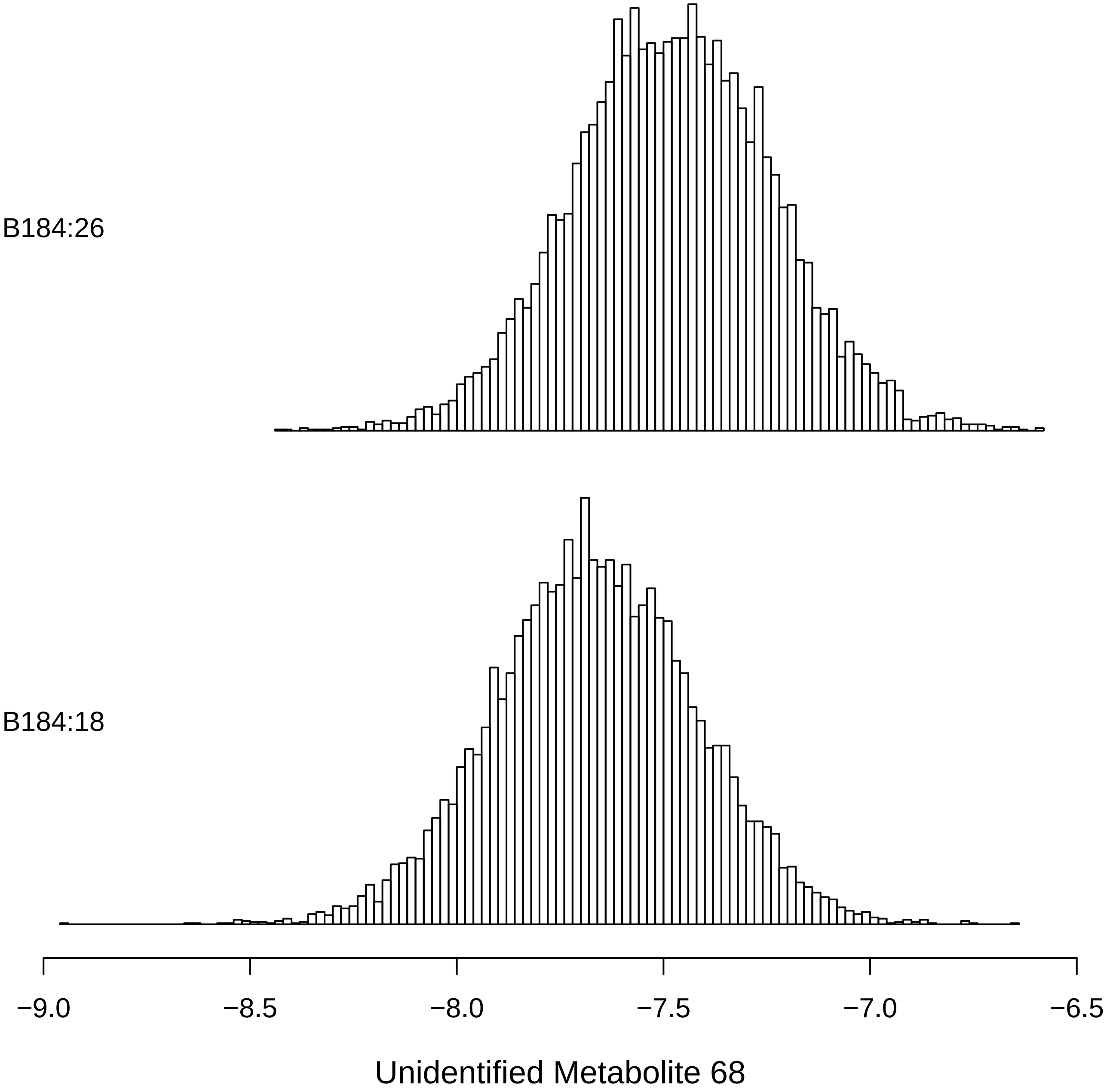
B184:26
B184:18
−9.0
−8.5
−8.0
−7.5
−7.0
−6.5
Unidentified Metabolite 68

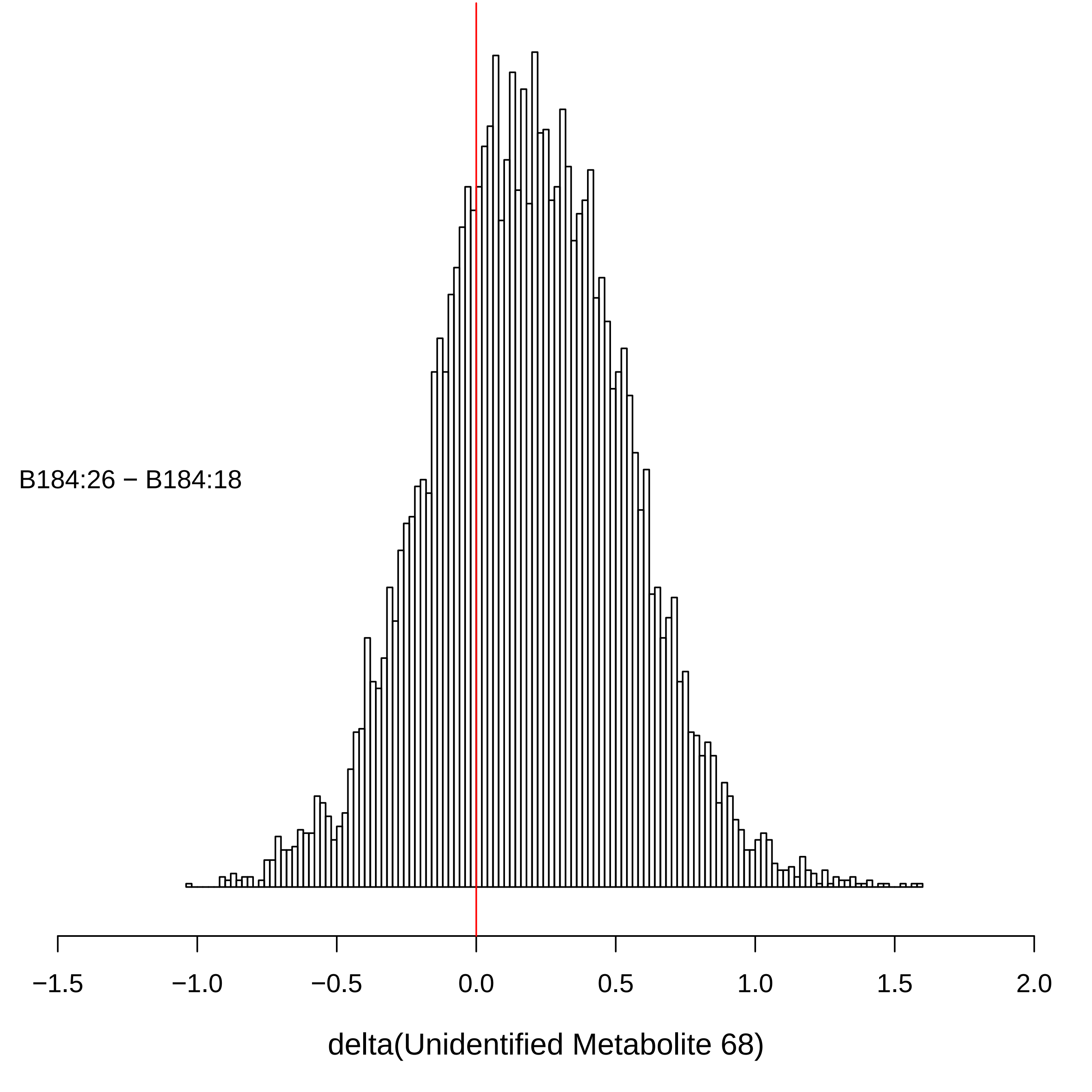
B184:26 − B184:18
−1.5
−1.0
−0.5
0.0
0.5
1.0
1.5
2.0
delta(Unidentified Metabolite 68)

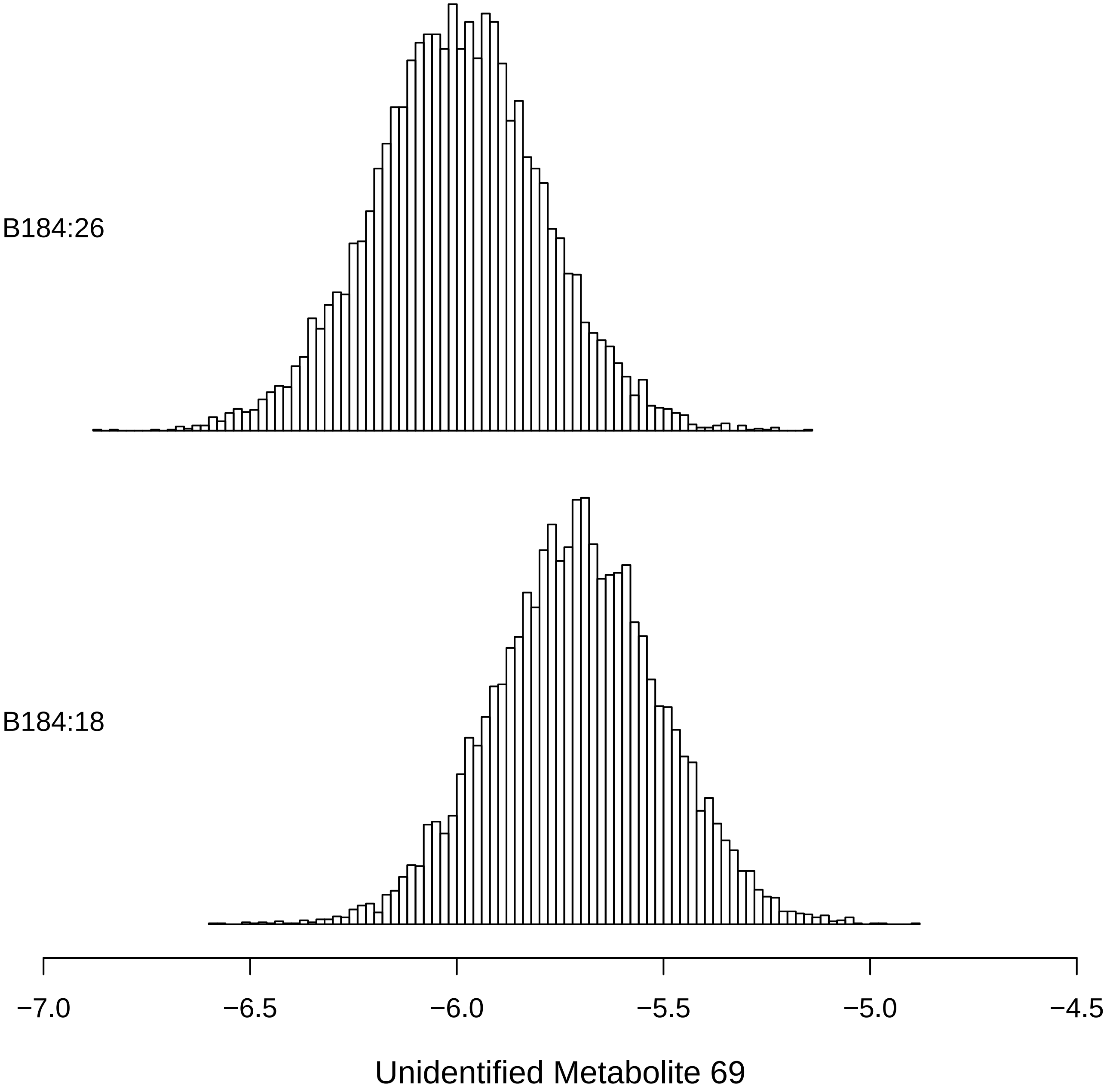
B184:26
B184:18
−7.0
−6.5
−6.0
−5.5
−5.0
−4.5
Unidentified Metabolite 69

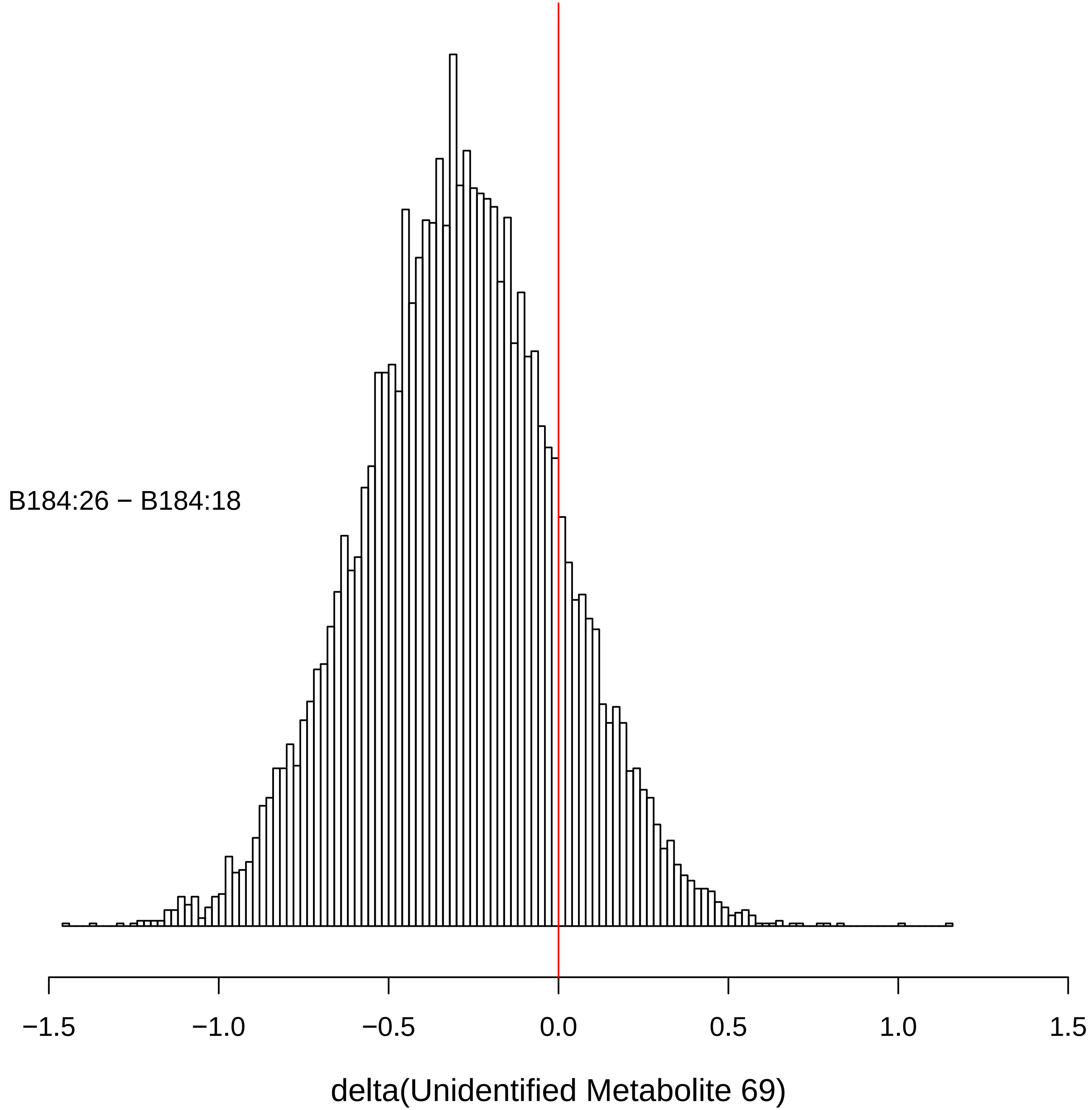
B184:26 − B184:18
−1.5
−1.0
−0.5
0.0
0.5
1.0
1.5
delta(Unidentified Metabolite 69)

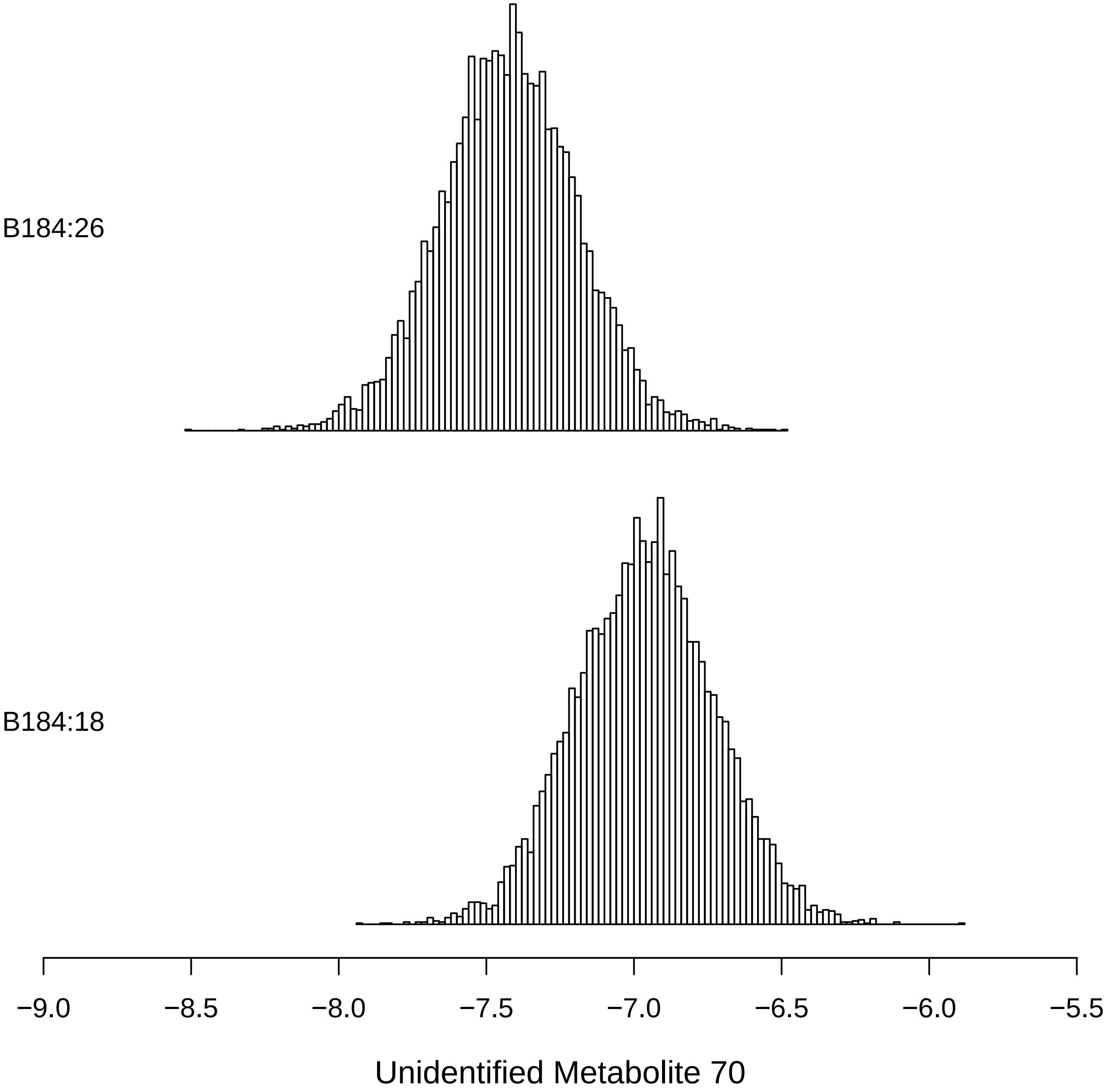
B184:26
B184:18
−9.0
−8.5
−8.0
−7.5
−7.0
−6.5
−6.0
−5.5
Unidentified Metabolite 70

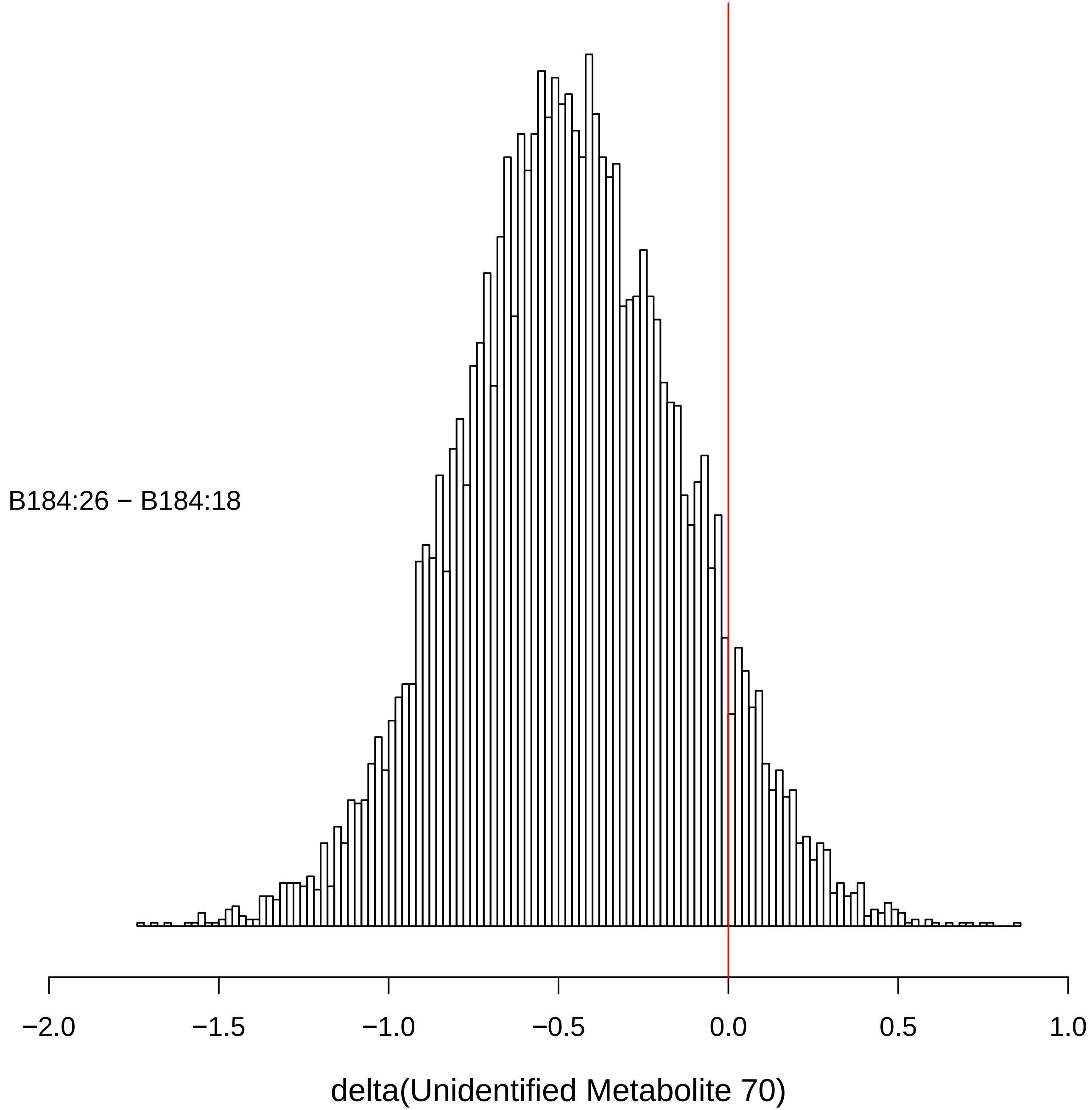
B184:26 − B184:18
−2.0
−1.5
−1.0
−0.5
0.0
0.5
1.0
delta(Unidentified Metabolite 70)

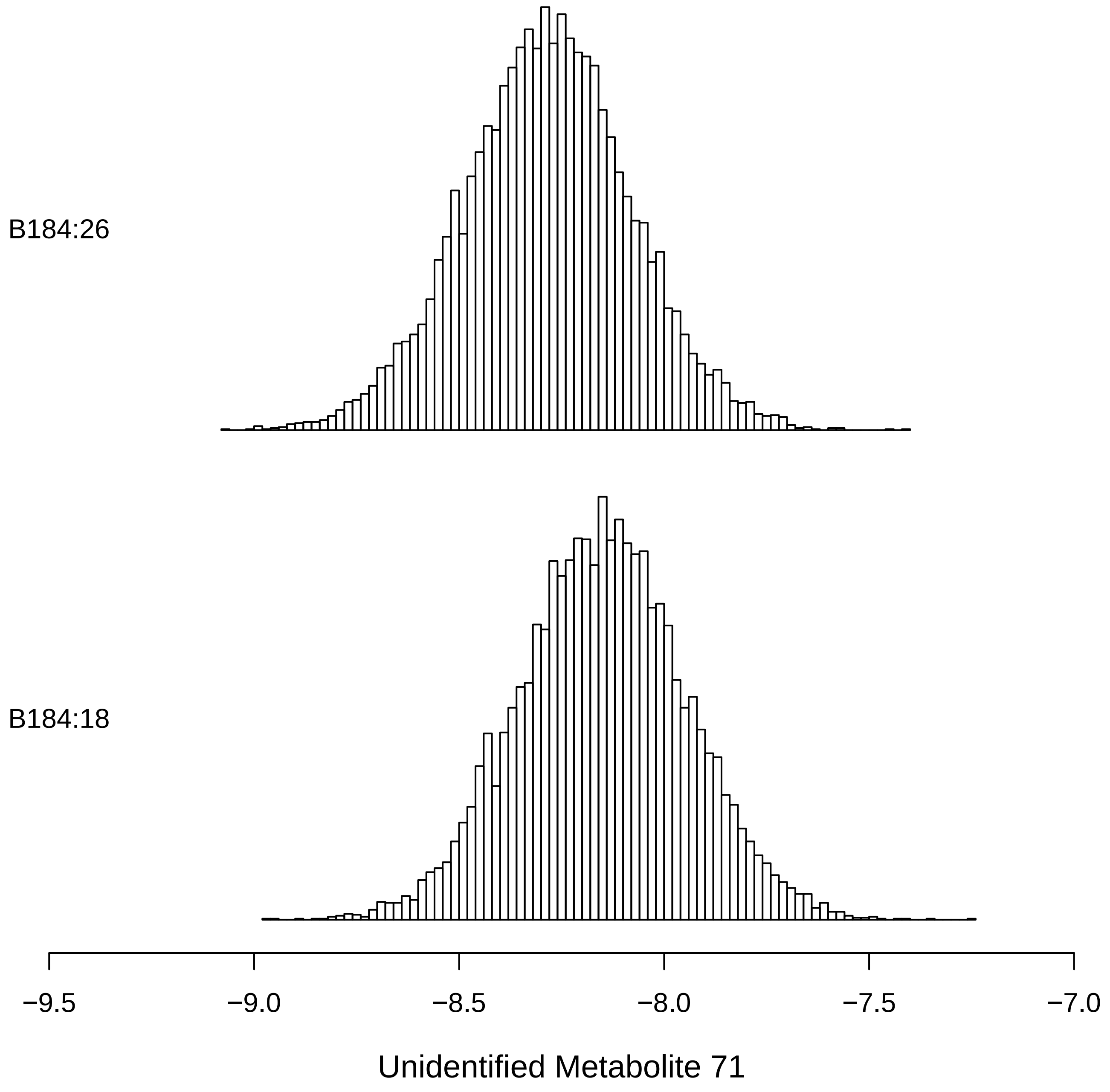
B184:26
B184:18
−9.5
−9.0
−8.5
−8.0
−7.5
−7.0
Unidentified Metabolite 71

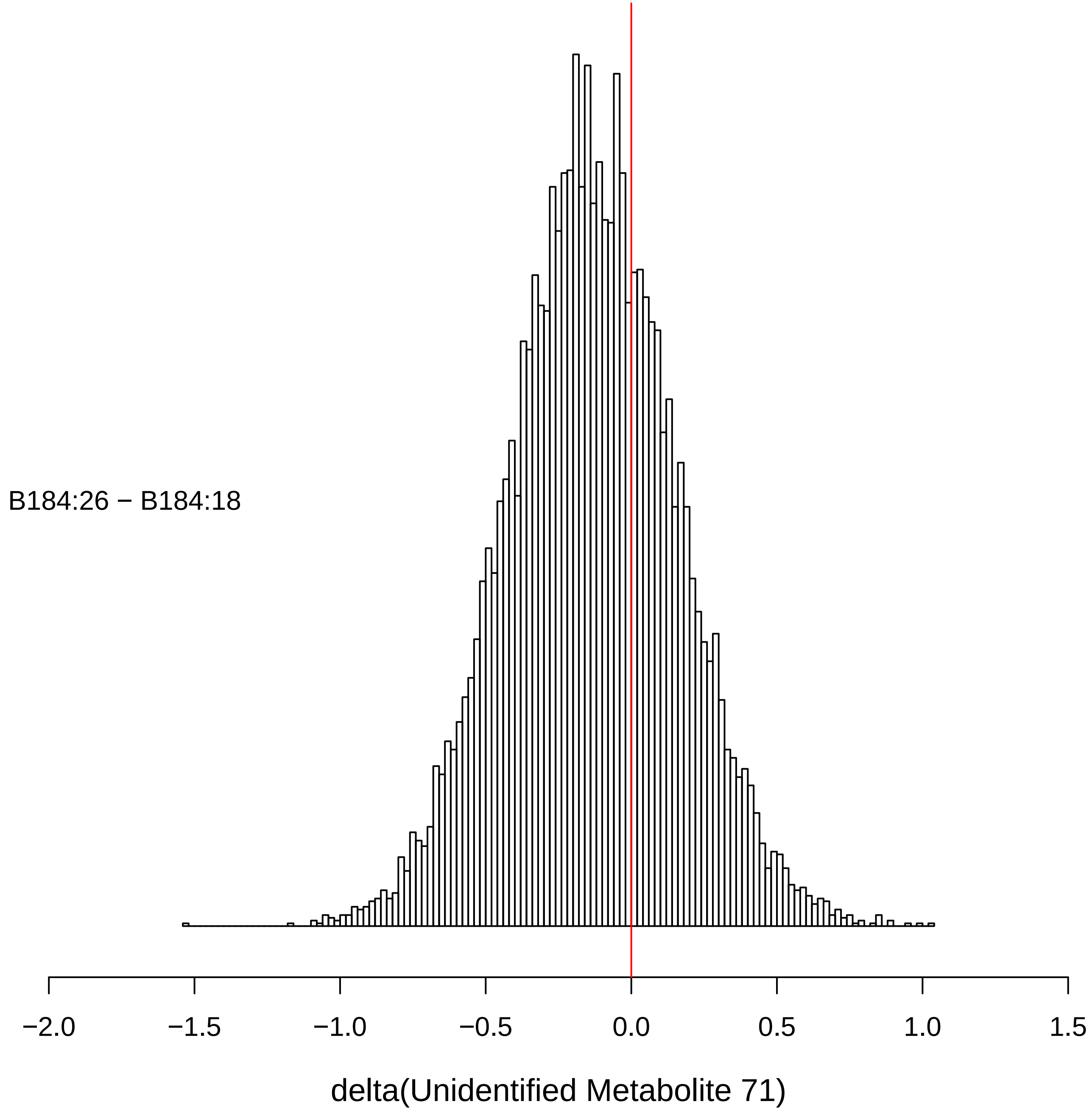
B184:26 − B184:18
−2.0
−1.5
−1.0
−0.5
0.0
0.5
1.0
1.5
delta(Unidentified Metabolite 71)

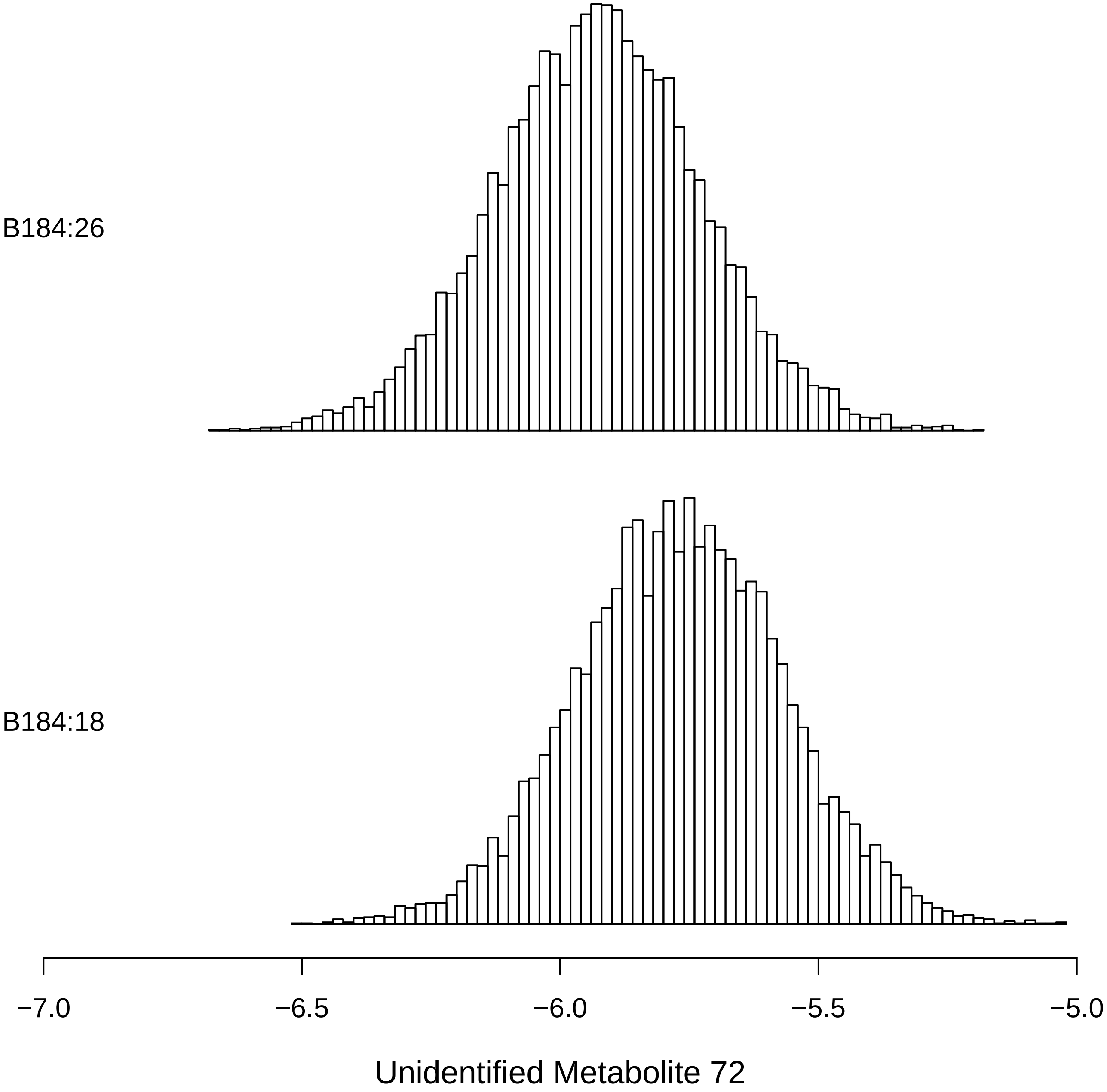
B184:26
B184:18
−7.0
−6.5
−6.0
−5.5
−5.0
Unidentified Metabolite 72

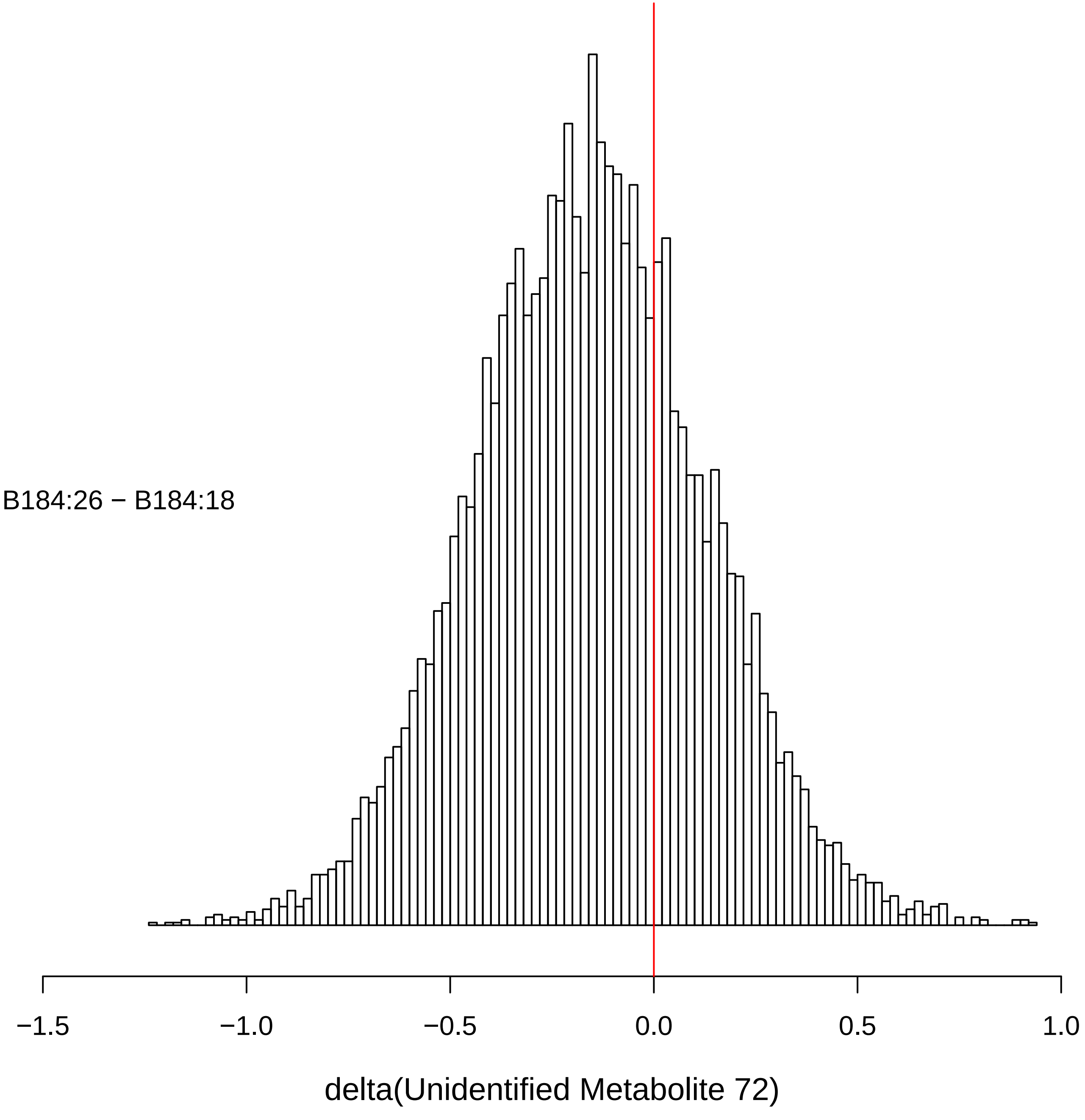

B184:26 − B184:18
−1.5
−1.0
−0.5
0.0
0.5
1.0
delta(Unidentified Metabolite 72)

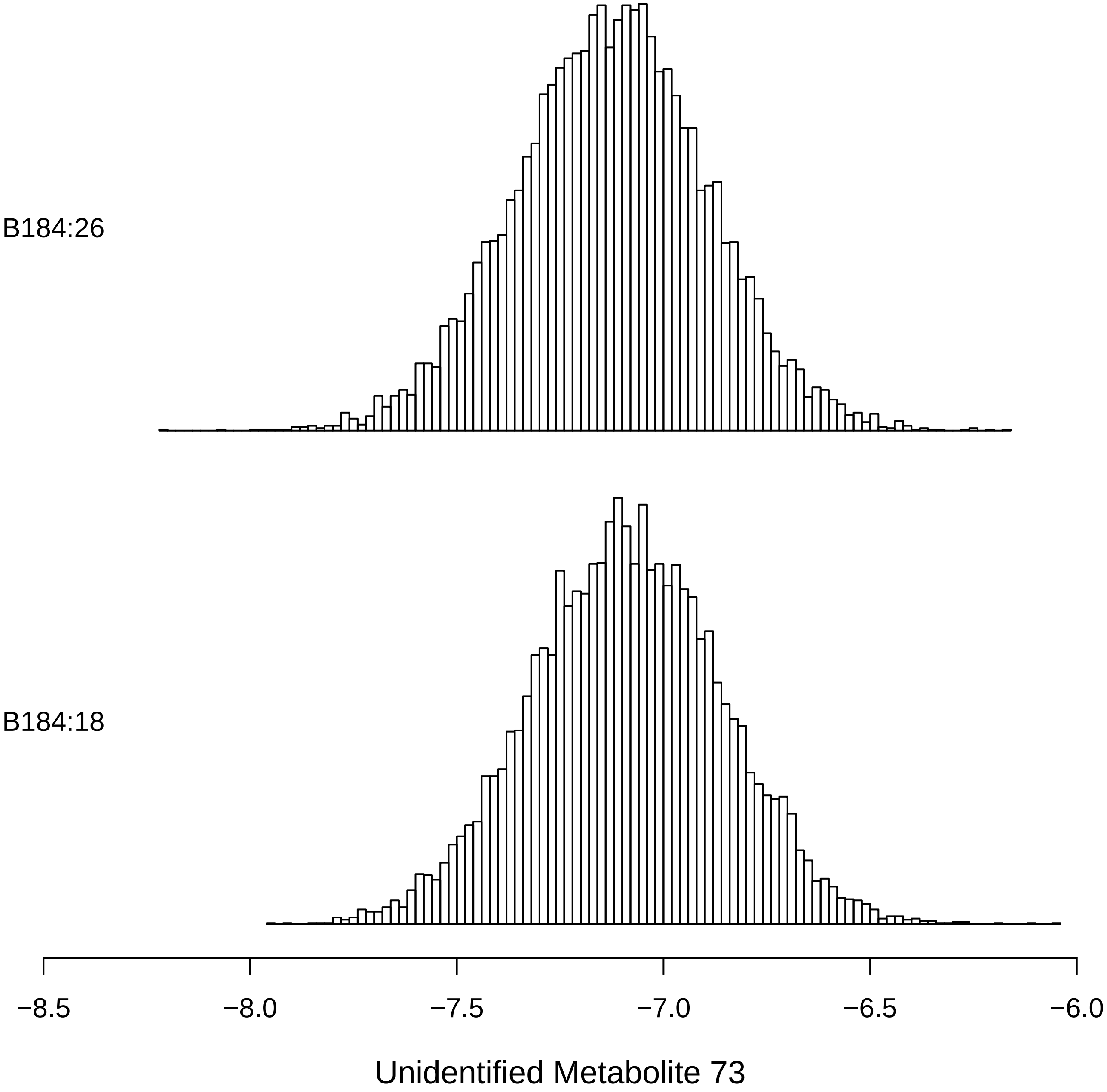
B184:26
B184:18
−8.5
−8.0
−7.5
−7.0
−6.5
−6.0
Unidentified Metabolite 73

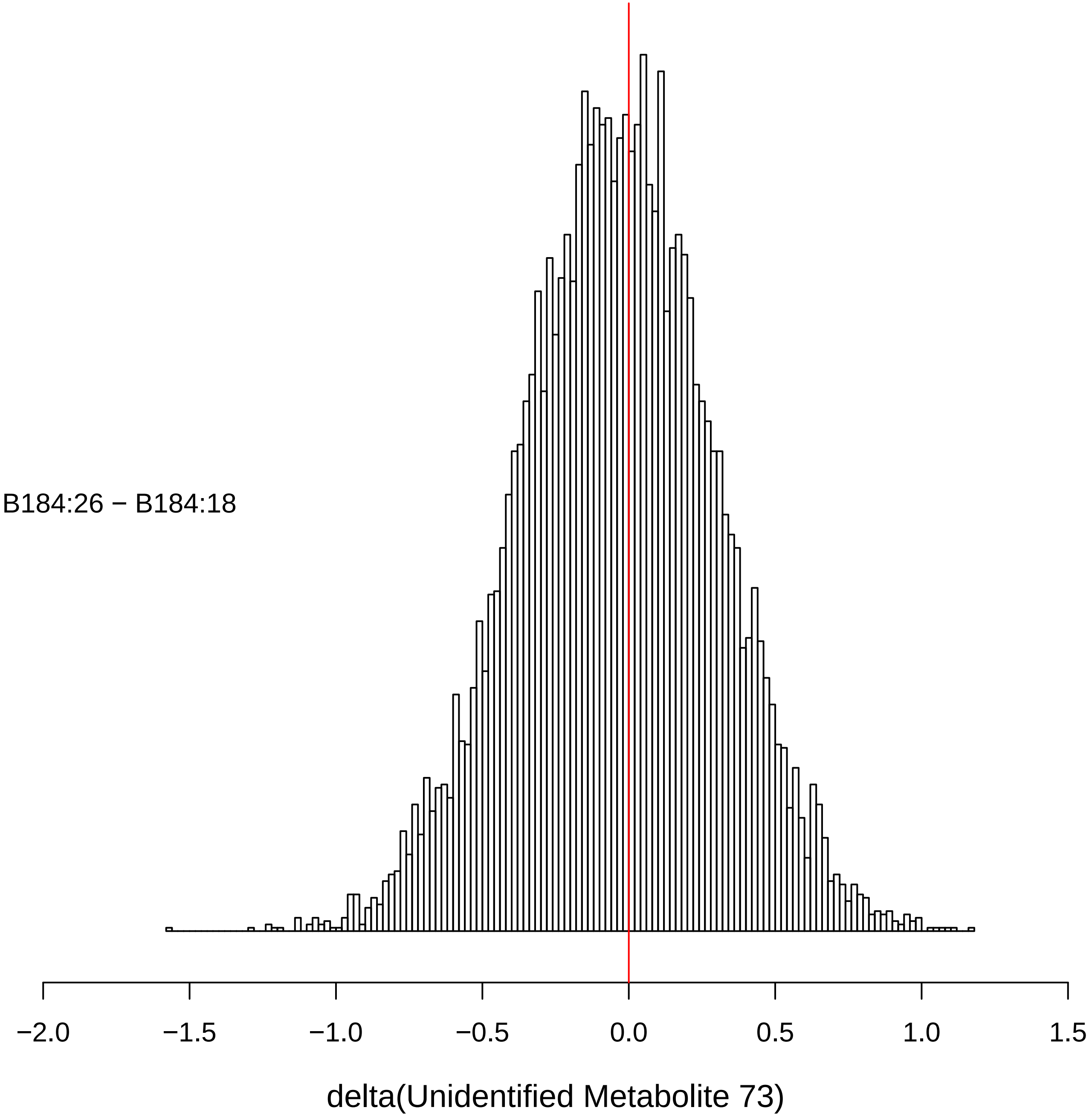
B184:26 − B184:18
−2.0
−1.5
−1.0
−0.5
0.0
0.5
1.0
1.5
delta(Unidentified Metabolite 73)

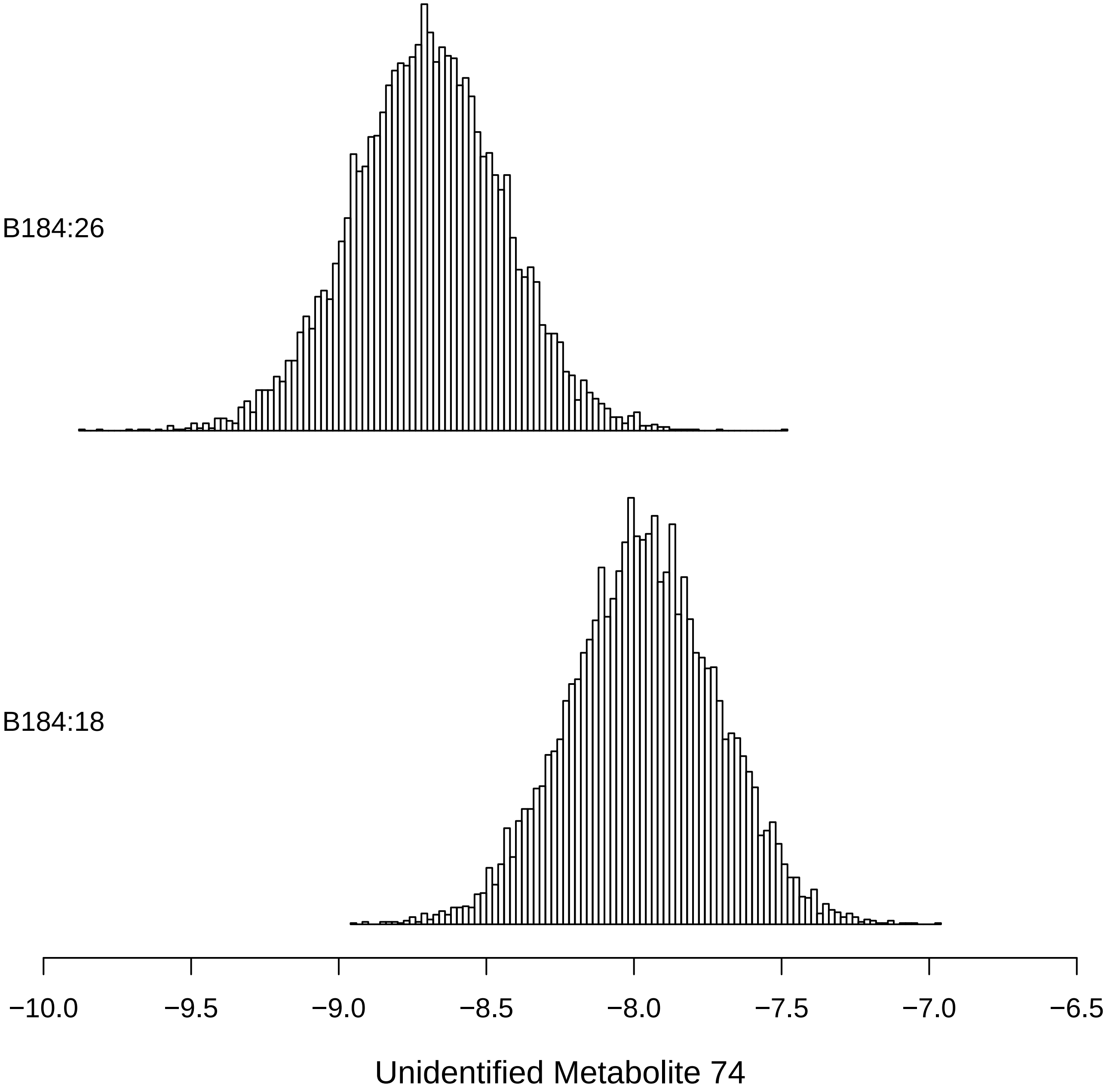

B184:26
B184:18
−10.0
−9.5
−9.0
−8.5
−8.0
−7.5
−7.0
−6.5
Unidentified Metabolite 74

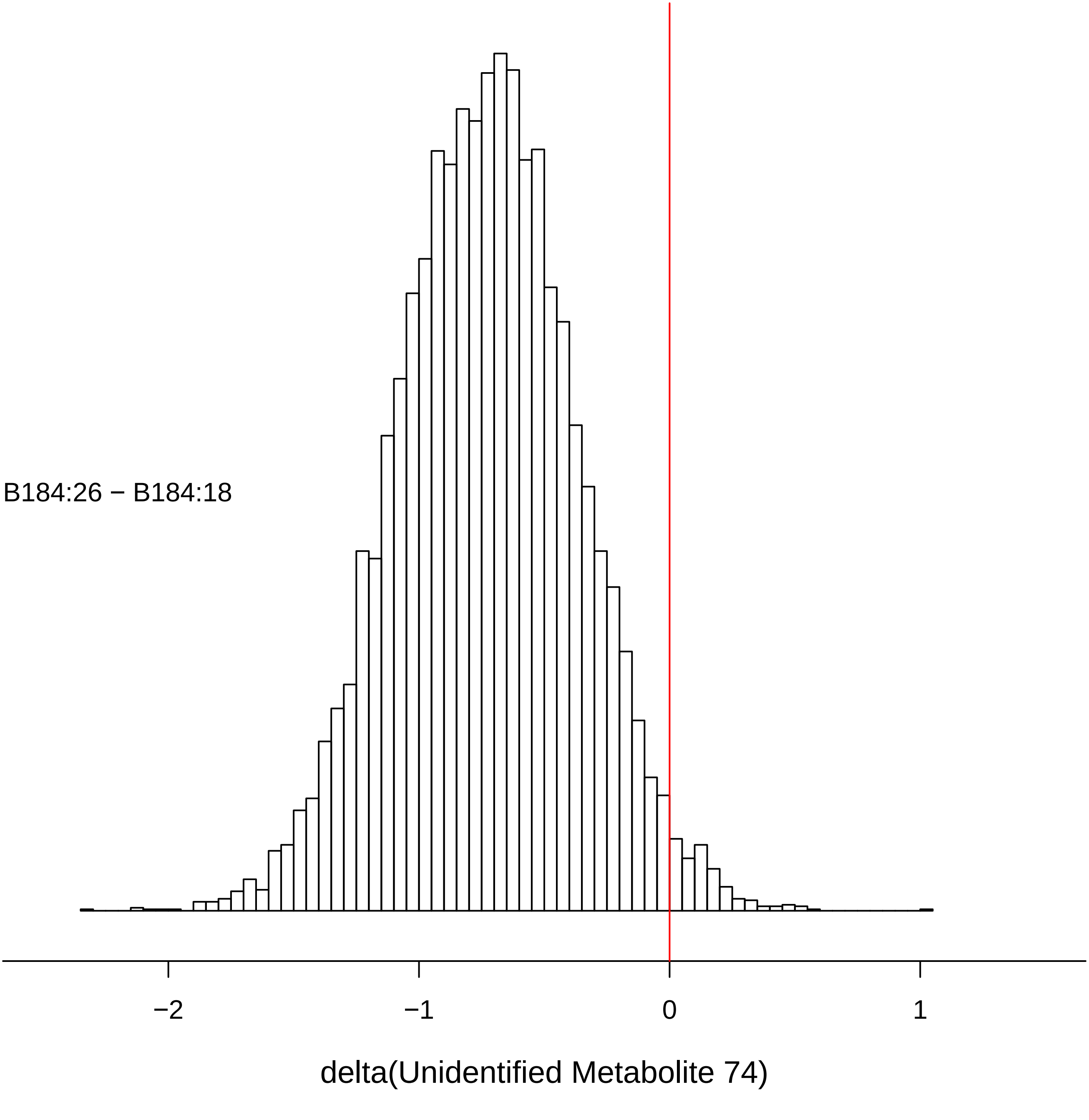

B184:26 − B184:18
−2
−1
0
1
delta(Unidentified Metabolite 74)

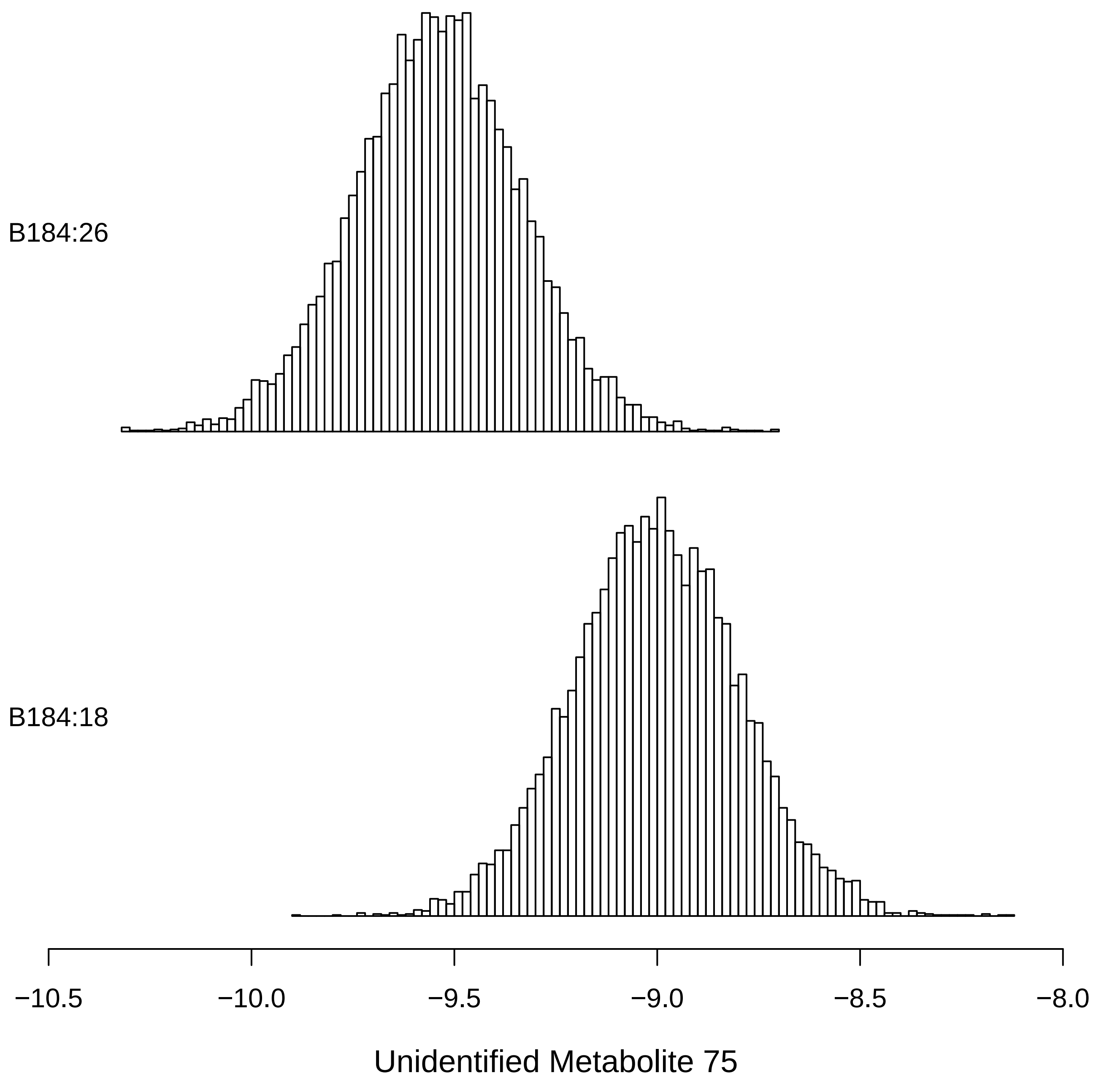
B184:26
B184:18
−10.5
−10.0
−9.5
−9.0
−8.5
−8.0
Unidentified Metabolite 75

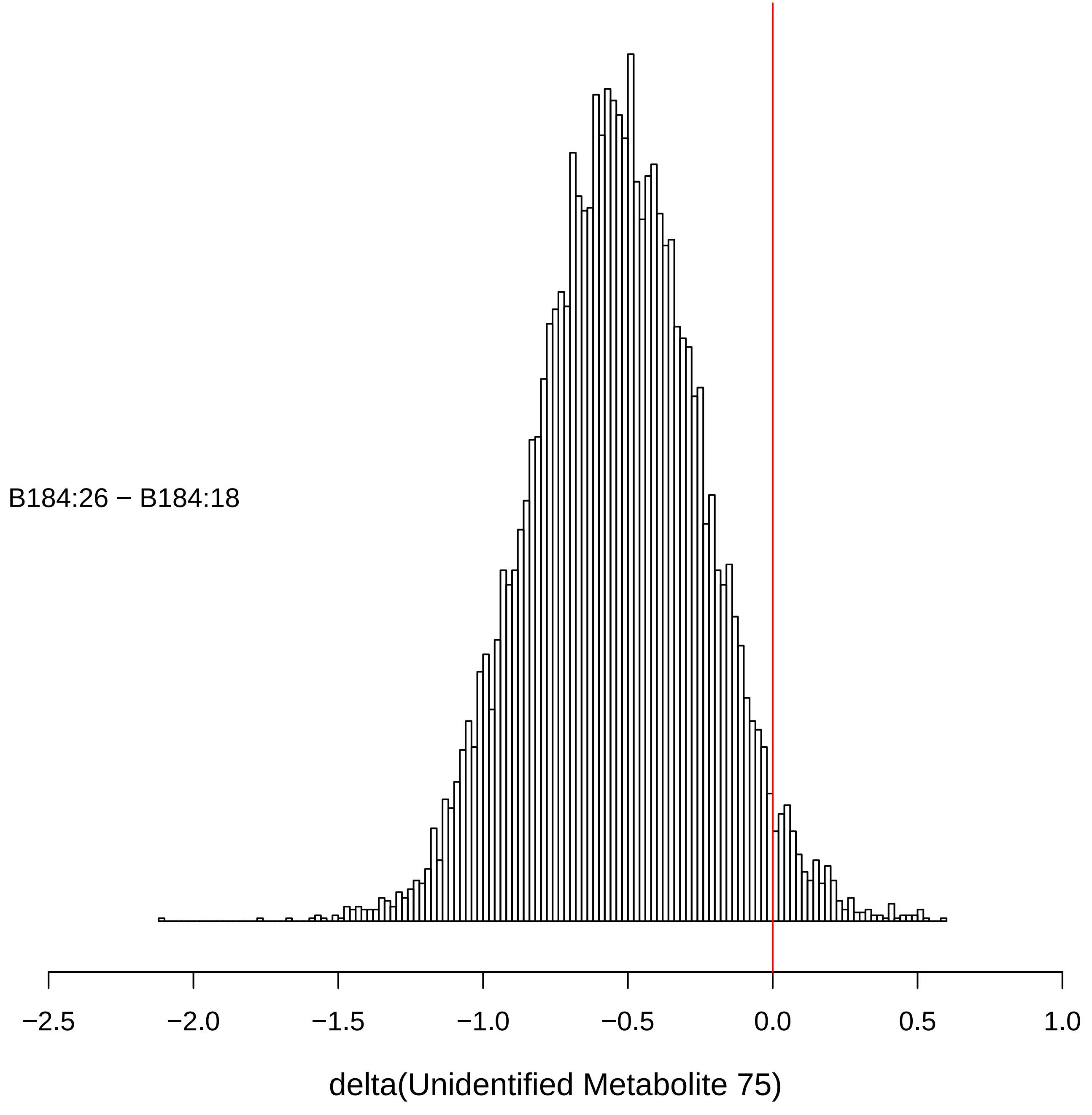
B184:26 − B184:18
−2.5
−2.0
−1.5
−1.0
−0.5
0.0
0.5
1.0
delta(Unidentified Metabolite 75)

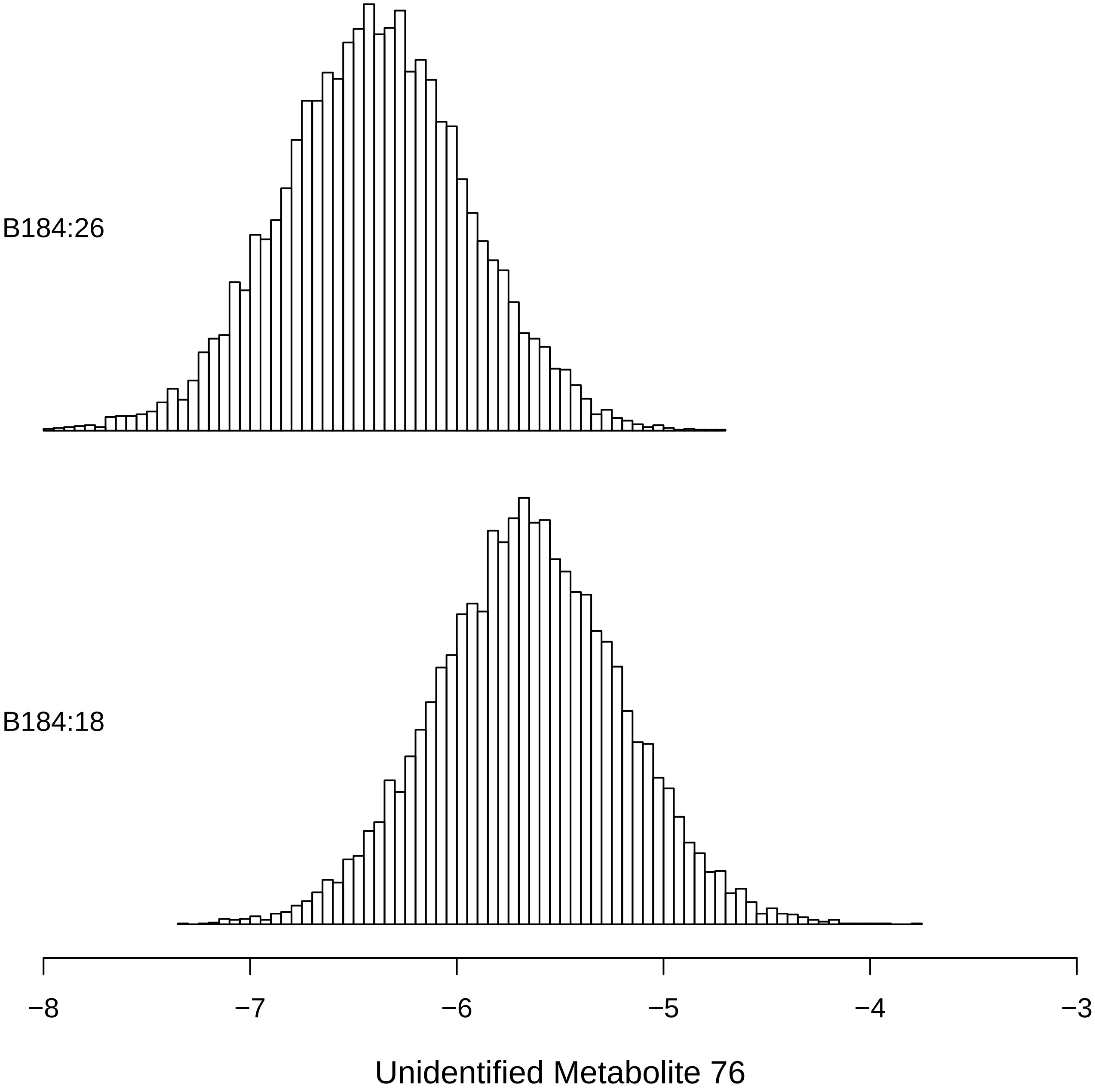
B184:26
B184:18
−8
−7
−6
−5
−4
−3
Unidentified Metabolite 76

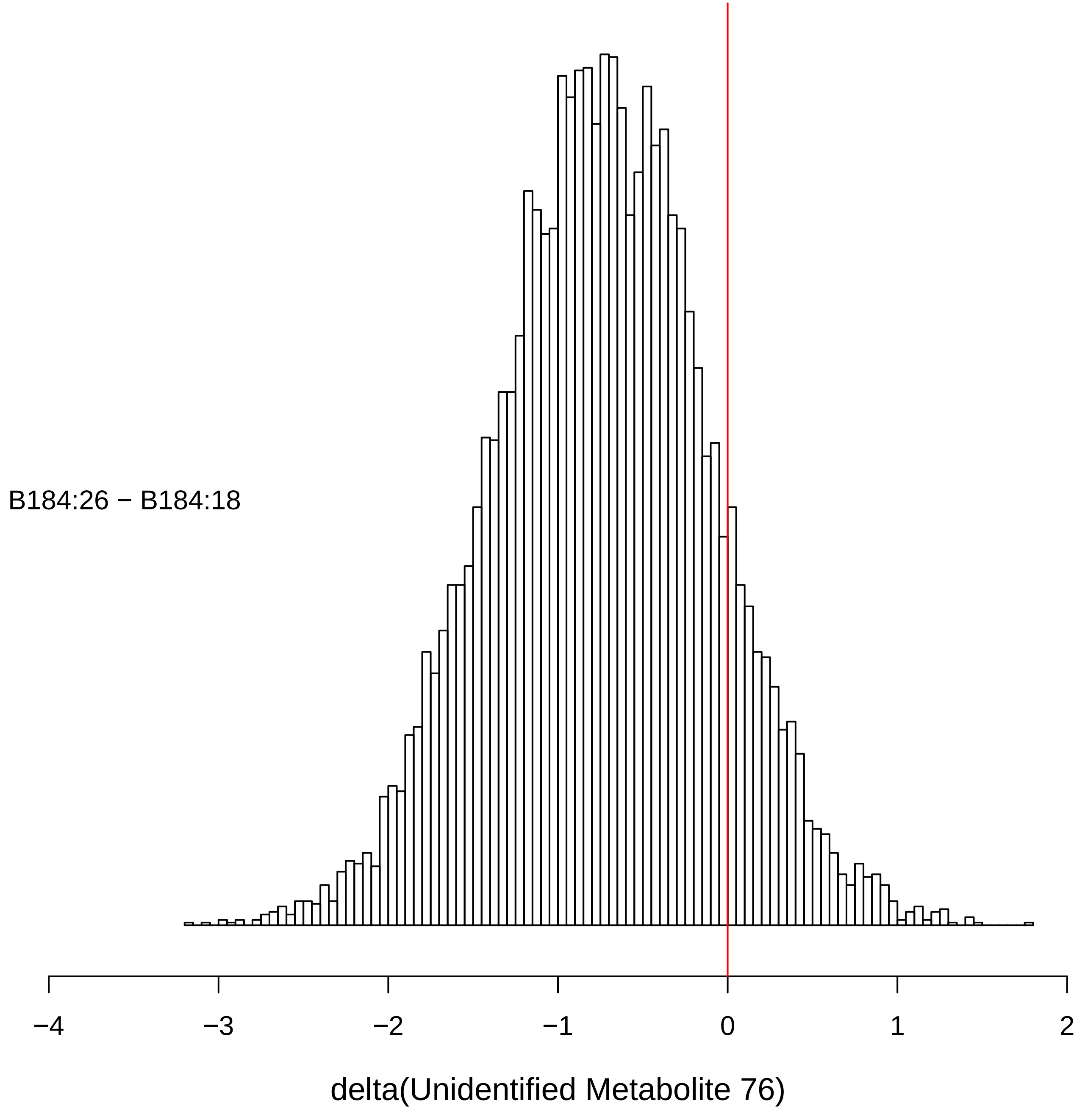
B184:26 − B184:18
−4
−3
−2
−1
0
1
2
delta(Unidentified Metabolite 76)

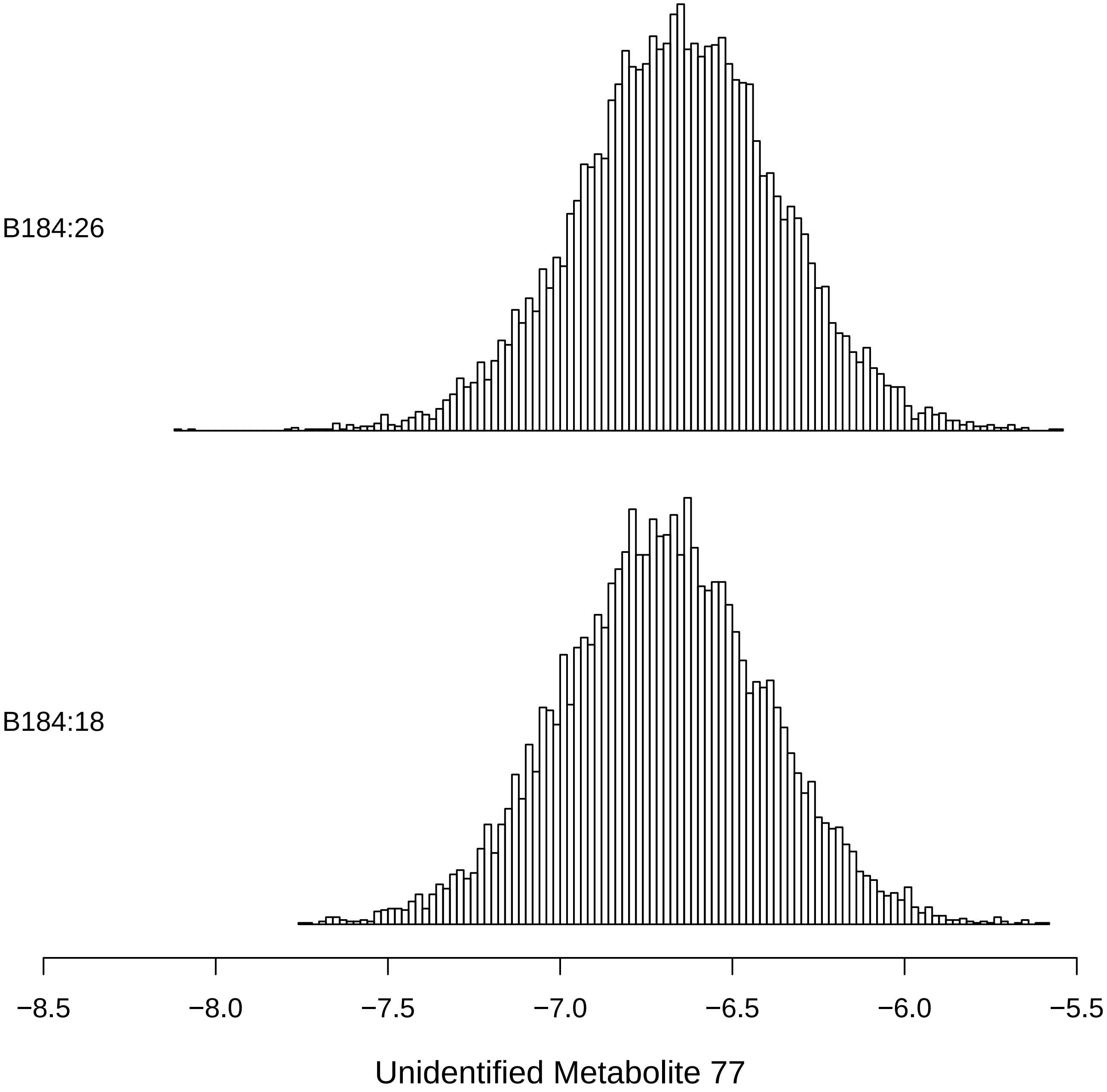

B184:26
B184:18
−8.5
−8.0
−7.5
−7.0
−6.5
−6.0
−5.5
Unidentified Metabolite 77

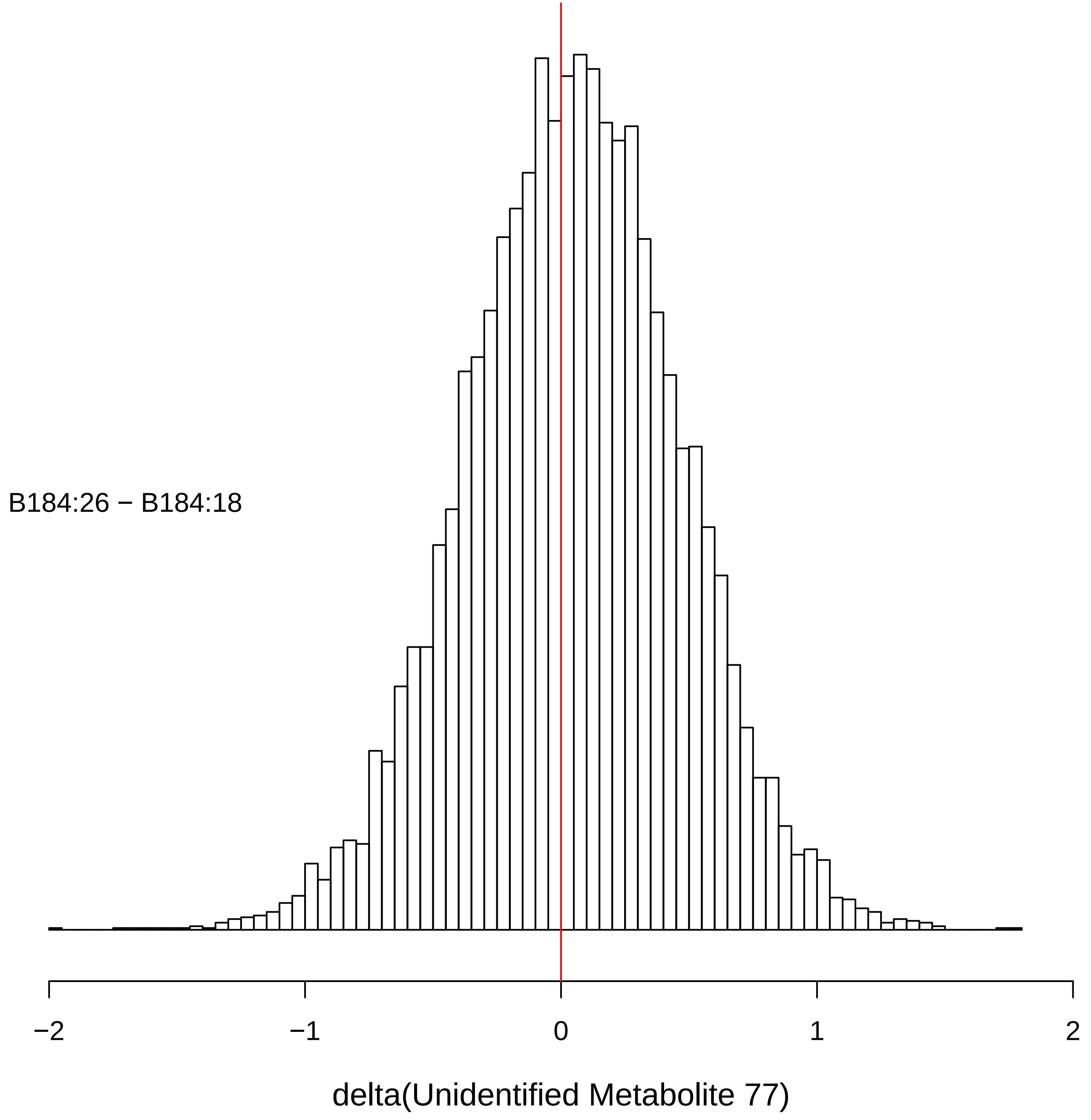
B184:26 − B184:18
−2
−1
0
1
2
delta(Unidentified Metabolite 77)

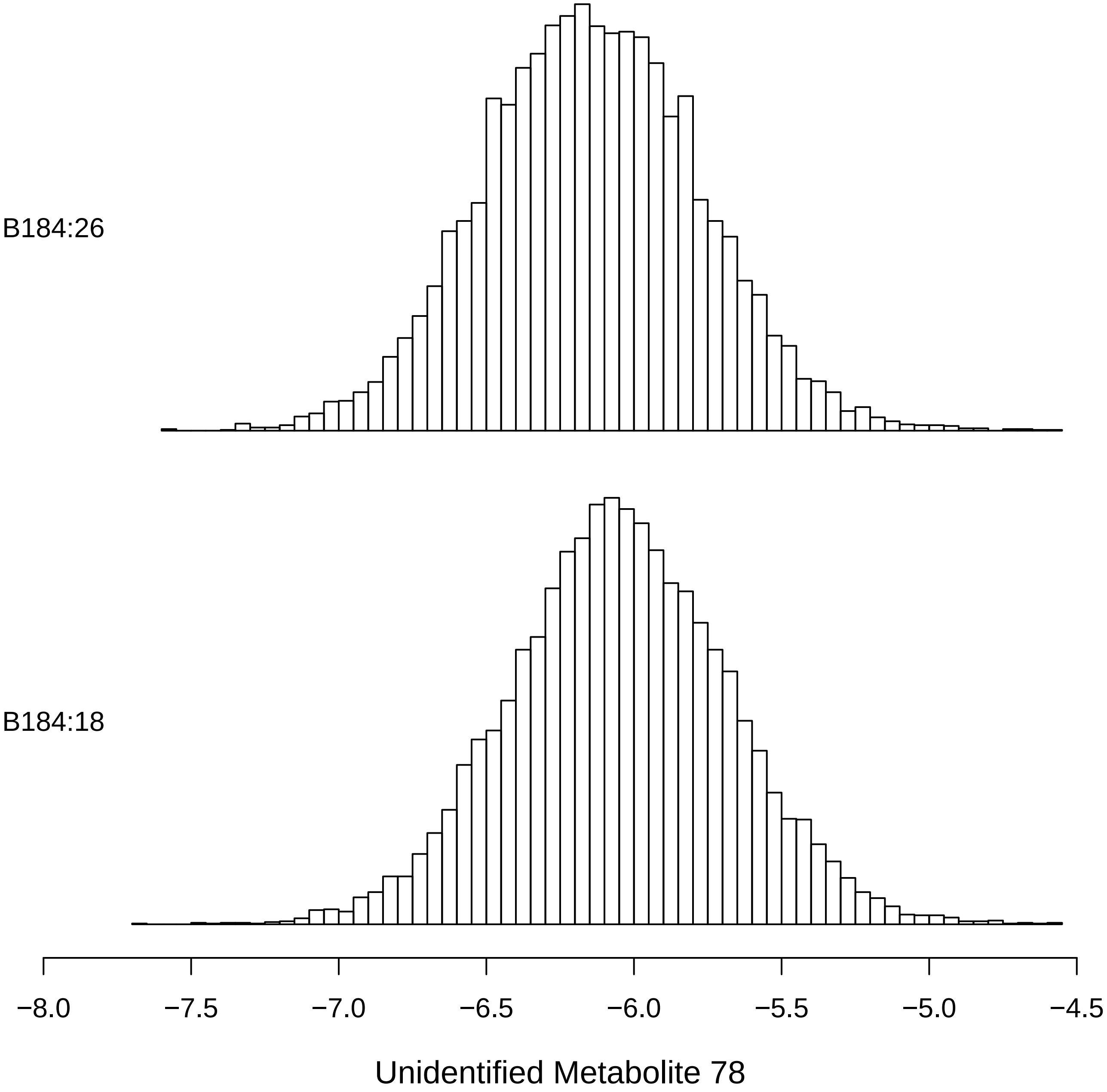

B184:26
B184:18
−8.0
−7.5
−7.0
−6.5
−6.0
−5.5
−5.0
−4.5
Unidentified Metabolite 78

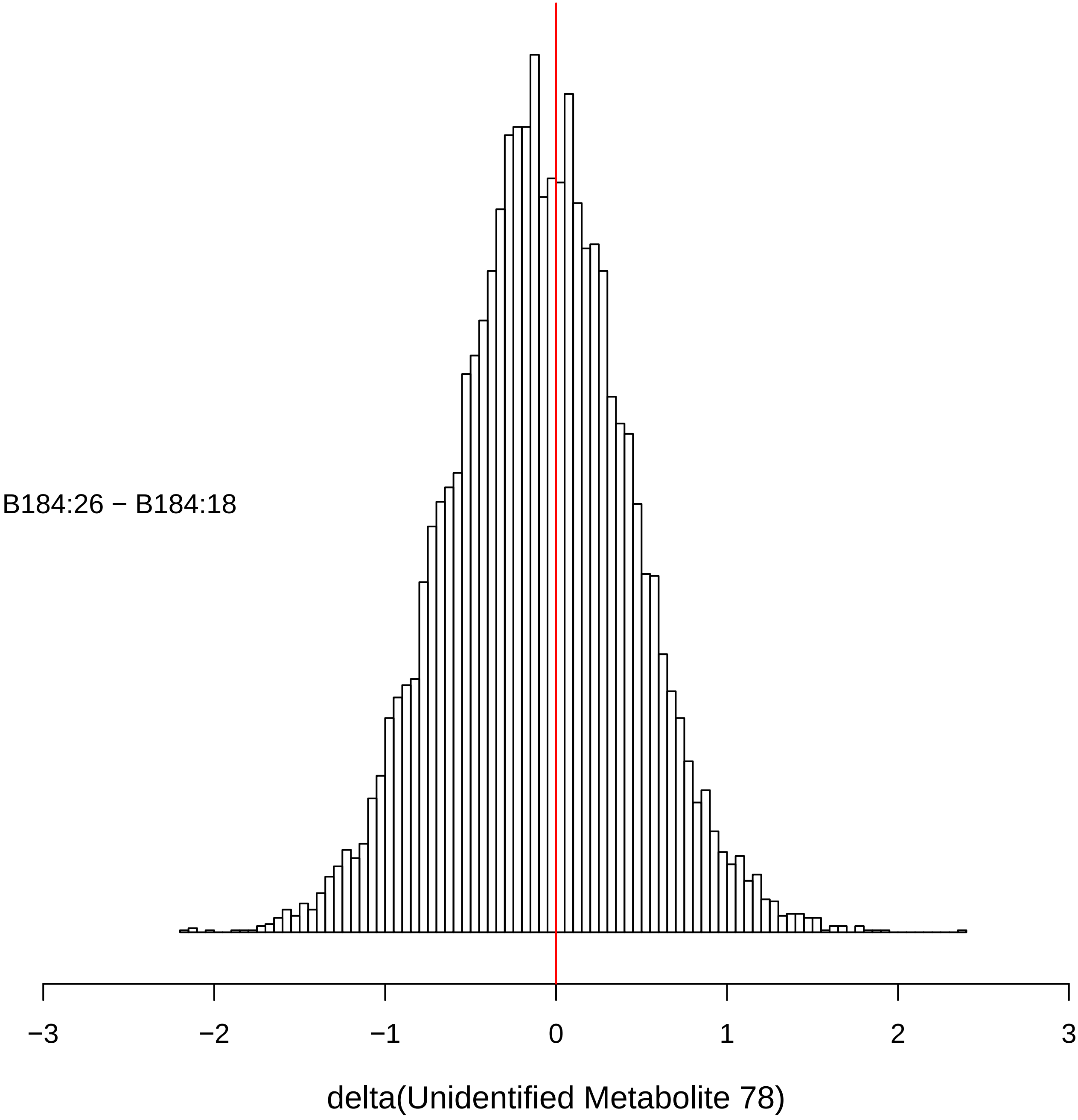

B184:26 − B184:18
−3
−2
−1
0
1
2
3
delta(Unidentified Metabolite 78)

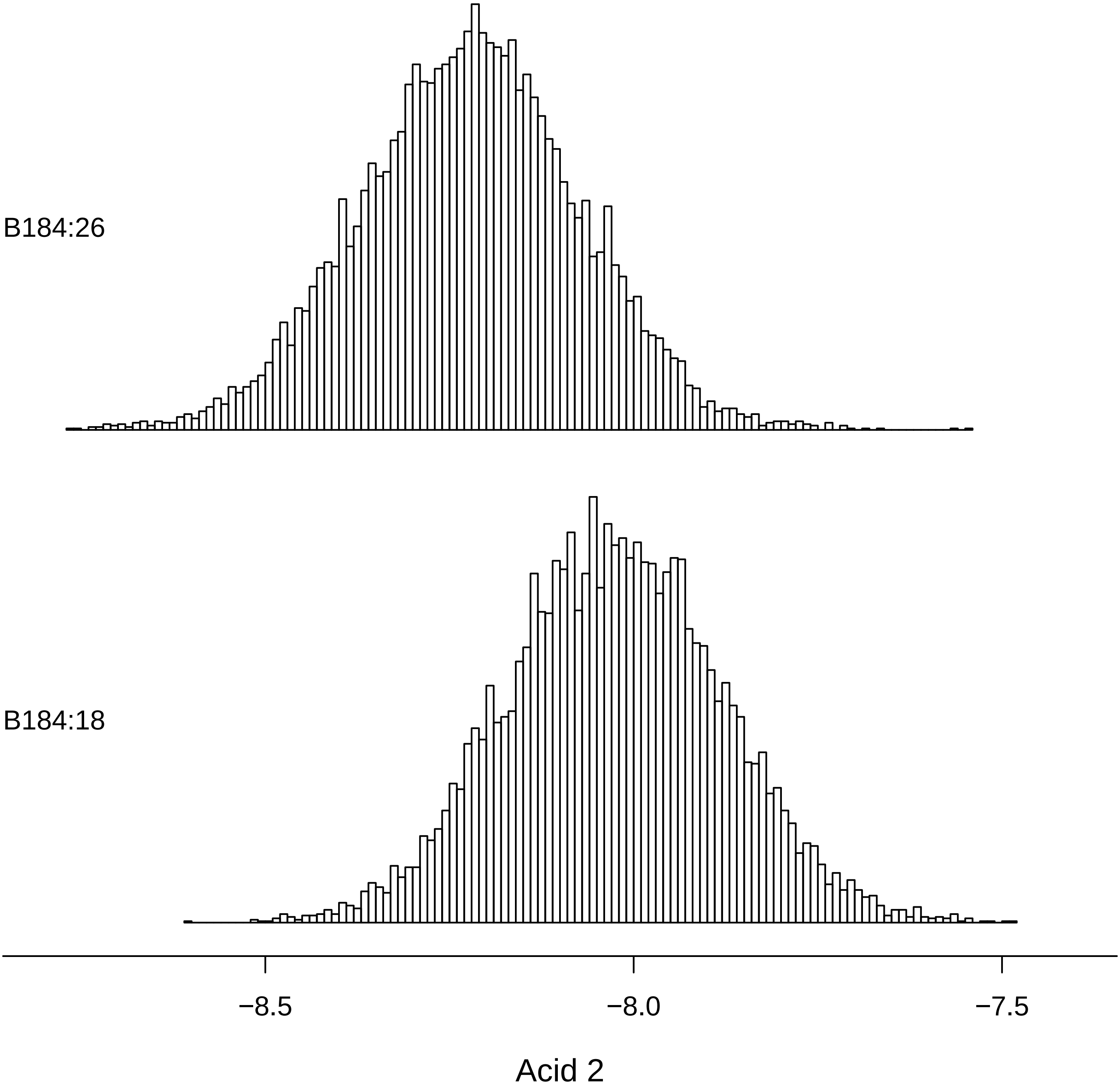
B184:26
B184:18
−8.5
−8.0
−7.5
Acid 2

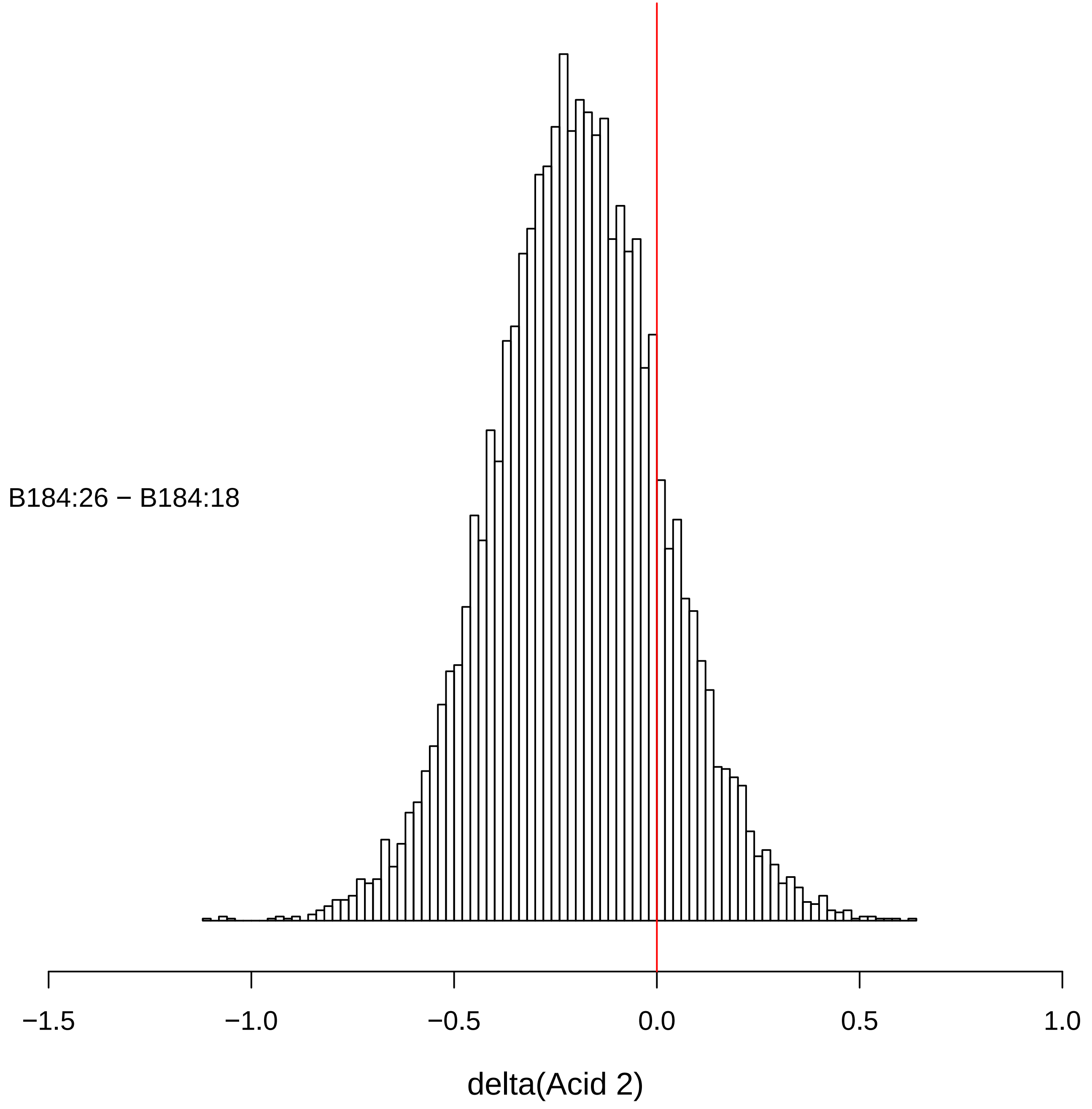
B184:26 − B184:18
−1.5
−1.0
−0.5
0.0
0.5
1.0
delta(Acid 2)

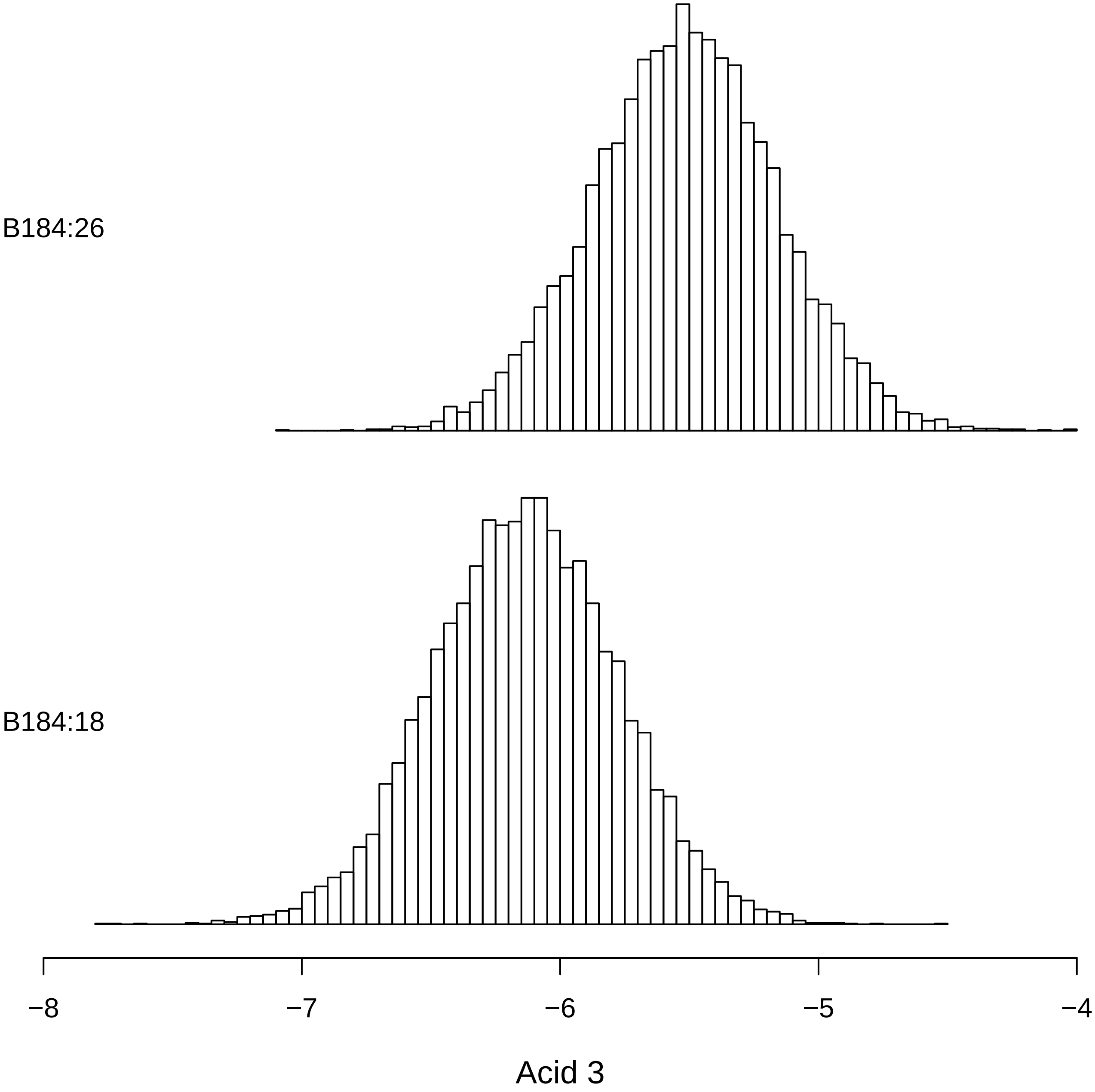

B184:26
B184:18
−8
−7
−6
−5
−4
Acid 3

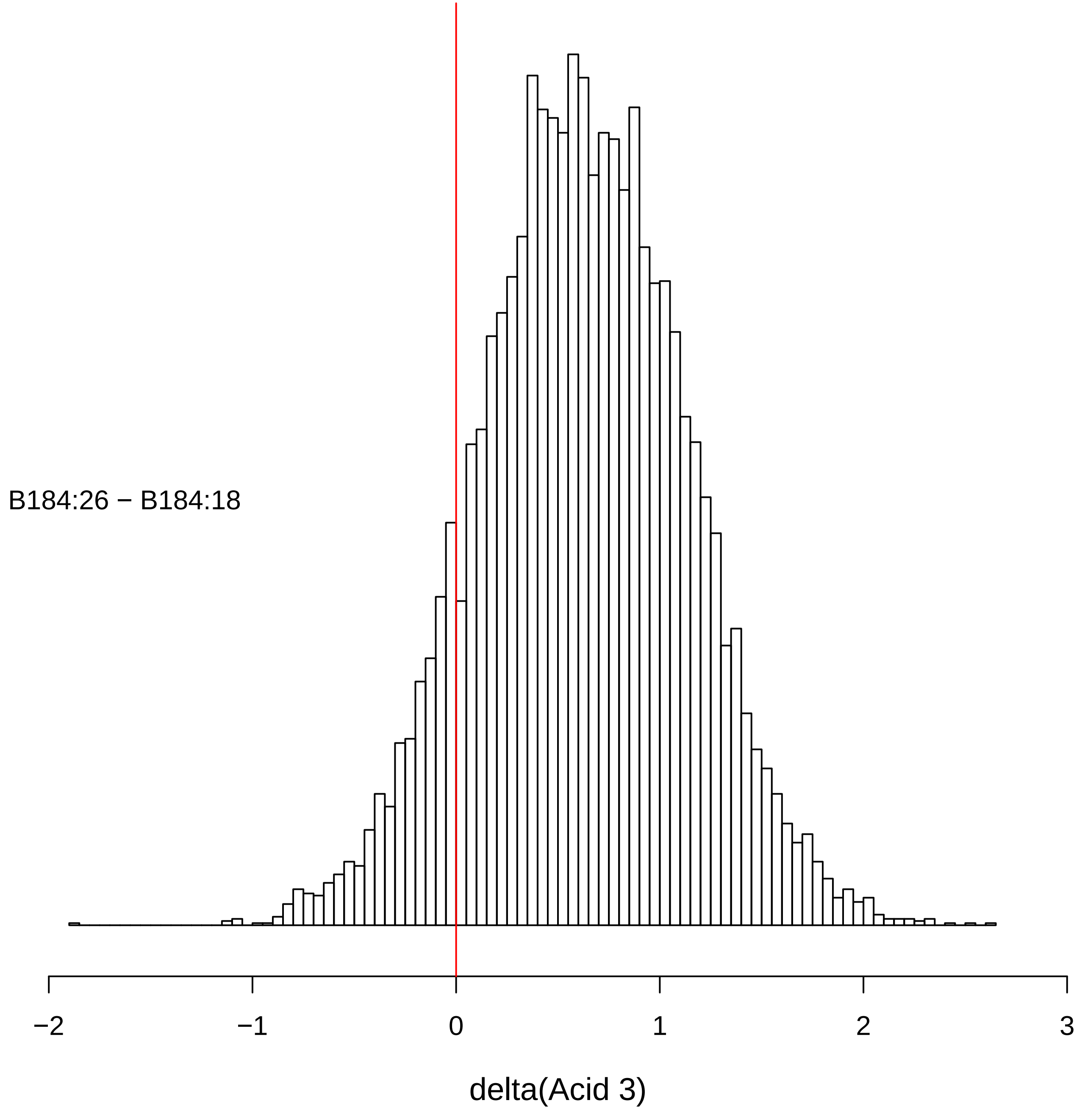

B184:26 − B184:18
−2
−1
0
1
2
3
delta(Acid 3)

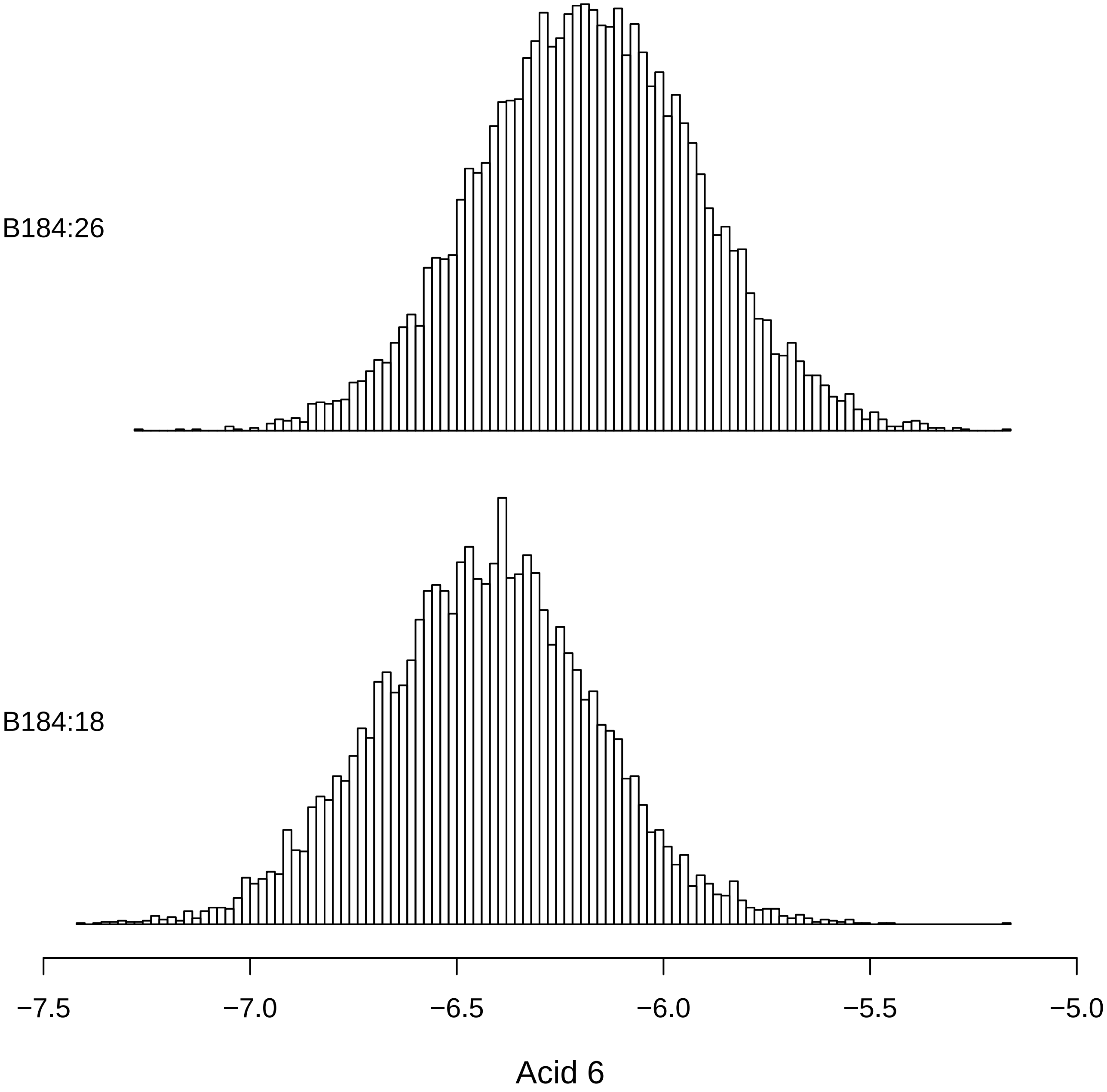
B184:26
B184:18
−7.5
−7.0
−6.5
−6.0
−5.5
−5.0
Acid 6

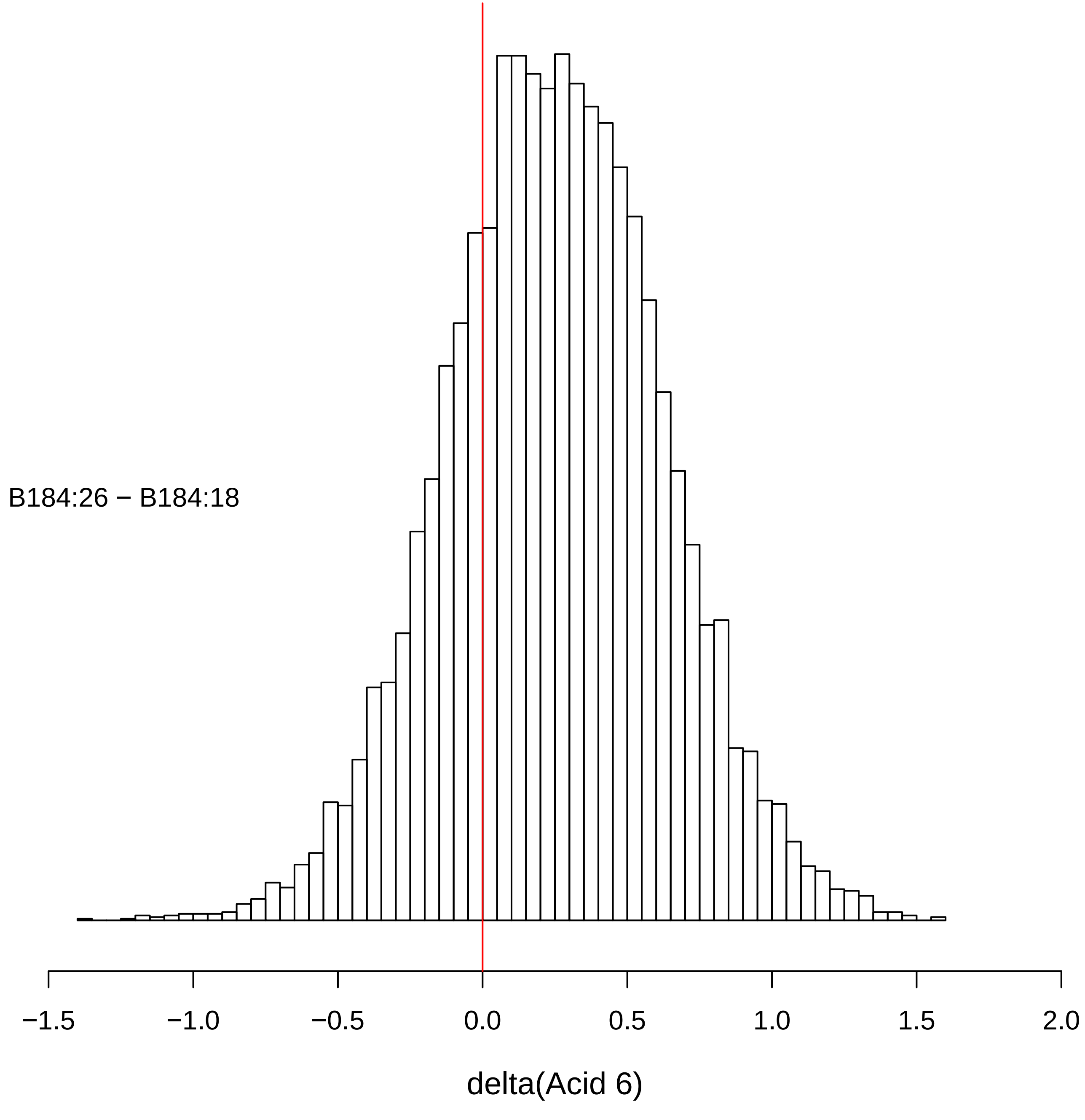

B184:26 − B184:18
−1.5
−1.0
−0.5
0.0
0.5
1.0
1.5
2.0
delta(Acid 6)

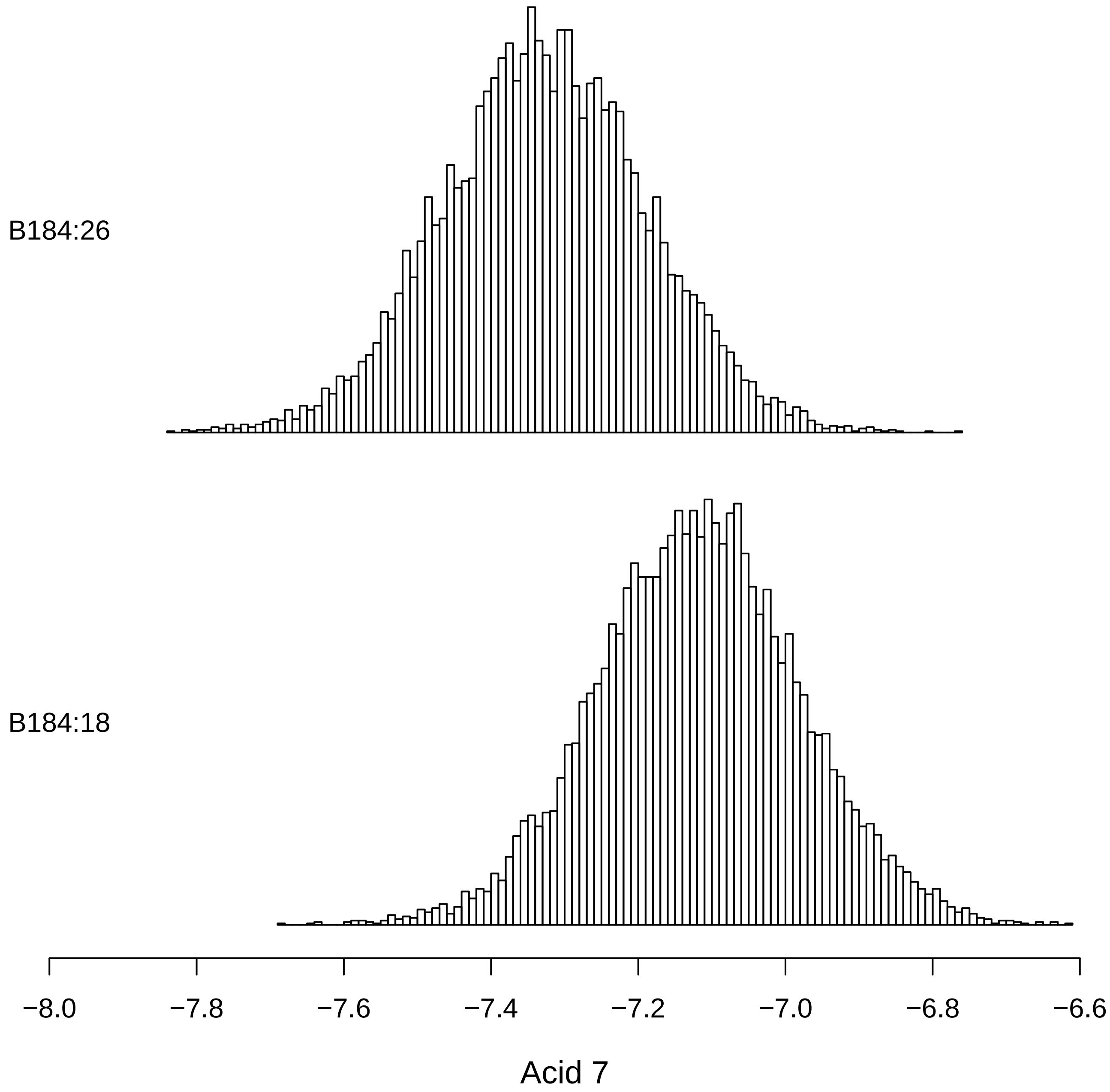
B184:26
B184:18
−8.0
−7.8
−7.6
−7.4
−7.2
−7.0
−6.8
−6.6
Acid 7

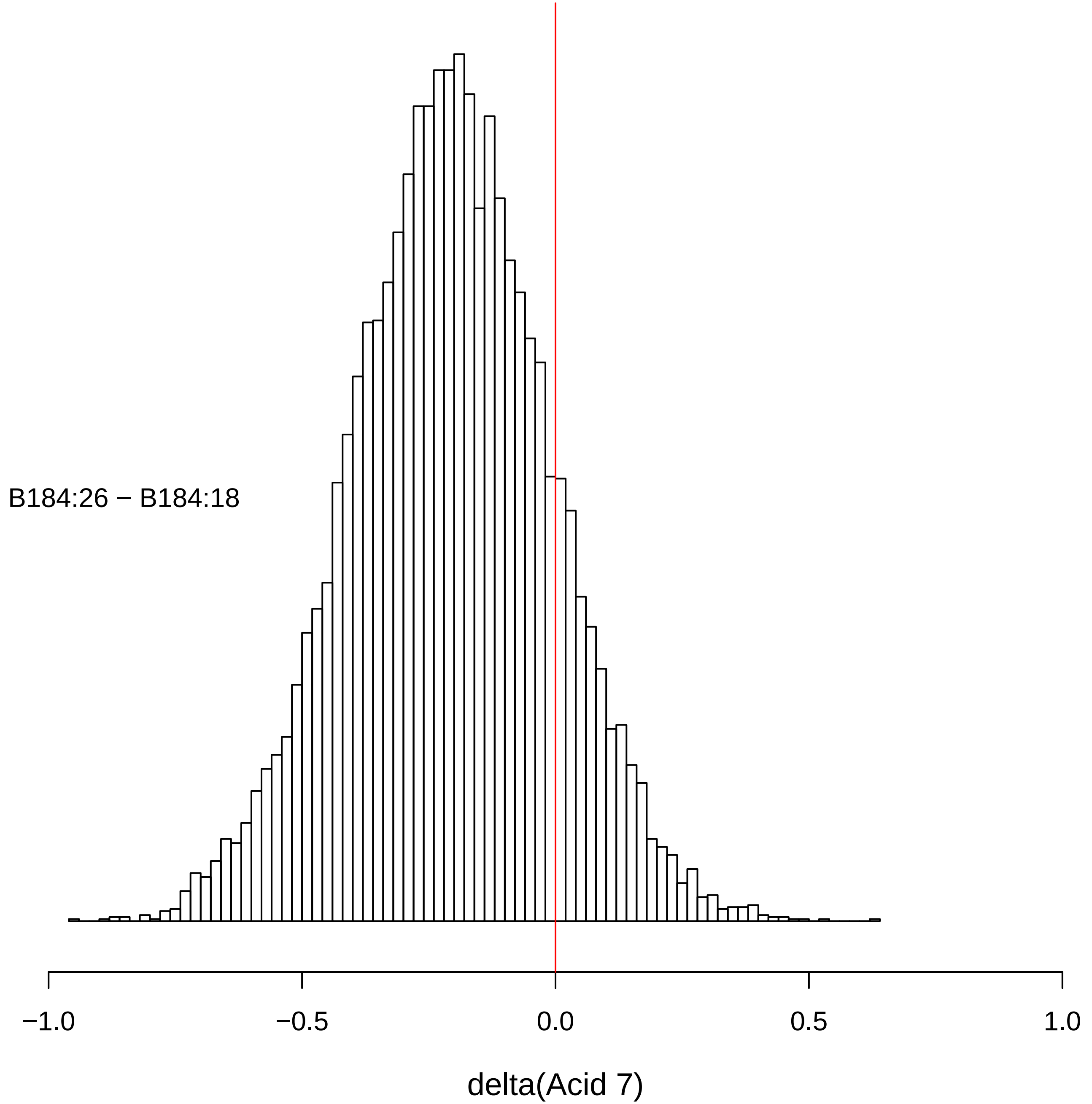
B184:26 − B184:18
−1.0
−0.5
0.0
0.5
1.0
delta(Acid 7)

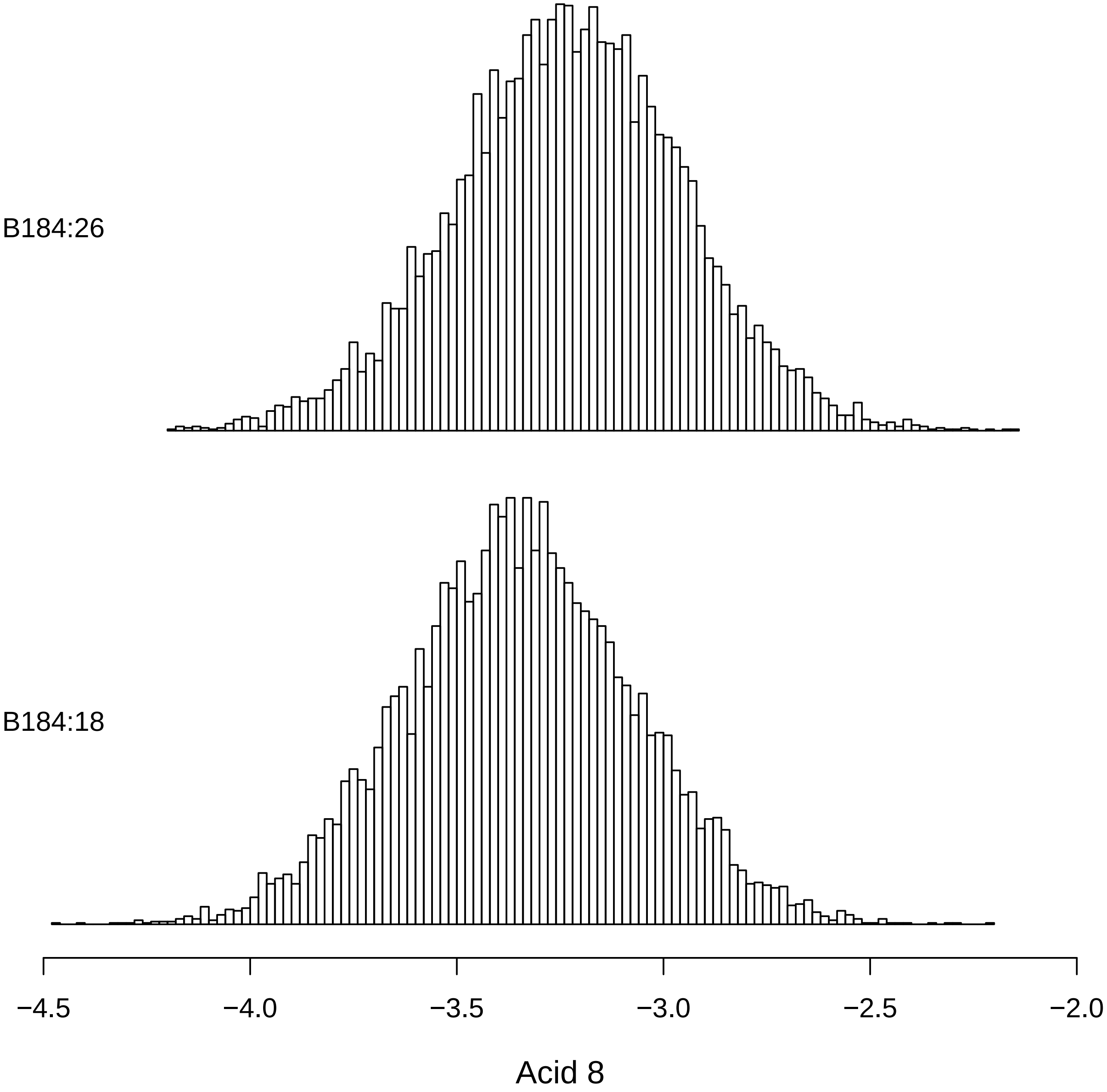
B184:26
B184:18
−4.5
−4.0
−3.5
−3.0
−2.5
−2.0
Acid 8

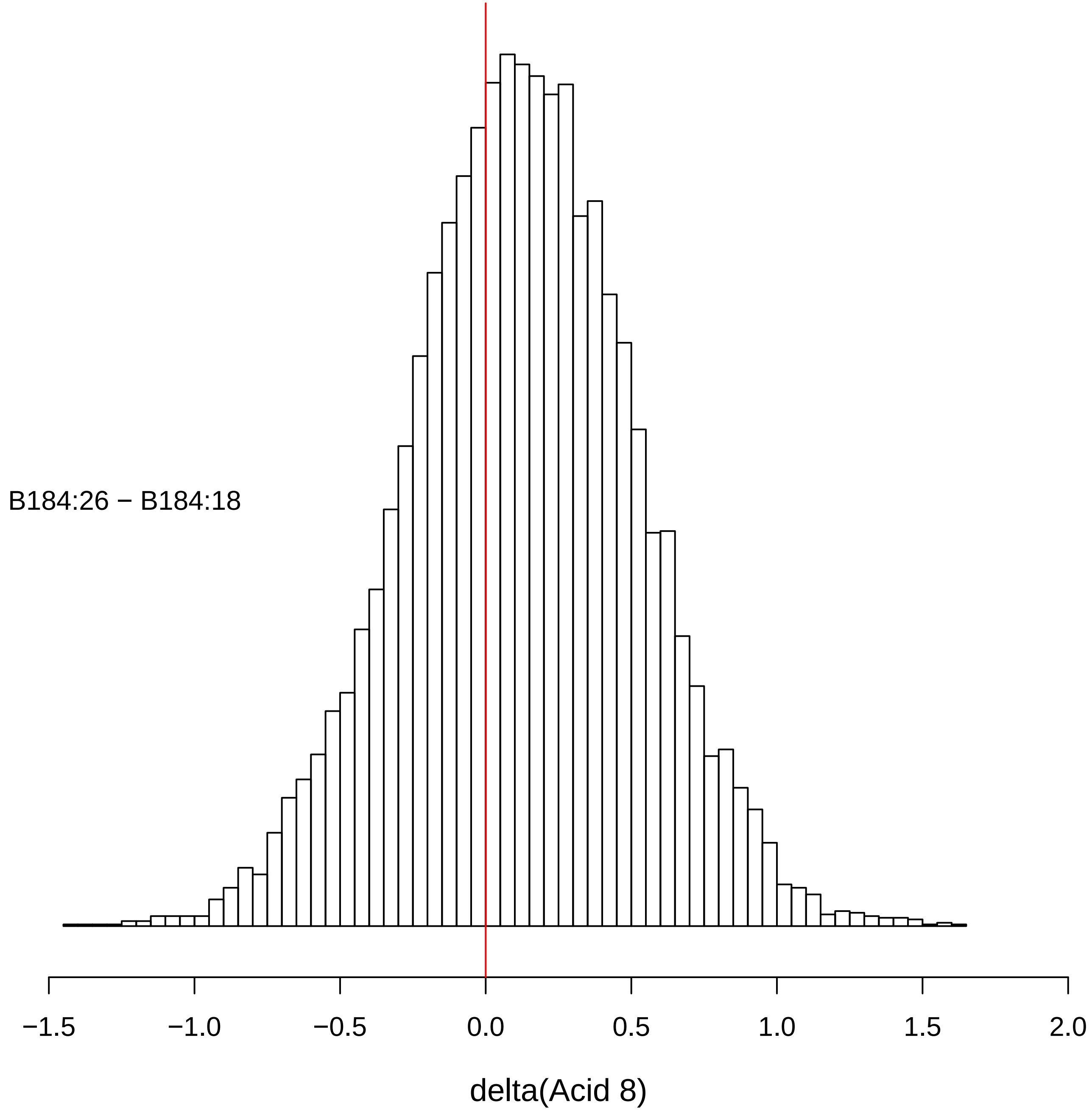
B184:26 − B184:18
−1.5
−1.0
−0.5
0.0
0.5
1.0
1.5
2.0
delta(Acid 8)

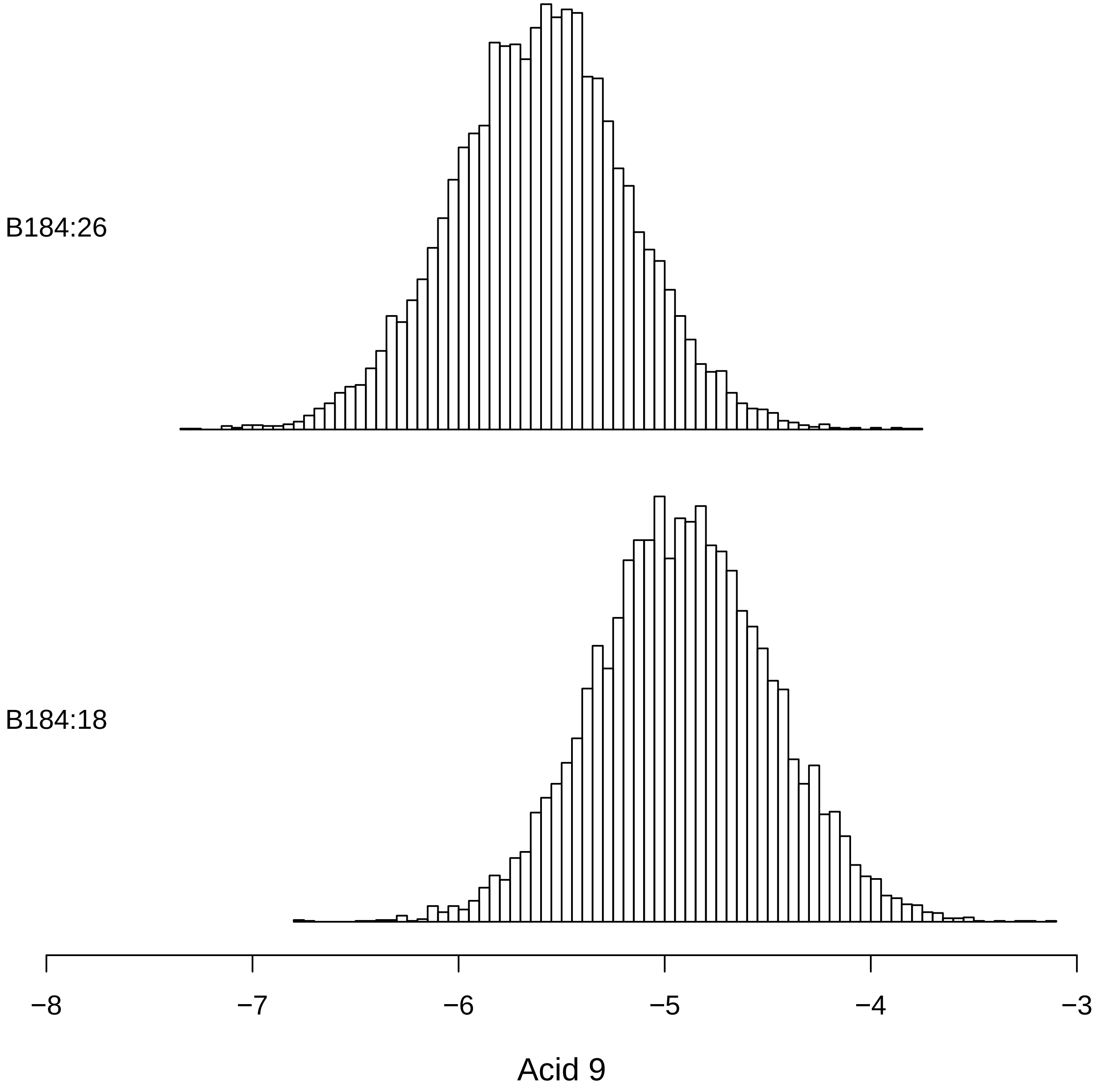

B184:26
B184:18
−8
−7
−6
−5
−4
−3
Acid 9

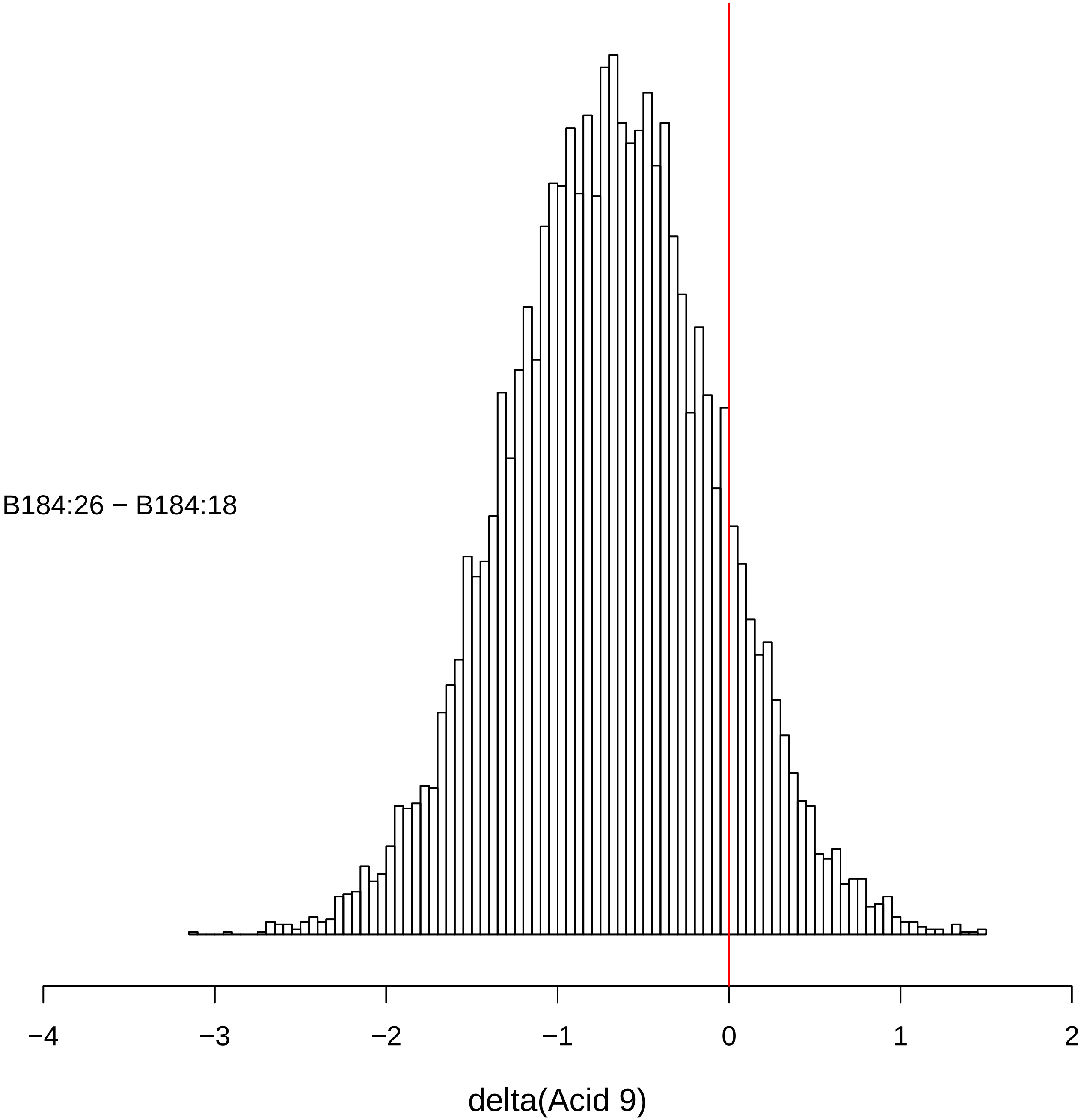

B184:26 − B184:18
−4
−3
−2
−1
0
1
2
delta(Acid 9)

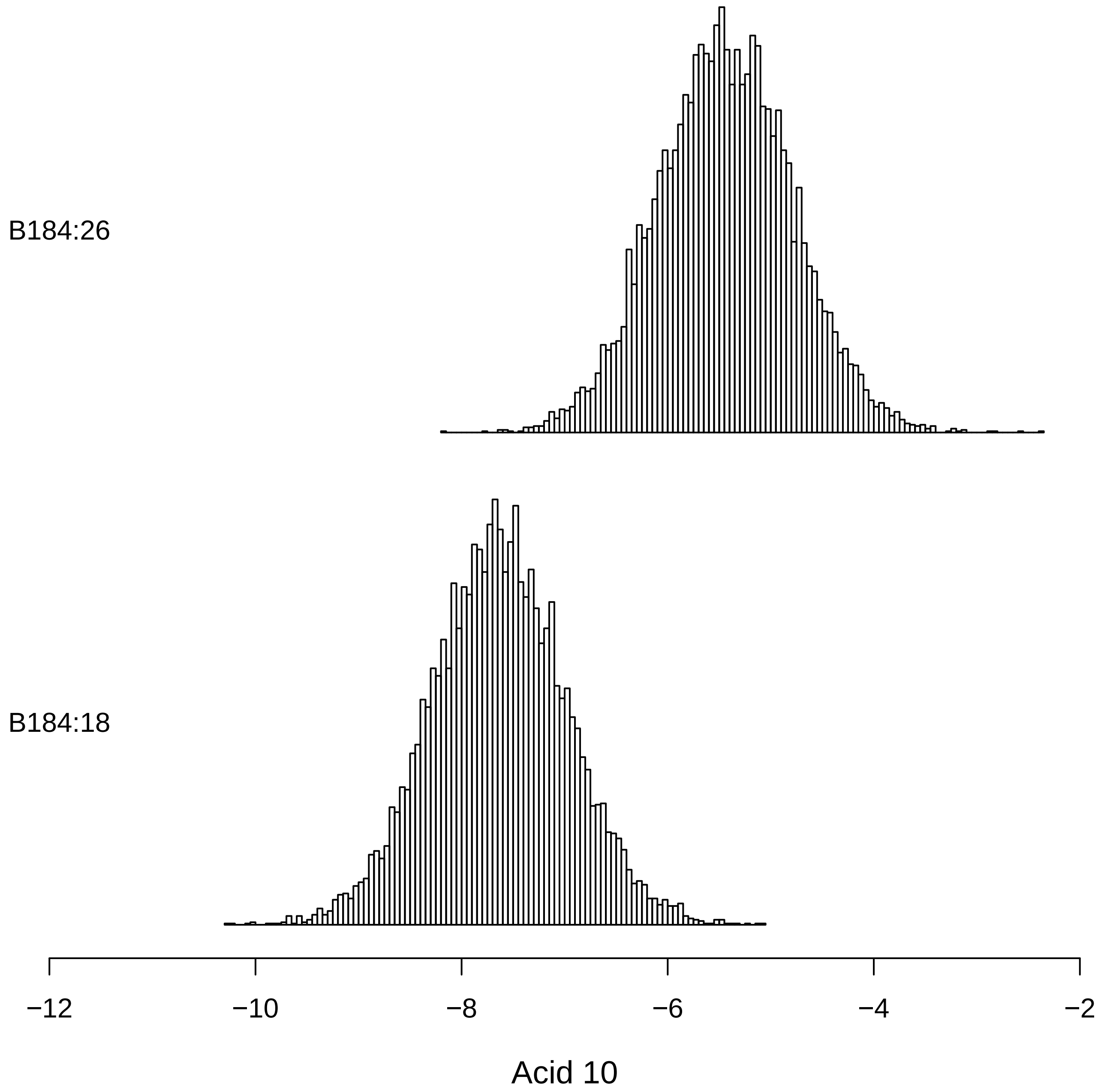
B184:26
B184:18
−12
−10
−8
−6
−4
−2
Acid 10

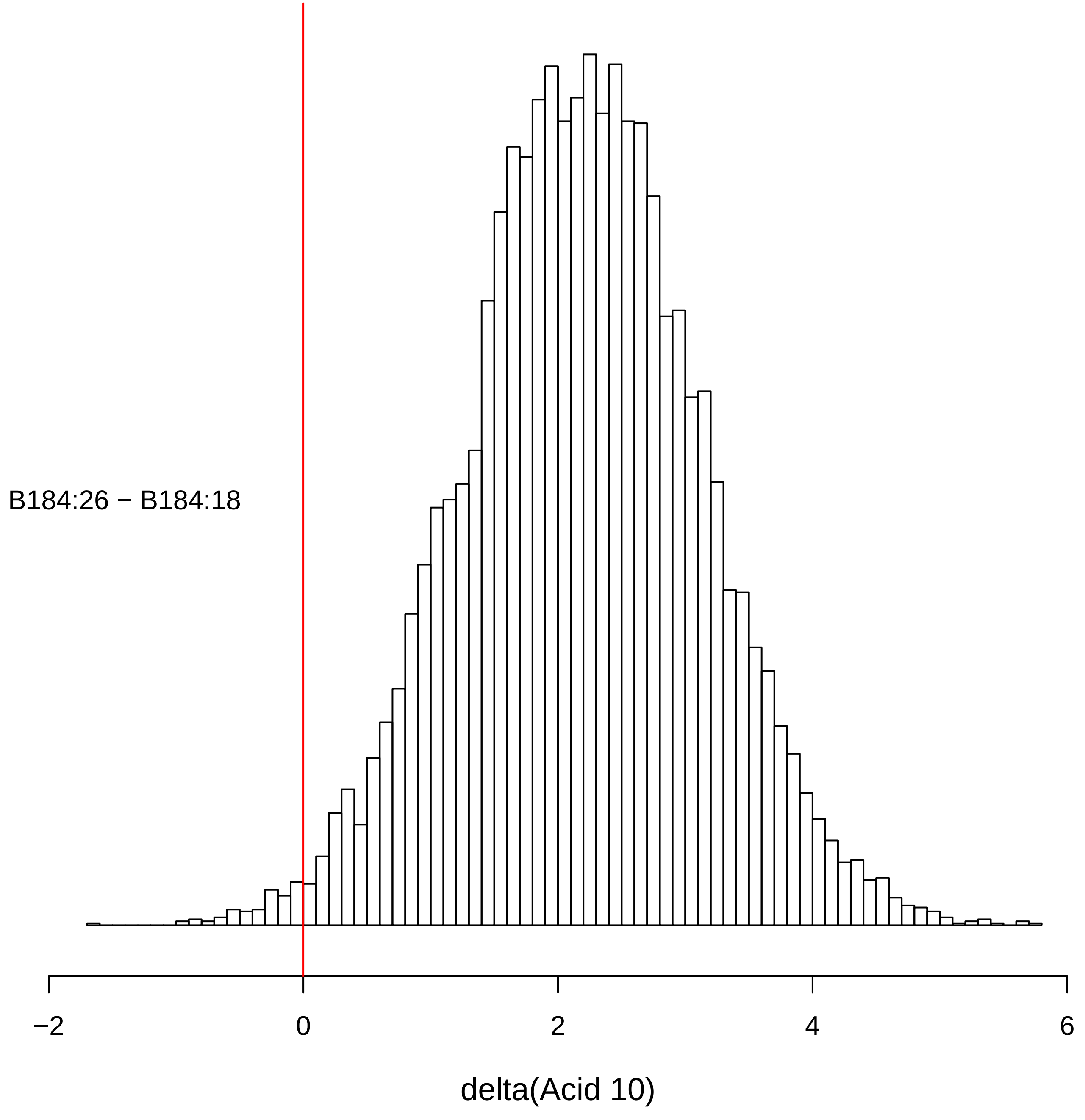
B184:26 − B184:18
−2
0
2
4
6
delta(Acid 10)

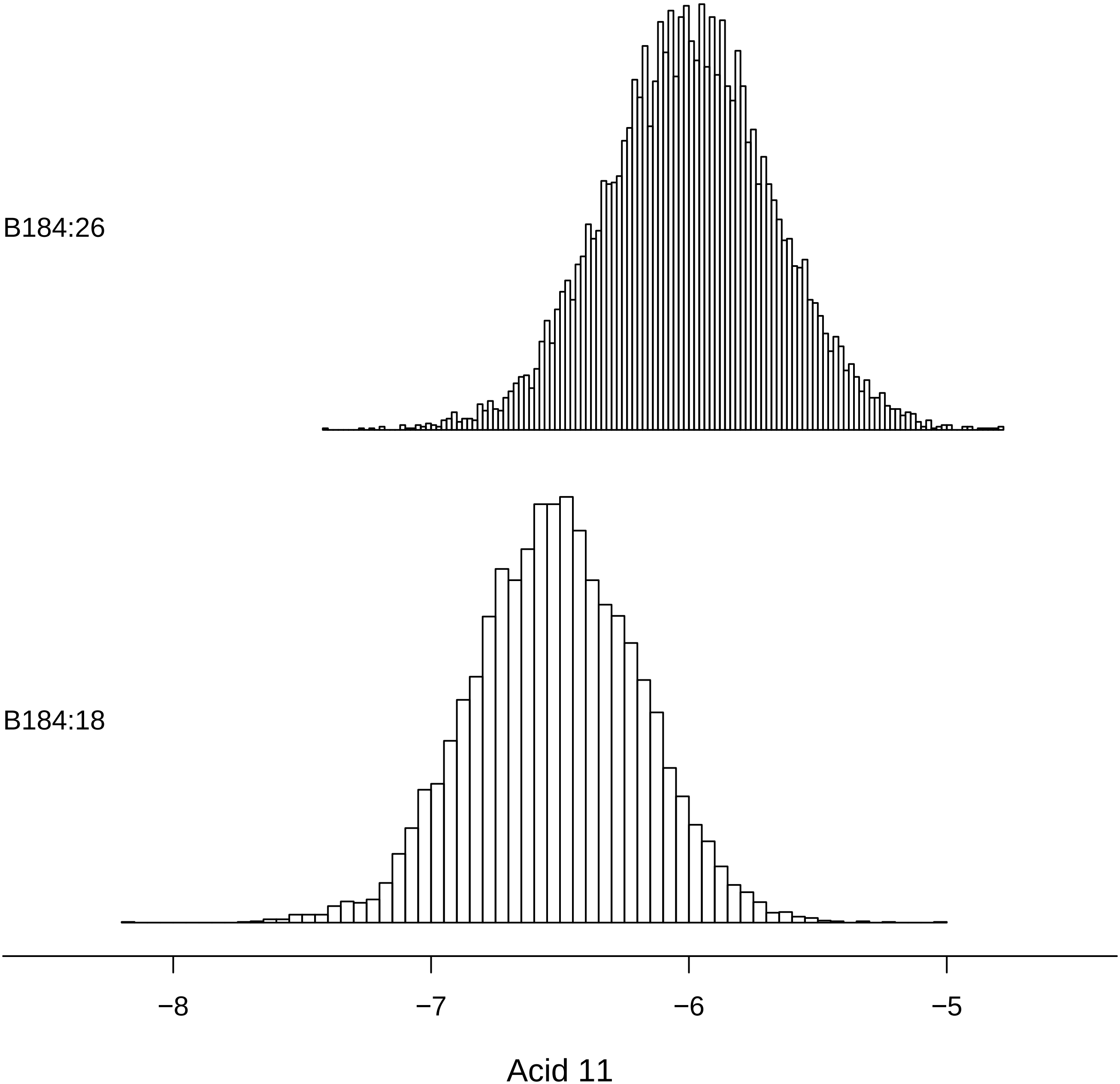

B184:26
B184:18
−8
−7
−6
−5
Acid 11

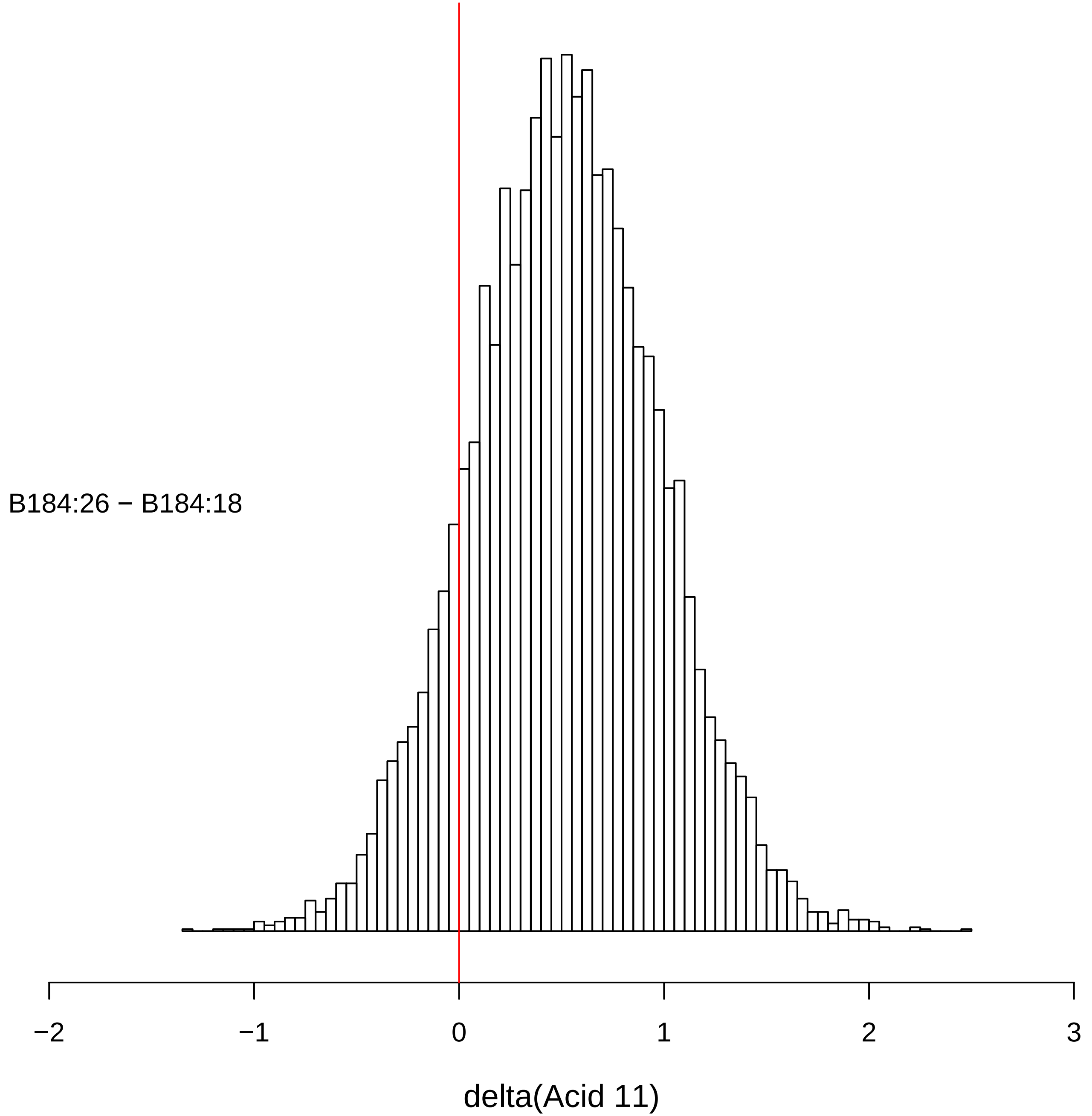
B184:26 − B184:18
−2
−1
0
1
2
3
delta(Acid 11)

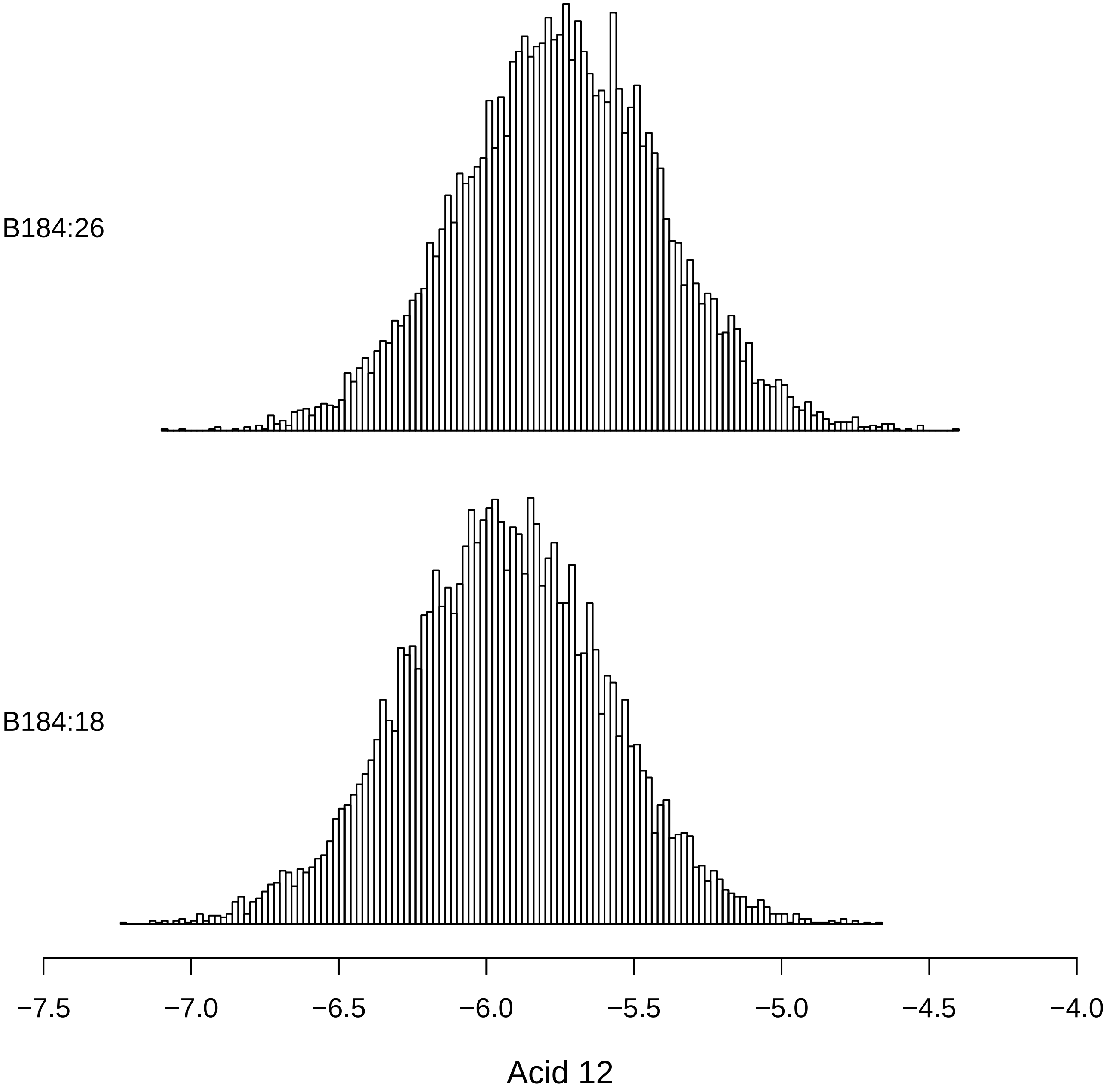
B184:26
B184:18
−7.5
−7.0
−6.5
−6.0
−5.5
−5.0
−4.5
−4.0
Acid 12

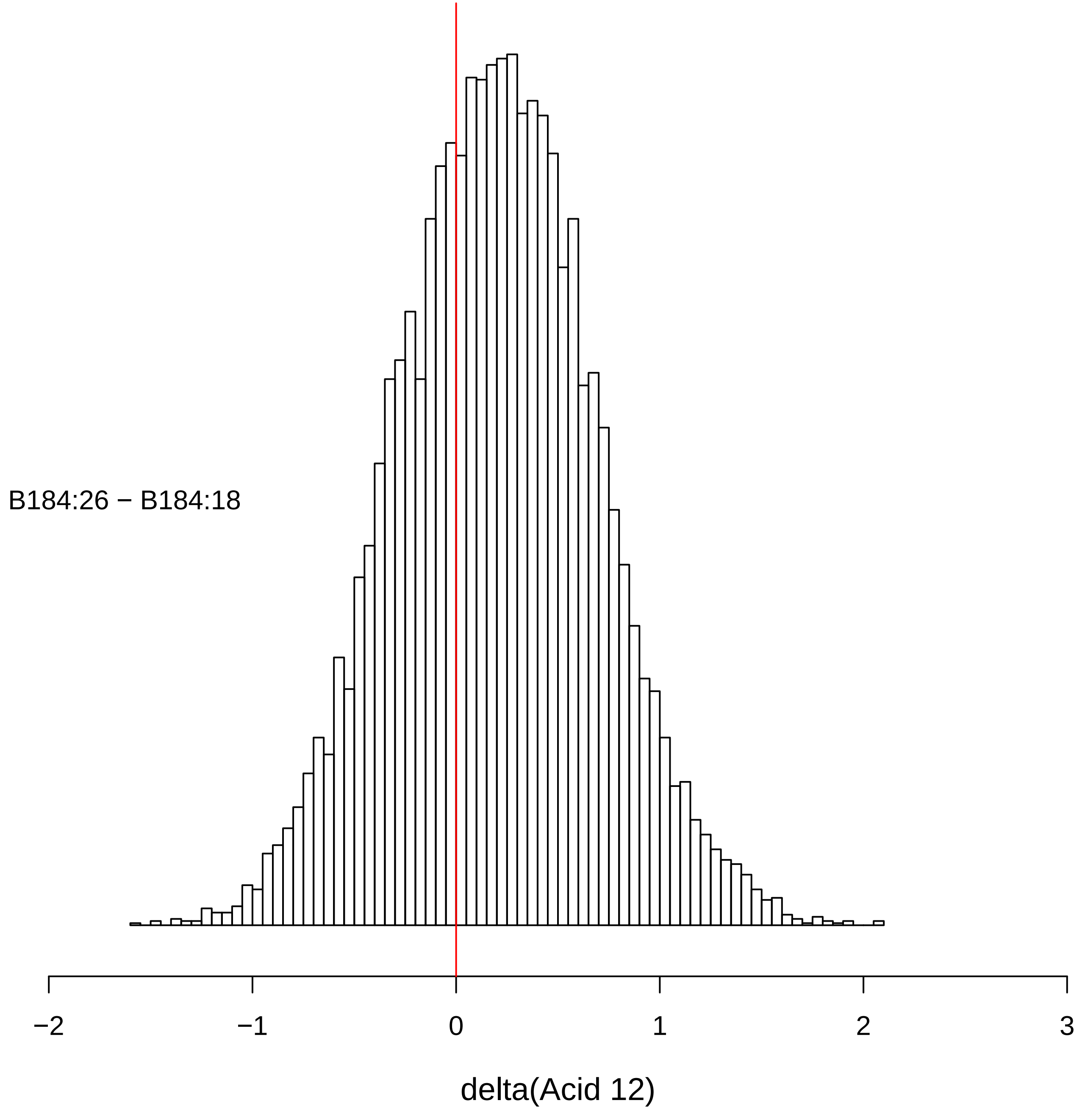
B184:26 − B184:18
−2
−1
0
1
2
3
delta(Acid 12)

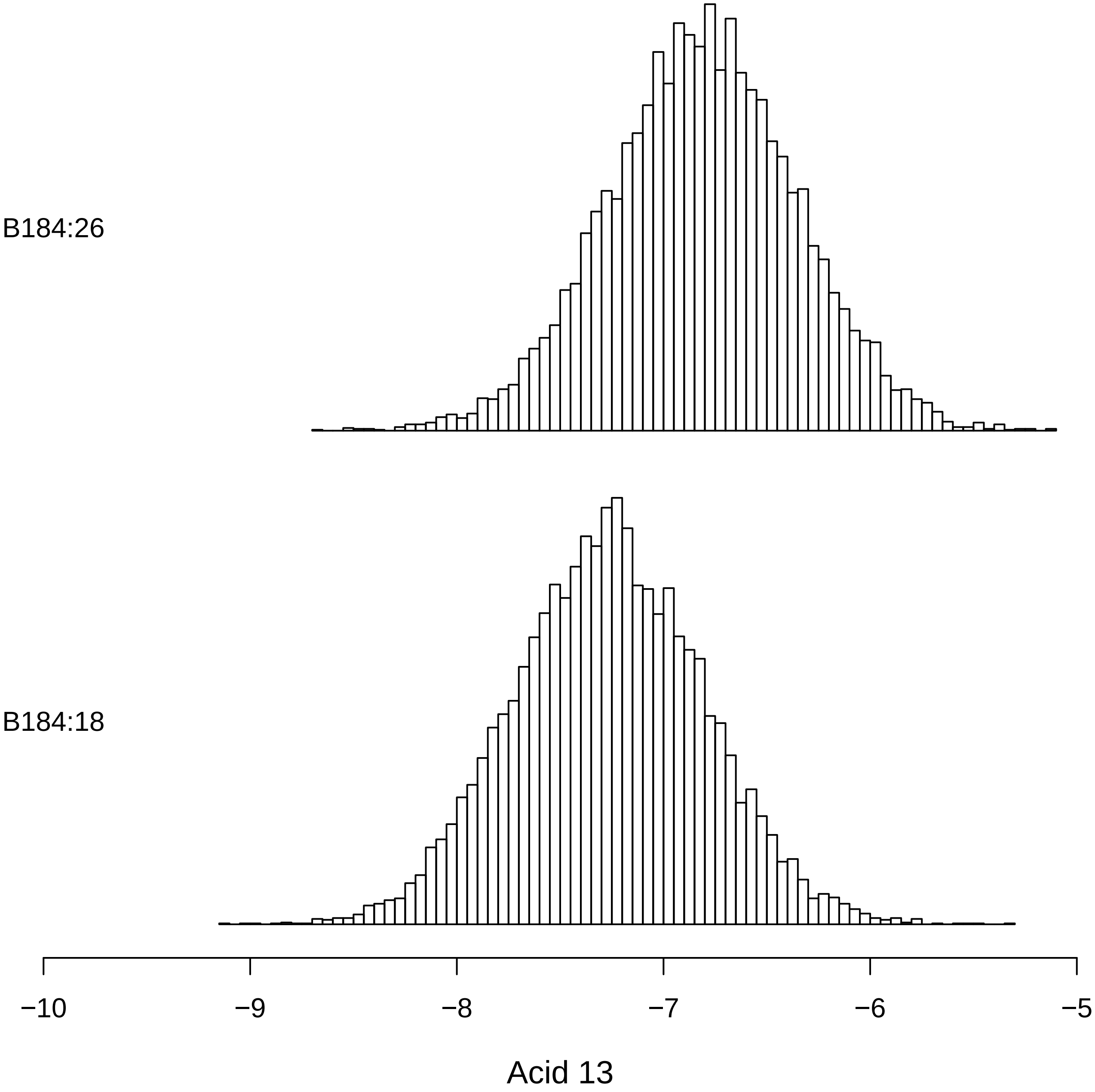
B184:26
B184:18
−10
−9
−8
−7
−6
−5
Acid 13

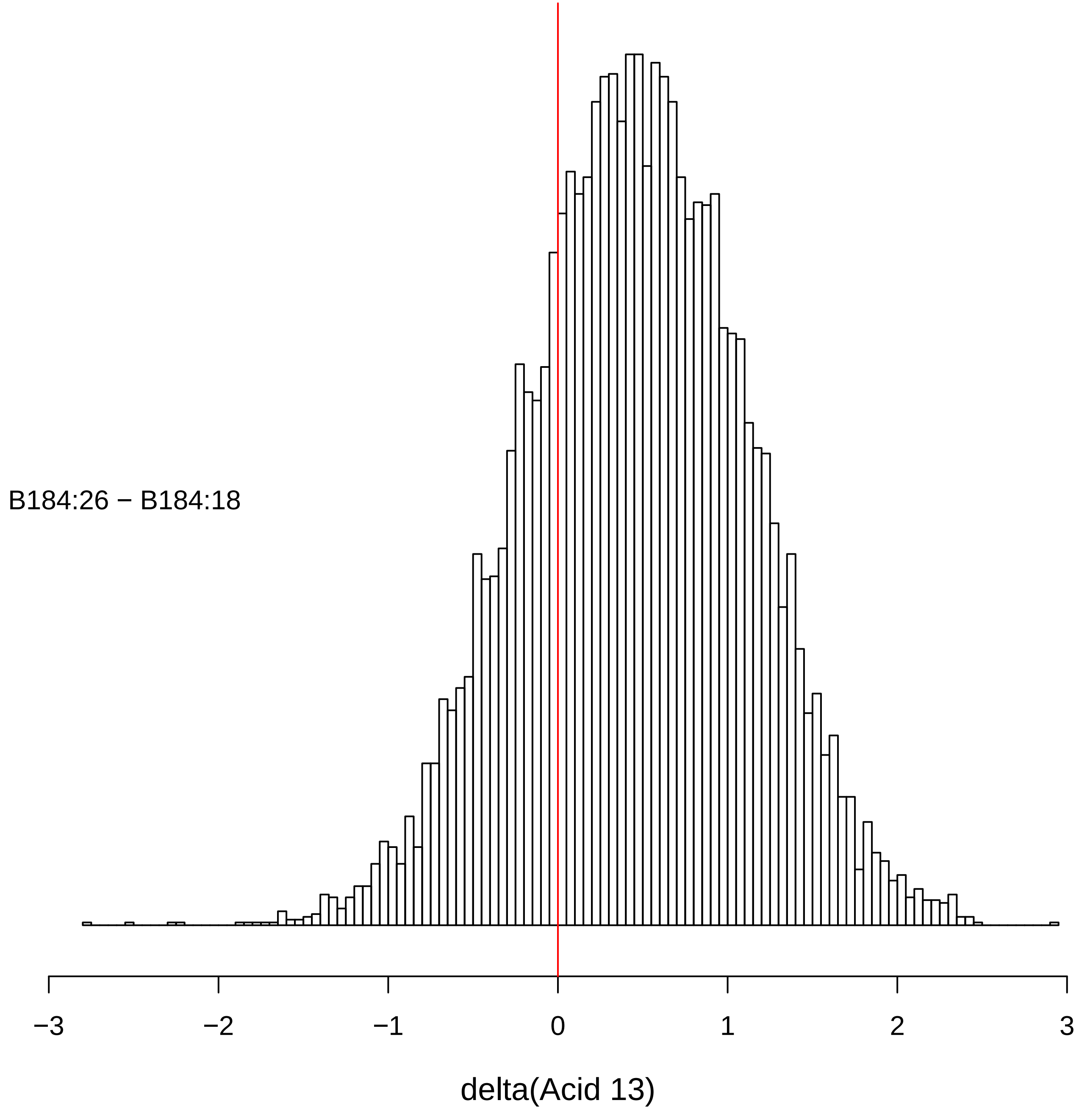
B184:26 − B184:18
−3
−2
−1
0
1
2
3
delta(Acid 13)

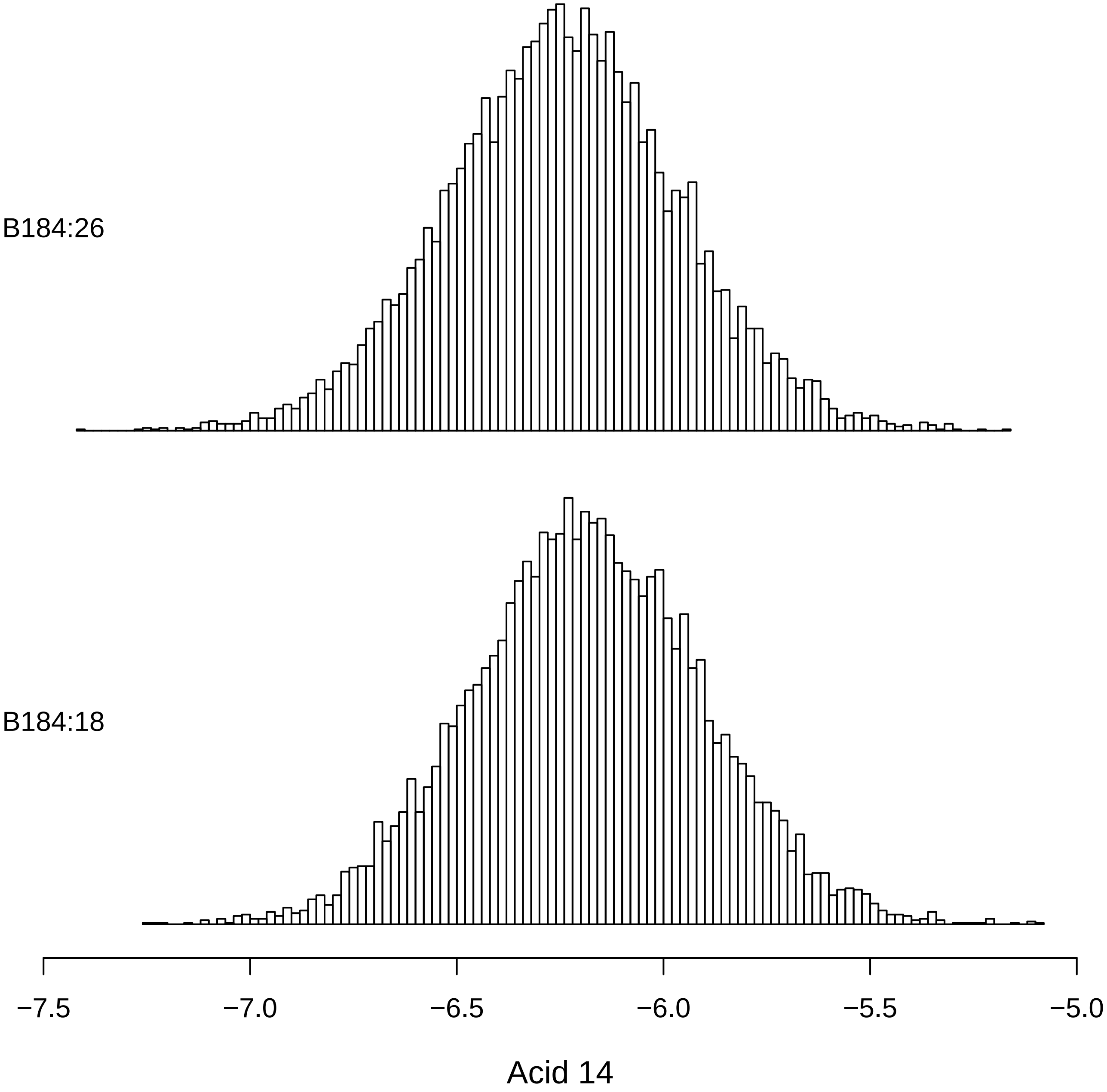
B184:26
B184:18
−7.5
−7.0
−6.5
−6.0
−5.5
−5.0
Acid 14

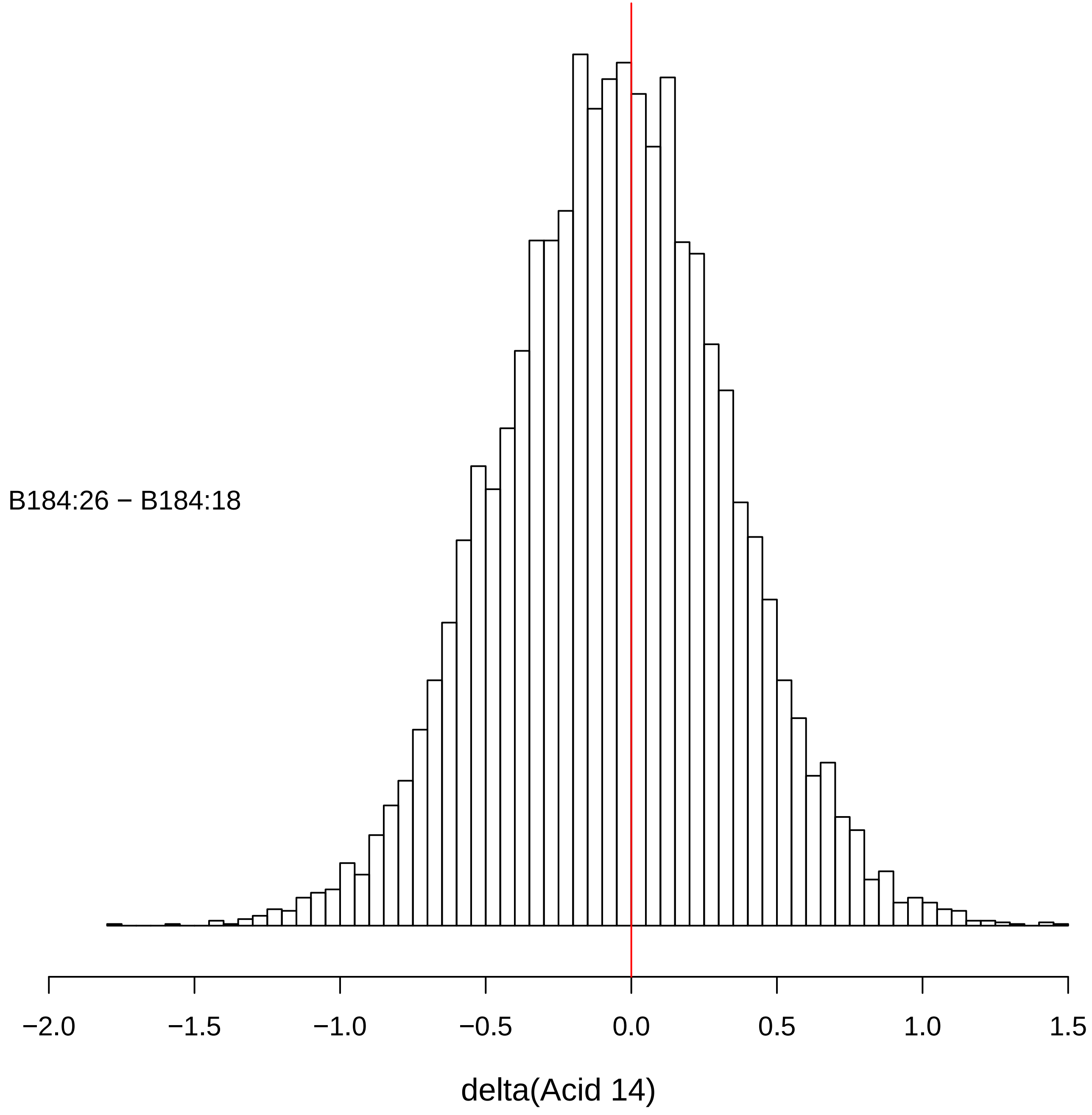
B184:26 − B184:18
−2.0
−1.5
−1.0
−0.5
0.0
0.5
1.0
1.5
delta(Acid 14)
