# Supplementary material for: Taxonomic and Environmental Variation of Metabolite Profiles in Marine Dinoflagellates of the Genus Symbiodinium
Source: Metabolites. 2015 Feb 16;5(1):74–99. doi: 10.3390/metabo5010074 (PMC4381291; doi:10.3390/metabo5010074)
Supplement: Supplementary File 1 [file metabolites-05-00074-s001.zip › Supplementary Information/Supplementary Information Figure S4d - temp.D206.pdf]

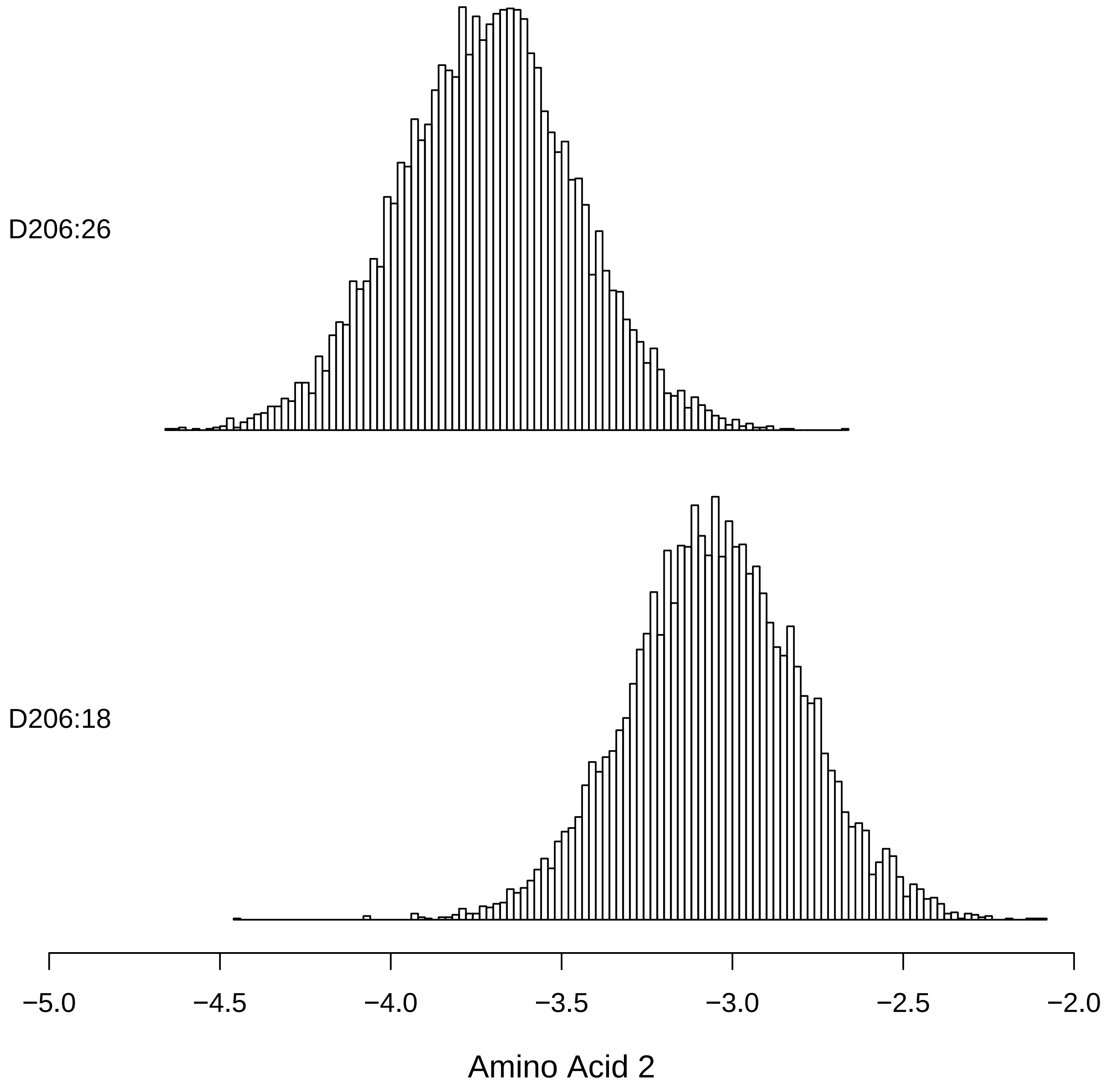
D206:26
D206:18
−5.0
−4.5
−4.0
−3.5
−3.0
−2.5
−2.0
Amino Acid 2

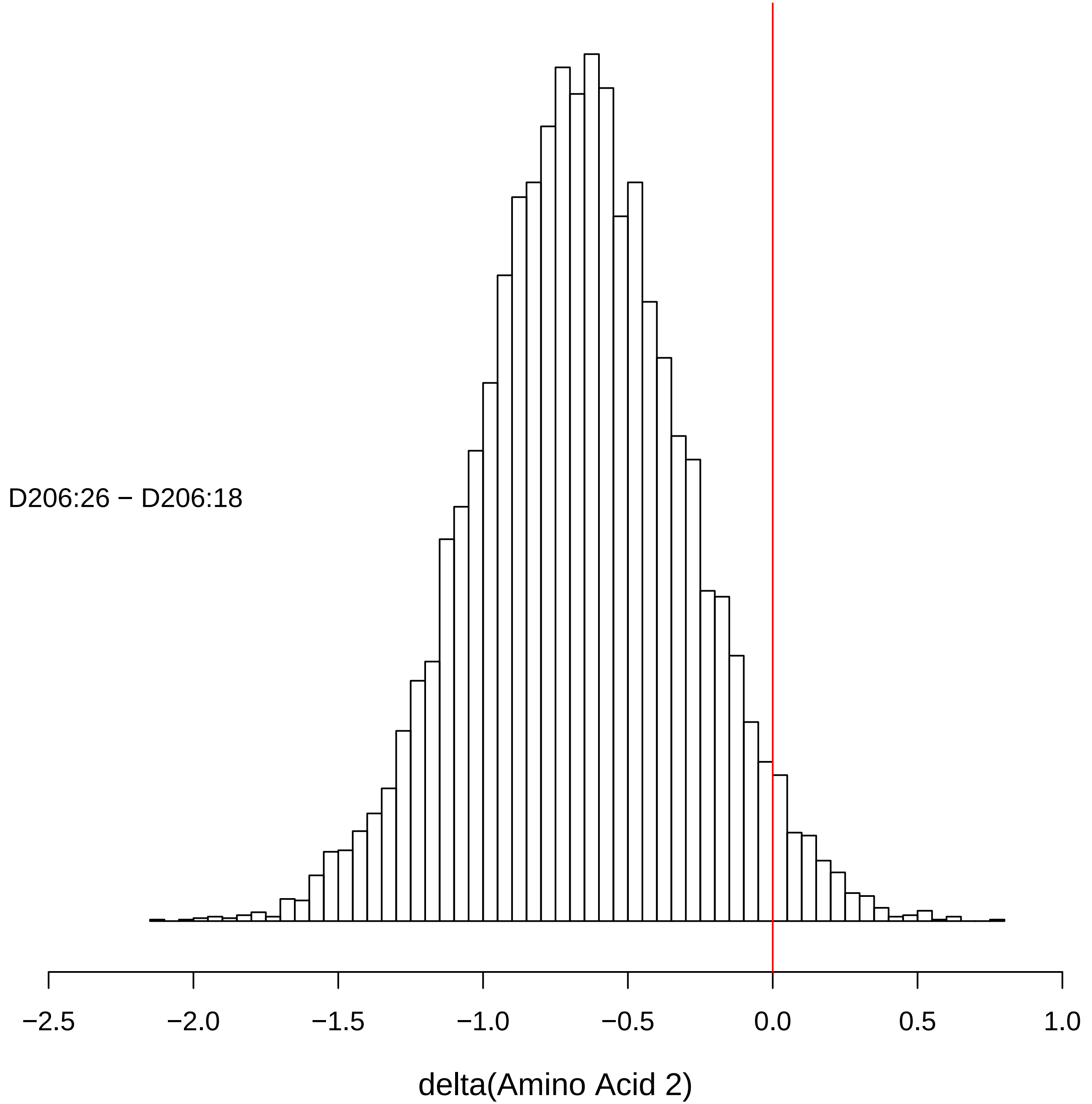
D206:26 − D206:18
−2.5
−2.0
−1.5
−1.0
−0.5
0.0
0.5
1.0
delta(Amino Acid 2)

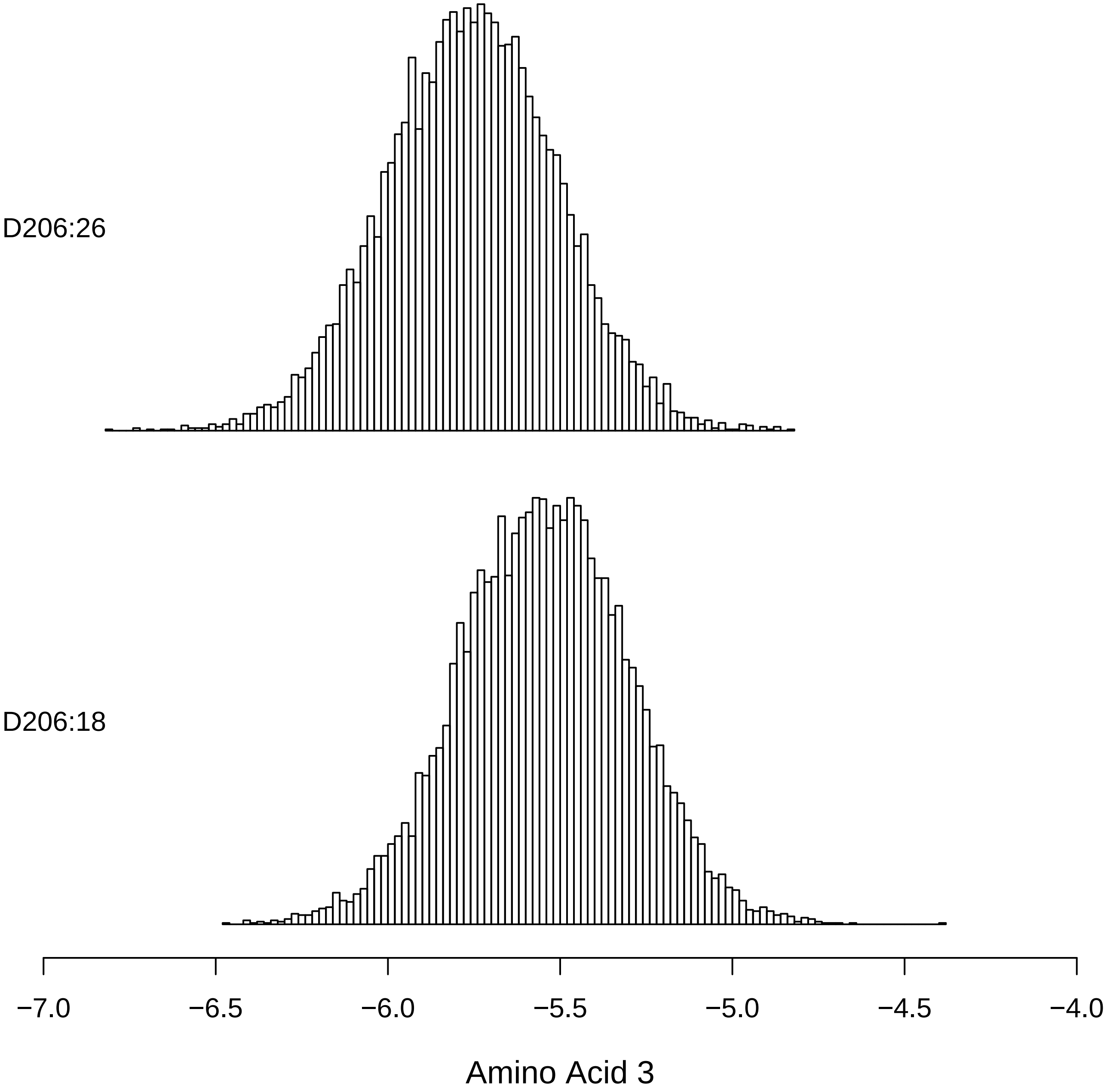
D206:26
D206:18
−7.0
−6.5
−6.0
−5.5
−5.0
−4.5
−4.0
Amino Acid 3

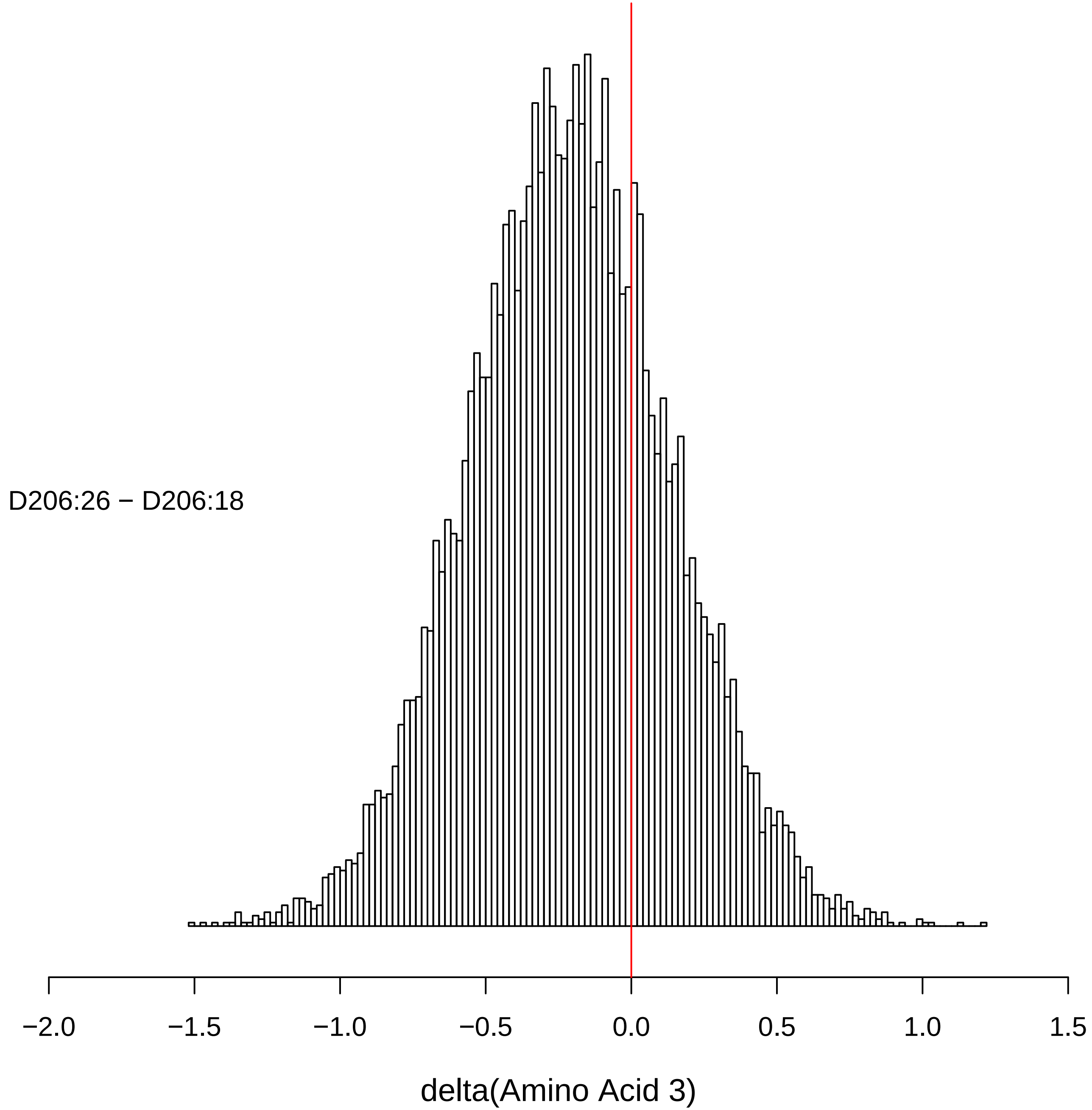
D206:26 − D206:18
−2.0
−1.5
−1.0
−0.5
0.0
0.5
1.0
1.5
delta(Amino Acid 3)

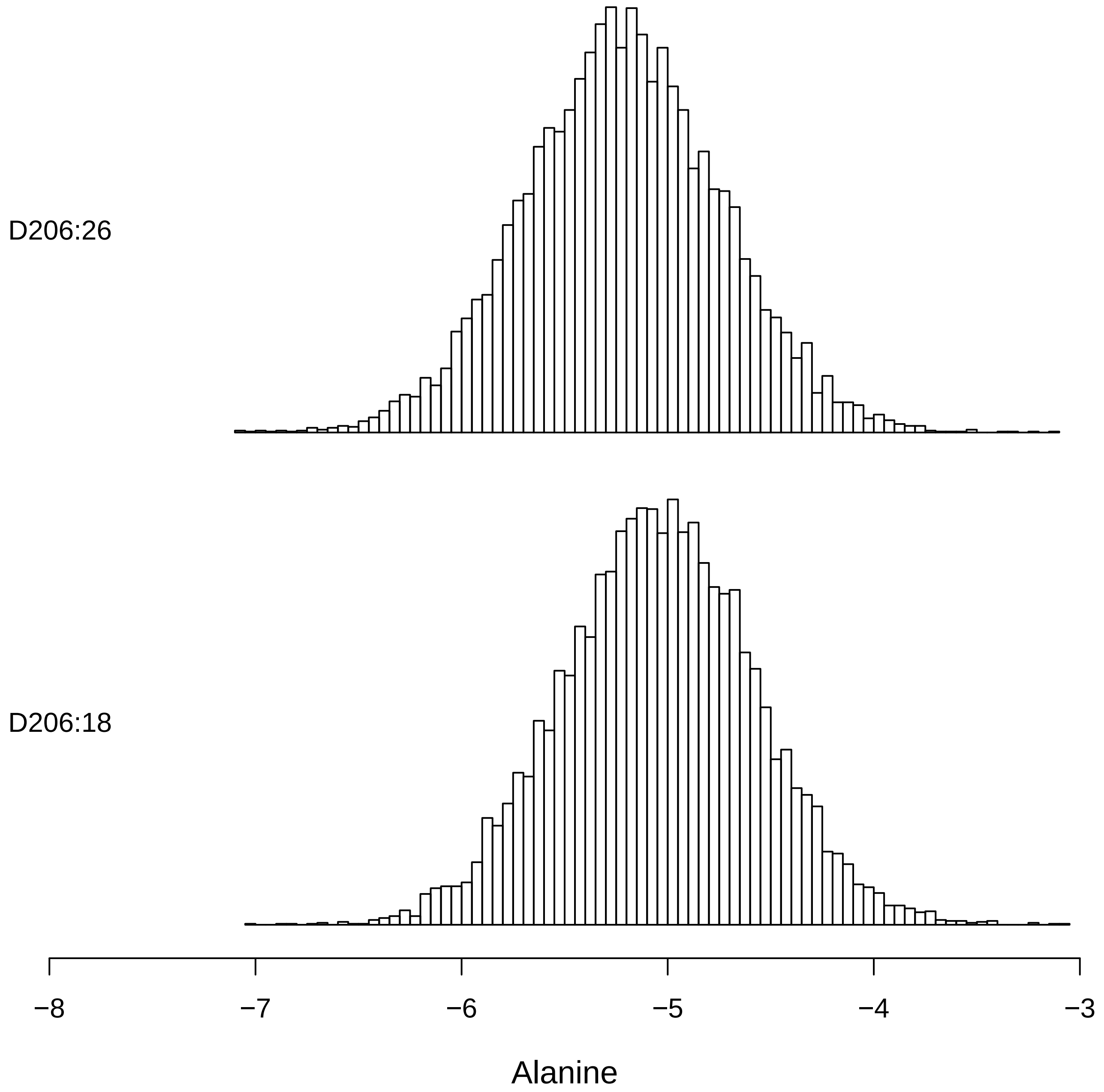
D206:26
D206:18
−8
−7
−6
−5
−4
−3
Alanine

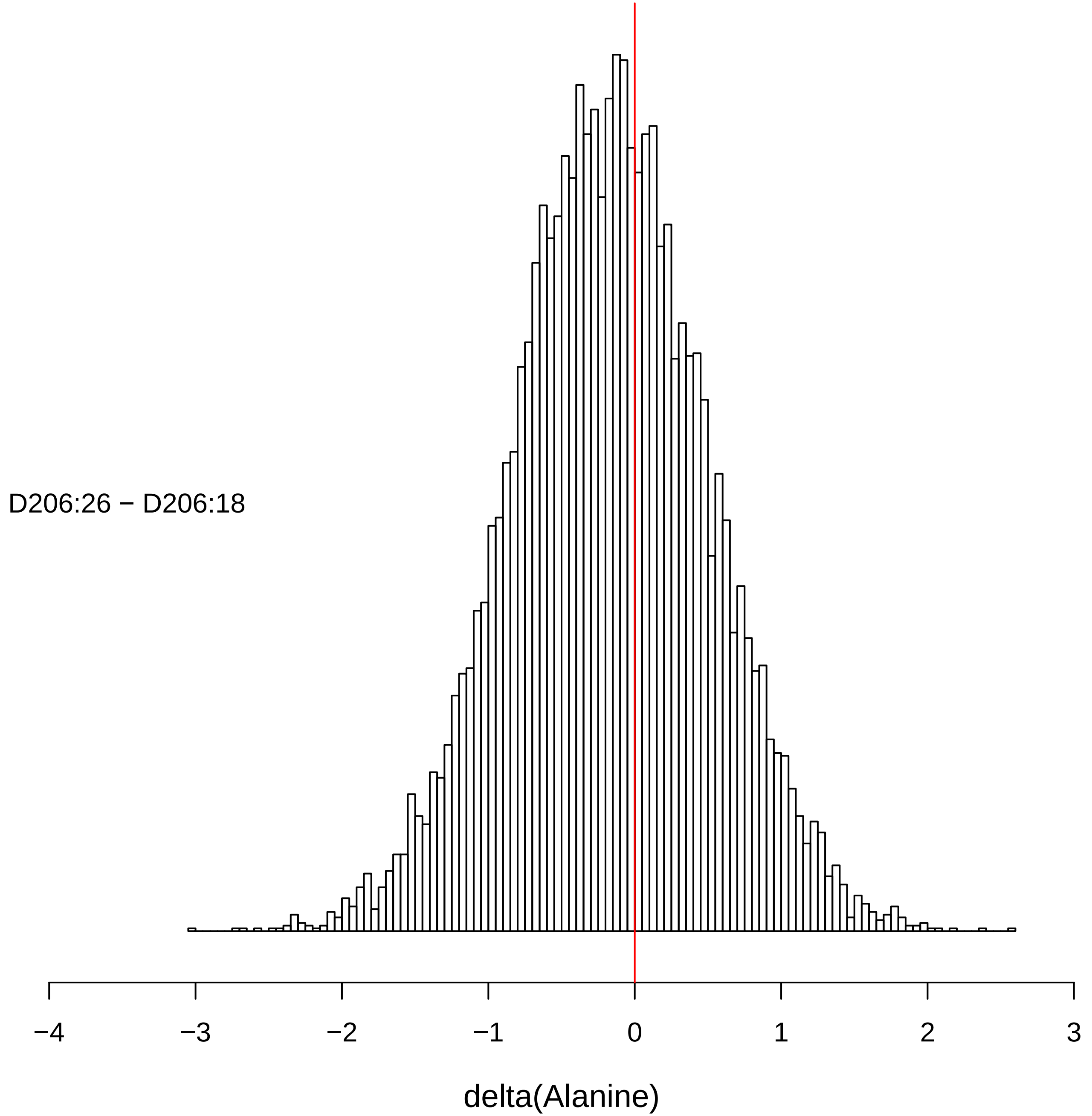
D206:26 − D206:18
−4
−3
−2
−1
0
1
2
3
delta(Alanine)

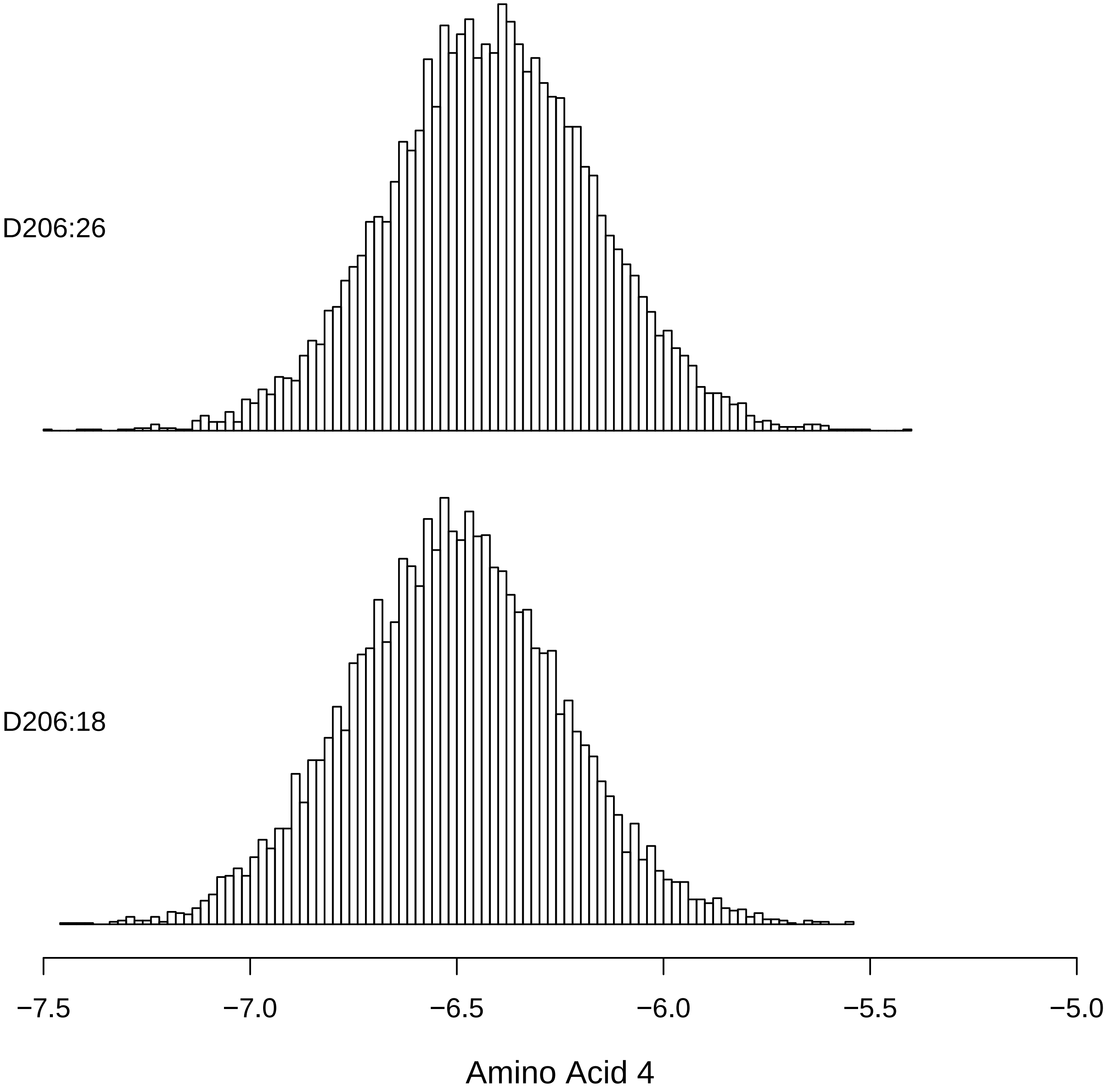
D206:26
D206:18
−7.5
−7.0
−6.5
−6.0
−5.5
−5.0
Amino Acid 4

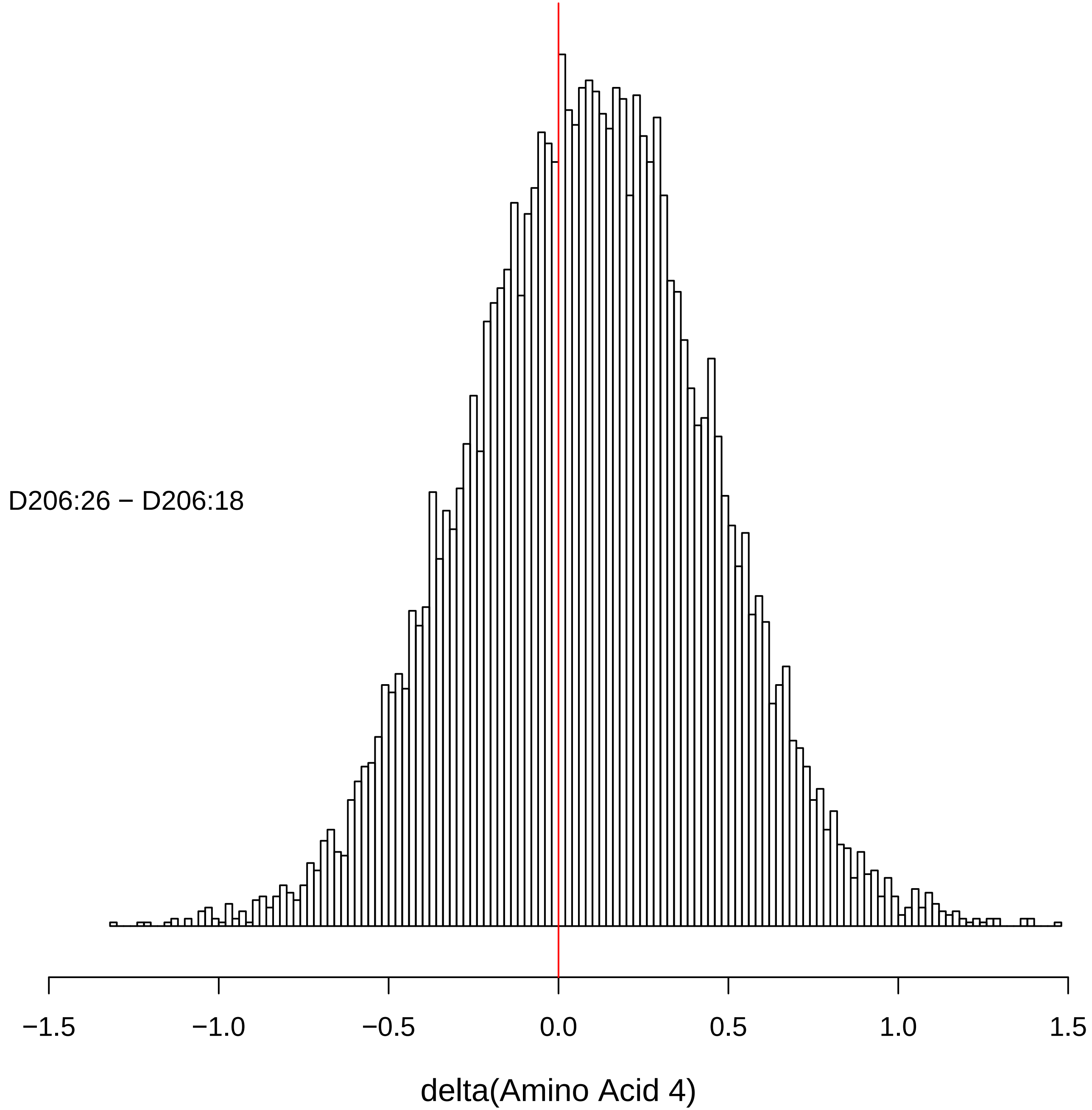
D206:26 − D206:18
−1.5
−1.0
−0.5
0.0
0.5
1.0
1.5
delta(Amino Acid 4)

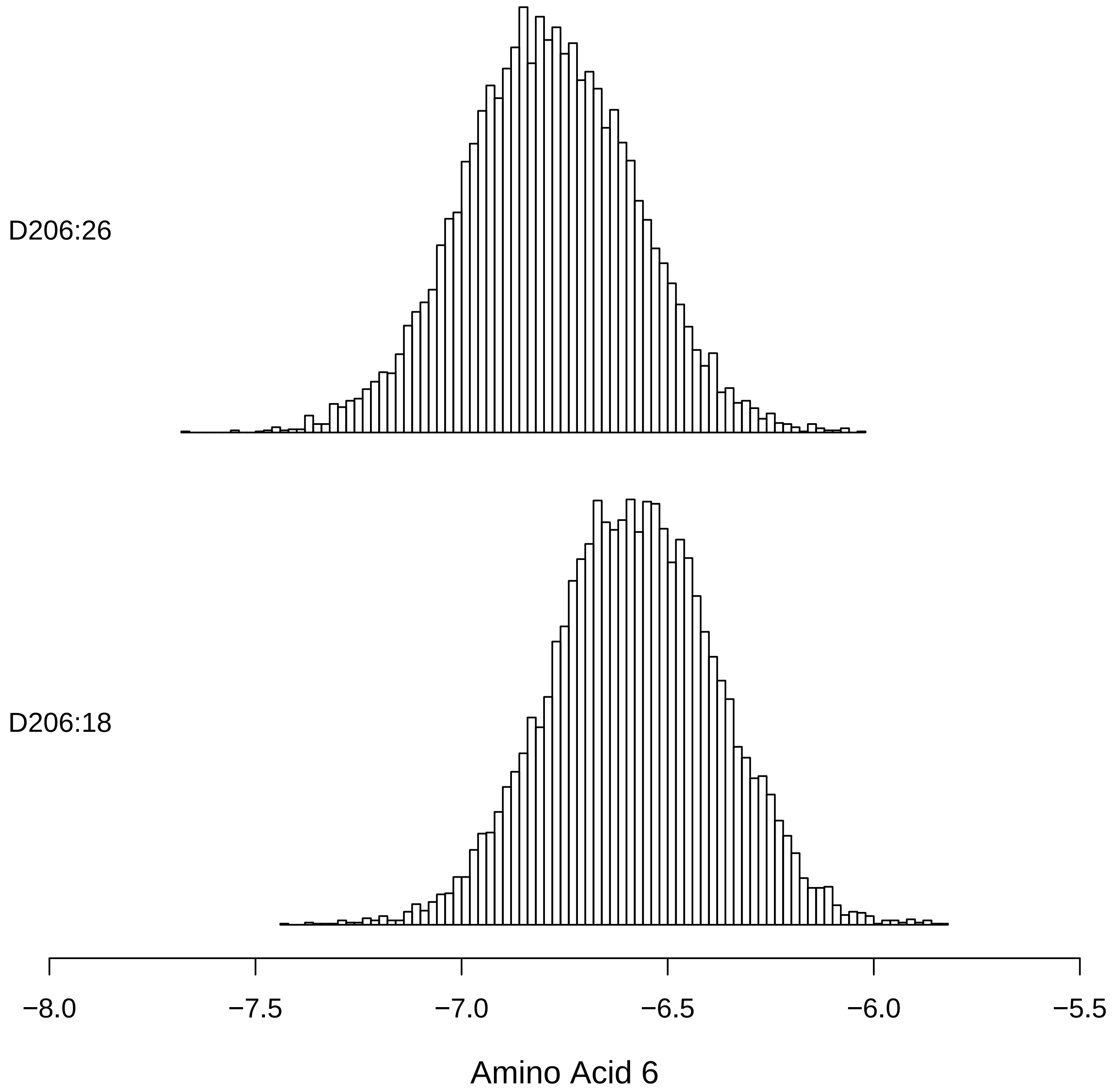
D206:26
D206:18
−8.0
−7.5
−7.0
−6.5
−6.0
−5.5
Amino Acid 6

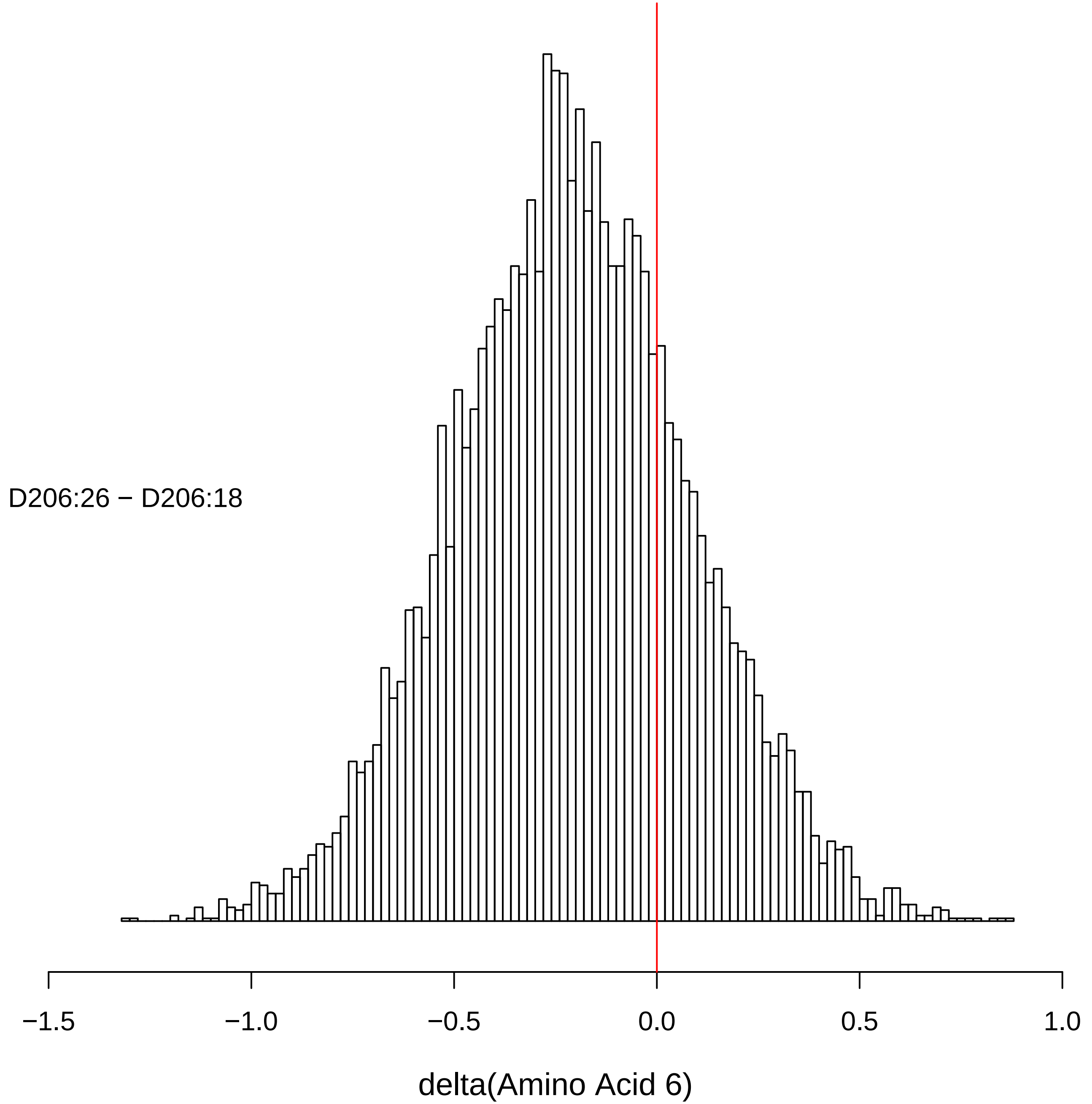
D206:26 − D206:18
−1.5
−1.0
−0.5
0.0
0.5
1.0
delta(Amino Acid 6)

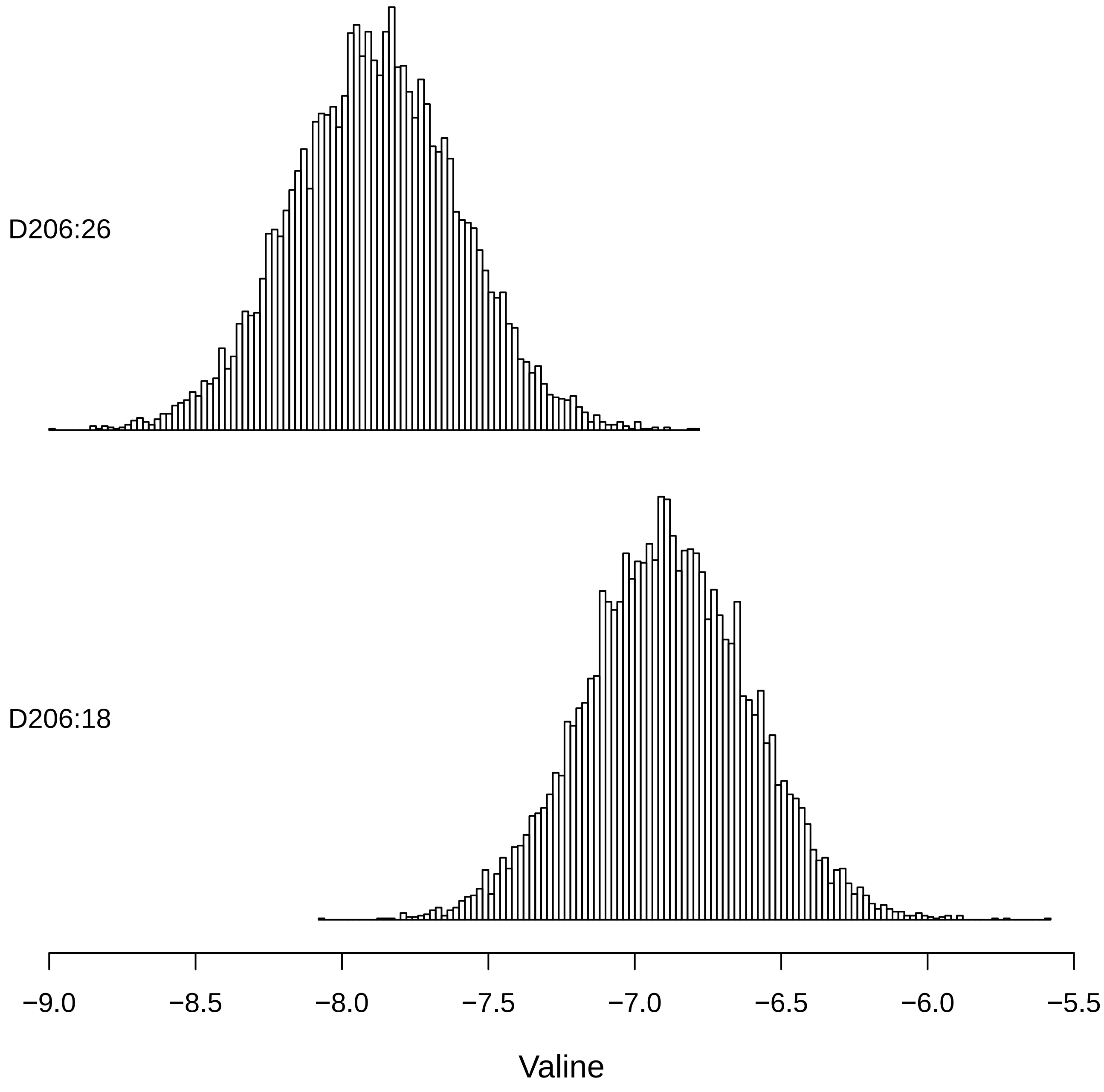
D206:26
D206:18
−9.0
−8.5
−8.0
−7.5
−7.0
−6.5
−6.0
−5.5
Valine

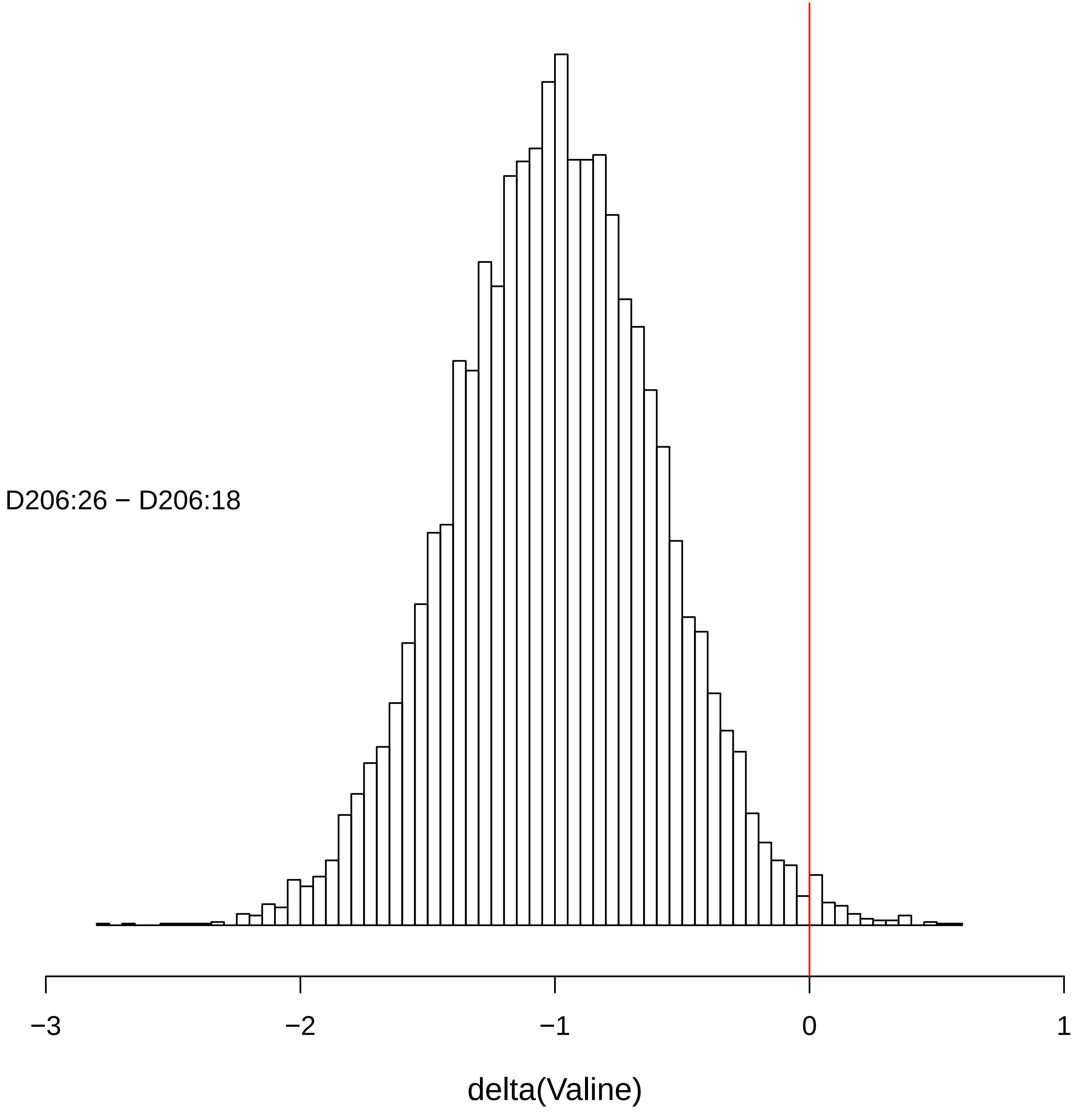
D206:26 − D206:18
−3
−2
−1
0
1
delta(Valine)

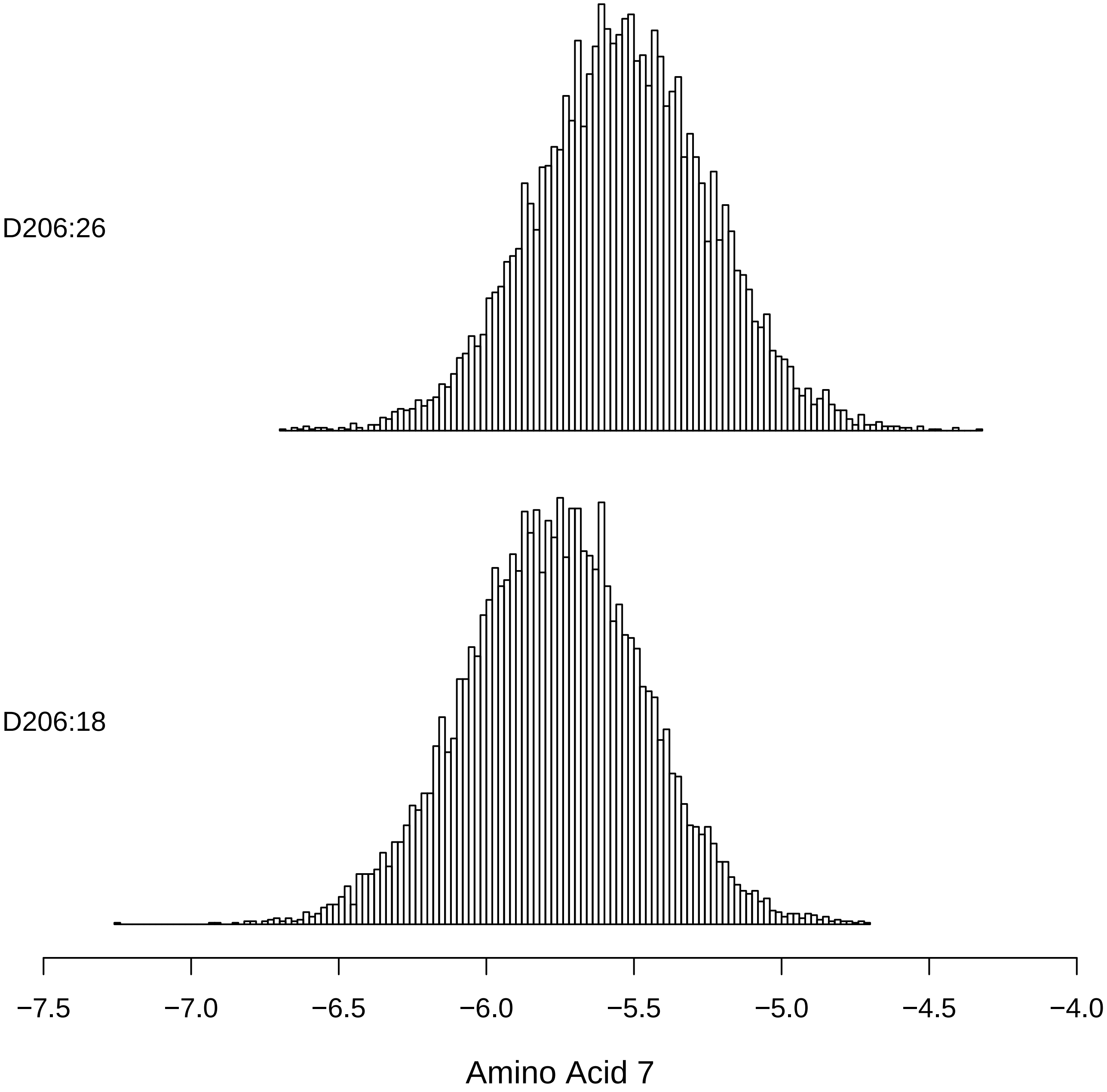

D206:26
D206:18
−7.5
−7.0
−6.5
−6.0
−5.5
−5.0
−4.5
−4.0
Amino Acid 7

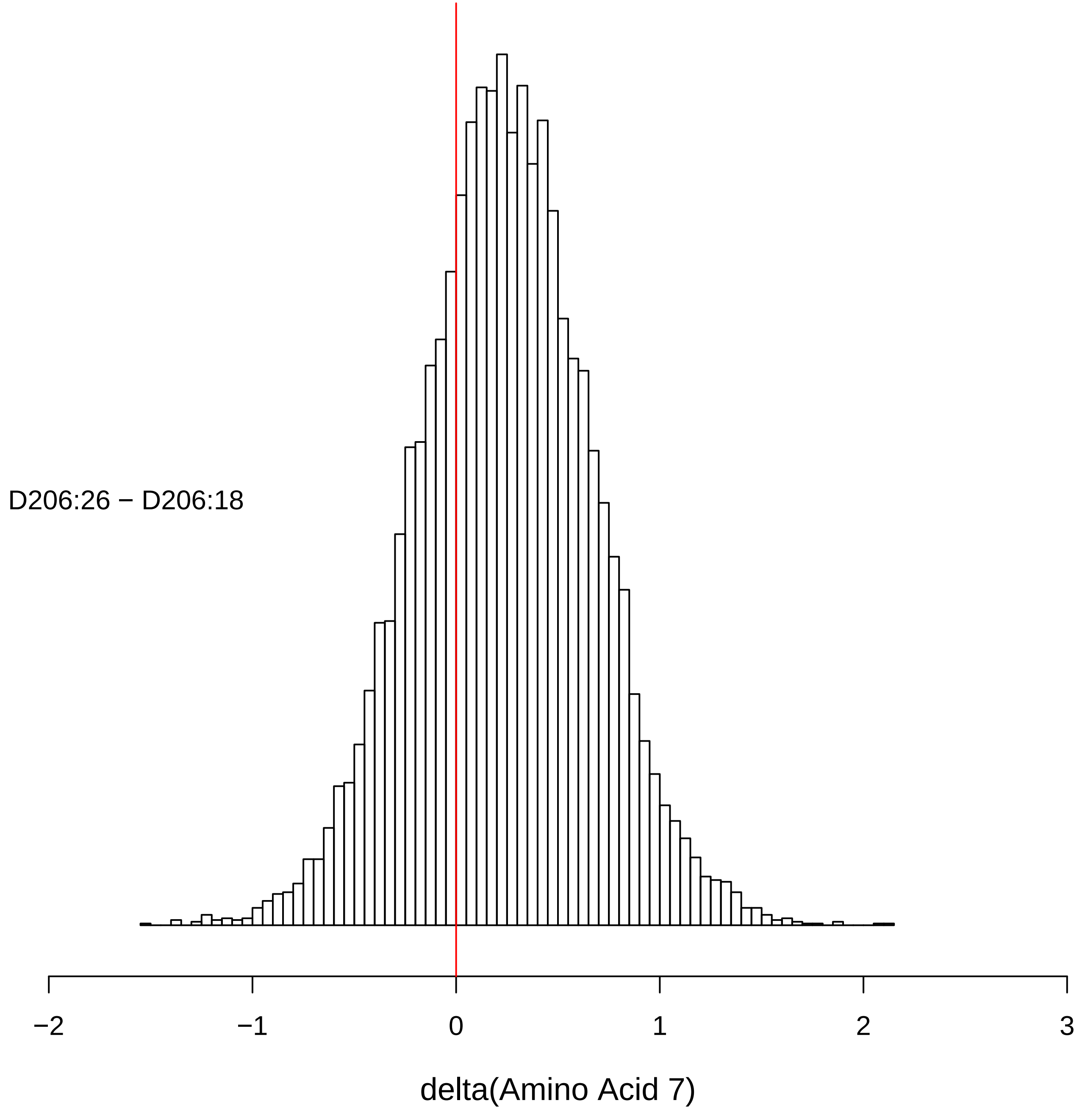
D206:26 − D206:18
−2
−1
0
1
2
3
delta(Amino Acid 7)

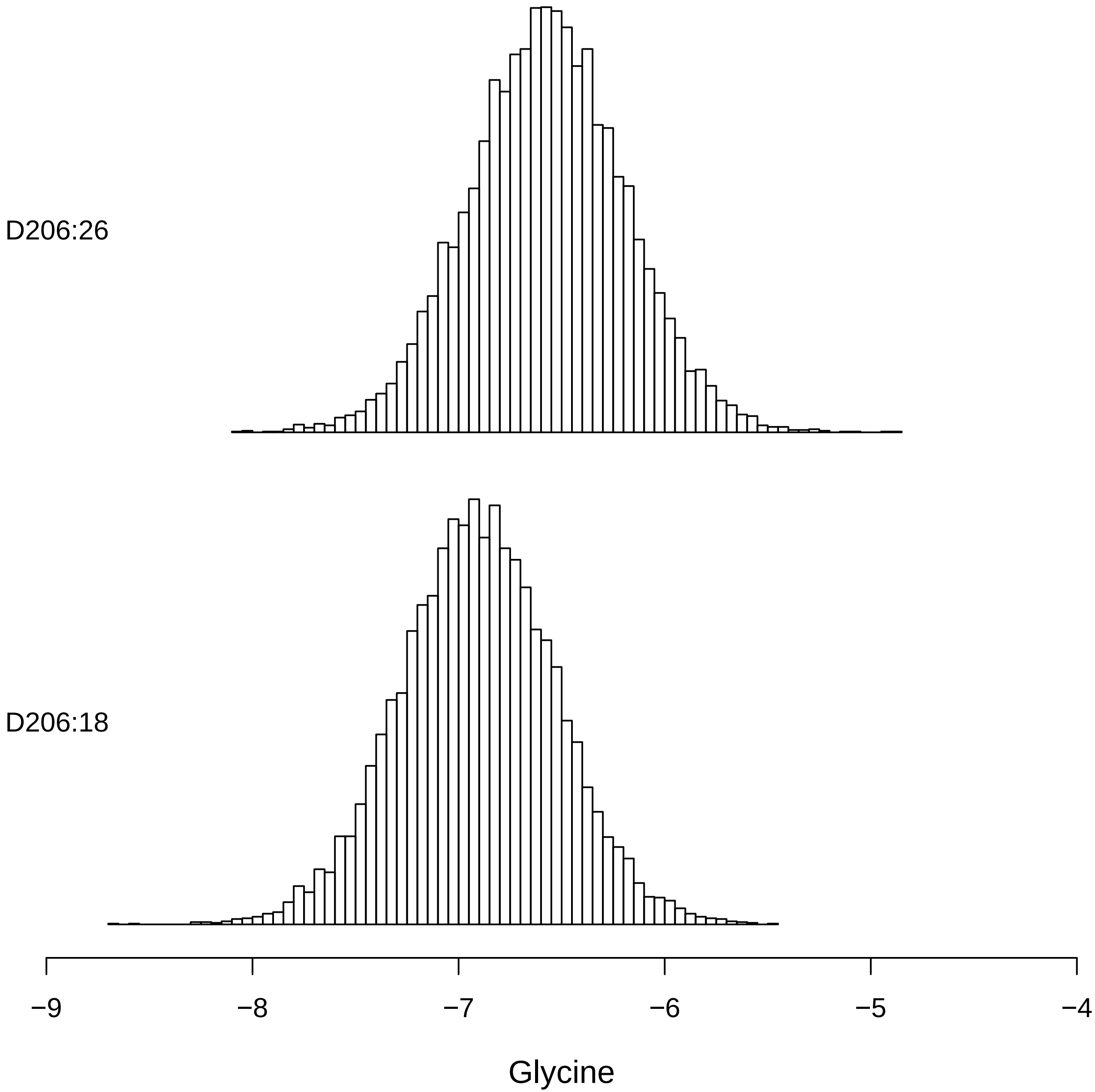

D206:26
D206:18
−9
−8
−7
−6
−5
−4
Glycine

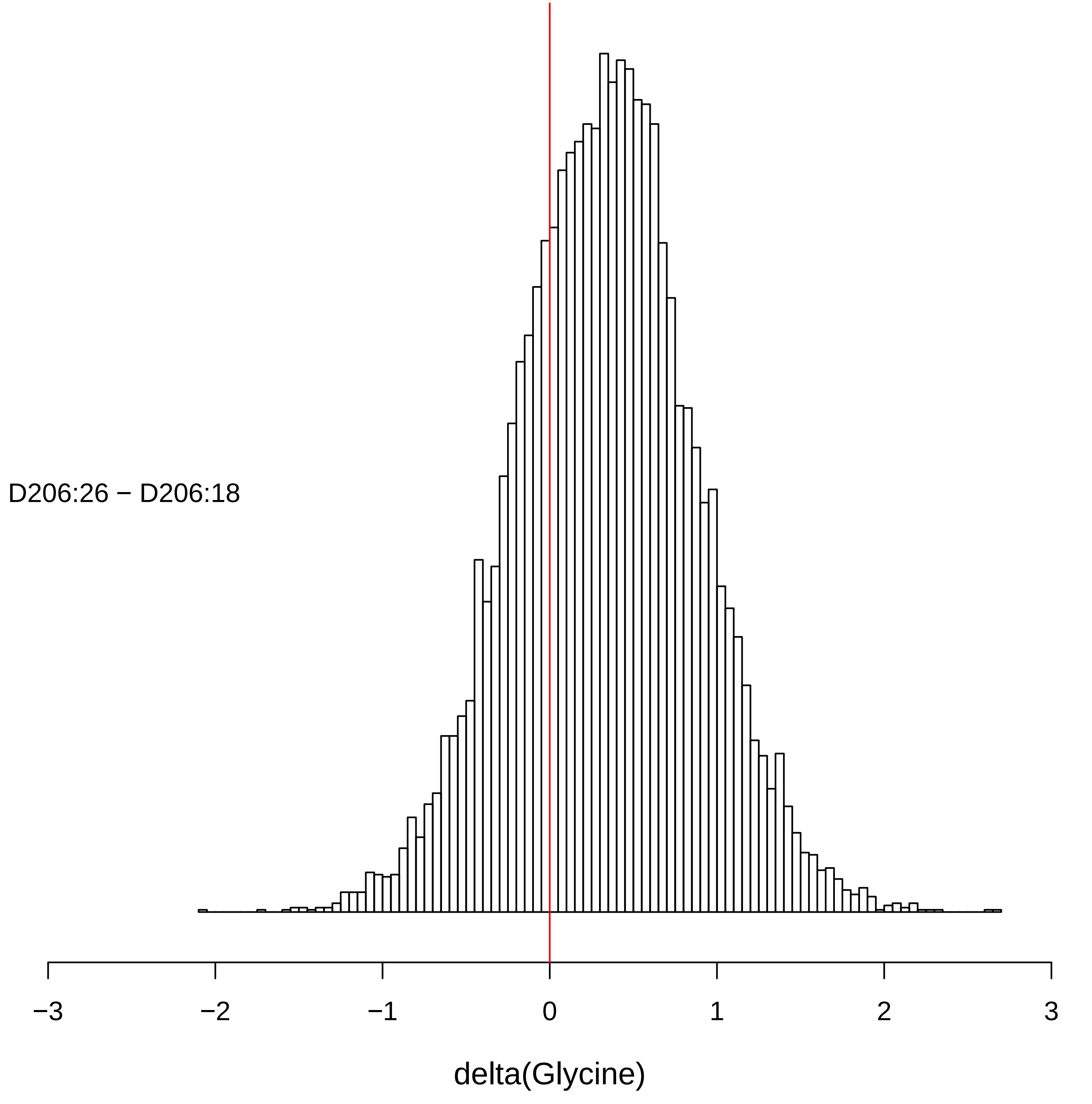
D206:26 − D206:18
−3
−2
−1
0
1
2
3
delta(Glycine)

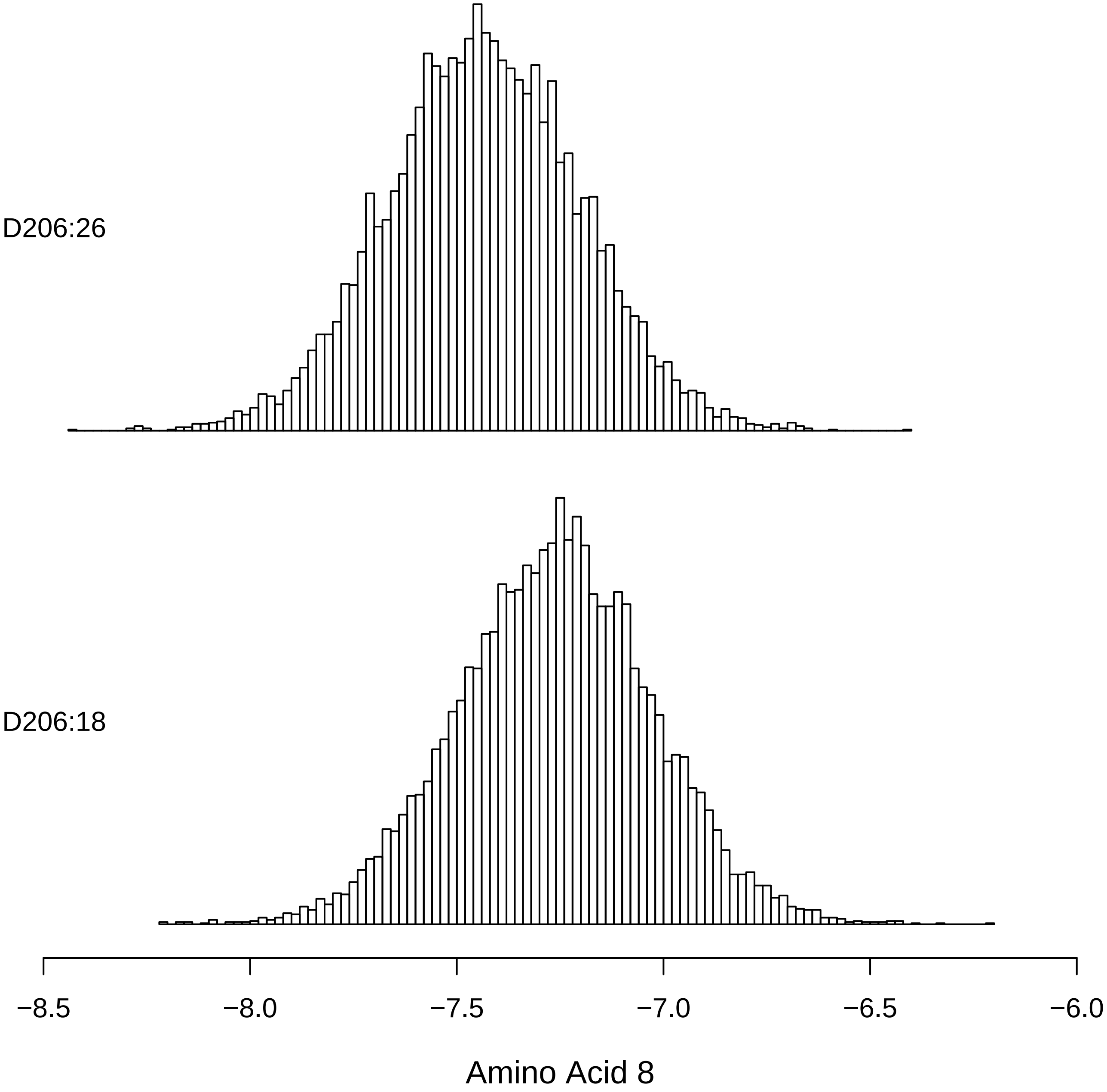

D206:26
D206:18
−8.5
−8.0
−7.5
−7.0
−6.5
−6.0
Amino Acid 8

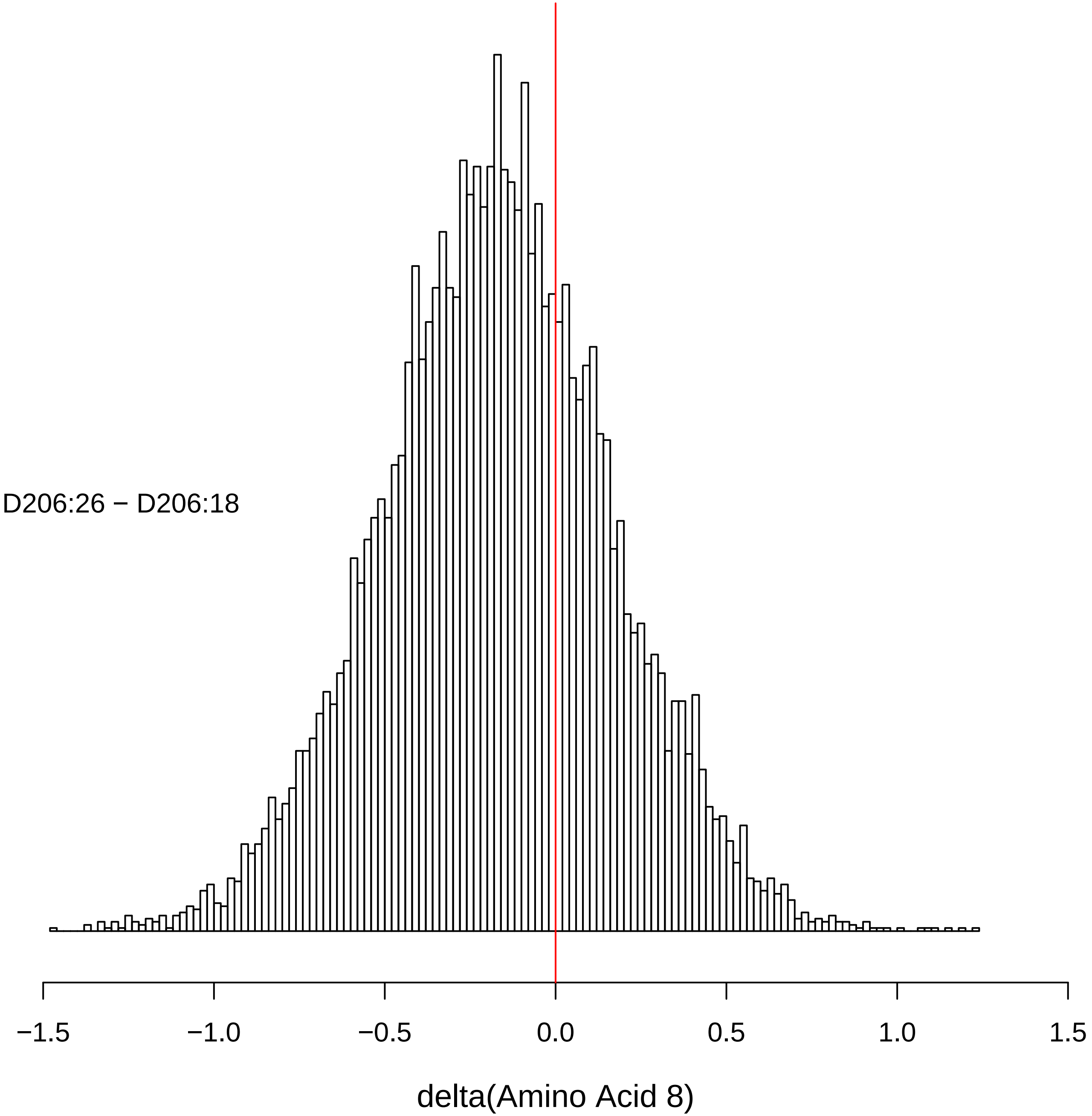
D206:26 − D206:18
−1.5
−1.0
−0.5
0.0
0.5
1.0
1.5
delta(Amino Acid 8)

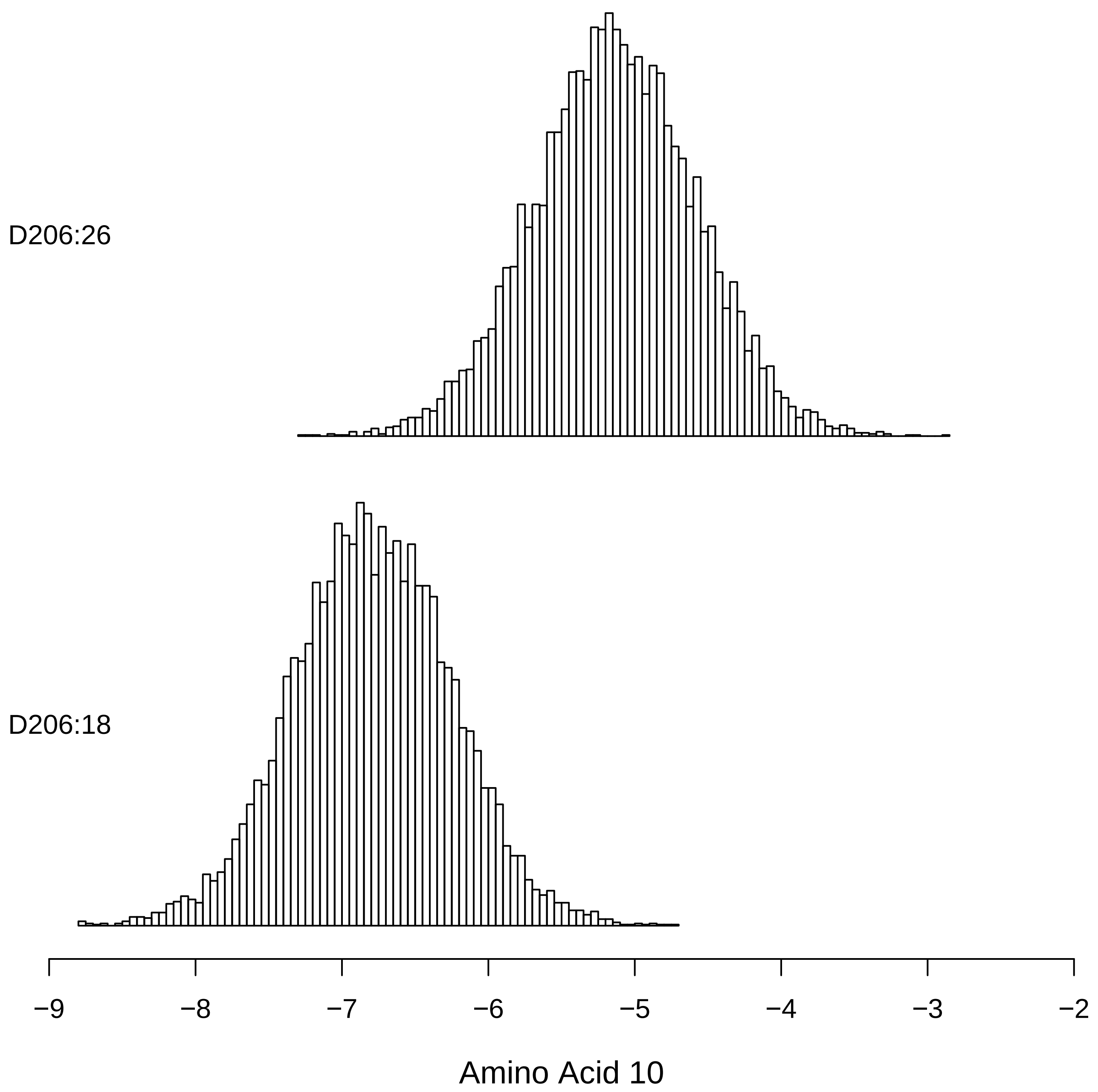

D206:26
D206:18
−9
−8
−7
−6
−5
−4
−3
−2
Amino Acid 10

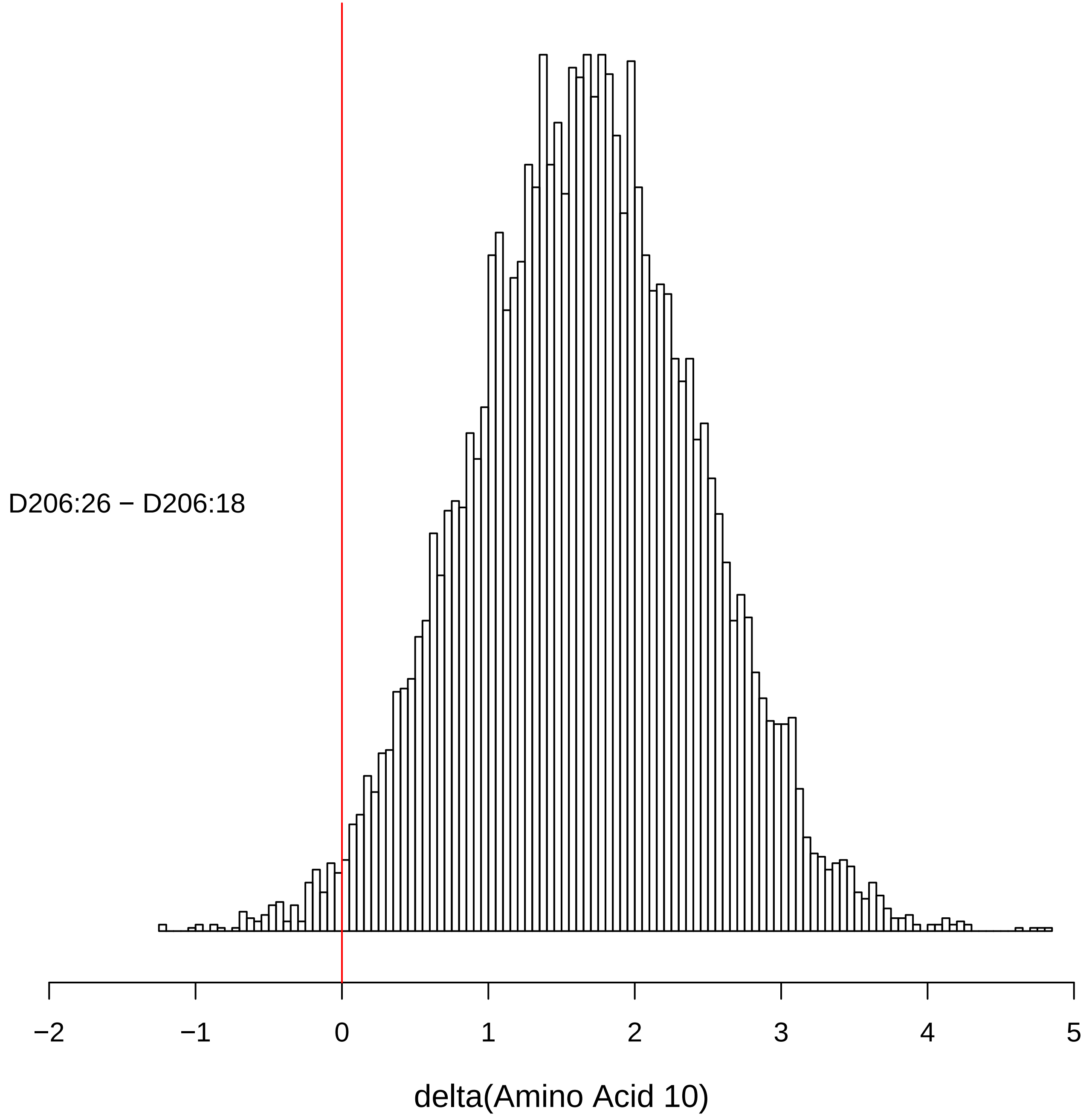

D206:26 − D206:18
−2
−1
0
1
2
3
4
5
delta(Amino Acid 10)

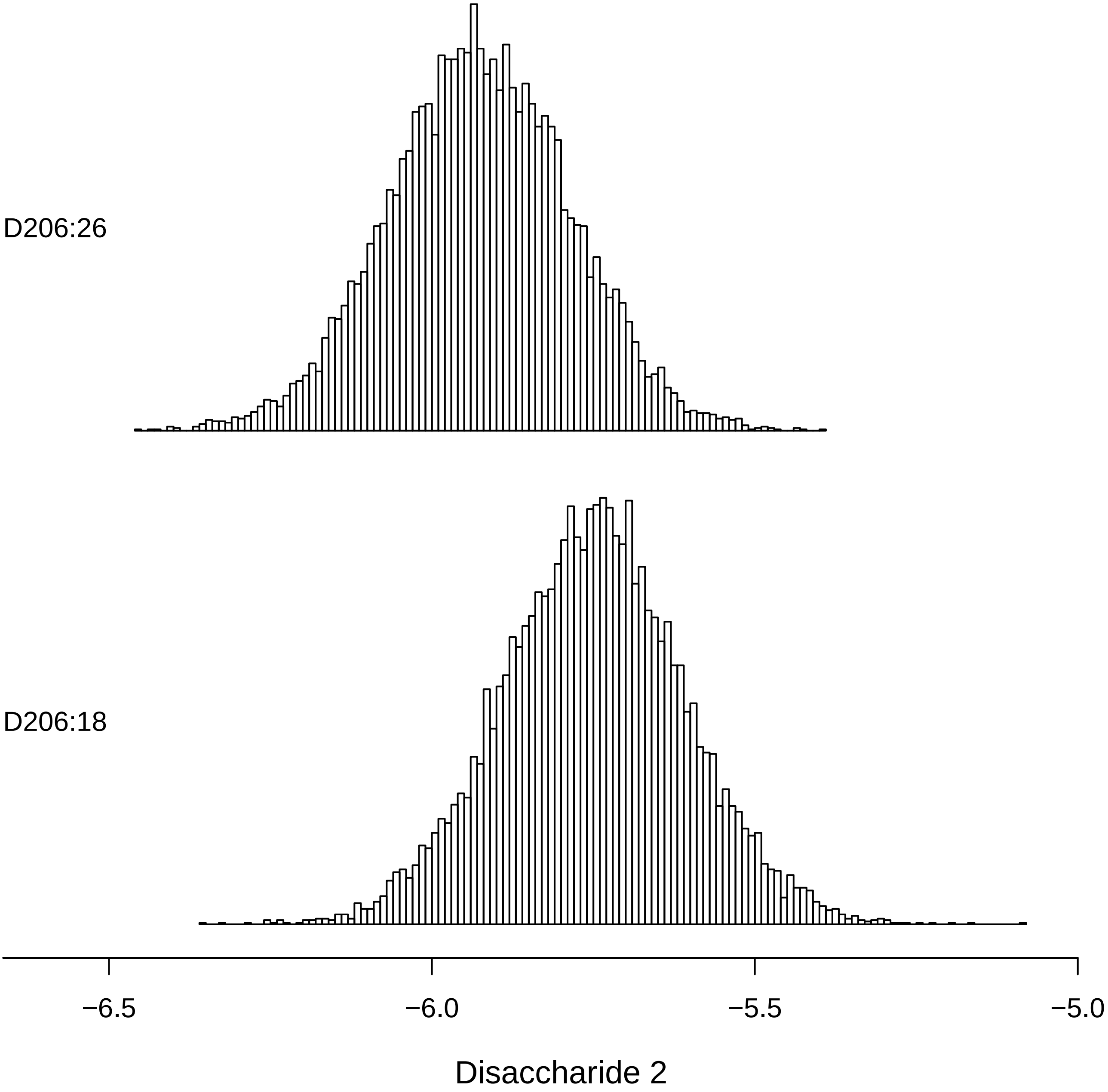
D206:26
D206:18
−6.5
−6.0
−5.5
−5.0
Disaccharide 2

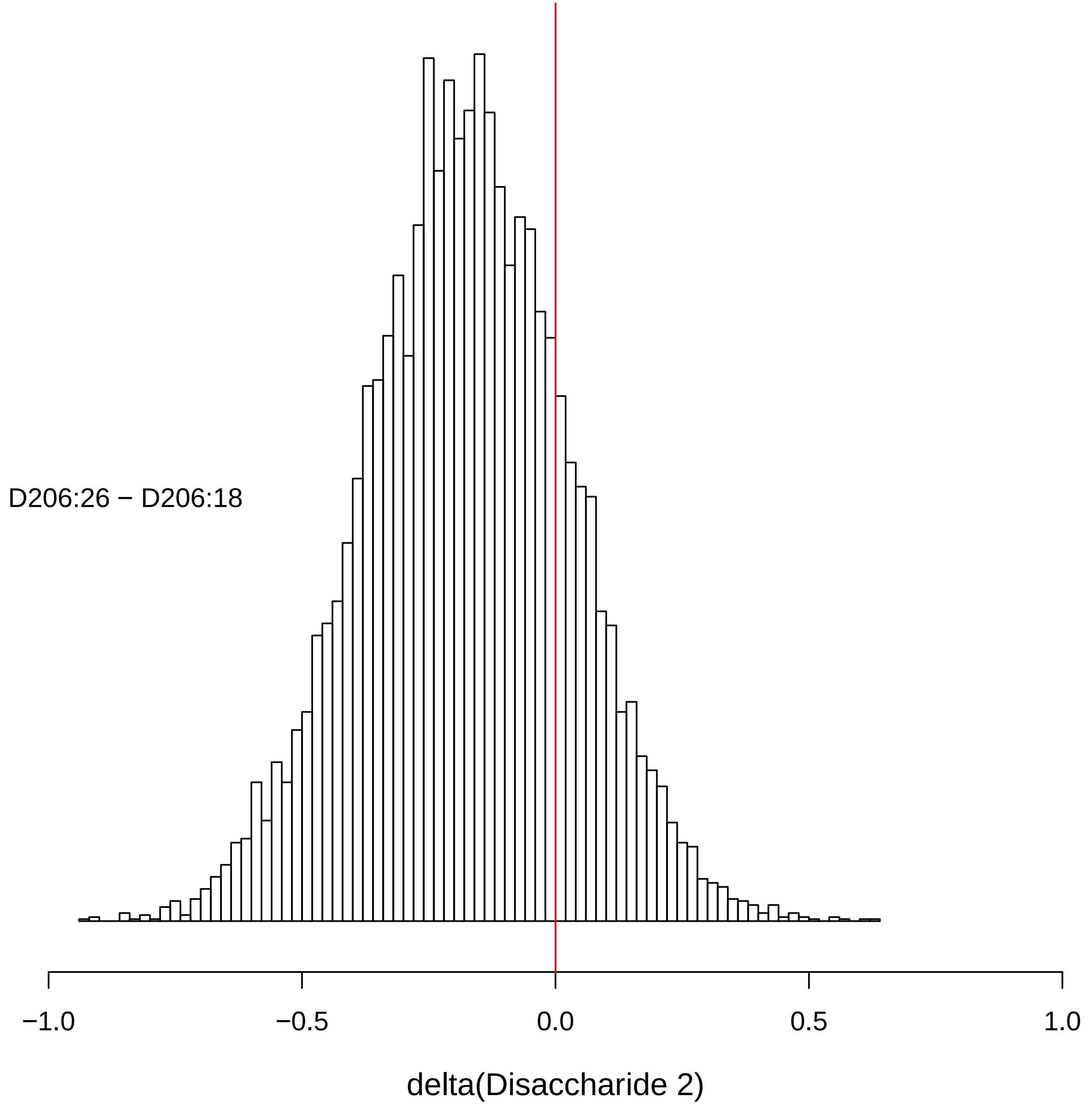
D206:26 − D206:18
−1.0
−0.5
0.0
0.5
1.0
delta(Disaccharide 2)

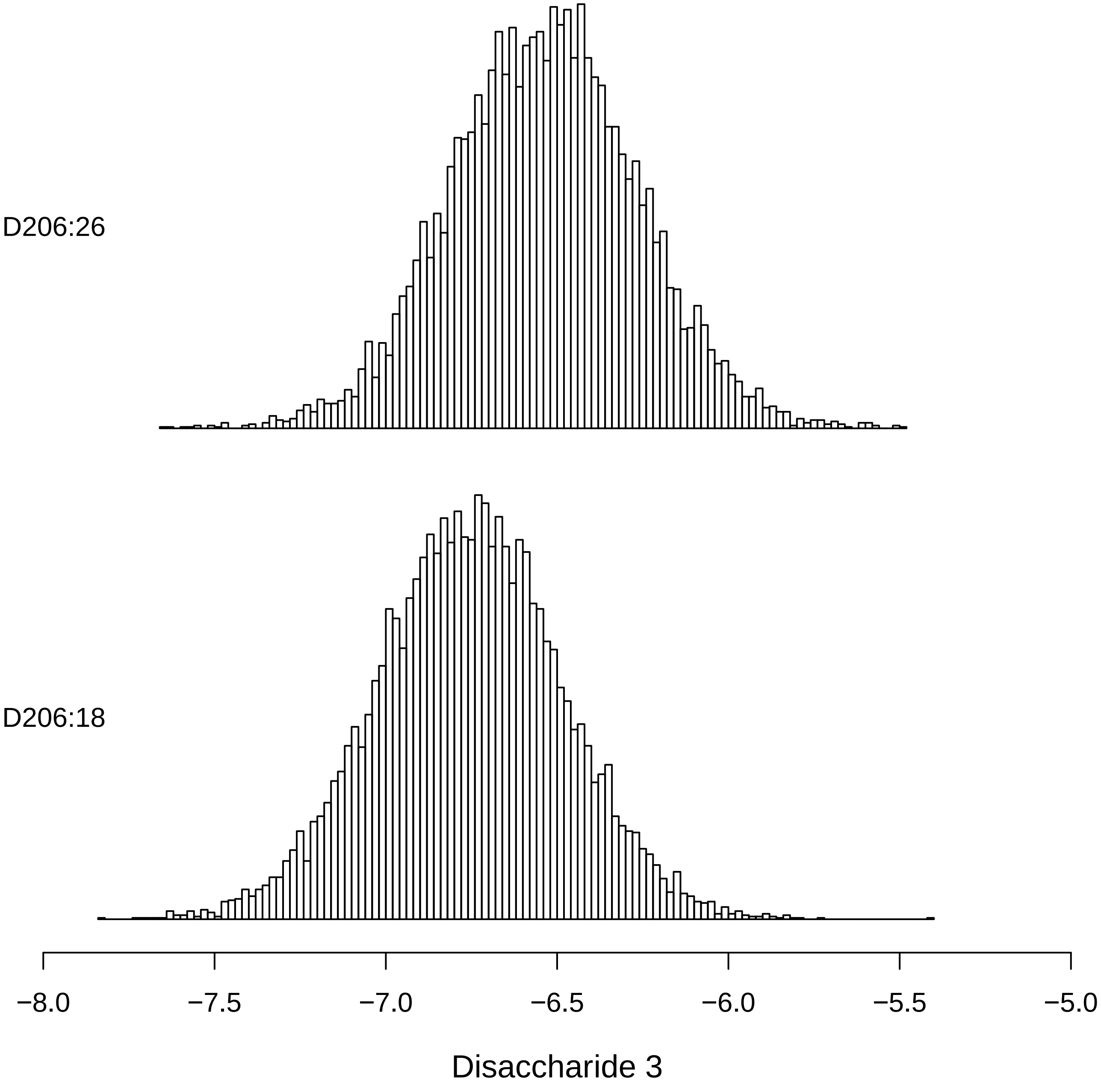
D206:26
D206:18
−8.0
−7.5
−7.0
−6.5
−6.0
−5.5
−5.0
Disaccharide 3

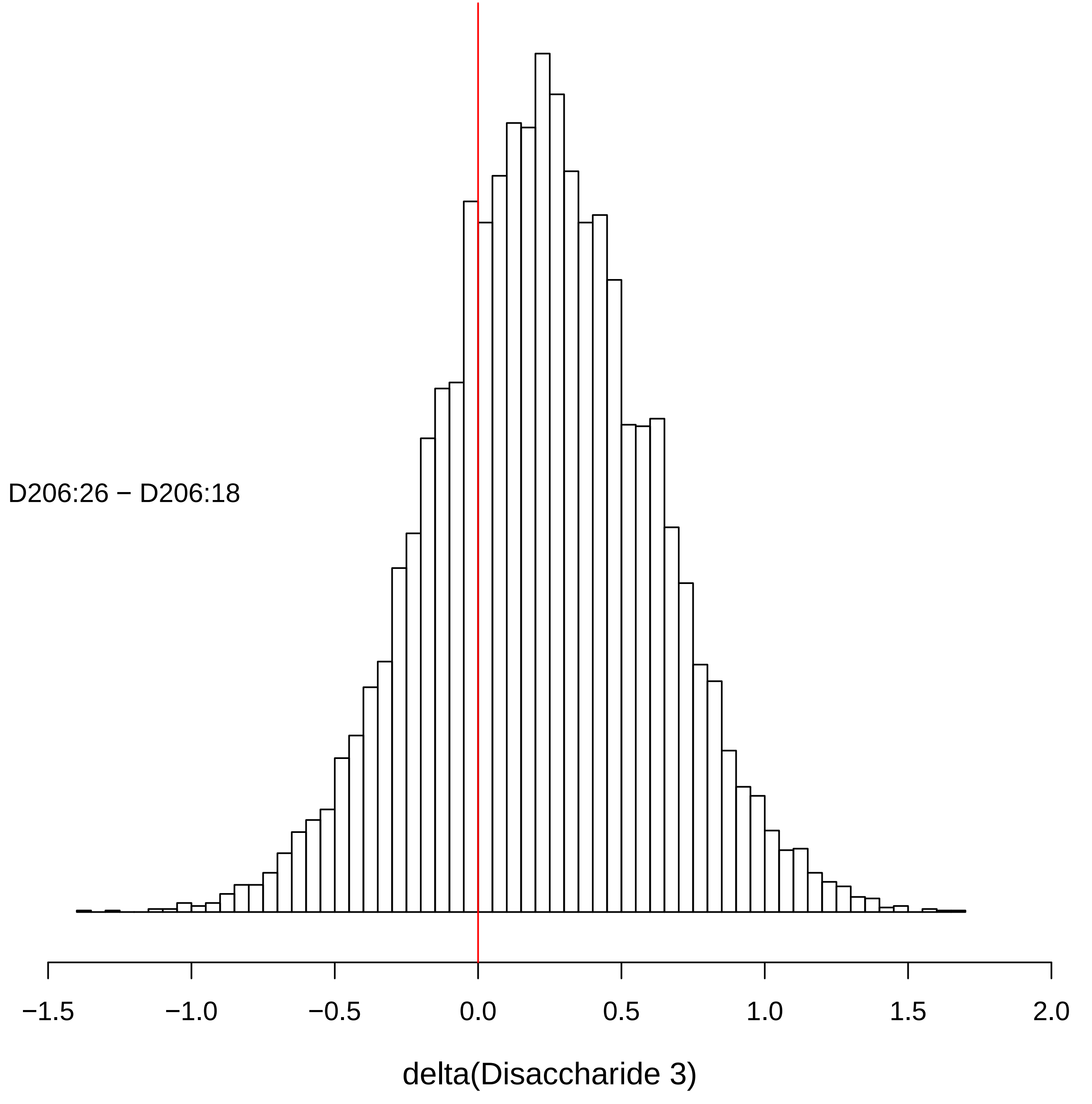
D206:26 − D206:18
−1.5
−1.0
−0.5
0.0
0.5
1.0
1.5
2.0
delta(Disaccharide 3)

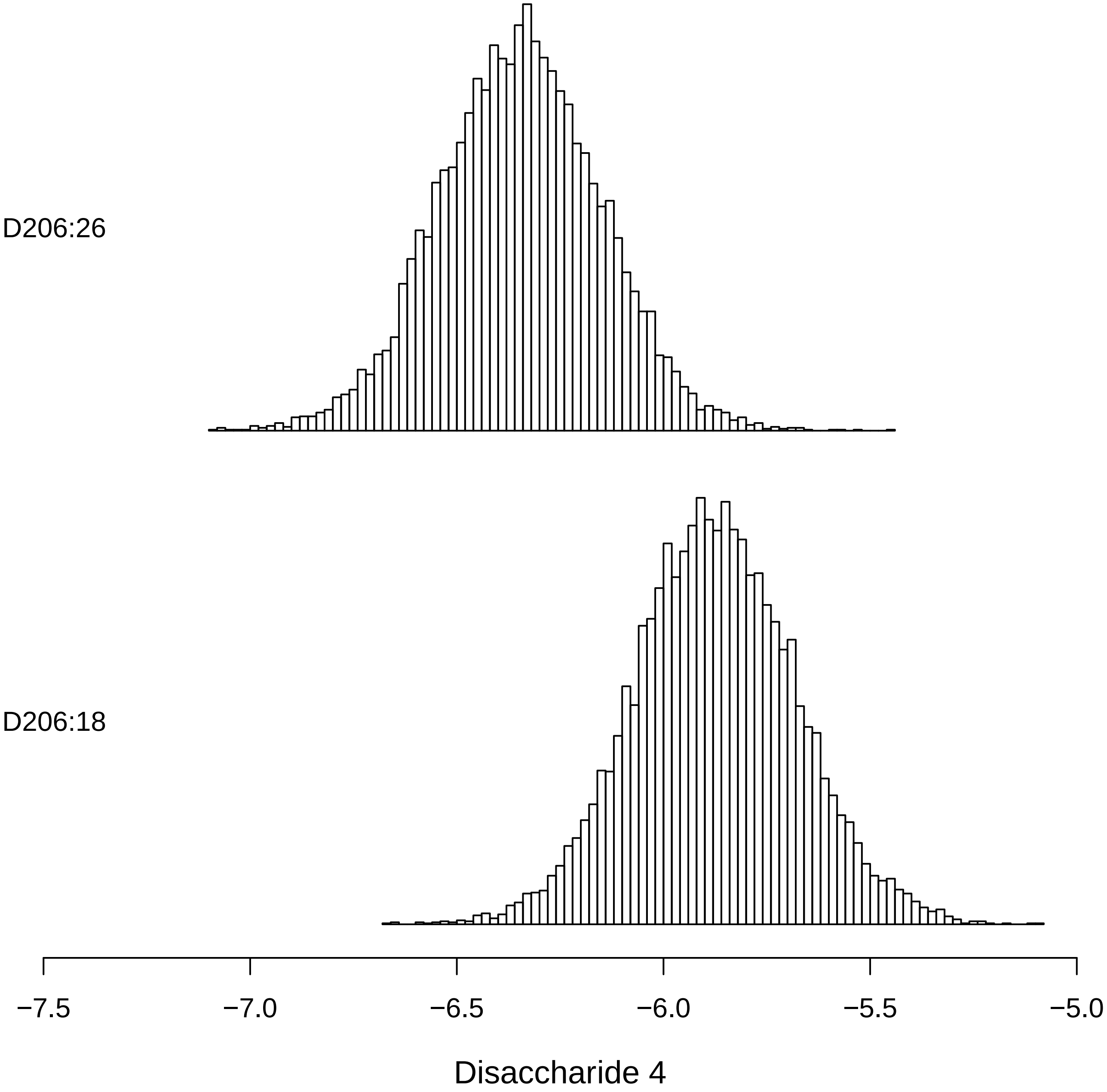

D206:26
D206:18
−7.5
−7.0
−6.5
−6.0
−5.5
−5.0
Disaccharide 4

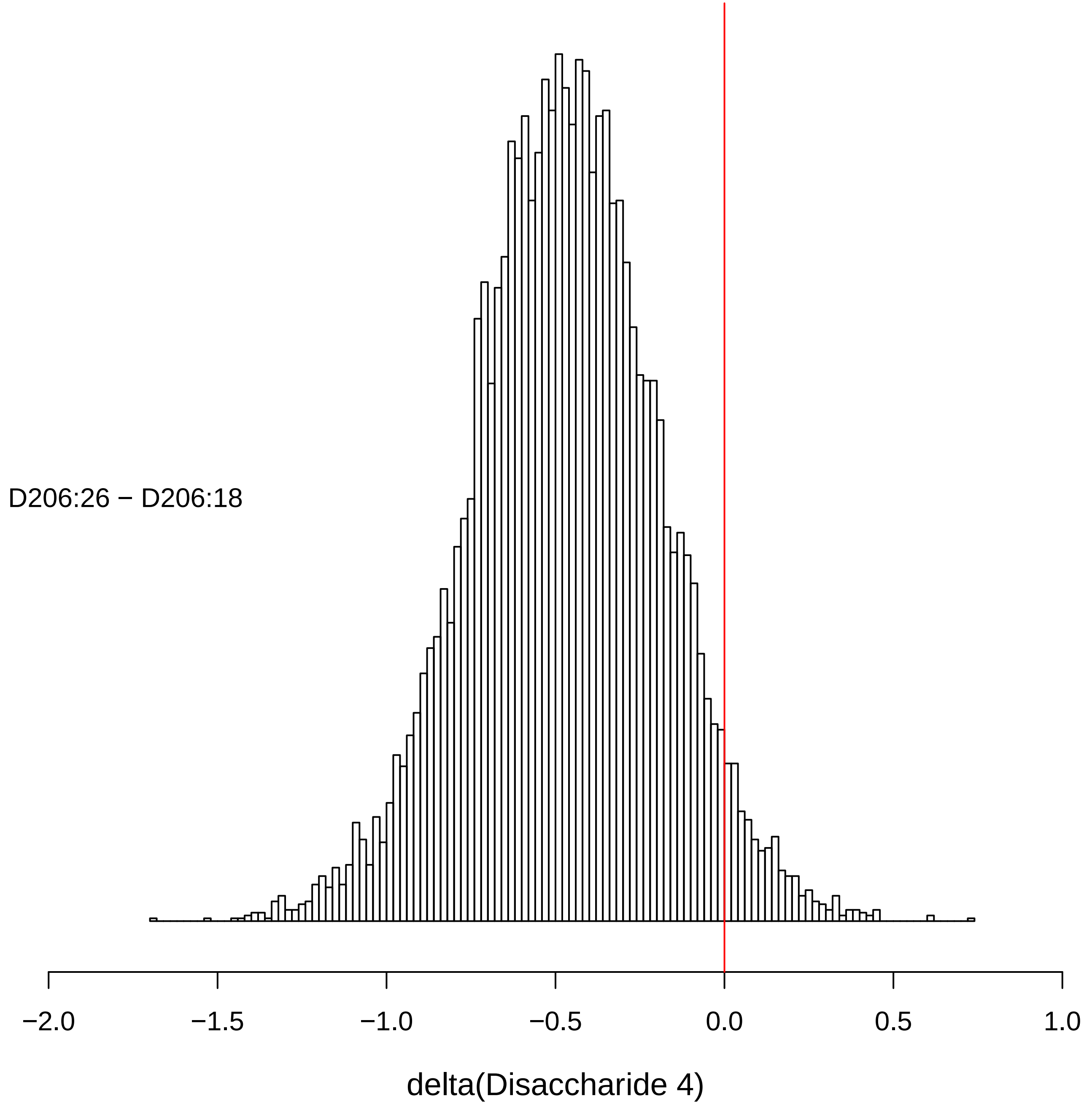
D206:26 − D206:18
−2.0
−1.5
−1.0
−0.5
0.0
0.5
1.0
delta(Disaccharide 4)

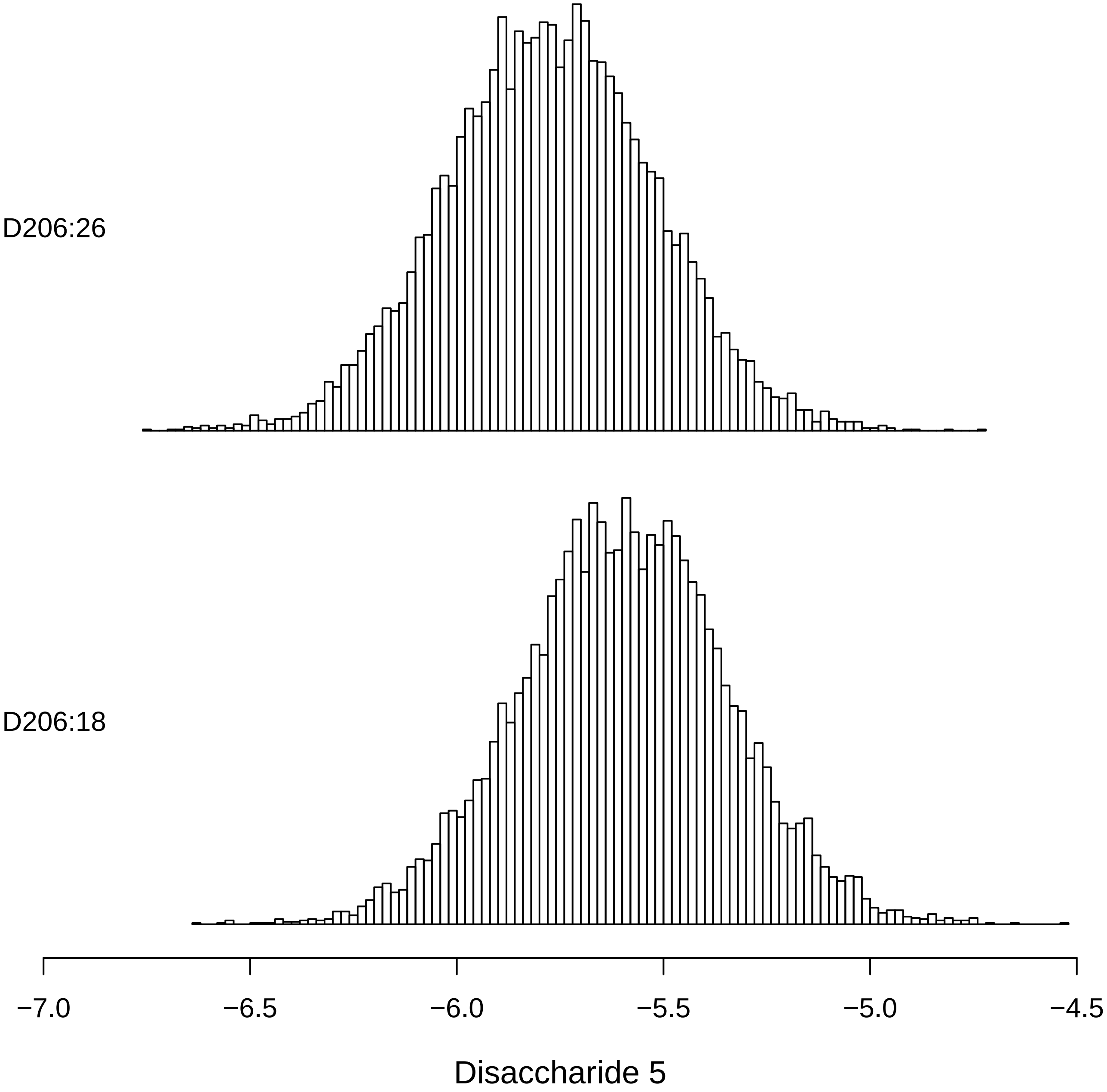
D206:26
D206:18
−7.0
−6.5
−6.0
−5.5
−5.0
−4.5
Disaccharide 5

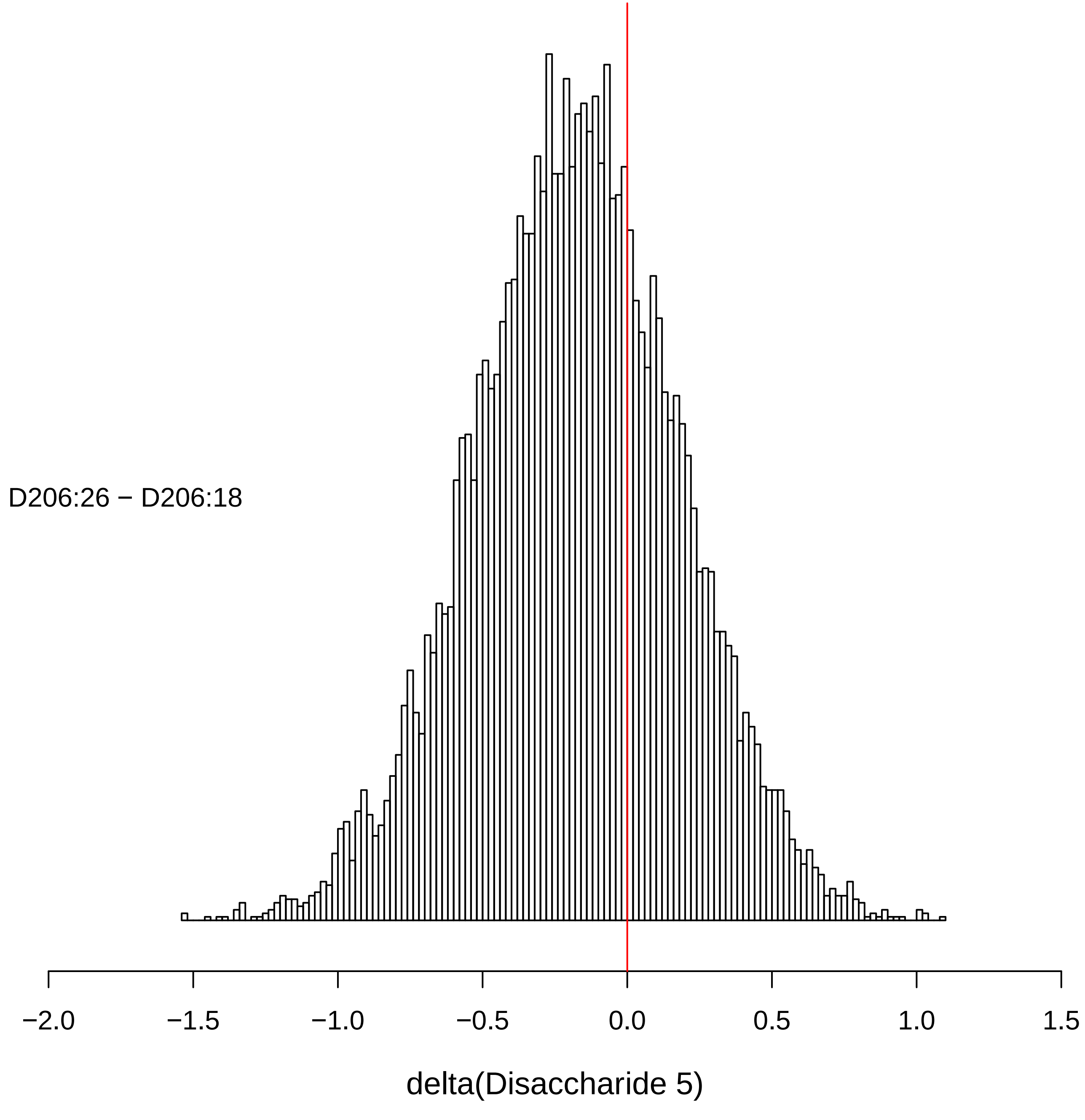
D206:26 − D206:18
−2.0
−1.5
−1.0
−0.5
0.0
0.5
1.0
1.5
delta(Disaccharide 5)

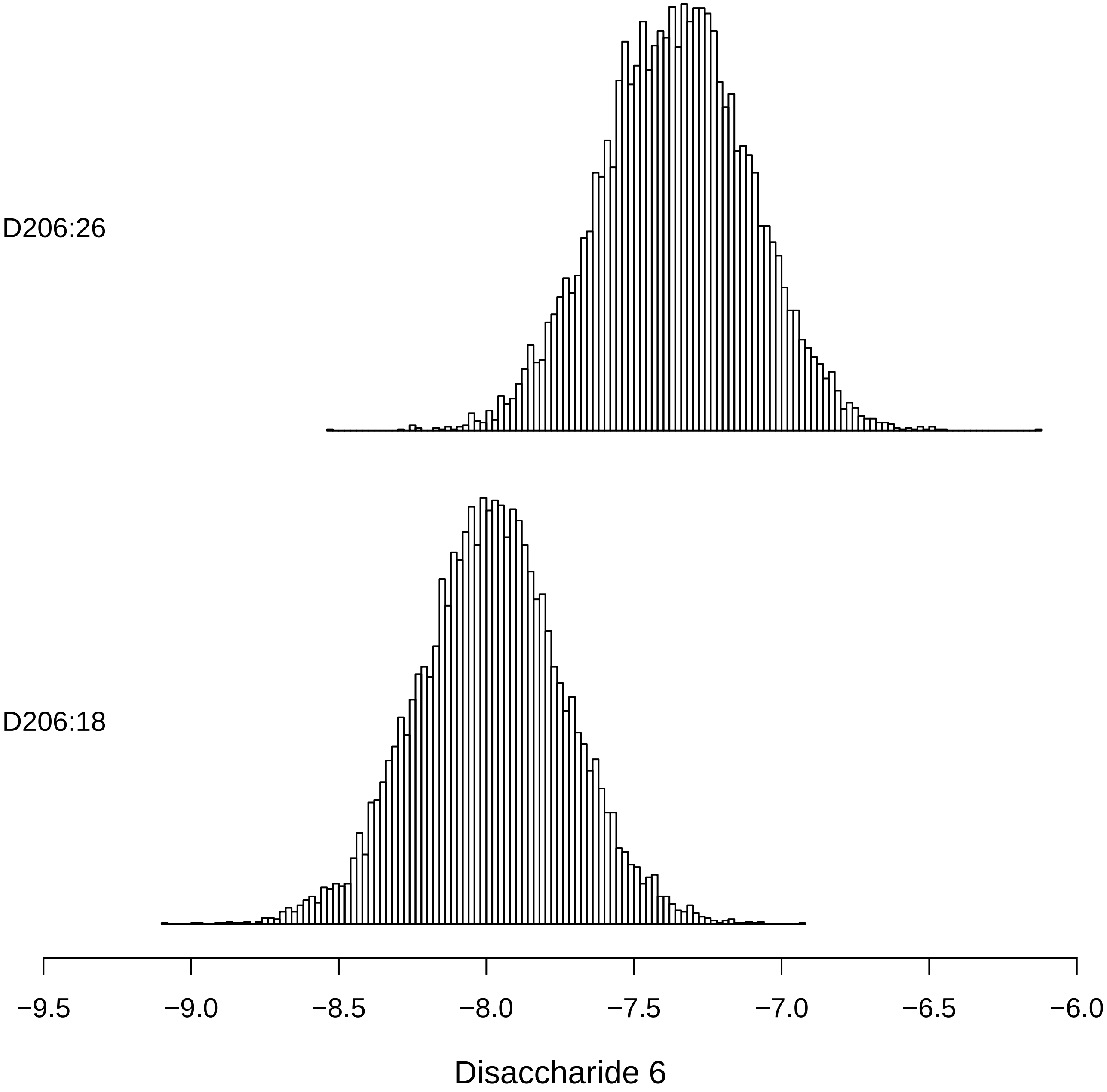
D206:26
D206:18
−9.5
−9.0
−8.5
−8.0
−7.5
−7.0
−6.5
−6.0
Disaccharide 6

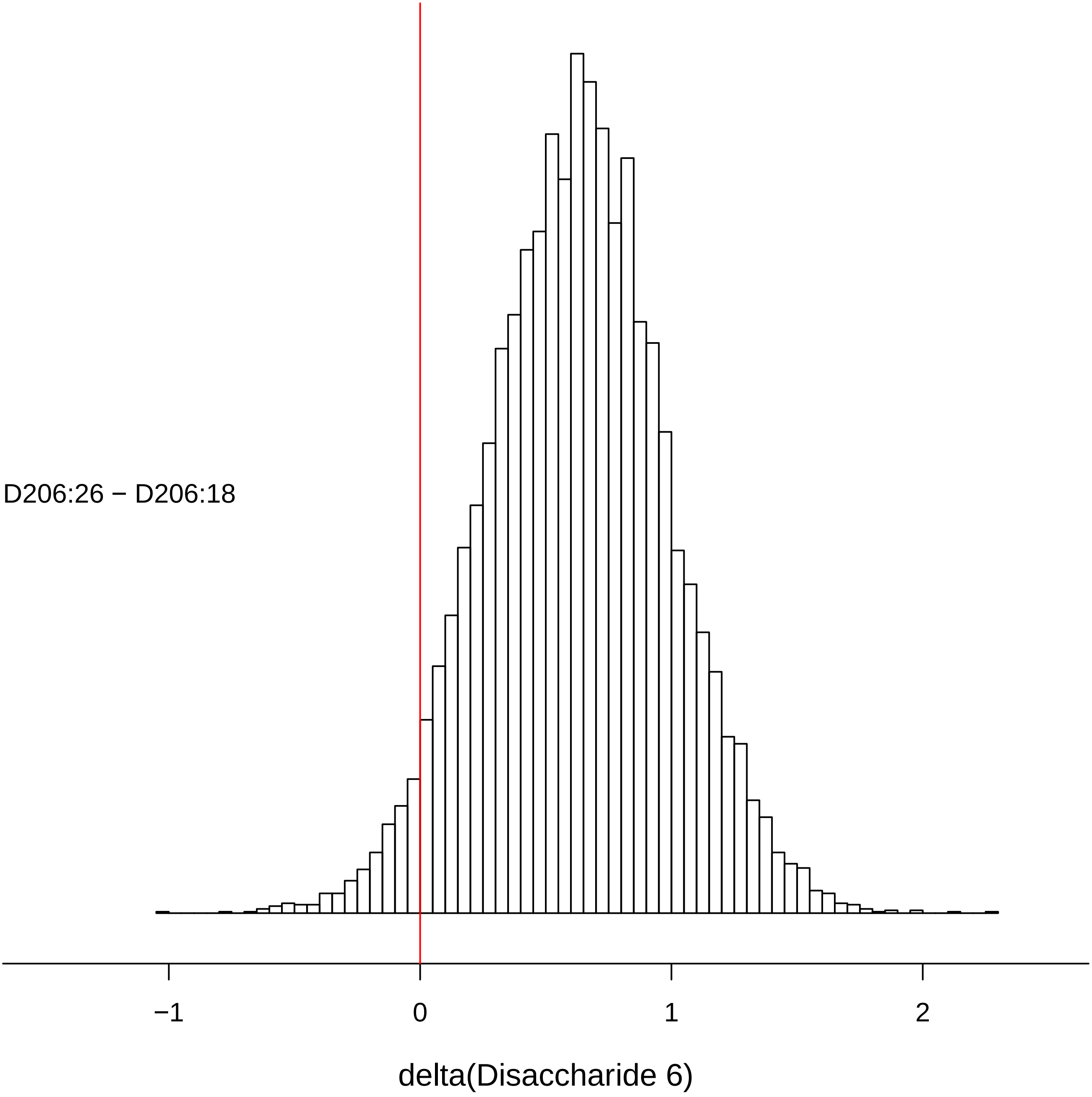
D206:26 − D206:18
−1
0
1
2
delta(Disaccharide 6)

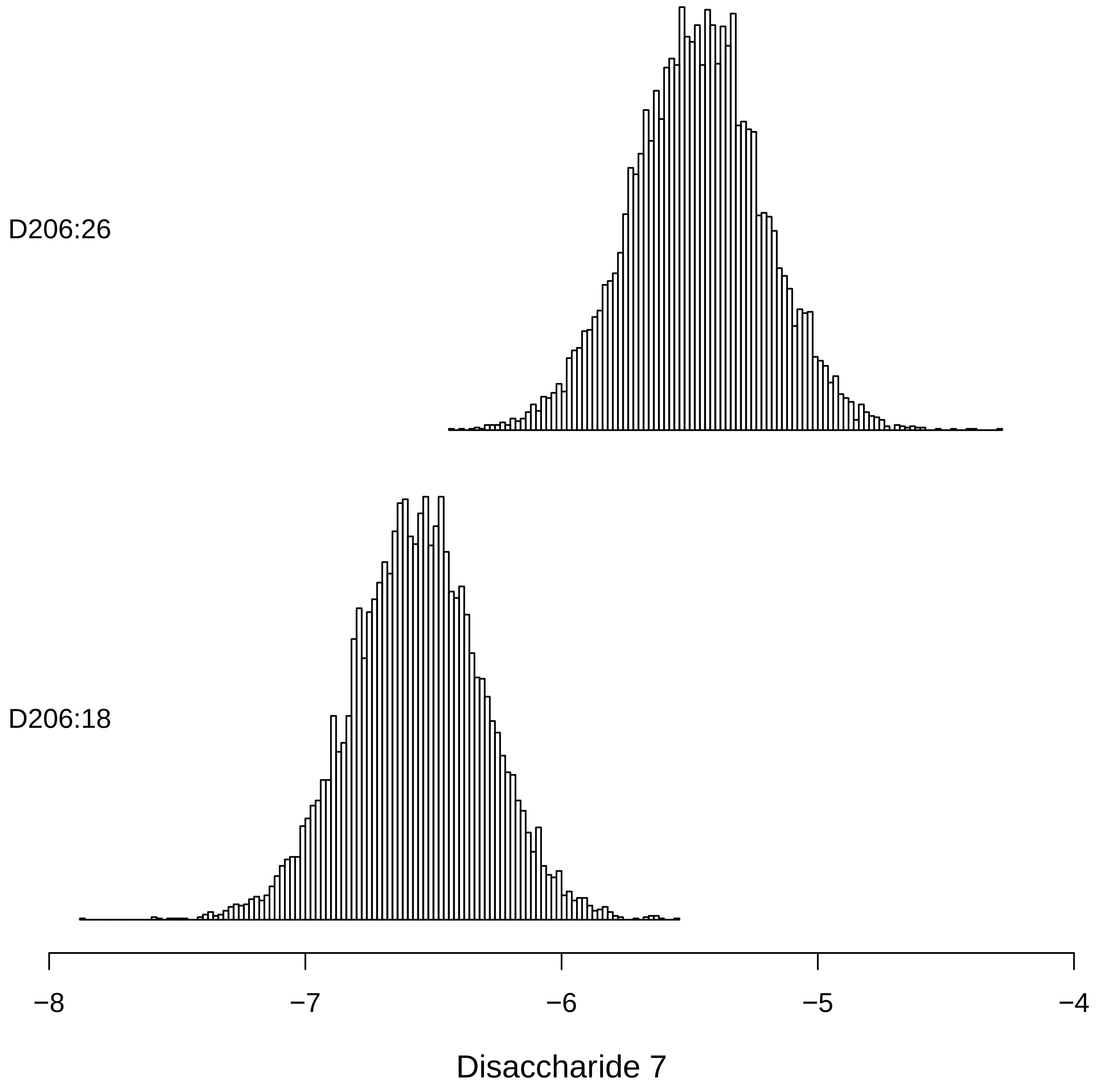

D206:26
D206:18
−8
−7
−6
−5
−4
Disaccharide 7

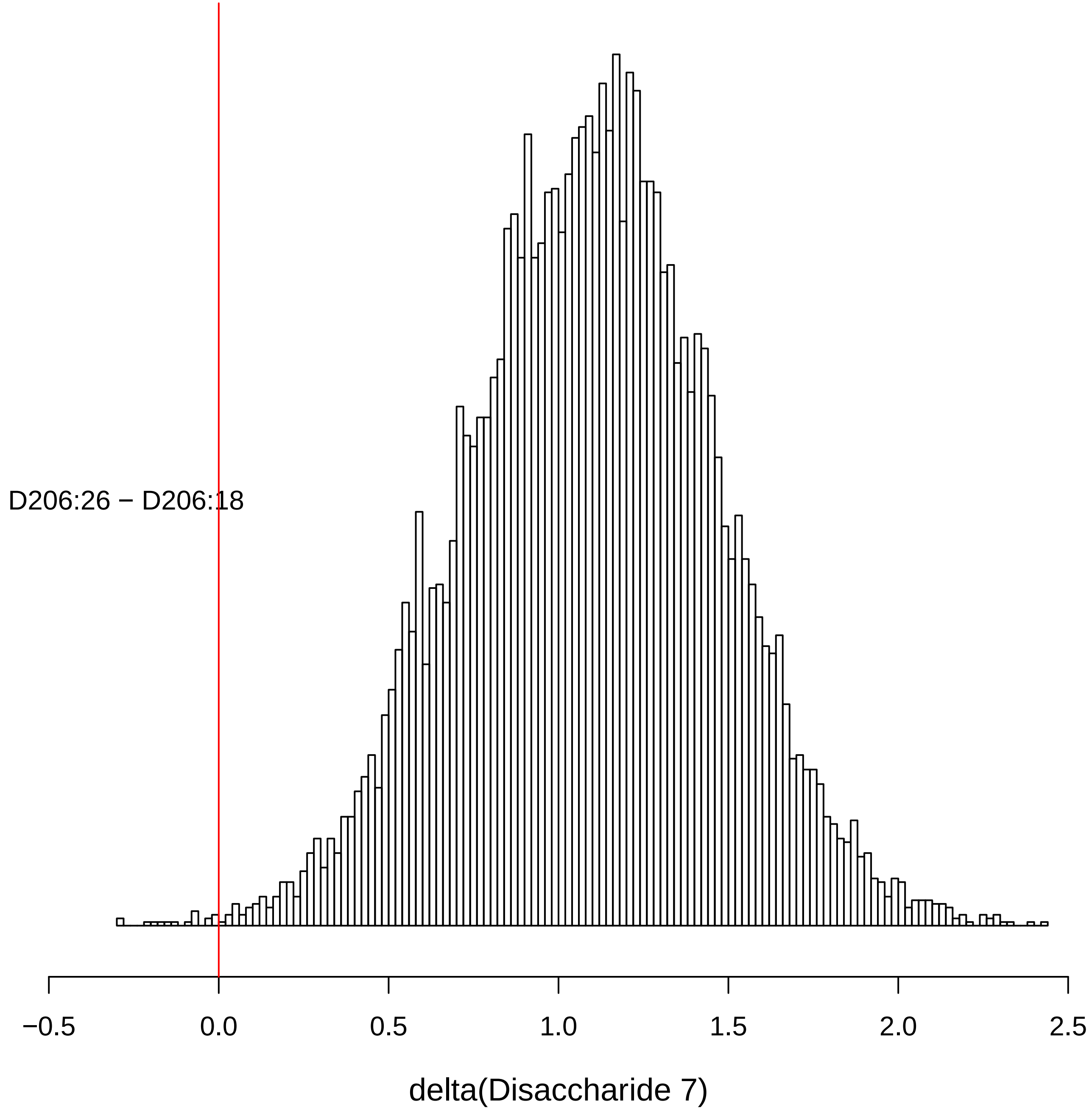
D206:26 − D206:18
−0.5
0.0
0.5
1.0
1.5
2.0
2.5
delta(Disaccharide 7)

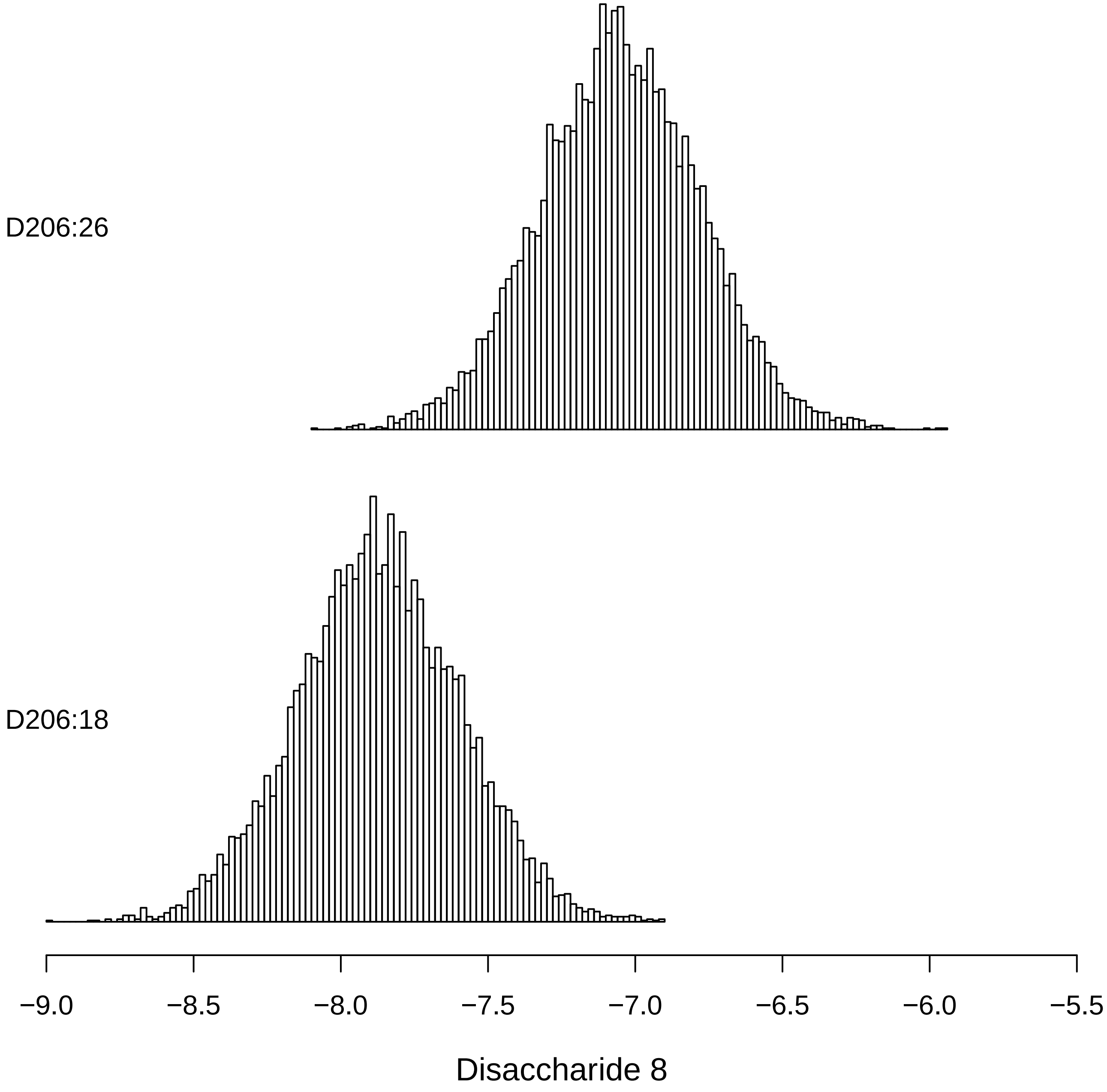
D206:26
D206:18
−9.0
−8.5
−8.0
−7.5
−7.0
−6.5
−6.0
−5.5
Disaccharide 8

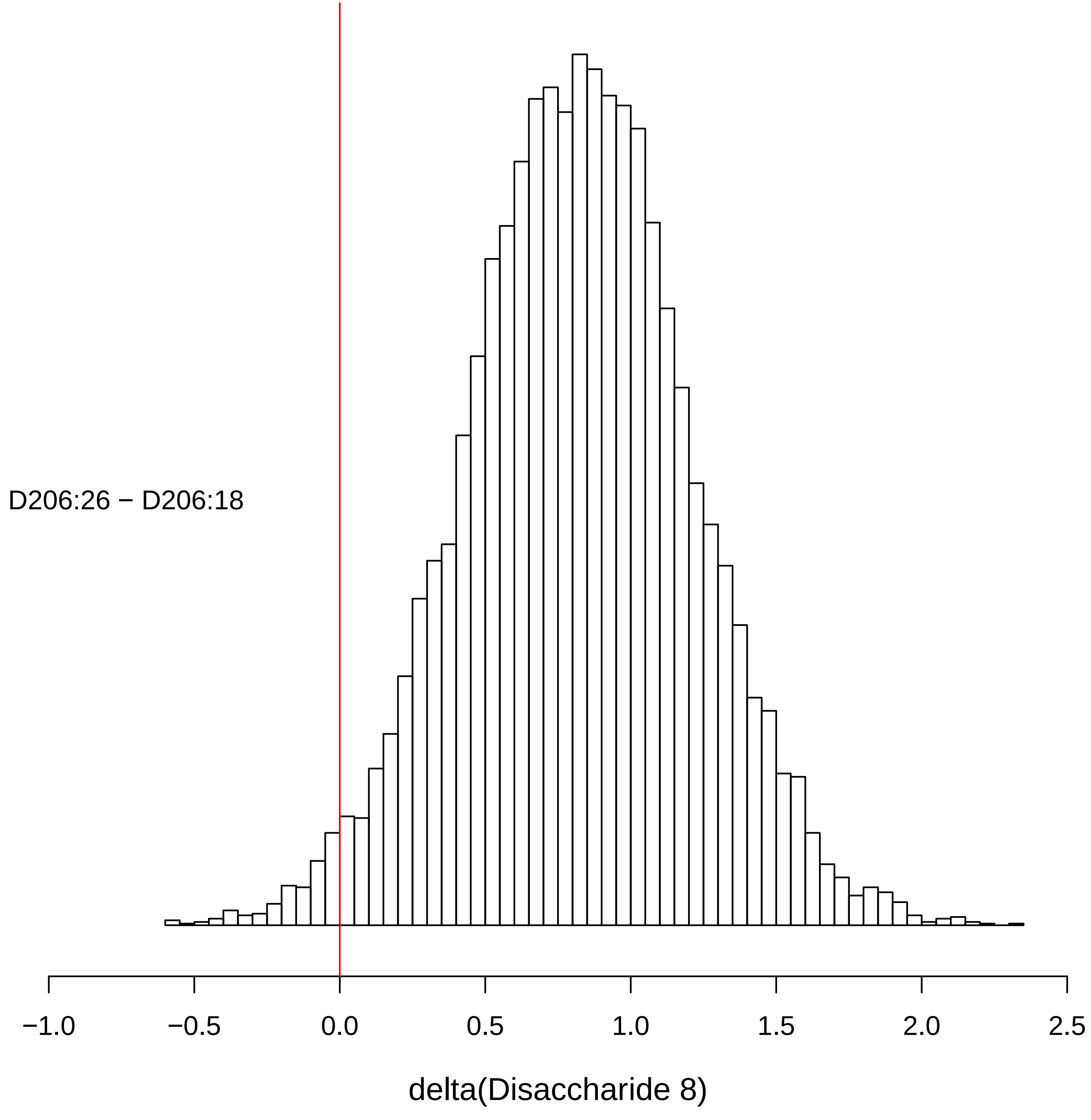
D206:26 − D206:18
−1.0
−0.5
0.0
0.5
1.0
1.5
2.0
2.5
delta(Disaccharide 8)

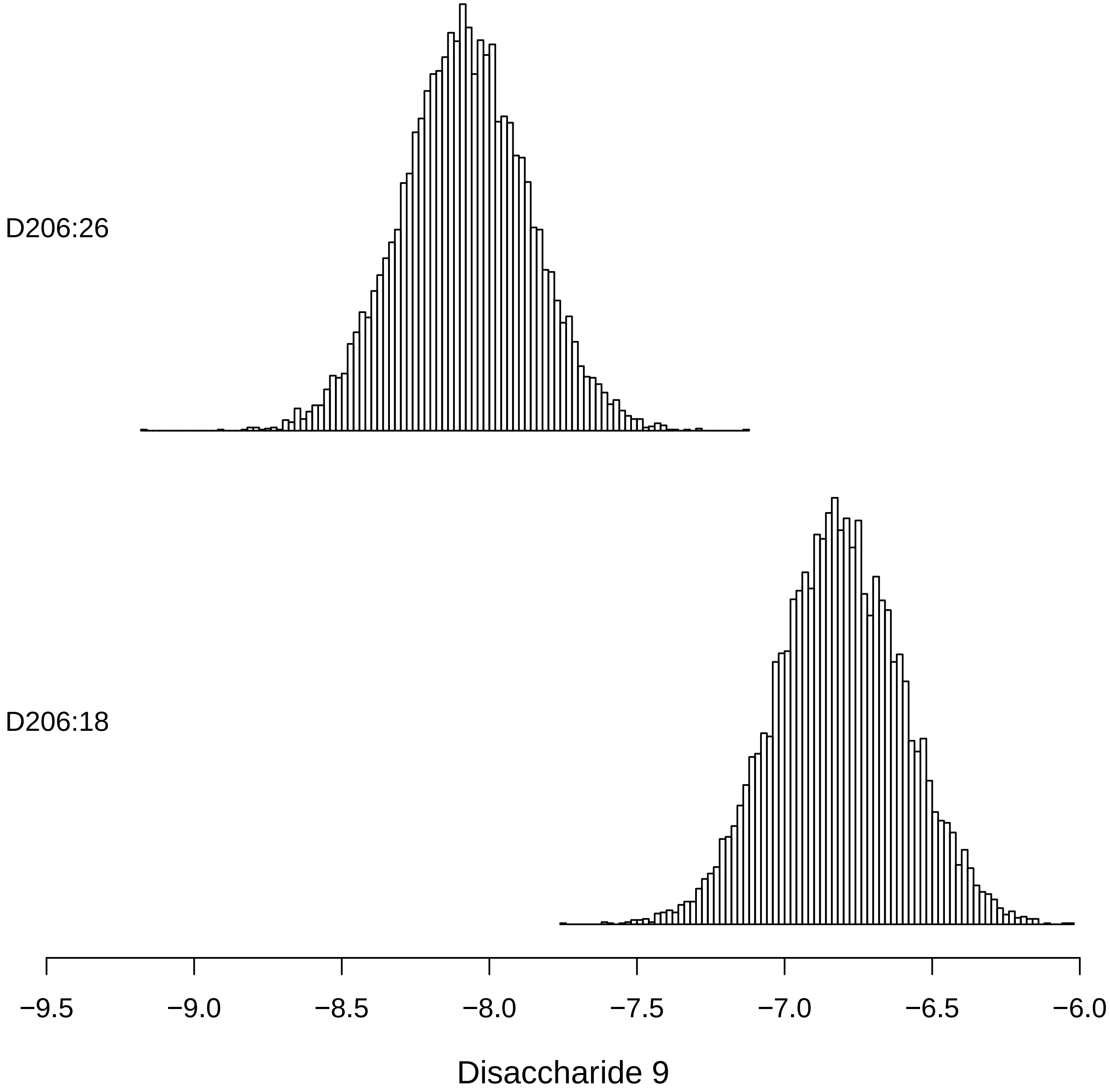
D206:26
D206:18
−9.5
−9.0
−8.5
−8.0
−7.5
−7.0
−6.5
−6.0
Disaccharide 9

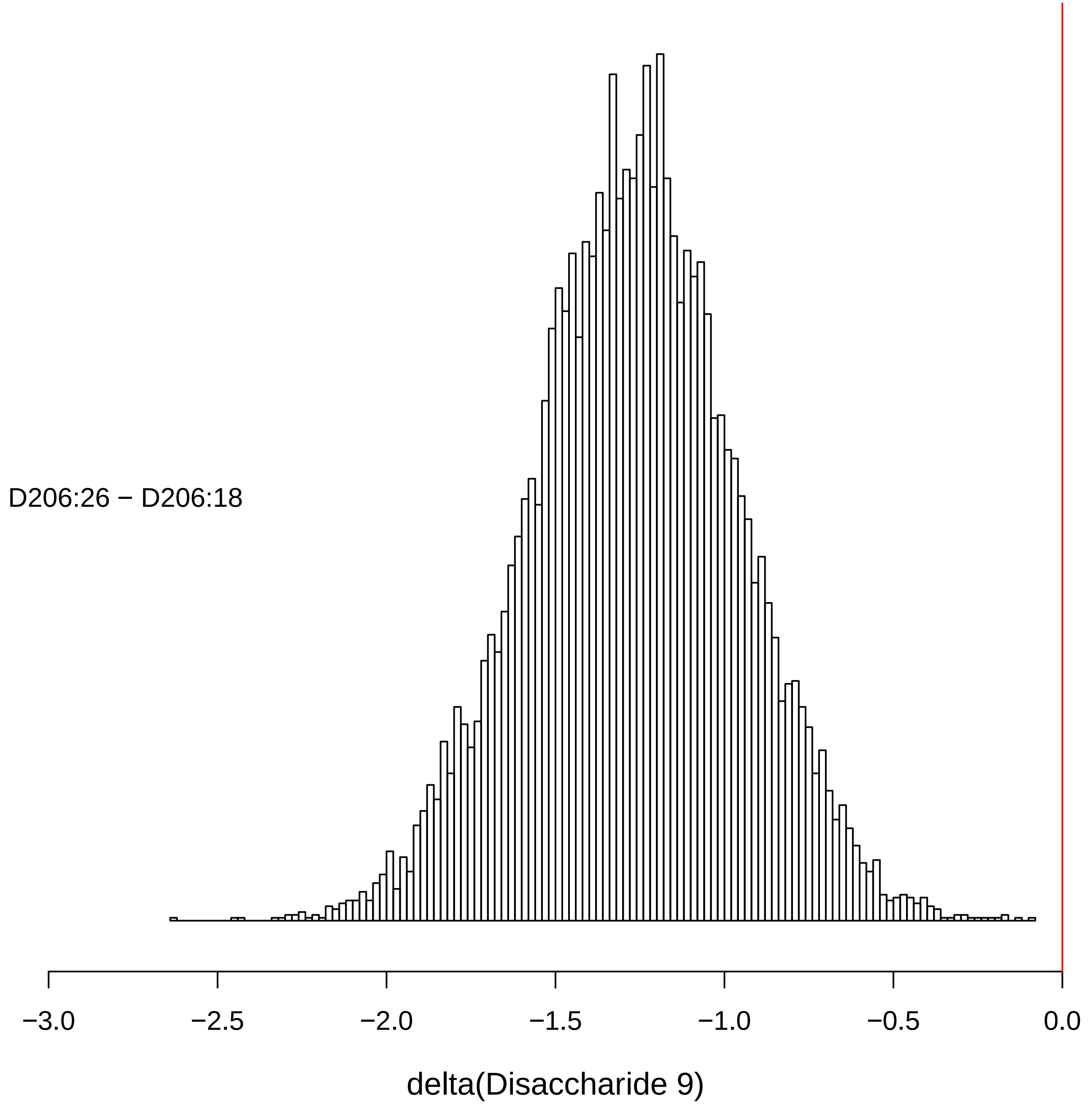
D206:26 − D206:18
−3.0
−2.5
−2.0
−1.5
−1.0
−0.5
0.0
delta(Disaccharide 9)

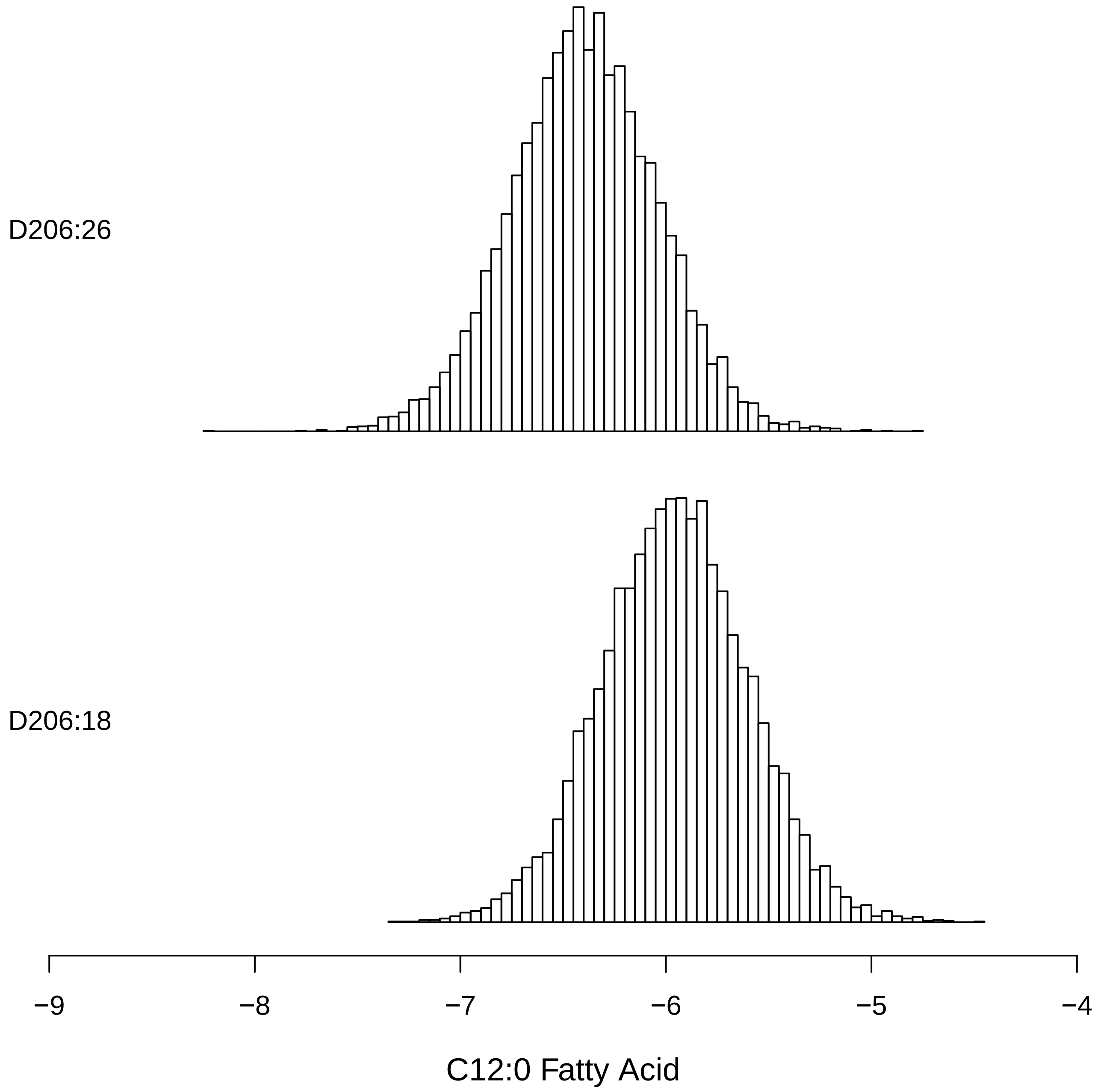

D206:26
D206:18
−9
−8
−7
−6
−5
−4
C12:0 Fatty Acid

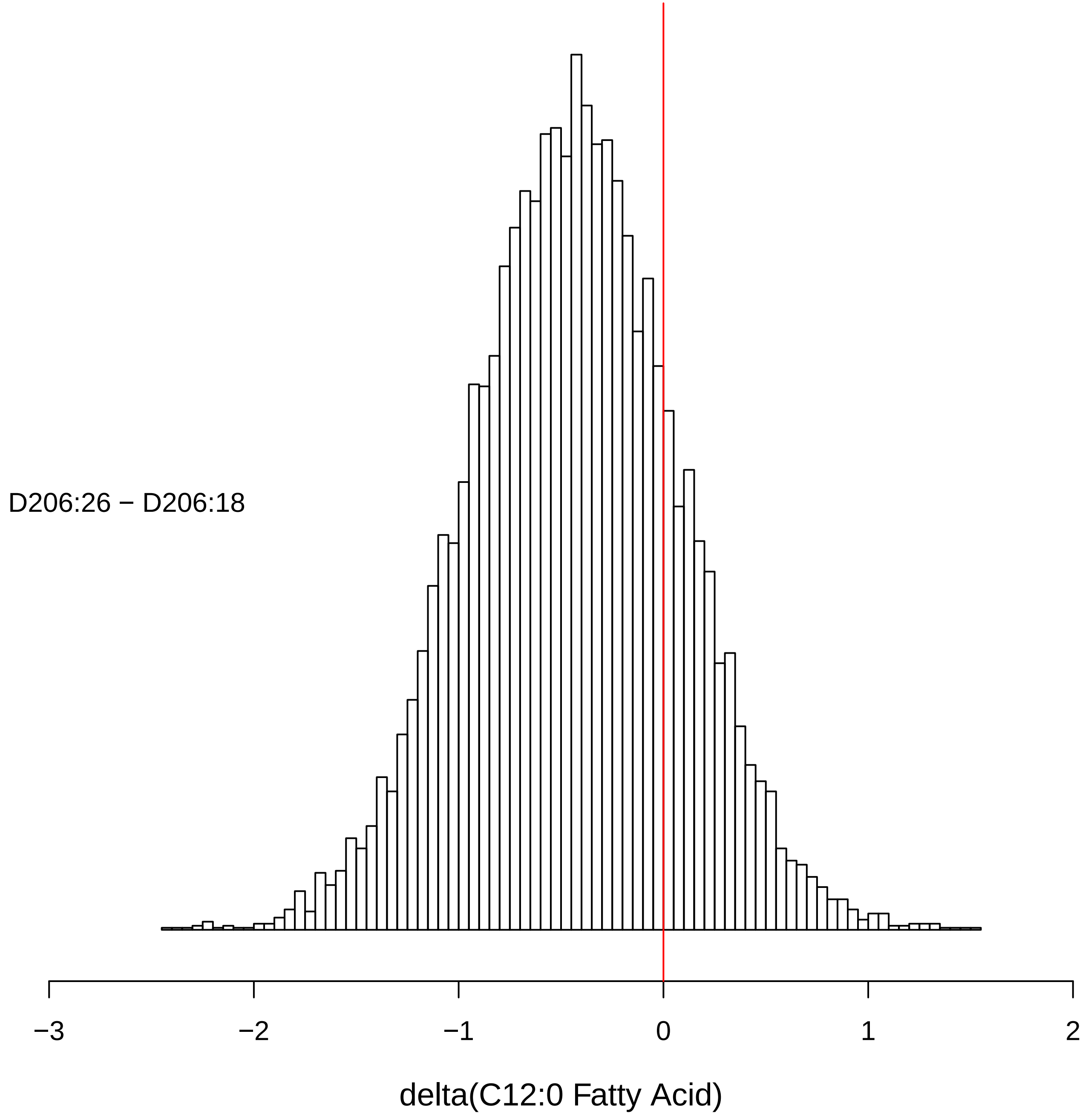
D206:26 − D206:18
−3
−2
−1
0
1
2
delta(C12:0 Fatty Acid)

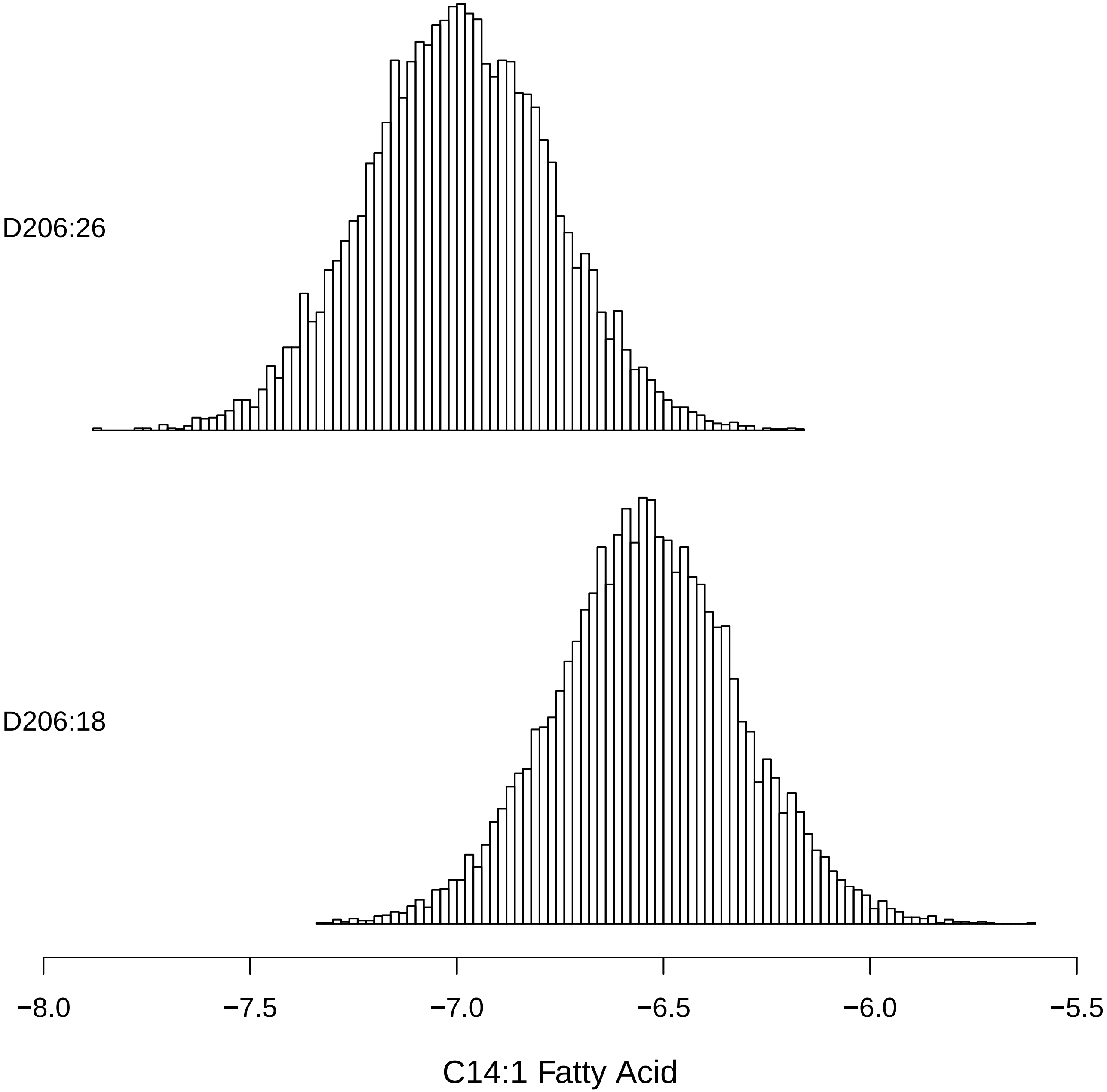
D206:26
D206:18
−8.0
−7.5
−7.0
−6.5
−6.0
−5.5
C14:1 Fatty Acid

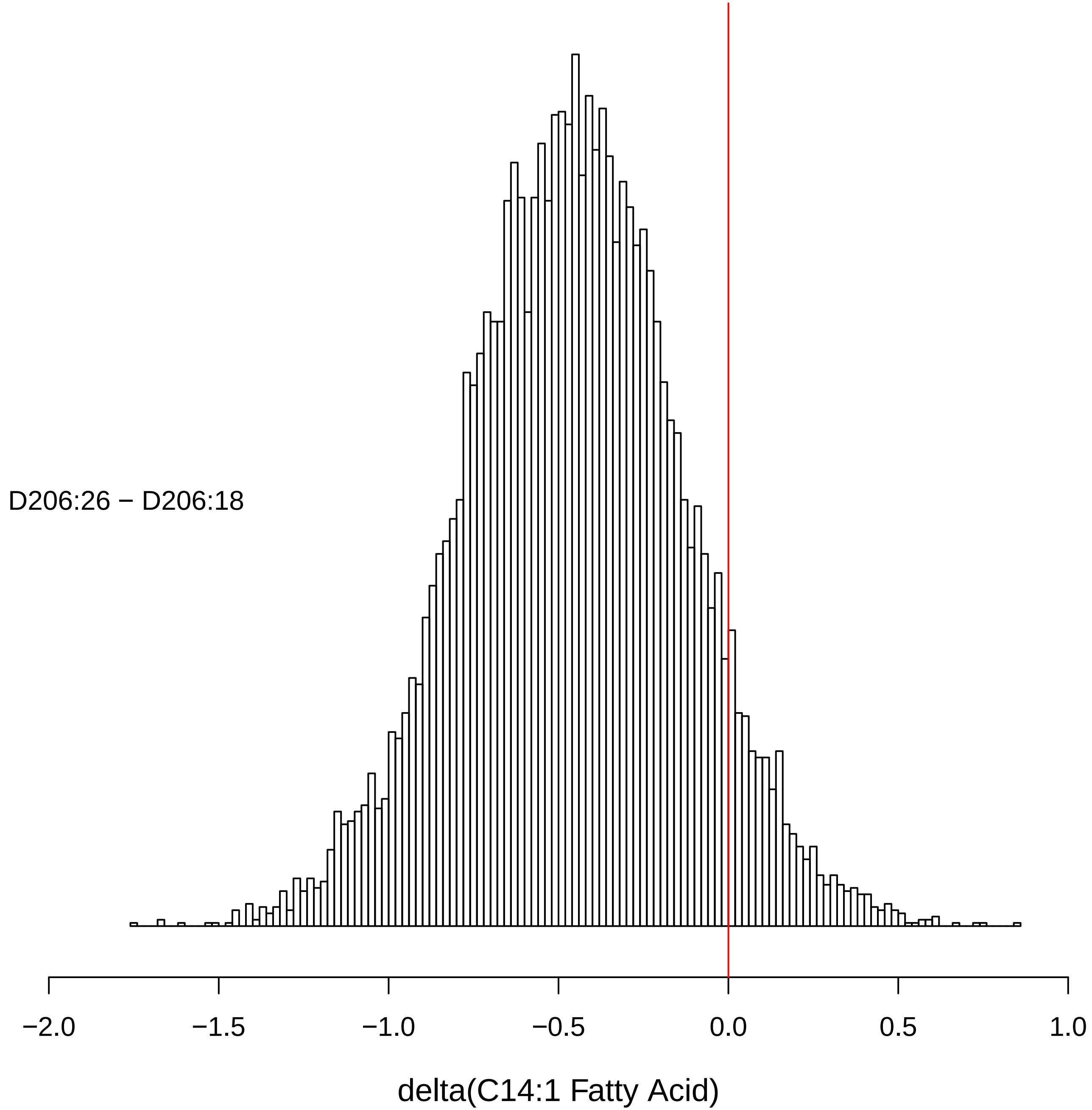
D206:26 − D206:18
−2.0
−1.5
−1.0
−0.5
0.0
0.5
1.0
delta(C14:1 Fatty Acid)

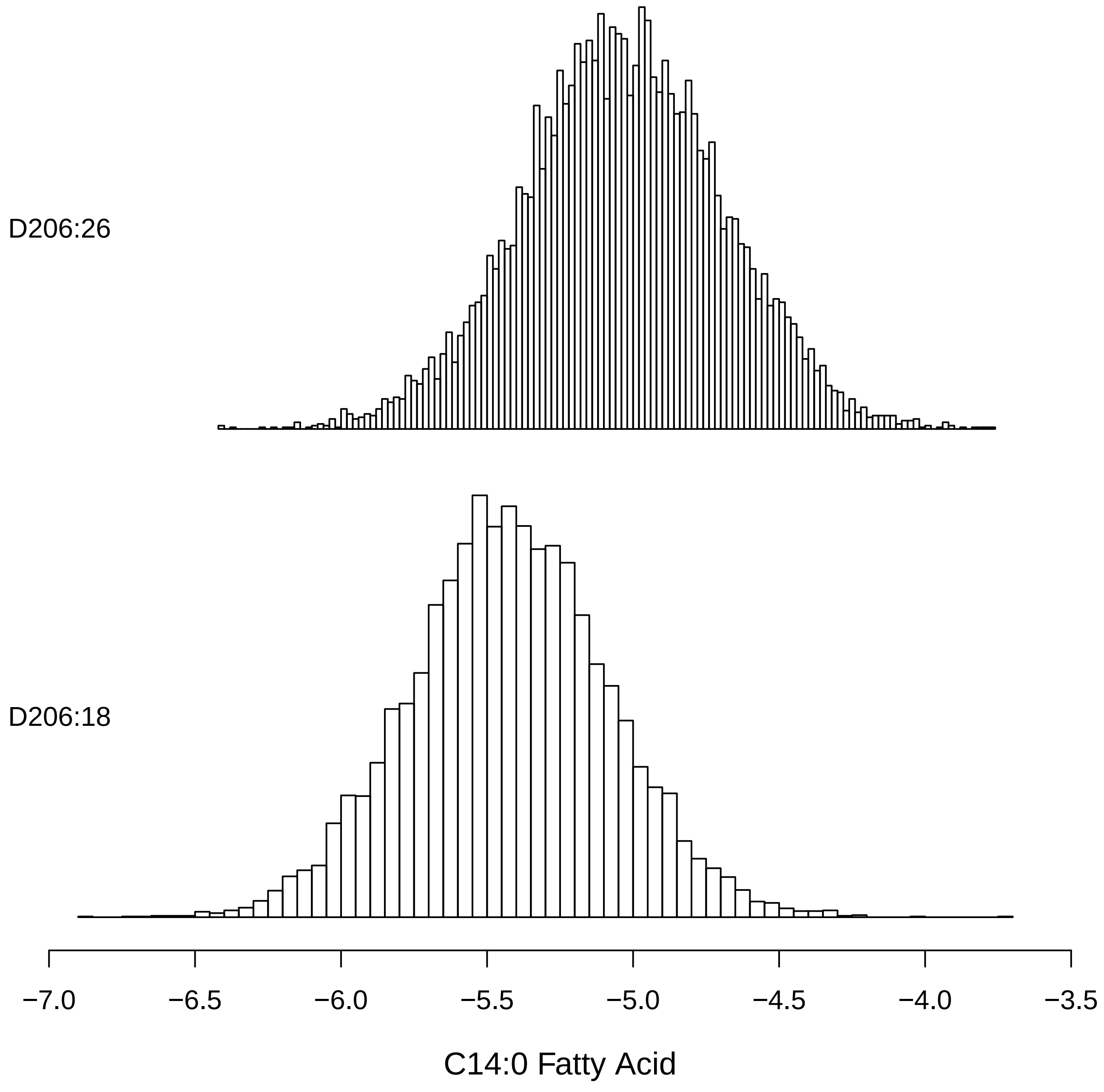
D206:26
D206:18
−7.0
−6.5
−6.0
−5.5
−5.0
−4.5
−4.0
−3.5
C14:0 Fatty Acid

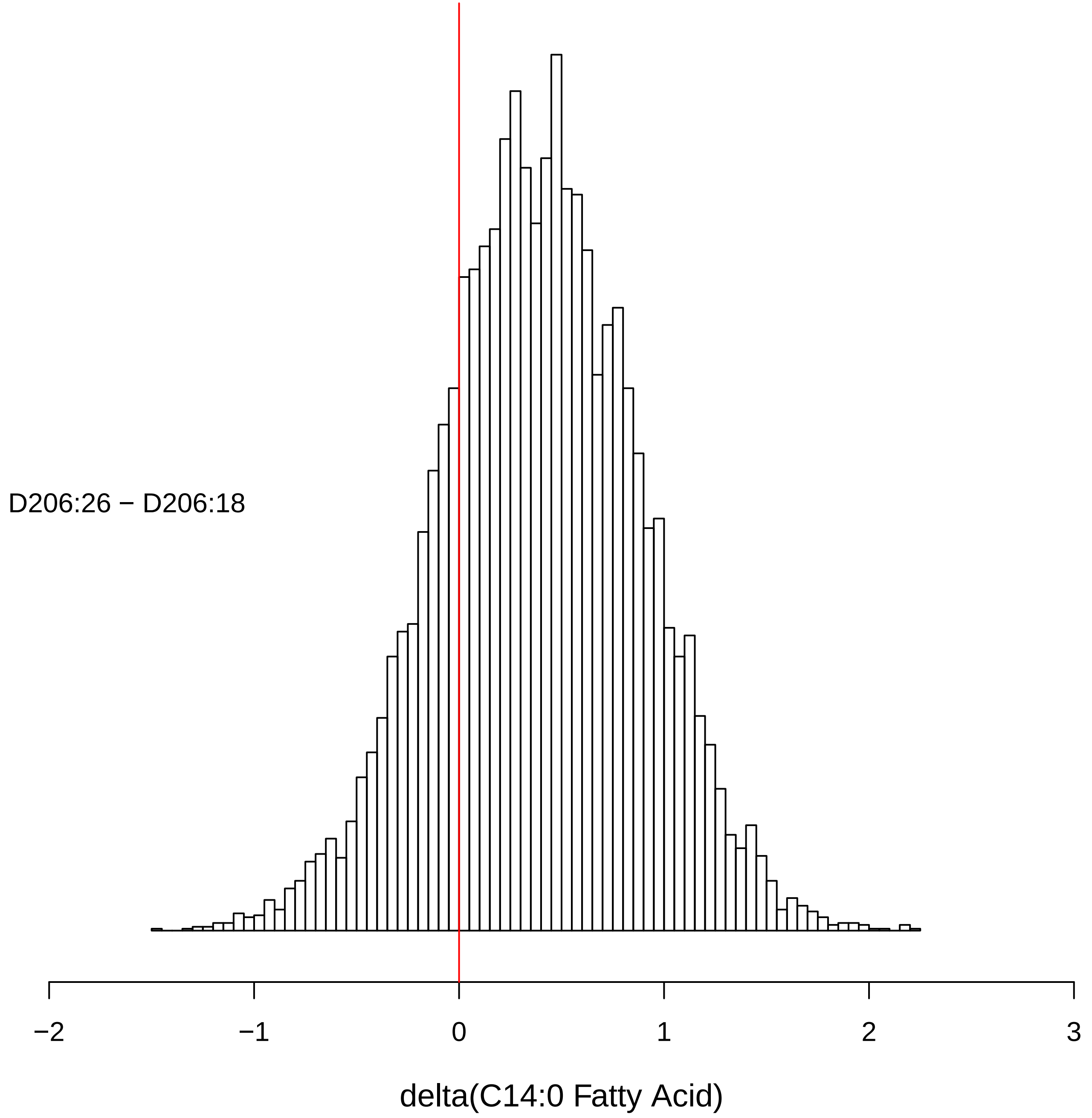
D206:26 − D206:18
−2
−1
0
1
2
3
delta(C14:0 Fatty Acid)

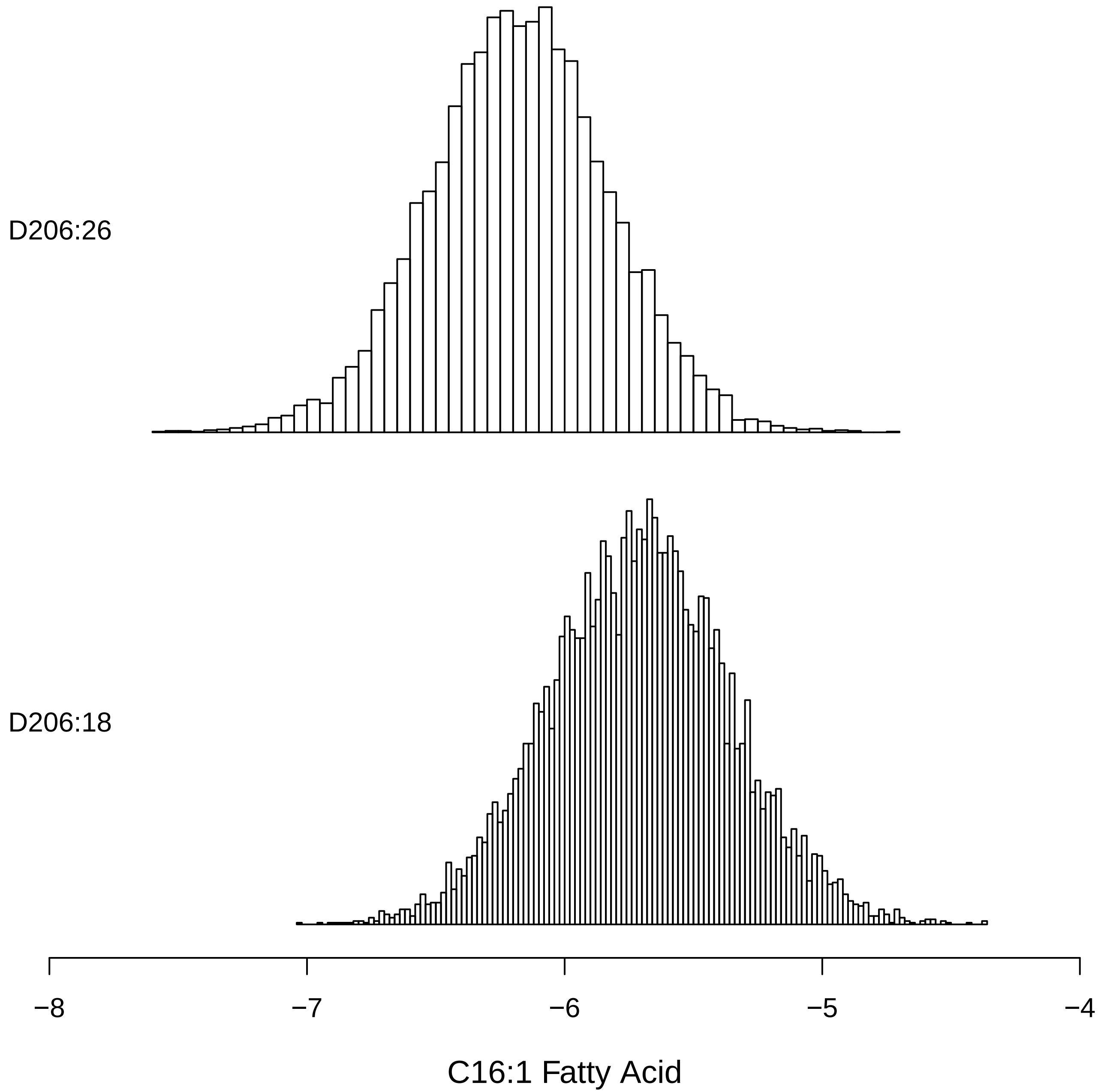
D206:26
D206:18
−8
−7
−6
−5
−4
C16:1 Fatty Acid

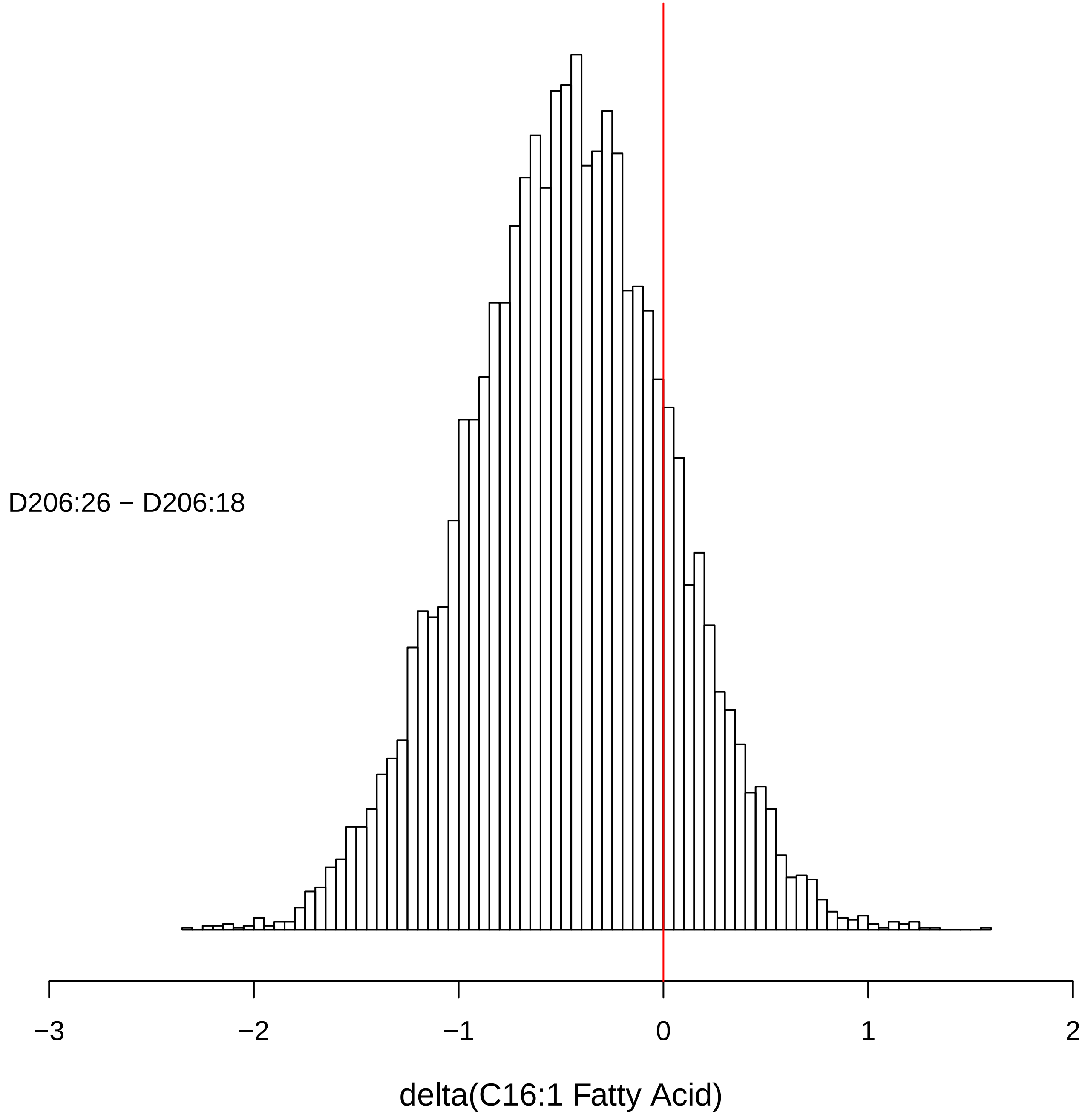

D206:26 − D206:18
−3
−2
−1
0
1
2
delta(C16:1 Fatty Acid)

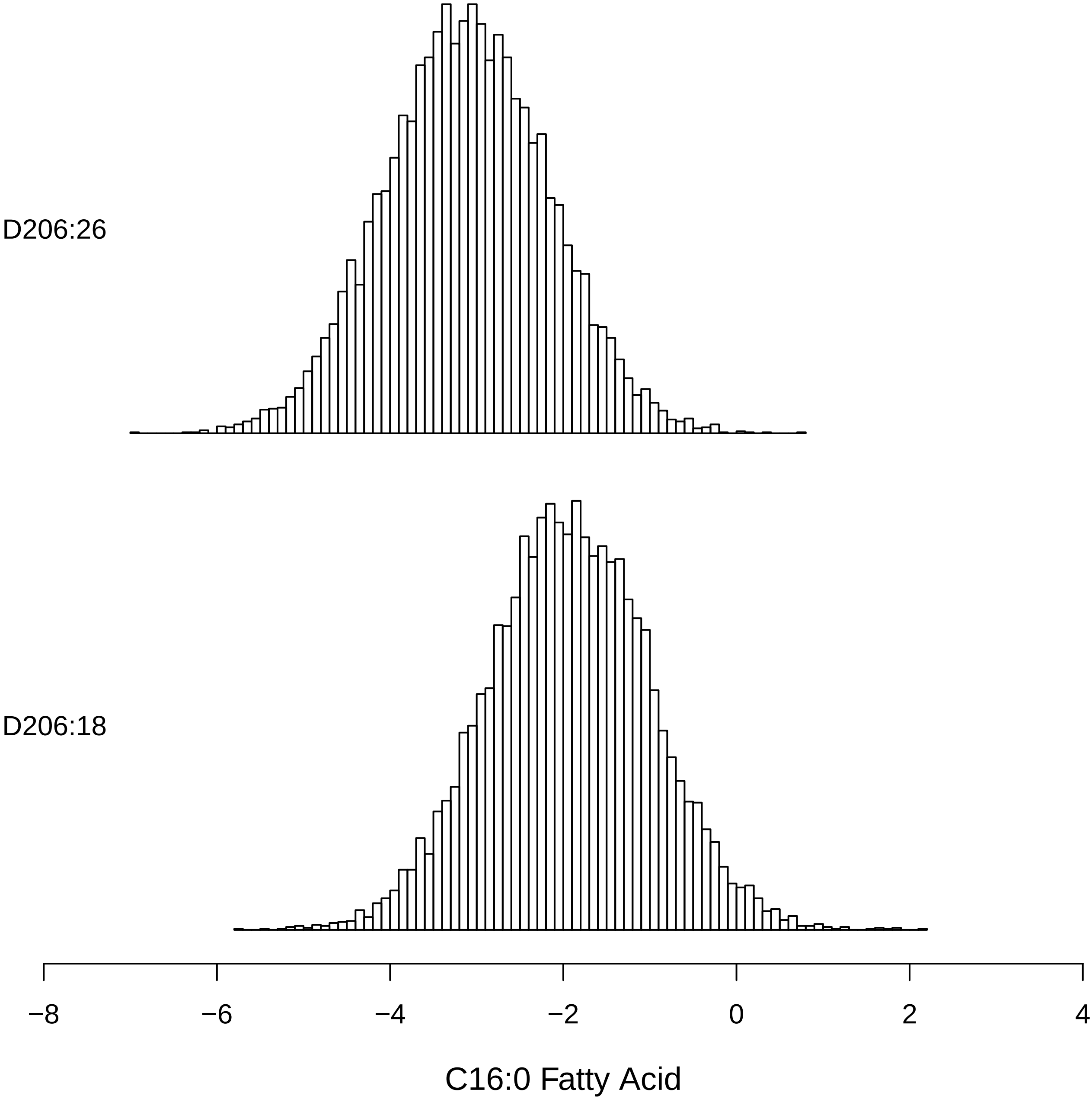
D206:26
D206:18
−8
−6
−4
−2
0
2
4
C16:0 Fatty Acid

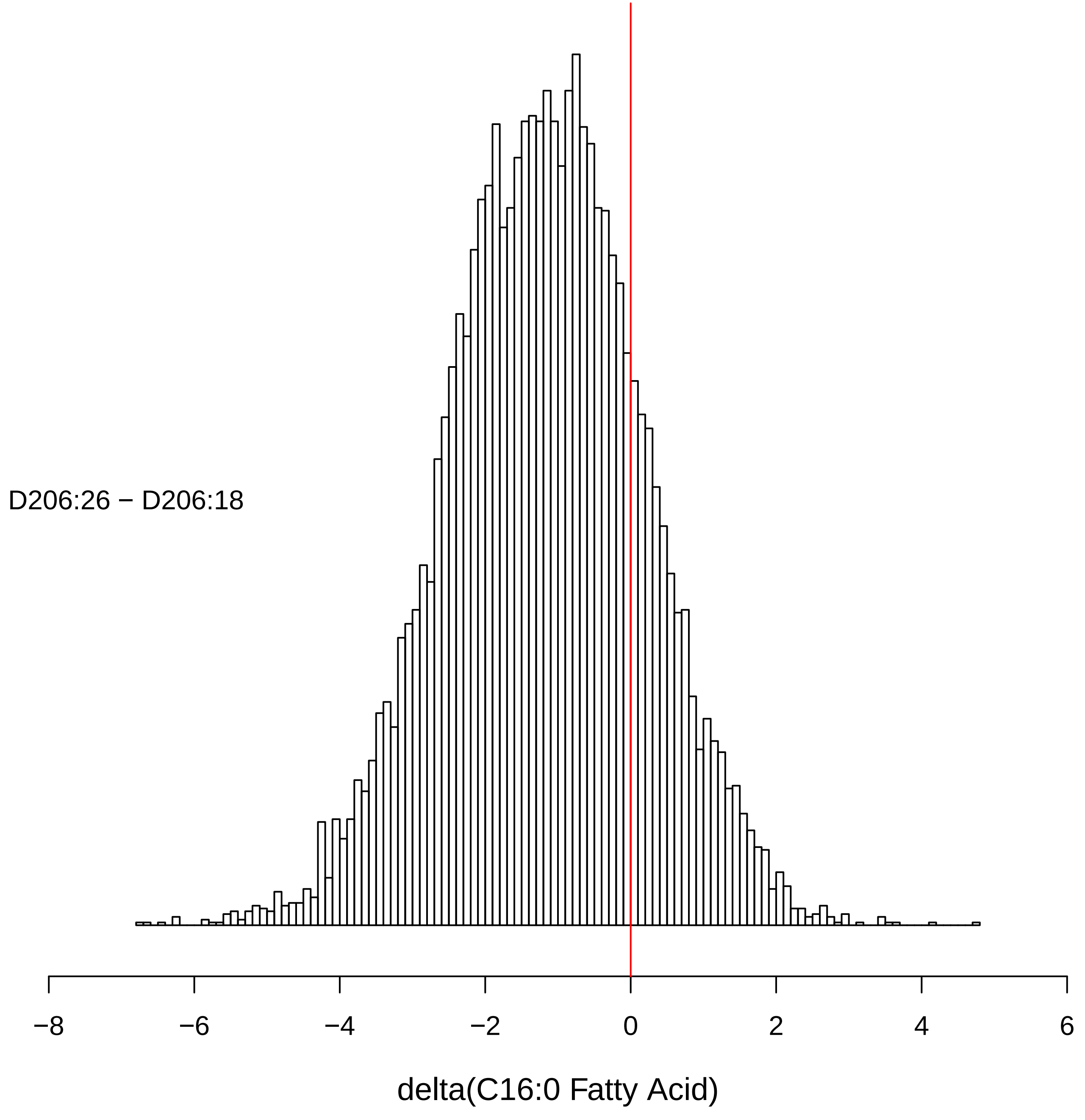
D206:26 − D206:18
−8
−6
−4
−2
0
2
4
6
delta(C16:0 Fatty Acid)

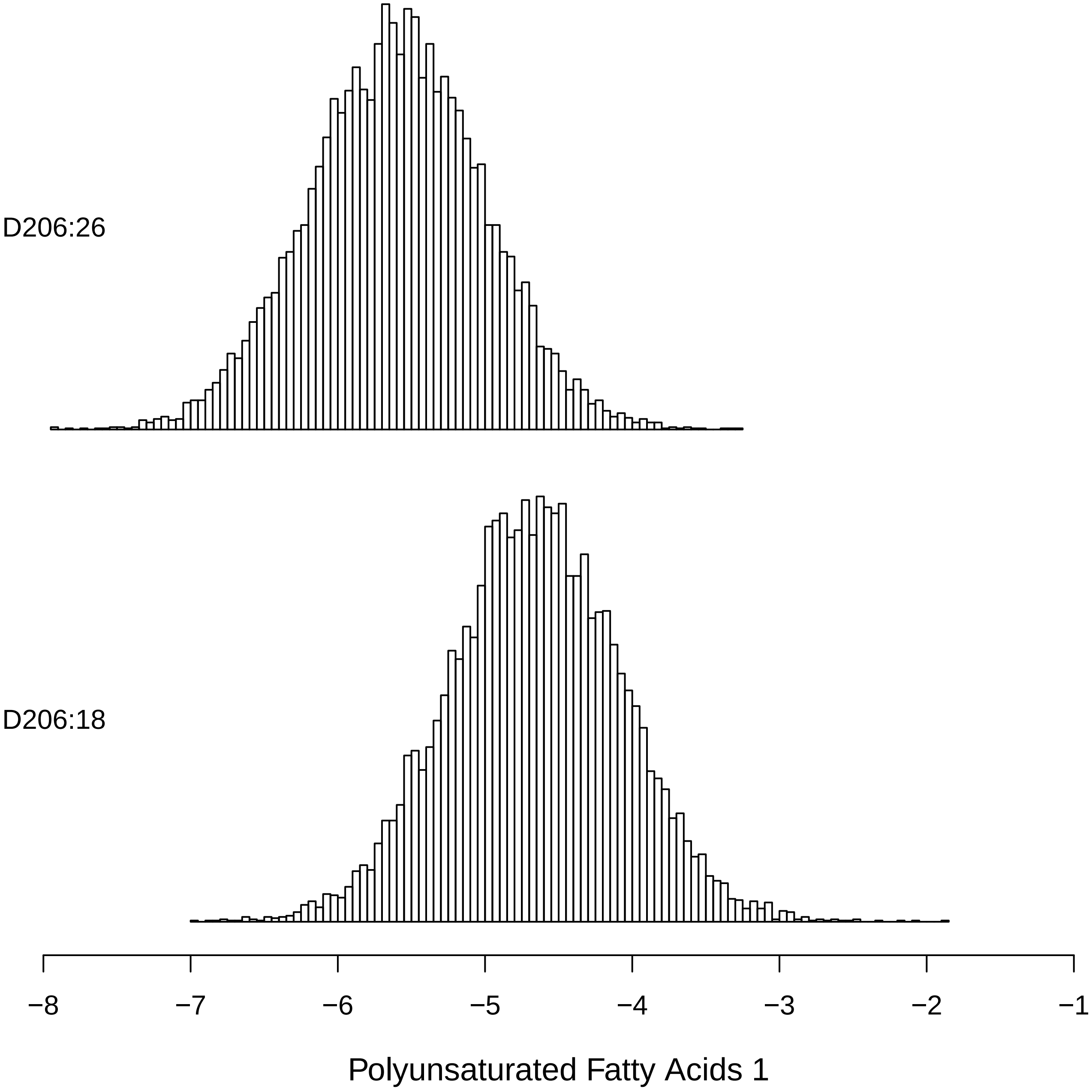

D206:26
D206:18
−8
−7
−6
−5
−4
−3
−2
−1
Polyunsaturated Fatty Acids 1

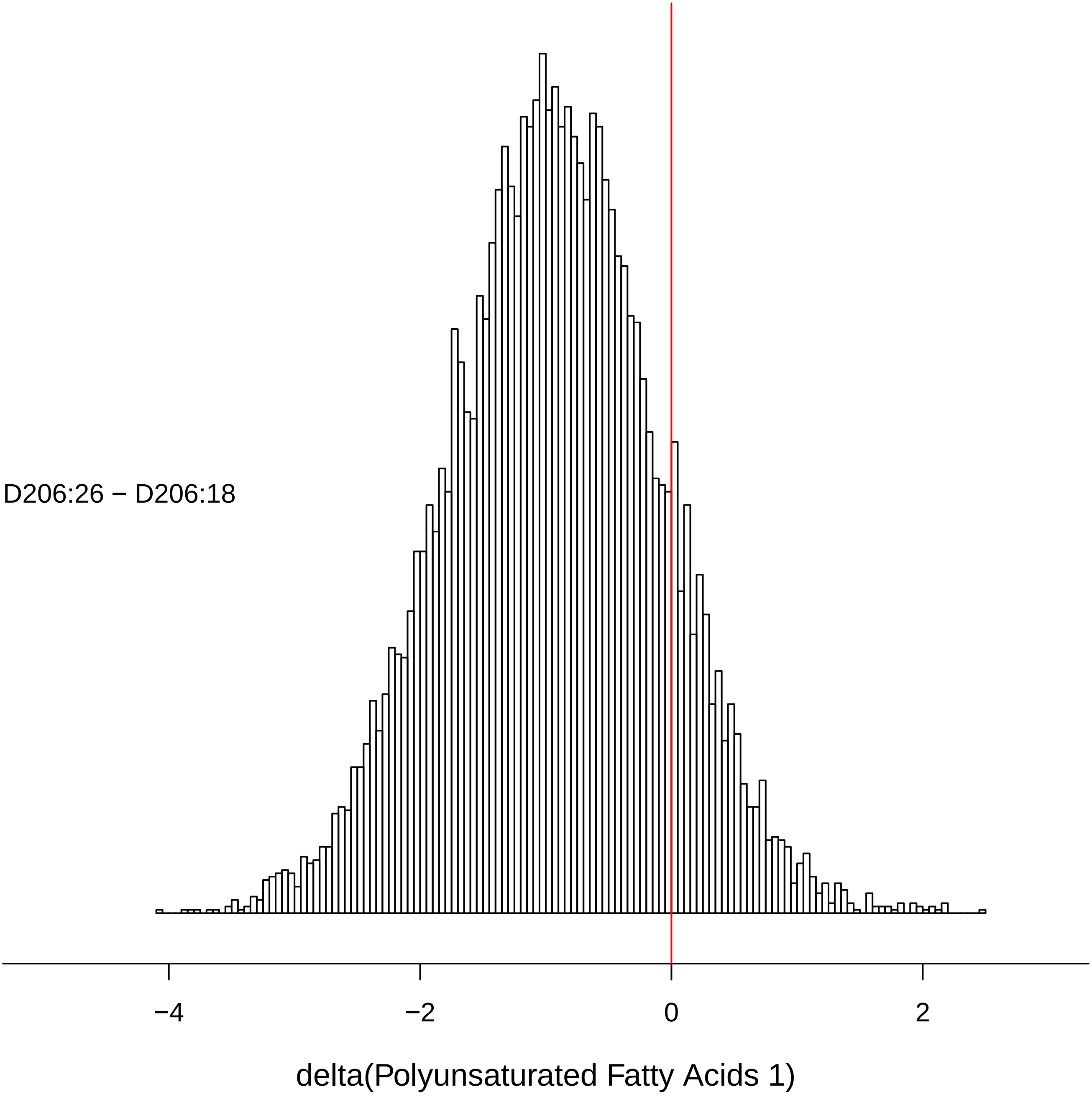
D206:26 − D206:18
−4
−2
0
2
delta(Polyunsaturated Fatty Acids 1)

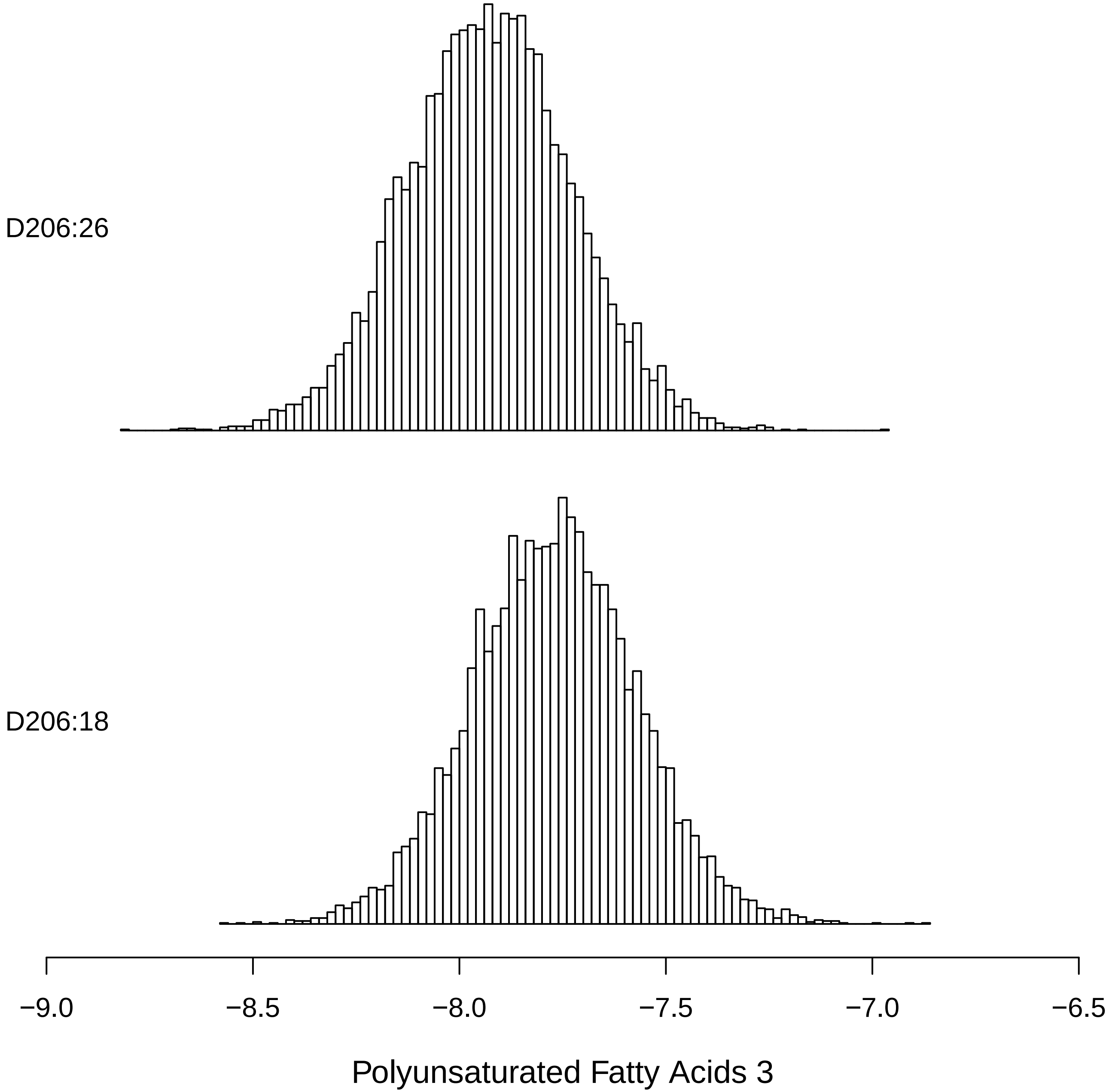
D206:26
D206:18
−9.0
−8.5
−8.0
−7.5
−7.0
−6.5
Polyunsaturated Fatty Acids 3

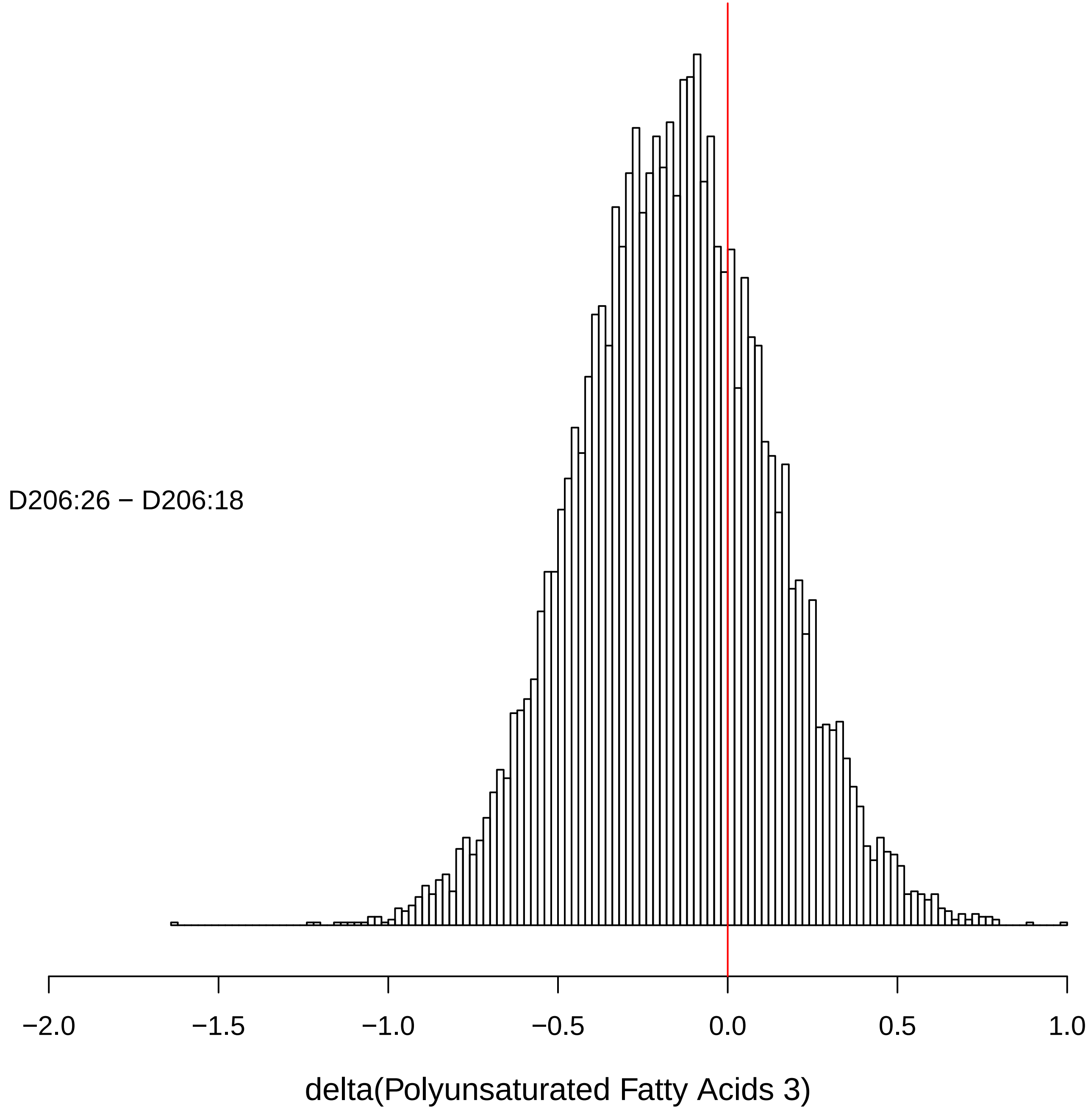
D206:26 − D206:18
−2.0
−1.5
−1.0
−0.5
0.0
0.5
1.0
delta(Polyunsaturated Fatty Acids 3)

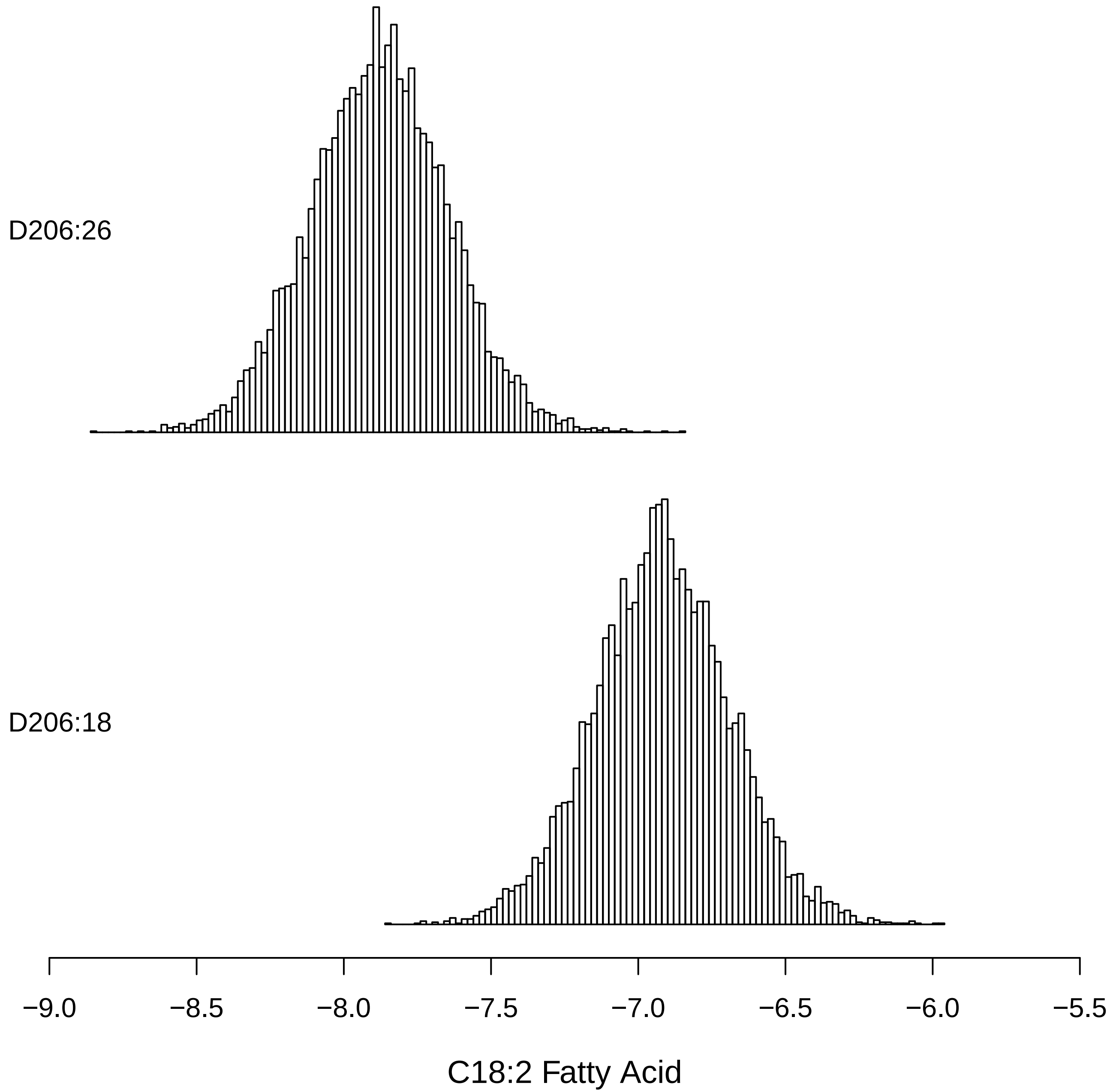
D206:26
D206:18
−9.0
−8.5
−8.0
−7.5
−7.0
−6.5
−6.0
−5.5
C18:2 Fatty Acid

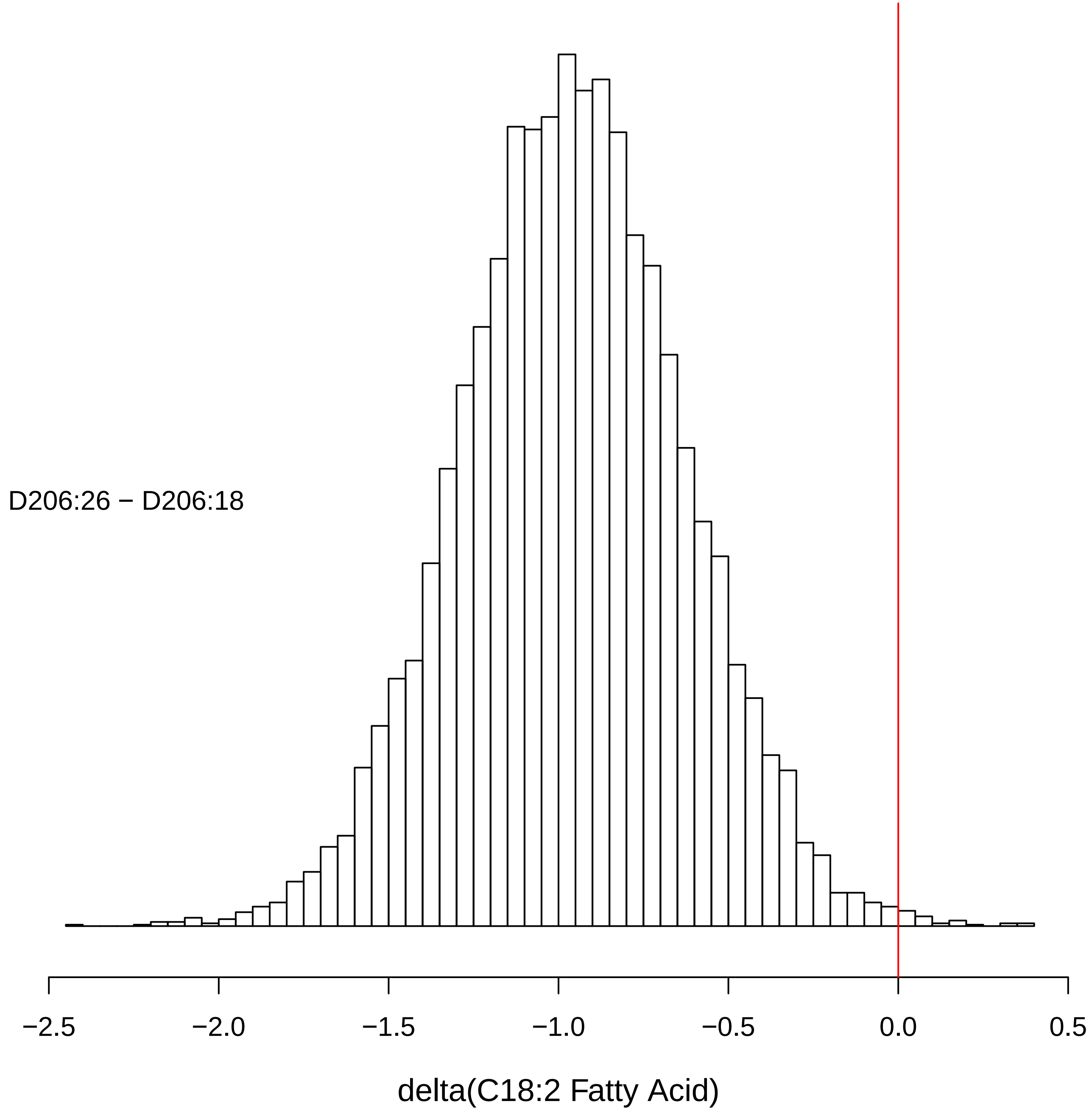
D206:26 − D206:18
−2.5
−2.0
−1.5
−1.0
−0.5
0.0
0.5
delta(C18:2 Fatty Acid)

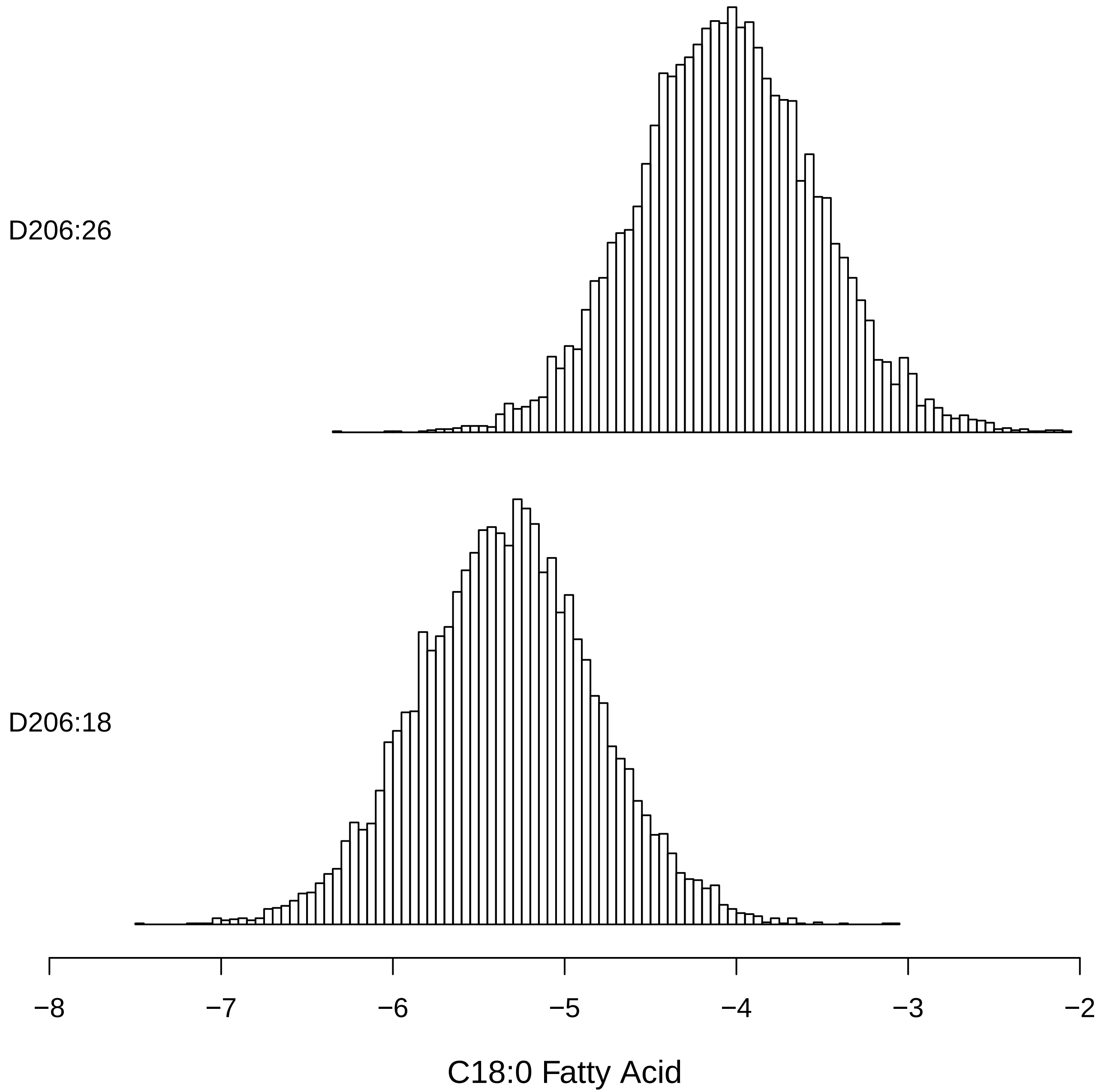
D206:26
D206:18
−8
−7
−6
−5
−4
−3
−2
C18:0 Fatty Acid

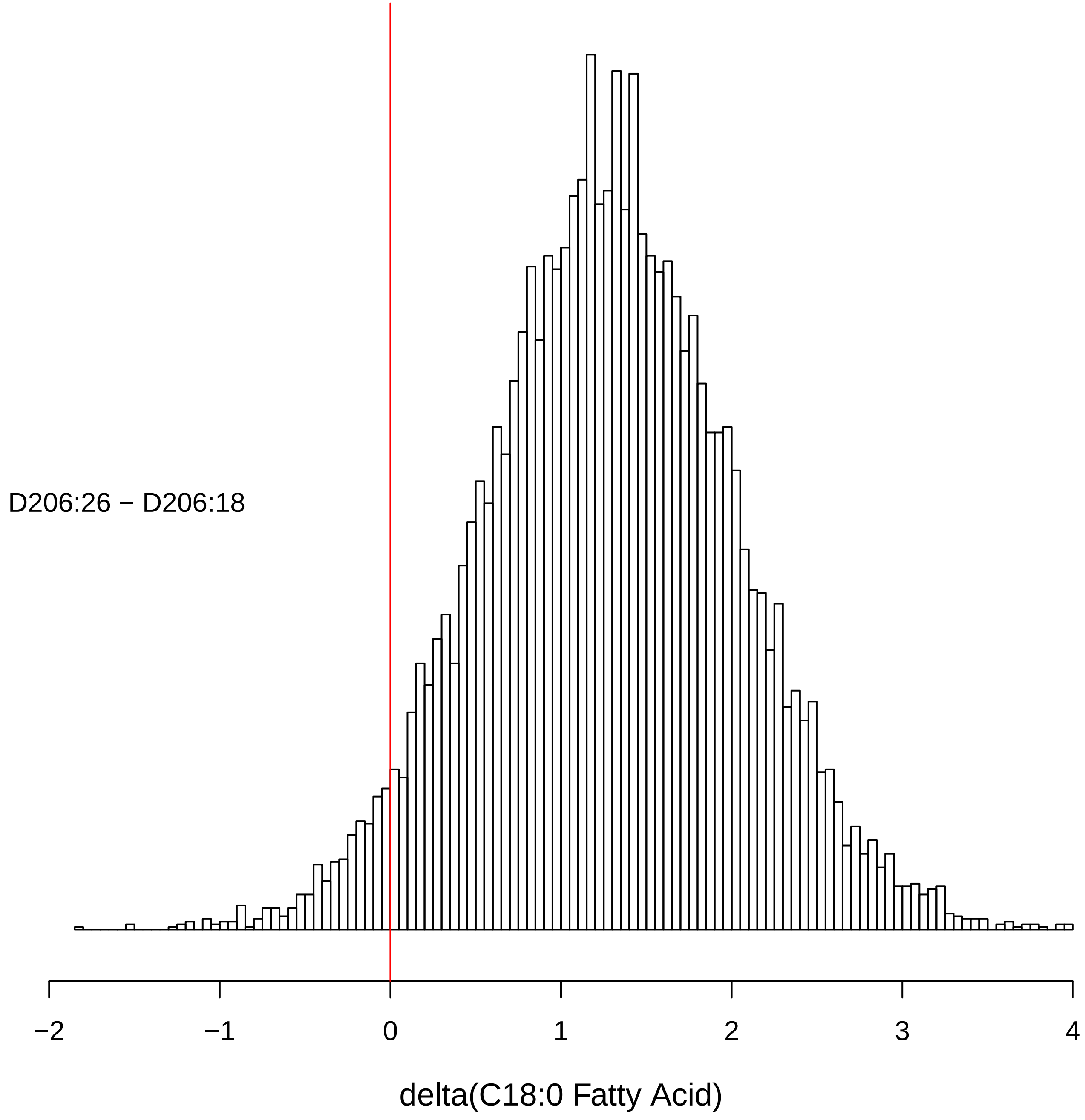
D206:26 − D206:18
−2
−1
0
1
2
3
4
delta(C18:0 Fatty Acid)

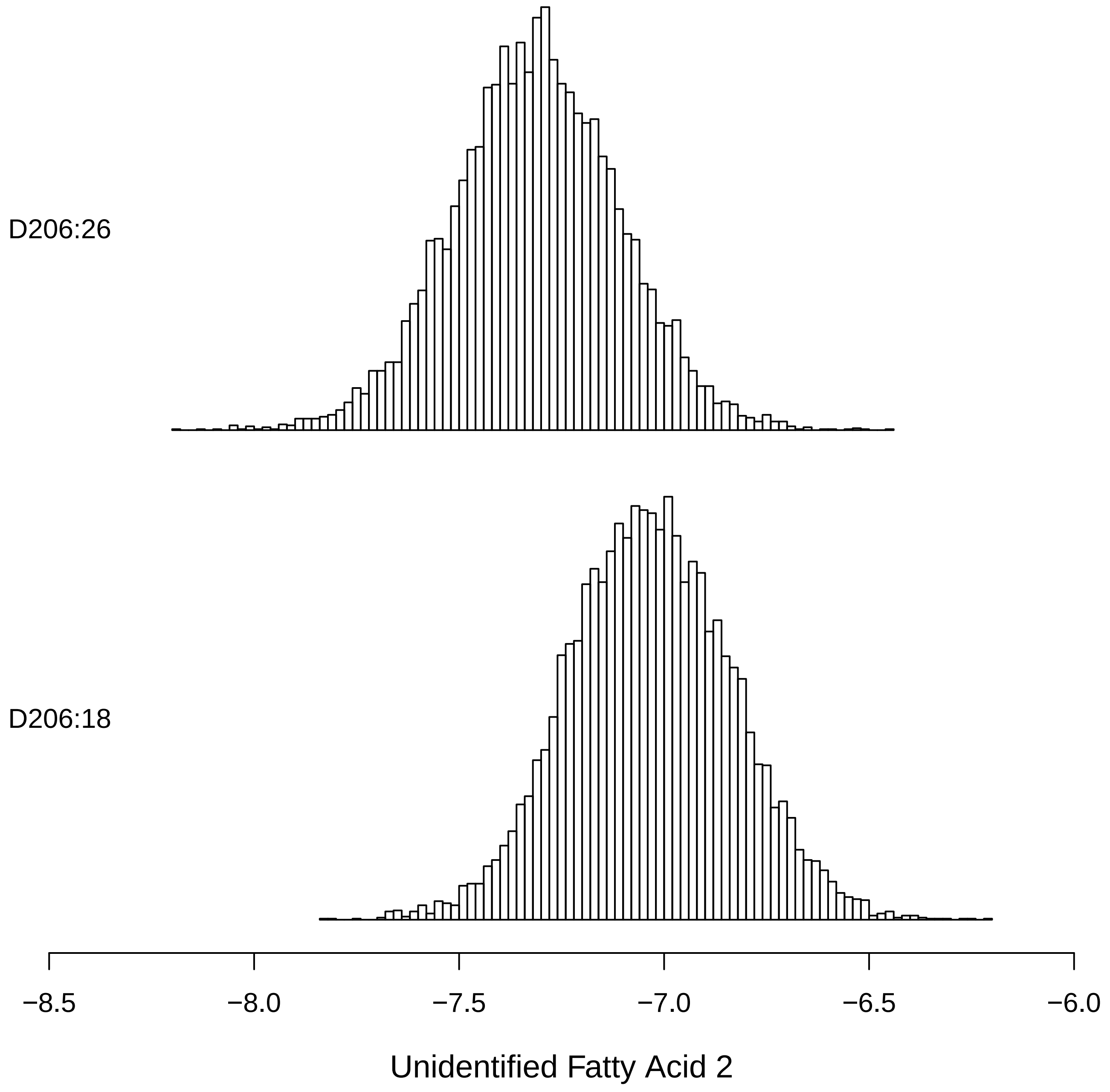
D206:26
D206:18
−8.5
−8.0
−7.5
−7.0
−6.5
−6.0
Unidentified Fatty Acid 2

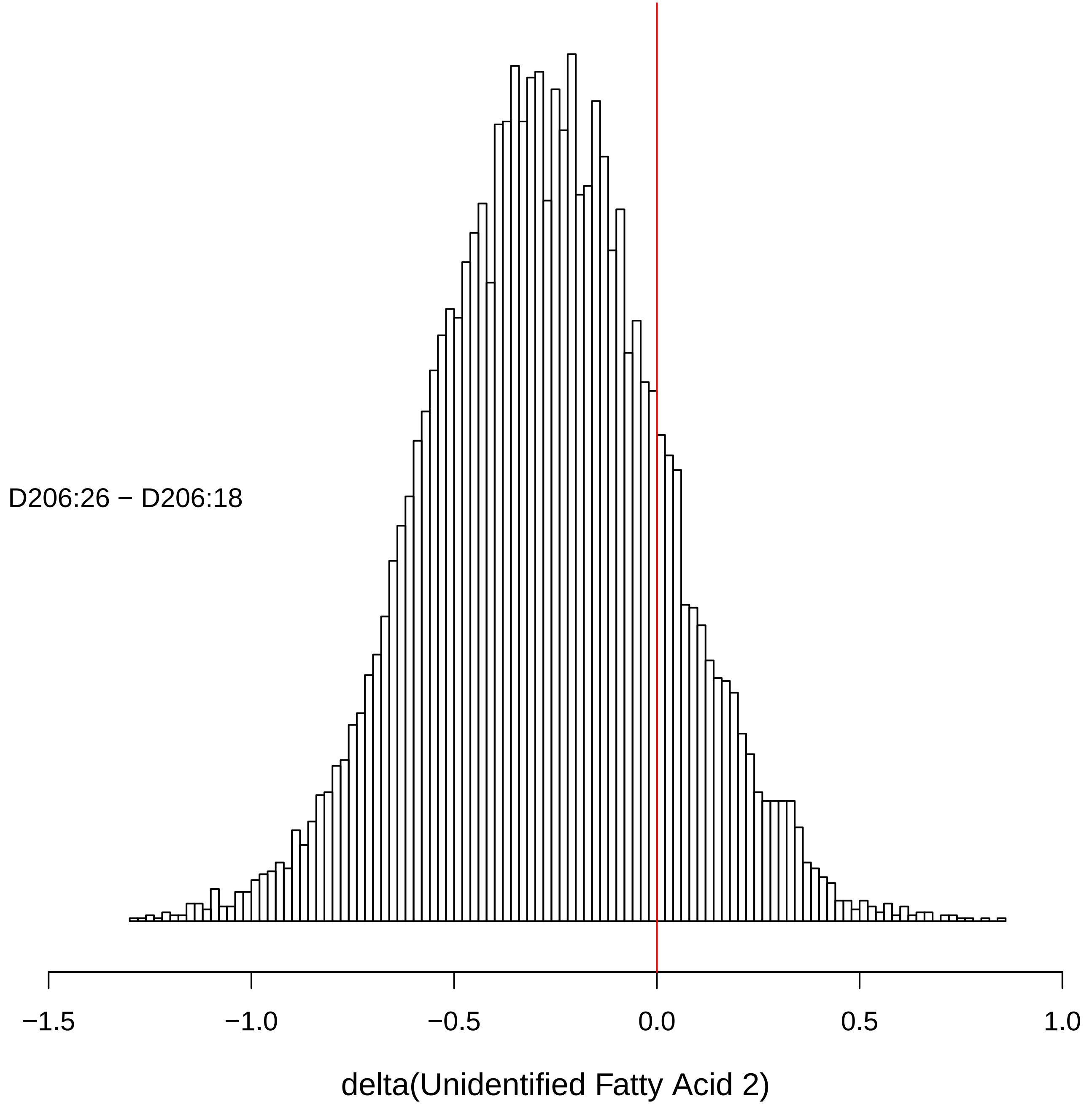
D206:26 − D206:18
−1.5
−1.0
−0.5
0.0
0.5
1.0
delta(Unidentified Fatty Acid 2)

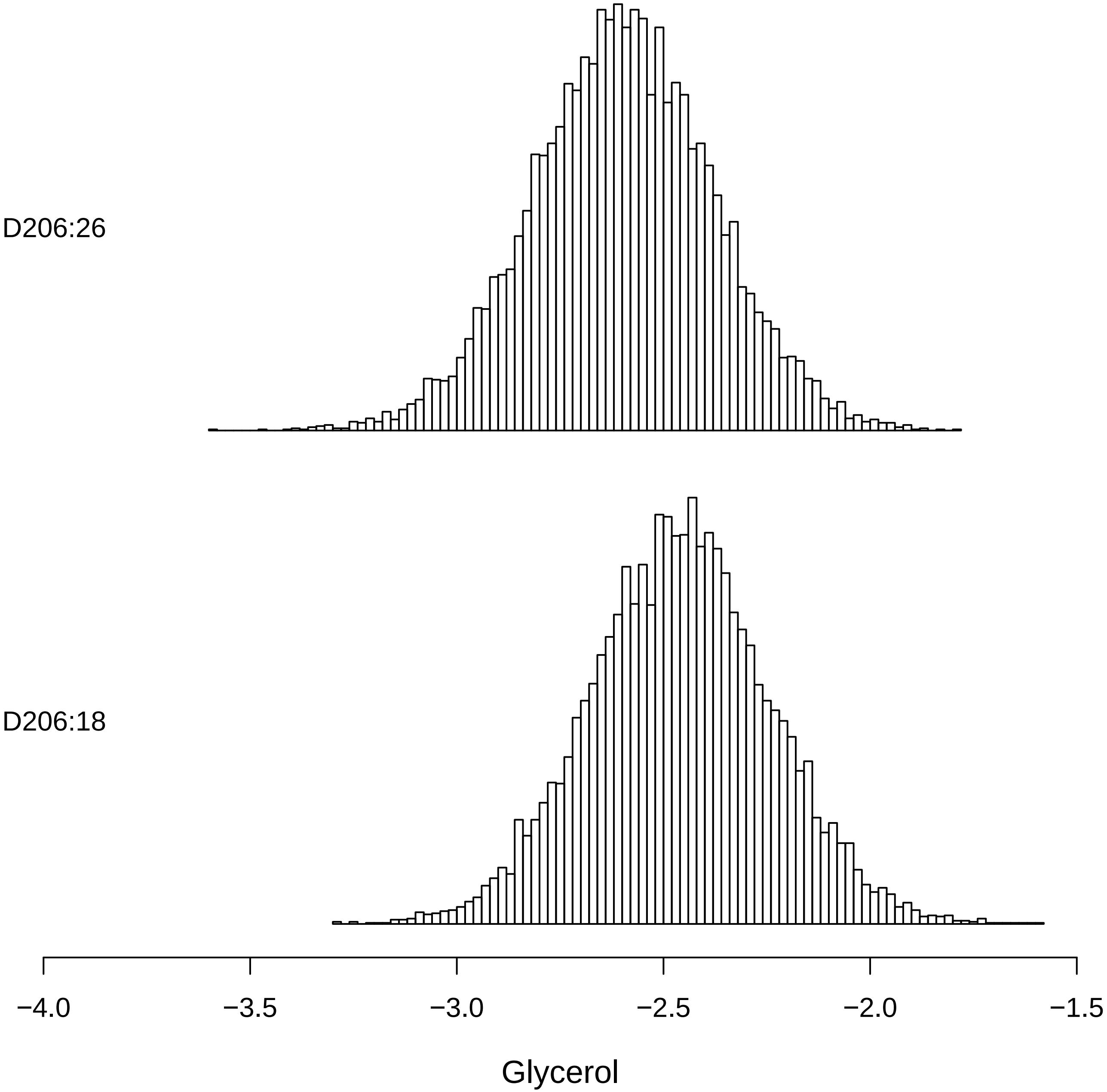
D206:26
D206:18
−4.0
−3.5
−3.0
−2.5
−2.0
−1.5
Glycerol

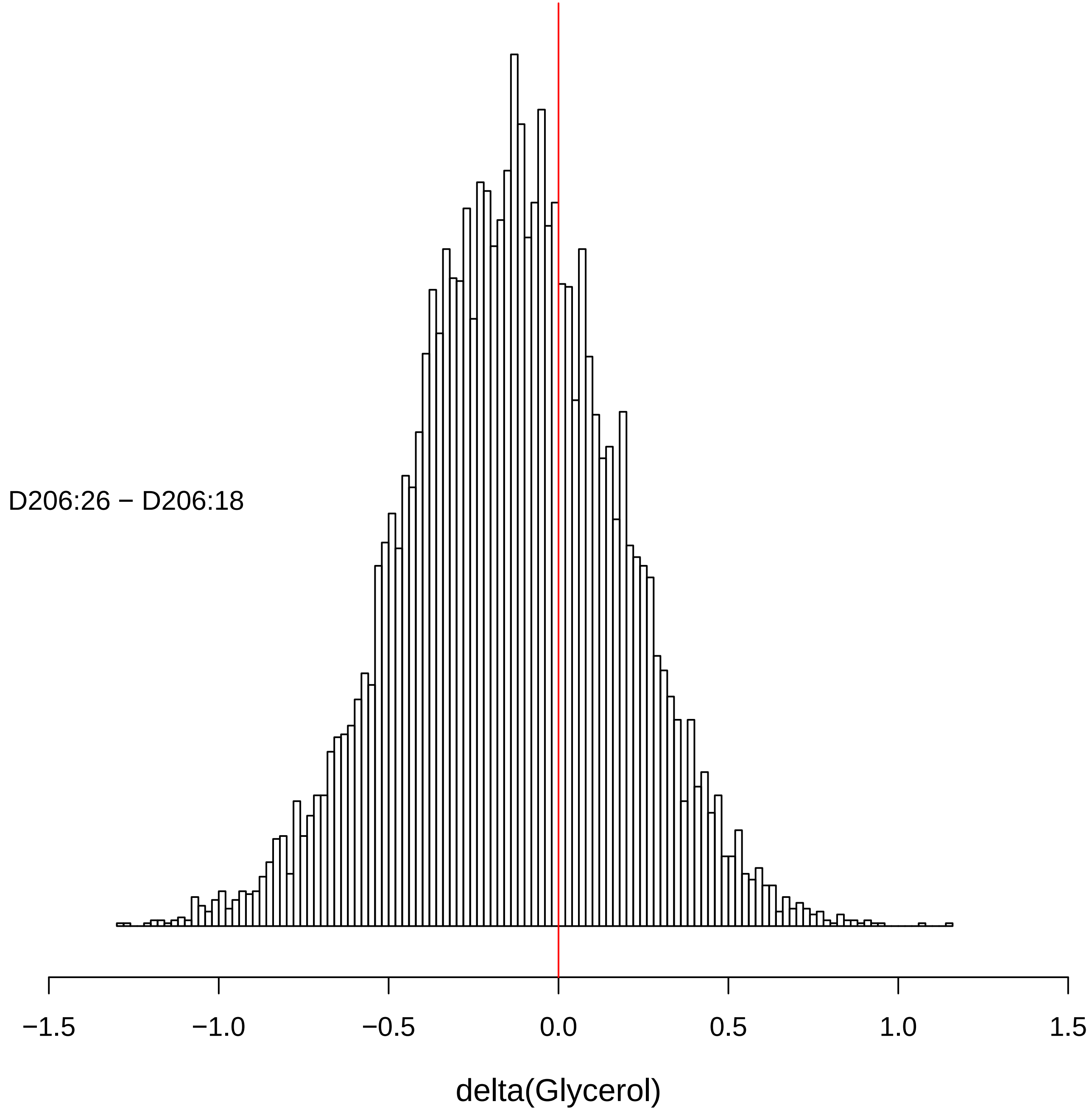
D206:26 − D206:18
−1.5
−1.0
−0.5
0.0
0.5
1.0
1.5
delta(Glycerol)

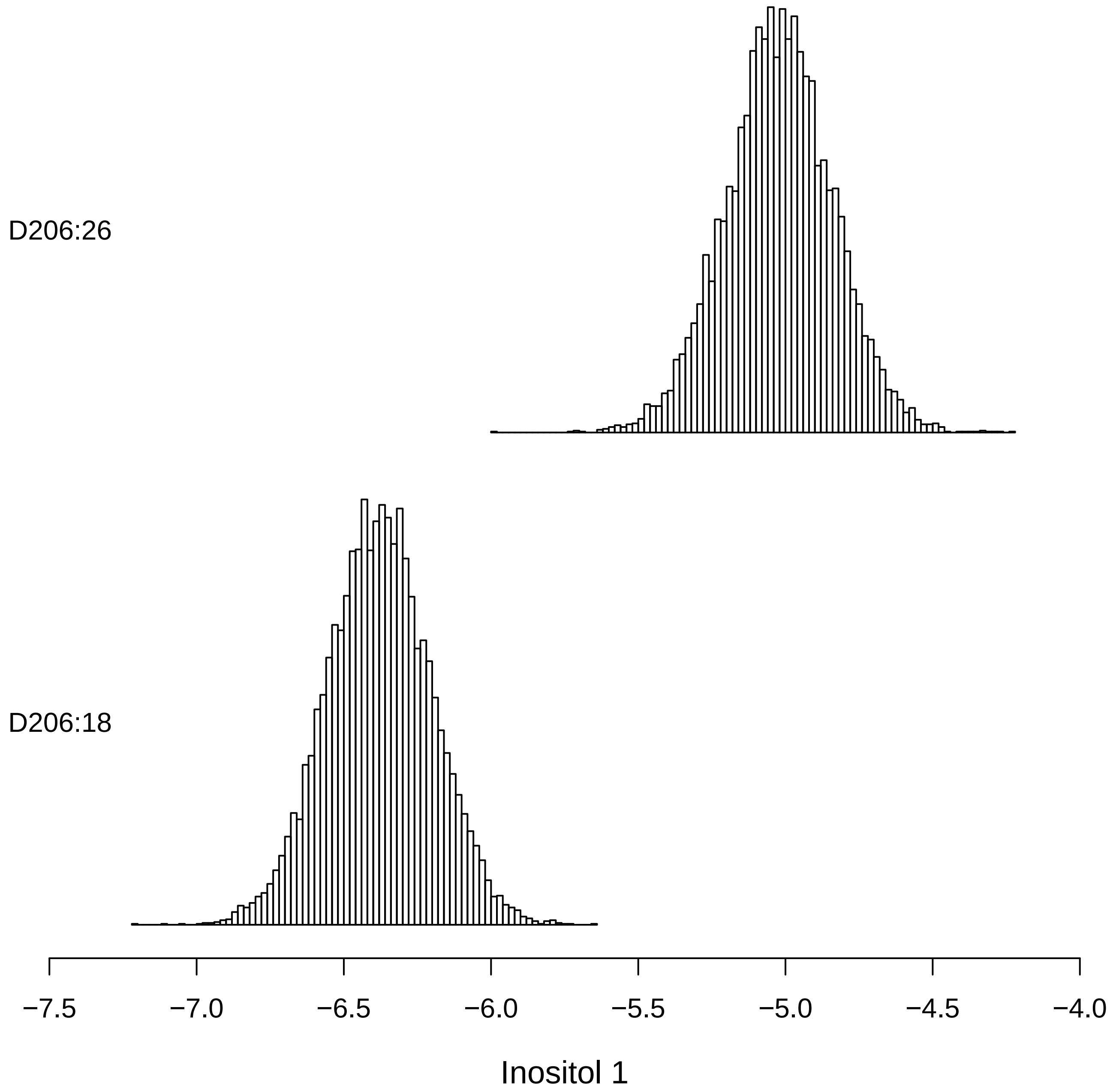

D206:26
D206:18
−7.5
−7.0
−6.5
−6.0
−5.5
−5.0
−4.5
−4.0
Inositol 1

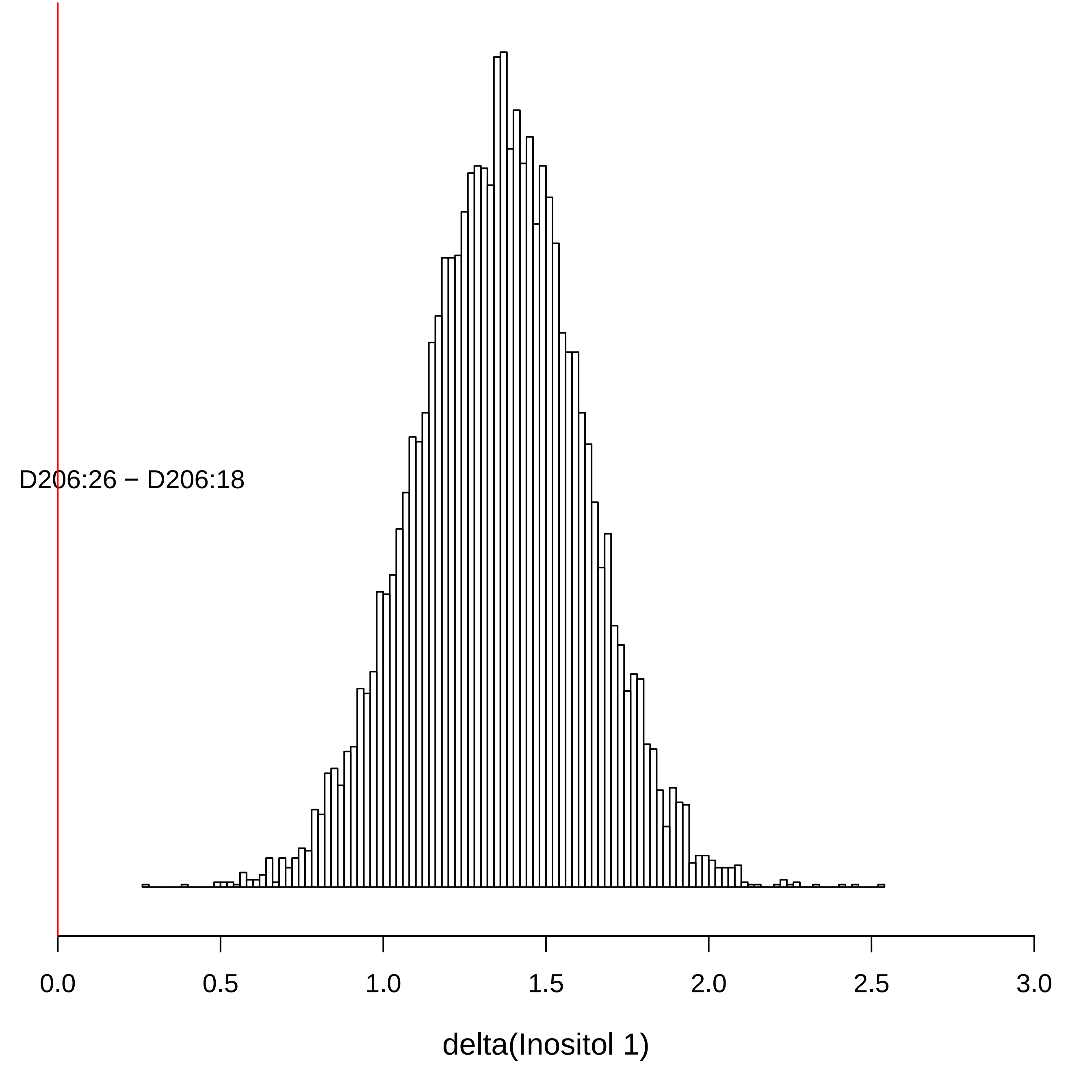
D206:26 − D206:18
0.0
0.5
1.0
1.5
2.0
2.5
3.0
delta(Inositol 1)

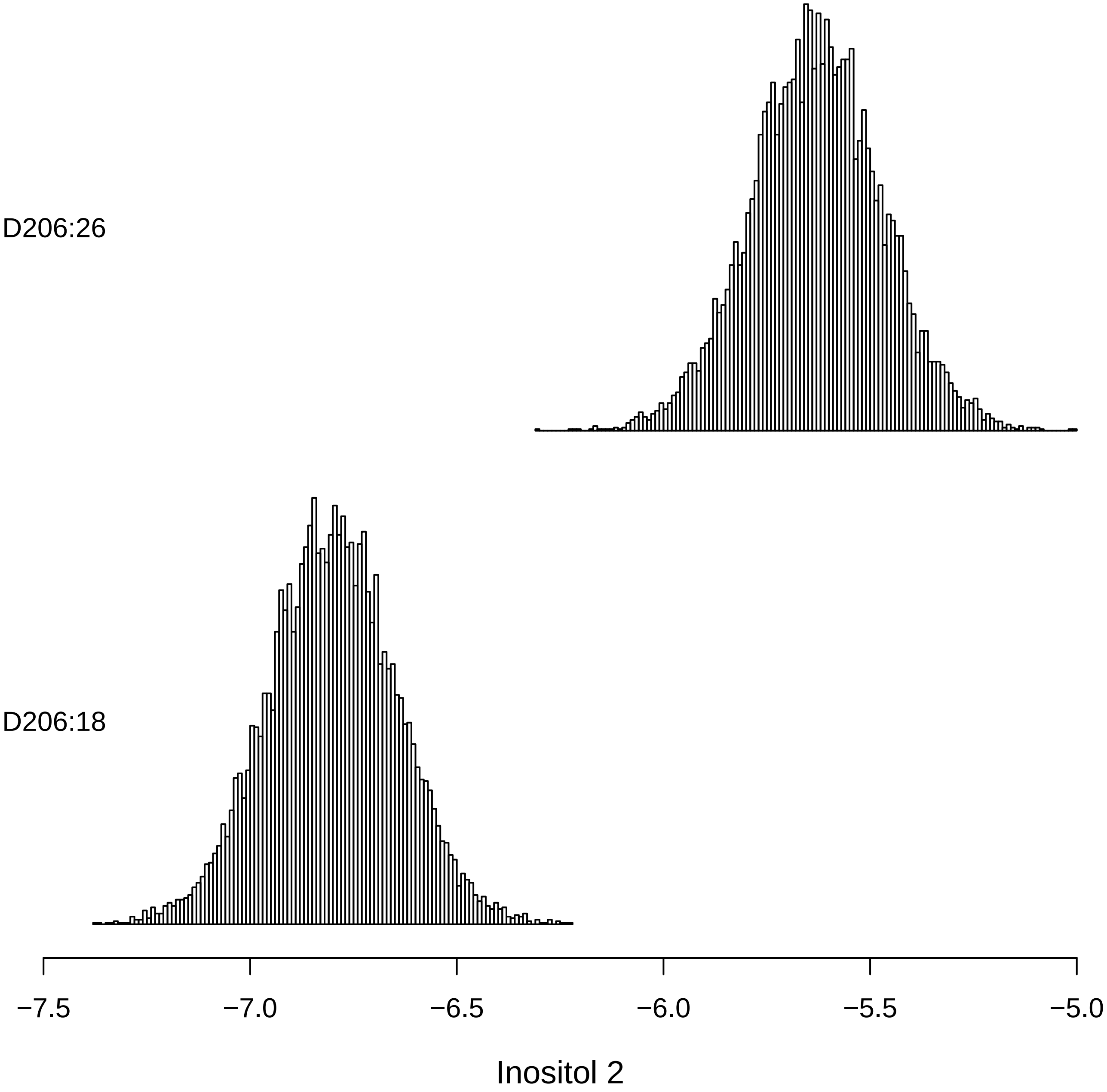

D206:26
D206:18
−7.5
−7.0
−6.5
−6.0
−5.5
−5.0
Inositol 2

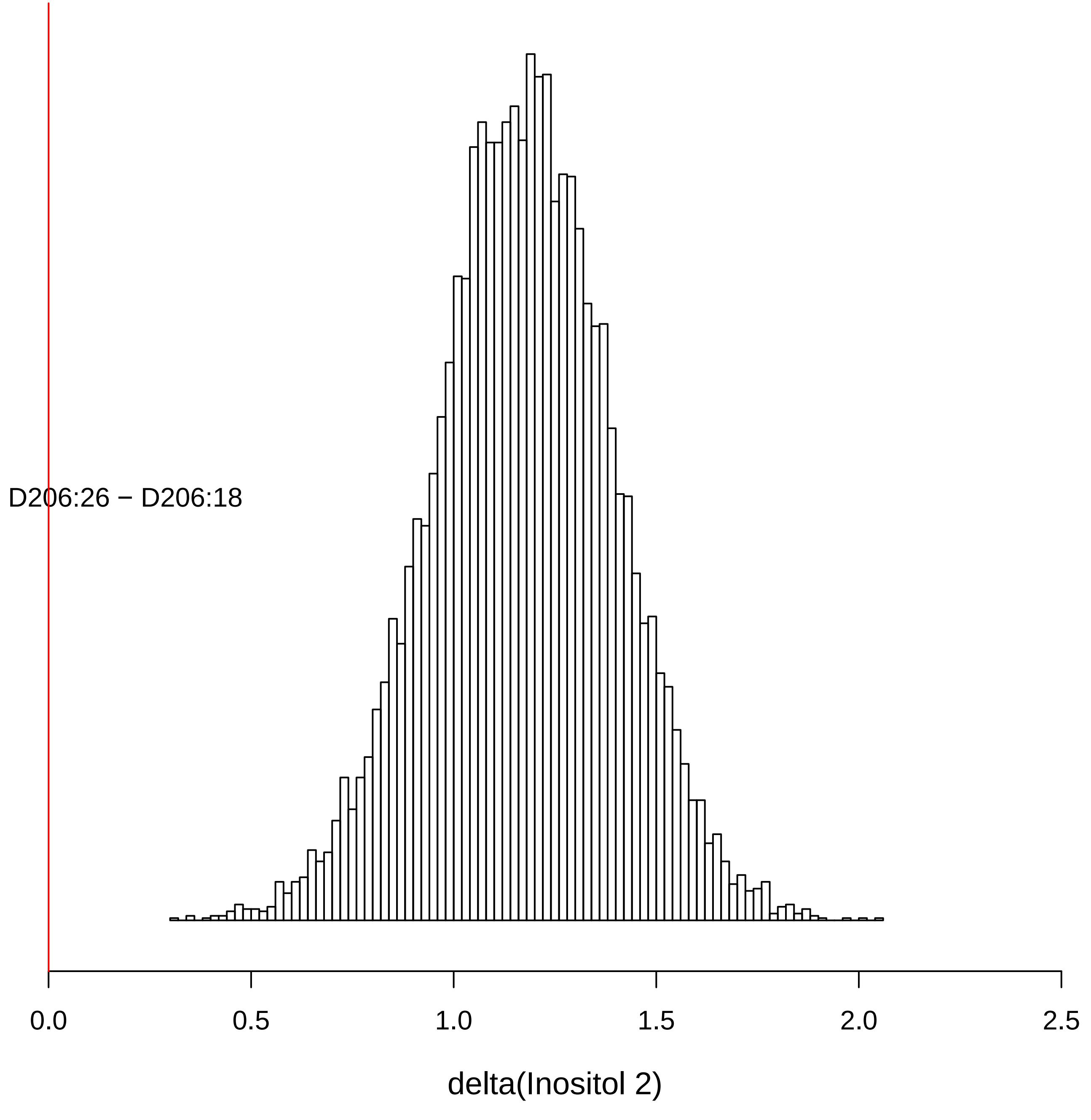
D206:26 − D206:18
0.0
0.5
1.0
1.5
2.0
2.5
delta(Inositol 2)

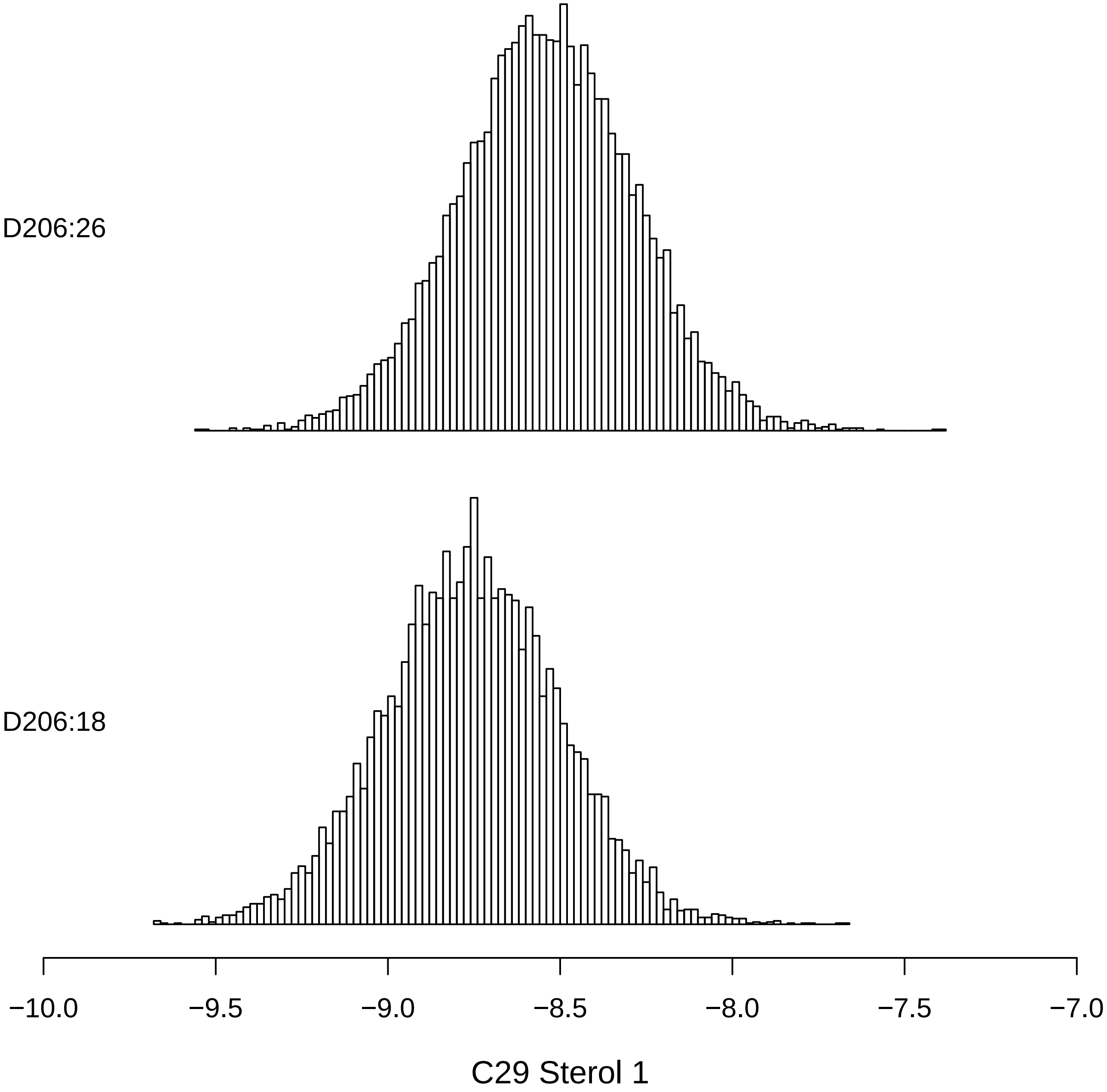
D206:26
D206:18
−10.0
−9.5
−9.0
−8.5
−8.0
−7.5
−7.0
C29 Sterol 1

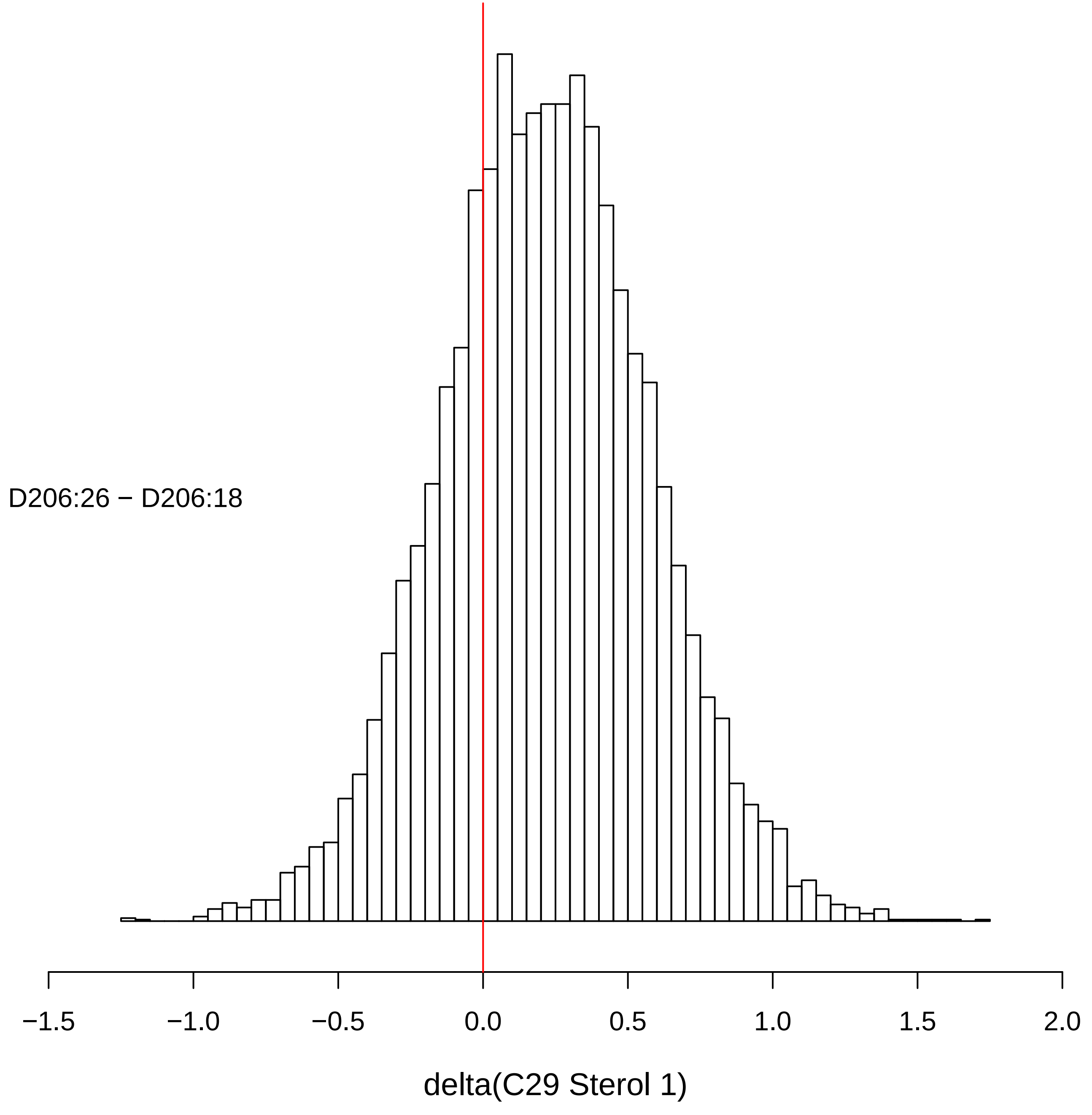
D206:26 − D206:18
−1.5
−1.0
−0.5
0.0
0.5
1.0
1.5
2.0
delta(C29 Sterol 1)

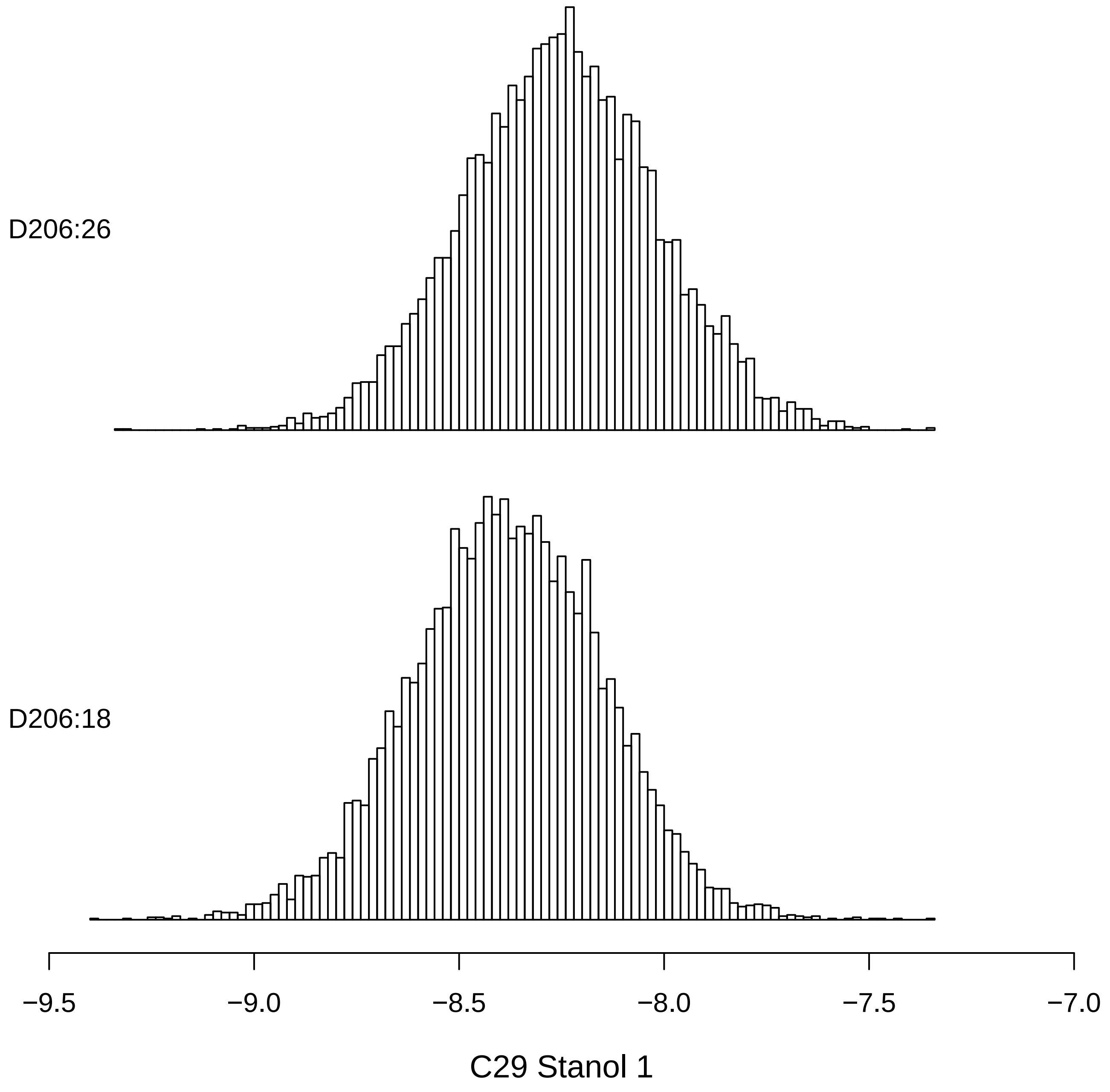
D206:26
D206:18
−9.5
−9.0
−8.5
−8.0
−7.5
−7.0
C29 Stanol 1

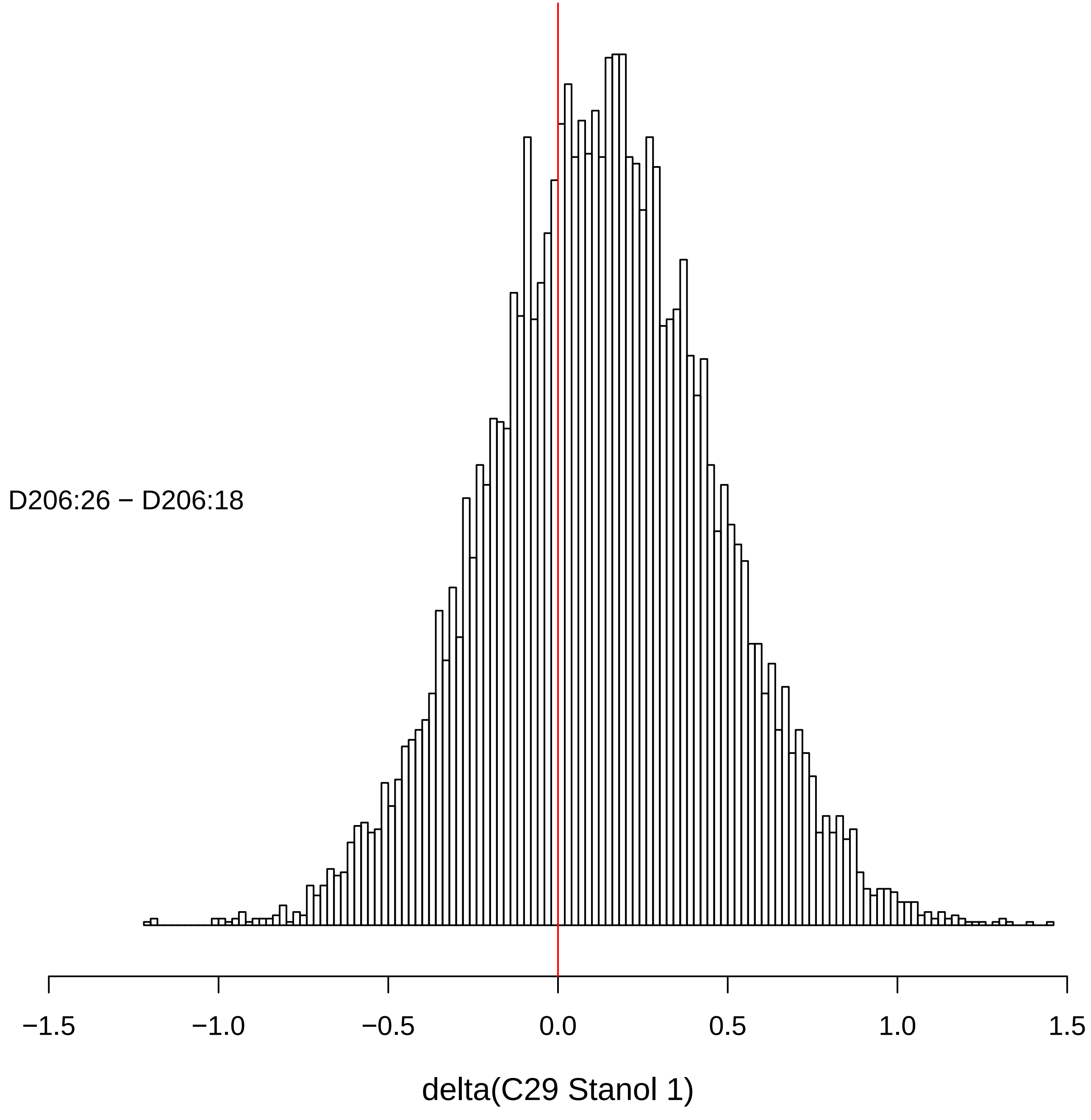
D206:26 − D206:18
−1.5
−1.0
−0.5
0.0
0.5
1.0
1.5
delta(C29 Stanol 1)

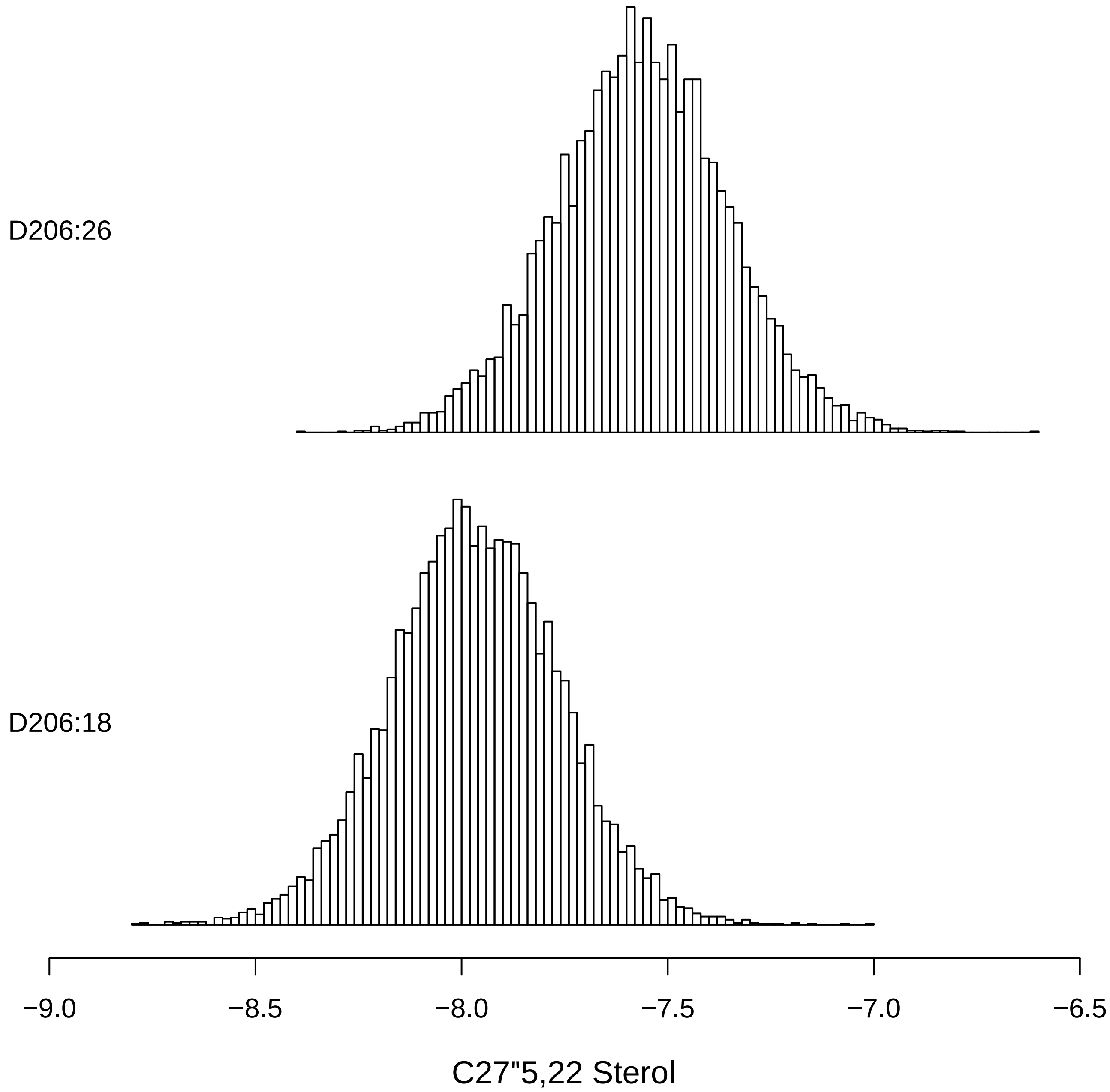
D206:26
D206:18
−9.0
−8.5
−8.0
−7.5
−7.0
−6.5
C27"5,22 Sterol

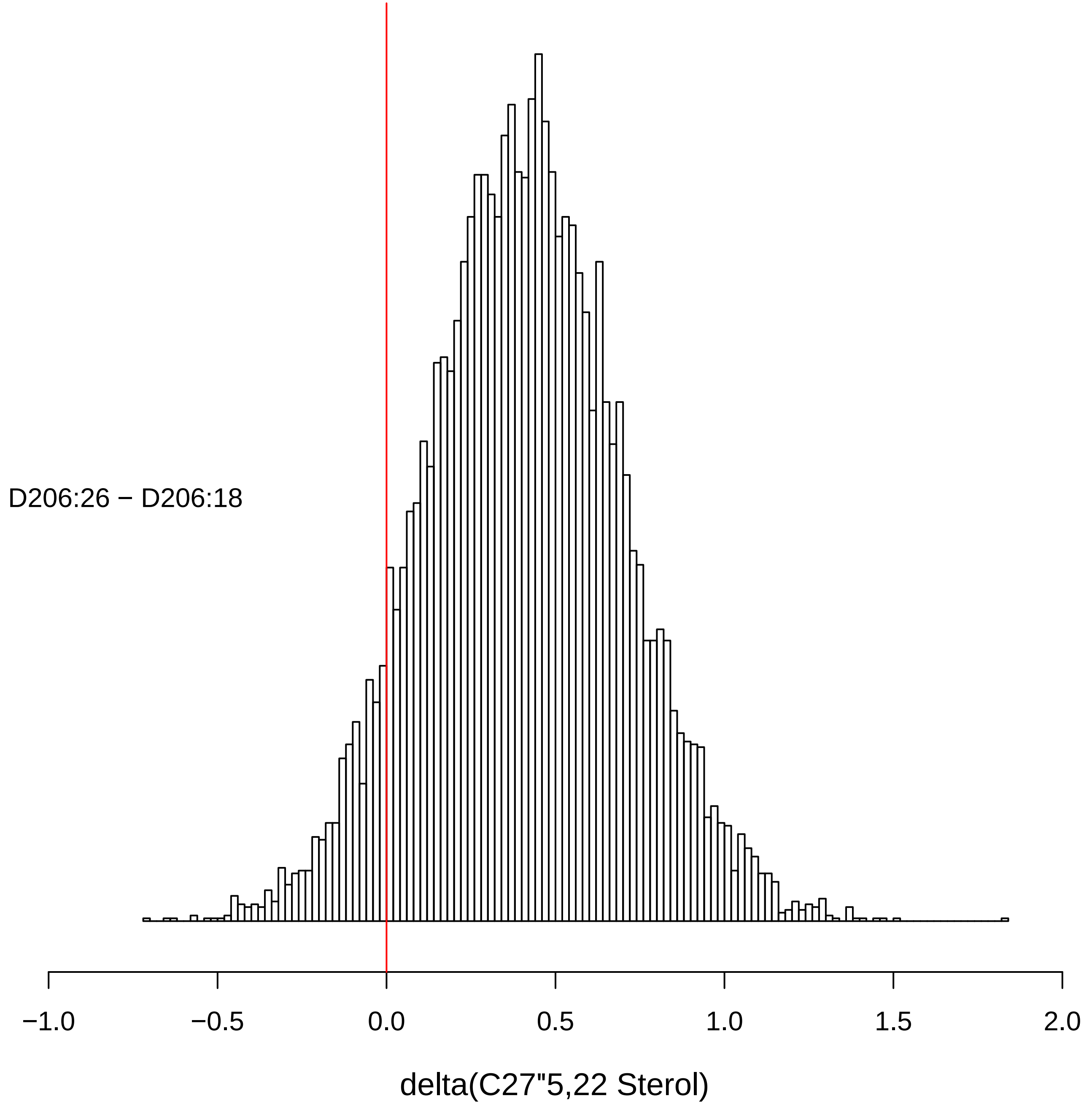
D206:26 − D206:18
−1.0
−0.5
0.0
0.5
1.0
1.5
2.0
delta(C27"5,22 Sterol)

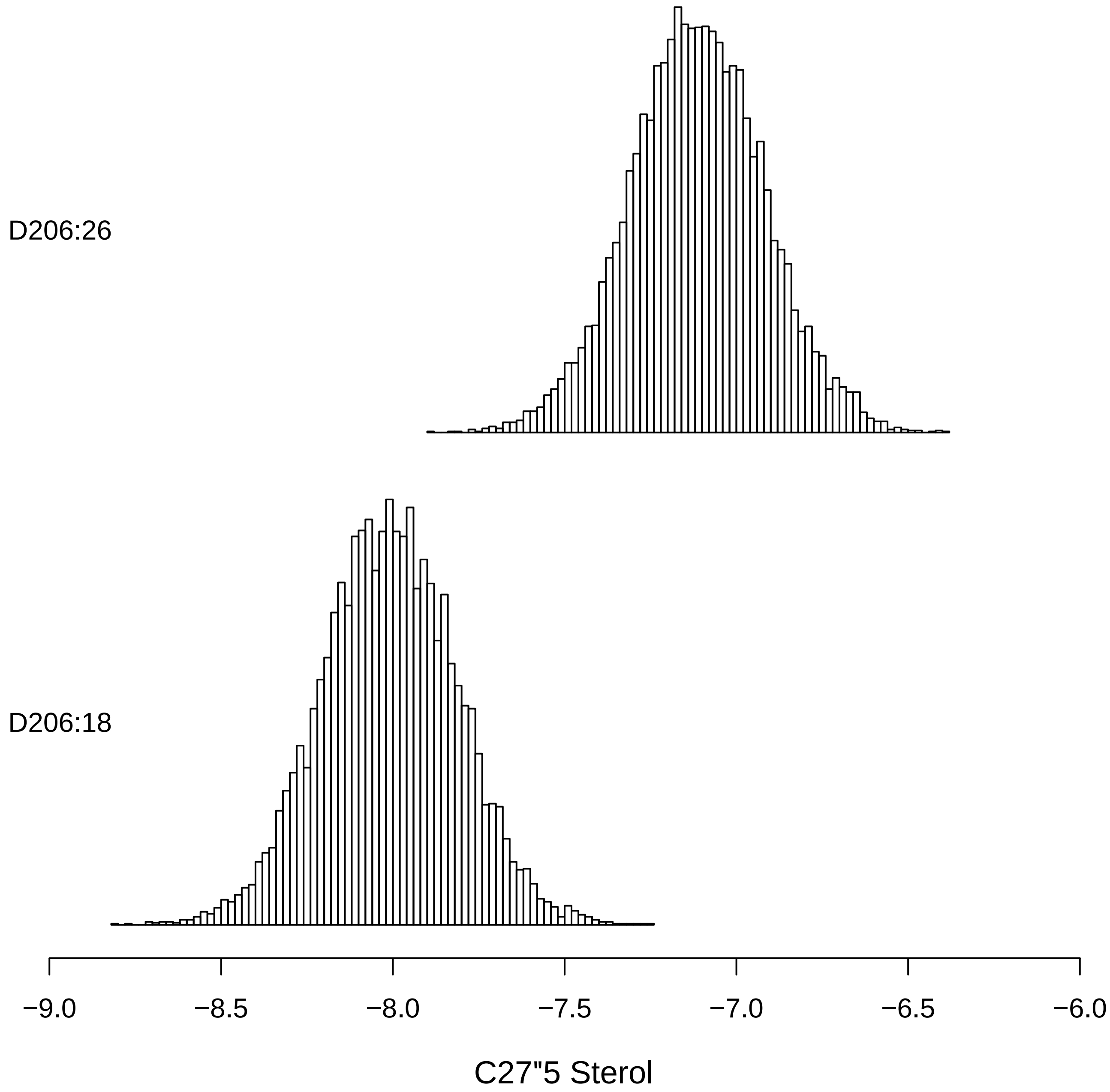
D206:26
D206:18
−9.0
−8.5
−8.0
−7.5
−7.0
−6.5
−6.0
C27"5 Sterol

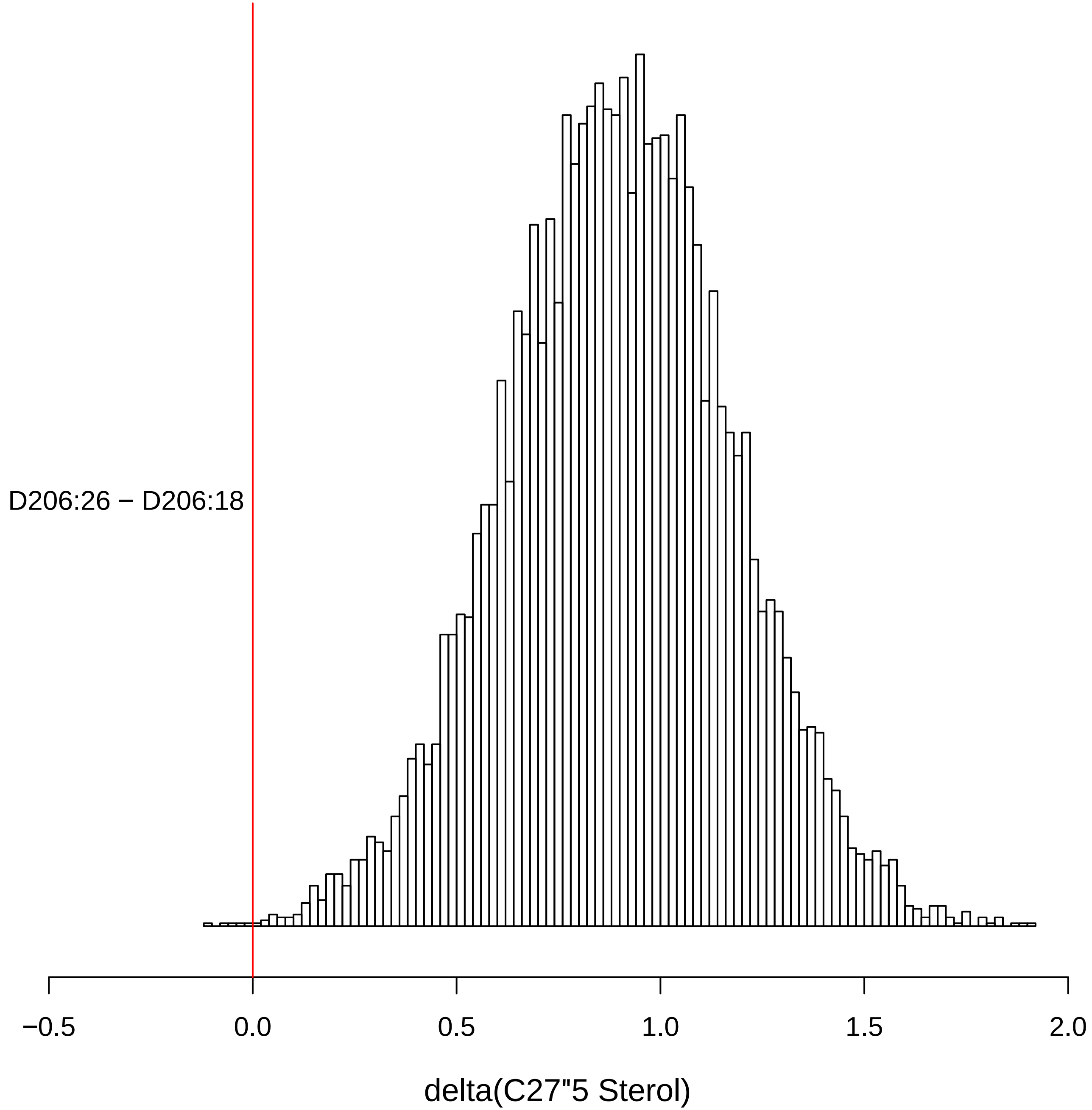
D206:26 − D206:18
−0.5
0.0
0.5
1.0
1.5
2.0
delta(C27"5 Sterol)

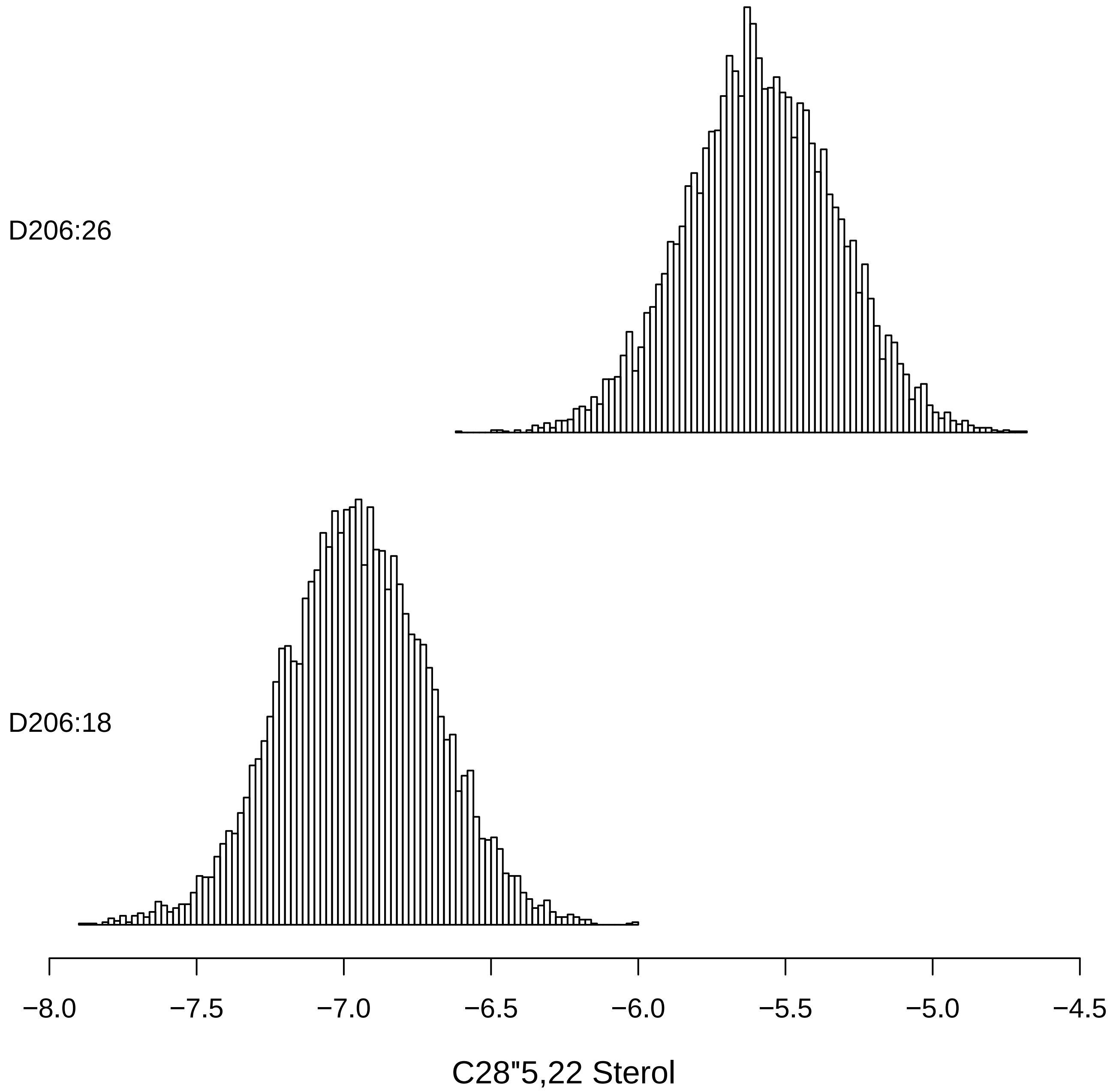
D206:26
D206:18
−8.0
−7.5
−7.0
−6.5
−6.0
−5.5
−5.0
−4.5
C28"5,22 Sterol

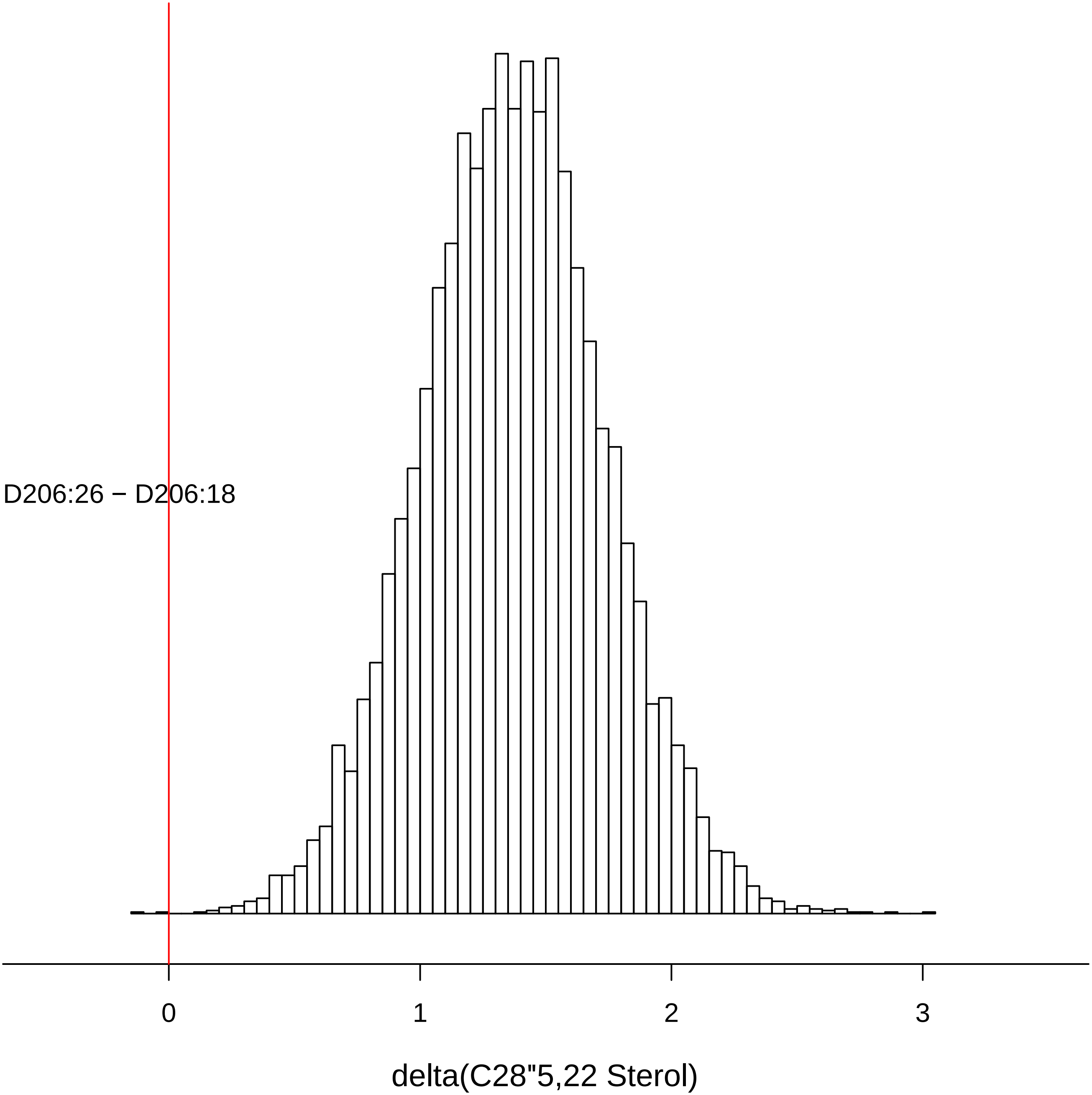
D206:26 − D206:18
0
1
2
3
delta(C28"5,22 Sterol)

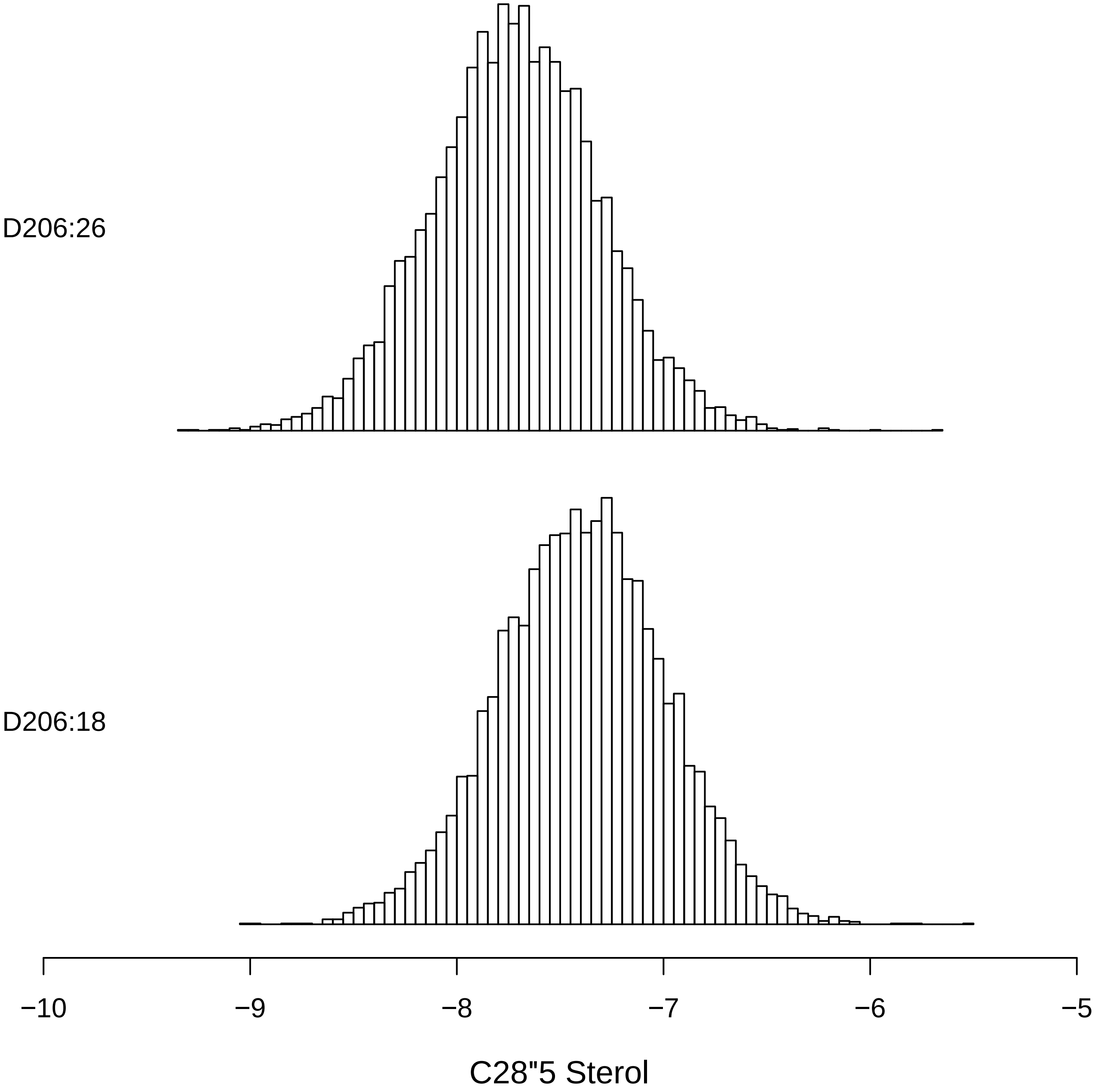
D206:26
D206:18
−10
−9
−8
−7
−6
−5
C28"5 Sterol

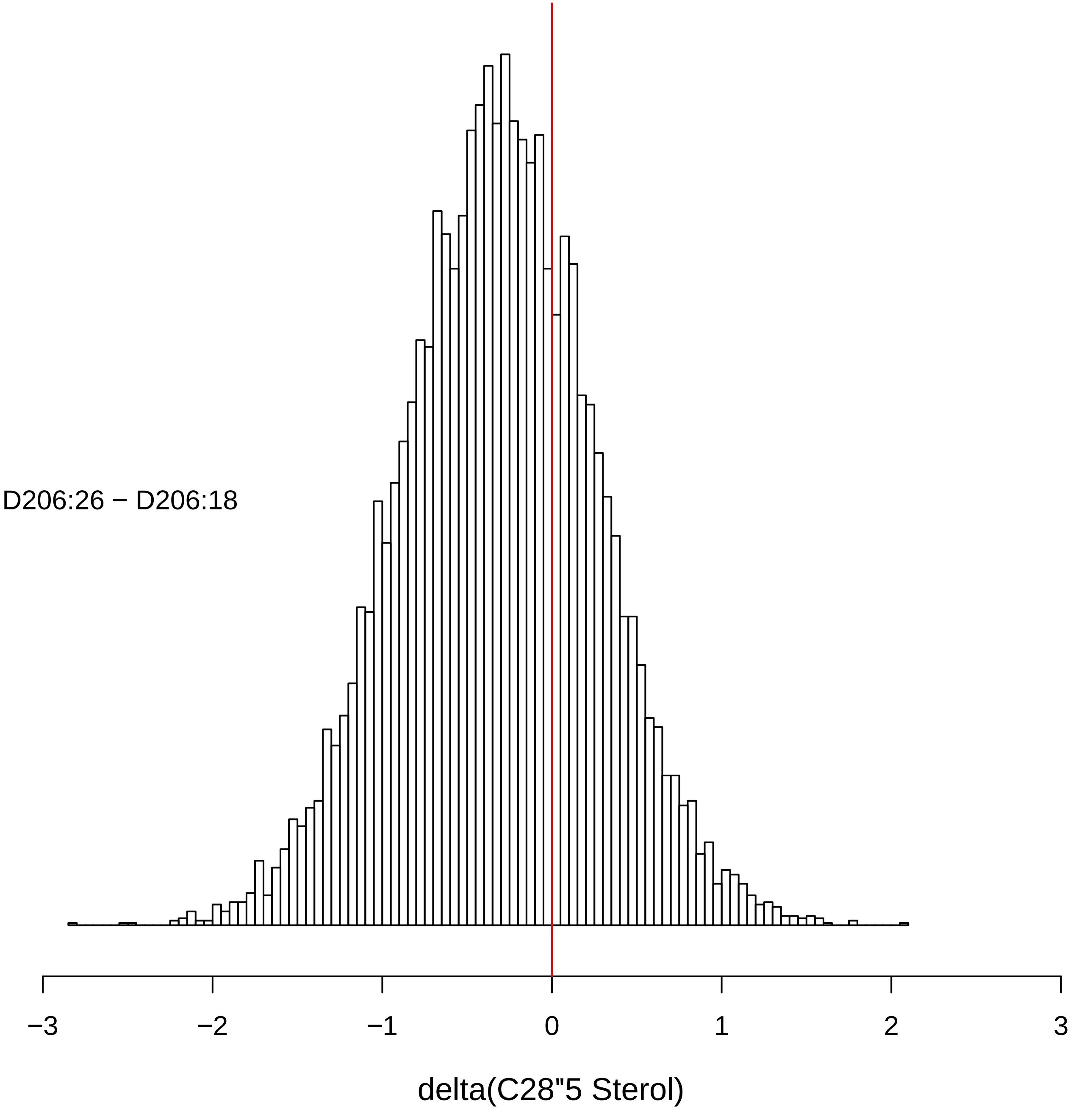
D206:26 − D206:18
−3
−2
−1
0
1
2
3
delta(C28"5 Sterol)

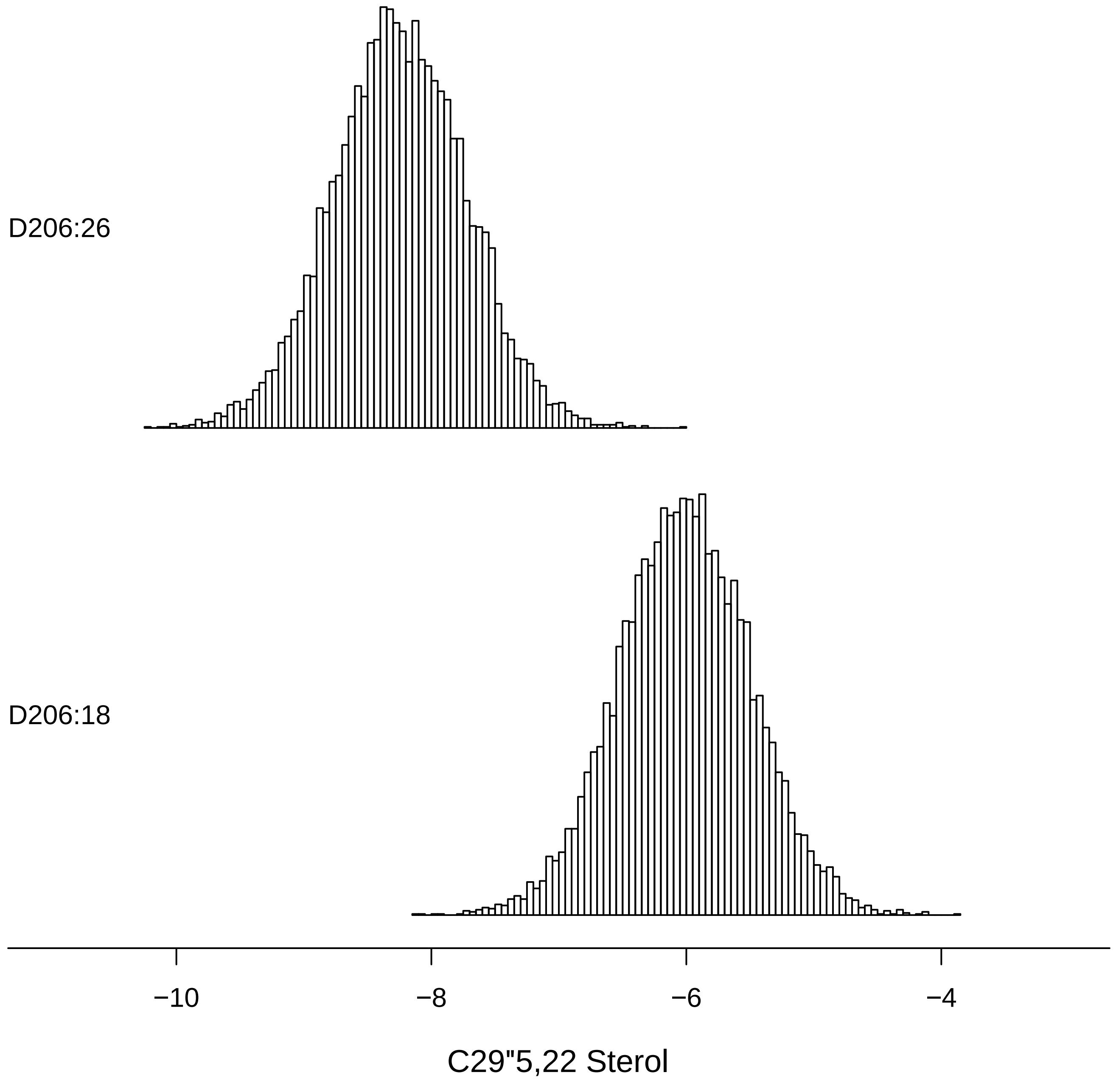
D206:26
D206:18
−10
−8
−6
−4
C29"5,22 Sterol

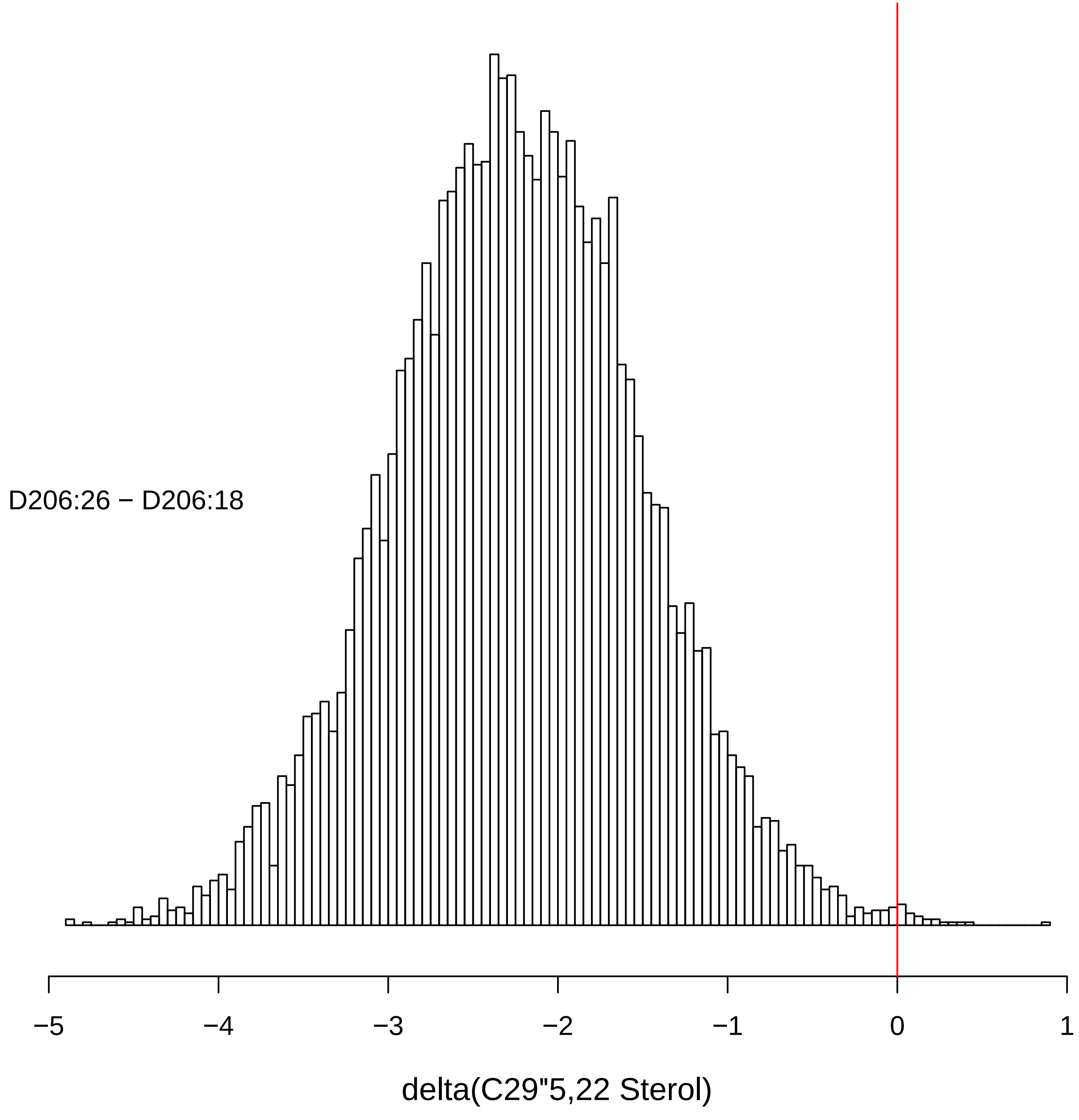
D206:26 − D206:18
−5
−4
−3
−2
−1
0
1
delta(C29"5,22 Sterol)

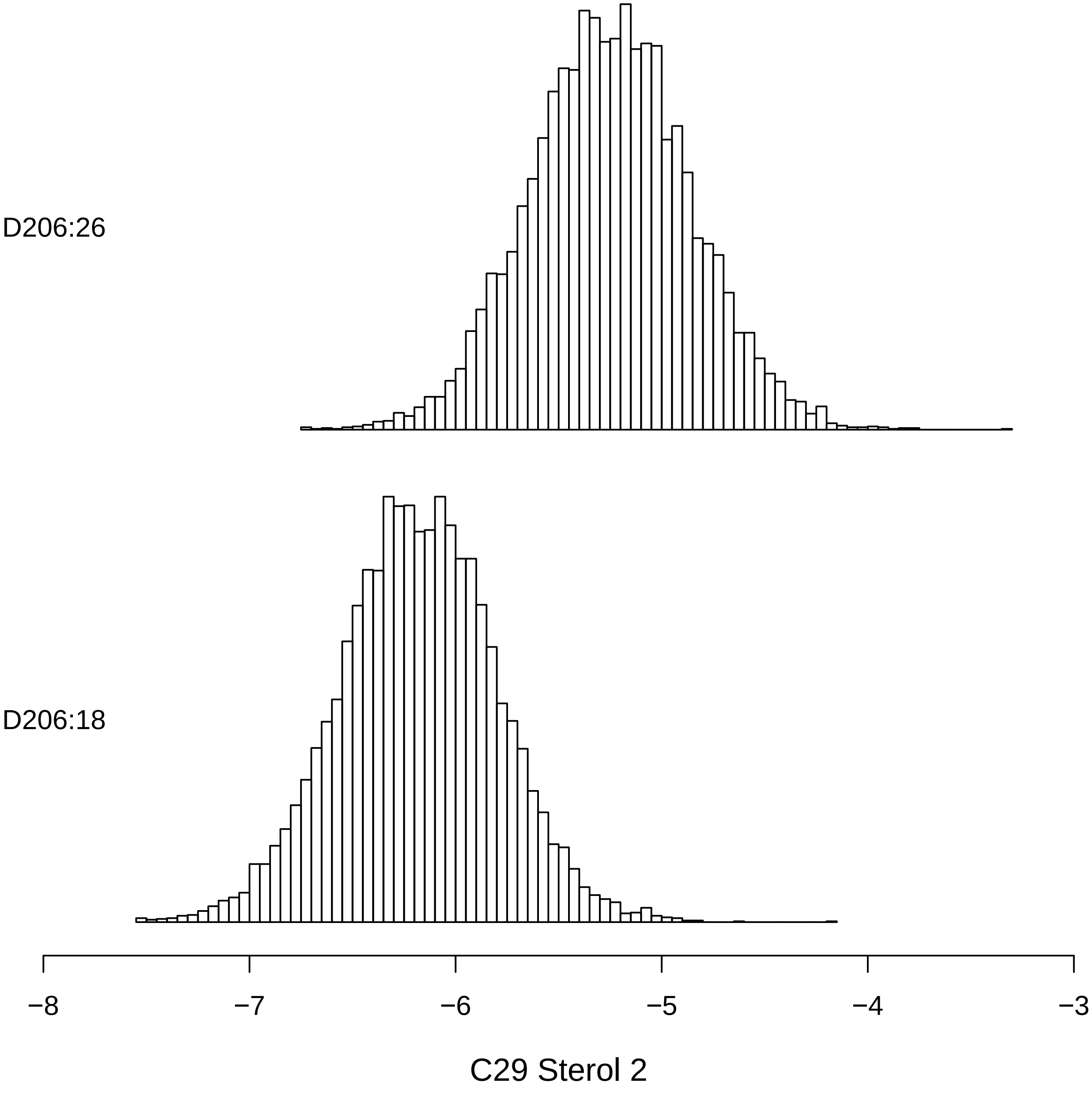
D206:26
D206:18
−8
−7
−6
−5
−4
−3
C29 Sterol 2

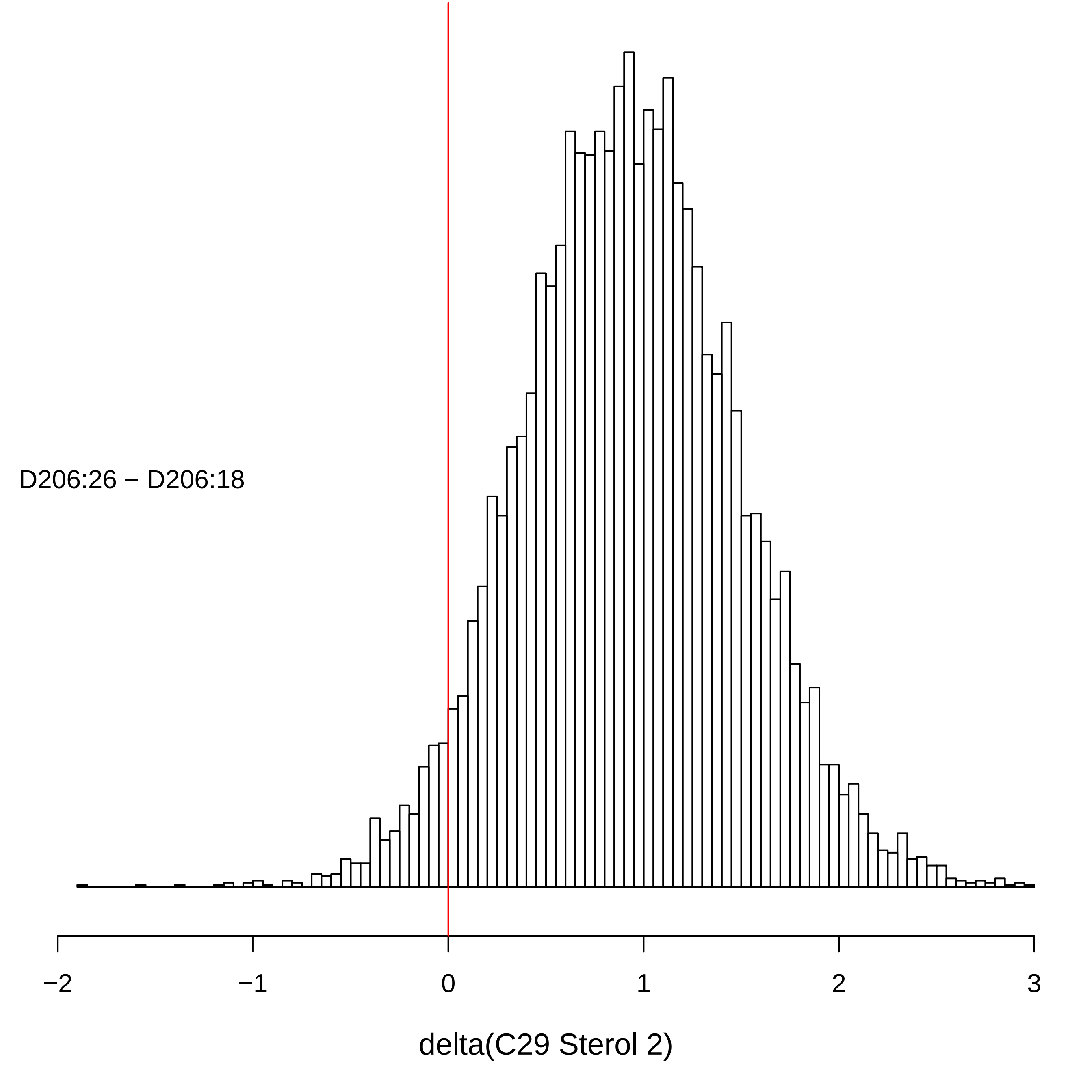
D206:26 − D206:18
−2
−1
0
1
2
3
delta(C29 Sterol 2)

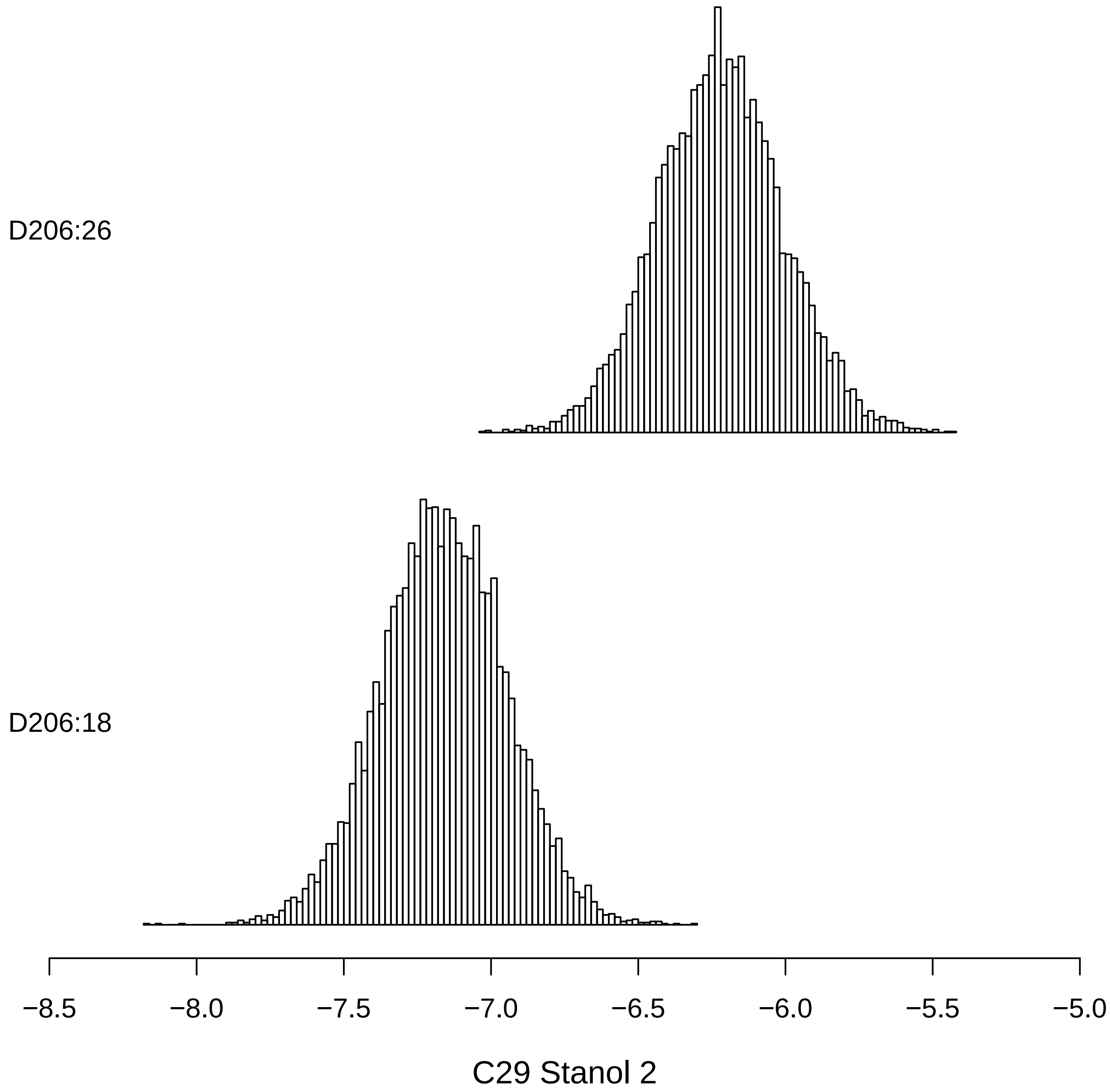
D206:26
D206:18
−8.5
−8.0
−7.5
−7.0
−6.5
−6.0
−5.5
−5.0
C29 Stanol 2

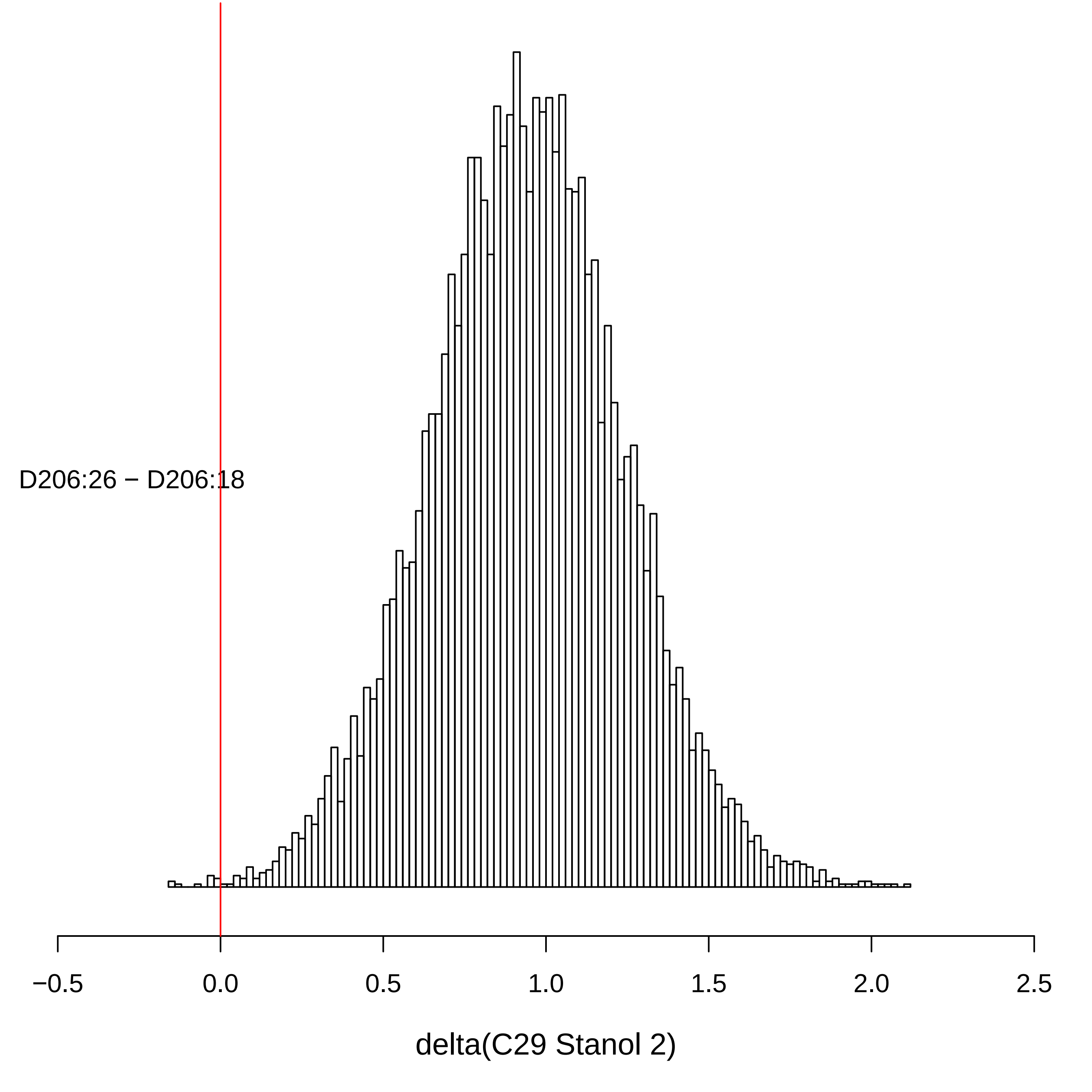

D206:26 − D206:18
−0.5
0.0
0.5
1.0
1.5
2.0
2.5
delta(C29 Stanol 2)

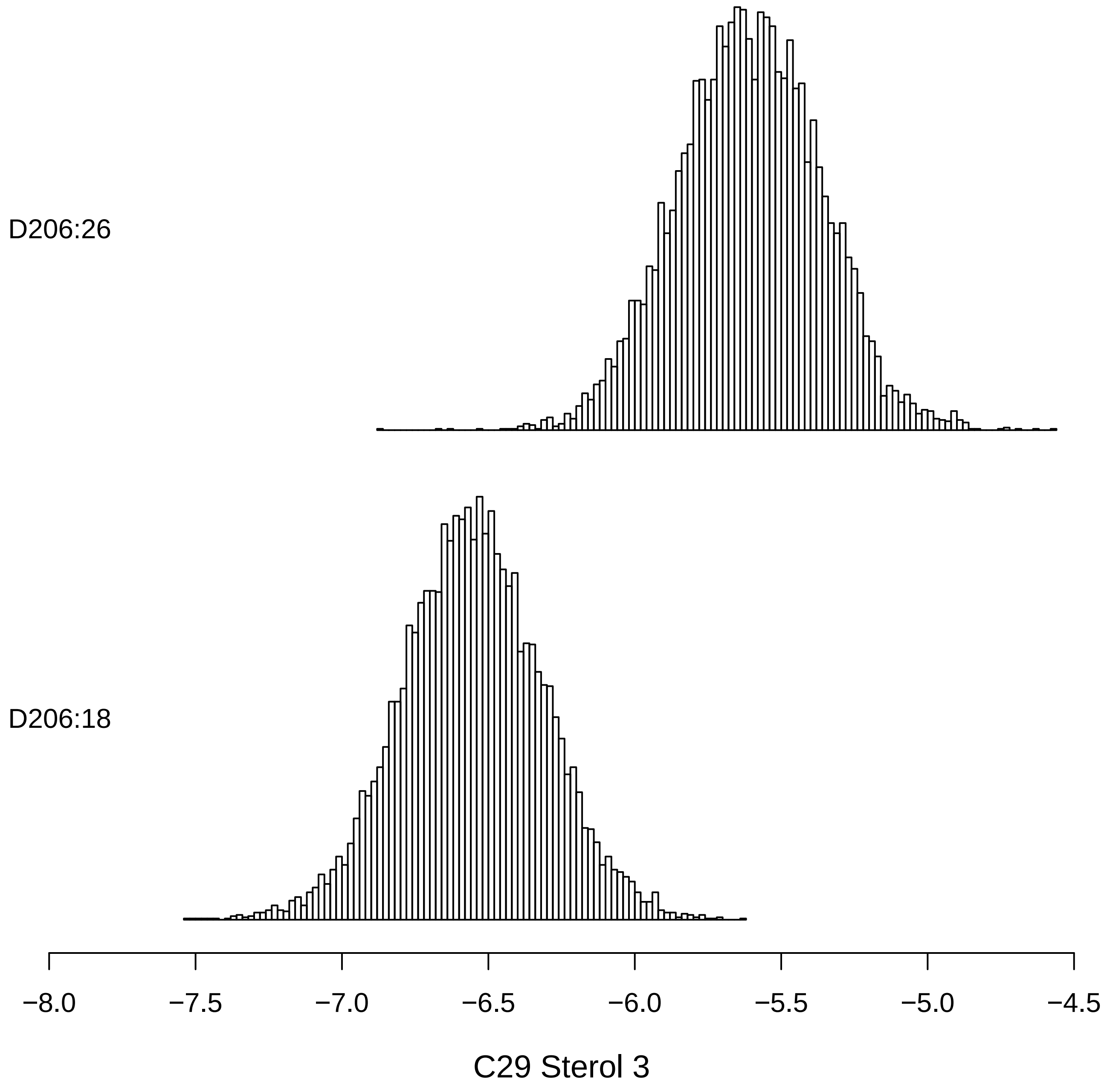

D206:26
D206:18
−8.0
−7.5
−7.0
−6.5
−6.0
−5.5
−5.0
−4.5
C29 Sterol 3

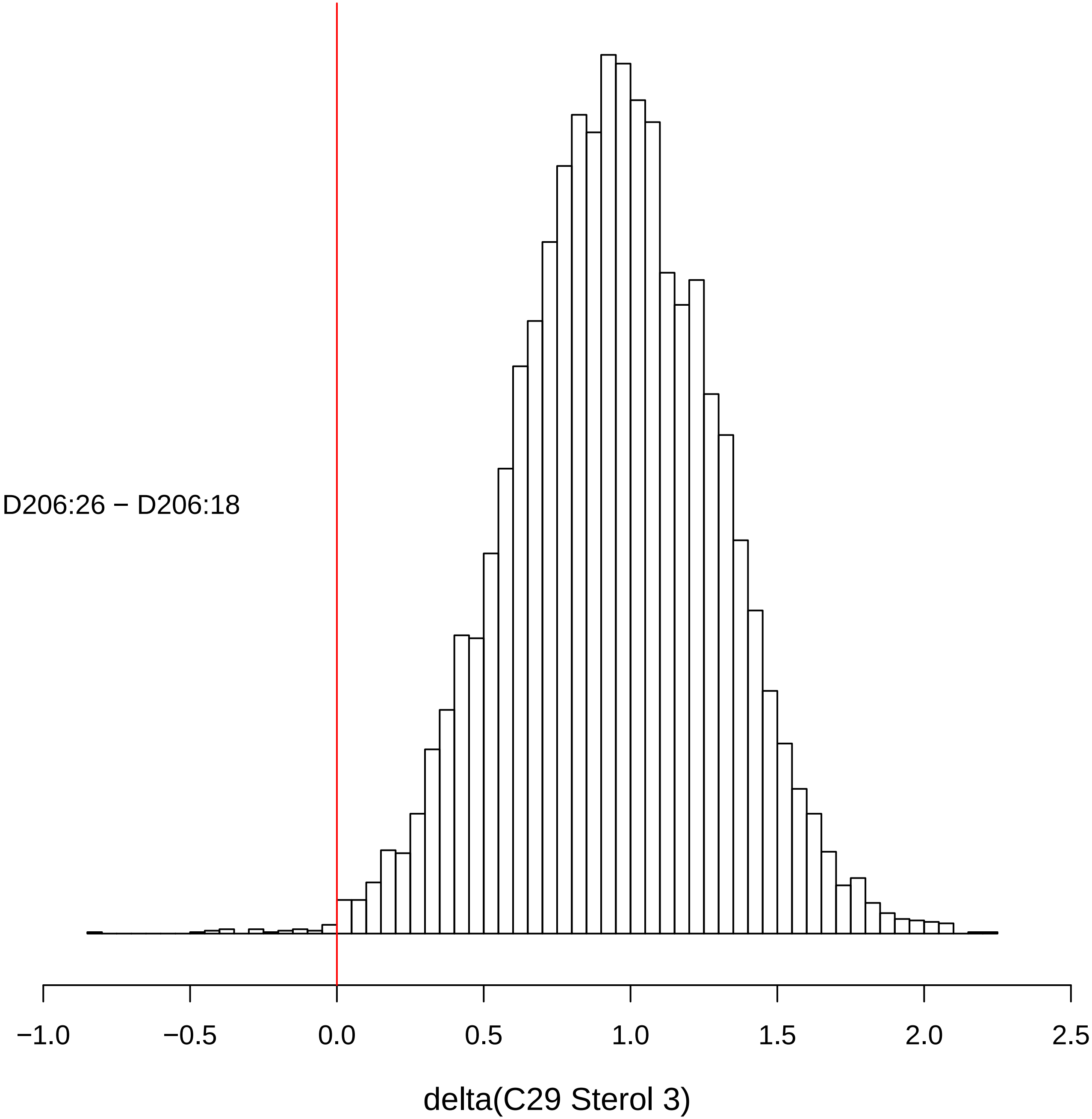

D206:26 − D206:18
−1.0
−0.5
0.0
0.5
1.0
1.5
2.0
2.5
delta(C29 Sterol 3)

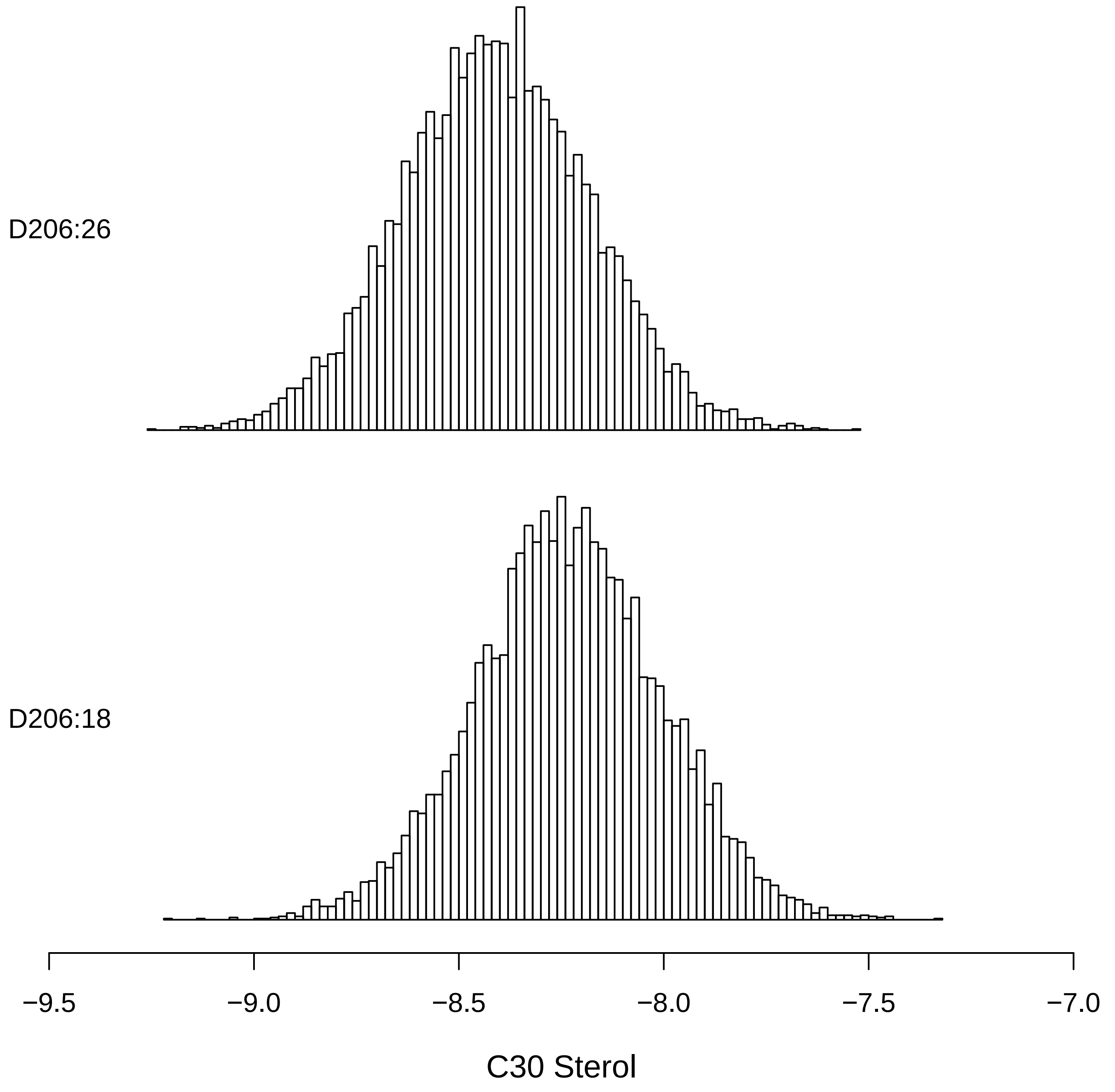

D206:26
D206:18
−9.5
−9.0
−8.5
−8.0
−7.5
−7.0
C30 Sterol

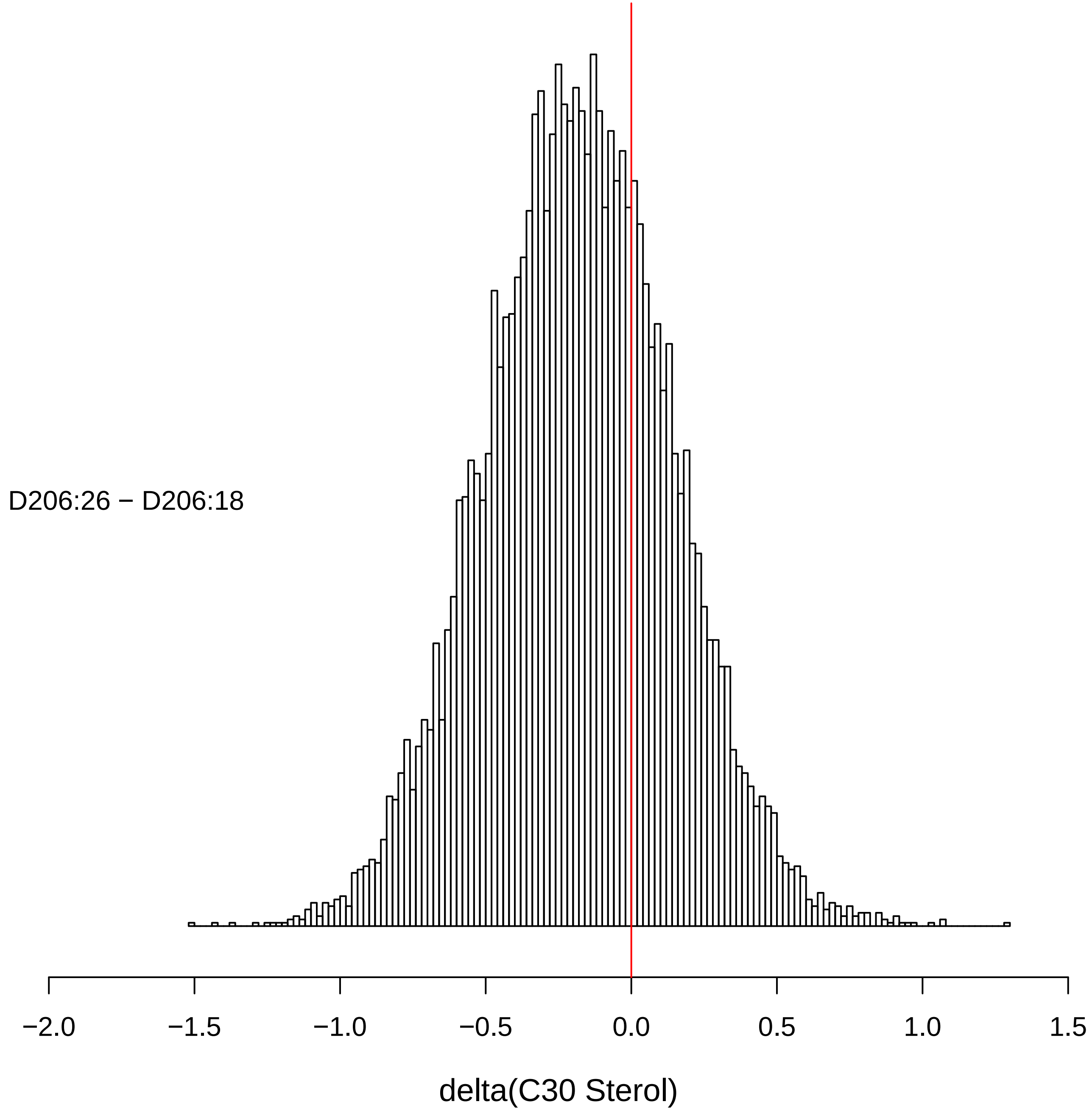
D206:26 − D206:18
−2.0
−1.5
−1.0
−0.5
0.0
0.5
1.0
1.5
delta(C30 Sterol)

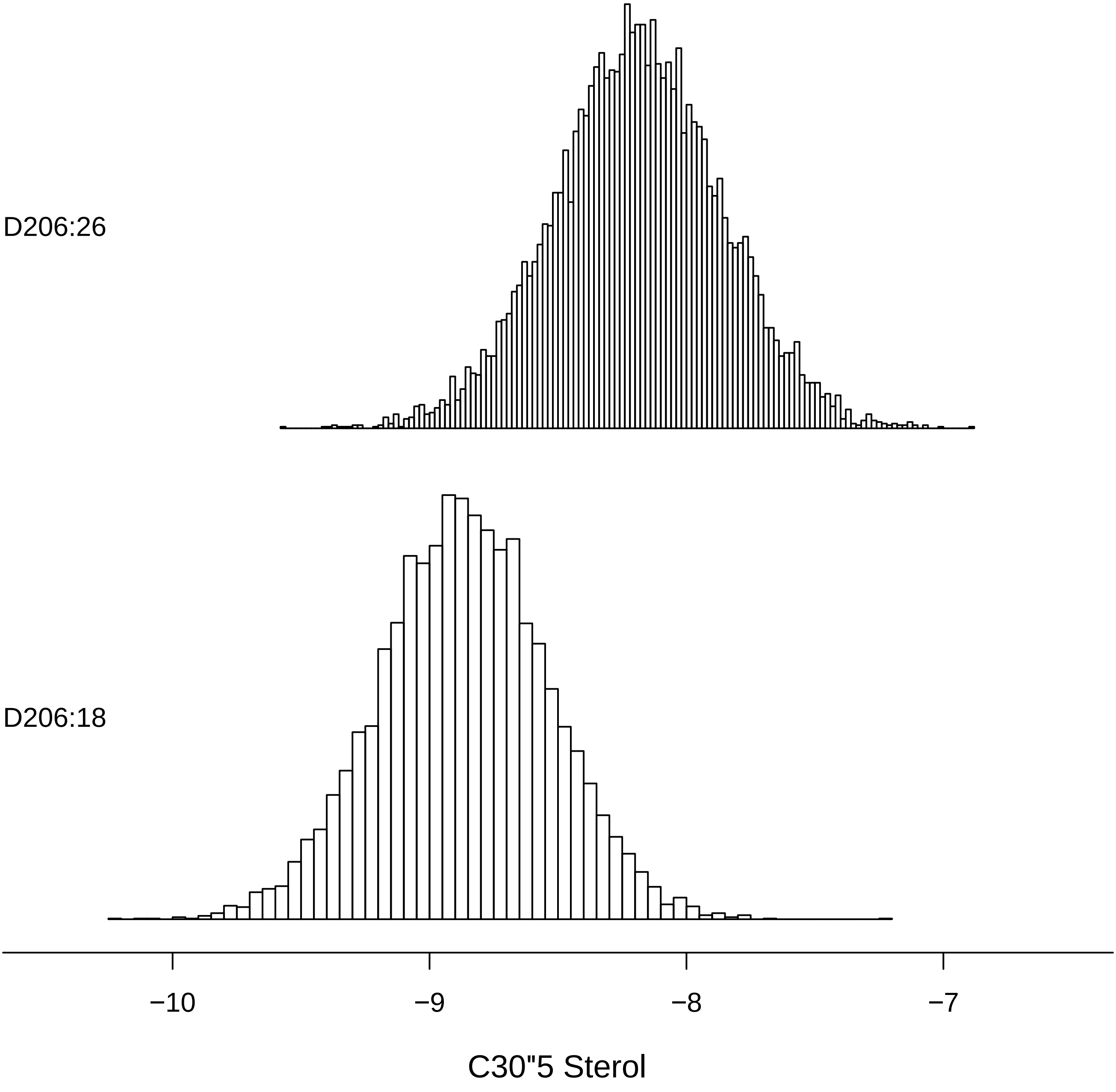
D206:26
D206:18
−10
−9
−8
−7
C30"5 Sterol

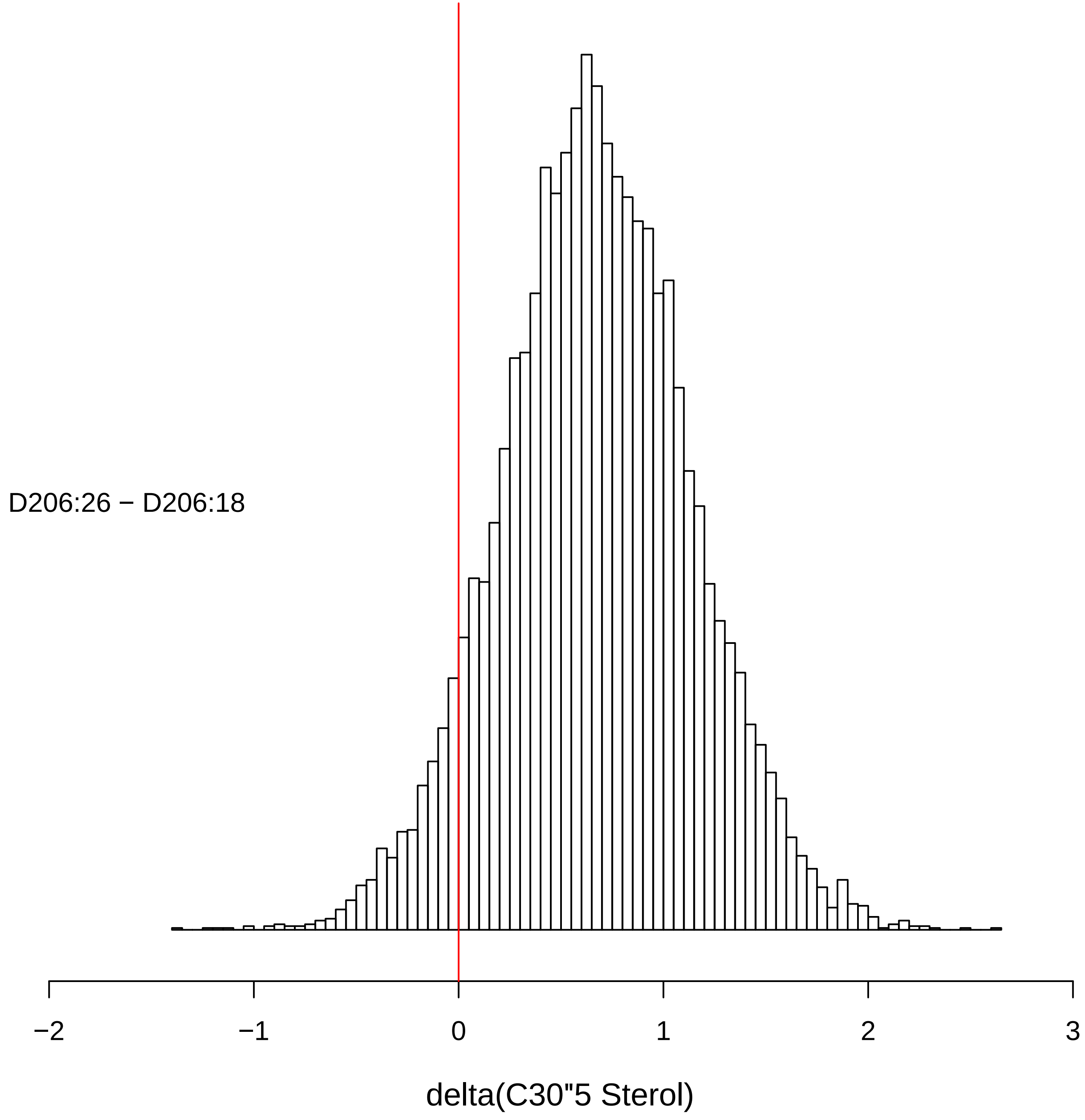
D206:26 − D206:18
−2
−1
0
1
2
3
delta(C30"5 Sterol)

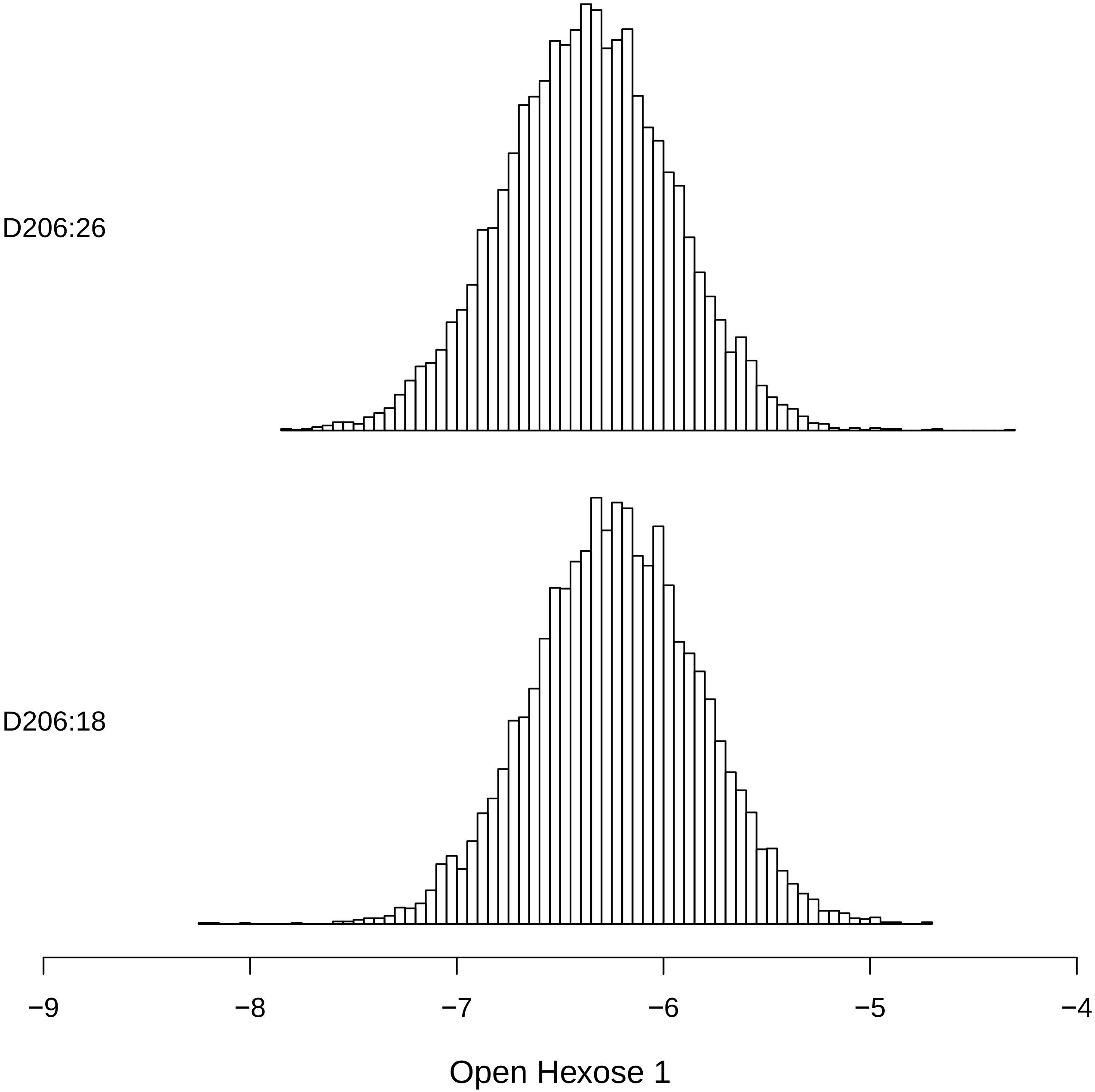

D206:26
D206:18
−9
−8
−7
−6
−5
−4
Open Hexose 1

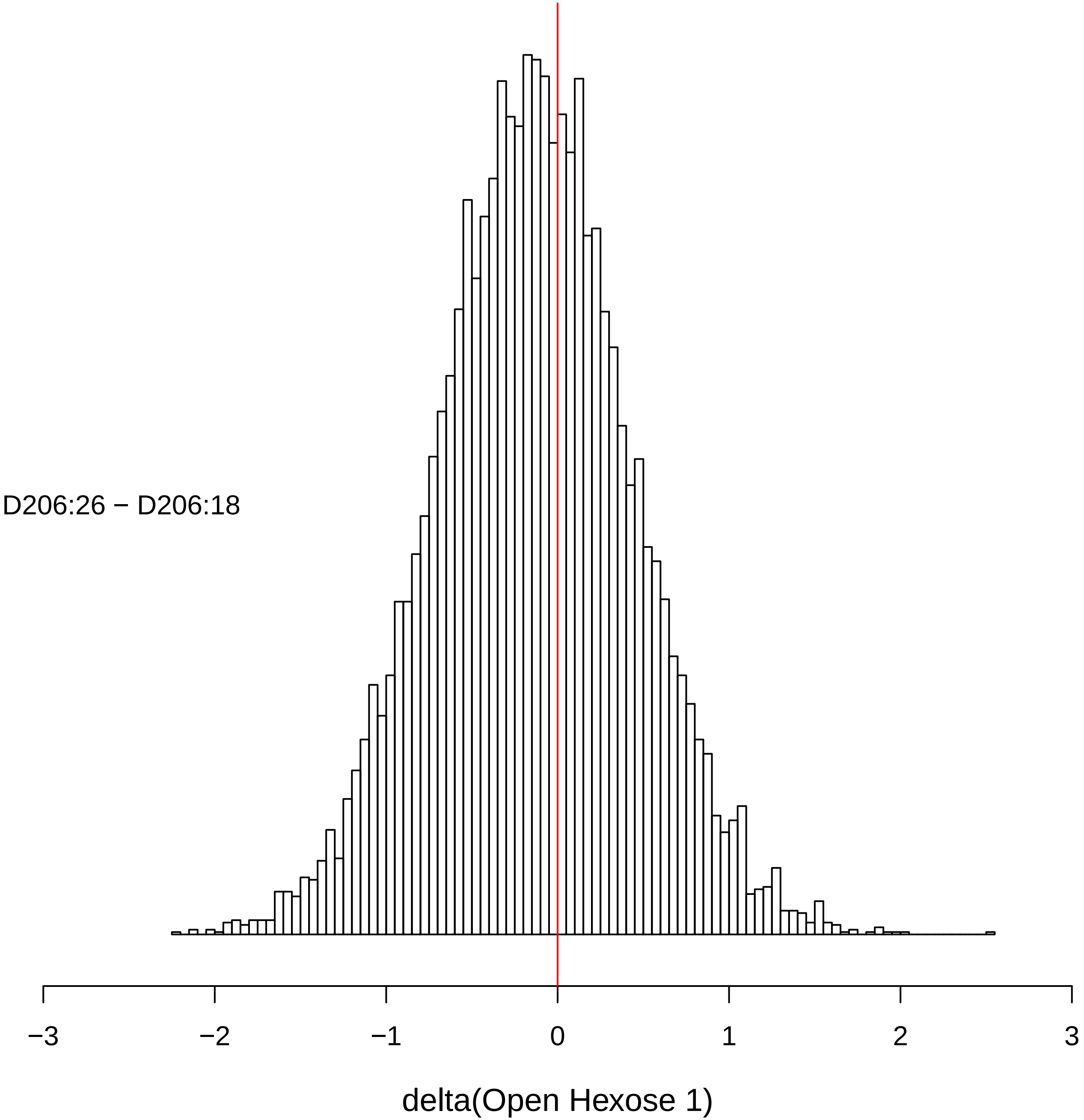
D206:26 − D206:18
−3
−2
−1
0
1
2
3
delta(Open Hexose 1)

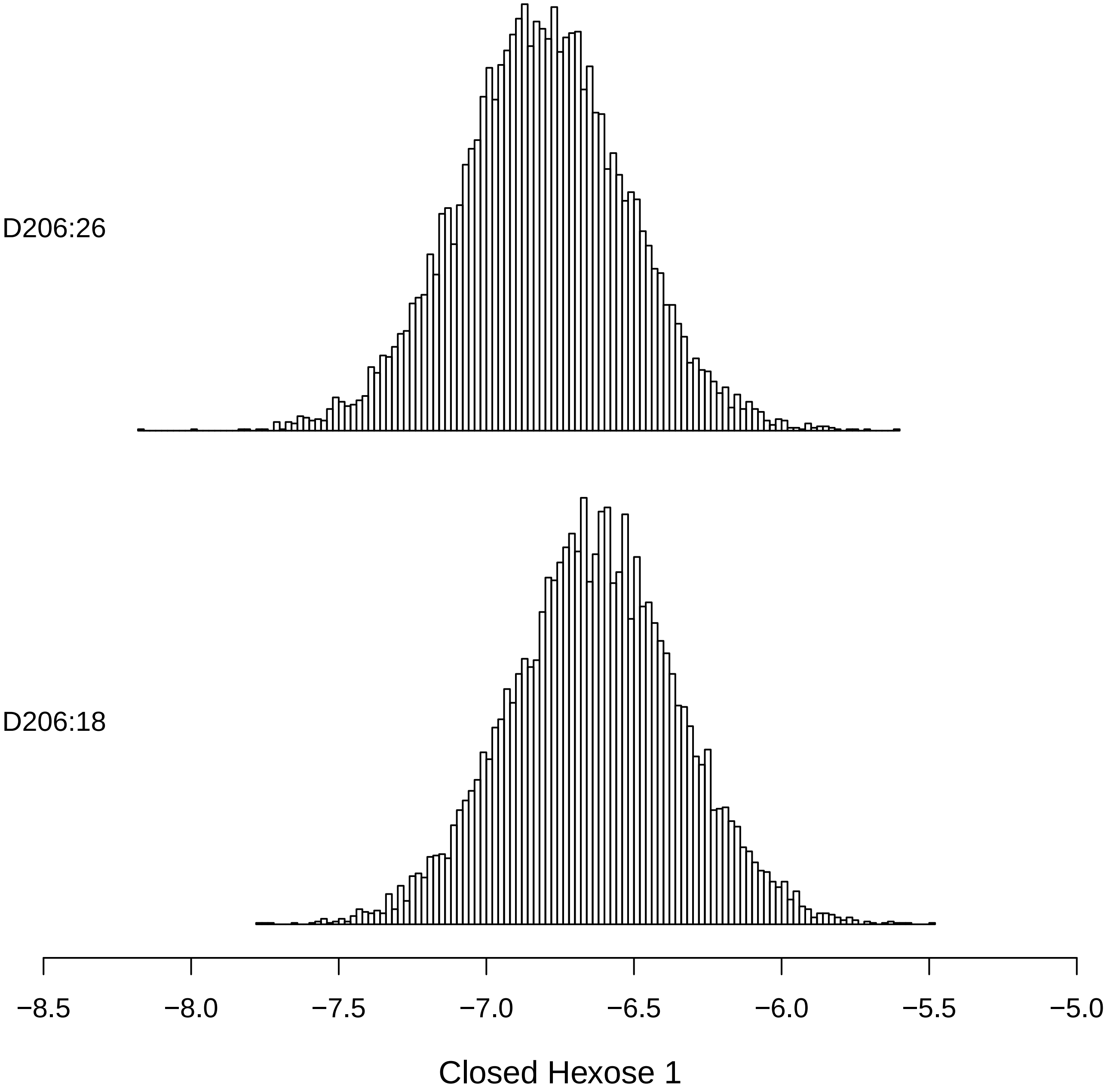
D206:26
D206:18
−8.5
−8.0
−7.5
−7.0
−6.5
−6.0
−5.5
−5.0
Closed Hexose 1

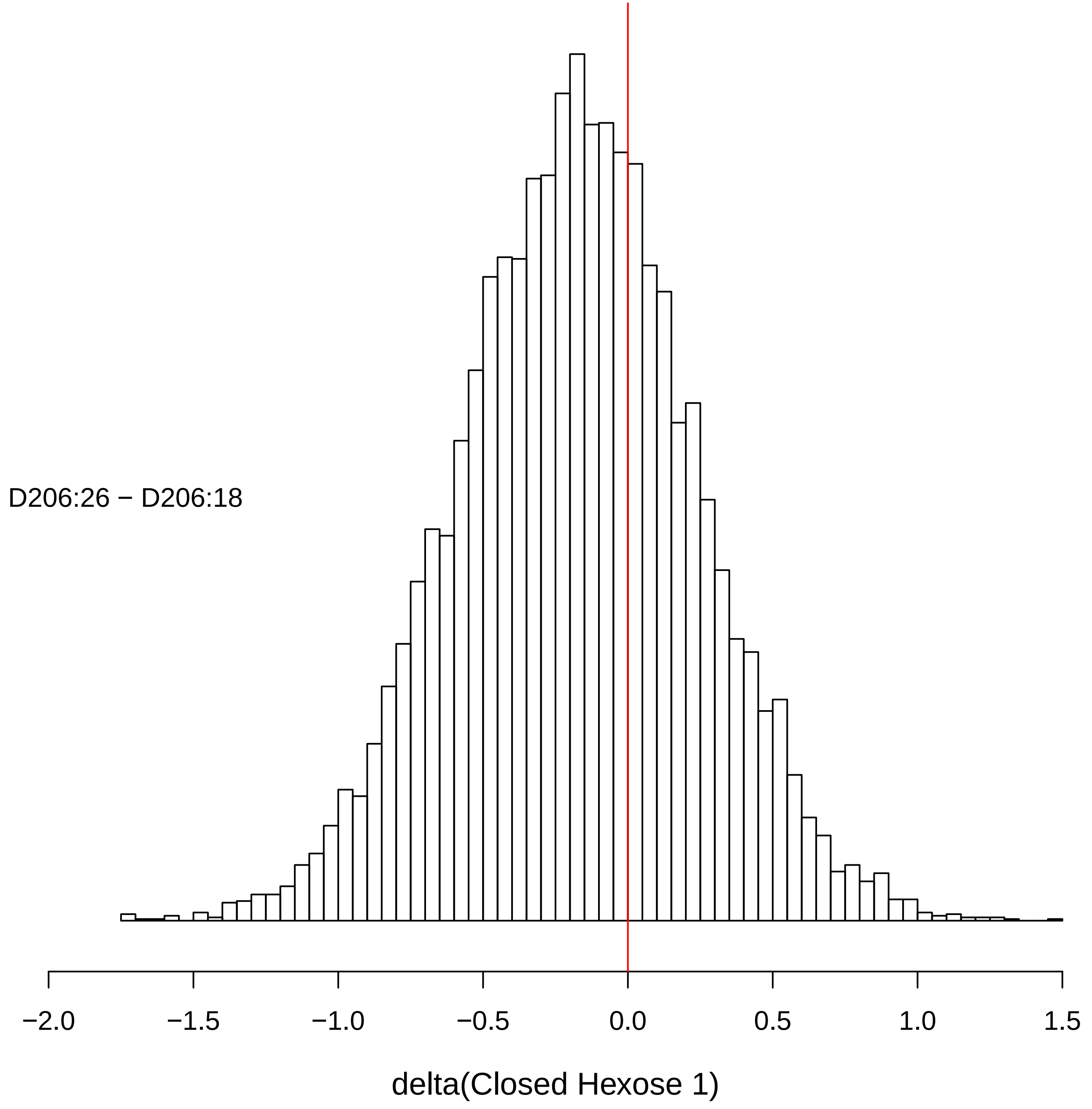

D206:26 − D206:18
−2.0
−1.5
−1.0
−0.5
0.0
0.5
1.0
1.5
delta(Closed Hexose 1)

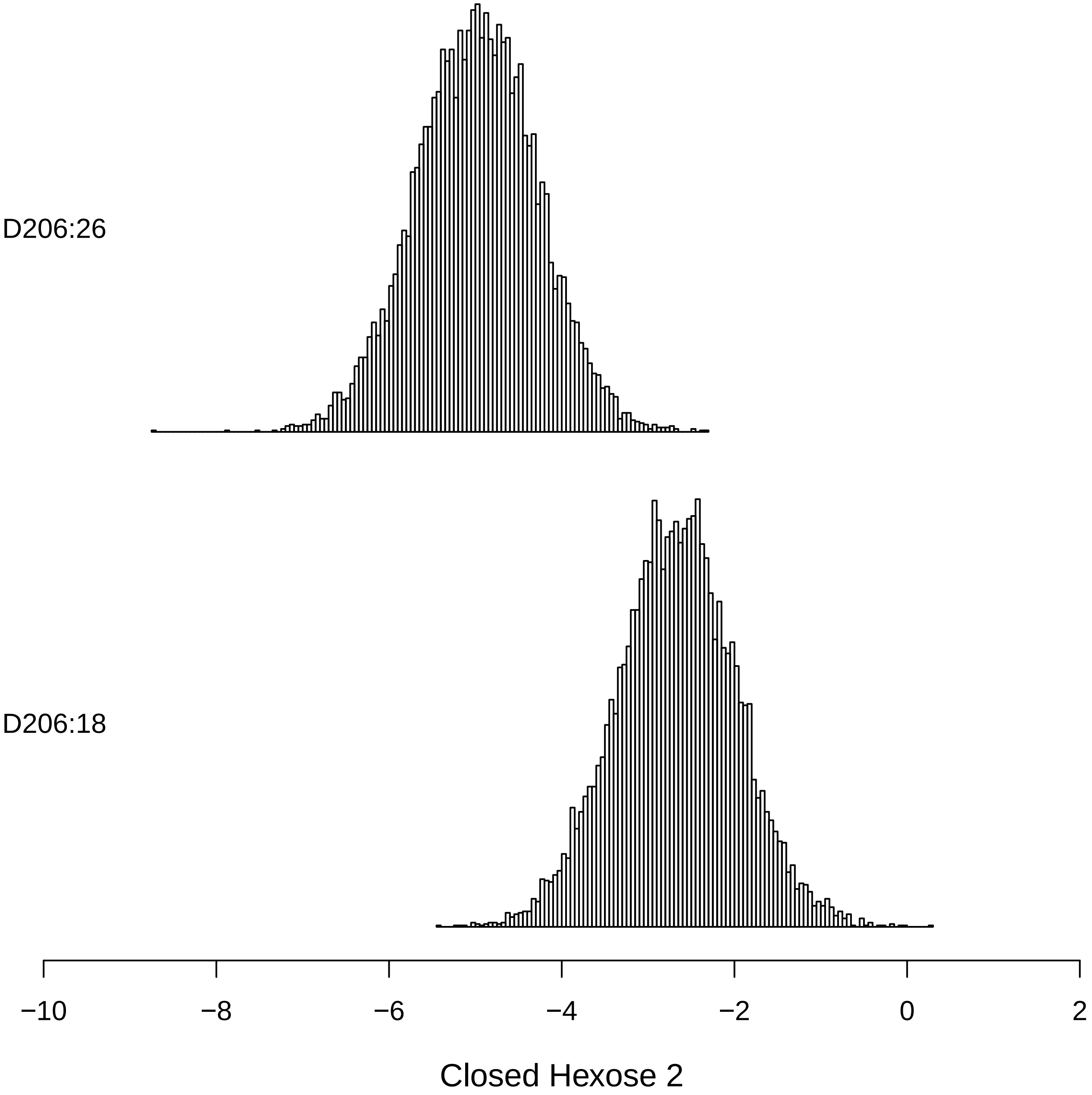

D206:26
D206:18
−10
−8
−6
−4
−2
0
2
Closed Hexose 2

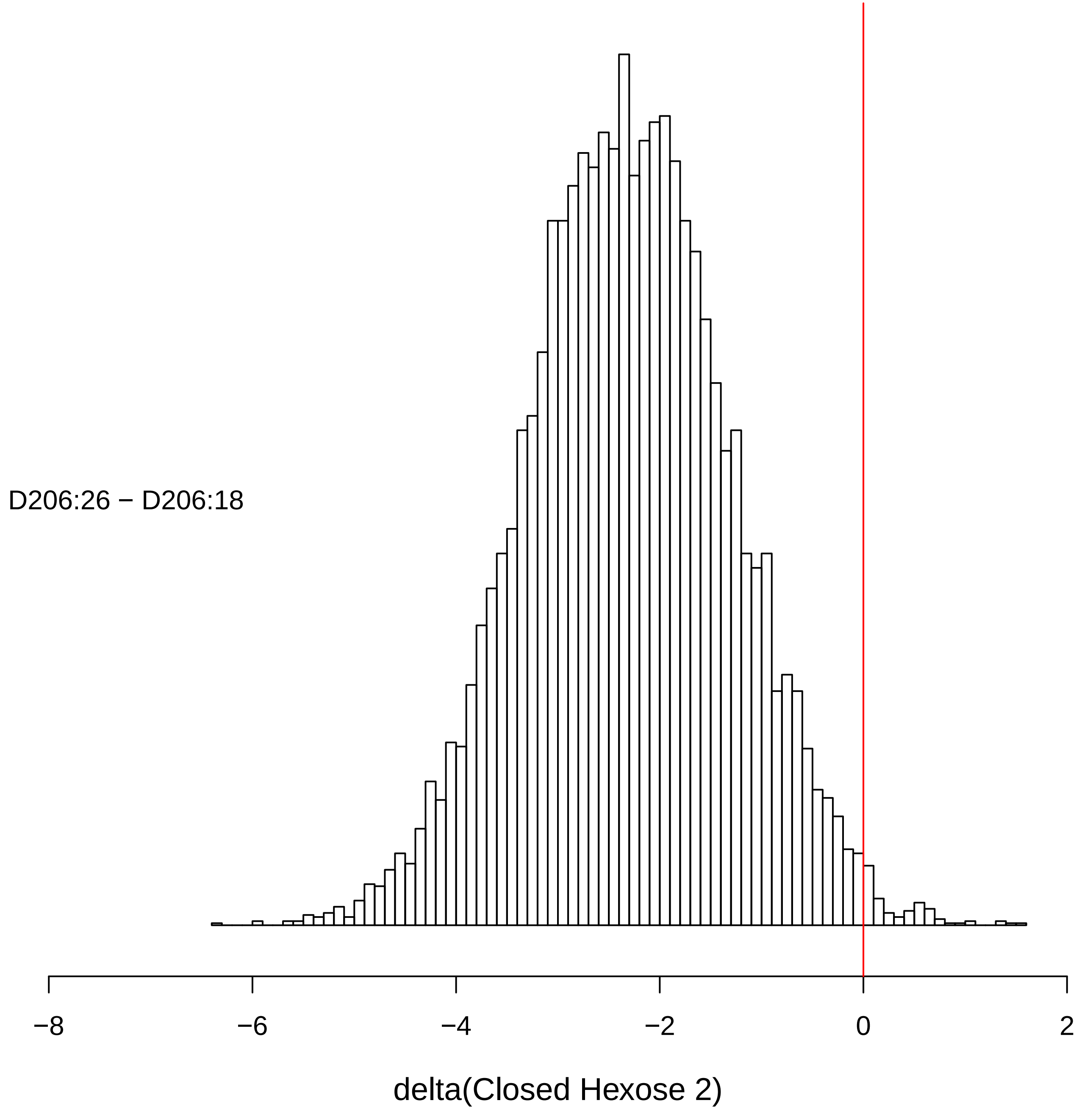
D206:26 − D206:18
−8
−6
−4
−2
0
2
delta(Closed Hexose 2)

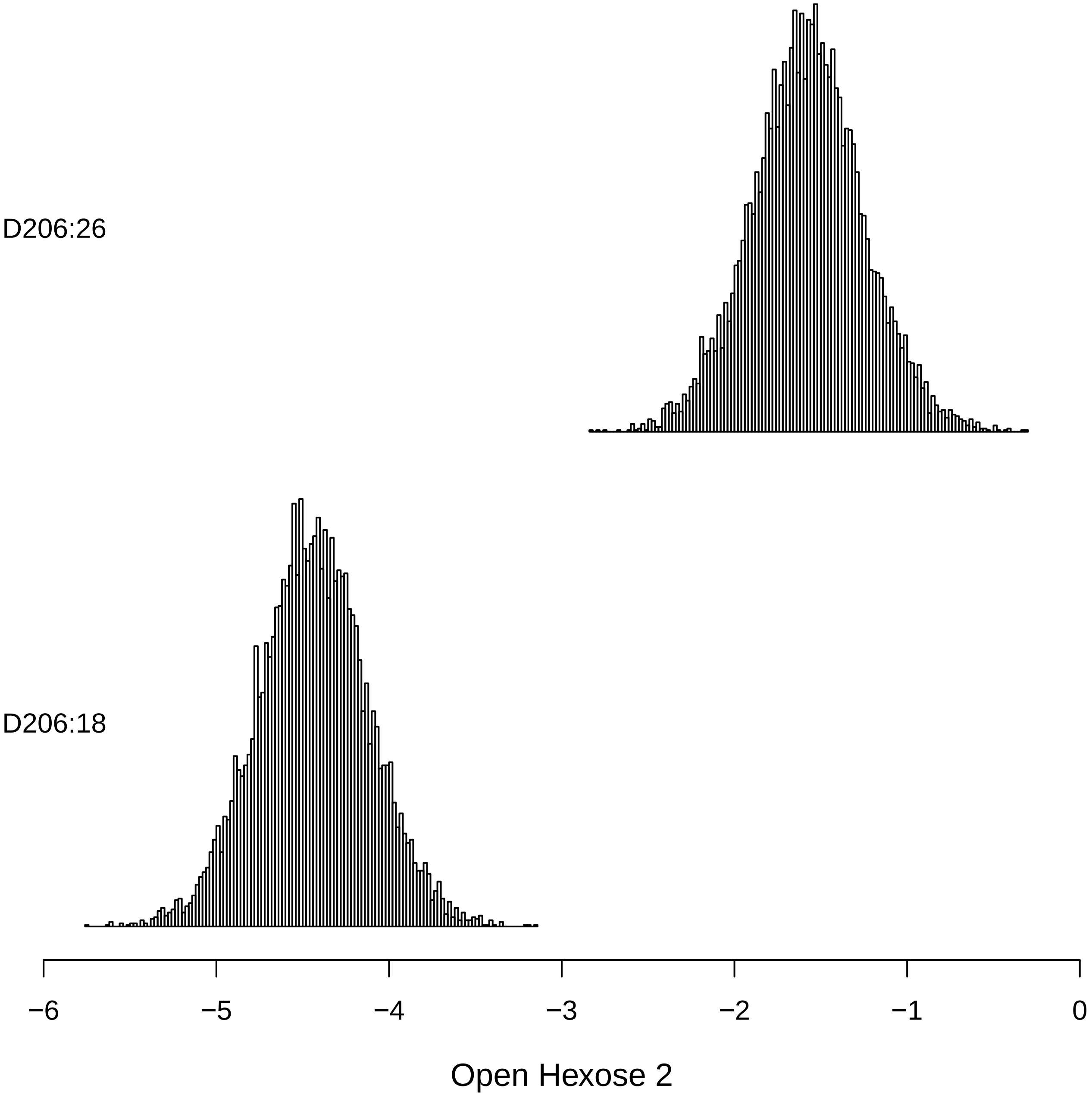
D206:26
D206:18
−6
−5
−4
−3
−2
−1
0
Open Hexose 2

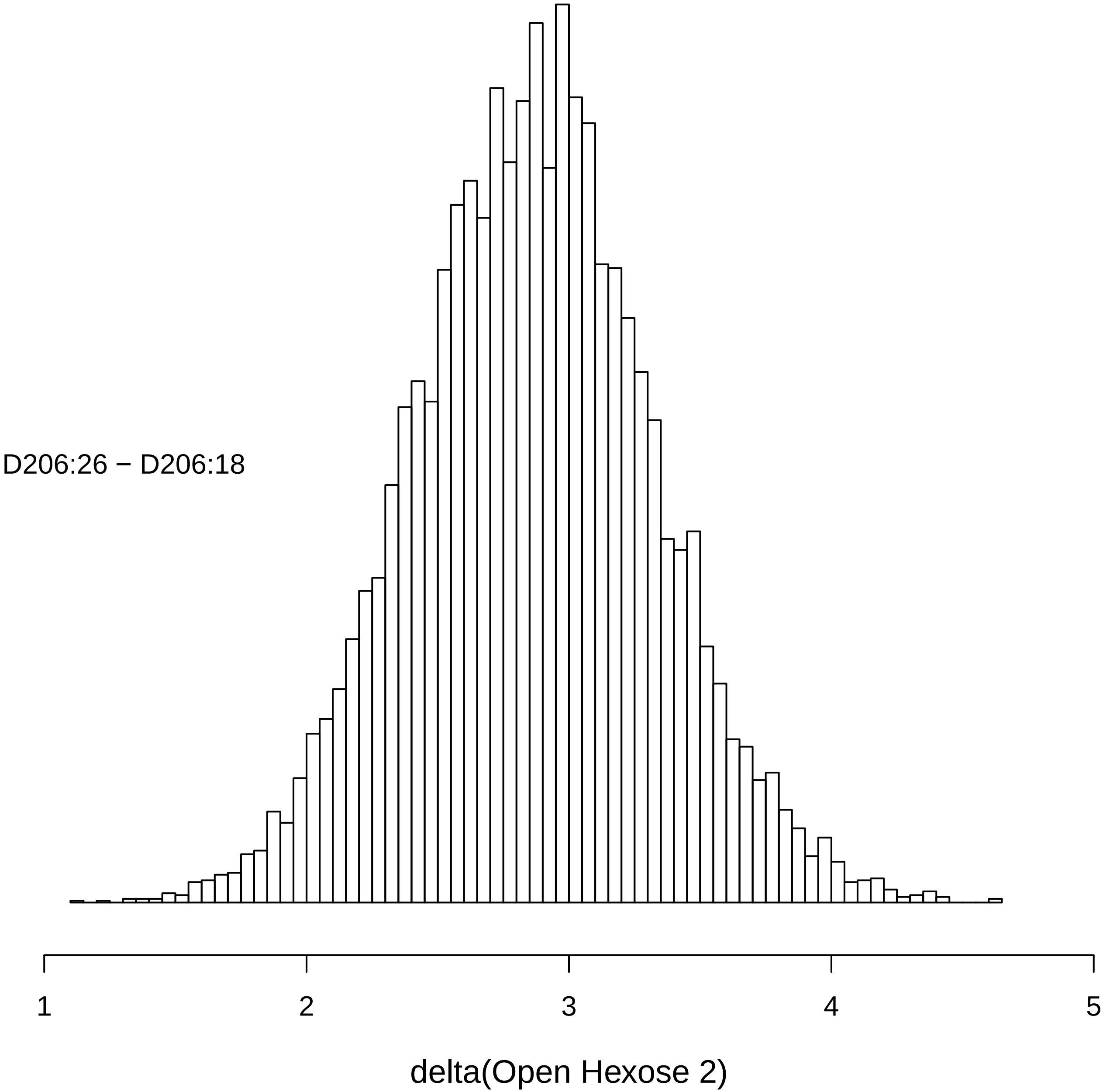
D206:26 − D206:18
1
2
3
4
5
delta(Open Hexose 2)

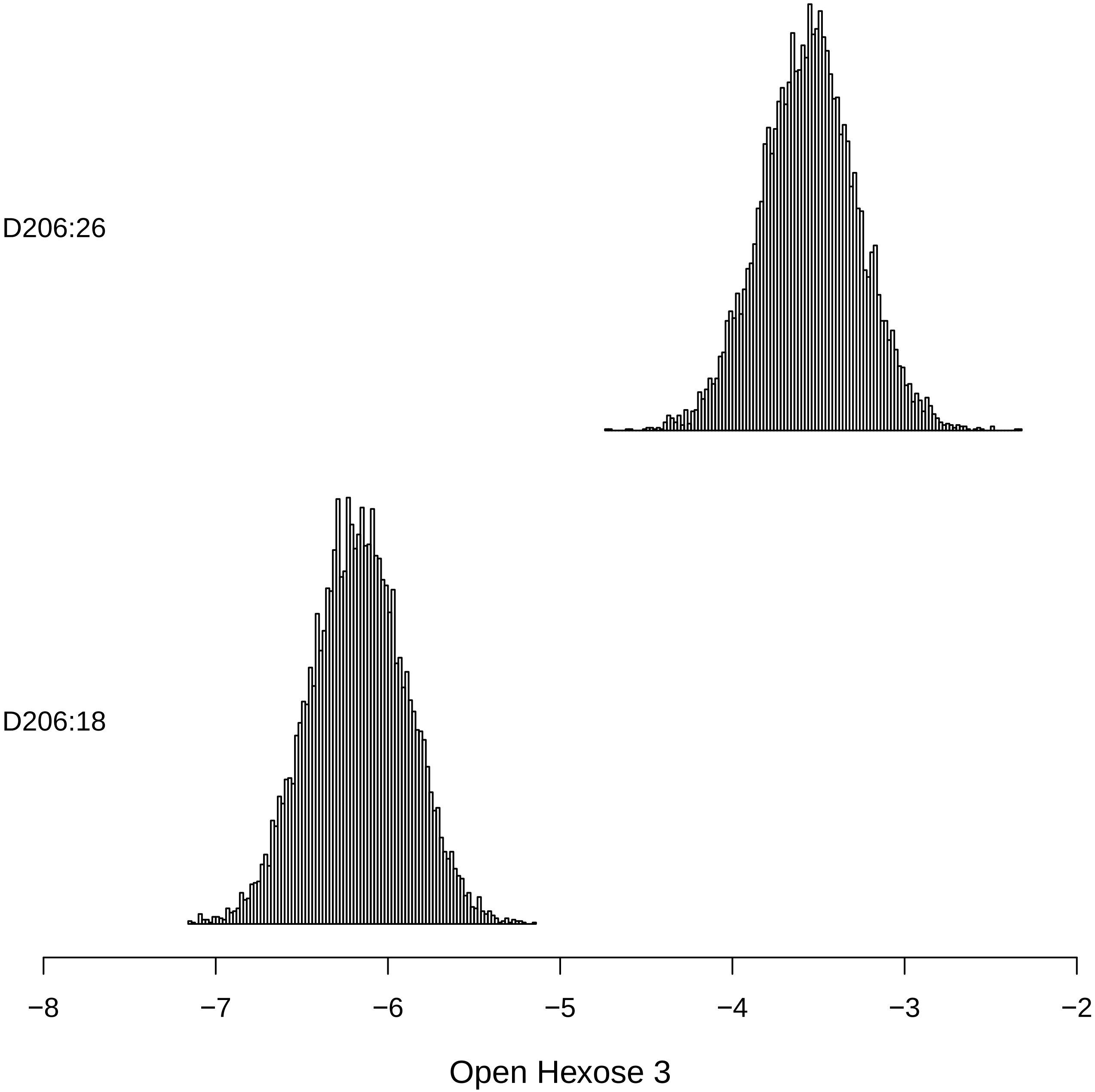
D206:26
D206:18
−8
−7
−6
−5
−4
−3
−2
Open Hexose 3

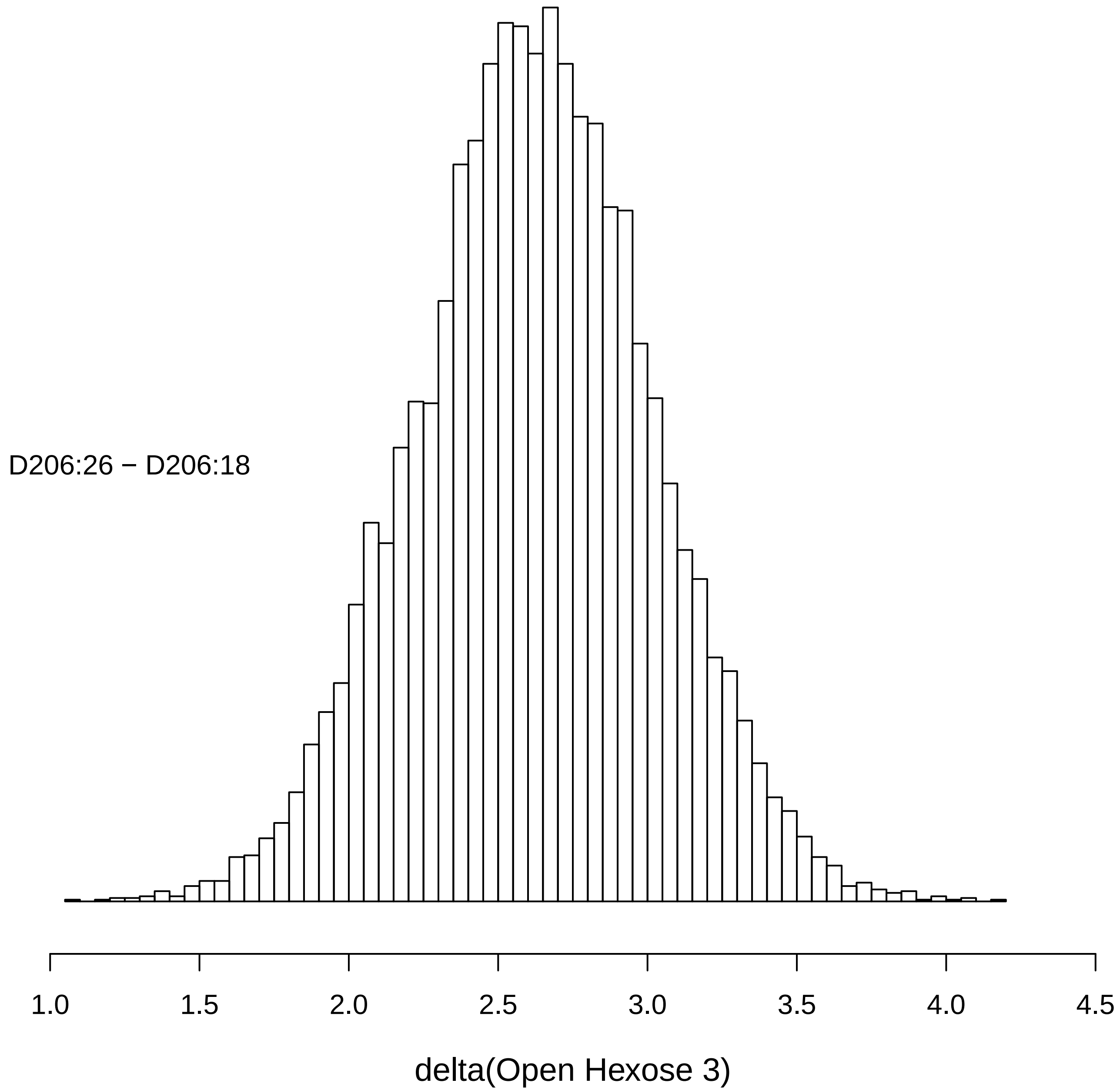
D206:26 − D206:18
1.0
1.5
2.0
2.5
3.0
3.5
4.0
4.5
delta(Open Hexose 3)

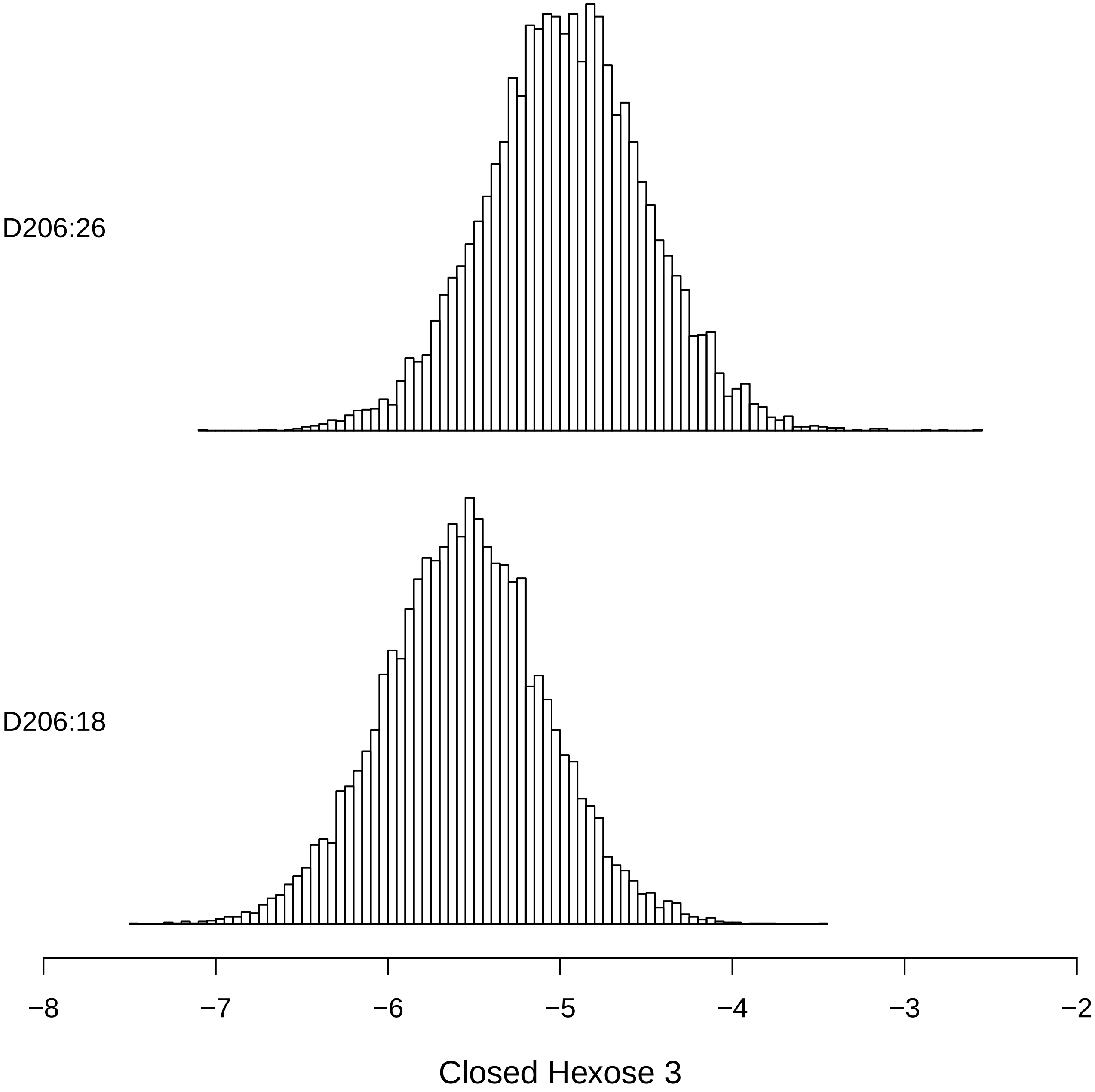
D206:26
D206:18
−8
−7
−6
−5
−4
−3
−2
Closed Hexose 3

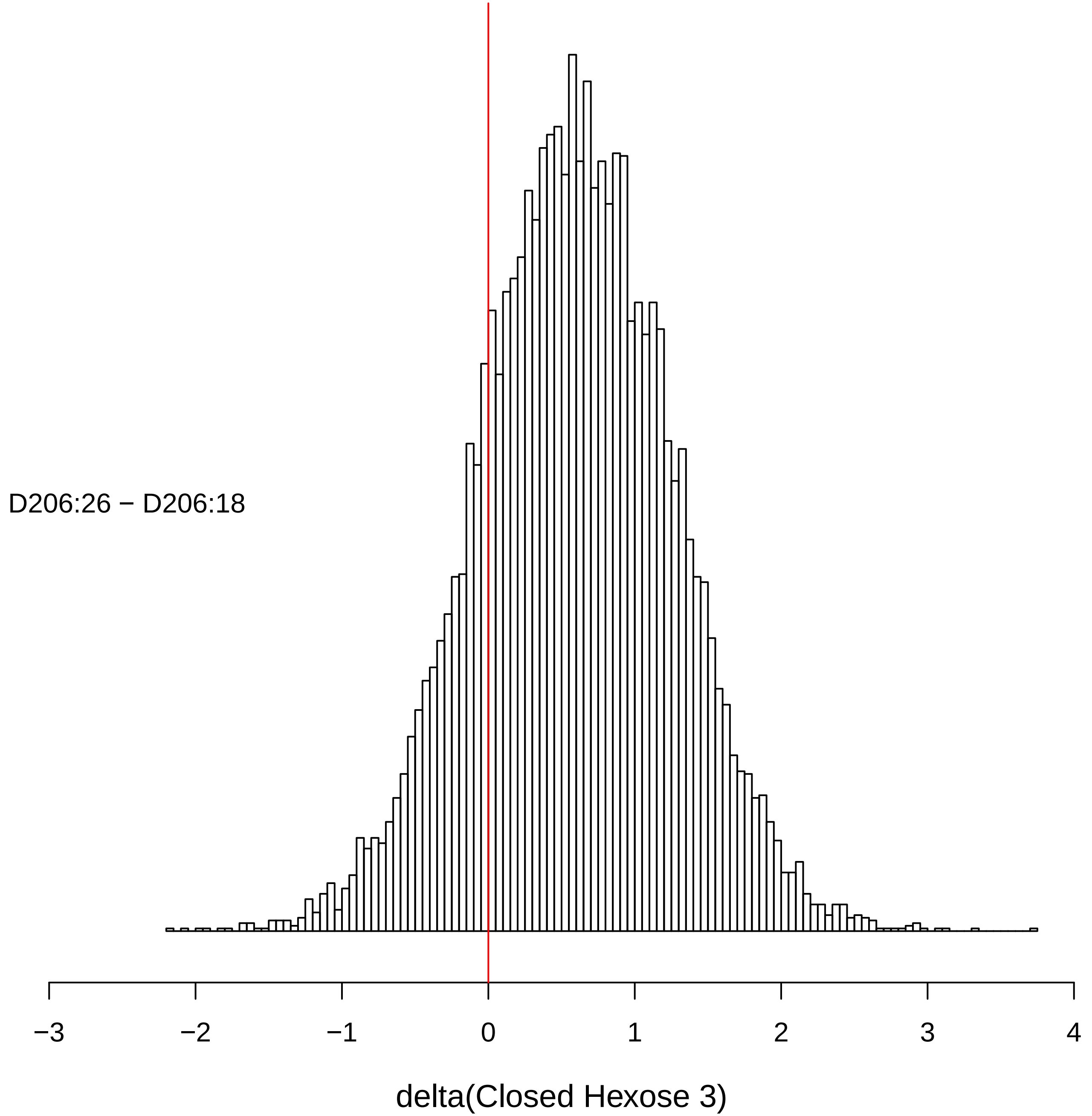
D206:26 − D206:18
−3
−2
−1
0
1
2
3
4
delta(Closed Hexose 3)

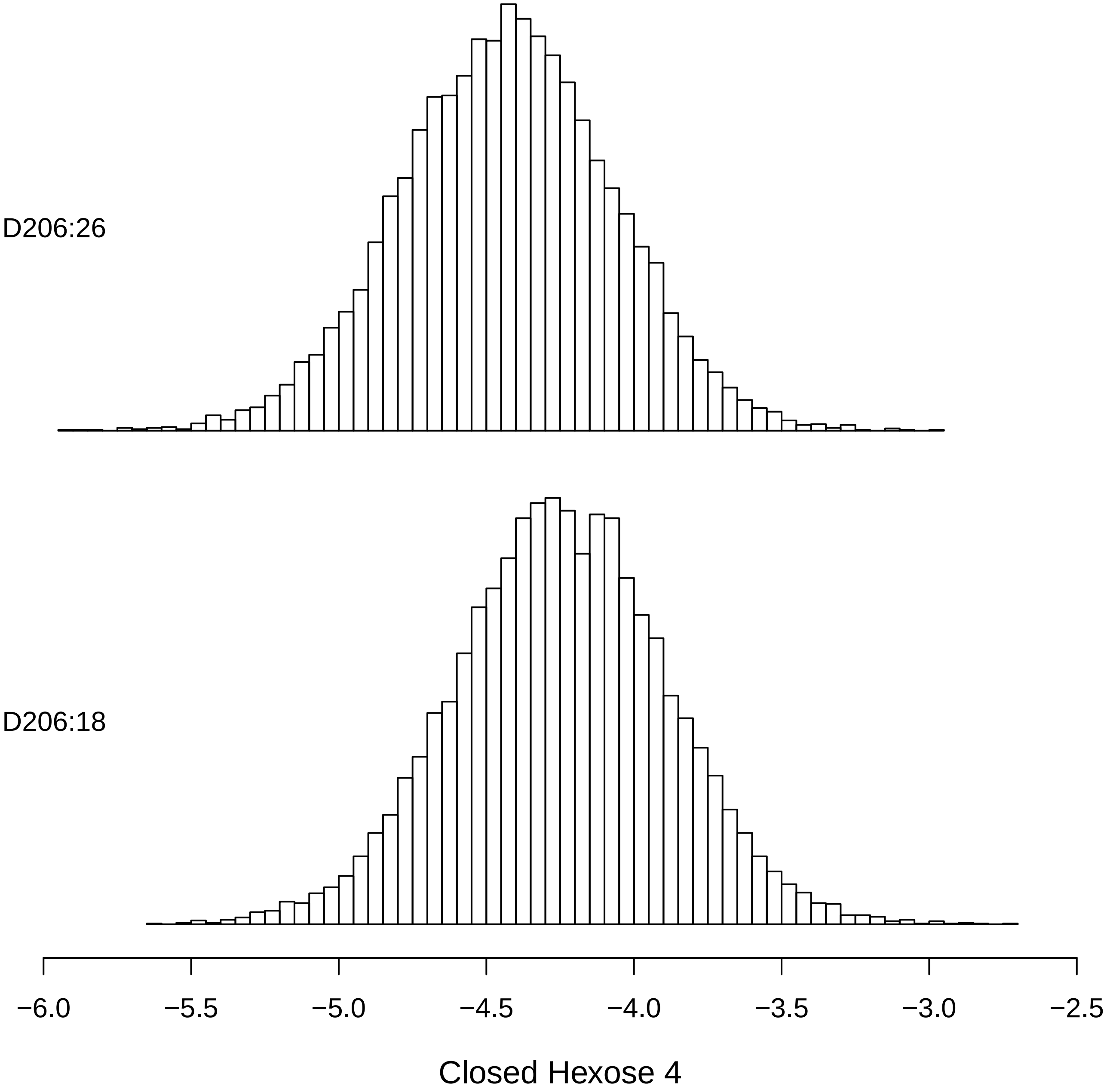
D206:26
D206:18
−6.0
−5.5
−5.0
−4.5
−4.0
−3.5
−3.0
−2.5
Closed Hexose 4

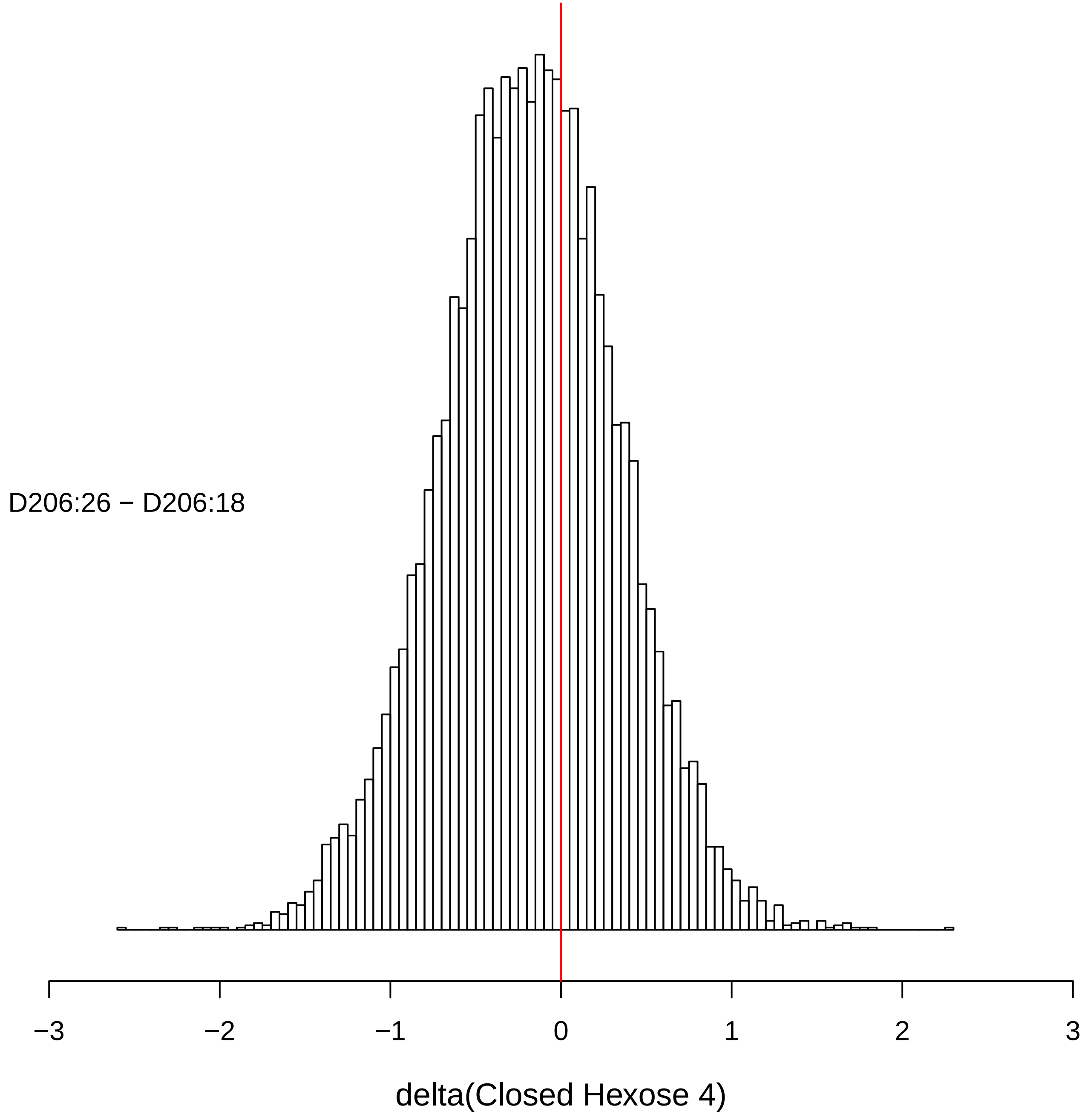

D206:26 − D206:18
−3
−2
−1
0
1
2
3
delta(Closed Hexose 4)

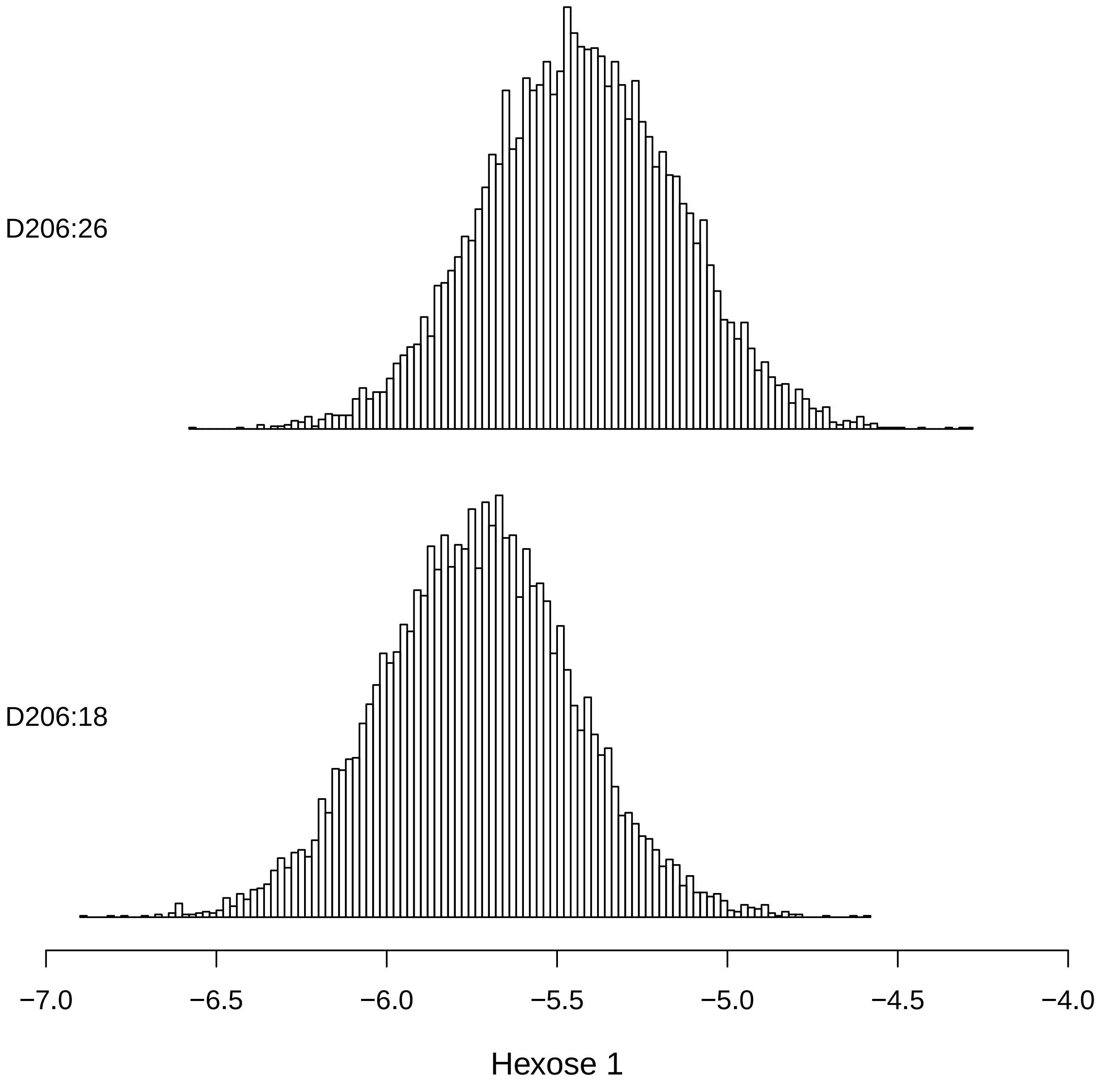
D206:26
D206:18
−7.0
−6.5
−6.0
−5.5
−5.0
−4.5
−4.0
Hexose 1

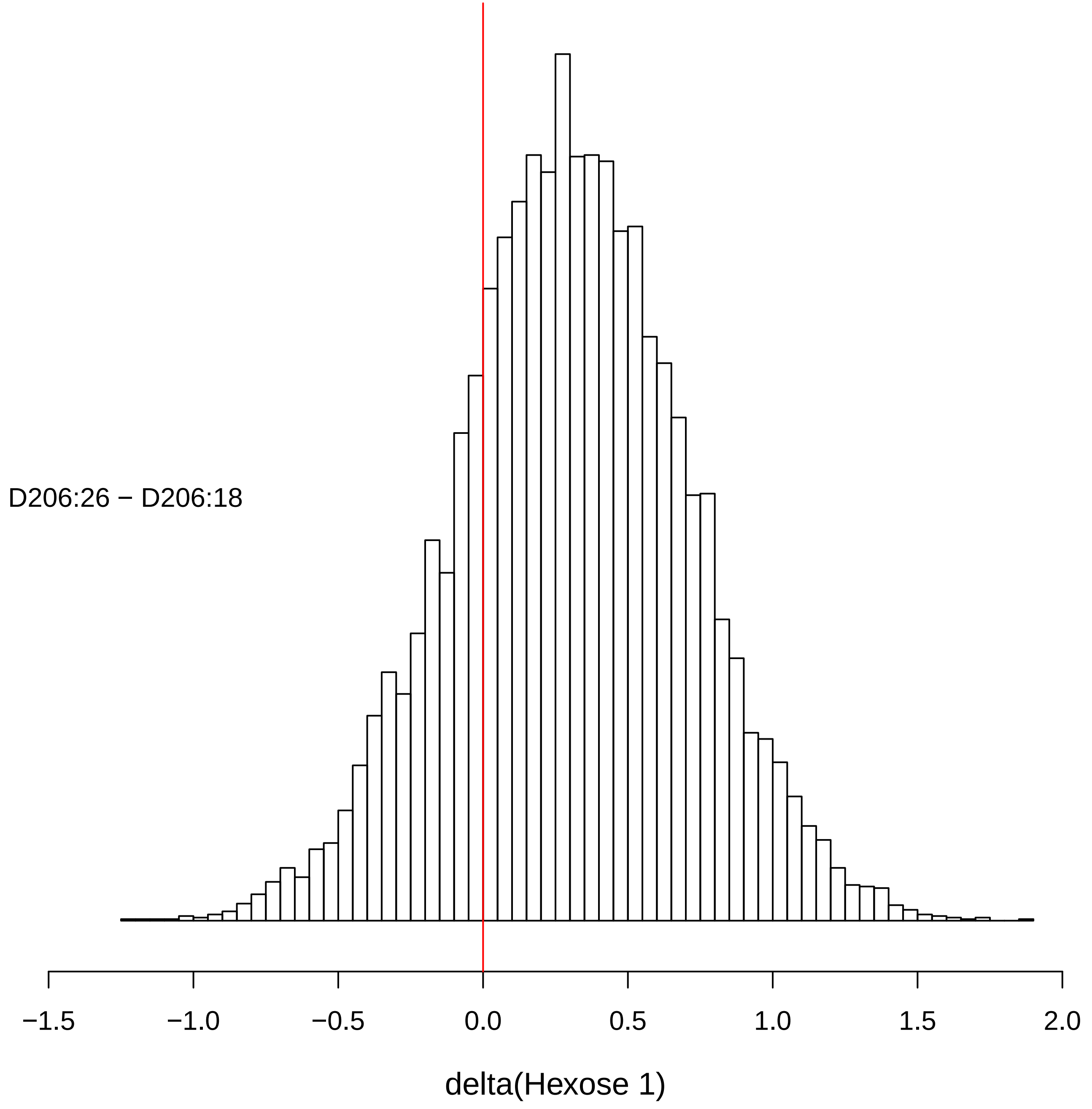
D206:26 − D206:18
−1.5
−1.0
−0.5
0.0
0.5
1.0
1.5
2.0
delta(Hexose 1)

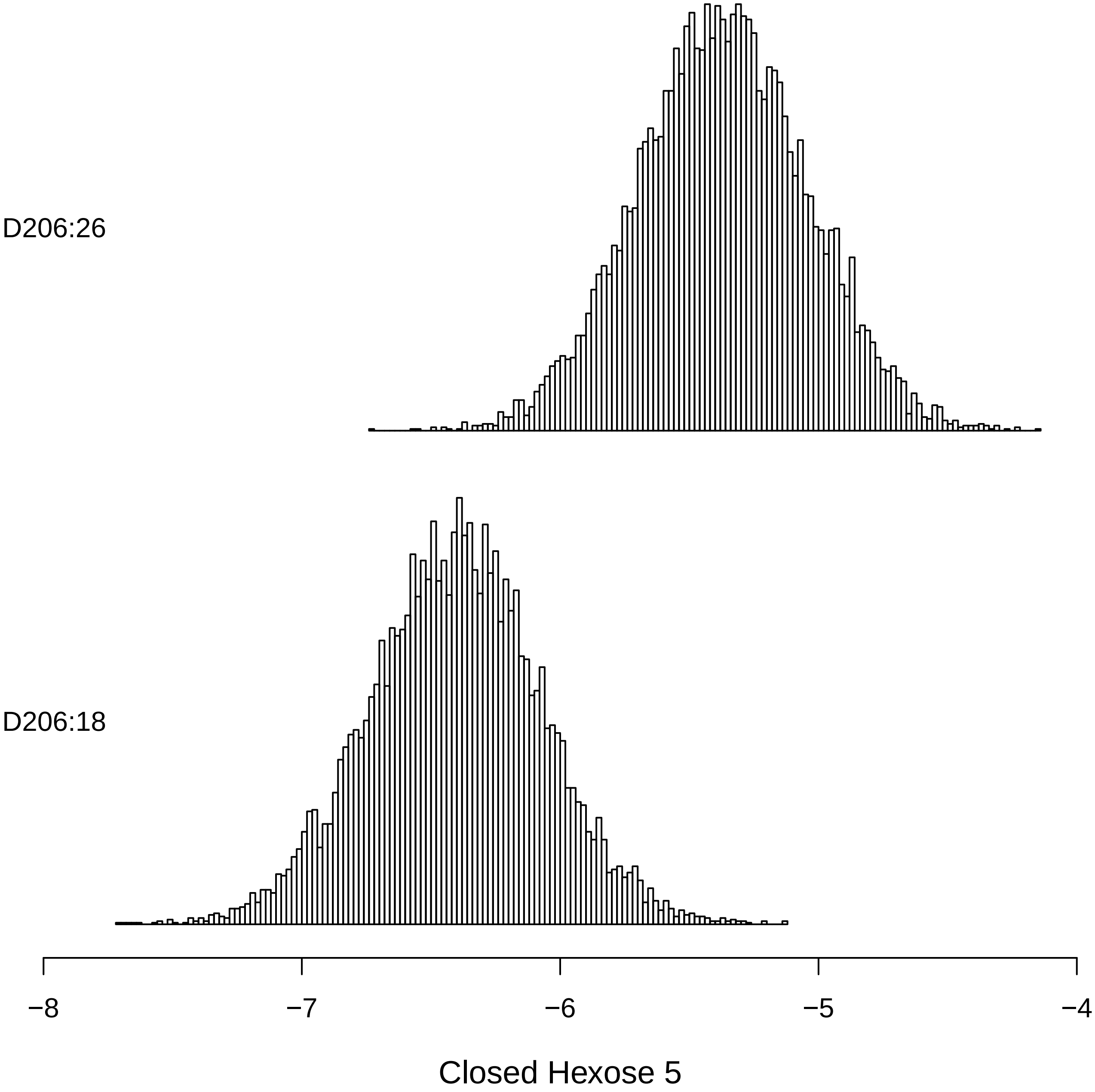
D206:26
D206:18
−8
−7
−6
−5
−4
Closed Hexose 5

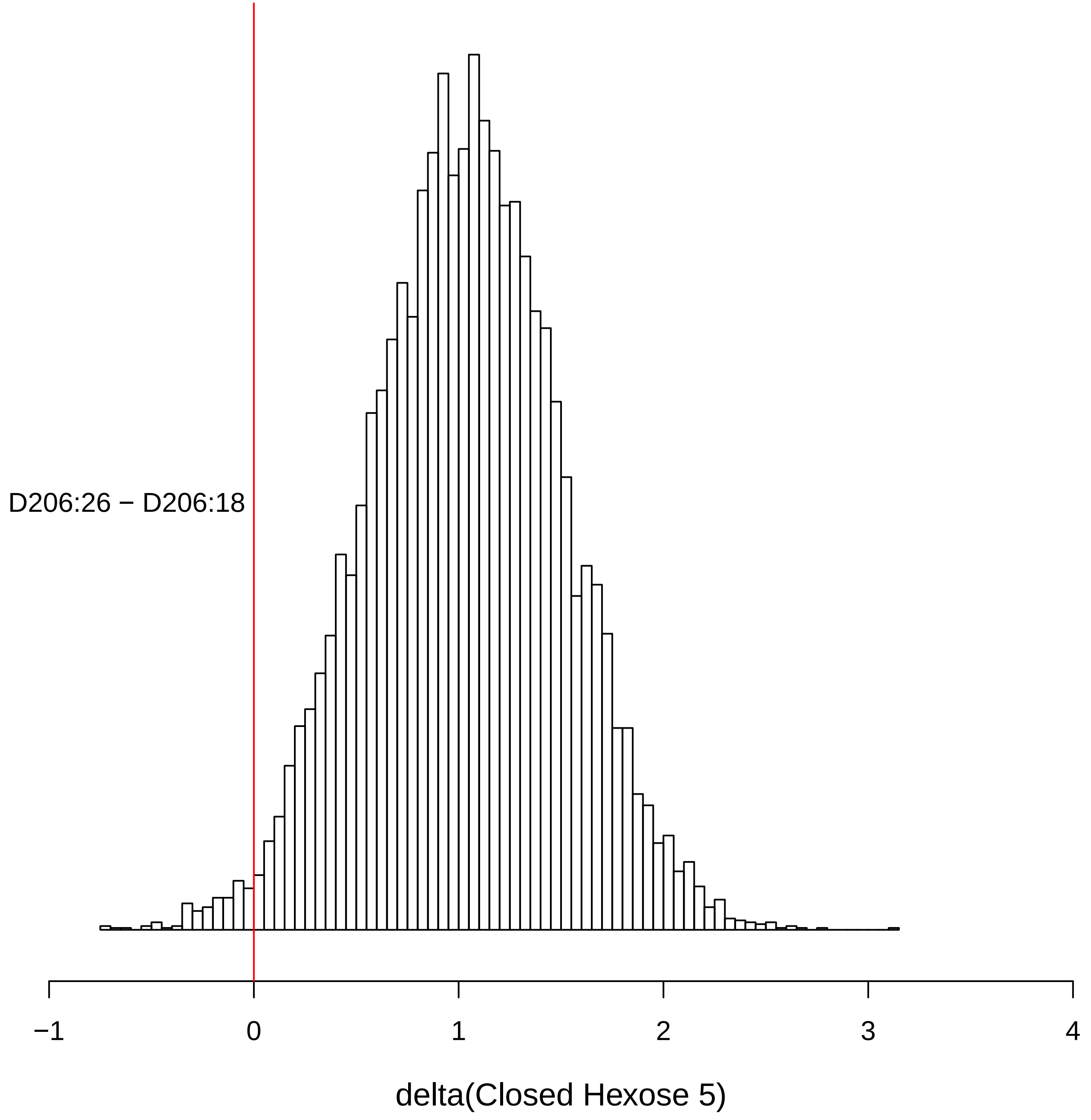
D206:26 − D206:18
−1
0
1
2
3
4
delta(Closed Hexose 5)

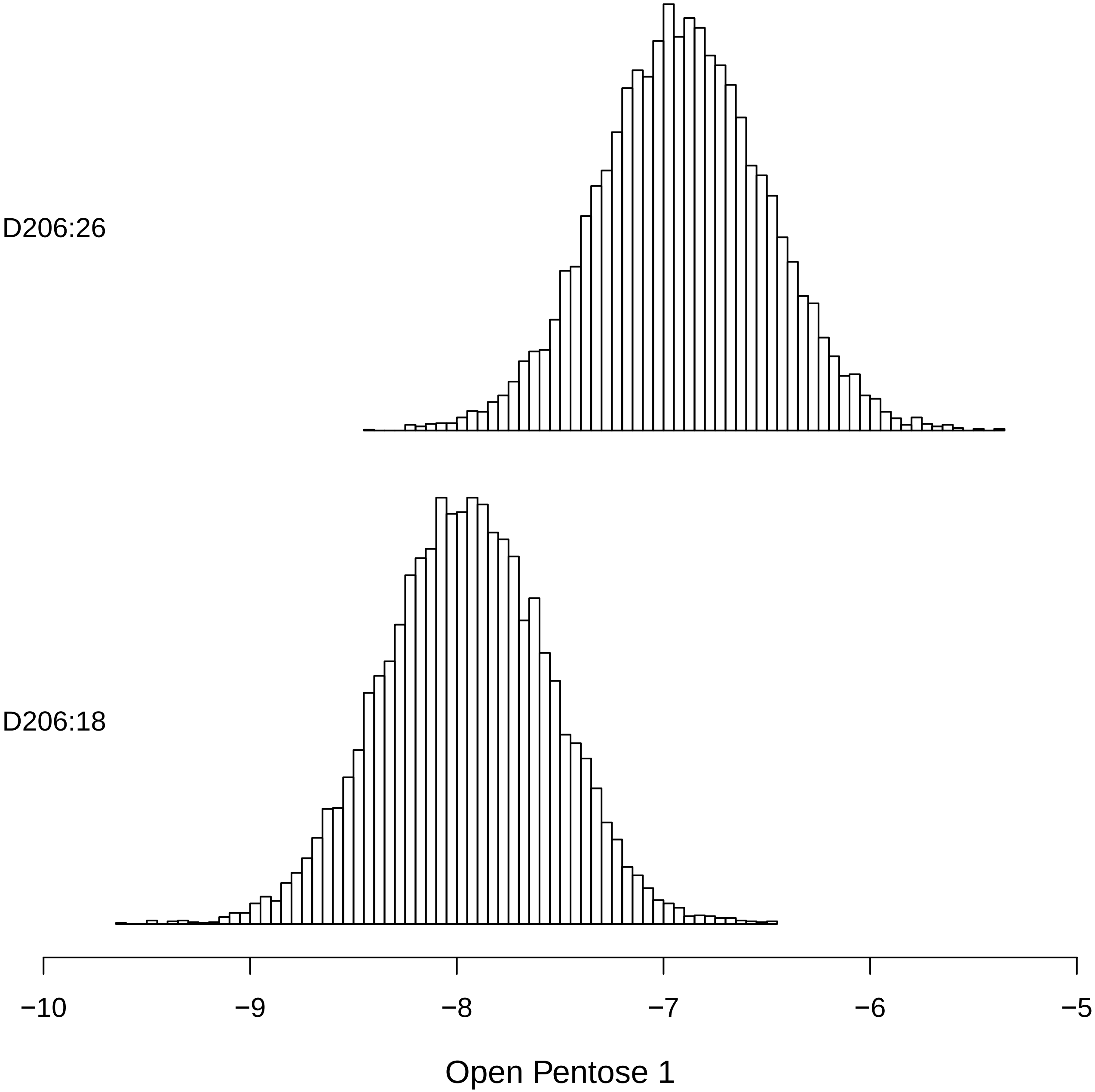
D206:26
D206:18
−10
−9
−8
−7
−6
−5
Open Pentose 1

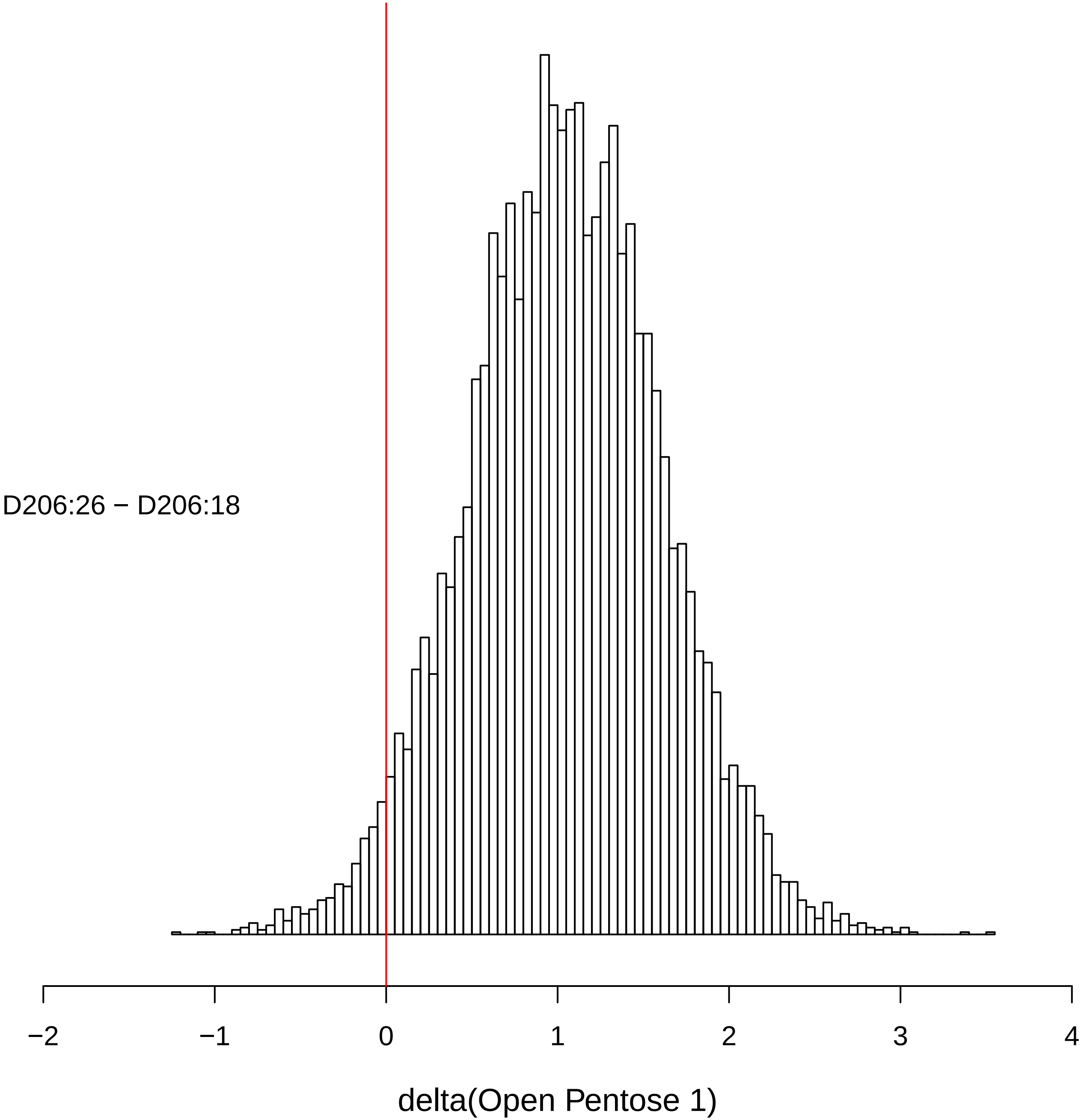
D206:26 − D206:18
−2
−1
0
1
2
3
4
delta(Open Pentose 1)

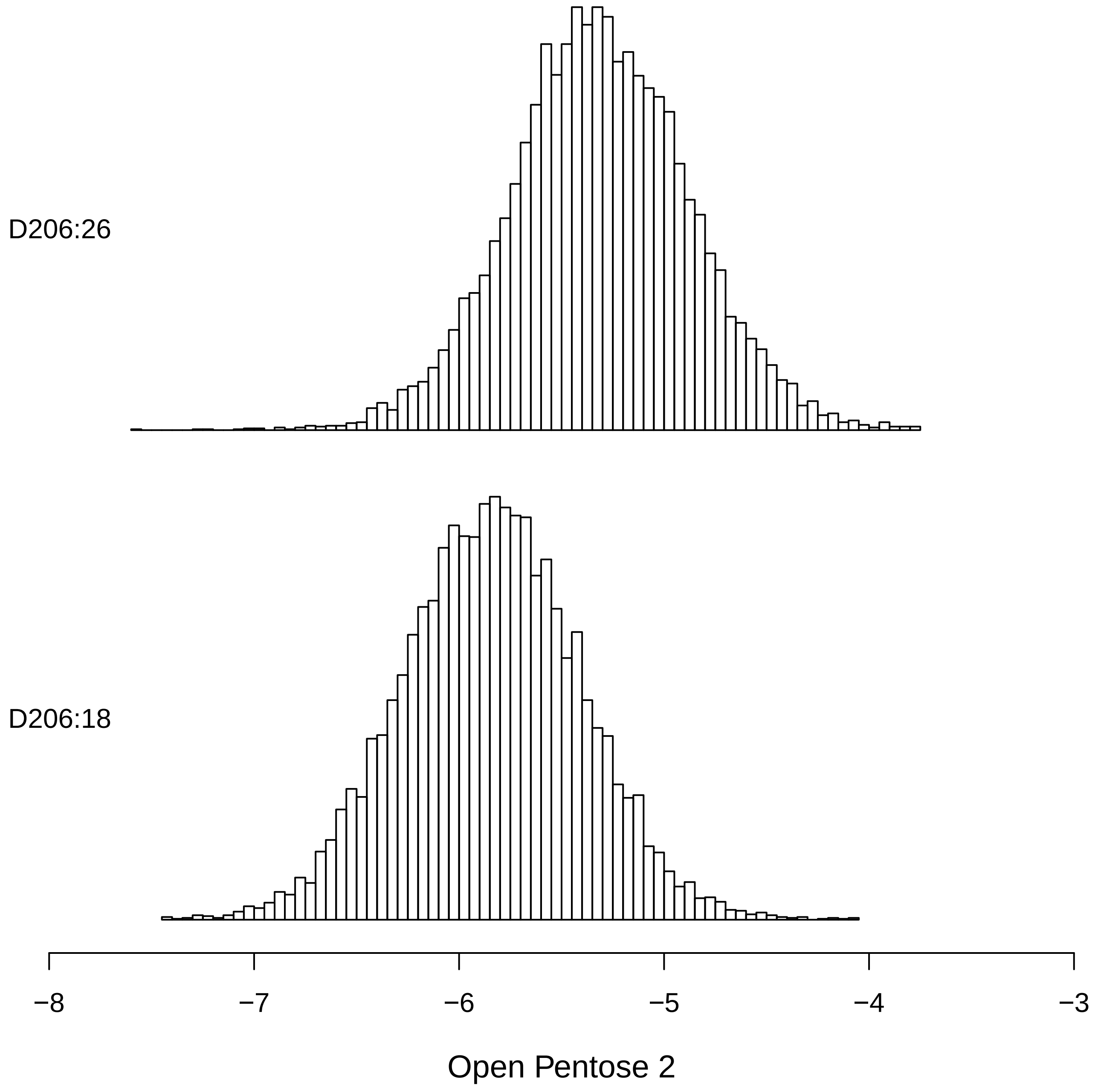
D206:26
D206:18
−8
−7
−6
−5
−4
−3
Open Pentose 2

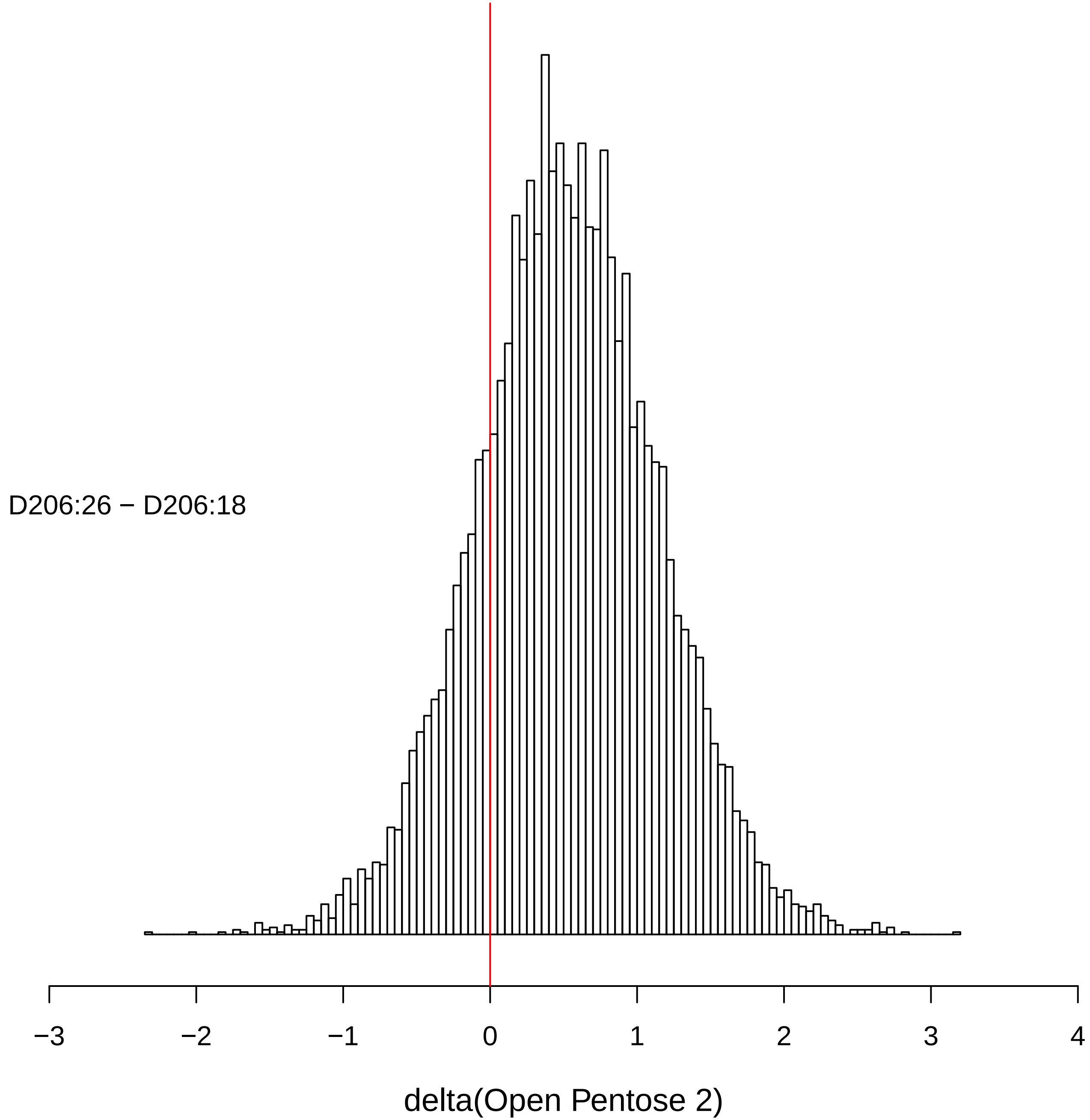
D206:26 − D206:18
−3
−2
−1
0
1
2
3
4
delta(Open Pentose 2)

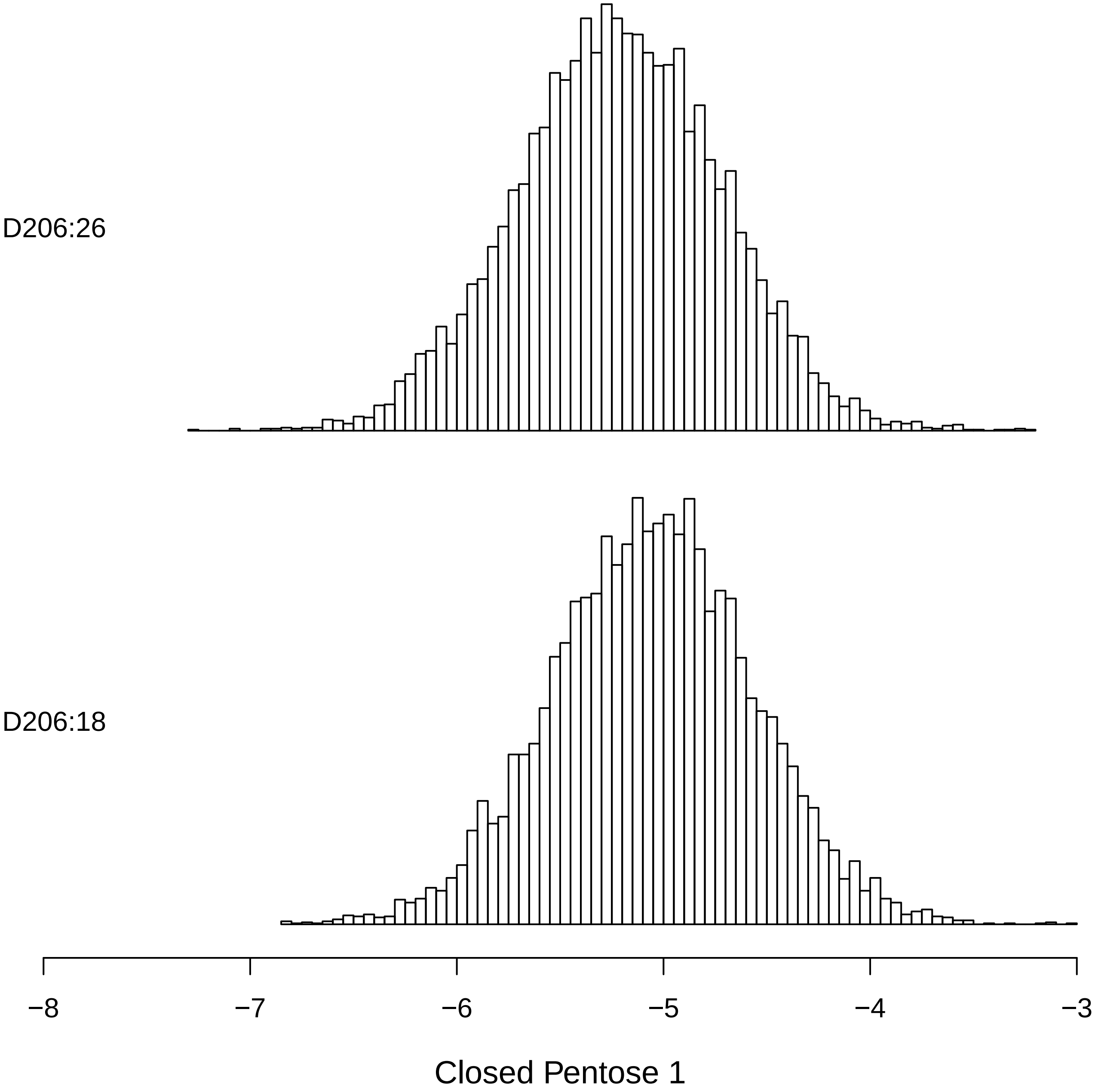
D206:26
D206:18
−8
−7
−6
−5
−4
−3
Closed Pentose 1

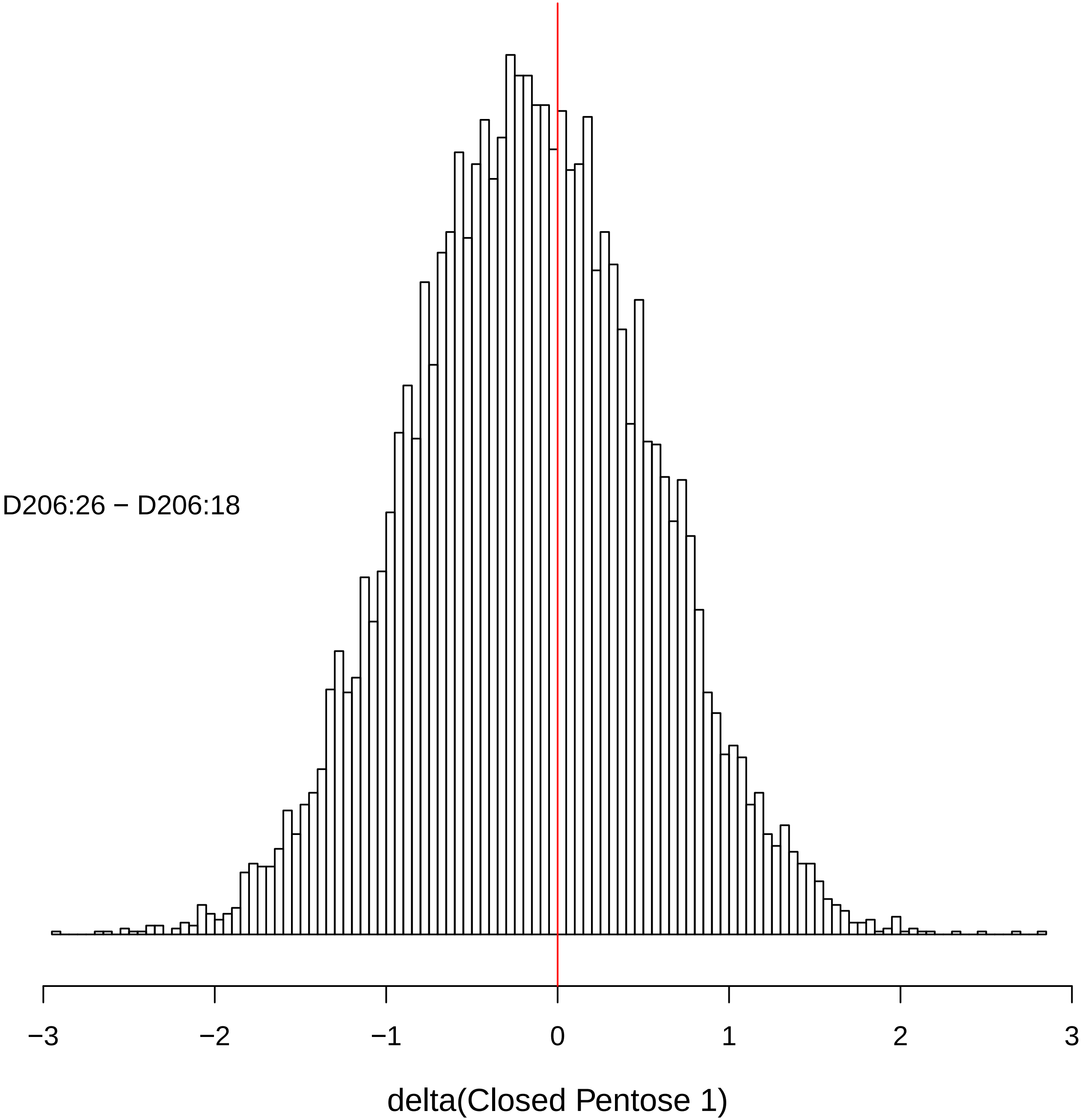
D206:26 − D206:18
−3
−2
−1
0
1
2
3
delta(Closed Pentose 1)

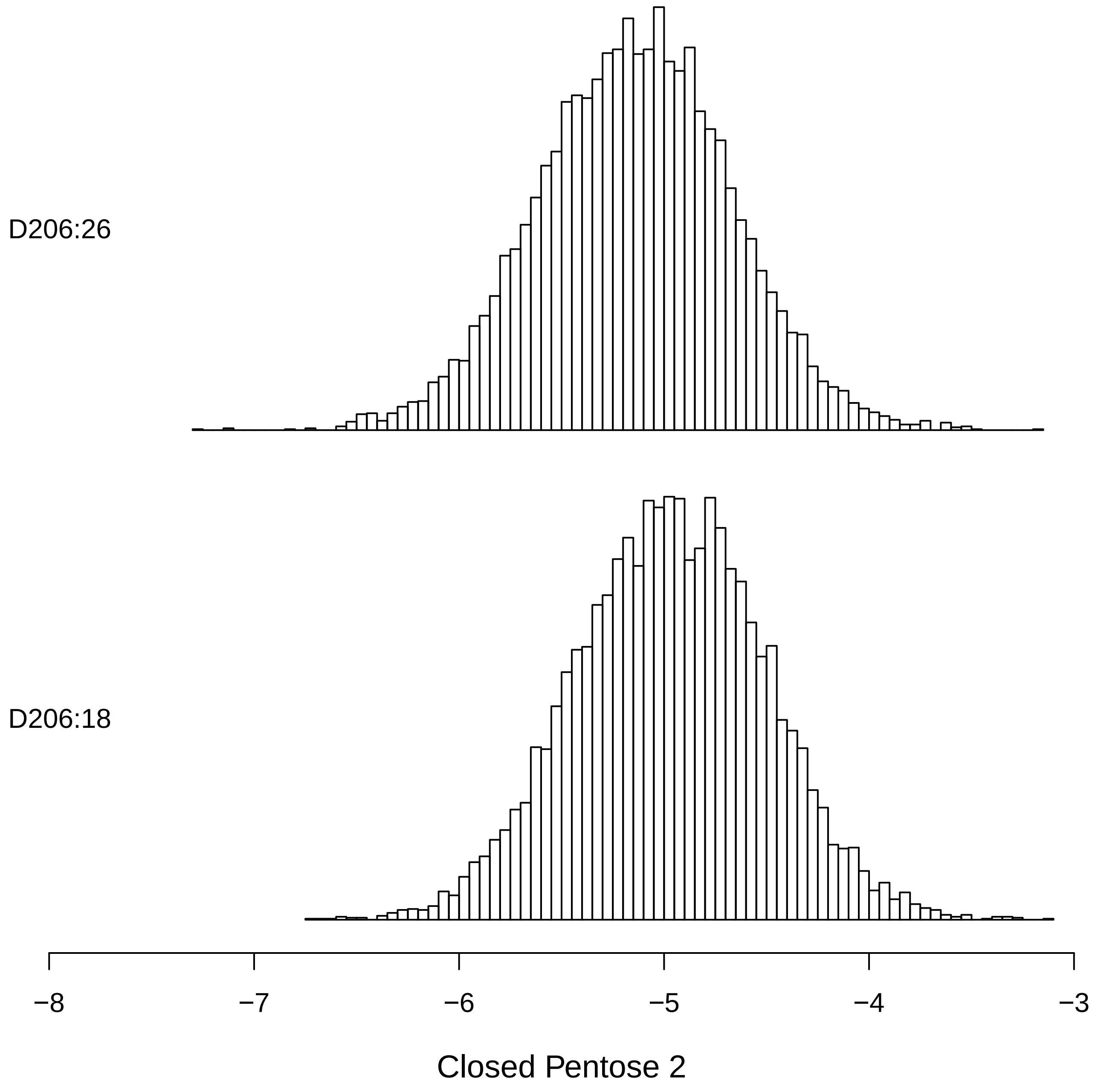
D206:26
D206:18
−8
−7
−6
−5
−4
−3
Closed Pentose 2

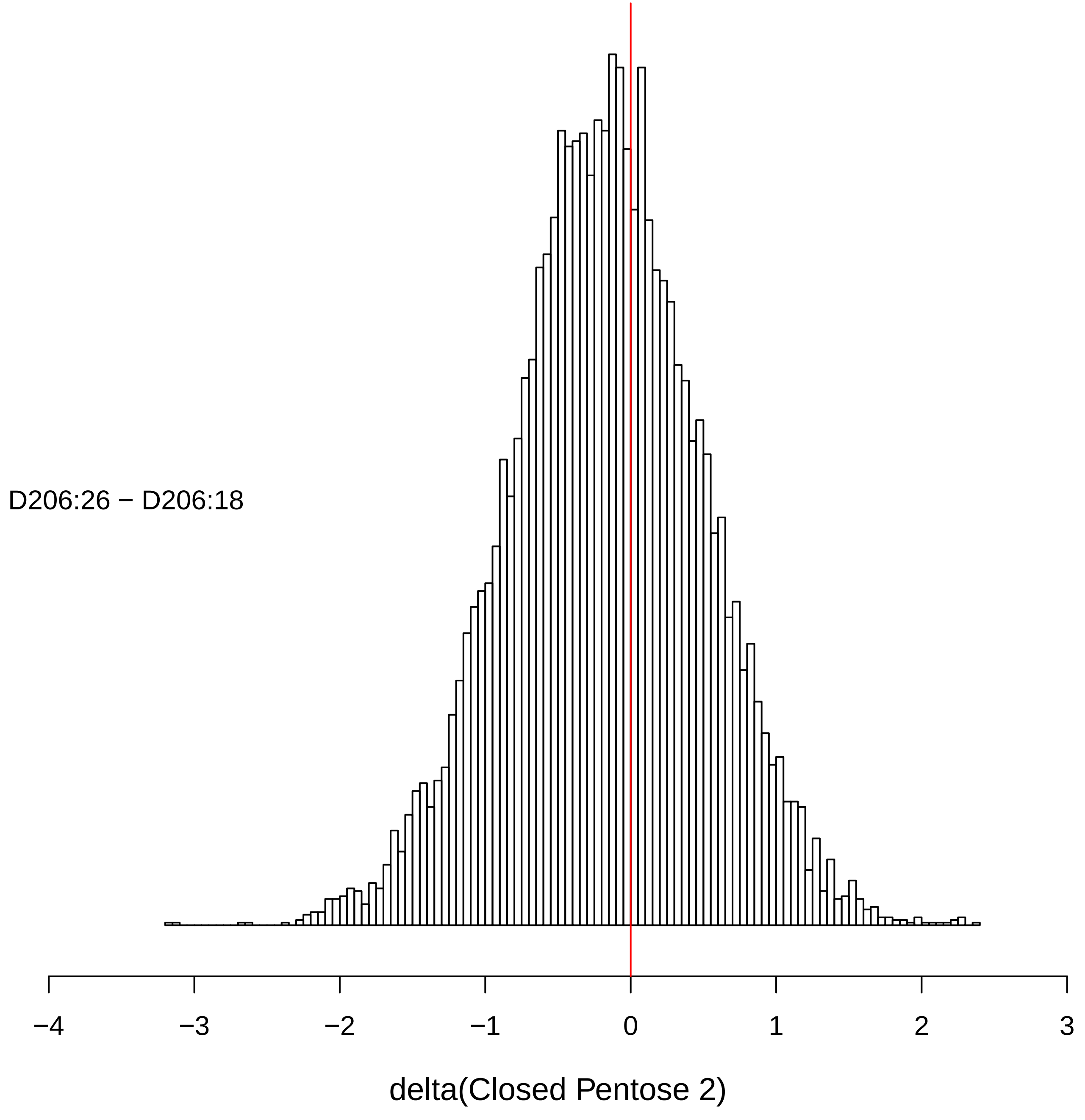
D206:26 − D206:18
−4
−3
−2
−1
0
1
2
3
delta(Closed Pentose 2)

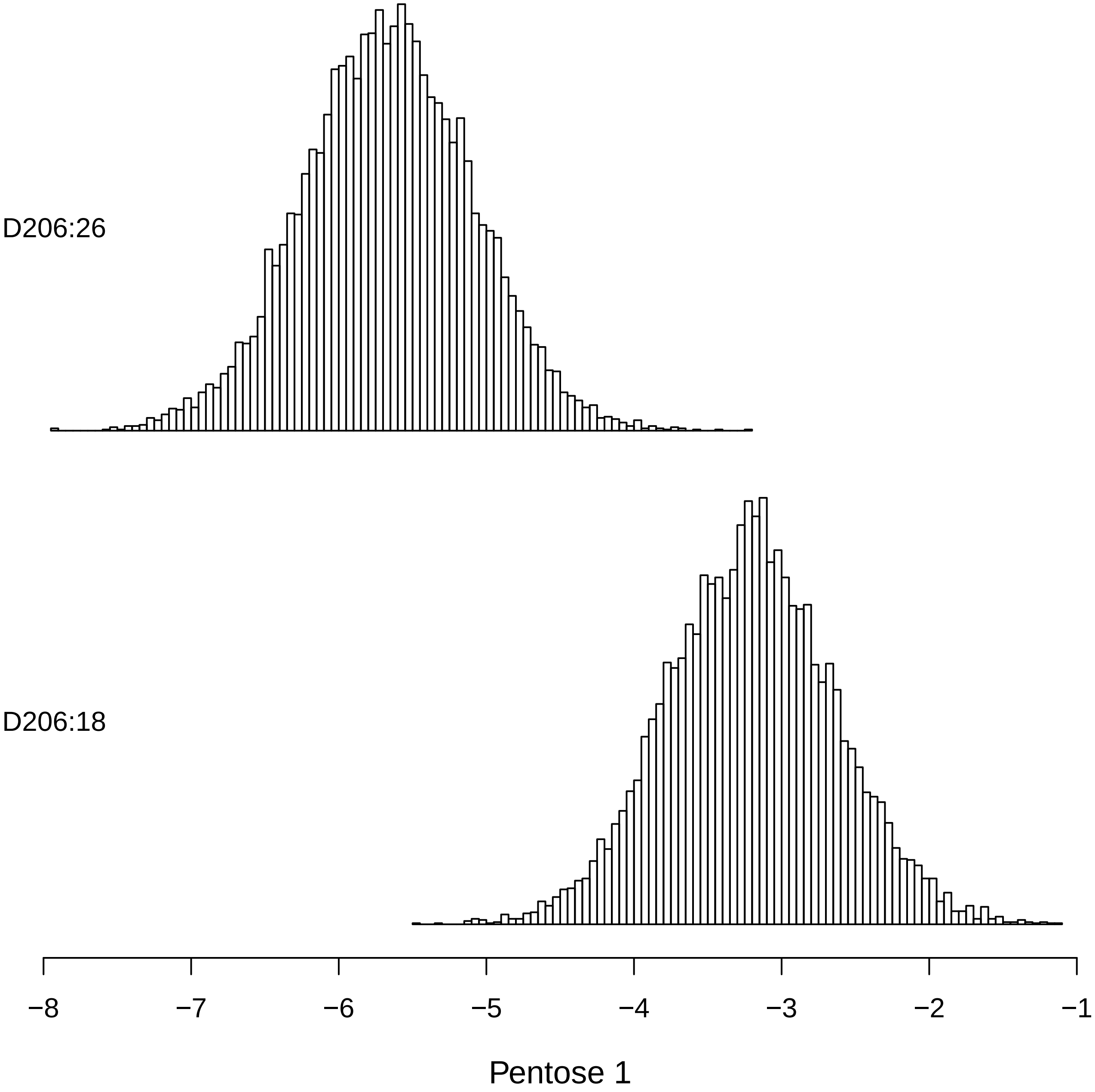
D206:26
D206:18
−8
−7
−6
−5
−4
−3
−2
−1
Pentose 1

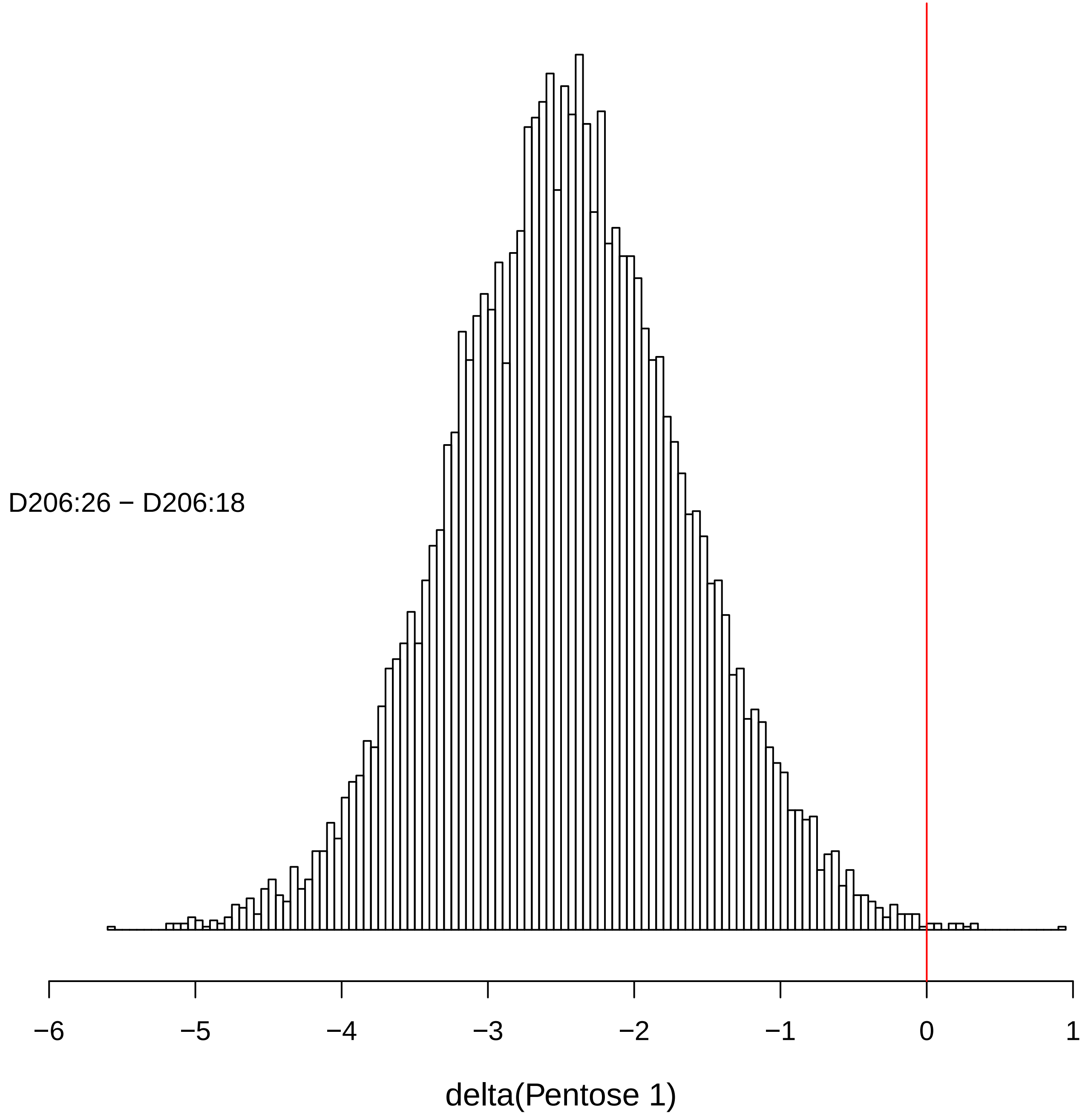
D206:26 − D206:18
−6
−5
−4
−3
−2
−1
0
1
delta(Pentose 1)

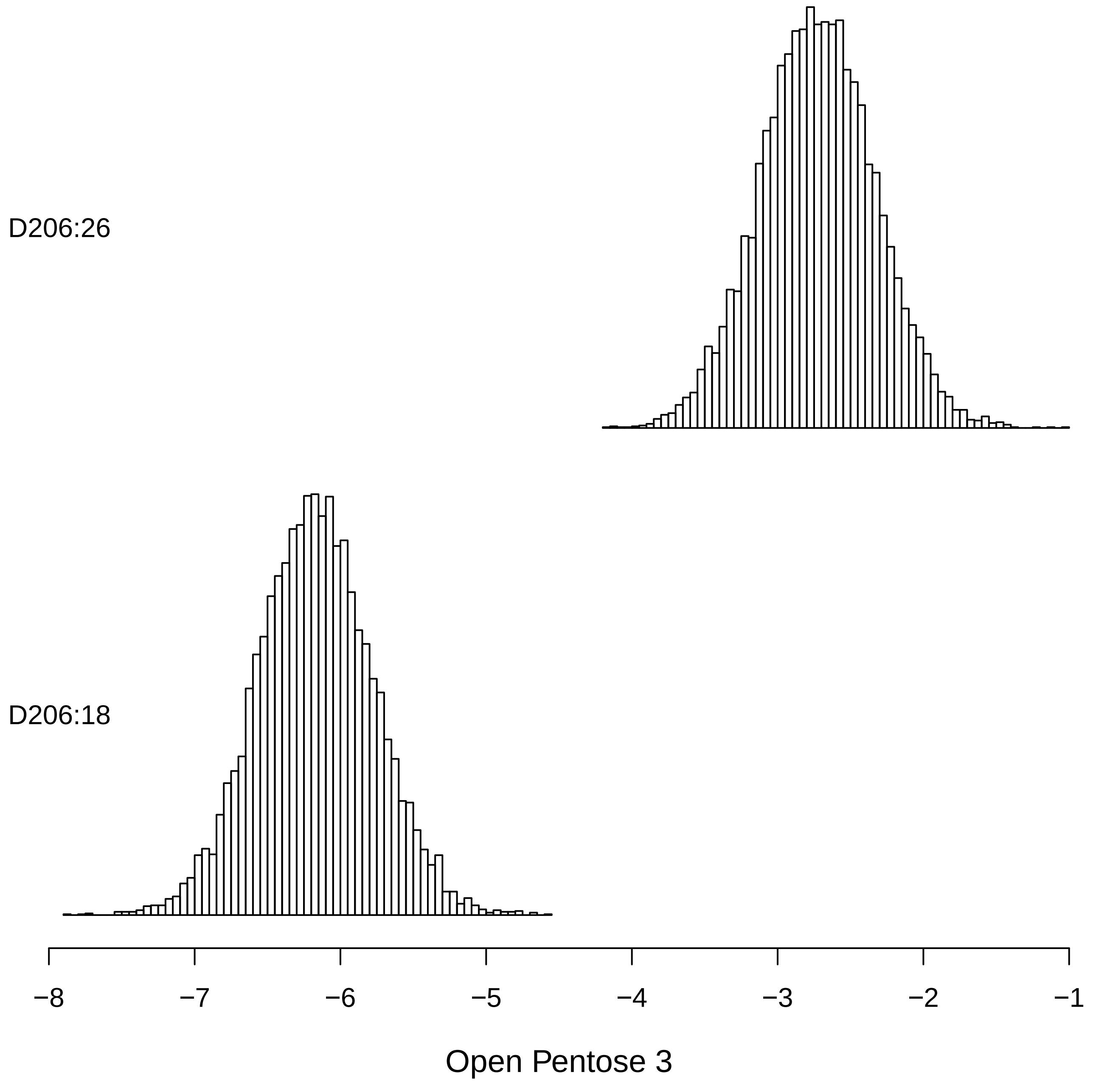

D206:26
D206:18
−8
−7
−6
−5
−4
−3
−2
−1
Open Pentose 3

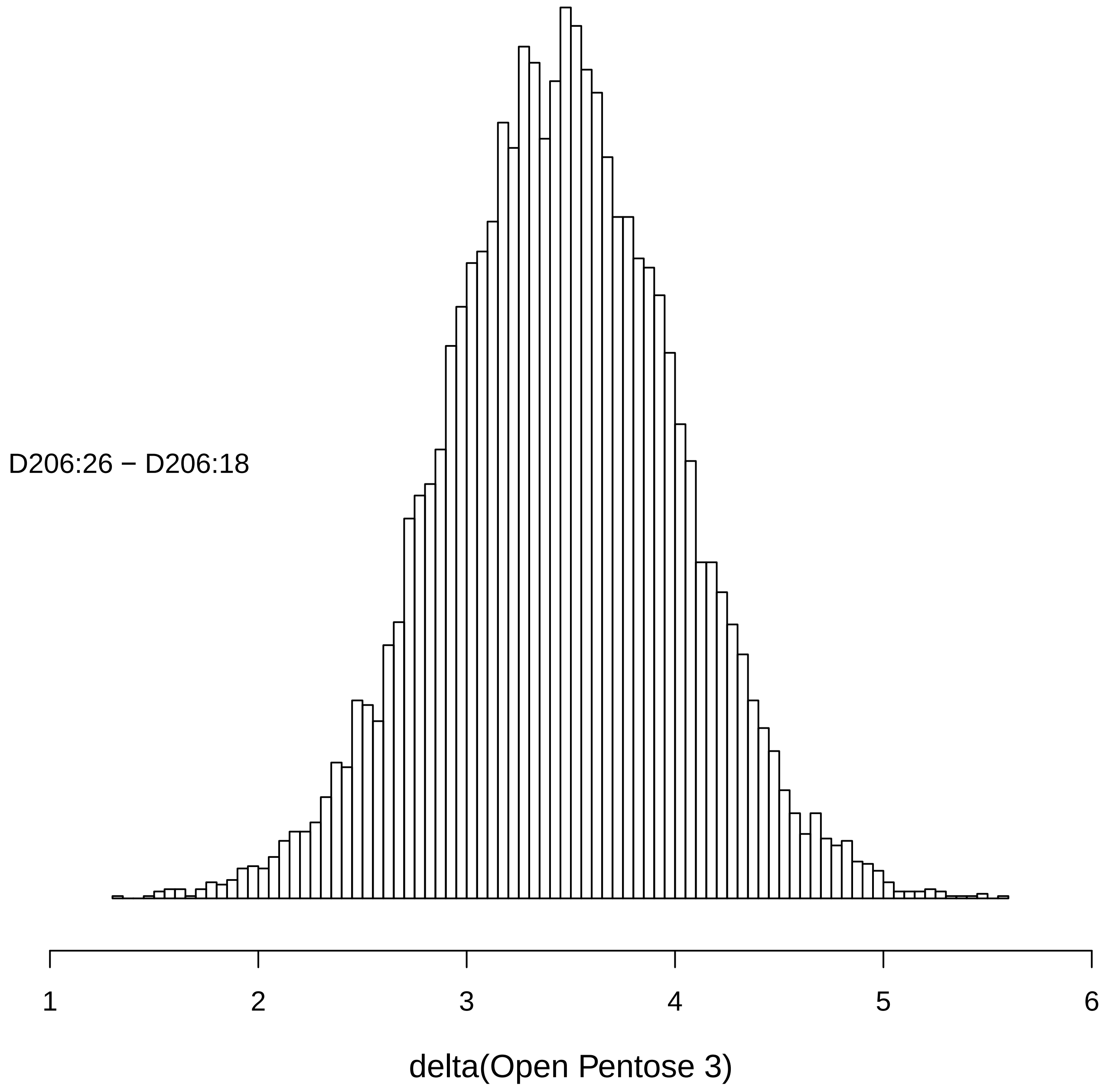
D206:26 − D206:18
1
2
3
4
5
6
delta(Open Pentose 3)

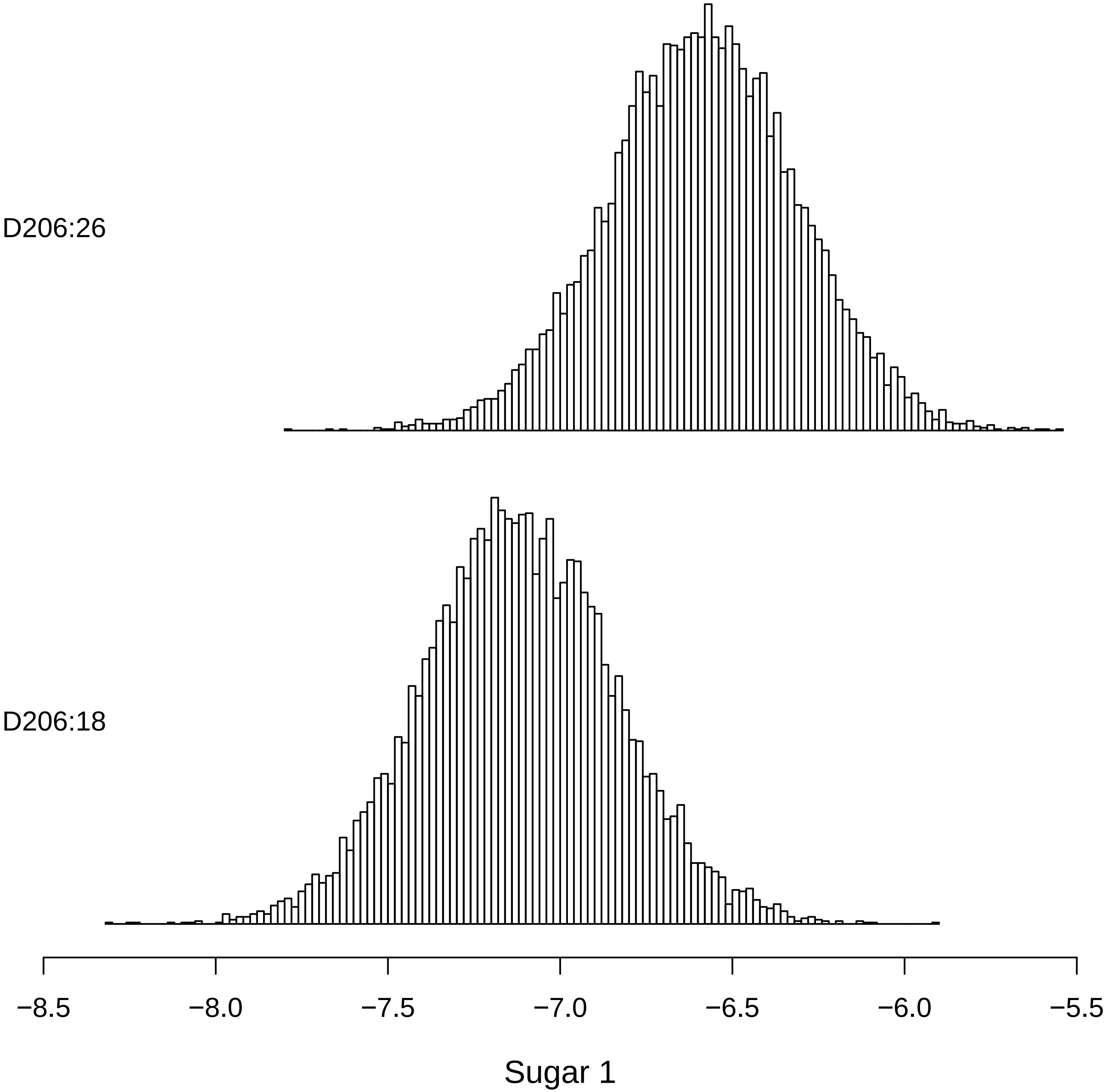

D206:26
D206:18
−8.5
−8.0
−7.5
−7.0
−6.5
−6.0
−5.5
Sugar 1

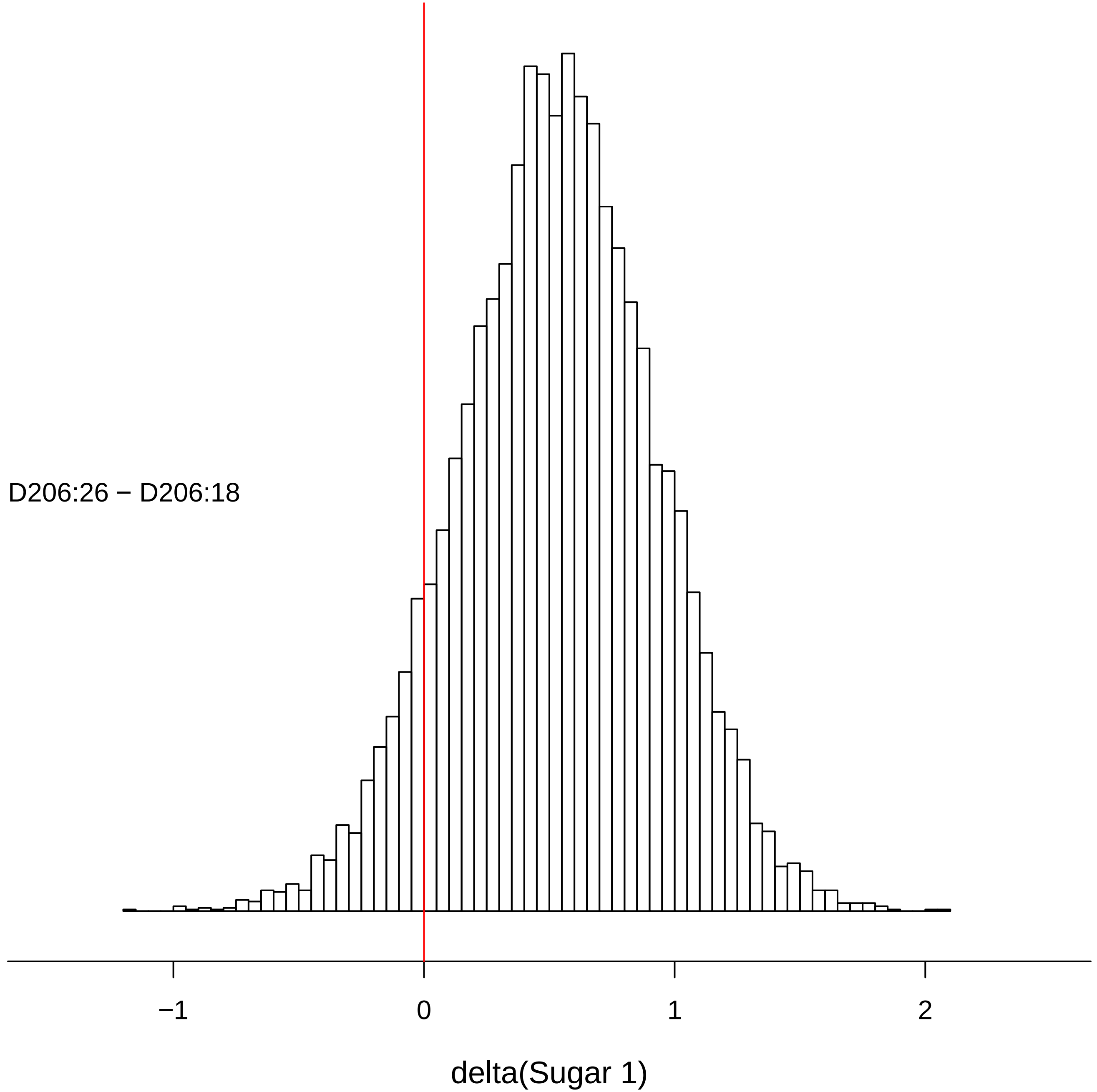
D206:26 − D206:18
−1
0
1
2
delta(Sugar 1)

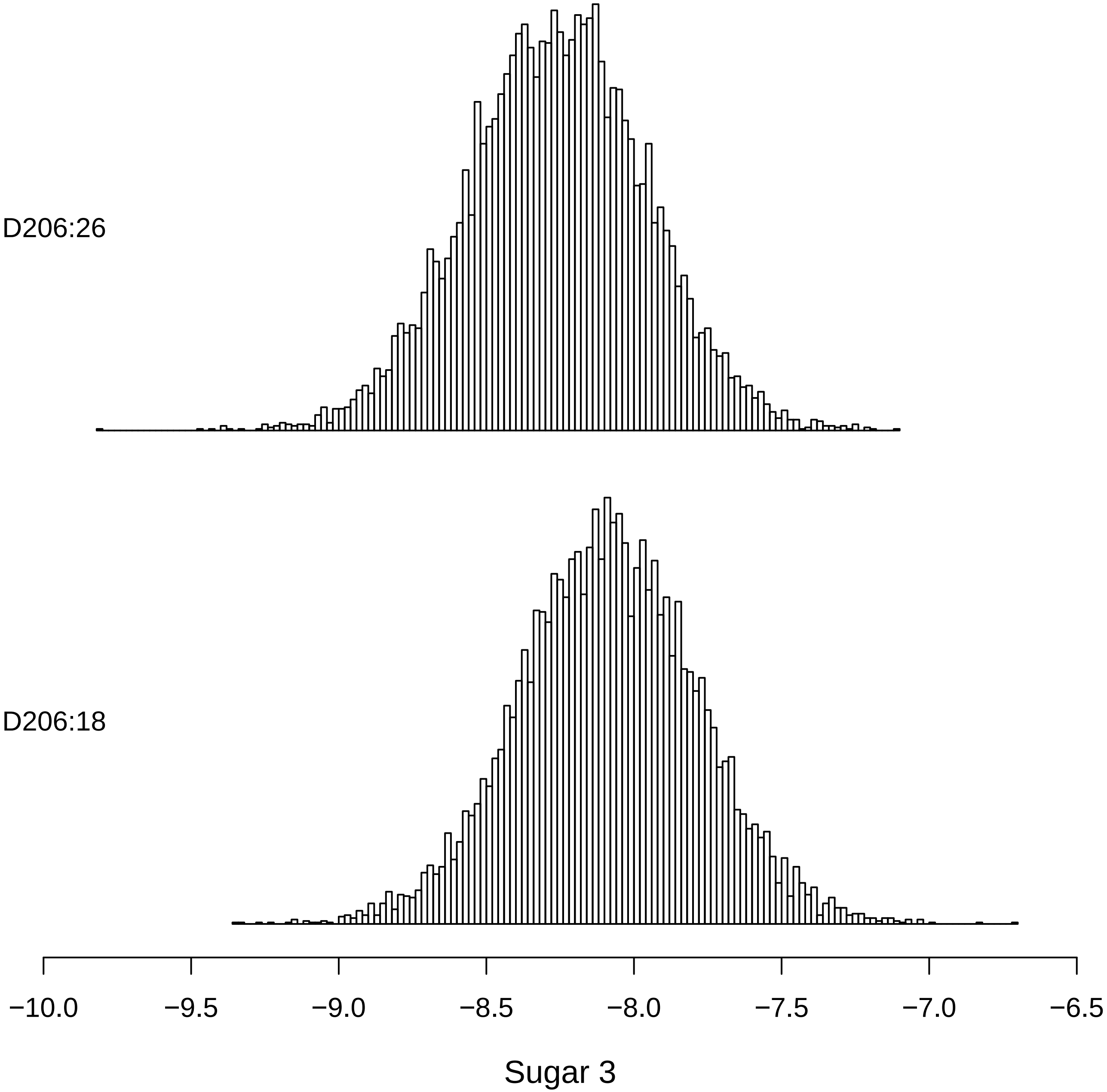
D206:26
D206:18
−10.0
−9.5
−9.0
−8.5
−8.0
−7.5
−7.0
−6.5
Sugar 3

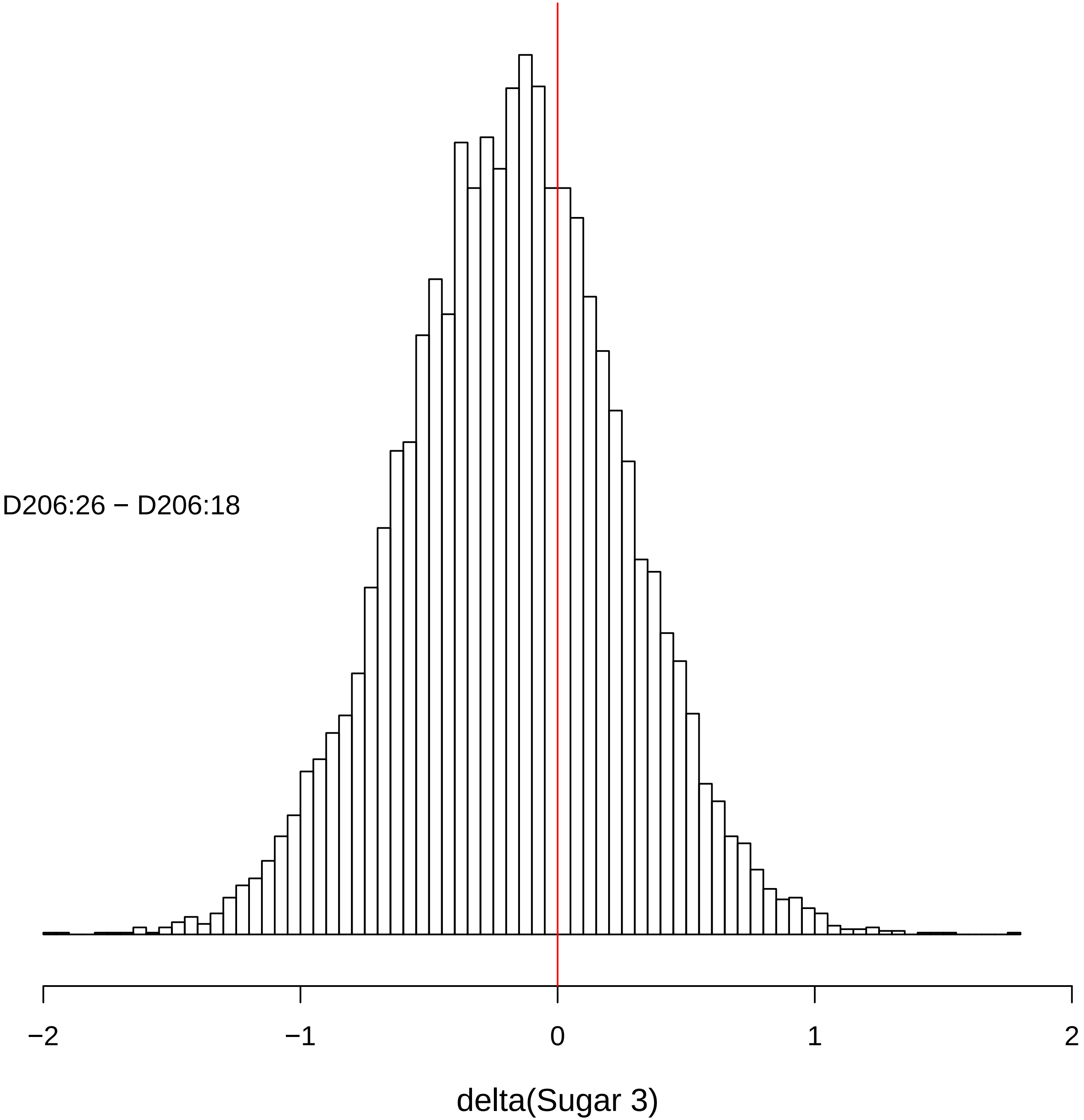
D206:26 − D206:18
−2
−1
0
1
2
delta(Sugar 3)

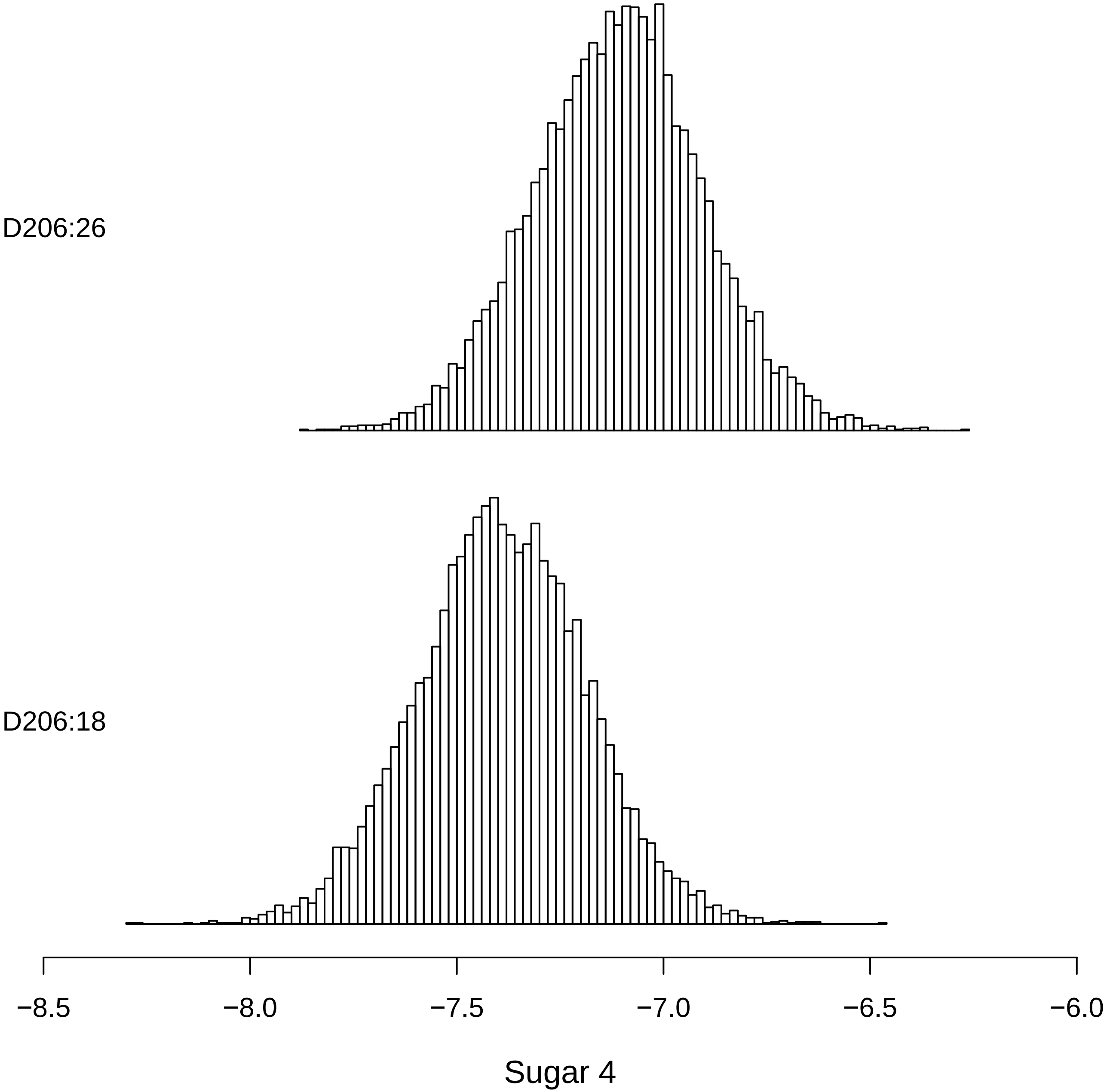
D206:26
D206:18
−8.5
−8.0
−7.5
−7.0
−6.5
−6.0
Sugar 4

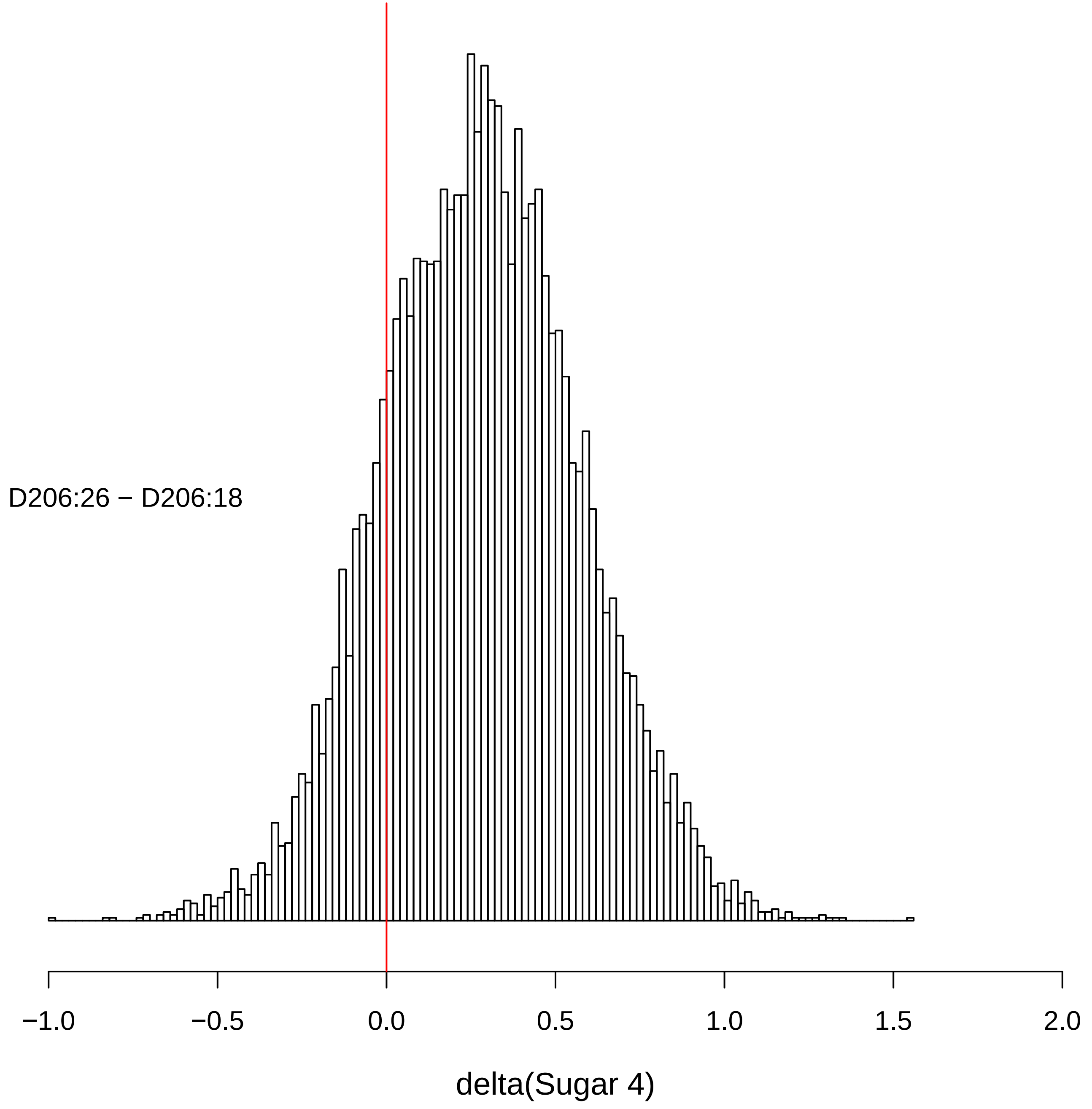
D206:26 − D206:18
−1.0
−0.5
0.0
0.5
1.0
1.5
2.0
delta(Sugar 4)

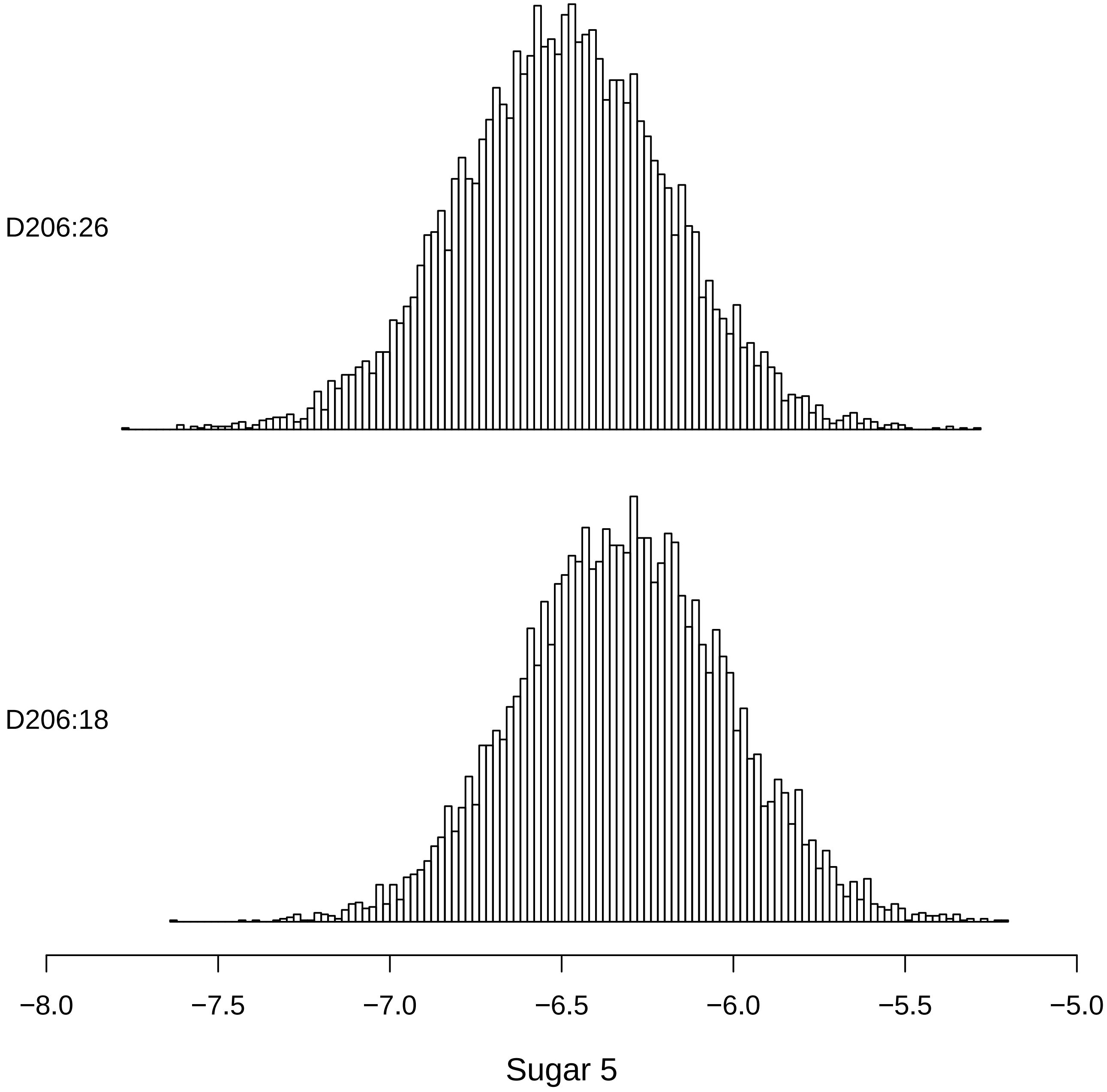

D206:26
D206:18
−8.0
−7.5
−7.0
−6.5
−6.0
−5.5
−5.0
Sugar 5

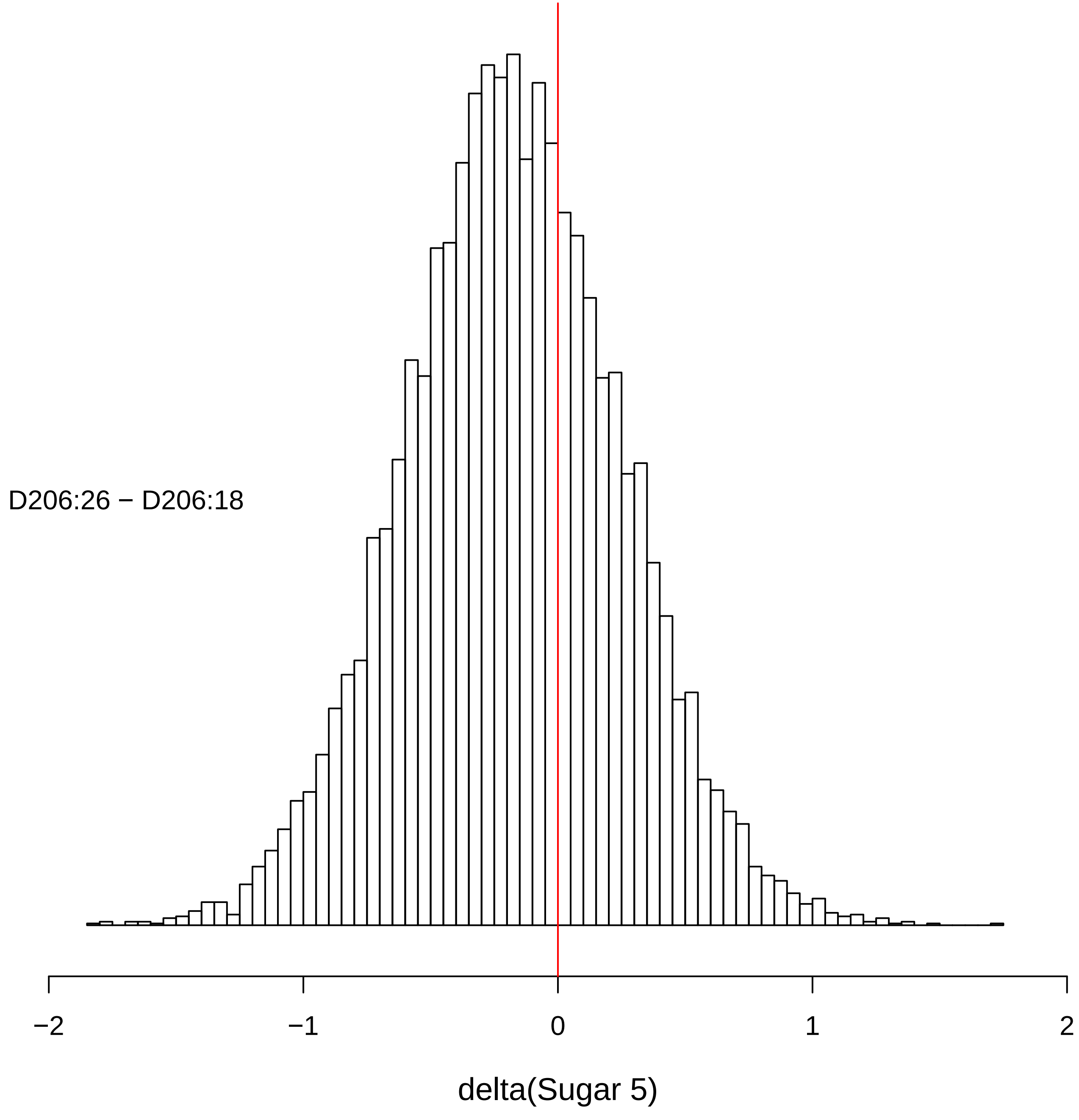
D206:26 − D206:18
−2
−1
0
1
2
delta(Sugar 5)

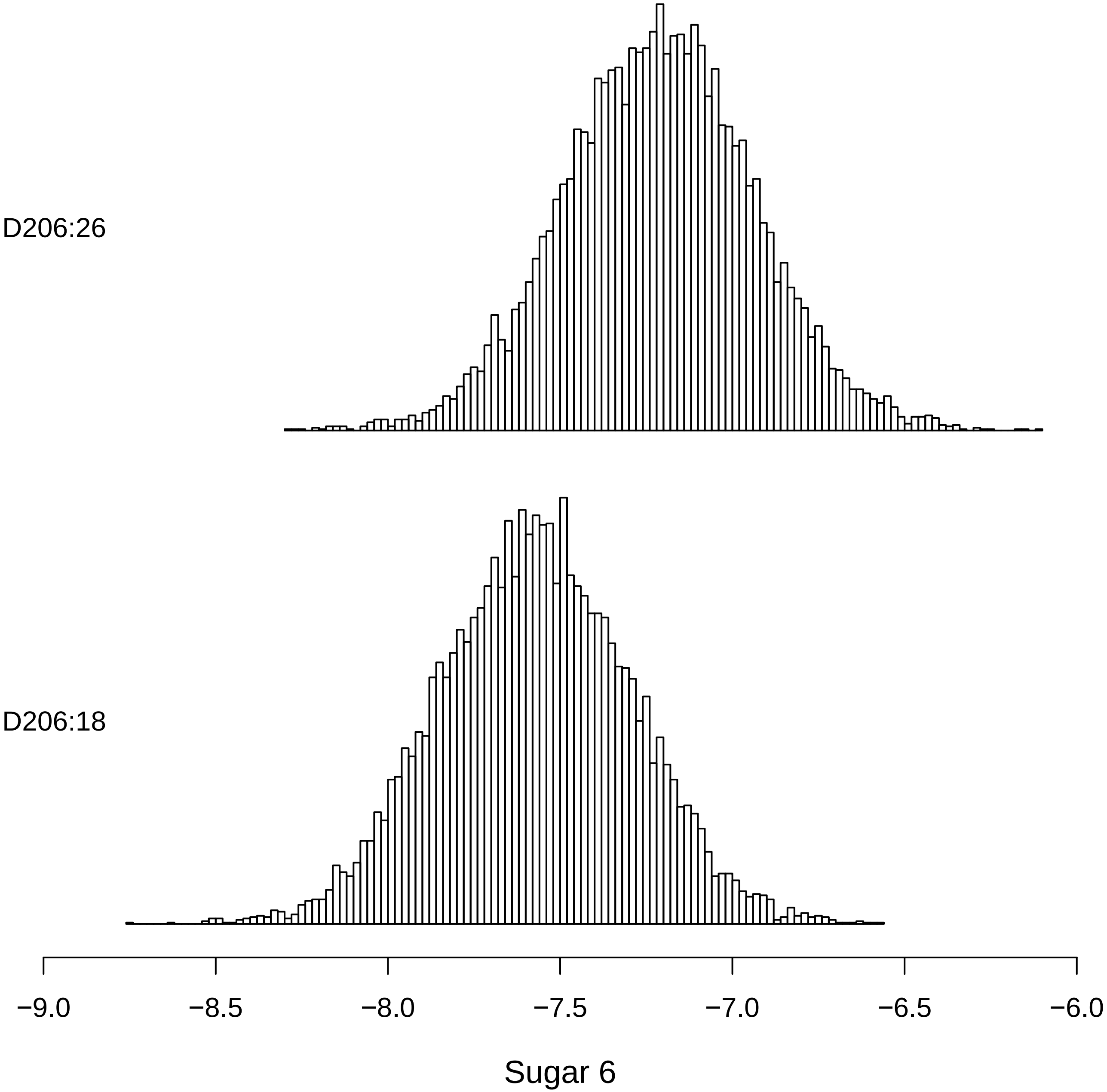
D206:26
D206:18
−9.0
−8.5
−8.0
−7.5
−7.0
−6.5
−6.0
Sugar 6

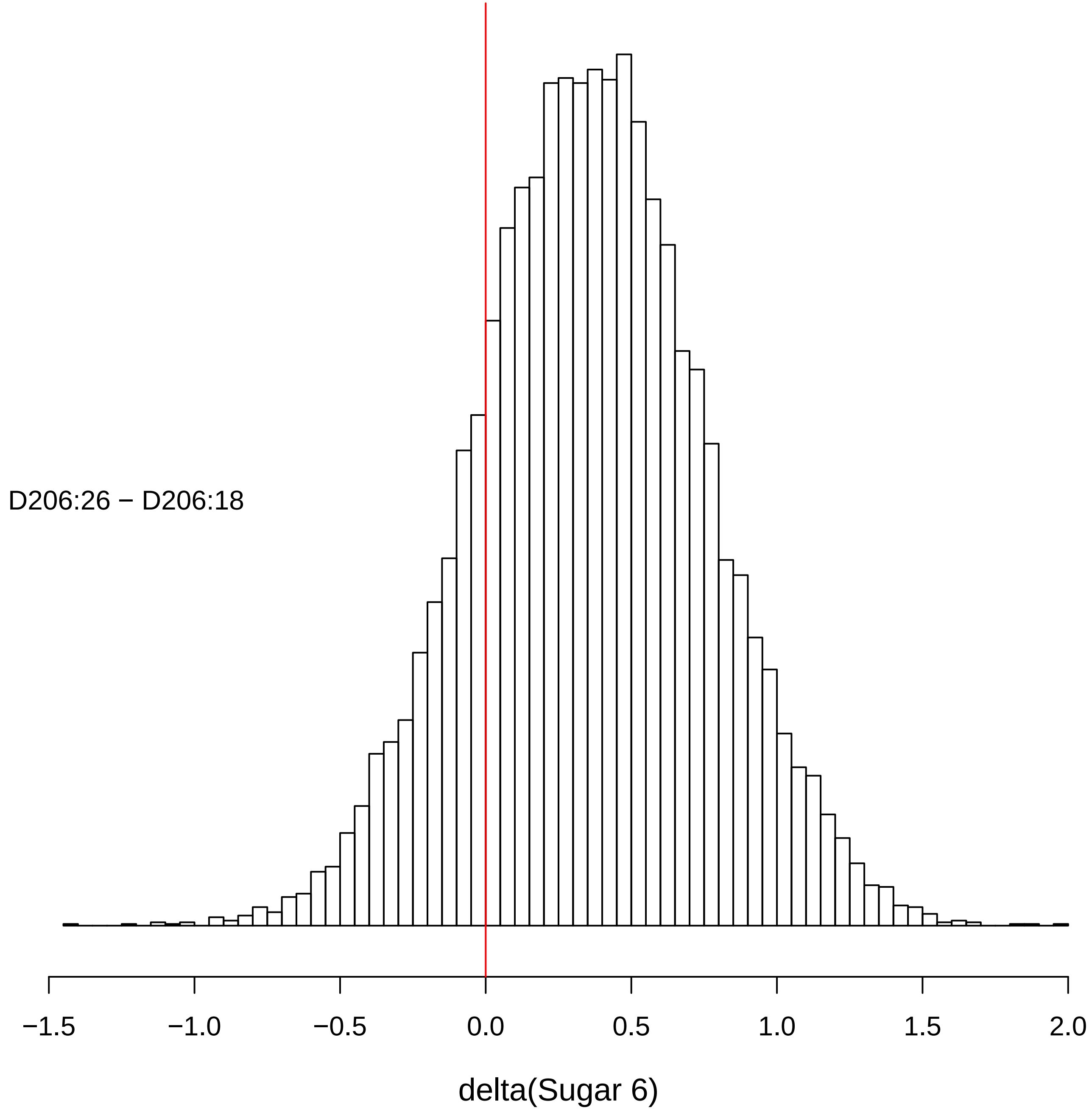
D206:26 − D206:18
−1.5
−1.0
−0.5
0.0
0.5
1.0
1.5
2.0
delta(Sugar 6)

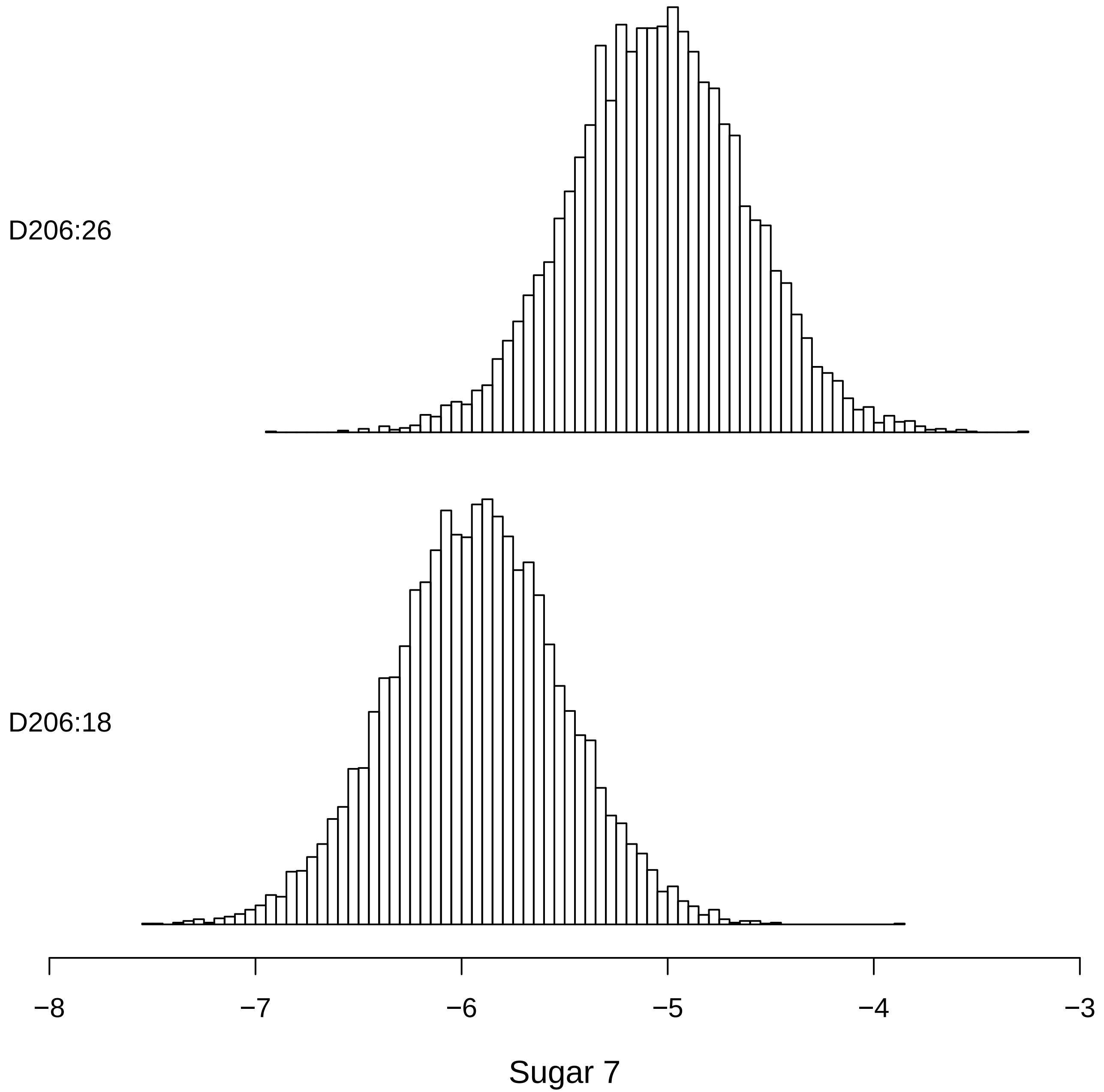
D206:26
D206:18
−8
−7
−6
−5
−4
−3
Sugar 7

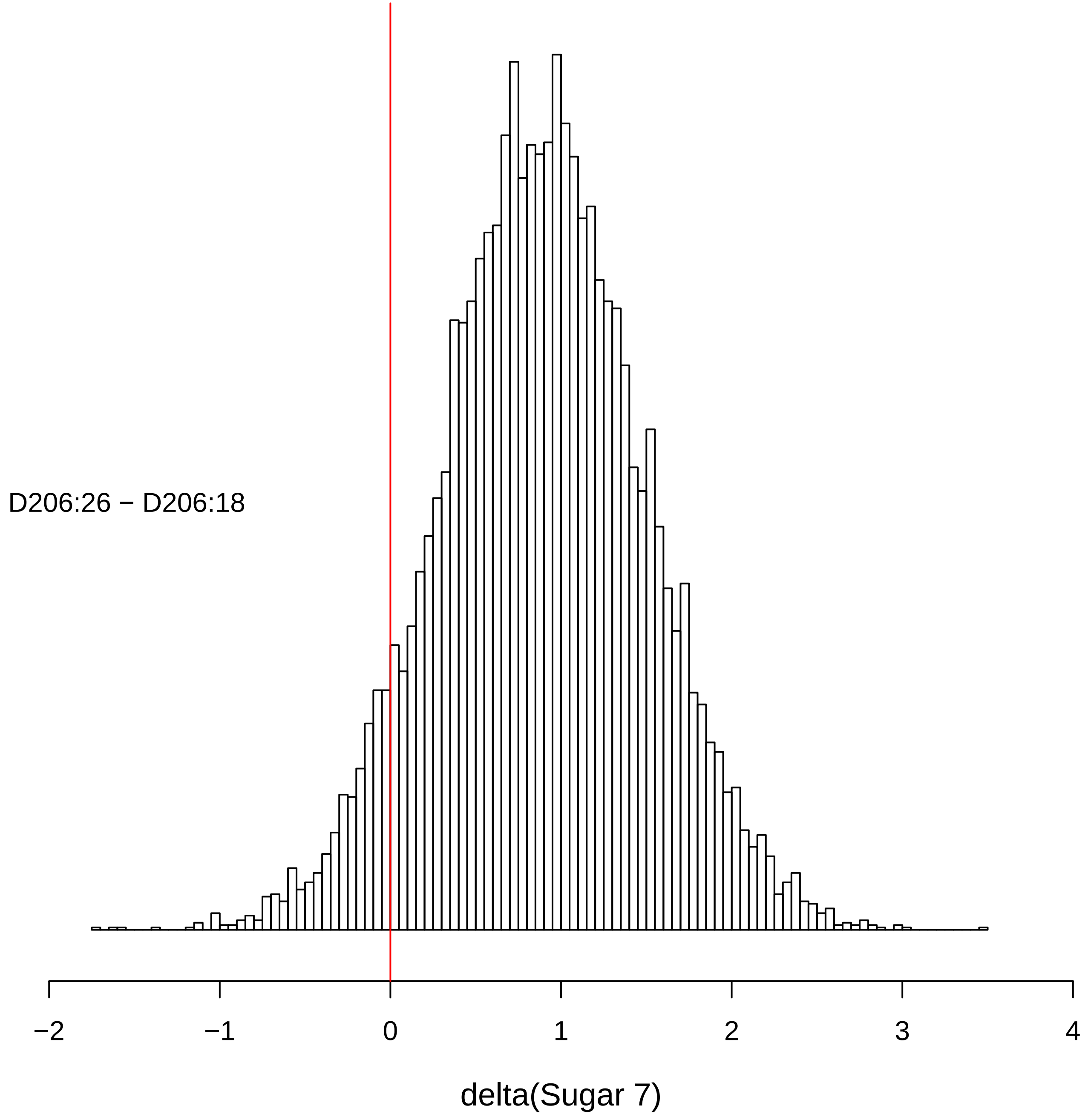
D206:26 − D206:18
−2
−1
0
1
2
3
4
delta(Sugar 7)

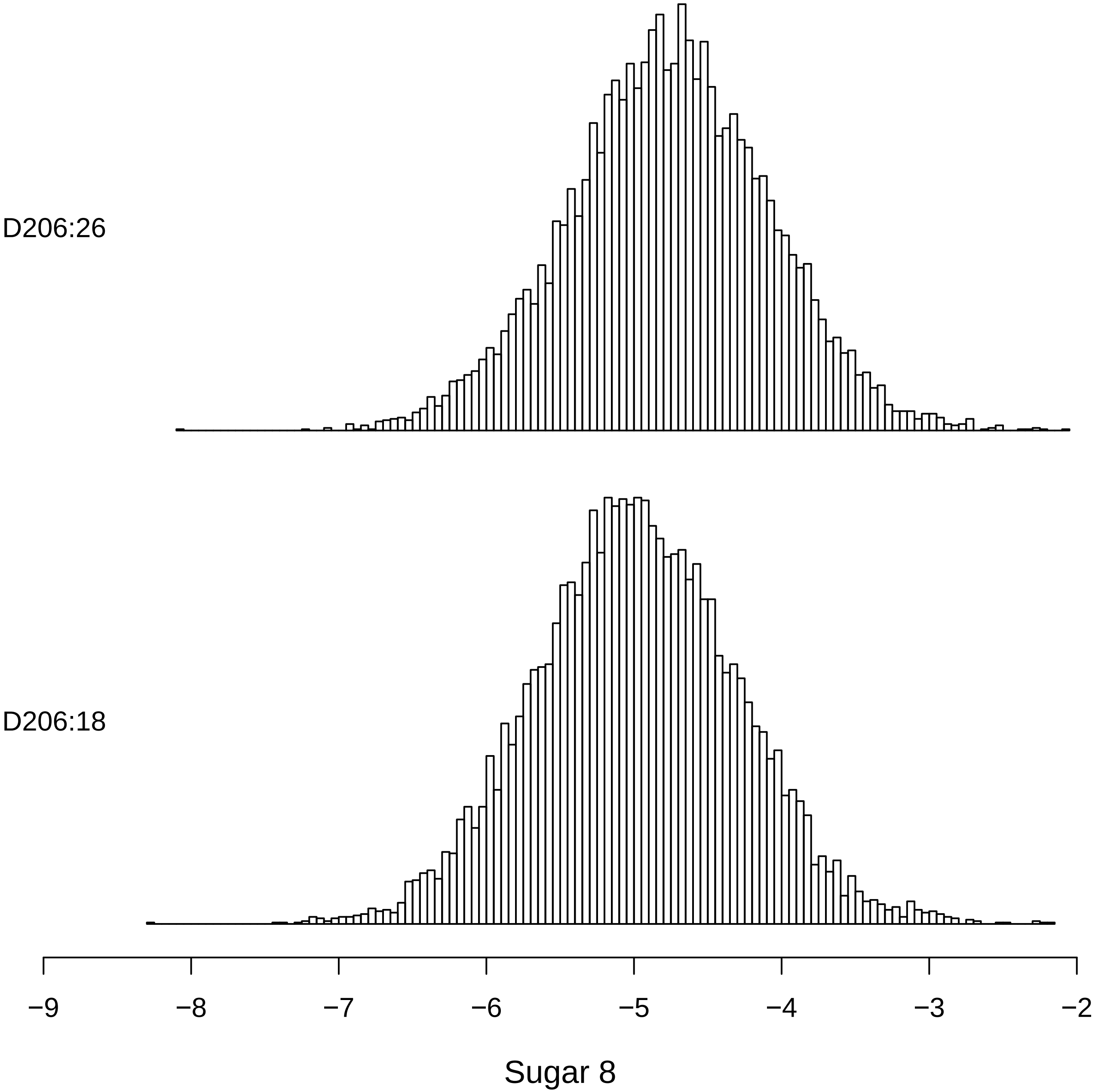
D206:26
D206:18
−9
−8
−7
−6
−5
−4
−3
−2
Sugar 8

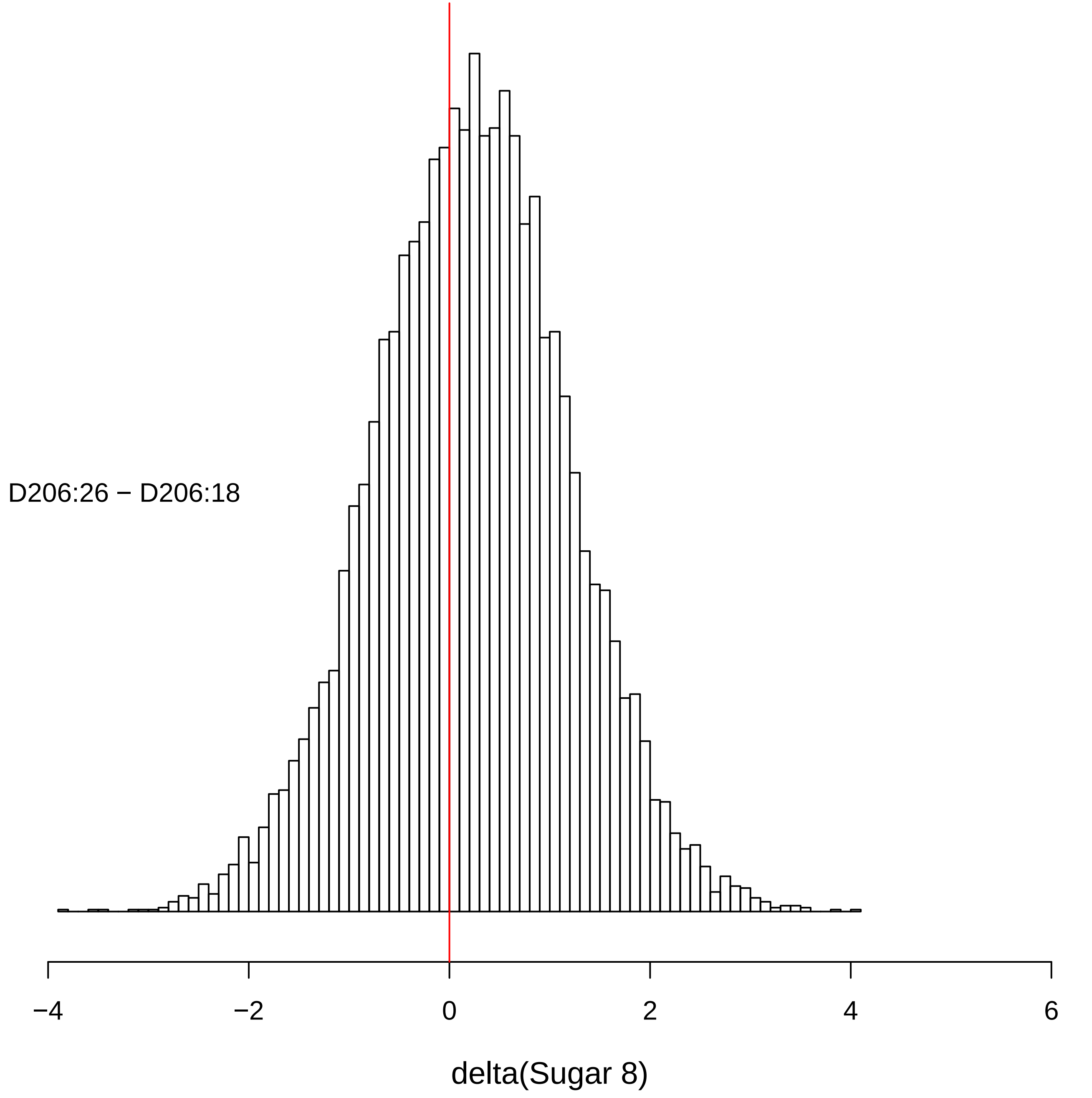
D206:26 − D206:18
−4
−2
0
2
4
6
delta(Sugar 8)

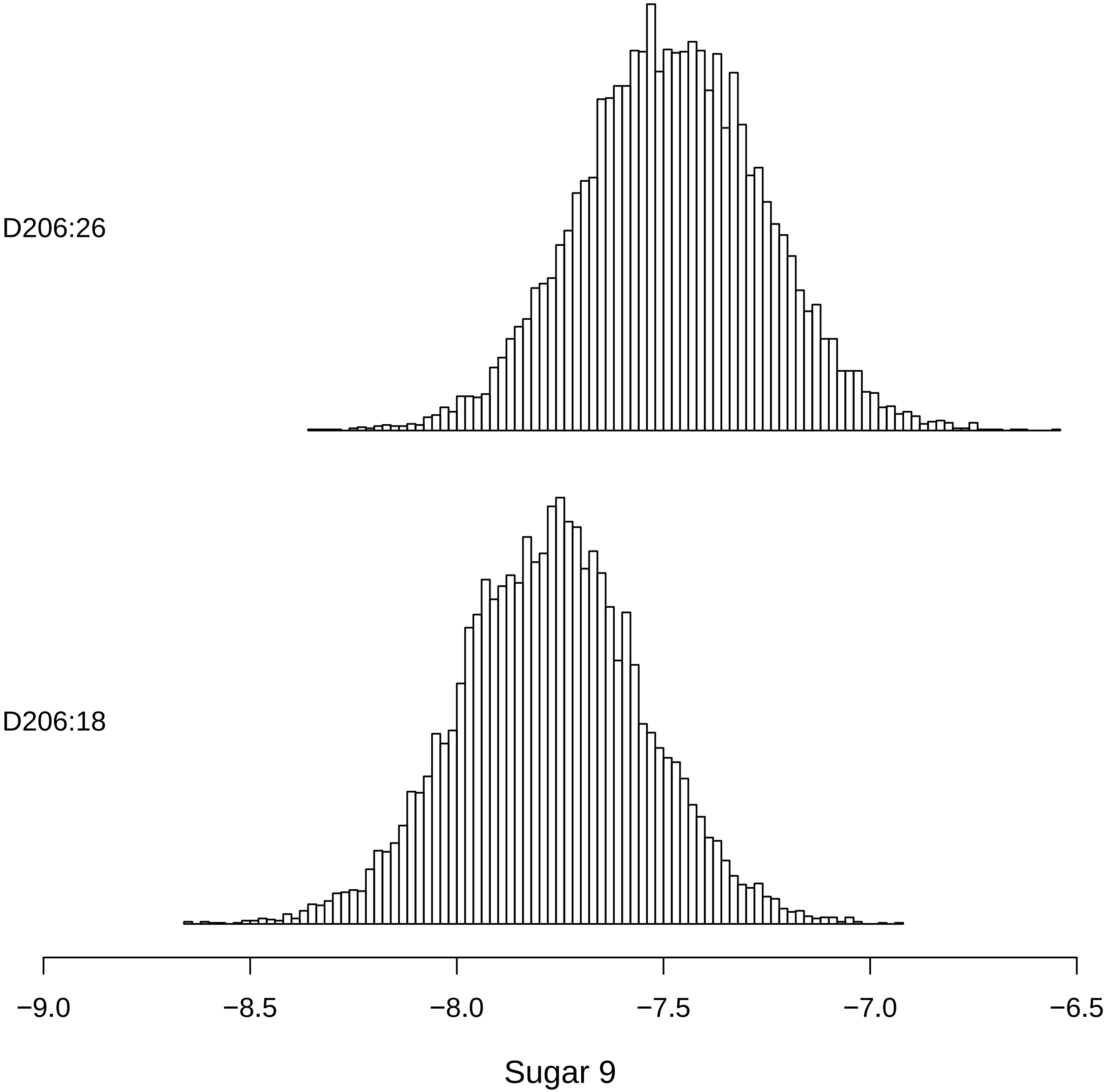
D206:26
D206:18
−9.0
−8.5
−8.0
−7.5
−7.0
−6.5
Sugar 9

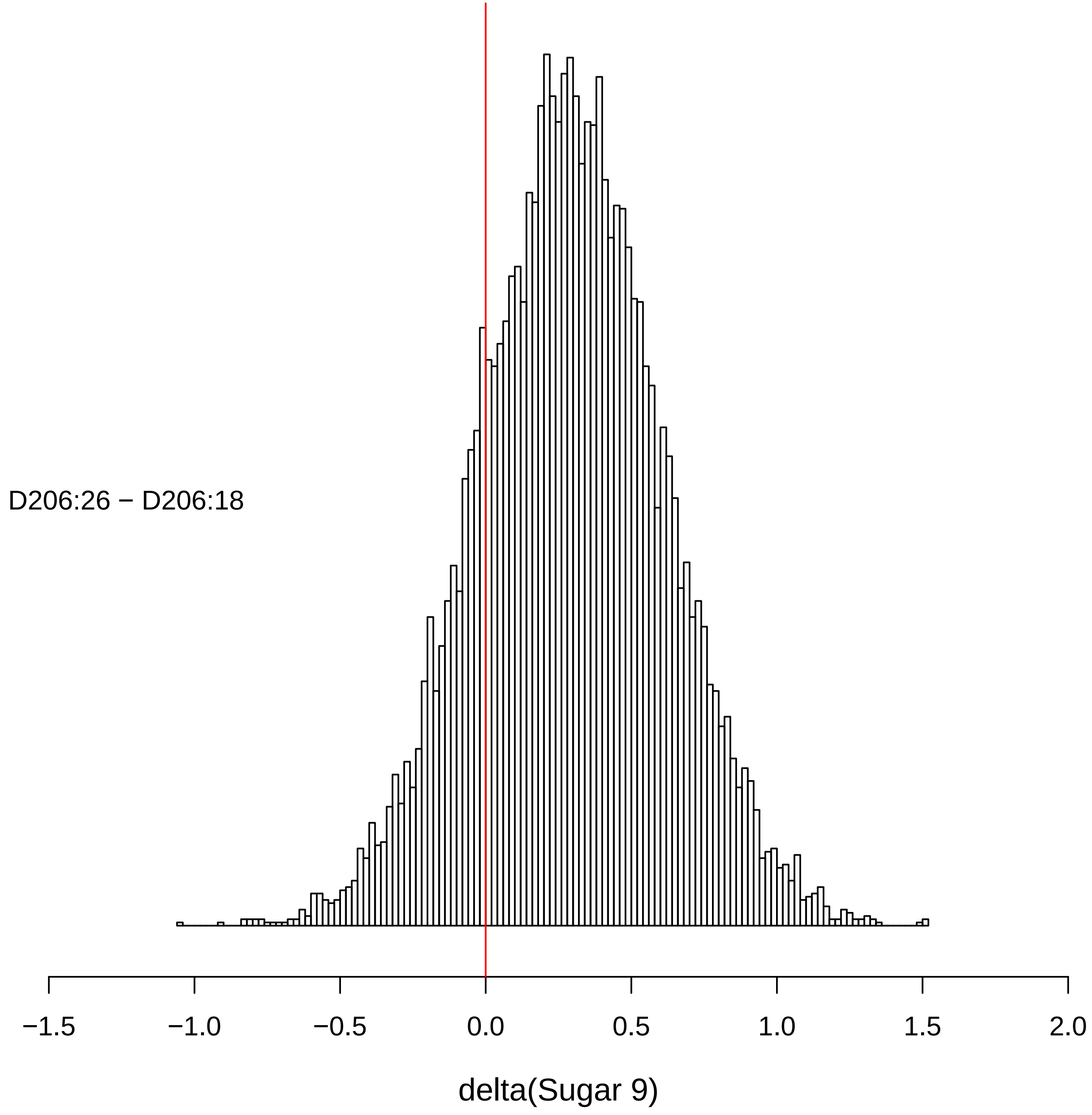
D206:26 − D206:18
−1.5
−1.0
−0.5
0.0
0.5
1.0
1.5
2.0
delta(Sugar 9)

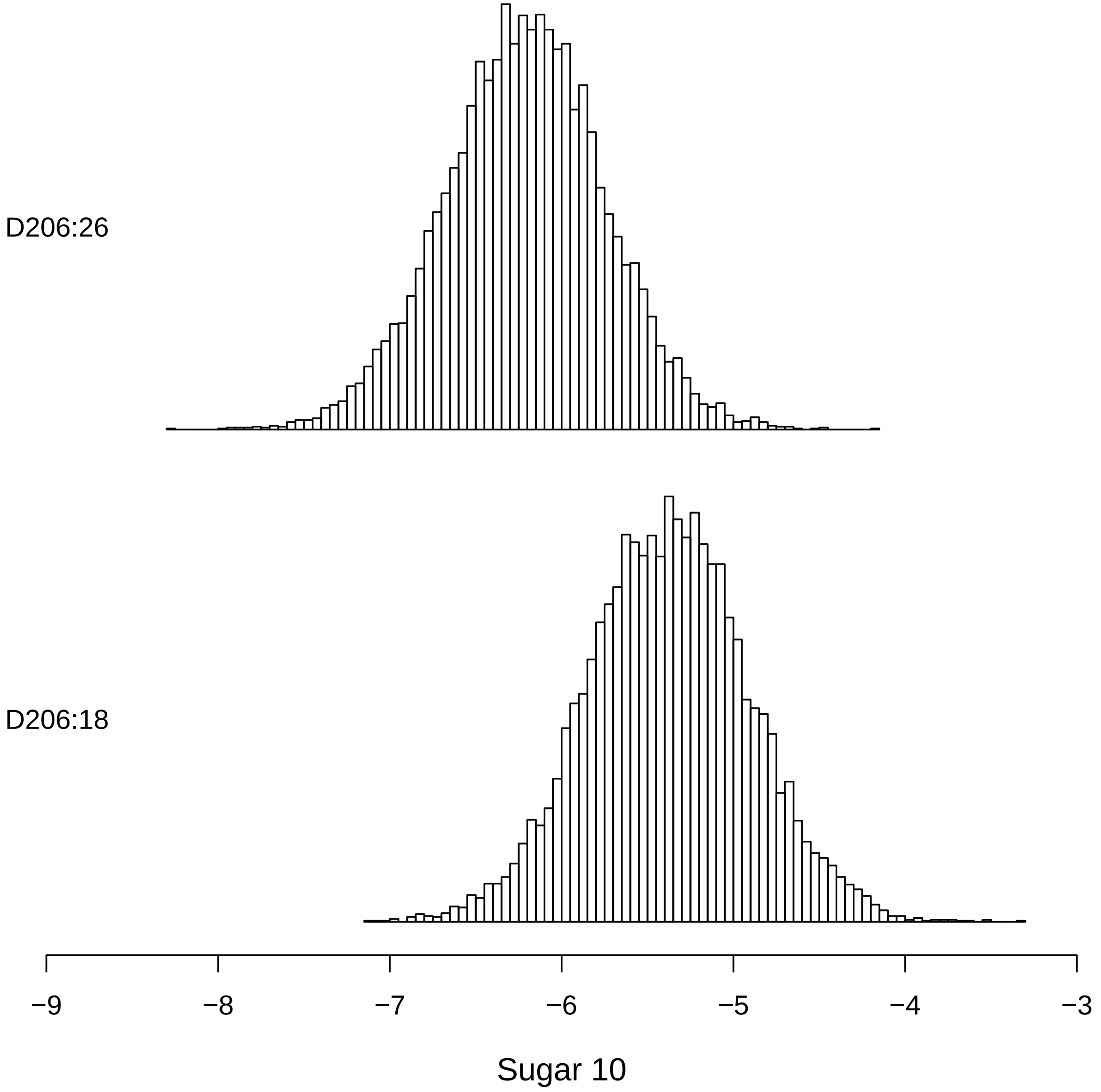
D206:26
D206:18
−9
−8
−7
−6
−5
−4
−3
Sugar 10

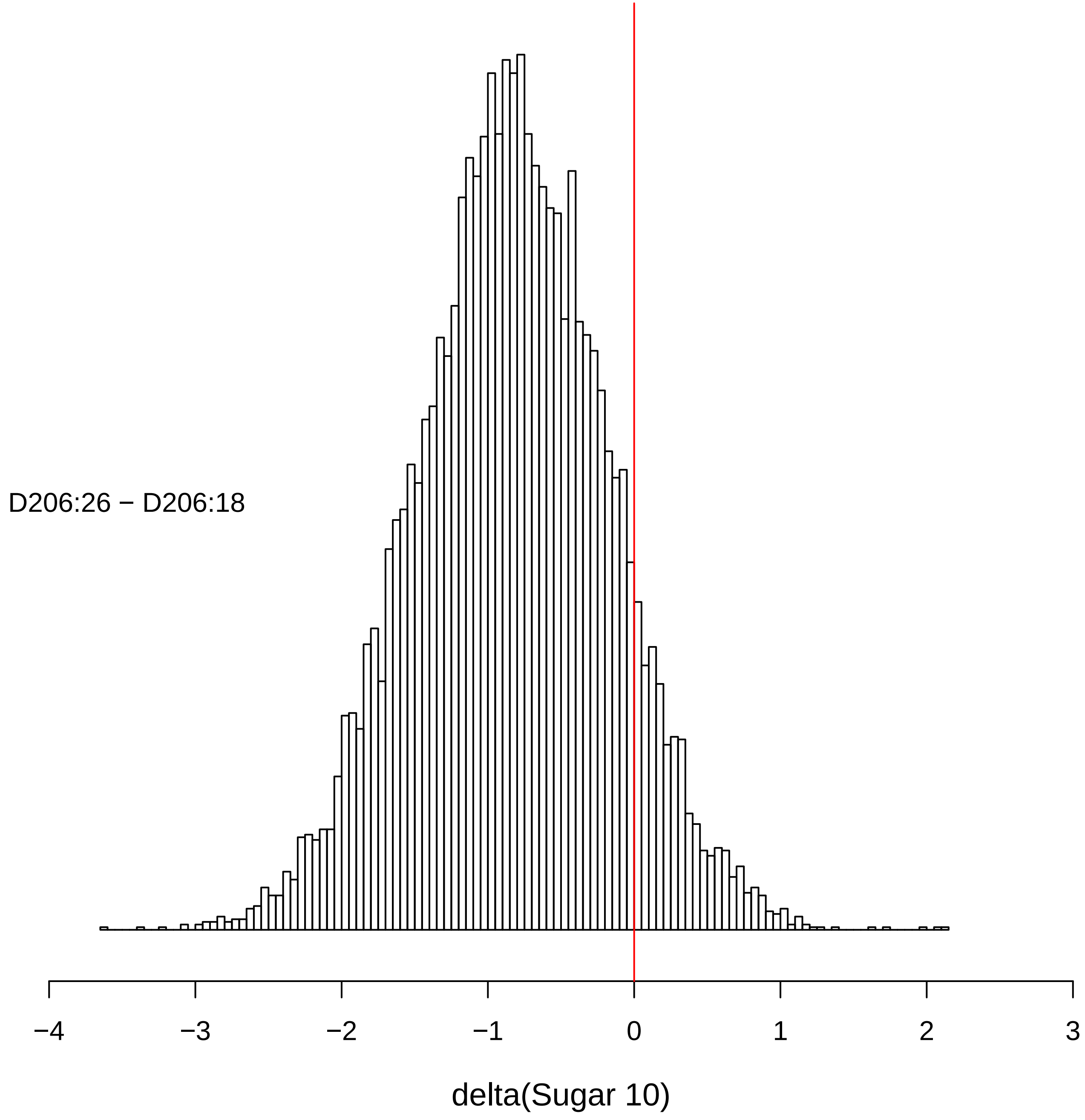
D206:26 − D206:18
−4
−3
−2
−1
0
1
2
3
delta(Sugar 10)

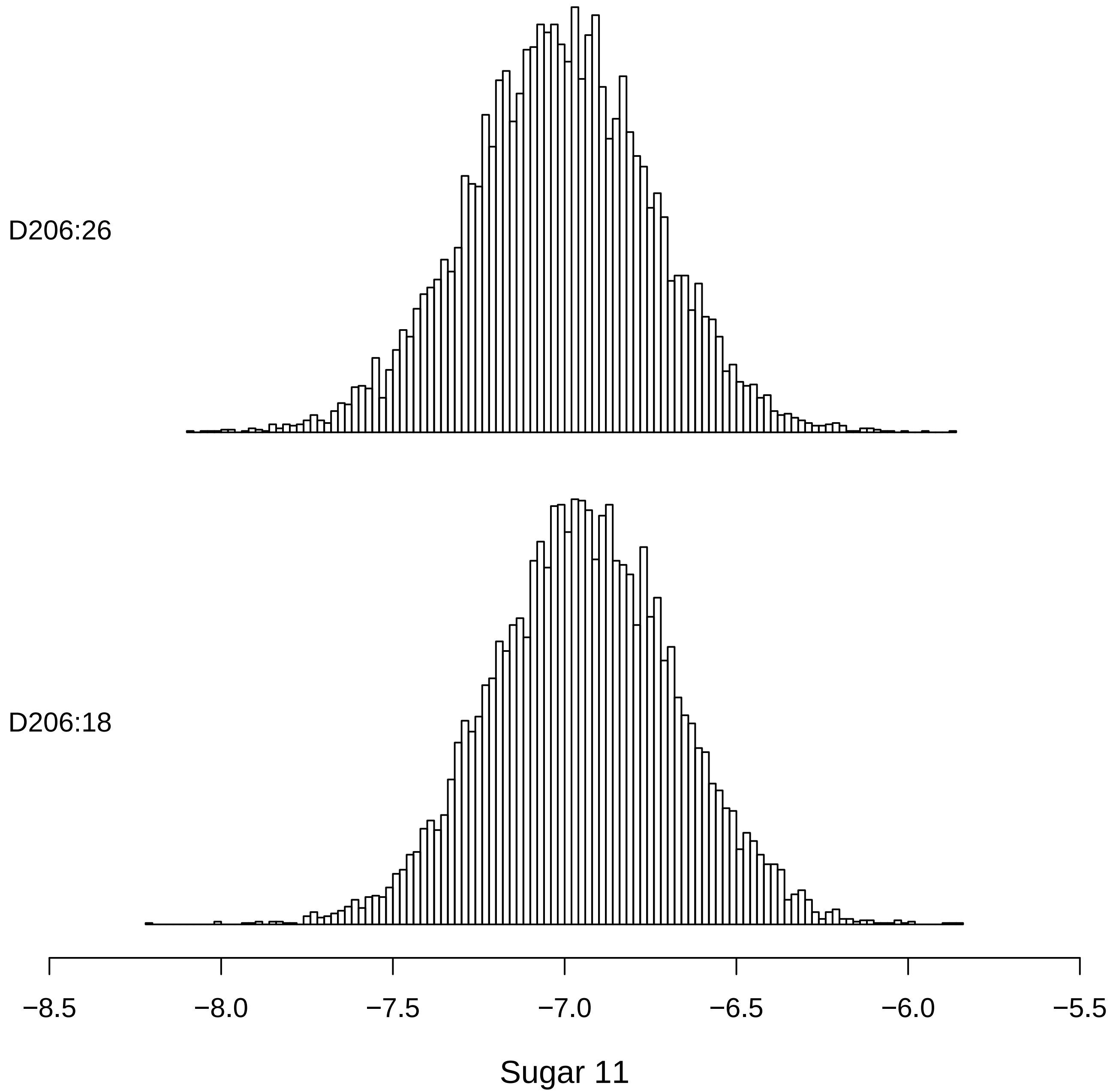
D206:26
D206:18
−8.5
−8.0
−7.5
−7.0
−6.5
−6.0
−5.5
Sugar 11

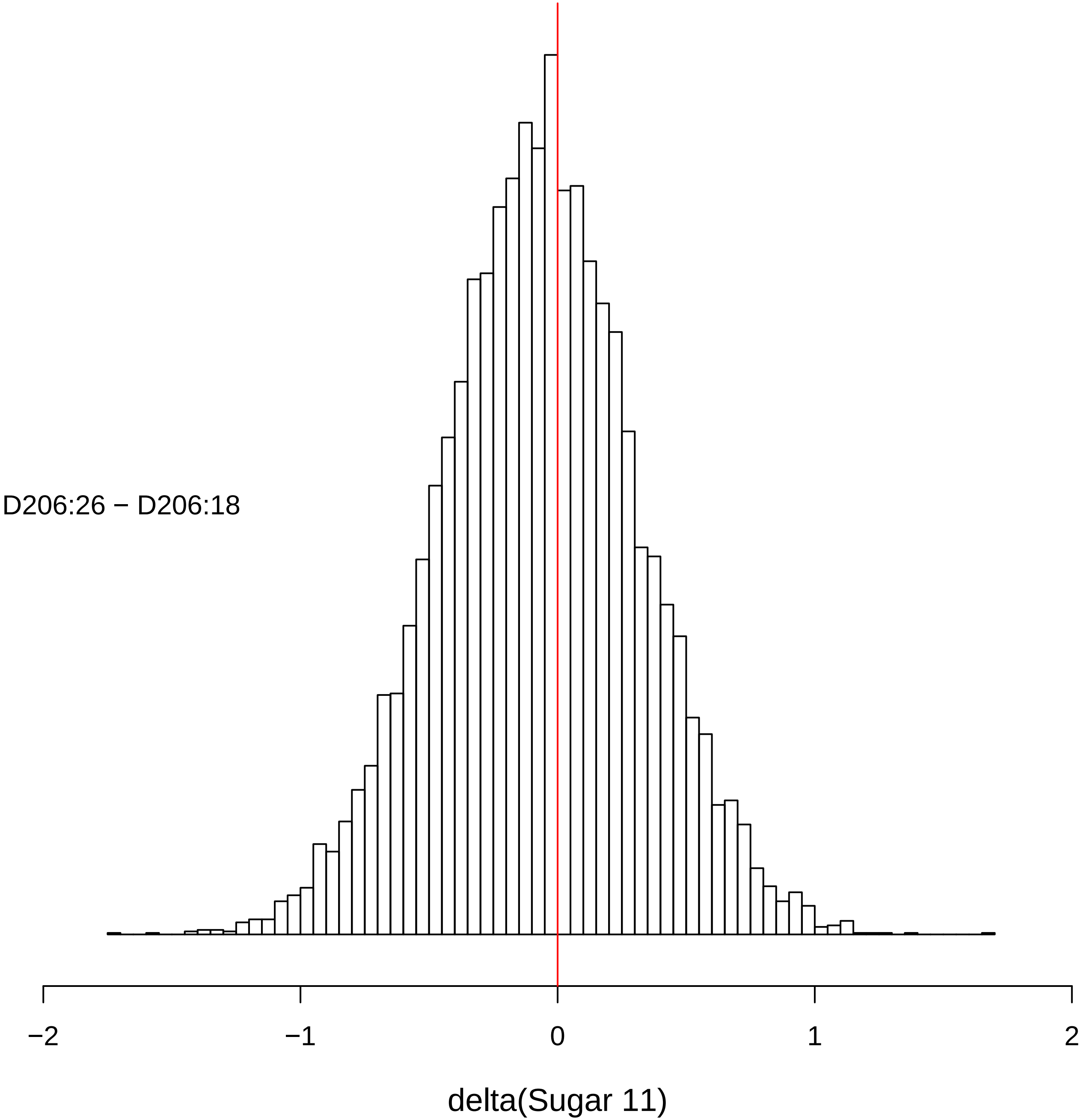
D206:26 − D206:18
−2
−1
0
1
2
delta(Sugar 11)

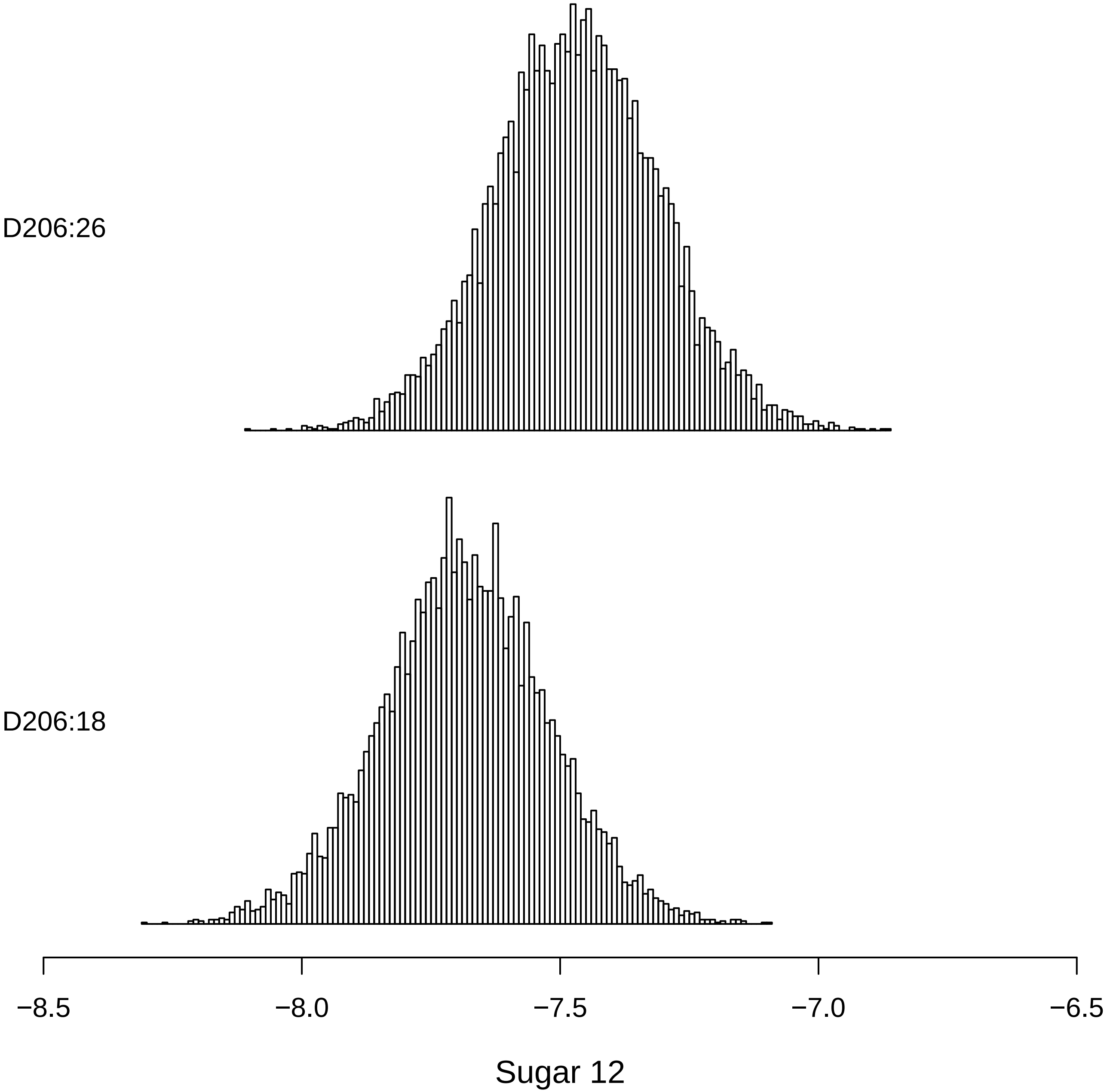
D206:26
D206:18
−8.5
−8.0
−7.5
−7.0
−6.5
Sugar 12

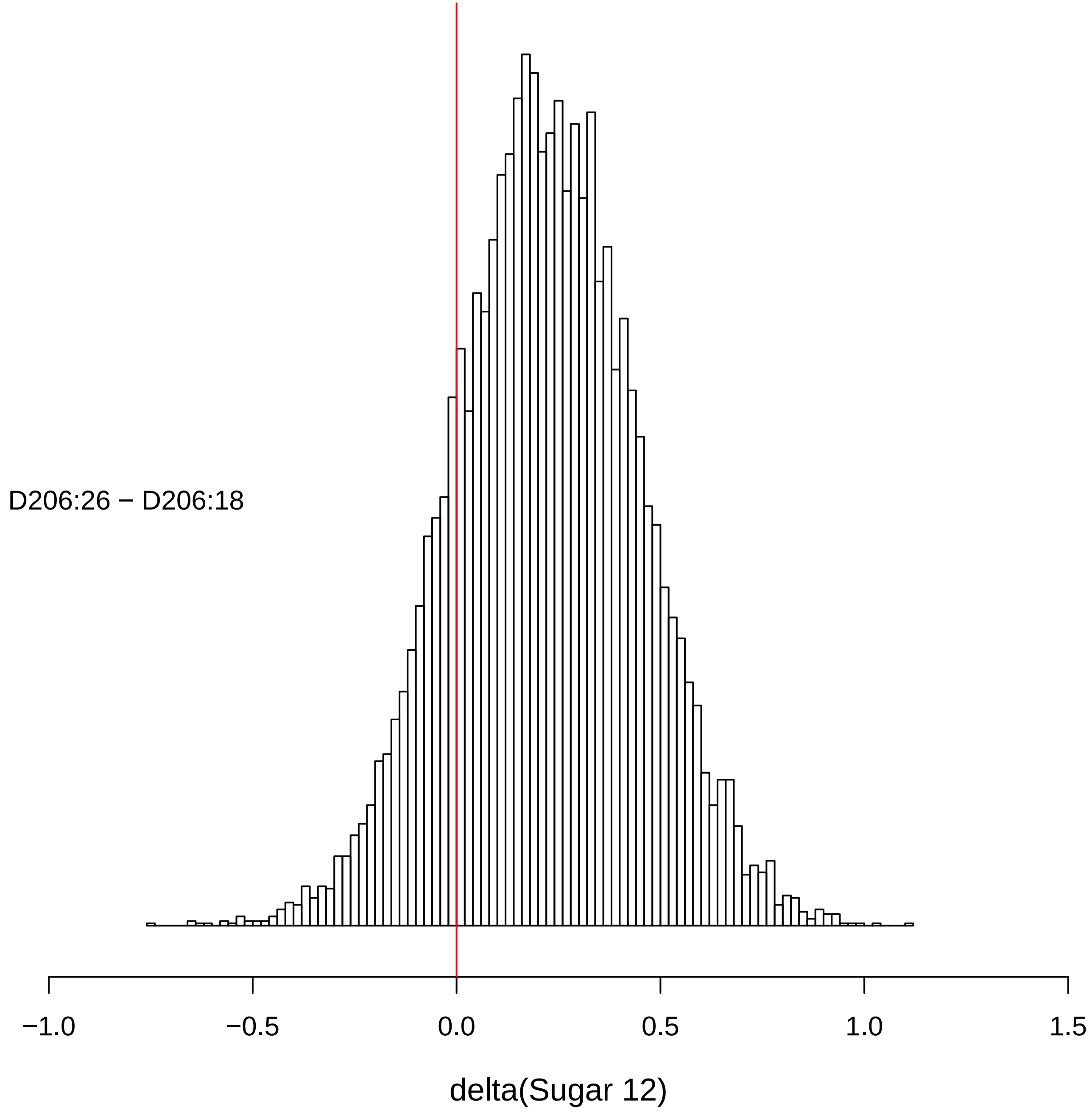
D206:26 − D206:18
−1.0
−0.5
0.0
0.5
1.0
1.5
delta(Sugar 12)

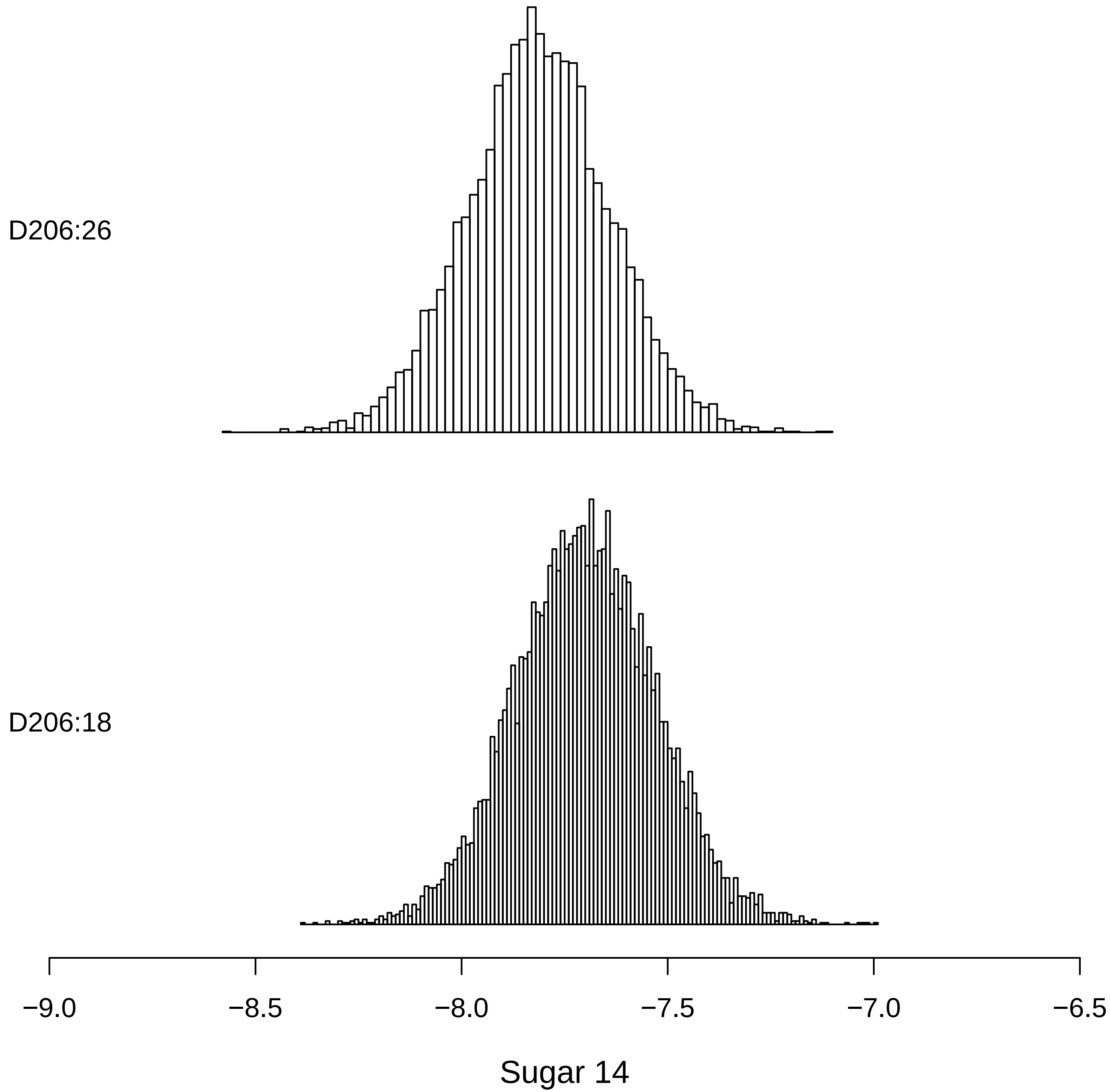
D206:26
D206:18
−9.0
−8.5
−8.0
−7.5
−7.0
−6.5
Sugar 14

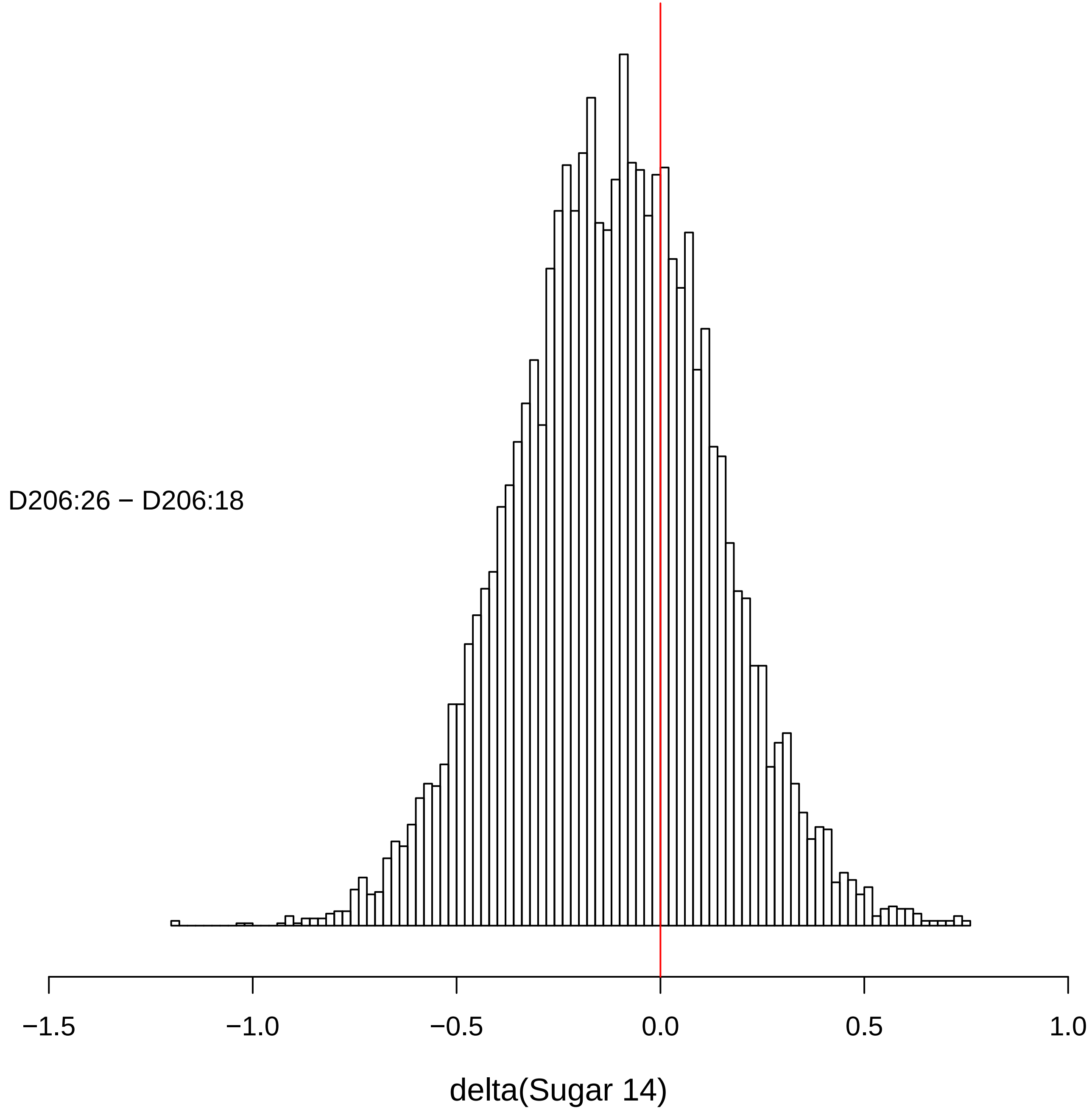
D206:26 − D206:18
−1.5
−1.0
−0.5
0.0
0.5
1.0
delta(Sugar 14)

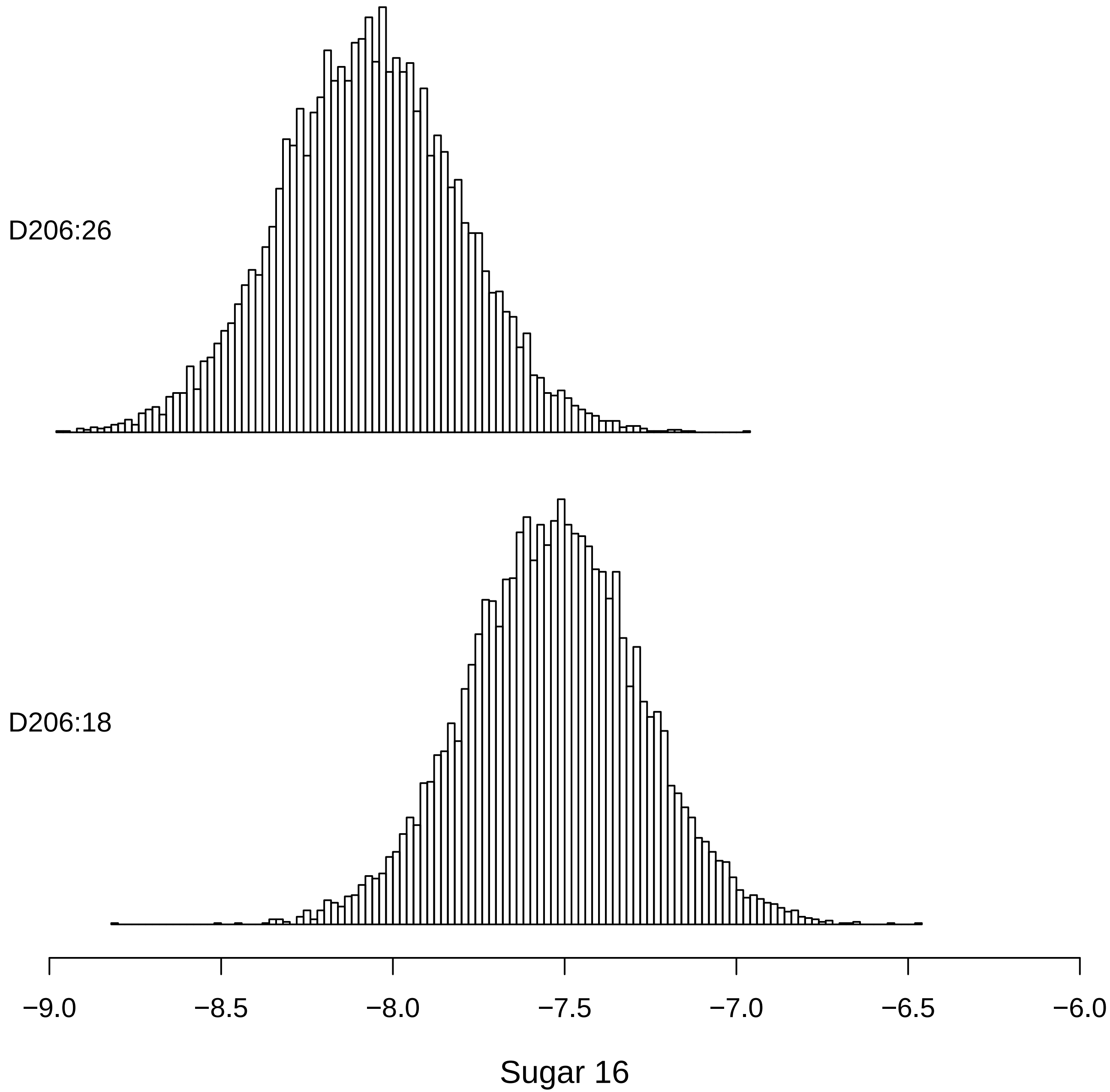
D206:26
D206:18
−9.0
−8.5
−8.0
−7.5
−7.0
−6.5
−6.0
Sugar 16

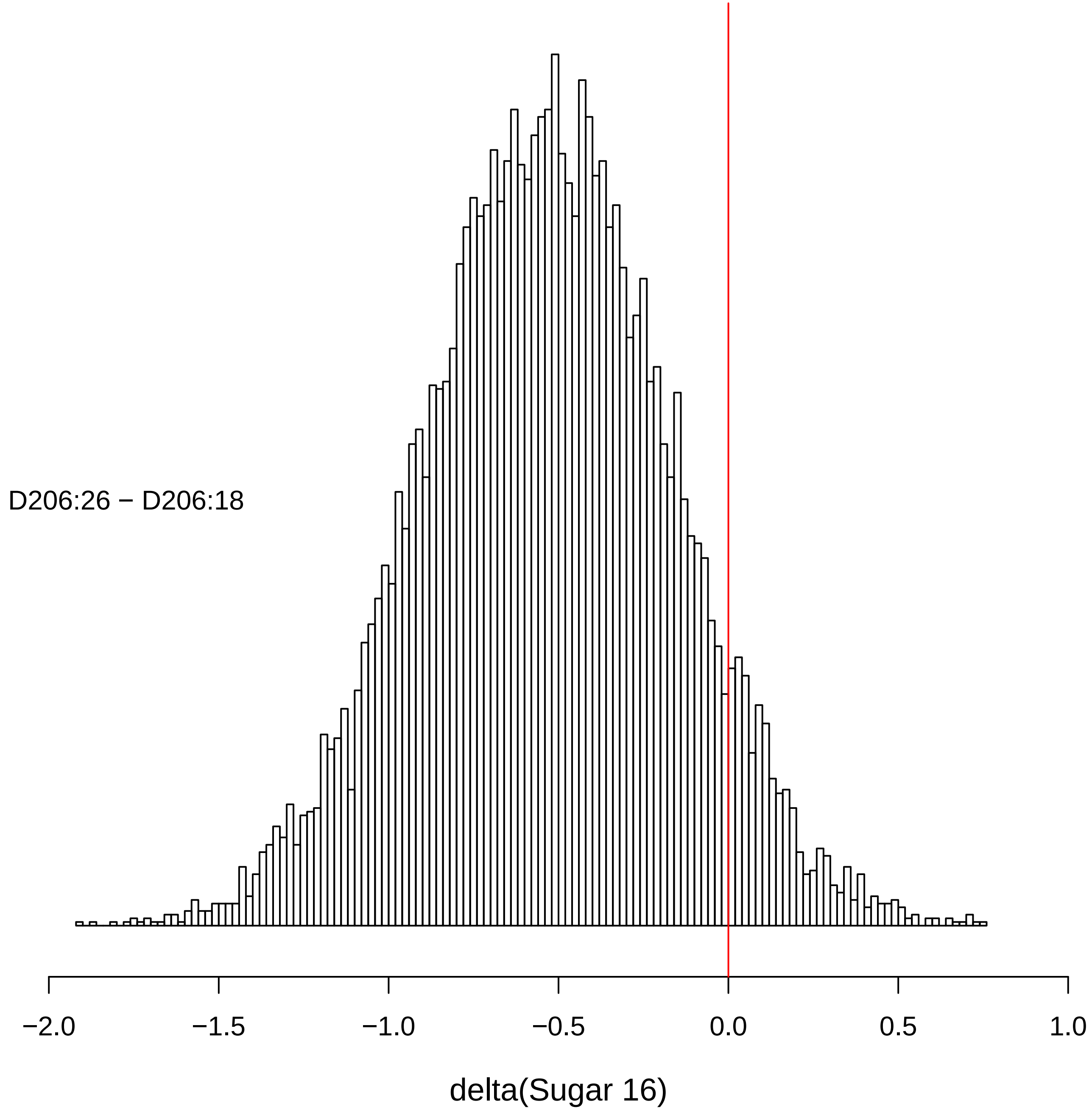
D206:26 − D206:18
−2.0
−1.5
−1.0
−0.5
0.0
0.5
1.0
delta(Sugar 16)

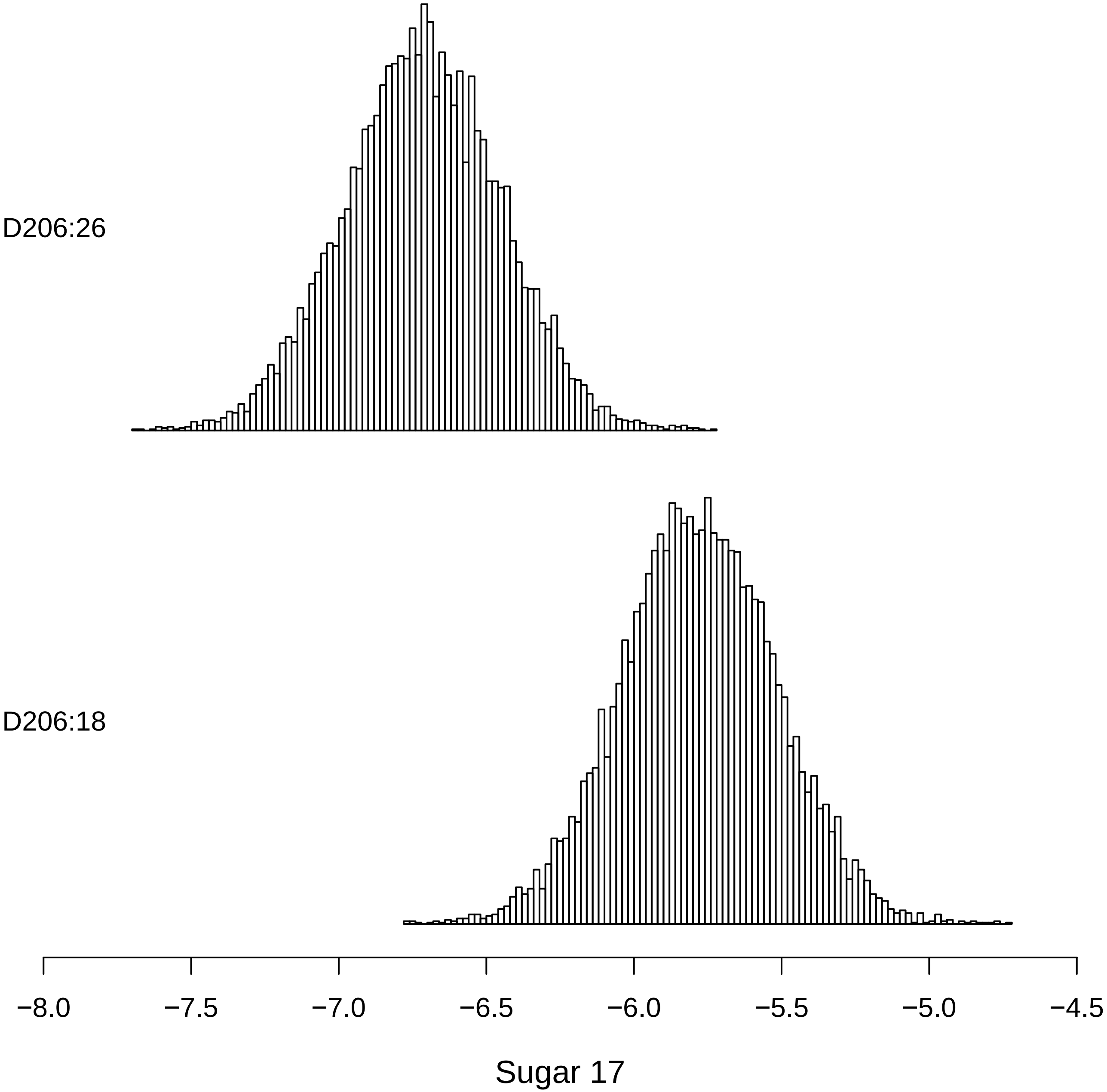

D206:26
D206:18
−8.0
−7.5
−7.0
−6.5
−6.0
−5.5
−5.0
−4.5
Sugar 17

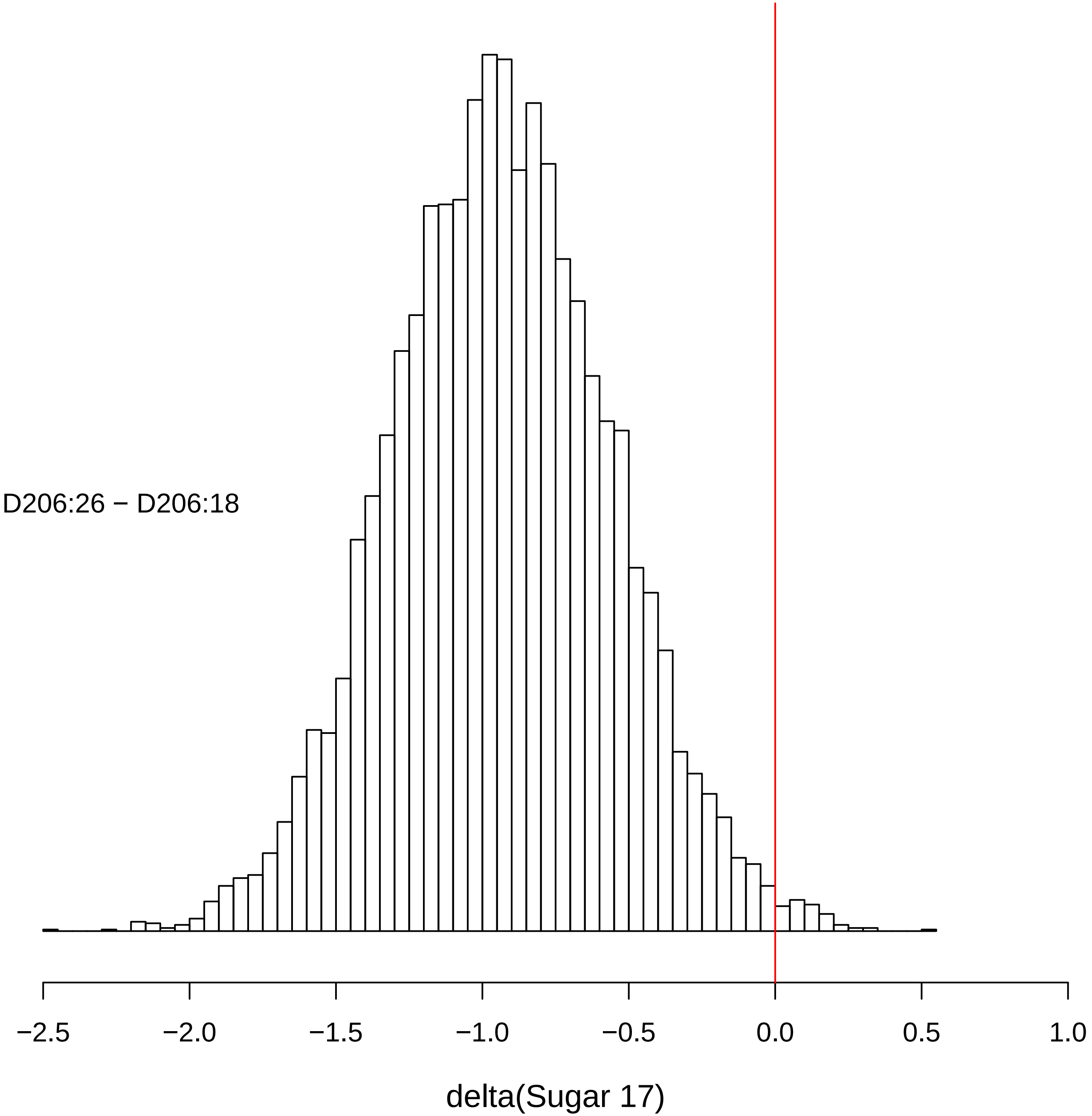
D206:26 − D206:18
−2.5
−2.0
−1.5
−1.0
−0.5
0.0
0.5
1.0
delta(Sugar 17)

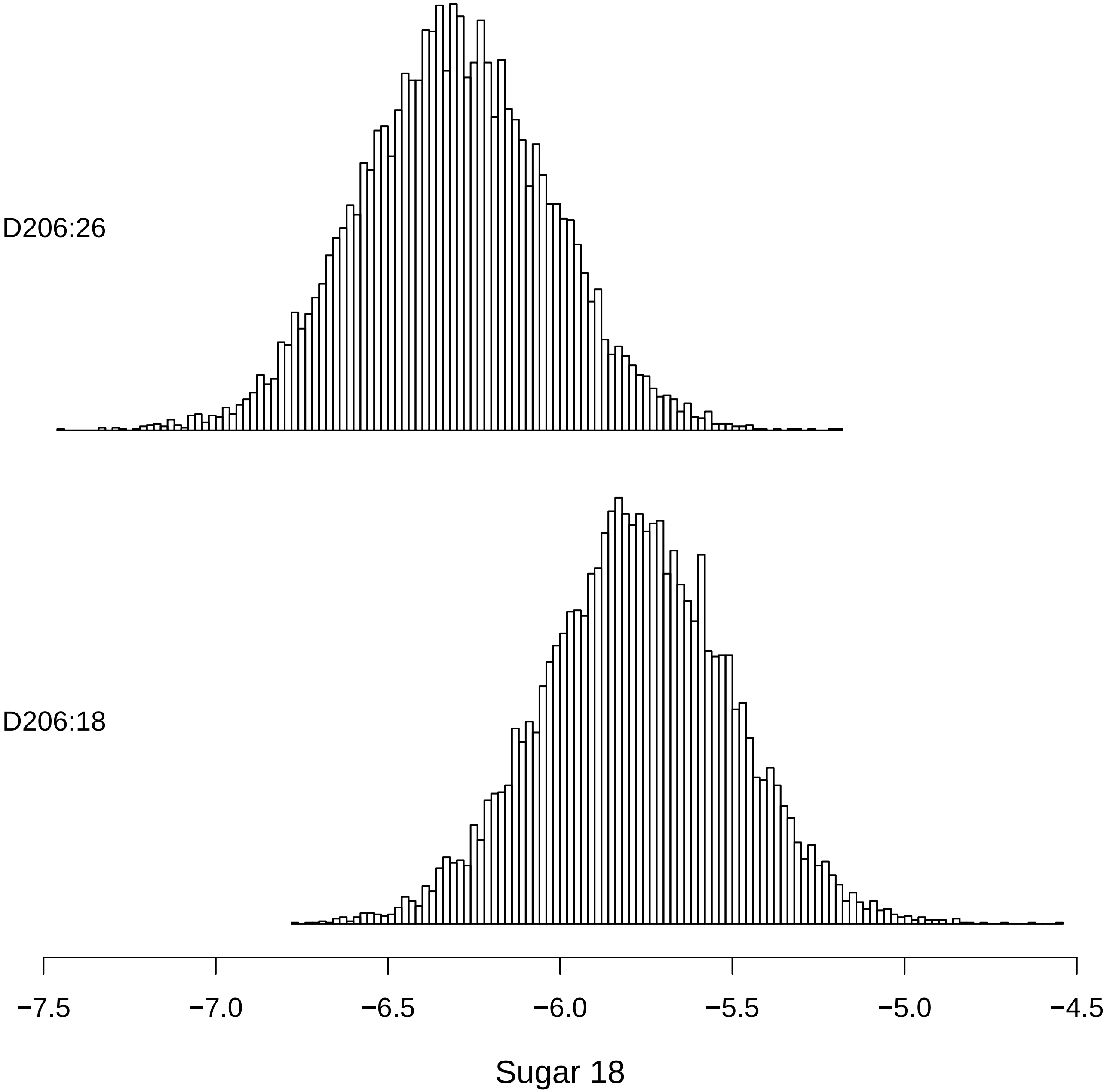
D206:26
D206:18
−7.5
−7.0
−6.5
−6.0
−5.5
−5.0
−4.5
Sugar 18

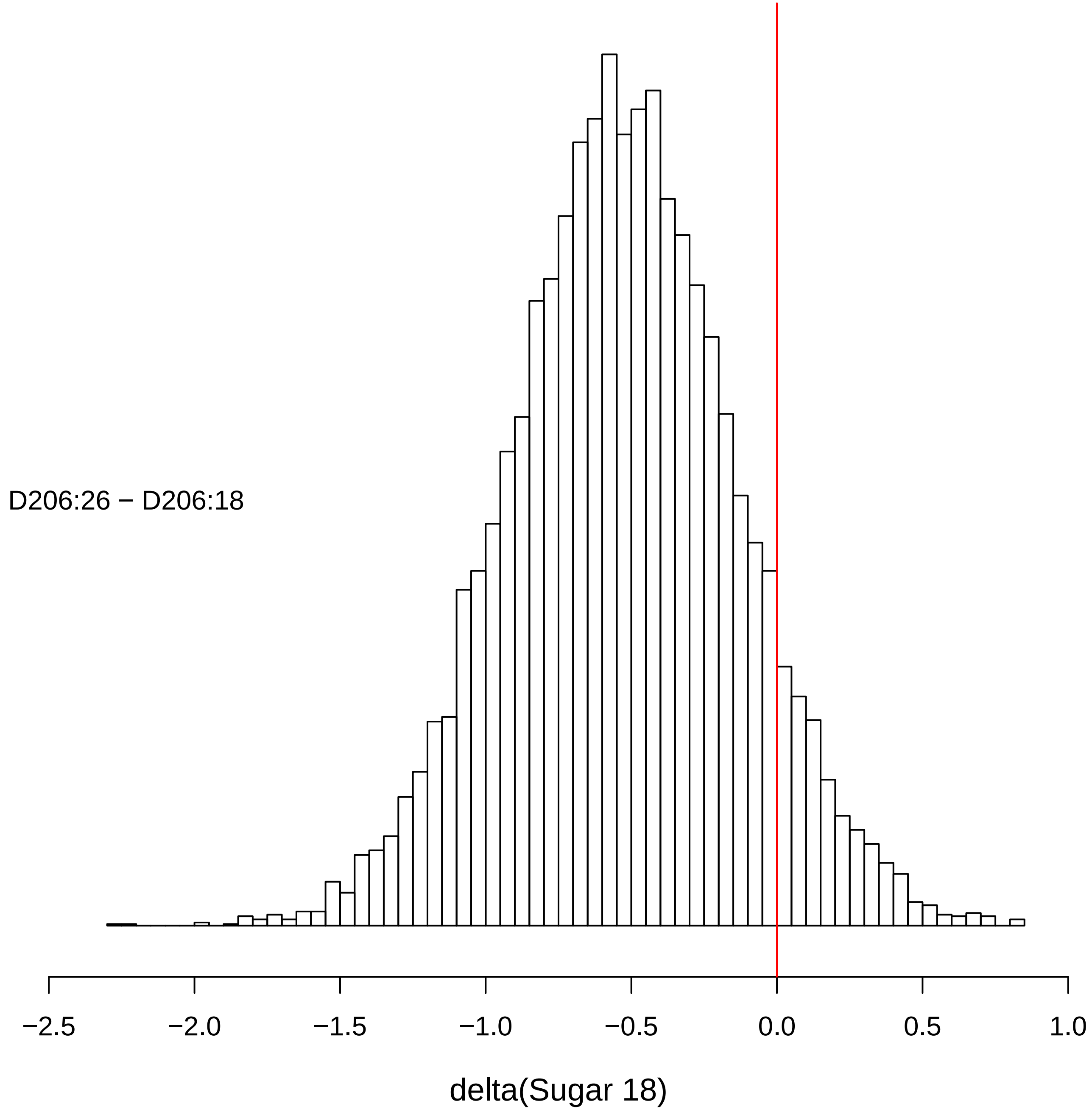
D206:26 − D206:18
−2.5
−2.0
−1.5
−1.0
−0.5
0.0
0.5
1.0
delta(Sugar 18)

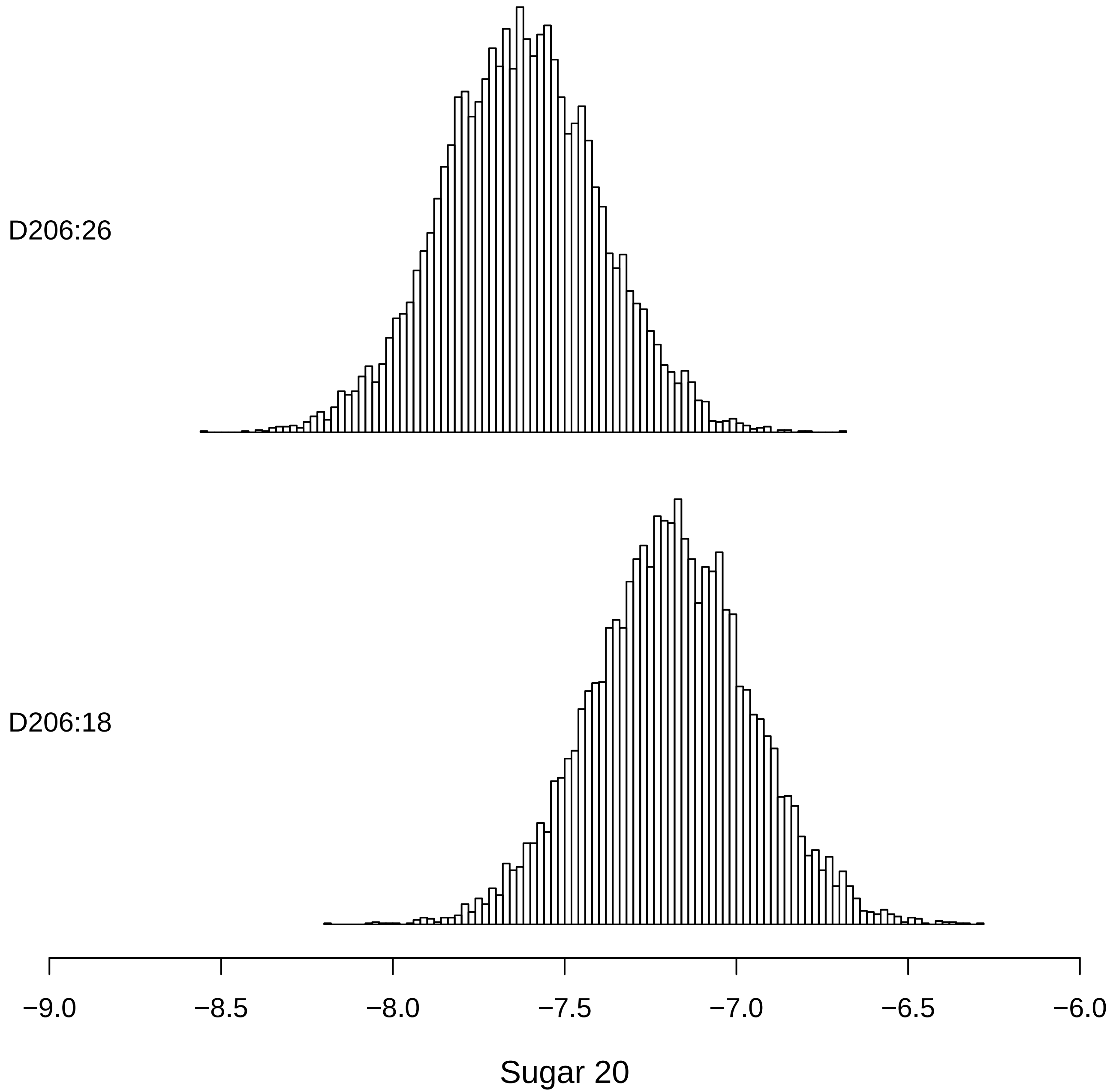

D206:26
D206:18
−9.0
−8.5
−8.0
−7.5
−7.0
−6.5
−6.0
Sugar 20

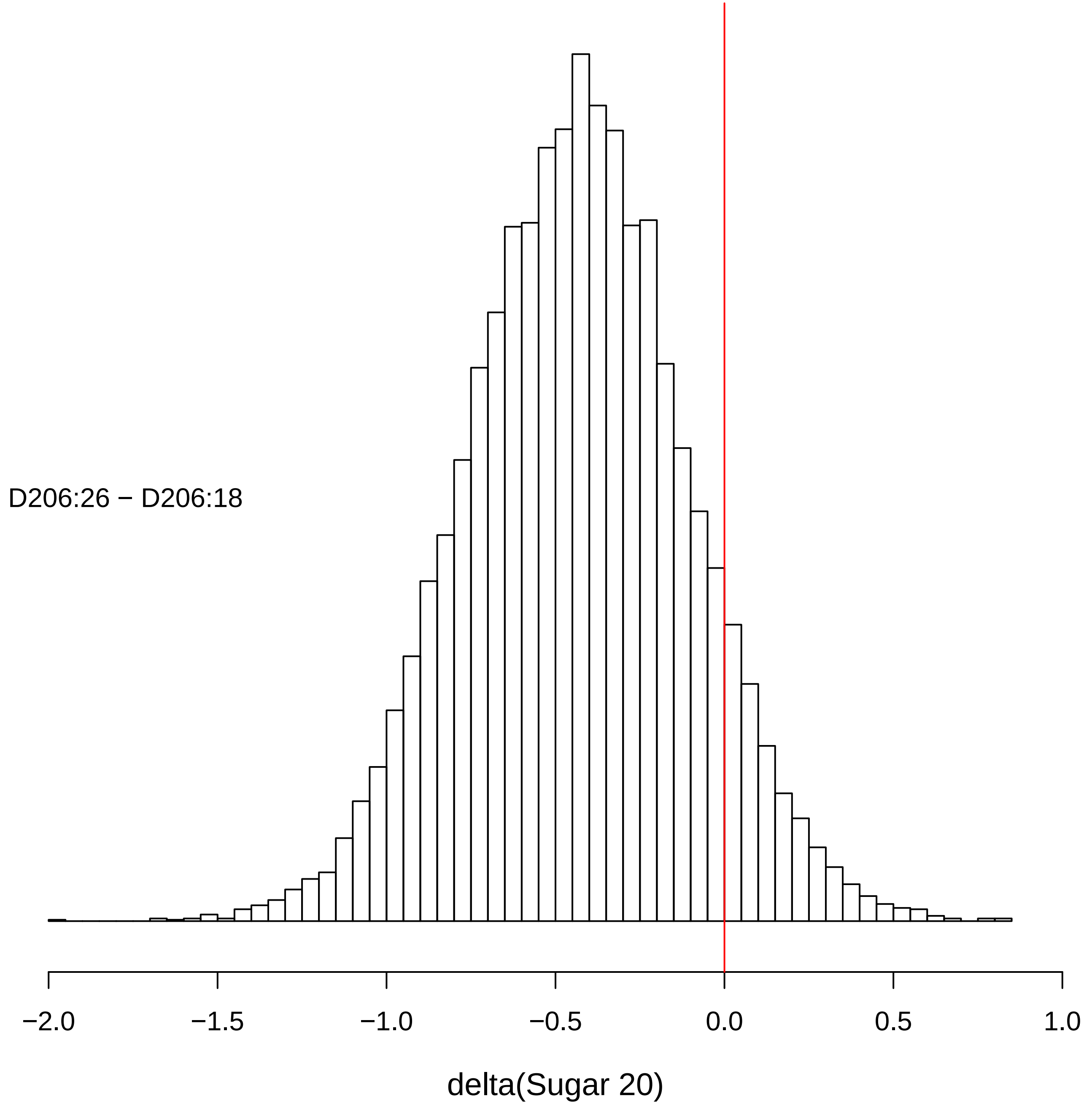
D206:26 − D206:18
−2.0
−1.5
−1.0
−0.5
0.0
0.5
1.0
delta(Sugar 20)

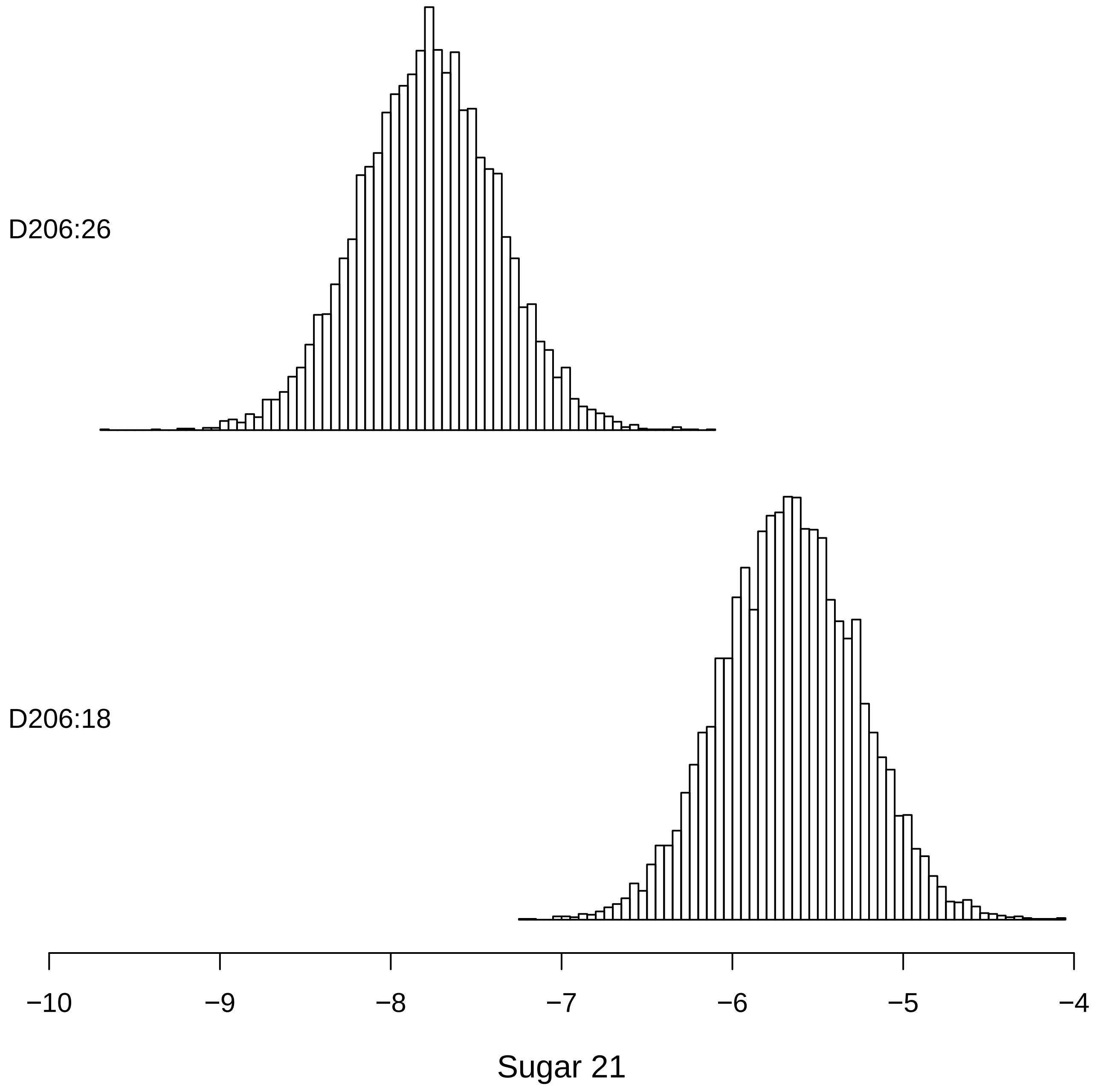
D206:26
D206:18
−10
−9
−8
−7
−6
−5
−4
Sugar 21

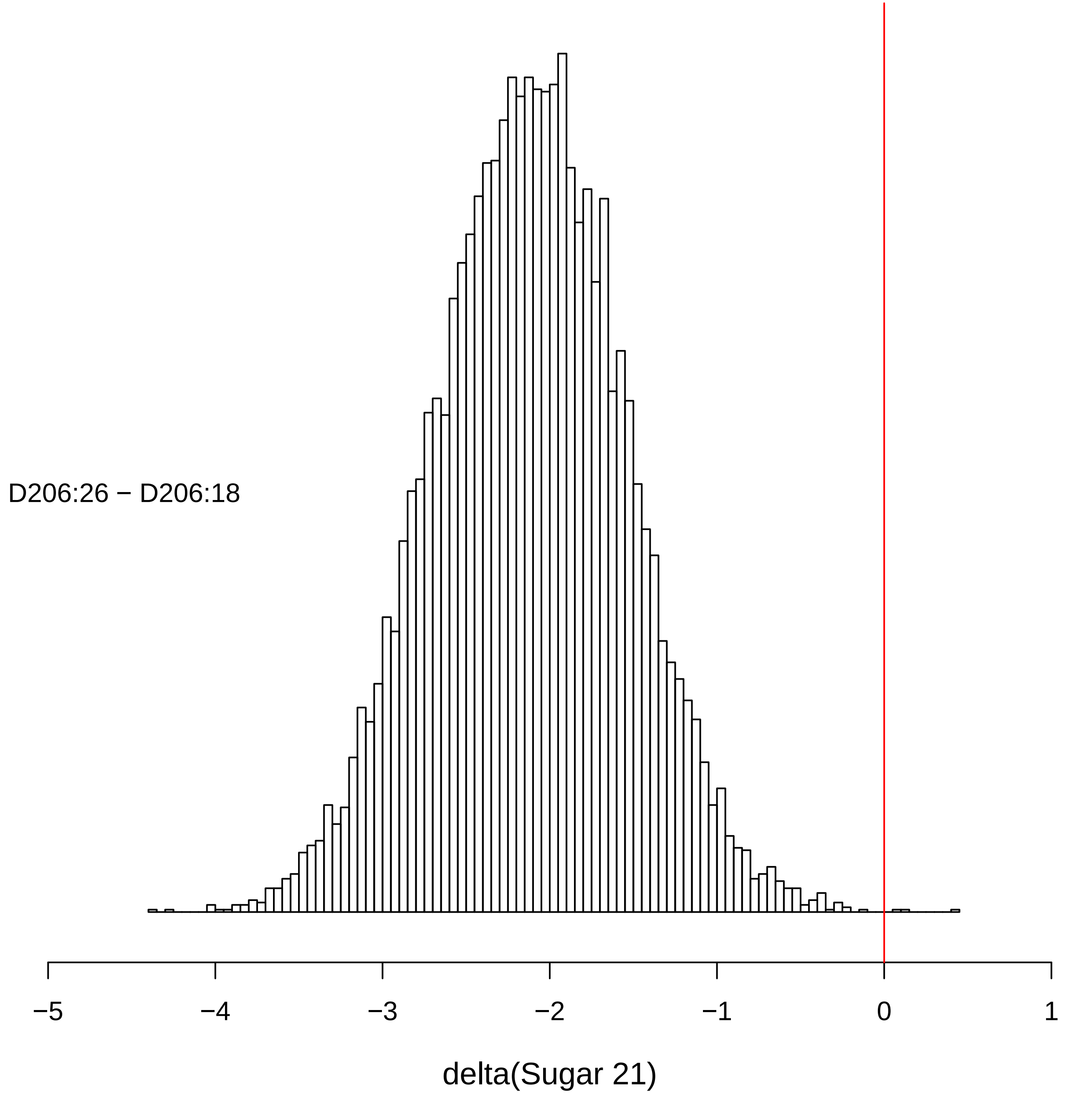
D206:26 − D206:18
−5
−4
−3
−2
−1
0
1
delta(Sugar 21)

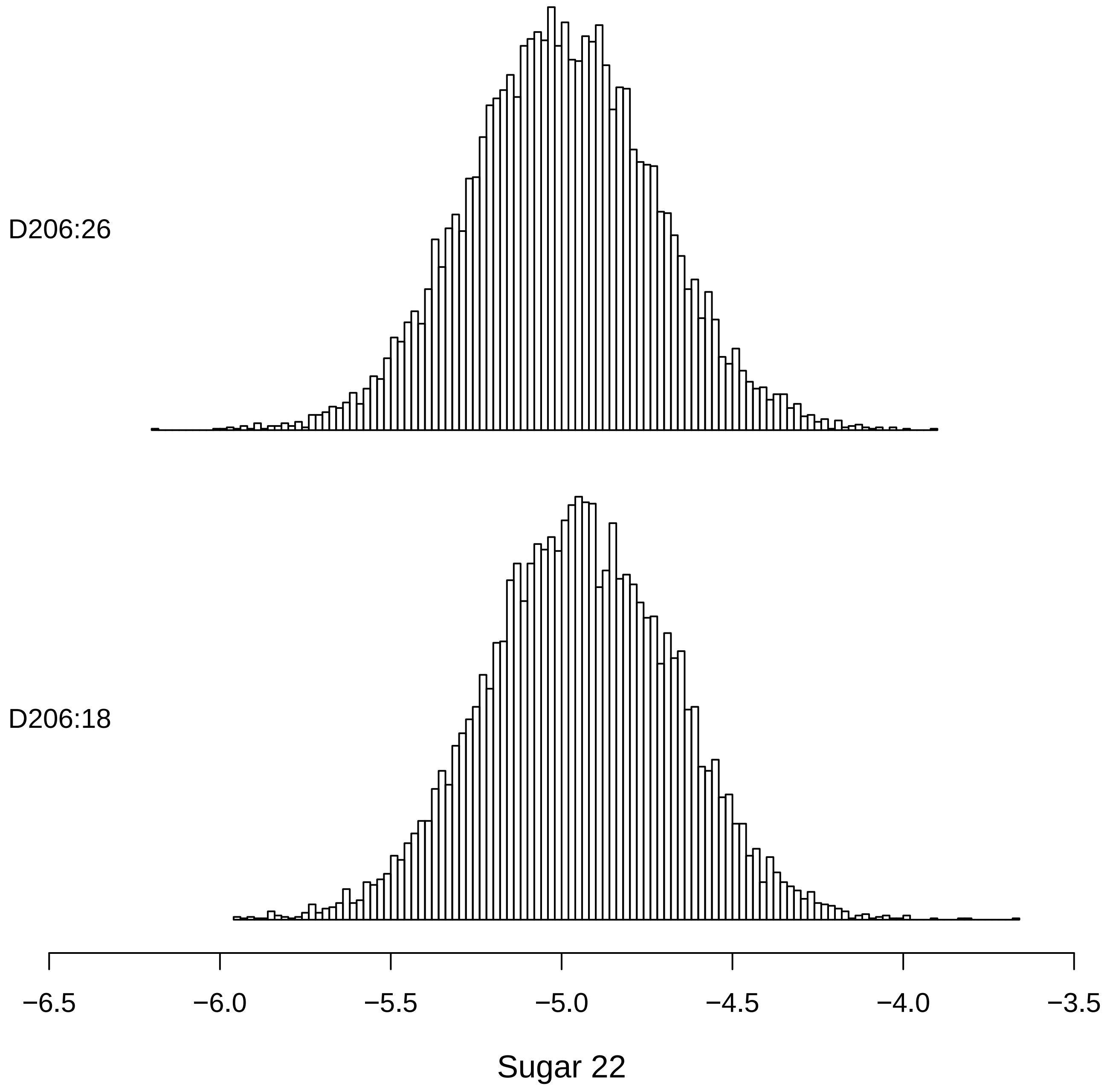
D206:26
D206:18
−6.5
−6.0
−5.5
−5.0
−4.5
−4.0
−3.5
Sugar 22

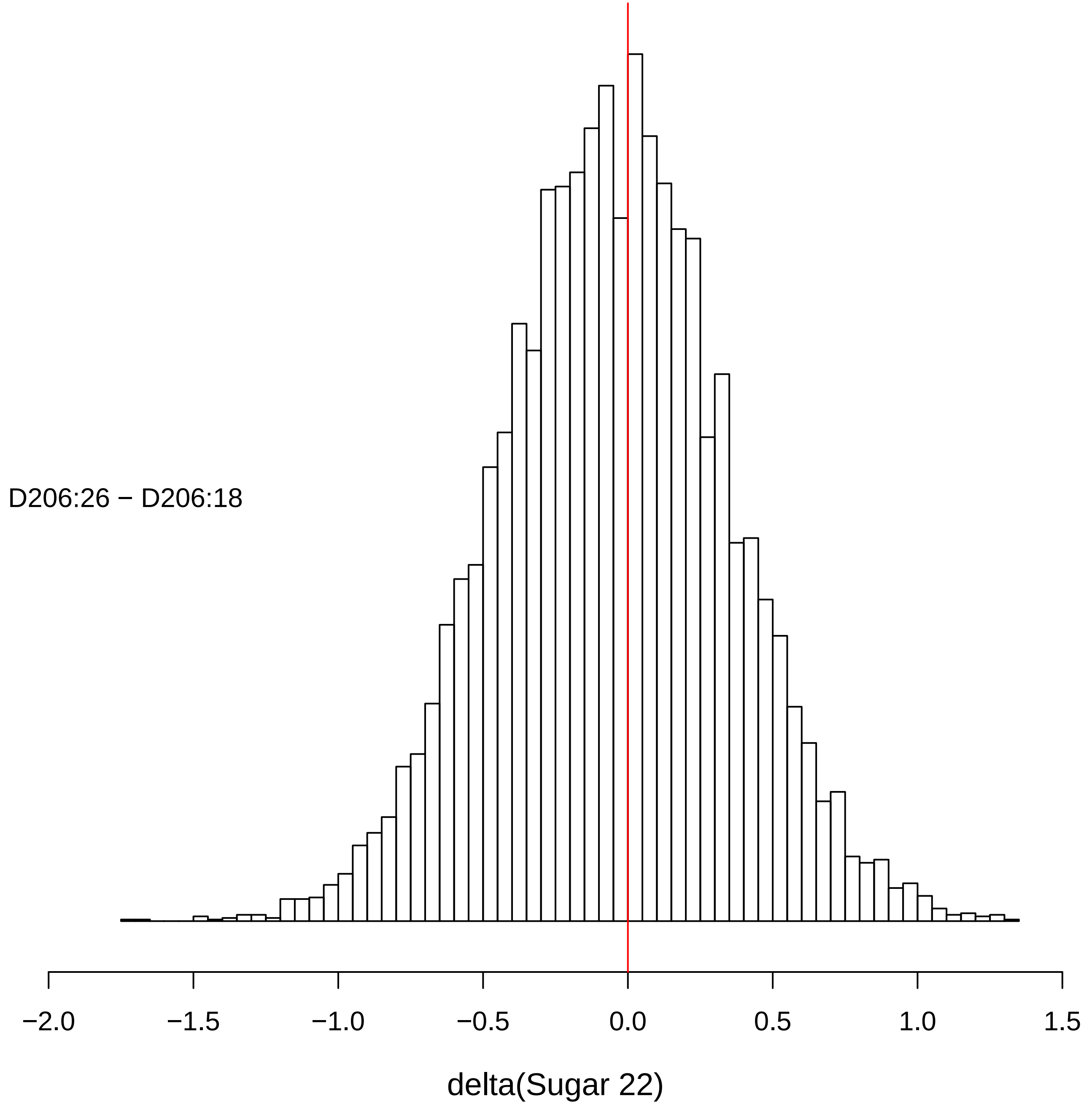

D206:26 − D206:18
−2.0
−1.5
−1.0
−0.5
0.0
0.5
1.0
1.5
delta(Sugar 22)

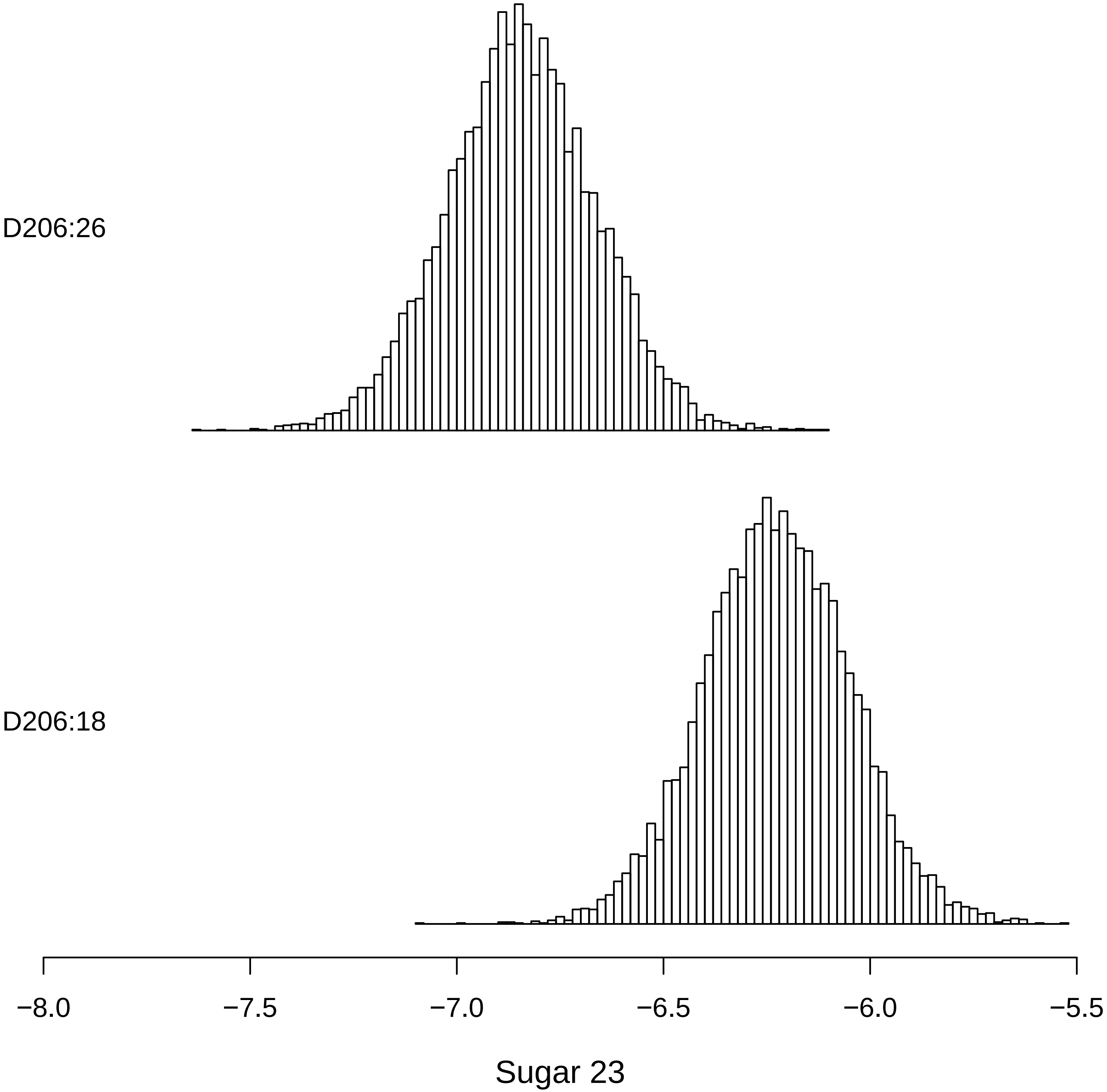
D206:26
D206:18
−8.0
−7.5
−7.0
−6.5
−6.0
−5.5
Sugar 23

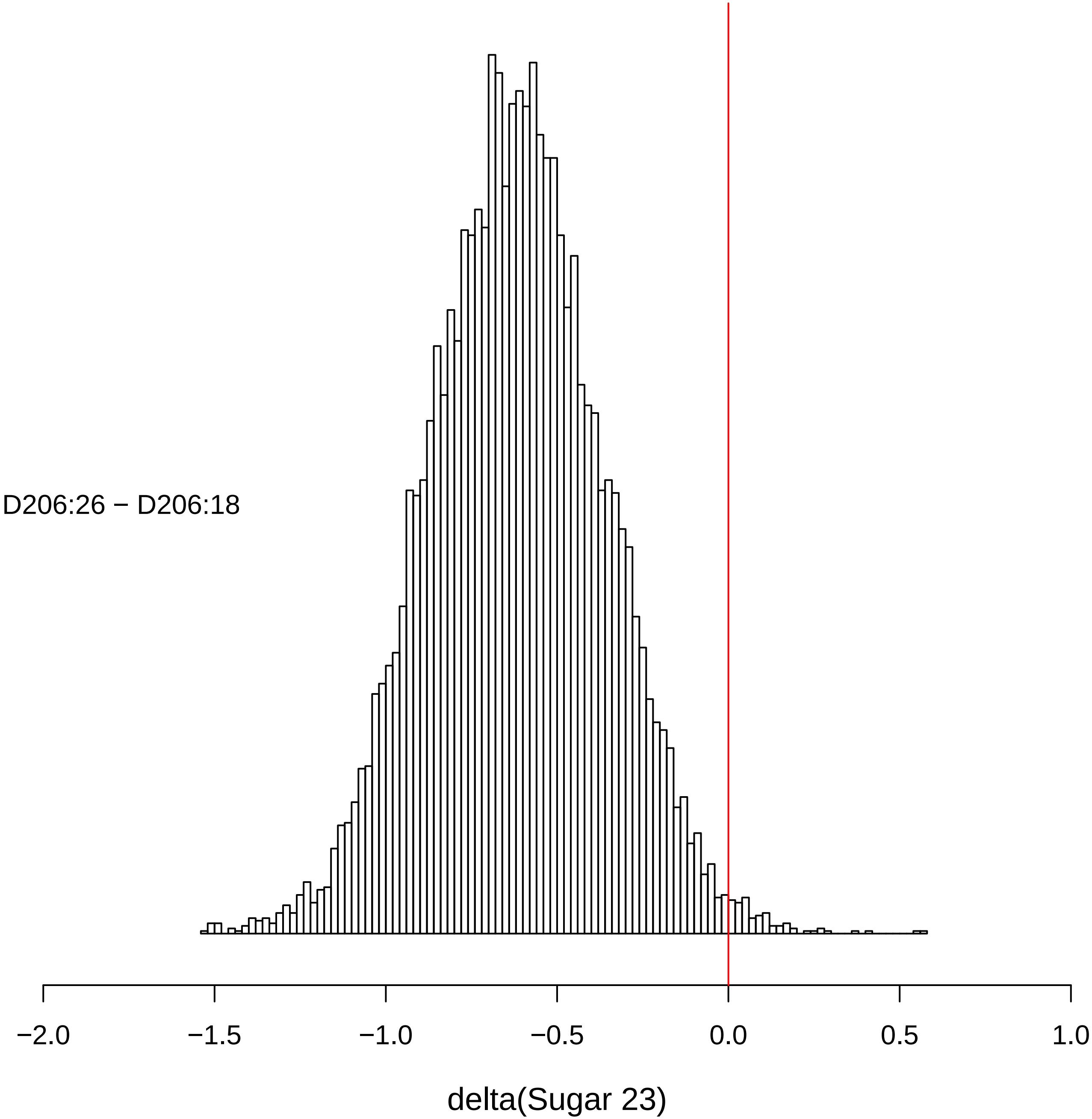
D206:26 − D206:18
−2.0
−1.5
−1.0
−0.5
0.0
0.5
1.0
delta(Sugar 23)

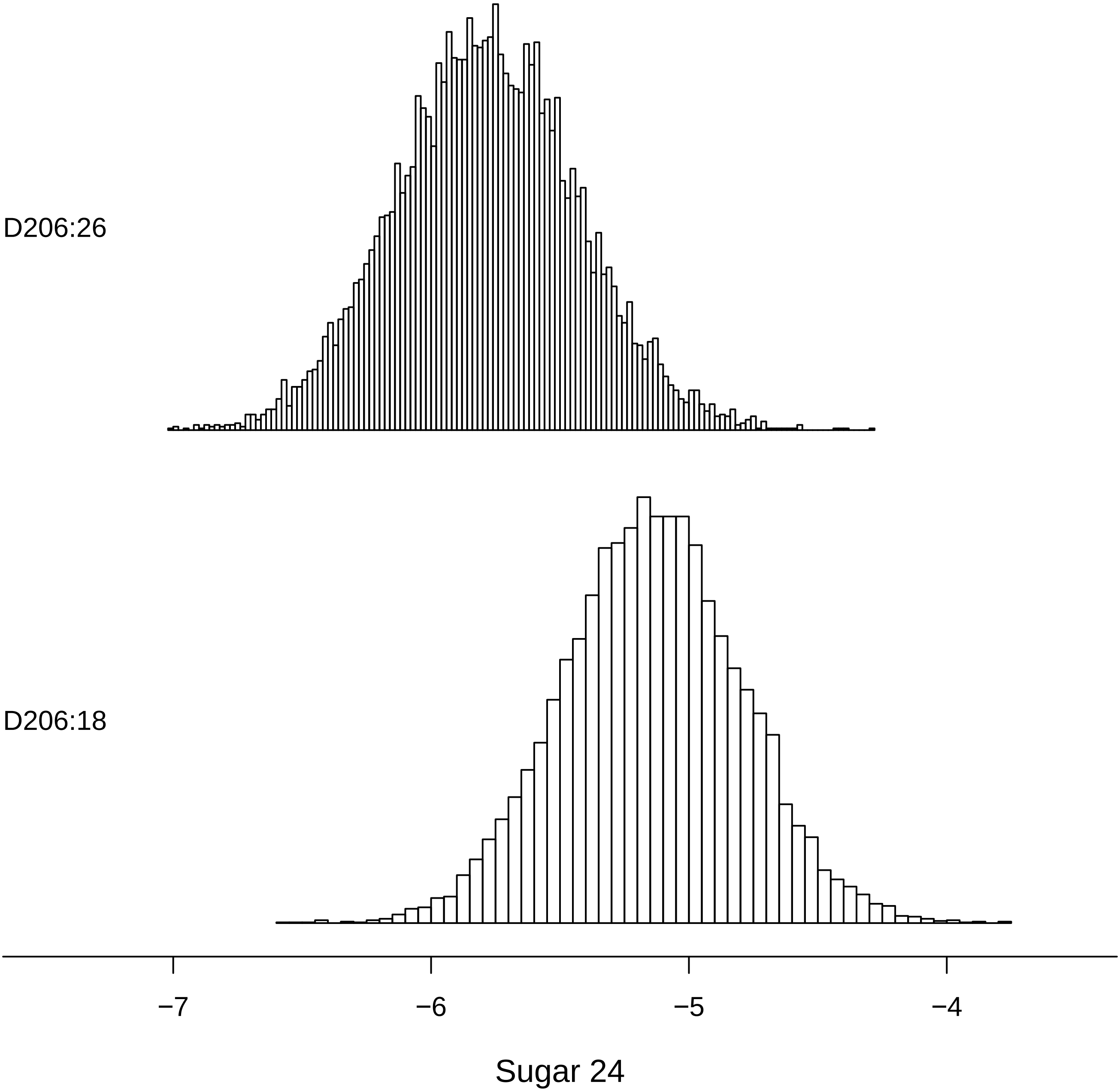
D206:26
D206:18
−7
−6
−5
−4
Sugar 24

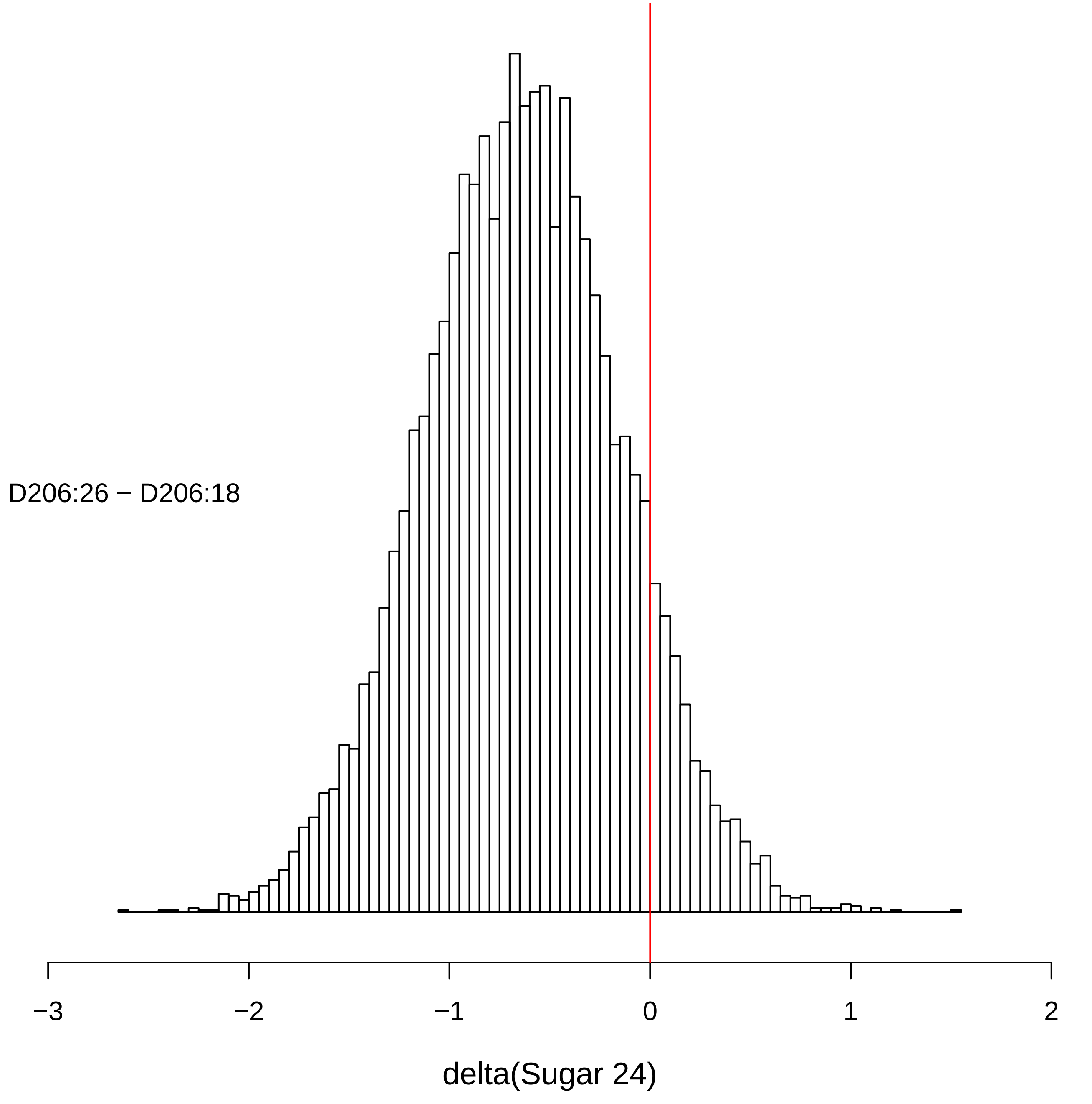

D206:26 − D206:18
−3
−2
−1
0
1
2
delta(Sugar 24)

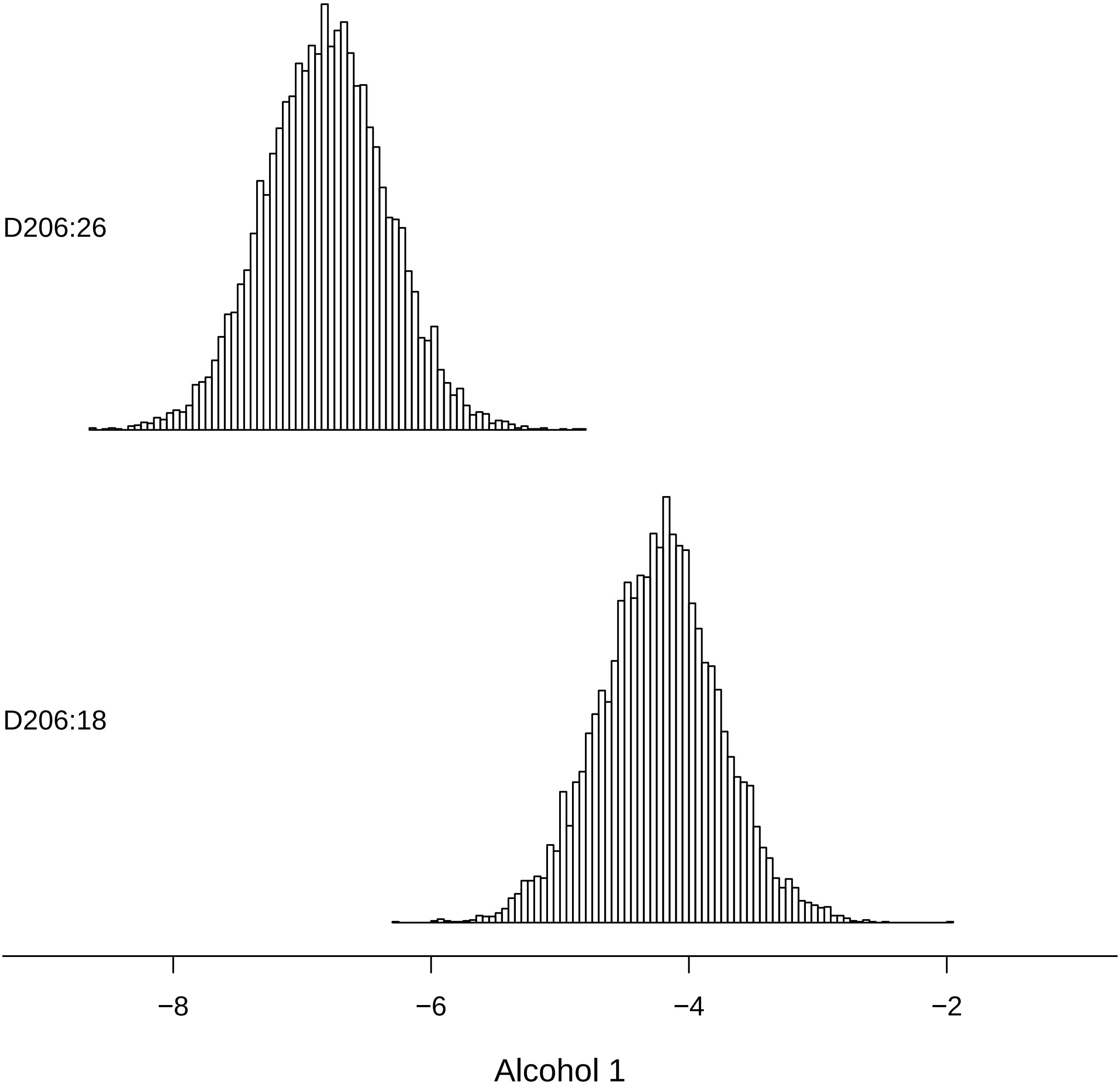
D206:26
D206:18
−8
−6
−4
−2
Alcohol 1

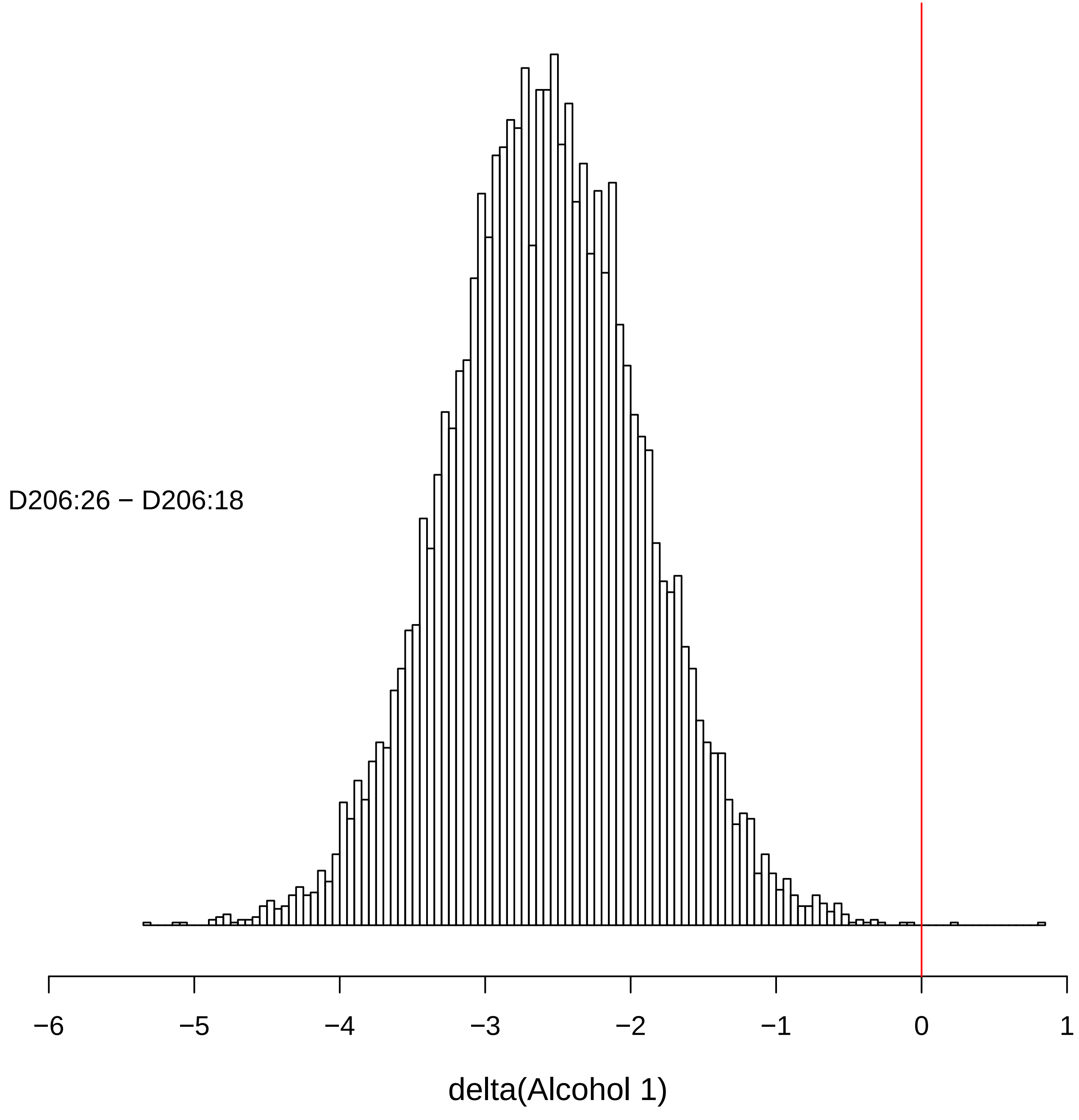
D206:26 − D206:18
−6
−5
−4
−3
−2
−1
0
1
delta(Alcohol 1)

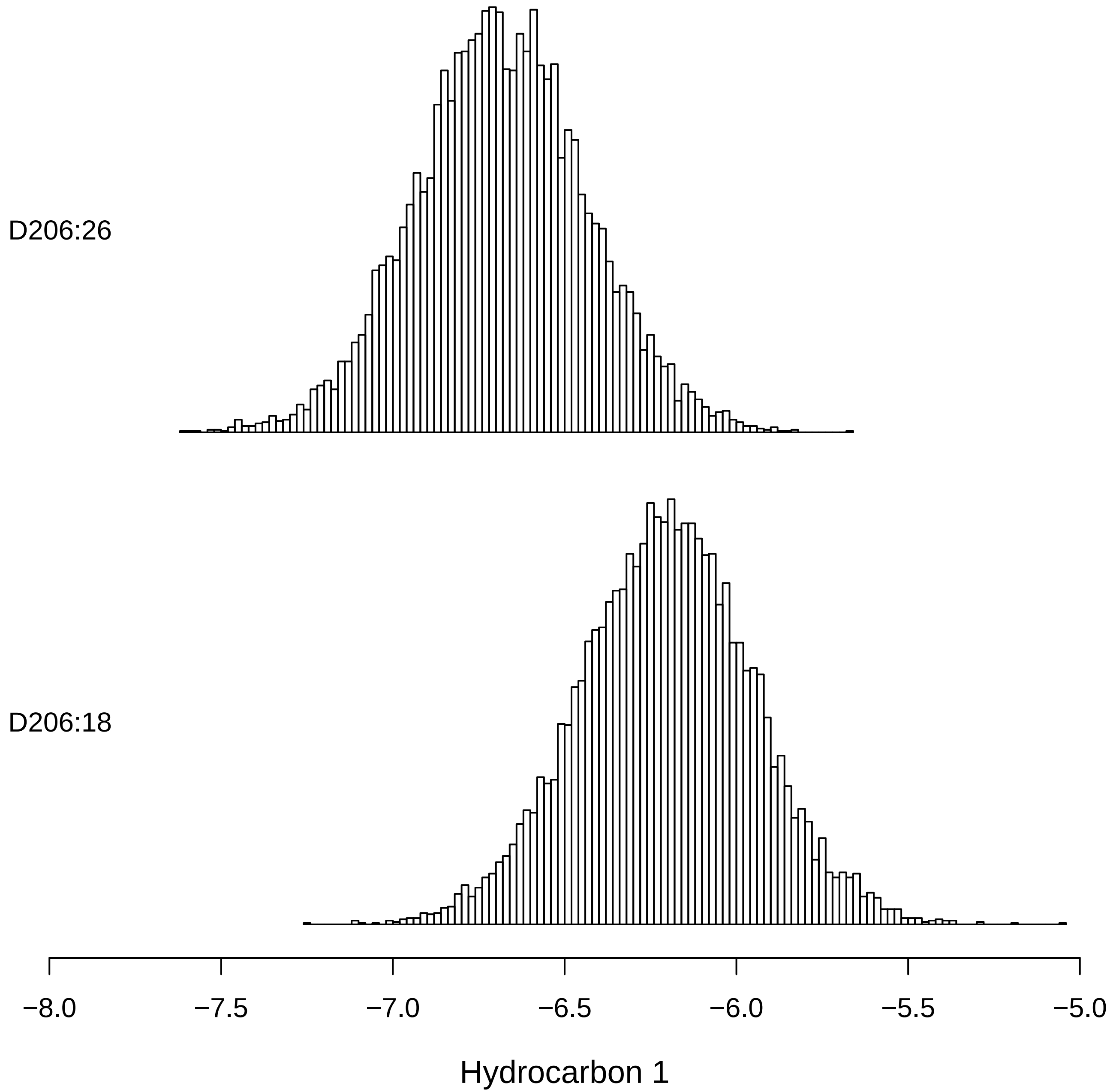
D206:26
D206:18
−8.0
−7.5
−7.0
−6.5
−6.0
−5.5
−5.0
Hydrocarbon 1

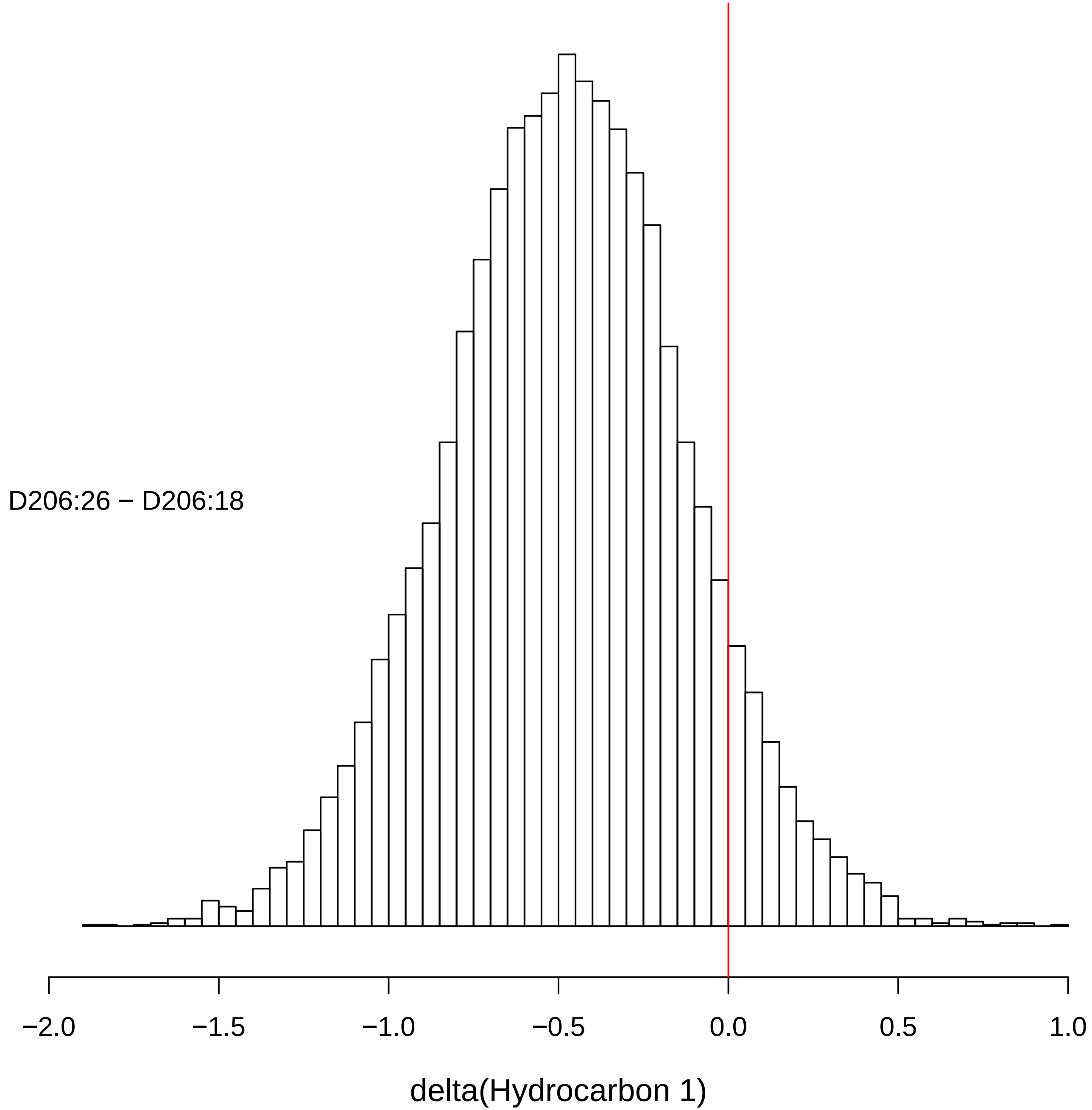
D206:26 − D206:18
−2.0
−1.5
−1.0
−0.5
0.0
0.5
1.0
delta(Hydrocarbon 1)

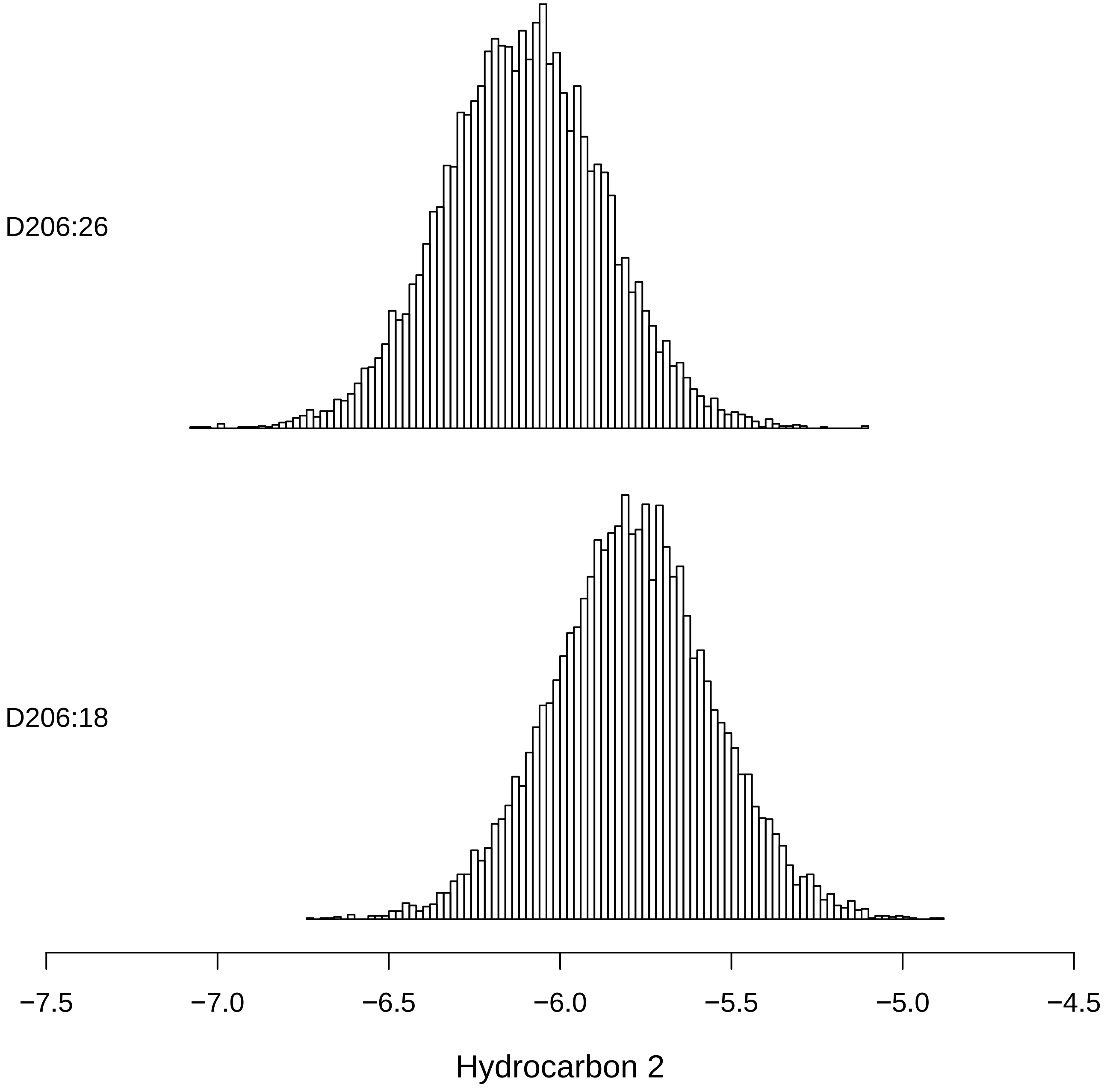
D206:26
D206:18
−7.5
−7.0
−6.5
−6.0
−5.5
−5.0
−4.5
Hydrocarbon 2

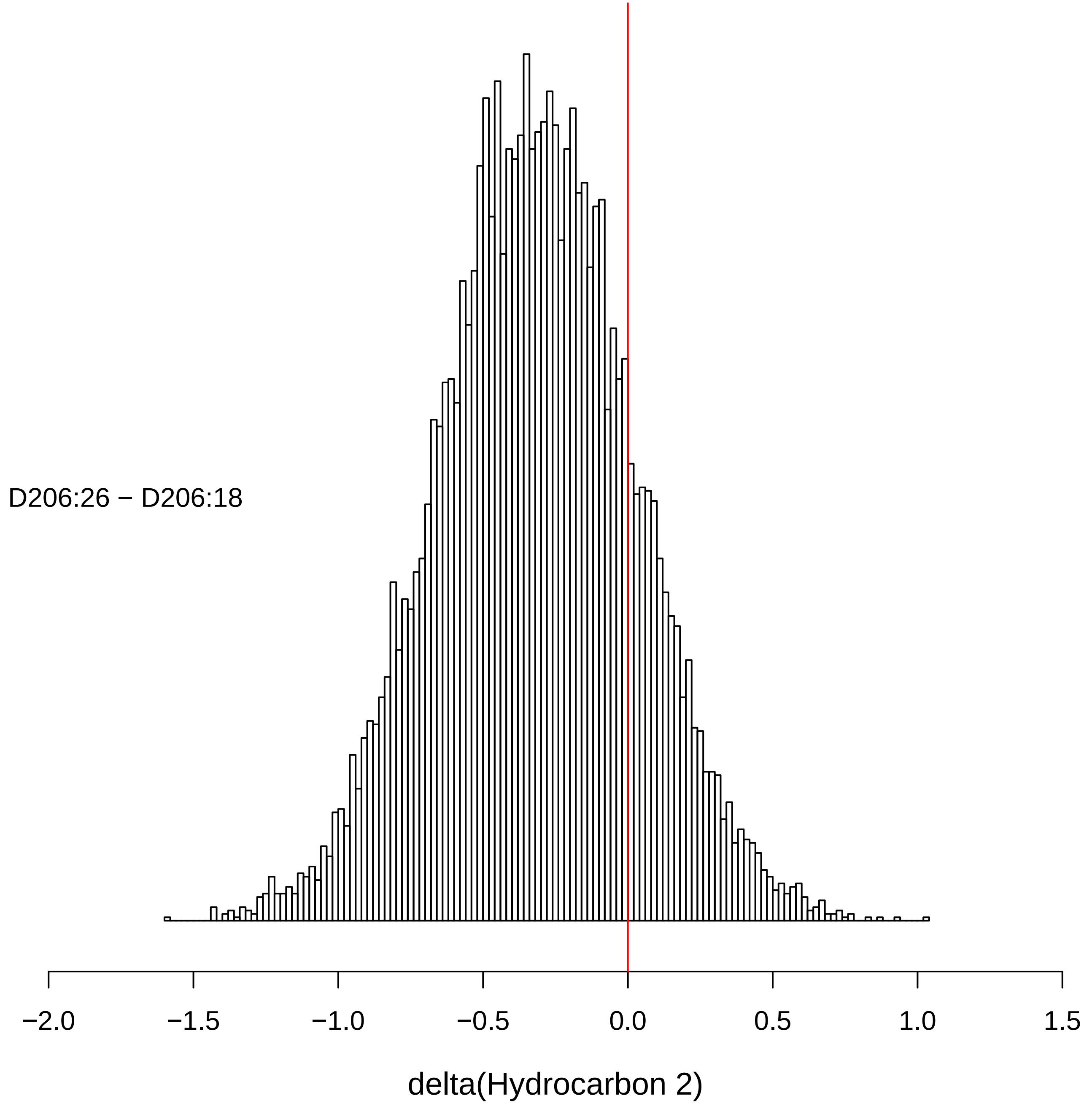
D206:26 − D206:18
−2.0
−1.5
−1.0
−0.5
0.0
0.5
1.0
1.5
delta(Hydrocarbon 2)

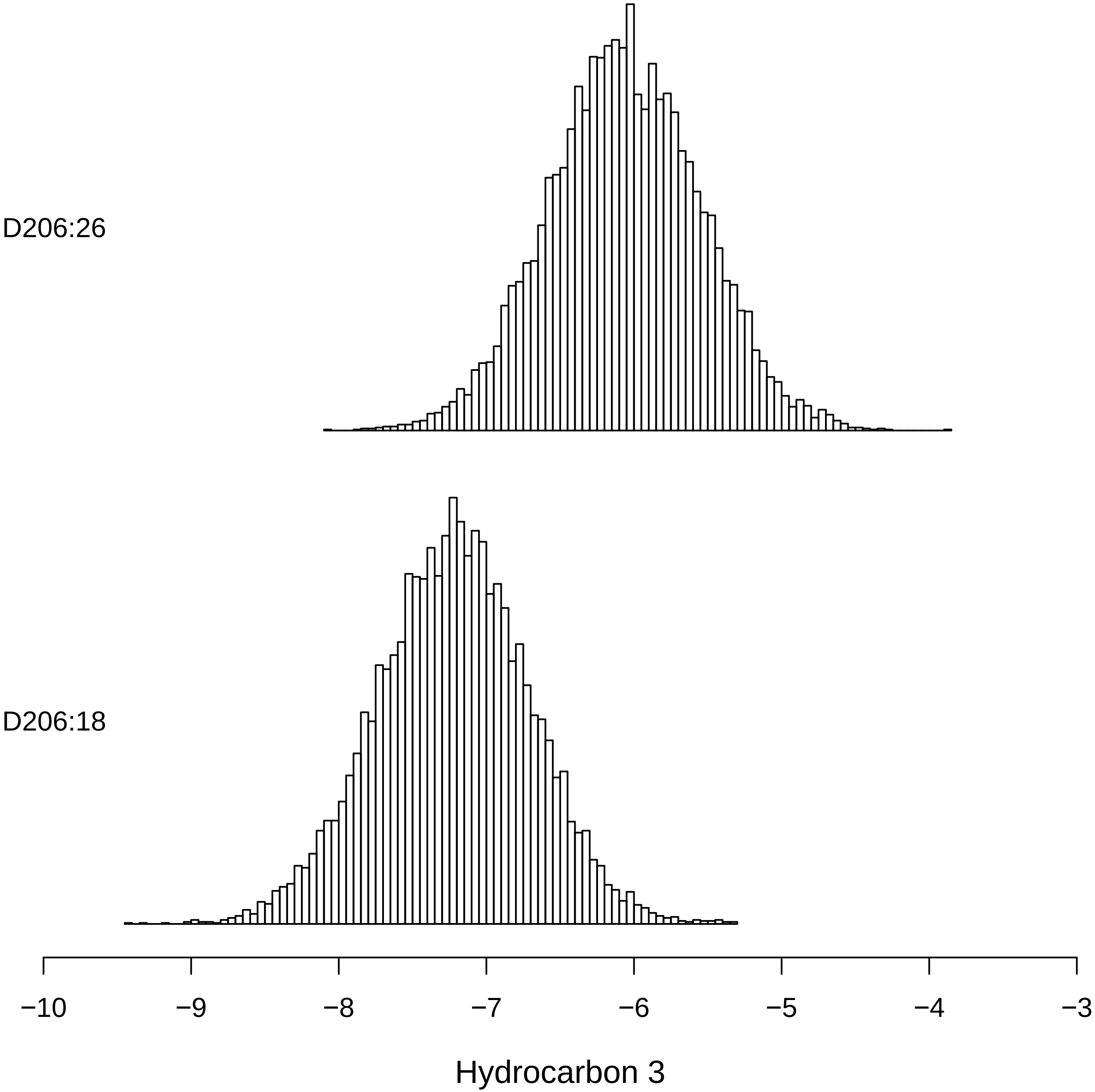
D206:26
D206:18
−10
−9
−8
−7
−6
−5
−4
−3
Hydrocarbon 3

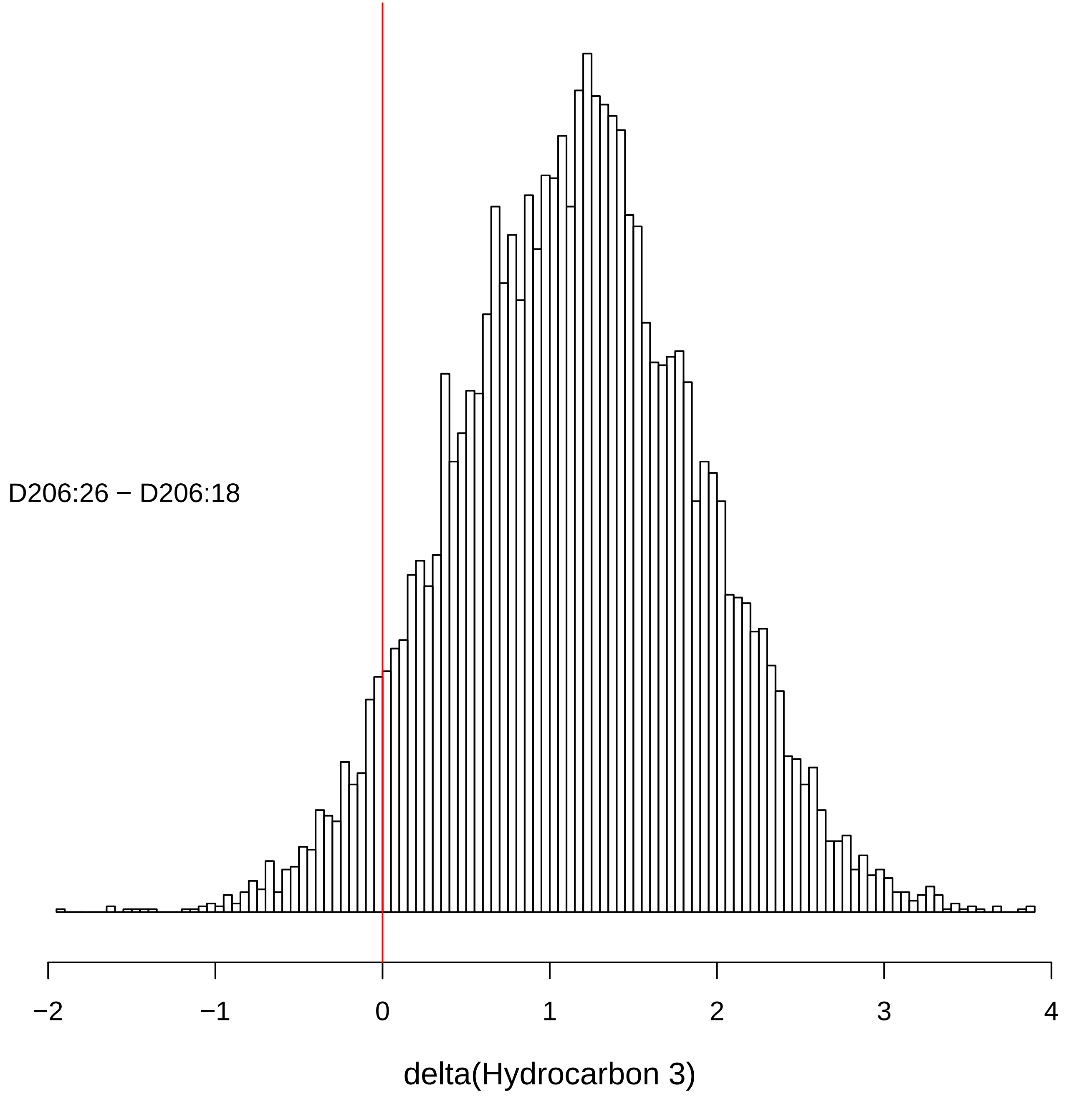
D206:26 − D206:18
−2
−1
0
1
2
3
4
delta(Hydrocarbon 3)

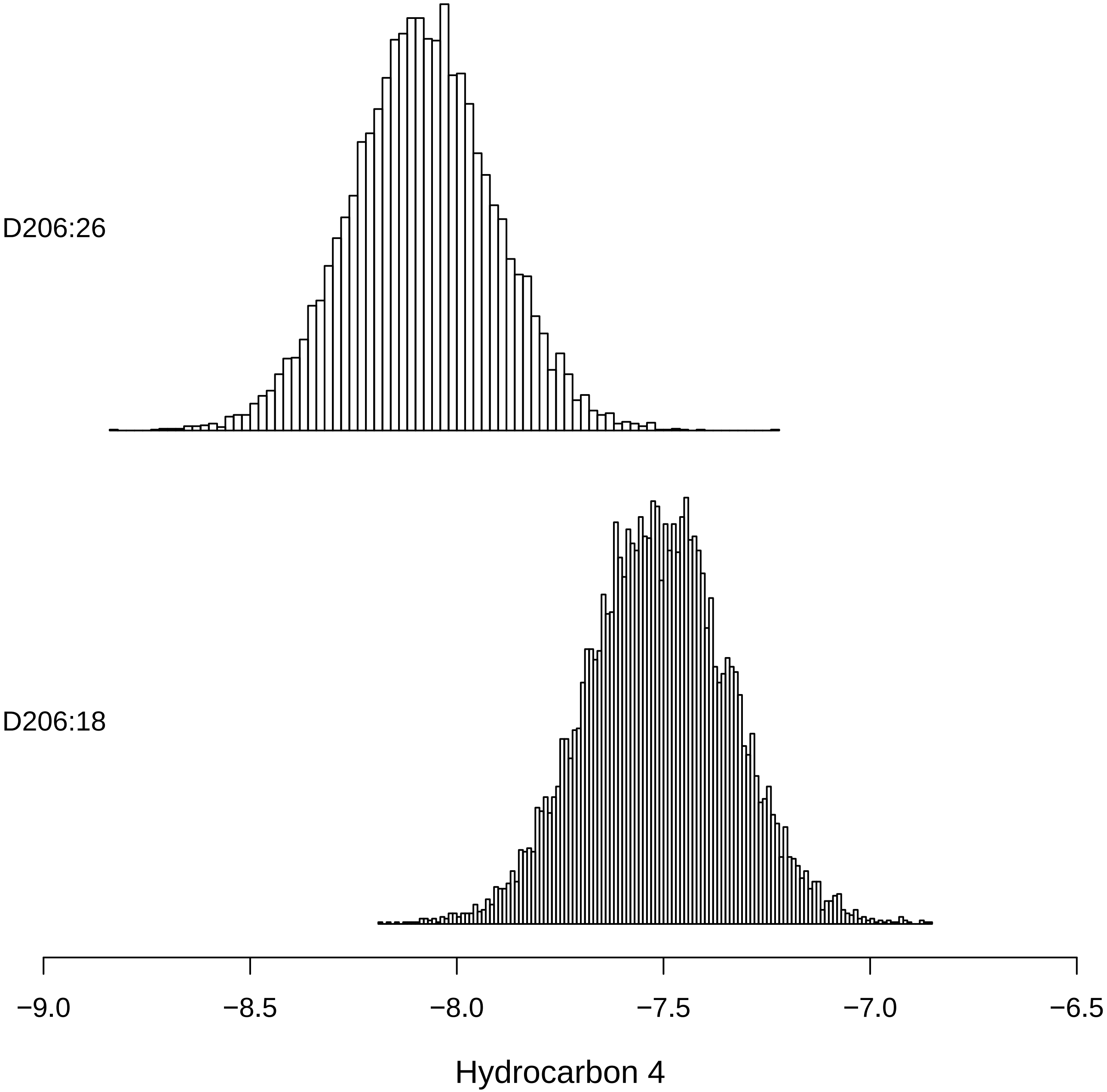
D206:26
D206:18
−9.0
−8.5
−8.0
−7.5
−7.0
−6.5
Hydrocarbon 4

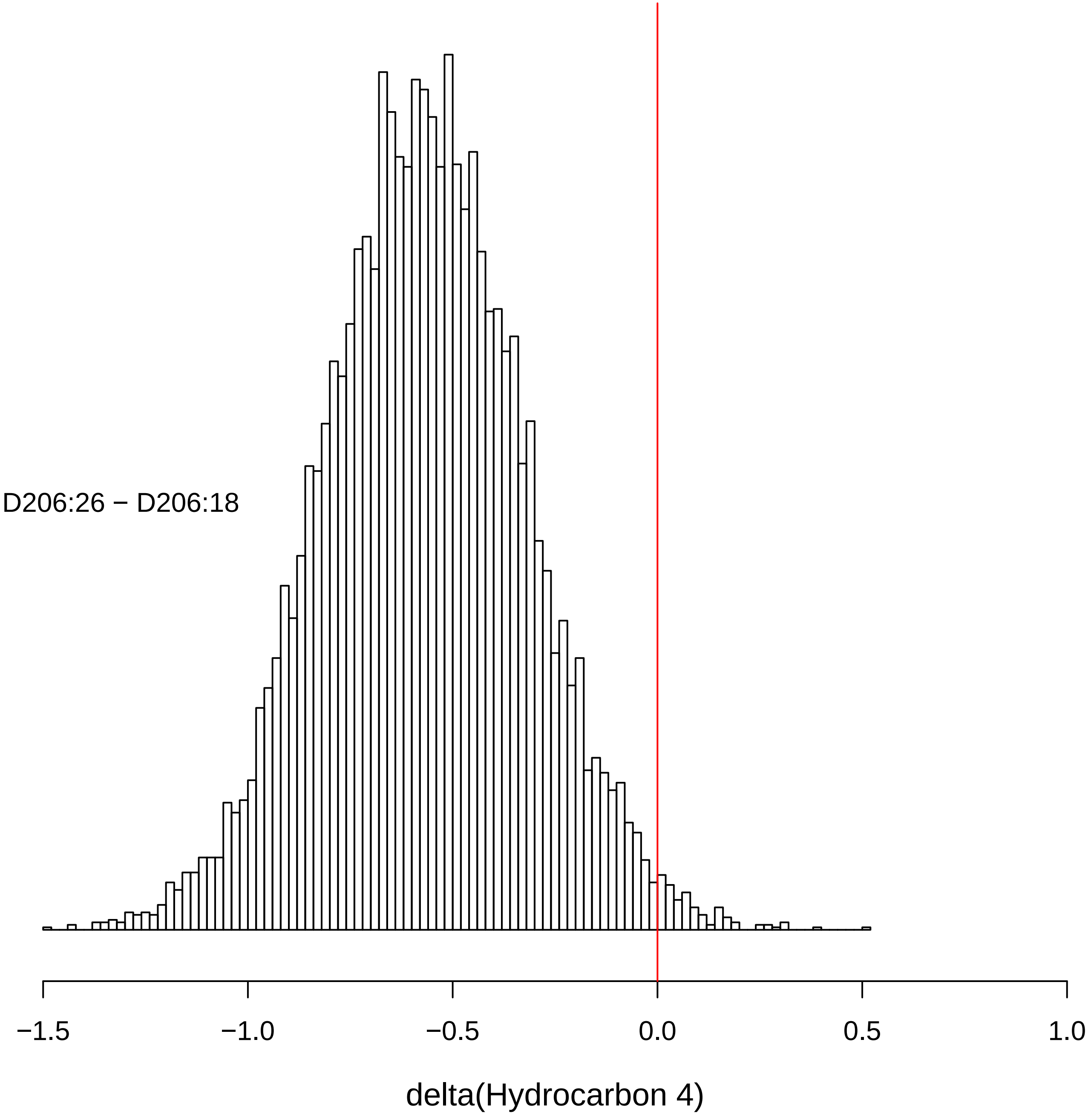

D206:26 − D206:18
−1.5
−1.0
−0.5
0.0
0.5
1.0
delta(Hydrocarbon 4)

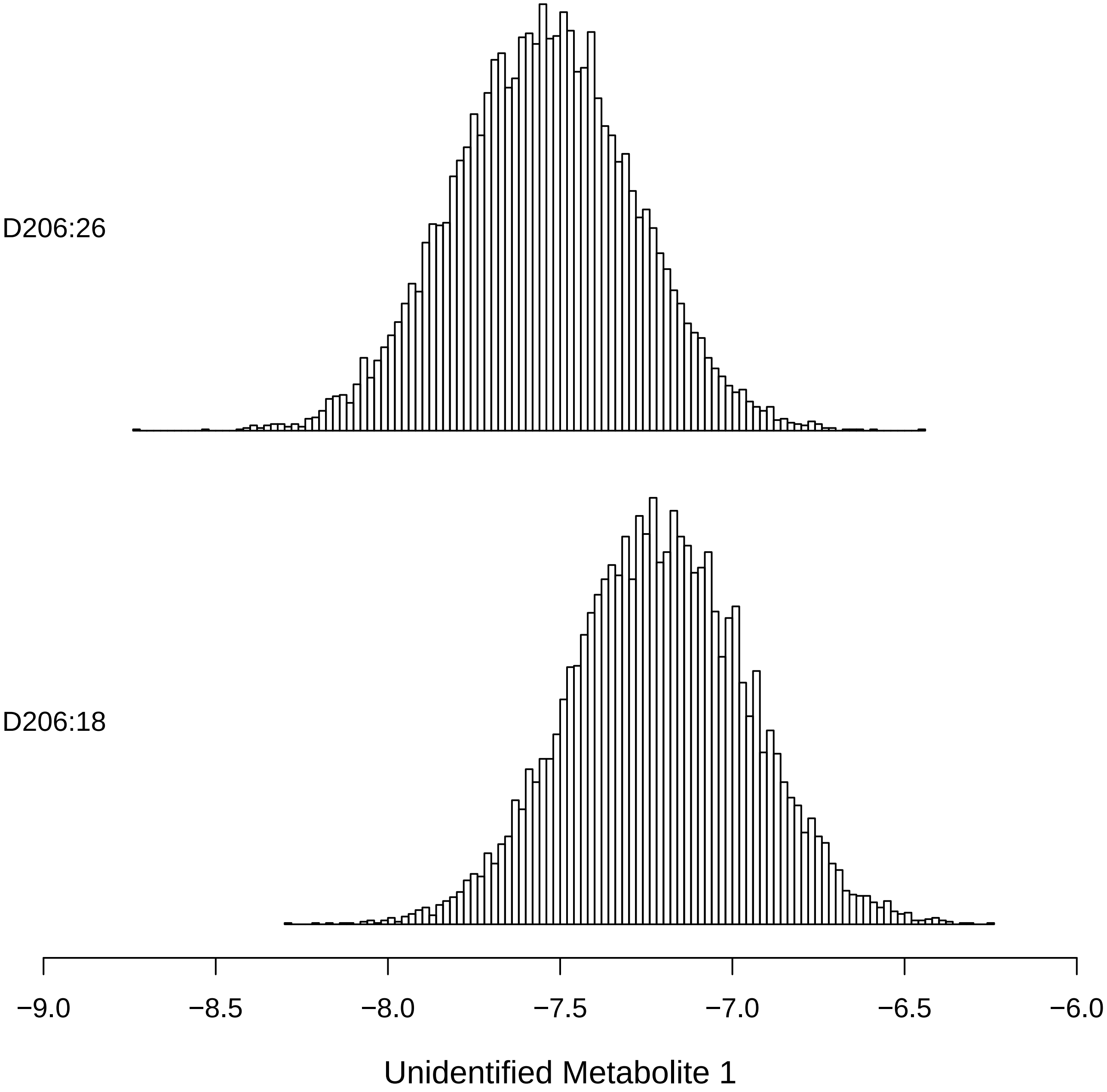
D206:26
D206:18
−9.0
−8.5
−8.0
−7.5
−7.0
−6.5
−6.0
Unidentified Metabolite 1

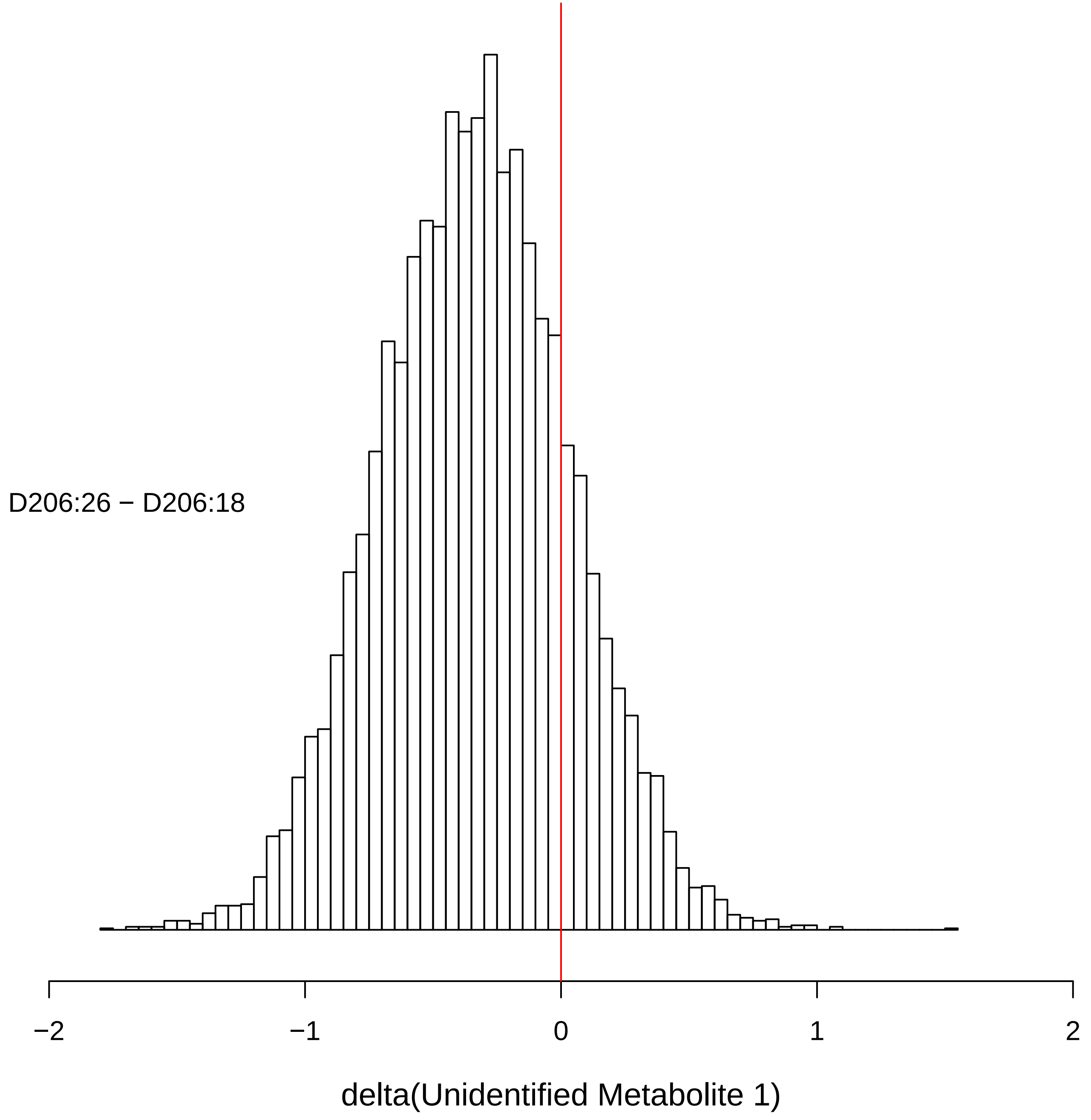
D206:26 − D206:18
−2
−1
0
1
2
delta(Unidentified Metabolite 1)

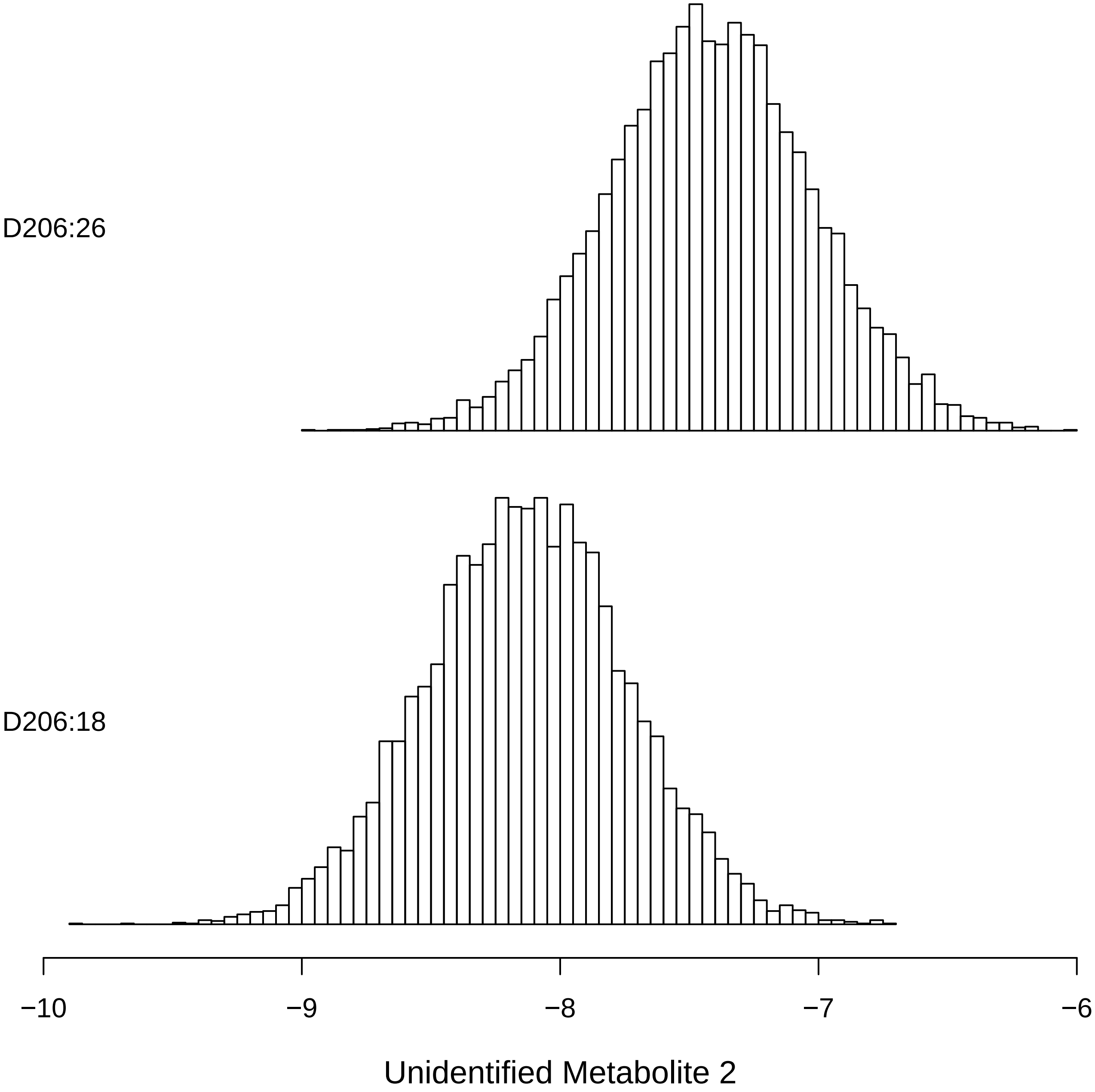
D206:26
D206:18
−10
−9
−8
−7
−6
Unidentified Metabolite 2

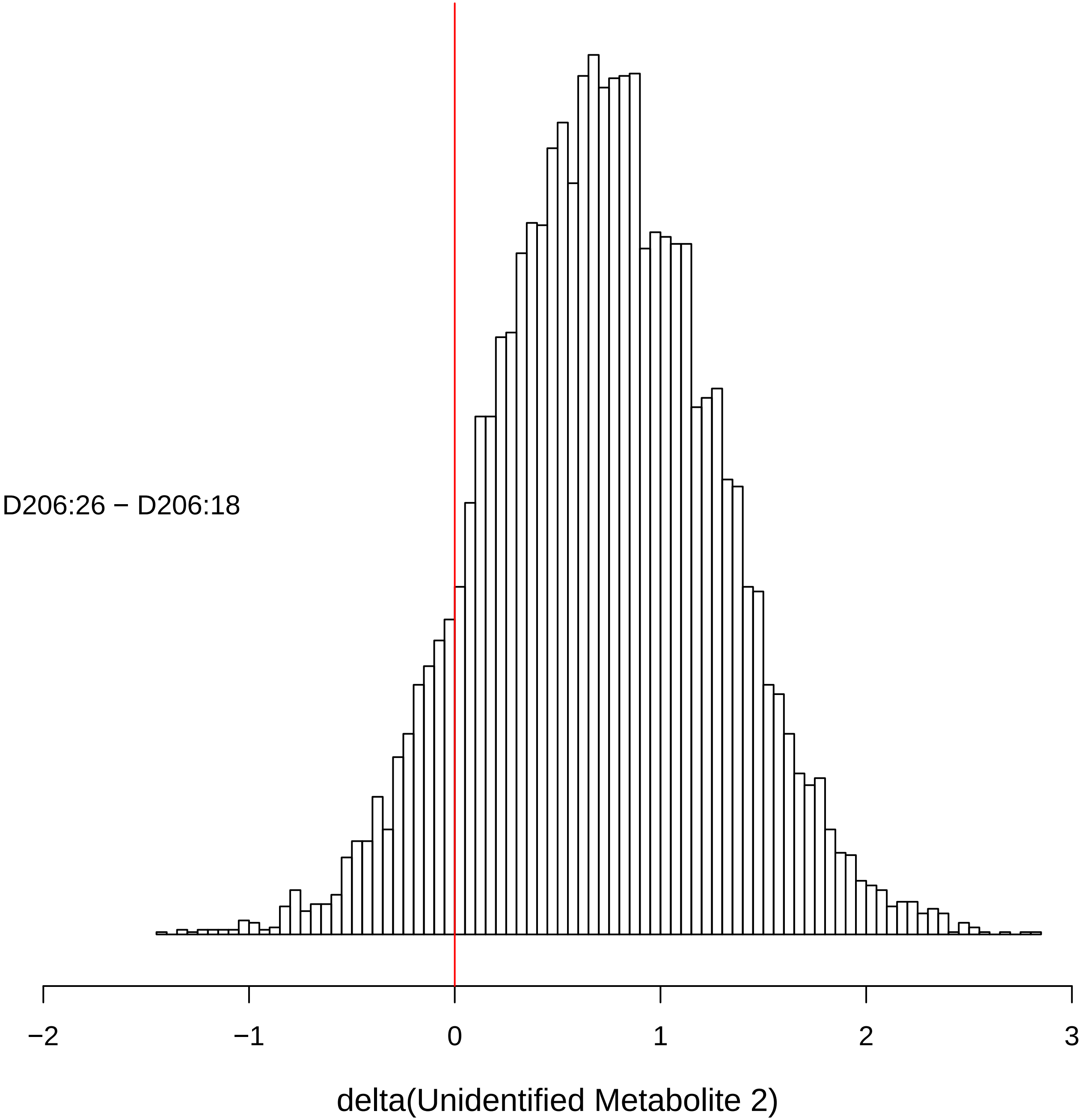
D206:26 − D206:18
−2
−1
0
1
2
3
delta(Unidentified Metabolite 2)

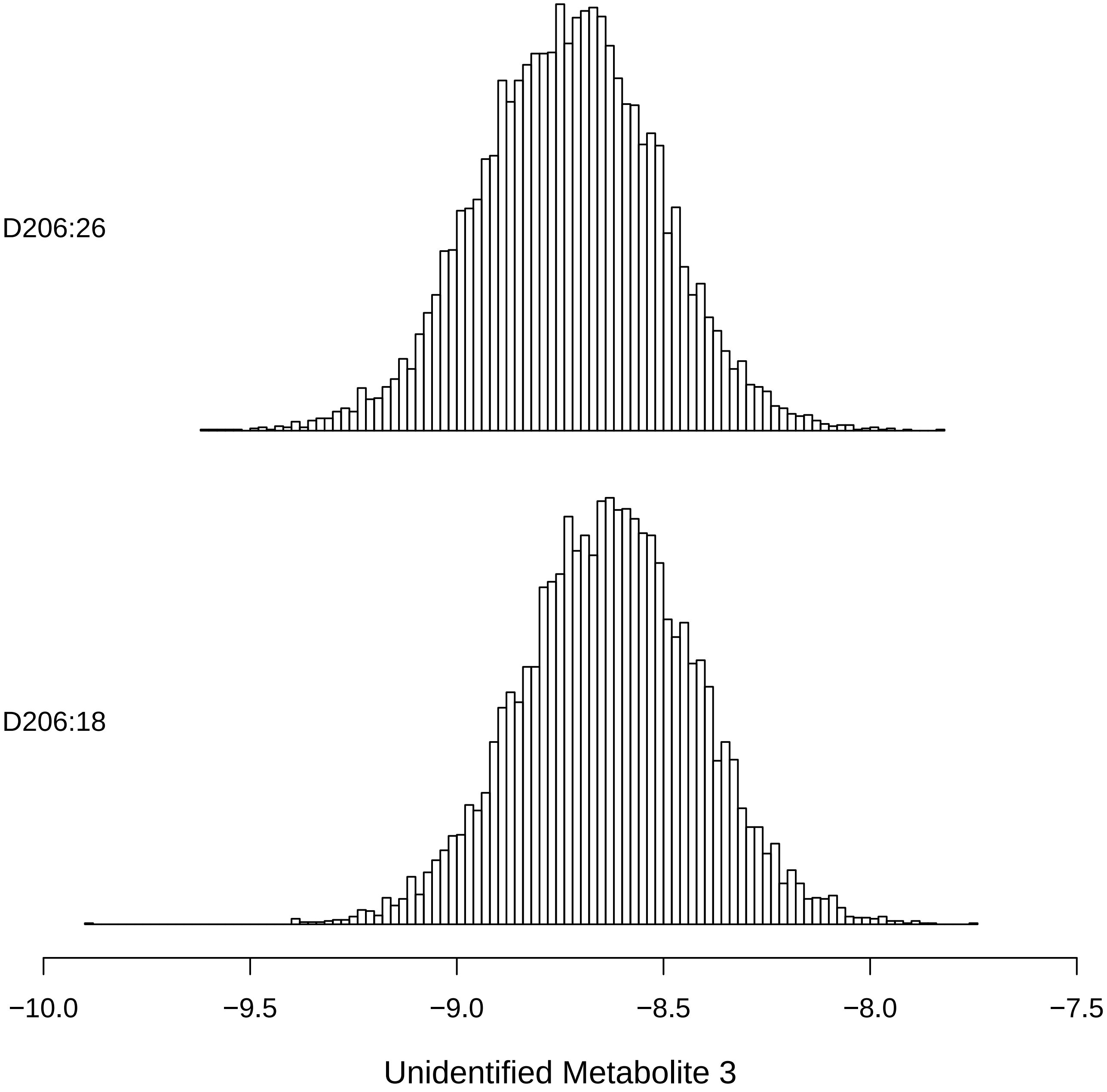

D206:26
D206:18
−10.0
−9.5
−9.0
−8.5
−8.0
−7.5
Unidentified Metabolite 3

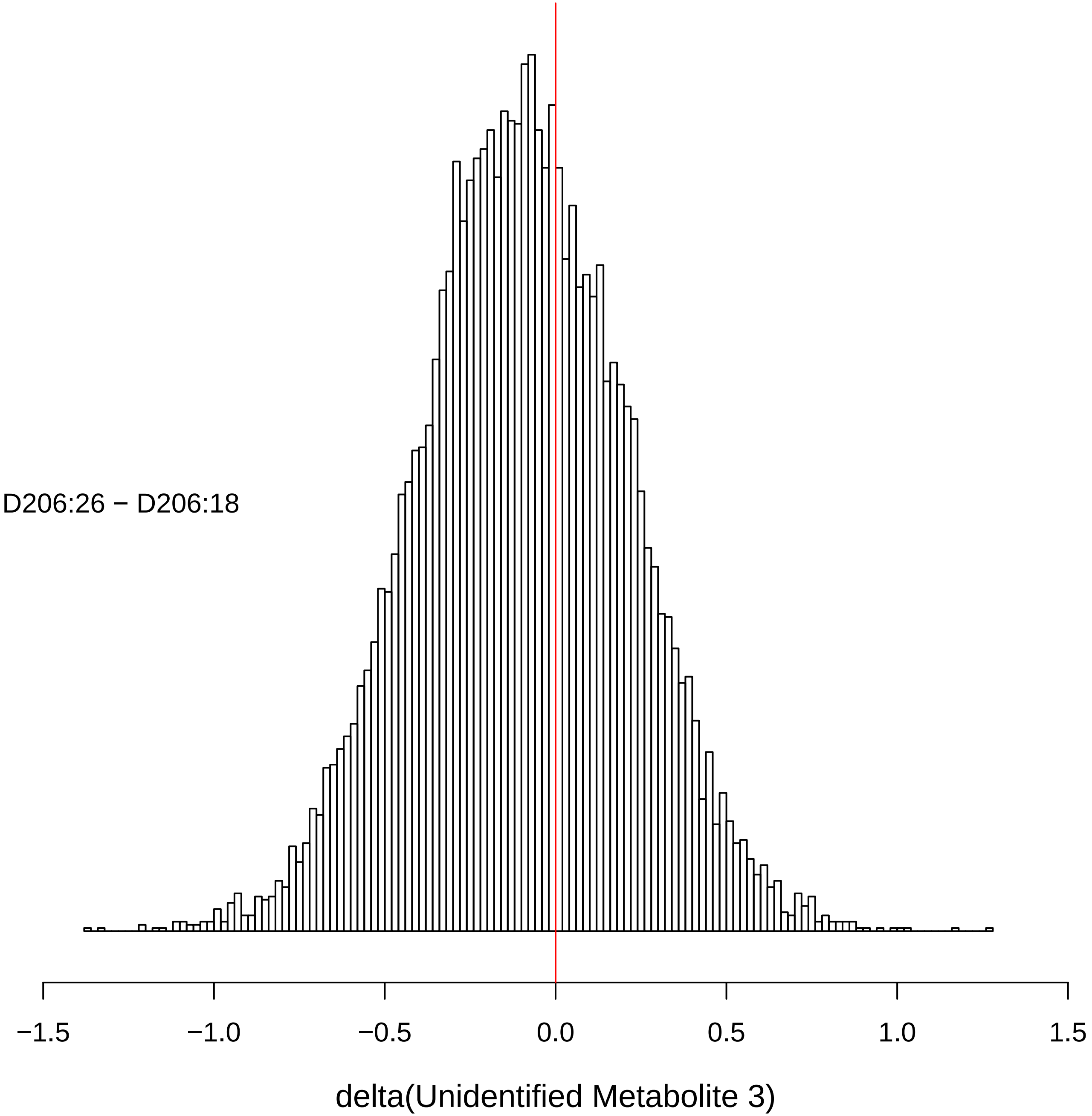
D206:26 − D206:18
−1.5
−1.0
−0.5
0.0
0.5
1.0
1.5
delta(Unidentified Metabolite 3)

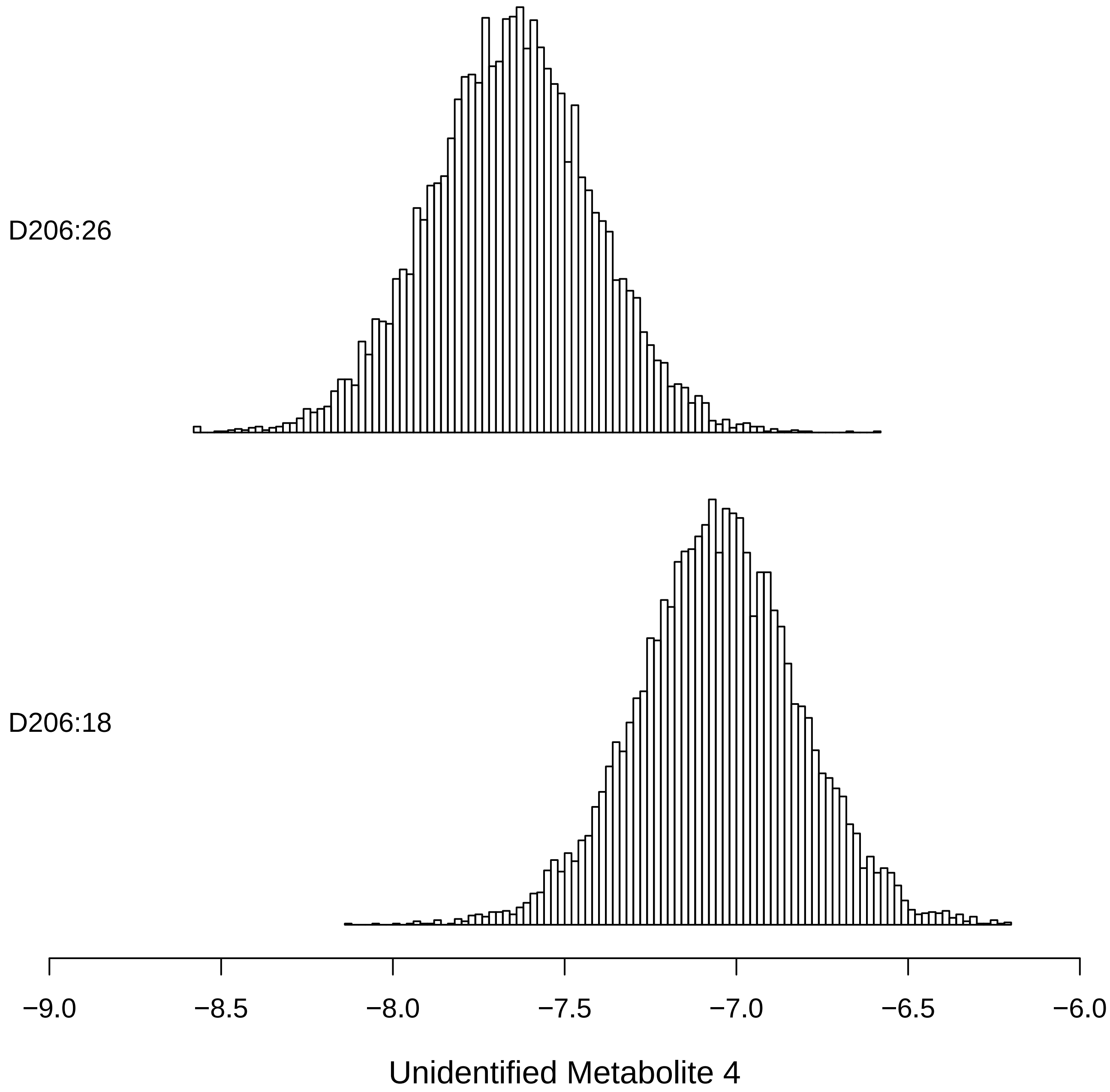
D206:26
D206:18
−9.0
−8.5
−8.0
−7.5
−7.0
−6.5
−6.0
Unidentified Metabolite 4

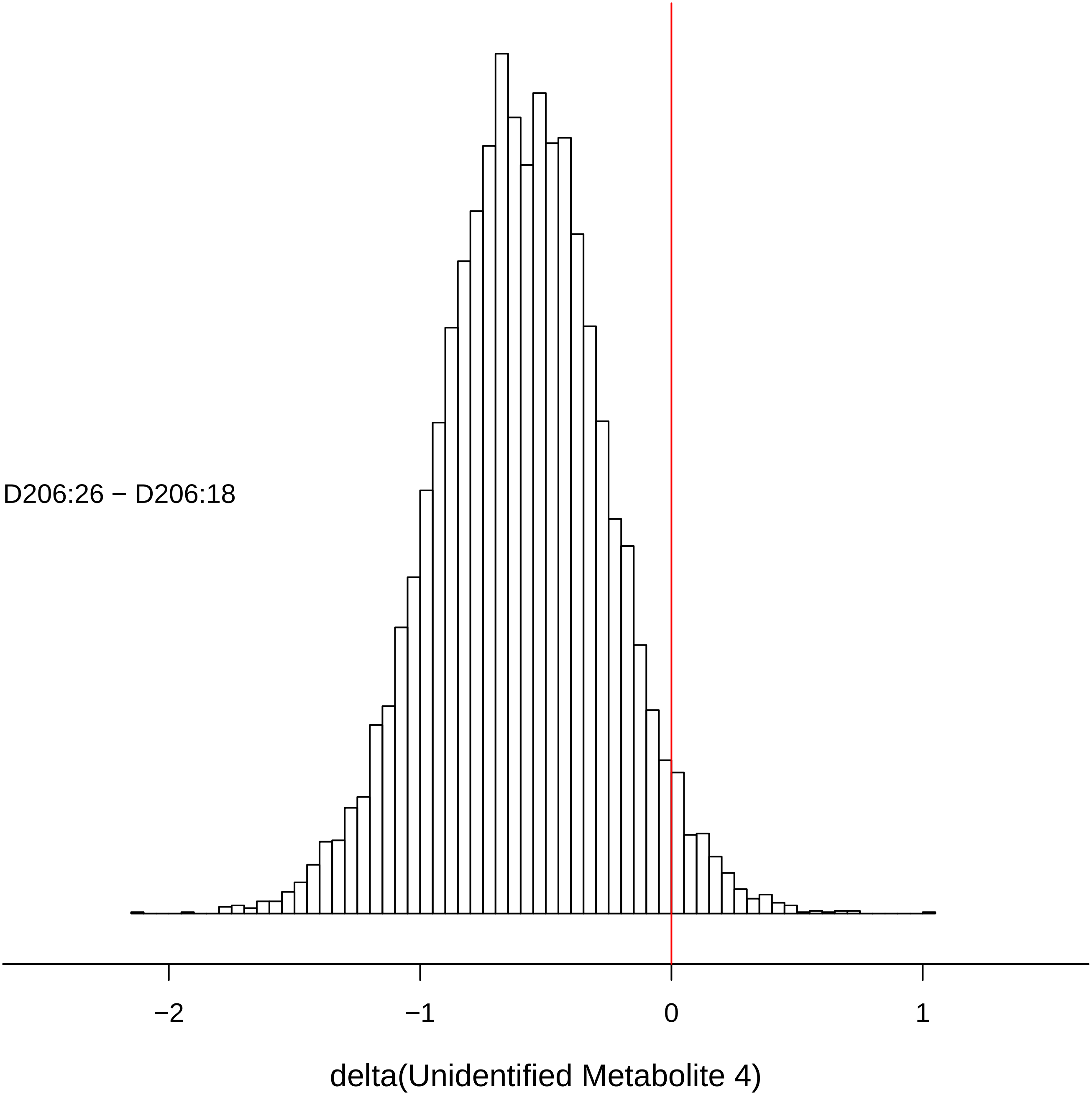
D206:26 − D206:18
−2
−1
0
1
delta(Unidentified Metabolite 4)

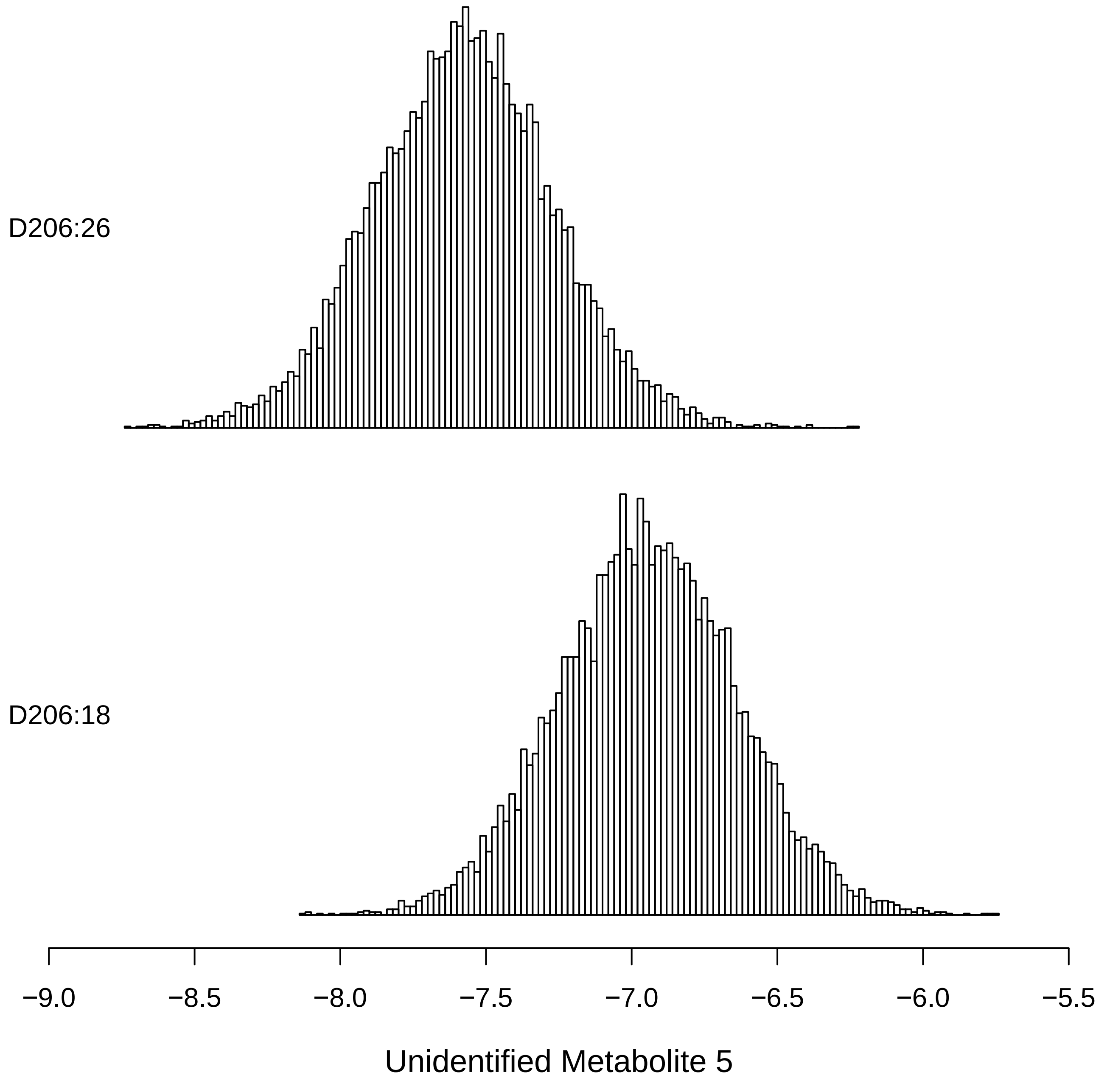
D206:26
D206:18
−9.0
−8.5
−8.0
−7.5
−7.0
−6.5
−6.0
−5.5
Unidentified Metabolite 5

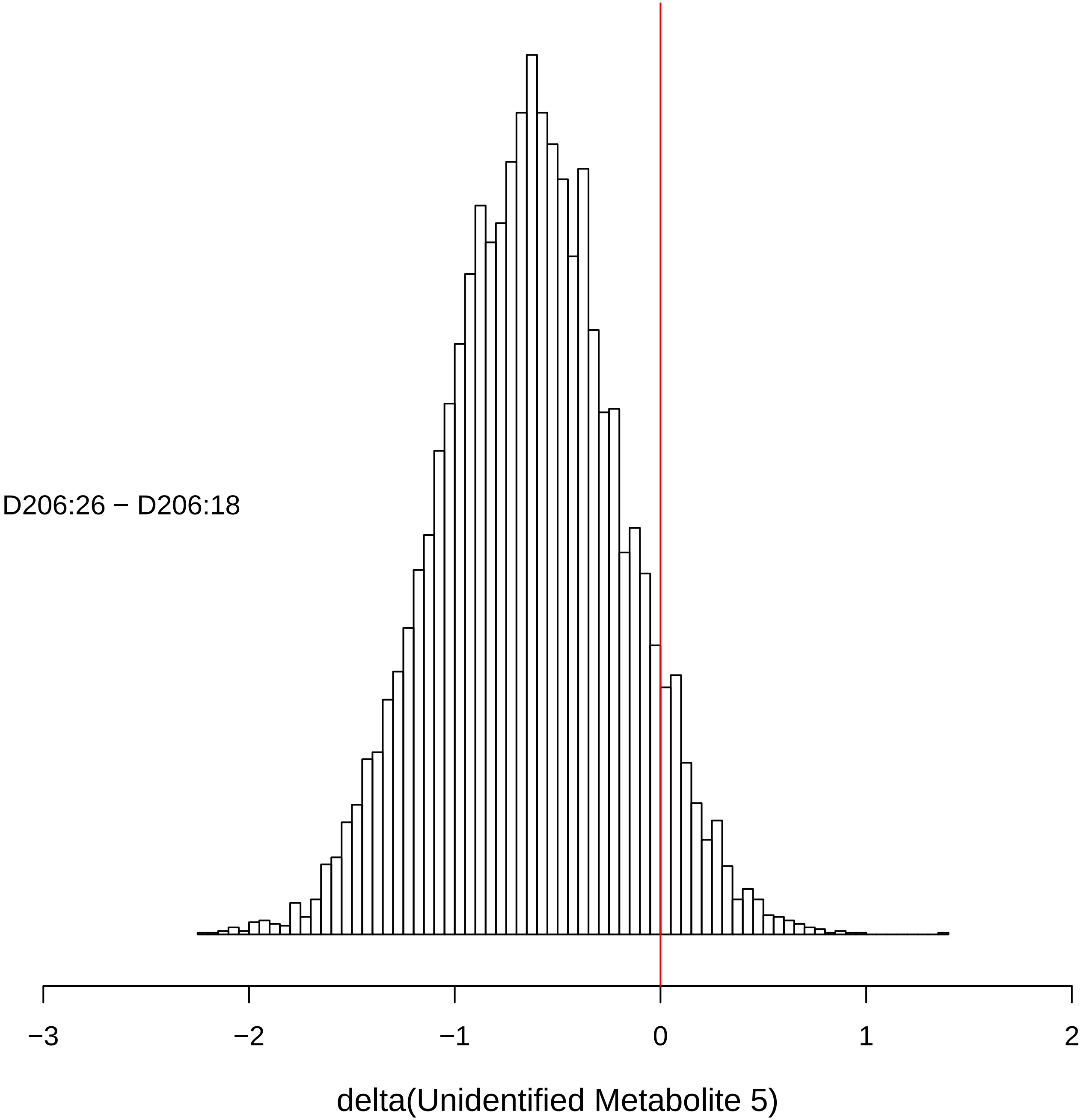
D206:26 − D206:18
−3
−2
−1
0
1
2
delta(Unidentified Metabolite 5)

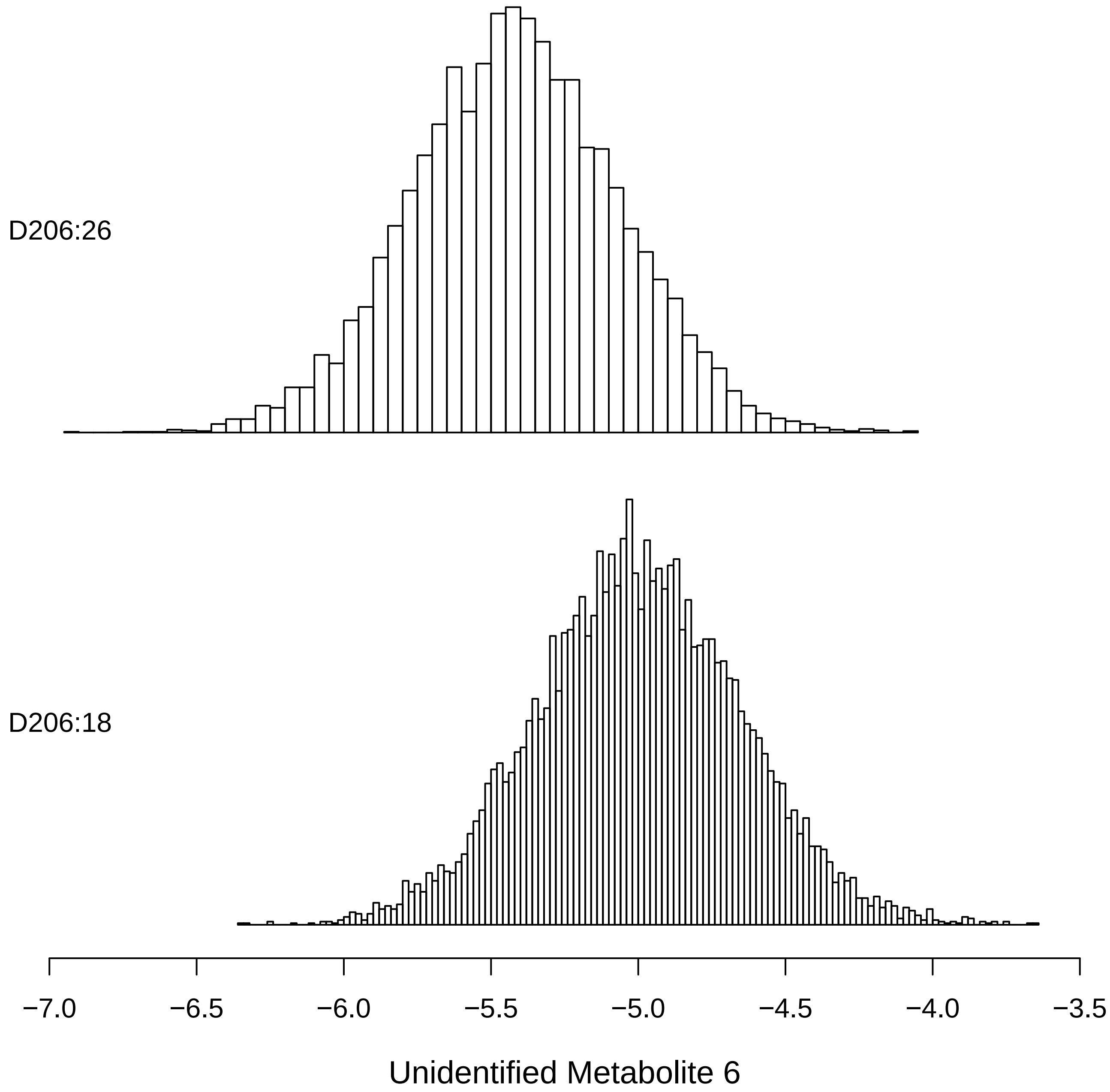
D206:26
D206:18
−7.0
−6.5
−6.0
−5.5
−5.0
−4.5
−4.0
−3.5
Unidentified Metabolite 6

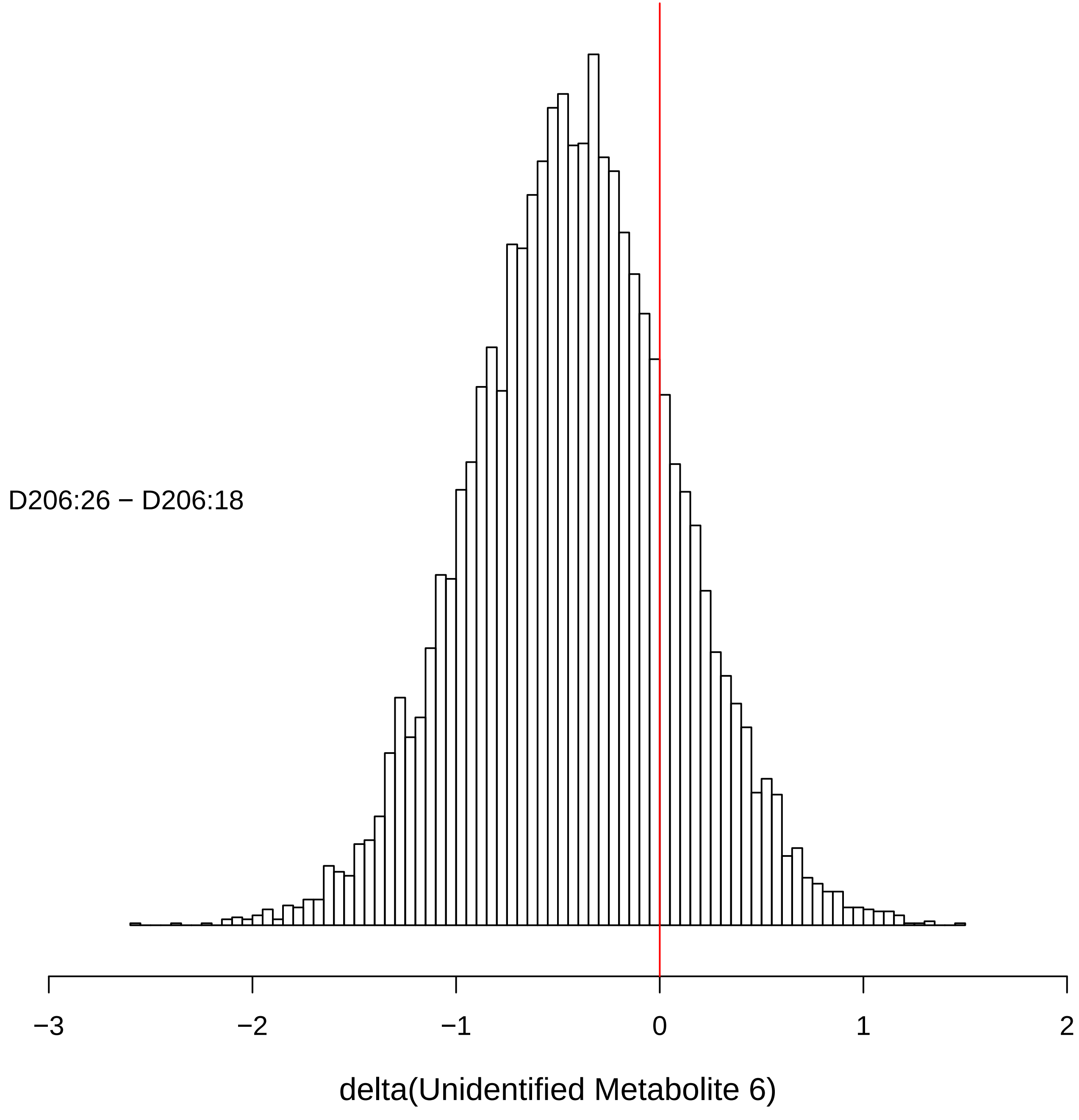

D206:26 − D206:18
−3
−2
−1
0
1
2
delta(Unidentified Metabolite 6)

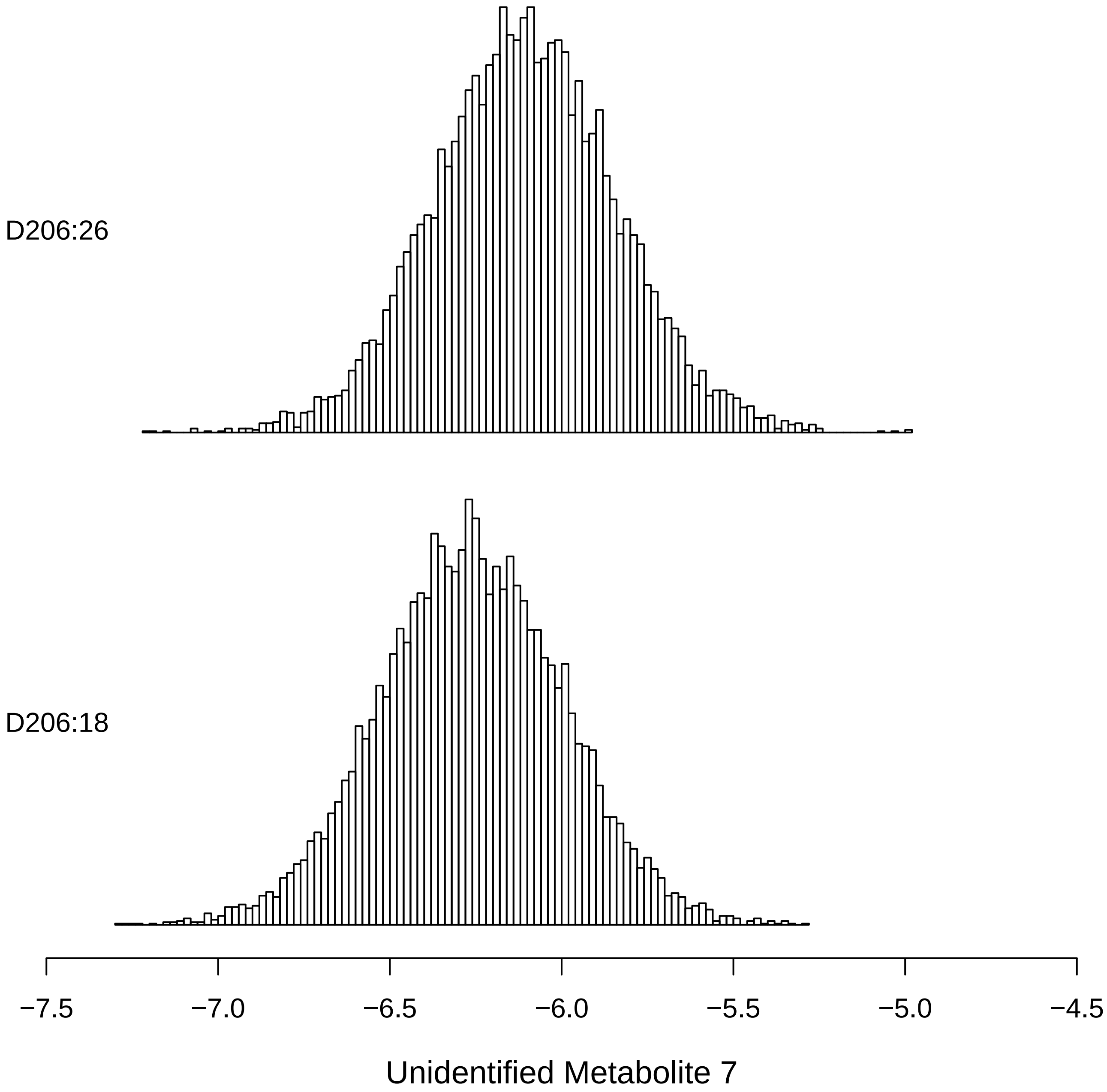

D206:26
D206:18
−7.5
−7.0
−6.5
−6.0
−5.5
−5.0
−4.5
Unidentified Metabolite 7

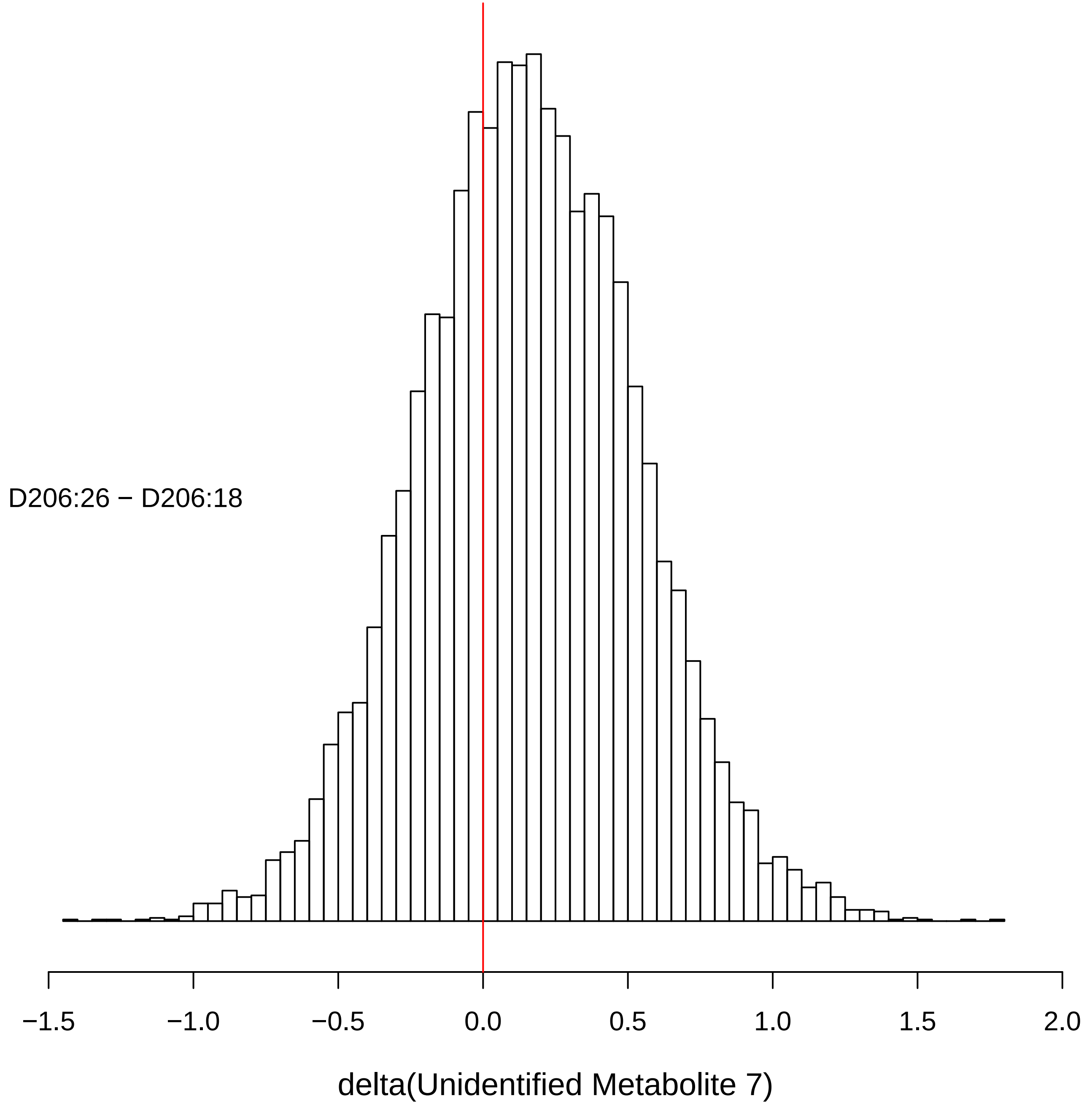
D206:26 − D206:18
−1.5
−1.0
−0.5
0.0
0.5
1.0
1.5
2.0
delta(Unidentified Metabolite 7)

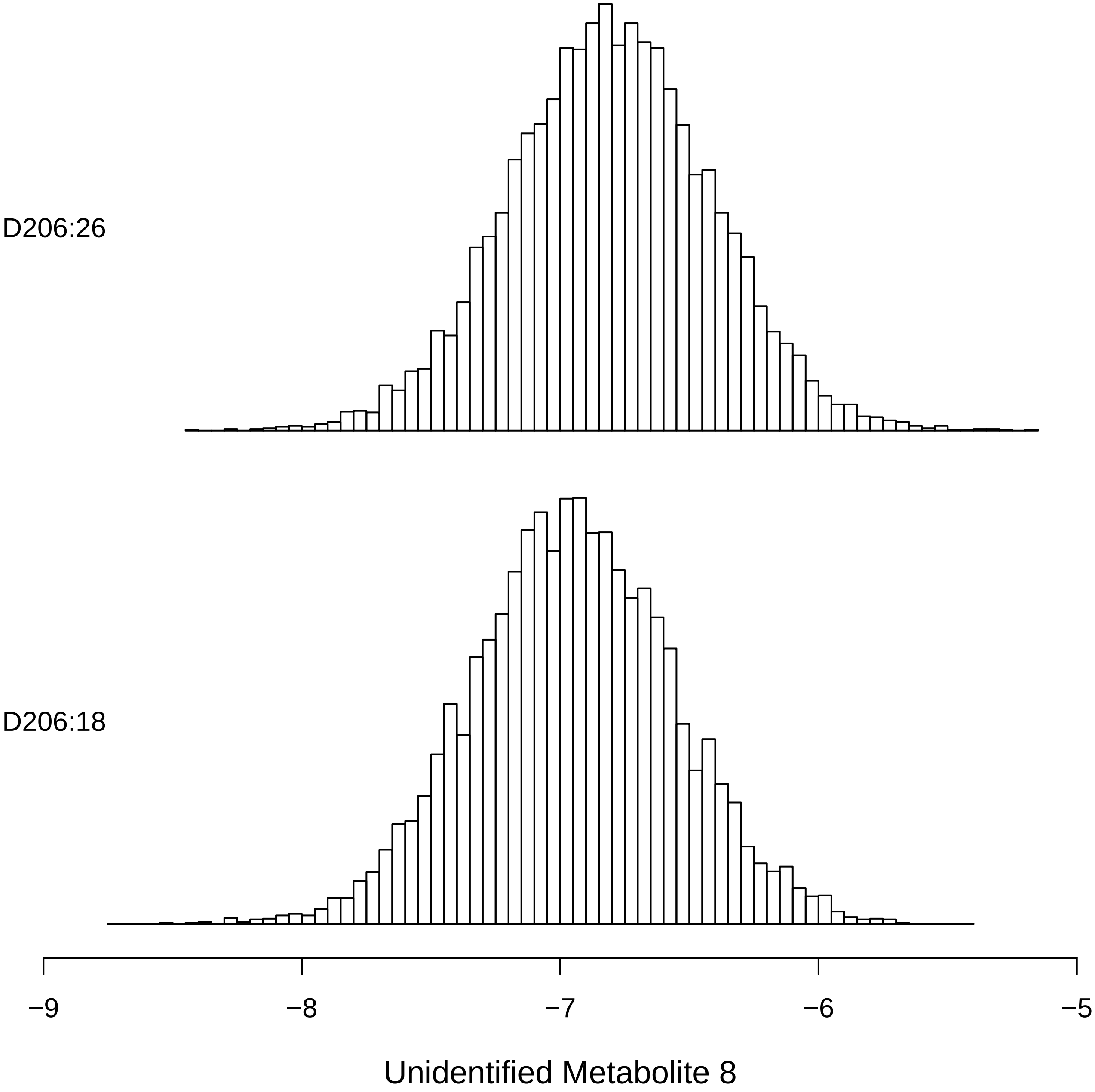
D206:26
D206:18
−9
−8
−7
−6
−5
Unidentified Metabolite 8

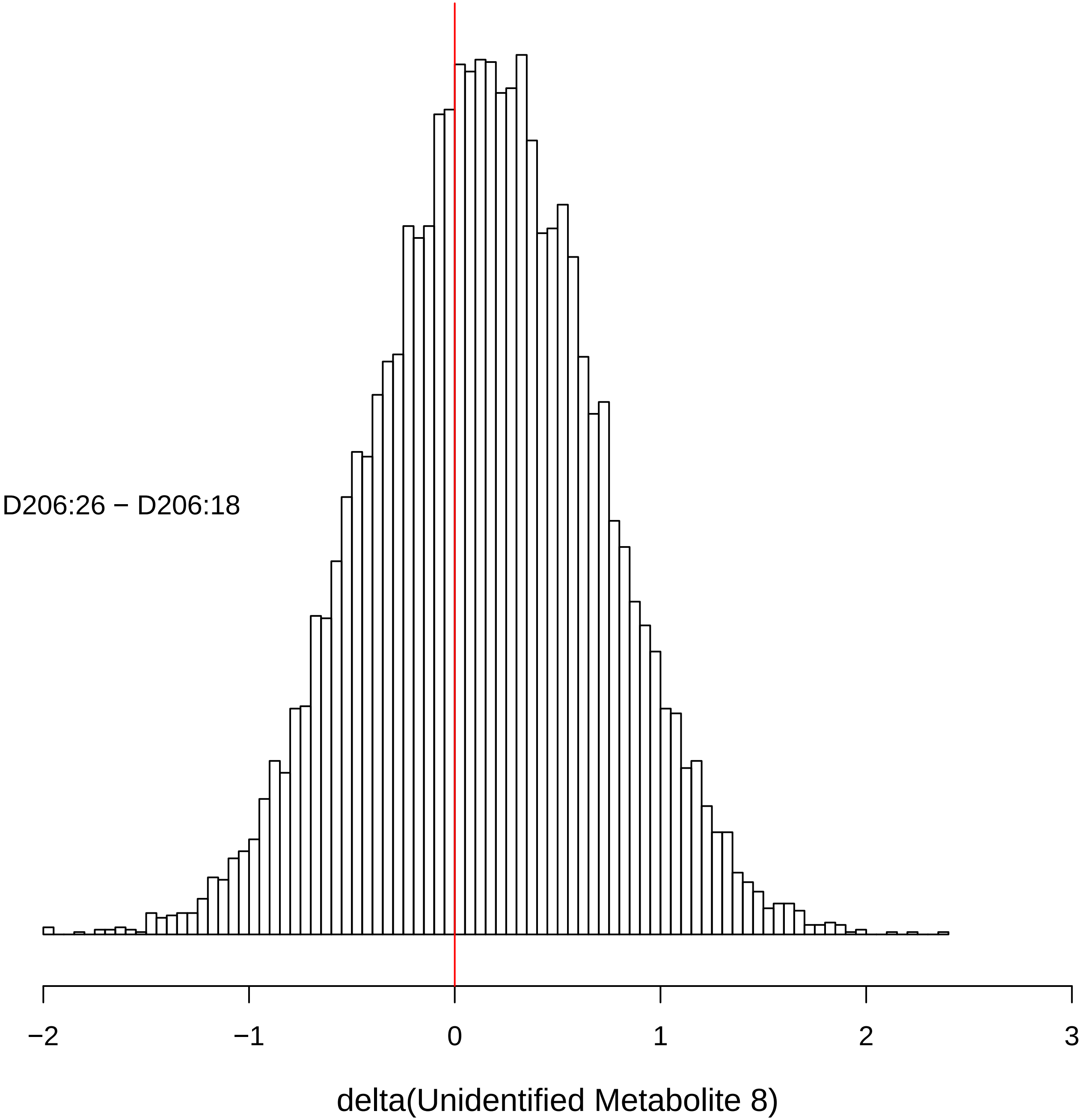
D206:26 − D206:18
−2
−1
0
1
2
3
delta(Unidentified Metabolite 8)

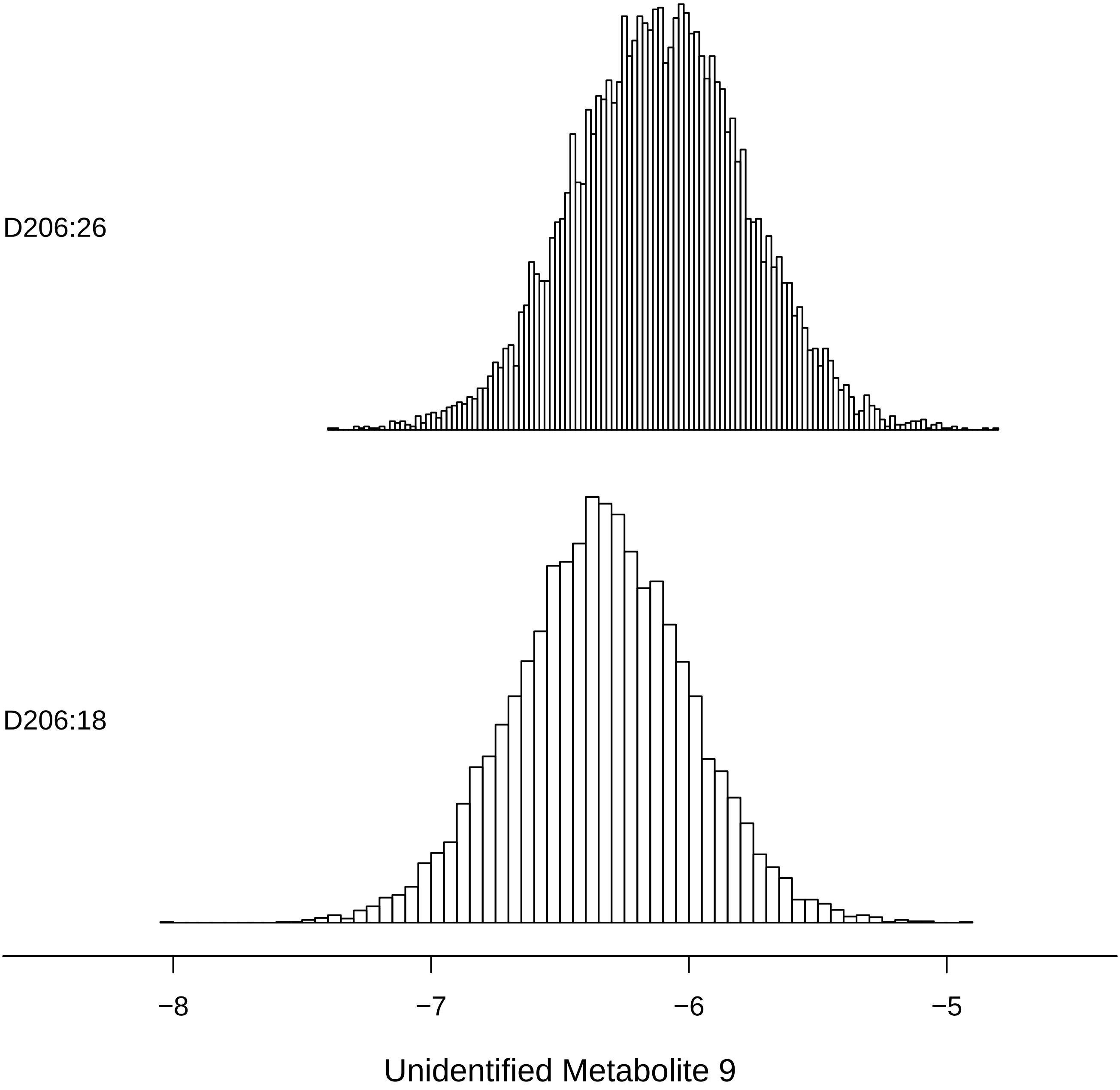
D206:26
D206:18
−8
−7
−6
−5
Unidentified Metabolite 9

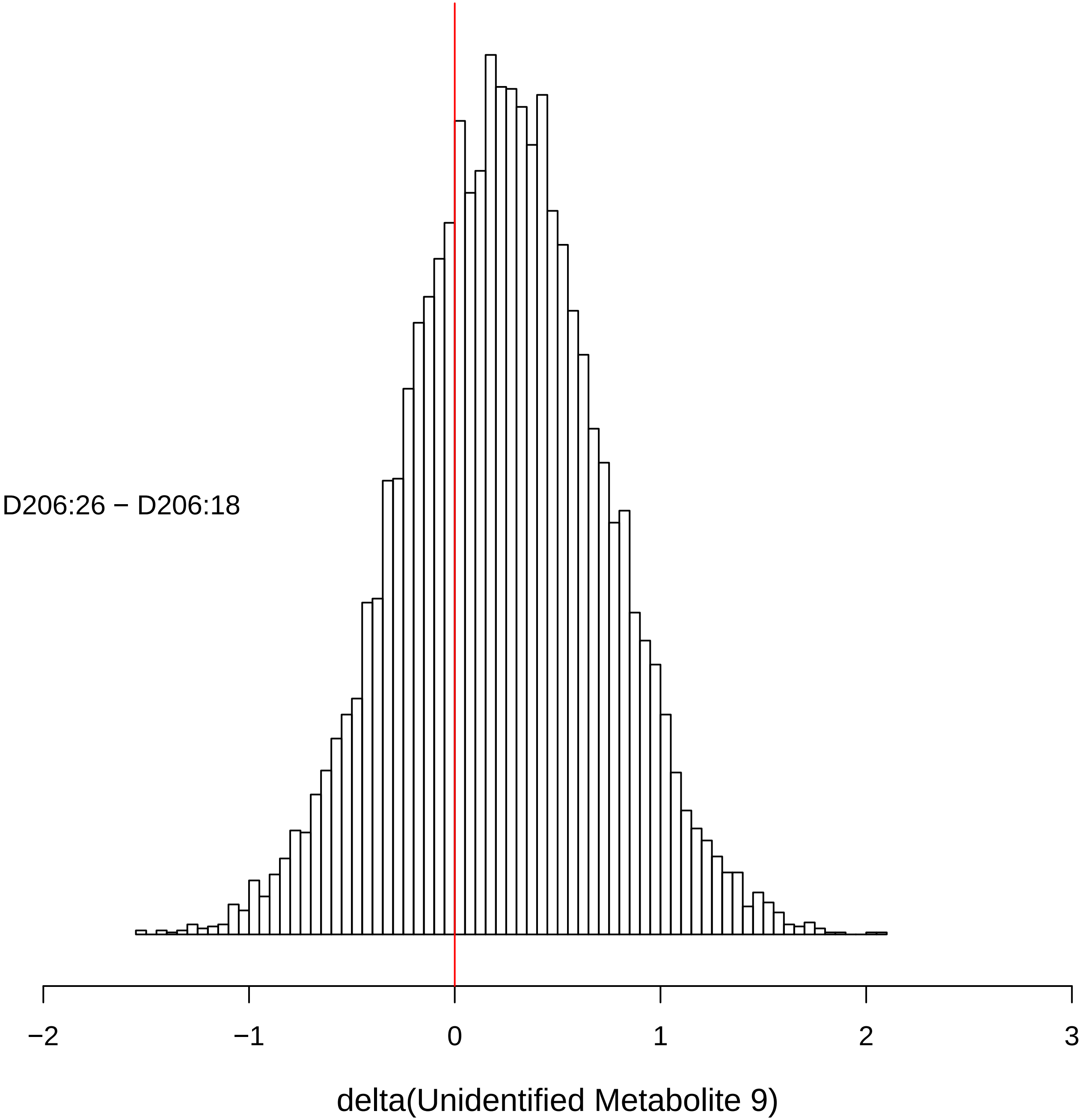
D206:26 − D206:18
−2
−1
0
1
2
3
delta(Unidentified Metabolite 9)

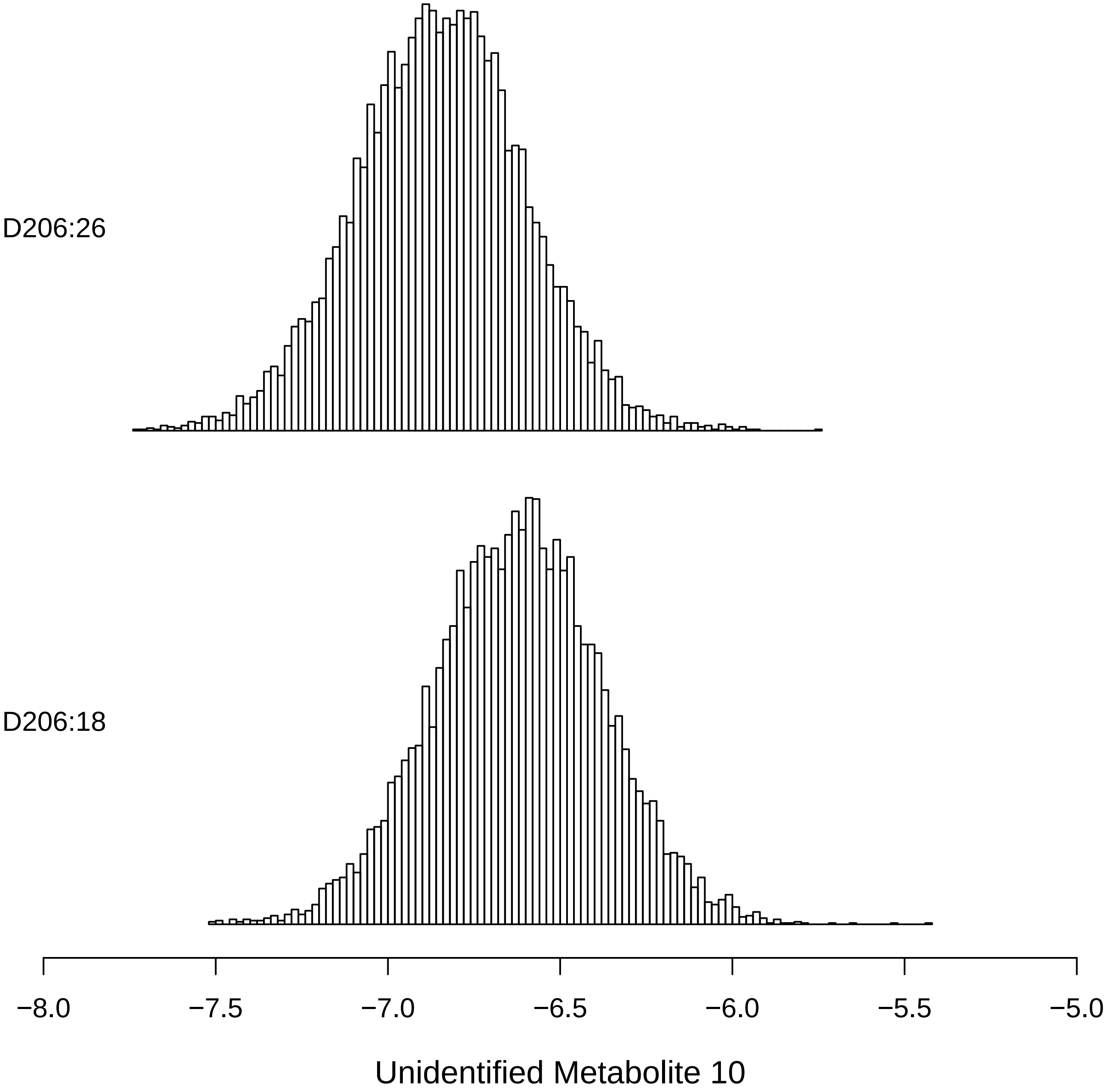
D206:26
D206:18
−8.0
−7.5
−7.0
−6.5
−6.0
−5.5
−5.0
Unidentified Metabolite 10

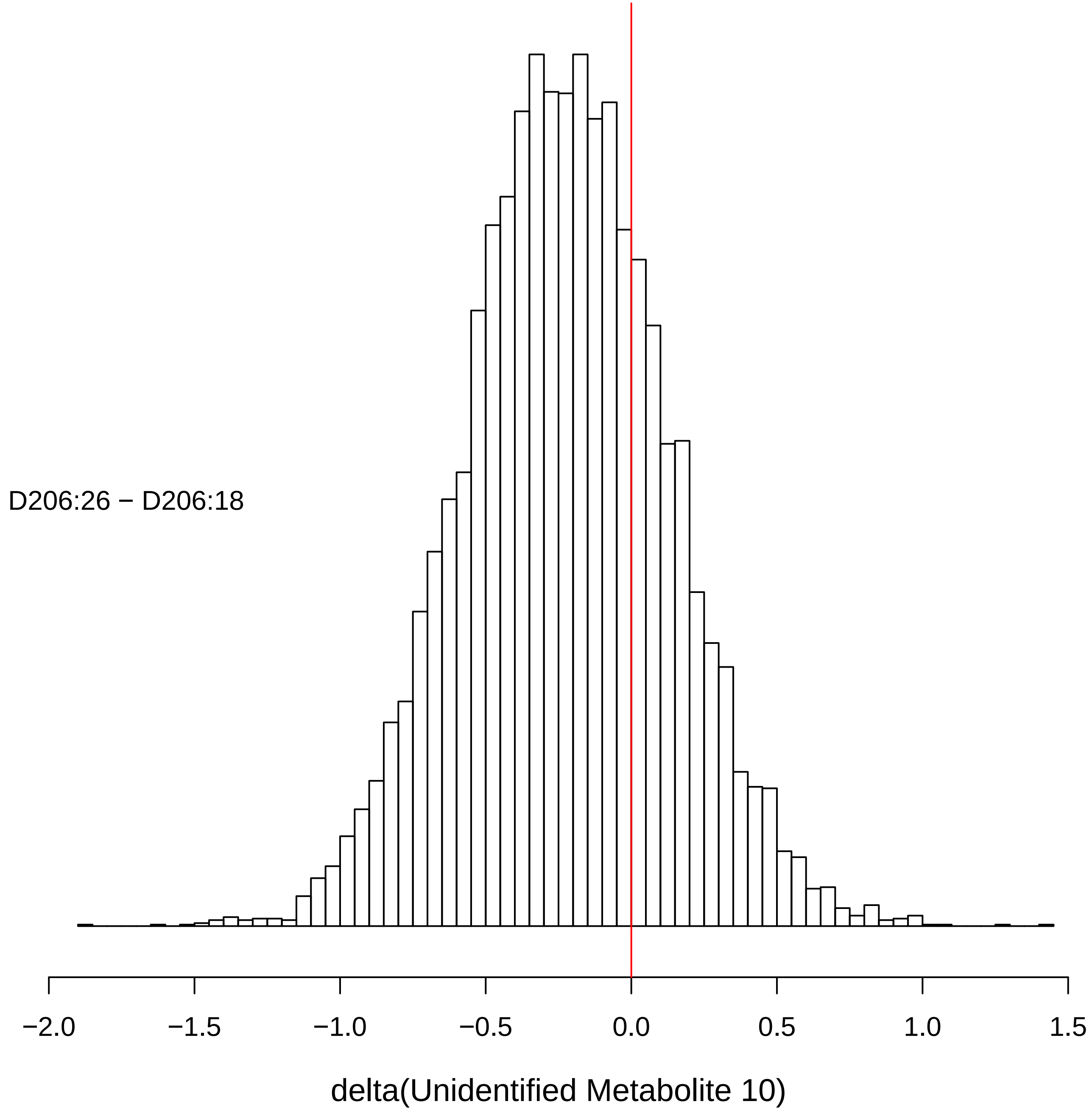
D206:26 − D206:18
−2.0
−1.5
−1.0
−0.5
0.0
0.5
1.0
1.5
delta(Unidentified Metabolite 10)

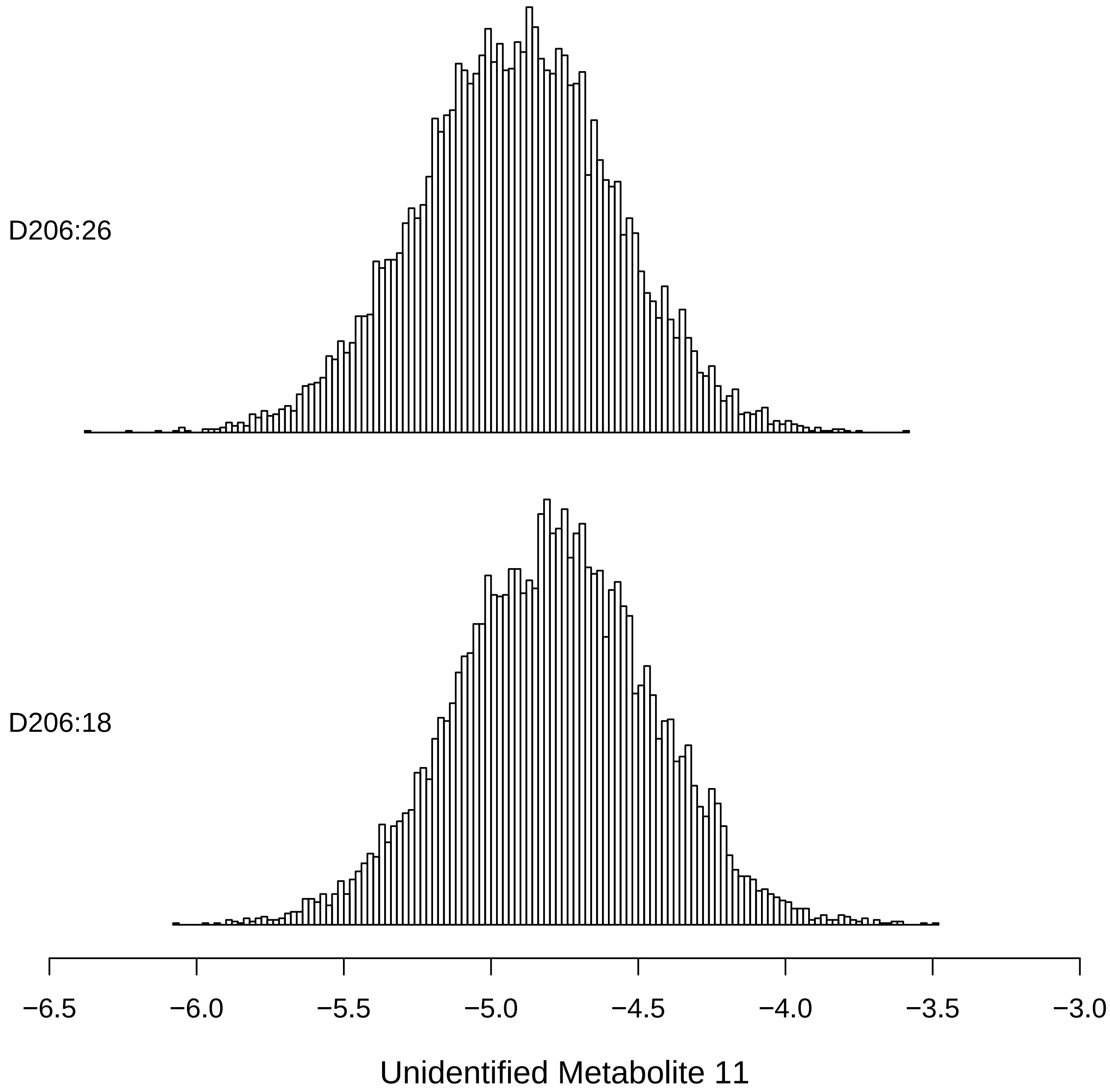
D206:26
D206:18
−6.5
−6.0
−5.5
−5.0
−4.5
−4.0
−3.5
−3.0
Unidentified Metabolite 11

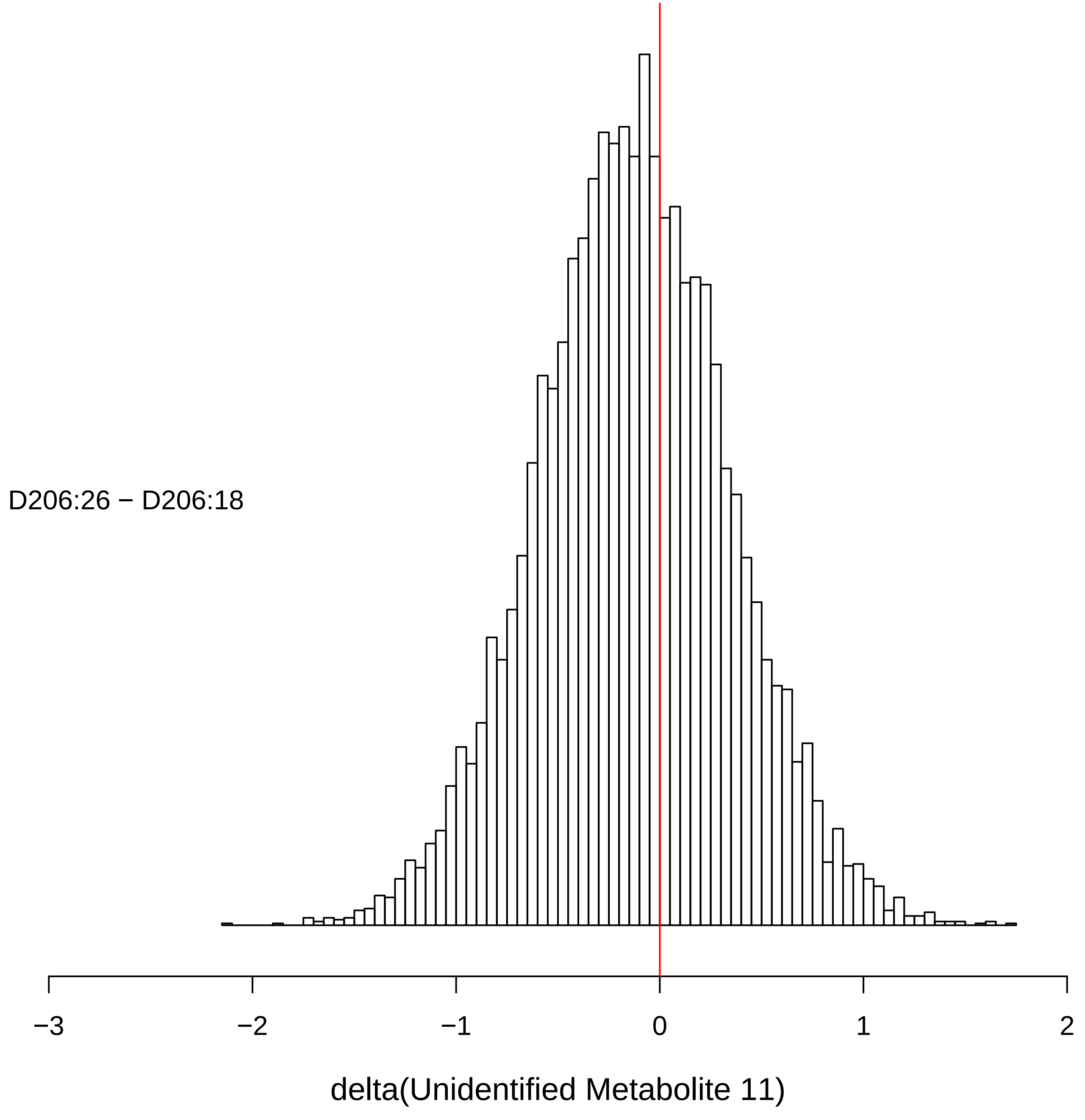
D206:26 − D206:18
−3
−2
−1
0
1
2
delta(Unidentified Metabolite 11)

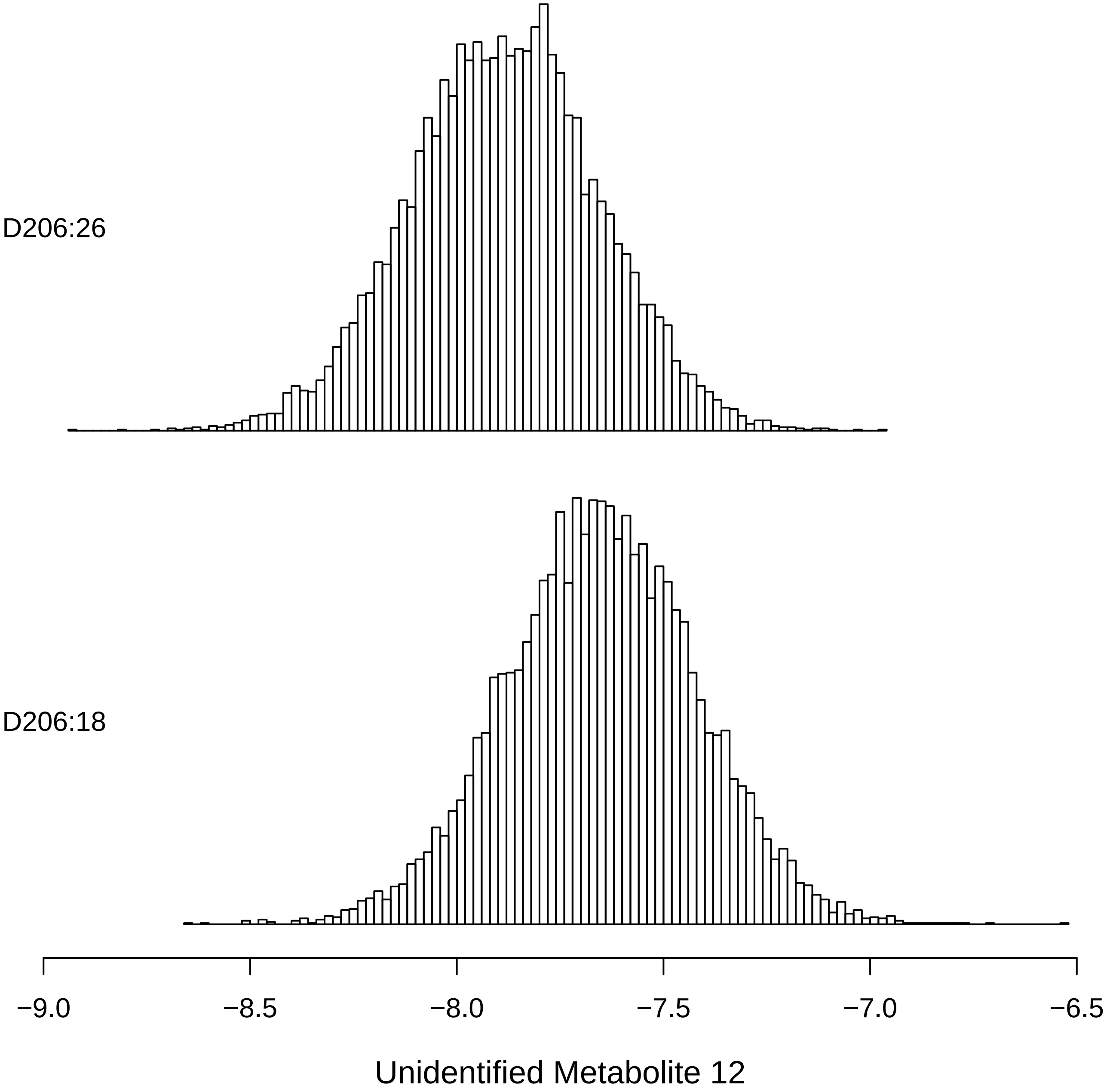
D206:26
D206:18
−9.0
−8.5
−8.0
−7.5
−7.0
−6.5
Unidentified Metabolite 12

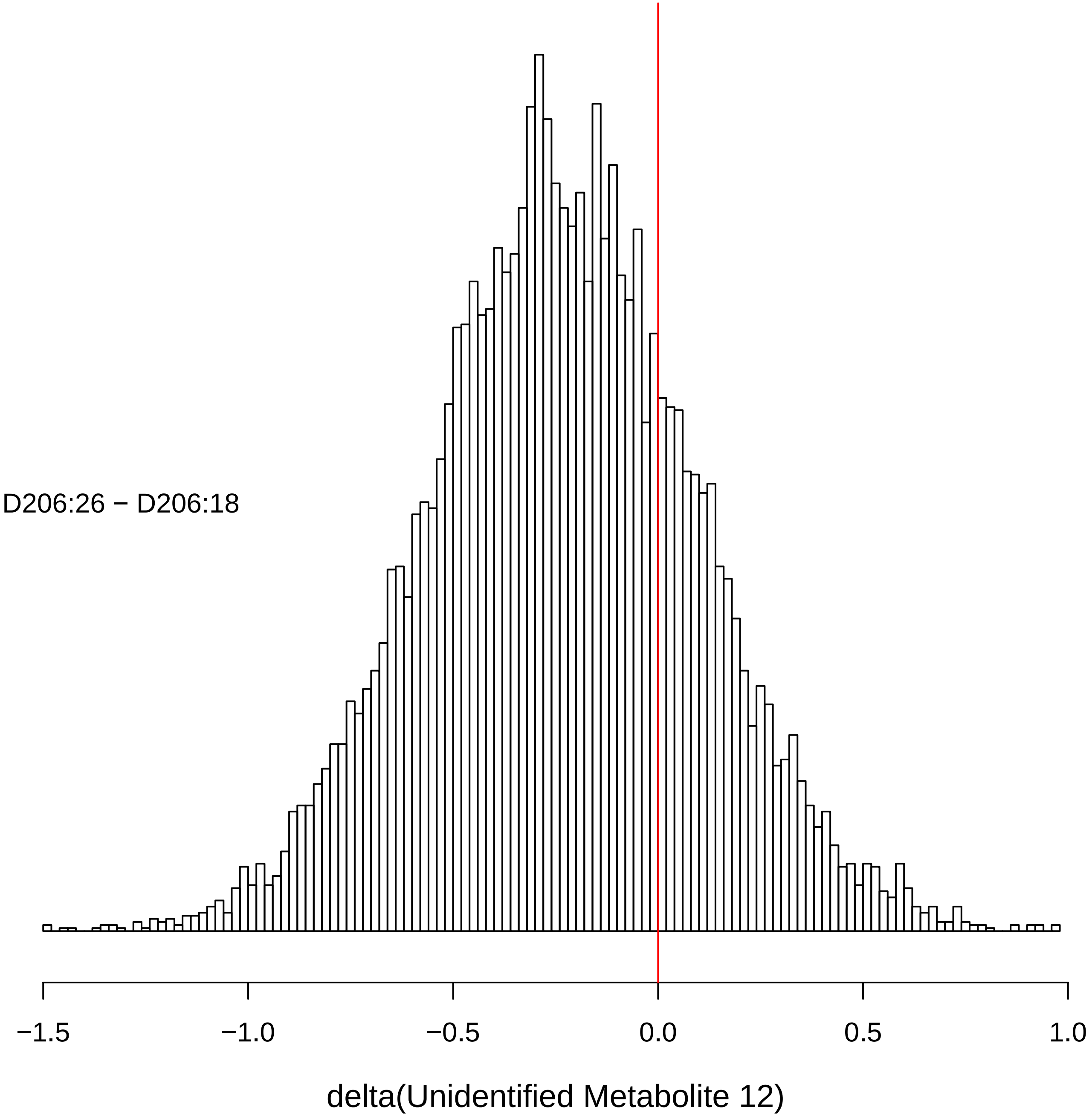
D206:26 − D206:18
−1.5
−1.0
−0.5
0.0
0.5
1.0
delta(Unidentified Metabolite 12)

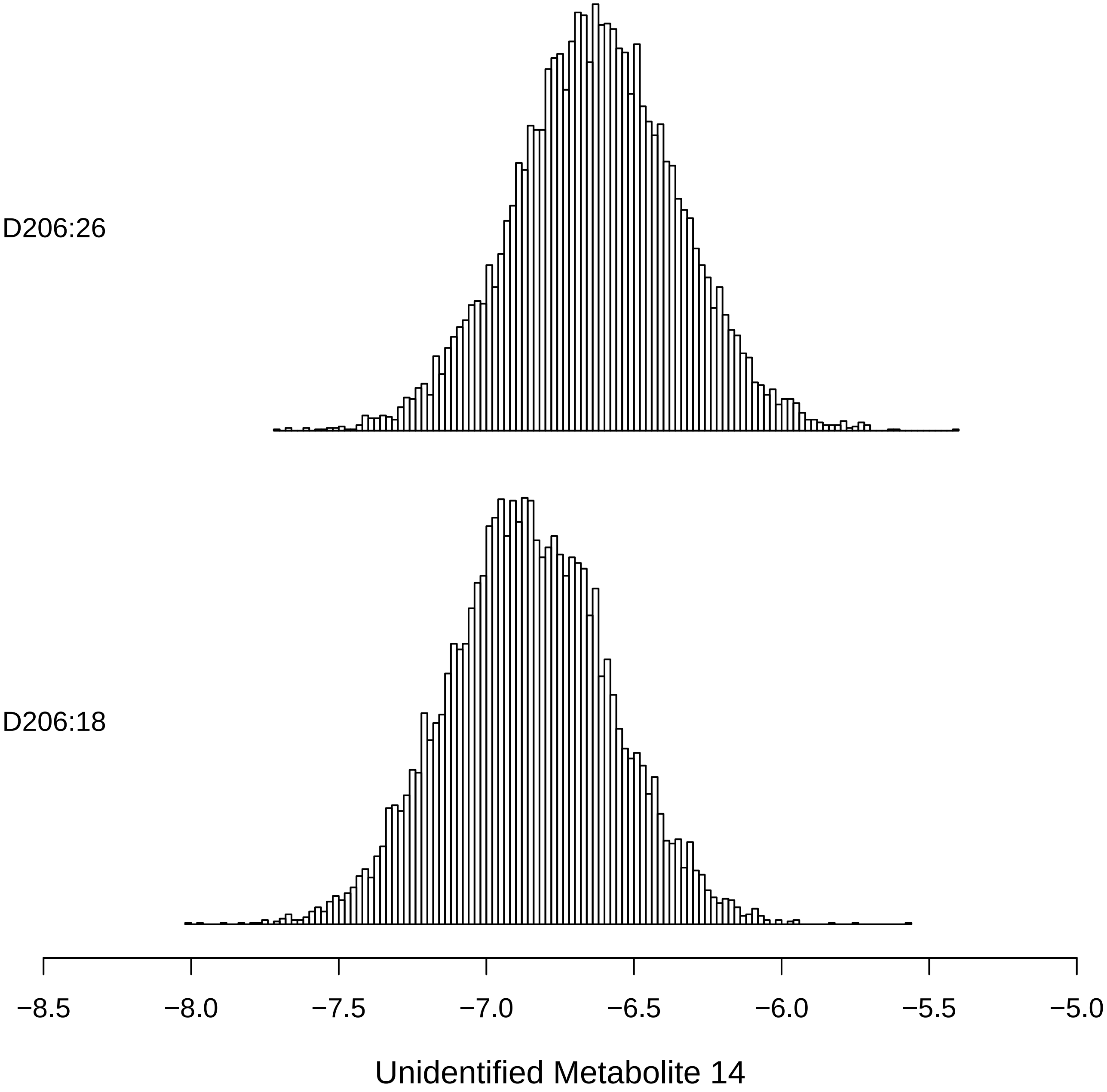
D206:26
D206:18
−8.5
−8.0
−7.5
−7.0
−6.5
−6.0
−5.5
−5.0
Unidentified Metabolite 14

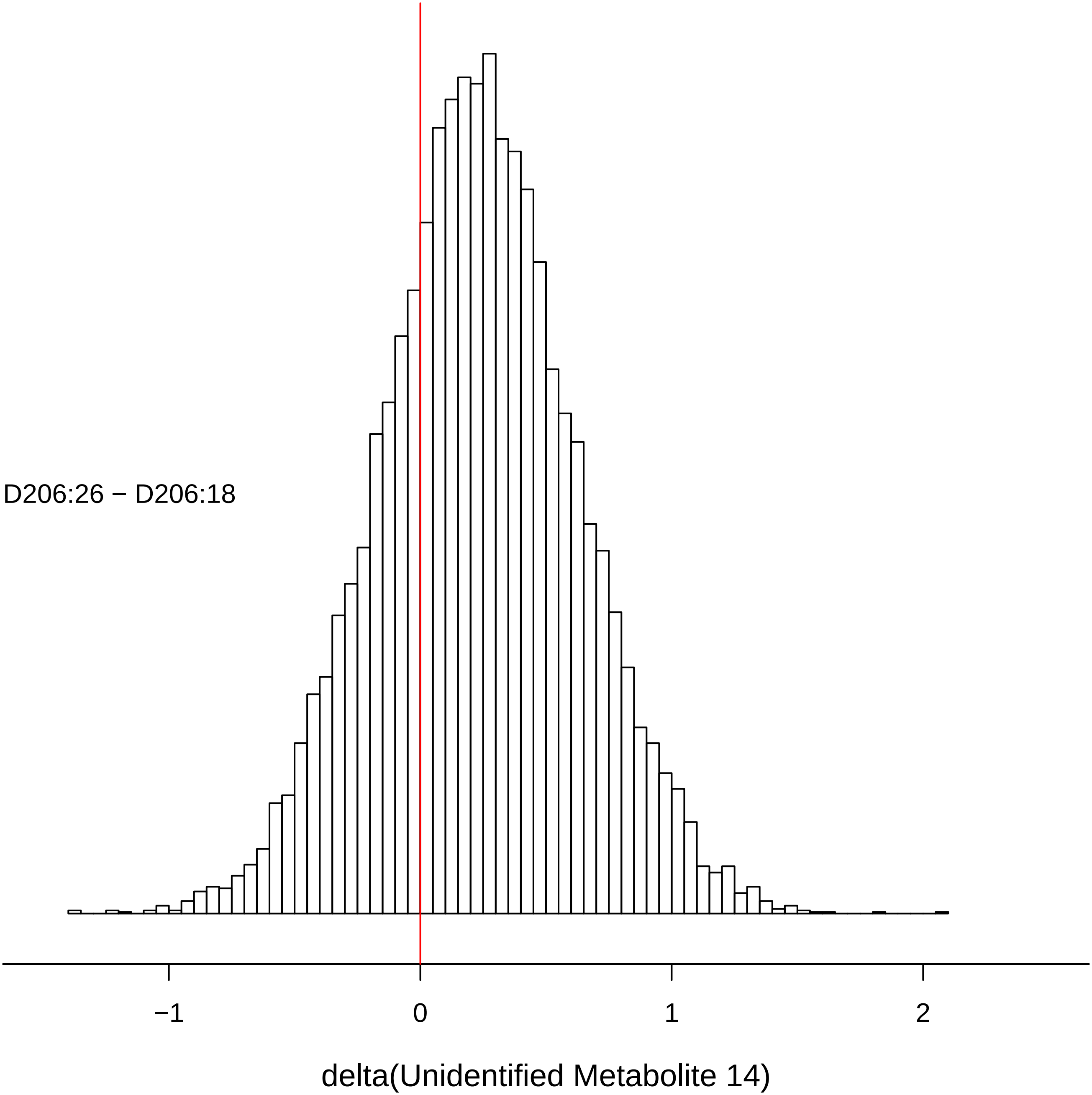
D206:26 − D206:18
−1
0
1
2
delta(Unidentified Metabolite 14)

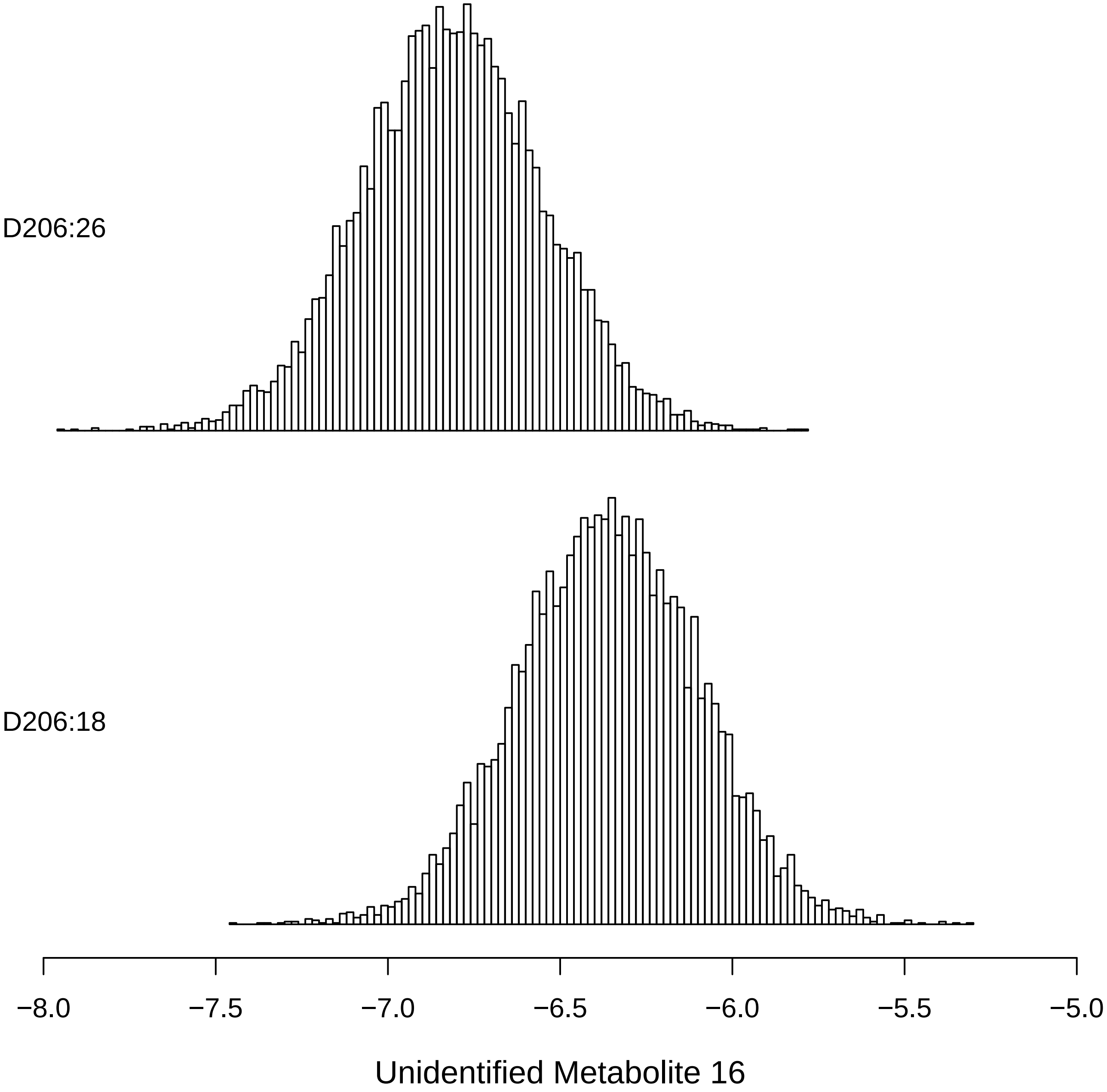
D206:26
D206:18
−8.0
−7.5
−7.0
−6.5
−6.0
−5.5
−5.0
Unidentified Metabolite 16

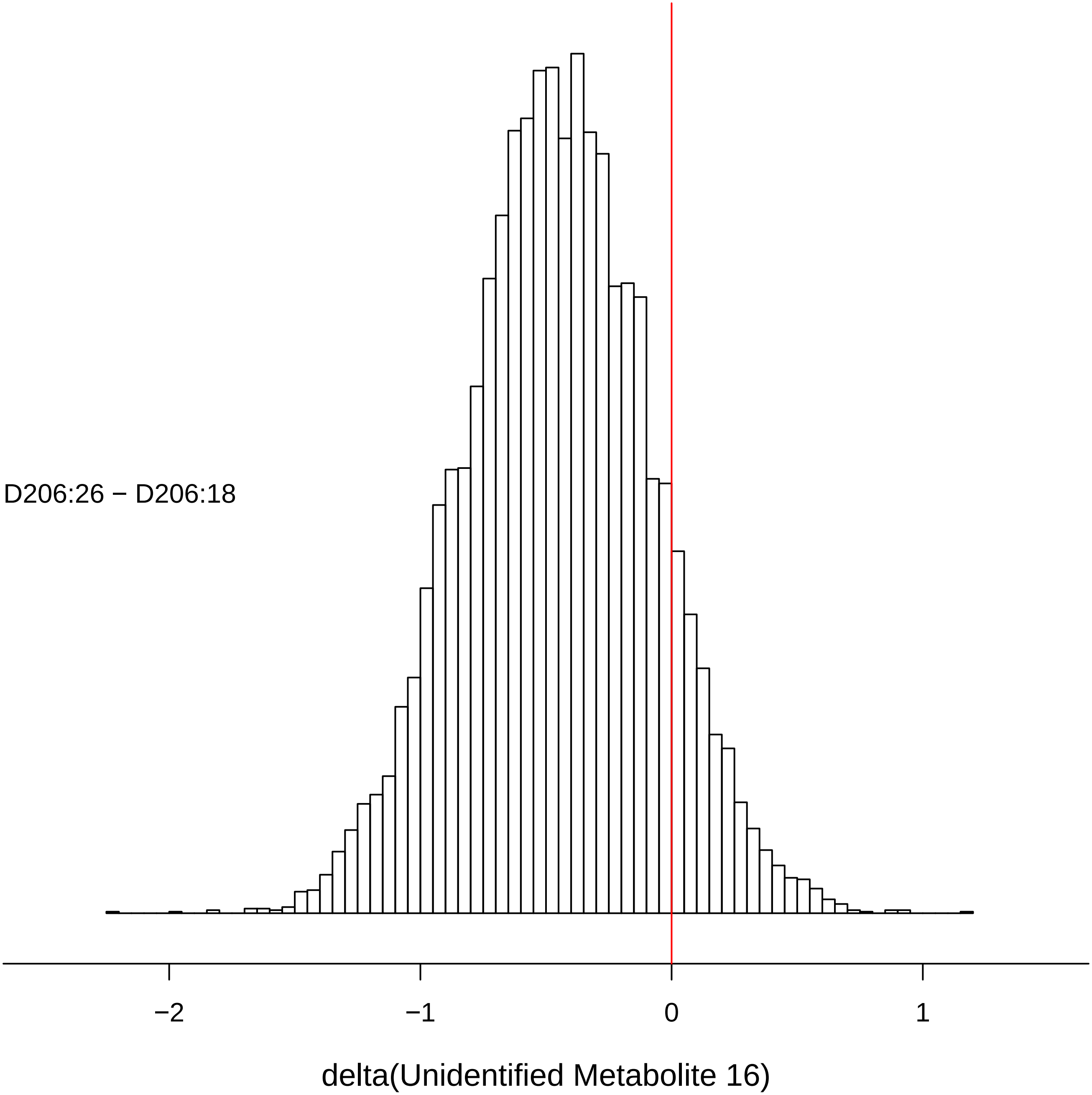
D206:26 − D206:18
−2
−1
0
1
delta(Unidentified Metabolite 16)

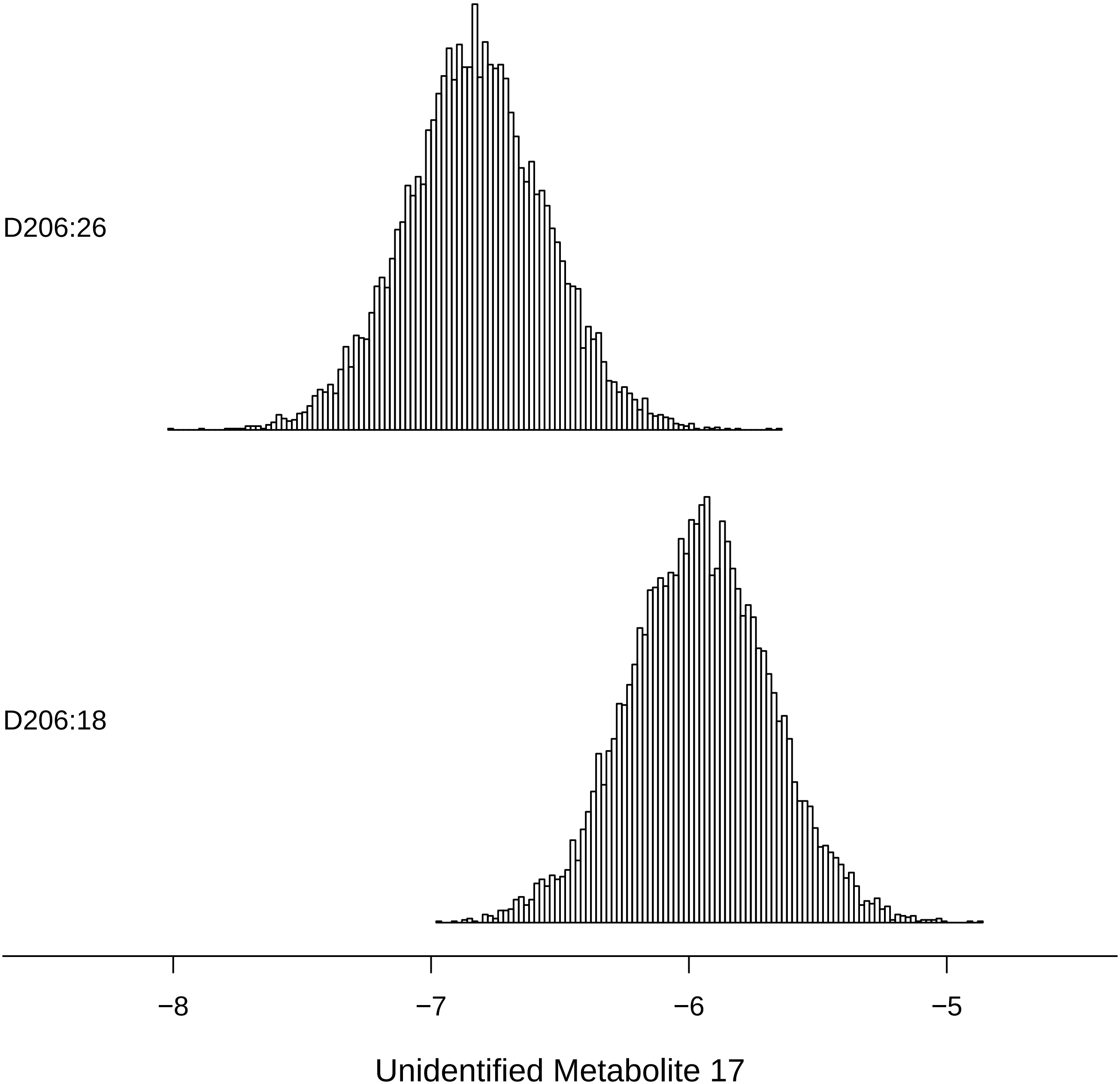
D206:26
D206:18
−8
−7
−6
−5
Unidentified Metabolite 17

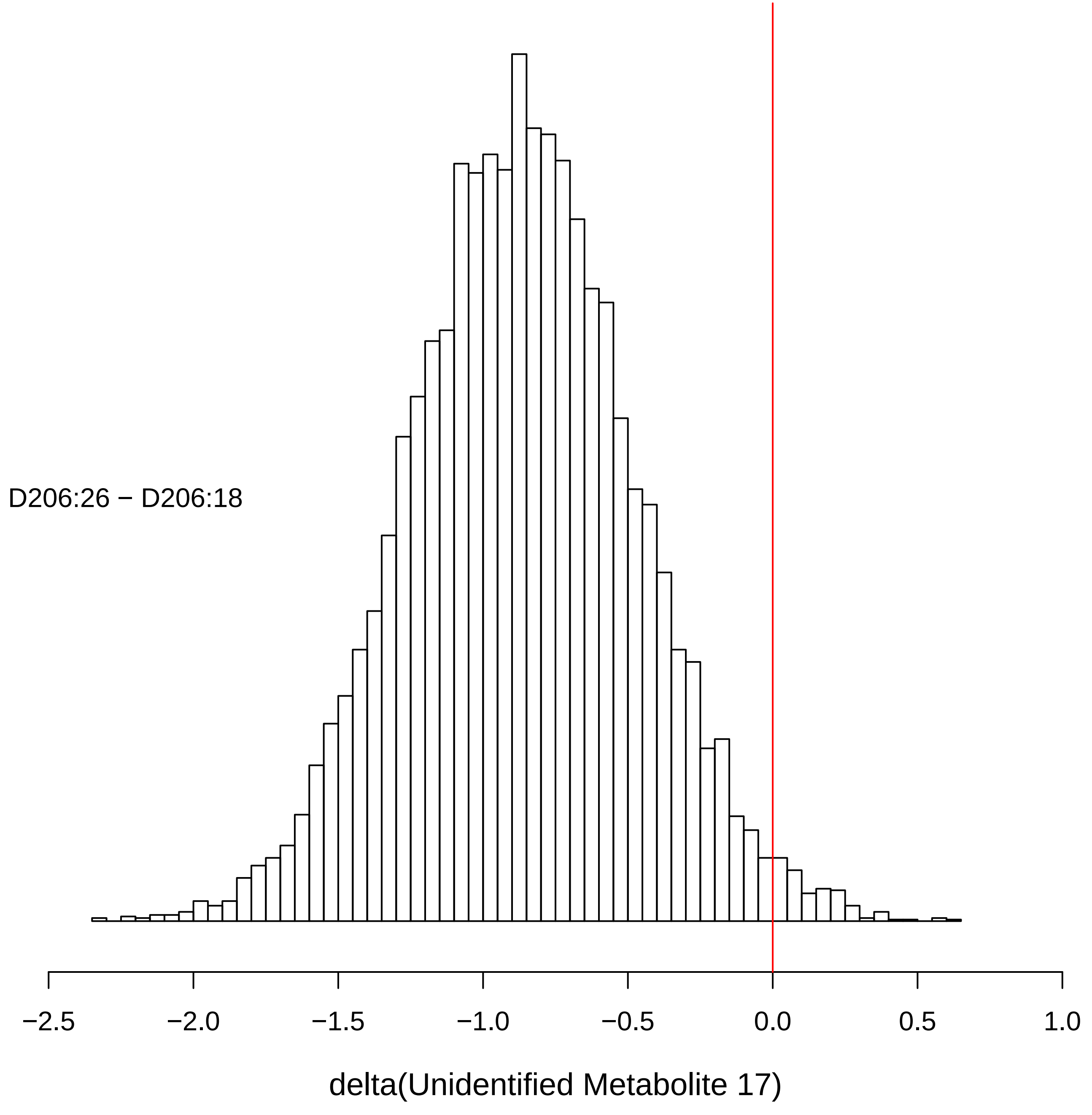
D206:26 − D206:18
−2.5
−2.0
−1.5
−1.0
−0.5
0.0
0.5
1.0
delta(Unidentified Metabolite 17)

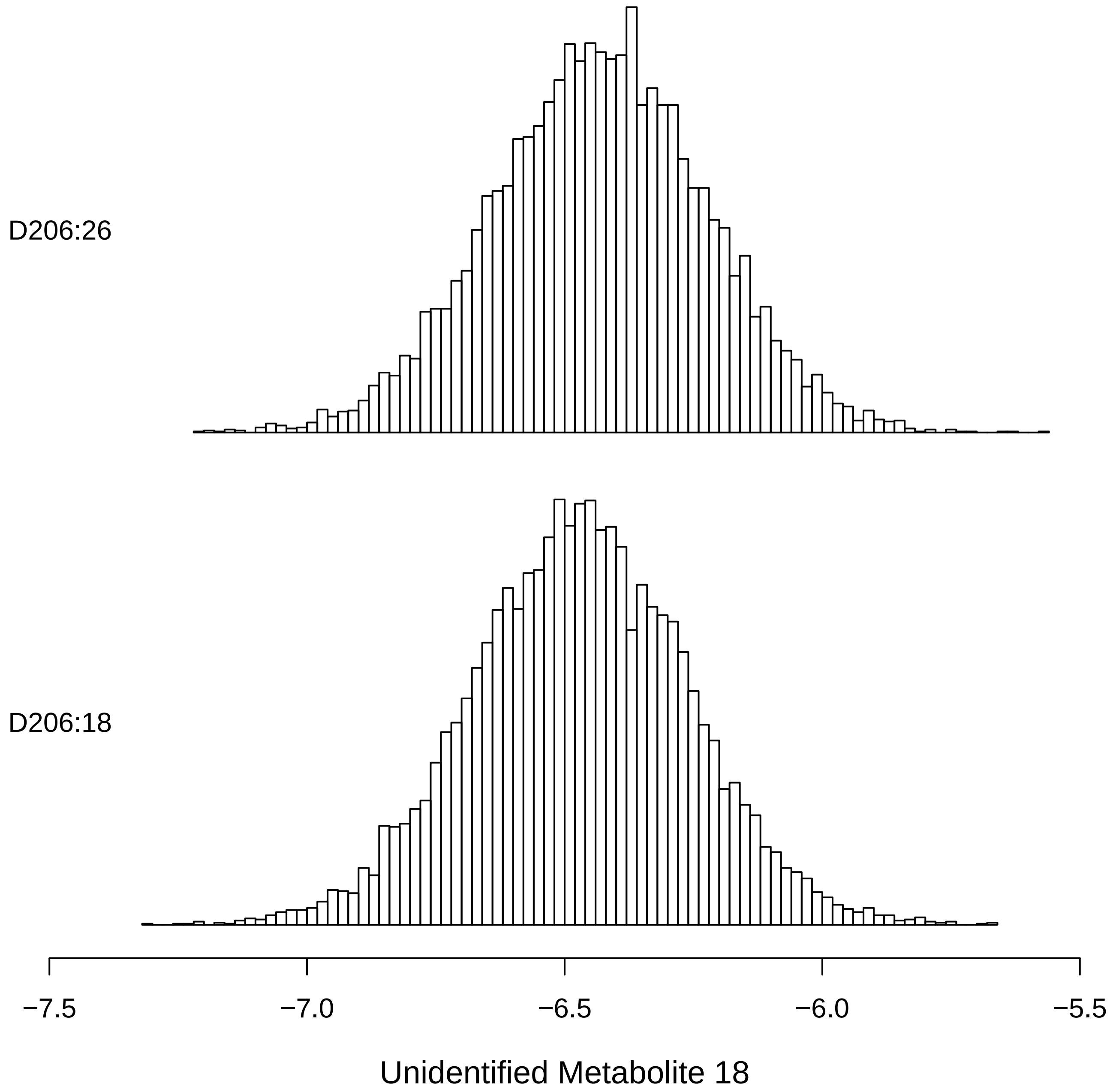
D206:26
D206:18
−7.5
−7.0
−6.5
−6.0
−5.5
Unidentified Metabolite 18

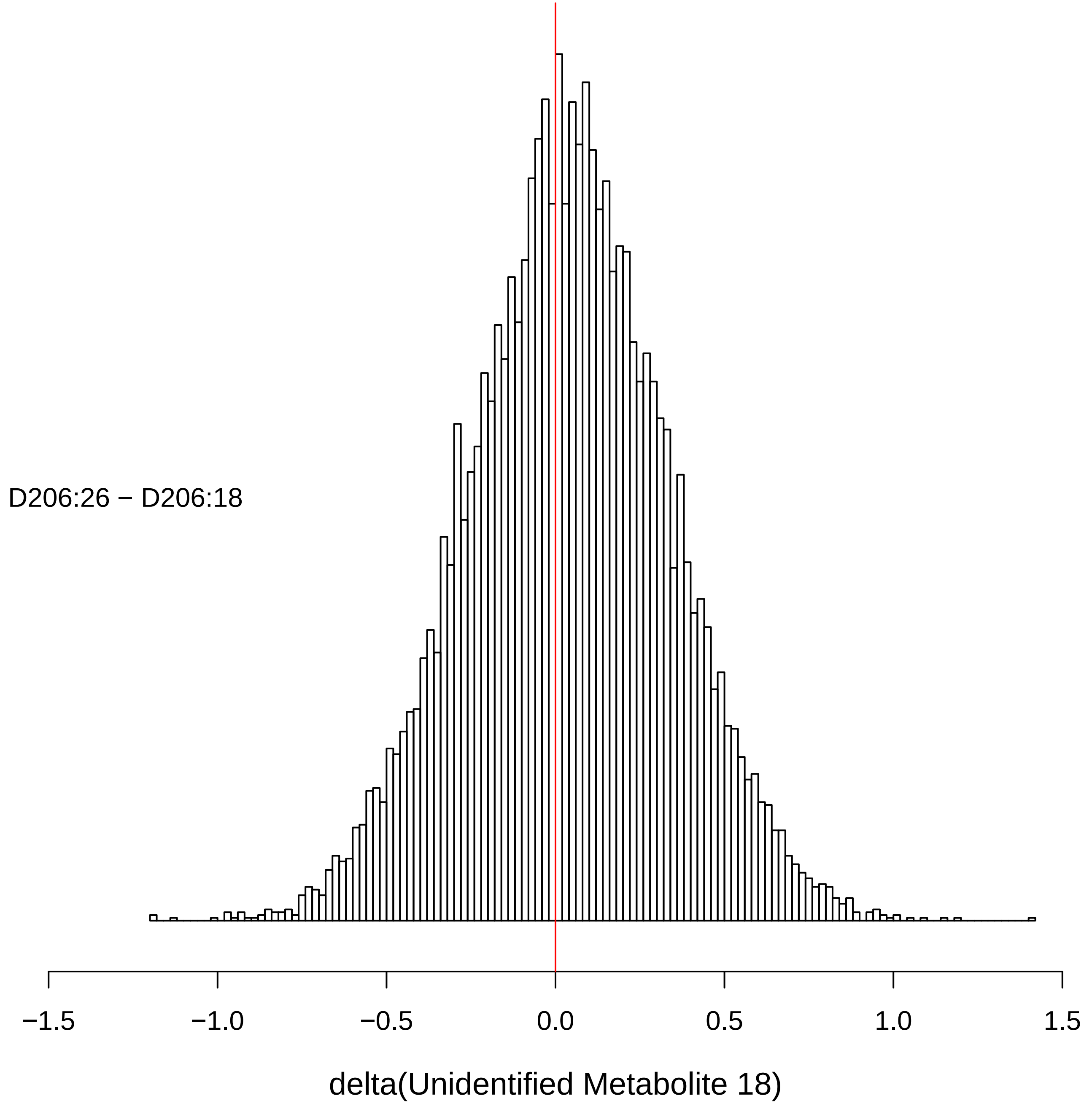
D206:26 − D206:18
−1.5
−1.0
−0.5
0.0
0.5
1.0
1.5
delta(Unidentified Metabolite 18)

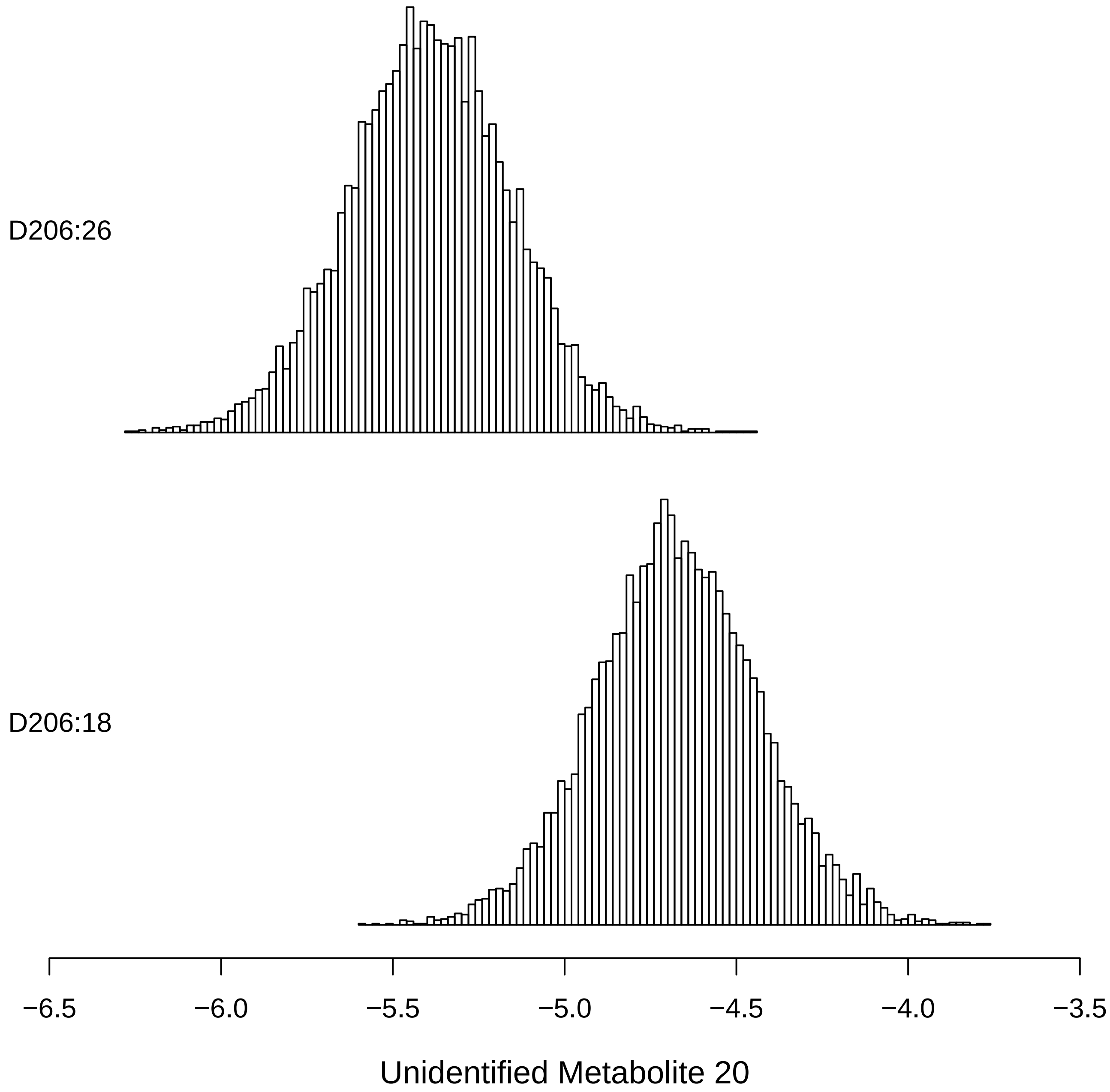
D206:26
D206:18
−6.5
−6.0
−5.5
−5.0
−4.5
−4.0
−3.5
Unidentified Metabolite 20

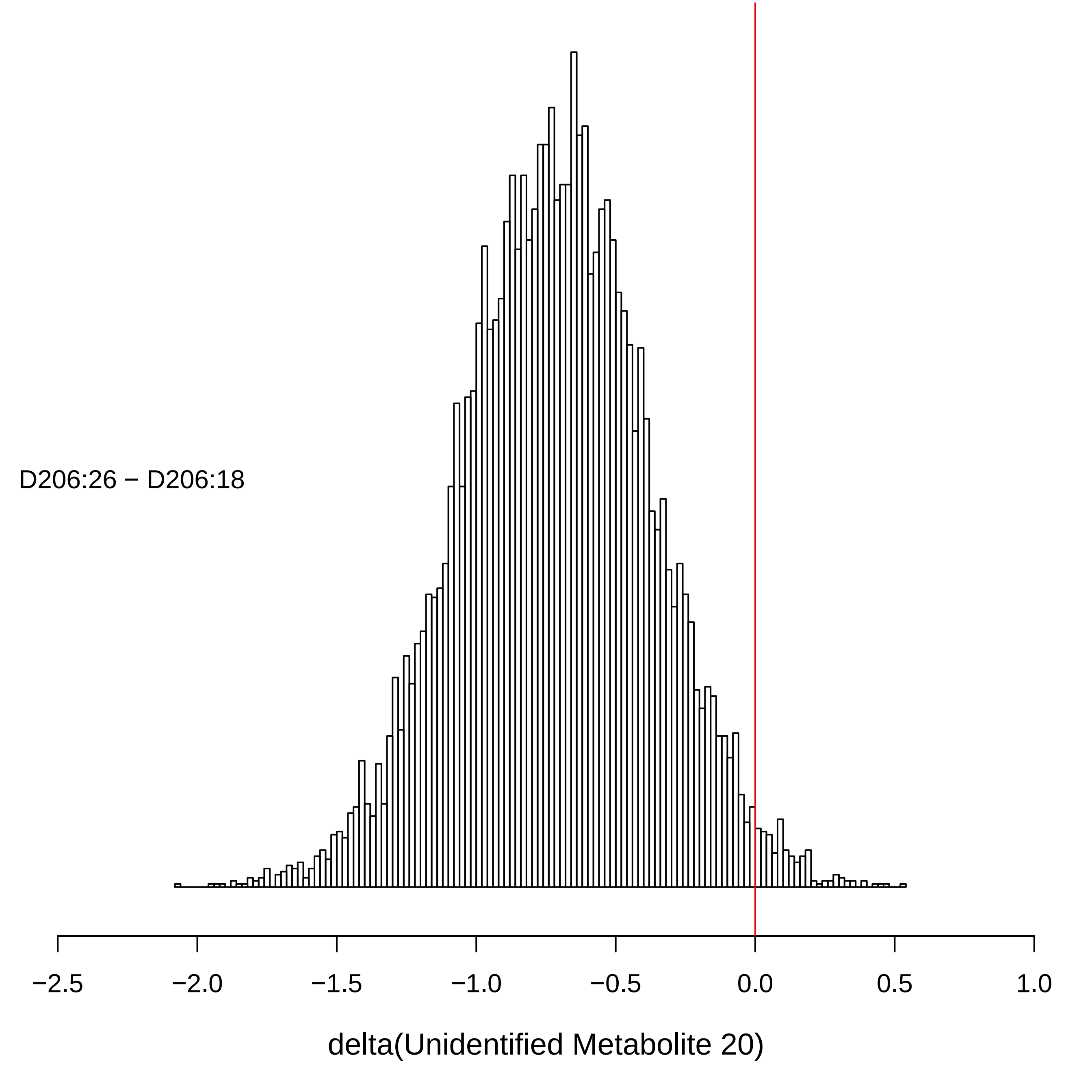
D206:26 − D206:18
−2.5
−2.0
−1.5
−1.0
−0.5
0.0
0.5
1.0
delta(Unidentified Metabolite 20)

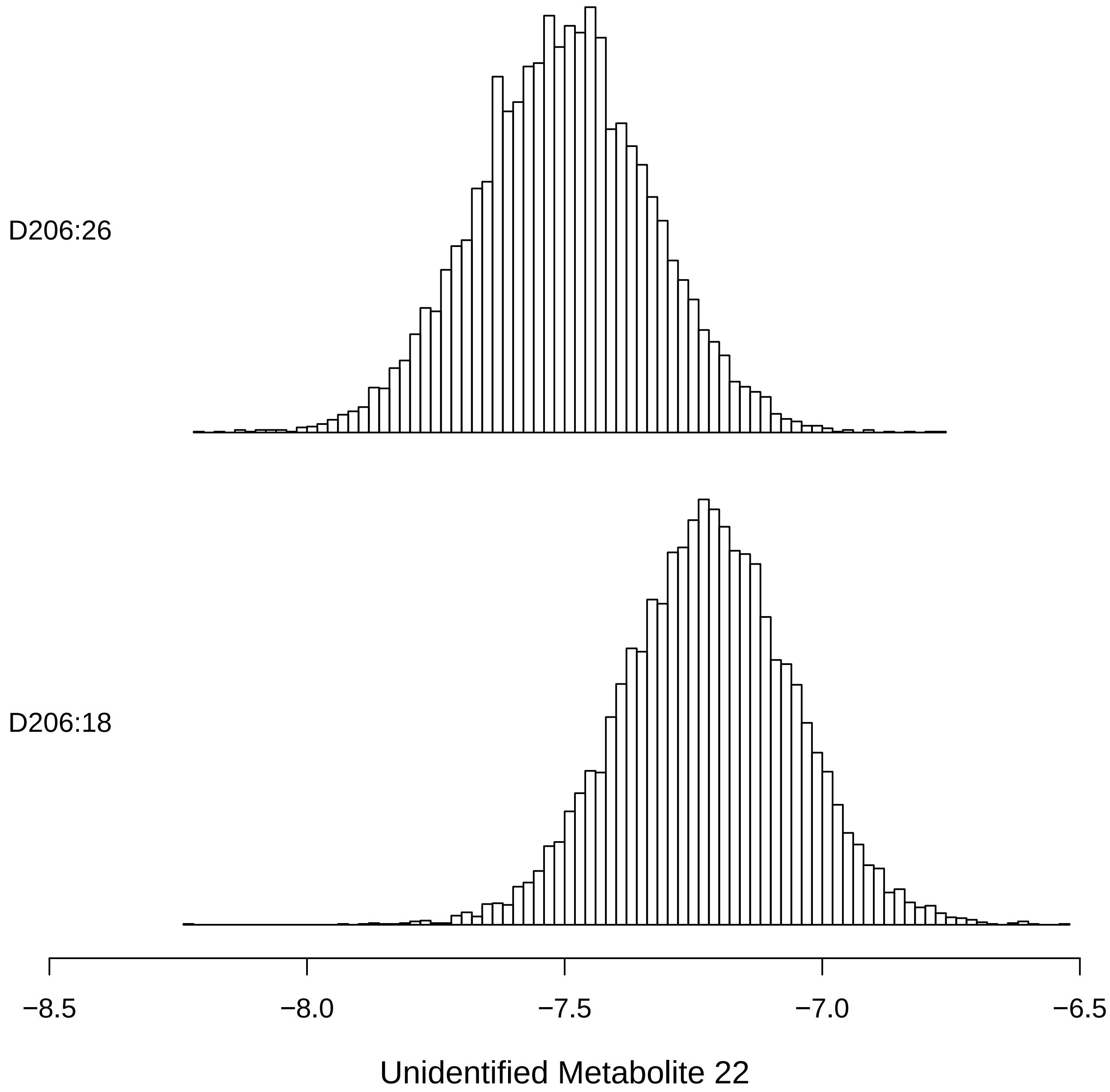
D206:26
D206:18
−8.5
−8.0
−7.5
−7.0
−6.5
Unidentified Metabolite 22

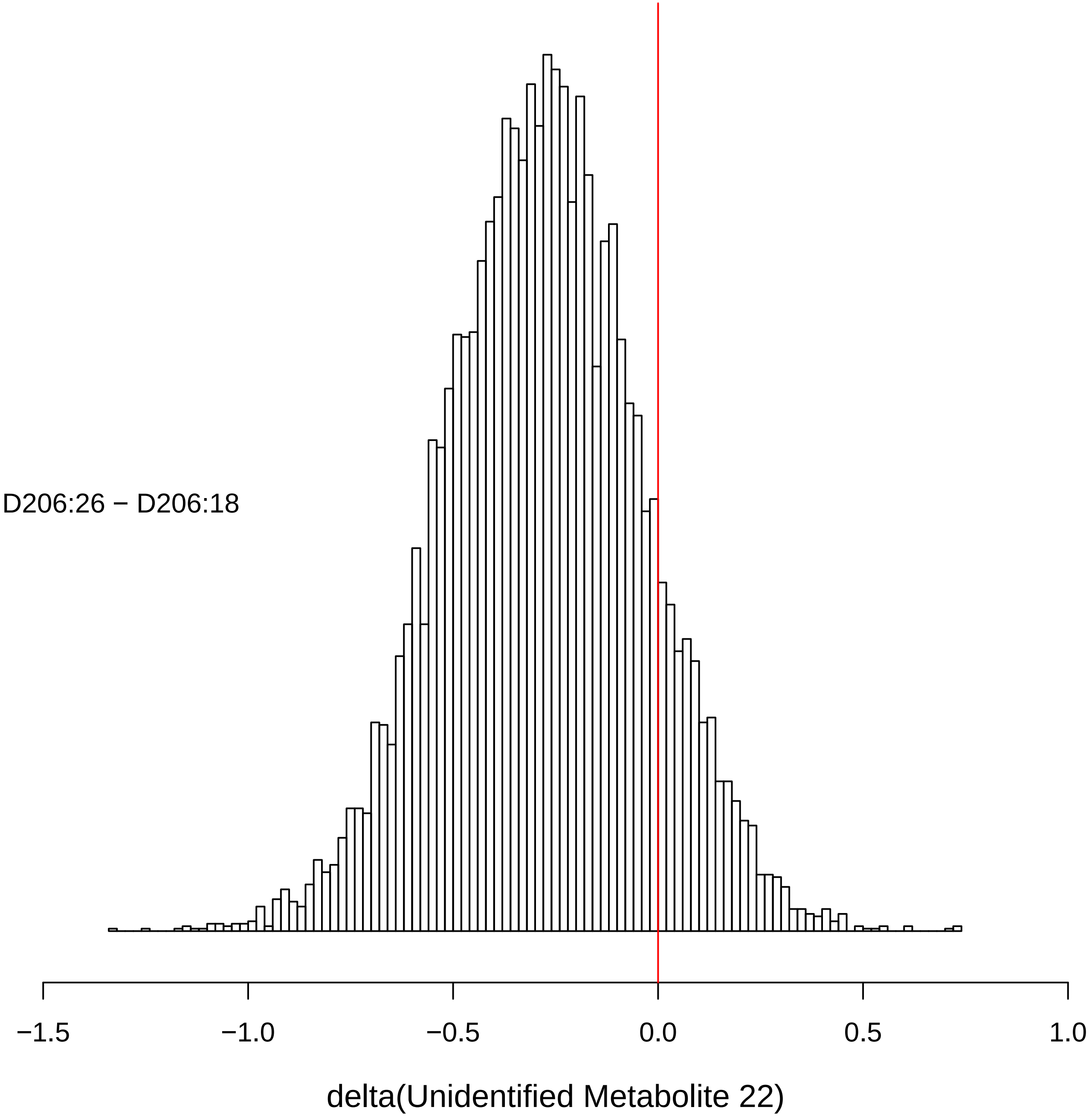
D206:26 − D206:18
−1.5
−1.0
−0.5
0.0
0.5
1.0
delta(Unidentified Metabolite 22)

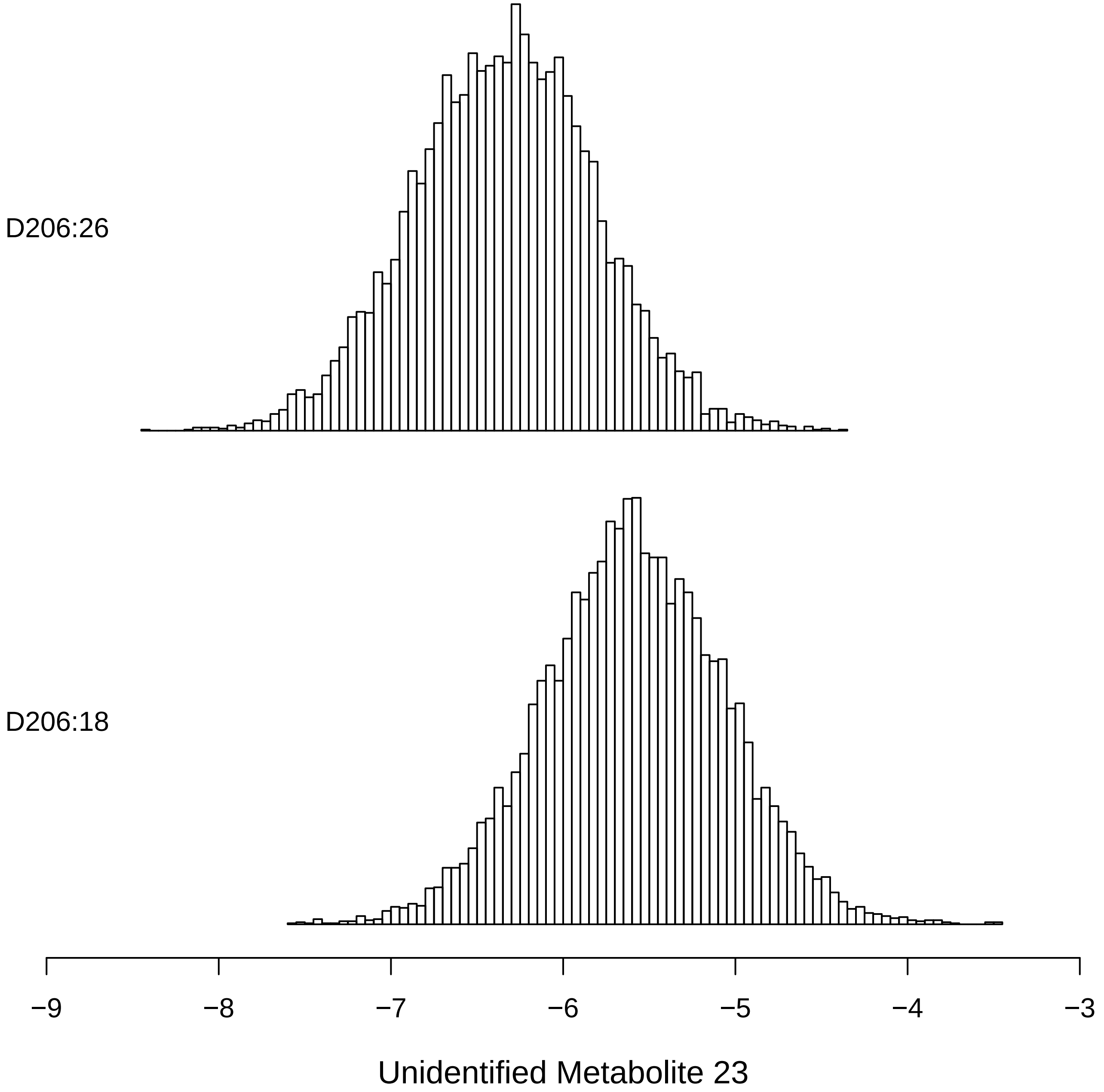
D206:26
D206:18
−9
−8
−7
−6
−5
−4
−3
Unidentified Metabolite 23

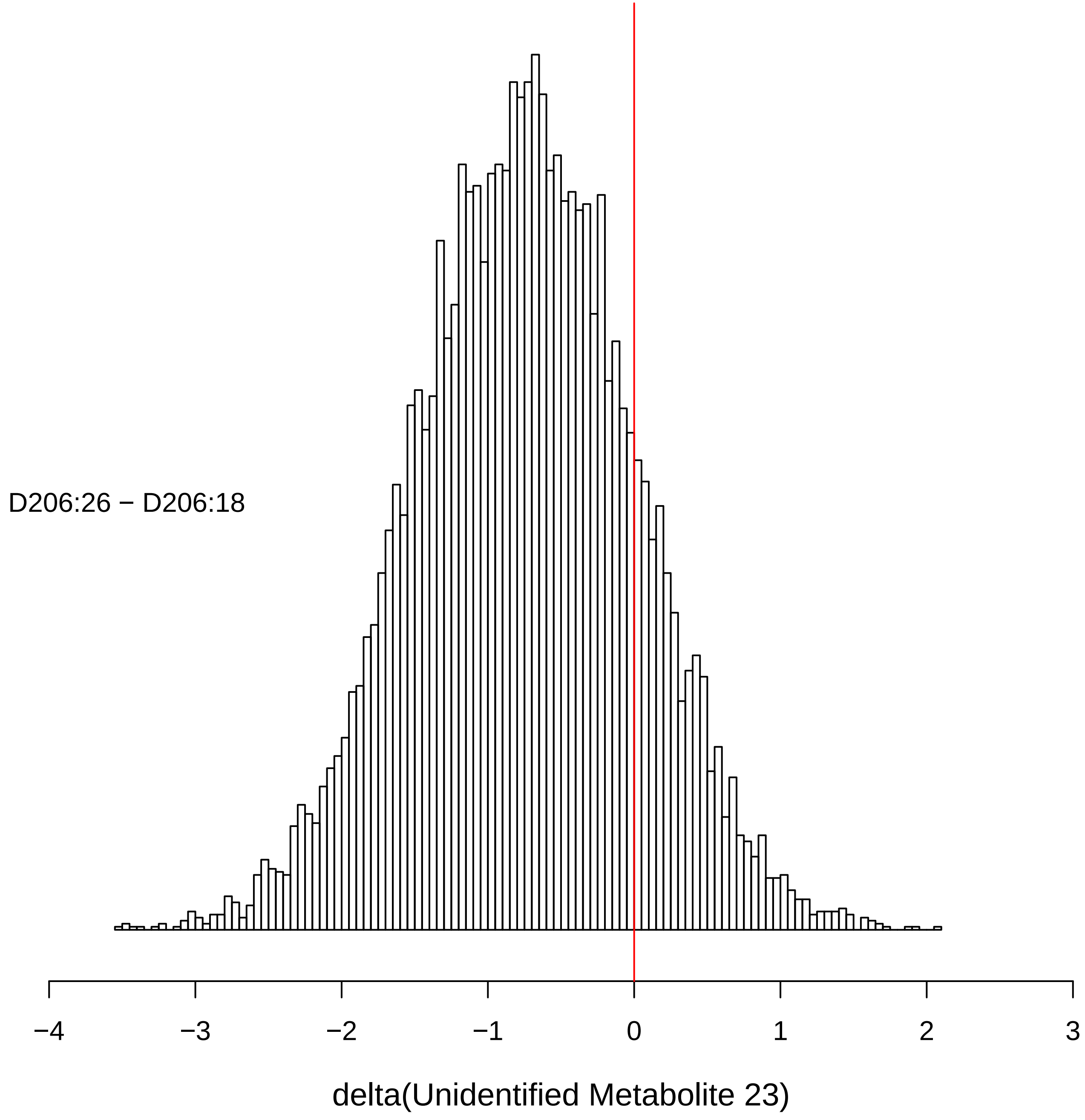
D206:26 − D206:18
−4
−3
−2
−1
0
1
2
3
delta(Unidentified Metabolite 23)

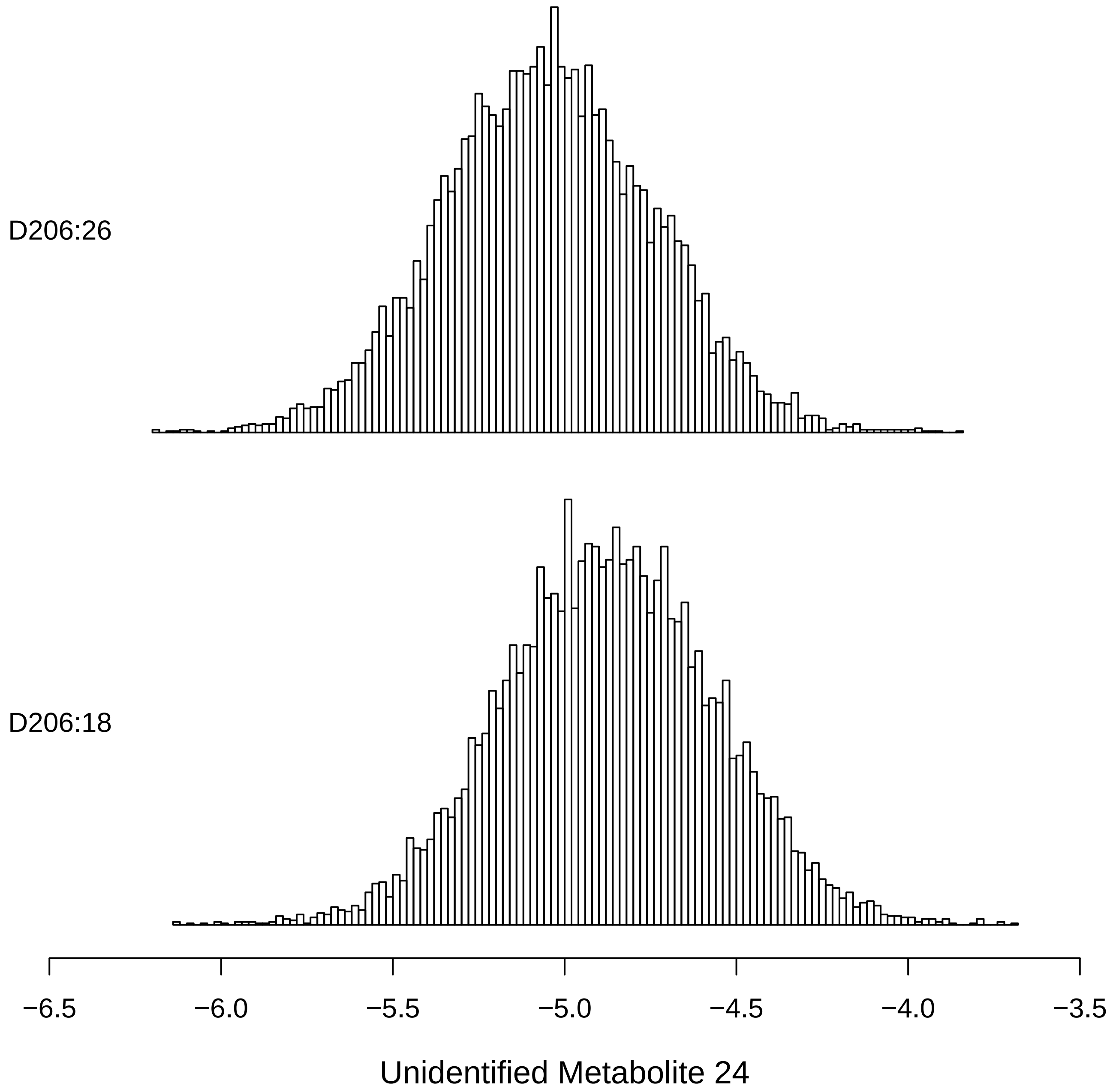
D206:26
D206:18
−6.5
−6.0
−5.5
−5.0
−4.5
−4.0
−3.5
Unidentified Metabolite 24

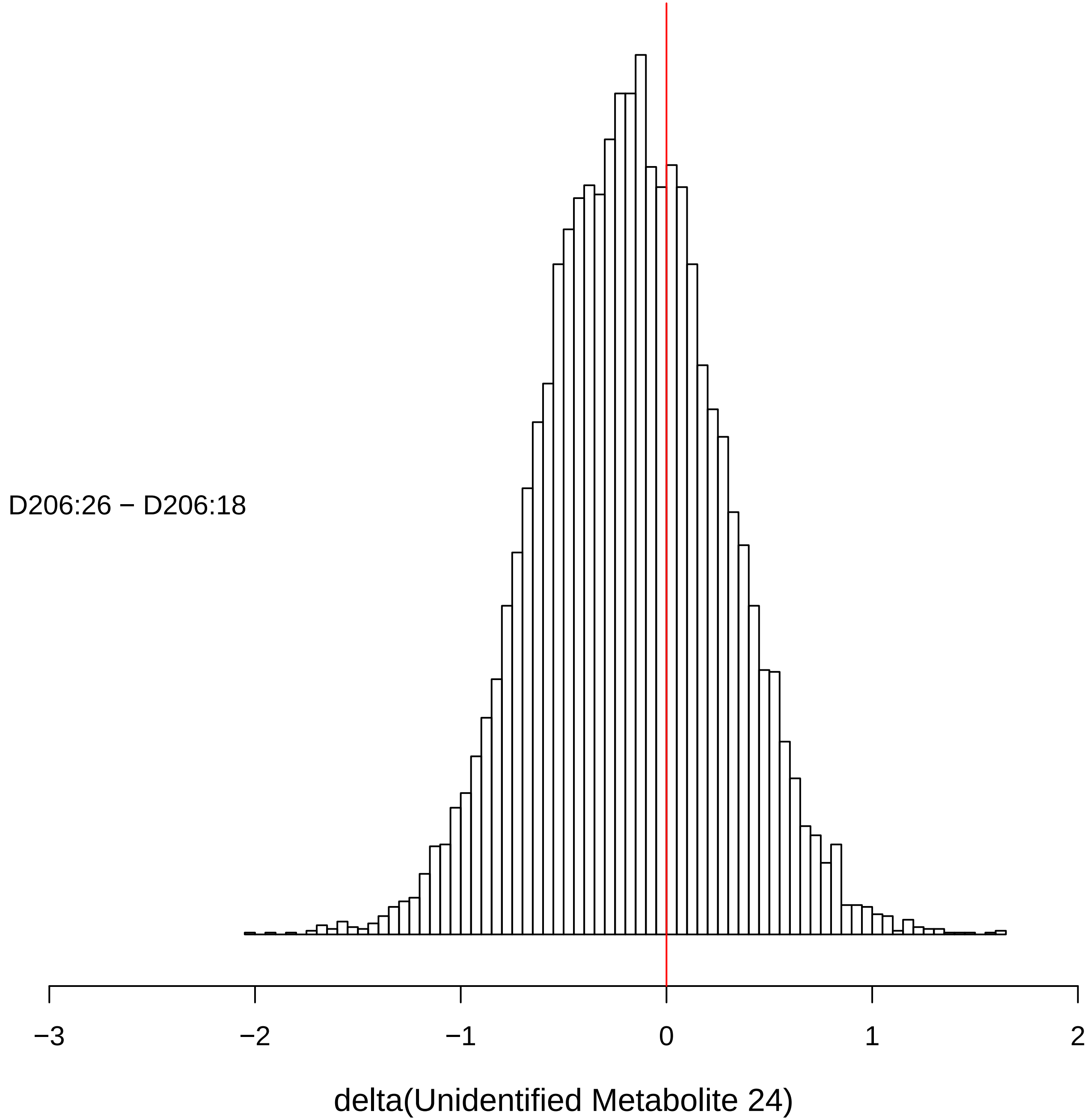
D206:26 − D206:18
−3
−2
−1
0
1
2
delta(Unidentified Metabolite 24)

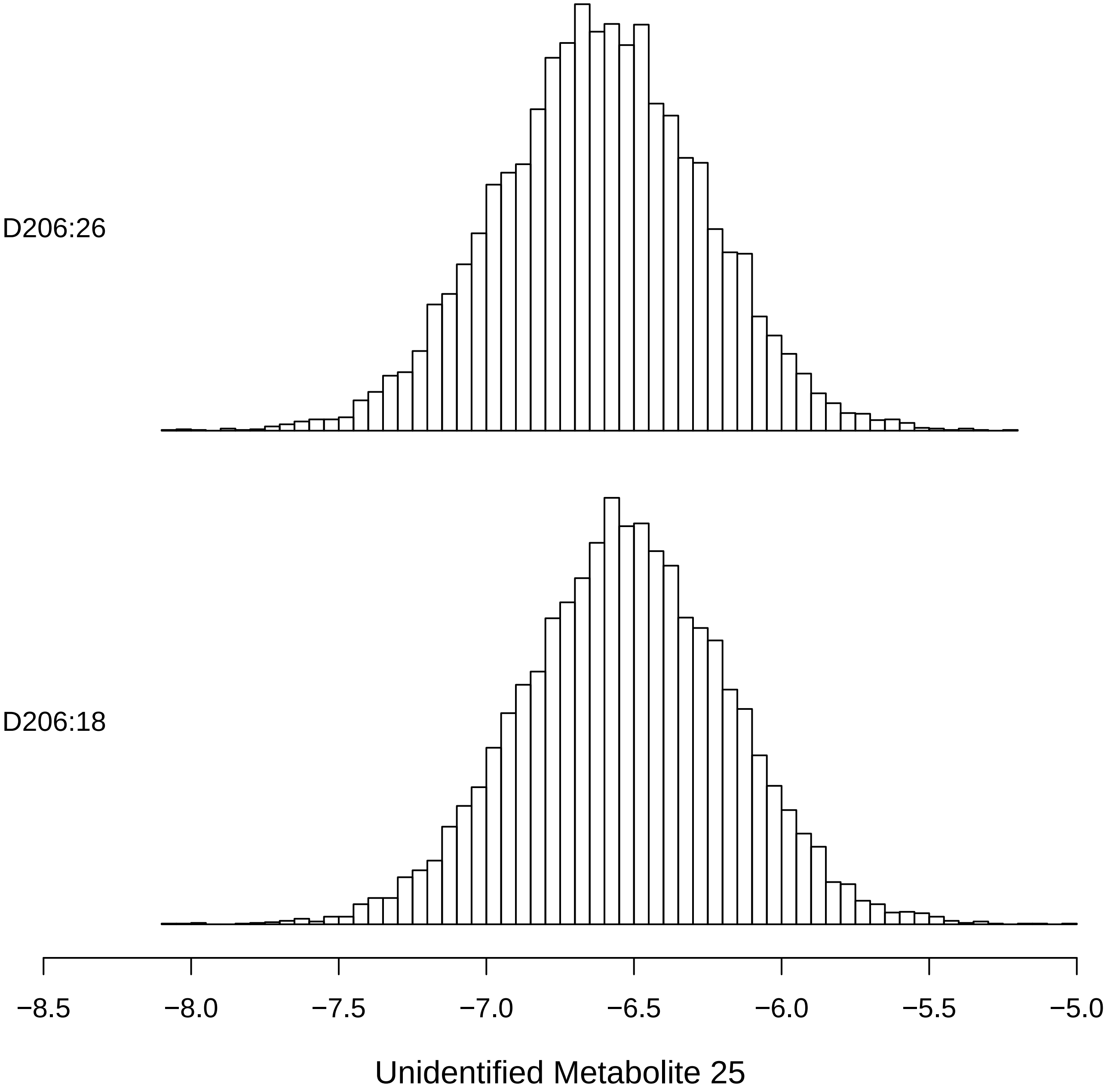
D206:26
D206:18
−8.5
−8.0
−7.5
−7.0
−6.5
−6.0
−5.5
−5.0
Unidentified Metabolite 25

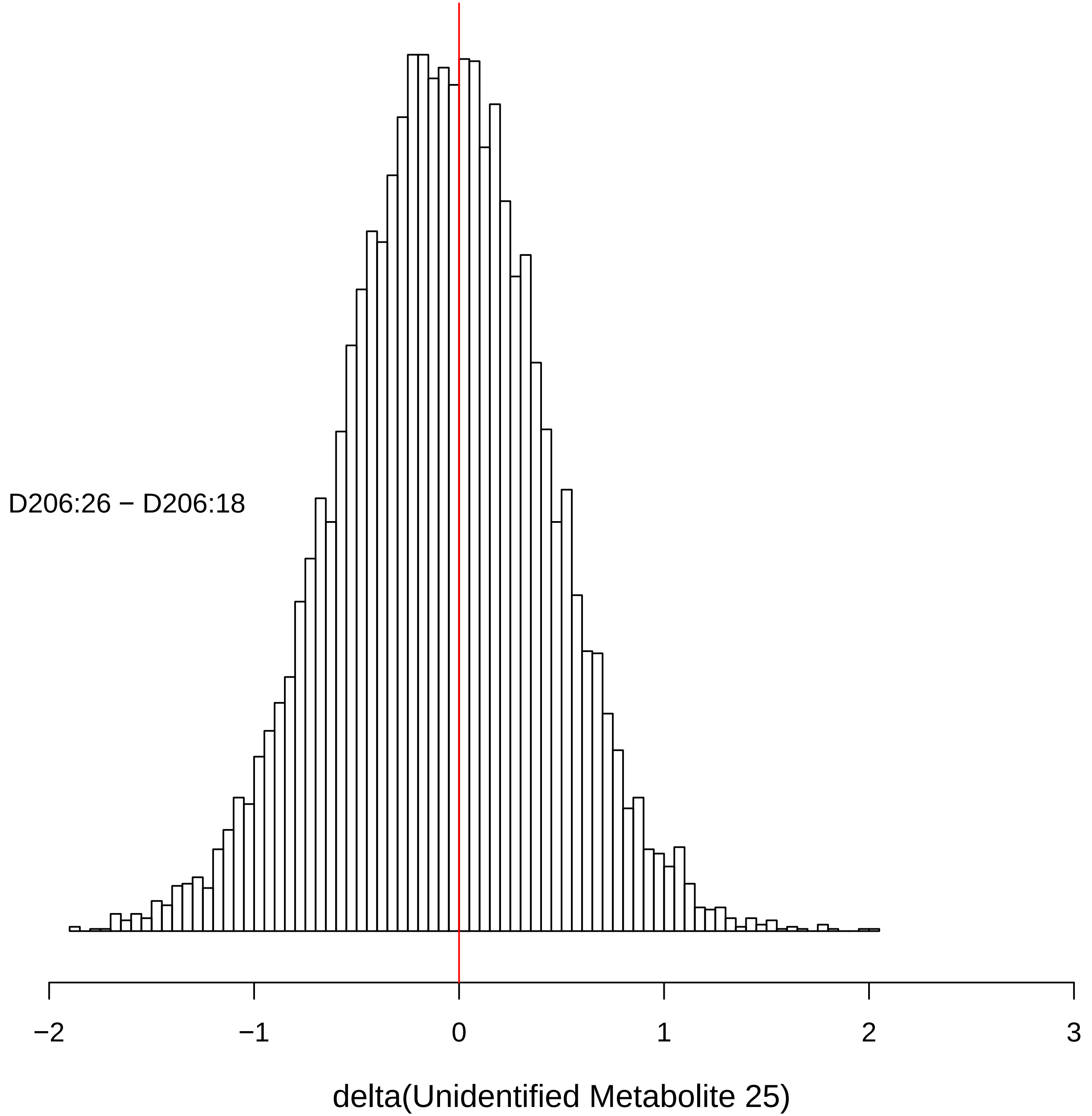
D206:26 − D206:18
−2
−1
0
1
2
3
delta(Unidentified Metabolite 25)

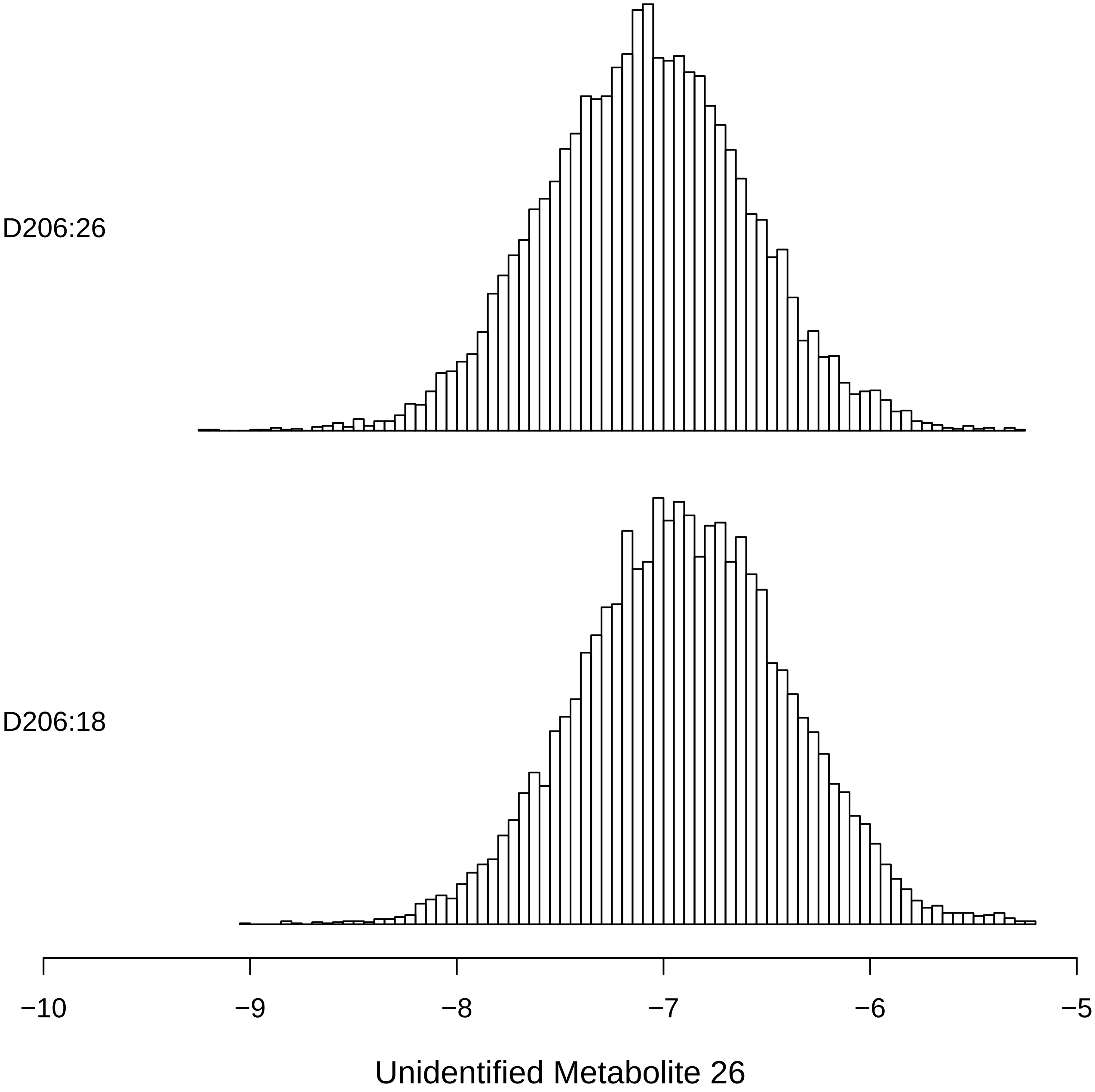

D206:26
D206:18
−10
−9
−8
−7
−6
−5
Unidentified Metabolite 26

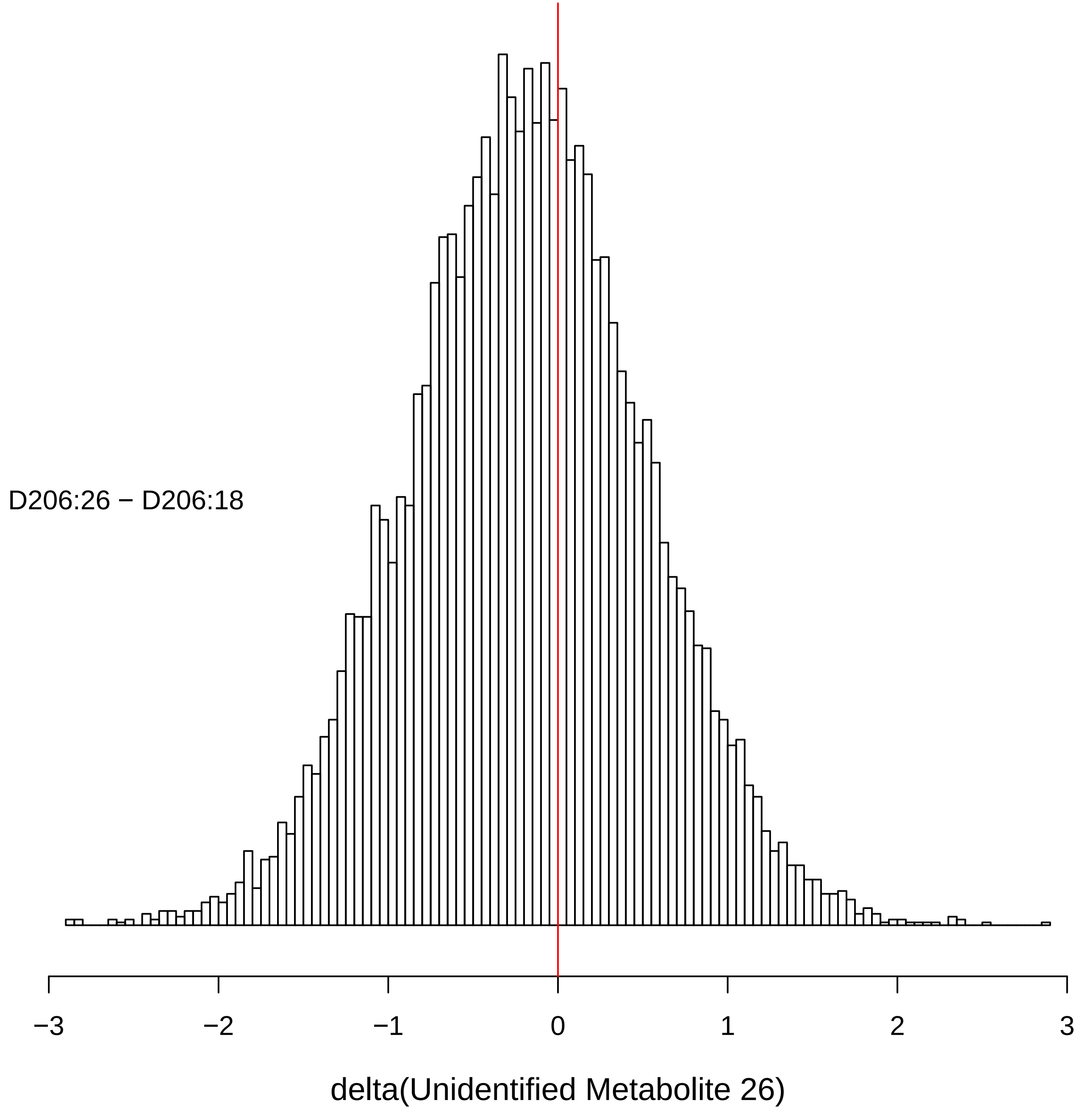
D206:26 − D206:18
−3
−2
−1
0
1
2
3
delta(Unidentified Metabolite 26)

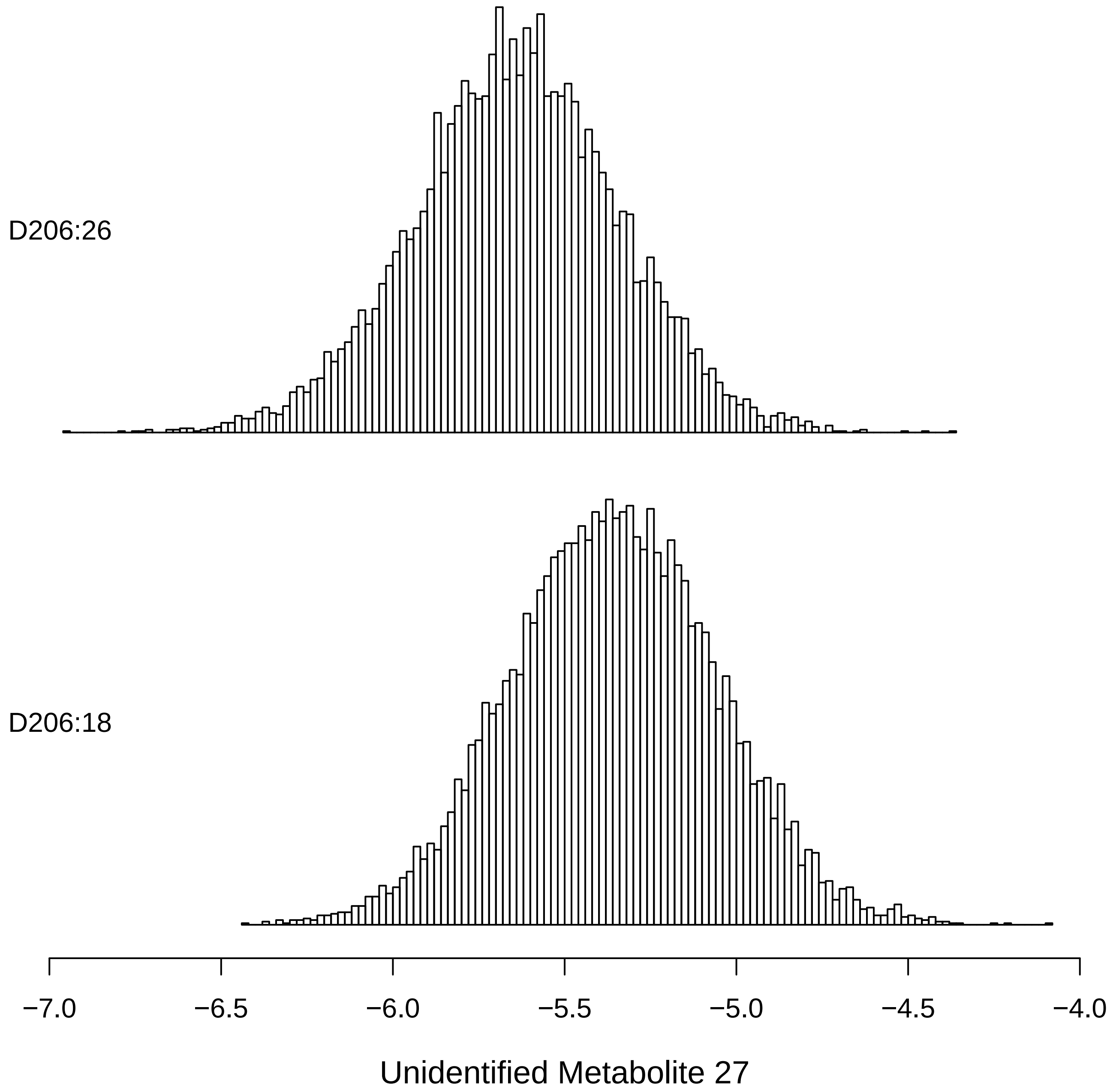
D206:26
D206:18
−7.0
−6.5
−6.0
−5.5
−5.0
−4.5
−4.0
Unidentified Metabolite 27

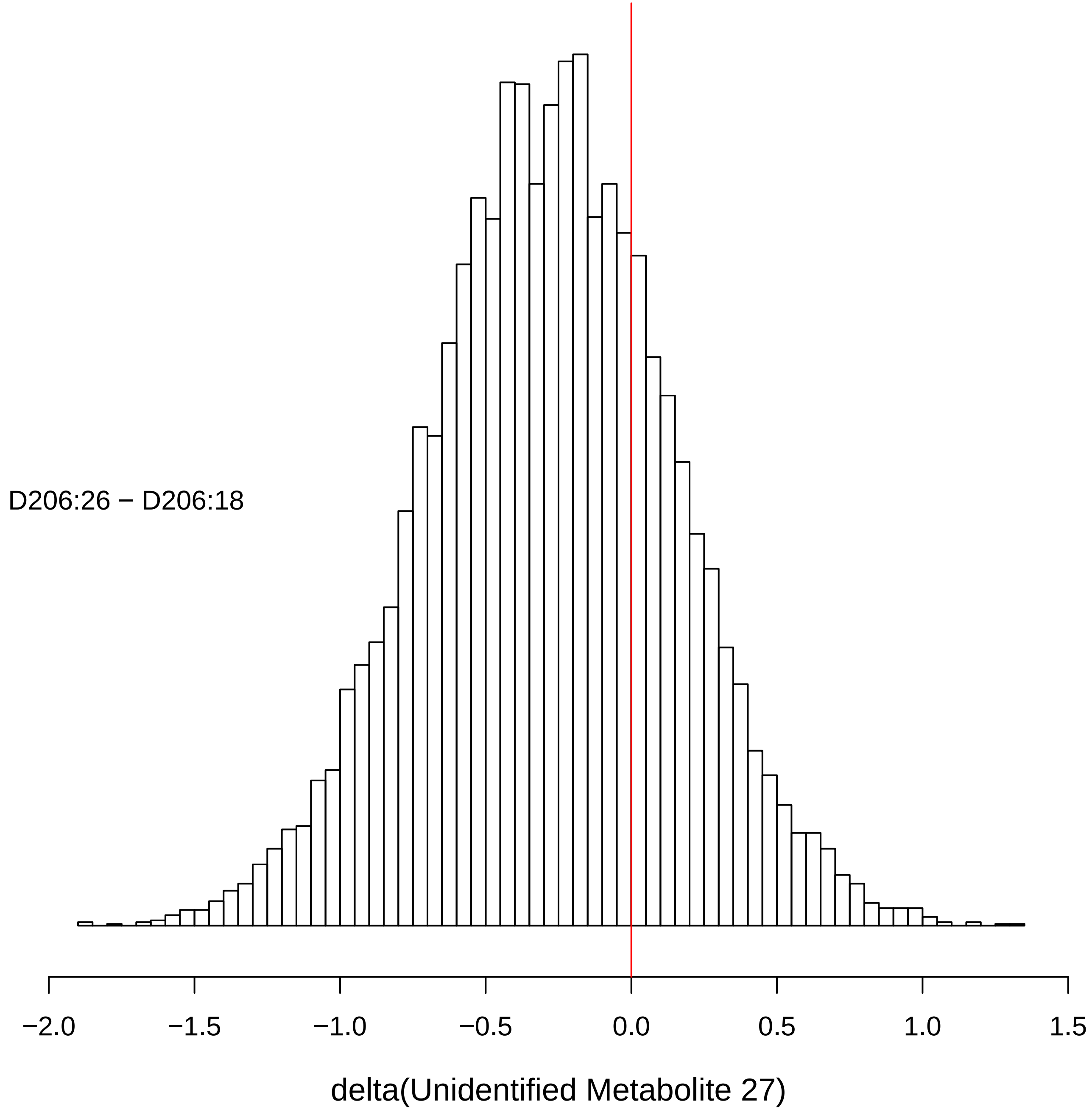
D206:26 − D206:18
−2.0
−1.5
−1.0
−0.5
0.0
0.5
1.0
1.5
delta(Unidentified Metabolite 27)

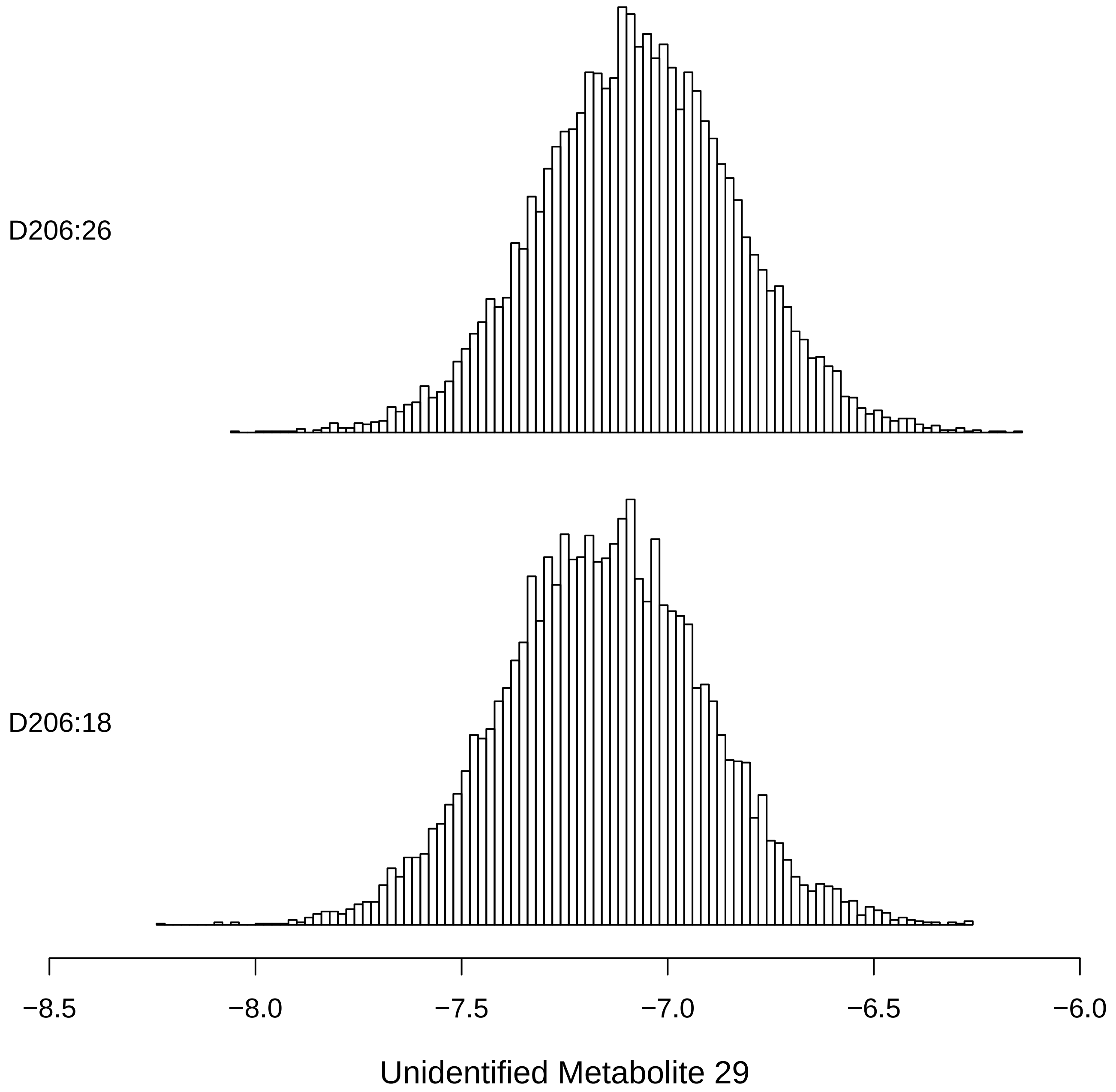
D206:26
D206:18
−8.5
−8.0
−7.5
−7.0
−6.5
−6.0
Unidentified Metabolite 29

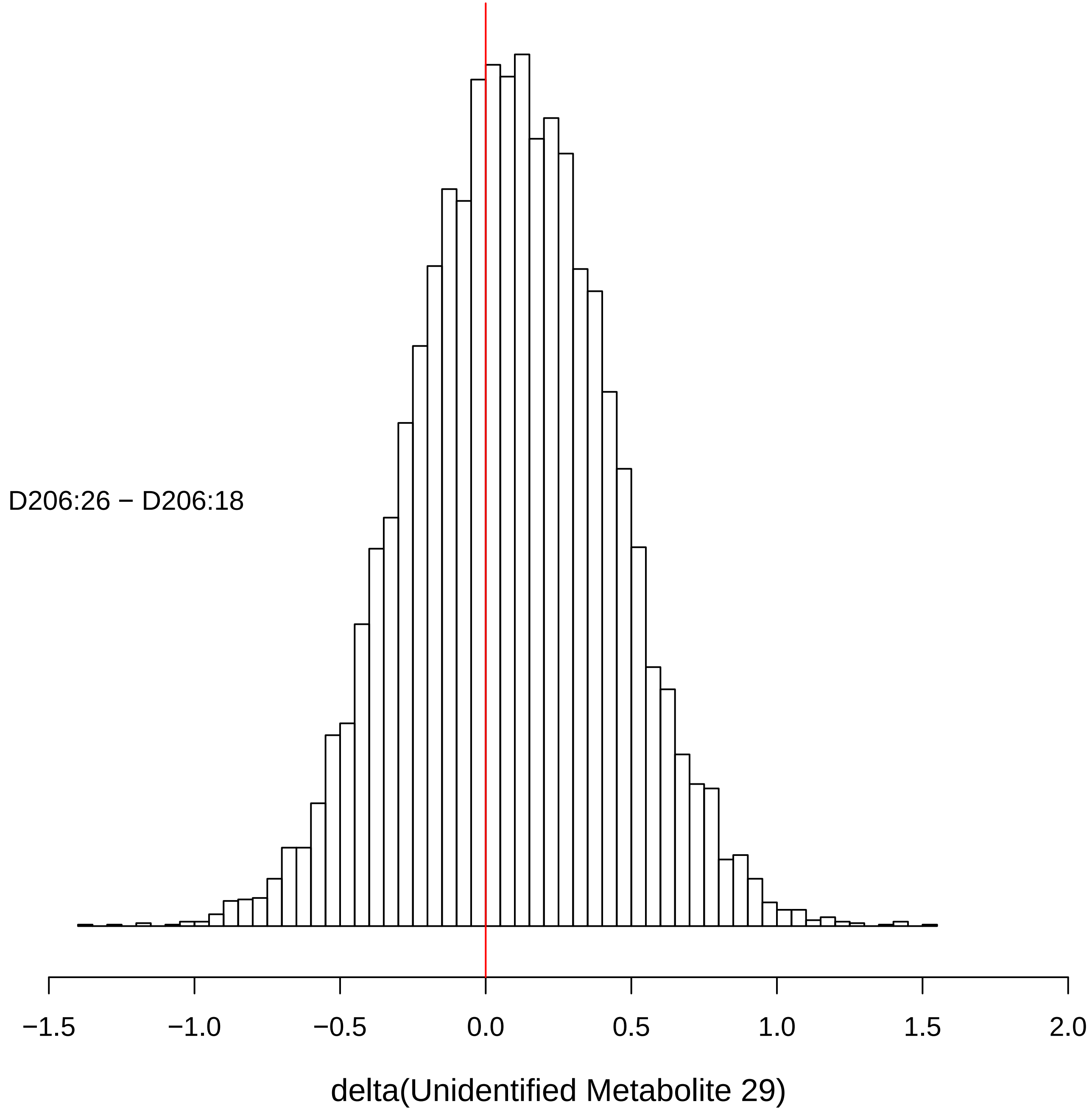
D206:26 − D206:18
−1.5
−1.0
−0.5
0.0
0.5
1.0
1.5
2.0
delta(Unidentified Metabolite 29)

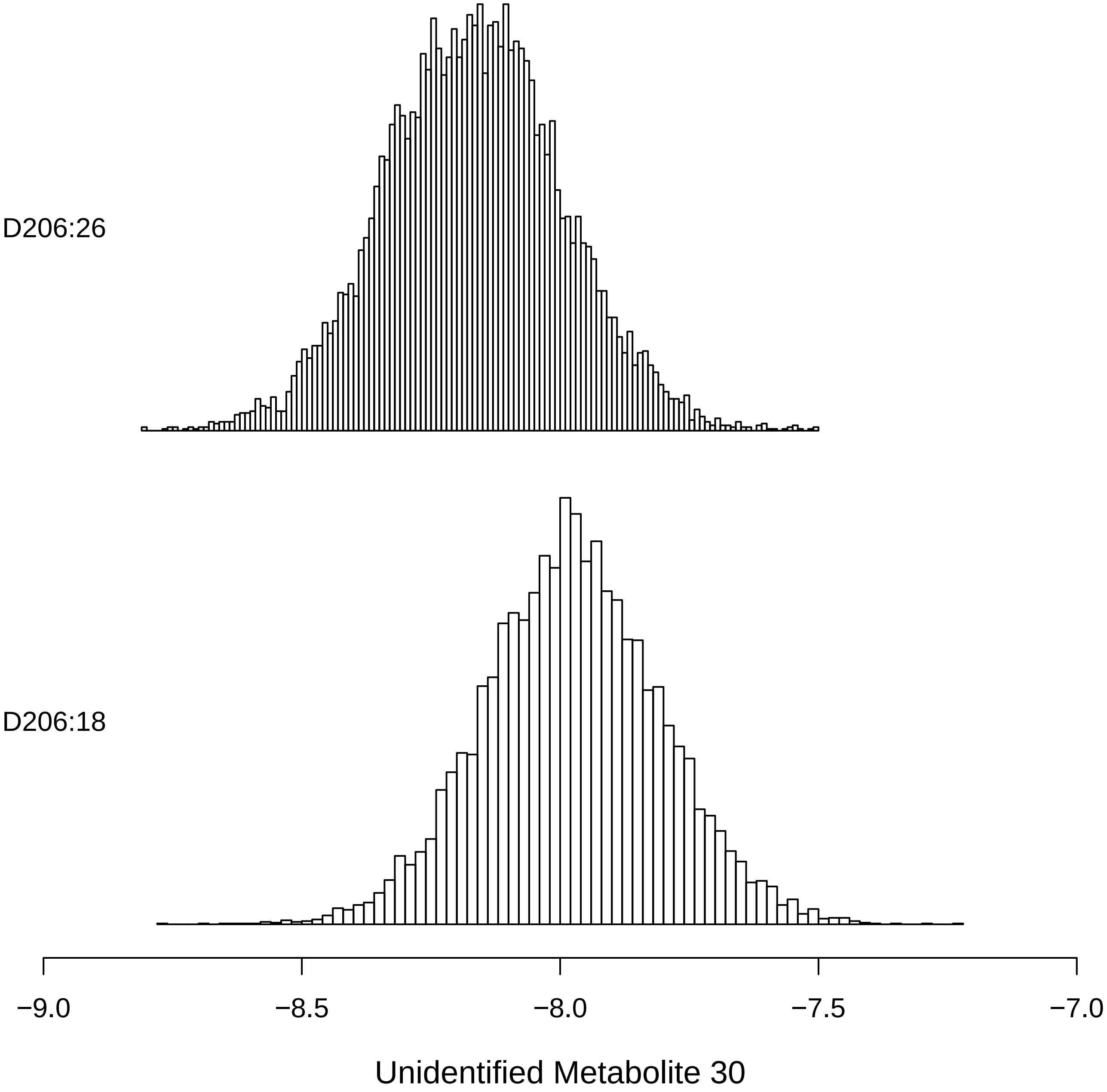
D206:26
D206:18
−9.0
−8.5
−8.0
−7.5
−7.0
Unidentified Metabolite 30

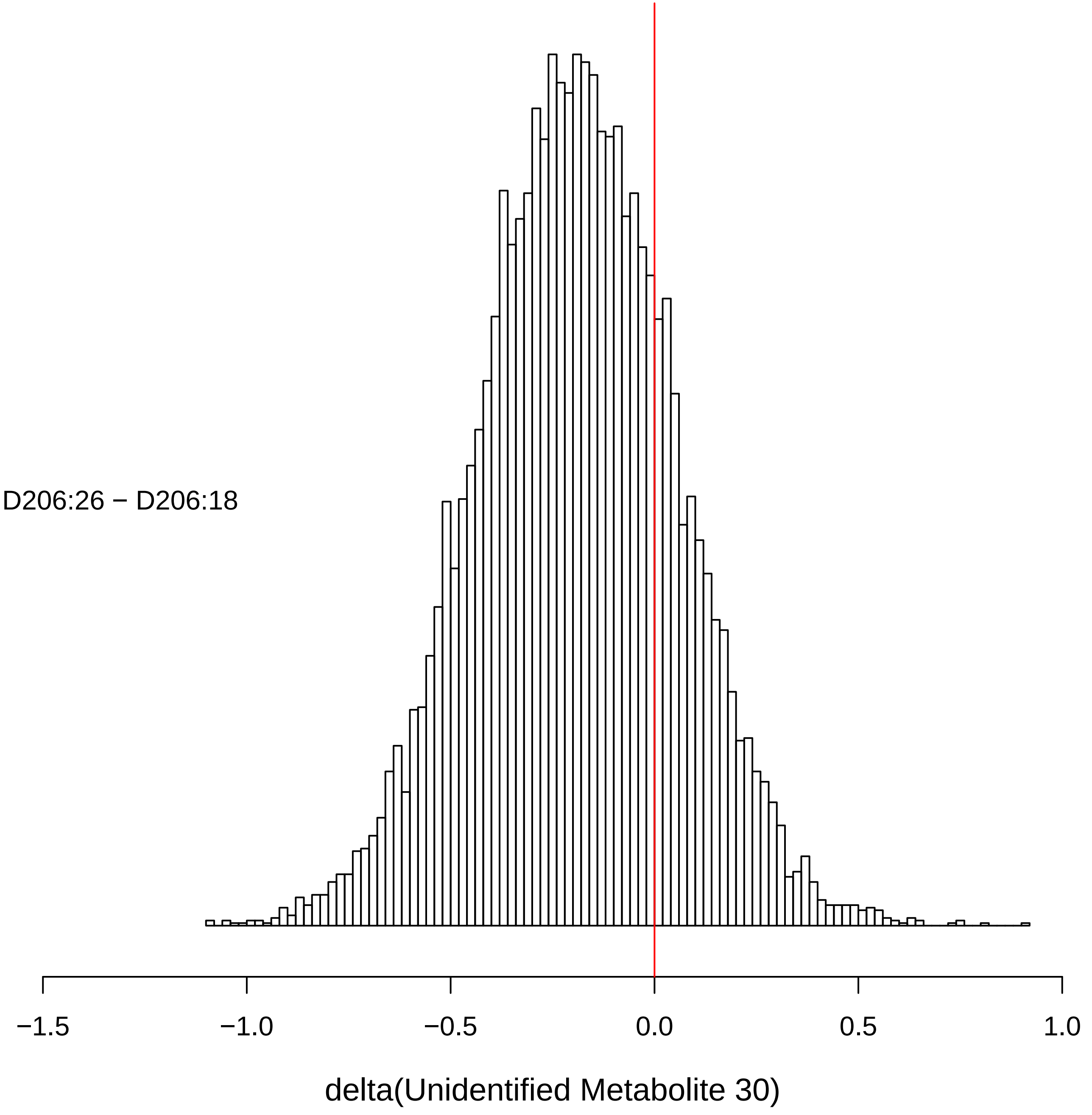
D206:26 − D206:18
−1.5
−1.0
−0.5
0.0
0.5
1.0
delta(Unidentified Metabolite 30)

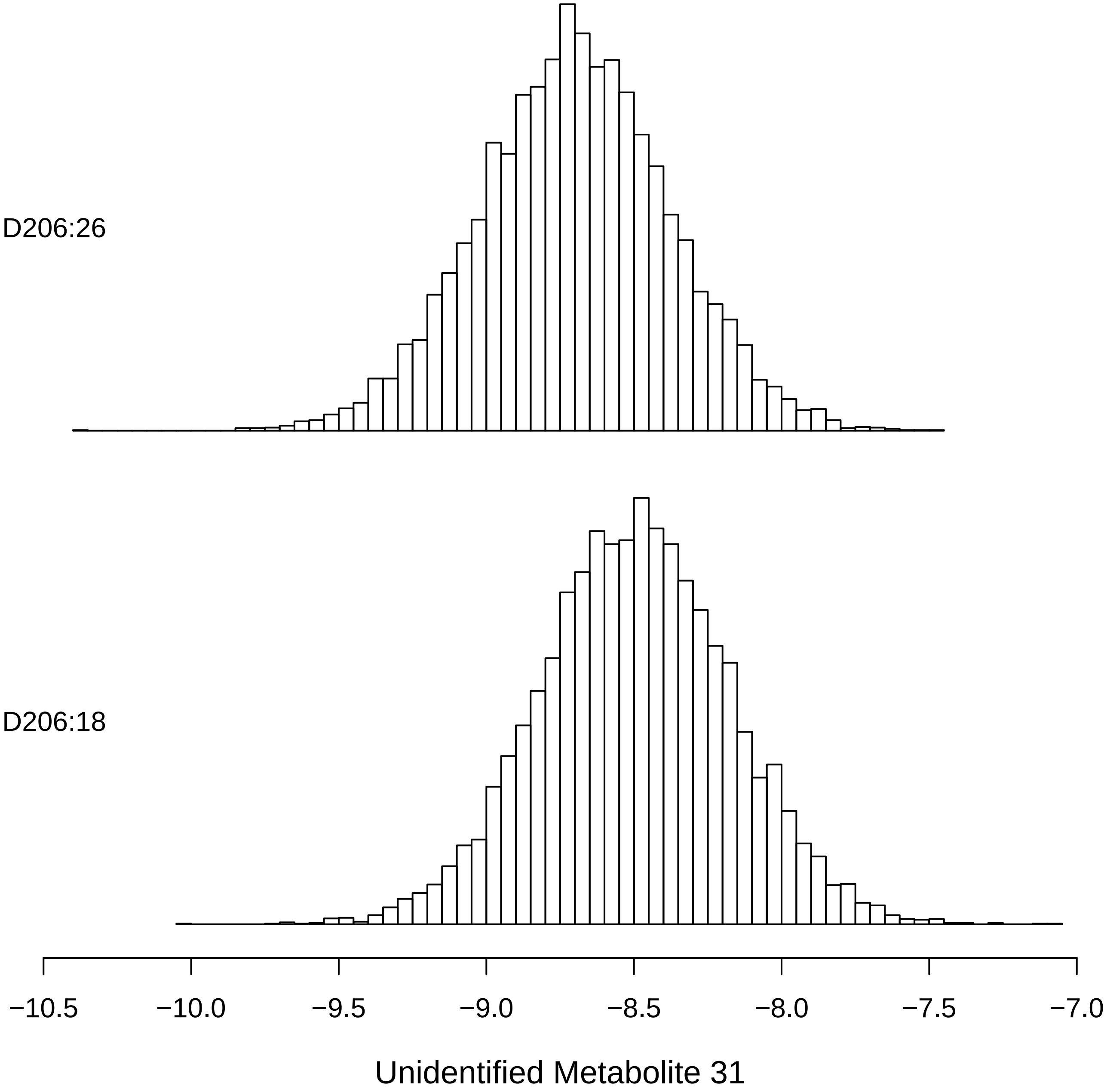
D206:26
D206:18
−10.5
−10.0
−9.5
−9.0
−8.5
−8.0
−7.5
−7.0
Unidentified Metabolite 31

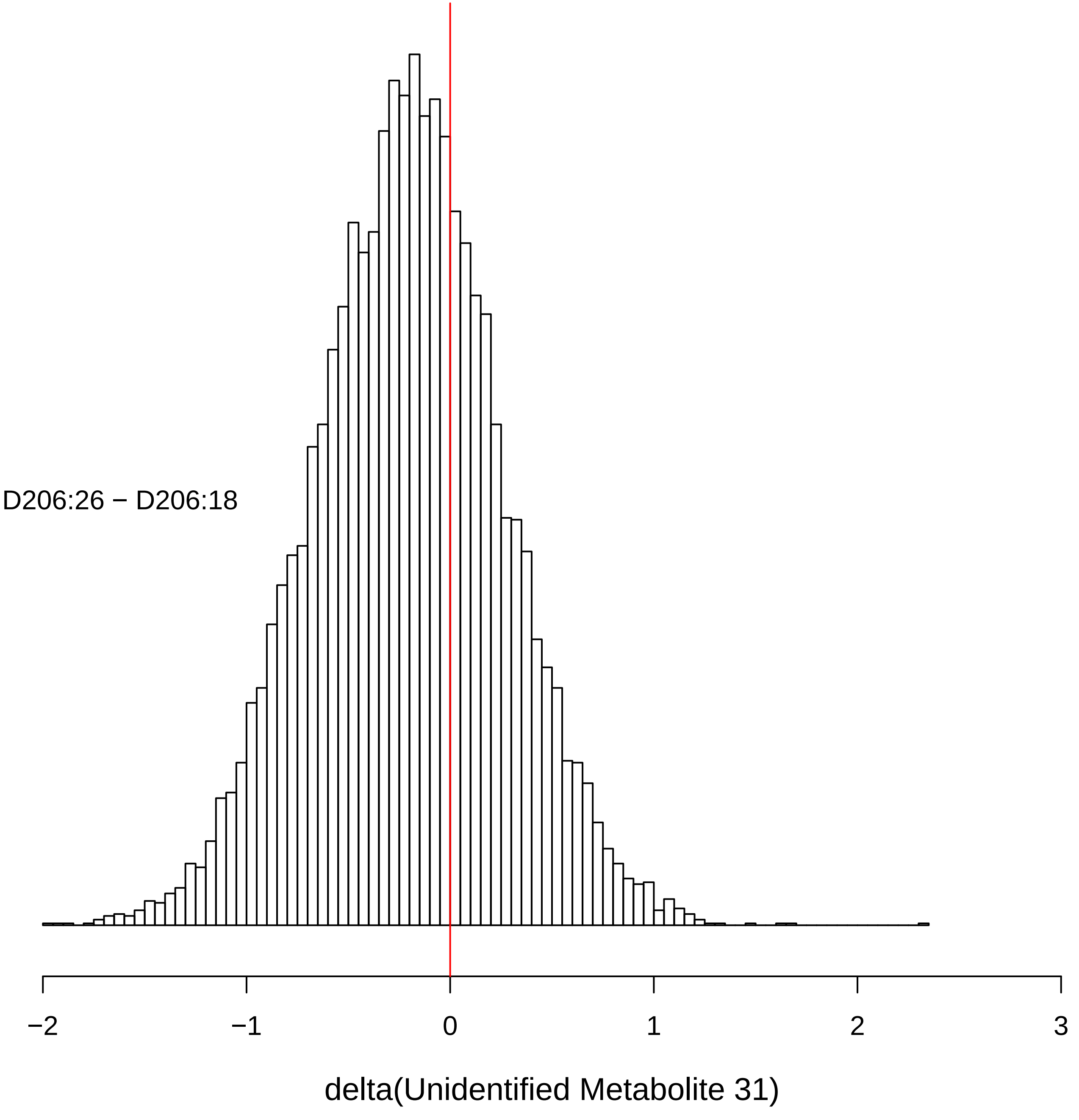
D206:26 − D206:18
−2
−1
0
1
2
3
delta(Unidentified Metabolite 31)

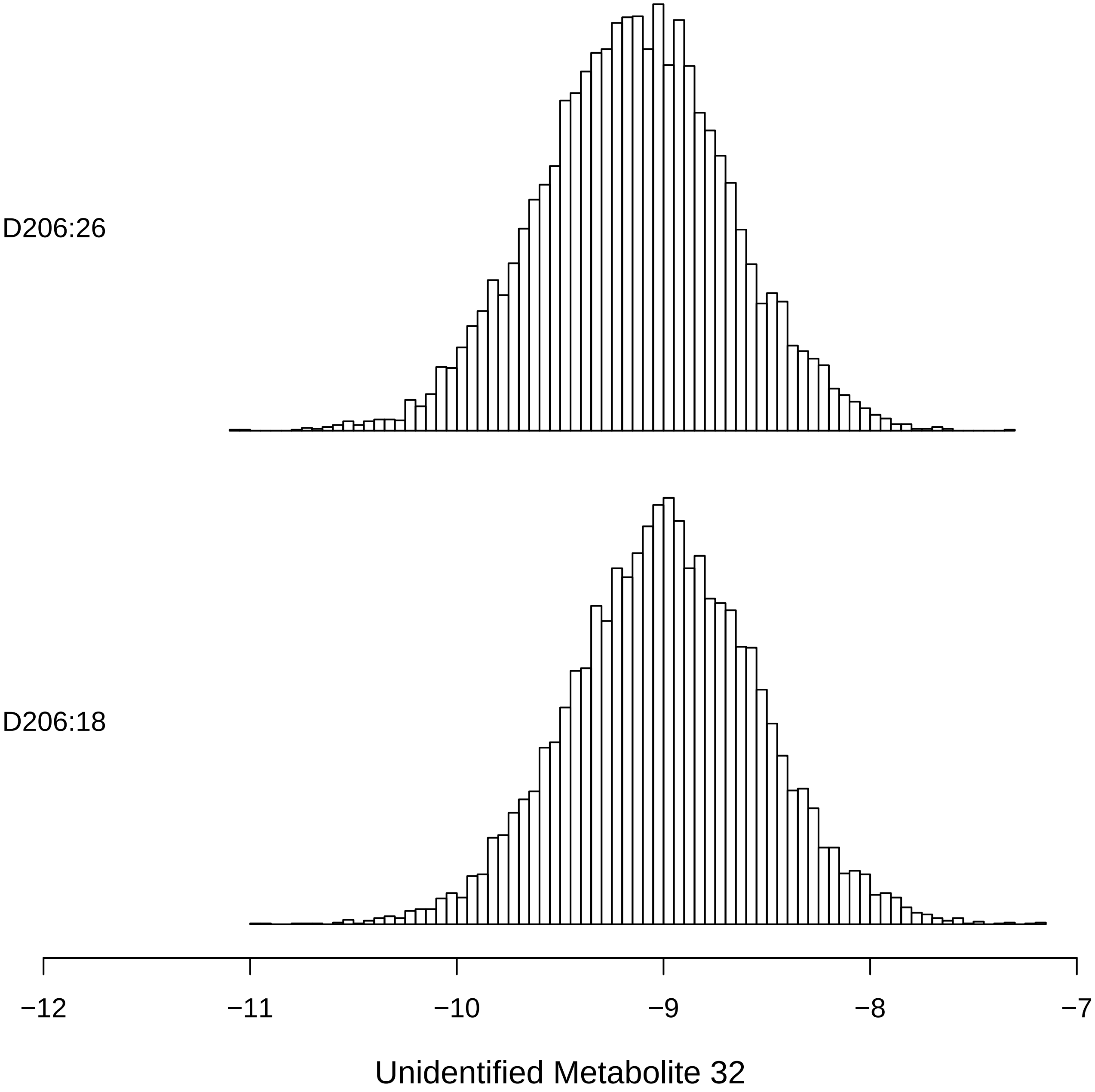
D206:26
D206:18
−12
−11
−10
−9
−8
−7
Unidentified Metabolite 32

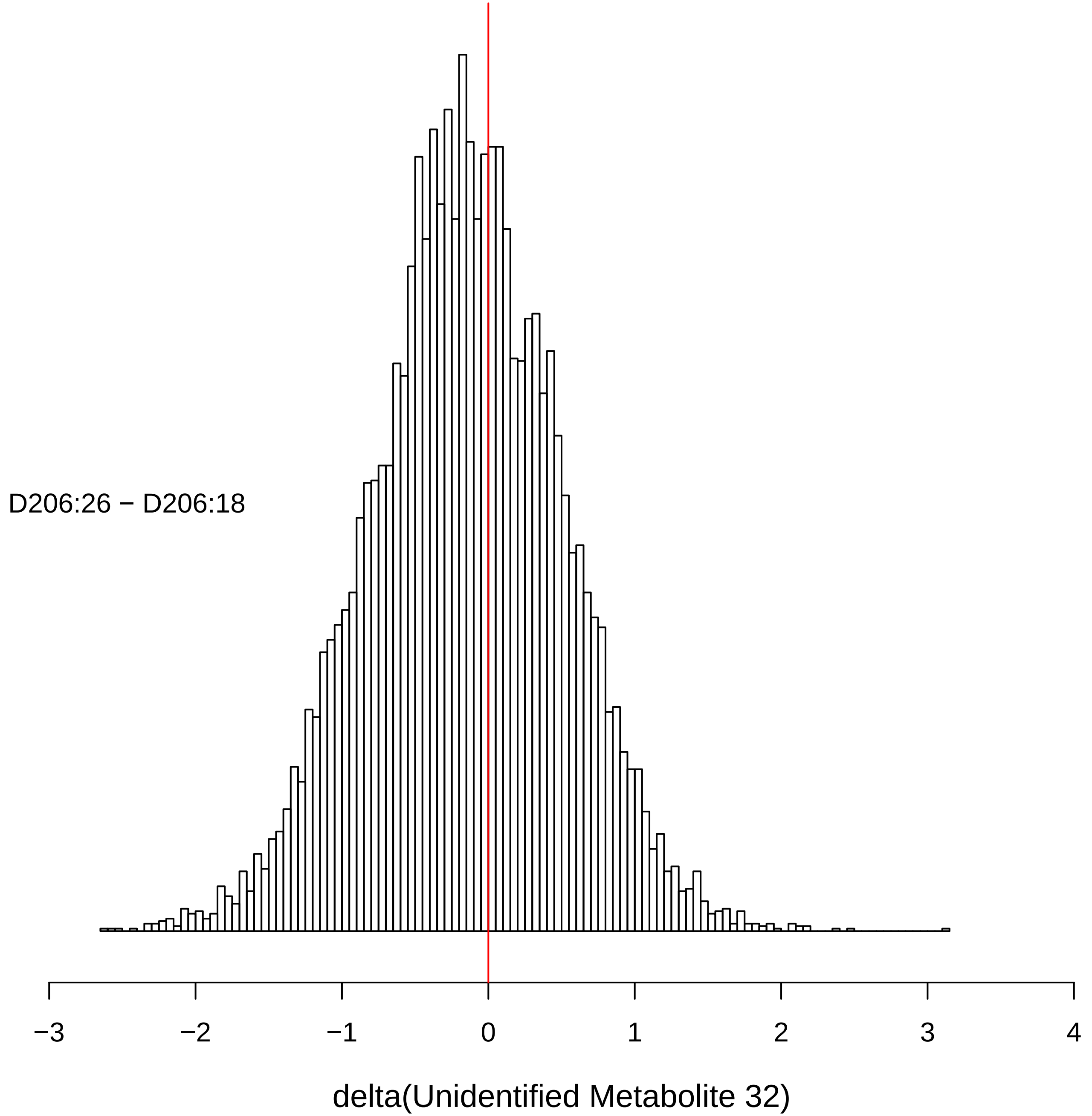
D206:26 − D206:18
−3
−2
−1
0
1
2
3
4
delta(Unidentified Metabolite 32)

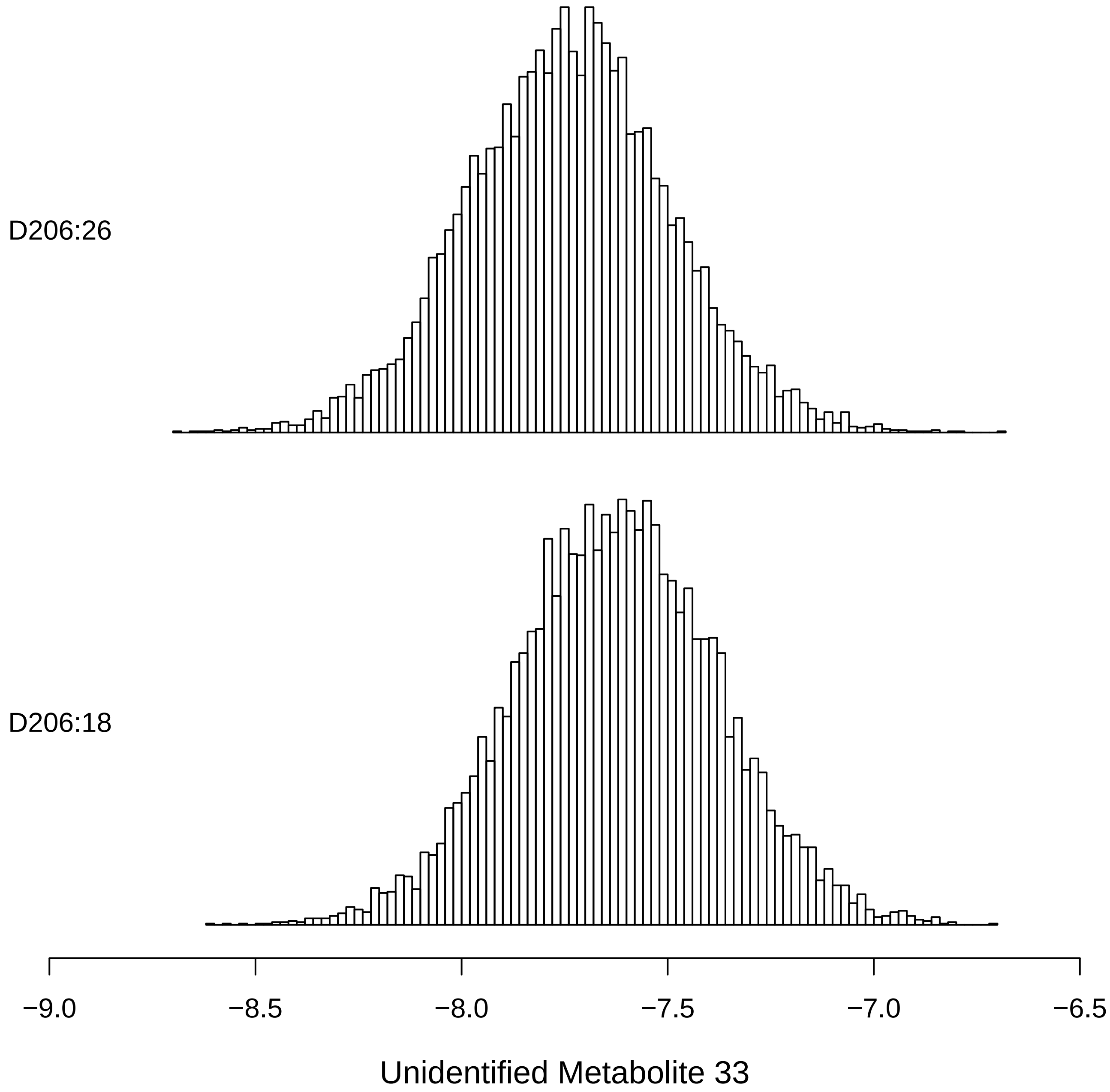
D206:26
D206:18
−9.0
−8.5
−8.0
−7.5
−7.0
−6.5
Unidentified Metabolite 33

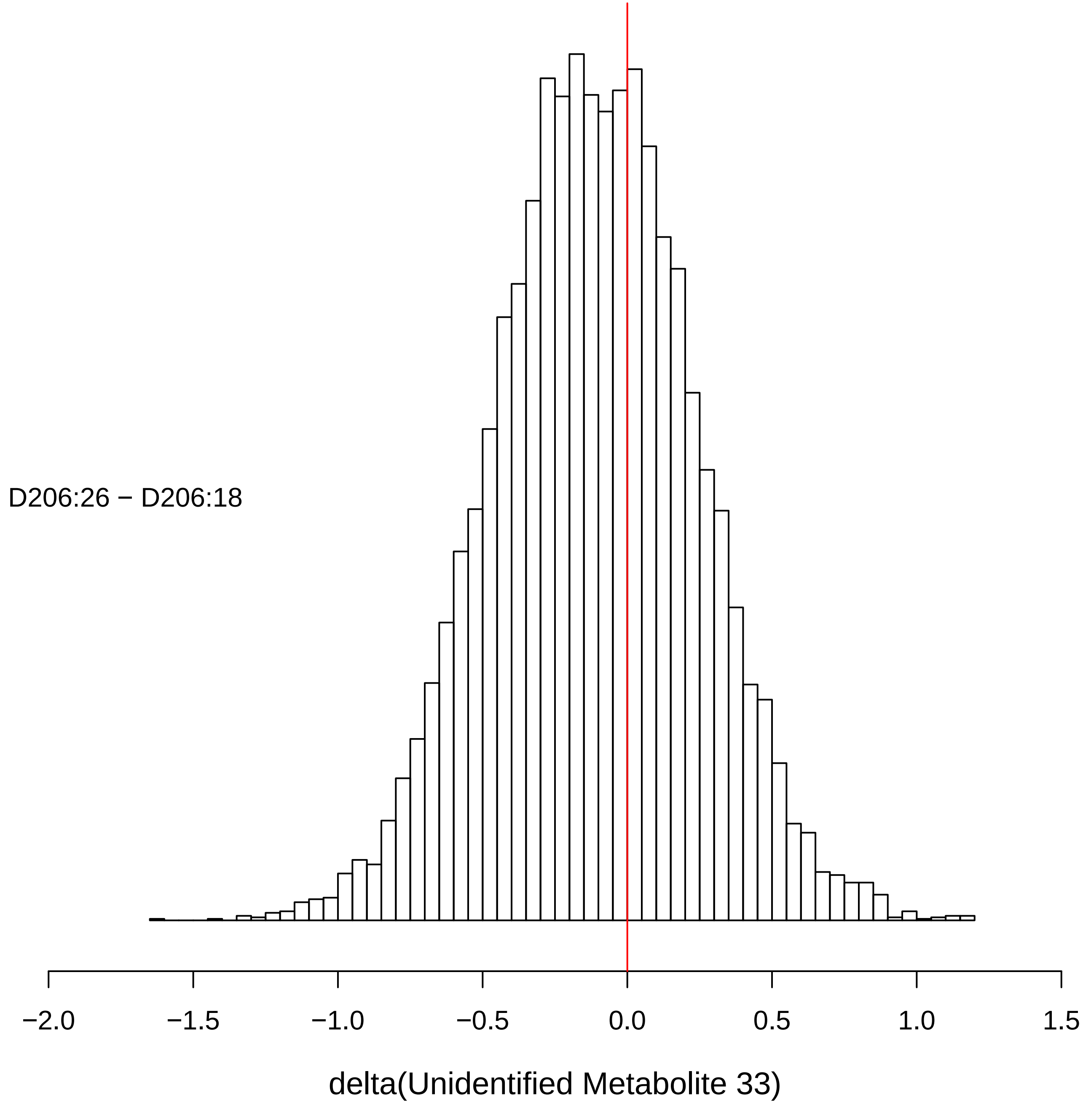
D206:26 − D206:18
−2.0
−1.5
−1.0
−0.5
0.0
0.5
1.0
1.5
delta(Unidentified Metabolite 33)

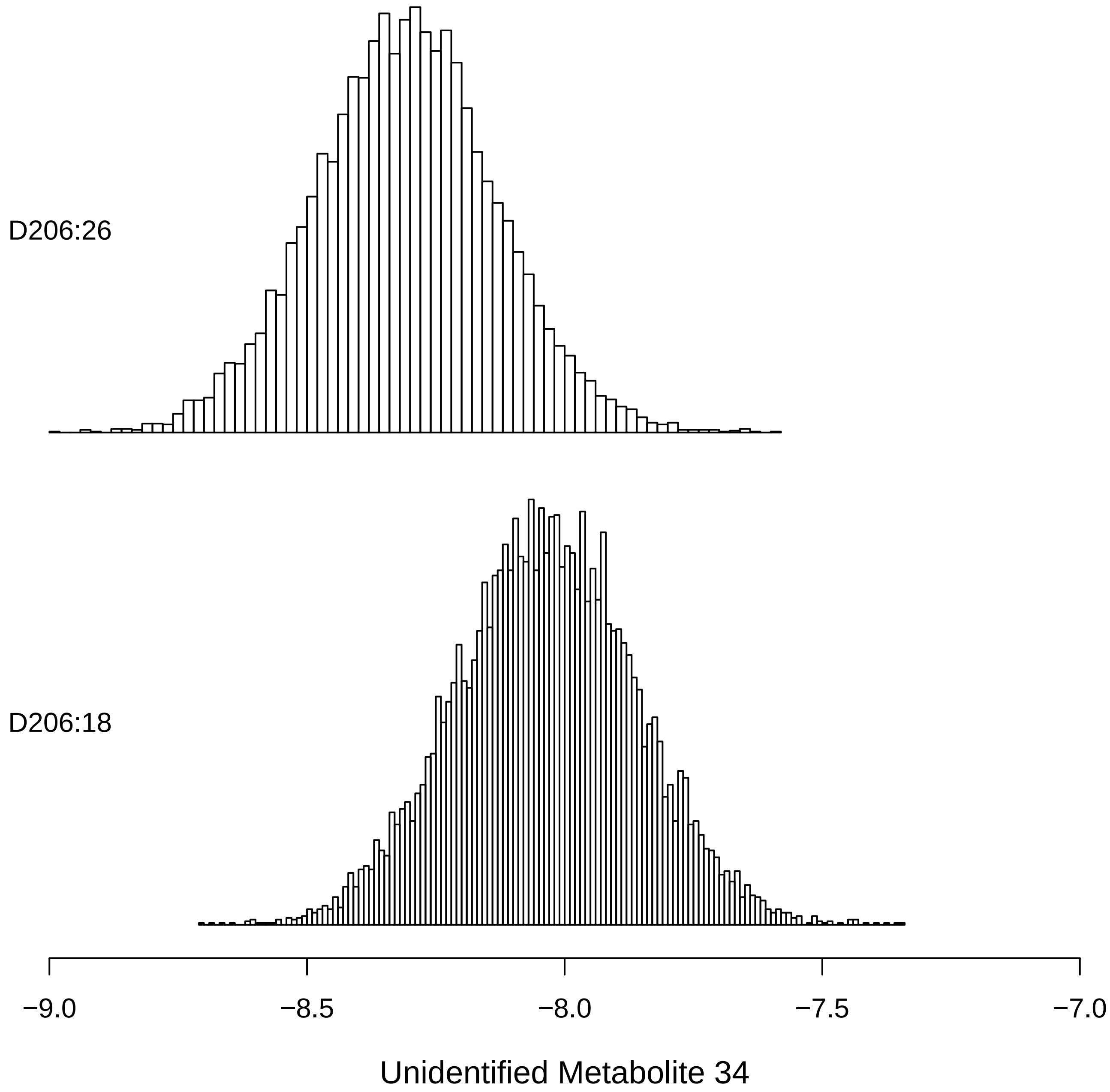
D206:26
D206:18
−9.0
−8.5
−8.0
−7.5
−7.0
Unidentified Metabolite 34

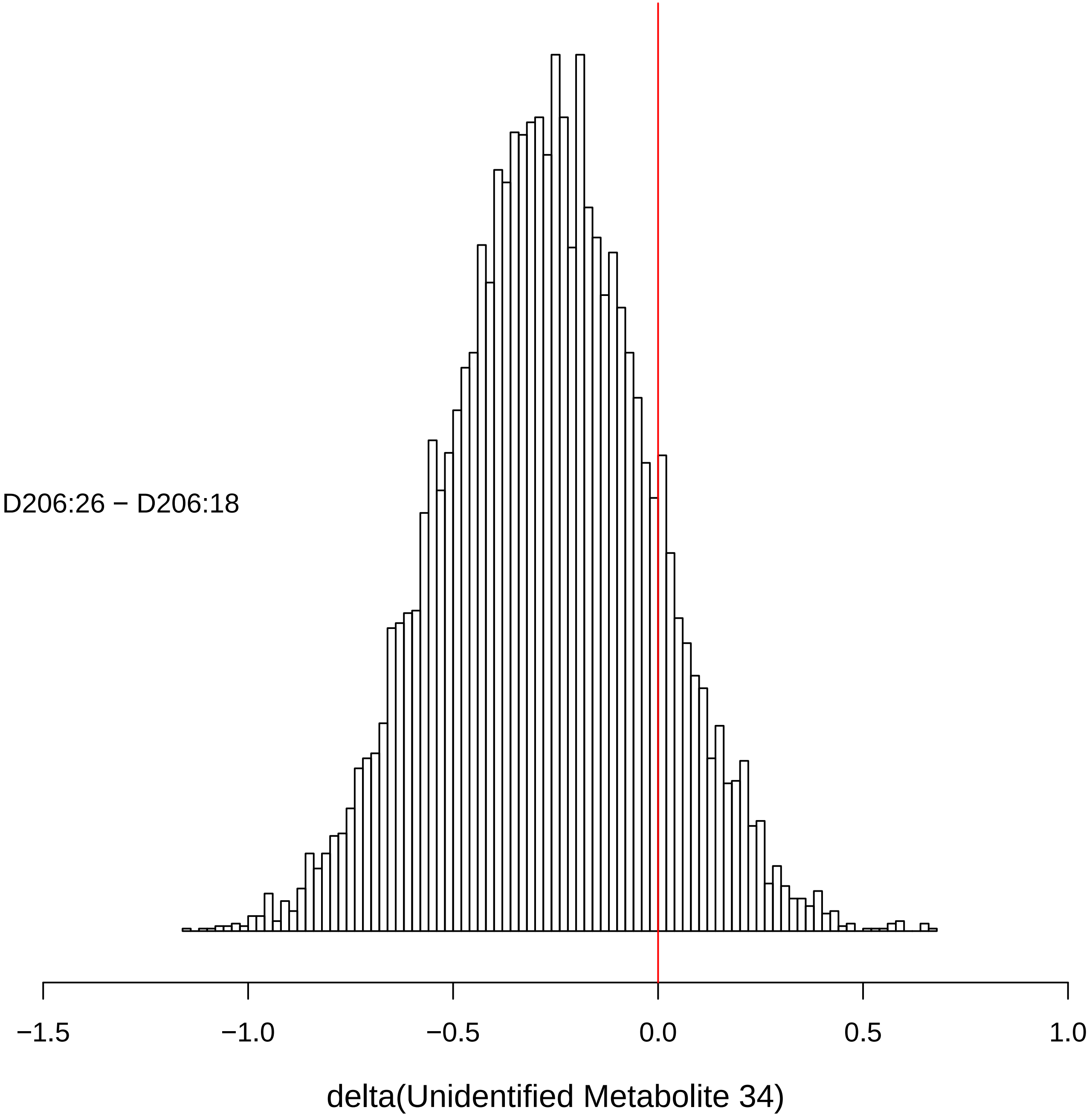
D206:26 − D206:18
−1.5
−1.0
−0.5
0.0
0.5
1.0
delta(Unidentified Metabolite 34)

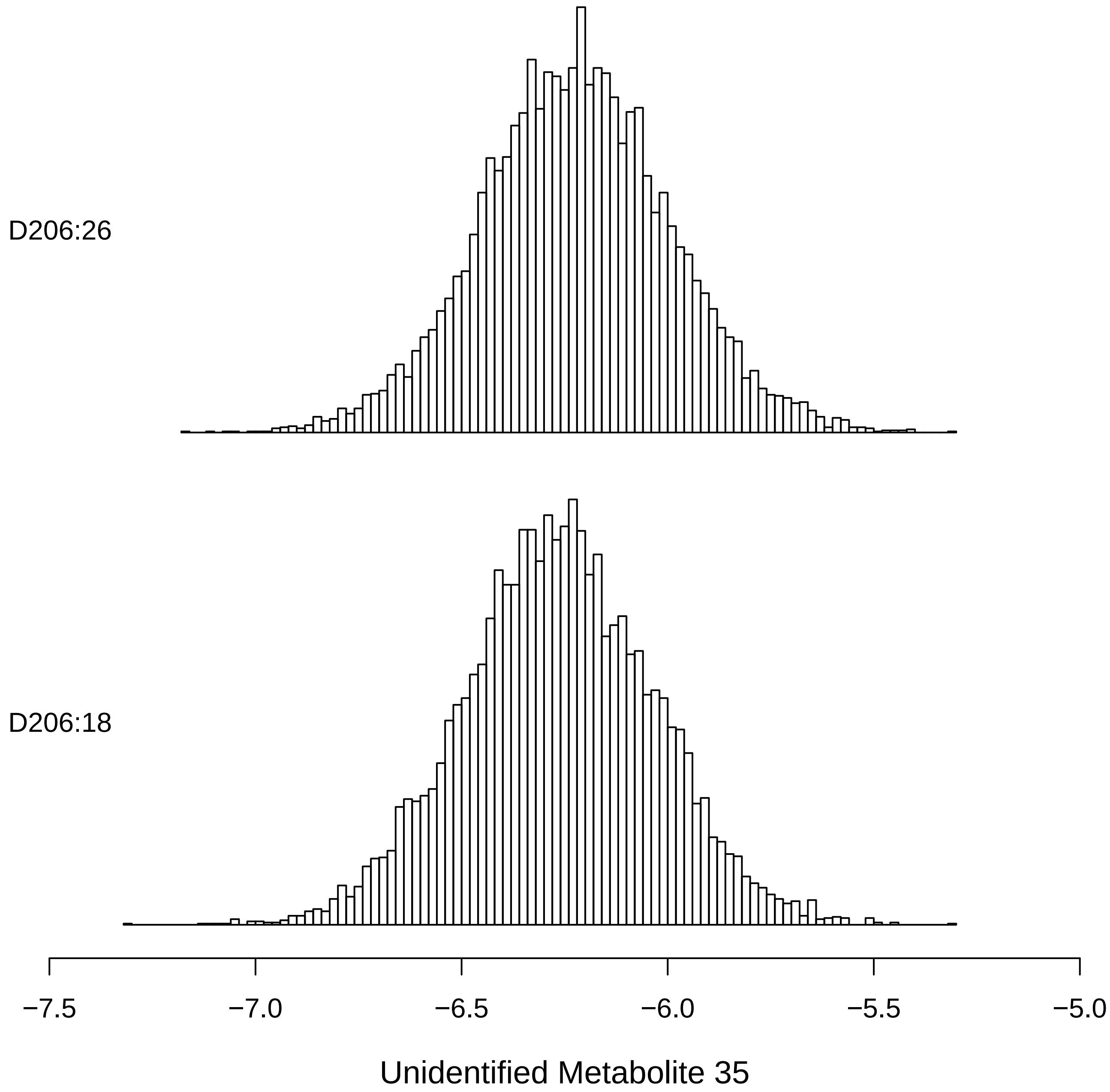
D206:26
D206:18
−7.5
−7.0
−6.5
−6.0
−5.5
−5.0
Unidentified Metabolite 35

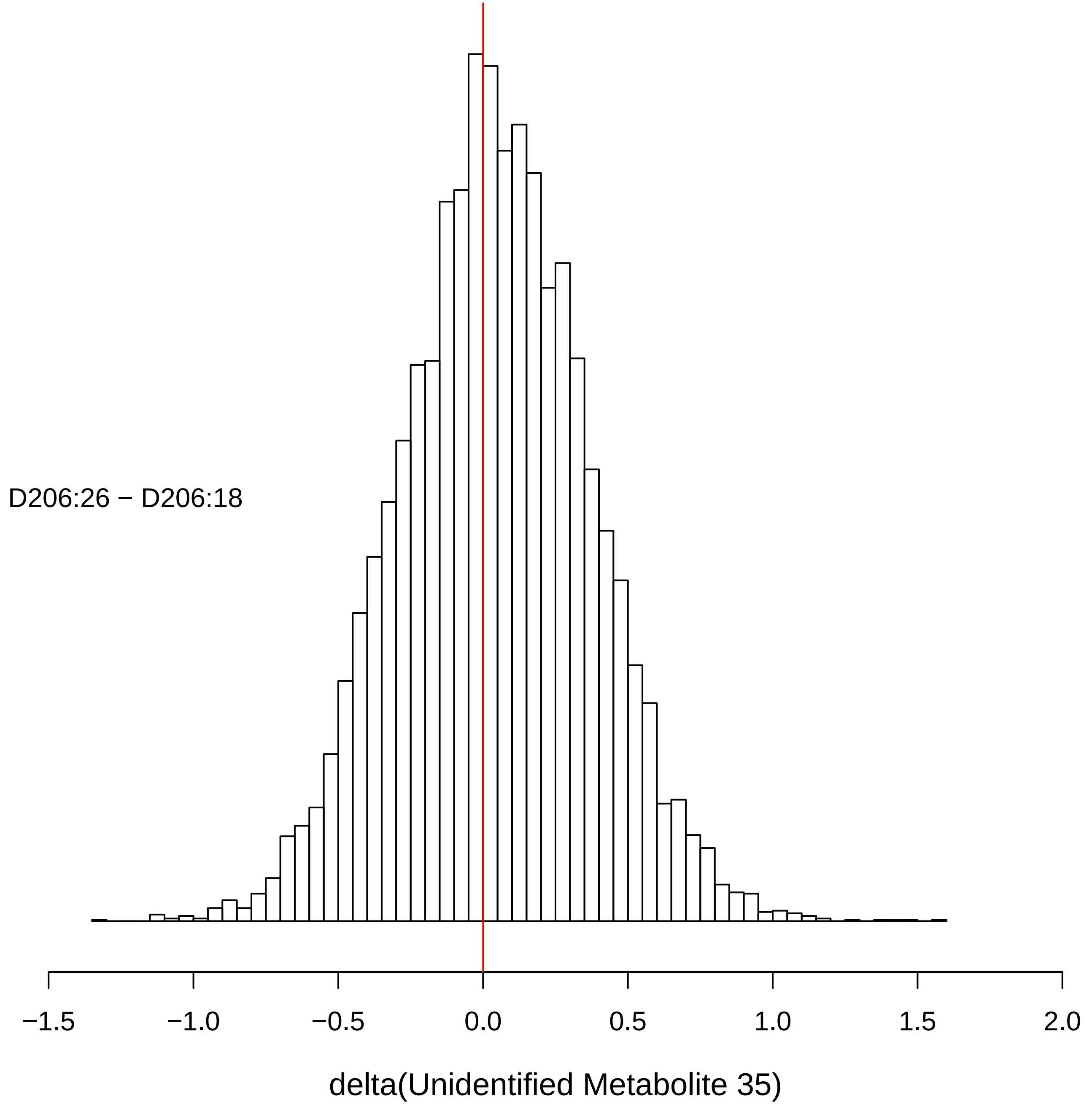
D206:26 − D206:18
−1.5
−1.0
−0.5
0.0
0.5
1.0
1.5
2.0
delta(Unidentified Metabolite 35)

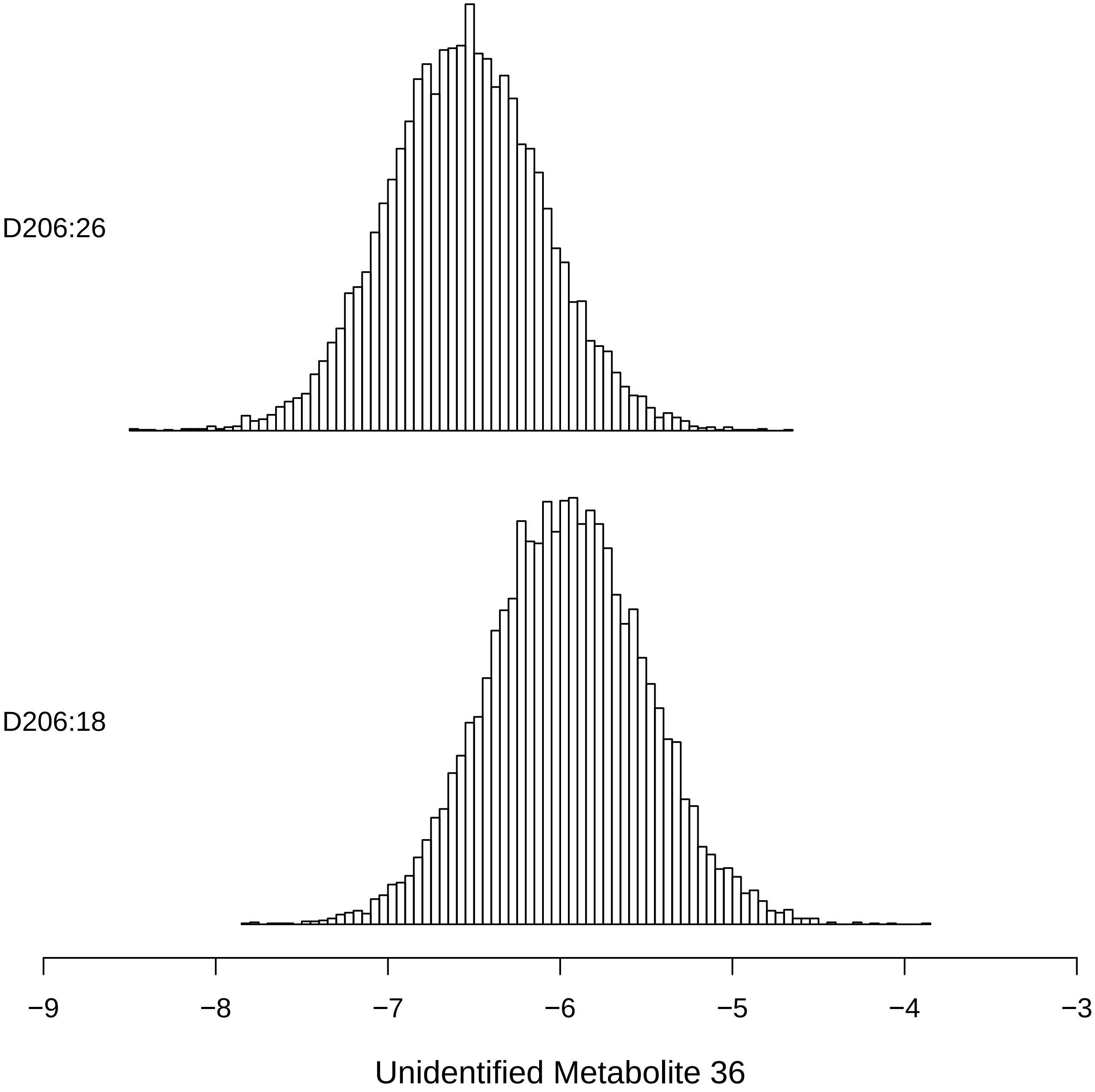

D206:26
D206:18
−9
−8
−7
−6
−5
−4
−3
Unidentified Metabolite 36

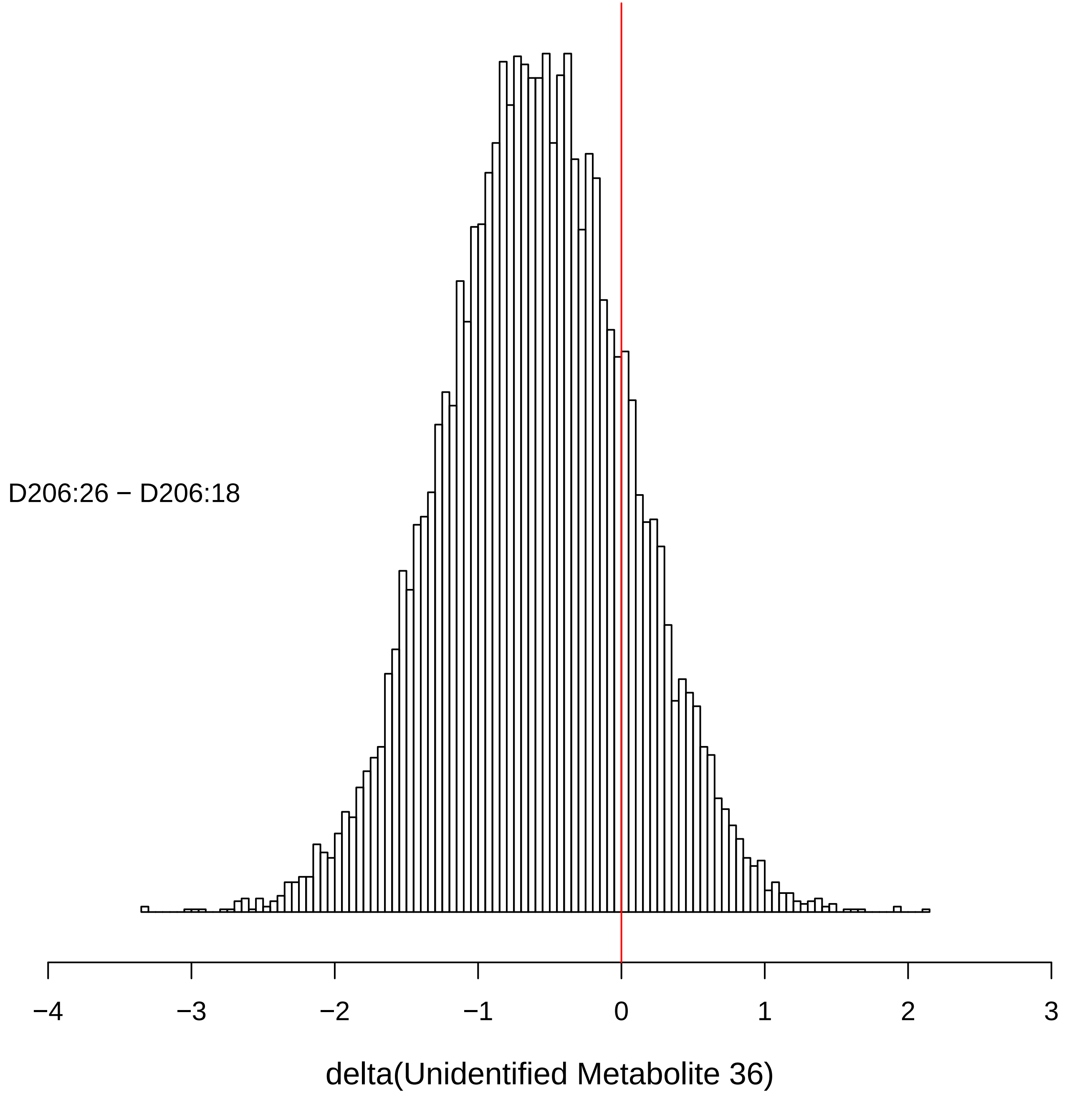

D206:26 − D206:18
−4
−3
−2
−1
0
1
2
3
delta(Unidentified Metabolite 36)

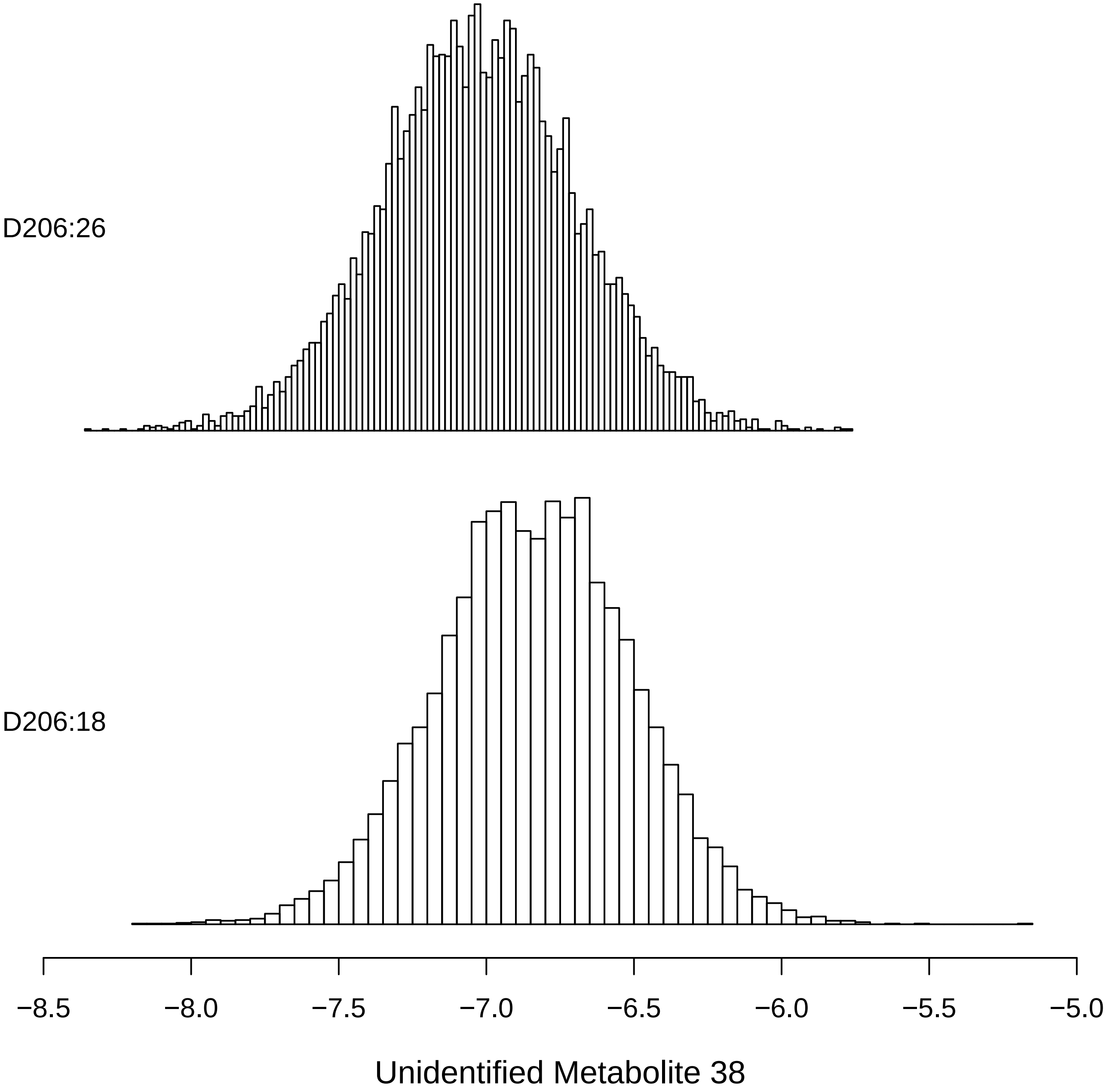
D206:26
D206:18
−8.5
−8.0
−7.5
−7.0
−6.5
−6.0
−5.5
−5.0
Unidentified Metabolite 38

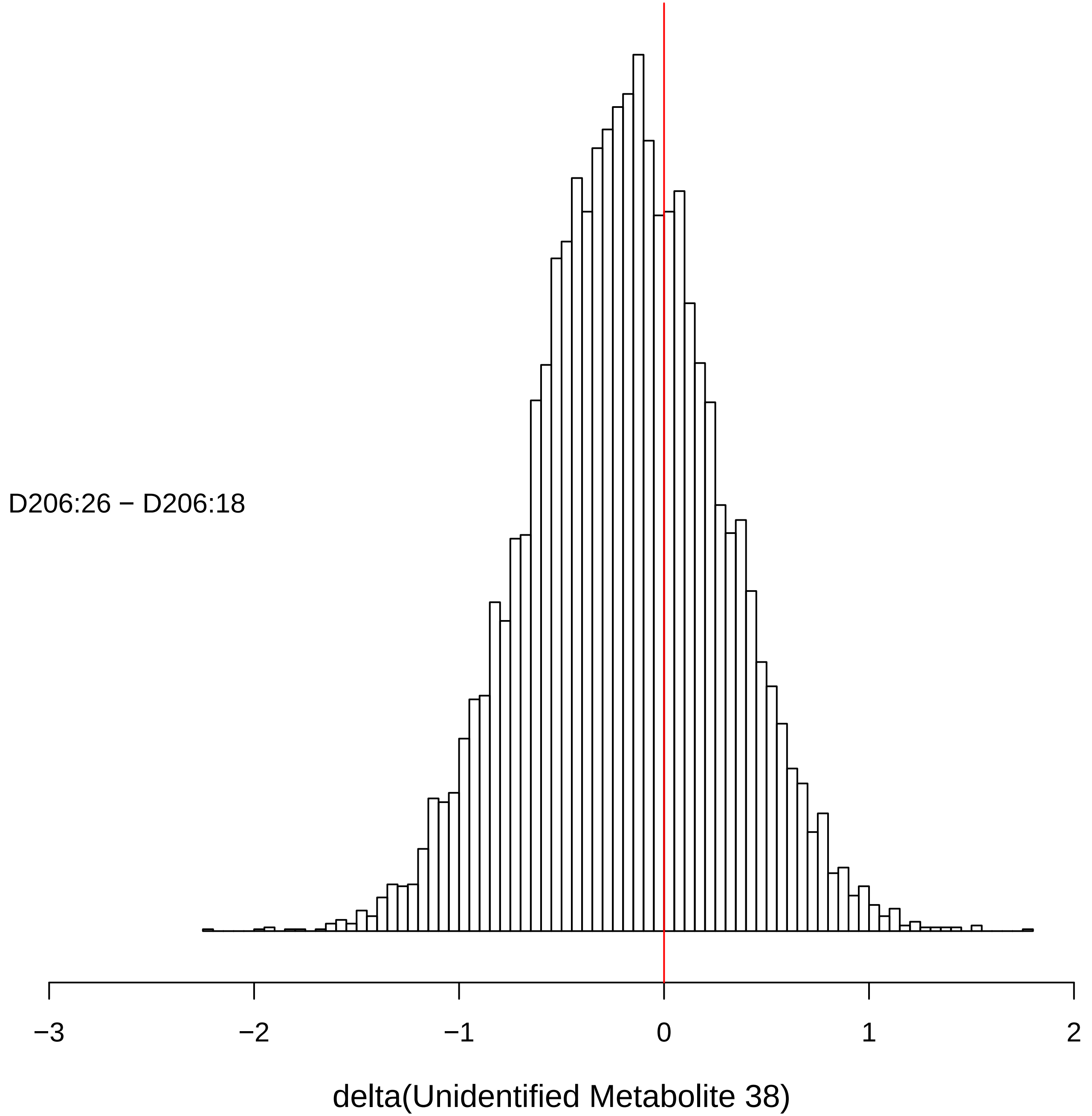
D206:26 − D206:18
−3
−2
−1
0
1
2
delta(Unidentified Metabolite 38)

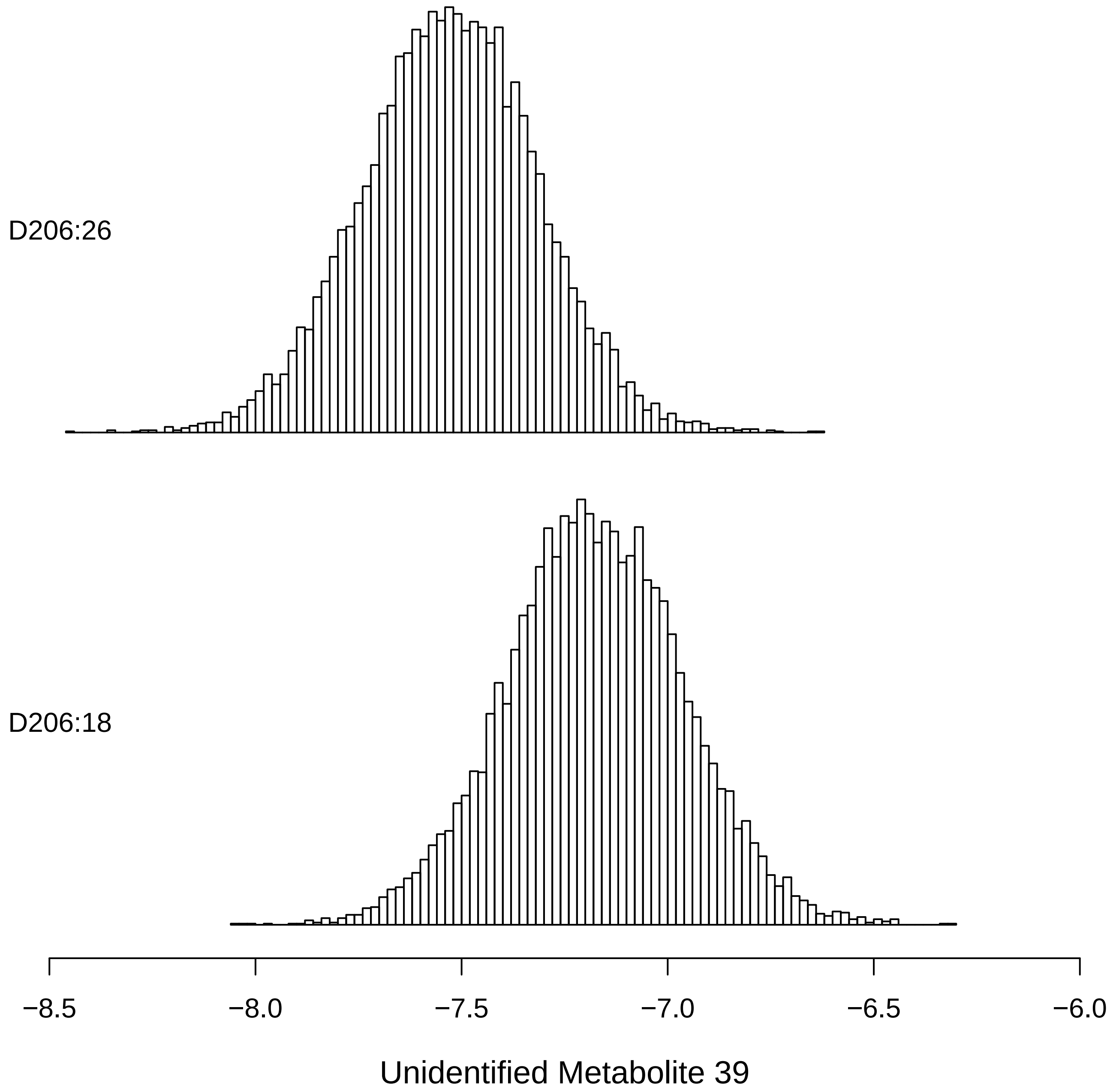

D206:26
D206:18
−8.5
−8.0
−7.5
−7.0
−6.5
−6.0
Unidentified Metabolite 39

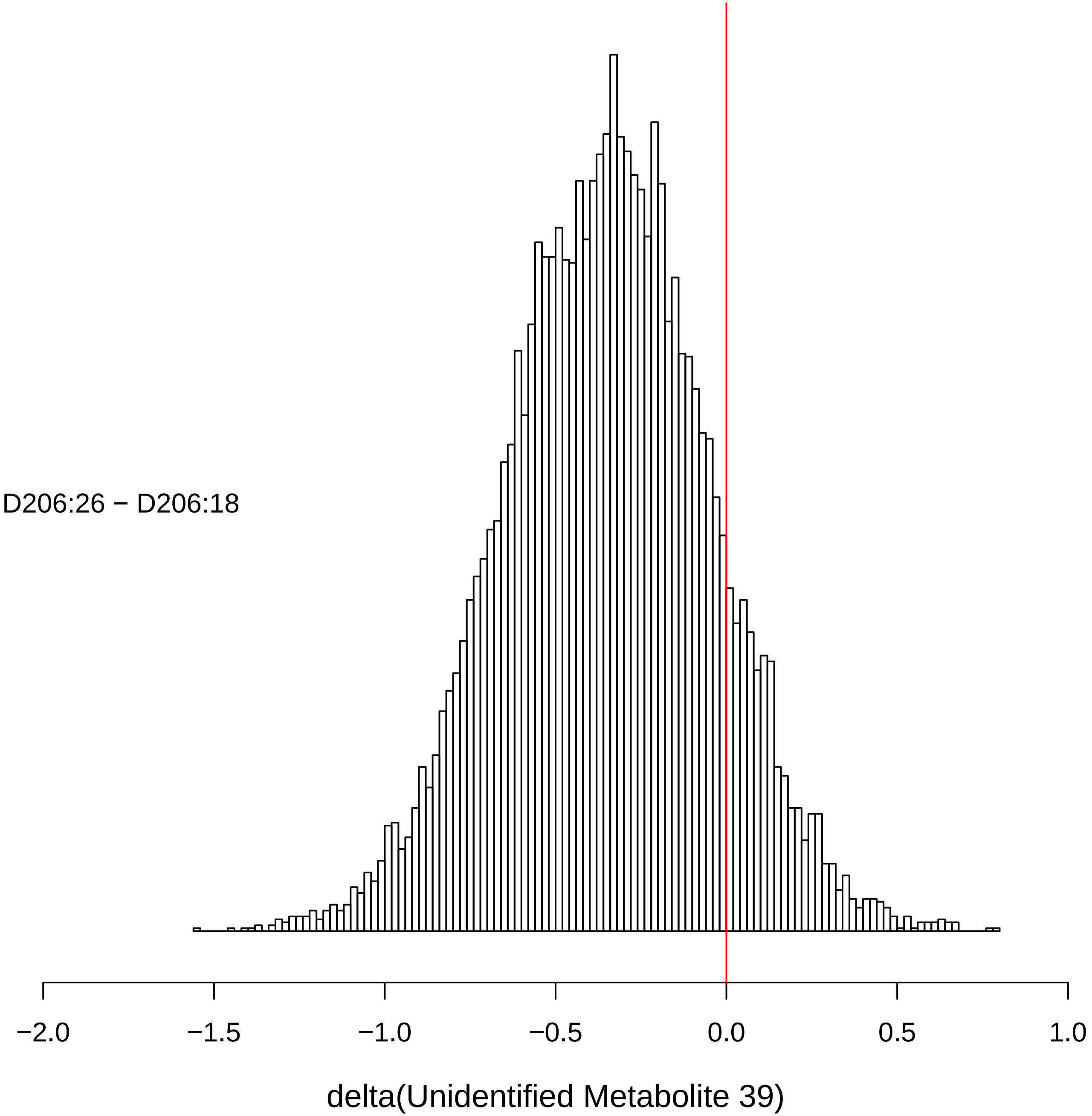
D206:26 − D206:18
−2.0
−1.5
−1.0
−0.5
0.0
0.5
1.0
delta(Unidentified Metabolite 39)

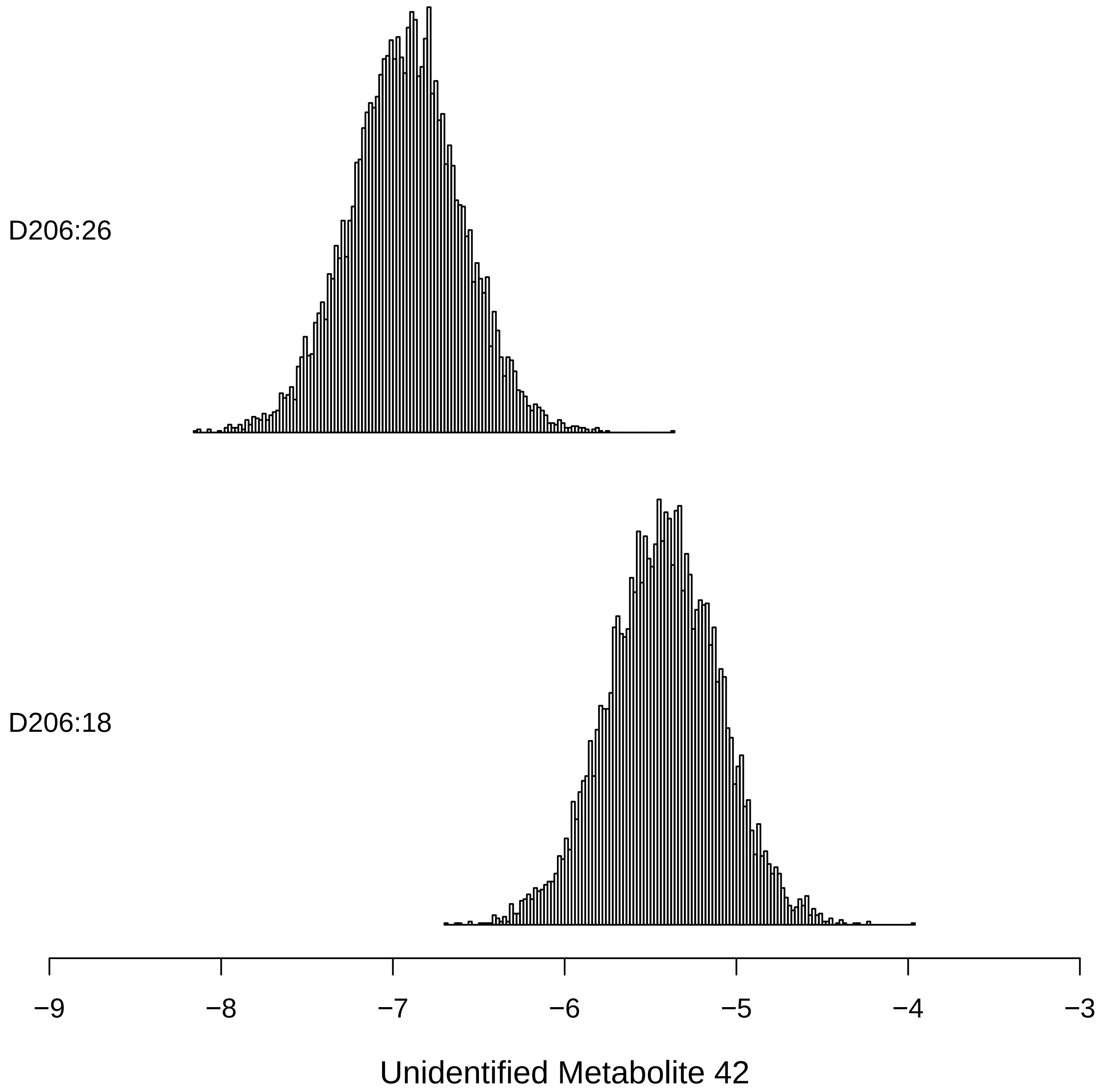
D206:26
D206:18
−9
−8
−7
−6
−5
−4
−3
Unidentified Metabolite 42

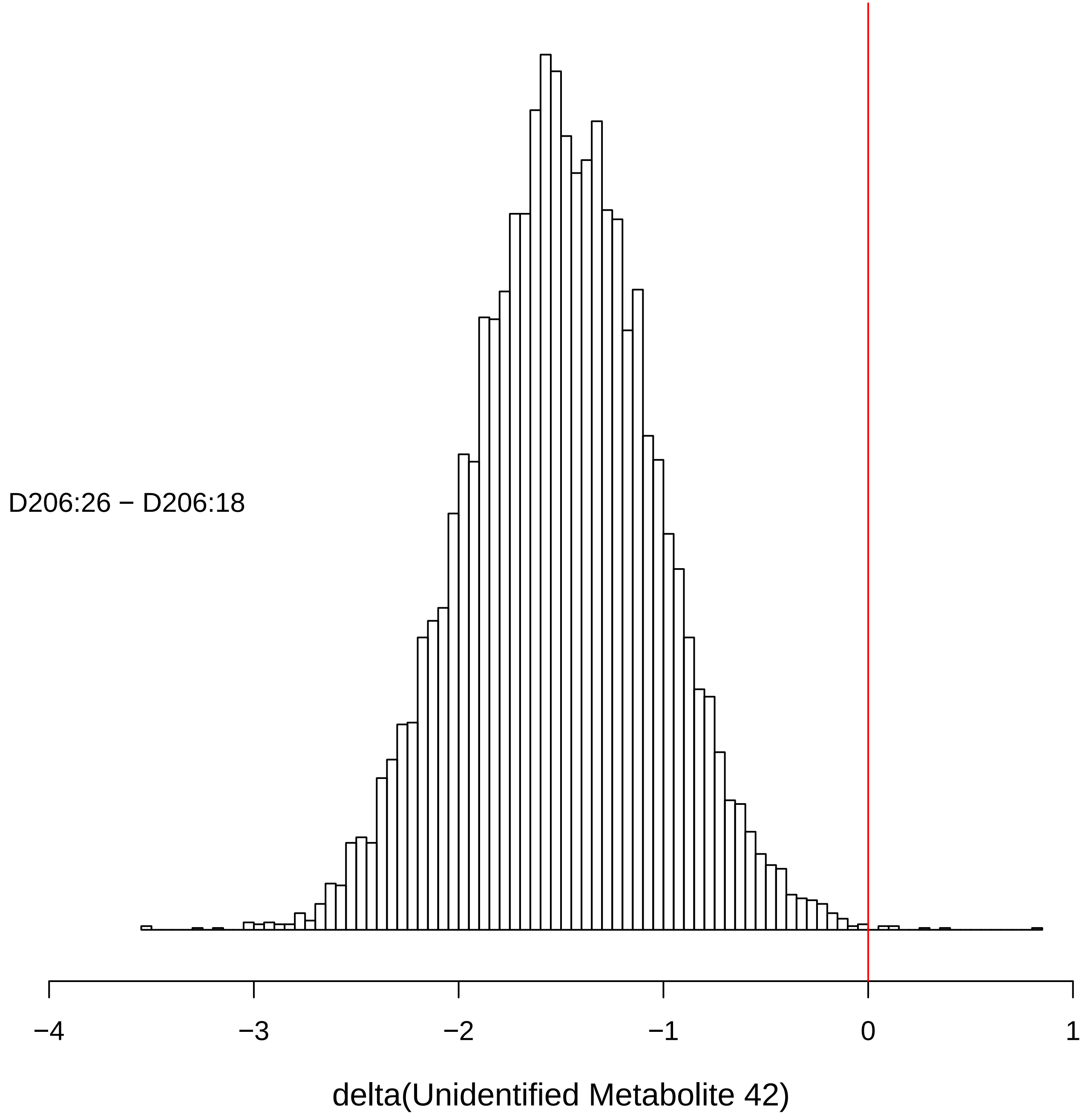
D206:26 − D206:18
−4
−3
−2
−1
0
1
delta(Unidentified Metabolite 42)

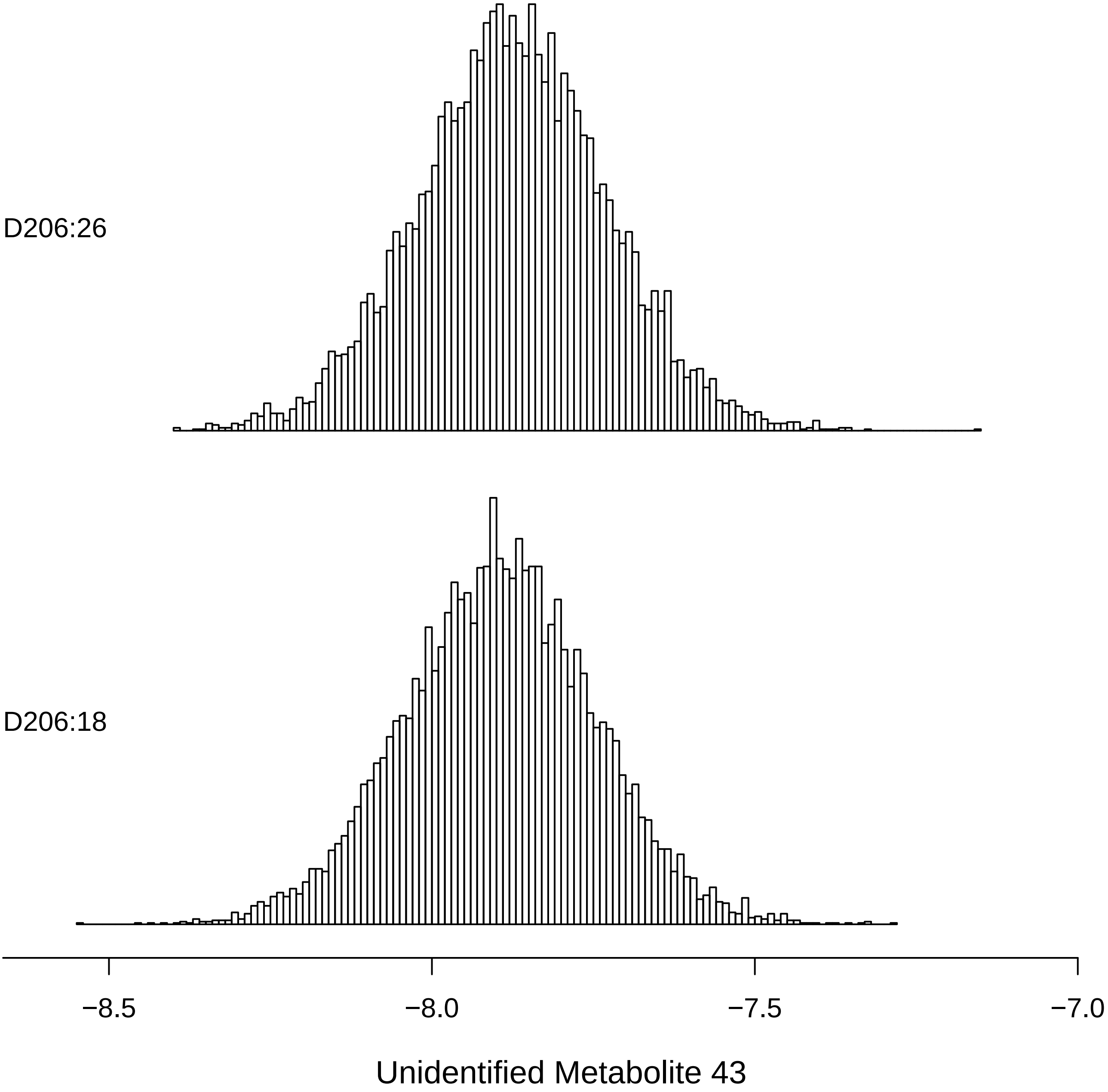
D206:26
D206:18
−8.5
−8.0
−7.5
−7.0
Unidentified Metabolite 43

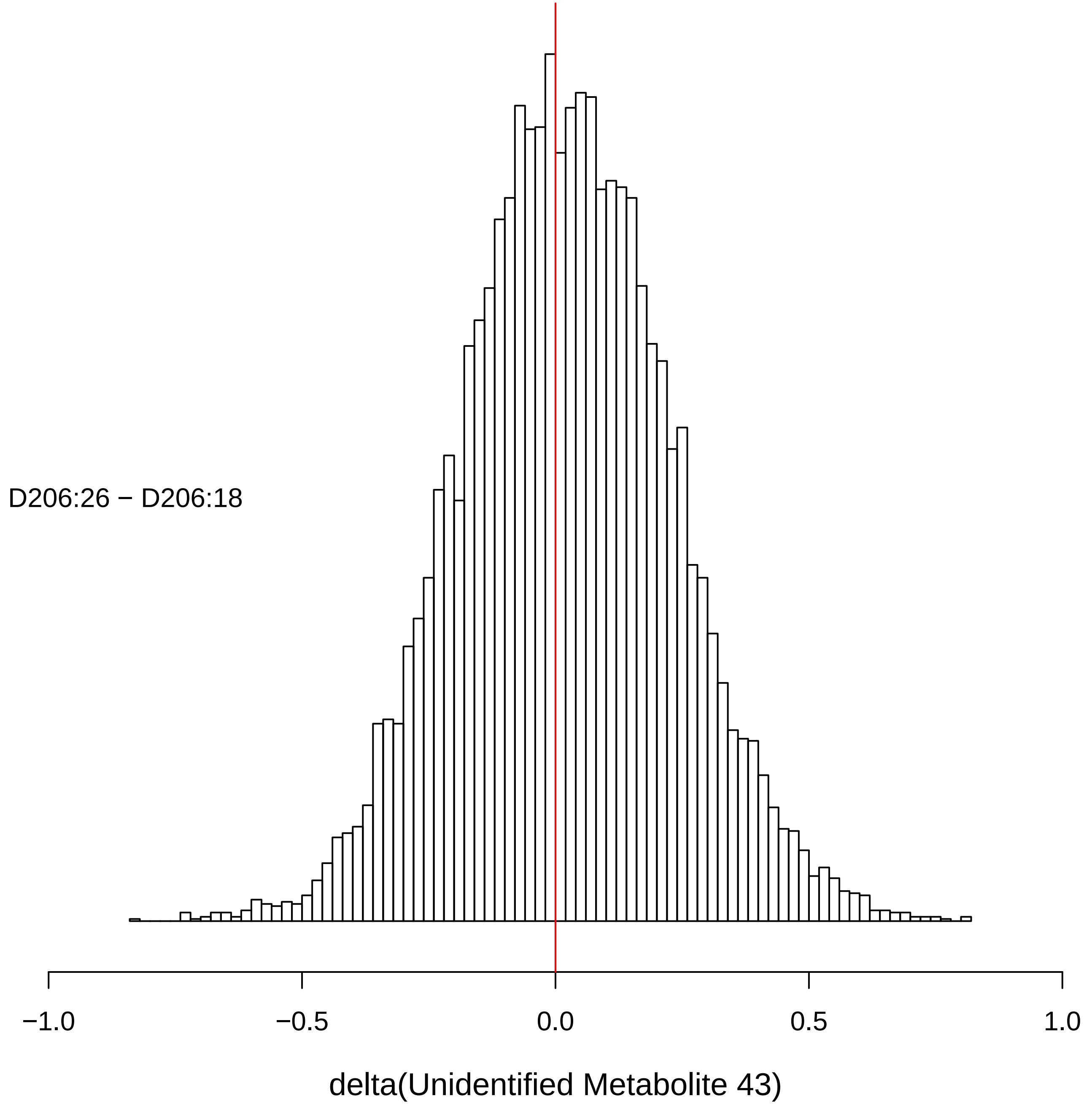
D206:26 − D206:18
−1.0
−0.5
0.0
0.5
1.0
delta(Unidentified Metabolite 43)

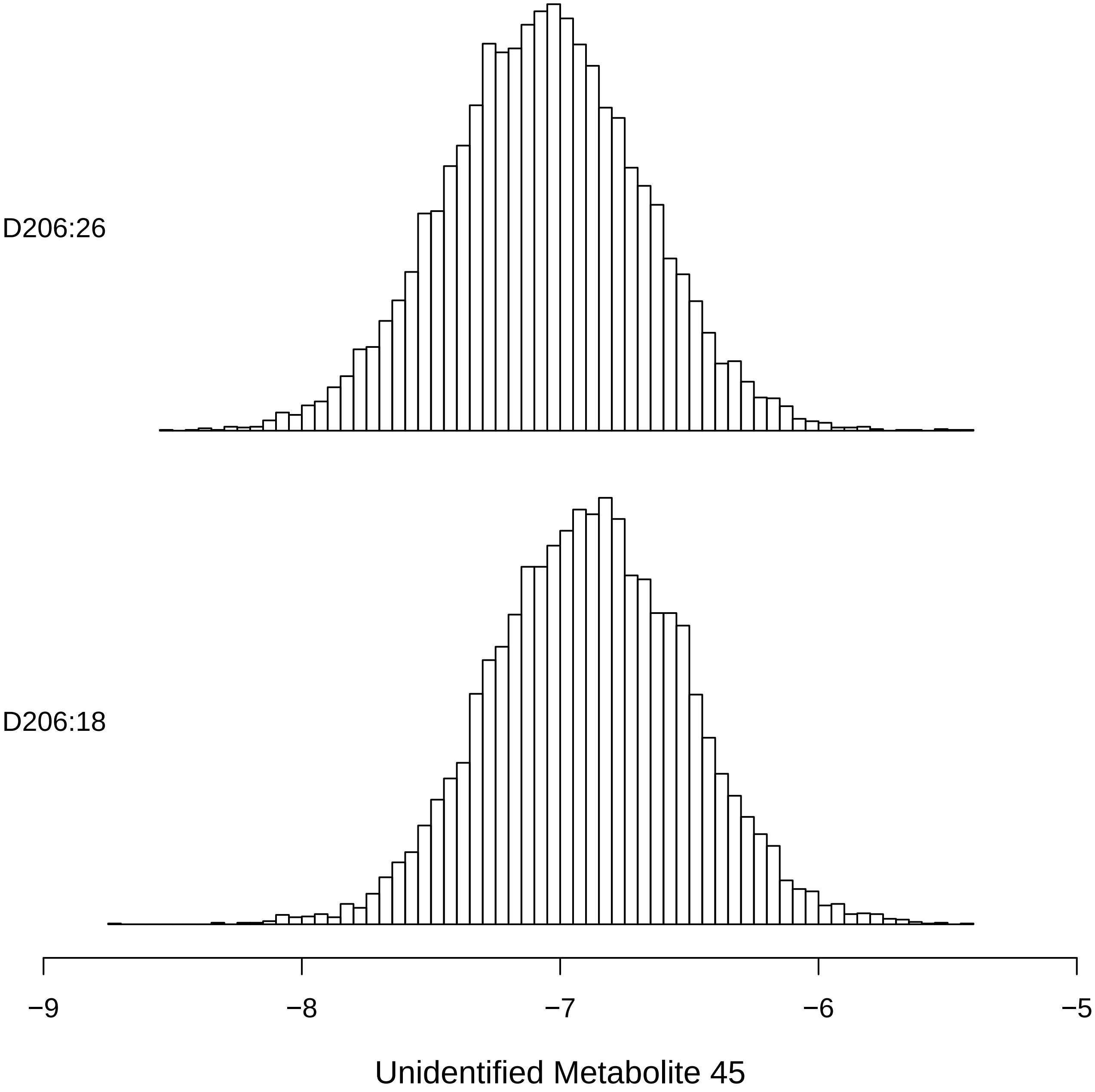
D206:26
D206:18
−9
−8
−7
−6
−5
Unidentified Metabolite 45

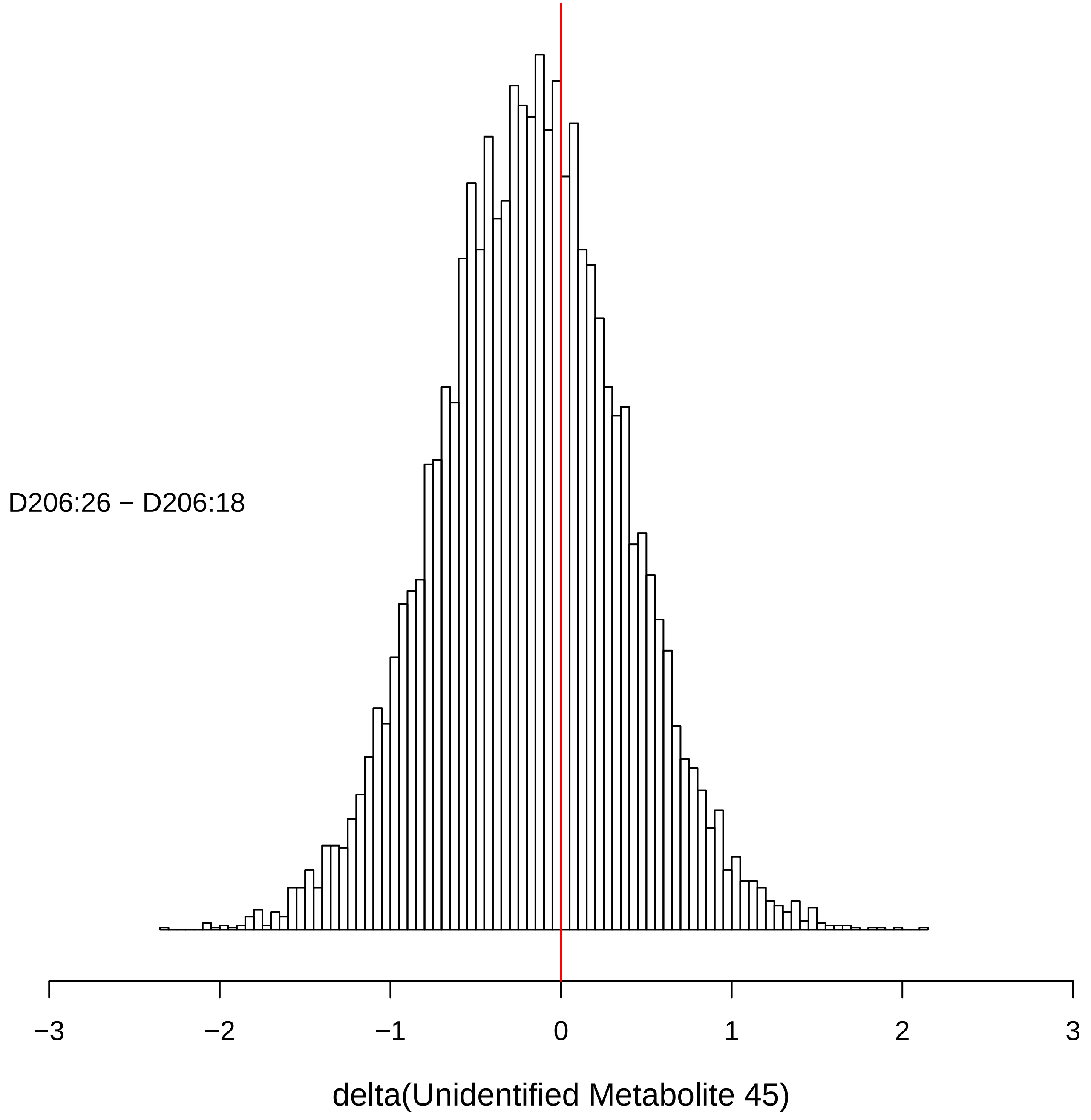
D206:26 − D206:18
−3
−2
−1
0
1
2
3
delta(Unidentified Metabolite 45)

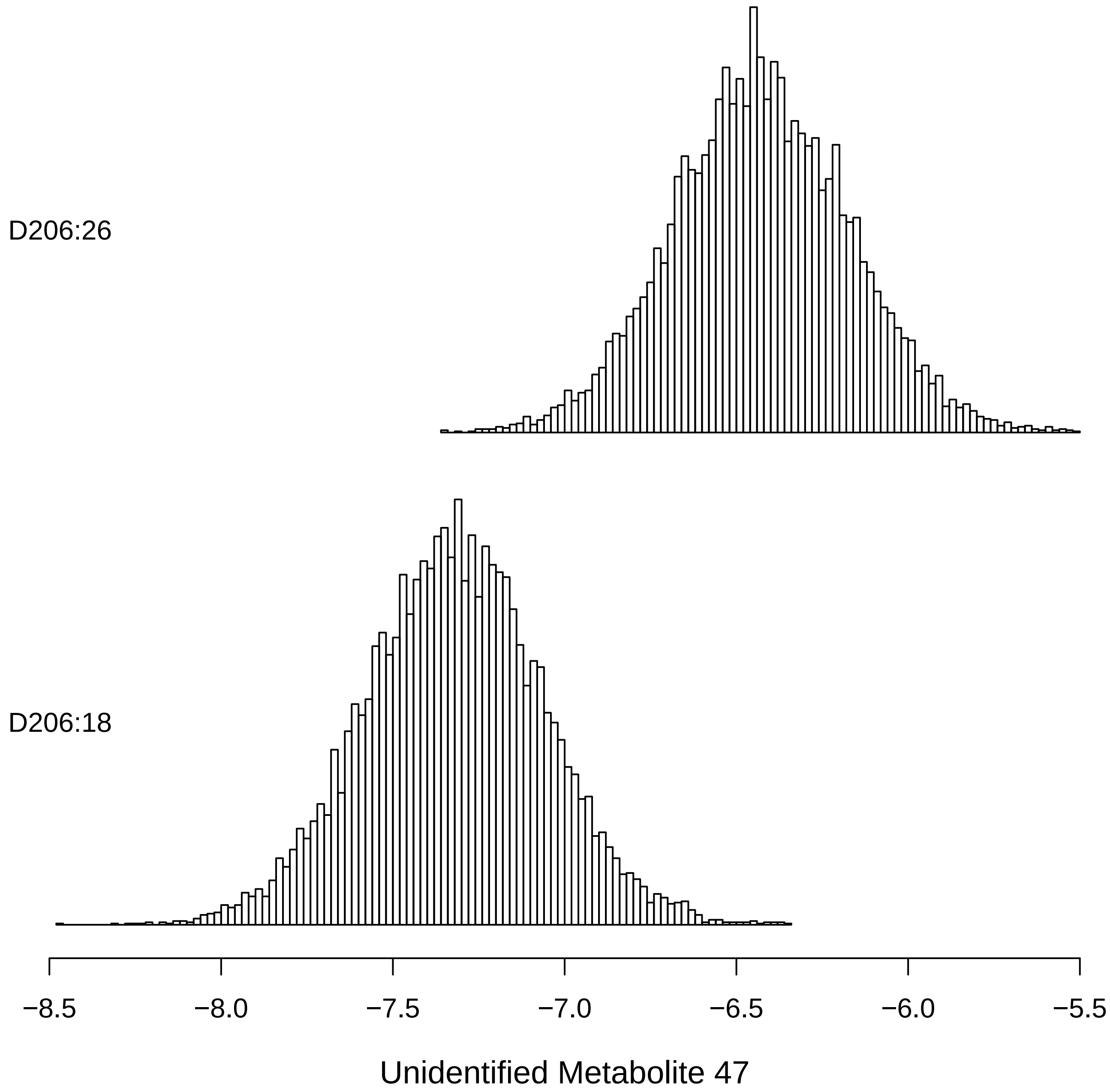
D206:26
D206:18
−8.5
−8.0
−7.5
−7.0
−6.5
−6.0
−5.5
Unidentified Metabolite 47

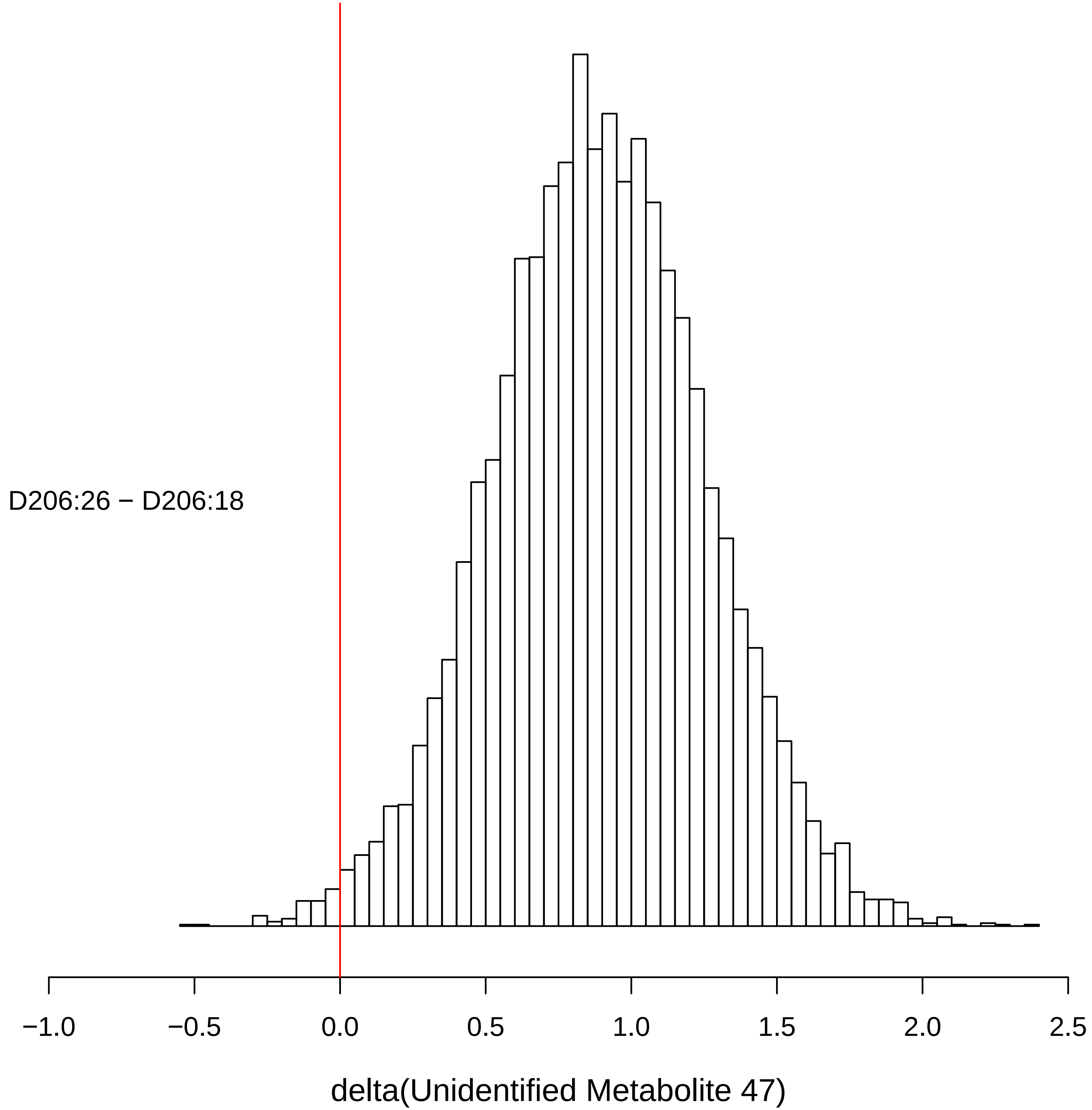
D206:26 − D206:18
−1.0
−0.5
0.0
0.5
1.0
1.5
2.0
2.5
delta(Unidentified Metabolite 47)

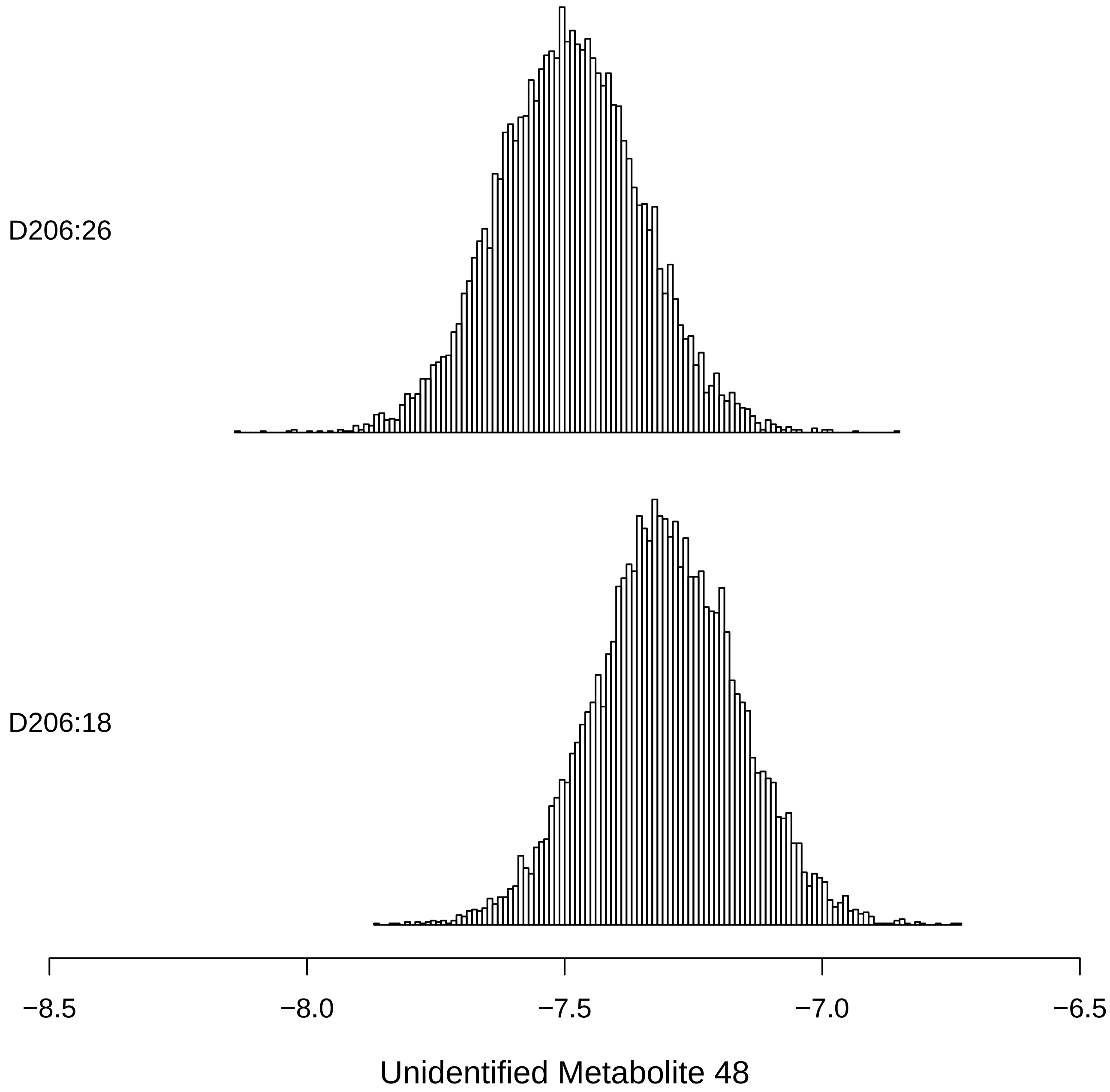
D206:26
D206:18
−8.5
−8.0
−7.5
−7.0
−6.5
Unidentified Metabolite 48

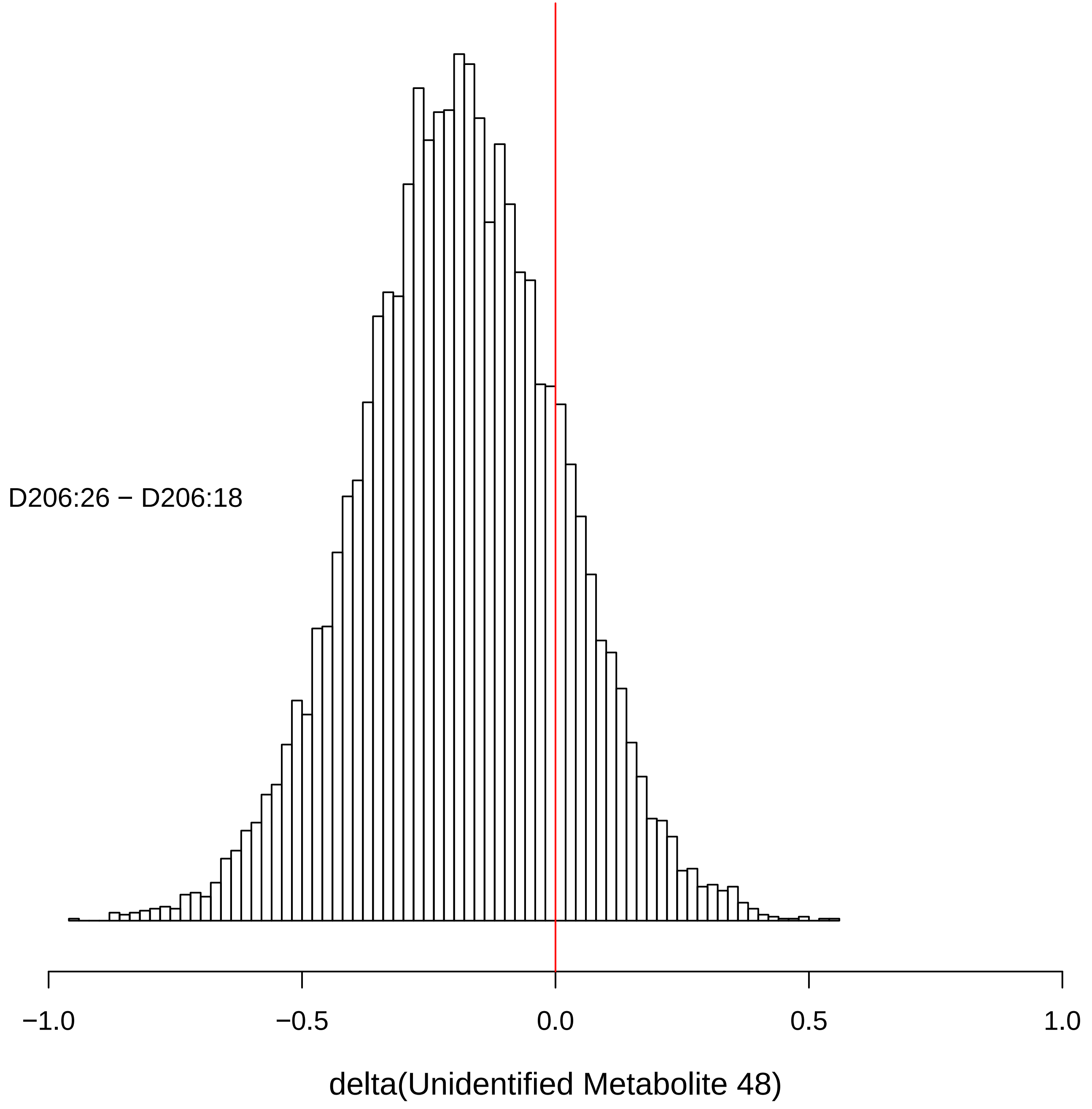
D206:26 − D206:18
−1.0
−0.5
0.0
0.5
1.0
delta(Unidentified Metabolite 48)

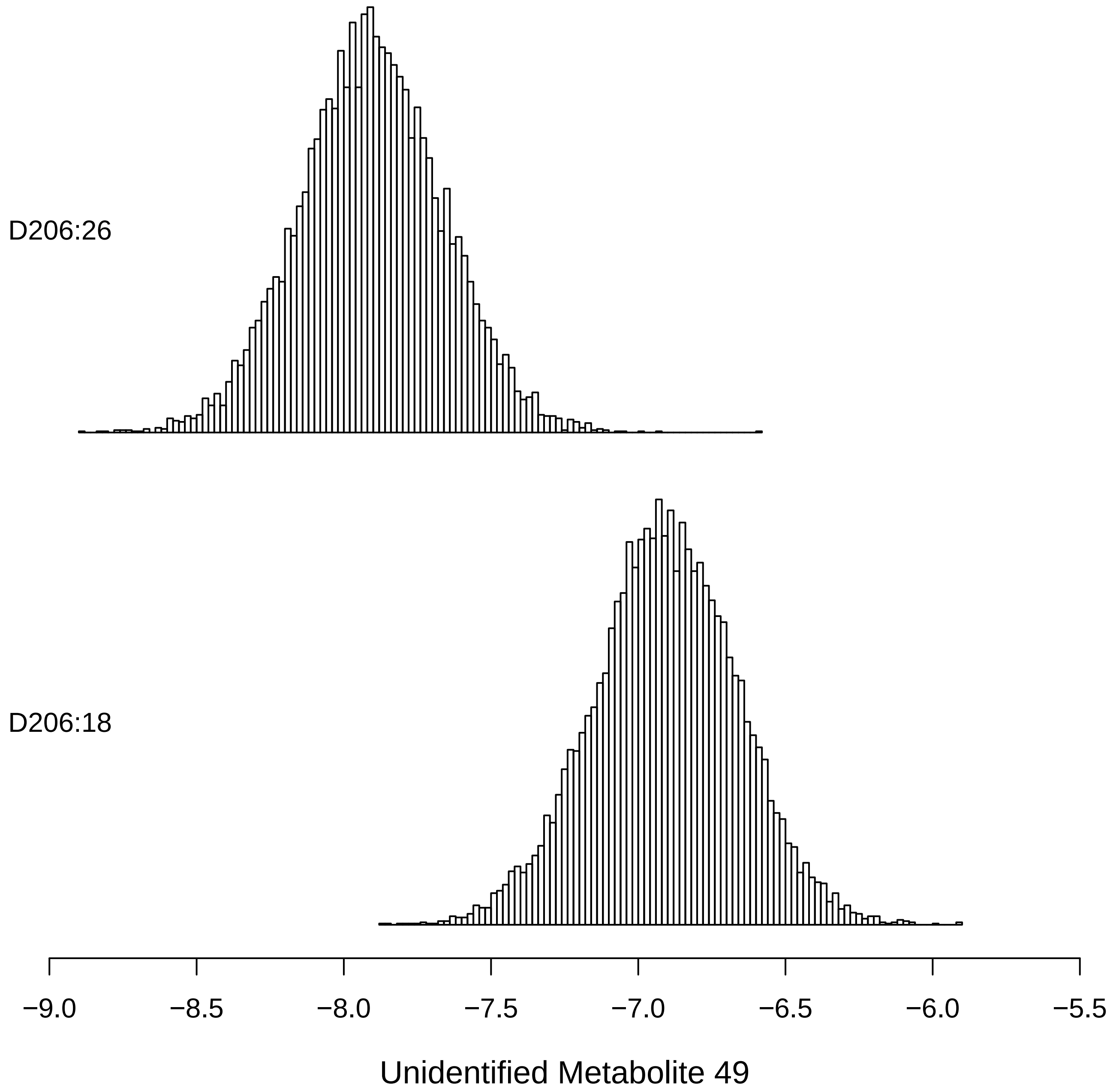
D206:26
D206:18
−9.0
−8.5
−8.0
−7.5
−7.0
−6.5
−6.0
−5.5
Unidentified Metabolite 49

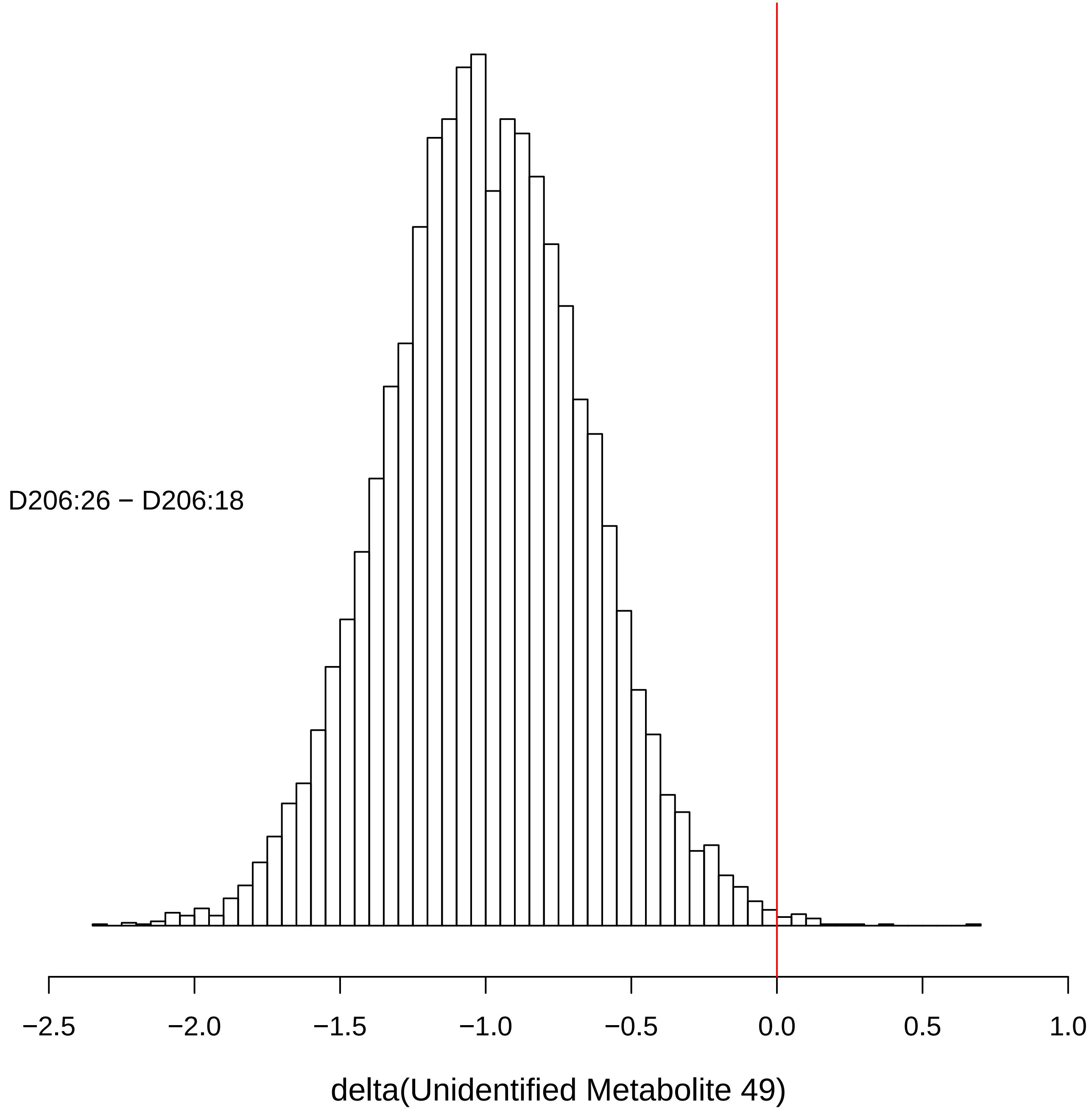
D206:26 − D206:18
−2.5
−2.0
−1.5
−1.0
−0.5
0.0
0.5
1.0
delta(Unidentified Metabolite 49)

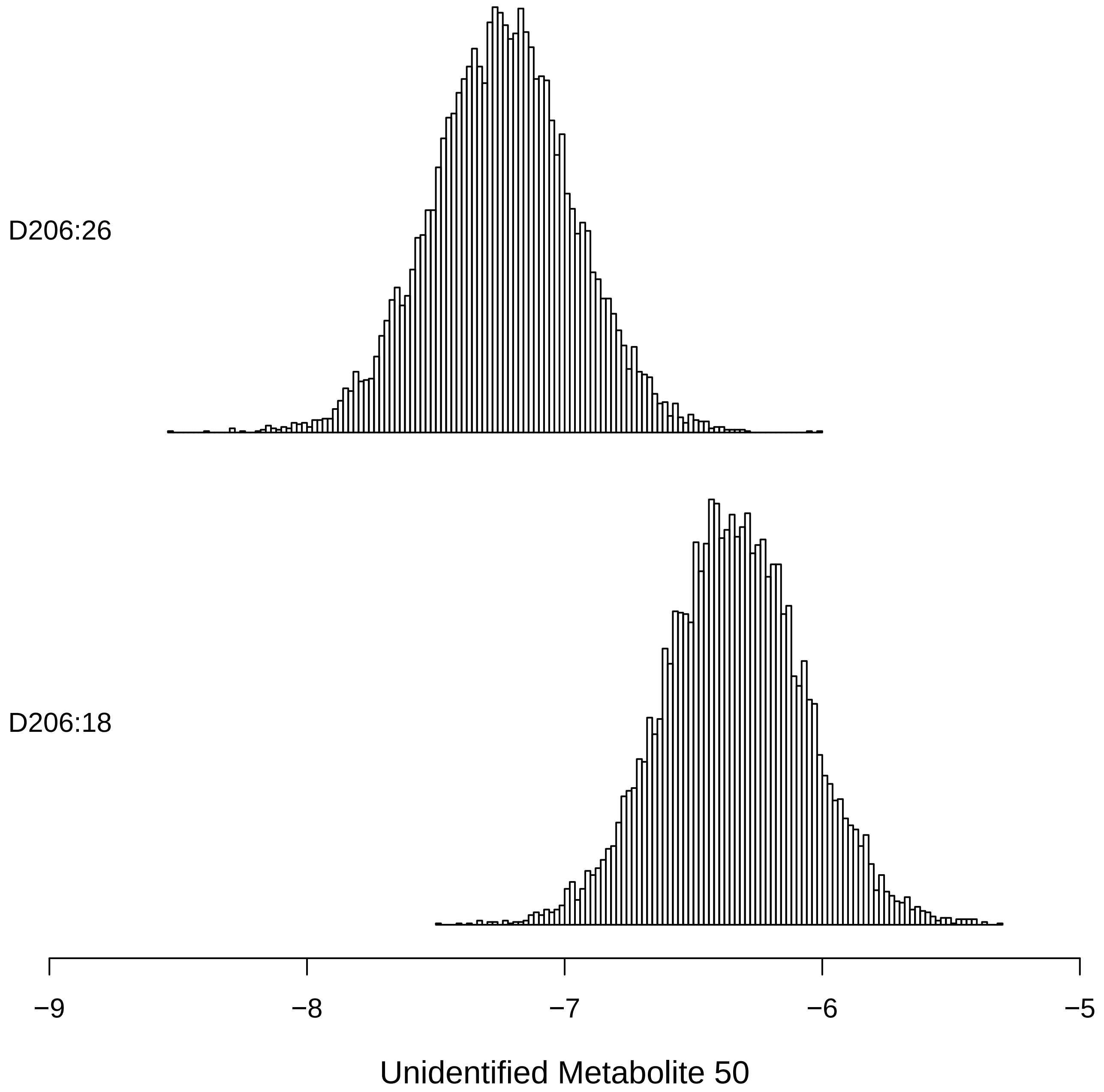
D206:26
D206:18
−9
−8
−7
−6
−5
Unidentified Metabolite 50

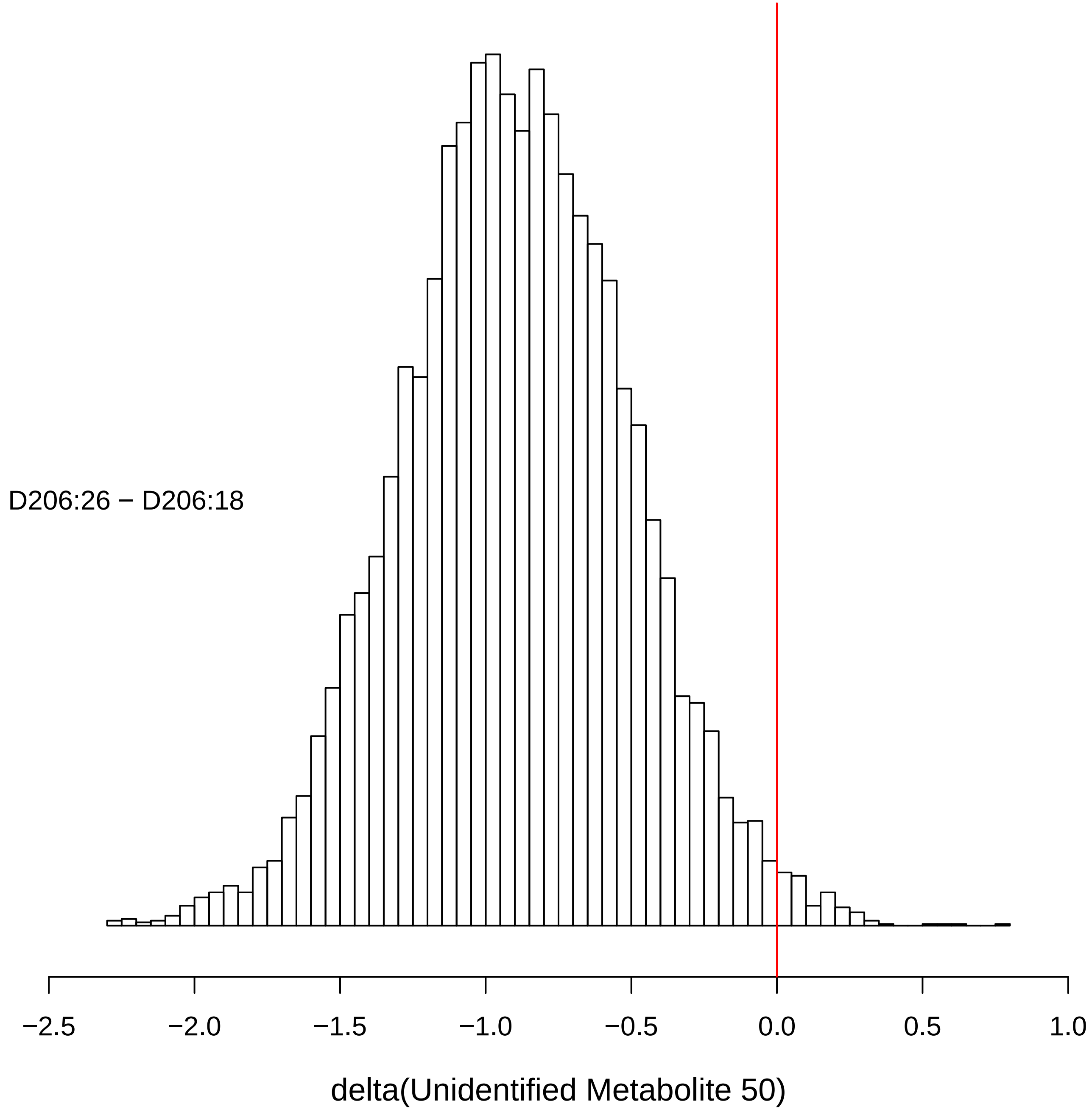

D206:26 − D206:18
−2.5
−2.0
−1.5
−1.0
−0.5
0.0
0.5
1.0
delta(Unidentified Metabolite 50)

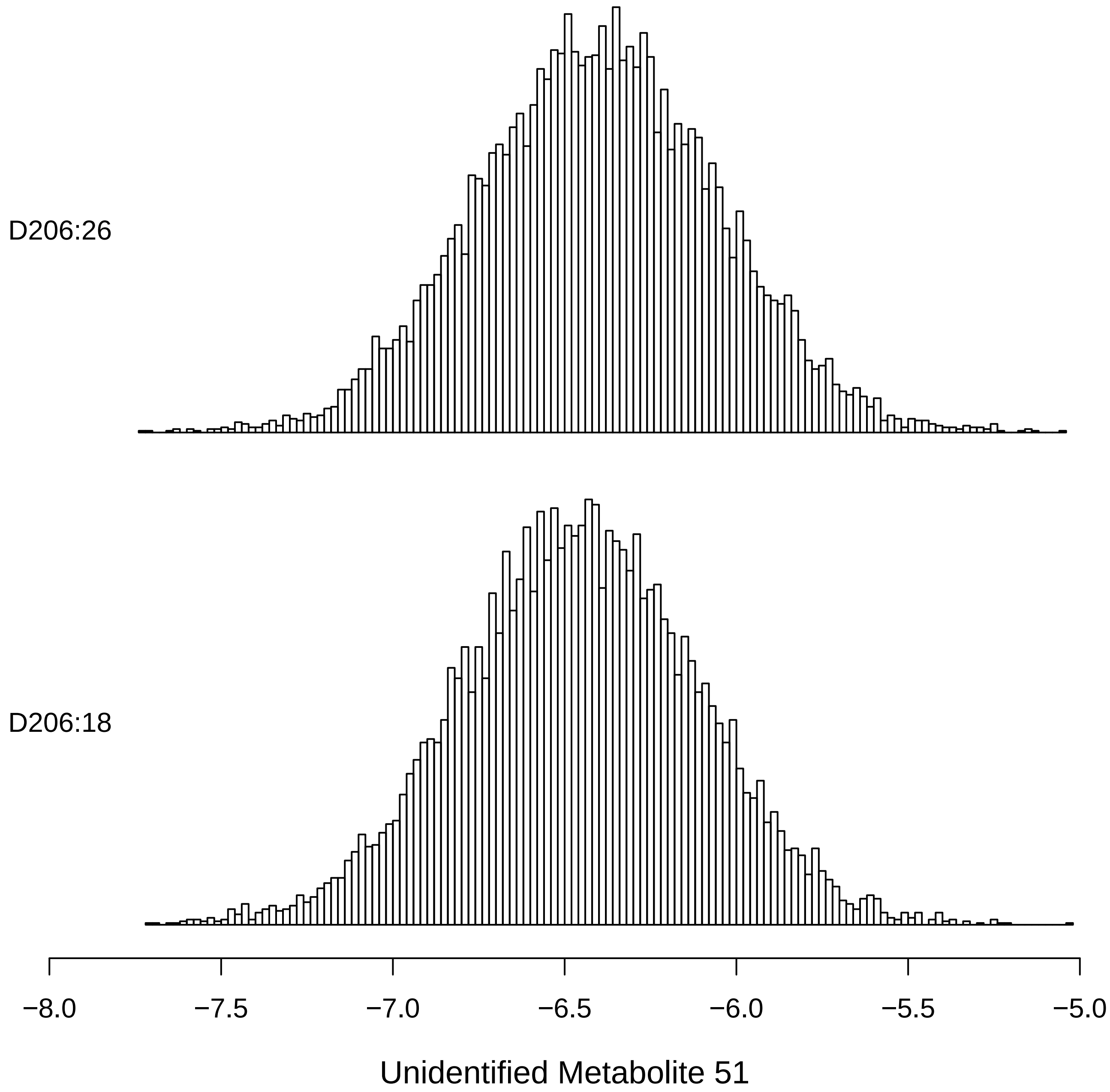
D206:26
D206:18
−8.0
−7.5
−7.0
−6.5
−6.0
−5.5
−5.0
Unidentified Metabolite 51

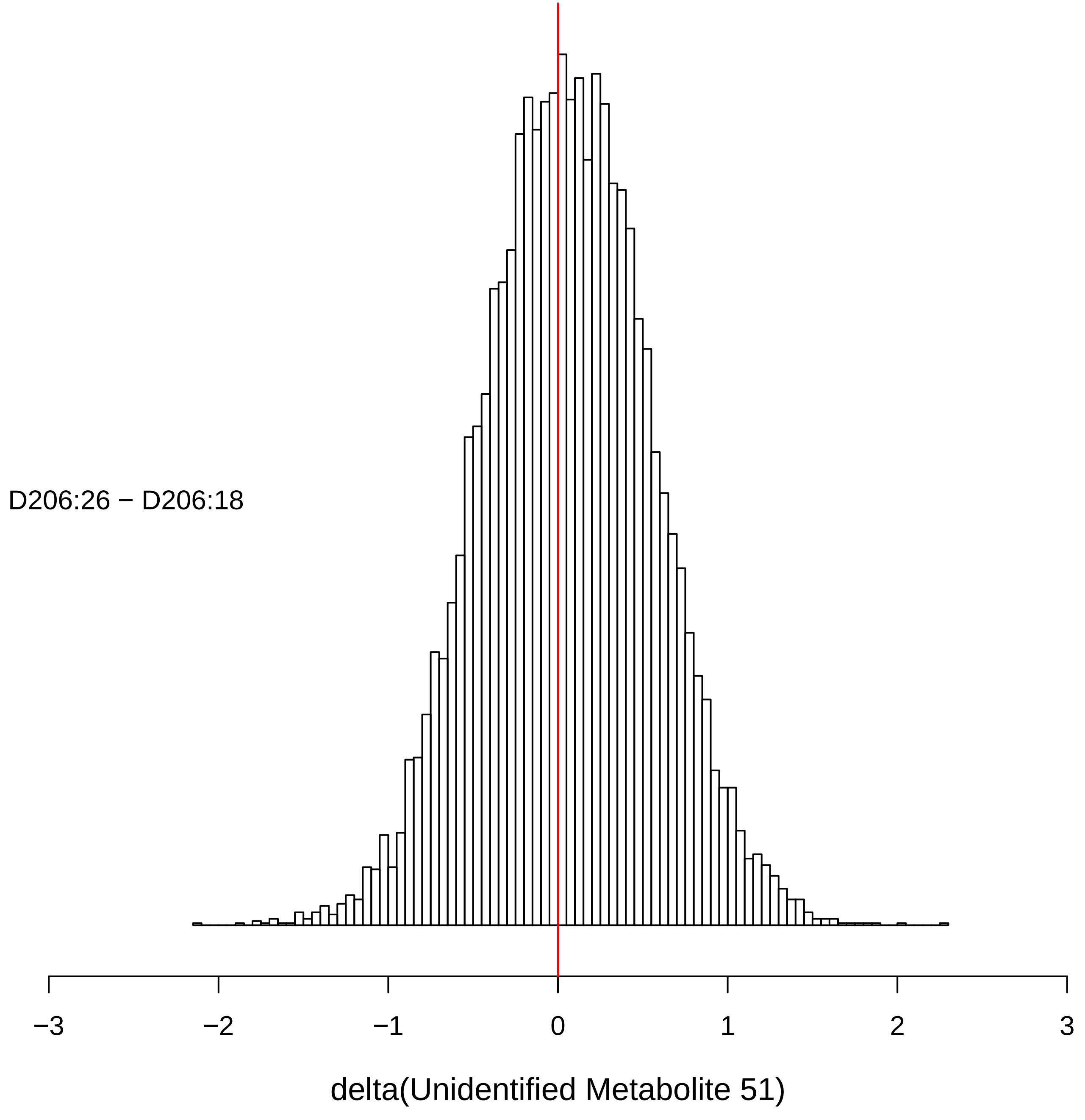
D206:26 − D206:18
−3
−2
−1
0
1
2
3
delta(Unidentified Metabolite 51)

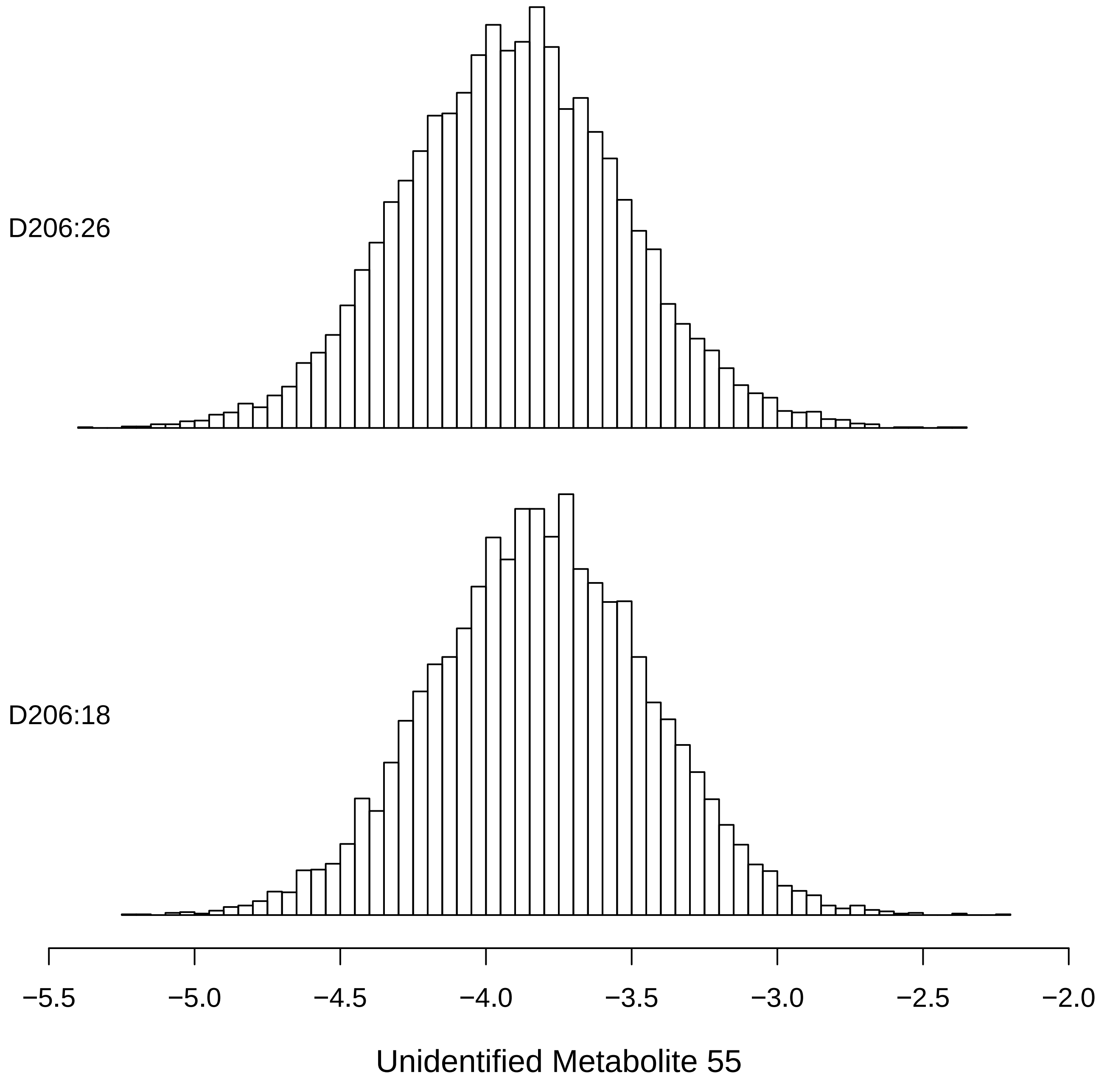

D206:26
D206:18
−5.5
−5.0
−4.5
−4.0
−3.5
−3.0
−2.5
−2.0
Unidentified Metabolite 55

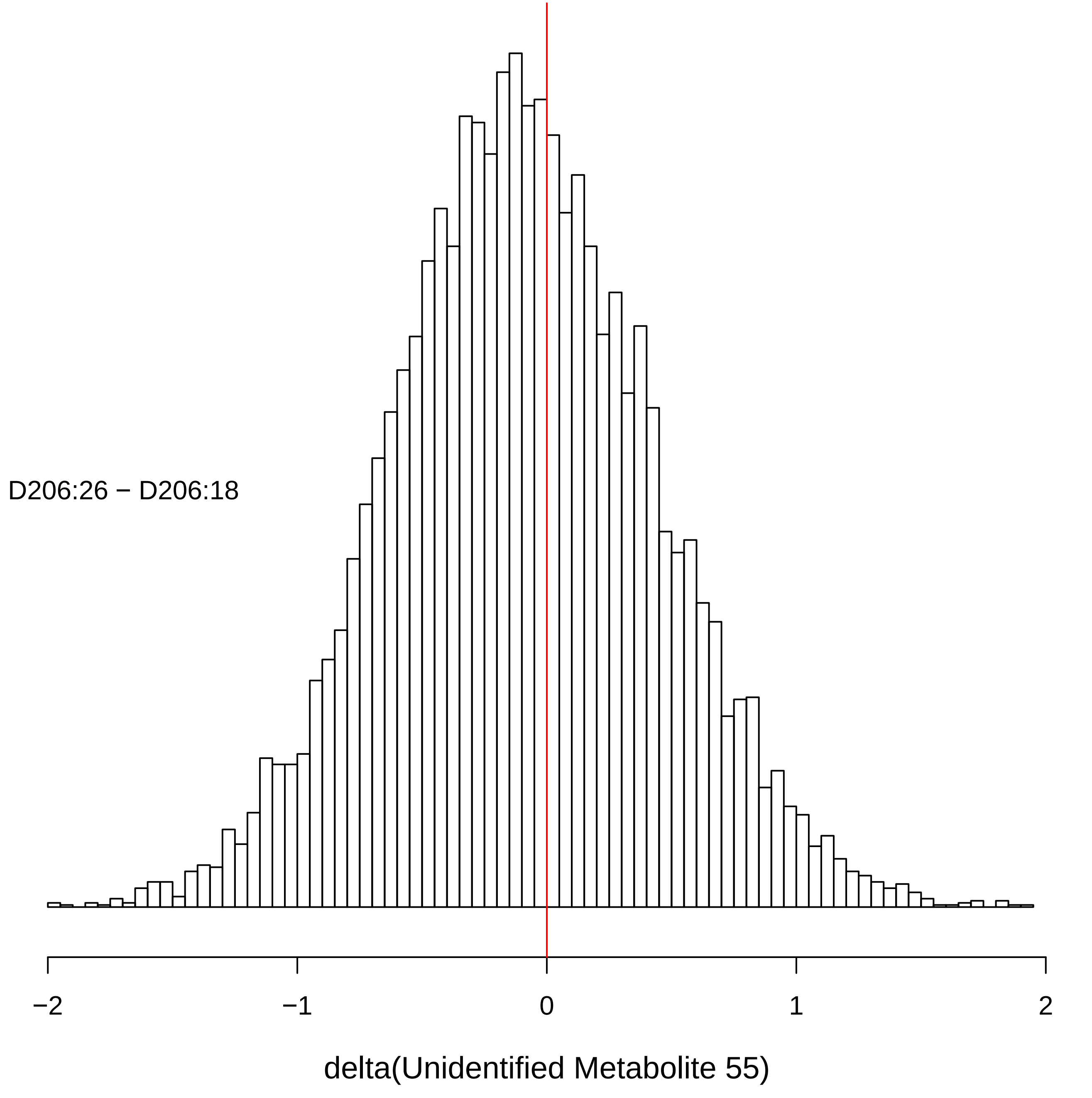
D206:26 − D206:18
−2
−1
0
1
2
delta(Unidentified Metabolite 55)

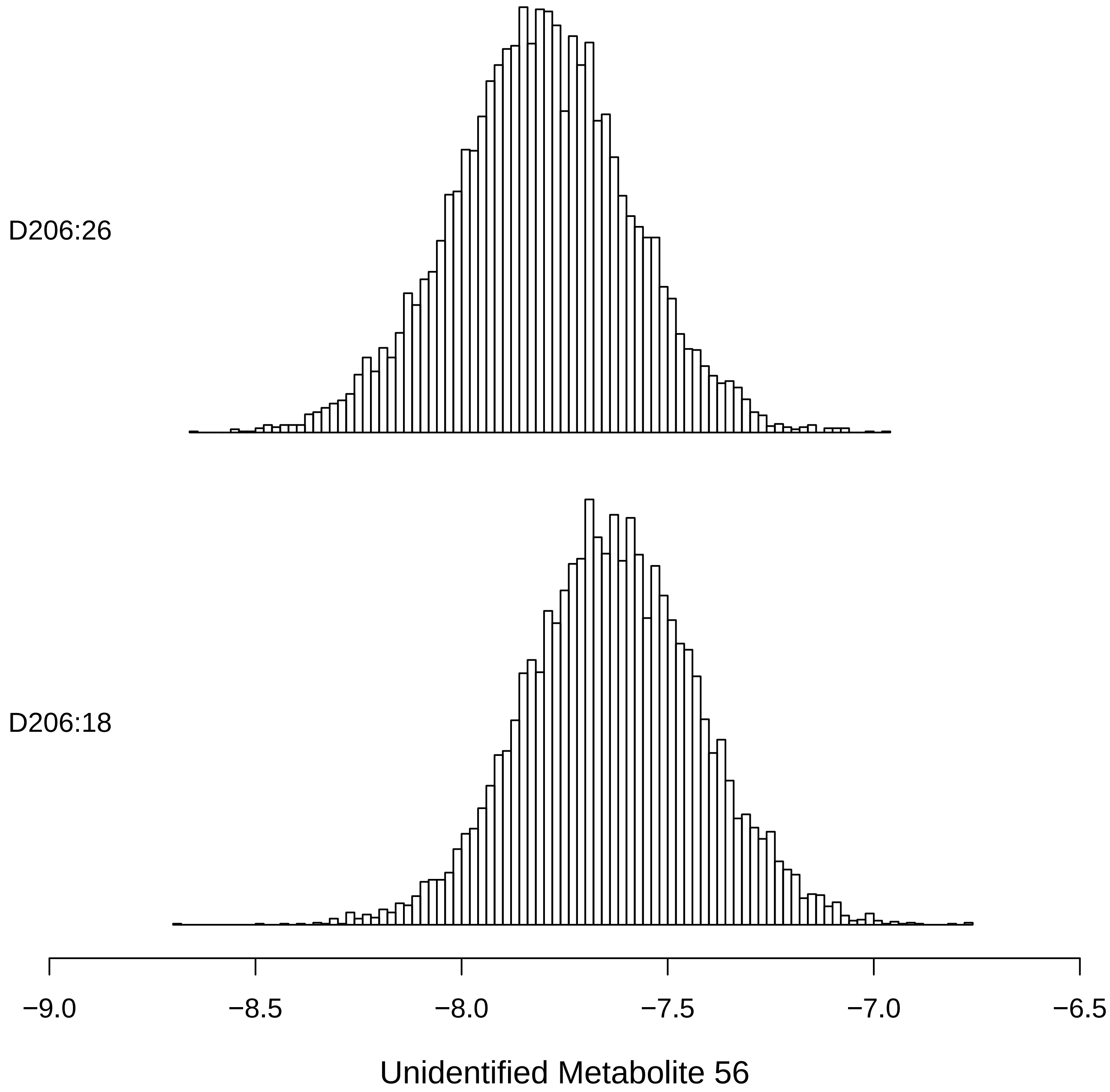
D206:26
D206:18
−9.0
−8.5
−8.0
−7.5
−7.0
−6.5
Unidentified Metabolite 56

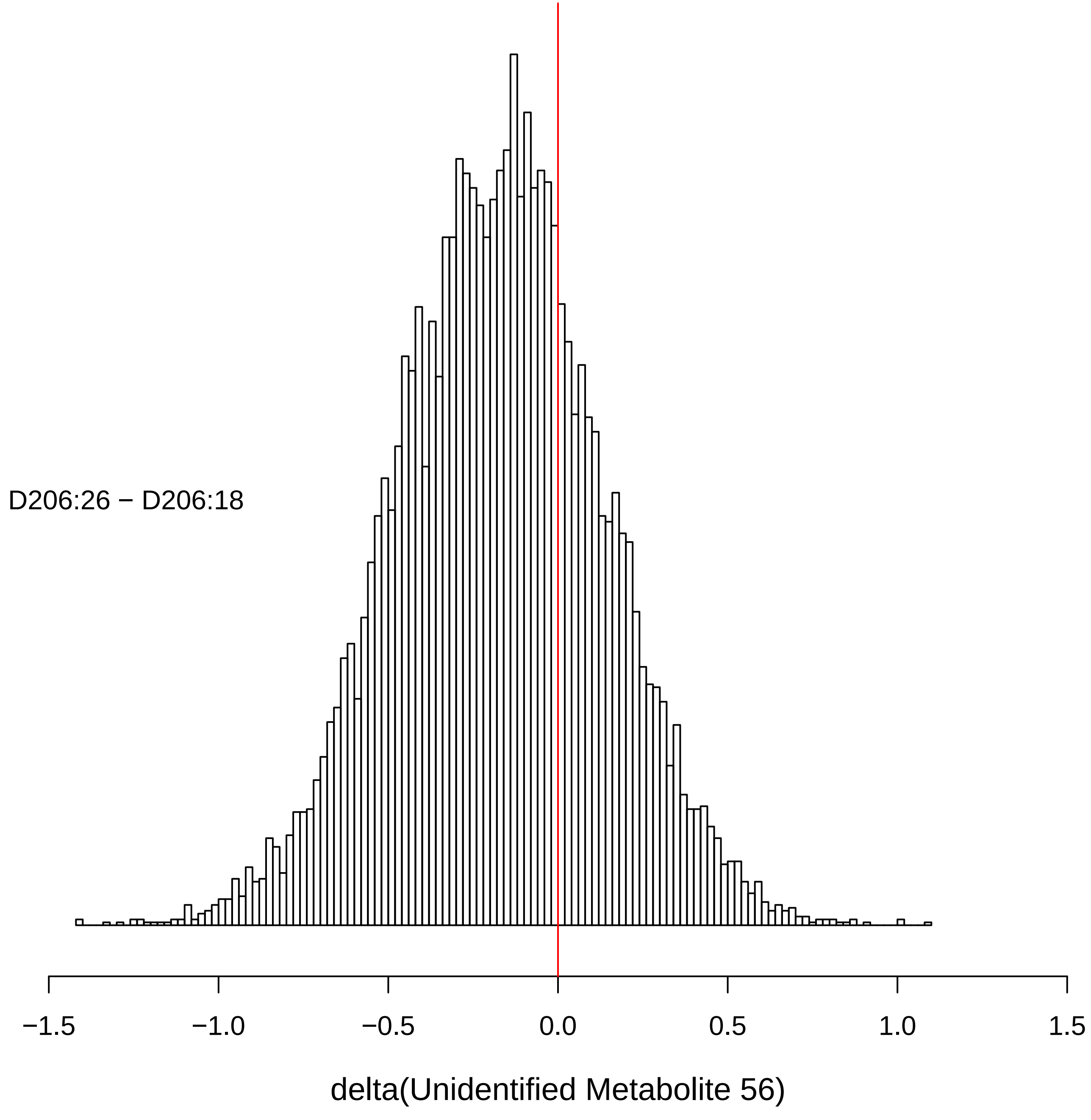
D206:26 − D206:18
−1.5
−1.0
−0.5
0.0
0.5
1.0
1.5
delta(Unidentified Metabolite 56)

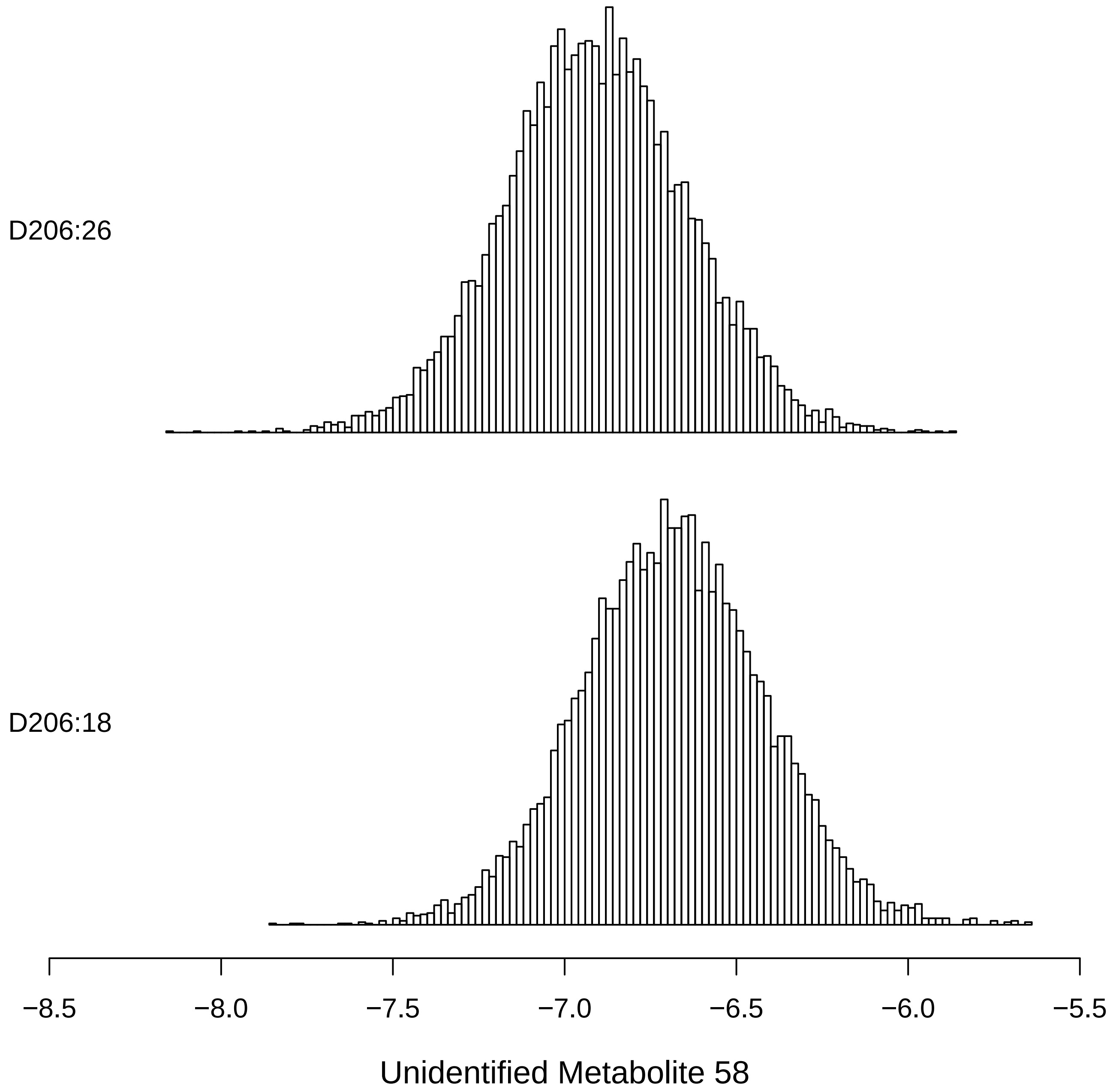

D206:26
D206:18
−8.5
−8.0
−7.5
−7.0
−6.5
−6.0
−5.5
Unidentified Metabolite 58

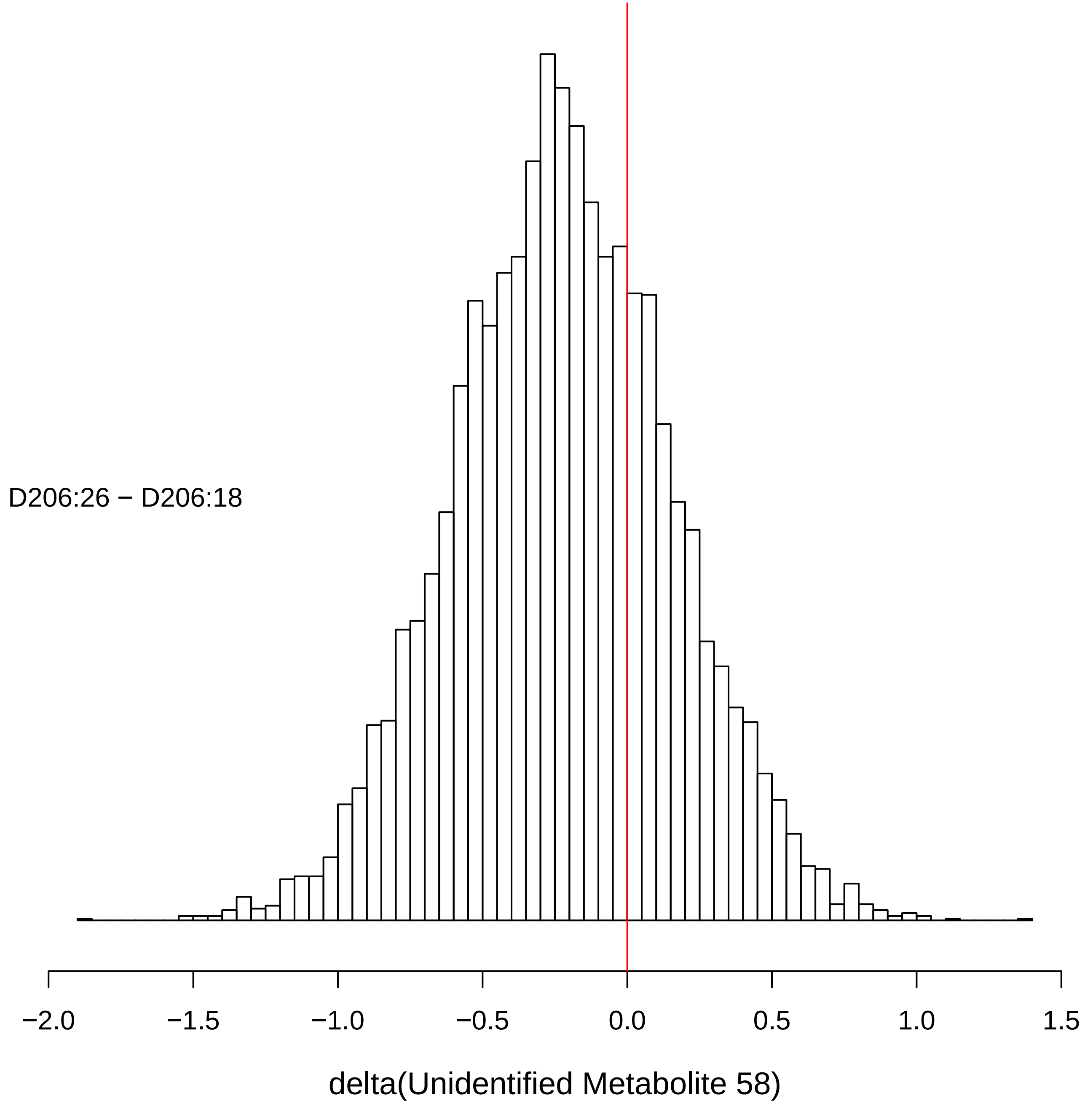
D206:26 − D206:18
−2.0
−1.5
−1.0
−0.5
0.0
0.5
1.0
1.5
delta(Unidentified Metabolite 58)

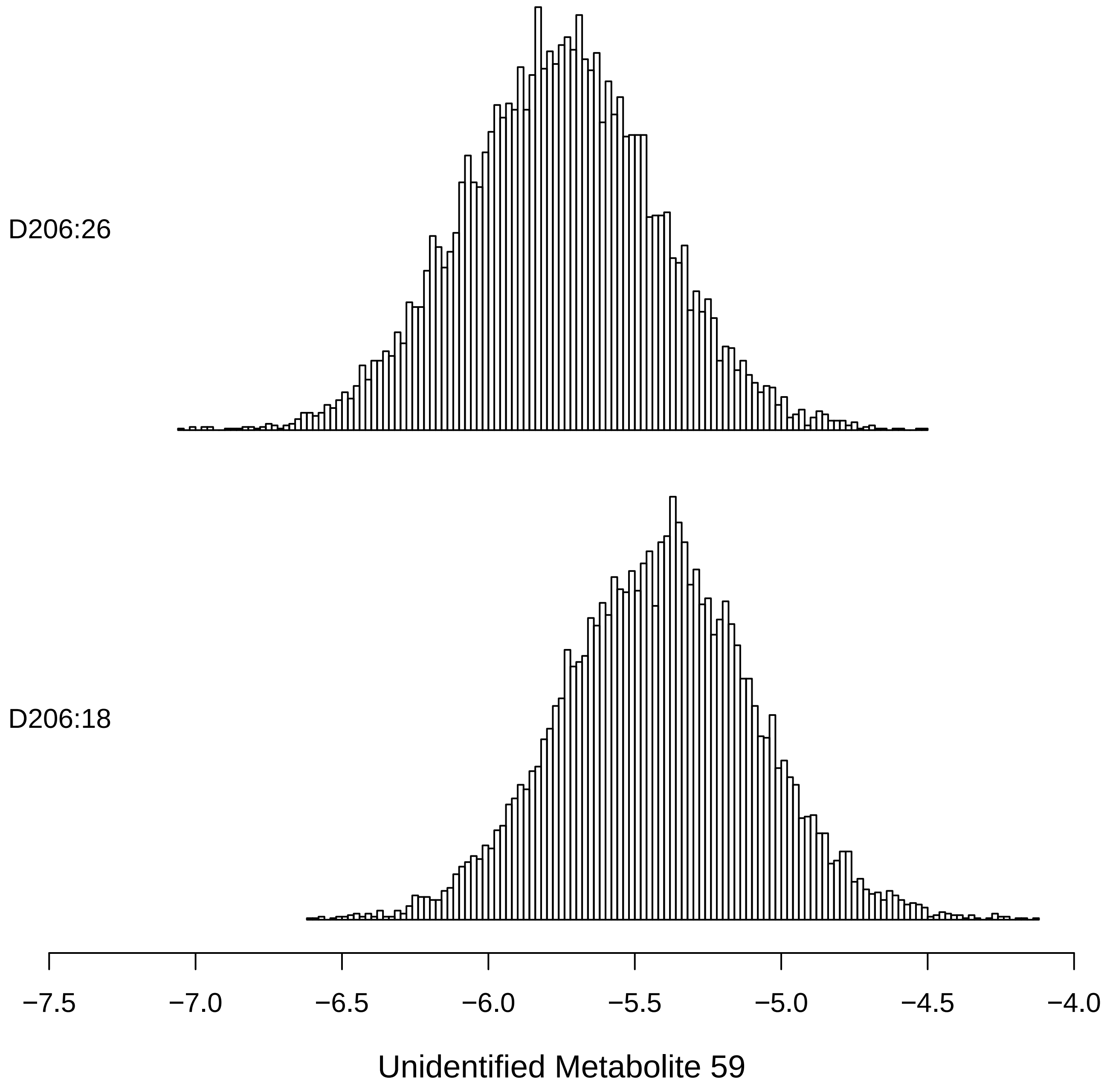
D206:26
D206:18
−7.5
−7.0
−6.5
−6.0
−5.5
−5.0
−4.5
−4.0
Unidentified Metabolite 59

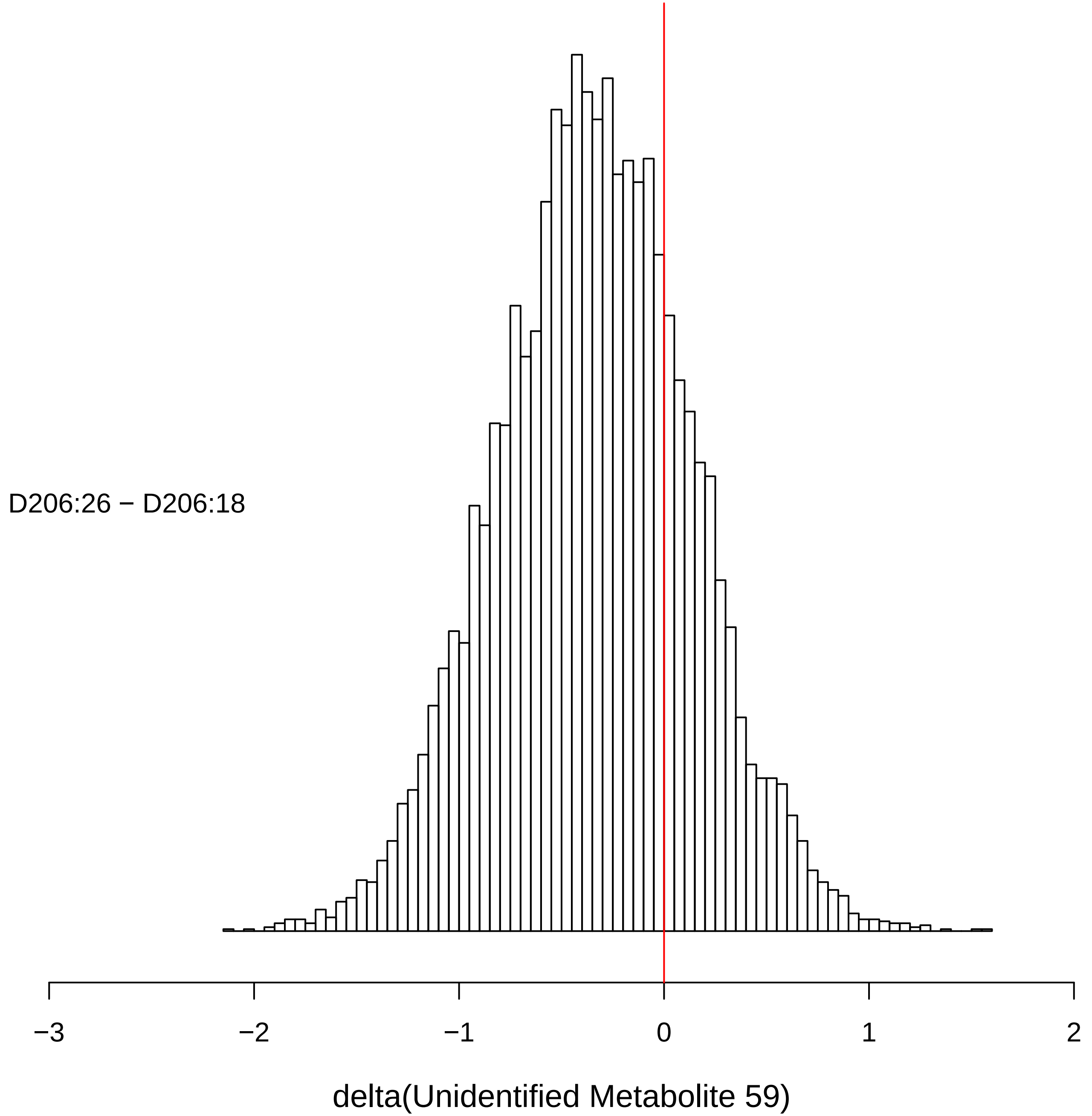
D206:26 − D206:18
−3
−2
−1
0
1
2
delta(Unidentified Metabolite 59)

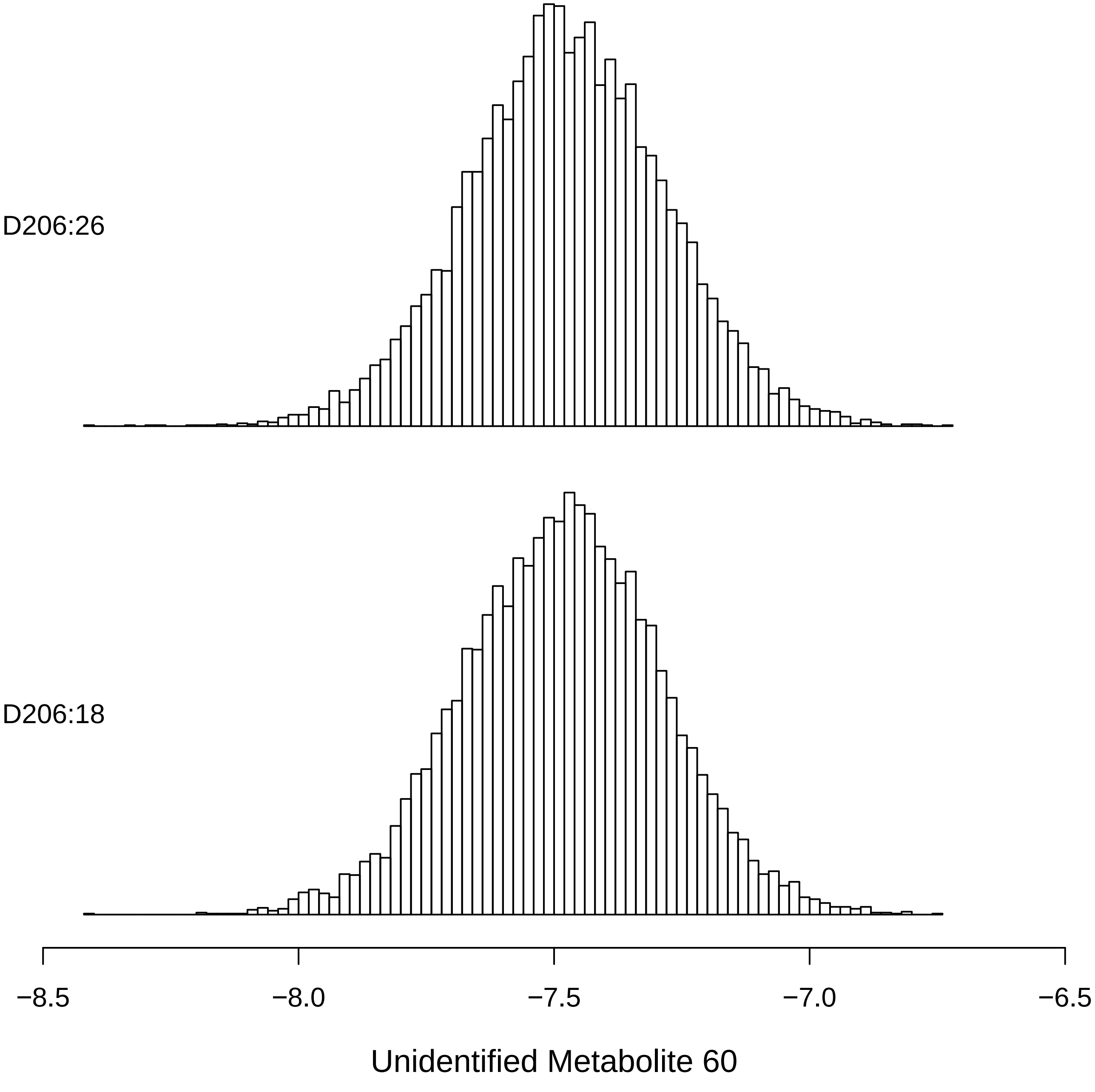
D206:26
D206:18
−8.5
−8.0
−7.5
−7.0
−6.5
Unidentified Metabolite 60

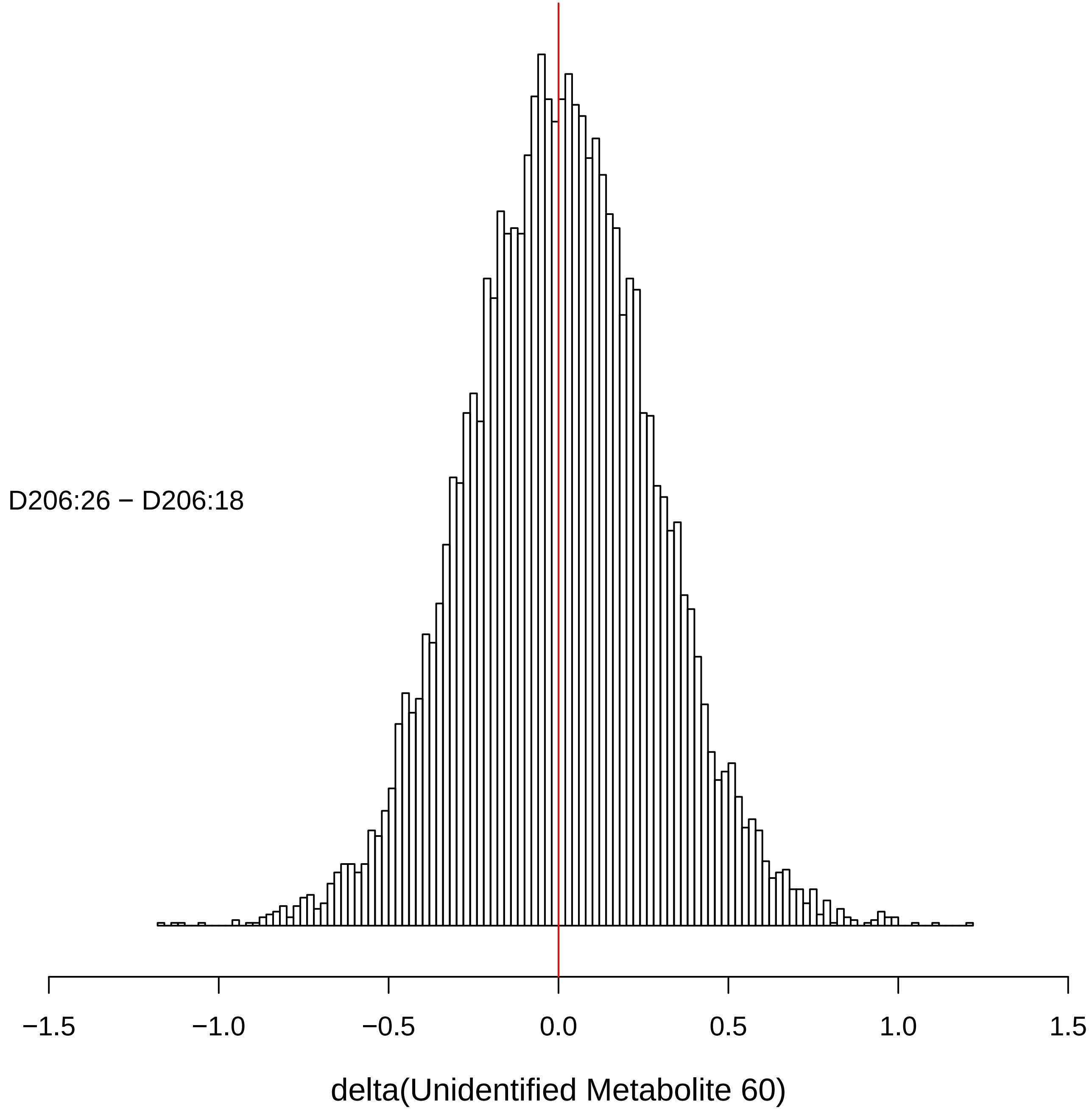
D206:26 − D206:18
−1.5
−1.0
−0.5
0.0
0.5
1.0
1.5
delta(Unidentified Metabolite 60)

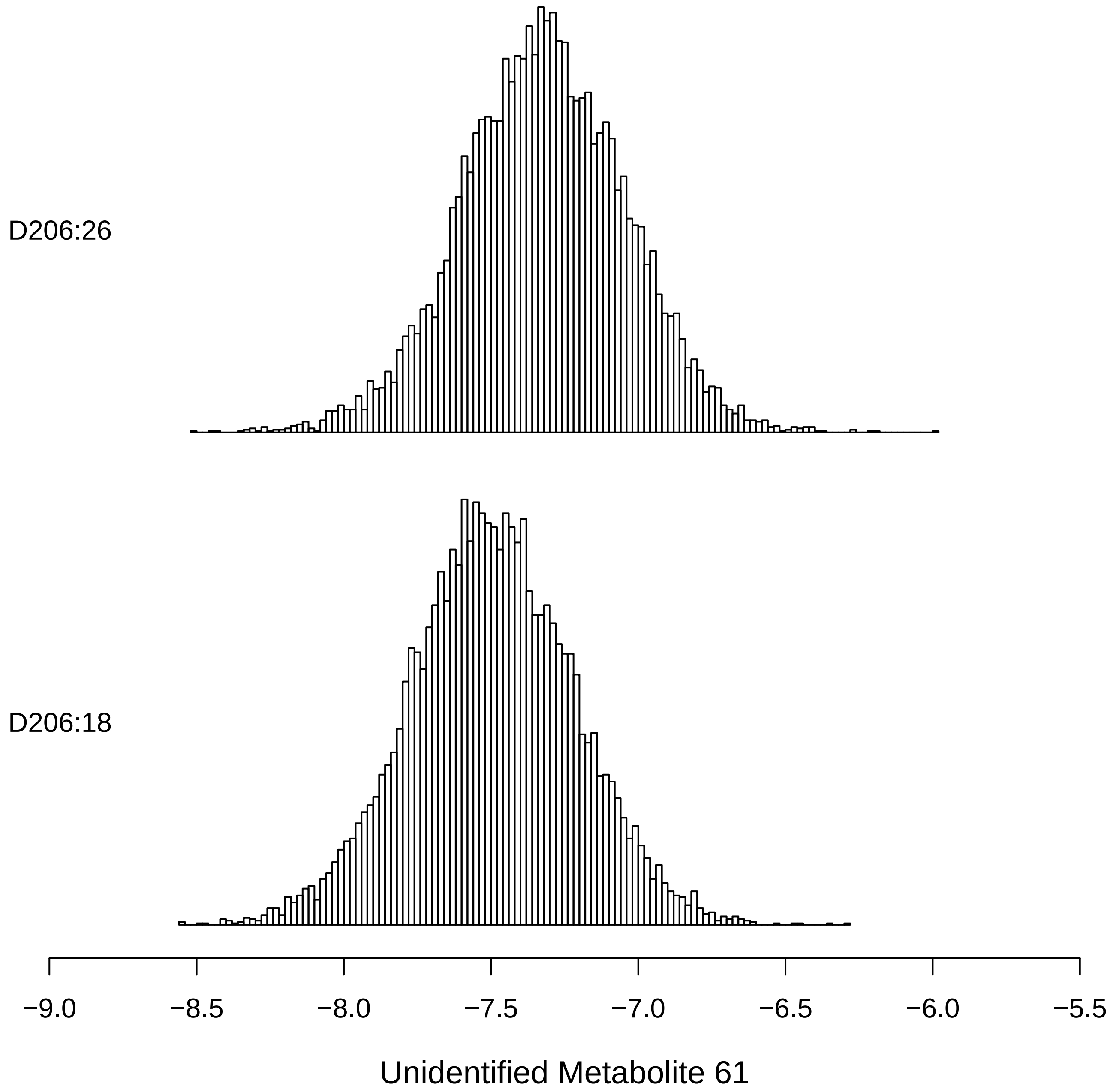
D206:26
D206:18
−9.0
−8.5
−8.0
−7.5
−7.0
−6.5
−6.0
−5.5
Unidentified Metabolite 61

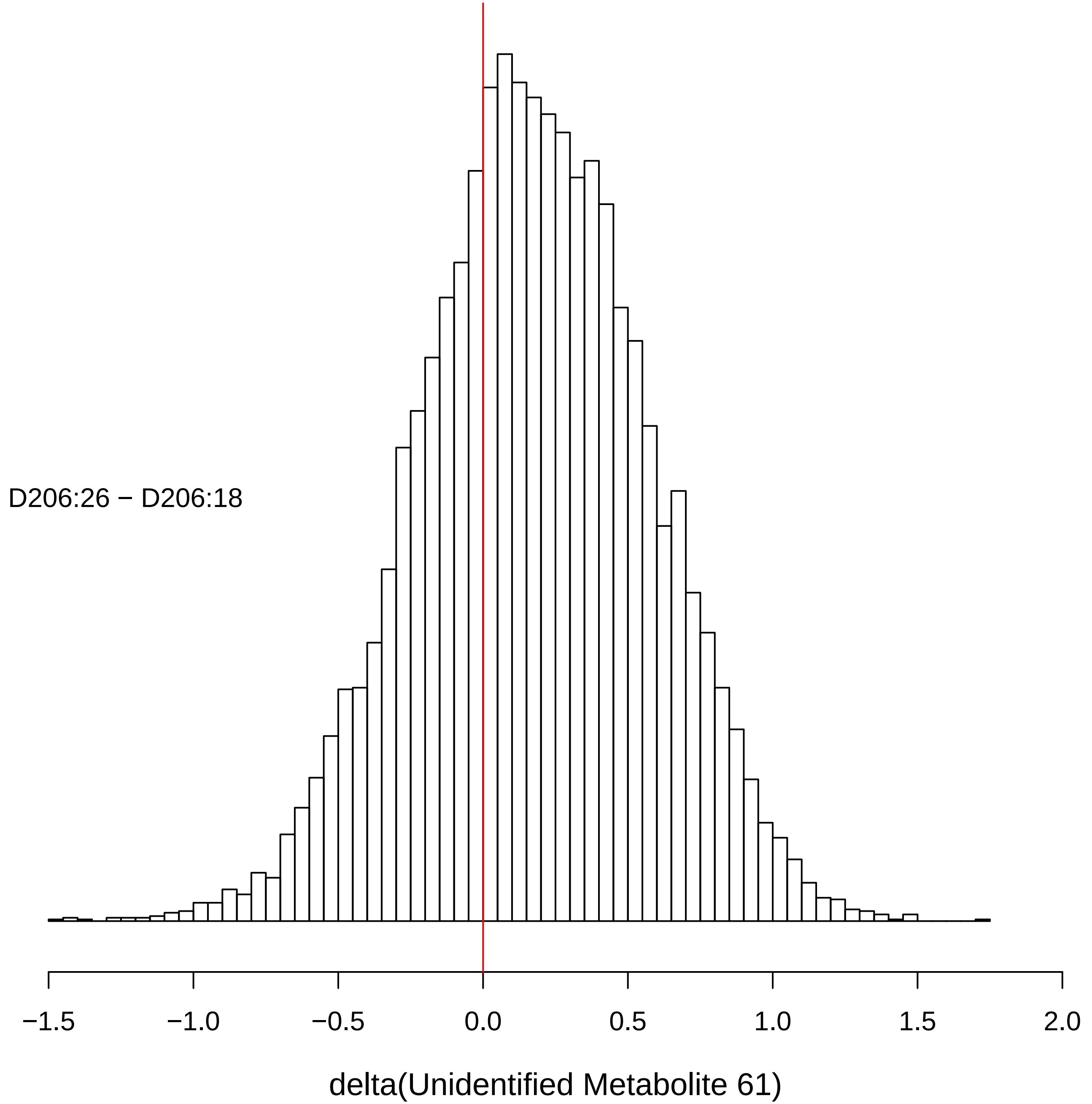
D206:26 − D206:18
−1.5
−1.0
−0.5
0.0
0.5
1.0
1.5
2.0
delta(Unidentified Metabolite 61)

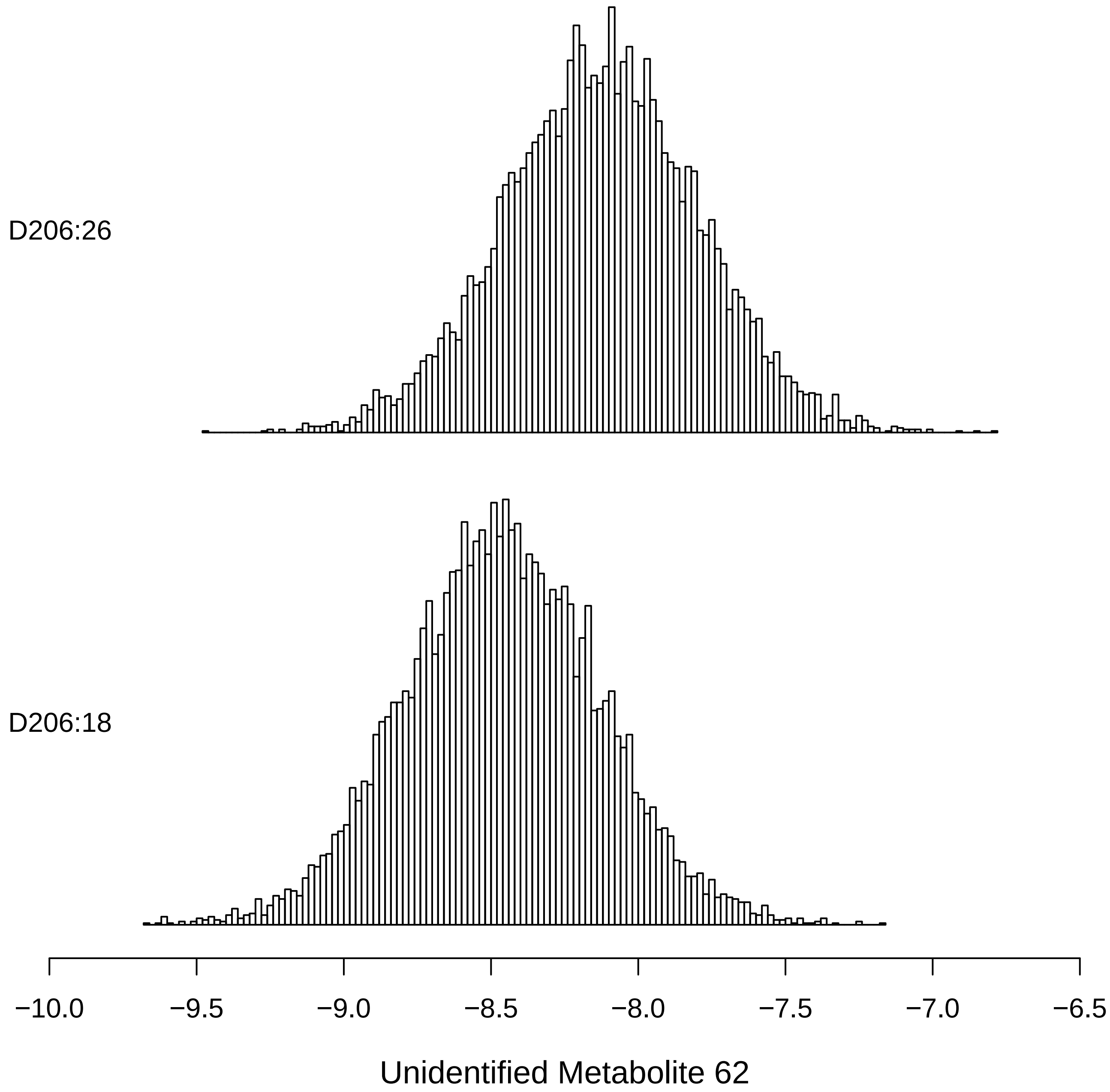
D206:26
D206:18
−10.0
−9.5
−9.0
−8.5
−8.0
−7.5
−7.0
−6.5
Unidentified Metabolite 62

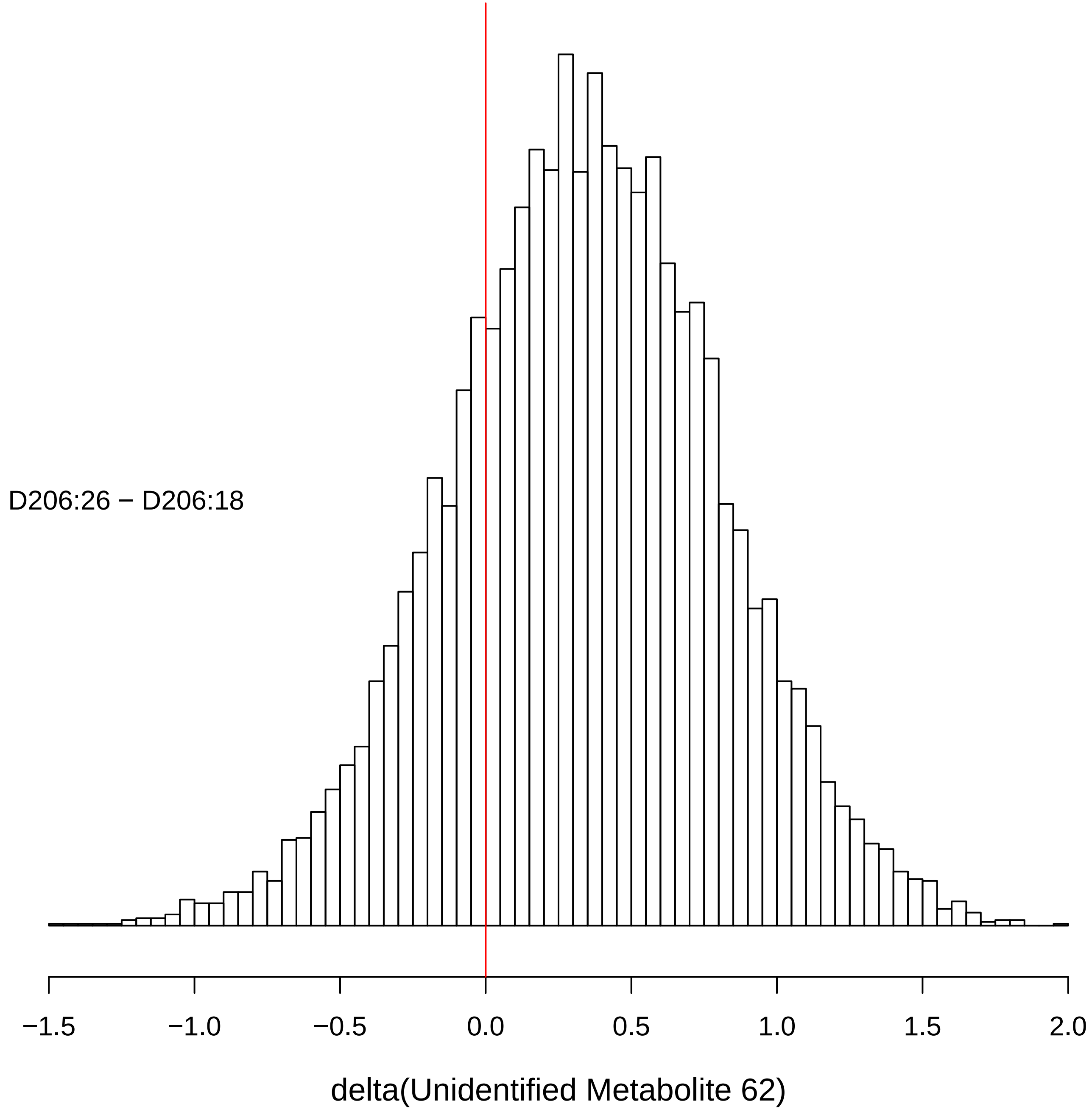
D206:26 − D206:18
−1.5
−1.0
−0.5
0.0
0.5
1.0
1.5
2.0
delta(Unidentified Metabolite 62)

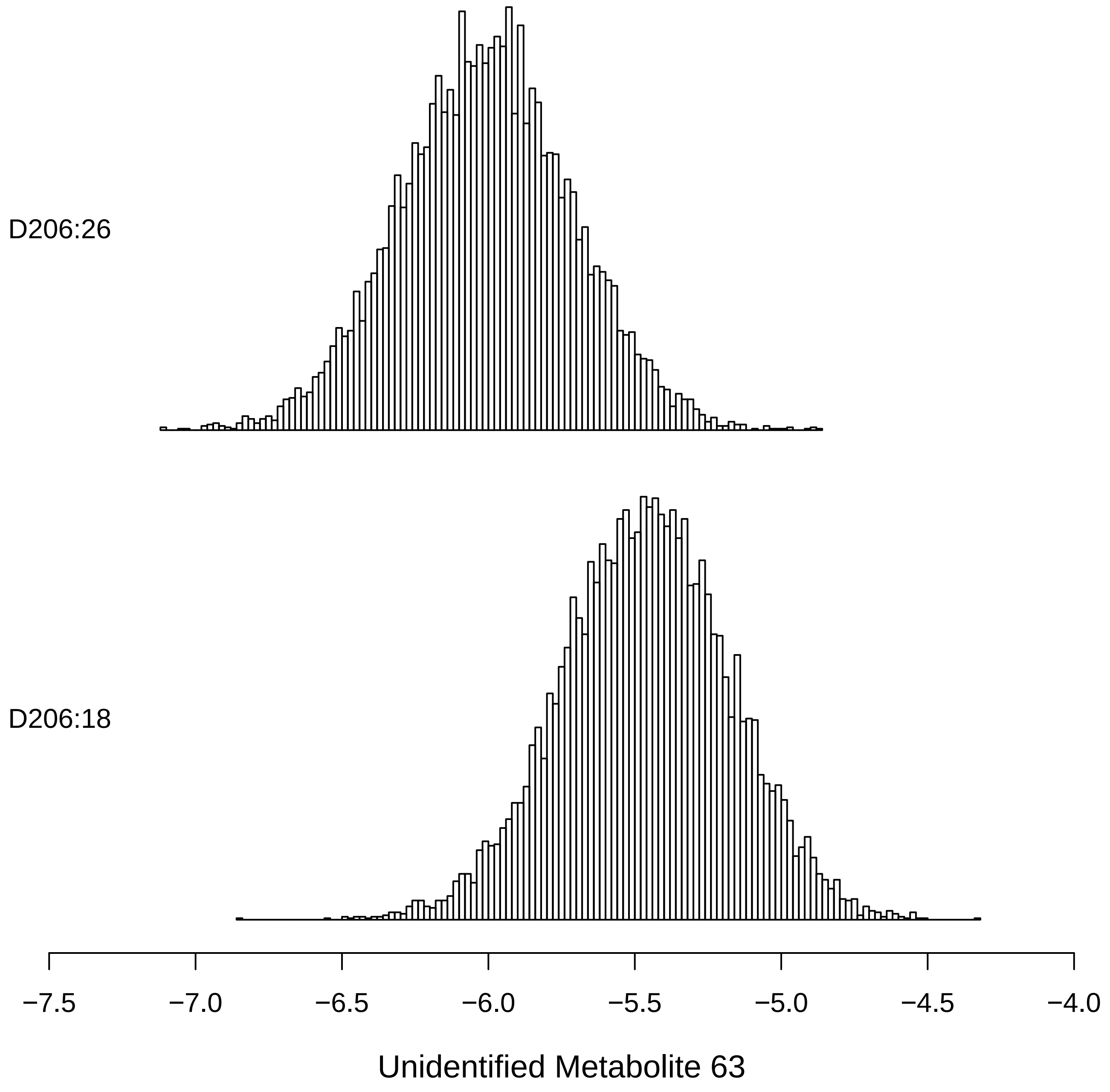
D206:26
D206:18
−7.5
−7.0
−6.5
−6.0
−5.5
−5.0
−4.5
−4.0
Unidentified Metabolite 63

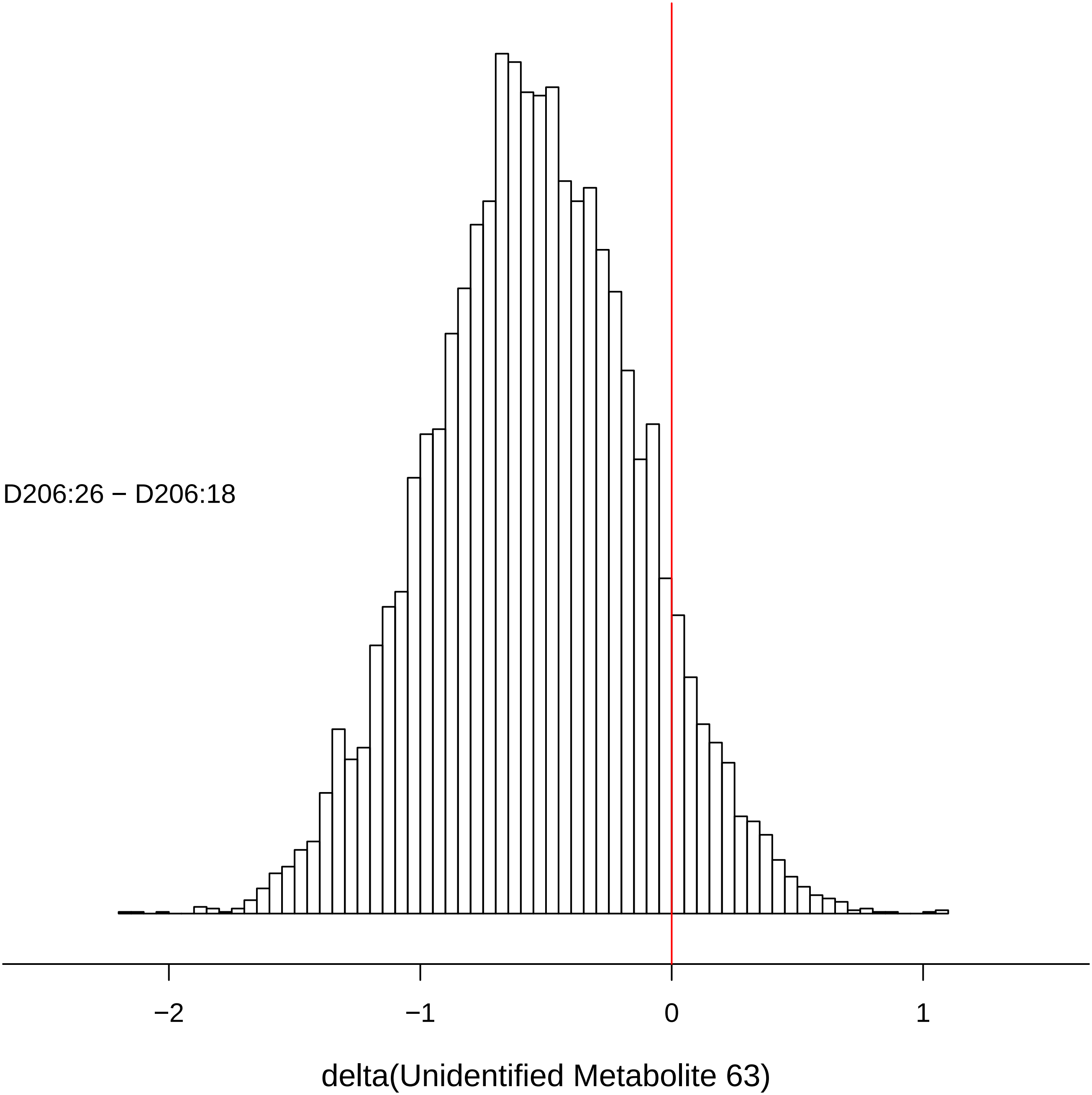
D206:26 − D206:18
−2
−1
0
1
delta(Unidentified Metabolite 63)

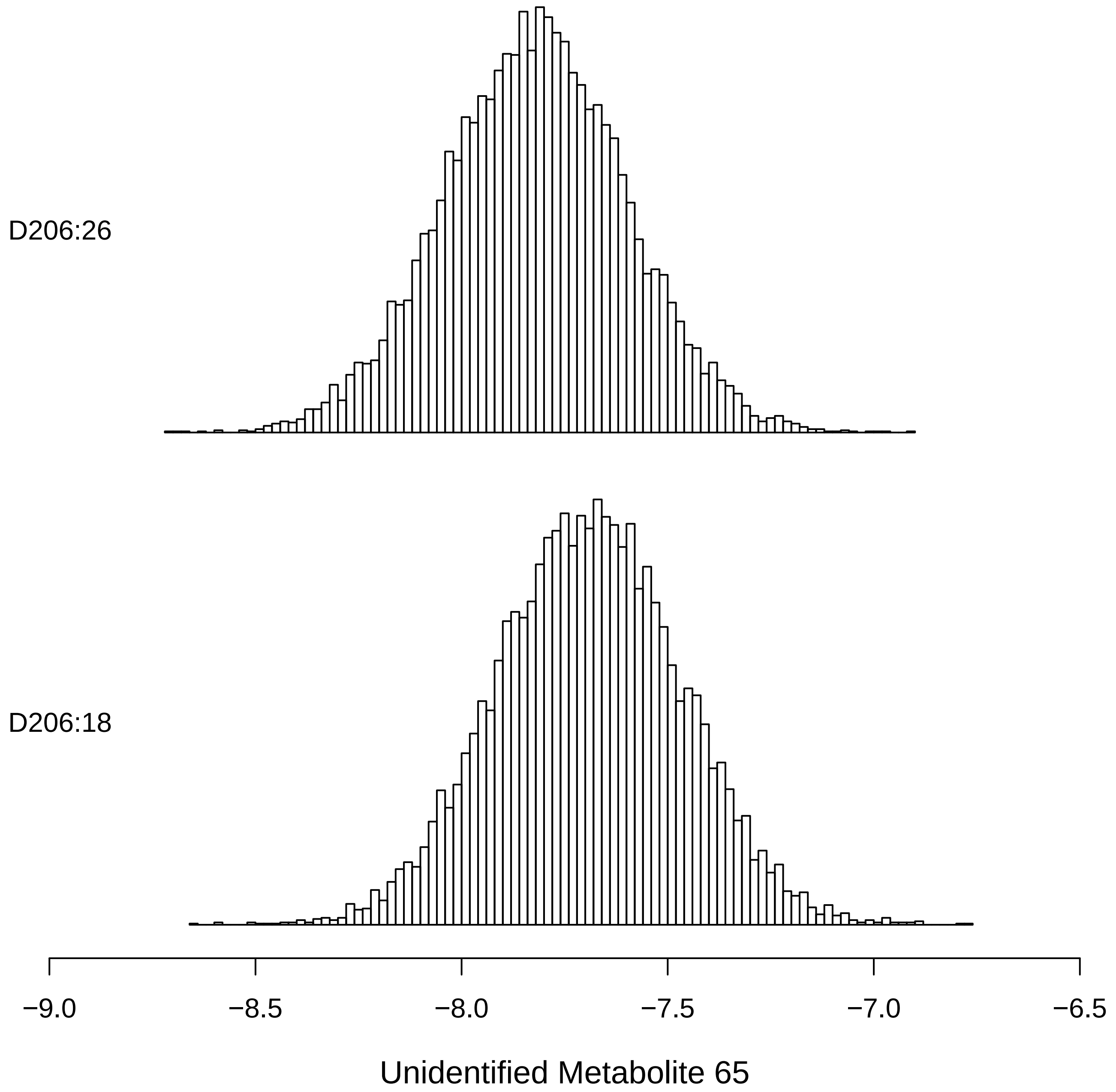
D206:26
D206:18
−9.0
−8.5
−8.0
−7.5
−7.0
−6.5
Unidentified Metabolite 65

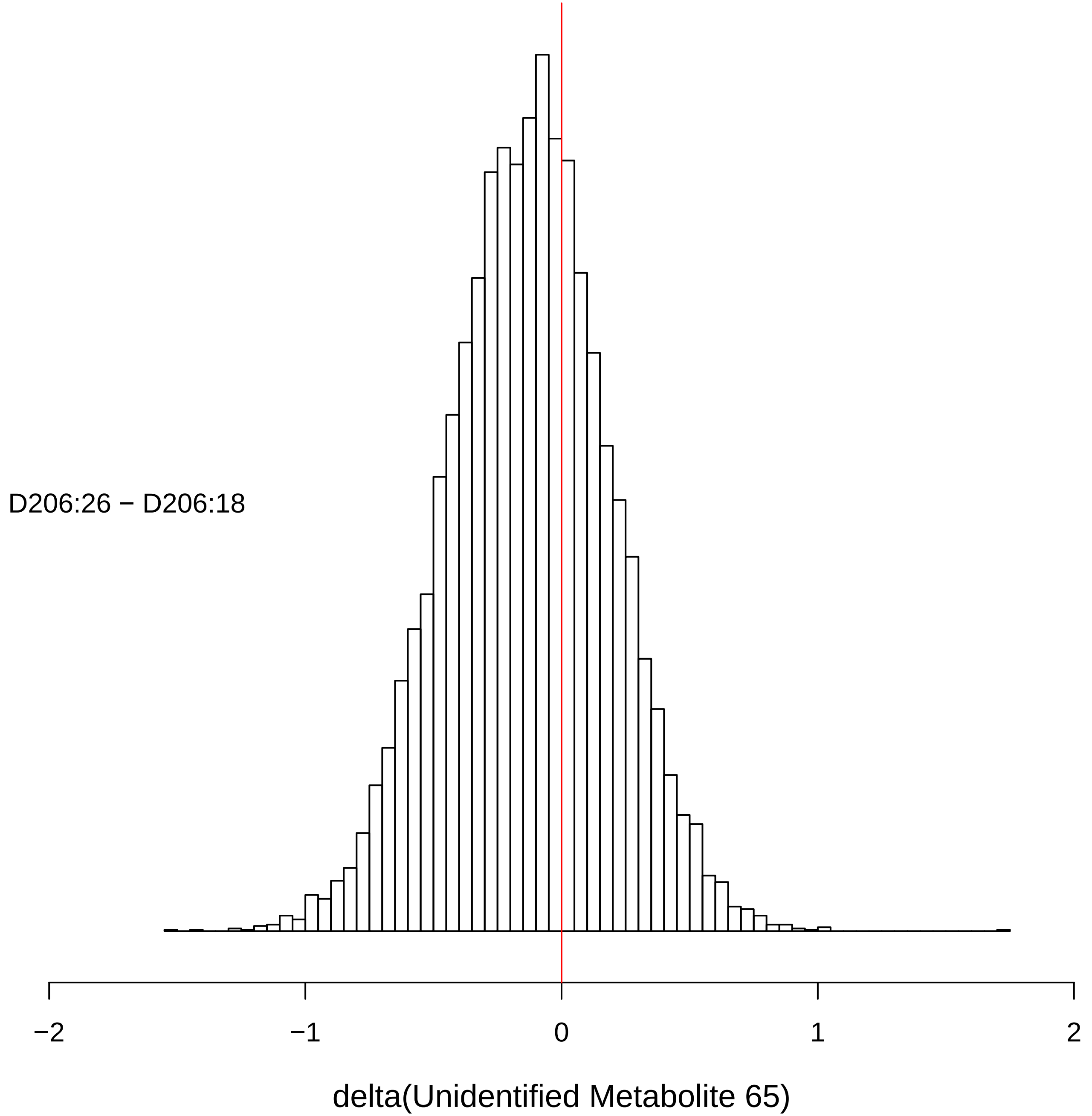
D206:26 − D206:18
−2
−1
0
1
2
delta(Unidentified Metabolite 65)

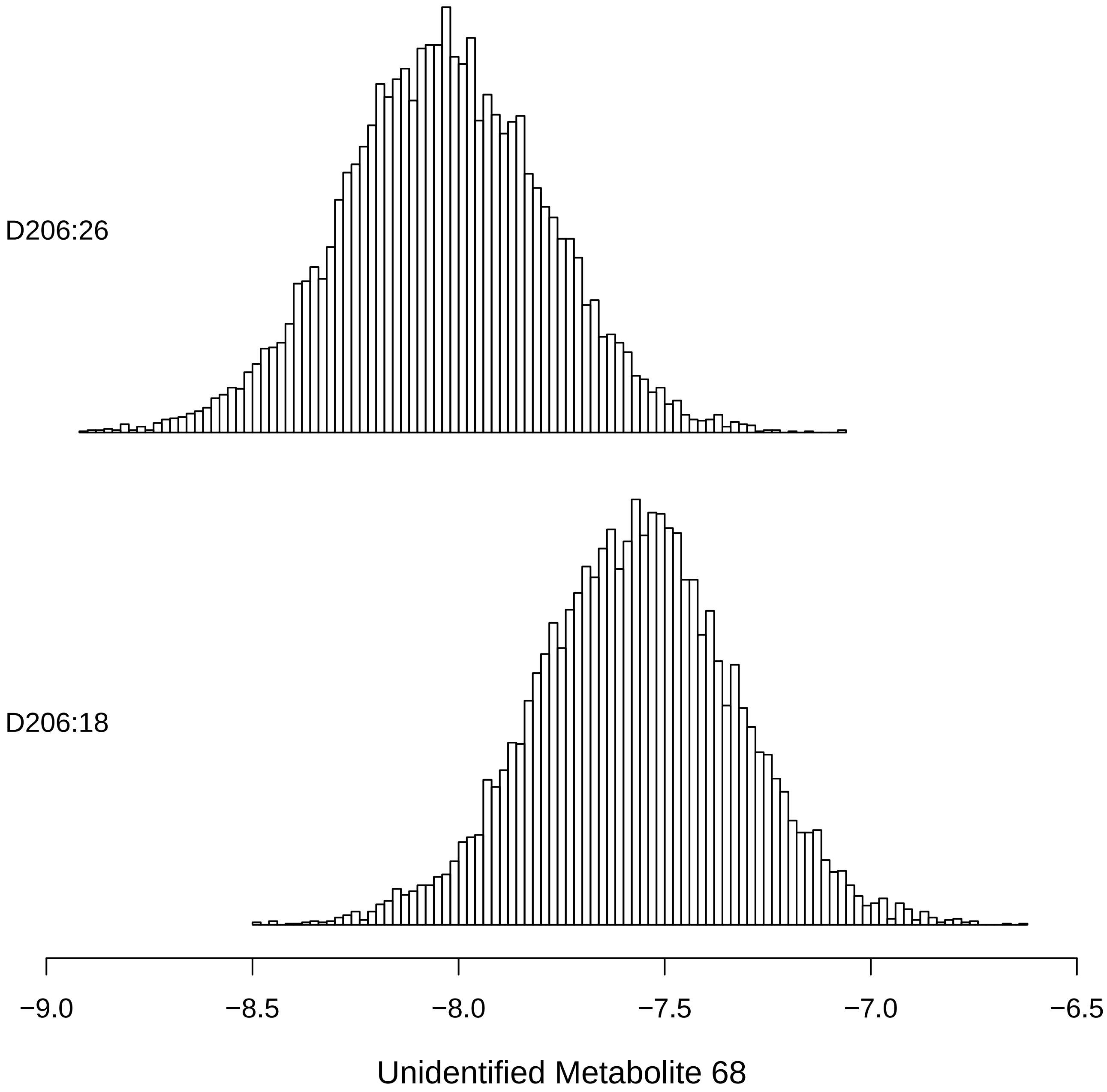
D206:26
D206:18
−9.0
−8.5
−8.0
−7.5
−7.0
−6.5
Unidentified Metabolite 68

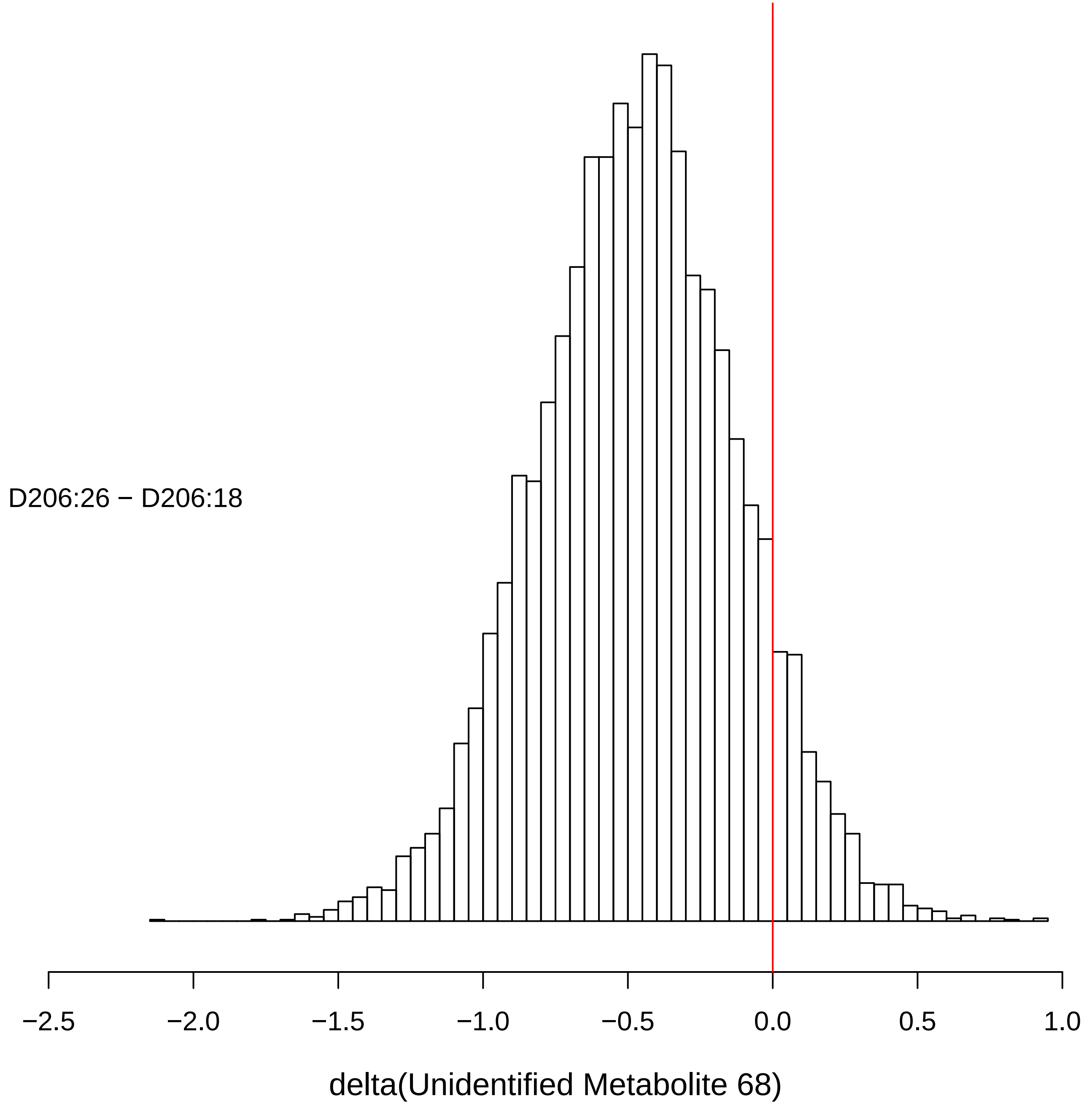
D206:26 − D206:18
−2.5
−2.0
−1.5
−1.0
−0.5
0.0
0.5
1.0
delta(Unidentified Metabolite 68)

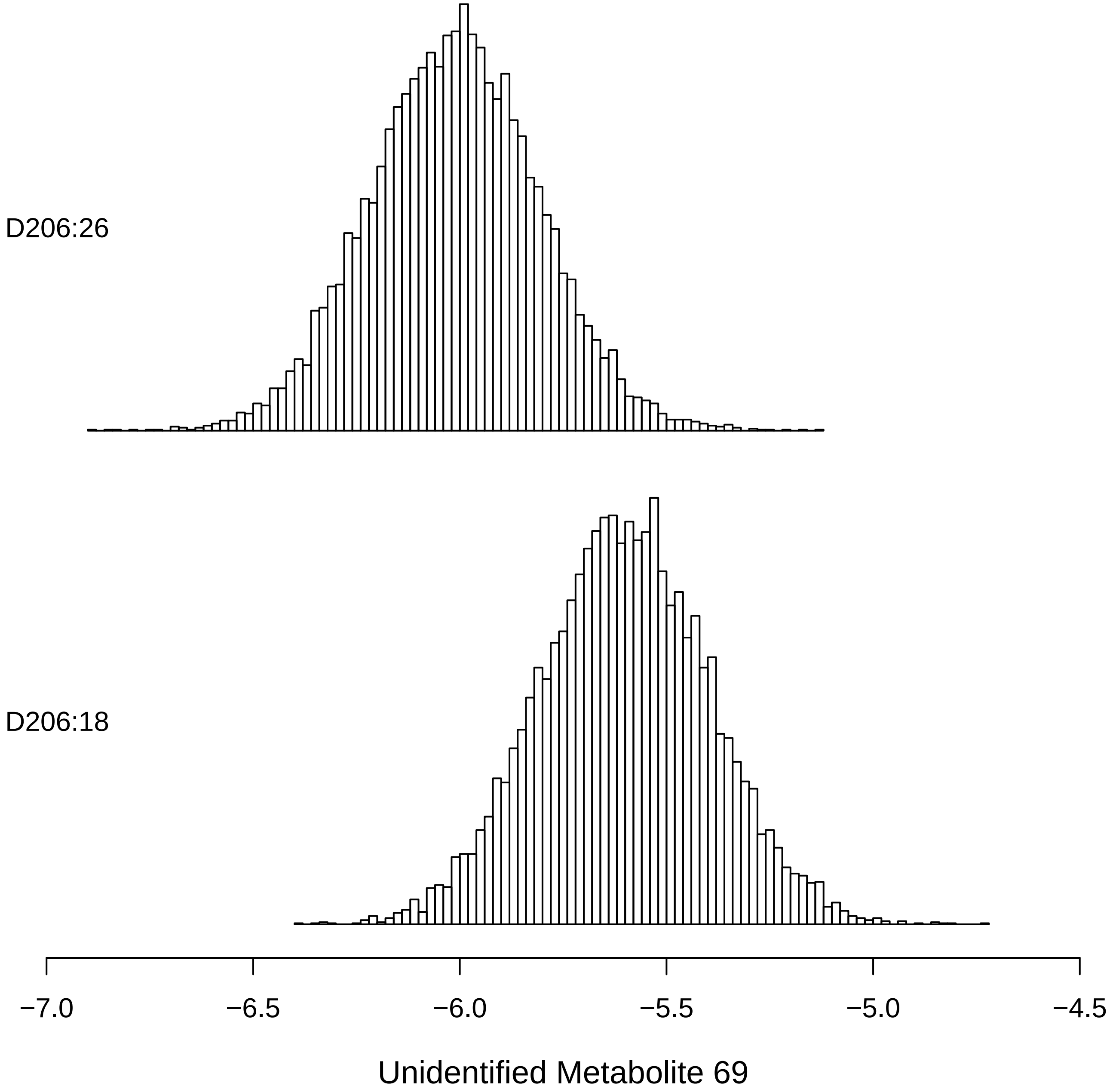
D206:26
D206:18
−7.0
−6.5
−6.0
−5.5
−5.0
−4.5
Unidentified Metabolite 69

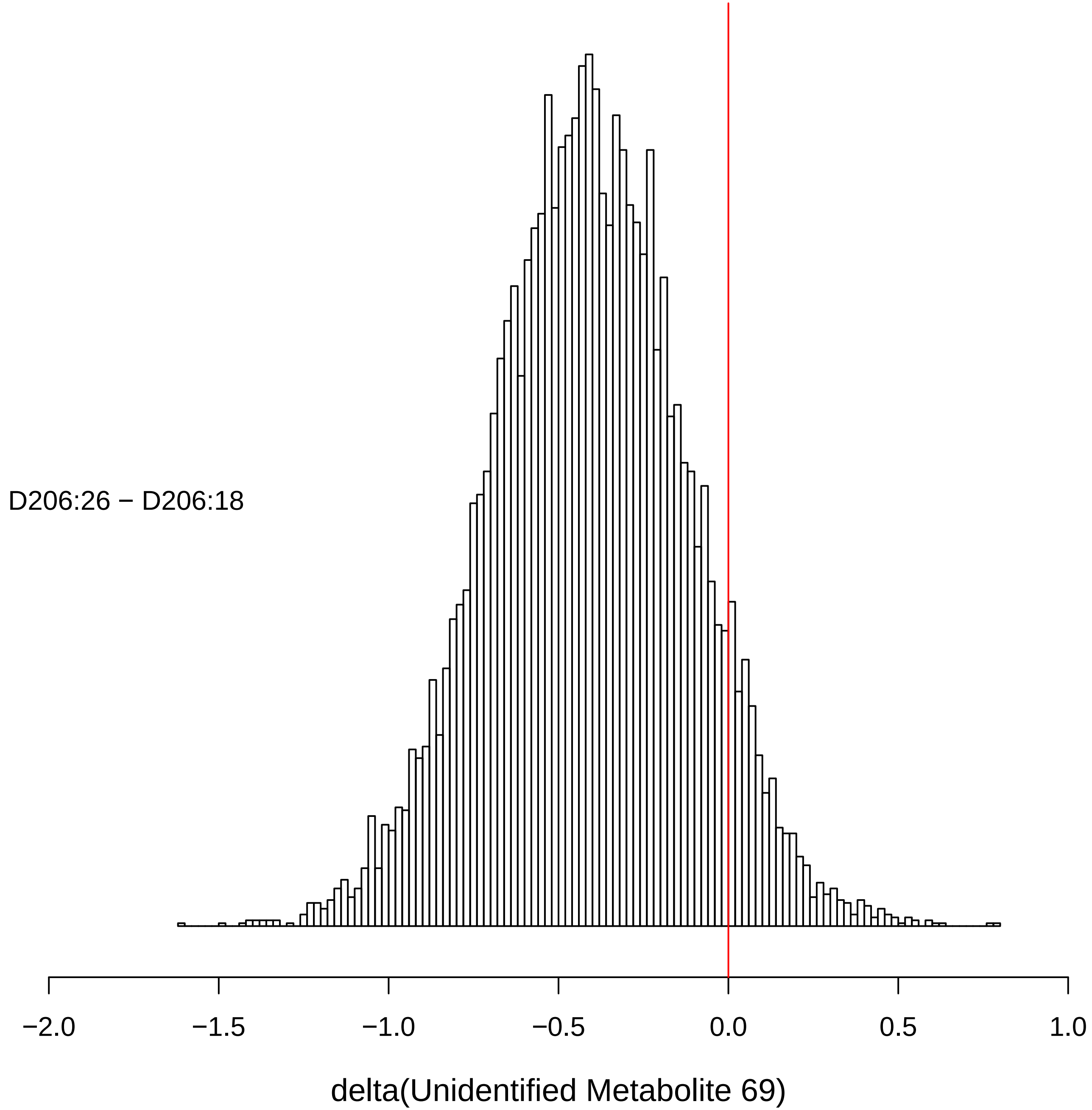
D206:26 − D206:18
−2.0
−1.5
−1.0
−0.5
0.0
0.5
1.0
delta(Unidentified Metabolite 69)

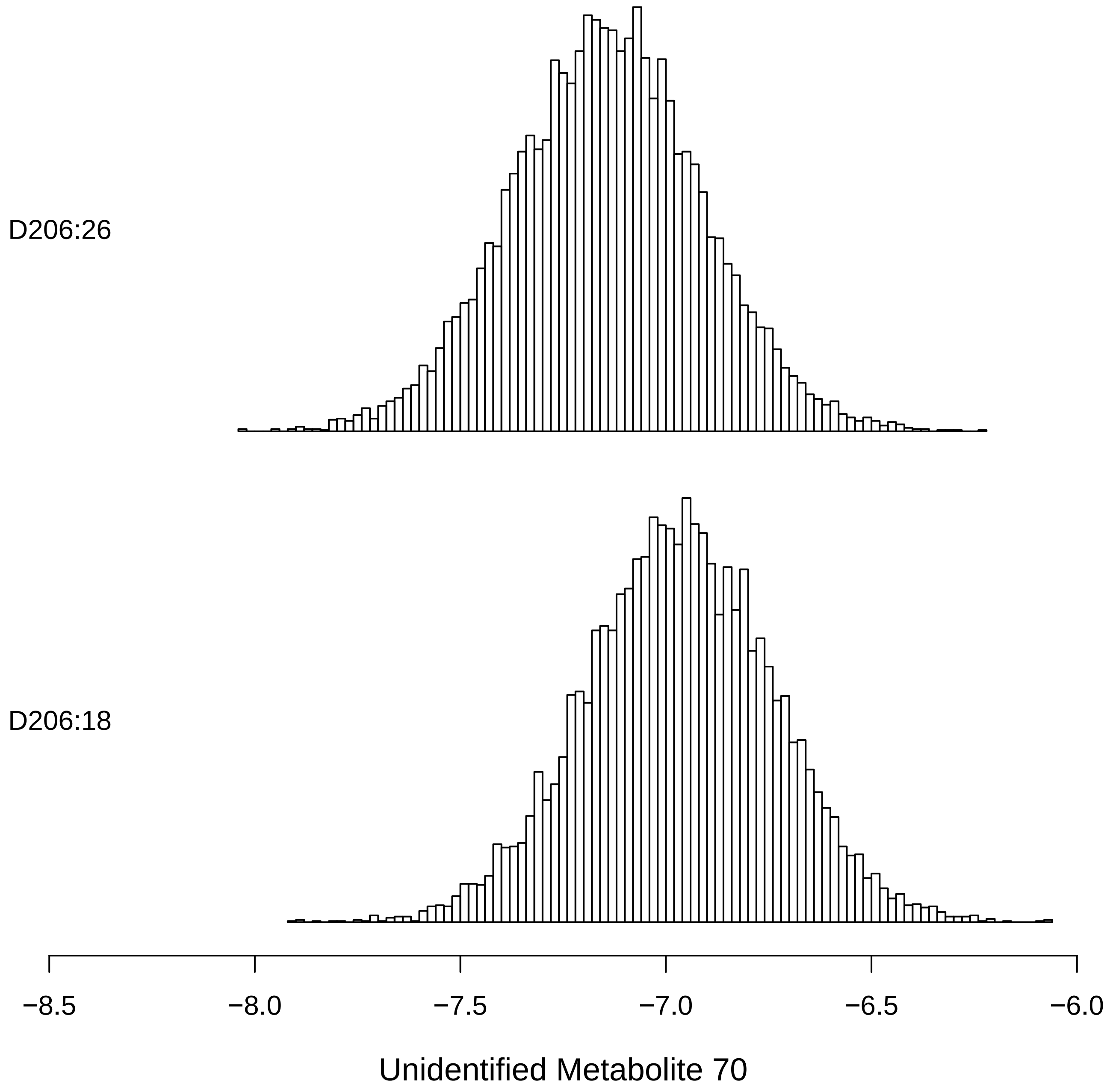

D206:26
D206:18
−8.5
−8.0
−7.5
−7.0
−6.5
−6.0
Unidentified Metabolite 70

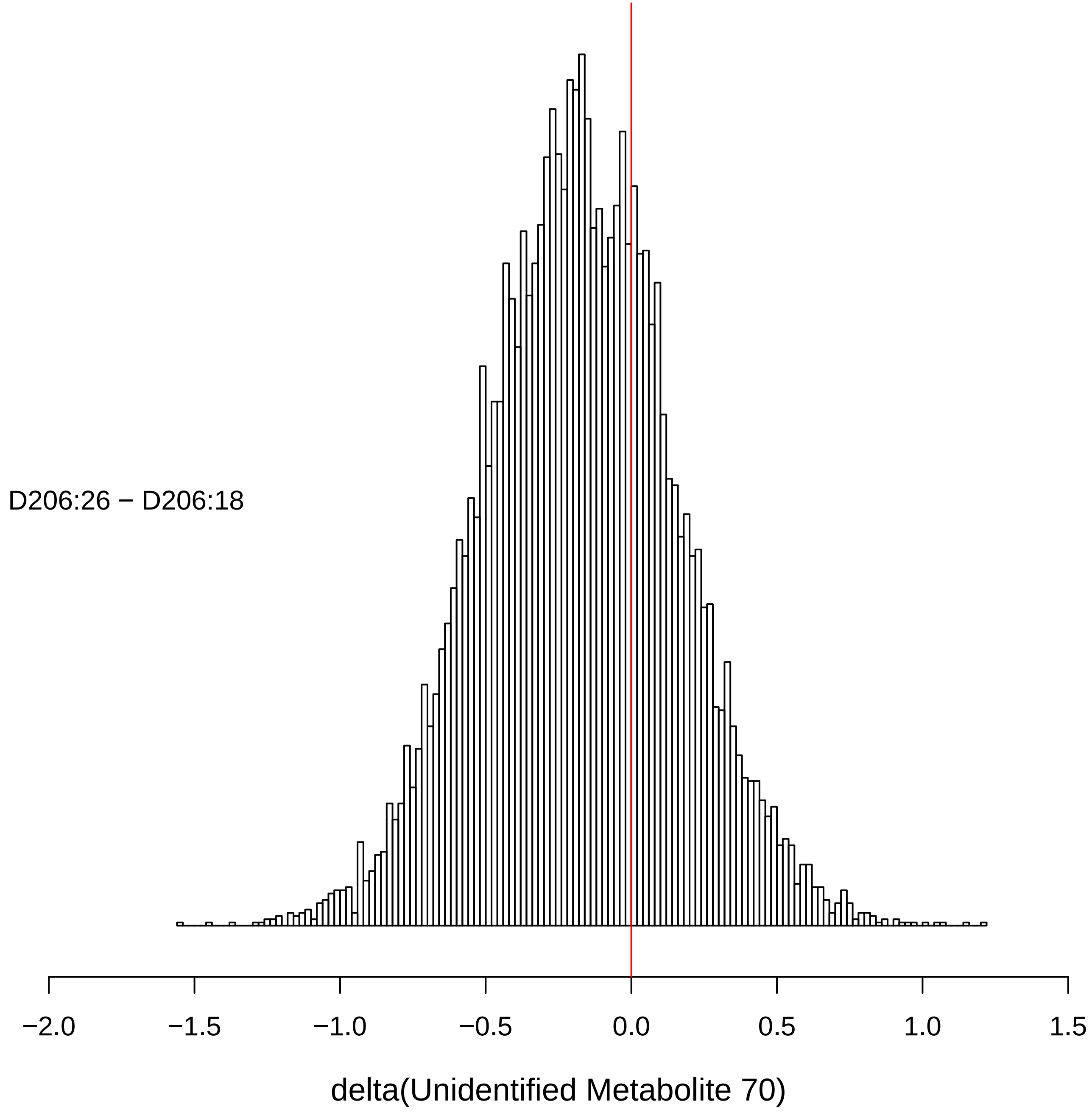
D206:26 − D206:18
−2.0
−1.5
−1.0
−0.5
0.0
0.5
1.0
1.5
delta(Unidentified Metabolite 70)

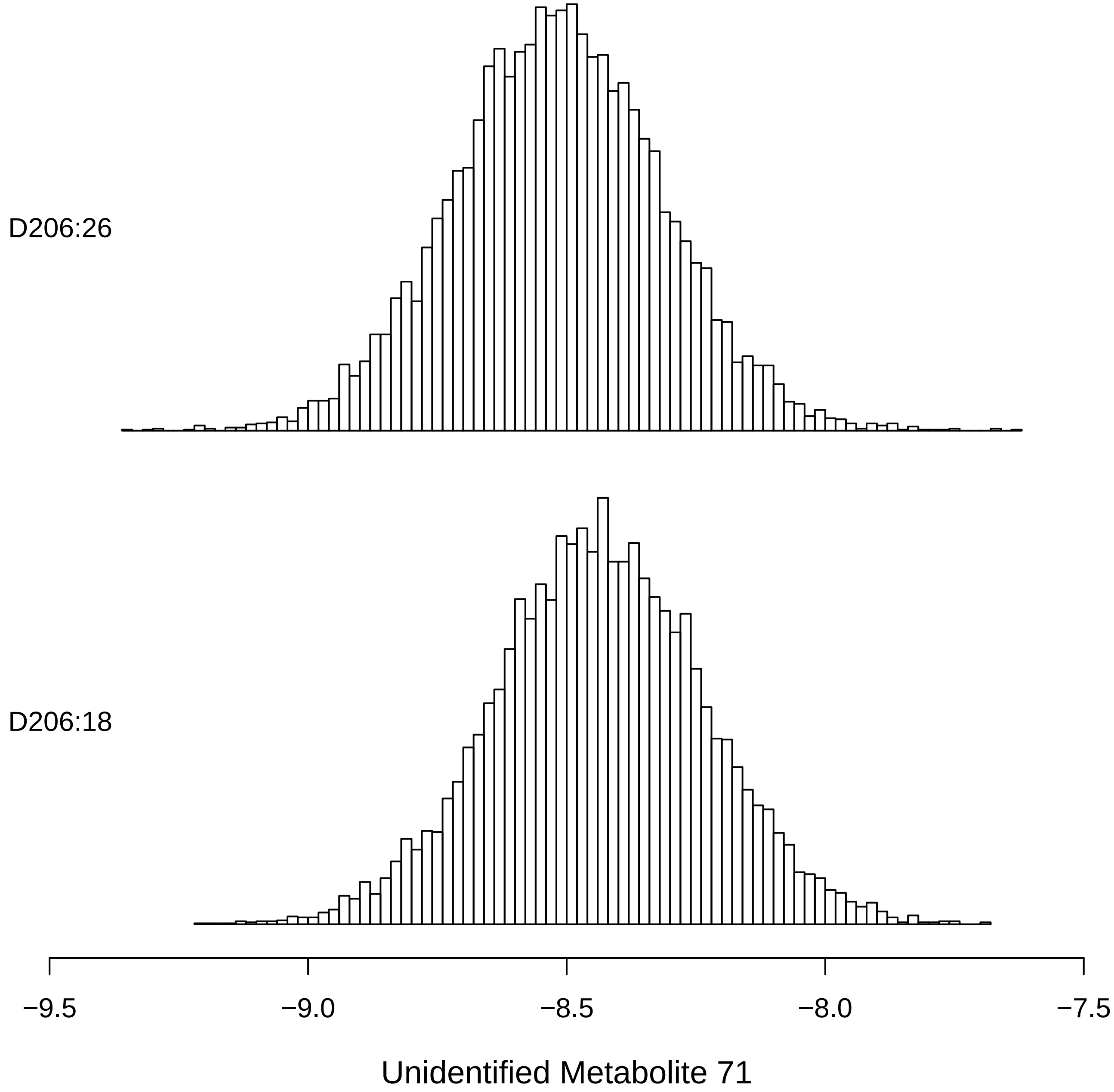
D206:26
D206:18
−9.5
−9.0
−8.5
−8.0
−7.5
Unidentified Metabolite 71

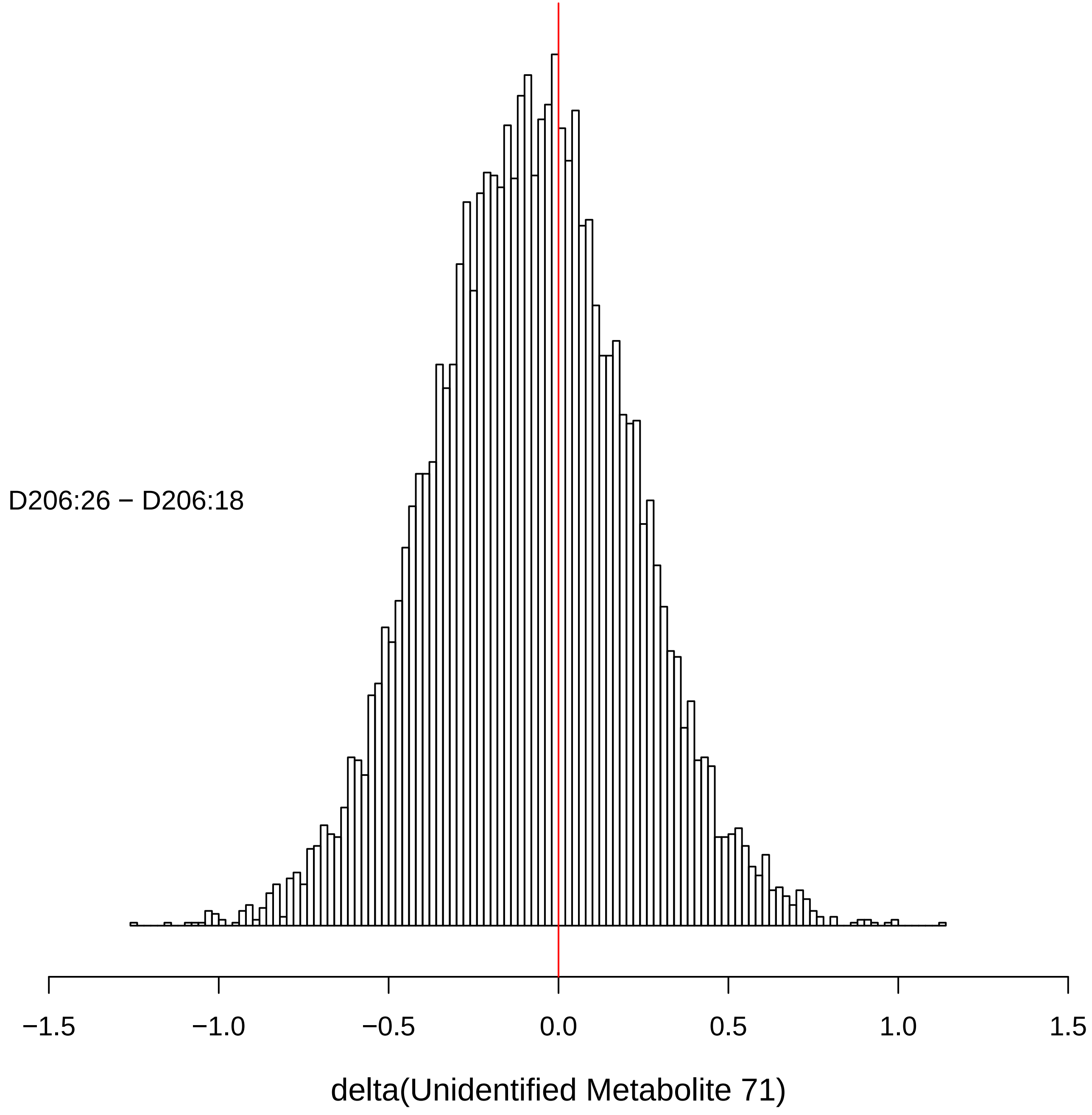
D206:26 − D206:18
−1.5
−1.0
−0.5
0.0
0.5
1.0
1.5
delta(Unidentified Metabolite 71)

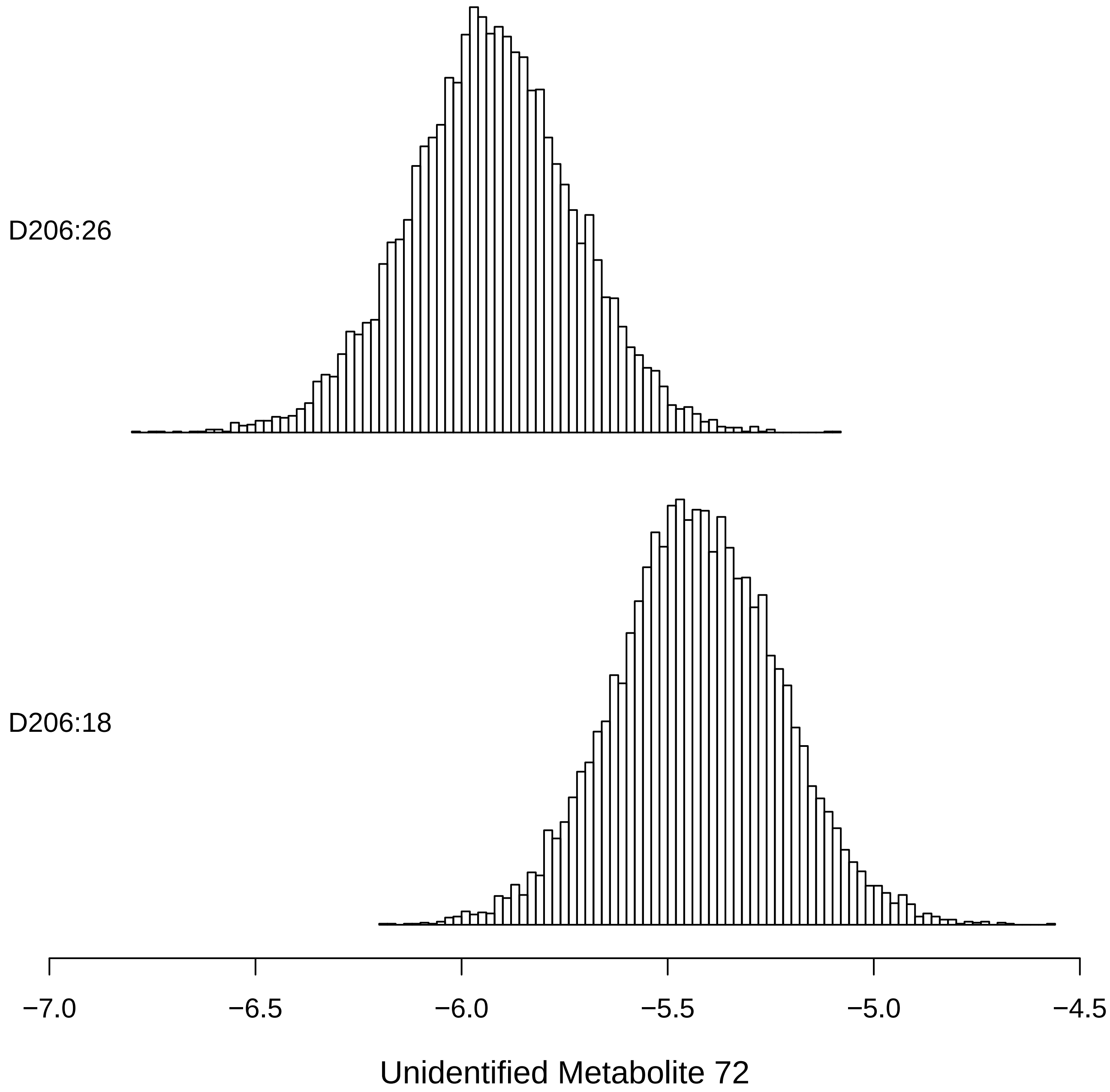

D206:26
D206:18
−7.0
−6.5
−6.0
−5.5
−5.0
−4.5
Unidentified Metabolite 72

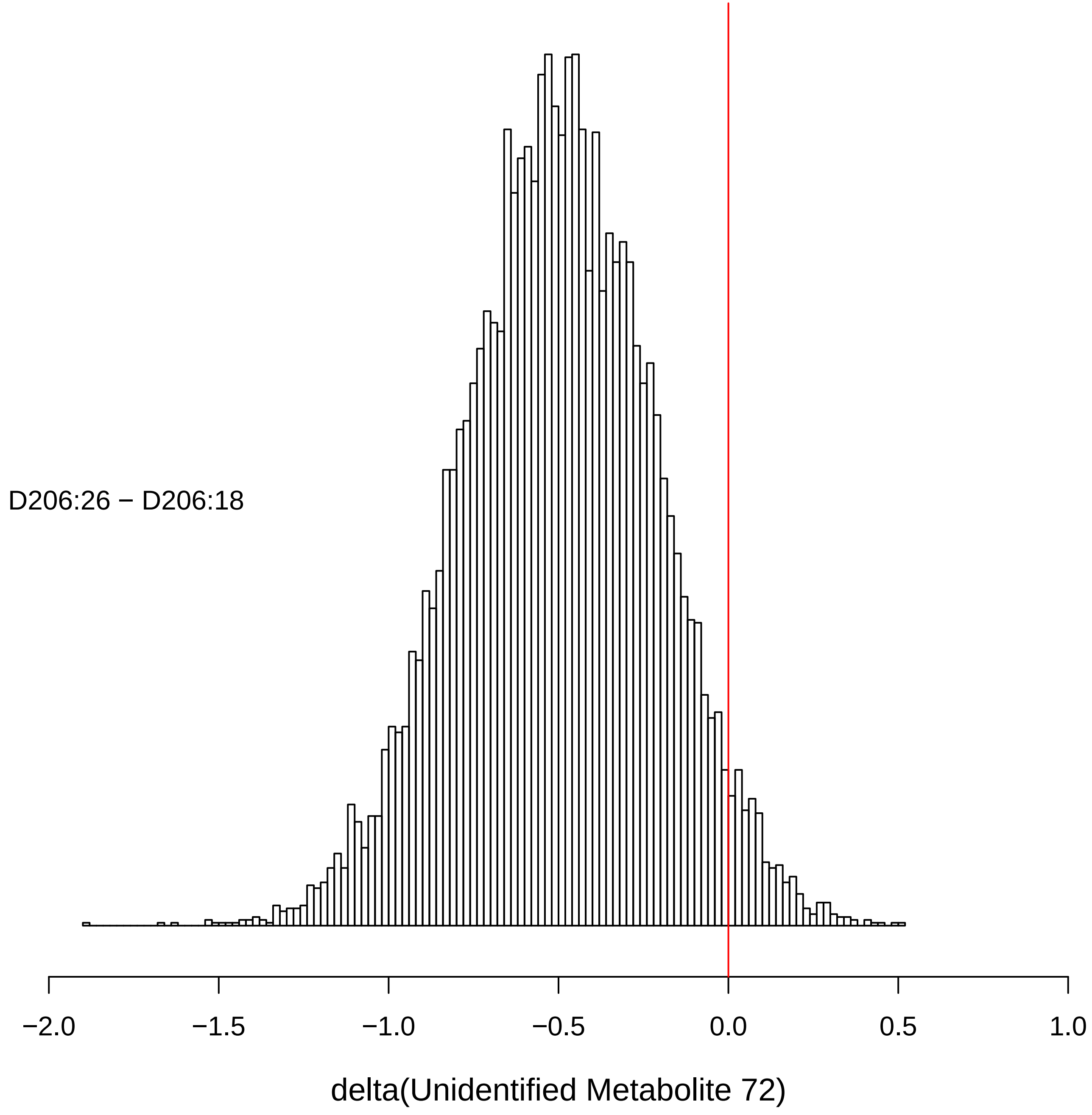
D206:26 − D206:18
−2.0
−1.5
−1.0
−0.5
0.0
0.5
1.0
delta(Unidentified Metabolite 72)

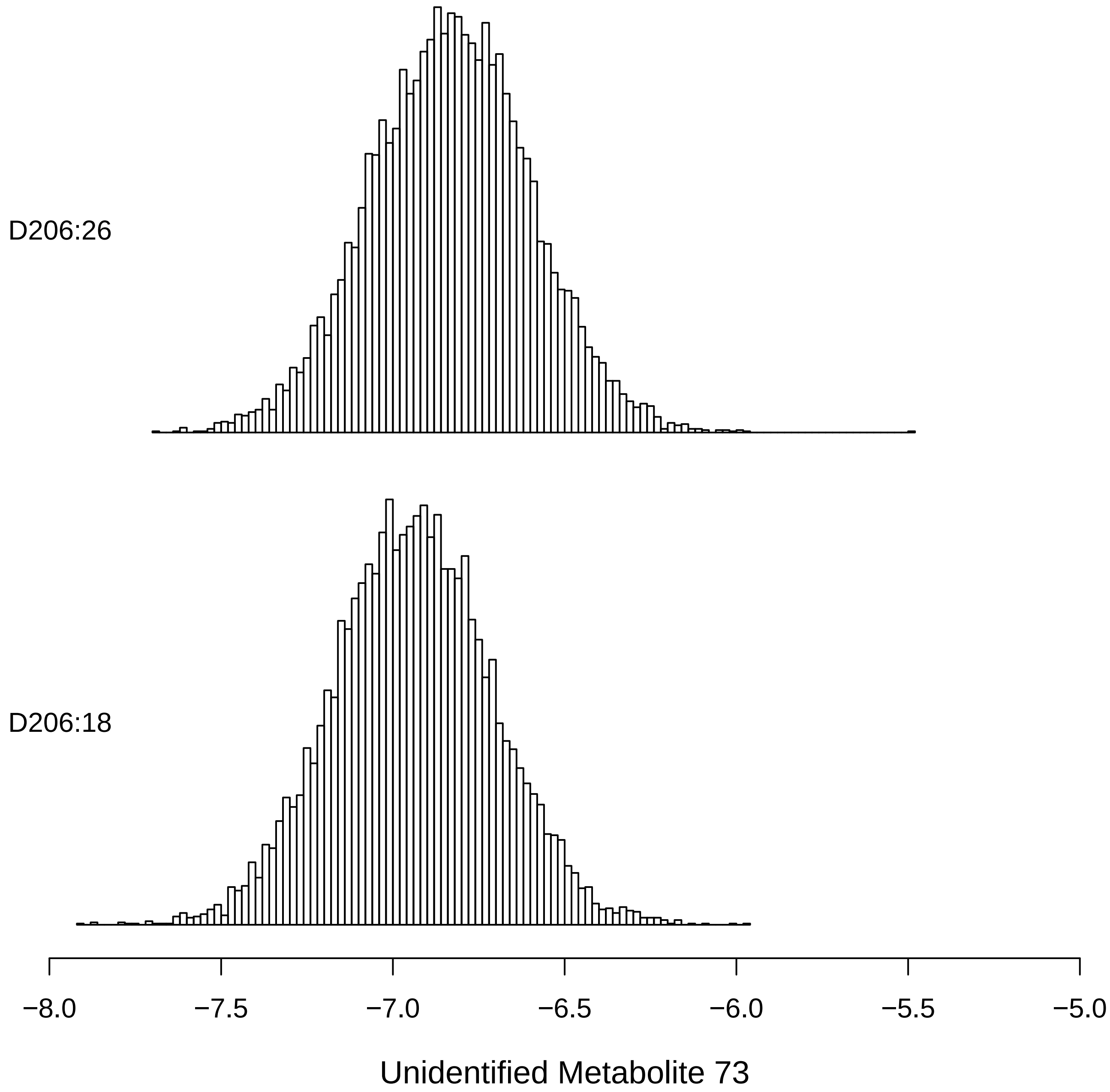
D206:26
D206:18
−8.0
−7.5
−7.0
−6.5
−6.0
−5.5
−5.0
Unidentified Metabolite 73

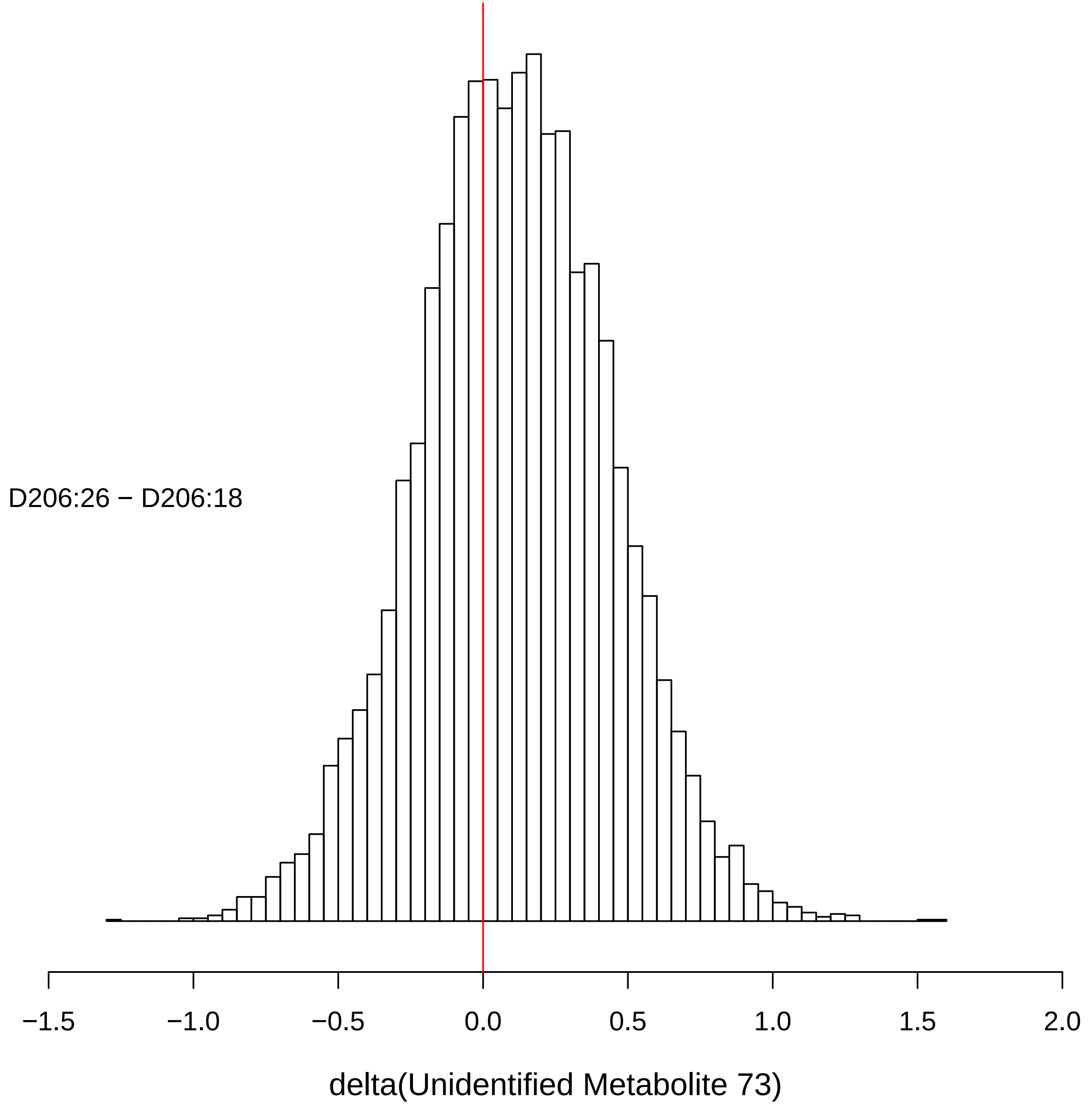
D206:26 − D206:18
−1.5
−1.0
−0.5
0.0
0.5
1.0
1.5
2.0
delta(Unidentified Metabolite 73)

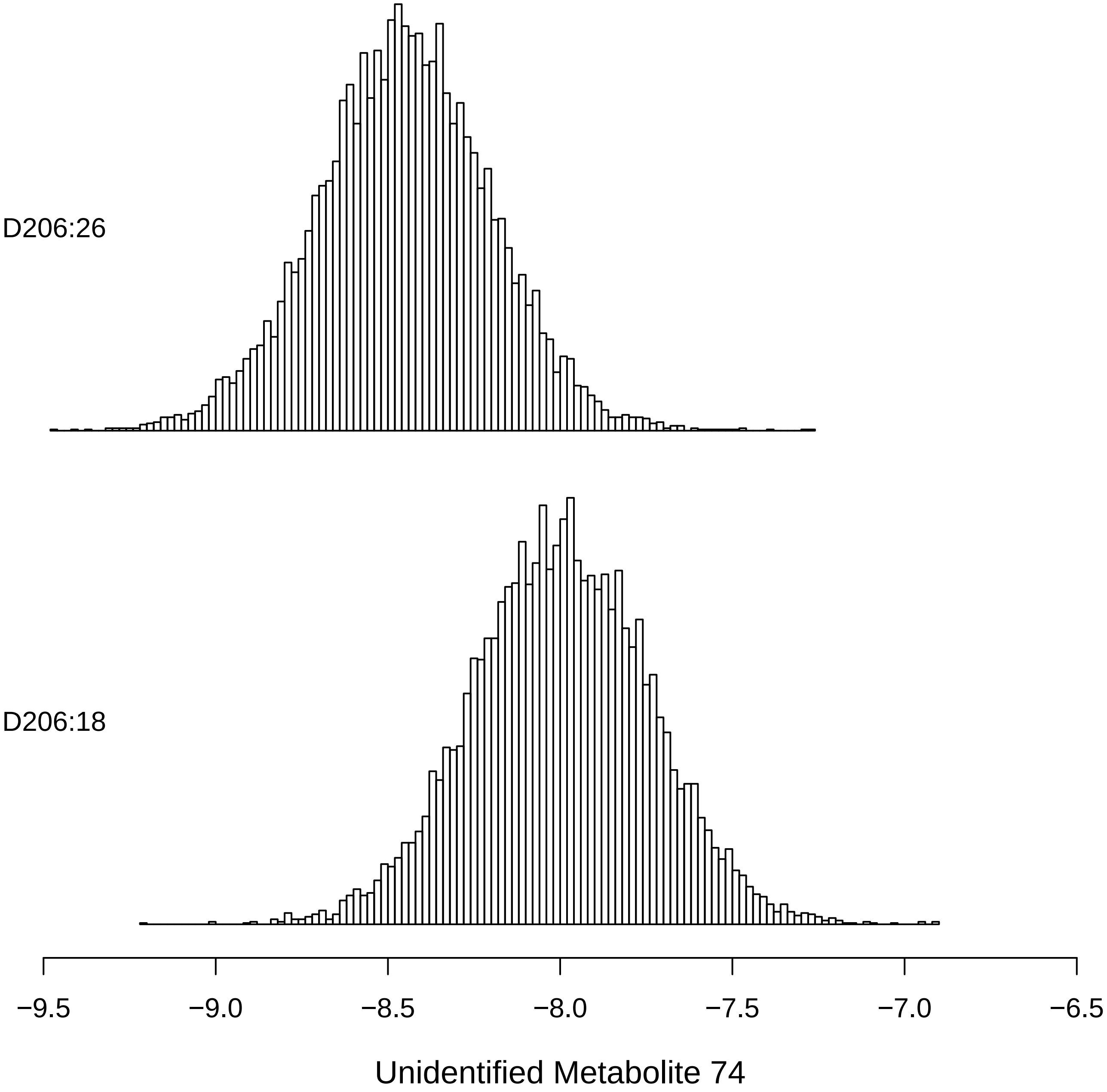
D206:26
D206:18
−9.5
−9.0
−8.5
−8.0
−7.5
−7.0
−6.5
Unidentified Metabolite 74

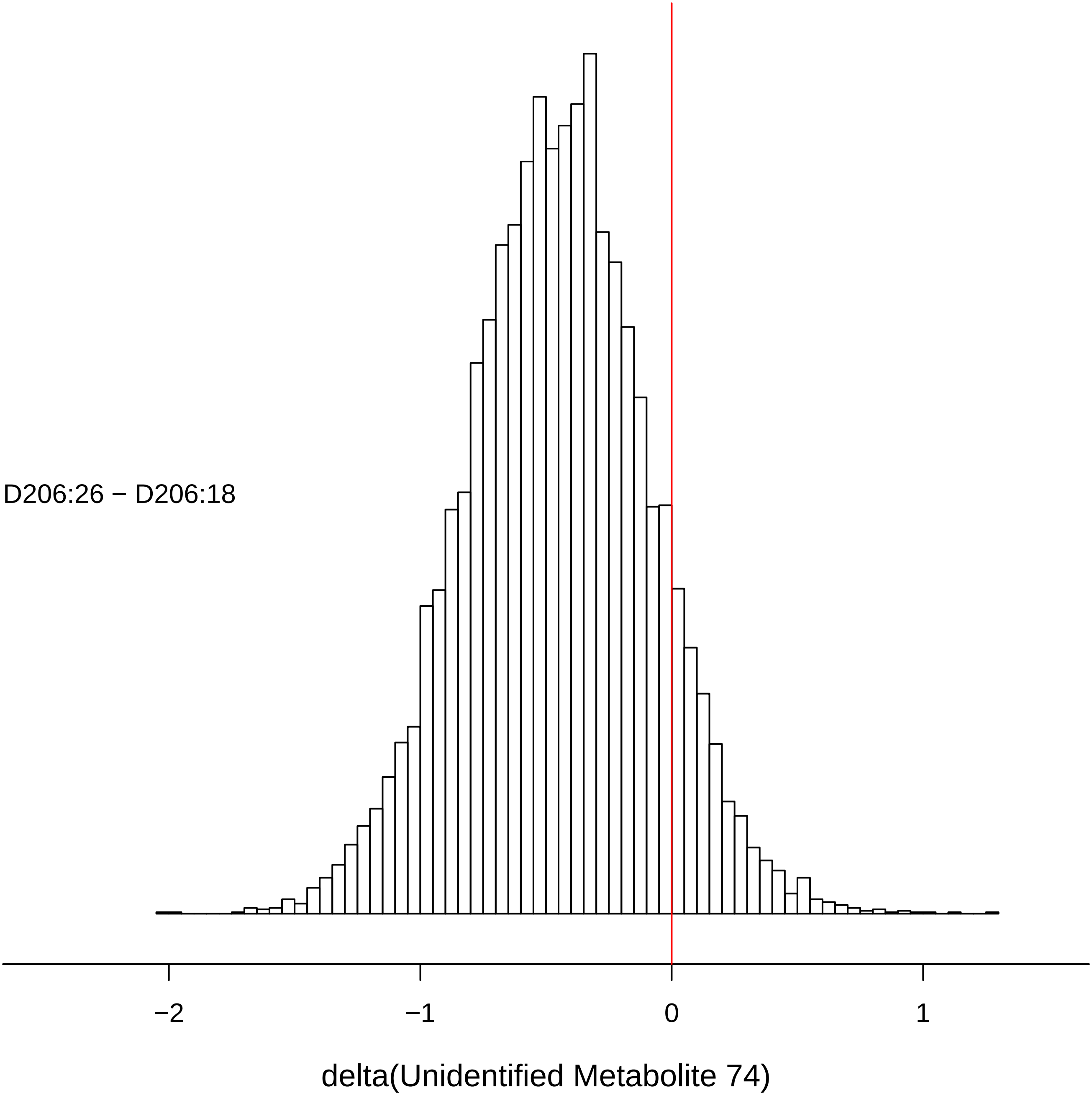
D206:26 − D206:18
−2
−1
0
1
delta(Unidentified Metabolite 74)

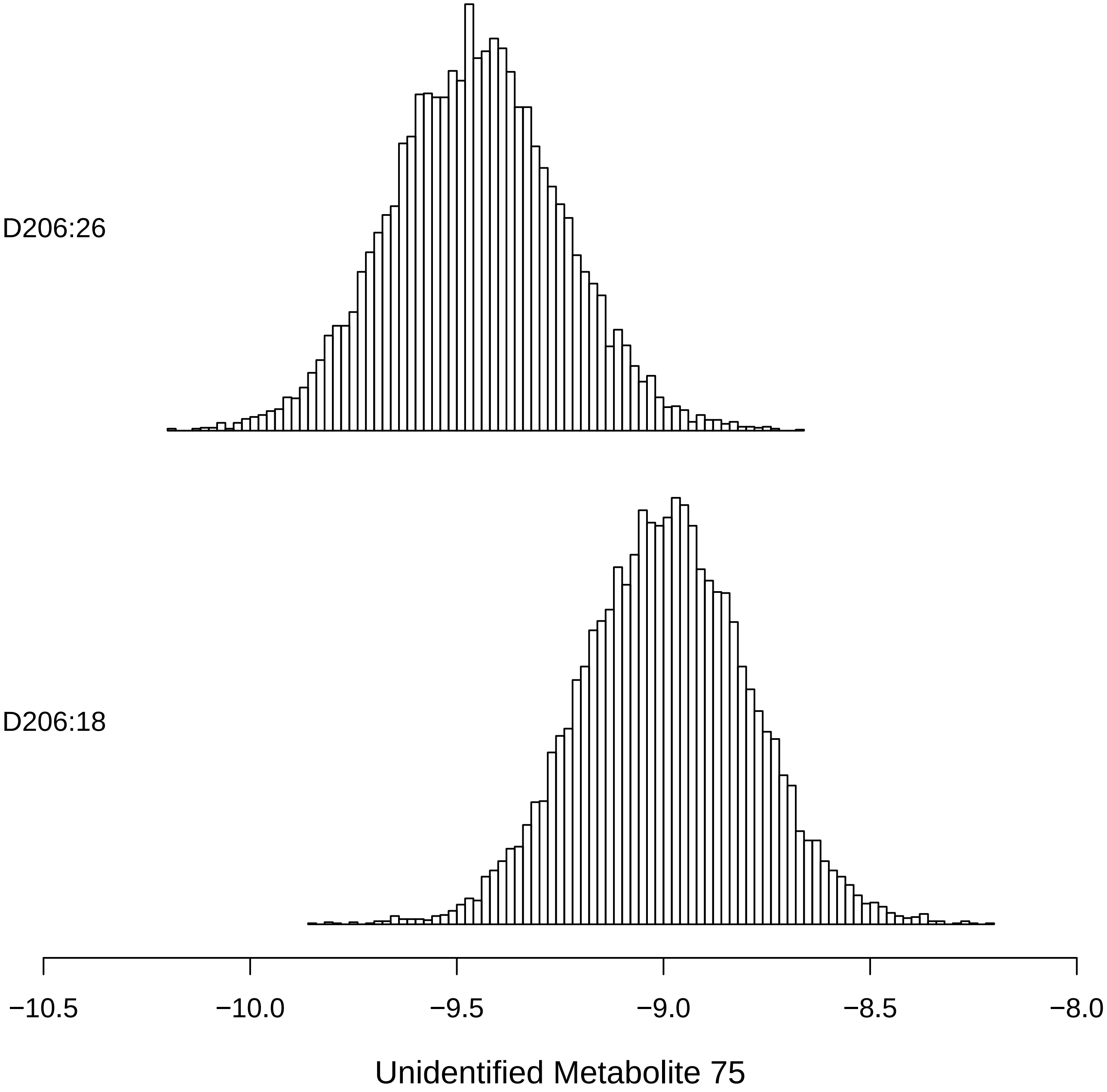
D206:26
D206:18
−10.5
−10.0
−9.5
−9.0
−8.5
−8.0
Unidentified Metabolite 75

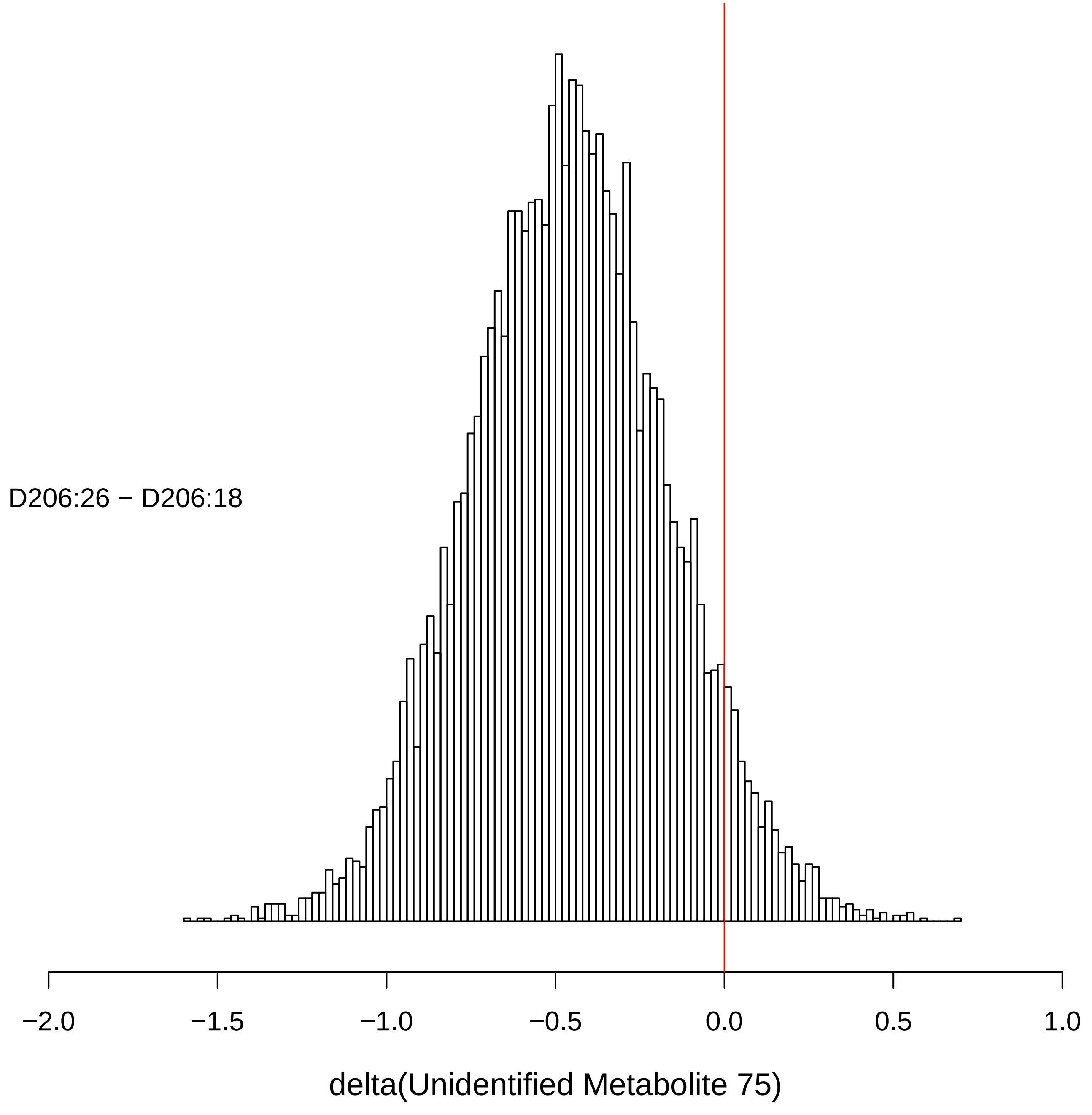
D206:26 − D206:18
−2.0
−1.5
−1.0
−0.5
0.0
0.5
1.0
delta(Unidentified Metabolite 75)

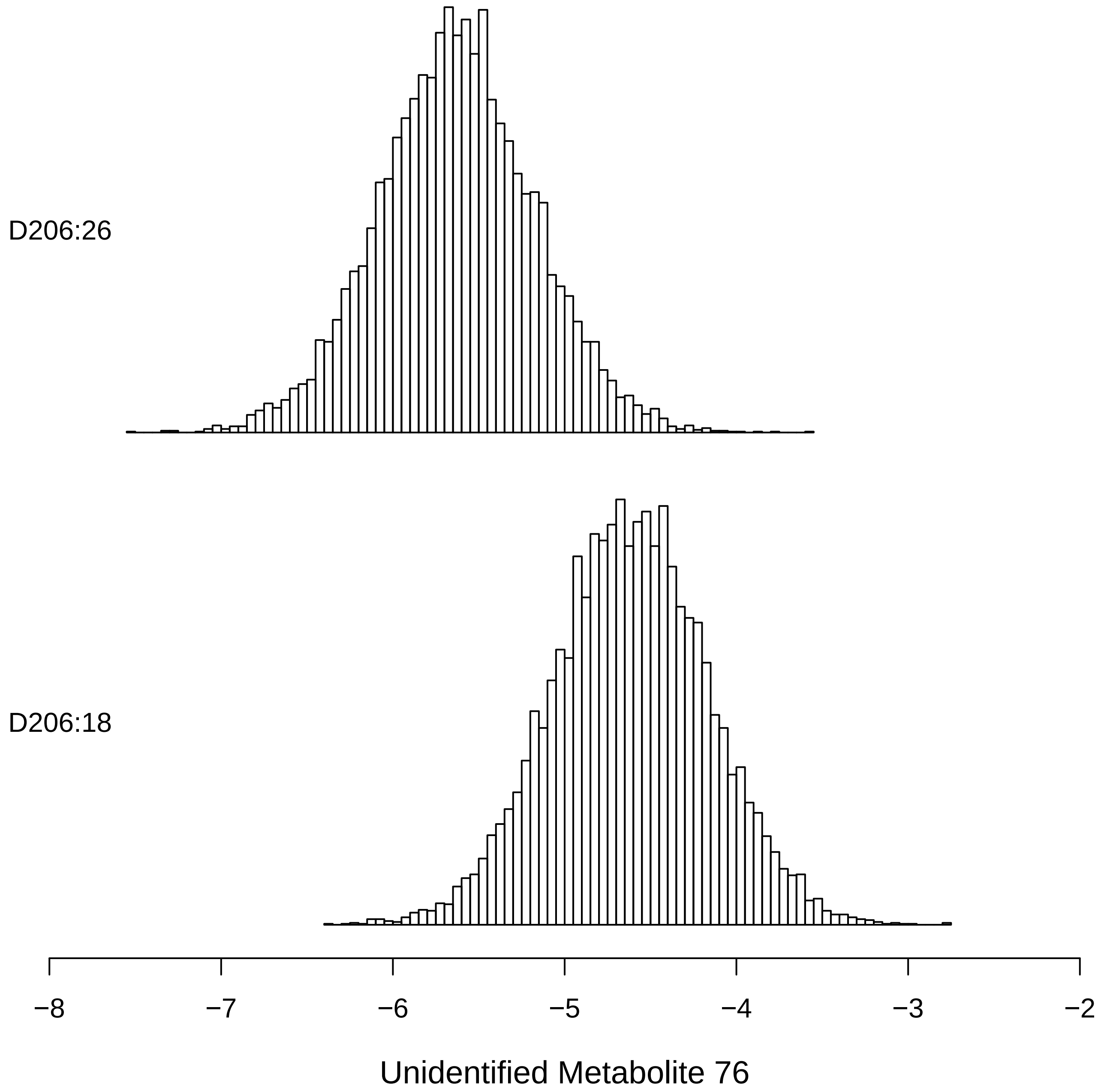
D206:26
D206:18
−8
−7
−6
−5
−4
−3
−2
Unidentified Metabolite 76

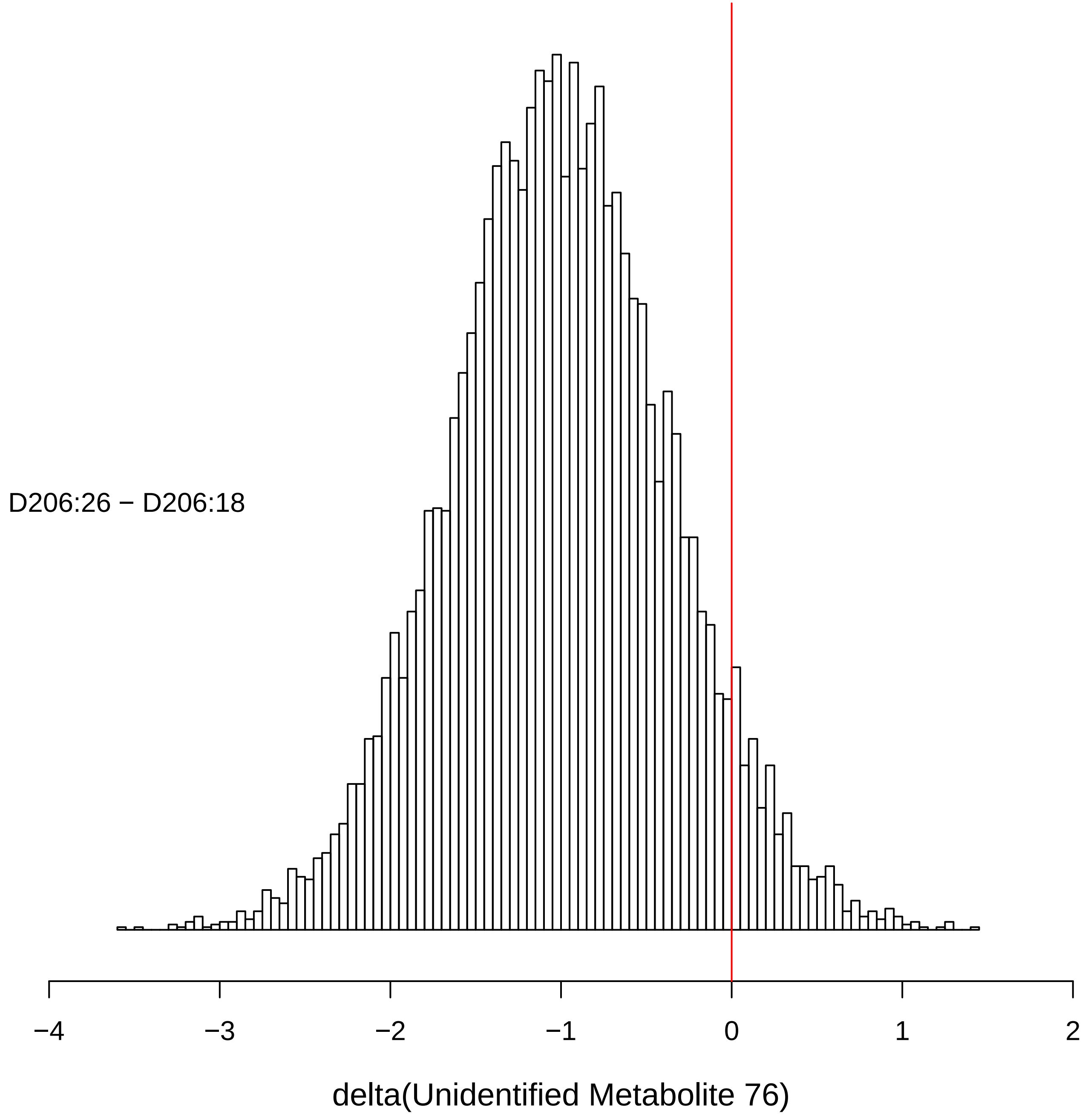
D206:26 − D206:18
−4
−3
−2
−1
0
1
2
delta(Unidentified Metabolite 76)

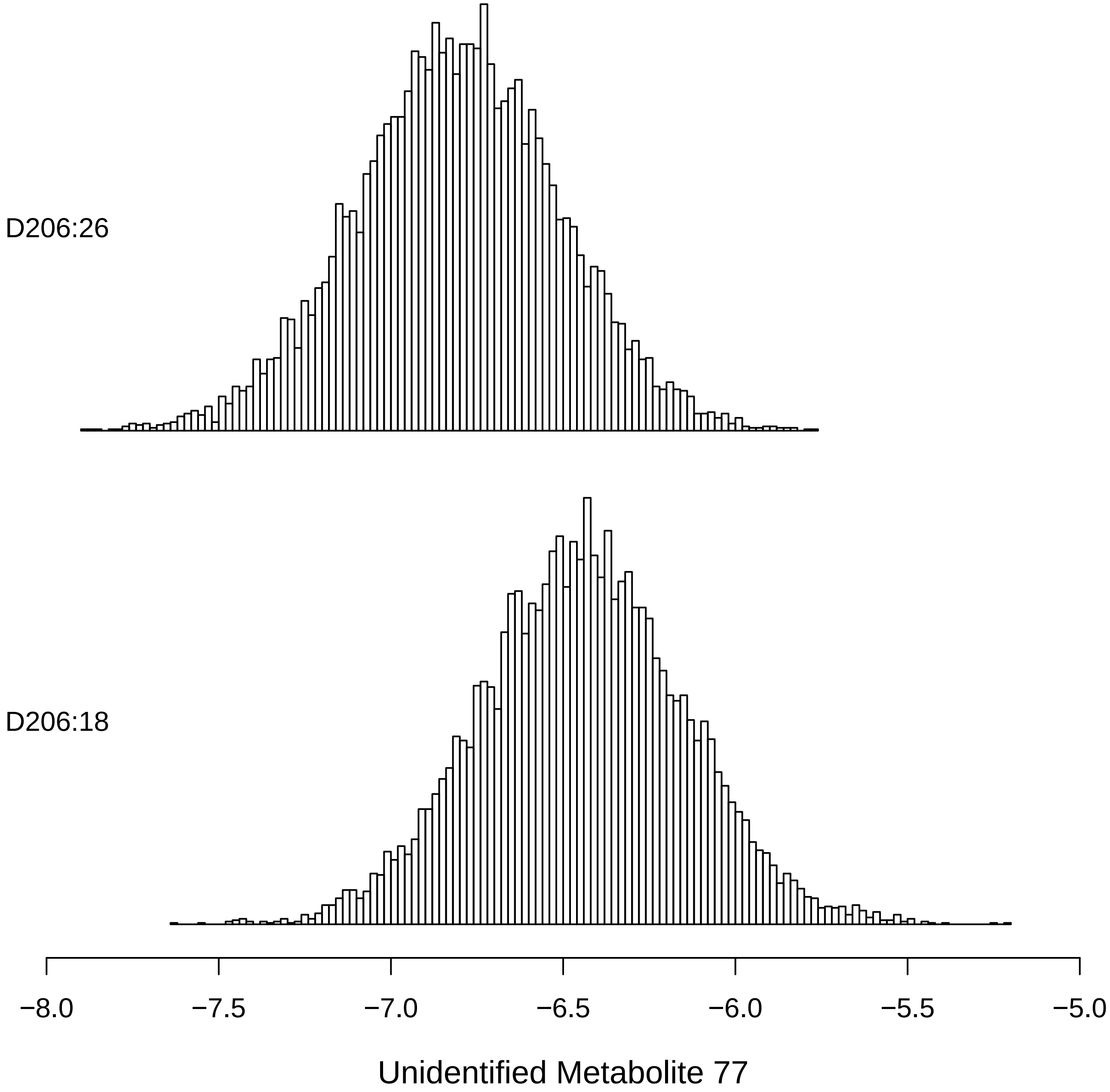
D206:26
D206:18
−8.0
−7.5
−7.0
−6.5
−6.0
−5.5
−5.0
Unidentified Metabolite 77

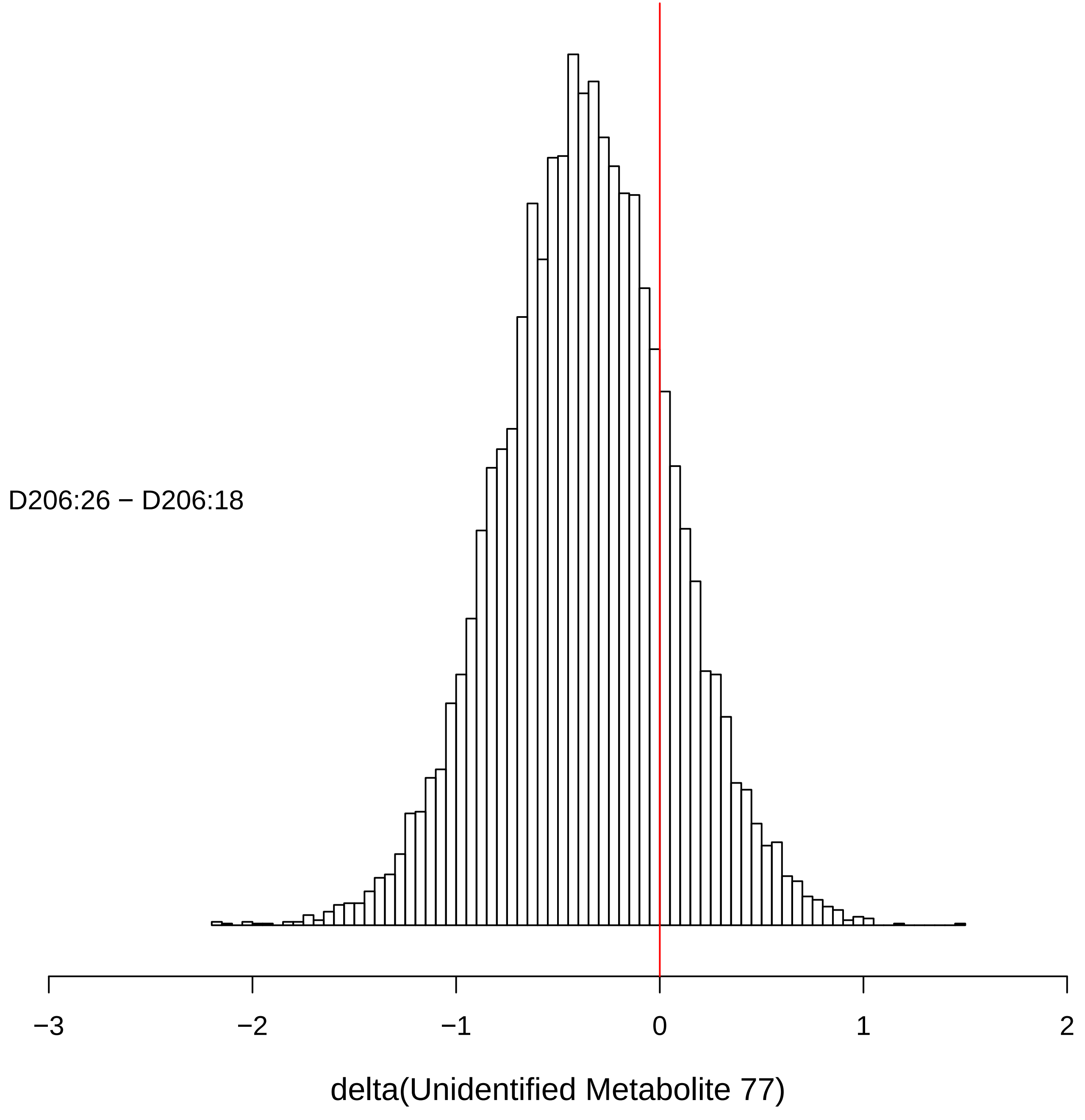

D206:26 − D206:18
−3
−2
−1
0
1
2
delta(Unidentified Metabolite 77)

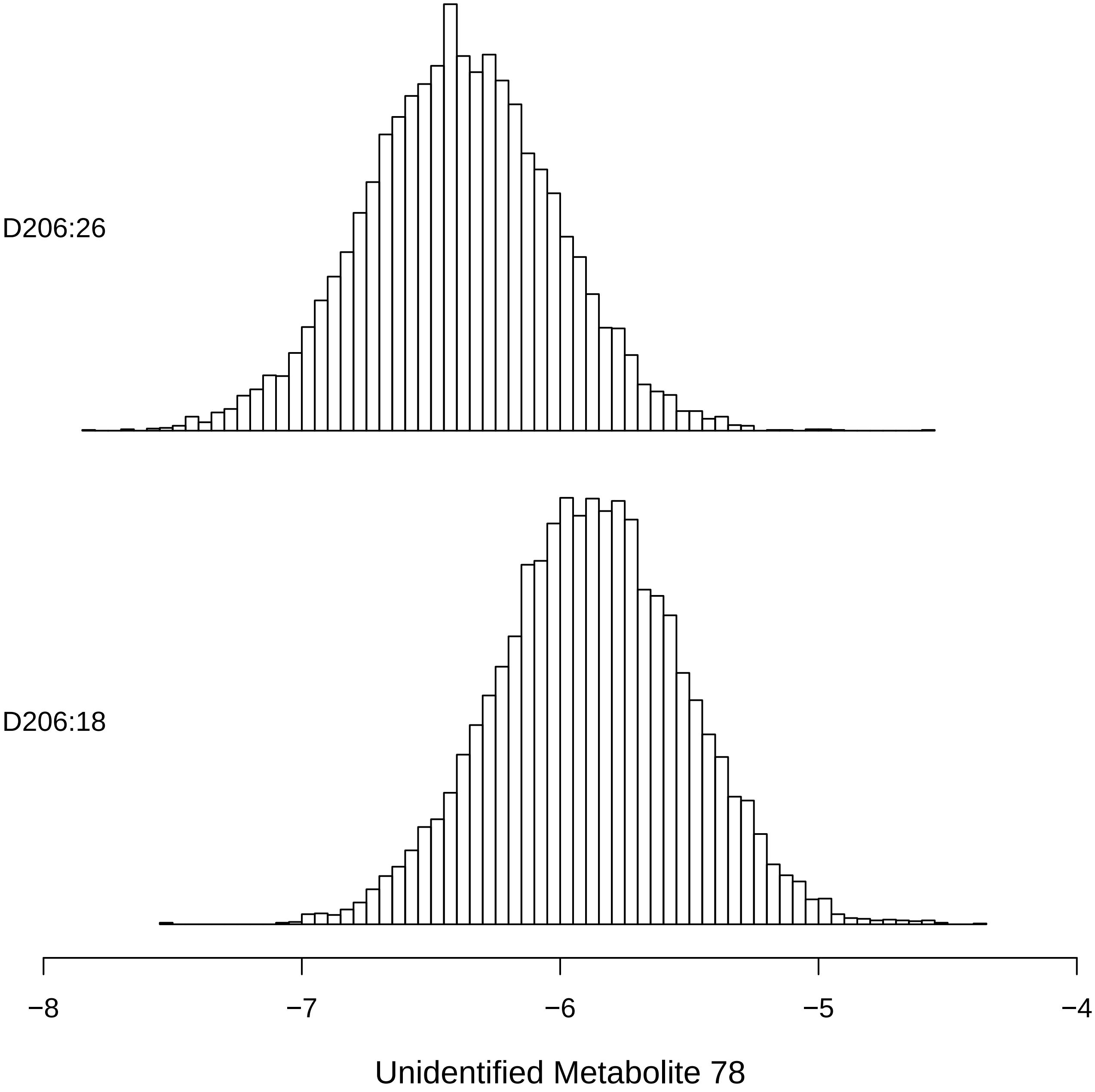
D206:26
D206:18
−8
−7
−6
−5
−4
Unidentified Metabolite 78

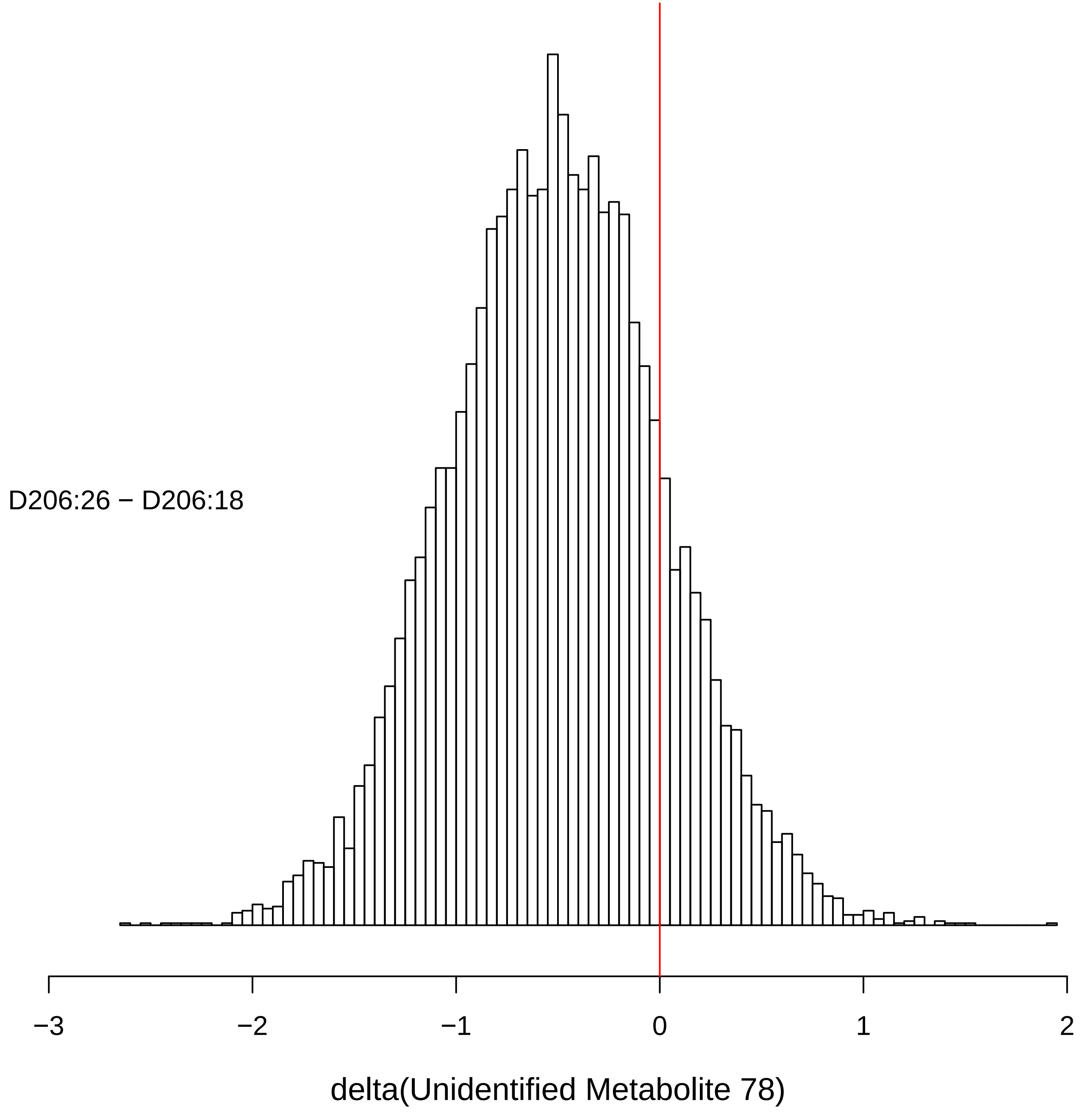
D206:26 − D206:18
−3
−2
−1
0
1
2
delta(Unidentified Metabolite 78)

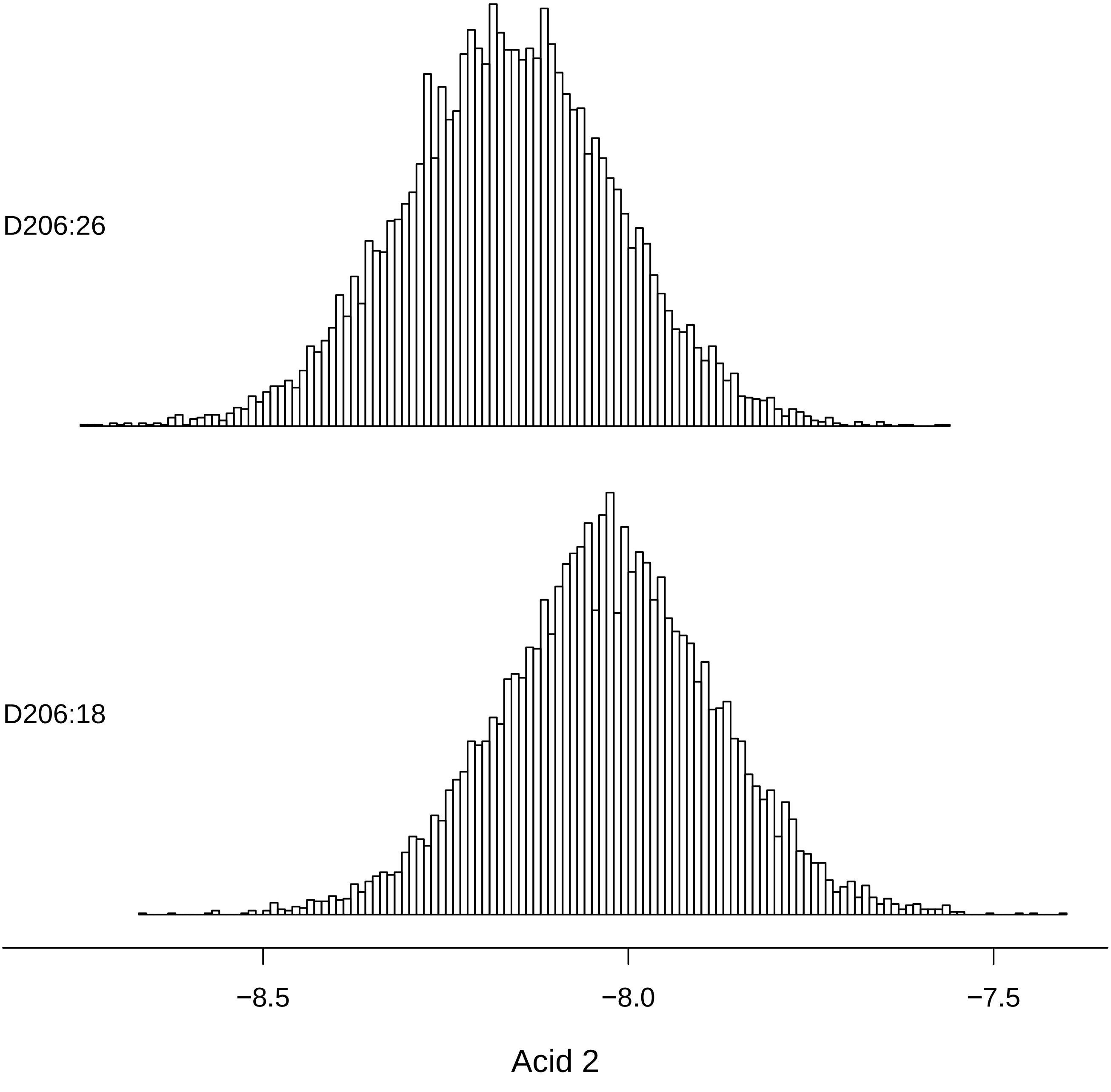
D206:26
D206:18
−8.5
−8.0
−7.5
Acid 2

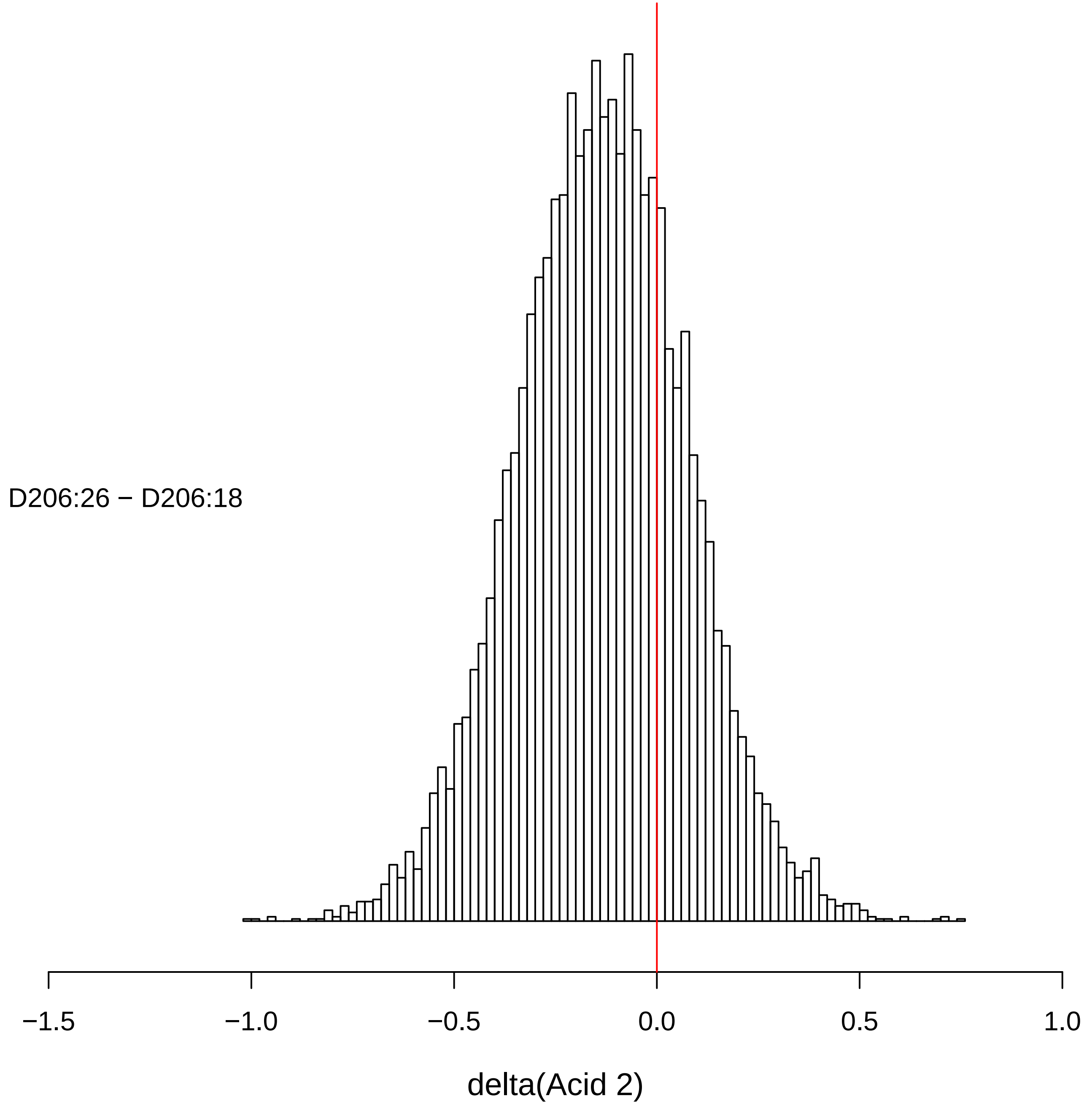
D206:26 − D206:18
−1.5
−1.0
−0.5
0.0
0.5
1.0
delta(Acid 2)

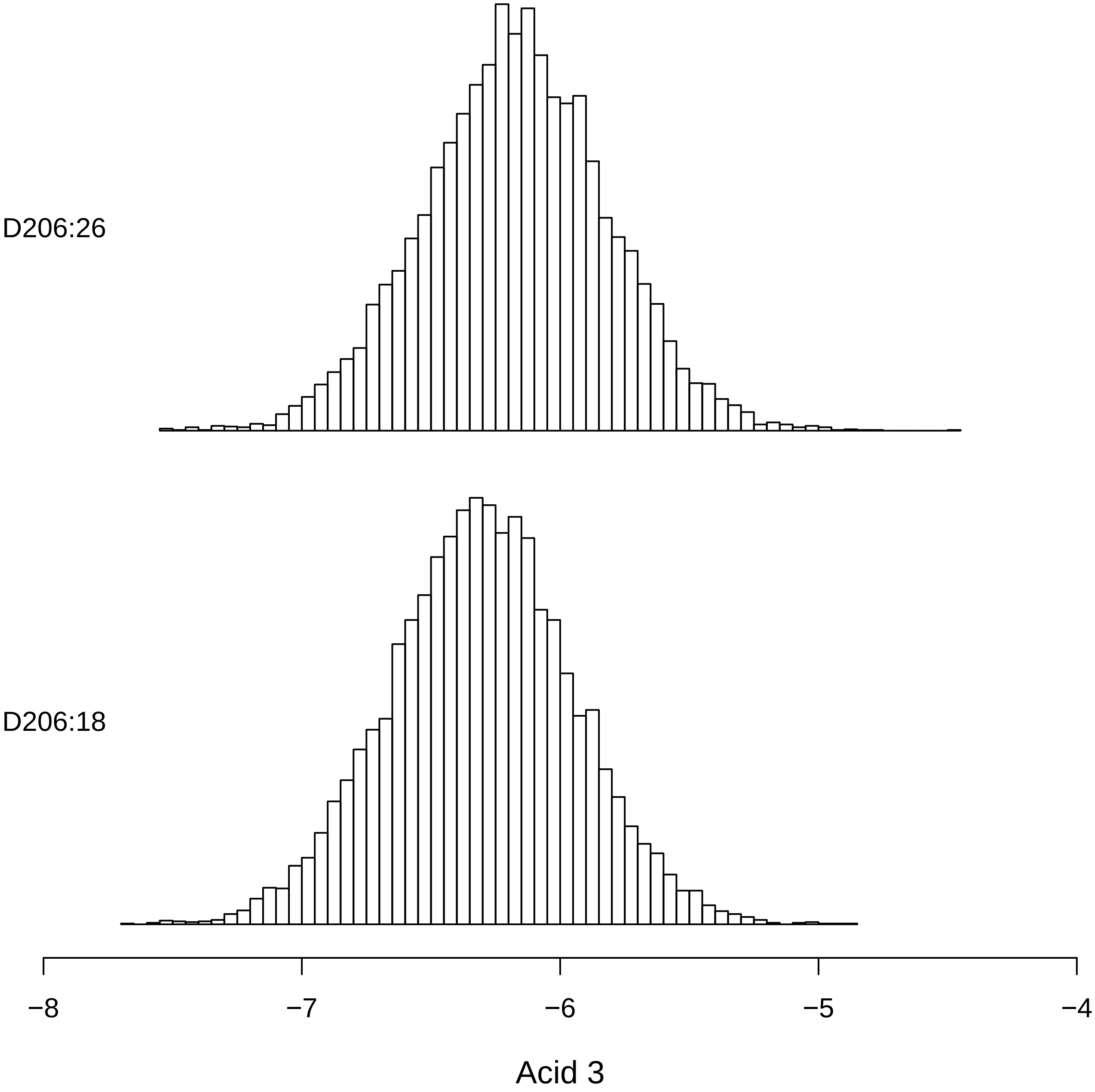
D206:26
D206:18
−8
−7
−6
−5
−4
Acid 3

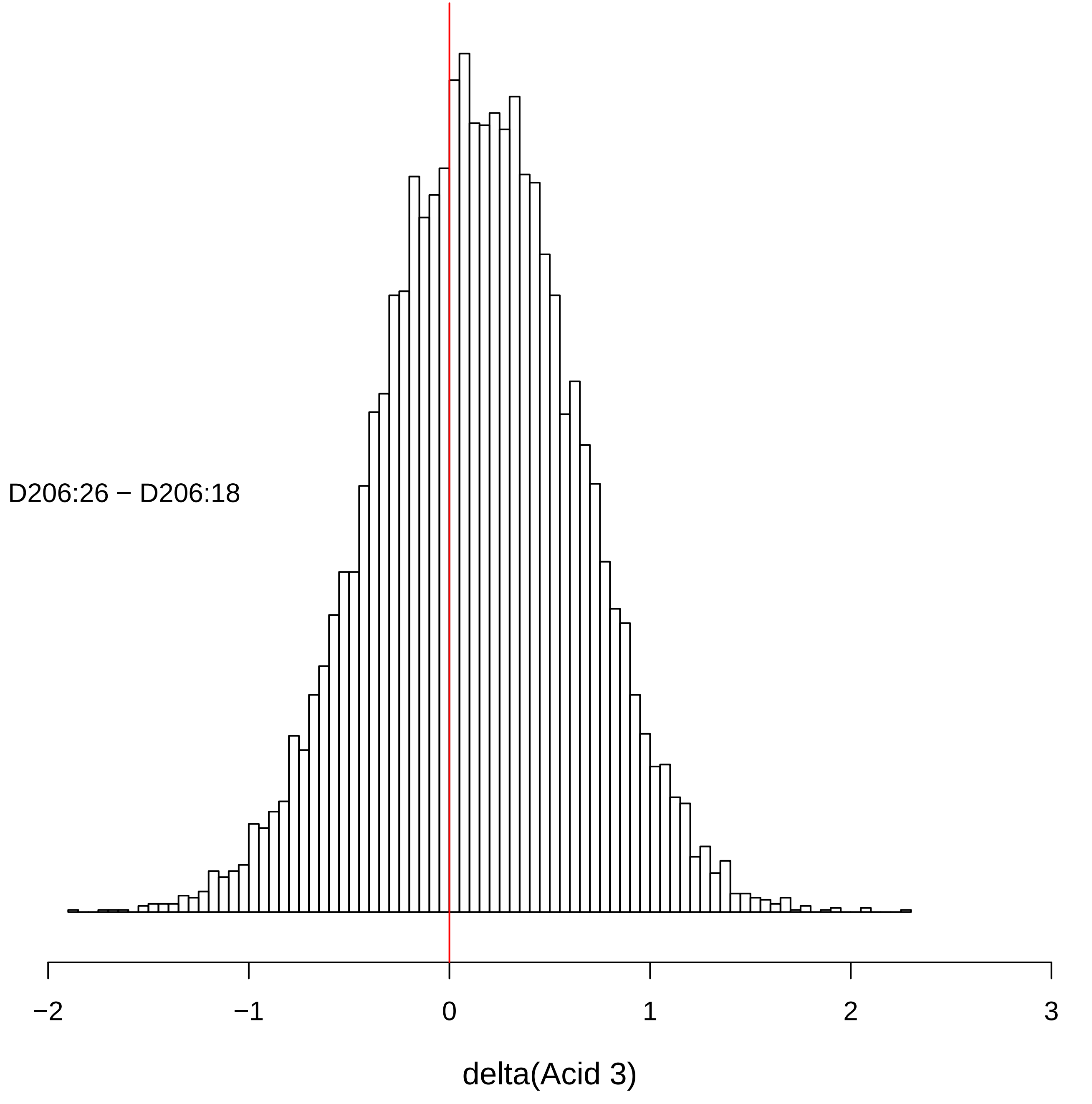
D206:26 − D206:18
−2
−1
0
1
2
3
delta(Acid 3)

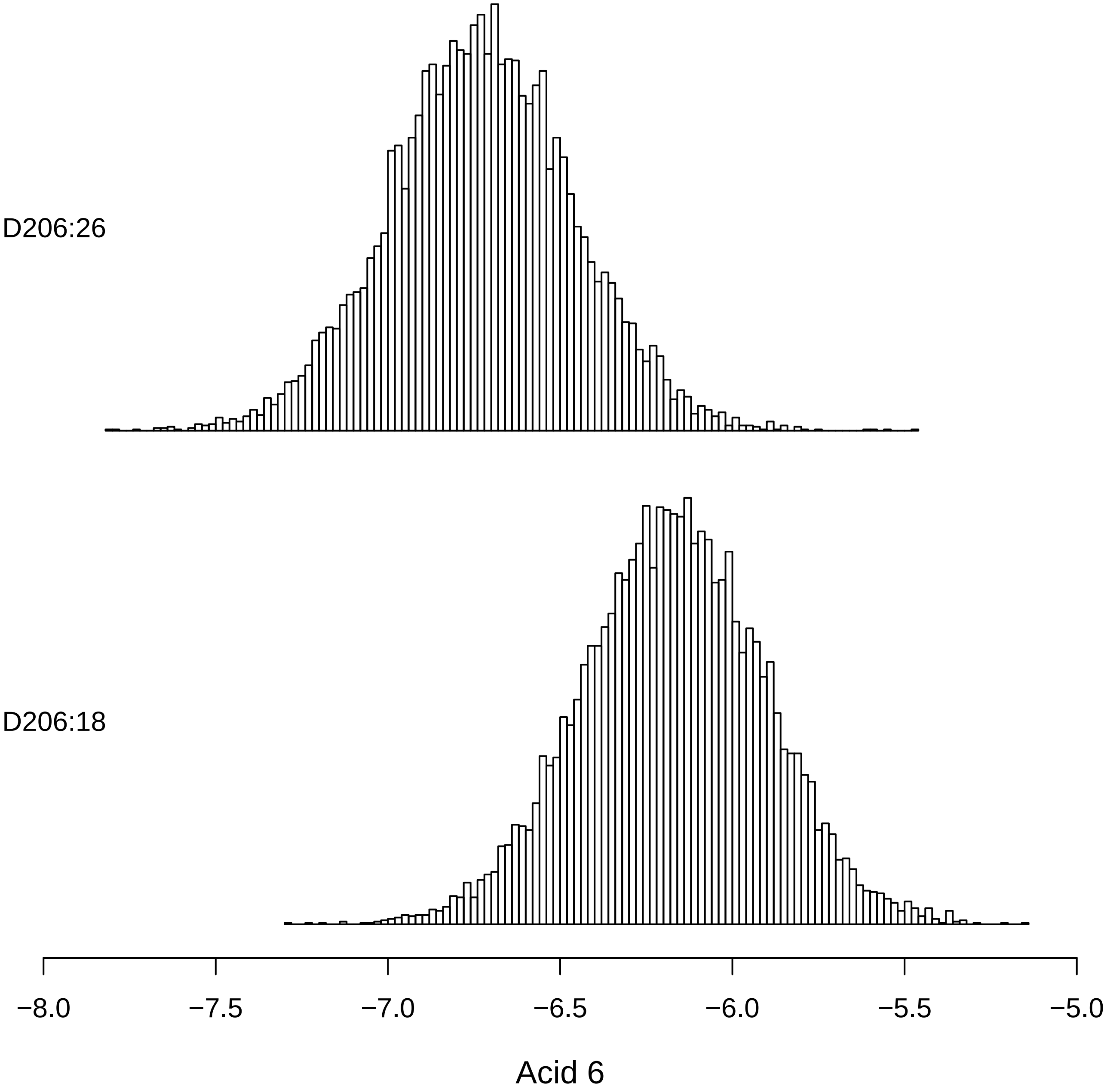

D206:26
D206:18
−8.0
−7.5
−7.0
−6.5
−6.0
−5.5
−5.0
Acid 6

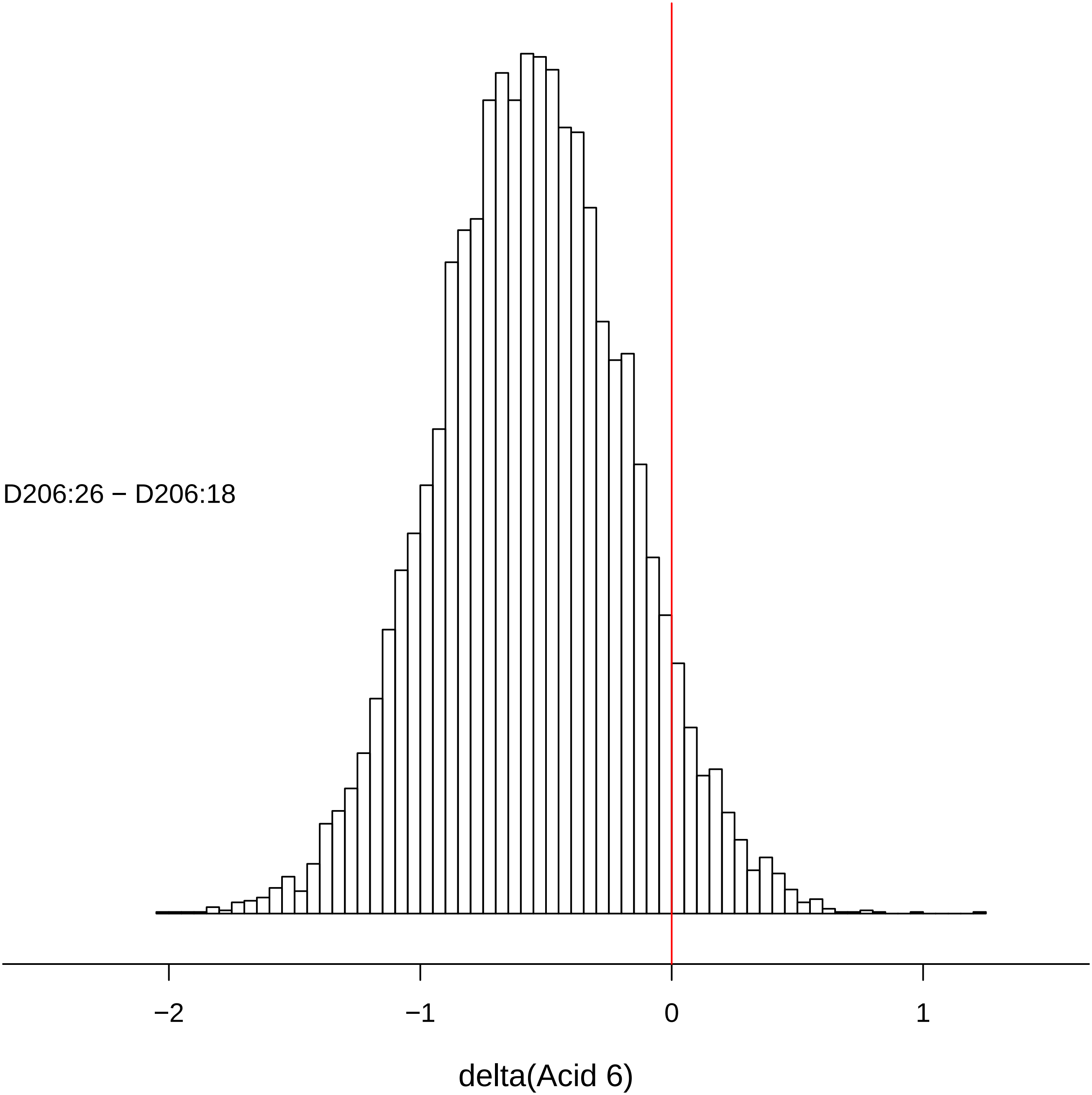
D206:26 − D206:18
−2
−1
0
1
delta(Acid 6)

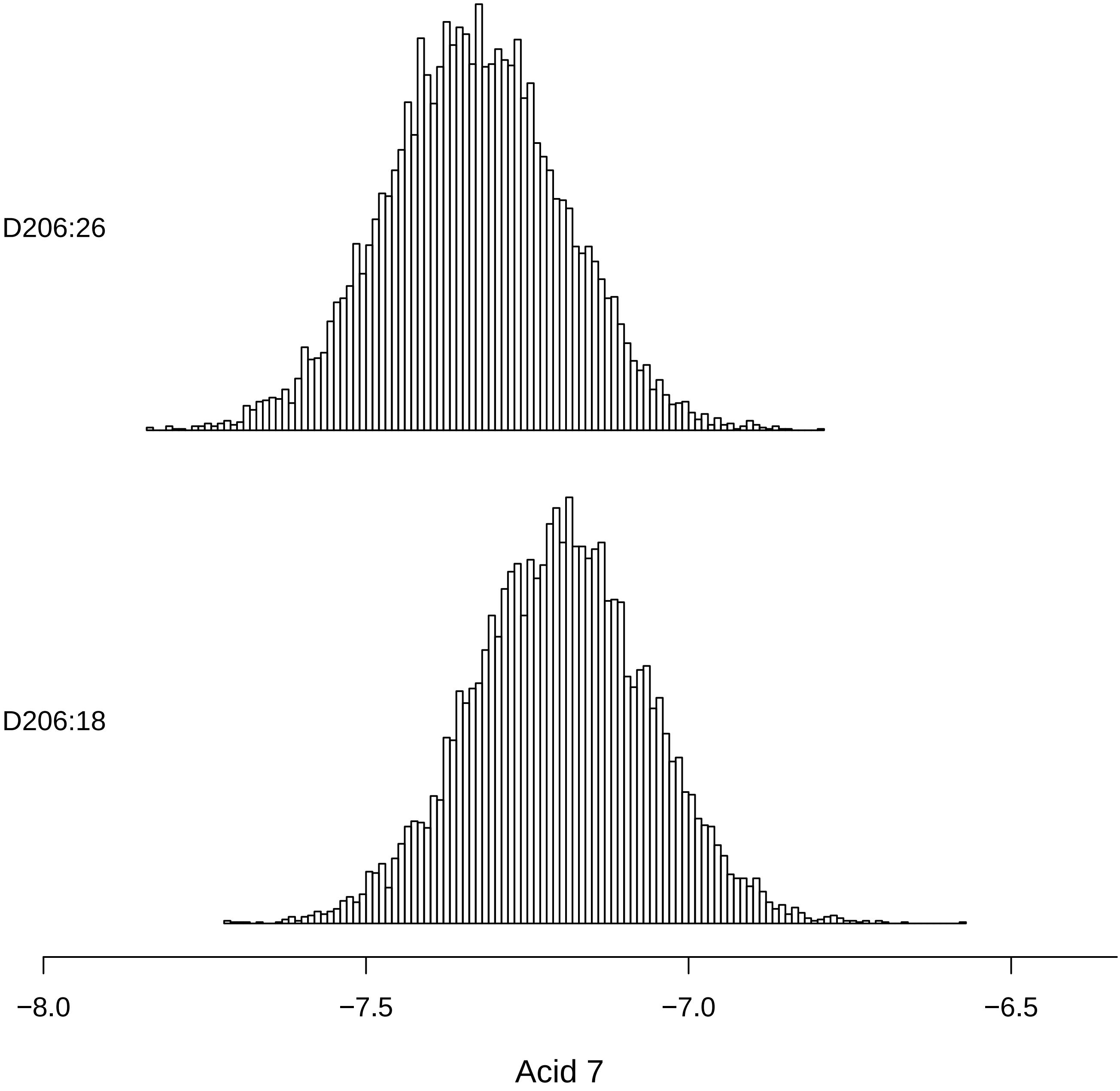
D206:26
D206:18
−8.0
−7.5
−7.0
−6.5
Acid 7

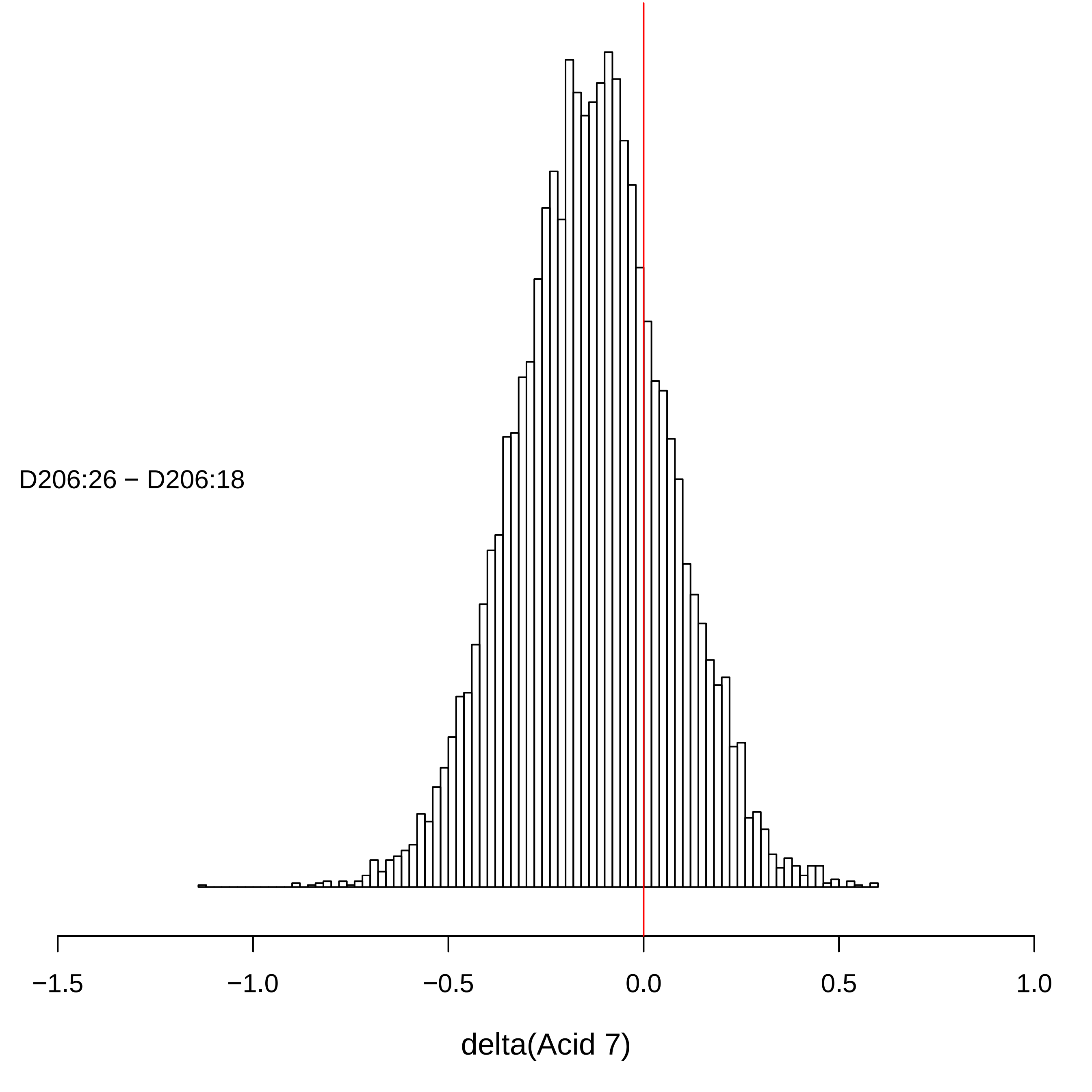
D206:26 − D206:18
−1.5
−1.0
−0.5
0.0
0.5
1.0
delta(Acid 7)

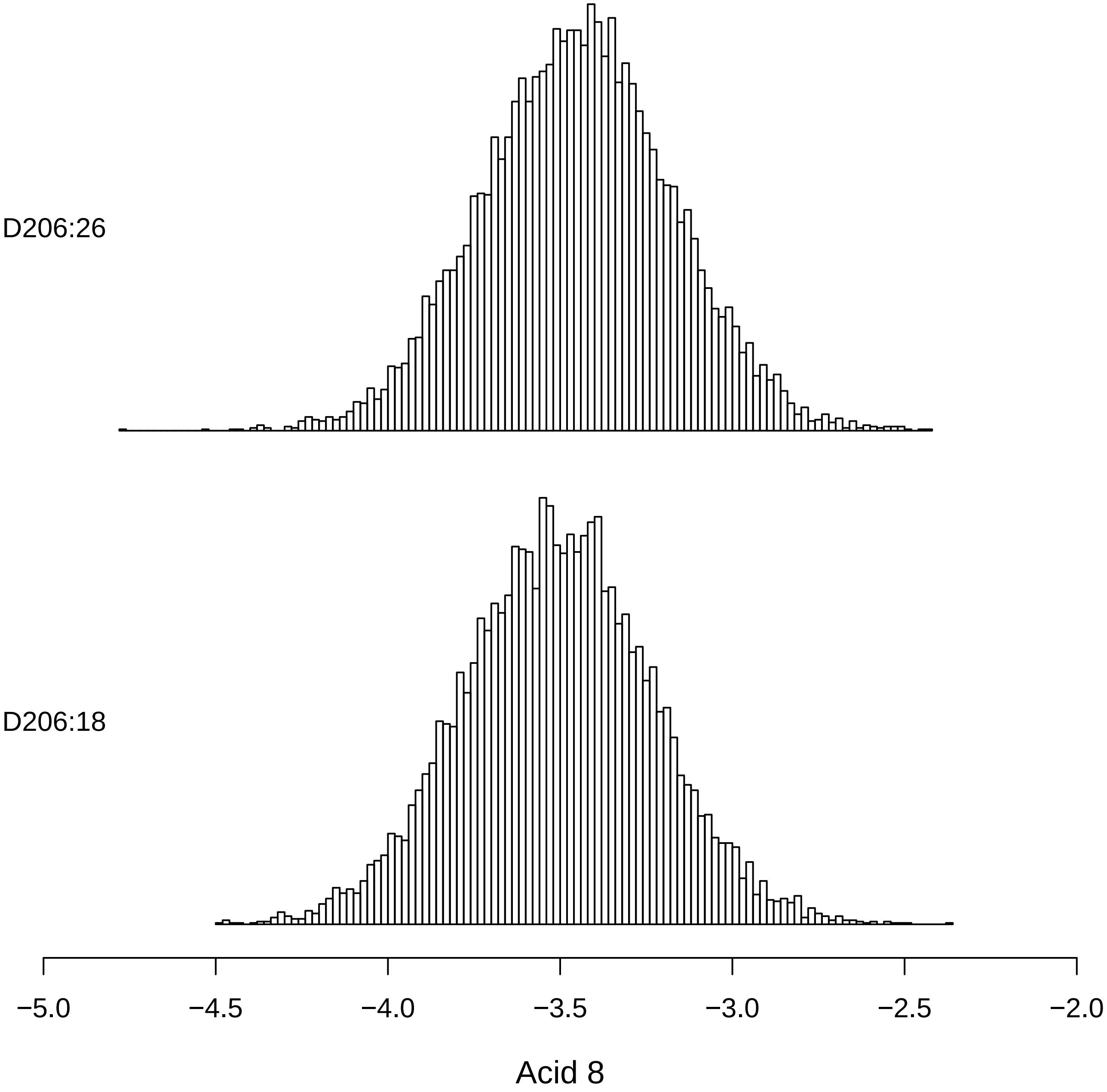
D206:26
D206:18
−5.0
−4.5
−4.0
−3.5
−3.0
−2.5
−2.0
Acid 8

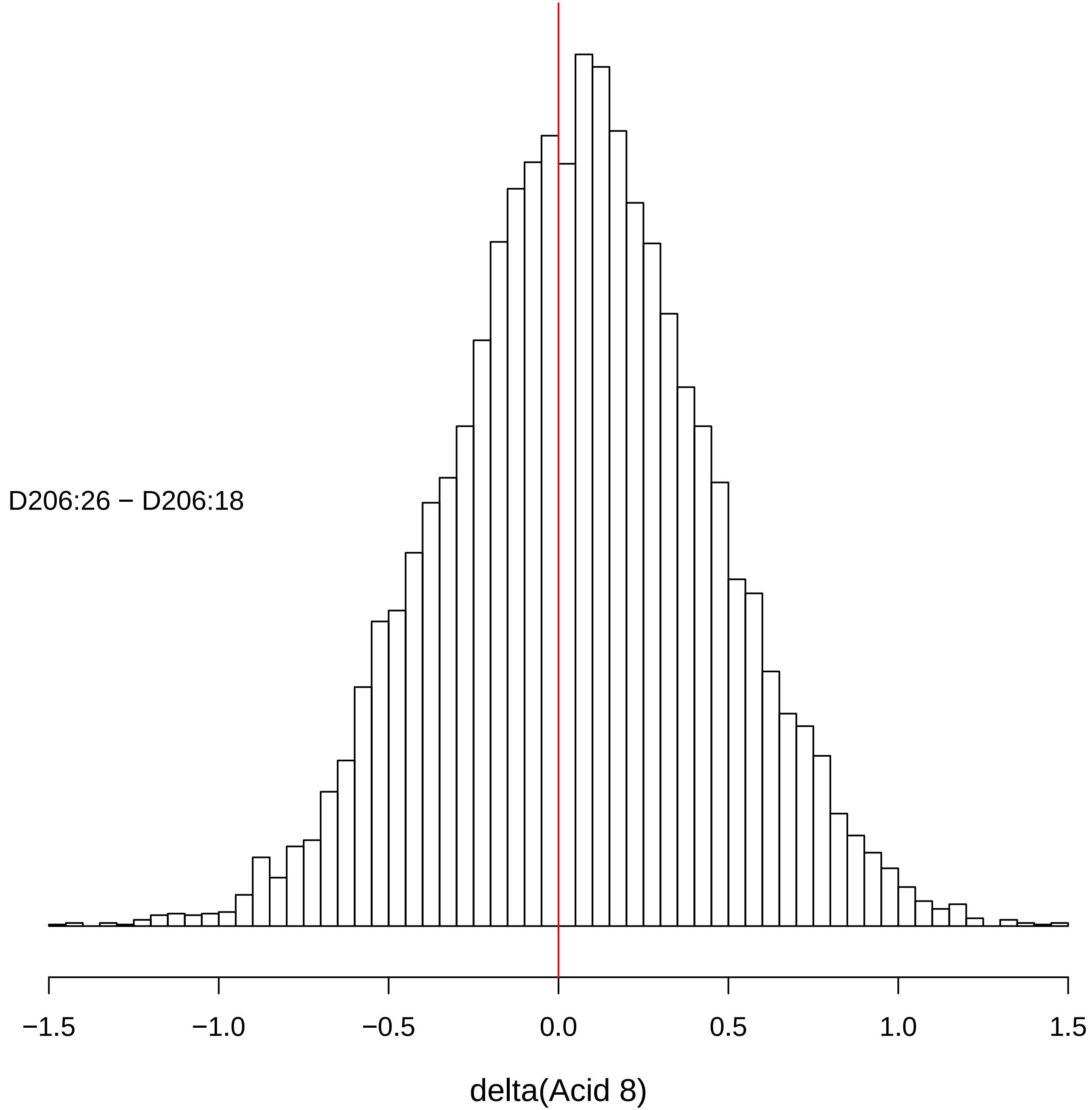

D206:26 − D206:18
−1.5
−1.0
−0.5
0.0
0.5
1.0
1.5
delta(Acid 8)

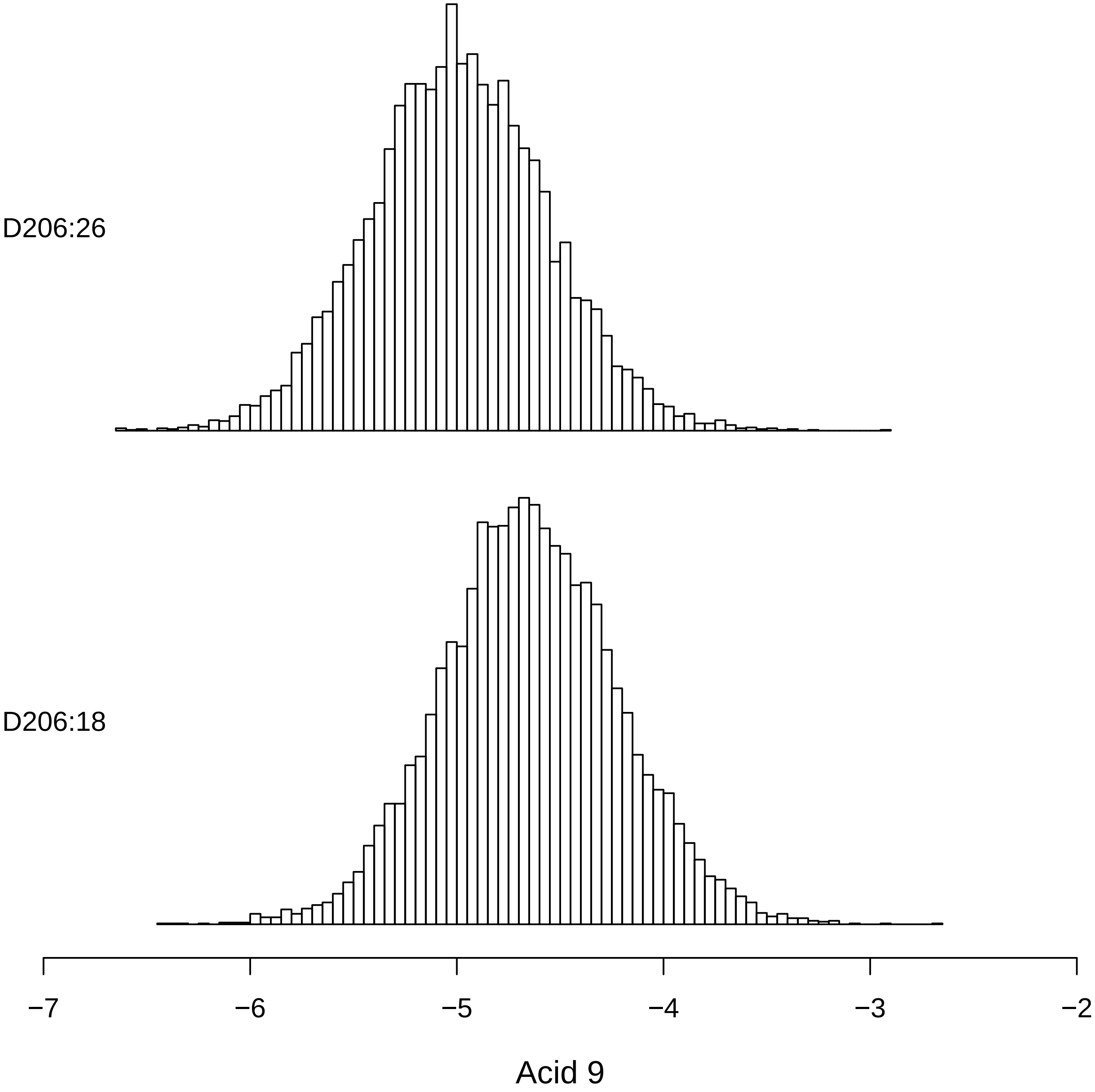

D206:26
D206:18
−7
−6
−5
−4
−3
−2
Acid 9

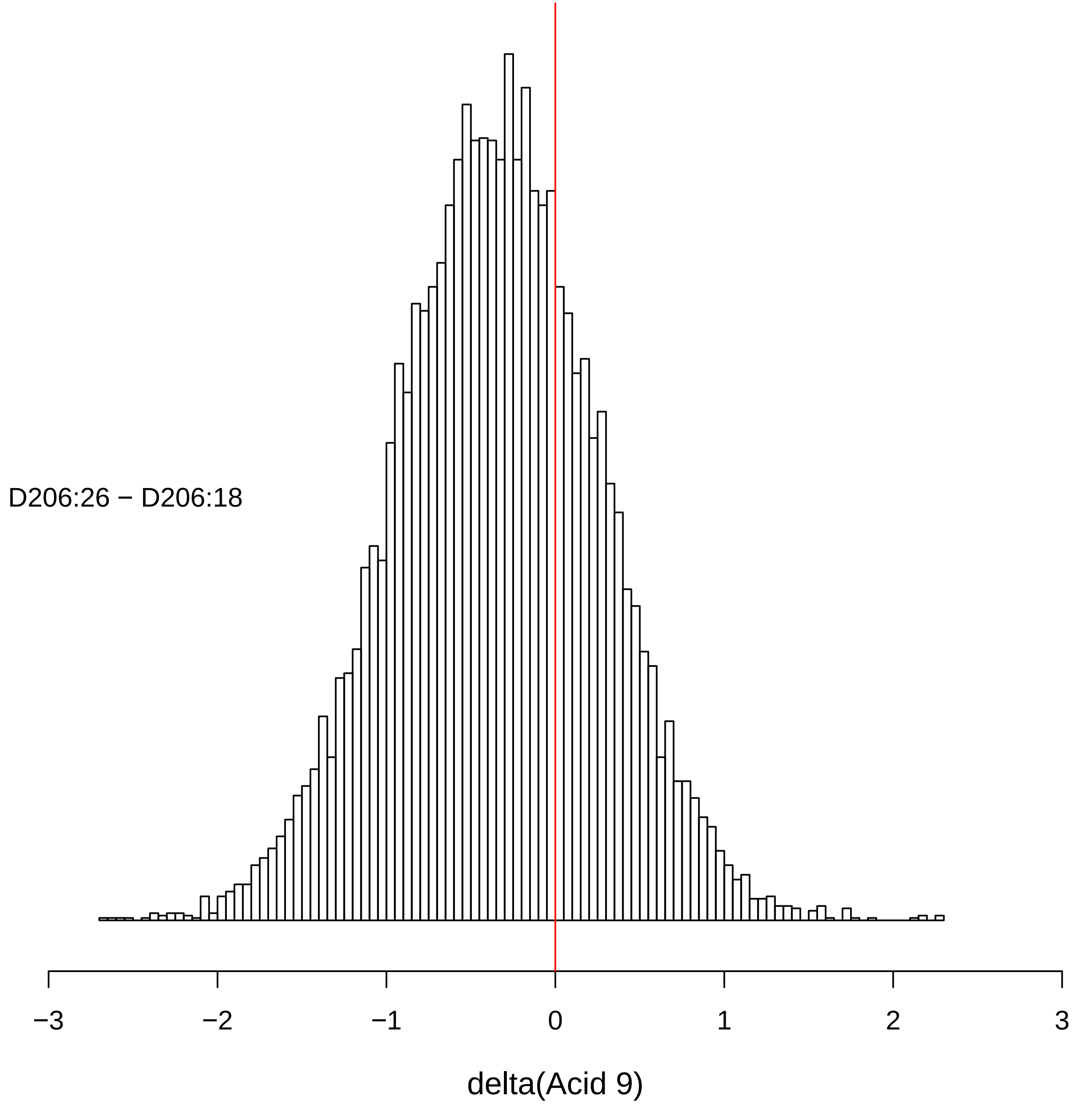
D206:26 − D206:18
−3
−2
−1
0
1
2
3
delta(Acid 9)

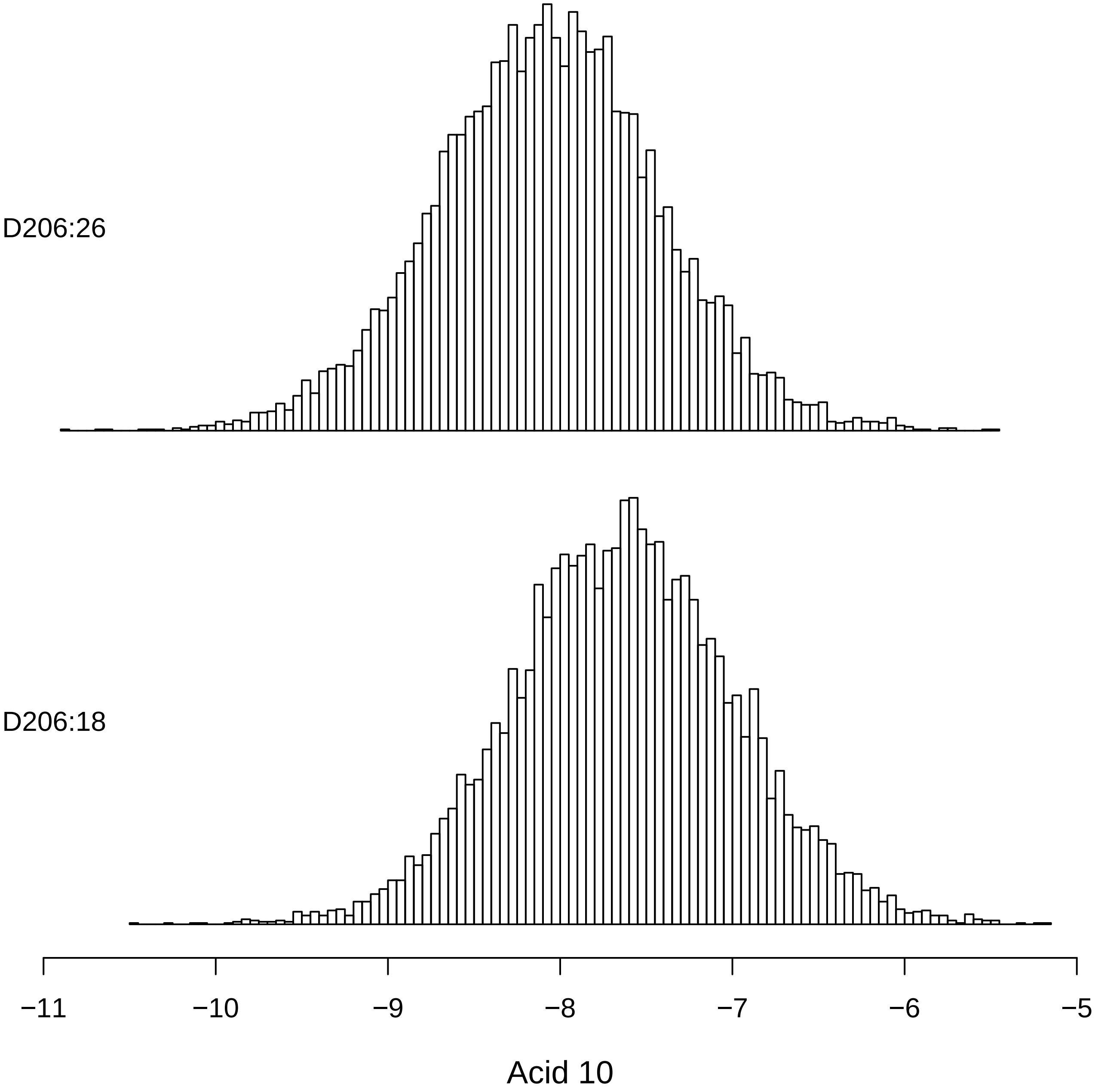
D206:26
D206:18
−11
−10
−9
−8
−7
−6
−5
Acid 10

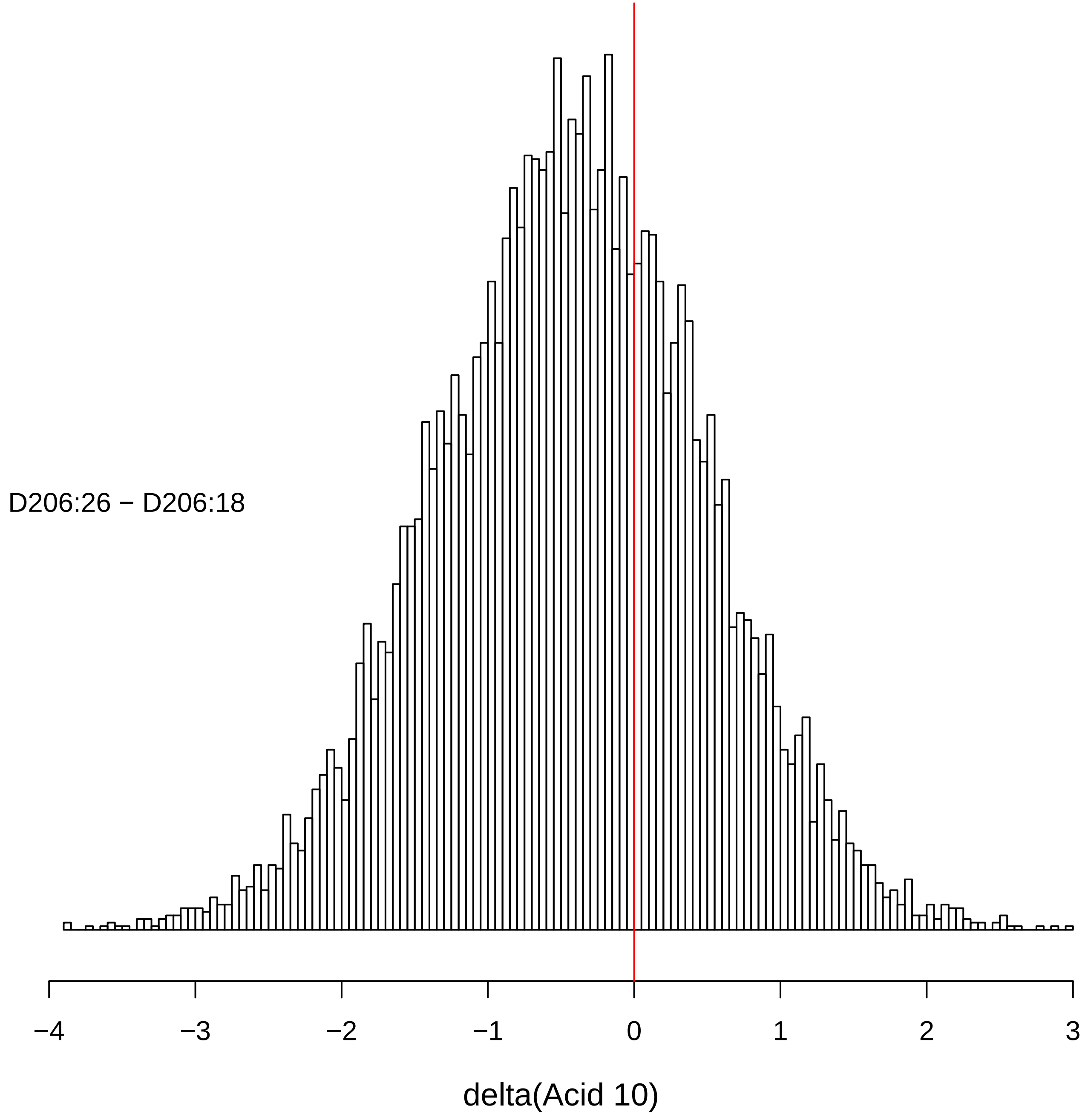
D206:26 − D206:18
−4
−3
−2
−1
0
1
2
3
delta(Acid 10)

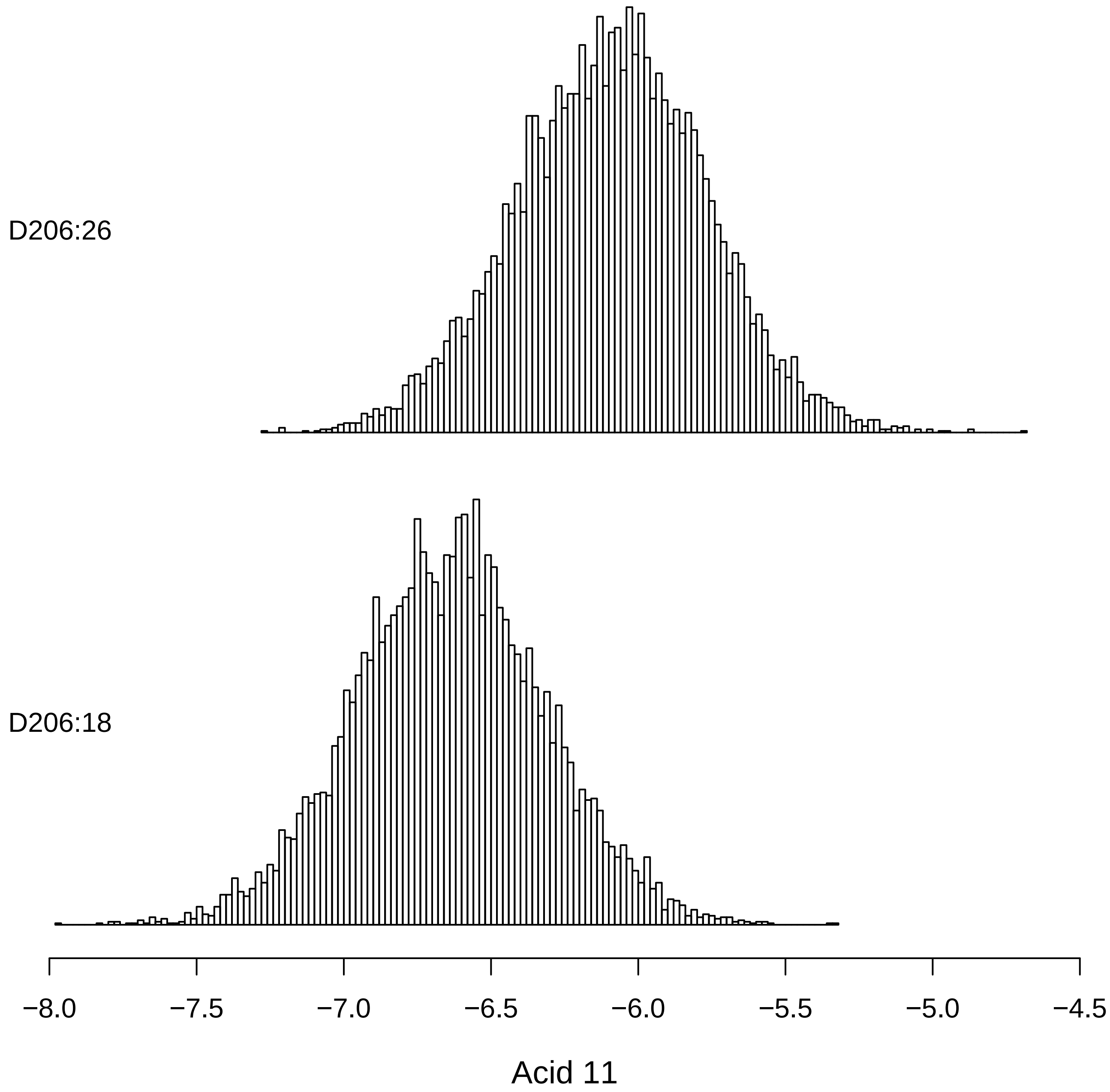
D206:26
D206:18
−8.0
−7.5
−7.0
−6.5
−6.0
−5.5
−5.0
−4.5
Acid 11

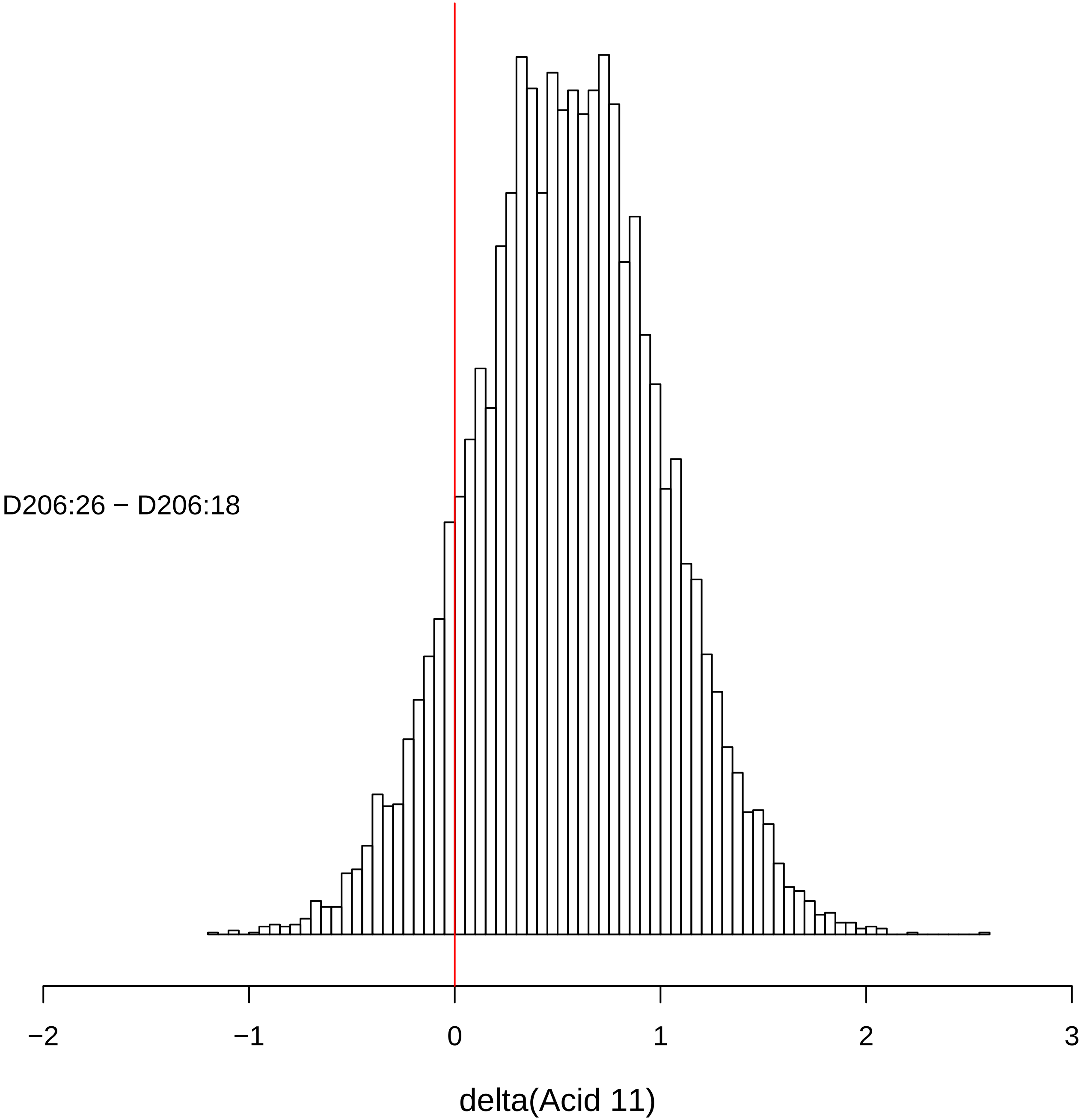
D206:26 − D206:18
−2
−1
0
1
2
3
delta(Acid 11)

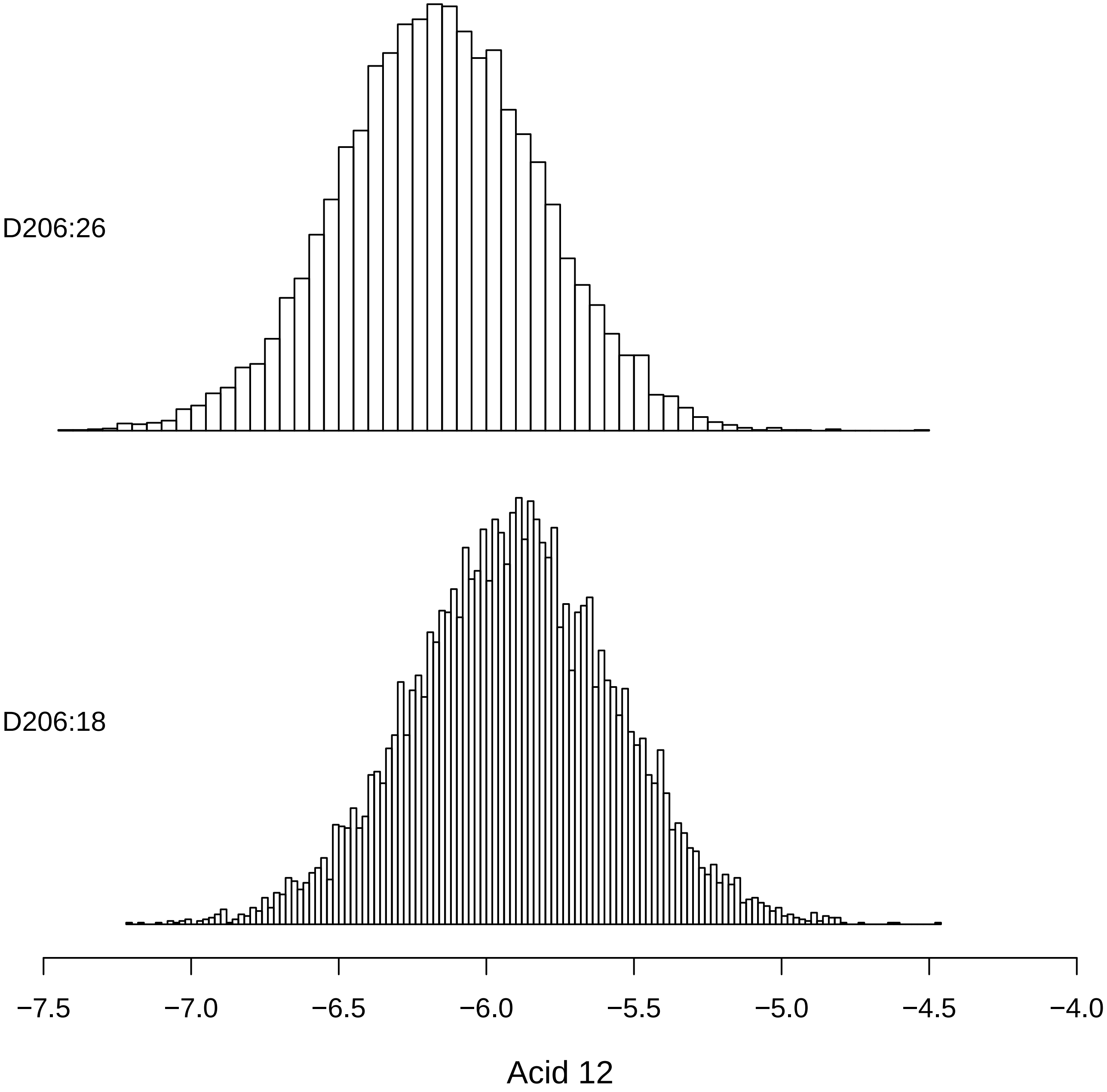
D206:26
D206:18
−7.5
−7.0
−6.5
−6.0
−5.5
−5.0
−4.5
−4.0
Acid 12

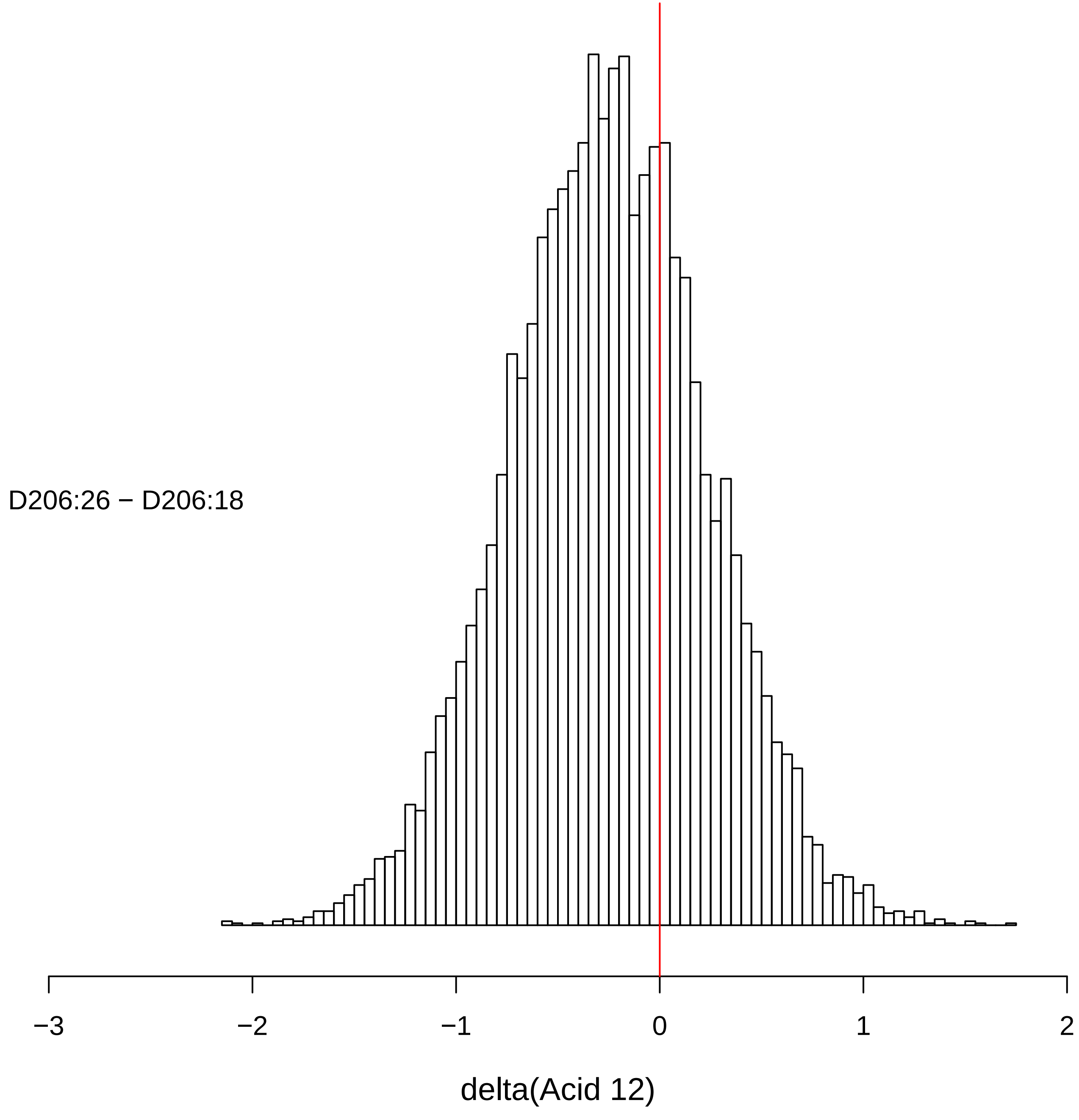
D206:26 − D206:18
−3
−2
−1
0
1
2
delta(Acid 12)

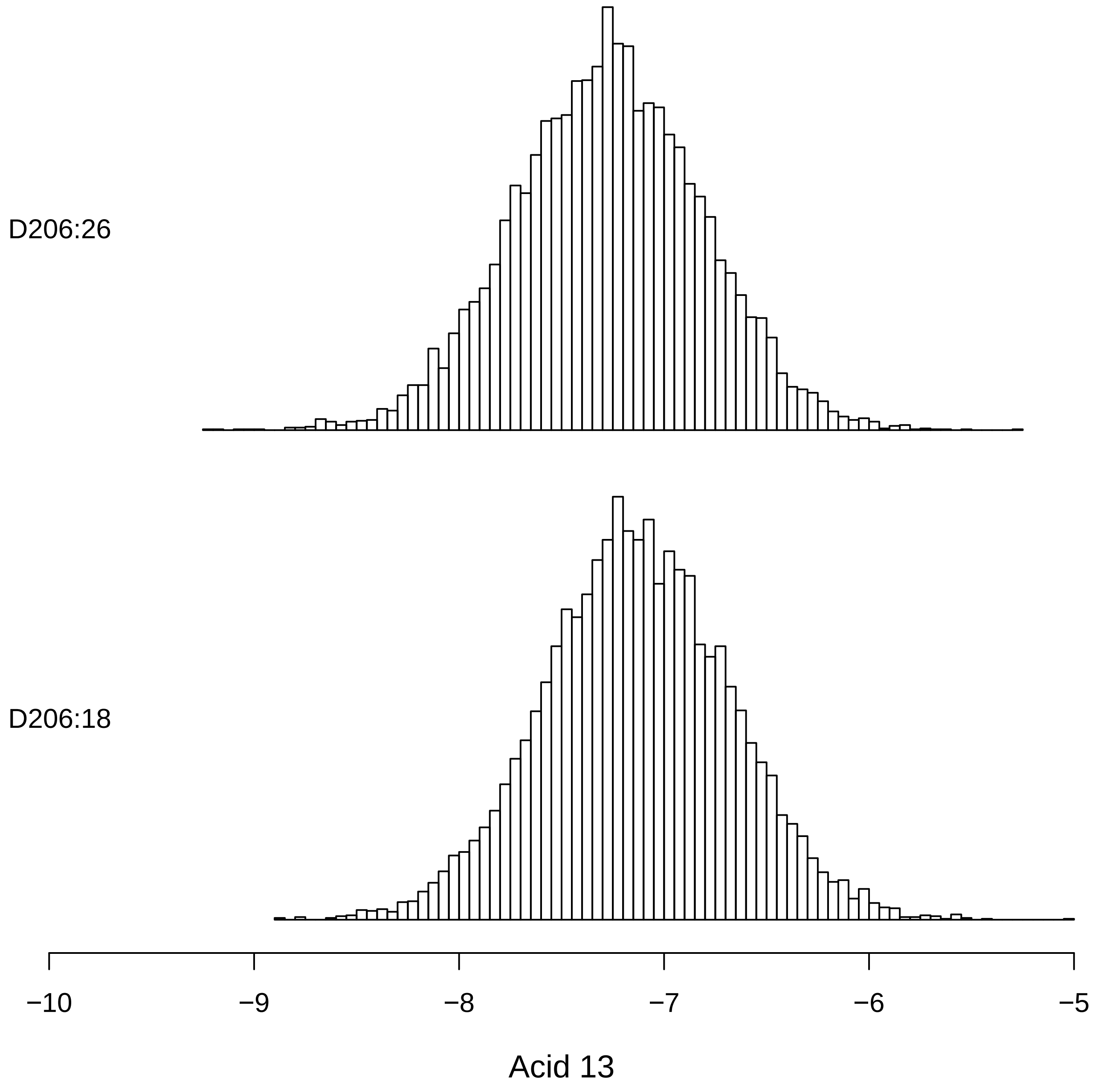
D206:26
D206:18
−10
−9
−8
−7
−6
−5
Acid 13

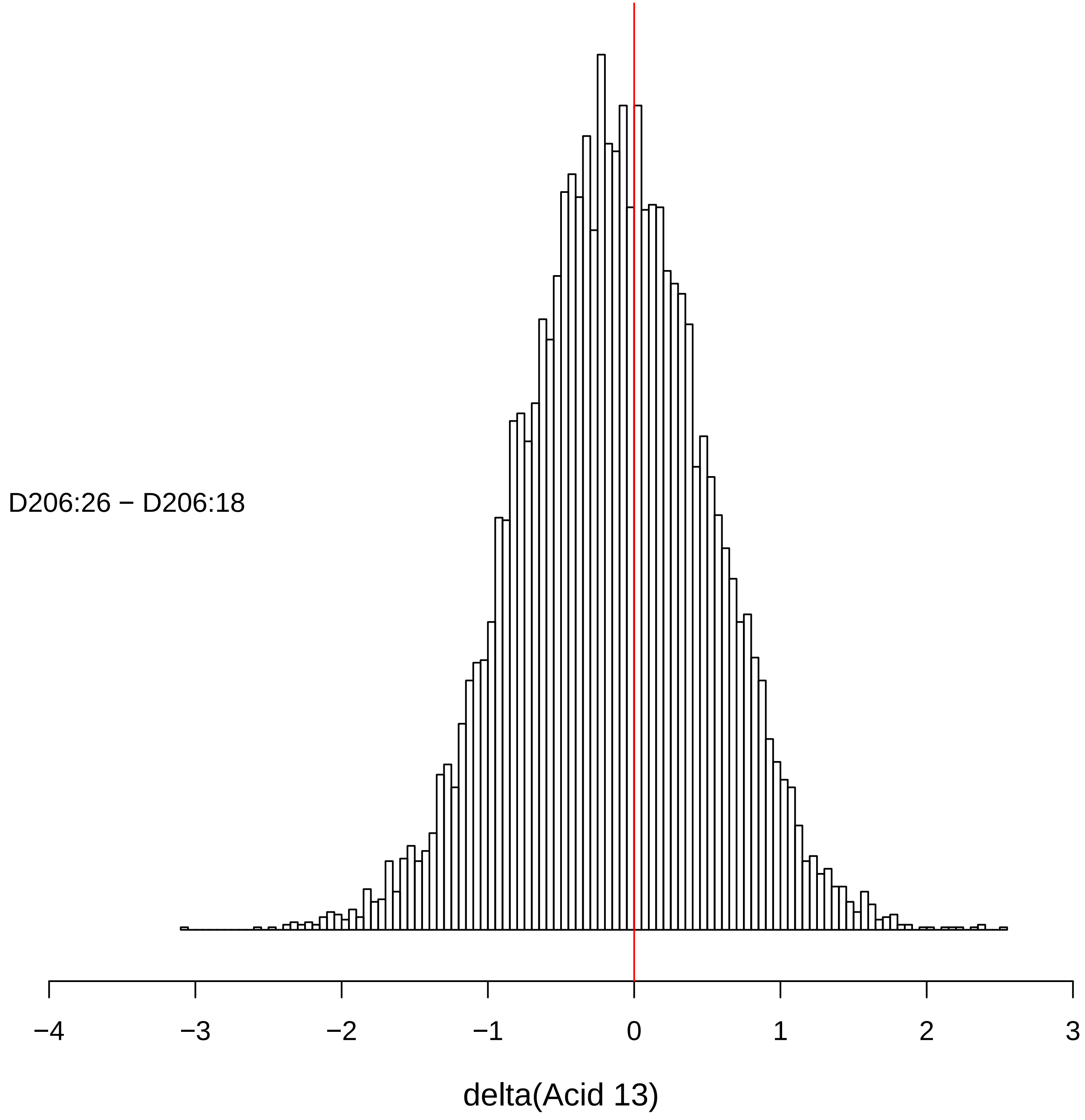
D206:26 − D206:18
−4
−3
−2
−1
0
1
2
3
delta(Acid 13)

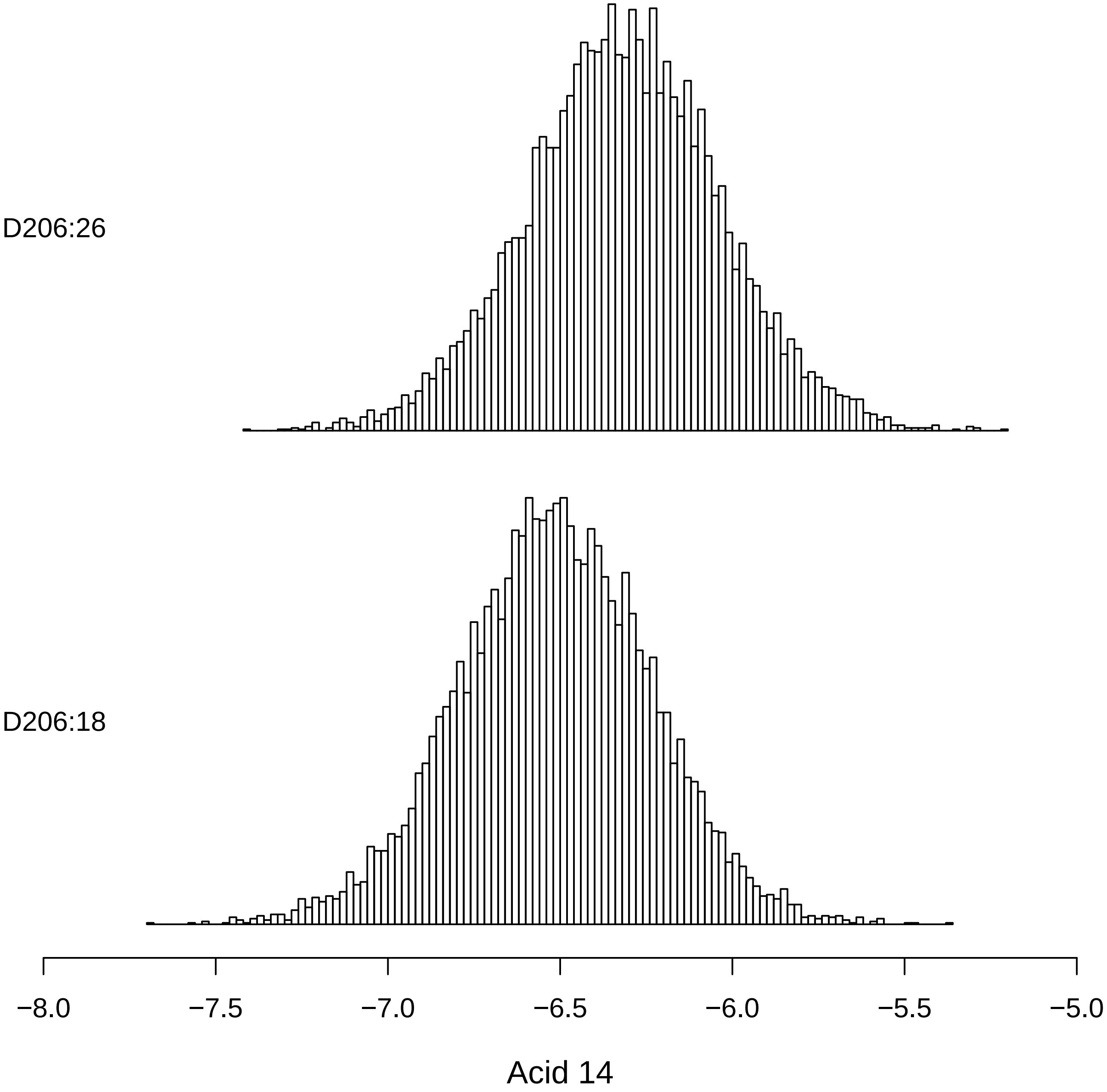
D206:26
D206:18
−8.0
−7.5
−7.0
−6.5
−6.0
−5.5
−5.0
Acid 14

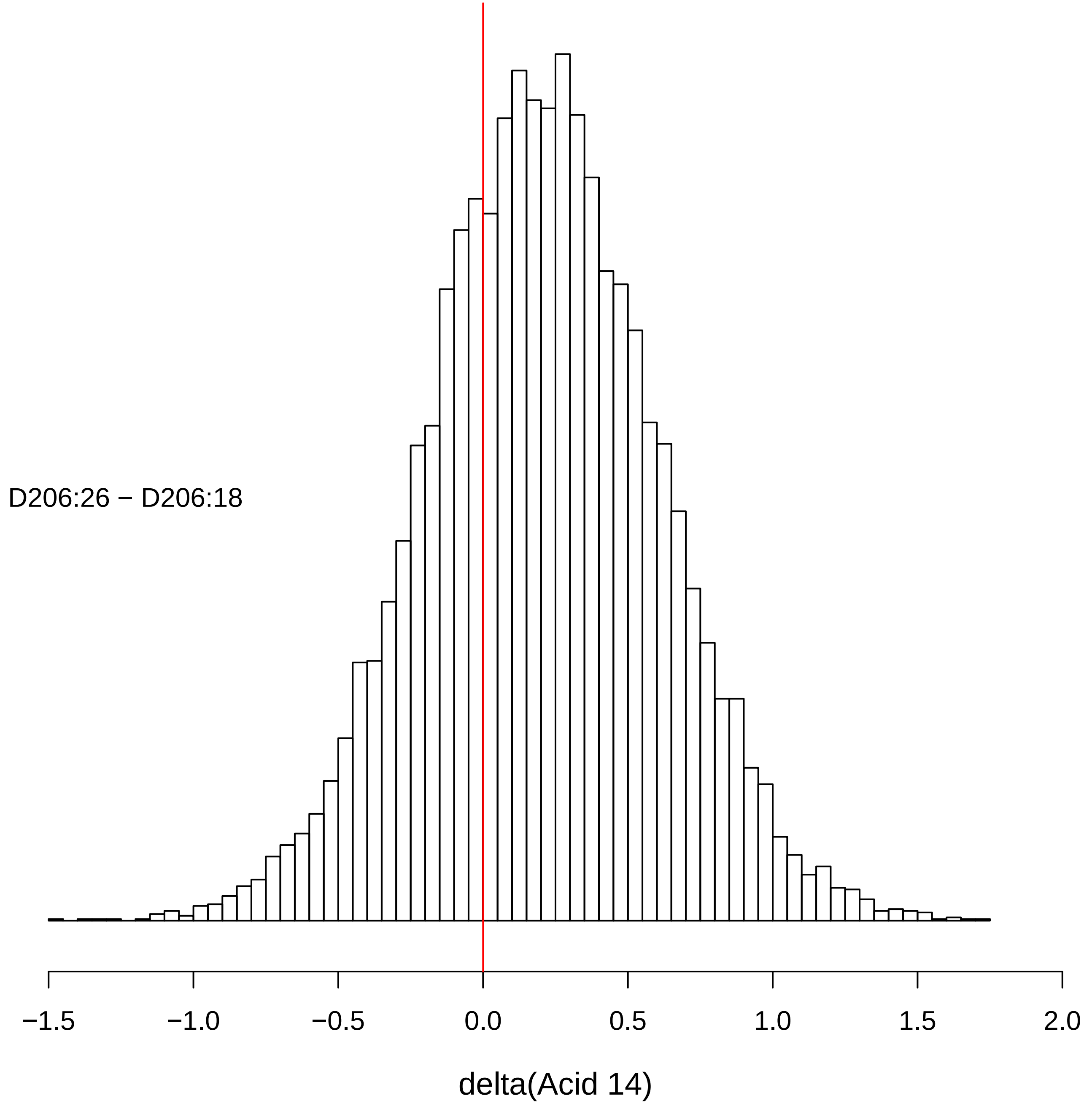
D206:26 − D206:18
−1.5
−1.0
−0.5
0.0
0.5
1.0
1.5
2.0
delta(Acid 14)
