# Supplementary material for: Taxonomic and Environmental Variation of Metabolite Profiles in Marine Dinoflagellates of the Genus Symbiodinium
Source: Metabolites. 2015 Feb 16;5(1):74–99. doi: 10.3390/metabo5010074 (PMC4381291; doi:10.3390/metabo5010074)
Supplement: Supplementary File 1 [file metabolites-05-00074-s001.zip › Supplementary Information/Supplementary Information Figure S5c - light.B224.pdf]

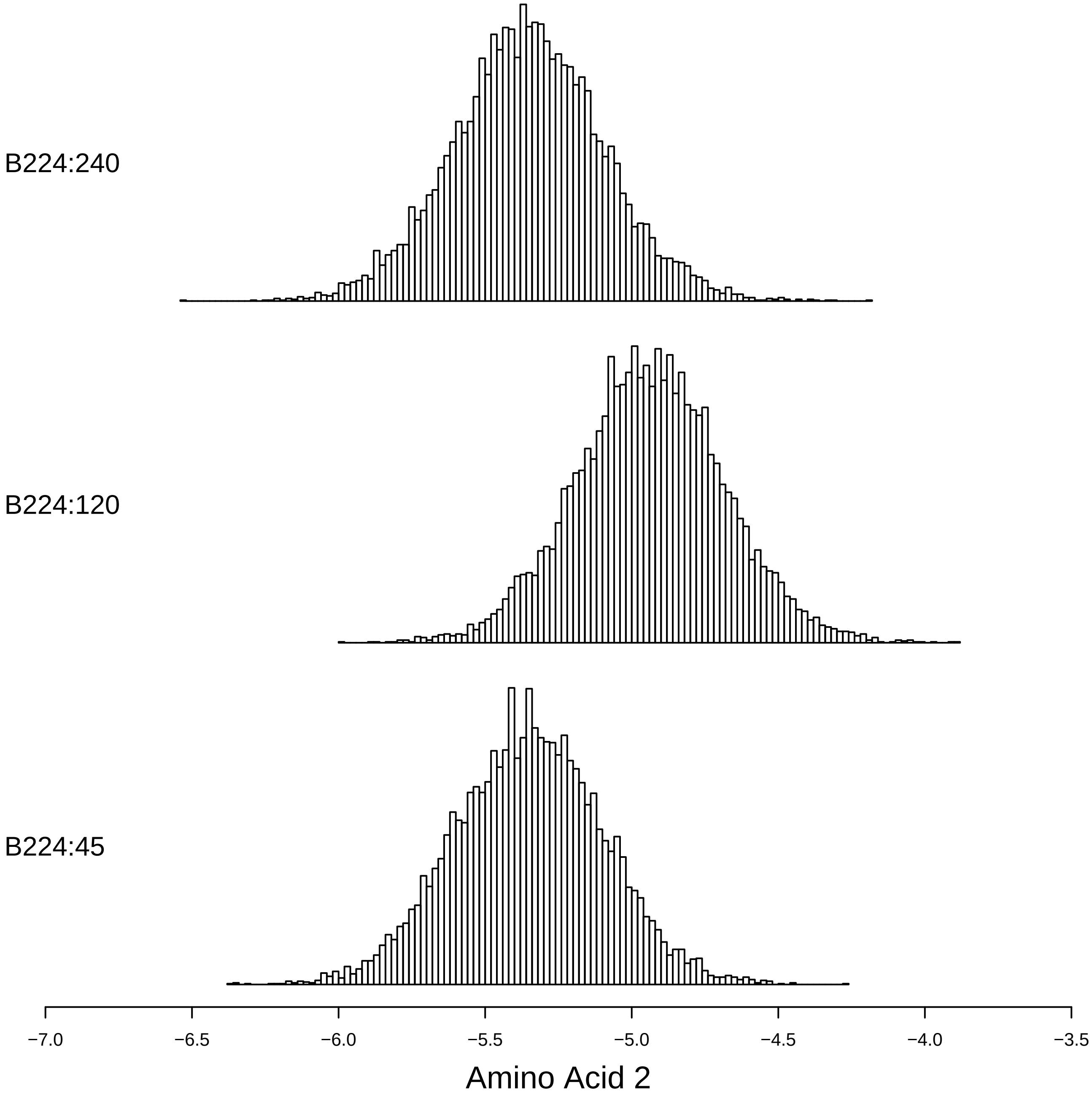
B224:240
B224:120
B224:45
−7.0
−6.5
−6.0
−5.5
−5.0
−4.5
−4.0
−3.5
Amino Acid 2

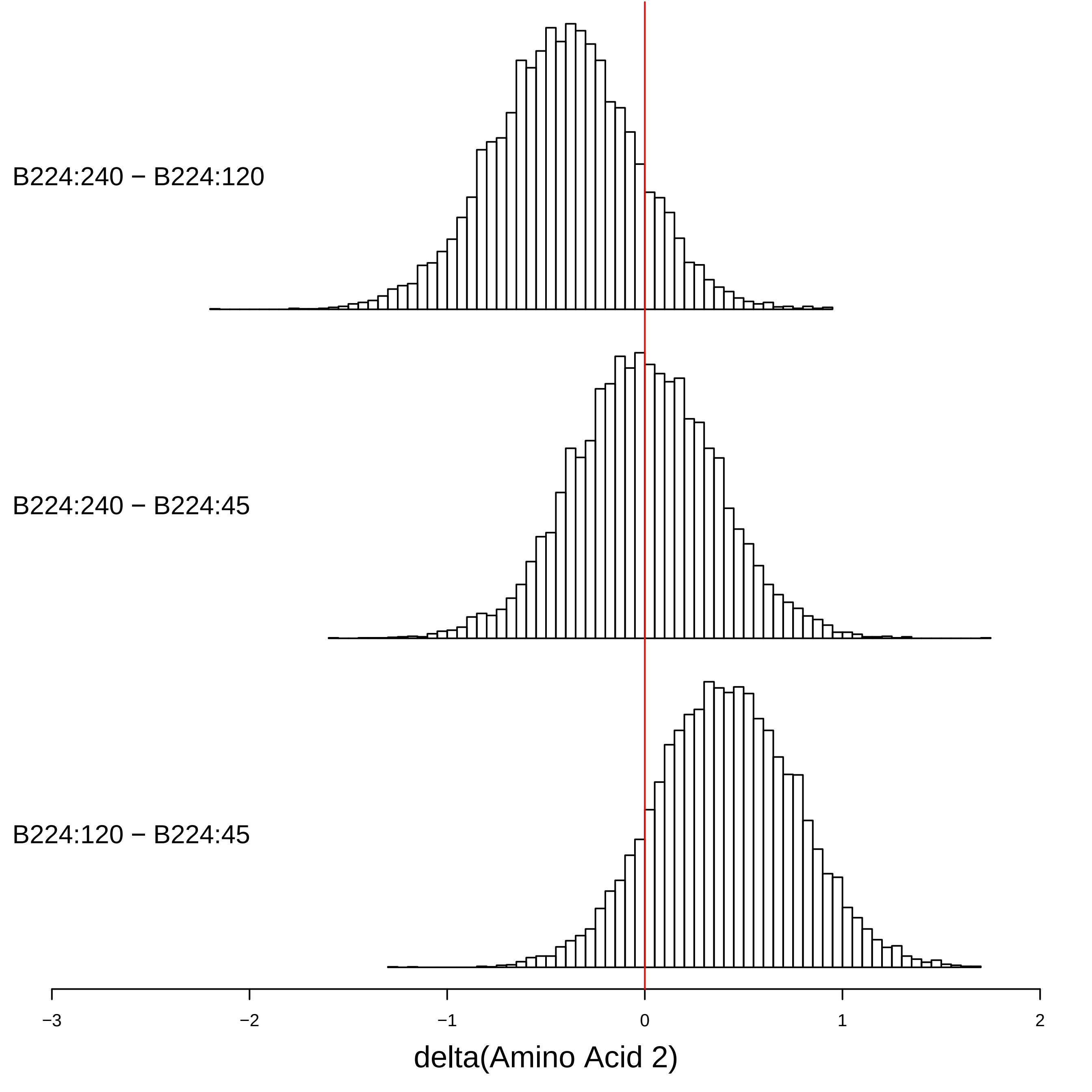
B224:240 − B224:120
B224:240 − B224:45
B224:120 − B224:45
−3
−2
−1
0
1
2
delta(Amino Acid 2)

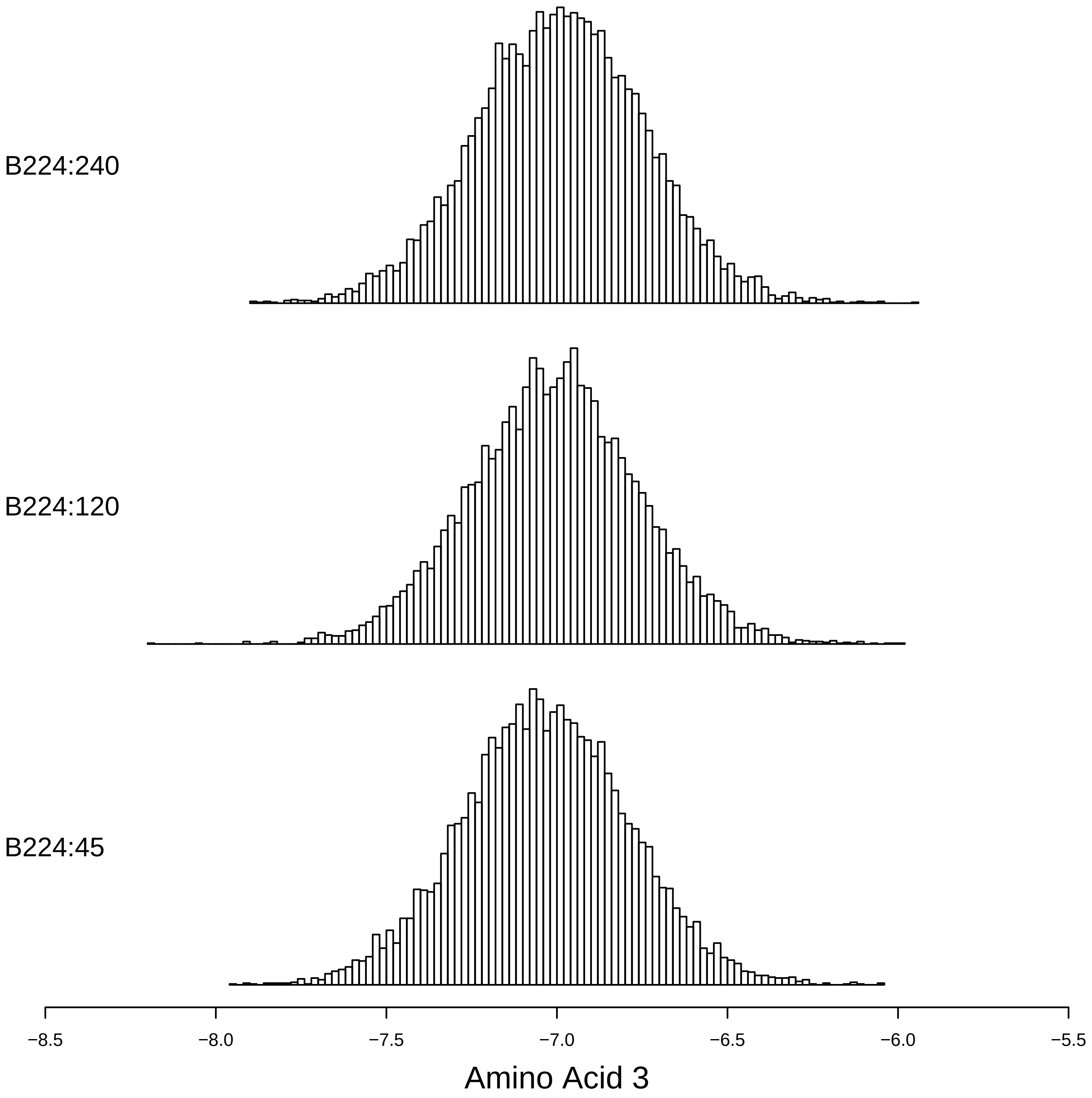

B224:240
B224:120
B224:45
−8.5
−8.0
−7.5
−7.0
−6.5
−6.0
−5.5
Amino Acid 3

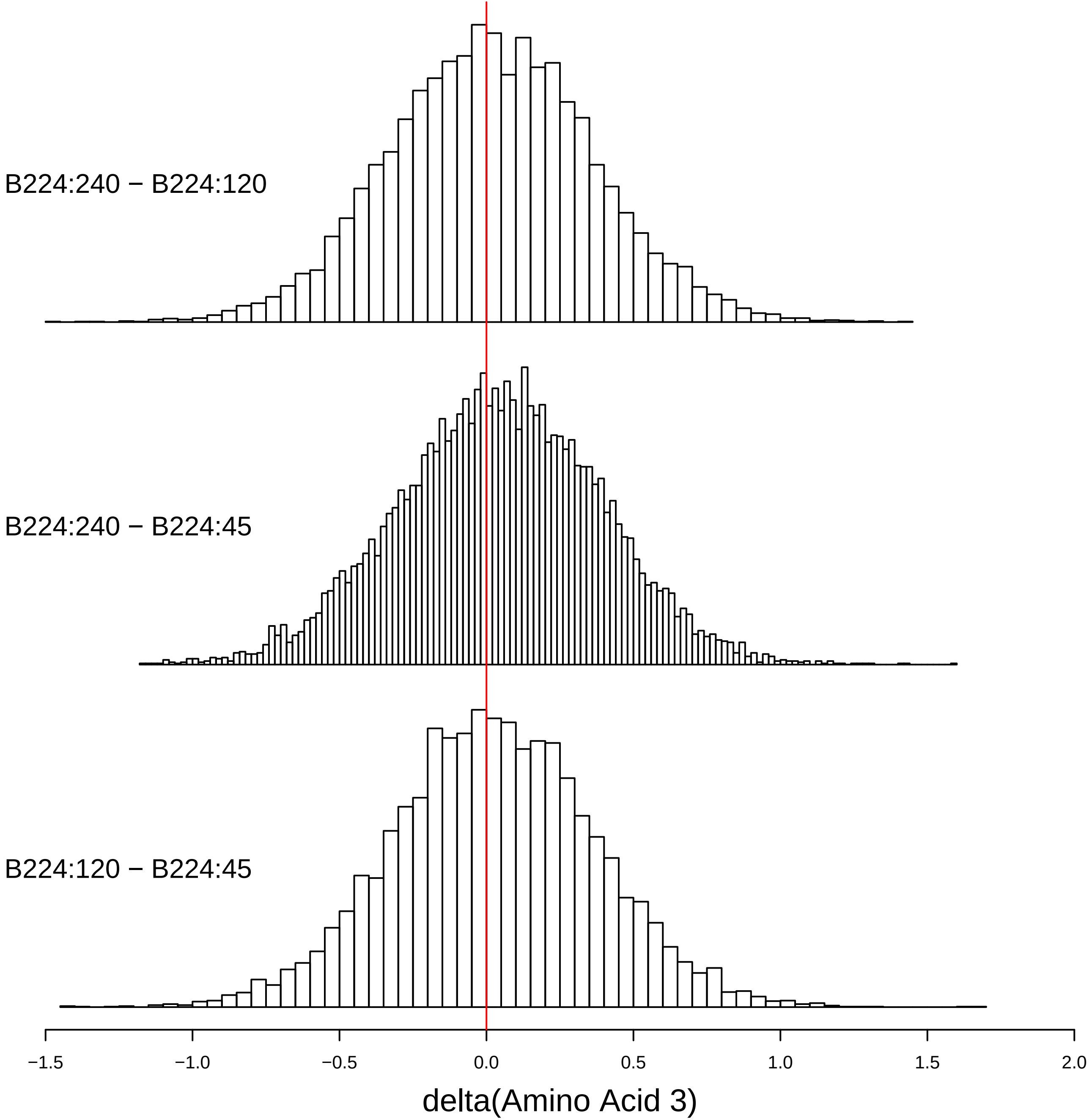
B224:240 − B224:120
B224:240 − B224:45
B224:120 − B224:45
−1.5
−1.0
−0.5
0.0
0.5
1.0
1.5
2.0
delta(Amino Acid 3)

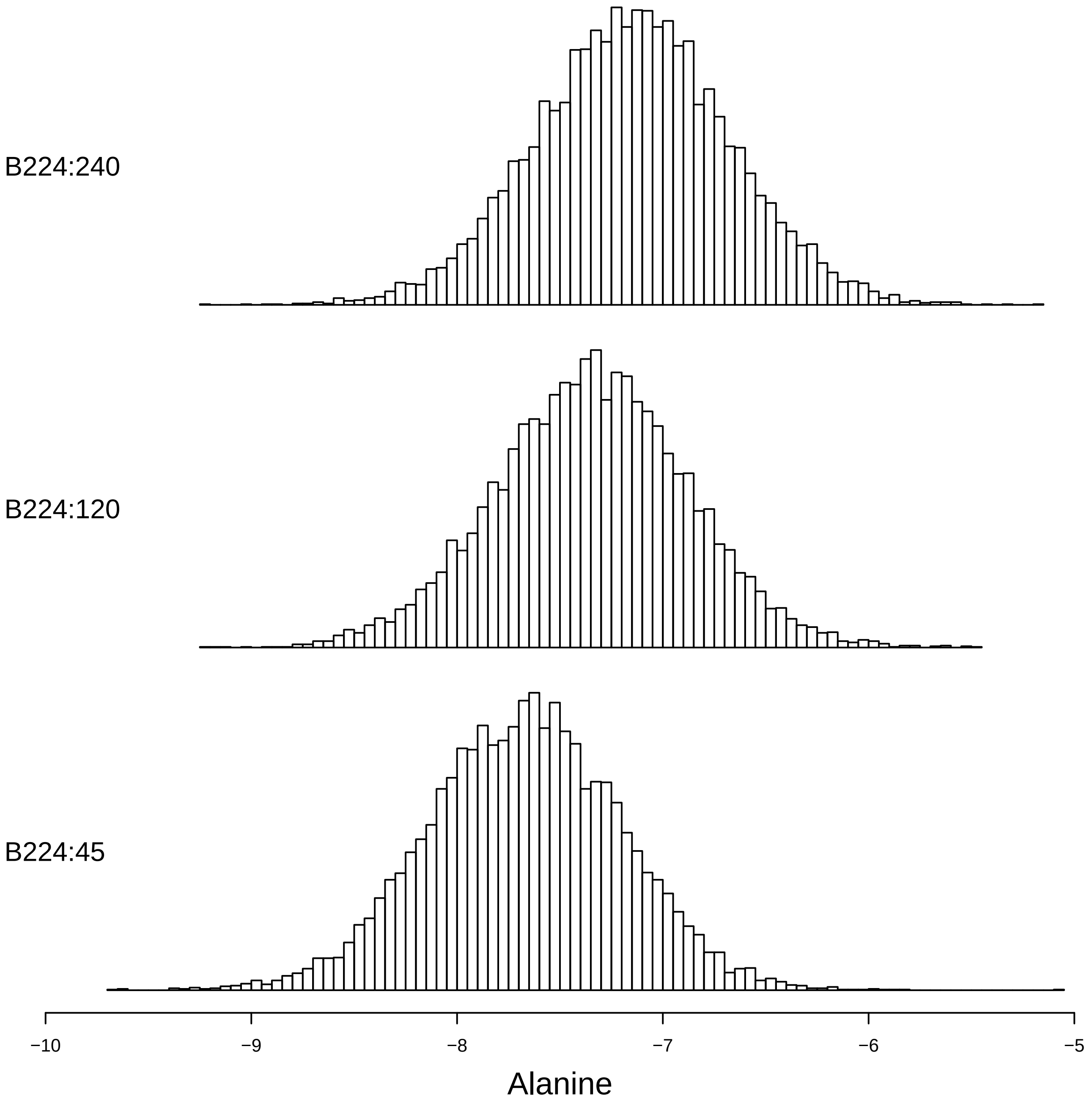
B224:240
B224:120
B224:45
−10
−9
−8
−7
−6
−5
Alanine

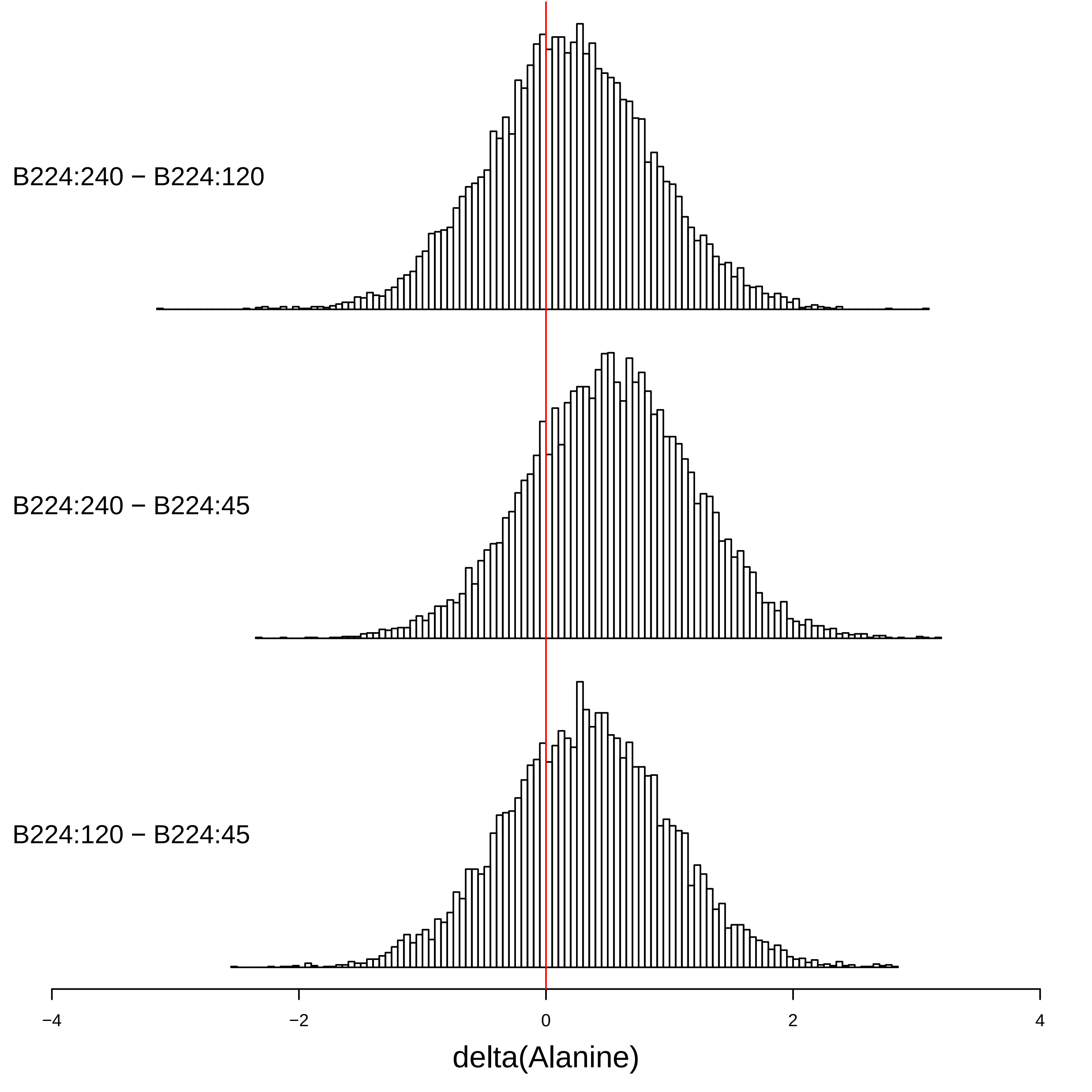
B224:240 − B224:120
B224:240 − B224:45
B224:120 − B224:45
−4
−2
0
2
4
delta(Alanine)

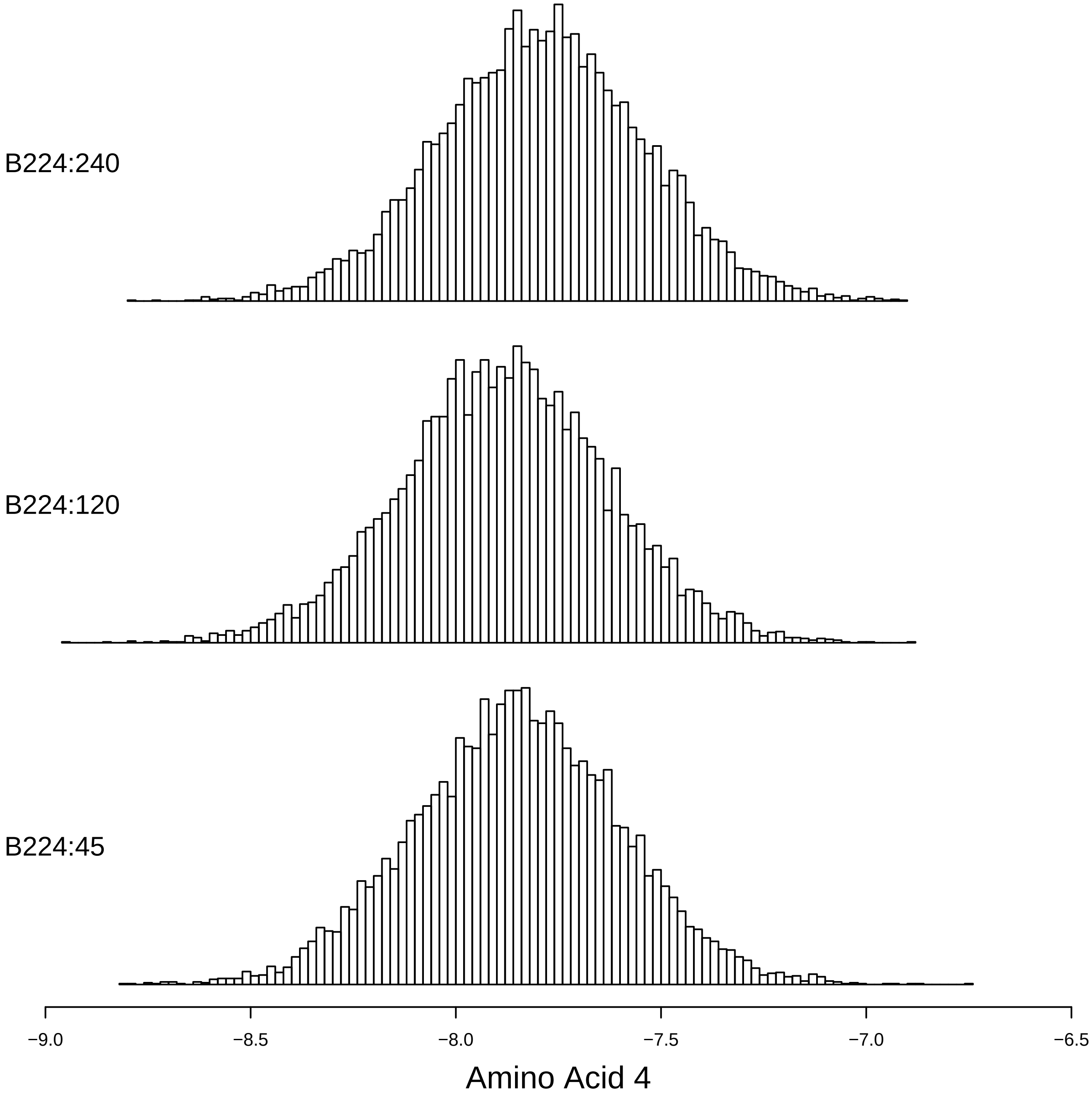

B224:240
B224:120
B224:45
−9.0
−8.5
−8.0
−7.5
−7.0
−6.5
Amino Acid 4

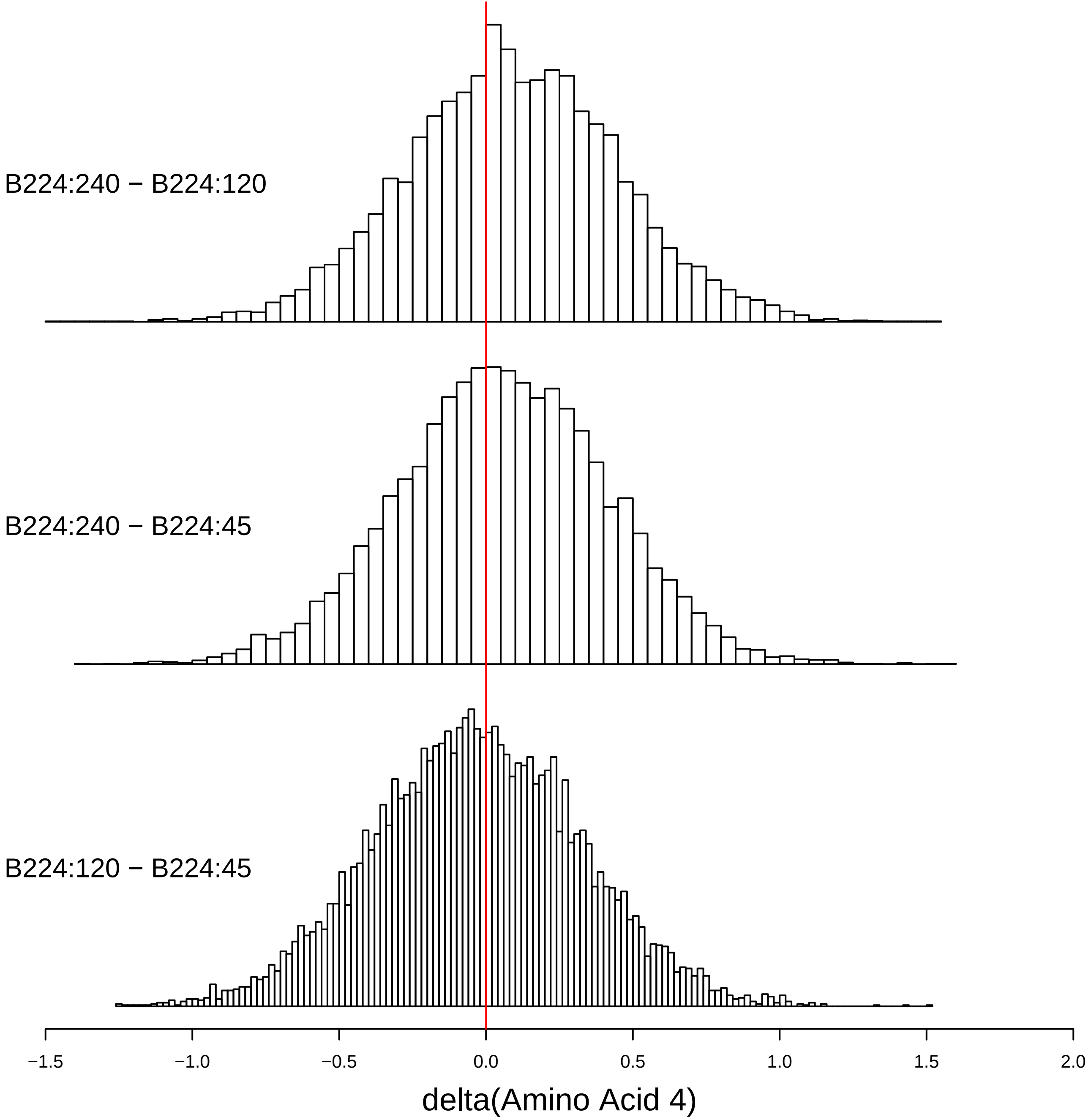
B224:240 − B224:120
B224:240 − B224:45
B224:120 − B224:45
−1.5
−1.0
−0.5
0.0
0.5
1.0
1.5
2.0
delta(Amino Acid 4)

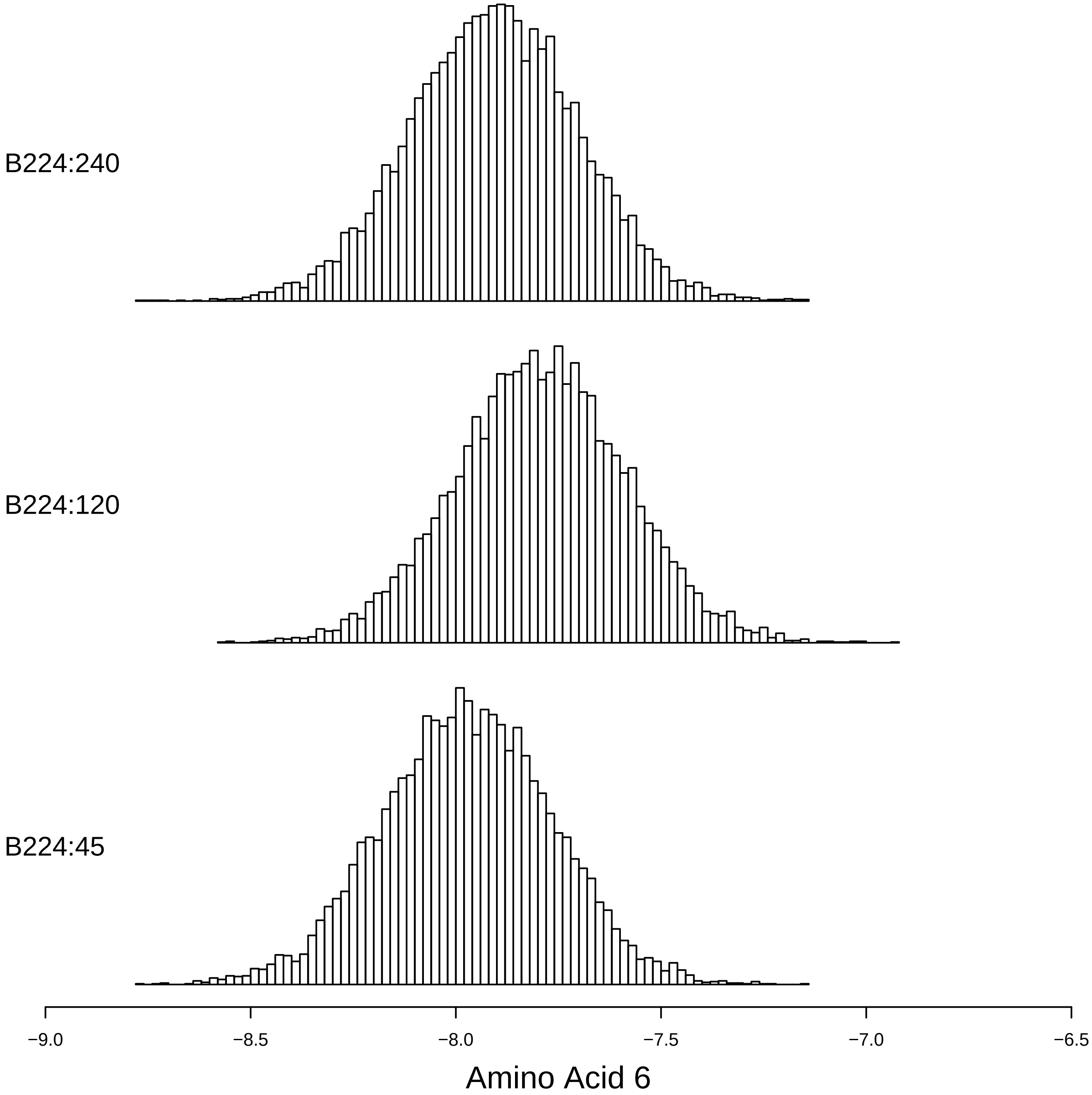

B224:240
B224:120
B224:45
−9.0
−8.5
−8.0
−7.5
−7.0
−6.5
Amino Acid 6

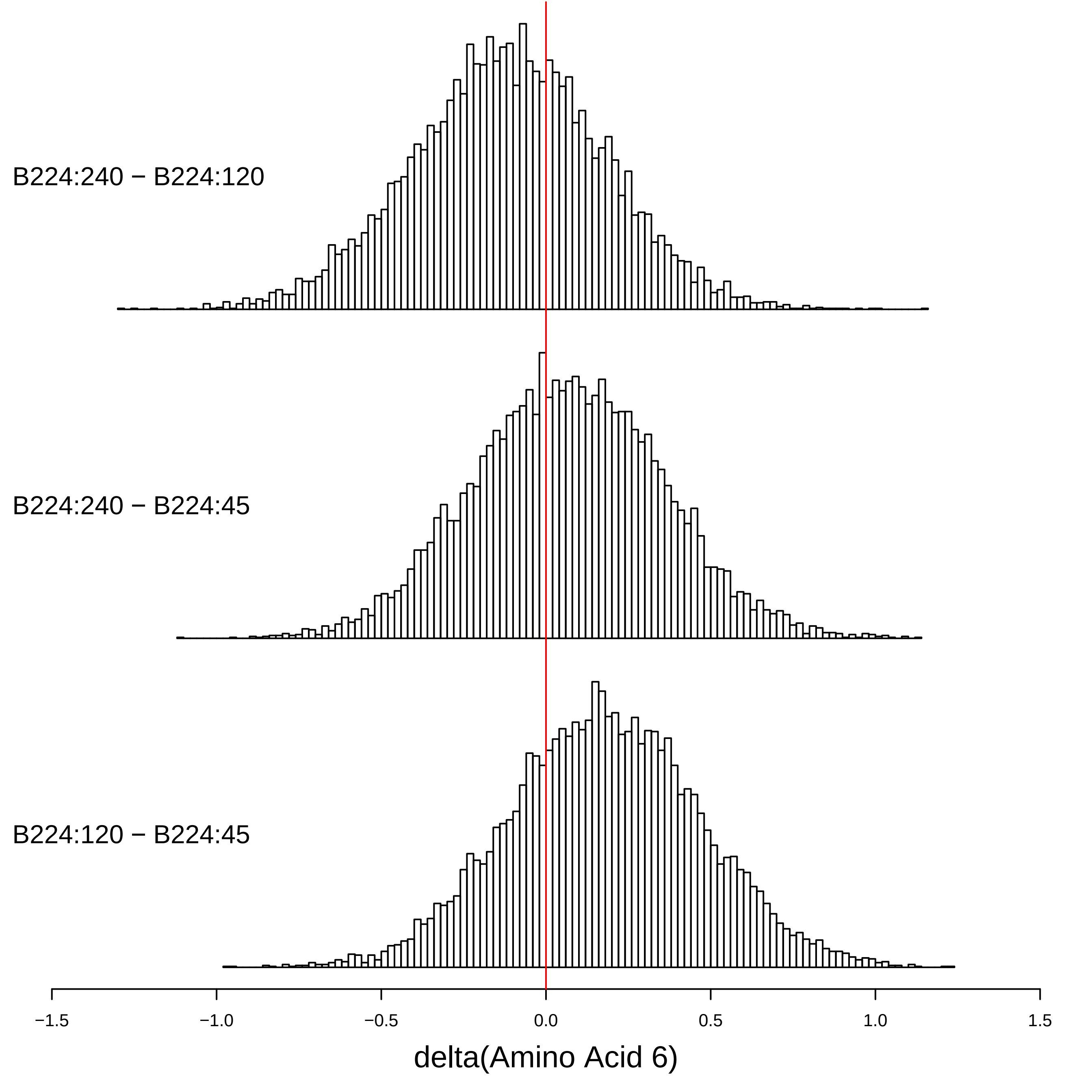
B224:240 − B224:120
B224:240 − B224:45
B224:120 − B224:45
−1.5
−1.0
−0.5
0.0
0.5
1.0
1.5
delta(Amino Acid 6)

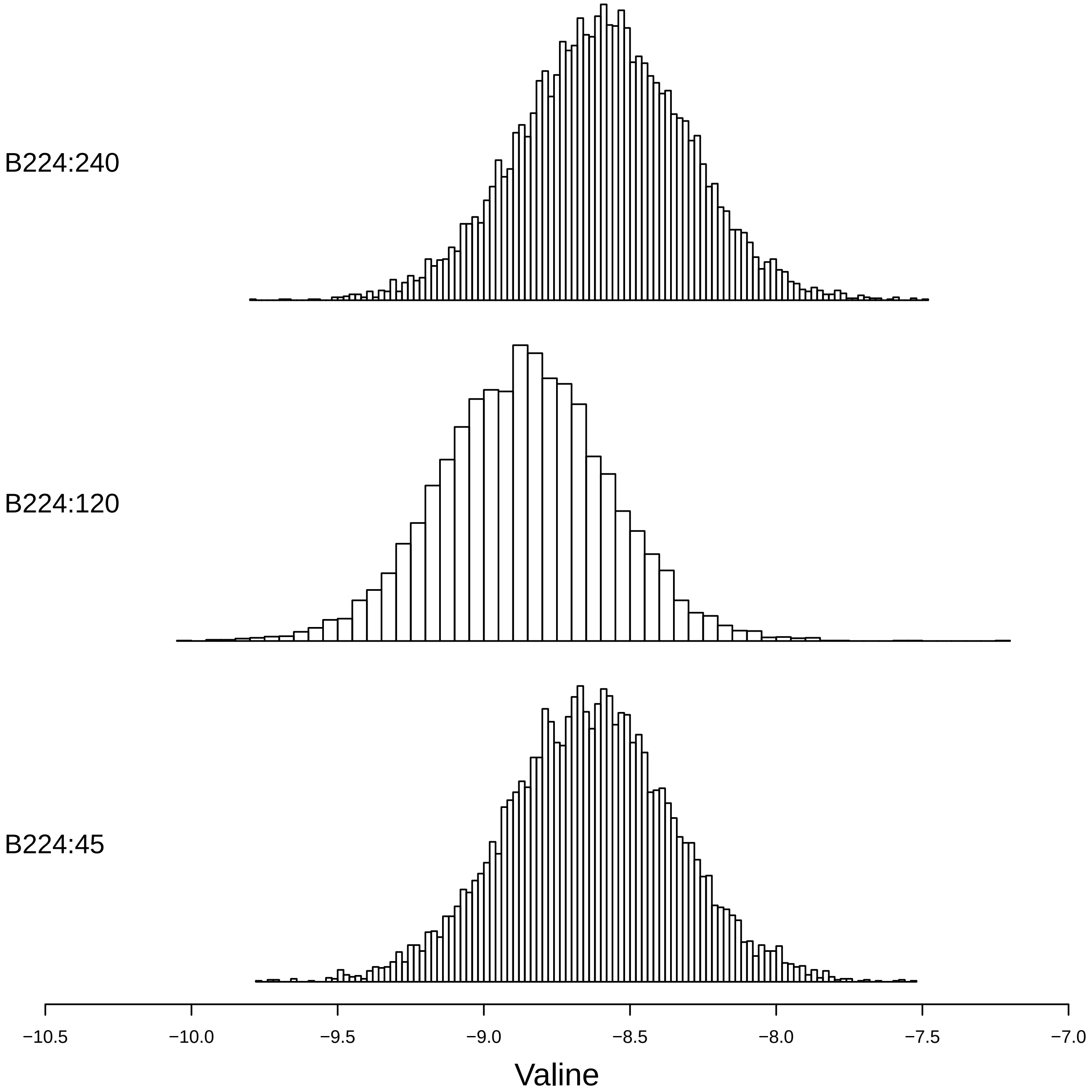

B224:240
B224:120
B224:45
−10.5
−10.0
−9.5
−9.0
−8.5
−8.0
−7.5
−7.0
Valine

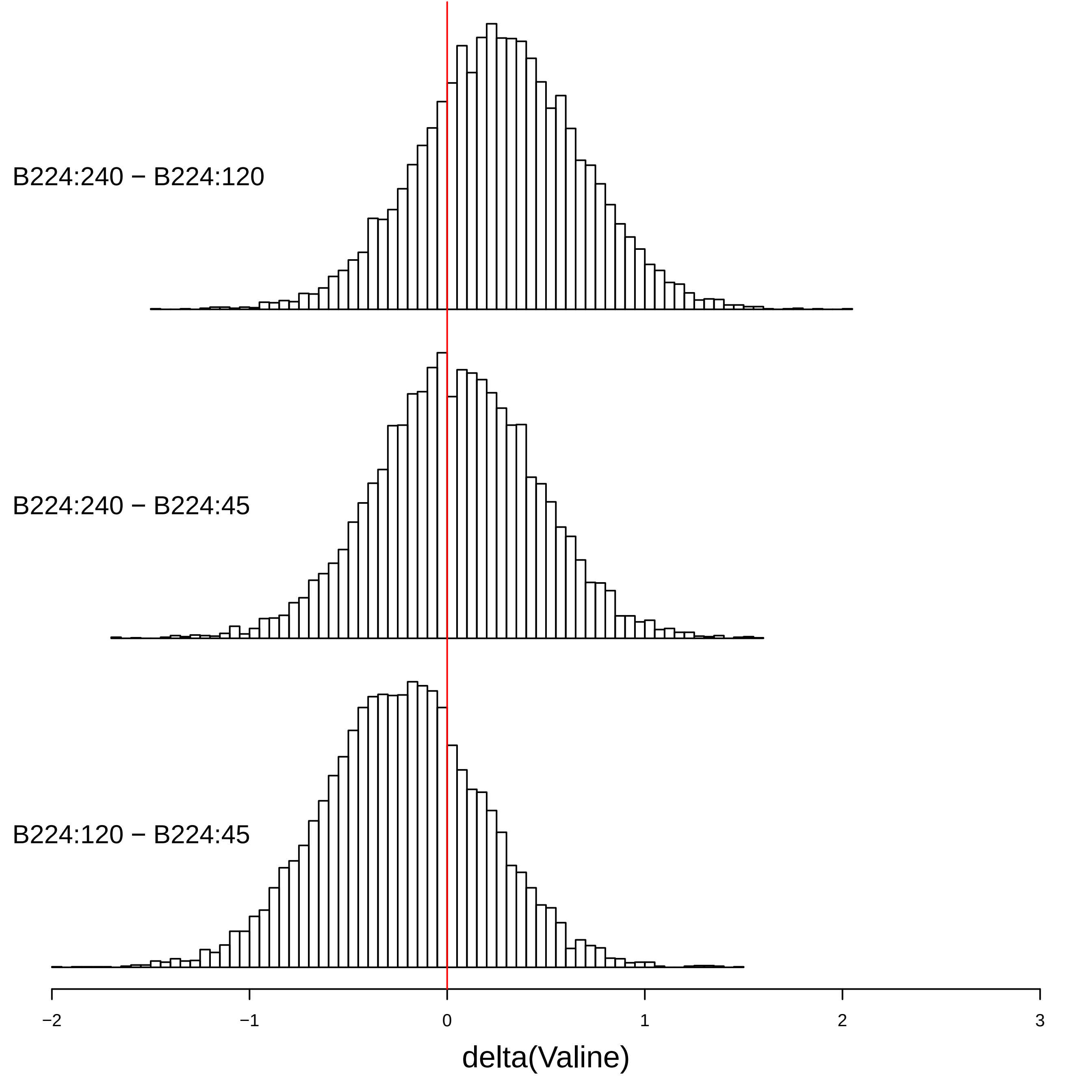
B224:240 − B224:120
B224:240 − B224:45
B224:120 − B224:45
−2
−1
0
1
2
3
delta(Valine)

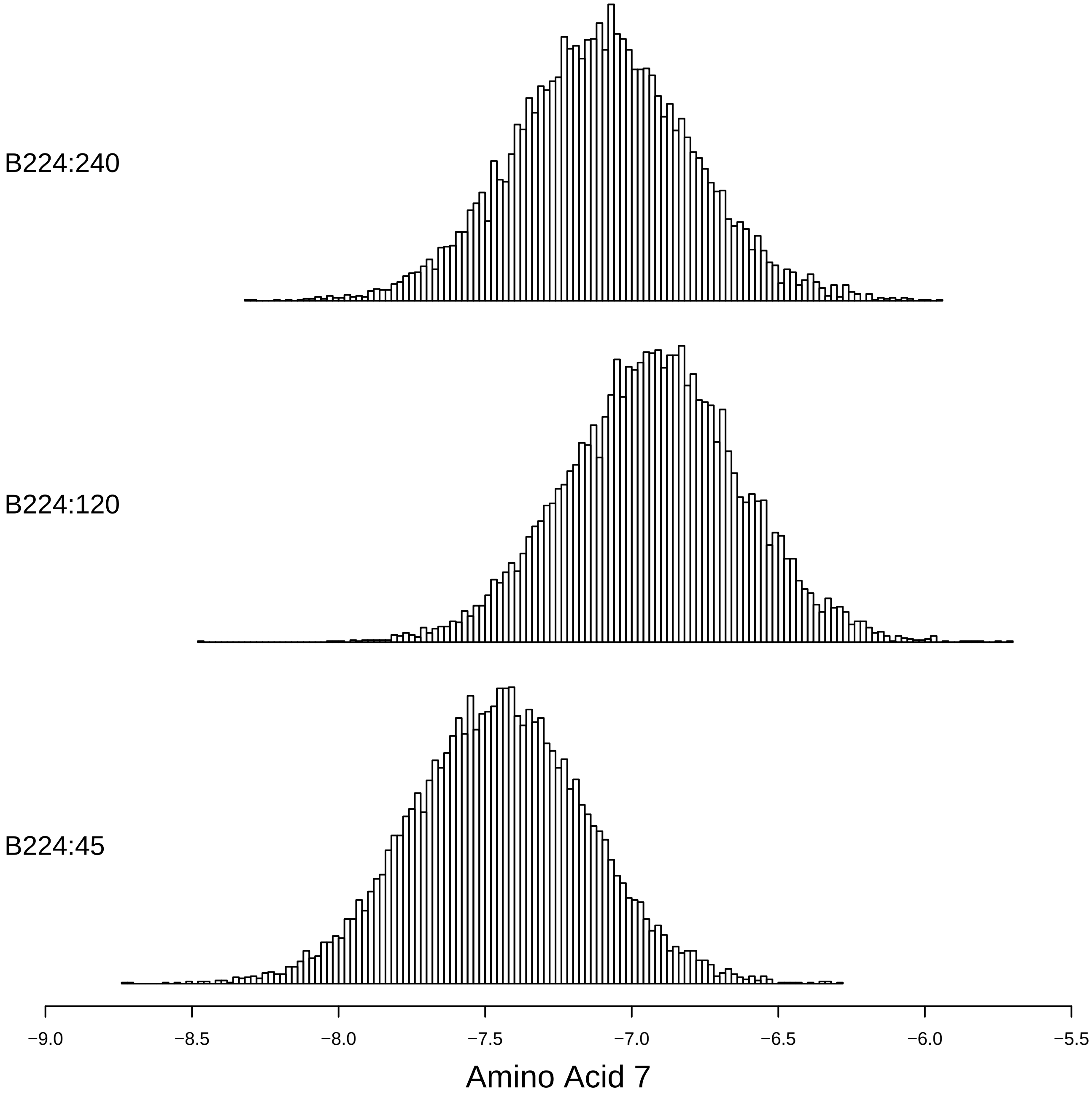
B224:240
B224:120
B224:45
−9.0
−8.5
−8.0
−7.5
−7.0
−6.5
−6.0
−5.5
Amino Acid 7

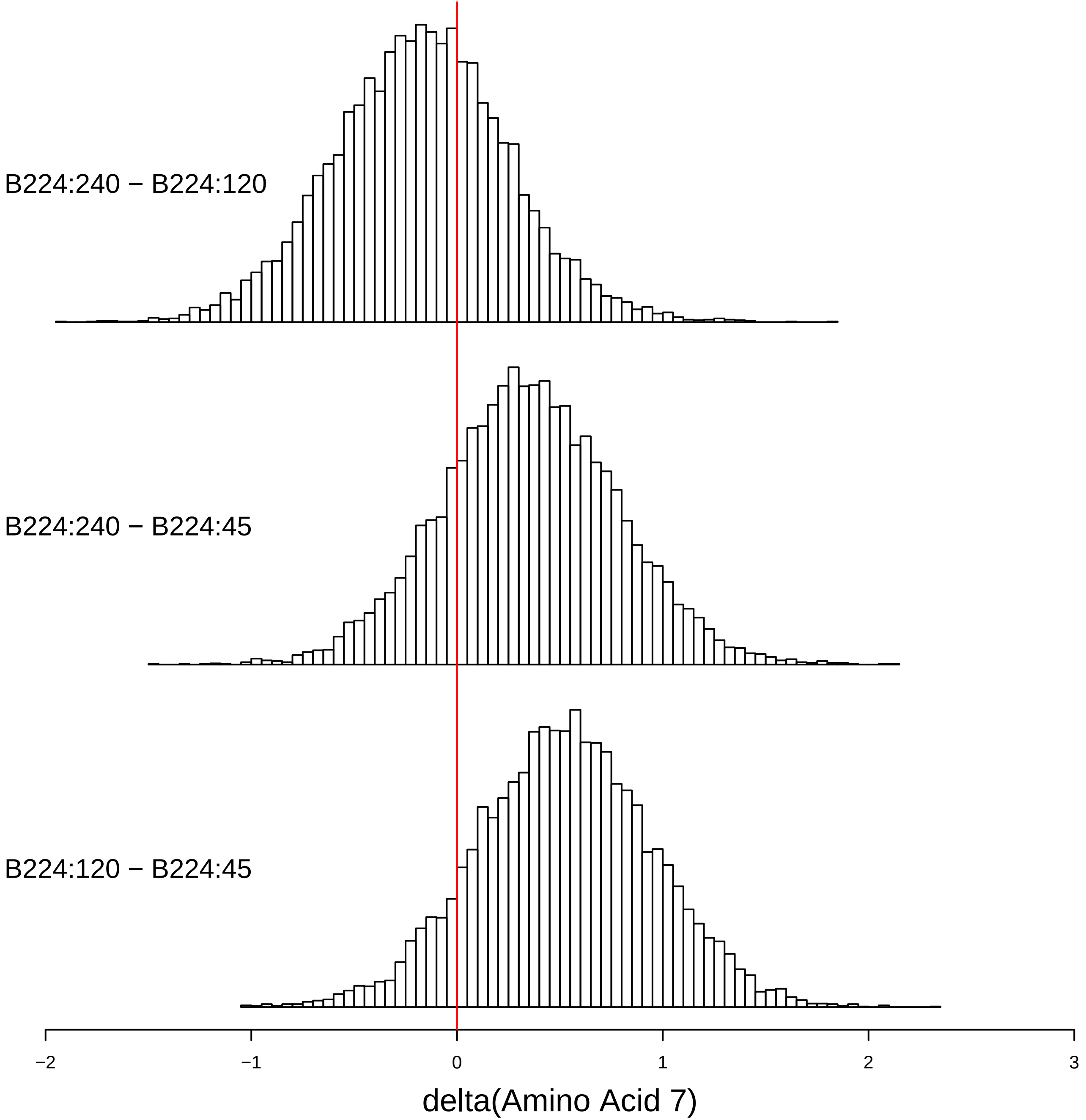

B224:240 − B224:120
B224:240 − B224:45
B224:120 − B224:45
−2
−1
0
1
2
3
delta(Amino Acid 7)

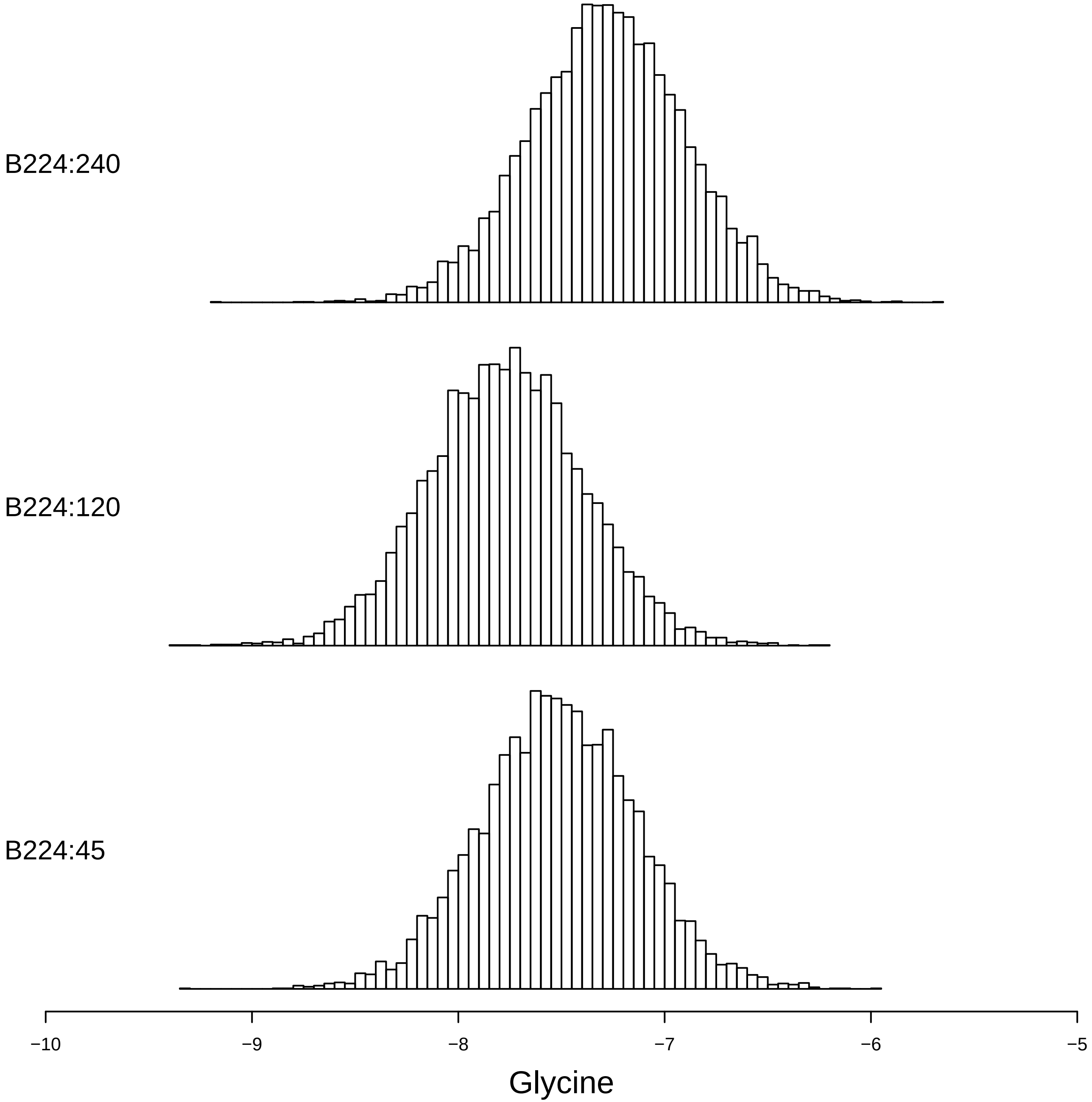
B224:240
B224:120
B224:45
−10
−9
−8
−7
−6
−5
Glycine

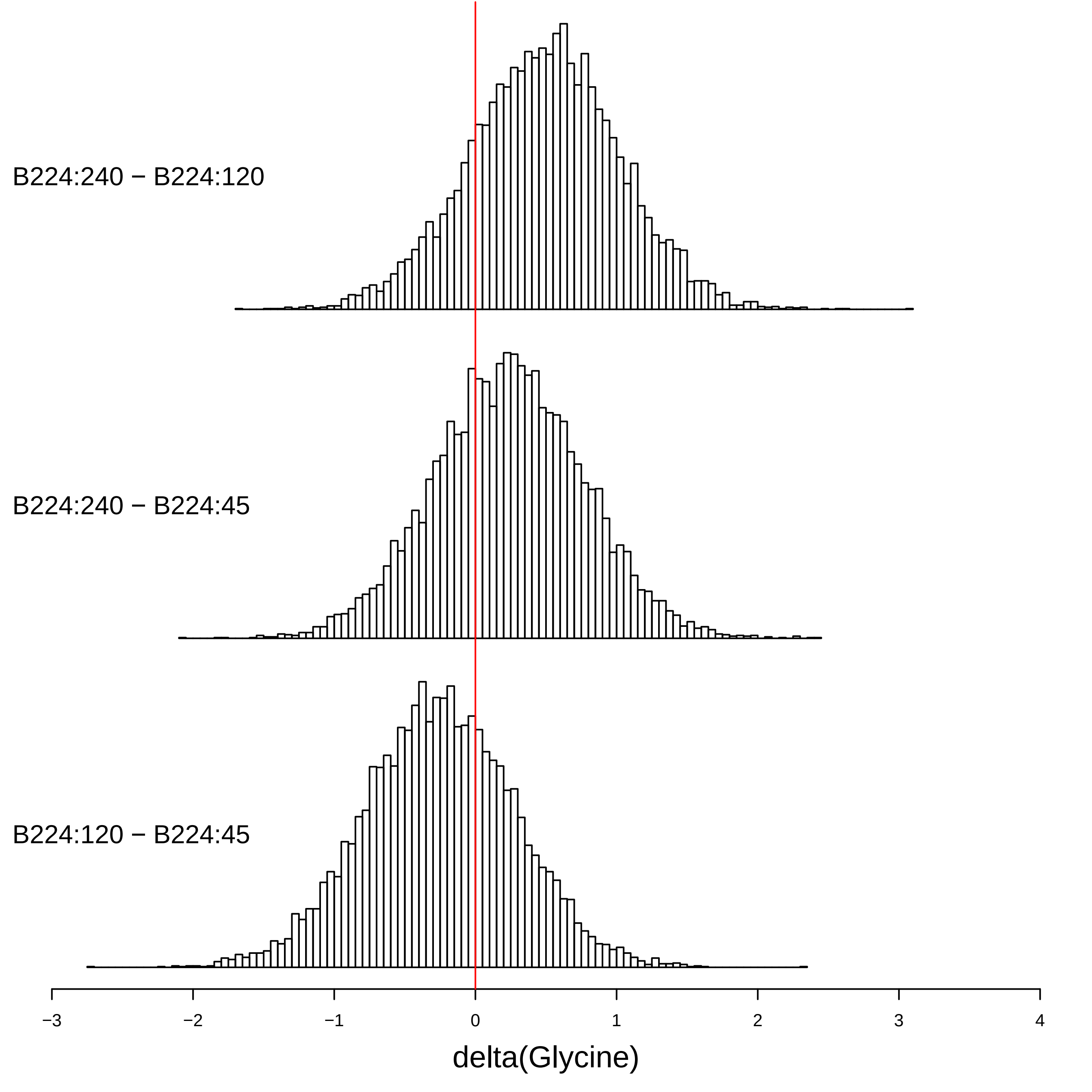

B224:240 − B224:120
B224:240 − B224:45
B224:120 − B224:45
−3
−2
−1
0
1
2
3
4
delta(Glycine)

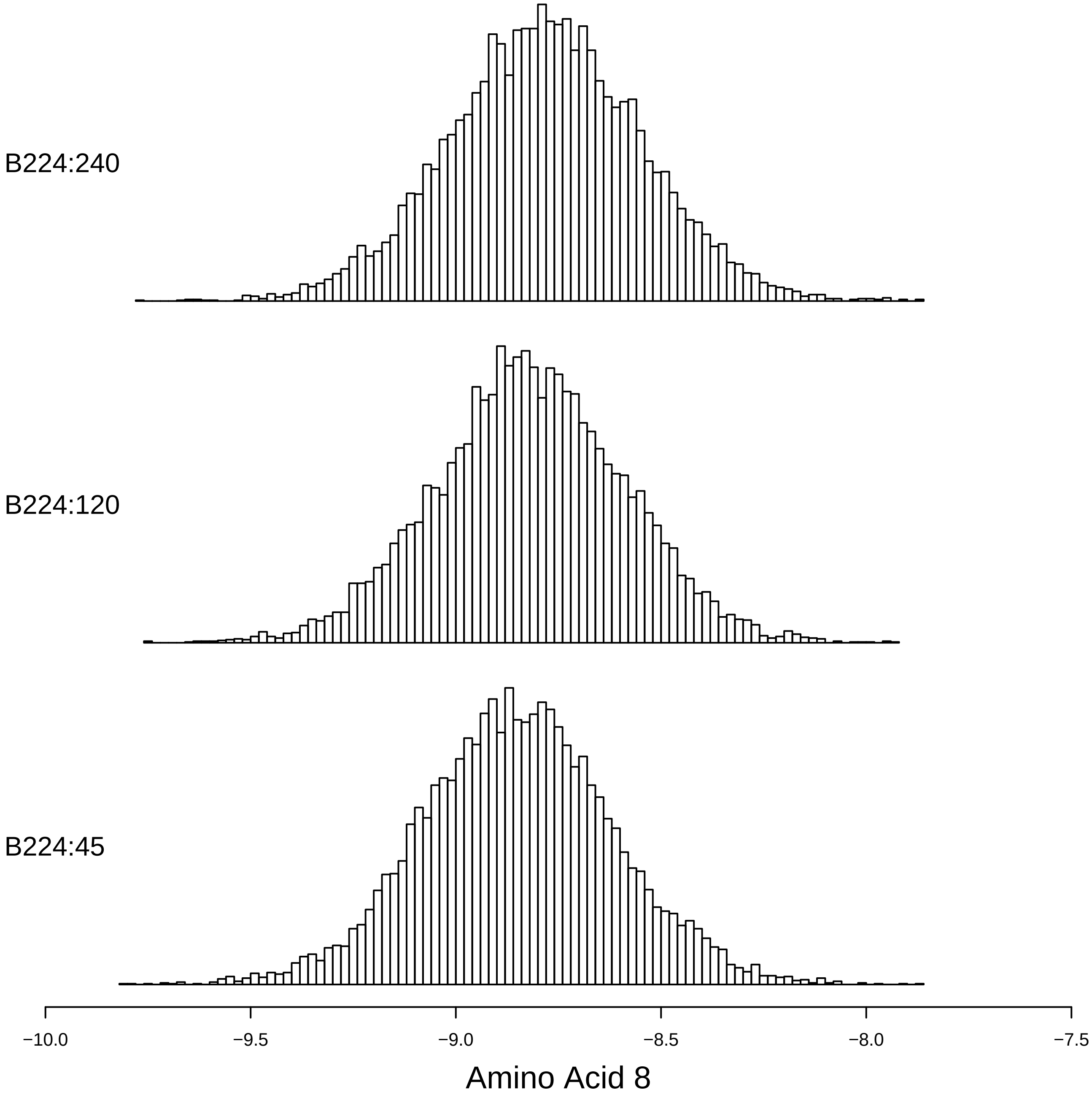
B224:240
B224:120
B224:45
−10.0
−9.5
−9.0
−8.5
−8.0
−7.5
Amino Acid 8

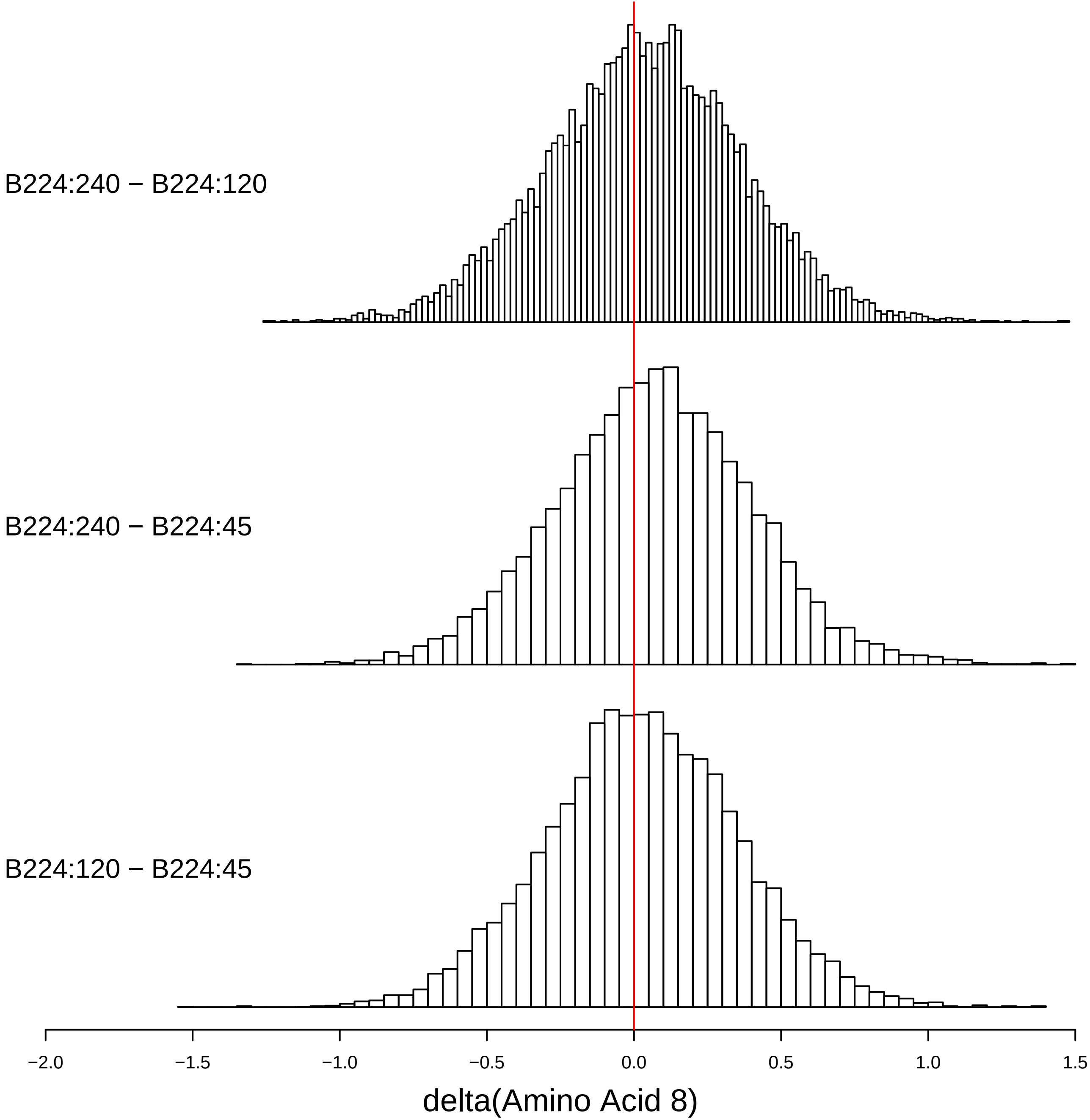

B224:240 − B224:120
B224:240 − B224:45
B224:120 − B224:45
−2.0
−1.5
−1.0
−0.5
0.0
0.5
1.0
1.5
delta(Amino Acid 8)

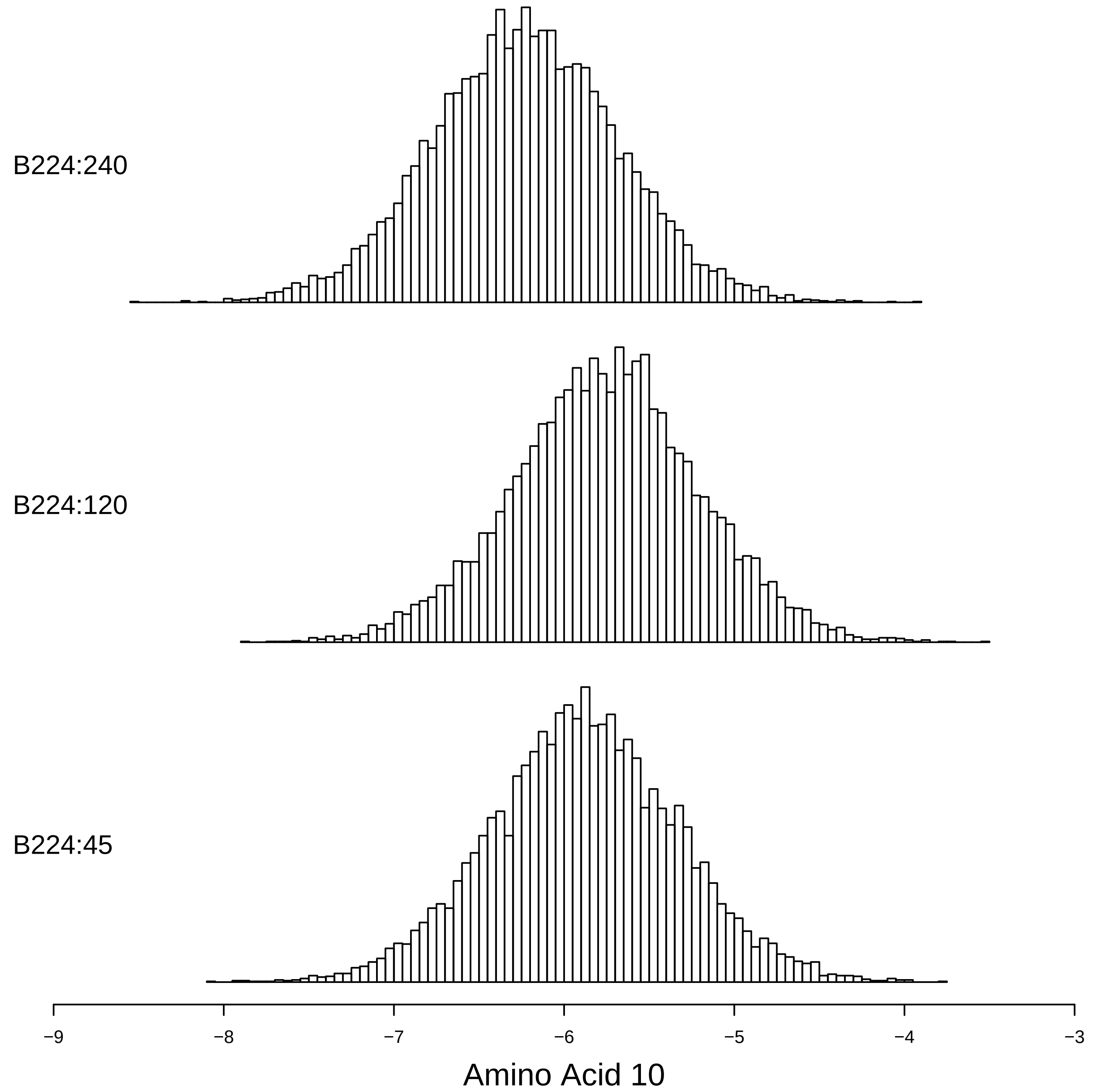
B224:240
B224:120
B224:45
−9
−8
−7
−6
−5
−4
−3
Amino Acid 10

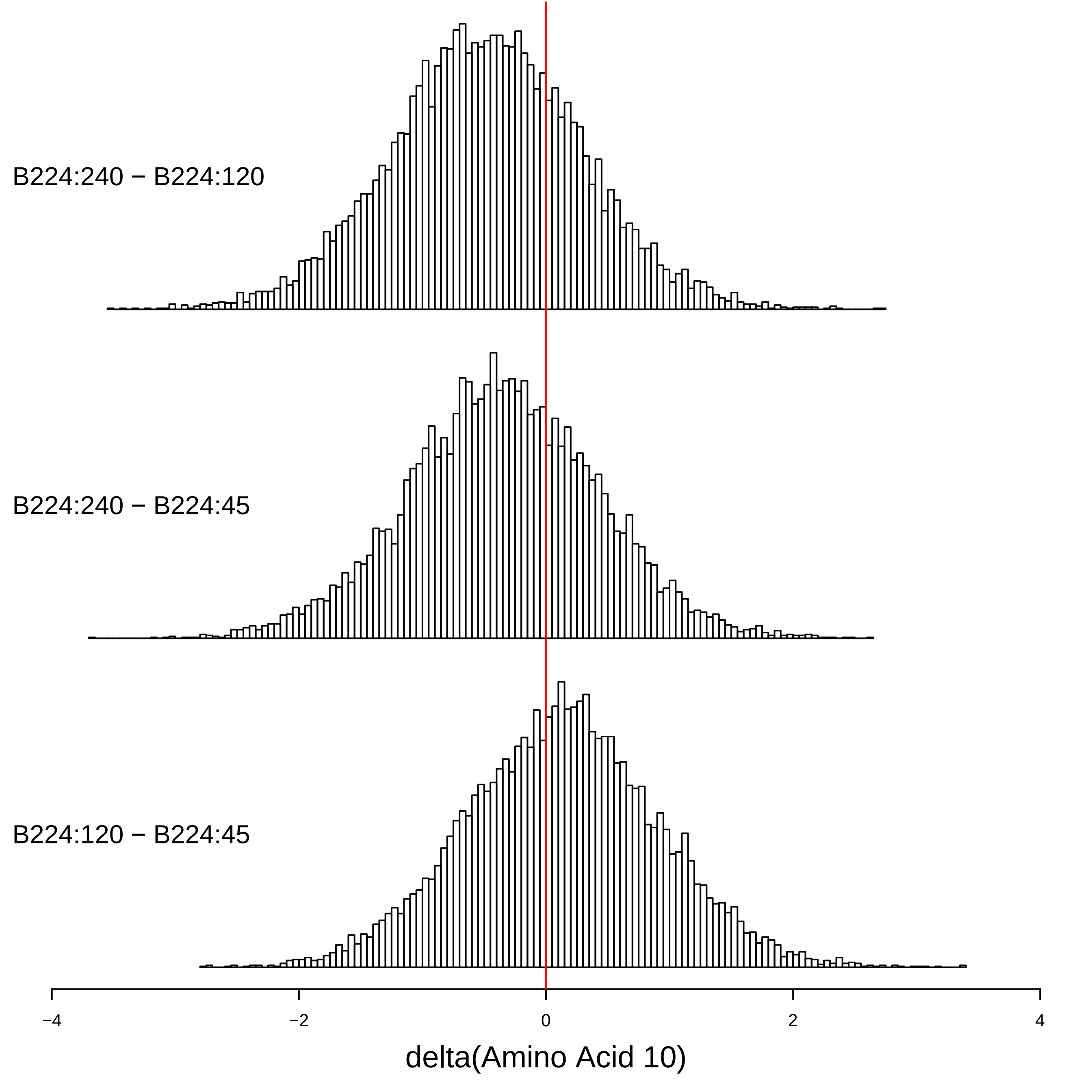

B224:240 − B224:120
B224:240 − B224:45
B224:120 − B224:45
−4
−2
0
2
4
delta(Amino Acid 10)

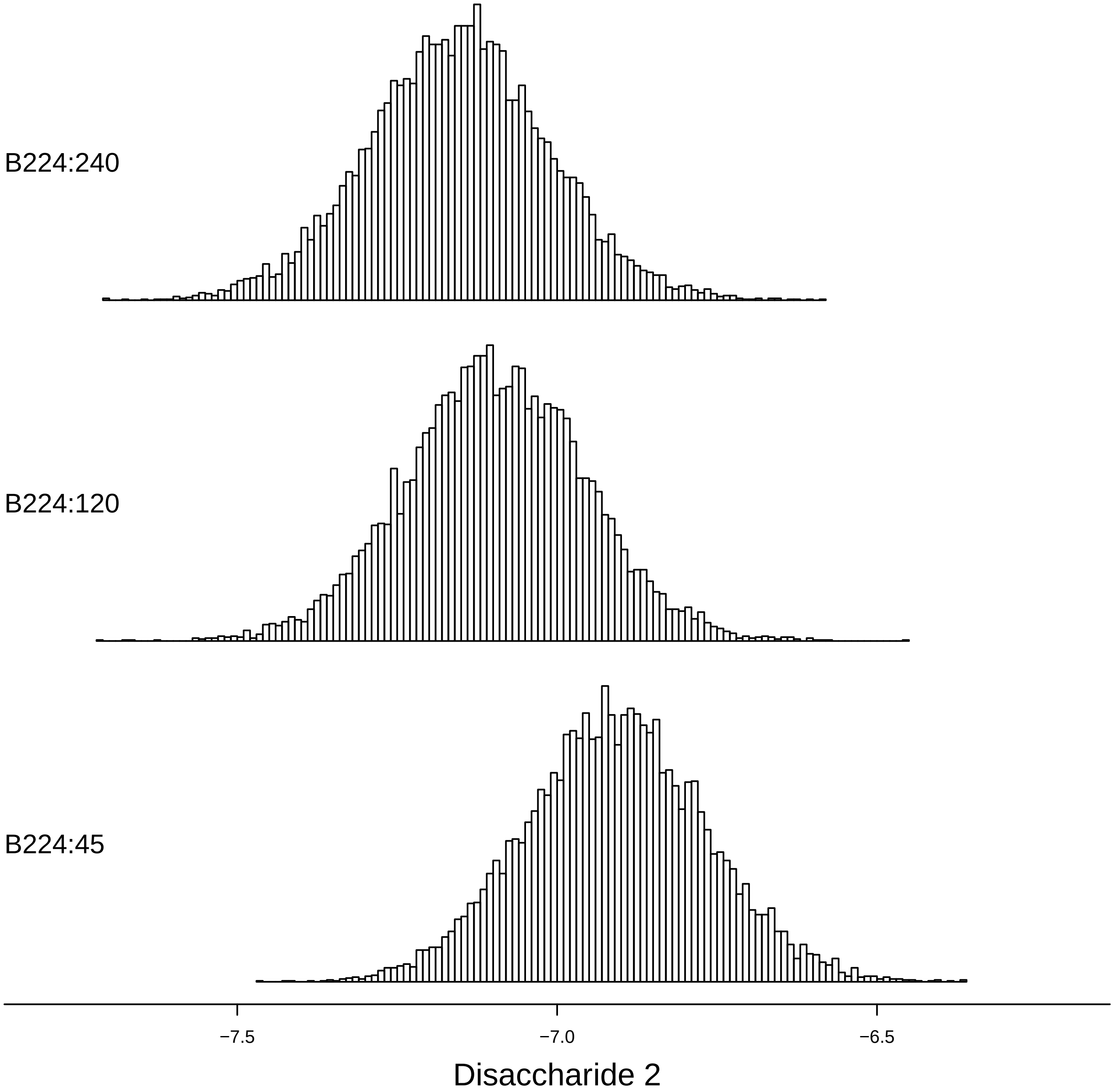

B224:240
B224:120
B224:45
−7.5
−7.0
−6.5
Disaccharide 2

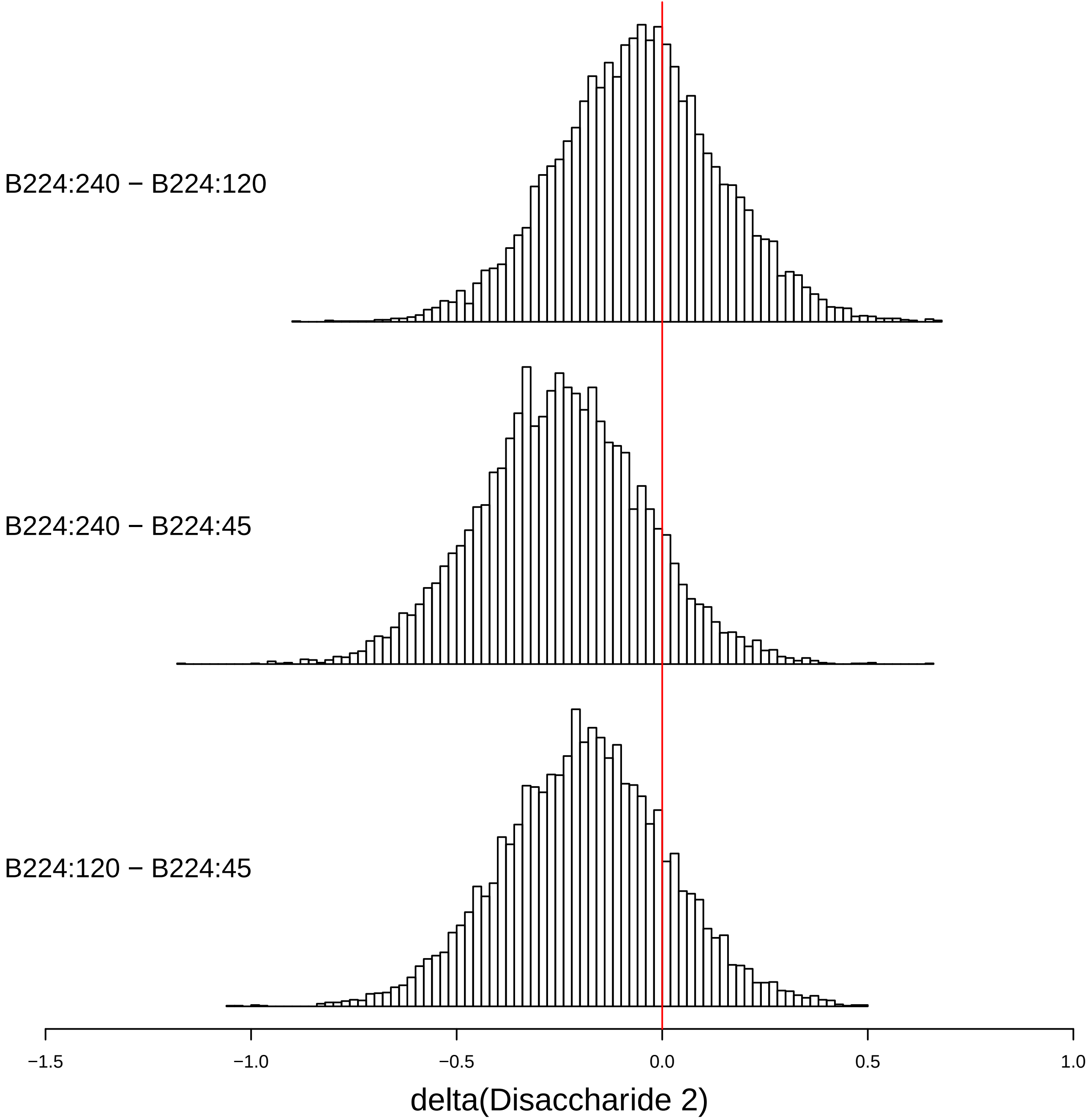
B224:240 − B224:120
B224:240 − B224:45
B224:120 − B224:45
−1.5
−1.0
−0.5
0.0
0.5
1.0
delta(Disaccharide 2)

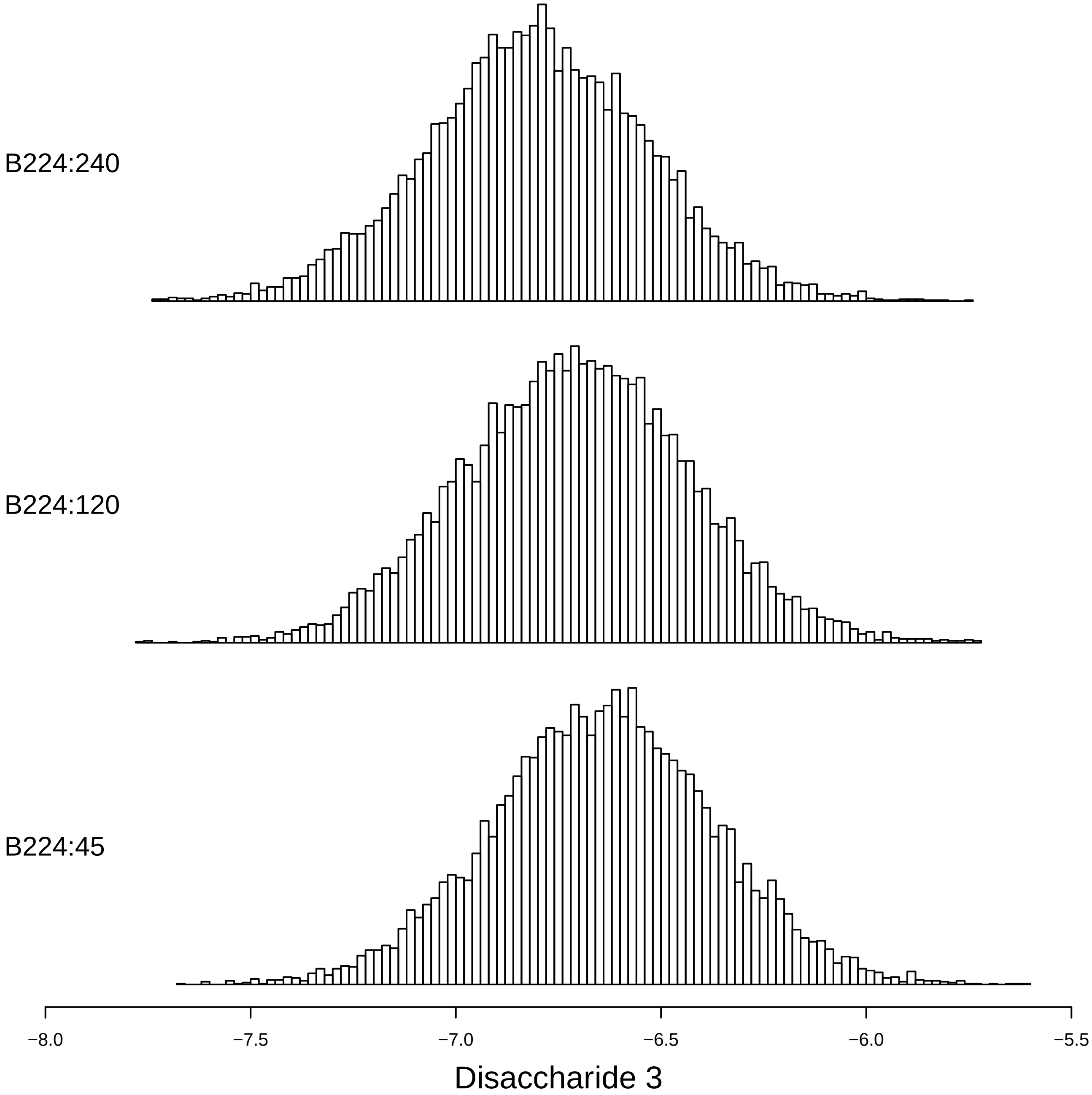

B224:240
B224:120
B224:45
−8.0
−7.5
−7.0
−6.5
−6.0
−5.5
Disaccharide 3

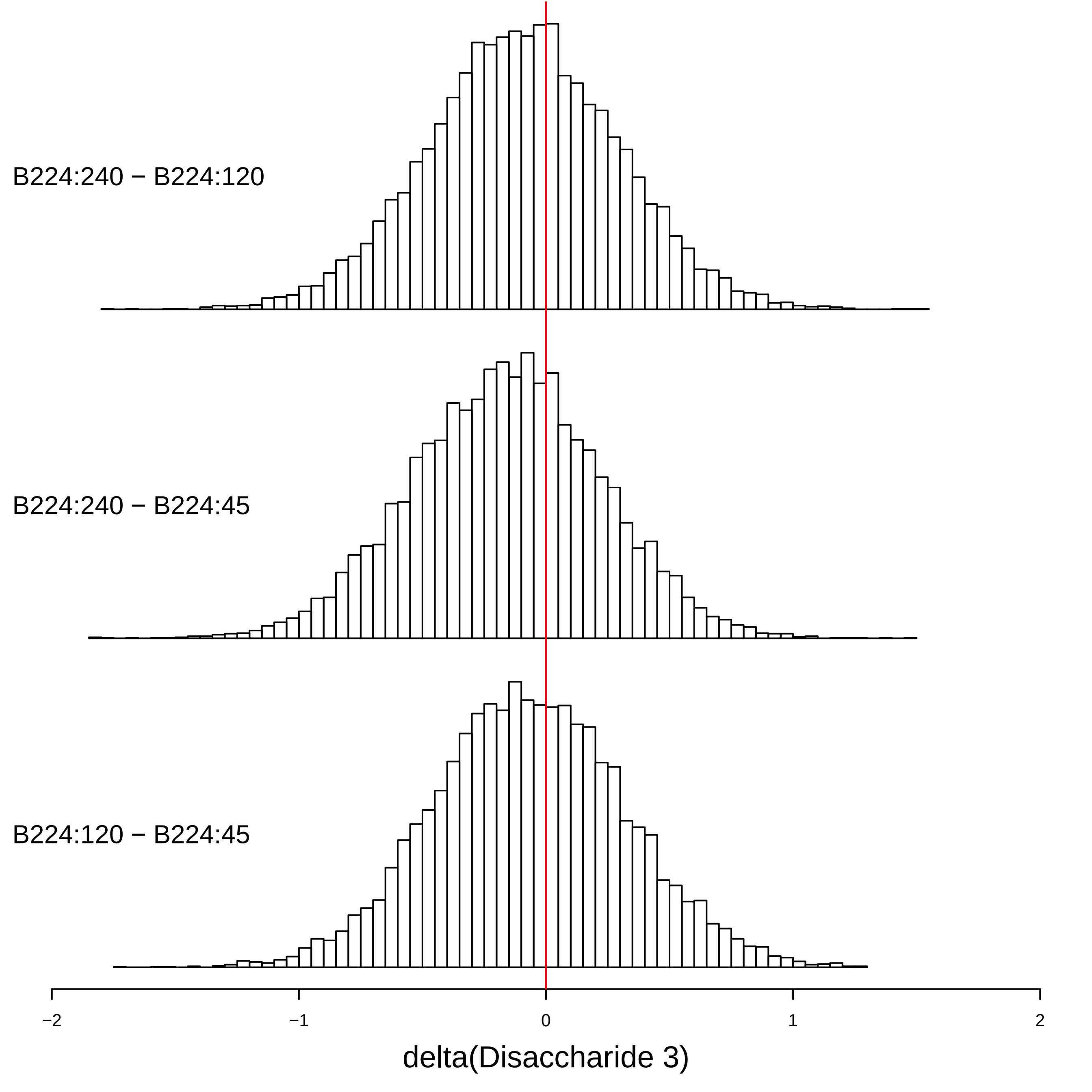
B224:240 − B224:120
B224:240 − B224:45
B224:120 − B224:45
−2
−1
0
1
2
delta(Disaccharide 3)

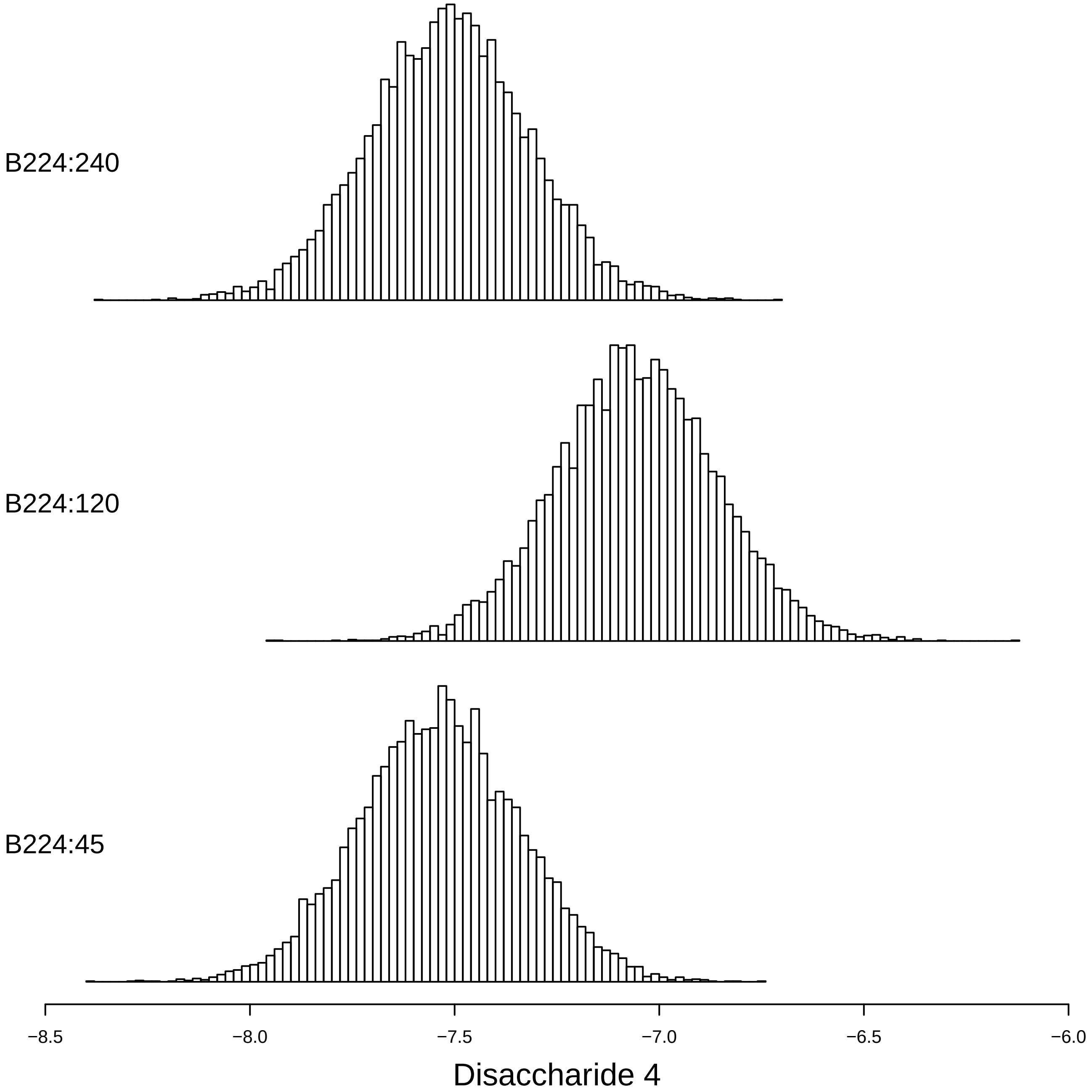
B224:240
B224:120
B224:45
−8.5
−8.0
−7.5
−7.0
−6.5
−6.0
Disaccharide 4

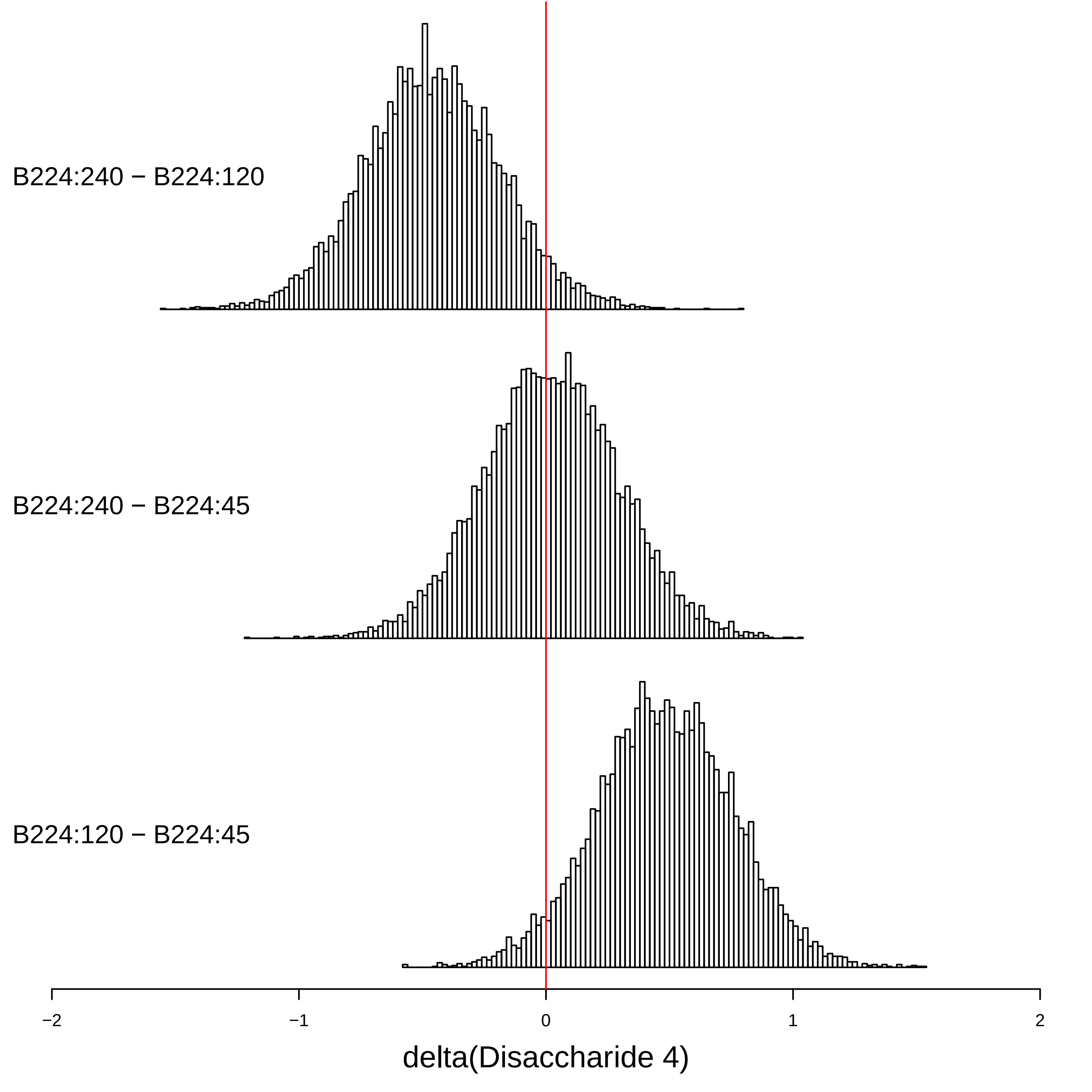
B224:240 − B224:120
B224:240 − B224:45
B224:120 − B224:45
−2
−1
0
1
2
delta(Disaccharide 4)

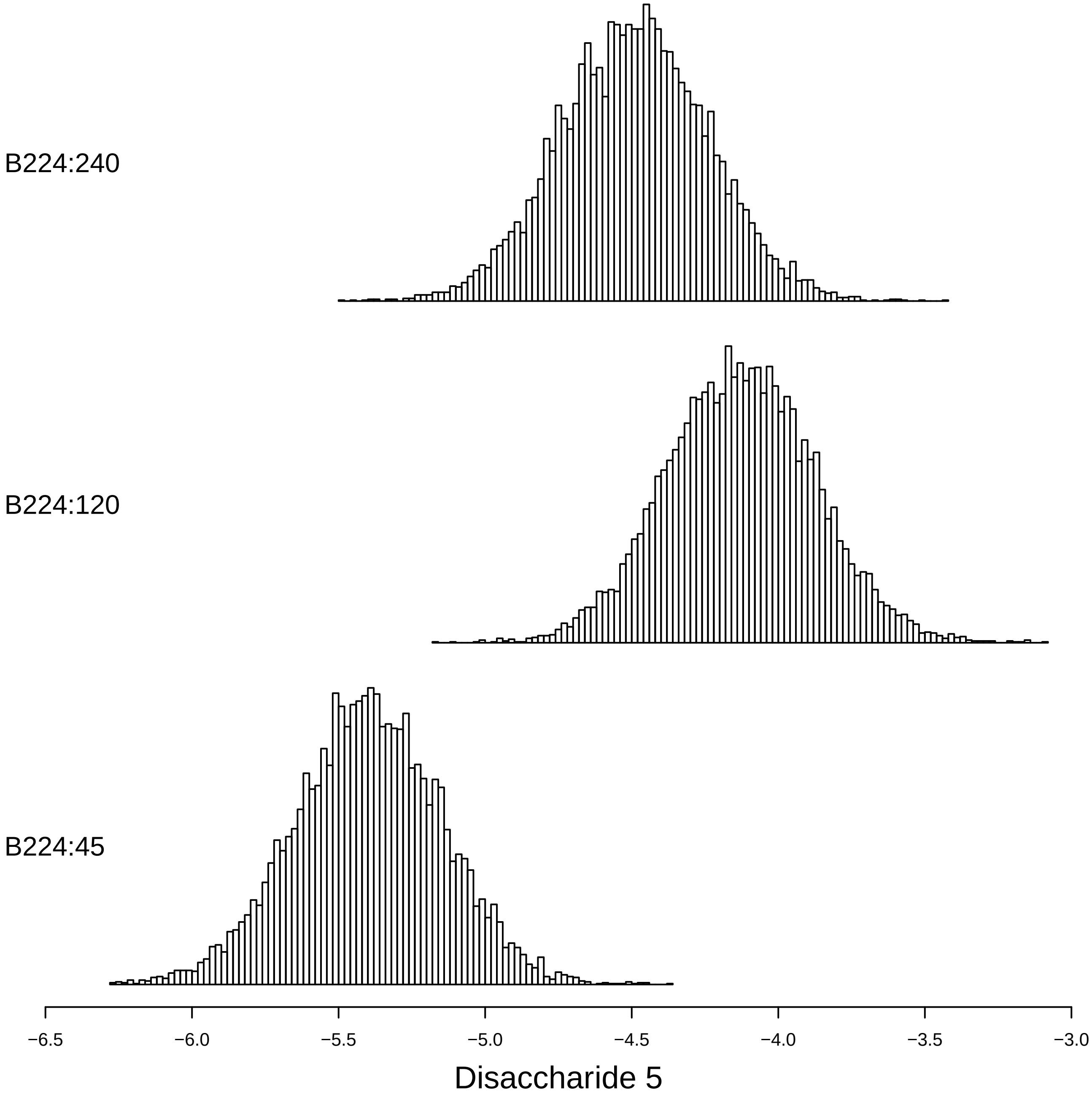

B224:240
B224:120
B224:45
−6.5
−6.0
−5.5
−5.0
−4.5
−4.0
−3.5
−3.0
Disaccharide 5

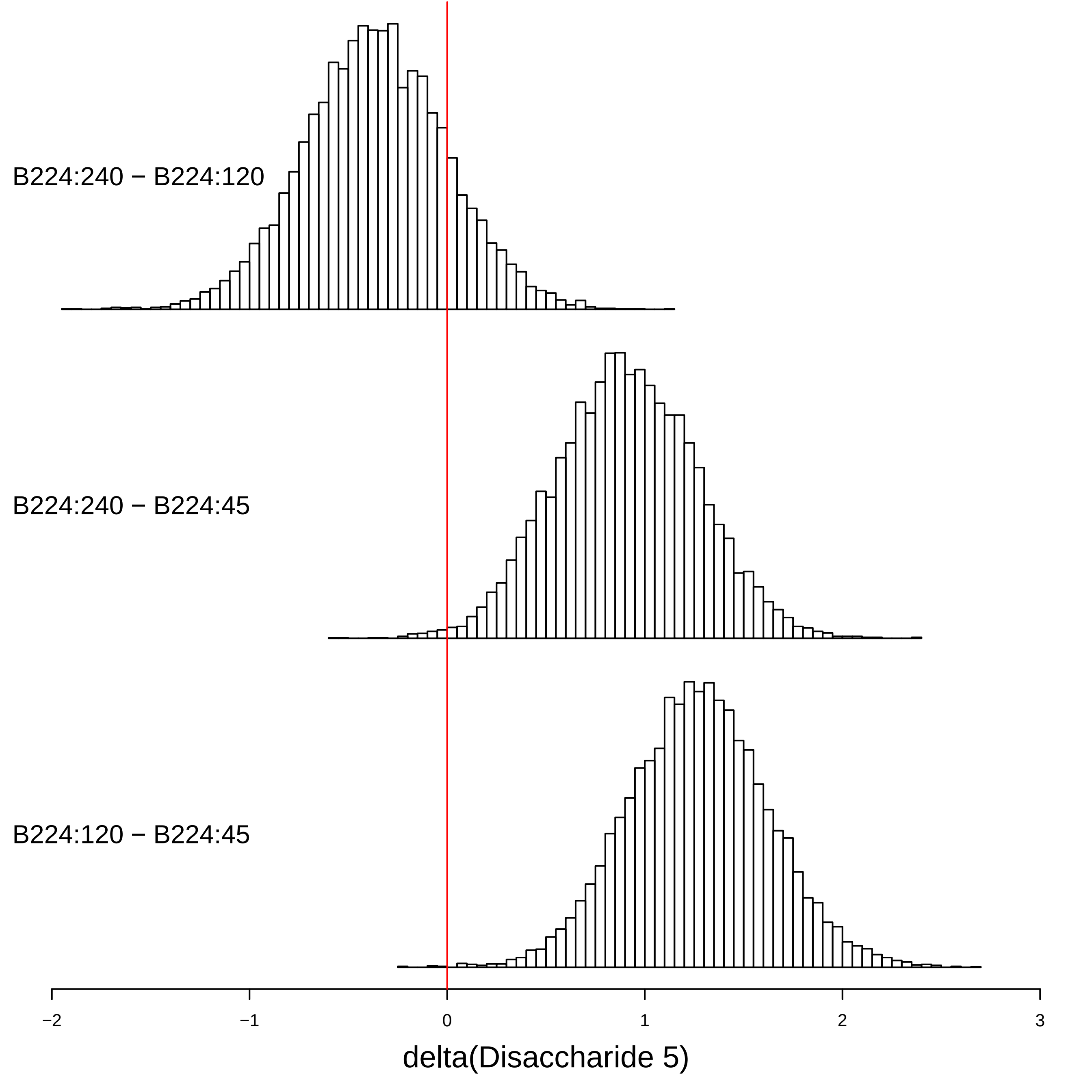

B224:240 − B224:120
B224:240 − B224:45
B224:120 − B224:45
−2
−1
0
1
2
3
delta(Disaccharide 5)

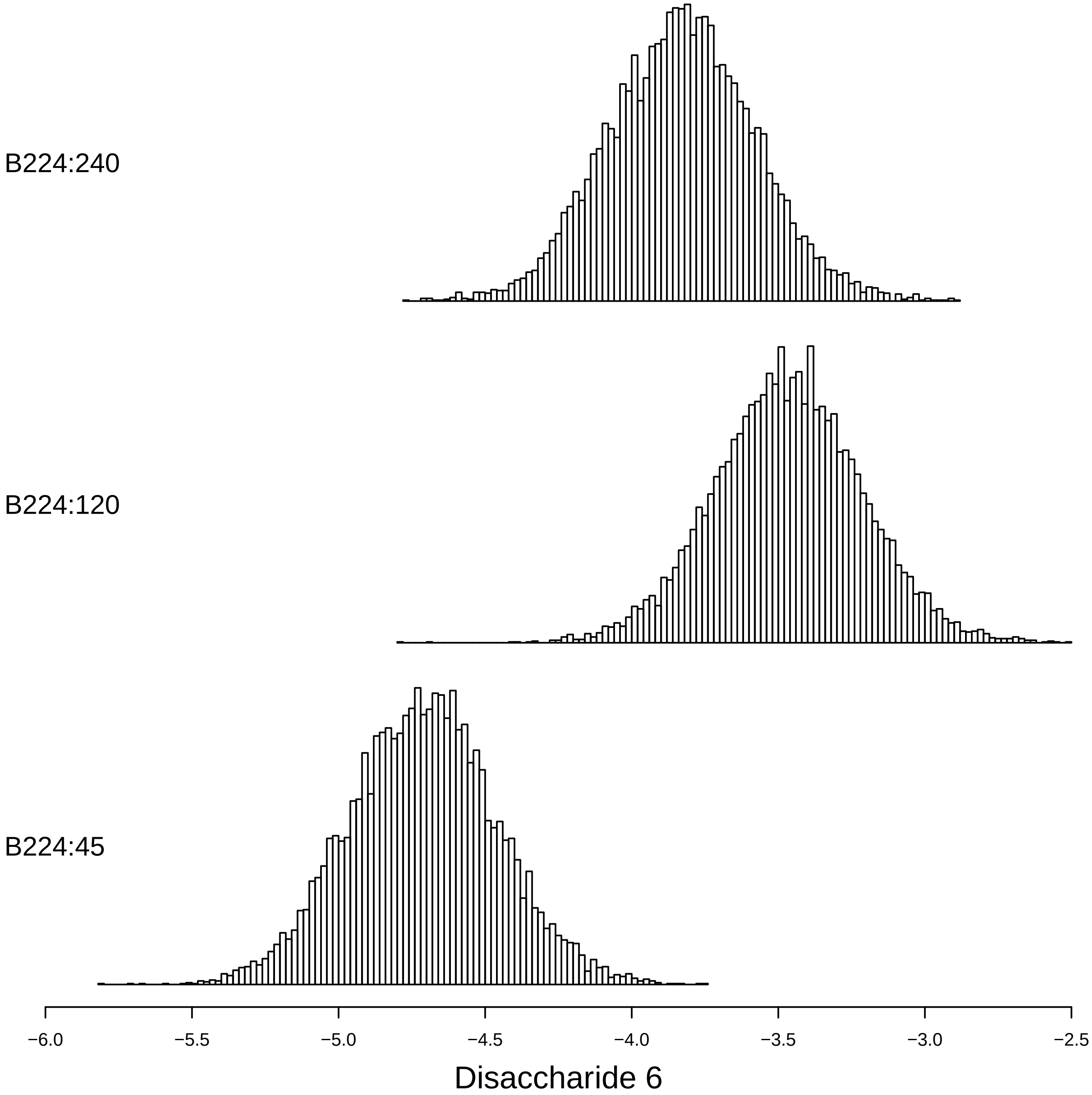

B224:240
B224:120
B224:45
−6.0
−5.5
−5.0
−4.5
−4.0
−3.5
−3.0
−2.5
Disaccharide 6

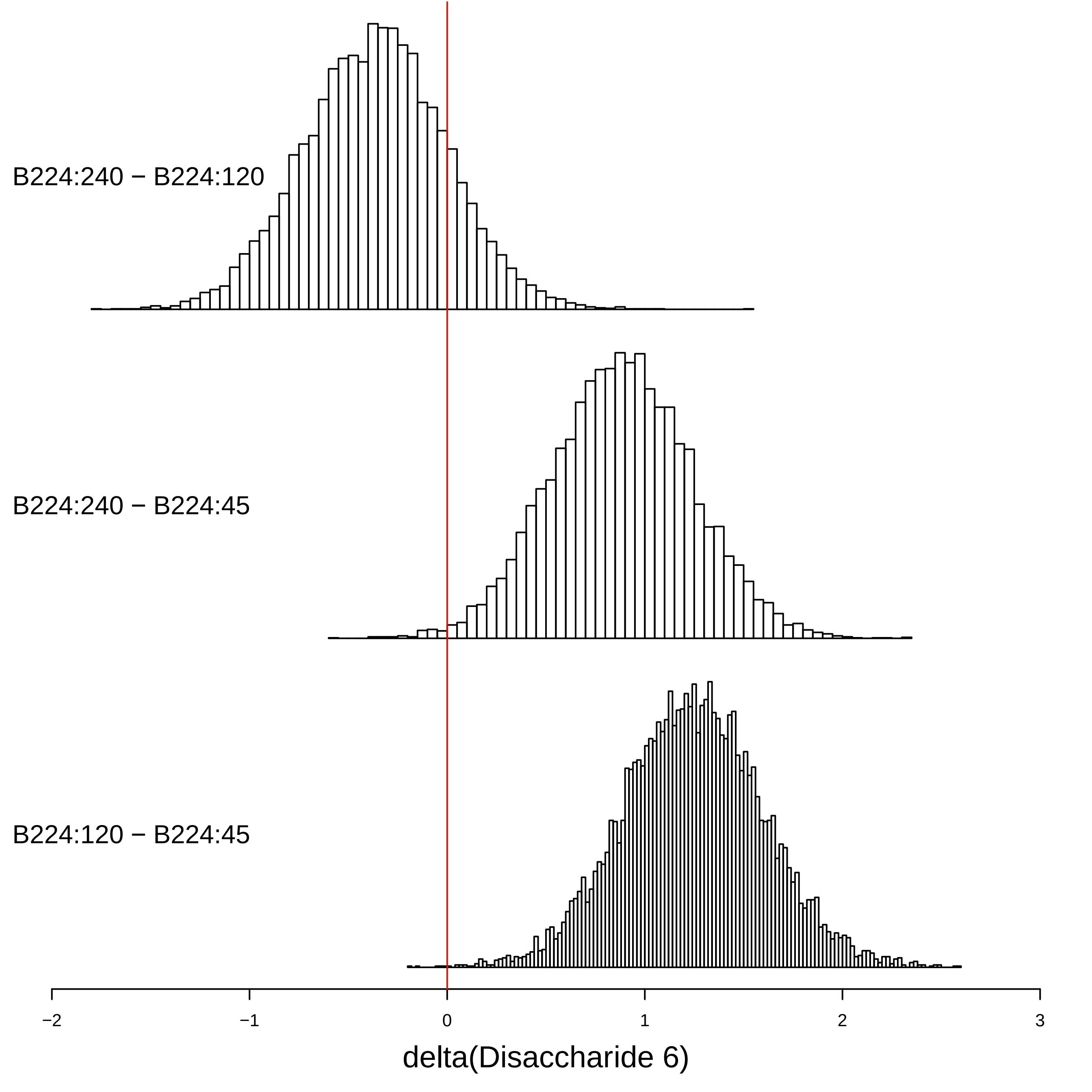

B224:240 − B224:120
B224:240 − B224:45
B224:120 − B224:45
−2
−1
0
1
2
3
delta(Disaccharide 6)

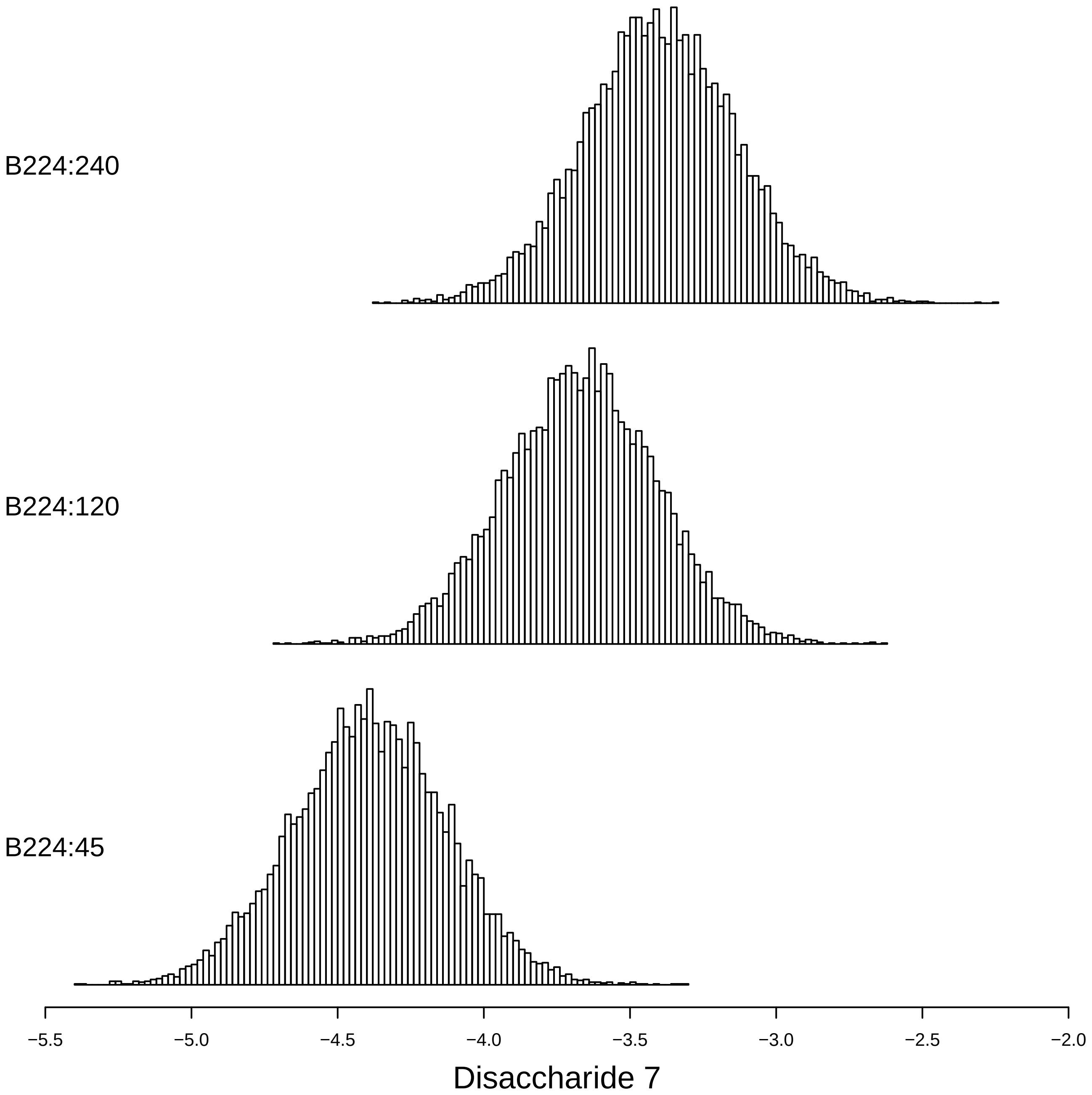
B224:240
B224:120
B224:45
−5.5
−5.0
−4.5
−4.0
−3.5
−3.0
−2.5
−2.0
Disaccharide 7

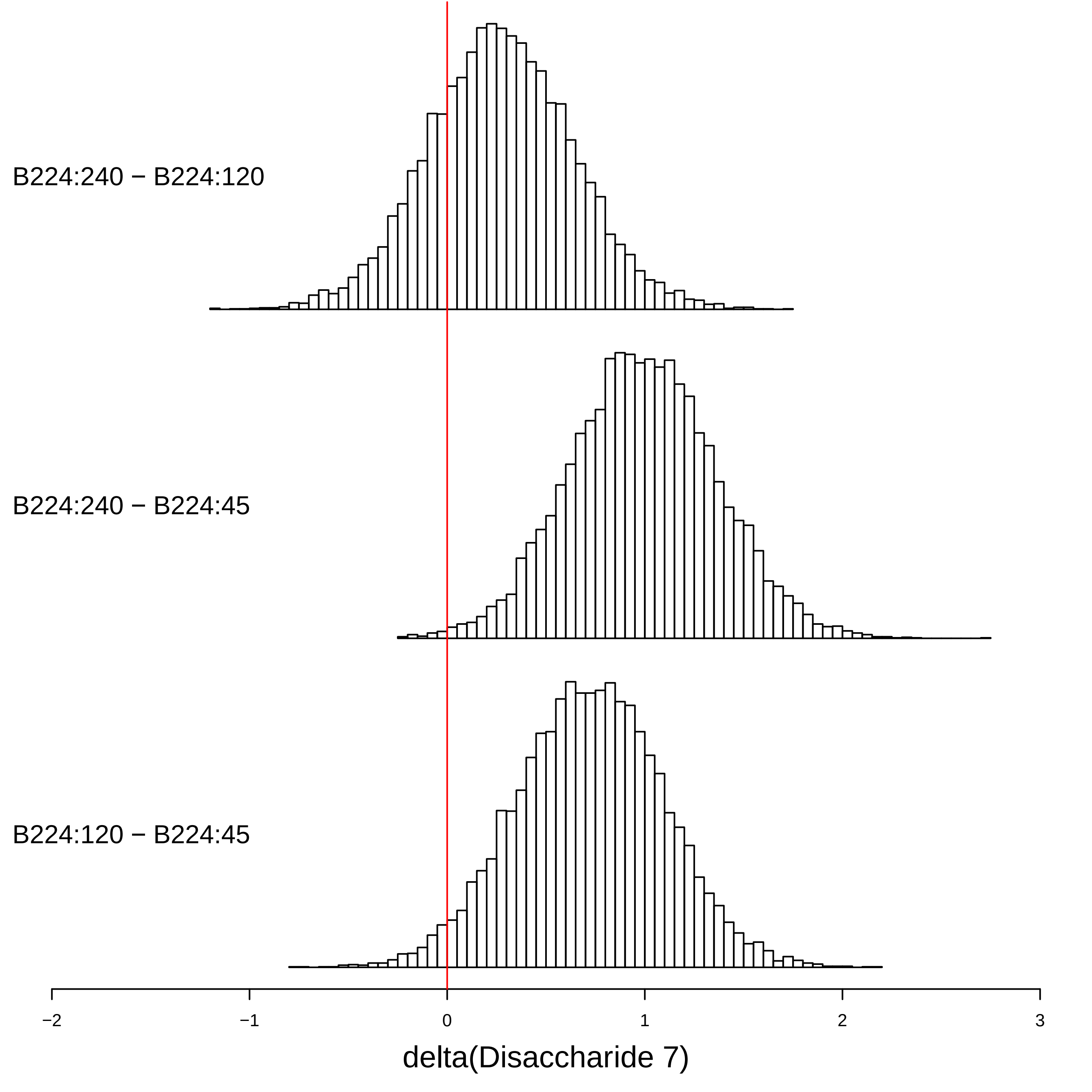
B224:240 − B224:120
B224:240 − B224:45
B224:120 − B224:45
−2
−1
0
1
2
3
delta(Disaccharide 7)

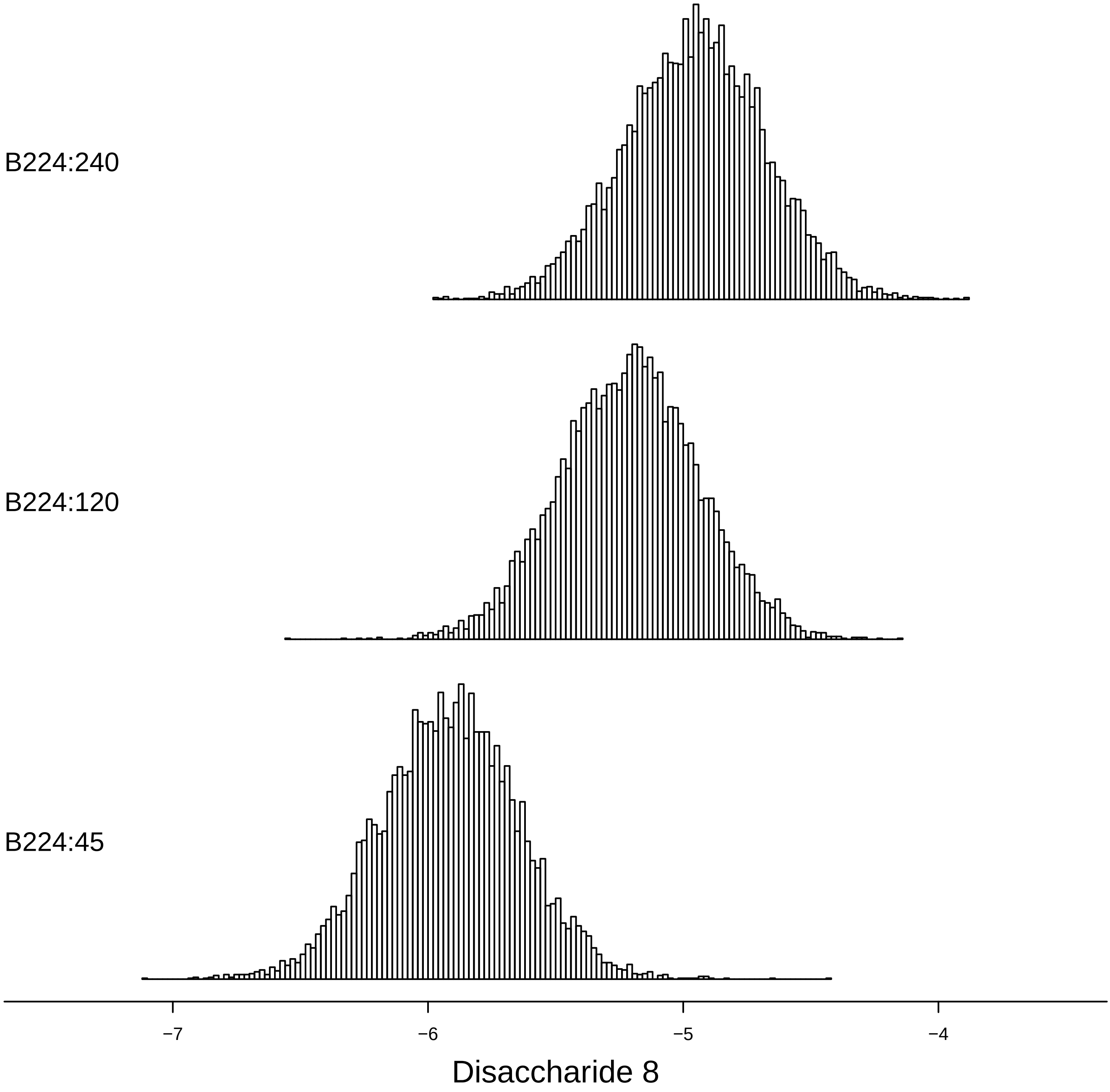

B224:240
B224:120
B224:45
−7
−6
−5
−4
Disaccharide 8

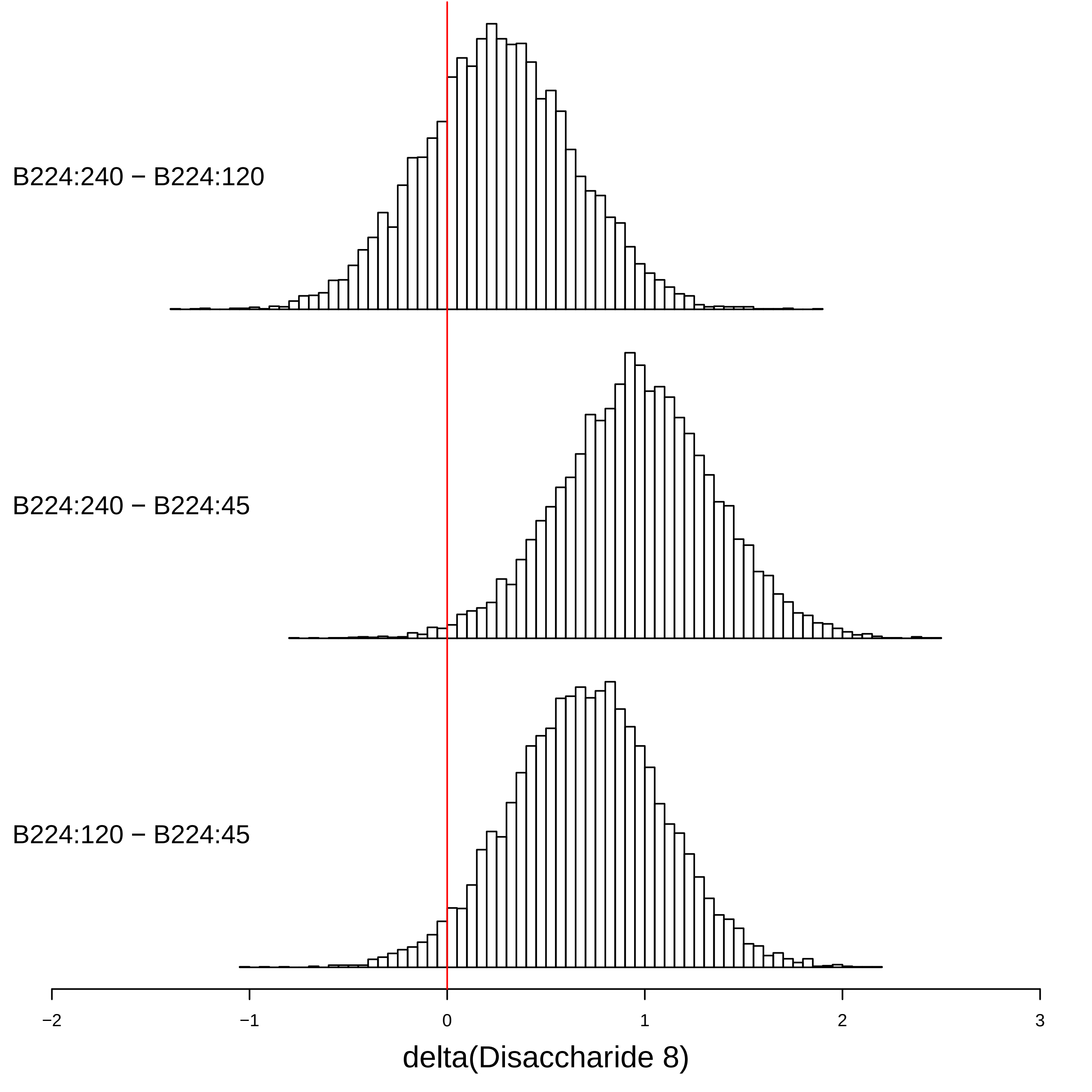

B224:240 − B224:120
B224:240 − B224:45
B224:120 − B224:45
−2
−1
0
1
2
3
delta(Disaccharide 8)

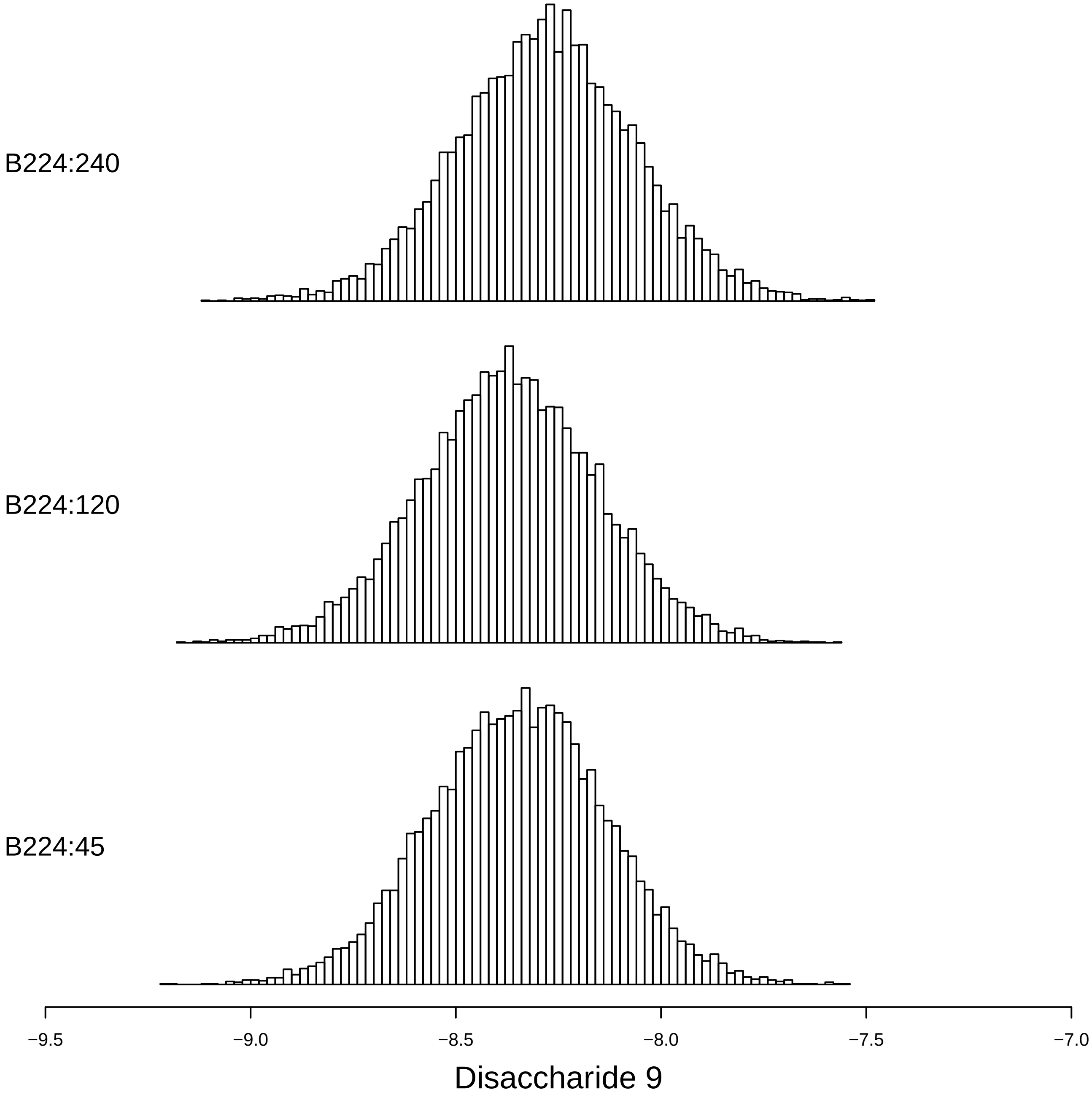
B224:240
B224:120
B224:45
−9.5
−9.0
−8.5
−8.0
−7.5
−7.0
Disaccharide 9

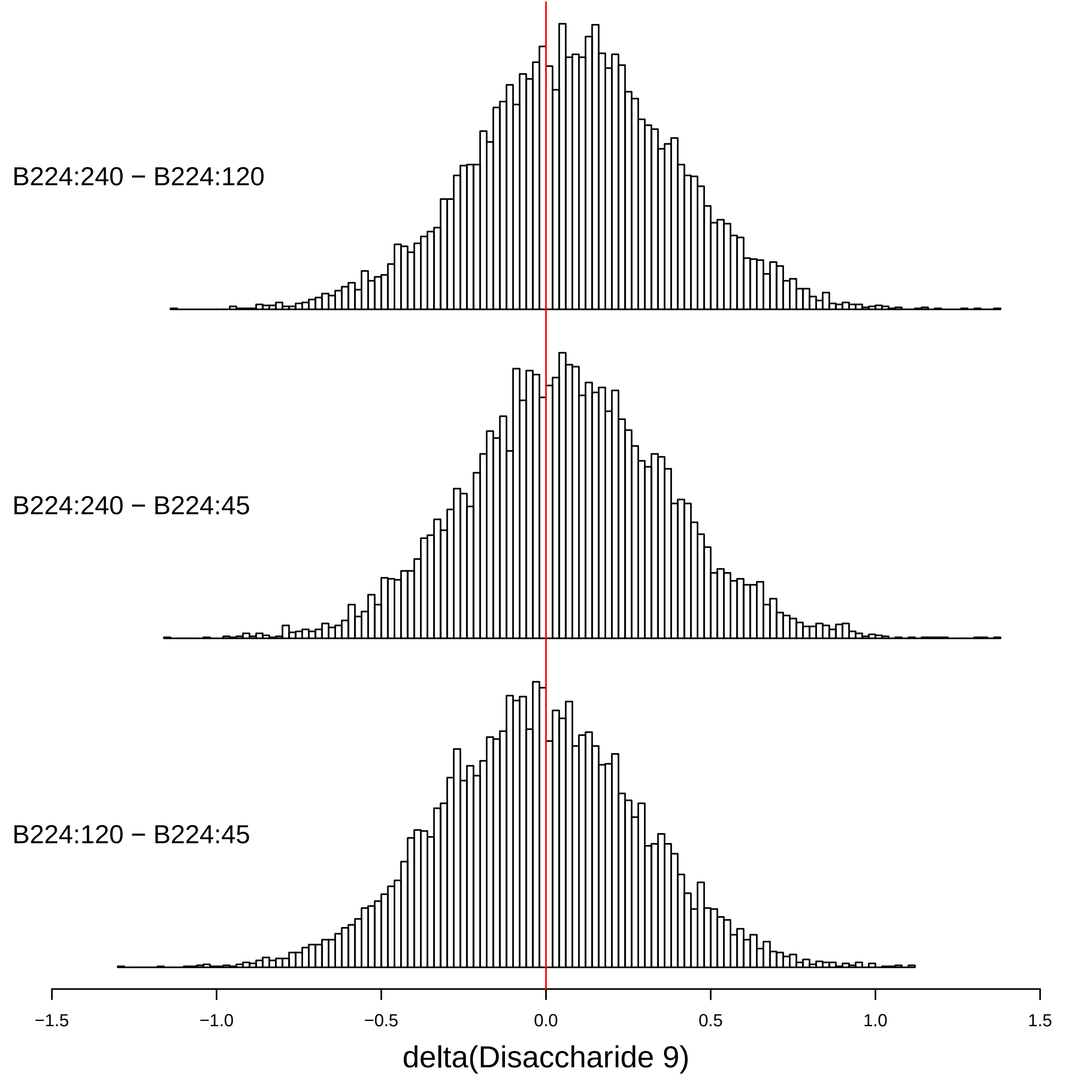
B224:240 − B224:120
B224:240 − B224:45
B224:120 − B224:45
−1.5
−1.0
−0.5
0.0
0.5
1.0
1.5
delta(Disaccharide 9)

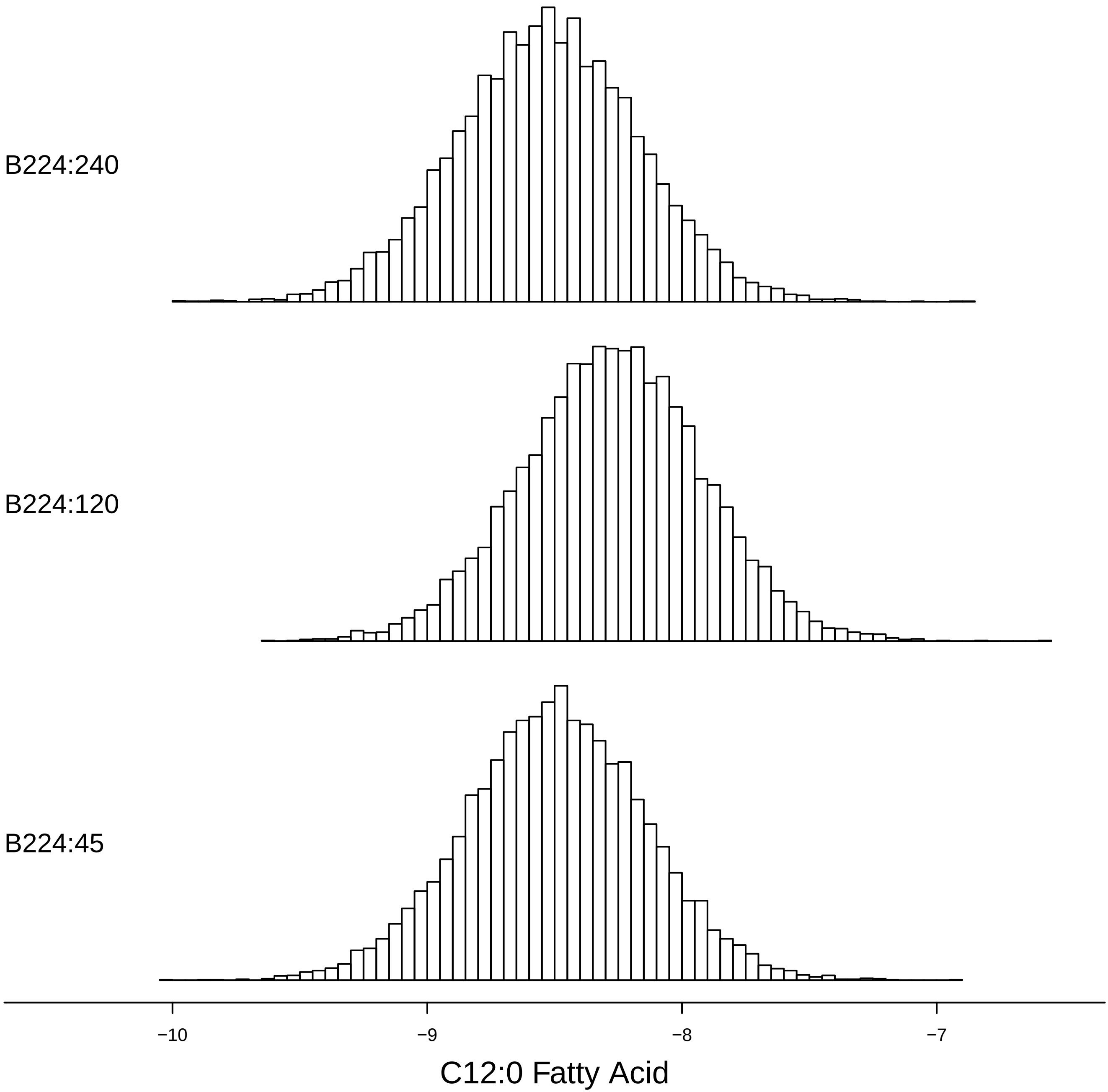
B224:240
B224:120
B224:45
−10
−9
−8
−7
C12:0 Fatty Acid

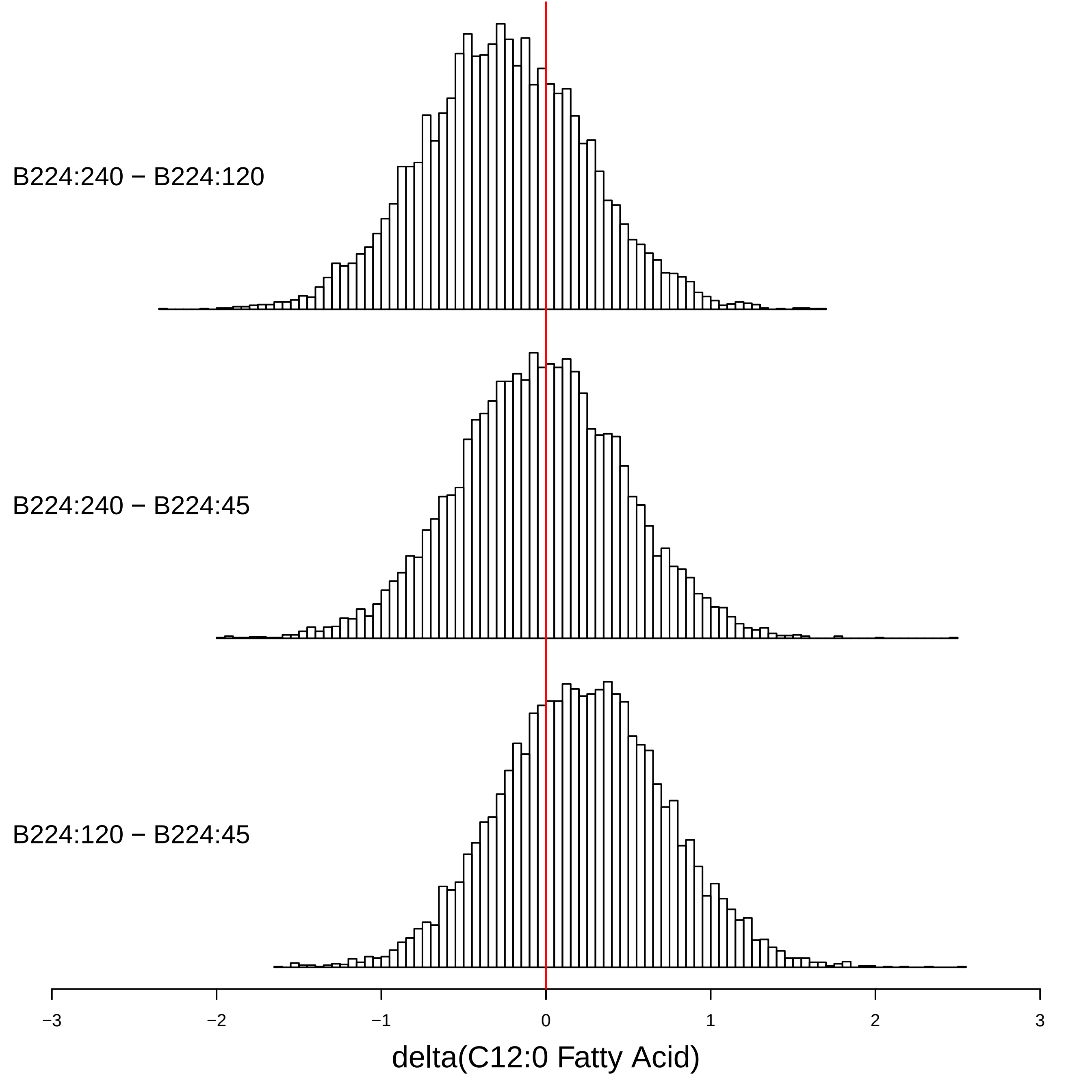

B224:240 − B224:120
B224:240 − B224:45
B224:120 − B224:45
−3
−2
−1
0
1
2
3
delta(C12:0 Fatty Acid)

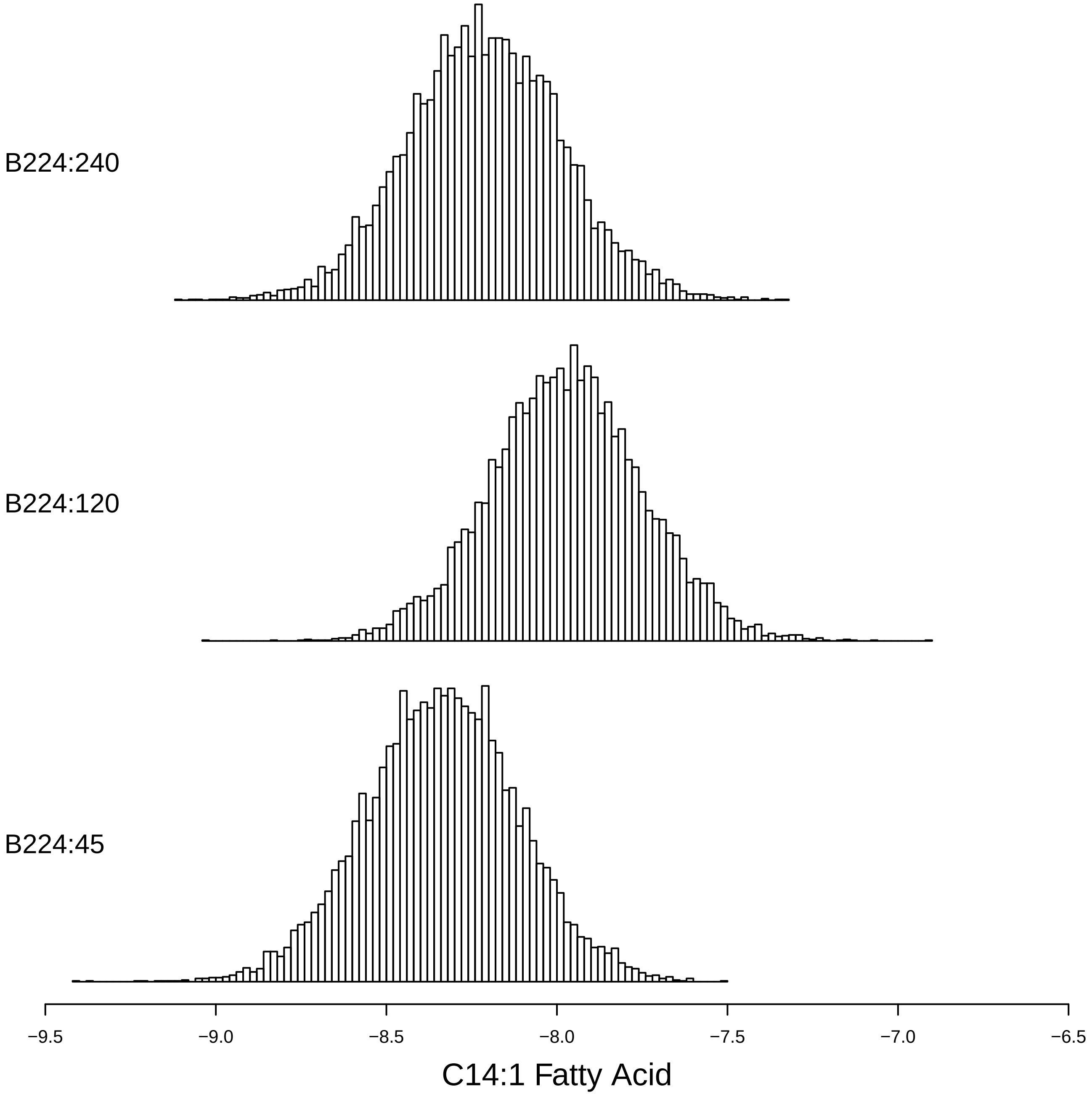
B224:240
B224:120
B224:45
−9.5
−9.0
−8.5
−8.0
−7.5
−7.0
−6.5
C14:1 Fatty Acid

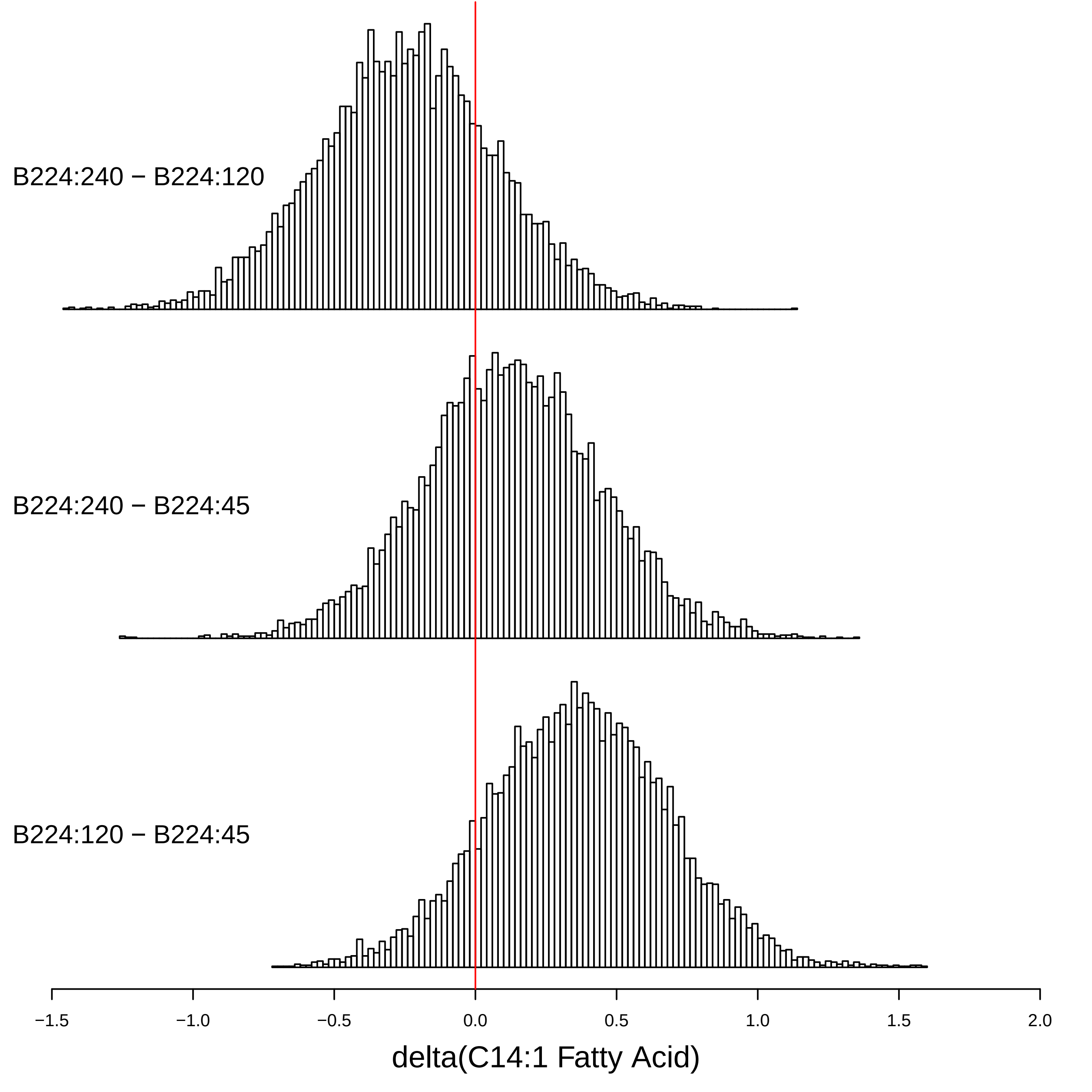

B224:240 − B224:120
B224:240 − B224:45
B224:120 − B224:45
−1.5
−1.0
−0.5
0.0
0.5
1.0
1.5
2.0
delta(C14:1 Fatty Acid)

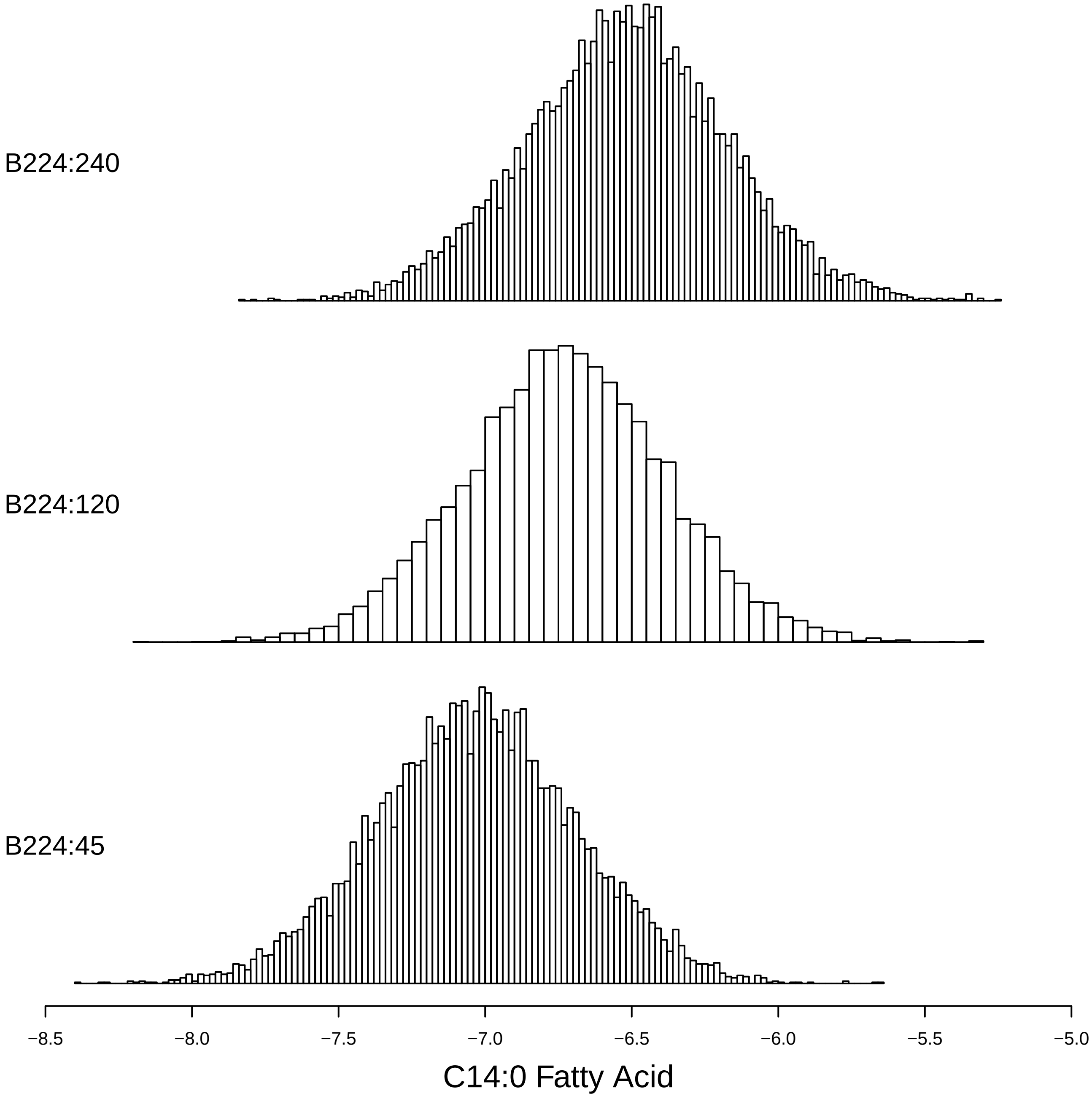

B224:240
B224:120
B224:45
−8.5
−8.0
−7.5
−7.0
−6.5
−6.0
−5.5
−5.0
C14:0 Fatty Acid

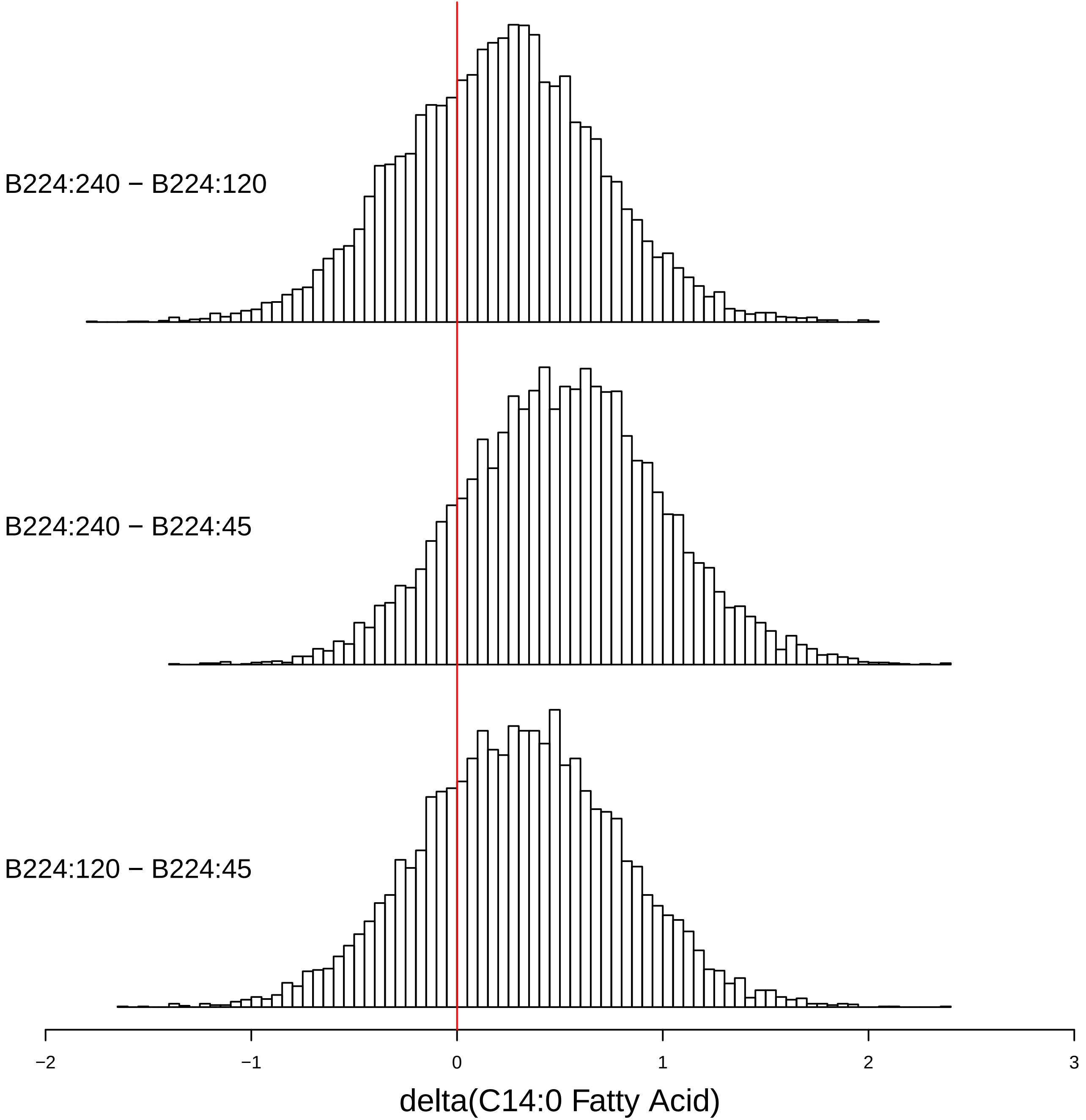
B224:240 − B224:120
B224:240 − B224:45
B224:120 − B224:45
−2
−1
0
1
2
3
delta(C14:0 Fatty Acid)

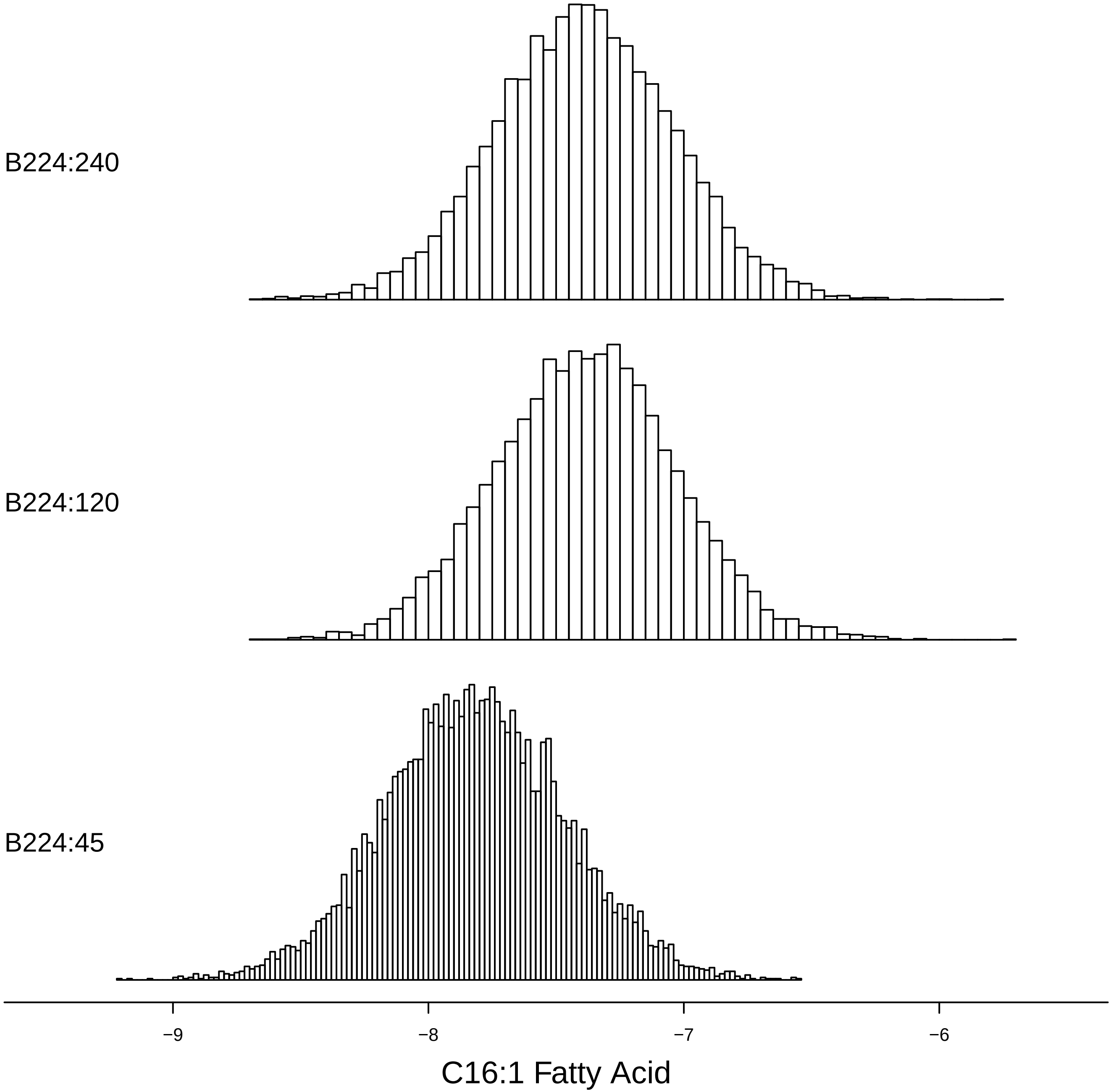
B224:240
B224:120
B224:45
−9
−8
−7
−6
C16:1 Fatty Acid

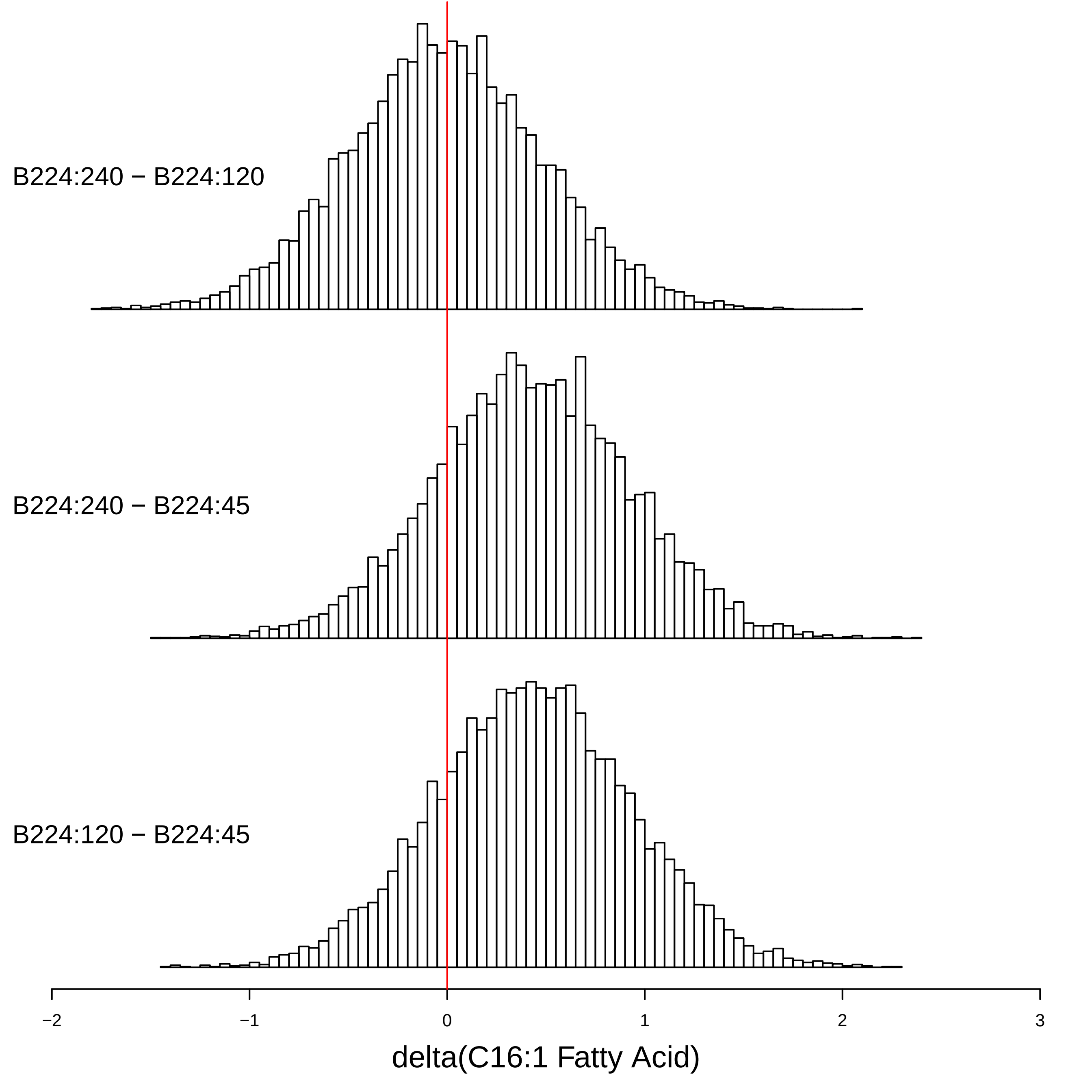

B224:240 − B224:120
B224:240 − B224:45
B224:120 − B224:45
−2
−1
0
1
2
3
delta(C16:1 Fatty Acid)

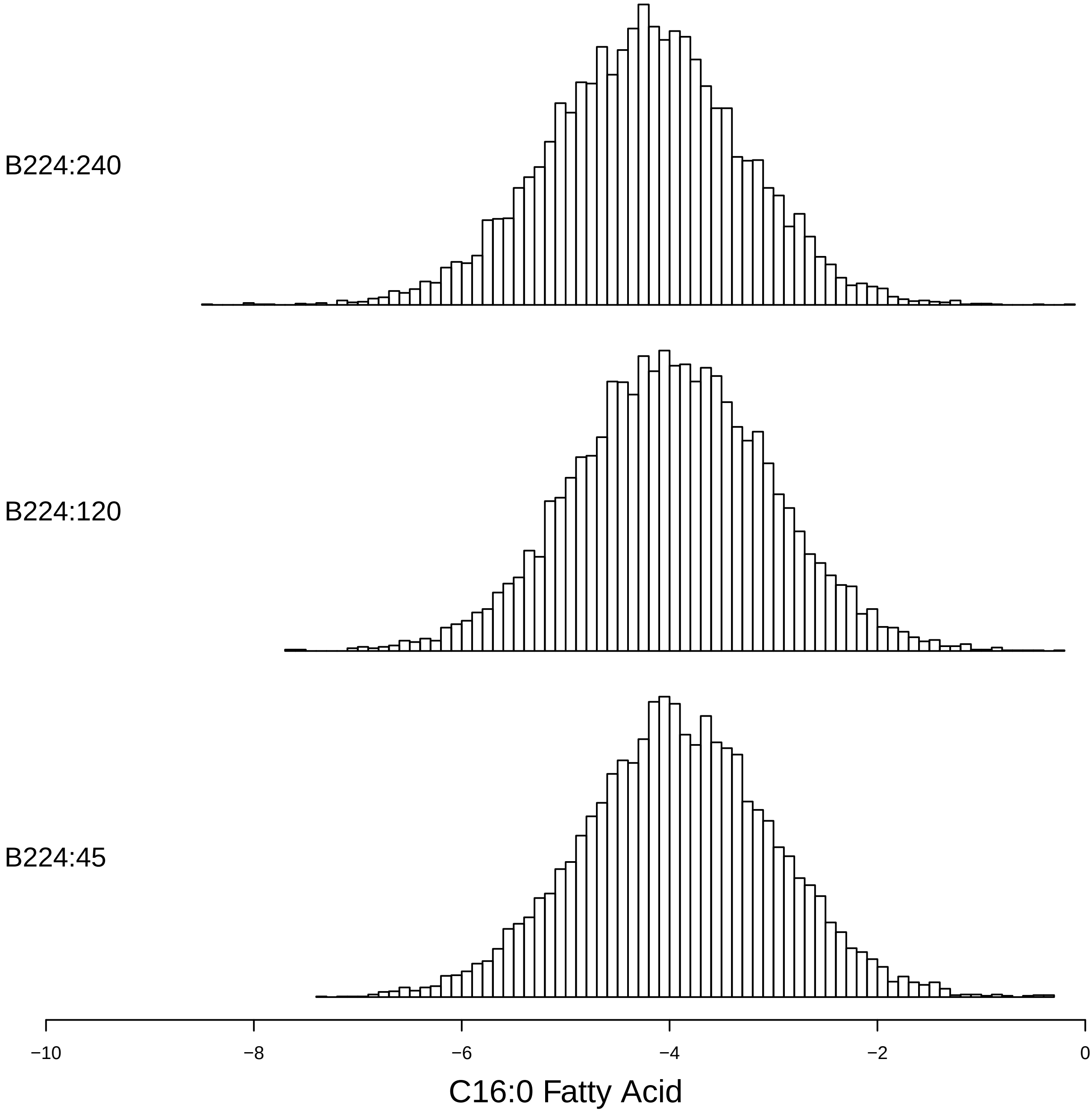
B224:240
B224:120
B224:45
−10
−8
−6
−4
−2
0
C16:0 Fatty Acid

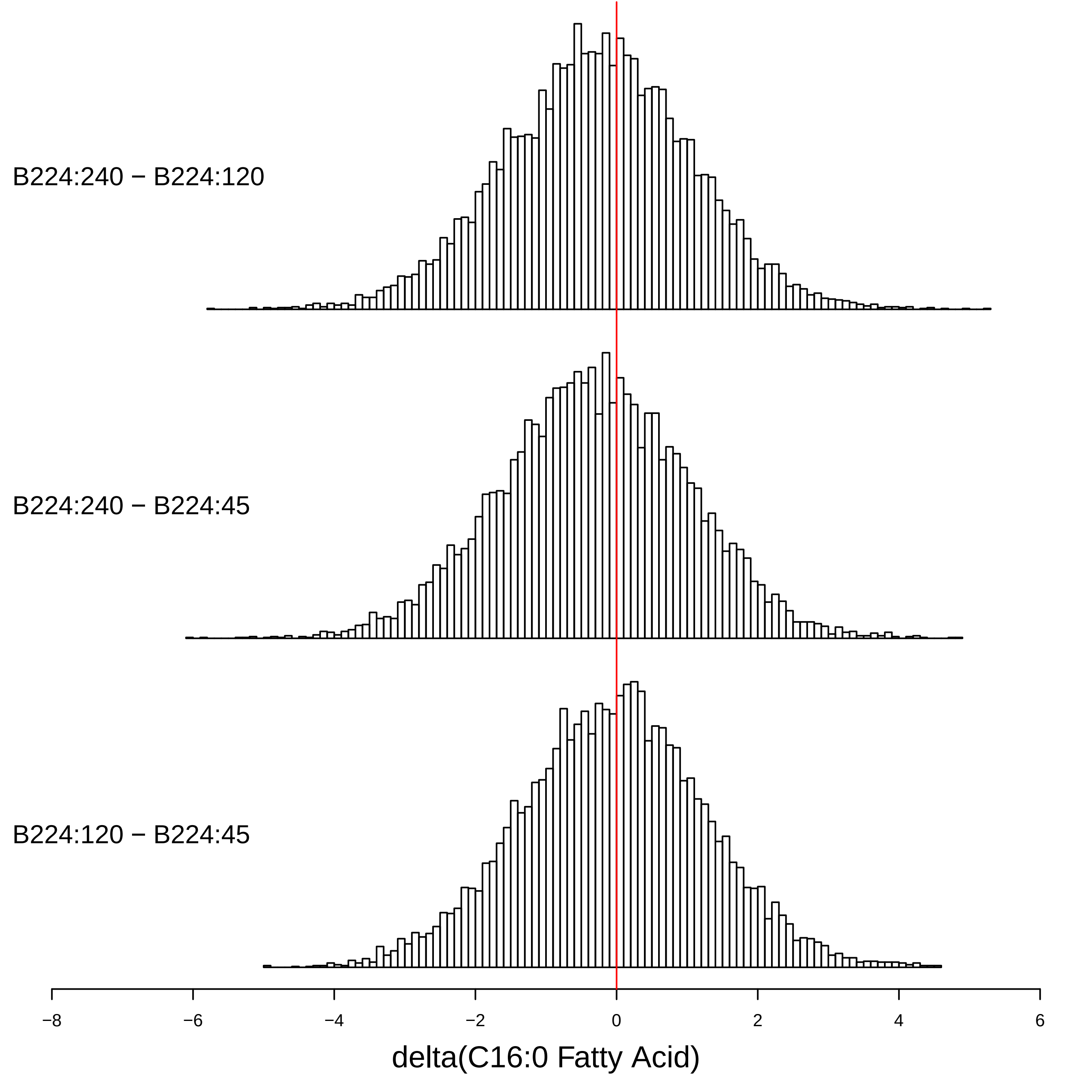
B224:240 − B224:120
B224:240 − B224:45
B224:120 − B224:45
−8
−6
−4
−2
0
2
4
6
delta(C16:0 Fatty Acid)

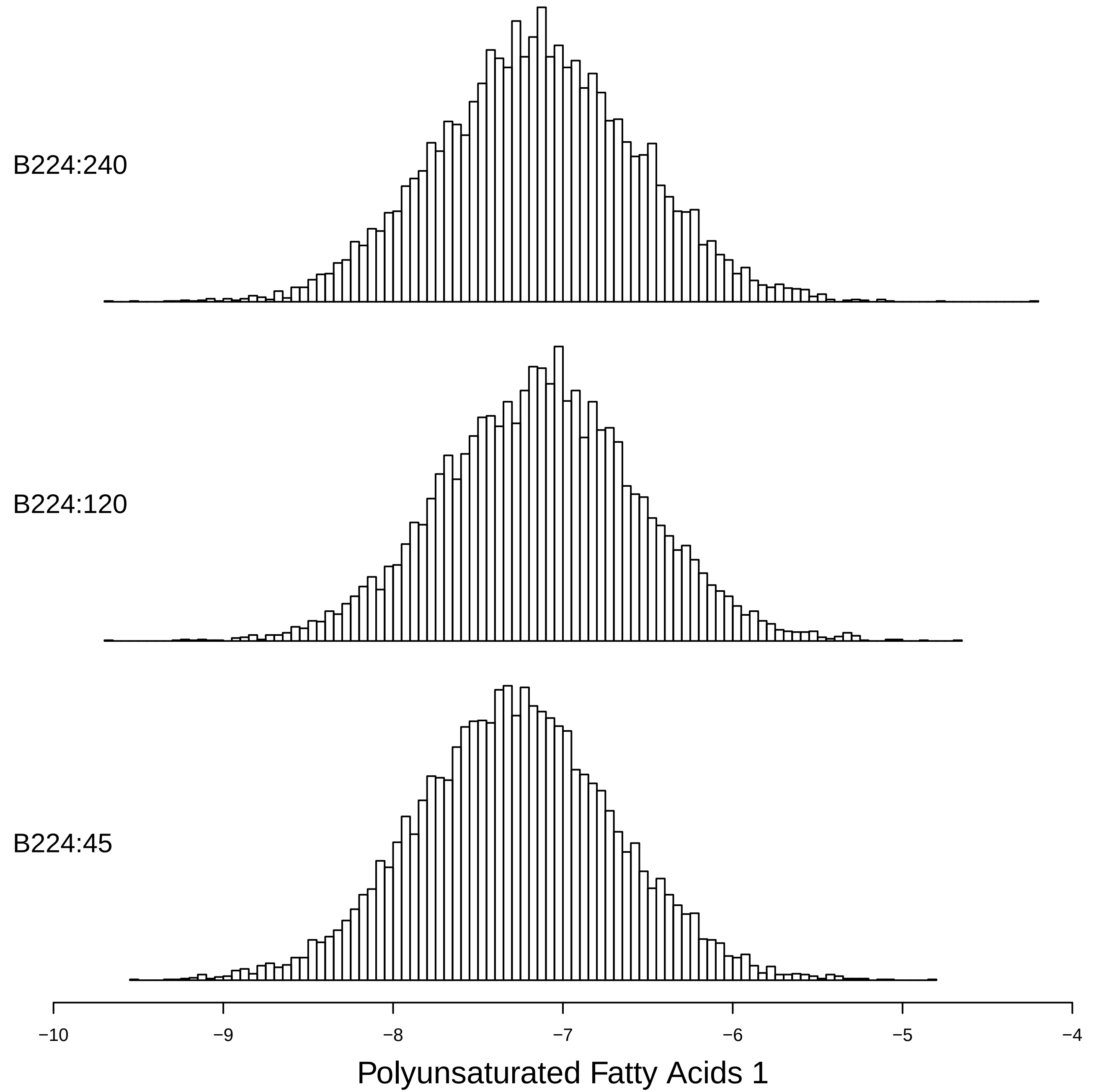
B224:240
B224:120
B224:45
−10
−9
−8
−7
−6
−5
−4
Polyunsaturated Fatty Acids 1

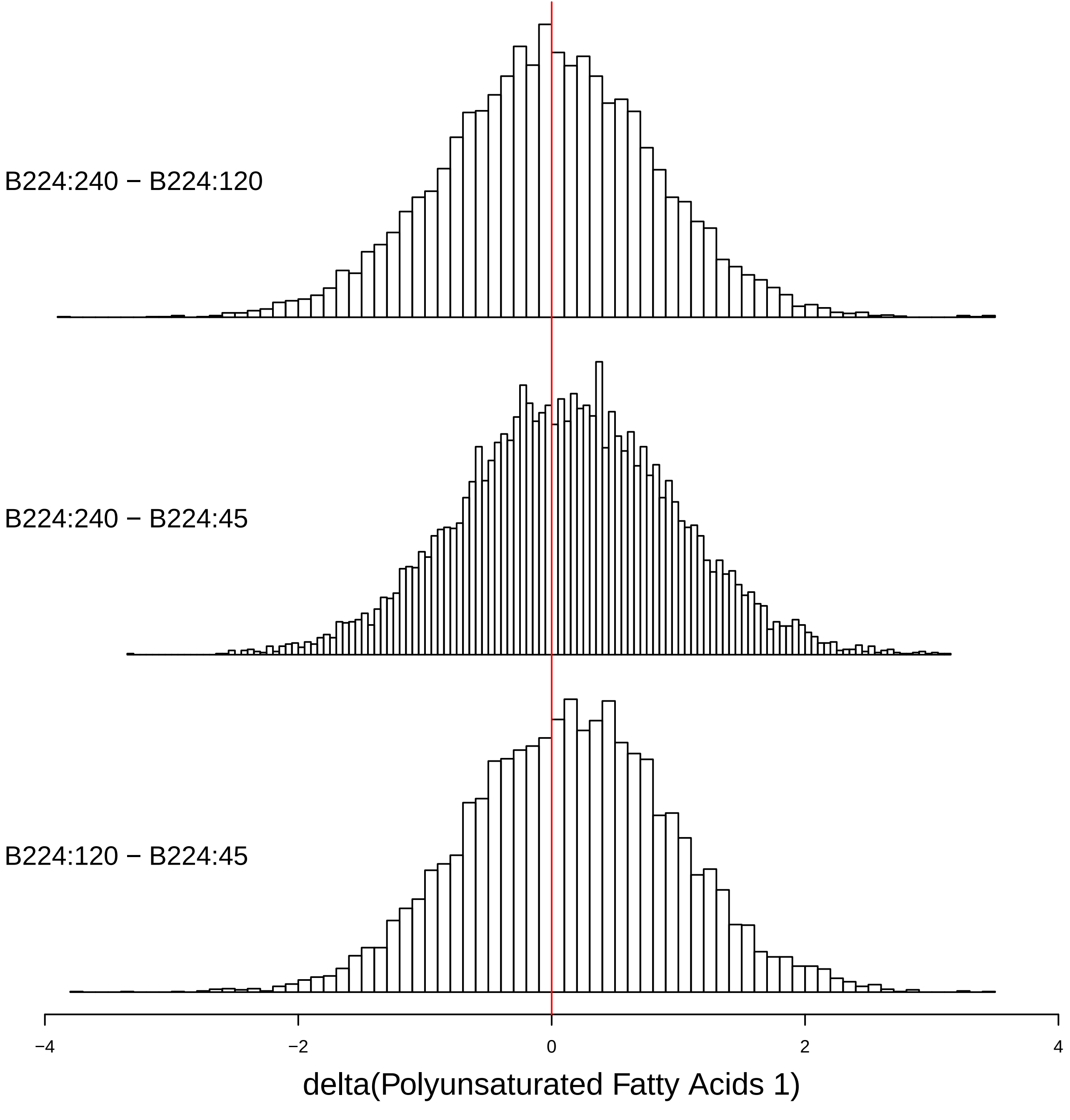
B224:240 − B224:120
B224:240 − B224:45
B224:120 − B224:45
−4
−2
0
2
4
delta(Polyunsaturated Fatty Acids 1)

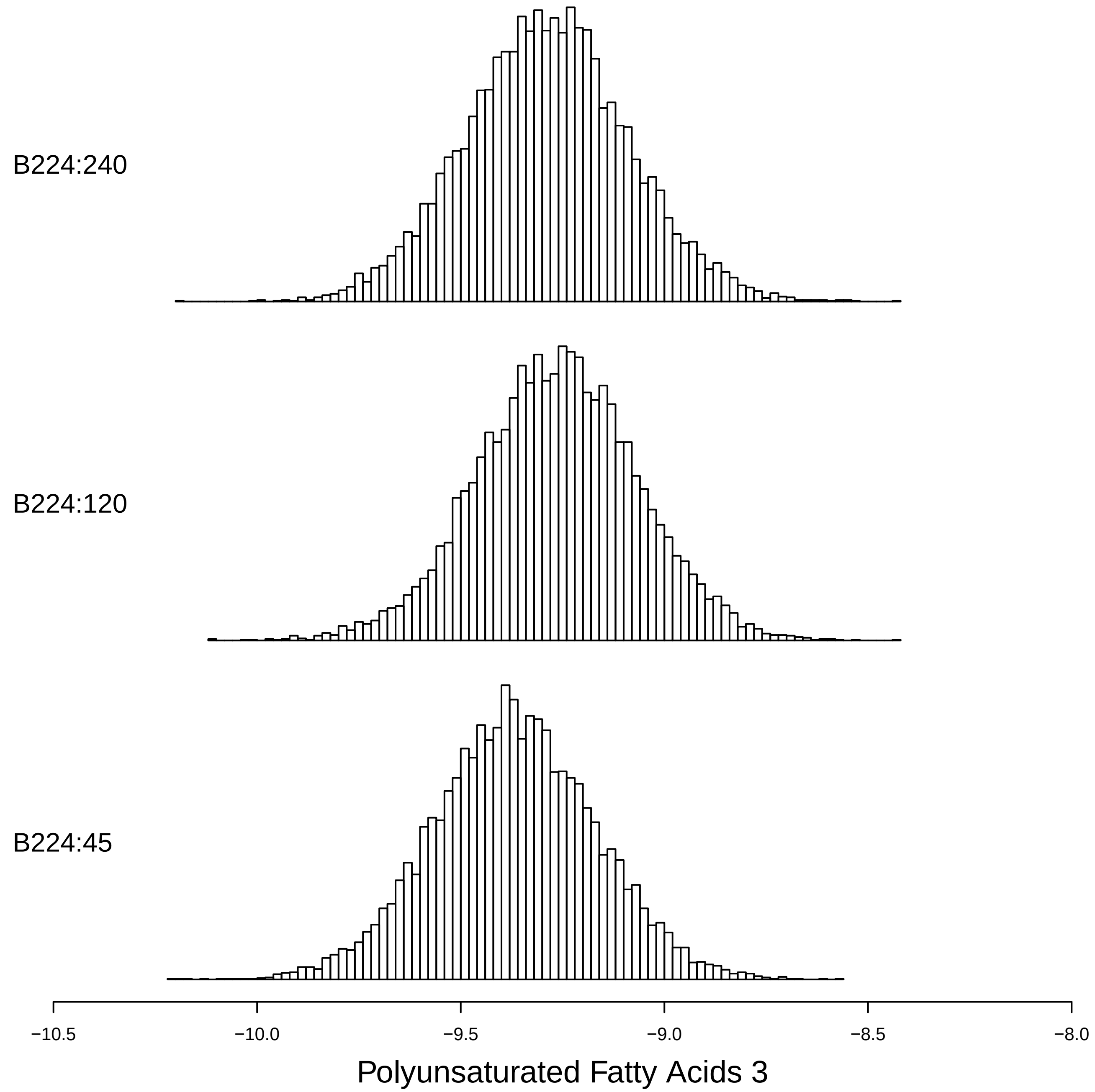
B224:240
B224:120
B224:45
−10.5
−10.0
−9.5
−9.0
−8.5
−8.0
Polyunsaturated Fatty Acids 3

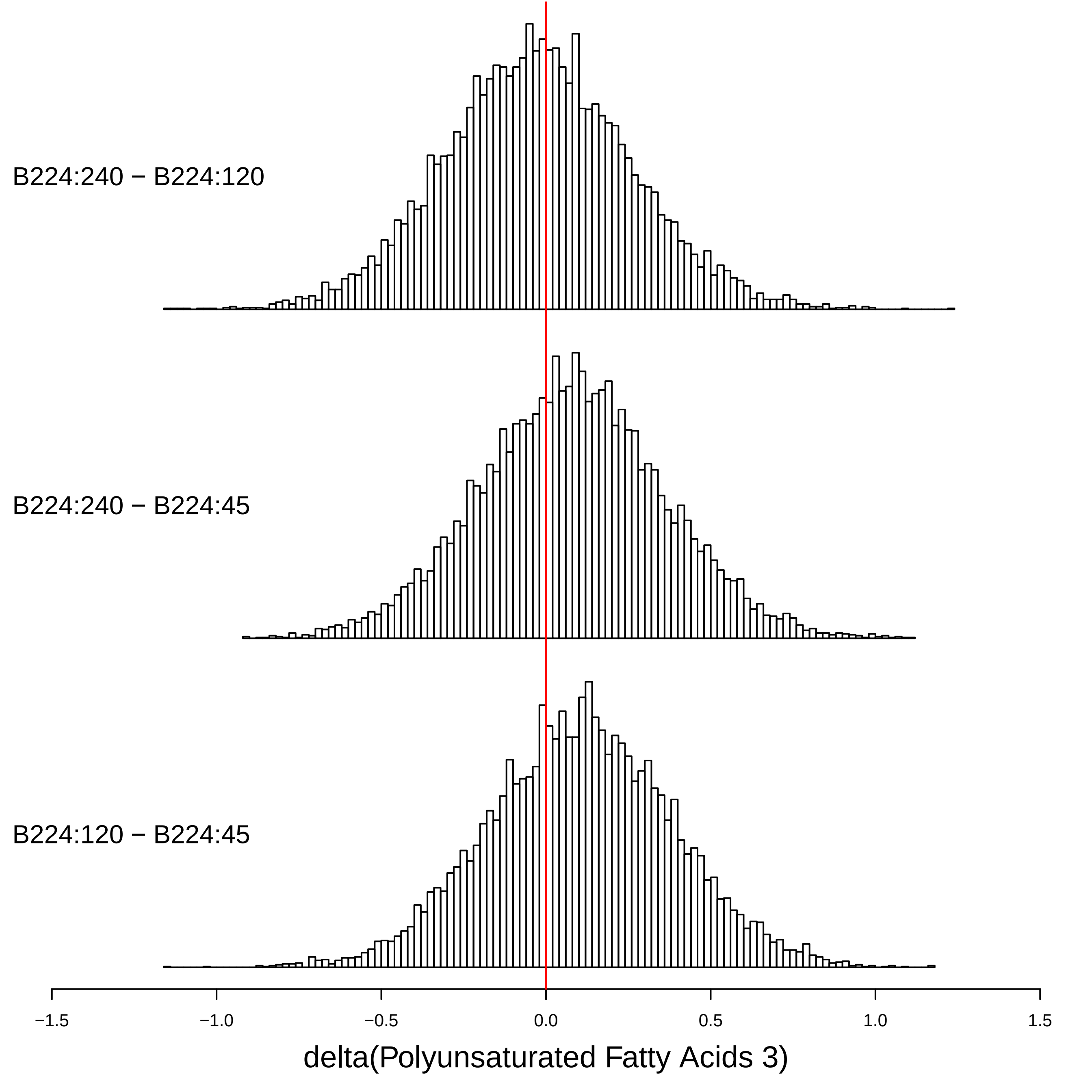

B224:240 − B224:120
B224:240 − B224:45
B224:120 − B224:45
−1.5
−1.0
−0.5
0.0
0.5
1.0
1.5
delta(Polyunsaturated Fatty Acids 3)

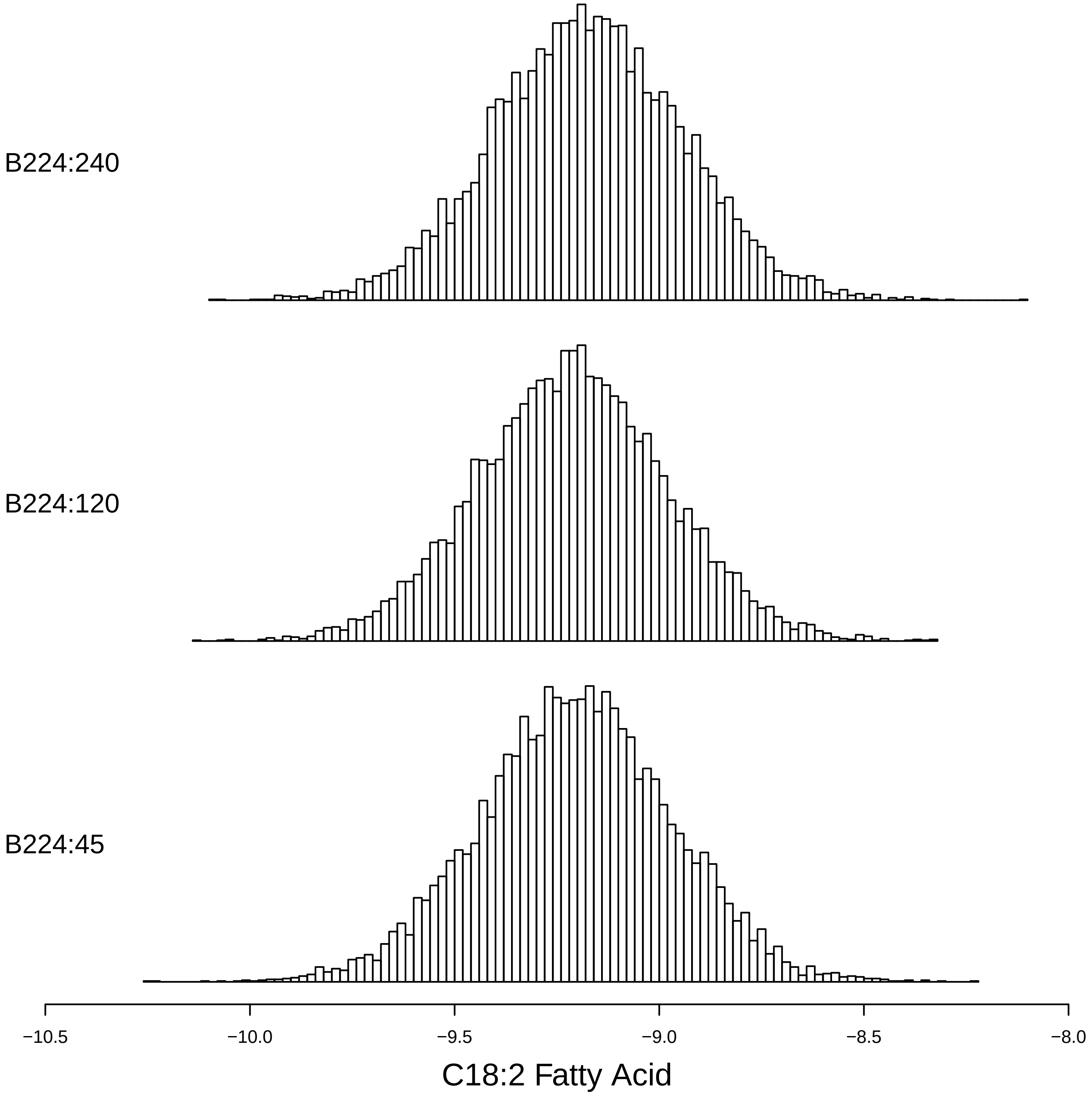
B224:240
B224:120
B224:45
−10.5
−10.0
−9.5
−9.0
−8.5
−8.0
C18:2 Fatty Acid

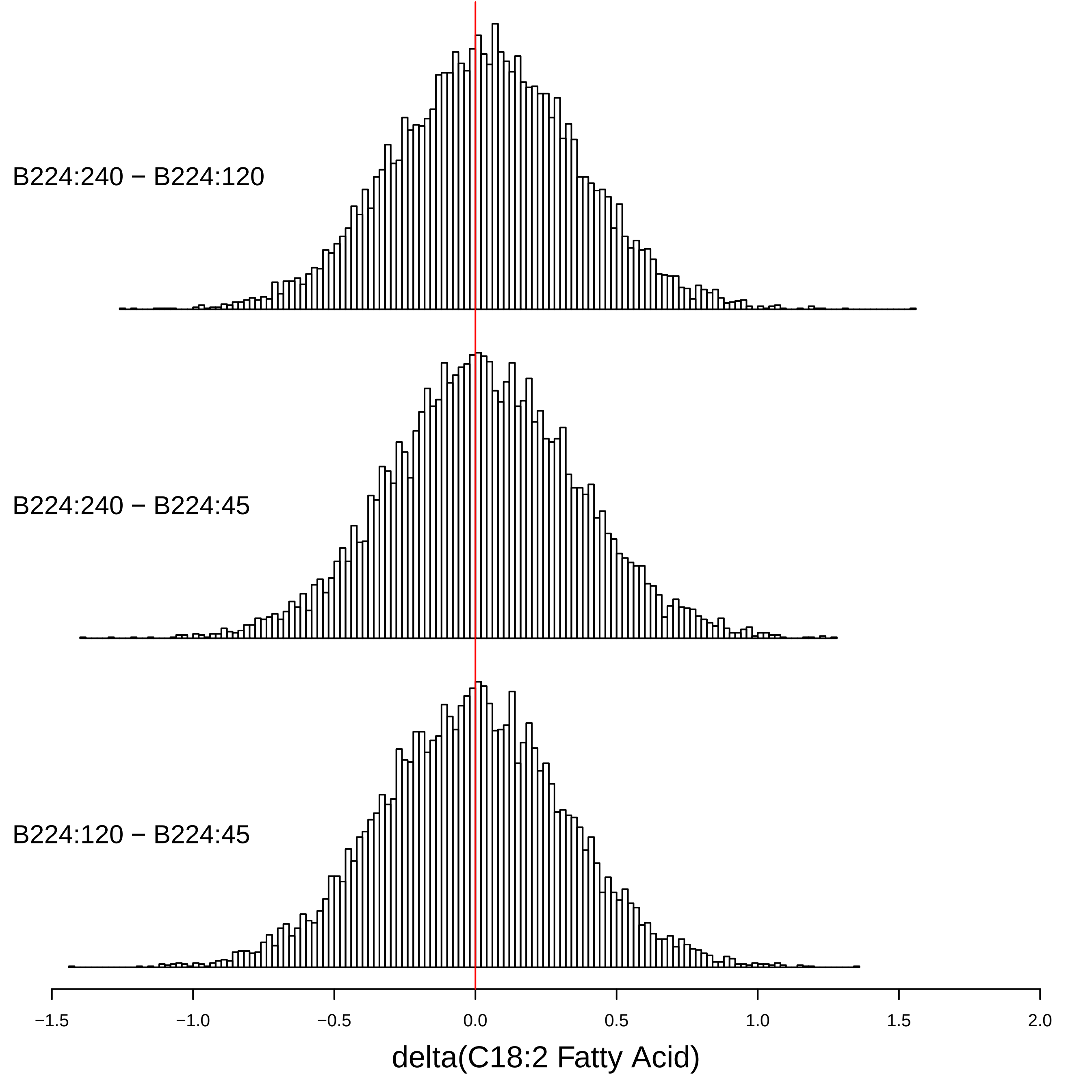

B224:240 − B224:120
B224:240 − B224:45
B224:120 − B224:45
−1.5
−1.0
−0.5
0.0
0.5
1.0
1.5
2.0
delta(C18:2 Fatty Acid)

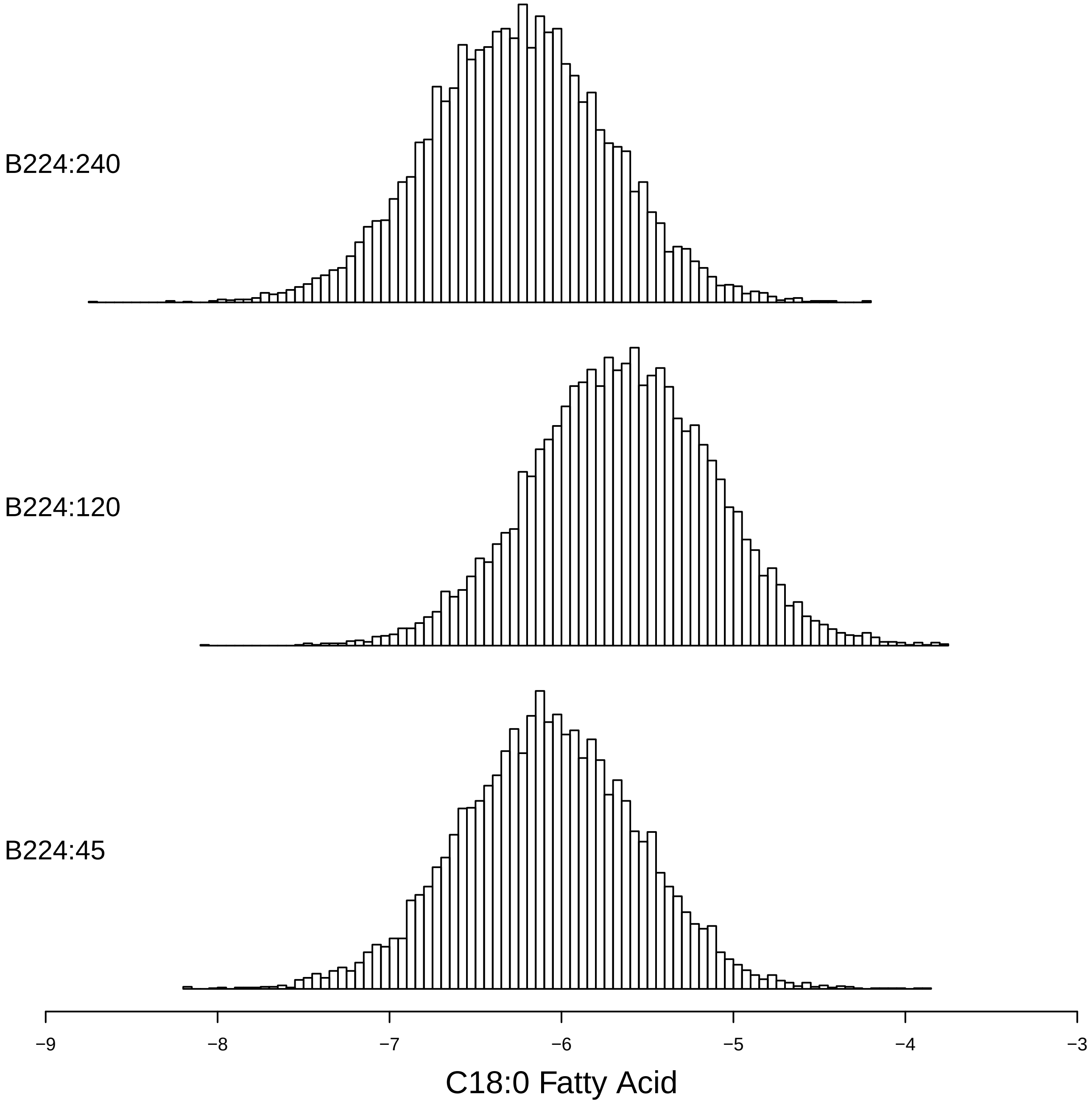
B224:240
B224:120
B224:45
−9
−8
−7
−6
−5
−4
−3
C18:0 Fatty Acid

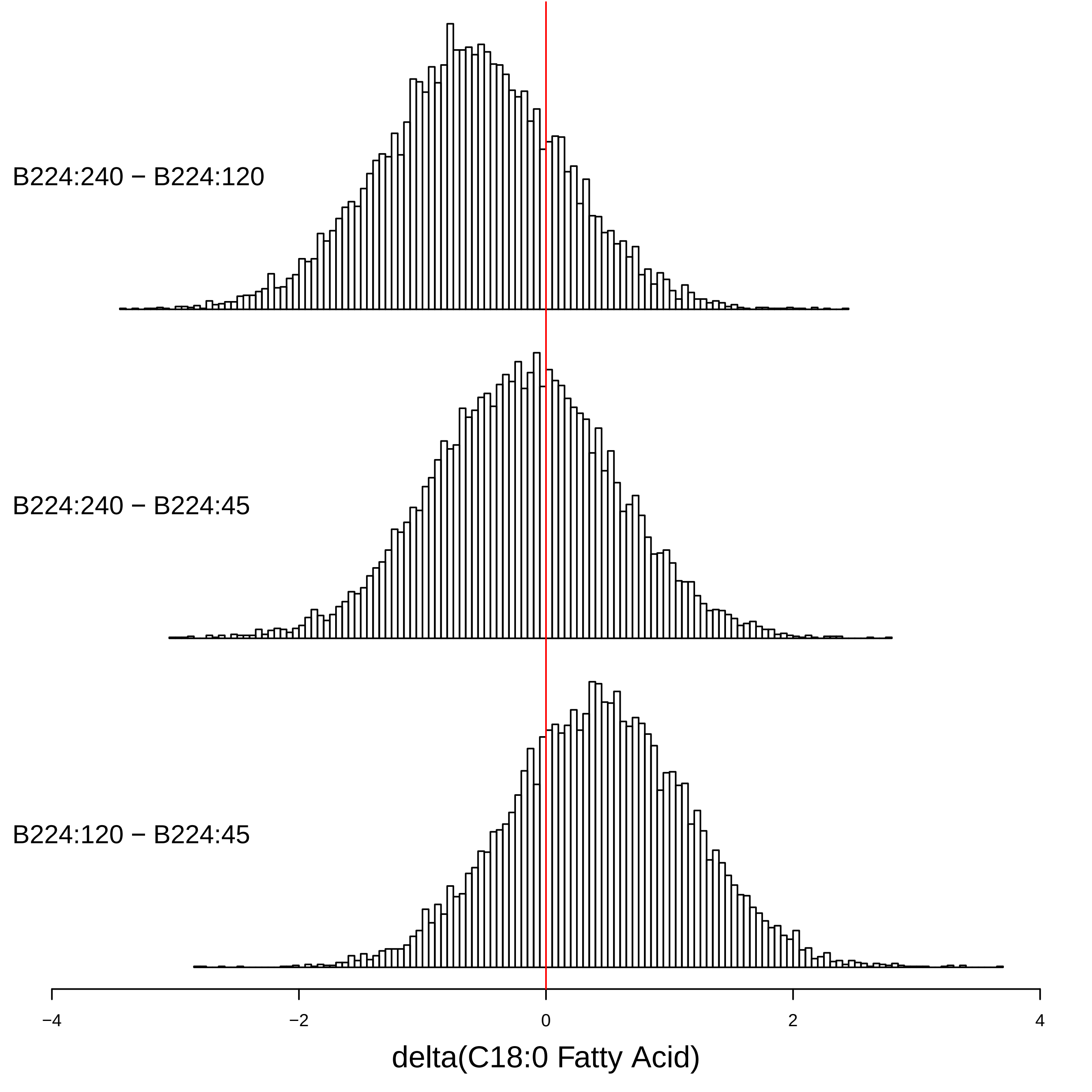
B224:240 − B224:120
B224:240 − B224:45
B224:120 − B224:45
−4
−2
0
2
4
delta(C18:0 Fatty Acid)

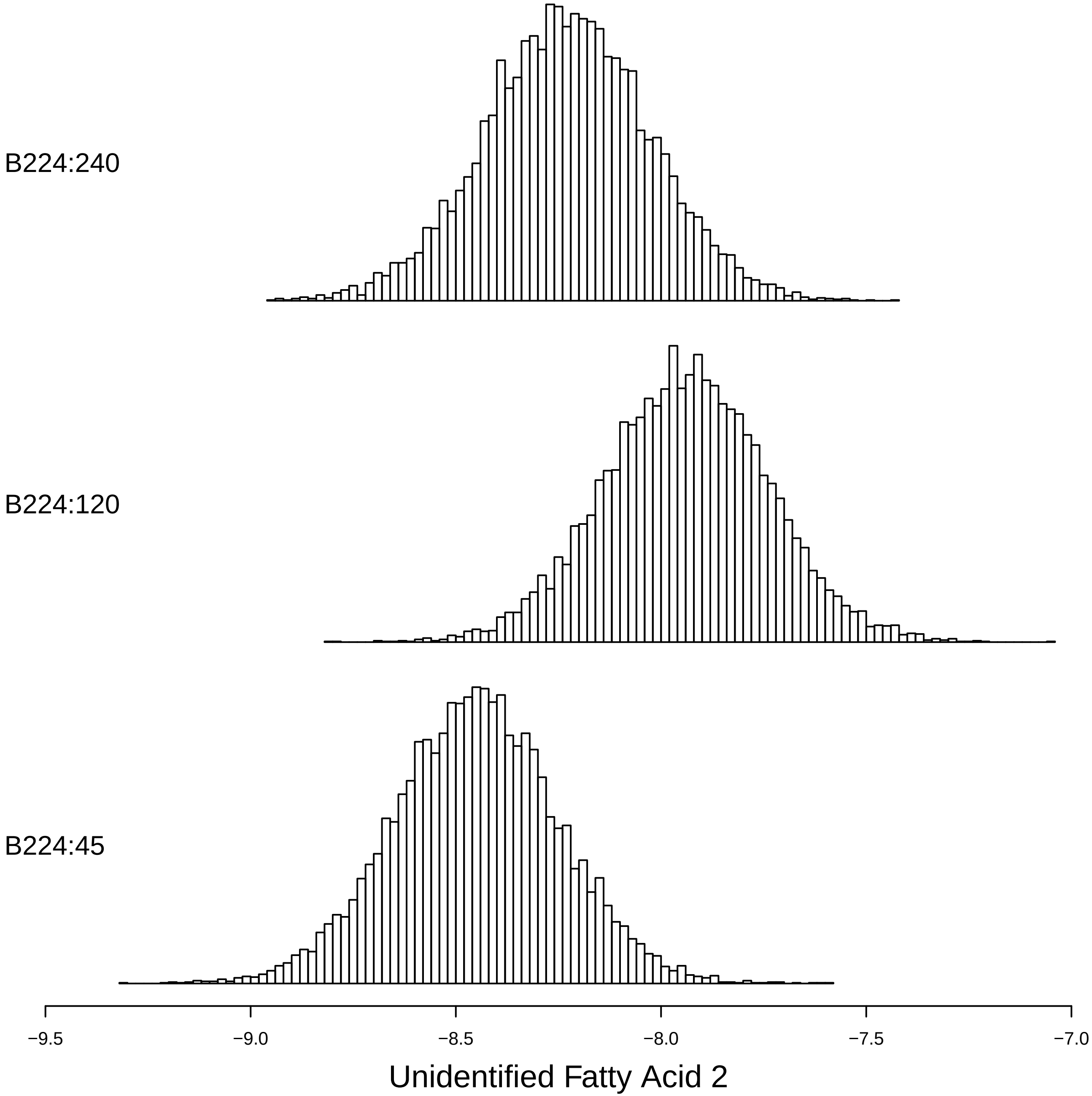

B224:240
B224:120
B224:45
−9.5
−9.0
−8.5
−8.0
−7.5
−7.0
Unidentified Fatty Acid 2

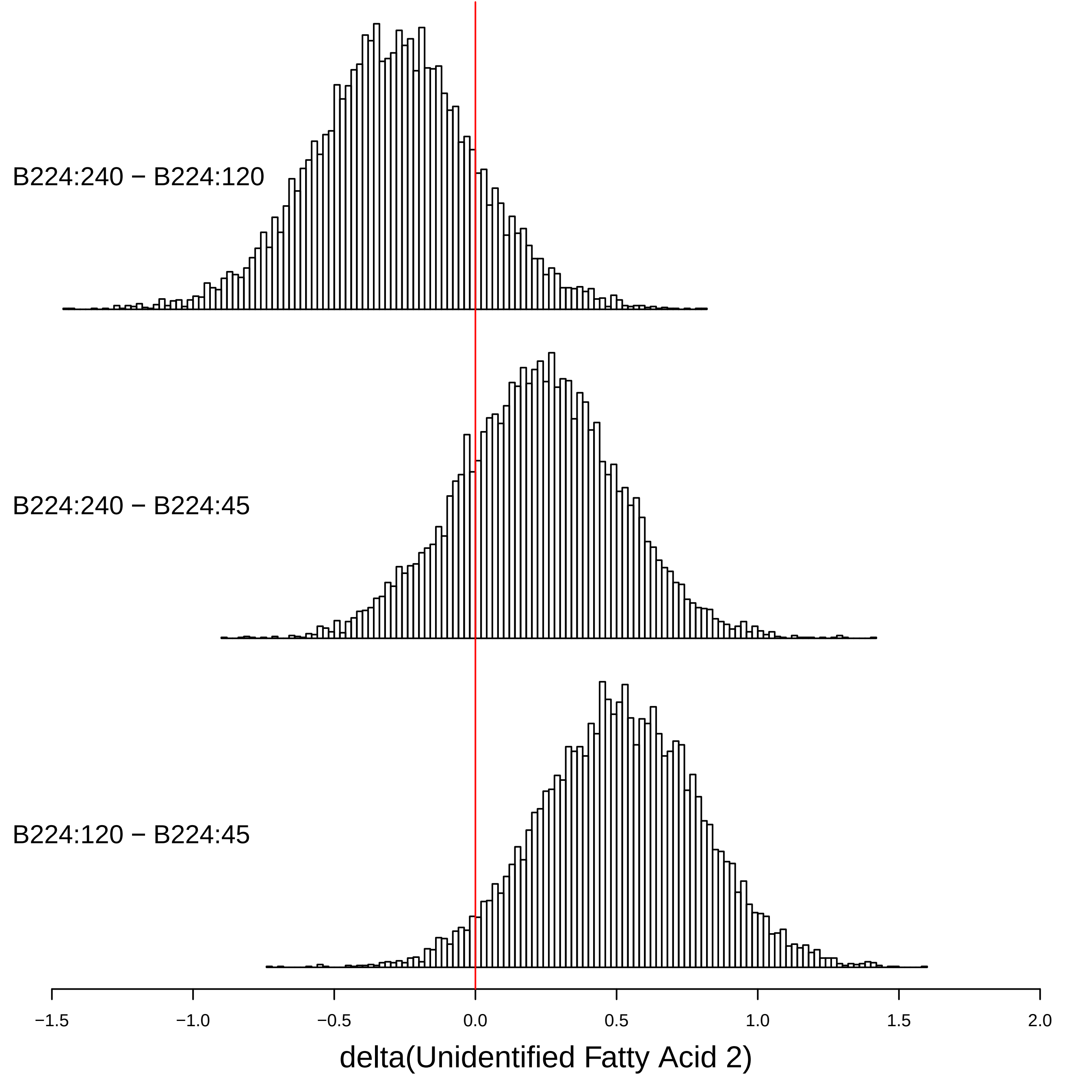
B224:240 − B224:120
B224:240 − B224:45
B224:120 − B224:45
−1.5
−1.0
−0.5
0.0
0.5
1.0
1.5
2.0
delta(Unidentified Fatty Acid 2)

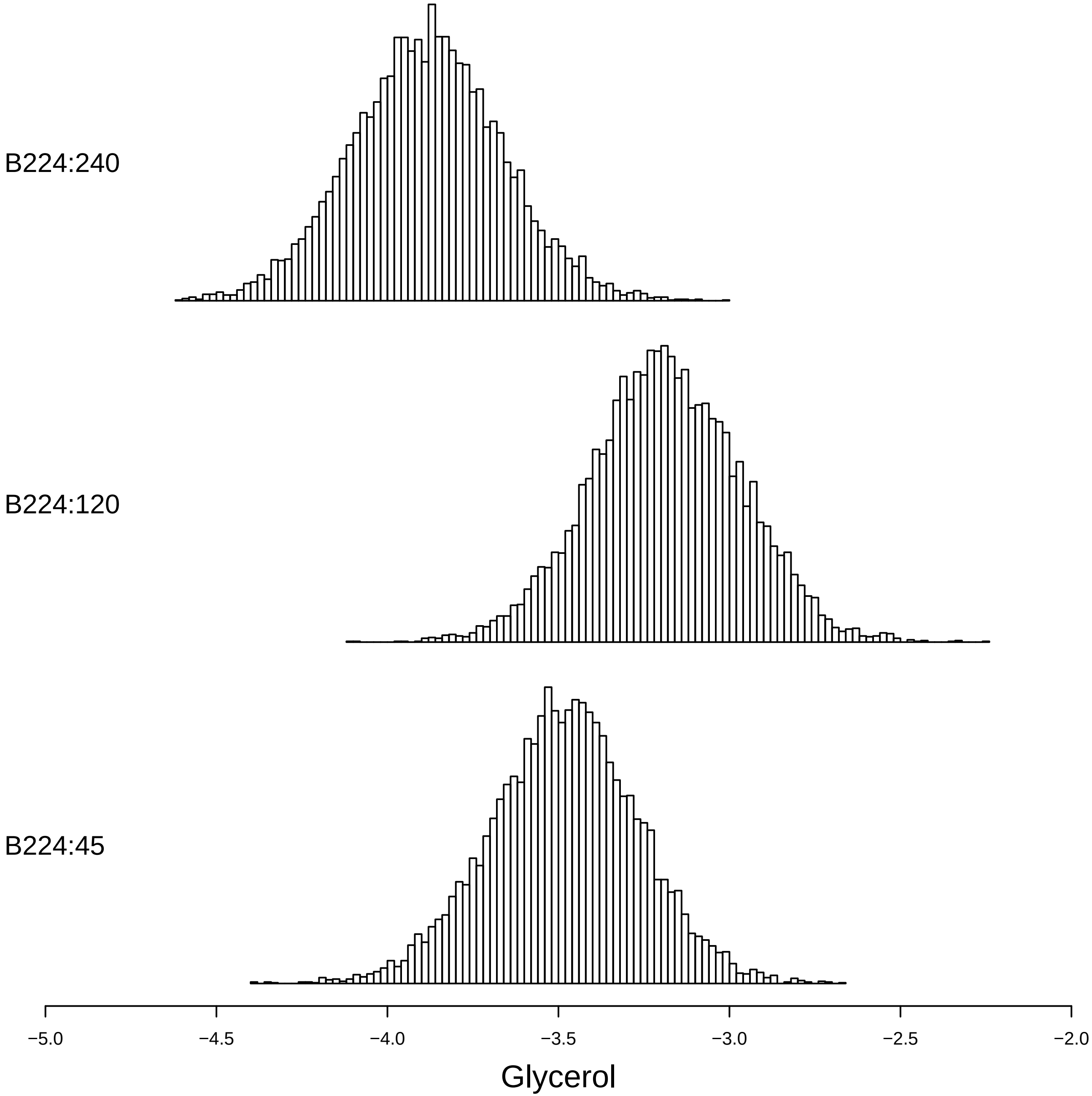
B224:240
B224:120
B224:45
−5.0
−4.5
−4.0
−3.5
−3.0
−2.5
−2.0
Glycerol

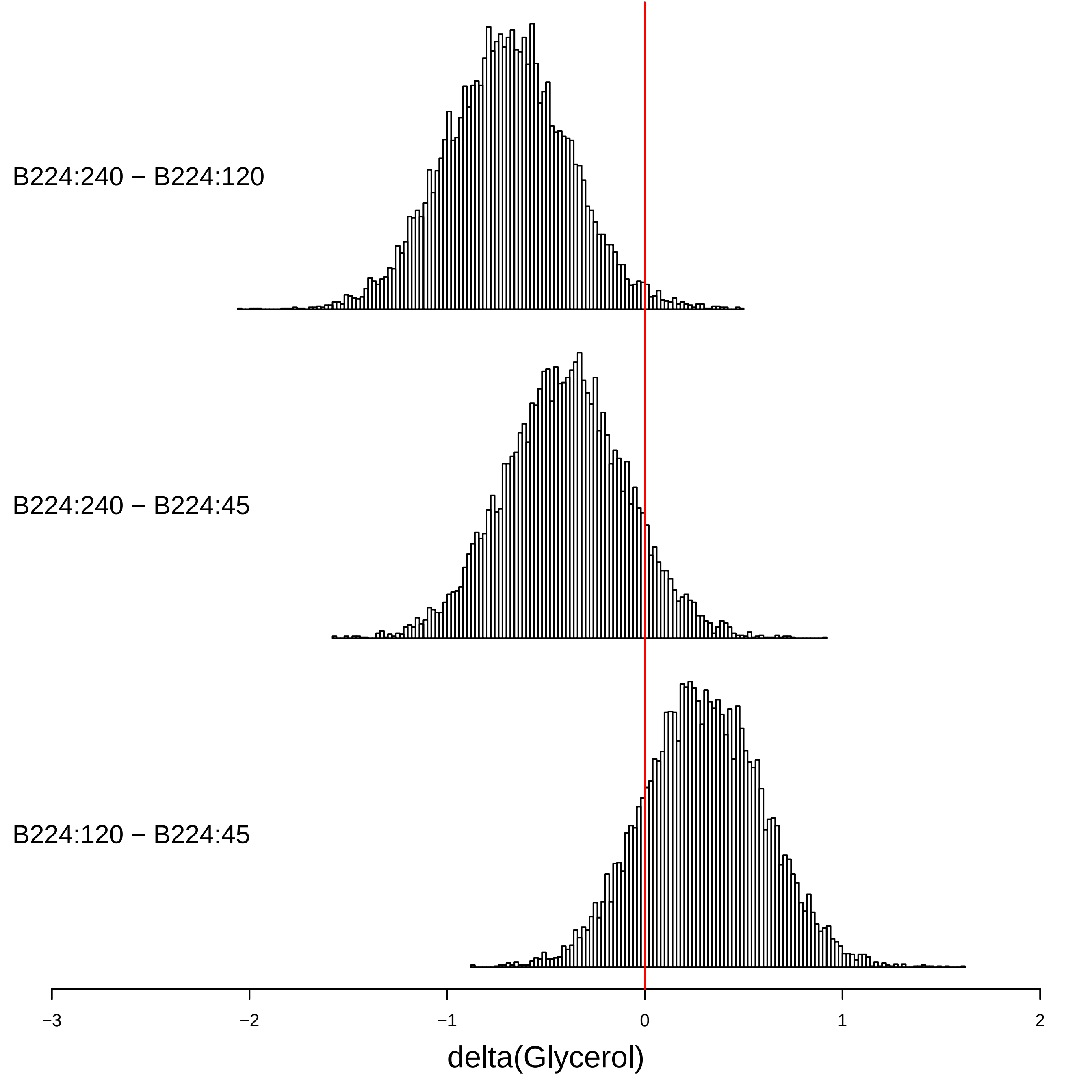

B224:240 − B224:120
B224:240 − B224:45
B224:120 − B224:45
−3
−2
−1
0
1
2
delta(Glycerol)

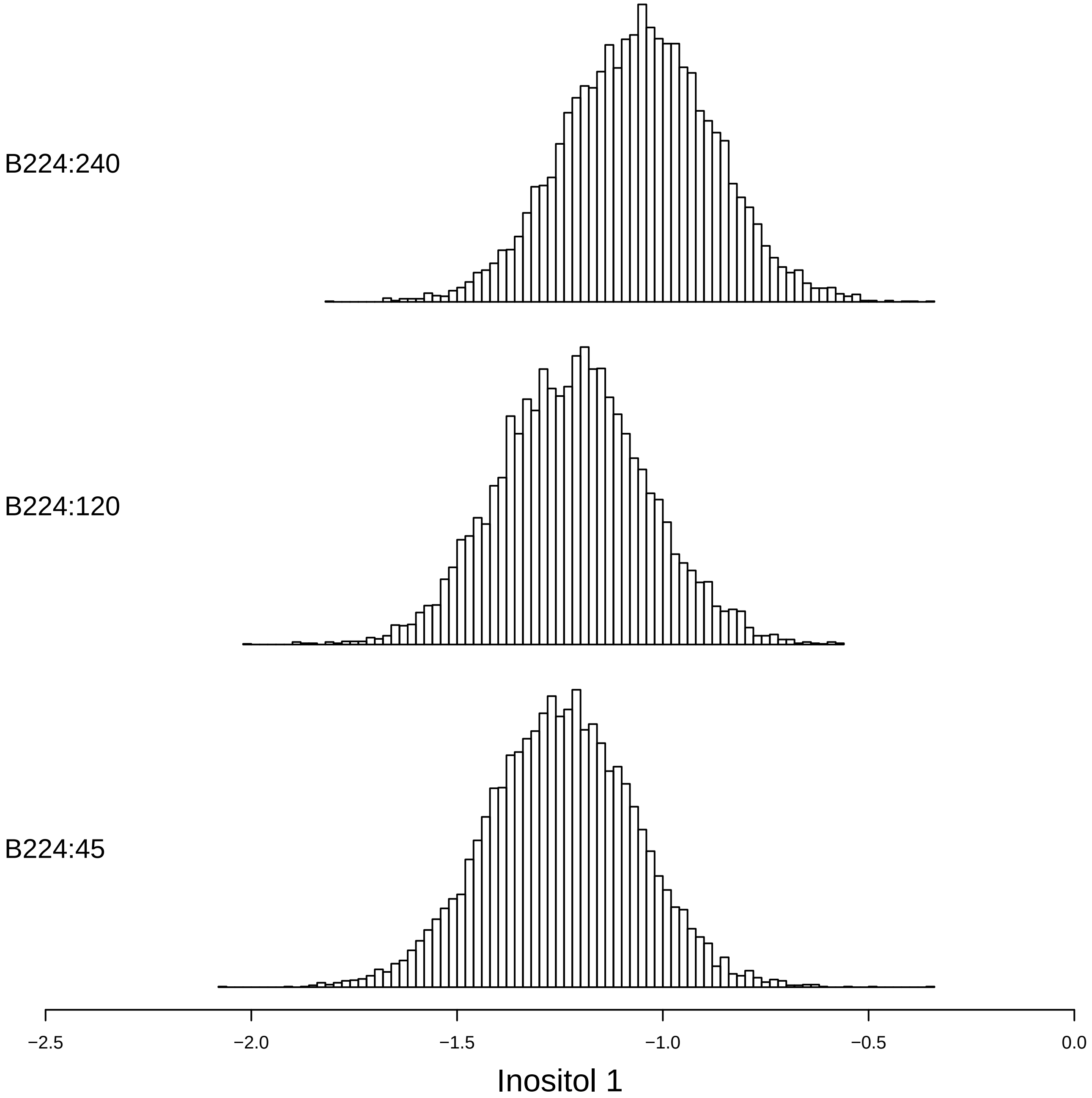

B224:240
B224:120
B224:45
−2.5
−2.0
−1.5
−1.0
−0.5
0.0
Inositol 1

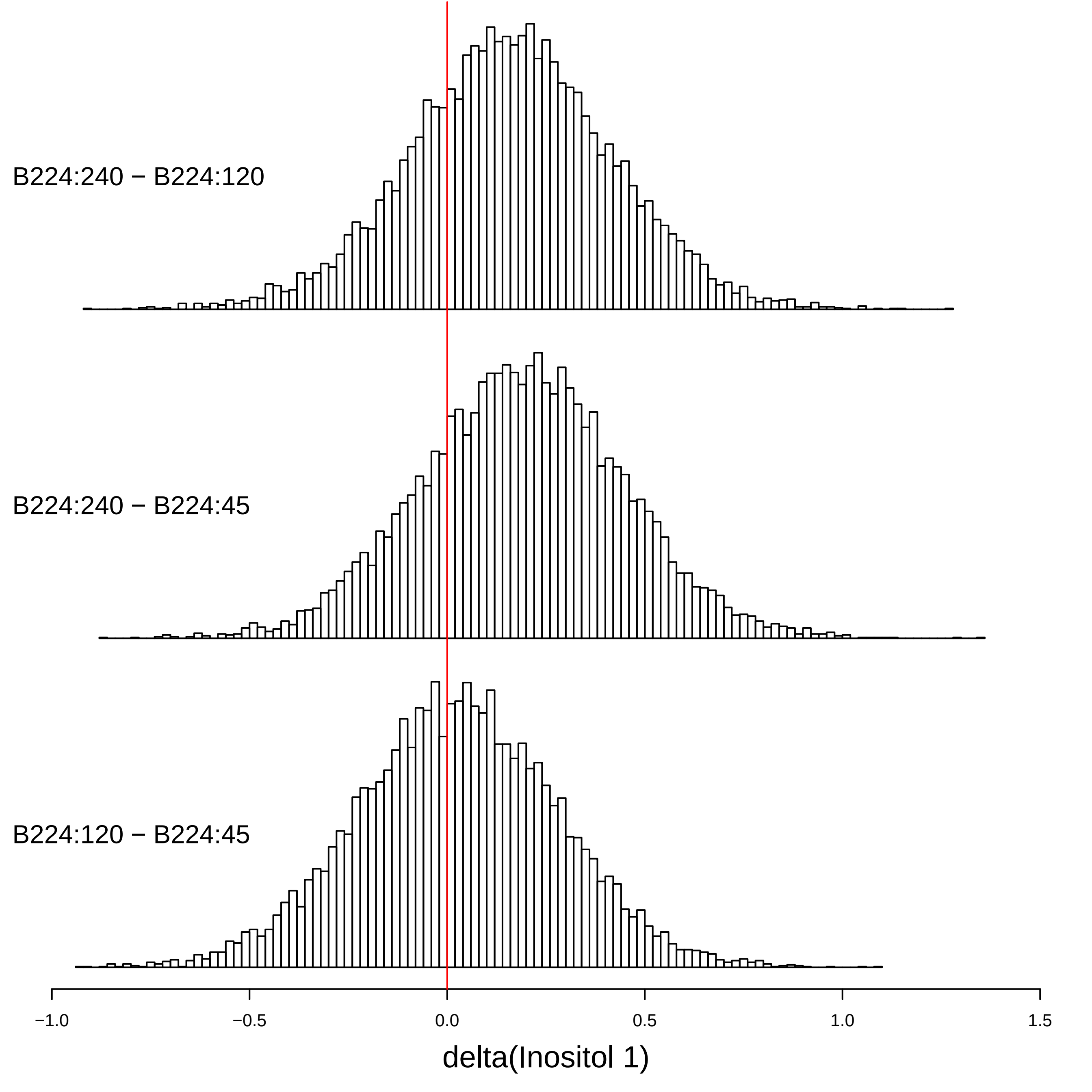

B224:240 − B224:120
B224:240 − B224:45
B224:120 − B224:45
−1.0
−0.5
0.0
0.5
1.0
1.5
delta(Inositol 1)

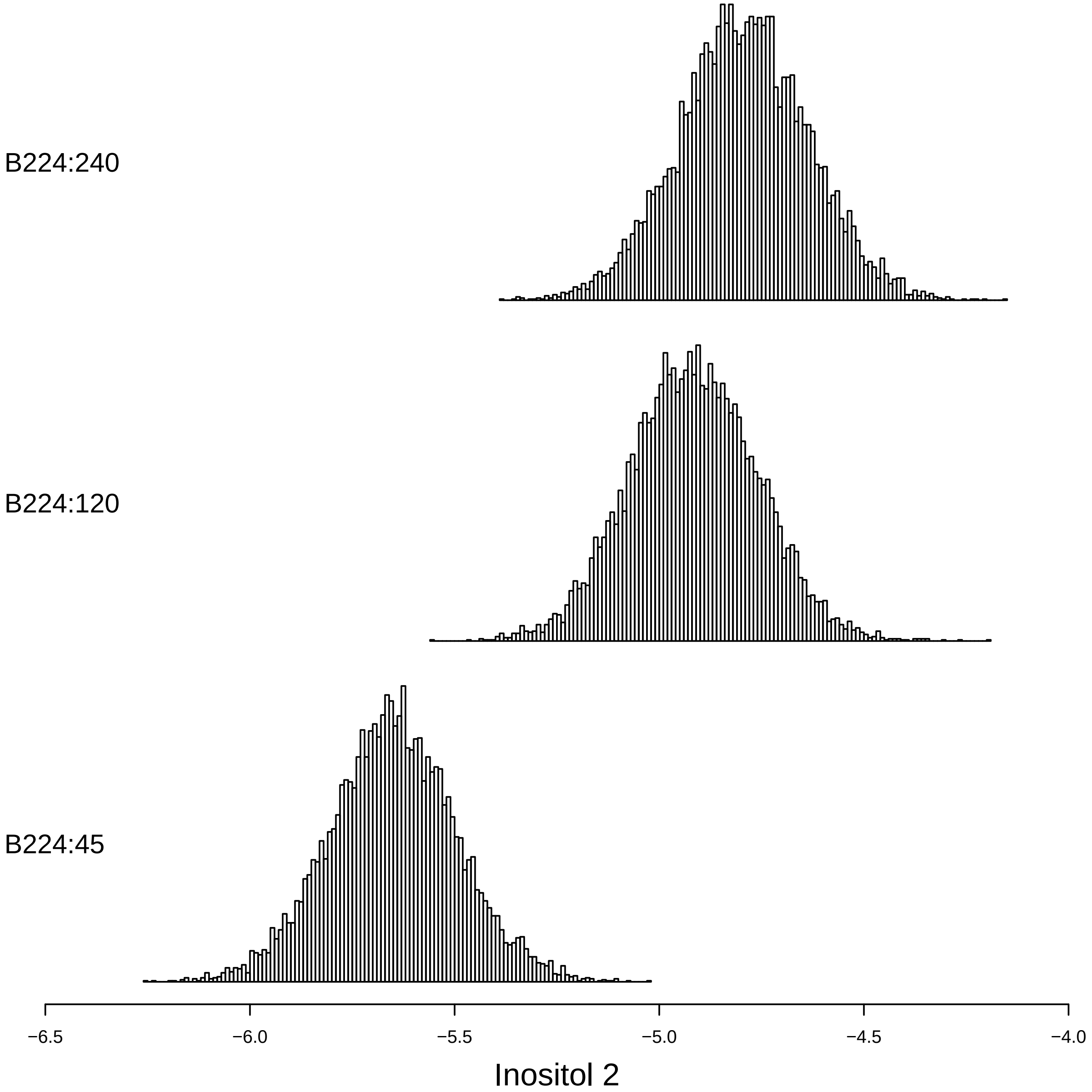
B224:240
B224:120
B224:45
−6.5
−6.0
−5.5
−5.0
−4.5
−4.0
Inositol 2

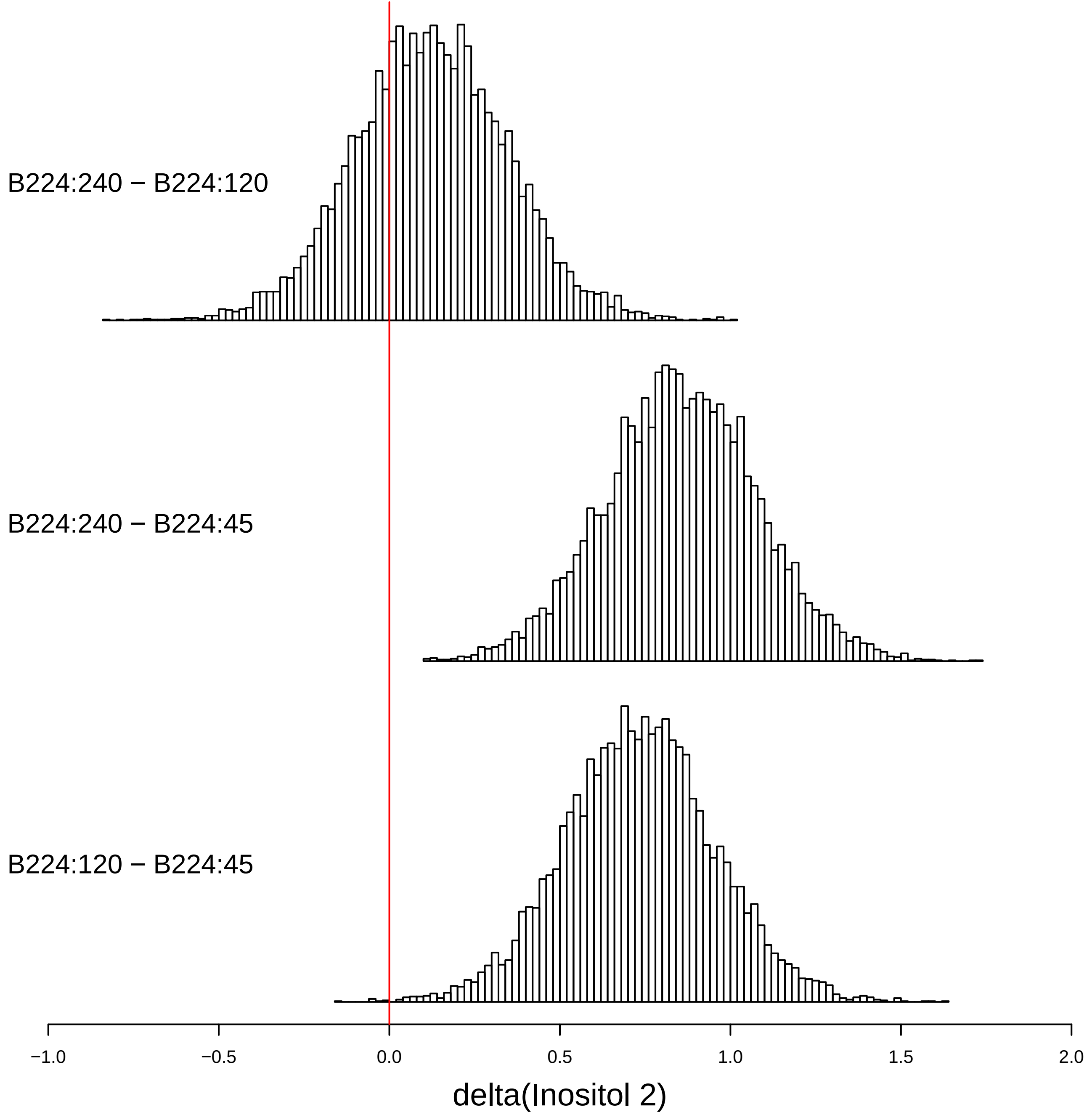
B224:240 − B224:120
B224:240 − B224:45
B224:120 − B224:45
−1.0
−0.5
0.0
0.5
1.0
1.5
2.0
delta(Inositol 2)

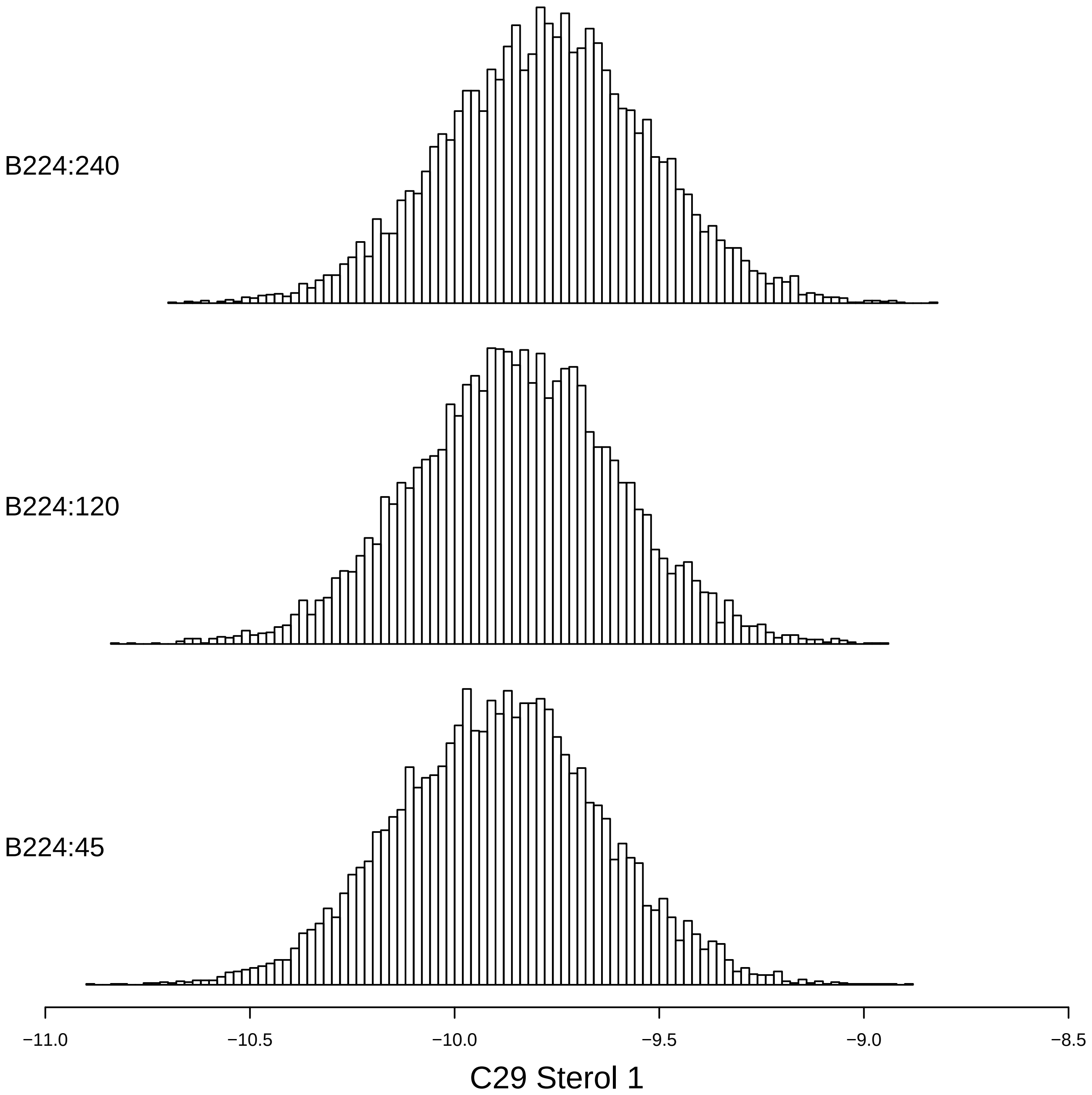
B224:240
B224:120
B224:45
−11.0
−10.5
−10.0
−9.5
−9.0
−8.5
C29 Sterol 1

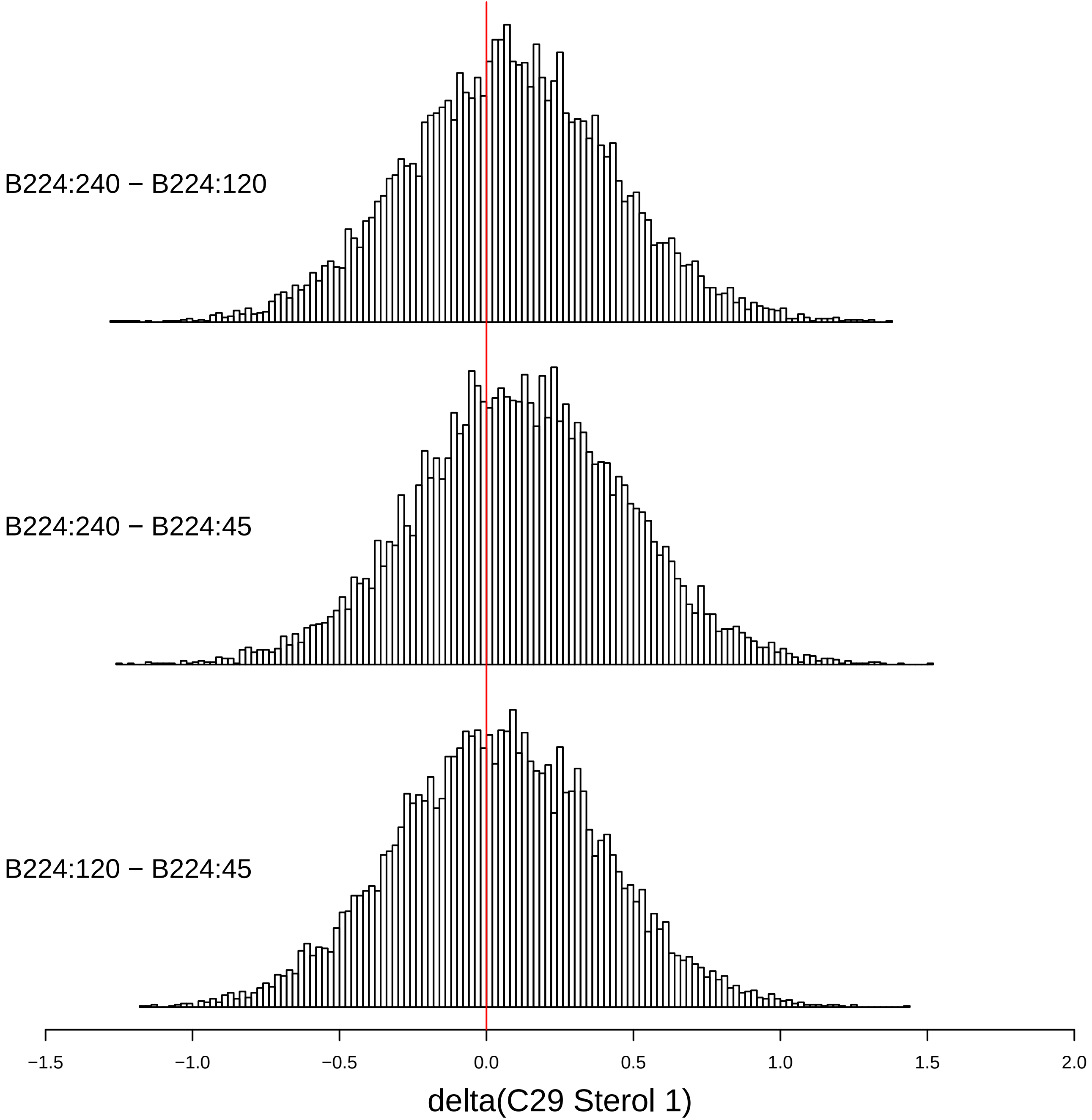
B224:240 − B224:120
B224:240 − B224:45
B224:120 − B224:45
−1.5
−1.0
−0.5
0.0
0.5
1.0
1.5
2.0
delta(C29 Sterol 1)

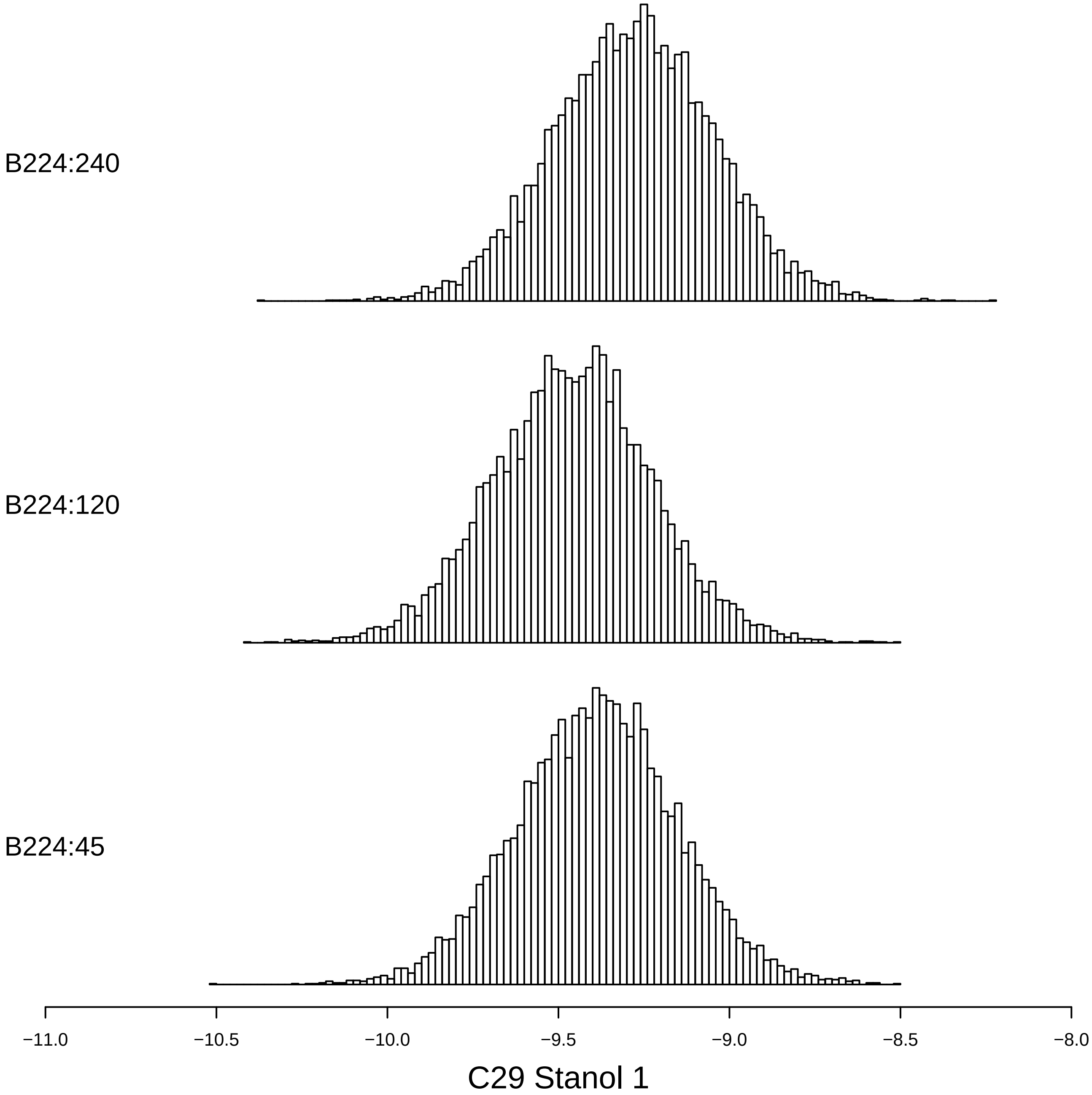

B224:240
B224:120
B224:45
−11.0
−10.5
−10.0
−9.5
−9.0
−8.5
−8.0
C29 Stanol 1

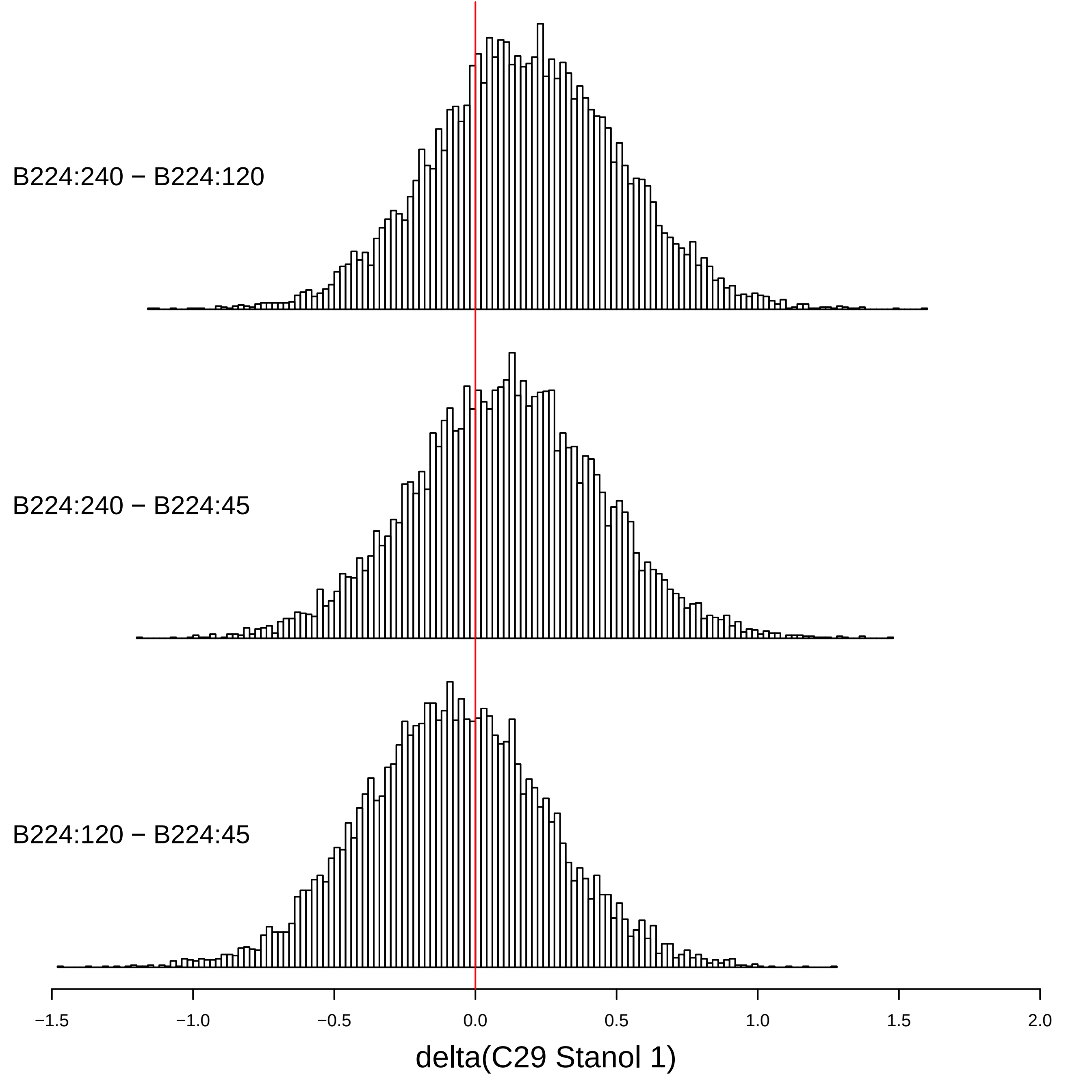

B224:240 − B224:120
B224:240 − B224:45
B224:120 − B224:45
−1.5
−1.0
−0.5
0.0
0.5
1.0
1.5
2.0
delta(C29 Stanol 1)

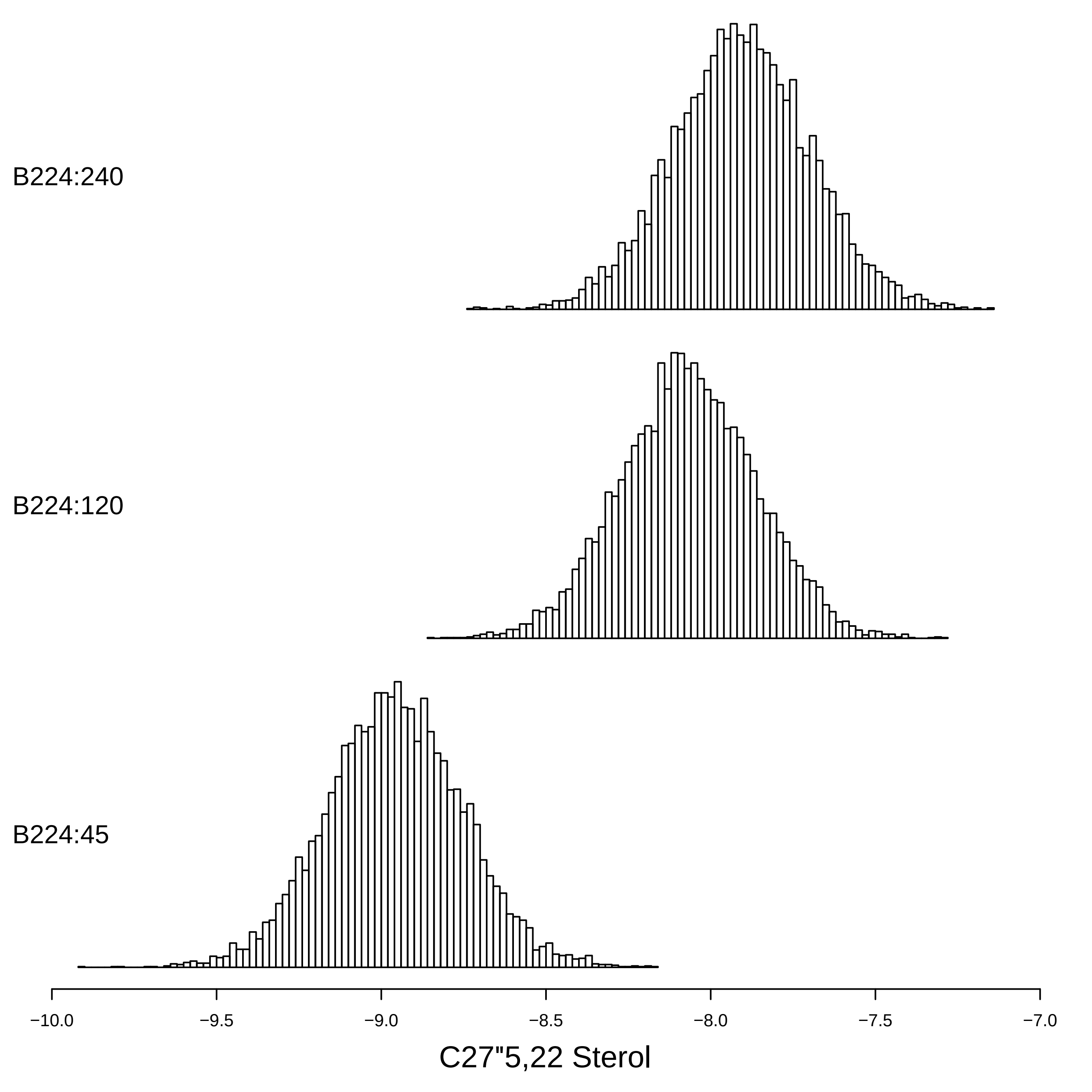
B224:240
B224:120
B224:45
−10.0
−9.5
−9.0
−8.5
−8.0
−7.5
−7.0
C27"5,22 Sterol

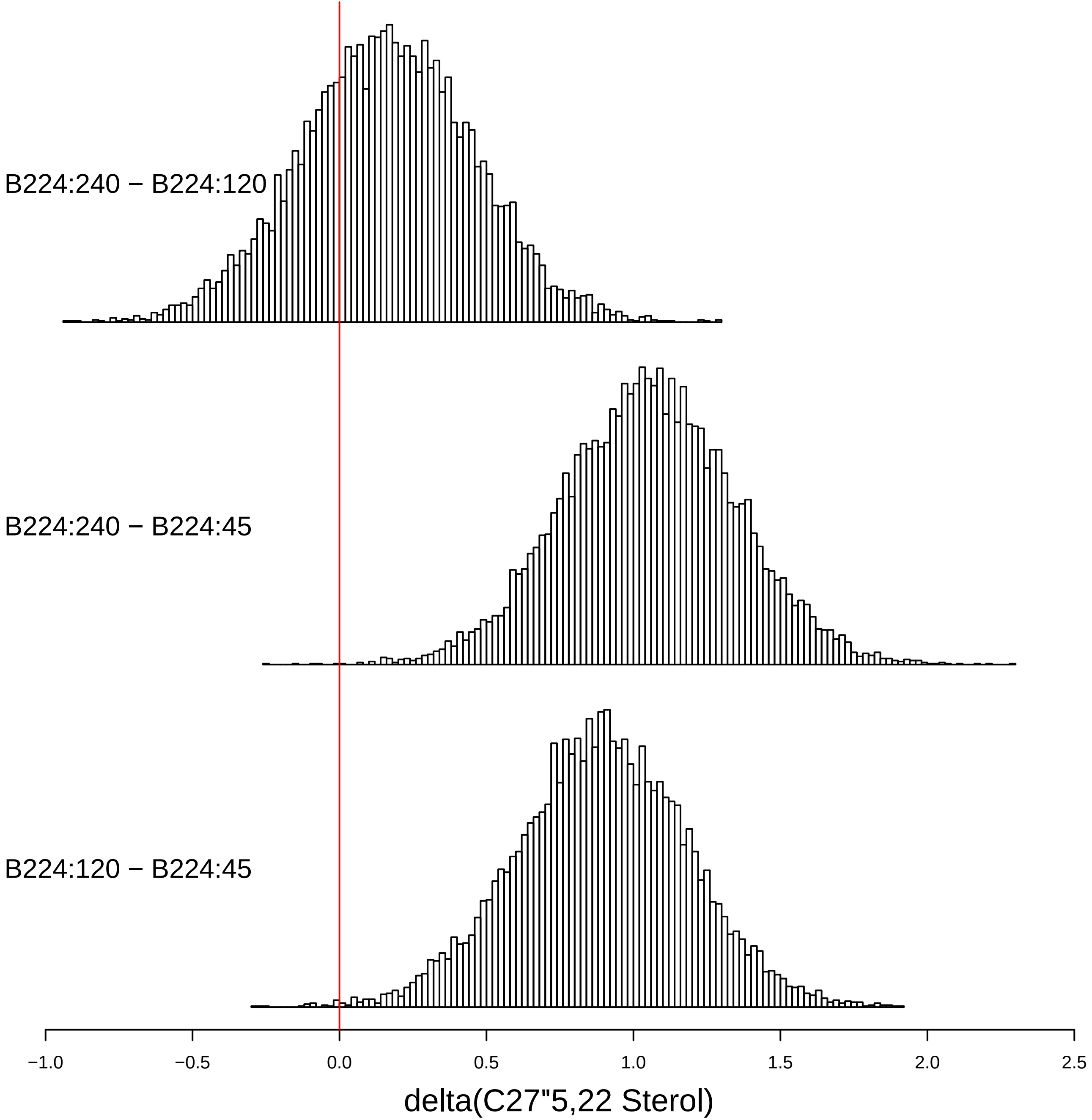
B224:240 − B224:120
B224:240 − B224:45
B224:120 − B224:45
−1.0
−0.5
0.0
0.5
1.0
1.5
2.0
2.5
delta(C27"5,22 Sterol)

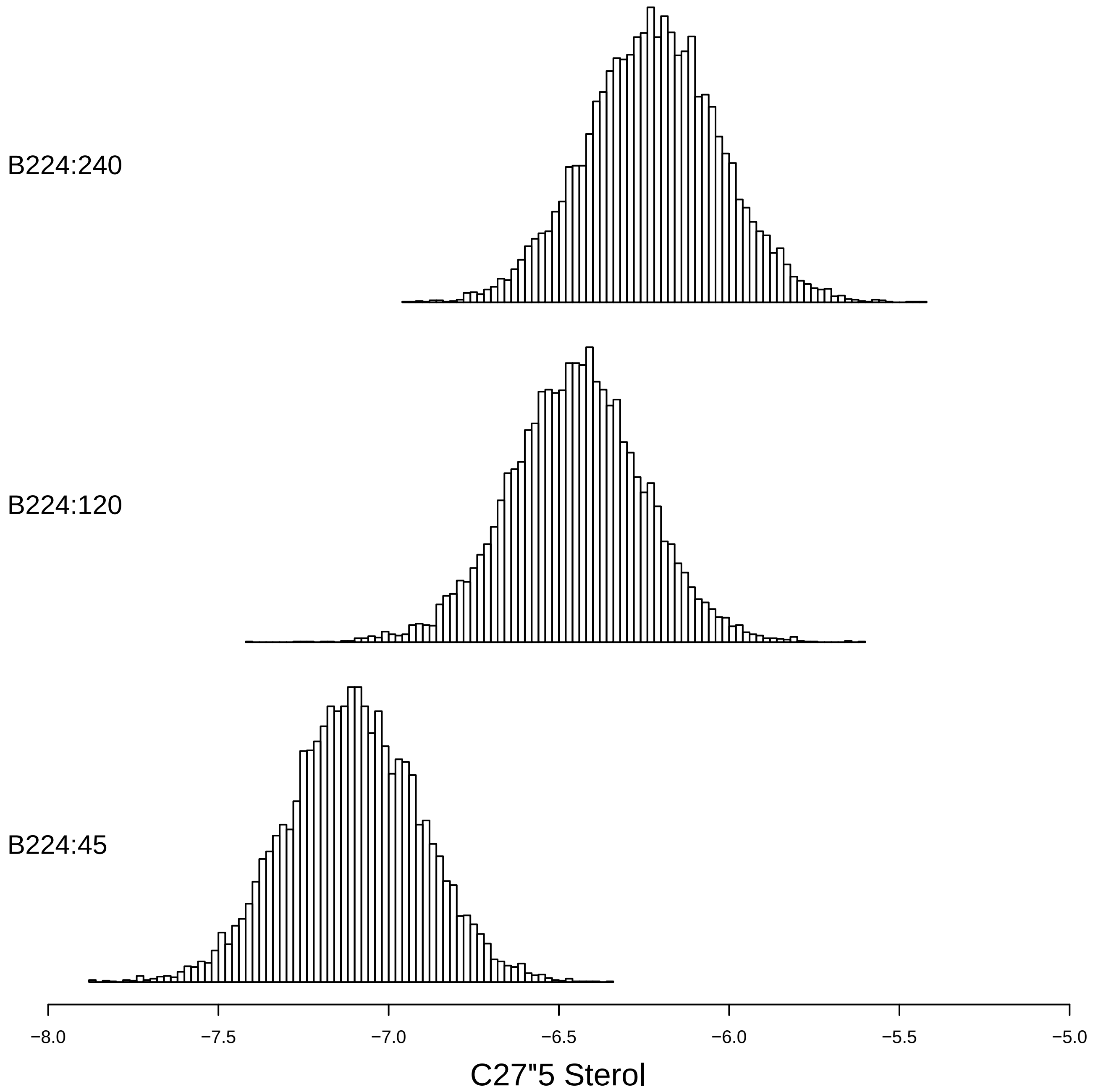
B224:240
B224:120
B224:45
−8.0
−7.5
−7.0
−6.5
−6.0
−5.5
−5.0
C27"5 Sterol

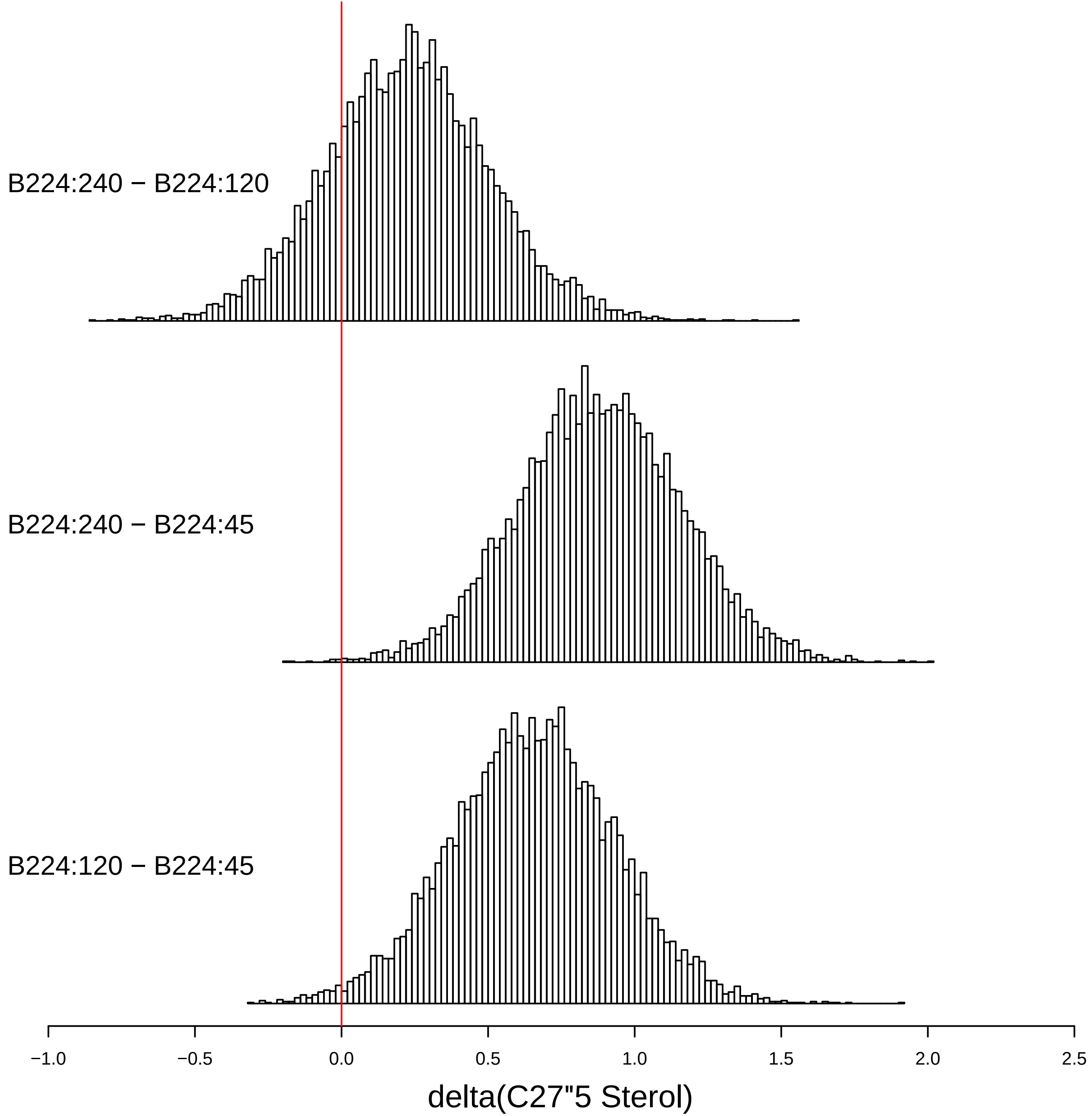

B224:240 − B224:120
B224:240 − B224:45
B224:120 − B224:45
−1.0
−0.5
0.0
0.5
1.0
1.5
2.0
2.5
delta(C27"5 Sterol)

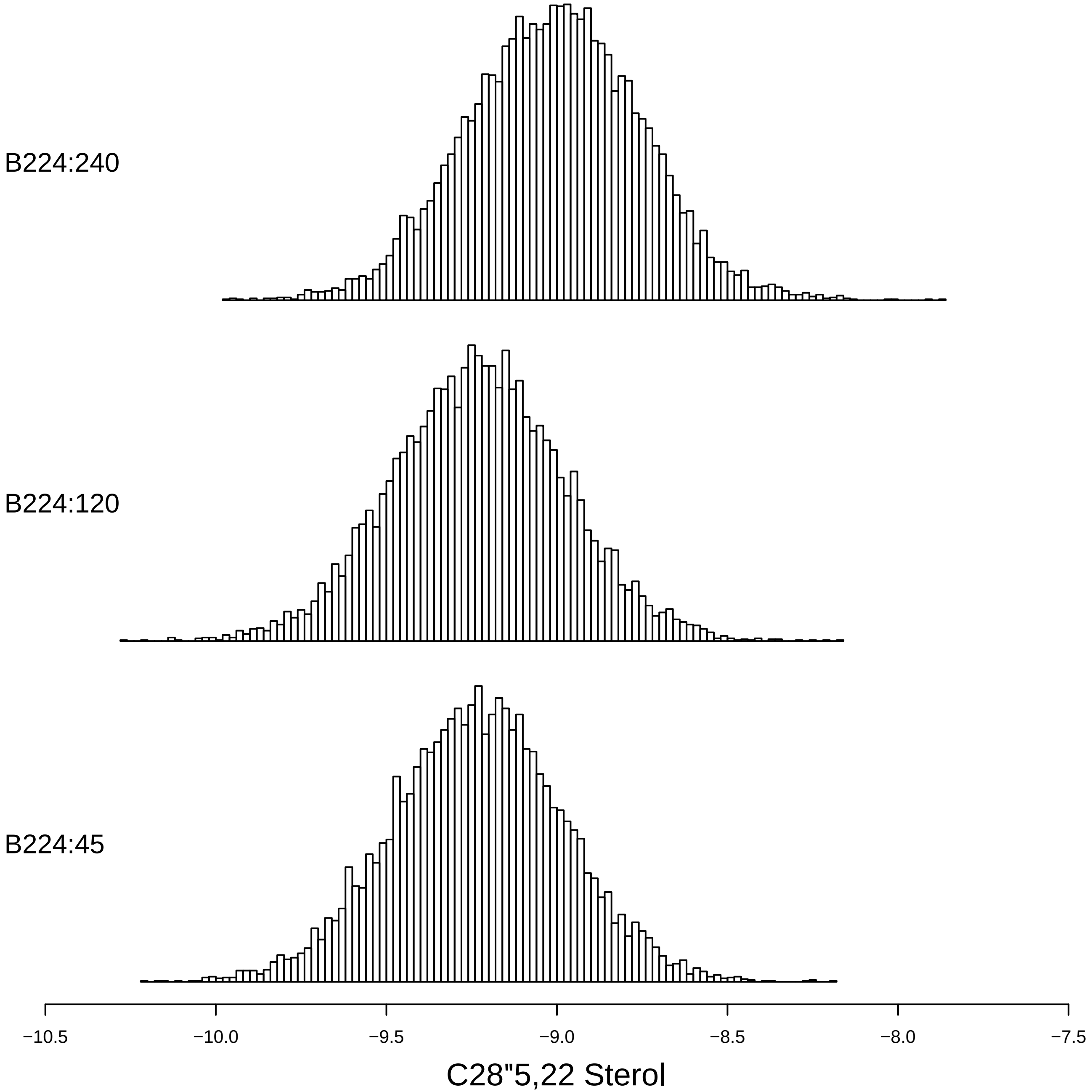

B224:240
B224:120
B224:45
−10.5
−10.0
−9.5
−9.0
−8.5
−8.0
−7.5
C28"5,22 Sterol

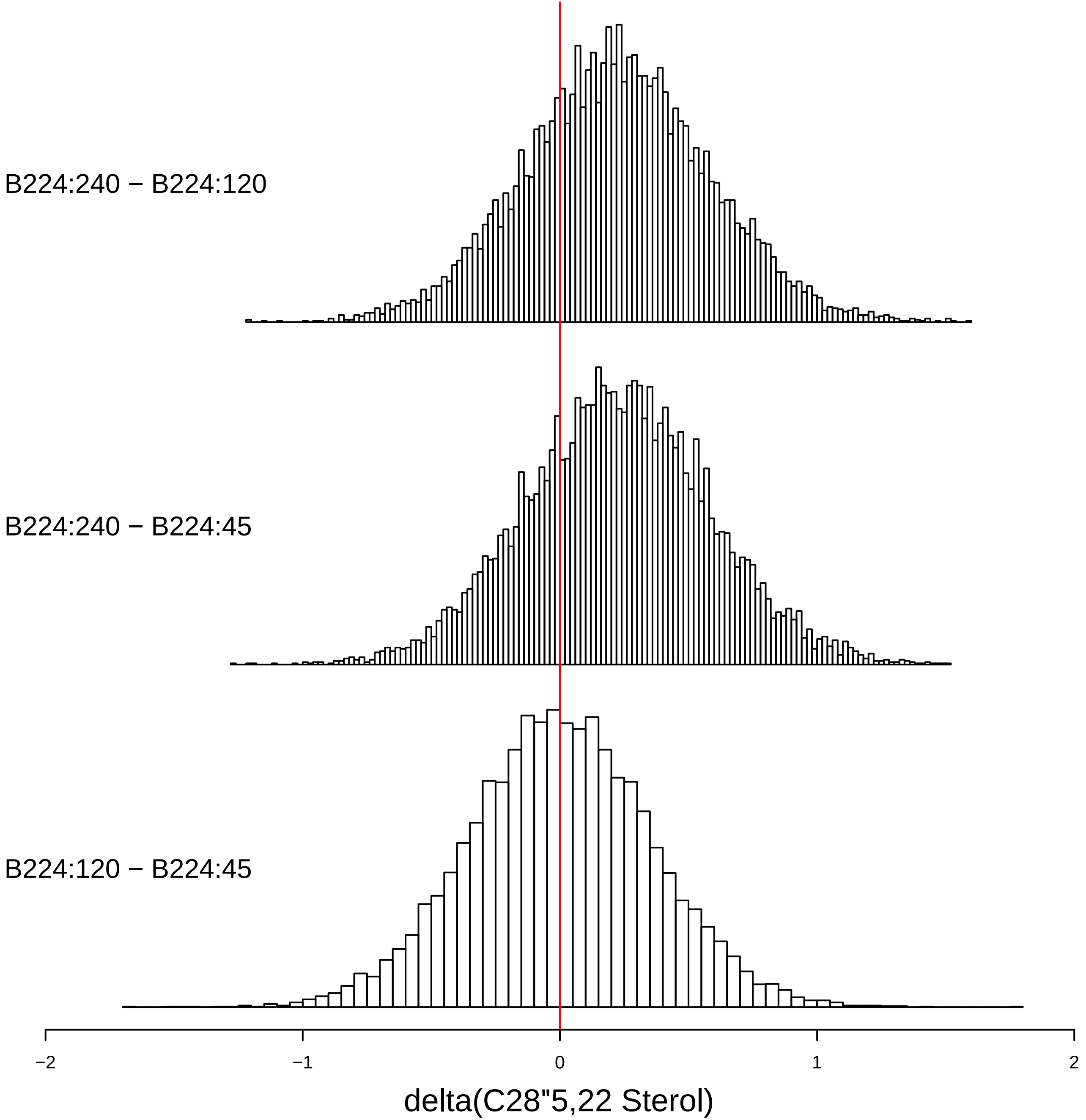
B224:240 − B224:120
B224:240 − B224:45
B224:120 − B224:45
−2
−1
0
1
2
delta(C28"5,22 Sterol)

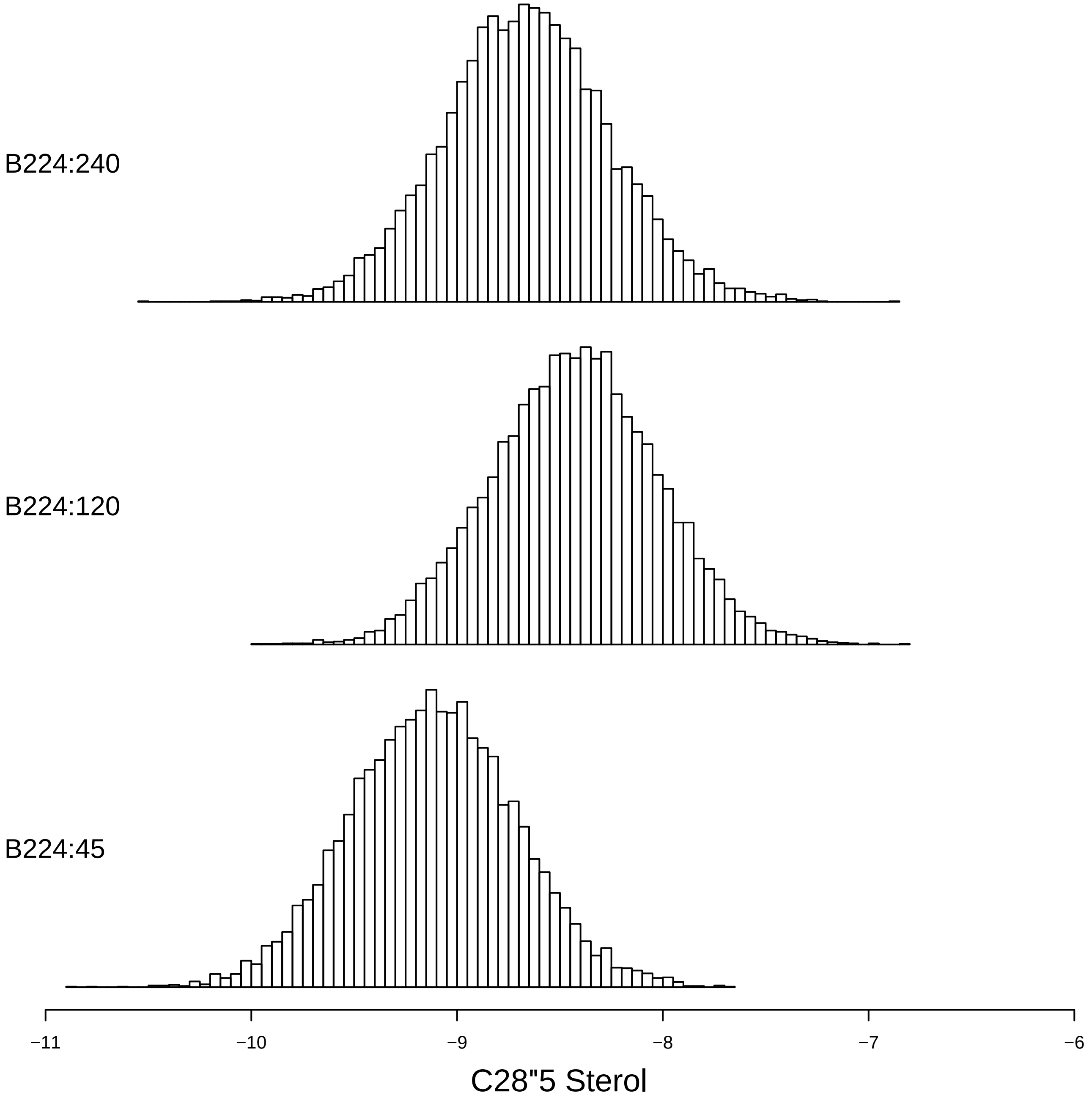

B224:240
B224:120
B224:45
−11
−10
−9
−8
−7
−6
C28"5 Sterol

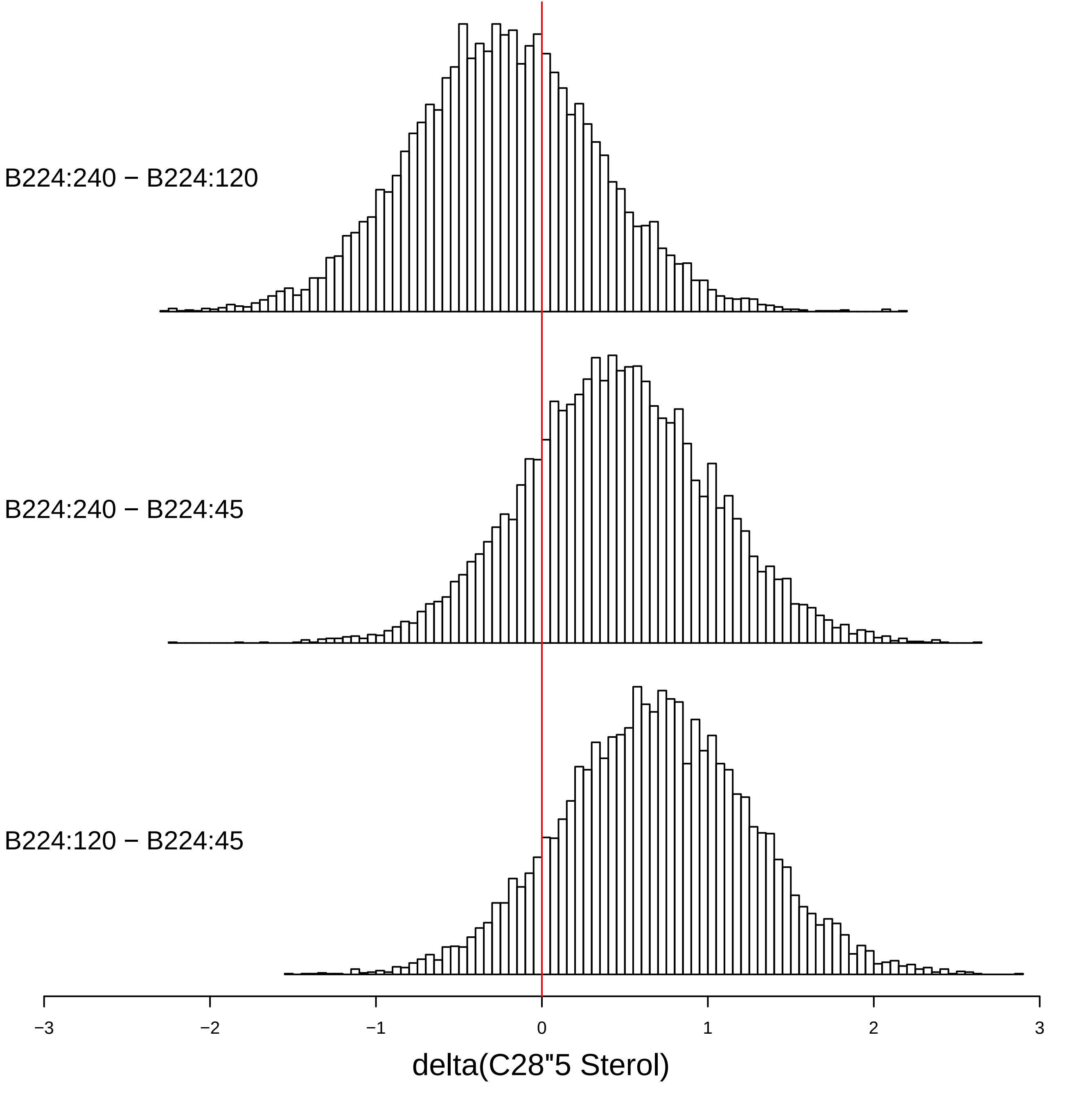
B224:240 − B224:120
B224:240 − B224:45
B224:120 − B224:45
−3
−2
−1
0
1
2
3
delta(C28"5 Sterol)

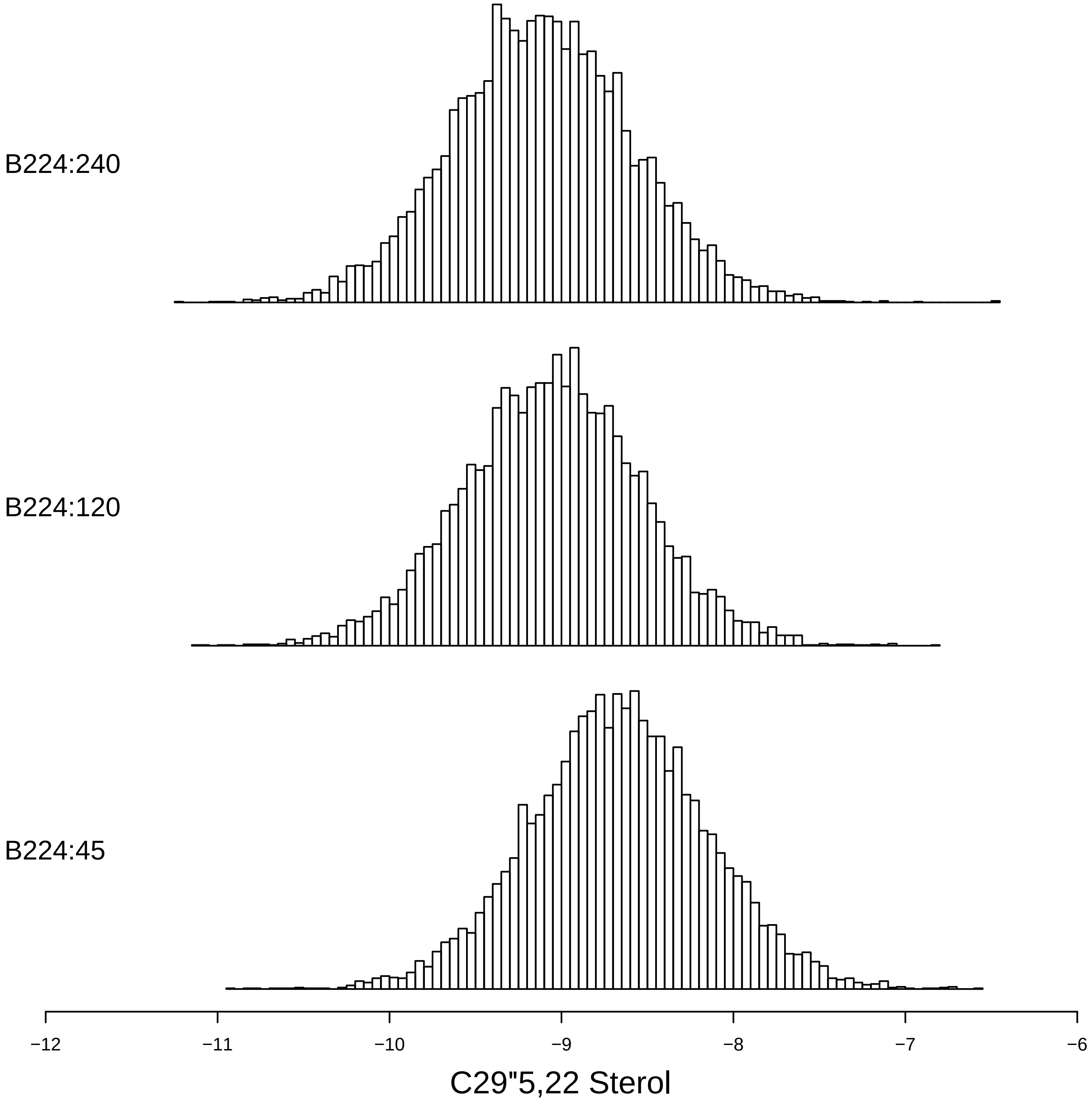
B224:240
B224:120
B224:45
−12
−11
−10
−9
−8
−7
−6
C29"5,22 Sterol

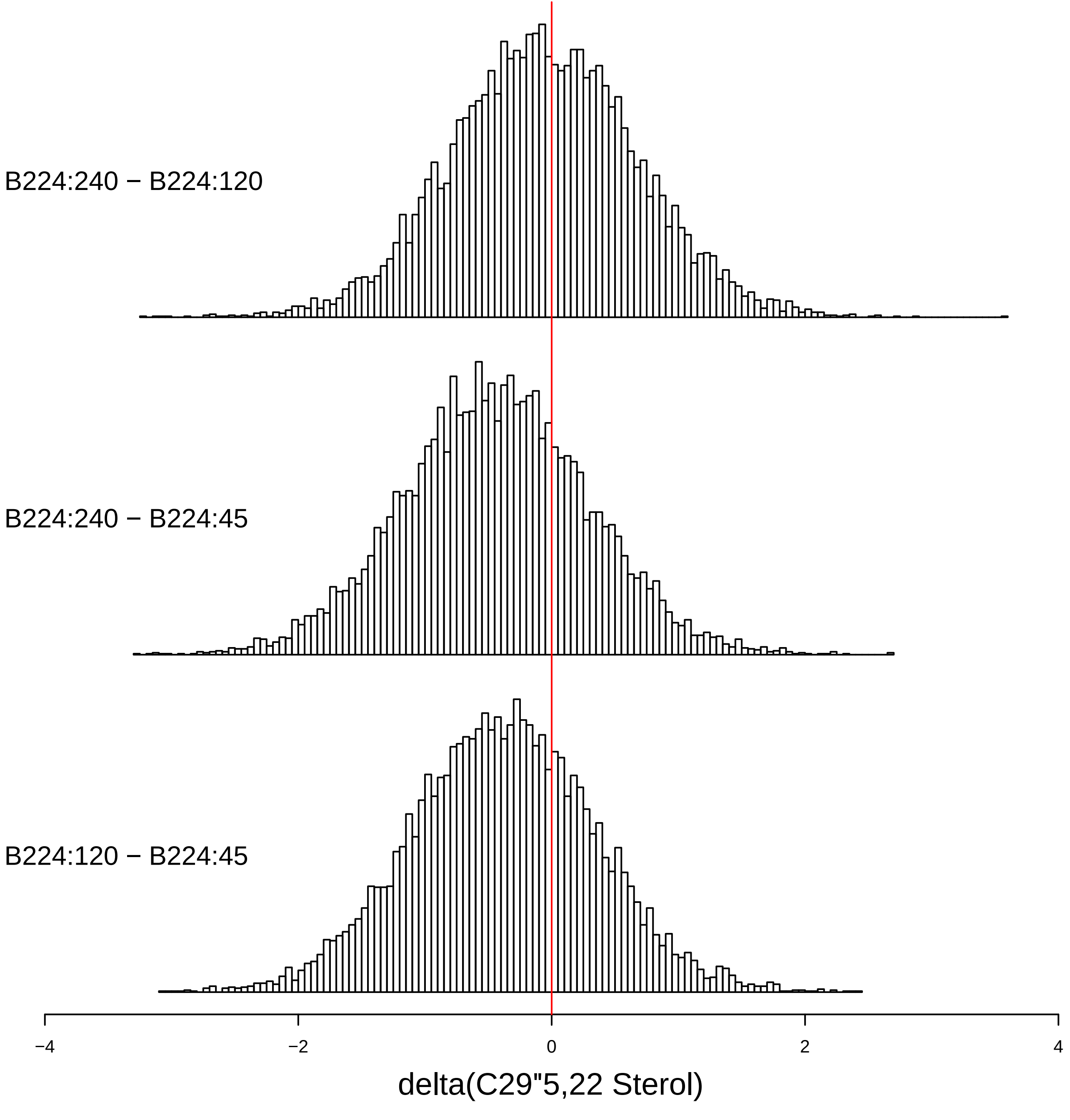

B224:240 − B224:120
B224:240 − B224:45
B224:120 − B224:45
−4
−2
0
2
4
delta(C29"5,22 Sterol)

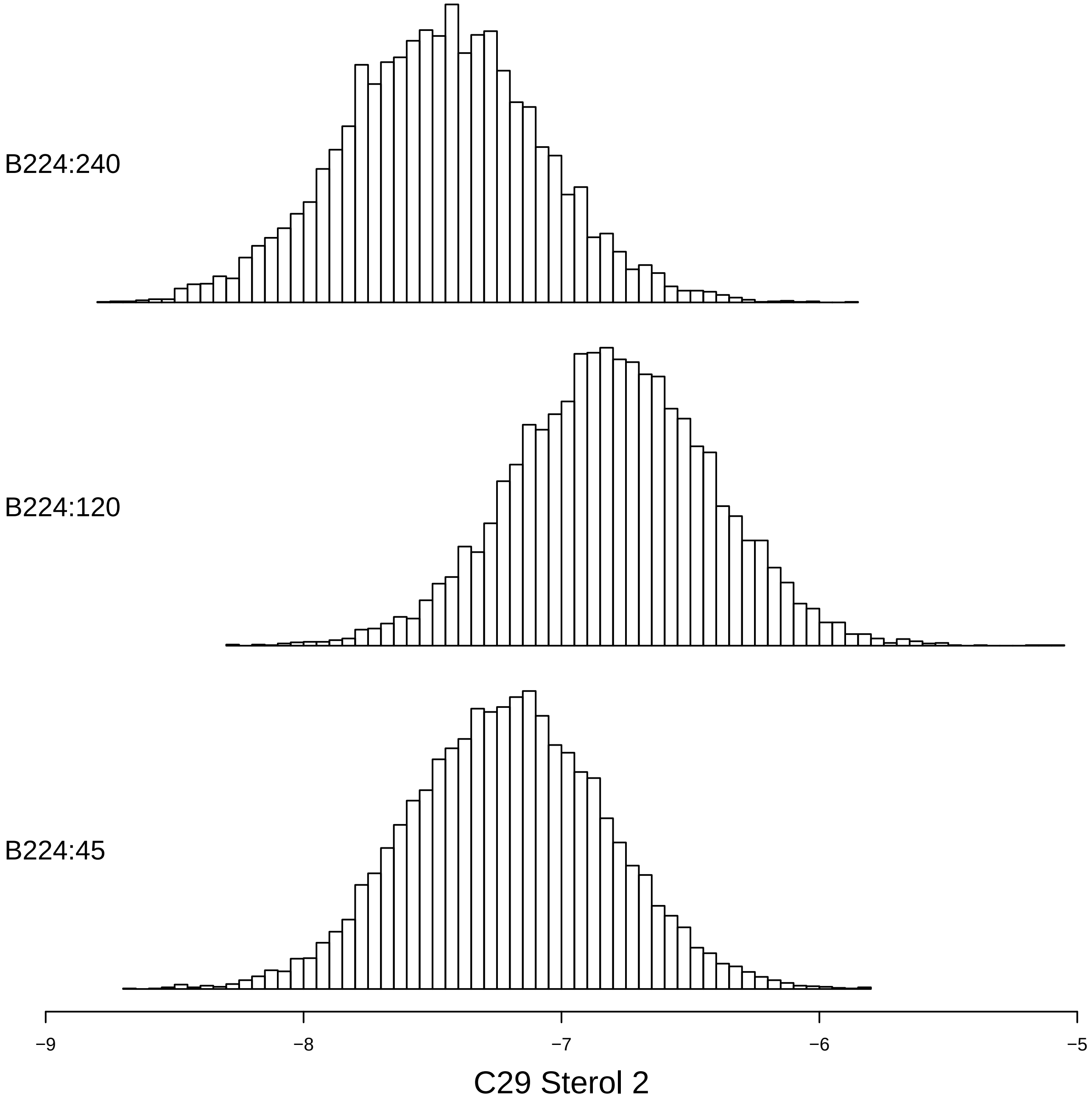
B224:240
B224:120
B224:45
−9
−8
−7
−6
−5
C29 Sterol 2

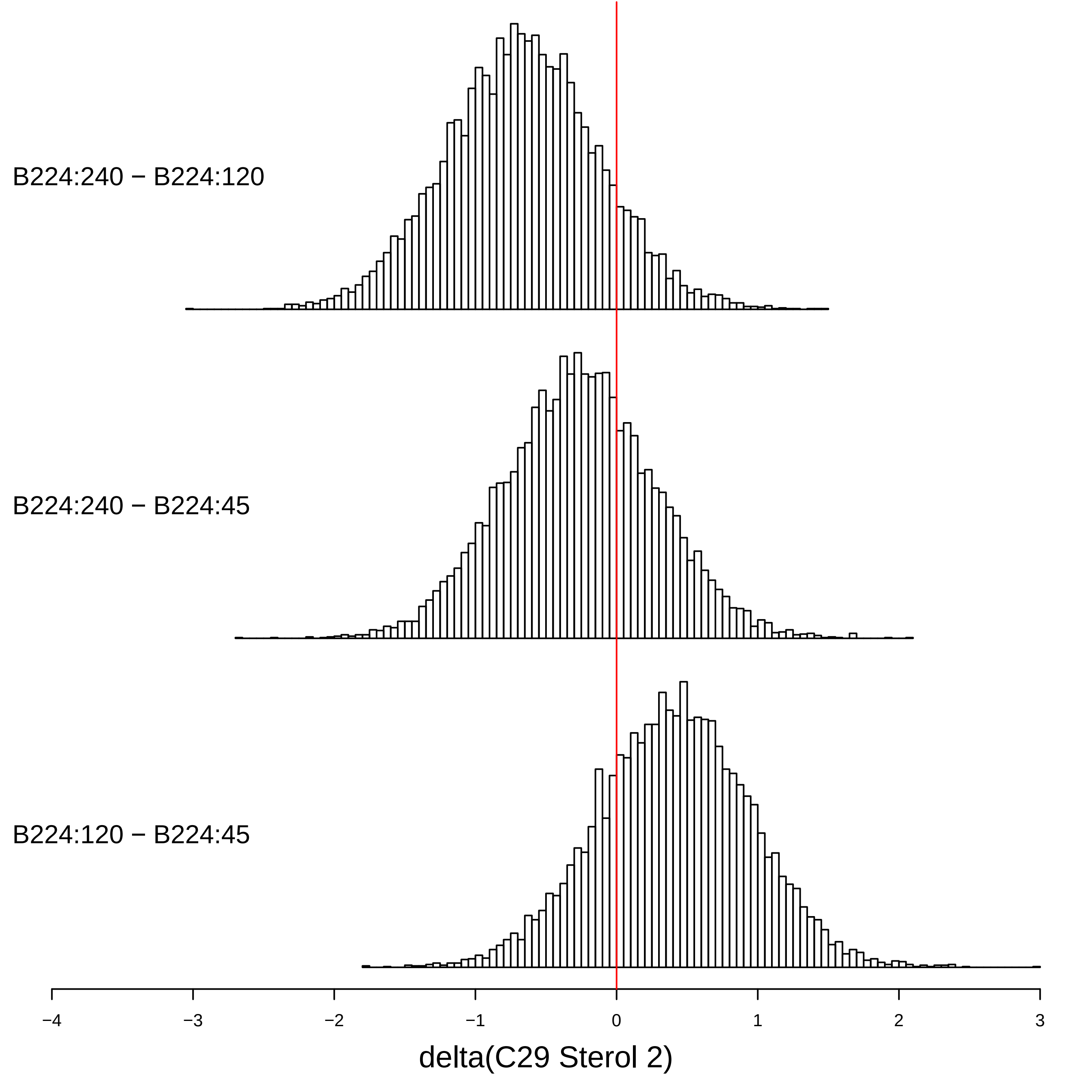

B224:240 − B224:120
B224:240 − B224:45
B224:120 − B224:45
−4
−3
−2
−1
0
1
2
3
delta(C29 Sterol 2)

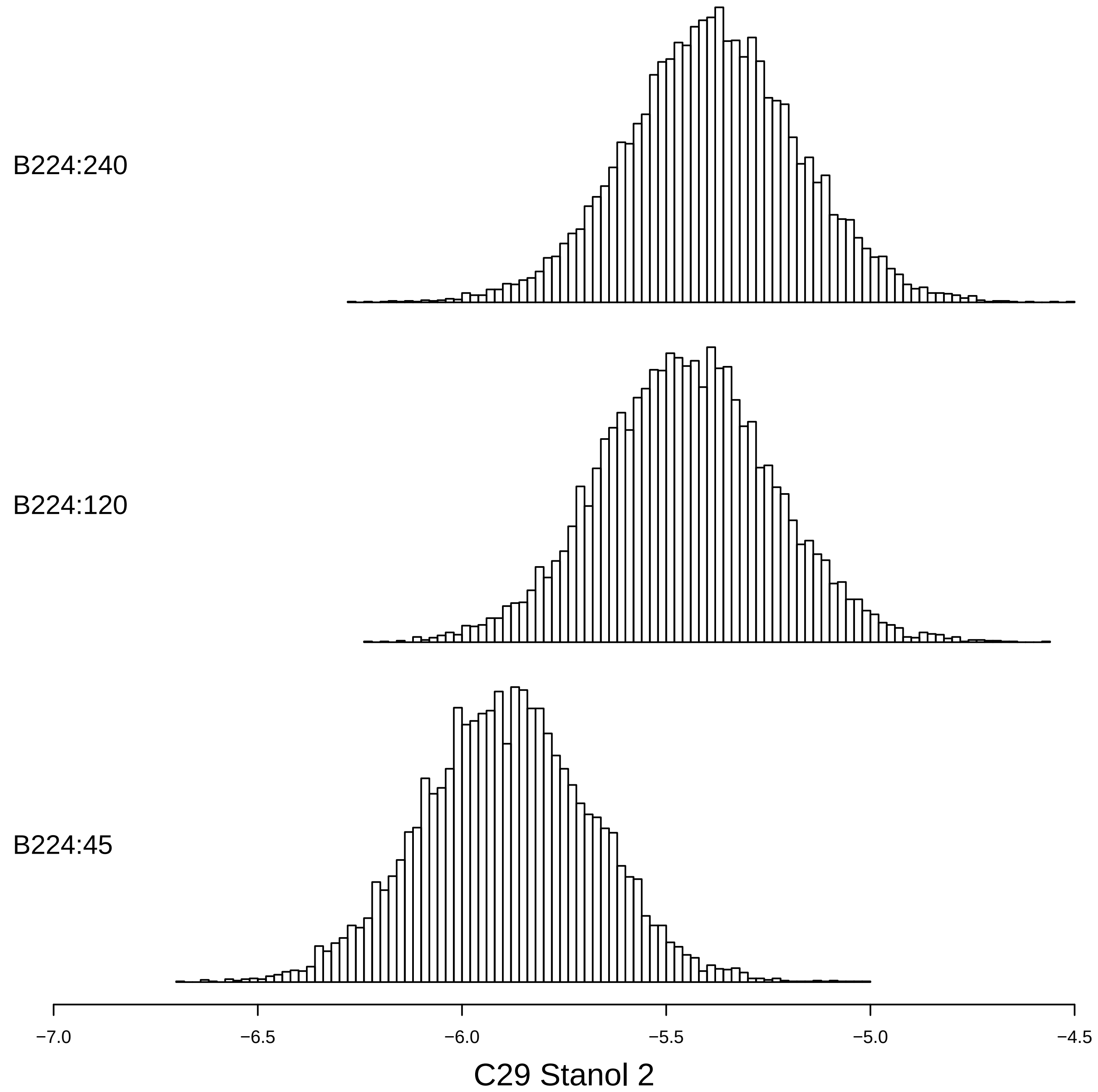
B224:240
B224:120
B224:45
−7.0
−6.5
−6.0
−5.5
−5.0
−4.5
C29 Stanol 2

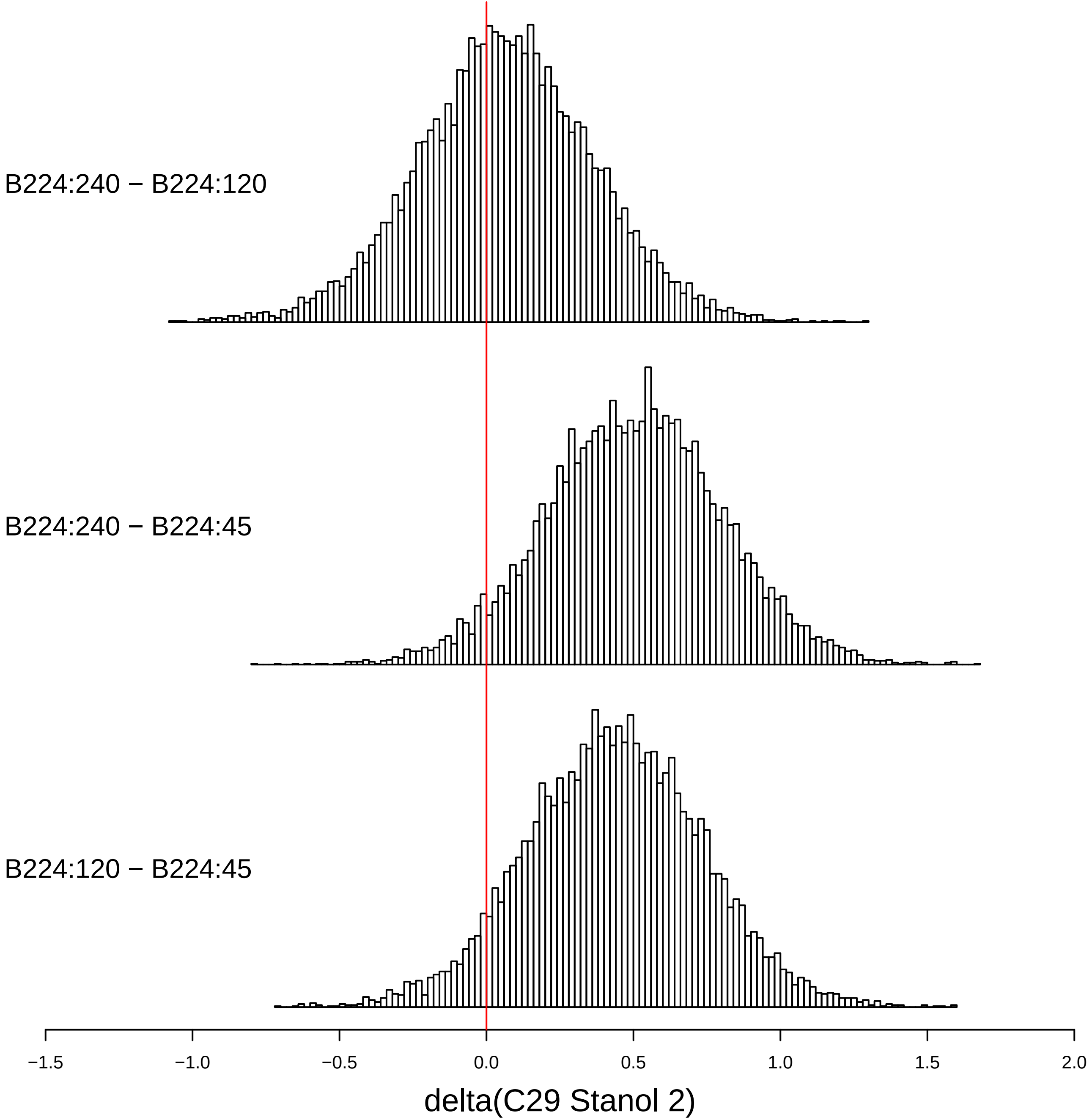

B224:240 − B224:120
B224:240 − B224:45
B224:120 − B224:45
−1.5
−1.0
−0.5
0.0
0.5
1.0
1.5
2.0
delta(C29 Stanol 2)

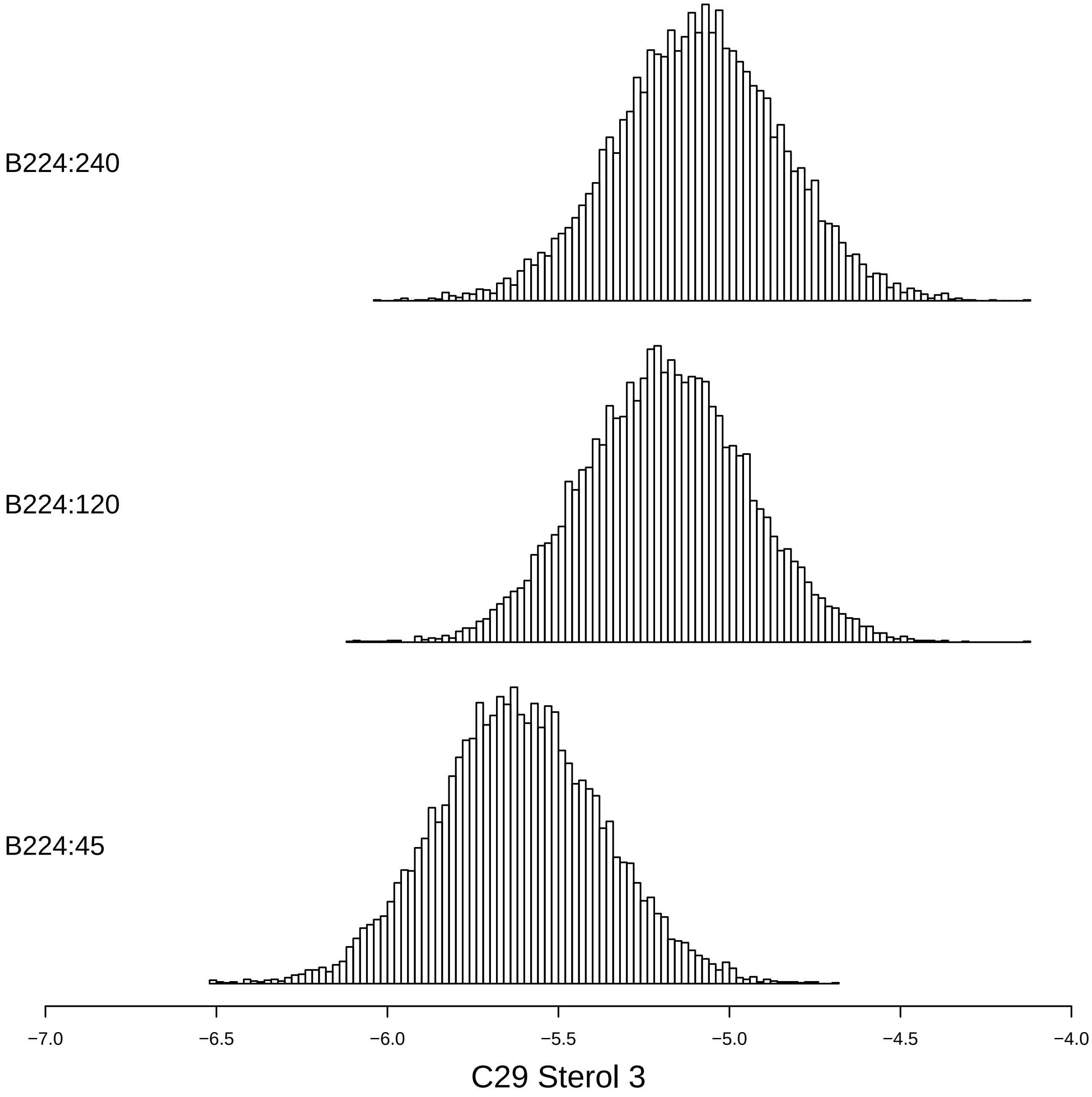
B224:240
B224:120
B224:45
−7.0
−6.5
−6.0
−5.5
−5.0
−4.5
−4.0
C29 Sterol 3

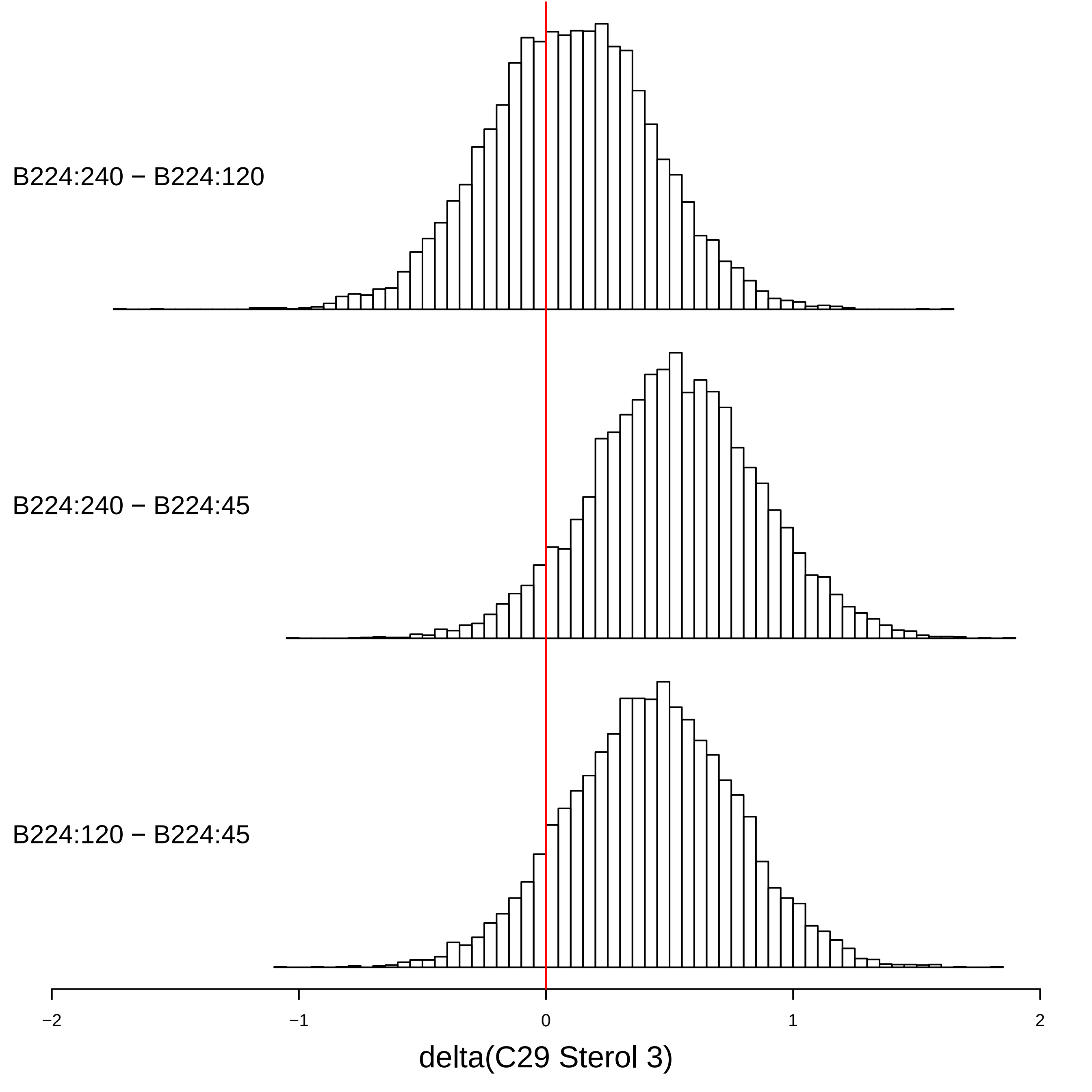

B224:240 − B224:120
B224:240 − B224:45
B224:120 − B224:45
−2
−1
0
1
2
delta(C29 Sterol 3)

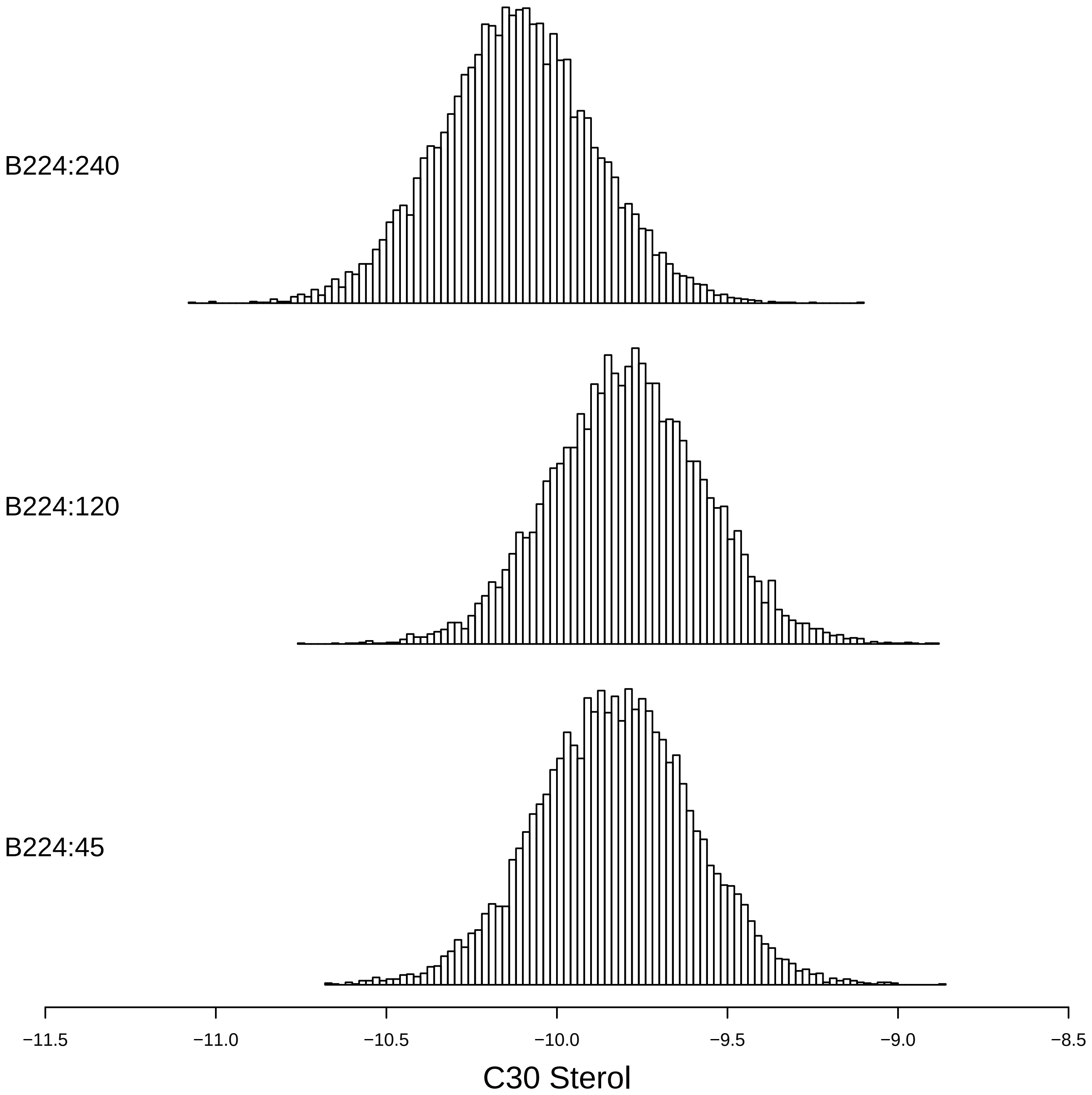
B224:240
B224:120
B224:45
−11.5
−11.0
−10.5
−10.0
−9.5
−9.0
−8.5
C30 Sterol

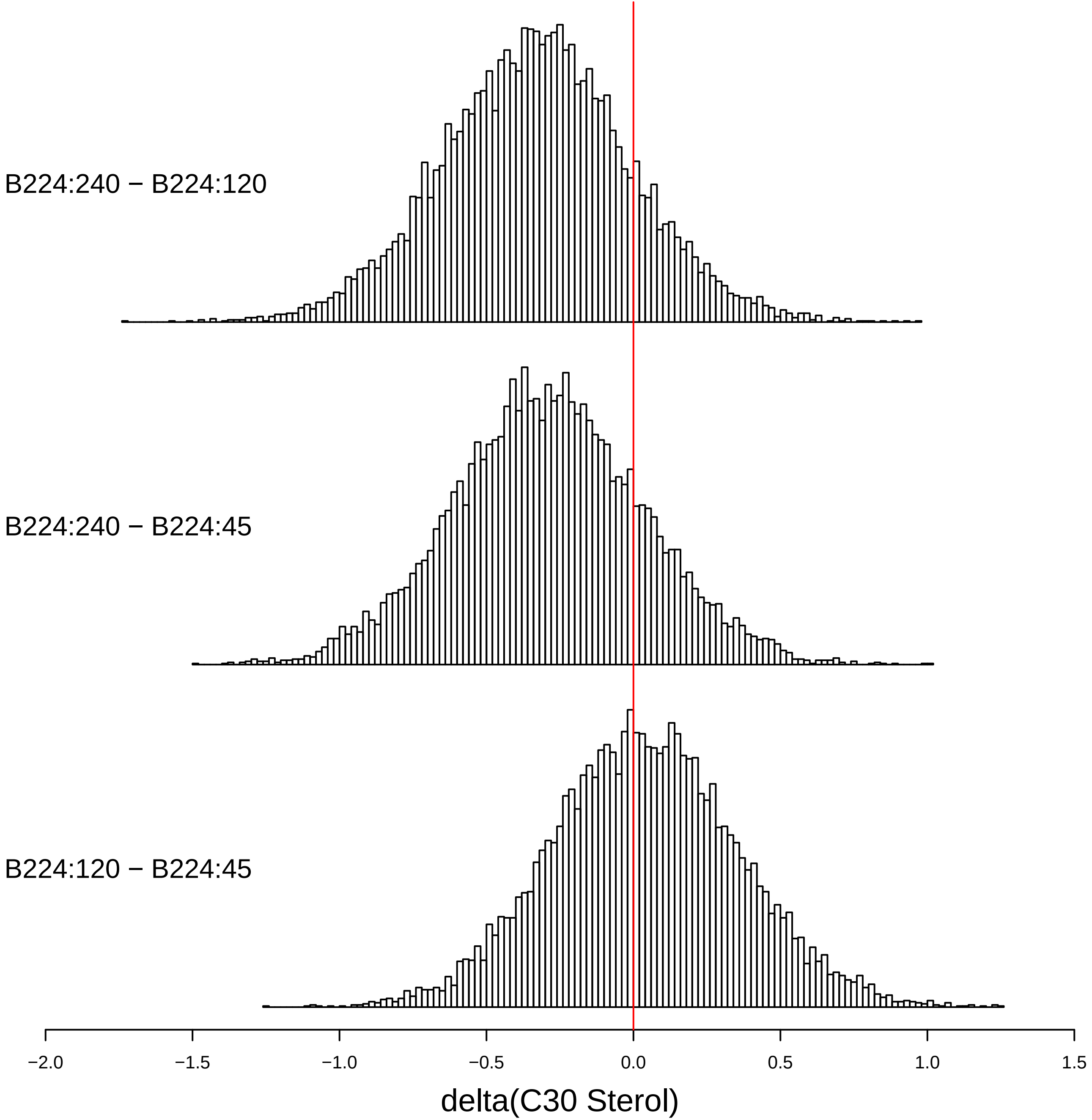

B224:240 − B224:120
B224:240 − B224:45
B224:120 − B224:45
−2.0
−1.5
−1.0
−0.5
0.0
0.5
1.0
1.5
delta(C30 Sterol)

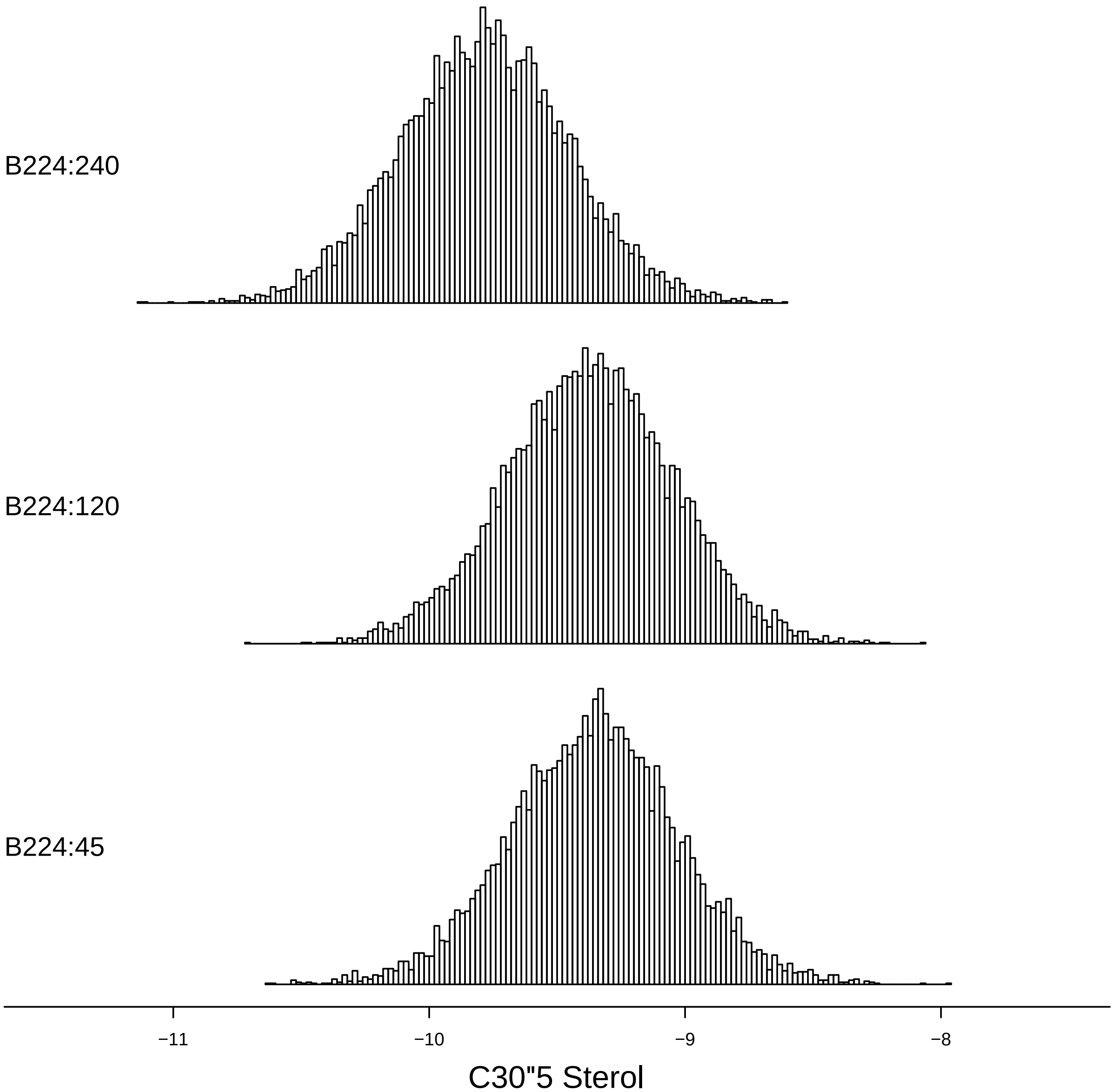
B224:240
B224:120
B224:45
−11
−10
−9
−8
C30"5 Sterol

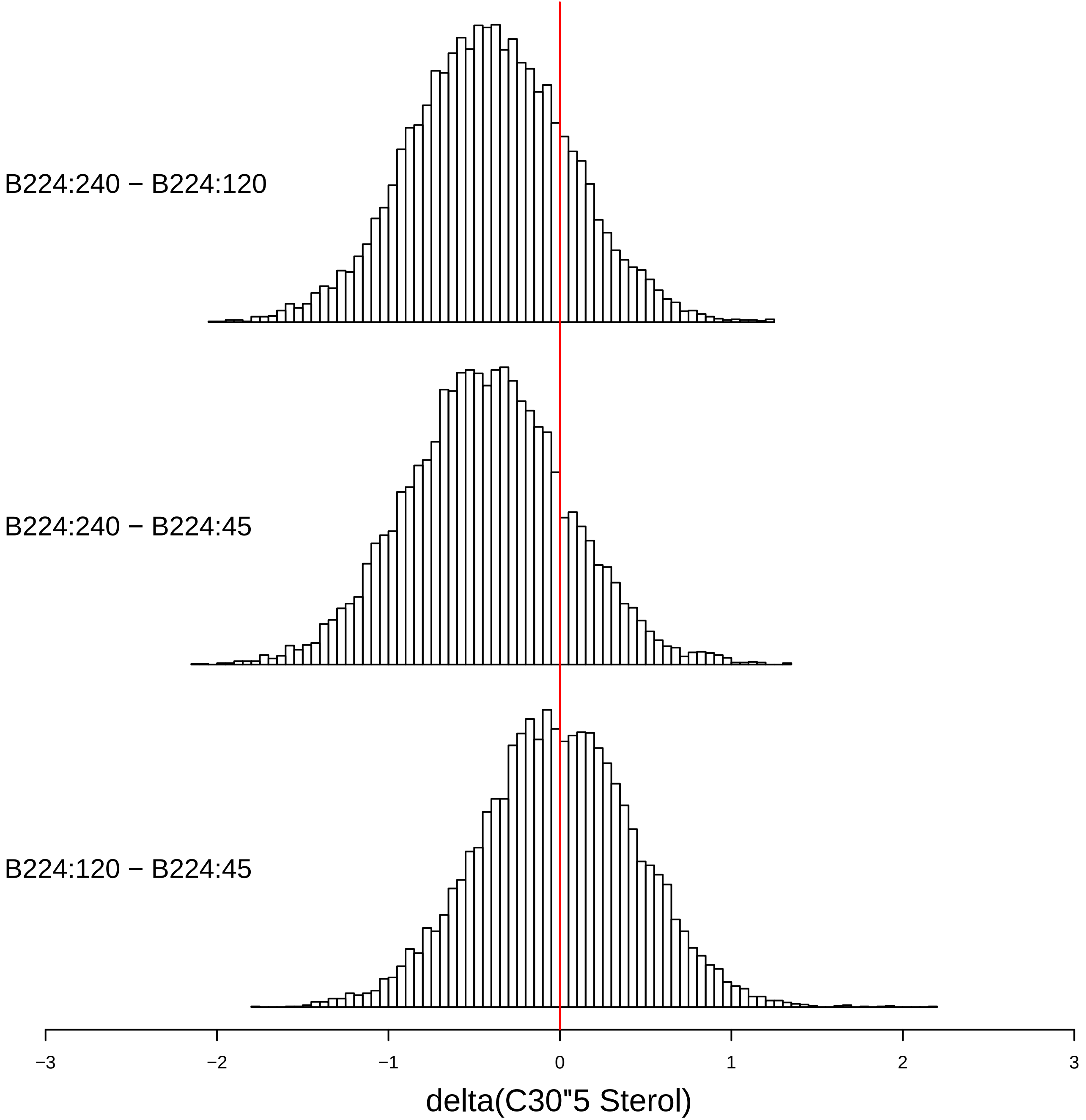
B224:240 − B224:120
B224:240 − B224:45
B224:120 − B224:45
−3
−2
−1
0
1
2
3
delta(C30"5 Sterol)

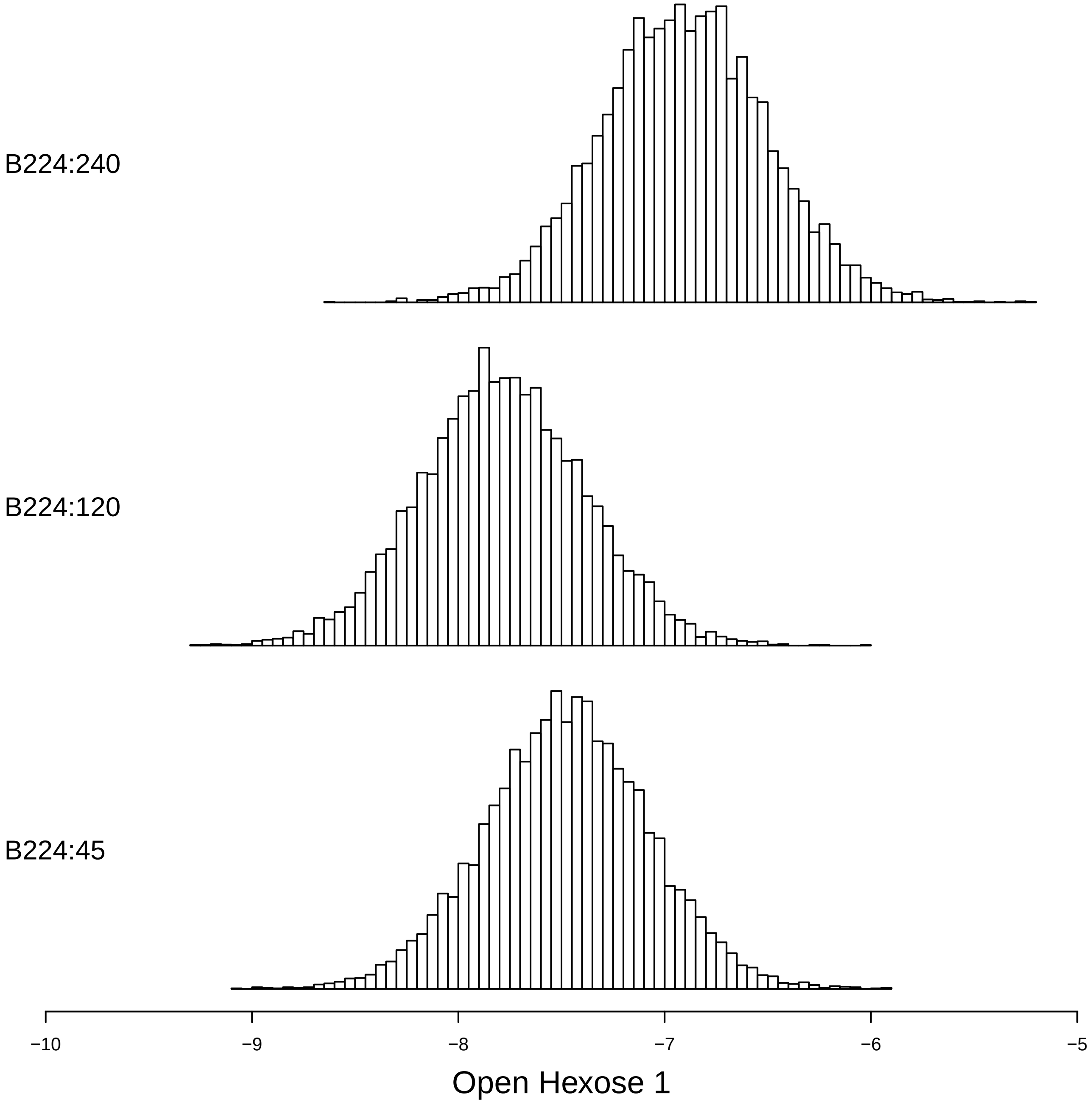

B224:240
B224:120
B224:45
−10
−9
−8
−7
−6
−5
Open Hexose 1

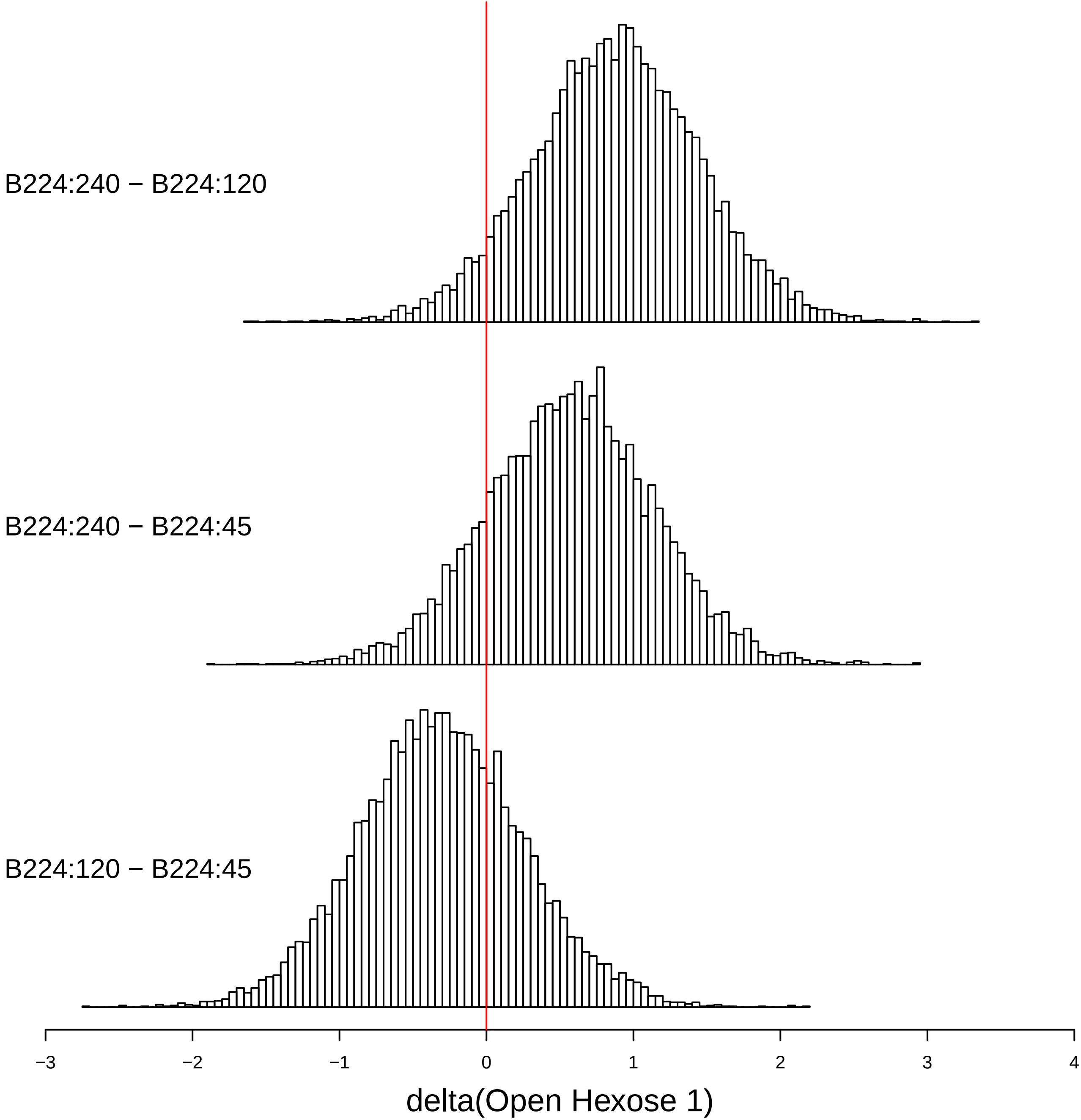
B224:240 − B224:120
B224:240 − B224:45
B224:120 − B224:45
−3
−2
−1
0
1
2
3
4
delta(Open Hexose 1)

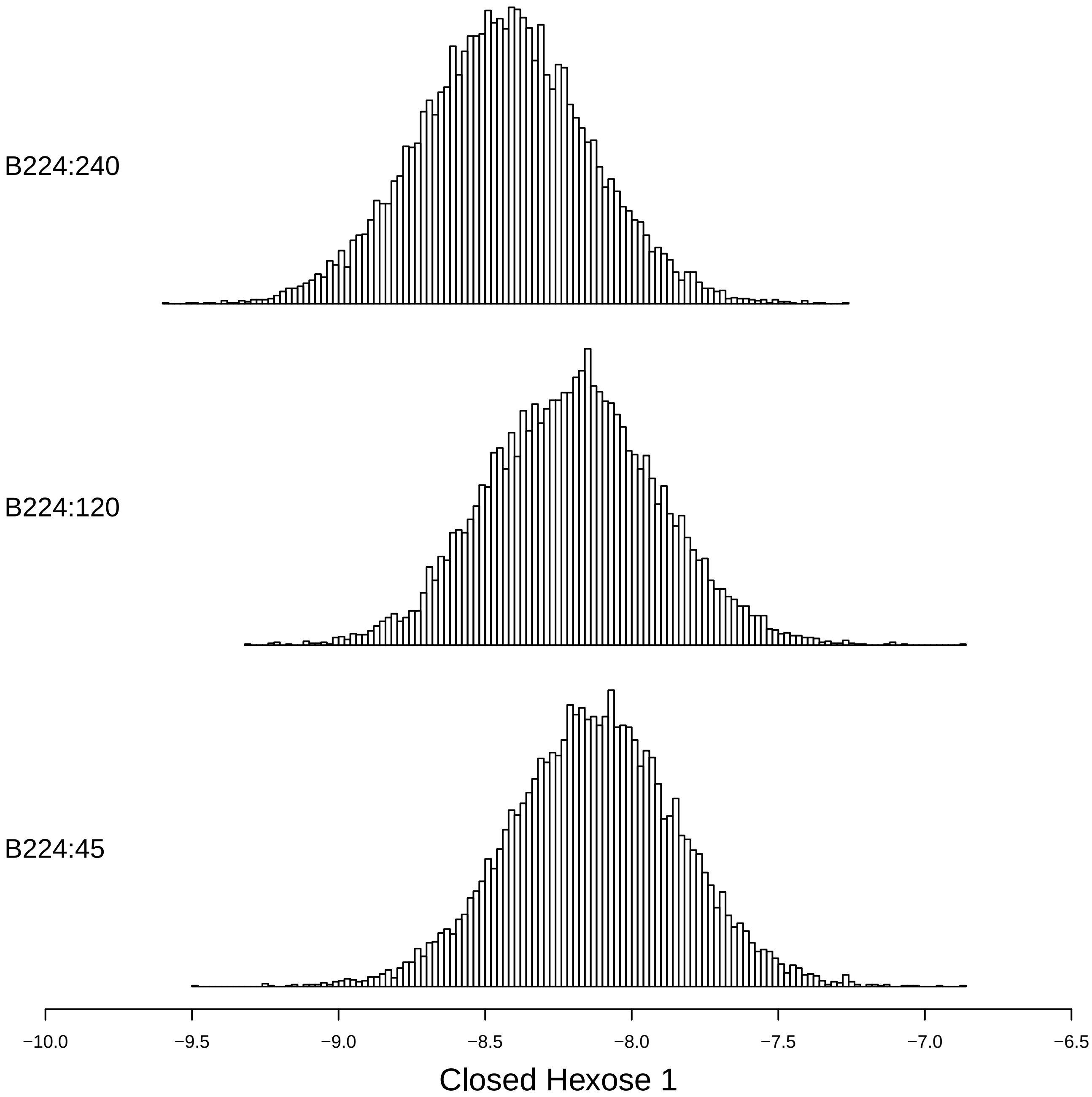
B224:240
B224:120
B224:45
−10.0
−9.5
−9.0
−8.5
−8.0
−7.5
−7.0
−6.5
Closed Hexose 1

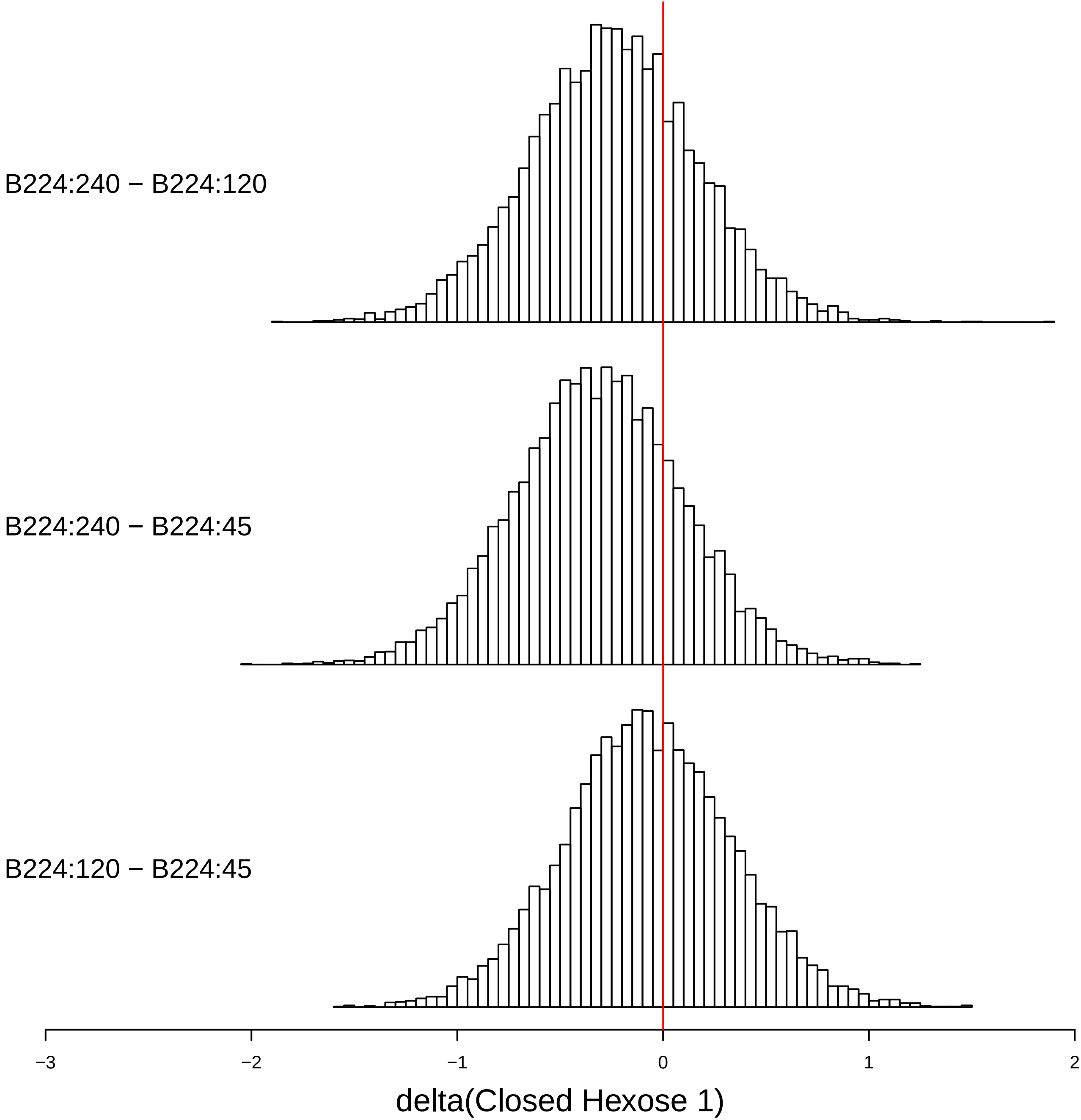

B224:240 − B224:120
B224:240 − B224:45
B224:120 − B224:45
−3
−2
−1
0
1
2
delta(Closed Hexose 1)

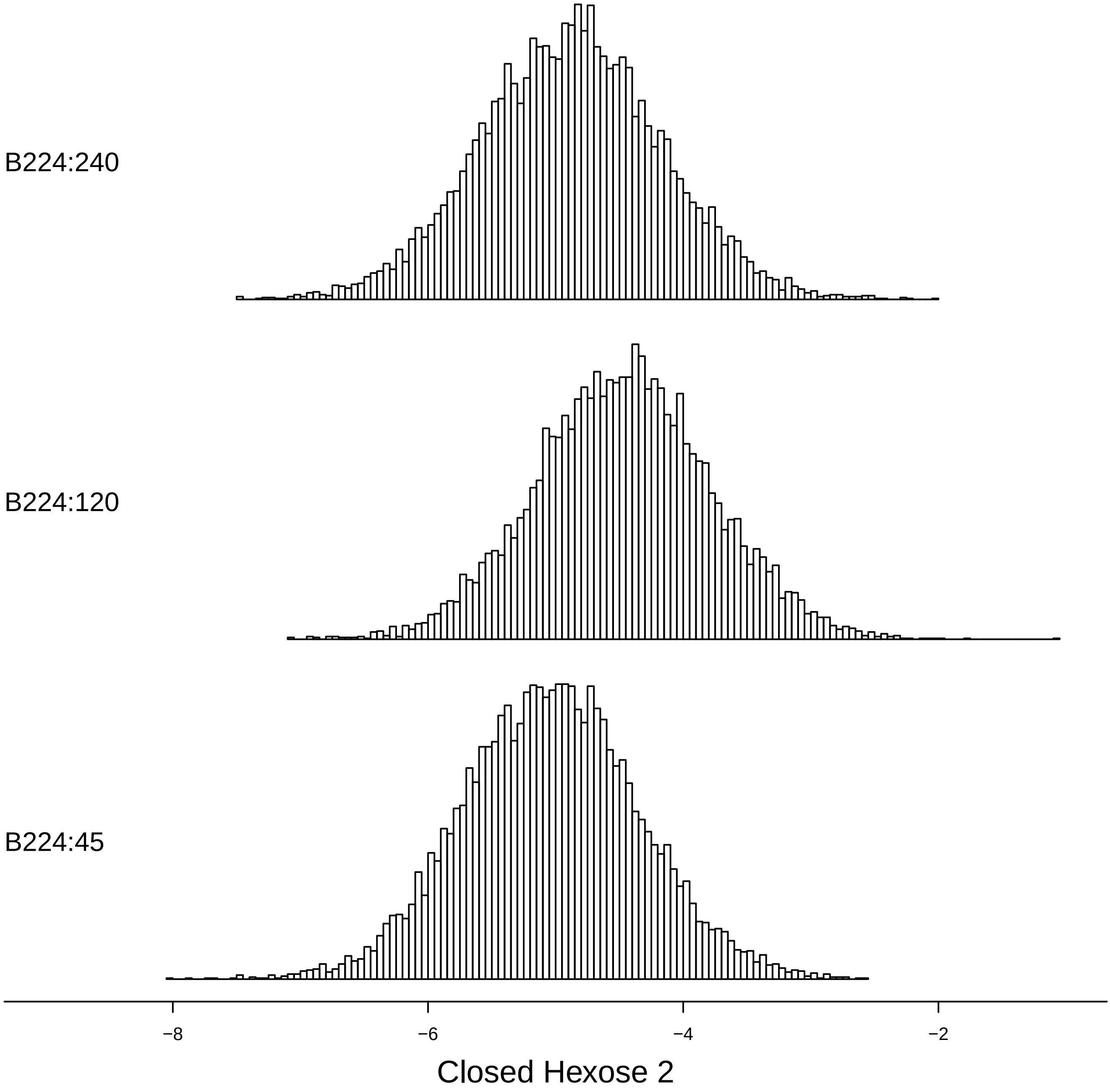
B224:240
B224:120
B224:45
−8
−6
−4
−2
Closed Hexose 2

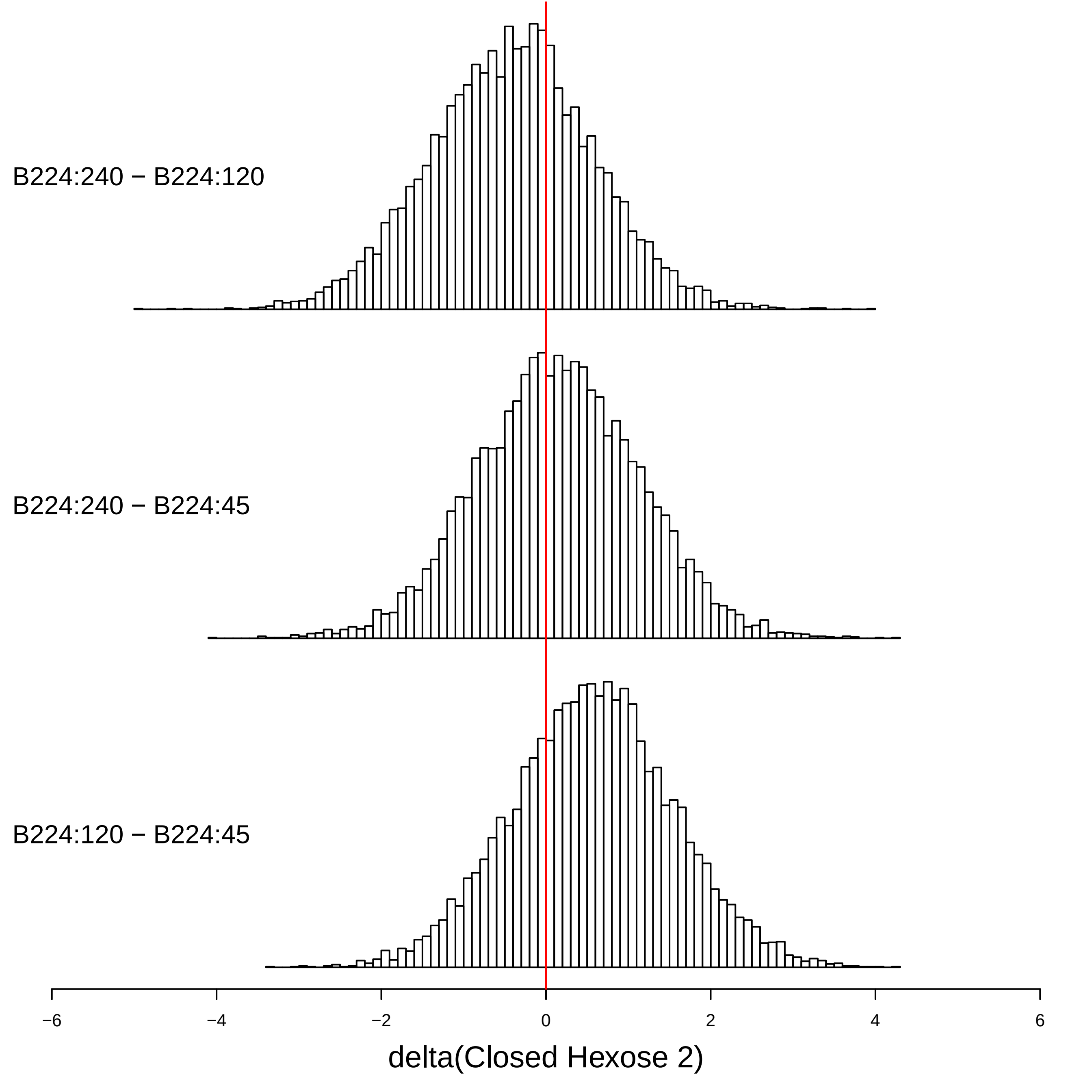

B224:240 − B224:120
B224:240 − B224:45
B224:120 − B224:45
−6
−4
−2
0
2
4
6
delta(Closed Hexose 2)

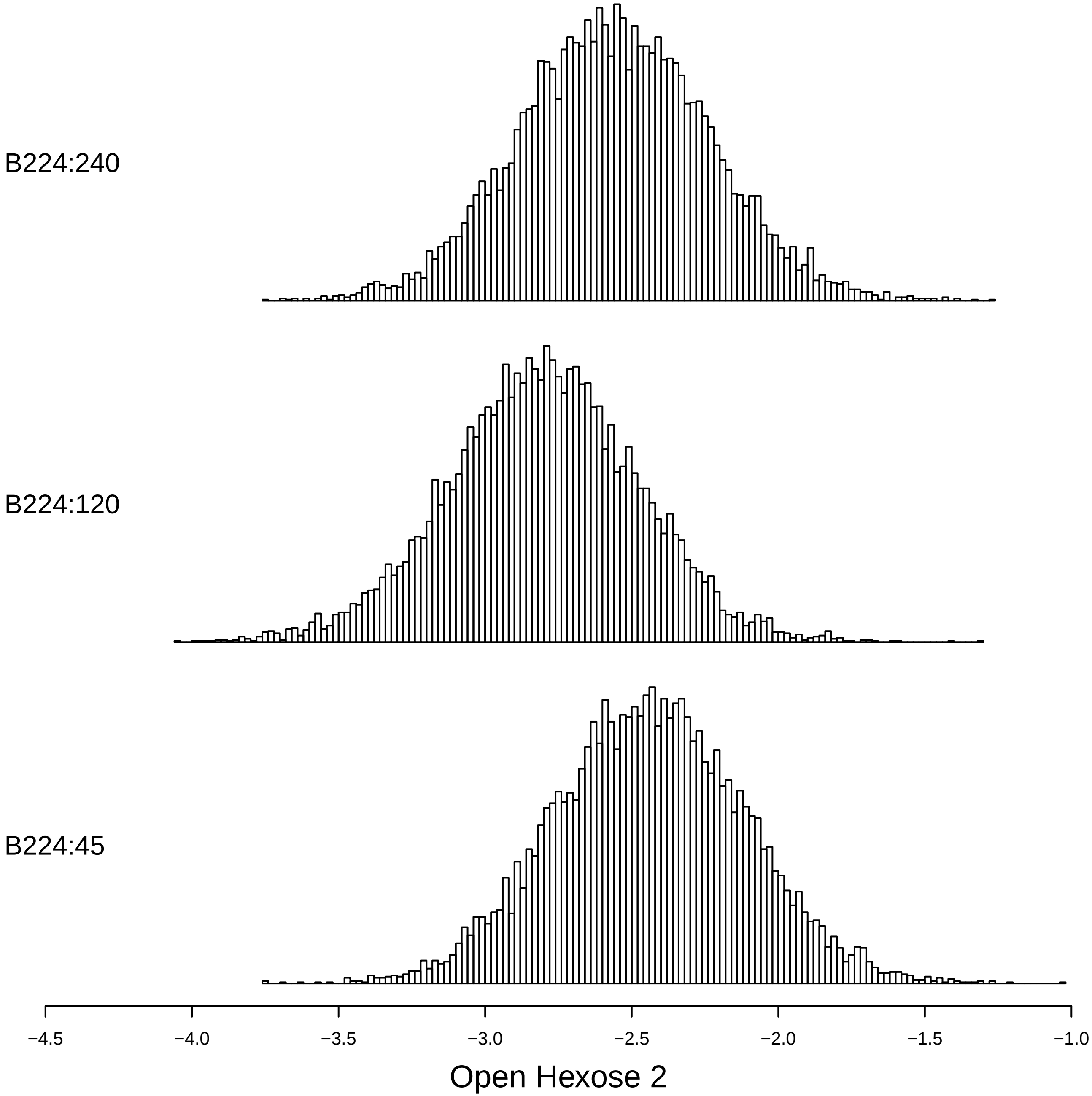

B224:240
B224:120
B224:45
−4.5
−4.0
−3.5
−3.0
−2.5
−2.0
−1.5
−1.0
Open Hexose 2

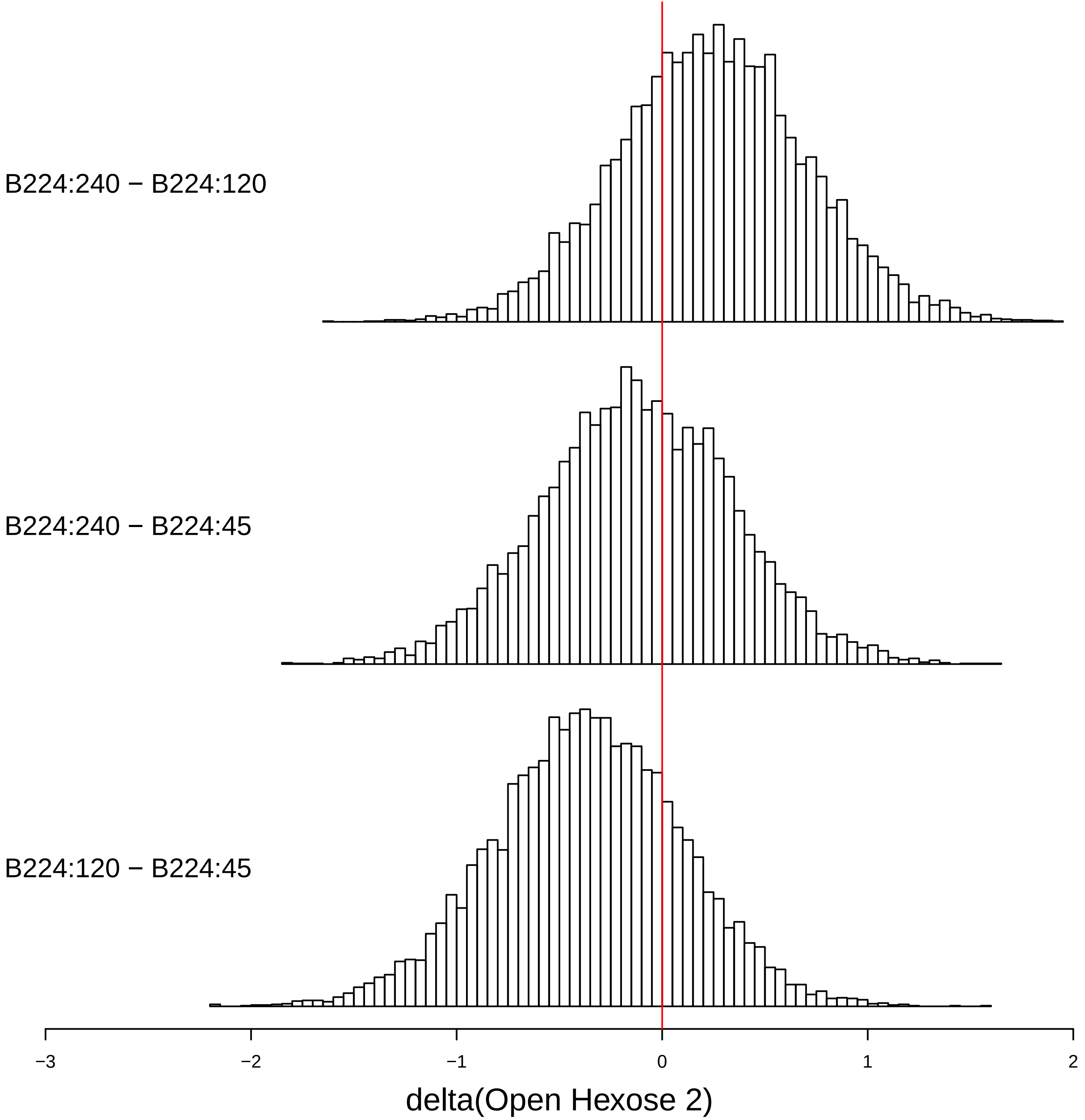
B224:240 − B224:120
B224:240 − B224:45
B224:120 − B224:45
−3
−2
−1
0
1
2
delta(Open Hexose 2)

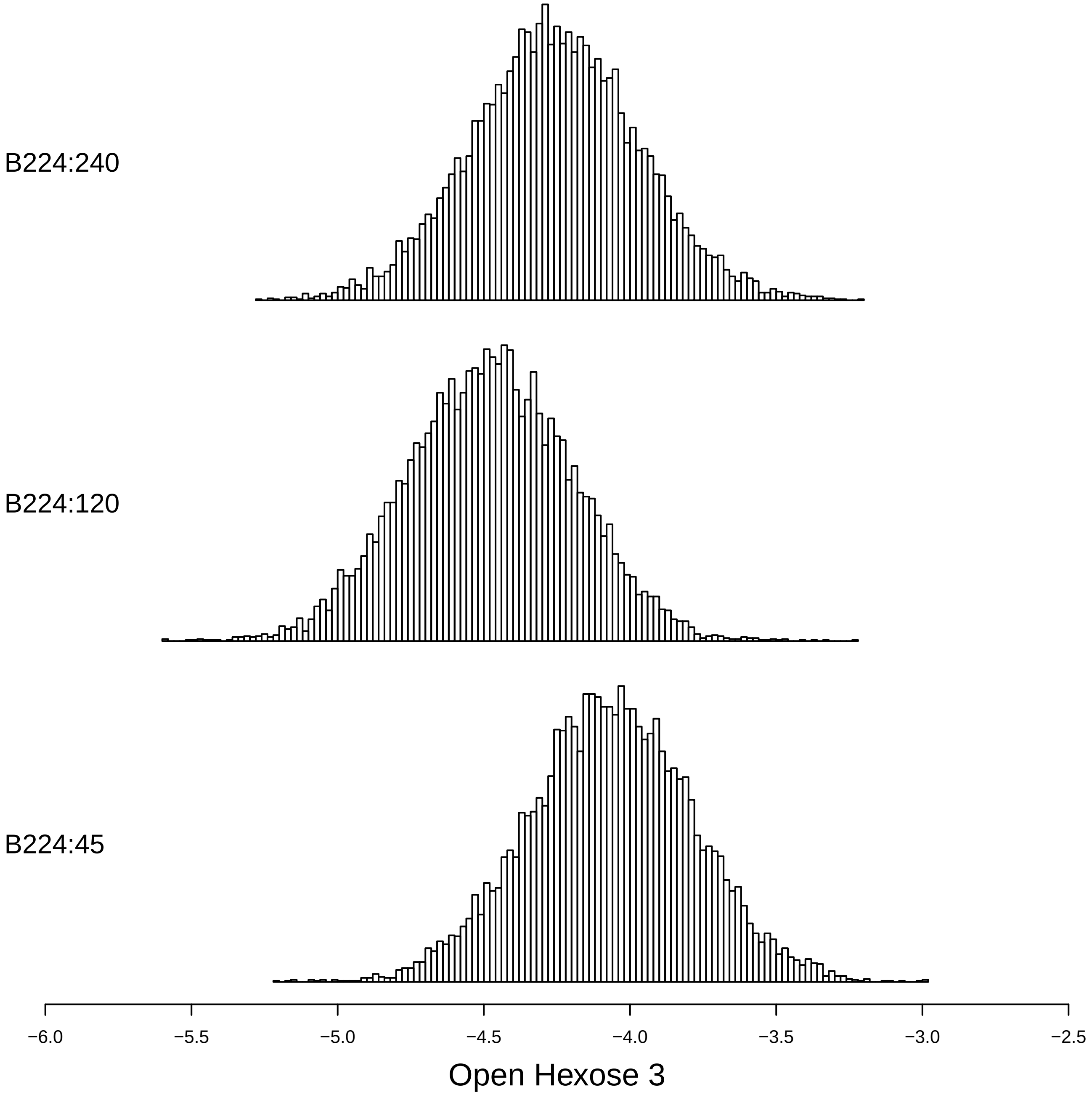

B224:240
B224:120
B224:45
−6.0
−5.5
−5.0
−4.5
−4.0
−3.5
−3.0
−2.5
Open Hexose 3

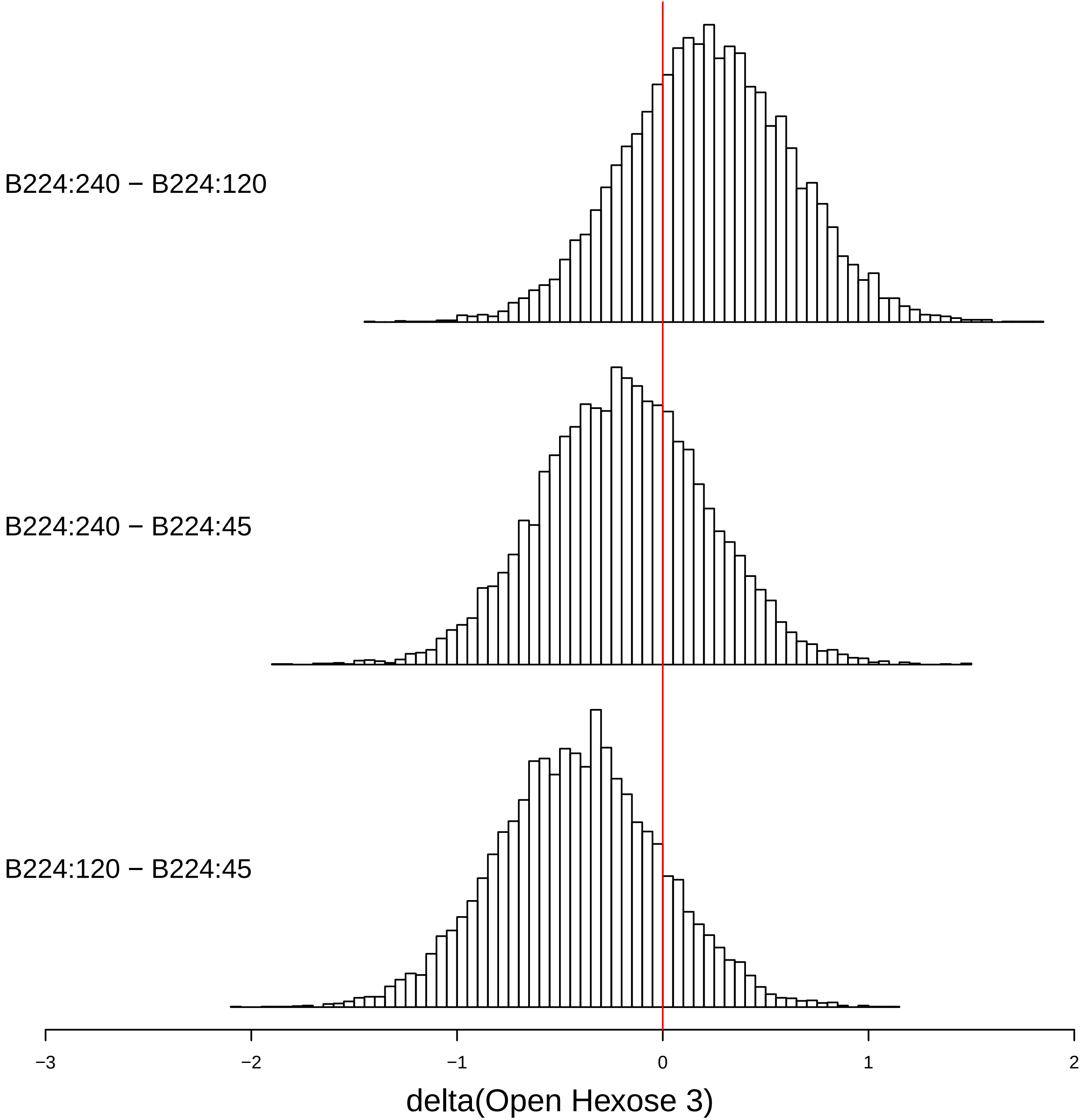

B224:240 − B224:120
B224:240 − B224:45
B224:120 − B224:45
−3
−2
−1
0
1
2
delta(Open Hexose 3)

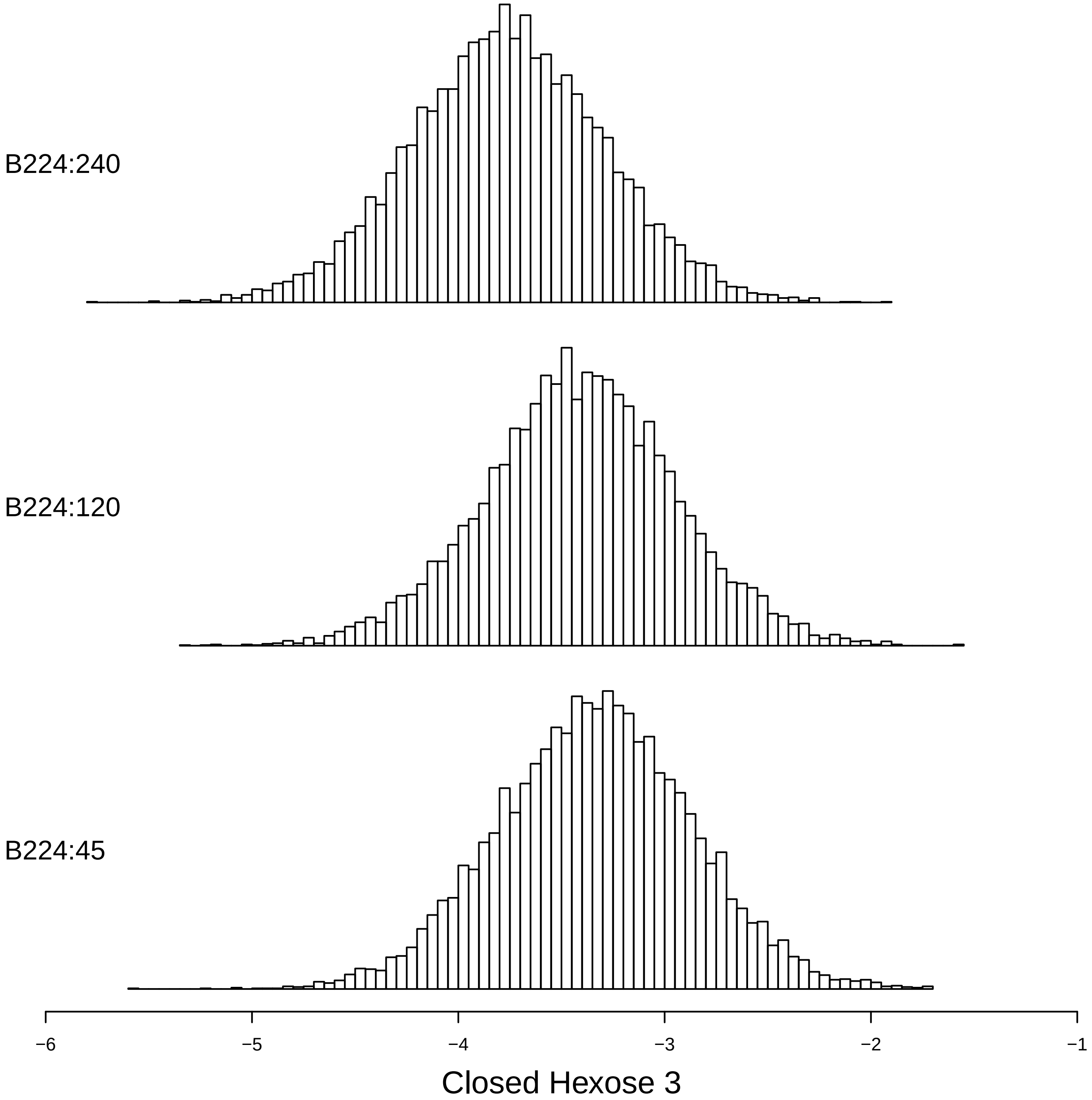

B224:240
B224:120
B224:45
−6
−5
−4
−3
−2
−1
Closed Hexose 3

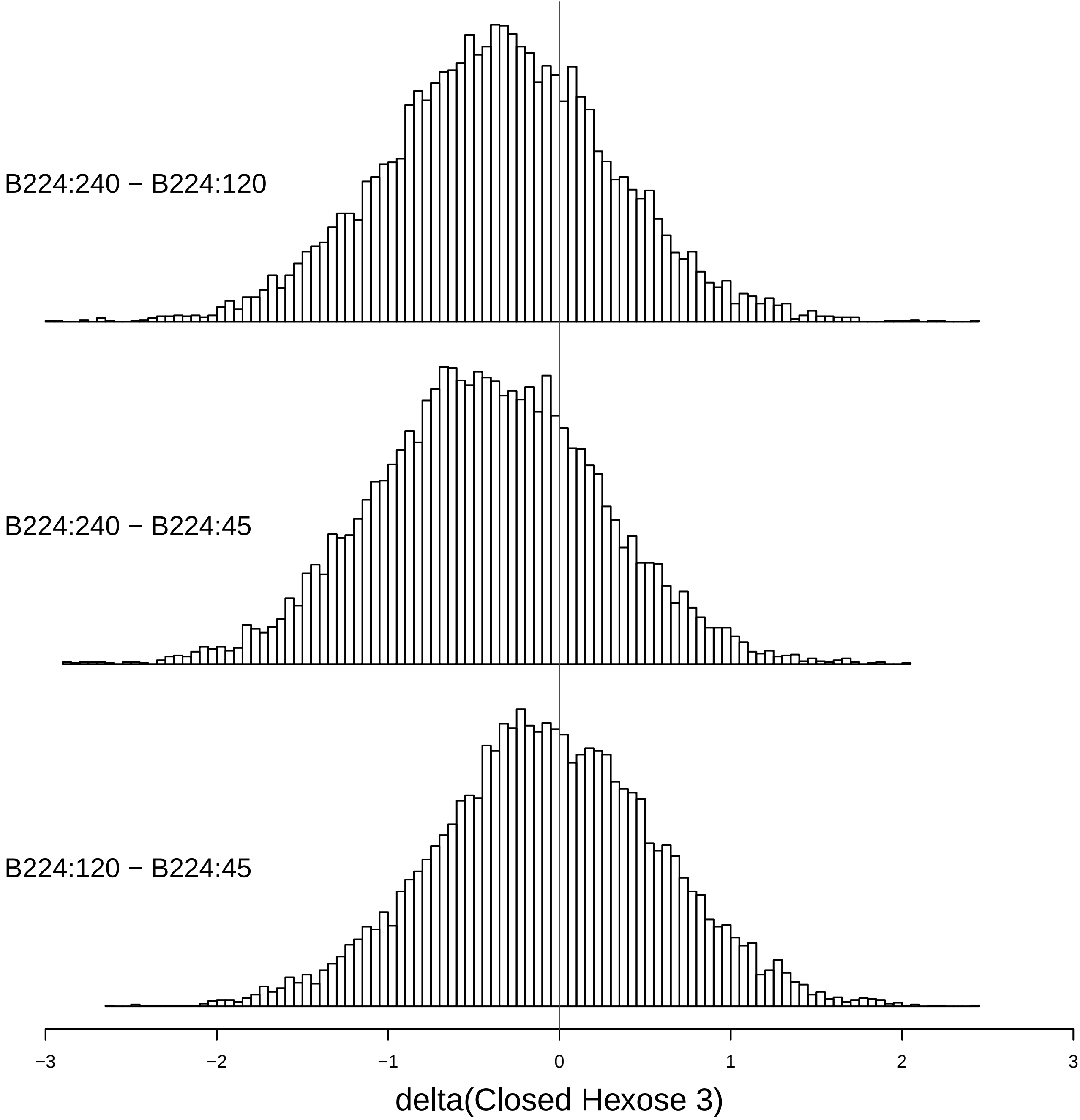

B224:240 − B224:120
B224:240 − B224:45
B224:120 − B224:45
−3
−2
−1
0
1
2
3
delta(Closed Hexose 3)

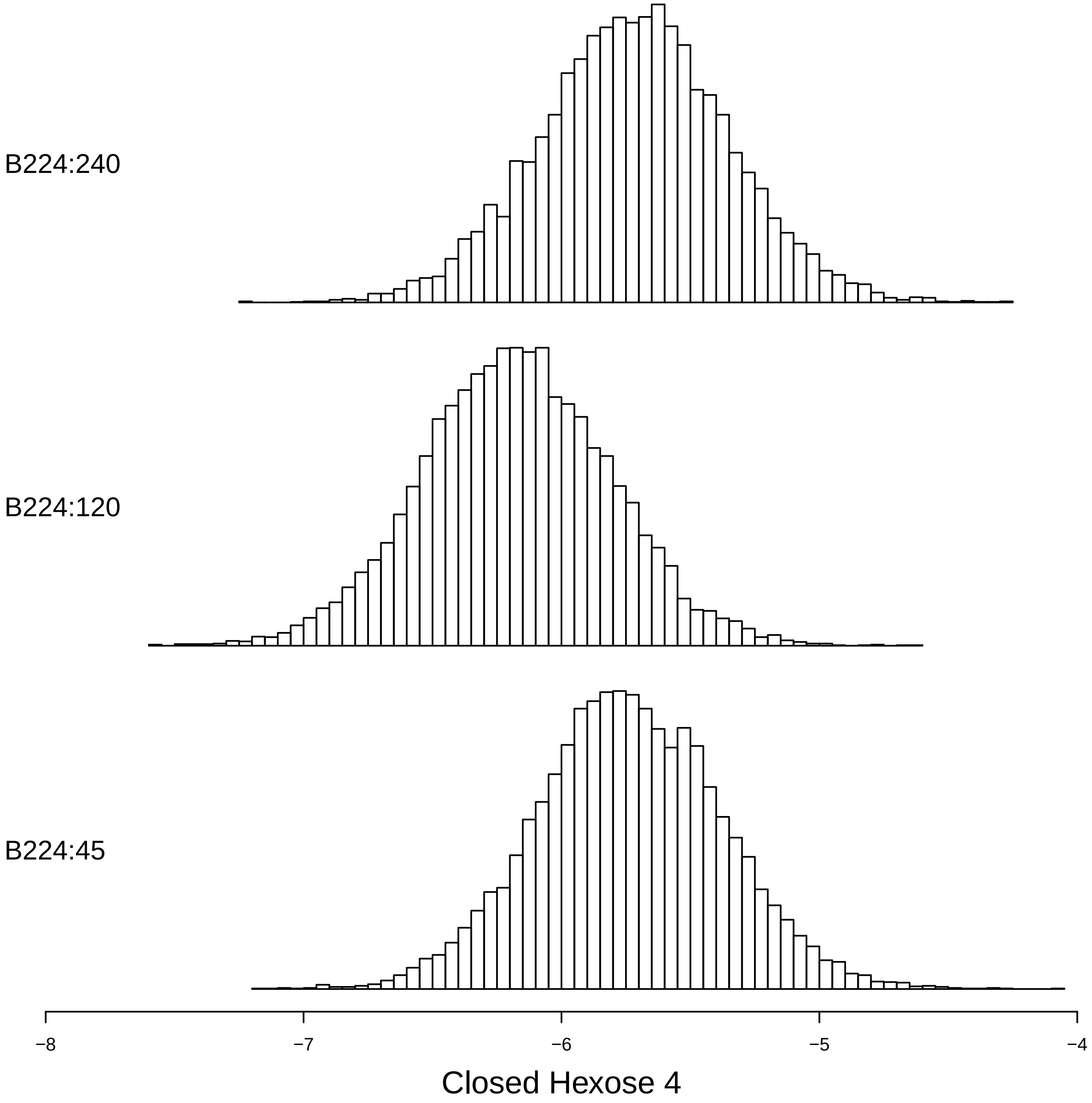
B224:240
B224:120
B224:45
−8
−7
−6
−5
−4
Closed Hexose 4

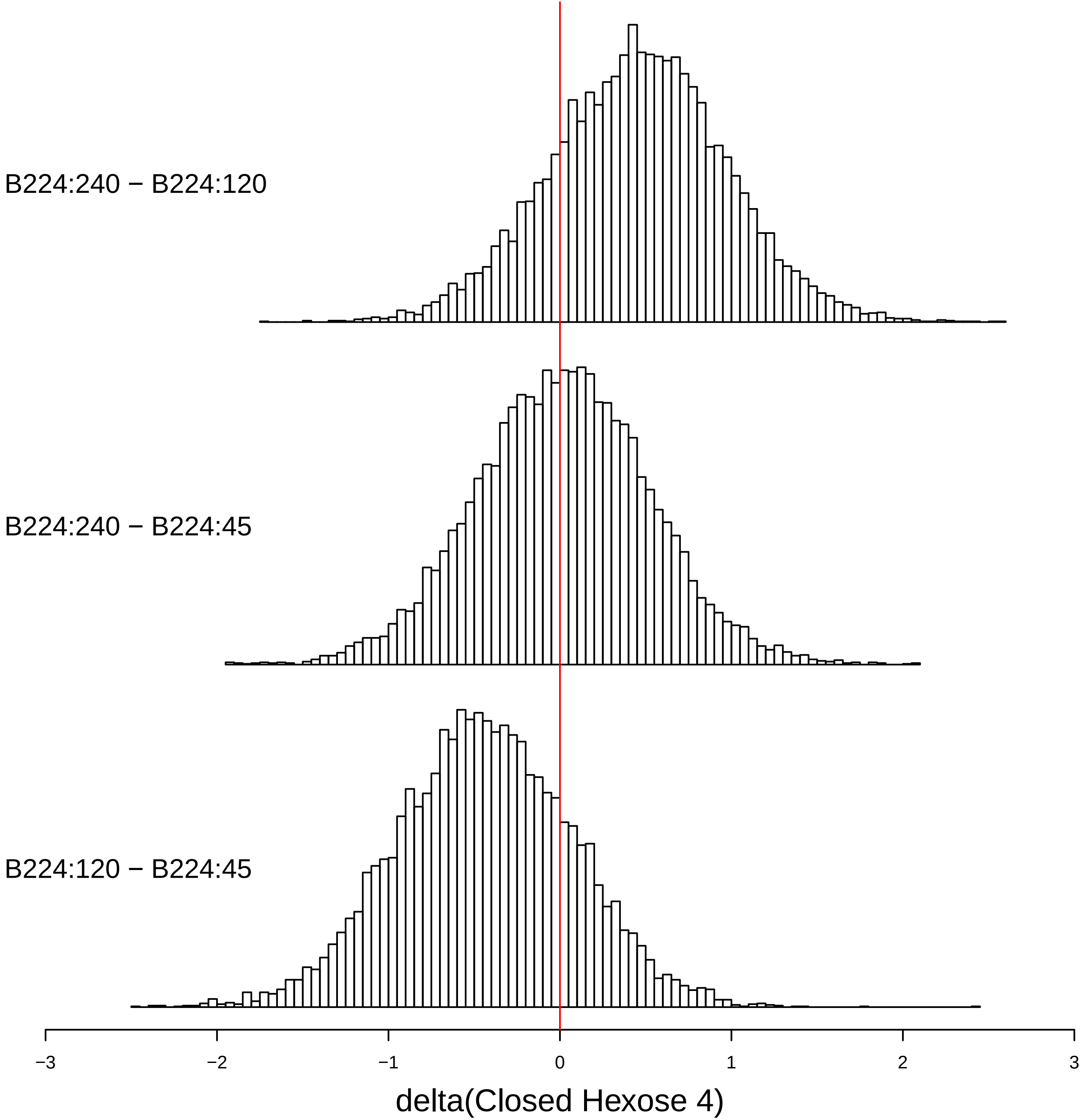
B224:240 − B224:120
B224:240 − B224:45
B224:120 − B224:45
−3
−2
−1
0
1
2
3
delta(Closed Hexose 4)

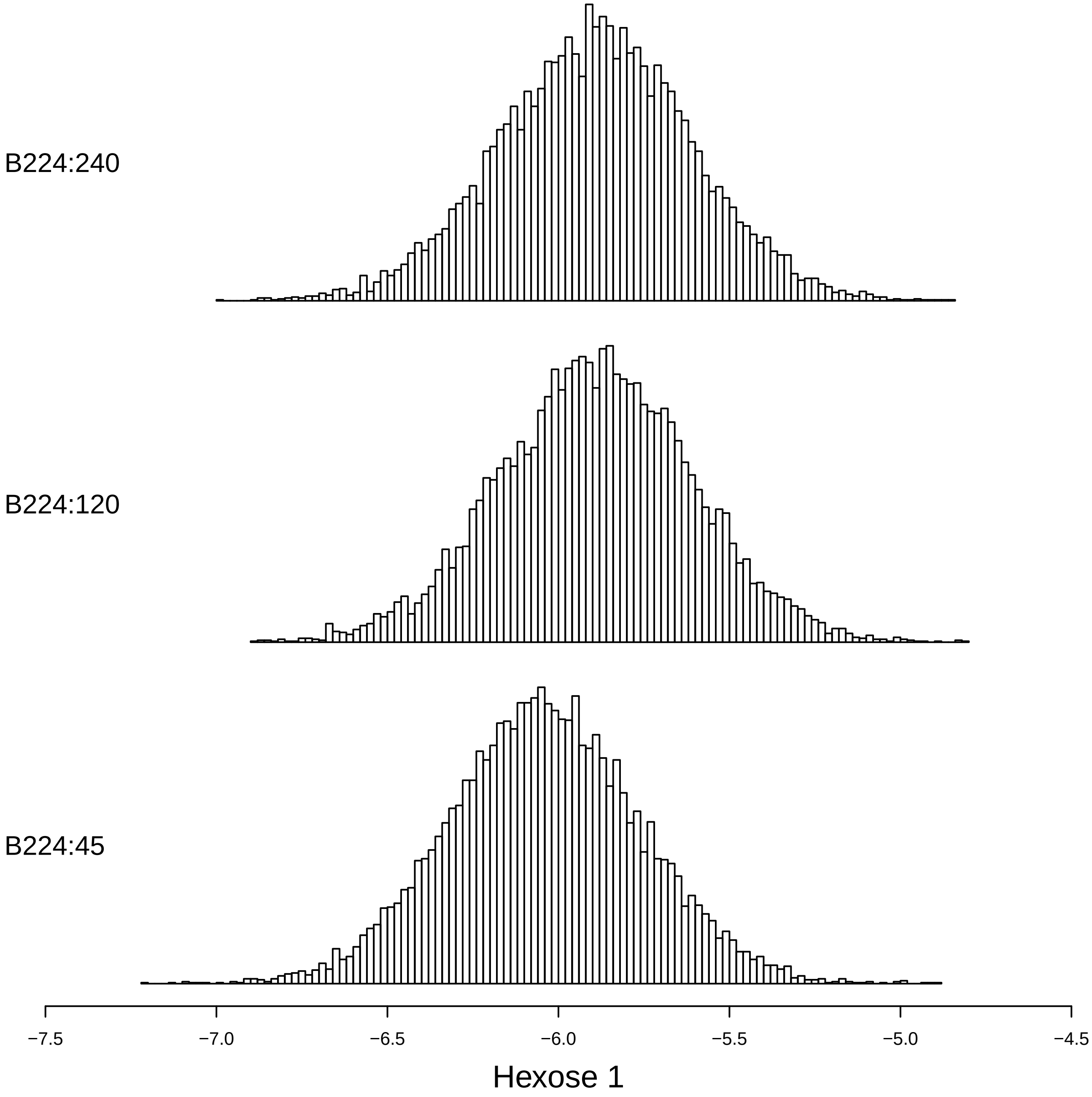
B224:240
B224:120
B224:45
−7.5
−7.0
−6.5
−6.0
−5.5
−5.0
−4.5
Hexose 1

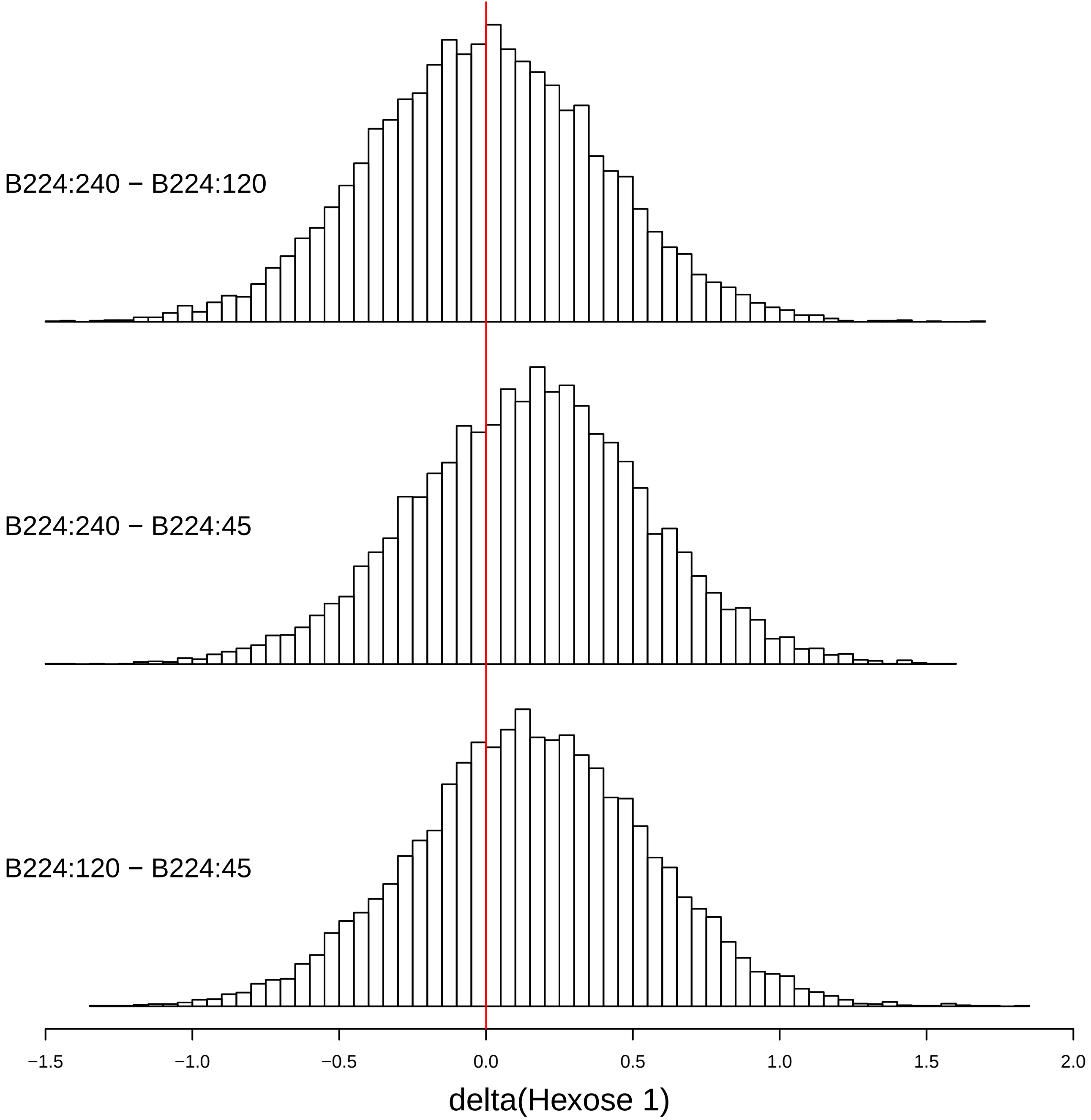
B224:240 − B224:120
B224:240 − B224:45
B224:120 − B224:45
−1.5
−1.0
−0.5
0.0
0.5
1.0
1.5
2.0
delta(Hexose 1)

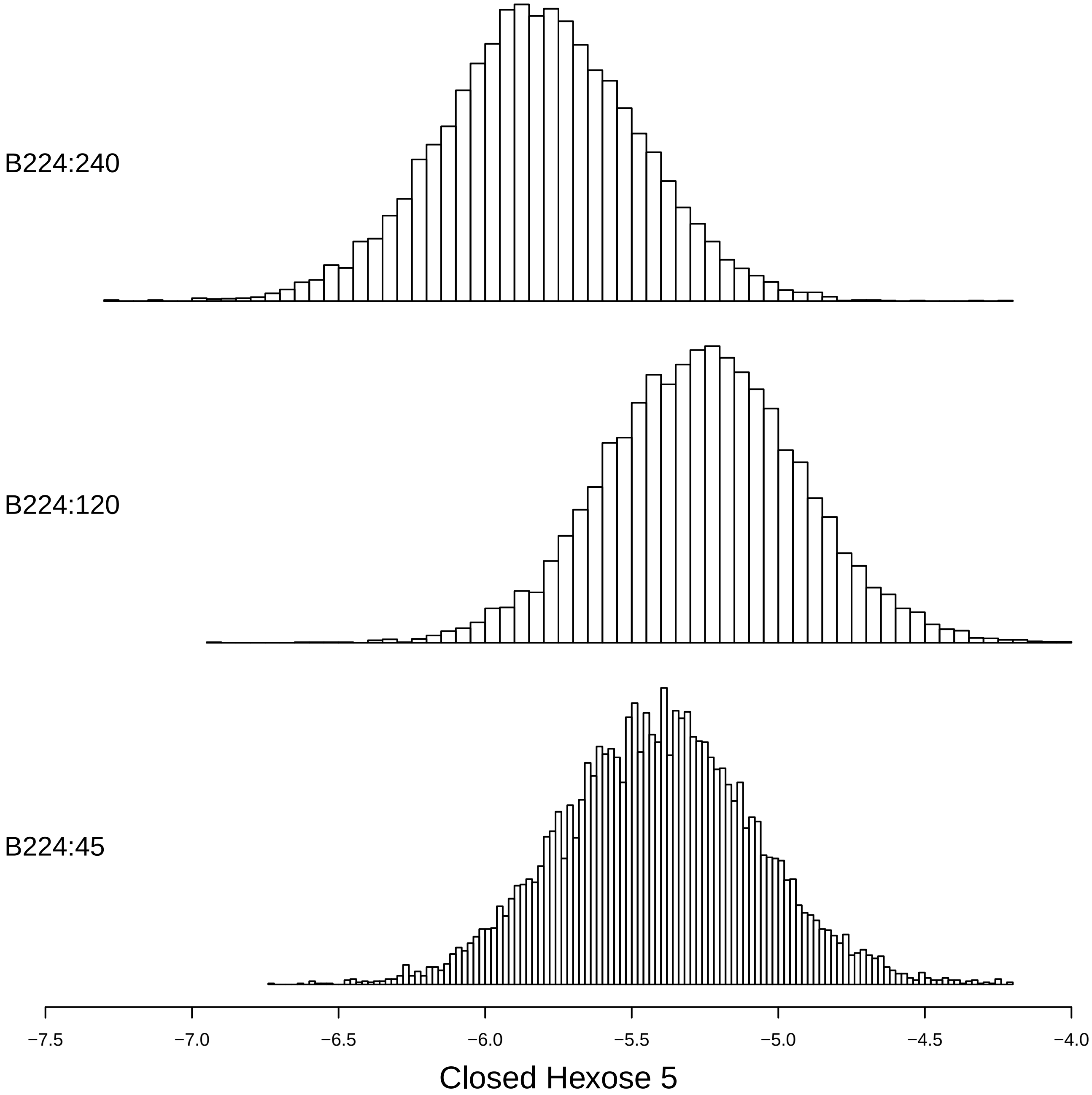

B224:240
B224:120
B224:45
−7.5
−7.0
−6.5
−6.0
−5.5
−5.0
−4.5
−4.0
Closed Hexose 5

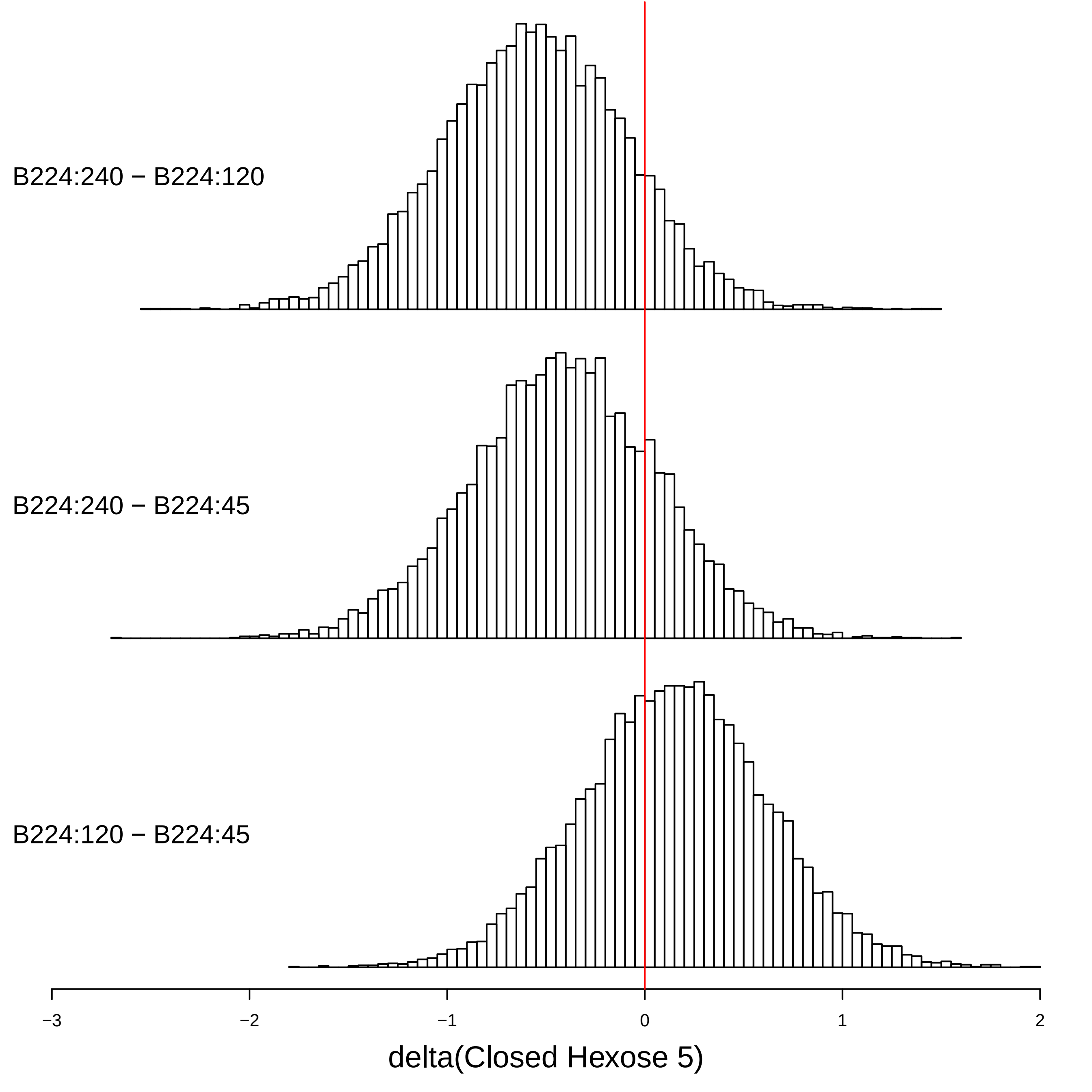
B224:240 − B224:120
B224:240 − B224:45
B224:120 − B224:45
−3
−2
−1
0
1
2
delta(Closed Hexose 5)

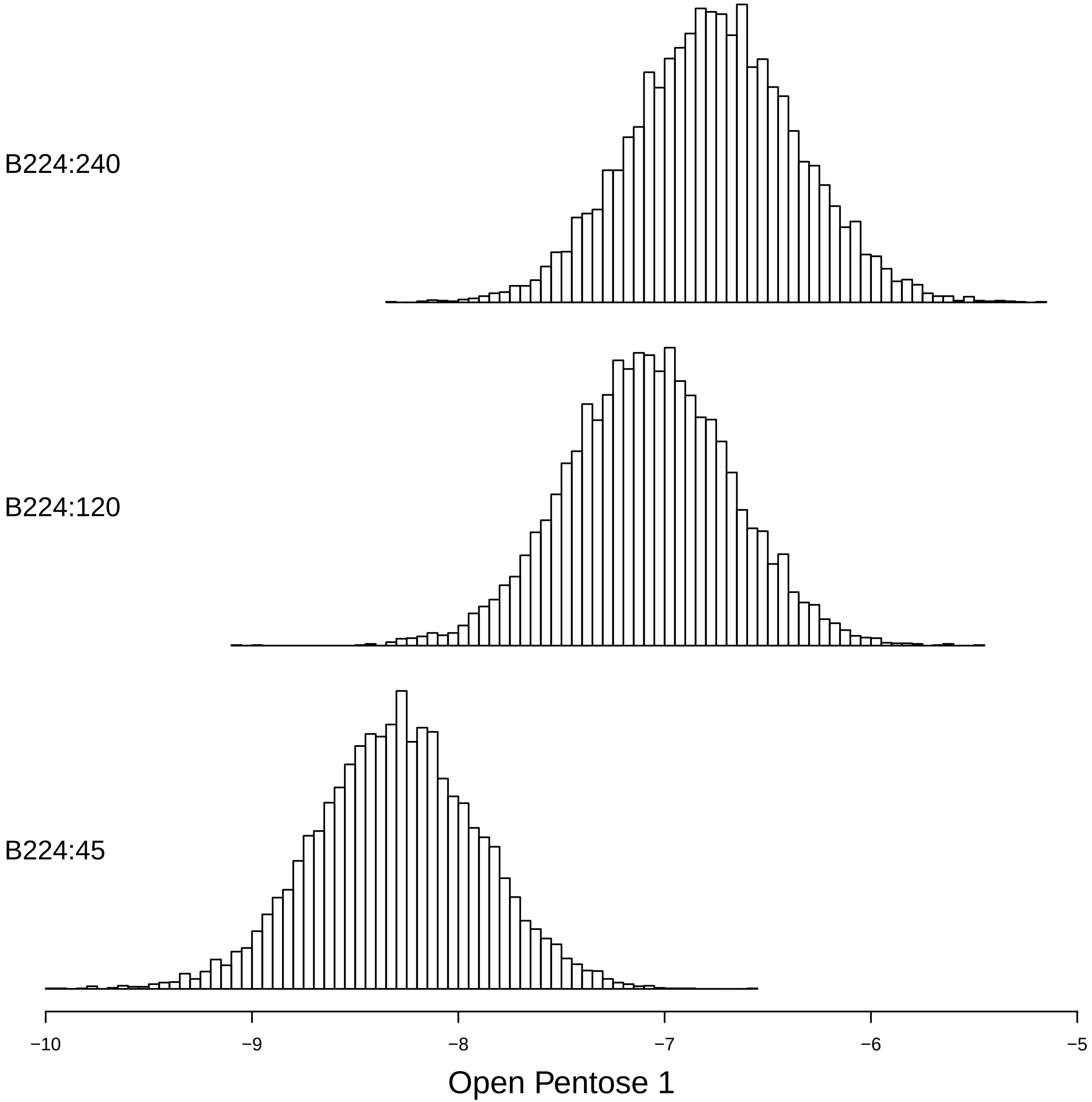
B224:240
B224:120
B224:45
−10
−9
−8
−7
−6
−5
Open Pentose 1

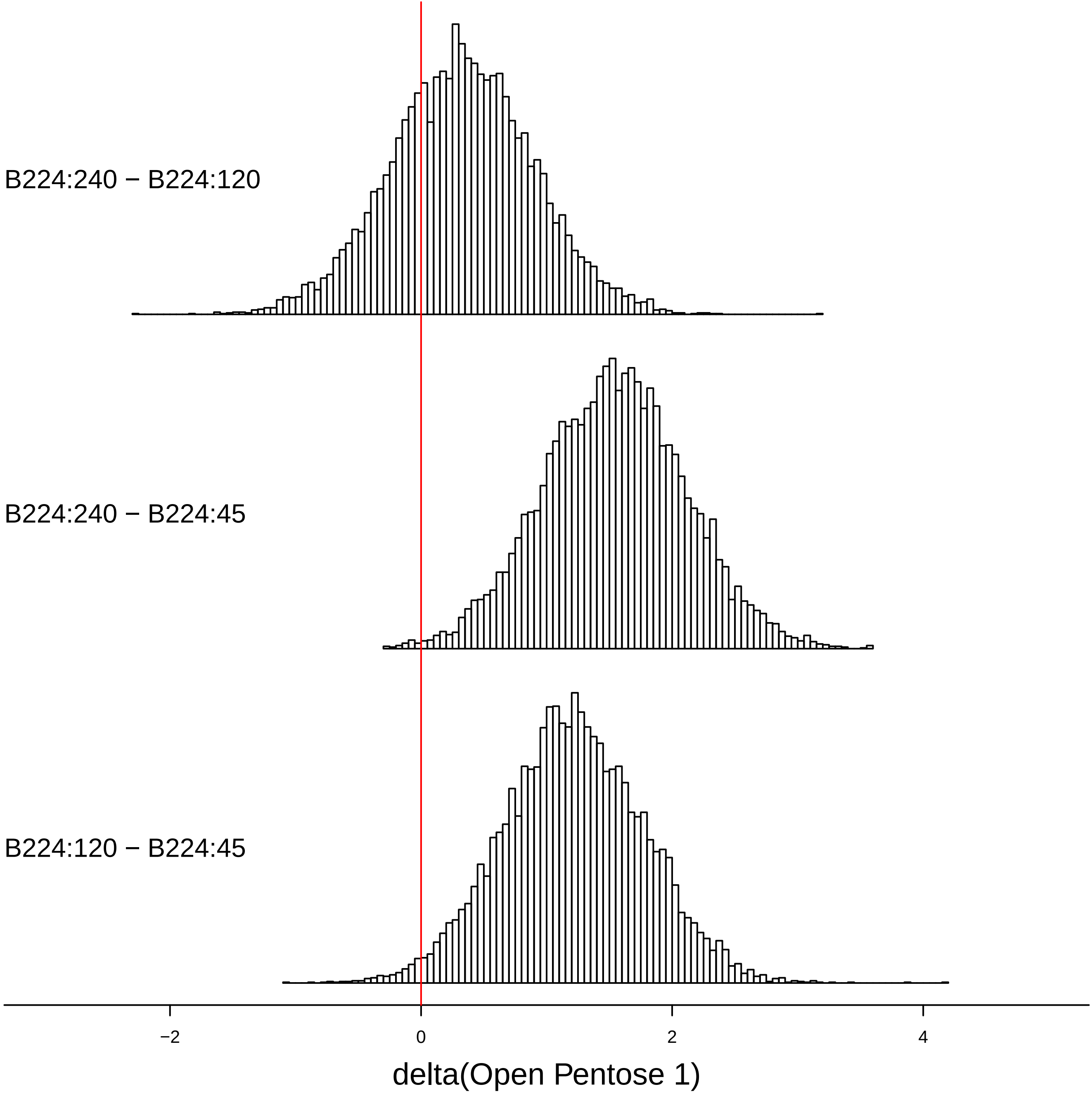
B224:240 − B224:120
B224:240 − B224:45
B224:120 − B224:45
−2
0
2
4
delta(Open Pentose 1)

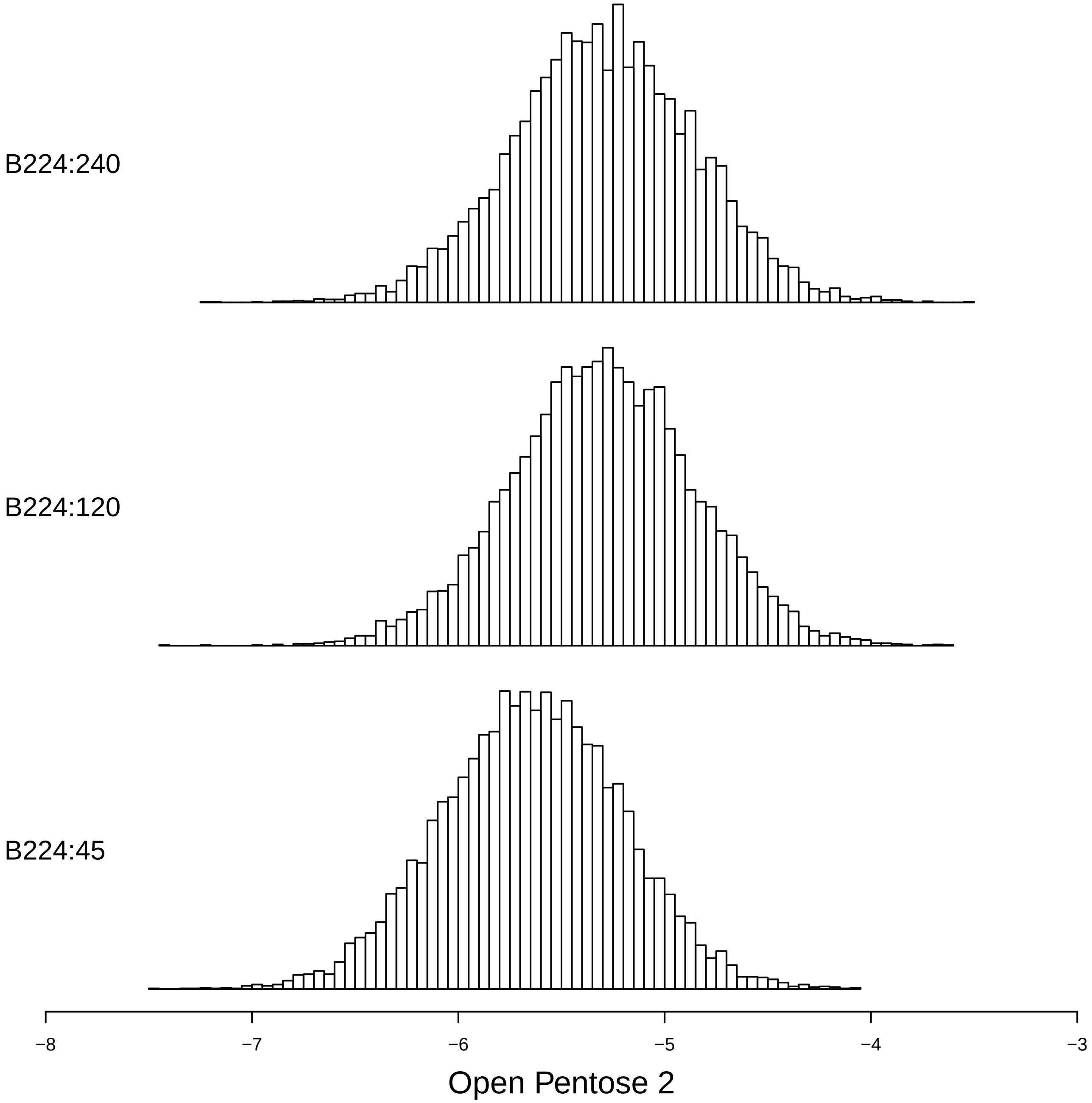
B224:240
B224:120
B224:45
−8
−7
−6
−5
−4
−3
Open Pentose 2

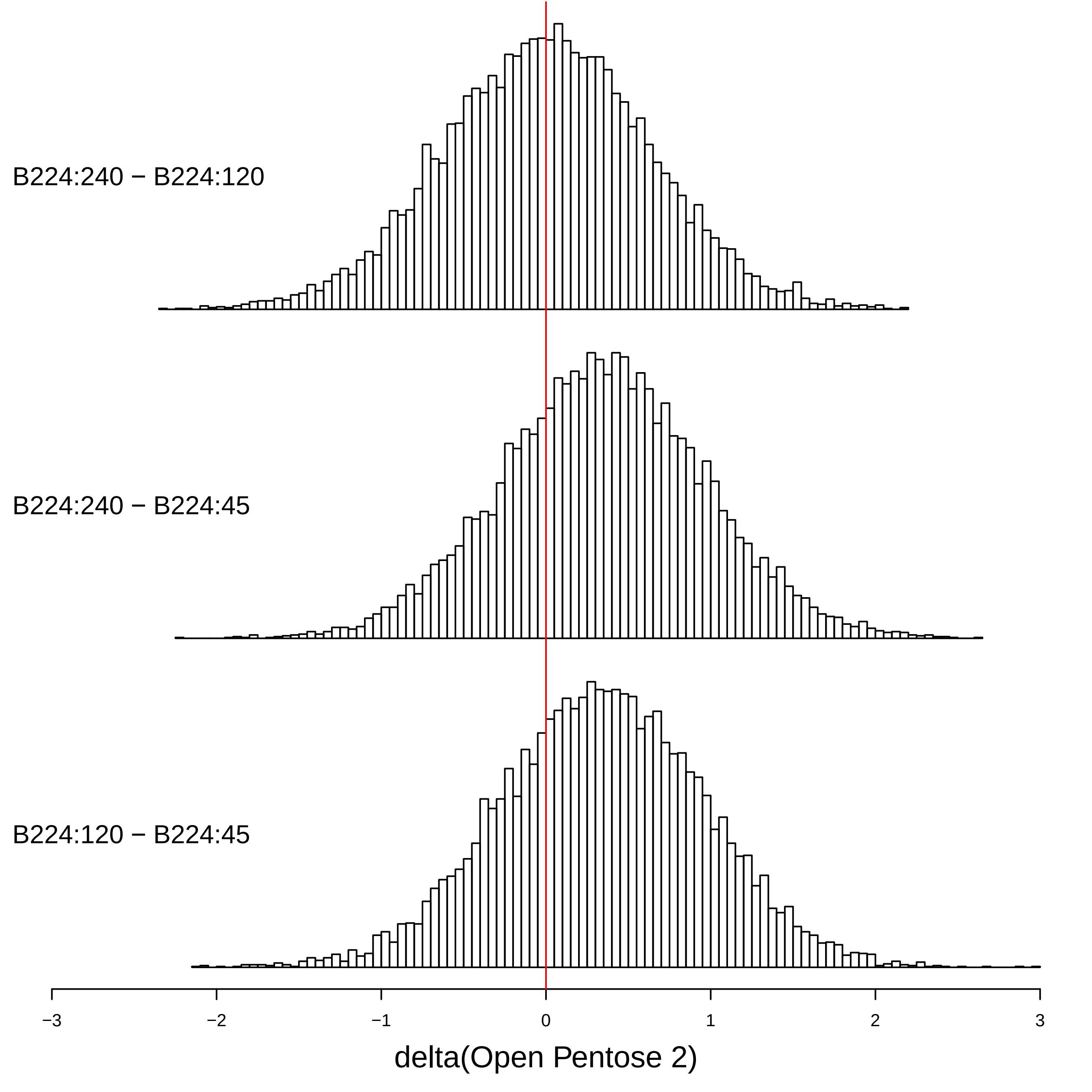
B224:240 − B224:120
B224:240 − B224:45
B224:120 − B224:45
−3
−2
−1
0
1
2
3
delta(Open Pentose 2)

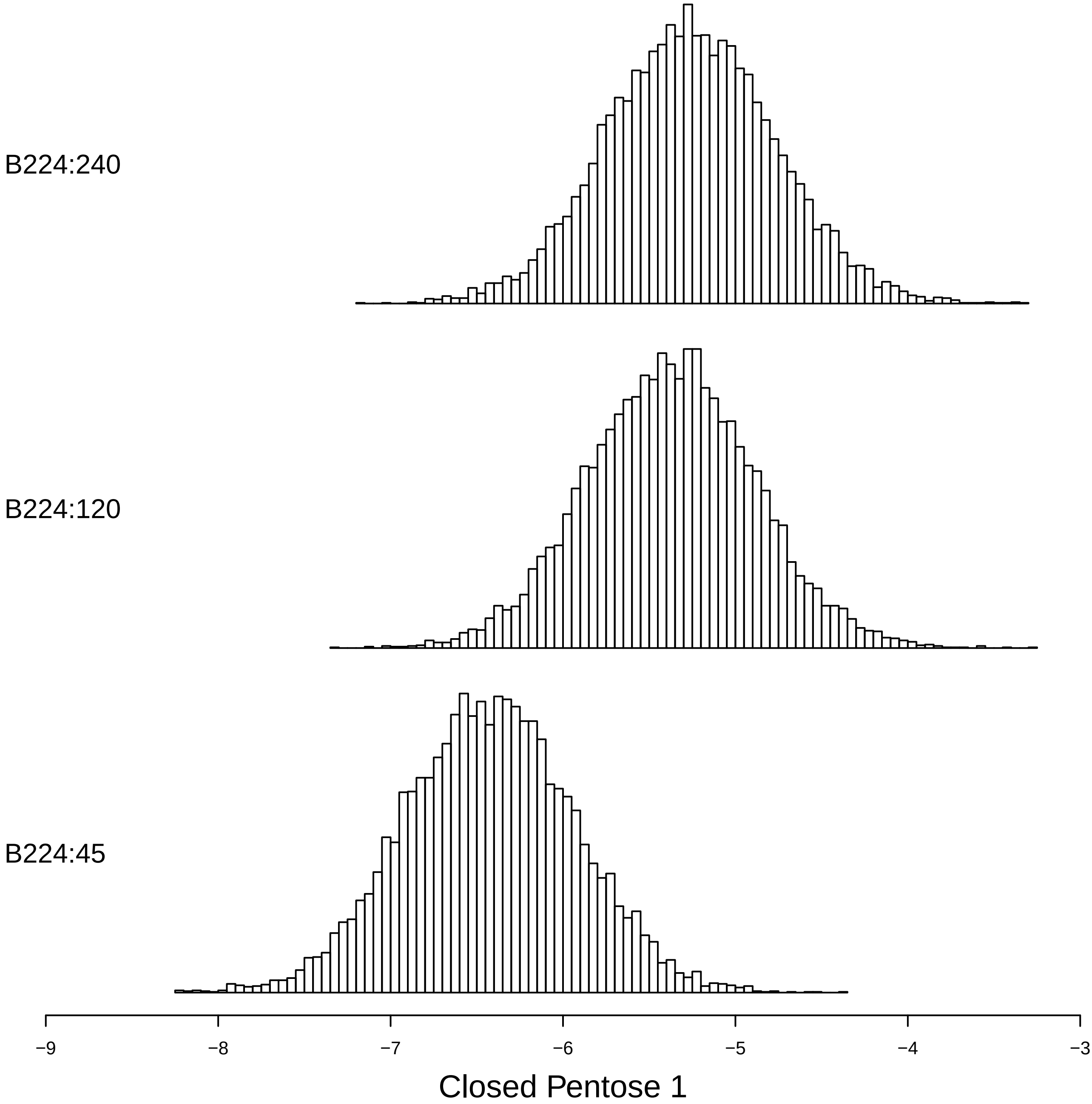
B224:240
B224:120
B224:45
−9
−8
−7
−6
−5
−4
−3
Closed Pentose 1

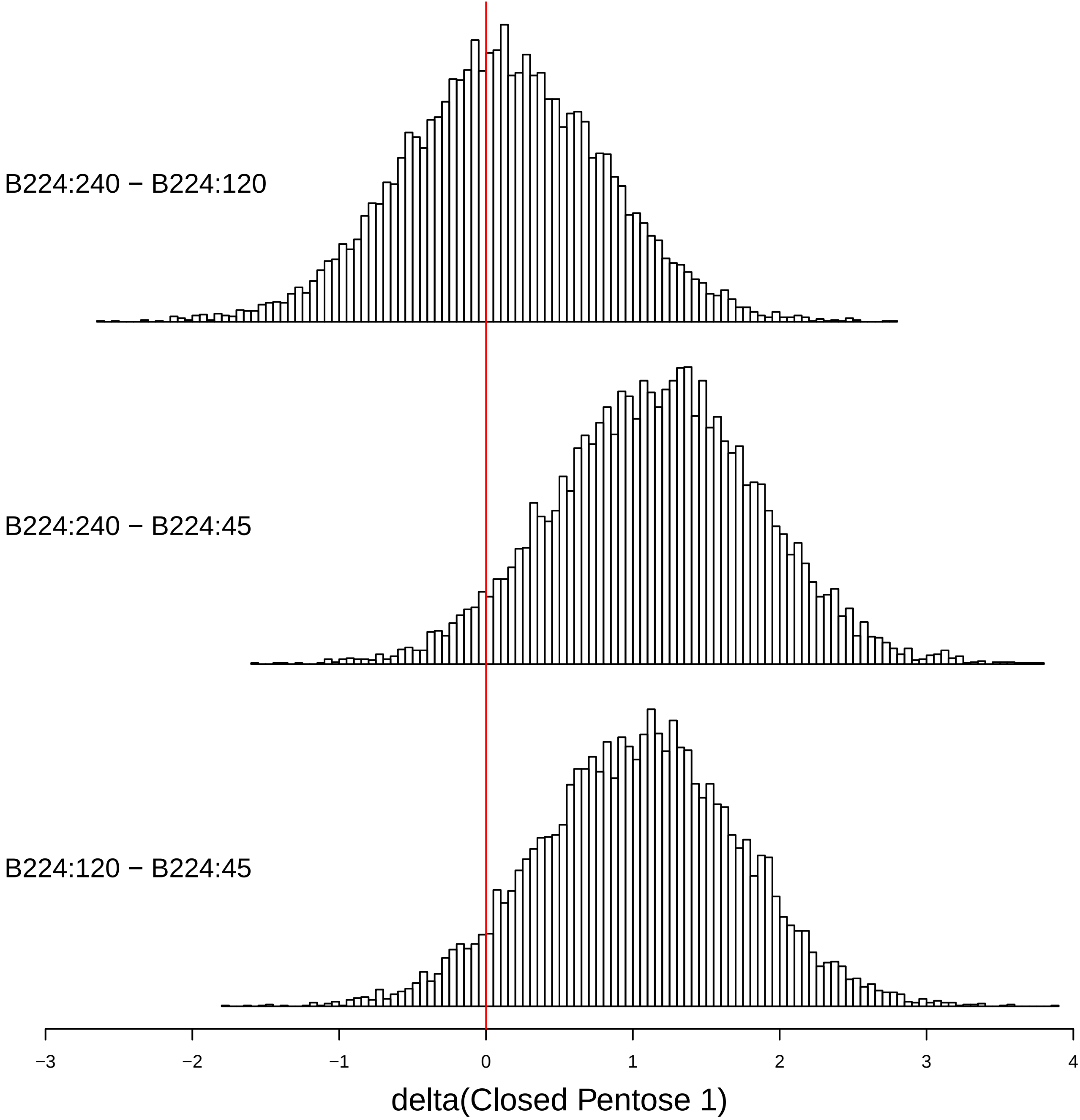

B224:240 − B224:120
B224:240 − B224:45
B224:120 − B224:45
−3
−2
−1
0
1
2
3
4
delta(Closed Pentose 1)

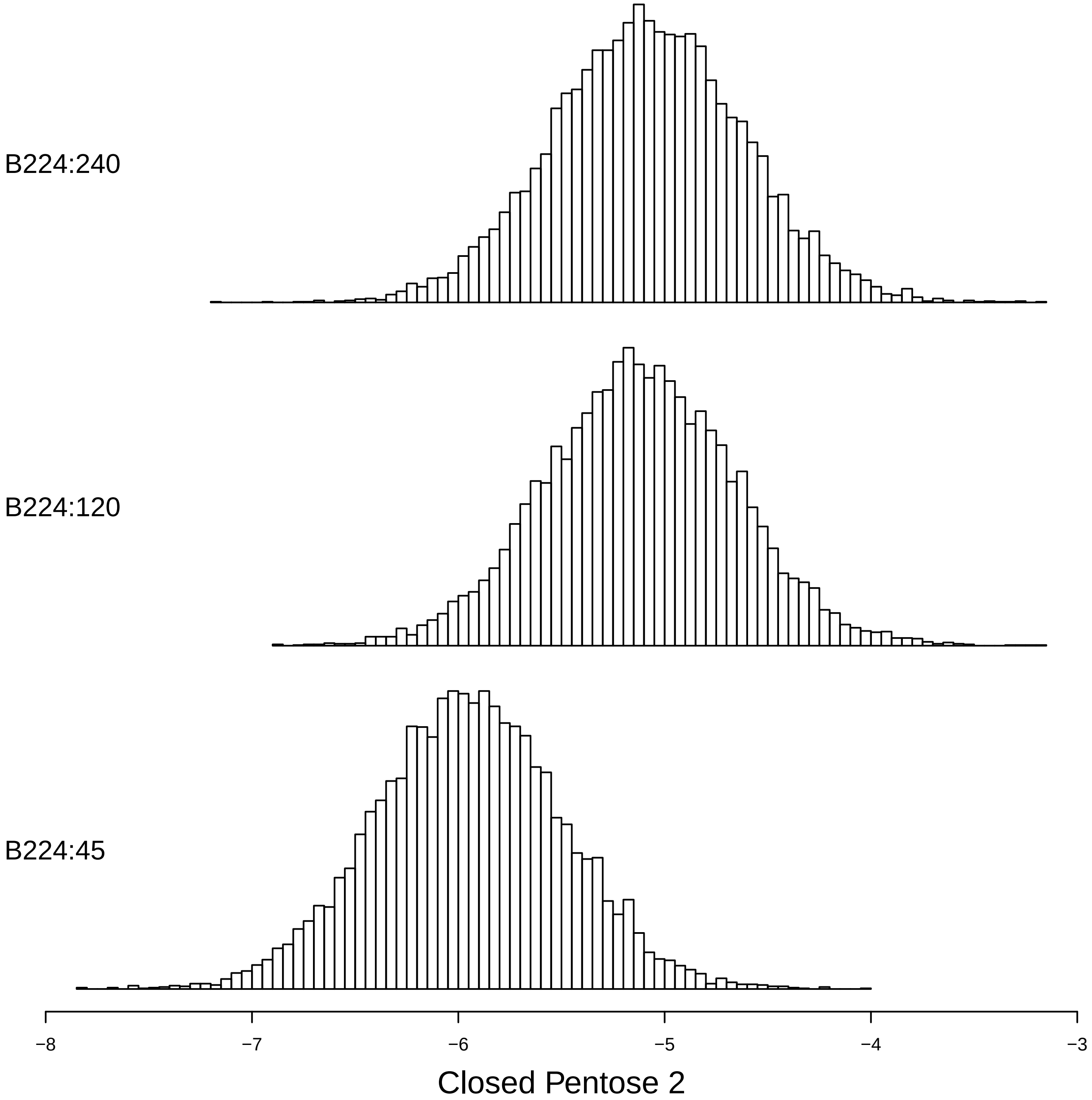
B224:240
B224:120
B224:45
−8
−7
−6
−5
−4
−3
Closed Pentose 2

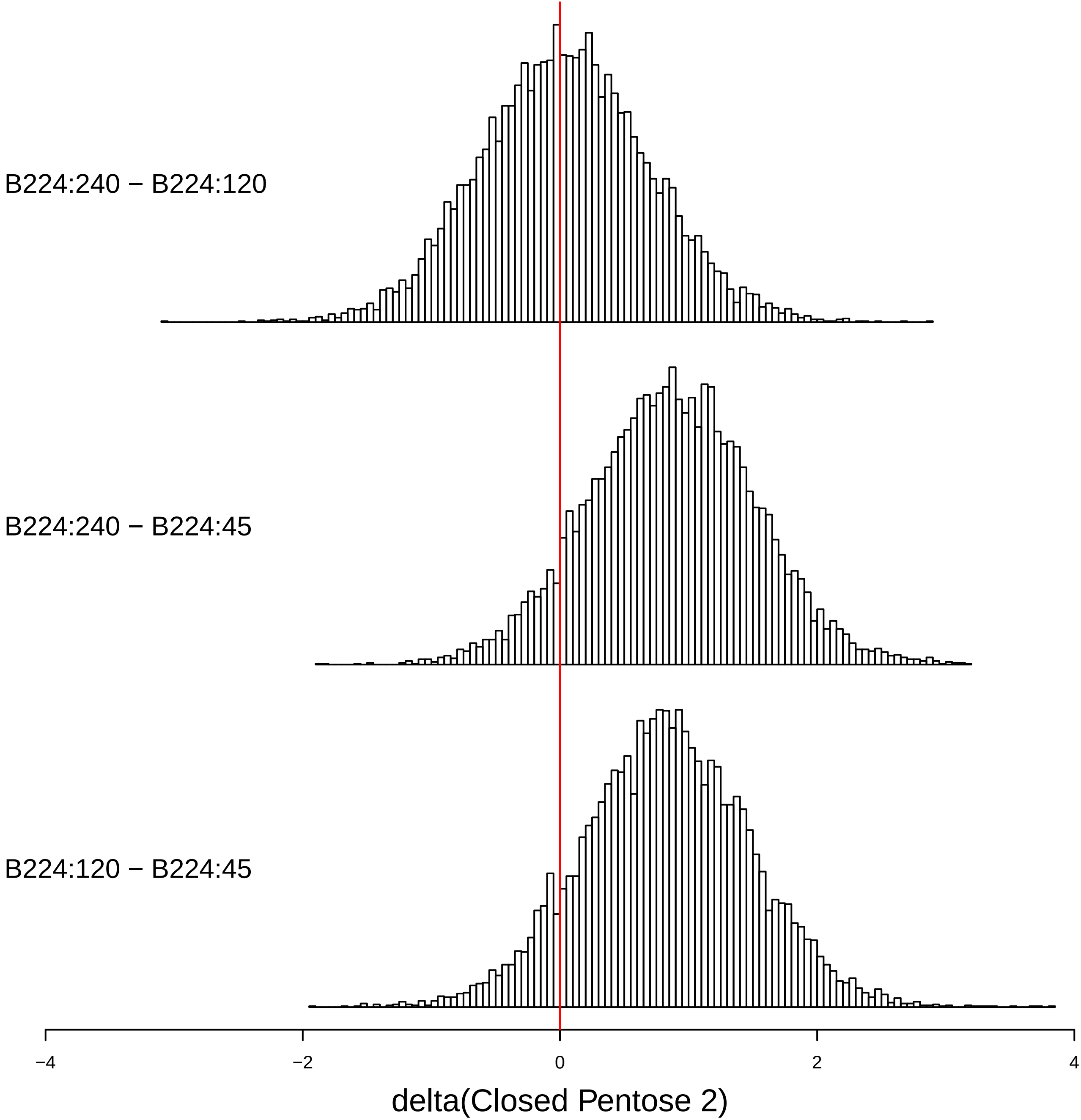
B224:240 − B224:120
B224:240 − B224:45
B224:120 − B224:45
−4
−2
0
2
4
delta(Closed Pentose 2)

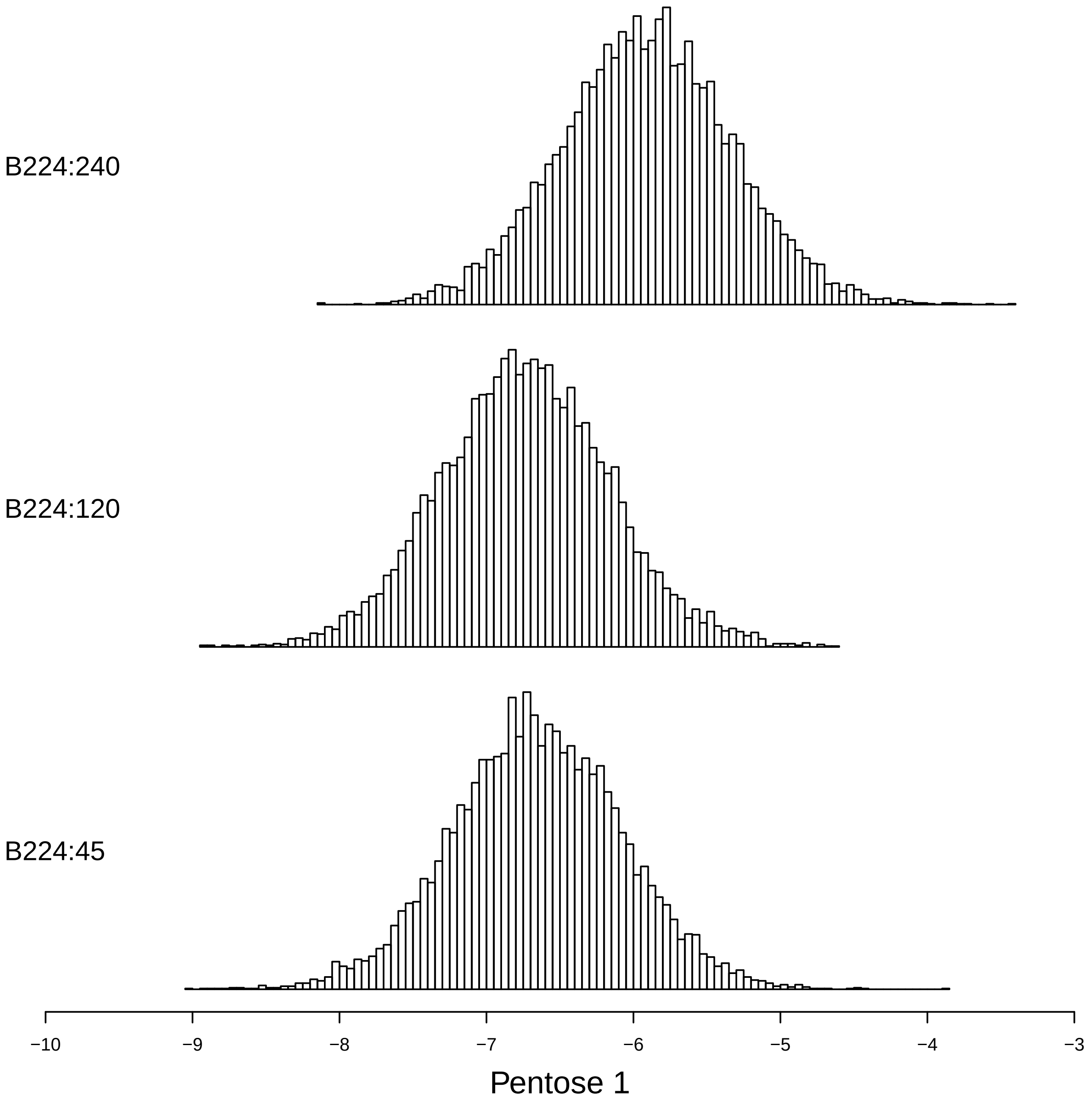

B224:240
B224:120
B224:45
−10
−9
−8
−7
−6
−5
−4
−3
Pentose 1

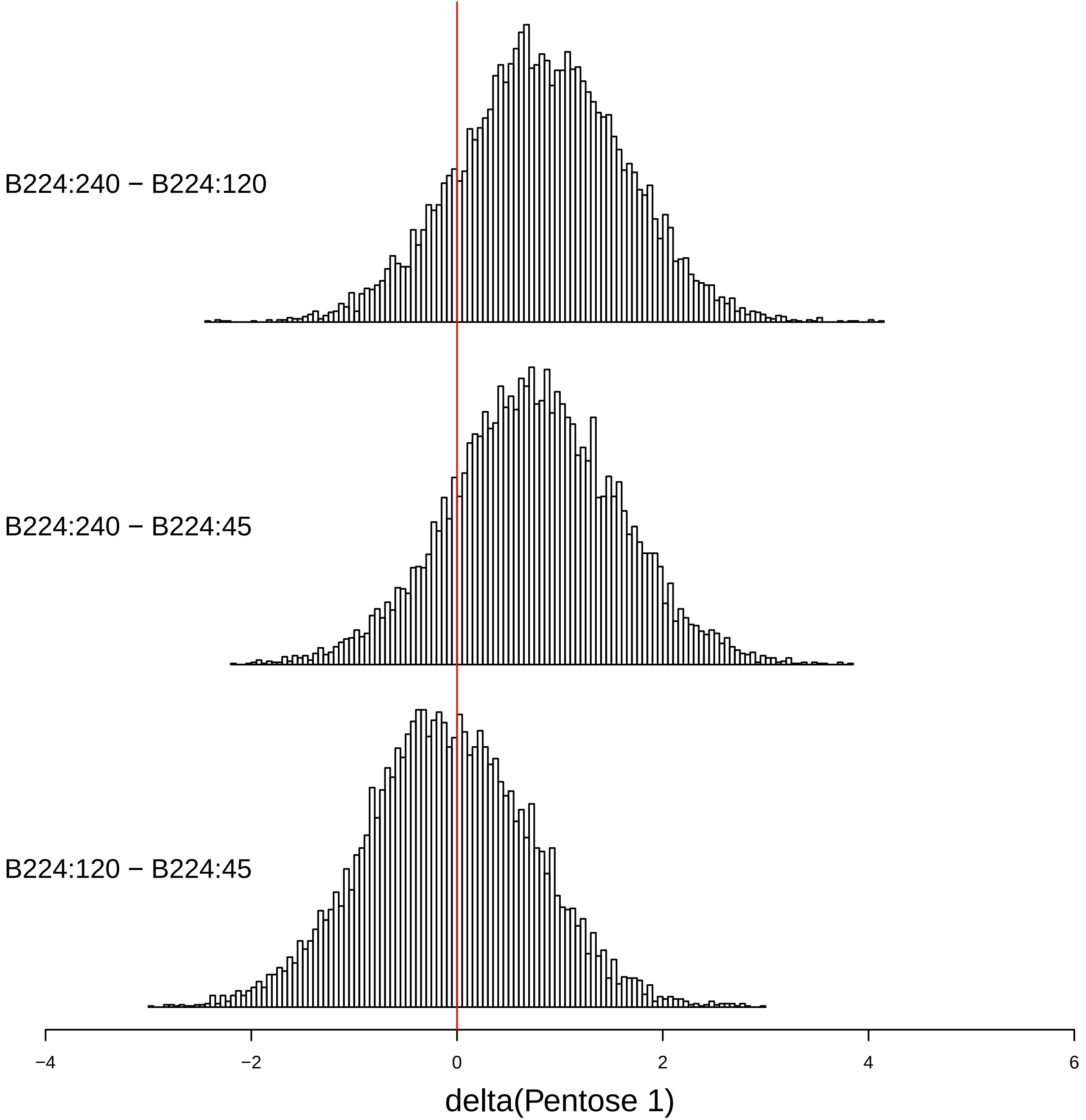
B224:240 − B224:120
B224:240 − B224:45
B224:120 − B224:45
−4
−2
0
2
4
6
delta(Pentose 1)

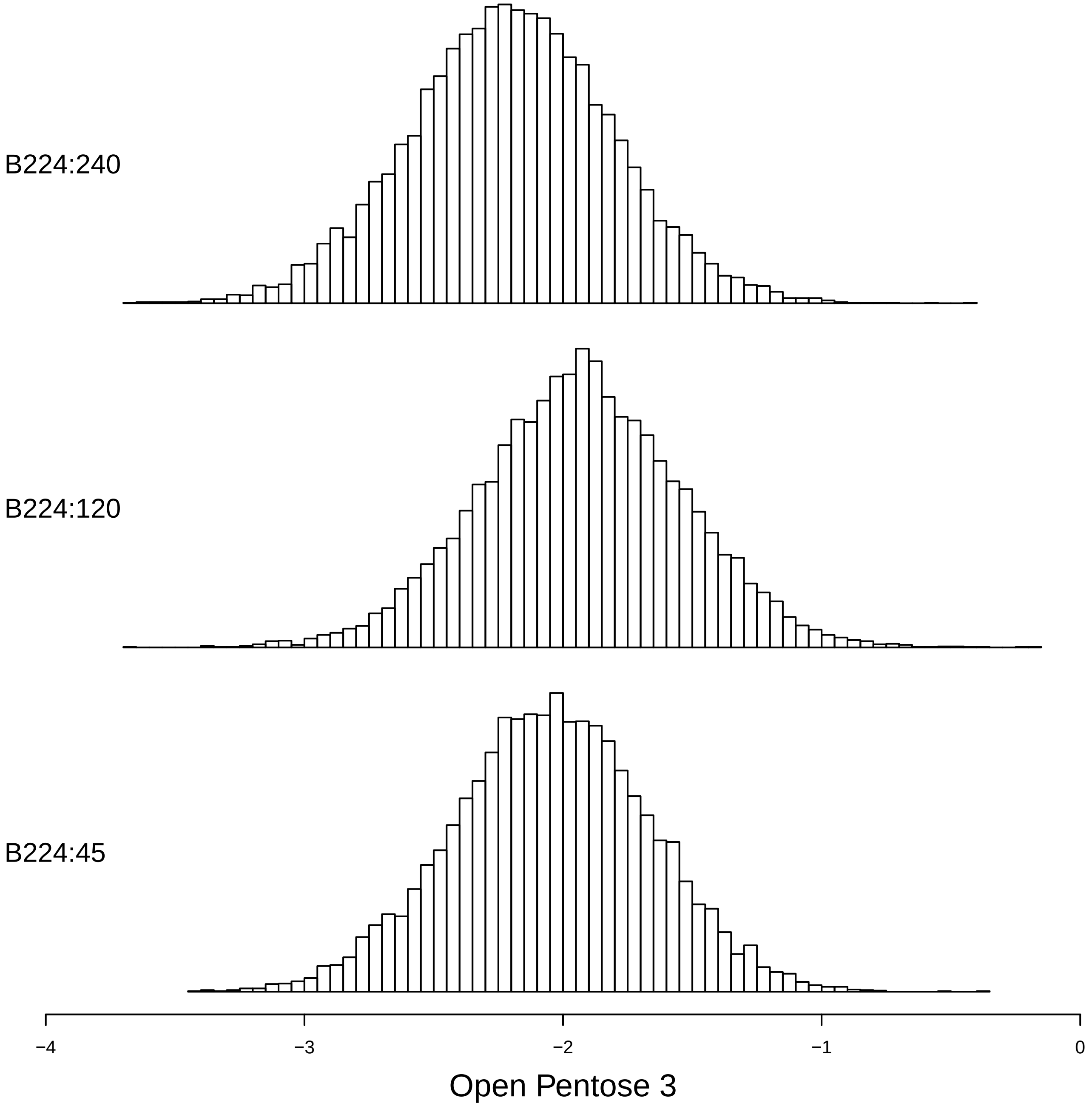

B224:240
B224:120
B224:45
−4
−3
−2
−1
0
Open Pentose 3

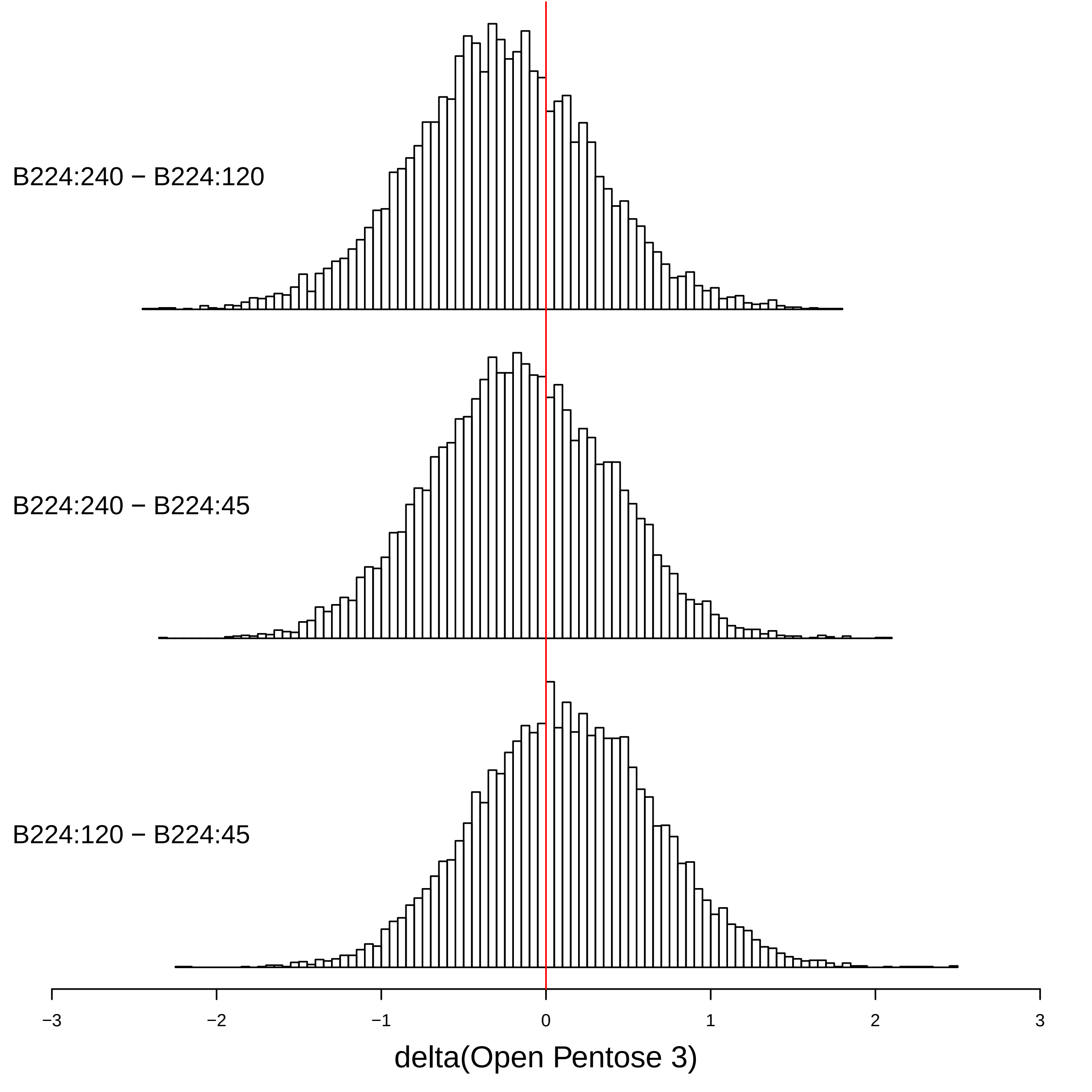
B224:240 − B224:120
B224:240 − B224:45
B224:120 − B224:45
−3
−2
−1
0
1
2
3
delta(Open Pentose 3)

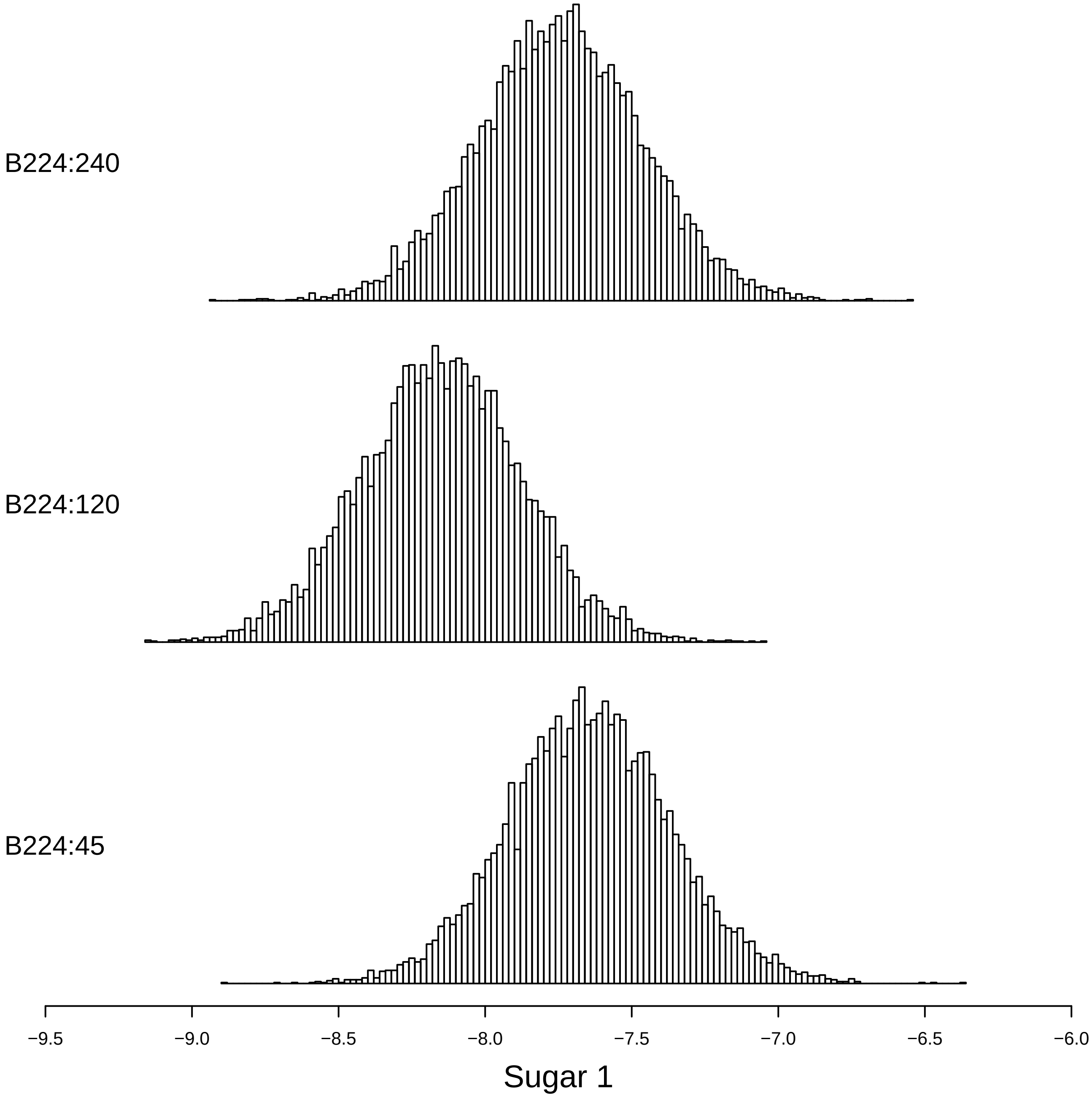
B224:240
B224:120
B224:45
−9.5
−9.0
−8.5
−8.0
−7.5
−7.0
−6.5
−6.0
Sugar 1

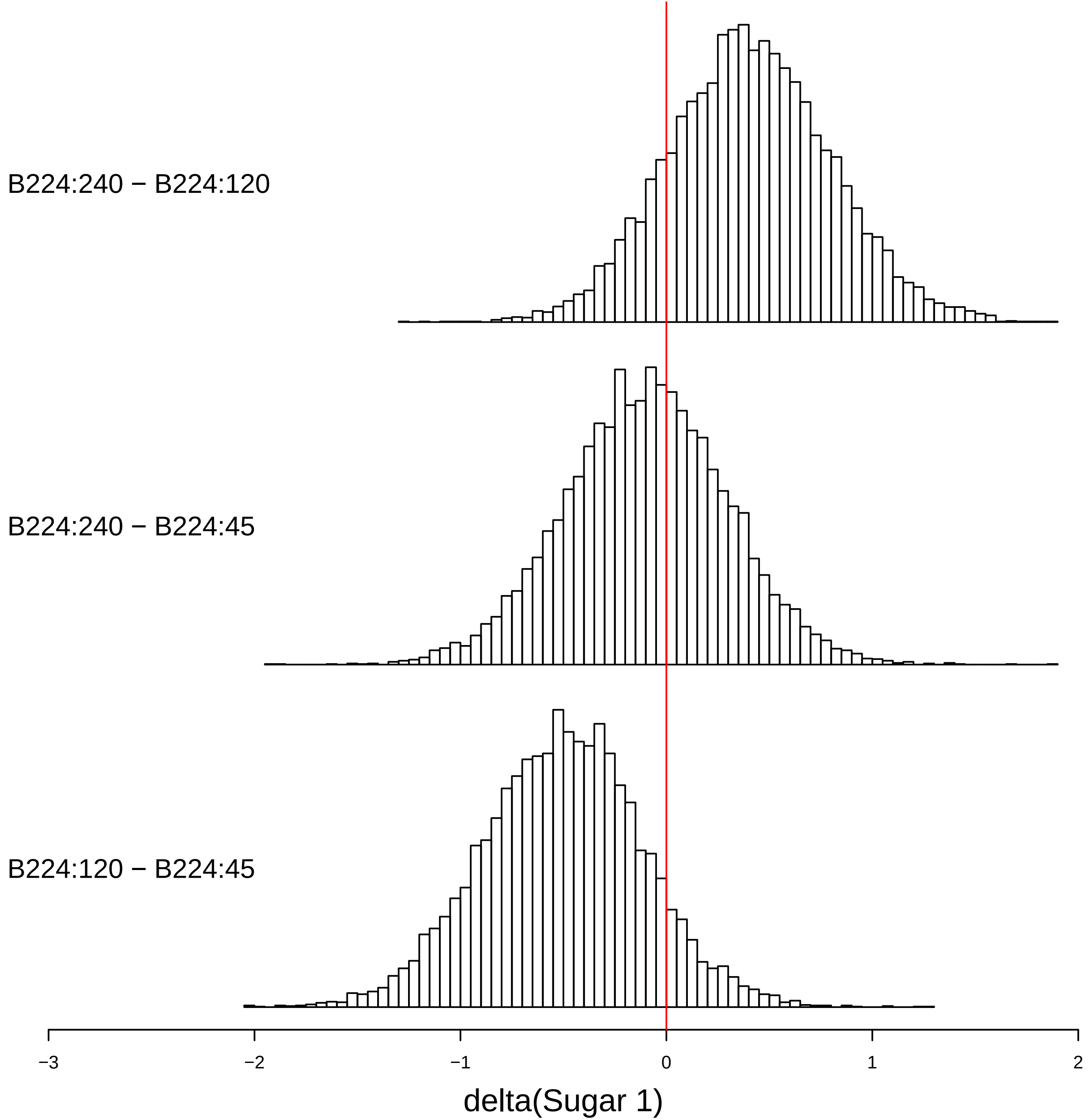

B224:240 − B224:120
B224:240 − B224:45
B224:120 − B224:45
−3
−2
−1
0
1
2
delta(Sugar 1)

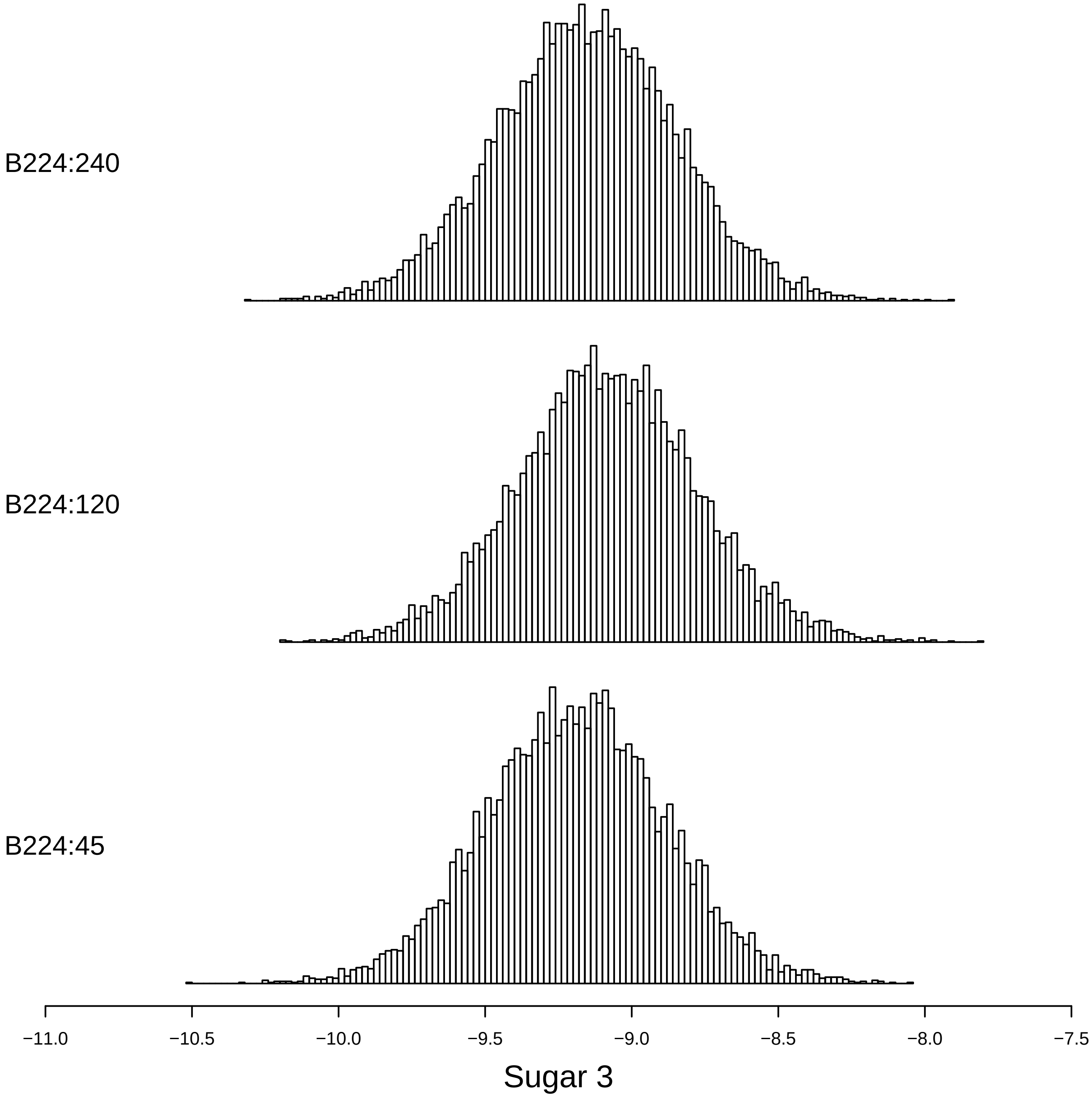
B224:240
B224:120
B224:45
−11.0
−10.5
−10.0
−9.5
−9.0
−8.5
−8.0
−7.5
Sugar 3

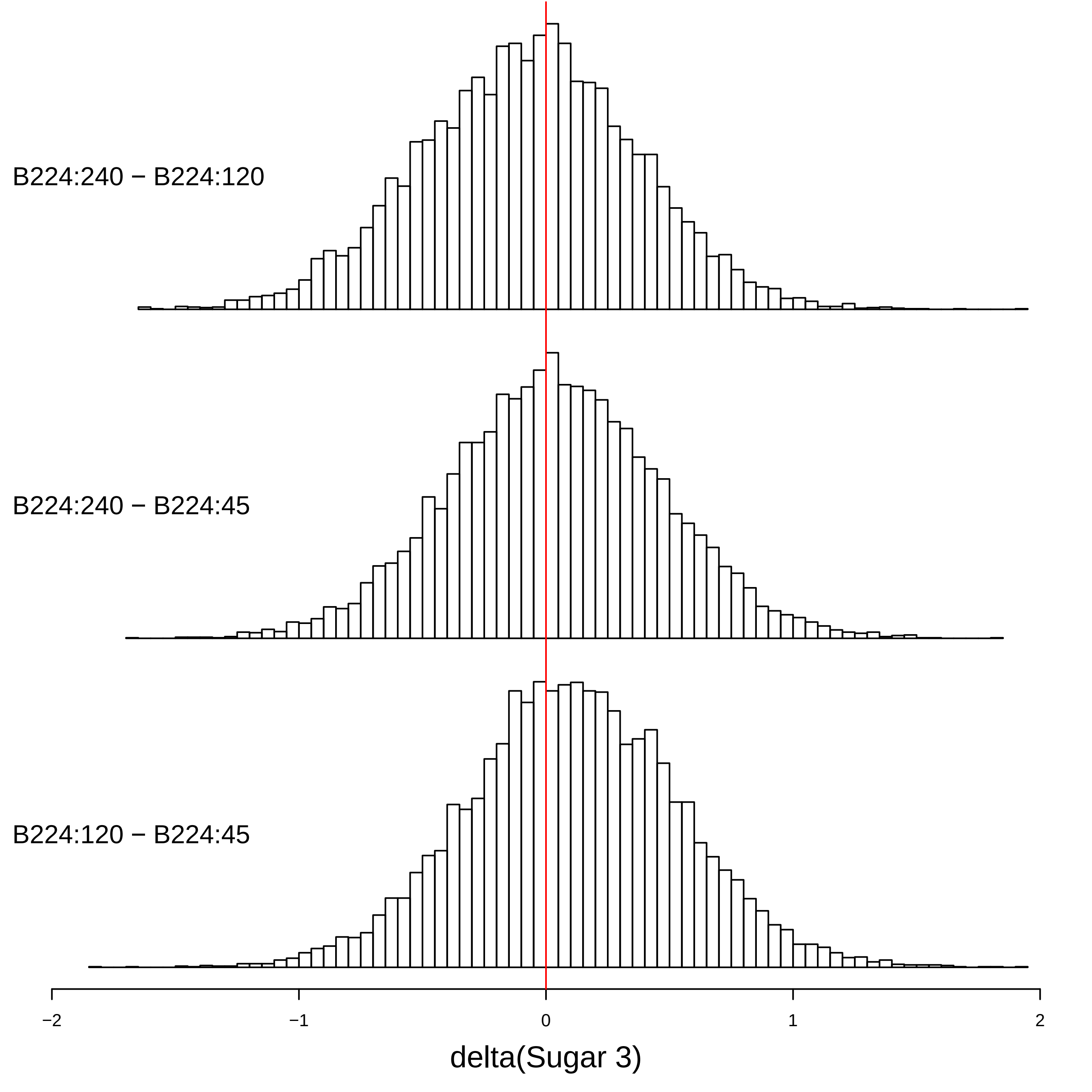
B224:240 − B224:120
B224:240 − B224:45
B224:120 − B224:45
−2
−1
0
1
2
delta(Sugar 3)

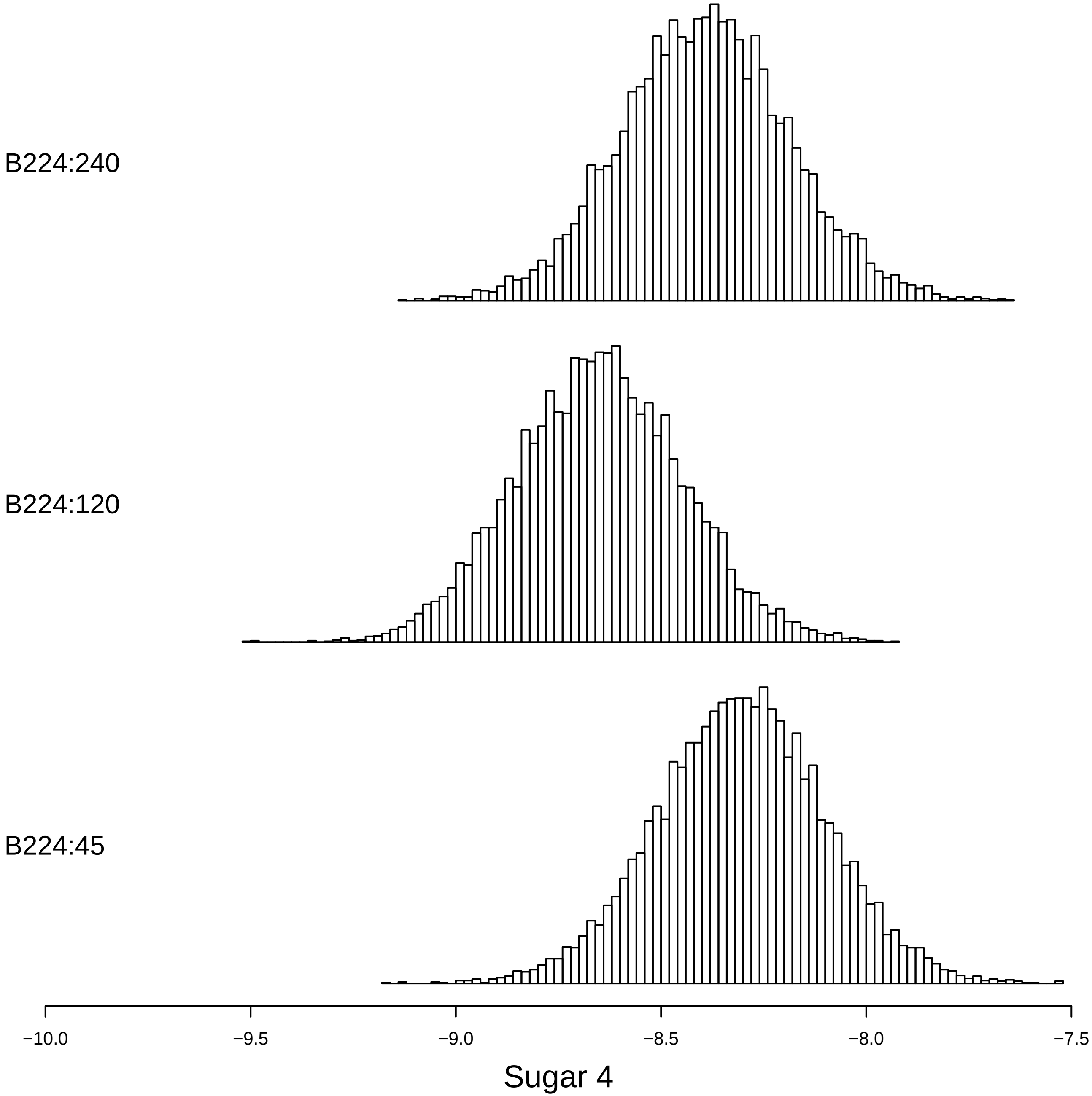

B224:240
B224:120
B224:45
−10.0
−9.5
−9.0
−8.5
−8.0
−7.5
Sugar 4

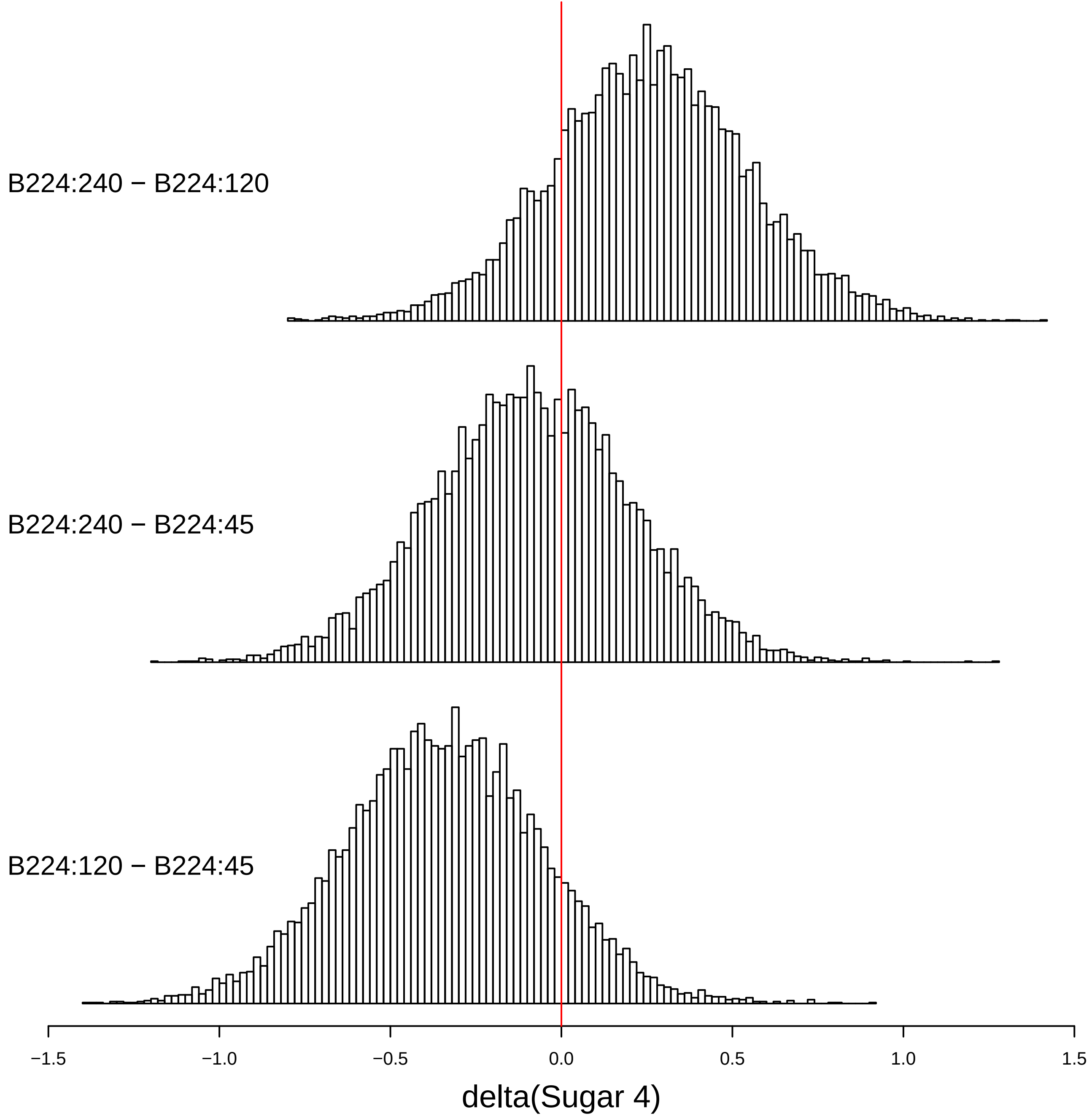
B224:240 − B224:120
B224:240 − B224:45
B224:120 − B224:45
−1.5
−1.0
−0.5
0.0
0.5
1.0
1.5
delta(Sugar 4)

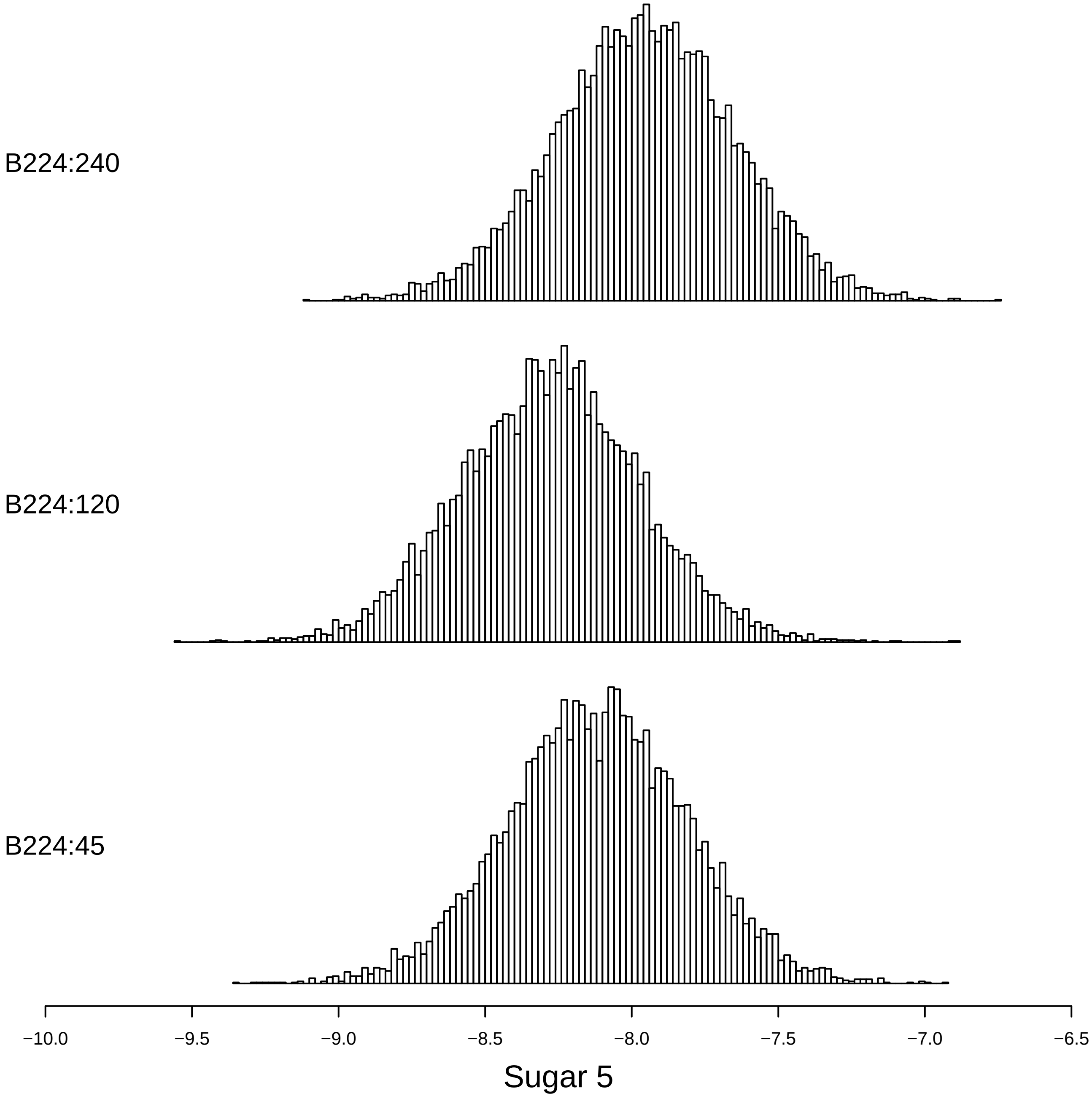
B224:240
B224:120
B224:45
−10.0
−9.5
−9.0
−8.5
−8.0
−7.5
−7.0
−6.5
Sugar 5

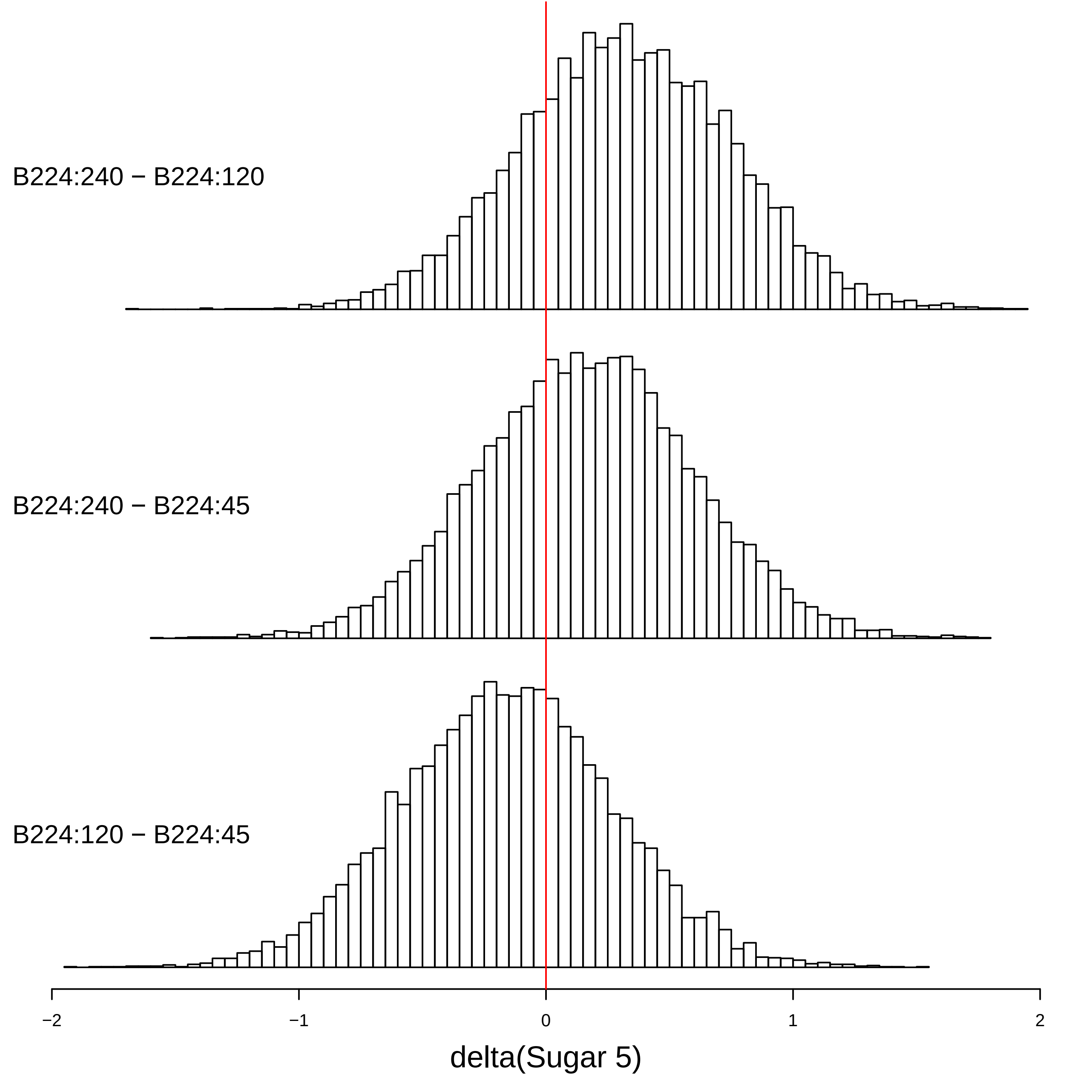
B224:240 − B224:120
B224:240 − B224:45
B224:120 − B224:45
−2
−1
0
1
2
delta(Sugar 5)

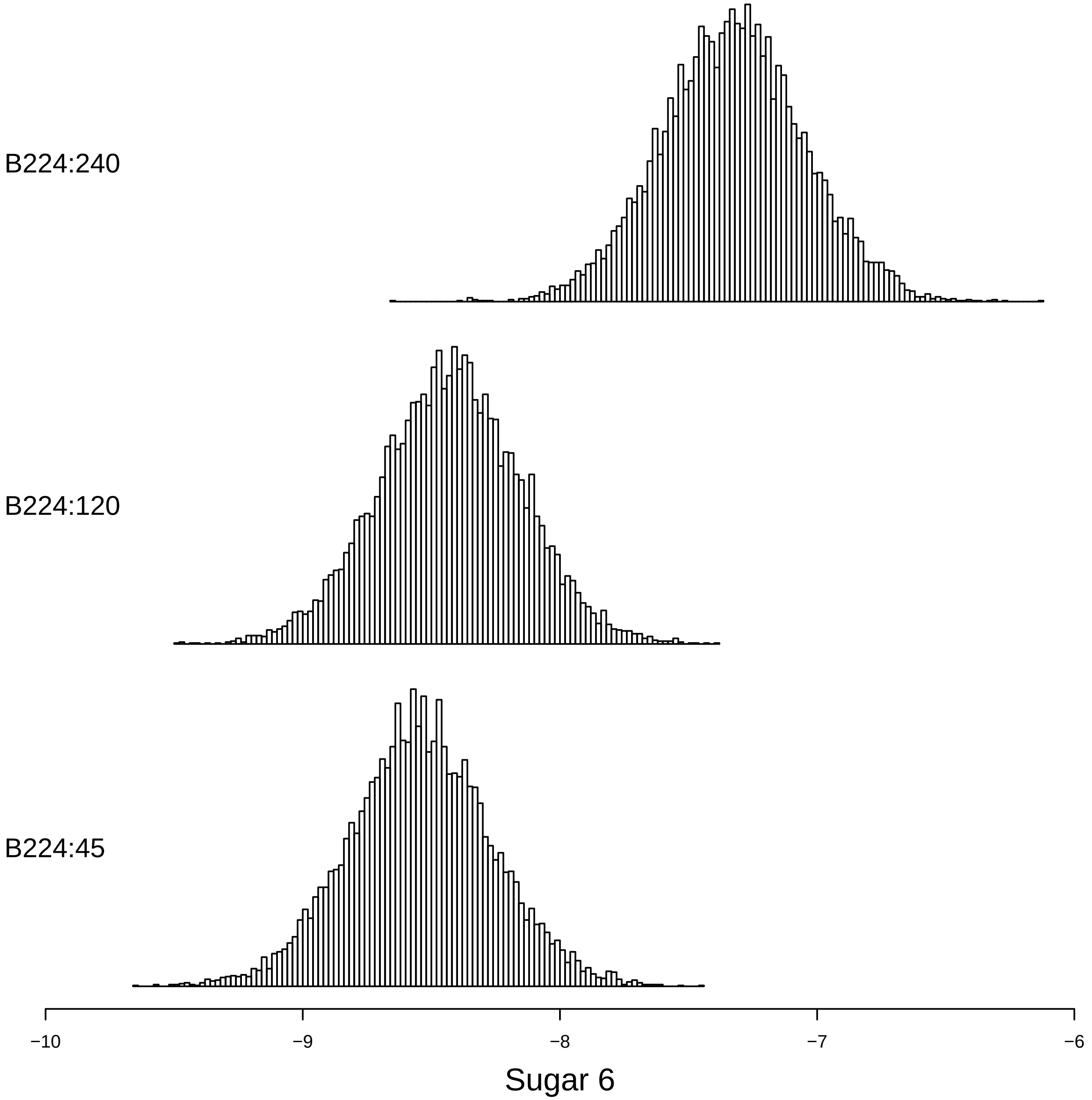
B224:240
B224:120
B224:45
−10
−9
−8
−7
−6
Sugar 6

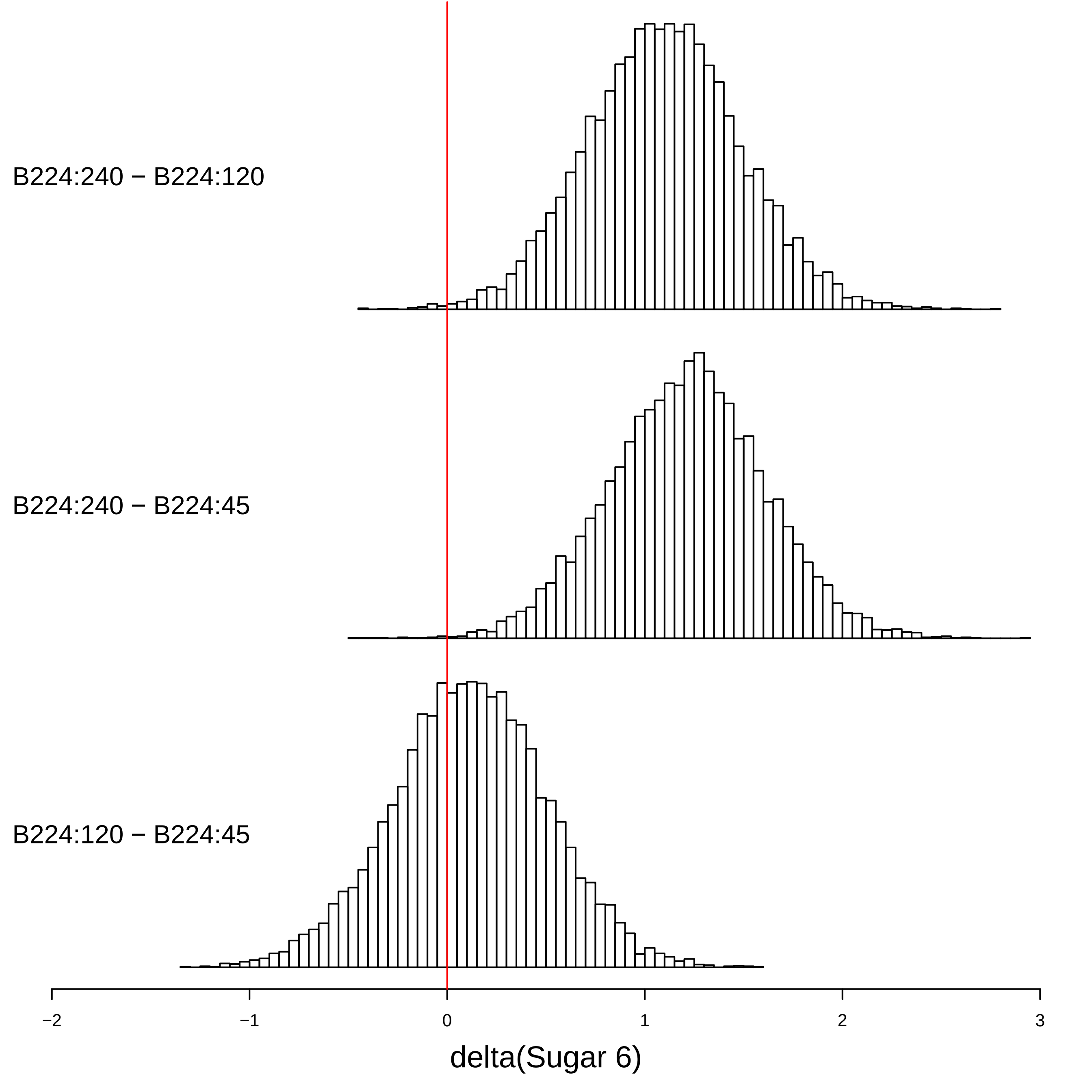
B224:240 − B224:120
B224:240 − B224:45
B224:120 − B224:45
−2
−1
0
1
2
3
delta(Sugar 6)

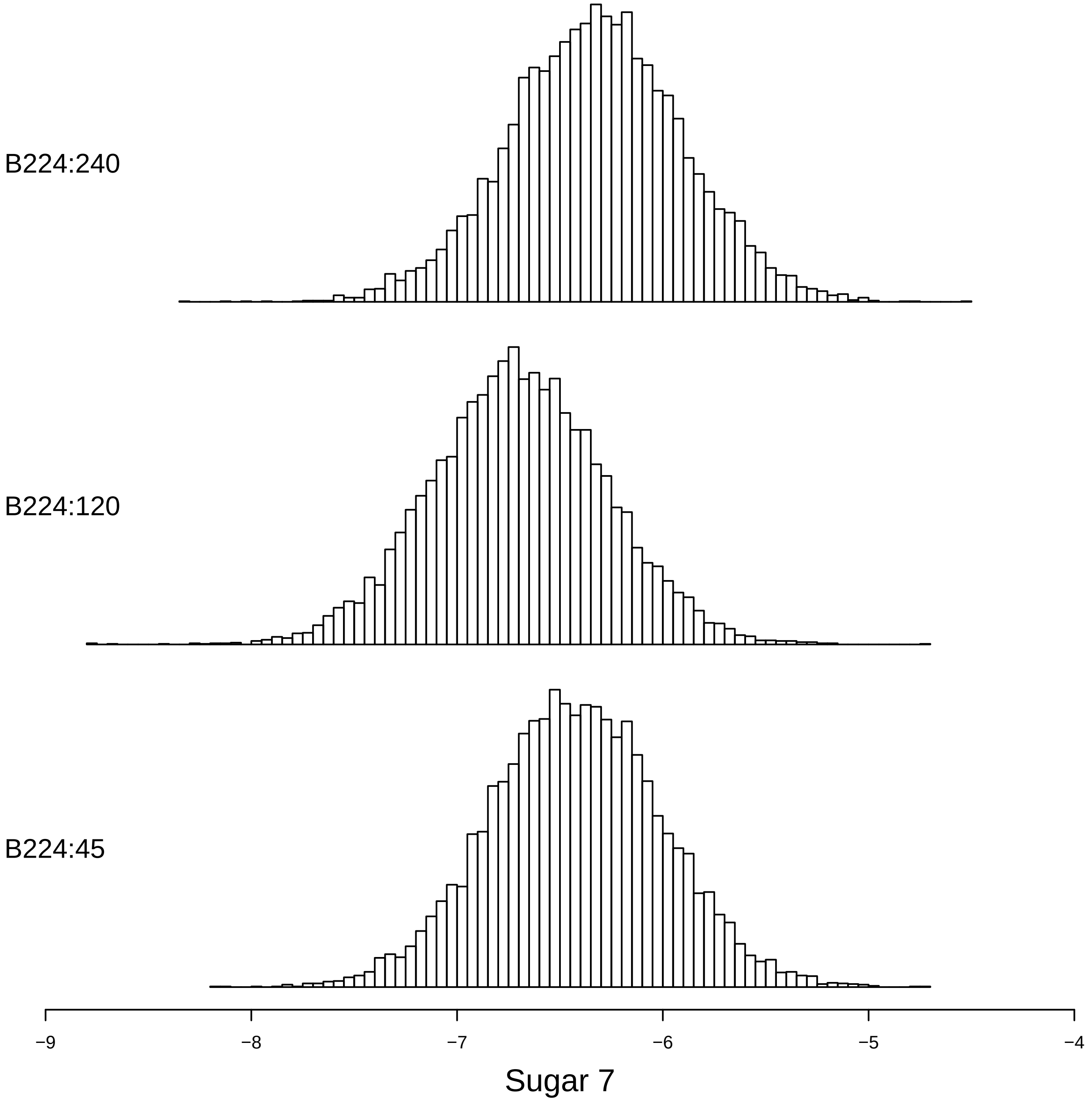
B224:240
B224:120
B224:45
−9
−8
−7
−6
−5
−4
Sugar 7

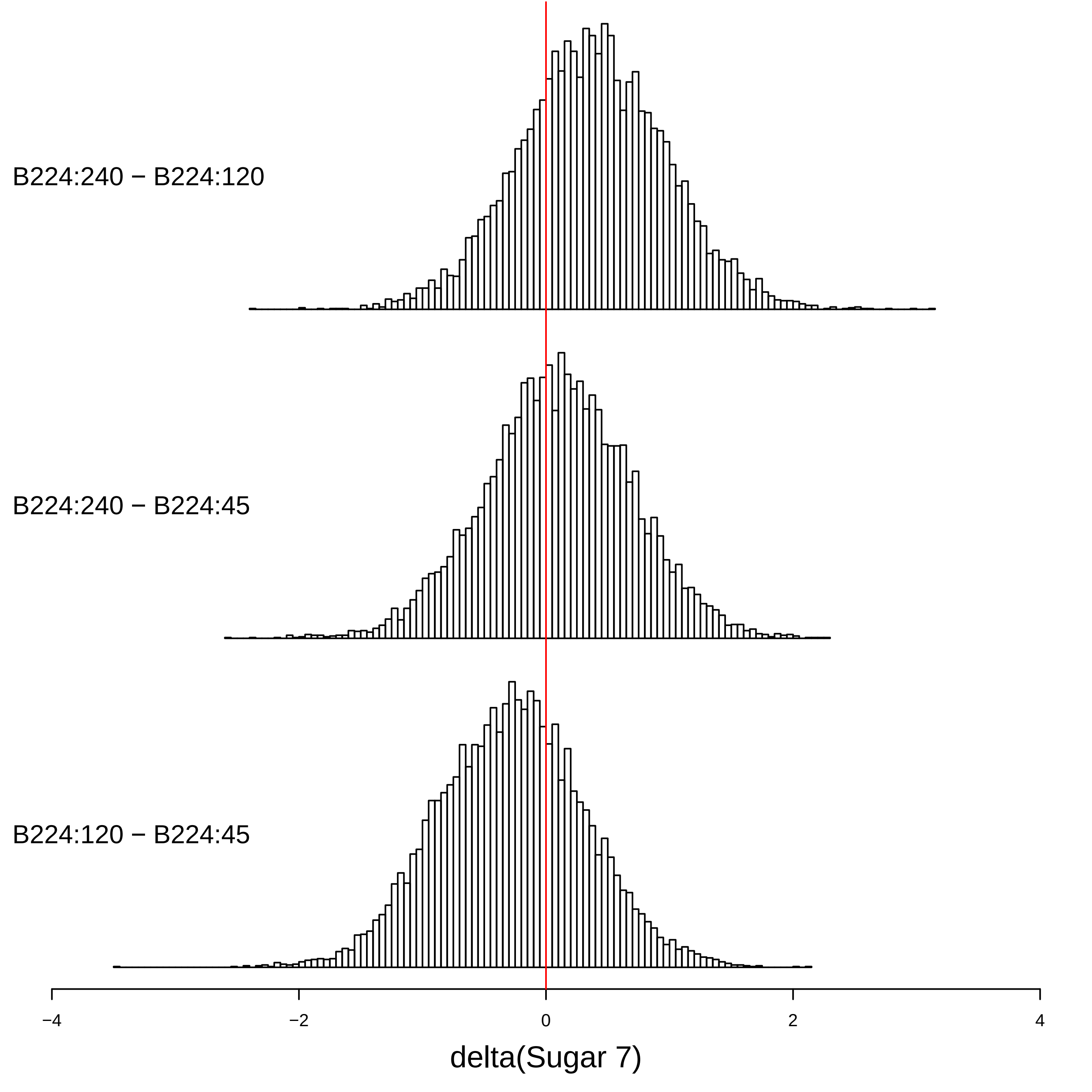

B224:240 − B224:120
B224:240 − B224:45
B224:120 − B224:45
−4
−2
0
2
4
delta(Sugar 7)

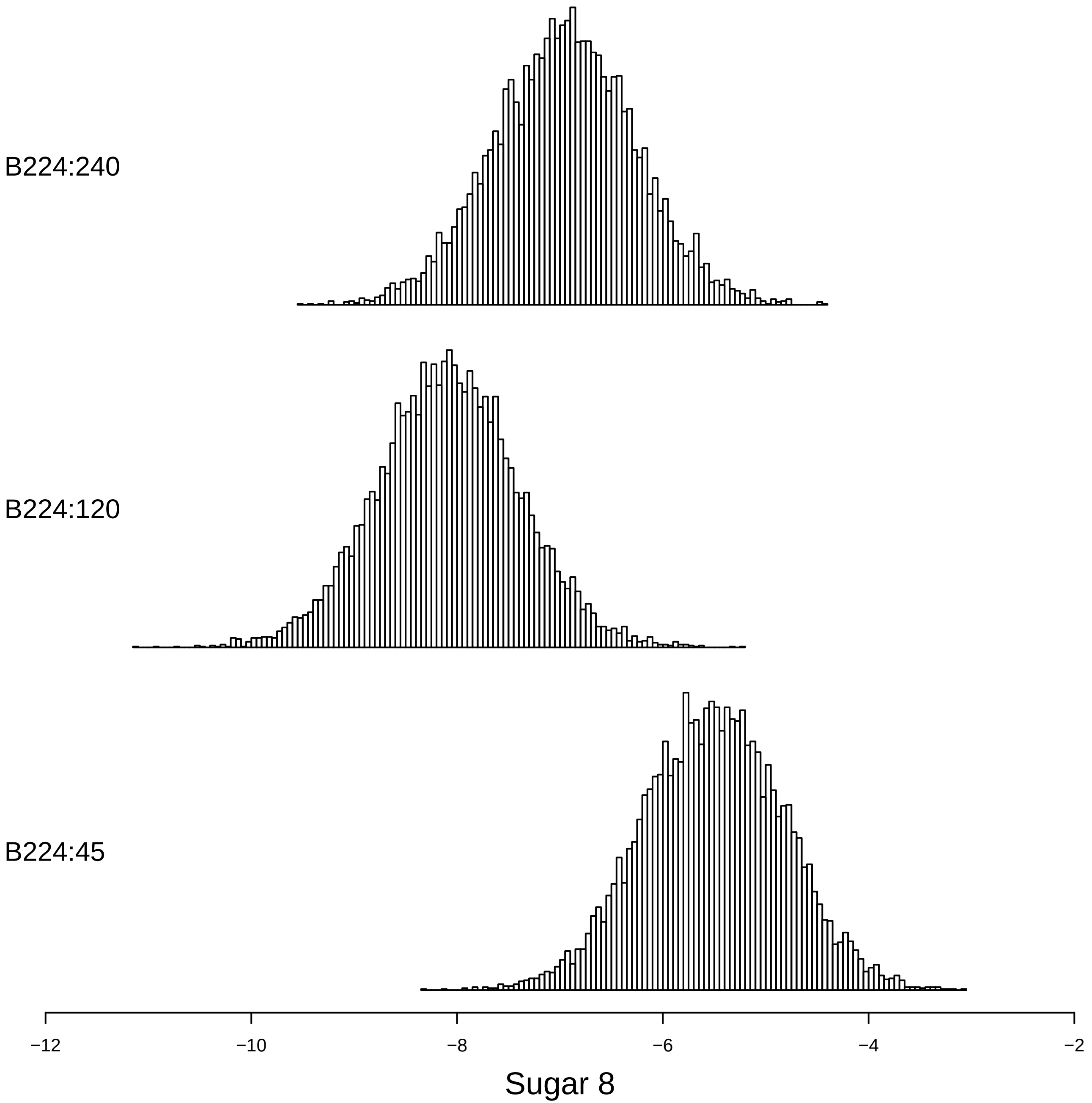

B224:240
B224:120
B224:45
−12
−10
−8
−6
−4
−2
Sugar 8

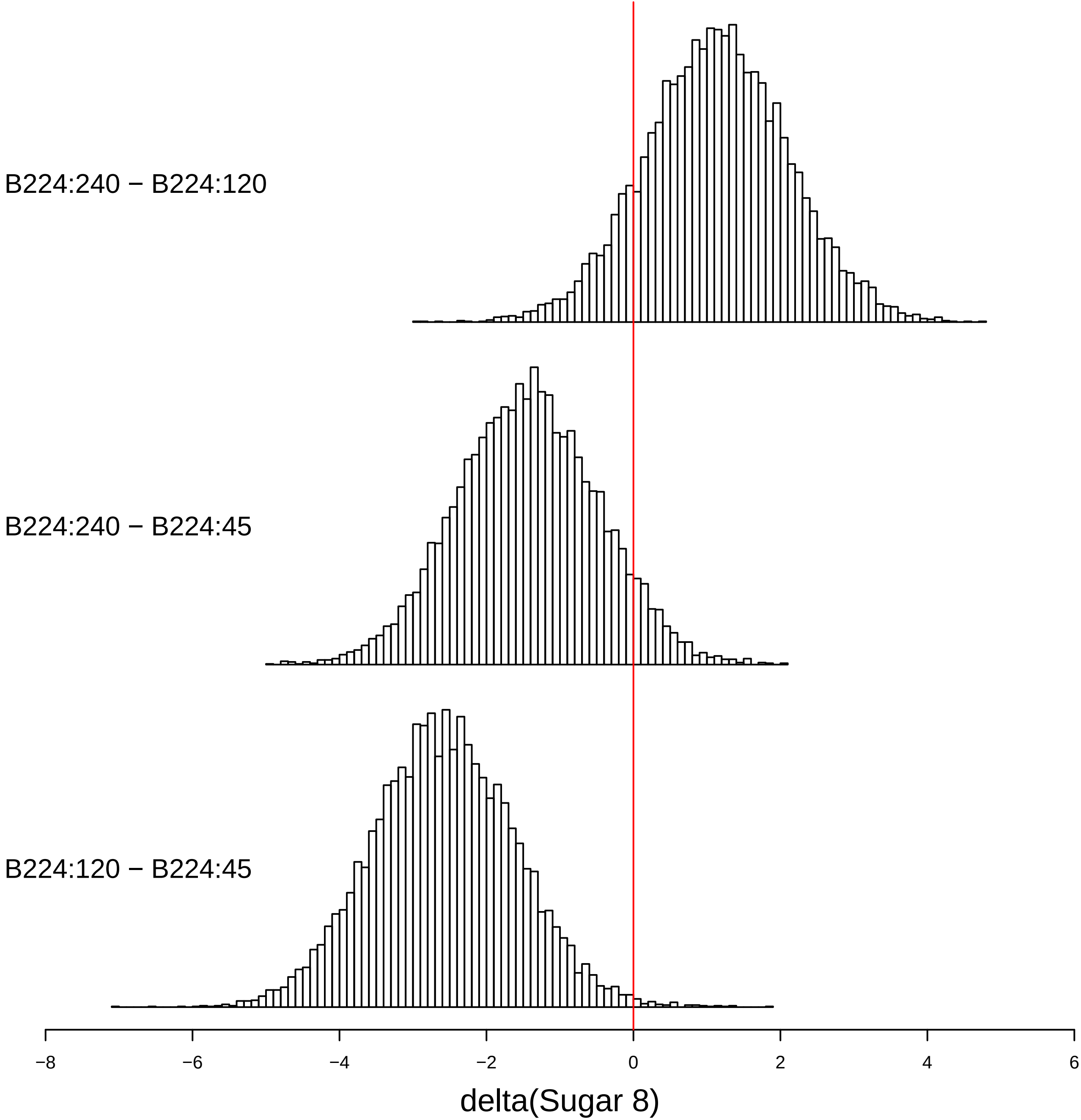
B224:240 − B224:120
B224:240 − B224:45
B224:120 − B224:45
−8
−6
−4
−2
0
2
4
6
delta(Sugar 8)

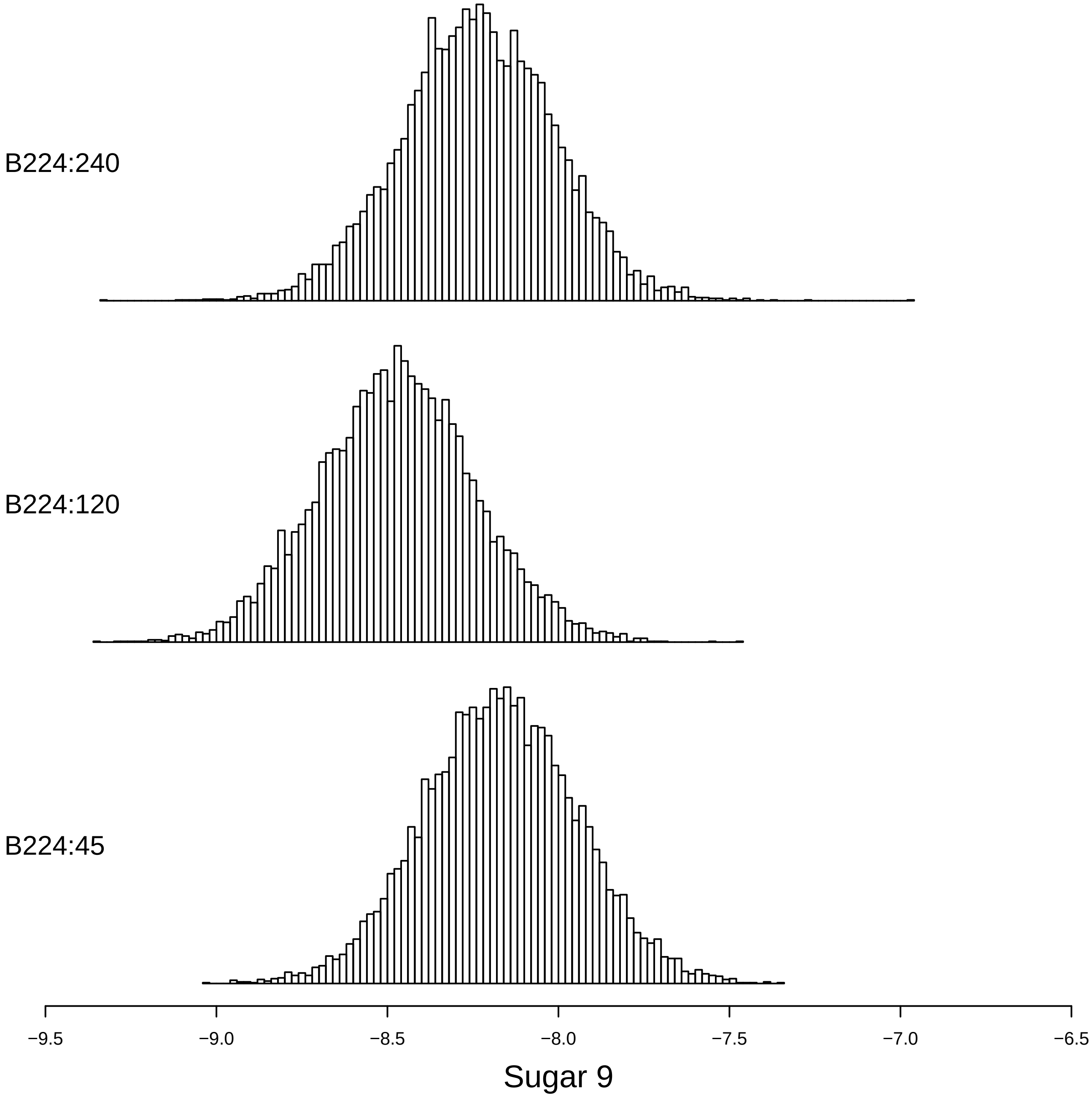

B224:240
B224:120
B224:45
−9.5
−9.0
−8.5
−8.0
−7.5
−7.0
−6.5
Sugar 9

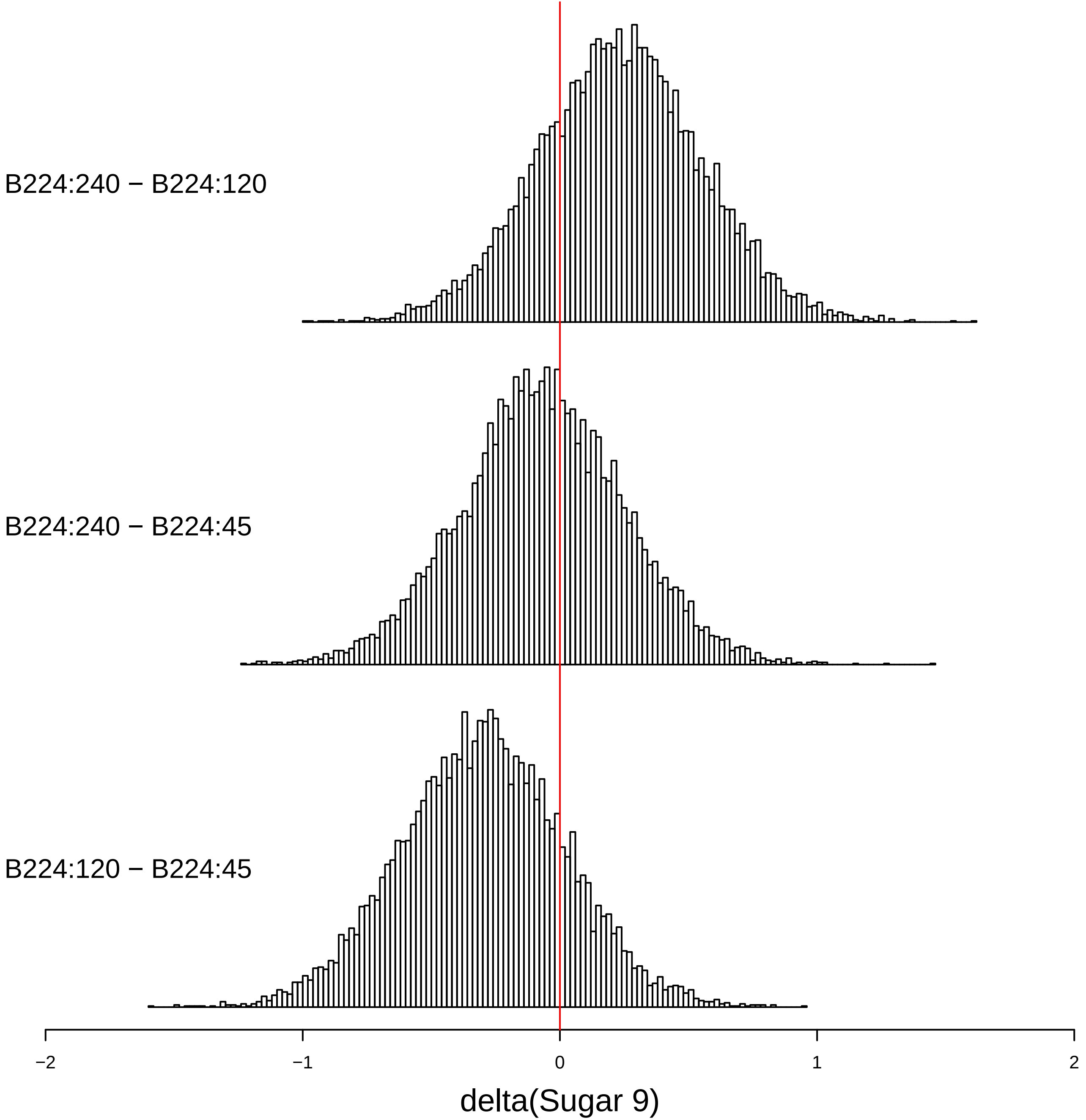

B224:240 − B224:120
B224:240 − B224:45
B224:120 − B224:45
−2
−1
0
1
2
delta(Sugar 9)

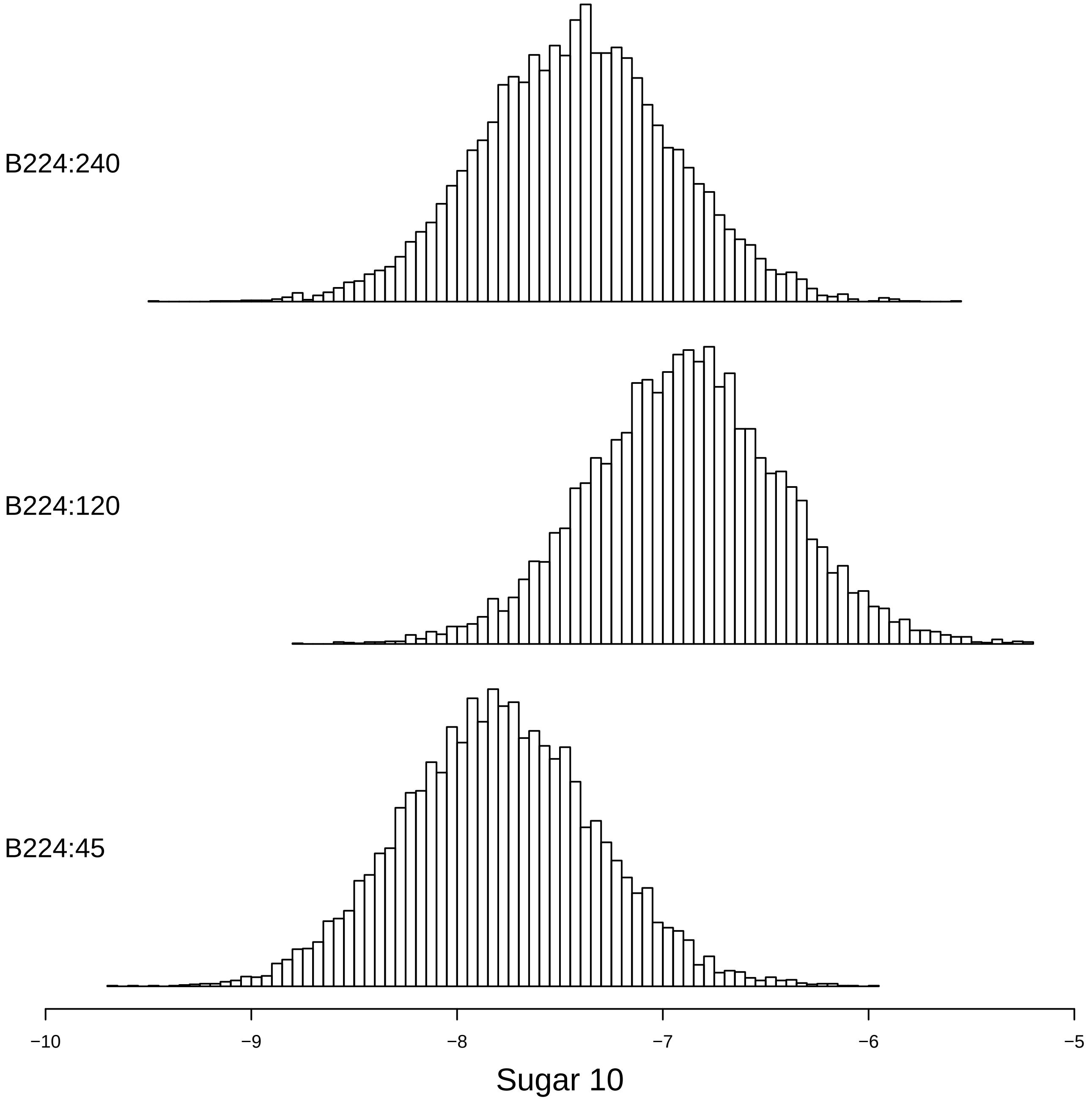
B224:240
B224:120
B224:45
−10
−9
−8
−7
−6
−5
Sugar 10

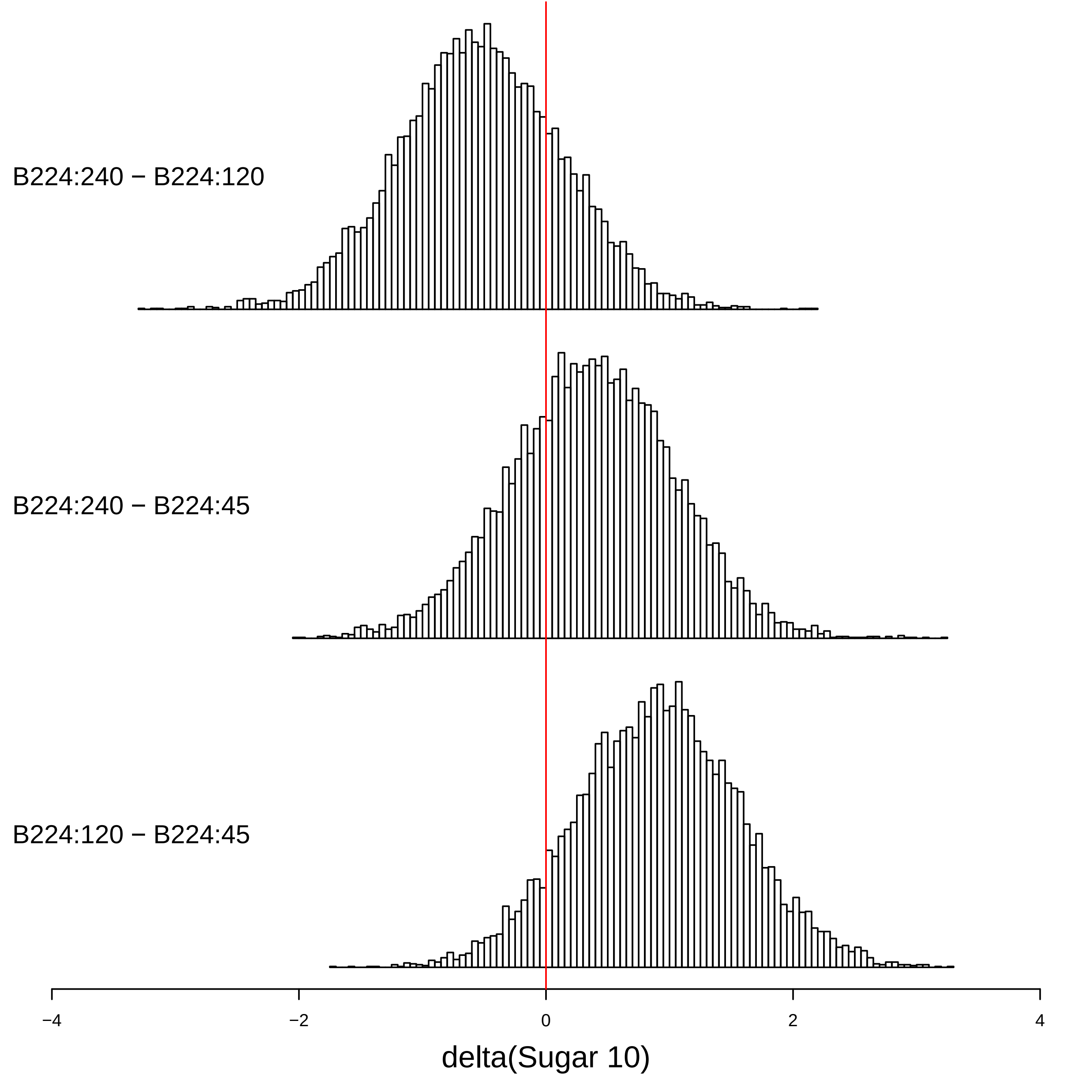
B224:240 − B224:120
B224:240 − B224:45
B224:120 − B224:45
−4
−2
0
2
4
delta(Sugar 10)

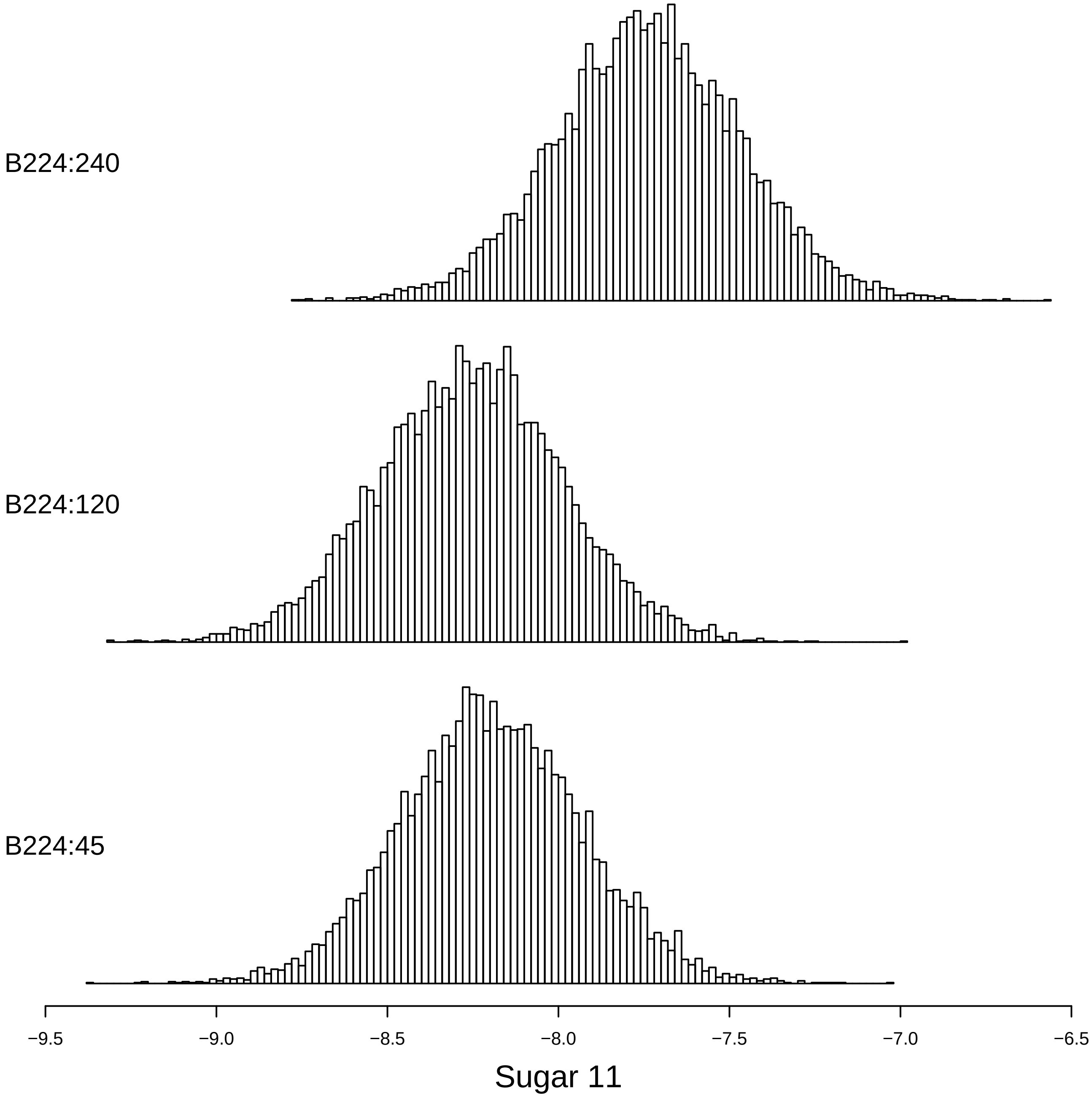
B224:240
B224:120
B224:45
−9.5
−9.0
−8.5
−8.0
−7.5
−7.0
−6.5
Sugar 11

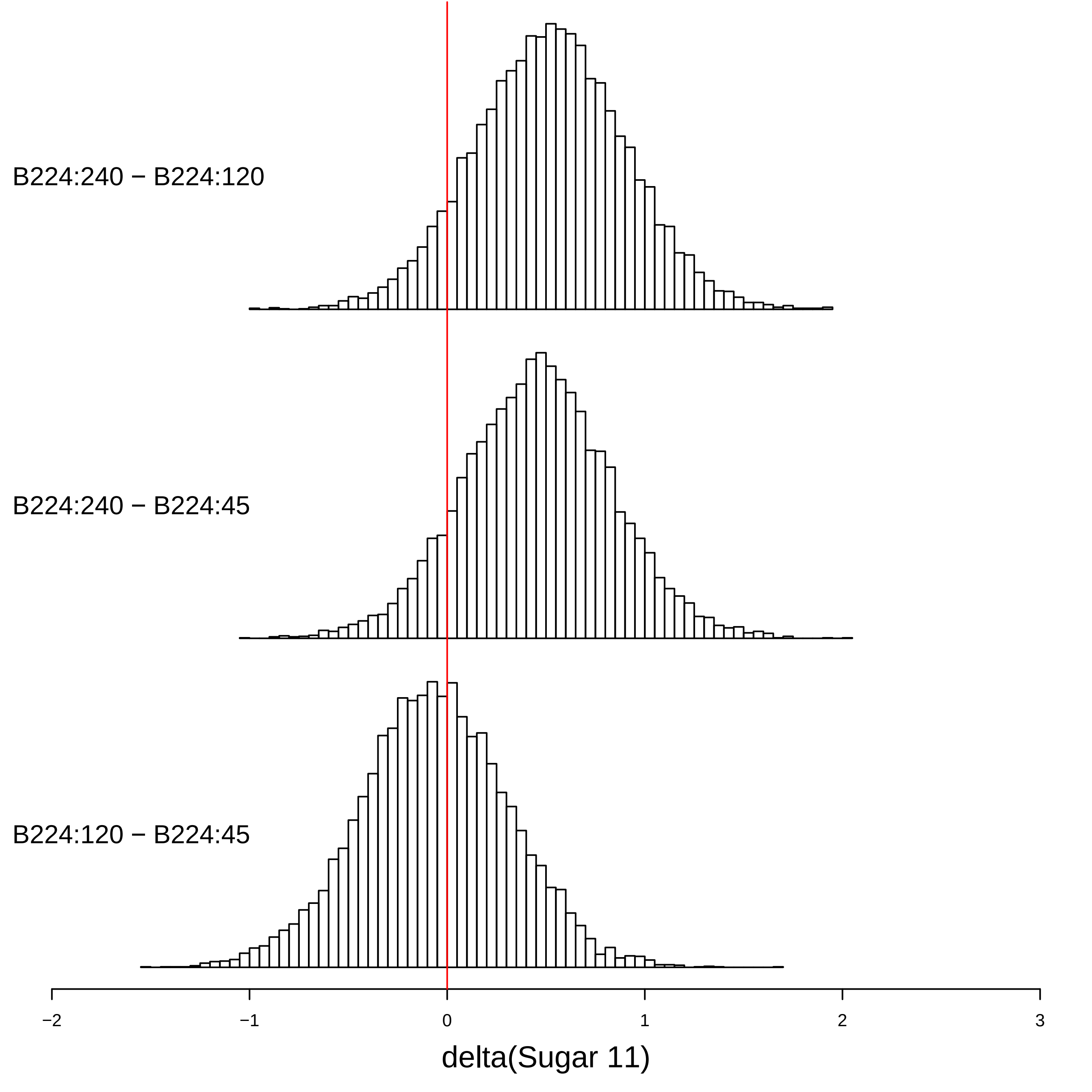
B224:240 − B224:120
B224:240 − B224:45
B224:120 − B224:45
−2
−1
0
1
2
3
delta(Sugar 11)

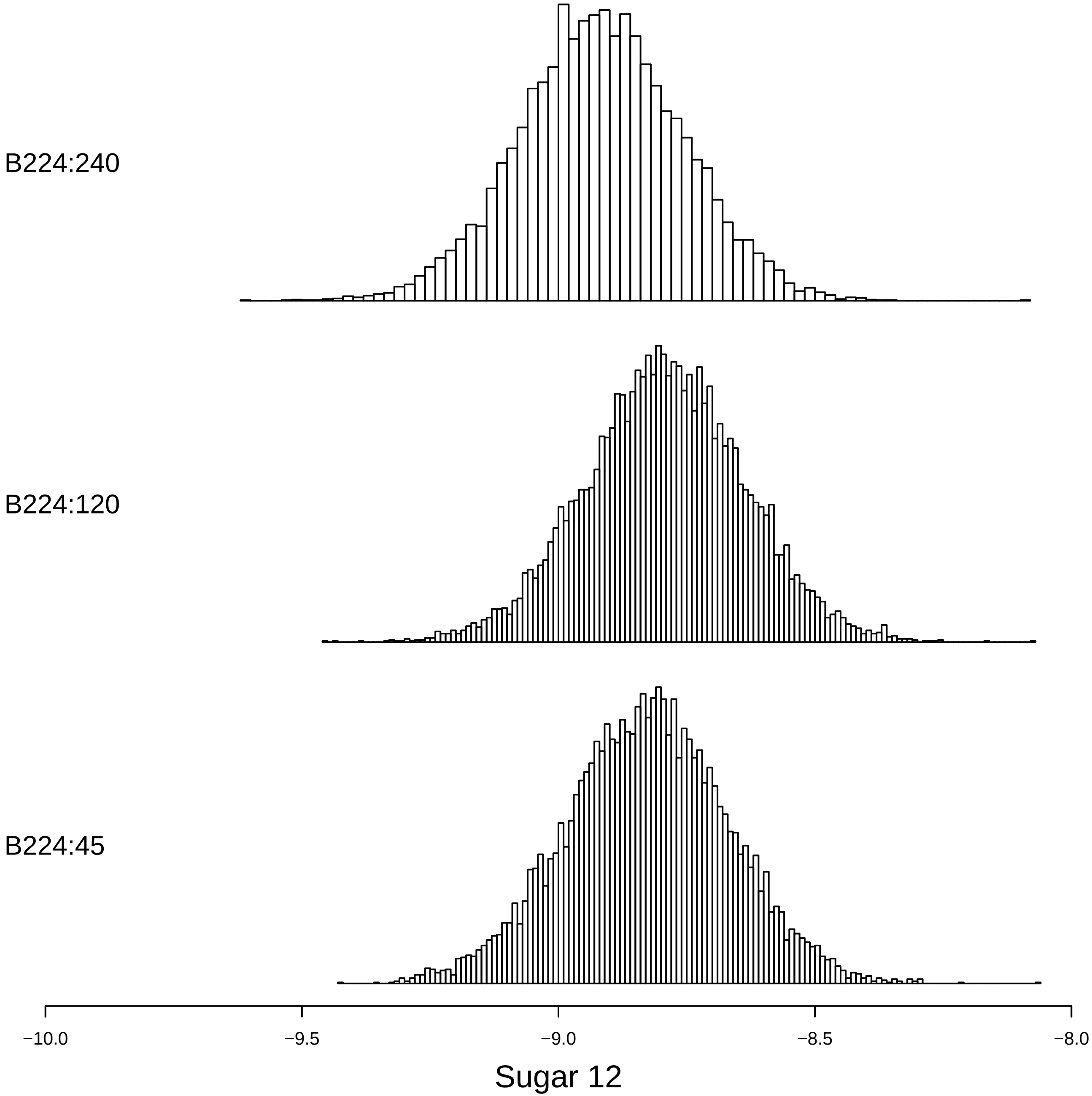

B224:240
B224:120
B224:45
−10.0
−9.5
−9.0
−8.5
−8.0
Sugar 12

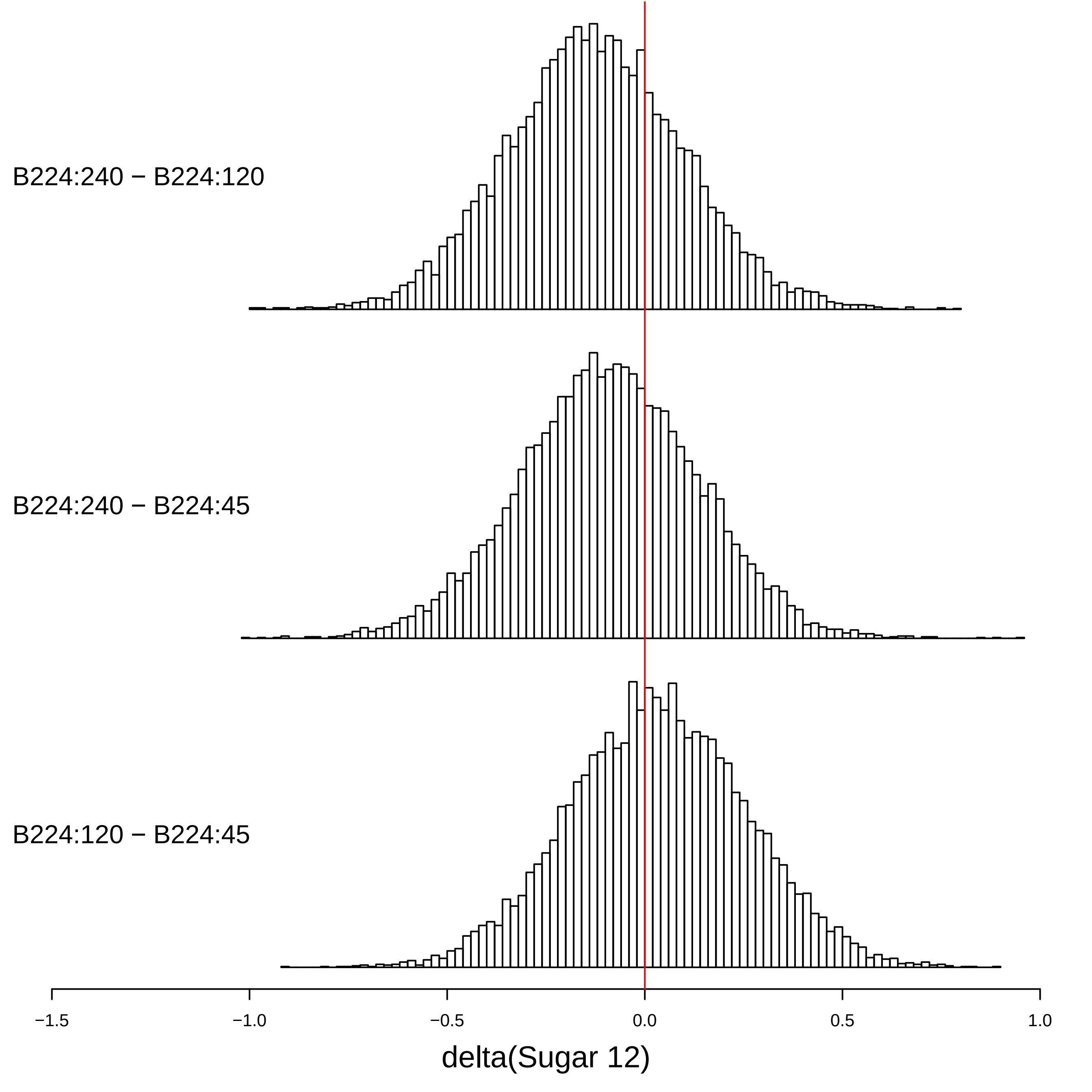

B224:240 − B224:120
B224:240 − B224:45
B224:120 − B224:45
−1.5
−1.0
−0.5
0.0
0.5
1.0
delta(Sugar 12)

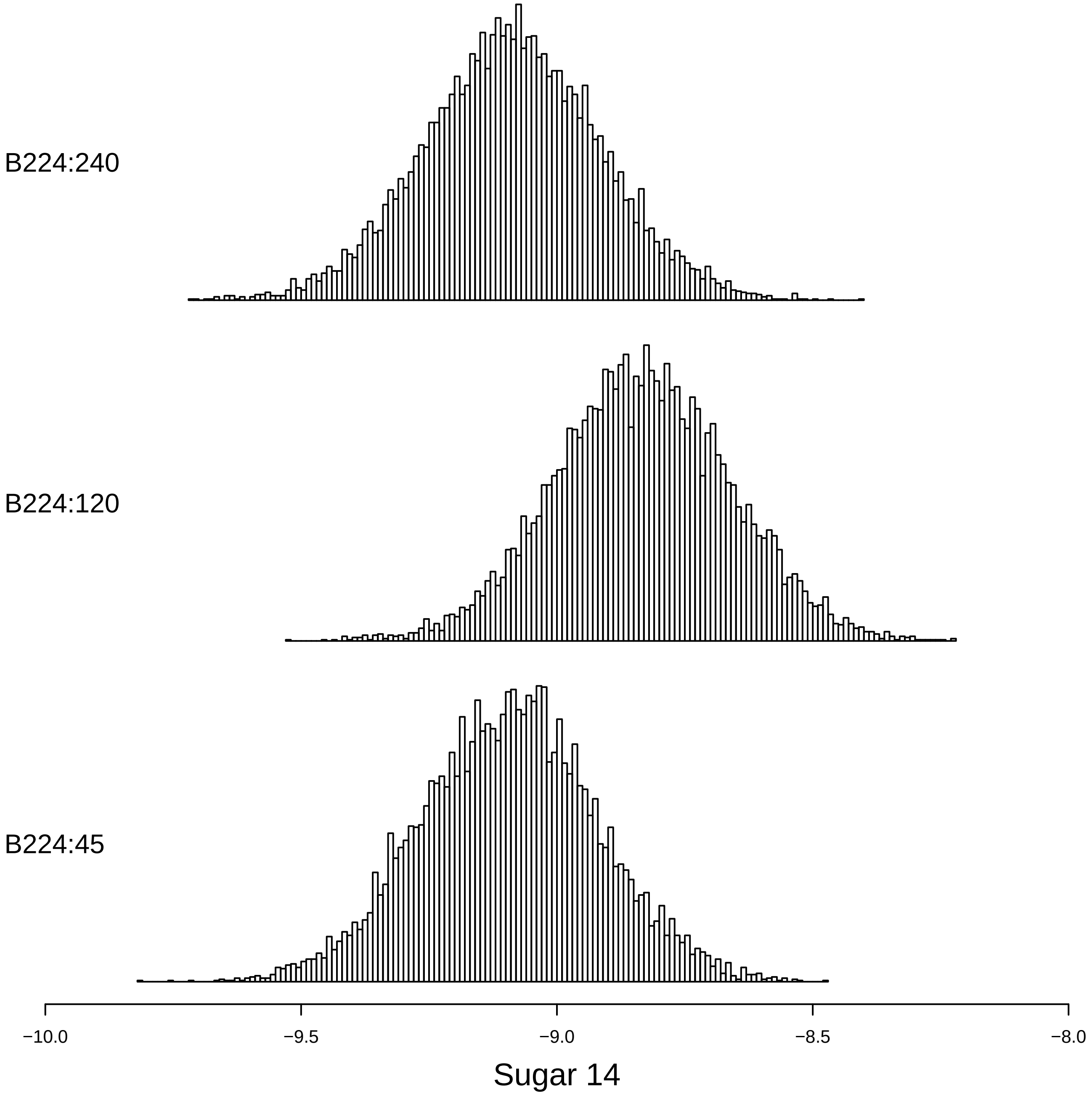

B224:240
B224:120
B224:45
−10.0
−9.5
−9.0
−8.5
−8.0
Sugar 14

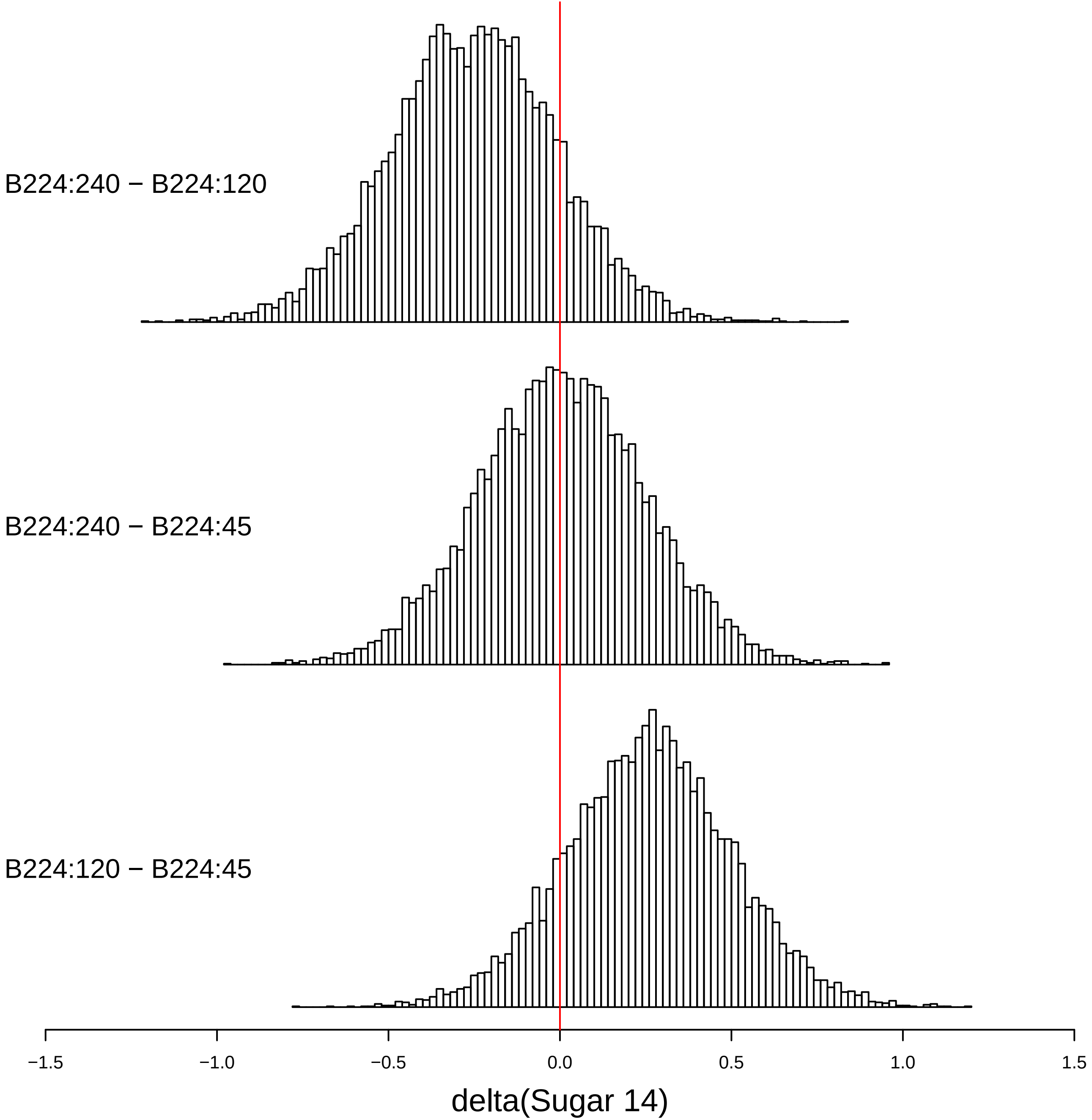
B224:240 − B224:120
B224:240 − B224:45
B224:120 − B224:45
−1.5
−1.0
−0.5
0.0
0.5
1.0
1.5
delta(Sugar 14)

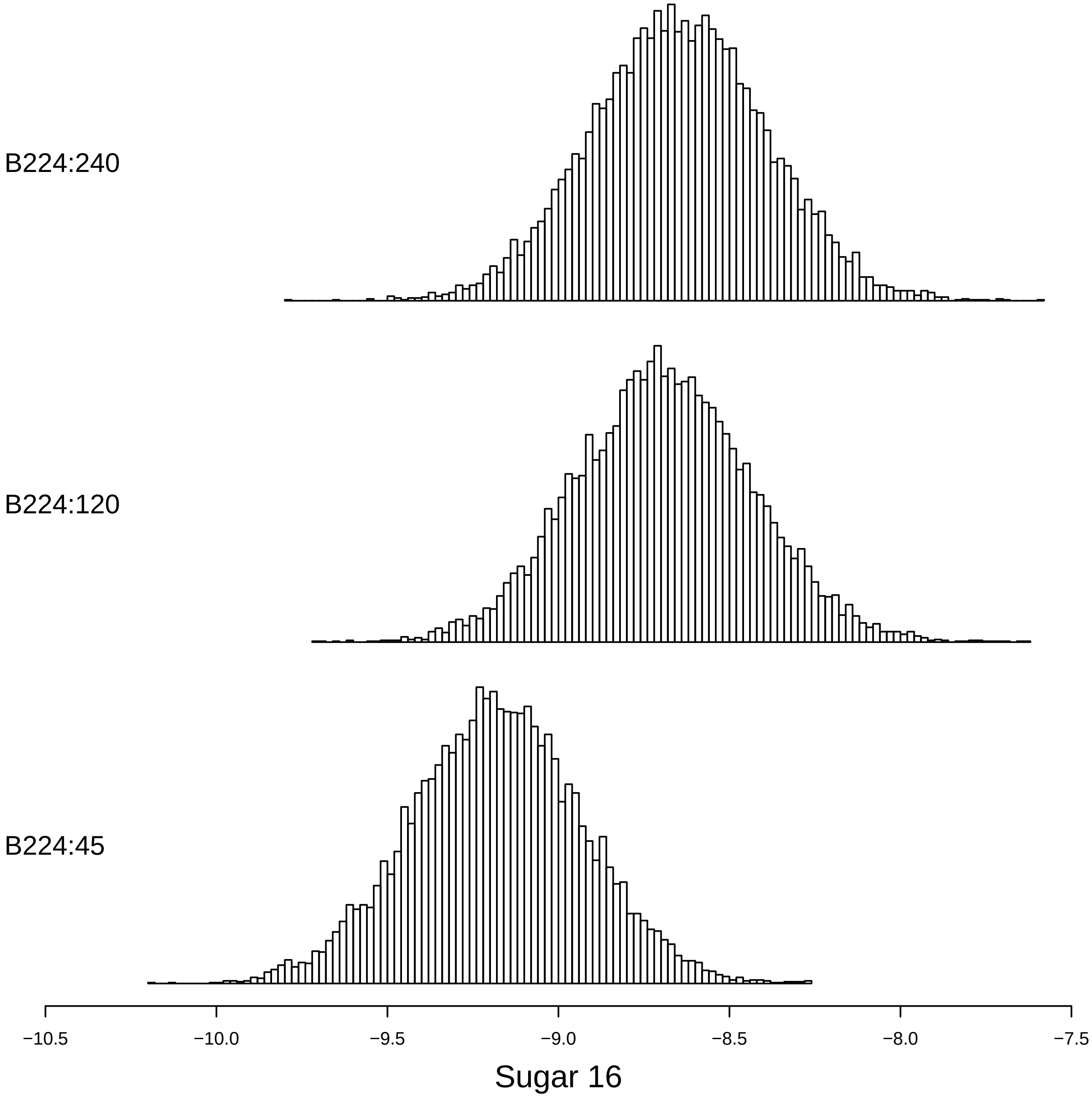

B224:240
B224:120
B224:45
−10.5
−10.0
−9.5
−9.0
−8.5
−8.0
−7.5
Sugar 16

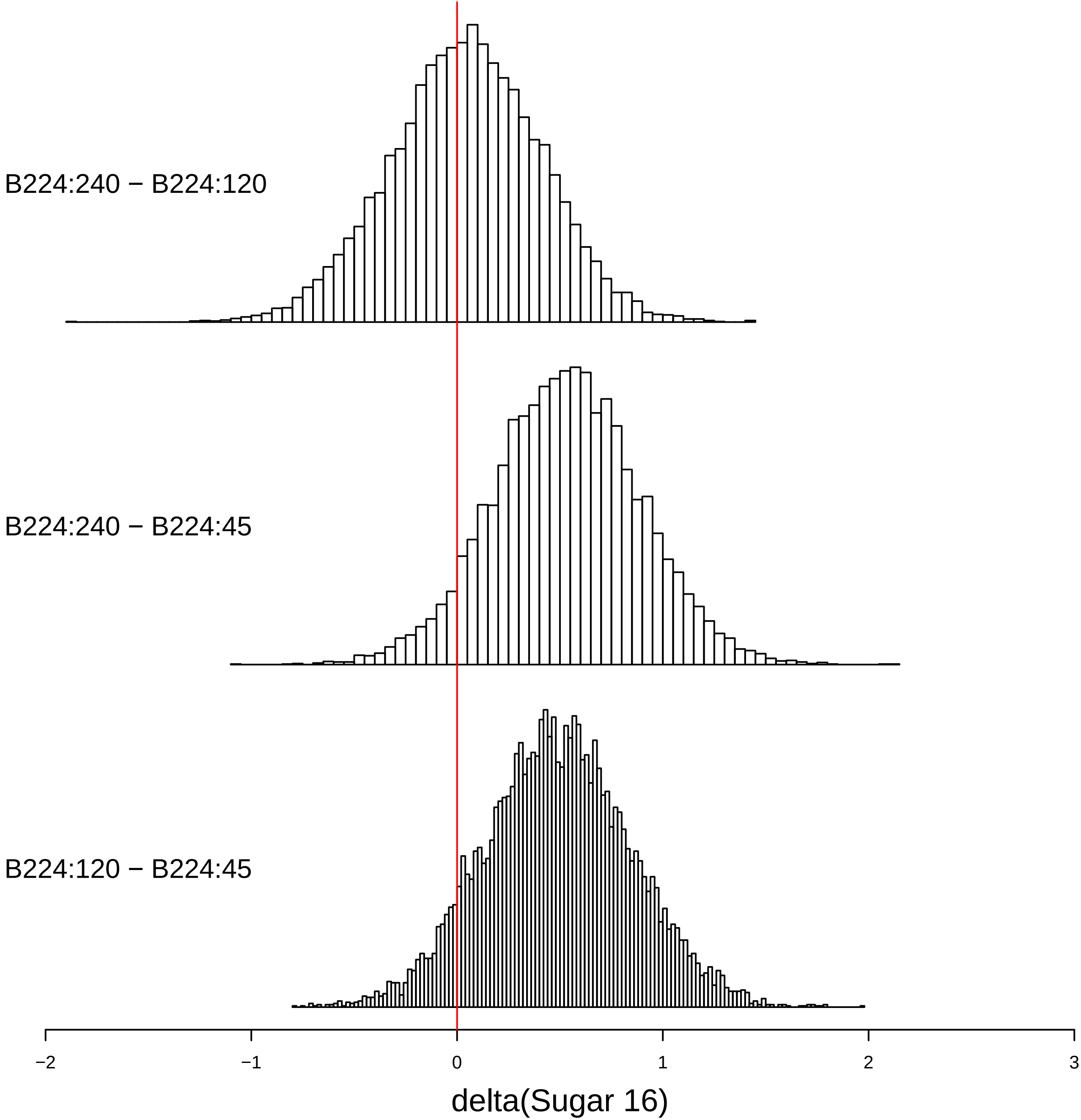
B224:240 − B224:120
B224:240 − B224:45
B224:120 − B224:45
−2
−1
0
1
2
3
delta(Sugar 16)

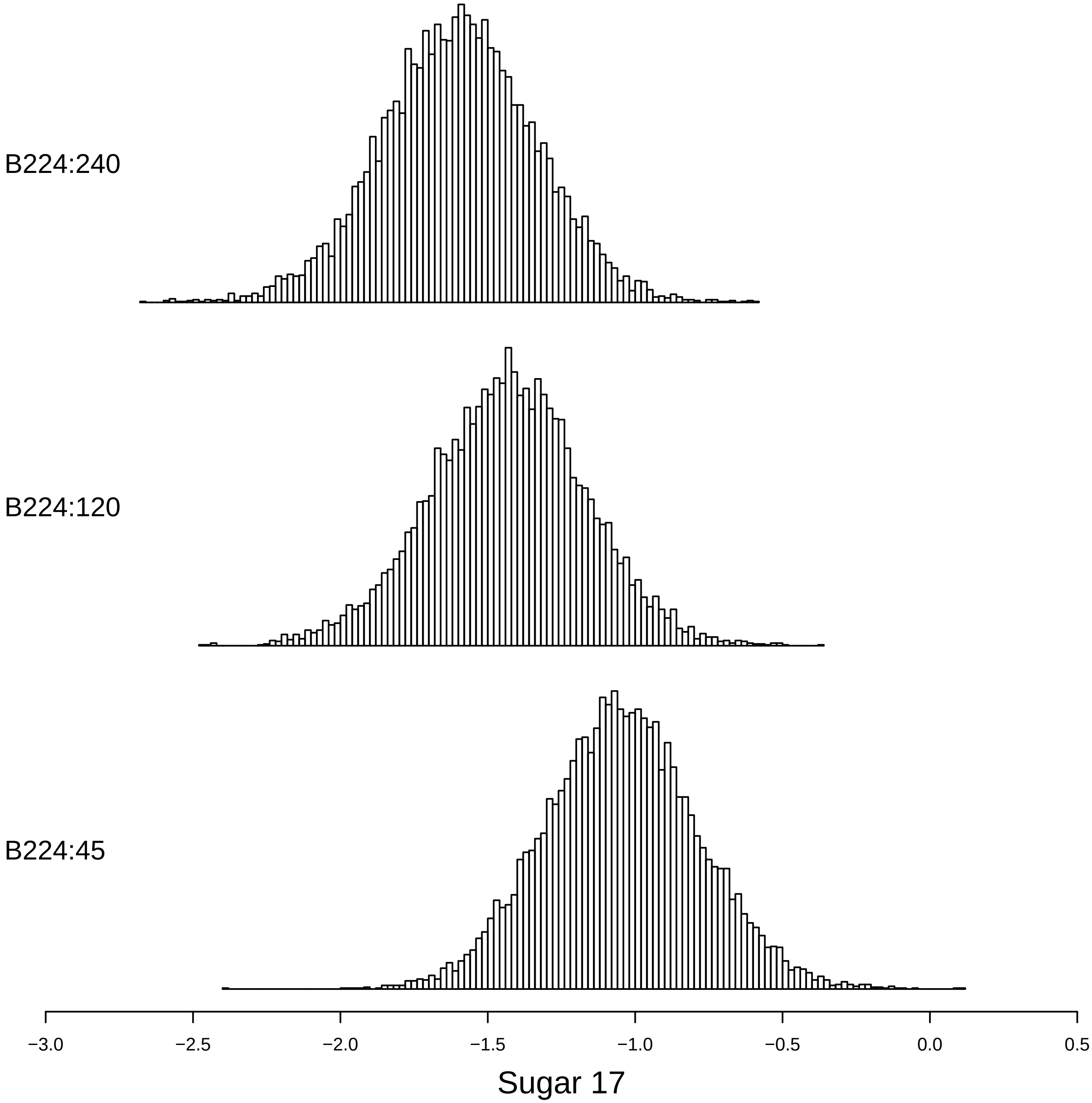
B224:240
B224:120
B224:45
−3.0
−2.5
−2.0
−1.5
−1.0
−0.5
0.0
0.5
Sugar 17

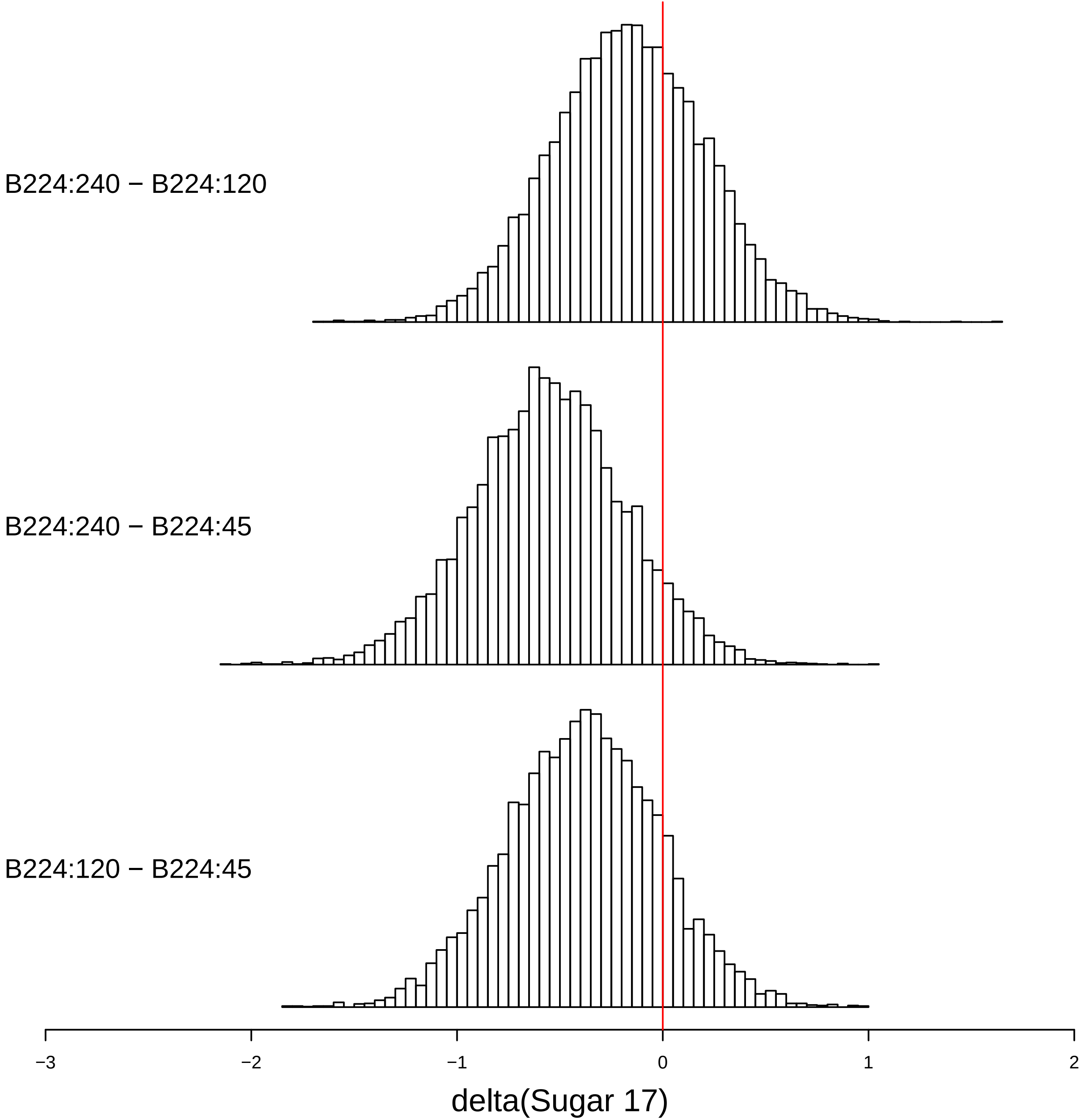

B224:240 − B224:120
B224:240 − B224:45
B224:120 − B224:45
−3
−2
−1
0
1
2
delta(Sugar 17)

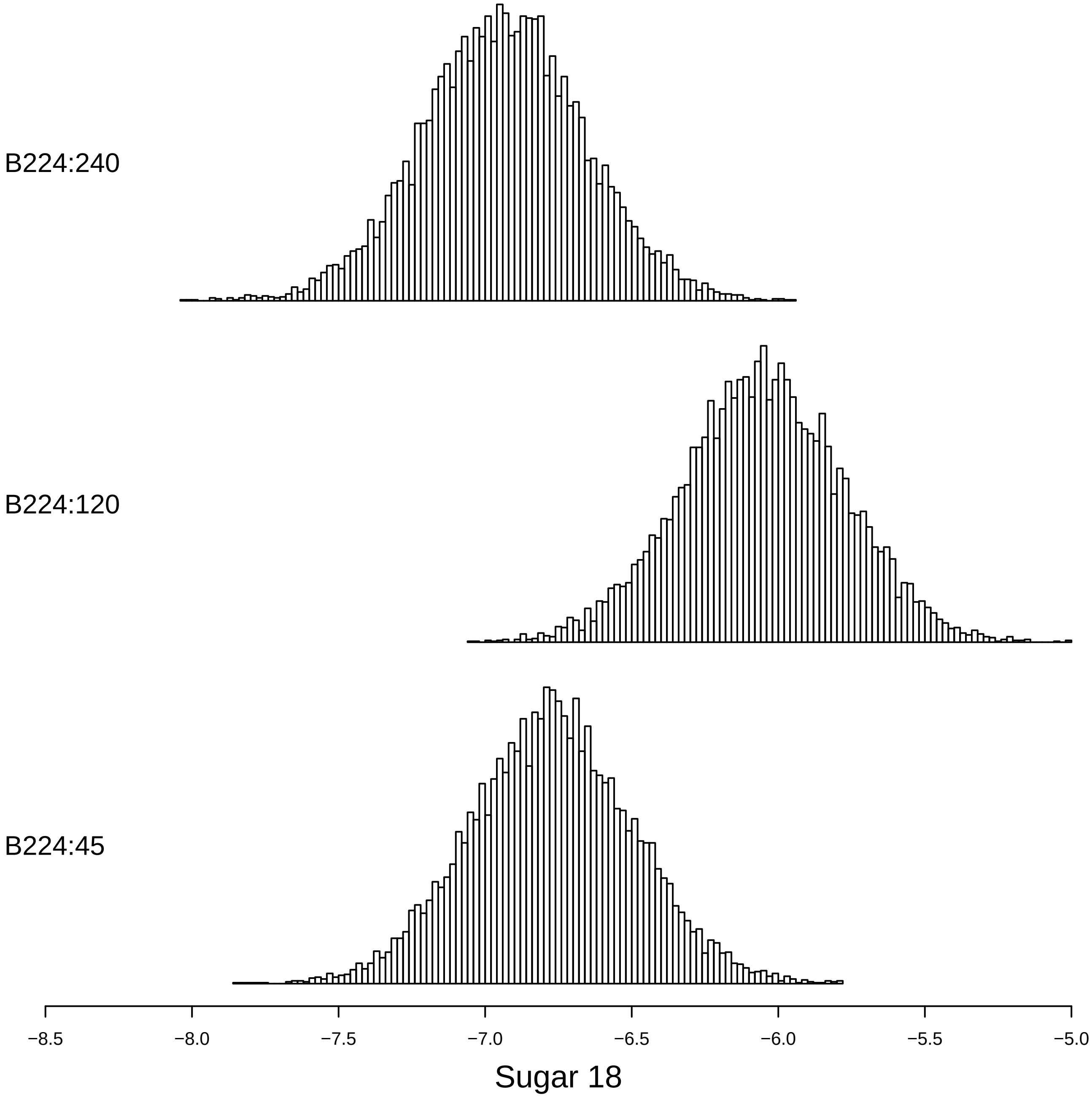

B224:240
B224:120
B224:45
−8.5
−8.0
−7.5
−7.0
−6.5
−6.0
−5.5
−5.0
Sugar 18

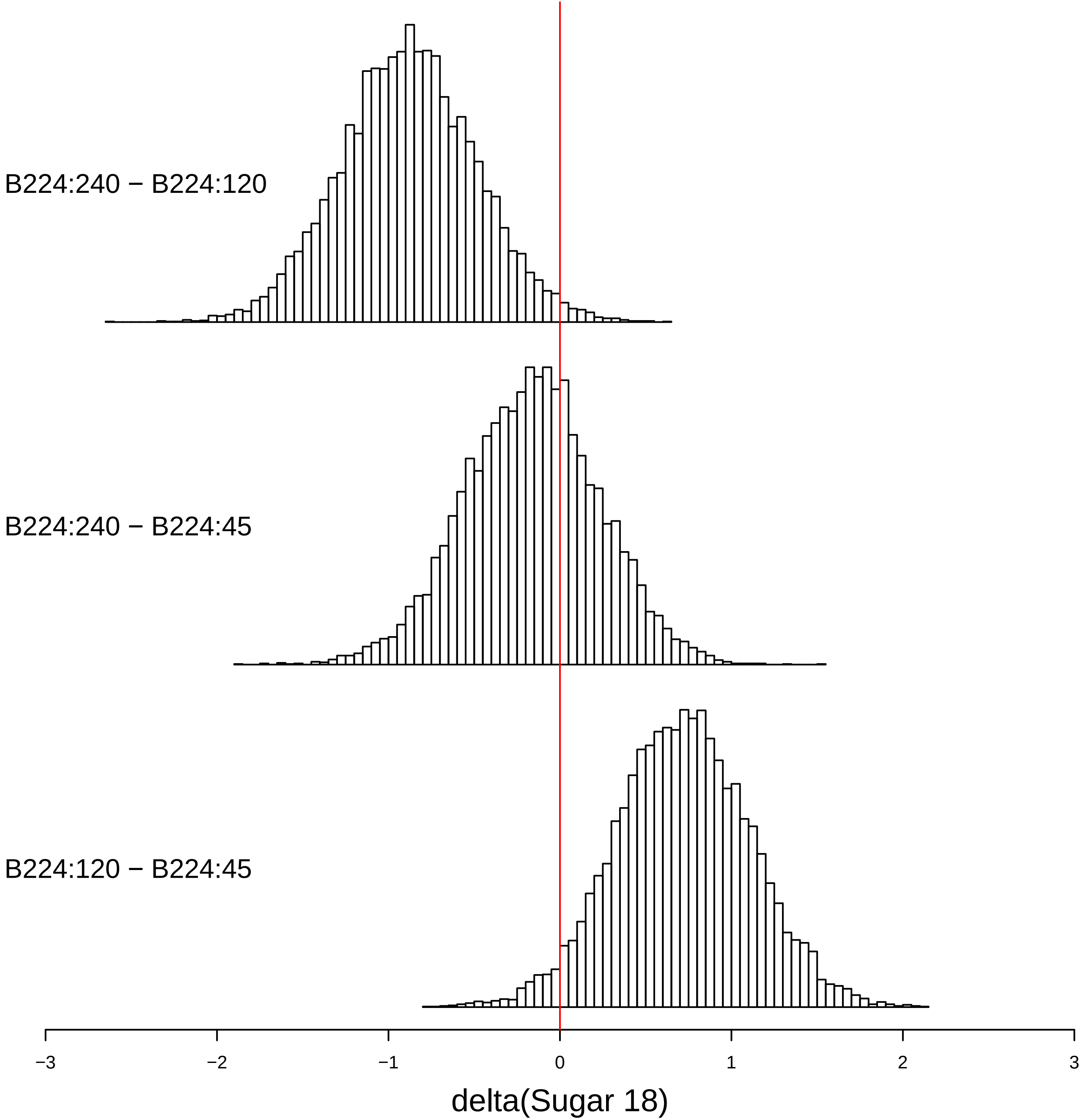
B224:240 − B224:120
B224:240 − B224:45
B224:120 − B224:45
−3
−2
−1
0
1
2
3
delta(Sugar 18)

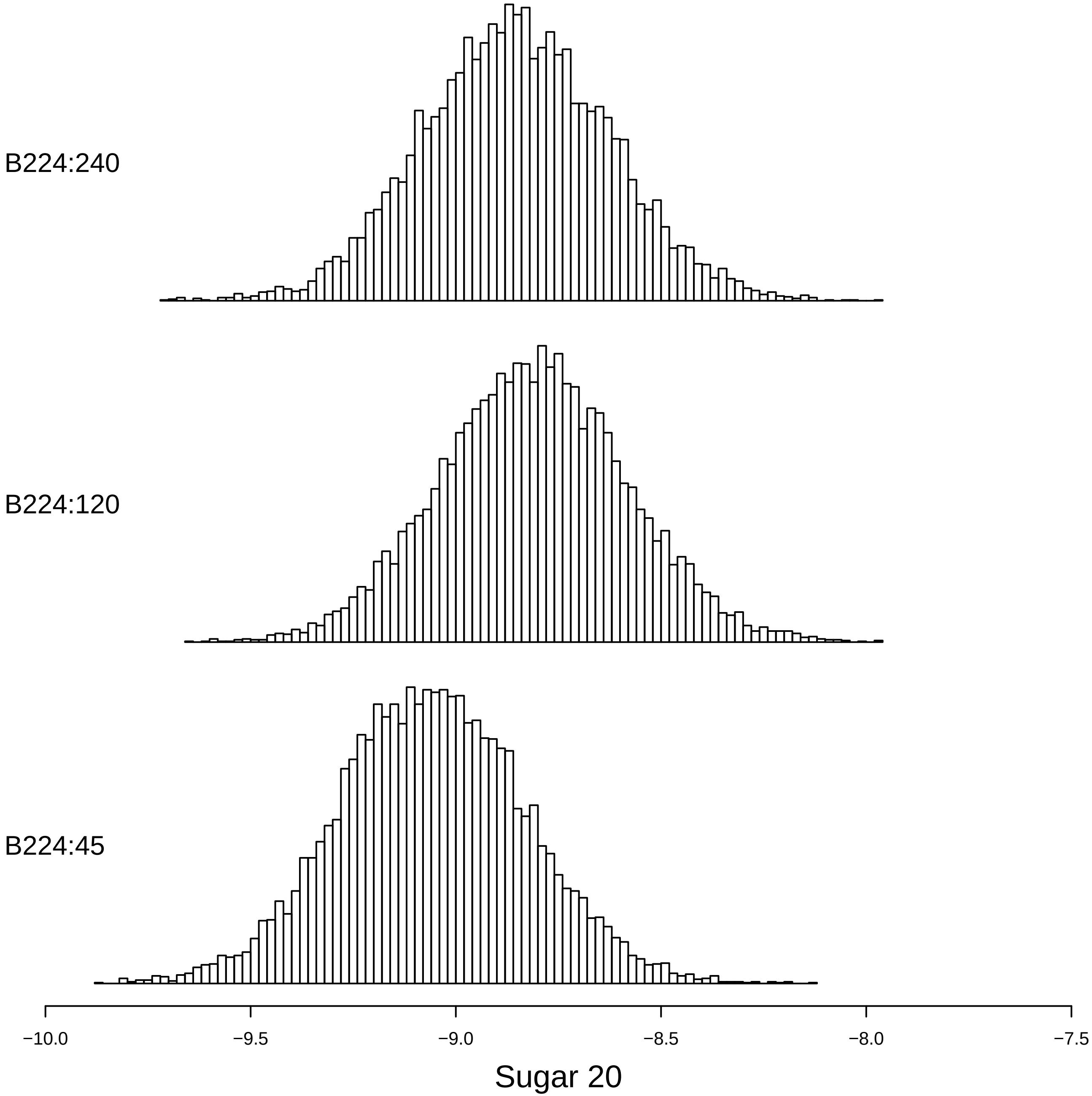
B224:240
B224:120
B224:45
−10.0
−9.5
−9.0
−8.5
−8.0
−7.5
Sugar 20

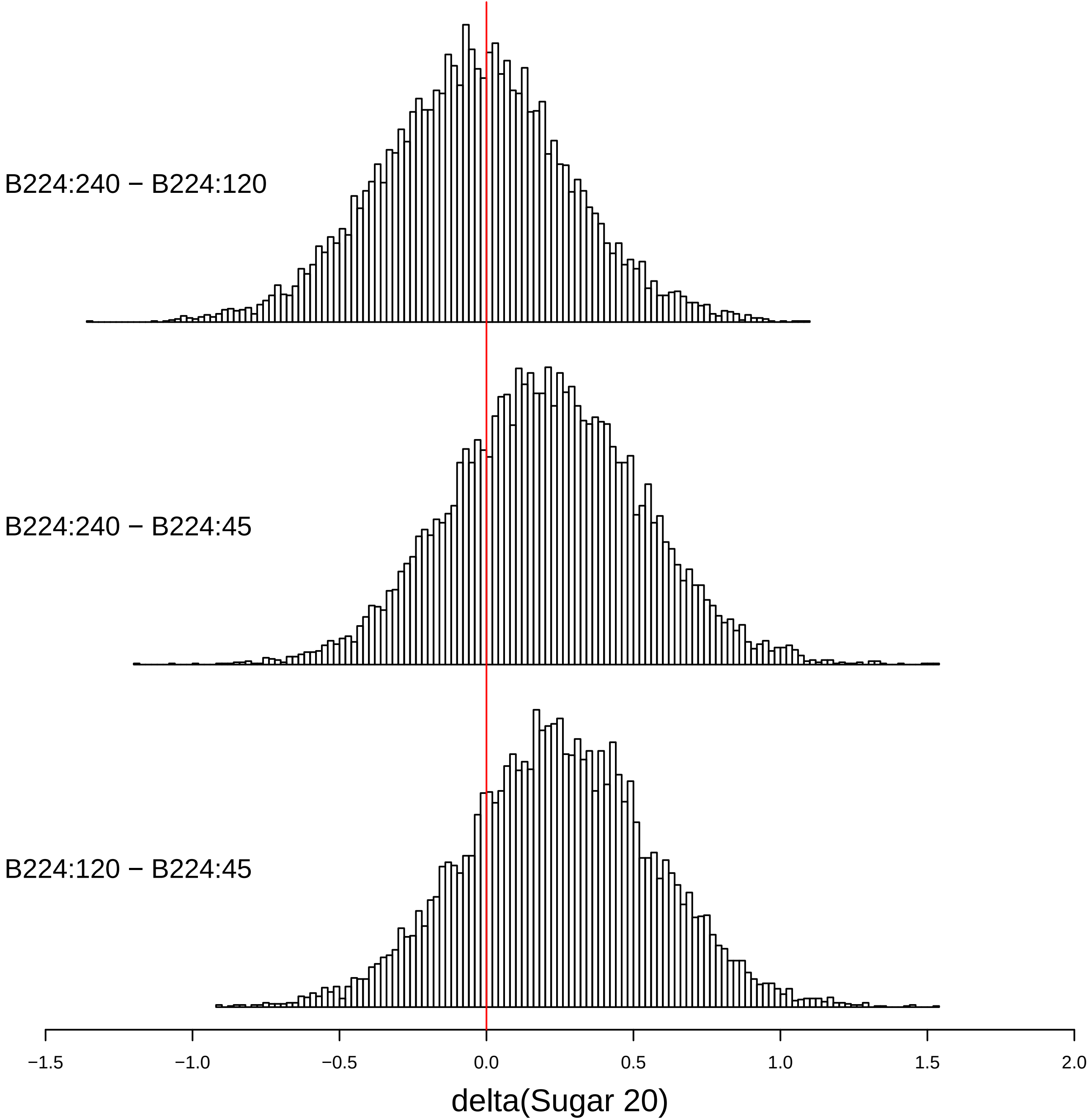
B224:240 − B224:120
B224:240 − B224:45
B224:120 − B224:45
−1.5
−1.0
−0.5
0.0
0.5
1.0
1.5
2.0
delta(Sugar 20)

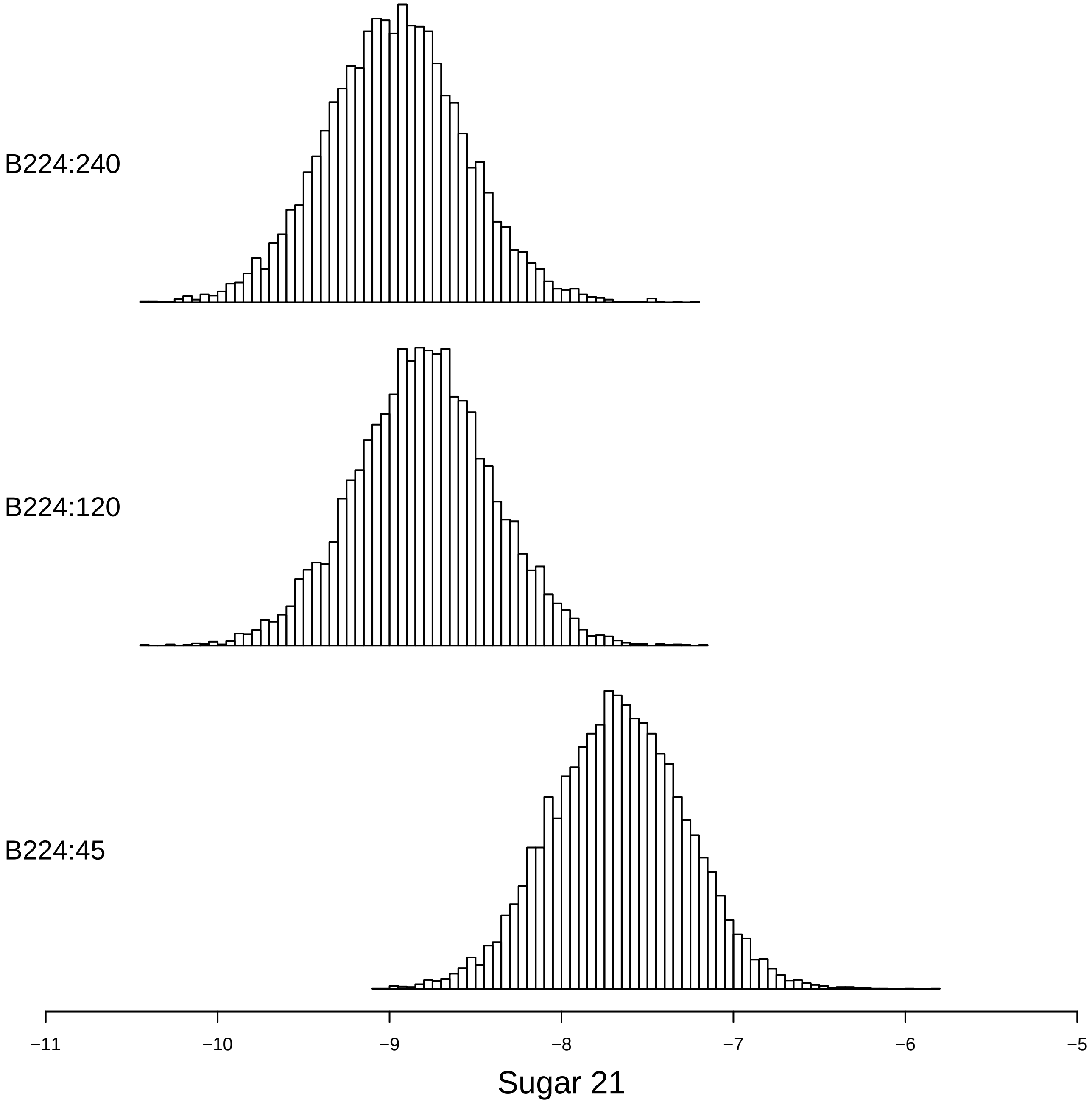
B224:240
B224:120
B224:45
−11
−10
−9
−8
−7
−6
−5
Sugar 21

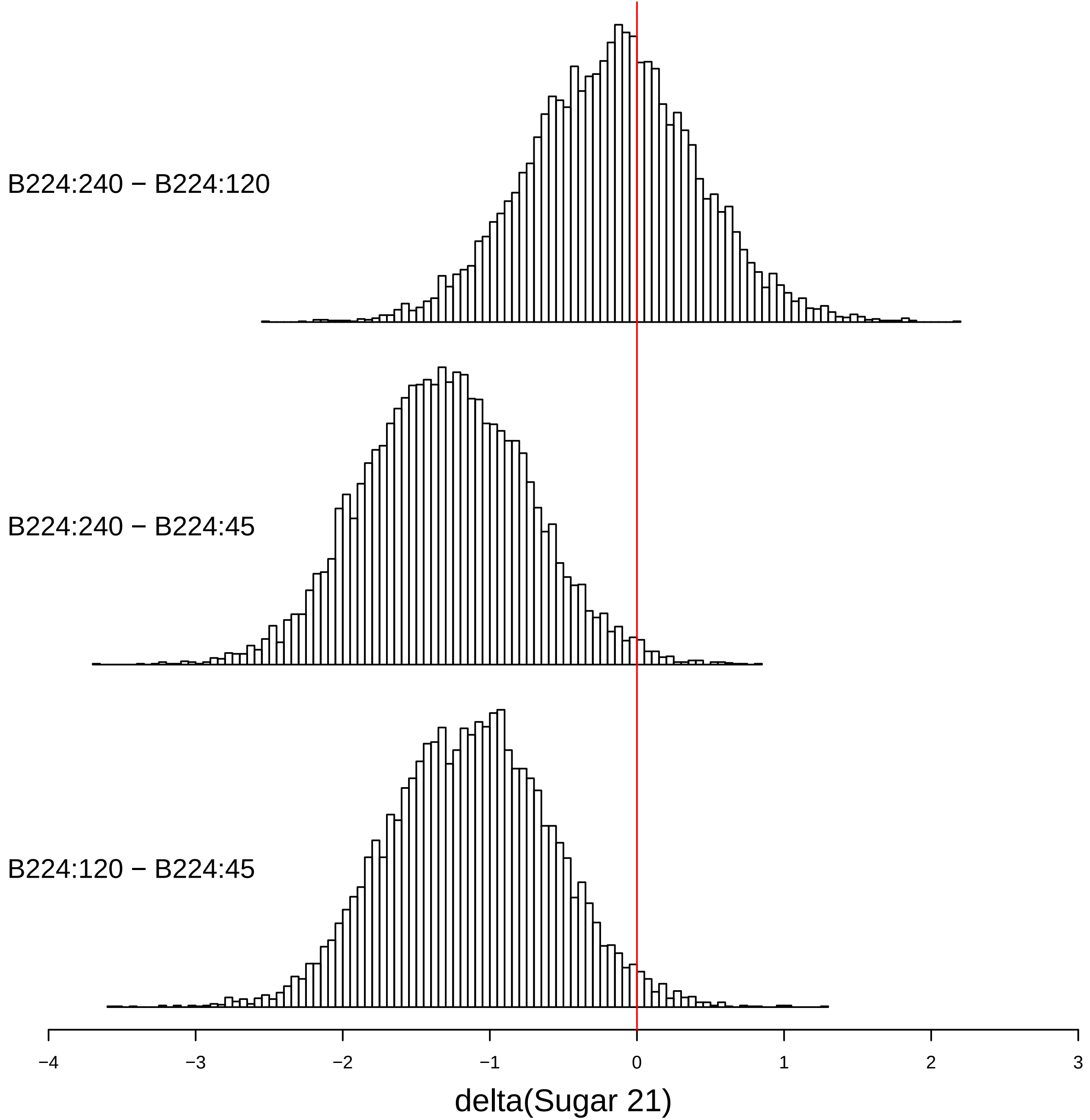

B224:240 − B224:120
B224:240 − B224:45
B224:120 − B224:45
−4
−3
−2
−1
0
1
2
3
delta(Sugar 21)

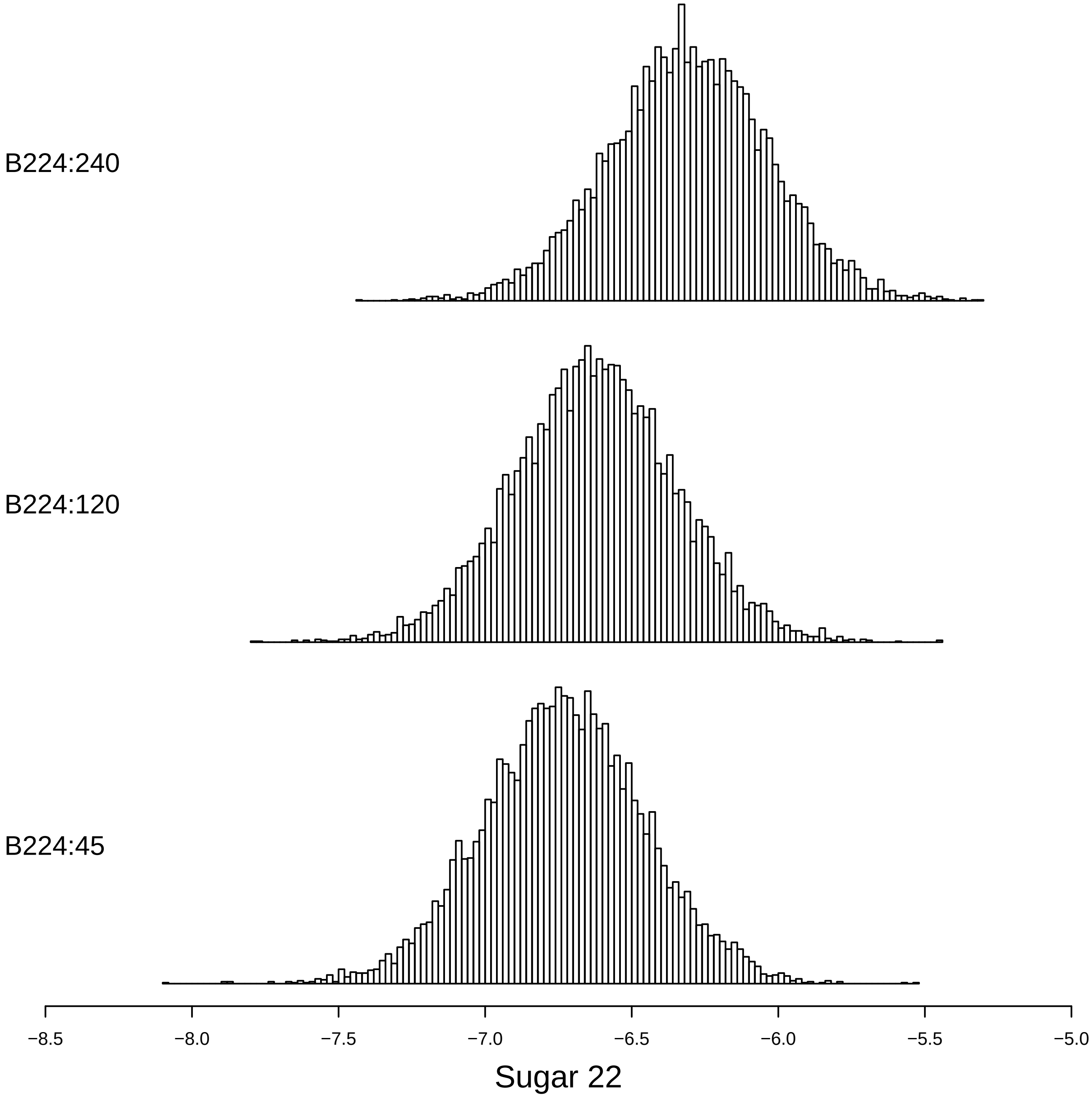
B224:240
B224:120
B224:45
−8.5
−8.0
−7.5
−7.0
−6.5
−6.0
−5.5
−5.0
Sugar 22

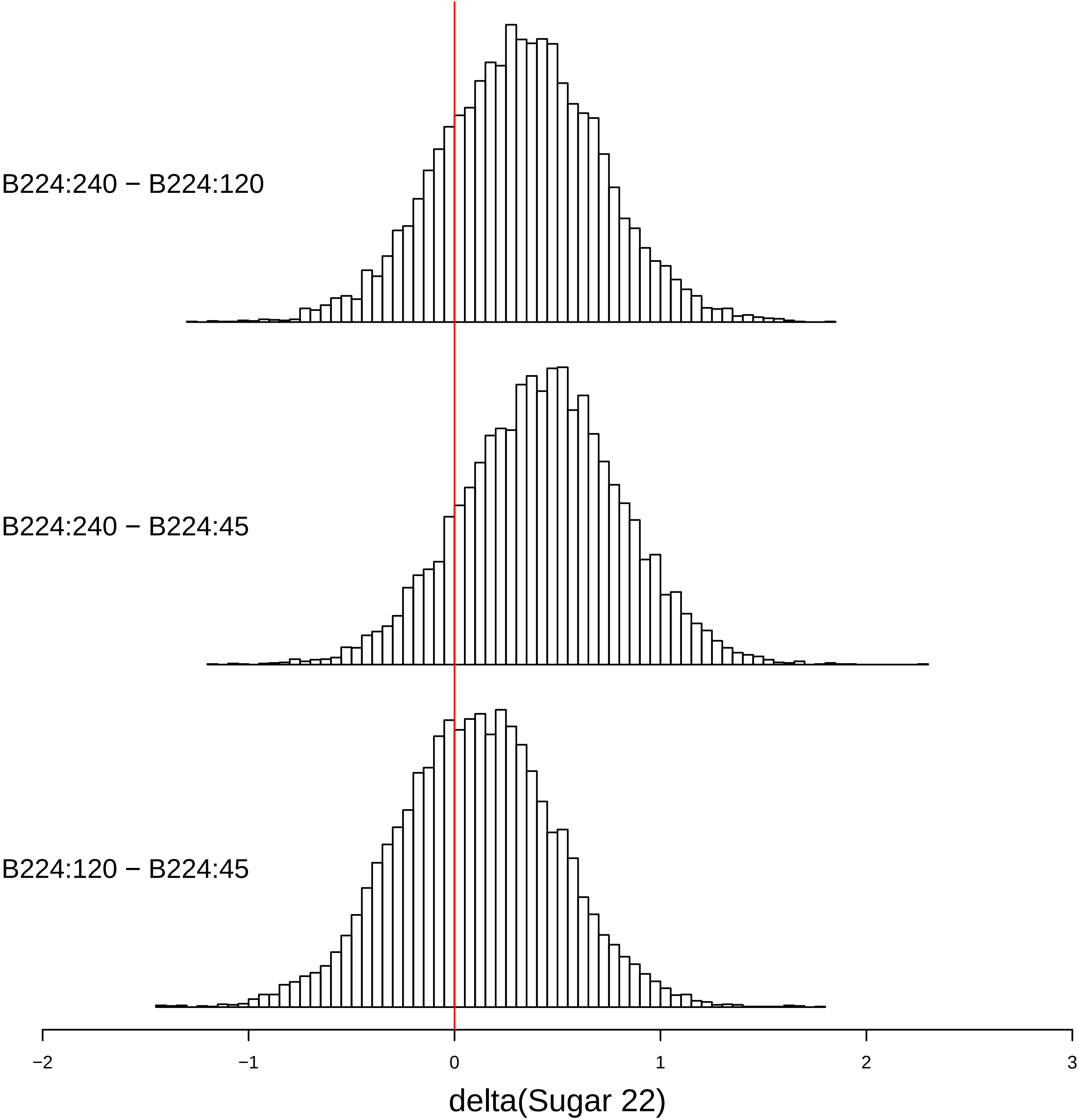

B224:240 − B224:120
B224:240 − B224:45
B224:120 − B224:45
−2
−1
0
1
2
3
delta(Sugar 22)

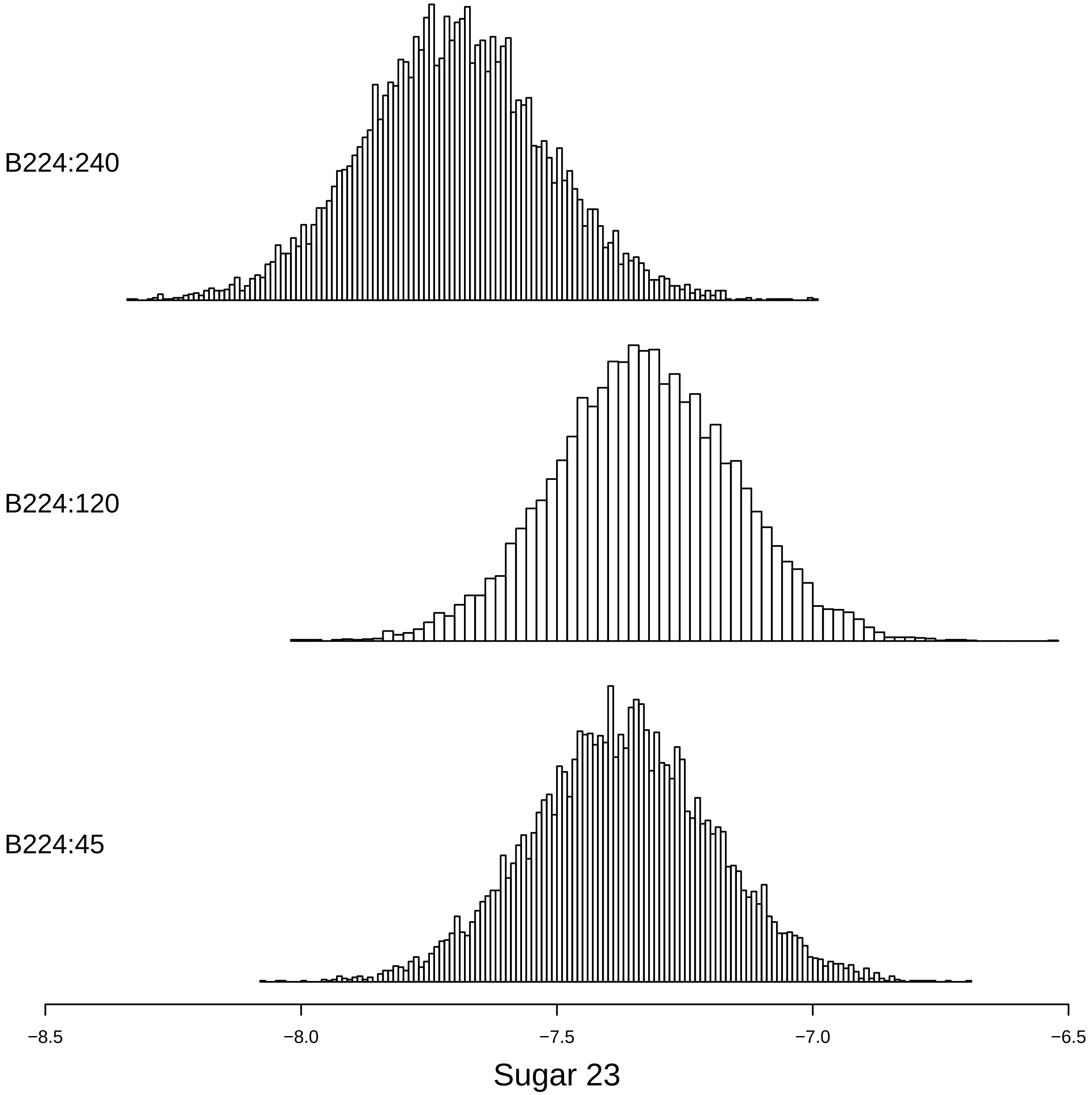

B224:240
B224:120
B224:45
−8.5
−8.0
−7.5
−7.0
−6.5
Sugar 23

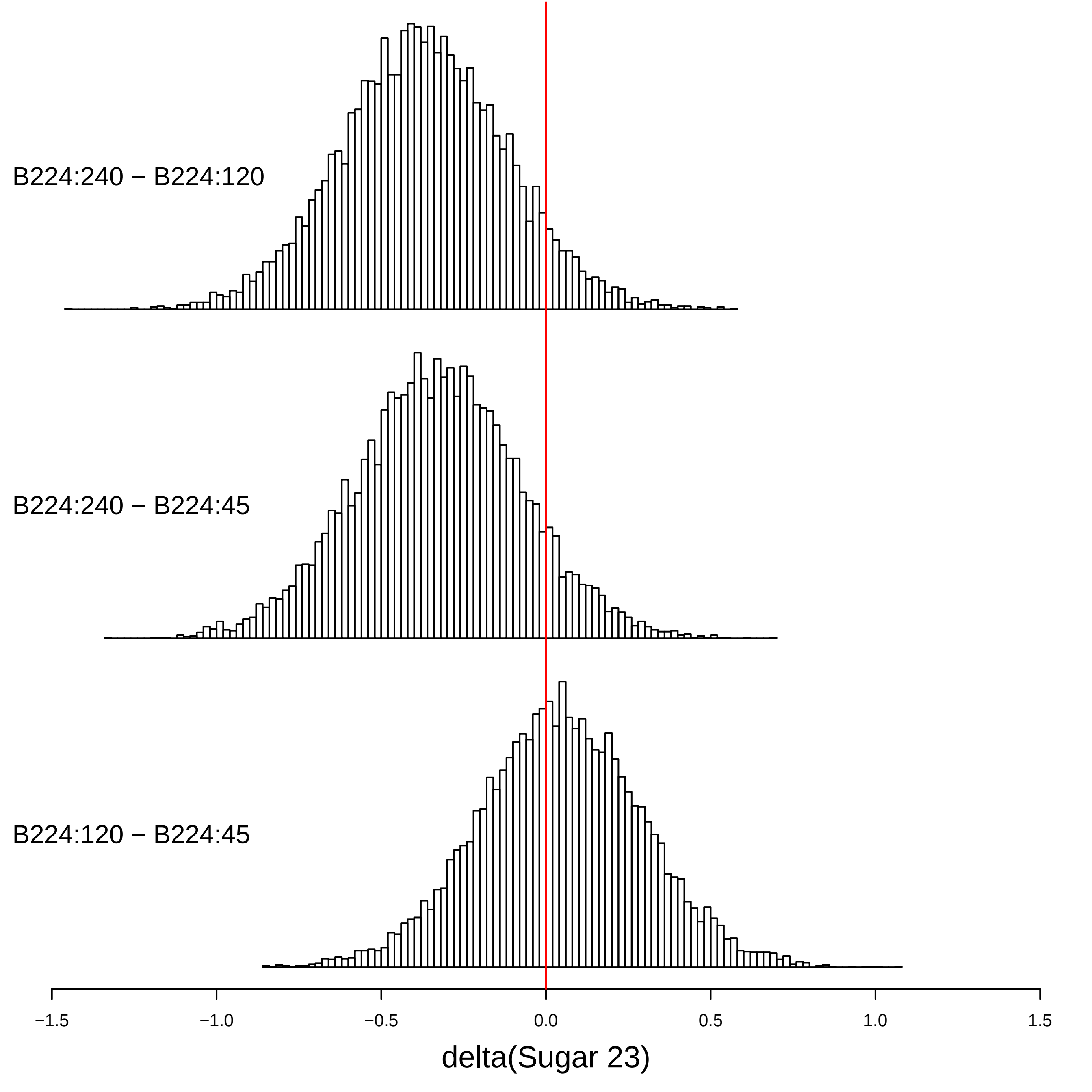
B224:240 − B224:120
B224:240 − B224:45
B224:120 − B224:45
−1.5
−1.0
−0.5
0.0
0.5
1.0
1.5
delta(Sugar 23)

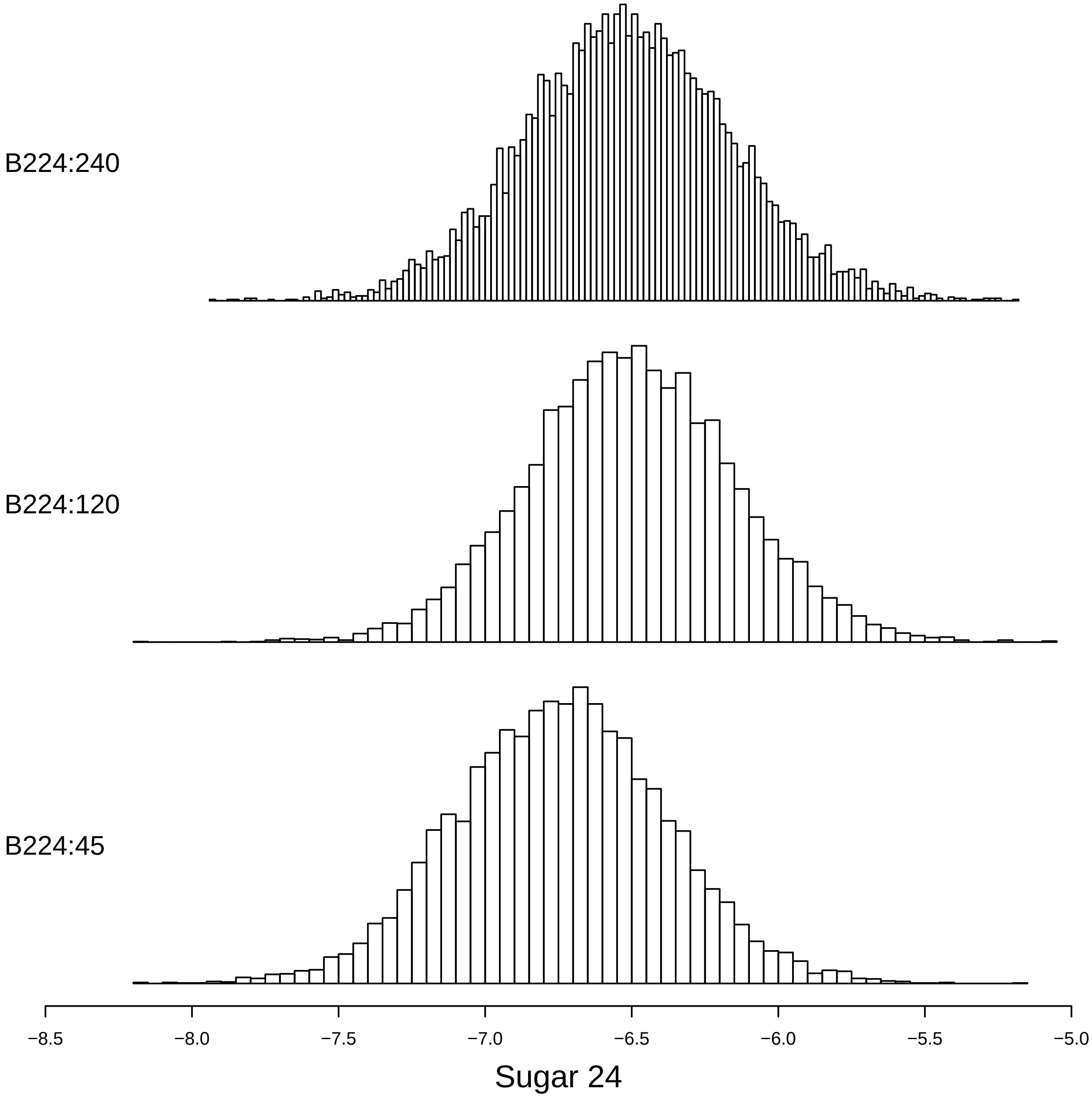

B224:240
B224:120
B224:45
−8.5
−8.0
−7.5
−7.0
−6.5
−6.0
−5.5
−5.0
Sugar 24

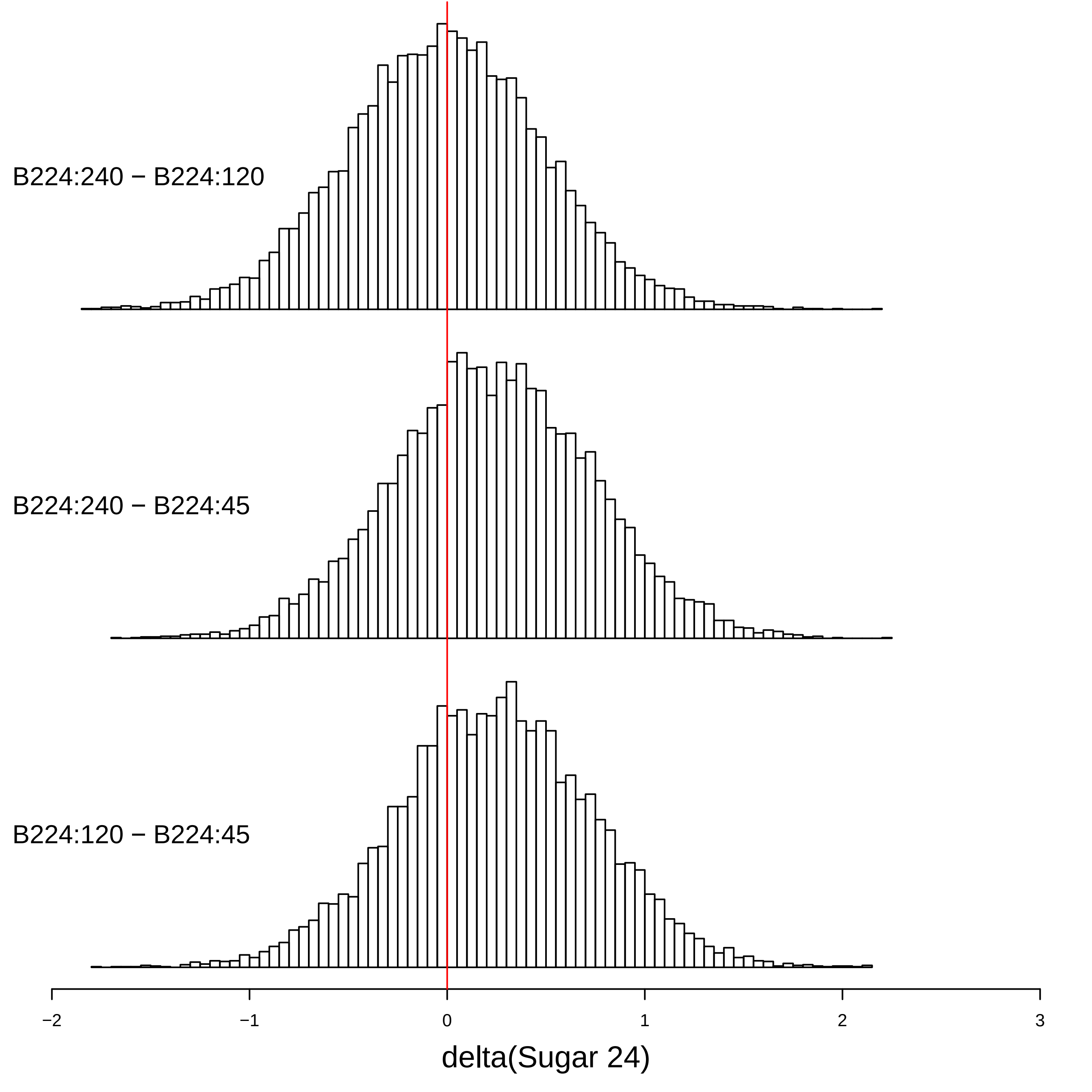

B224:240 − B224:120
B224:240 − B224:45
B224:120 − B224:45
−2
−1
0
1
2
3
delta(Sugar 24)

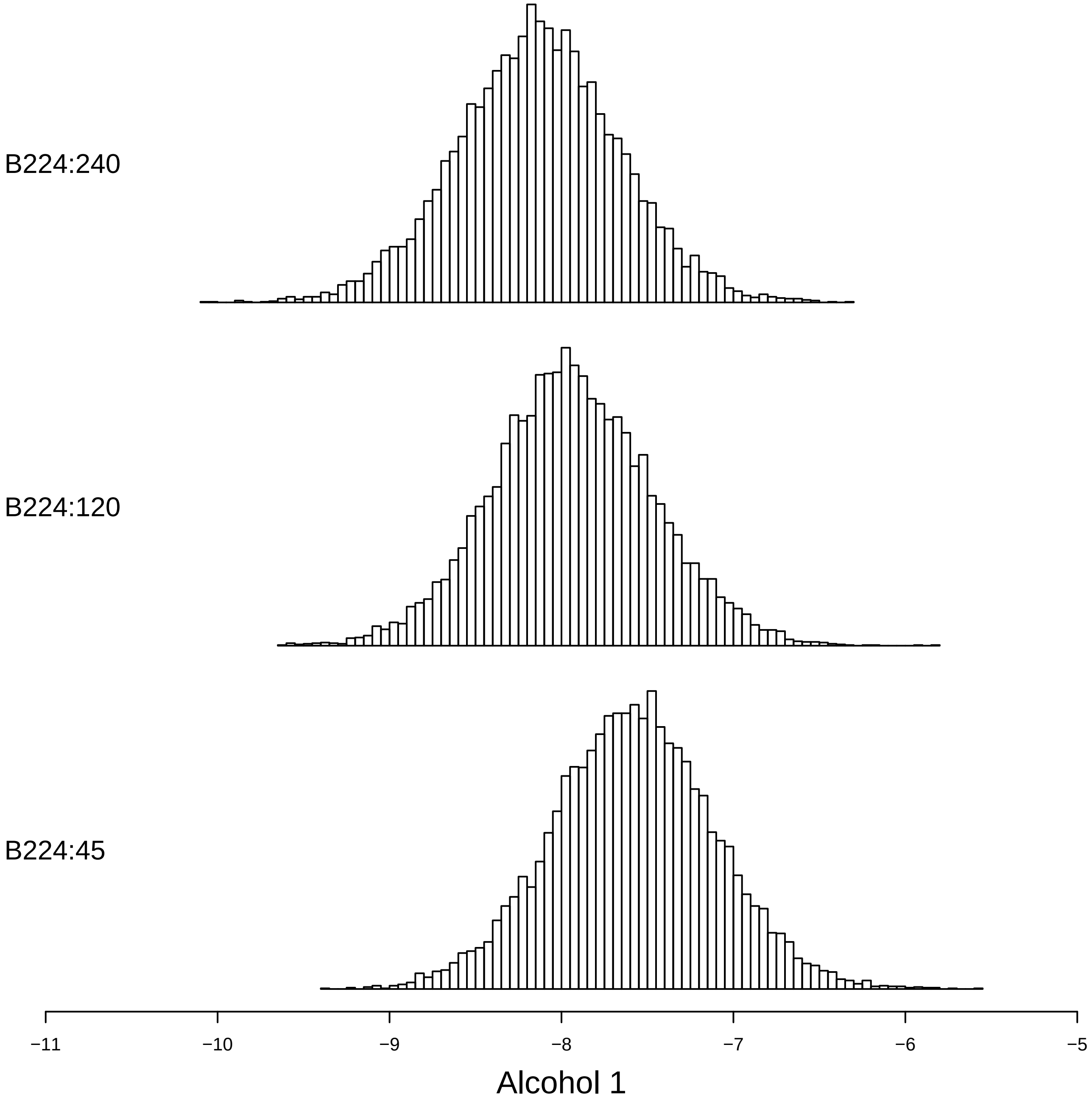
B224:240
B224:120
B224:45
−11
−10
−9
−8
−7
−6
−5
Alcohol 1

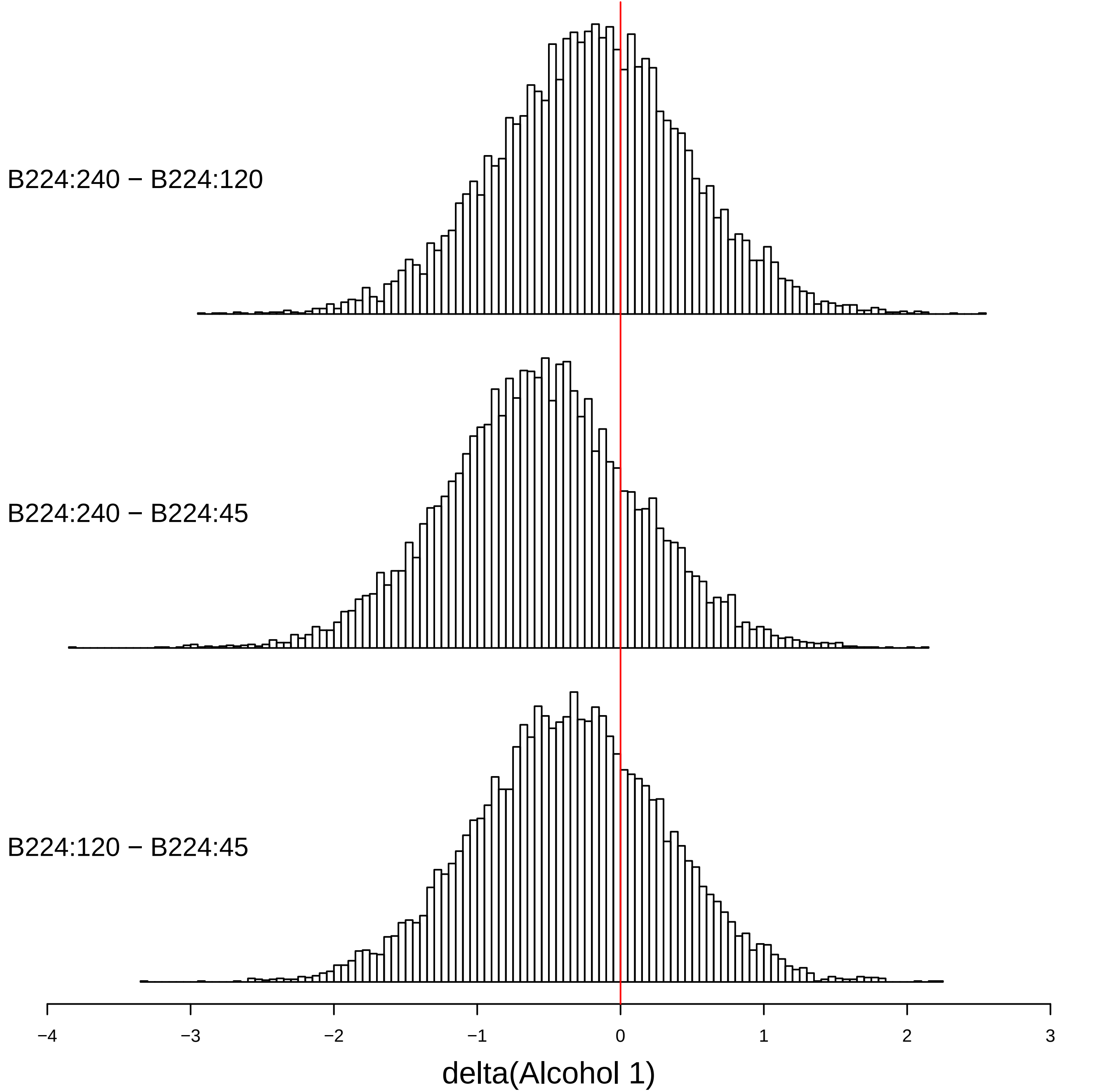
B224:240 − B224:120
B224:240 − B224:45
B224:120 − B224:45
−4
−3
−2
−1
0
1
2
3
delta(Alcohol 1)

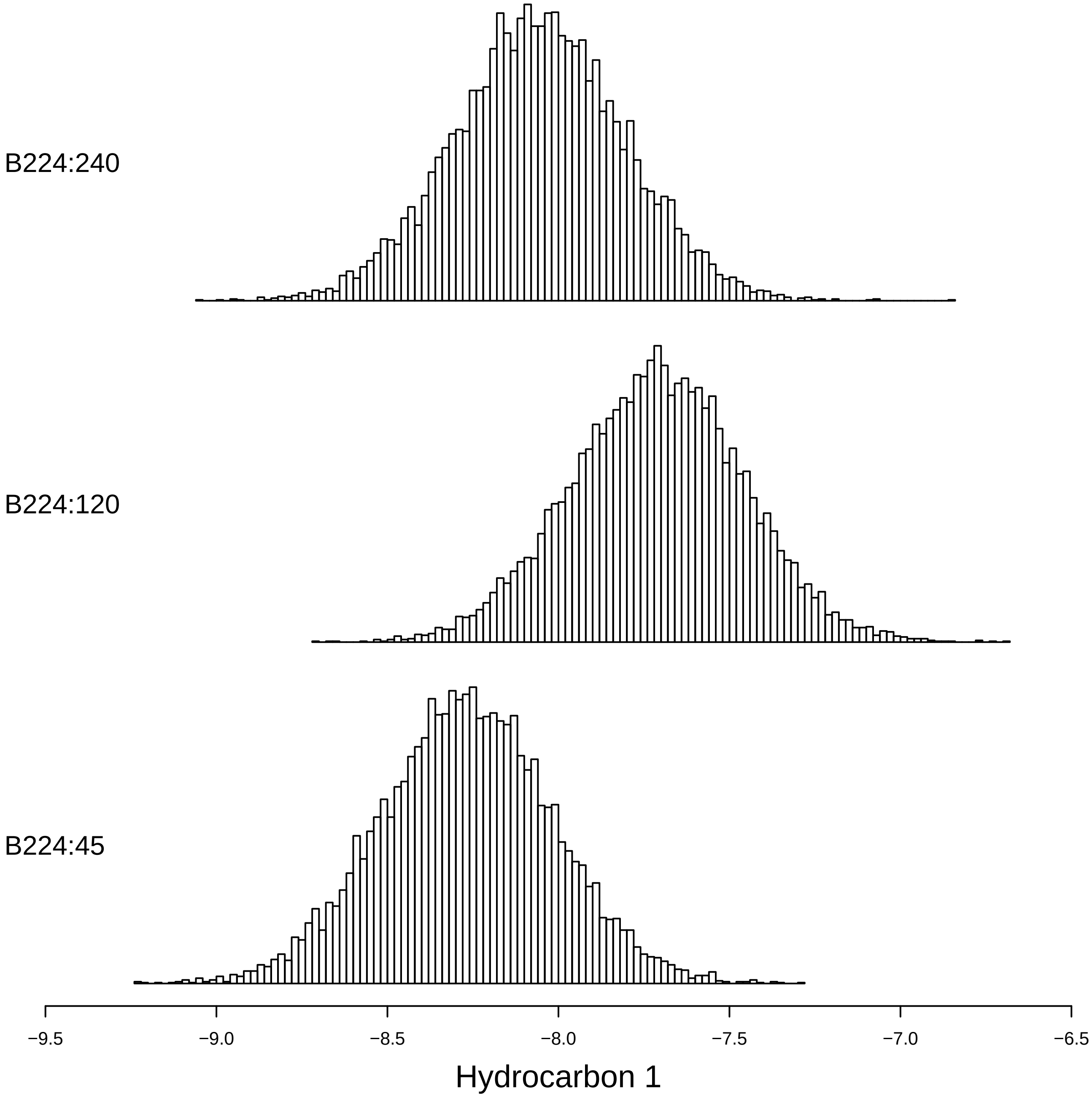

B224:240
B224:120
B224:45
−9.5
−9.0
−8.5
−8.0
−7.5
−7.0
−6.5
Hydrocarbon 1

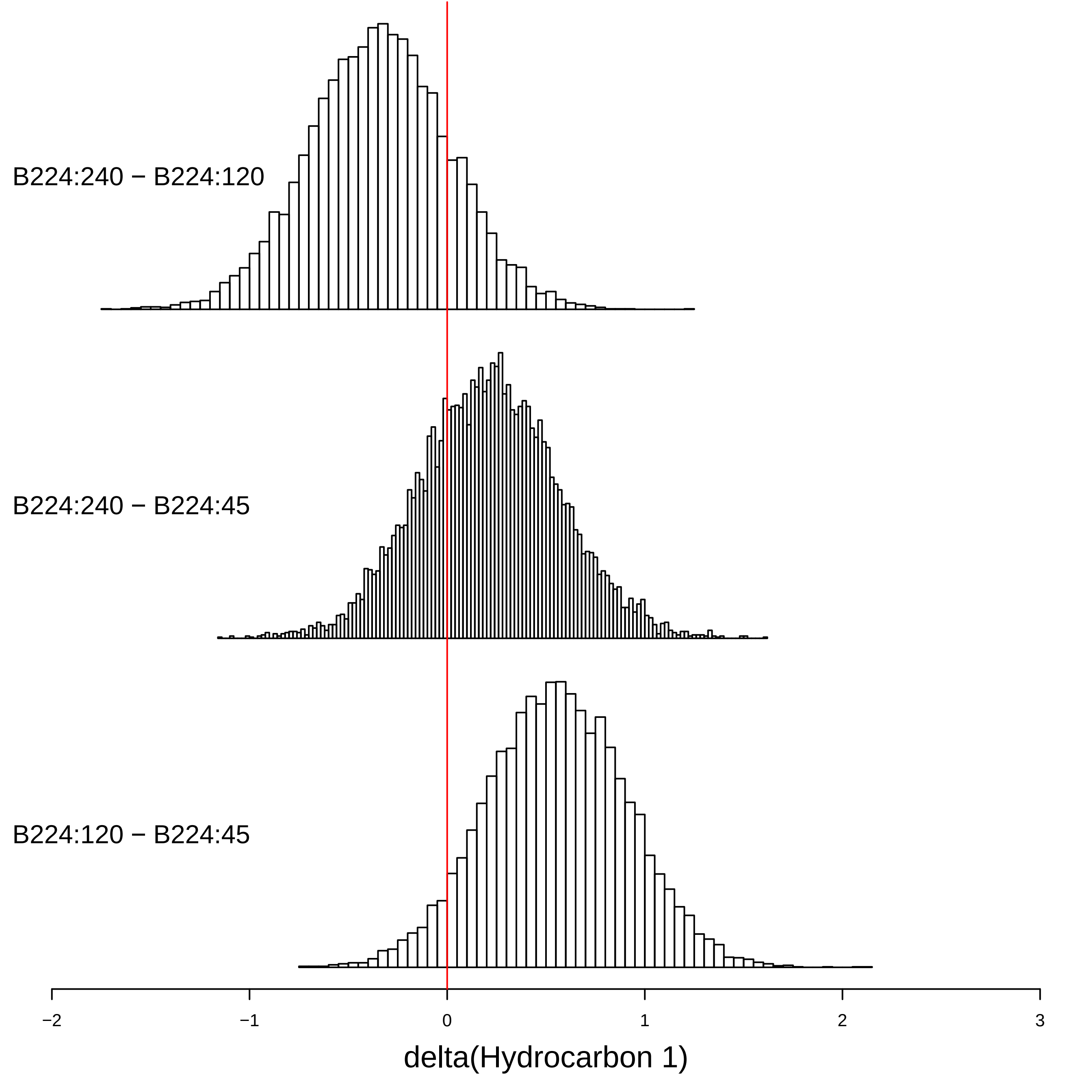
B224:240 − B224:120
B224:240 − B224:45
B224:120 − B224:45
−2
−1
0
1
2
3
delta(Hydrocarbon 1)

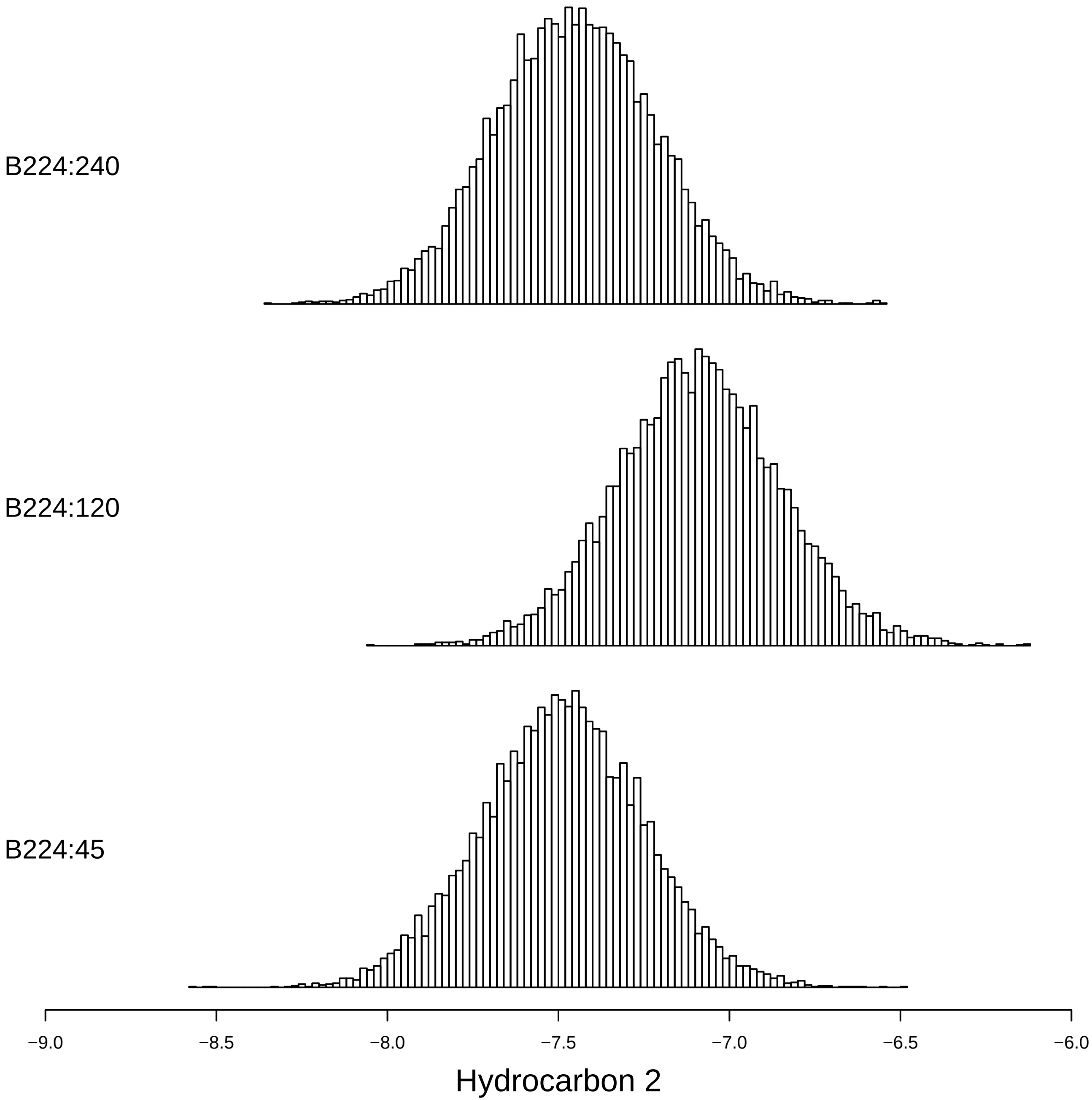

B224:240
B224:120
B224:45
−9.0
−8.5
−8.0
−7.5
−7.0
−6.5
−6.0
Hydrocarbon 2

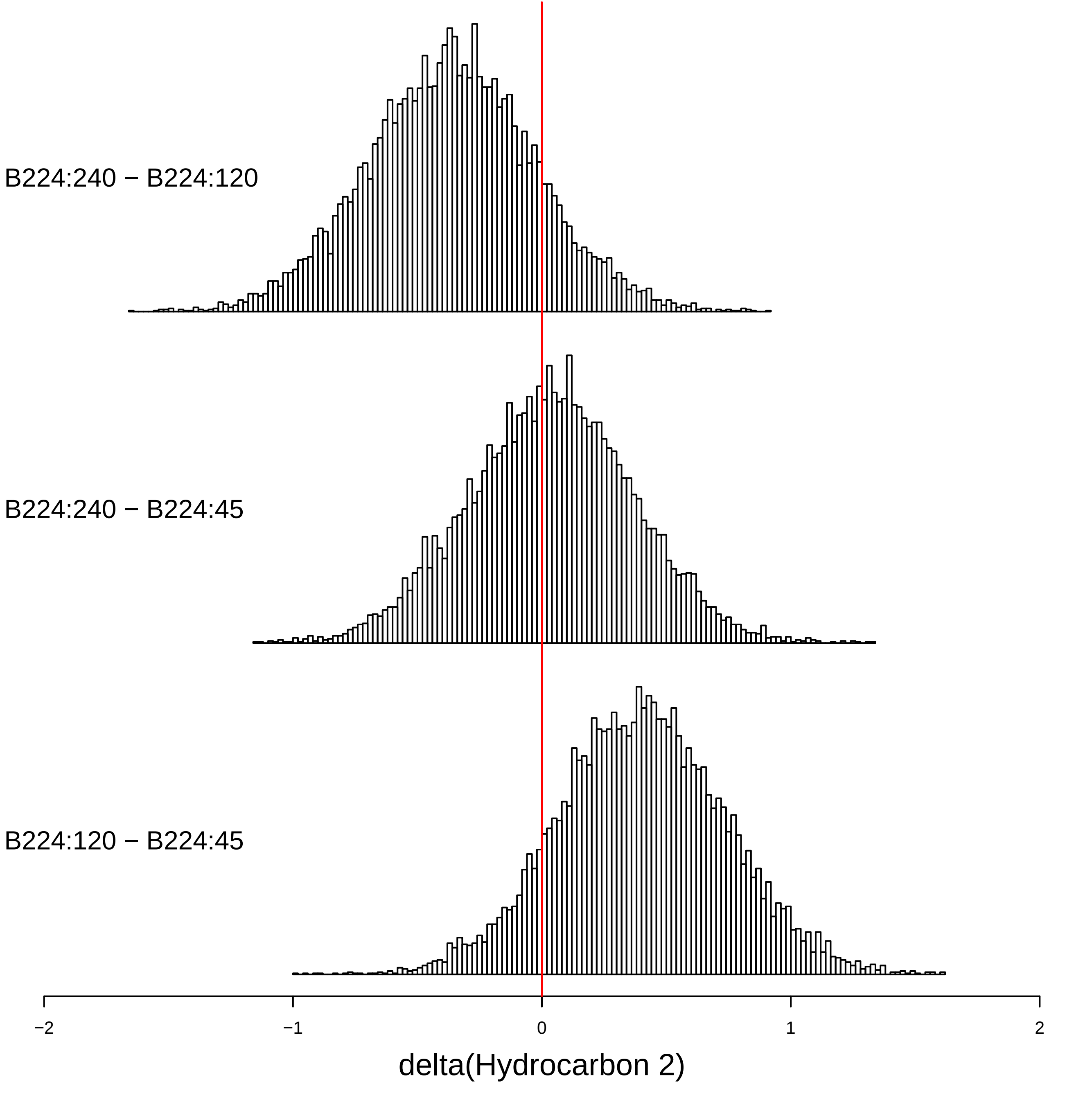
B224:240 − B224:120
B224:240 − B224:45
B224:120 − B224:45
−2
−1
0
1
2
delta(Hydrocarbon 2)

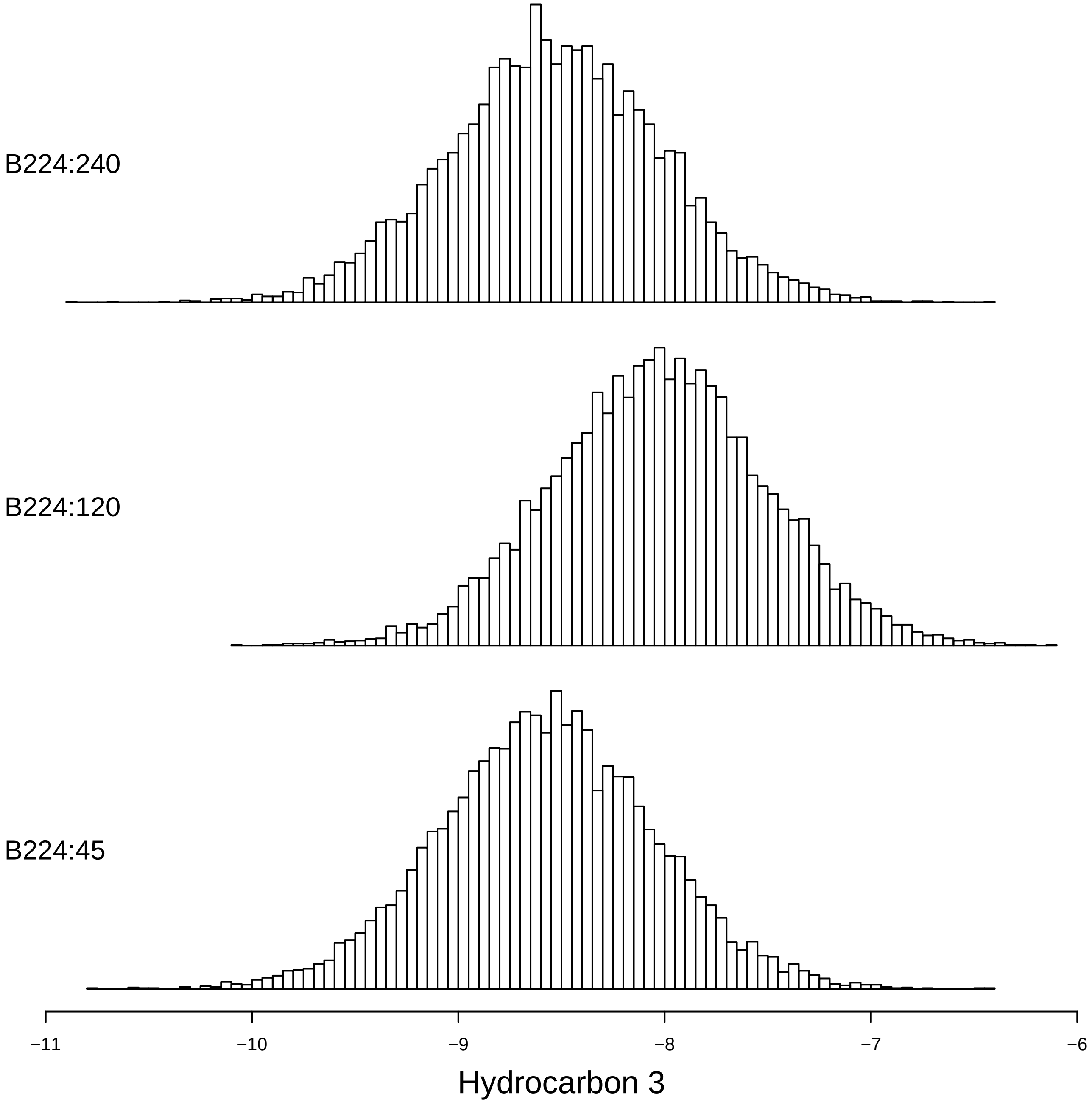

B224:240
B224:120
B224:45
−11
−10
−9
−8
−7
−6
Hydrocarbon 3

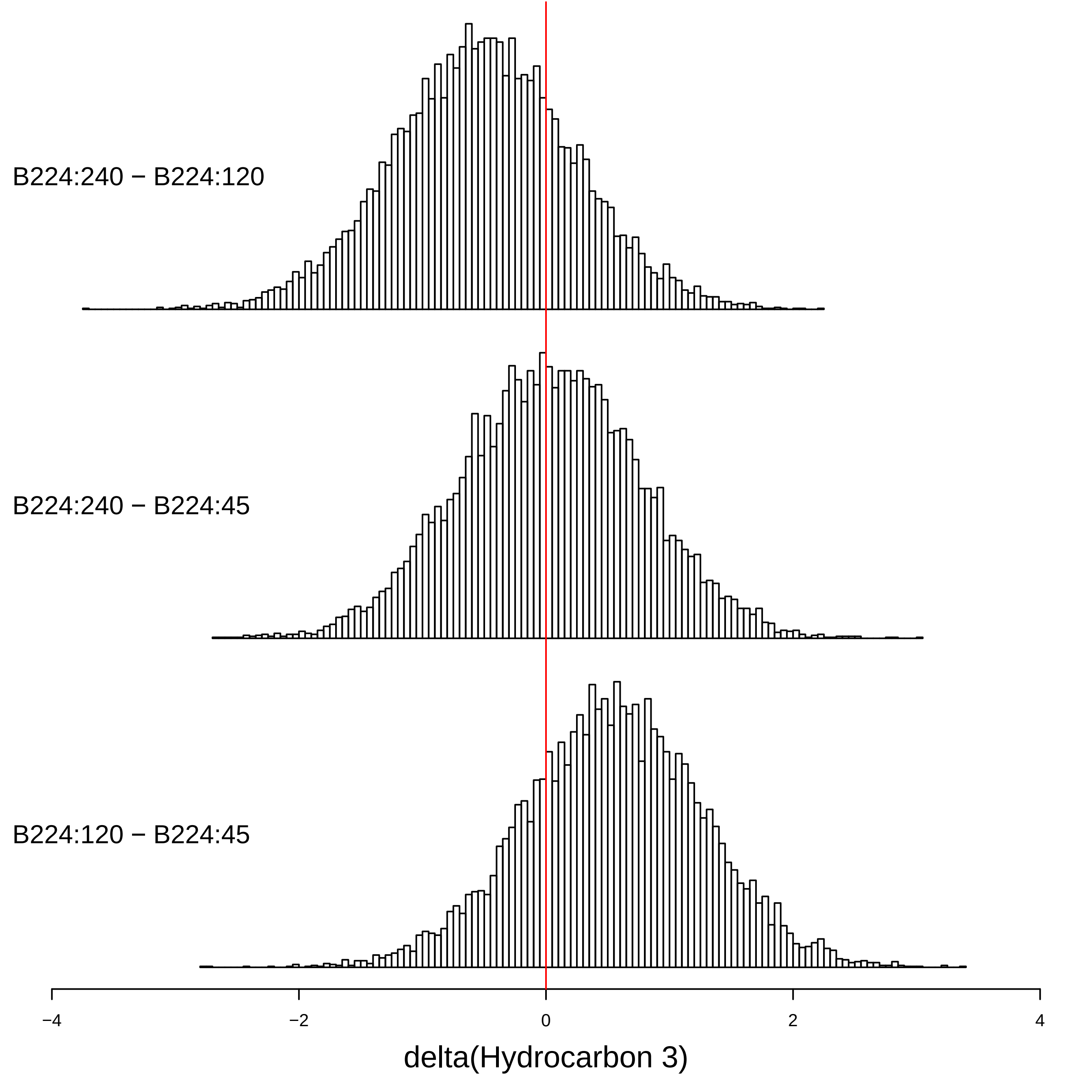

B224:240 − B224:120
B224:240 − B224:45
B224:120 − B224:45
−4
−2
0
2
4
delta(Hydrocarbon 3)

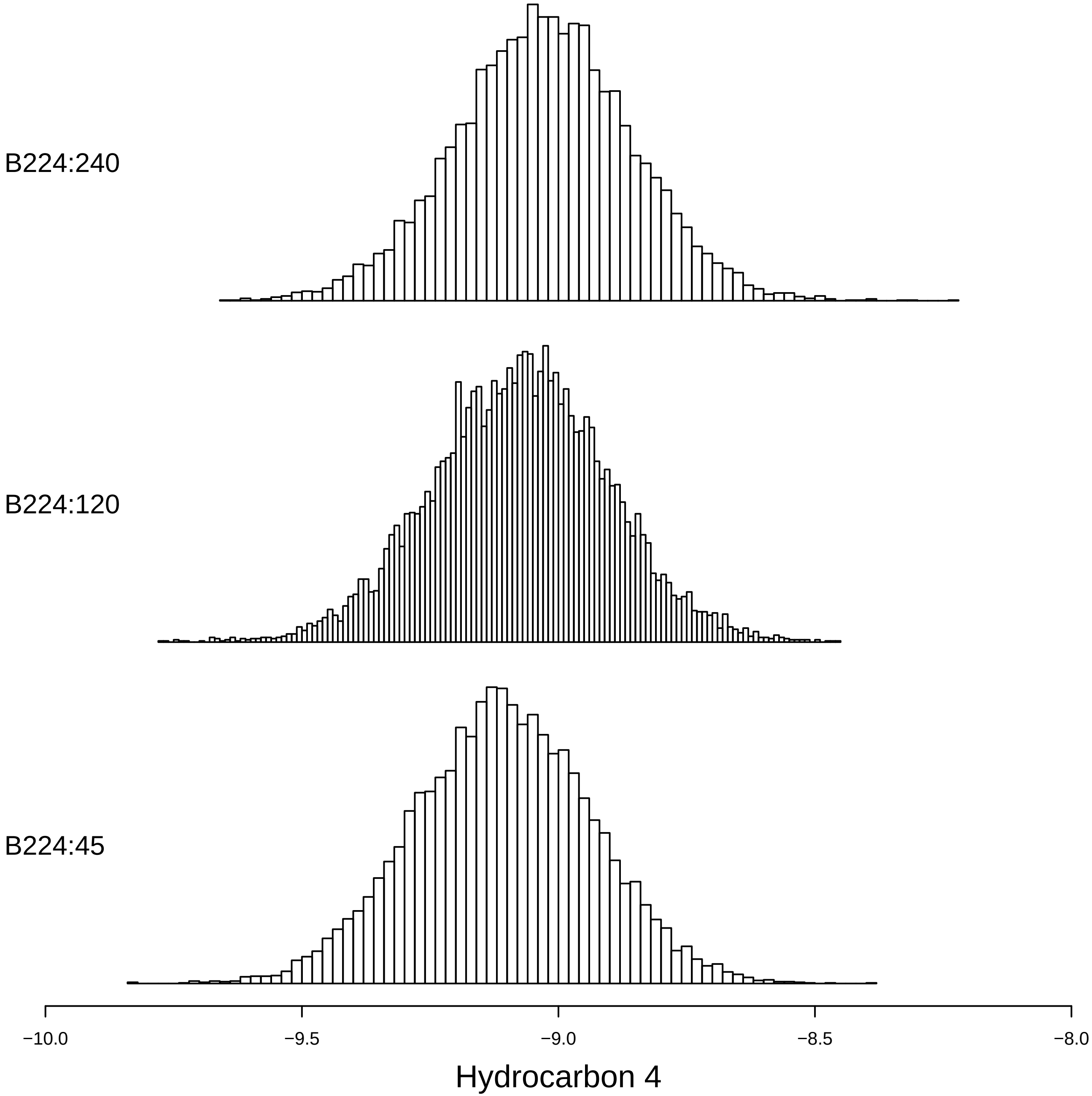
B224:240
B224:120
B224:45
−10.0
−9.5
−9.0
−8.5
−8.0
Hydrocarbon 4

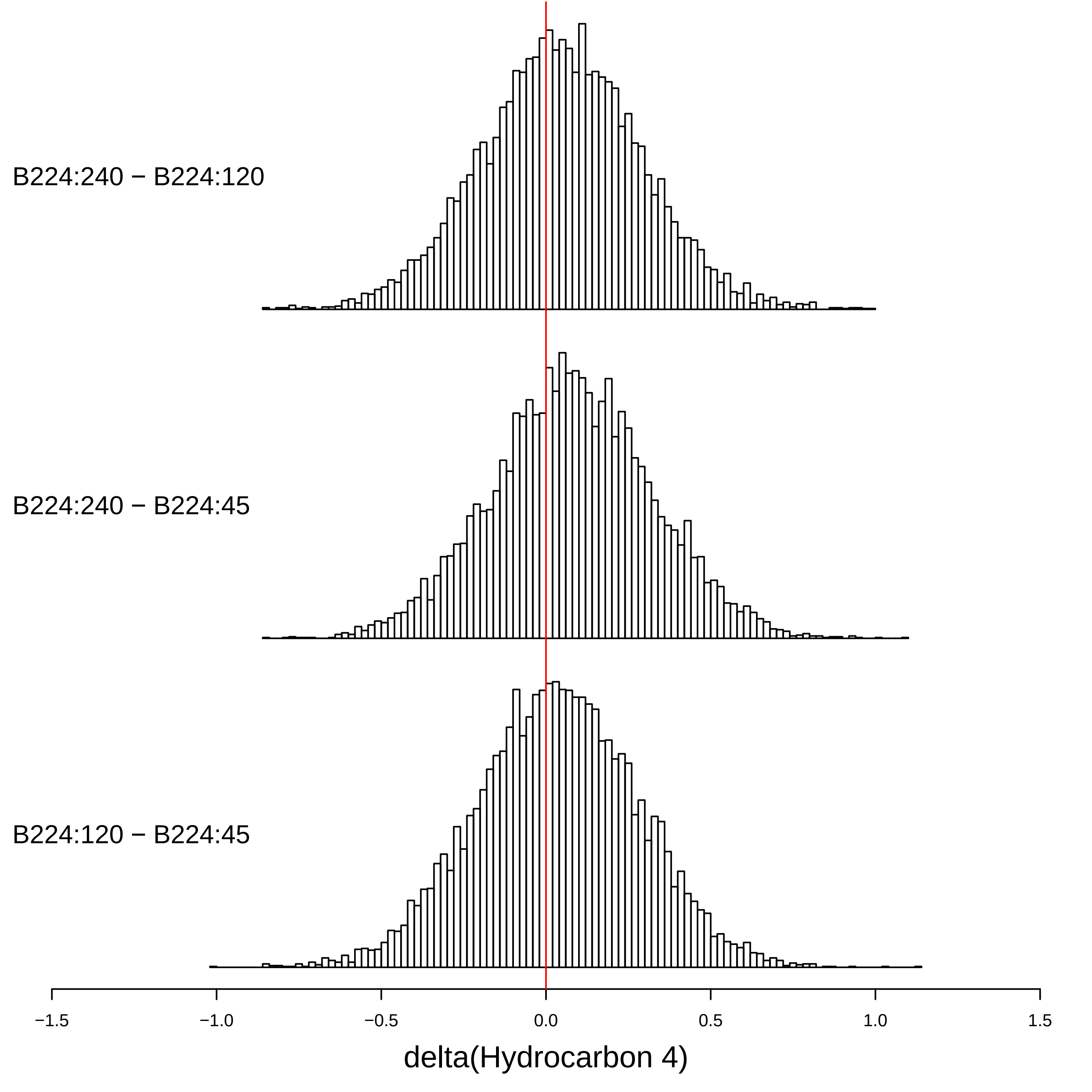
B224:240 − B224:120
B224:240 − B224:45
B224:120 − B224:45
−1.5
−1.0
−0.5
0.0
0.5
1.0
1.5
delta(Hydrocarbon 4)

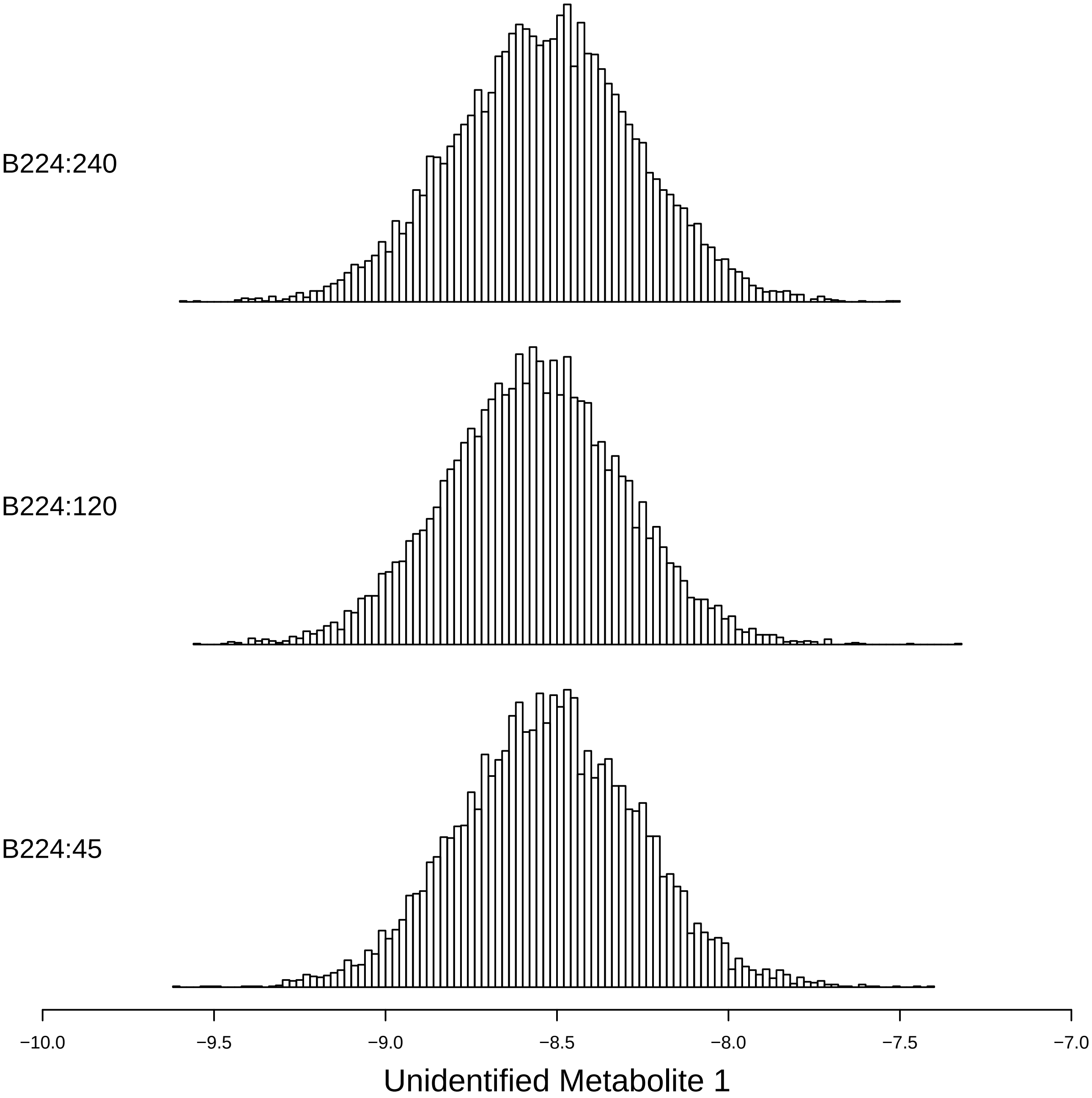
B224:240
B224:120
B224:45
−10.0
−9.5
−9.0
−8.5
−8.0
−7.5
−7.0
Unidentified Metabolite 1

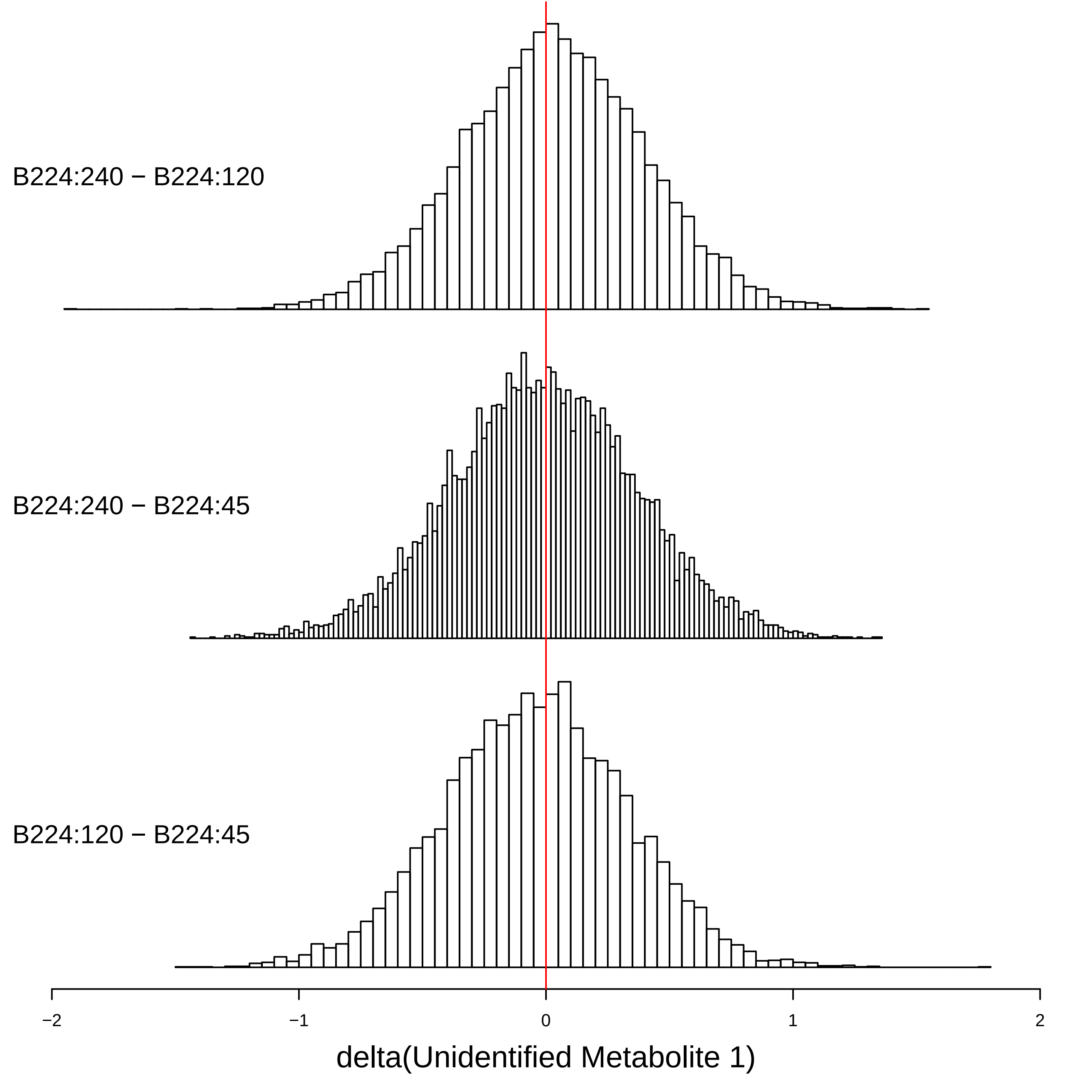

B224:240 − B224:120
B224:240 − B224:45
B224:120 − B224:45
−2
−1
0
1
2
delta(Unidentified Metabolite 1)

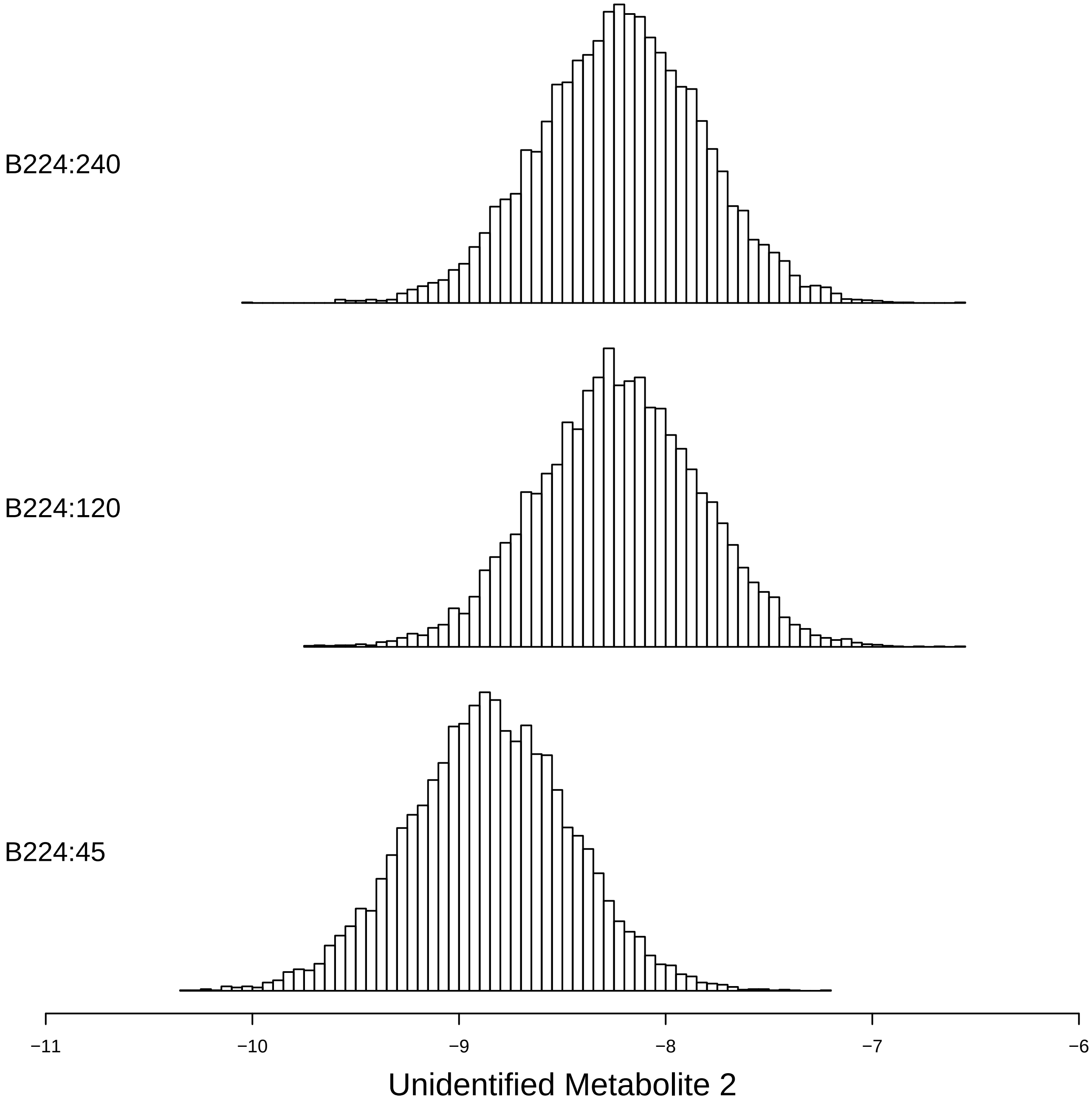
B224:240
B224:120
B224:45
−11
−10
−9
−8
−7
−6
Unidentified Metabolite 2

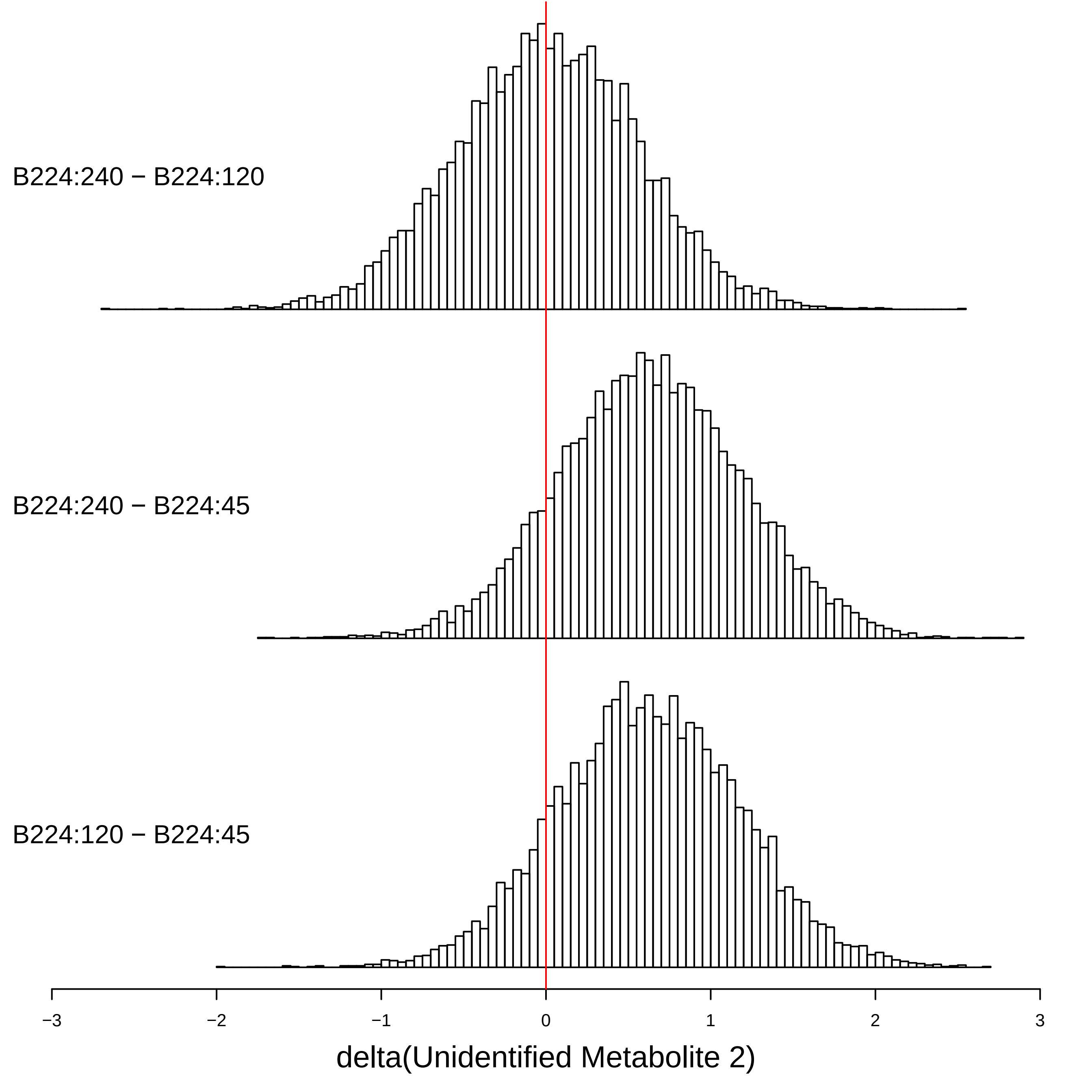
B224:240 − B224:120
B224:240 − B224:45
B224:120 − B224:45
−3
−2
−1
0
1
2
3
delta(Unidentified Metabolite 2)

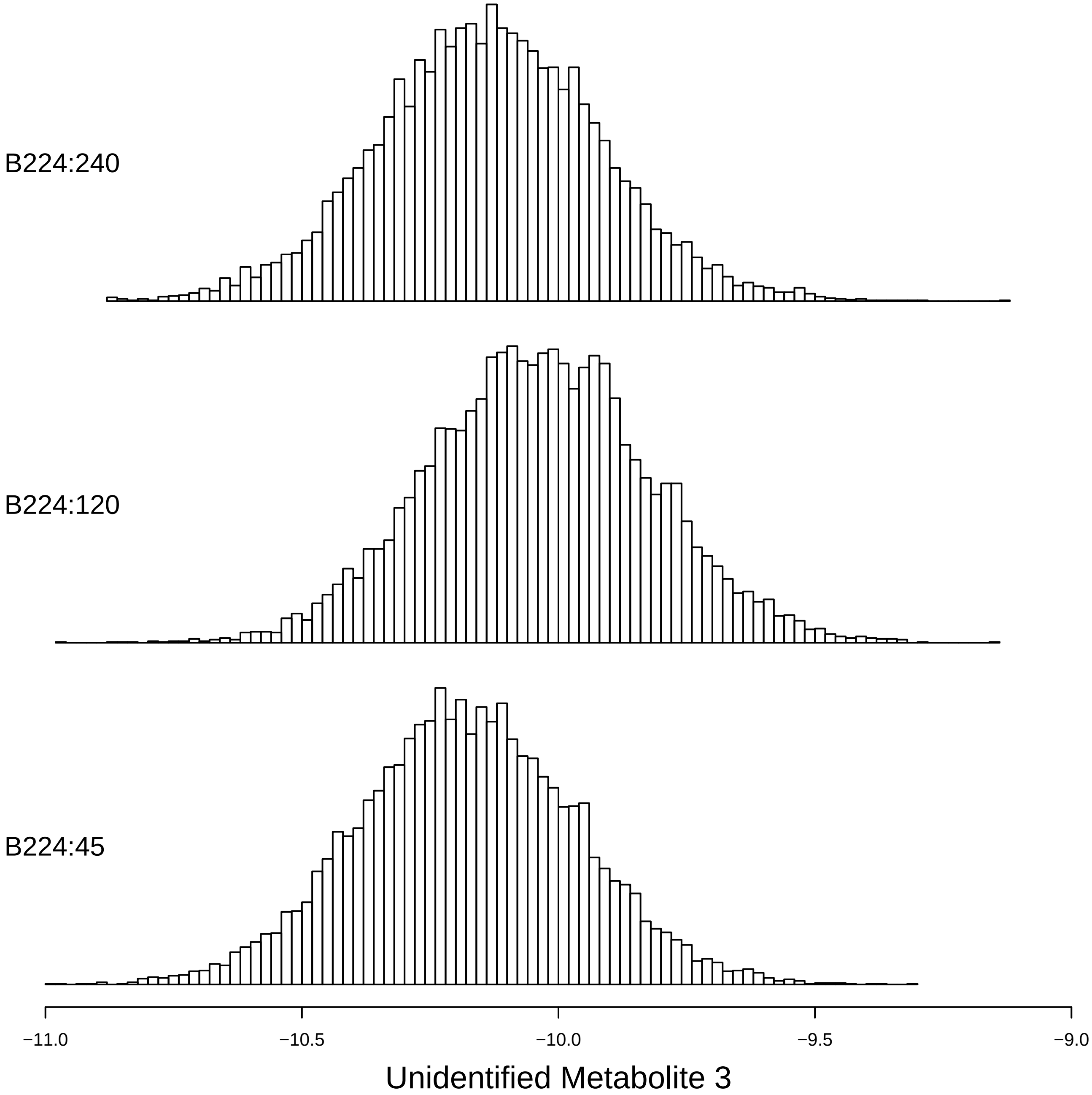

B224:240
B224:120
B224:45
−11.0
−10.5
−10.0
−9.5
−9.0
Unidentified Metabolite 3

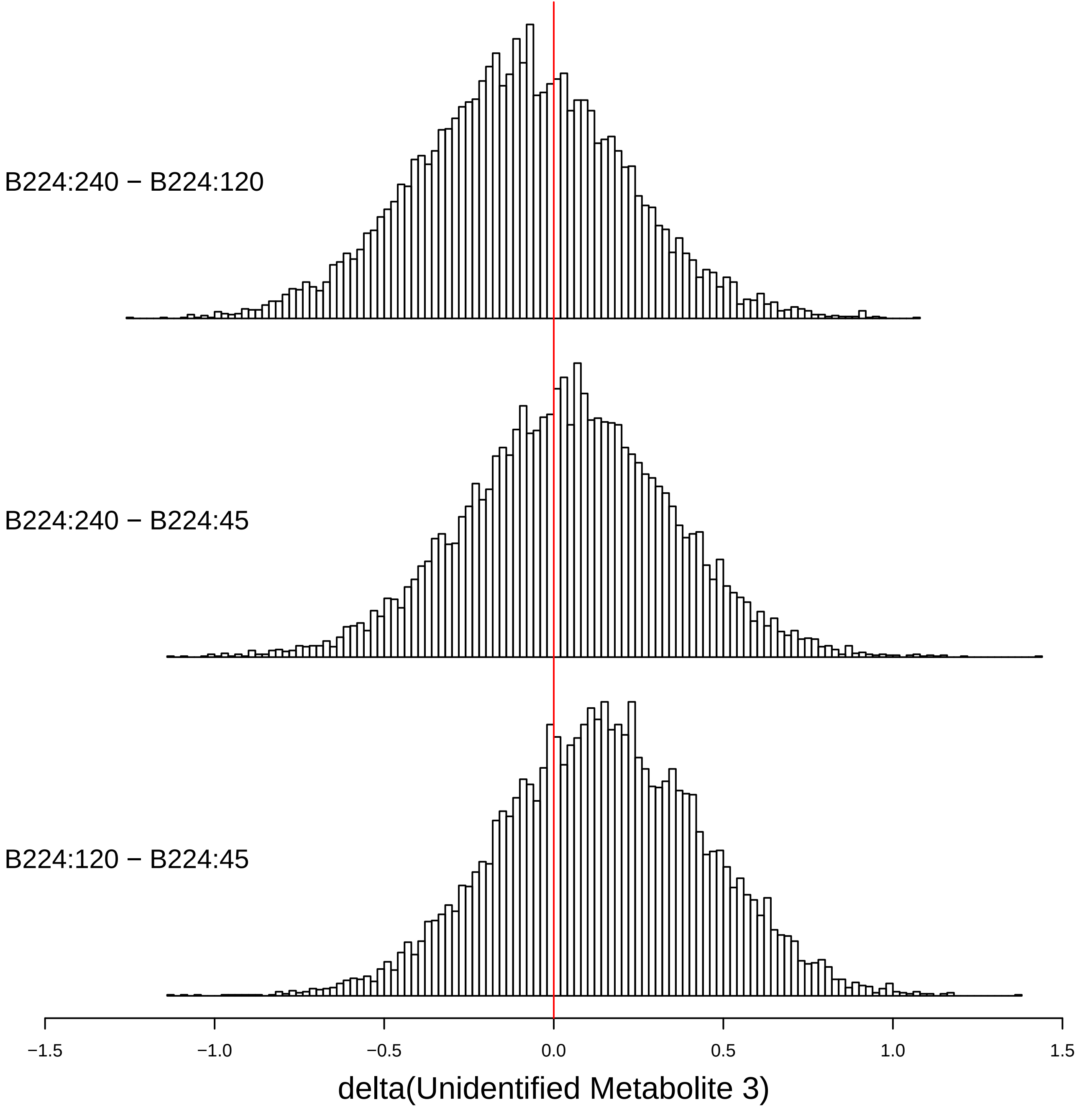

B224:240 − B224:120
B224:240 − B224:45
B224:120 − B224:45
−1.5
−1.0
−0.5
0.0
0.5
1.0
1.5
delta(Unidentified Metabolite 3)

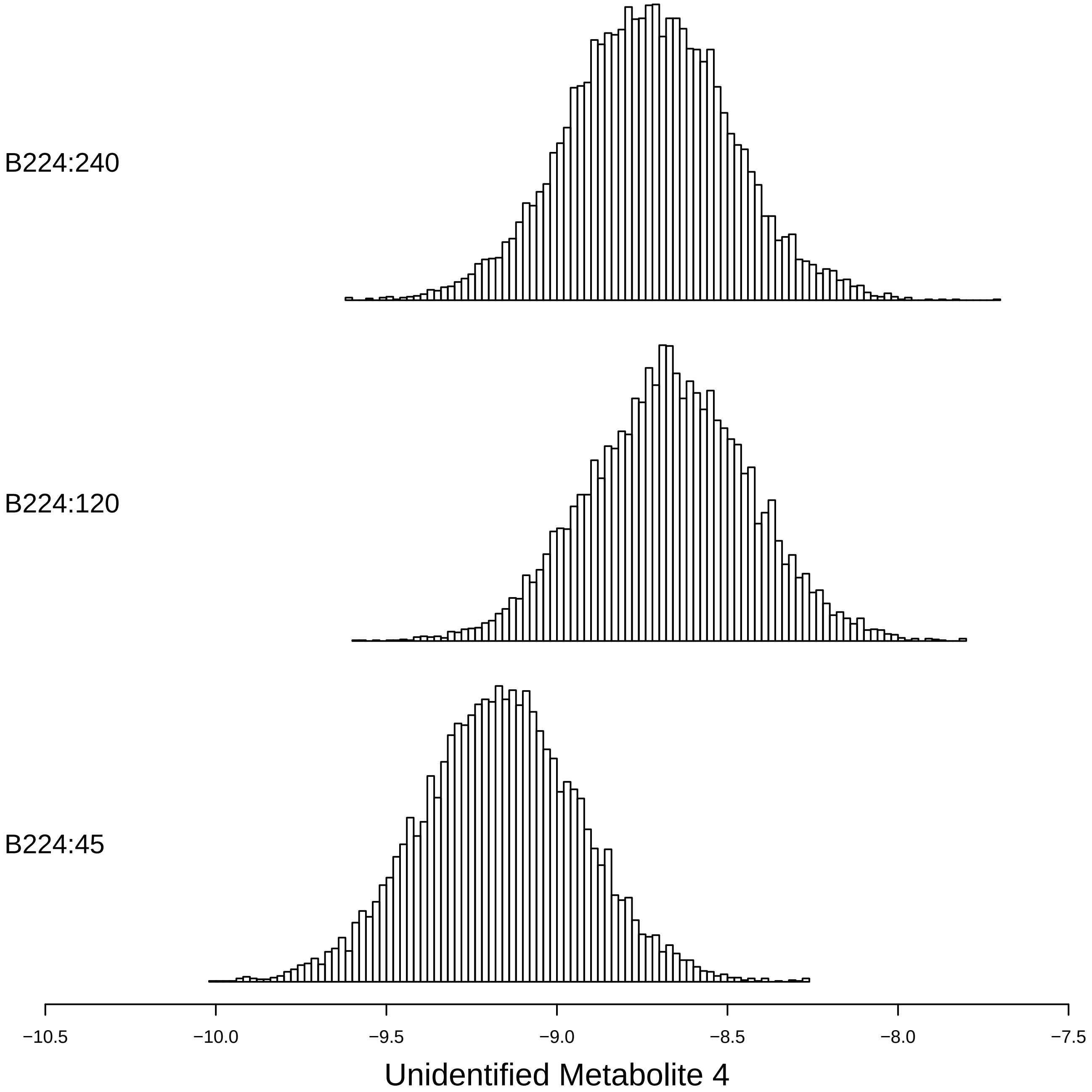

B224:240
B224:120
B224:45
−10.5
−10.0
−9.5
−9.0
−8.5
−8.0
−7.5
Unidentified Metabolite 4

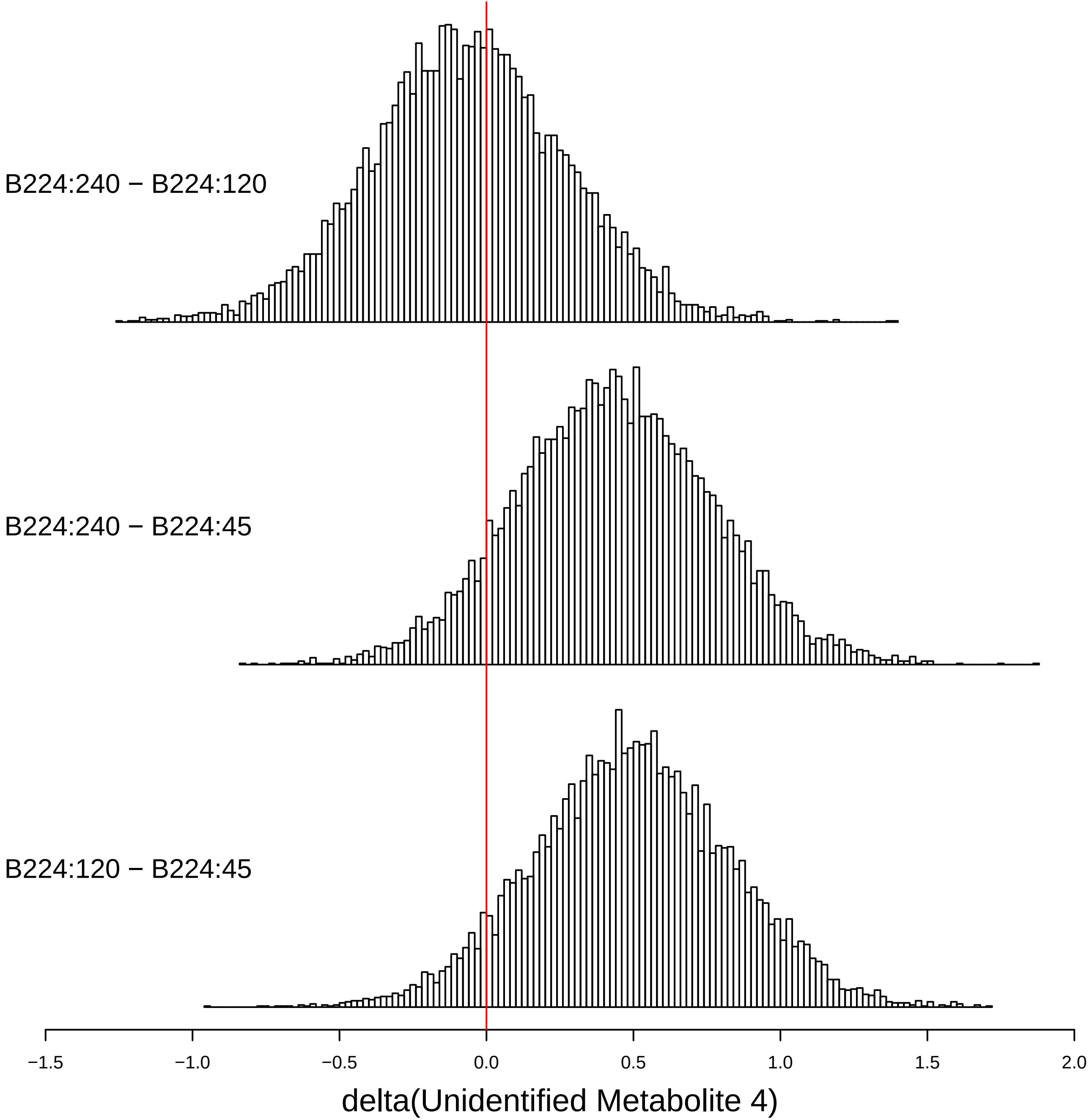

B224:240 − B224:120
B224:240 − B224:45
B224:120 − B224:45
−1.5
−1.0
−0.5
0.0
0.5
1.0
1.5
2.0
delta(Unidentified Metabolite 4)

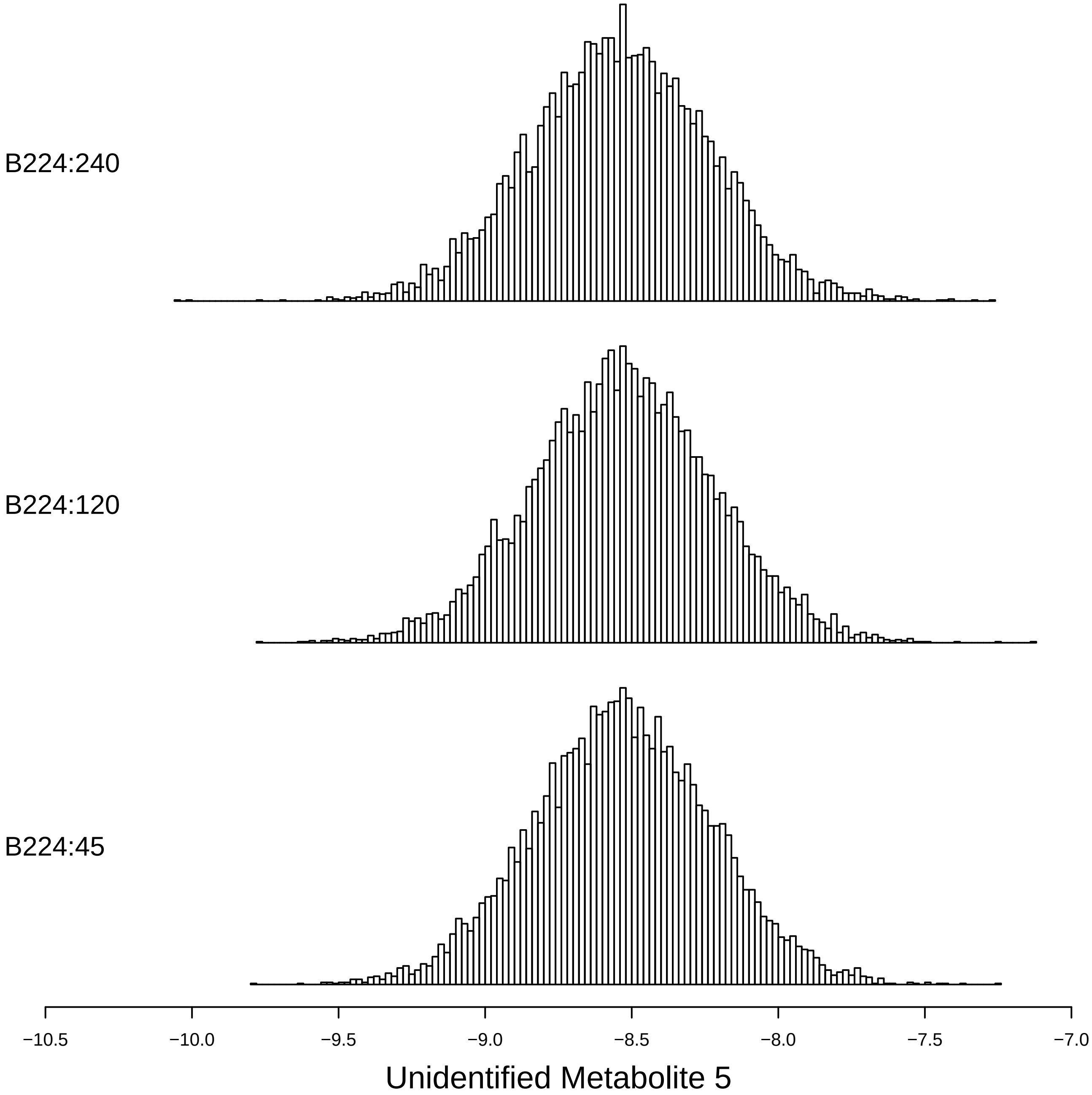

B224:240
B224:120
B224:45
−10.5
−10.0
−9.5
−9.0
−8.5
−8.0
−7.5
−7.0
Unidentified Metabolite 5

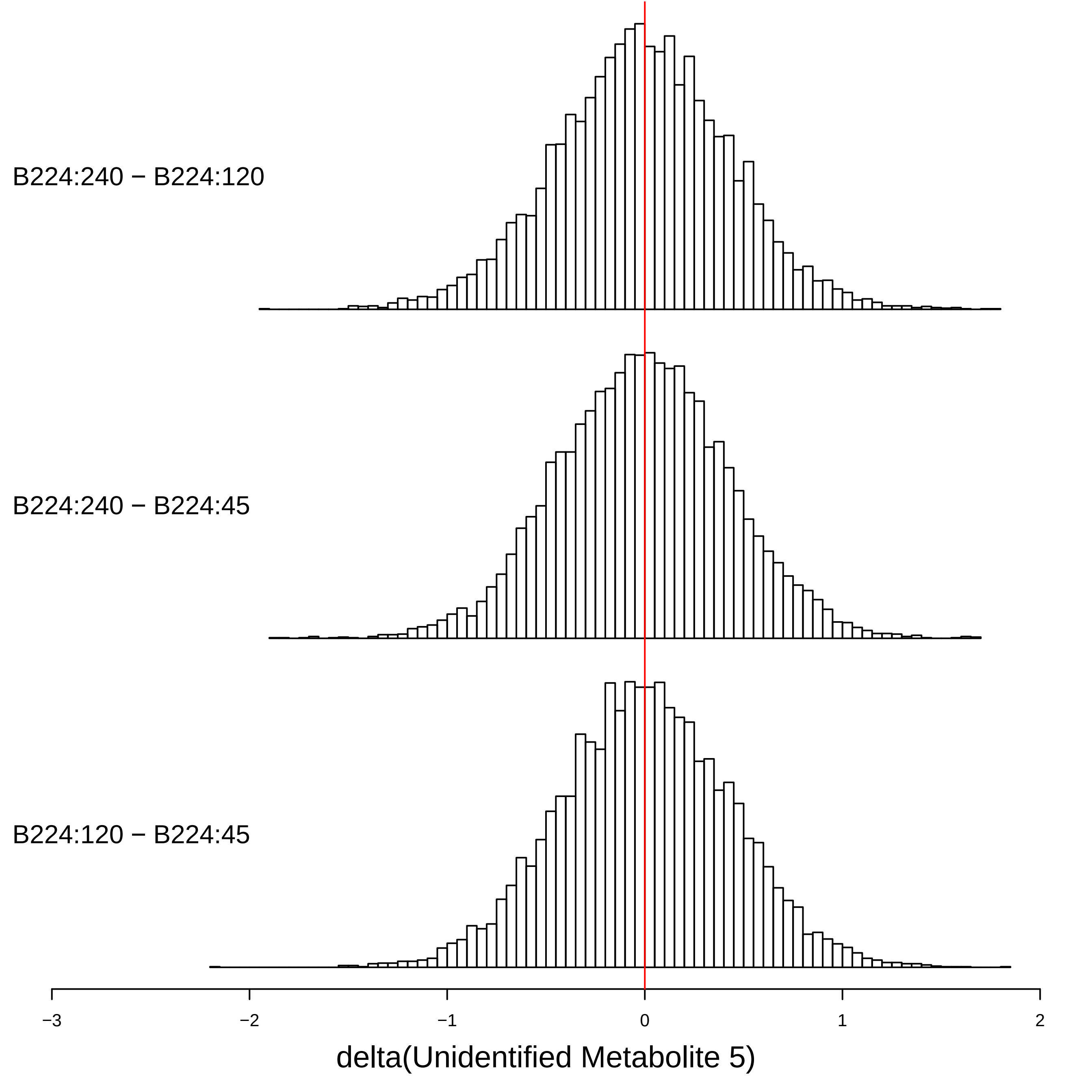
B224:240 − B224:120
B224:240 − B224:45
B224:120 − B224:45
−3
−2
−1
0
1
2
delta(Unidentified Metabolite 5)

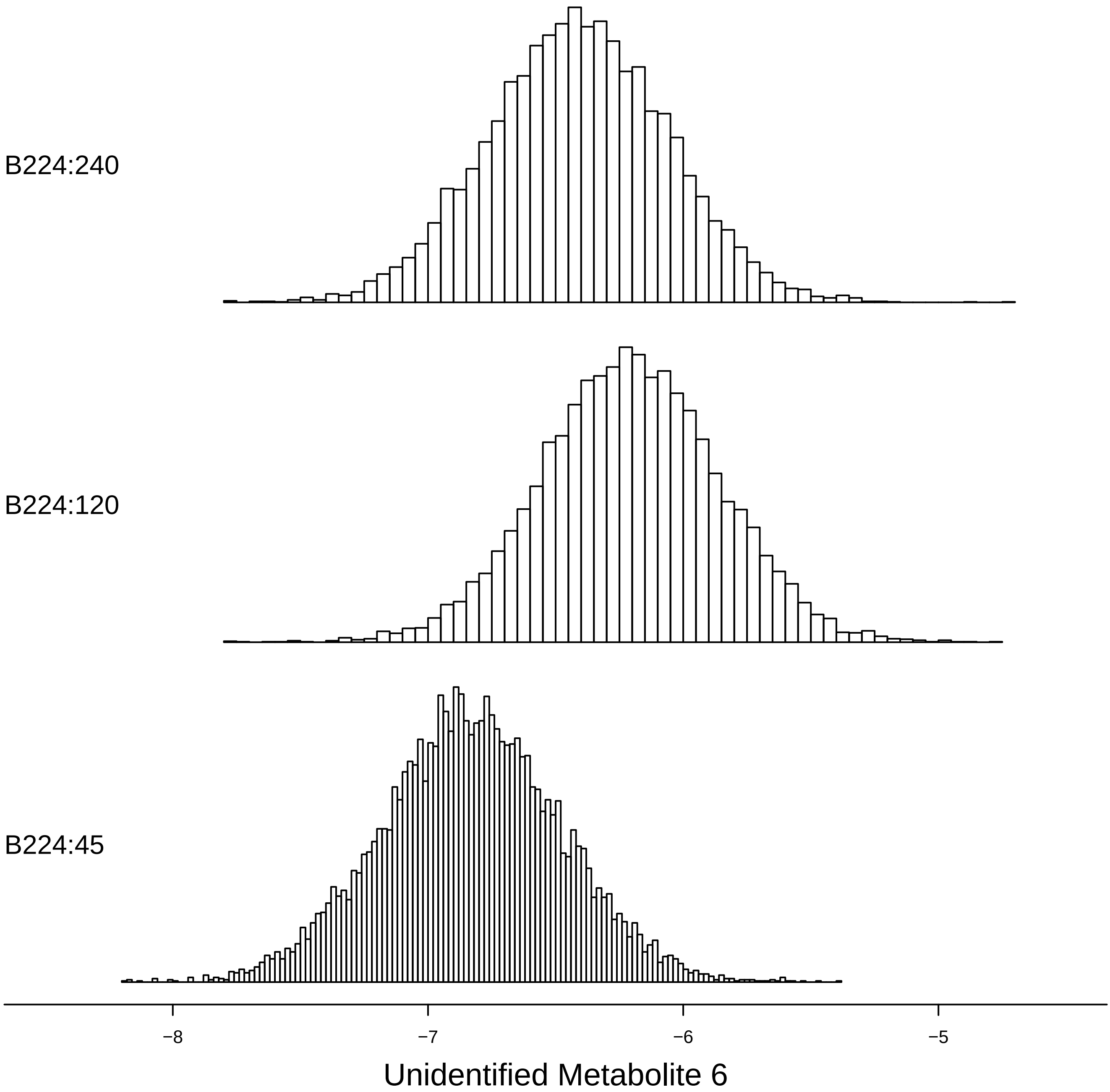
B224:240
B224:120
B224:45
−8
−7
−6
−5
Unidentified Metabolite 6

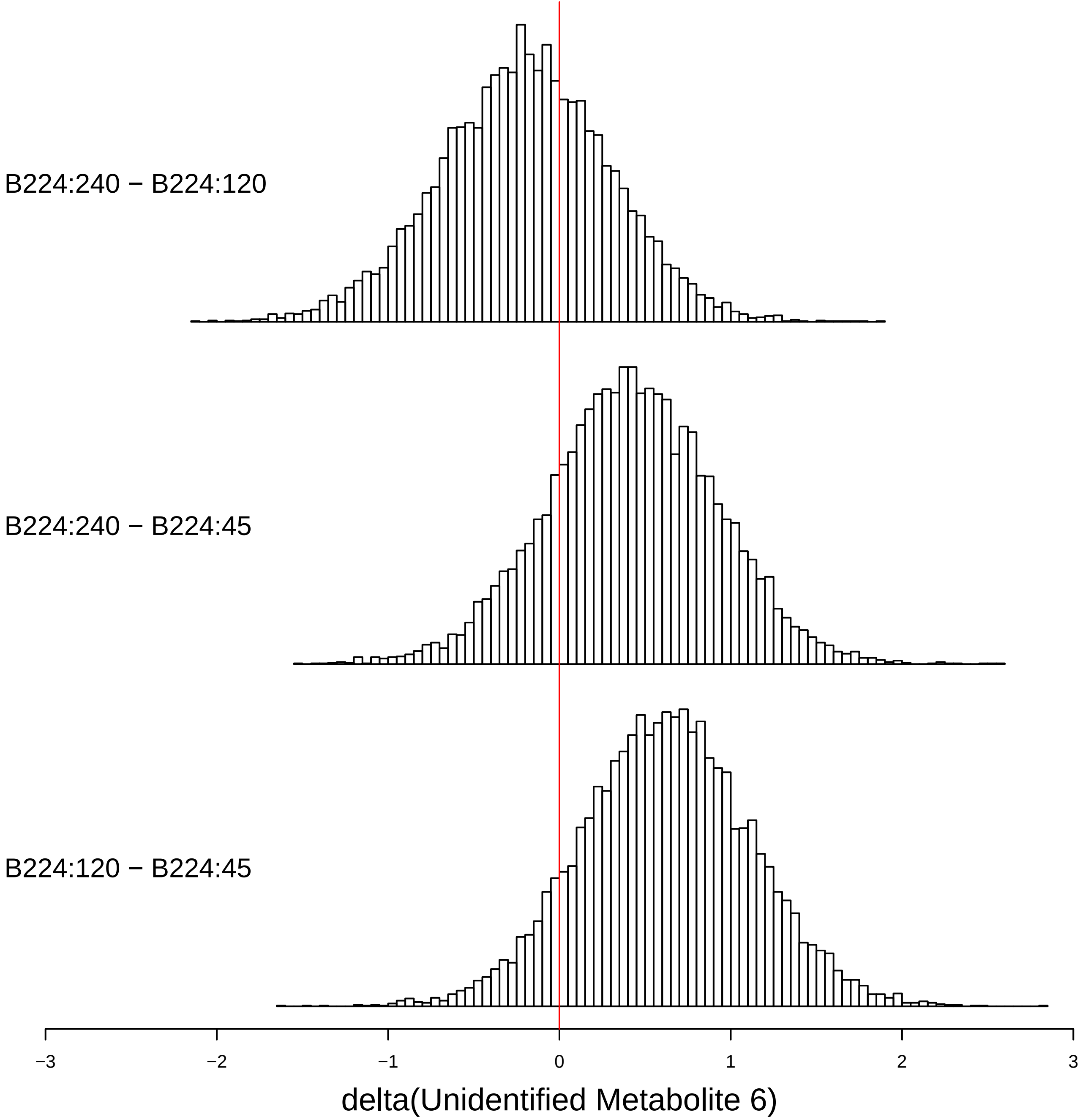

B224:240 − B224:120
B224:240 − B224:45
B224:120 − B224:45
−3
−2
−1
0
1
2
3
delta(Unidentified Metabolite 6)

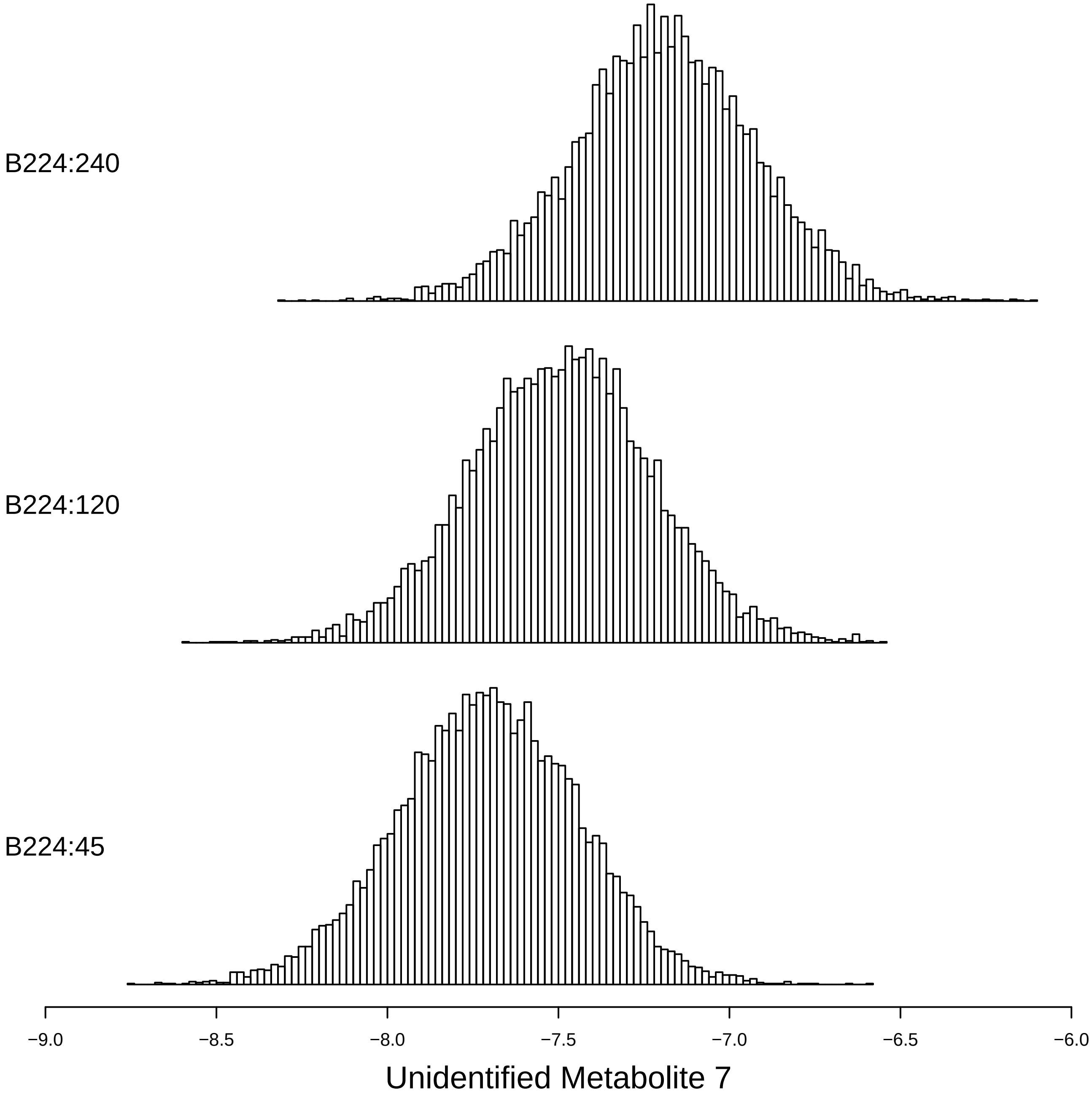

B224:240
B224:120
B224:45
−9.0
−8.5
−8.0
−7.5
−7.0
−6.5
−6.0
Unidentified Metabolite 7

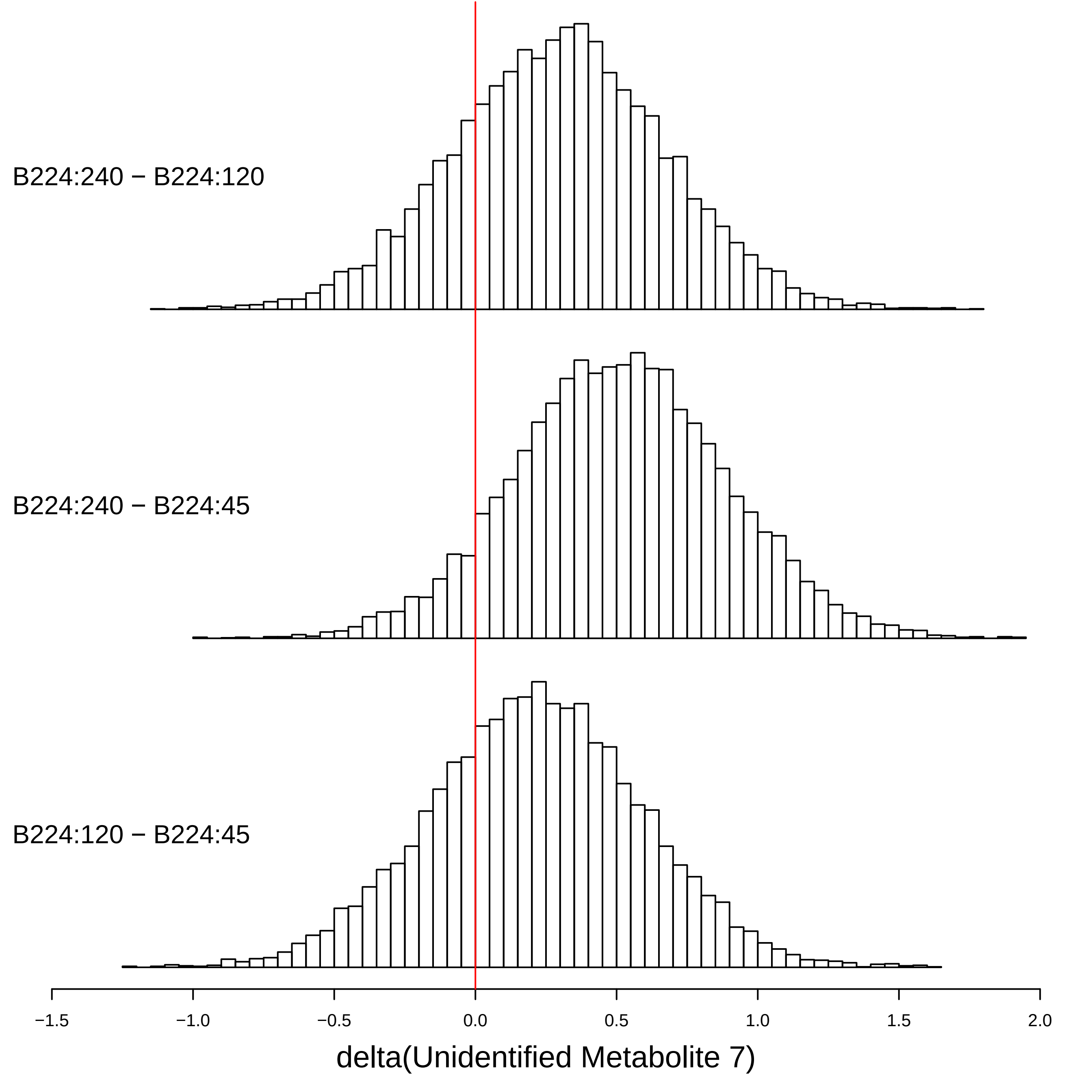
B224:240 − B224:120
B224:240 − B224:45
B224:120 − B224:45
−1.5
−1.0
−0.5
0.0
0.5
1.0
1.5
2.0
delta(Unidentified Metabolite 7)

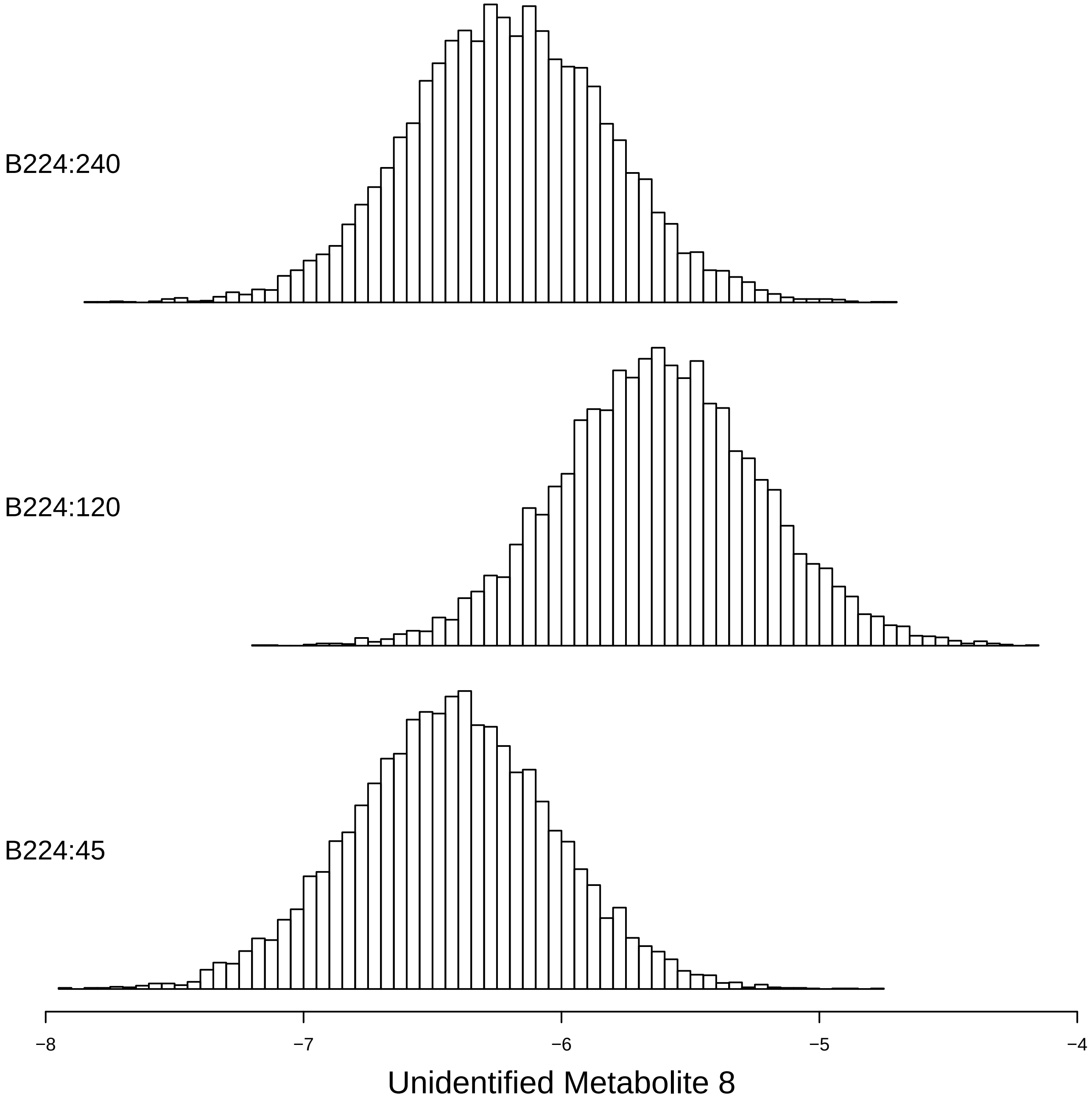
B224:240
B224:120
B224:45
−8
−7
−6
−5
−4
Unidentified Metabolite 8

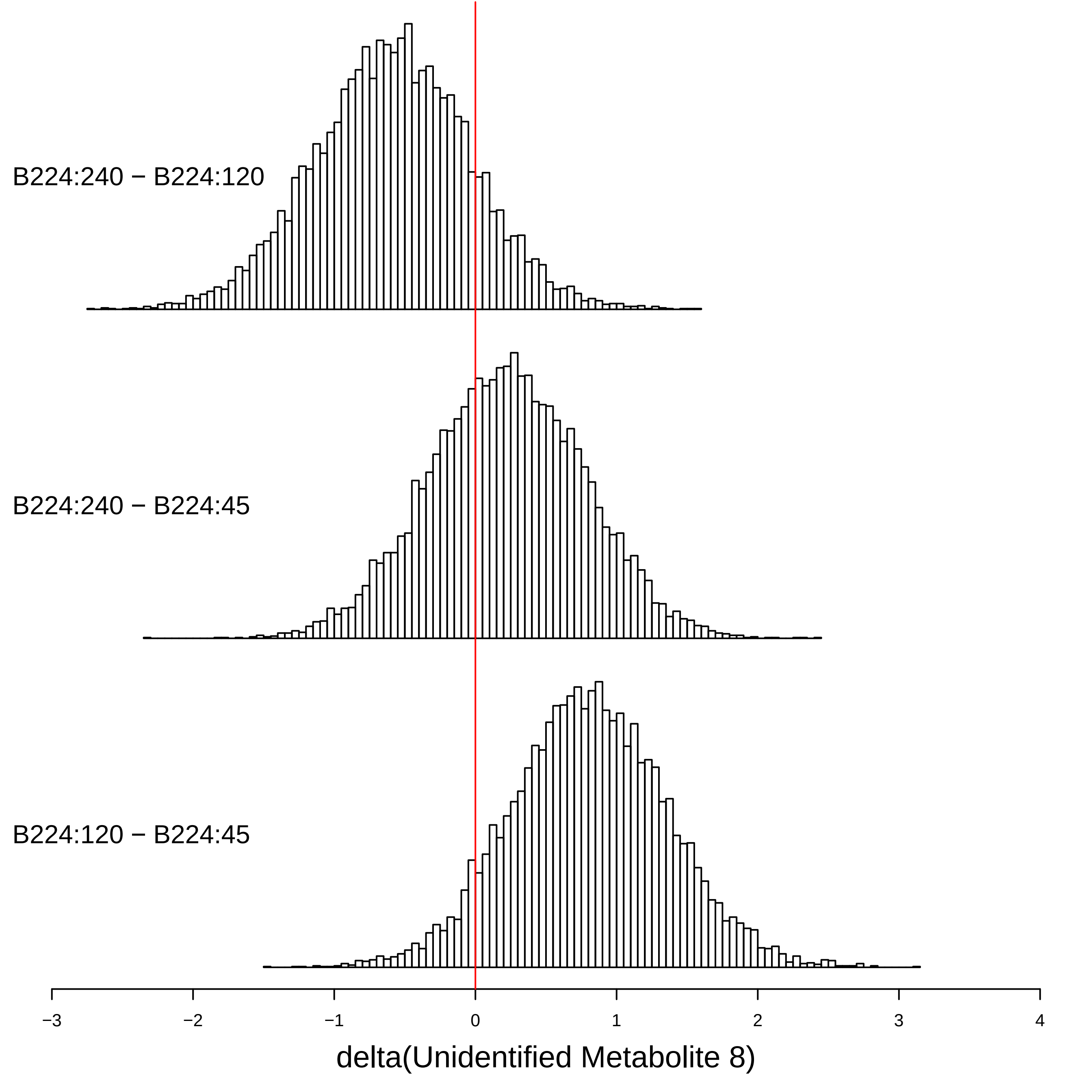

B224:240 − B224:120
B224:240 − B224:45
B224:120 − B224:45
−3
−2
−1
0
1
2
3
4
delta(Unidentified Metabolite 8)

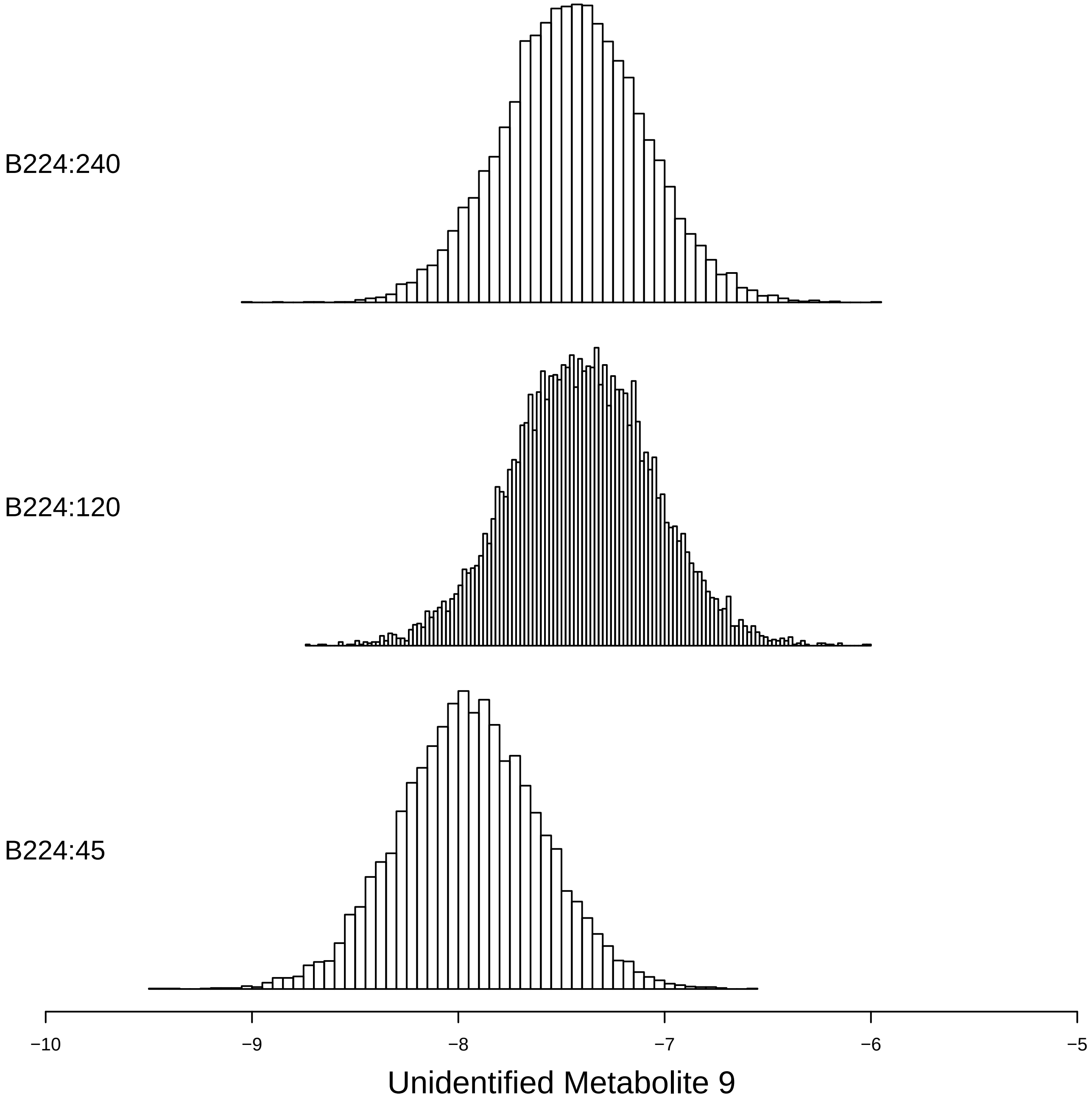
B224:240
B224:120
B224:45
−10
−9
−8
−7
−6
−5
Unidentified Metabolite 9

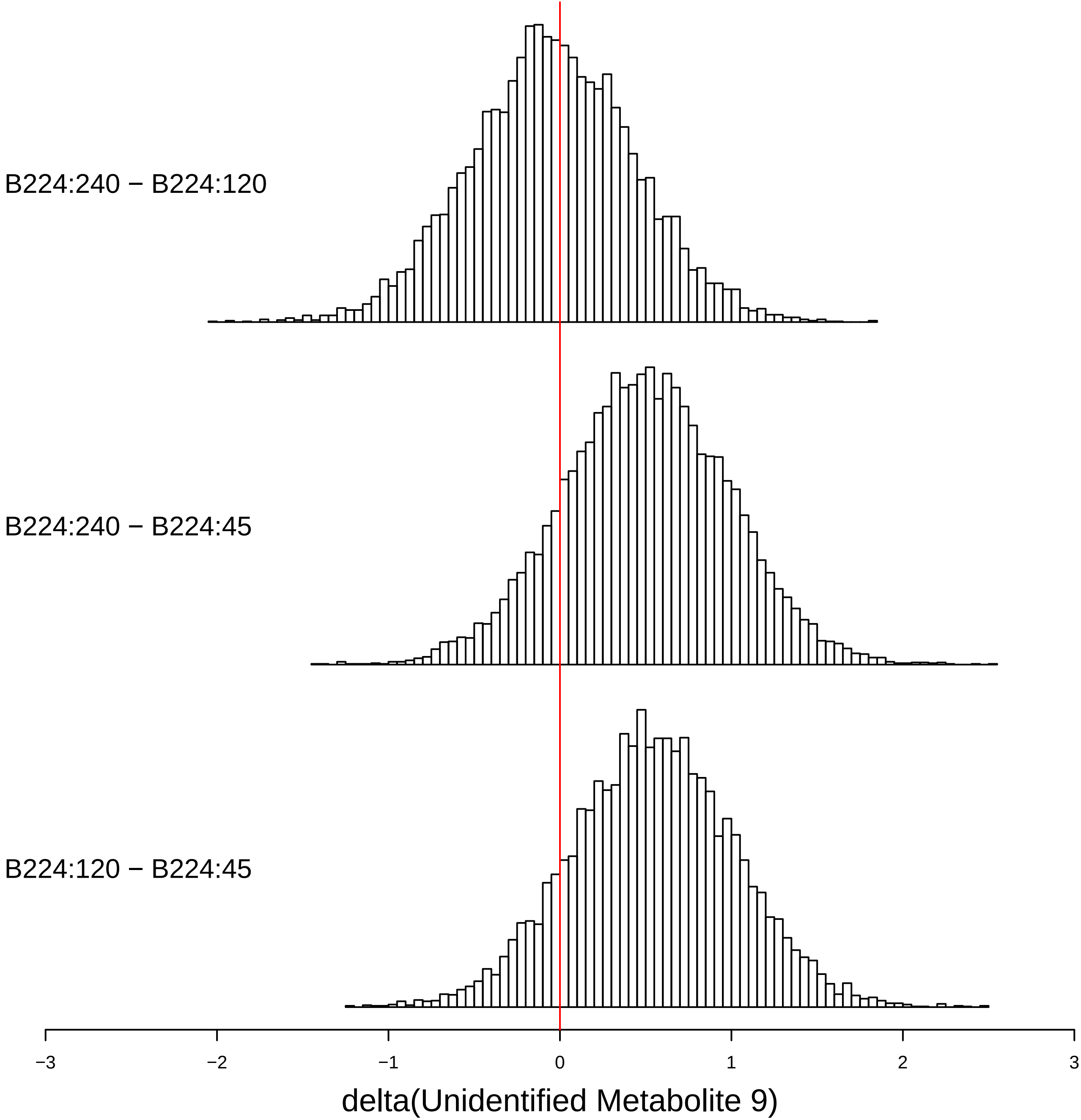

B224:240 − B224:120
B224:240 − B224:45
B224:120 − B224:45
−3
−2
−1
0
1
2
3
delta(Unidentified Metabolite 9)

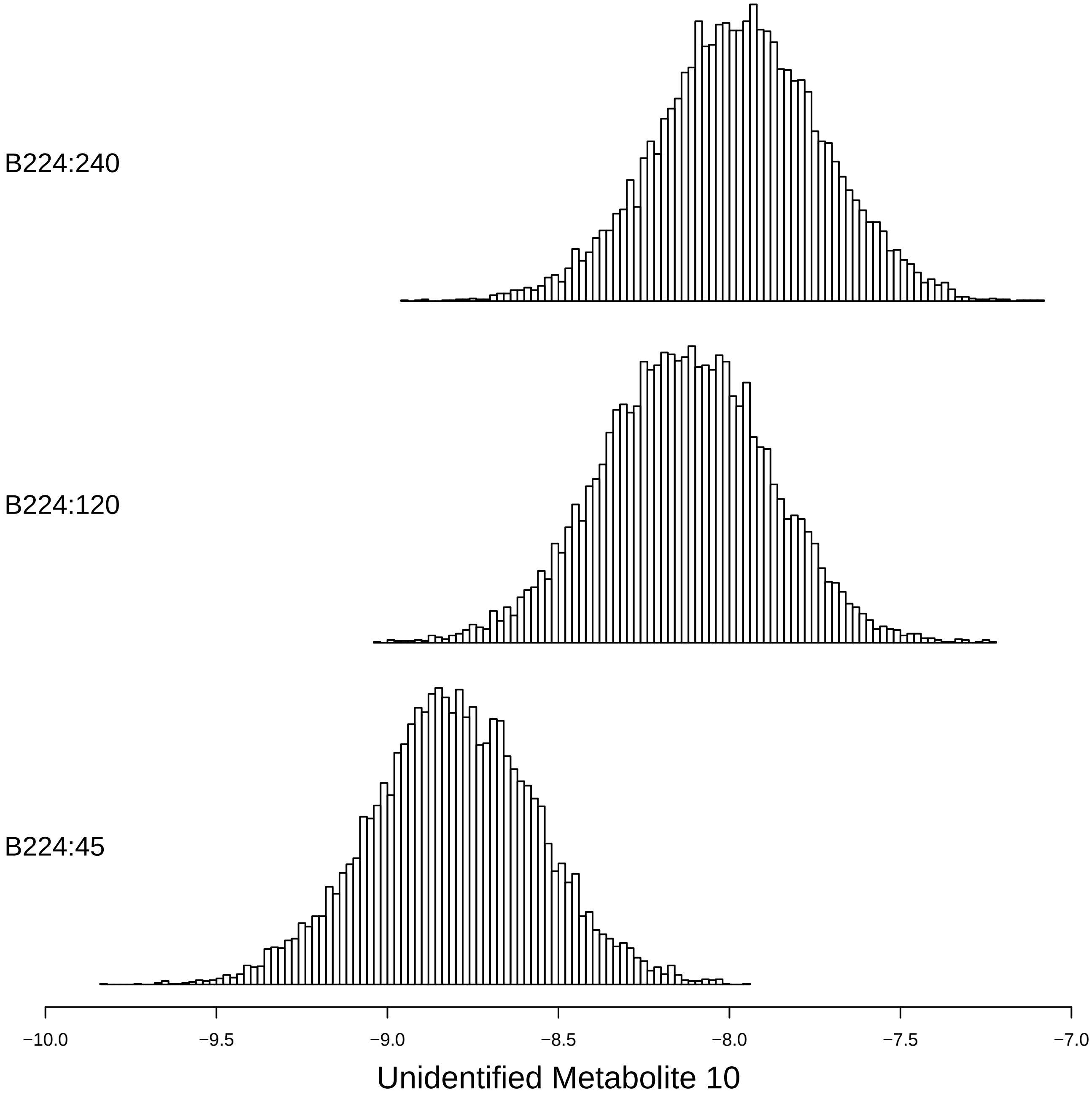
B224:240
B224:120
B224:45
−10.0
−9.5
−9.0
−8.5
−8.0
−7.5
−7.0
Unidentified Metabolite 10

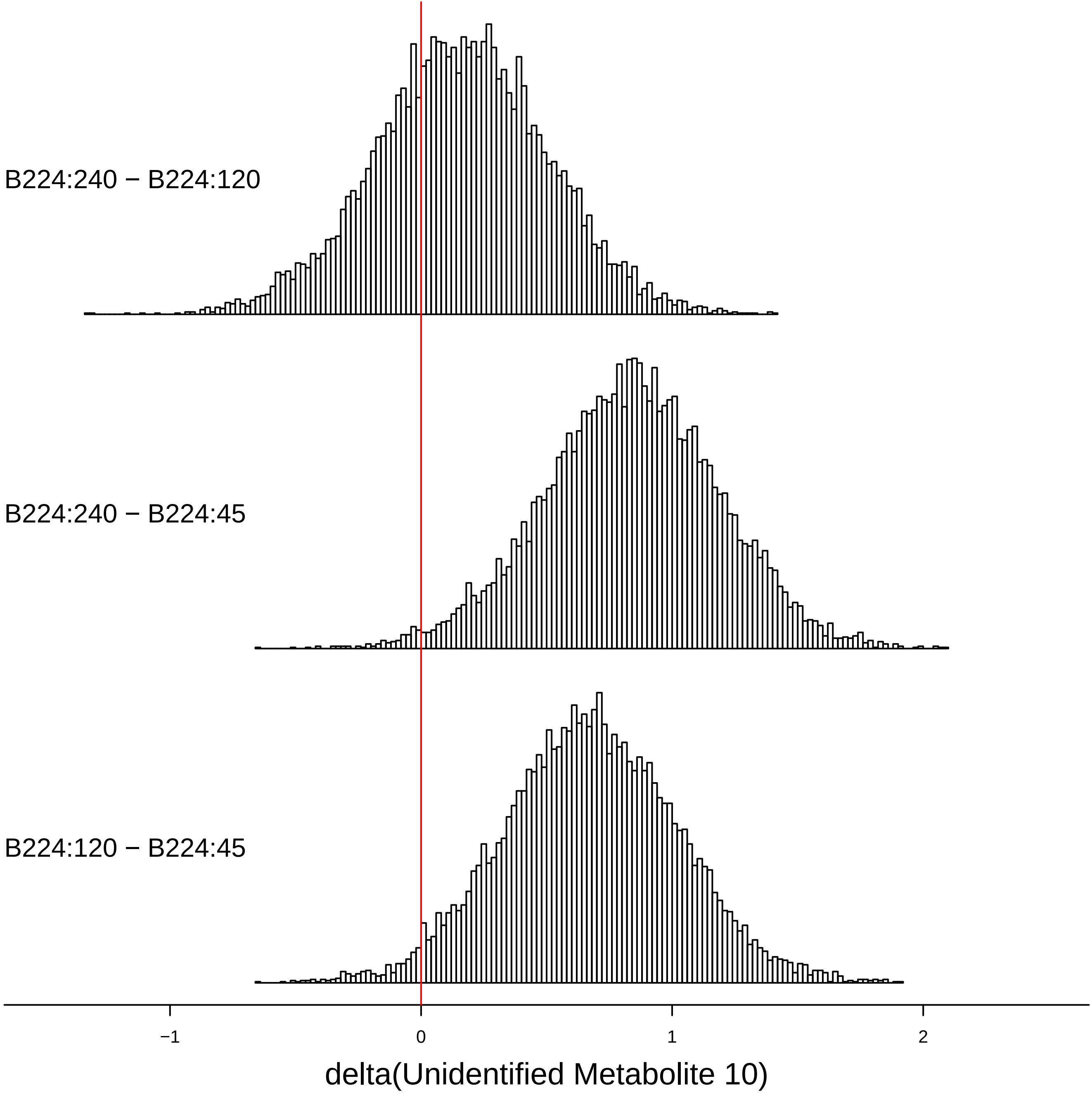

B224:240 − B224:120
B224:240 − B224:45
B224:120 − B224:45
−1
0
1
2
delta(Unidentified Metabolite 10)

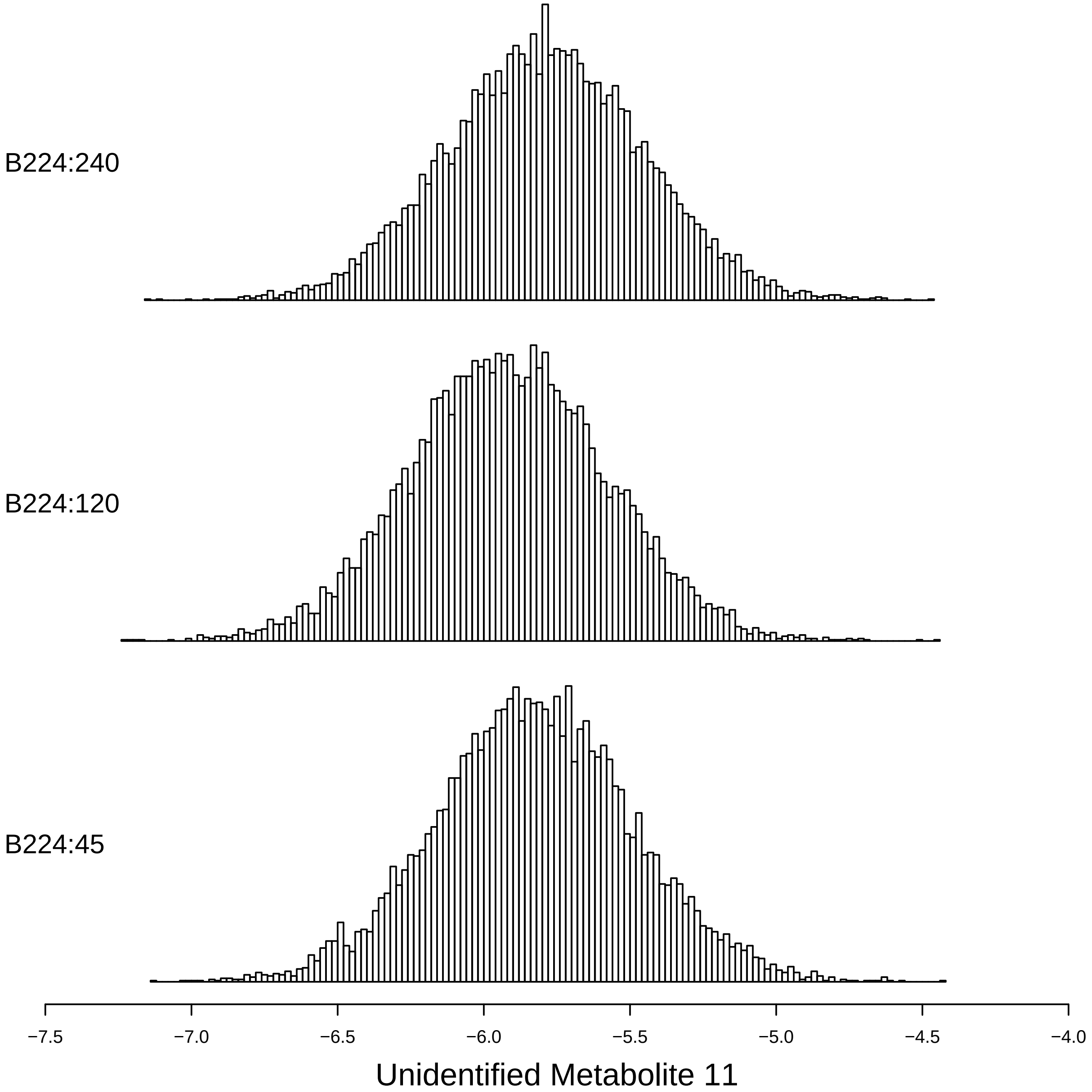
B224:240
B224:120
B224:45
−7.5
−7.0
−6.5
−6.0
−5.5
−5.0
−4.5
−4.0
Unidentified Metabolite 11

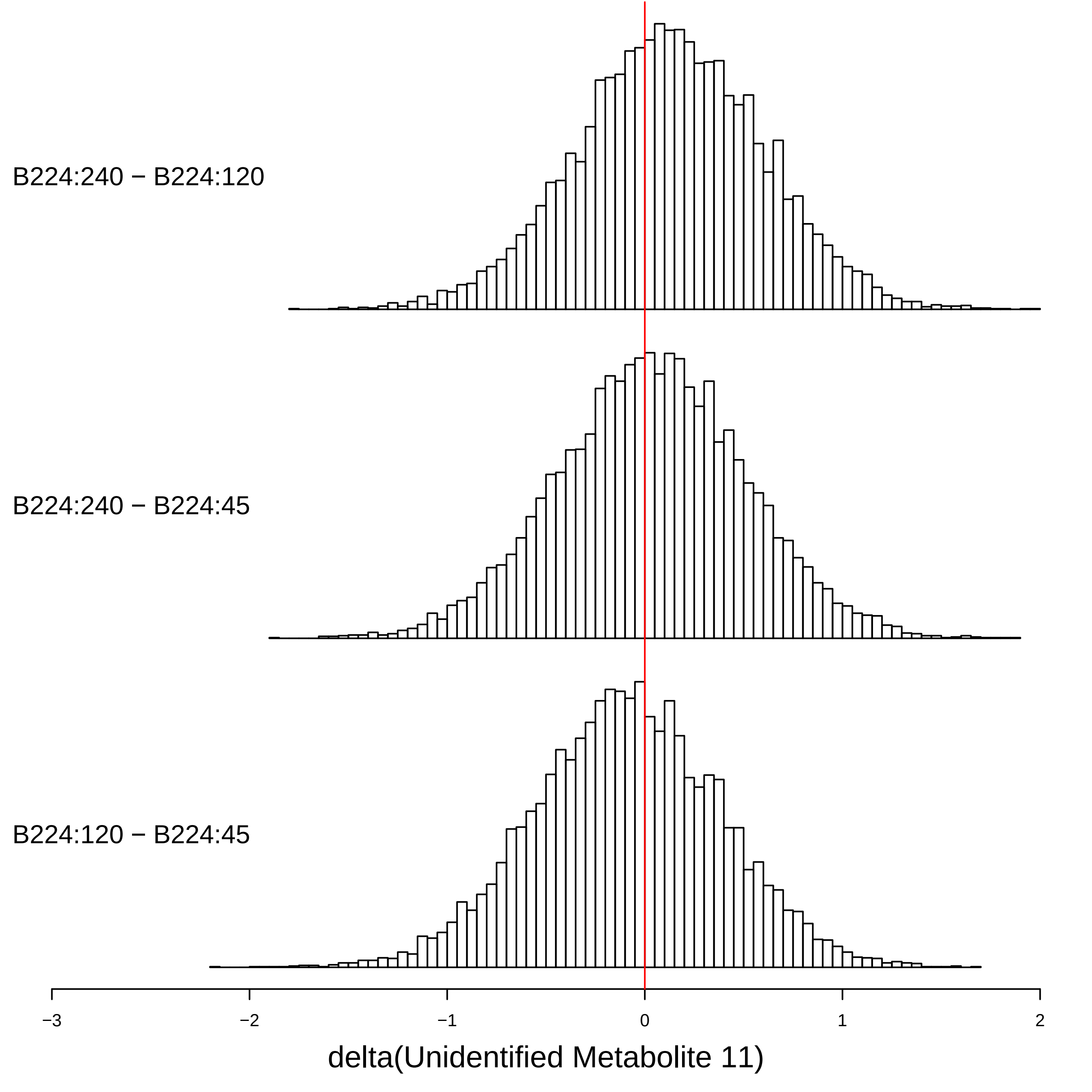
B224:240 − B224:120
B224:240 − B224:45
B224:120 − B224:45
−3
−2
−1
0
1
2
delta(Unidentified Metabolite 11)

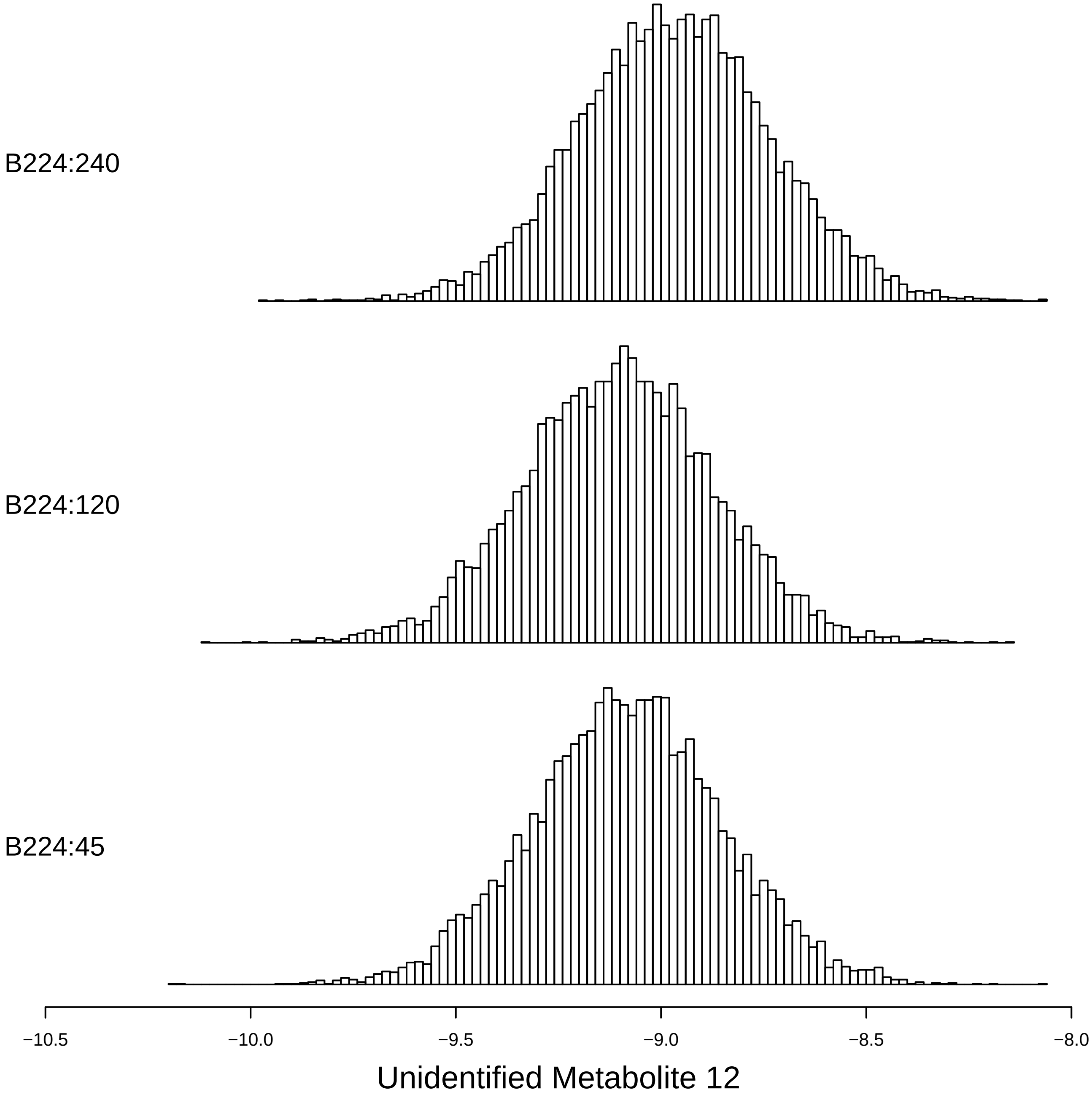
B224:240
B224:120
B224:45
−10.5
−10.0
−9.5
−9.0
−8.5
−8.0
Unidentified Metabolite 12

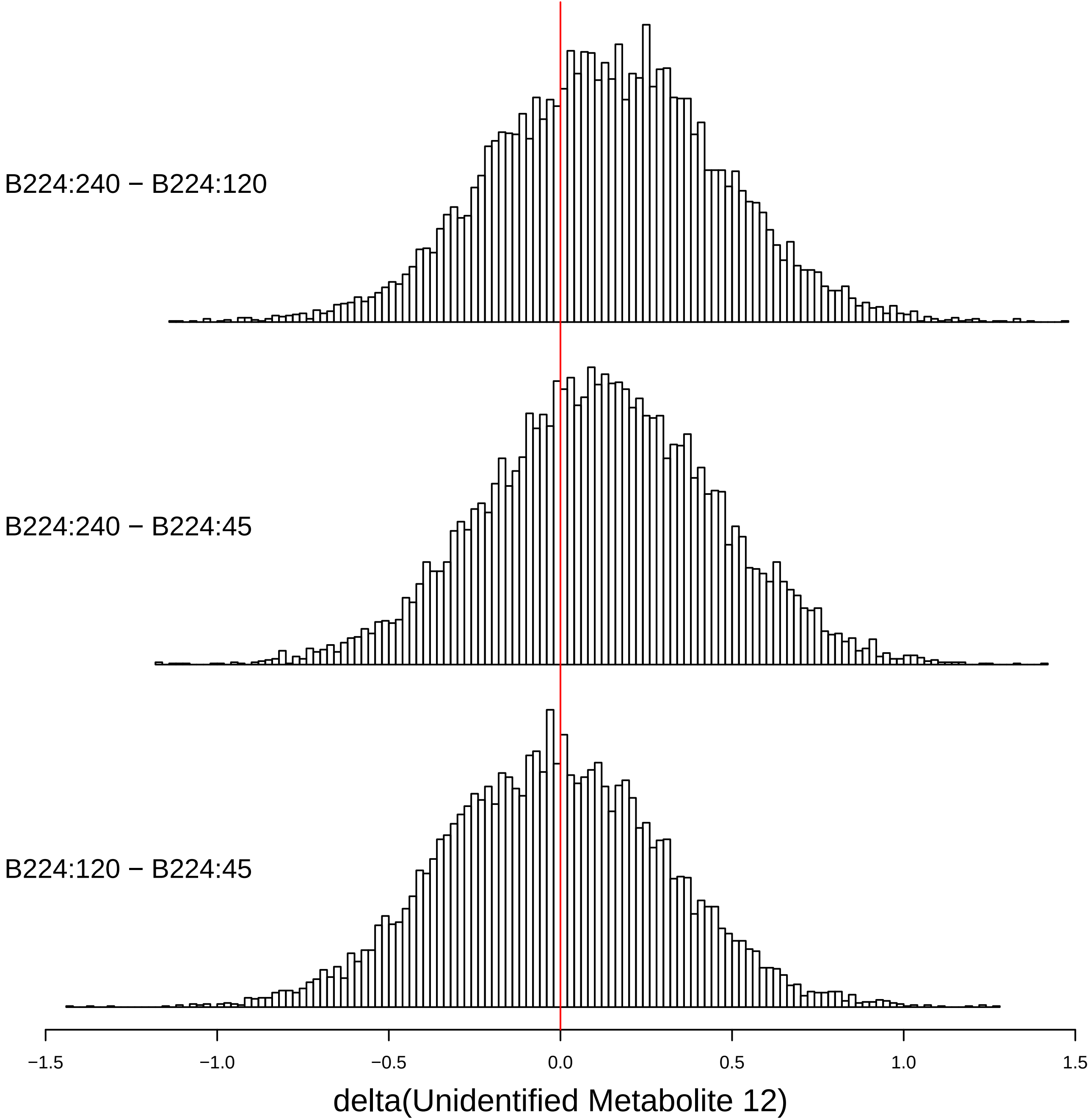

B224:240 − B224:120
B224:240 − B224:45
B224:120 − B224:45
−1.5
−1.0
−0.5
0.0
0.5
1.0
1.5
delta(Unidentified Metabolite 12)

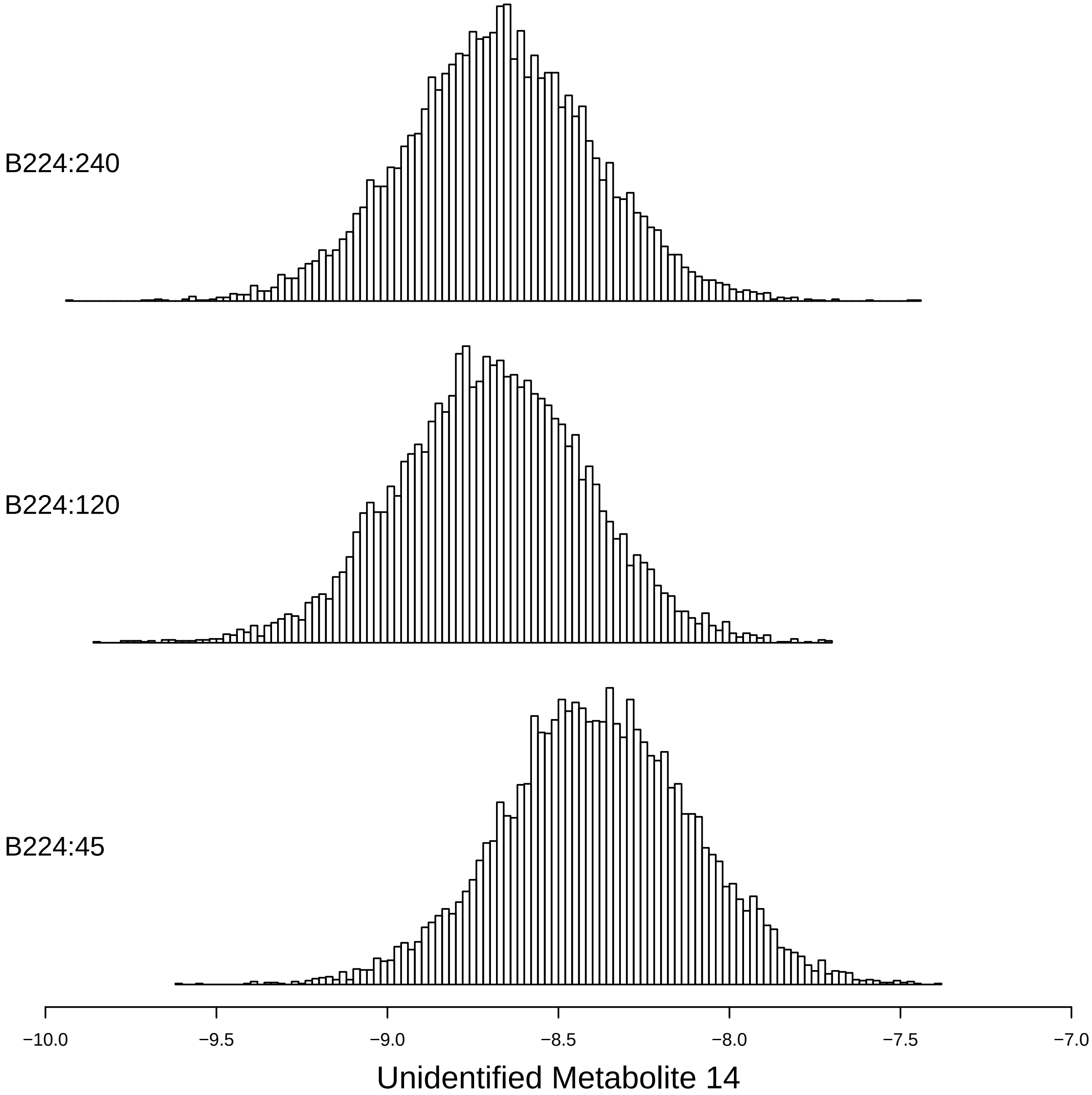

B224:240
B224:120
B224:45
−10.0
−9.5
−9.0
−8.5
−8.0
−7.5
−7.0
Unidentified Metabolite 14

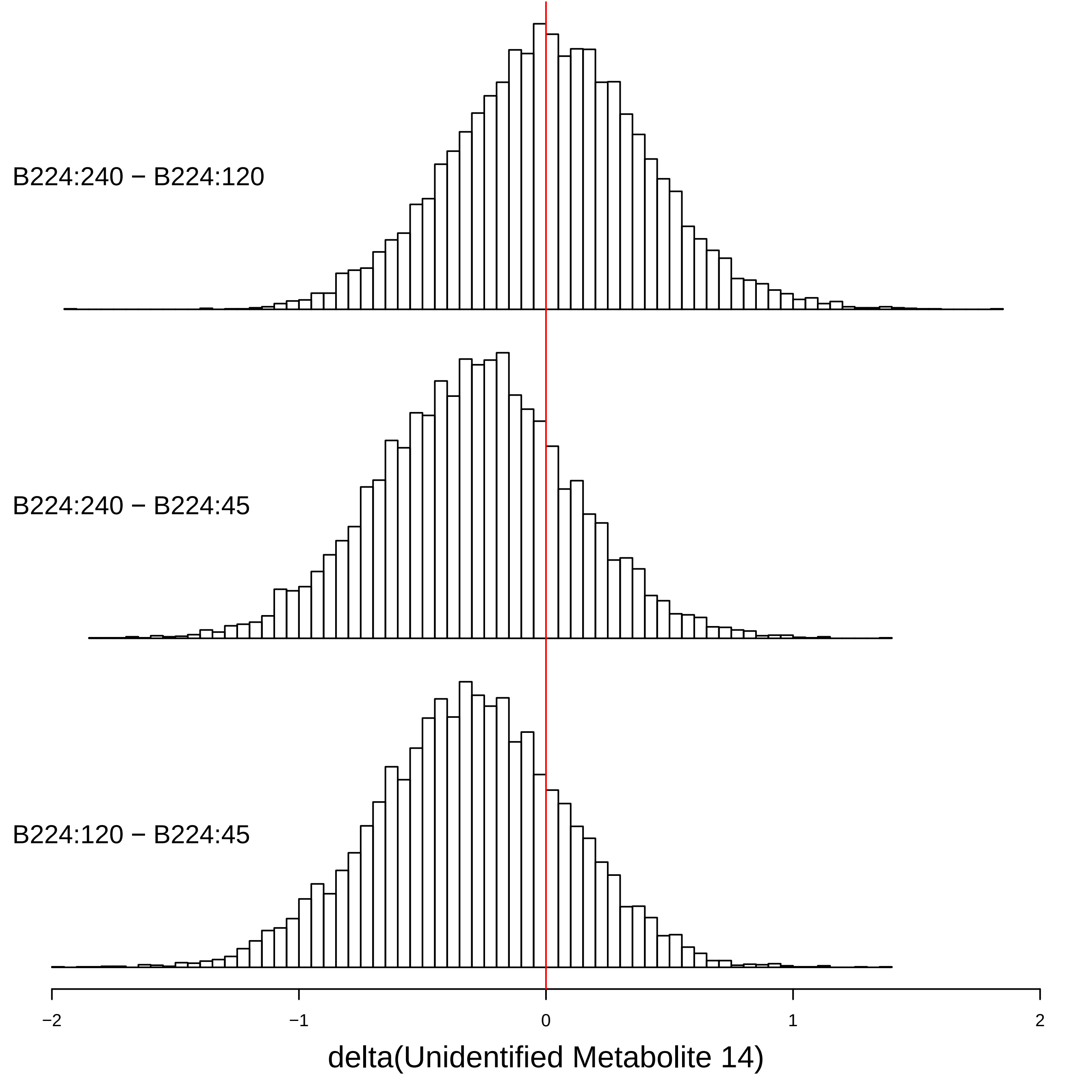
B224:240 − B224:120
B224:240 − B224:45
B224:120 − B224:45
−2
−1
0
1
2
delta(Unidentified Metabolite 14)

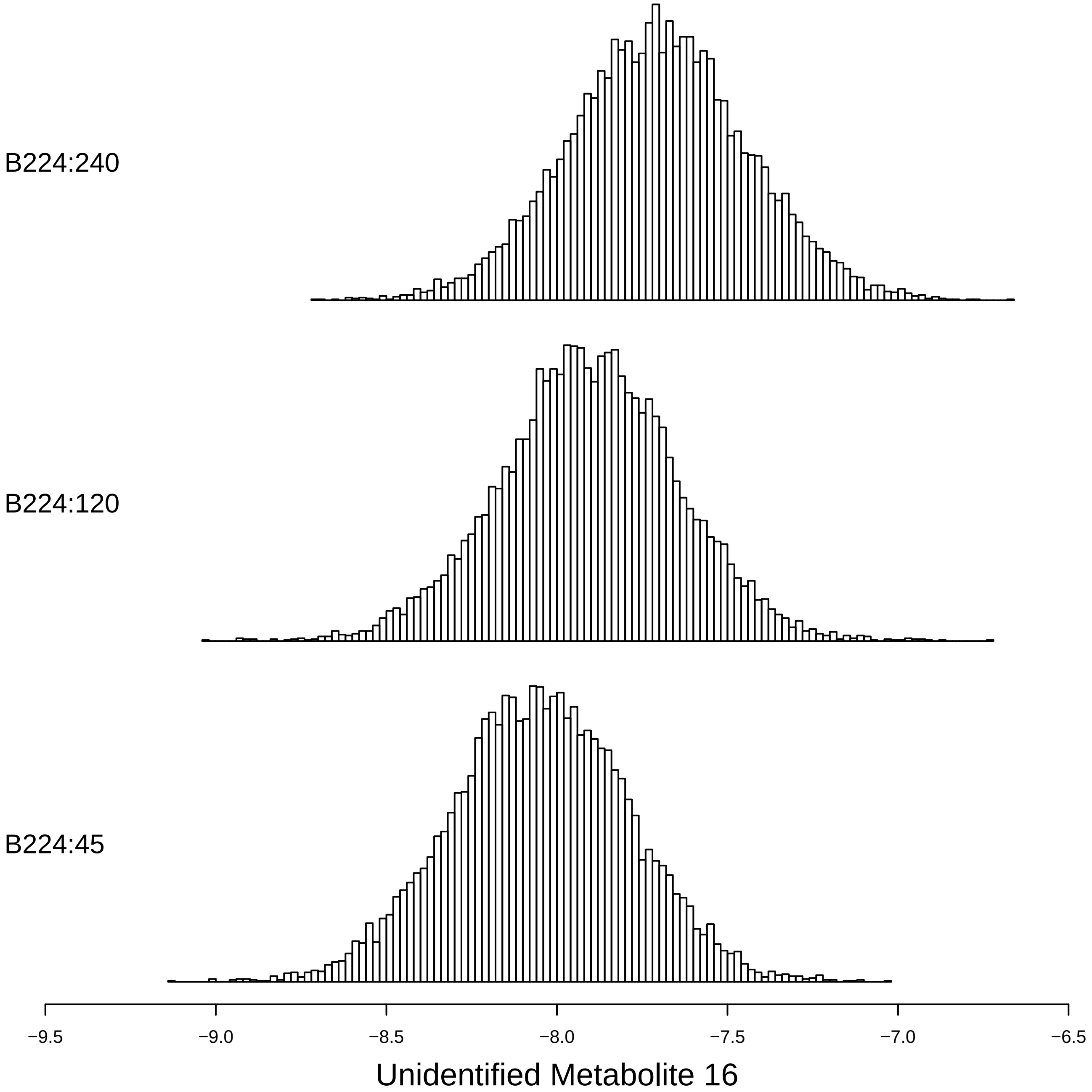
B224:240
B224:120
B224:45
−9.5
−9.0
−8.5
−8.0
−7.5
−7.0
−6.5
Unidentified Metabolite 16

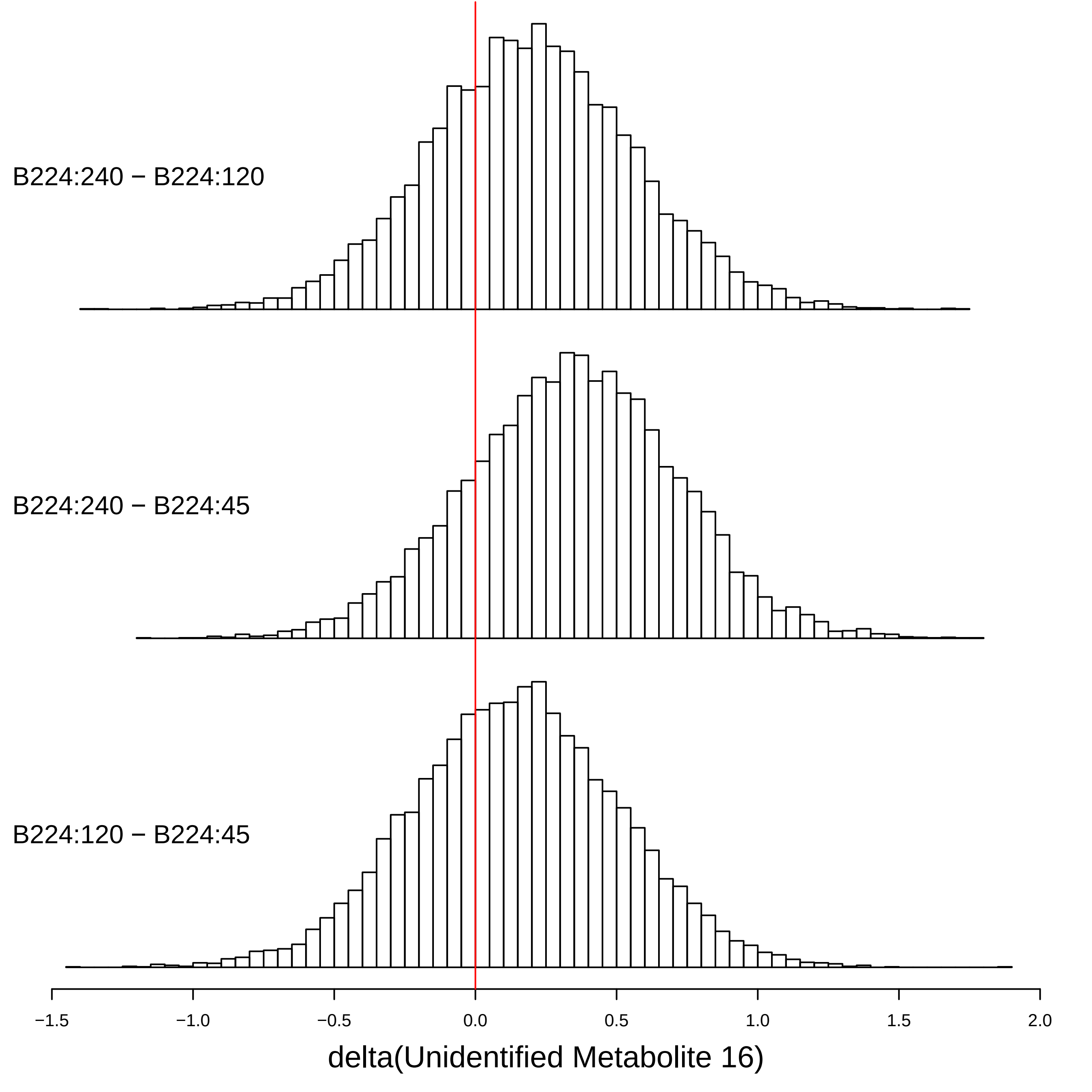

B224:240 − B224:120
B224:240 − B224:45
B224:120 − B224:45
−1.5
−1.0
−0.5
0.0
0.5
1.0
1.5
2.0
delta(Unidentified Metabolite 16)

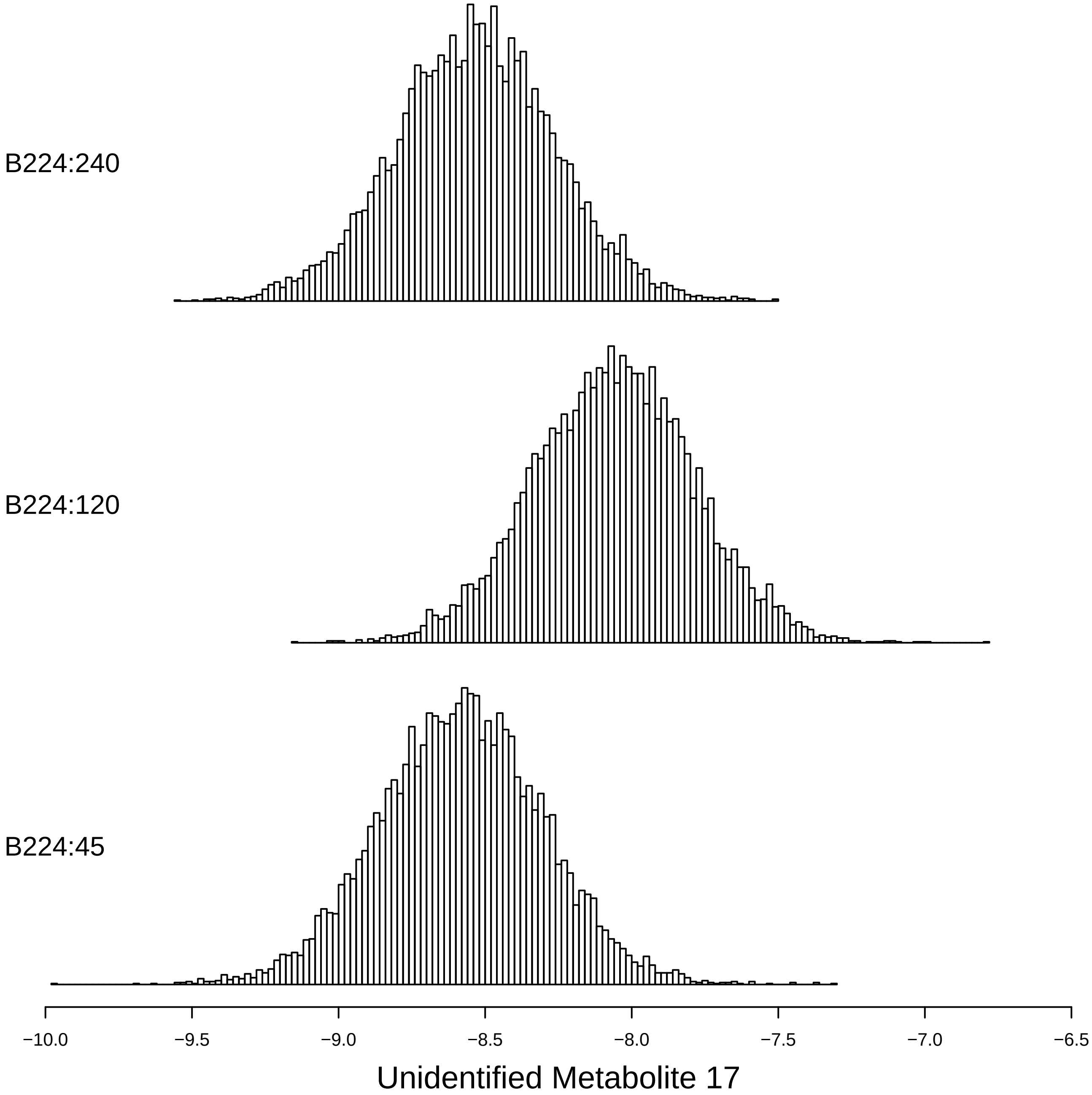
B224:240
B224:120
B224:45
−10.0
−9.5
−9.0
−8.5
−8.0
−7.5
−7.0
−6.5
Unidentified Metabolite 17

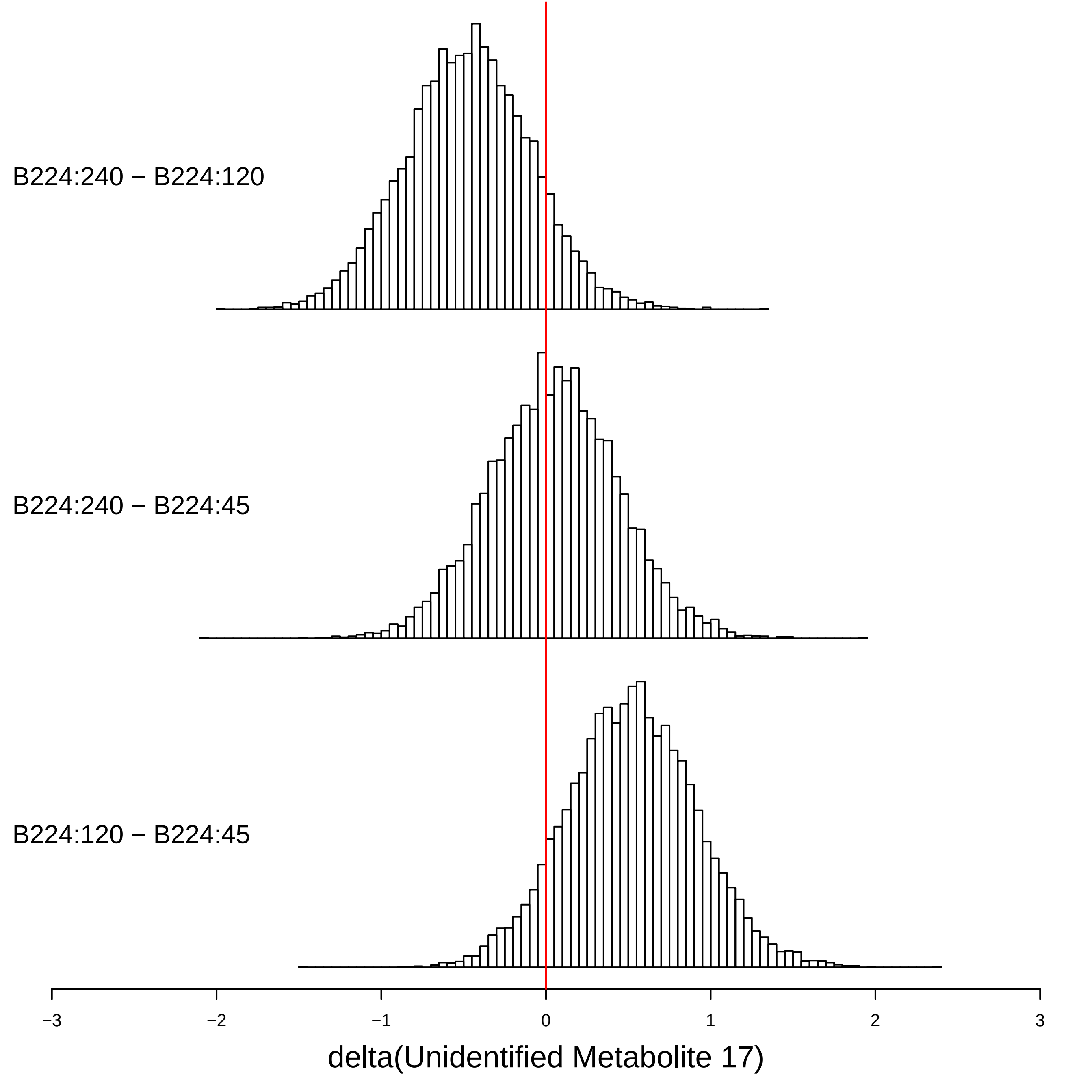

B224:240 − B224:120
B224:240 − B224:45
B224:120 − B224:45
−3
−2
−1
0
1
2
3
delta(Unidentified Metabolite 17)

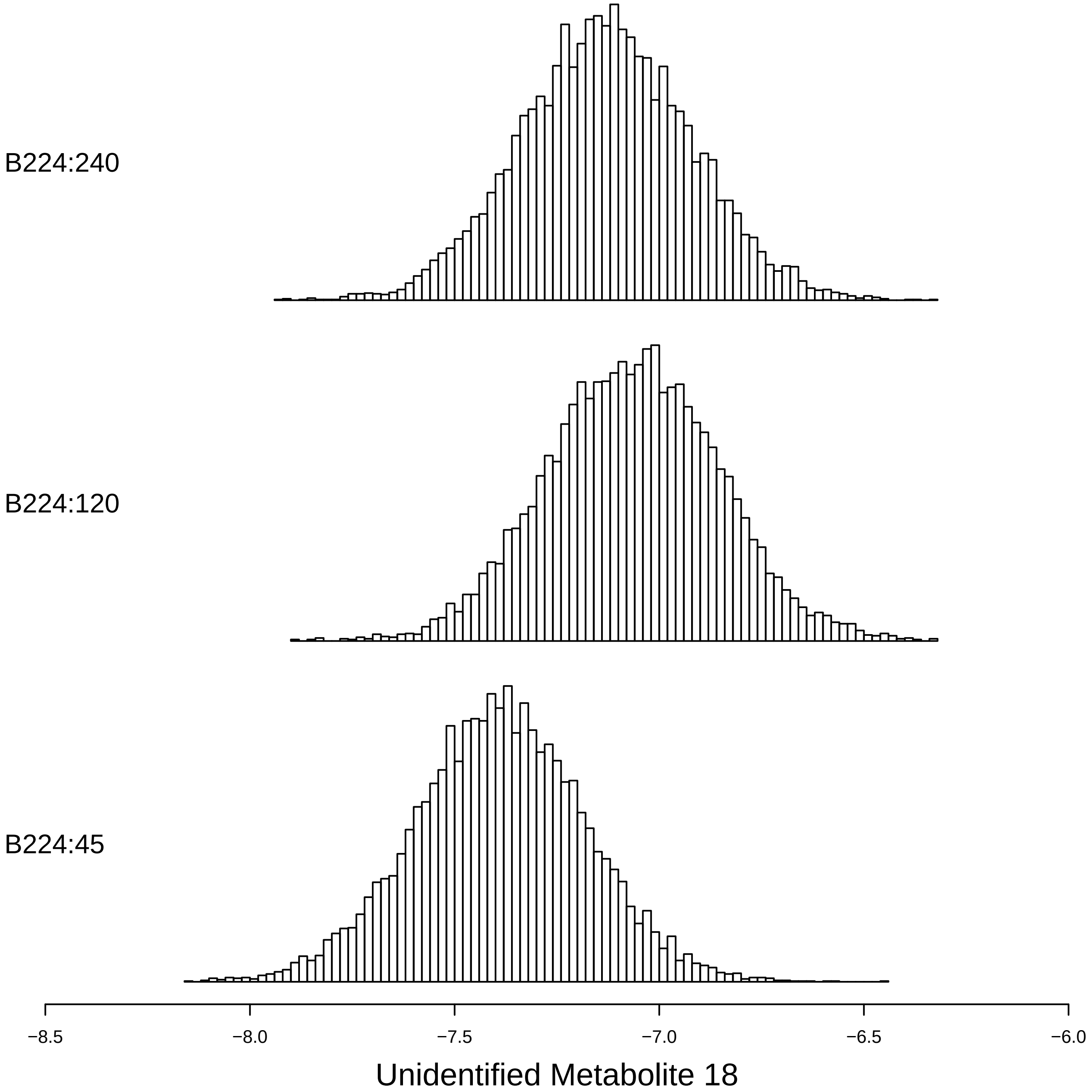

B224:240
B224:120
B224:45
−8.5
−8.0
−7.5
−7.0
−6.5
−6.0
Unidentified Metabolite 18

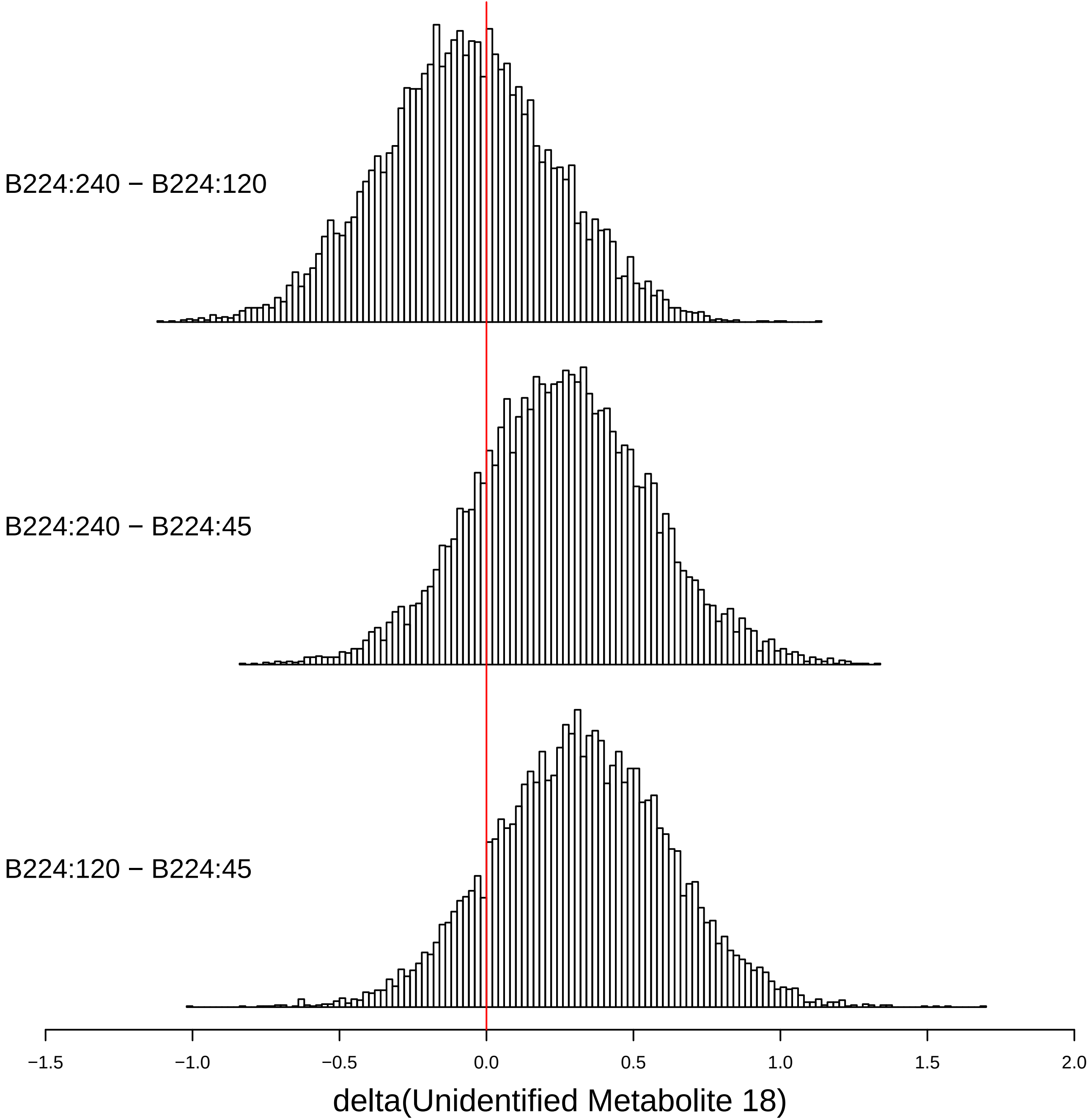

B224:240 − B224:120
B224:240 − B224:45
B224:120 − B224:45
−1.5
−1.0
−0.5
0.0
0.5
1.0
1.5
2.0
delta(Unidentified Metabolite 18)

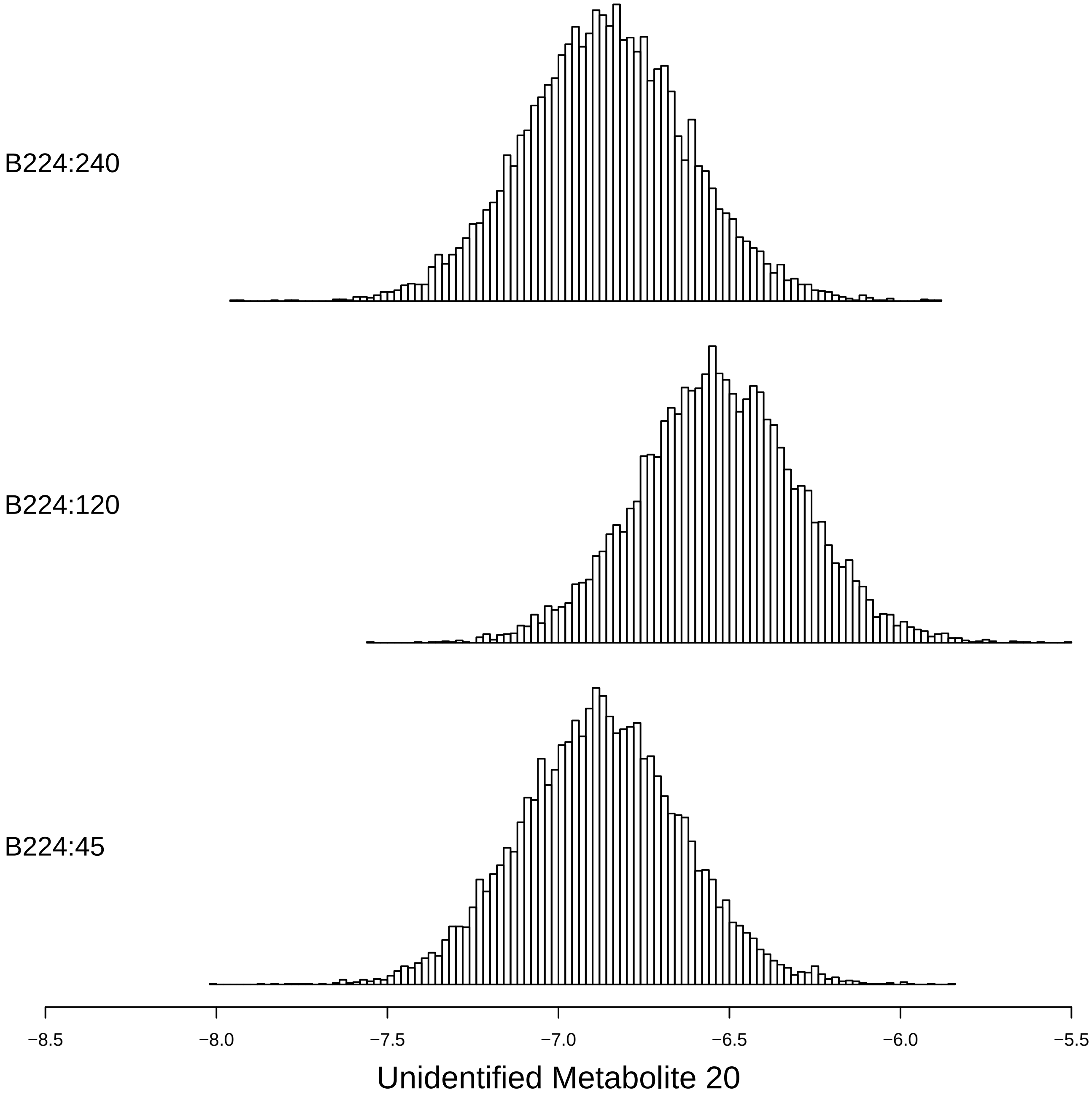

B224:240
B224:120
B224:45
−8.5
−8.0
−7.5
−7.0
−6.5
−6.0
−5.5
Unidentified Metabolite 20

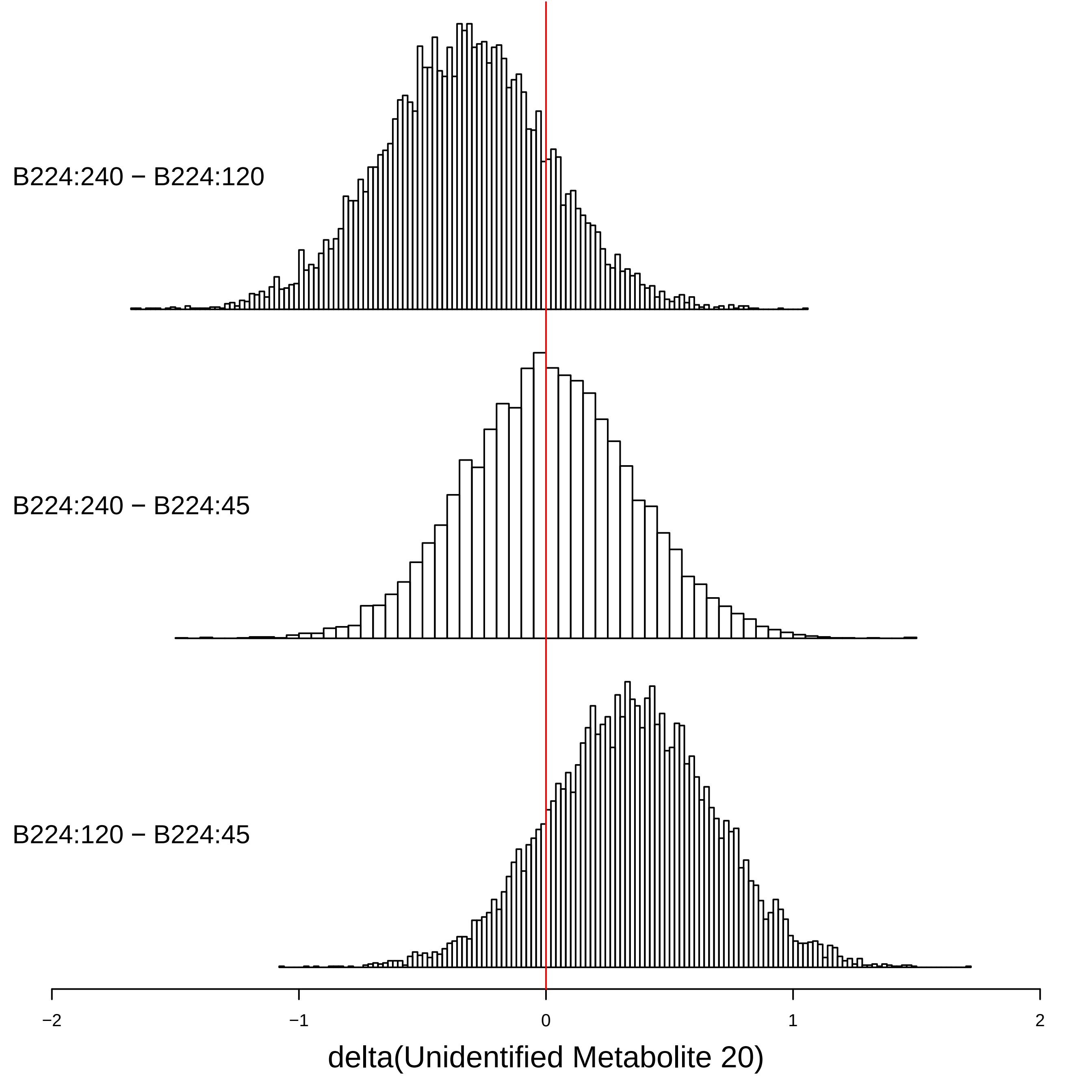
B224:240 − B224:120
B224:240 − B224:45
B224:120 − B224:45
−2
−1
0
1
2
delta(Unidentified Metabolite 20)

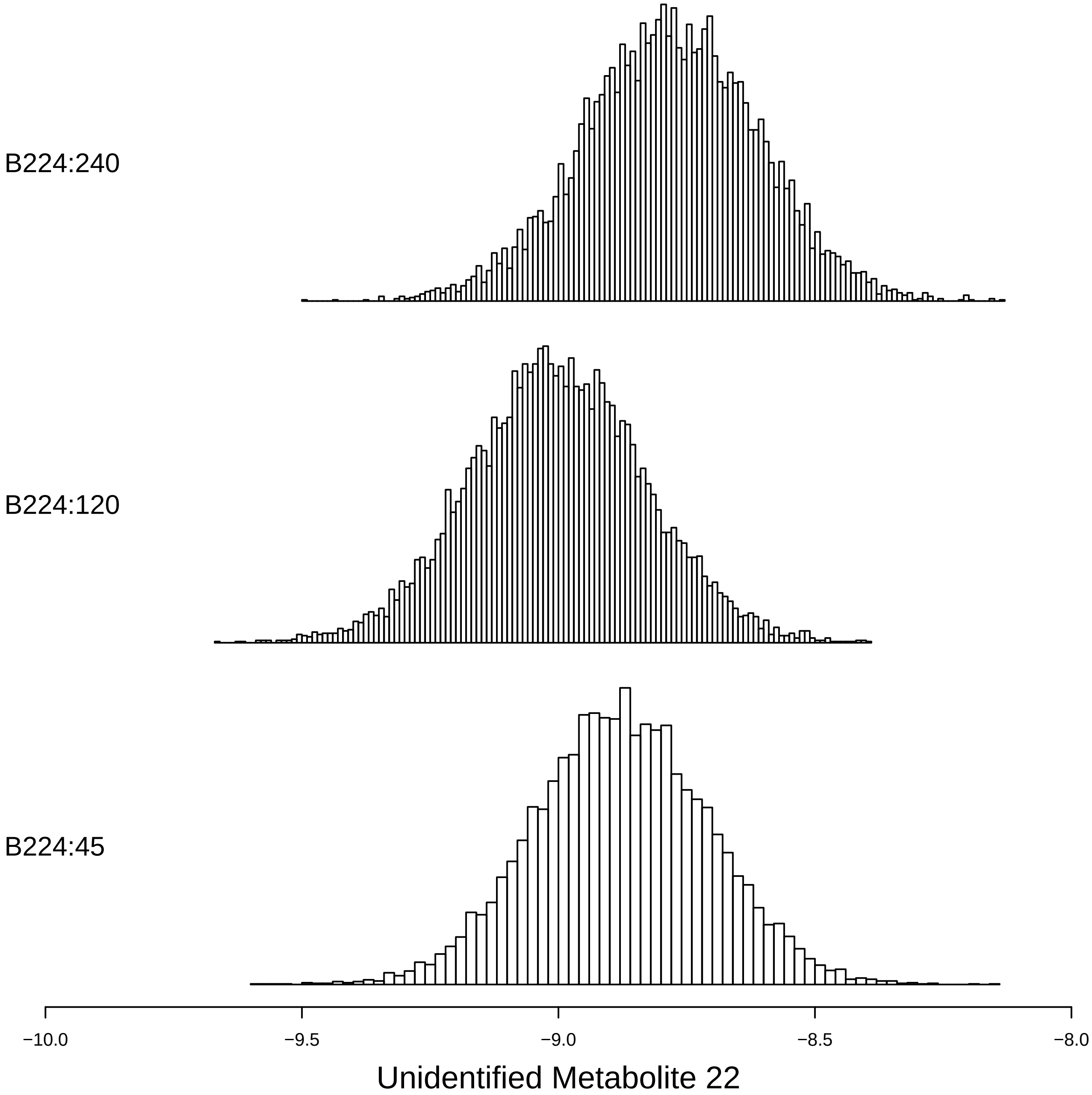
B224:240
B224:120
B224:45
−10.0
−9.5
−9.0
−8.5
−8.0
Unidentified Metabolite 22

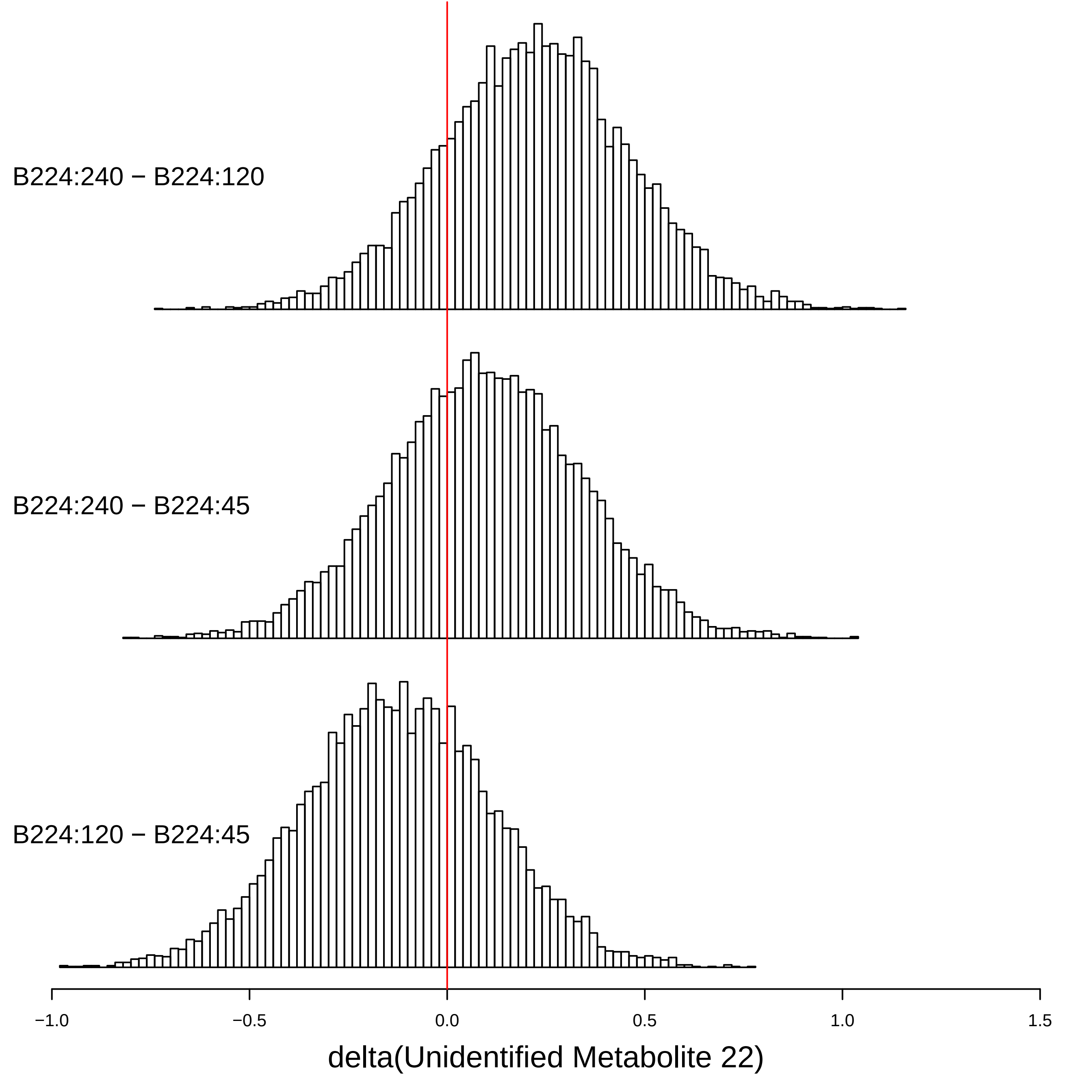
B224:240 − B224:120
B224:240 − B224:45
B224:120 − B224:45
−1.0
−0.5
0.0
0.5
1.0
1.5
delta(Unidentified Metabolite 22)

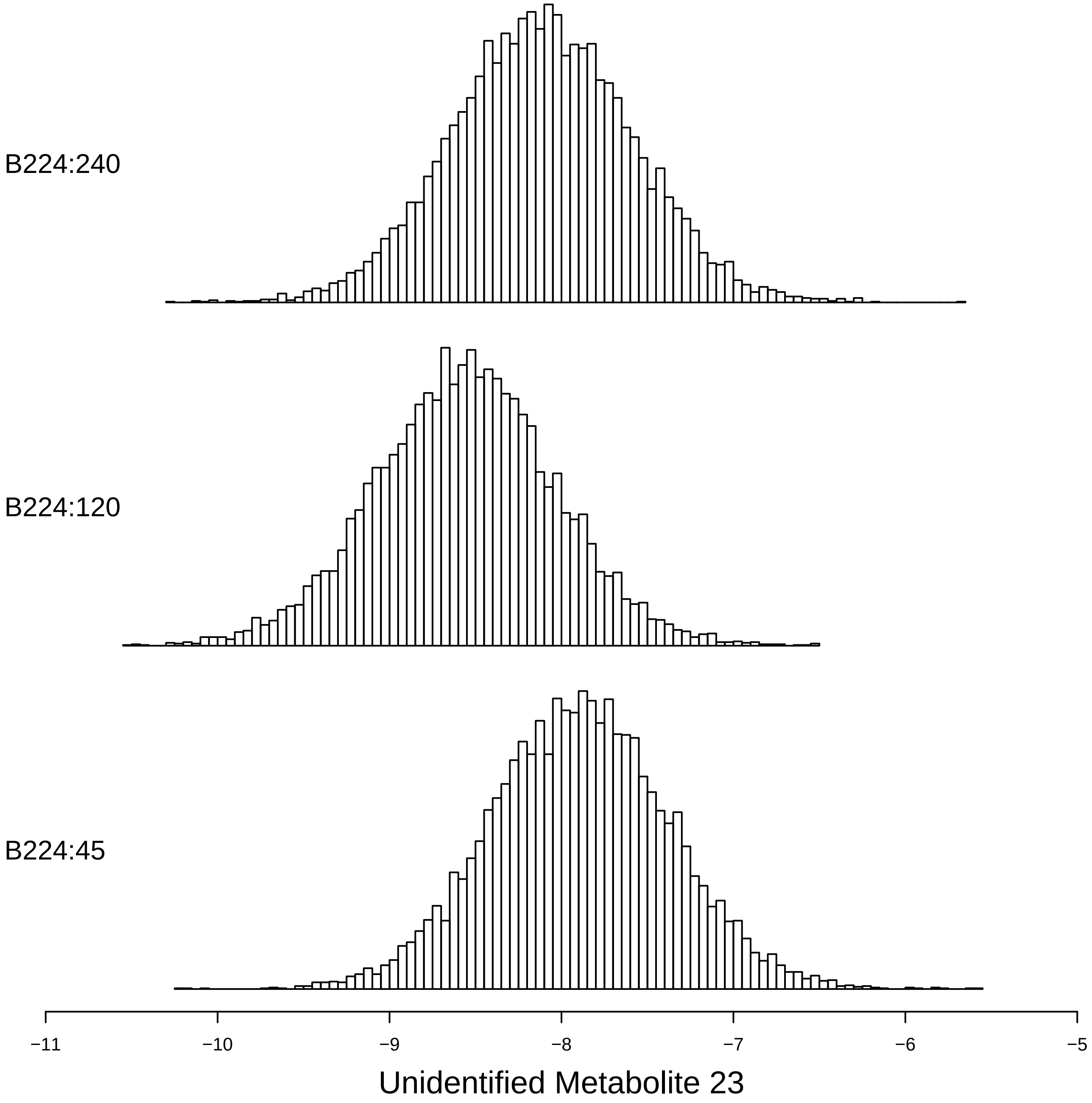

B224:240
B224:120
B224:45
−11
−10
−9
−8
−7
−6
−5
Unidentified Metabolite 23

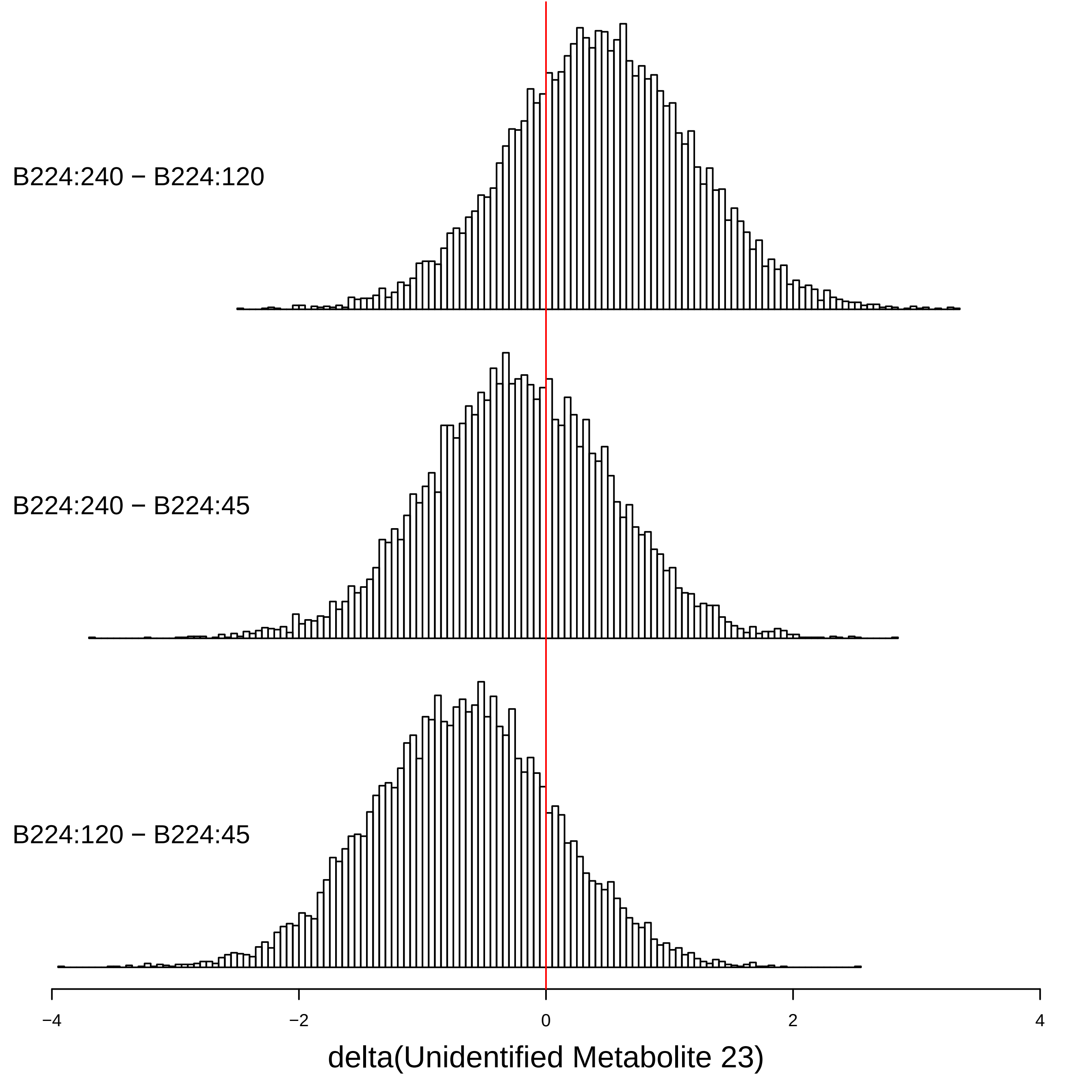

B224:240 − B224:120
B224:240 − B224:45
B224:120 − B224:45
−4
−2
0
2
4
delta(Unidentified Metabolite 23)

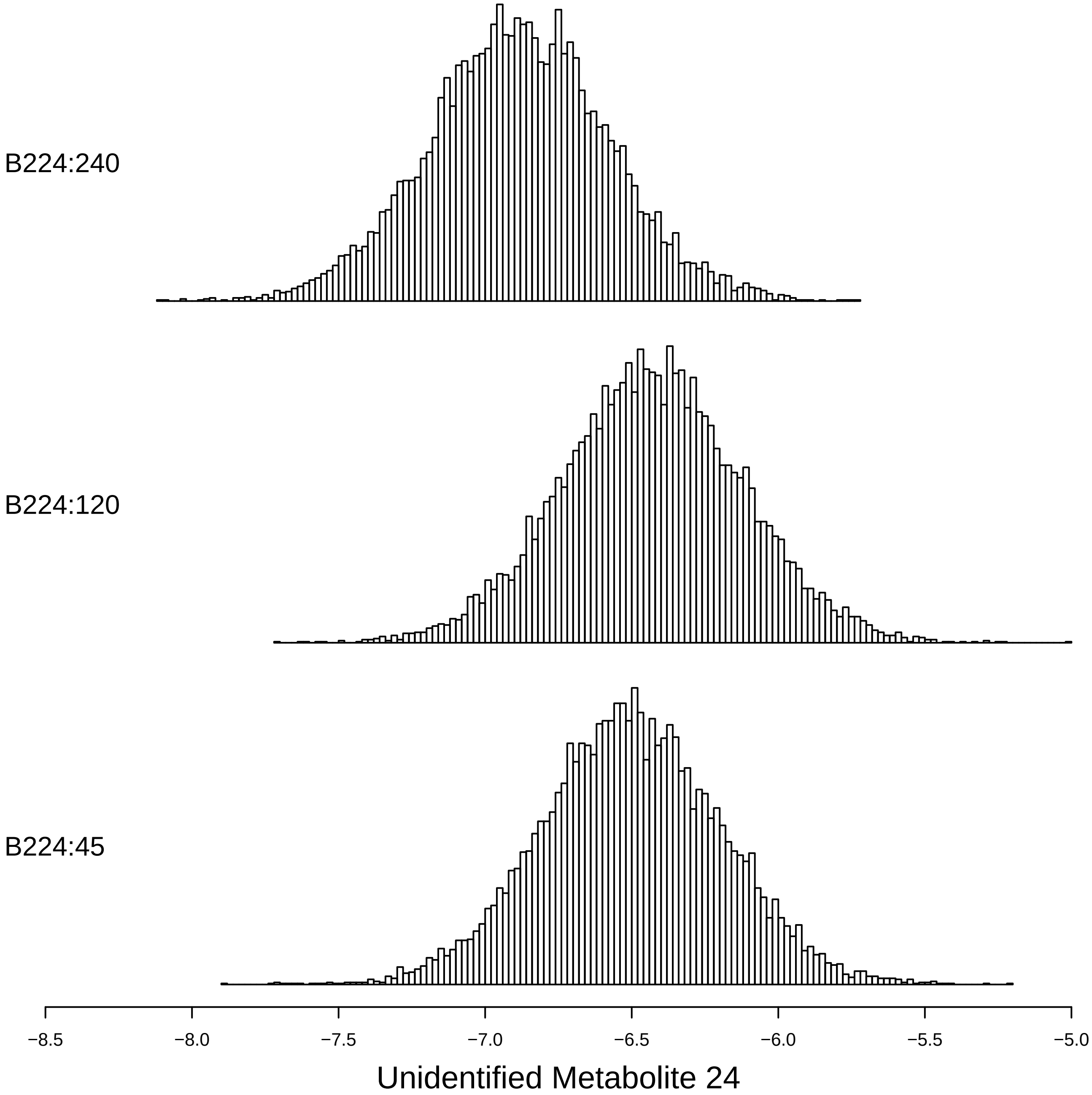
B224:240
B224:120
B224:45
−8.5
−8.0
−7.5
−7.0
−6.5
−6.0
−5.5
−5.0
Unidentified Metabolite 24

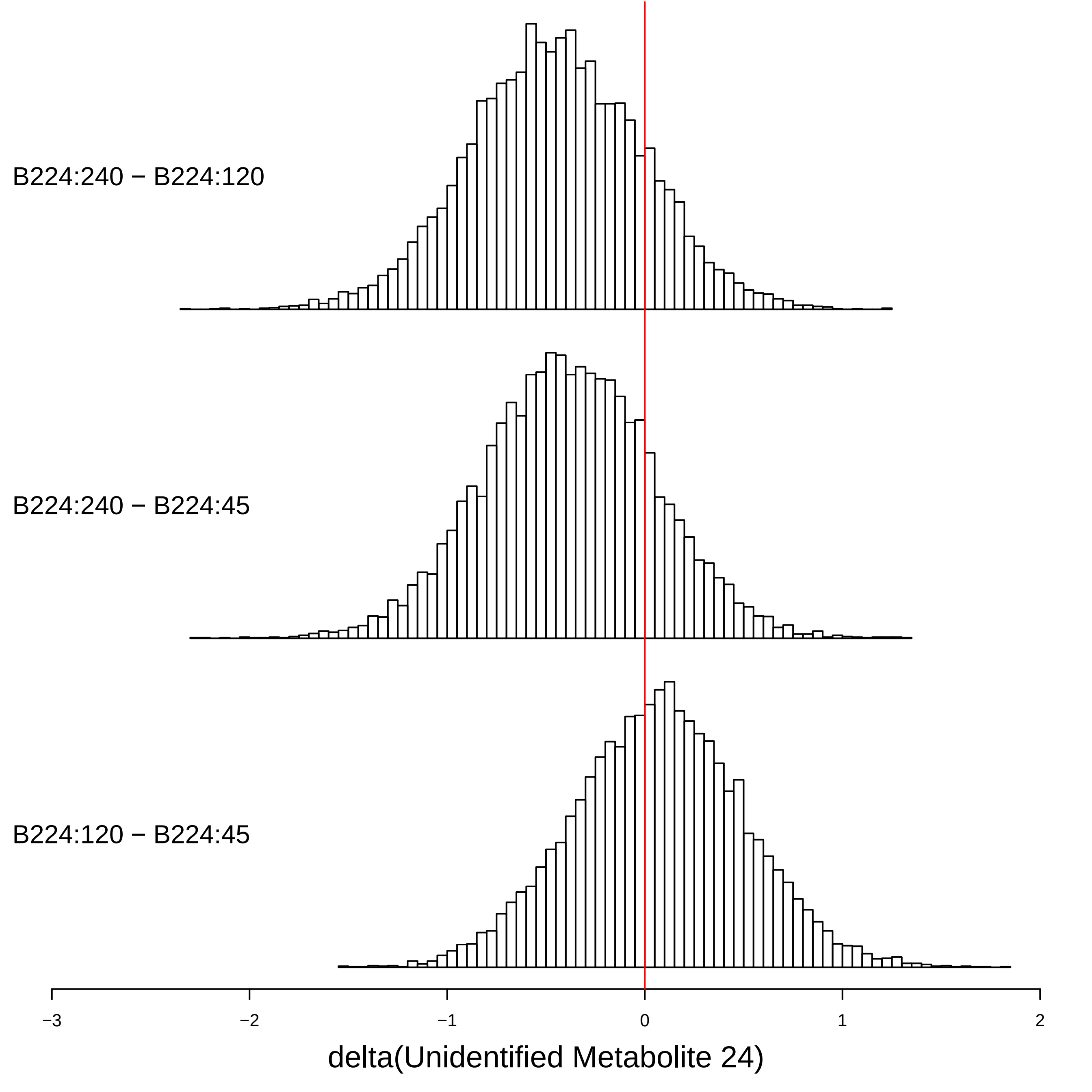

B224:240 − B224:120
B224:240 − B224:45
B224:120 − B224:45
−3
−2
−1
0
1
2
delta(Unidentified Metabolite 24)

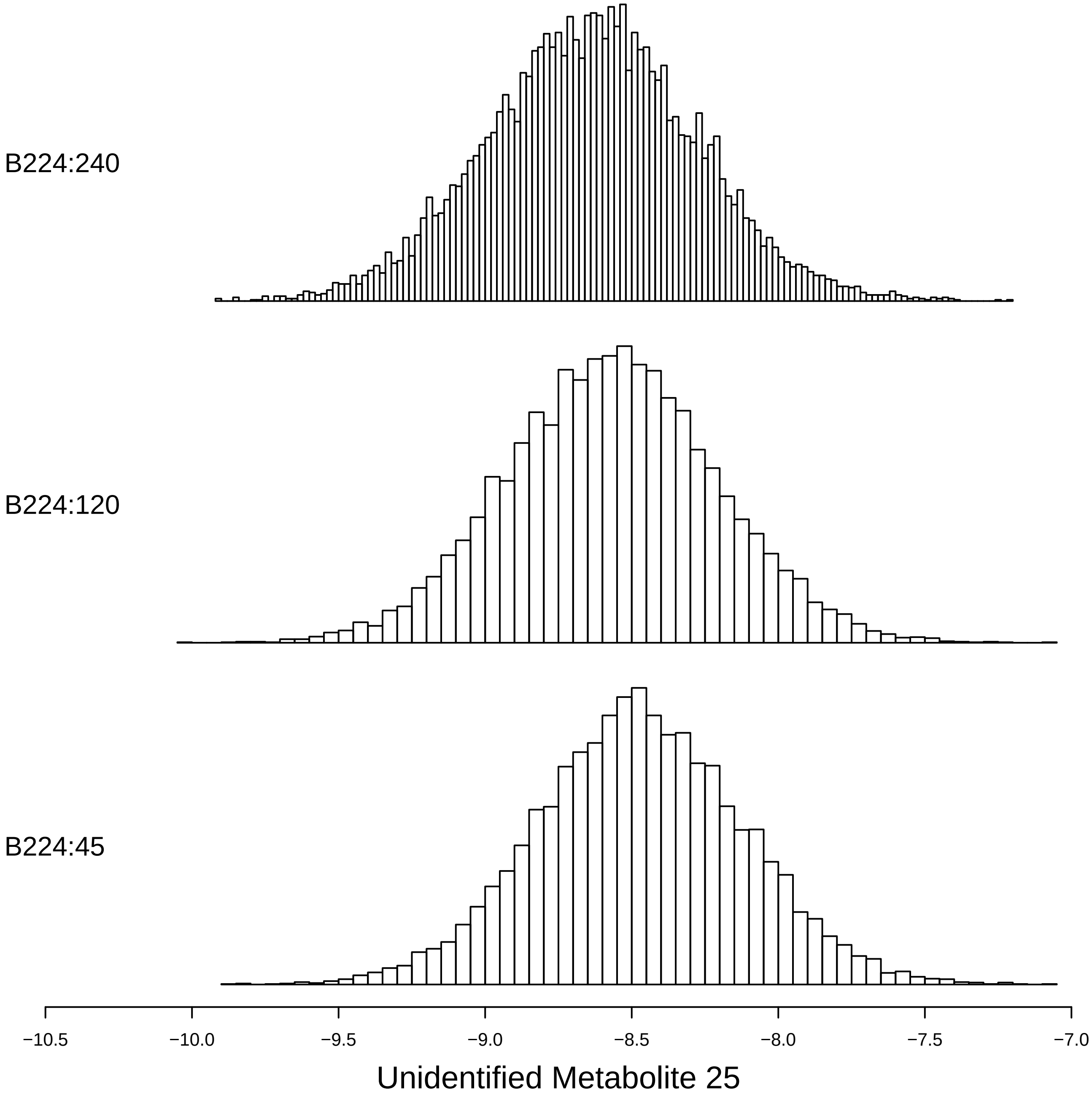
B224:240
B224:120
B224:45
−10.5
−10.0
−9.5
−9.0
−8.5
−8.0
−7.5
−7.0
Unidentified Metabolite 25

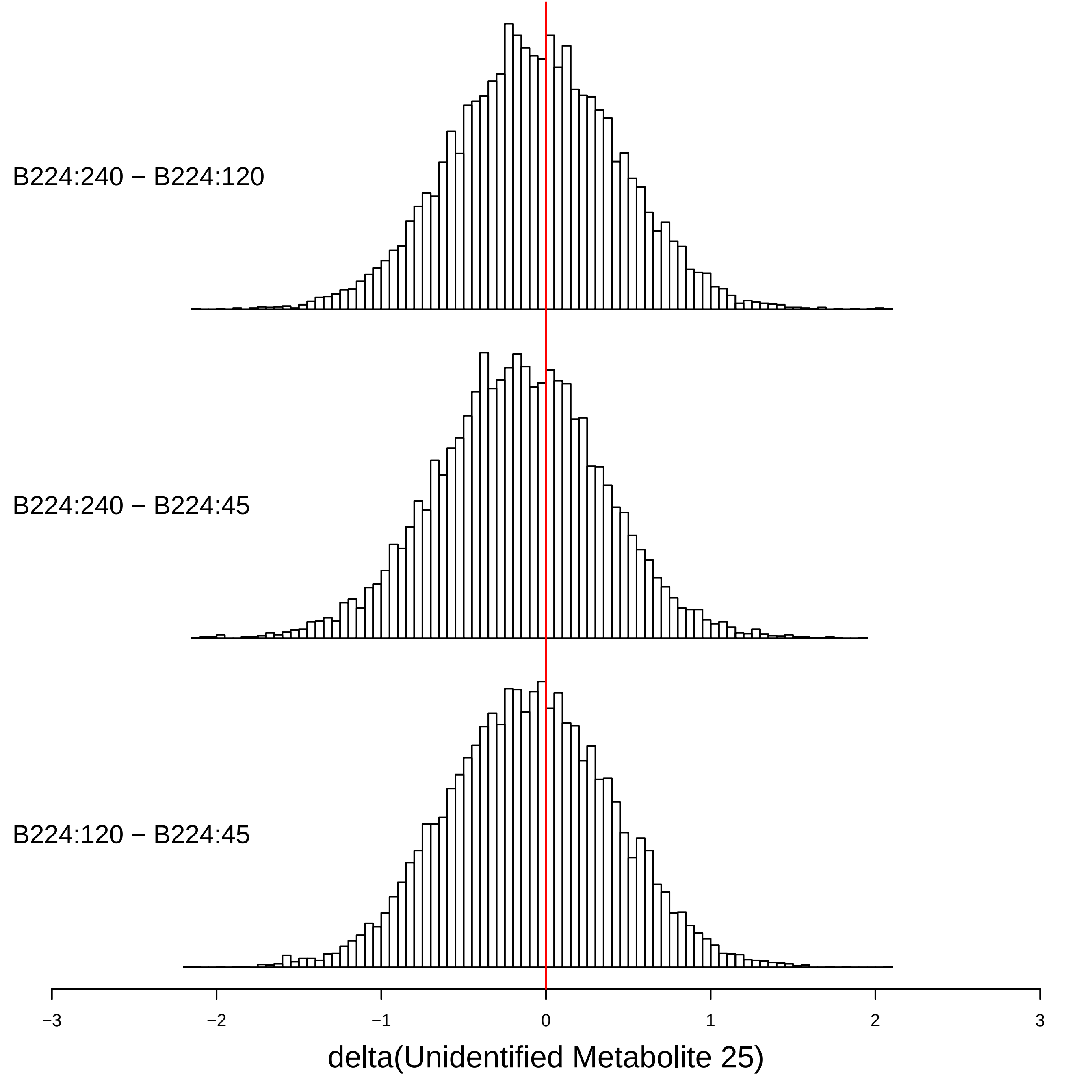

B224:240 − B224:120
B224:240 − B224:45
B224:120 − B224:45
−3
−2
−1
0
1
2
3
delta(Unidentified Metabolite 25)

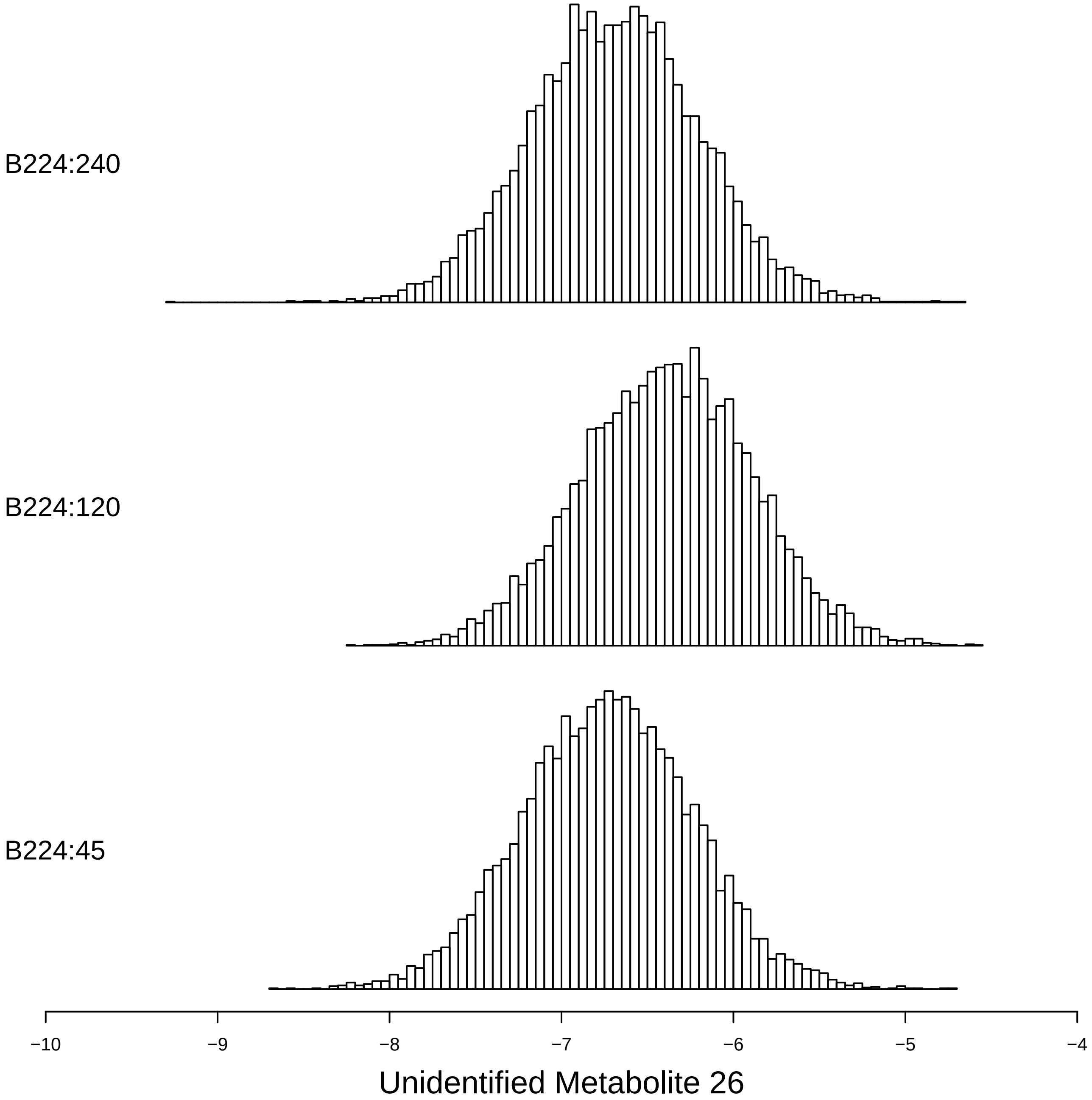
B224:240
B224:120
B224:45
−10
−9
−8
−7
−6
−5
−4
Unidentified Metabolite 26

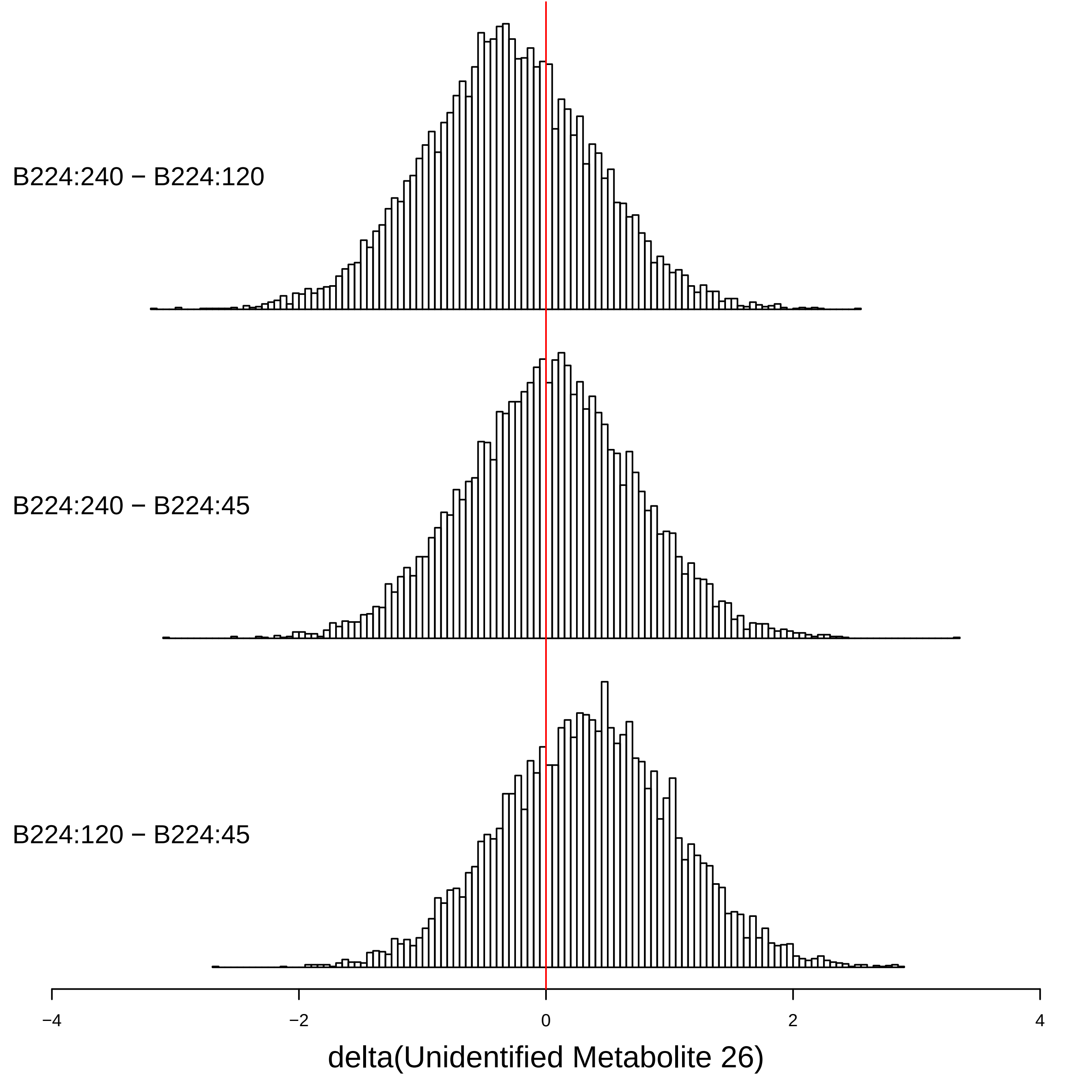

B224:240 − B224:120
B224:240 − B224:45
B224:120 − B224:45
−4
−2
0
2
4
delta(Unidentified Metabolite 26)

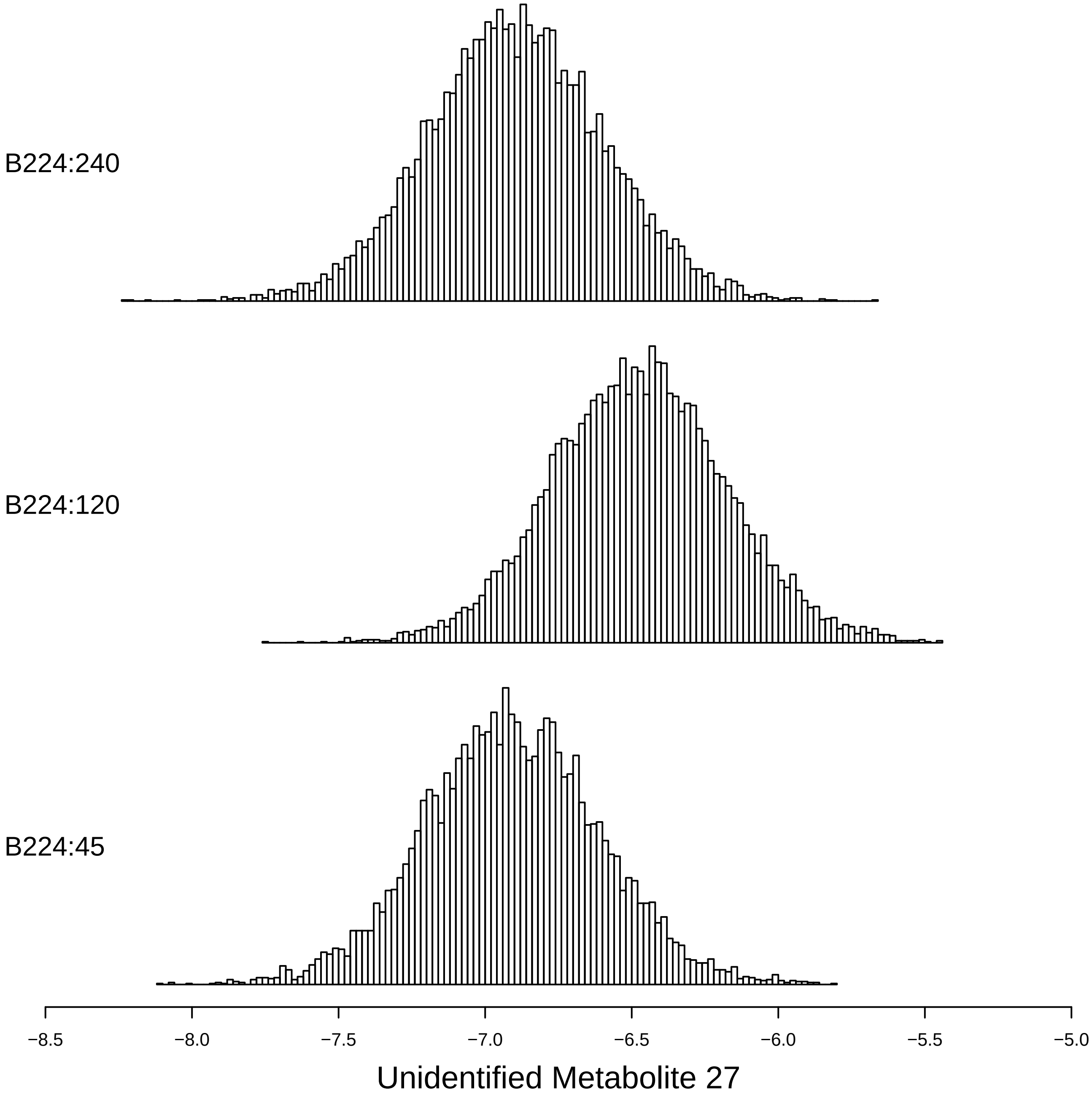

B224:240
B224:120
B224:45
−8.5
−8.0
−7.5
−7.0
−6.5
−6.0
−5.5
−5.0
Unidentified Metabolite 27

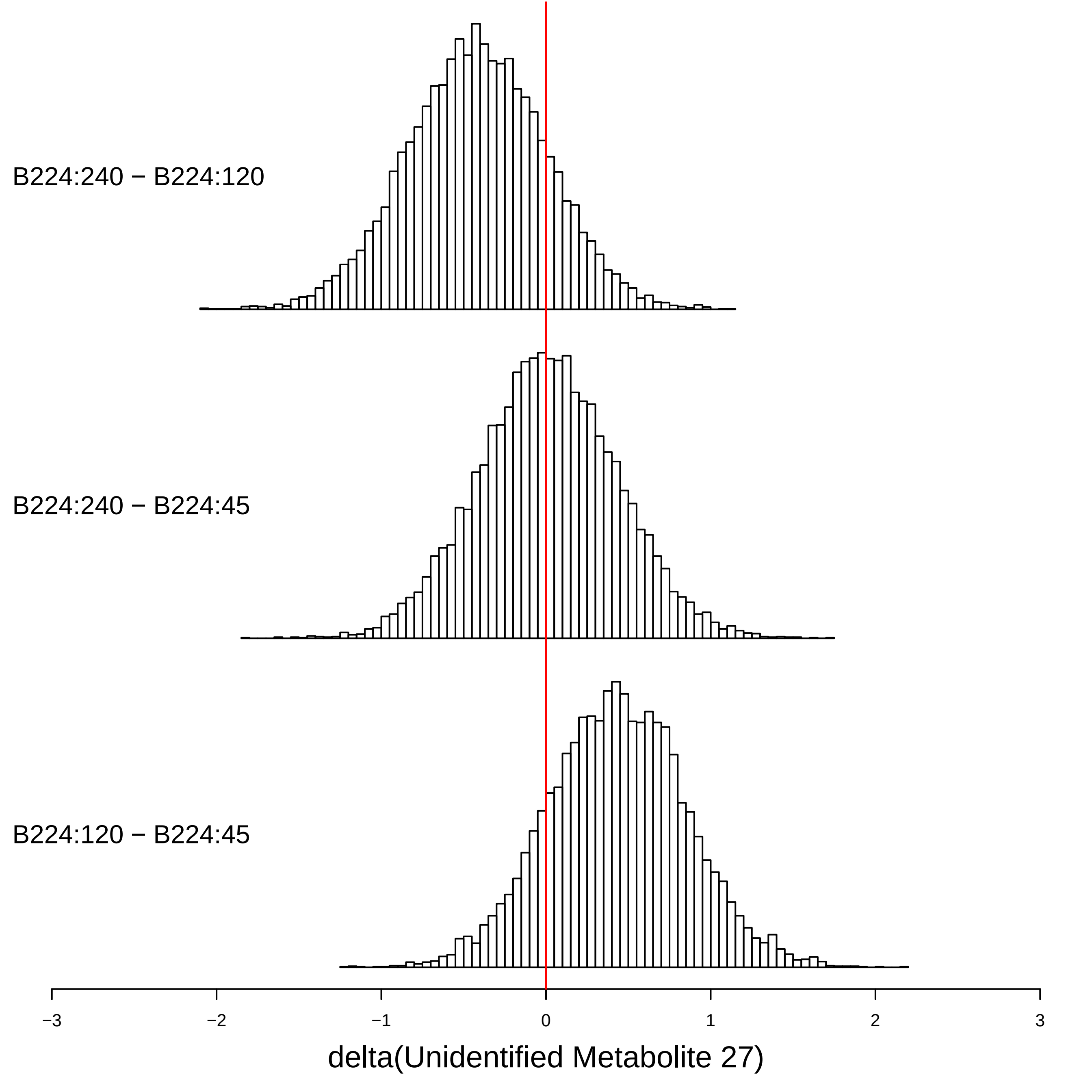
B224:240 − B224:120
B224:240 − B224:45
B224:120 − B224:45
−3
−2
−1
0
1
2
3
delta(Unidentified Metabolite 27)

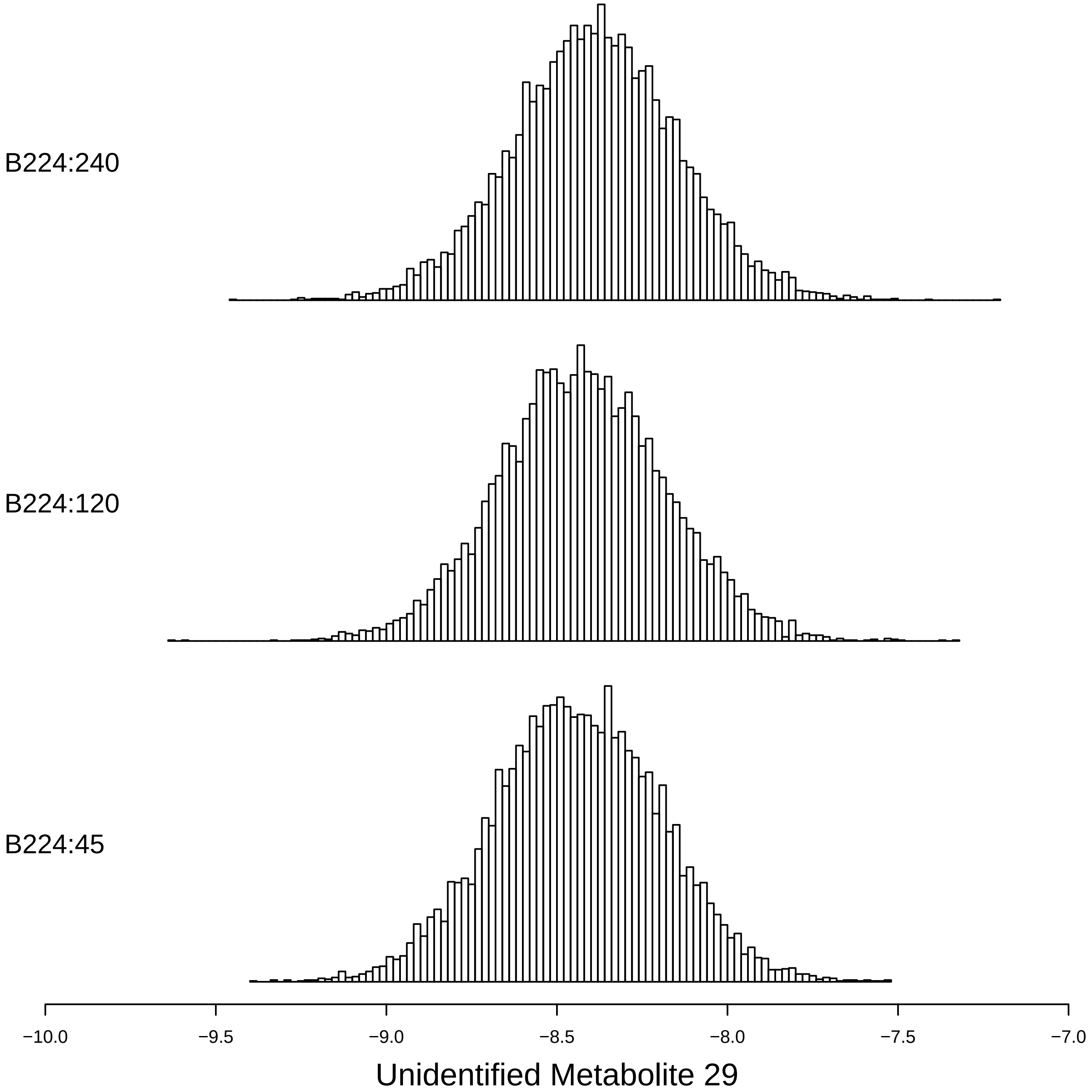

B224:240
B224:120
B224:45
−10.0
−9.5
−9.0
−8.5
−8.0
−7.5
−7.0
Unidentified Metabolite 29

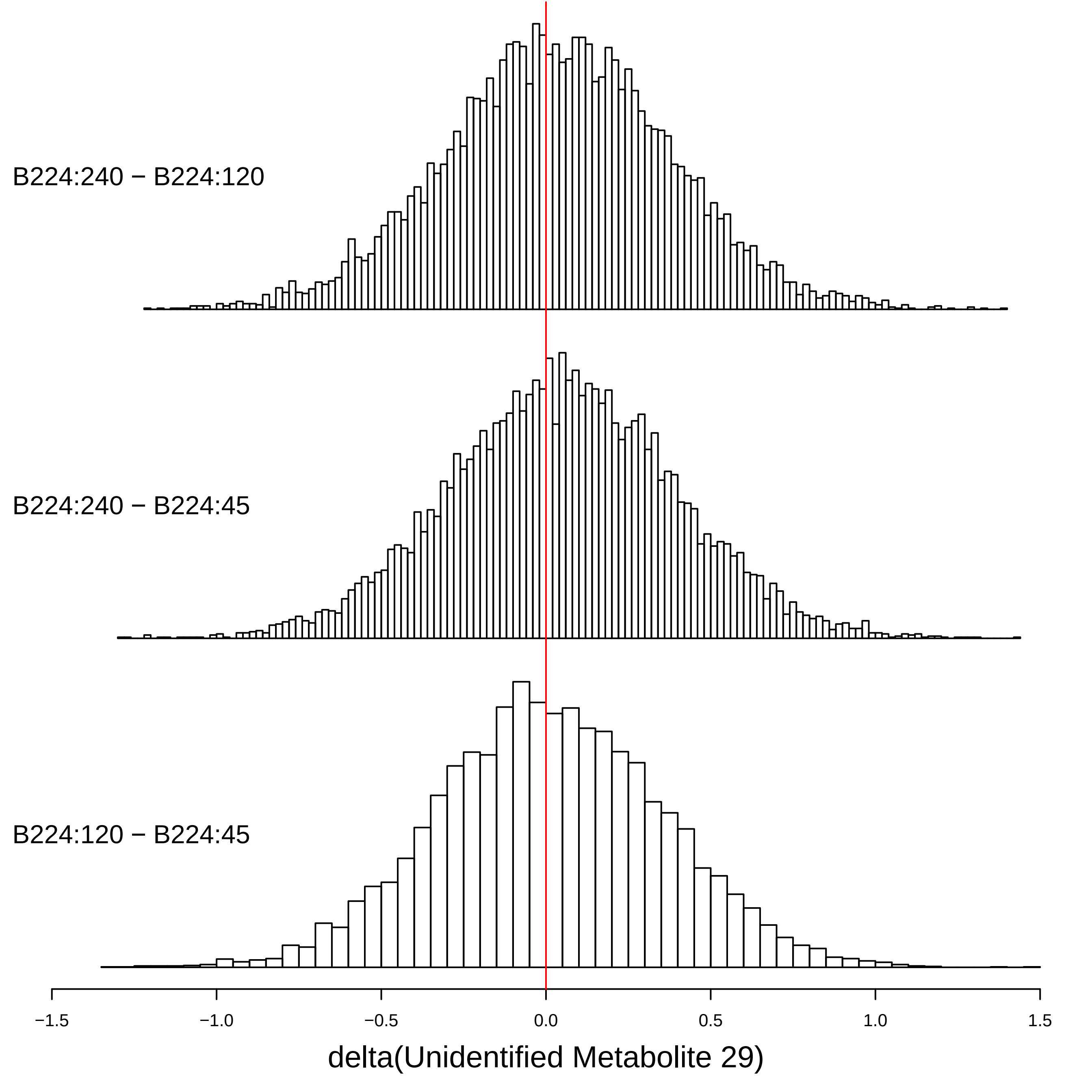
B224:240 − B224:120
B224:240 − B224:45
B224:120 − B224:45
−1.5
−1.0
−0.5
0.0
0.5
1.0
1.5
delta(Unidentified Metabolite 29)

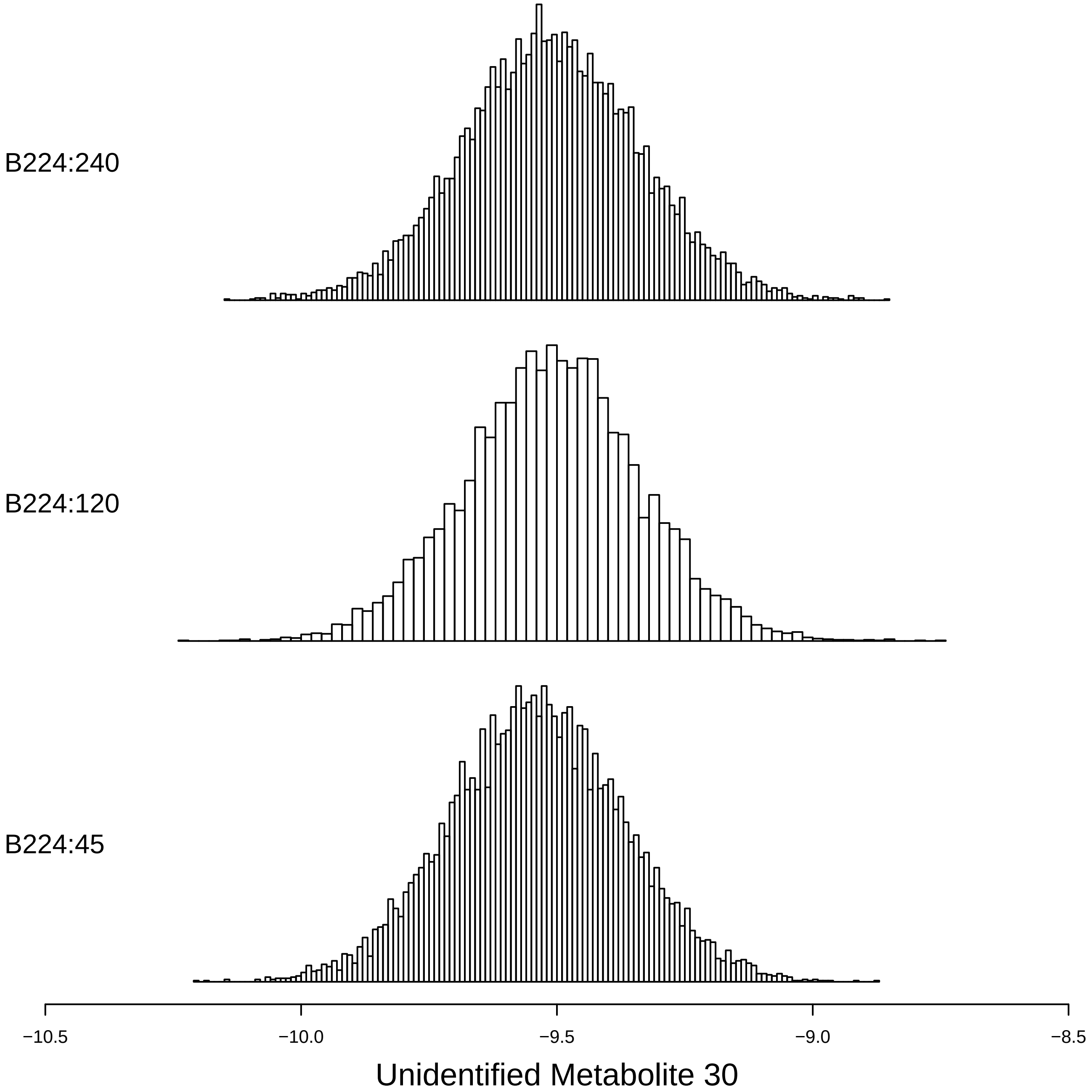
B224:240
B224:120
B224:45
−10.5
−10.0
−9.5
−9.0
−8.5
Unidentified Metabolite 30

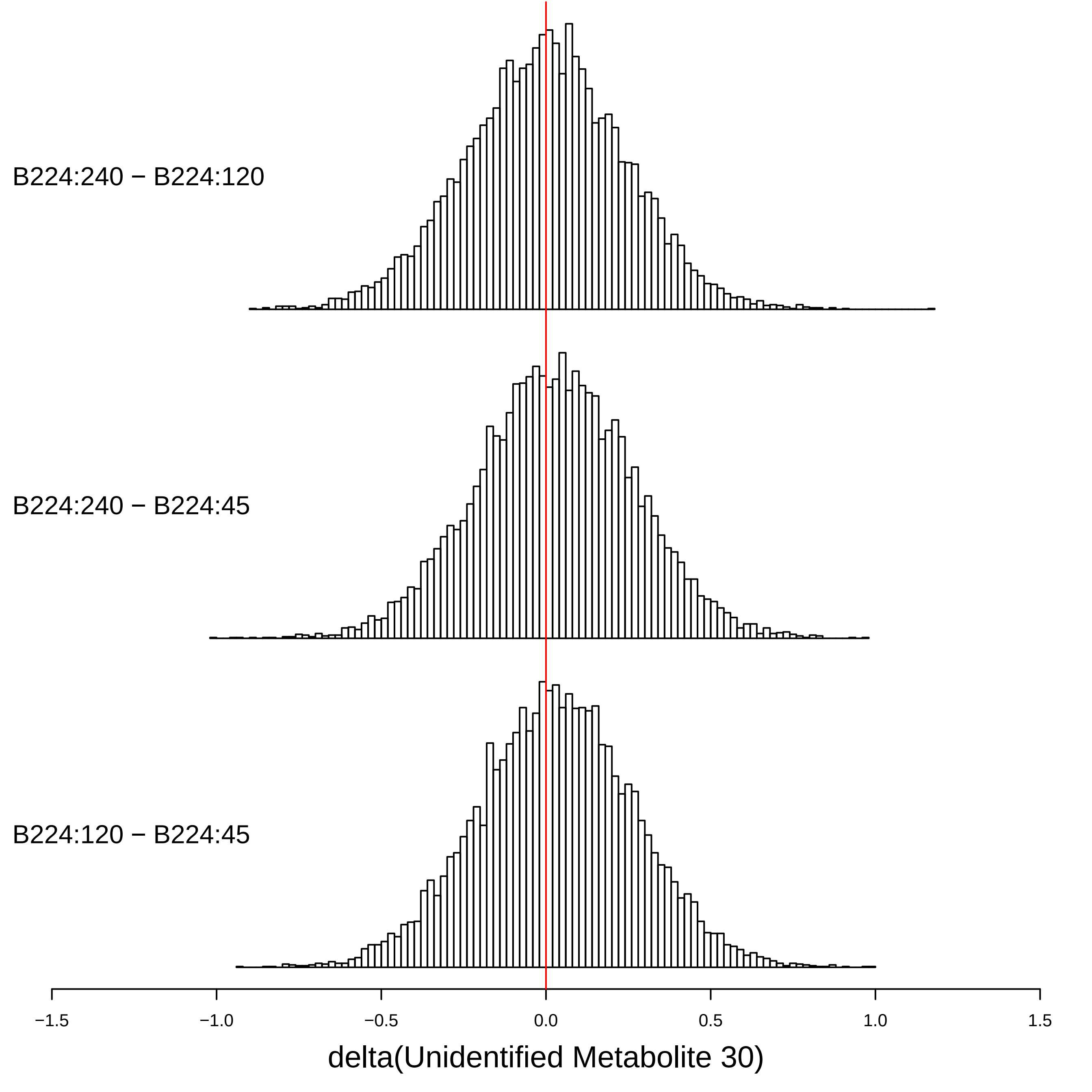
B224:240 − B224:120
B224:240 − B224:45
B224:120 − B224:45
−1.5
−1.0
−0.5
0.0
0.5
1.0
1.5
delta(Unidentified Metabolite 30)

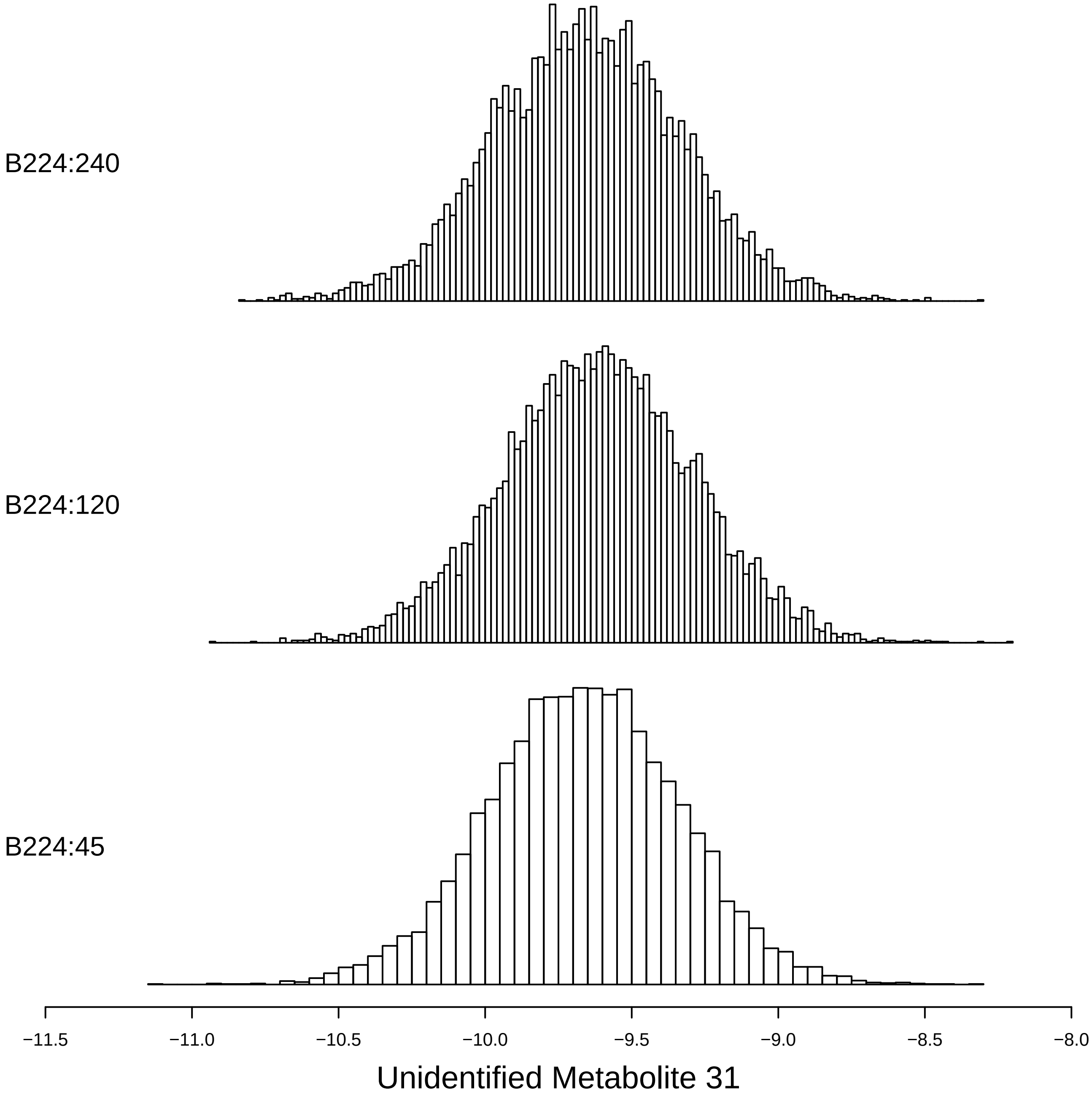
B224:240
B224:120
B224:45
−11.5
−11.0
−10.5
−10.0
−9.5
−9.0
−8.5
−8.0
Unidentified Metabolite 31

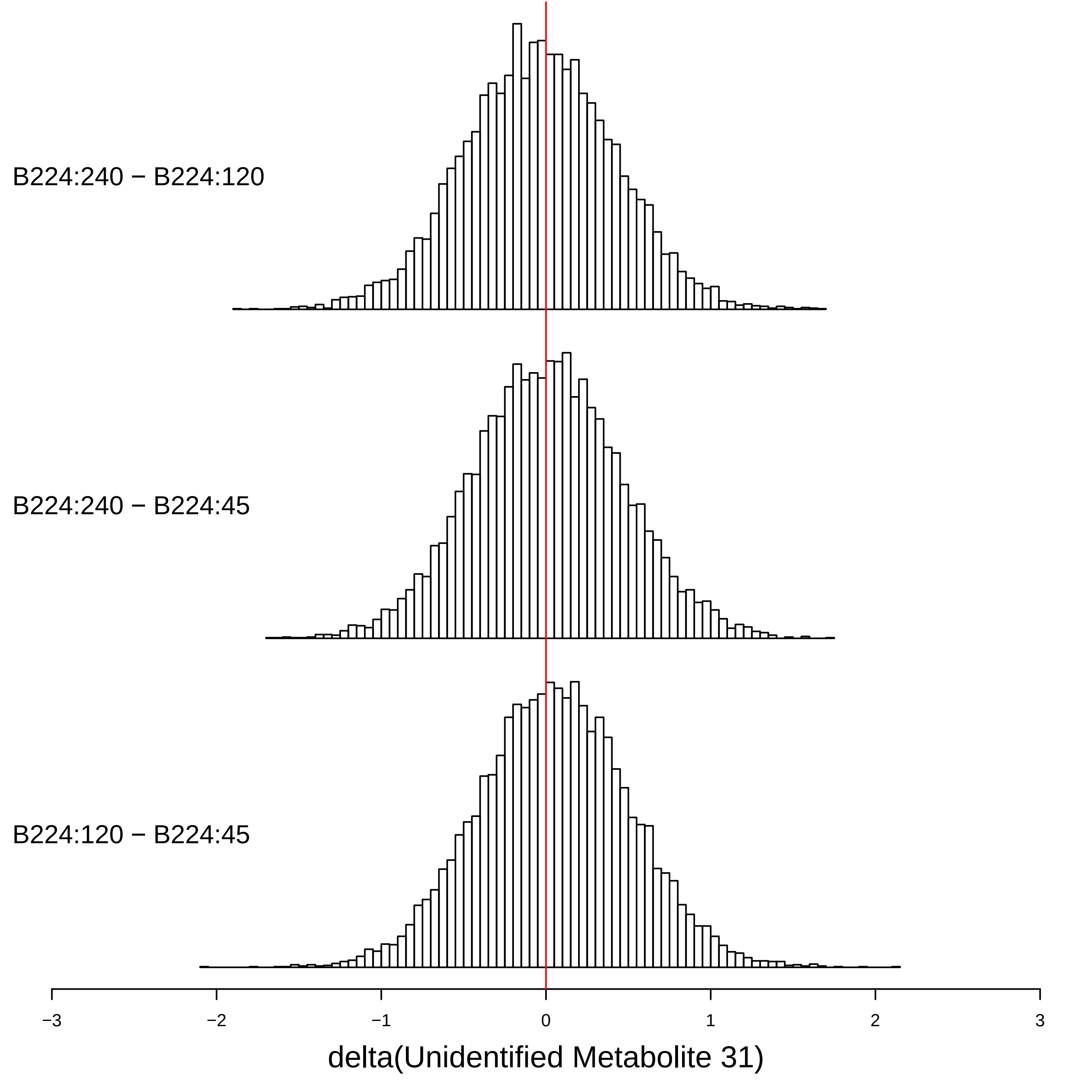

B224:240 − B224:120
B224:240 − B224:45
B224:120 − B224:45
−3
−2
−1
0
1
2
3
delta(Unidentified Metabolite 31)

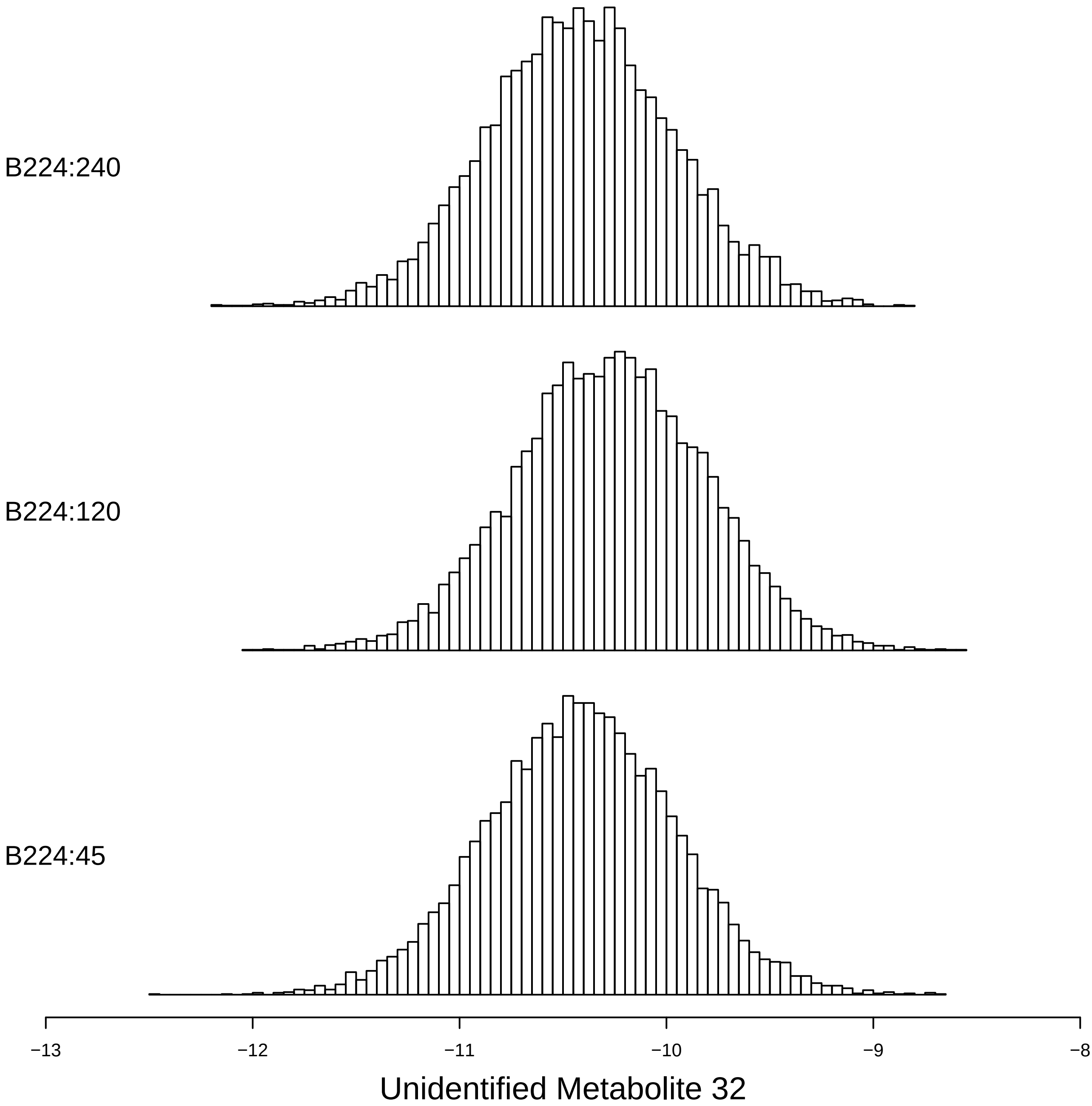
B224:240
B224:120
B224:45
−13
−12
−11
−10
−9
−8
Unidentified Metabolite 32

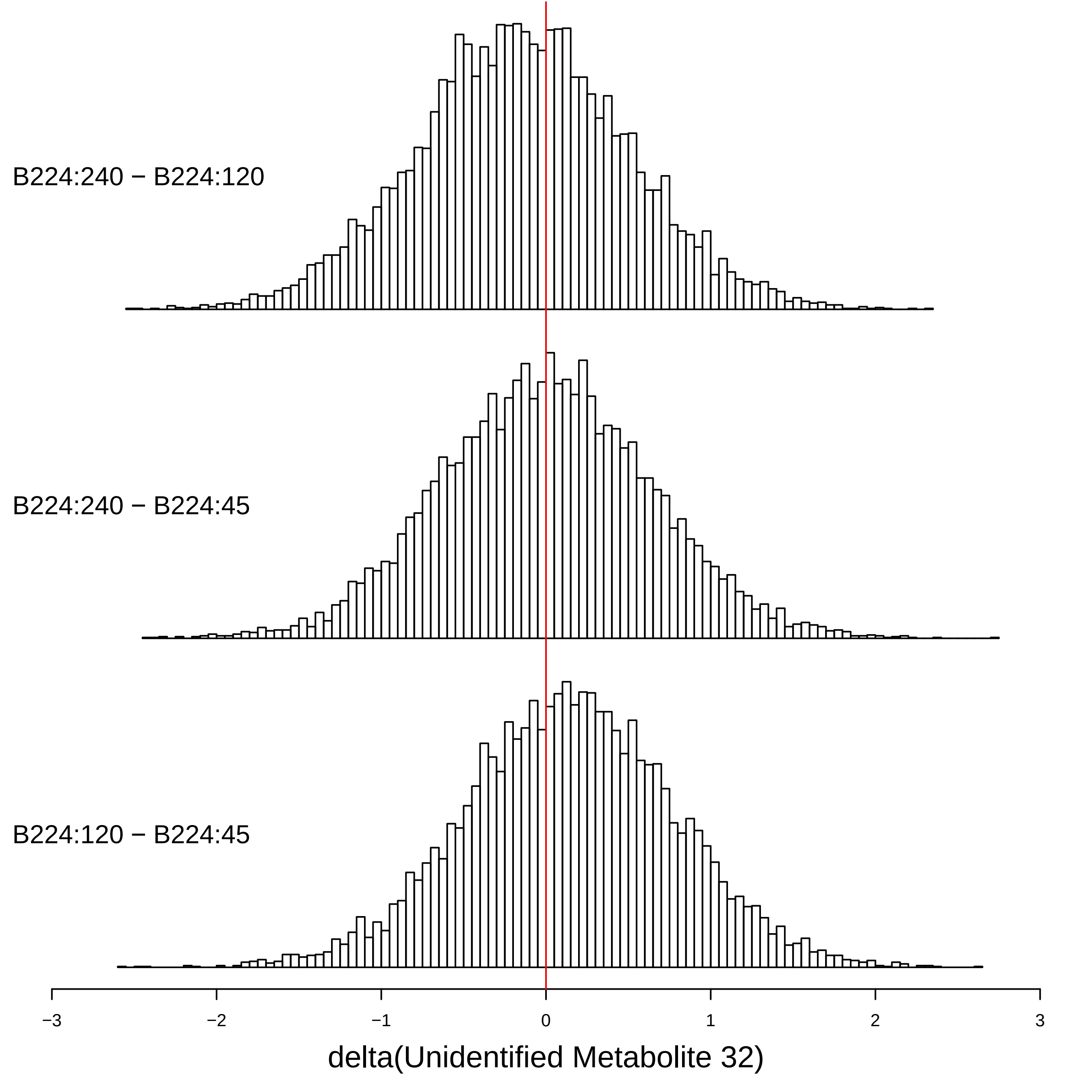

B224:240 − B224:120
B224:240 − B224:45
B224:120 − B224:45
−3
−2
−1
0
1
2
3
delta(Unidentified Metabolite 32)

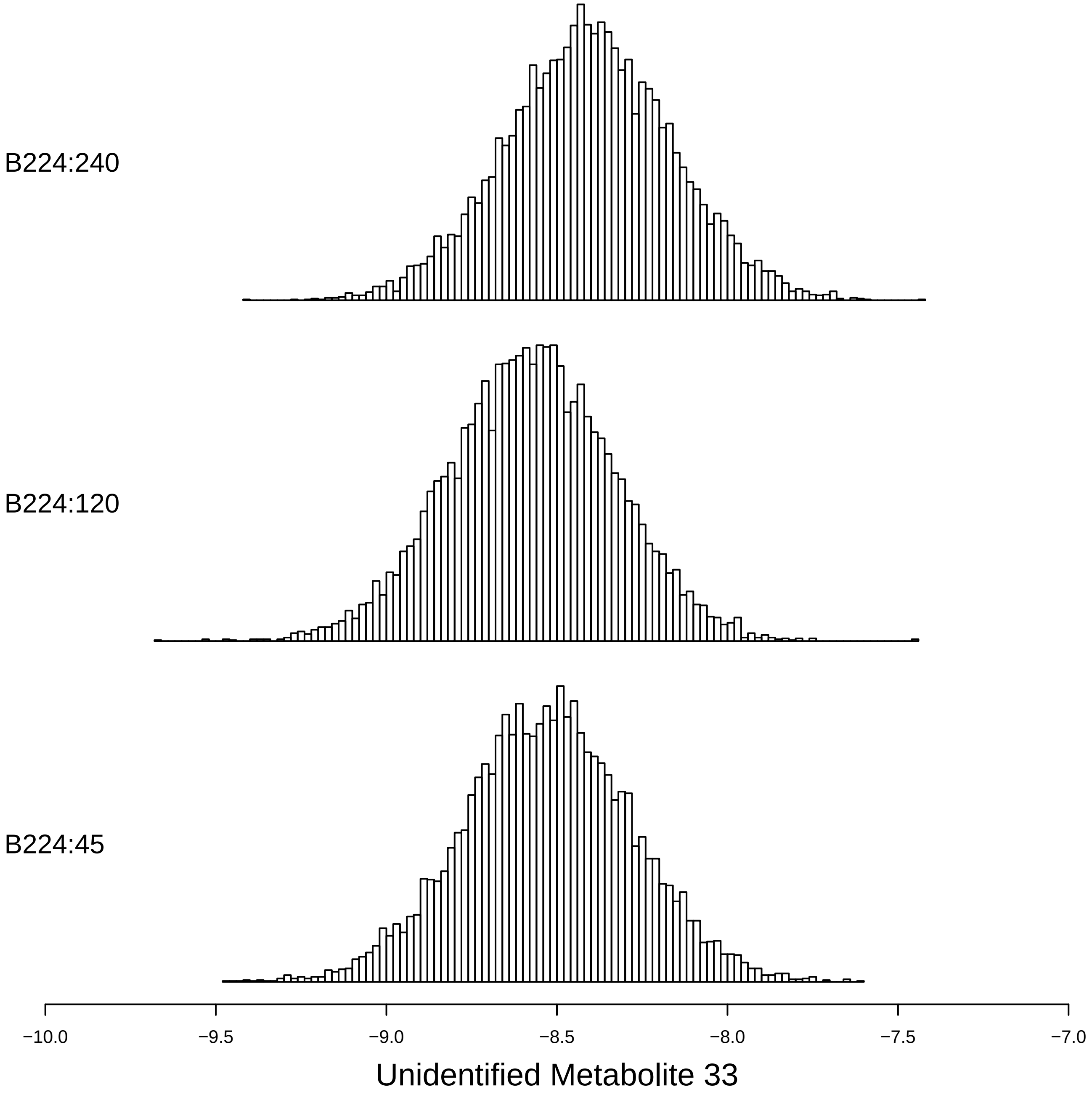
B224:240
B224:120
B224:45
−10.0
−9.5
−9.0
−8.5
−8.0
−7.5
−7.0
Unidentified Metabolite 33

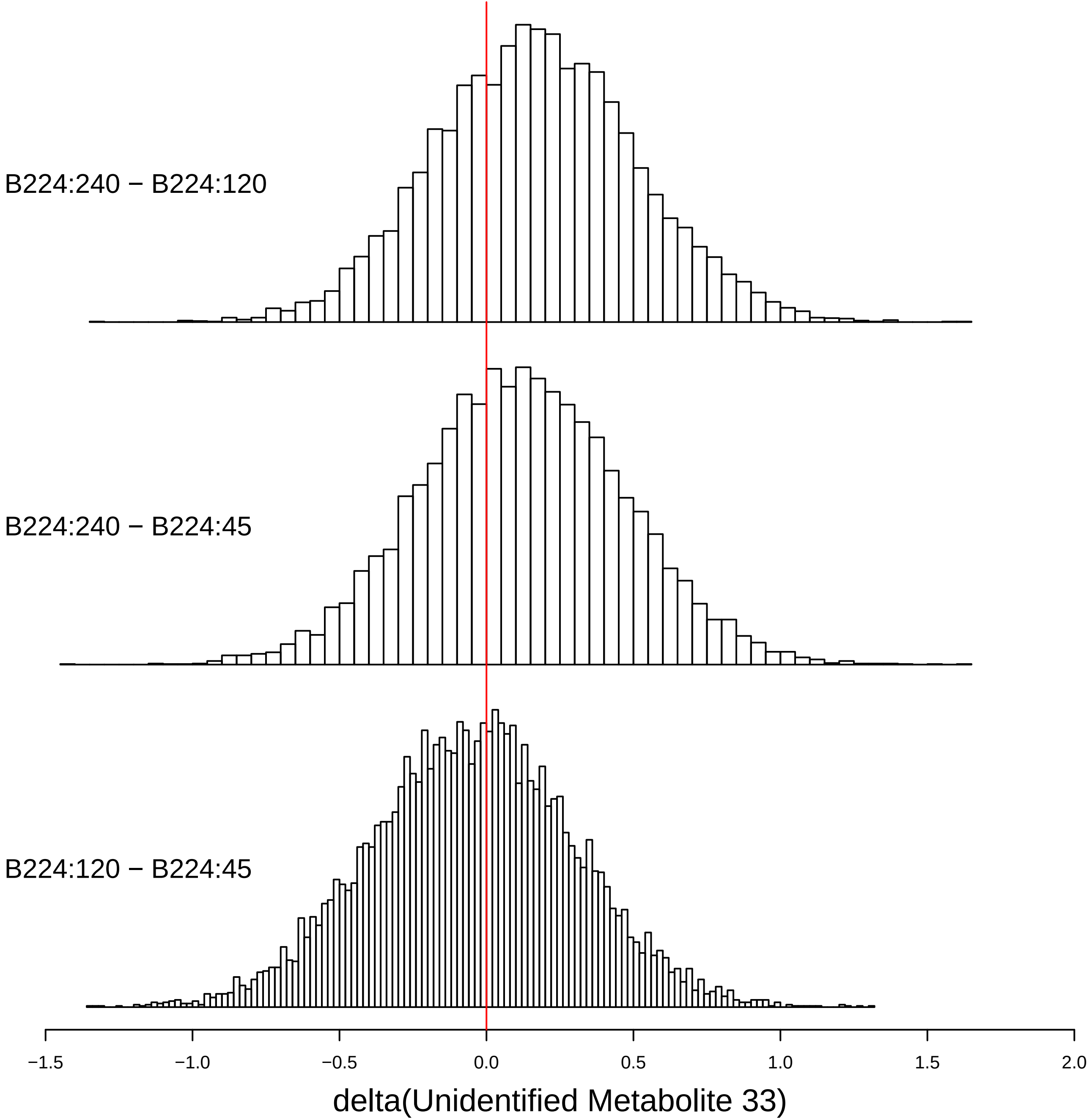

B224:240 − B224:120
B224:240 − B224:45
B224:120 − B224:45
−1.5
−1.0
−0.5
0.0
0.5
1.0
1.5
2.0
delta(Unidentified Metabolite 33)

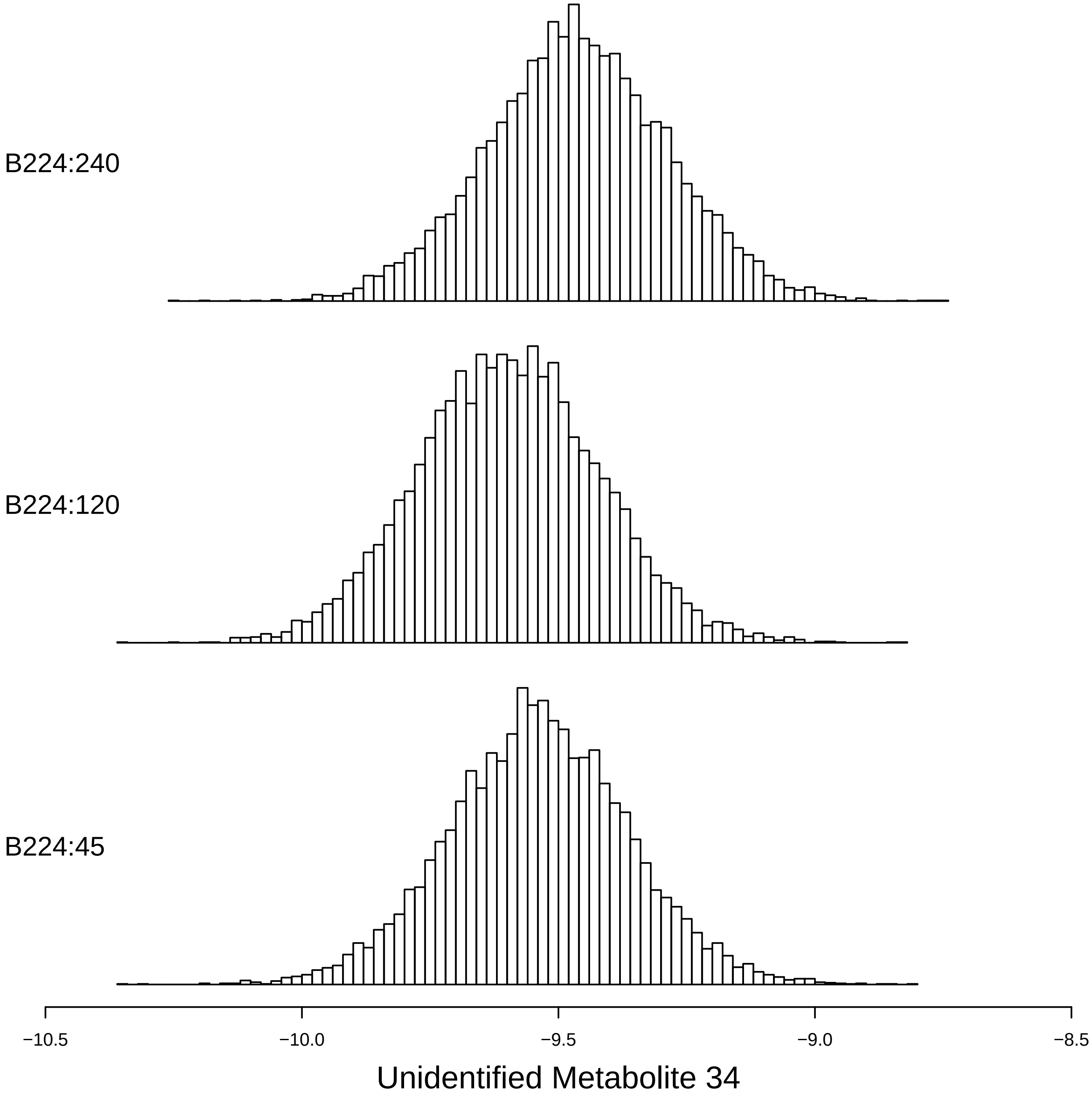

B224:240
B224:120
B224:45
−10.5
−10.0
−9.5
−9.0
−8.5
Unidentified Metabolite 34

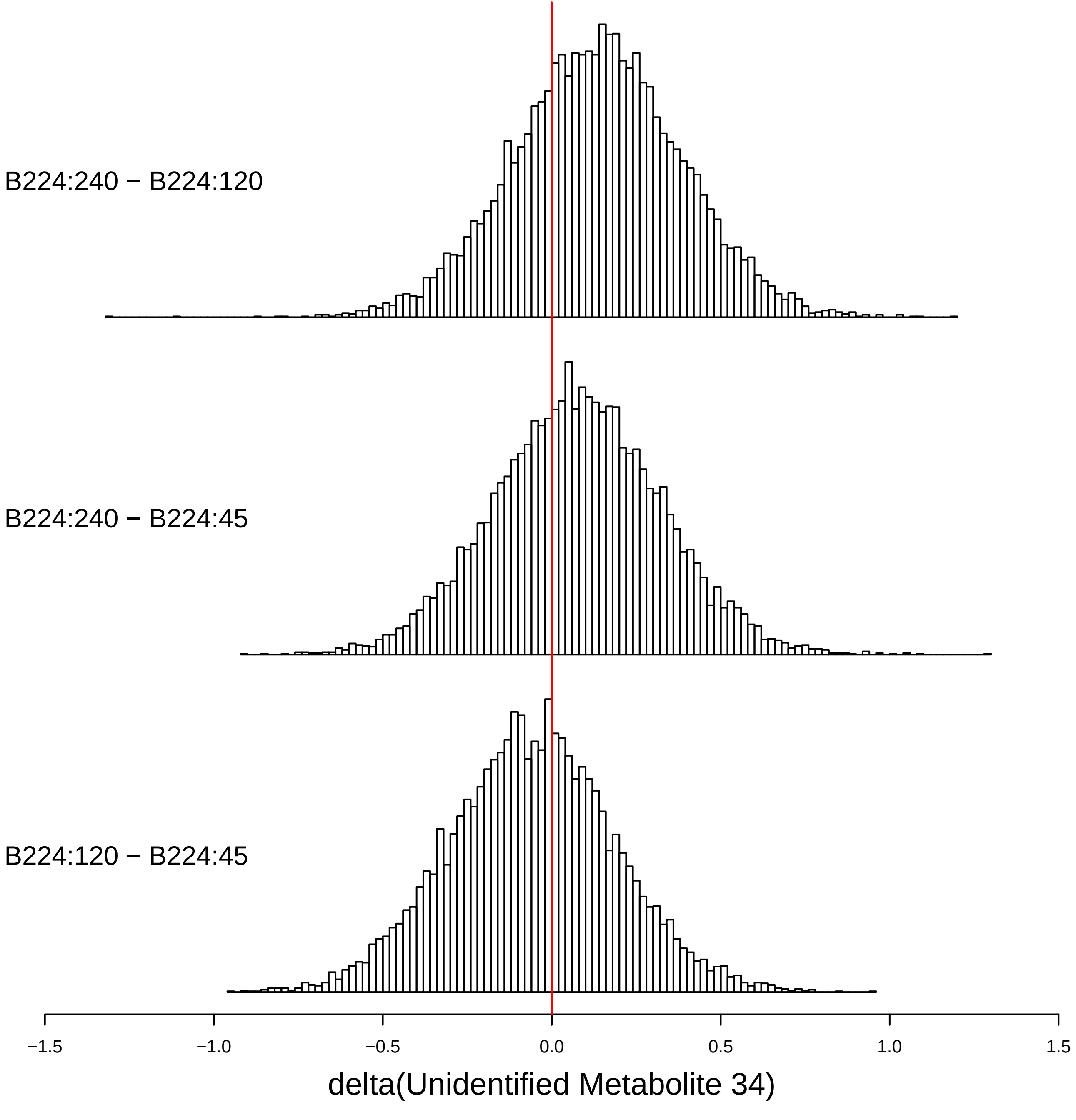
B224:240 − B224:120
B224:240 − B224:45
B224:120 − B224:45
−1.5
−1.0
−0.5
0.0
0.5
1.0
1.5
delta(Unidentified Metabolite 34)

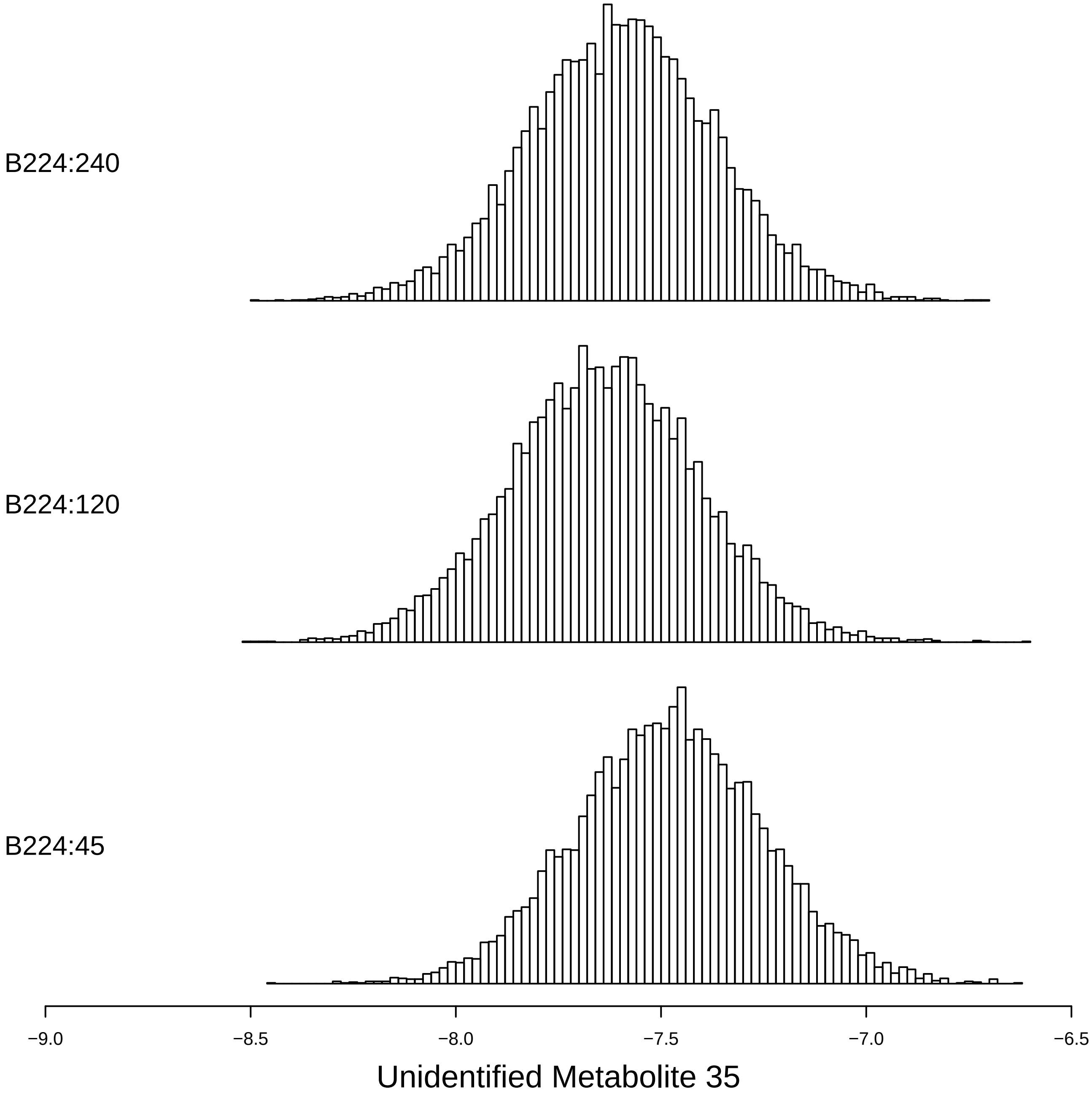

B224:240
B224:120
B224:45
−9.0
−8.5
−8.0
−7.5
−7.0
−6.5
Unidentified Metabolite 35

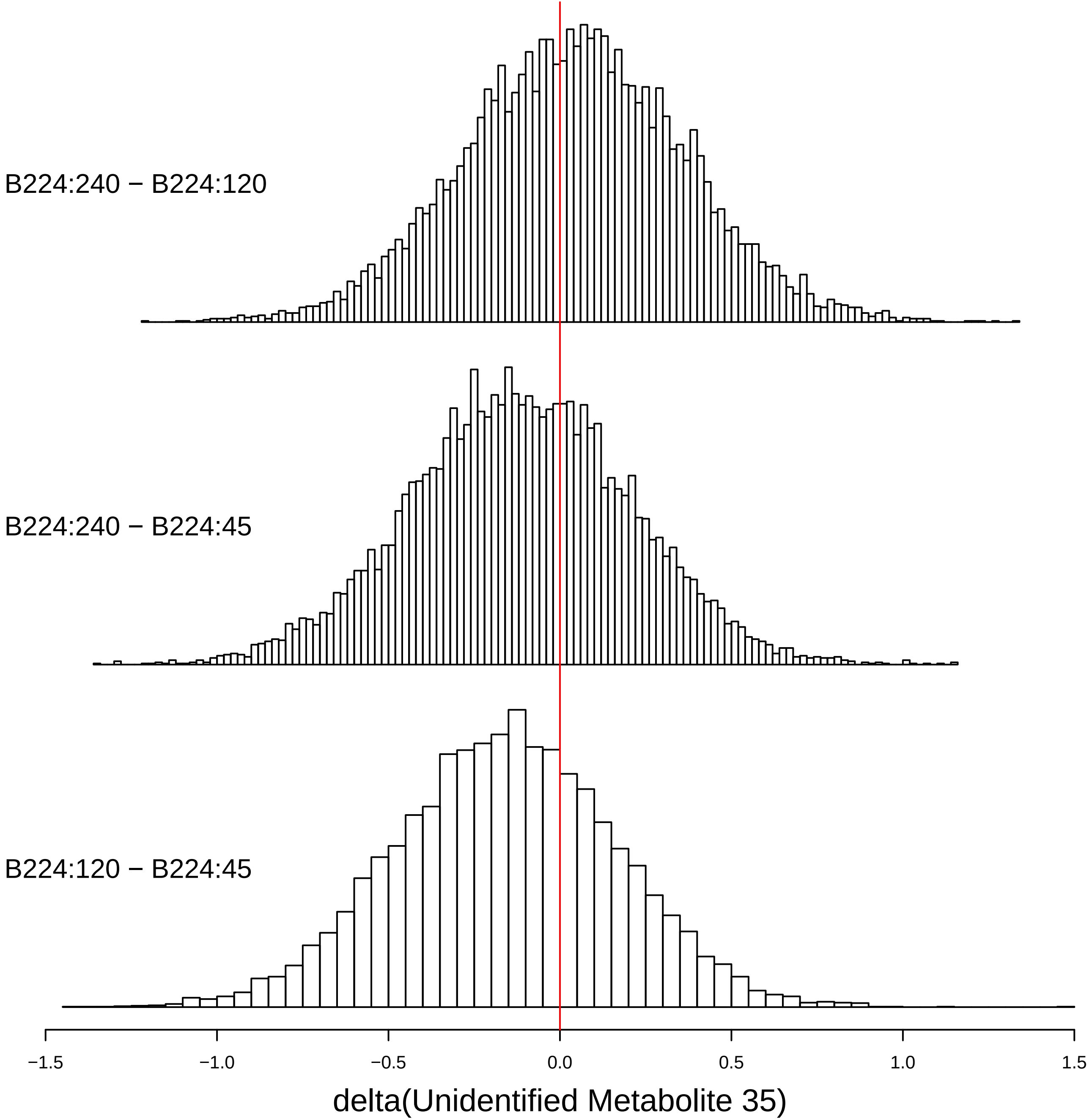

B224:240 − B224:120
B224:240 − B224:45
B224:120 − B224:45
−1.5
−1.0
−0.5
0.0
0.5
1.0
1.5
delta(Unidentified Metabolite 35)

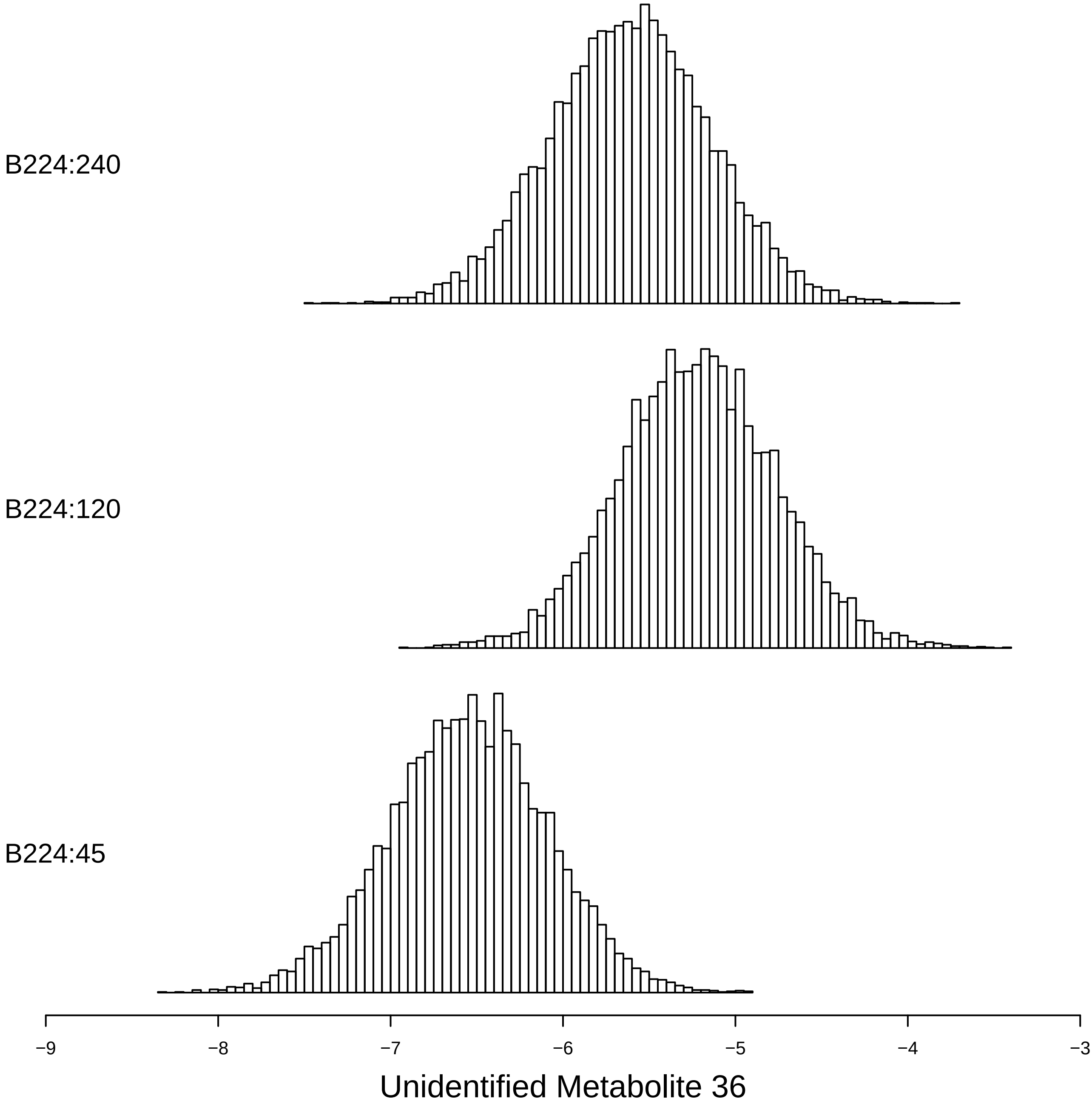
B224:240
B224:120
B224:45
−9
−8
−7
−6
−5
−4
−3
Unidentified Metabolite 36

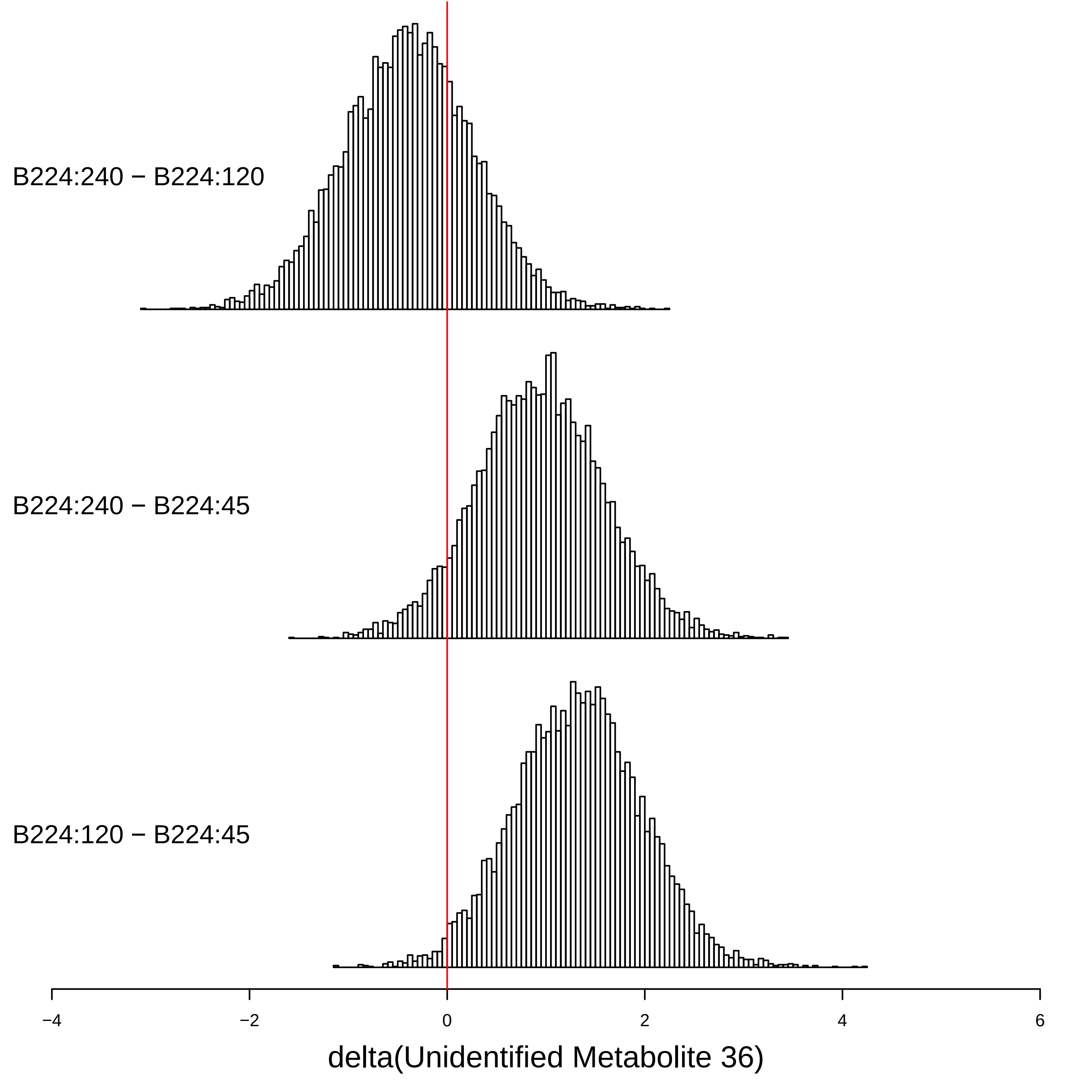

B224:240 − B224:120
B224:240 − B224:45
B224:120 − B224:45
−4
−2
0
2
4
6
delta(Unidentified Metabolite 36)

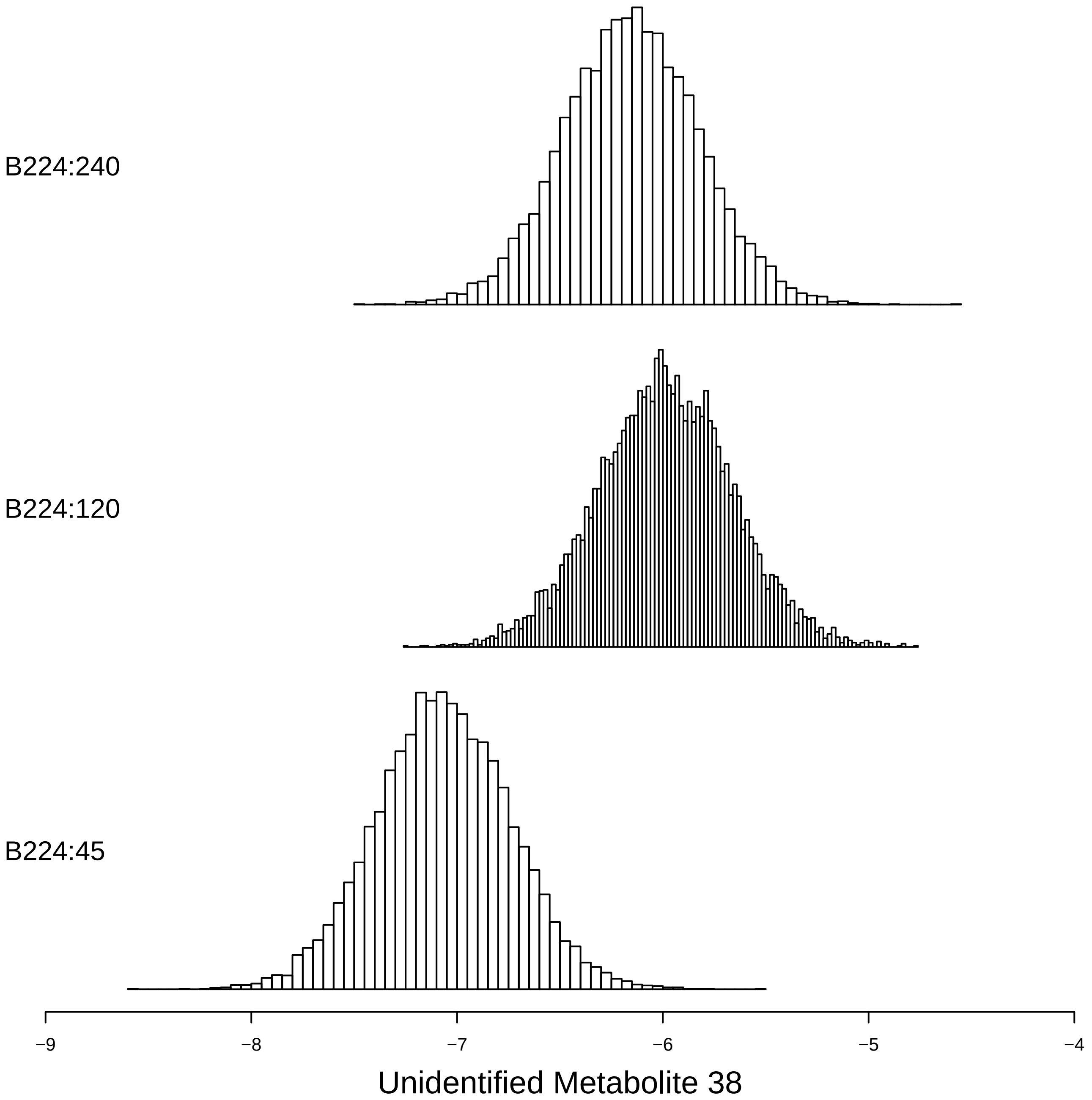
B224:240
B224:120
B224:45
−9
−8
−7
−6
−5
−4
Unidentified Metabolite 38

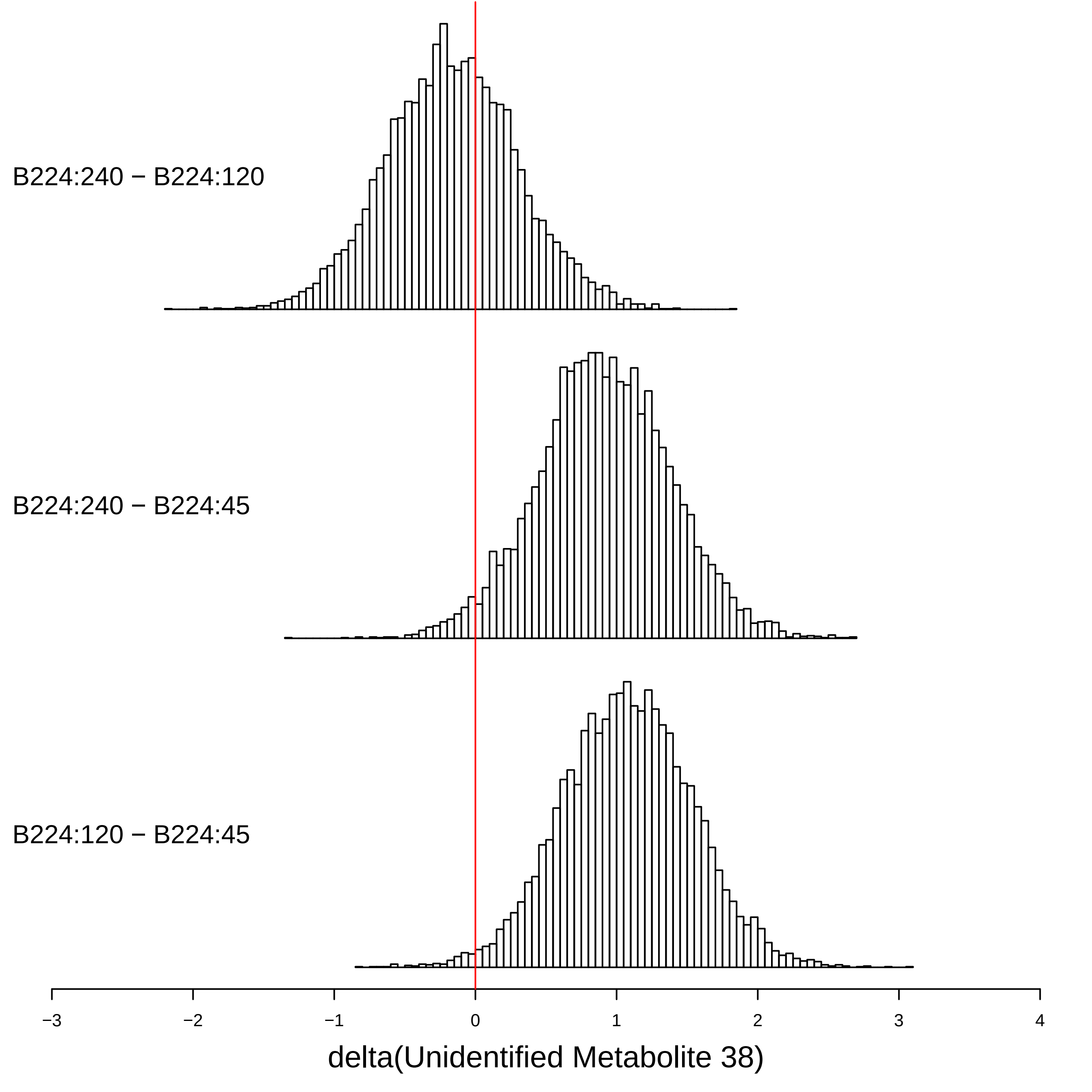
B224:240 − B224:120
B224:240 − B224:45
B224:120 − B224:45
−3
−2
−1
0
1
2
3
4
delta(Unidentified Metabolite 38)

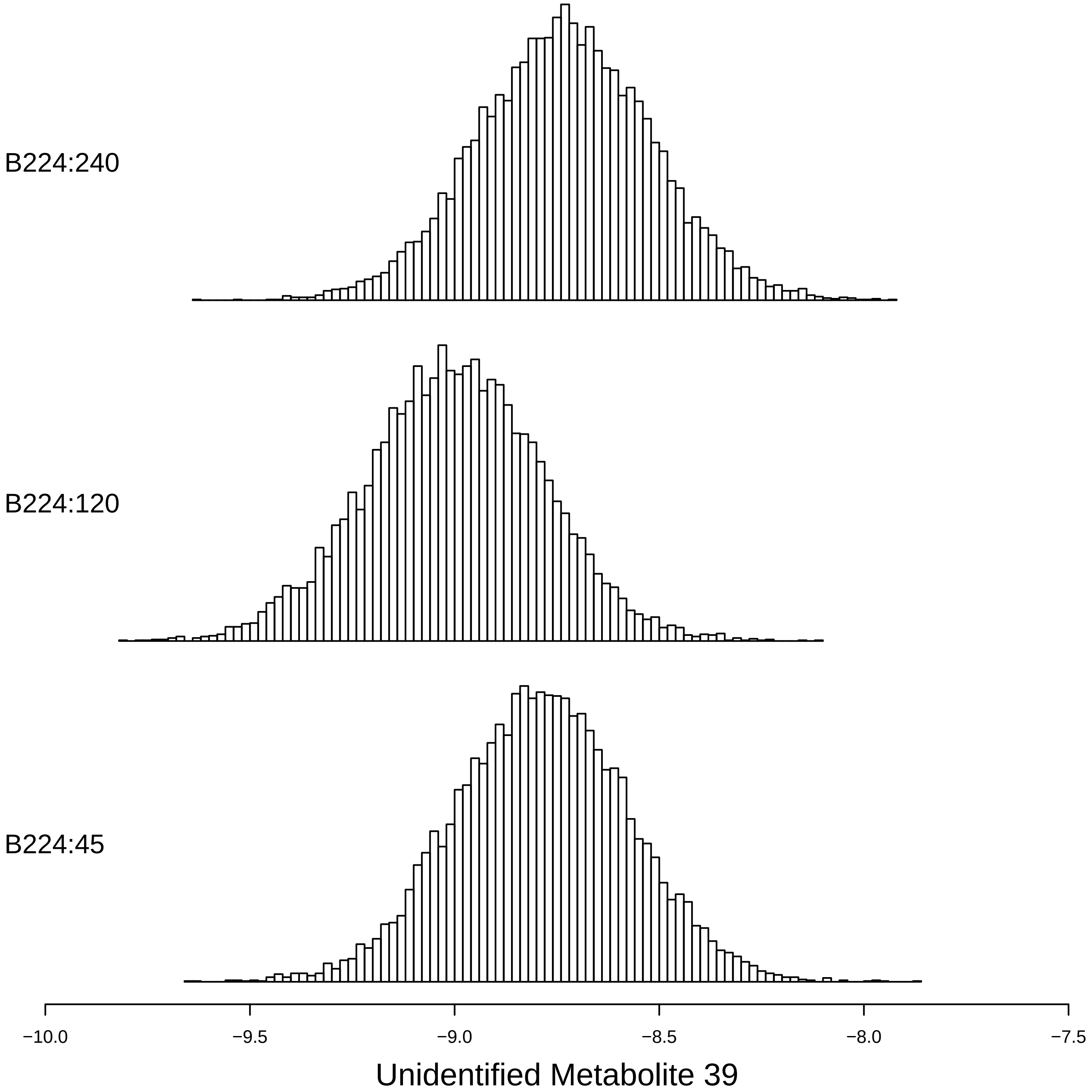
B224:240
B224:120
B224:45
−10.0
−9.5
−9.0
−8.5
−8.0
−7.5
Unidentified Metabolite 39

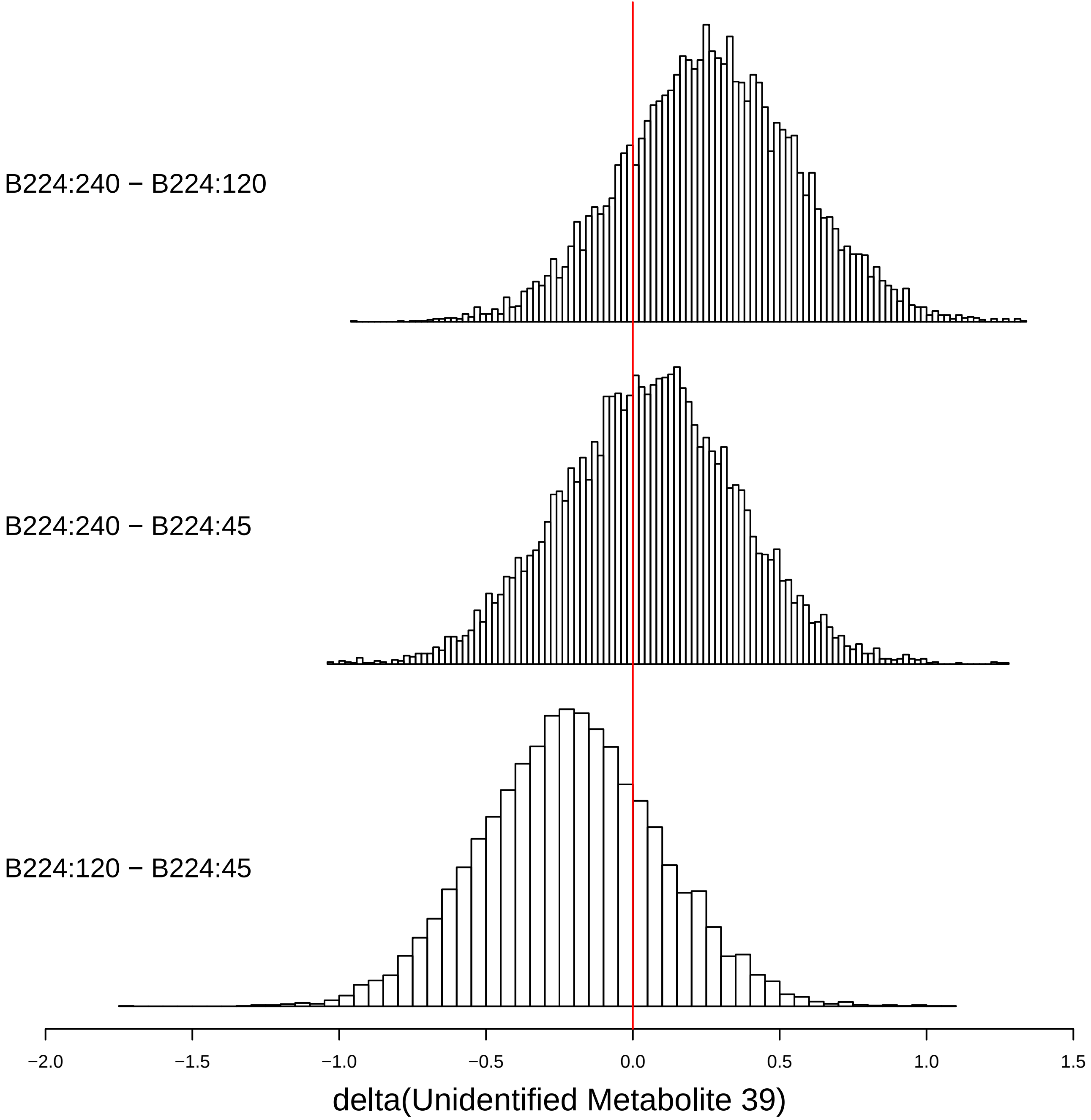

B224:240 − B224:120
B224:240 − B224:45
B224:120 − B224:45
−2.0
−1.5
−1.0
−0.5
0.0
0.5
1.0
1.5
delta(Unidentified Metabolite 39)

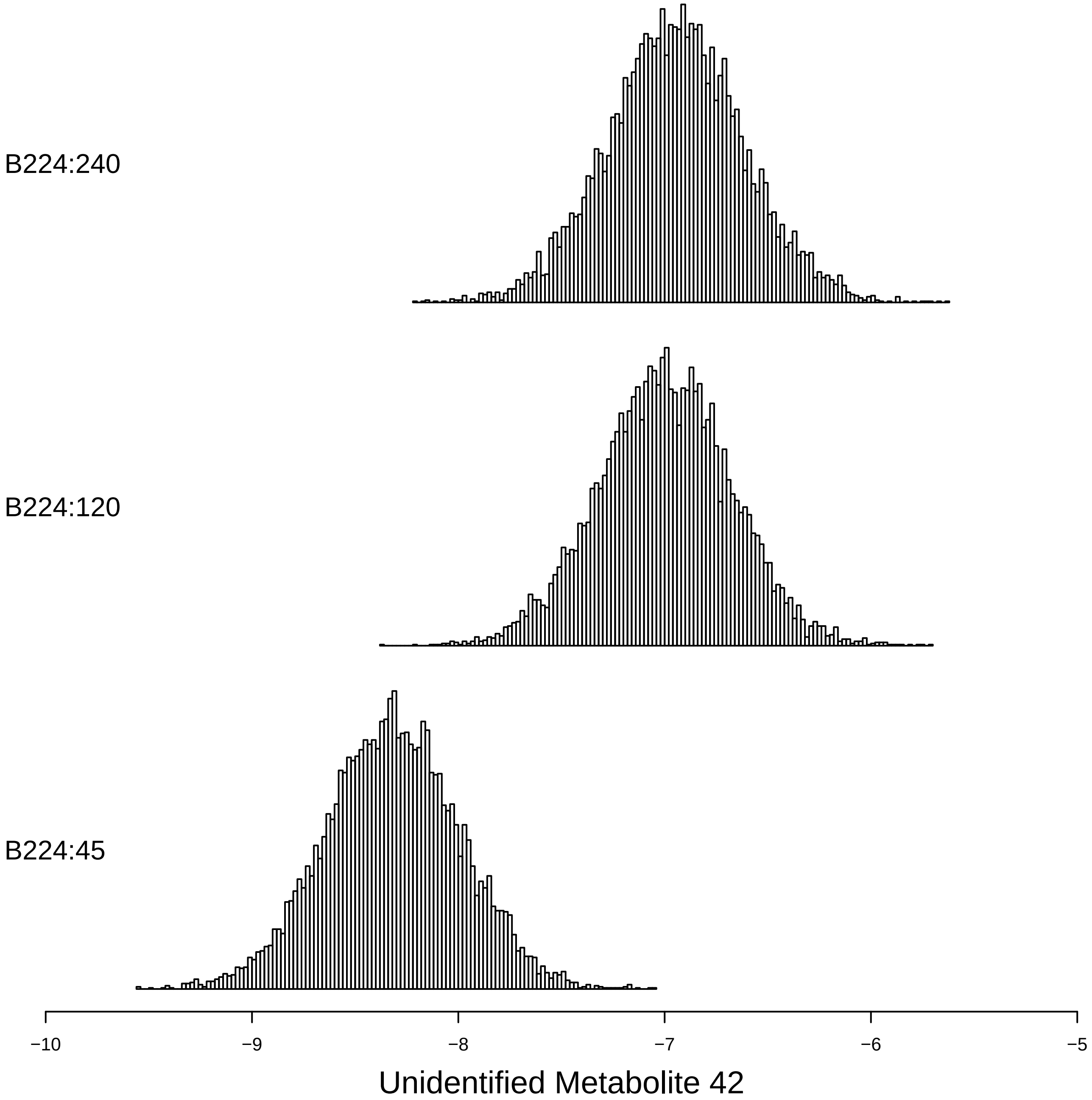
B224:240
B224:120
B224:45
−10
−9
−8
−7
−6
−5
Unidentified Metabolite 42

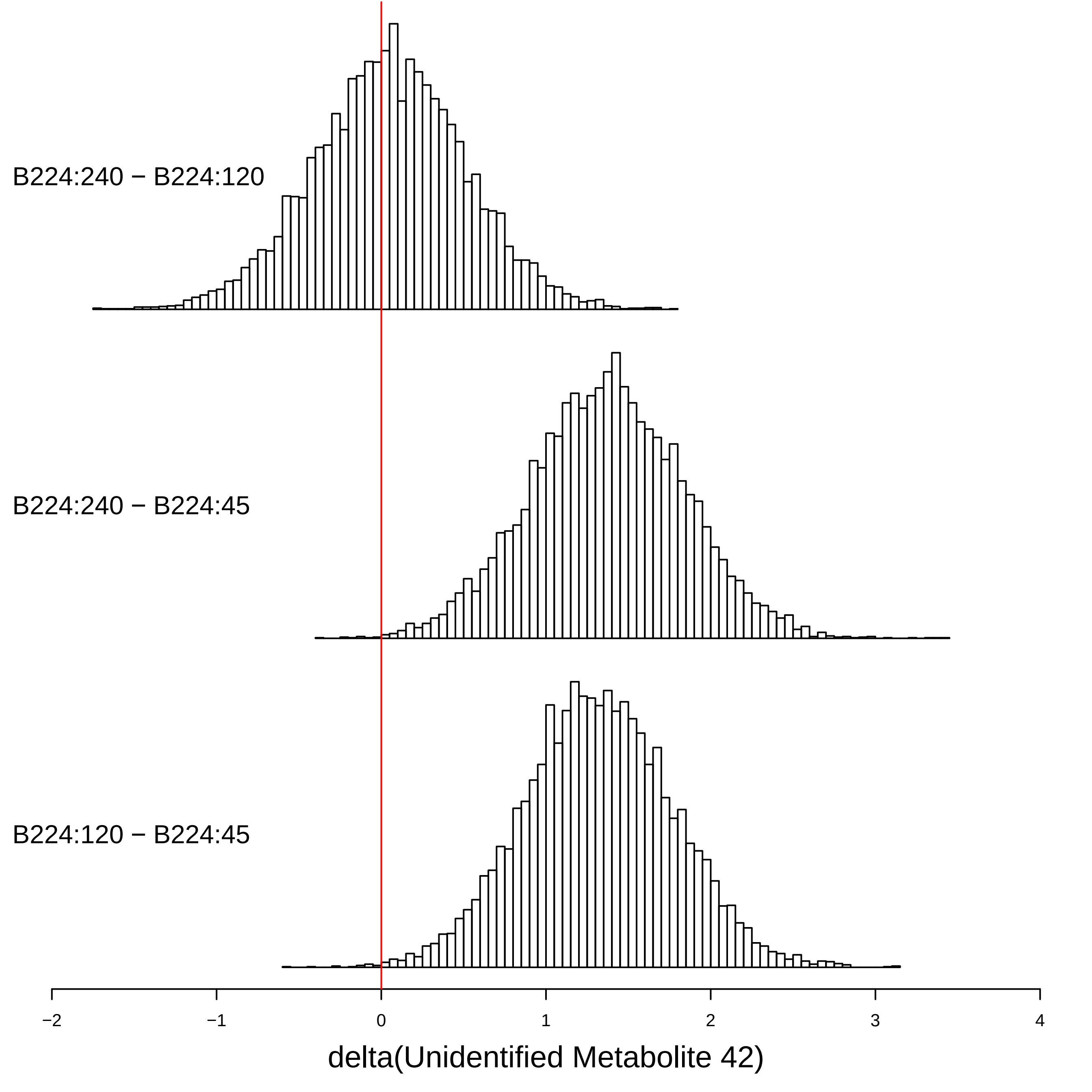

B224:240 − B224:120
B224:240 − B224:45
B224:120 − B224:45
−2
−1
0
1
2
3
4
delta(Unidentified Metabolite 42)

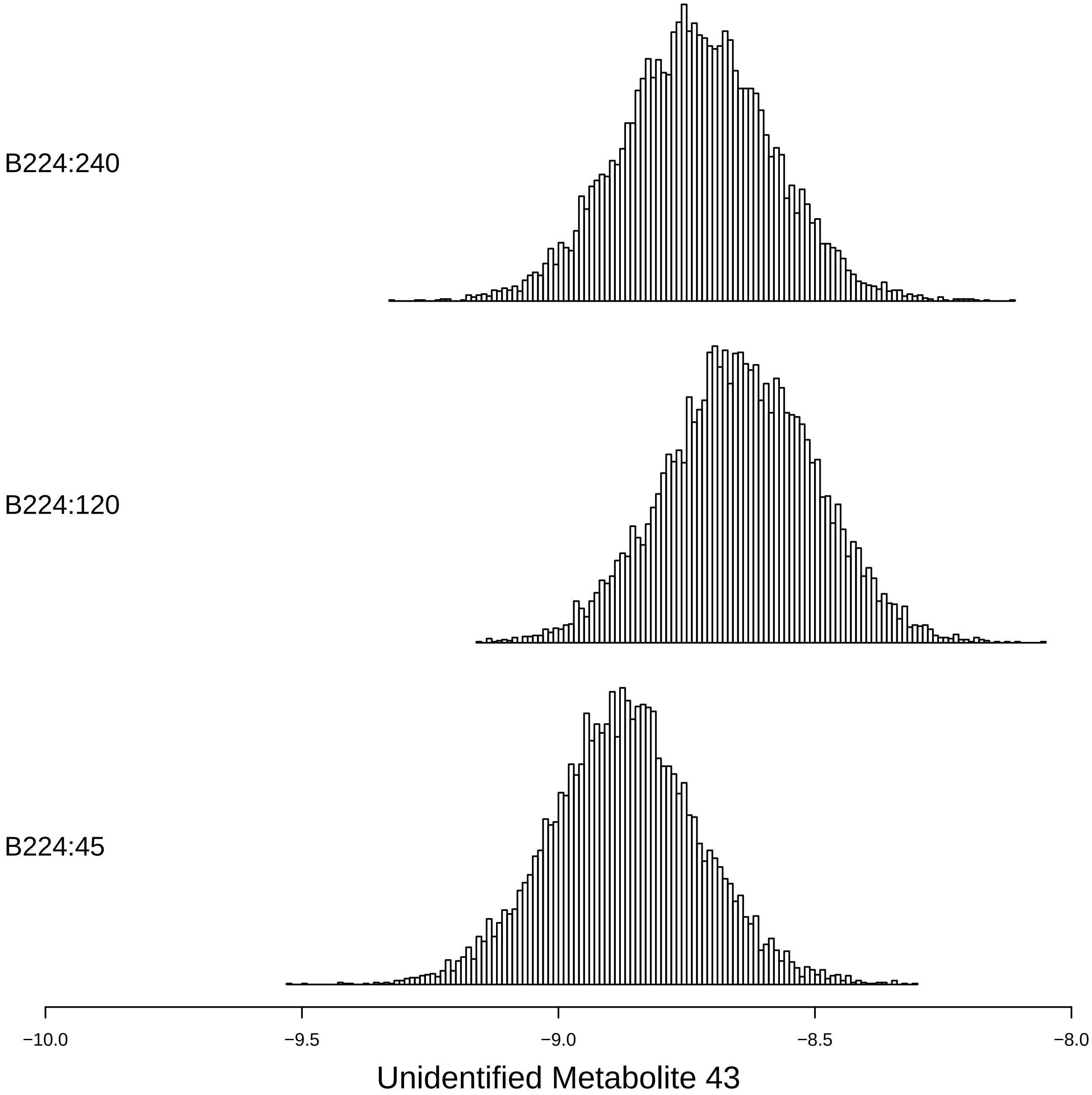

B224:240
B224:120
B224:45
−10.0
−9.5
−9.0
−8.5
−8.0
Unidentified Metabolite 43

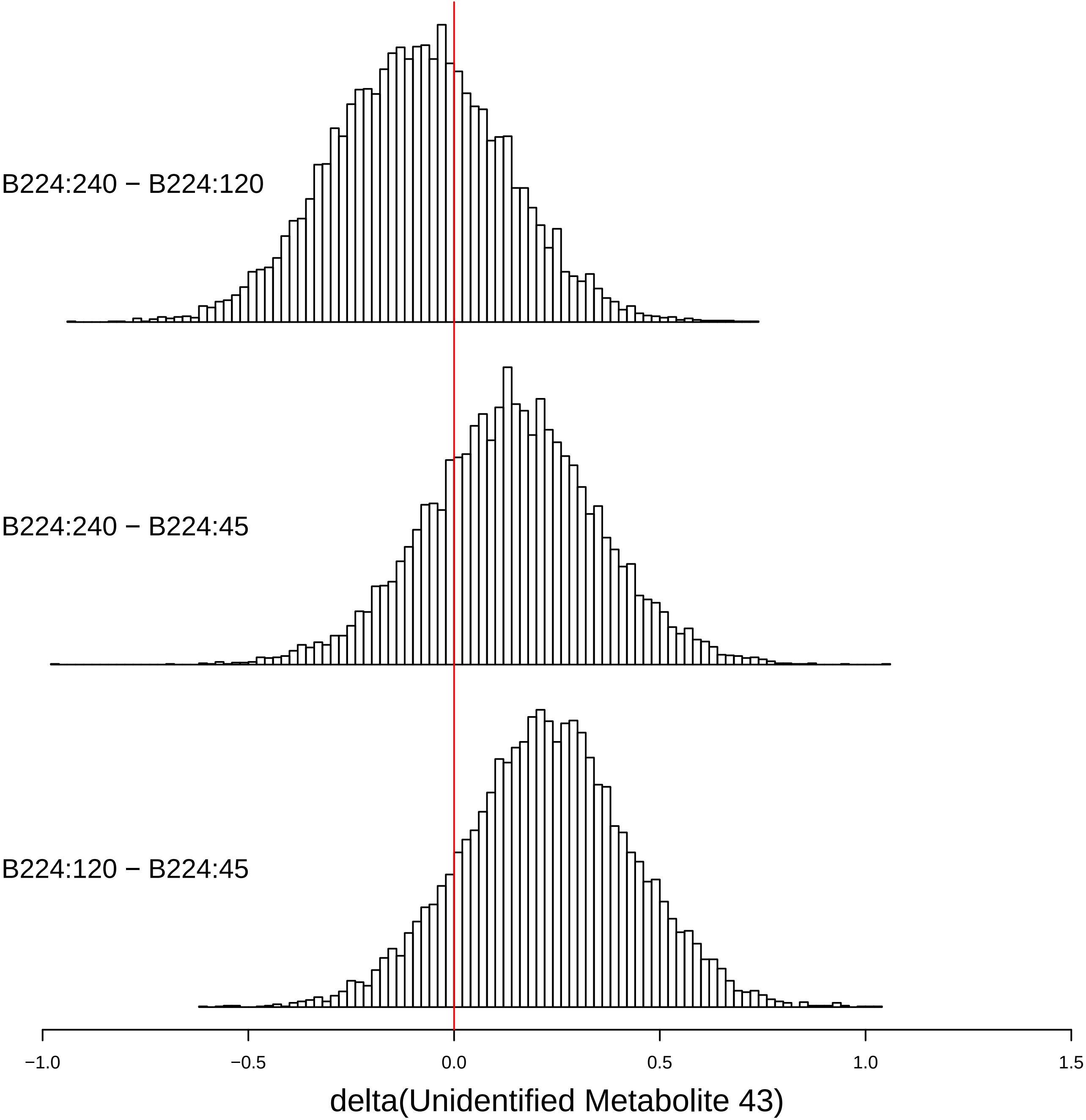
B224:240 − B224:120
B224:240 − B224:45
B224:120 − B224:45
−1.0
−0.5
0.0
0.5
1.0
1.5
delta(Unidentified Metabolite 43)

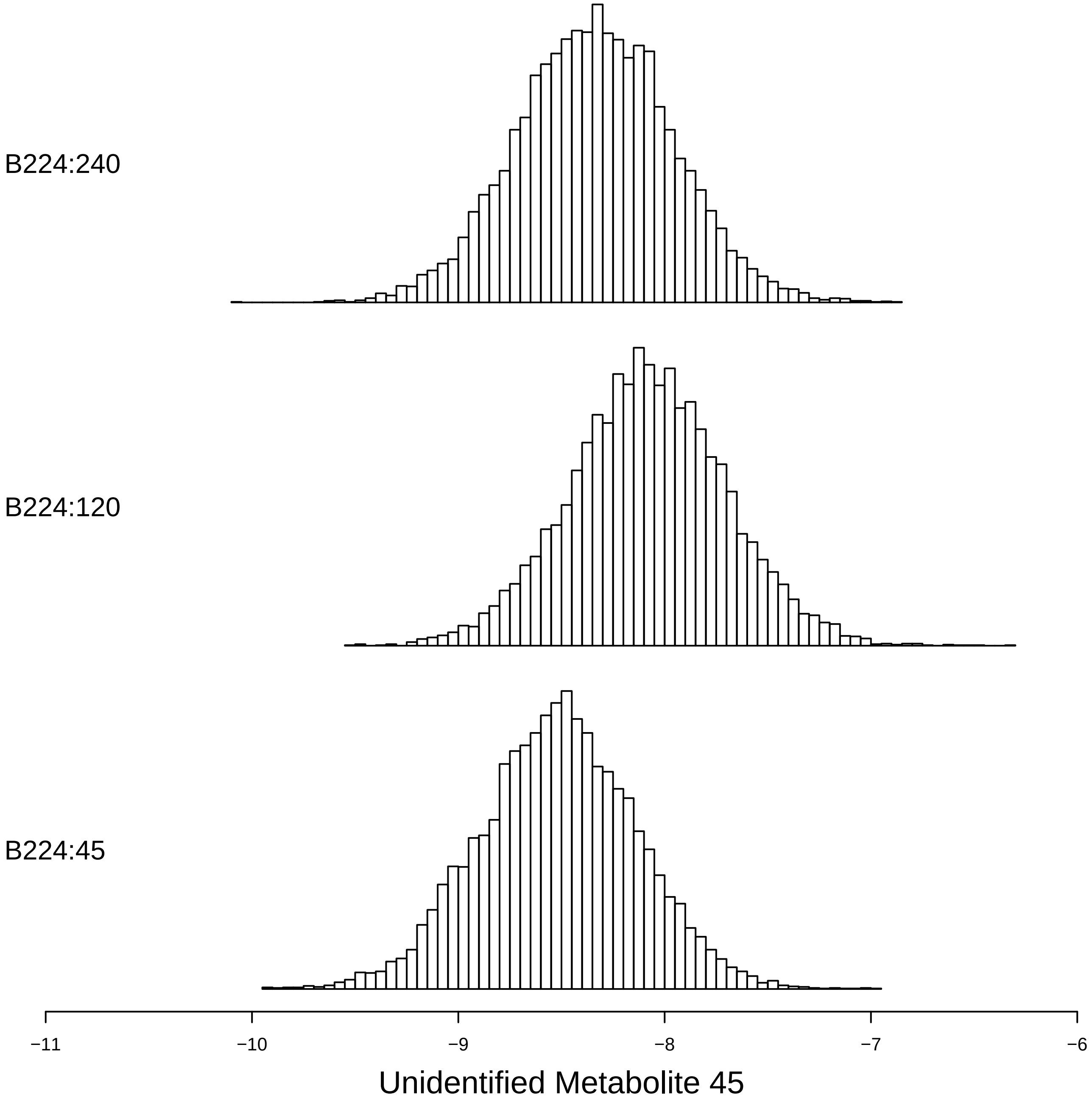
B224:240
B224:120
B224:45
−11
−10
−9
−8
−7
−6
Unidentified Metabolite 45

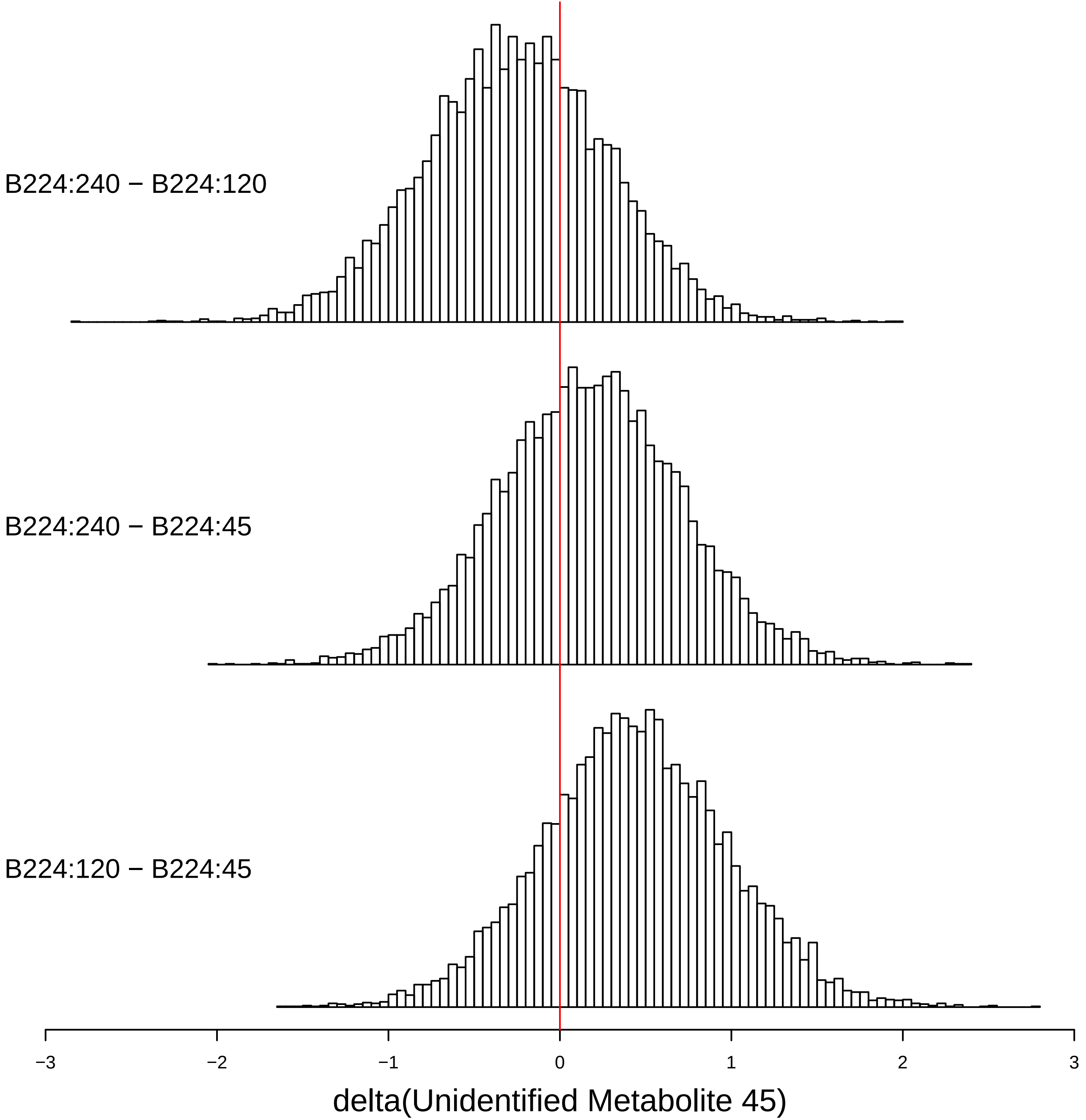

B224:240 − B224:120
B224:240 − B224:45
B224:120 − B224:45
−3
−2
−1
0
1
2
3
delta(Unidentified Metabolite 45)

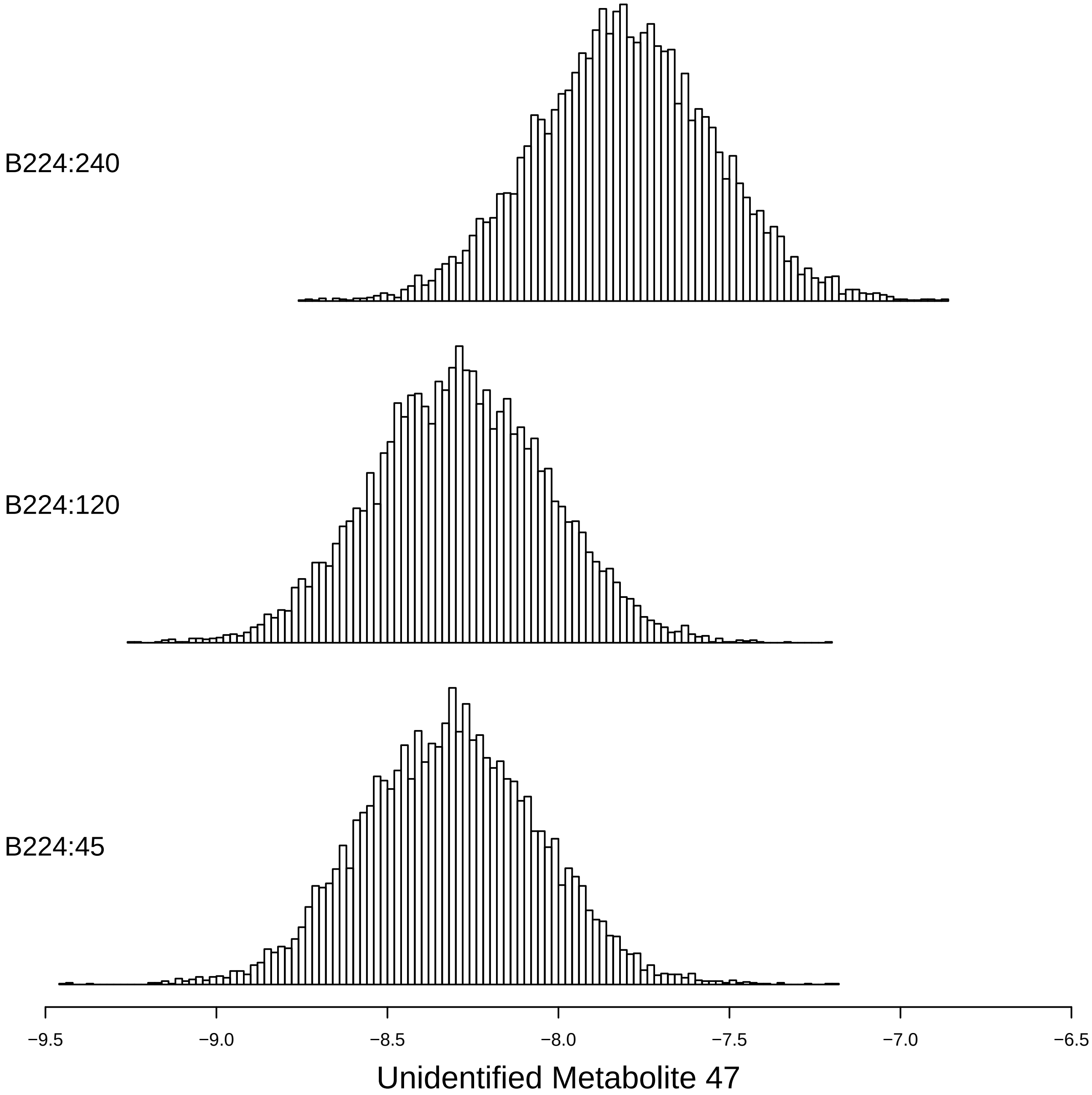
B224:240
B224:120
B224:45
−9.5
−9.0
−8.5
−8.0
−7.5
−7.0
−6.5
Unidentified Metabolite 47

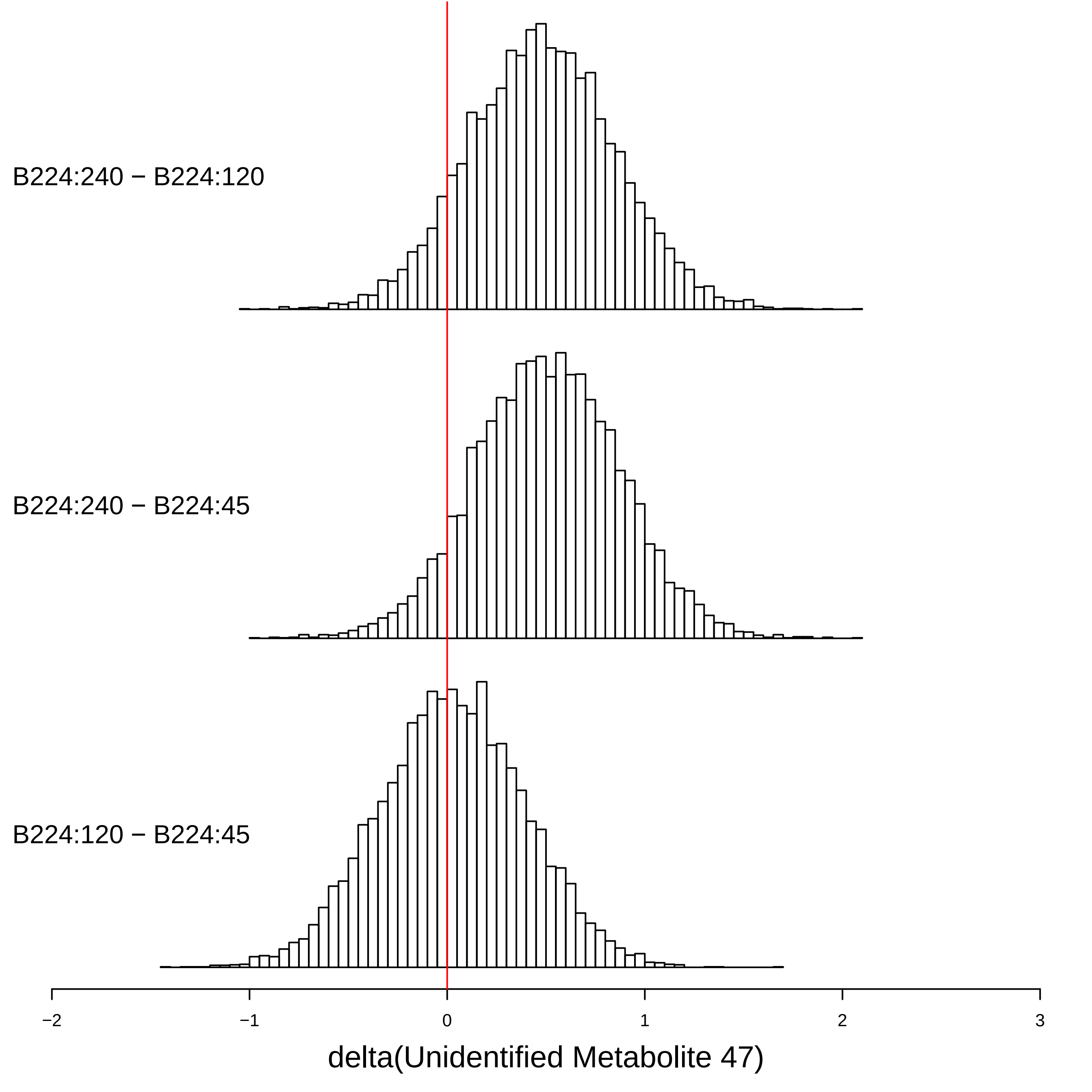
B224:240 − B224:120
B224:240 − B224:45
B224:120 − B224:45
−2
−1
0
1
2
3
delta(Unidentified Metabolite 47)

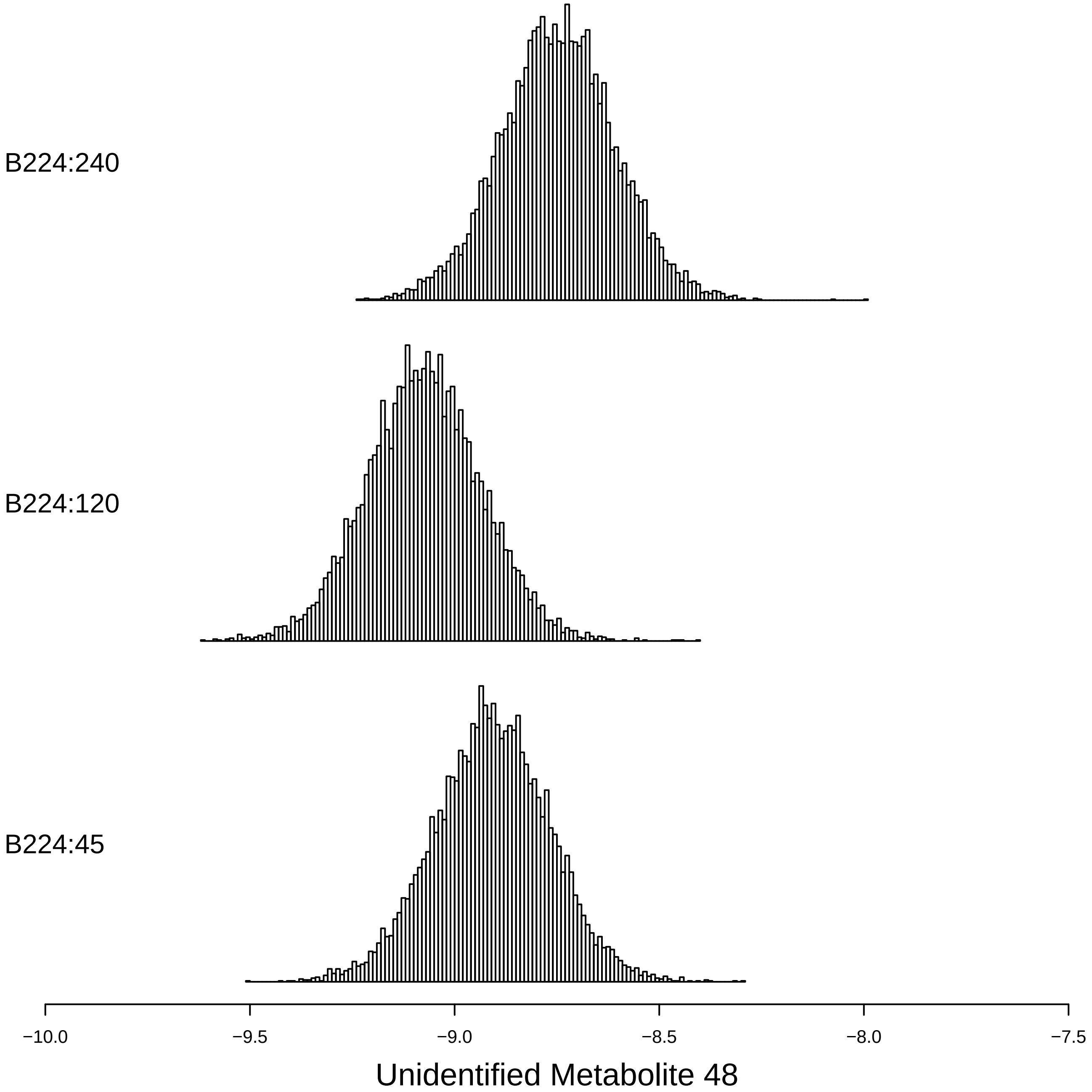
B224:240
B224:120
B224:45
−10.0
−9.5
−9.0
−8.5
−8.0
−7.5
Unidentified Metabolite 48

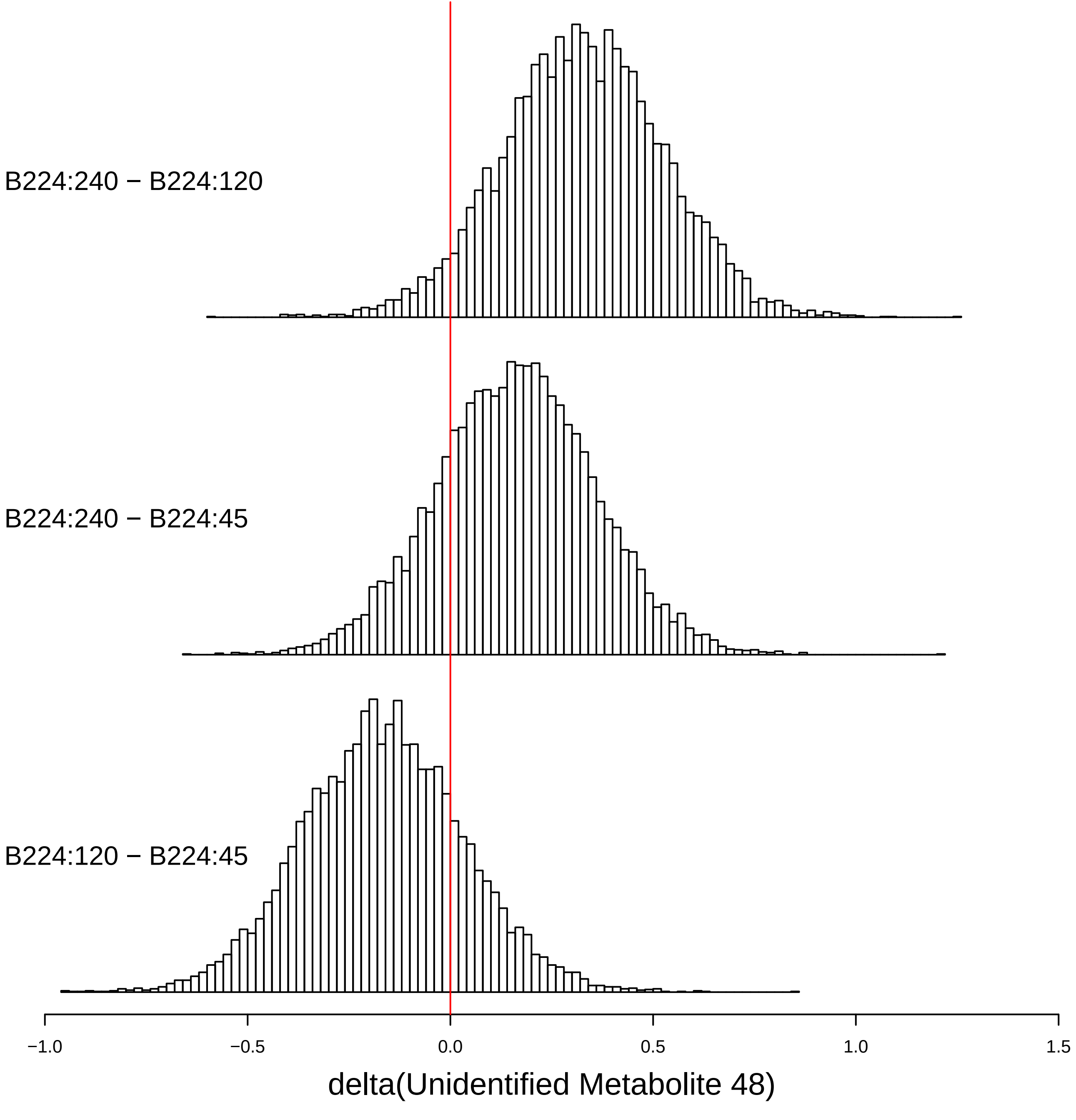
B224:240 − B224:120
B224:240 − B224:45
B224:120 − B224:45
−1.0
−0.5
0.0
0.5
1.0
1.5
delta(Unidentified Metabolite 48)

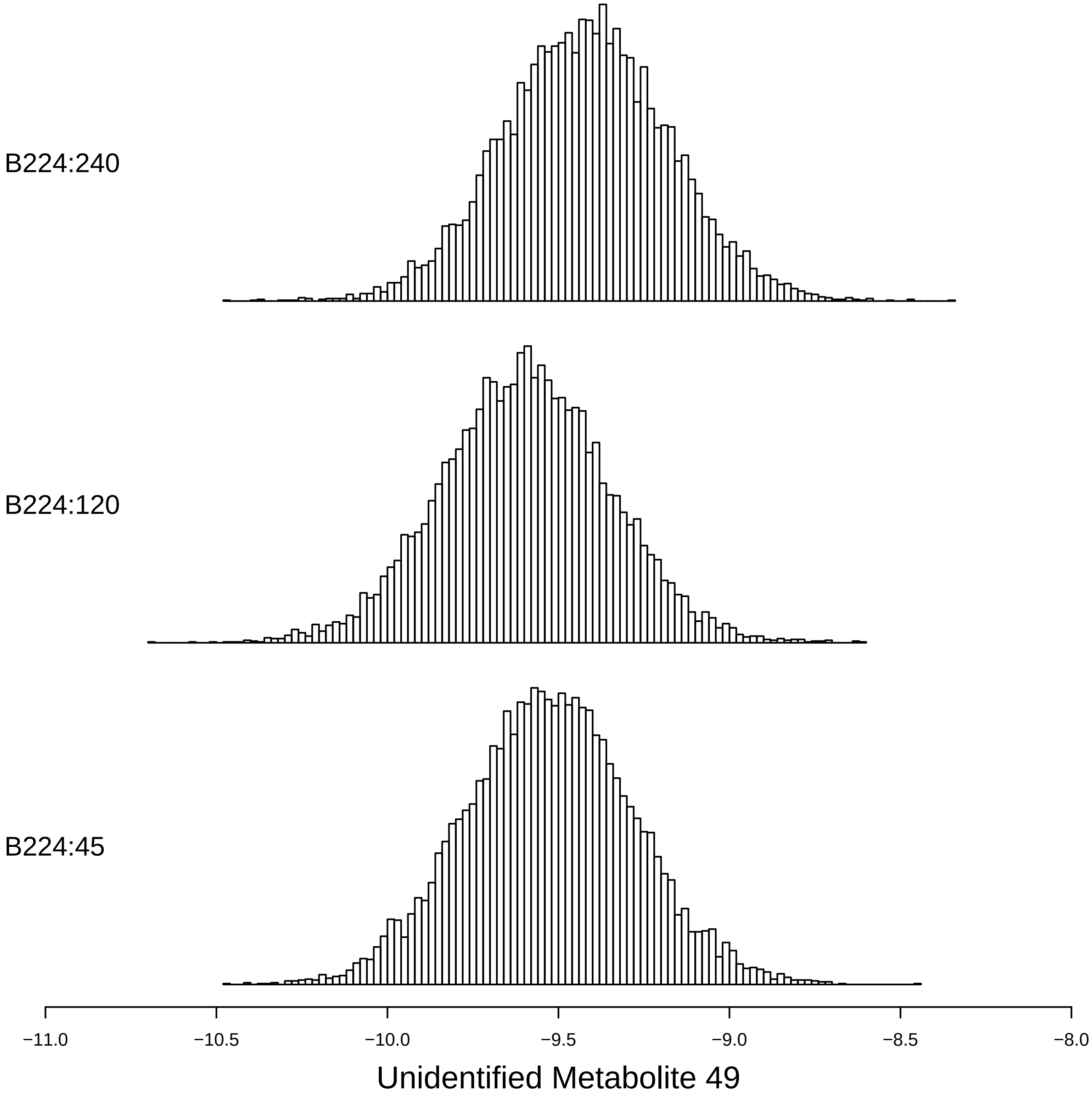
B224:240
B224:120
B224:45
−11.0
−10.5
−10.0
−9.5
−9.0
−8.5
−8.0
Unidentified Metabolite 49

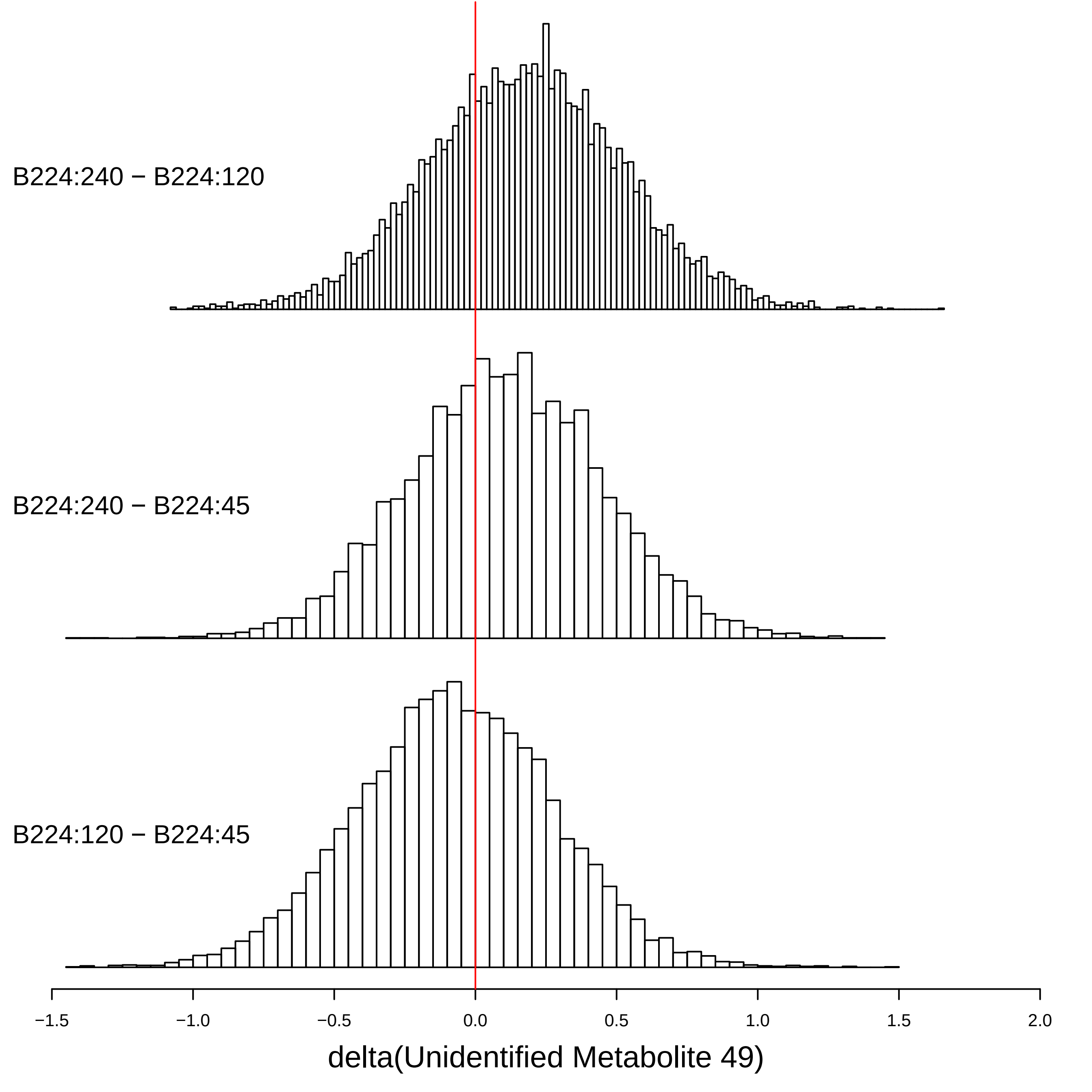
B224:240 − B224:120
B224:240 − B224:45
B224:120 − B224:45
−1.5
−1.0
−0.5
0.0
0.5
1.0
1.5
2.0
delta(Unidentified Metabolite 49)

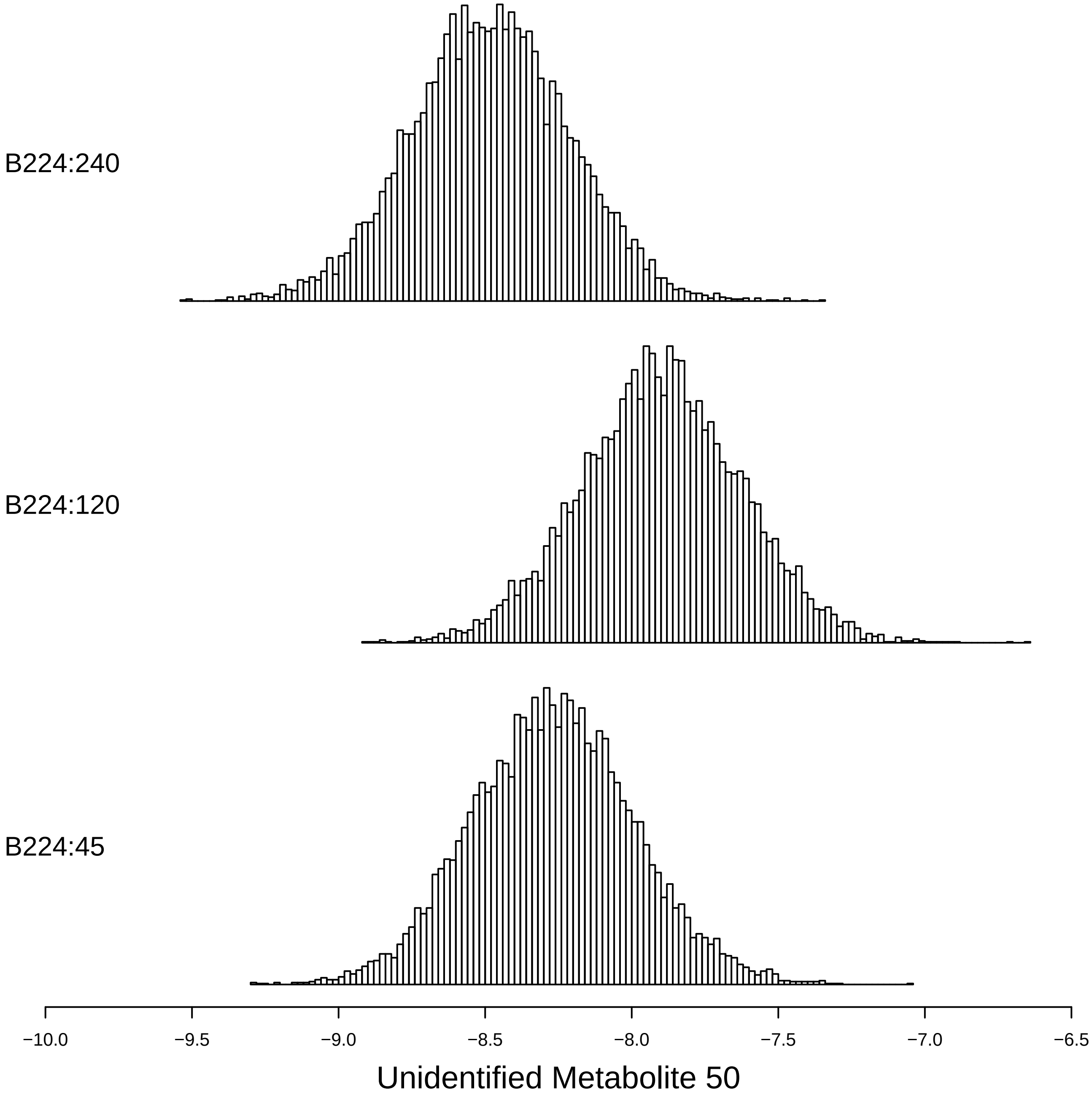

B224:240
B224:120
B224:45
−10.0
−9.5
−9.0
−8.5
−8.0
−7.5
−7.0
−6.5
Unidentified Metabolite 50

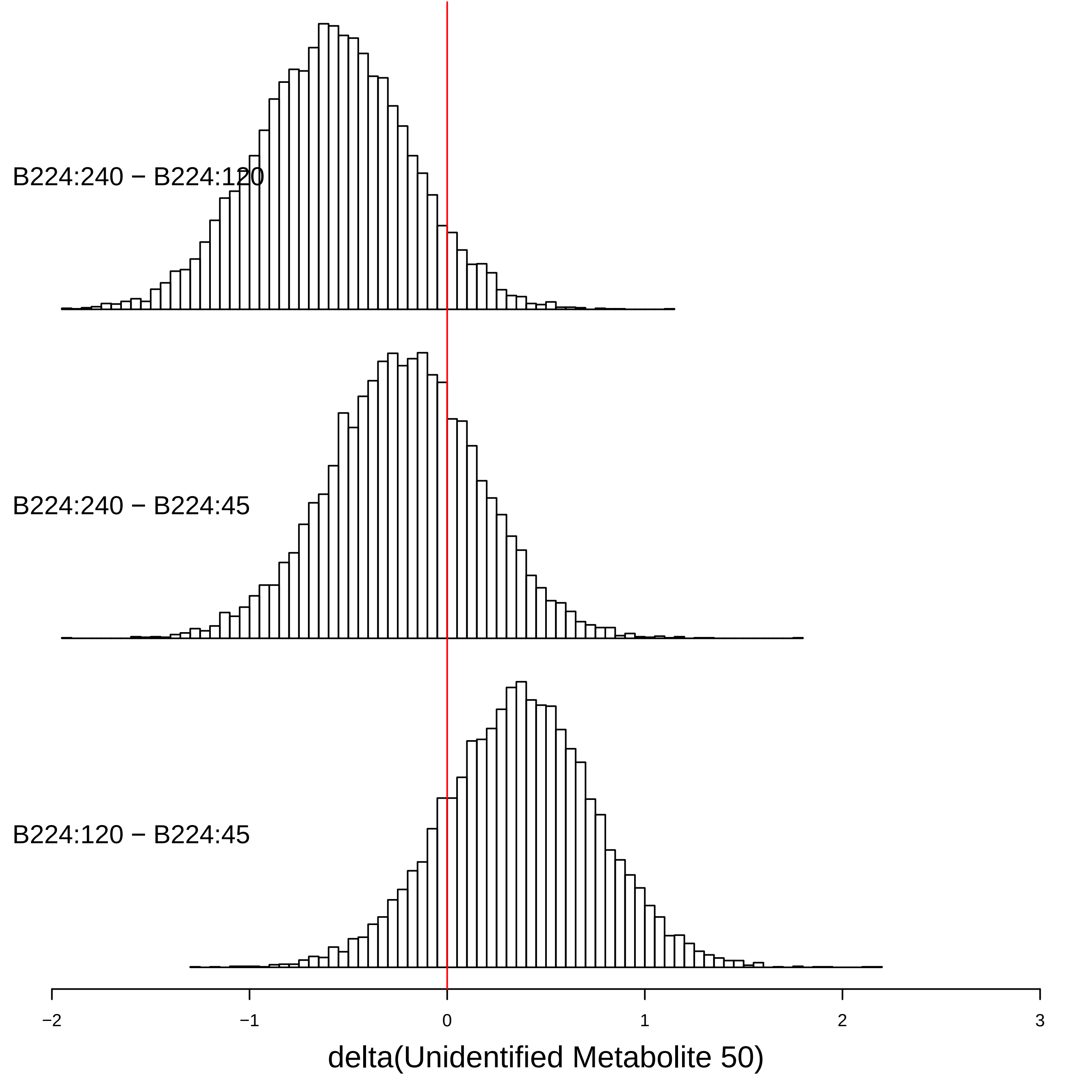
B224:240 − B224:120
B224:240 − B224:45
B224:120 − B224:45
−2
−1
0
1
2
3
delta(Unidentified Metabolite 50)

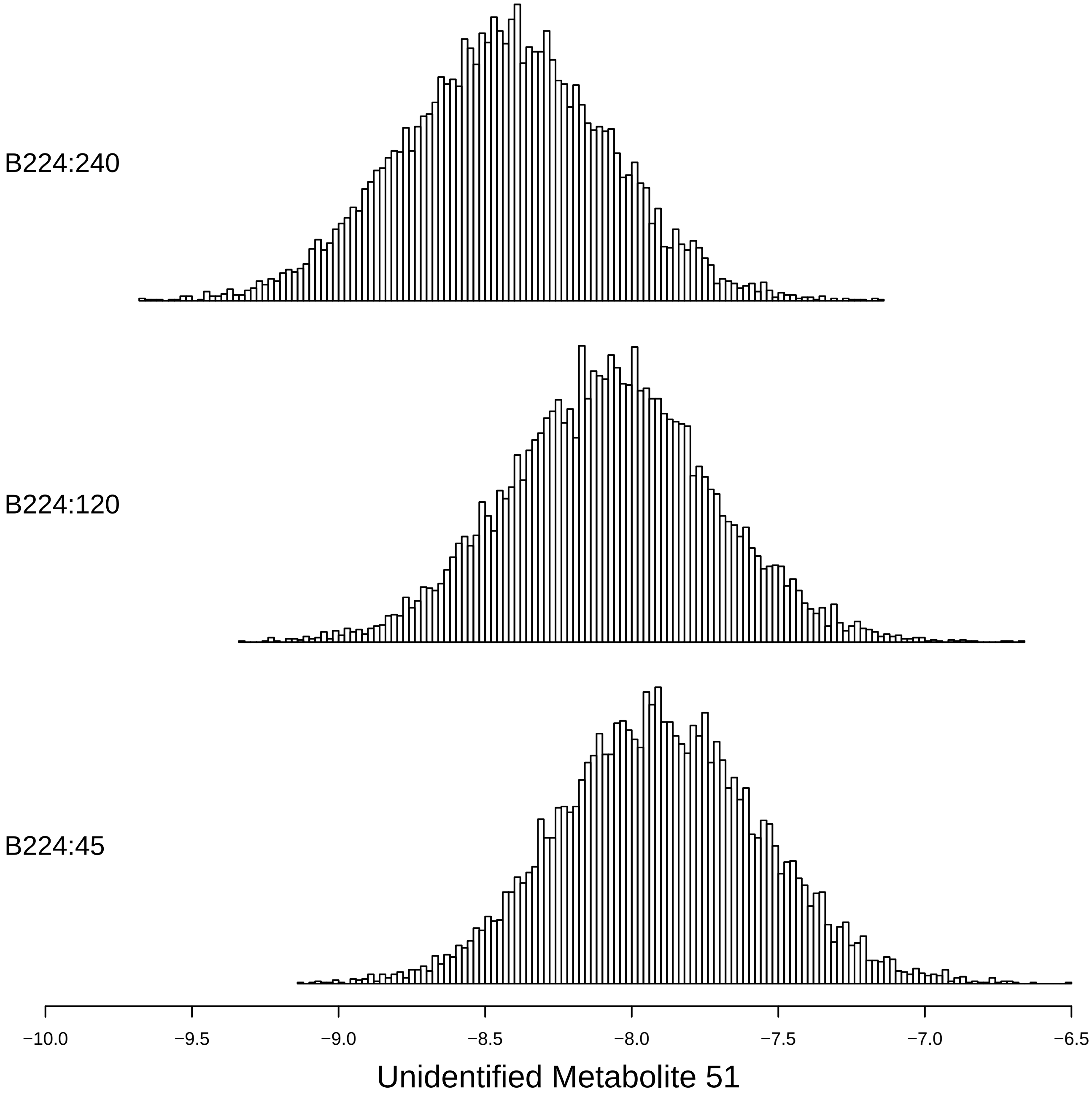
B224:240
B224:120
B224:45
−10.0
−9.5
−9.0
−8.5
−8.0
−7.5
−7.0
−6.5
Unidentified Metabolite 51

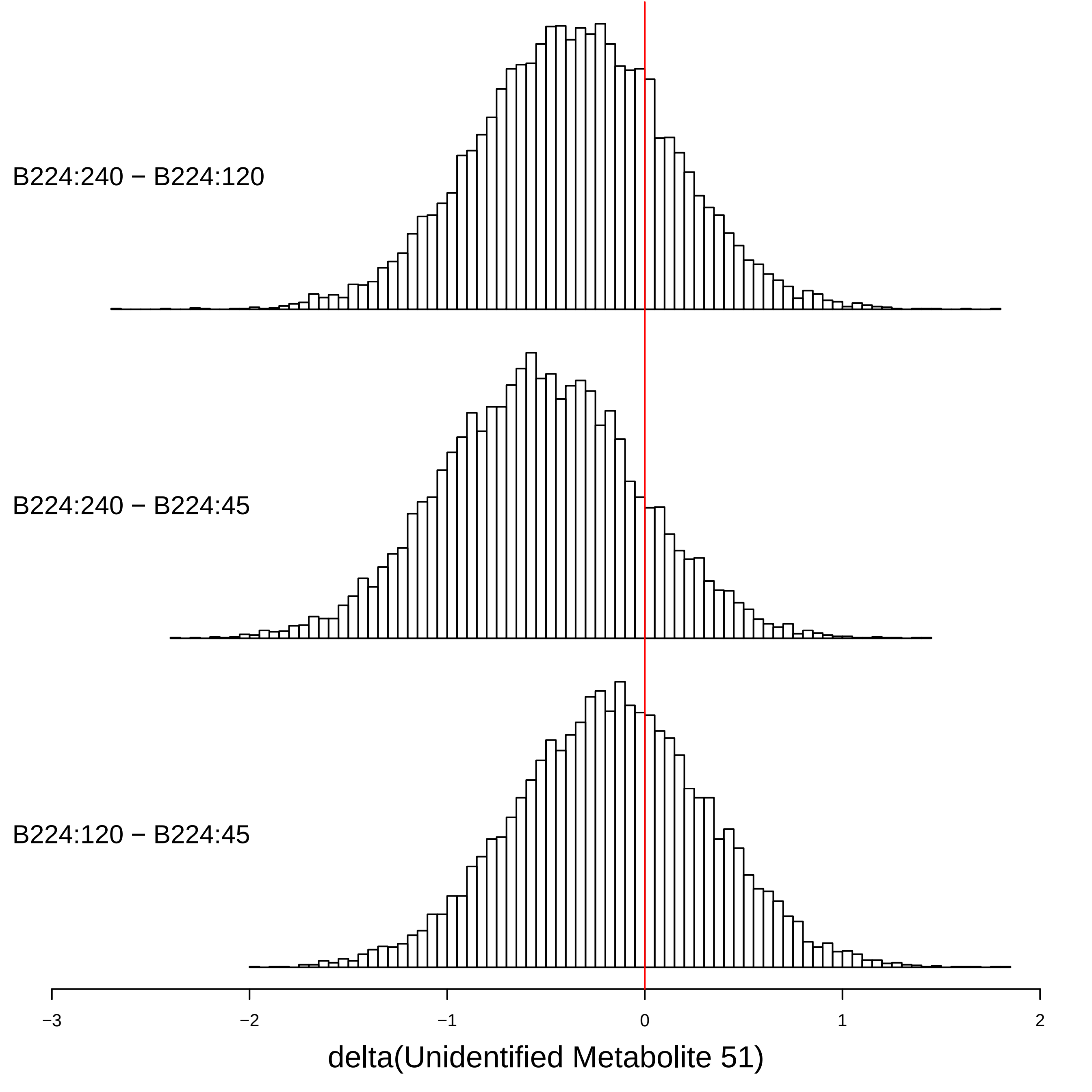

B224:240 − B224:120
B224:240 − B224:45
B224:120 − B224:45
−3
−2
−1
0
1
2
delta(Unidentified Metabolite 51)

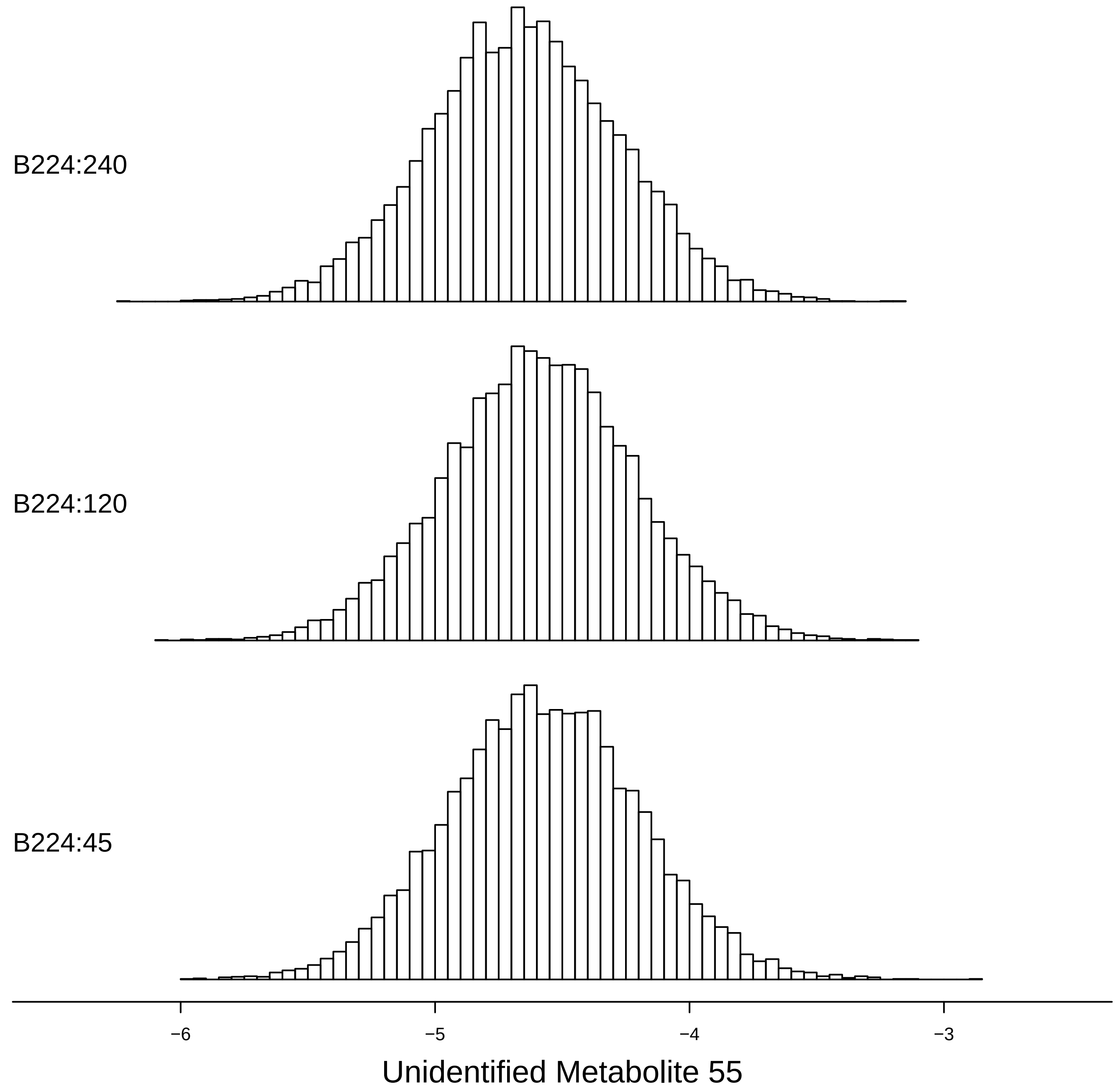
B224:240
B224:120
B224:45
−6
−5
−4
−3
Unidentified Metabolite 55

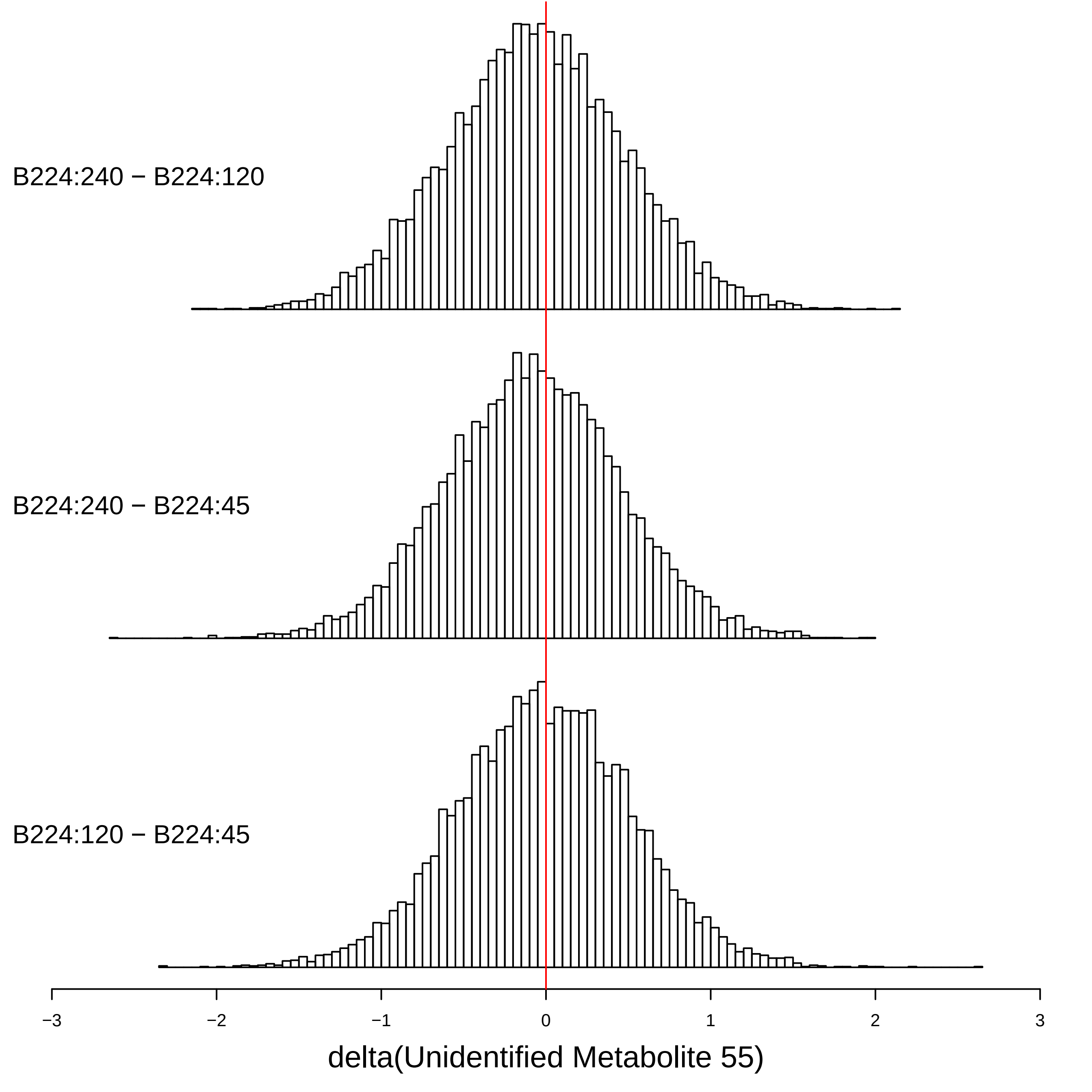

B224:240 − B224:120
B224:240 − B224:45
B224:120 − B224:45
−3
−2
−1
0
1
2
3
delta(Unidentified Metabolite 55)

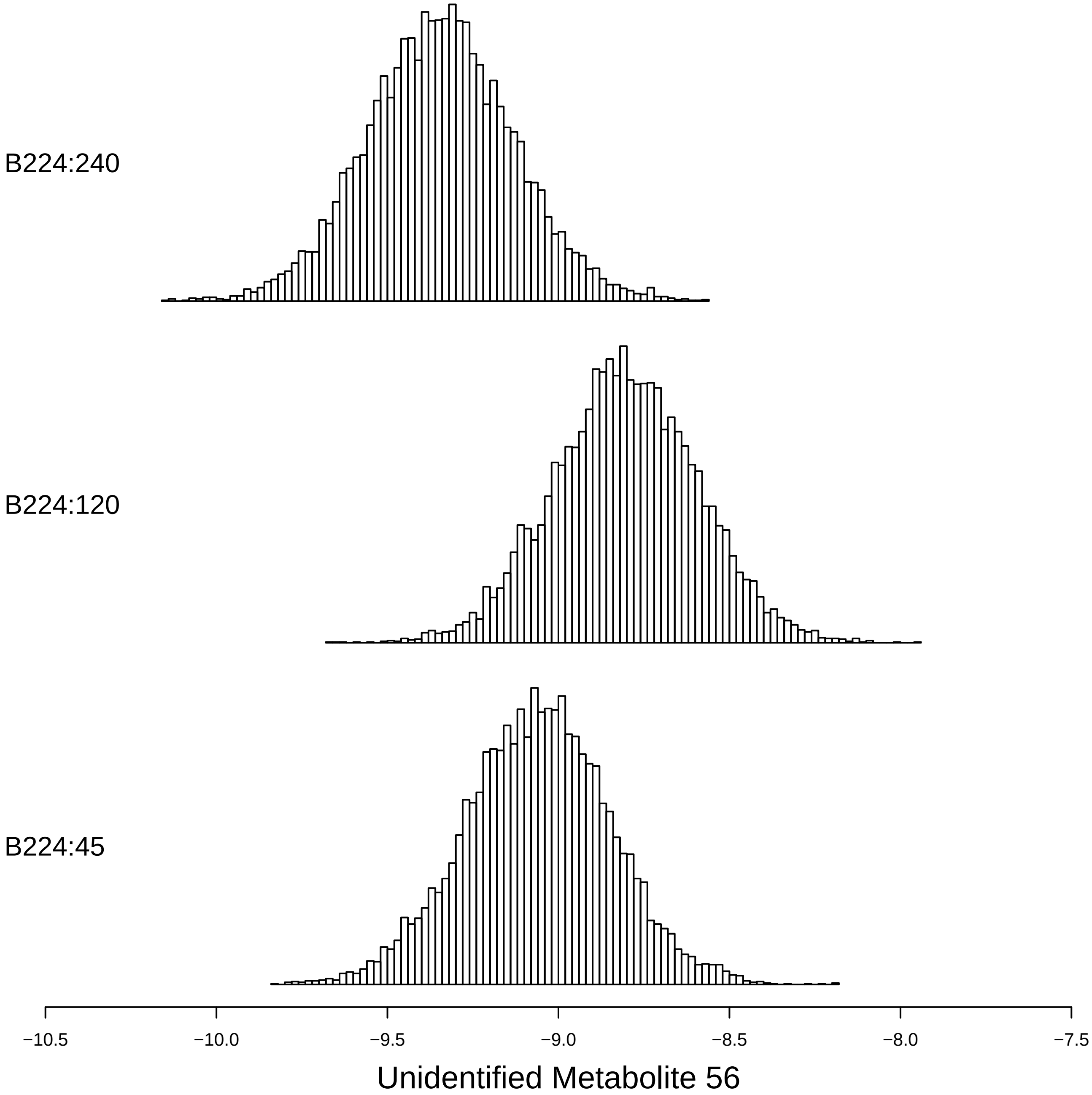
B224:240
B224:120
B224:45
−10.5
−10.0
−9.5
−9.0
−8.5
−8.0
−7.5
Unidentified Metabolite 56

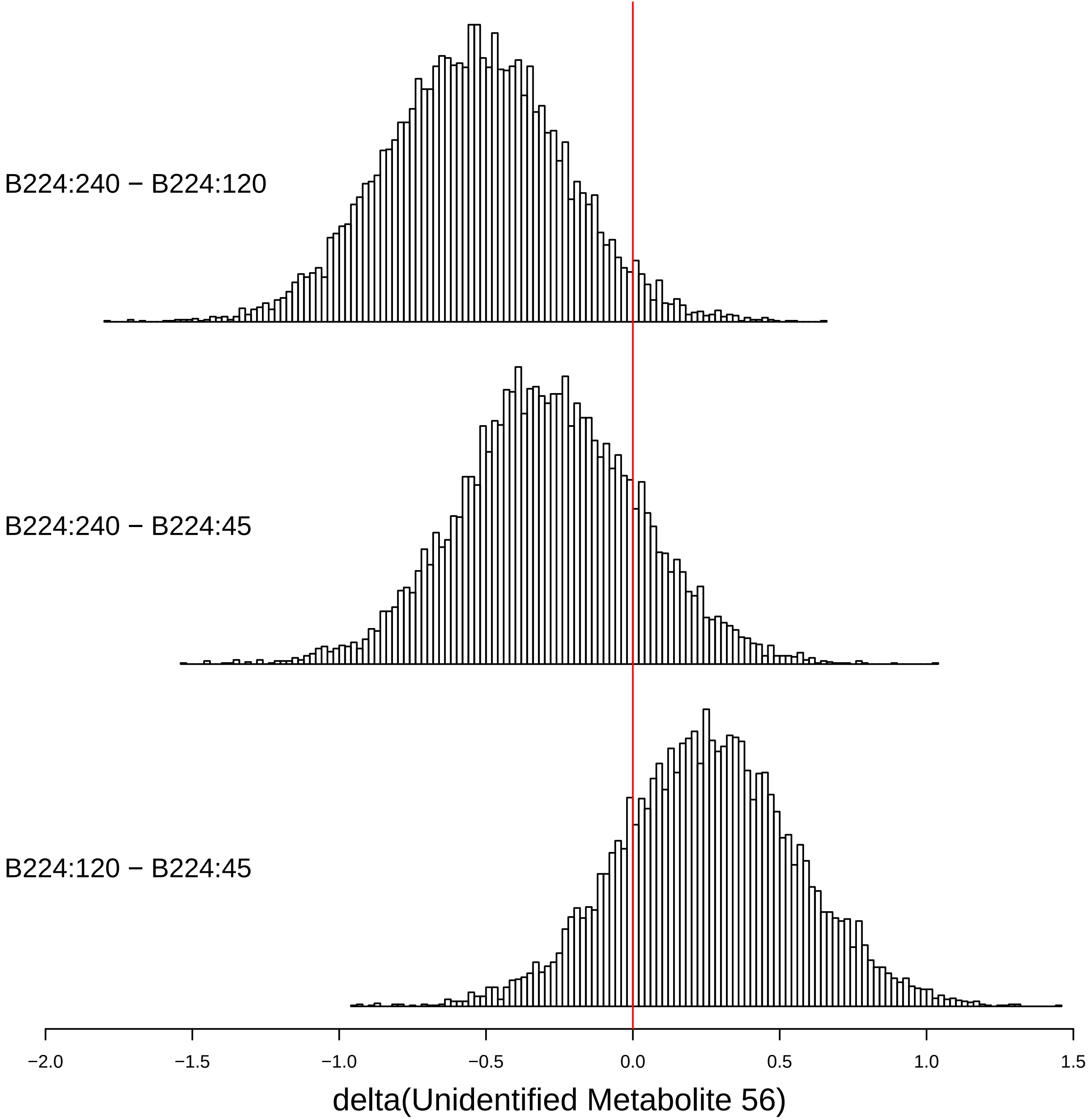
B224:240 − B224:120
B224:240 − B224:45
B224:120 − B224:45
−2.0
−1.5
−1.0
−0.5
0.0
0.5
1.0
1.5
delta(Unidentified Metabolite 56)

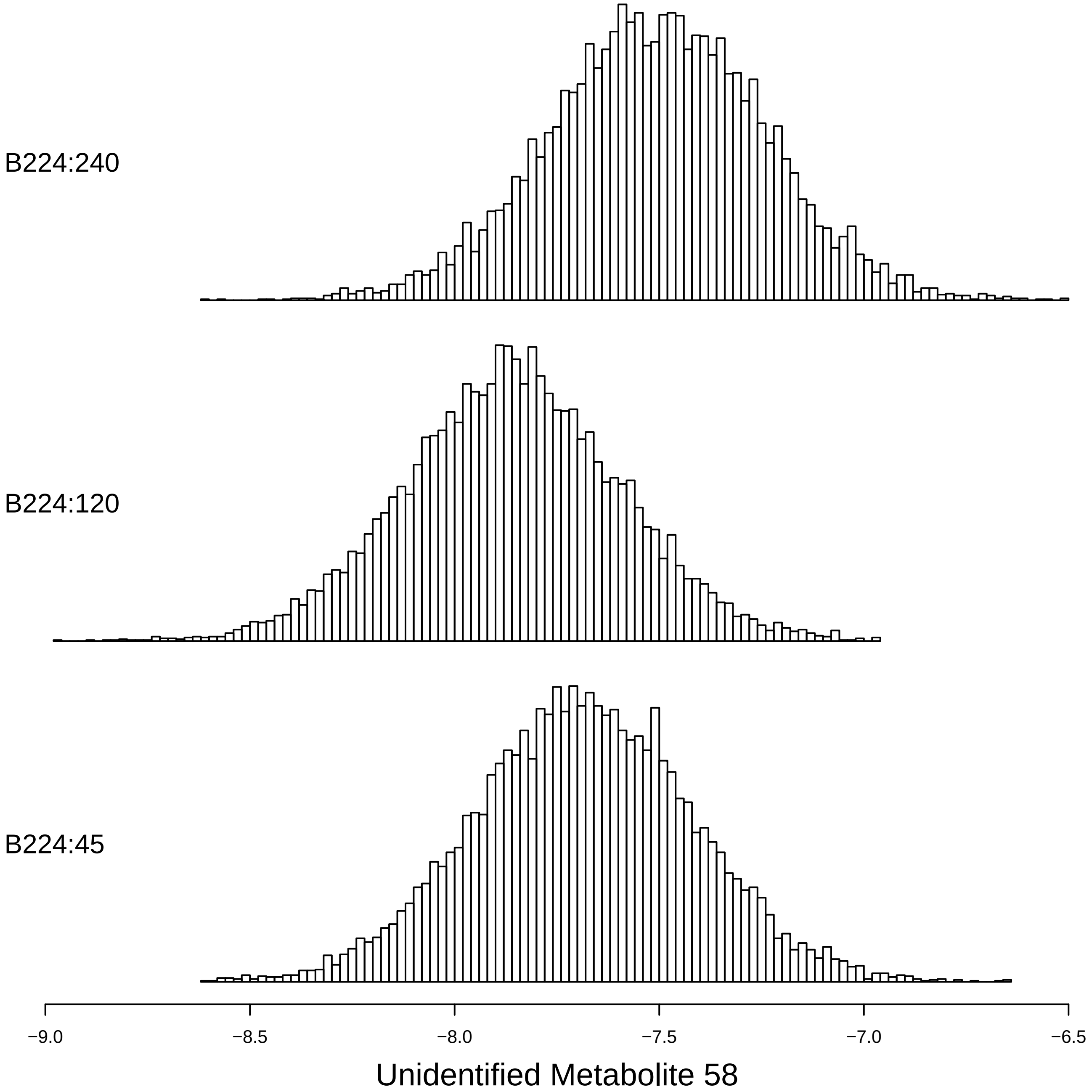

B224:240
B224:120
B224:45
−9.0
−8.5
−8.0
−7.5
−7.0
−6.5
Unidentified Metabolite 58

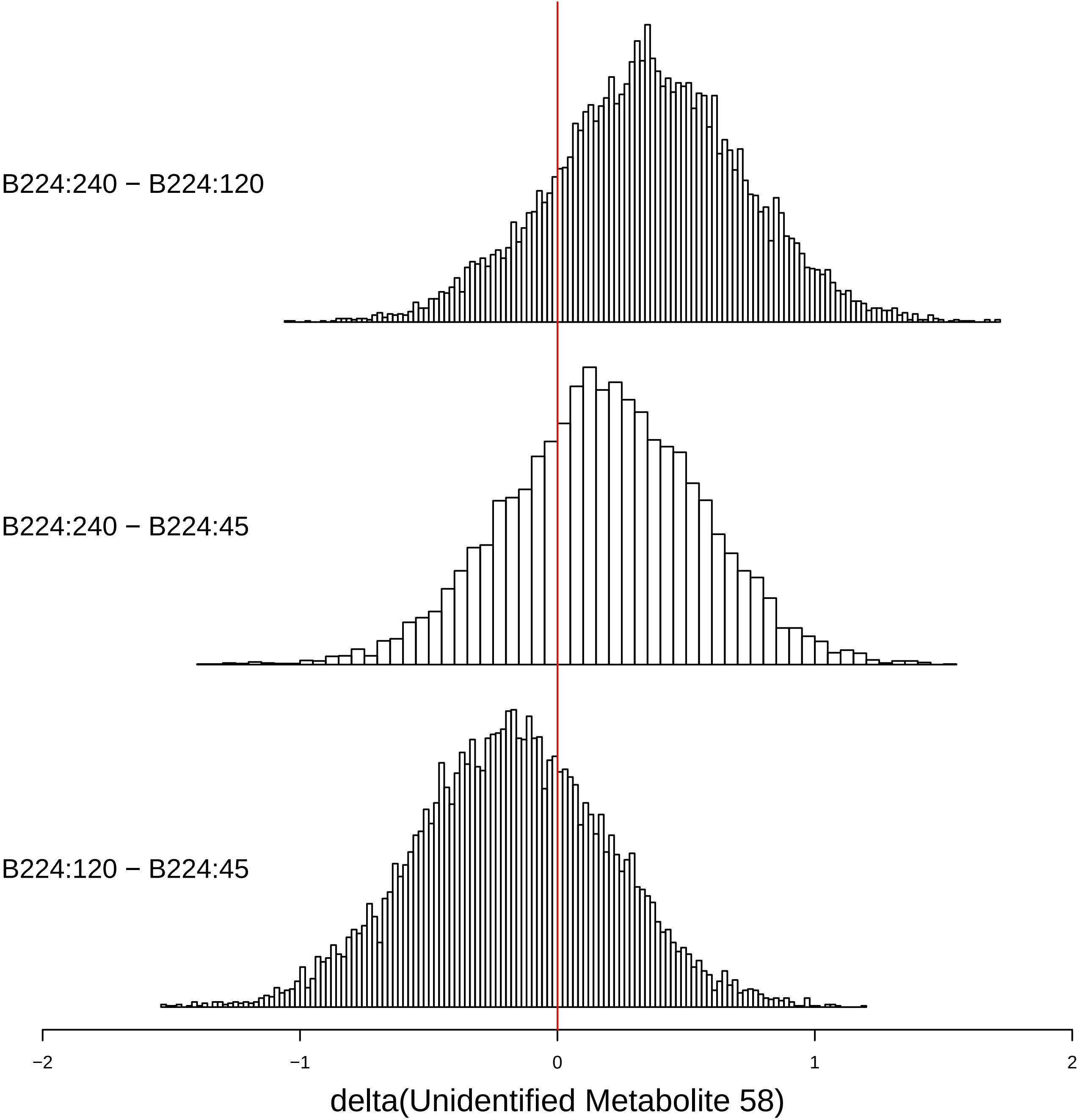

B224:240 − B224:120
B224:240 − B224:45
B224:120 − B224:45
−2
−1
0
1
2
delta(Unidentified Metabolite 58)

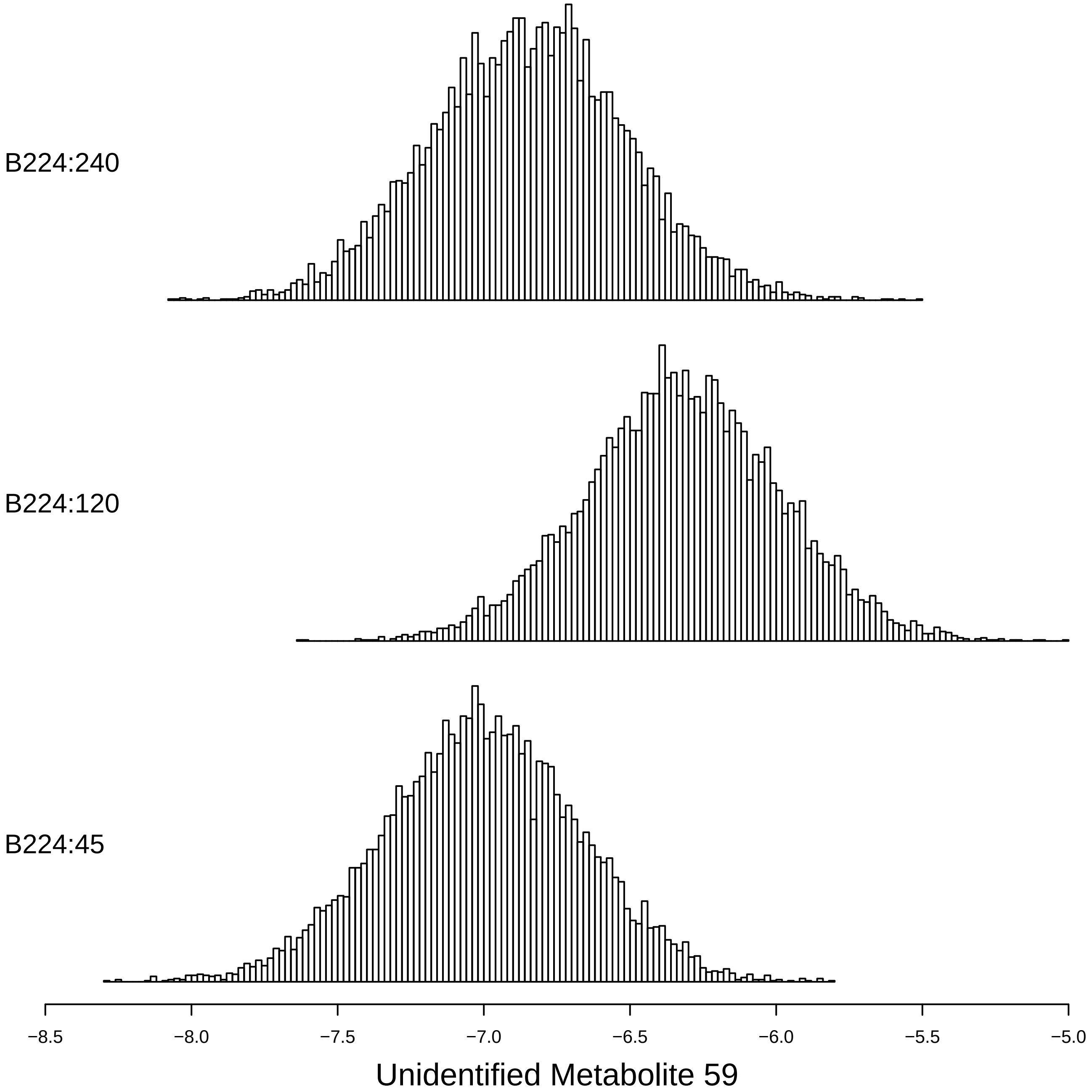
B224:240
B224:120
B224:45
−8.5
−8.0
−7.5
−7.0
−6.5
−6.0
−5.5
−5.0
Unidentified Metabolite 59

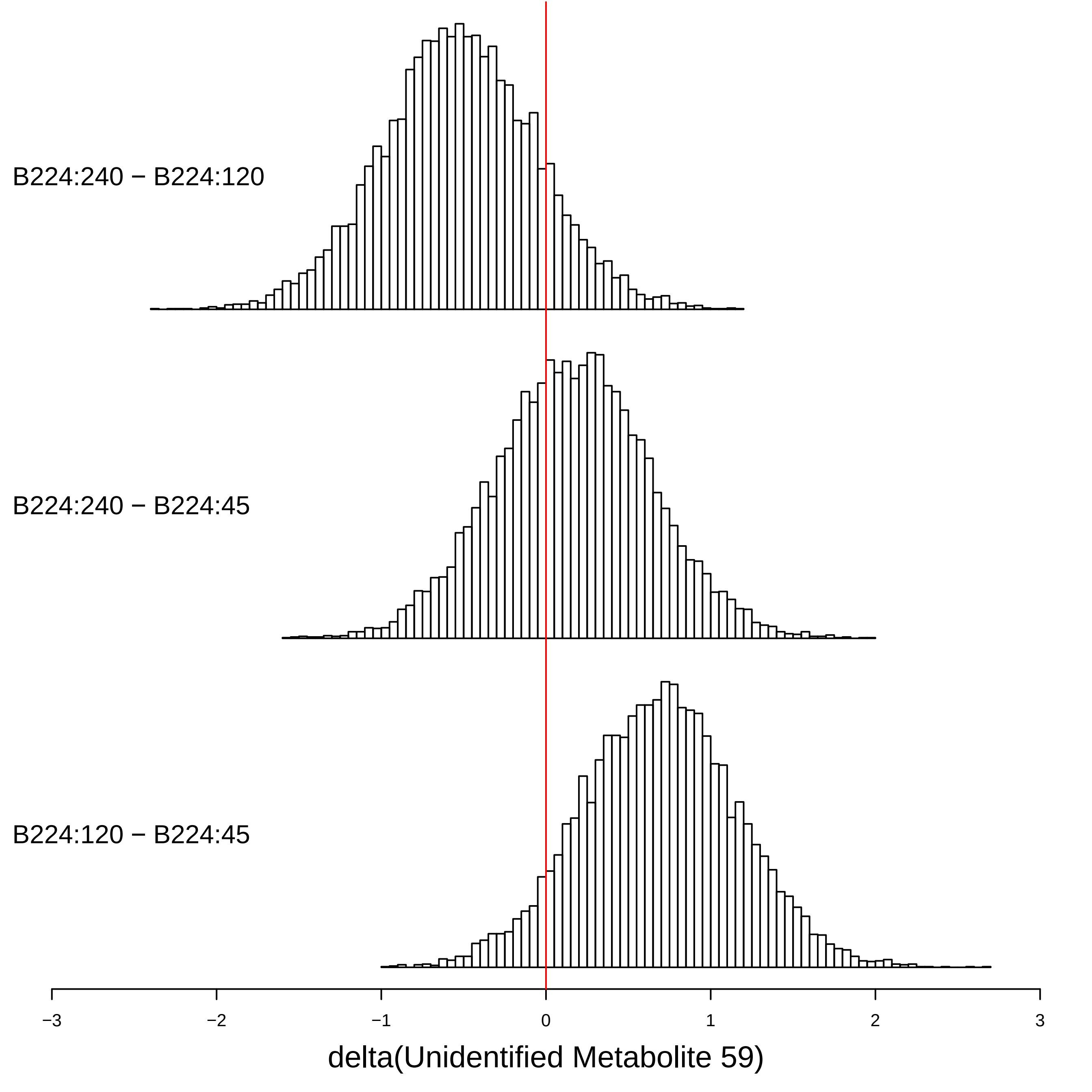
B224:240 − B224:120
B224:240 − B224:45
B224:120 − B224:45
−3
−2
−1
0
1
2
3
delta(Unidentified Metabolite 59)

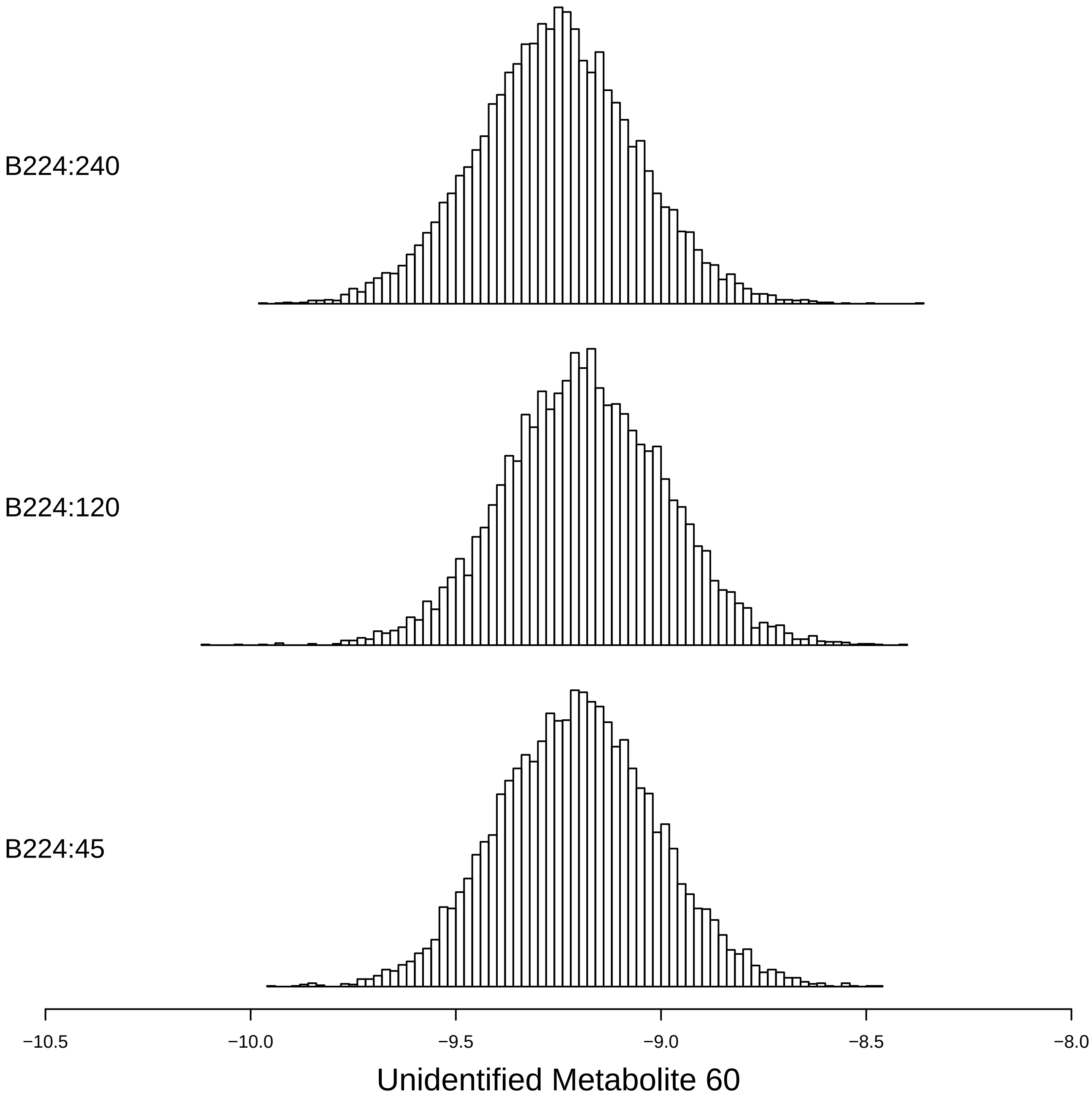
B224:240
B224:120
B224:45
−10.5
−10.0
−9.5
−9.0
−8.5
−8.0
Unidentified Metabolite 60

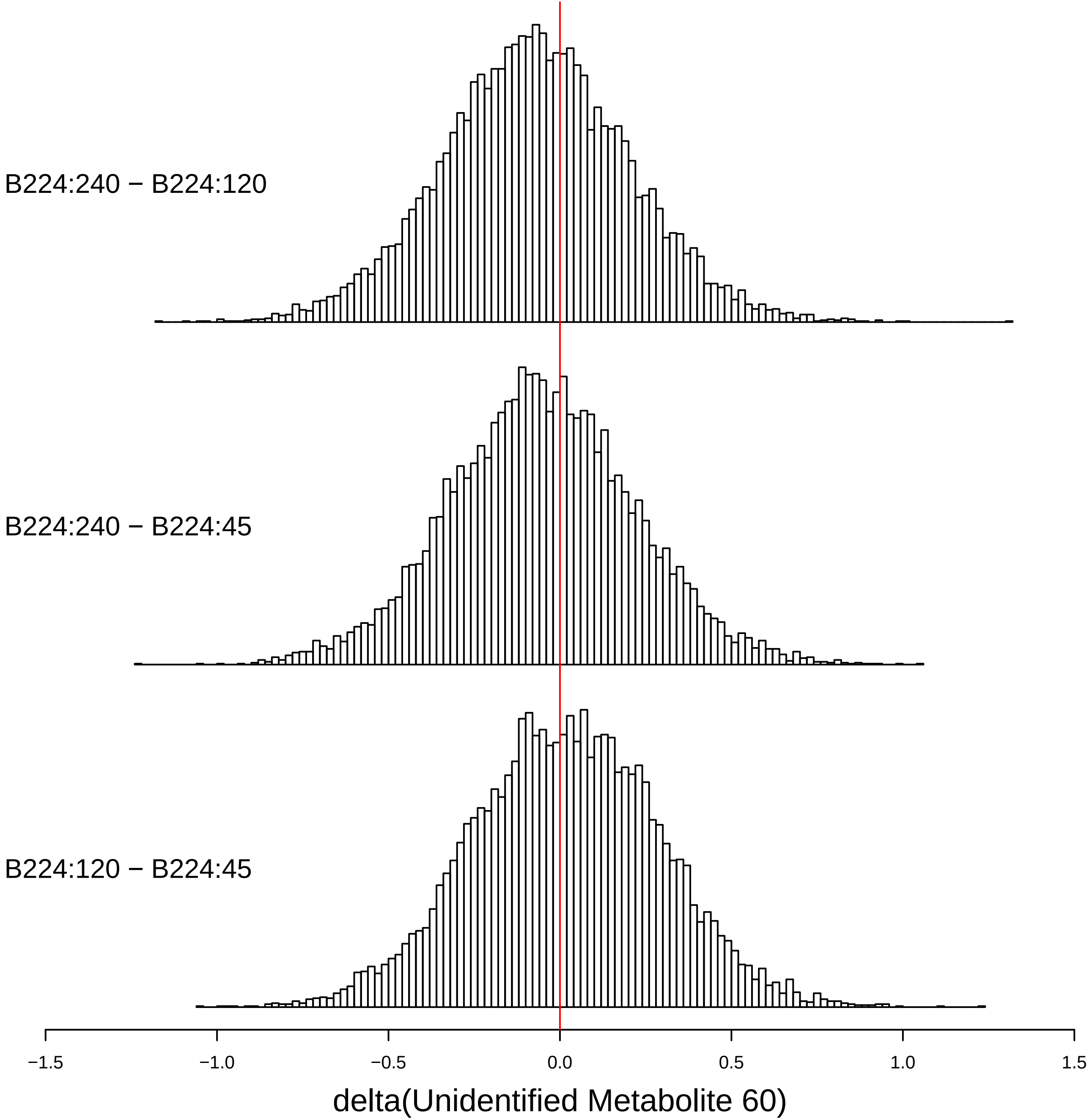
B224:240 − B224:120
B224:240 − B224:45
B224:120 − B224:45
−1.5
−1.0
−0.5
0.0
0.5
1.0
1.5
delta(Unidentified Metabolite 60)

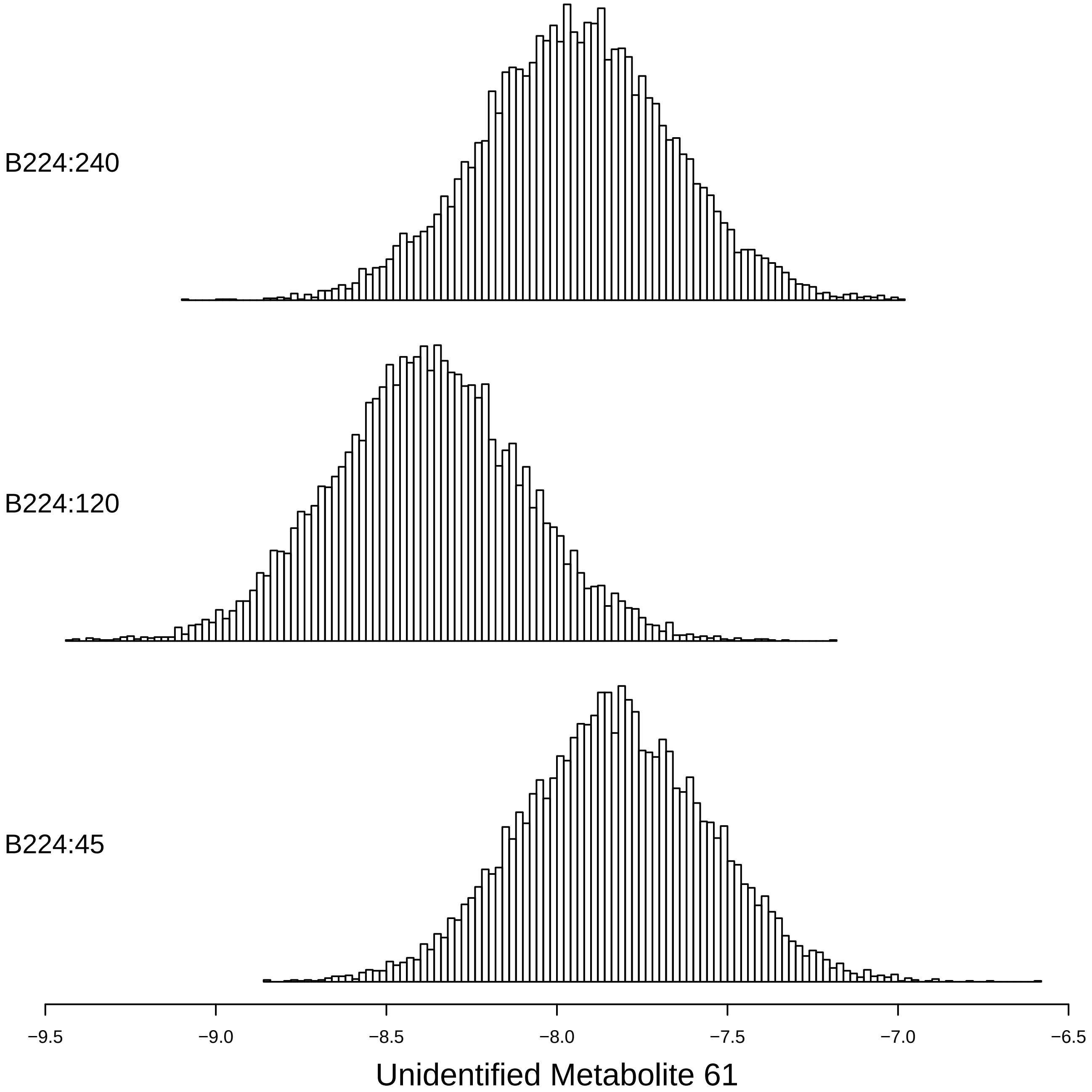

B224:240
B224:120
B224:45
−9.5
−9.0
−8.5
−8.0
−7.5
−7.0
−6.5
Unidentified Metabolite 61

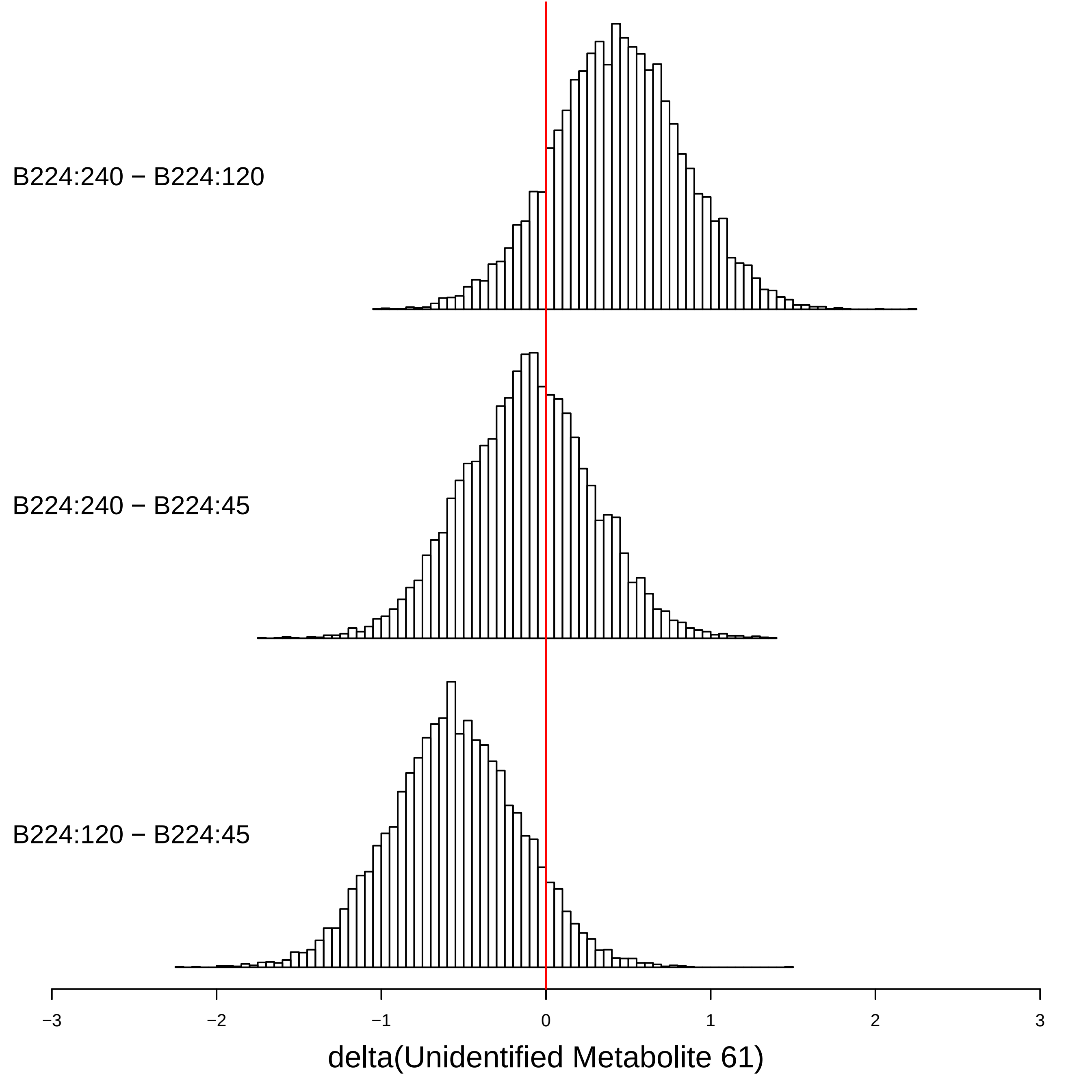
B224:240 − B224:120
B224:240 − B224:45
B224:120 − B224:45
−3
−2
−1
0
1
2
3
delta(Unidentified Metabolite 61)

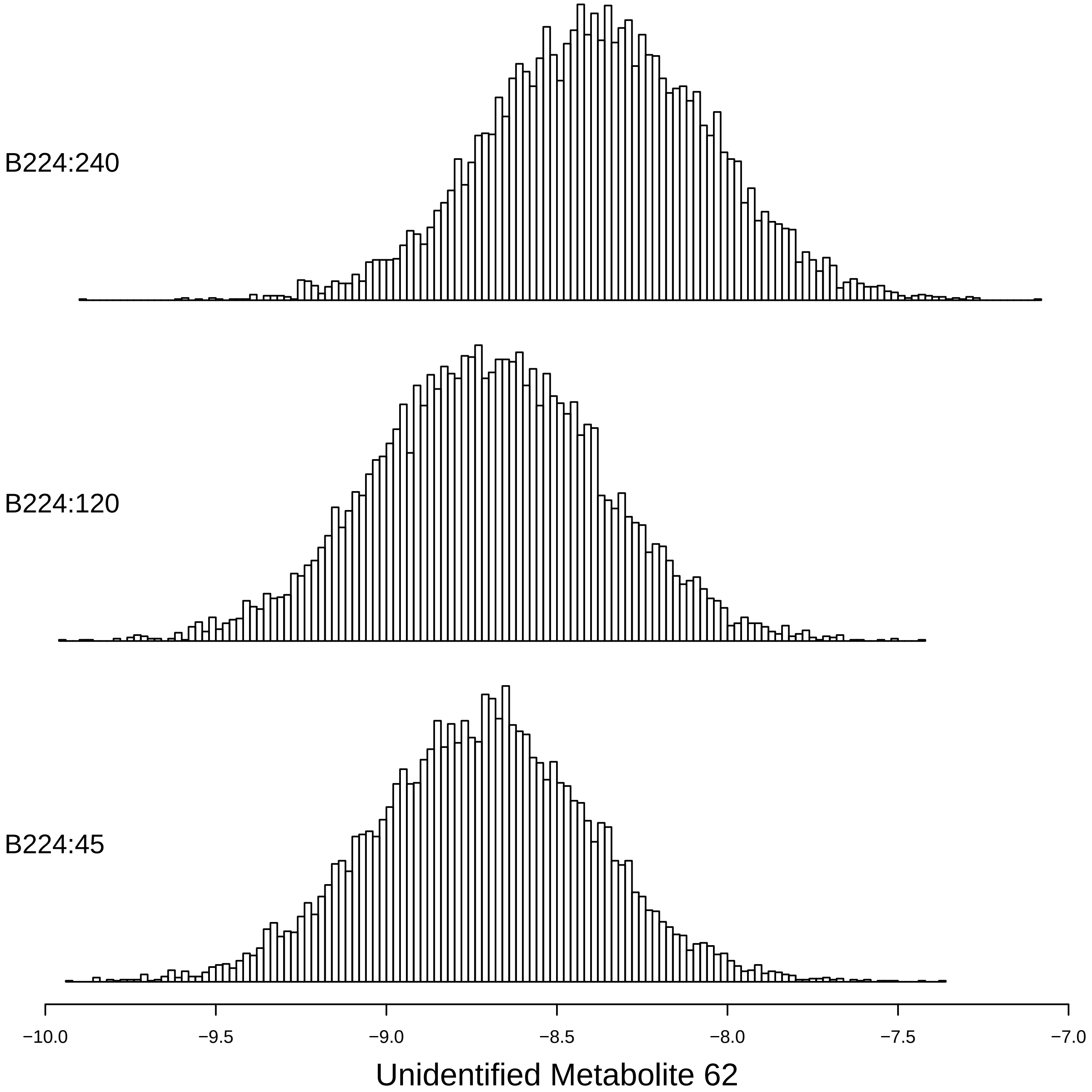
B224:240
B224:120
B224:45
−10.0
−9.5
−9.0
−8.5
−8.0
−7.5
−7.0
Unidentified Metabolite 62

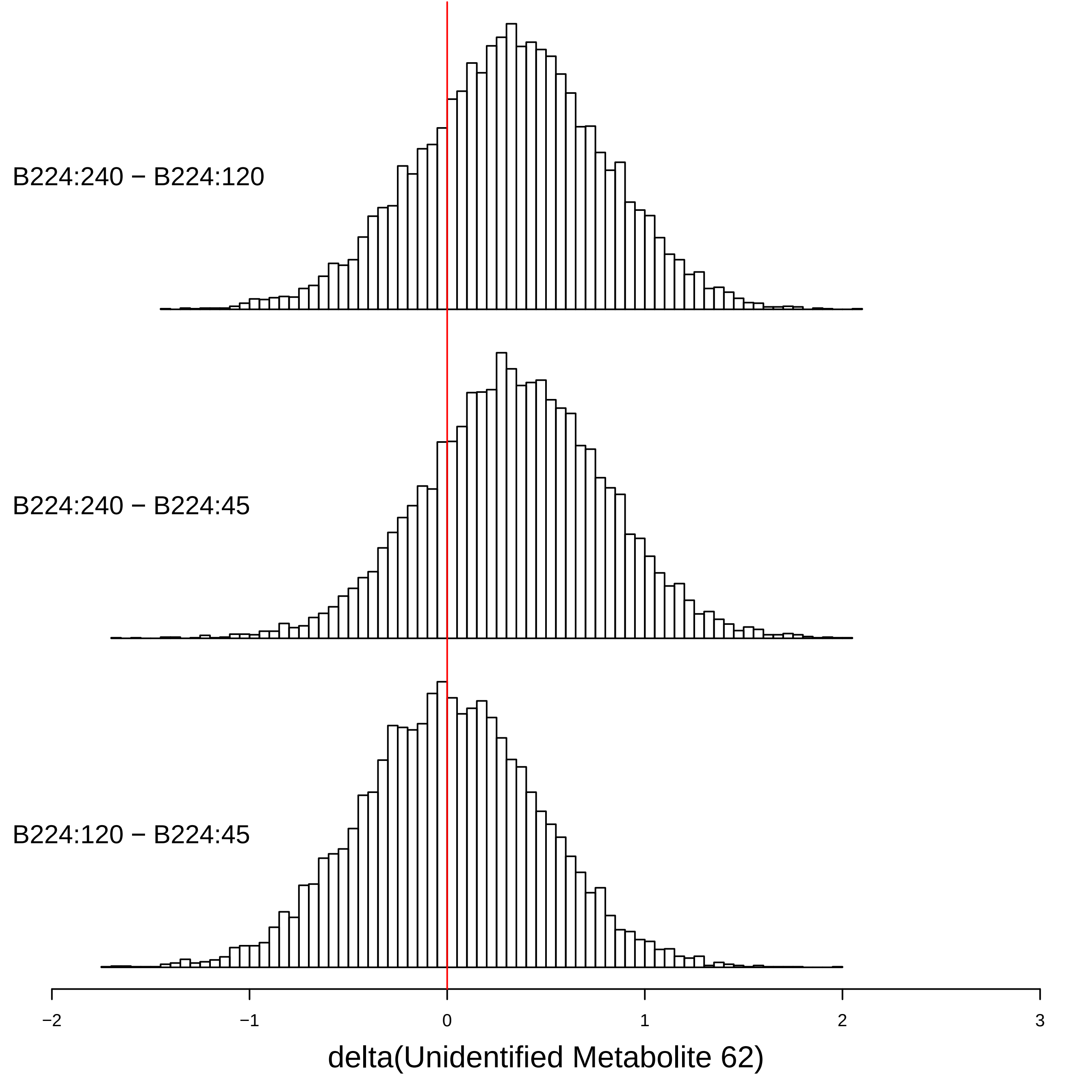

B224:240 − B224:120
B224:240 − B224:45
B224:120 − B224:45
−2
−1
0
1
2
3
delta(Unidentified Metabolite 62)

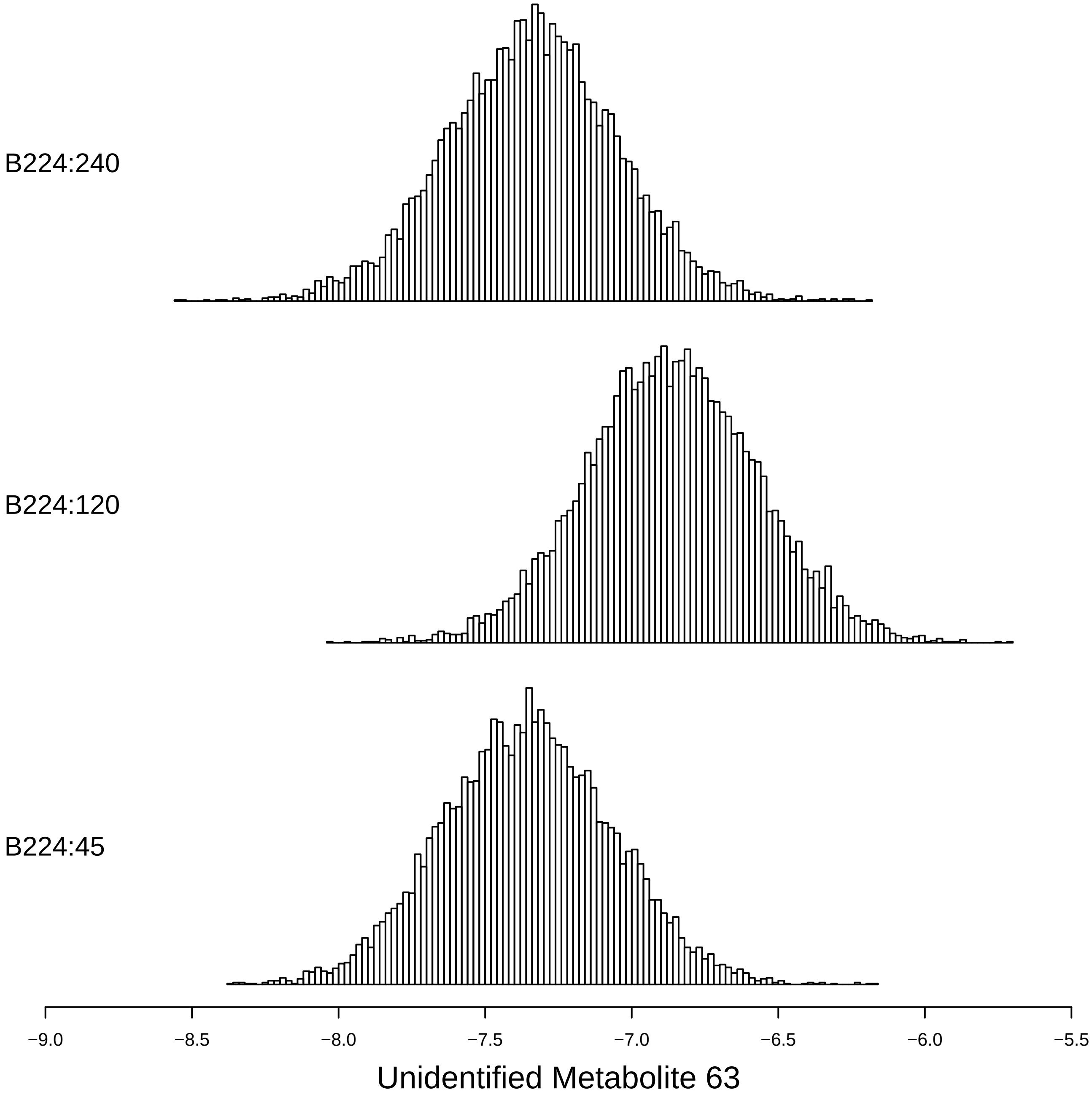
B224:240
B224:120
B224:45
−9.0
−8.5
−8.0
−7.5
−7.0
−6.5
−6.0
−5.5
Unidentified Metabolite 63

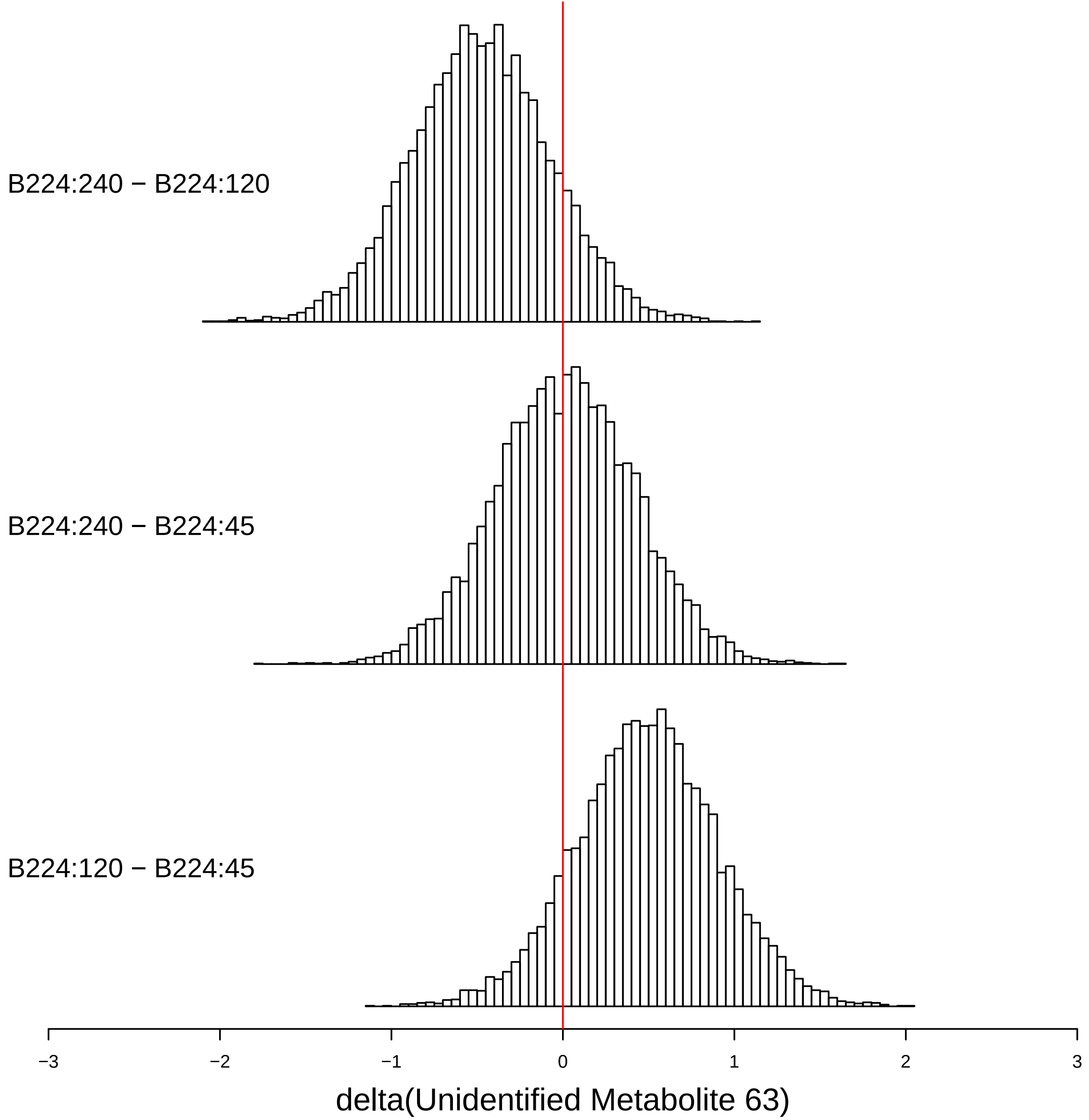
B224:240 − B224:120
B224:240 − B224:45
B224:120 − B224:45
−3
−2
−1
0
1
2
3
delta(Unidentified Metabolite 63)

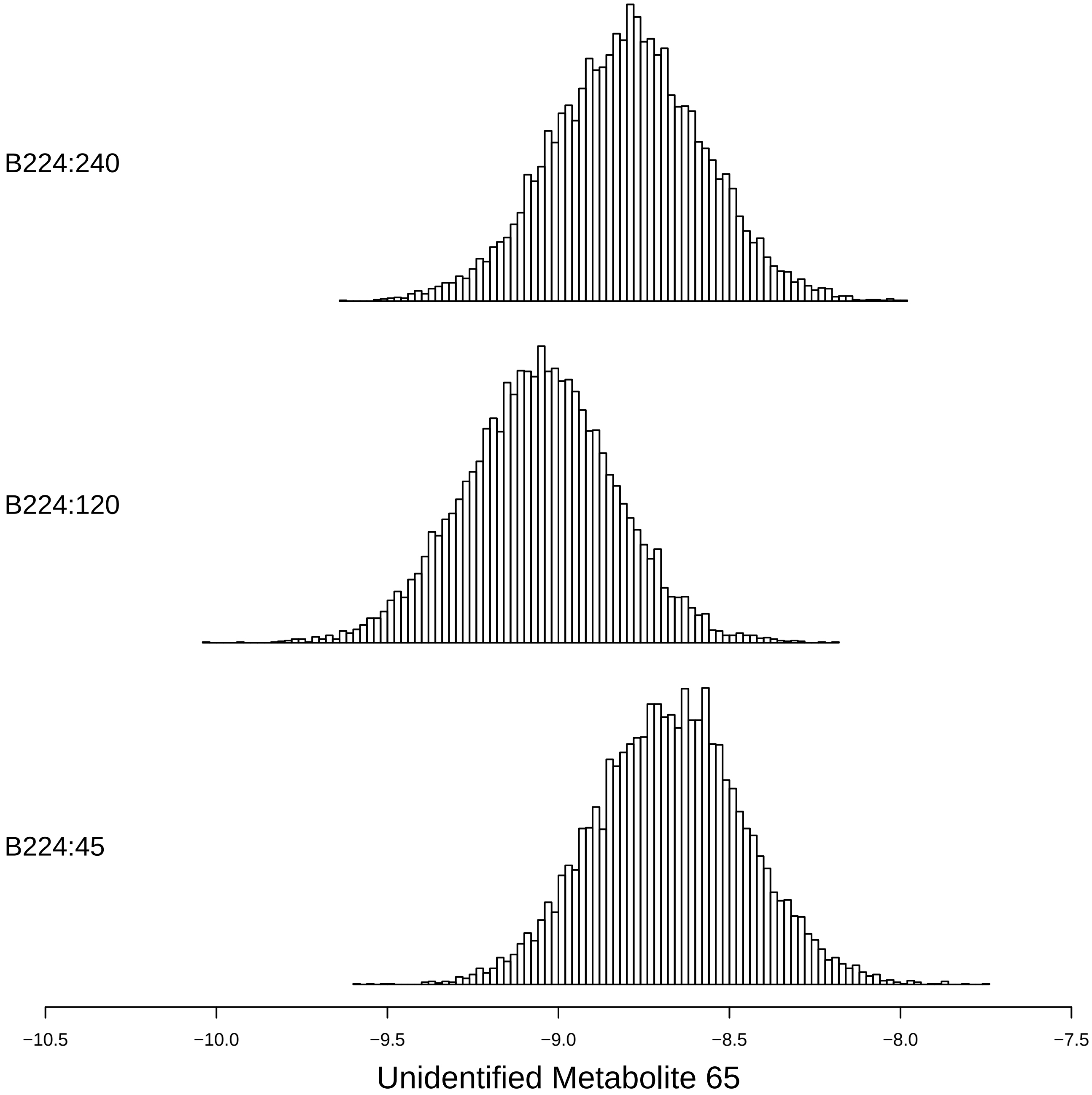

B224:240
B224:120
B224:45
−10.5
−10.0
−9.5
−9.0
−8.5
−8.0
−7.5
Unidentified Metabolite 65

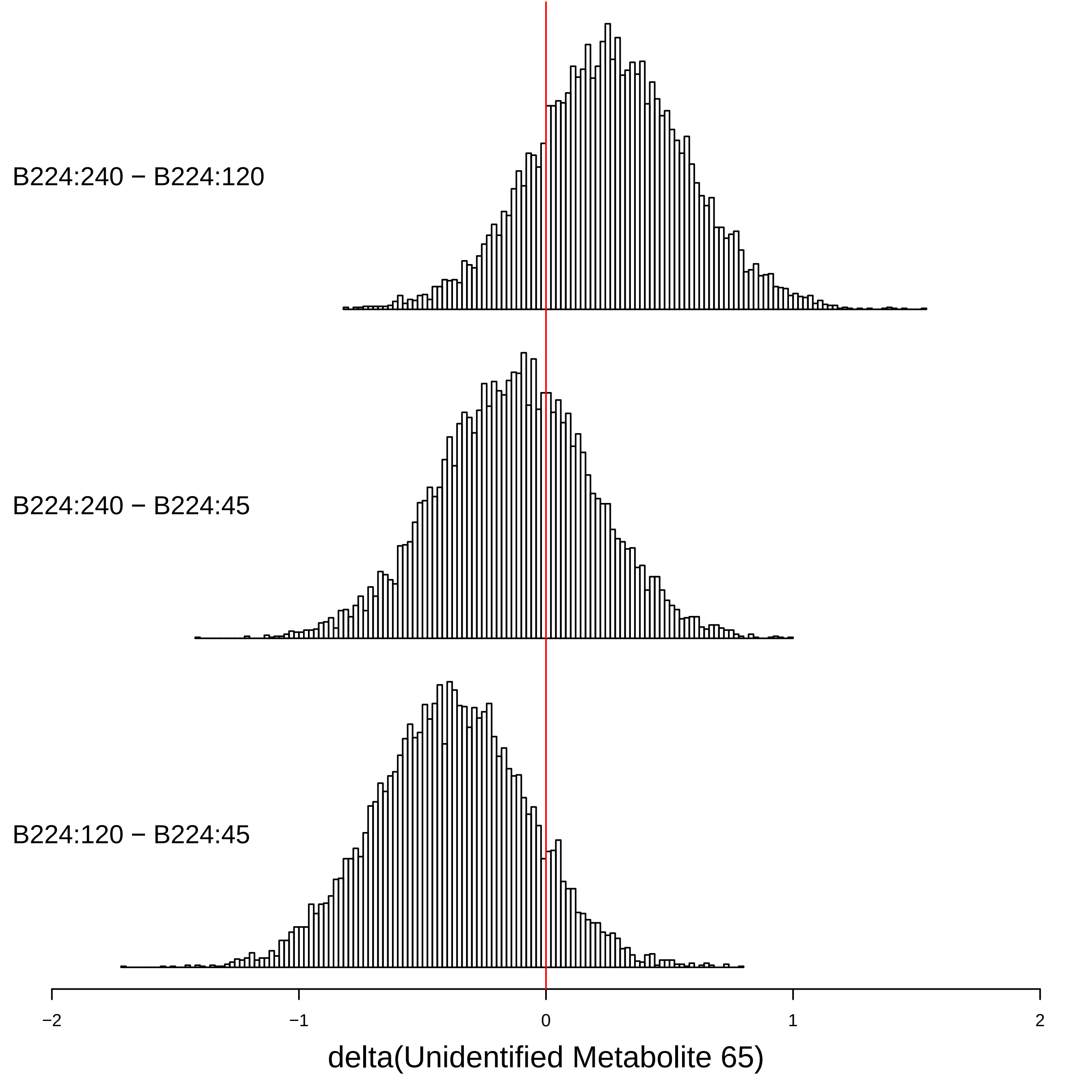
B224:240 − B224:120
B224:240 − B224:45
B224:120 − B224:45
−2
−1
0
1
2
delta(Unidentified Metabolite 65)

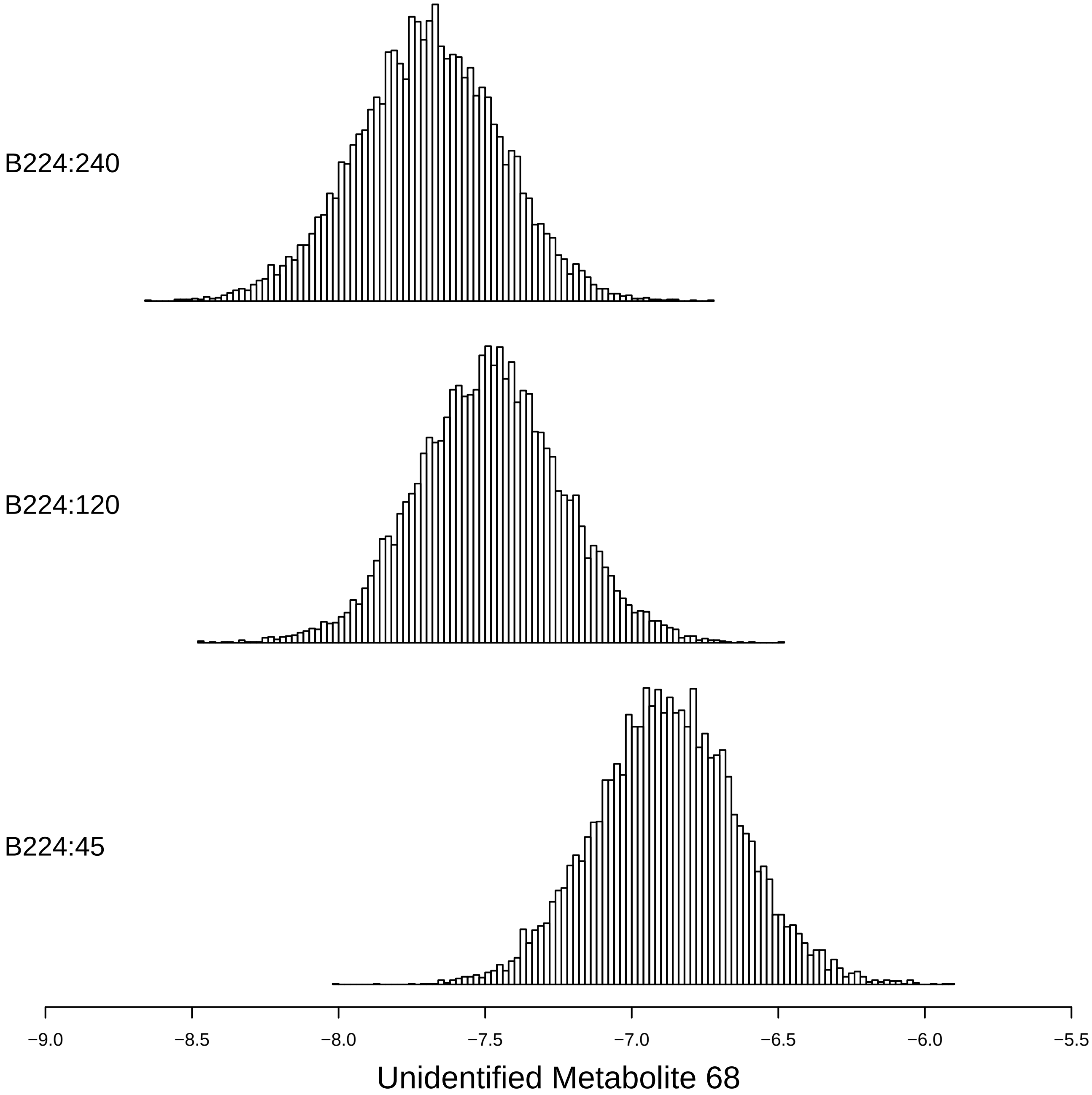
B224:240
B224:120
B224:45
−9.0
−8.5
−8.0
−7.5
−7.0
−6.5
−6.0
−5.5
Unidentified Metabolite 68

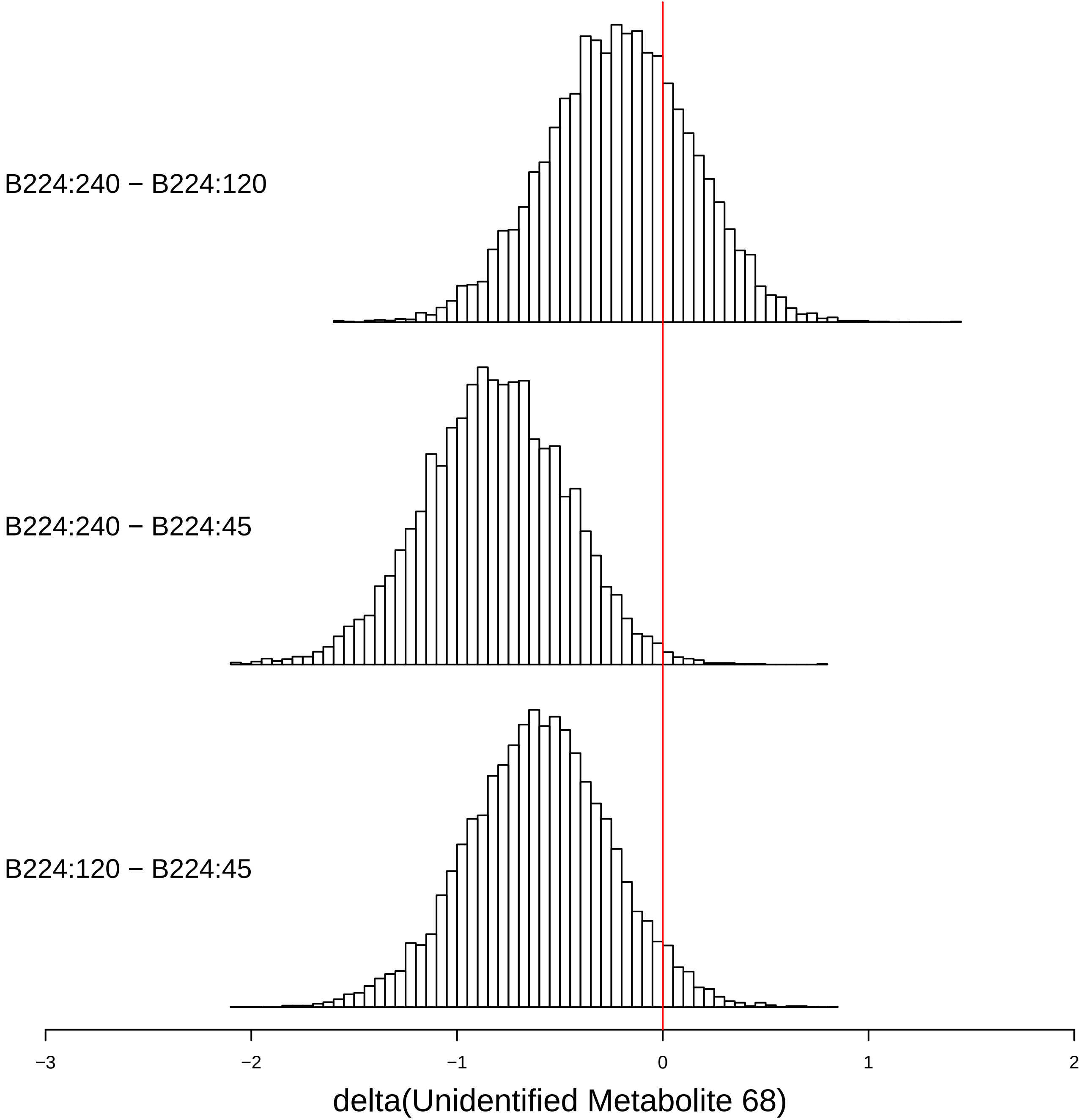
B224:240 − B224:120
B224:240 − B224:45
B224:120 − B224:45
−3
−2
−1
0
1
2
delta(Unidentified Metabolite 68)

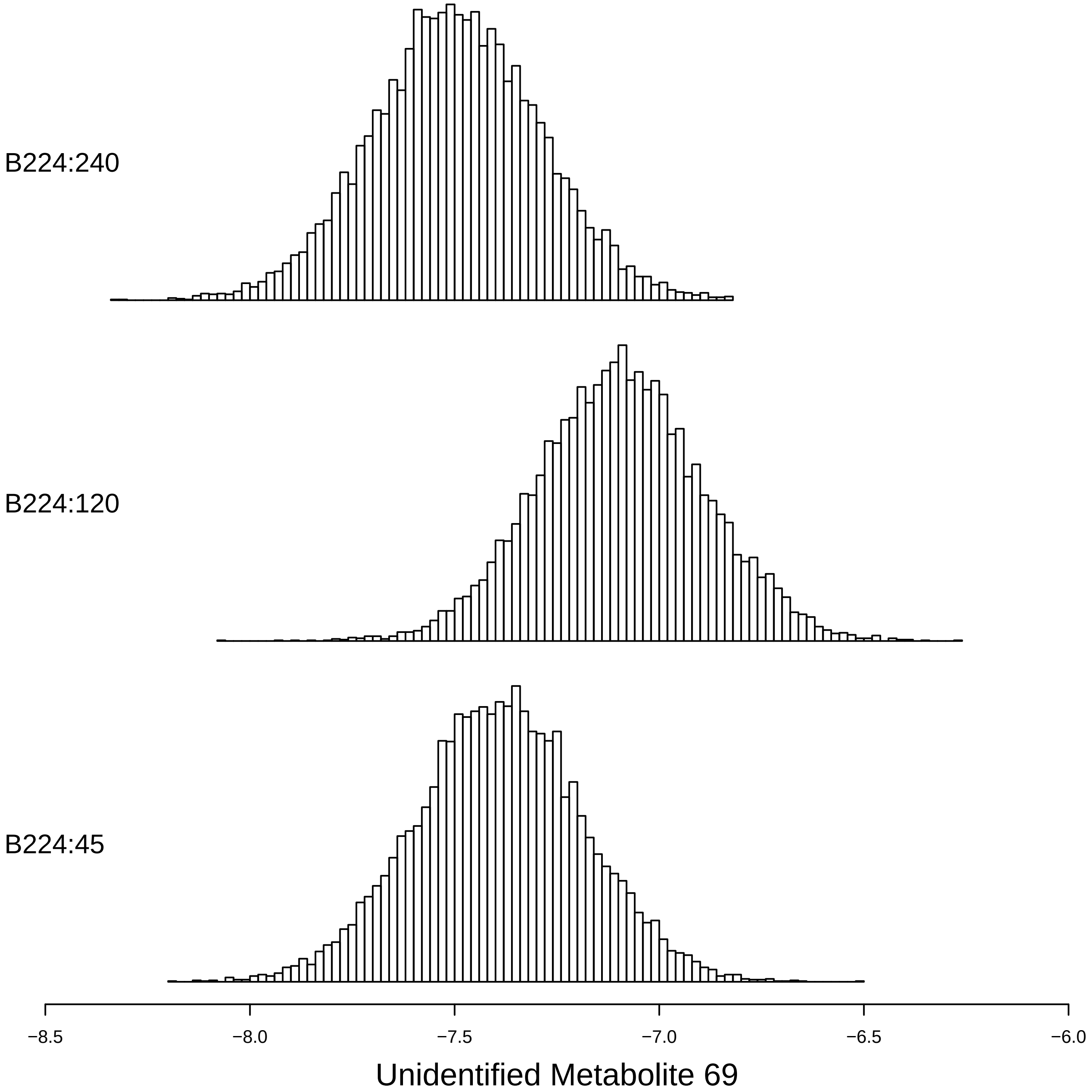
B224:240
B224:120
B224:45
−8.5
−8.0
−7.5
−7.0
−6.5
−6.0
Unidentified Metabolite 69

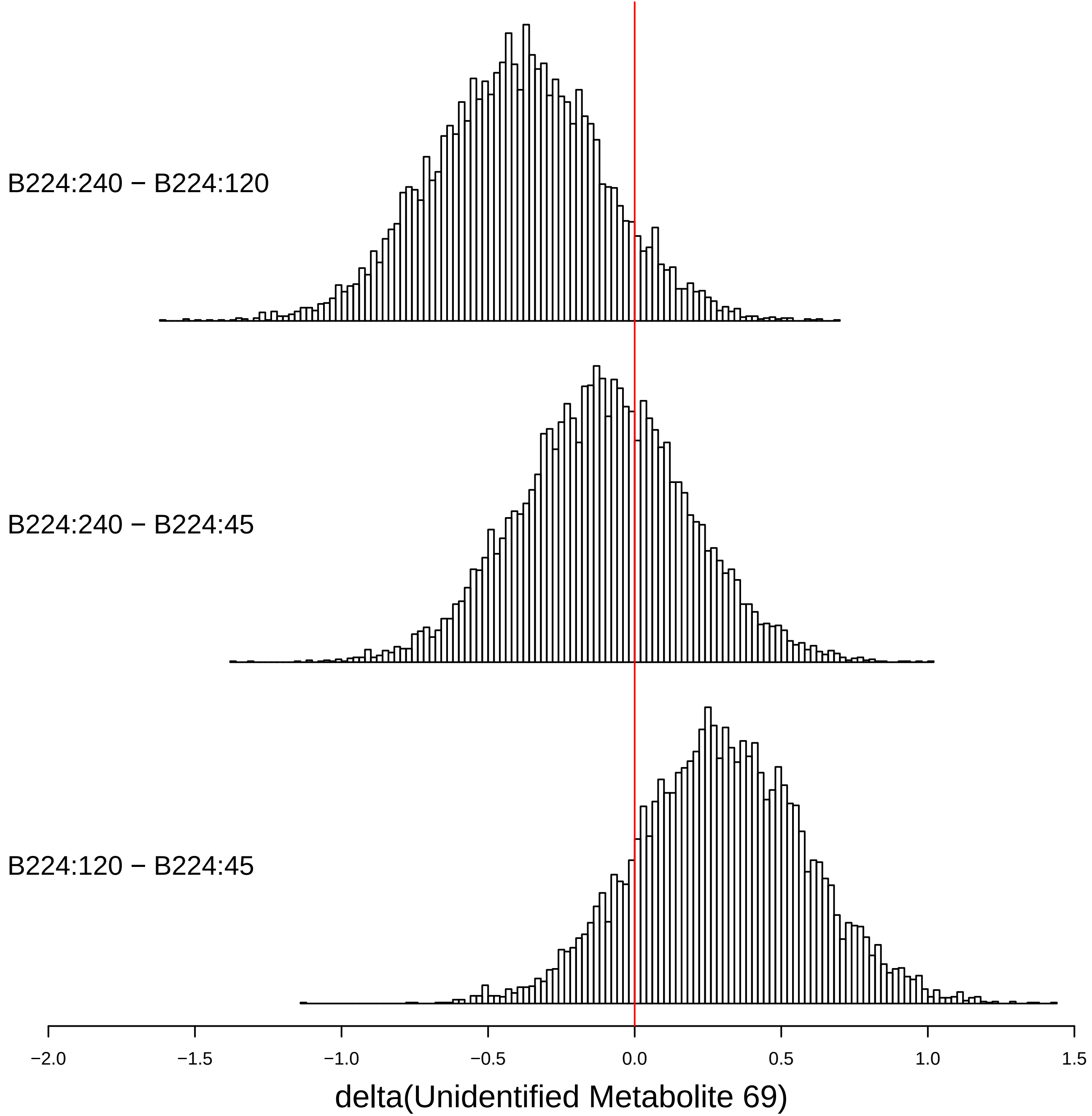
B224:240 − B224:120
B224:240 − B224:45
B224:120 − B224:45
−2.0
−1.5
−1.0
−0.5
0.0
0.5
1.0
1.5
delta(Unidentified Metabolite 69)

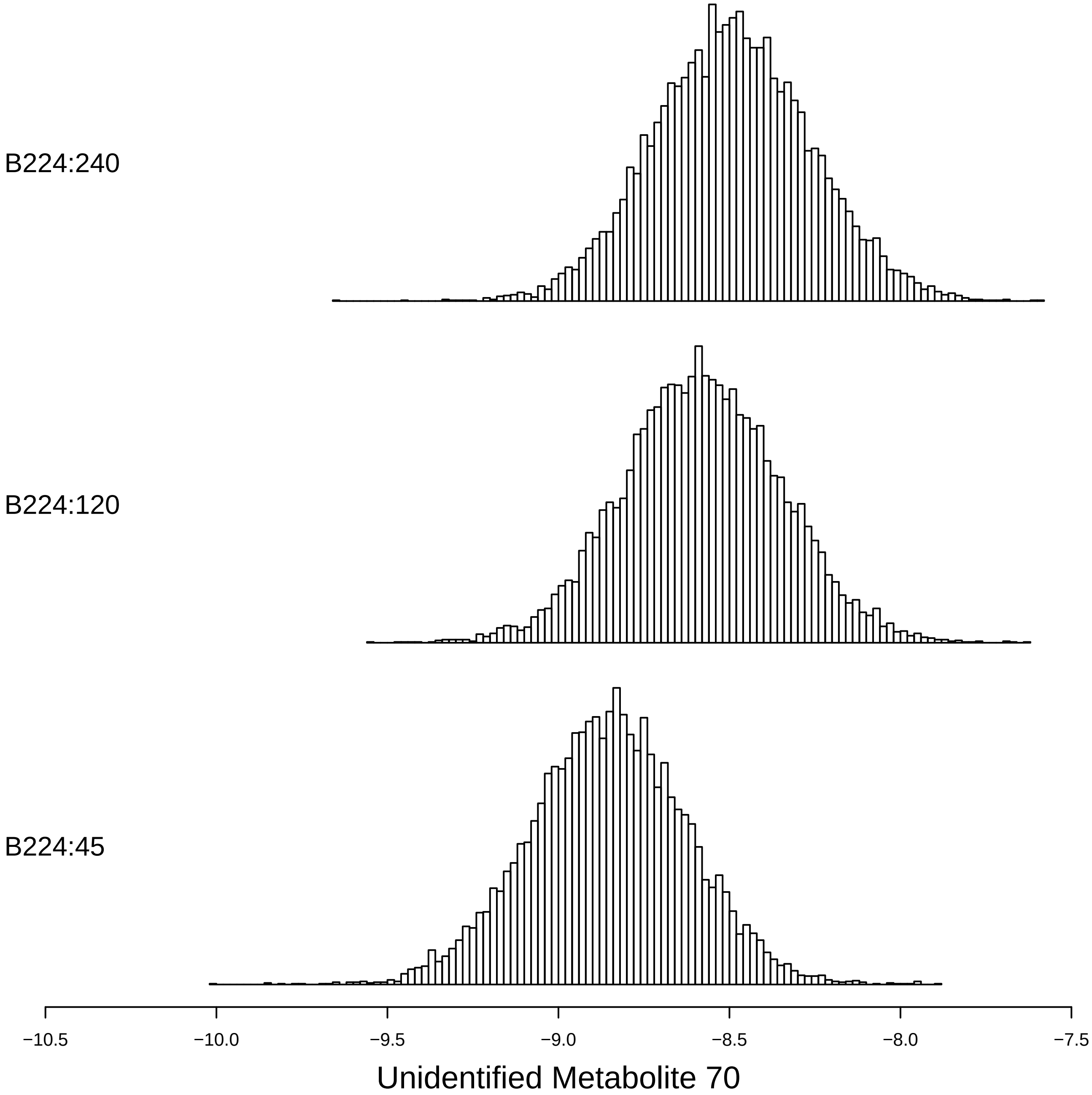

B224:240
B224:120
B224:45
−10.5
−10.0
−9.5
−9.0
−8.5
−8.0
−7.5
Unidentified Metabolite 70

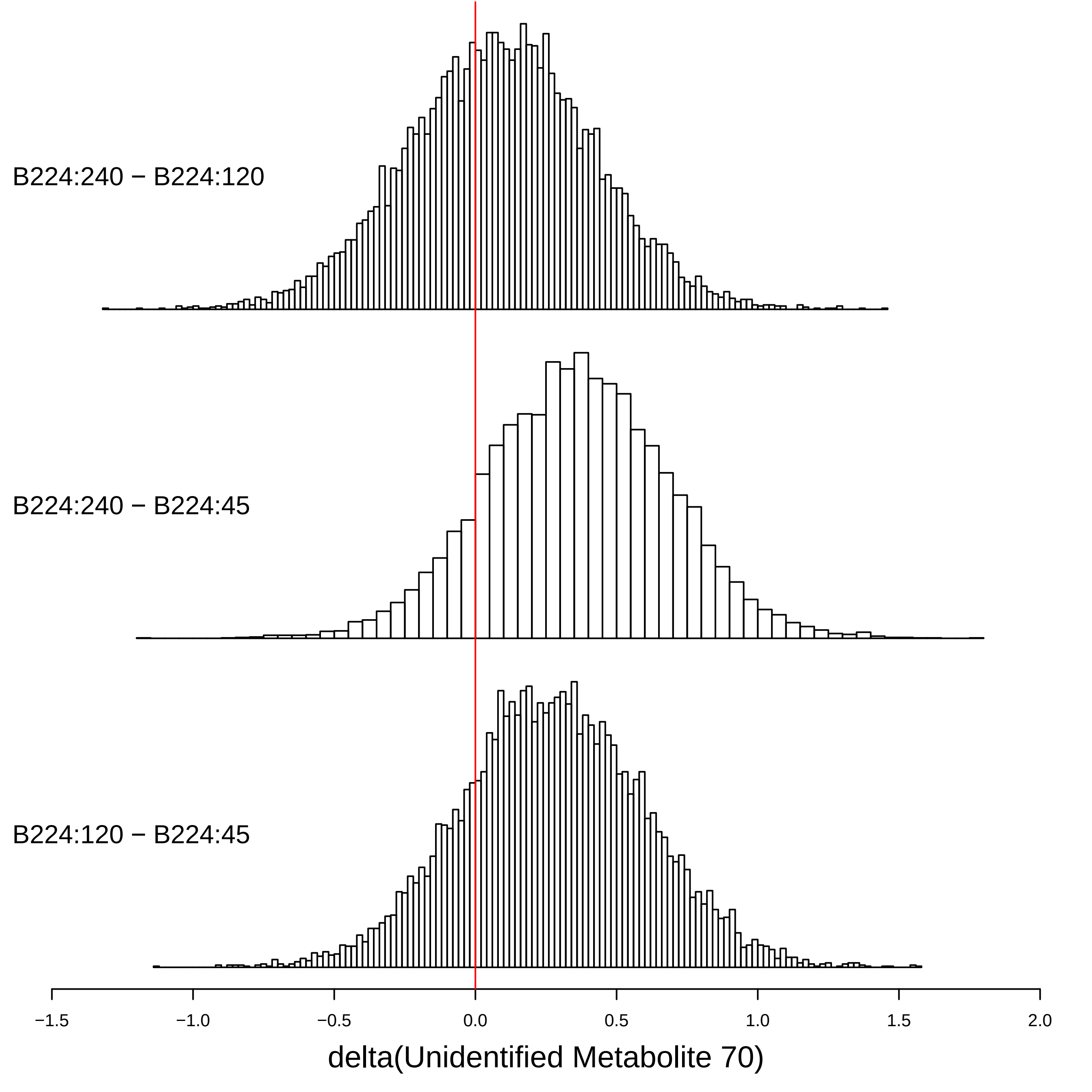

B224:240 − B224:120
B224:240 − B224:45
B224:120 − B224:45
−1.5
−1.0
−0.5
0.0
0.5
1.0
1.5
2.0
delta(Unidentified Metabolite 70)

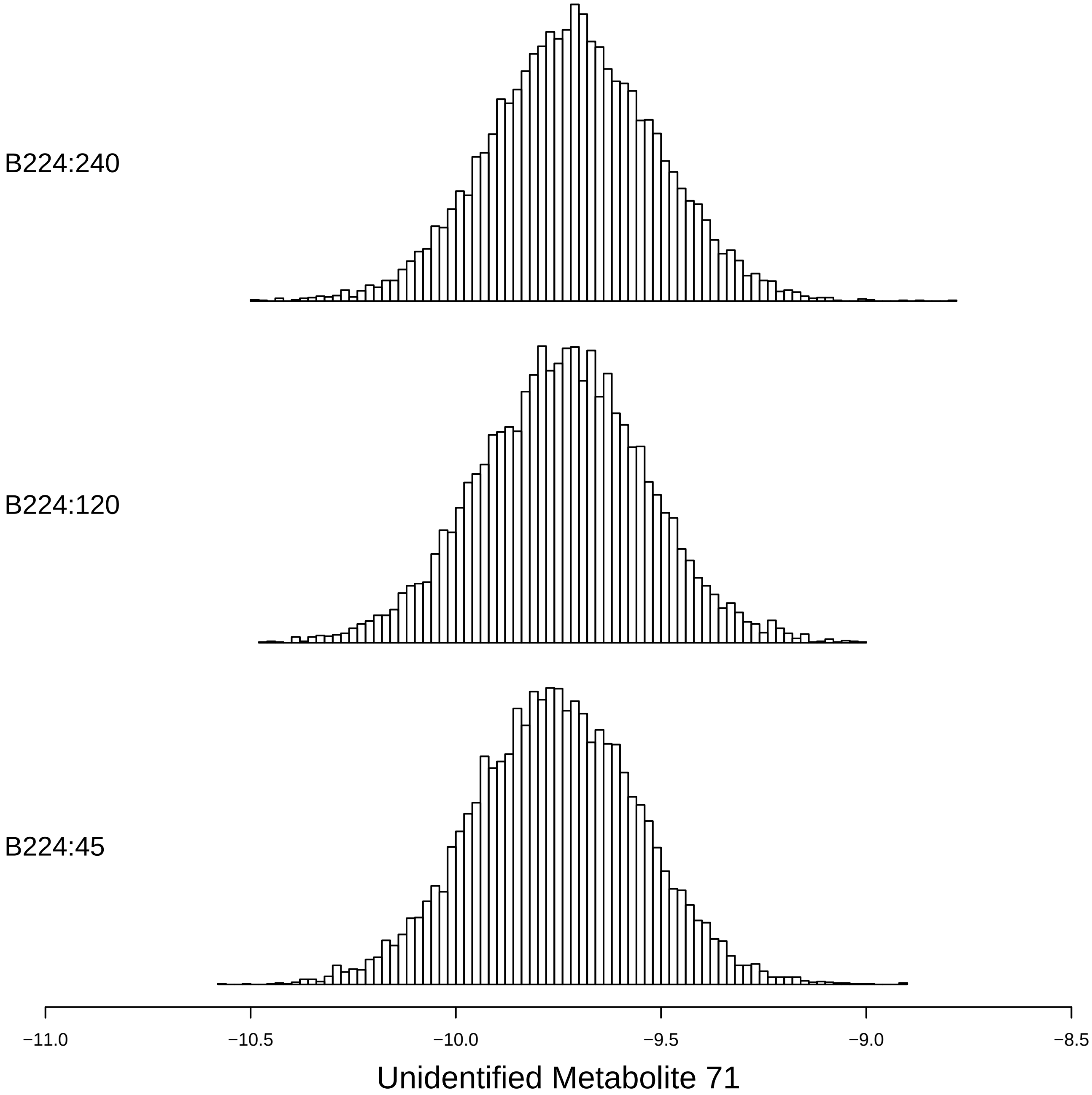

B224:240
B224:120
B224:45
−11.0
−10.5
−10.0
−9.5
−9.0
−8.5
Unidentified Metabolite 71

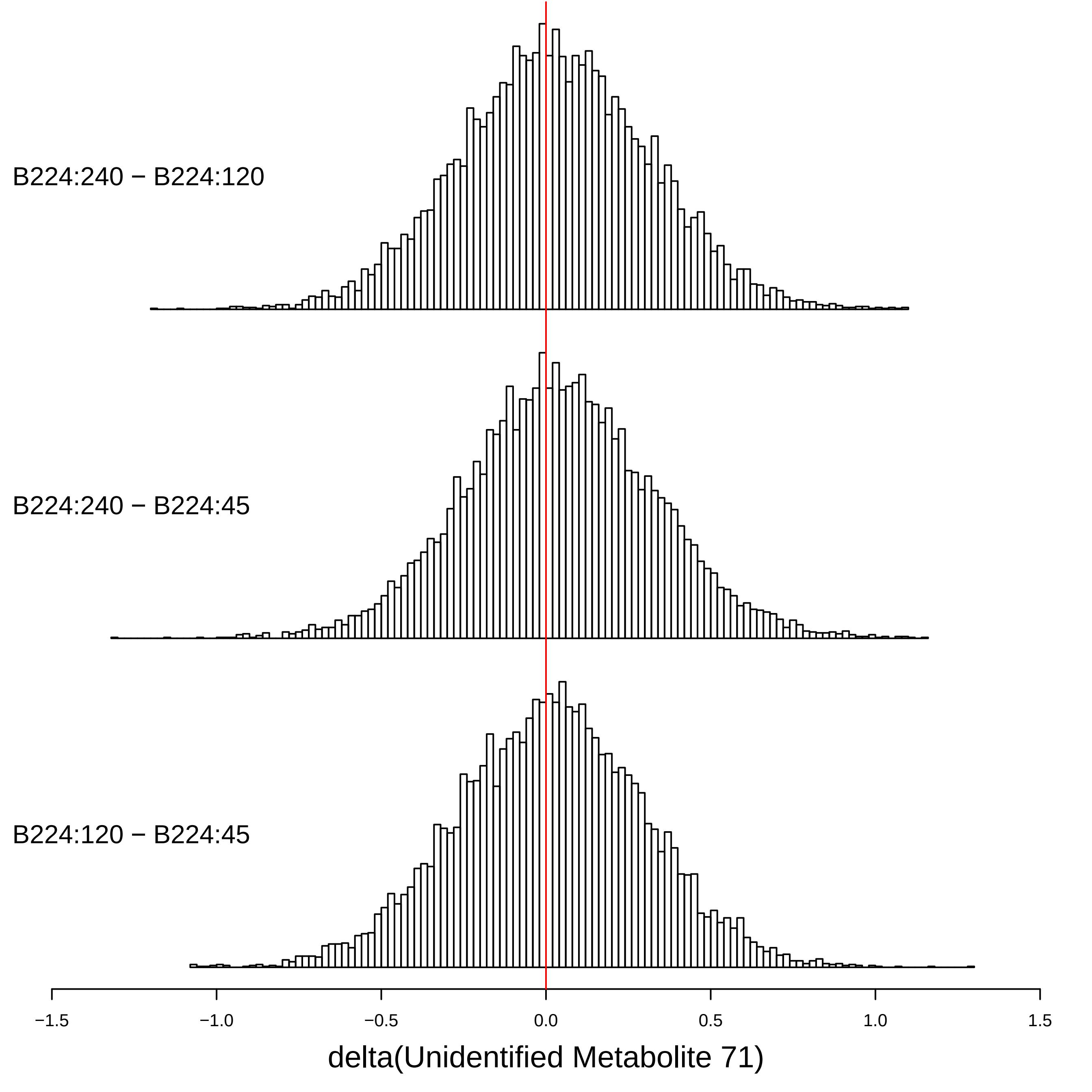
B224:240 − B224:120
B224:240 − B224:45
B224:120 − B224:45
−1.5
−1.0
−0.5
0.0
0.5
1.0
1.5
delta(Unidentified Metabolite 71)

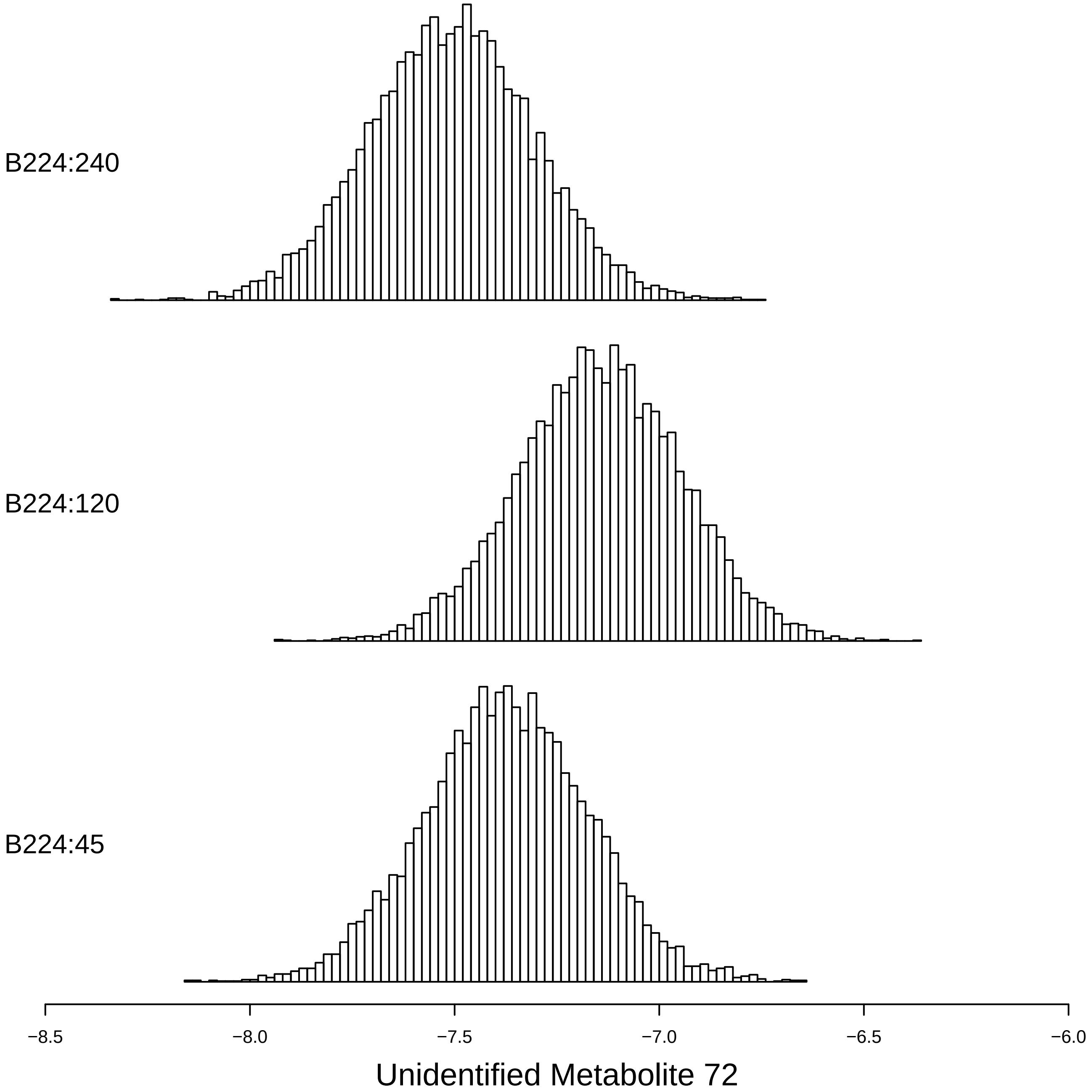
B224:240
B224:120
B224:45
−8.5
−8.0
−7.5
−7.0
−6.5
−6.0
Unidentified Metabolite 72

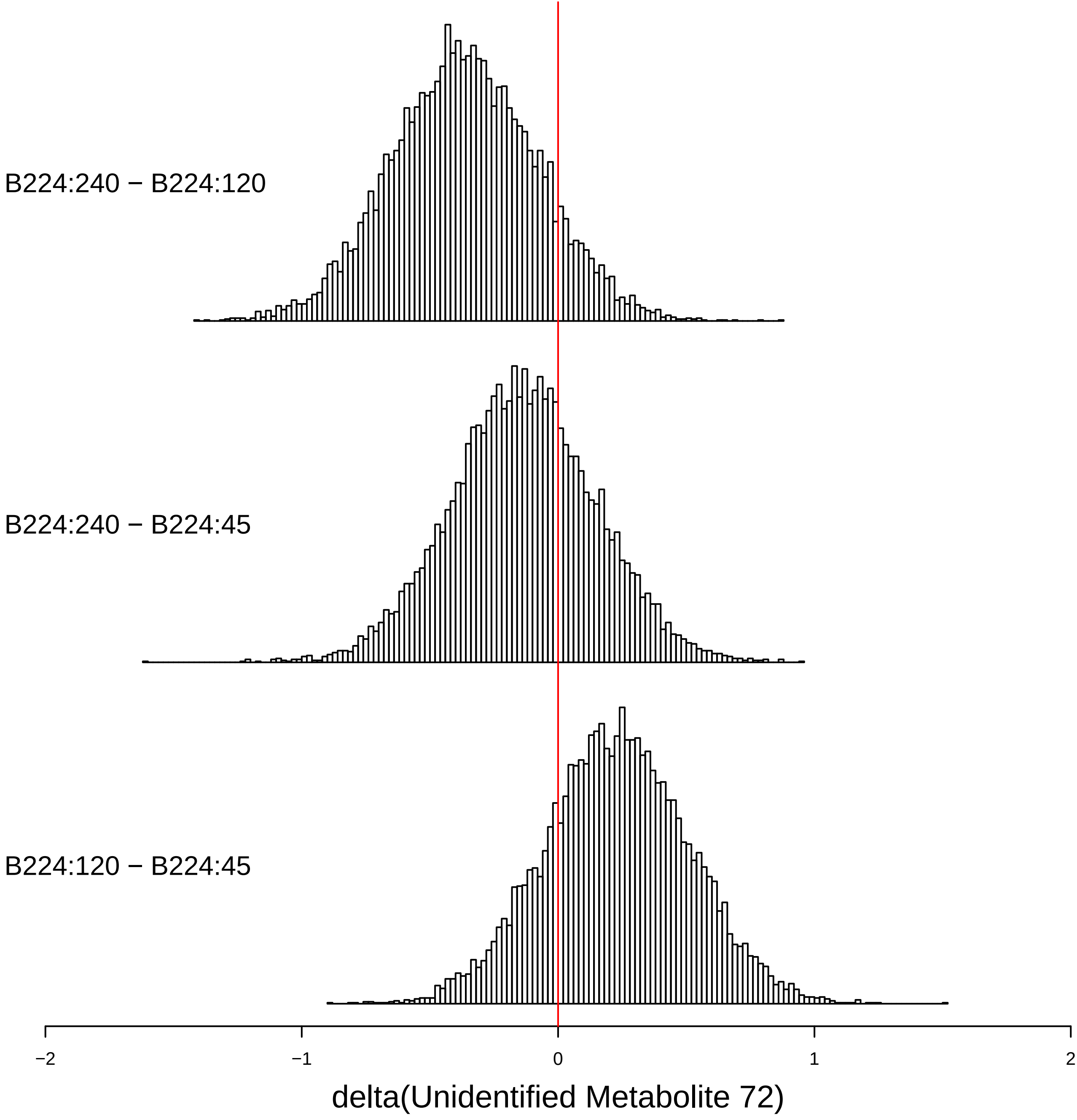

B224:240 − B224:120
B224:240 − B224:45
B224:120 − B224:45
−2
−1
0
1
2
delta(Unidentified Metabolite 72)

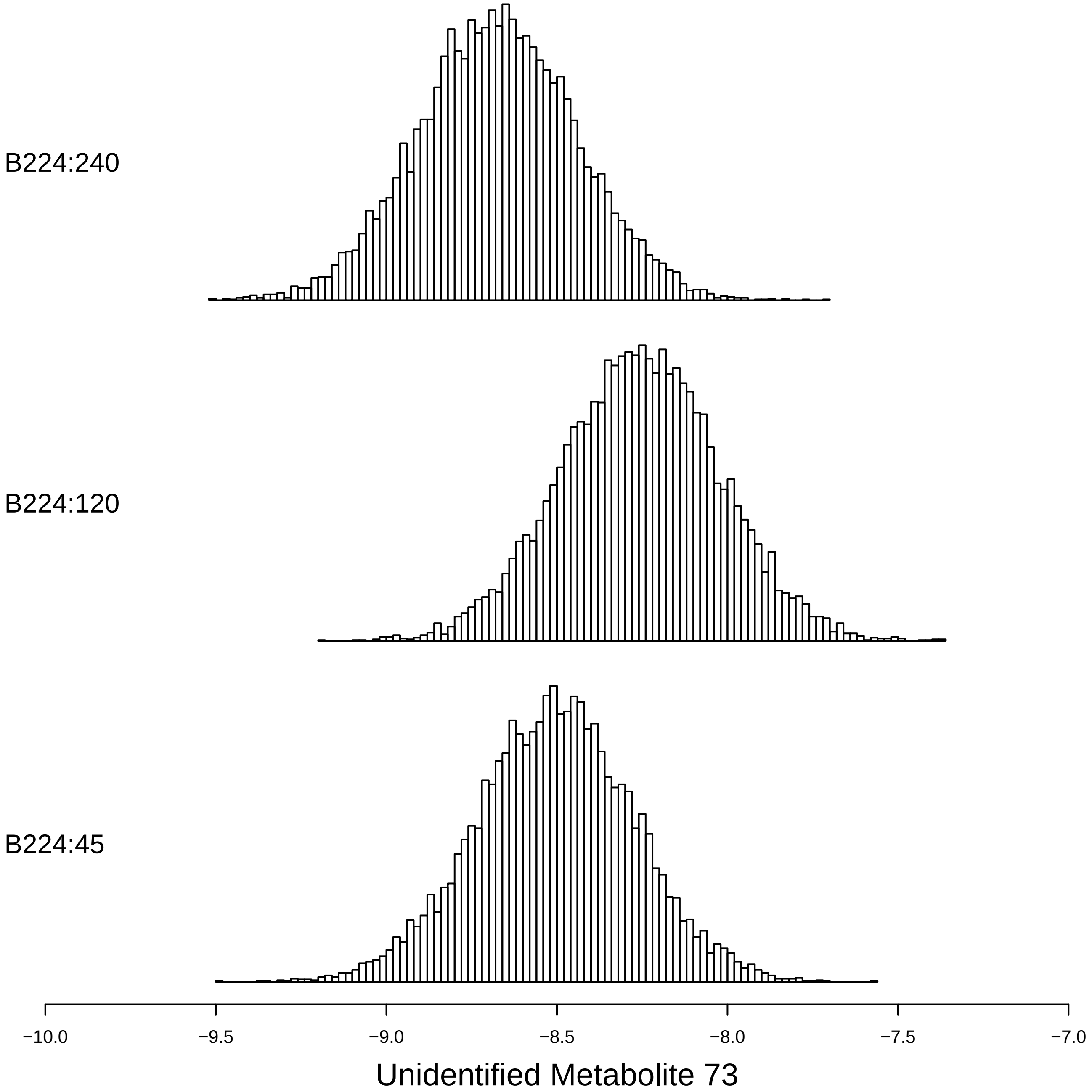

B224:240
B224:120
B224:45
−10.0
−9.5
−9.0
−8.5
−8.0
−7.5
−7.0
Unidentified Metabolite 73

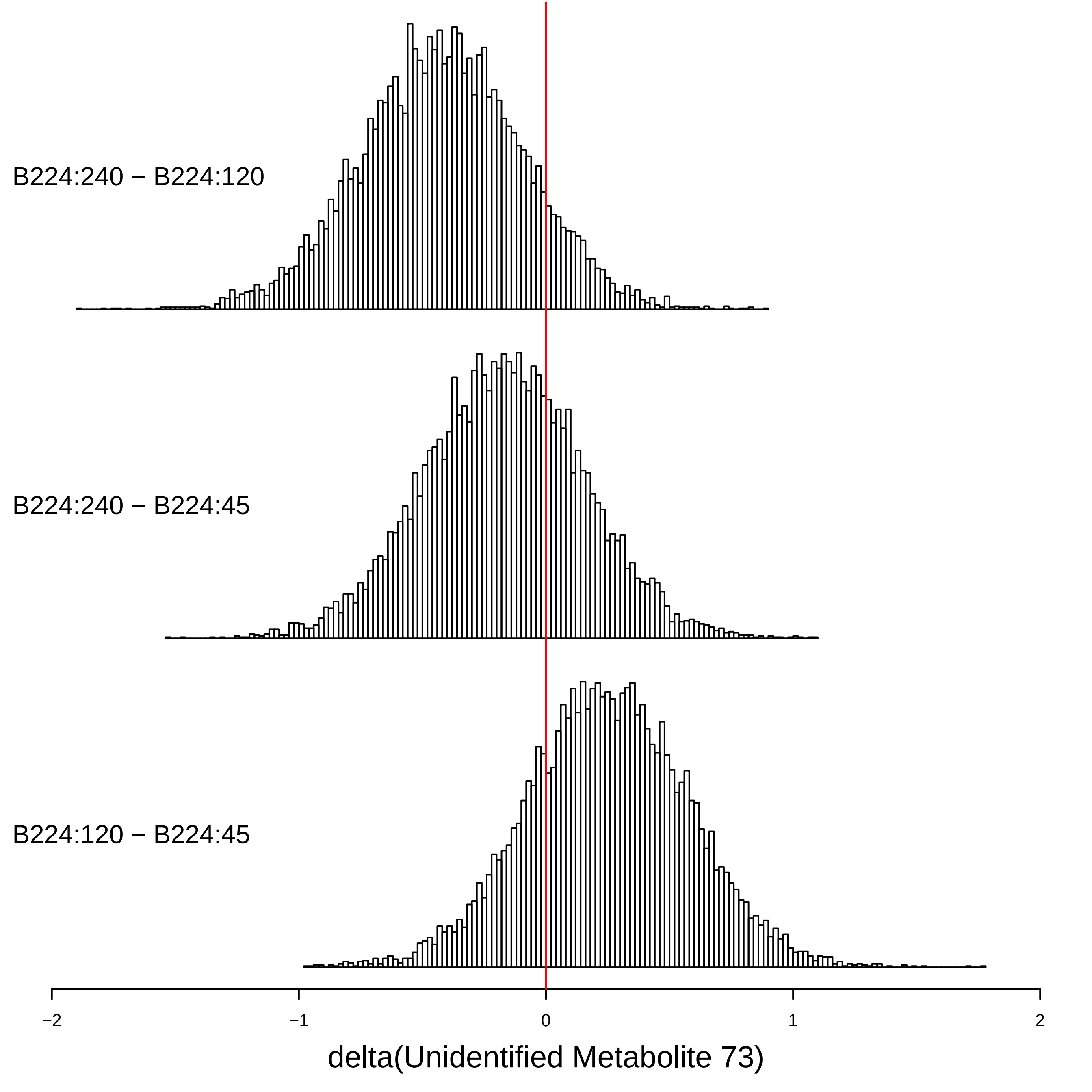

B224:240 − B224:120
B224:240 − B224:45
B224:120 − B224:45
−2
−1
0
1
2
delta(Unidentified Metabolite 73)

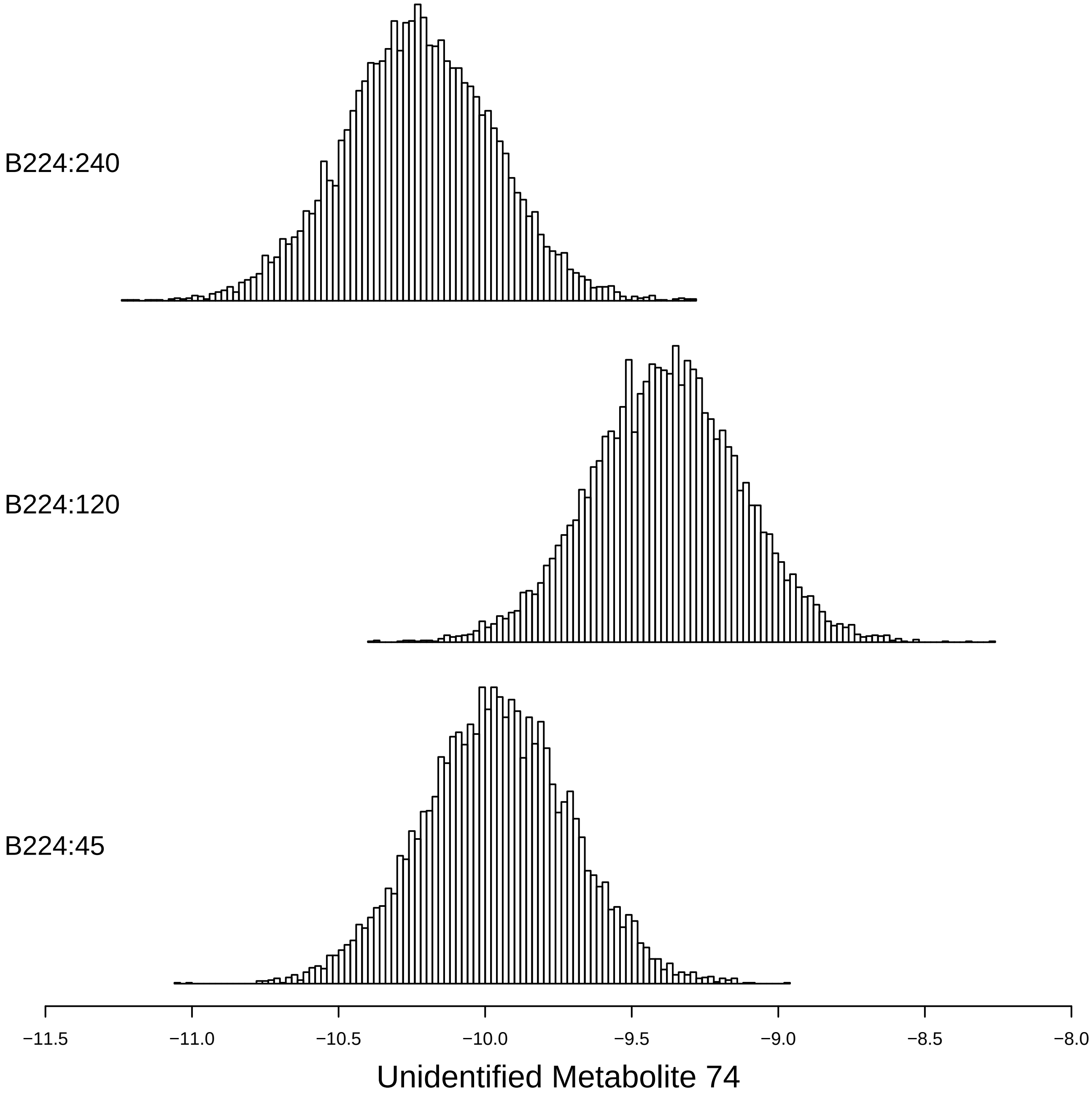
B224:240
B224:120
B224:45
−11.5
−11.0
−10.5
−10.0
−9.5
−9.0
−8.5
−8.0
Unidentified Metabolite 74

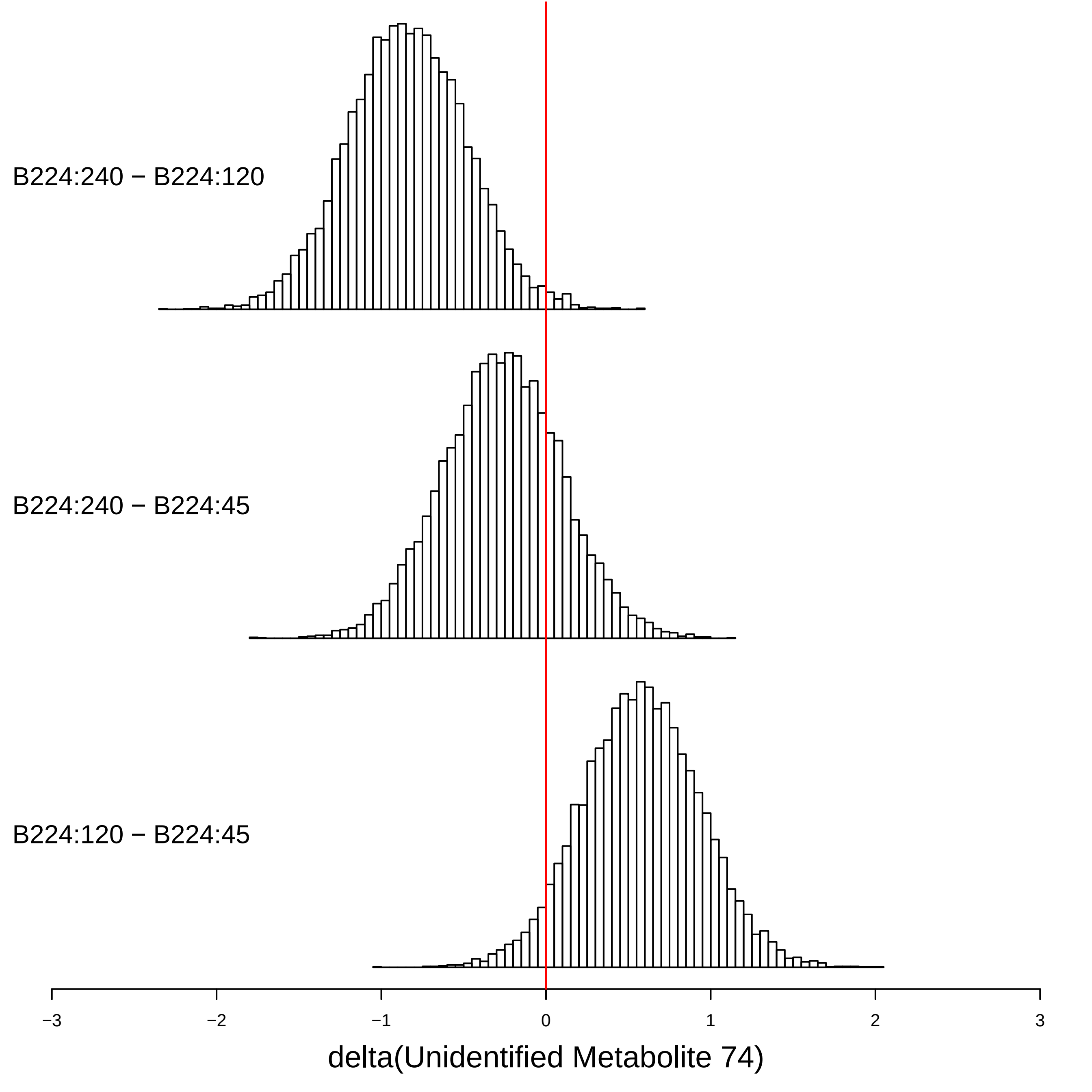
B224:240 − B224:120
B224:240 − B224:45
B224:120 − B224:45
−3
−2
−1
0
1
2
3
delta(Unidentified Metabolite 74)

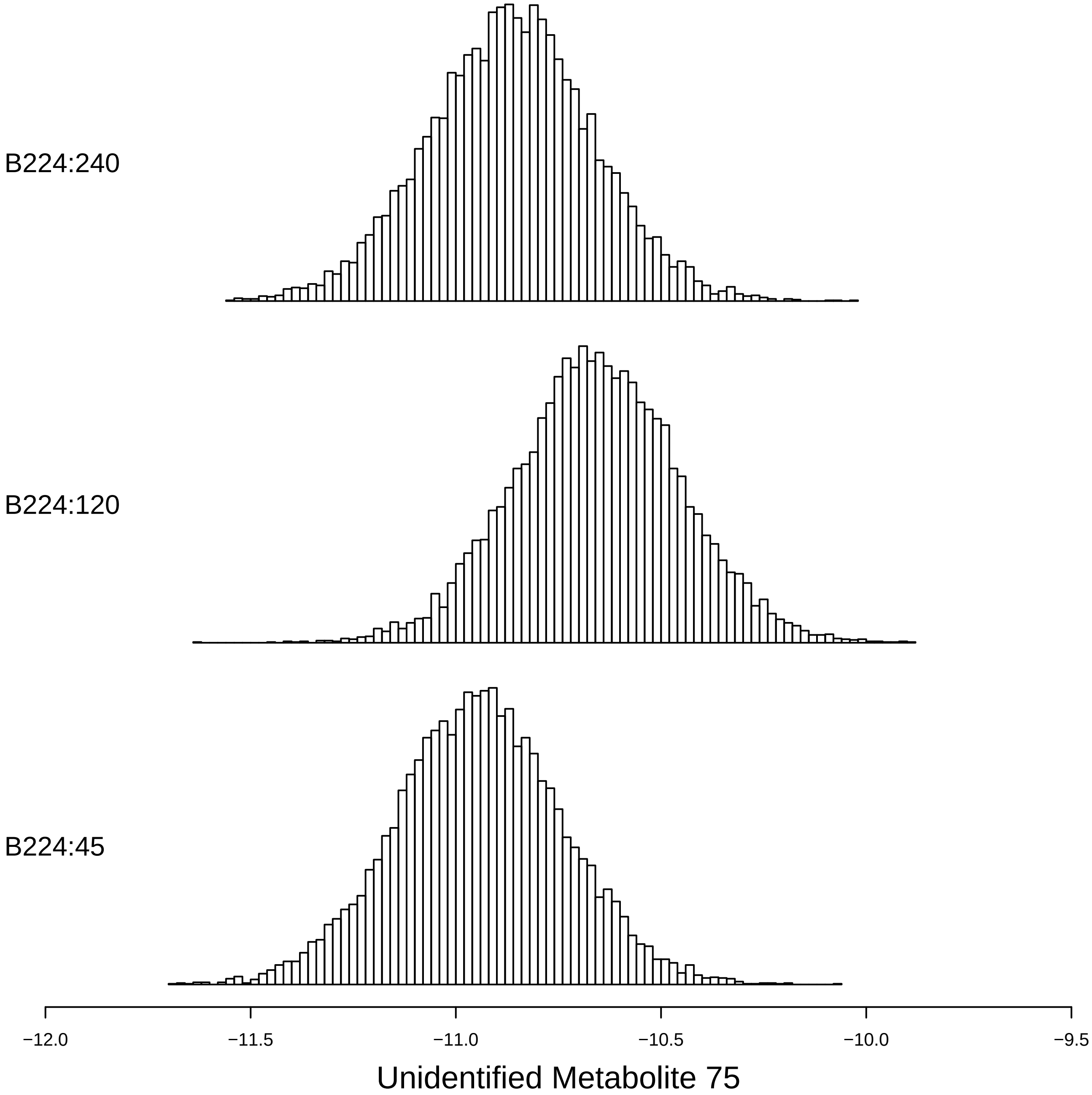
B224:240
B224:120
B224:45
−12.0
−11.5
−11.0
−10.5
−10.0
−9.5
Unidentified Metabolite 75

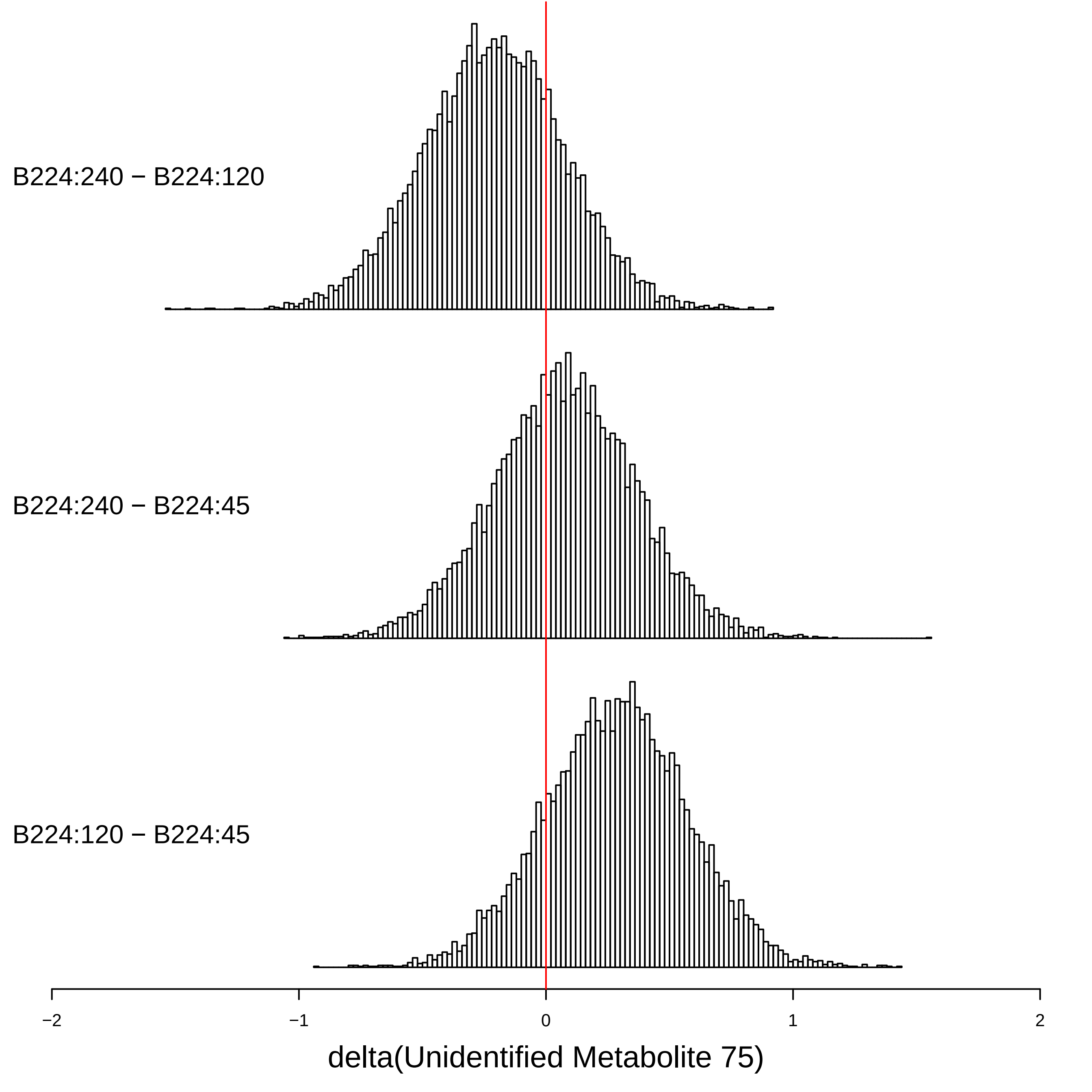
B224:240 − B224:120
B224:240 − B224:45
B224:120 − B224:45
−2
−1
0
1
2
delta(Unidentified Metabolite 75)

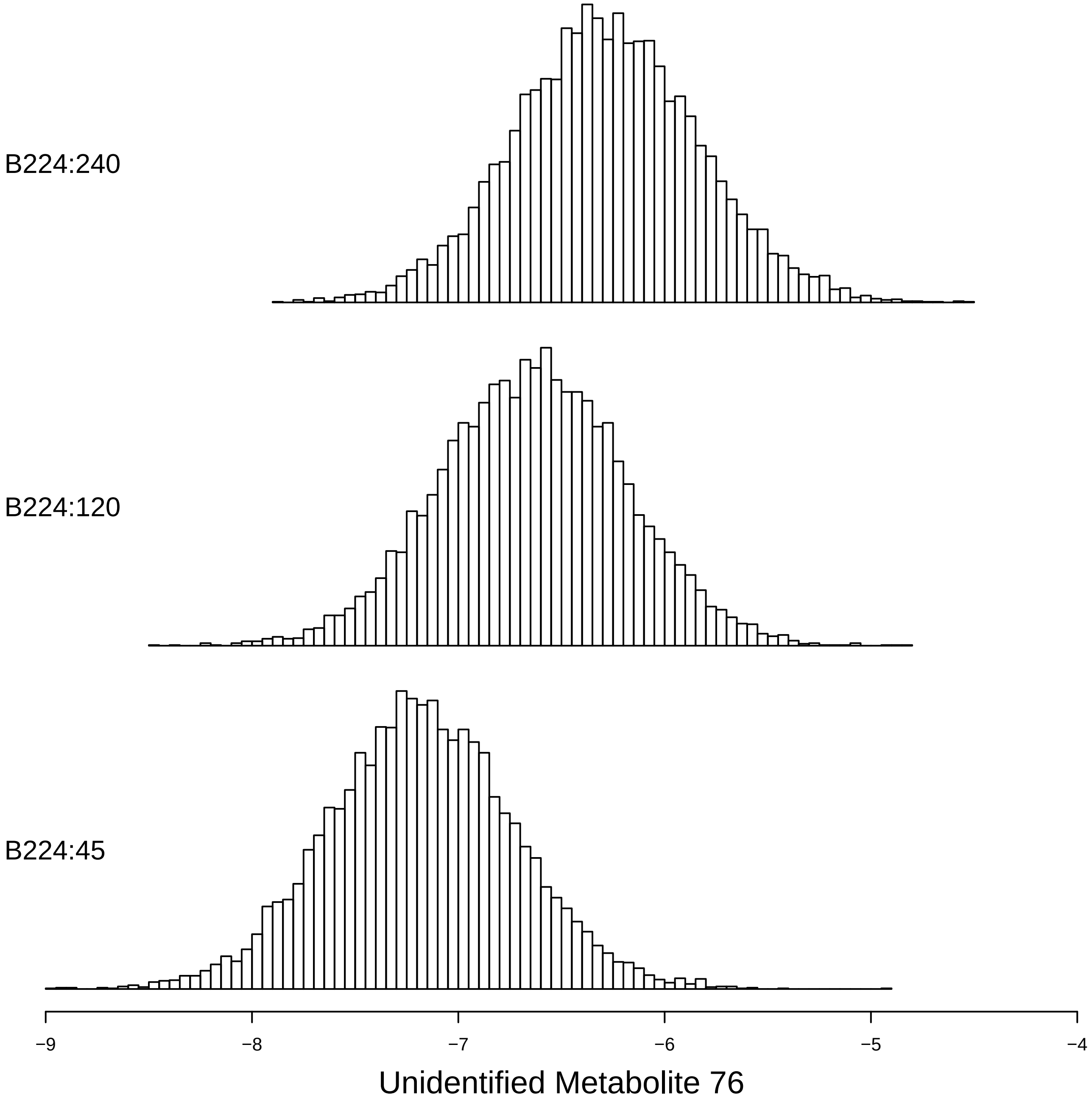
B224:240
B224:120
B224:45
−9
−8
−7
−6
−5
−4
Unidentified Metabolite 76

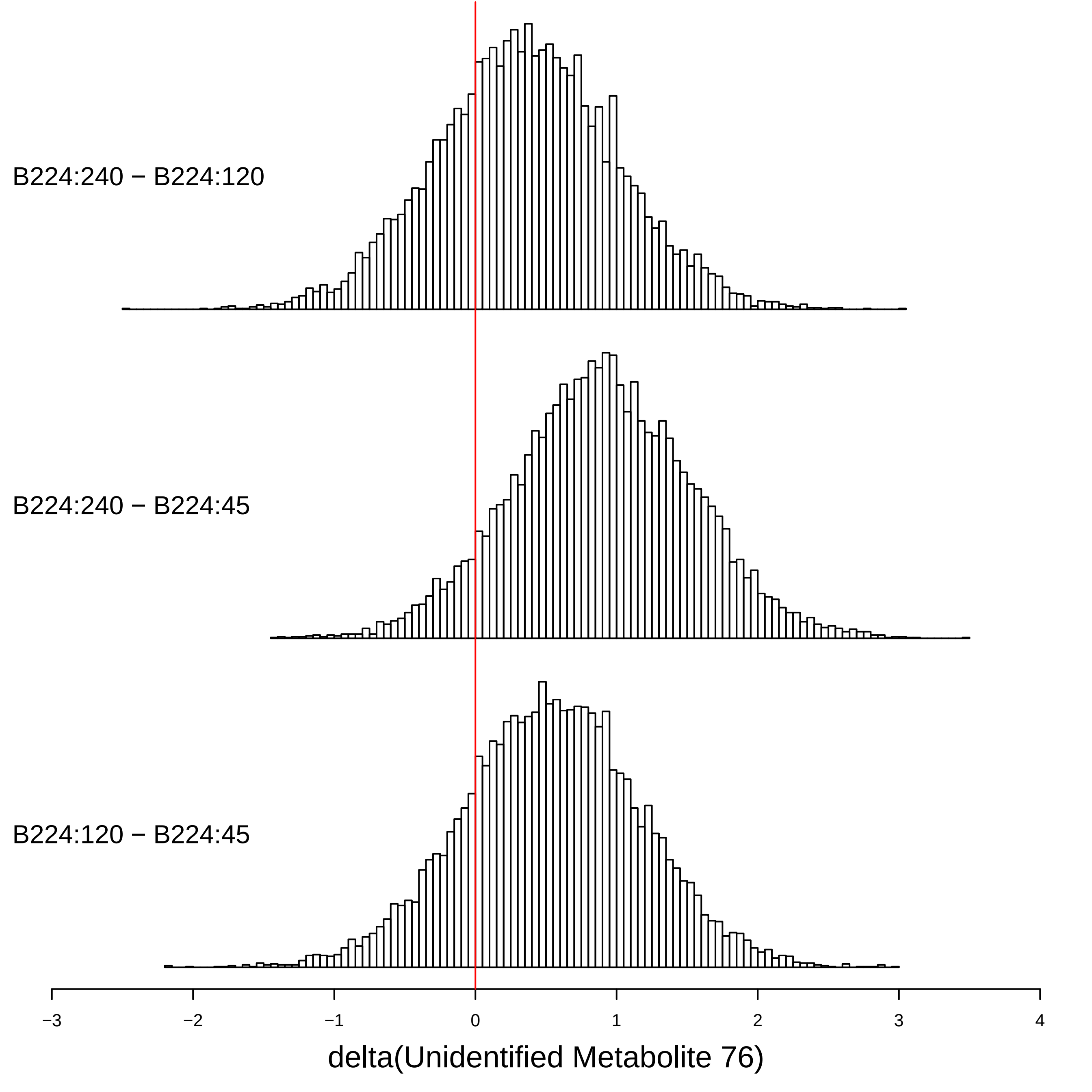

B224:240 − B224:120
B224:240 − B224:45
B224:120 − B224:45
−3
−2
−1
0
1
2
3
4
delta(Unidentified Metabolite 76)

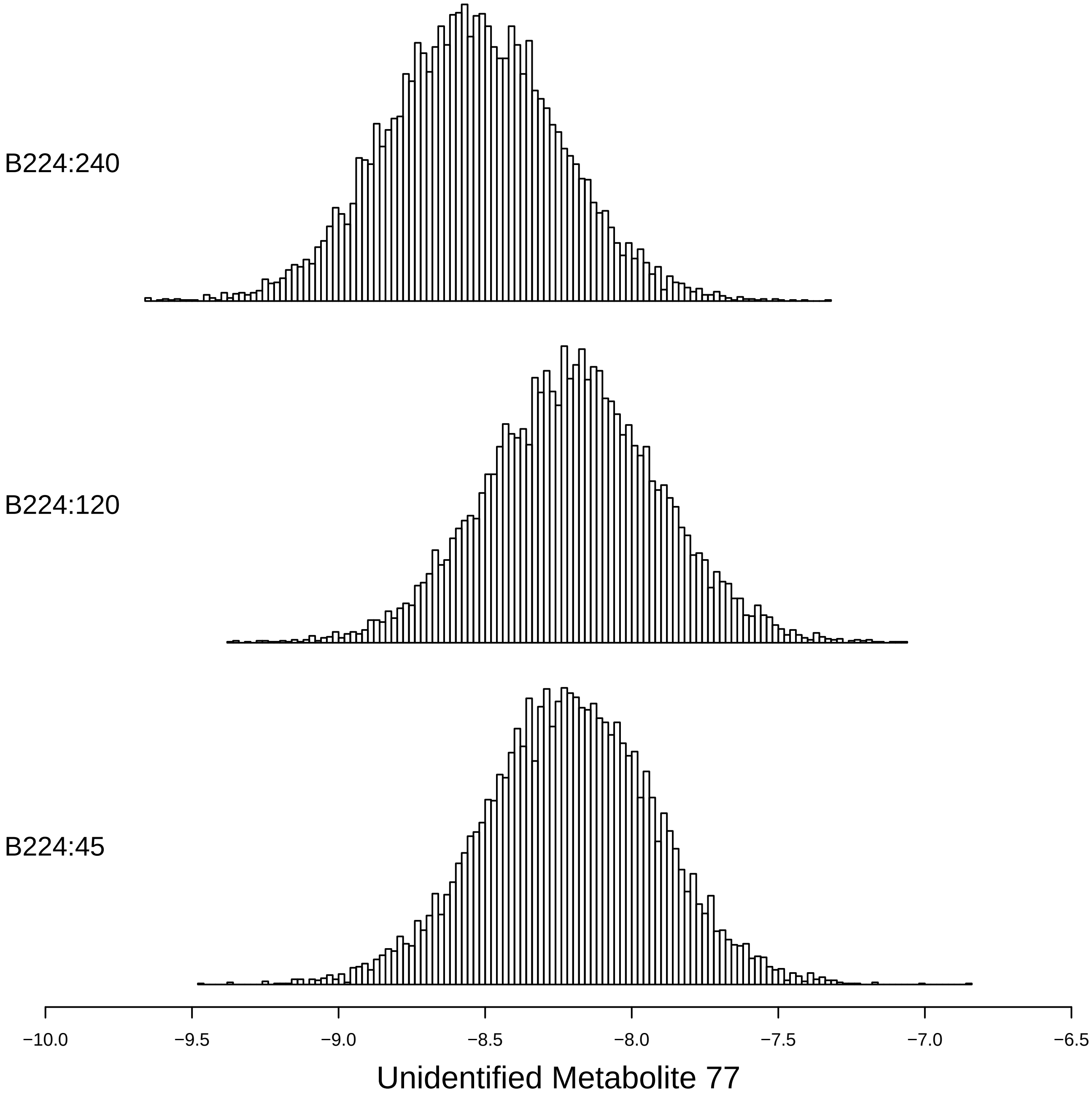
B224:240
B224:120
B224:45
−10.0
−9.5
−9.0
−8.5
−8.0
−7.5
−7.0
−6.5
Unidentified Metabolite 77

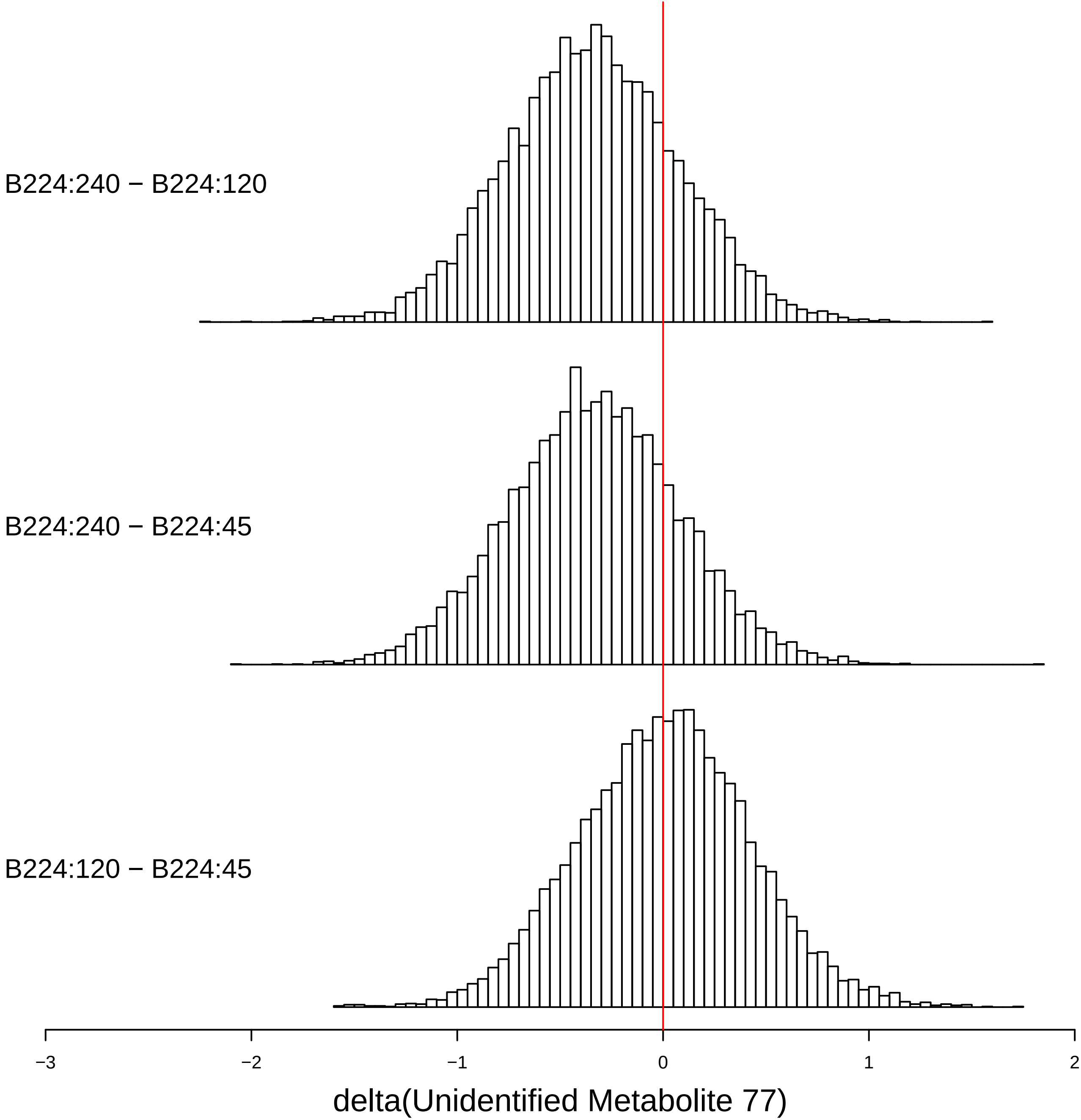

B224:240 − B224:120
B224:240 − B224:45
B224:120 − B224:45
−3
−2
−1
0
1
2
delta(Unidentified Metabolite 77)

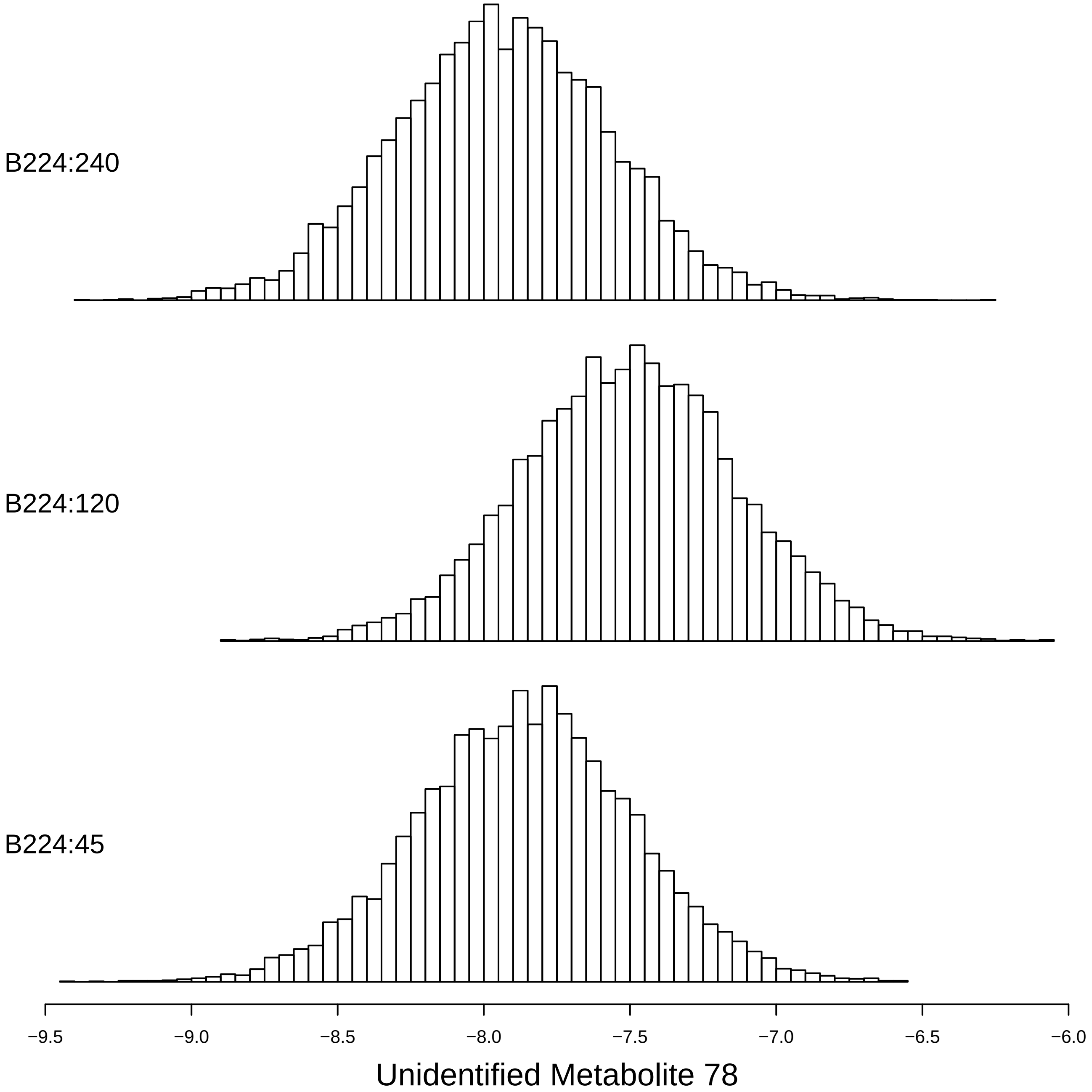
B224:240
B224:120
B224:45
−9.5
−9.0
−8.5
−8.0
−7.5
−7.0
−6.5
−6.0
Unidentified Metabolite 78

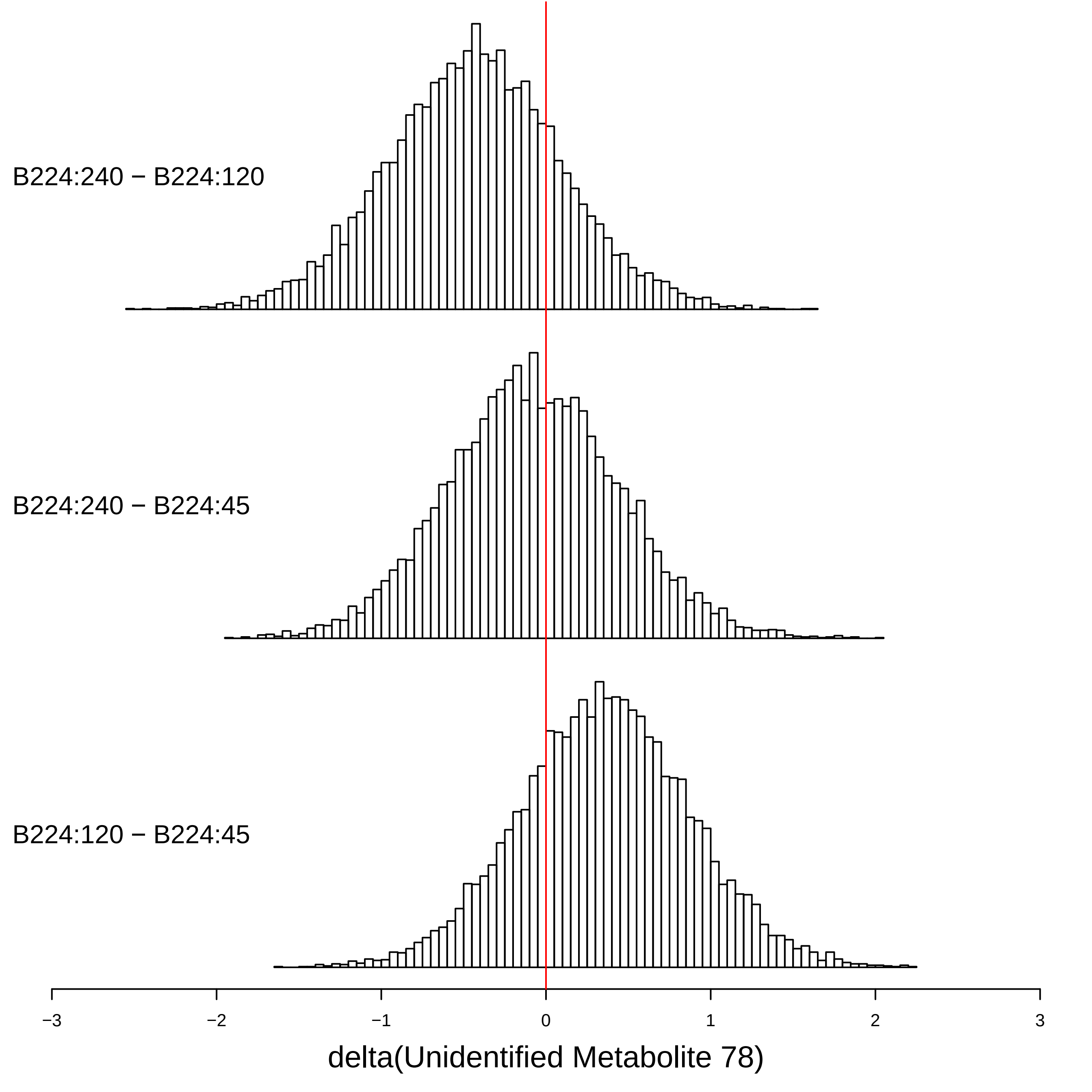

B224:240 − B224:120
B224:240 − B224:45
B224:120 − B224:45
−3
−2
−1
0
1
2
3
delta(Unidentified Metabolite 78)

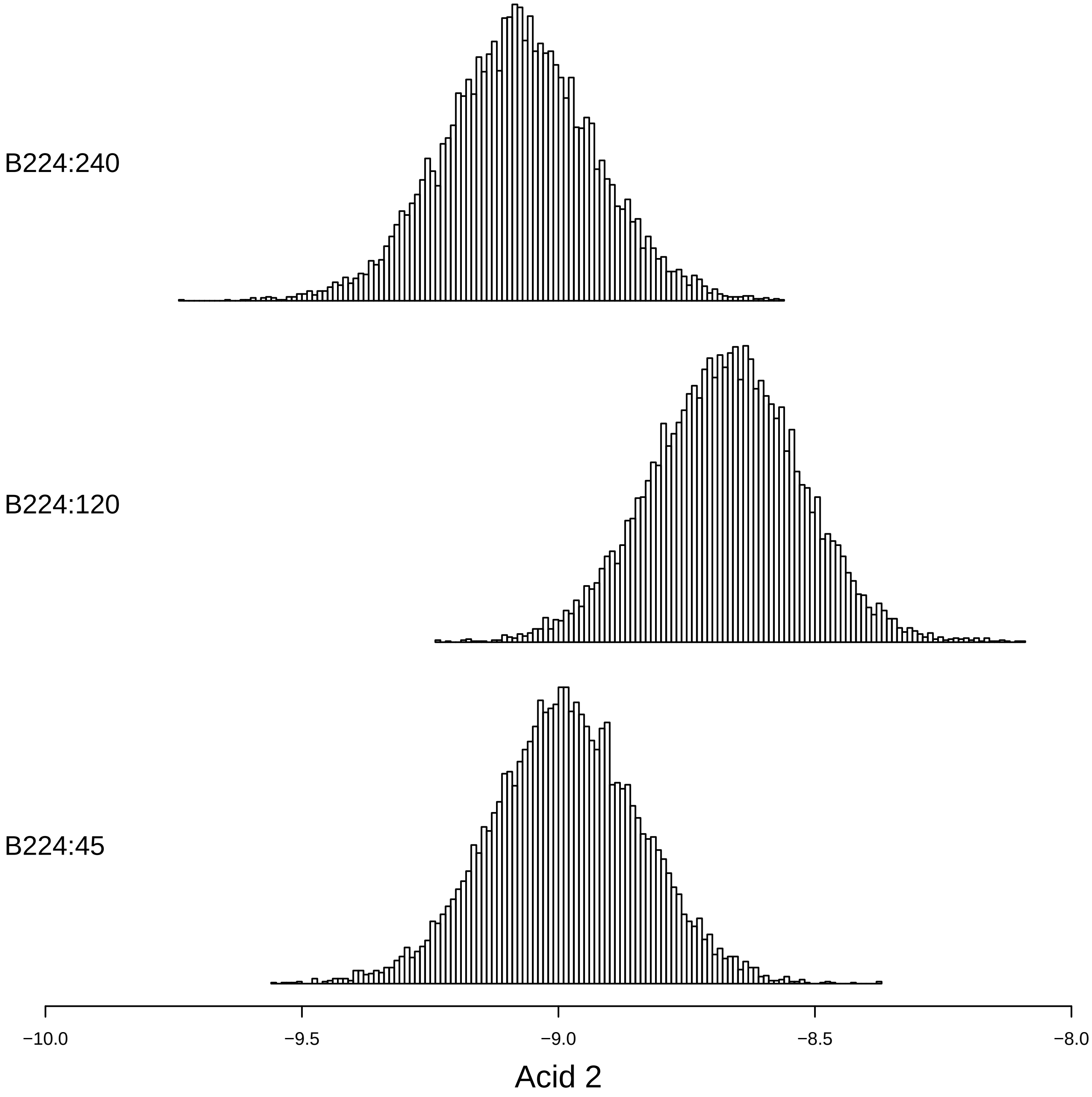
B224:240
B224:120
B224:45
−10.0
−9.5
−9.0
−8.5
−8.0
Acid 2

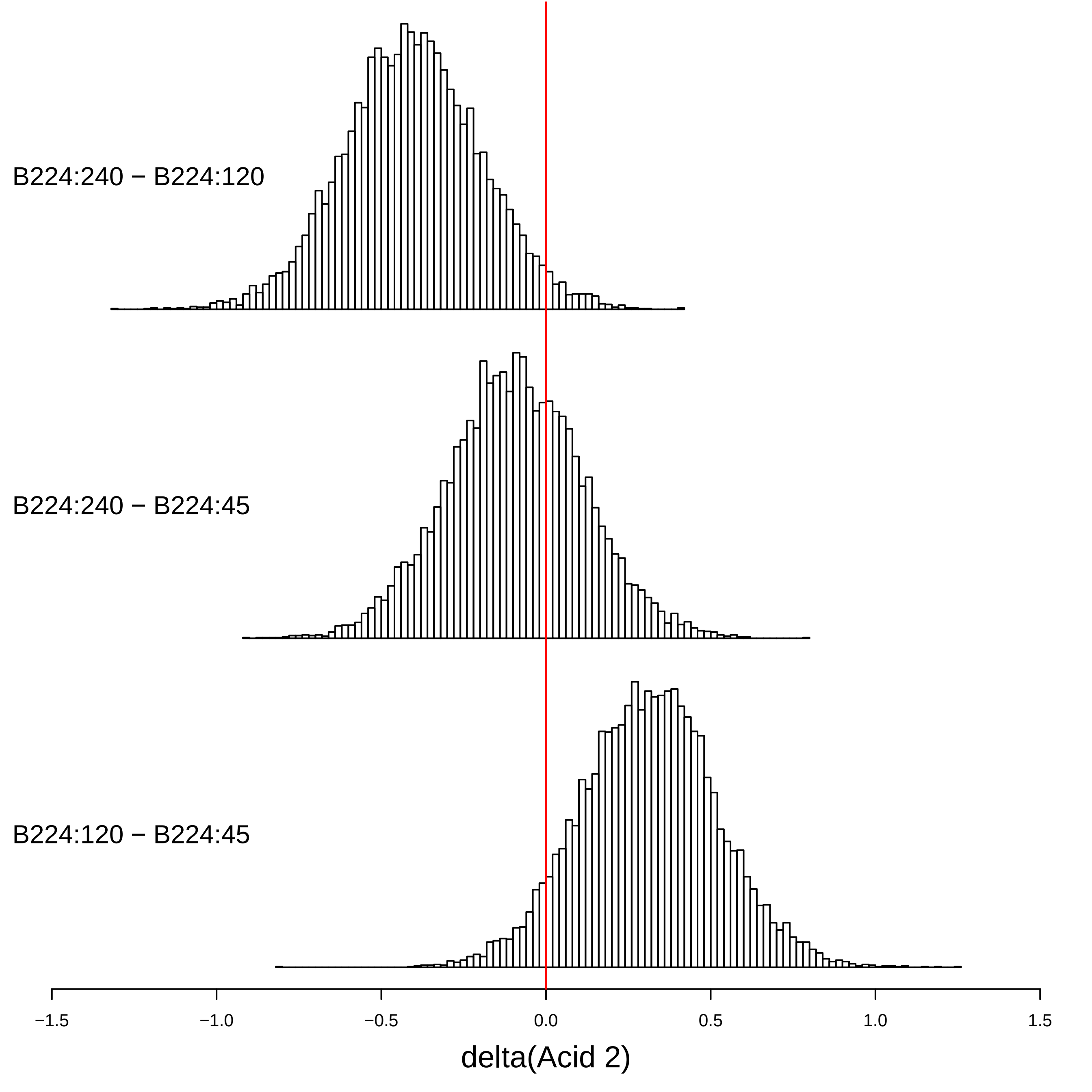
B224:240 − B224:120
B224:240 − B224:45
B224:120 − B224:45
−1.5
−1.0
−0.5
0.0
0.5
1.0
1.5
delta(Acid 2)

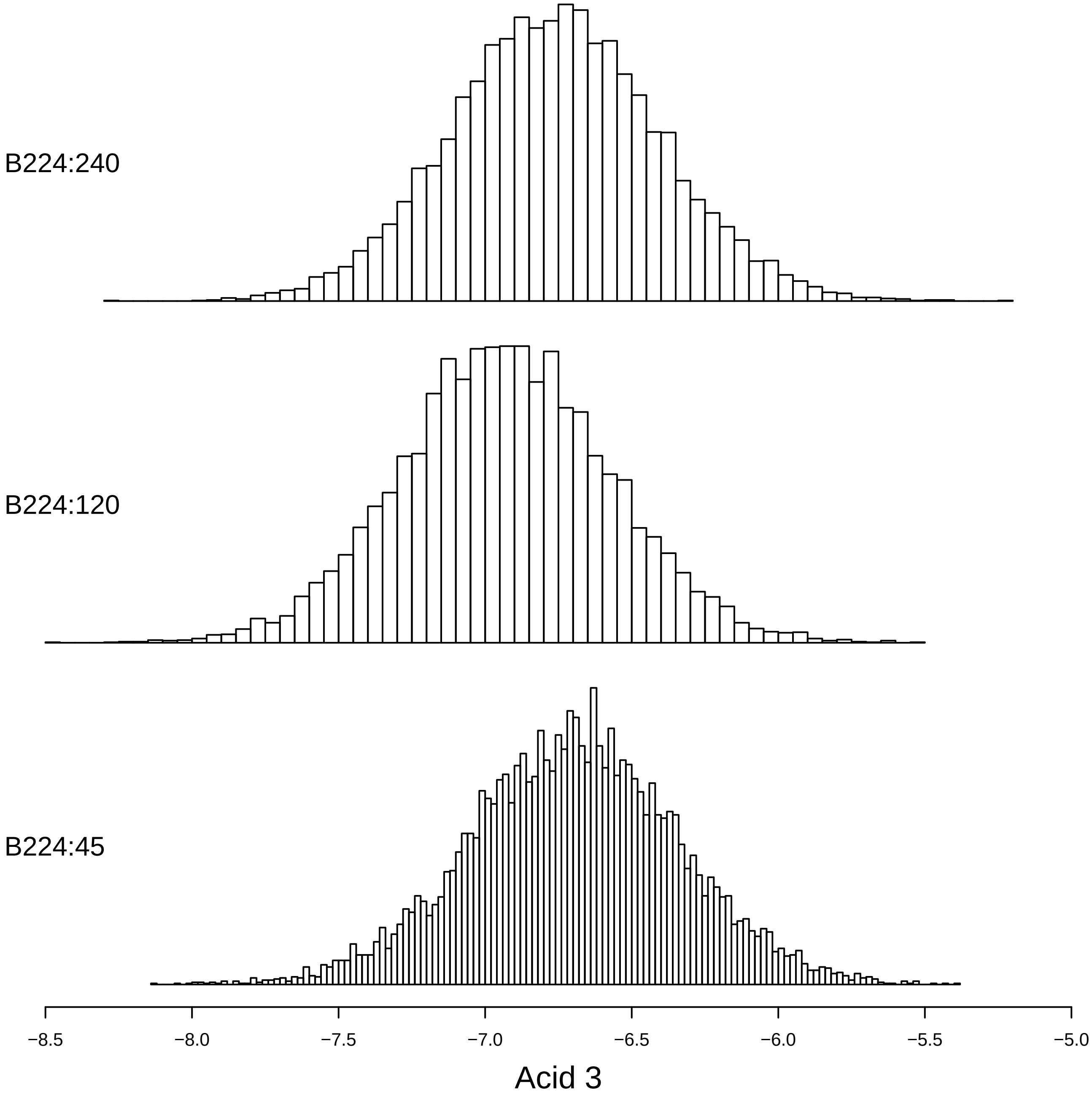

B224:240
B224:120
B224:45
−8.5
−8.0
−7.5
−7.0
−6.5
−6.0
−5.5
−5.0
Acid 3

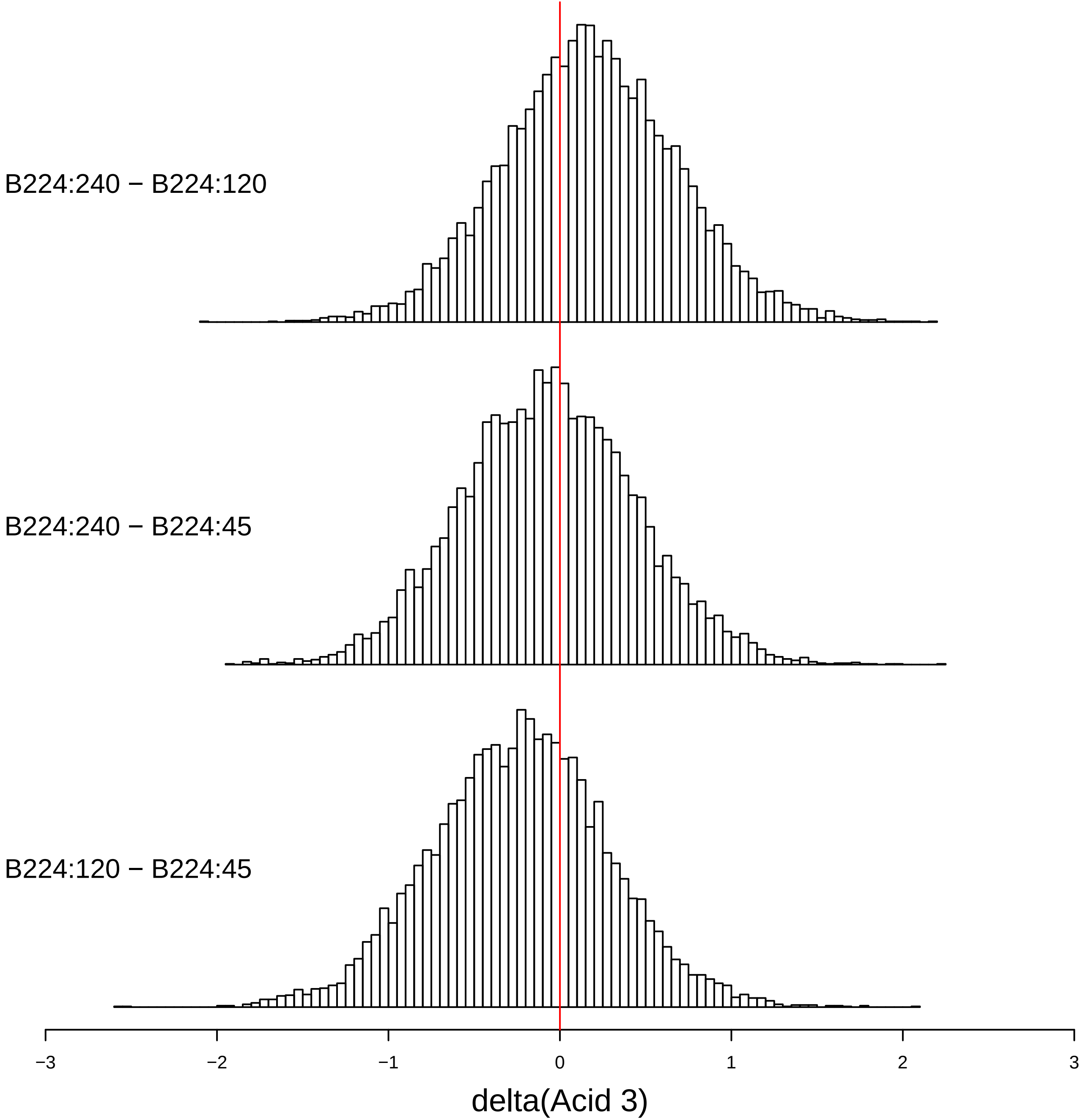

B224:240 − B224:120
B224:240 − B224:45
B224:120 − B224:45
−3
−2
−1
0
1
2
3
delta(Acid 3)

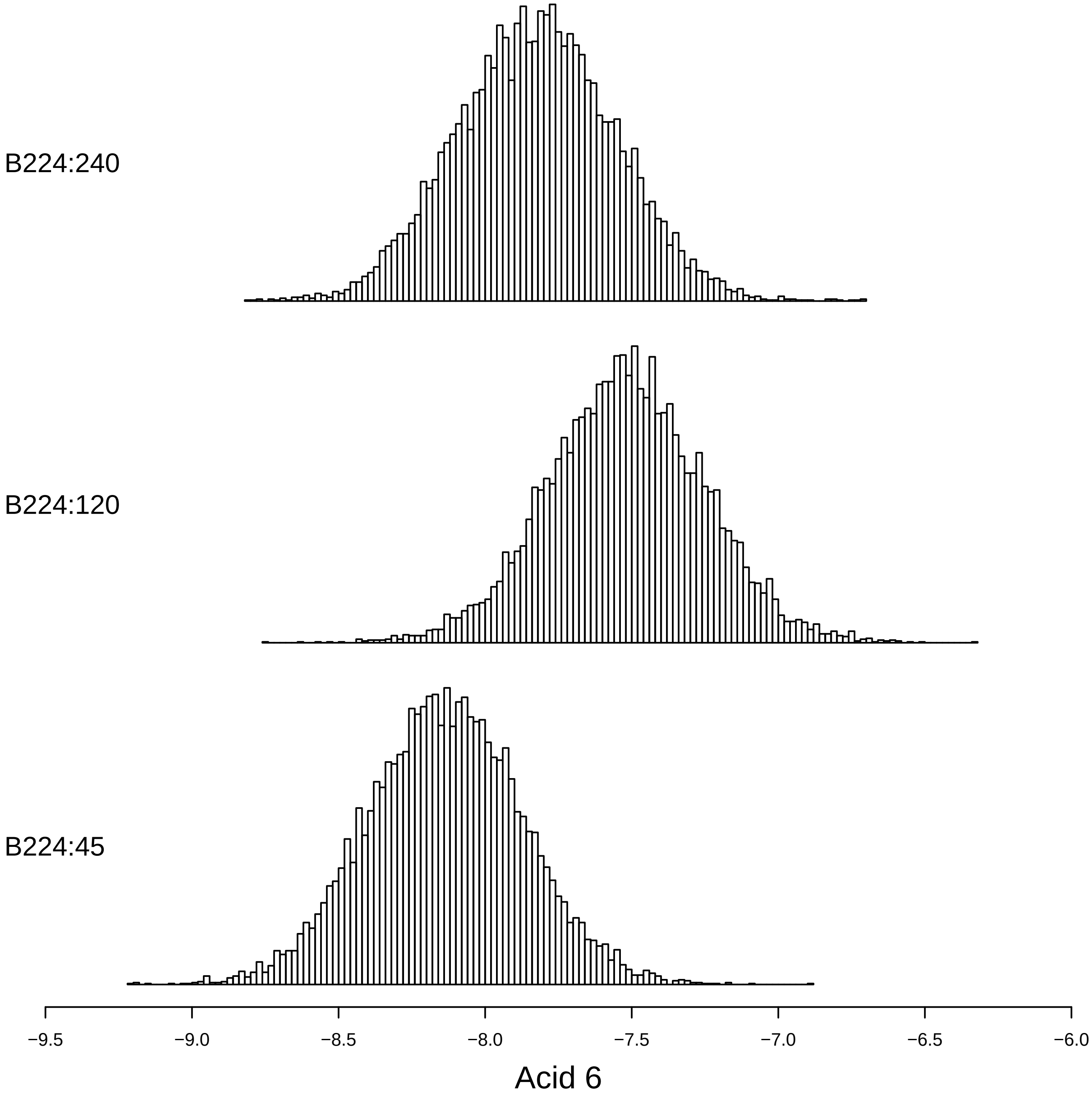
B224:240
B224:120
B224:45
−9.5
−9.0
−8.5
−8.0
−7.5
−7.0
−6.5
−6.0
Acid 6

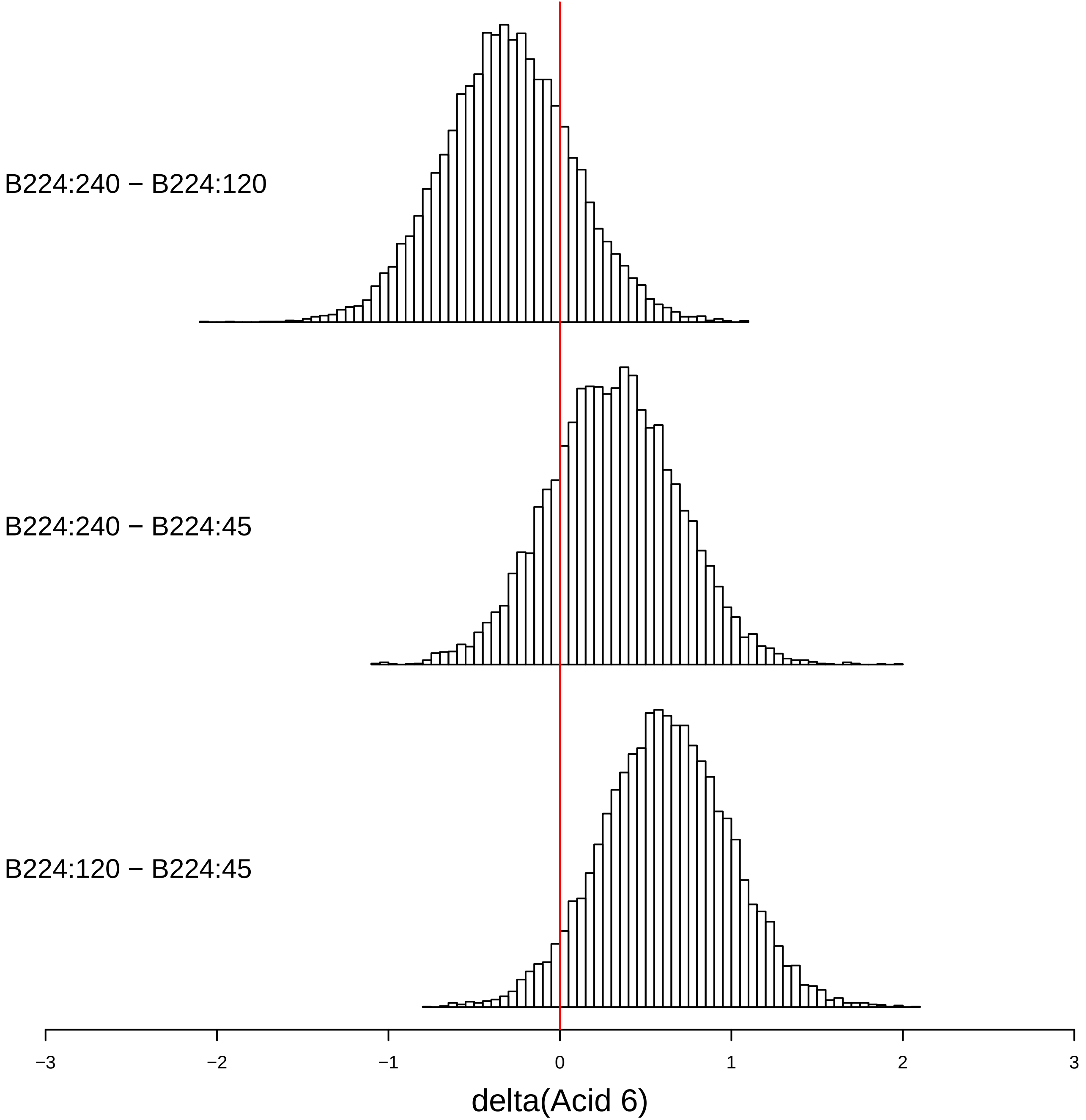

B224:240 − B224:120
B224:240 − B224:45
B224:120 − B224:45
−3
−2
−1
0
1
2
3
delta(Acid 6)

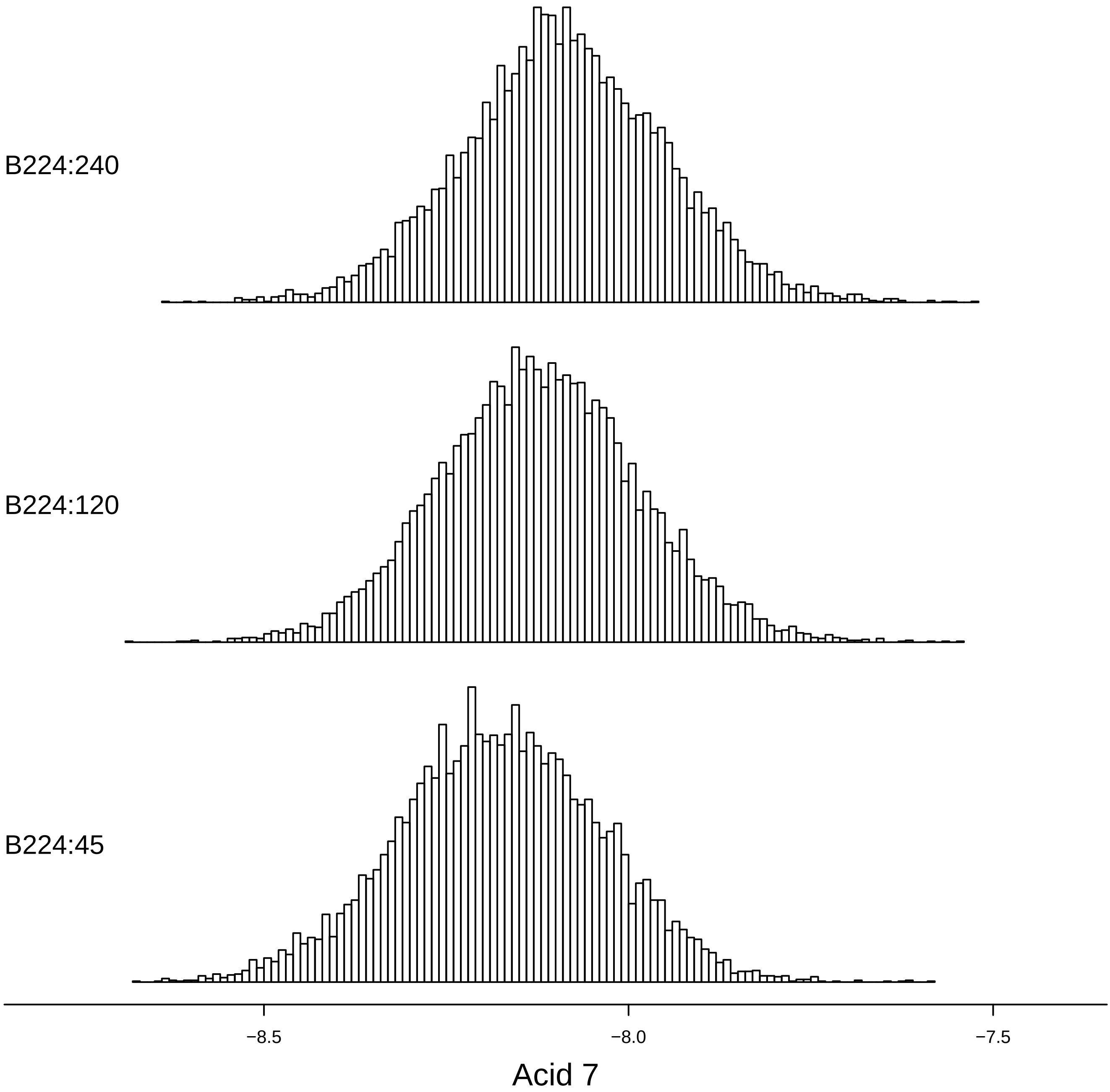
B224:240
B224:120
B224:45
−8.5
−8.0
−7.5
Acid 7

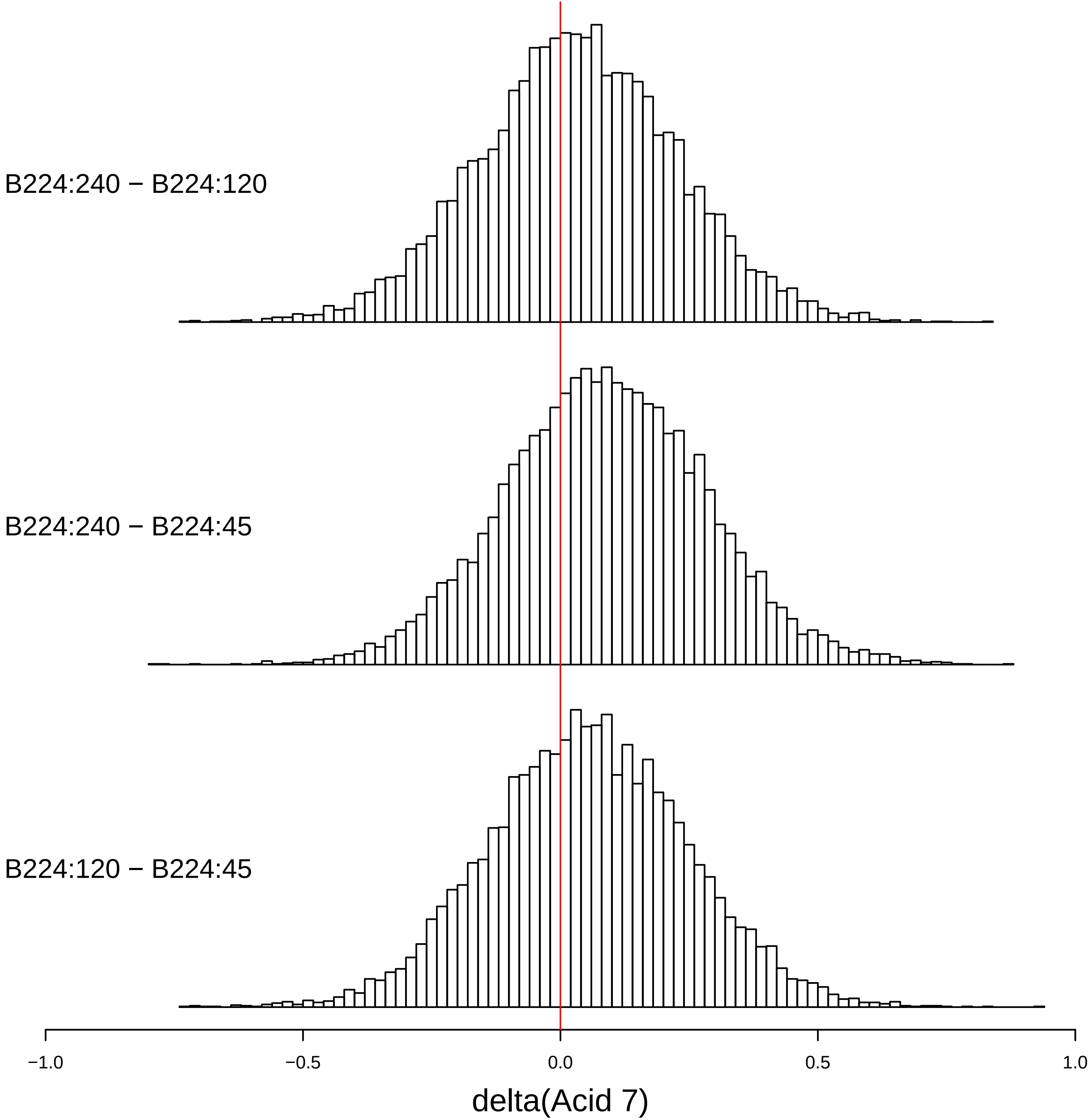

B224:240 − B224:120
B224:240 − B224:45
B224:120 − B224:45
−1.0
−0.5
0.0
0.5
1.0
delta(Acid 7)

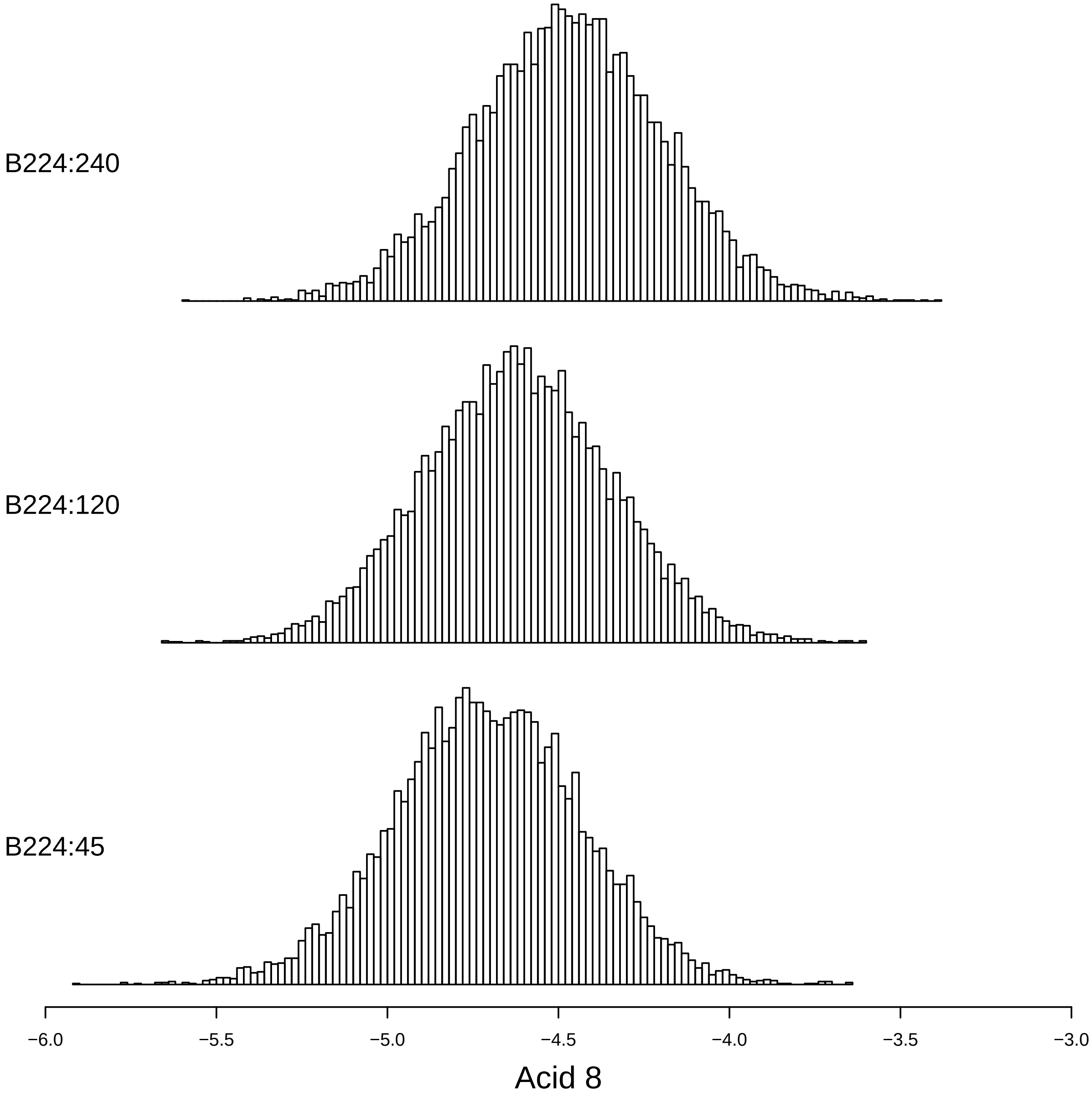

B224:240
B224:120
B224:45
−6.0
−5.5
−5.0
−4.5
−4.0
−3.5
−3.0
Acid 8

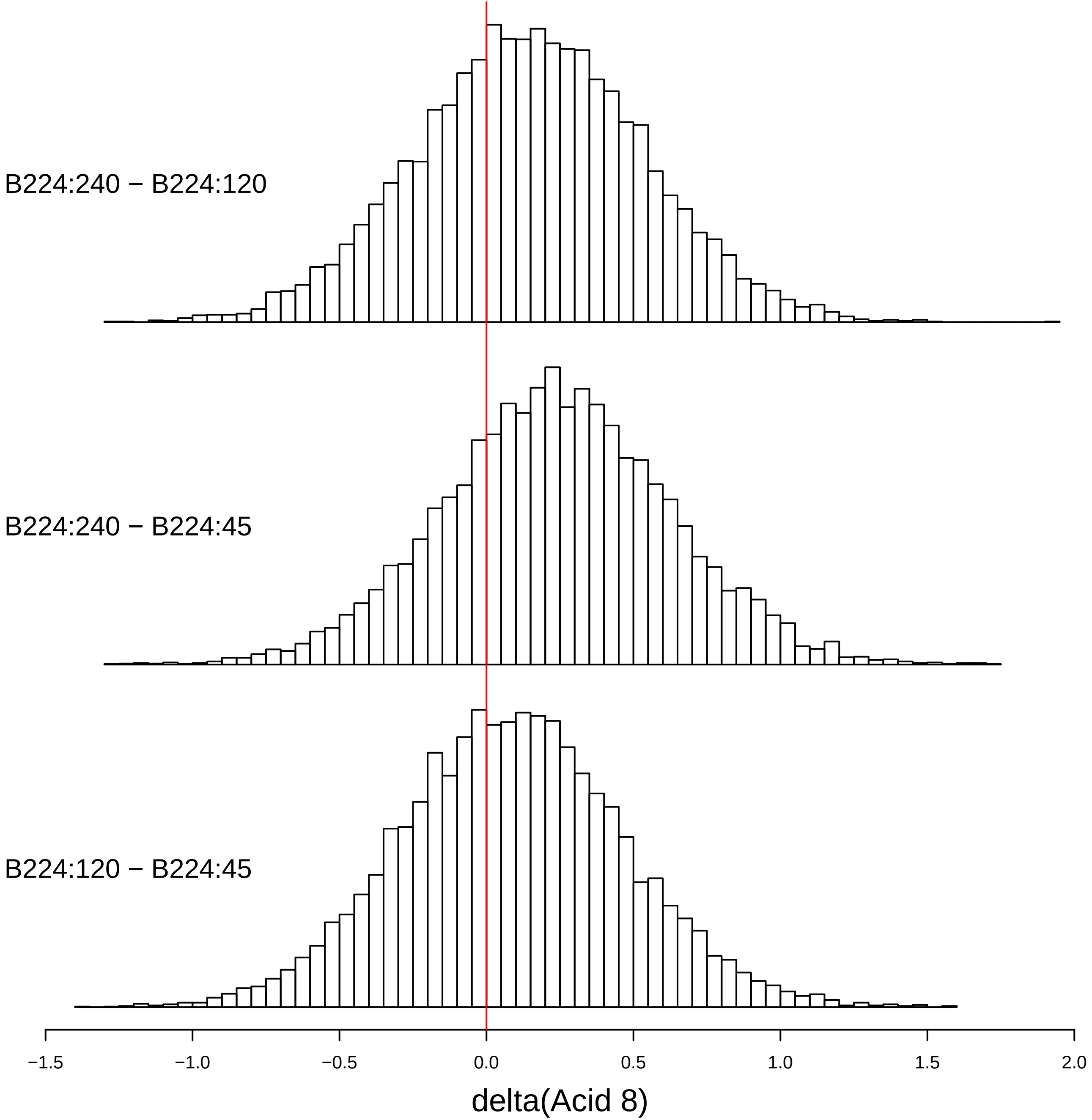
B224:240 − B224:120
B224:240 − B224:45
B224:120 − B224:45
−1.5
−1.0
−0.5
0.0
0.5
1.0
1.5
2.0
delta(Acid 8)

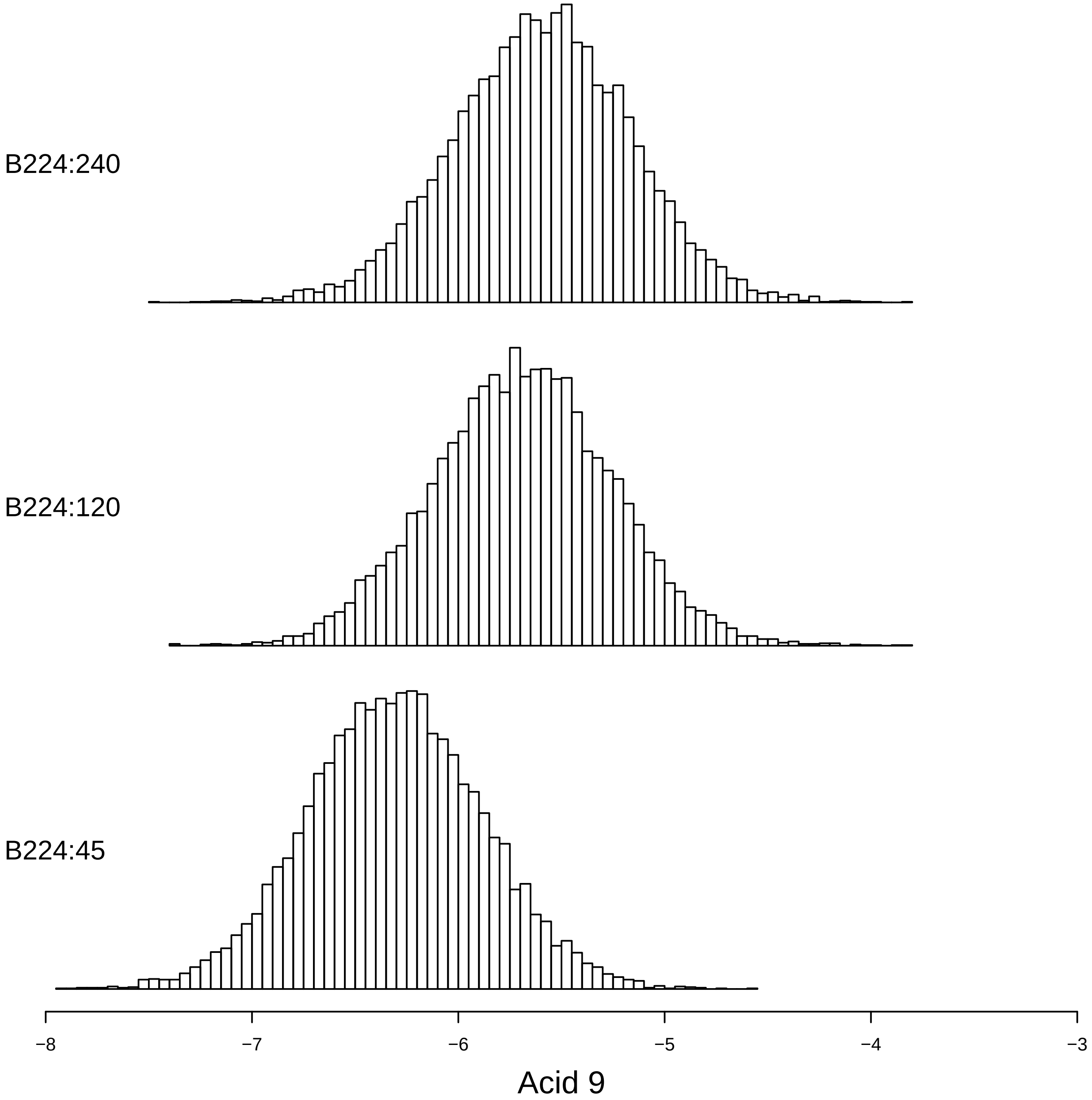
B224:240
B224:120
B224:45
−8
−7
−6
−5
−4
−3
Acid 9

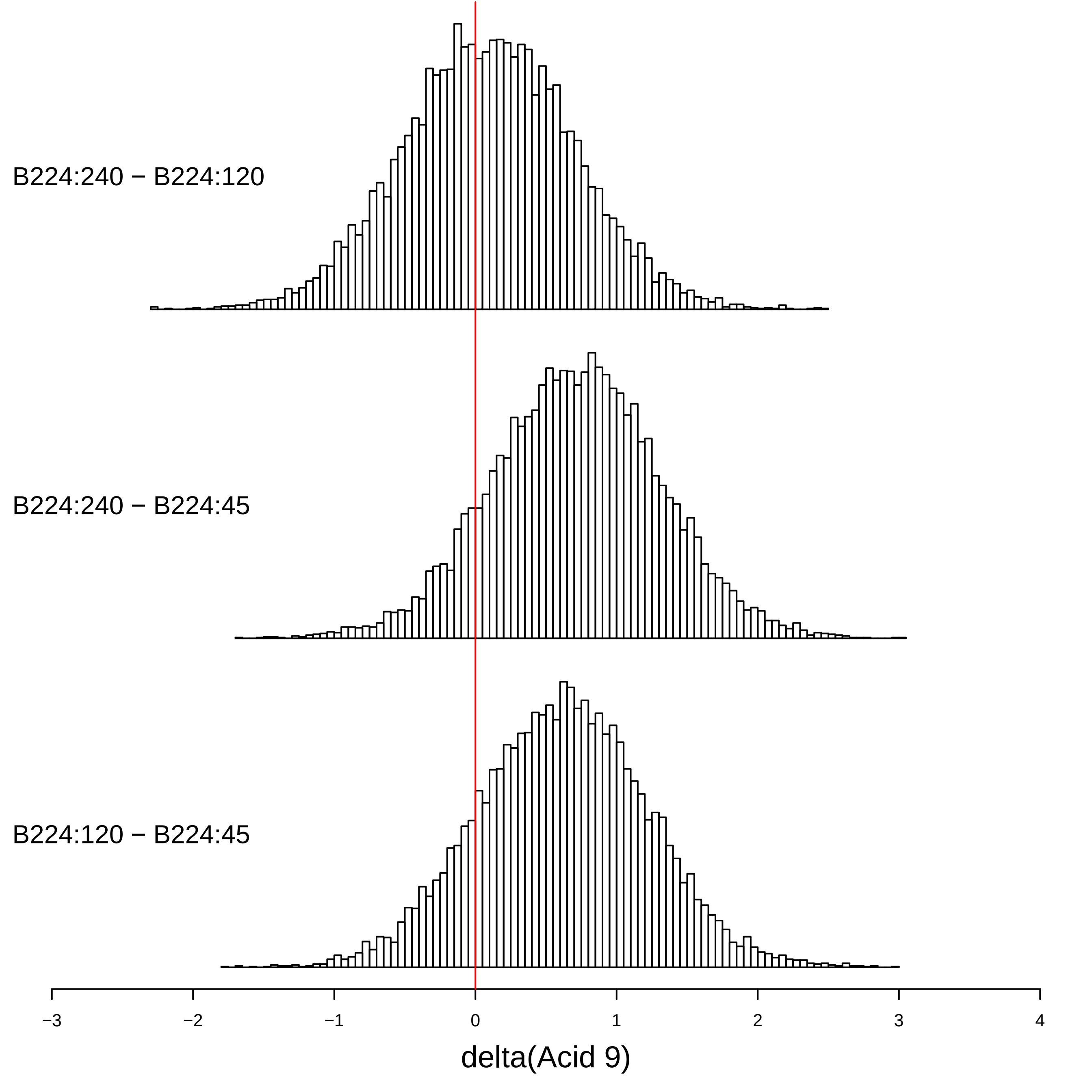
B224:240 − B224:120
B224:240 − B224:45
B224:120 − B224:45
−3
−2
−1
0
1
2
3
4
delta(Acid 9)

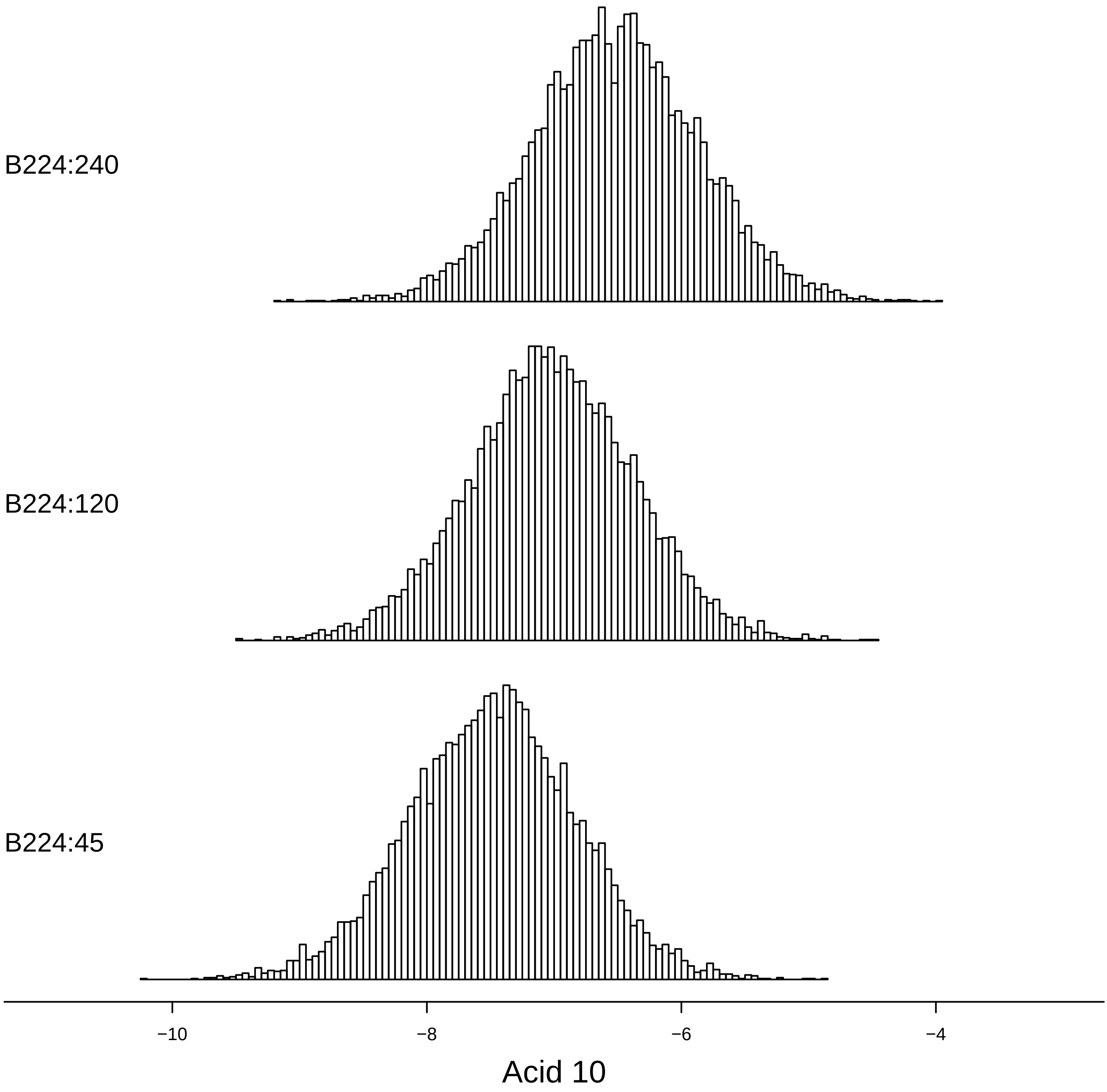

B224:240
B224:120
B224:45
−10
−8
−6
−4
Acid 10

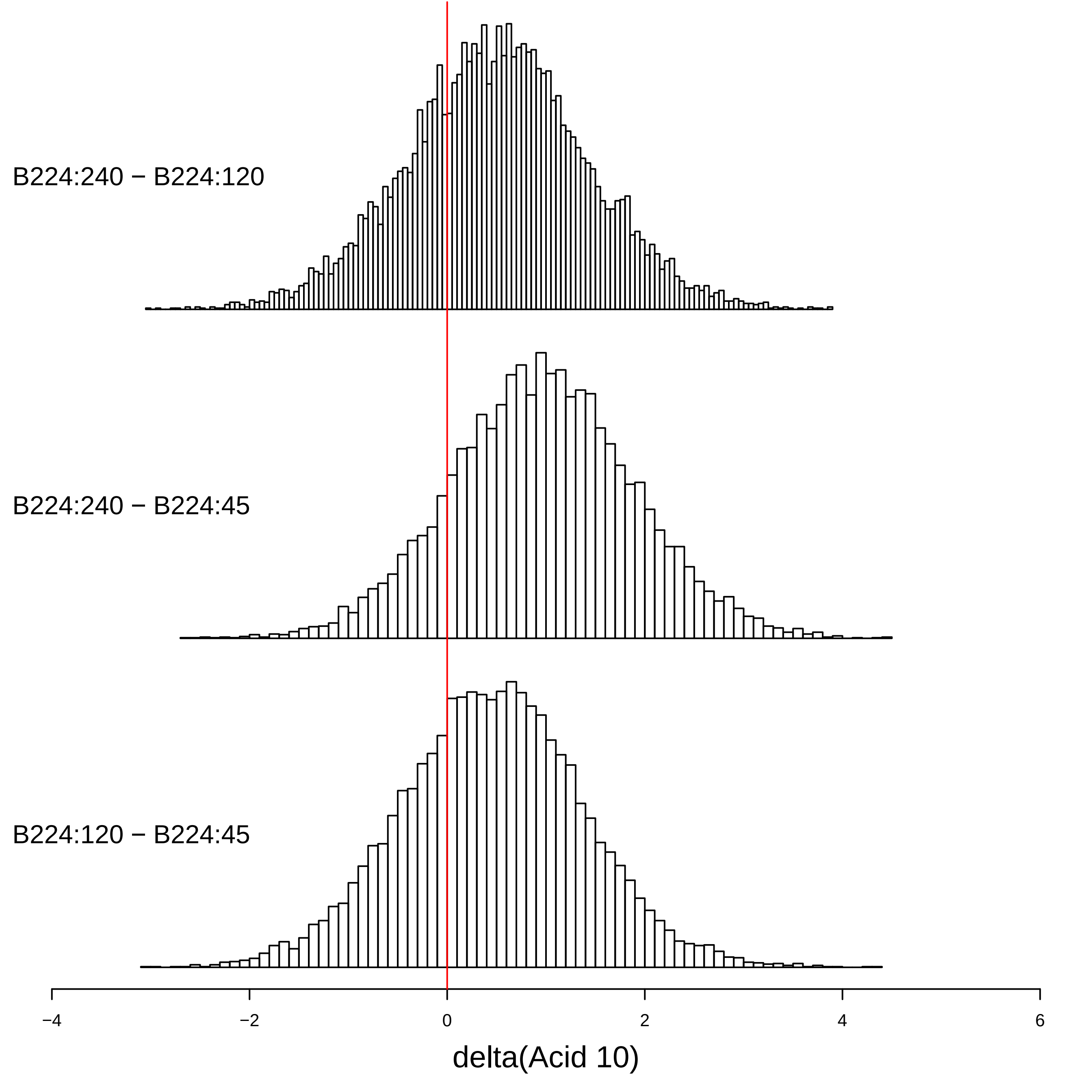

B224:240 − B224:120
B224:240 − B224:45
B224:120 − B224:45
−4
−2
0
2
4
6
delta(Acid 10)

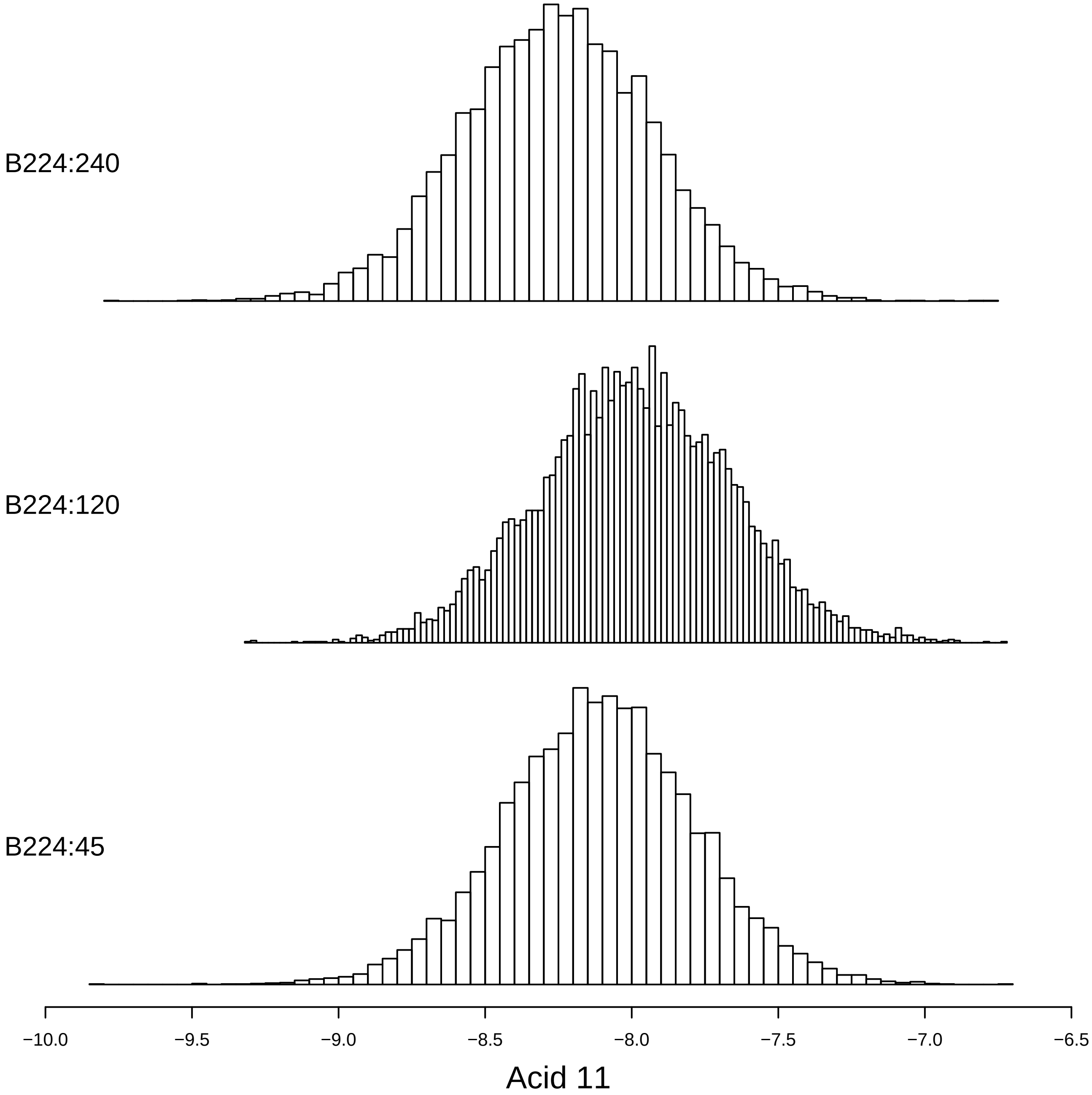

B224:240
B224:120
B224:45
−10.0
−9.5
−9.0
−8.5
−8.0
−7.5
−7.0
−6.5
Acid 11

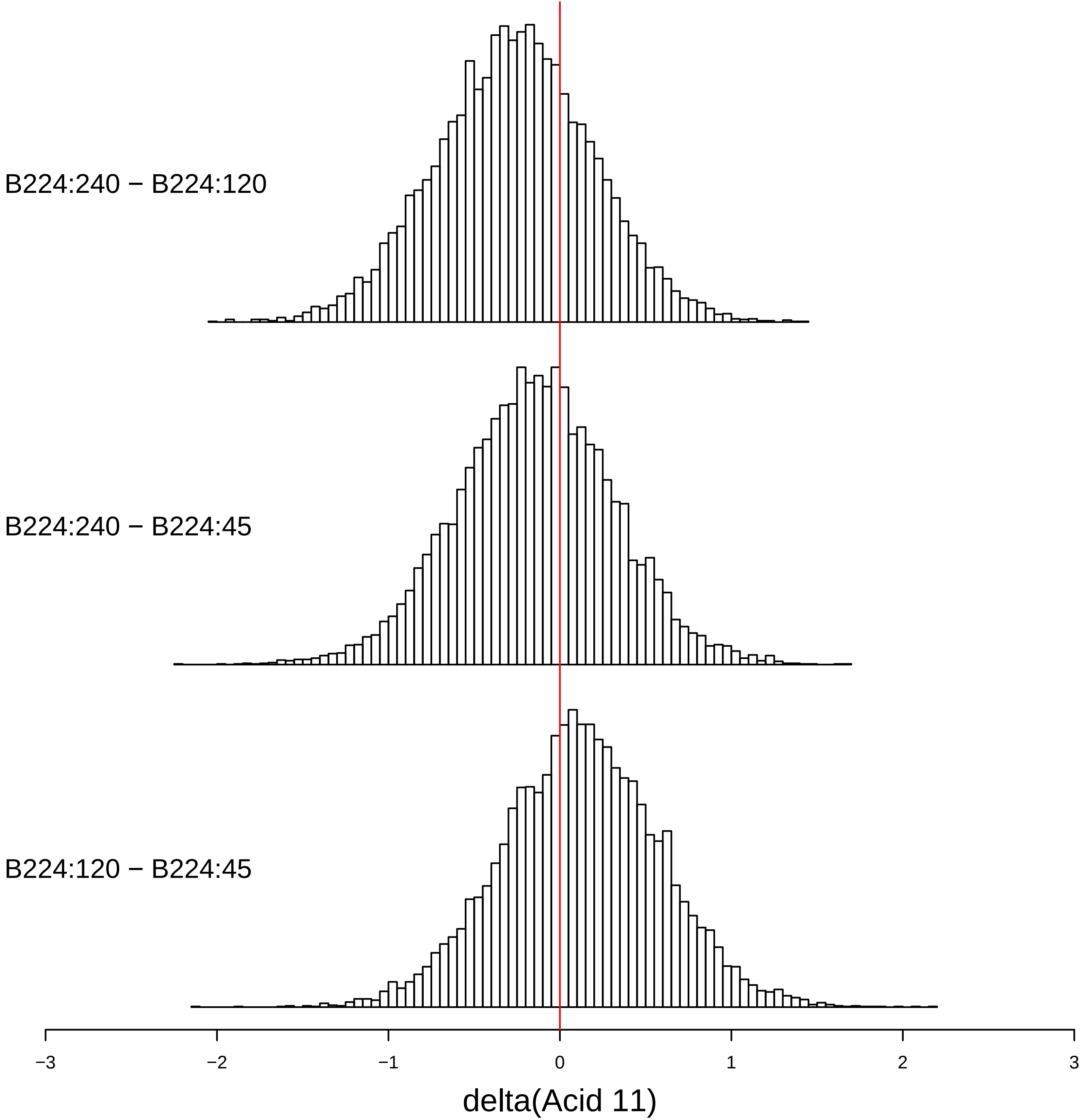

B224:240 − B224:120
B224:240 − B224:45
B224:120 − B224:45
−3
−2
−1
0
1
2
3
delta(Acid 11)

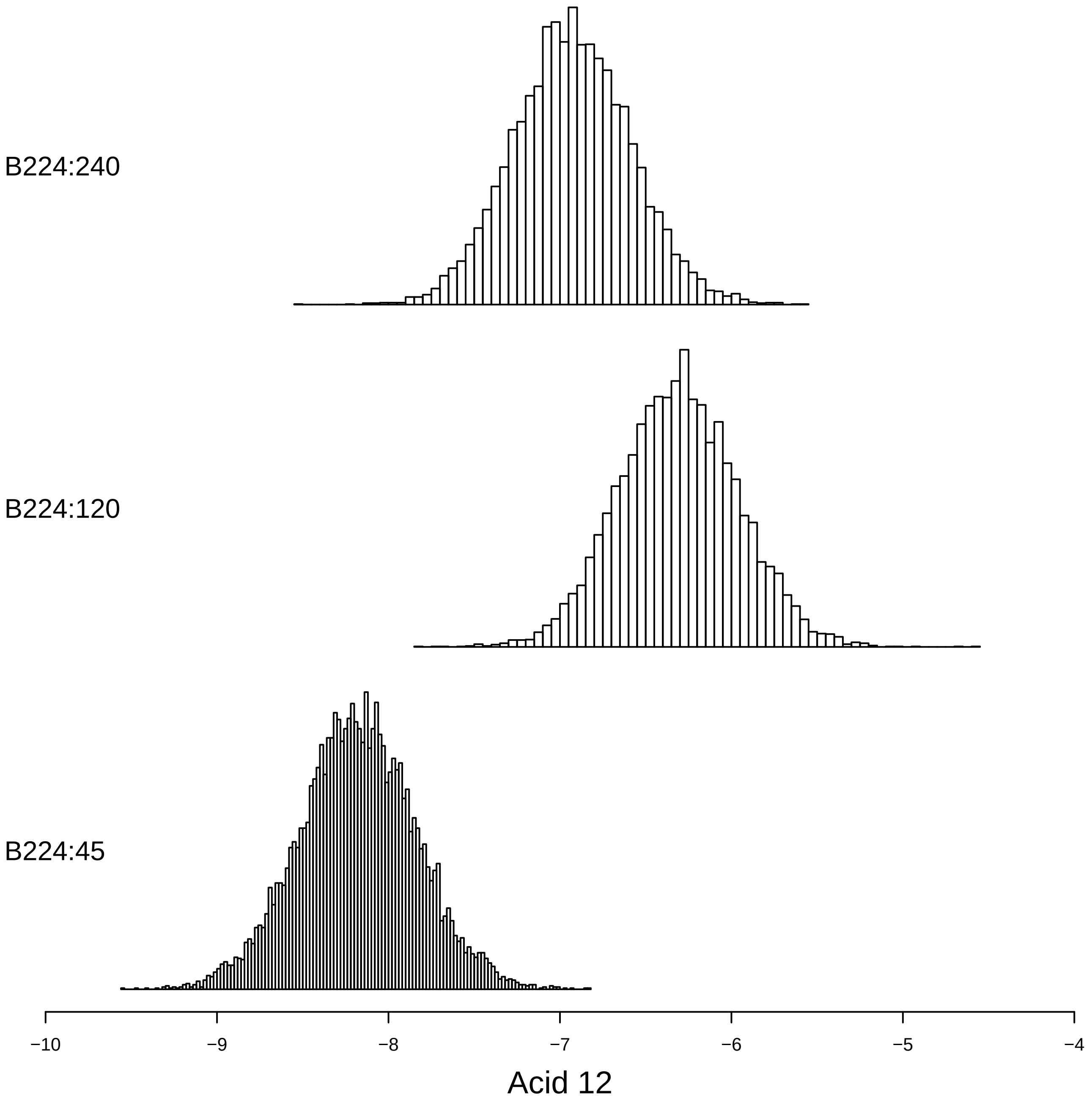

B224:240
B224:120
B224:45
−10
−9
−8
−7
−6
−5
−4
Acid 12

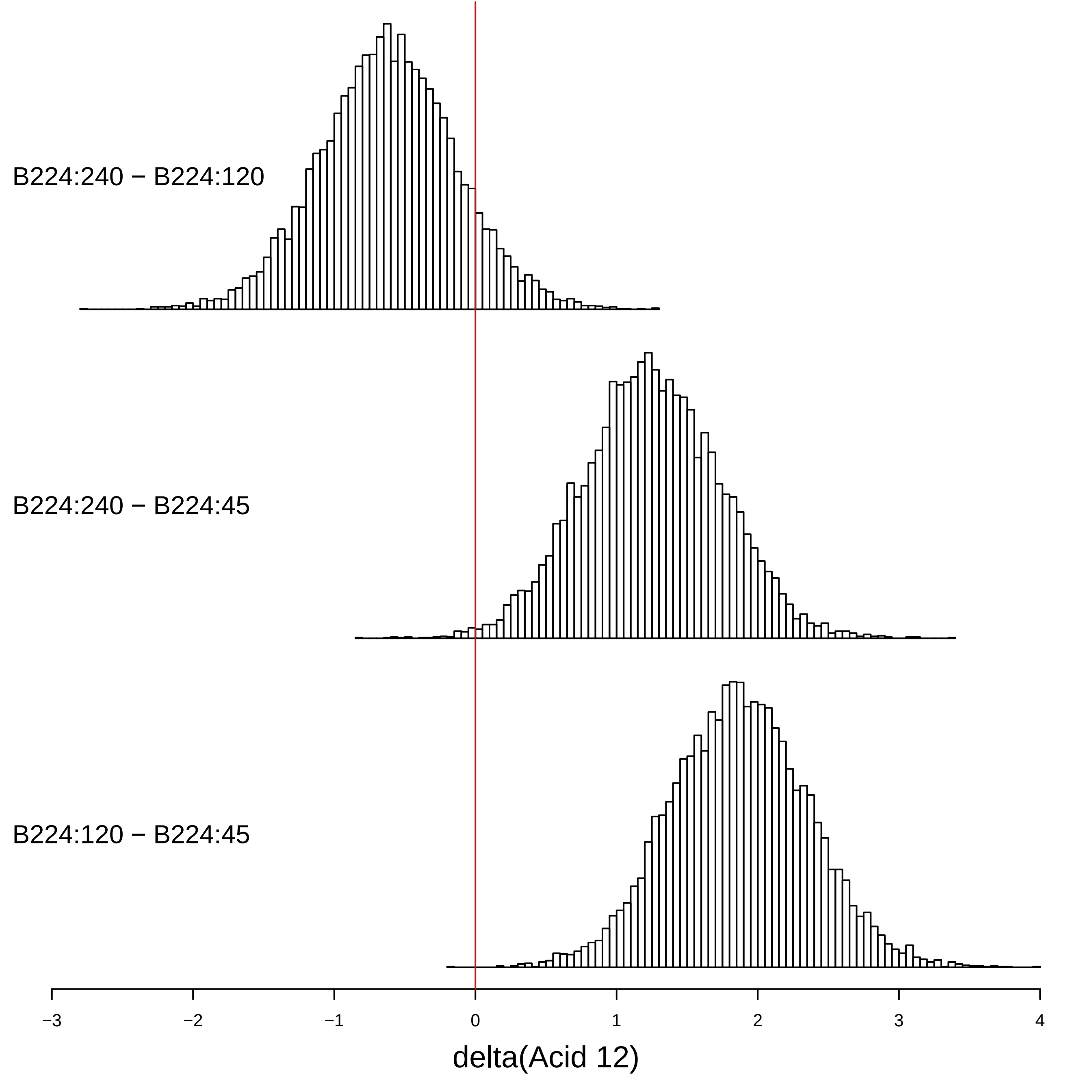
B224:240 − B224:120
B224:240 − B224:45
B224:120 − B224:45
−3
−2
−1
0
1
2
3
4
delta(Acid 12)

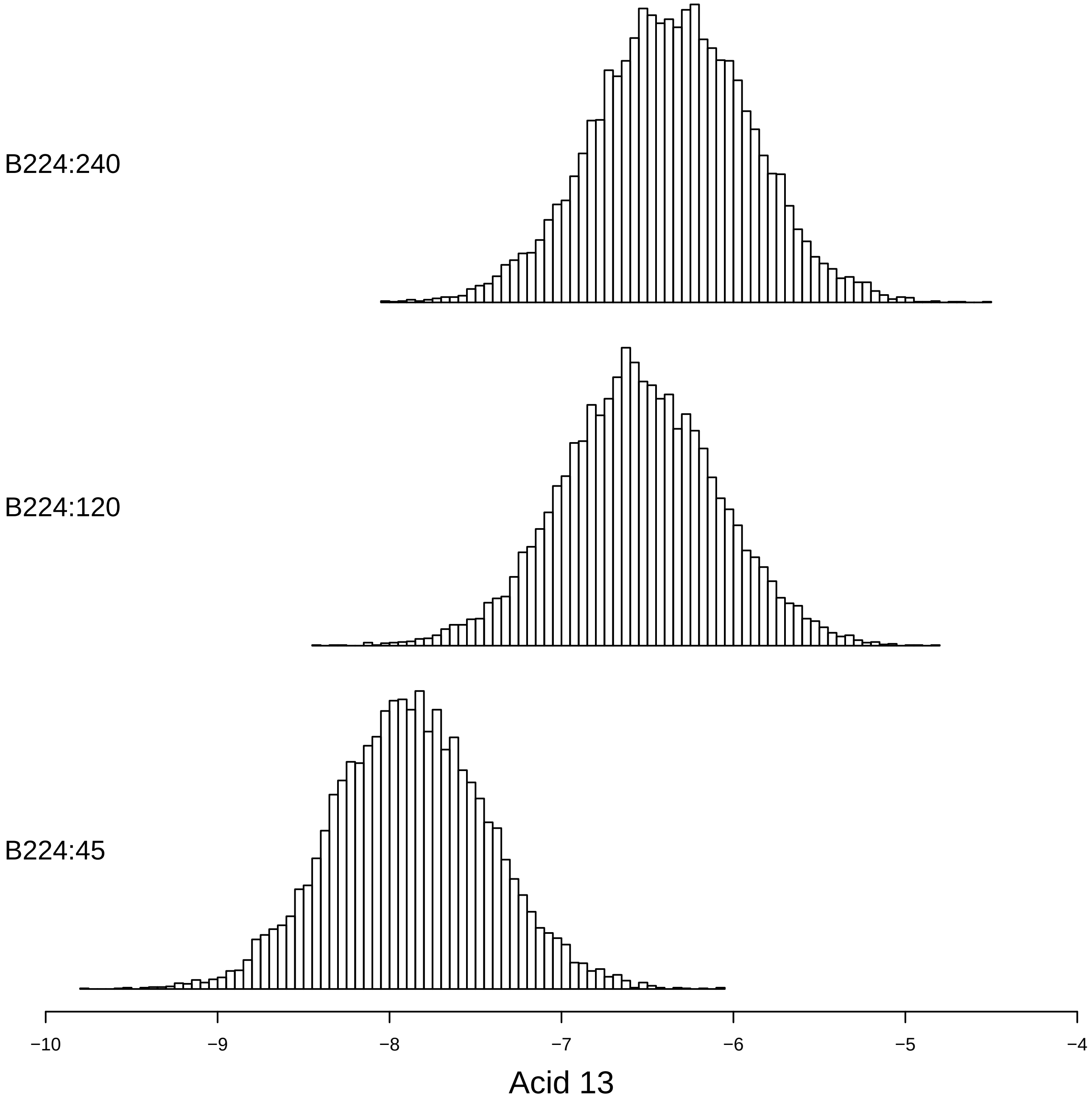
B224:240
B224:120
B224:45
−10
−9
−8
−7
−6
−5
−4
Acid 13

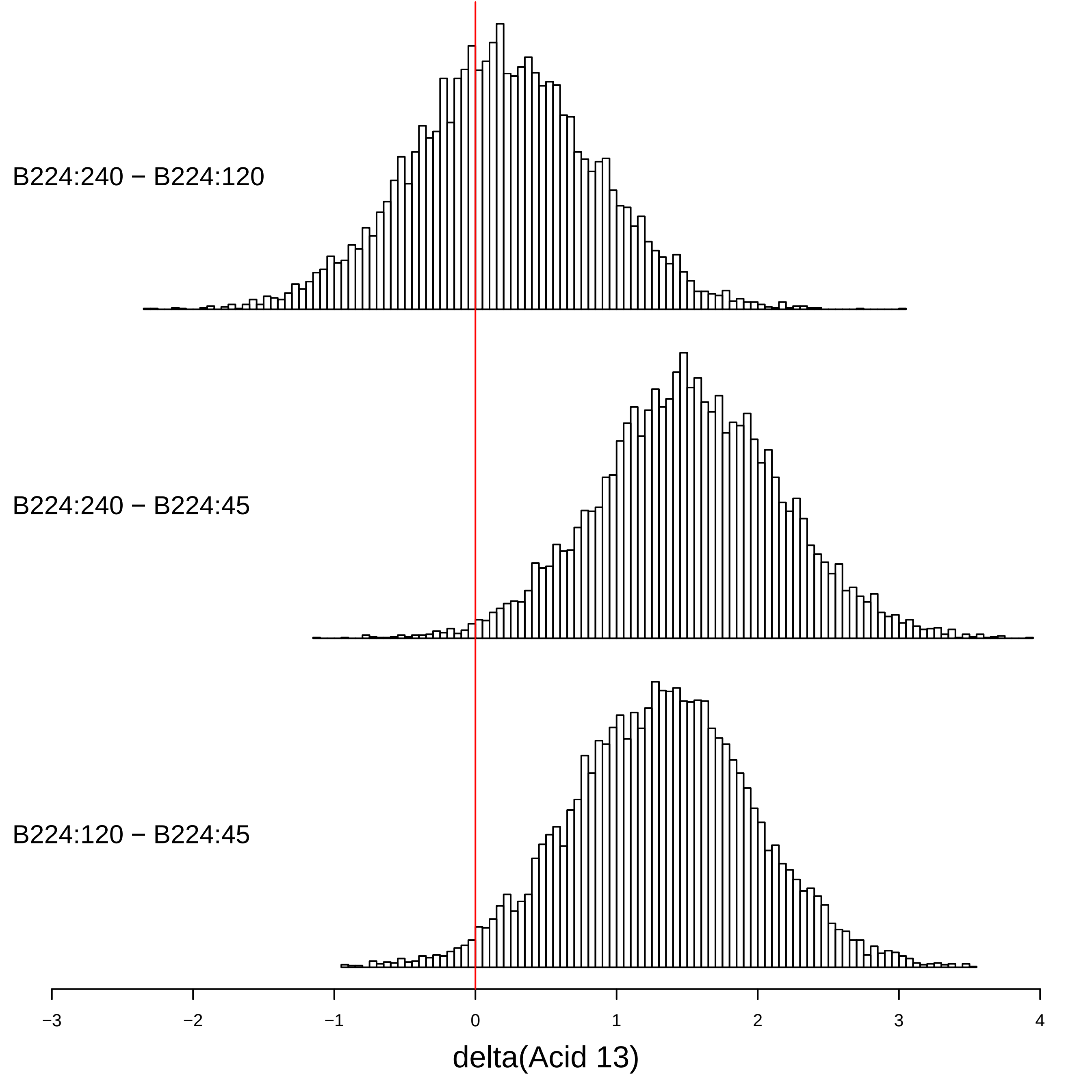

B224:240 − B224:120
B224:240 − B224:45
B224:120 − B224:45
−3
−2
−1
0
1
2
3
4
delta(Acid 13)

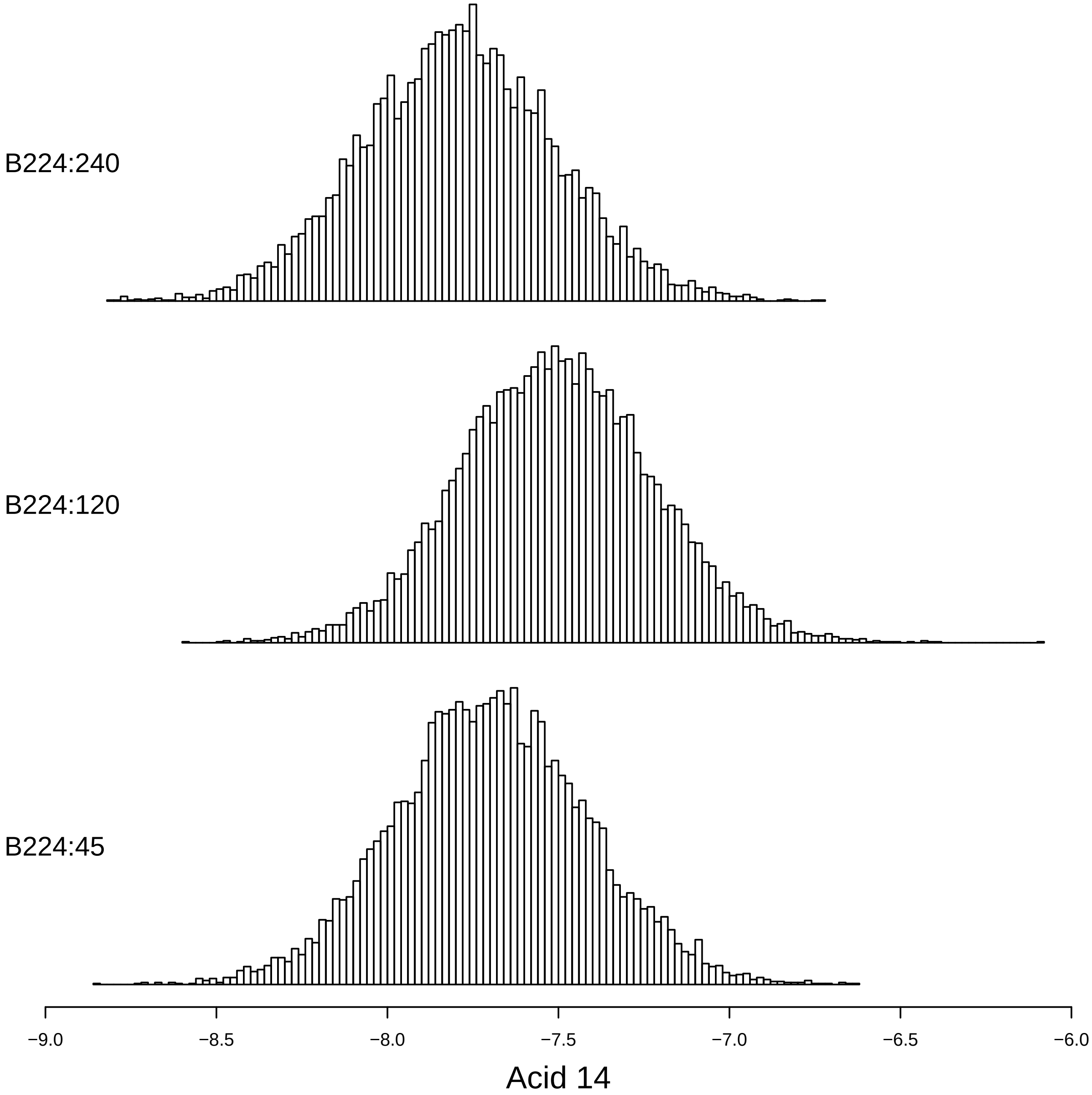
B224:240
B224:120
B224:45
−9.0
−8.5
−8.0
−7.5
−7.0
−6.5
−6.0
Acid 14

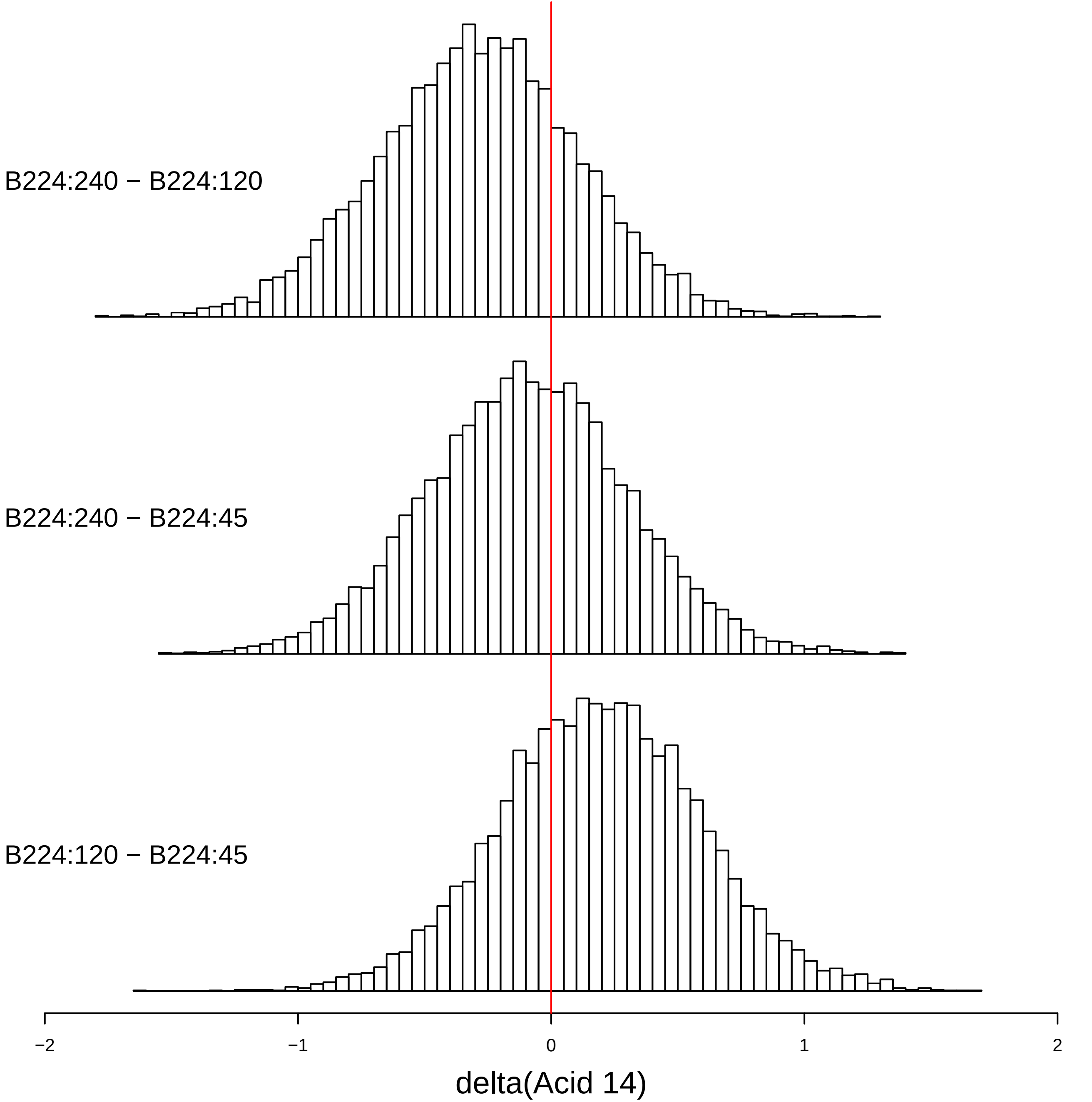

B224:240 − B224:120
B224:240 − B224:45
B224:120 − B224:45
−2
−1
0
1
2
delta(Acid 14)
