# Supplementary material for: Taxonomic and Environmental Variation of Metabolite Profiles in Marine Dinoflagellates of the Genus Symbiodinium
Source: Metabolites. 2015 Feb 16;5(1):74–99. doi: 10.3390/metabo5010074 (PMC4381291; doi:10.3390/metabo5010074)
Supplement: Supplementary File 1 [file metabolites-05-00074-s001.zip › Supplementary Information/Supplementary Information Figure S8 - Metabolite concentration posteriors.pdf]

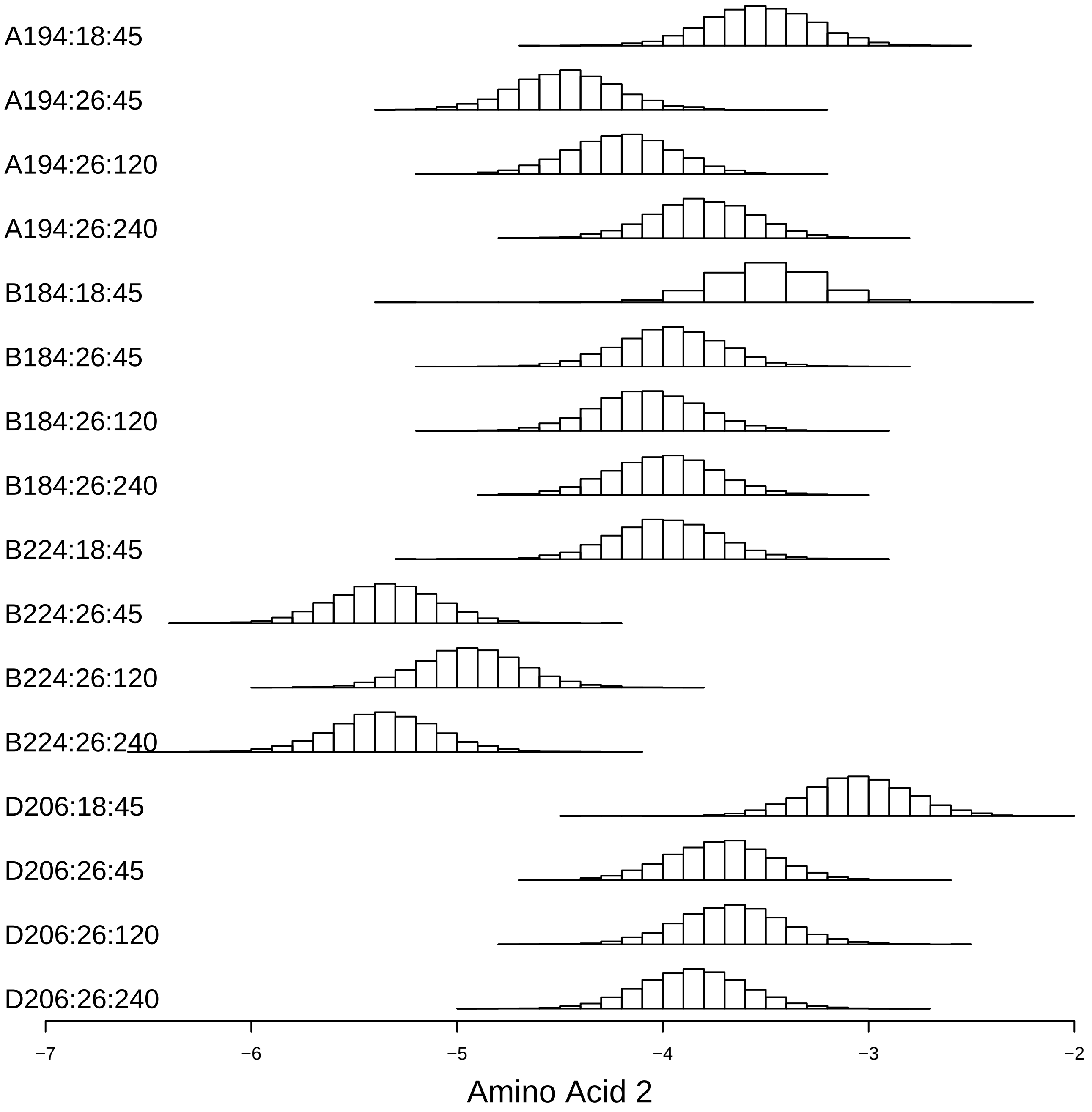
A194:18:45
A194:26:45
A194:26:120
A194:26:240
B184:18:45
B184:26:45
B184:26:120
B184:26:240
B224:18:45
B224:26:45
B224:26:120
B224:26:240
D206:18:45
D206:26:45
D206:26:120
D206:26:240
−7
−6
−5
−4
−3
−2
Amino Acid 2

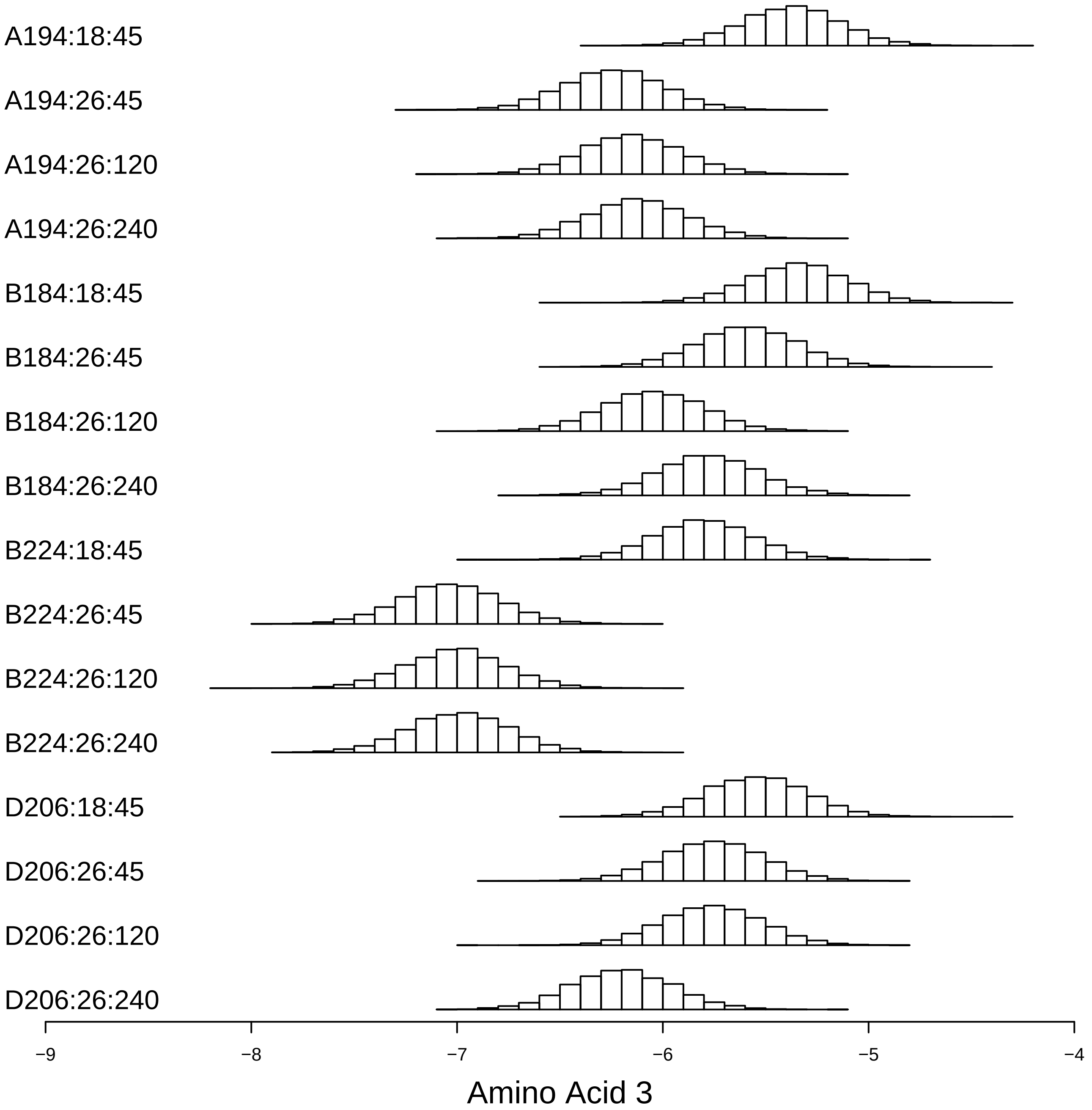

A194:18:45
A194:26:45
A194:26:120
A194:26:240
B184:18:45
B184:26:45
B184:26:120
B184:26:240
B224:18:45
B224:26:45
B224:26:120
B224:26:240
D206:18:45
D206:26:45
D206:26:120
D206:26:240
−9
−8
−7
−6
−5
−4
Amino Acid 3

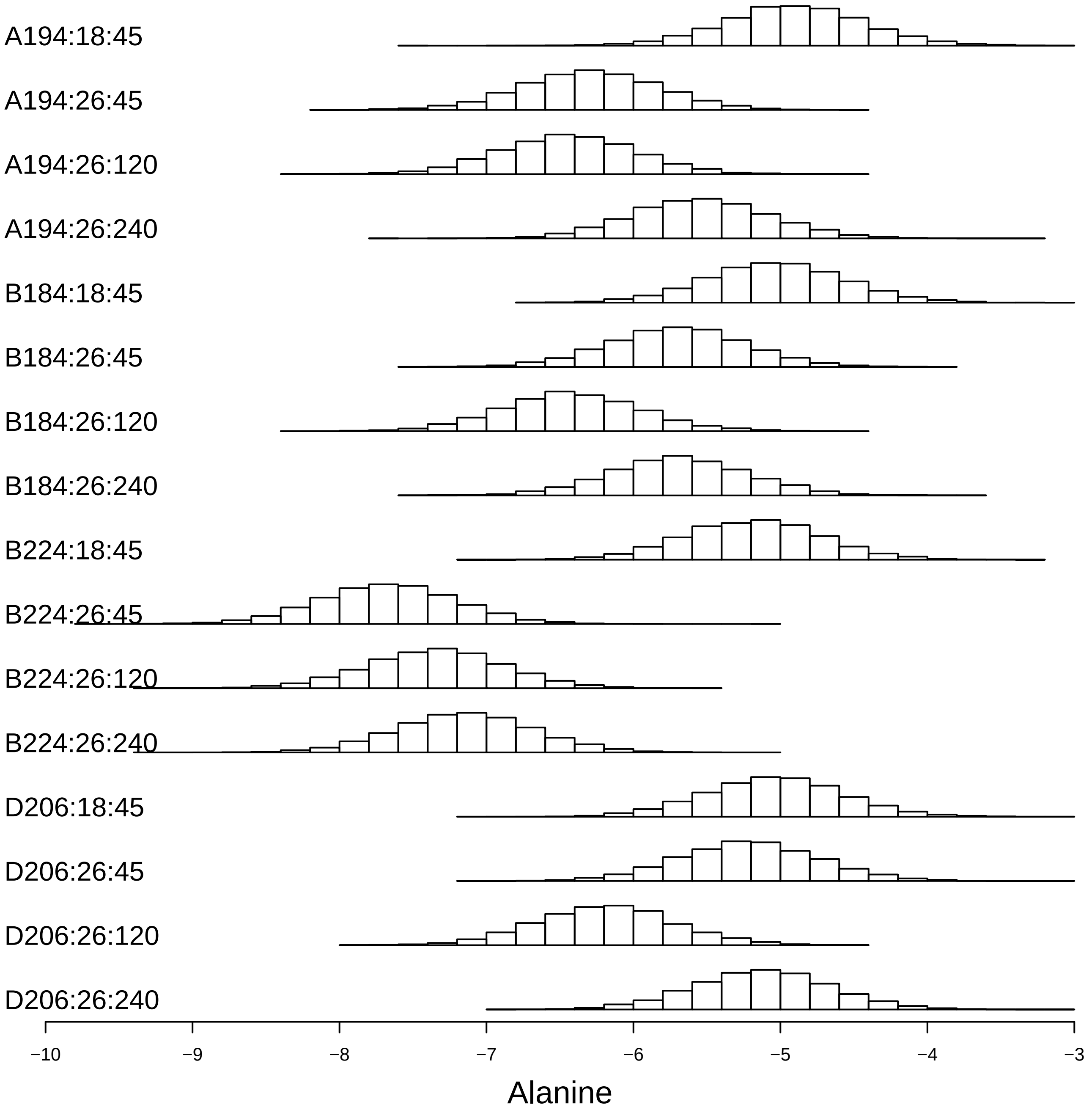
A194:18:45
A194:26:45
A194:26:120
A194:26:240
B184:18:45
B184:26:45
B184:26:120
B184:26:240
B224:18:45
B224:26:45
B224:26:120
B224:26:240
D206:18:45
D206:26:45
D206:26:120
D206:26:240
−10
−9
−8
−7
−6
−5
−4
−3
Alanine

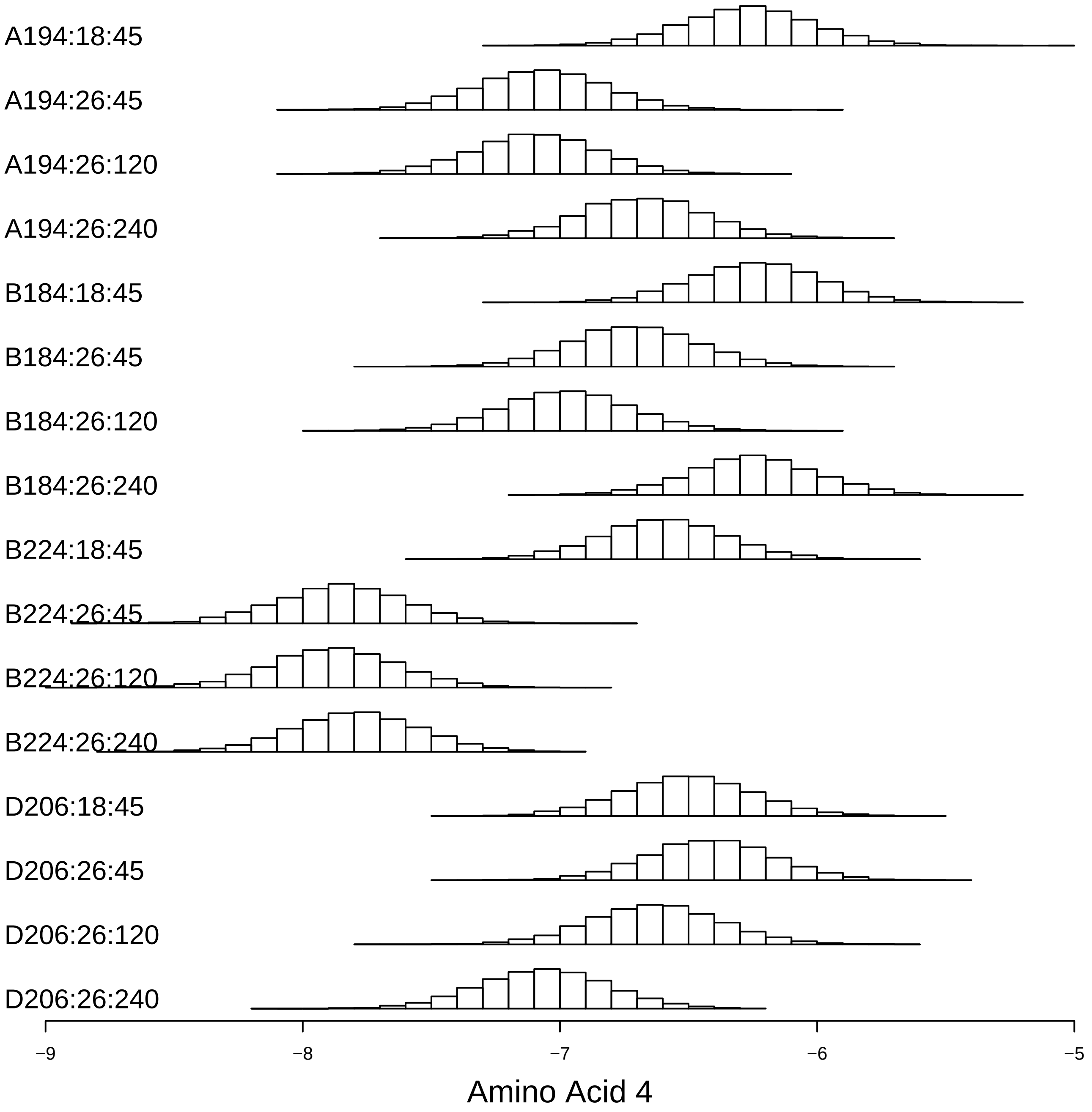

A194:18:45
A194:26:45
A194:26:120
A194:26:240
B184:18:45
B184:26:45
B184:26:120
B184:26:240
B224:18:45
B224:26:45
B224:26:120
B224:26:240
D206:18:45
D206:26:45
D206:26:120
D206:26:240
−9
−8
−7
−6
−5
Amino Acid 4

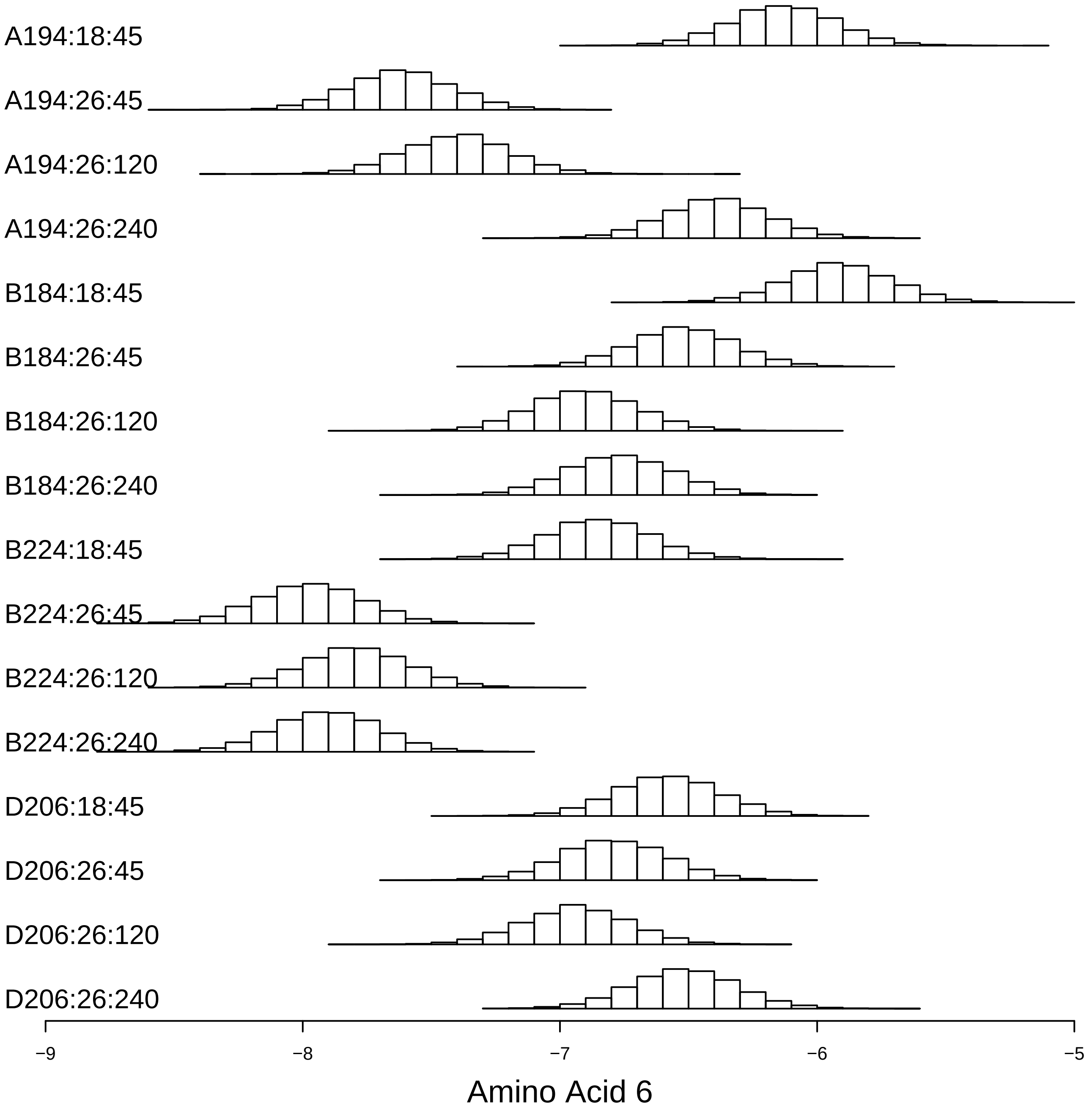
A194:18:45
A194:26:45
A194:26:120
A194:26:240
B184:18:45
B184:26:45
B184:26:120
B184:26:240
B224:18:45
B224:26:45
B224:26:120
B224:26:240
D206:18:45
D206:26:45
D206:26:120
D206:26:240
−9
−8
−7
−6
−5
Amino Acid 6

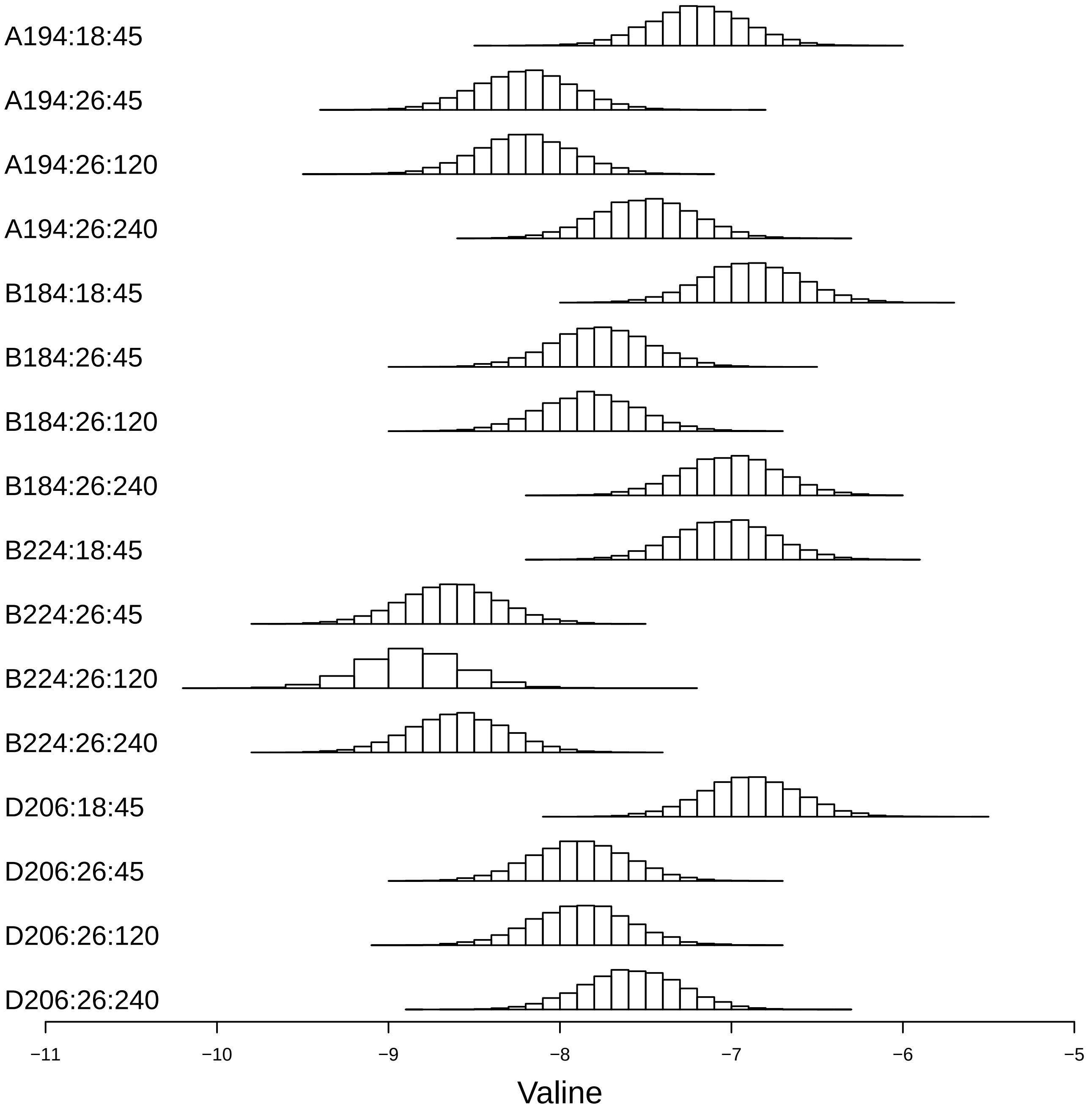

A194:18:45
A194:26:45
A194:26:120
A194:26:240
B184:18:45
B184:26:45
B184:26:120
B184:26:240
B224:18:45
B224:26:45
B224:26:120
B224:26:240
D206:18:45
D206:26:45
D206:26:120
D206:26:240
−11
−10
−9
−8
−7
−6
−5
Valine

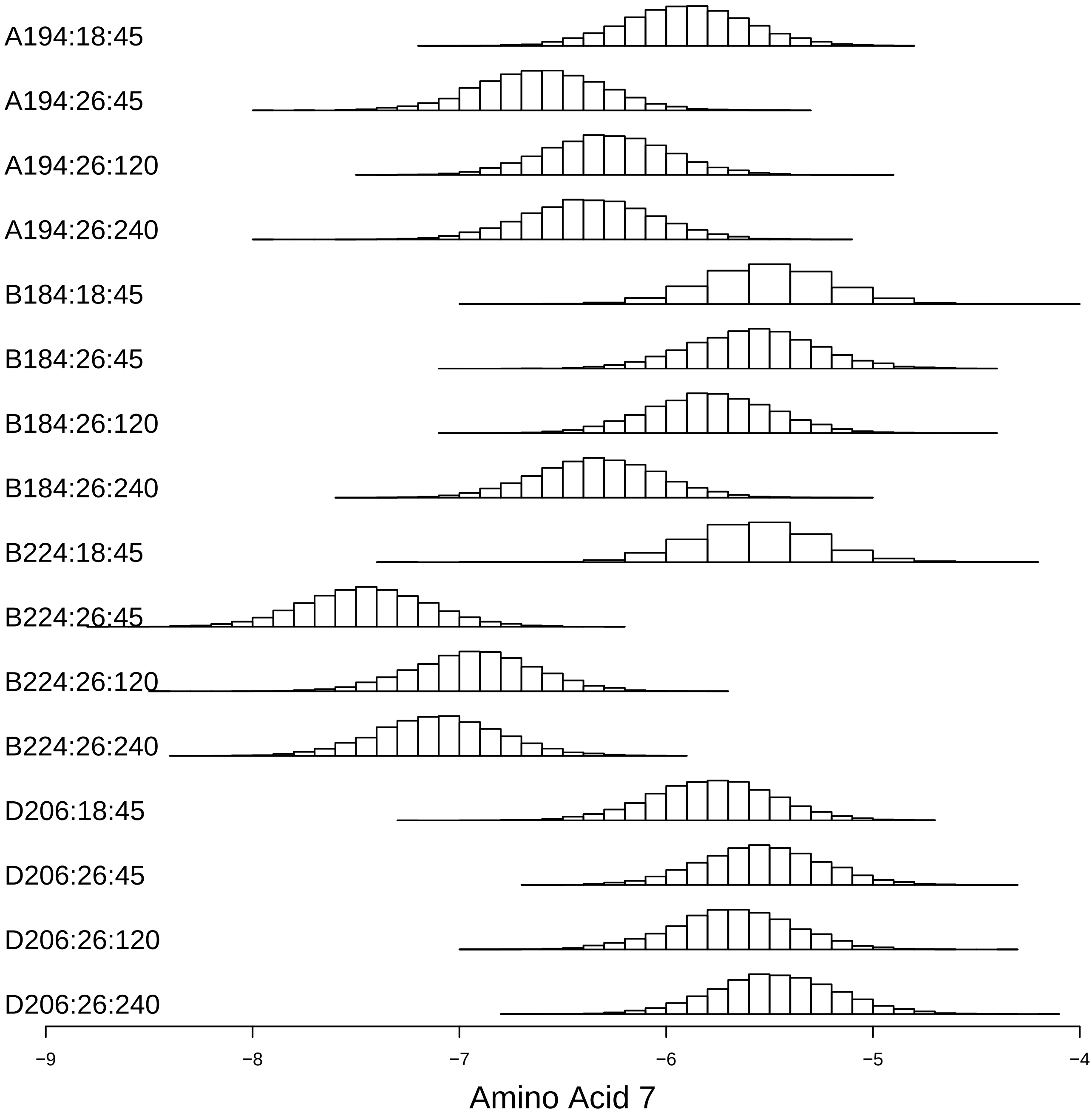

A194:18:45
A194:26:45
A194:26:120
A194:26:240
B184:18:45
B184:26:45
B184:26:120
B184:26:240
B224:18:45
B224:26:45
B224:26:120
B224:26:240
D206:18:45
D206:26:45
D206:26:120
D206:26:240
−9
−8
−7
−6
−5
−4
Amino Acid 7

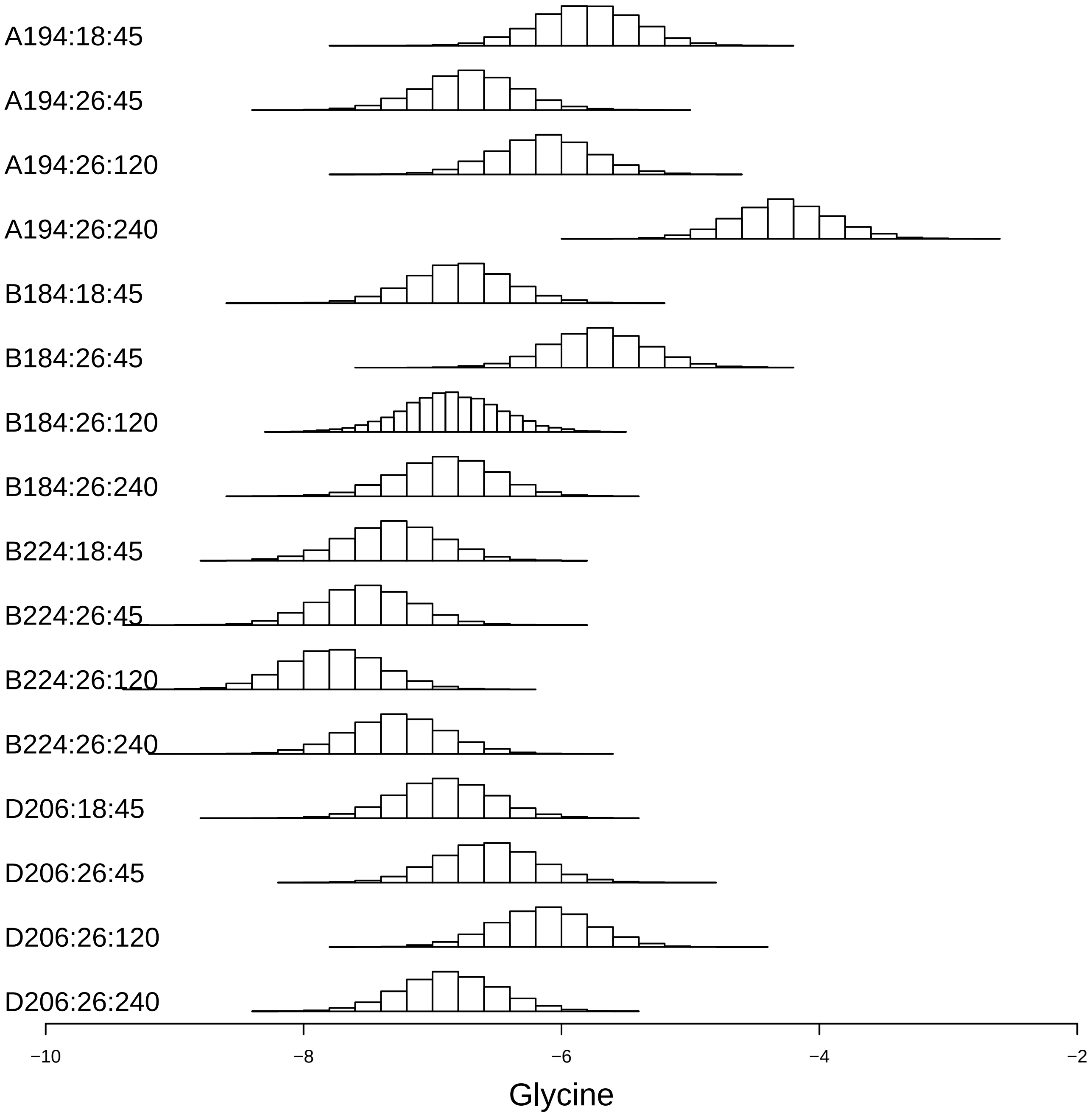

A194:18:45
A194:26:45
A194:26:120
A194:26:240
B184:18:45
B184:26:45
B184:26:120
B184:26:240
B224:18:45
B224:26:45
B224:26:120
B224:26:240
D206:18:45
D206:26:45
D206:26:120
D206:26:240
−10
−8
−6
−4
−2
Glycine

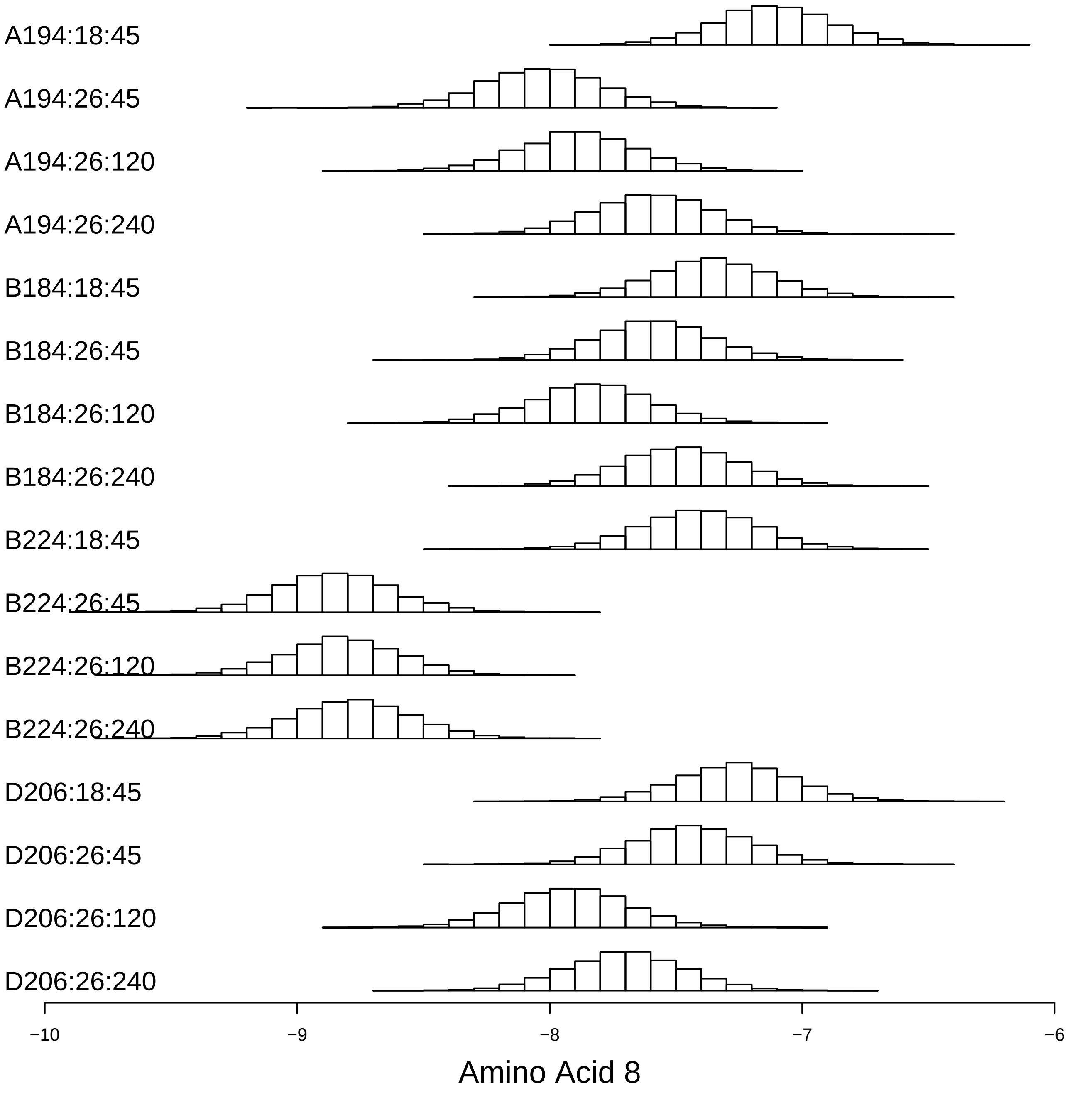
A194:18:45
A194:26:45
A194:26:120
A194:26:240
B184:18:45
B184:26:45
B184:26:120
B184:26:240
B224:18:45
B224:26:45
B224:26:120
B224:26:240
D206:18:45
D206:26:45
D206:26:120
D206:26:240
−10
−9
−8
−7
−6
Amino Acid 8

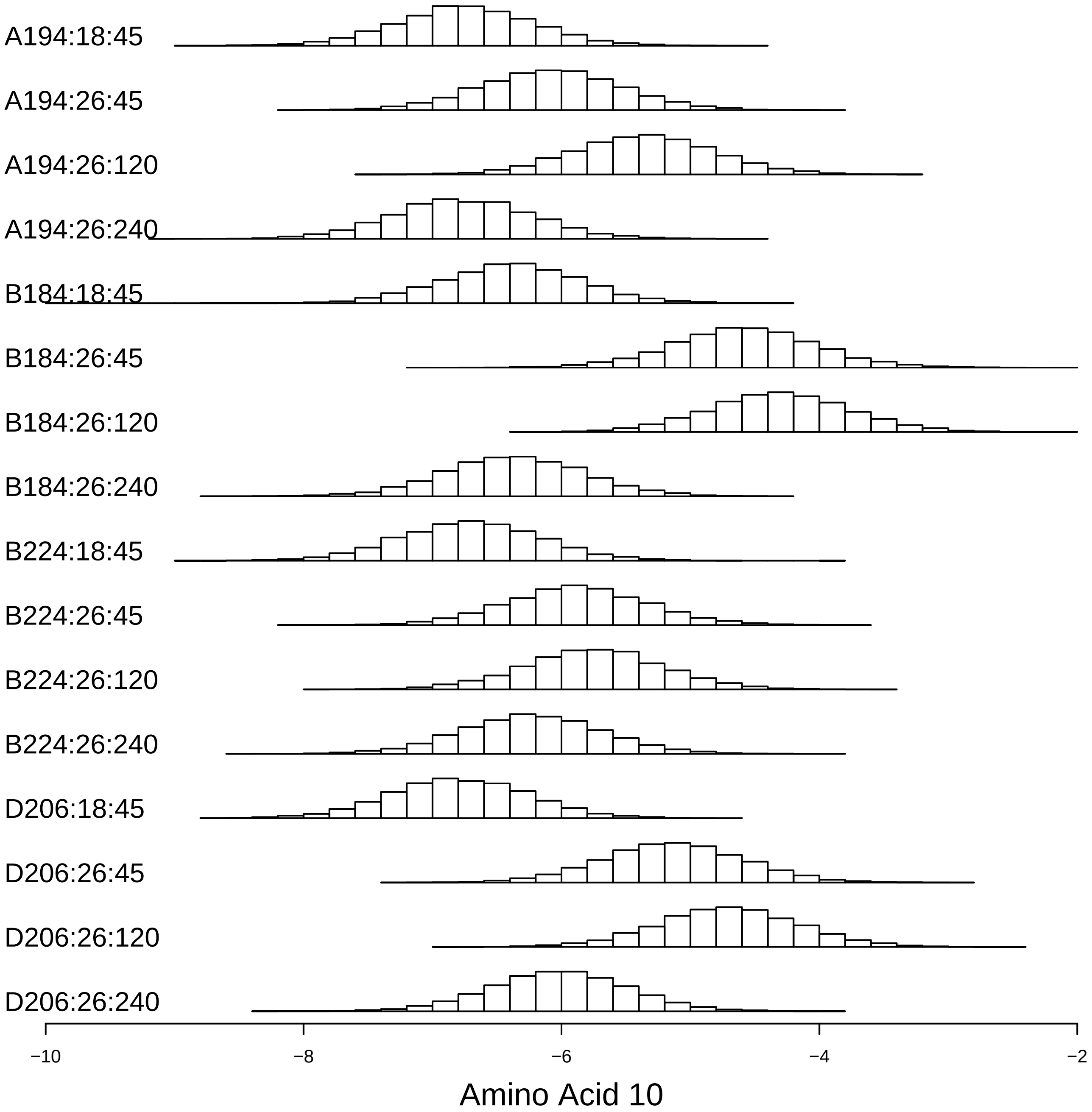
A194:18:45
A194:26:45
A194:26:120
A194:26:240
B184:18:45
B184:26:45
B184:26:120
B184:26:240
B224:18:45
B224:26:45
B224:26:120
B224:26:240
D206:18:45
D206:26:45
D206:26:120
D206:26:240
−10
−8
−6
−4
−2
Amino Acid 10

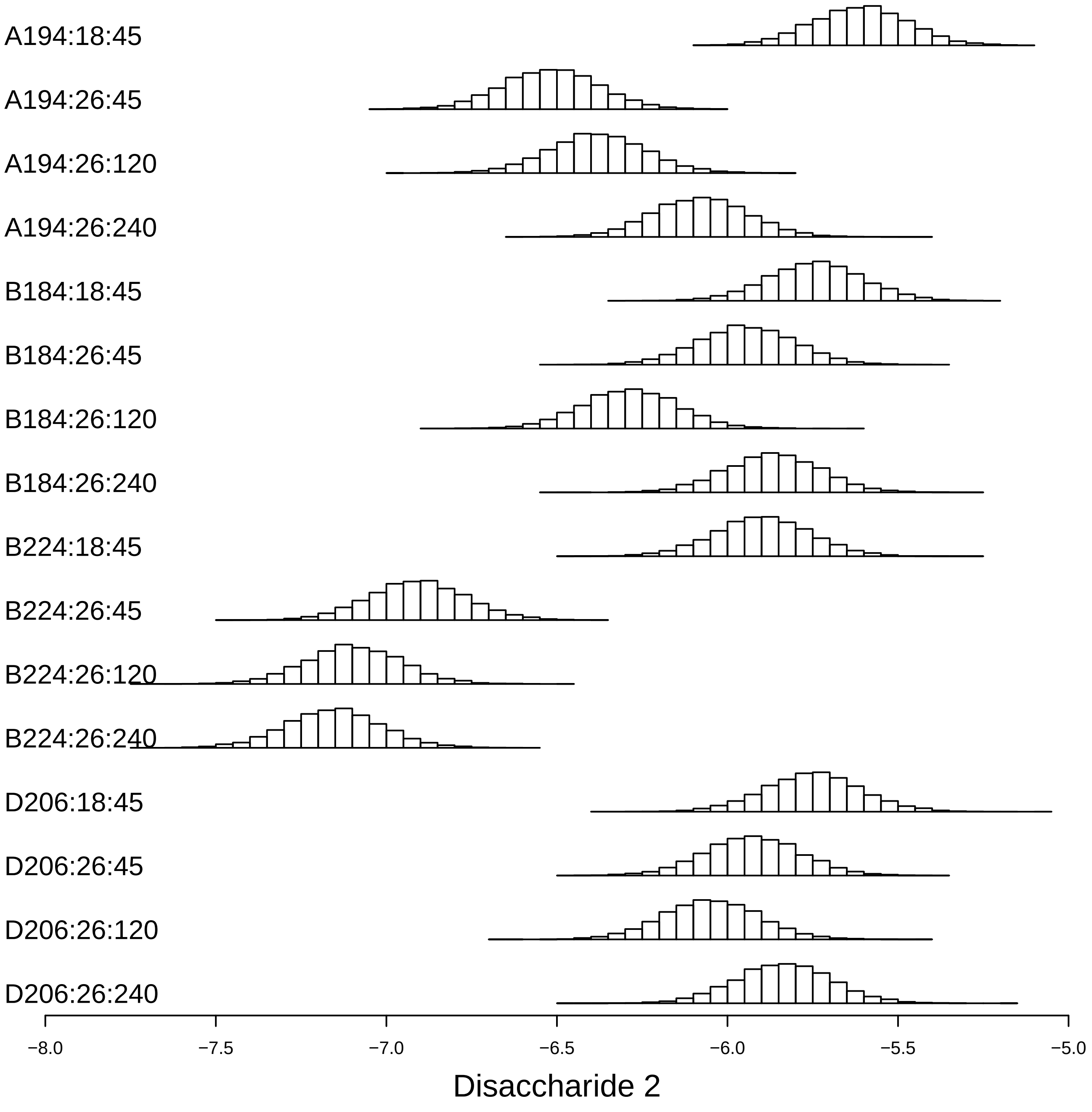
A194:18:45
A194:26:45
A194:26:120
A194:26:240
B184:18:45
B184:26:45
B184:26:120
B184:26:240
B224:18:45
B224:26:45
B224:26:120
B224:26:240
D206:18:45
D206:26:45
D206:26:120
D206:26:240
−8.0
−7.5
−7.0
−6.5
−6.0
−5.5
−5.0
Disaccharide 2

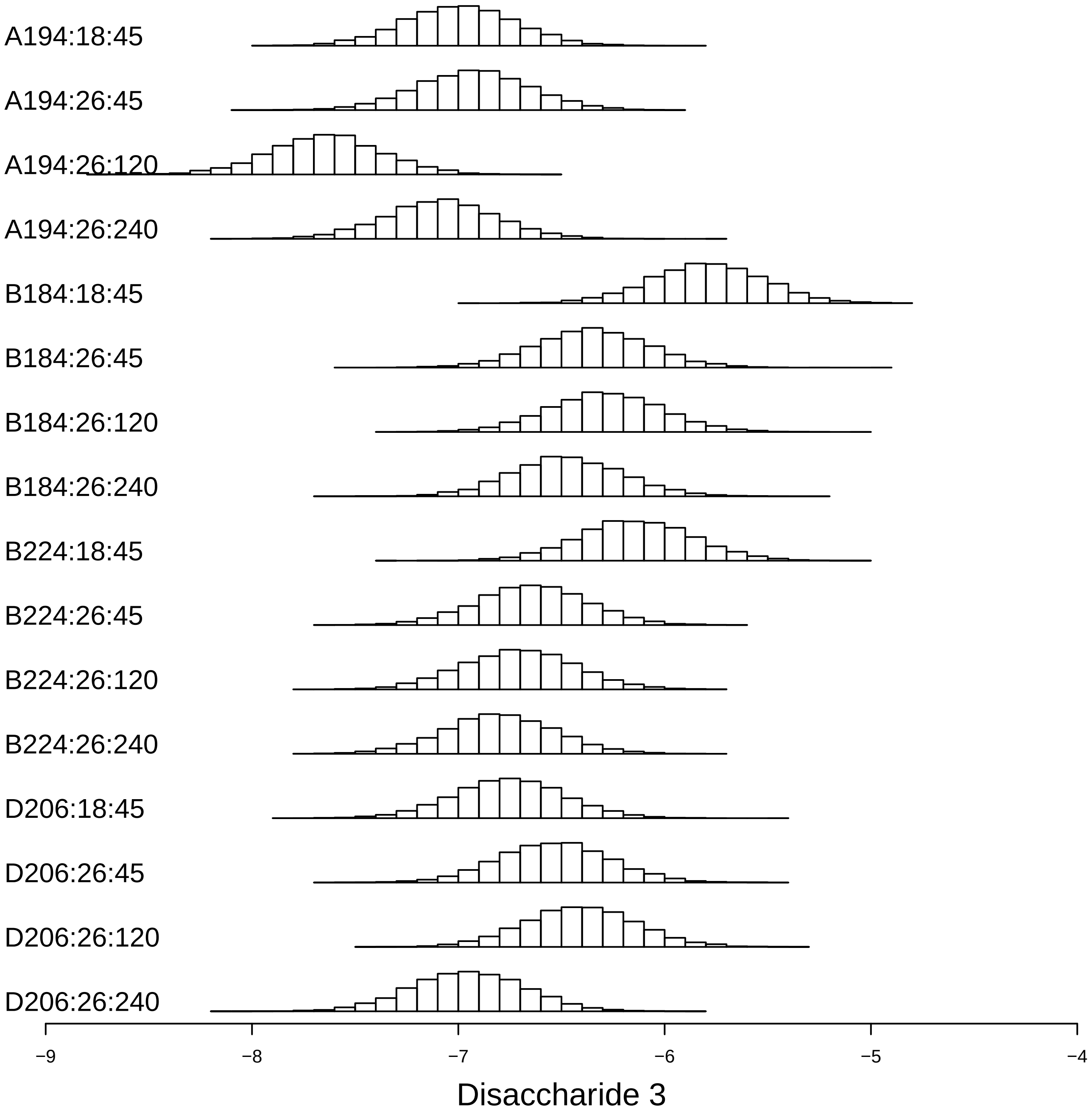
A194:18:45
A194:26:45
A194:26:120
A194:26:240
B184:18:45
B184:26:45
B184:26:120
B184:26:240
B224:18:45
B224:26:45
B224:26:120
B224:26:240
D206:18:45
D206:26:45
D206:26:120
D206:26:240
−9
−8
−7
−6
−5
−4
Disaccharide 3

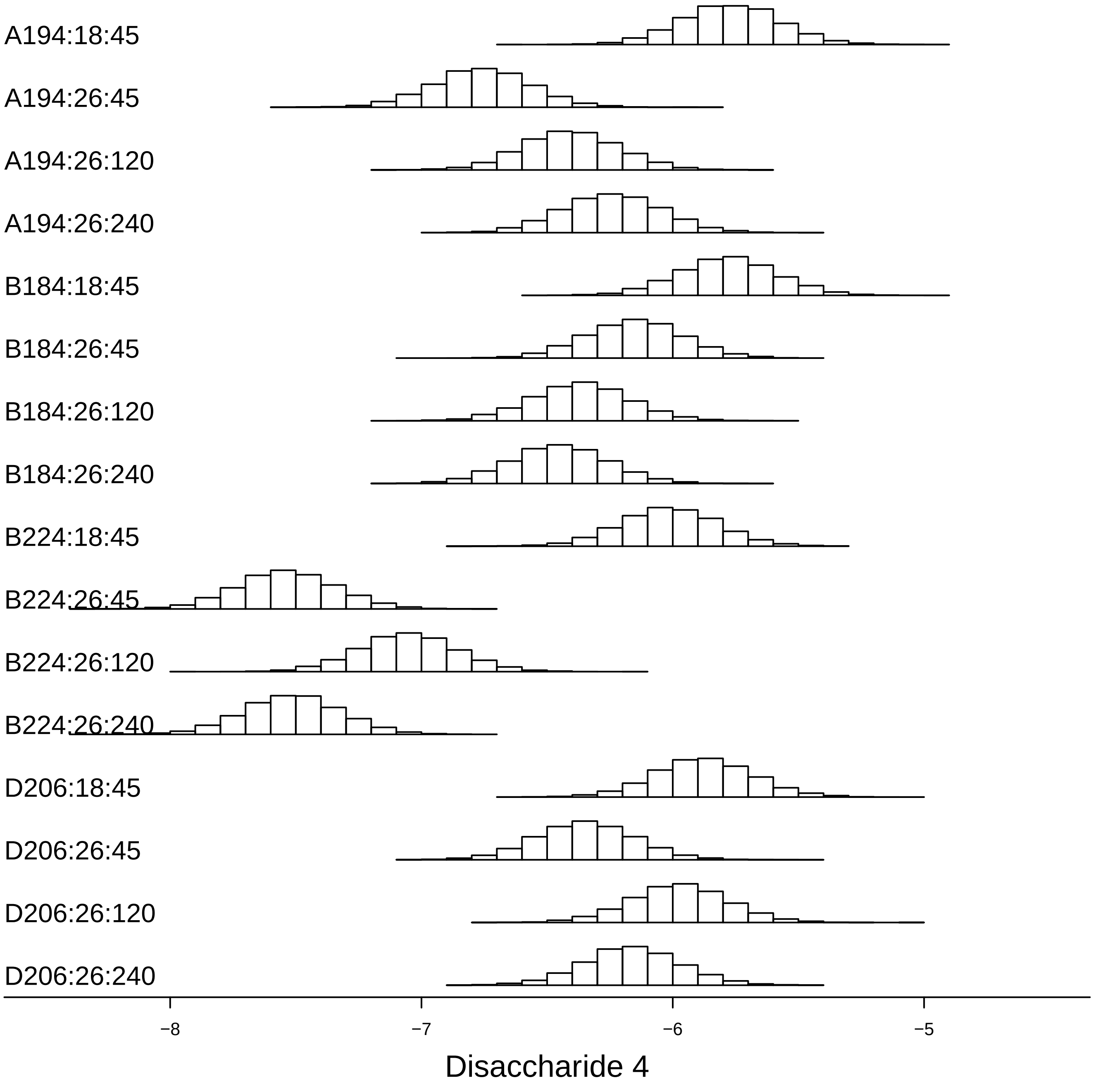
A194:18:45
A194:26:45
A194:26:120
A194:26:240
B184:18:45
B184:26:45
B184:26:120
B184:26:240
B224:18:45
B224:26:45
B224:26:120
B224:26:240
D206:18:45
D206:26:45
D206:26:120
D206:26:240
−8
−7
−6
−5
Disaccharide 4

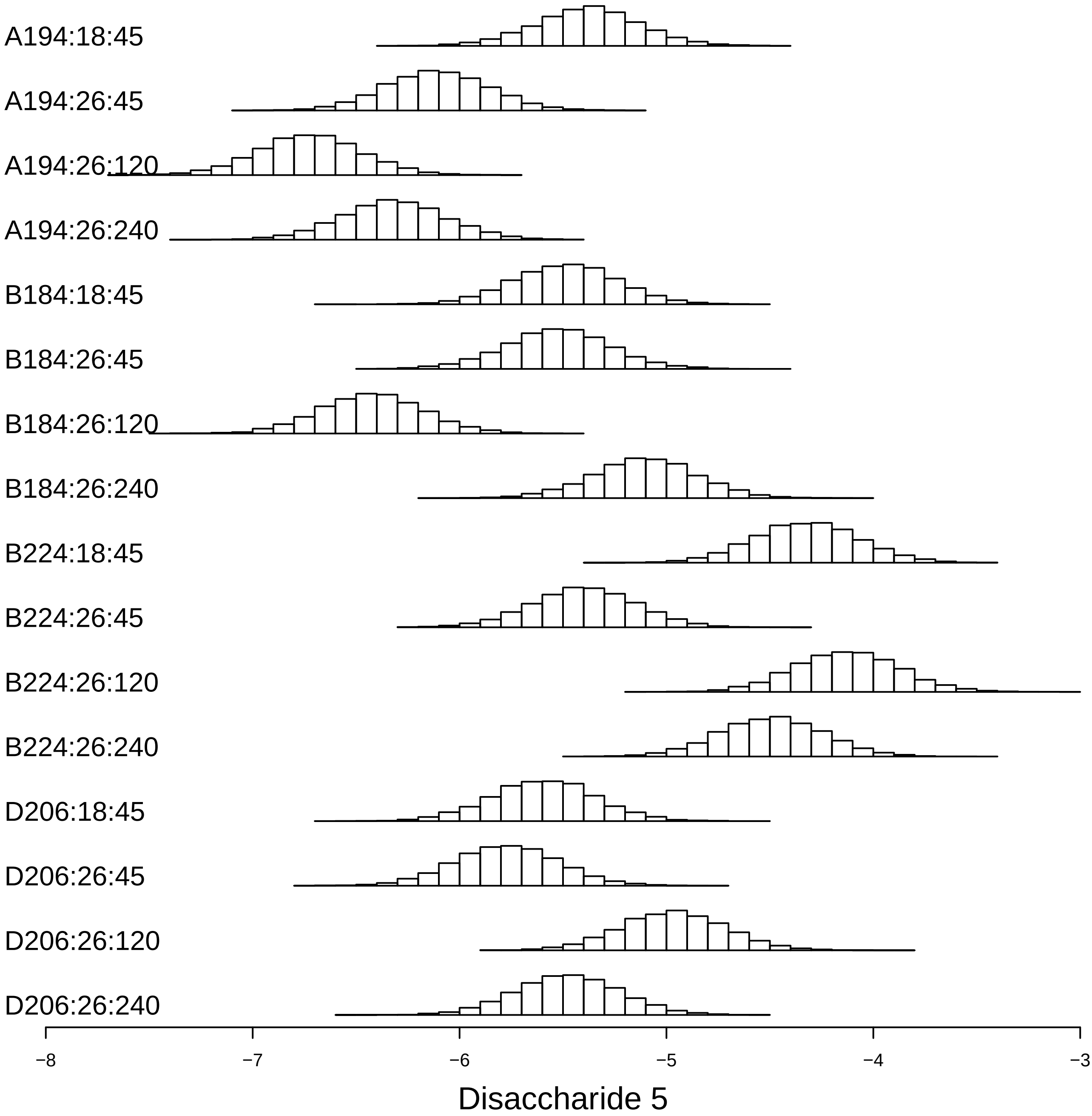
A194:18:45
A194:26:45
A194:26:120
A194:26:240
B184:18:45
B184:26:45
B184:26:120
B184:26:240
B224:18:45
B224:26:45
B224:26:120
B224:26:240
D206:18:45
D206:26:45
D206:26:120
D206:26:240
−8
−7
−6
−5
−4
−3
Disaccharide 5

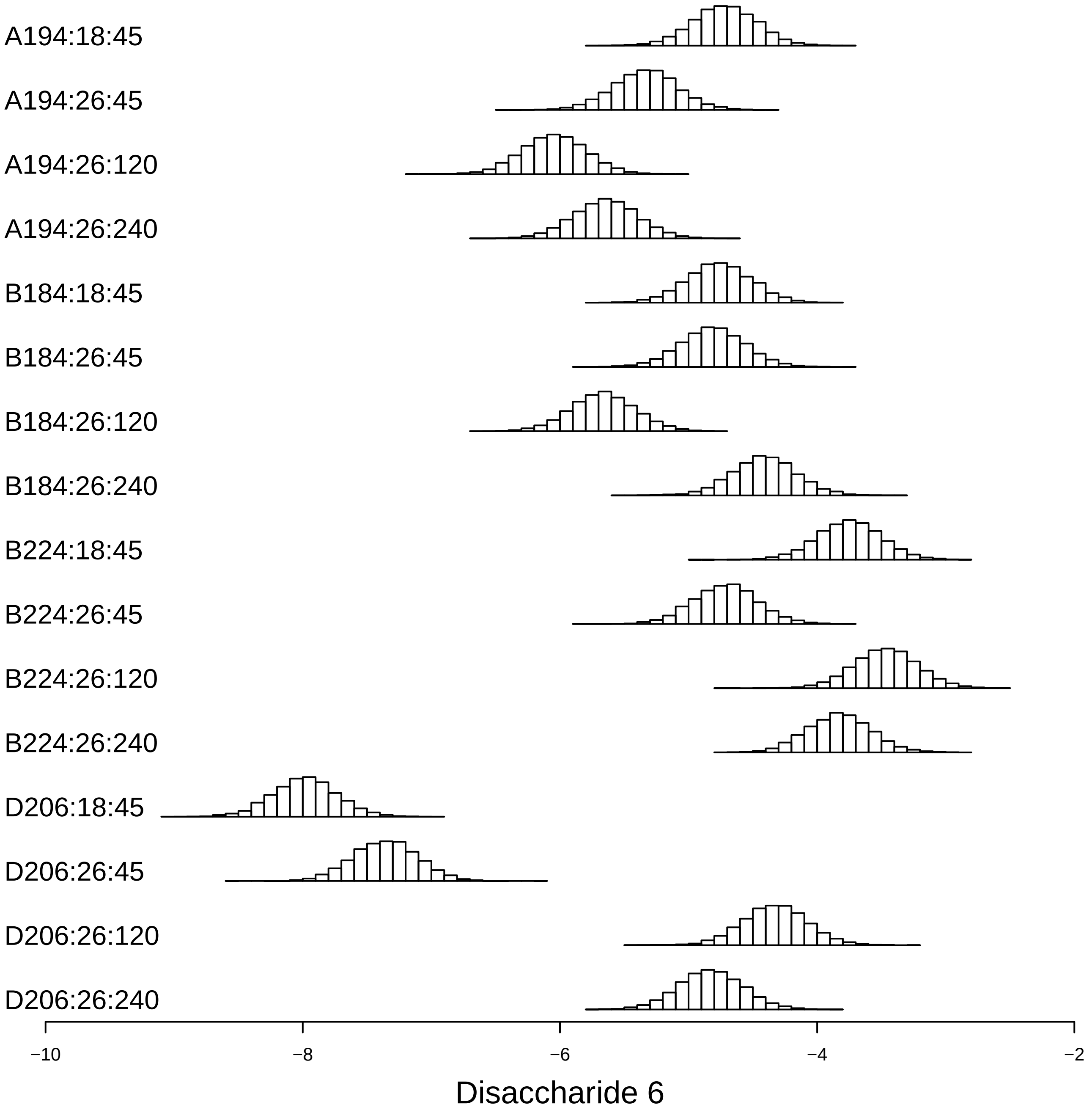
A194:18:45
A194:26:45
A194:26:120
A194:26:240
B184:18:45
B184:26:45
B184:26:120
B184:26:240
B224:18:45
B224:26:45
B224:26:120
B224:26:240
D206:18:45
D206:26:45
D206:26:120
D206:26:240
−10
−8
−6
−4
−2
Disaccharide 6

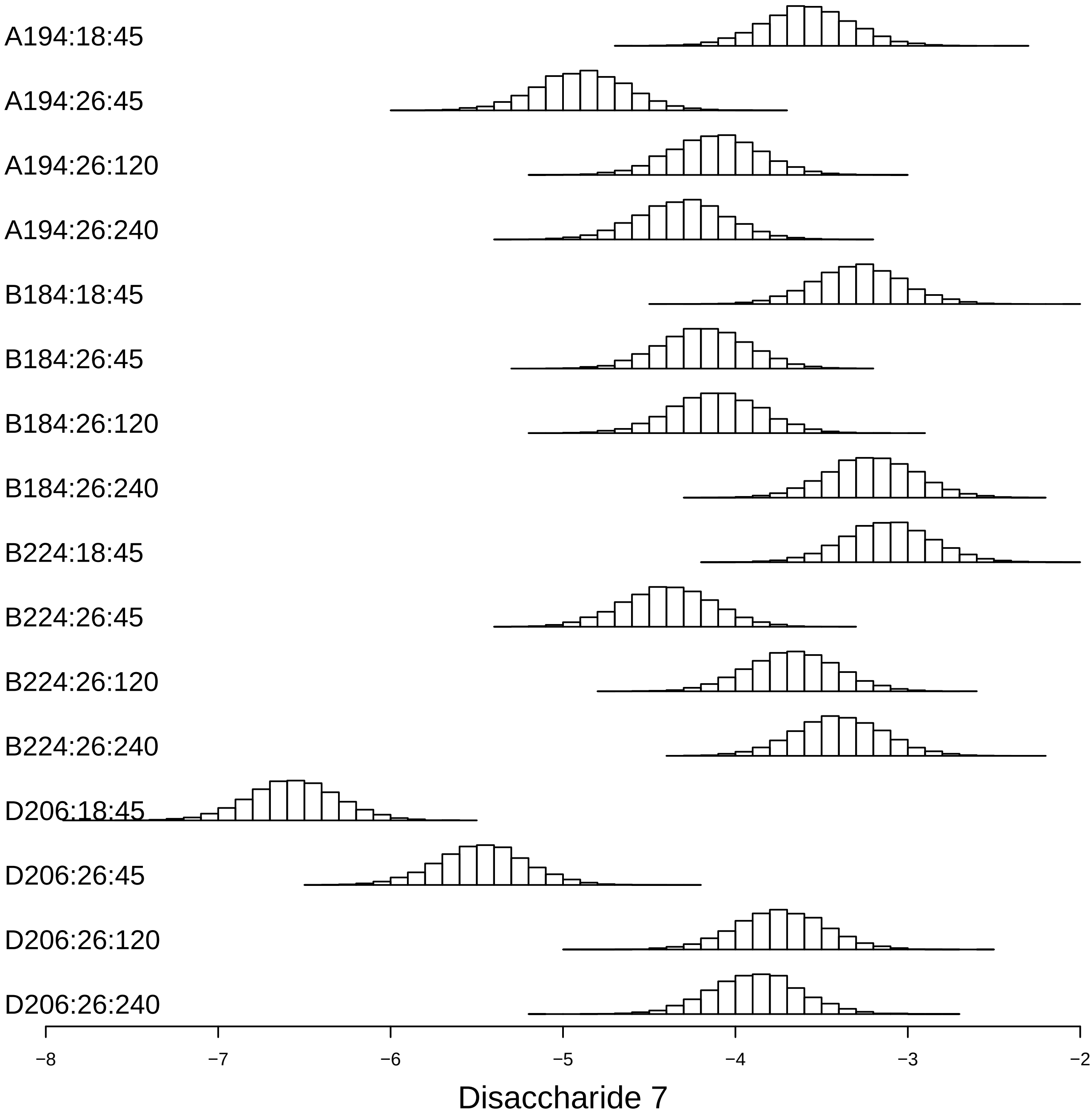
A194:18:45
A194:26:45
A194:26:120
A194:26:240
B184:18:45
B184:26:45
B184:26:120
B184:26:240
B224:18:45
B224:26:45
B224:26:120
B224:26:240
D206:18:45
D206:26:45
D206:26:120
D206:26:240
−8
−7
−6
−5
−4
−3
−2
Disaccharide 7

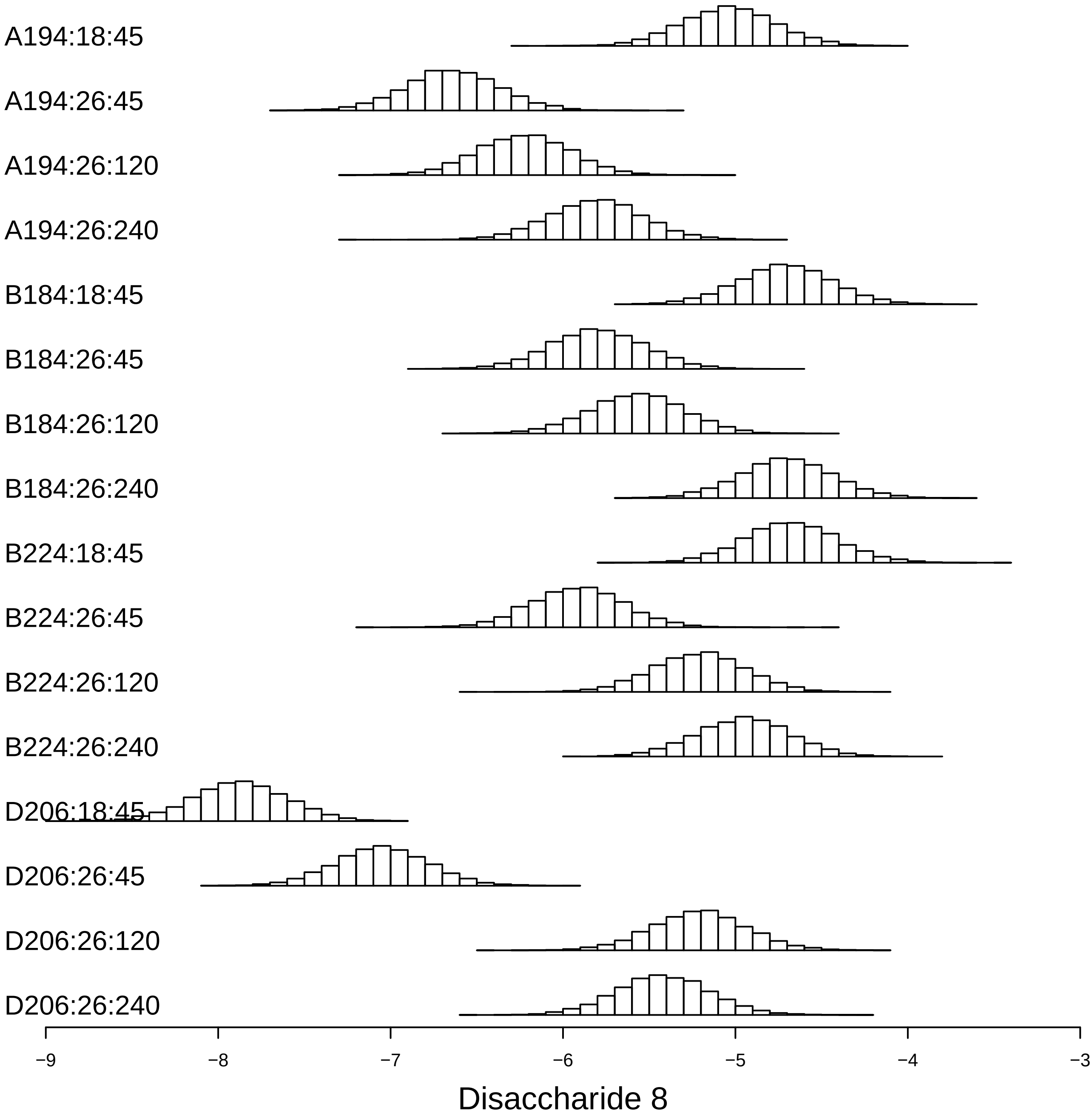

A194:18:45
A194:26:45
A194:26:120
A194:26:240
B184:18:45
B184:26:45
B184:26:120
B184:26:240
B224:18:45
B224:26:45
B224:26:120
B224:26:240
D206:18:45
D206:26:45
D206:26:120
D206:26:240
−9
−8
−7
−6
−5
−4
−3
Disaccharide 8

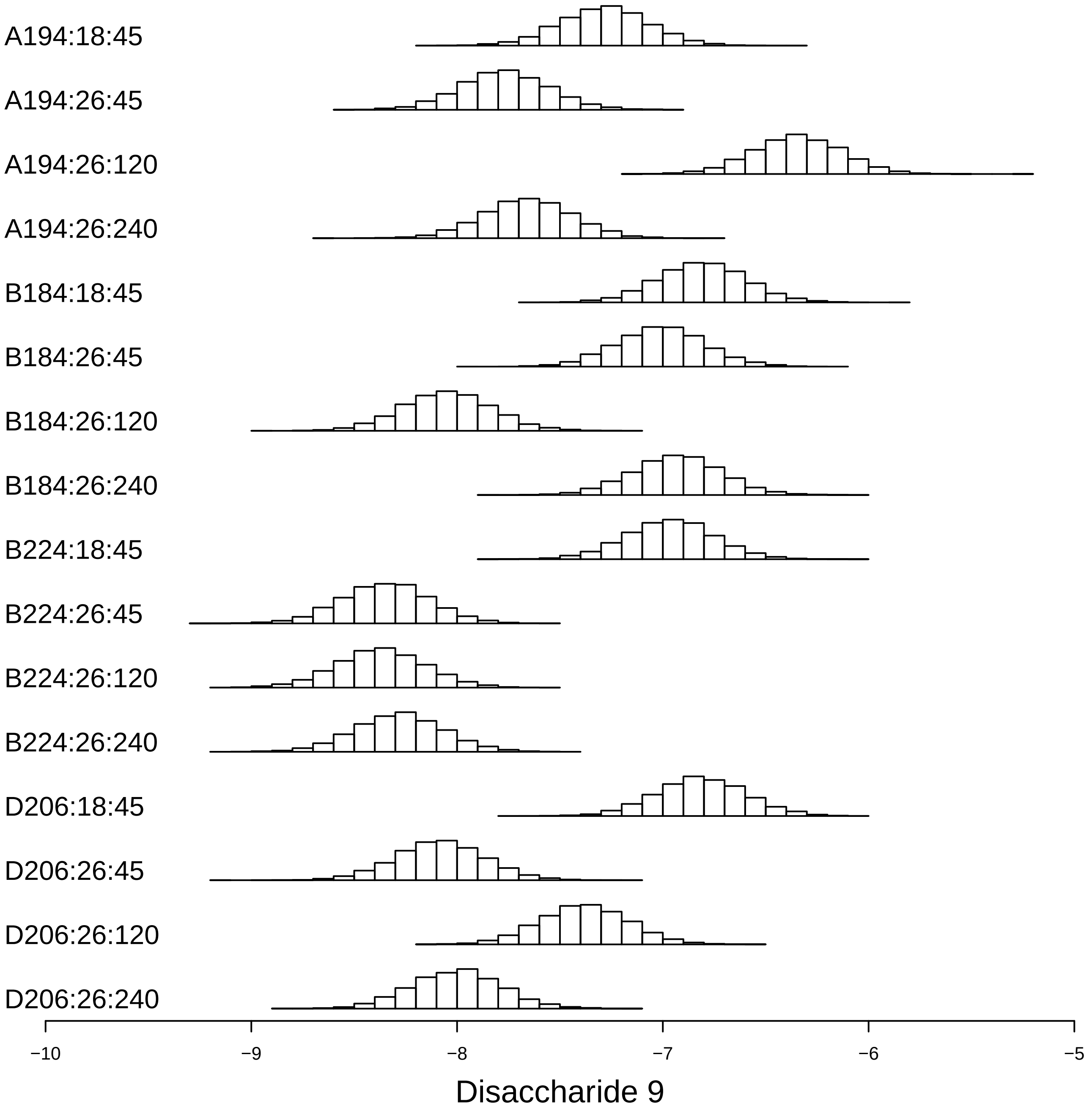
A194:18:45
A194:26:45
A194:26:120
A194:26:240
B184:18:45
B184:26:45
B184:26:120
B184:26:240
B224:18:45
B224:26:45
B224:26:120
B224:26:240
D206:18:45
D206:26:45
D206:26:120
D206:26:240
−10
−9
−8
−7
−6
−5
Disaccharide 9

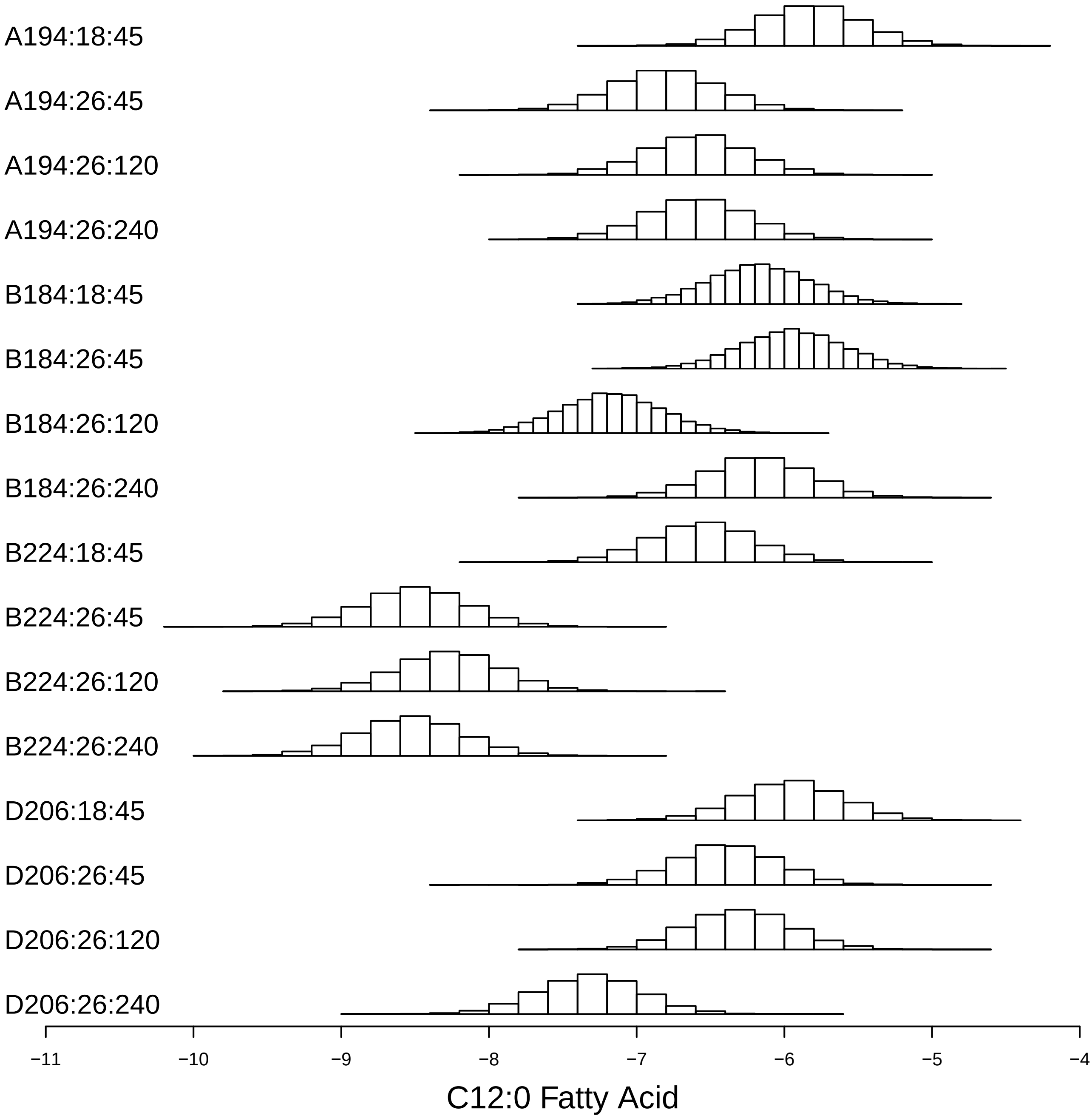
A194:18:45
A194:26:45
A194:26:120
A194:26:240
B184:18:45
B184:26:45
B184:26:120
B184:26:240
B224:18:45
B224:26:45
B224:26:120
B224:26:240
D206:18:45
D206:26:45
D206:26:120
D206:26:240
−11
−10
−9
−8
−7
−6
−5
−4
C12:0 Fatty Acid

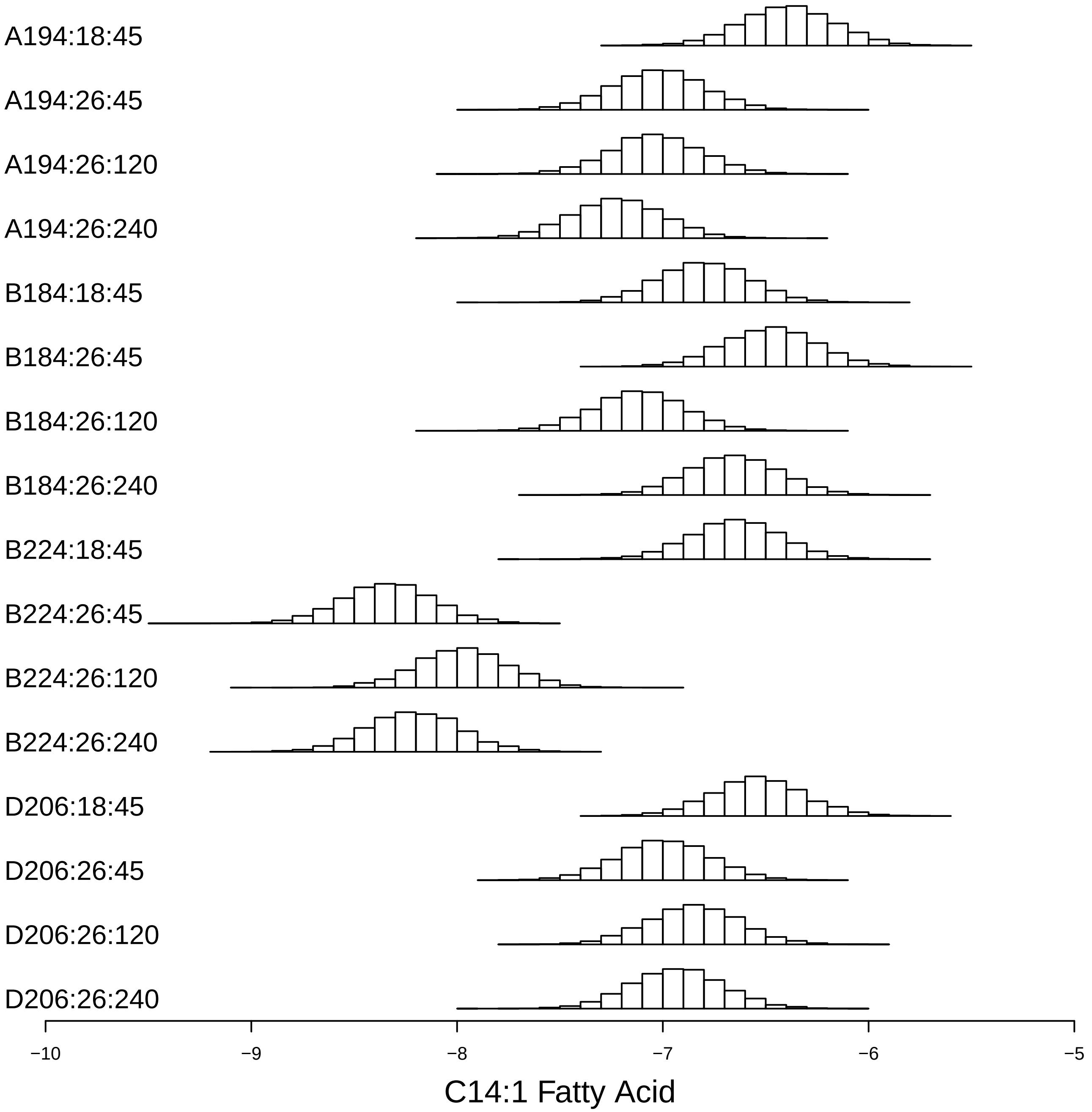

A194:18:45
A194:26:45
A194:26:120
A194:26:240
B184:18:45
B184:26:45
B184:26:120
B184:26:240
B224:18:45
B224:26:45
B224:26:120
B224:26:240
D206:18:45
D206:26:45
D206:26:120
D206:26:240
−10
−9
−8
−7
−6
−5
C14:1 Fatty Acid

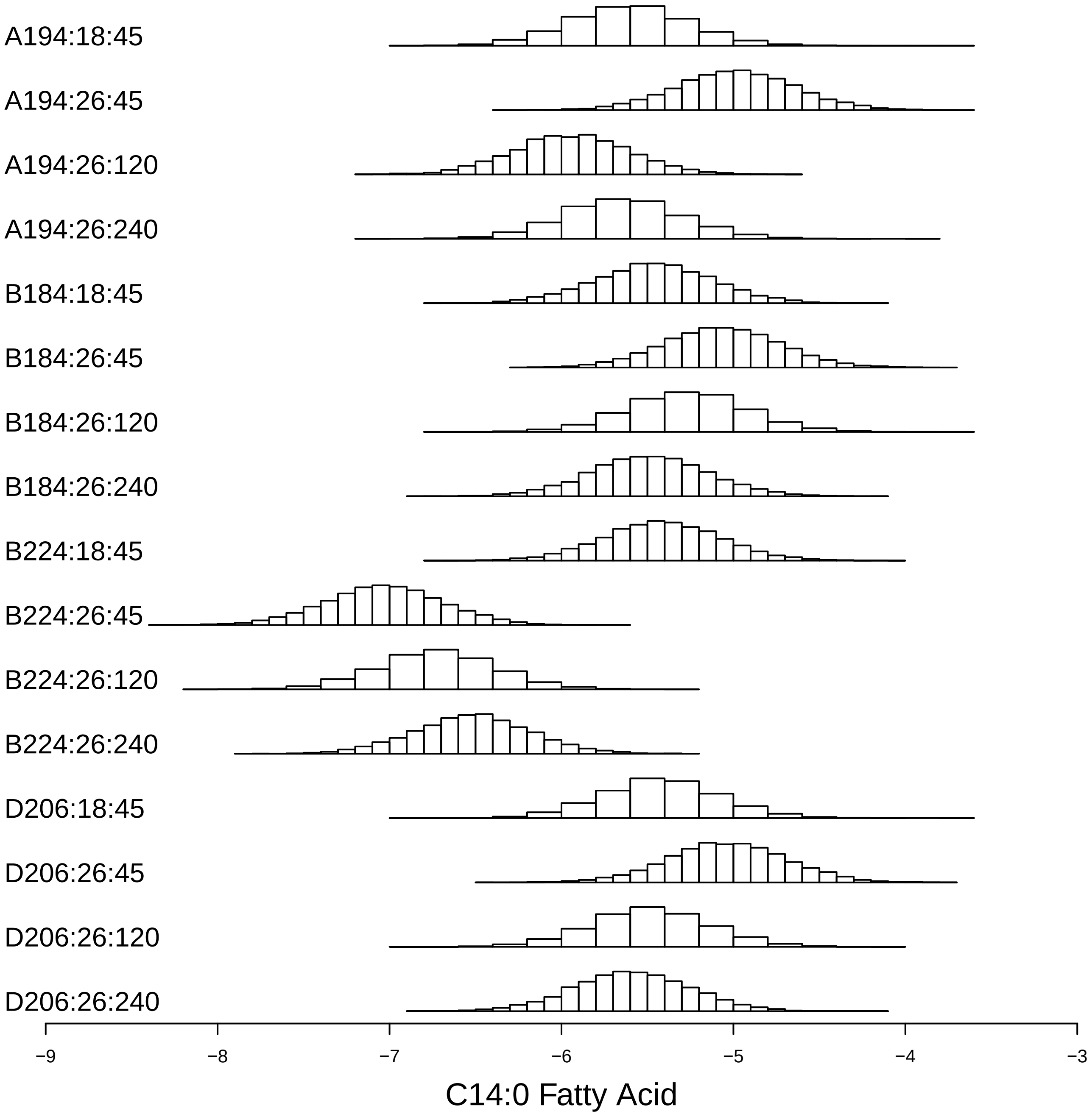
A194:18:45
A194:26:45
A194:26:120
A194:26:240
B184:18:45
B184:26:45
B184:26:120
B184:26:240
B224:18:45
B224:26:45
B224:26:120
B224:26:240
D206:18:45
D206:26:45
D206:26:120
D206:26:240
−9
−8
−7
−6
−5
−4
−3
C14:0 Fatty Acid

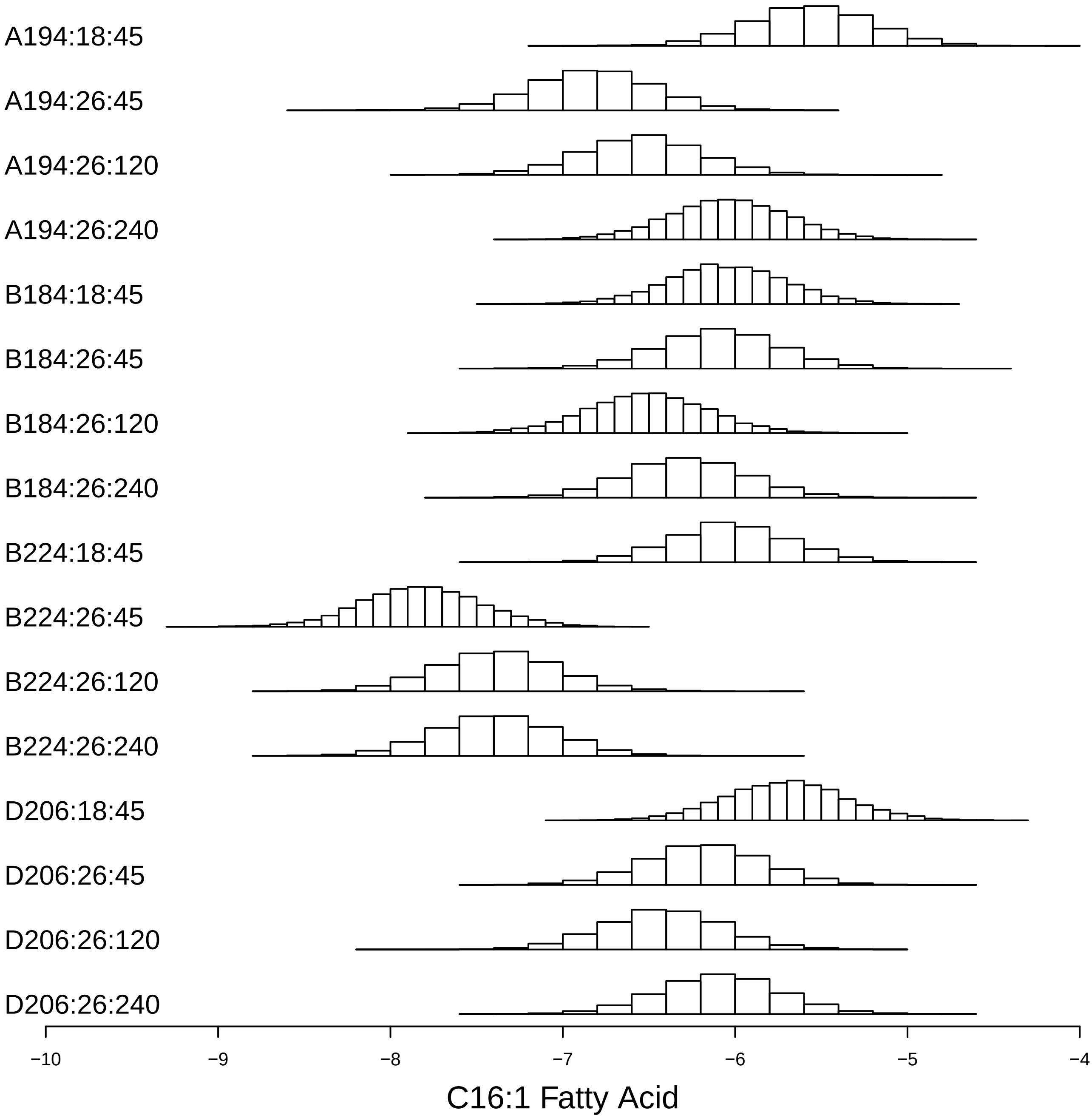
A194:18:45
A194:26:45
A194:26:120
A194:26:240
B184:18:45
B184:26:45
B184:26:120
B184:26:240
B224:18:45
B224:26:45
B224:26:120
B224:26:240
D206:18:45
D206:26:45
D206:26:120
D206:26:240
−10
−9
−8
−7
−6
−5
−4
C16:1 Fatty Acid

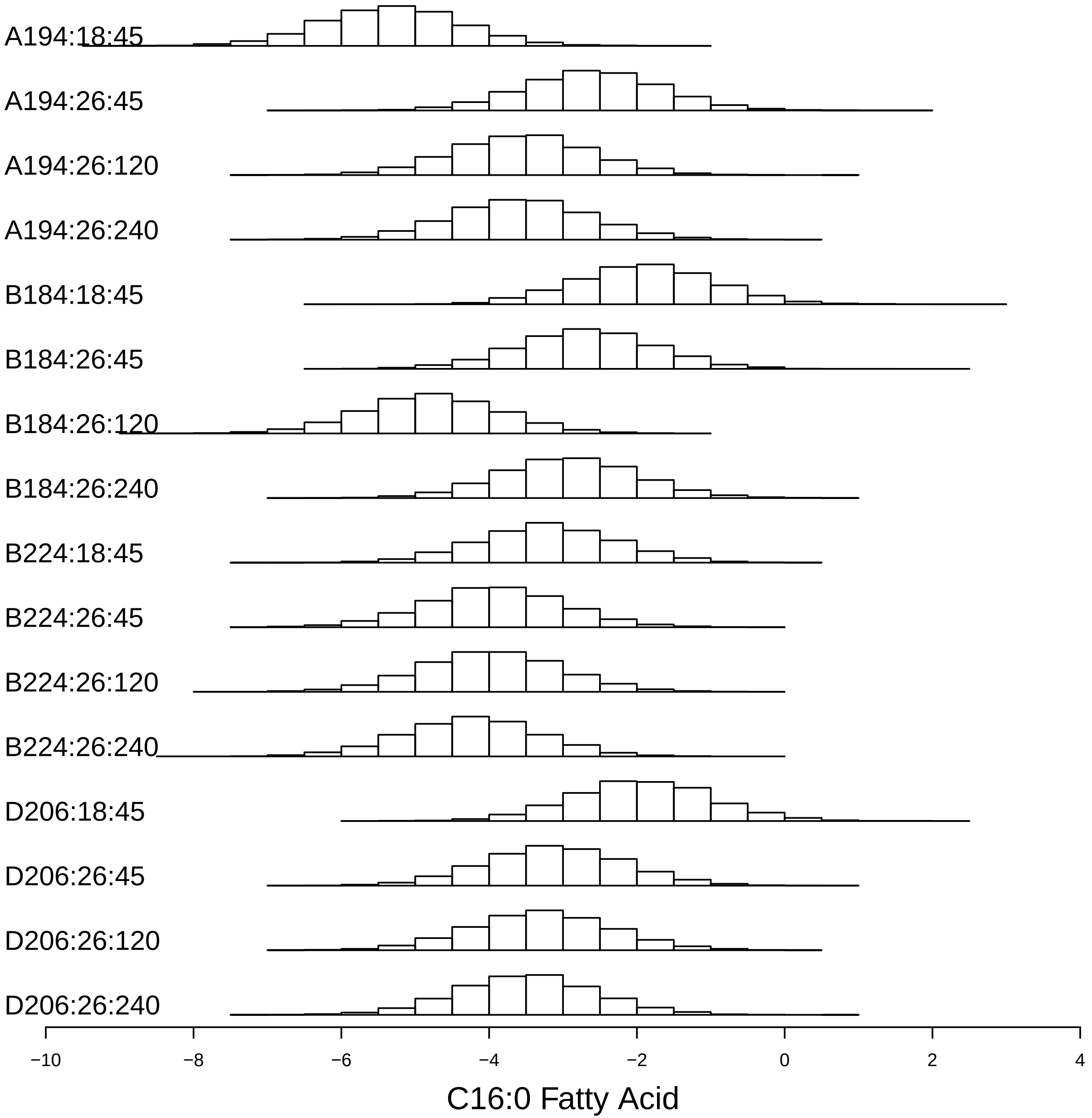
A194:18:45
A194:26:45
A194:26:120
A194:26:240
B184:18:45
B184:26:45
B184:26:120
B184:26:240
B224:18:45
B224:26:45
B224:26:120
B224:26:240
D206:18:45
D206:26:45
D206:26:120
D206:26:240
−10
−8
−6
−4
−2
0
2
4
C16:0 Fatty Acid

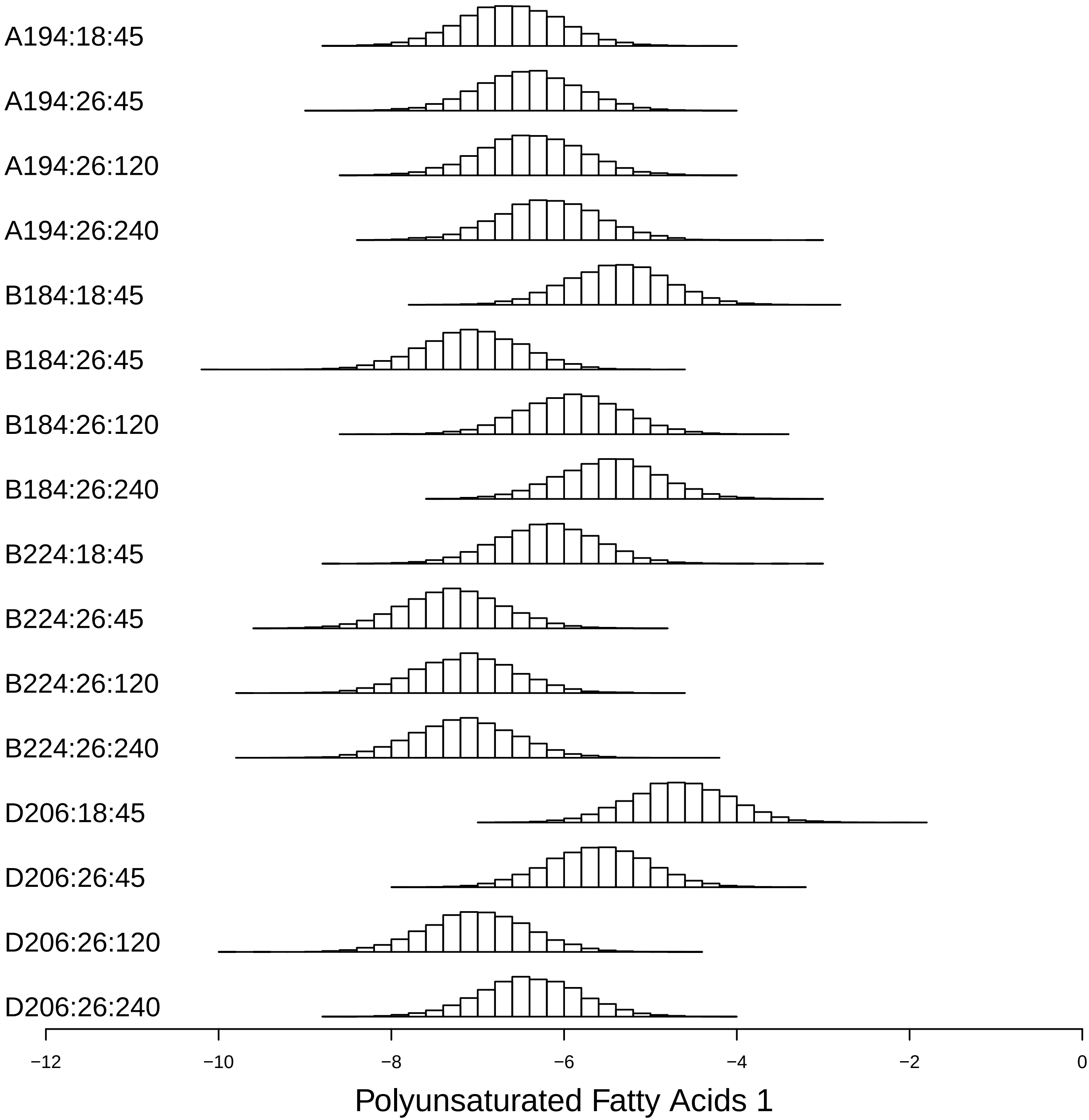
A194:18:45
A194:26:45
A194:26:120
A194:26:240
B184:18:45
B184:26:45
B184:26:120
B184:26:240
B224:18:45
B224:26:45
B224:26:120
B224:26:240
D206:18:45
D206:26:45
D206:26:120
D206:26:240
−12
−10
−8
−6
−4
−2
0
Polyunsaturated Fatty Acids 1

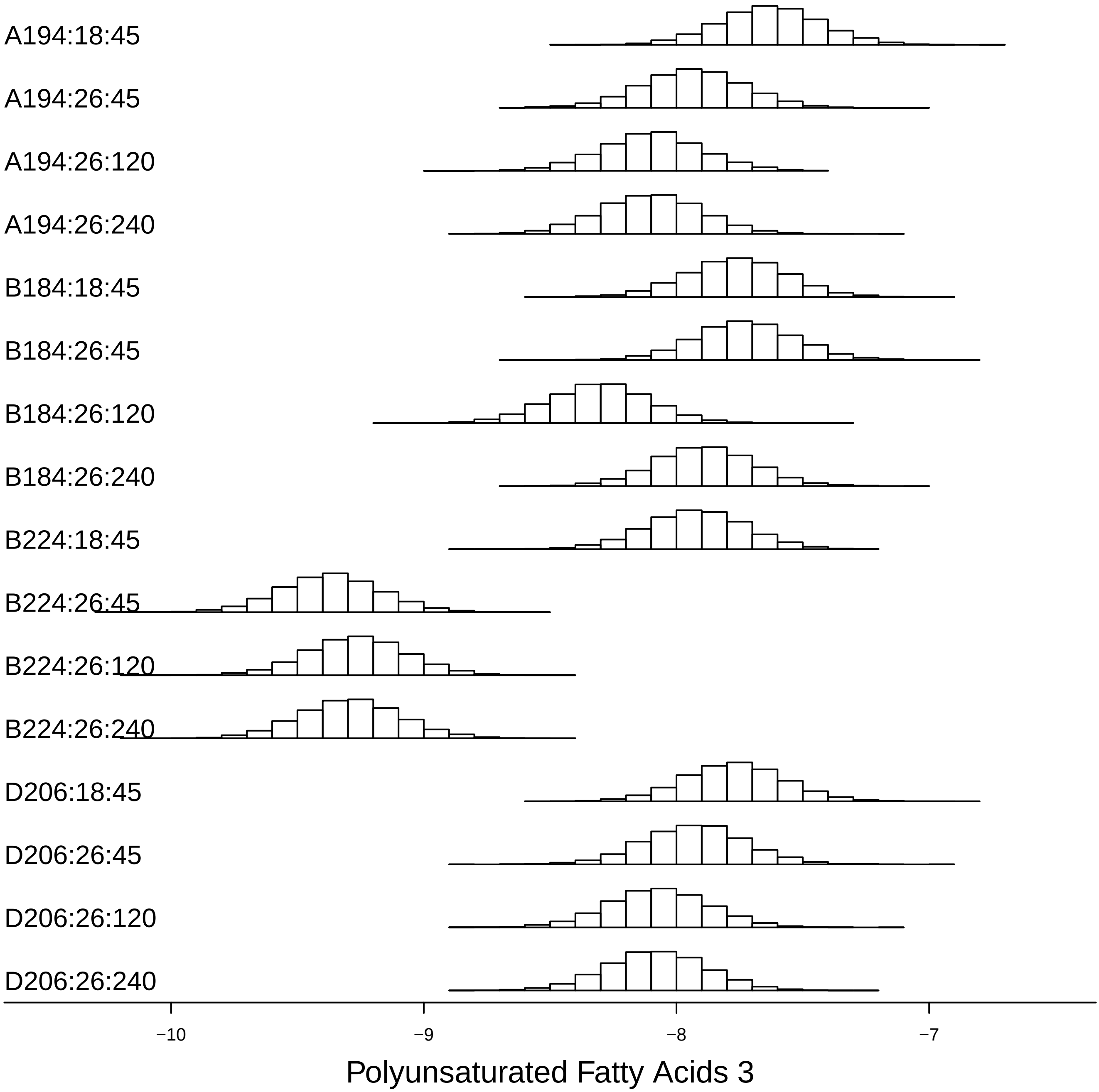

A194:18:45
A194:26:45
A194:26:120
A194:26:240
B184:18:45
B184:26:45
B184:26:120
B184:26:240
B224:18:45
B224:26:45
B224:26:120
B224:26:240
D206:18:45
D206:26:45
D206:26:120
D206:26:240
−10
−9
−8
−7
Polyunsaturated Fatty Acids 3

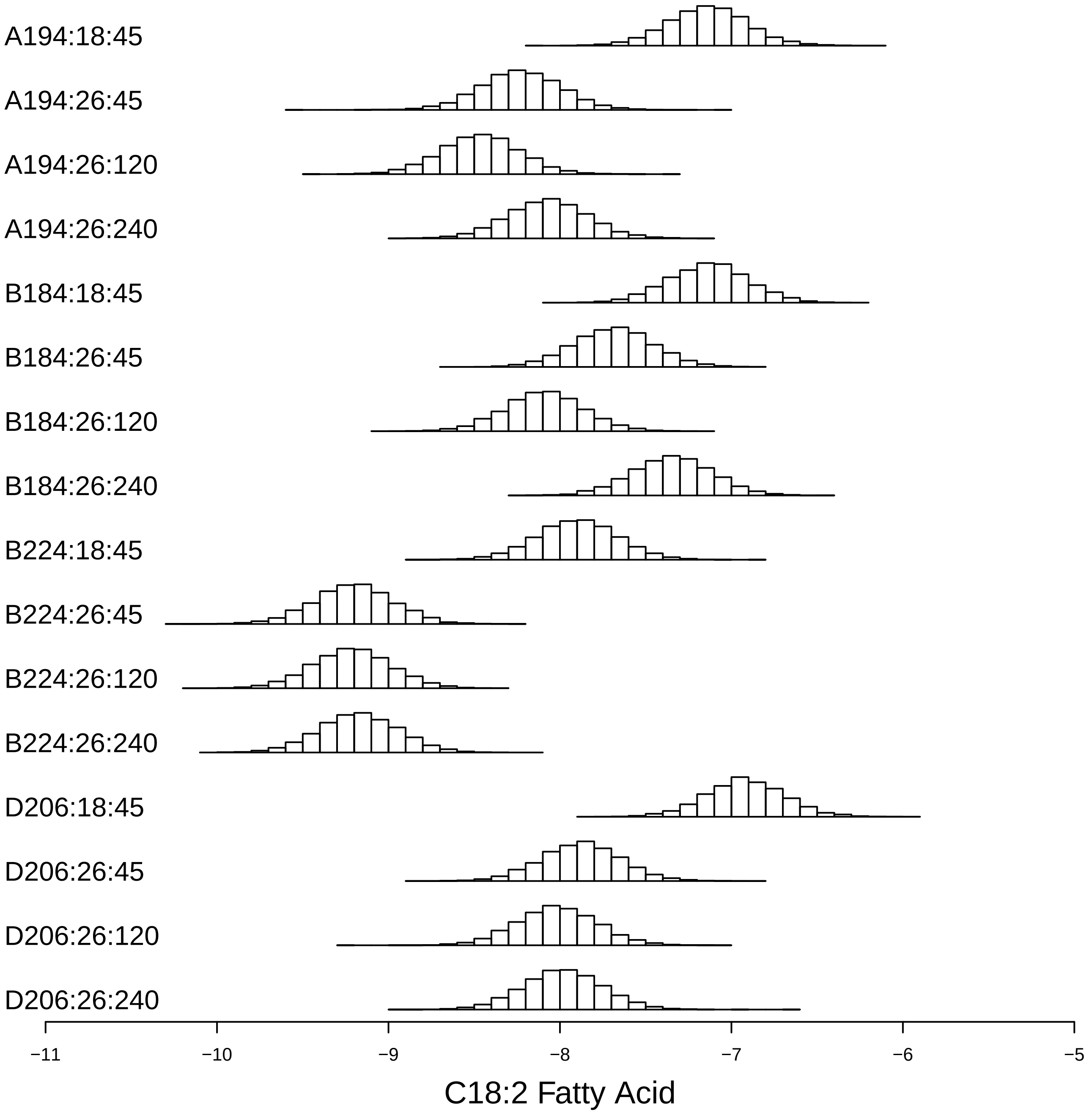

A194:18:45
A194:26:45
A194:26:120
A194:26:240
B184:18:45
B184:26:45
B184:26:120
B184:26:240
B224:18:45
B224:26:45
B224:26:120
B224:26:240
D206:18:45
D206:26:45
D206:26:120
D206:26:240
−11
−10
−9
−8
−7
−6
−5
C18:2 Fatty Acid

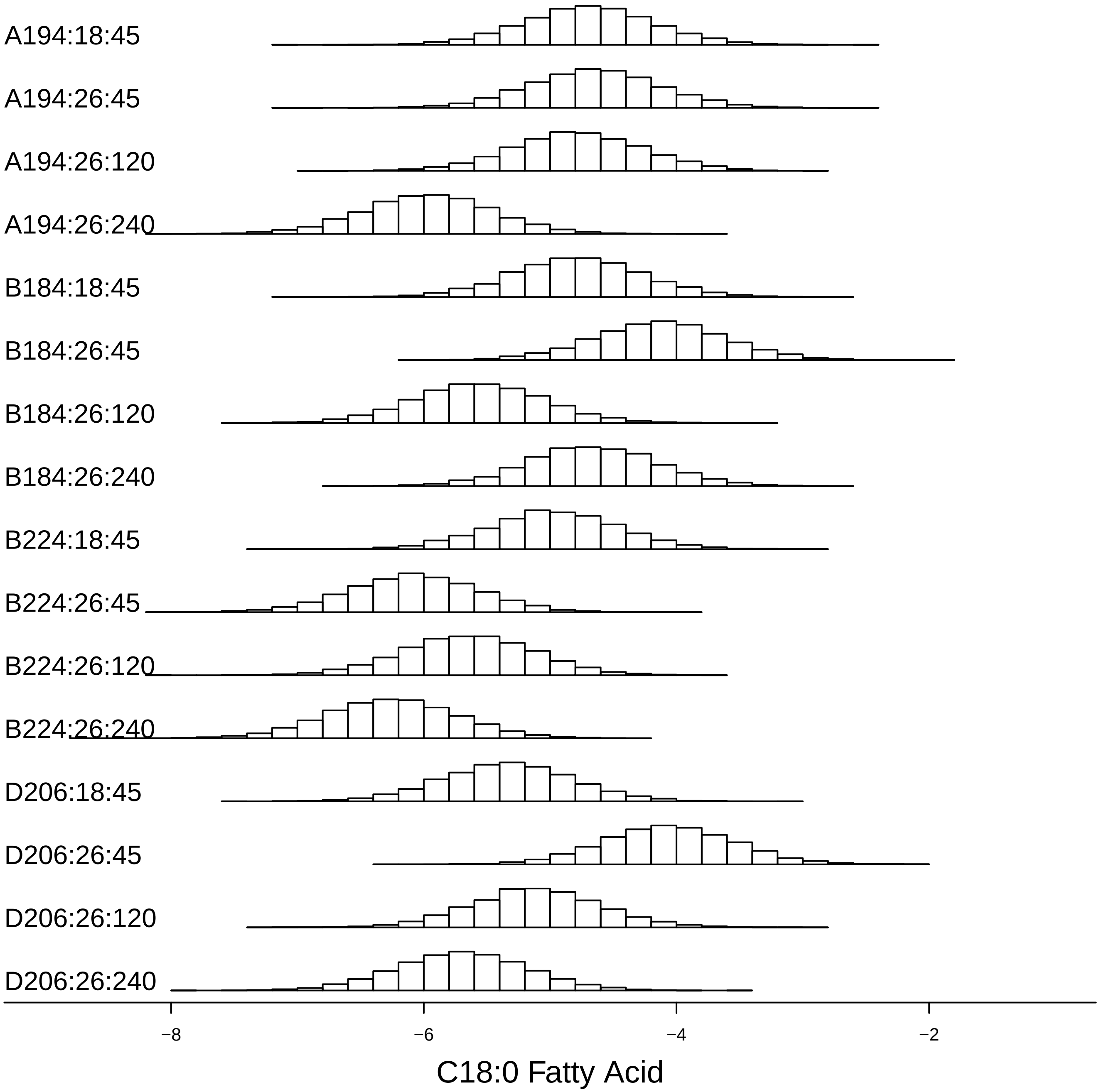

A194:18:45
A194:26:45
A194:26:120
A194:26:240
B184:18:45
B184:26:45
B184:26:120
B184:26:240
B224:18:45
B224:26:45
B224:26:120
B224:26:240
D206:18:45
D206:26:45
D206:26:120
D206:26:240
−8
−6
−4
−2
C18:0 Fatty Acid

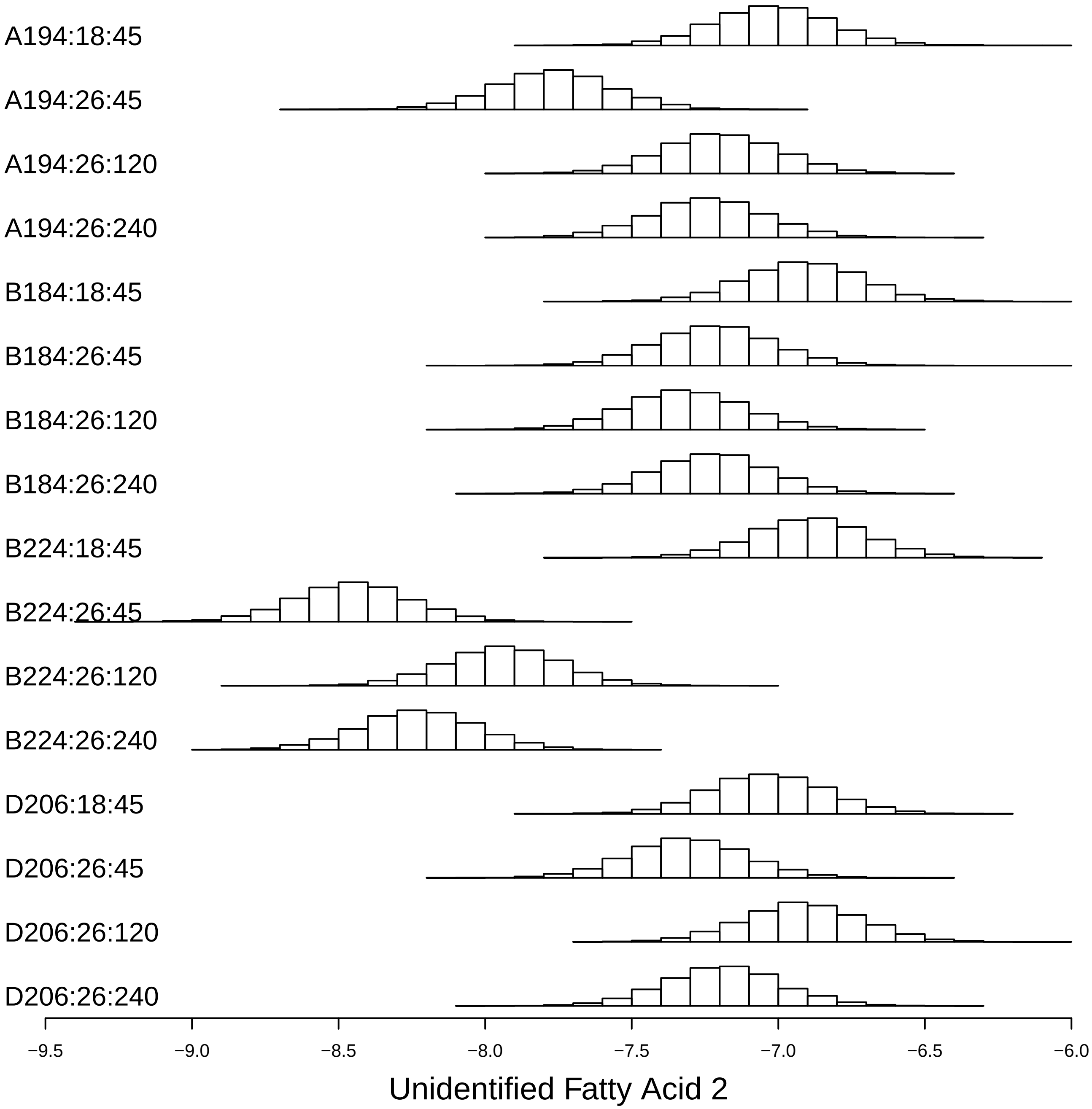
A194:18:45
A194:26:45
A194:26:120
A194:26:240
B184:18:45
B184:26:45
B184:26:120
B184:26:240
B224:18:45
B224:26:45
B224:26:120
B224:26:240
D206:18:45
D206:26:45
D206:26:120
D206:26:240
−9.5
−9.0
−8.5
−8.0
−7.5
−7.0
−6.5
−6.0
Unidentified Fatty Acid 2

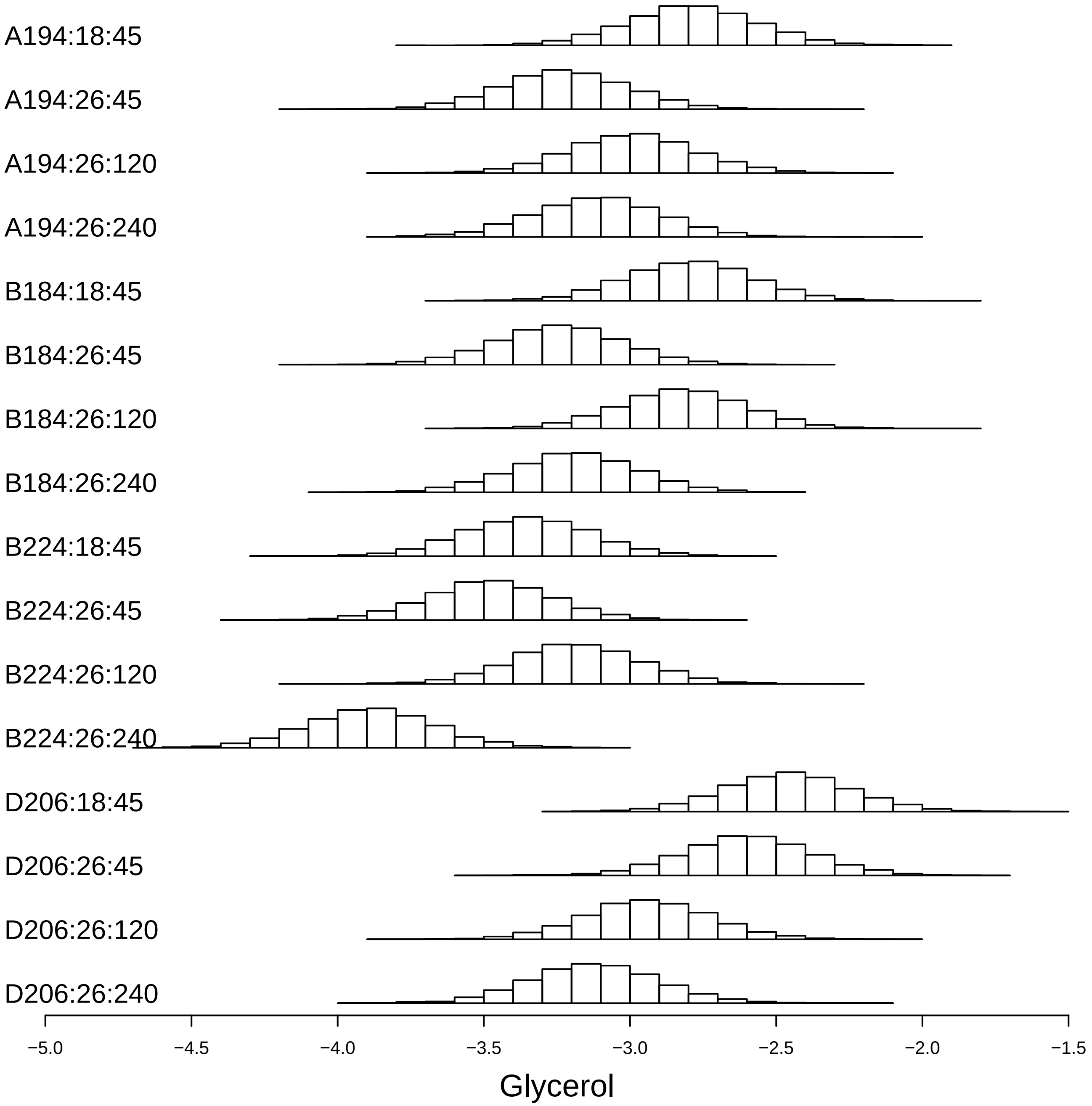

A194:18:45
A194:26:45
A194:26:120
A194:26:240
B184:18:45
B184:26:45
B184:26:120
B184:26:240
B224:18:45
B224:26:45
B224:26:120
B224:26:240
D206:18:45
D206:26:45
D206:26:120
D206:26:240
−5.0
−4.5
−4.0
−3.5
−3.0
−2.5
−2.0
−1.5
Glycerol

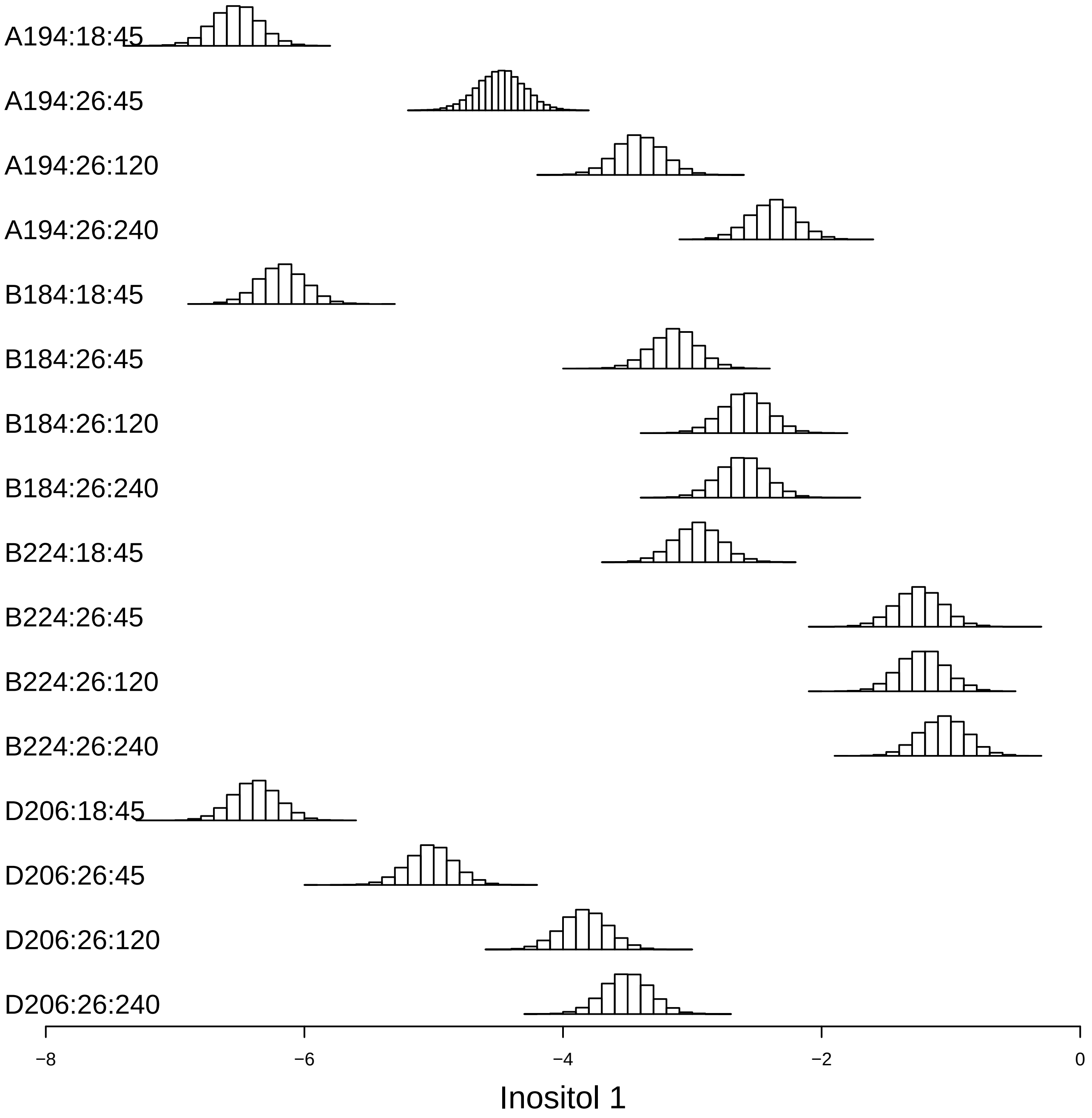
A194:18:45
A194:26:45
A194:26:120
A194:26:240
B184:18:45
B184:26:45
B184:26:120
B184:26:240
B224:18:45
B224:26:45
B224:26:120
B224:26:240
D206:18:45
D206:26:45
D206:26:120
D206:26:240
−8
−6
−4
−2
0
Inositol 1

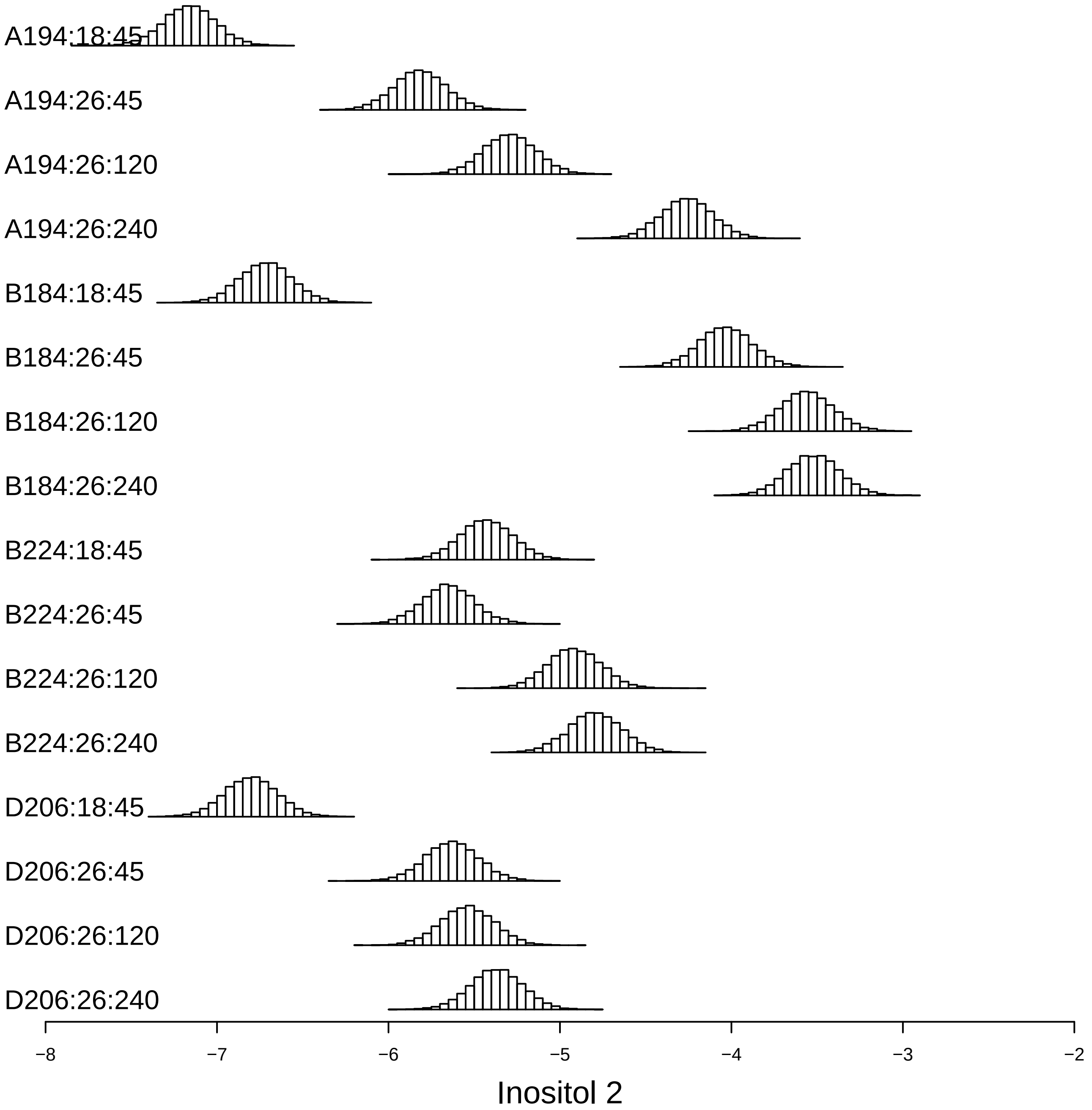
A194:18:45
A194:26:45
A194:26:120
A194:26:240
B184:18:45
B184:26:45
B184:26:120
B184:26:240
B224:18:45
B224:26:45
B224:26:120
B224:26:240
D206:18:45
D206:26:45
D206:26:120
D206:26:240
−8
−7
−6
−5
−4
−3
−2
Inositol 2

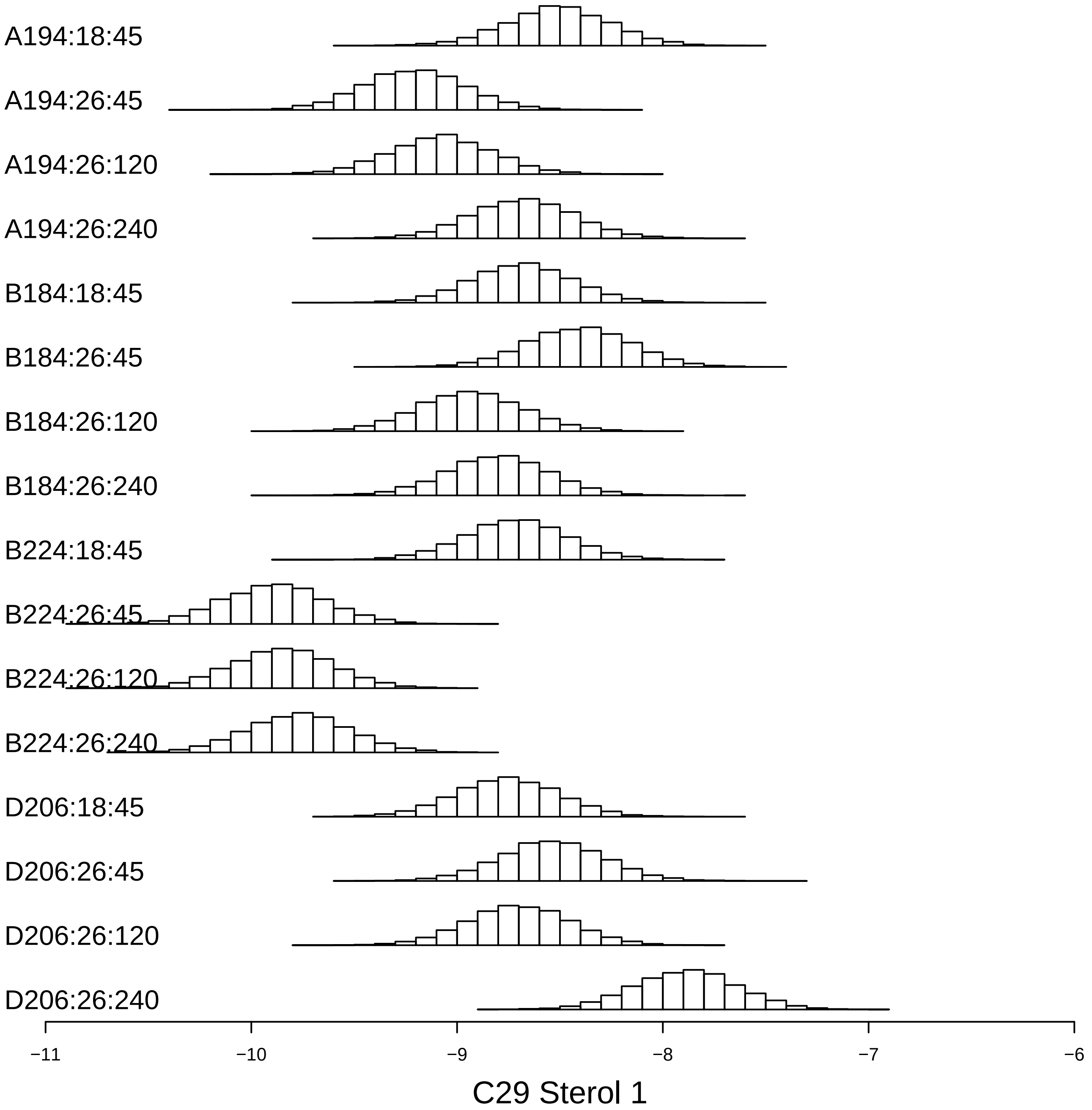

A194:18:45
A194:26:45
A194:26:120
A194:26:240
B184:18:45
B184:26:45
B184:26:120
B184:26:240
B224:18:45
B224:26:45
B224:26:120
B224:26:240
D206:18:45
D206:26:45
D206:26:120
D206:26:240
−11
−10
−9
−8
−7
−6
C29 Sterol 1

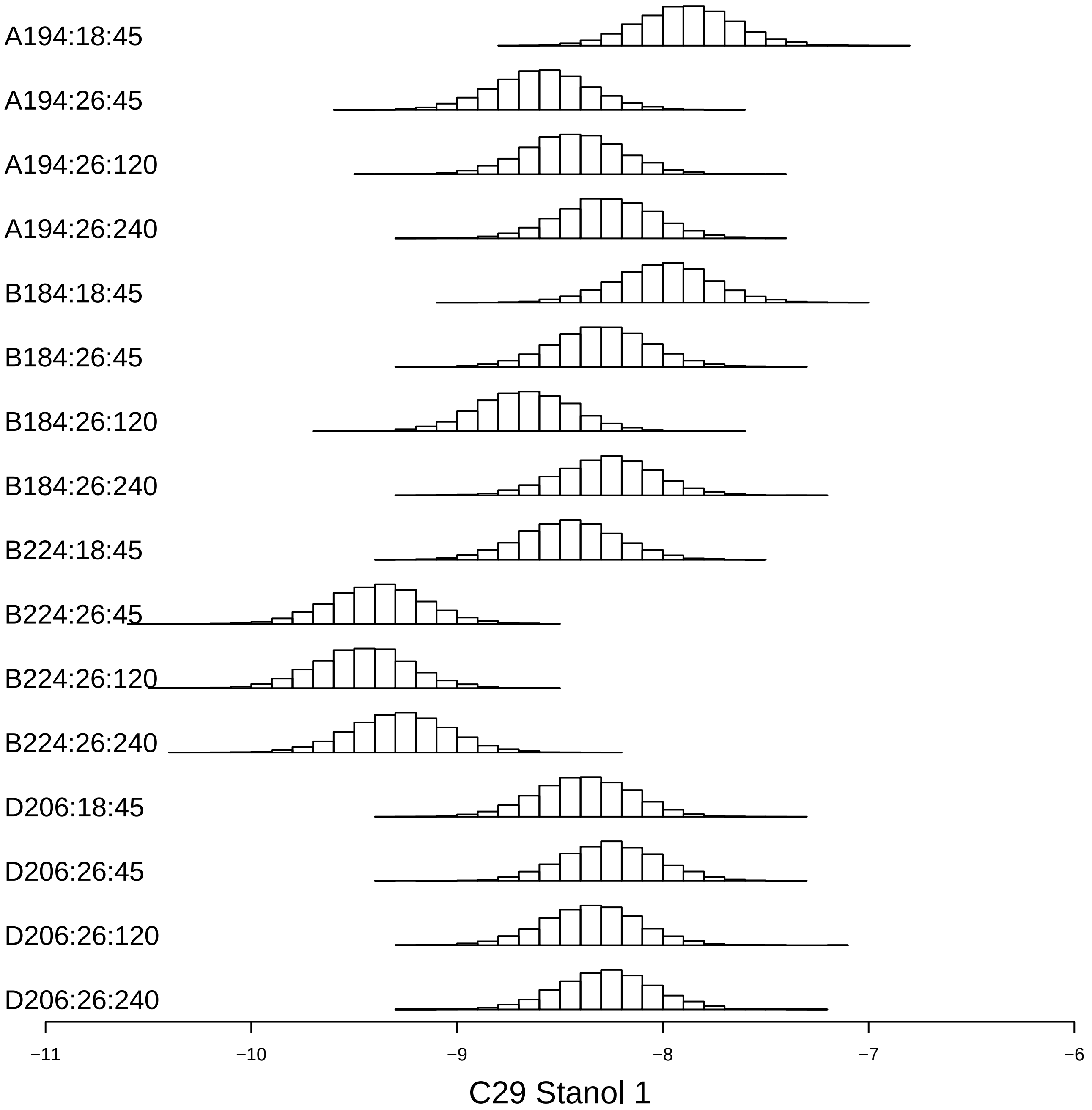

A194:18:45
A194:26:45
A194:26:120
A194:26:240
B184:18:45
B184:26:45
B184:26:120
B184:26:240
B224:18:45
B224:26:45
B224:26:120
B224:26:240
D206:18:45
D206:26:45
D206:26:120
D206:26:240
−11
−10
−9
−8
−7
−6
C29 Stanol 1

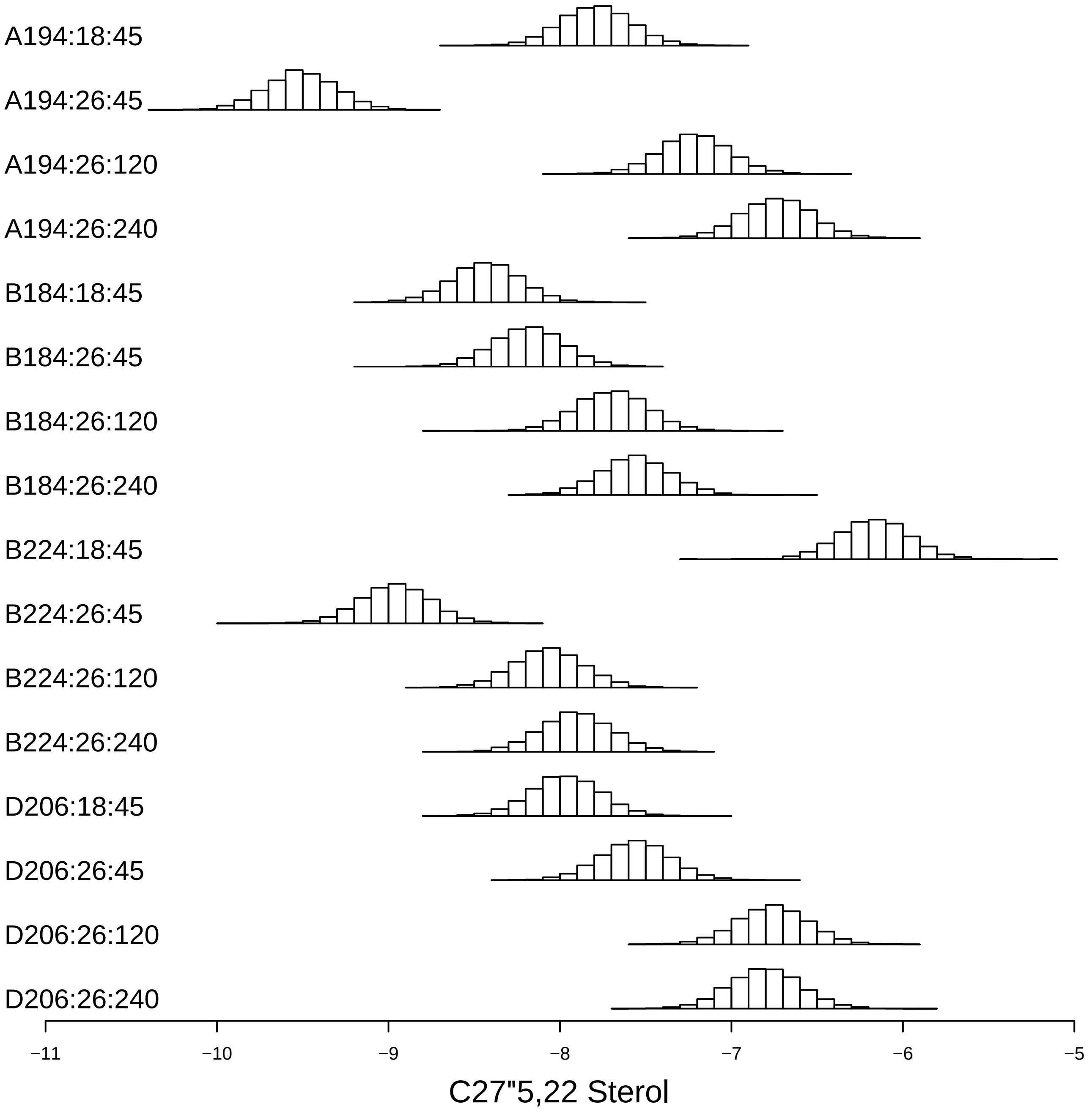
A194:18:45
A194:26:45
A194:26:120
A194:26:240
B184:18:45
B184:26:45
B184:26:120
B184:26:240
B224:18:45
B224:26:45
B224:26:120
B224:26:240
D206:18:45
D206:26:45
D206:26:120
D206:26:240
−11
−10
−9
−8
−7
−6
−5
C27"5,22 Sterol

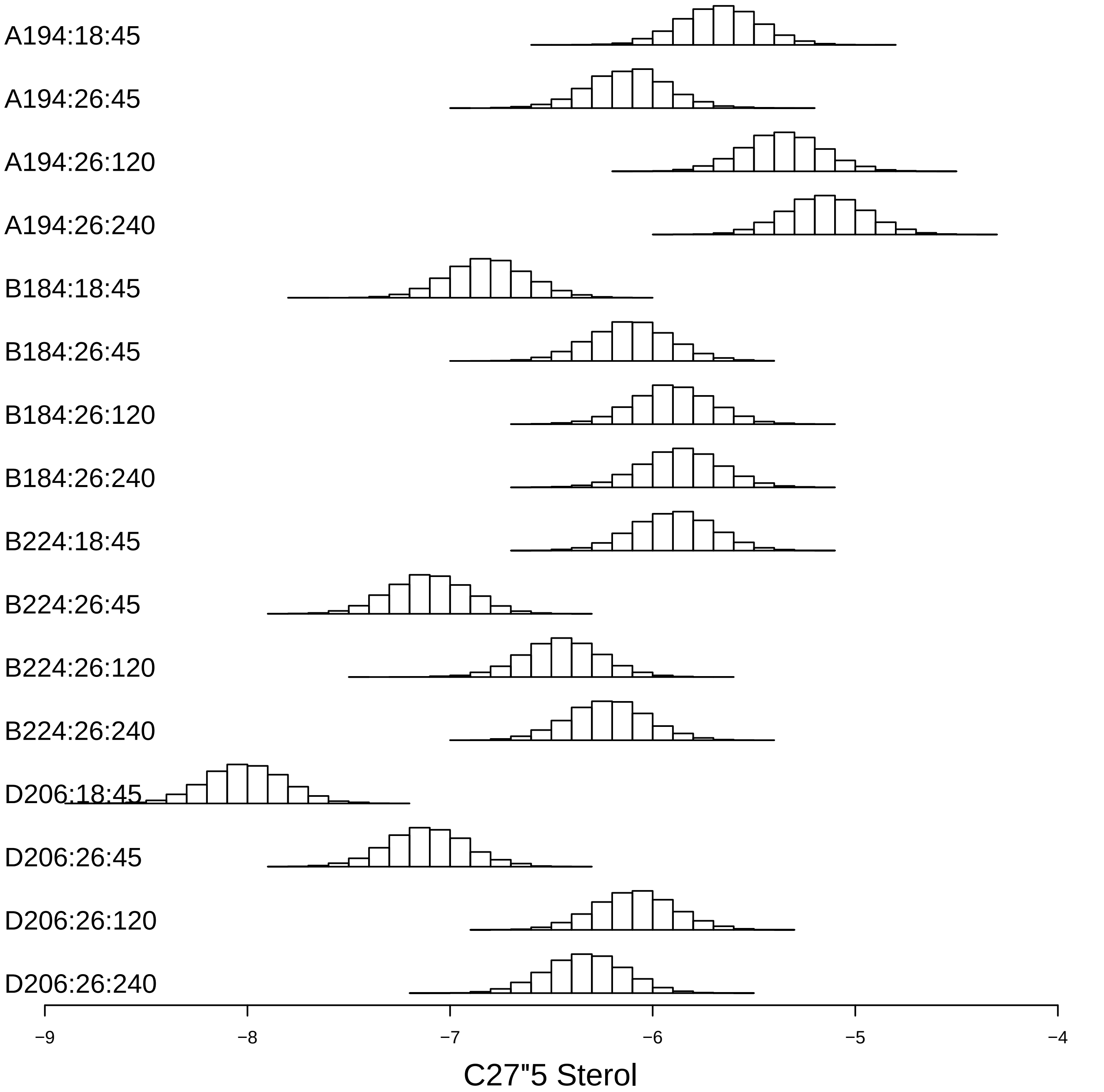
A194:18:45
A194:26:45
A194:26:120
A194:26:240
B184:18:45
B184:26:45
B184:26:120
B184:26:240
B224:18:45
B224:26:45
B224:26:120
B224:26:240
D206:18:45
D206:26:45
D206:26:120
D206:26:240
−9
−8
−7
−6
−5
−4
C27"5 Sterol

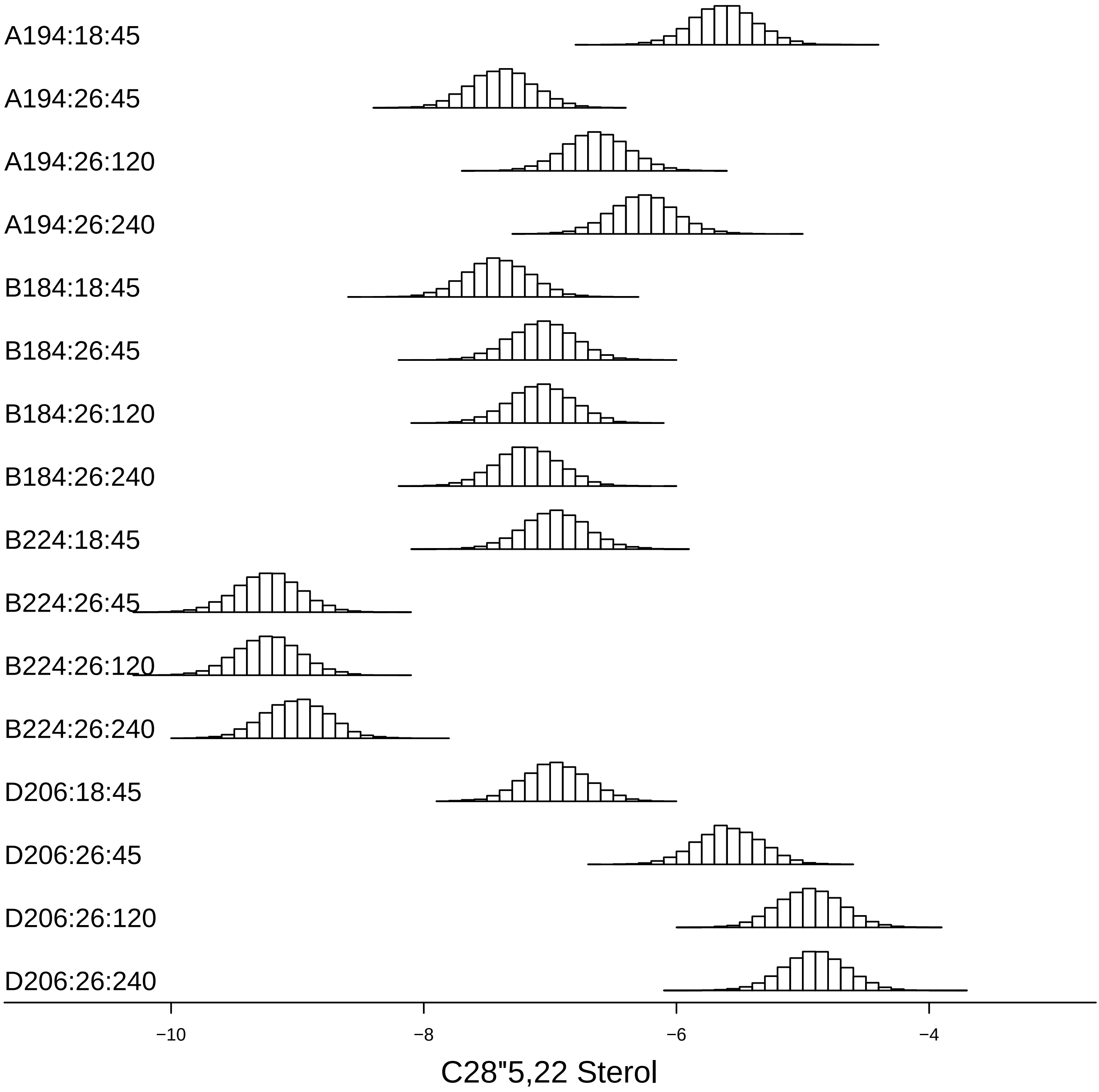
A194:18:45
A194:26:45
A194:26:120
A194:26:240
B184:18:45
B184:26:45
B184:26:120
B184:26:240
B224:18:45
B224:26:45
B224:26:120
B224:26:240
D206:18:45
D206:26:45
D206:26:120
D206:26:240
−10
−8
−6
−4
C28"5,22 Sterol

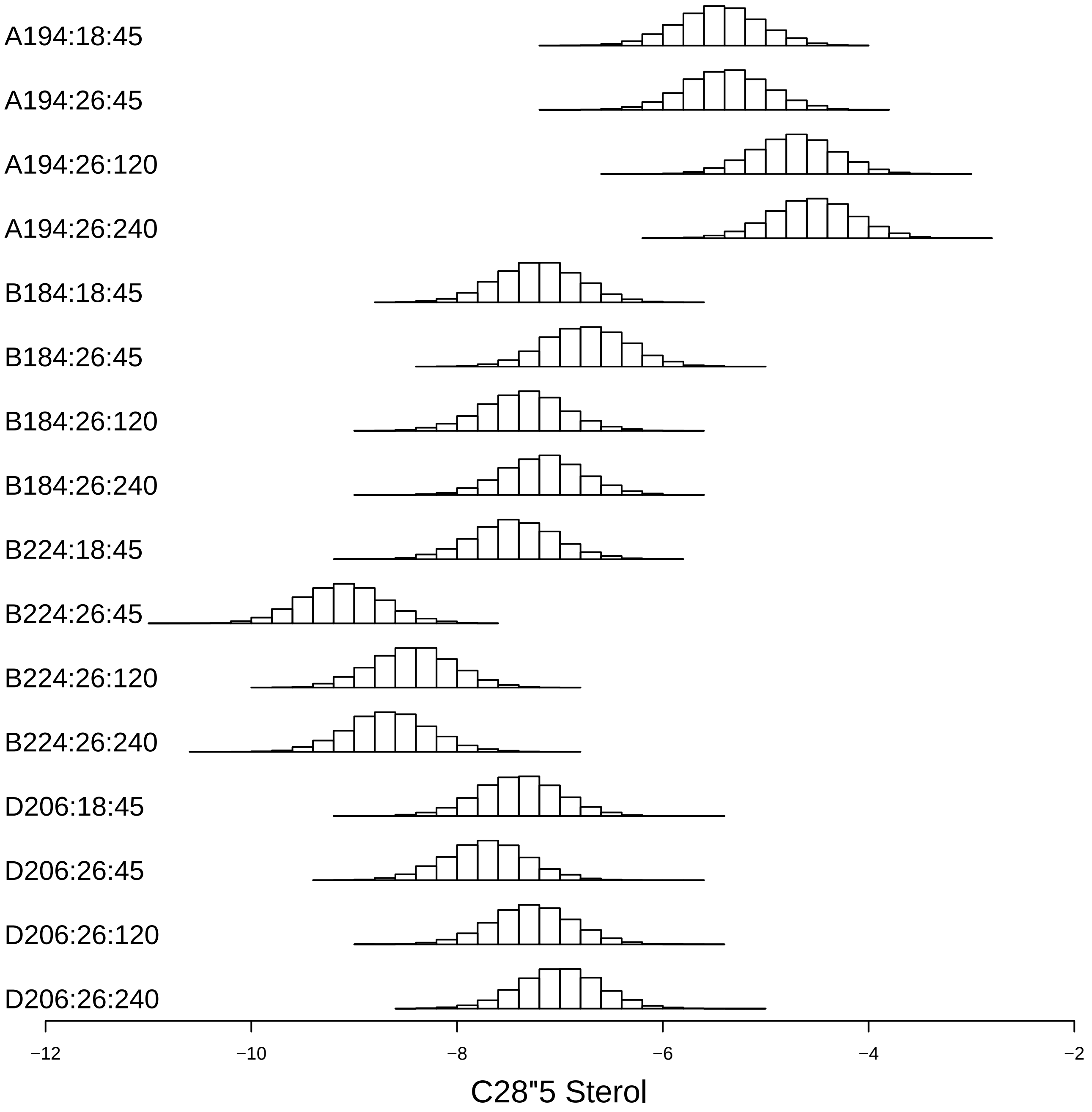
A194:18:45
A194:26:45
A194:26:120
A194:26:240
B184:18:45
B184:26:45
B184:26:120
B184:26:240
B224:18:45
B224:26:45
B224:26:120
B224:26:240
D206:18:45
D206:26:45
D206:26:120
D206:26:240
−12
−10
−8
−6
−4
−2
C28"5 Sterol

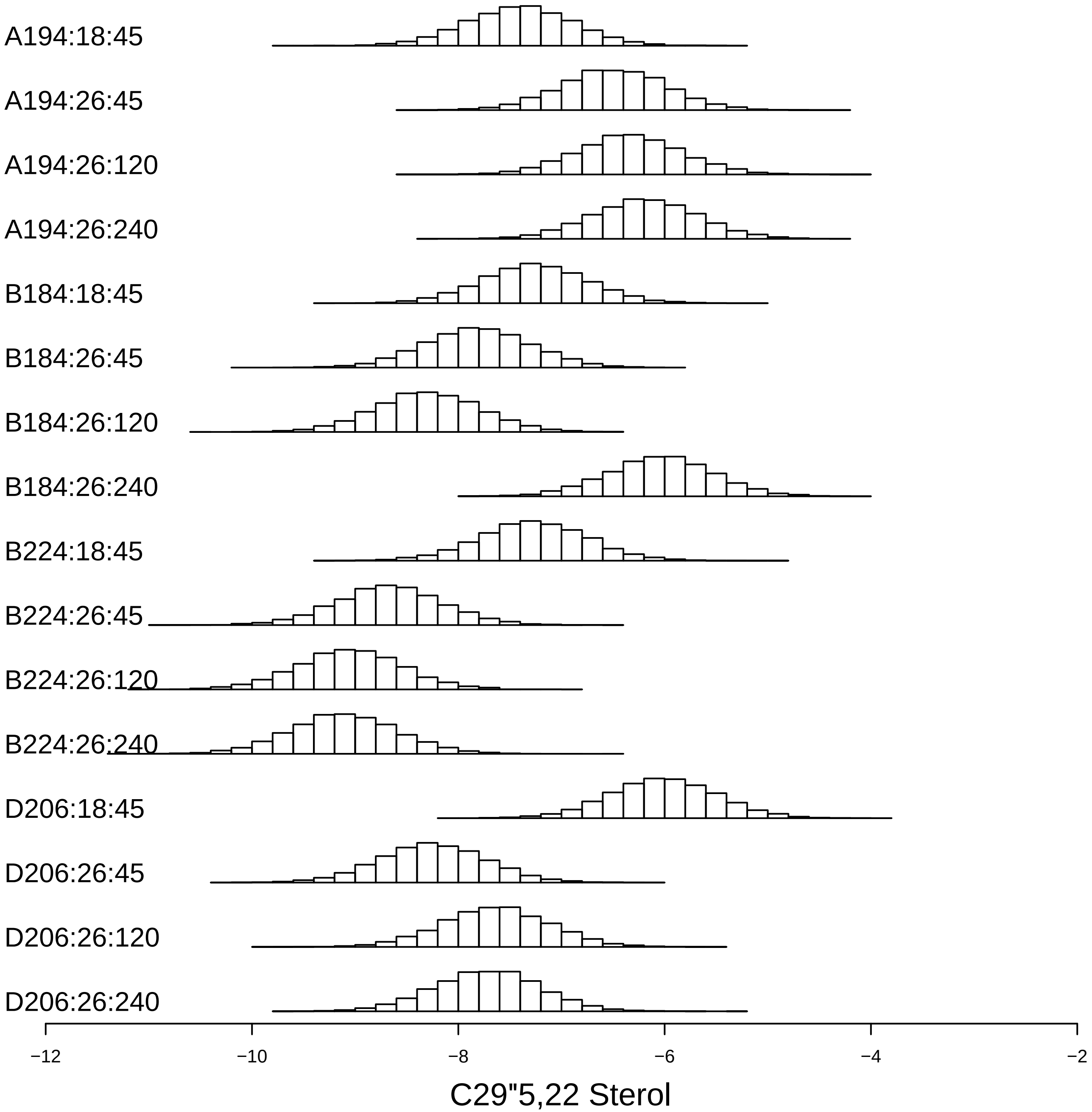

A194:18:45
A194:26:45
A194:26:120
A194:26:240
B184:18:45
B184:26:45
B184:26:120
B184:26:240
B224:18:45
B224:26:45
B224:26:120
B224:26:240
D206:18:45
D206:26:45
D206:26:120
D206:26:240
−12
−10
−8
−6
−4
−2
C29"5,22 Sterol

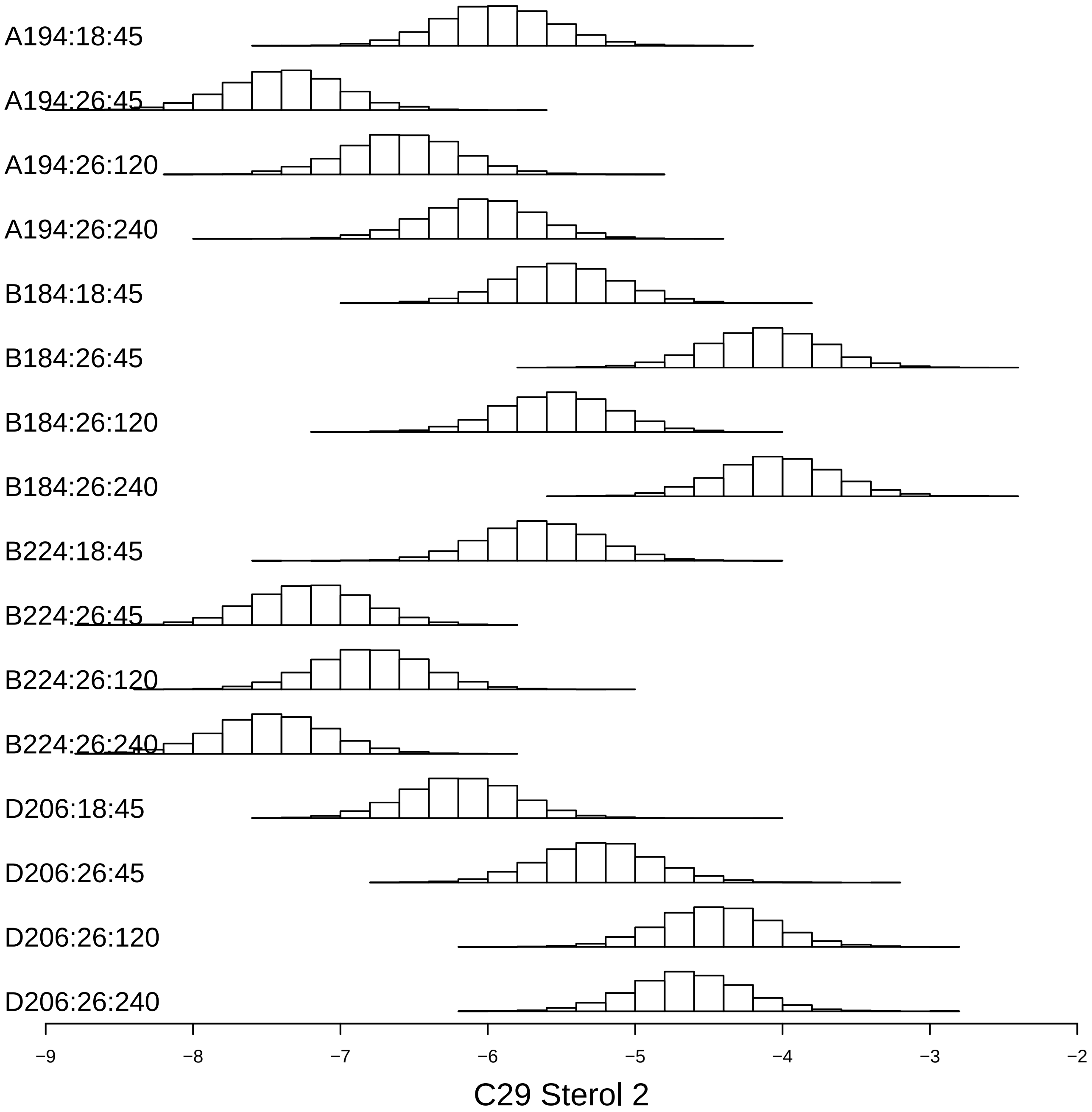
A194:18:45
A194:26:45
A194:26:120
A194:26:240
B184:18:45
B184:26:45
B184:26:120
B184:26:240
B224:18:45
B224:26:45
B224:26:120
B224:26:240
D206:18:45
D206:26:45
D206:26:120
D206:26:240
−9
−8
−7
−6
−5
−4
−3
−2
C29 Sterol 2

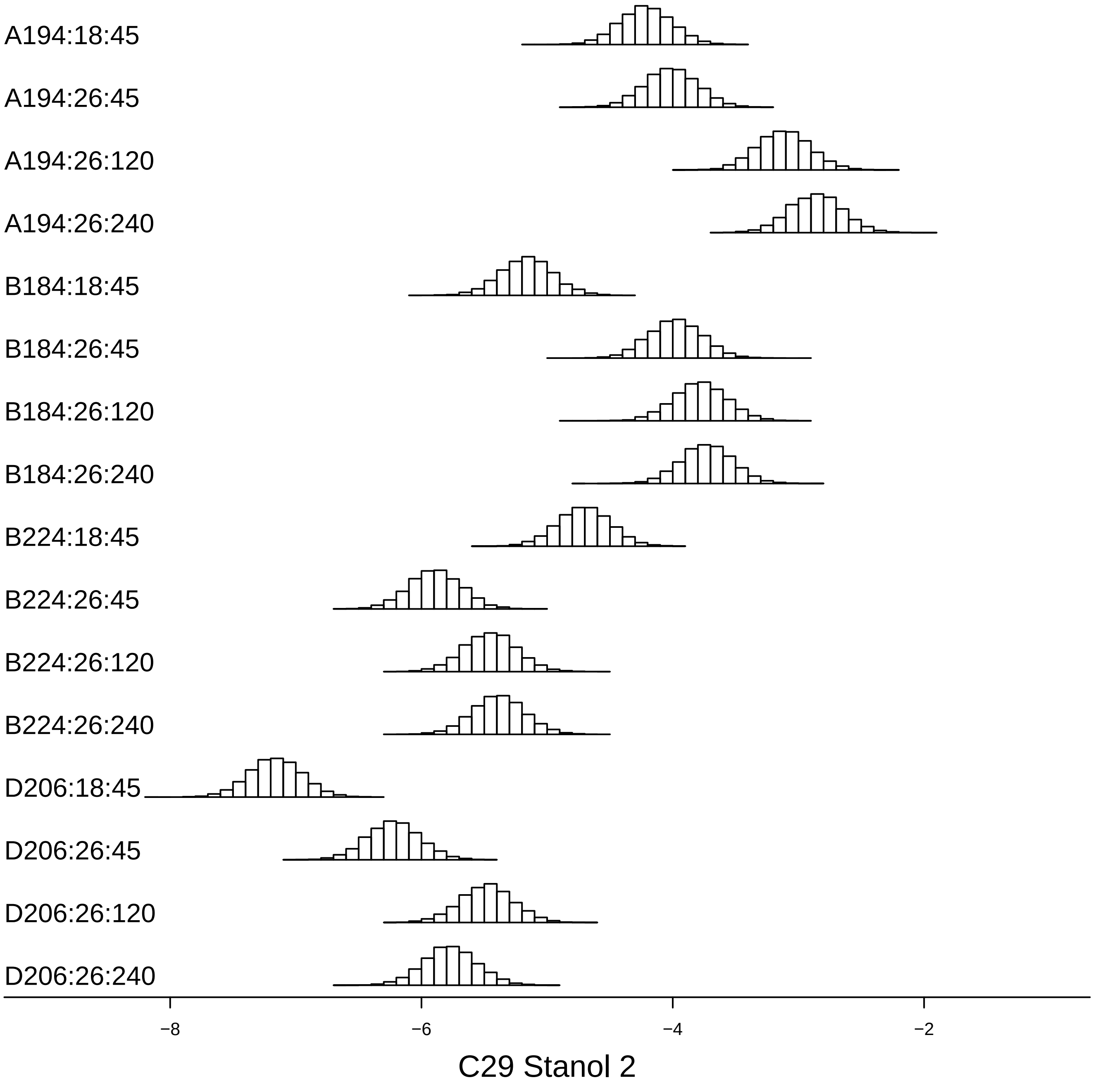

A194:18:45
A194:26:45
A194:26:120
A194:26:240
B184:18:45
B184:26:45
B184:26:120
B184:26:240
B224:18:45
B224:26:45
B224:26:120
B224:26:240
D206:18:45
D206:26:45
D206:26:120
D206:26:240
−8
−6
−4
−2
C29 Stanol 2

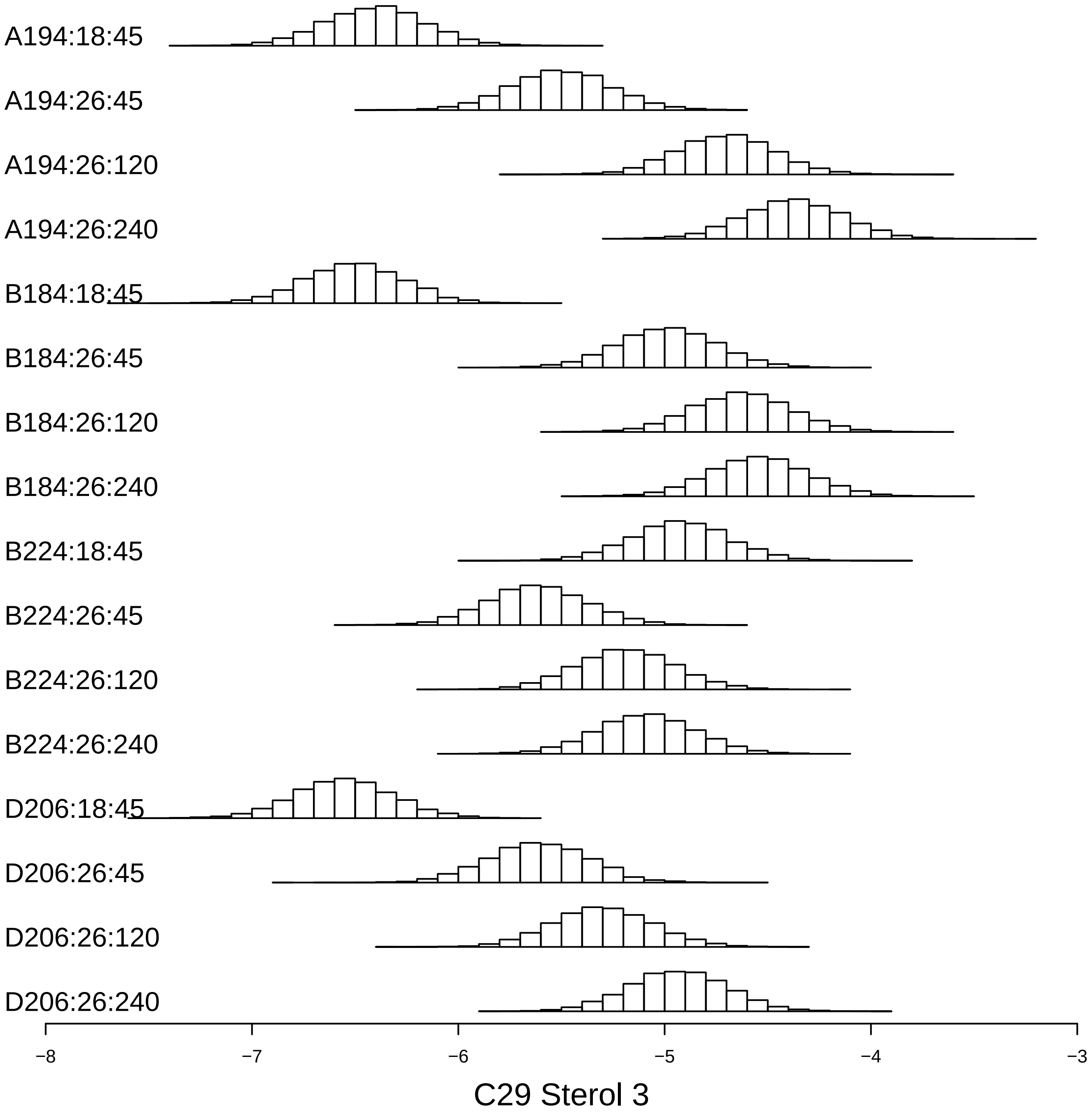
A194:18:45
A194:26:45
A194:26:120
A194:26:240
B184:18:45
B184:26:45
B184:26:120
B184:26:240
B224:18:45
B224:26:45
B224:26:120
B224:26:240
D206:18:45
D206:26:45
D206:26:120
D206:26:240
−8
−7
−6
−5
−4
−3
C29 Sterol 3

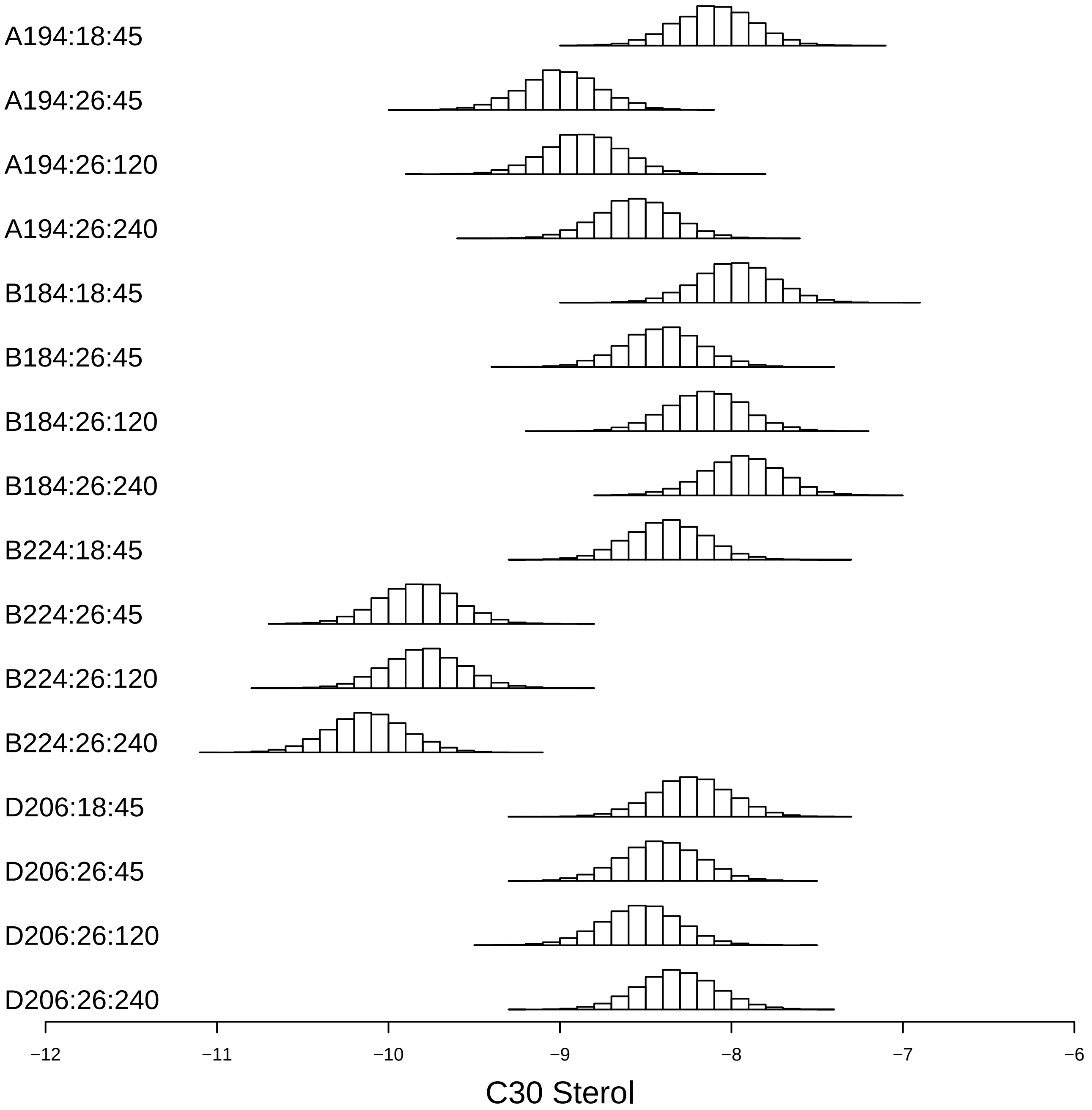
A194:18:45
A194:26:45
A194:26:120
A194:26:240
B184:18:45
B184:26:45
B184:26:120
B184:26:240
B224:18:45
B224:26:45
B224:26:120
B224:26:240
D206:18:45
D206:26:45
D206:26:120
D206:26:240
−12
−11
−10
−9
−8
−7
−6
C30 Sterol

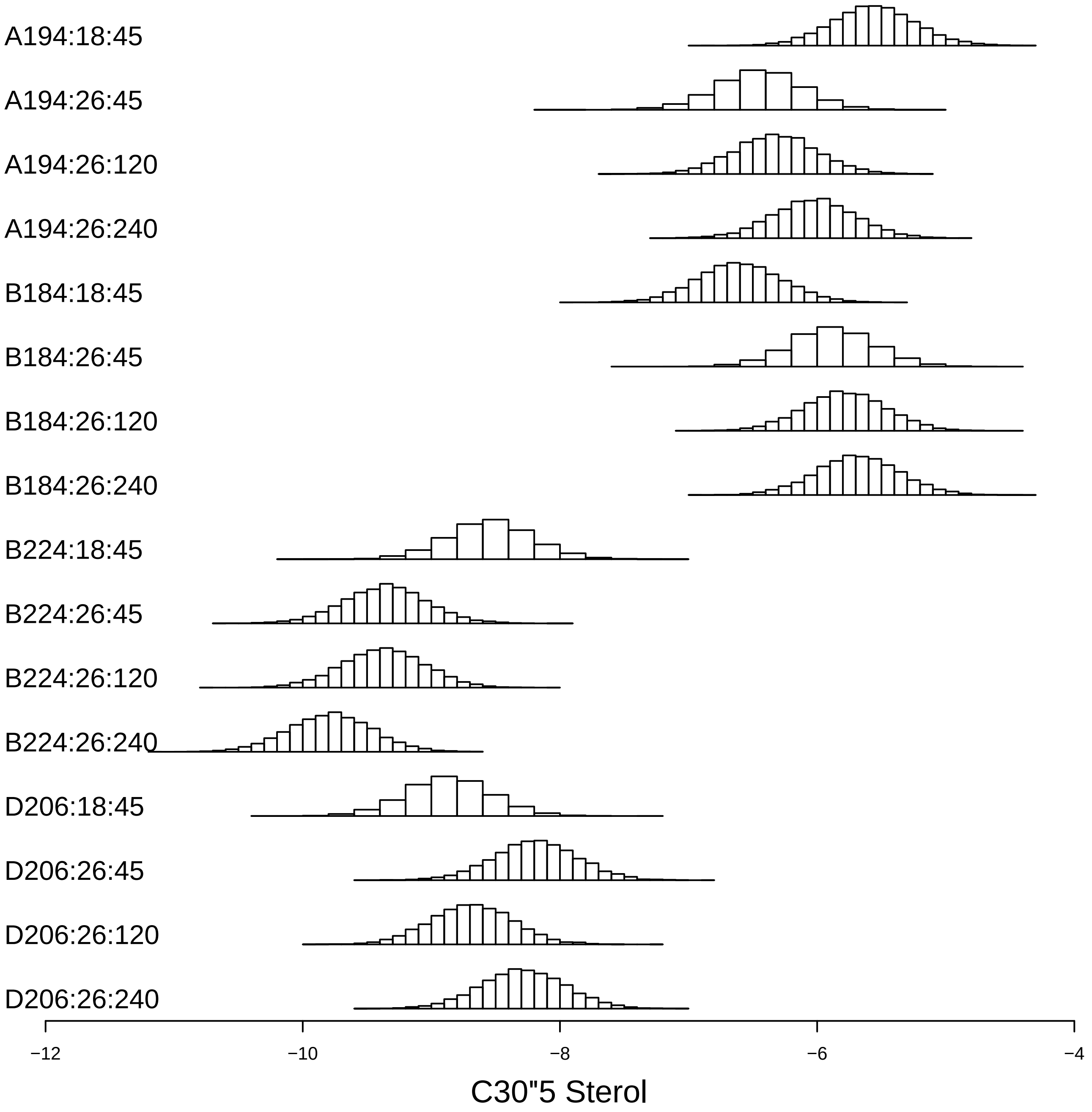
A194:18:45
A194:26:45
A194:26:120
A194:26:240
B184:18:45
B184:26:45
B184:26:120
B184:26:240
B224:18:45
B224:26:45
B224:26:120
B224:26:240
D206:18:45
D206:26:45
D206:26:120
D206:26:240
−12
−10
−8
−6
−4
C30"5 Sterol

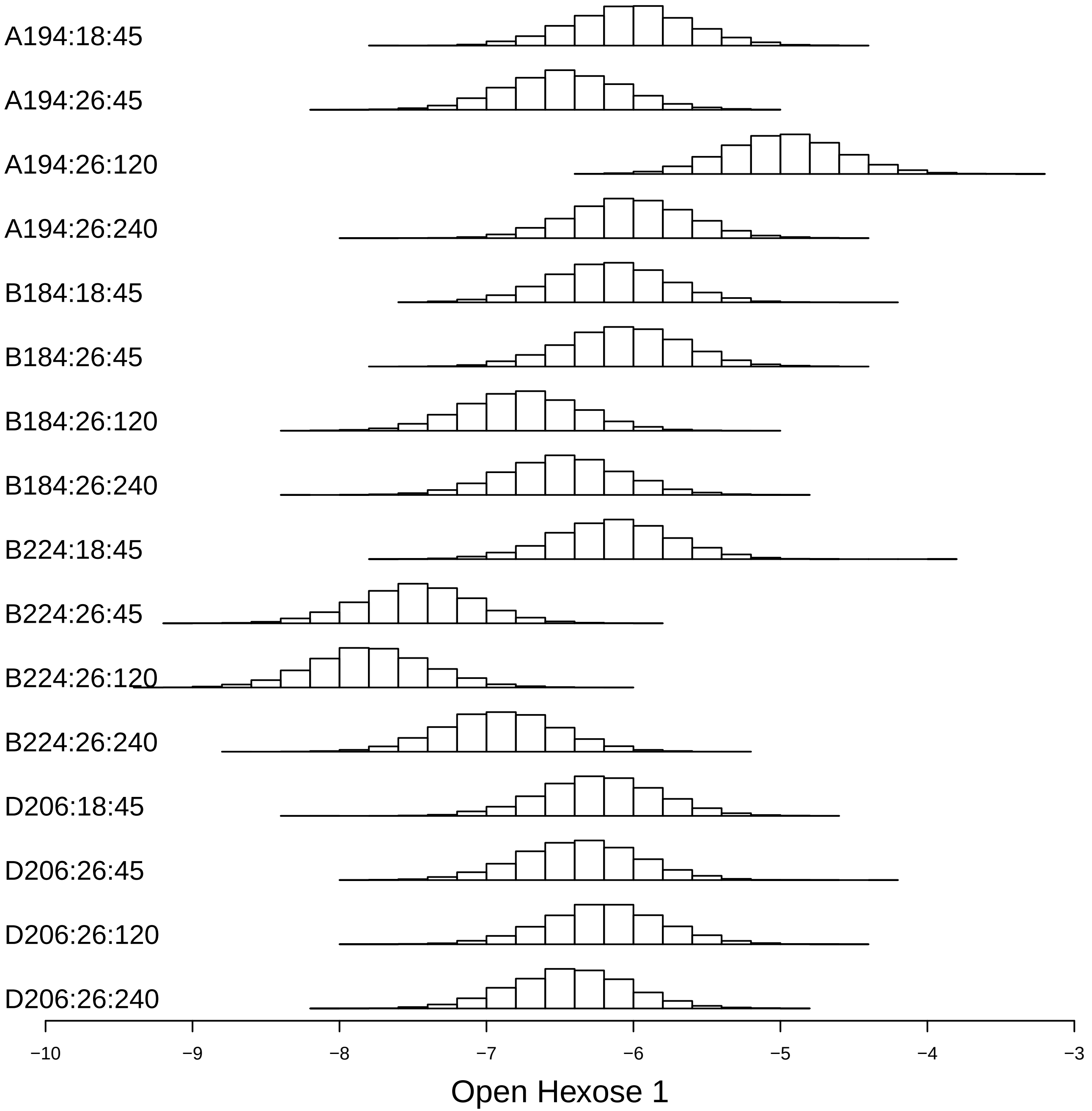
A194:18:45
A194:26:45
A194:26:120
A194:26:240
B184:18:45
B184:26:45
B184:26:120
B184:26:240
B224:18:45
B224:26:45
B224:26:120
B224:26:240
D206:18:45
D206:26:45
D206:26:120
D206:26:240
−10
−9
−8
−7
−6
−5
−4
−3
Open Hexose 1

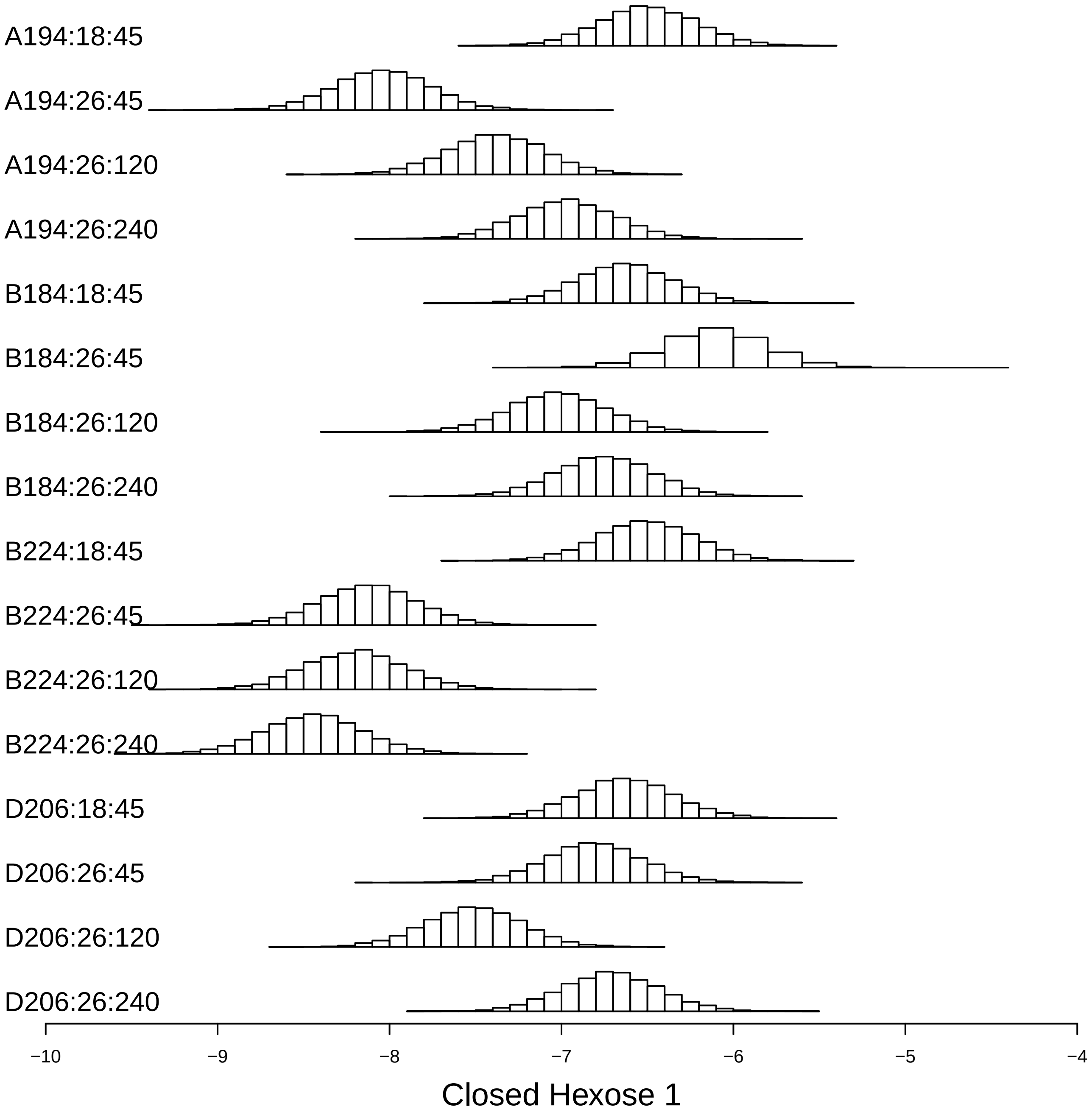
A194:18:45
A194:26:45
A194:26:120
A194:26:240
B184:18:45
B184:26:45
B184:26:120
B184:26:240
B224:18:45
B224:26:45
B224:26:120
B224:26:240
D206:18:45
D206:26:45
D206:26:120
D206:26:240
−10
−9
−8
−7
−6
−5
−4
Closed Hexose 1

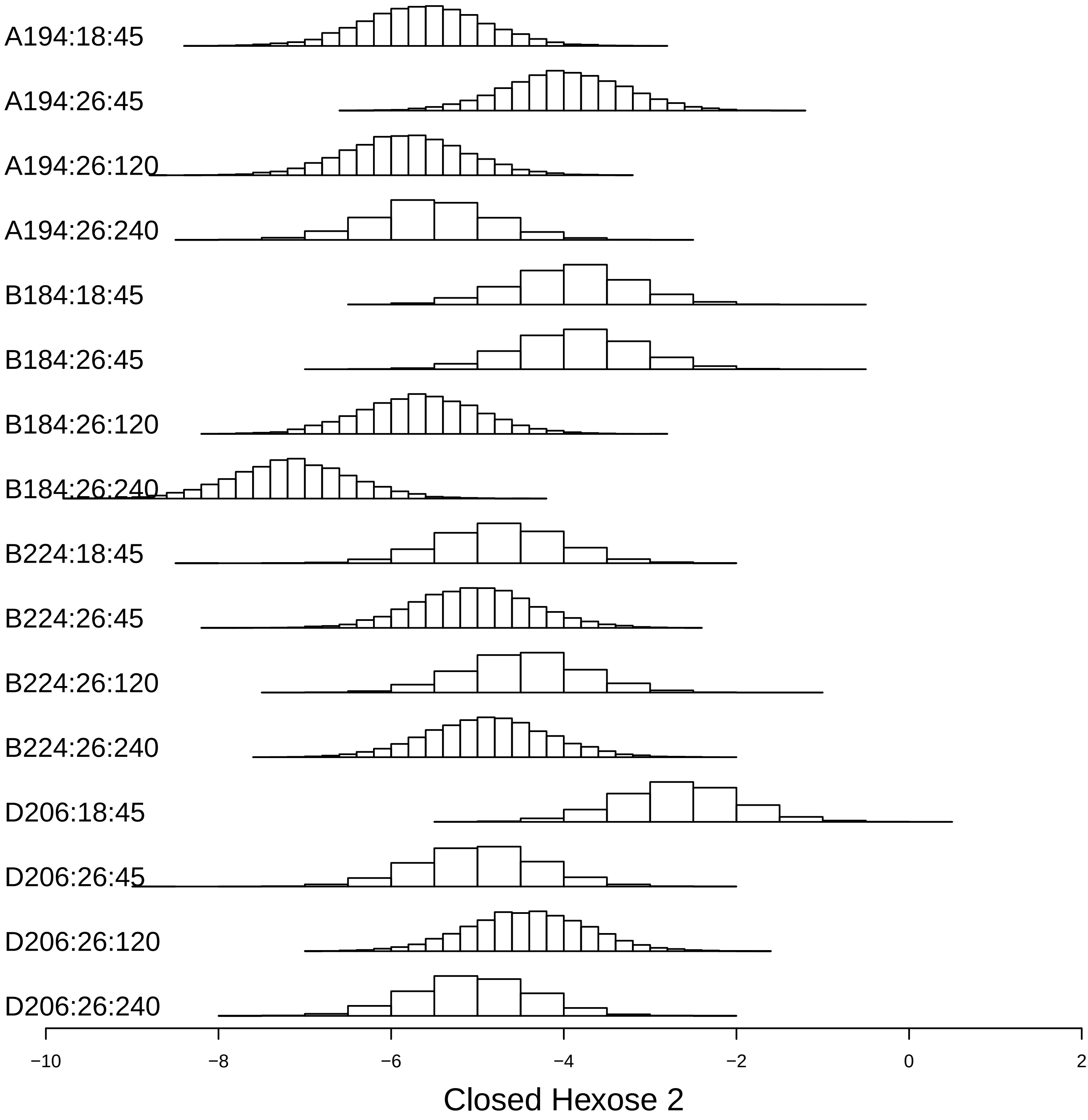
A194:18:45
A194:26:45
A194:26:120
A194:26:240
B184:18:45
B184:26:45
B184:26:120
B184:26:240
B224:18:45
B224:26:45
B224:26:120
B224:26:240
D206:18:45
D206:26:45
D206:26:120
D206:26:240
−10
−8
−6
−4
−2
0
2
Closed Hexose 2

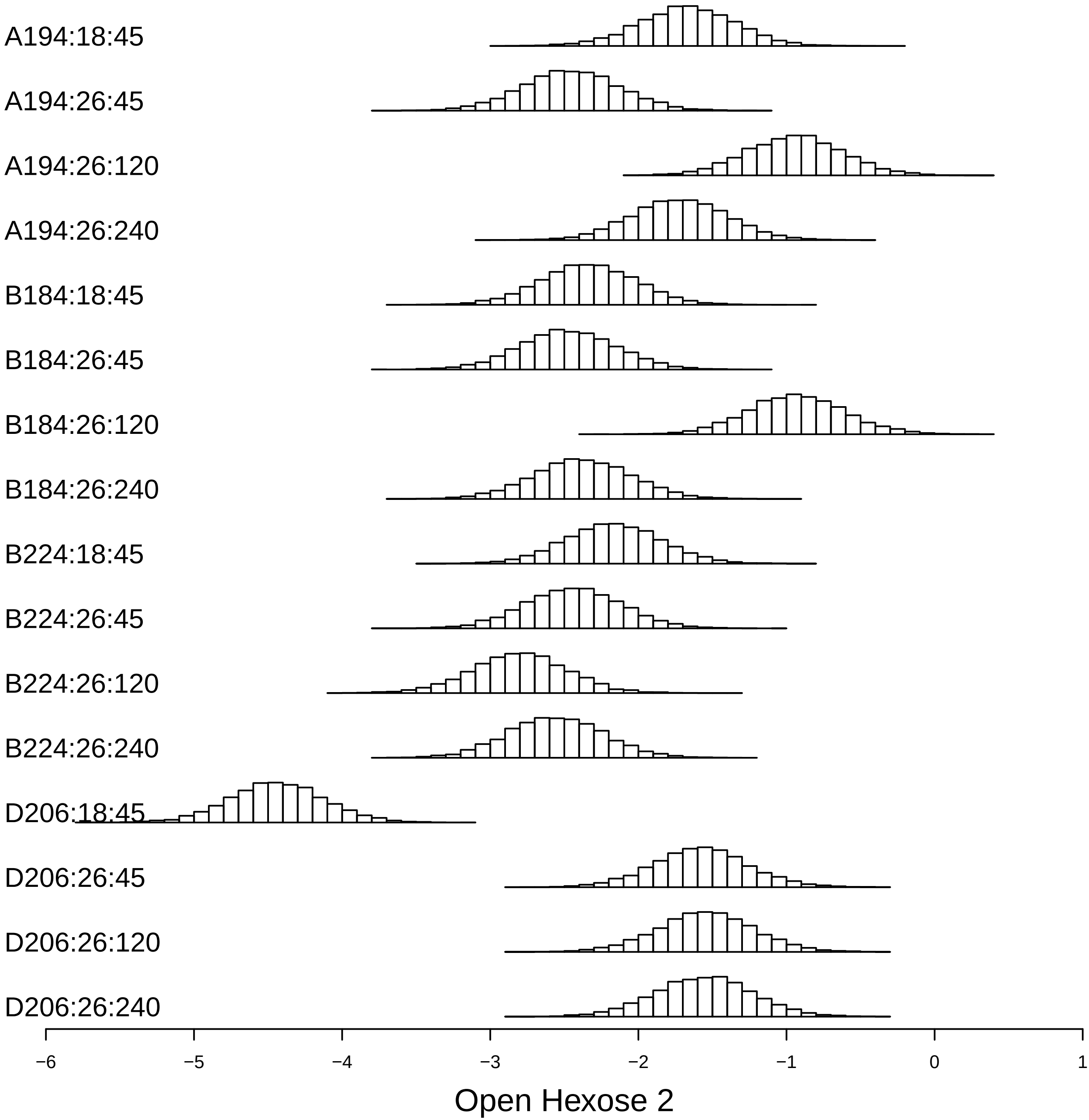

A194:18:45
A194:26:45
A194:26:120
A194:26:240
B184:18:45
B184:26:45
B184:26:120
B184:26:240
B224:18:45
B224:26:45
B224:26:120
B224:26:240
D206:18:45
D206:26:45
D206:26:120
D206:26:240
−6
−5
−4
−3
−2
−1
0
1
Open Hexose 2

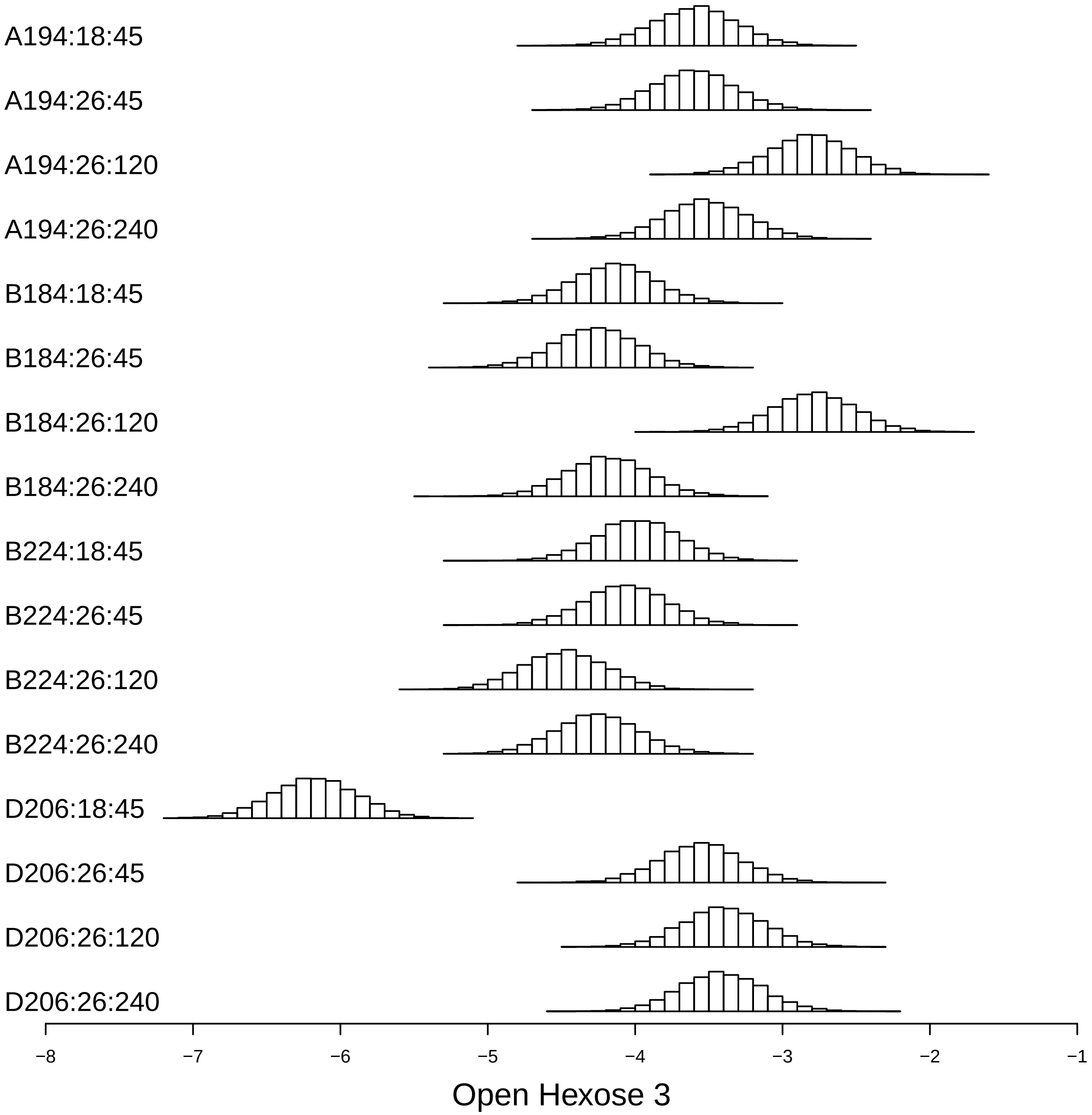
A194:18:45
A194:26:45
A194:26:120
A194:26:240
B184:18:45
B184:26:45
B184:26:120
B184:26:240
B224:18:45
B224:26:45
B224:26:120
B224:26:240
D206:18:45
D206:26:45
D206:26:120
D206:26:240
−8
−7
−6
−5
−4
−3
−2
−1
Open Hexose 3

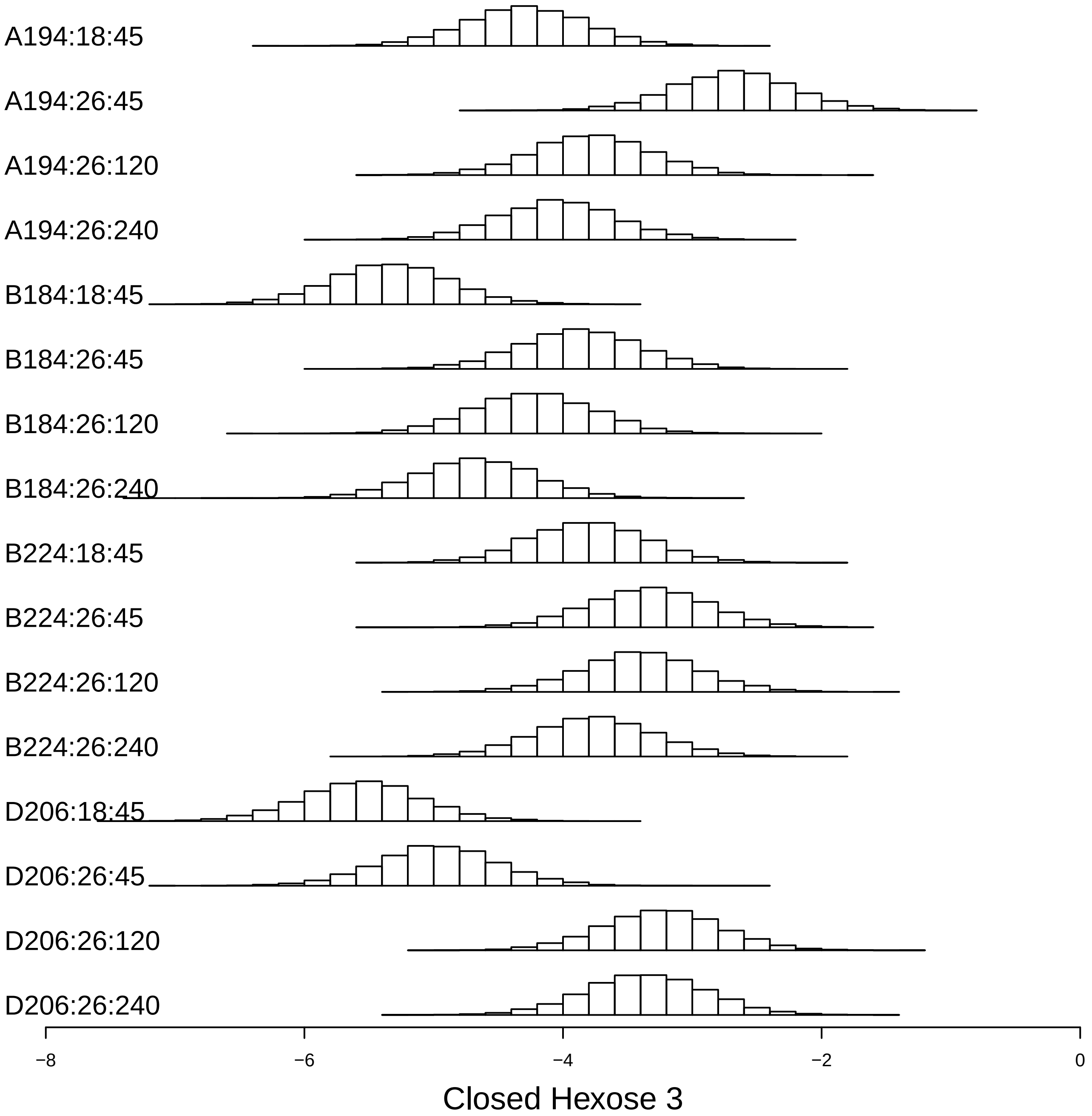
A194:18:45
A194:26:45
A194:26:120
A194:26:240
B184:18:45
B184:26:45
B184:26:120
B184:26:240
B224:18:45
B224:26:45
B224:26:120
B224:26:240
D206:18:45
D206:26:45
D206:26:120
D206:26:240
−8
−6
−4
−2
0
Closed Hexose 3

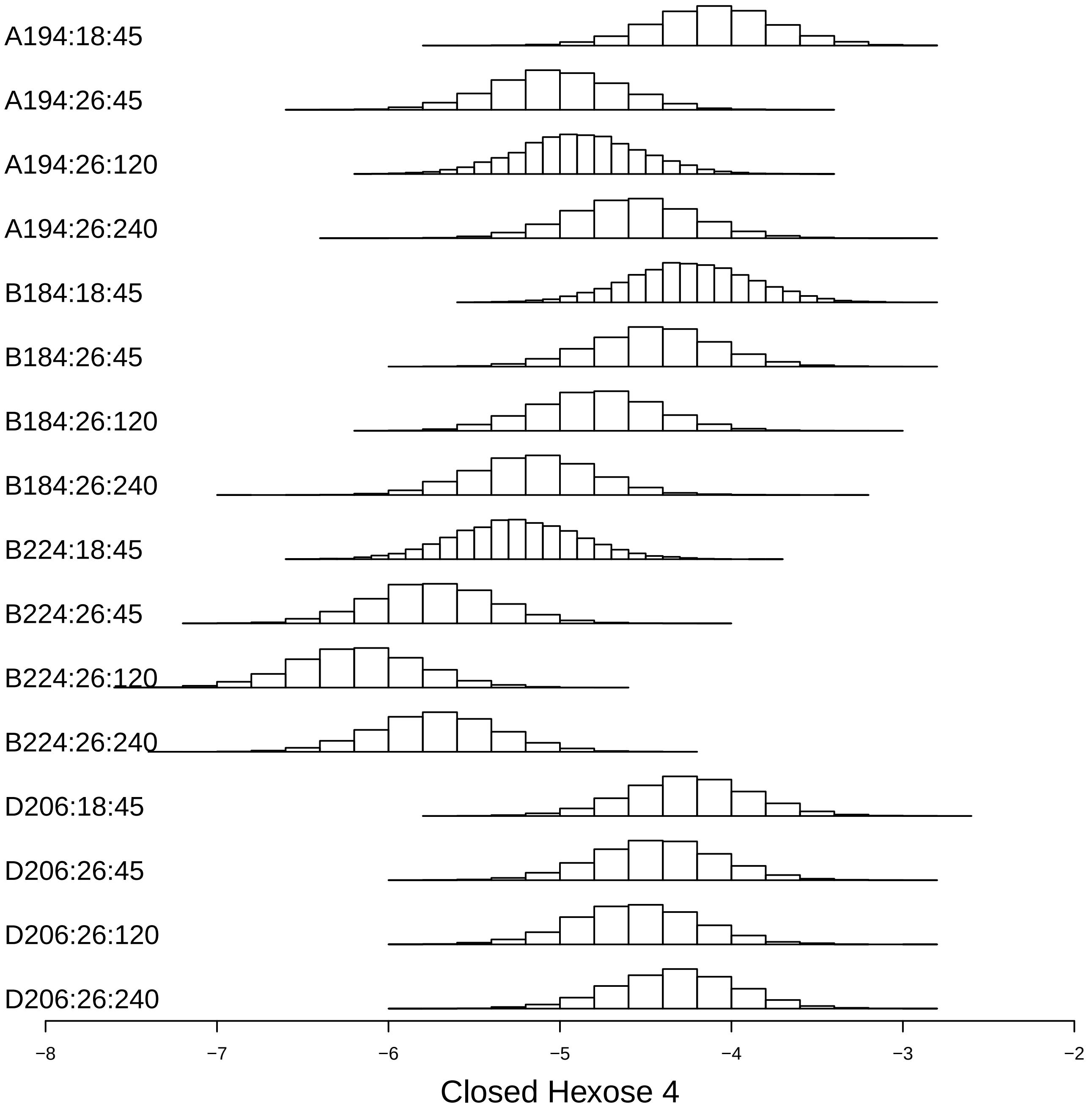
A194:18:45
A194:26:45
A194:26:120
A194:26:240
B184:18:45
B184:26:45
B184:26:120
B184:26:240
B224:18:45
B224:26:45
B224:26:120
B224:26:240
D206:18:45
D206:26:45
D206:26:120
D206:26:240
−8
−7
−6
−5
−4
−3
−2
Closed Hexose 4

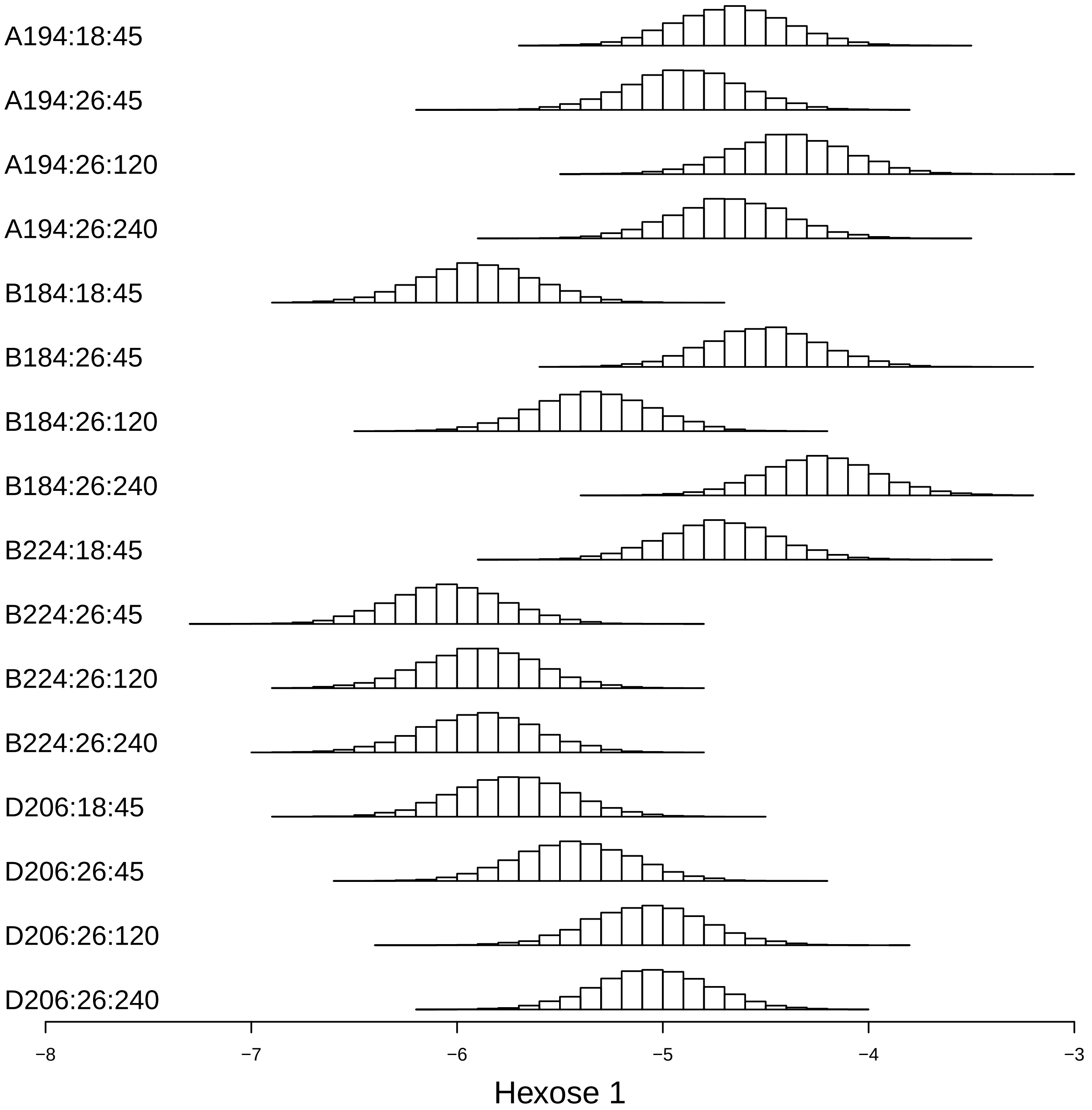
A194:18:45
A194:26:45
A194:26:120
A194:26:240
B184:18:45
B184:26:45
B184:26:120
B184:26:240
B224:18:45
B224:26:45
B224:26:120
B224:26:240
D206:18:45
D206:26:45
D206:26:120
D206:26:240
−8
−7
−6
−5
−4
−3
Hexose 1

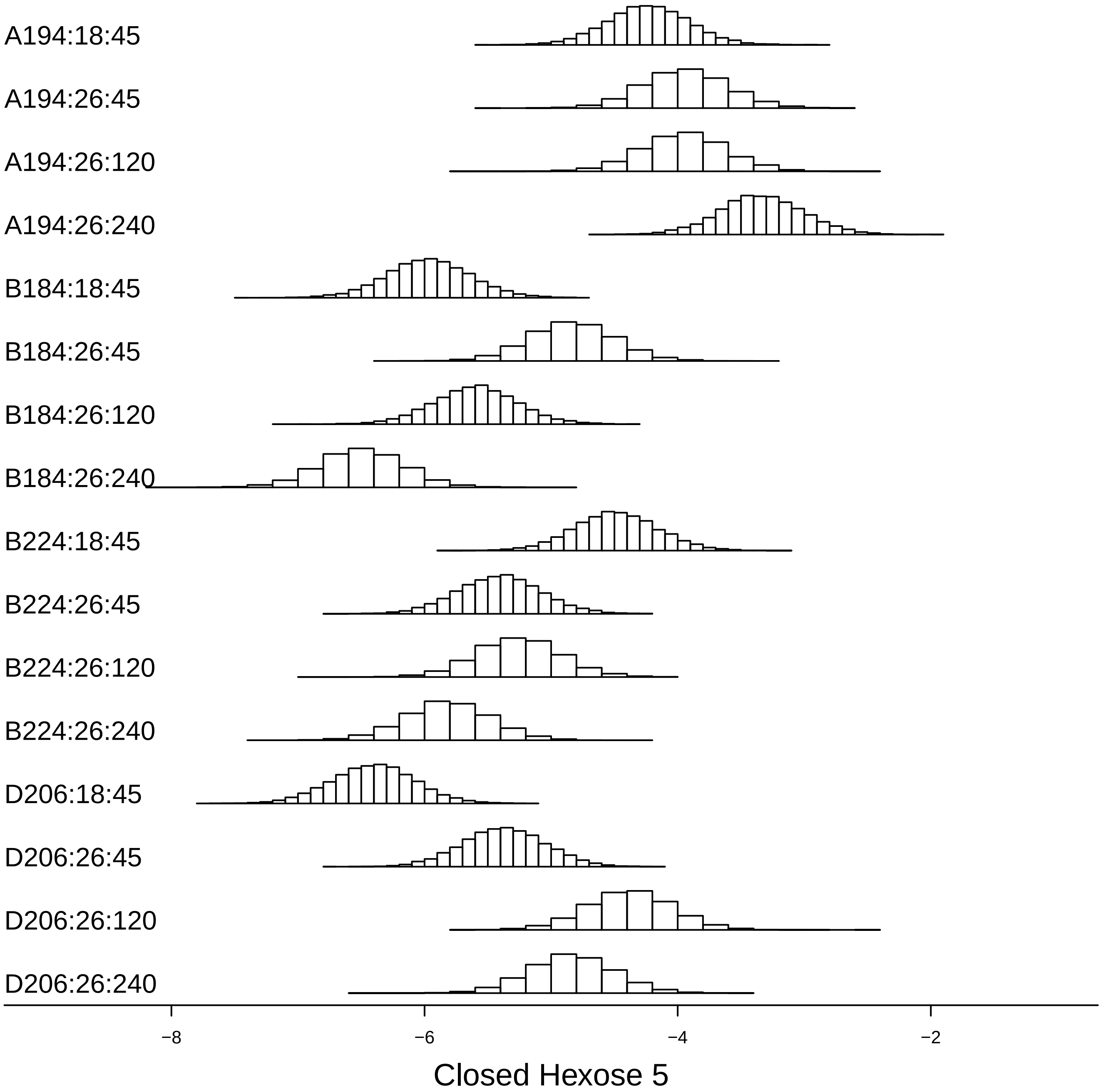
A194:18:45
A194:26:45
A194:26:120
A194:26:240
B184:18:45
B184:26:45
B184:26:120
B184:26:240
B224:18:45
B224:26:45
B224:26:120
B224:26:240
D206:18:45
D206:26:45
D206:26:120
D206:26:240
−8
−6
−4
−2
Closed Hexose 5

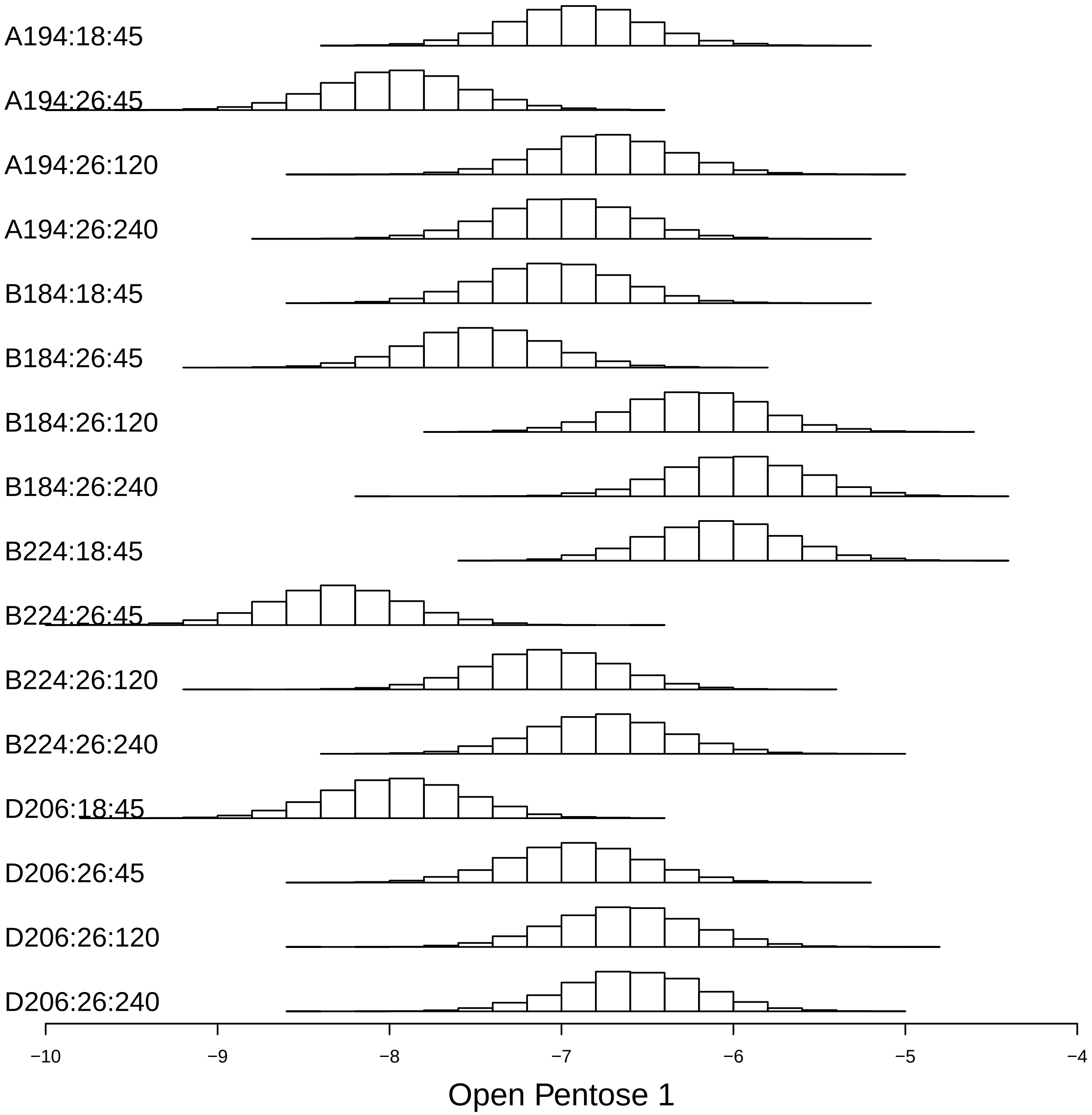

A194:18:45
A194:26:45
A194:26:120
A194:26:240
B184:18:45
B184:26:45
B184:26:120
B184:26:240
B224:18:45
B224:26:45
B224:26:120
B224:26:240
D206:18:45
D206:26:45
D206:26:120
D206:26:240
−10
−9
−8
−7
−6
−5
−4
Open Pentose 1

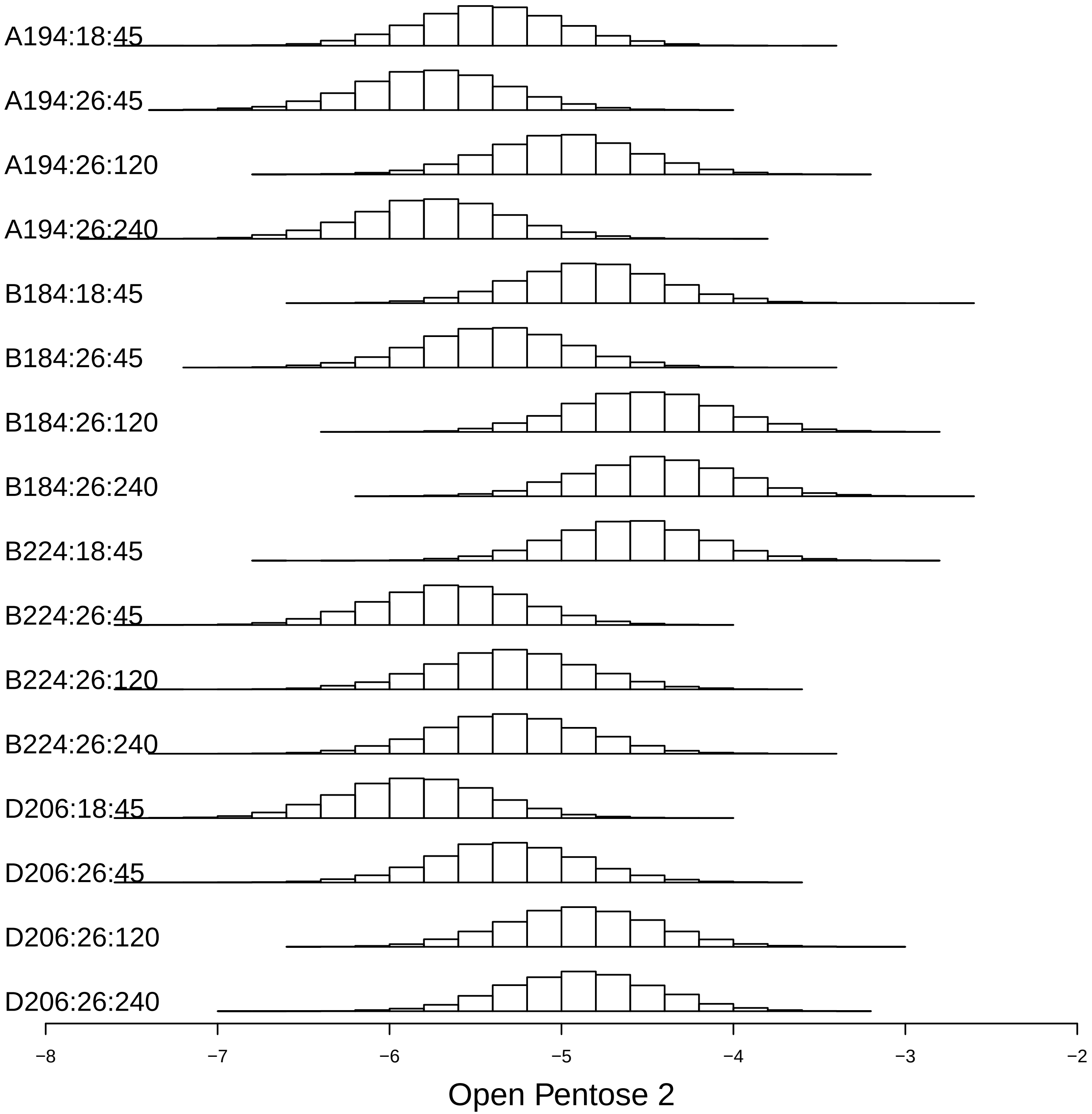
A194:18:45
A194:26:45
A194:26:120
A194:26:240
B184:18:45
B184:26:45
B184:26:120
B184:26:240
B224:18:45
B224:26:45
B224:26:120
B224:26:240
D206:18:45
D206:26:45
D206:26:120
D206:26:240
−8
−7
−6
−5
−4
−3
−2
Open Pentose 2

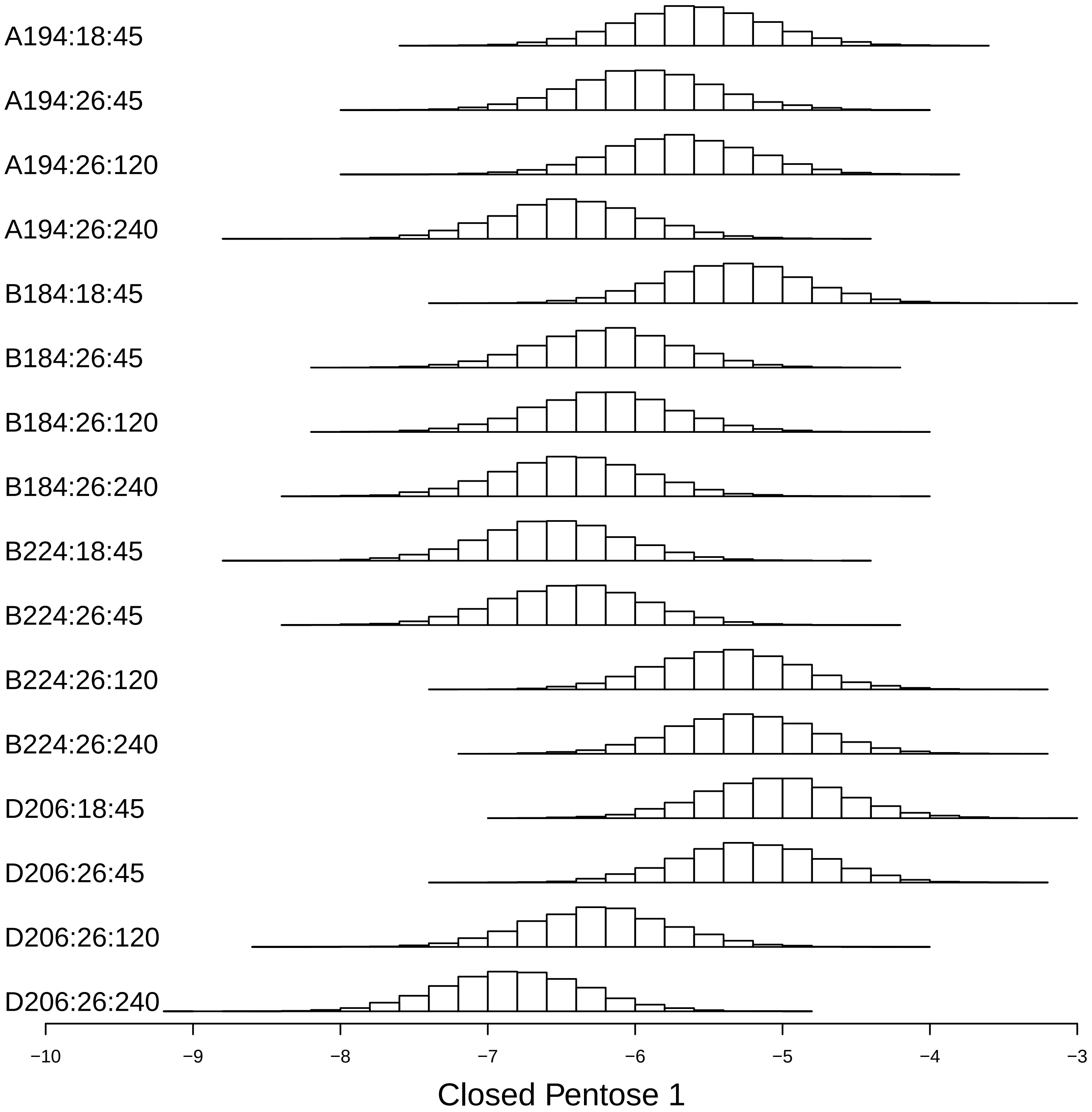
A194:18:45
A194:26:45
A194:26:120
A194:26:240
B184:18:45
B184:26:45
B184:26:120
B184:26:240
B224:18:45
B224:26:45
B224:26:120
B224:26:240
D206:18:45
D206:26:45
D206:26:120
D206:26:240
−10
−9
−8
−7
−6
−5
−4
−3
Closed Pentose 1

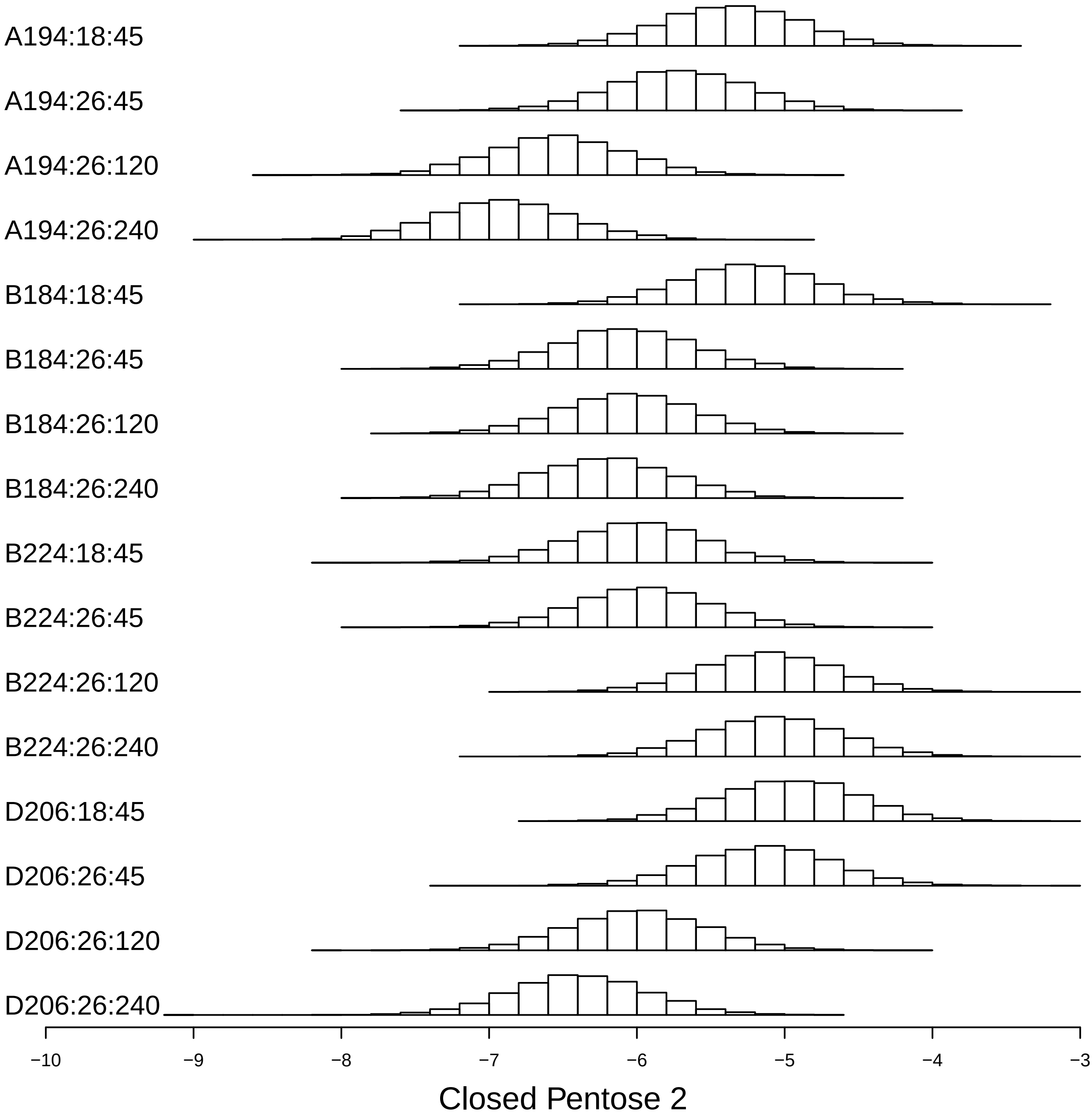
A194:18:45
A194:26:45
A194:26:120
A194:26:240
B184:18:45
B184:26:45
B184:26:120
B184:26:240
B224:18:45
B224:26:45
B224:26:120
B224:26:240
D206:18:45
D206:26:45
D206:26:120
D206:26:240
−10
−9
−8
−7
−6
−5
−4
−3
Closed Pentose 2

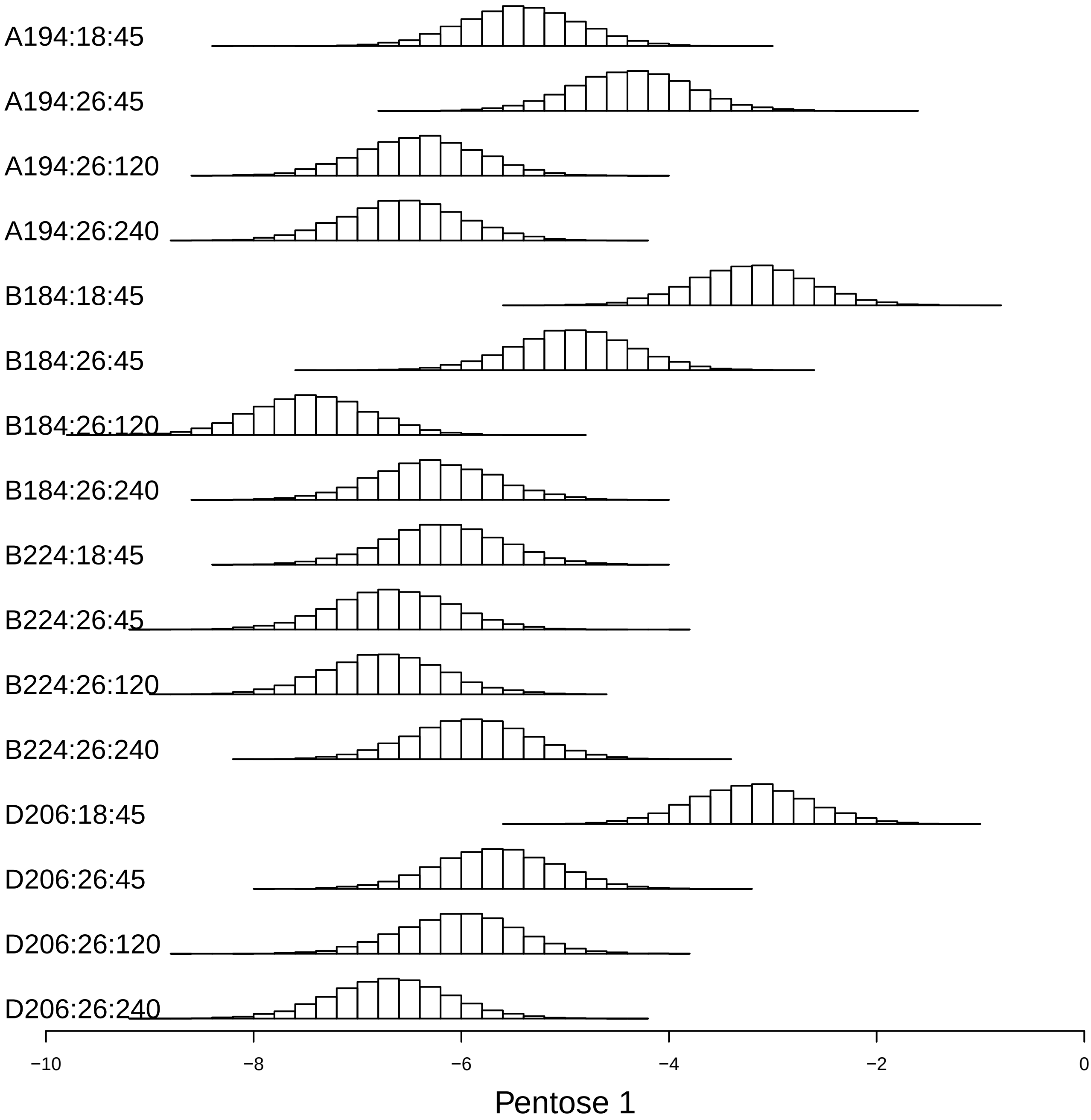
A194:18:45
A194:26:45
A194:26:120
A194:26:240
B184:18:45
B184:26:45
B184:26:120
B184:26:240
B224:18:45
B224:26:45
B224:26:120
B224:26:240
D206:18:45
D206:26:45
D206:26:120
D206:26:240
−10
−8
−6
−4
−2
0
Pentose 1

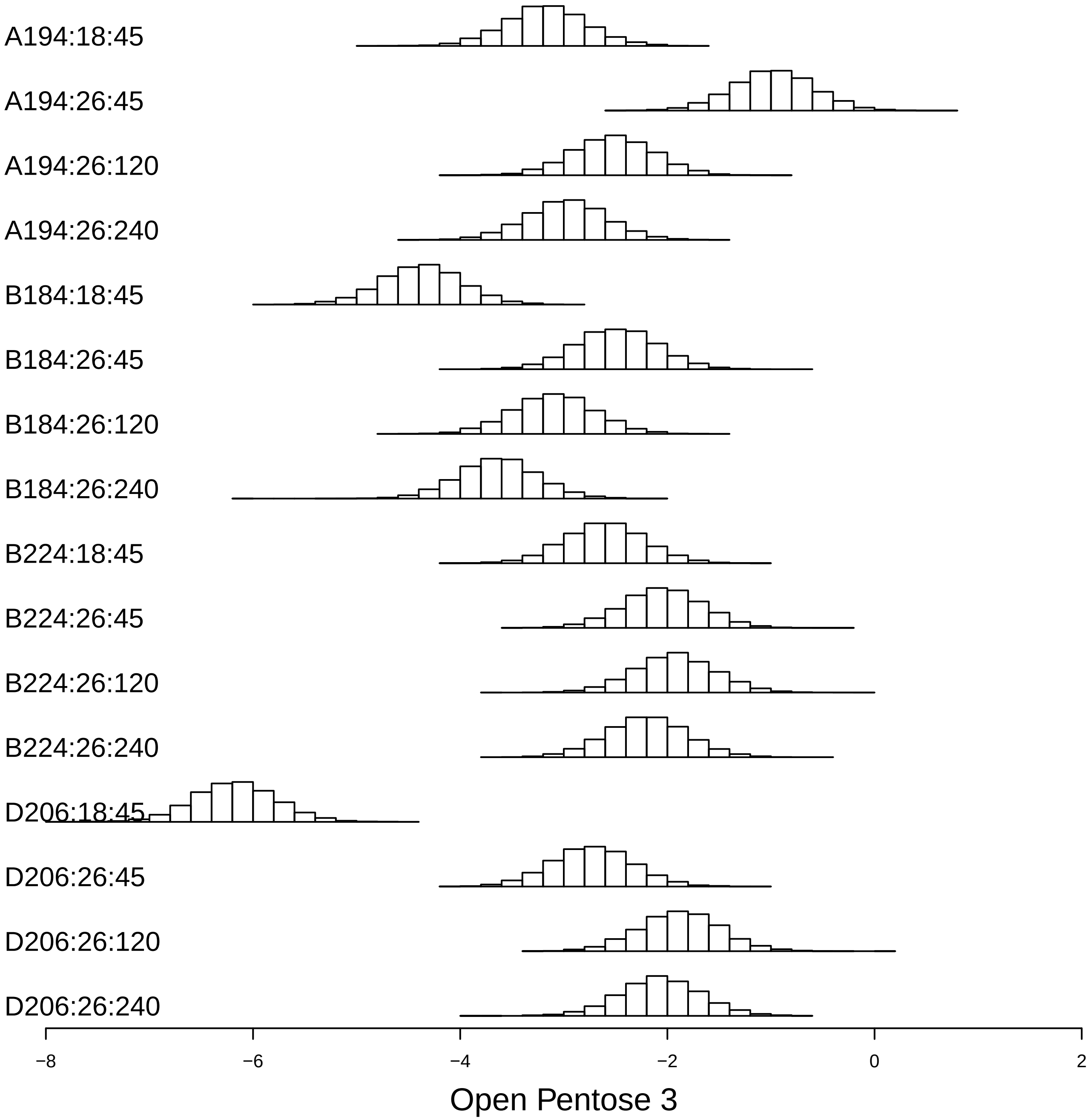

A194:18:45
A194:26:45
A194:26:120
A194:26:240
B184:18:45
B184:26:45
B184:26:120
B184:26:240
B224:18:45
B224:26:45
B224:26:120
B224:26:240
D206:18:45
D206:26:45
D206:26:120
D206:26:240
−8
−6
−4
−2
0
2
Open Pentose 3

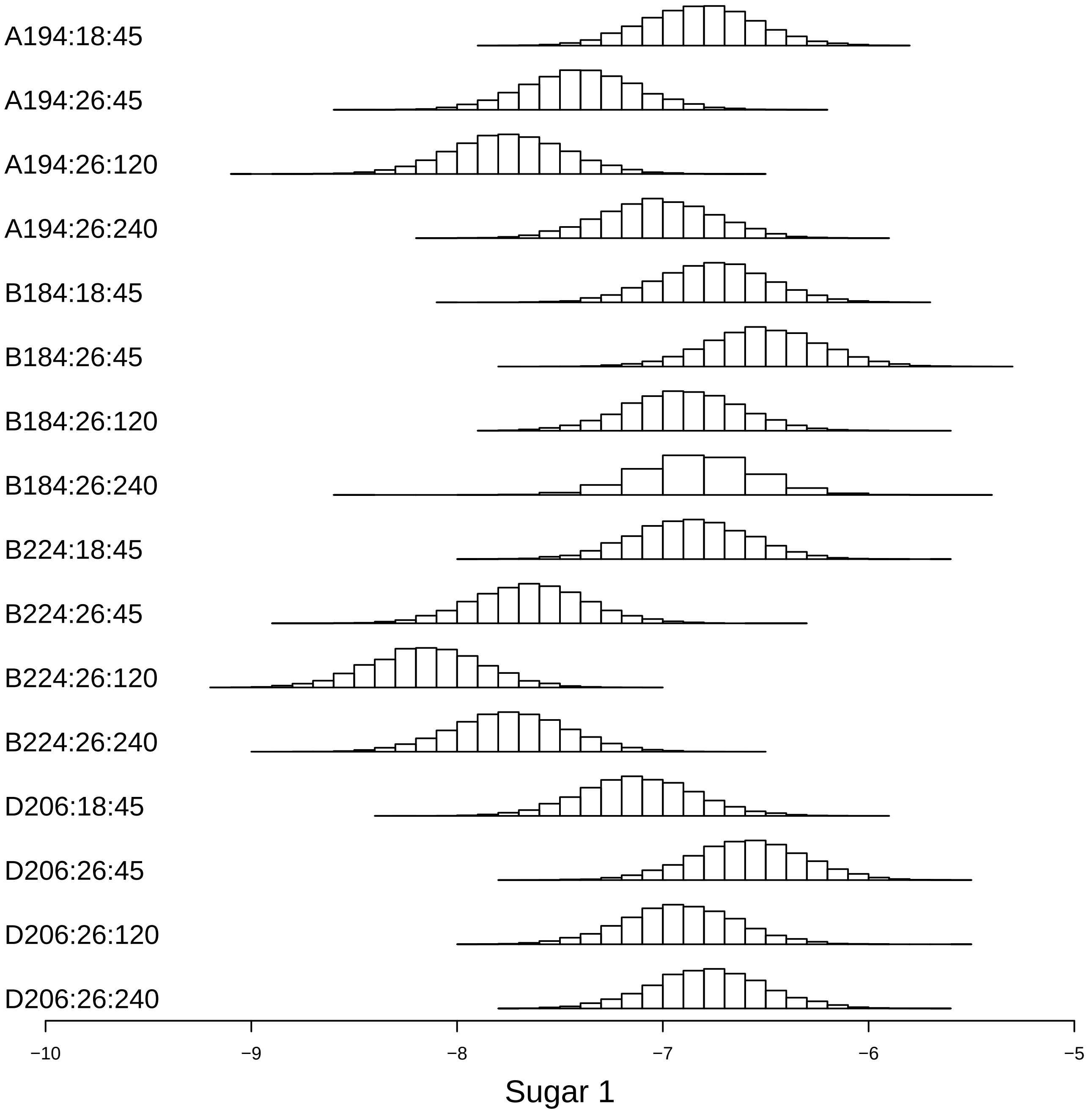
A194:18:45
A194:26:45
A194:26:120
A194:26:240
B184:18:45
B184:26:45
B184:26:120
B184:26:240
B224:18:45
B224:26:45
B224:26:120
B224:26:240
D206:18:45
D206:26:45
D206:26:120
D206:26:240
−10
−9
−8
−7
−6
−5
Sugar 1

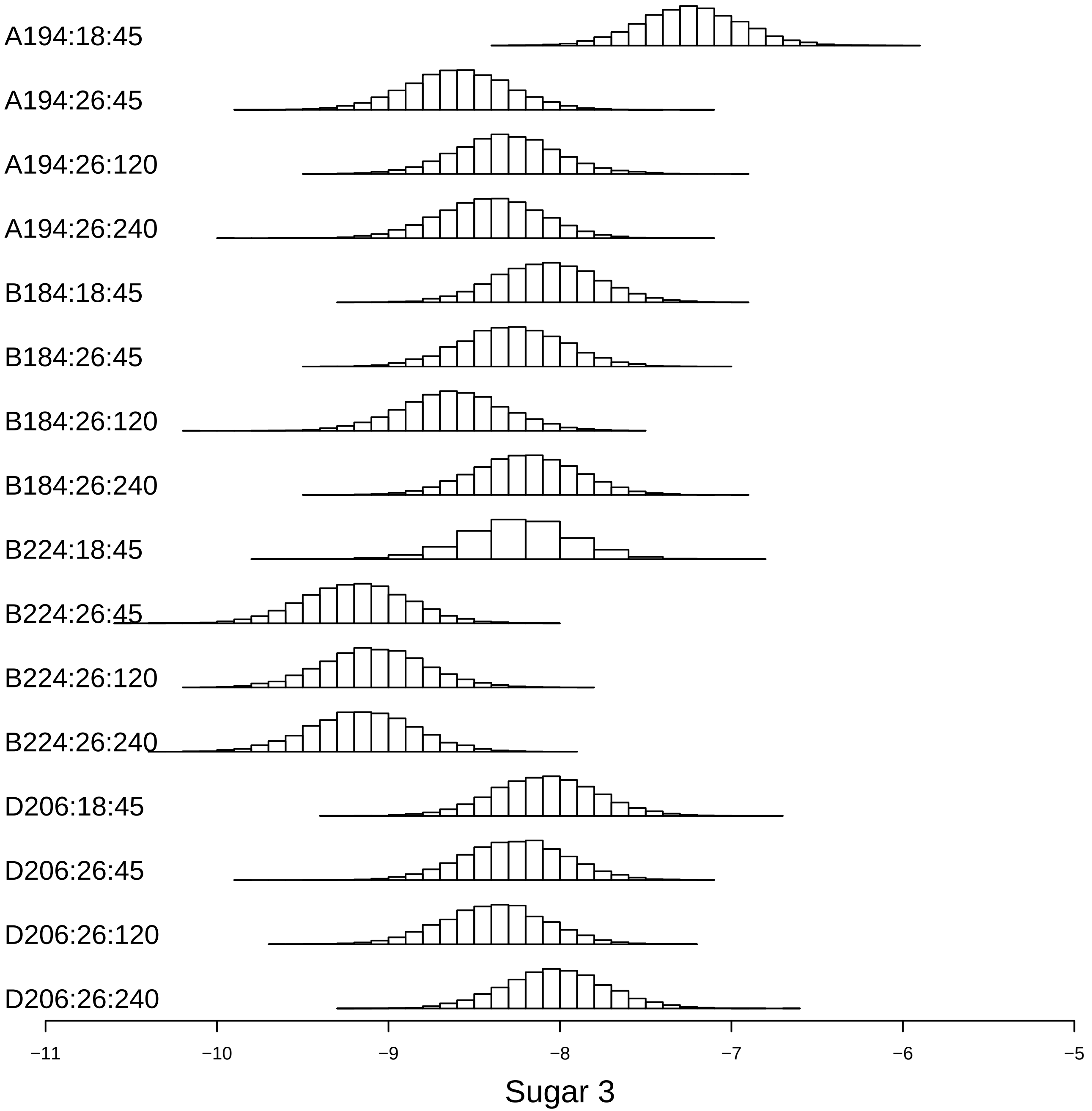

A194:18:45
A194:26:45
A194:26:120
A194:26:240
B184:18:45
B184:26:45
B184:26:120
B184:26:240
B224:18:45
B224:26:45
B224:26:120
B224:26:240
D206:18:45
D206:26:45
D206:26:120
D206:26:240
−11
−10
−9
−8
−7
−6
−5
Sugar 3

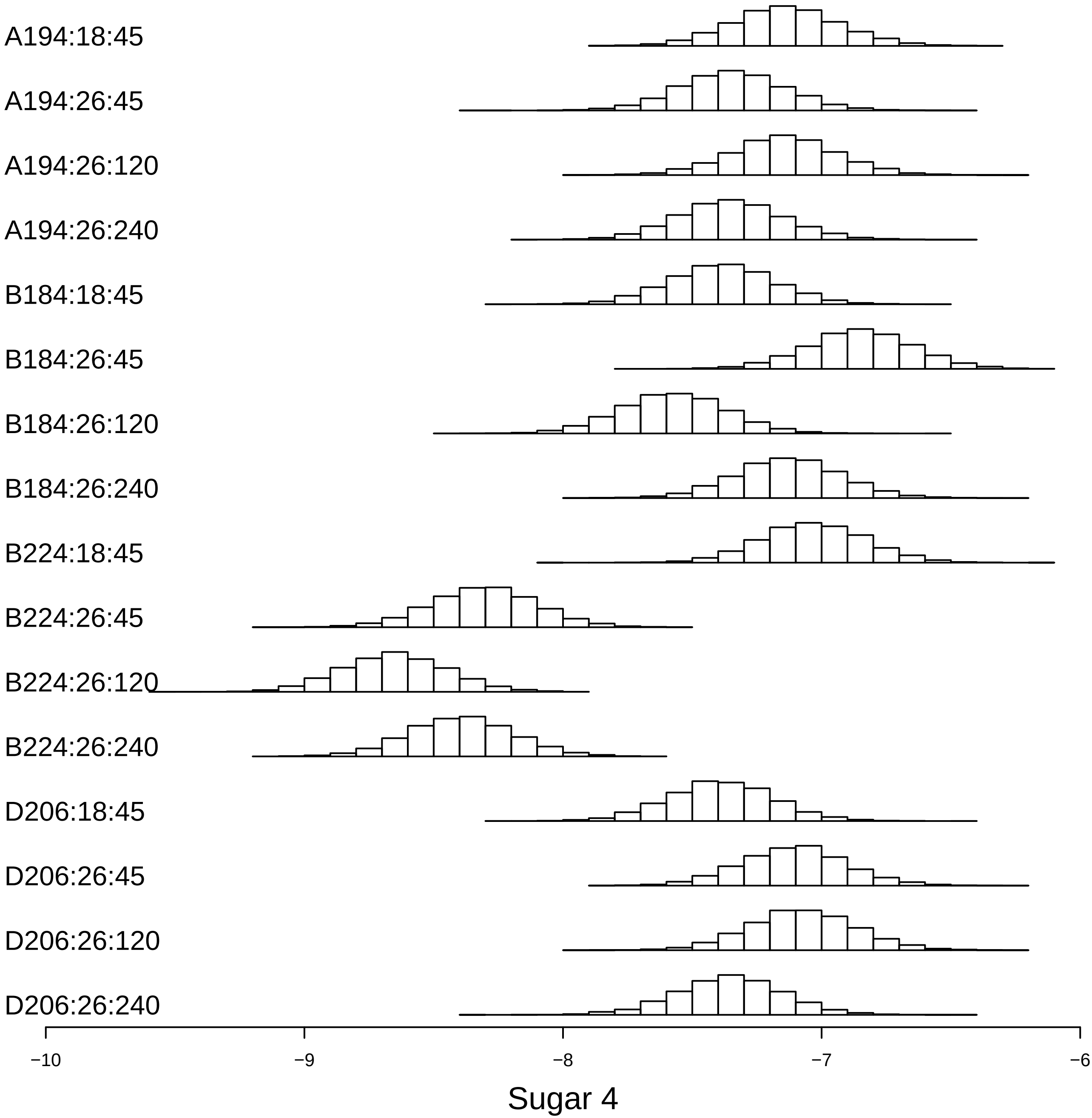
A194:18:45
A194:26:45
A194:26:120
A194:26:240
B184:18:45
B184:26:45
B184:26:120
B184:26:240
B224:18:45
B224:26:45
B224:26:120
B224:26:240
D206:18:45
D206:26:45
D206:26:120
D206:26:240
−10
−9
−8
−7
−6
Sugar 4

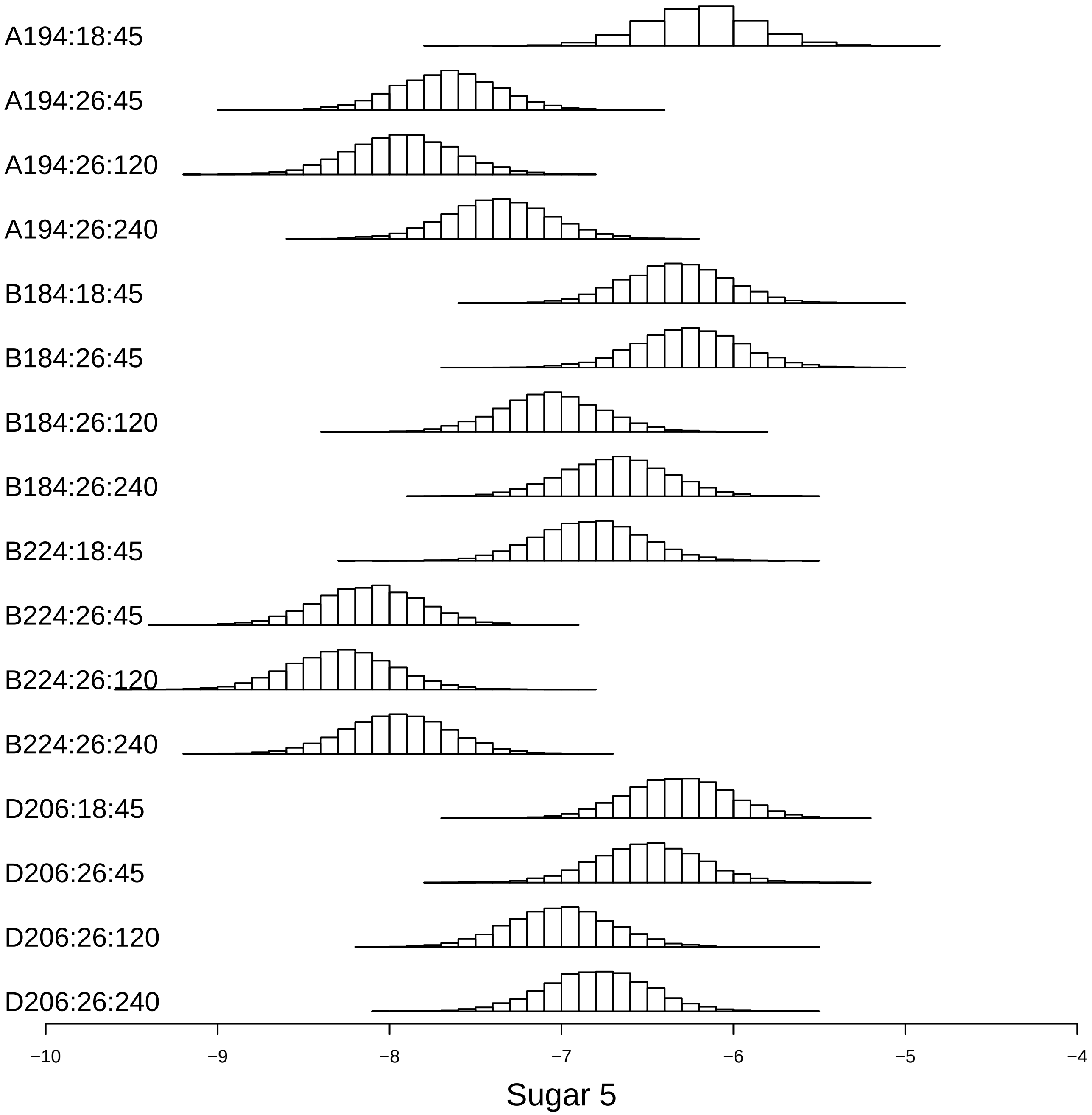
A194:18:45
A194:26:45
A194:26:120
A194:26:240
B184:18:45
B184:26:45
B184:26:120
B184:26:240
B224:18:45
B224:26:45
B224:26:120
B224:26:240
D206:18:45
D206:26:45
D206:26:120
D206:26:240
−10
−9
−8
−7
−6
−5
−4
Sugar 5

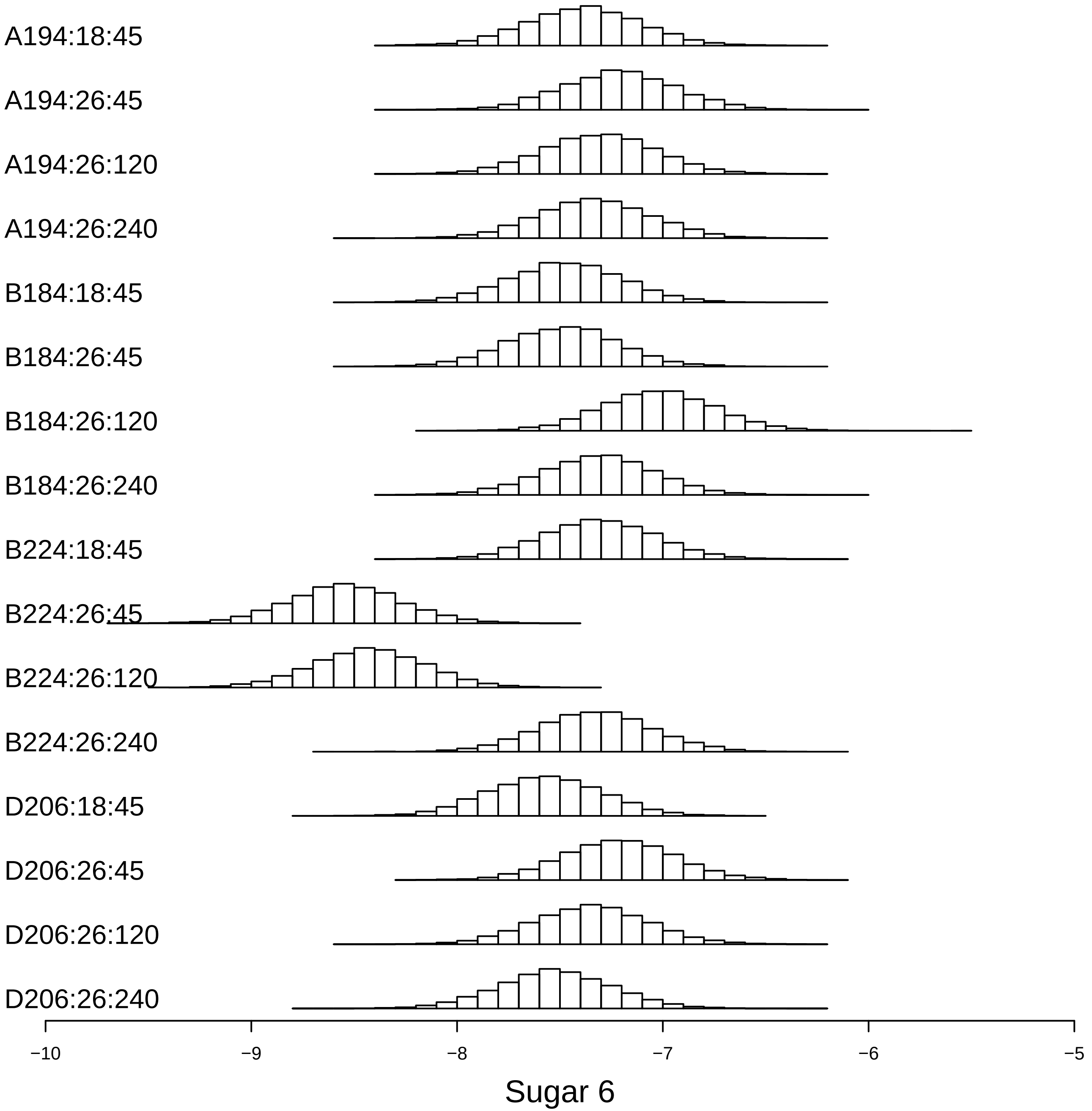
A194:18:45
A194:26:45
A194:26:120
A194:26:240
B184:18:45
B184:26:45
B184:26:120
B184:26:240
B224:18:45
B224:26:45
B224:26:120
B224:26:240
D206:18:45
D206:26:45
D206:26:120
D206:26:240
−10
−9
−8
−7
−6
−5
Sugar 6

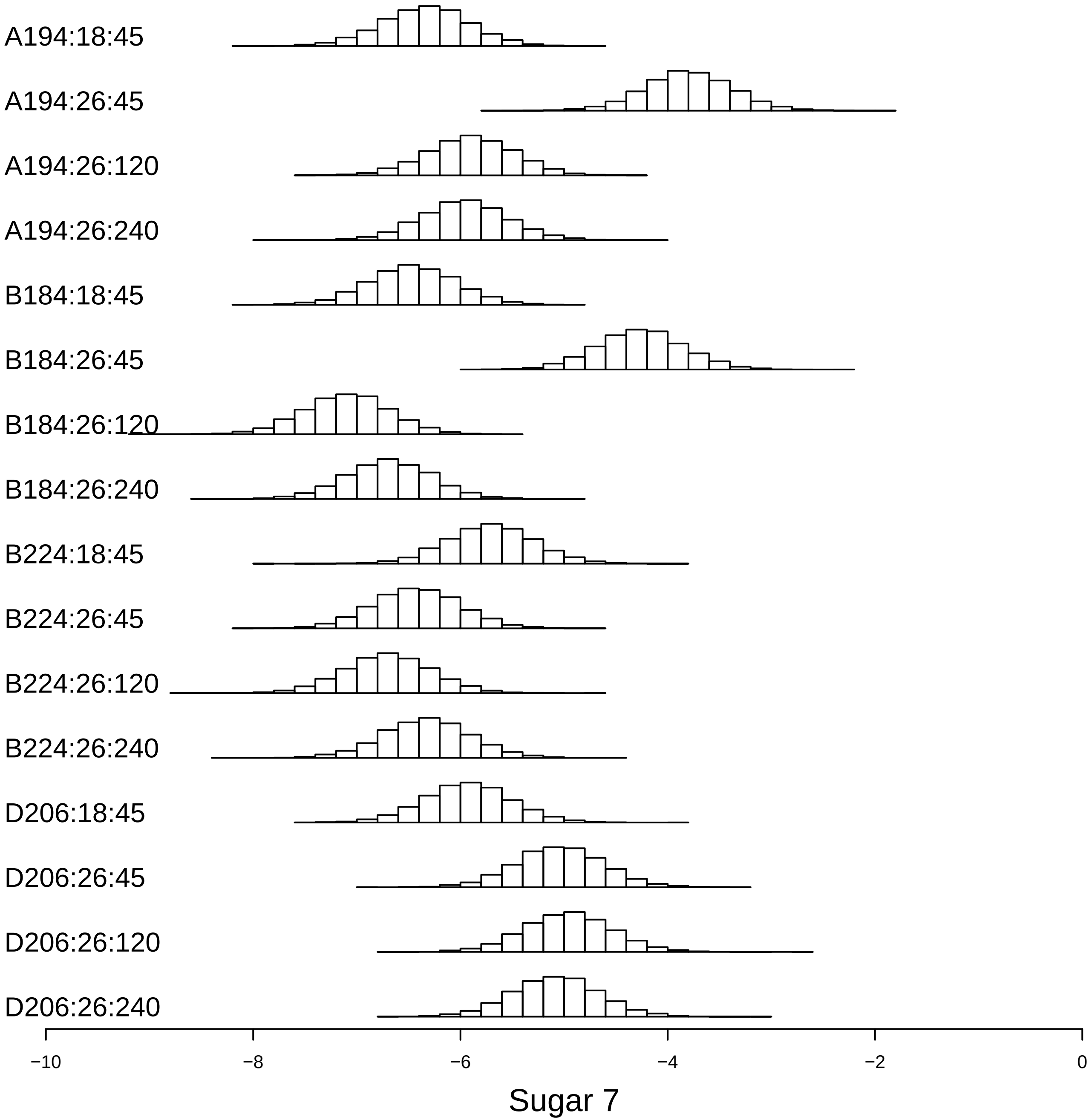
A194:18:45
A194:26:45
A194:26:120
A194:26:240
B184:18:45
B184:26:45
B184:26:120
B184:26:240
B224:18:45
B224:26:45
B224:26:120
B224:26:240
D206:18:45
D206:26:45
D206:26:120
D206:26:240
−10
−8
−6
−4
−2
0
Sugar 7

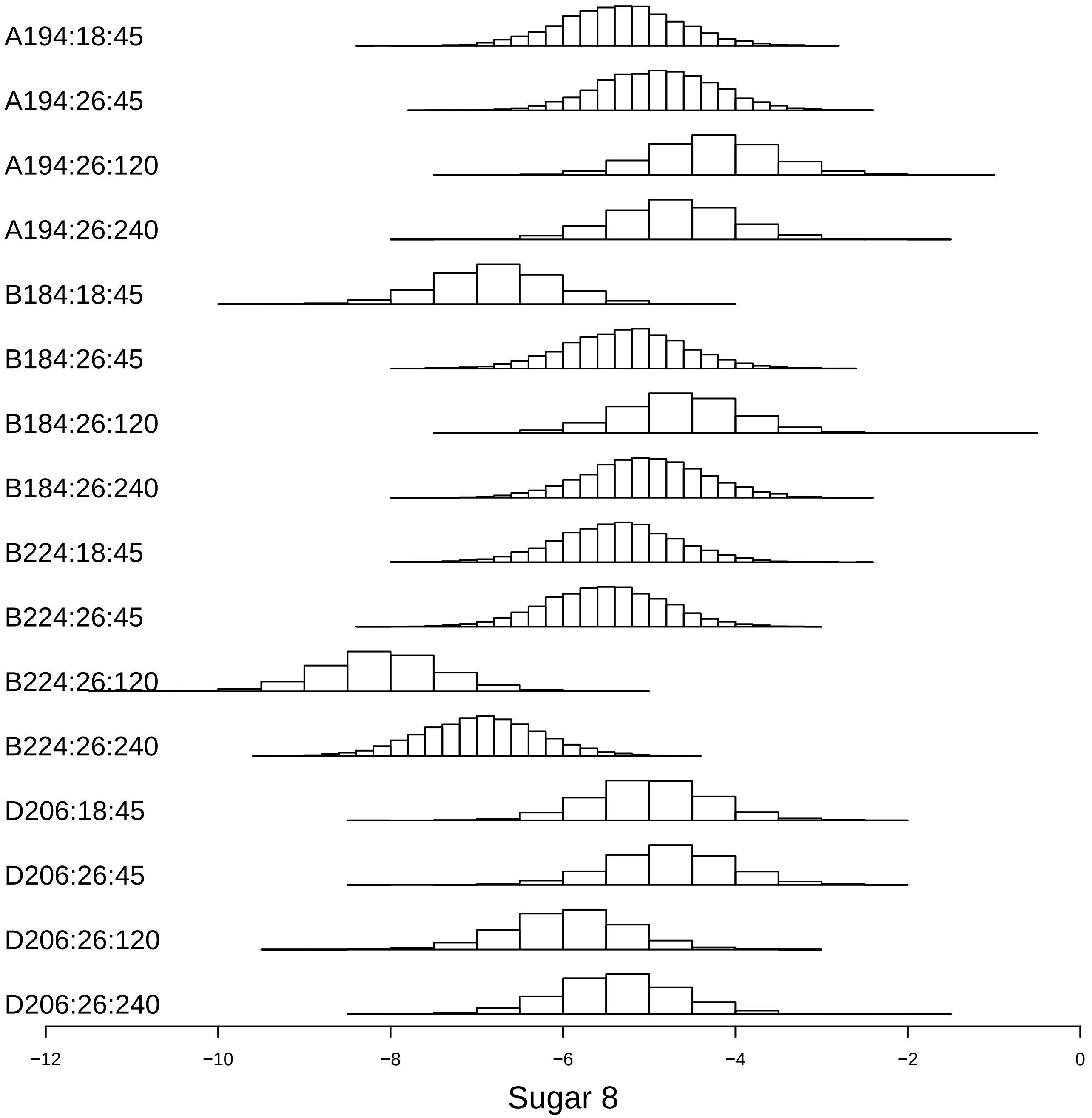
A194:18:45
A194:26:45
A194:26:120
A194:26:240
B184:18:45
B184:26:45
B184:26:120
B184:26:240
B224:18:45
B224:26:45
B224:26:120
B224:26:240
D206:18:45
D206:26:45
D206:26:120
D206:26:240
−12
−10
−8
−6
−4
−2
0
Sugar 8

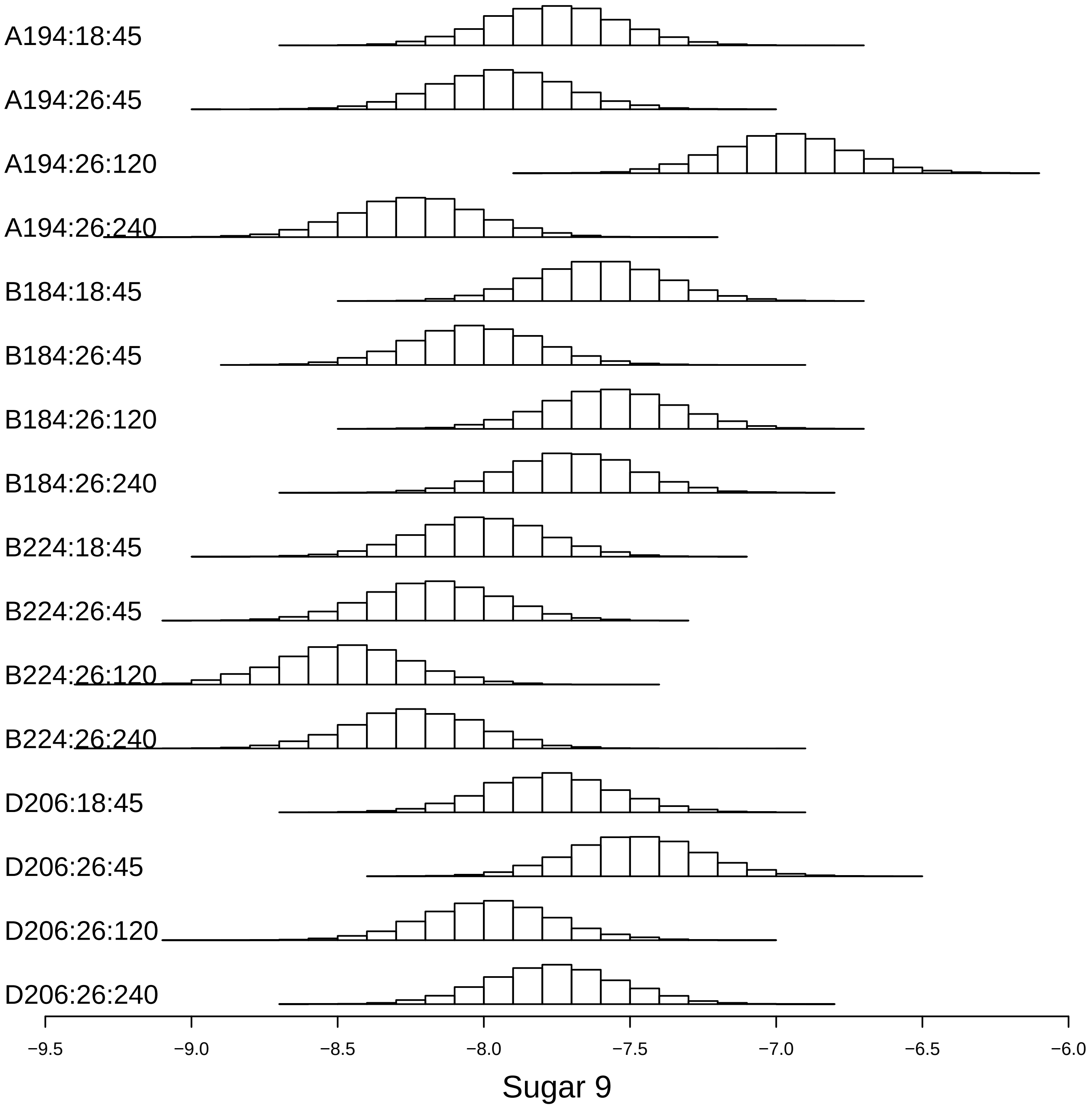

A194:18:45
A194:26:45
A194:26:120
A194:26:240
B184:18:45
B184:26:45
B184:26:120
B184:26:240
B224:18:45
B224:26:45
B224:26:120
B224:26:240
D206:18:45
D206:26:45
D206:26:120
D206:26:240
−9.5
−9.0
−8.5
−8.0
−7.5
−7.0
−6.5
−6.0
Sugar 9

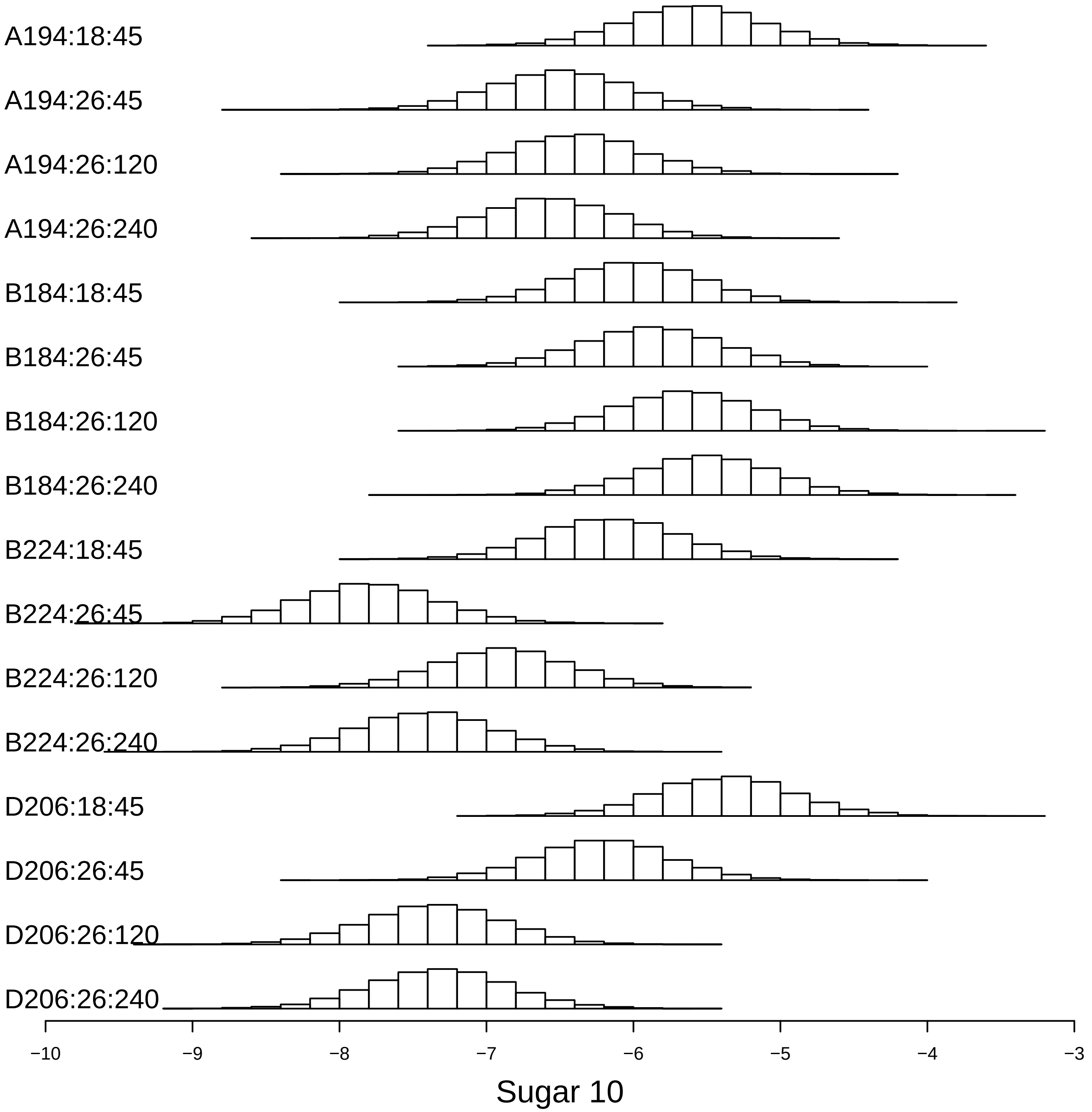
A194:18:45
A194:26:45
A194:26:120
A194:26:240
B184:18:45
B184:26:45
B184:26:120
B184:26:240
B224:18:45
B224:26:45
B224:26:120
B224:26:240
D206:18:45
D206:26:45
D206:26:120
D206:26:240
−10
−9
−8
−7
−6
−5
−4
−3
Sugar 10

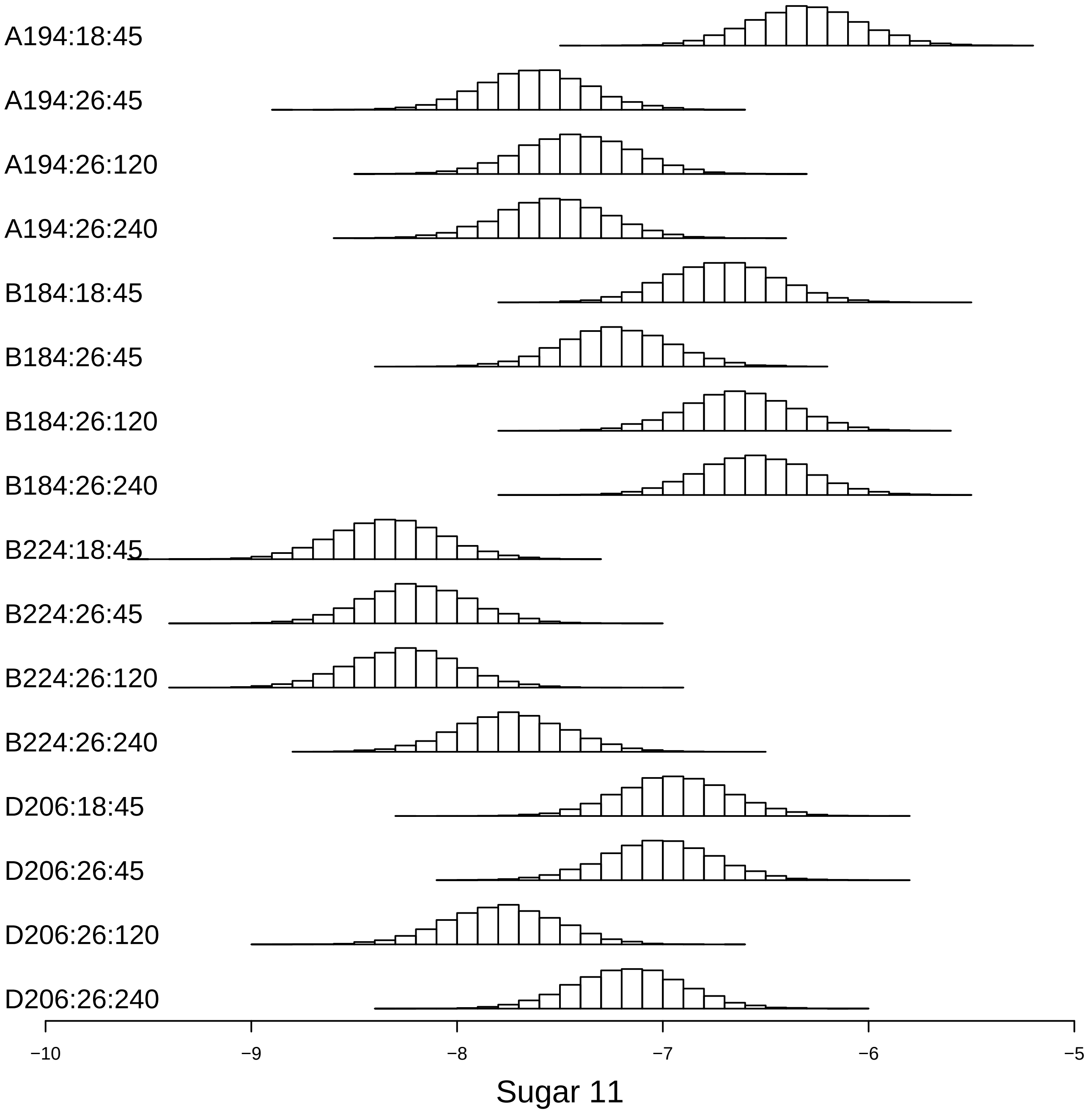

A194:18:45
A194:26:45
A194:26:120
A194:26:240
B184:18:45
B184:26:45
B184:26:120
B184:26:240
B224:18:45
B224:26:45
B224:26:120
B224:26:240
D206:18:45
D206:26:45
D206:26:120
D206:26:240
−10
−9
−8
−7
−6
−5
Sugar 11

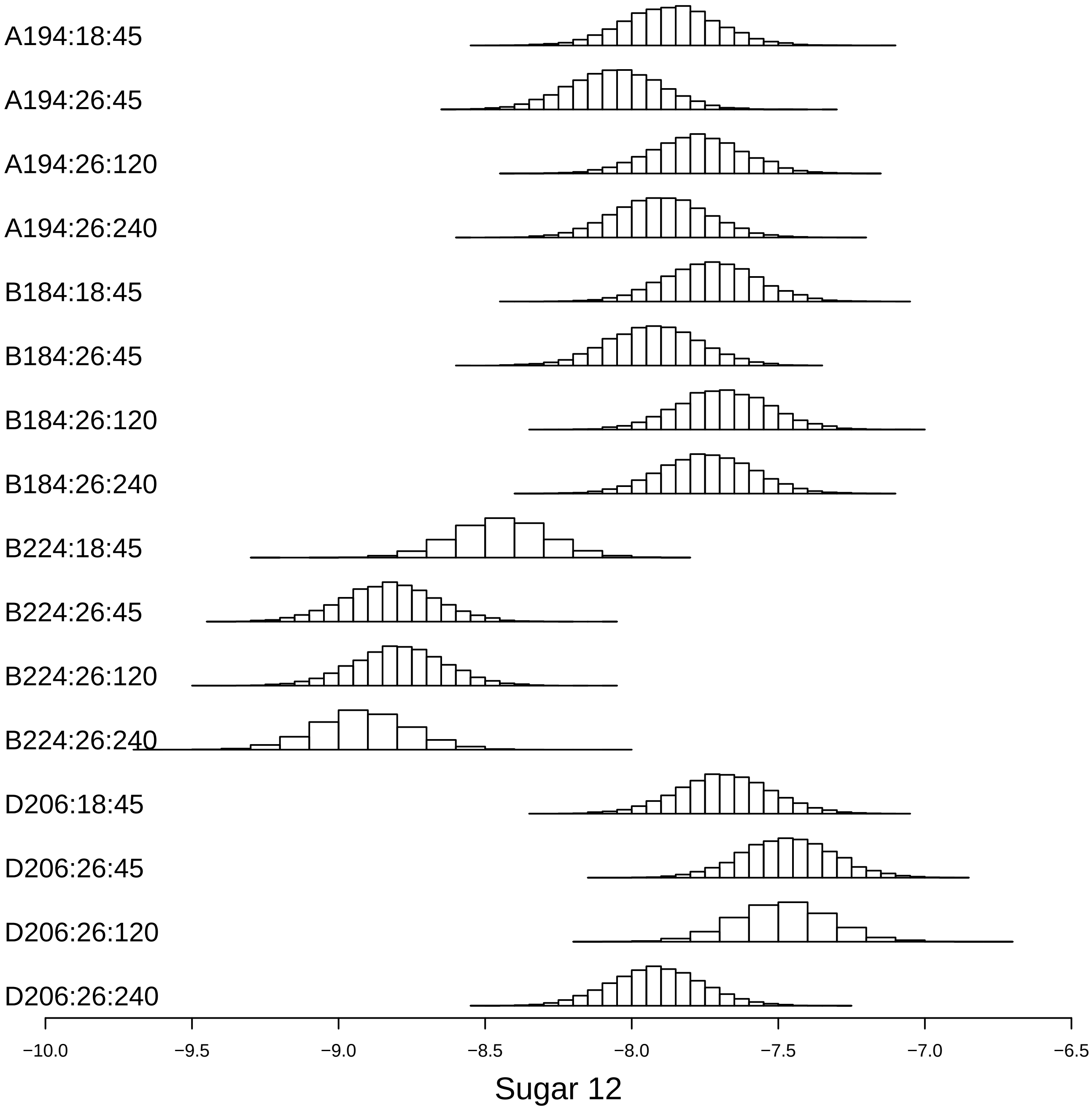
A194:18:45
A194:26:45
A194:26:120
A194:26:240
B184:18:45
B184:26:45
B184:26:120
B184:26:240
B224:18:45
B224:26:45
B224:26:120
B224:26:240
D206:18:45
D206:26:45
D206:26:120
D206:26:240
−10.0
−9.5
−9.0
−8.5
−8.0
−7.5
−7.0
−6.5
Sugar 12

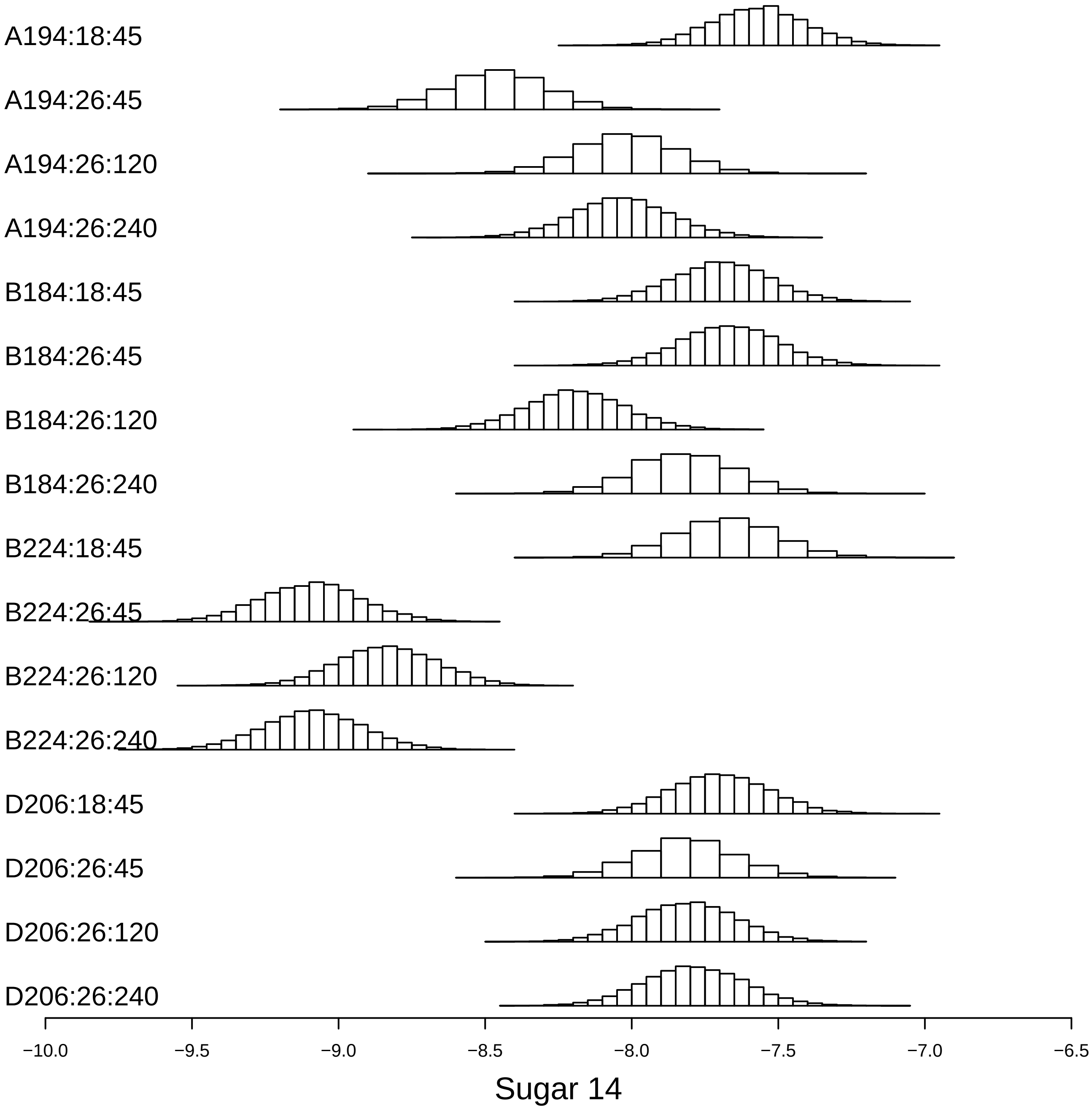
A194:18:45
A194:26:45
A194:26:120
A194:26:240
B184:18:45
B184:26:45
B184:26:120
B184:26:240
B224:18:45
B224:26:45
B224:26:120
B224:26:240
D206:18:45
D206:26:45
D206:26:120
D206:26:240
−10.0
−9.5
−9.0
−8.5
−8.0
−7.5
−7.0
−6.5
Sugar 14

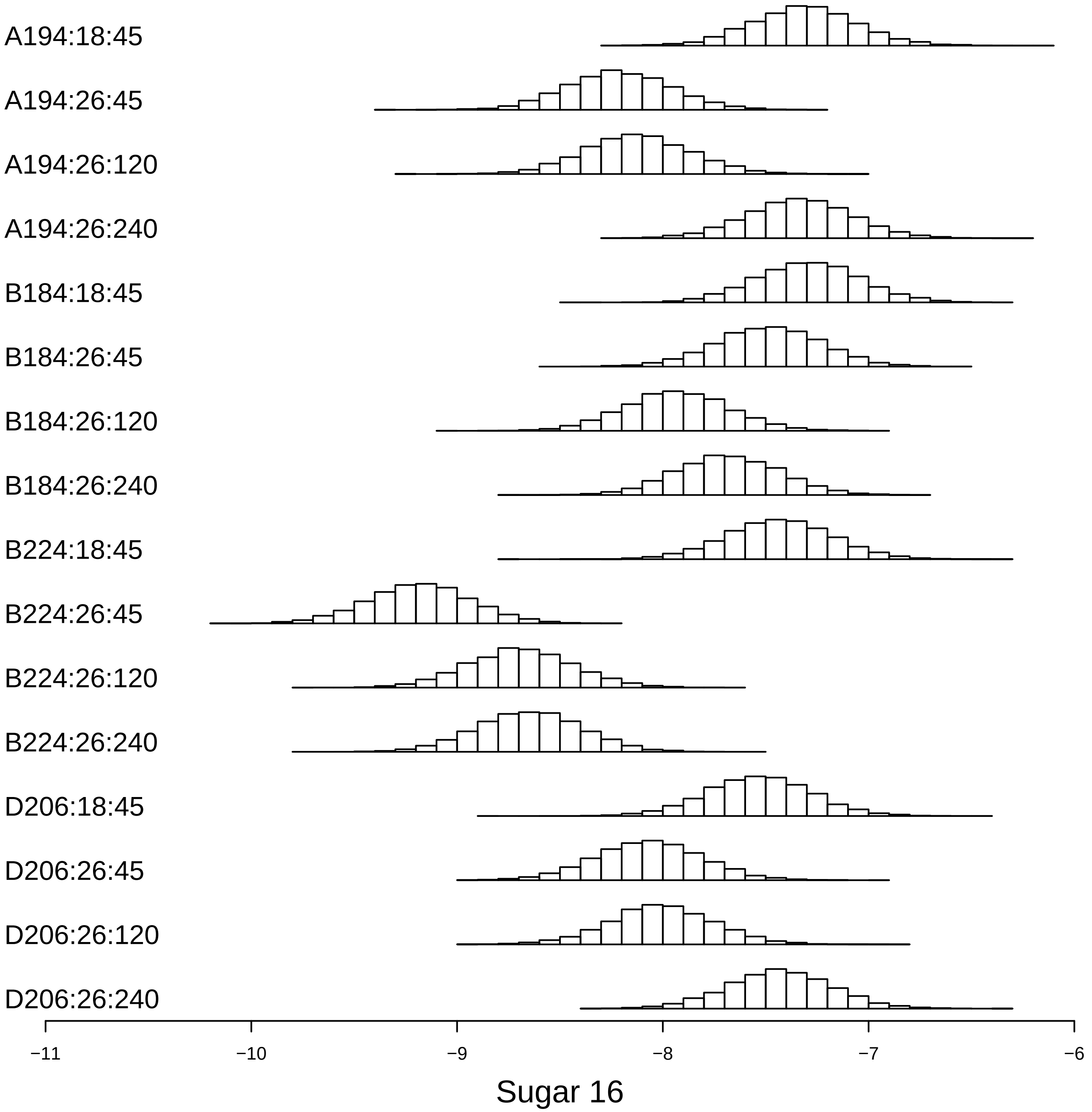
A194:18:45
A194:26:45
A194:26:120
A194:26:240
B184:18:45
B184:26:45
B184:26:120
B184:26:240
B224:18:45
B224:26:45
B224:26:120
B224:26:240
D206:18:45
D206:26:45
D206:26:120
D206:26:240
−11
−10
−9
−8
−7
−6
Sugar 16

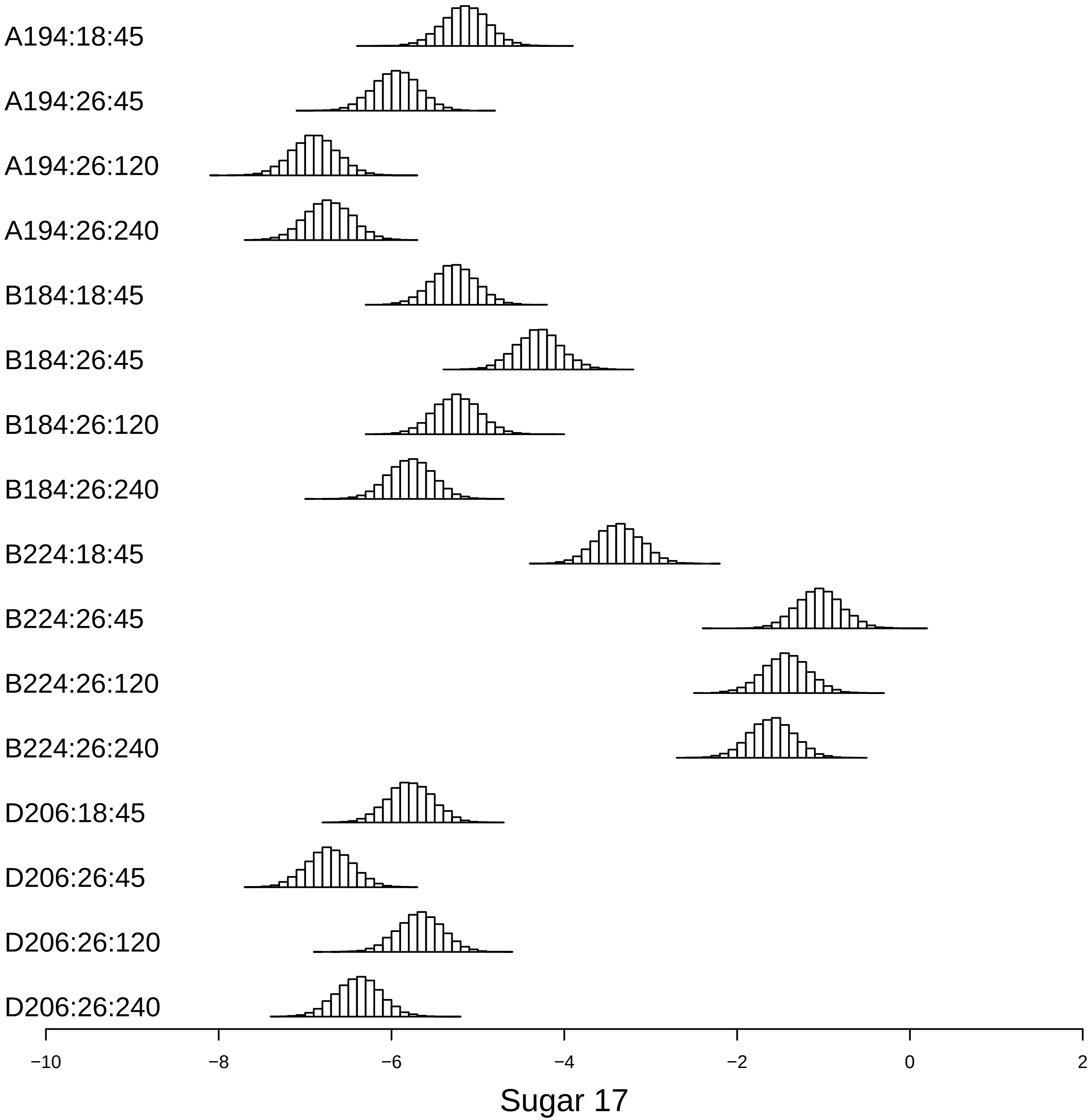
A194:18:45
A194:26:45
A194:26:120
A194:26:240
B184:18:45
B184:26:45
B184:26:120
B184:26:240
B224:18:45
B224:26:45
B224:26:120
B224:26:240
D206:18:45
D206:26:45
D206:26:120
D206:26:240
−10
−8
−6
−4
−2
0
2
Sugar 17

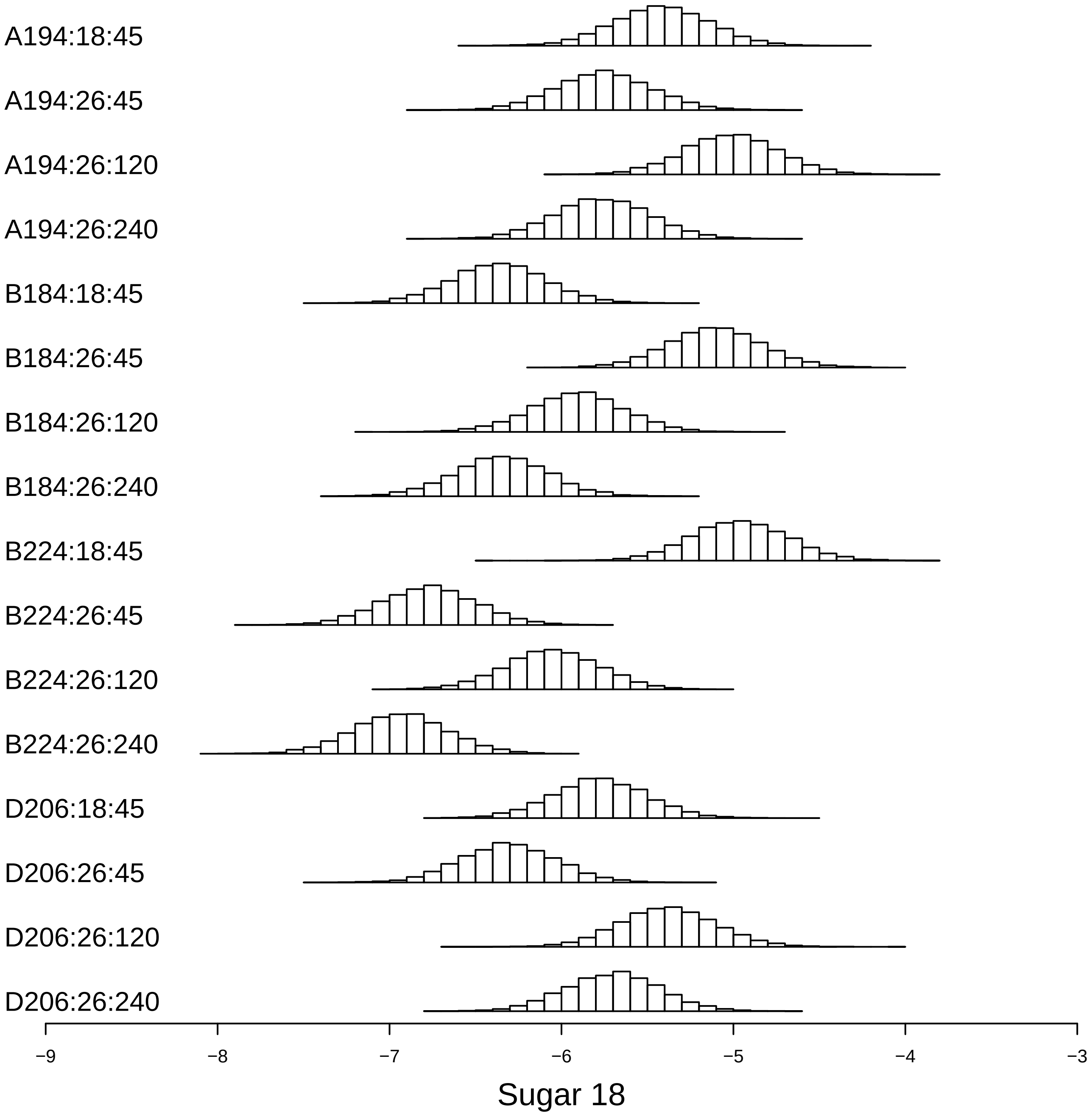
A194:18:45
A194:26:45
A194:26:120
A194:26:240
B184:18:45
B184:26:45
B184:26:120
B184:26:240
B224:18:45
B224:26:45
B224:26:120
B224:26:240
D206:18:45
D206:26:45
D206:26:120
D206:26:240
−9
−8
−7
−6
−5
−4
−3
Sugar 18

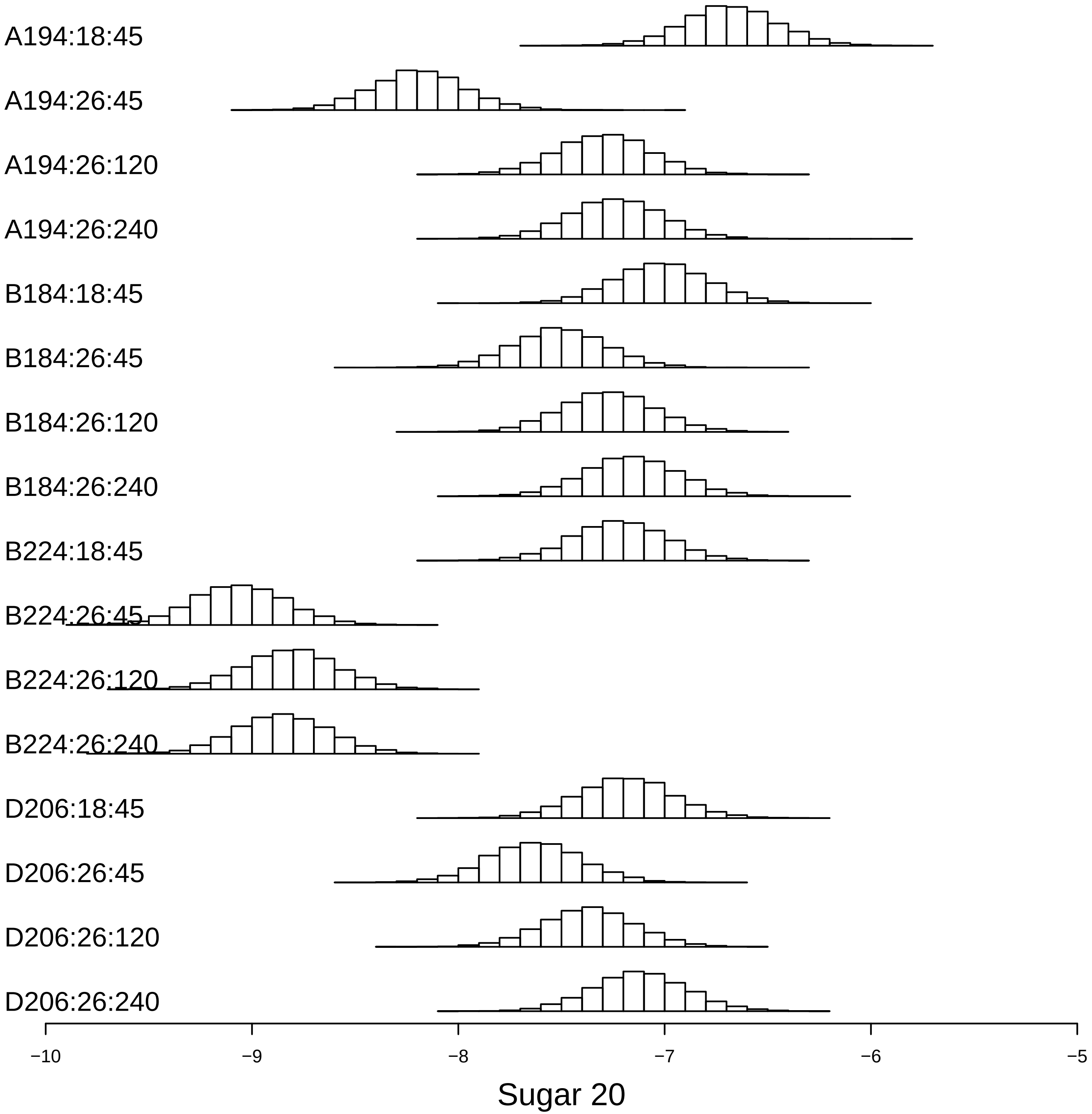
A194:18:45
A194:26:45
A194:26:120
A194:26:240
B184:18:45
B184:26:45
B184:26:120
B184:26:240
B224:18:45
B224:26:45
B224:26:120
B224:26:240
D206:18:45
D206:26:45
D206:26:120
D206:26:240
−10
−9
−8
−7
−6
−5
Sugar 20

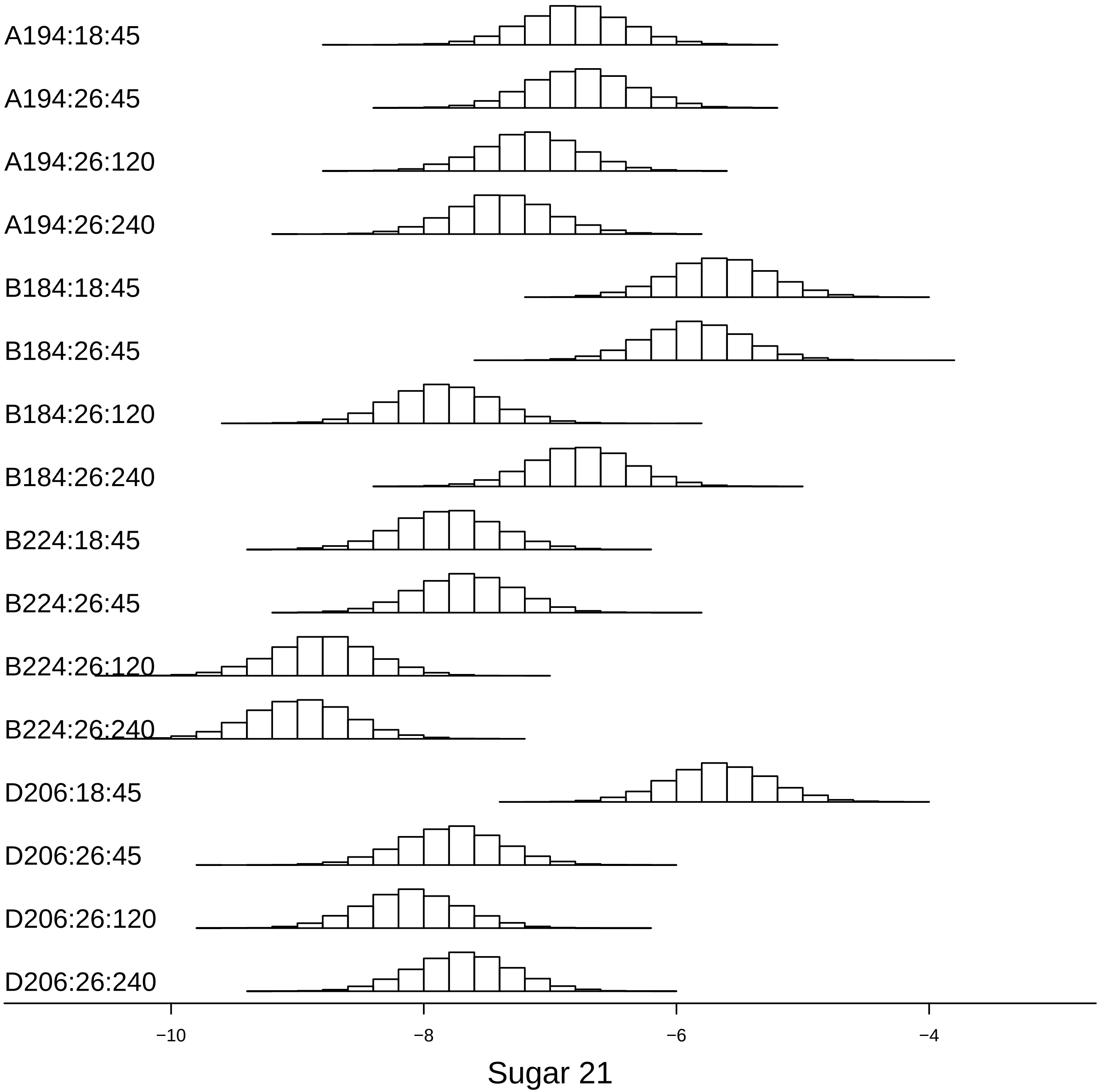

A194:18:45
A194:26:45
A194:26:120
A194:26:240
B184:18:45
B184:26:45
B184:26:120
B184:26:240
B224:18:45
B224:26:45
B224:26:120
B224:26:240
D206:18:45
D206:26:45
D206:26:120
D206:26:240
−10
−8
−6
−4
Sugar 21

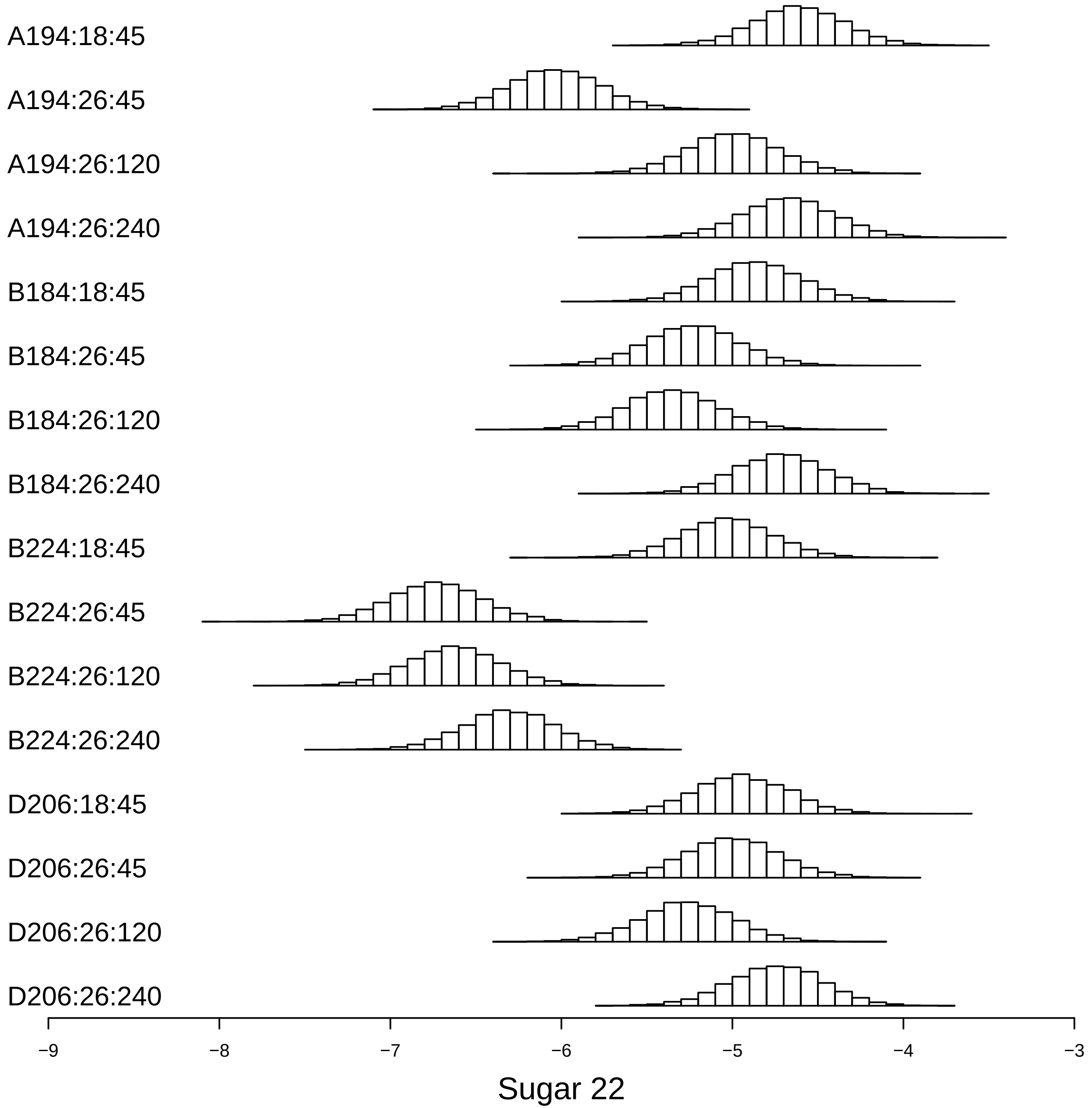

A194:18:45
A194:26:45
A194:26:120
A194:26:240
B184:18:45
B184:26:45
B184:26:120
B184:26:240
B224:18:45
B224:26:45
B224:26:120
B224:26:240
D206:18:45
D206:26:45
D206:26:120
D206:26:240
−9
−8
−7
−6
−5
−4
−3
Sugar 22

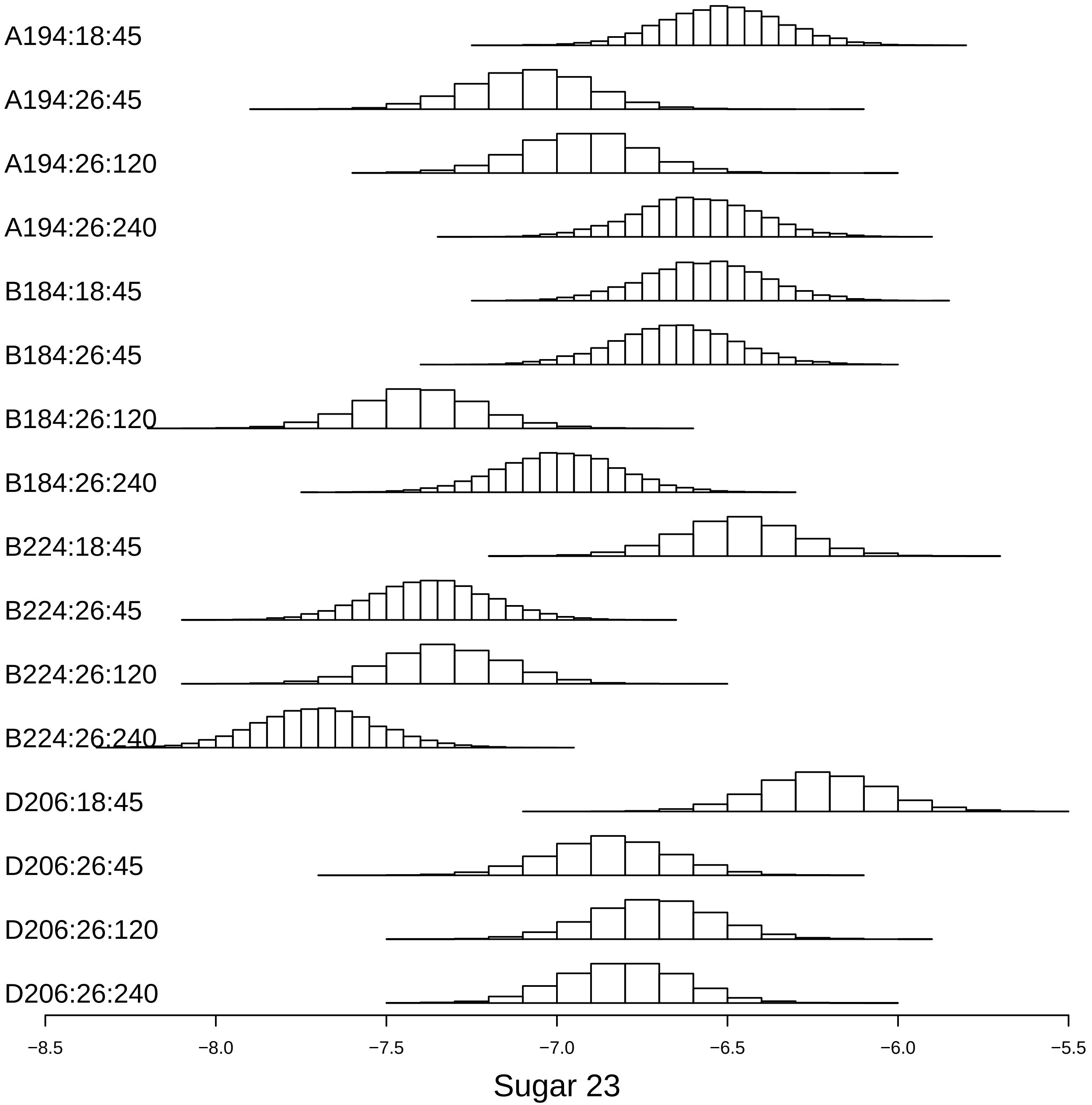

A194:18:45
A194:26:45
A194:26:120
A194:26:240
B184:18:45
B184:26:45
B184:26:120
B184:26:240
B224:18:45
B224:26:45
B224:26:120
B224:26:240
D206:18:45
D206:26:45
D206:26:120
D206:26:240
−8.5
−8.0
−7.5
−7.0
−6.5
−6.0
−5.5
Sugar 23

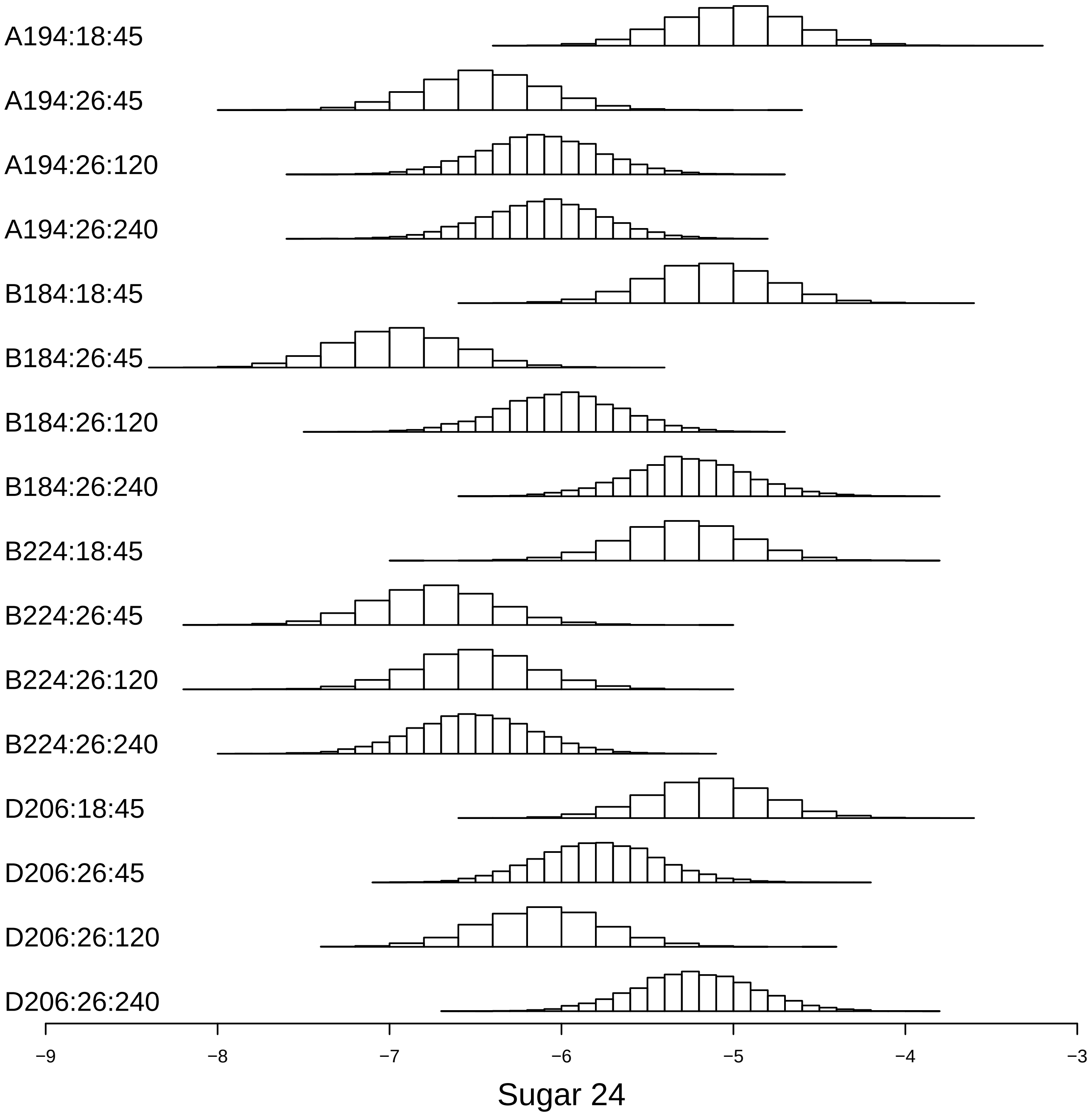
A194:18:45
A194:26:45
A194:26:120
A194:26:240
B184:18:45
B184:26:45
B184:26:120
B184:26:240
B224:18:45
B224:26:45
B224:26:120
B224:26:240
D206:18:45
D206:26:45
D206:26:120
D206:26:240
−9
−8
−7
−6
−5
−4
−3
Sugar 24

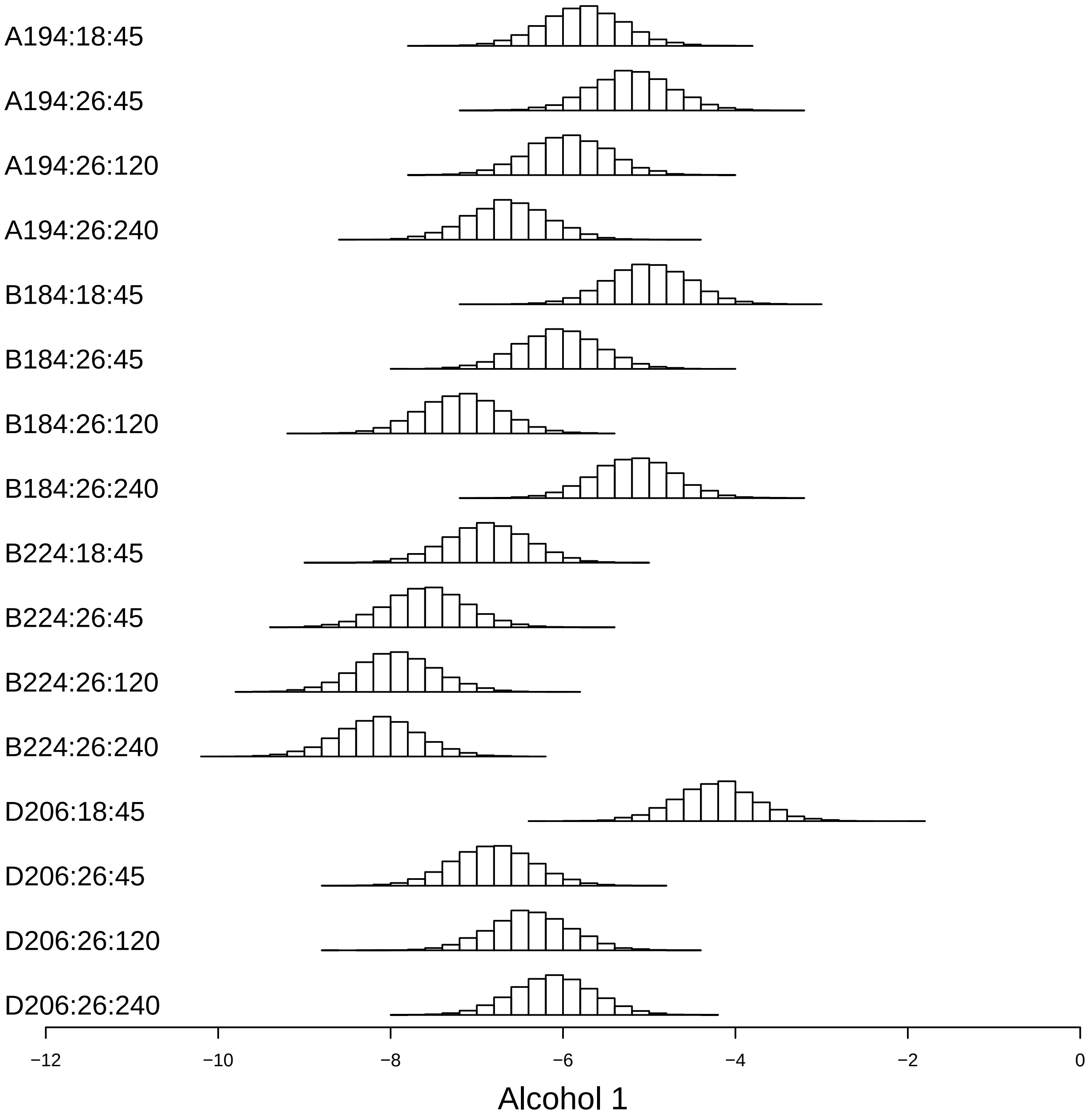
A194:18:45
A194:26:45
A194:26:120
A194:26:240
B184:18:45
B184:26:45
B184:26:120
B184:26:240
B224:18:45
B224:26:45
B224:26:120
B224:26:240
D206:18:45
D206:26:45
D206:26:120
D206:26:240
−12
−10
−8
−6
−4
−2
0
Alcohol 1

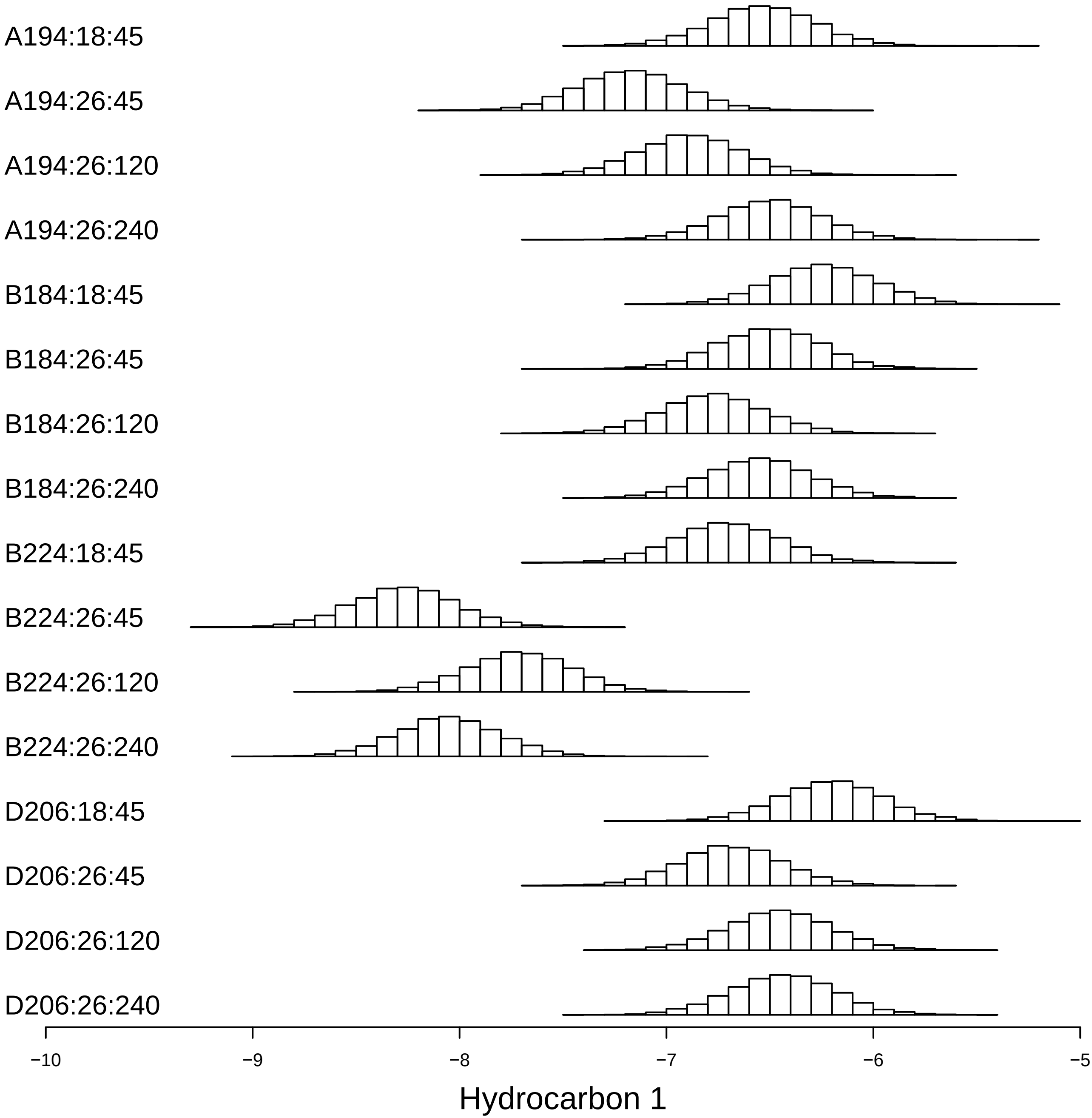
A194:18:45
A194:26:45
A194:26:120
A194:26:240
B184:18:45
B184:26:45
B184:26:120
B184:26:240
B224:18:45
B224:26:45
B224:26:120
B224:26:240
D206:18:45
D206:26:45
D206:26:120
D206:26:240
−10
−9
−8
−7
−6
−5
Hydrocarbon 1

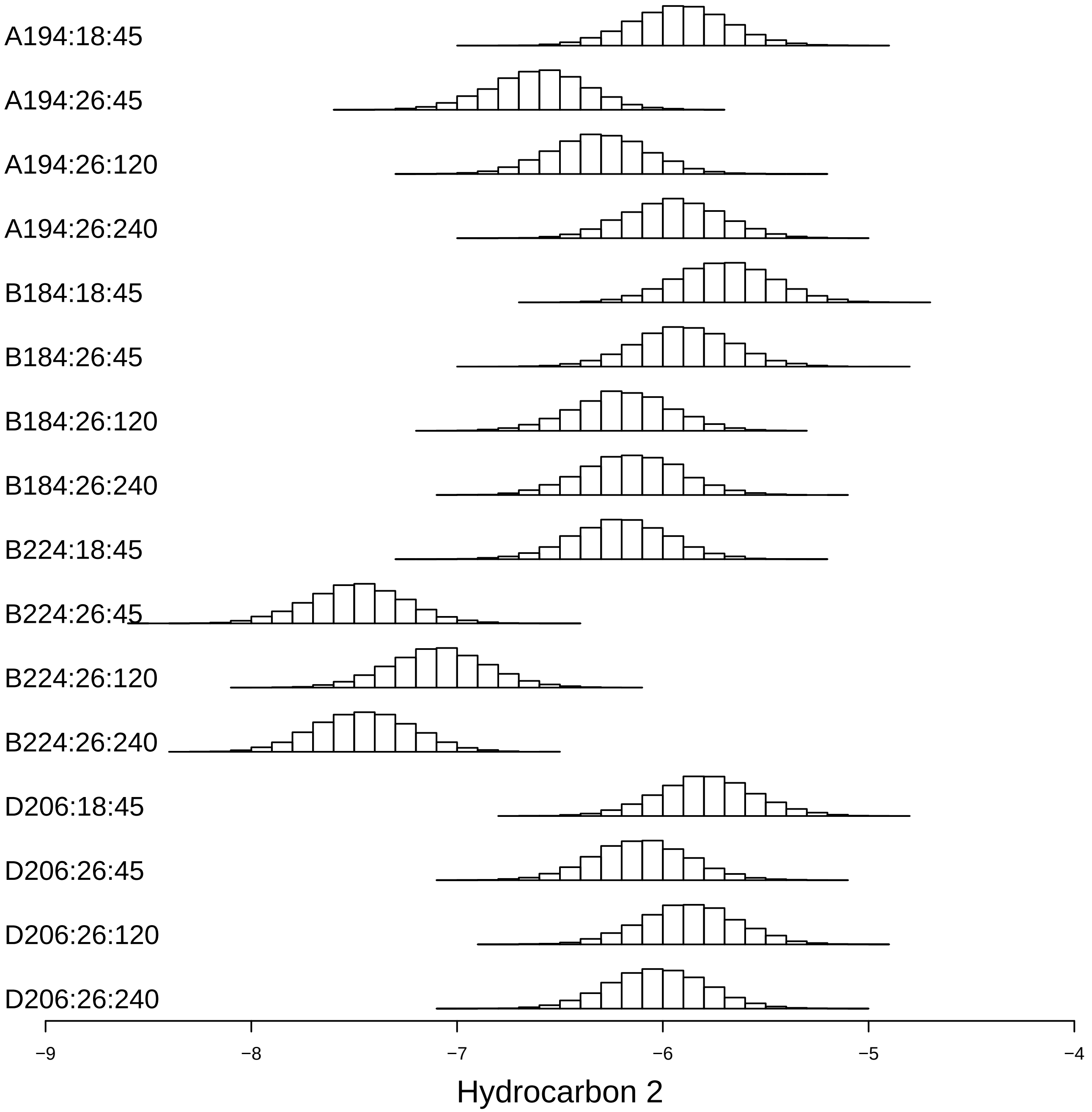
A194:18:45
A194:26:45
A194:26:120
A194:26:240
B184:18:45
B184:26:45
B184:26:120
B184:26:240
B224:18:45
B224:26:45
B224:26:120
B224:26:240
D206:18:45
D206:26:45
D206:26:120
D206:26:240
−9
−8
−7
−6
−5
−4
Hydrocarbon 2

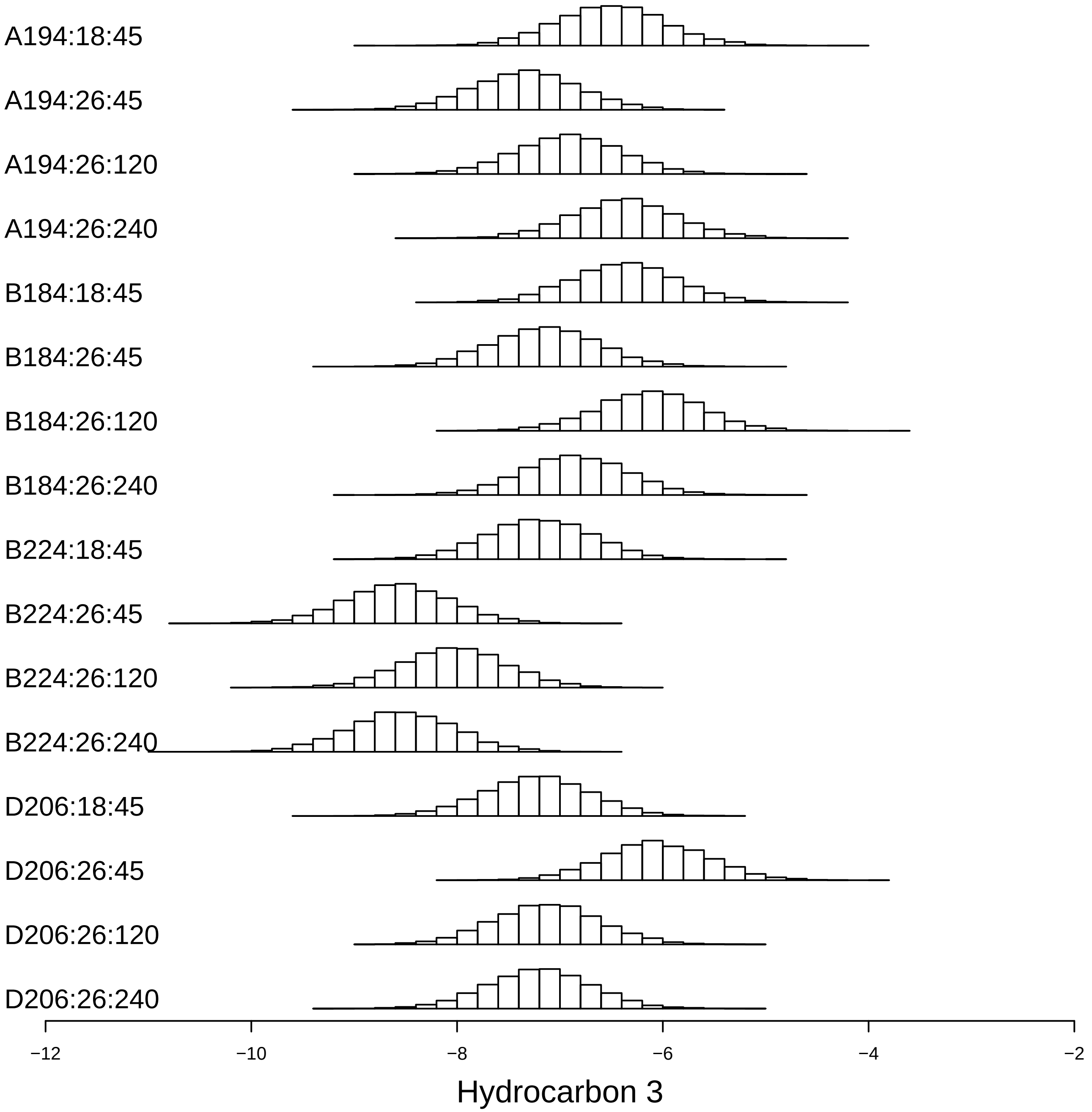
A194:18:45
A194:26:45
A194:26:120
A194:26:240
B184:18:45
B184:26:45
B184:26:120
B184:26:240
B224:18:45
B224:26:45
B224:26:120
B224:26:240
D206:18:45
D206:26:45
D206:26:120
D206:26:240
−12
−10
−8
−6
−4
−2
Hydrocarbon 3

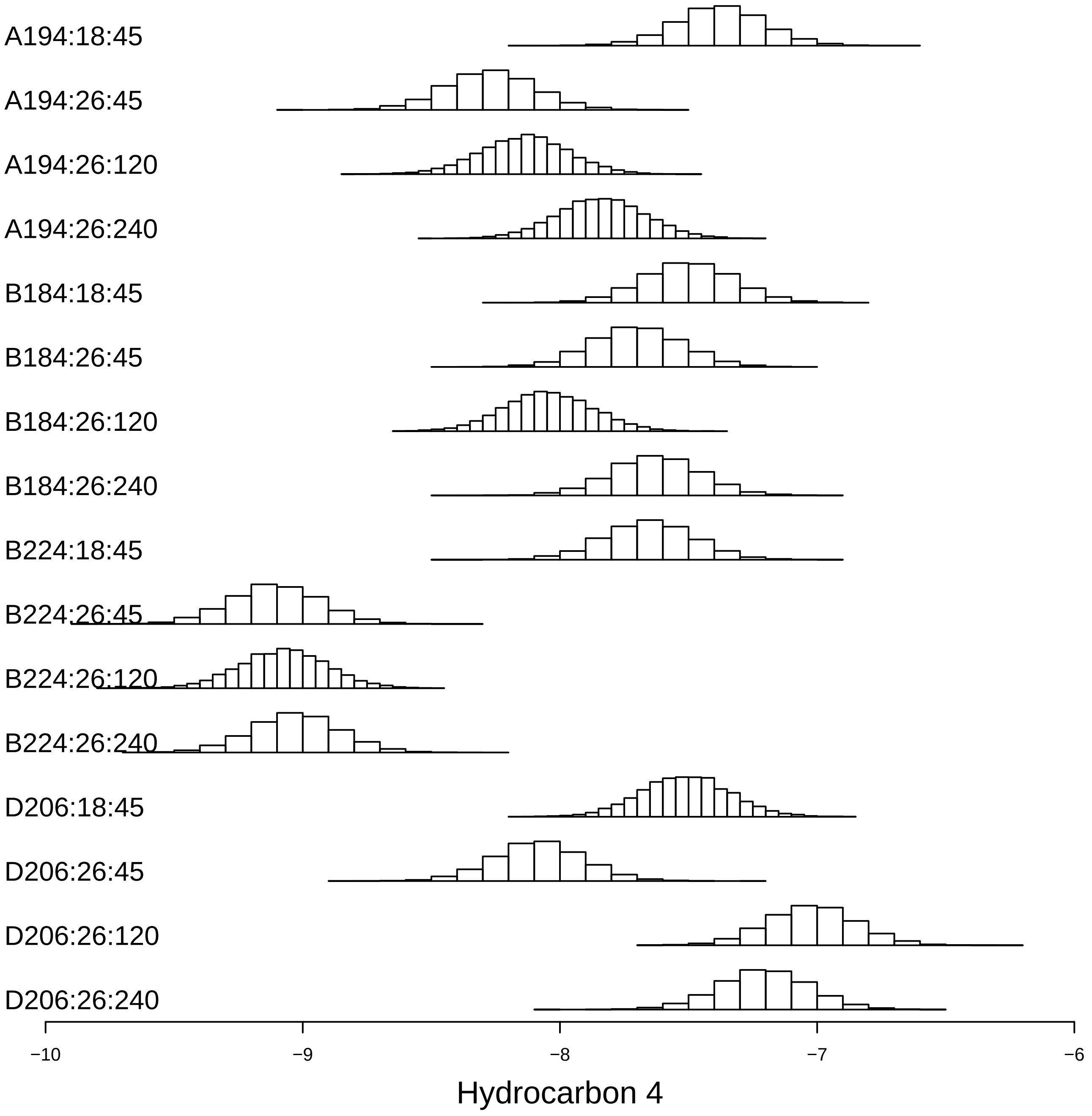
A194:18:45
A194:26:45
A194:26:120
A194:26:240
B184:18:45
B184:26:45
B184:26:120
B184:26:240
B224:18:45
B224:26:45
B224:26:120
B224:26:240
D206:18:45
D206:26:45
D206:26:120
D206:26:240
−10
−9
−8
−7
−6
Hydrocarbon 4

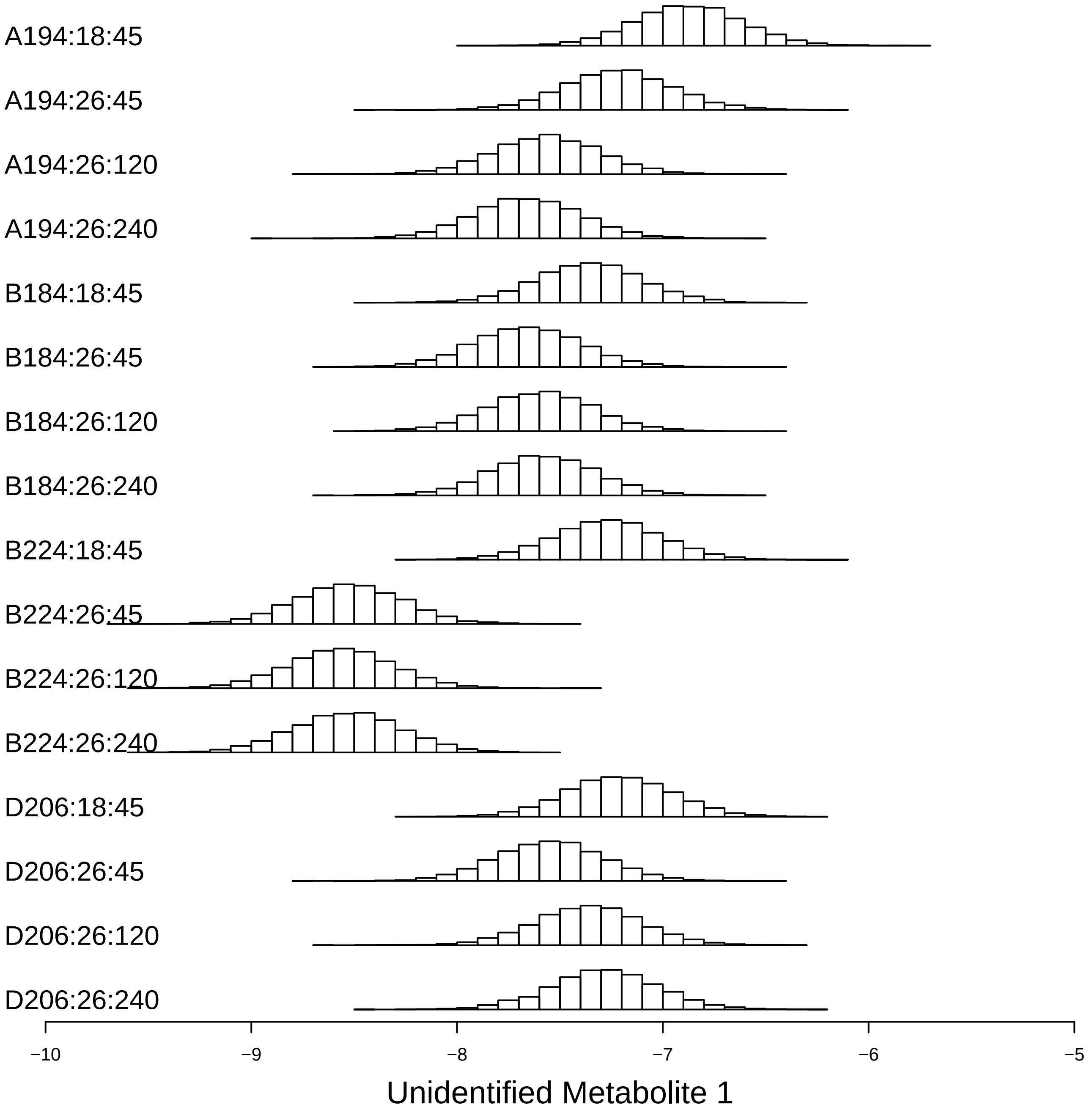

A194:18:45
A194:26:45
A194:26:120
A194:26:240
B184:18:45
B184:26:45
B184:26:120
B184:26:240
B224:18:45
B224:26:45
B224:26:120
B224:26:240
D206:18:45
D206:26:45
D206:26:120
D206:26:240
−10
−9
−8
−7
−6
−5
Unidentified Metabolite 1

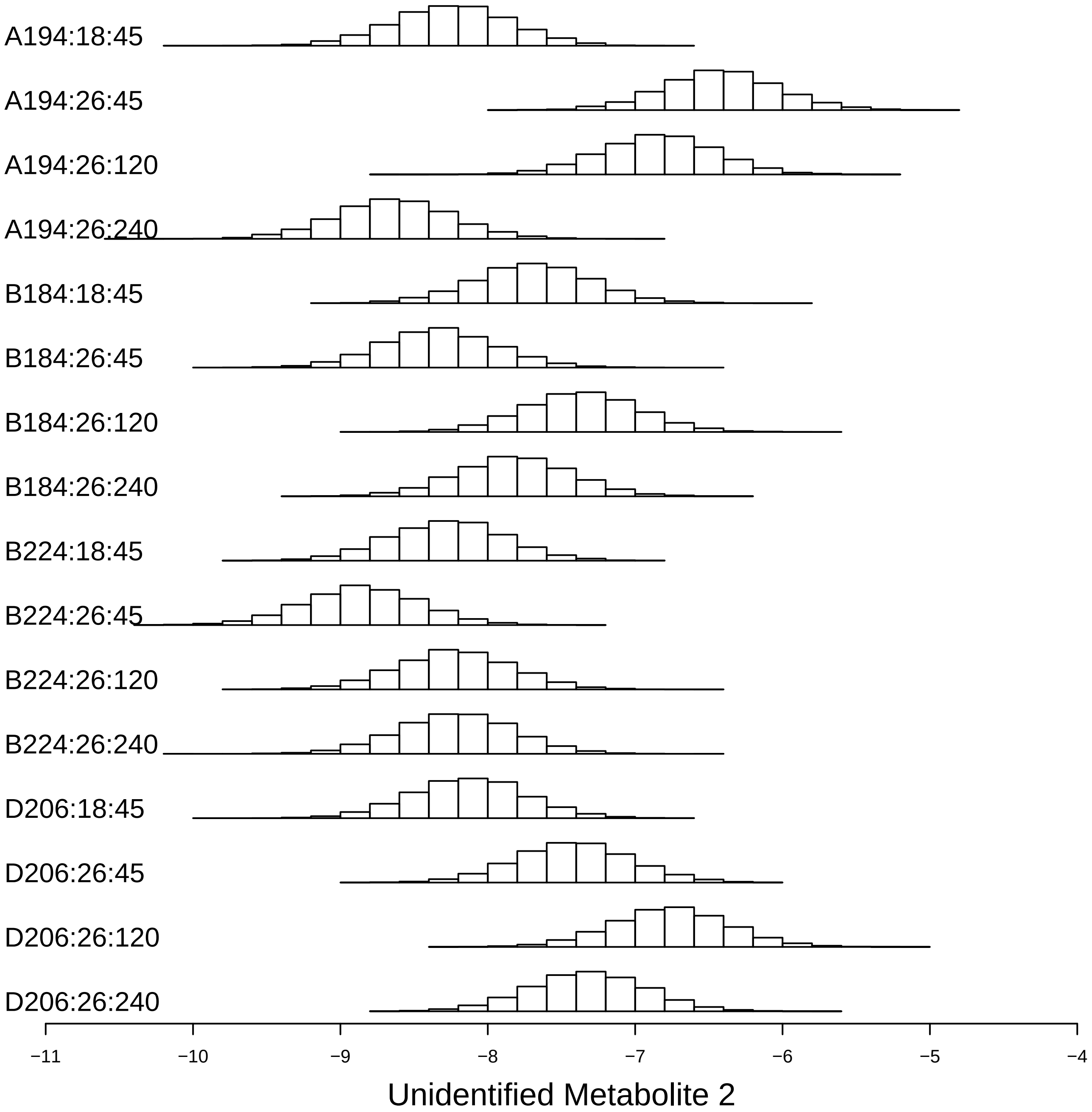
A194:18:45
A194:26:45
A194:26:120
A194:26:240
B184:18:45
B184:26:45
B184:26:120
B184:26:240
B224:18:45
B224:26:45
B224:26:120
B224:26:240
D206:18:45
D206:26:45
D206:26:120
D206:26:240
−11
−10
−9
−8
−7
−6
−5
−4
Unidentified Metabolite 2

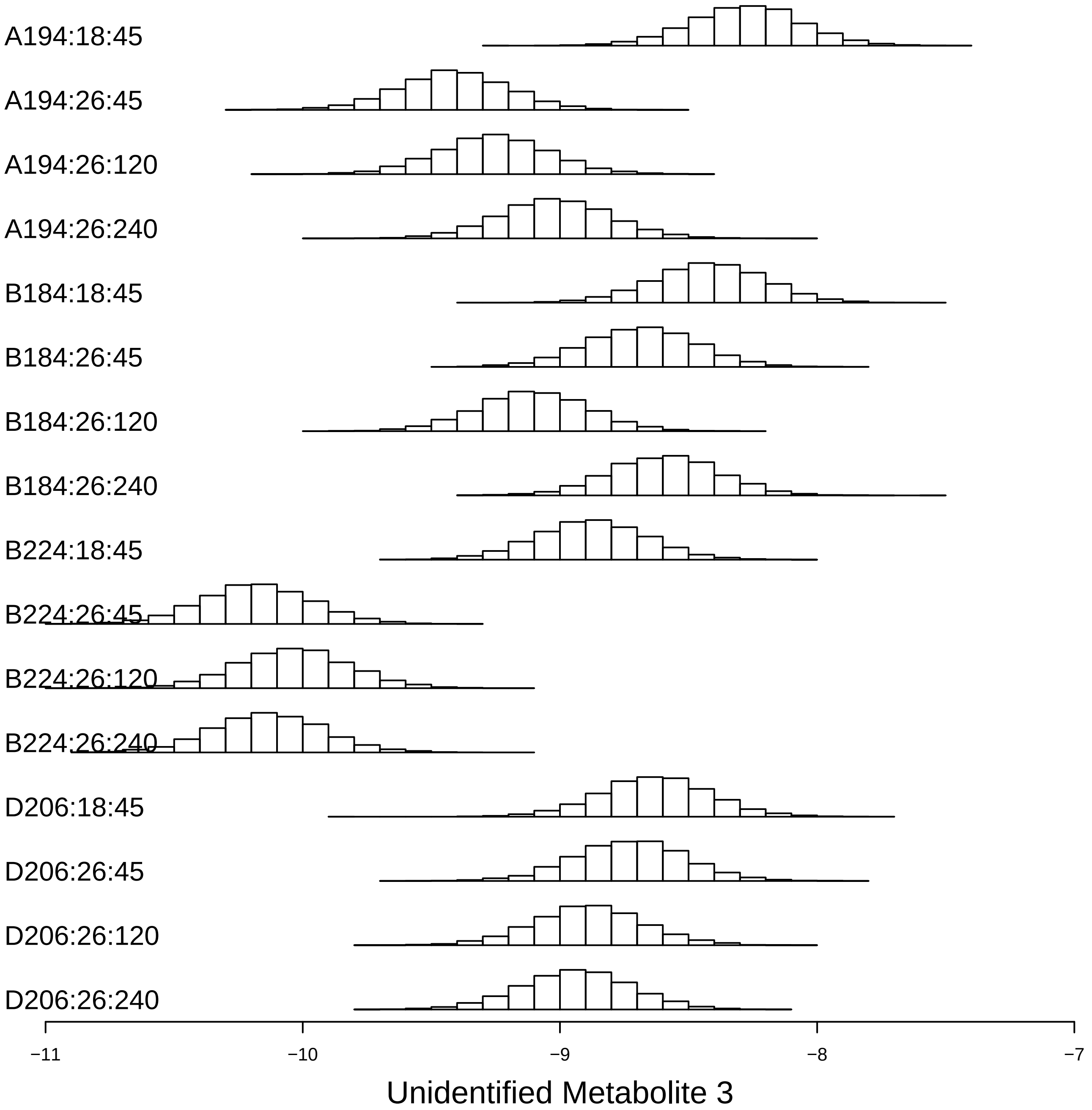
A194:18:45
A194:26:45
A194:26:120
A194:26:240
B184:18:45
B184:26:45
B184:26:120
B184:26:240
B224:18:45
B224:26:45
B224:26:120
B224:26:240
D206:18:45
D206:26:45
D206:26:120
D206:26:240
−11
−10
−9
−8
−7
Unidentified Metabolite 3

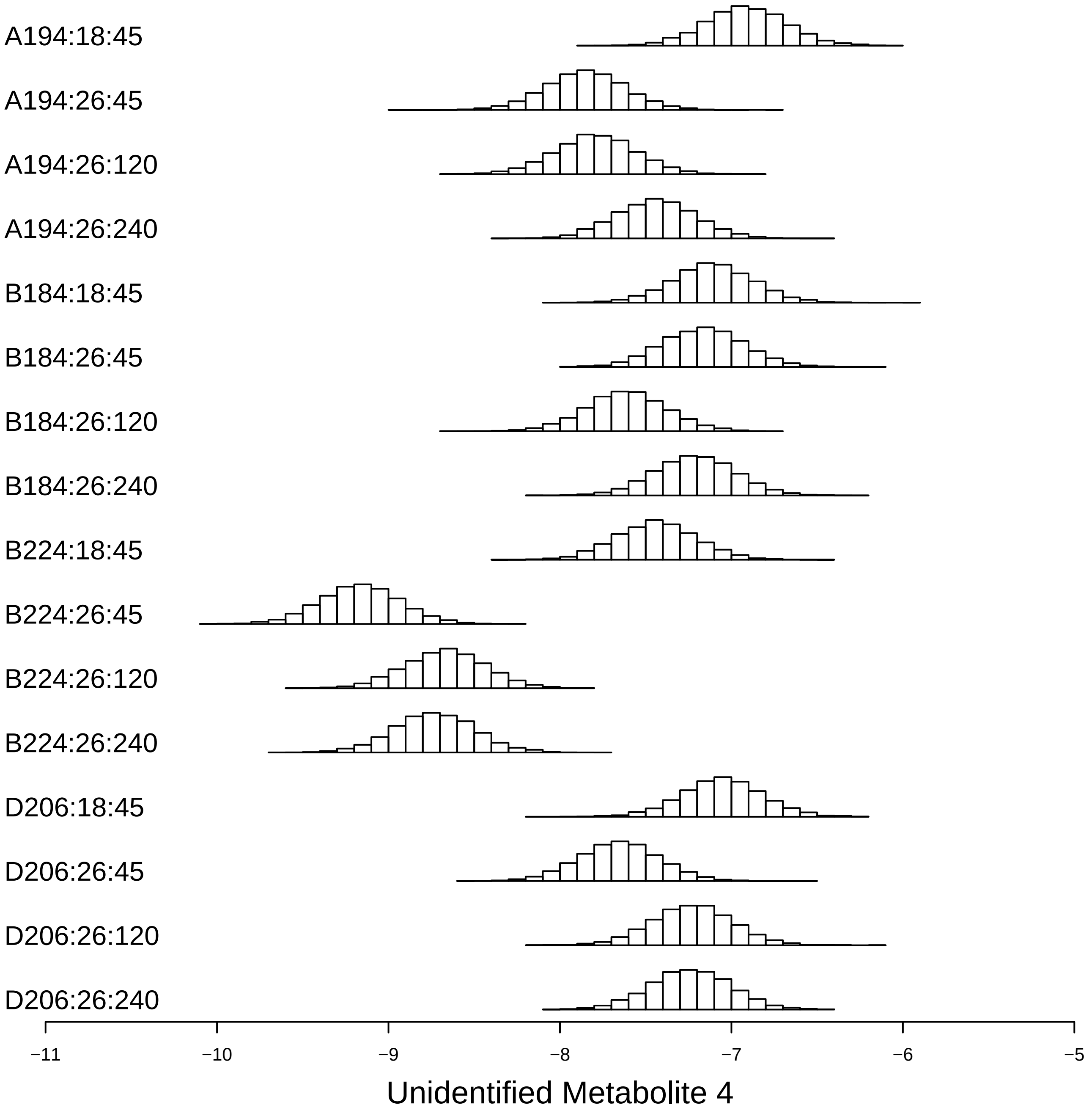
A194:18:45
A194:26:45
A194:26:120
A194:26:240
B184:18:45
B184:26:45
B184:26:120
B184:26:240
B224:18:45
B224:26:45
B224:26:120
B224:26:240
D206:18:45
D206:26:45
D206:26:120
D206:26:240
−11
−10
−9
−8
−7
−6
−5
Unidentified Metabolite 4

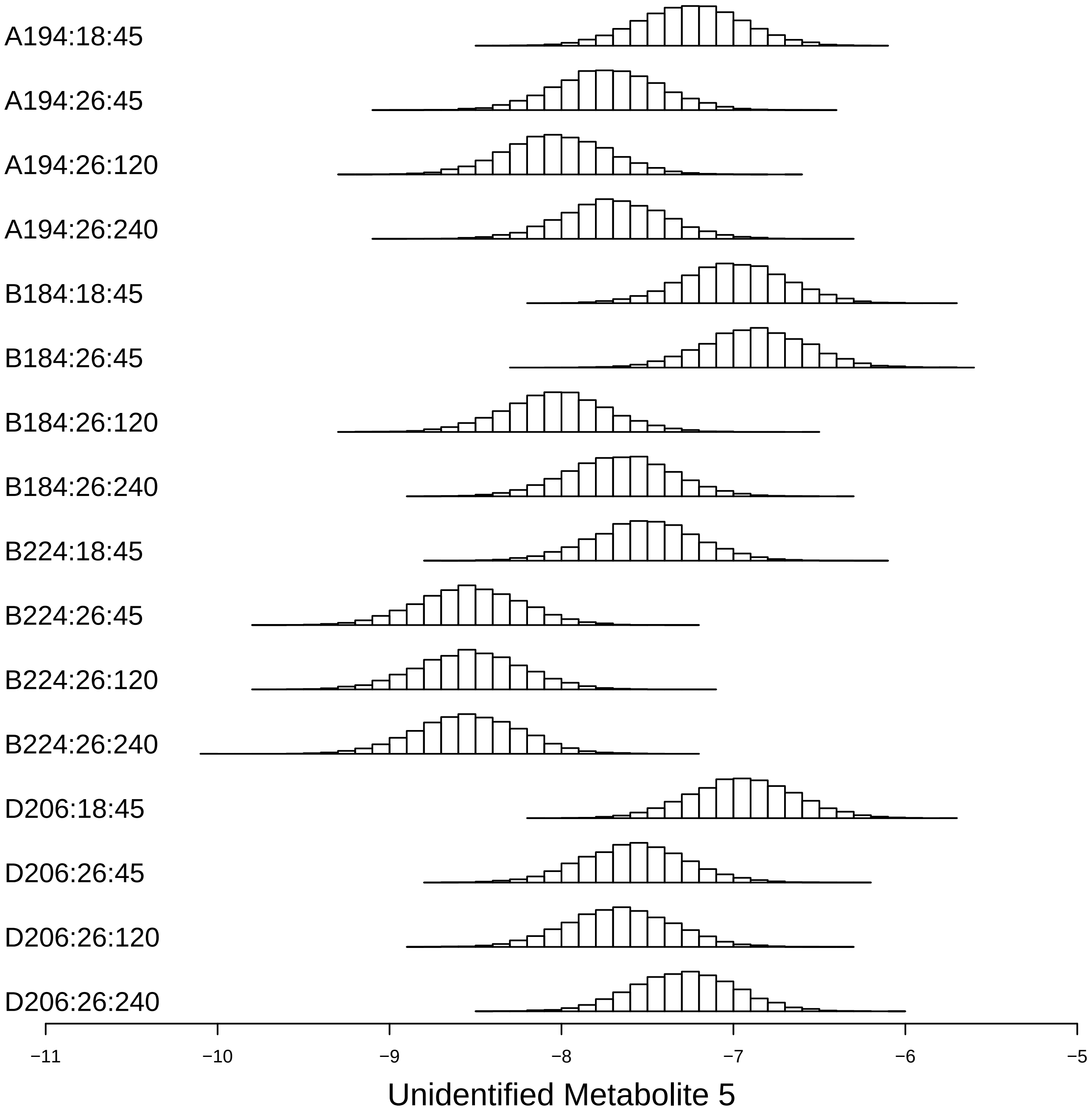
A194:18:45
A194:26:45
A194:26:120
A194:26:240
B184:18:45
B184:26:45
B184:26:120
B184:26:240
B224:18:45
B224:26:45
B224:26:120
B224:26:240
D206:18:45
D206:26:45
D206:26:120
D206:26:240
−11
−10
−9
−8
−7
−6
−5
Unidentified Metabolite 5

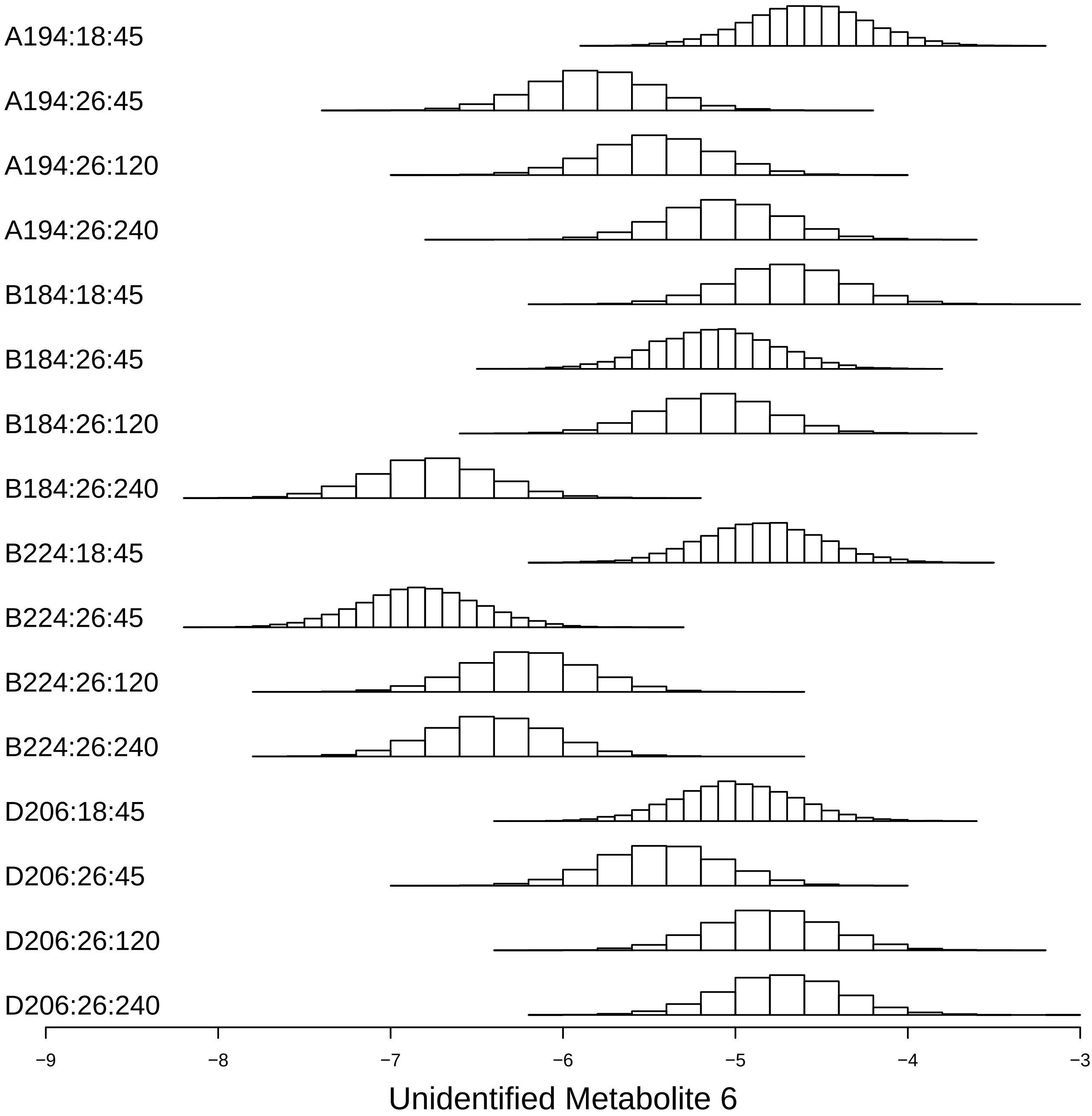
A194:18:45
A194:26:45
A194:26:120
A194:26:240
B184:18:45
B184:26:45
B184:26:120
B184:26:240
B224:18:45
B224:26:45
B224:26:120
B224:26:240
D206:18:45
D206:26:45
D206:26:120
D206:26:240
−9
−8
−7
−6
−5
−4
−3
Unidentified Metabolite 6

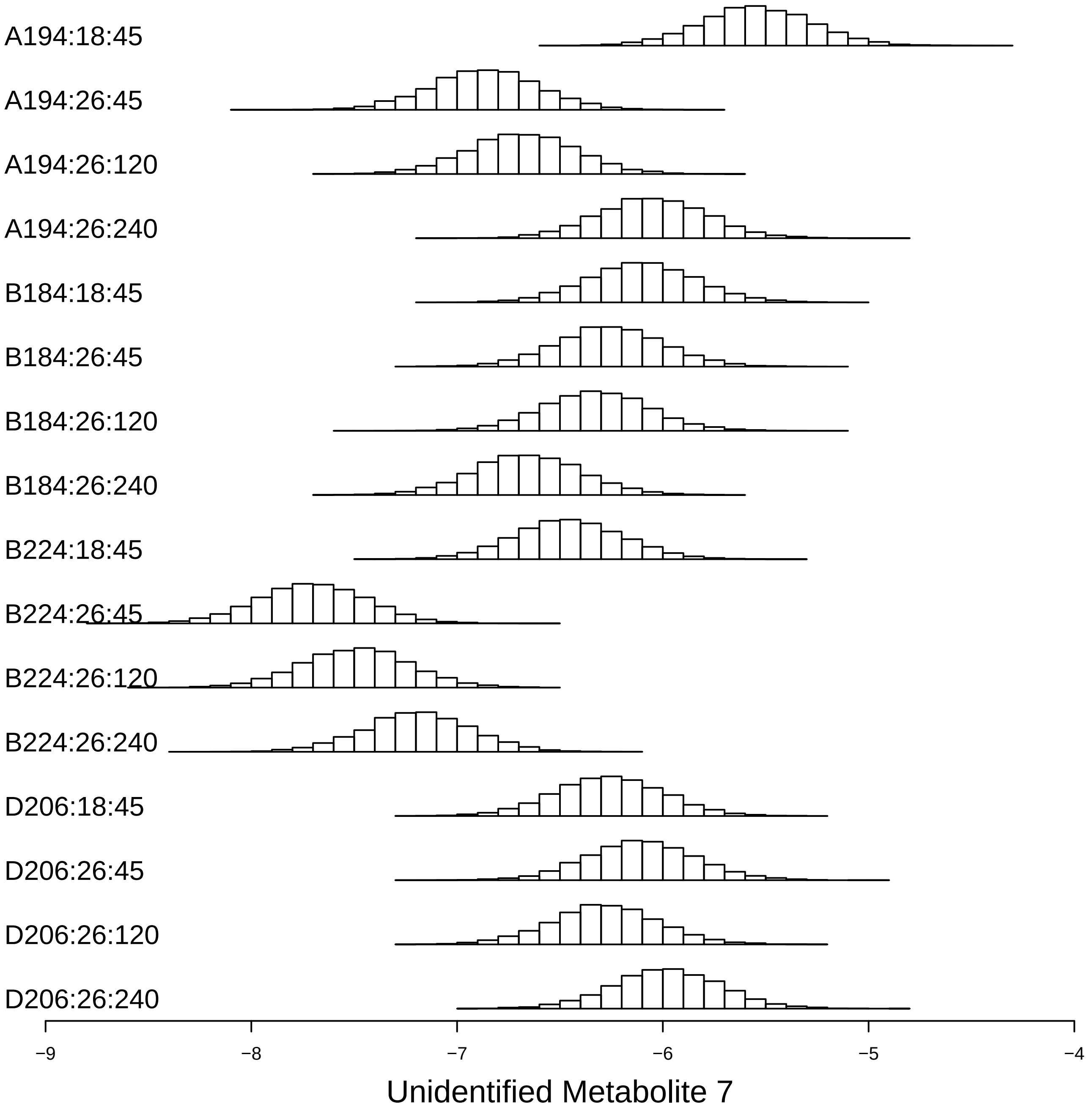
A194:18:45
A194:26:45
A194:26:120
A194:26:240
B184:18:45
B184:26:45
B184:26:120
B184:26:240
B224:18:45
B224:26:45
B224:26:120
B224:26:240
D206:18:45
D206:26:45
D206:26:120
D206:26:240
−9
−8
−7
−6
−5
−4
Unidentified Metabolite 7

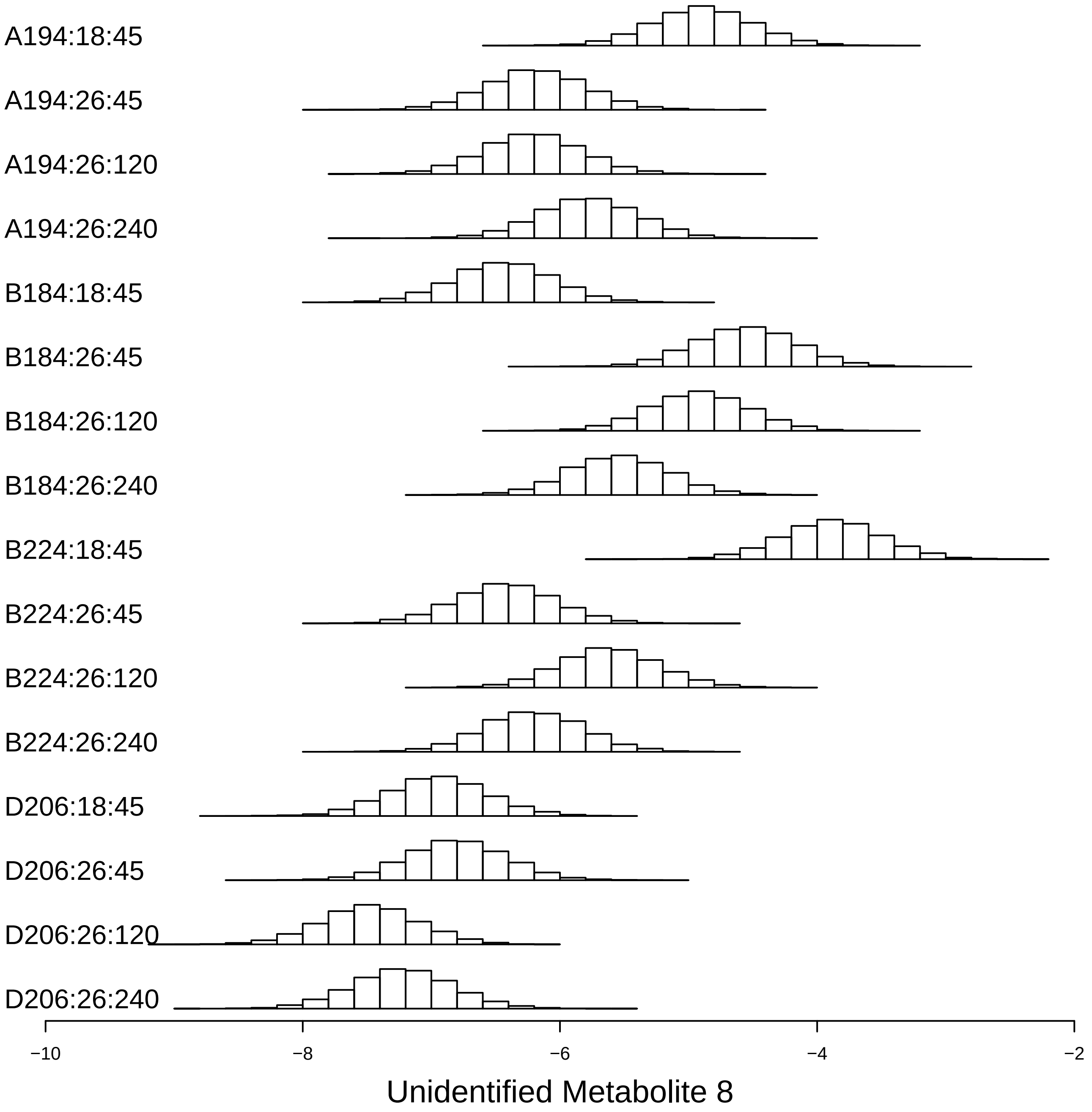
A194:18:45
A194:26:45
A194:26:120
A194:26:240
B184:18:45
B184:26:45
B184:26:120
B184:26:240
B224:18:45
B224:26:45
B224:26:120
B224:26:240
D206:18:45
D206:26:45
D206:26:120
D206:26:240
−10
−8
−6
−4
−2
Unidentified Metabolite 8

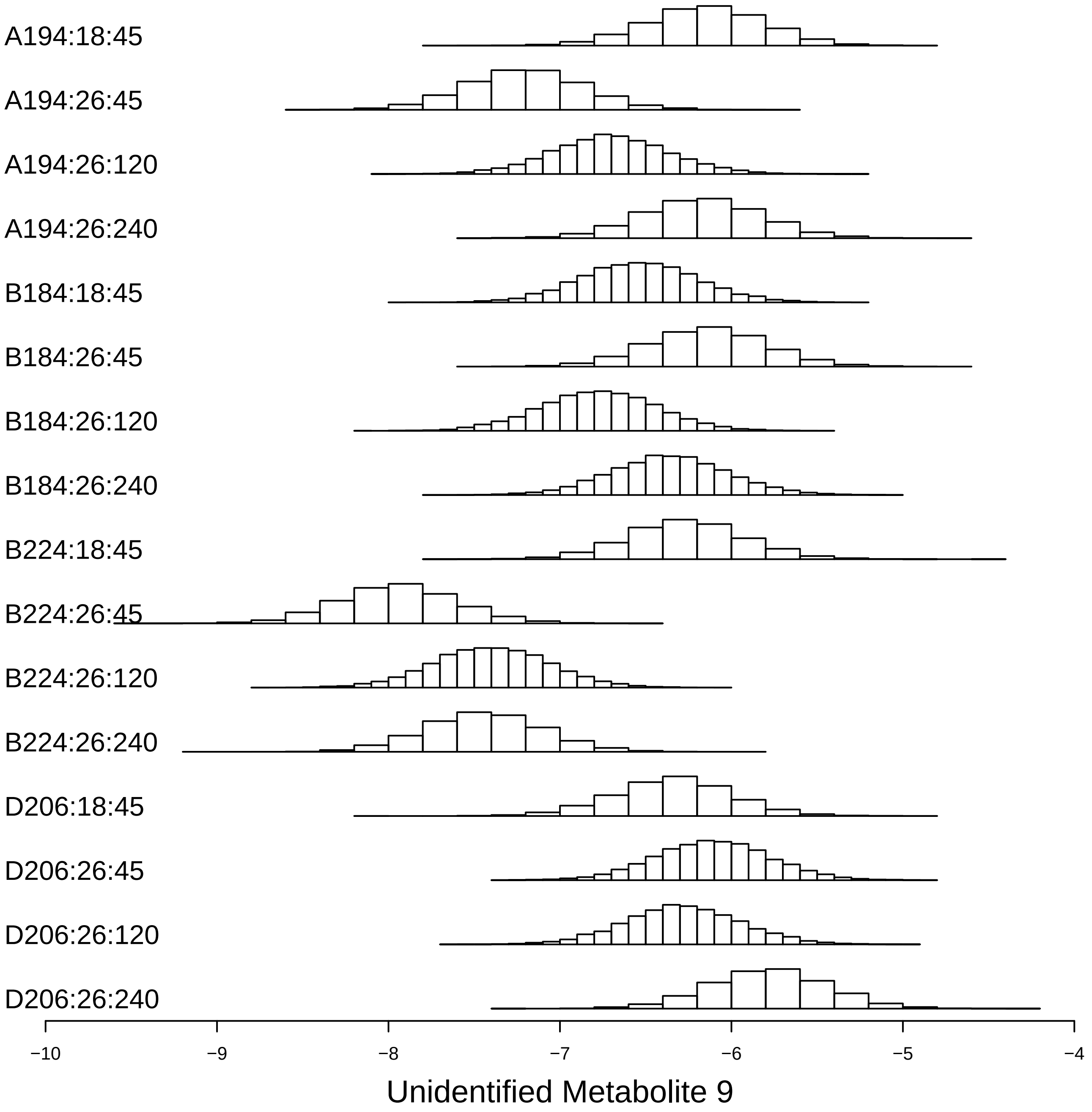
A194:18:45
A194:26:45
A194:26:120
A194:26:240
B184:18:45
B184:26:45
B184:26:120
B184:26:240
B224:18:45
B224:26:45
B224:26:120
B224:26:240
D206:18:45
D206:26:45
D206:26:120
D206:26:240
−10
−9
−8
−7
−6
−5
−4
Unidentified Metabolite 9

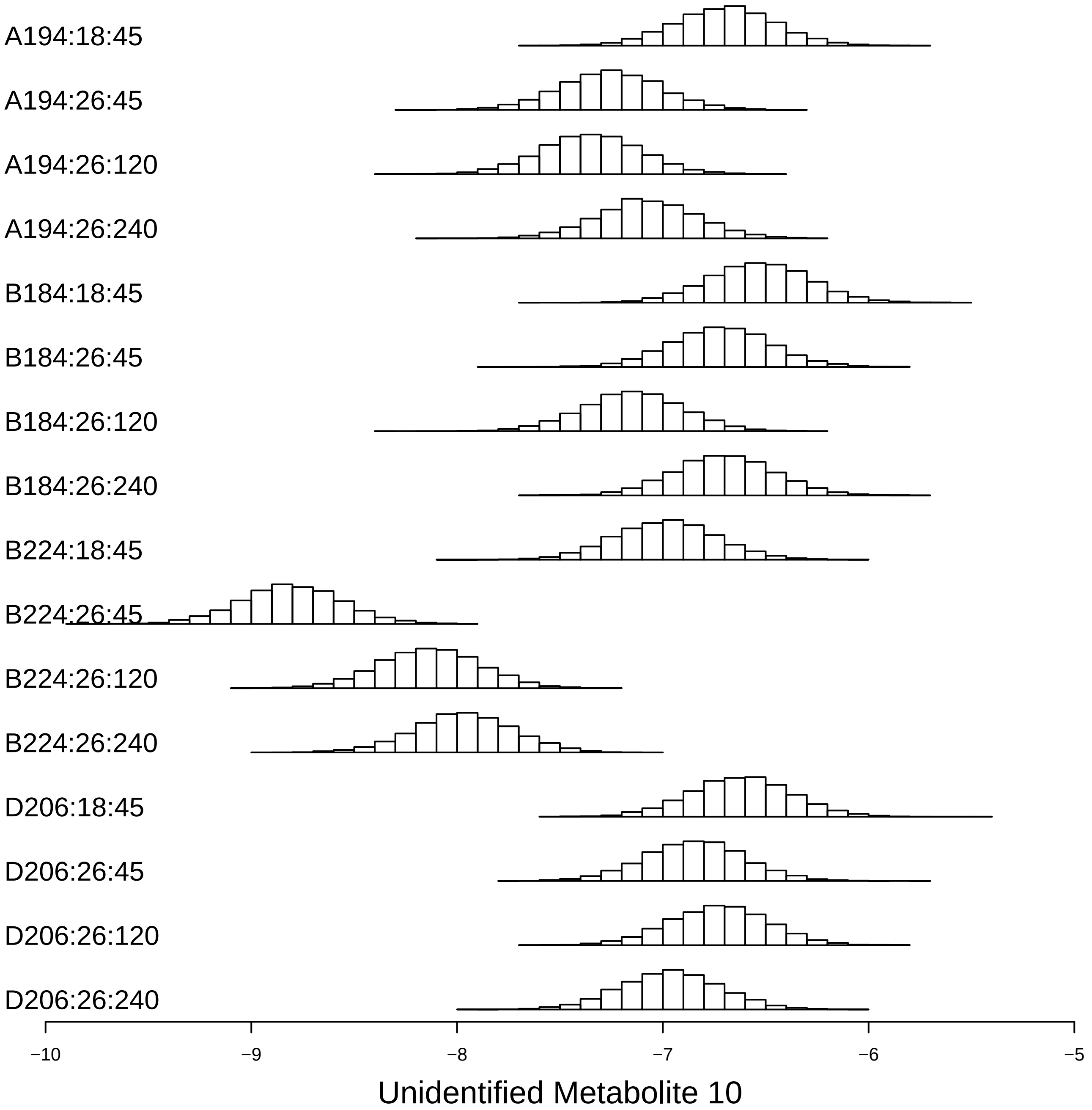

A194:18:45
A194:26:45
A194:26:120
A194:26:240
B184:18:45
B184:26:45
B184:26:120
B184:26:240
B224:18:45
B224:26:45
B224:26:120
B224:26:240
D206:18:45
D206:26:45
D206:26:120
D206:26:240
−10
−9
−8
−7
−6
−5
Unidentified Metabolite 10

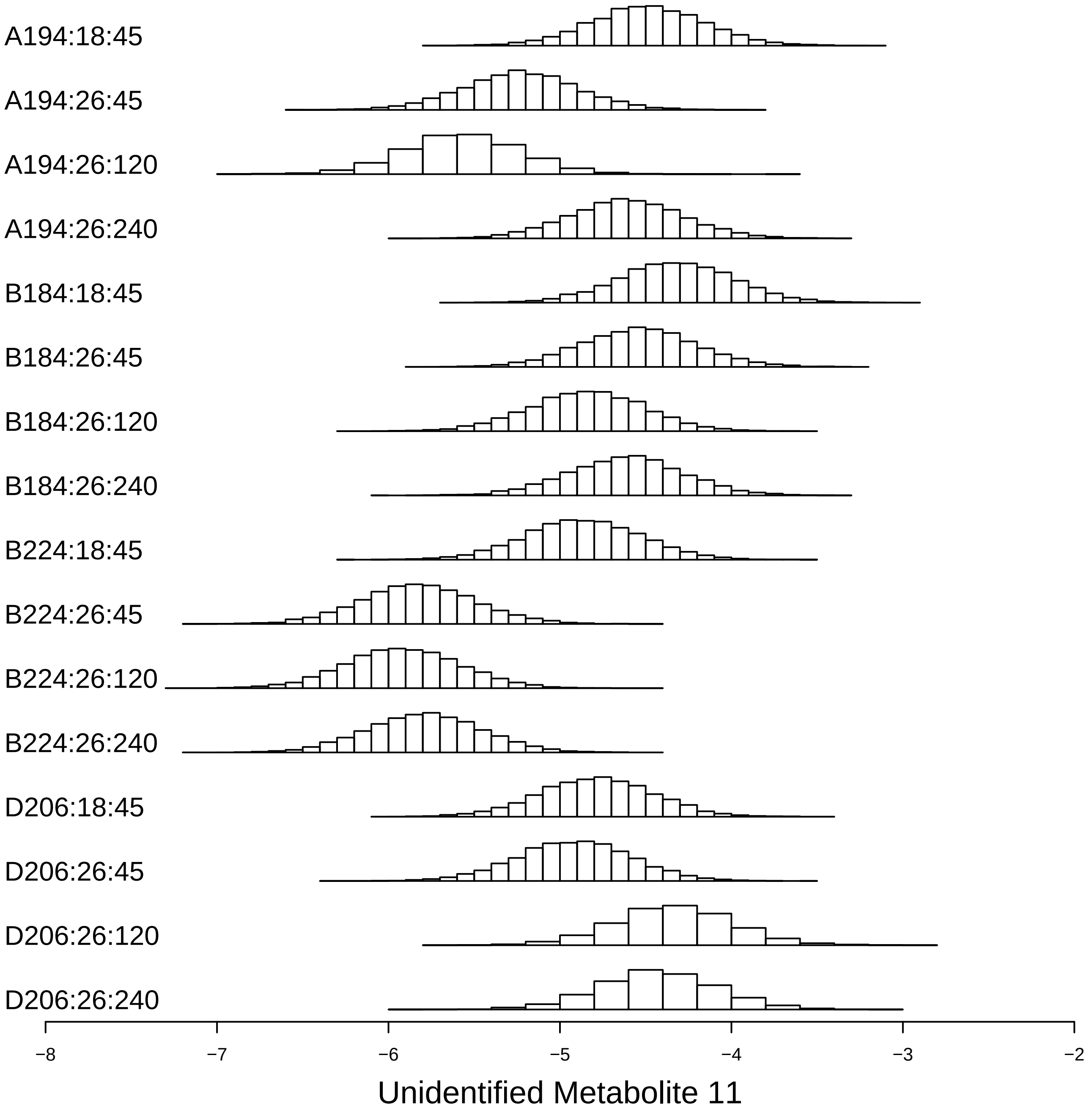
A194:18:45
A194:26:45
A194:26:120
A194:26:240
B184:18:45
B184:26:45
B184:26:120
B184:26:240
B224:18:45
B224:26:45
B224:26:120
B224:26:240
D206:18:45
D206:26:45
D206:26:120
D206:26:240
−8
−7
−6
−5
−4
−3
−2
Unidentified Metabolite 11

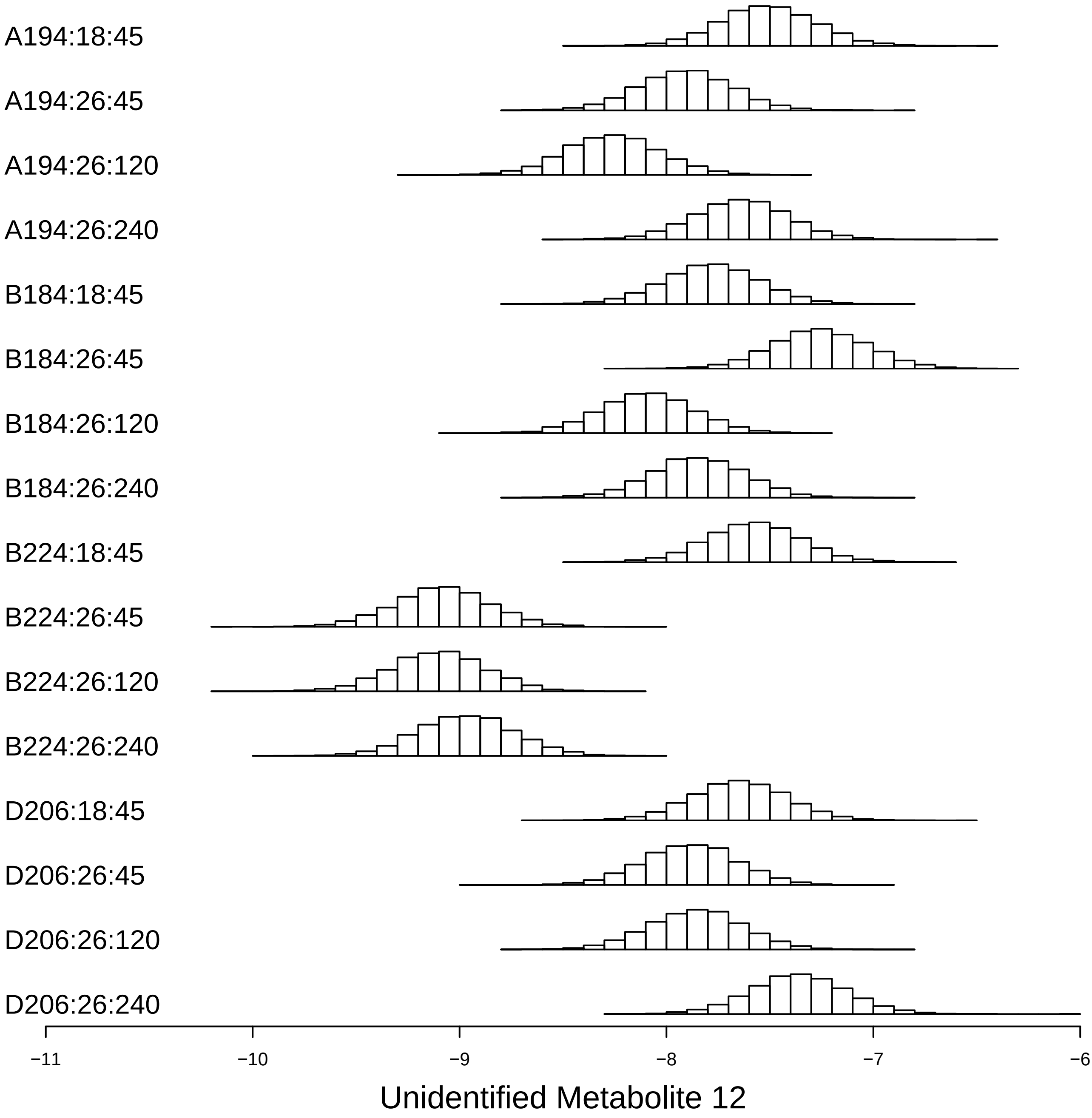
A194:18:45
A194:26:45
A194:26:120
A194:26:240
B184:18:45
B184:26:45
B184:26:120
B184:26:240
B224:18:45
B224:26:45
B224:26:120
B224:26:240
D206:18:45
D206:26:45
D206:26:120
D206:26:240
−11
−10
−9
−8
−7
−6
Unidentified Metabolite 12

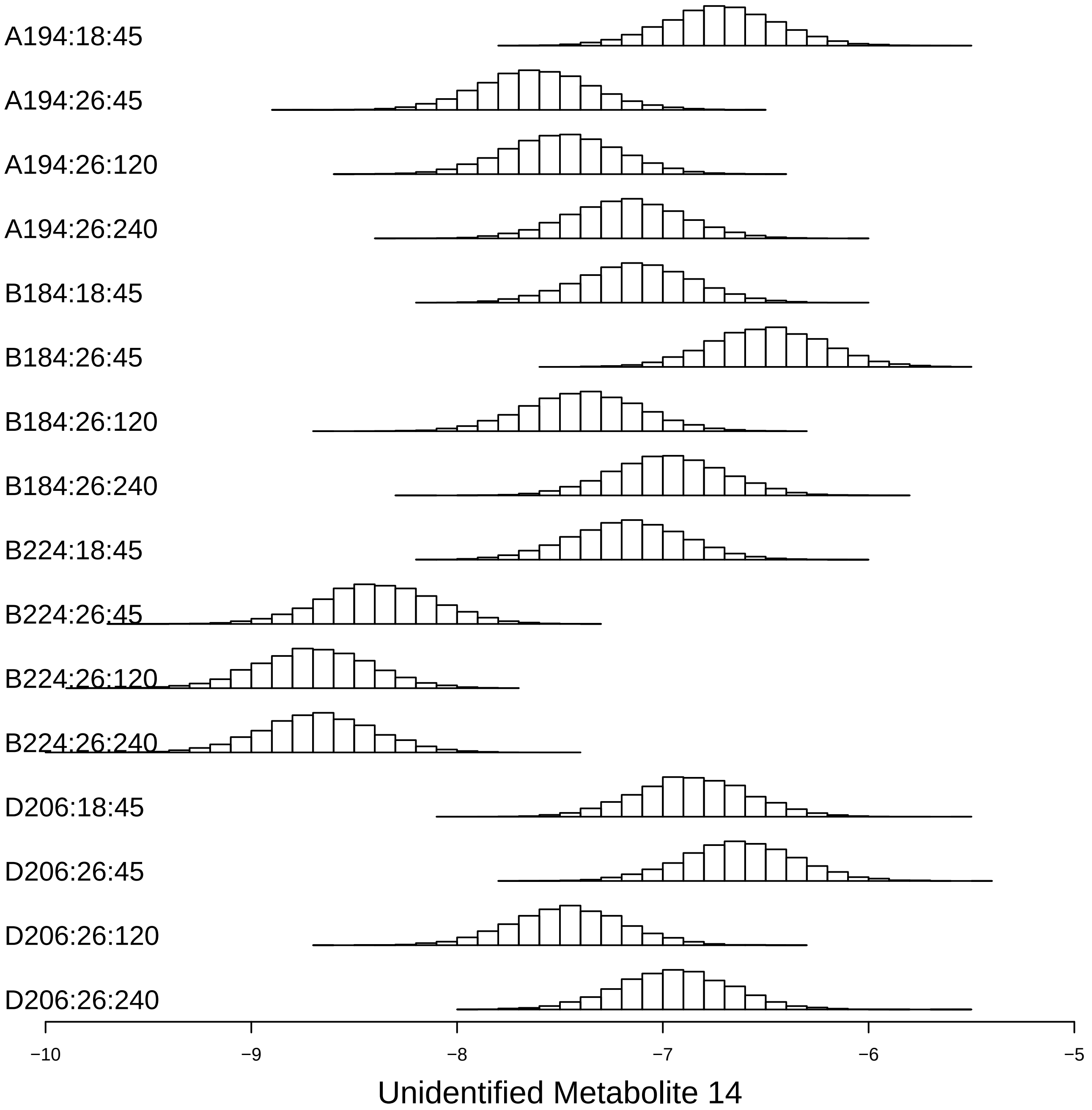
A194:18:45
A194:26:45
A194:26:120
A194:26:240
B184:18:45
B184:26:45
B184:26:120
B184:26:240
B224:18:45
B224:26:45
B224:26:120
B224:26:240
D206:18:45
D206:26:45
D206:26:120
D206:26:240
−10
−9
−8
−7
−6
−5
Unidentified Metabolite 14

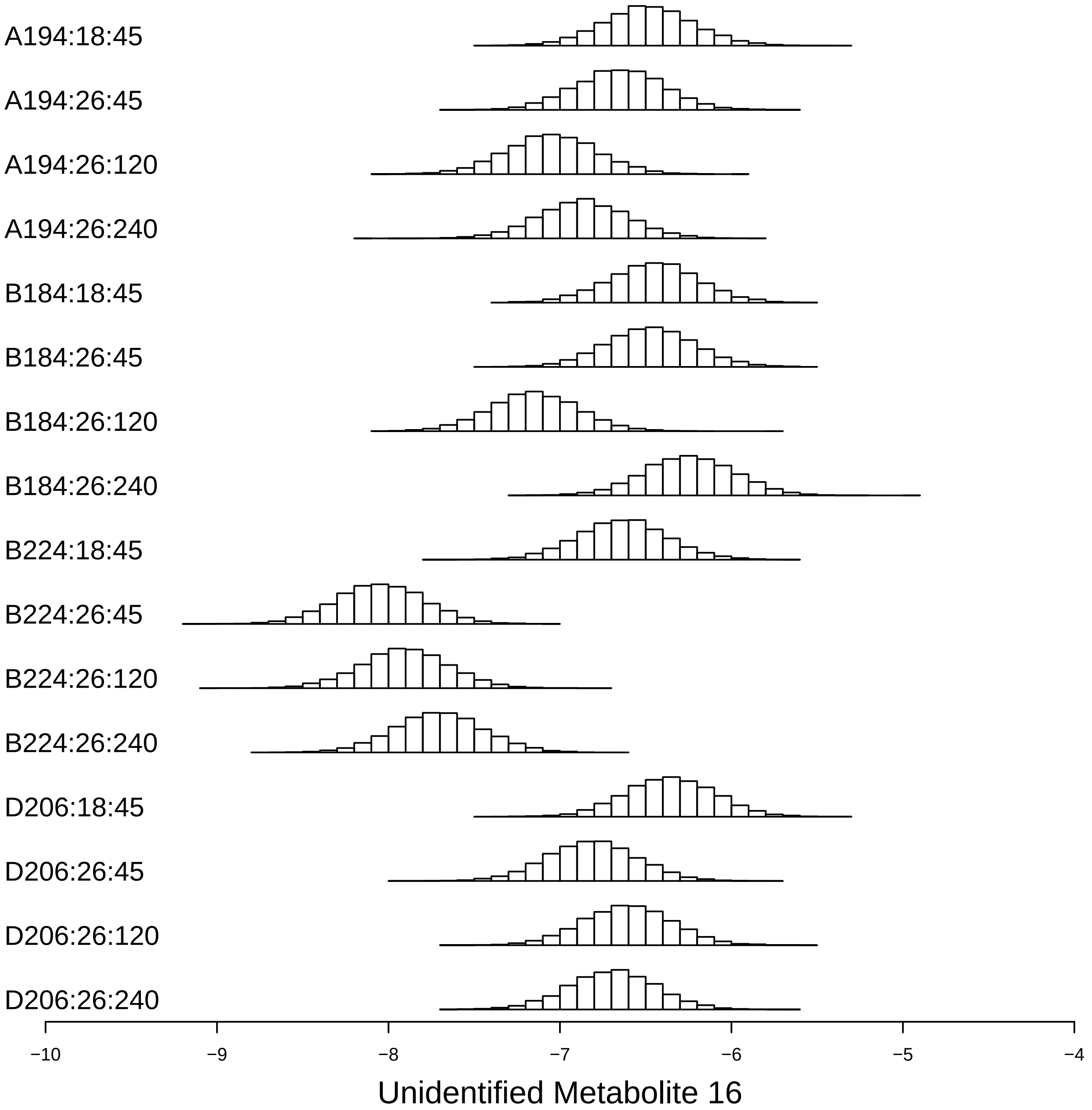

A194:18:45
A194:26:45
A194:26:120
A194:26:240
B184:18:45
B184:26:45
B184:26:120
B184:26:240
B224:18:45
B224:26:45
B224:26:120
B224:26:240
D206:18:45
D206:26:45
D206:26:120
D206:26:240
−10
−9
−8
−7
−6
−5
−4
Unidentified Metabolite 16

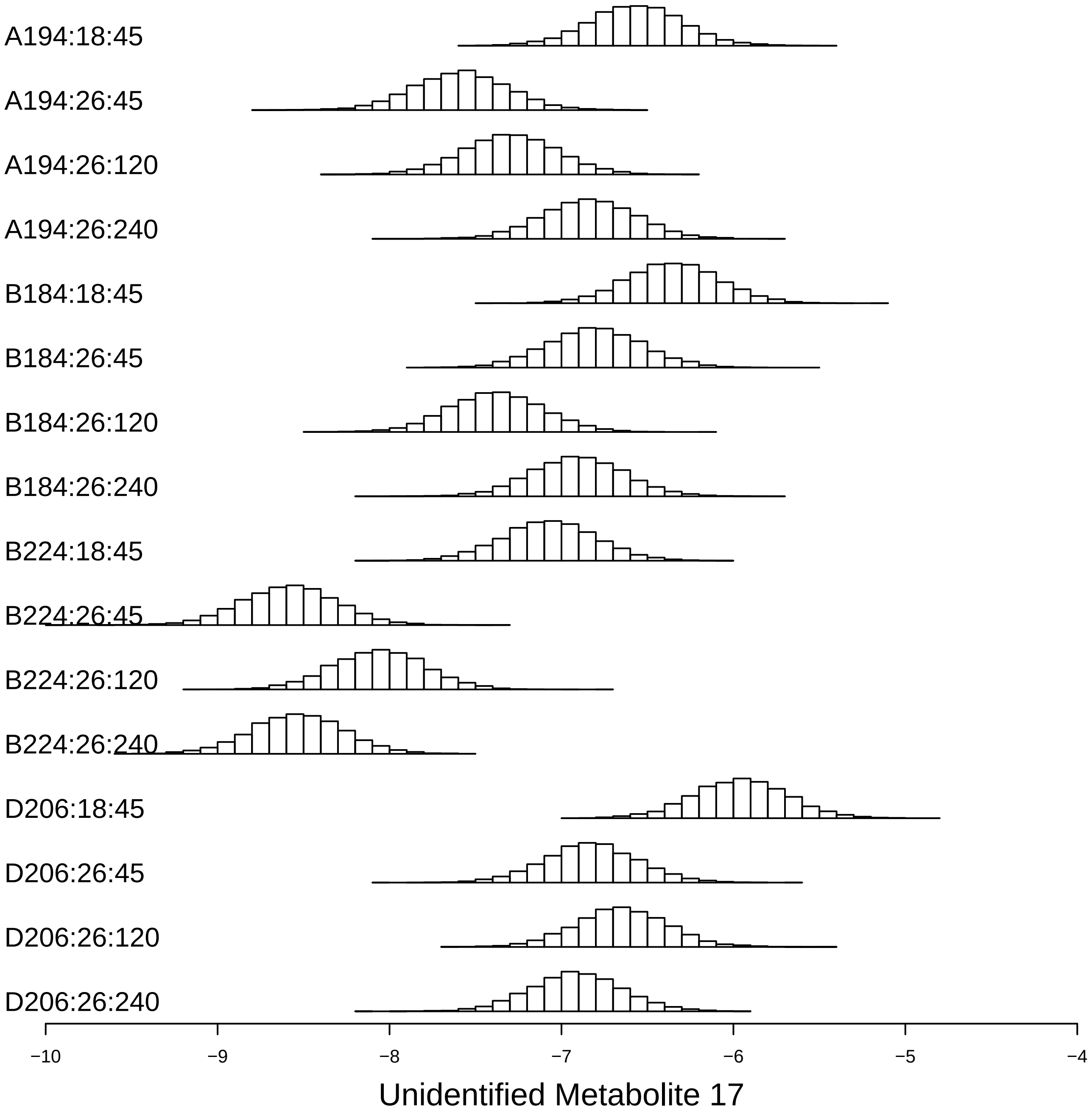
A194:18:45
A194:26:45
A194:26:120
A194:26:240
B184:18:45
B184:26:45
B184:26:120
B184:26:240
B224:18:45
B224:26:45
B224:26:120
B224:26:240
D206:18:45
D206:26:45
D206:26:120
D206:26:240
−10
−9
−8
−7
−6
−5
−4
Unidentified Metabolite 17

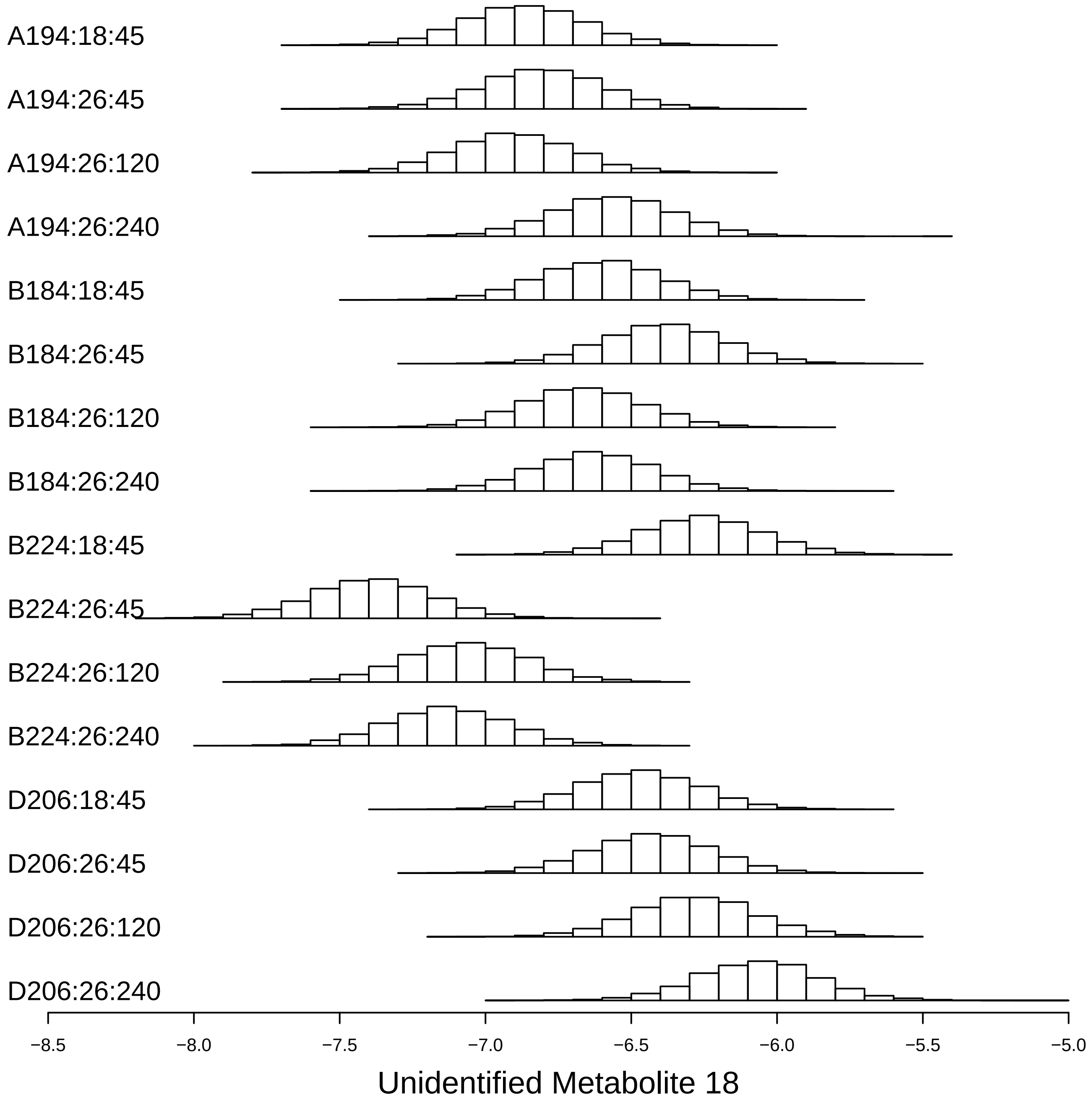
A194:18:45
A194:26:45
A194:26:120
A194:26:240
B184:18:45
B184:26:45
B184:26:120
B184:26:240
B224:18:45
B224:26:45
B224:26:120
B224:26:240
D206:18:45
D206:26:45
D206:26:120
D206:26:240
−8.5
−8.0
−7.5
−7.0
−6.5
−6.0
−5.5
−5.0
Unidentified Metabolite 18

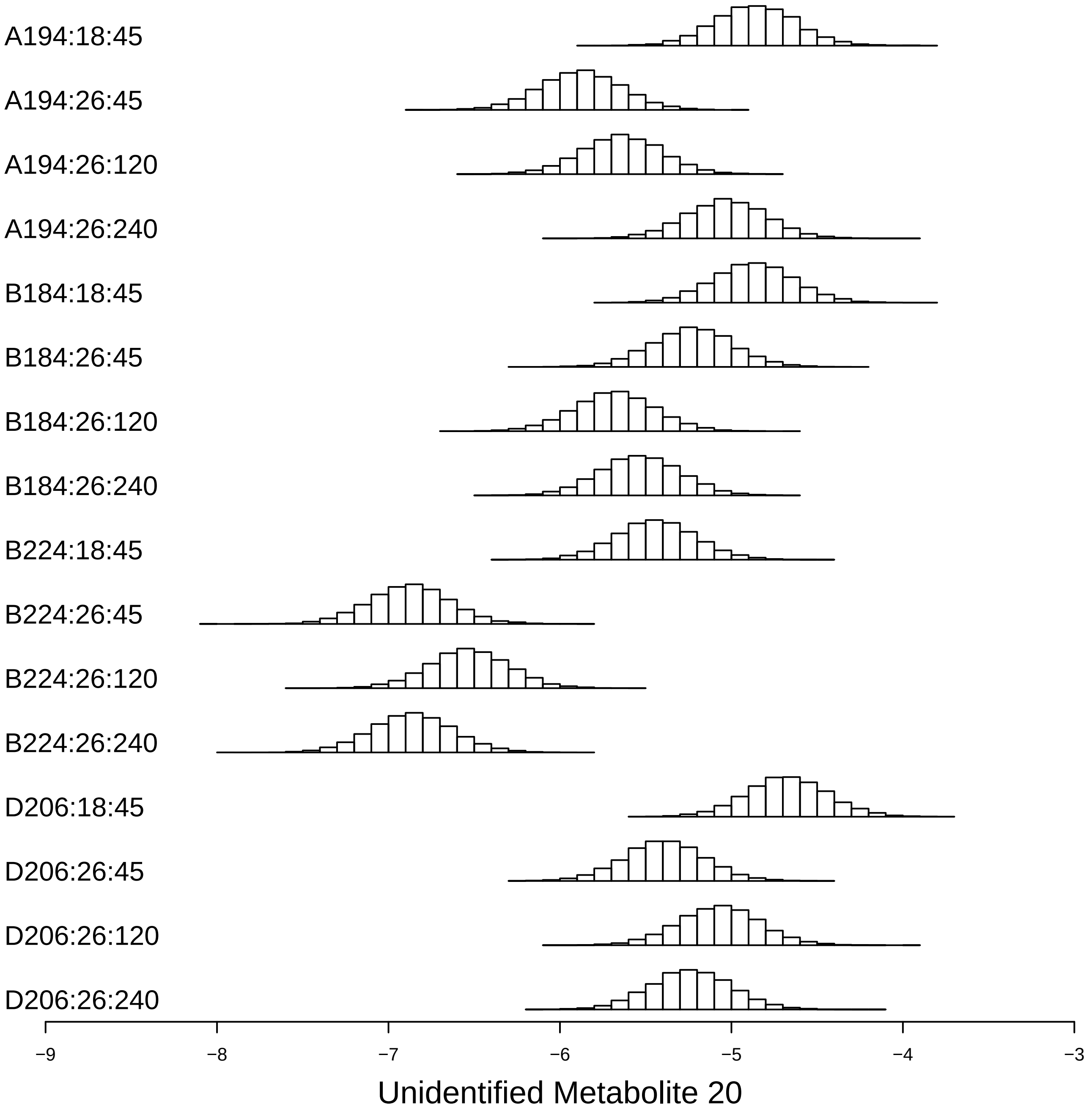
A194:18:45
A194:26:45
A194:26:120
A194:26:240
B184:18:45
B184:26:45
B184:26:120
B184:26:240
B224:18:45
B224:26:45
B224:26:120
B224:26:240
D206:18:45
D206:26:45
D206:26:120
D206:26:240
−9
−8
−7
−6
−5
−4
−3
Unidentified Metabolite 20

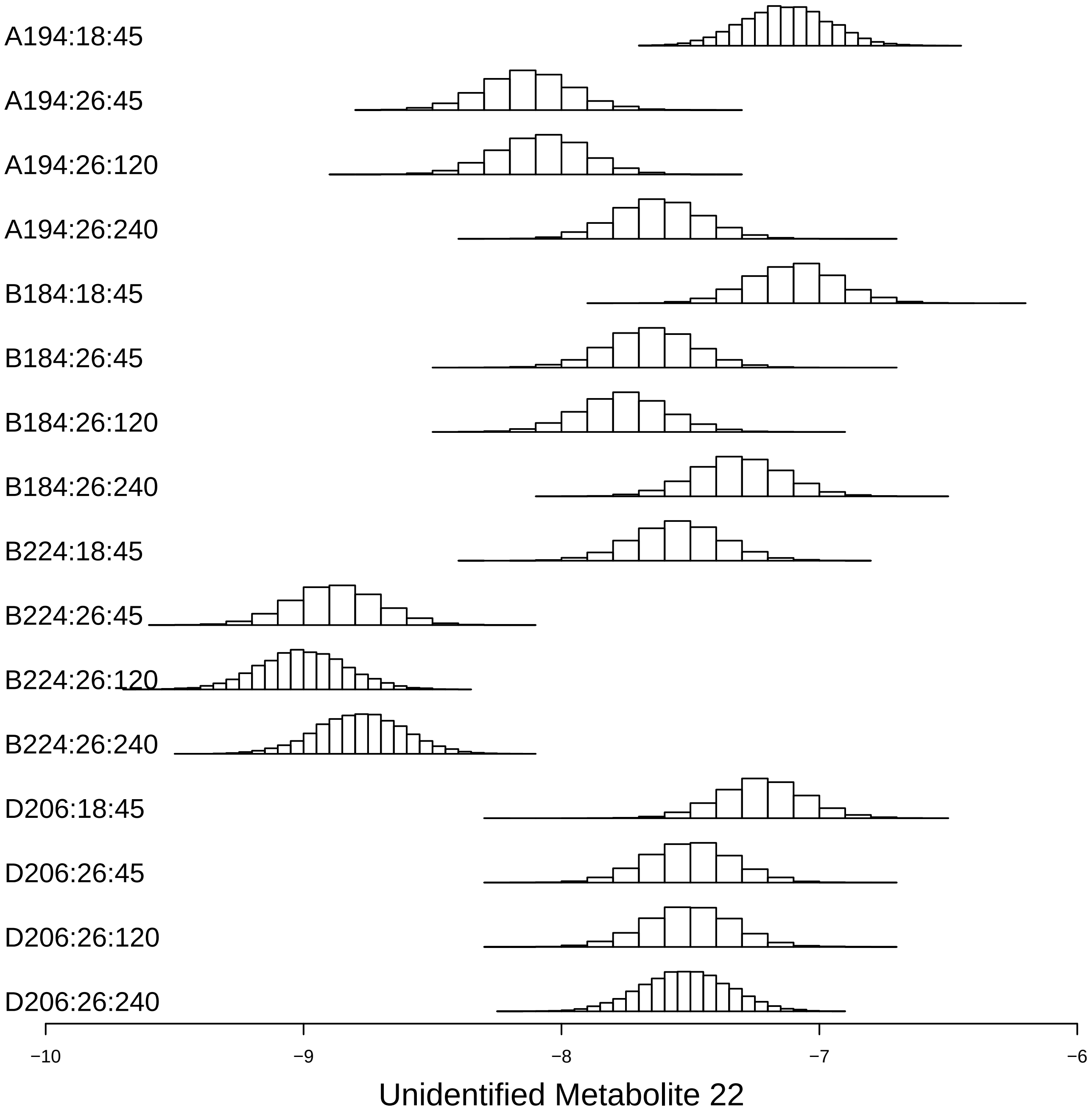
A194:18:45
A194:26:45
A194:26:120
A194:26:240
B184:18:45
B184:26:45
B184:26:120
B184:26:240
B224:18:45
B224:26:45
B224:26:120
B224:26:240
D206:18:45
D206:26:45
D206:26:120
D206:26:240
−10
−9
−8
−7
−6
Unidentified Metabolite 22

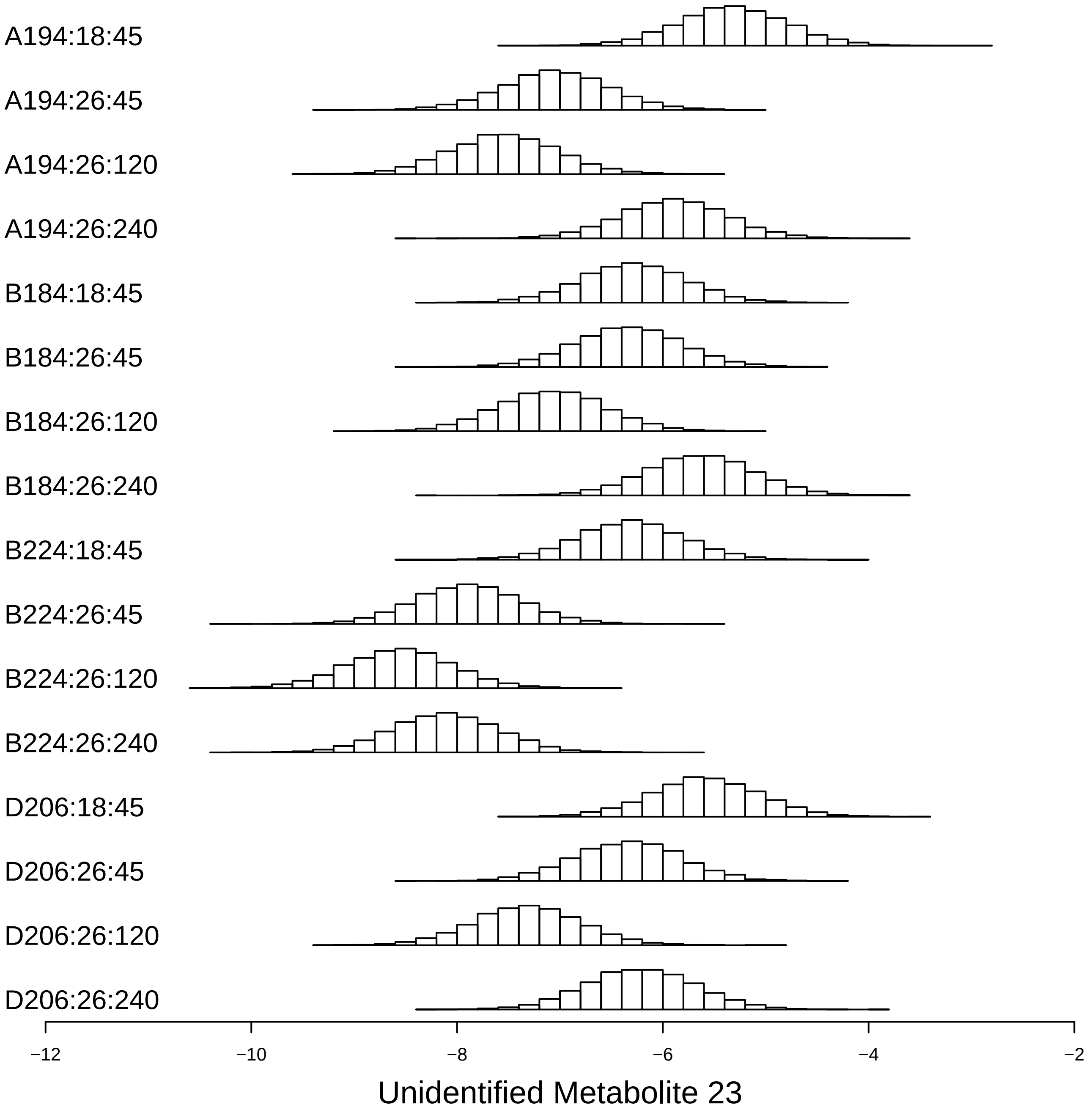
A194:18:45
A194:26:45
A194:26:120
A194:26:240
B184:18:45
B184:26:45
B184:26:120
B184:26:240
B224:18:45
B224:26:45
B224:26:120
B224:26:240
D206:18:45
D206:26:45
D206:26:120
D206:26:240
−12
−10
−8
−6
−4
−2
Unidentified Metabolite 23

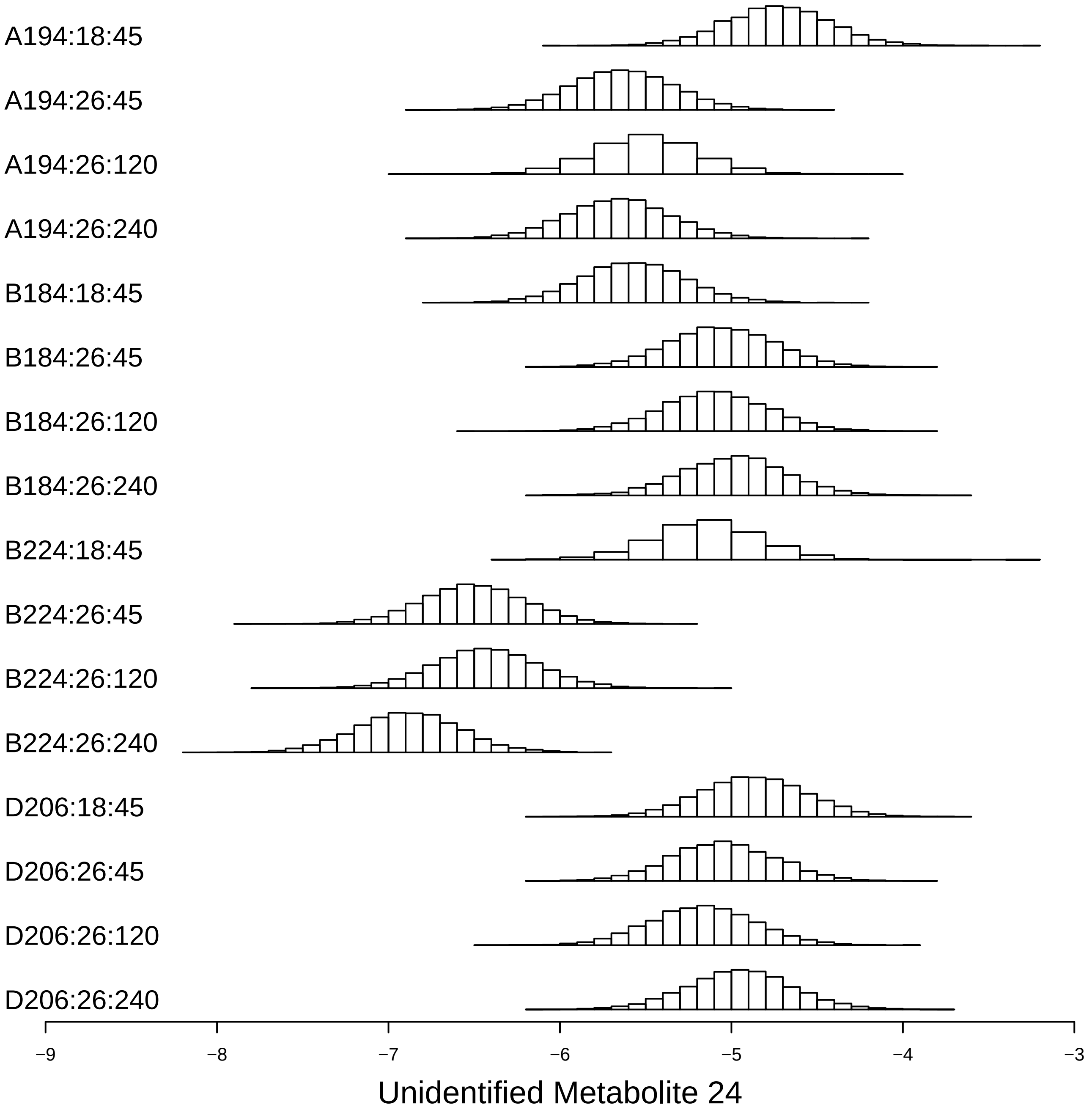
A194:18:45
A194:26:45
A194:26:120
A194:26:240
B184:18:45
B184:26:45
B184:26:120
B184:26:240
B224:18:45
B224:26:45
B224:26:120
B224:26:240
D206:18:45
D206:26:45
D206:26:120
D206:26:240
−9
−8
−7
−6
−5
−4
−3
Unidentified Metabolite 24

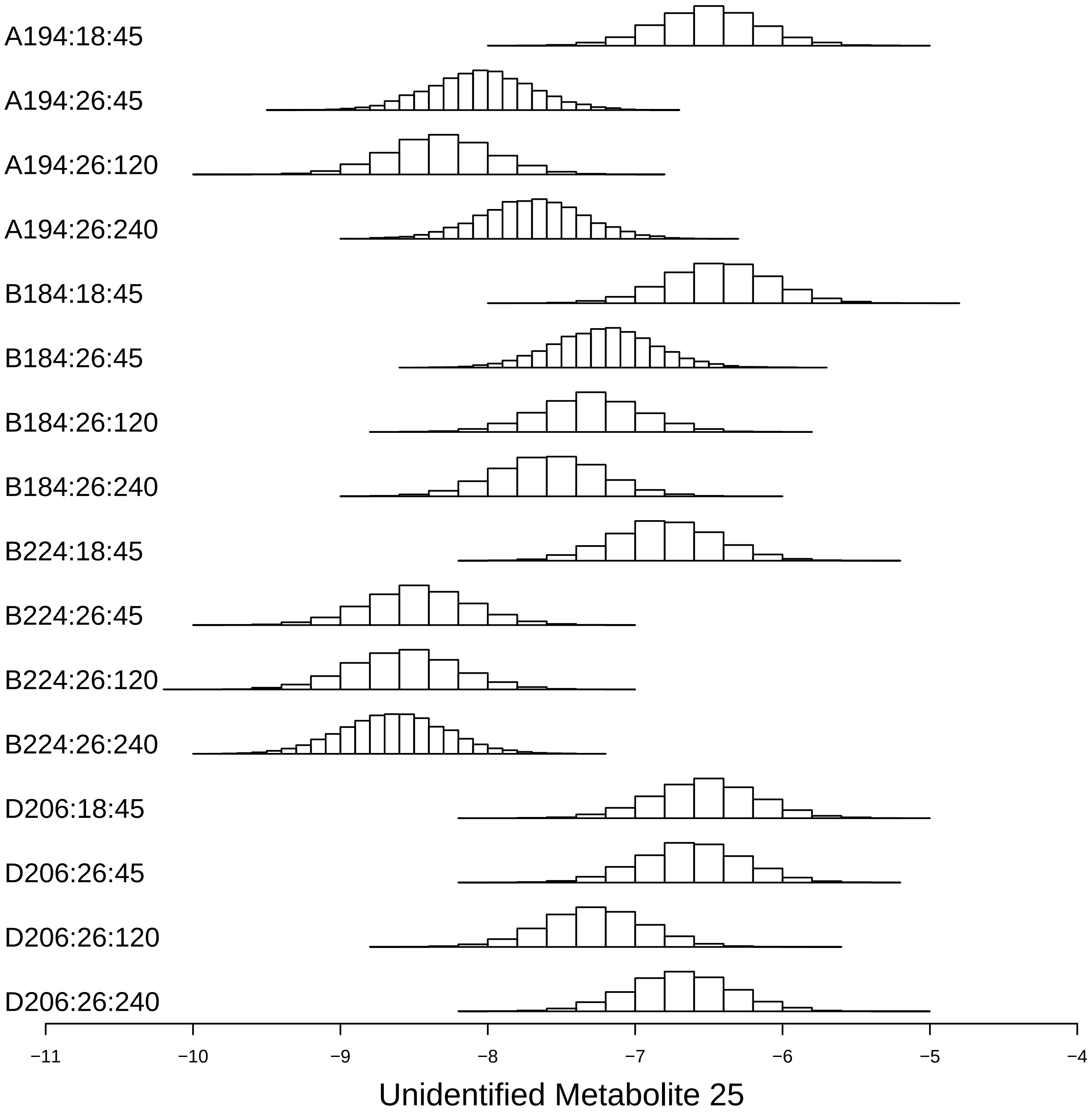
A194:18:45
A194:26:45
A194:26:120
A194:26:240
B184:18:45
B184:26:45
B184:26:120
B184:26:240
B224:18:45
B224:26:45
B224:26:120
B224:26:240
D206:18:45
D206:26:45
D206:26:120
D206:26:240
−11
−10
−9
−8
−7
−6
−5
−4
Unidentified Metabolite 25

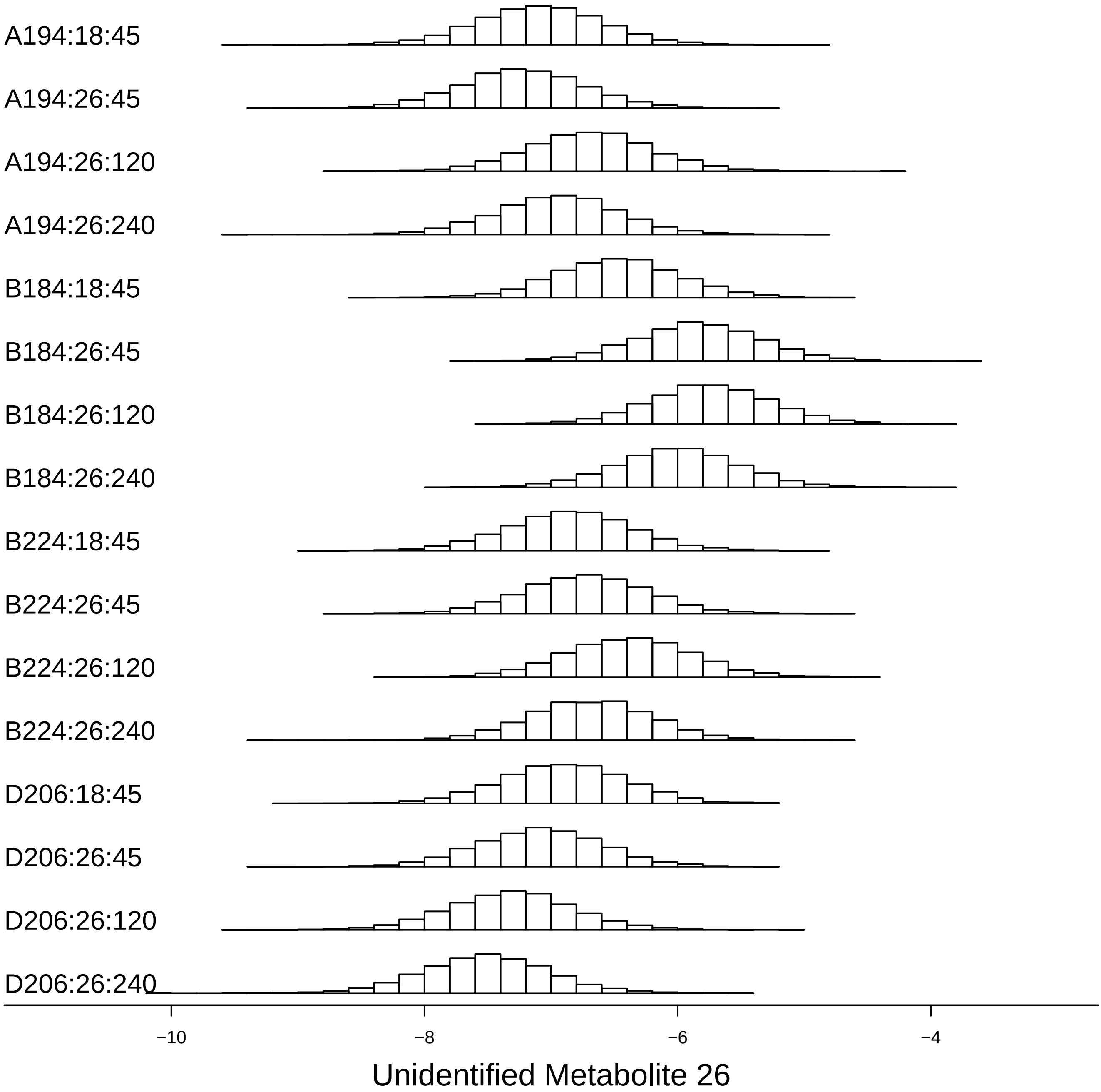

A194:18:45
A194:26:45
A194:26:120
A194:26:240
B184:18:45
B184:26:45
B184:26:120
B184:26:240
B224:18:45
B224:26:45
B224:26:120
B224:26:240
D206:18:45
D206:26:45
D206:26:120
D206:26:240
−10
−8
−6
−4
Unidentified Metabolite 26

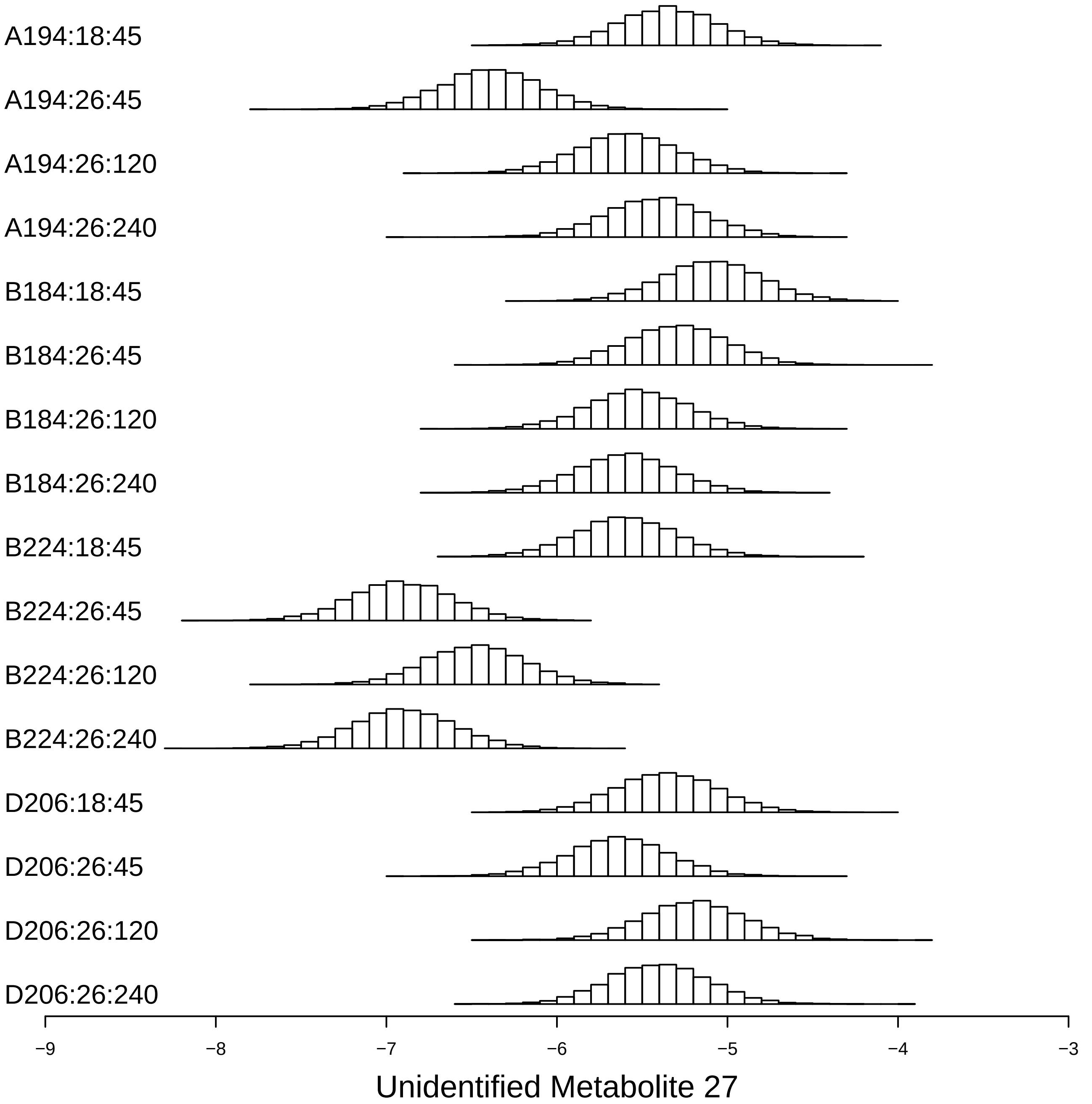

A194:18:45
A194:26:45
A194:26:120
A194:26:240
B184:18:45
B184:26:45
B184:26:120
B184:26:240
B224:18:45
B224:26:45
B224:26:120
B224:26:240
D206:18:45
D206:26:45
D206:26:120
D206:26:240
−9
−8
−7
−6
−5
−4
−3
Unidentified Metabolite 27

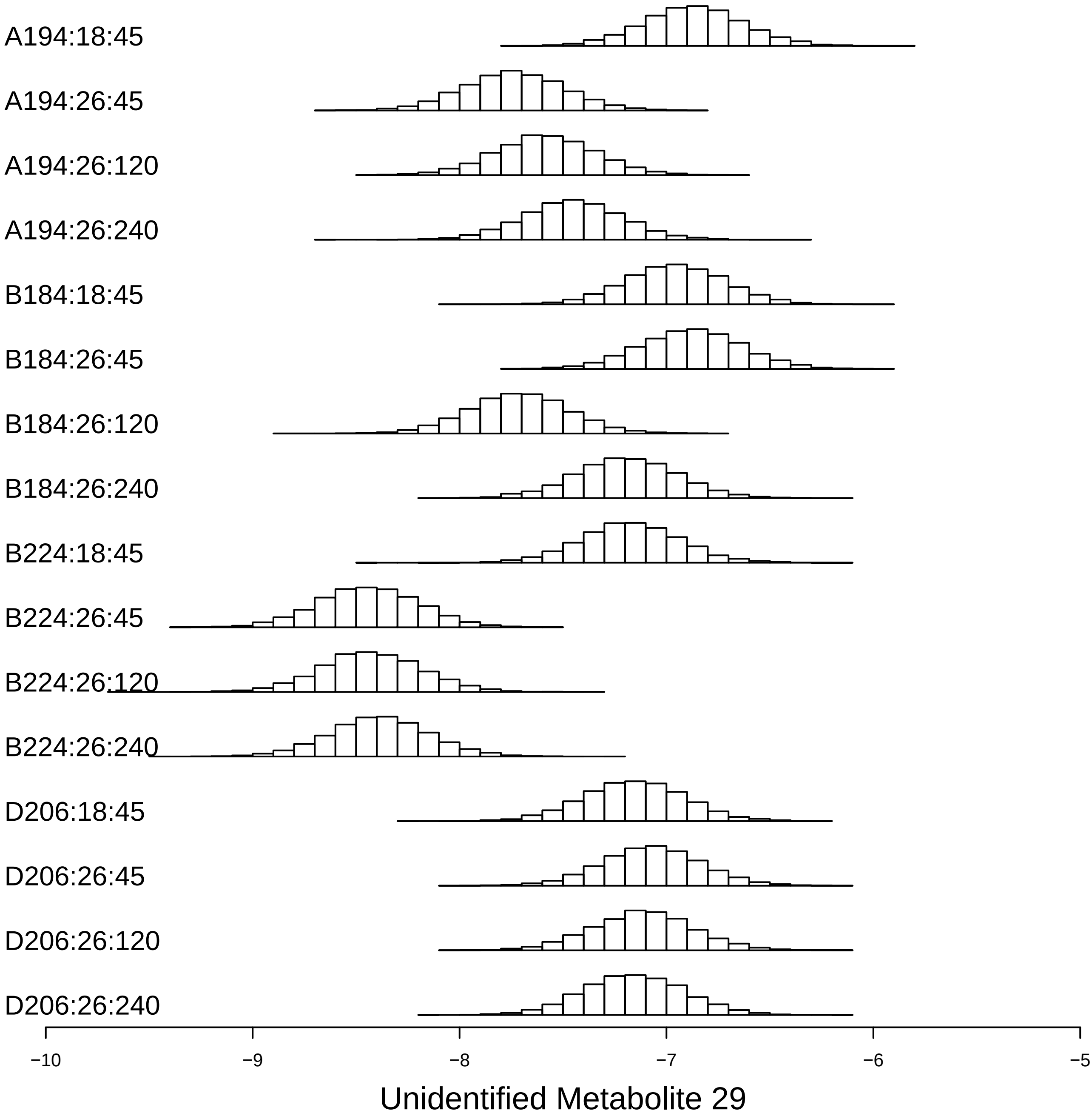
A194:18:45
A194:26:45
A194:26:120
A194:26:240
B184:18:45
B184:26:45
B184:26:120
B184:26:240
B224:18:45
B224:26:45
B224:26:120
B224:26:240
D206:18:45
D206:26:45
D206:26:120
D206:26:240
−10
−9
−8
−7
−6
−5
Unidentified Metabolite 29

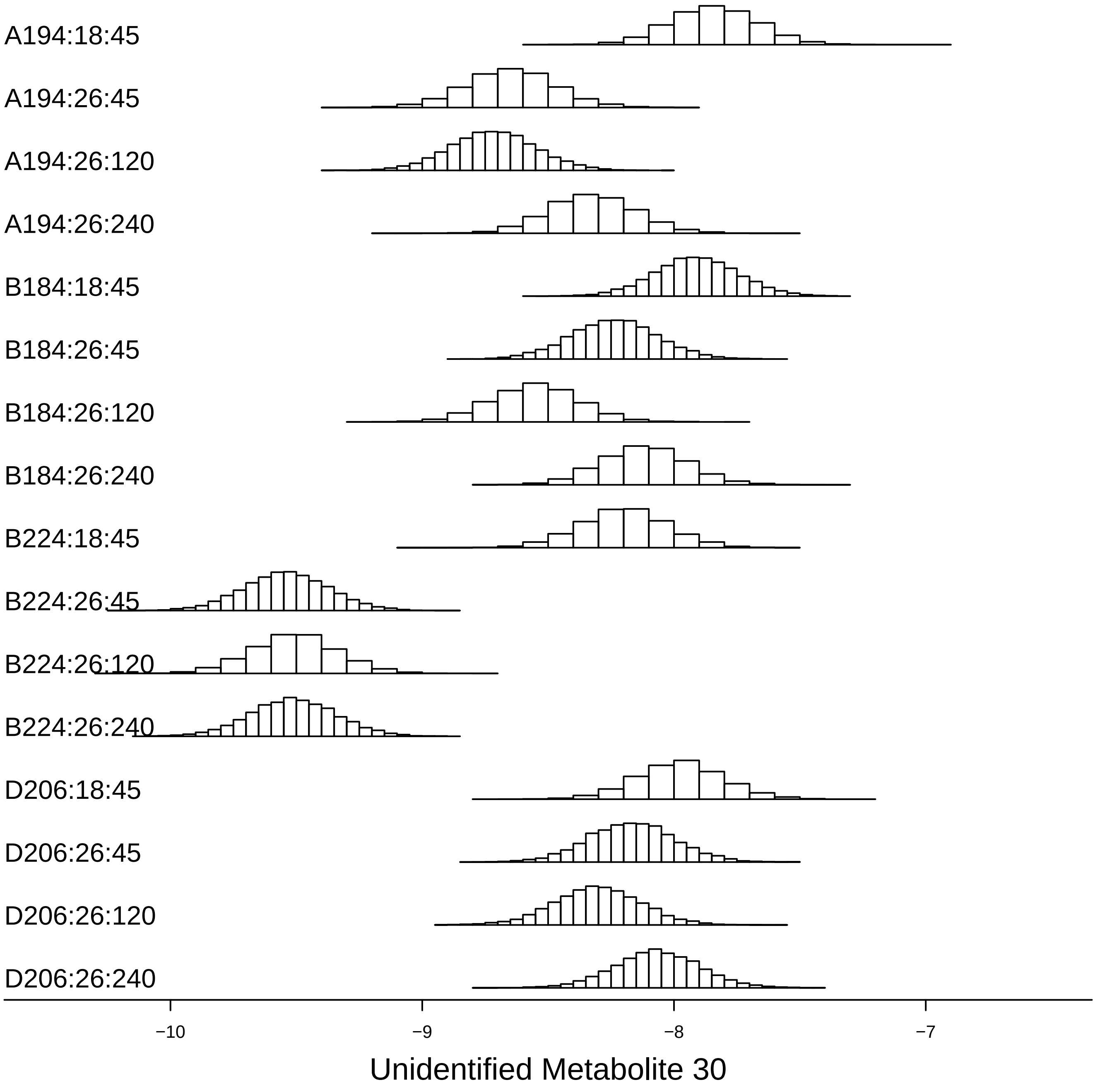

A194:18:45
A194:26:45
A194:26:120
A194:26:240
B184:18:45
B184:26:45
B184:26:120
B184:26:240
B224:18:45
B224:26:45
B224:26:120
B224:26:240
D206:18:45
D206:26:45
D206:26:120
D206:26:240
−10
−9
−8
−7
Unidentified Metabolite 30

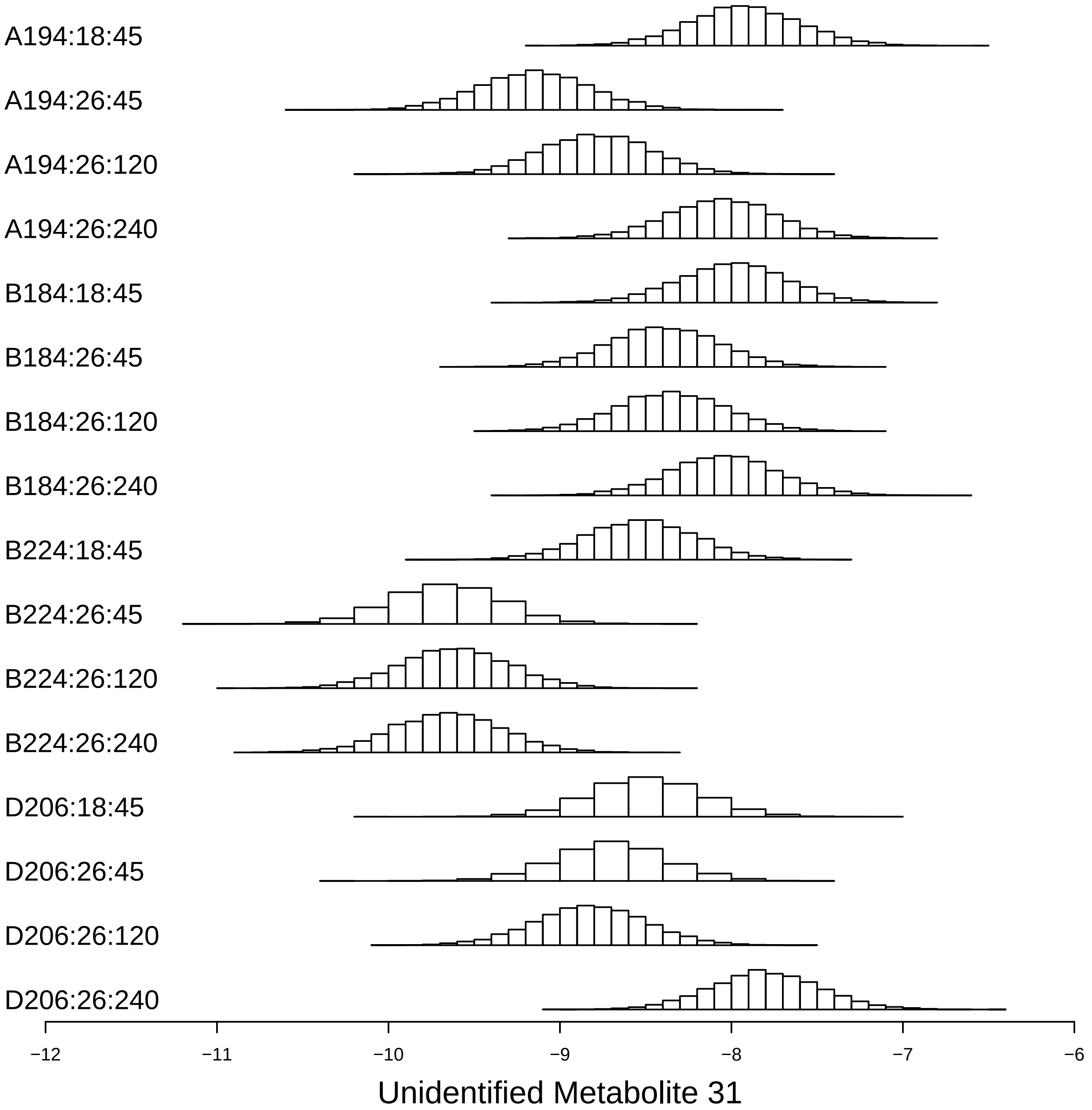
A194:18:45
A194:26:45
A194:26:120
A194:26:240
B184:18:45
B184:26:45
B184:26:120
B184:26:240
B224:18:45
B224:26:45
B224:26:120
B224:26:240
D206:18:45
D206:26:45
D206:26:120
D206:26:240
−12
−11
−10
−9
−8
−7
−6
Unidentified Metabolite 31

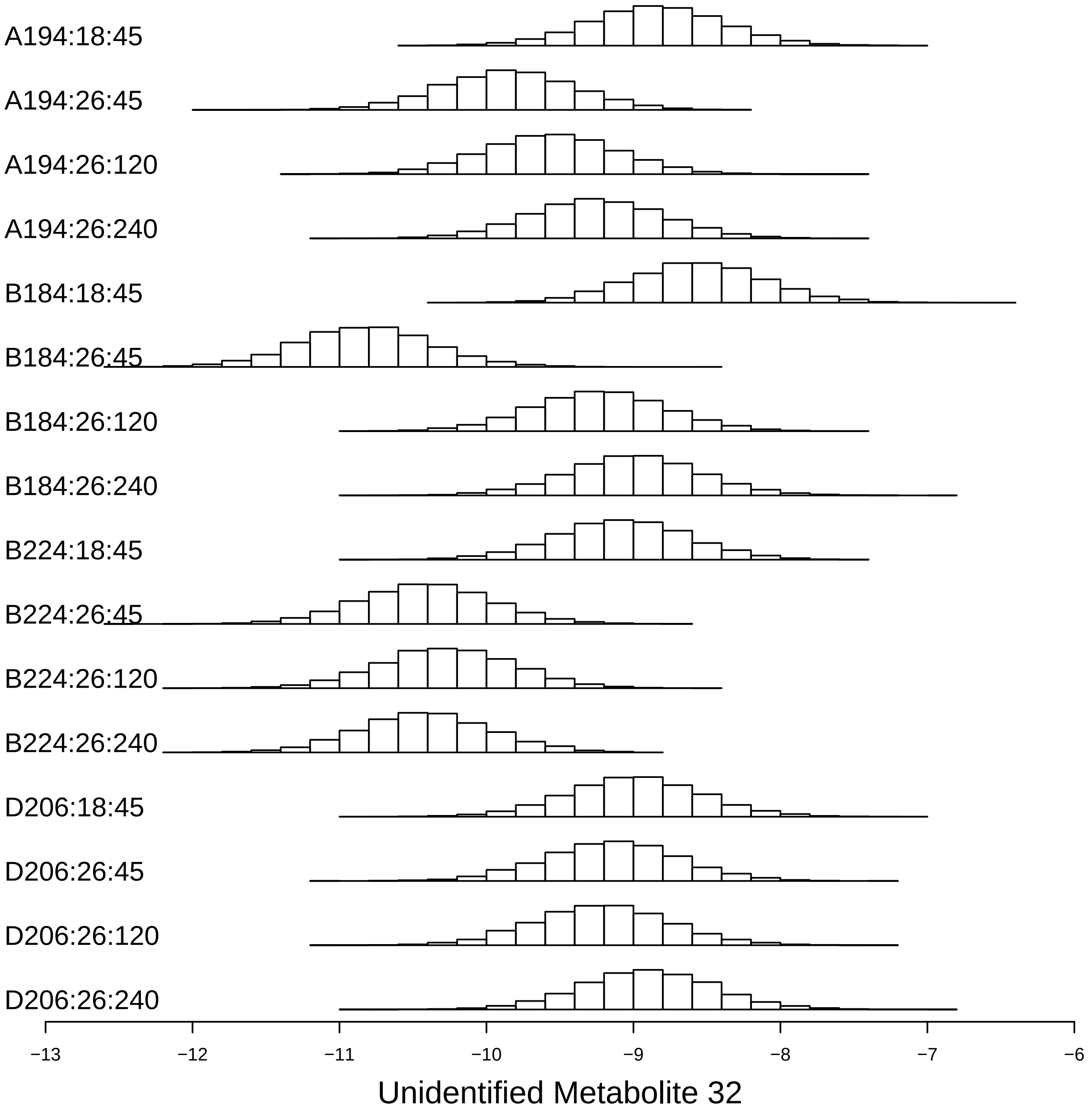
A194:18:45
A194:26:45
A194:26:120
A194:26:240
B184:18:45
B184:26:45
B184:26:120
B184:26:240
B224:18:45
B224:26:45
B224:26:120
B224:26:240
D206:18:45
D206:26:45
D206:26:120
D206:26:240
−13
−12
−11
−10
−9
−8
−7
−6
Unidentified Metabolite 32

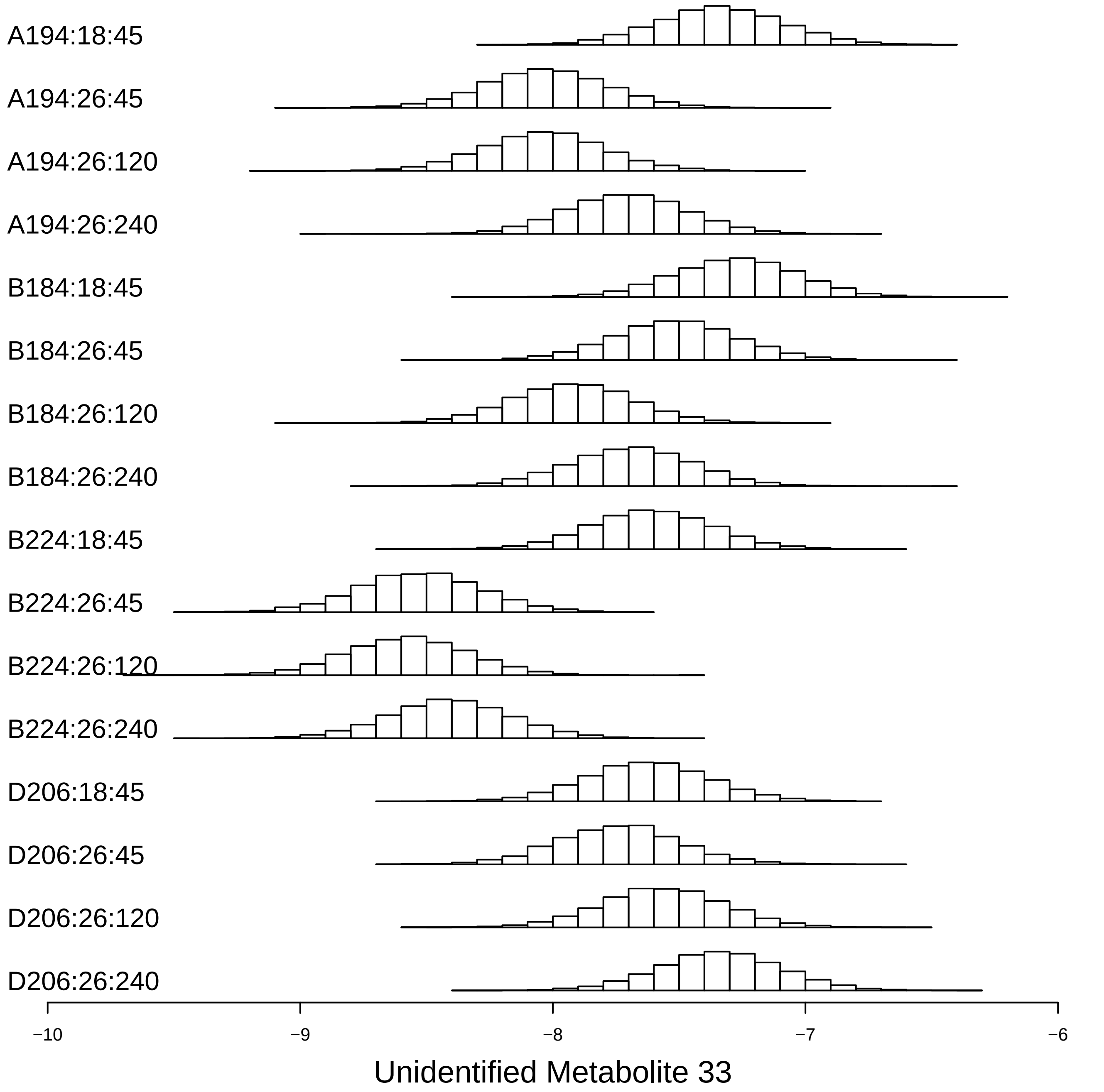
A194:18:45
A194:26:45
A194:26:120
A194:26:240
B184:18:45
B184:26:45
B184:26:120
B184:26:240
B224:18:45
B224:26:45
B224:26:120
B224:26:240
D206:18:45
D206:26:45
D206:26:120
D206:26:240
−10
−9
−8
−7
−6
Unidentified Metabolite 33

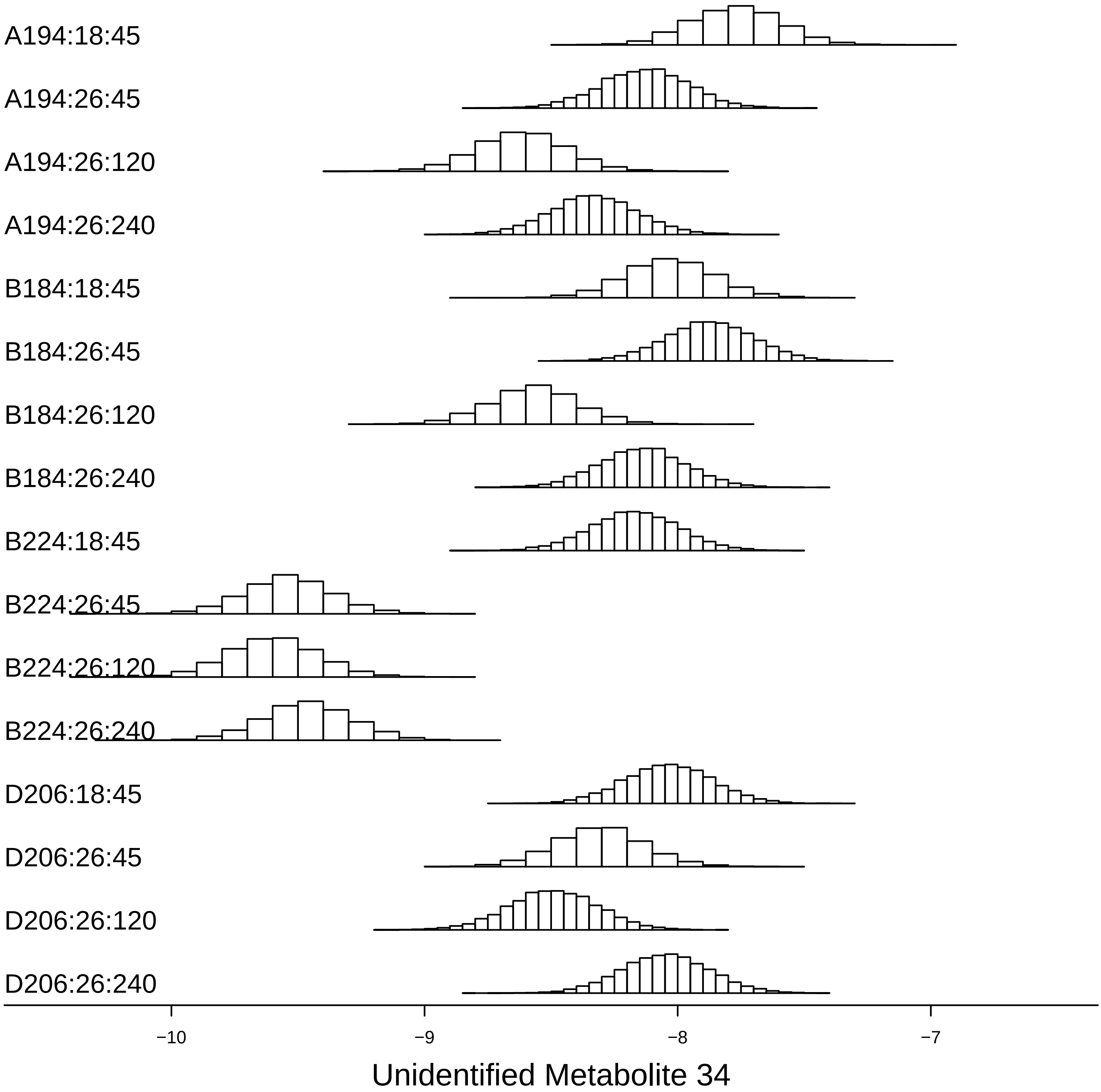
A194:18:45
A194:26:45
A194:26:120
A194:26:240
B184:18:45
B184:26:45
B184:26:120
B184:26:240
B224:18:45
B224:26:45
B224:26:120
B224:26:240
D206:18:45
D206:26:45
D206:26:120
D206:26:240
−10
−9
−8
−7
Unidentified Metabolite 34

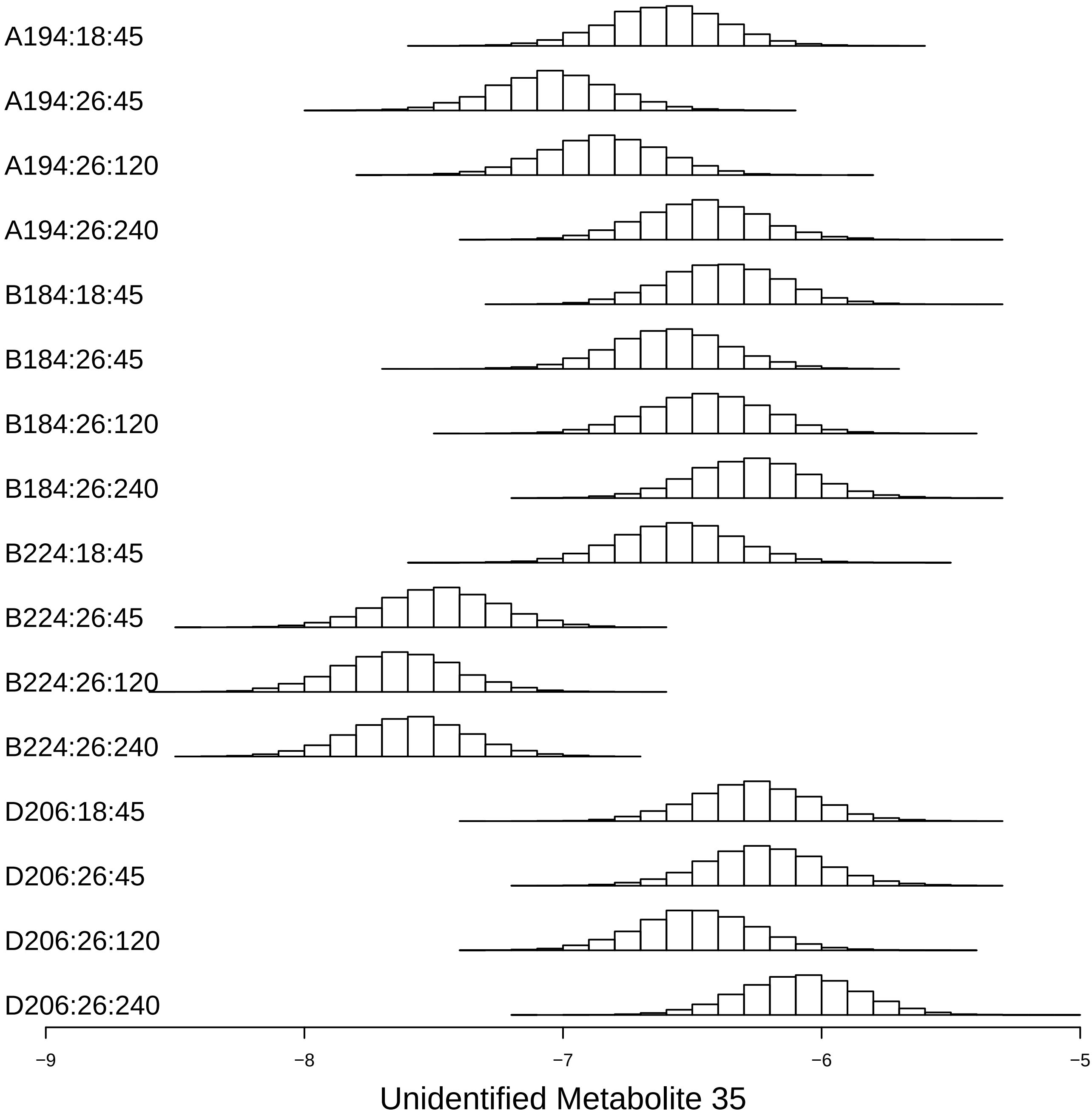
A194:18:45
A194:26:45
A194:26:120
A194:26:240
B184:18:45
B184:26:45
B184:26:120
B184:26:240
B224:18:45
B224:26:45
B224:26:120
B224:26:240
D206:18:45
D206:26:45
D206:26:120
D206:26:240
−9
−8
−7
−6
−5
Unidentified Metabolite 35

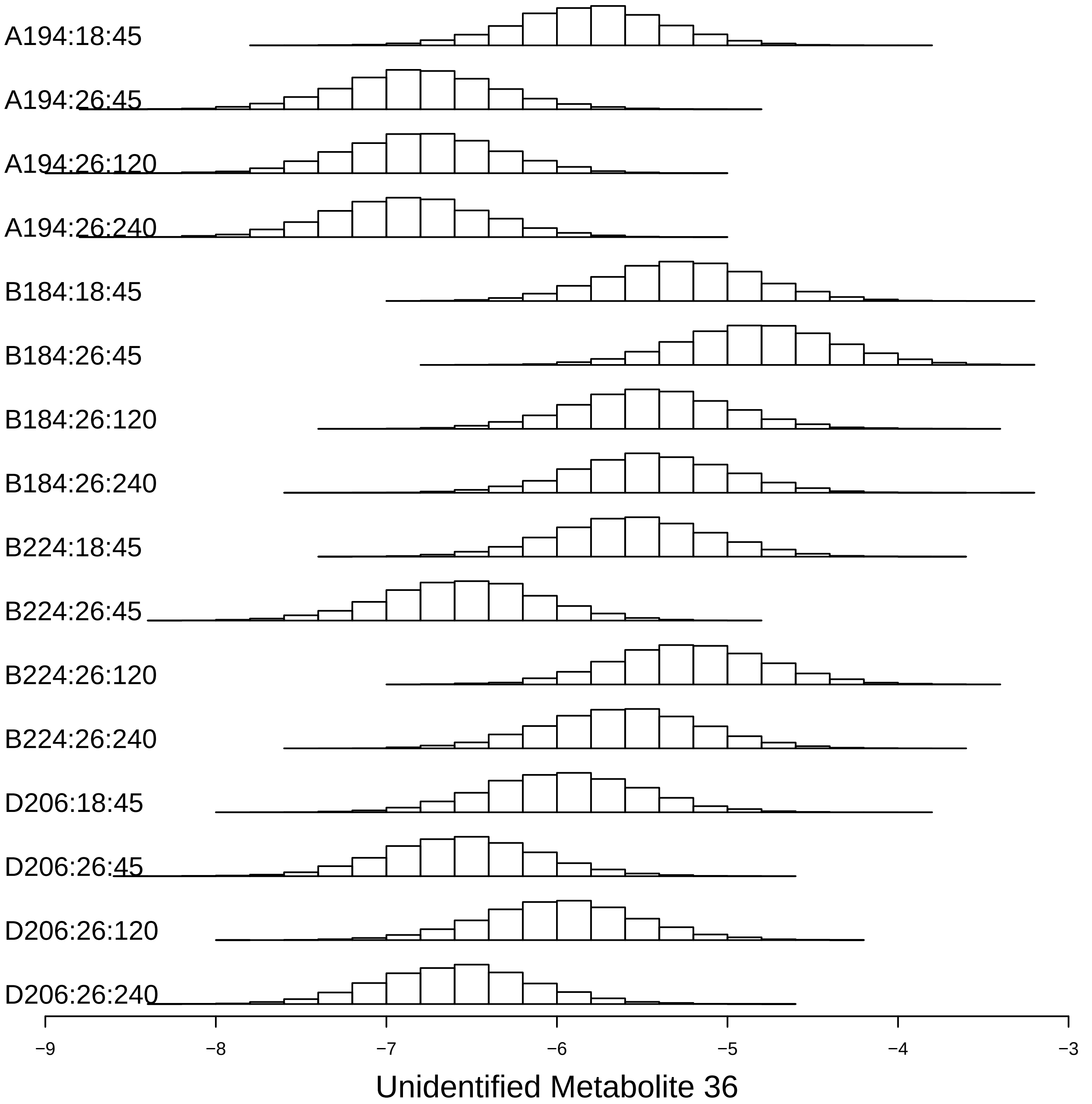

A194:18:45
A194:26:45
A194:26:120
A194:26:240
B184:18:45
B184:26:45
B184:26:120
B184:26:240
B224:18:45
B224:26:45
B224:26:120
B224:26:240
D206:18:45
D206:26:45
D206:26:120
D206:26:240
−9
−8
−7
−6
−5
−4
−3
Unidentified Metabolite 36

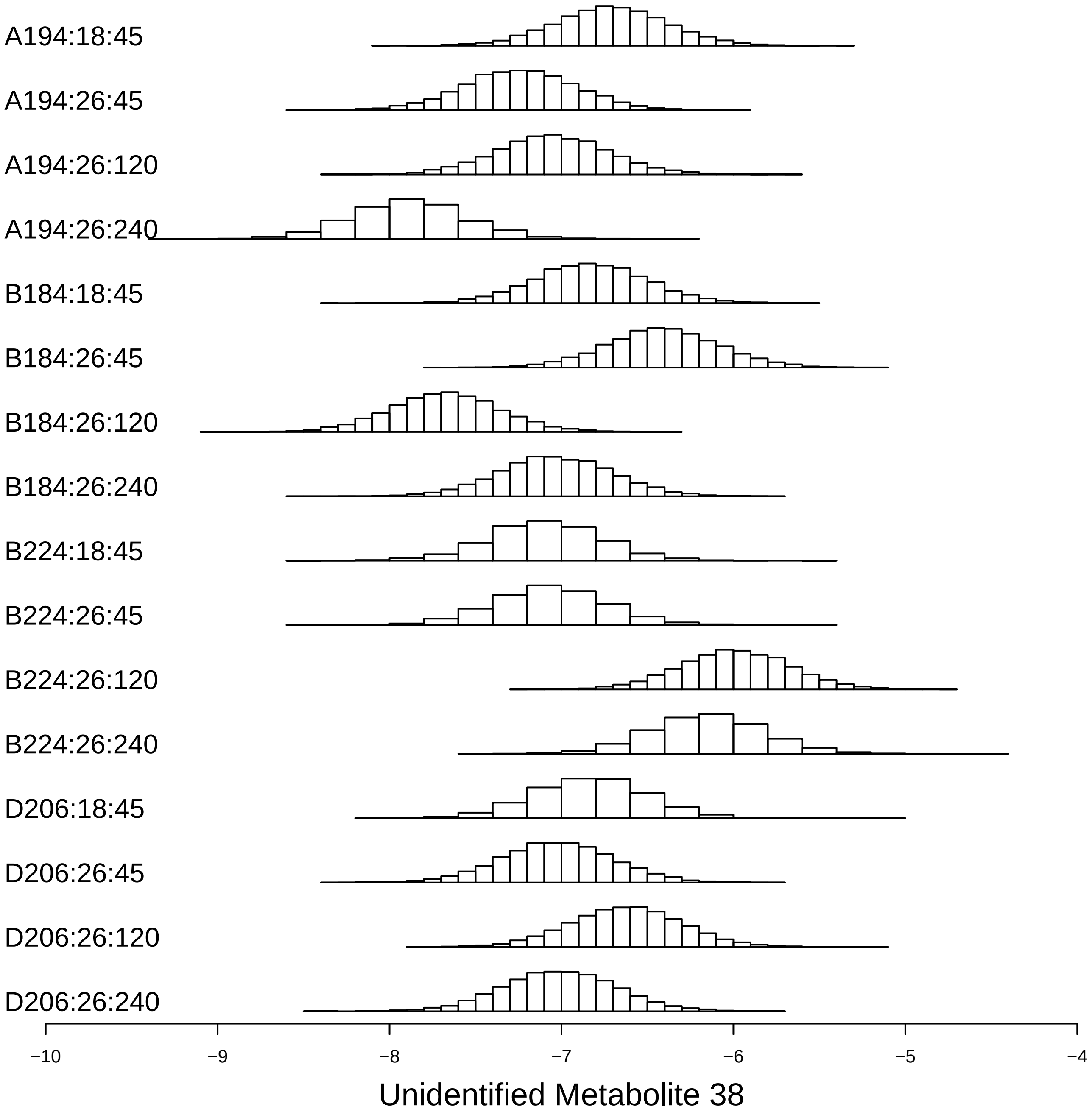
A194:18:45
A194:26:45
A194:26:120
A194:26:240
B184:18:45
B184:26:45
B184:26:120
B184:26:240
B224:18:45
B224:26:45
B224:26:120
B224:26:240
D206:18:45
D206:26:45
D206:26:120
D206:26:240
−10
−9
−8
−7
−6
−5
−4
Unidentified Metabolite 38

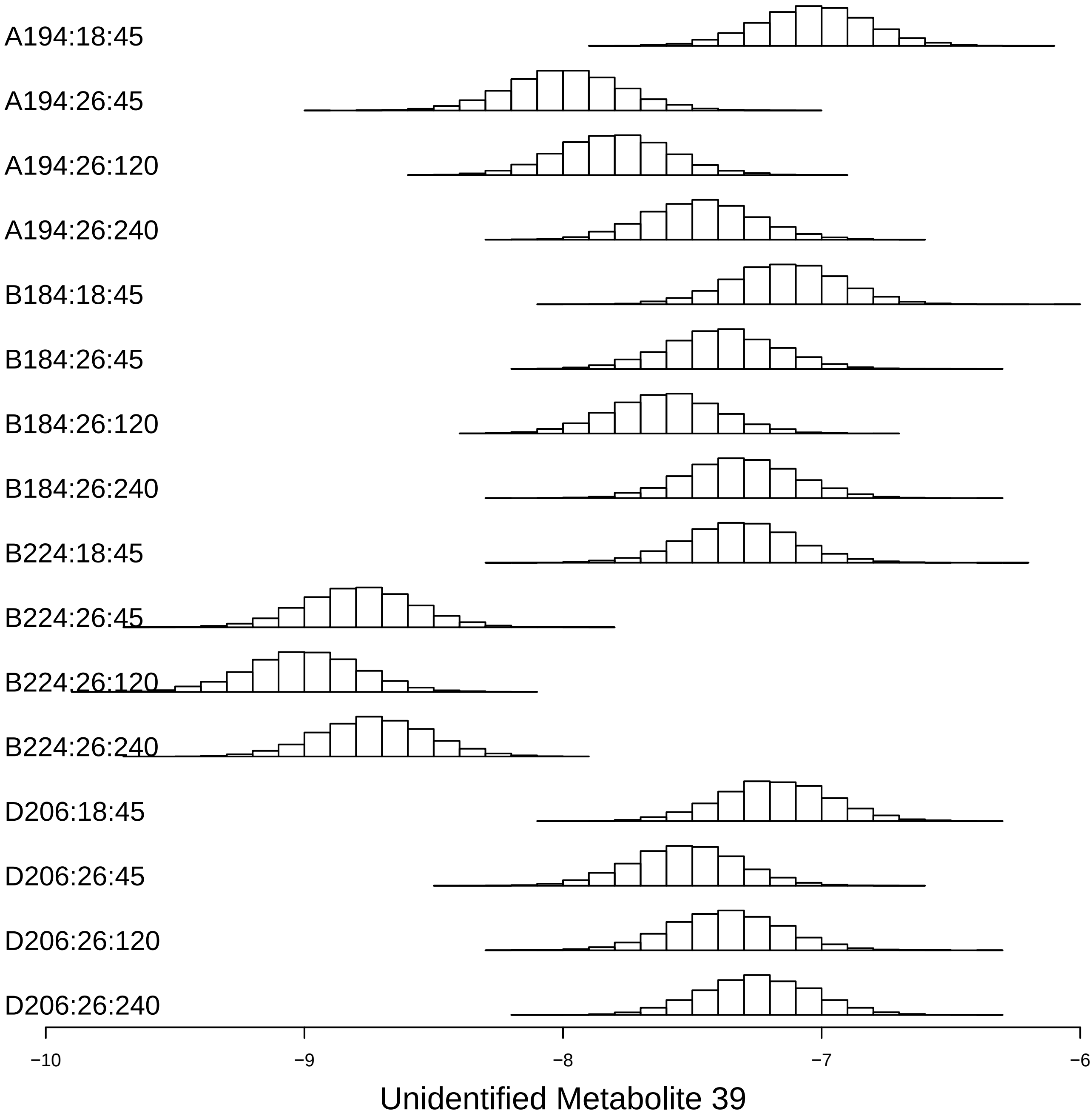
A194:18:45
A194:26:45
A194:26:120
A194:26:240
B184:18:45
B184:26:45
B184:26:120
B184:26:240
B224:18:45
B224:26:45
B224:26:120
B224:26:240
D206:18:45
D206:26:45
D206:26:120
D206:26:240
−10
−9
−8
−7
−6
Unidentified Metabolite 39

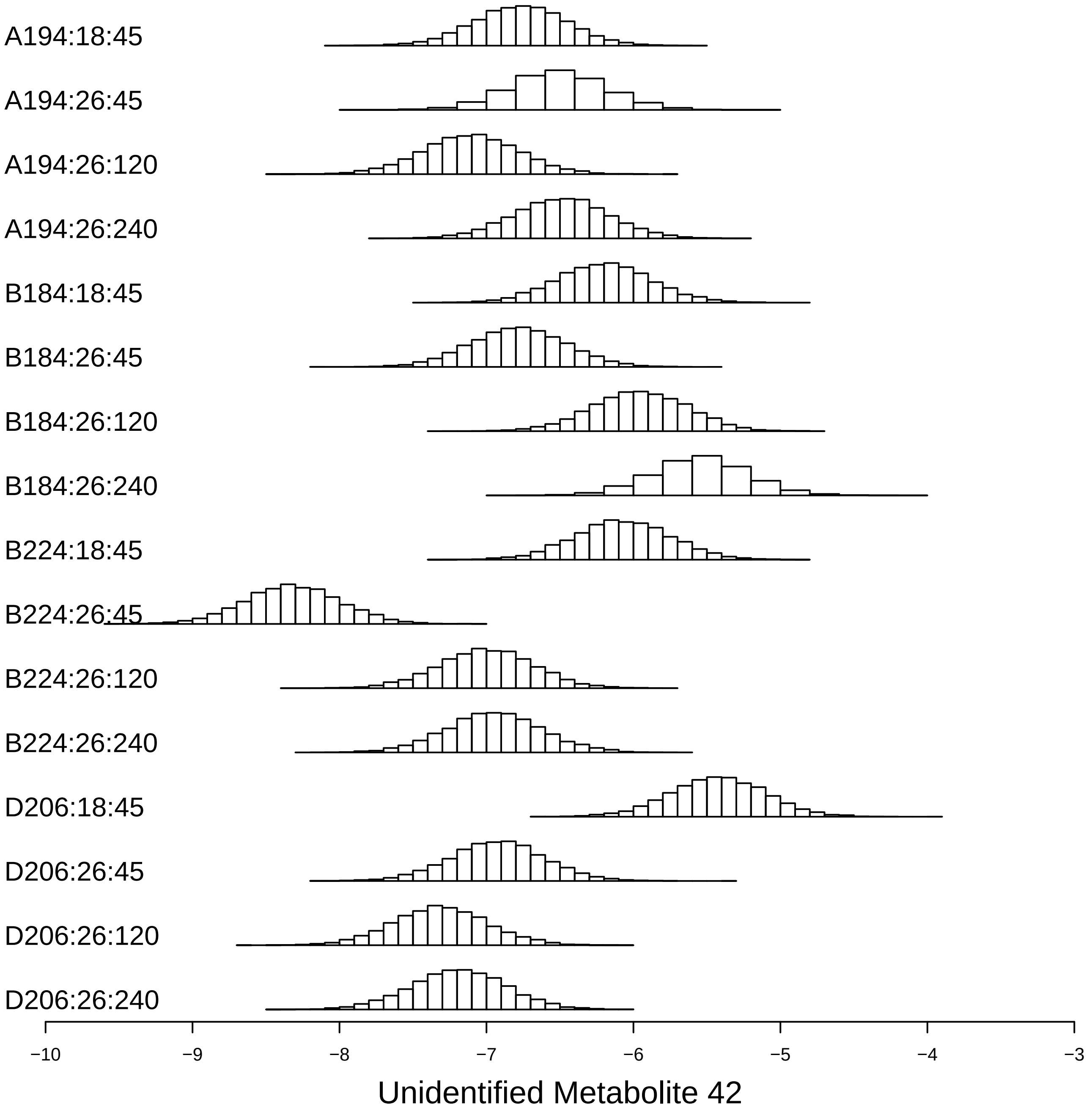
A194:18:45
A194:26:45
A194:26:120
A194:26:240
B184:18:45
B184:26:45
B184:26:120
B184:26:240
B224:18:45
B224:26:45
B224:26:120
B224:26:240
D206:18:45
D206:26:45
D206:26:120
D206:26:240
−10
−9
−8
−7
−6
−5
−4
−3
Unidentified Metabolite 42

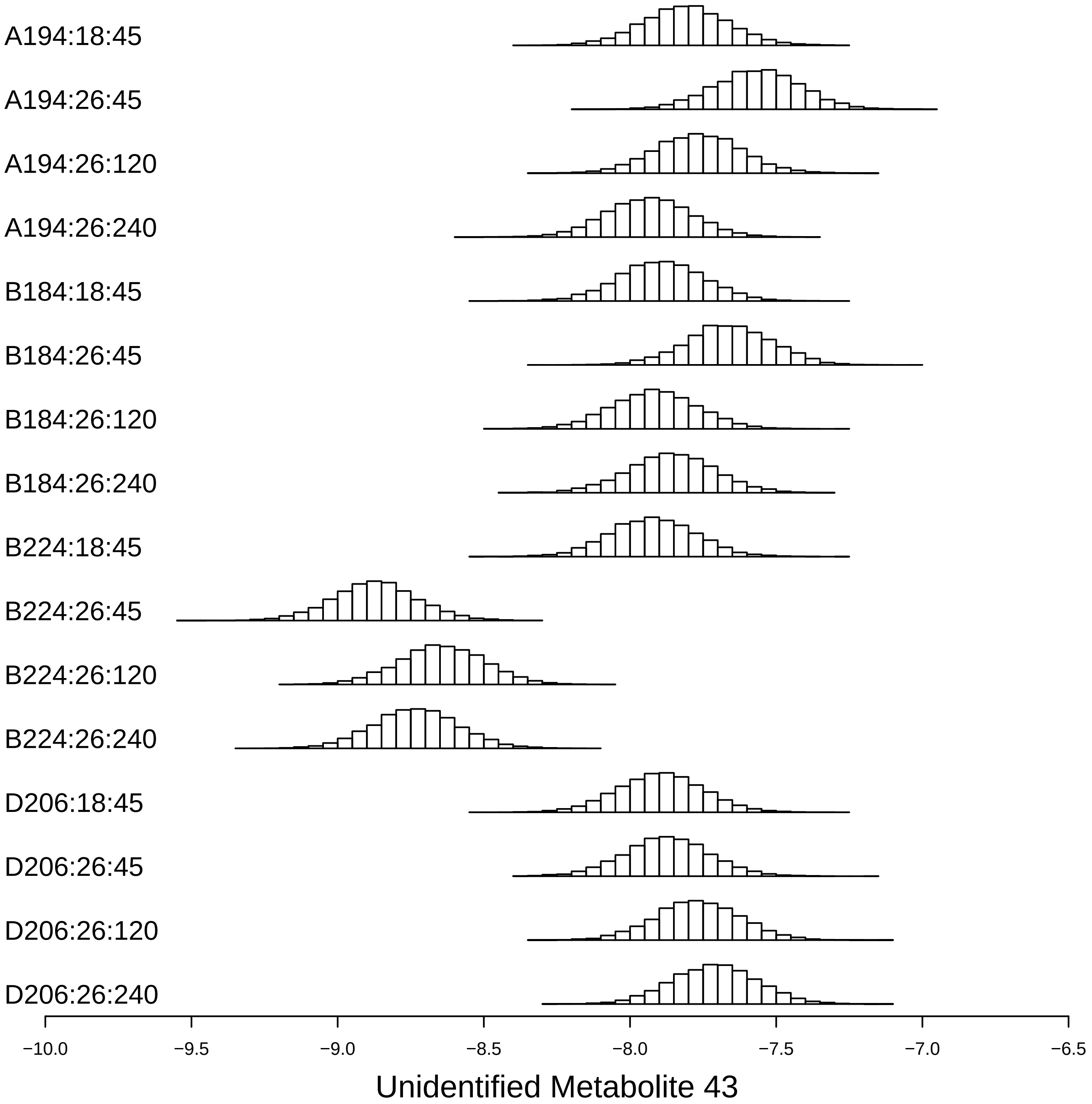
A194:18:45
A194:26:45
A194:26:120
A194:26:240
B184:18:45
B184:26:45
B184:26:120
B184:26:240
B224:18:45
B224:26:45
B224:26:120
B224:26:240
D206:18:45
D206:26:45
D206:26:120
D206:26:240
−10.0
−9.5
−9.0
−8.5
−8.0
−7.5
−7.0
−6.5
Unidentified Metabolite 43

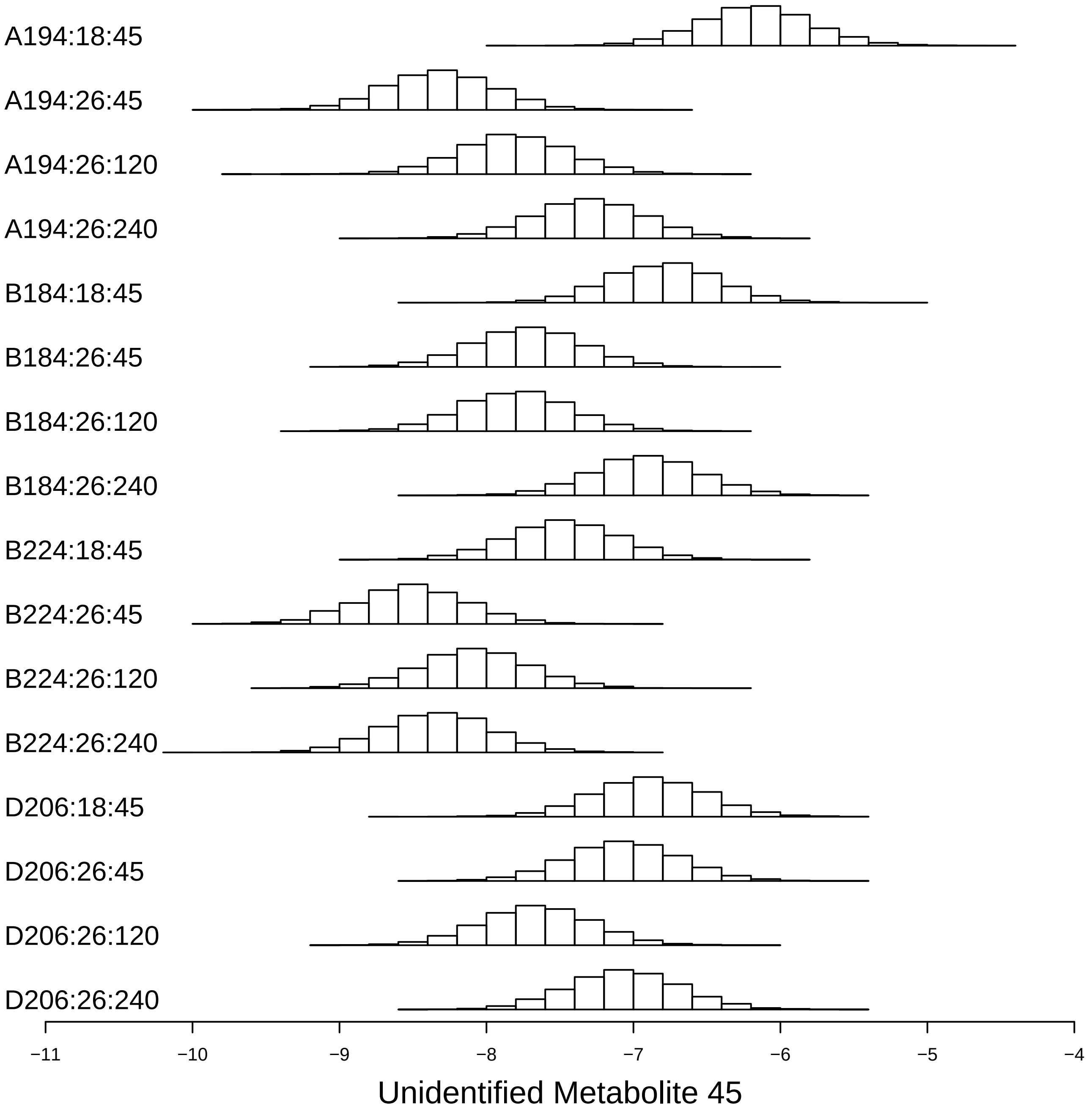

A194:18:45
A194:26:45
A194:26:120
A194:26:240
B184:18:45
B184:26:45
B184:26:120
B184:26:240
B224:18:45
B224:26:45
B224:26:120
B224:26:240
D206:18:45
D206:26:45
D206:26:120
D206:26:240
−11
−10
−9
−8
−7
−6
−5
−4
Unidentified Metabolite 45

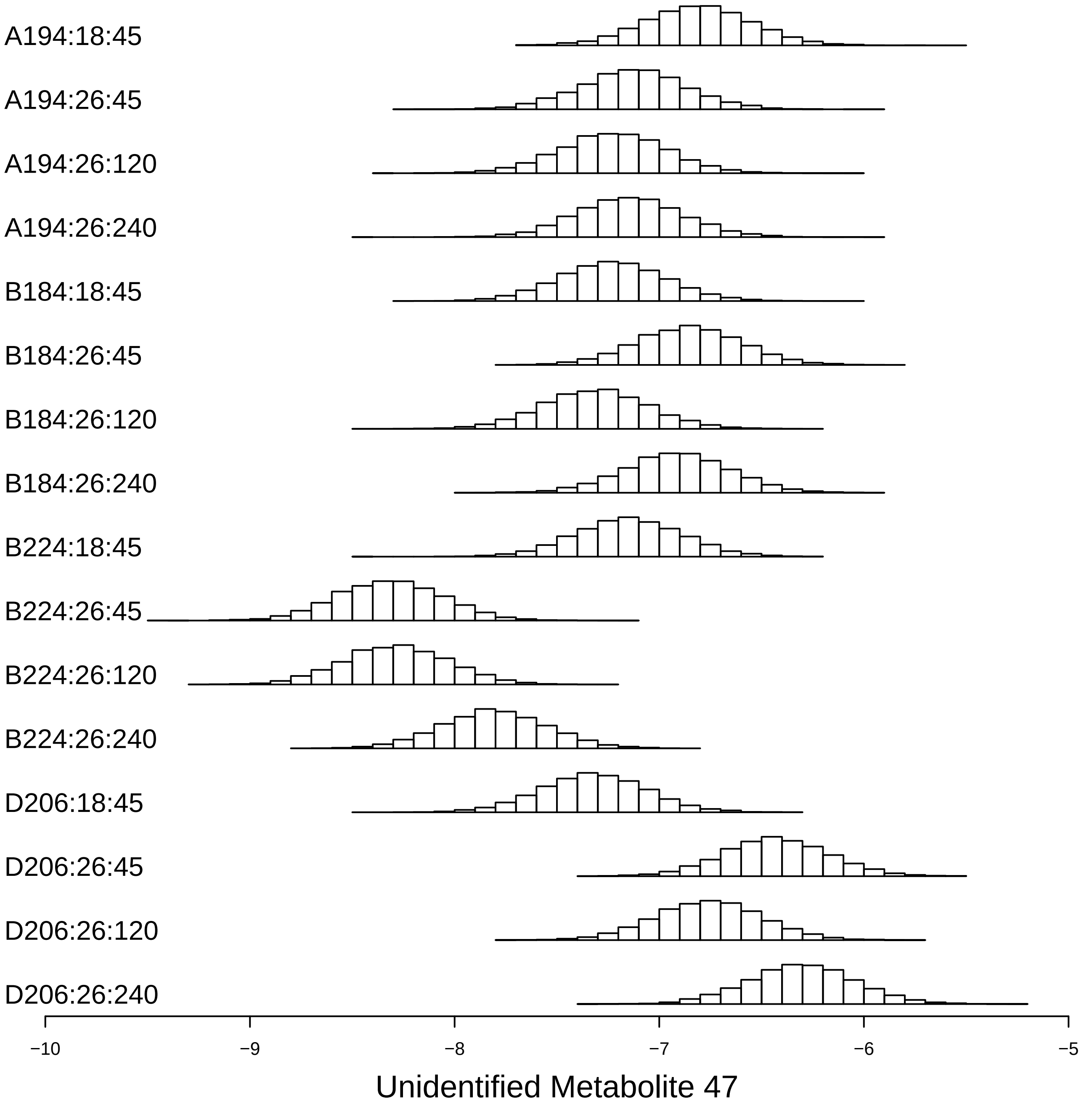
A194:18:45
A194:26:45
A194:26:120
A194:26:240
B184:18:45
B184:26:45
B184:26:120
B184:26:240
B224:18:45
B224:26:45
B224:26:120
B224:26:240
D206:18:45
D206:26:45
D206:26:120
D206:26:240
−10
−9
−8
−7
−6
−5
Unidentified Metabolite 47

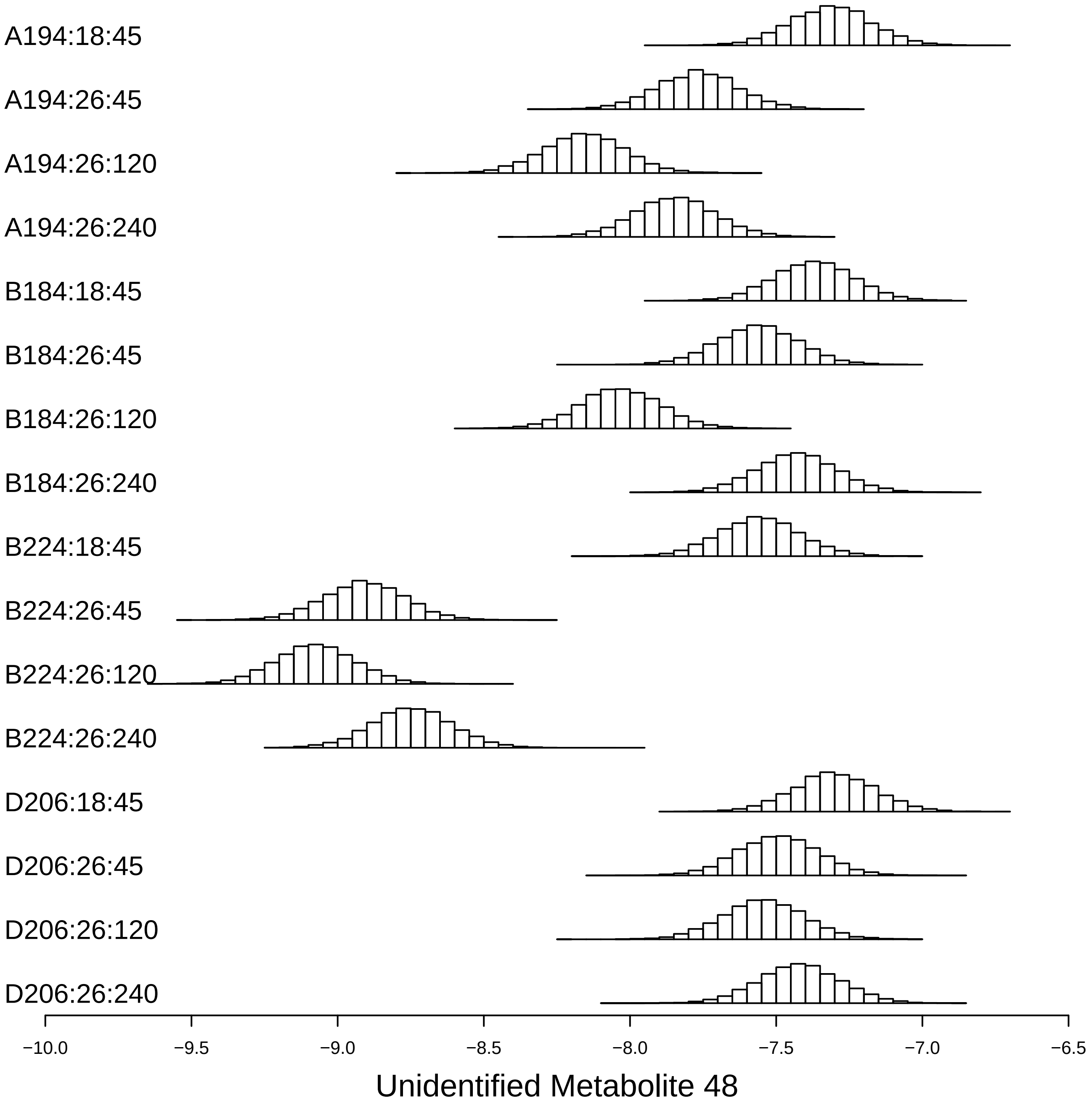
A194:18:45
A194:26:45
A194:26:120
A194:26:240
B184:18:45
B184:26:45
B184:26:120
B184:26:240
B224:18:45
B224:26:45
B224:26:120
B224:26:240
D206:18:45
D206:26:45
D206:26:120
D206:26:240
−10.0
−9.5
−9.0
−8.5
−8.0
−7.5
−7.0
−6.5
Unidentified Metabolite 48

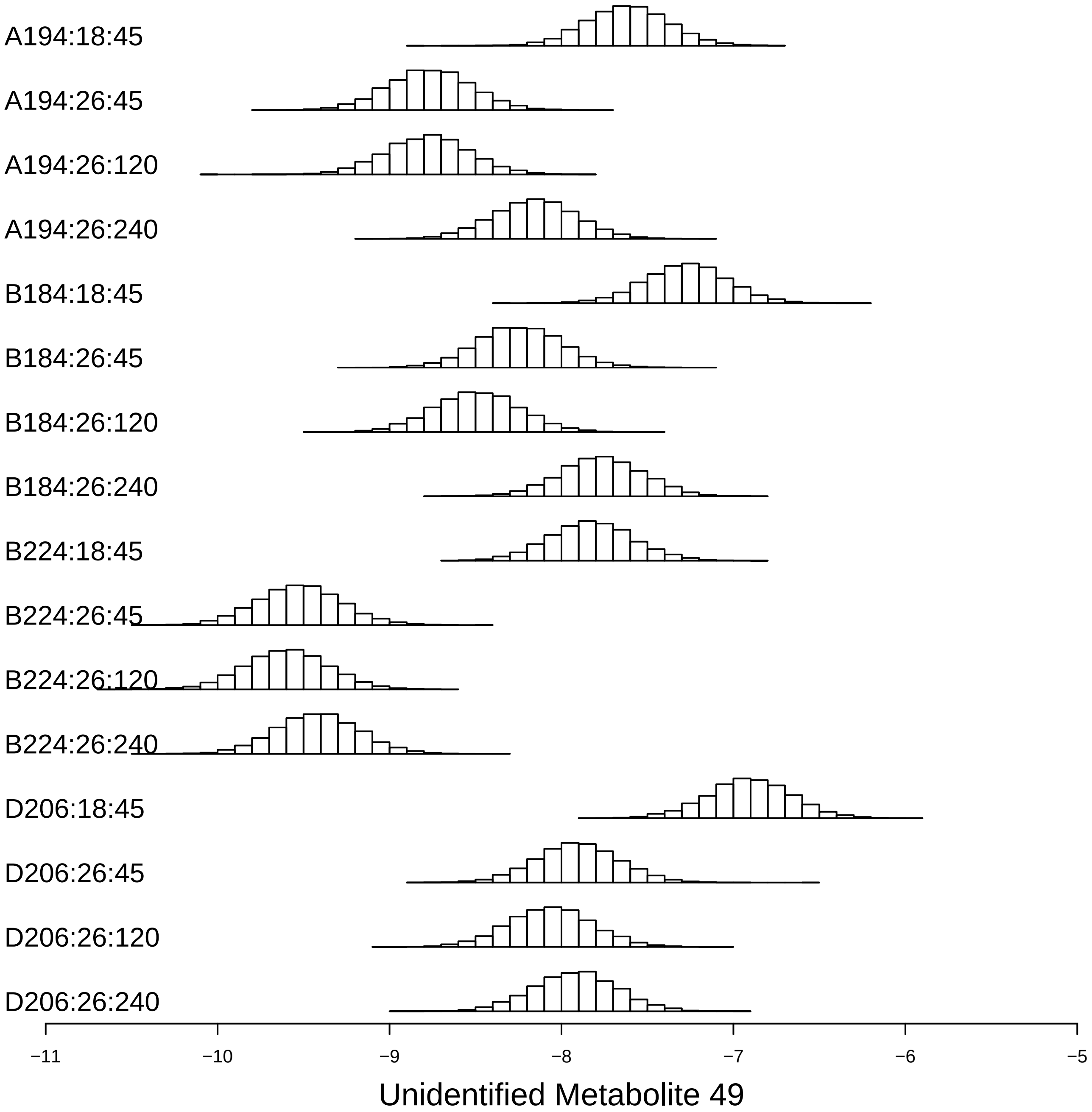
A194:18:45
A194:26:45
A194:26:120
A194:26:240
B184:18:45
B184:26:45
B184:26:120
B184:26:240
B224:18:45
B224:26:45
B224:26:120
B224:26:240
D206:18:45
D206:26:45
D206:26:120
D206:26:240
−11
−10
−9
−8
−7
−6
−5
Unidentified Metabolite 49

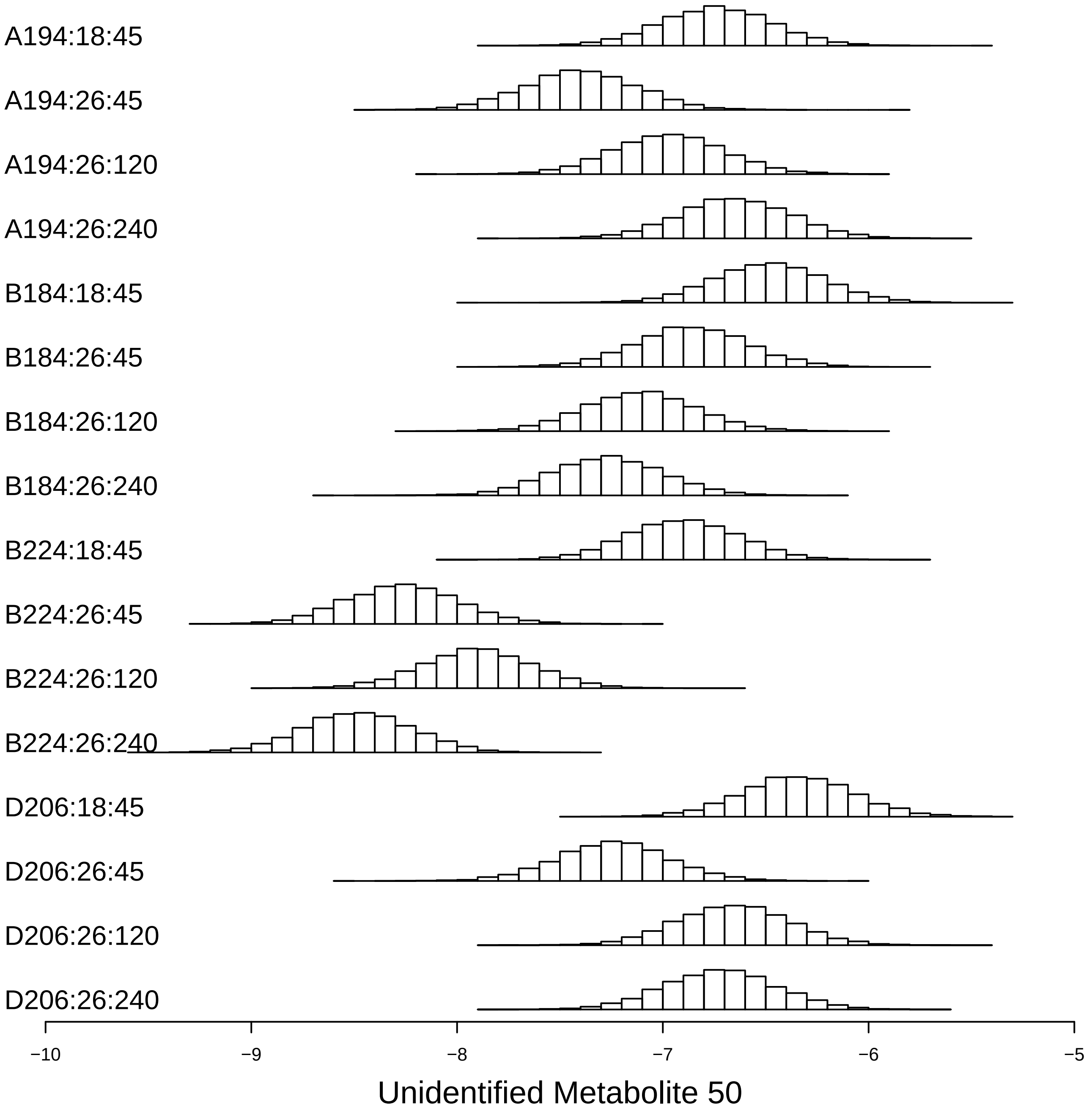
A194:18:45
A194:26:45
A194:26:120
A194:26:240
B184:18:45
B184:26:45
B184:26:120
B184:26:240
B224:18:45
B224:26:45
B224:26:120
B224:26:240
D206:18:45
D206:26:45
D206:26:120
D206:26:240
−10
−9
−8
−7
−6
−5
Unidentified Metabolite 50

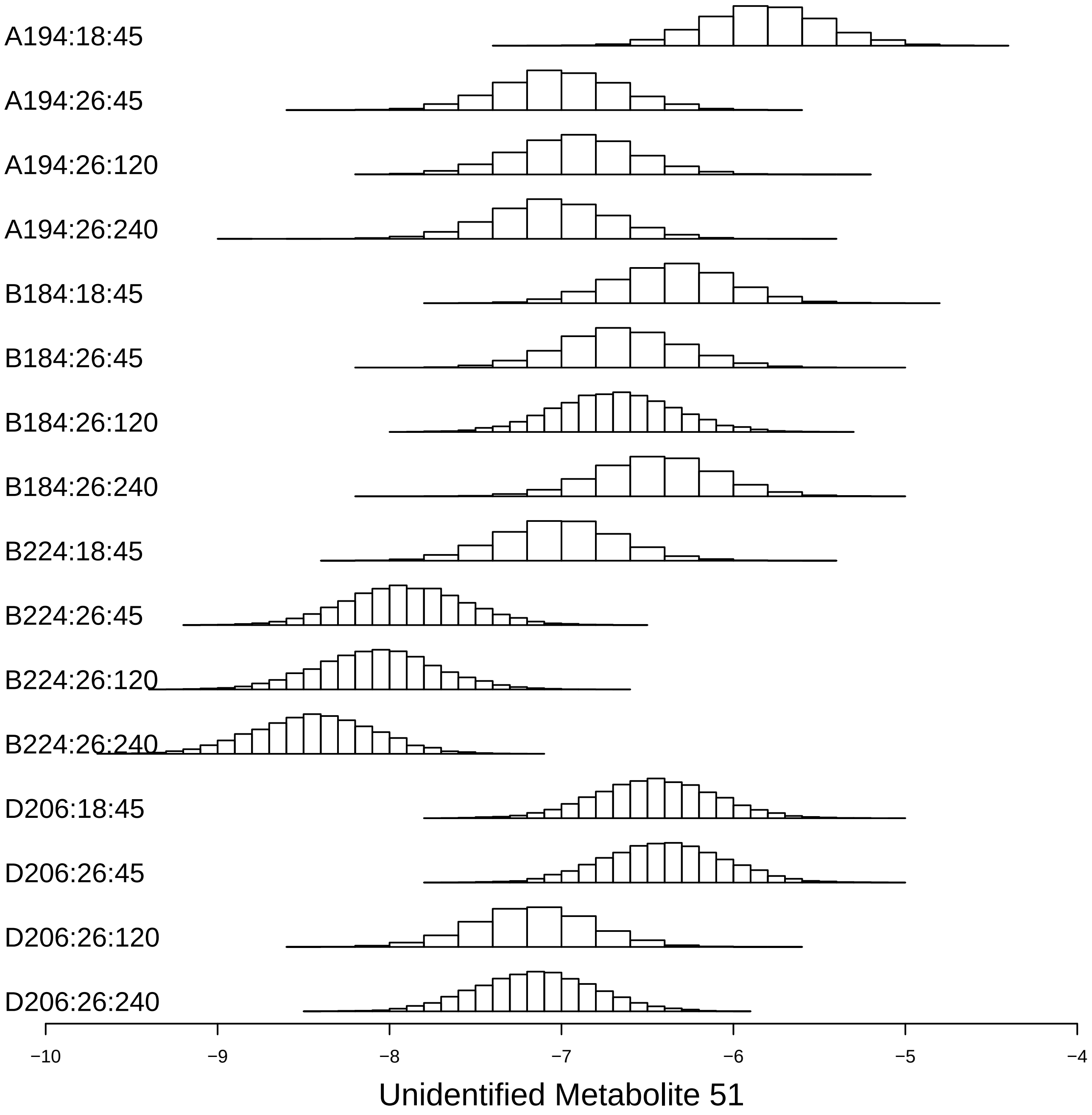
A194:18:45
A194:26:45
A194:26:120
A194:26:240
B184:18:45
B184:26:45
B184:26:120
B184:26:240
B224:18:45
B224:26:45
B224:26:120
B224:26:240
D206:18:45
D206:26:45
D206:26:120
D206:26:240
−10
−9
−8
−7
−6
−5
−4
Unidentified Metabolite 51

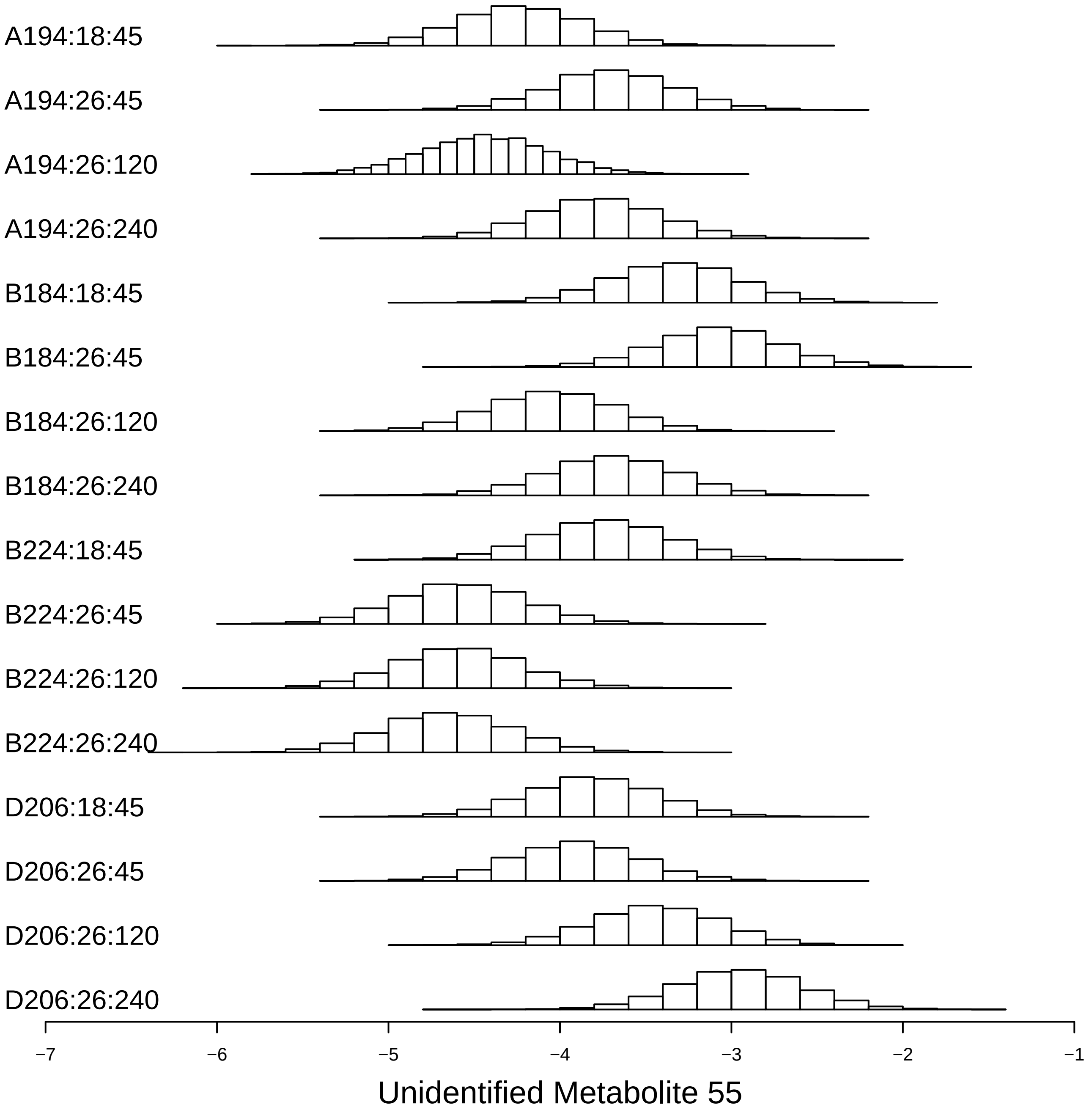
A194:18:45
A194:26:45
A194:26:120
A194:26:240
B184:18:45
B184:26:45
B184:26:120
B184:26:240
B224:18:45
B224:26:45
B224:26:120
B224:26:240
D206:18:45
D206:26:45
D206:26:120
D206:26:240
−7
−6
−5
−4
−3
−2
−1
Unidentified Metabolite 55

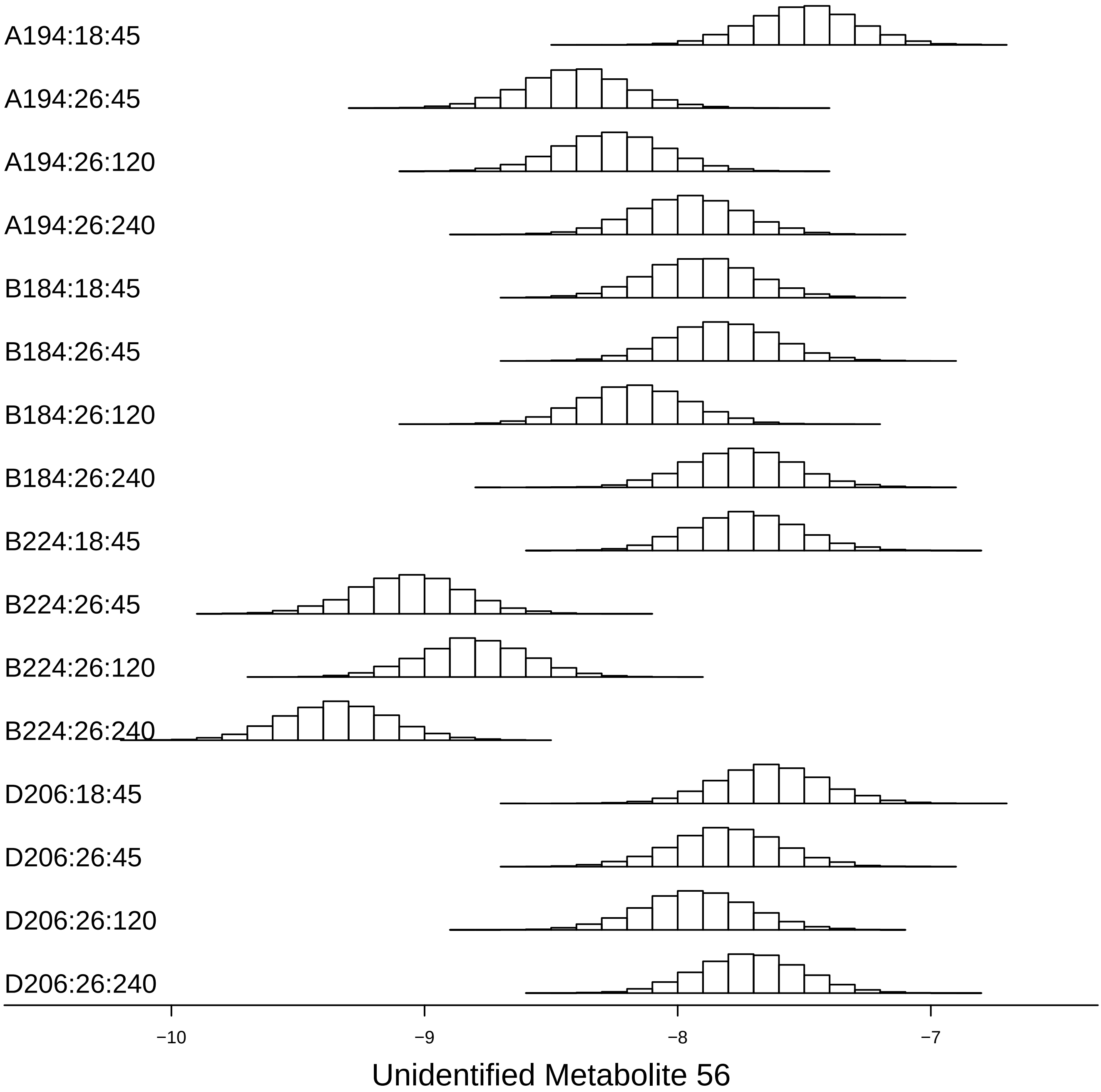
A194:18:45
A194:26:45
A194:26:120
A194:26:240
B184:18:45
B184:26:45
B184:26:120
B184:26:240
B224:18:45
B224:26:45
B224:26:120
B224:26:240
D206:18:45
D206:26:45
D206:26:120
D206:26:240
−10
−9
−8
−7
Unidentified Metabolite 56

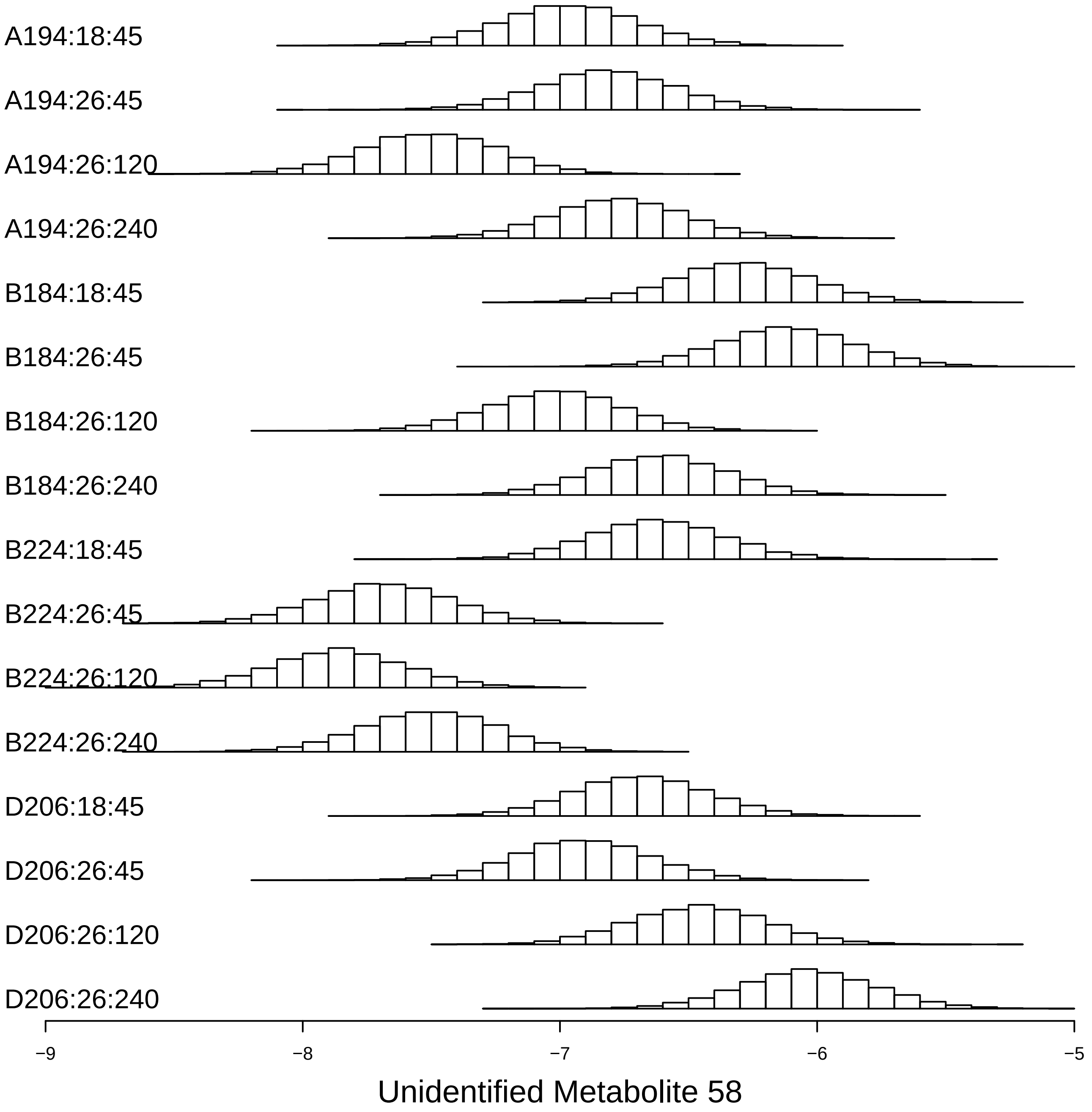
A194:18:45
A194:26:45
A194:26:120
A194:26:240
B184:18:45
B184:26:45
B184:26:120
B184:26:240
B224:18:45
B224:26:45
B224:26:120
B224:26:240
D206:18:45
D206:26:45
D206:26:120
D206:26:240
−9
−8
−7
−6
−5
Unidentified Metabolite 58

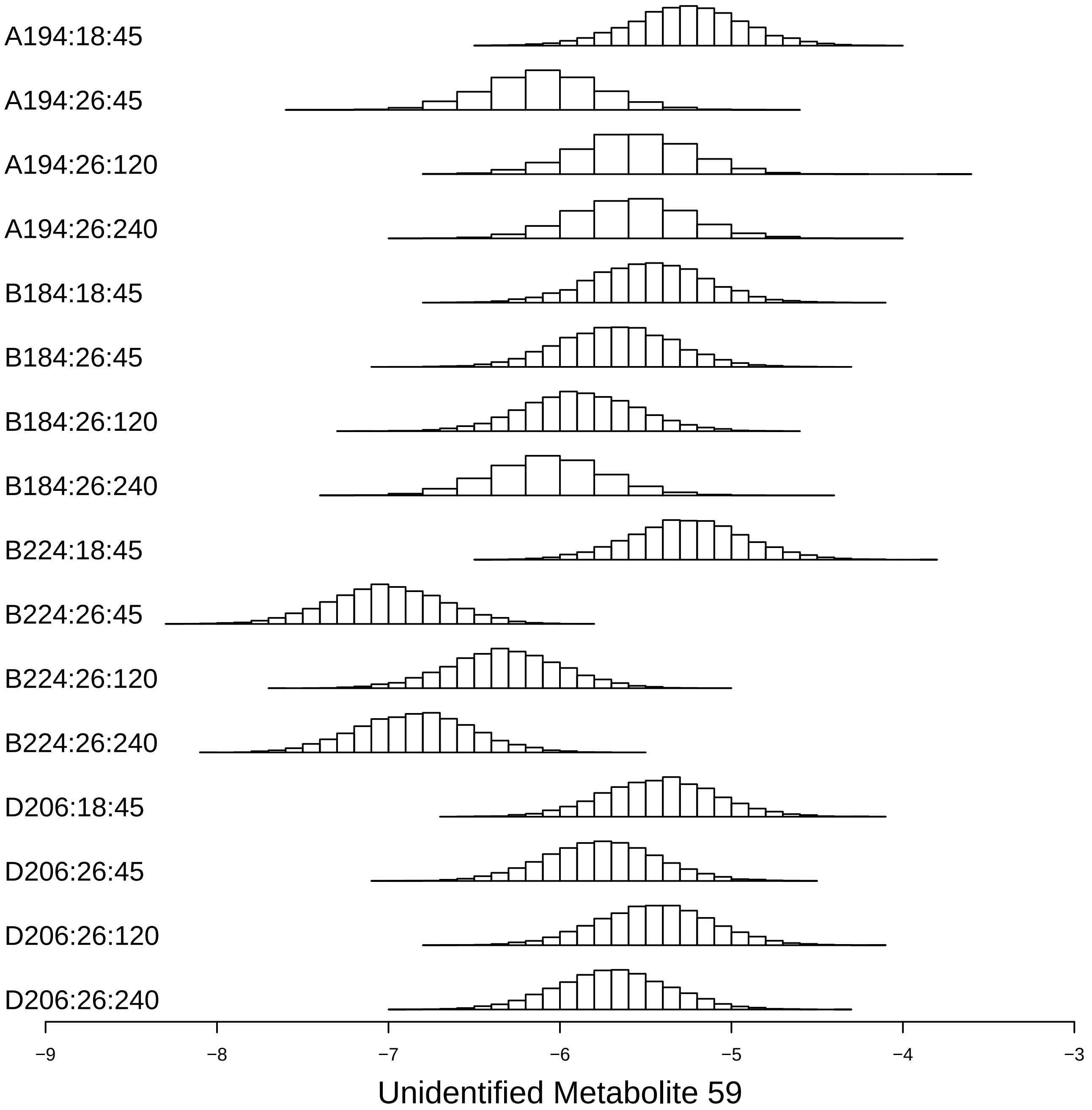
A194:18:45
A194:26:45
A194:26:120
A194:26:240
B184:18:45
B184:26:45
B184:26:120
B184:26:240
B224:18:45
B224:26:45
B224:26:120
B224:26:240
D206:18:45
D206:26:45
D206:26:120
D206:26:240
−9
−8
−7
−6
−5
−4
−3
Unidentified Metabolite 59

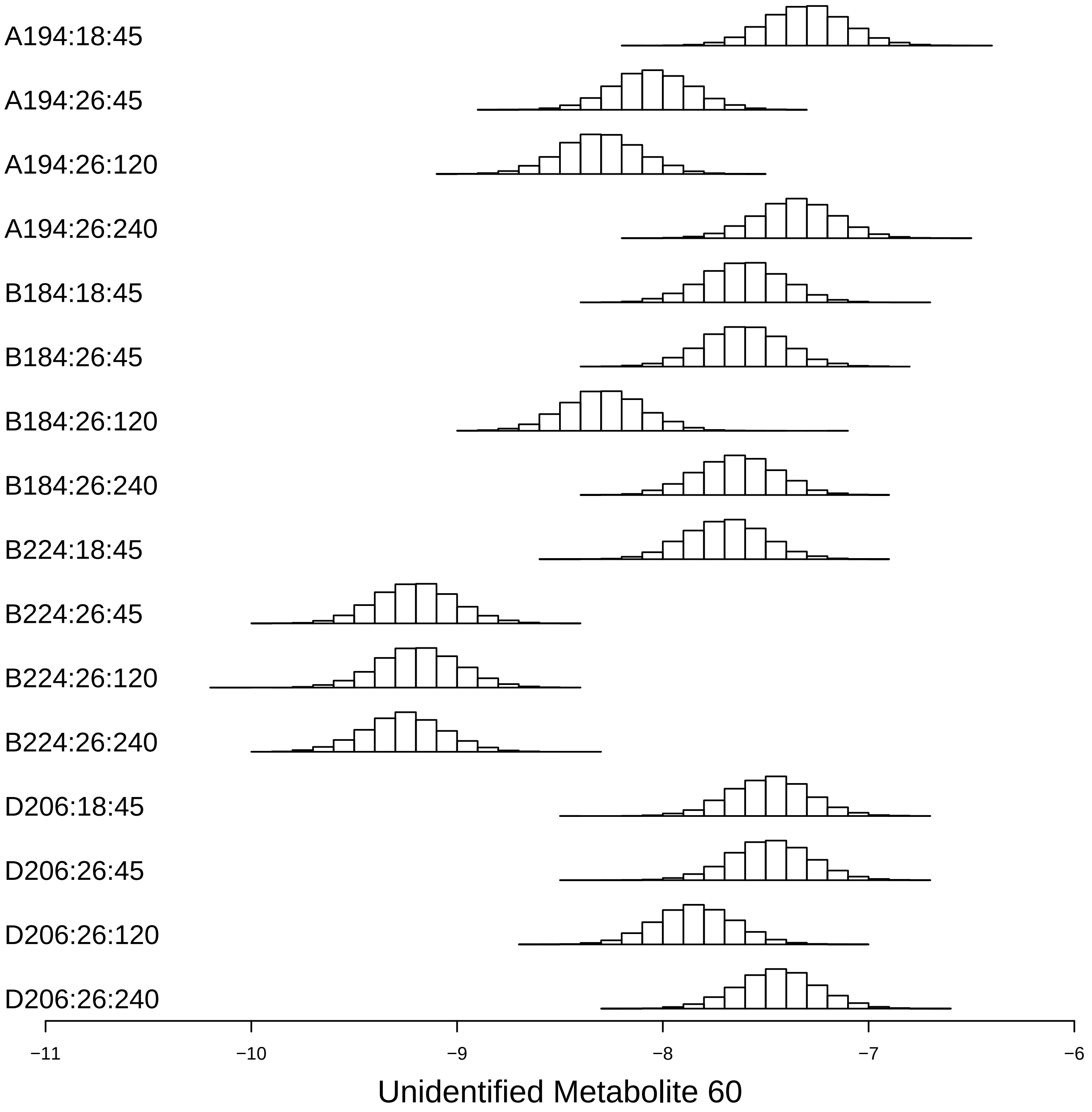
A194:18:45
A194:26:45
A194:26:120
A194:26:240
B184:18:45
B184:26:45
B184:26:120
B184:26:240
B224:18:45
B224:26:45
B224:26:120
B224:26:240
D206:18:45
D206:26:45
D206:26:120
D206:26:240
−11
−10
−9
−8
−7
−6
Unidentified Metabolite 60

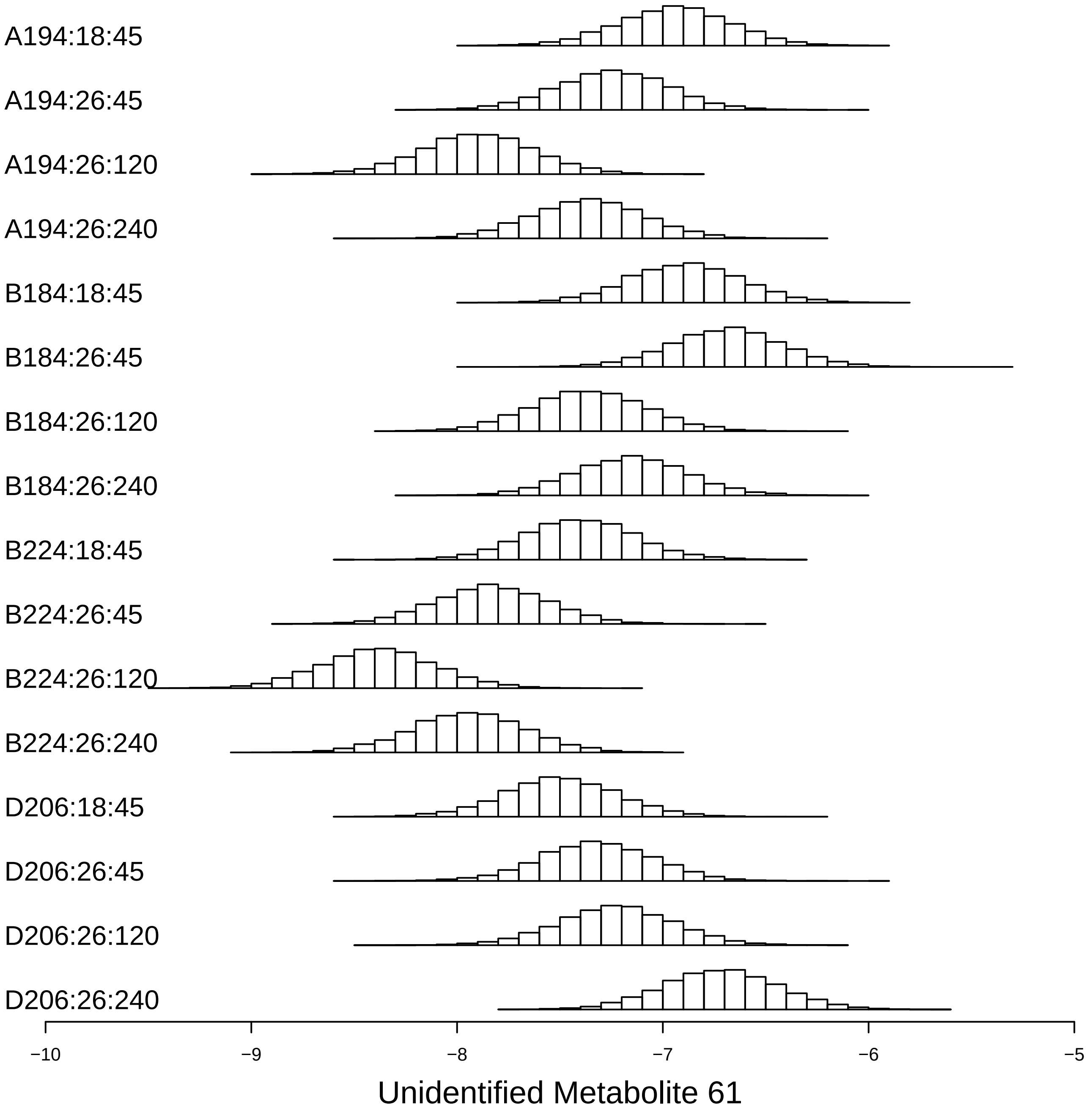

A194:18:45
A194:26:45
A194:26:120
A194:26:240
B184:18:45
B184:26:45
B184:26:120
B184:26:240
B224:18:45
B224:26:45
B224:26:120
B224:26:240
D206:18:45
D206:26:45
D206:26:120
D206:26:240
−10
−9
−8
−7
−6
−5
Unidentified Metabolite 61

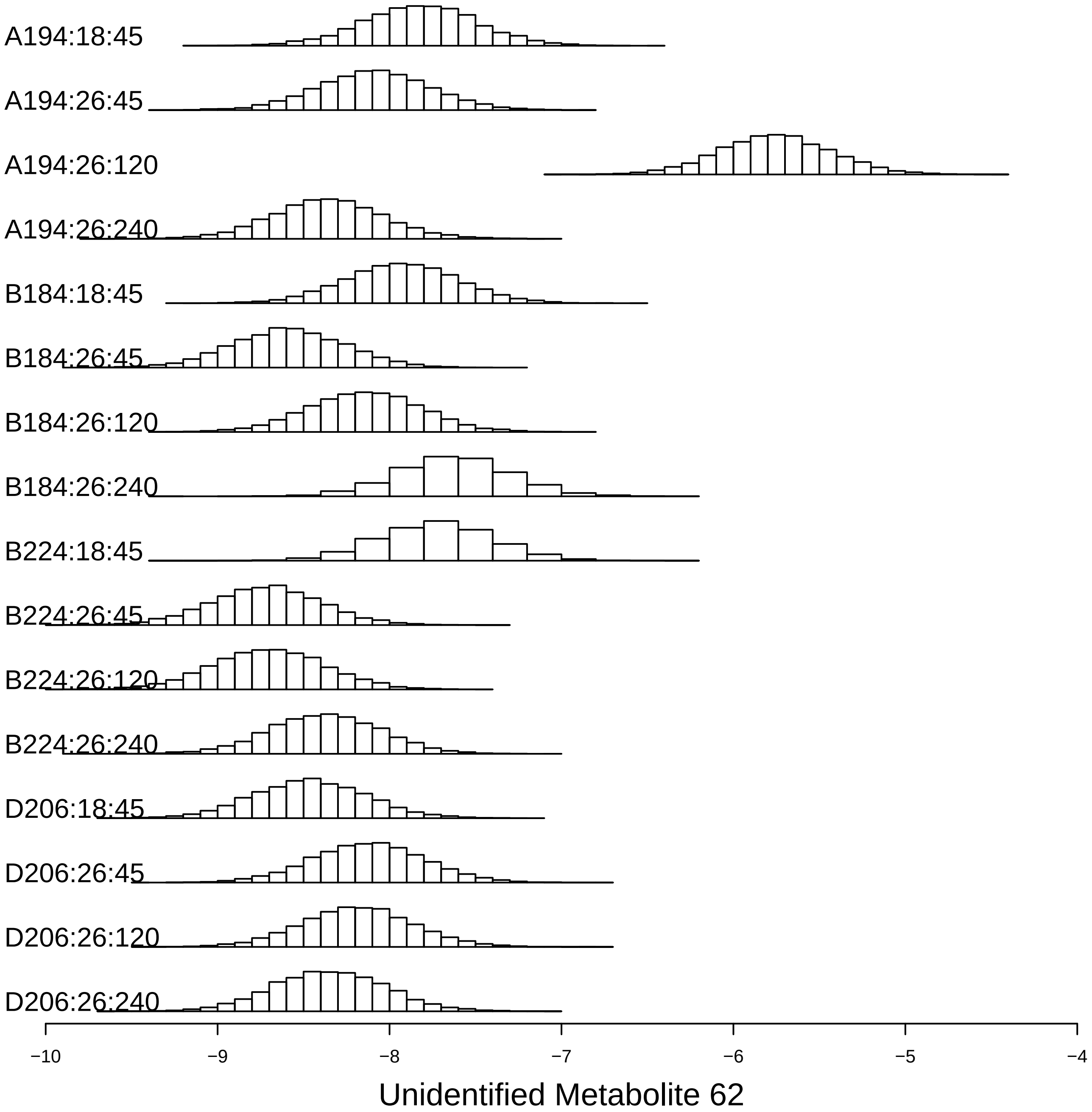
A194:18:45
A194:26:45
A194:26:120
A194:26:240
B184:18:45
B184:26:45
B184:26:120
B184:26:240
B224:18:45
B224:26:45
B224:26:120
B224:26:240
D206:18:45
D206:26:45
D206:26:120
D206:26:240
−10
−9
−8
−7
−6
−5
−4
Unidentified Metabolite 62

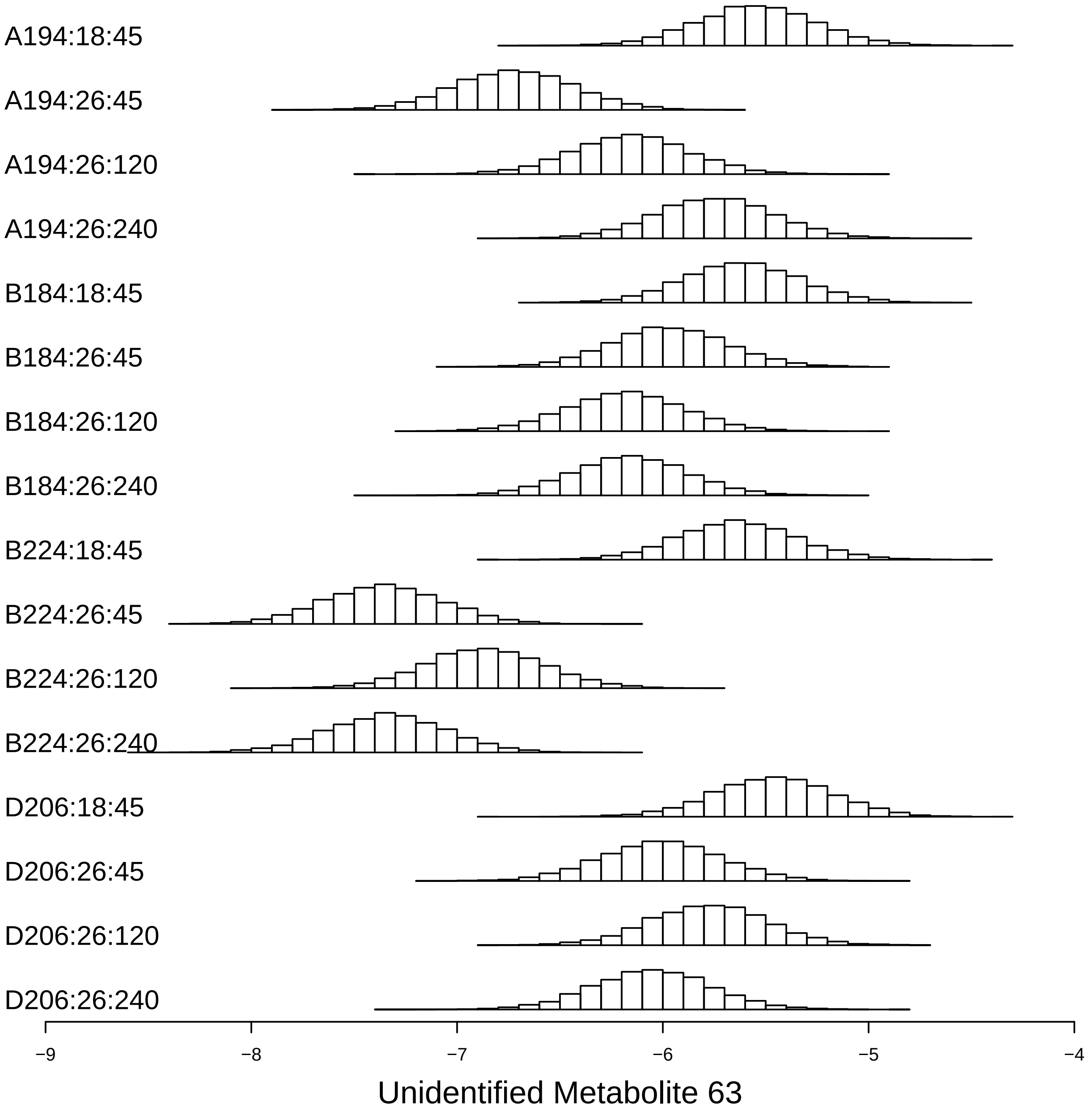
A194:18:45
A194:26:45
A194:26:120
A194:26:240
B184:18:45
B184:26:45
B184:26:120
B184:26:240
B224:18:45
B224:26:45
B224:26:120
B224:26:240
D206:18:45
D206:26:45
D206:26:120
D206:26:240
−9
−8
−7
−6
−5
−4
Unidentified Metabolite 63

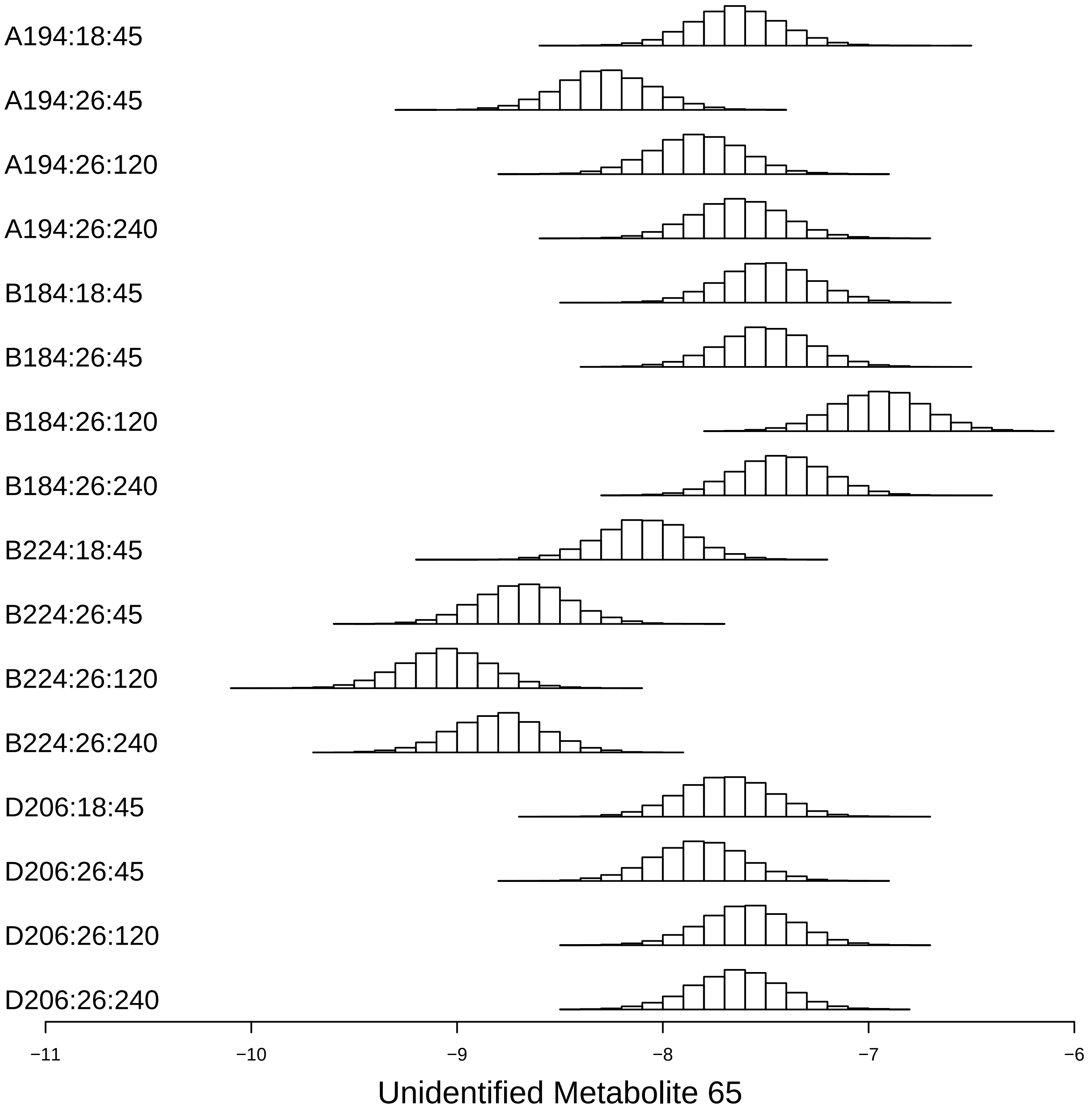
A194:18:45
A194:26:45
A194:26:120
A194:26:240
B184:18:45
B184:26:45
B184:26:120
B184:26:240
B224:18:45
B224:26:45
B224:26:120
B224:26:240
D206:18:45
D206:26:45
D206:26:120
D206:26:240
−11
−10
−9
−8
−7
−6
Unidentified Metabolite 65

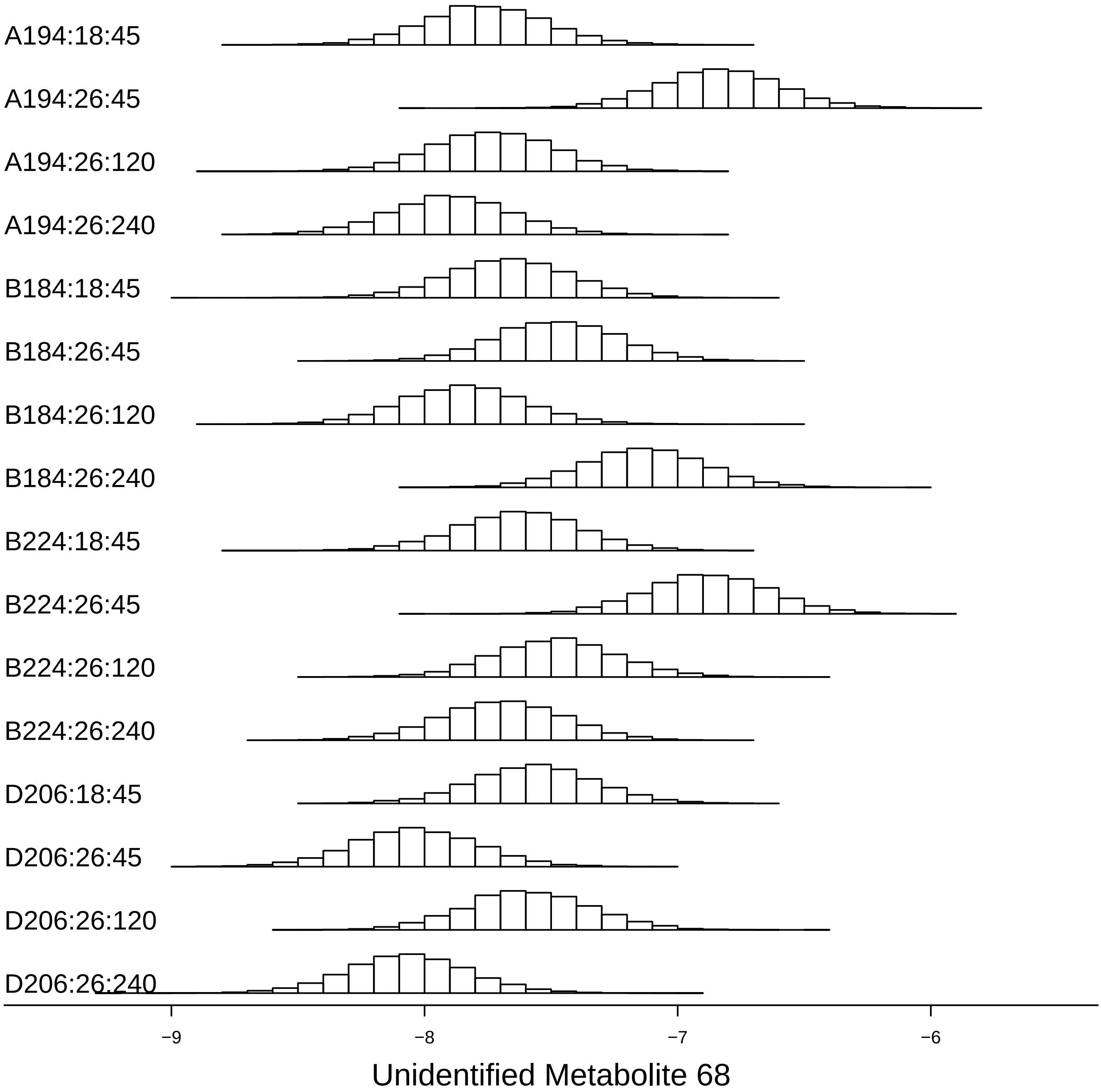
A194:18:45
A194:26:45
A194:26:120
A194:26:240
B184:18:45
B184:26:45
B184:26:120
B184:26:240
B224:18:45
B224:26:45
B224:26:120
B224:26:240
D206:18:45
D206:26:45
D206:26:120
D206:26:240
−9
−8
−7
−6
Unidentified Metabolite 68

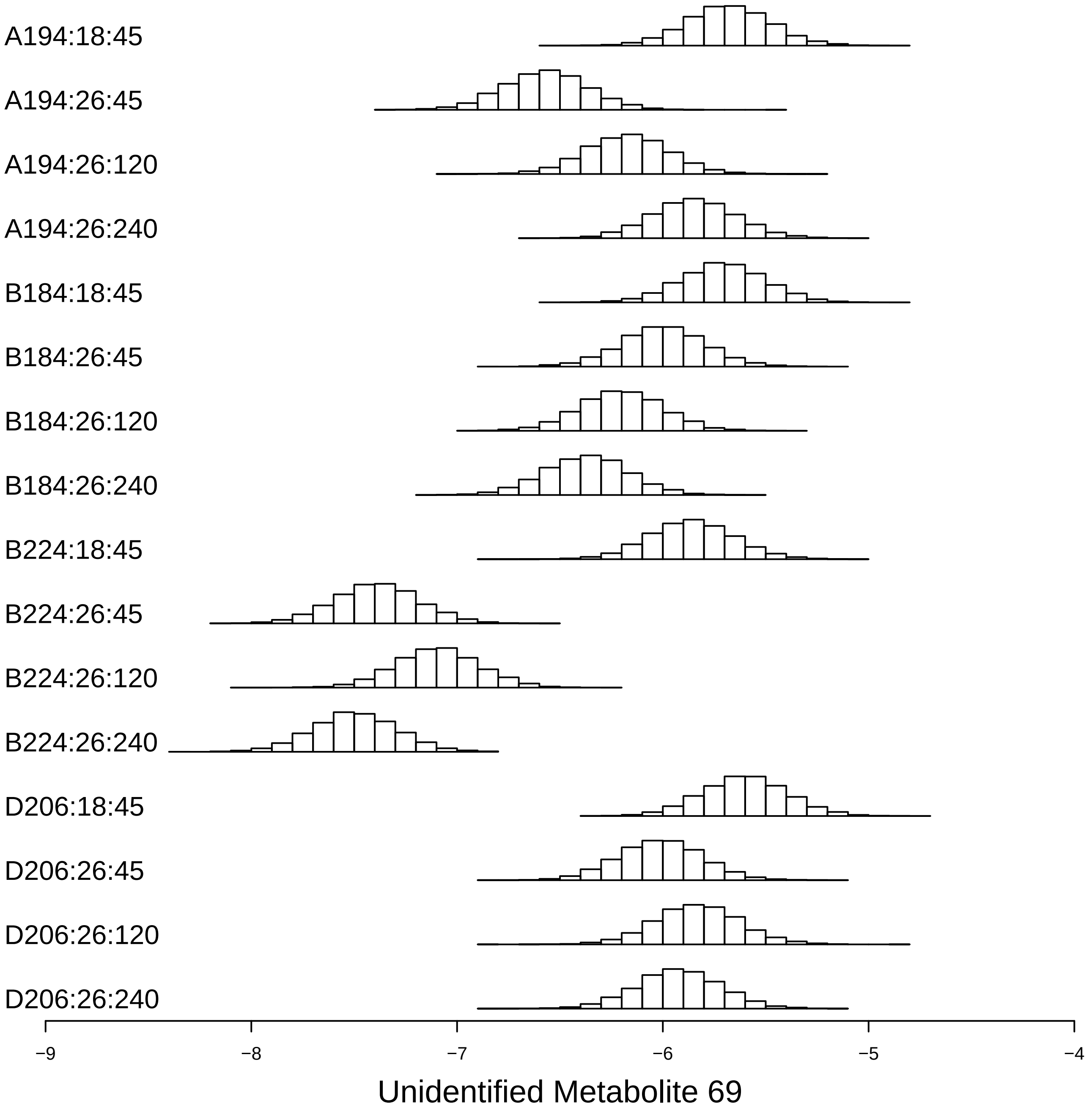
A194:18:45
A194:26:45
A194:26:120
A194:26:240
B184:18:45
B184:26:45
B184:26:120
B184:26:240
B224:18:45
B224:26:45
B224:26:120
B224:26:240
D206:18:45
D206:26:45
D206:26:120
D206:26:240
−9
−8
−7
−6
−5
−4
Unidentified Metabolite 69

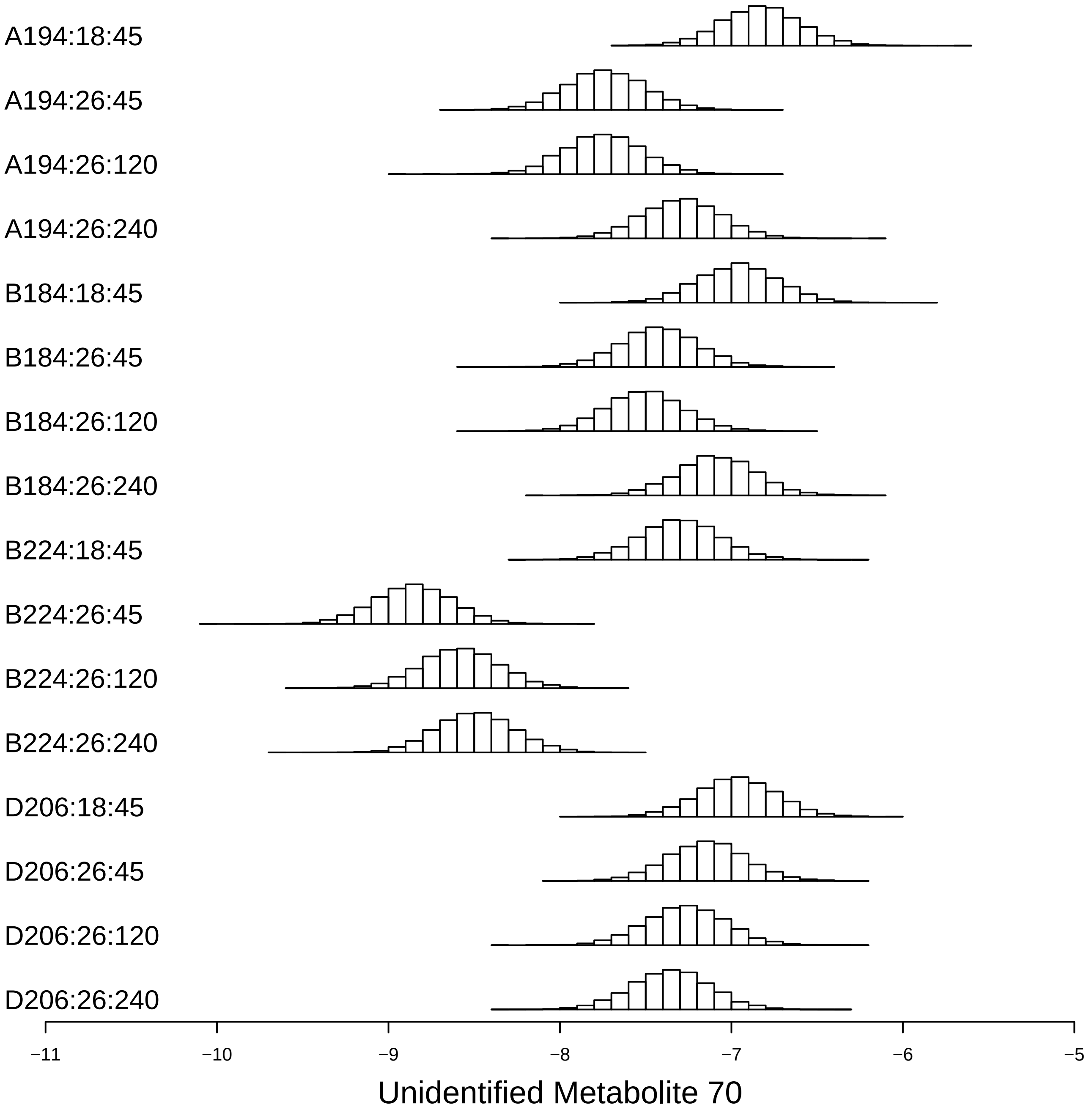

A194:18:45
A194:26:45
A194:26:120
A194:26:240
B184:18:45
B184:26:45
B184:26:120
B184:26:240
B224:18:45
B224:26:45
B224:26:120
B224:26:240
D206:18:45
D206:26:45
D206:26:120
D206:26:240
−11
−10
−9
−8
−7
−6
−5
Unidentified Metabolite 70

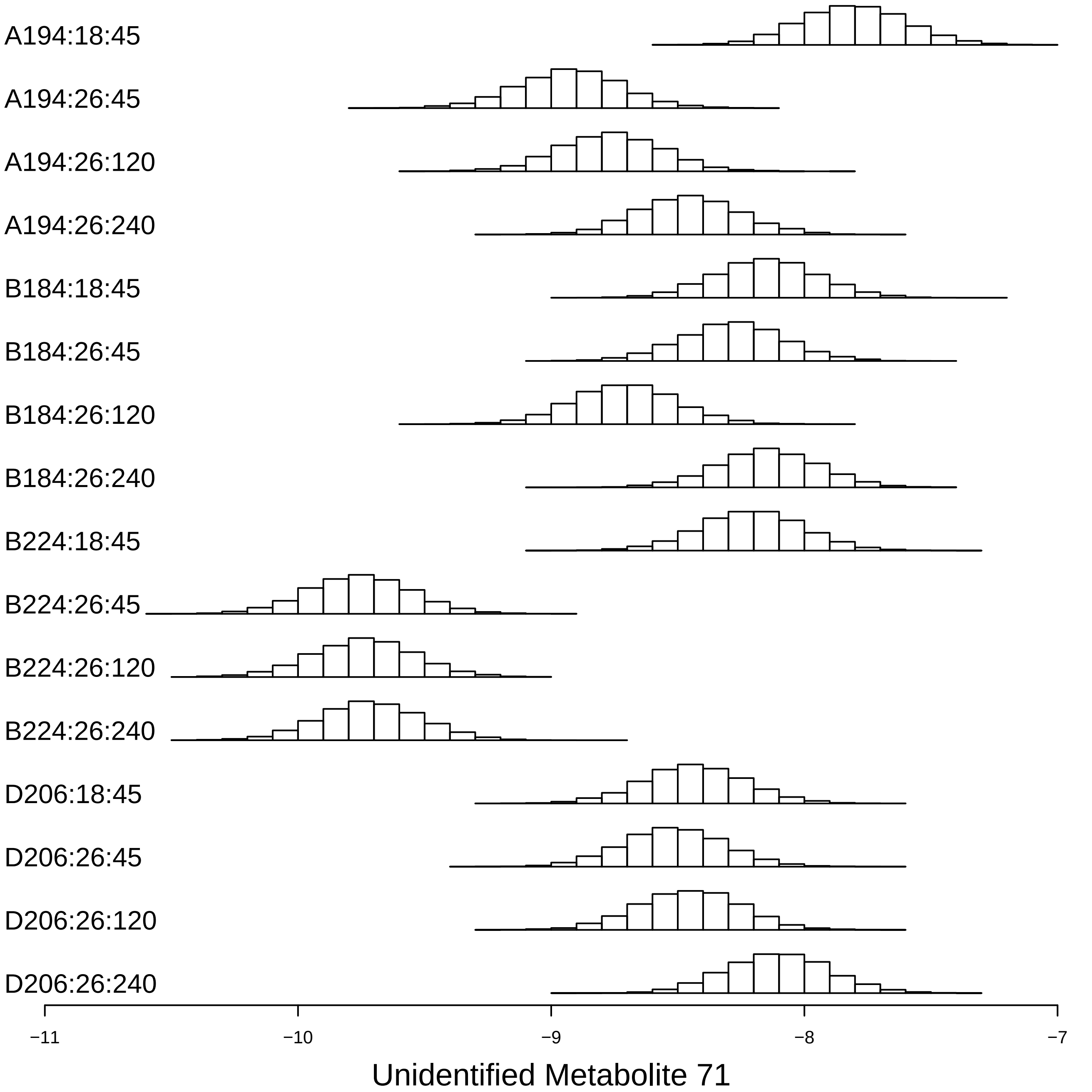
A194:18:45
A194:26:45
A194:26:120
A194:26:240
B184:18:45
B184:26:45
B184:26:120
B184:26:240
B224:18:45
B224:26:45
B224:26:120
B224:26:240
D206:18:45
D206:26:45
D206:26:120
D206:26:240
−11
−10
−9
−8
−7
Unidentified Metabolite 71

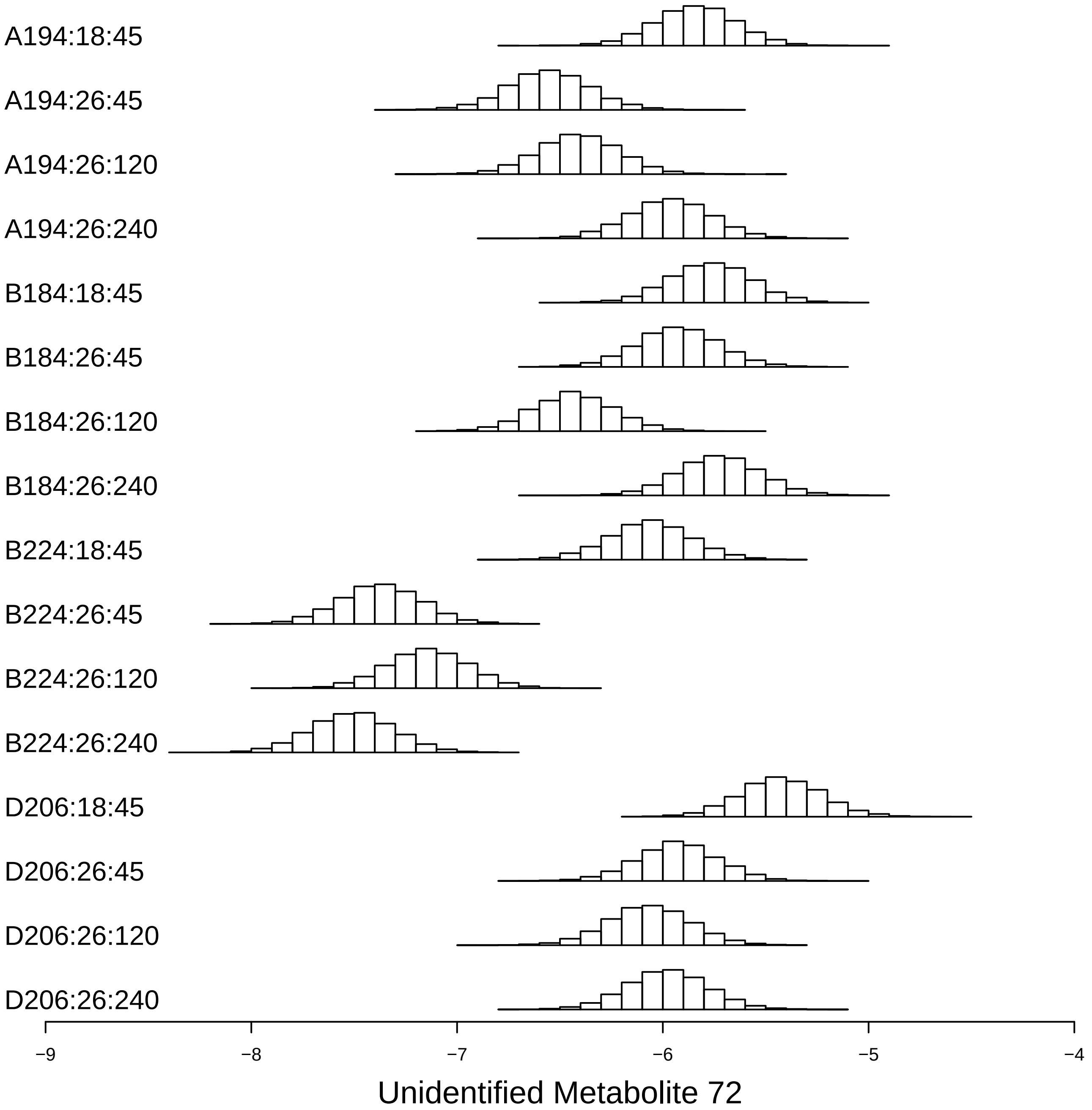

A194:18:45
A194:26:45
A194:26:120
A194:26:240
B184:18:45
B184:26:45
B184:26:120
B184:26:240
B224:18:45
B224:26:45
B224:26:120
B224:26:240
D206:18:45
D206:26:45
D206:26:120
D206:26:240
−9
−8
−7
−6
−5
−4
Unidentified Metabolite 72

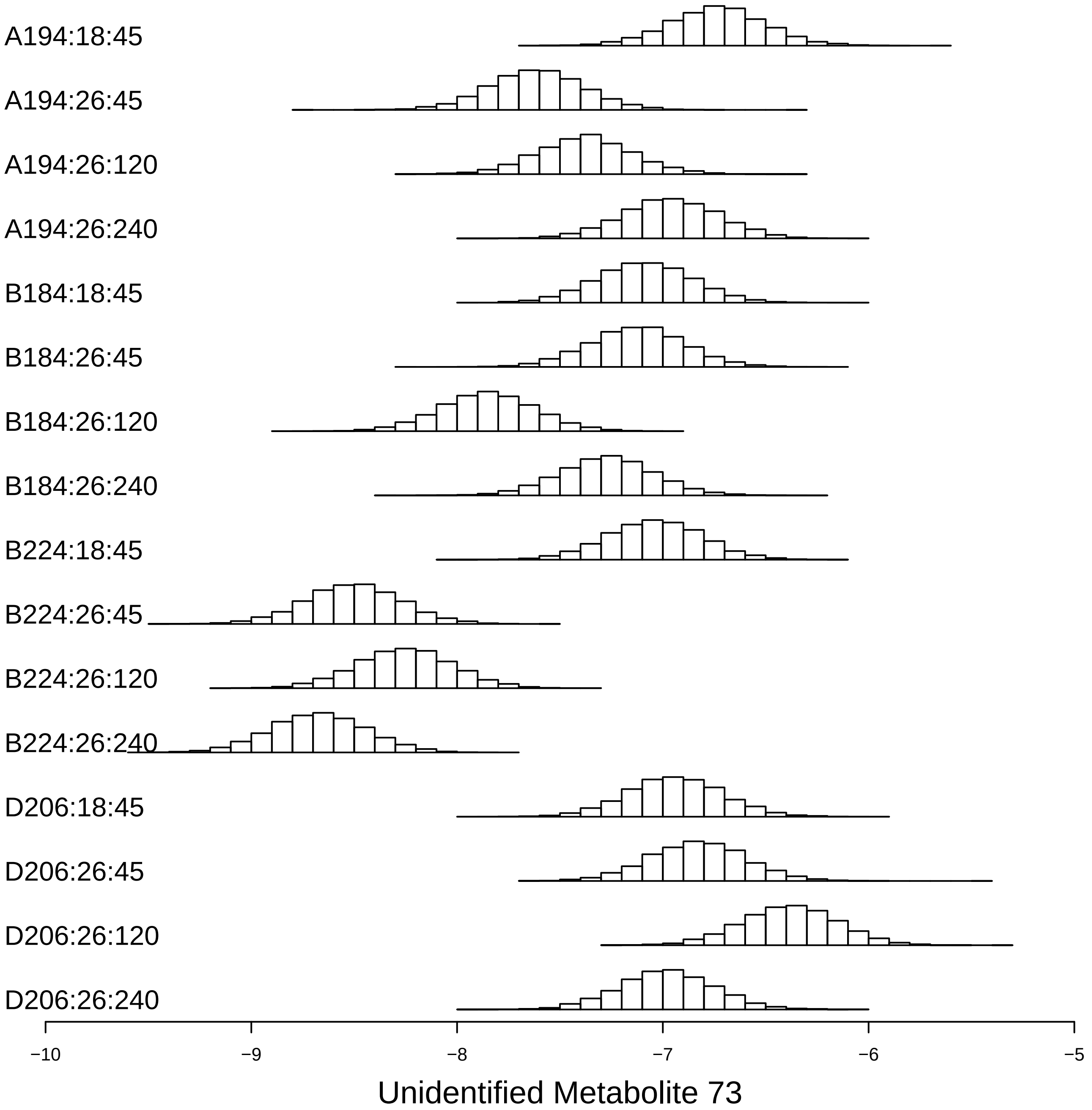
A194:18:45
A194:26:45
A194:26:120
A194:26:240
B184:18:45
B184:26:45
B184:26:120
B184:26:240
B224:18:45
B224:26:45
B224:26:120
B224:26:240
D206:18:45
D206:26:45
D206:26:120
D206:26:240
−10
−9
−8
−7
−6
−5
Unidentified Metabolite 73

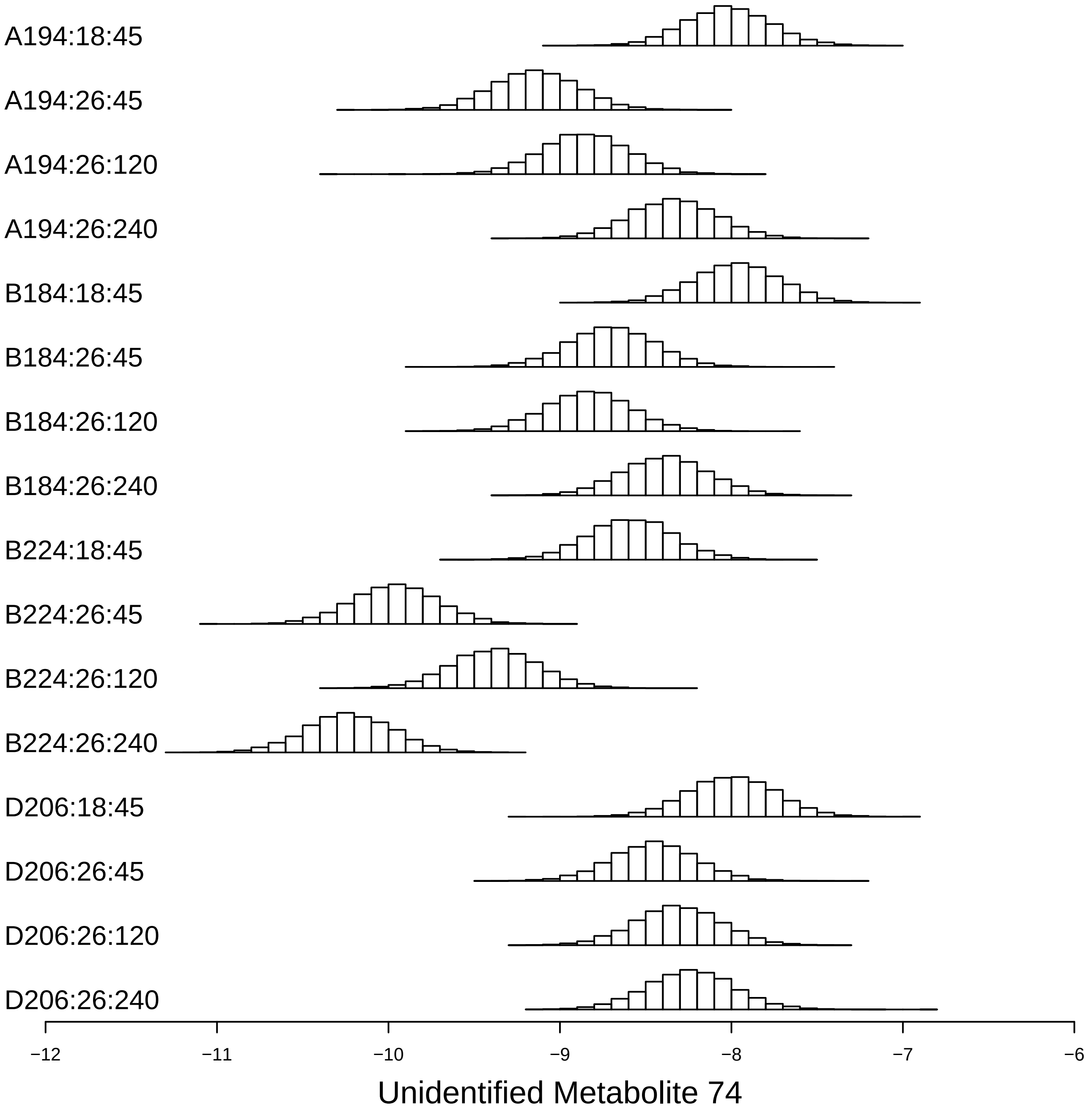
A194:18:45
A194:26:45
A194:26:120
A194:26:240
B184:18:45
B184:26:45
B184:26:120
B184:26:240
B224:18:45
B224:26:45
B224:26:120
B224:26:240
D206:18:45
D206:26:45
D206:26:120
D206:26:240
−12
−11
−10
−9
−8
−7
−6
Unidentified Metabolite 74

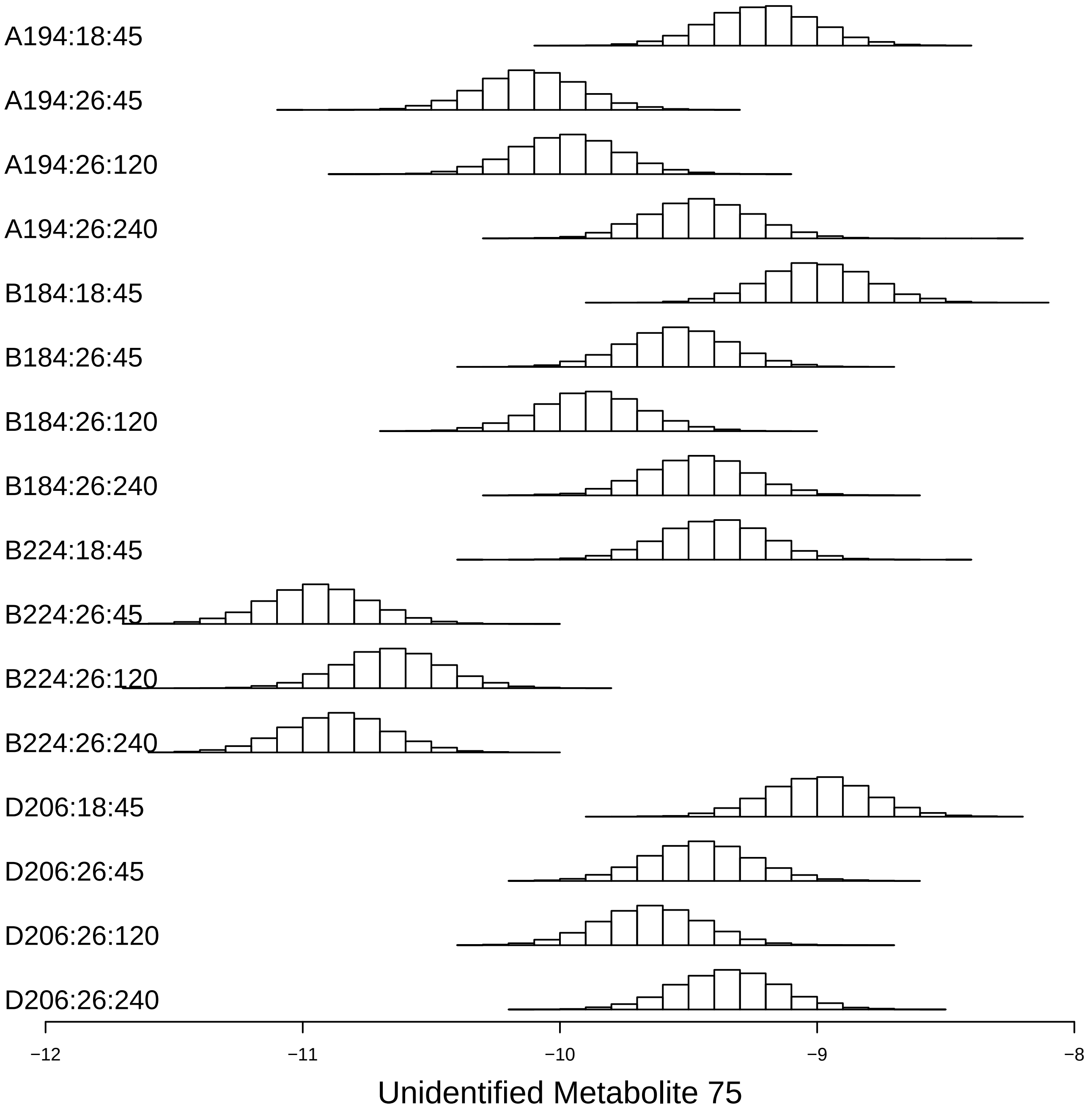
A194:18:45
A194:26:45
A194:26:120
A194:26:240
B184:18:45
B184:26:45
B184:26:120
B184:26:240
B224:18:45
B224:26:45
B224:26:120
B224:26:240
D206:18:45
D206:26:45
D206:26:120
D206:26:240
−12
−11
−10
−9
−8
Unidentified Metabolite 75

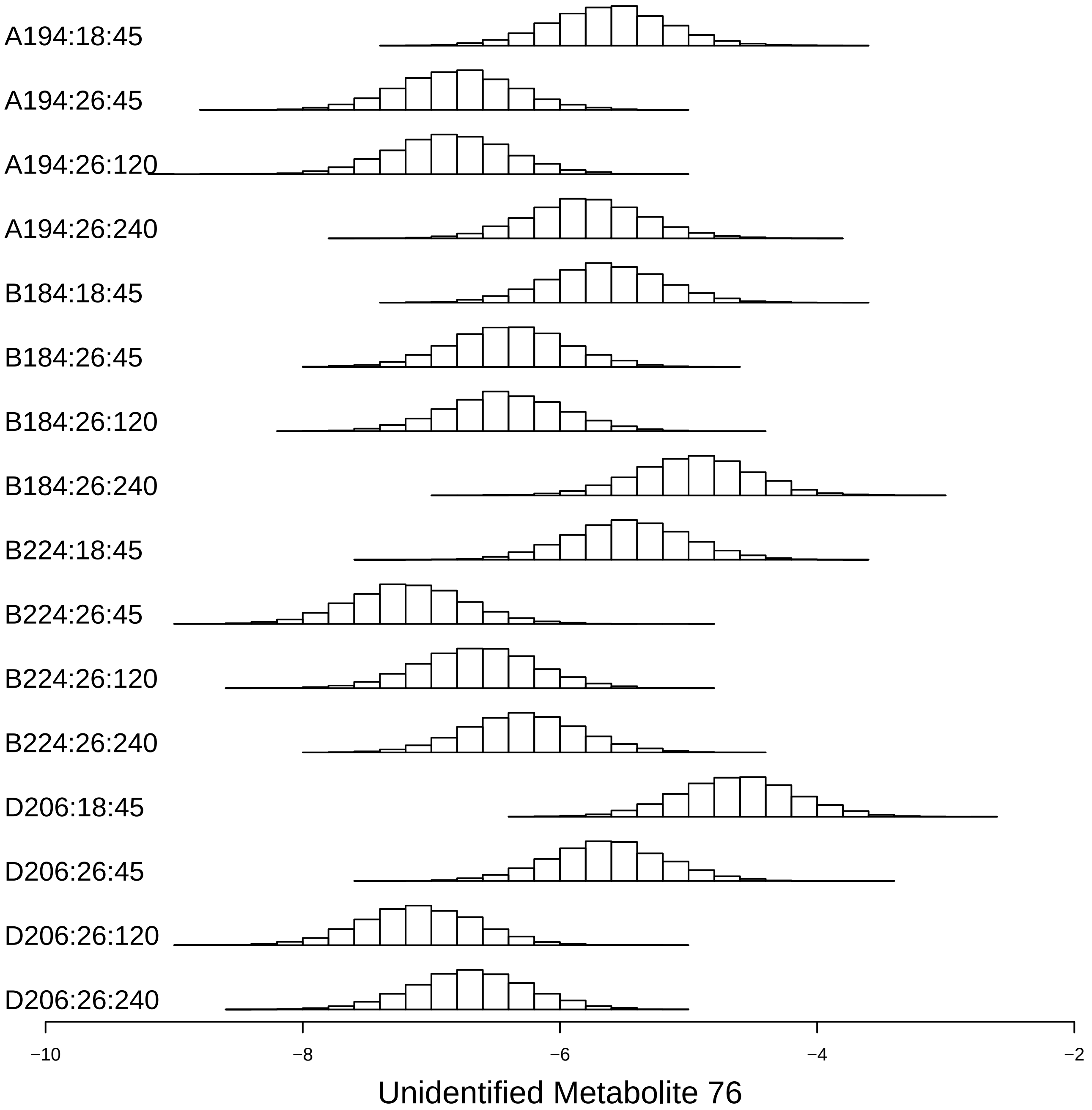
A194:18:45
A194:26:45
A194:26:120
A194:26:240
B184:18:45
B184:26:45
B184:26:120
B184:26:240
B224:18:45
B224:26:45
B224:26:120
B224:26:240
D206:18:45
D206:26:45
D206:26:120
D206:26:240
−10
−8
−6
−4
−2
Unidentified Metabolite 76

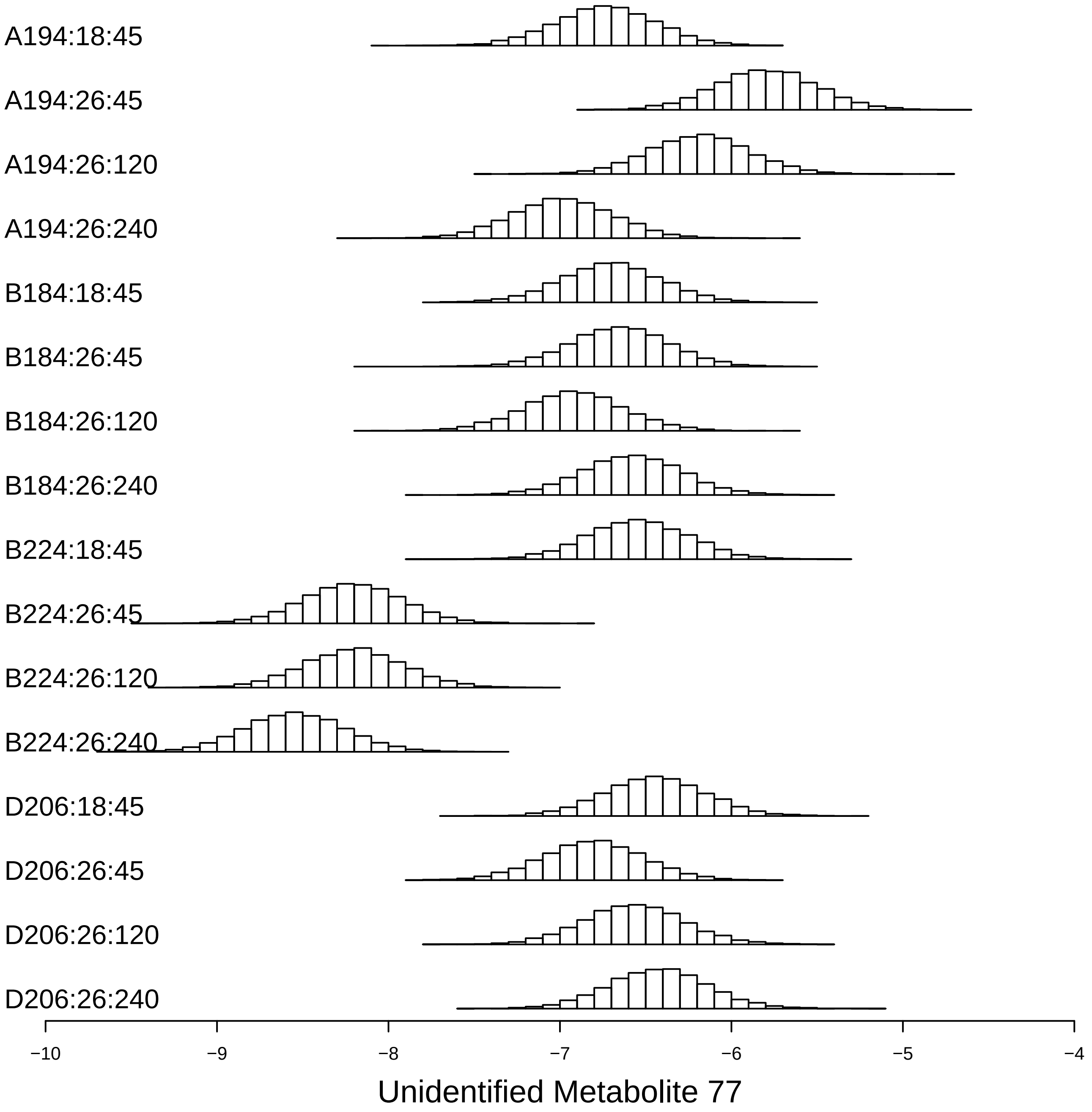
A194:18:45
A194:26:45
A194:26:120
A194:26:240
B184:18:45
B184:26:45
B184:26:120
B184:26:240
B224:18:45
B224:26:45
B224:26:120
B224:26:240
D206:18:45
D206:26:45
D206:26:120
D206:26:240
−10
−9
−8
−7
−6
−5
−4
Unidentified Metabolite 77

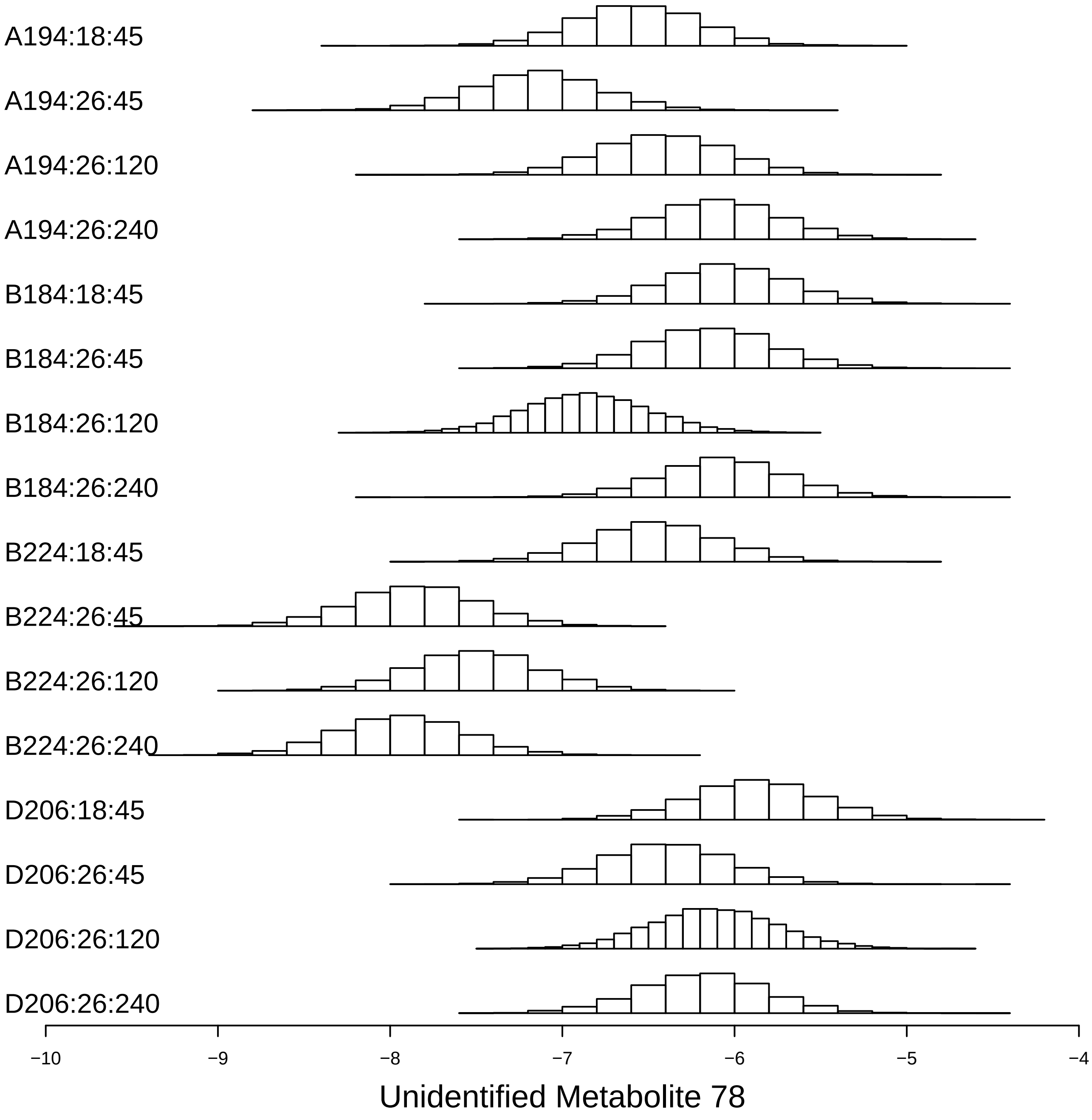

A194:18:45
A194:26:45
A194:26:120
A194:26:240
B184:18:45
B184:26:45
B184:26:120
B184:26:240
B224:18:45
B224:26:45
B224:26:120
B224:26:240
D206:18:45
D206:26:45
D206:26:120
D206:26:240
−10
−9
−8
−7
−6
−5
−4
Unidentified Metabolite 78

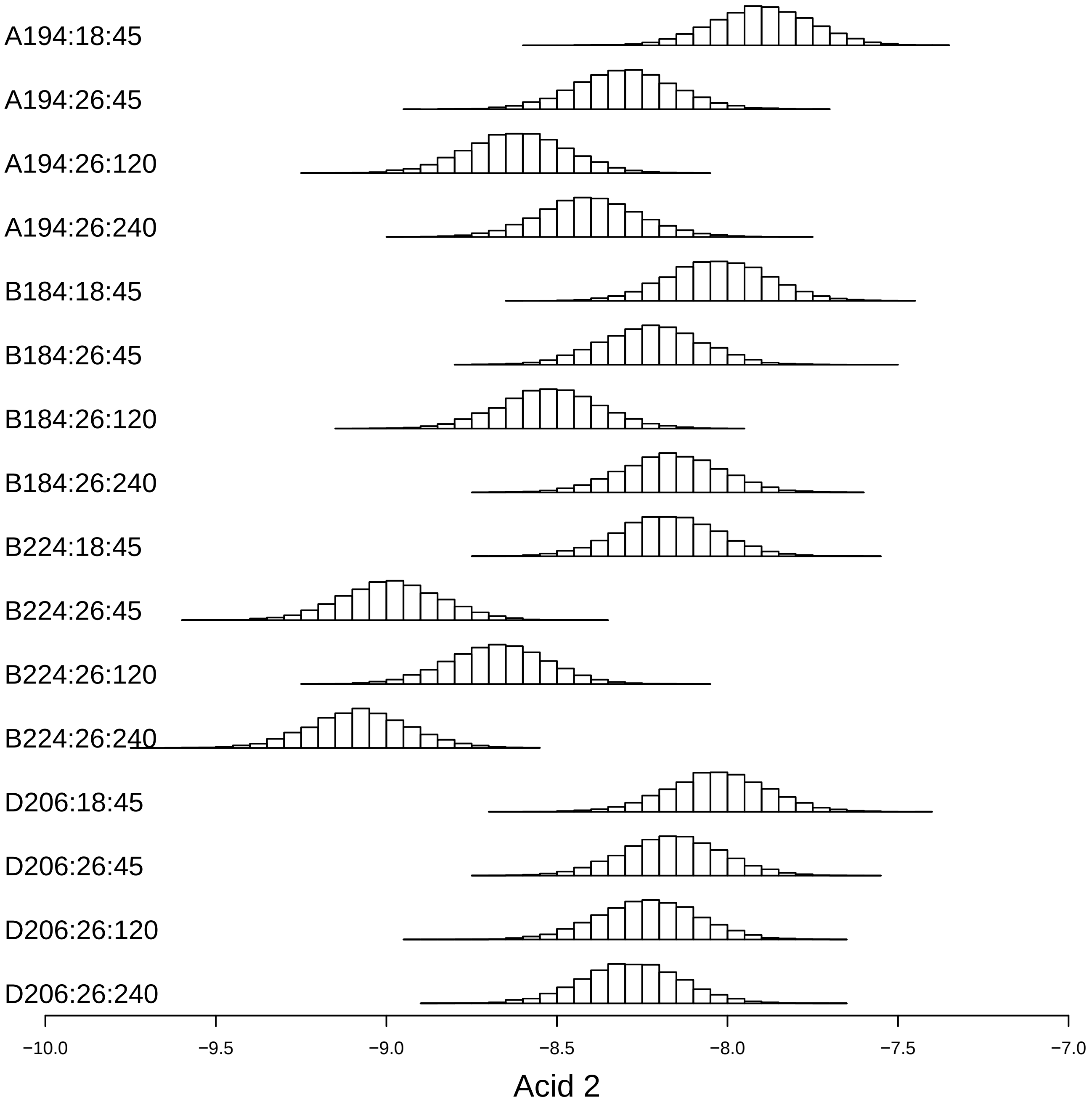

A194:18:45
A194:26:45
A194:26:120
A194:26:240
B184:18:45
B184:26:45
B184:26:120
B184:26:240
B224:18:45
B224:26:45
B224:26:120
B224:26:240
D206:18:45
D206:26:45
D206:26:120
D206:26:240
−10.0
−9.5
−9.0
−8.5
−8.0
−7.5
−7.0
Acid 2

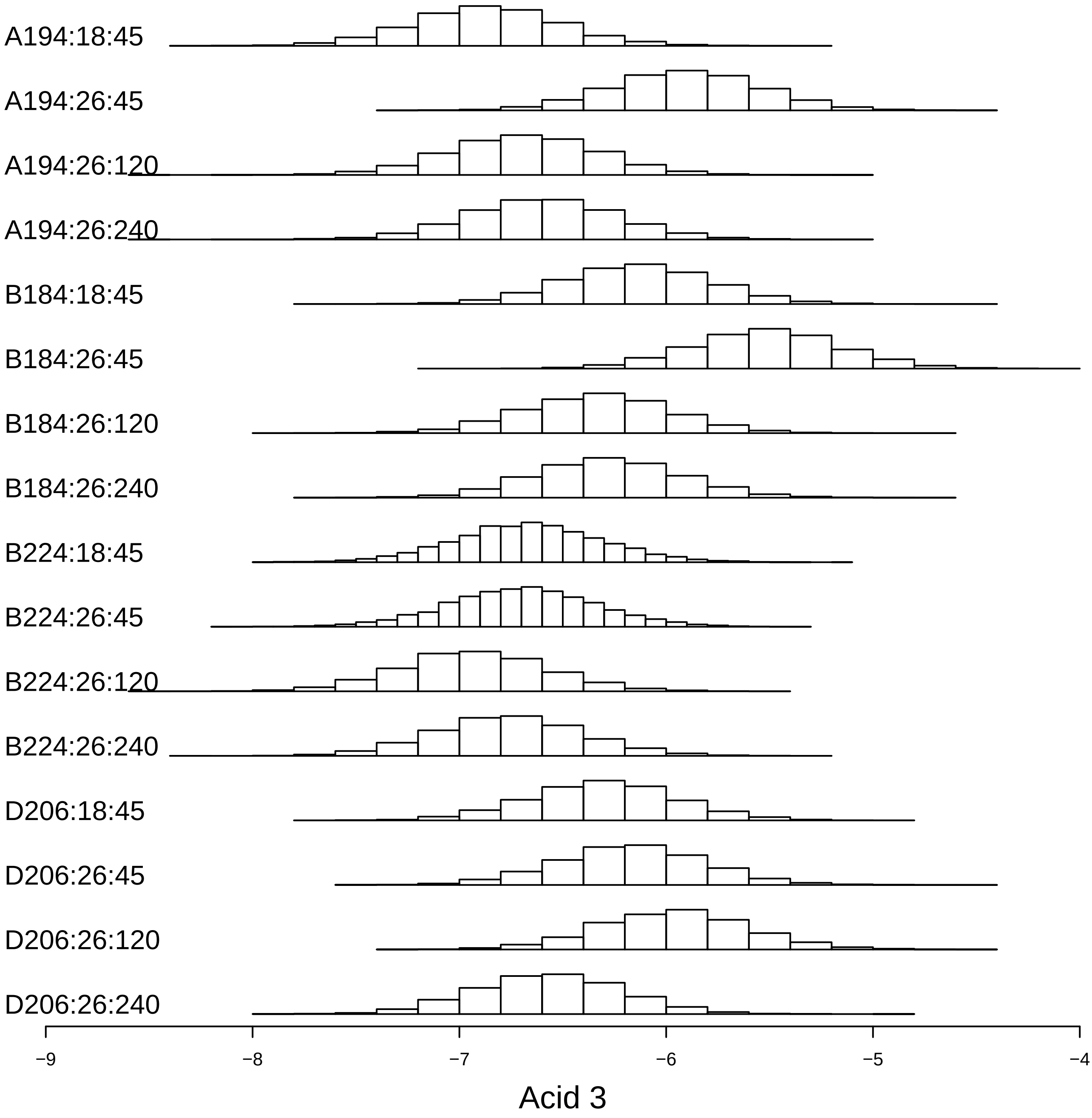
A194:18:45
A194:26:45
A194:26:120
A194:26:240
B184:18:45
B184:26:45
B184:26:120
B184:26:240
B224:18:45
B224:26:45
B224:26:120
B224:26:240
D206:18:45
D206:26:45
D206:26:120
D206:26:240
−9
−8
−7
−6
−5
−4
Acid 3

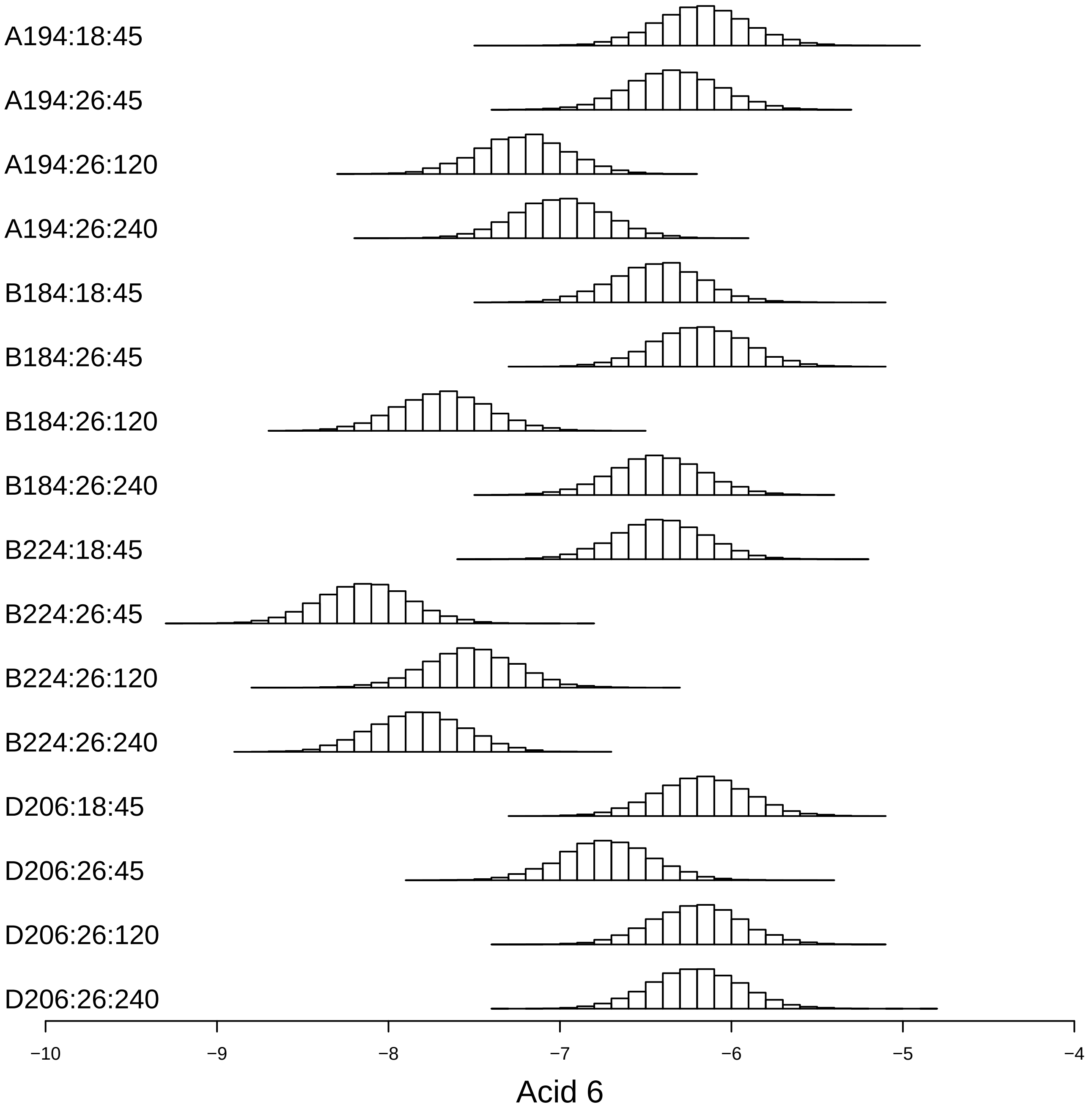
A194:18:45
A194:26:45
A194:26:120
A194:26:240
B184:18:45
B184:26:45
B184:26:120
B184:26:240
B224:18:45
B224:26:45
B224:26:120
B224:26:240
D206:18:45
D206:26:45
D206:26:120
D206:26:240
−10
−9
−8
−7
−6
−5
−4
Acid 6

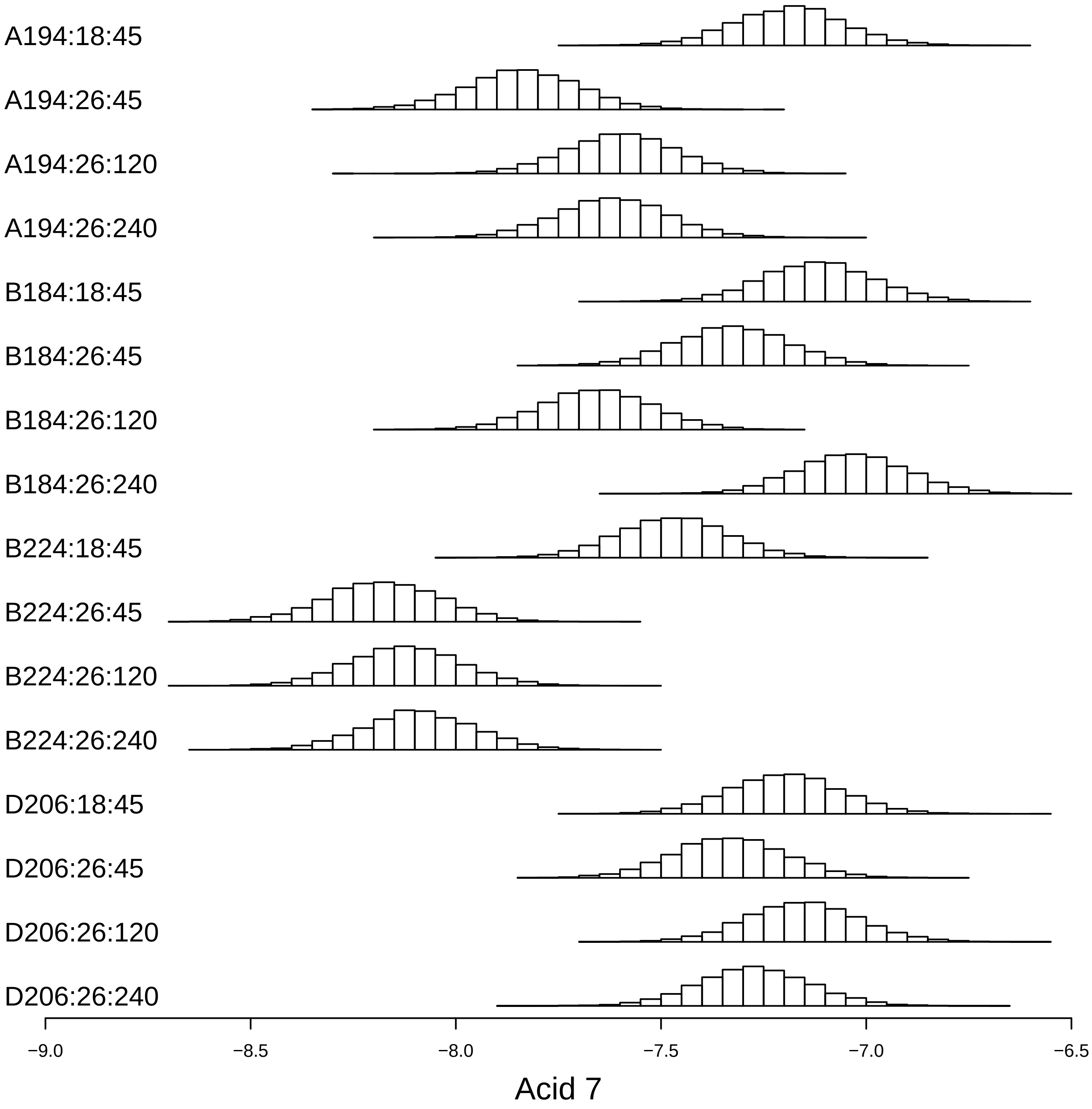
A194:18:45
A194:26:45
A194:26:120
A194:26:240
B184:18:45
B184:26:45
B184:26:120
B184:26:240
B224:18:45
B224:26:45
B224:26:120
B224:26:240
D206:18:45
D206:26:45
D206:26:120
D206:26:240
−9.0
−8.5
−8.0
−7.5
−7.0
−6.5
Acid 7

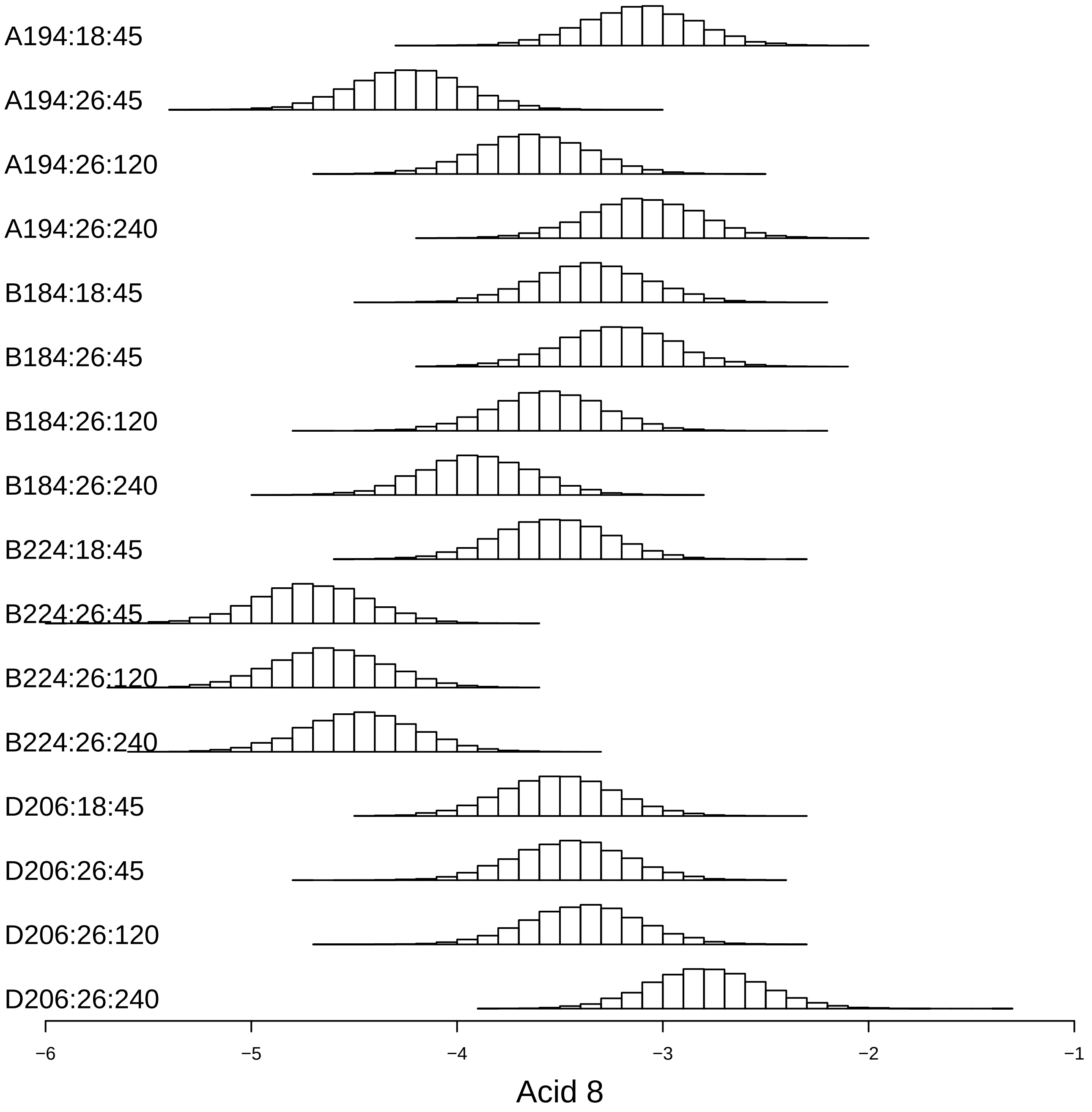
A194:18:45
A194:26:45
A194:26:120
A194:26:240
B184:18:45
B184:26:45
B184:26:120
B184:26:240
B224:18:45
B224:26:45
B224:26:120
B224:26:240
D206:18:45
D206:26:45
D206:26:120
D206:26:240
−6
−5
−4
−3
−2
−1
Acid 8

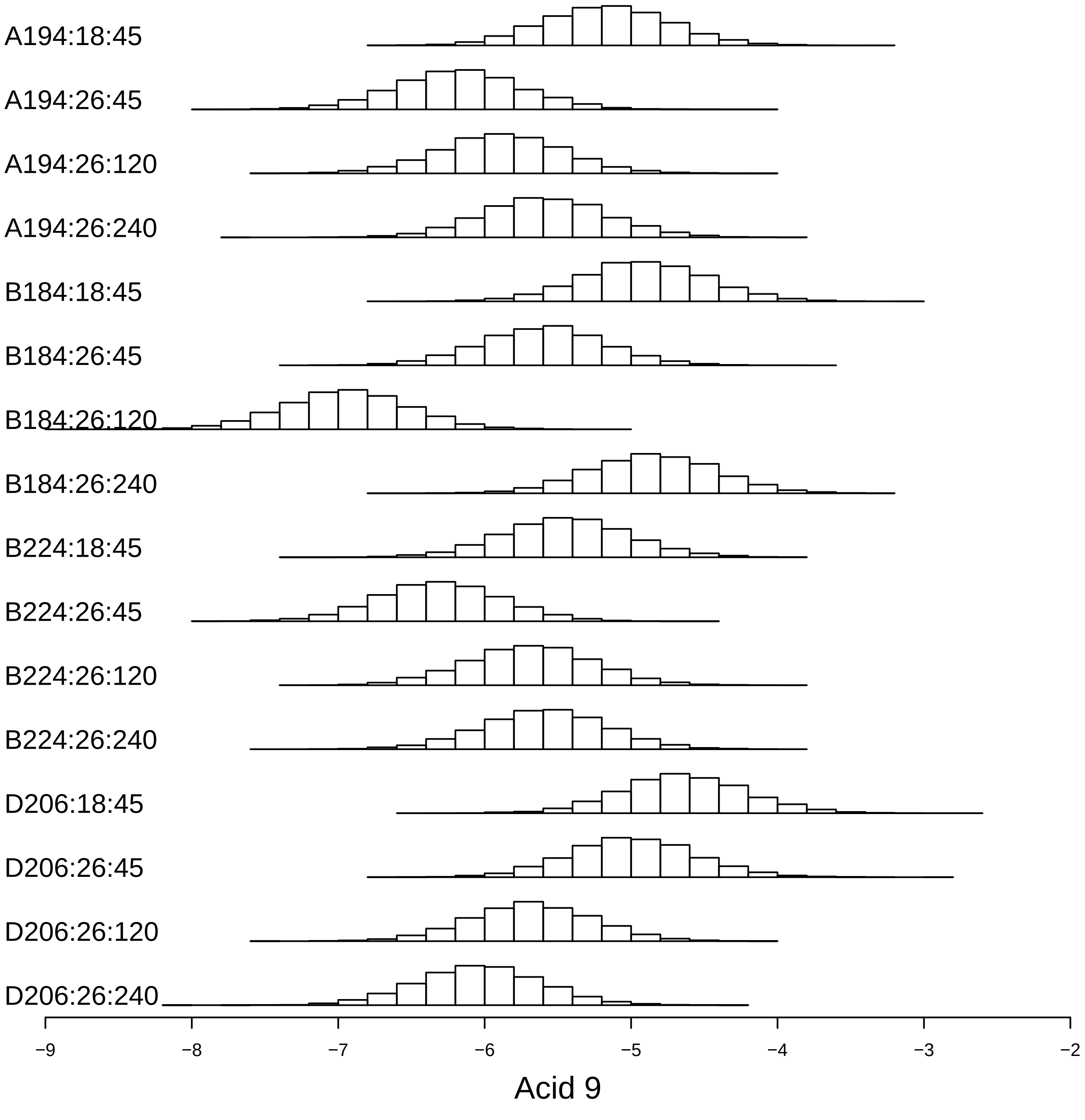
A194:18:45
A194:26:45
A194:26:120
A194:26:240
B184:18:45
B184:26:45
B184:26:120
B184:26:240
B224:18:45
B224:26:45
B224:26:120
B224:26:240
D206:18:45
D206:26:45
D206:26:120
D206:26:240
−9
−8
−7
−6
−5
−4
−3
−2
Acid 9

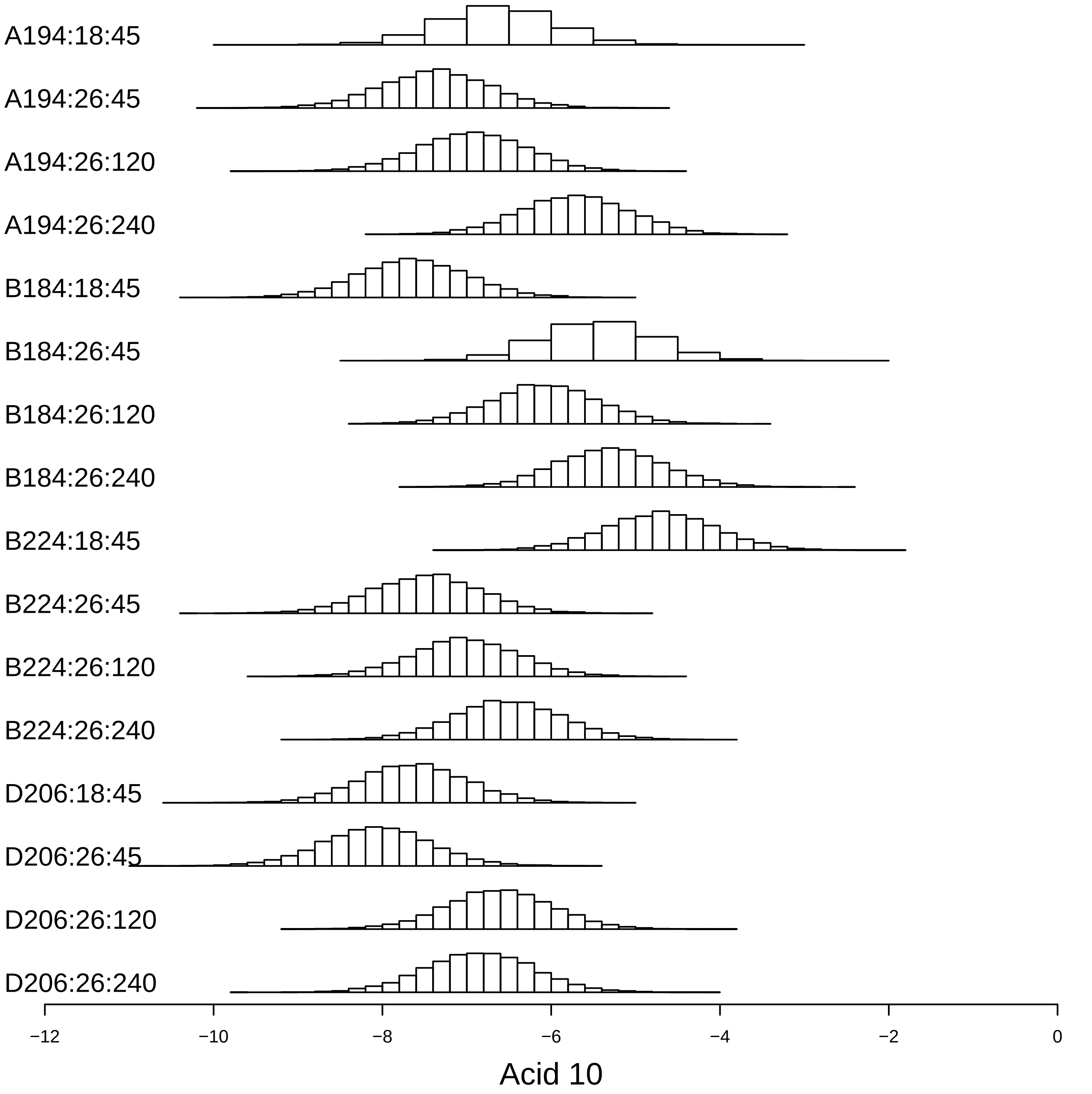

A194:18:45
A194:26:45
A194:26:120
A194:26:240
B184:18:45
B184:26:45
B184:26:120
B184:26:240
B224:18:45
B224:26:45
B224:26:120
B224:26:240
D206:18:45
D206:26:45
D206:26:120
D206:26:240
−12
−10
−8
−6
−4
−2
0
Acid 10

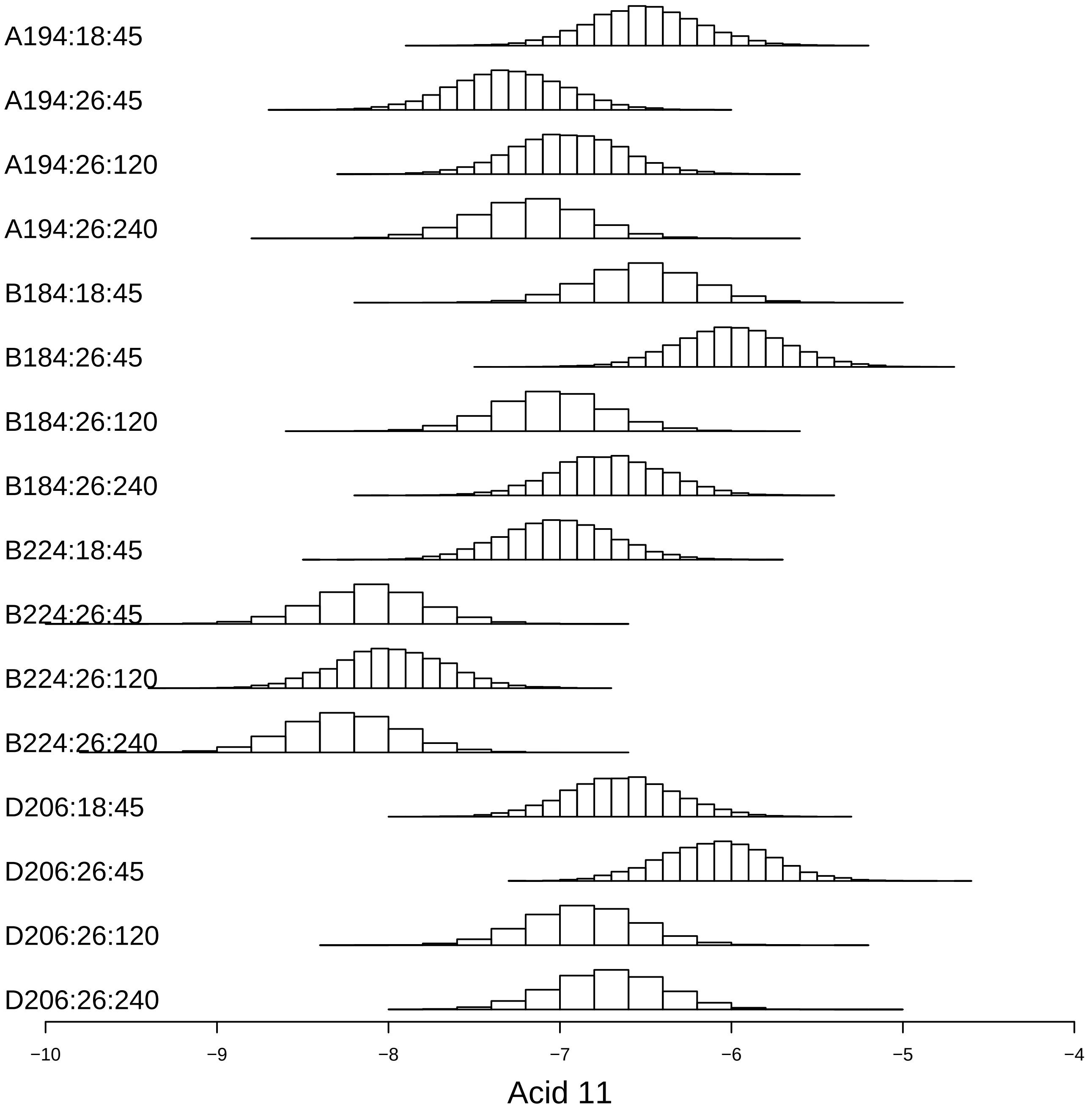
A194:18:45
A194:26:45
A194:26:120
A194:26:240
B184:18:45
B184:26:45
B184:26:120
B184:26:240
B224:18:45
B224:26:45
B224:26:120
B224:26:240
D206:18:45
D206:26:45
D206:26:120
D206:26:240
−10
−9
−8
−7
−6
−5
−4
Acid 11

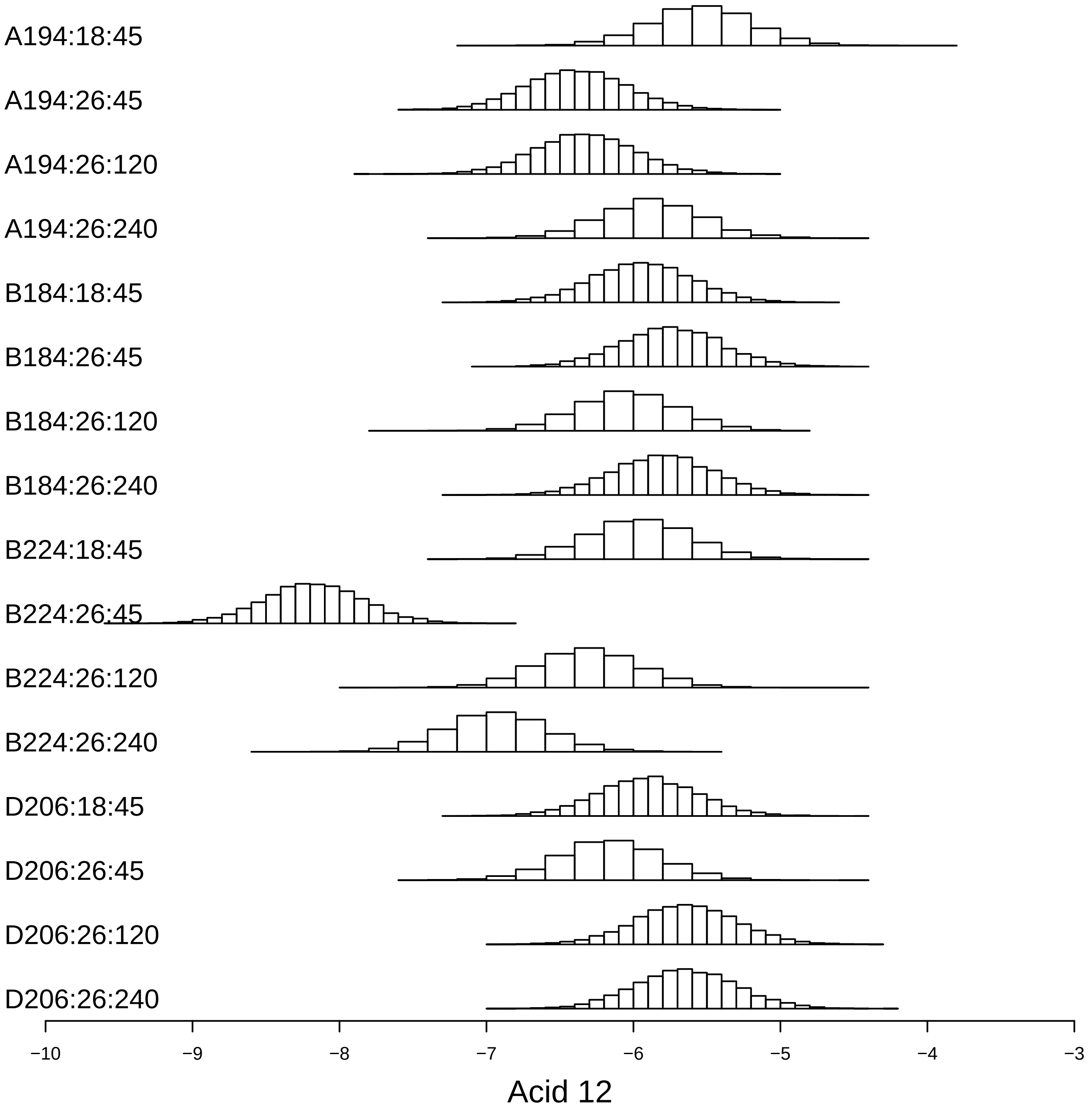

A194:18:45
A194:26:45
A194:26:120
A194:26:240
B184:18:45
B184:26:45
B184:26:120
B184:26:240
B224:18:45
B224:26:45
B224:26:120
B224:26:240
D206:18:45
D206:26:45
D206:26:120
D206:26:240
−10
−9
−8
−7
−6
−5
−4
−3
Acid 12

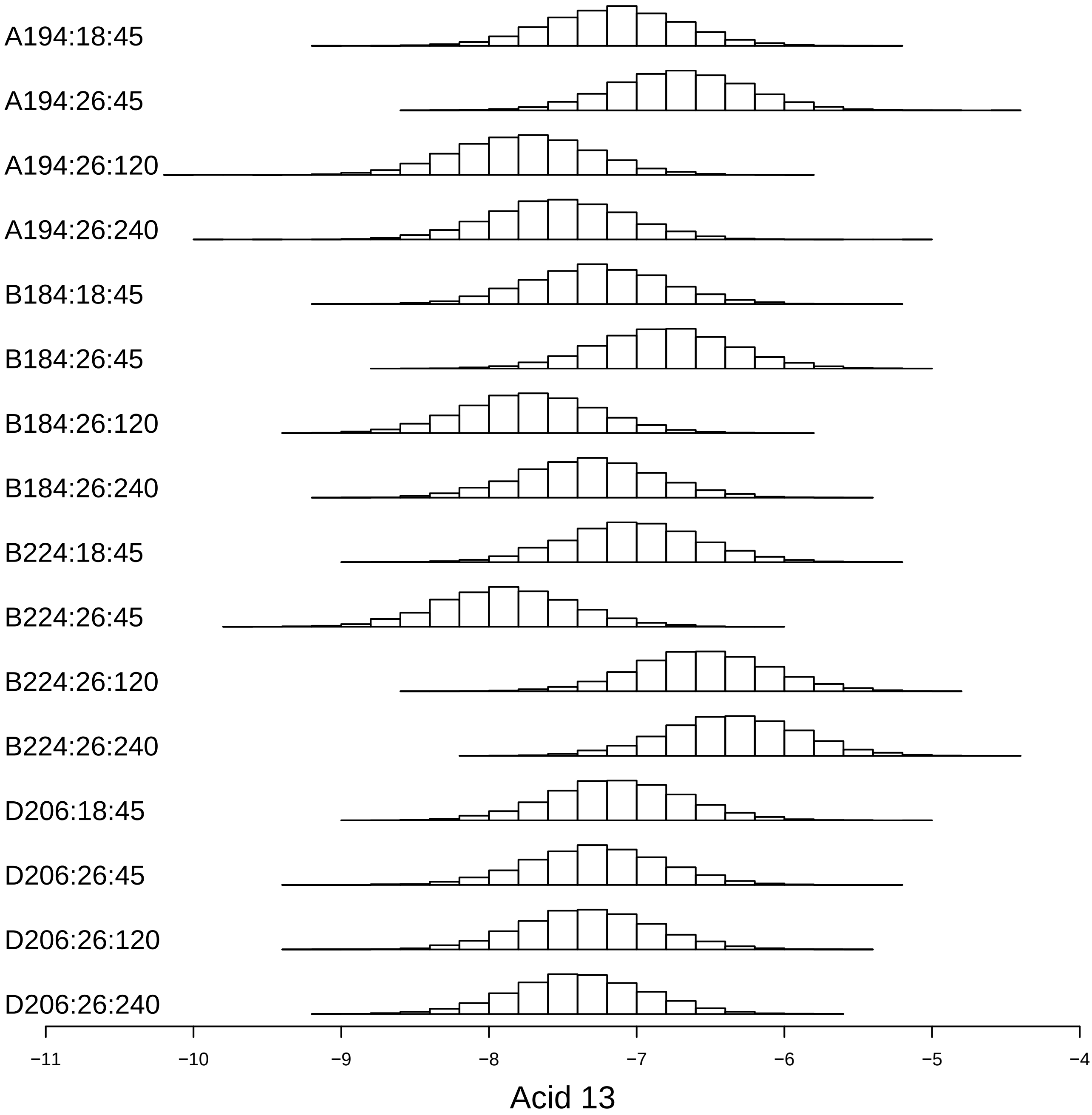
A194:18:45
A194:26:45
A194:26:120
A194:26:240
B184:18:45
B184:26:45
B184:26:120
B184:26:240
B224:18:45
B224:26:45
B224:26:120
B224:26:240
D206:18:45
D206:26:45
D206:26:120
D206:26:240
−11
−10
−9
−8
−7
−6
−5
−4
Acid 13

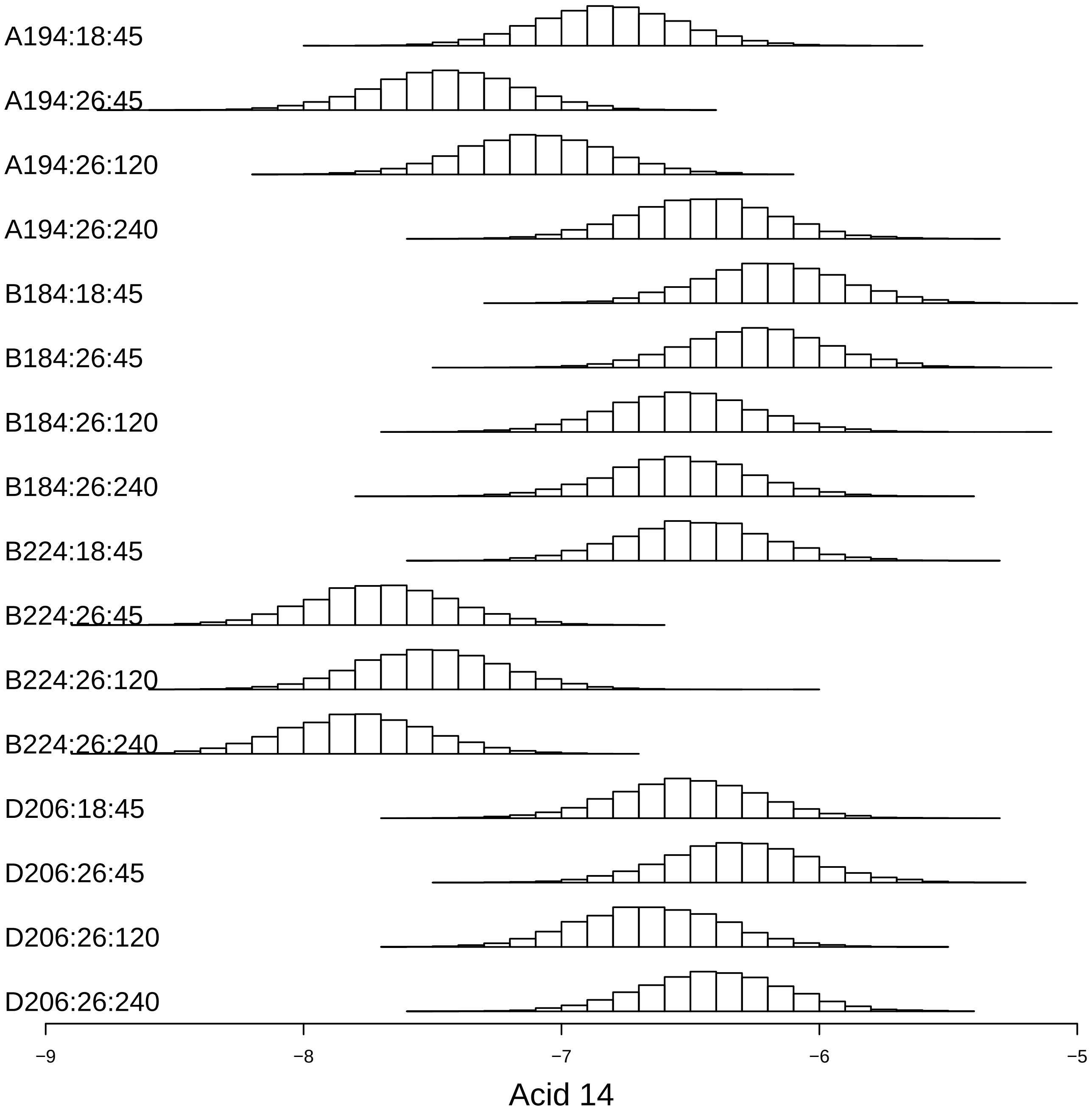
A194:18:45
A194:26:45
A194:26:120
A194:26:240
B184:18:45
B184:26:45
B184:26:120
B184:26:240
B224:18:45
B224:26:45
B224:26:120
B224:26:240
D206:18:45
D206:26:45
D206:26:120
D206:26:240
−9
−8
−7
−6
−5
Acid 14
