# Supplementary figures and images for: Taxonomic and Environmental Variation of Metabolite Profiles in Marine Dinoflagellates of the Genus Symbiodinium
Source: Metabolites. 2015 Feb 16;5(1):74–99. doi: 10.3390/metabo5010074 (PMC4381291; doi:10.3390/metabo5010074)

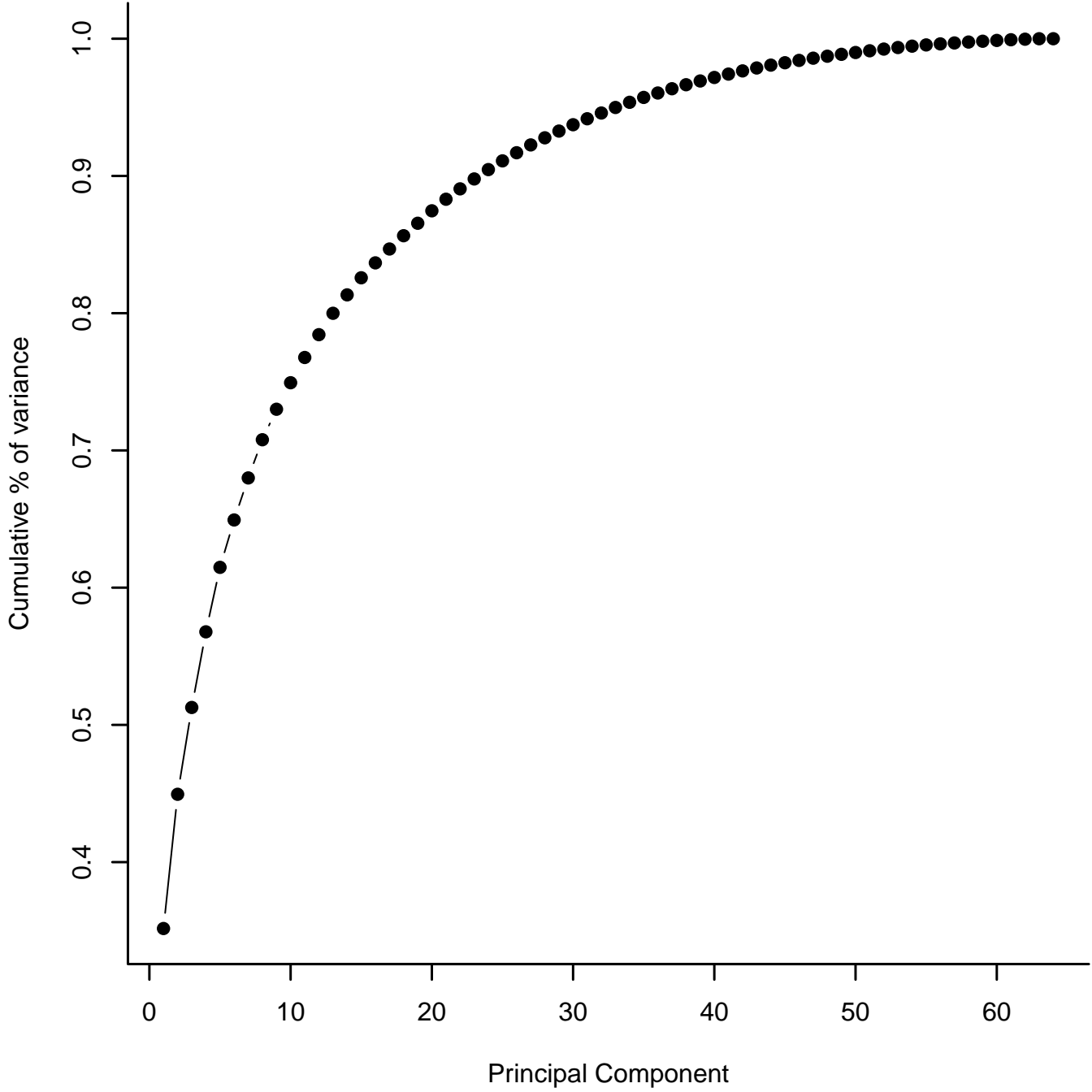

Supplement: Supplementary File 1 [file metabolites-05-00074-s001.zip › Supplementary Information/Supplementary Information Figure S2 - PCA Component variance.pdf]

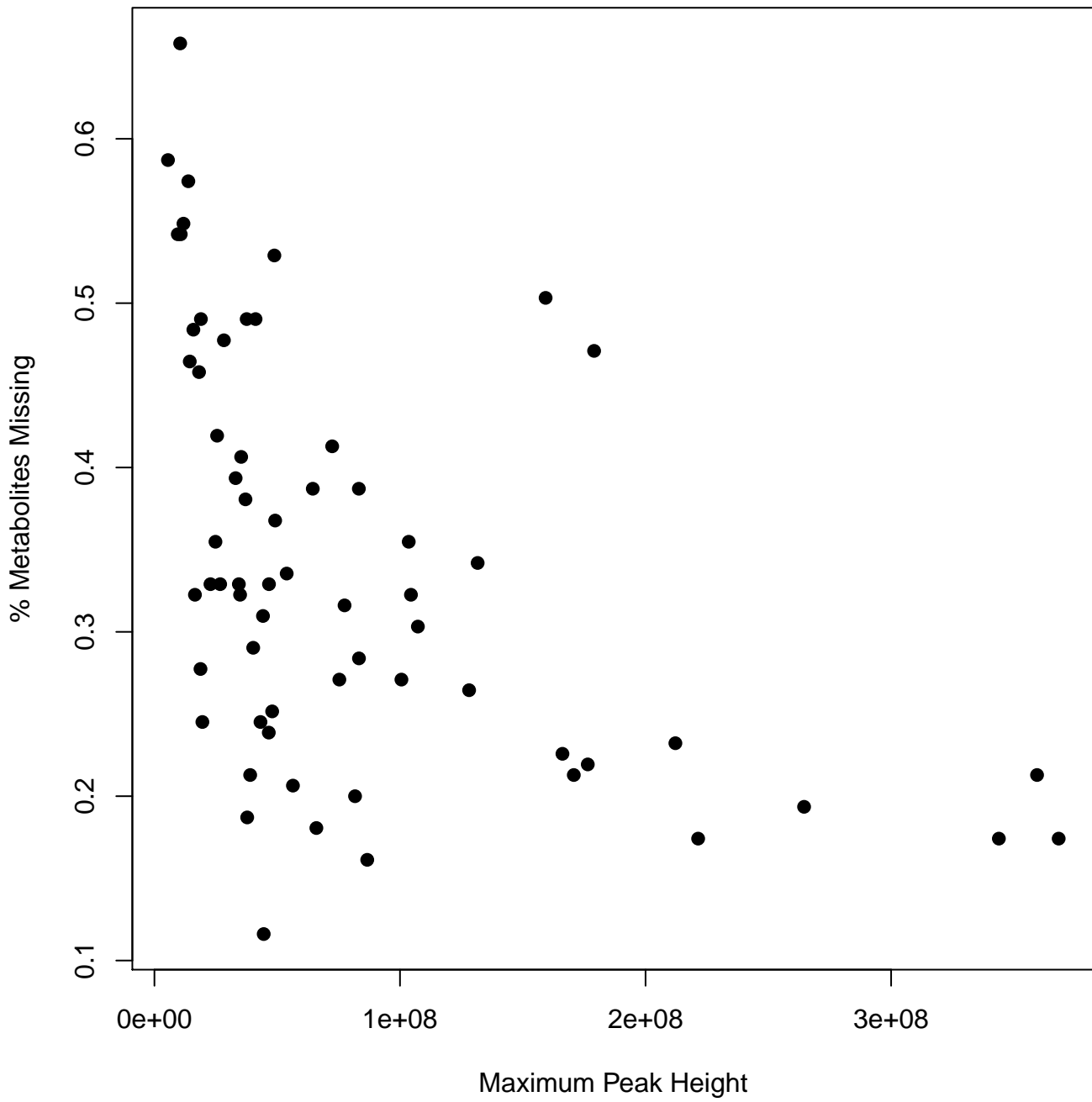

Supplement: Supplementary File 1 [file metabolites-05-00074-s001.zip › Supplementary Information/Supplementary Information Figure S6 - Missing vs. peak height.pdf]

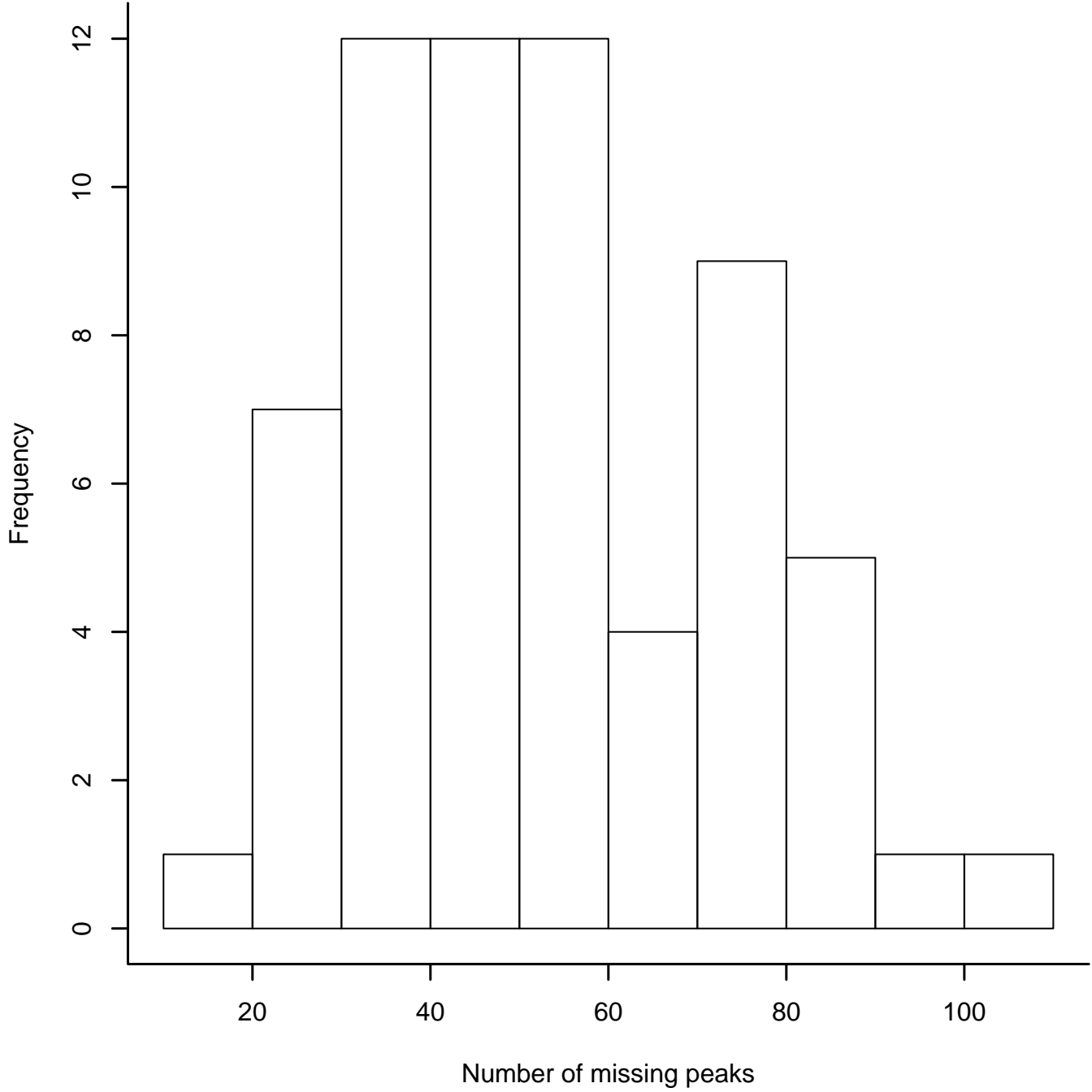

Supplement: Supplementary File 1 [file metabolites-05-00074-s001.zip › Supplementary Information/Supplementary Information Figure S7 - Missing Data histogram.pdf]
